# Interplay of telomerase non-canonical functions in NK cell resistance to iCasp9-mediated apoptosis

**DOI:** 10.1038/s41420-026-03183-y

**Published:** 2026-06-12

**Authors:** Anastasia I. Palamarchuk, Maria O. Ustiuzhanina, Rodion A. Velichinskii, Julia D. Vavilova, Maria V. Grechikhina, Elena I. Kovalenko, Maria A. Streltsova

**Affiliations:** 1 Shemyakin–Ovchinnikov Institute of Bioorganic Chemistry RAS, Moscow, Russia; 2 Pirogov Russian National Research Medical University, Moscow, Russia

**Keywords:** Transcriptional regulatory elements, Immunotherapy, RNA splicing, Gene regulation, Gene regulation in immune cells

## Abstract

NK cells are promising candidates for adoptive cell therapy; however, their proliferative capacity and functional persistence may be limited. Genetic modification with *hTERT* enhances their proliferative potential, while co-expression of the *iCASP9* suicide gene provides a safety mechanism based on late-stage apoptosis induction by chemical dimerizer (CID). Whether hTERT overexpression interferes with iCasp9-mediated cell death remains unclear, and the non-canonical functions of telomerase in this context are poorly understood. This study served a dual purpose: to assess the efficacy of the iCasp9 “suicide switch” in NK cells, and to investigate a non-canonical role of telomerase in NK cell-mediated evasion from cell death. Here, we demonstrate that hTERT-modified NK cells exhibit significant resistance to CID-induced apoptosis, an effect independent of telomerase catalytic activity, as confirmed using a dominant-negative hTERT (DN-hTERT) mutant. Transcriptomic profiling revealed that both CID-resistant iCasp9-NK cells and hTERT-iCasp9-NK cells share common gene expression signatures: upregulation of cell cycle-associated genes and downregulation of splicing-related factors, including *HNRNPH1* and *SNRPD3*, accompanied by shared patterns of alternative splicing. Among apoptosis-related transcripts, *BIRC3, which* encodes c-IAP-2, a direct inhibitor of caspase 9, was consistently elevated in both “resistant” and “survived” NK cells. However, shRNA-mediated knockdown of *BIRC3* failed to restore sensitivity to CID, indicating that *BIRC3* upregulation is not the unique determinant of resistance and suggesting involvement of additional compensatory pathways. Overall, our findings define specific transcriptional signatures associated with evasion of NK cells from iCasp9-mediated apoptosis, implying the contribution of cell cycle progression, enhanced anti-apoptotic signaling, and alterations in splicing regulation, and highlighting the complex role of non-canonical hTERT functions in these adaptations. In the rational design of next-generation gene-modified NK cell therapies with improved safety and persistence, the uncovered insights should be considered.

## Introduction

NK cells are a promising platform for cancer immunotherapy due to their ability to eliminate malignant cells without prior sensitization. While allogeneic application is possible [1], obtaining therapeutic numbers of cells remains challenging [2–4]. Genetic modification to enforce sustained expression of the catalytic subunit of human telomerase (hTERT) extends NK cell replicative capacity, preserves effector function, and enhances persistence in vitro and in vivo [5–7]. Beyond telomere maintenance [8–10], hTERT exerts non-canonical functions affecting transcription [11, 12], splicing, mitochondrial and endoplasmic reticulum stress responses [13–15], intracellular signaling [16, 17], and has been associated with enhanced cell survival [18, 19]. Although hTERT is typically downregulated in resting lymphocytes, it can be timely induced following cytokine stimulation or target recognition [20–22].

Enhanced function of engineered cells may increase risks of unintended toxicity [23] and malignant transformation, as reported for mesenchymal and induced pluripotent stem cells [24]. The inducible caspase-9 (iCasp9) suicide switch provides a clinically validated elimination mechanism [25–27]. Upon exposure to a chemical inducer of dimerization (CID), iCasp9 rapidly activates apoptosis [28–30]. However, incomplete elimination has been observed, notably in NK cells [31–33], potentially compromising safety. Since hTERT enhances pro-survival pathways, it may attenuate iCasp9-mediated apoptosis by elevating Bcl-2 [32] and Bcl‑X_L_ expression in mature NK cells [34]. Here, we examined whether hTERT overexpression impairs iCasp9-mediated apoptosis in primary human NK cells. Co-expressing hTERT and iCasp9, we assessed CID-induced elimination and identified determinants of incomplete cell death. Integrative analyses revealed convergent pro-survival signatures shared between CID-resistant iCasp9-NK cells and hTERT-iCasp9-NK cells. Our findings demonstrate that hTERT confers resistance to iCasp9-mediated apoptosis independently of telomerase activity, uncovering a non-canonical pro-survival function that undermines this safety switch.

## Results

### hTERT-iCasp9-NK cells are more resistant to apoptosis induction upon CID treatment compared to iCasp9-NK cells

**Fig. 1:**
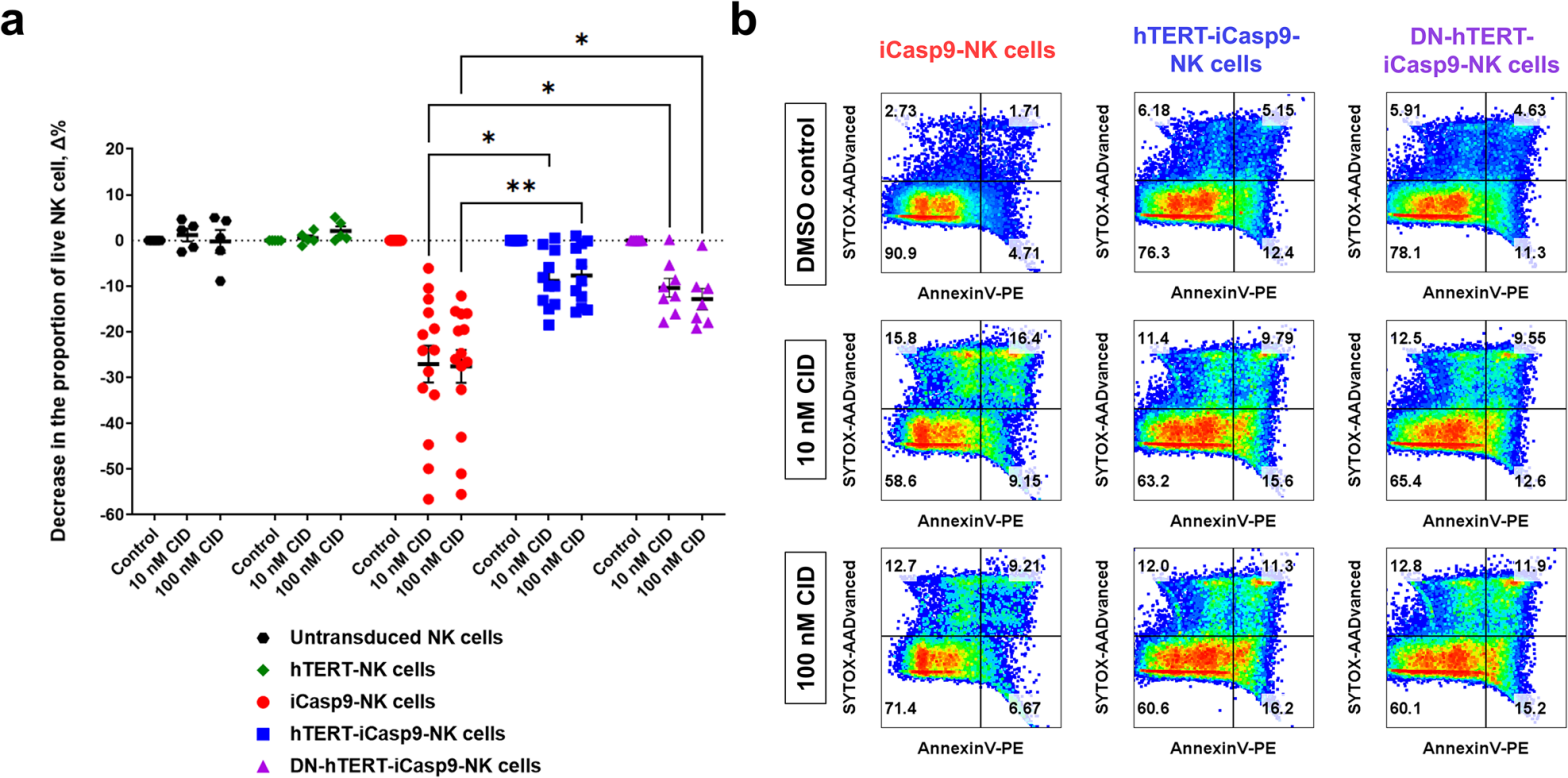
Evaluation of the effectiveness of cell death induction by iCasp9 suicide construct in NK cells transduced with *iCASP9, hTERT* and *DN-hTERT* genes. **a** Reduction in the proportion of live NK cells after treatment with 10 nM and 100 nM CID for 24 h, normalized on the percentage of live NK cells in the control group incubated in the presence of DMSO, the CID solvent. Tukey’s multiple comparisons test was used to compare untransduced NK cells, *n* = 5; hTERT-NK cells, n = 5; iCasp9-NK cells, *n* = 14; hTERT-iCasp9-NK cells *n* = 11; DN-hTERT-iCasp9-NK cells, *n* = 8. Means +/- SEM. **b** Representative plots of NK cells stained with AnnexinV-PE and SYTOX-AADvanced after 24 h of CID treatment.

NK cells retrovirally modified for stable expression of *hTERT* and/or *iCasp9* were tested for the effectiveness of iCasp9-mediated cell elimination. Even though hTERT-iCasp9-NK cells and iCasp9-NK cells demonstrated comparable levels of the *iCASP9* transgene expression, hTERT-iCasp9-NK cells were more resistant to apoptosis upon 10 nM or 100 nM CID treatment for 24 h (less than 19%, mean 10%) in comparison with iCasp9-NK cells (up to 56%, mean 28.6%) (Fig. 1 and Supplementary Fig. 2).

*hTERT* overexpression may counteract cell death induction by its non-canonical functions [35, 36]. DN-hTERT-iCasp9-NK cells, as well as hTERT-iCasp9-NK cells, showed resistance to cell death induction upon 10 nM or 100 nM CID treatment (Fig. 1).

### NK cells with different types of transgenes (*hTERT, hTERT* + *iCASP9, iCASP9*) regulate apoptotic signaling differently

**Fig. 2:**
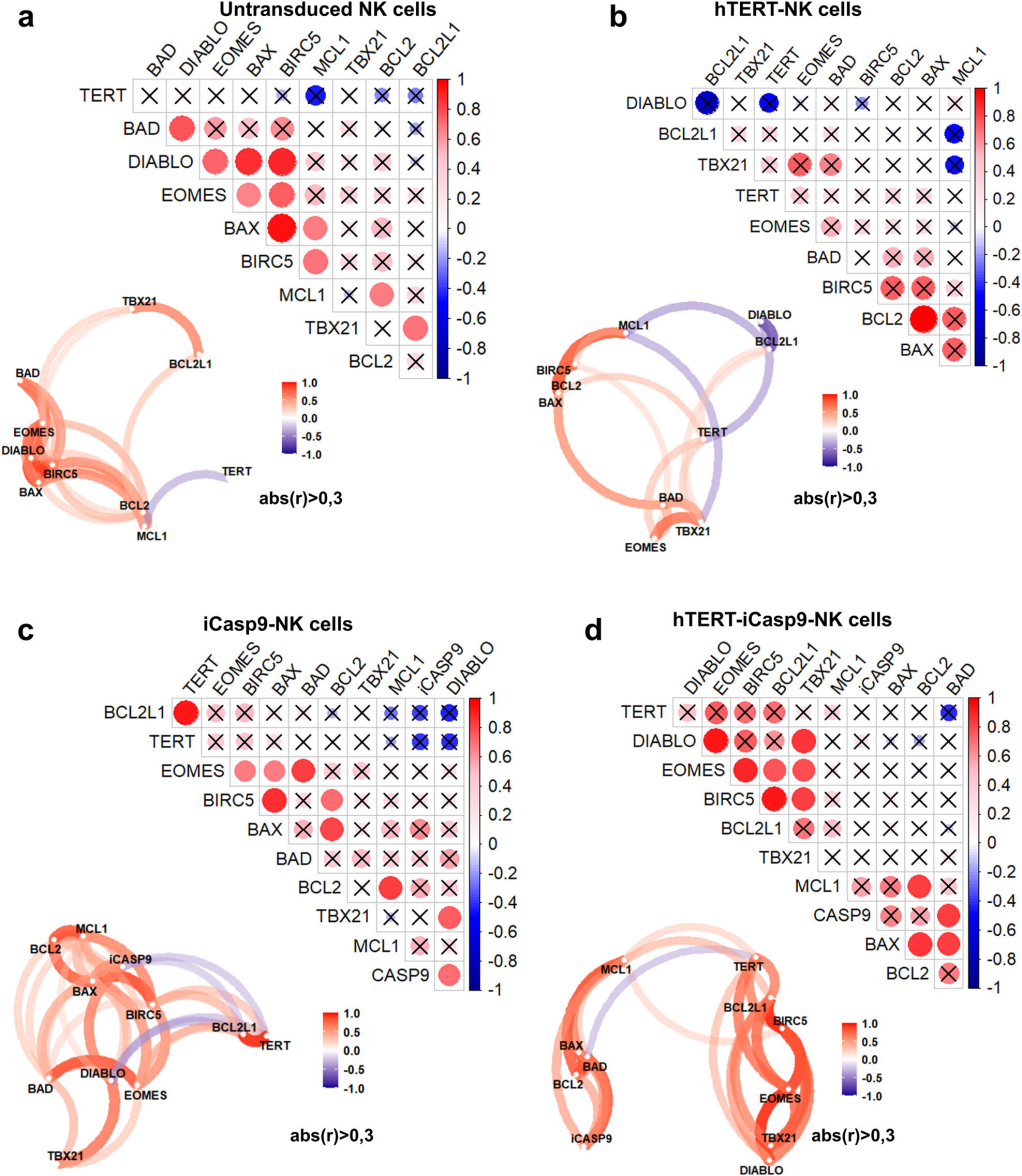
Interrelations of the expression levels of *BAD, BAX, DIABLO, BCL2, BCL2L1* (Bcl-X_L_), *MCL1, BIRC5* apoptotic genes, *hTERT* and *iCASP9* transgenes and *EOMES* and *TBX21* genes encoding transcriptional factors Eomes and T-bet in NK cells. The upper right corner of each plot represents clustered correlation matrices, and the lower left corner stands for a graphic representation of data (abs(r)>0.3). Correlations of gene expression levels are shown for: **a** untransduced NK cell, *n* = 10; **b** hTERT-NK cells, *n* = 7; **c** iCasp9-NK cells, *n* = 9; **d** hTERT-iCasp9-NK cells, *n* = 7. Spearman’s correlation test. *P*-value > 0.05 is signed by a cross on the correlation plot.

mRNA levels of: (1) *hTERT* and *iCASP9* transgenes, (2) *EOMES* and *TBX21* encoding the Eomes and T-bet transcription factors, (3) *DIABLO, BAX*, and *BAD* pro-apoptotic genes, (4) *BIRC5, MCL1, BCL2* and *BCL2L1* pro-survival genes were analyzed in NK cells *via* qPCR. However, no statistical differences were observed between four groups of NK cells: untransduced, hTERT-NK cells, hTERT-iCasp9-NK cells and iCasp9-NK cells (Supplementary Fig. 2). As transgene levels and apoptotic gene expression levels can be dependent on each other, we searched for associations between pro-survival and pro-apoptotic genes by correlational analysis. (Fig. 2). Untransduced NK cells formed a cluster of positively associated genes, including *DIABLO, EOMES, BIRC5, BAX*, and *MCL1* (associated with *BAX* and *BIRC5*). This cluster likely describes the life-death balance of pro-survival and pro-apoptotic factors determined by environmental conditions, including the availability of cytokines and growth factors (Fig. 2a). In hTERT-NK cells, correlational analysis revealed a positive relationship between *BCL2* and *BAX* mRNA levels, while *hTERT* showed a tendency to negatively correlate with *DIABLO* (Fig. 2b). Within iCasp9-NK cells, the gene cluster observed in untransduced NK cells was partially preserved and included *EOMES, BIRC5, BAD and BCL2*. In addition, *iCASP9* was positively associated with *DIABLO*, which in turn positively correlated with *TBX21*. We also noticed a strong positive correlation for *hTERT* and *BCL2L1*, and separately for *BCL2* correlated with *MCL1* (Fig. 2c). Within hTERT-iCasp9-NK cells, *hTERT* and *iCASP9* showed mutual influence, revealing two clusters: one included *iCASP9, BAD, BAX, BCL2*, and *MCL1*; the second included *DIABLO, EOMES, BCL2L1, BIRC5, and TBX21* with a tendency for positive association with *hTERT* (Fig. 2d).

Thus, introduction of *iCASP9* generally increased pro-apoptotic gene expression, such as *DIABLO* or *BAD*, whereas *hTERT* expression rose simultaneously with pro-survival factors expression (*BCL2L1* and *BIRC5)* and negatively correlated with *DIABLO*. For hTERT-iCasp9-NK cells, we noticed the most evident separation of transcript levels of studied genes into two clusters, indicating an independent effect of each of the transgenes in tuning NK cell survival.

### (CID)-iCasp9-NK cells and hTERT-iCasp9-NK cells had altered transcriptional profiles compared to iCasp9-NK cells, characterized by activation of pro-survival genes

We investigated transgene-related transcriptomic changes in NK cells using RNAseq. Additional characteristics of NK cells that underwent RNAseq analysis are presented in Supplementary Fig. 3. We studied 3 sets of modified NK cells: hTERT-iCasp9-NK cells, iCasp9-NK cells and (CID)-iCasp9-NK cells (iCasp9-NK cells survived after 24 h 100 nM CID exposure) to reveal factors providing enhanced resistance toward iCasp9-induced apoptosis.

**Fig. 3:**
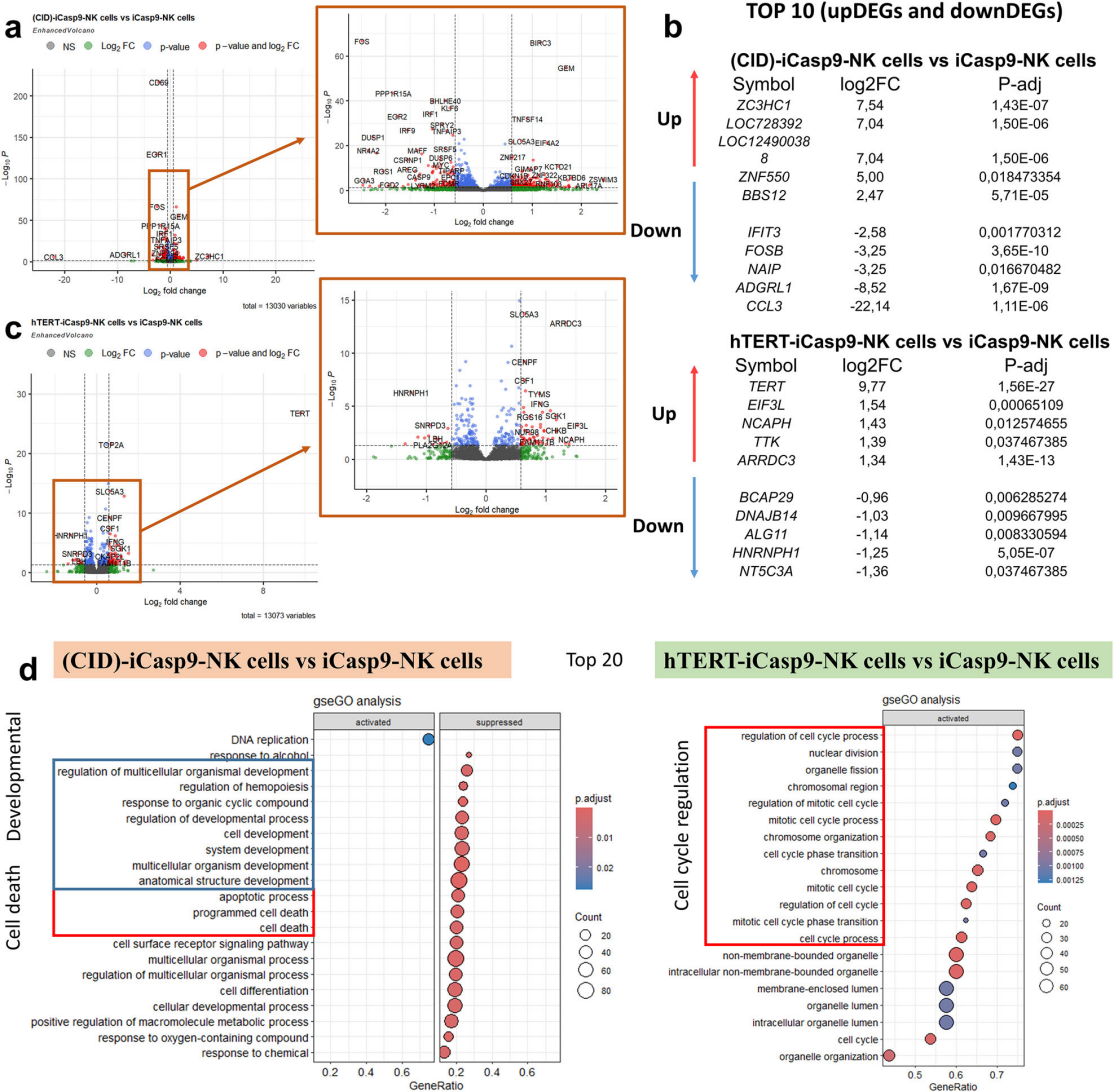
Transcriptional profiles of three groups of comparison. Enhanced volcano plot for comparisons: **a** “survived” iCasp9-NK cells that underwent incubation with 100 nM CID for 24 h compared *vs* “susceptible” iCasp9-NK cells, **c** “resistant” hTERT-iCasp9-NK cells *vs* “susceptible” iCasp9-NK cells. P-adj<0.05 and abs(Log2FC)>0.58. **b** The table representing Top 10 (5 upDEGs and 5 downDEGs) for both comparisons presented on the volcano plot. **d** The representation of RNA seq data for iCasp9-NK cells, iCasp9-hTERT-NK cells and “survived” iCasp9-NK cells that underwent incubation with 100 nM CID for 24 h. DEGs (p-adj<0.05 and abs(Log2FC)>0.58). Dotplot of GeneOntology gene set enrichment analysis, where the y-axis represents GO molecular pathways, namely, Biological Processes (BP). The greater the size of a circle, the greater the number of genes involved in a pathway. The circles are colored based on p-adjusted values.

Comparison of (CID)-iCasp9-NK cells with iCasp9-NK cells revealed 402 differentially expressed genes (DEGs) (p-adj<0.05 and abs(Log_2_FC)>0.58), of which 251 DEGs were upregulated (upDEGs) and 151 DEGs were downregulated (downDEGs) (Fig. 3a, b). *ZC3HC1, CDC25A, CCNE2, ESCO2* associated with cell division were among the most significantly upregulated genes. We also observed downregulation of *IFIT3*, whose protein product protects from mitochondria-mediated apoptosis [37]. *ZNF550*, encoding the transcription factor with predicted DNA-binding activity, was also significantly elevated, whereas *FOSB* was downregulated. Among apoptosis-associated factors, *BIRC3* and *FADD* were upregulated, whereas *NAIP, FOS, CTSK, iCASP9, NFKBIA and DDIT3* were downregulated. Thus, “survived” (CID)-iCasp9-NK cells showed a large number of DEGs in intracellular signaling pathways capable of compensating the destructive effects of iCasp9 (Fig. 3b and Supplementary Fig. 4).

When comparing hTERT-iCasp9-NK cells and iCasp9-NK cells, we determined 63 upDEGs and 13 downDEGs (Fig. 3b, c). Among upDEGs, we confirmed the overexpression of *TERT* and observed several genes associated with the cell cycle: *NCAPH, TTK*, and *CDCA5*. The *IFNG* transcripts were also accumulated within hTERT-iCasp9-NK cells*. EIF3L,* encoding a factor linked to translation initiation, was upregulated. Among downDEGs, we observed *HNRNPH1* and *SNRPD3* encoding proteins participating in RNA splicing. *DNAJB14*, encoding a protein involved in chaperone protein refolding, was downregulated in hTERT-iCasp9-NK cells (Fig. 3b and Supplementary Fig. 5). Therefore, hTERT overexpression is associated with upregulation of cell cycle-promoting genes, transcription activity and modulation of splicing within NK cells.

We conducted parallel comparisons of transcriptomic data of (CID)-iCasp9-NK cells *vs* iCasp9-NK cells and hTERT-iCasp-NK cells *vs* iCasp9-NK cells. (CID)-iCasp9-NK cells upregulated a multitude of genes involved in DNA binding and DNA replication. For hTERT-iCasp9-NK cells, a large number of DEGs corresponded to pathways regulating cell cycle and cell division. Altogether, most of the DEGs observed in both groups refer to intranuclei protein-encoding transcripts (Fig. 3d). Using Gene Ontology gene set enrichment analysis we found significant suppression of cell developmental programs and cell death regulation pathways in (CID)-iCasp9-NK cells and activation of pathways involved in cell cycle regulation in hTERT-iCasp9-NK cells. So, (CID)-iCasp9-NK cells and hTERT-iCasp9-NK cells were characterized by differently directed transcriptomic changes relative to iCasp9-NK cells (Fig. 3d and Supplementary Figs. 6, 7).

**Fig. 4:**
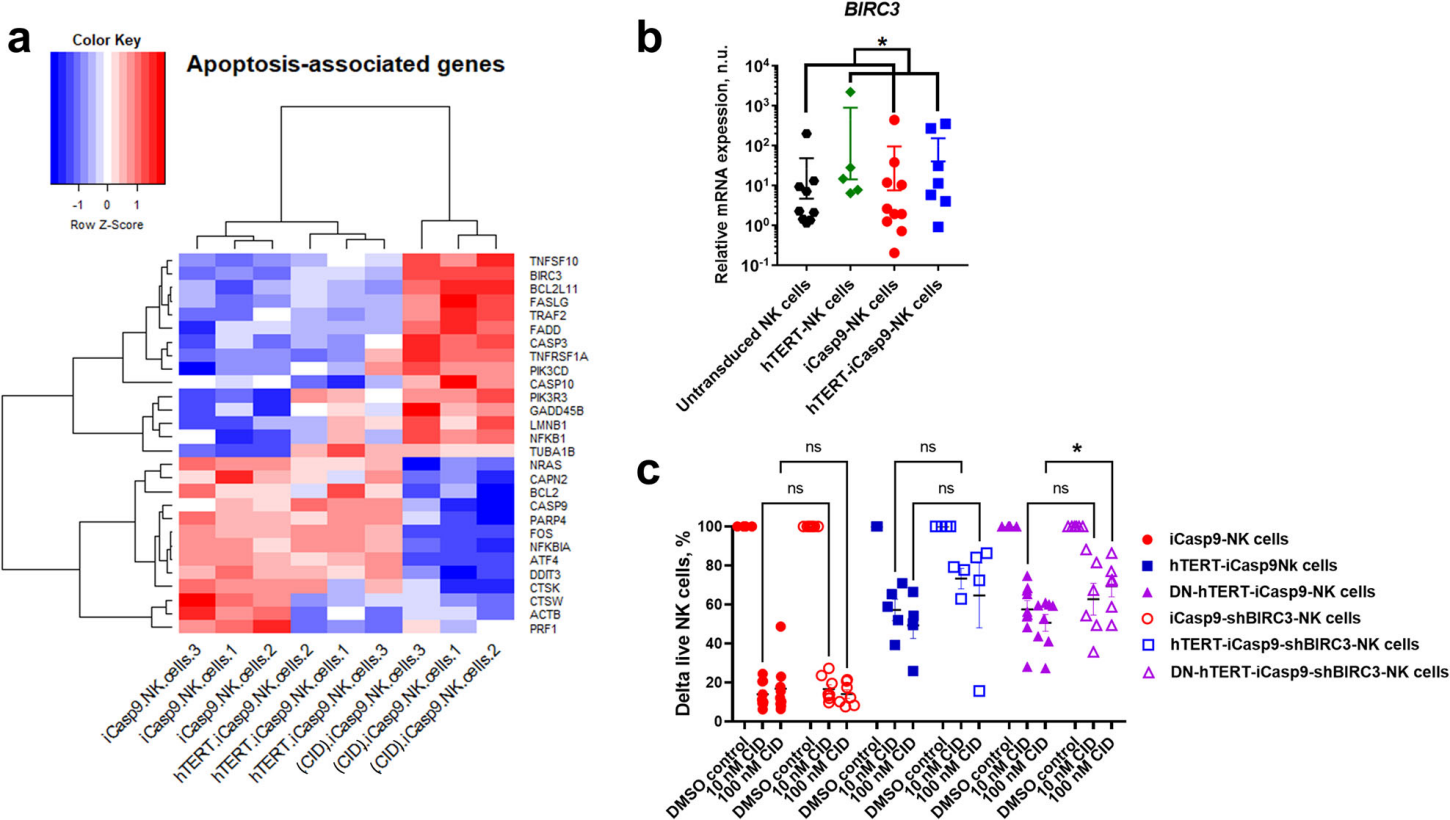
The investigation of iCasp9-mediated cell death induction in NK cells with *BIRC3* knockdown. **a** Evaluation of the effectiveness of iCasp9-mediated cell death in NK cells modified with *hTERT, DN-hTERT, iCASP9* genes and shBIRC3 construct. Using the Kyoto Encyclopedia of Genes and Genomes (KEGG) database, normalized counts of genes corresponding to the apoptosis pathway “Hsa04210” with p-adj<0.05 from (CID)-iCasp9-NK cells *vs* iCasp9-NK cells comparison were extracted for all NK cell populations and plotted on a heatmap. Row Z-score normalized data is presented. Red stands for transcripts with increased expression and blue for decreased expression. **b** Relative mRNA expression of *BIRC3* measured by qPCR in untransduced NK cells and transduced with *hTERT, hTERT* + *iCASP9* and *iCASP9*. Mann-Whitney test for merged data from untransduced + iCasp9-NK cells (*n* = 19), compared to hTERT-NK cells + hTERT-iCasp9-NK cells (*n* = 12). The groups were defined regarding hTERT expression within cells. Untransduced and iCasp9-NK cells are characterised with low hTERT expression compared to hTERT-overexpressing hTERT-NK cells and hTERT-iCasp9-NK cells. Median +/- SEM. **c** Decrease in the proportion of live NK cells after 10 nM and 100 nM CID treatment for 24 h normalized on the counts and percentage of live NK cells in the control group incubated in the presence of DMSO, CID solvent. Tukey’s multiple comparisons test. 10 populations of transduced cells from 8 donors (iCasp9-NK cells, *n* = 9; hTERT-iCasp9-NK cells, *n* = 5; DN-hTERT-iCasp9-NK cells, *n* = 9; iCasp9-shBIRC3-NK cells, *n* = 8; hTERT-iCasp9-shBIRC3-NK cells, *n* = 3; DN-hTERT-iCasp9-shBIRC3-NK cells, *n* = 6). Mean +/- SEM.

We precisely studied the transcript levels of genes linked to apoptotic pathways in these three cell types. (CID)-iCasp9-NK cells had altered expression levels of both pro-survival and pro-apoptotic genes compared to iCasp9-NK cells. Among cell death-associated genes, TNF receptor signaling genes and genes encoding BIM (*BCL2L11*), caspases 3, 2, and 10 were upregulated, while caspase 9 showed a reduced expression level. Among pro-survival genes, the *NFKB1* gene and its target *BIRC3* and *TRAF2* were upregulated (Fig. 4a and Supplementary Fig. 8). In the comparison of hTERT-iCasp9-NK cells *vs* iCasp9-NK cells, *BIRC3* was also enhanced. We also verified the levels of *BIRC3* transcripts in NK cells by qPCR. *BIRC3* expression was elevated in NK cells with *hTERT* overexpression (hTERT- and hTERT-iCasp9-NK cells) compared with untransduced and iCasp9-modified NK cells (Fig. 4b). Since iCasp9 acts at late stages of apoptosis, the upregulation of *BIRC3* (encoding c-IAP2) can mitigate caspase 9-induced apoptosis and may contribute to the NK cell resistance to iCasp9-mediated cell death. We incorporated the *U6-shBIRC3-cPPT* sequence into existing vectors to reduce *BIRC3* transcript levels in modified NK cells. The shBIRC3-modified iCasp9-, hTERT-iCasp9-, and DN-hTERT-iCasp9-NK cells were treated with CID and evaluated for cell death induction. We did not detect any significant changes in the proportions of dead cells between the shBIRC3-modified cells and corresponding controls (Fig. 4c and Supplementary Fig. 9). These results suggest that a variety of hTERT non-canonical functions modulate cell survival by protecting NK cells in an indirect manner.

### hTERT-iCasp9-NK cells and CID-treated iCasp9-NK cells share common transcriptomic patterns

We investigated common signatures among (CID)-iCasp9-NK cells and hTERT-iCasp9-NK cells *via* RNAseq analysis. Pathways acting in a similar way (activation or downregulation) were extracted from the data obtained from (CID)-iCasp9-NK cells *vs* iCasp9-NK cells and hTERT-iCasp9-NK cells *vs* iCasp9-NK cells comparisons.

**Fig. 5:**
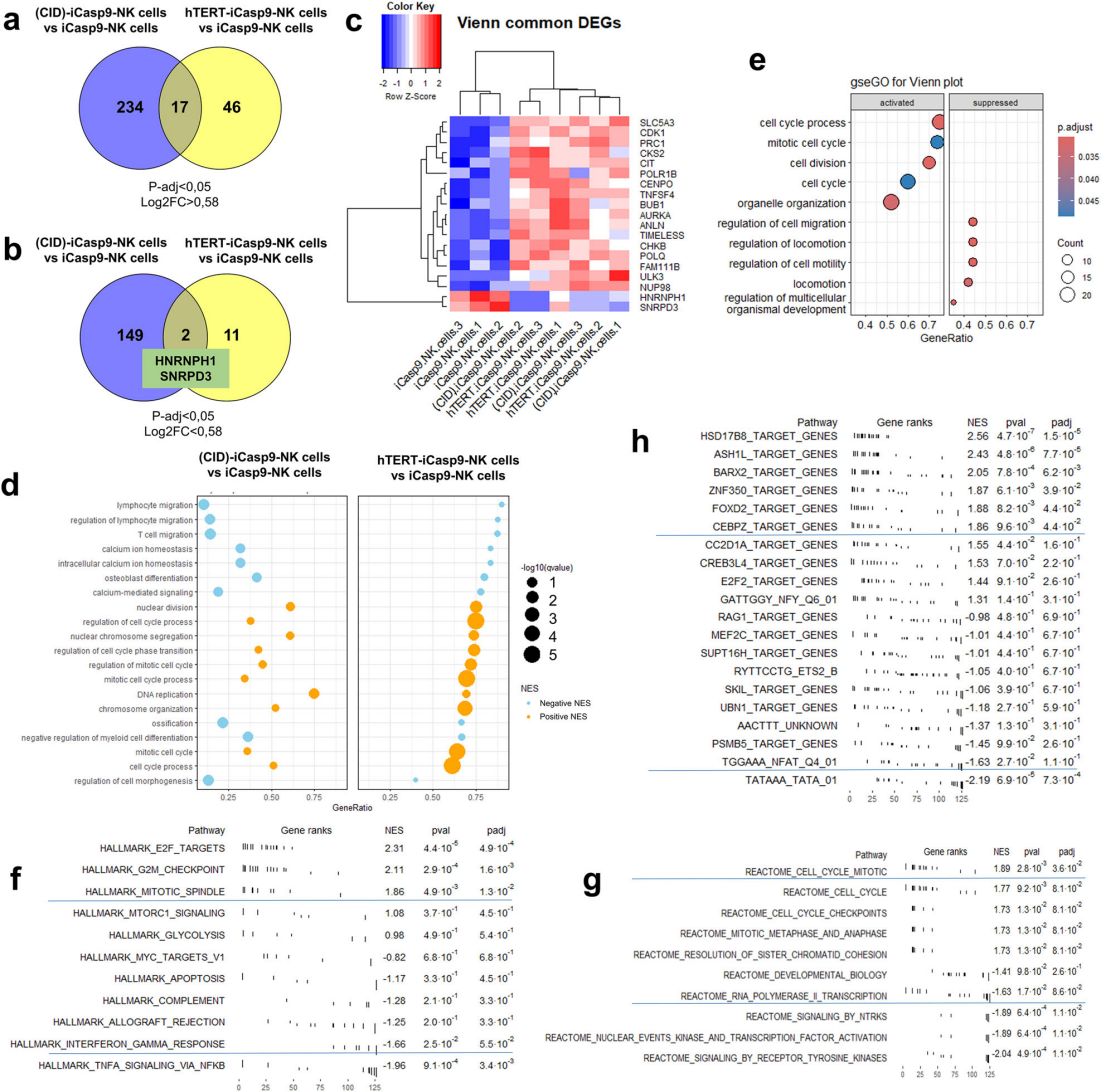
The representation of RNA seq data of “resistant” iCasp9-hTERT-NK cells and “survived” iCasp9-NK cells that underwent incubation with 100 nM CID for 24 h, compared to “susceptible” iCasp9-NK cells. DEGs (p-adj<0.05 and abs(Log2FC)>0.58) where upDEGs (p-adj<0.05 and Log2FC > 0.58) and downDEGs (p-adj<0.05 and Log2FC < 0.58). Two-circle Venn diagrams for common **a** upDEGs and **b** downDEGs. **c** Normalized counts for common upDEGs and downDEGS were extracted for all NK cell populations and plotted on a heatmap. Row Z-score normalized data is presented. Red stands for transcripts with increased expression and blue – for decreased expression. **d** GSEA comparison dotplot of GeneOntology gene set enrichment analysis of GO molecular pathways, namely, Biological Processes (BP). For (CID)-iCasp9-NK cells *vs* iCasp9-NK cells comparison and for hTERT-iCasp9-NK cells *vs* iCasp9-NK cells comparison Top 10 common pathways of both comparisons with NES > 0.9 percentile. Pathways with negative NES are blue and with positive NES are yellow. The greater the size of a circle, the greater significance. **e** The representation of RNA seq data of “resistant” iCasp9-hTERT-NK cells and “survived” iCasp9-NK cells that underwent incubation with 100 nM CID for 24 h compared *vs* “susceptible” iCasp9-NK cells. Common 126 DE genes with p-adj<0.05 from both comparisons were analyzed using gene set enrichment analysis (GSEA) and GeneOntology gene set enrichment analysis (gseGO). The corresponding Log2FC values were taken from “survived” (CID)-iCasp9-NK cells *vs* “susceptible” iCasp9-NK cells. gseGO represents GO molecular pathways, namely, Biological Processes (BP). The greater the size of a circle, the greater the number of genes involved in a pathway. The circles are colored based on p-adjusted values. On the X-axis, suppressed and activated pathways are shown. Bar plots represent normalized enrichment scores (NES) of GSEA for f Hallmark pathways, g Reactome pathways, h transcription factor targets (TFT). For plots (**f, g, h**) blue lines crop significantly c**h**anged pathways in accordance with p-adj.

We compared the obtained DEGs using a Venn diagram. 1096 genes with p-adj<0.05 from (CID)-iCasp9-NK cells *vs* iCasp9-NK cells comparison were opposed to 266 genes with p-adj<0.05 from comparison hTERT-iCasp9-NK cells *vs* iCasp9-NK cells. 126 genes were shared by NK cells susceptible to iCasp9-mediated apoptosis induction. We determined 17 genes upregulated in both comparisons (Fig. 5a, c) and 2 common downDEGs that stand for RNA processing in ribonucleoprotein particles (Fig. 5b, c). The upregulated common genes are described to be involved in biological processes such as cell division, immunologic processes, and cell migration; and their products possess serine-threonine kinase, histone-modifying and histone kinase, or protein serine kinase activities (Supplementary Fig. 10). 126 common genes (p-adj<0.05) from the Venn diagram were subjected to analysis (Fig. 5e and Supplementary Fig. 10), ranked on corresponding Log2FC levels from (CID)-iCasp9-NK cells *vs* iCasp9-NK cells comparison (Fig. 5e and Supplementary Fig. 10). Common pathways related to cell migration and calcium homeostasis were suppressed, whereas pathways associated with cell cycle progression were enhanced (Fig. 5d, e).

Hallmark pathways showed enrichment in E2F target genes that are responsible for cell cycle regulation and involved in DNA replication (Fig. 5f and supplementary Fig. 11). Similar results were observed when examining Reactome pathways, where an increase in “cell cycle mitotic” was revealed (Fig. 5g and Supplementary Fig. 11). The “Hallmark TNFa signaling *via* NFkB” pathway along with “Reactome signaling via NTRKS”, “Reactome nuclear events kinase and transcription factor activation”, and “Reactome signaling by receptor tyrosine kinase” were significantly reduced (Fig. 5f, g). We also identified involvement of a number of transcription factors by their upregulated or downregulated target genes. Thus, the activation of genes involved in transcriptional regulation, such as *HSD17D8, ASH1L, BARX2, FOXD2, ZNF350* and *CEBPZ*, and the suppression of TATAAA TATA 01 may orchestrate the NK cell intrinsic processes responsible for their survival (Fig. 5h and Supplementary Fig. 12).

### NK cells resistant to CID-mediated apoptosis induction showed upregulation of cell cycle-promoting genes

**Fig. 6:**
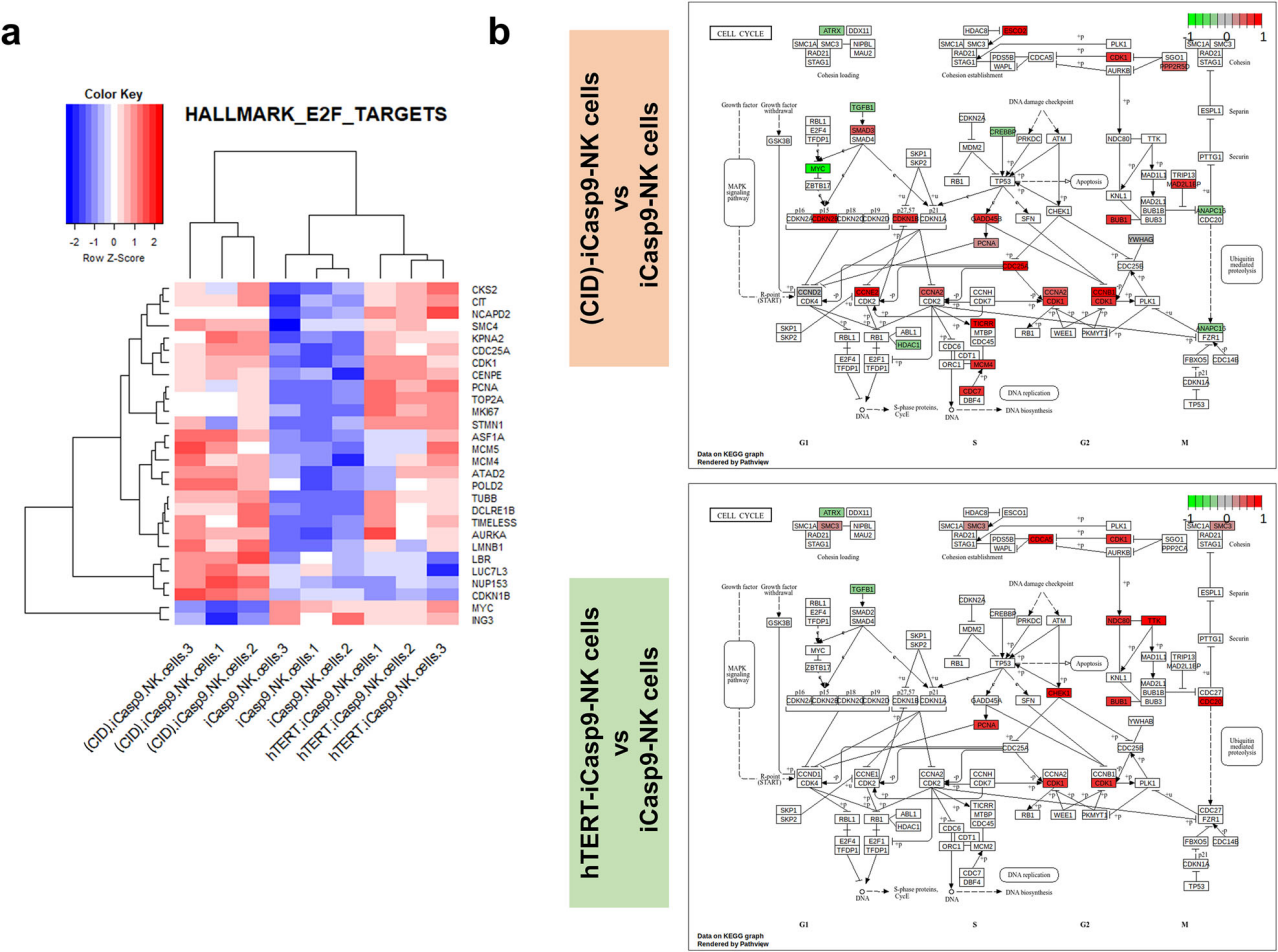
The representation of transcriptomic changes of cell cycle related genes for “resistant” hTERT-iCasp9-NK cells and “survived” iCasp9-NK cells that underwent incubation with 100 nM CID for 24 h compared with “susceptible” iCasp9-NK cells. **a** Using Kyoto Encyclopedia of Genes and Genomes (KEGG) database normalized counts of genes corresponding to cell cycle pathway “Hsa04110” with p-adj<0.05 from (CID)-iCasp9-NK cells *vs* iCasp9-NK cells comparison were extracted for all NK cell populations and plotted on a heatmap. Row Z-score normalized data is presented. Red stands for transcripts with increased transcripts and blue – for decreased expression. **b** KEGG pathview plot for cell cycle pathway “Hsa04110” for “survived” (CID)-iCasp9-NK cells *vs* “susceptible” iCasp9-NK cells. c KEGG pathview plot for cell cycle pathway “Hsa04110” for“resistant” iCasp9-hTERT-NK cells *vs* “susceptible” iCasp9-NK cells.

“Survived” (CID)-iCasp9-NK cells upregulated cell cycle genes throughout the whole cell cycle, while hTERT-iCasp9-NK cells were more precisely regulated in the G2 stage. Regarding E2F targets, a vast majority of genes changed their expression in the same direction compared to (CID)-iCasp9-NK cells and hTERT-iCasp9-NK cells. However, genes such as *LRB, LUC7L3, NUP153, CDKN1B, MYC* and *ING3*, which are involved in the regulation of cell proliferation and nuclear processes, changed their mRNA expression differently in hTERT-iCasp9-NK cells and iCasp9-NK cells compared to (CID)-iCasp9-NK cells (Fig. 6).

### “Survived” and “resistant” NK cells share several alternatively spliced transcripts

**Fig. 7:**
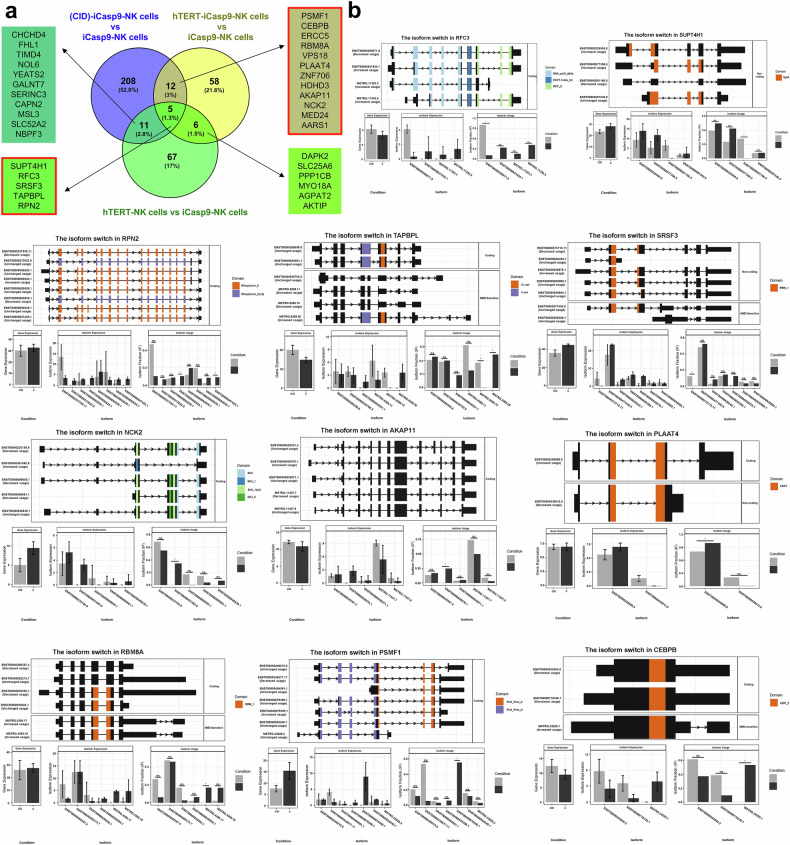
The alternatively spliced transcripts were evaluated for NK cell populations of “resistant” hTERT-overexpressing hTERT-NK cells, hTERT-iCasp9-NK cells and “survived” iCasp9-NK cells that underwent incubation with 100 nM CID for 24 h compared *vs* “susceptible” iCasp9-NK cells. **a** The common splicing events were analyzed using Venn diagrams. **b** Representative plots for genes listed in red are presented in the counter boxes of the Venn diagram. Upper graphs represent the most abundant isoforms detected, with some highlighted domains. The lower line shows from left to right: Gene expression, Isoform expression and Isoform usage. Gray bars stand for (CID)-iCasp9-NK cells and hTERT-iCasp9-NK cells and black bars reflect iCasp9-NK cells. The analysis was performed on the Galaxy platform following the manual https://training.galaxyproject.org/training-material/topics/transcriptomics/tutorials/differential-isoform-expression/tutorial.html.

To investigate splicing-related changes that can mediate cell survival, we applied analysis of splicing variants in 3 groups of comparison: (CID)-iCasp9-NK cells *vs* iCasp9-NK cells, hTERT-iCasp9-NK cells *vs* iCasp9-NK cells and hTERT-NK cells *vs* iCasp9-NK cells. We also applied hTERT-NK cells *vs* iCasp9-NK cells comparison to highlight the role of hTERT overexpression in splicing changes. For all three comparison groups, we defined 5 common alternatively spliced transcripts (*SUPT4H1, RFC3, SRSF3, TAPBPL, RPN2*) compared to iCasp9-NK cells, which may be attributed to the increased TERT levels. The products of these genes are primarily involved in fundamental processes of gene expression and cellular metabolism. Next, we observed 12 transcripts (*PSMF1, CEBPB, ERCC5, RBM8A, VPS18, PLAAT4, ZNF706, HDHD3, AKAP11, NCK2, MED24, AARS1*) with splicing changes common among “survived” and “resistant” NK cells and are associated with a lower tendency for these cells to undergo apoptosis (Fig. 7 and Supplementary Fig. 13). Collectively, they play a key role in maintaining cellular homeostasis, stress response, and genome integrity. We also observed NMD-sensitive (Nonsense Mediated Decay) isoforms that are susceptible to degradation (Fig. 7 and Supplementary Fig. 13). Thus, we specified a profile of differently expressed splicing isoforms, the balance of which determines the expression levels of functional proteins.

## Discussion

Although NK cells are promising for immunotherapy, expanding them without functional loss remains difficult. We generated hTERT-overexpressing NK cells with an iCasp9 suicide switch, but hTERT-iCasp9 cells showed incomplete CID-induced apoptosis. Similar incomplete elimination was reported in iCasp9-HeLa cells [31]. Despite 10 nM CID being sufficient to trigger iCasp9 cell death [32], even 100 nM CID failed to fully eliminate hTERT-iCasp9-NK cells. Catalytically inactive DN-hTERT conferred similar resistance, indicating a telomere-independent, non-canonical TERT effect [38, 39].

To elucidate the reasons for the partial resistance of hTERT-iCasp9-cells to apoptosis, we studied associations of various pro- and anti-apoptotic factors in cells transduced with *iCASP9* and/or *hTERT* genes. Notably, in hTERT‑iCasp9‑NK cells, the level of *hTERT* correlated positively with *BCL2L1* (Bcl‑X_L_), *BIRC5* (survivin), and *EOMES*, whereas iCasp9 correlated positively with *BAX, BAD*, and *DIABLO. DIABLO* is a direct p53 target and, together with *BAD*, participates in cellular stress responses [40, 41]. These correlation patterns suggest that hTERT‑iCasp9‑NK and iCasp9‑NK cells experienced elevated cellular stress compared to untransduced or hTERT‑NK cells.

Both “resistant” and “survived” cells exhibited elevated *BIRC3* (c‑IAP2), but not *BIRC4* or *BIRC5* transcripts, confirmed by RNAseq and qPCR. c‑IAP2 is a direct caspase‑9 inhibitor [42, 43] and an NF‑κB/Rel-responsive gene; its upregulation may reflect pro‑survival NF‑κB pathway activation [44]. In agreement with Mishima et al. [45], we observed that *hTERT* upregulation induced TNF-NFκB signaling and anti-apoptotic genes. However, shRNA-mediated *BIRC3* knockdown failed to restore CID sensitivity, suggesting redundant or parallel pathways in primary NK cells compensate for *BIRC3* loss *via BIRC4*- and *BIRC5*-independent mechanisms. “Survived” cells uniquely suppressed multiple developmental programs, possibly reflecting selection for cells with enhanced survival capabilities. Moreover, since both catalytically active *hTERT* and inactive *DN‑hTERT* conferred similar resistance, this effect is attributable to a non‑canonical function of TERT.

One of telomerase's non-canonical functions is the ability to modulate transcription activity [12, 46, 47]. This TERT function is highlighted by alterations in several pathways in both “survived” (CID)-iCasp9-NK cells and “resistant” hTERT-iCasp9-NK cells, linked to cell cycle progression and DNA replication (upregulated), and homeostasis and migration (downregulated). *STMN1* activates the PI3K/Akt pathway to suppress apoptosis, whereas its knockdown elevates caspase‑3 levels and susceptibility to apoptosis [48]; *PIK3R3* silencing reduces Akt phosphorylation and thus chemoresistance in sarcoma stem‑like cells [49]. Importantly, Akt directly phosphorylates pro‑caspase‑9 at Ser196, inhibiting its protease activity, and a Ser196Ala mutation renders caspase‑9 resistant to Akt‑mediated suppression [50]. Our observation of increased *PIK3R3* and *STMN1* suggests that signaling through PI3K/Akt may dampen iCasp9 effector function. Introducing a mutation at the corresponding Ser196Ala site of caspase 9 in the iCasp9 construct might potentially restore CID sensitivity in hTERT‑overexpressing therapeutic NK cells.

According to the transcription target database, we noted enrichment in genes associated with the *ASH1L, BARX2, FOXD2, ZNF350*, and *CEBPZ* transcription factors and TATA-box-associated factors linked to apoptosis and proliferation among both “survived” and “resistant’ NK cells. *ASH1L*, a histone methyltransferase maintaining active chromatin, suppresses apoptosis *via* downregulation of *CASP3* and *BAX*; its knockdown increases apoptotic rates [51]. *FOXD2* facilitates MLL4-dependent enhancer reprogramming *via* chromatin remodeling and modulates p53-responsive gene expression [52]. *BARX2-*related genes are implicated in immune cell function and tissue development [53]. *CEBPZ* promotes S-phase entry by suppressing C/EBPα and enhancing Pol II recruitment [54, 55]. *ZNF350 (ZBRK1)* functions within BRCA1-regulated transcriptional repression complexes controlling cell cycle checkpoints [56]. We previously reported that hTERT‑modified NK cells are enriched in the T‑BET⁺ subset, whereas iCasp9‑modified cells contain a larger EOMES⁺ fraction [57]. EOMES is linked to hTERT *via* the β‑catenin/Wnt pathway and can regulate apoptosis‑related genes; T‑bet modulates cell cycle through mTORC signaling [12, 34, 58]. Thus, positive cross‑regulation between *EOMES* and *TBX21* may contribute to the attenuated apoptosis observed in iCasp9‑expressing NK cells upon CID challenge. Collectively, these data demonstrate overlapping transcriptional programs for “survived” and “resistant” NK cells, favoring proliferation and survival and potentially influenced by chromatin-remodeling pathways.

Another common feature for these cells was downregulation of *HNRNPH1* and *SNRPD3*, encoding core splicing regulators whose knockdown impairs proliferation and induces cell death [59–61]. We identified a set of transcripts coherently spliced in “survived”, “resistant”, and hTERT‑NK cells compared to “susceptible” iCasp9‑NK control: *SUPT4H1, RFC3, SRSF3, TAPBPL*, and *RPN2. SRSF3* promotes growth and DNA repair [62–64]. *SUPT4H1* regulates transcription activity by tuning Pol II [65]. *RFC3* is a replication factor C subunit essential for DNA replication [66] that regulates the homologous recombination repair (HRR) pathway [64]. The knockdown of *RFC3* halts cell viability and proliferation [66]. *RPN2* maintains ER homeostasis and correlates with drug resistance [67]. *TAPBPL* influences antigen presentation [68]. The appearance of identical isoforms in both hTERT‑NK cells and CID‑selected survivors implicates hTERT in orchestrating a pro‑survival splicing programme independent of its catalytic activity.

A broader set of alternatively spliced genes, including *PSMF1 (PI31), CEBPB, ERCC5, RBM8A, VPS18*, and *PLAAT4,* was common to “survived” (CID)-iCasp9-NK cells and “resistant” hTERT-iCasp9-NK cells compared against “susceptible” iCasp9-NK cells. Overall, transcripts of *RBM8A* [69], *NCK2* [70], *CEBPB* [71] are known to encode prosurvival factors. *NCK2* adaptor proteins enhance proliferation [70]. For some transcripts, namely *PLAAT4*, the enrichment in NMD-sensitive isoform was shown, which may reflect a regulative mechanism of fine-tuning of intracellular protein concentrations [72]. These differences may display compensatory responses to apoptotic pressure caused by basal spontaneous iCasp9 dimerization rather than by direct hTERT activity. Still, we cannot exclude the possible role of telomerase in these splicing changes, since iCasp9-NK cells might obtain nonzero levels of endogenous telomerase activity.

While our data align with reports linking PI3K/Akt signaling to caspase-9 inhibition and splicing dysregulation to survival, several findings diverged from expectations. shRNA-mediated *BIRC3* knockdown failed to restore CID sensitivity. *SNRPD3* downregulation assumes a regulatory axis possibly involving hTERT itself or upstream stress signaling. Telomerase itself is regulated by splicing, and it is possible that the effects we observe are part of a compensatory effect on telomerase overexpression. Finally, our observation that both *hTERT-* and *DN‑hTERT* confer equivalent resistance, together with the correlation and splicing data, provides compelling evidence that hTERT modulates NK cell survival through non‑canonical, transcription‑related and splicing‑related mechanisms independent of telomere elongation.

In summary, CID-survived iCasp9-NK cells and hTERT-iCasp9-NK cells share extensive transcriptional and splicing signatures centered on cell cycle activation, PI3K/Akt signaling, and altered DNA/RNA processing. Some of these changes are consistent with known hTERT non-canonical functions. Others likely represent convergent adaptations to apoptotic stress. These findings highlight the complexity of apoptosis regulation in gene-modified NK cells and underscore the need to consider chromatin remodeling, kinase-mediated post-translational modulation and splicing rewiring when designing suicide switch-equipped cellular therapies.

## Methods

### Cell lines

K562-mbIL21 cells (expressing mbIL-21, CD64, CD86, CD137L, and CD19 fragment) [21] were provided by Dr. Dean Lee (MD Anderson Cancer Center, Houston, TX, USA). Raji was obtained from ATCC (Manassas, VA, USA). Both cell lines were cultured in RPMI-1640 complete medium (PanEco, Russia) with 10% FCS (HyClone, Logan, UT, USA), 2mM L-glutamine (PanEco), and 2 mM antibiotic‑antimycotic (Sigma-Aldrich, St. Louis, MO, USA). Phoenix Ampho (HEK293‑derived; ATCC) cells were maintained in DMEM complete medium (PanEco) with 10% FCS, 2mM L-glutamine, 2 mM sodium pyruvate (PanEco), and 2 mM antibiotic‑antimycotic.

### NK cells

Peripheral blood from healthy volunteers was obtained with informed written consent approved by the Ethics Committee of Pirogov Russian National Research Medical University. Blood from 15 healthy volunteers was applied in order to get at least 3 replicates in each experiment condition. NK cells were isolated from PBMC by negative magnetic separation (NK Cell Isolation Kit, Miltenyi Biotec, Germany). Cells were stimulated with γ‑irradiated K562‑mbIL21 feeders and 100 U/mL IL‑2 (Sci-Store, Russian Federation) [21] and cultured in medium: 10% FCS, 45% NK MACS (with supplement, Miltenyi Biotec), and 45% complete DMEM.

### Plasmids

Plasmids used: hTERT (xlox(GFP)hTERT, Addgene #69809) and iCasp9 (pMSCV‑F‑del‑Casp9.IRES.GFP, Addgene #15567). We made xlox‑TERT‑PGK-iCasp9‑IRES‑GFP [57] and DN‑hTERT constructs. For cloning strategy see Supplementary methods. shBIRC3 was assembled in pLKO‑1‑TRC. Primer design used Primer‑BLAST (NCBI, MD, USA) and SnapGene v3.2.1 (GSL Biotech, IL, USA).

### Transfection and concentration of viral particles

Phoenix Ampho cells were transfected using the calcium phosphate transfection kit (Biospecifica, Russian Federation). Viral supernatant was harvested at 48 h, 72 h and 96 h post-transfection and concentrated by centrifugation (21000 × *g*, 2.5 h, 4°C).

### Transduction and cell sorting

The viral suspension was centrifuged (2–4 h, 1800 × *g*, 4 °С) in the retronectin-coated (Clontech/Takara, CA, USA) plate. Transduction efficiency was assessed by GFP fluorescence using MACSQuant 10 (Miltenyi Biotec, 405/488/635 nm lasers) and sorting on Sony SH800S (Sony Biothechnology, SJ, USA; 405/488/643 nm lasers). Sorting purity was verified (Supplementary Fig. 1).

### Cell death induction in NK cells by a chemical inductor of dimerization

Сells were treated for 24 h with 10 nM or 100 nM of CID AP20187 (MedChemExpress, NJ, USA). Control cells were incubated with DMSO at an equivalent of 100 nM CID concentration. Cells were stained with AnnexinV-PE (Invitrogen, San Jose, CA, USA) and SYTOX AADvanced (Invitrogen, San Jose, CA, USA).

### qPCR analysis

Total RNA was extracted using ExtractRNA reagent (Evrogen, Russian Federation) and purified using TURBO DNA-free™ Kit (Invitrogen). cDNA was obtained with MMLV revertase (Evrogen).

Expression of *ACTB, BAD, BAX, BCL2, BCL2L1, BIRC5, BIRC3, DIABLO, EOMES, iCASP9, MCL1, TBX21 and hTERT* was quantified by CFX Connect Real-Time System (Bio-Rad Laboratories, CA, USA) using the 2-ΔΔCq method with *ACTB* normalization.

### RNAseq analysis

One month after isolation, modified NK cells were harvested in the RLT buffer. Surviving AnnexinV-PE^–^ SYTOX-AADvanced^–^ GFP^+^ (CID)-iCasp9-NK cells were sorted. RNAseq libraries were prepared with KAPA RNA HyperPrep Kit and KAPA UDI Primer Mixes (Roche) and sequenced on Illumina NovaSeq X (paired‑end). Analysis used the UseGalaxy platform (https://usegalaxy.eu) with alignment to hgGRCh38, and pipeline: trimmomatic, salmon, DESeq2. Splicing analysis followed the Galaxy tutorial (https://training.galaxyproject.org/training-material/topics/transcriptomics/tutorials/differential-isoform-expression/tutorial.html).

### Statistical analysis

Statistical analysis was carried out using GraphPad Prism 8.4 (StatSoft Inc., OK, USA) software, Python and R packages. For multiple comparisons, 2-ANOVA analysis with Tukey’s multiple comparisons was applied. For data that did not pass, the normality test was subjected to Spearman’s correlation test and the Mann-Whitney test. P-value: * *p* < 0.05; ** *p* < 0.01; *** *p* < 0.001. For RNA seq data, DEGs (P-adj<0.05, Log2FC > 0.58) were defined.

## Supplementary information

Supplementary methods and materials

Comparison hTERT-iCasp9-NK cells vs iCasp9-NK cells

Comparison CID-iCasp9-NK cells vs iCasp9-NK cells

## Data Availability

The dataset supporting the conclusions of this article are openly available under the BioProject ID PRJNA1423915 at https://www.ncbi.nlm.nih.gov/bioproject.
