# Supplementary material for: Interplay of telomerase non-canonical functions in NK cell resistance to iCasp9-mediated apoptosis
Source: Cell Death Discov. 2026 Jun 12;12:340. doi: 10.1038/s41420-026-03183-y (PMC13487172; doi:10.1038/s41420-026-03183-y)
Supplement: Supplementary file 1 [file 41420_2026_3183_MOESM1_ESM.pdf]

Supplementary methods

1. Cell lines

K562-mbIL21 is cell line derived from K562 cells that were genetically modified to express membrane-bound IL-21, CD64, CD86, CD137L and the fragment of the CD19 receptor (10.1371/journal.pone.0030264) and kindly provided by Dean Lee (MD Anderson Cancer Center, Houston, TX, USA). K562 was obtained from ATCC (Manassass, VA, USA).

K562-mbIL21 was cultured in a RPMI-1640 medium (PanEco, Russian Federation) containing 10% fetal calf serum (FCS, HyClone Labs, Logan, UT, USA) and supplemented with 2mM L-glutamine (PanEco, Russian Federation) and 2mM antibiotic-antimycotic (Sigma-Aldrich, St. Louis, MO, USA).

Phoenix Ampho cell line derived from HEK293 cells (ATCC, Manassass, VA, USA) constitutively expresses *Gag-Pol/Tat/Env/Rev* viral genes. Phoenix Ampho cells were maintained in DMEM medium (PanEco, Russian Federation) containing 10% FCS, 2mM L-glutamine (PanEco, Russian Federation), 2mM sodium pyruvate (PanEco, Russian Federation), 2mM antibiotic-antimycotic (Sigma-Aldrich, St. Louis, MO, USA).

2. NK cells

2.1. Isolation of NK cell from peripheral blood

Peripheral blood was obtained from 15 healthy volunteers with their informed consent (approved by the local ethics committee). PBMC were separated by Ficoll (ρ=1.077 g/ml, PanEco, Russian Federation) gradient centrifugation. NK cells were purified from PBMC by negative magnetic separation. NK cell isolation kit (Miltenyi Biotec, Germany) was applied in accordance with the manufacturer’s protocol.

2.2. Stimulation of NK cell

In order to enhance transduction efficiency and overall lifespan of NK cells (10.1371/journal.pone.0030264), freshly isolated NK cells were stimulated with γ-irridated K562-mbIL21 feeder cell line in proportion 2*NK / 1*K562-mbIL21 and 100U/ml of IL-2 (Hoffmann La-Roche, Basel, Switzerland). The cultivation at all stages was performed in medium mixed of 10% fetal calf serum (FCS, HyClone Labs, Logan, UT, USA), 45% NK MACS medium with its supplement (Miltenyi Biotec, Germany) and 45% DMEM medium containing 2mM L-glutamine (PanEco, Russian Federation), 2mM sodium pyruvate (PanEco, Russian Federation) with addition of 2mM antimycotic-antibiotic (Sigma-Aldrich, St. Louis, MO, USA). The medium was refreshed by a half/whole volume medium change every 3-4 days. The concentration of IL-2 was adjusted up to 100U/mL. NK cells were maintained in the concentration 0.6*10^6 to 0.9*10^6 cells/ml.

3. Plasmids assembly

3.1. Plasmid constructs and assembly

We used 8 plasmid retroviral vectors for simultaneous delivery of *hTERT, DN-hTERT, iCASP9* genes and *U6-shBIRC3-cPPT* construct in NK cells (Fig. 1). In order to identify the contribution of non-telomeric effects of telomerase, site-directed mutagenesis of the *hTERT* gene region in xlox(GFP)hTERT (Addgene #69809) was performed. The *DN-hTERT* gene from pBABE-puro-*DN-hTERT* (Plasmid #1775) was used as a reference sequence to clone xlox(GFP)DN-hTERT.

Plasmids bearing *hTERT* gene - xlox(GFP)hTERT (Addgene #69809), *DN-hTERT* gene - xlox(GFP)DN-hTERT and iCasp9 gene - pMSCV-F-del Casp9.IRES.GFP (Addgene #15567) were used to deliver *hTERT/DN-hTERT* and *iCASP9* genes into the cells. The plasmid *xlox TERT PGK iCasp9 IRES GFP* and *xlox DN-TERT PGK iCasp9 IRES GFP* consisting of both genes was assembled. Thus, both genes, *hTERT/DN-hTERT* and *iCASP9*, were simultaneously transduced to NK cells.

Sequence containing *iCASP9-IRES-GFP* was amplified by Encyclo polymerase (Evrogen, Russian Federation) and primers NotI iCasp9 F (5’-ctttcggcggccgcaatgctcgagggagtgcag-3’) and

iCasp9 BFP R (5’- gcctgcaggtcgactctag-3’). Then, the restriction of a target plasmid *xlox(GFP)hTERT* (Addgene #69809) and PCR-fragment fragments was carried out by endonuclease NotI (isoshizomer CciNI, SibEnzyme, Russian Federation) in SE-buffer Y (SibEnzyme, Russian Federation). Fragments were purified with the Cleanup Mini kit (Eurogen, Russian Federation) on each step according to the manufacturer’s instructions.

Retroviral constructs *pMSCV-F-del Casp9.IRES.GFP U6-shBIRC3-cPPT*, *xlox TERT PGK iCasp9 IRES GFP U6-shBIRC3-cPPT*, *xlox DN-TERT PGK iCasp9 IRES GFP U6-shBIRC3-cPPT* were assembled. The *shBIRC3* (short-hairpin BIRC3) region was assembled in plasmid pLKO-1 using oligonucleotides 5'-CCGGACACATGCAGCCCGCTTTAAACTCGAGTTTAAAGCGGGCTGCATGTGTTTTG-3' and 5'-AATTCAAAAAACACATGCAGCCCGTTTAAACTCGAGTTTAAAGCGGGCTGCATGTGT-3 ' (Clone ID: TRCN0000355771 https://portals.broadinstitute.org). Next, the *iCASP9-IRES-GFP U6-sh BIRC3-cPPT* regions were merged via overlap PCR was transferred to the target plasmids and inserted at the HindIII site.

In order to avoid self-closure of the plasmid fragment at the NotI site as a result of ligation, it was pretreated with Shrimp Alkaline Phosphatase (rSAP) (New England Biolabs, United Kingdom) in the rCutSmart™ Buffer (New England Biolabs, United Kingdom), which removes phosphate groups from the 5’ ends. The fragment treated with phosphatase was cleaned with a Cleanup Mini kit (Eurogen, Russian Federation) and then mixed for subsequent ligation with T4 DNA Ligase (SibEnzyme, Russian Federation) in a 5x Quick Ligation Buffer (SibEnzyme, Russian Federation) for 15 minutes according to the manufacturer's recommendations.

Amplification of plasmid DNA was conducted in *E.Coli XL1-Blue* bacteria cell line cultivated in selective LB+ampicillin medium. Plasmid was extracted by Plasmid Miniprep and Plasmid Midiprep 2.0 kits (Eurogen, Russian Federation) in accordance with the manufacturer’s protocol.

The assembled plasmid was verified by Sanger sequencing provided by Eurogen (Russian Federation). DNA concentration was measured by Biodrop (Innovative Solutions, USA) on each step. The primers and regents used are listed in table 1.

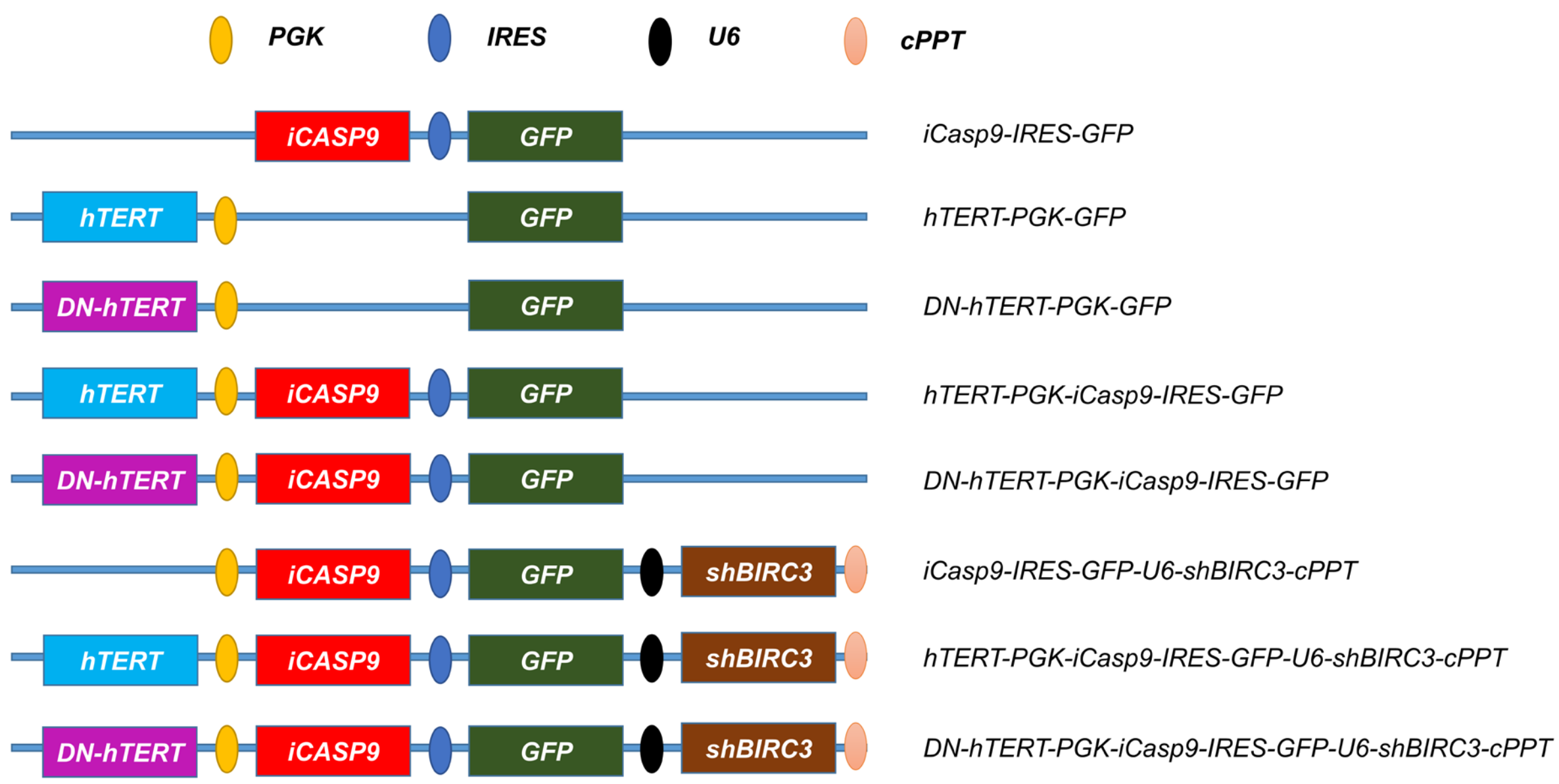

Fig. 1 The representation of plasmid constructs applied to obtain retroviral particles bearing *hTERT, iCASP9* genes and *shBIRC3* construct.

3.2. Electronic resources

Primers were selected with the use of Primer BLAST (NCBI, MD, USA) web-tool. The work on planning, modeling of the plasmid reassembly and sequence alignment were carried out in the SnapGene v3.2.1. (GSL Biotech LLC, Chicago, IL, USA) program.

4. Transfection and concentration of viral particles

Phoenix Ampho cells were placed in 90mm and 150mm Petri dishes covered with poly-L-lysine solution (Sigma-Aldrich, St. Louis, MO, USA). In order to produce RD114-pseudotyped retroviral particles pLTR-RD114A #17576 and target-gene bearing plasmid were mixed in the ratio ½. Then, the mix was transfected using a calcium phosphate transfection kit (Biospecifica, Russian

Federation) according to the manufacturer's instructions into Phoenix Ampho line cells. Viral supernatant was harvested at 48 h, 72 h and 96 h post-transfection and then filtered through Millex-HV-0.45 μm PES filter (Millipore, Burlington, MA, USA). Viral particles were concentrated by centrifugation of viral supernatant (21000g, 2.5h, 4°C).

5. Transduction and cell sorting

Prior transduction procedure 24-well and 96-well flat-bottom plates were coated with 20μg/mL retronectin (Clontech/Takara, Mountain View, CA, USA) solution in PBS (PanEco, Russian Federation) to enhance transduction efficiency. The viral suspension was centrifuged (2-4h, 1800g, 4°C) in the retronectin-coated plate. NK cells stimulated for 4-5 days were added to the plates in their normal concentrations and centrifuged (30 min, 200g, room temperature). On the day 3 cells were replaced from retronectin-coated wells. The transduction efficiency was firstly verified by the GFP fluorescence.

Genetically modified cells were then separated from unmodified ones based on the presence of the GFP fluorescent reporter. The FACSVantageDiVa cell sorter (Becton Dickinson, NJ, USA) equipped with 405, 488, and 643nm lasers was used for cell sorting. Purity of cell sorting was verified (Supplementary Fig. 1).

6. Flow cytometry

MACSQuant 10 cytometer (Miltenyi Biotech, Bergisch Gladbach, Germany) equipped with 405 nm, 488 nm, and 635 nm lasers was used for checking of transduction efficiency on day 3 after transduction.

7. Cell death induction in iCasp9-NK cells by chemical inductor of dimerization

Cells were maintained for 24h in cell medium with addition of PBS, DMSO (PanReac AppliChem, Germany) or 10nM and 100nM of CID AP20187 (MedChemExpress, Monmouth Junction, NJ, USA). DMSO was taken at a concentration corresponding to its content in a sample with 100nM

CID. Cell death induction rate was examined by AnnexinV-PE (Invitrogen, San Jose, CA, USA) and SYTOX AADvanced Dead Cell Stain Kit (Invitrogen, San Jose, CA, USA) staining according to the manufacturer's instructions.

8. Gene expression analysis

Preparation of cell samples and isolation of total RNA was performed with ExtractRNA reagent (Eurogene, Russian Federation), following the manufacturer's recommendations. The samples were also purified from genomic DNA using TURBO DNA-free™ Kit (Invitrogen, San Jose, CA, USA) according to the manufacturer's instructions. The MMLV revertase and the standard 18dT primer were used to obtain cDNA by reverse transcription of the total RNA. The protocol for MMLV reverse transcriptase (Eurogene, Russian Federation) was followed.

9. Quantitative polymerase chain reaction (qPCR)

The expression level of *ACTB, BAD, BAX, BCL2, BCL2L1, BIRC5, BIRC3, DIABLO, EOMES, iCASP9, MCL1, TBX21, hTERT* genes in modified and control cells was studied using qPCR (Quantitative polymerase chain reaction). To do this, cDNA samples were amplified with a set of primers performed in table 1 using CFX Connect Real-Time System (Bio-Rad Laboratories, Hercules, CA, USA). Intercalating dye qPCRmix-HS SYBR (Eurogen, Russian Federation) was used for detection. Data was analyzed in BioRad CFX Maestro program (Bio-Rad Laboratories, Hercules, CA, USA). The 2-ΔΔCq method was used to calculate the relative expression of the target genes, *ACTB* was used as the internal reference gene for normalization.

10. RNAseq analysis

hTERT-NK cells, hTERT-iCasp9-NK cells and iCasp9-NK cells were harvested for RNAseq analysis in one month after isolation. Each sample (hTERT-NK cells, hTERT-iCasp9-NK cells, iCasp9-NK cells) was harvested in triplicates in the RLT buffer. (CID)-iCasp9-NK cells were washed 3 times stained with AnnexinV-PE and SYTOX-AADvanced and then sorted as AnnexinV-PE- SYTOX-AADvanced- GFP+ cells. KAPA RNA HyperPrep Kit (Roche, Basel,

Switzerland) and KAPA Unique Dual-Indexed (UDI) Primer Mixes (Roche, Basel, Switzerland) were applied for RNAseq library preparation according to manufacturer's instructions. The data was obtained using Illumina NovaSeq X in paired-end mode. Data was analyzed on UseGalaxy platform (10.1093/nar/gkae410) and aligned on hgGRCh38 assembly. Pipeline was used: trimmomatic, salmon, DESeq2. DESeq2 results were analyzed using Cluster Profiler R library (10.1089/omi.2011.0118) based on Gene Onthology (GO), KEGG (10.1093/nar/gkw1092) and GSEA (10.1073/pnas.0506580102S, 10.1038/ng1180) databases. Genes with P-adj < 0.05 were subjected to gseGO, gseKEGG and fgsea analysis. Genes with P-adj < 0.05 and absolute Log2FC>0.58 were considered as DEGs and were analyzed with enrichGO and enrichKEGG. Splicing analysis was performed on usegalaxy platform by following manual https://training.galaxyproject.org/training-material/topics/transcriptomics/tutorials/differential-isoform-expression/tutorial.html.

11. Statistical analysis

Statistical analysis was carried out using GraphPad Prism 8.4 (StatSoft Inc., Tulsa, OK, USA) software, Python and R packages. The normalized qPCR data was analyzed by Spearman's correlation test. P-value: * $p < 0.05$; ** $p < 0.01$; *** $p< 0.001$.

Table 1 Primers and reagents used for plasmid assembly.

**Primer**	**Primer sequence**
NotI iCasp9 F	ctttcggcggccgcaatgctcgagggagtgcag
iCasp9 BFP R	gcctgcaggtcgactctag
shBIRC3-F	5'-CCGGACACATGCAGCCCGCTTTAAACTCGAGTTTAAAGCGGGCTGCATGTGTTTTG-3'

shBIRC3-R	5'-AATTCAAAAAACACATGCAGCCCGTTTAAACTCGAGTTTAAAGCGGGCTGCATGTGT-3 '
EGFP-U6	GGAAATAGGCCCTCgctgcaggtcgattgcg
U6-EGFP	CAATCGACCTGCAGCgagggcctatttcccatgat
Apa-IRES-DIR	gcaatgtgagggcccggaaa
HindIII-cPPT-2	TCGATAAGCTTtgctgtccctgtaataaacccg
DN TERT BsiW1 R	ggtgtcgtacgcgcccgtgatcgcgaccttgacaaagtacagctc
**Plasmid**	**Addgene #**
xlox(GFP)hTERT	69809
pMSCV-F-del Casp9.IRES.GFP	15567
pBABE-puro-DN-hTERT	1775
**Enzyme**	**Enzyme buffer**
NotI (isoshizomer CciNI, SibEnzyme, Russian Federation)	SE-buffer Y (SibEnzyme, Russian Federation)
HindIII (SibEnzyme, Russian Federation)	SE-buffer W (SibEnzyme, Russian Federation)
EcoRI (SibEnzyme, Russian Federation)	SE-buffer O (SibEnzyme, Russian Federation)
AgeI (isoshizomerAsiGI, SibEnzyme, Russian Federation)	

Shrimp Alkaline Phosphatase (rSAP) (New England Biolabs, United Kingdom)	rCutSmart™ Buffer (New England Biolabs, United Kingdom)
DNA Ligase (SibEnzyme, Russian Federation)	5x Quick Ligation Buffer (SibEnzyme, Russian Federation)
Encyclo polymerase (Evrogen, Russian Federation)	
**Kit**	
Cleanup Mini kit (Eurogen, Russian Federation)	
Midiprep 2.0 kits (Eurogen, Russian Federation)	

Supplementary Materials

Supplementary Tables

Supplementary Table 1. DEseq2 results data obtained for comparison (CID)-iCasp9-NK cells *vs* iCasp9-NK cells

Supplementary Table 2. DEseq2 results data obtained for comparison hTERT-iCasp9-NK cells *vs* iCasp9-NK cells

Supplementary Figures

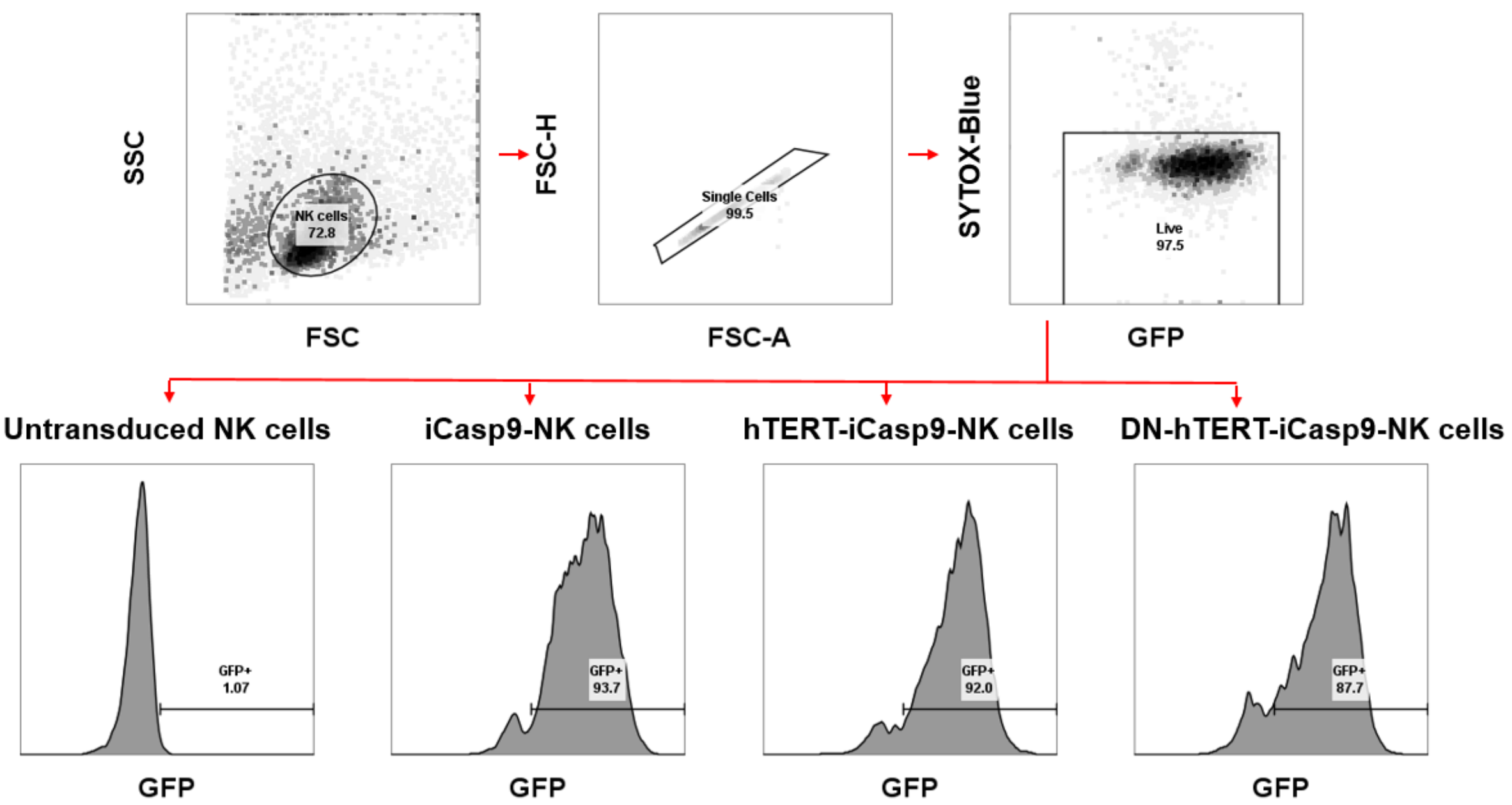

Supplementary Fig. 1 The purity of NK cell sorting verified in 1-2 weeks after sorting. Gating strategy is performed. NK cells were stained with SYTOX-AADvanced to determine dead cells.

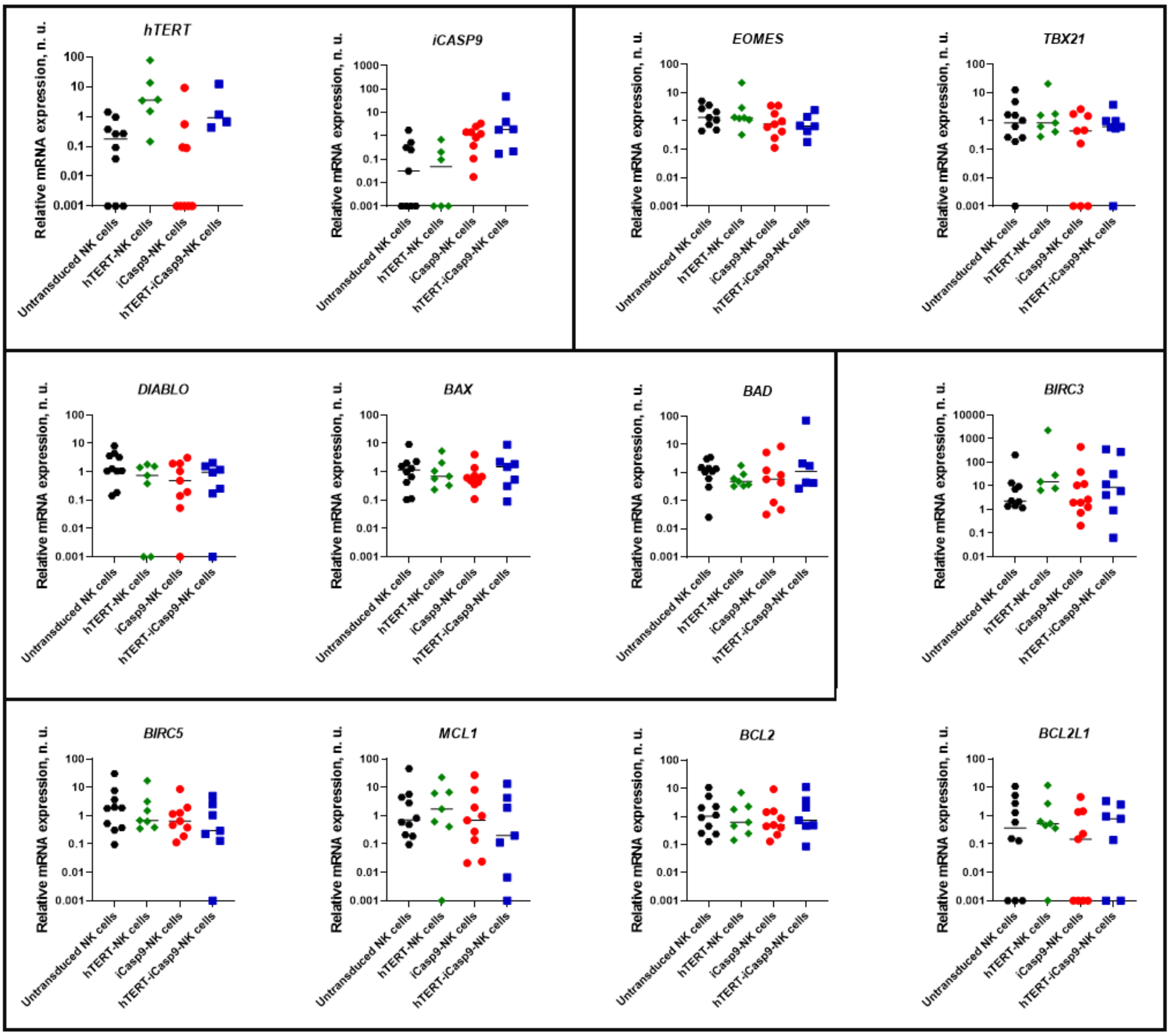

Supplementary Fig. 2 Relative mRNAexpression of 1) *hTERT, iCASP9* transgenes, 2) *EOMES* and *TBX21* encoding transcription factors Eomes and T-bet, 3) *DIABLO, BAX, BAD* pro-apoptotic genes, 4) *BIRC3, BIRC5, MCL1, BCL2, BCL2L1* pro-survival genes. in NK cell populations untransduced and transduced with *hTERT, hTERT+iCASP9* and *iCASP9* transgenes. Untransduced NK cells, n=10; hTERT-NK cells, n=7; hTERT-iCasp9-NK cells, n=7; iCasp9NK cells, n=9. Median.

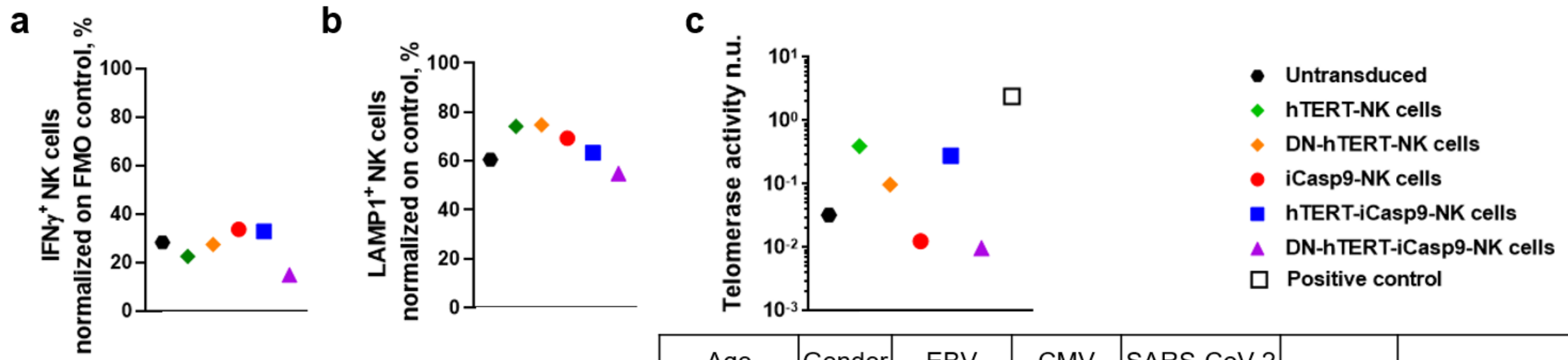

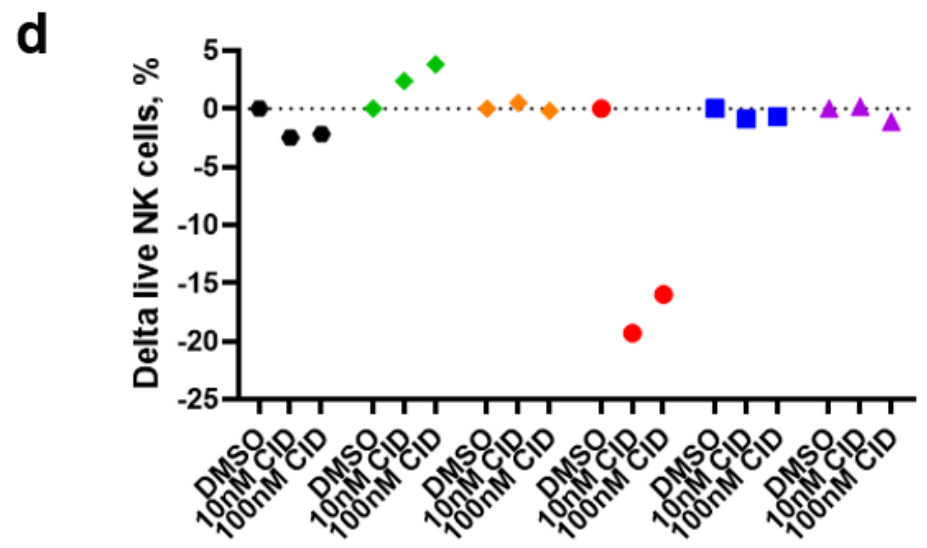

Age	Gender	EBV	CMV	SARS-CoV-2		
25 years	Female	positive	positive	positive		
HLA genotyping						
A_1	A_2	B_1	B_2	C_1	C_2	
A*03:01:01	NA	B*07:02:01	B*35:01:01	C*04:01:01	C*07:02:01	
DPB1_1	DPB1_2	DQA1_1	DQA1_2	DQB1_1	DQB1_2	
DPB1*04:01:01	NA	NA	NA	DQB1*03:01:01	DQB1*06:03:01	
DRB1_1	DRB1_2	DRB3_1	DRB3_2	DRB4_1	DRB4_2	DRB5_1
DRB1*11:01:01	DRB1*13:01:01	DRB3*02:02:01	NA	NA	NA	NA

Supplementary Fig. 3 The investigation of functional activity of NK cells in a one month after NK cell isolation from peripheral blood. The applied methods were previously described in detail in 10.3390/biomedicines12030650. The data obtained from donor whose NK cells were studied using RNAseq. **a** The proportion of IFNγ$^+$ NK cells in response to IL2+IL12+IL18 is presented for populations transduced with *iCASP9, hTERT* and *DN-hTERT* genes. Data is normalized on FMO control. **b** The fraction of degranulating LAMP-1+ (CD107a+) NK cells in populations of modified cells in response to K562 cell recognition. The experiment performed on unsorted NK cell. **c** Telomerase activity assay evolved by protocol for Real-time Q-TRAP in modified NK cells. Telomerase activity of K562 cells is taken for positive control. **d** Evaluation of the effectiveness of cell death induction by iCasp9 suicide construct in NK cells transduced with *iCASP9, hTERT* and *DN-hTERT* genes. Decrease in the proportion of live NK cells after treatment with 10nM and 100nM CID for 24h normalized on the percentage of live NK cells in the control group incubated in the presence of DMSO, CID solvent.

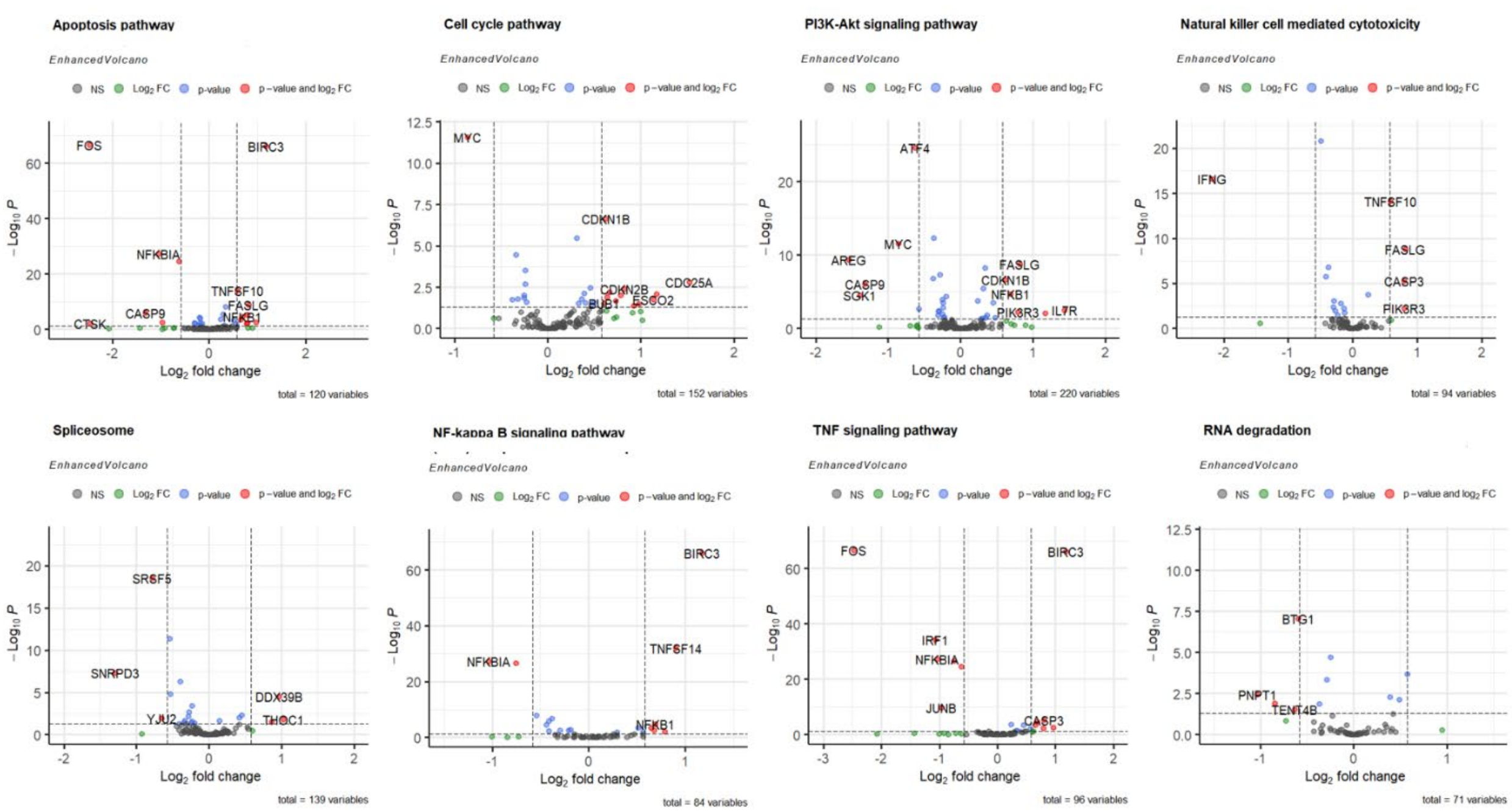

Supplementary Fig. 4 Enhanced volcano plot for comparison “survived” iCasp9-NK cells that underwent incubation with 100nM CID for 24h compared versus “susceptible” iCasp9-NK cells. P-adj<0.05 and abs(Log2FC)>0.58. The data was sorted on the basis of KEGG database. From right to left the genes associated with Apoptosis pathway “hsa04210”, Cell cycle pathway “hsa04110”, PI3K-Akt signaling pathway “hsa04151”, Natural killer cell mediated cytotoxicity “hsa04650”, Spliceosome “03040”, NF-kappa B signaling pathway “hsa04064”, TNF signaling pathway “hsa04668”, RNA degradation “hsa03018”.

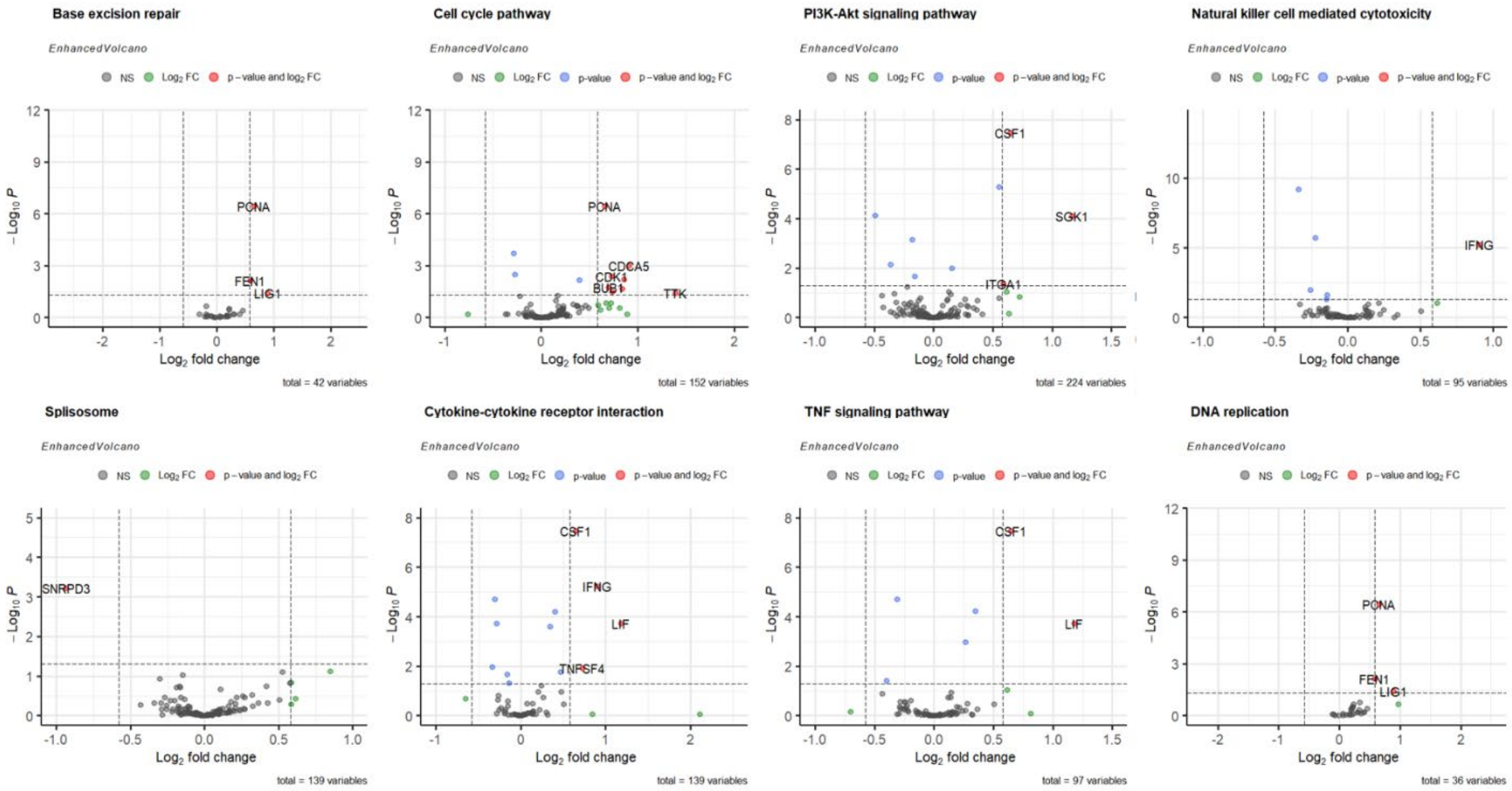

Supplementary Fig. 5 Enhanced volcano plot for comparisons "resistant" hTERT-iCasp9-NK cells versus "susceptible" iCasp9-NK cells. P-adj<0.05 and abs(Log2FC)>0.58. The data was sorted on the basis of KEGG database. From right to left the genes associated with Base excision repair "hsa03410", Cell cycle pathway "hsa04110", PI3K-Akt signaling pathway "hsa04151", Natural killer cell mediated cytotoxicity "hsa04650", Spliceosome "03040", Cytokine-cytokine receptor interaction "hsa04060", TNF signaling pathway "hsa04668", DNA replication "hsa03030".

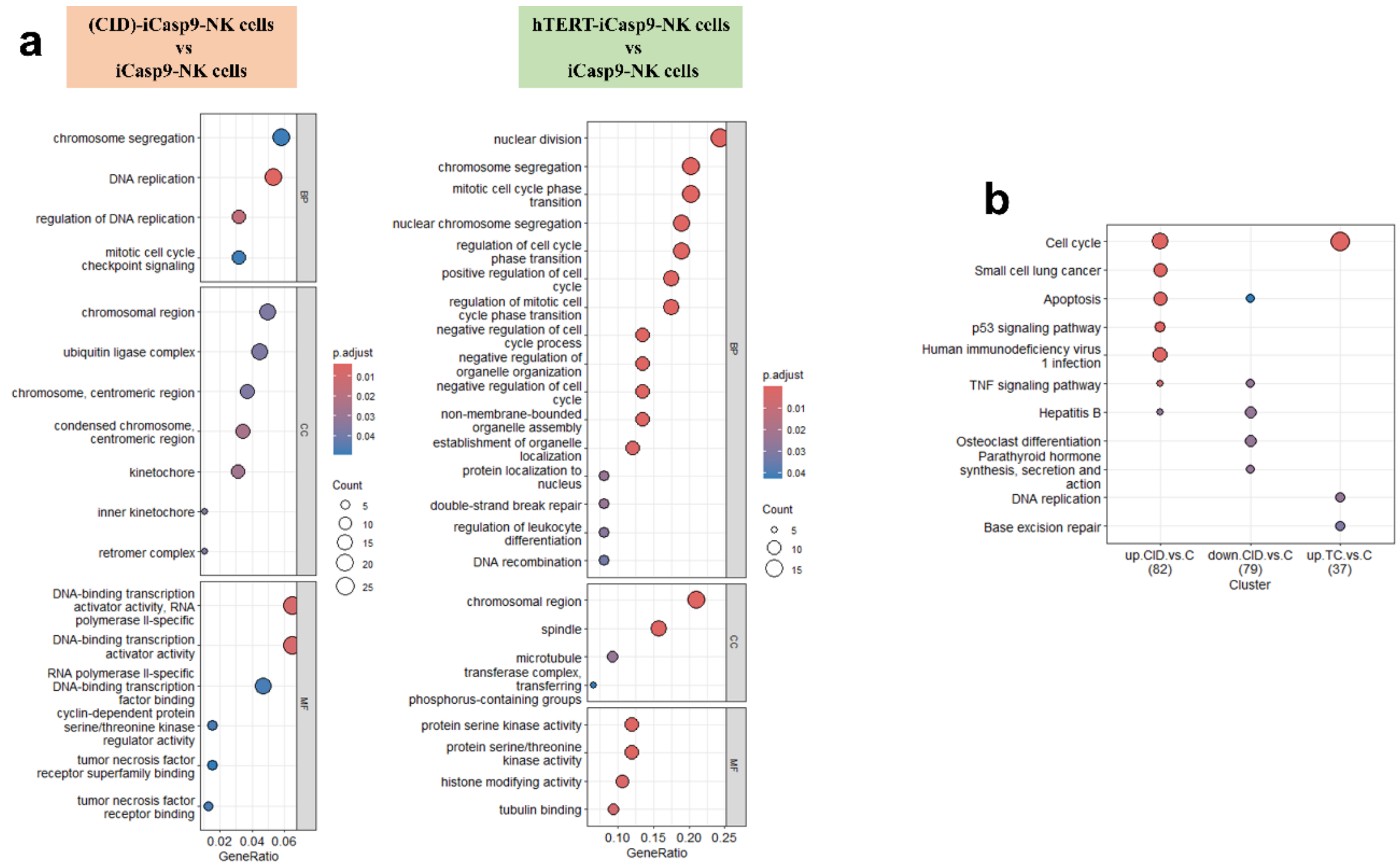

Supplementary Fig. 6 The representation of RNA seq data for iCasp9-NK cells, hTERT- iCasp9-NK cells and “survived” (CID)-iCasp9-NK cells that underwent incubation with 100nM CID for 24h. **a** Dotplot of GeneOntology gene set enrichment analysis where the y-axis represents GO molecular pathways (Biological Processes (BP), Molecular Functions (MF), Cellular components (CC)). The greater the size of a circle, the greater the number of genes involved in a pathway. The circles are colored based on p-adjusted values. **b** The representation of upDEGs p-adj<0.05 and Log2FC>0.58) and downDEGs (p-adj<0.05 and Log2FC<0.58) by Cluster compare. “CID” standa for (CID)-iCasp9-NK cells, C – iCasp9-NK cells, TC - hTERT- iCasp9-NK cells.

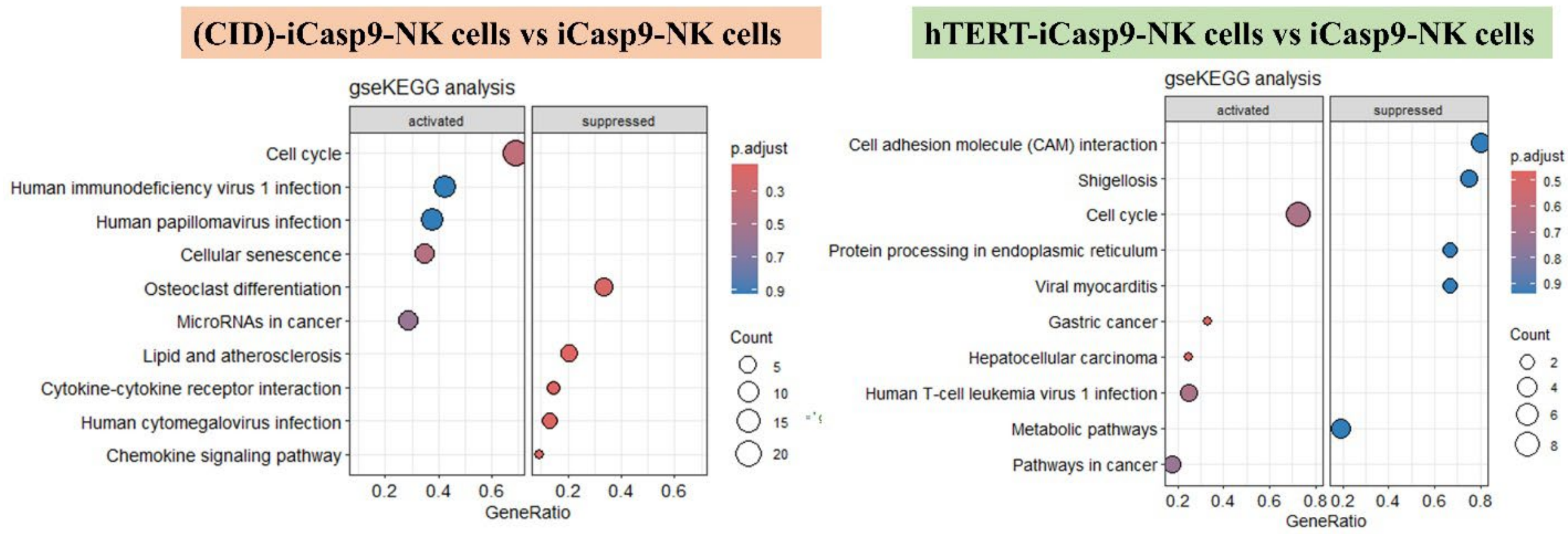

Supplementary Fig. 7 The representation of RNAseq data for iCasp9-NK cells, hTERT-iCasp9-NK cells and "survived" iCasp9-NK cells that underwent incubation with 100nM CID for 24h. DEGs (p-adj<0.05 and abs(Log2FC)>0.58). gseKEGG shows enriched pathways of Kyoto Encyclopedia of Genes and Genomes (KEGG). The greater the size of a circle, the greater the number of genes involved in a pathway. The circles are colored based on p-adjusted values.

a **(CID)-iCasp9-NK cells vs iCasp9-NK cells**

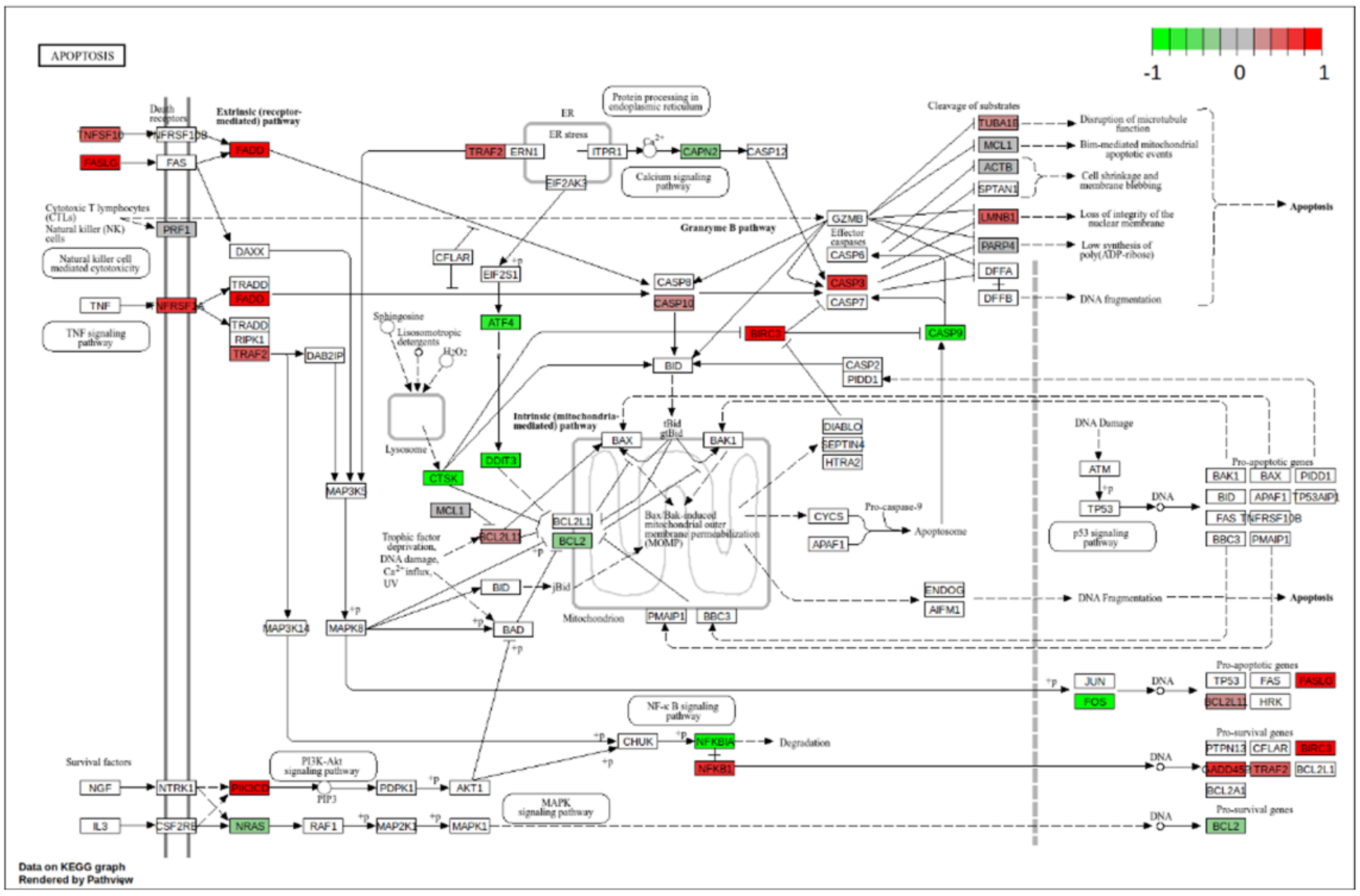

hTERT-iCasp9-NK cells vs iCasp9-NK cells

b

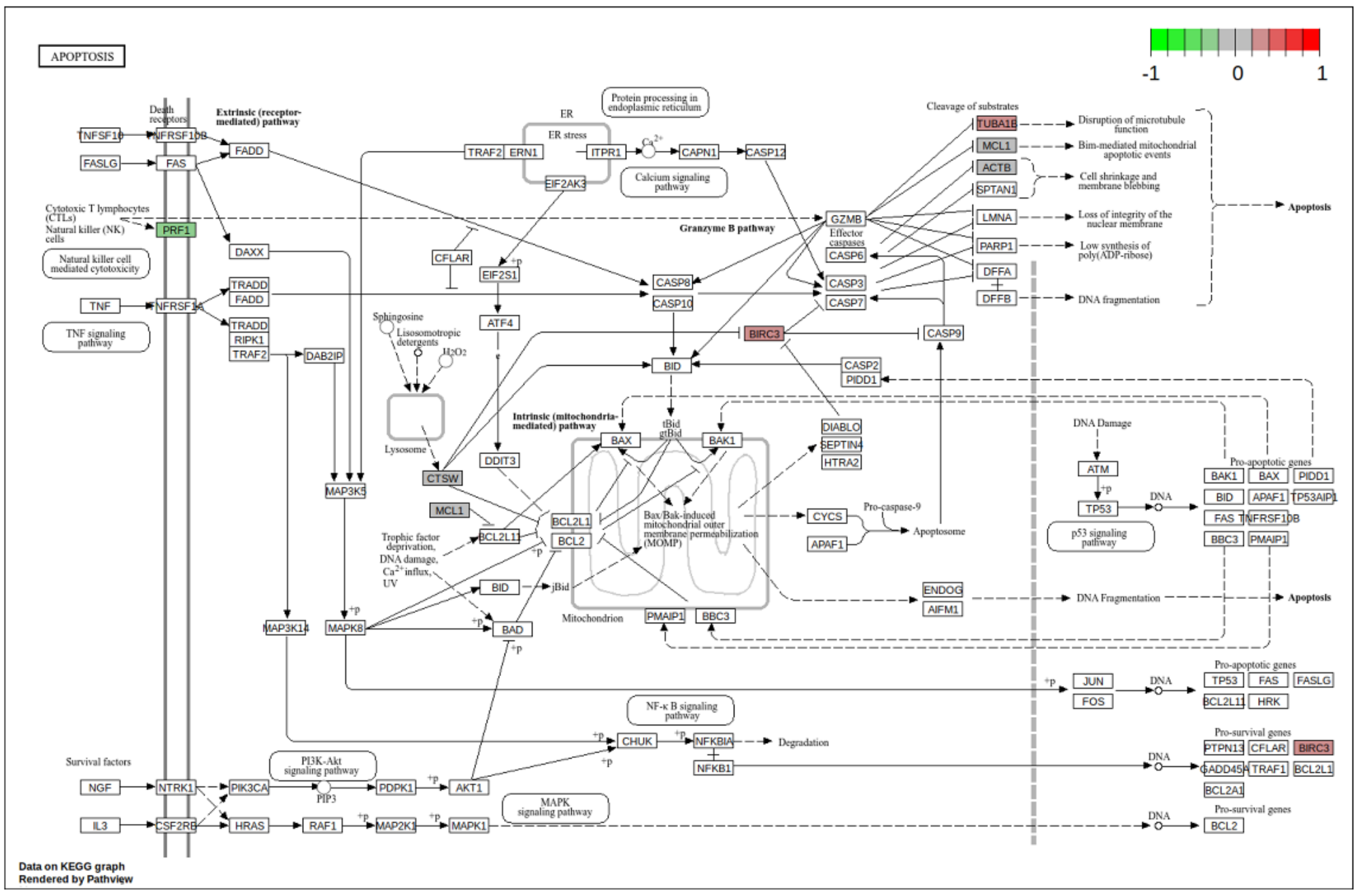

Supplementary Fig. 8 KEGG pathview plot representing apoptosis pathway “Hsa04210” **a** for “survived” (CID)-iCasp9-NK cells versus “susceptible” iCasp9-NK cells and **b** for “resistant” iCasp9-hTERT-NK cells versus “susceptible” iCasp9-NK cells.

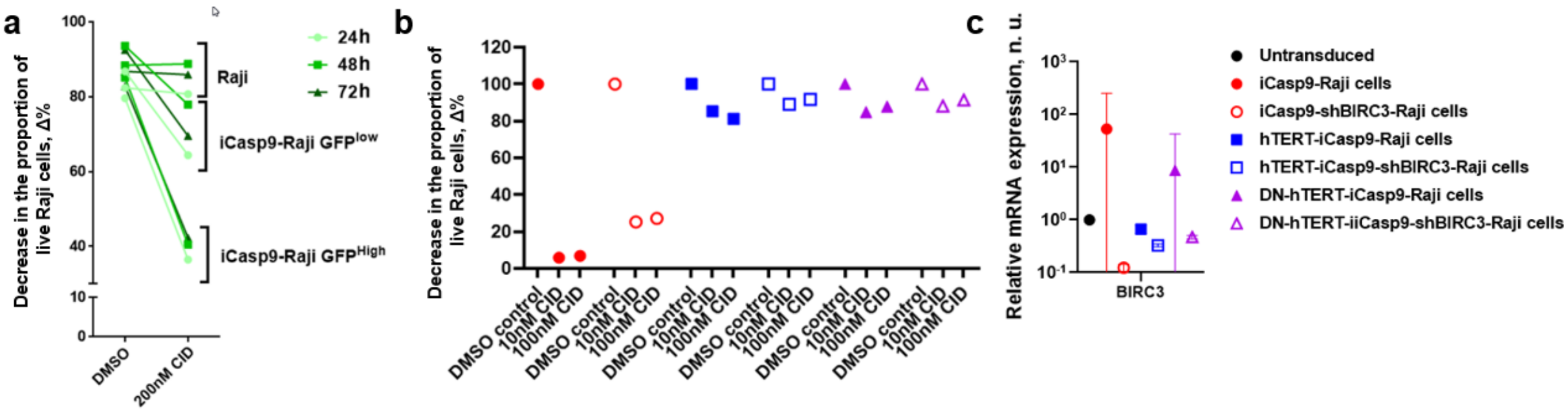

Supplementary Fig. 9 Evaluation cell death induction in Raji cells that were modified with *iCASP9, hTERT, DN-hTERT* genes and *shBIRC3* construct. **a** Decrease in the proportion of live Raji cells normalized on DMSO control. iCasp9-Raji cells were sorted on two subsets “low”, “High” based on the GFP MFI level. Cells were treated with DMSO (CID solvent), 200nM CID for 24h. **b** Decrease in the proportion of live Raji cells normalized on DMSO control. Cells were treated with DMSO (CID solvent), 10nM CID, 100nM CID for 24h. **c** Relative mRNA expression of BIRC3 transcript levels in modified Raji cells.

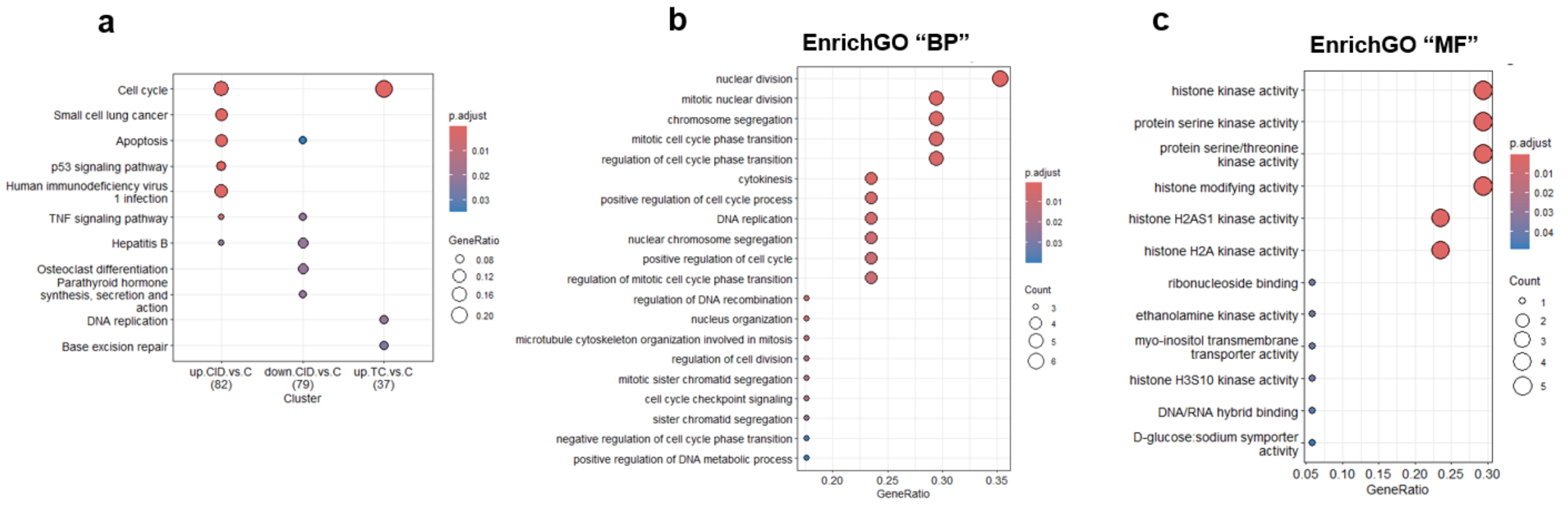

Supplementary Fig. 10 The representation of RNA seq data of "resistant" hTERT-iCasp9-NK cells and "survived" iCasp9-NK cells that underwent incubation with 100nM CID for 24h compared versus "susceptible" iCasp9-NK cells. **a** Pathways that changed in a common way were defined by ClusterCompare package. upDEGs and downDEGs were compared for hTERT-iCasp9-NK cells (TC) vs iCasp9-NK cells (C) and (CID)-iCasp9-NK cells (CID) vs iCasp9-NK cells (C). DEGs that changed mutually in both comparisons were also suspected for enrichment analysis using **b** "Biological Process" ("BP") and **c** "Molecular Function" ("MF") ontology.

126 DE genes with p-adj<0.05 from both comparisons were analyzed using gene set enrichment analysis (GSEA) gseKEGG. The corresponding Log2FC values were taken from "survived" (CID)-iCasp9-NK cells versus "susceptible" iCasp9-NK cells. gseKEGG shows enriched pathways of Kyoto Encyclopedia of Genes and Genomes (KEGG). The greater the size of a circle, the greater the number of genes involved in a pathway. The circles are colored based on p-adjusted values. On the X-axis suppressed and activated genes are shown.

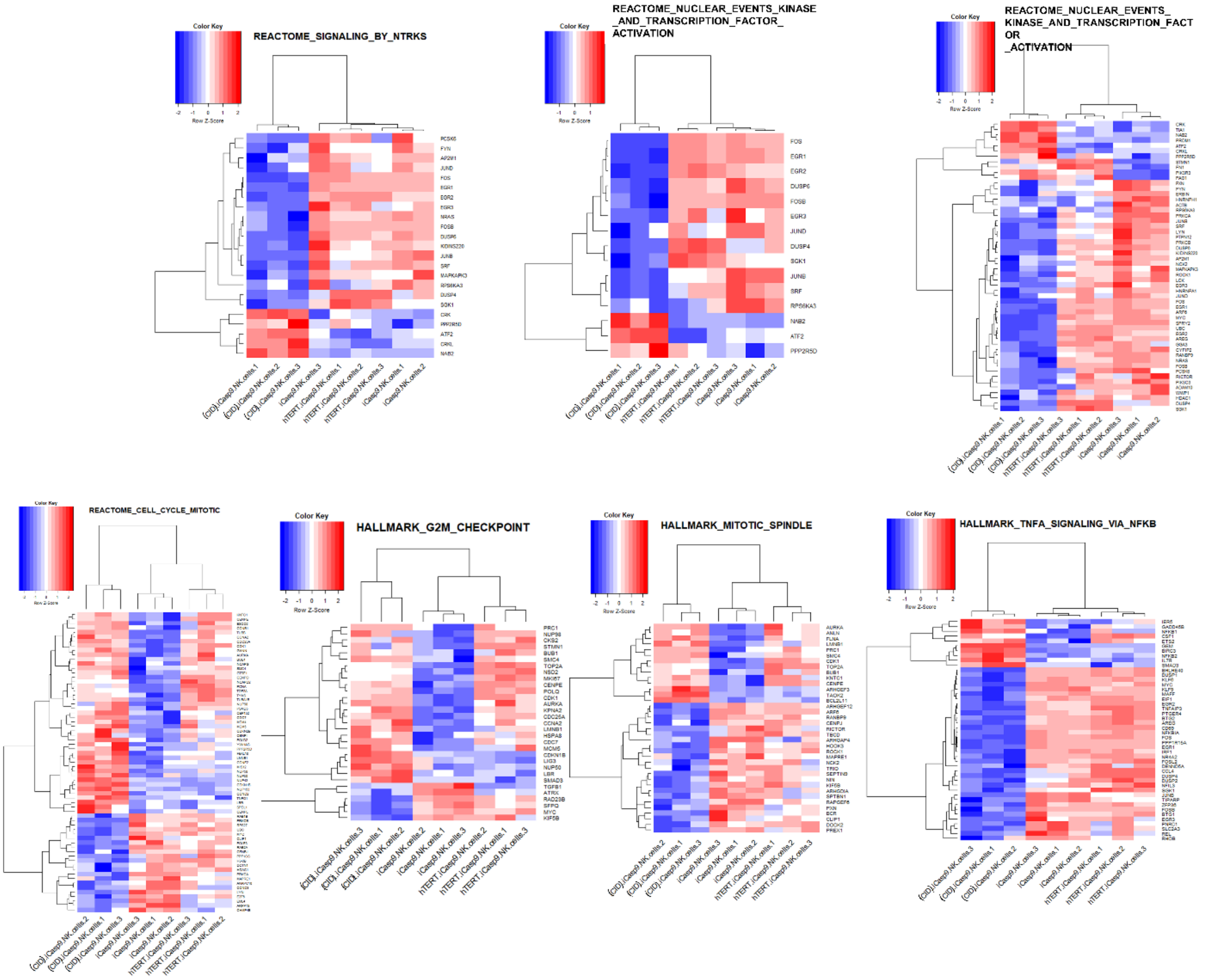

Supplementary Fig. 10 For set of genes (p-adj<0.05) common in (CID)-iCasp9-NK cells vs iCasp9-NK cells and hTERT-iCasp9-NK cells vs iCasp9-NK cells, the most significantly changed pathways (p-adj<0.05) from Reactome and Hallmark databases were defined based on (CID)-iCasp9-NK cells vs iCasp9-NK cells comparison. Normalized counts of genes corresponding to the significantly changed pathways were extracted for all NK cell populations and plotted on a heatmap. Row Z-score normalized data is presented. Red stands for transcripts with increased transcripts and blue – for decreased expression.

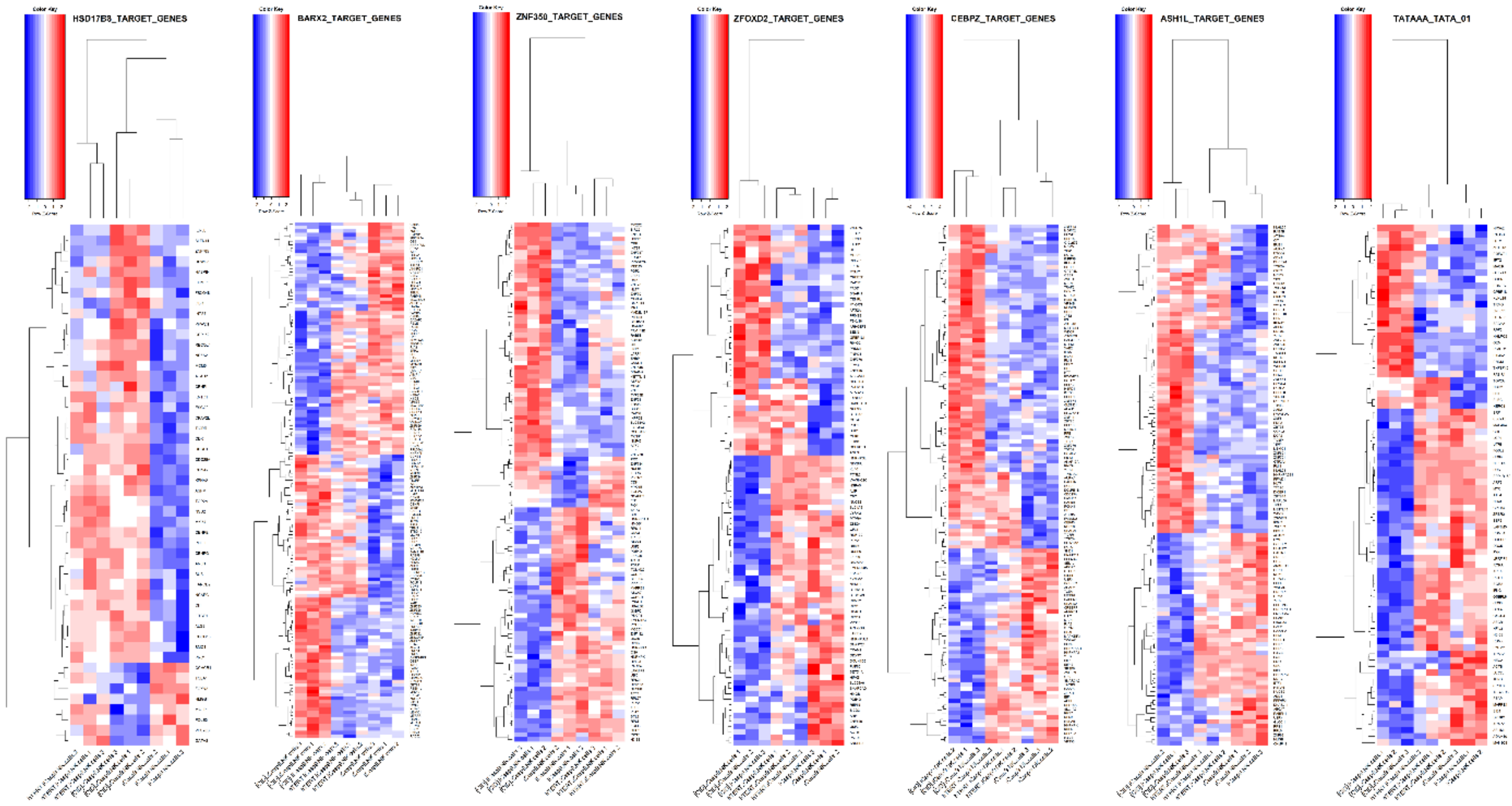

Supplementary Fig. 11 For set of genes (p-adj<0.05) common in (CID)-iCasp9-NK cells vs iCasp9-NK cells and hTERT-iCasp9-NK cells vs iCasp9-NK cells, the most significantly changed pathways (p-adj<0.05) from TFT database were defined based on (CID)-iCasp9-NK cells vs iCasp9-NK cells comparison. Normalized counts of genes corresponding to the significantly changed pathways were extracted for all NK cell populations and plotted on a heatmap. Row Z-score normalized data is presented. Red stands for transcripts with increased transcripts and blue – for decreased expression.

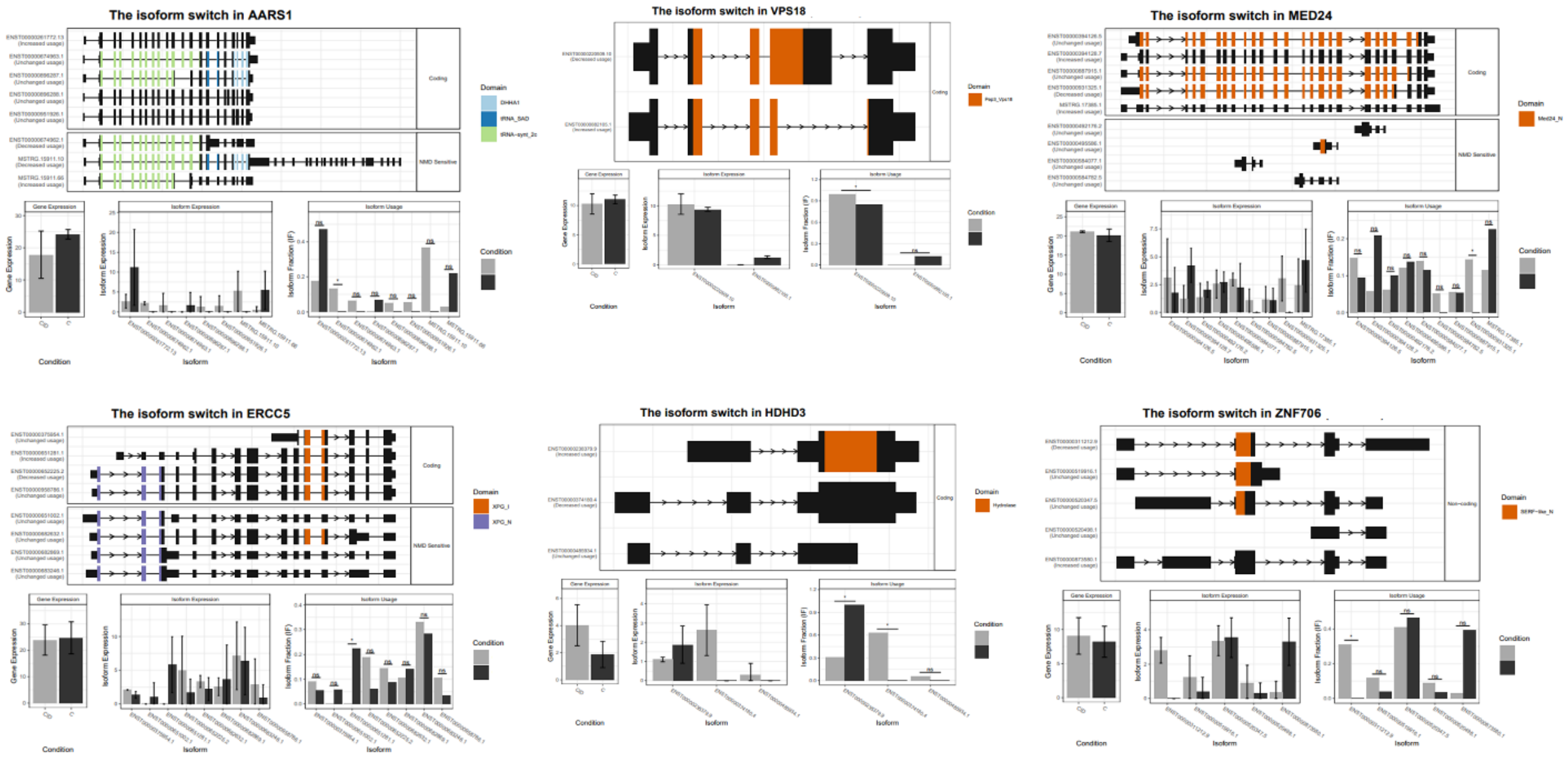

Supplementary Fig. 13 The alternatively spliced transcripts were evaluated for NK cell populations. “Resistant” hTERT-iCasp9-NK cells and “survived” iCasp9-NK cells that underwent incubation with 100nM CID for 24h compared versus “susceptible” iCasp9-NK cells. Representative plots for genes listed in red countered boxes of Vienn diagram are presented. Upper graphs represent the most abundant isoforms detected with some highlighted domains. The lower line shows from left to right: Gene expression, Isoform expression and Isoform usage. Gray bars stand for (CID)-iCasp9-NK cells and hTERT-iCasp9-NK cells and black bars reflect iCasp9-NK cells. The analysis was performed on the Galaxy platform following the manual https://training.galaxyproject.org/training-material/topics/transcriptomics/tutorials/differential-isoform-expression/tutorial.html
