# Supplementary material for: Interplay of telomerase non-canonical functions in NK cell resistance to iCasp9-mediated apoptosis
Source: Cell Death Discov. 2026 Jun 12;12:340. doi: 10.1038/s41420-026-03183-y (PMC13487172; doi:10.1038/s41420-026-03183-y)
Supplement: Supplementary file 2 [file 41420_2026_3183_MOESM2_ESM.pdf]

ENSEMBL	SYMBOL	GENENAME	ENTREZID	Base mean	log2FC	StdErr	Wald-Stats	P-value	P-adj
ENSG00000110848	CD69	CD69 molecule	969	1090,62395	0,260713635	0,049386	5,279058659	1,30E-07	2,55E-05
ENSG00000120738	EGR1	early growth response 1	1958	732,5324227	-0,000774294	0,082365	-0,0094008	0,992499357	0,998500439
ENSG00000170345	FOS	Fos proto-oncogene, AP-1 transcription factor subunit	2353	354,2896367	0,023061193	0,098987	0,232972442	0,815782805	0,959707099
ENSG00000023445	BIRC3	baculoviral IAP repeat containing 3	330	870,8201533	0,348001734	0,068352	5,091309333	3,56E-07	6,07E-05
ENSG00000164949	GEM	GTP binding protein overexpressed in skeletal muscle	2669	289,6676954	0,168891772	0,114272	1,477975643	0,139414298	0,581497554
ENSG00000087074	PPP1R15A	protein phosphatase 1 regulatory subunit 15A	23645	348,3772269	-0,054107446	0,107462	-0,503502799	0,614610813	0,901765987
ENSG00000134107	BHLHE40	basic helix-loop-helix family member e40	8553	2346,336901	0,066908921	0,053225	1,257089688	0,208721161	0,662366797
ENSG00000164691	TAGAP	T cell activation RhoGTPase activating protein	117289	763,3719928	0,05693281	0,06754	0,842953734	0,39925432	0,814478813
ENSG00000067082	KLF6	KLF transcription factor 6	1316	1997,456715	0,036459441	0,048518	0,751462777	0,452374195	0,843234809
ENSG00000125347	IRF1	interferon regulatory factor 1	3659	1166,116273	-0,032896479	0,078961	-0,416615203	0,676959887	0,924186156
ENSG00000122877	EGR2	early growth response 2	1959	280,9159112	0,510124247	0,110712	4,607670018	4,07E-06	0,00049203
ENSG00000125735	TNFSF14	TNF superfamily member 14	8740	642,5994981	0,205276708	0,074125	2,76932867	0,005617194	0,107603514
ENSG00000136158	SPRY2	sprouty RTK signaling antagonist 2	10253	719,2756238	-0,102277313	0,064183	-1,593532157	0,11104086	0,53211348
ENSG00000100906	NFKBIA	NFKB inhibitor alpha	4792	637,0716181	0,051241954	0,082109	0,624073969	0,532579008	0,873069408
ENSG00000213928	IRF9	interferon regulatory factor 9	10379	210,7129232	-0,339968145	0,110734	-3,070120989	0,002139721	0,055967203
ENSG00000118503	TNFAIP3	TNF alpha induced protein 3	7128	1041,224446	0,26576501	0,060437	4,397401002	1,10E-05	0,001062276
ENSG00000128272	ATF4	activating transcription factor 4	468	1446,302467	-0,06800841	0,053433	-1,272769634	0,2030998	0,660954946
ENSG00000120129	DUSP1	dual specificity phosphatase 1	1843	129,571738	0,083222944	0,159003	0,523403496	0,600693492	0,896014669
ENSG00000150991	UBC	ubiquitin C	7316	4130,661114	0,053903575	0,035124	1,534671525	0,124864534	0,5624706
ENSG00000198743	SLC5A3	solute carrier family 5 member 3	6526	646,7489295	0,640959624	0,075013	8,544674067	1,29E-17	2,02E-14
ENSG00000155926	SLA	Src like adaptor	6503	1034,578096	-0,010324972	0,059287	-0,174152613	0,861745526	0,970863509
ENSG00000156976	EIF4A2	eukaryotic translation initiation factor 4A2	1974	355,2281166	-0,039414757	0,132956	-0,296450701	0,766885911	0,944698783
ENSG00000213809	KLRK1	killer cell lectin like receptor K1	22914	1607,173829	-0,145743249	0,04616	-3,157364967	0,00159202	0,047362581
ENSG00000213809	KLRC4-KLRK1	KLRC4-KLRK1 readthrough	100528032	1607,173829	-0,145743249	0,04616	-3,157364967	0,00159202	0,047362581
ENSG00000265972	TXNIP	thioredoxin interacting protein	10628	3175,429791	-0,057352503	0,044506	-1,28864066	0,197523041	0,65618735
ENSG00000100650	SRSF5	serine and arginine rich splicing factor 5	6430	1012,77535	-0,188491086	0,077667	-2,426901971	0,01522837	0,19384703
ENSG00000188042	ARL4C	ADP ribosylation factor like GTPase 4C	10123	2126,312761	-0,26439214	0,046591	-5,674794512	1,39E-08	4,36E-06
ENSG00000153234	NR4A2	nuclear receptor subfamily 4 group A member 2	4929	105,2490351	0,252527609	0,188617	1,338839915	0,180622793	0,634062228
ENSG00000185022	MAFF	MAF bZIP transcription factor F	23764	189,1755813	-0,06024242	0,122002	-0,493782014	0,621460137	0,904764752
ENSG00000152795	HNRNPDL	heterogeneous nuclear ribonucleoprotein D like	9987	963,7711397	0,027964396	0,058791	0,475654562	0,634320497	0,908608932
ENSG00000111537	IFNG	interferon gamma	3458	104,1649192	0,904464213	0,161535	5,599168943	2,15E-08	5,83E-06
ENSG00000275023	MLLT6	MLLT6, PHD finger containing	4302	1990,442276	-0,218704541	0,040848	-5,354058599	8,60E-08	1,88E-05
ENSG00000119138	KLF9	KLF transcription factor 9	687	188,9357488	-0,272772386	0,123648	-2,206039925	0,027381204	0,268479374
ENSG00000171940	ZNF217	zinc finger protein 217	7764	651,6963782	0,205693614	0,070011	2,937997635	0,003303395	0,075727344
ENSG00000139318	DUSP6	dual specificity phosphatase 6	1848	449,4621377	-0,163387738	0,094547	-1,728110125	0,083968483	0,471737097
ENSG00000196230	TUBB	tubulin beta class I	203068	2068,700938	0,432079384	0,056469	7,651571996	1,99E-14	2,23E-11
ENSG00000121858	TNFSF10	TNF superfamily member 10	8743	844,9827138	0,139897986	0,069336	2,017694809	0,043623056	0,333843576
ENSG00000029363	BCLAF1	BCL2 associated transcription factor 1	9774	1178,215428	0,103444967	0,055603	1,860421629	0,062825897	0,404317327
ENSG00000144655	CSRNP1	cysteine and serine rich nuclear protein 1	64651	155,8541559	-0,028687654	0,161287	-0,177867543	0,858827001	0,969699147
ENSG00000196865	NHLRC2	NHL repeat containing 2	374354	208,8968089	-0,033272146	0,130685	-0,254598653	0,799033099	0,954319642
ENSG00000123066	MED13L	mediator complex subunit 13L	23389	417,7863997	0,01680022	0,098287	0,170930183	0,864278663	0,971561202
ENSG00000127334	DYRK2	dual specificity tyrosine phosphorylation regulated kinase 2	8445	760,5883927	-0,367220129	0,08119	-4,522960812	6,10E-06	0,000665196
ENSG00000100385	IL2RB	interleukin 2 receptor subunit beta	3560	8191,834545	-0,160643696	0,046296	-3,469890916	0,00052067	0,021410159
ENSG00000149294	NCAM1	neural cell adhesion molecule 1	4684	3545,699333	-0,237304749	0,042747	-5,551325228	2,84E-08	6,96E-06
ENSG00000152291	TGOLN2	trans-golgi network protein 2	10618	2179,666147	-0,080074235	0,041412	-1,933615825	0,05316038	0,372224799
ENSG00000117868	ESYT2	extended synaptotagmin 2	57488	952,699705	-0,14891992	0,061689	-2,414042903	0,015776605	0,19762273
ENSG00000136997	MYC	MYC proto-oncogene, bHLH transcription factor	4609	304,6659503	-0,060868065	0,101025	-0,602507841	0,546836145	0,878739902
ENSG00000137193	PIM1	Pim-1 proto-oncogene, serine/threonine kinase	5292	982,6981282	-0,148502447	0,053957	-2,752227972	0,00591913	0,110163144

ENSG00000124193	SRSF6	serine and arginine rich splicing factor 6	6431	678,8005392	-0,167757969	0,067687	-2,478452888	0,013195354	0,180237056
ENSG00000148600	CDHR1	cadherin related family member 1	92211	227,4459192	-0,426744473	0,139006	-3,069980681	0,002140726	0,055967203
ENSG00000160908	ZNF394	zinc finger protein 394	84124	204,8048671	-0,125736287	0,118283	-1,063013898	0,287775649	0,739107242
ENSG00000188997	KCTD21	potassium channel tetramerization domain containing 21	283219	73,35855984	0,245116557	0,220027	1,114030226	0,26526622	0,720899962
ENSG00000215301	DDX3X	DEAD-box helicase 3 X-linked	1654	2489,690917	-0,070757978	0,061904	-1,143030807	0,2530258	0,708754466
ENSG00000144802	NFKBIZ	NFKB inhibitor zeta	64332	178,6982042	0,125080345	0,121632	1,028350152	0,303785148	0,751870158
ENSG00000128016	ZFP36	ZFP36 ring finger protein	7538	382,7770708	-0,066507726	0,115108	-0,577786689	0,563408143	0,884816202
ENSG00000157514	TSC22D3	TSC22 domain family member 3	1831	464,8659506	-0,14915111	0,07838	-1,902927269	0,057050036	0,38821726
ENSG00000196405	EVL	Enah/Vasp-like	51466	3651,815026	-0,137484569	0,042189	-3,258766638	0,001118977	0,037467385
ENSG00000132680	KHDC4	KH domain containing 4, pre-mRNA splicing factor	22889	180,3599807	-0,158221142	0,15427	-1,02560887	0,305075985	0,751870158
ENSG00000171223	JUNB	JunB proto-oncogene, AP-1 transcription factor subunit	3726	165,8126999	-0,398921869	0,123088	-3,240957975	0,001191287	0,038345785
ENSG00000179144	GIMAP7	GTPase, IMAP family member 7	168537	179,0664189	-0,042266024	0,136487	-0,309670828	0,756811289	0,942901768
ENSG00000125740	FOSB	FosB proto-oncogene, AP-1 transcription factor subunit	2354	444,9091429	-0,403355874	0,451431	-0,893504447	0,371587092	0,800231702
ENSG00000173762	CD7	CD7 molecule	924	2032,17267	-0,436888204	0,063469	-6,88348633	5,84E-12	4,17E-09
ENSG00000109321	AREG	amphiregulin	374	97,88695186	0,336996792	0,182462	1,846939521	0,064755906	0,410155553
ENSG00000140678	ITGAX	integrin subunit alpha X	3687	4940,033112	-0,264124157	0,04156	-6,355247463	2,08E-10	1,17E-07
ENSG00000185885	IFITM1	interferon induced transmembrane protein 1	8519	592,351307	-0,153461355	0,079824	-1,922503166	0,054542476	0,378695505
ENSG00000004399	PLXND1	plexin D1	23129	423,6327298	-0,500951203	0,079062	-6,336195471	2,36E-10	1,23E-07
ENSG00000196914	ARHGEF12	Rho guanine nucleotide exchange factor 12	23365	1794,235941	-0,008940396	0,046218	-0,193438865	0,84661528	0,967231052
ENSG00000080546	SESN1	sestrin 1	27244	234,6605943	0,179238618	0,145137	1,234960382	0,216845249	0,672891335
ENSG00000163659	TIPARP	TCDD inducible poly(ADP-ribose) polymerase	25976	456,568339	-0,244757861	0,089825	-2,724819684	0,006433661	0,11589444
ENSG00000117560	FASLG	Fas ligand	356	291,8325711	0,117335279	0,122339	0,959098211	0,337509271	0,77693253
ENSG00000072071	ADGRL1	adhesion G protein-coupled receptor L1	22859	55,15872261	0,222720674	0,444719	0,500811846	0,616503548	0,902090873
ENSG00000100664	EIF5	eukaryotic translation initiation factor 5	1983	752,2305066	-0,046228663	0,063873	-0,723756751	0,469215085	0,852144526
ENSG00000132819	RBM38	RNA binding motif protein 38	55544	146,4591031	-0,292955624	0,139335	-2,102531434	0,035506749	0,300111909
ENSG00000090104	RGS1	regulator of G protein signaling 1	5996	83,75215486	0,732106841	0,247047	2,963426554	0,003042347	0,072162376
ENSG00000169045	HNRNPH1	heterogeneous nuclear ribonucleoprotein H1	3187	212,7186974	-1,252830364	0,20615	-6,077282404	1,22E-09	5,05E-07
ENSG00000148841	ITPRIP	inositol 1,4,5-trisphosphate receptor interacting protein	85450	293,450279	-0,063578014	0,11263	-0,564487298	0,572422542	0,886795904
ENSG00000153094	BCL2L11	BCL2 like 11	10018	1958,002531	0,048131453	0,051654	0,931800139	0,351439823	0,785712602
ENSG00000110321	EIF4G2	eukaryotic translation initiation factor 4 gamma 2	1982	3123,614055	-0,087999545	0,041132	-2,139451048	0,032399156	0,287528785
ENSG00000198876	DCAF12	DDB1 and CUL4 associated factor 12	25853	239,6597821	0,197626621	0,122201	1,617223549	0,105830035	0,522649494
ENSG00000165650	PDZD8	PDZ domain containing 8	118987	346,2603078	-0,172831515	0,096037	-1,799629243	0,0719192	0,435172111
ENSG00000113719	ERGIC1	endoplasmic reticulum-golgi intermediate compartment 1	57222	236,9051576	-0,373265995	0,150828	-2,474785506	0,013331622	0,180367712
ENSG00000102317	RBM3	RNA binding motif protein 3	5935	683,252303	0,046402096	0,08437	0,549982919	0,582331089	0,888501395
ENSG00000196924	FLNA	filamin A	2316	6503,779478	0,188344259	0,037929	4,965766945	6,84E-07	0,000101289
ENSG00000275302	CCL4	C-C motif chemokine ligand 4	6351	826,4565583	0,402233617	0,079136	5,082795379	3,72E-07	6,22E-05
ENSG00000183508	TENT5C	terminal nucleotidyltransferase 5C	54855	164,9104047	0,022038588	0,147329	0,149587888	0,881089765	0,976300778
ENSG00000007944	MYLIP	myosin regulatory light chain interacting protein	29116	241,4360828	-0,263613935	0,129458	-2,036291089	0,041721134	0,325303563
ENSG00000099622	CIRBP	cold inducible RNA binding protein	1153	429,6597815	-0,456017144	0,104828	-4,350139562	1,36E-05	0,001287402
ENSG00000148154	UGCG	UDP-glucose ceramide glucosyltransferase	7357	347,1345243	-0,190841761	0,09069	-2,104321636	0,0353504	0,299505978
ENSG00000185262	UBALD2	UBA like domain containing 2	283991	367,0313643	-0,338672433	0,109682	-3,087754446	0,00201675	0,054949576
ENSG00000173812	EIF1	eukaryotic translation initiation factor 1	10209	683,954871	0,048952117	0,075629	0,647266142	0,51745971	0,868791049
ENSG00000063046	EIF4B	eukaryotic translation initiation factor 4B	1975	2125,272372	-0,084950825	0,044876	-1,893019156	0,058355322	0,3920639
ENSG00000100028	SNRPD3	small nuclear ribonucleoprotein D3 polypeptide	6634	275,9560912	-0,934755702	0,204999	-4,559810674	5,12E-06	0,000591357
ENSG00000136536	MARCHF7	membrane associated ring-CH-type finger 7	64844	452,539554	-0,225166064	0,075855	-2,968385784	0,002993683	0,071684113
ENSG00000122566	HNRNPA2B1	heterogeneous nuclear ribonucleoprotein A2/B1	3181	1558,372573	0,209627412	0,069858	3,00076264	0,002693044	0,066513105
ENSG00000003756	RBM5	RNA binding motif protein 5	10181	479,8084089	-0,038247922	0,089964	-0,425147607	0,670729075	0,922641254
ENSG00000163508	EOMES	eomesodermin	8320	429,7271259	0,148502857	0,084501	1,757405076	0,078848789	0,455017184
ENSG00000184897	H1-10	H1,10 linker histone	8971	242,2637467	-0,019156672	0,108889	-0,175928385	0,860350205	0,97061627

ENSG00000133639	BTG1	BTG anti-proliferation factor 1	694	800,372714	-0,023346937	0,094479	-0,247113639	0,804820278	0,957328863
ENSG00000181315	ZNF322	zinc finger protein 322	79692	91,1048845	0,078811647	0,218397	0,360863477	0,718201508	0,932668593
ENSG00000145649	GZMA	granzyme A	3001	1238,173058	-0,228666018	0,051318	-4,455902669	8,35E-06	0,000852117
ENSG00000091732	ZC3HC1	zinc finger C3HC-type containing 1	51530	18,58329942	6,99119912	1,247125	5,60585478	2,07E-08	NA
ENSG00000143184	XCL1	X-C motif chemokine ligand 1	6375	1103,885204	0,344806767	0,072536	4,753572172	2,00E-06	0,000249151
ENSG00000196542	SPTSSB	serine palmitoyltransferase small subunit B	165679	406,365203	-0,472526175	0,084366	-5,600874786	2,13E-08	5,83E-06
ENSG00000254772	EEF1G	eukaryotic translation elongation factor 1 gamma	1937	2771,837694	0,027234891	0,042116	0,646660522	0,517851679	0,868791049
ENSG00000165025	SYK	spleen associated tyrosine kinase	6850	920,466905	-0,074321961	0,059669	-1,245565523	0,212923946	0,667215123
ENSG00000181163	NPM1	nucleophosmin 1	4869	2102,79717	0,026744328	0,049091	0,544795427	0,585894224	0,890759735
ENSG00000120253	NUP43	nucleoporin 43	348995	128,4102819	-0,104008244	0,160085	-0,64970545	0,515882503	0,868791049
ENSG00000171522	PTGER4	prostaglandin E receptor 4	5734	377,9410334	0,165114456	0,085075	1,940812088	0,052281074	0,36949064
ENSG00000178917	ZNF852	zinc finger protein 852	285346	88,30674591	0,416456623	0,194809	2,137773858	0,032535102	0,288320967
ENSG00000128989	ARPP19	cAMP regulated phosphoprotein 19	10776	916,6233422	-0,334035649	0,069084	-4,835211592	1,33E-06	0,000186538
ENSG00000111276	CDKN1B	cyclin dependent kinase inhibitor 1B	1027	274,402737	-0,131982394	0,10833	-1,218332822	0,223097522	0,678694696
ENSG00000152518	ZFP36L2	ZFP36 ring finger protein like 2	678	1332,507345	0,022252261	0,050561	0,440108011	0,65985888	0,919368176
ENSG00000105939	ZC3HAV1	zinc finger CCCH-type containing, antiviral 1	56829	1010,4366	-0,100616211	0,071166	-1,413831445	0,157411399	0,61428162
ENSG00000110934	BIN2	bridging integrator 2	51411	867,7312335	-0,178806548	0,066348	-2,694970194	0,007039493	0,12313625
ENSG00000102241	HTATSF1	HIV-1 Tat specific factor 1	27336	782,5169289	0,12492823	0,068172	1,832537894	0,066871321	0,417162318
ENSG00000156508	EEF1A1	eukaryotic translation elongation factor 1 alpha 1	1915	26626,11125	0,028456191	0,041949	0,678350876	0,497549248	0,861283987
ENSG00000133112	TPT1	tumor protein, translationally-controlled 1	7178	11229,8619	0,054208332	0,046864	1,156716193	0,247388332	0,705258787
ENSG00000139496	NUP58	nucleoporin 58	9818	173,7114616	0,278722348	0,157208	1,772957542	0,076235743	0,447360046
ENSG00000184007	PTP4A2	protein tyrosine phosphatase 4A2	8073	1211,28483	-0,089658667	0,055919	-1,603360587	0,108855067	0,52908614
ENSG00000145012	LPP	LIM domain containing preferred translocation partner in lipoma	4026	492,0088799	-0,273132243	0,087324	-3,127789291	0,001761264	0,05104416
ENSG00000100316	RPL3	ribosomal protein L3	6122	3952,874847	-0,009974857	0,050894	-0,19599211	0,844616341	0,967231052
ENSG00000099204	ABLIM1	actin binding LIM protein 1	3983	962,4389574	-0,138161434	0,065606	-2,105938375	0,035209706	0,299241865
ENSG00000135486	HNRNPA1	heterogeneous nuclear ribonucleoprotein A1	3178	2790,392151	-0,053773519	0,06671	-0,806077251	0,420198308	0,826919947
ENSG00000167202	TBC1D2B	TBC1 domain family member 2B	23102	621,1599065	0,02426034	0,084642	0,286622883	0,774401091	0,946448613
ENSG00000176018	LYSMD3	LysM domain containing 3	116068	145,7758696	-0,255422616	0,146193	-1,74716215	0,080609215	0,4605948
ENSG00000180229	NA	NA	NA	272,322668	0,085109744	0,12613	0,674779893	0,499815617	0,86161226
ENSG00000132906	CASP9	caspase 9	842	222,0592799	0,553692138	0,217622	2,544279505	0,010950342	0,163343491
ENSG00000243335	KCTD7	potassium channel tetramerization domain containing 7	154881	272,580377	-0,037122848	0,108522	-0,342075711	0,732293916	0,93912057
ENSG00000120616	EPC1	enhancer of polycomb homolog 1	80314	329,1569026	-0,055224026	0,112935	-0,488989994	0,624848781	0,90479929
ENSG00000156030	MIDEAS	mitotic deacetylase associated SANT domain protein	91748	737,3591983	-0,040255829	0,098983	-0,406695665	0,684231525	0,925586358
ENSG00000178209	PLEC	plectin	5339	1190,661168	0,326848298	0,052734	6,198042515	5,72E-10	2,64E-07
ENSG00000167460	TPM4	tropomyosin 4	7171	755,6003279	-0,246229791	0,129573	-1,900316197	0,057391637	0,38821726
ENSG00000277632	CCL3	C-C motif chemokine ligand 3	6348	96,99920581	2,106073343	3,726978	0,565088826	0,572013348	0,886795904
ENSG00000266094	RASSF5	Ras association domain family member 5	83593	1400,700088	-0,150036563	0,054759	-2,739947979	0,006144891	0,113291018
ENSG00000165572	KBTBD6	kelch repeat and BTB domain containing 6	89890	37,39555746	0,297967477	0,345173	0,86324075	0,388005113	NA
ENSG00000286190	LOC728392	uncharacterized LOC728392	728392	13,46879175	6,557759837	1,256949	5,217203372	1,82E-07	NA
ENSG00000286190	LOC124900388	uncharacterized LOC124900388	124900388	13,46879175	6,557759837	1,256949	5,217203372	1,82E-07	NA
ENSG00000167232	ZNF91	zinc finger protein 91	7644	156,7546355	0,034541061	0,189119	0,182642	0,855078927	0,968377739
ENSG00000166501	PRKCB	protein kinase C beta	5579	675,2151441	-0,060933095	0,0718	-0,848656104	0,396072678	0,812799303
ENSG00000126860	EVI2A	ecotropic viral integration site 2A	2123	384,7089403	0,0167525	0,088334	0,189649897	0,849583485	0,967902179
ENSG00000116580	GON4L	gon-4 like	54856	436,1479714	0,387307469	0,112863	3,431669534	0,000599878	0,023440011
ENSG00000172840	PDP2	pyruvate dehydrogenase phosphatase catalytic subunit 2	57546	146,8806956	0,018320052	0,163935	0,111752235	0,911019861	0,979595261
ENSG00000080822	CLDND1	claudin domain containing 1	56650	490,0701454	-0,051017141	0,084585	-0,603144875	0,546412315	0,878739902
ENSG00000131747	TOP2A	DNA topoisomerase II alpha	7153	757,1095999	0,719934495	0,068811	10,46251023	1,28E-25	3,36E-22
ENSG00000138326	RPS24	ribosomal protein S24	6229	1554,756581	0,074118366	0,04856	1,526328454	0,126928086	0,563689485
ENSG00000108298	RPL19	ribosomal protein L19	6143	2026,639044	0,103973148	0,0537	1,936185235	0,052845018	0,371734827
ENSG00000165527	ARF6	ADP ribosylation factor 6	382	1408,940049	-0,009001368	0,048485	-0,185651765	0,852717857	0,968229875
ENSG00000131469	RPL27	ribosomal protein L27	6155	1165,597089	-0,081925427	0,050069	-1,636250866	0,101787095	0,514437479
ENSG00000138413	IDH1	isocitrate dehydrogenase (NADP(+)) 1	3417	84,40411268	0,415896204	0,198587	2,094274043	0,036235575	0,302116994

ENSG00000132670	PTPRA	protein tyrosine phosphatase receptor type A	5786	452,8389092	-0,153718897	0,079155	-1,941998756	0,052137251	0,369238926
ENSG00000077147	TM9SF3	transmembrane 9 superfamily member 3	56889	675,7343795	-0,275469299	0,064466	-4,273071743	1,93E-05	0,001740504
ENSG00000123416	TUBA1B	tubulin alpha 1b	10376	1773,956957	0,371183326	0,052032	7,133732737	9,77E-13	7,67E-10
ENSG00000184640	SEPTIN9	septin 9	10801	2230,479741	-0,059874018	0,060674	-0,986814271	0,323733697	0,767603859
ENSG00000206053	JPT2	Jupiter microtubule associated homolog 2	90861	106,9519842	0,424606081	0,206865	2,052574907	0,040113823	0,319386212
ENSG00000198624	CCDC69	coiled-coil domain containing 69	26112	804,6835402	-0,222923269	0,060775	-3,668032654	0,000244424	0,012305807
ENSG00000116001	TIA1	TIA1 cytotoxic granule associated RNA binding protein	7072	368,3091567	0,112268663	0,101188	1,10950534	0,267212242	0,722449525
ENSG00000118971	CCND2	cyclin D2	894	3160,179597	-0,029850384	0,058039	-0,514313773	0,607032634	0,897481254
ENSG00000059804	SLC2A3	solute carrier family 2 member 3	6515	625,4644023	-0,122612387	0,078905	-1,553933439	0,120200294	0,554021779
ENSG00000150045	KLRF1	killer cell lectin like receptor F1	51348	406,251331	-0,203119192	0,093139	-2,180812418	0,029197294	0,275445021
ENSG00000108788	MLX	MAX dimerization protein MLX	6945	103,1901304	0,419686165	0,201449	2,083332356	0,037220939	0,306351862
ENSG00000119714	GPR68	G protein-coupled receptor 68	8111	623,2865639	-0,009899261	0,075967	-0,130310238	0,89632098	0,977990819
ENSG00000135048	CEMIP2	cell migration inducing hyaluronidase 2	23670	546,7404847	-0,070840565	0,081647	-0,867644227	0,385589127	0,809653368
ENSG00000198034	RPS4X	ribosomal protein S4 X-linked	6191	2918,992304	0,13633573	0,03926	3,472599331	0,000515444	0,021306837
ENSG00000204977	TRIM13	tripartite motif containing 13	10206	103,230221	0,132361861	0,195316	0,677681305	0,497973782	0,861283987
ENSG00000127947	PTPN12	protein tyrosine phosphatase non-receptor type 12	5782	703,8175992	-0,199082379	0,079177	-2,514381085	0,011924151	0,17121075
ENSG00000177409	SAMD9L	sterile alpha motif domain containing 9 like	219285	800,9955963	0,152241572	0,060365	2,522024671	0,011668152	0,169255469
ENSG00000183283	DAZAP2	DAZ associated protein 2	9802	1285,808644	-0,007752106	0,065296	-0,118723349	0,905494537	0,978460866
ENSG00000164305	CASP3	caspase 3	836	197,9499489	0,158069503	0,151069	1,046338705	0,295404681	0,747362888
ENSG00000205765	RIMOC1	RAB7A interacting MON1-CCZ1 complex subunit 1	285636	121,257637	-0,030666466	0,187689	-0,163389516	0,870211757	0,973873345
ENSG00000101751	POLI	DNA polymerase iota	11201	32,29667759	0,703904721	0,355649	1,979211814	0,047792163	NA
ENSG00000137692	DCUN1D5	defective in cullin neddylation 1 domain containing 5	84259	75,80203113	-0,594904887	0,245727	-2,42100122	0,015477825	0,195124944
ENSG00000005379	TSPOAP1	TSPO associated protein 1	9256	300,4243626	-0,297619167	0,095439	-3,118410744	0,001818292	0,051326425
ENSG00000189057	FAM111B	FAM111 trypsin like peptidase B	374393	67,24143911	0,852007084	0,24627	3,459641598	0,000540895	0,022011336
ENSG00000171988	JMJD1C	jumonji domain containing 1C	221037	347,2028157	0,446706248	0,111408	4,009629111	6,08E-05	0,004257158
ENSG00000132801	ZSWIM3	zinc finger SWIM-type containing 3	140831	19,70190538	0,866121472	0,498144	1,738698534	0,082087804	NA
ENSG00000183161	FANCF	FA complementation group F	2188	63,41150353	0,235772504	0,255215	0,923819796	0,355580147	0,788460325
ENSG00000161813	LARP4	La ribonucleoprotein 4	113251	162,9873906	0,457400704	0,148183	3,086722399	0,002023765	0,054949576
ENSG00000057657	PRDM1	PR/SET domain 1	639	326,9500581	0,064528242	0,098241	0,65683609	0,511286316	0,866432075
ENSG00000122406	RPL5	ribosomal protein L5	6125	1469,485582	0,051686227	0,050611	1,021244328	0,307138695	0,754087936
ENSG00000143185	XCL2	X-C motif chemokine ligand 2	6846	671,6875899	0,24257798	0,079805	3,039649784	0,002368534	0,059432797
ENSG00000184730	APOBR	apolipoprotein B receptor	55911	1015,083535	-0,408150335	0,069767	-5,850207667	4,91E-09	1,84E-06
ENSG00000044574	HSPA5	heat shock protein family A (Hsp70) member 5	3309	2402,35529	0,01671304	0,061465	0,271909417	0,78569167	0,950624564
ENSG00000124789	NUP153	nucleoporin 153	9972	571,4103669	-0,002469811	0,075975	-0,032508366	0,974066644	0,994534085
ENSG00000129128	SPCS3	signal peptidase complex subunit 3	60559	1067,048365	-0,28444913	0,060678	-4,687864265	2,76E-06	0,000338791
ENSG00000125384	PTGER2	prostaglandin E receptor 2	5732	625,2220841	0,064493748	0,07006	0,920544896	0,357288083	0,790004408
ENSG00000108654	DDX5	DEAD-box helicase 5	1655	950,0281791	-0,128391041	0,102904	-1,247678415	0,212148849	0,666699723
ENSG00000145990	GFOD1	Gfo/Idh/MocA-like oxidoreductase domain containing 1	54438	776,6079238	-0,261607375	0,084264	-3,104620082	0,001905236	0,053441862
ENSG00000197183	NOL4L	nucleolar protein 4 like	140688	610,6487227	-0,120528513	0,073643	-1,636662998	0,101700904	0,514437479
ENSG00000125691	RPL23	ribosomal protein L23	9349	1530,150527	0,003427059	0,049036	0,069888094	0,94428273	0,989126091
ENSG00000130175	PRKCSH	protein kinase C substrate 80K-H	5589	554,7017737	-0,302350568	0,096851	-3,121810773	0,001797424	0,051326425
ENSG00000168724	DNAJC21	DnaJ heat shock protein family (Hsp40) member C21	134218	147,5936198	-0,37523071	0,176556	-2,125280552	0,03356321	0,293547274
ENSG00000082074	FYB1	FYN binding protein 1	2533	1104,422562	-0,128310086	0,066332	-1,934348348	0,053070312	0,372224799
ENSG00000052802	MSMO1	methylsterol monooxygenase 1	6307	111,6426308	-0,277241996	0,1771	-1,565451089	0,117477177	0,546602932
ENSG00000159388	BTG2	BTG anti-proliferation factor 2	7832	1459,816187	0,065769693	0,046127	1,425836993	0,1539154	0,611457538
ENSG00000145741	BTF3	basic transcription factor 3	689	824,5374353	-0,017979486	0,061015	-0,294672136	0,76824435	0,944698783
ENSG00000109320	NFKB1	nuclear factor kappa B subunit 1	4790	452,8985592	0,280611825	0,136449	2,056531198	0,039731342	0,317468275
ENSG00000116560	SFPQ	splicing factor proline and glutamine rich	6421	938,5244893	-0,074984916	0,0662	-1,132694763	0,257342458	0,713362633

ENSG00000254087	LYN	LYN proto-oncogene, Src family tyrosine kinase	4067	436,0083145	-0,216758962	0,083231	-2,604312628	0,009205874	0,146957187
ENSG00000143970	ASXL2	ASXL transcriptional regulator 2	55252	906,8420469	0,108274354	0,071982	1,504188728	0,132532779	0,569738615
ENSG00000152558	TMEM123	transmembrane protein 123	114908	1687,488154	-0,038623352	0,045401	-0,850717659	0,394926211	0,812498986
ENSG00000198563	DDX39B	DExD-box helicase 39B	7919	153,9474293	0,09101317	0,197627	0,460529973	0,645135863	0,912530858
ENSG00000140474	ULK3	unc-51 like kinase 3	25989	79,49111808	0,91598184	0,264938	3,457343089	0,00054553	0,022085522
ENSG00000168172	HOOK3	hook microtubule tethering protein 3	84376	744,0719861	-0,109972139	0,118695	-0,926508081	0,354182	0,788228969
ENSG00000118515	SGK1	serum/glucocorticoid regulated kinase 1	6446	91,15786768	1,169743157	0,23257	5,029649146	4,91E-07	7,74E-05
ENSG00000125447	GGA3	golgi associated, gamma adaptin ear containing, ARF binding protein 3	23163	142,2064919	0,409369852	0,45952	0,890864925	0,373001639	0,800776142
ENSG00000147138	GPR174	G protein-coupled receptor 174	84636	322,1322298	0,046754044	0,097659	0,478745589	0,632119629	0,907119965
ENSG00000180626	ZNF594	zinc finger protein 594	84622	42,03411514	0,196894044	0,366288	0,537539306	0,590895142	NA
ENSG00000085224	ATRX	ATRX chromatin remodeler	546	788,6350494	-0,274956182	0,067181	-4,092756293	4,26E-05	0,003250455
ENSG00000180353	HCLS1	hematopoietic cell-specific Lyn substrate 1	3059	1288,253806	-0,221370938	0,054069	-4,094195115	4,24E-05	0,003250455
ENSG00000163947	ARHGEF3	Rho guanine nucleotide exchange factor 3	50650	656,3792435	0,07961638	0,076936	1,034832505	0,30074714	0,751870158
ENSG00000143384	MCL1	MCL1 apoptosis regulator, BCL2 family member	4170	2295,306357	-0,177656158	0,039499	-4,497738153	6,87E-06	0,000719219
ENSG00000143384	ADAMTSL4-AS1	ADAMTSL4 antisense RNA 1	574406	2295,306357	-0,177656158	0,039499	-4,497738153	6,87E-06	0,000719219
ENSG00000132196	HSD17B7	hydroxysteroid 17-beta dehydrogenase 7	51478	59,64712801	0,050416174	0,239229	0,210744743	0,833086458	0,963446071
ENSG00000167395	ZNF646	zinc finger protein 646	9726	234,56109	0,009701942	0,132203	0,073386628	0,941498458	0,988441237
ENSG00000067225	PKM	pyruvate kinase M1/2	5315	1584,358883	0,495700539	0,047275	10,4854357	1,01E-25	3,36E-22
ENSG00000176095	IP6K1	inositol hexakisphosphate kinase 1	9807	175,4586821	0,122312606	0,148844	0,821748217	0,411220211	0,821394592
ENSG00000172716	SLFN11	schlafen family member 11	91607	241,6340895	-0,035757067	0,118096	-0,302778718	0,762058503	0,943004836
ENSG00000172315	TP53RK	TP53 regulating kinase	112858	148,8965804	0,220003185	0,147507	1,491478718	0,135835855	0,576449438
ENSG00000181004	BBS12	Bardet-Biedl syndrome 12	166379	17,2955637	0,870966646	0,539102	1,615587843	0,106183454	NA
ENSG00000125827	TMX4	thioredoxin related transmembrane protein 4	56255	764,8530628	-0,162861373	0,072484	-2,246846309	0,024649851	0,252419853
ENSG00000196684	HSH2D	hematopoietic SH2 domain containing	84941	284,5402839	0,338808472	0,173013	1,958279427	0,050197234	0,359061086
ENSG00000166886	NAB2	NGFI-A binding protein 2	4665	76,69696658	0,023995594	0,208968	0,114829136	0,908580555	0,97930822
ENSG00000166750	SLFN5	schlafen family member 5	162394	3214,143402	-0,040347977	0,059034	-0,68347281	0,494308126	0,860513824
ENSG00000047365	ARAP2	ArfGAP with RhoGAP domain, ankyrin repeat and PH domain 2	116984	1040,87083	-0,121577652	0,061865	-1,965212717	0,049389623	0,356859335
ENSG00000232119	MCTS1	MCTS1 re-initiation and release factor	28985	123,4389076	0,368567578	0,198386	1,857830714	0,063193058	0,404317327
ENSG00000172757	CFL1	cofilin 1	1072	2496,588592	-0,066738855	0,042877	-1,556532203	0,119581591	0,552466951
ENSG00000075426	FOSL2	FOS like 2, AP-1 transcription factor subunit	2355	771,3998294	0,05540646	0,062878	0,881179762	0,378220531	0,804187219
ENSG00000156482	RPL30	ribosomal protein L30	6156	921,7650651	-0,05291898	0,062211	-0,850632723	0,394973406	0,812498986
ENSG00000146278	PNRC1	proline rich nuclear receptor coactivator 1	10957	477,8027236	-0,177615242	0,095473	-1,860371495	0,062832985	0,404317327
ENSG00000185219	ZNF445	zinc finger protein 445	353274	284,3356944	0,046441295	0,099244	0,467950994	0,639819635	0,910851161
ENSG00000163026	WDCP	WD repeat and coiled coil containing	80304	43,98784525	0,710329947	0,323094	2,198526827	0,027911585	0,269753518
ENSG00000149930	TAOK2	TAO kinase 2	9344	223,8705667	0,044092884	0,114782	0,384143004	0,700872459	0,930783276
ENSG00000165355	FBXO33	F-box protein 33	254170	214,5335686	-0,226882484	0,116305	-1,9507475	0,05108709	0,364671481
ENSG00000163219	ARHGAP25	Rho GTPase activating protein 25	9938	328,7591939	0,290442202	0,129993	2,234287592	0,025464156	0,257063603
ENSG00000142937	RPS8	ribosomal protein S8	6202	1639,142889	-0,041993136	0,050923	-0,824633638	0,409579618	0,82130194
ENSG00000164463	CREBRF	CREB3 regulatory factor	153222	263,481016	-0,239364705	0,161622	-1,481012483	0,138603247	0,580290876
ENSG00000164134	NAA15	N-alpha-acetyltransferase 15, NatA auxiliary subunit	80155	204,4516829	0,034883784	0,121766	0,286483167	0,774508084	0,946448613
ENSG00000111912	NCOA7	nuclear receptor coactivator 7	135112	190,312133	-0,272610658	0,138296	-1,97120546	0,04870038	0,354159983
ENSG00000143740	SNAP47	synaptosome associated protein 47	116841	256,4143618	0,144607063	0,114459	1,263395426	0,206447107	0,661359894
ENSG00000172543	CTSW	cathepsin W	1521	6146,006472	-0,166954701	0,039317	-4,246426631	2,17E-05	0,001922139
ENSG00000179388	EGR3	early growth response 3	1960	334,3693278	-0,154479933	0,171281	-0,901910633	0,367104342	0,795235567
ENSG00000167658	EEF2	eukaryotic translation elongation factor 2	1938	4865,63901	-0,067295416	0,035612	-1,889686137	0,058799949	0,39312401
ENSG00000170871	KIAA0232	KIAA0232	9778	301,3603079	-0,136373812	0,098125	-1,389791511	0,164592197	0,624374678
ENSG00000162434	JAK1	Janus kinase 1	3716	3644,770885	-0,127548059	0,053234	-2,395978785	0,01657605	0,203098821
ENSG00000088179	PTPN4	protein tyrosine phosphatase non-receptor type 4	5775	848,4475594	-0,07642976	0,080071	-0,954527865	0,339816511	0,777431905
ENSG00000244165	P2RY11	purinergic receptor P2Y11	5032	88,04327736	-0,382450522	0,193384	-1,977676743	0,047965186	0,350971037
ENSG00000165802	NSMF	NMDA receptor synaptonuclear signaling and neuronal migration factor	26012	311,6329235	-0,276289357	0,093916	-2,94188642	0,003262196	0,075374002

ENSG00000161970	RPL26	ribosomal protein L26	6154	1406,455337	0,0836713	0,053168	1,573713121	0,115553787	0,541856098
ENSG00000143376	SNX27	sorting nexin 27	81609	105,4880913	0,236927238	0,170652	1,388362453	0,165026704	0,624454568
ENSG00000120696	KBTBD7	kelch repeat and BTB domain containing 7	84078	45,36680572	0,415290922	0,302503	1,372848989	0,169799297	0,629558603
ENSG00000170915	PAQR8	progestin and adipoQ receptor family member 8	85315	190,0922429	0,006210812	0,127504	0,048710614	0,961149917	0,992235217
ENSG00000148773	MKI67	marker of proliferation Ki-67	4288	1863,047439	0,561271431	0,063172	8,88475496	6,41E-19	1,26E-15
ENSG00000138061	CYP1B1	cytochrome P450 family 1 subfamily B member 1	1545	696,7148587	0,164255725	0,07744	2,121083142	0,033914806	0,294612488
ENSG00000008083	JARID2	jumonji and AT-rich interaction domain containing 2	3720	607,993801	-0,161788968	0,071476	-2,26355751	0,023601344	0,245726955
ENSG00000234851	RPL23AP42	ribosomal protein L23a pseudogene 42	647099	997,0171615	-0,203195009	0,076472	-2,657115653	0,00788124	0,132558158
ENSG00000183530	PRR14L	proline rich 14 like	253143	447,4711229	0,013061865	0,084822	0,153990977	0,87761685	0,976179399
ENSG00000106560	GIMAP2	GTPase, IMAP family member 2	26157	167,0359765	-0,178791485	0,139341	-1,283122598	0,199449128	0,658208139
ENSG00000167193	CRK	CRK proto-oncogene, adaptor protein	1398	85,43902387	0,233830175	0,19489	1,199805351	0,230214945	0,684730271
ENSG00000144711	IQSEC1	IQ motif and Sec7 domain ArfGEF 1	9922	611,5360179	-0,196823308	0,070601	-2,787830671	0,005306227	0,103412177
ENSG00000171608	PIK3CD	phosphatidylinositol-4,5-bisphosphate 3-kinase catalytic subunit delta	5293	2077,249176	0,141549377	0,051739	2,735855985	0,006221825	0,114173394
ENSG00000077044	DGKD	diacylglycerol kinase delta	8527	1071,321525	-0,144561777	0,064075	-2,256144919	0,024061557	0,247679511
ENSG00000066279	ASPM	assembly factor for spindle microtubules	259266	477,5541604	0,471857062	0,088487	5,332524552	9,69E-08	2,00E-05
ENSG00000065308	TRAM2	translocation associated membrane protein 2	9697	114,2196807	-0,413774256	0,16967	-2,438699594	0,014740216	0,190724312
ENSG00000124126	PREX1	phosphatidylinositol-3,4,5-trisphosphate dependent Rac exchange factor 1	57580	4571,338789	-0,031787952	0,040207	-0,790607678	0,429172965	0,832060923
ENSG00000183864	TOB2	transducer of ERBB2, 2	10766	164,8424925	-0,185814097	0,132526	-1,402096711	0,160886369	0,618147537
ENSG00000169490	TM2D2	TM2 domain containing 2	83877	59,62417683	0,344524715	0,236066	1,459444555	0,14444279	0,59024645
ENSG00000152601	MBNL1	muscleblind like splicing regulator 1	4154	2101,798482	-0,258527877	0,061765	-4,185672233	2,84E-05	0,002375613
ENSG00000114737	CISH	cytokine inducible SH2 containing protein	1154	430,5349633	-0,00530284	0,112059	-0,047321832	0,962256728	0,992328441
ENSG00000198851	CD3E	CD3 epsilon subunit of T-cell receptor complex	916	1679,247854	-0,257685258	0,069779	-3,692857775	0,000221748	0,011545615
ENSG00000122545	SEPTIN7	septin 7	989	1326,665056	-0,085824019	0,065491	-1,310466121	0,190038198	0,647812504
ENSG00000136807	CDK9	cyclin dependent kinase 9	1025	505,7312484	-0,267867458	0,078799	-3,399383964	0,000675378	0,025875225
ENSG00000111540	RAB5B	RAB5B, member RAS oncogene family	5869	495,6091322	0,299955196	0,092324	3,248936492	0,001158373	0,037594481
ENSG00000163875	MEAF6	MYST/Esa1 associated factor 6	64769	135,1615782	-0,342242243	0,223245	-1,533031321	0,125268138	0,5624706
ENSG00000240053	LY6G5B	lymphocyte antigen 6 family member G5B	58496	181,8296939	-0,32787576	0,133782	-2,450828094	0,0142528	0,186258726
ENSG00000177954	RPS27	ribosomal protein S27	6232	2078,043373	-0,001259007	0,067504	-0,01865088	0,985119614	0,996185513
ENSG00000198836	OPA1	OPA1 mitochondrial dynamin like GTPase	4976	304,8481725	-0,172925464	0,148531	-1,164238293	0,244327456	0,701113569
ENSG00000108370	RGS9	regulator of G protein signaling 9	8787	205,3380868	-0,235973911	0,115655	-2,0403326	0,04131721	0,323534763
ENSG00000055070	SZRD1	SUZ RNA binding domain containing 1	26099	560,8436488	-0,086667597	0,084401	-1,026850877	0,304490688	0,751870158
ENSG00000170004	CHD3	chromodomain helicase DNA binding protein 3	1107	1731,807647	-0,014696448	0,054886	-0,267764291	0,788880759	0,95148885
ENSG00000154451	GBP5	guanylate binding protein 5	115362	878,9689803	0,257484279	0,073115	3,521628869	0,000428904	0,018508863
ENSG00000099991	CABIN1	calcineurin binding protein 1	23523	779,7131013	-0,195393532	0,070665	-2,765079775	0,005690888	0,10771718
ENSG00000156802	ATAD2	ATPase family AAA domain containing 2	29028	297,316084	0,435172197	0,126761	3,433023654	0,00059689	0,023439871
ENSG00000184203	PPP1R2	protein phosphatase 1 regulatory inhibitor subunit 2	5504	273,3036314	-0,324016969	0,099491	-3,25675012	0,001126956	0,037467385
ENSG00000164924	YWHAZ	tyrosine 3-monooxygenase/tryptophan 5-monooxygenase activation protein zeta	7534	3315,147761	-0,028091968	0,05355	-0,524592475	0,599866519	0,895523976
ENSG00000121966	CXCR4	C-X-C motif chemokine receptor 4	7852	231,2467404	0,467131954	0,131233	3,559552277	0,000371488	0,016865107
ENSG00000184371	CSF1	colony stimulating factor 1	1435	322,2700612	0,64384455	0,098156	6,559426955	5,40E-11	3,54E-08
ENSG00000035720	STAP1	signal transducing adaptor family member 1	26228	375,7271936	-0,053797863	0,099143	-0,54262758	0,587386246	0,891455115
ENSG00000153815	CMIP	c-Maf inducing protein	80790	851,4326902	-0,222318008	0,061123	-3,637200087	0,000275618	0,012885133
ENSG00000116754	SRSF11	serine and arginine rich splicing factor 11	9295	348,46156	-0,061645418	0,087008	-0,708504247	0,478632176	0,854008769
ENSG00000112406	HECA	hdc homolog, cell cycle regulator	51696	429,16067	-0,42863668	0,085922	-4,988663386	6,08E-07	9,36E-05
ENSG00000265681	RPL17	ribosomal protein L17	6139	1535,159769	-0,012576637	0,052952	-0,237508684	0,812262183	0,959038743
ENSG00000067182	TNFRSF1A	TNF receptor superfamily member 1A	7132	166,1400656	0,135671875	0,148666	0,91259568	0,361455227	0,791408368

ENSG00000141522	ARHGDIA	Rho GDP dissociation inhibitor alpha	396	1111,912944	-0,145749395	0,061119	-2,384687282	0,017093645	0,204616219
ENSG00000077150	NFKB2	nuclear factor kappa B subunit 2	4791	227,9413668	0,086196792	0,116666	0,73883252	0,460008702	0,846622987
ENSG00000132153	DHX30	DExH-box helicase 30	22907	194,5413963	0,390750779	0,138514	2,821011523	0,004787248	0,096161249
ENSG00000187605	TET3	tet methylcytosine dioxygenase 3	200424	497,2679434	-0,211915966	0,080799	-2,622766863	0,008721893	0,14257795
ENSG00000162783	IER5	immediate early response 5	51278	349,1418014	0,146148969	0,115375	1,266727661	0,205252686	0,660954946
ENSG00000143924	EML4	EMAP like 4	27436	561,4930431	-0,226158579	0,069364	-3,26047963	0,00111224	0,037467385
ENSG00000120705	ETF1	eukaryotic translation termination factor 1	2107	564,4769381	0,005336905	0,088759	0,060127955	0,952053725	0,991212866
ENSG00000187514	PTMA	prothymosin alpha	5757	3035,768597	-0,060232113	0,057673	-1,044364134	0,296316951	0,747862492
ENSG00000115306	SPTBN1	spectrin beta, non-erythrocytic 1	6711	720,3817848	-0,187548566	0,079942	-2,346053801	0,018973369	0,214721666
ENSG00000174197	MGA	MAX dimerization protein MGA	23269	519,4849806	0,039403605	0,081884	0,481210807	0,630366681	0,905960607
ENSG00000170144	HNRNPA3	heterogeneous nuclear ribonucleoprotein A3	220988	1566,533637	-0,149407546	0,052744	-2,832686258	0,004615867	0,094406576
ENSG00000140299	BNIP2	BCL2 interacting protein 2	663	507,1369336	-0,103802584	0,116647	-0,889885576	0,37352733	0,800776142
ENSG00000174738	NR1D2	nuclear receptor subfamily 1 group D member 2	9975	119,0946516	-0,279642026	0,176824	-1,581467513	0,113771181	0,539588682
ENSG00000198799	LRIG2	leucine rich repeats and immunoglobulin like domains 2	9860	94,94582119	-0,146671693	0,205588	-0,713425296	0,475582625	0,853811879
ENSG00000158987	RAPGEF6	Rap guanine nucleotide exchange factor 6	51735	690,2557286	-0,111591904	0,073684	-1,514466385	0,129907593	0,565894171
ENSG00000070756	PABPC1	poly(A) binding protein cytoplasmic 1	26986	6382,923439	-0,264395302	0,066531	-3,974028198	7,07E-05	0,004784656
ENSG00000182481	KPNA2	karyopherin subunit alpha 2	3838	325,8804896	0,376491113	0,102265	3,681540323	0,000231829	0,011900561
ENSG00000079112	CDH17	cadherin 17	1015	1067,411547	-0,272824891	0,067863	-4,020213569	5,81E-05	0,00411418
ENSG00000112658	SRF	serum response factor	6722	271,8664211	-0,136501489	0,105692	-1,291508433	0,196527433	0,655659726
ENSG00000105732	ZNF574	zinc finger protein 574	64763	42,07273872	-0,368995814	0,316026	-1,167612287	0,242963192	NA
ENSG00000255302	EID1	EP300 interacting inhibitor of differentiation 1	23741	402,1454625	-0,190852652	0,088441	-2,157965033	0,030930551	0,281657534
ENSG00000171467	ZNF318	zinc finger protein 318	24149	332,7786954	-0,189588998	0,096219	-1,970380907	0,048794732	0,354517878
ENSG00000130921	MTRFR	mitochondrial translation release factor in rescue	91574	60,38061715	-0,111799693	0,250012	-0,447177507	0,654746901	0,91762708
ENSG00000163961	RNF168	ring finger protein 168	165918	188,3139366	-0,337209331	0,124766	-2,702744759	0,006876953	0,121374361
ENSG00000150938	CRIM1	cysteine rich transmembrane BMP regulator 1	51232	536,2924421	-0,409750118	0,08579	-4,776200874	1,79E-06	0,000233837
ENSG00000188215	DCUN1D3	defective in cullin neddylation 1 domain containing 3	123879	56,41880829	0,090585433	0,278728	0,324995337	0,745184601	0,939745744
ENSG00000122122	SASH3	SAM and SH3 domain containing 3	54440	1181,246689	-0,0021385	0,053281	-0,040136012	0,967984691	0,993537868
ENSG00000118579	MED28	mediator complex subunit 28	80306	320,9856225	-0,081428582	0,110663	-0,73582546	0,461836925	0,846765498
ENSG00000159873	CCDC117	coiled-coil domain containing 117	150275	200,1358445	-0,229809391	0,122887	-1,870084925	0,061472026	0,402334407
ENSG00000113368	LMNB1	lamin B1	4001	281,7148649	0,267427066	0,118179	2,262899623	0,023641878	0,245726955
ENSG00000129245	FXR2	FMR1 autosomal homolog 2	9513	107,0658981	0,244329905	0,176385	1,38521148	0,165987811	0,624933502
ENSG00000162894	FCMR	Fc mu receptor	9214	340,0363595	-0,45120741	0,160892	-2,804404241	0,005040966	0,099979158
ENSG00000175414	ARL10	ADP ribosylation factor like GTPase 10	285598	248,9750508	0,076419312	0,116354	0,656782643	0,511320686	0,866432075
ENSG00000088970	KIZ	kizuna centrosomal protein	55857	105,5562867	-0,304108422	0,162864	-1,867258963	0,061865433	0,402854784
ENSG00000167378	IRGQ	immunity related GTPase Q	126298	86,65387789	0,251394775	0,217608	1,155263086	0,247982707	0,706134779
ENSG00000105281	SLC1A5	solute carrier family 1 member 5	6510	2098,405478	0,149462428	0,040856	3,658263468	0,00025393	0,012374745
ENSG00000176986	SEC24C	SEC24 homolog C, COPII coat complex component	9632	448,5222635	0,308654647	0,112869	2,734636345	0,006244923	0,114330122
ENSG00000073861	TBX21	T-box transcription factor 21	30009	503,6135151	-0,24022061	0,088845	-2,703809094	0,006854966	0,12125879
ENSG00000196693	ZNF33B	zinc finger protein 33B	7582	151,2941705	0,016361499	0,141158	0,115908734	0,907724874	0,979026525
ENSG00000114302	PRKAR2A	protein kinase cAMP-dependent type II regulatory subunit alpha	5576	318,3978591	-0,168048142	0,10631	-1,580739051	0,113937716	0,539617633
ENSG00000010810	FYN	FYN proto-oncogene, Src family tyrosine kinase	2534	1132,70912	-0,157299439	0,06795	-2,314916316	0,020617512	0,225859999
ENSG00000109111	SUPT6H	SPT6 homolog, histone chaperone and transcription elongation factor	6830	917,4293211	-0,076317282	0,06615	-1,153701784	0,248622452	0,706724843
ENSG00000101695	RNF125	ring finger protein 125	54941	838,2064857	-0,160321351	0,061322	-2,614403622	0,008938337	0,14473205
ENSG00000111602	TIMELESS	timeless circadian regulator	8914	140,1628458	0,616683565	0,167844	3,674150049	0,000238642	0,012170766
ENSG00000186812	ZNF397	zinc finger protein 397	84307	88,06566176	-0,196462664	0,206933	-0,949400068	0,342417176	0,780064992
ENSG00000115419	GLS	glutaminase	2744	542,5639695	-0,123376962	0,127339	-0,968883738	0,3326032	0,773425407
ENSG00000150867	PIP4K2A	phosphatidylinositol-5-phosphate 4-kinase type 2 alpha	5305	1533,032519	-0,242239865	0,061262	-3,954170634	7,68E-05	0,005155486
ENSG00000091527	CDV3	CDV3 homolog	55573	1033,498309	-0,208217565	0,067215	-3,097791766	0,001949684	0,054300765

ENSG00000149313	AASDHPPT	aminoadipate-semialdehyde dehydrogenase-phosphopantetheinyl transferase	60496	154,7230045	0,194862444	0,148246	1,314457379	0,188692332	0,64659231
ENSG00000173517	PEAK1	pseudopodium enriched atypical kinase 1	79834	229,9076417	-0,135884459	0,112388	-1,209068797	0,226636416	0,681621061
ENSG00000082153	BZW1	basic leucine zipper and W2 domains 1	9689	1068,508594	-0,042129659	0,080528	-0,523167102	0,600857973	0,896014669
ENSG00000136436	CALCOCO2	calcium binding and coiled-coil domain 2	10241	743,3865052	-0,079304204	0,089612	-0,88497669	0,376169203	0,803373958
ENSG00000100503	NIN	ninein	51199	947,3993275	-0,058618743	0,088126	-0,665167644	0,505943273	0,864630986
ENSG00000185811	IKZF1	IKAROS family zinc finger 1	10320	1631,230694	-0,187071203	0,060874	-3,073093387	0,002118522	0,05587092
ENSG00000119318	RAD23B	RAD23 homolog B, nucleotide excision repair protein	5887	470,1023896	-0,063416236	0,079216	-0,800549445	0,423392528	0,828638155
ENSG00000177189	RPS6KA3	ribosomal protein S6 kinase A3	6197	1304,806797	-0,208611674	0,065256	-3,19683667	0,001389436	0,043476608
ENSG00000087460	GNAS	GNAS complex locus	2778	1324,239457	-0,121726141	0,051536	-2,361950325	0,018179078	0,211210766
ENSG00000239305	RNF103	ring finger protein 103	7844	41,42969954	0,151817723	0,339342	0,447388746	0,6545944	NA
ENSG00000134758	RNF138	ring finger protein 138	51444	164,6626974	0,137614037	0,137221	1,002862248	0,31592733	0,76048697
ENSG00000150637	CD226	CD226 molecule	10666	782,583411	-0,212526136	0,088906	-2,390461043	0,016827234	0,204616219
ENSG00000167522	ANKRD11	ankyrin repeat domain containing 11	29123	1378,017667	-0,148334729	0,070229	-2,112171266	0,034671762	0,297161096
ENSG00000076928	ARHGEF1	Rho guanine nucleotide exchange factor 1	9138	1301,790594	-0,182133054	0,075071	-2,42614679	0,015260097	0,193936567
ENSG00000134294	SLC38A2	solute carrier family 38 member 2	54407	1232,37913	-0,0199134	0,071298	-0,279297341	0,780016647	0,947749187
ENSG00000153310	CYRIB	CYFIP related Rac1 interactor B	51571	846,6476583	0,017634559	0,062703	0,281241383	0,778525266	0,947109268
ENSG00000215252	GOLGA8B	golgin A8 family member B	440270	1290,414097	-0,162125552	0,175392	-0,924363037	0,355297333	0,788341988
ENSG00000196670	ZFP62	ZFP62 zinc finger protein	643836	80,78226757	0,125239429	0,234123	0,534930351	0,59269802	0,893312272
ENSG00000067900	ROCK1	Rho associated coiled-coil containing protein kinase 1	6093	977,1442729	-0,038251489	0,056404	-0,678174198	0,49766125	0,861283987
ENSG00000105323	HNRNPUL1	heterogeneous nuclear ribonucleoprotein U like 1	11100	1475,151615	-0,094341172	0,063263	-1,491255317	0,135894476	0,576449438
ENSG00000179152	TCAIM	T cell activation inhibitor, mitochondrial	285343	48,57415141	0,874628553	0,329932	2,650934771	0,008026934	0,133850406
ENSG00000174748	RPL15	ribosomal protein L15	6138	1462,994668	-0,030924176	0,073406	-0,421276757	0,673553001	0,923602509
ENSG00000170759	KIF5B	kinesin family member 5B	3799	744,3787494	-0,027547591	0,071474	-0,385422376	0,699924507	0,930783276
ENSG00000140691	ARMC5	armadillo repeat containing 5	79798	322,5495847	-0,074683813	0,131064	-0,569827234	0,568794882	0,886263374
ENSG00000186088	GSAP	gamma-secretase activating protein	54103	66,77596217	-0,098632119	0,234192	-0,421159839	0,673638368	0,923602509
ENSG00000113742	CPEB4	cytoplasmic polyadenylation element binding protein 4	80315	221,037682	0,0358123	0,132242	0,27080941	0,786537622	0,950818299
ENSG00000272333	KMT2B	lysine methyltransferase 2B	9757	421,6933015	-0,100984698	0,105823	-0,954278055	0,339942913	0,777431905
ENSG00000137154	RPS6	ribosomal protein S6	6194	2266,786593	-0,013985157	0,05889	-0,237481093	0,812283586	0,959038743
ENSG00000142541	RPL13A	ribosomal protein L13a	23521	3954,678088	-0,011269408	0,05145	-0,219036097	0,826621929	0,961917534
ENSG00000176049	JAKMIP2	janus kinase and microtubule interacting protein 2	9832	195,2109565	-0,05311201	0,133441	-0,398017288	0,690617442	0,927252461
ENSG00000153029	MR1	major histocompatibility complex, class I-related	3140	326,4854758	0,138925566	0,114751	1,210674633	0,226020128	0,681621061
ENSG00000198755	RPL10A	ribosomal protein L10a	4736	1213,137088	-0,064850894	0,054136	-1,197933252	0,230943003	0,685179309
ENSG00000164045	CDC25A	cell division cycle 25A	993	27,25371519	1,196112175	0,377998	3,164335402	0,001554376	NA
ENSG00000166848	TERF2IP	TERF2 interacting protein	54386	465,2840246	-0,127959028	0,076177	-1,679756042	0,093004791	0,495562842
ENSG00000166848	LOC105371348	uncharacterized LOC105371348	105371348	465,2840246	-0,127959028	0,076177	-1,679756042	0,093004791	0,495562842
ENSG00000182866	LCK	LCK proto-oncogene, Src family tyrosine kinase	3932	1539,080696	-0,063983544	0,044921	-1,424359366	0,154342471	0,611607348
ENSG00000179820	MYADM	myeloid associated differentiation marker	91663	112,4360179	-0,40213122	0,236634	-1,6993816	0,089247307	0,488176025
ENSG00000091039	OSBPL8	oxysterol binding protein like 8	114882	1112,166564	0,059762973	0,055461	1,07756695	0,281227058	0,734459547
ENSG00000196323	ZBTB44	zinc finger and BTB domain containing 44	29068	429,0085362	-0,234869788	0,094935	-2,474016011	0,013360371	0,180367712
ENSG00000113161	HMGCR	3-hydroxy-3-methylglutaryl-CoA reductase	3156	410,1432667	0,177646564	0,09055	1,961858198	0,049778996	0,357759312
ENSG00000120948	TARDBP	TAR DNA binding protein	23435	570,8830424	0,052299412	0,095134	0,549746012	0,582493593	0,888501395
ENSG00000135842	NIBAN1	niban apoptosis regulator 1	116496	1417,327666	-0,160245094	0,049246	-3,253961502	0,001138077	0,037467385
ENSG00000183655	KLHL25	kelch like family member 25	64410	70,94217377	0,58224299	0,246299	2,363963862	0,018080576	0,210377549
ENSG00000102572	STK24	serine/threonine kinase 24	8428	538,1165009	0,069503728	0,068422	1,015805289	0,309722117	0,755959614
ENSG00000131845	ZNF304	zinc finger protein 304	57343	60,46926813	0,160792234	0,247321	0,650135906	0,515604438	0,868791049
ENSG00000135439	AGAP2	ArfGAP with GTPase domain, ankyrin repeat and PH domain 2	116986	476,0714608	-0,175115555	0,087396	-2,003705223	0,045101646	0,340276976
ENSG00000173575	CHD2	chromodomain helicase DNA binding protein 2	1106	974,7277776	0,039244747	0,07964	0,492776099	0,6221708	0,904764752
ENSG00000117281	CD160	CD160 molecule	11126	56,35709227	-0,569736048	0,218571	-2,60664148	0,009143504	0,146613742
ENSG00000125970	RALY	RALY heterogeneous nuclear ribonucleoprotein	22913	347,3898503	-0,089380304	0,104188	-0,857877255	0,390960245	0,811337525

ENSG00000135916	ITM2C	integral membrane protein 2C	81618	233,048928	-0,235673119	0,107713	-2,187981598	0,028670943	0,272623046
ENSG00000187626	ZKSCAN4	zinc finger with KRAB and SCAN domains 4	387032	33,74034465	-0,267932078	0,373174	-0,717981292	0,472768822	NA
ENSG00000137845	ADAM10	ADAM metallopeptidase domain 10	102	733,6205055	-0,201574894	0,112487	-1,791979973	0,073136174	0,439488532
ENSG00000197162	ZNF785	zinc finger protein 785	146540	63,1122303	-0,105313798	0,227874	-0,46215814	0,64396792	0,912530858
ENSG00000159882	ZNF230	zinc finger protein 230	7773	28,32529988	0,05609299	0,383473	0,146276222	0,883703336	NA
ENSG00000168421	RHOH	ras homolog family member H	399	307,6881225	-0,058930022	0,152052	-0,387564262	0,69833852	0,930783276
ENSG00000119917	IFIT3	interferon induced protein with tetratricopeptide repeats 3	3437	17,95399114	0,851780479	0,457008	1,863820381	0,062346933	NA
ENSG00000166441	RPL27A	ribosomal protein L27a	6157	1786,224274	0,012552423	0,05296	0,237016481	0,812644004	0,959054247
ENSG00000121989	ACVR2A	activin A receptor type 2A	92	105,6166397	-0,024815621	0,199101	-0,124638182	0,900809999	0,977990819
ENSG00000174946	GPR171	G protein-coupled receptor 171	29909	597,5011518	0,005837426	0,073646	0,079263419	0,936823102	0,987098279
ENSG00000108107	RPL28	ribosomal protein L28	6158	1549,678834	-0,387119577	0,081348	-4,758822216	1,95E-06	0,000246674
ENSG00000156675	RAB11FIP1	RAB11 family interacting protein 1	80223	500,7741831	-0,16888115	0,093279	-1,810491496	0,070219602	0,430243114
ENSG00000117602	RCAN3	RCAN family member 3	11123	369,5827933	-0,476880107	0,126901	-3,757893982	0,000171349	0,009752019
ENSG00000179583	CIITA	class II major histocompatibility complex transactivator	4261	270,8292435	-0,120233385	0,102239	-1,176006706	0,239592196	0,694482777
ENSG00000292334	SLC25A6	solute carrier family 25 member 6	293	688,0723866	-0,181357789	0,070885	-2,558486522	0,01051289	0,158898917
ENSG00000015479	MATR3	matrin 3	9782	1136,195363	0,004408415	0,058292	0,075626588	0,939716183	0,987813564
ENSG00000184634	MED12	mediator complex subunit 12	9968	306,3803006	0,437512986	0,153321	2,853583697	0,004322914	0,090819559
ENSG00000105329	TGFB1	transforming growth factor beta 1	7040	940,7341579	-0,285007485	0,059019	-4,829061856	1,37E-06	0,000189017
ENSG00000114391	RPL24	ribosomal protein L24	6152	806,7019019	-0,101281026	0,06539	-1,548864768	0,121414232	0,556676811
ENSG00000121931	LRIF1	ligand dependent nuclear receptor interacting factor 1	55791	152,8214581	0,088041394	0,150177	0,586251124	0,557706771	0,883921037
ENSG00000124201	ZNFX1	zinc finger NFX1-type containing 1	57169	452,5861981	0,077662287	0,085368	0,909732818	0,362963432	0,791865221
ENSG00000197713	RPE	ribulose-5-phosphate-3-epimerase	6120	187,6597446	-0,095598628	0,154974	-0,616867112	0,537322381	0,874923109
ENSG00000237440	ZNF737	zinc finger protein 737	100129842	59,12660902	0,423767144	0,396853	1,067819233	0,285602061	0,737867955
ENSG00000196954	CASP4	caspase 4	837	352,1320454	-0,00823241	0,095153	-0,086517445	0,931055089	0,986491322
ENSG00000115524	SF3B1	splicing factor 3b subunit 1	23451	2505,142553	-0,134482499	0,070106	-1,918282411	0,055075211	0,380440379
ENSG00000100426	ZBED4	zinc finger BED-type containing 4	9889	433,0480637	-0,077182565	0,083512	-0,924209476	0,355377263	0,788341988
ENSG00000085563	ABCB1	ATP binding cassette subfamily B member 1	5243	513,3096238	0,100095772	0,078475	1,275514258	0,202127281	0,660119983
ENSG00000089820	ARHGAP4	Rho GTPase activating protein 4	393	526,5017993	-0,302662707	0,087619	-3,454293217	0,000551737	0,022222275
ENSG00000154889	MPPE1	metallophosphoesterase 1	65258	54,73236391	0,164290988	0,250687	0,655363973	0,512233442	0,867417303
ENSG00000143815	LBR	lamin B receptor	3930	323,7518212	-0,005142714	0,11328	-0,045398304	0,963789833	0,992694268
ENSG00000168040	FADD	Fas associated via death domain	8772	52,5422558	0,045654425	0,257461	0,177325797	0,859252489	0,969900697
ENSG00000108175	ZMIZ1	zinc finger MIZ-type containing 1	57178	1152,004	-0,123682684	0,055488	-2,229012685	0,025813061	0,259252915
ENSG00000051341	POLQ	DNA polymerase theta	10721	92,61157419	0,948085054	0,182521	5,194401873	2,05E-07	3,75E-05
ENSG00000075131	TIPIN	TIMELESS interacting protein	54962	20,32304674	2,097015479	0,558991	3,751429166	0,000175829	NA
ENSG00000162543	UBXN10	UBX domain protein 10	127733	99,24198322	-0,042173633	0,1896	-0,222434744	0,823975468	0,961917534
ENSG00000135999	EPC2	enhancer of polycomb homolog 2	26122	200,8020673	-0,195972175	0,116488	-1,682333315	0,092504224	0,4945733
ENSG00000159592	GPBP1L1	GC-rich promoter binding protein 1 like 1	60313	485,028389	-0,028353187	0,083307	-0,340345818	0,733596117	0,939157822
ENSG00000204843	DCTN1	dynactin subunit 1	1639	389,8561357	-0,011772573	0,145568	-0,080873382	0,935542649	0,987098279
ENSG00000103197	TSC2	TSC complex subunit 2	7249	255,6426903	-0,133570449	0,142794	-0,935408344	0,349577899	0,784901319
ENSG00000174903	RAB1B	RAB1B, member RAS oncogene family	81876	915,8634529	-0,054403317	0,06884	-0,79028753	0,429359868	0,832060923
ENSG00000109079	TNFAIP1	TNF alpha induced protein 1	7126	72,5722868	-0,331011604	0,242043	-1,367573045	0,171445759	0,629558603
ENSG00000106261	ZKSCAN1	zinc finger with KRAB and SCAN domains 1	7586	705,1943437	-0,076675873	0,069605	-1,101584343	0,270642418	0,725477256
ENSG00000164631	ZNF12	zinc finger protein 12	7559	148,6103188	0,089201471	0,168771	0,528536199	0,597127229	0,894257182
ENSG00000183340	JRKL	JRK like	8690	51,65020441	-0,116114702	0,305959	-0,379510616	0,704308723	0,930846779
ENSG00000229106	NA	NA	NA	67,02782366	0,812007391	0,241615	3,360745706	0,000777324	0,029211001
ENSG00000037749	MFAP3	microfibril associated protein 3	4238	183,6908642	0,257322849	0,1639	1,569999388	0,116415254	0,543917551
ENSG00000093072	ADA2	adenosine deaminase 2	51816	864,1616081	-0,113069294	0,059857	-1,888996772	0,058892261	0,39312401
ENSG00000110713	NUP98	nucleoporin 98 and 96 precursor	4928	295,7019438	0,68505697	0,164292	4,169755179	3,05E-05	0,002468966
ENSG00000099290	WASHC2A	WASH complex subunit 2A	387680	421,6506772	0,047086407	0,085025	0,553796609	0,579718049	0,887548253
ENSG00000146192	FGD2	FYVE, RhoGEF and PH domain containing 2	221472	42,7947358	-0,666906022	0,466813	-1,428637806	0,153108362	0,609793649
ENSG00000179454	KLHL28	kelch like family member 28	54813	101,9635897	0,286701914	0,232854	1,231249903	0,218229412	0,673519155
ENSG00000112282	MED23	mediator complex subunit 23	9439	401,8022794	0,033327317	0,105484	0,315947137	0,752042633	0,941832449
ENSG00000135535	CD164	CD164 molecule	8763	1025,068515	-0,095521147	0,069324	-1,377886304	0,168238394	0,627733684

ENSG00000275111	ZNF2	zinc finger protein 2	7549	22,29649941	-0,482206352	0,584138	-0,825500193	0,409087673	NA
ENSG00000105373	NOP53	NOP53 ribosome biogenesis factor	29997	740,7007913	-0,229356361	0,093573	-2,451093256	0,014242305	0,186258726
ENSG00000107719	PALD1	phosphatase domain containing paladin 1	27143	143,9382758	-0,393562913	0,184373	-2,134596039	0,032794021	0,289398026
ENSG00000130522	JUND	JunD proto-oncogene, AP-1 transcription factor subunit	3727	380,4926725	-0,160813103	0,117583	-1,367658756	0,171418916	0,629558603
ENSG00000070081	NUCB2	nucleobindin 2	4925	198,841836	0,186620195	0,130311	1,43211282	0,152111536	0,608564843
ENSG00000182774	RPS17	ribosomal protein S17	6218	1036,995929	0,013055142	0,054306	0,240400995	0,810019407	0,959038743
ENSG00000163600	ICOS	inducible T cell costimulator	29851	180,962084	0,079536817	0,122638	0,648549137	0,51662984	0,868791049
ENSG00000146223	RPL7L1	ribosomal protein L7 like 1	285855	434,9360491	0,221179499	0,088362	2,50310266	0,012310983	0,174531515
ENSG00000141582	CBX4	chromobox 4	8535	188,9992777	-0,412607315	0,150033	-2,750103099	0,005957652	0,110617958
ENSG00000143507	DUSP10	dual specificity phosphatase 10	11221	383,310109	0,24540322	0,086457	2,838454404	0,00453326	0,093204766
ENSG00000084112	SSH1	slingshot protein phosphatase 1	54434	613,895798	0,139300513	0,08132	1,712983803	0,086715519	0,481317095
ENSG00000167625	ZNF526	zinc finger protein 526	116115	58,55180865	-0,002248951	0,249219	-0,009023986	0,992799998	0,998500439
ENSG00000134419	RPS15A	ribosomal protein S15a	6210	1831,117374	0,143417977	0,074718	1,919449401	0,054927484	0,379868172
ENSG00000004534	RBM6	RNA binding motif protein 6	10180	553,1276819	-0,061705172	0,096888	-0,636873066	0,524207528	0,870612376
ENSG00000198840	ND3	NADH dehydrogenase subunit 3	4537	1864,79341	-0,076167867	0,089972	-0,846572166	0,397233634	0,813102153
ENSG00000120686	UFM1	ubiquitin fold modifier 1	51569	305,1128318	-0,284192944	0,110769	-2,565636566	0,010298667	0,157671992
ENSG00000151849	CENPJ	centromere protein J	55835	66,80399772	0,151517379	0,375338	0,403682349	0,686446321	0,926667137
ENSG00000163840	DTX3L	deltex E3 ubiquitin ligase 3L	151636	522,1765309	0,049217683	0,076509	0,64329627	0,520031876	0,86944974
ENSG00000101346	POFUT1	protein O-fucosyltransferase 1	23509	101,9728549	0,318374757	0,187961	1,693831774	0,090297254	0,489623111
ENSG00000025800	KPNA6	karyopherin subunit alpha 6	23633	421,8460927	0,011774972	0,091845	0,128204141	0,897987423	0,977990819
ENSG00000084676	NCOA1	nuclear receptor coactivator 1	8648	393,8495264	-0,09599965	0,093133	-1,030775405	0,302646154	0,751870158
ENSG00000155034	FBXL18	F-box and leucine rich repeat protein 18	80028	99,92403354	0,195683658	0,19695	0,993568131	0,320433166	0,76448423
ENSG00000130653	PNPLA7	patatin like phospholipase domain containing 7	375775	169,0989534	-0,426768472	0,123964	-3,442669151	0,000576004	0,022961201
ENSG00000067066	SP100	SP100 nuclear antigen	6672	842,6095792	0,051881192	0,064412	0,805458769	0,42055499	0,827182516
ENSG00000204084	INPP5B	inositol polyphosphate-5-phosphatase B	3633	580,4399995	-0,0696116	0,074916	-0,929193692	0,352788716	0,787159822
ENSG00000175197	DDIT3	DNA damage inducible transcript 3	1649	53,57393313	-0,041704985	0,223854	-0,186304719	0,852205807	0,968180755
ENSG00000084652	TXLNA	taxilin alpha	200081	463,556523	-0,031941987	0,085766	-0,372432252	0,709571044	0,930846779
ENSG00000168685	IL7R	interleukin 7 receptor	3575	32,05620411	-0,265044009	0,402298	-0,658825504	0,510007828	NA
ENSG00000165671	NSD1	nuclear receptor binding SET domain protein 1	64324	923,0649848	0,093112547	0,078205	1,190617681	0,233803707	0,688858646
ENSG00000186660	ZFP91	ZFP91 zinc finger protein, atypical E3 ubiquitin ligase	80829	661,2724849	-0,051682657	0,07693	-0,671816155	0,501700743	0,862227298
ENSG00000198286	CARD11	caspase recruitment domain family member 11	84433	1561,265276	-0,149697337	0,053017	-2,823582454	0,004749021	0,096039621
ENSG00000118816	CCNI	cyclin I	10983	949,1060364	-0,155860756	0,054113	-2,880273138	0,003973308	0,086684333
ENSG00000047849	MAP4	microtubule associated protein 4	4134	1434,877706	-0,033689846	0,059272	-0,568398198	0,569764614	0,886300511
ENSG00000142534	RPS11	ribosomal protein S11	6205	2601,191149	-0,135197287	0,045319	-2,983231081	0,002852226	0,069661304
ENSG00000184787	UBE2G2	ubiquitin conjugating enzyme E2 G2	7327	299,2445593	-0,163214658	0,158337	-1,03080536	0,302632104	0,751870158
ENSG00000142867	BCL10	BCL10 immune signaling adaptor	8915	111,0454084	0,138235426	0,184843	0,747851749	0,454549584	0,843234809
ENSG00000166949	SMAD3	SMAD family member 3	4088	333,3970771	-0,100511595	0,123795	-0,811918968	0,41683813	0,82446781
ENSG00000140471	LINS1	lines homolog 1	55180	56,37798099	0,343484936	0,270573	1,269472505	0,204272591	0,660954946
ENSG00000126653	NSRP1	nuclear speckle splicing regulatory protein 1	84081	153,2763516	-0,182232758	0,172158	-1,058517388	0,289819628	0,741447348
ENSG00000143761	ARF1	ADP ribosylation factor 1	375	1082,25533	-0,12740515	0,057419	-2,218875304	0,026495209	0,263409332
ENSG00000108819	PPP1R9B	protein phosphatase 1 regulatory subunit 9B	84687	834,9955529	-0,289662381	0,071577	-4,046856949	5,19E-05	0,003752175
ENSG00000134516	DOCK2	dedicator of cytokinesis 2	1794	1715,775951	0,040166406	0,055558	0,722966185	0,469700659	0,852363442
ENSG00000124831	LRRFIP1	LRR binding FLII interacting protein 1	9208	999,733842	-0,049378442	0,067831	-0,727962541	0,466636517	0,850142242
ENSG00000075624	ACTB	actin beta	60	18587,27204	-0,194390306	0,048277	-4,026570376	5,66E-05	0,004040976
ENSG00000142765	SYTL1	synaptotagmin like 1	84958	149,8908262	-0,171960504	0,138989	-1,237224995	0,216003565	0,672410621
ENSG00000104388	RAB2A	RAB2A, member RAS oncogene family	5862	152,2346742	-0,161085275	0,138564	-1,162530172	0,245020176	0,701819279
ENSG00000257103	LSM14A	LSM14A mRNA processing body assembly factor	26065	482,9273039	0,030717539	0,084374	0,364063075	0,71581091	0,932040329
ENSG00000182670	TTC3	tetratricopeptide repeat domain 3	7267	987,4604817	-0,230221717	0,075331	-3,056139982	0,002242066	0,057304094
ENSG00000171791	BCL2	BCL2 apoptosis regulator	596	1704,269835	-0,017119788	0,079008	-0,216685523	0,828453431	0,962723654
ENSG00000131263	RLIM	ring finger protein, LIM domain interacting	51132	305,3467169	-0,266624929	0,096742	-2,756031589	0,005850735	0,109148859
ENSG00000134313	KIDINS220	kinase D interacting substrate 220	57498	690,6176207	-0,074995821	0,071877	-1,043392144	0,29676671	0,748371252
ENSG00000138035	PNPT1	polyribonucleotide nucleotidyltransferase 1	87178	69,60907016	-0,170406342	0,257467	-0,661857014	0,508062863	0,864738156
ENSG00000198538	ZNF28	zinc finger protein 28	7576	81,21260167	-0,032383862	0,235459	-0,1375353	0,890607693	0,977990819

ENSG00000057663	ATG5	autophagy related 5	9474	61,16986563	0,215226233	0,266091	0,808844884	0,418604375	0,825023528
ENSG00000172175	MALT1	MALT1 paracaspase	10892	488,7922722	0,093664034	0,107498	0,871312676	0,383583451	0,808586507
ENSG00000198408	OGA	O-GlcNAcase	10724	1267,9684	-0,091627426	0,055331	-1,6559924	0,097723358	0,505612156
ENSG00000081320	STK17B	serine/threonine kinase 17b	9262	2576,608272	-0,066483308	0,048814	-1,361958981	0,173210831	0,629558603
ENSG00000060140	STYK1	serine/threonine/tyrosine kinase 1	55359	243,7412522	0,307099957	0,109774	2,797575737	0,005148769	0,100843978
ENSG00000089159	PXN	paxillin	5829	657,2767057	-0,161584804	0,073867	-2,187496516	0,028706297	0,272623046
ENSG00000100647	SUSD6	sushi domain containing 6	9766	860,0738077	-0,140758811	0,057038	-2,467828213	0,013593554	0,181356713
ENSG00000170802	FOXN2	forkhead box N2	3344	484,0571712	-0,18051145	0,077823	-2,319525971	0,020366535	0,224346091
ENSG00000198901	PRC1	protein regulator of cytokinesis 1	9055	86,41015163	0,786386386	0,239533	3,283002349	0,001027078	0,035851873
ENSG00000156886	ITGAD	integrin subunit alpha D	3681	144,4273563	-0,234827716	0,15174	-1,547567498	0,121726462	0,556808172
ENSG00000153250	RBMS1	RNA binding motif single stranded interacting protein 1	5937	403,0776178	-0,168405163	0,128371	-1,311862814	0,189566426	0,647463823
ENSG00000169660	HEXD	hexosaminidase D	284004	74,70944704	-0,46533014	0,209138	-2,224995439	0,026081544	0,260750739
ENSG00000125354	SEPTIN6	septin 6	23157	927,6760911	-0,11798375	0,097673	-1,207943374	0,227069044	0,682242748
ENSG00000147065	MSN	moesin	4478	7266,520334	-0,13150354	0,045281	-2,904144189	0,003682585	0,081473307
ENSG00000185085	INTS5	integrator complex subunit 5	80789	91,01652977	0,151714987	0,218676	0,693788757	0,487814695	0,859420505
ENSG00000147883	CDKN2B	cyclin dependent kinase inhibitor 2B	1030	95,10606854	0,492347239	0,224706	2,191077095	0,028446214	0,271466056
ENSG00000171115	GIMAP8	GTPase, IMAP family member 8	155038	113,0926509	0,147952243	0,177372	0,8341342	0,404205365	0,817992511
ENSG00000221963	APOL6	apolipoprotein L6	80830	2238,061186	0,085303565	0,039592	2,154568484	0,031195618	0,281657534
ENSG00000169155	ZBTB43	zinc finger and BTB domain containing 43	23099	190,3256407	-0,087730249	0,130976	-0,669817368	0,502974221	0,863089257
ENSG00000010017	RANBP9	RAN binding protein 9	10048	332,5178061	-0,060616212	0,092107	-0,658108272	0,510468561	0,866180216
ENSG00000213281	NRAS	NRAS proto-oncogene, GTPase	4893	452,6485336	-0,062777735	0,078099	-0,803823787	0,42149875	0,827612795
ENSG00000168806	LCMT2	leucine carboxyl methyltransferase 2	9836	46,9182237	0,200431883	0,293589	0,68269518	0,494799476	0,860513824
ENSG00000089009	RPL6	ribosomal protein L6	6128	2252,098114	-0,087096889	0,044962	-1,937141281	0,052728076	0,371734827
ENSG00000008513	ST3GAL1	ST3 beta-galactoside alpha-2,3-sialyltransferase 1	6482	1055,673009	-0,075685526	0,062056	-1,219640987	0,222601002	0,678694696
ENSG00000099942	CRKL	CRK like proto-oncogene, adaptor protein	1399	531,3927962	-0,142279687	0,08029	-1,772071361	0,076382712	0,447360046
ENSG00000273136	NBPF26	NBPF member 26	101060684	121,0866961	-0,054452212	0,261498	-0,208231978	0,835047841	0,964065248
ENSG00000198431	TXNRD1	thioredoxin reductase 1	7296	479,8750772	0,15827839	0,082672	1,914529367	0,05555255	0,382157692
ENSG00000196776	CD47	CD47 molecule	961	412,8012331	-0,172656809	0,124203	-1,390122421	0,164491706	0,624374678
ENSG00000075151	EIF4G3	eukaryotic translation initiation factor 4 gamma 3	8672	547,6529534	0,052488833	0,080374	0,653060991	0,51371696	0,868514045
ENSG00000213995	NAXD	NAD(P)HX dehydratase	55739	71,22603873	0,236030431	0,269459	0,875942589	0,381061245	0,806699466
ENSG00000197136	PCNX3	pecanex 3	399909	307,503956	0,289269754	0,115754	2,499009992	0,01245408	0,175323113
ENSG00000184014	DENND5A	DENN domain containing 5A	23258	208,5765804	0,001258207	0,162712	0,00773272	0,993830243	0,998500439
ENSG00000136897	MRPL50	mitochondrial ribosomal protein L50	54534	59,823219	0,125195379	0,239835	0,52200716	0,601665345	0,896505335
ENSG00000177125	ZBTB34	zinc finger and BTB domain containing 34	403341	84,57873883	0,071797219	0,19214	0,373670821	0,708649237	0,930846779
ENSG00000107738	VSIR	V-set immunoregulatory receptor	64115	345,0149583	0,007846989	0,097449	0,080523983	0,935820523	0,987098279
ENSG00000076924	XAB2	XPA binding protein 2	56949	182,4192017	0,057750646	0,124521	0,463782843	0,642803337	0,912118773
ENSG00000173064	HECTD4	HECT domain E3 ubiquitin protein ligase 4	283450	1129,004433	0,318617859	0,082937	3,841678265	0,000122196	0,00761688
ENSG00000130935	NOL11	nucleolar protein 11	25926	151,5469621	-0,054574488	0,137218	-0,397722326	0,690834879	0,927252461
ENSG00000107581	EIF3A	eukaryotic translation initiation factor 3 subunit A	8661	1614,277498	-0,085341483	0,064463	-1,323875383	0,185544435	0,642967931
ENSG00000196189	SEMA4A	semaphorin 4A	64218	244,123655	0,280712977	0,112832	2,487875723	0,012850863	0,177810208
ENSG00000110048	OSBP	oxysterol binding protein	5007	319,311489	0,066290386	0,100615	0,658852028	0,509990794	0,866180216
ENSG00000144381	HSPD1	heat shock protein family D (Hsp60) member 1	3329	327,5518806	0,175248265	0,106749	1,641688848	0,100654504	0,51200808
ENSG00000079332	SAR1A	secretion associated Ras related GTPase 1A	56681	766,5535312	-0,095941392	0,067873	-1,413542417	0,157496299	0,61428162
ENSG00000064102	INTS13	integrator complex subunit 13	55726	134,4298463	0,286735983	0,16099	1,781080844	0,074899245	0,44463996
ENSG00000066135	KDM4A	lysine demethylase 4A	9682	265,9765335	0,202296962	0,102265	1,978162441	0,047910384	0,350971037
ENSG00000117461	PIK3R3	phosphoinositide-3-kinase regulatory subunit 3	8503	65,00207076	0,615752611	0,216748	2,840865886	0,004499123	0,092745699
ENSG00000172661	WASHC2C	WASH complex subunit 2C	253725	521,6811654	0,047901259	0,075752	0,632339337	0,527165164	0,871653726
ENSG00000134884	ARGLU1	arginine and glutamate rich 1	55082	121,9685831	0,127548159	0,222364	0,573600469	0,566238194	0,88521771
ENSG00000227191	TRGC2	T cell receptor gamma constant 2	6967	103,5540562	-0,046885738	0,199681	-0,234803585	0,814361185	0,959207071
ENSG00000121486	TRMT1L	tRNA methyltransferase 1 like	81627	102,9520735	0,40094631	0,173677	2,308579408	0,020966932	0,226641441
ENSG00000115993	TRAK2	trafficking kinesin protein 2	66008	545,7087048	-0,334557983	0,08722	-3,835783923	0,000125164	0,00768001
ENSG00000186399	GOLGA8R	golgin A8 family member R	101059918	551,2139277	-0,099923098	0,079447	-1,257737641	0,208486655	0,662366797
ENSG00000071243	ING3	inhibitor of growth family member 3	54556	114,5777239	-0,160996652	0,17049	-0,944314836	0,345008787	0,781625815
ENSG00000102178	UBL4A	ubiquitin like 4A	8266	108,8694183	0,337450952	0,162803	2,072750774	0,03819548	0,310224714
ENSG00000161203	AP2M1	adaptor related protein complex 2 subunit mu 1	1173	657,2433783	-0,08612233	0,077664	-1,108909638	0,267469166	0,722449525

ENSG00000114331	ACAP2	ArfGAP with coiled-coil, ankyrin repeat and PH domains 2	23527	510,8711332	-0,137711351	0,080126	-1,718681265	0,085672425	0,477891497
ENSG00000083099	LYRM2	LYR motif containing 2	57226	65,00137494	0,417906021	0,336002	1,24375997	0,213587917	0,668600838
ENSG00000122966	CIT	citron rho-interacting serine/threonine kinase	11113	81,15353732	0,807614311	0,213701	3,77918674	0,000157341	0,009361813
ENSG00000151502	VPS26B	VPS26 retromer complex component B	112936	361,5896312	-0,072582647	0,091932	-0,789529154	0,429802797	0,832060923
ENSG00000175115	PACS1	phosphofurin acidic cluster sorting protein 1	55690	1054,749685	-0,167525478	0,059571	-2,812209922	0,004920238	0,098329643
ENSG00000159792	PSKH1	protein serine kinase H1	5681	95,64946948	0,489448013	0,187257	2,613782886	0,008954592	0,14473205
ENSG00000084070	SMAP2	small ArfGAP2	64744	1074,917709	-0,087788491	0,079663	-1,102002839	0,270460437	0,725477256
ENSG00000176890	TYMS	thymidylate synthetase	7298	207,7063327	0,895605842	0,148697	6,023036589	1,71E-09	6,72E-07
ENSG00000066422	ZBTB11	zinc finger and BTB domain containing 11	27107	92,77534575	0,31747287	0,352164	0,901492159	0,367326701	0,795318634
ENSG00000163346	PBXIP1	PBX homeobox interacting protein 1	57326	560,201274	-0,185714567	0,090881	-2,04349549	0,041003413	0,322789747
ENSG00000127191	TRAF2	TNF receptor associated factor 2	7186	120,8570084	0,013898686	0,160554	0,086567302	0,931015458	0,986491322
ENSG00000180644	PRF1	perforin 1	5551	6874,748479	-0,22517647	0,038638	-5,827857864	5,61E-09	2,00E-06
ENSG00000023330	ALAS1	5'-aminolevulinate synthase 1	211	204,5544188	0,077176655	0,120865	0,638534186	0,523126008	0,870221868
ENSG00000198833	UBE2J1	ubiquitin conjugating enzyme E2 J1	51465	473,4206193	0,070670161	0,079983	0,883561149	0,376933159	0,803373958
ENSG00000176542	USF3	upstream transcription factor family member 3	205717	451,0018705	-0,337355044	0,107414	-3,140699531	0,001685449	0,049578699
ENSG00000204536	CCHCR1	coiled-coil alpha-helical rod protein 1	54535	26,78779897	-0,597612803	0,325278	-1,837239605	0,066174531	NA
ENSG00000172575	RASGRP1	RAS guanyl releasing protein 1	10125	1143,027671	0,026051965	0,073772	0,353143085	0,723981174	0,934902053
ENSG00000163902	RPN1	ribophorin I	6184	567,1925543	0,193840182	0,07685	2,522331404	0,011657982	0,169255469
ENSG00000170571	EMB	embigin	133418	1158,67821	-0,064849315	0,073577	-0,881383295	0,378110396	0,804187219
ENSG00000186130	ZBTB6	zinc finger and BTB domain containing 6	10773	97,14359448	-0,059803439	0,175185	-0,341372733	0,732822999	0,93912057
ENSG00000254004	ZNF260	zinc finger protein 260	339324	84,31379607	-0,10100444	0,259235	-0,389624579	0,696814174	0,930422107
ENSG00000188033	ZNF490	zinc finger protein 490	57474	93,89541206	-0,252498151	0,213703	-1,181540163	0,237388208	0,692443518
ENSG00000169607	CKAP2L	cytoskeleton associated protein 2 like	150468	146,3066889	0,646483998	0,152264	4,245801883	2,18E-05	0,001922139
ENSG00000170312	CDK1	cyclin dependent kinase 1	983	88,95310987	0,731479755	0,182508	4,007941962	6,13E-05	0,004257158
ENSG00000100813	ACIN1	apoptotic chromatin condensation inducer 1	22985	894,2295294	-0,158296849	0,079555	-1,989785487	0,046614571	0,345654018
ENSG00000187257	RSBN1L	round spermatid basic protein 1 like	222194	246,2194178	-0,001761948	0,116394	-0,015137796	0,987922248	0,996894778
ENSG00000161405	IKZF3	IKAROS family zinc finger 3	22806	1111,082108	0,19384955	0,080115	2,419637976	0,015535966	0,195544028
ENSG00000161526	SAP30BP	SAP30 binding protein	29115	101,4976403	0,264506687	0,181187	1,45985382	0,144330253	0,59024645
ENSG00000135090	TAOK3	TAO kinase 3	51347	500,8198551	0,015663005	0,091526	0,171132571	0,864119526	0,971561202
ENSG00000172005	MAL	mal, T cell differentiation protein	4118	258,6774942	0,39102261	0,108735	3,596116163	0,000323004	0,014854173
ENSG00000167978	SRRM2	serine/arginine repetitive matrix 2	23524	4917,435103	-0,057948347	0,048882	-1,185469847	0,23583173	0,691328984
ENSG00000100697	DICER1	dicer 1, ribonuclease III	23405	212,4894379	0,472946183	0,495898	0,953716714	0,340227057	0,777431905
ENSG00000112182	BACH2	BTB domain and CNC homolog 2	60468	349,1844927	-0,191334215	0,139931	-1,36735012	0,171515589	0,629558603
ENSG00000172795	DCP2	decapping mRNA 2	167227	354,1875444	-0,03228116	0,1415	-0,228135208	0,81954113	0,961128271
ENSG00000165417	GTF2A1	general transcription factor IIA subunit 1	2957	251,4288885	0,027644243	0,122627	0,225433572	0,821641997	0,961898134
ENSG00000145287	PLAC8	placenta associated 8	51316	97,9039523	-0,190223498	0,171816	-1,107133268	0,268236314	0,723761767
ENSG00000132646	PCNA	proliferating cell nuclear antigen	5111	345,3650731	0,658165105	0,107208	6,13914357	8,30E-10	3,62E-07
ENSG00000178229	ZNF543	zinc finger protein 543	125919	27,98808597	0,296194041	0,400115	0,740273109	0,459134295	NA
ENSG00000143387	CTSK	cathepsin K	1513	11,8287067	-0,608620259	0,588962	-1,033377036	0,301427489	NA
ENSG00000119778	ATAD2B	ATPase family AAA domain containing 2B	54454	229,531545	0,256638436	0,152142	1,686830562	0,091635937	0,492276778
ENSG00000148229	POLE3	DNA polymerase epsilon 3, accessory subunit	54107	260,5130747	-0,088926574	0,109773	-0,810091922	0,417887346	0,82446781
ENSG00000075218	GTSE1	G2 and S-phase expressed 1	51512	87,74185029	0,605956035	0,207972	2,913640703	0,003572408	0,080139791
ENSG00000125772	GPCPD1	glycerophosphocholine phosphodiesterase 1	56261	161,5003904	-0,202532391	0,147831	-1,370030102	0,170677505	0,629558603
ENSG00000228696	ARL17A	ADP ribosylation factor like GTPase 17A	51326	22,50719557	-0,328586816	0,648522	-0,506670533	0,612386009	NA
ENSG00000228696	ARL17B	ADP ribosylation factor like GTPase 17B	100506084	22,50719557	-0,328586816	0,648522	-0,506670533	0,612386009	NA
ENSG00000228696	LOC100996709	ADP-ribosylation factor-like protein 17	100996709	22,50719557	-0,328586816	0,648522	-0,506670533	0,612386009	NA
ENSG00000152102	FAM168B	family with sequence similarity 168 member B	130074	583,1843123	-0,059207361	0,073306	-0,807677817	0,419276077	0,825672195
ENSG00000136870	ZNF189	zinc finger protein 189	7743	61,86817548	0,405505473	0,256946	1,578170971	0,114526337	0,54024053
ENSG00000197930	ERO1A	endoplasmic reticulum oxidoreductase 1 alpha	30001	605,3723626	-0,210856541	0,102647	-2,054200291	0,03995631	0,318595798

ENSG00000138757	G3BP2	G3BP stress granule assembly factor 2	9908	716,1616134	-0,109737789	0,064101	-1,711955667	0,086904839	0,481411407
ENSG00000106733	NMRK1	nicotinamide riboside kinase 1	54981	161,2133337	0,077339138	0,164397	0,470441228	0,638039815	0,909829037
ENSG00000177058	SLC38A9	solute carrier family 38 member 9	153129	61,74141543	0,31941666	0,256025	1,247598886	0,212177987	0,666699723
ENSG00000276043	UHRF1	ubiquitin like with PHD and ring finger domains 1	29128	120,5120085	0,416846632	0,169701	2,45636314	0,014035125	0,185037772
ENSG00000146067	FAM193B	family with sequence similarity 193 member B	54540	138,3851983	-0,116109852	0,139415	-0,832836313	0,404937052	0,818437414
ENSG00000068724	TTC7A	tetratricopeptide repeat domain 7A	57217	145,4403391	0,388882304	0,166235	2,339345764	0,019317546	0,217675763
ENSG00000007202	BLTP2	bridge-like lipid transfer protein family member 2	9703	1096,578187	0,179974829	0,051029	3,526892509	0,000420467	0,018310702
ENSG00000092871	RFFL	ring finger and FYVE like domain containing E3 ubiquitin protein ligase	117584	241,9272354	-0,093992517	0,13115	-0,716679558	0,473571842	0,853811879
ENSG00000175305	CCNE2	cyclin E2	9134	31,71331897	0,523897076	0,34332	1,525974638	0,127016181	NA
ENSG00000184922	FMNL1	formin like 1	752	1270,900083	-0,09007375	0,0562	-1,602723259	0,108995766	0,52908614
ENSG00000123728	RAP2C	RAP2C, member of RAS oncogene family	57826	290,7473199	-0,269672676	0,109284	-2,467642726	0,013600599	0,181356713
ENSG00000232653	GOLGA8N	golgin A8 family member N	643699	425,1668737	-0,327223112	0,10474	-3,124136921	0,001783275	0,051326425
ENSG00000087274	ADD1	adducin 1	118	1067,937897	-0,015312319	0,075651	-0,202407757	0,839597964	0,96627649
ENSG00000172215	CXCR6	C-X-C motif chemokine receptor 6	10663	257,5649286	0,036667214	0,126062	0,290867507	0,771152653	0,945905503
ENSG00000196656	NA	NA	NA	426,2824921	-0,191639058	0,079453	-2,411976524	0,015866303	0,198429848
ENSG00000173011	TADA2B	transcriptional adaptor 2B	93624	243,8800262	-0,196053815	0,12875	-1,522743336	0,127822931	0,563689485
ENSG00000140006	WDR89	WD repeat domain 89	112840	61,42228158	-0,158535186	0,225311	-0,703627716	0,481664646	0,855577353
ENSG00000113269	RNF130	ring finger protein 130	55819	138,9265462	-0,066148212	0,211719	-0,312433904	0,754710788	0,942901768
ENSG00000131127	ZNF141	zinc finger protein 141	7700	163,287704	-0,0222105	0,159124	-0,139579819	0,888991986	0,977990819
ENSG00000173744	AGFG1	ArfGAP with FG repeats 1	3267	469,8760271	0,1047851	0,092539	1,132335681	0,257493335	0,713362633
ENSG00000083312	TNPO1	transportin 1	3842	421,4996565	0,013830647	0,126466	0,109362958	0,912914613	0,980488351
ENSG00000128487	SPECC1	sperm antigen with calponin homology and coiled-coil domains 1	92521	169,8202671	0,051432996	0,178448	0,288224255	0,773175083	0,946448613
ENSG00000056586	RC3H2	ring finger and CCCH-type domains 2	54542	525,8731618	0,089548853	0,098579	0,908396065	0,363669004	0,792858313
ENSG00000154814	OXNAD1	oxidoreductase NAD binding domain containing 1	92106	438,1730822	0,055301254	0,084747	0,65254514	0,514049563	0,868514045
ENSG00000266302	NA	NA	NA	192,6408099	-0,323568824	0,129965	-2,489669622	0,012786189	0,177739342
ENSG00000136167	LCP1	lymphocyte cytosolic protein 1	3936	8101,500934	0,005399299	0,036018	0,14990416	0,880840229	0,976300778
ENSG00000204560	DHX16	DEAH-box helicase 16	8449	217,1572986	-0,00153354	0,120518	-0,012724607	0,989847506	0,997211687
ENSG00000188483	IER5L	immediate early response 5 like	389792	47,98699092	0,057063904	0,258581	0,220681178	0,825340688	0,961917534
ENSG00000007923	DNAJC11	DnaJ heat shock protein family (Hsp40) member C11	55735	121,9716117	-0,326269948	0,17056	-1,912936872	0,055756135	0,382350454
ENSG00000197157	SND1	staphylococcal nuclease and tudor domain containing 1	27044	753,3272951	-0,131473079	0,064186	-2,048323497	0,040528312	0,321200165
ENSG00000108669	CYTH1	cytohesin 1	9267	581,8222187	-0,106645311	0,074347	-1,434418657	0,151452827	0,608445268
ENSG00000150347	ARID5B	AT-rich interaction domain 5B	84159	133,2089487	-0,170698787	0,180988	-0,943148543	0,345604929	0,782042713
ENSG00000099860	GADD45B	growth arrest and DNA damage inducible beta	4616	69,99017327	0,388635414	0,229458	1,693710625	0,090320284	0,489623111
ENSG00000117643	MAN1C1	mannosidase alpha class 1C member 1	57134	152,1763231	-0,486922812	0,167539	-2,906332084	0,003656931	0,081134286
ENSG00000171101	NA	NA	NA	52,81275187	-0,498713561	0,237317	-2,101468618	0,03559985	0,300111909
ENSG00000185963	BICD2	BICD cargo adaptor 2	23299	367,6516469	-0,242223851	0,091172	-2,656783805	0,007889002	0,132558158
ENSG00000134308	YWHAQ	tyrosine 3-monooxygenase/tryptophan 5-monooxygenase activation protein theta	10971	609,6205763	-0,06765653	0,071502	-0,946214616	0,344039133	0,781132432
ENSG00000139233	LLPH	LLP homolog, long-term synaptic facilitation factor	84298	146,2426253	-0,291630402	0,15732	-1,853744117	0,063775777	0,406570577
ENSG00000113851	CRBN	cereblon	51185	116,8802368	0,257567321	0,195489	1,317552154	0,187653611	0,645758518
ENSG00000113387	SUB1	SUB1 regulator of transcription	10923	330,2745705	-0,199683296	0,141363	-1,412560285	0,157785053	0,61428162
ENSG00000071054	MAP4K4	mitogen-activated protein kinase kinase kinase kinase 4	9448	451,9870902	0,04440299	0,090371	0,491343412	0,62318358	0,90479929
ENSG00000132424	PNISR	PNN interacting serine and arginine rich protein	25957	880,8579243	-0,066777063	0,085242	-0,783386022	0,433400462	0,835093272
ENSG00000123684	LPGAT1	lysophosphatidylglycerol acyltransferase 1	9926	496,6459107	-0,12468014	0,09006	-1,38441956	0,166230023	0,625273276
ENSG00000142207	URB1	URB1 ribosome biogenesis homolog	9875	187,5751868	0,209910919	0,132063	1,589472115	0,111953845	0,534832035
ENSG00000182700	IGIP	IgA inducing protein	492311	26,5395914	0,067226658	0,369767	0,181808129	0,855733305	NA
ENSG00000183049	CAMK1D	calcium/calmodulin dependent protein kinase ID	57118	64,20946463	-0,207466924	0,264054	-0,785699889	0,432043328	0,834341849
ENSG00000158869	FCER1G	Fc epsilon receptor Ig	2207	801,9514375	-0,145822445	0,078123	-1,866568452	0,061961877	0,402854784

ENSG00000182318	ZSCAN22	zinc finger and SCAN domain containing 22	342945	23,96568392	0,110375721	0,402299	0,274362504	0,783806055	NA
ENSG00000147654	EBAG9	estrogen receptor binding site associated antigen 9	9166	33,77834843	-0,401509956	0,350534	-1,145423212	0,252033887	NA
ENSG00000163348	PYGO2	pygopus family PHD finger 2	90780	224,0661676	-0,081907288	0,115082	-0,71172913	0,476632525	0,853811879
ENSG00000110075	PPP6R3	protein phosphatase 6 regulatory subunit 3	55291	662,0983709	-0,17922729	0,065313	-2,744119338	0,006067348	0,11238903
ENSG00000100836	PABPN1	poly(A) binding protein nuclear 1	8106	693,5099385	-0,239880344	0,069172	-3,46790516	0,000524532	0,021456652
ENSG00000115165	CYTIP	cytohesin 1 interacting protein	9595	454,8319927	0,168642533	0,094985	1,775473309	0,075819769	0,446229554
ENSG00000139163	ETNK1	ethanolamine kinase 1	55500	404,8448483	0,18625555	0,100009	1,8623927	0,062547757	0,404317327
ENSG00000105248	YJU2	YJU2 splicing factor homolog	55702	90,01076534	-0,214492174	0,181199	-1,183738612	0,236516549	0,691328984
ENSG00000142188	TMEM50B	transmembrane protein 50B	757	111,5963474	-0,251537197	0,178089	-1,412420739	0,157826114	0,61428162
ENSG00000152147	GEMIN6	gem nuclear organelle associated protein 6	79833	38,16293735	0,72131106	0,394881	1,826652844	0,067751983	NA
ENSG00000100796	PPP4R3A	protein phosphatase 4 regulatory subunit 3A	55671	494,1282049	-0,214551123	0,081283	-2,639547317	0,008301683	0,13755574
ENSG00000103126	AXIN1	axin 1	8312	167,4205813	-0,297715235	0,129076	-2,306510229	0,021082139	0,227443848
ENSG00000142303	ADAMTS10	ADAM metallopeptidase with thrombospondin type 1 motif 10	81794	59,91392154	-0,698411693	0,240899	-2,899186551	0,003741322	0,082540294
ENSG00000163682	RPL9	ribosomal protein L9	6133	947,2628769	-0,03781835	0,064942	-0,582338518	0,560338686	0,883921037
ENSG00000146828	SLC12A9	solute carrier family 12 member 9	56996	24,45372165	-0,121798606	0,62632	-0,194467082	0,845810168	NA
ENSG00000111348	ARHGDIB	Rho GDP dissociation inhibitor beta	397	2268,580885	-0,034988166	0,075835	-0,461370856	0,644532557	0,912530858
ENSG00000152492	CCDC50	coiled-coil domain containing 50	152137	183,8582398	-0,244333339	0,126505	-1,931420073	0,053431125	0,373020492
ENSG00000284024	MSANTD7	Myb/SANT DNA binding domain containing 7	100421372	74,31909625	0,196945241	0,212607	0,926335818	0,354271487	0,788228969
ENSG00000160785	SLC25A44	solute carrier family 25 member 44	9673	182,6818509	-0,073008978	0,143535	-0,508650075	0,610997523	0,899995226
ENSG00000069667	RORA	RAR related orphan receptor A	6095	355,7407353	-0,142140288	0,12142	-1,170650715	0,241739203	0,696802579
ENSG00000087586	AURKA	aurora kinase A	6790	53,41179119	0,943567467	0,281518	3,351714688	0,000803127	0,029613909
ENSG00000178115	GOLGA8Q	golgin A8 family member Q	727909	707,6557467	-0,20838114	0,102397	-2,03503007	0,041847847	0,325741322
ENSG00000178115	GOLGA8O	golgin A8 family member O	728047	707,6557467	-0,20838114	0,102397	-2,03503007	0,041847847	0,325741322
ENSG00000197283	SYNGAP1	synaptic Ras GTPase activating protein 1	8831	59,779793	0,300245852	0,239697	1,252605452	0,21034933	0,664554962
ENSG00000107140	TESK1	testis associated actin remodelling kinase 1	7016	151,4700131	0,266024459	0,166015	1,602413372	0,10906423	0,52908614
ENSG00000197223	C1D	C1D nuclear receptor corepressor	10438	57,68823711	-0,415737332	0,244439	-1,700781845	0,08898396	0,488044705
ENSG00000097046	CDC7	cell division cycle 7	8317	83,93857118	0,456632031	0,189598	2,408418648	0,016021797	0,19984025
ENSG00000148925	BTBD10	BTB domain containing 10	84280	71,90418052	0,689992559	0,290811	2,372647297	0,01766112	0,20765035
ENSG00000135828	RNASEL	ribonuclease L	6041	102,7294712	0,110651239	0,18365	0,602512516	0,546833034	0,878739902
ENSG00000113448	PDE4D	phosphodiesterase 4D	5144	136,1601288	-0,537117303	0,173741	-3,091487811	0,001991562	0,054949576
ENSG00000127084	FGD3	FYVE, RhoGEF and PH domain containing 3	89846	603,7651676	-0,152695934	0,088008	-1,735031951	0,082735146	0,468169196
ENSG00000132825	PPP1R3D	protein phosphatase 1 regulatory subunit 3D	5509	52,07839941	0,069461166	0,271231	0,256096321	0,797876463	0,95354564
ENSG00000140836	ZFHX3	zinc finger homeobox 3	463	1559,876167	-0,050841118	0,057228	-0,888396924	0,374327279	0,801403701
ENSG00000116833	NR5A2	nuclear receptor subfamily 5 group A member 2	2494	26,12529582	0,673663552	0,373947	1,801493658	0,071625106	NA
ENSG00000075415	SLC25A3	solute carrier family 25 member 3	5250	626,5925065	0,424897311	0,079116	5,370529293	7,85E-08	1,76E-05
ENSG00000182141	ZNF708	zinc finger protein 708	7562	70,32608467	-0,070310599	0,244481	-0,28759099	0,773659842	0,946448613
ENSG00000004468	CD38	CD38 molecule	952	286,406142	-0,093273495	0,10796	-0,863963168	0,387608124	0,809653368
ENSG00000005339	CREBBP	CREB binding protein	1387	1051,610471	-0,007235797	0,074171	-0,097556058	0,922284818	0,984284131
ENSG00000064726	BTBD1	BTB domain containing 1	53339	399,5545545	-0,011453176	0,082367	-0,139050381	0,889410337	0,977990819
ENSG00000124217	MOCS3	molybdenum cofactor synthesis 3	27304	34,40549682	0,243517743	0,366051	0,665255517	0,505887077	NA
ENSG00000101082	SLA2	Src like adaptor 2	84174	836,9642217	-0,017366488	0,060462	-0,28723099	0,773935459	0,946448613
ENSG00000130695	CEP85	centrosomal protein 85	64793	117,7381151	0,521751529	0,173485	3,007469171	0,002634328	0,065268188
ENSG00000152193	OBI1	ORC ubiquitin ligase 1	79596	89,84743295	-0,206029058	0,210091	-0,980667433	0,326756769	0,769687654
ENSG00000011426	ANLN	anillin, actin binding protein	54443	83,70612458	0,638815146	0,200254	3,190029868	0,001422581	0,043903611
ENSG00000133574	GIMAP4	GTPase, IMAP family member 4	55303	389,5877511	-0,142422001	0,093873	-1,517178173	0,129221705	0,564778673
ENSG00000171928	TVP23B	trans-golgi network vesicle protein 23 homolog B	51030	96,59184064	0,068470559	0,22248	0,307760518	0,758264566	0,942901768
ENSG00000111530	CAND1	cullin associated and neddylation dissociated 1	55832	287,8789831	0,285645583	0,142391	2,00606262	0,044849567	0,339916761
ENSG00000107960	STN1	STN1 subunit of CST complex	79991	387,116284	-0,256222994	0,09825	-2,607868994	0,009110782	0,146613742
ENSG00000140259	MFAP1	microfibril associated protein 1	4236	104,3602253	0,094803454	0,170385	0,556407396	0,577932384	0,887069818
ENSG00000198837	DENND4B	DENN domain containing 4B	9909	486,4711709	0,120806949	0,080036	1,509405607	0,131195161	0,567474728
ENSG00000256087	ZNF432	zinc finger protein 432	9668	129,4147484	-0,071974716	0,21895	-0,328726398	0,742362499	0,939193793
ENSG00000198791	CNOT7	CCR4-NOT transcription complex subunit 7	29883	270,8107362	0,064403531	0,106833	0,602843373	0,54661289	0,878739902

ENSG00000138834	MAPK8IP3	mitogen-activated protein kinase 8 interacting protein 3	23162	391,6579924	-0,262639479	0,095809	-2,741267979	0,006120257	0,113102347
ENSG00000170684	ZNF296	zinc finger protein 296	162979	31,75951165	-0,655825031	0,333365	-1,967286932	0,049150141	NA
ENSG00000008516	MMP25	matrix metallopeptidase 25	64386	1104,726158	-0,30788868	0,058276	-5,283275565	1,27E-07	2,55E-05
ENSG00000169249	ZRSR2	zinc finger CCCH-type, RNA binding motif and serine/arginine rich 2	8233	109,1575955	-0,059609632	0,155629	-0,383025025	0,701701209	0,930846779
ENSG00000122483	CCDC18	coiled-coil domain containing 18	343099	65,10638985	0,548365258	0,254057	2,158432364	0,030894232	0,281657534
ENSG00000118058	KMT2A	lysine methyltransferase 2A	4297	1299,316998	0,124775948	0,104345	1,195801942	0,231773858	0,686407194
ENSG00000181751	MACIR	macrophage immunometabolism regulator	90355	15,63643359	0,736214837	0,561592	1,310942602	0,189877156	NA
ENSG00000162511	LAPTM5	lysosomal protein transmembrane 5	7805	2874,411416	-0,030584885	0,045189	-0,676814799	0,498523467	0,86161226
ENSG00000146757	ZNF92	zinc finger protein 92	168374	161,9159205	-0,359785157	0,144825	-2,484278161	0,012981436	0,178245106
ENSG00000137478	FCHSD2	FCH and double SH3 domains 2	9873	251,1266341	-0,136069996	0,122186	-1,11362902	0,265438371	0,721118288
ENSG00000145216	FIP1L1	factor interacting with PAPOLA and CPSF1	81608	218,4223697	0,039544262	0,133688	0,295795545	0,767386226	0,944698783
ENSG00000115232	ITGA4	integrin subunit alpha 4	3676	447,5887064	-0,365148611	0,094717	-3,855154581	0,000115657	0,007325535
ENSG00000055163	CYFIP2	cytoplasmic FMR1 interacting protein 2	26999	2061,169111	-0,026348368	0,039418	-0,668433657	0,503856817	0,863283473
ENSG00000103995	CEP152	centrosomal protein 152	22995	262,5254293	0,165015391	0,122803	1,343741711	0,179031924	0,633385915
ENSG00000162924	REL	REL proto-oncogene, NF-kB subunit	5966	801,2070308	-0,124507954	0,077052	-1,615903751	0,106115125	0,522853317
ENSG00000165502	RPL36AL	ribosomal protein L36a like	6166	411,0837417	0,007591186	0,095186	0,079750776	0,936435474	0,987098279
ENSG00000111796	KLRB1	killer cell lectin like receptor B1	3820	365,7176964	-0,255246158	0,08913	-2,863741537	0,004186695	0,089354079
ENSG00000071794	HLTF	helicase like transcription factor	6596	175,3587361	0,167644359	0,137087	1,22290659	0,221364979	0,677288877
ENSG00000132357	CARD6	caspase recruitment domain family member 6	84674	58,28291089	0,619240632	0,255753	2,421240714	0,015467631	0,195124944
ENSG00000109805	NCAPG	non-SMC condensin I complex subunit G	64151	89,47940534	0,687980252	0,265227	2,593928141	0,009488632	0,150249421
ENSG00000140479	PCSK6	proprotein convertase subtilisin/kexin type 6	5046	15,89510613	-0,523778783	0,563245	-0,929931331	0,35240664	NA
ENSG00000152219	ARL14EP	ADP ribosylation factor like GTPase 14 effector protein	120534	125,983714	0,171536898	0,174984	0,980298926	0,326938584	0,769687654
ENSG00000214022	REPIN1	replication initiator 1	29803	243,2774304	-0,409610297	0,112598	-3,637815698	0,00027496	0,012885133
ENSG00000185670	ZBTB3	zinc finger and BTB domain containing 3	79842	18,87418525	-0,147726004	0,470219	-0,314164147	0,753396371	NA
ENSG00000100225	FBXO7	F-box protein 7	25793	264,7558125	-0,062719058	0,137475	-0,456220793	0,648231214	0,914533708
ENSG00000134717	BTF3L4	basic transcription factor 3 like 4	91408	156,618023	-0,523826784	0,160507	-3,263580957	0,001100137	0,037467385
ENSG00000054277	OPN3	opsin 3	23596	22,7700442	1,025046144	0,469786	2,181940447	0,029113928	NA
ENSG00000197746	PSAP	prosaposin	5660	925,5251875	-0,089877732	0,056556	-1,589182539	0,112019187	0,534832035
ENSG00000079134	THOC1	THO complex subunit 1	9984	43,34570838	0,614102076	0,317367	1,93499045	0,052991468	0,372224799
ENSG00000132613	MTSS2	MTSS I-BAR domain containing 2	92154	13,10587526	-0,535026635	0,505476	-1,058460476	0,289845561	NA
ENSG00000170027	YWHAG	tyrosine 3-monooxygenase/tryptophan 5-monooxygenase activation protein gamma	7532	340,7235194	0,111821098	0,111827	0,999948612	0,317335377	0,762292941
ENSG00000038382	TRIO	trio Rho guanine nucleotide exchange factor	7204	263,2852149	-0,066445766	0,18613	-0,356985746	0,721102477	0,933829316
ENSG00000116273	PHF13	PHD finger protein 13	148479	51,64061745	-0,086166077	0,254759	-0,338225955	0,735192918	0,939157822
ENSG00000189403	HMGB1	high mobility group box 1	3146	1659,494182	-0,178048928	0,075731	-2,351069552	0,018719534	0,213533226
ENSG00000134086	VHL	von Hippel-Lindau tumor suppressor	7428	569,4201429	-0,327262622	0,08388	-3,901542492	9,56E-05	0,006255821
ENSG00000184916	JAG2	jagged canonical Notch ligand 2	3714	232,7796022	-0,286262478	0,117954	-2,426906271	0,01522819	0,19384703
ENSG00000137876	RSL24D1	ribosomal L24 domain containing 1	51187	256,2345055	0,085885639	0,113265	0,758269535	0,448289641	0,84263906
ENSG00000134852	CLOCK	clock circadian regulator	9575	245,7335915	-0,088924463	0,114516	-0,776523126	0,437440172	0,837249449
ENSG00000166326	TRIM44	tripartite motif containing 44	54765	609,2340627	-0,049169363	0,067608	-0,727274066	0,467058082	0,850515691
ENSG00000249437	NAIP	NLR family apoptosis inhibitory protein	4671	14,24839659	-1,143223078	0,892842	-1,28043158	0,200393393	NA
ENSG00000078589	P2RY10	P2Y receptor family member 10	27334	134,7221428	0,226370429	0,189712	1,193229384	0,23277955	0,6881557
ENSG00000135966	TGFBRAP1	transforming growth factor beta receptor associated protein 1	9392	222,4965128	-0,073099396	0,141499	-0,516608088	0,605429769	0,897347689
ENSG00000227507	LTB	lymphotoxin beta	4050	631,8238807	0,081922986	0,089939	0,910876237	0,362360587	0,791409024
ENSG00000177663	IL17RA	interleukin 17 receptor A	23765	584,9267319	0,047005882	0,07214	0,651591513	0,514664724	0,868514045
ENSG00000167895	TMC8	transmembrane channel like 8	147138	1287,539581	0,048765216	0,05874	0,830188307	0,406432325	0,819098895
ENSG00000127328	RAB3IP	RAB3A interacting protein	117177	71,63349501	0,151407696	0,241717	0,626383528	0,531063411	0,872770879
ENSG00000187239	FNBP1	formin binding protein 1	23048	712,944633	-0,169300976	0,072137	-2,346939303	0,018928338	0,214721666

ENSG00000064687	ABCA7	ATP binding cassette subfamily A member 7	10347	733,5226488	-0,151434821	0,070096	-2,16040159	0,030741594	0,281403826
ENSG00000143153	ATP1B1	ATPase Na+/K+ transporting subunit beta 1	481	28,84345674	0,071401894	0,318103	0,224461602	0,822398142	NA
ENSG00000198763	ND2	NADH dehydrogenase subunit 2	4536	12326,2793	0,19904701	0,091742	2,169648128	0,030033512	0,279077601
ENSG00000076641	PAG1	phosphoprotein membrane anchor with glycosphingolipid microdomains 1	55824	513,2278116	0,257688669	0,084903	3,035099065	0,002404567	0,060144804
ENSG00000153936	HS2ST1	heparan sulfate 2-O-sulfotransferase 1	9653	102,2218279	-0,208452901	0,209183	-0,996510138	0,319002344	0,763389521
ENSG00000102580	DNAJC3	DnaJ heat shock protein family (Hsp40) member C3	5611	530,7591681	-0,186883319	0,07479	-2,498758021	0,012462938	0,175323113
ENSG00000113595	TRIM23	tripartite motif containing 23	373	48,29814844	0,009677117	0,302504	0,031990059	0,974479979	0,994534085
ENSG00000196843	ARID5A	AT-rich interaction domain 5A	10865	159,1479825	0,334195845	0,221877	1,50621965	0,132010797	0,568919111
ENSG00000116478	HDAC1	histone deacetylase 1	3065	441,4250139	0,027072434	0,09493	0,285183223	0,775503773	0,946448613
ENSG00000197386	HTT	huntingtin	3064	701,2493068	0,172206817	0,077973	2,208538484	0,027206758	0,268107753
ENSG00000249978	TRGV7	T cell receptor gamma variable 7 (pseudogene)	6981	20,67285162	-0,971635087	0,489642	-1,984377218	0,047213804	NA
ENSG00000153283	CD96	CD96 molecule	10225	5606,05207	0,038504132	0,036462	1,056009335	0,29096395	0,743239565
ENSG00000276550	NA	NA	NA	124,4051982	-0,038870428	0,205769	-0,188902932	0,850168895	0,967902179
ENSG00000005844	ITGAL	integrin subunit alpha L	3683	3869,073076	0,021536201	0,040123	0,536756234	0,591436006	0,892834002
ENSG00000158050	DUSP2	dual specificity phosphatase 2	1844	219,6471918	0,201818226	0,11906	1,695097942	0,090056843	0,489389871
ENSG00000151239	TWF1	twinfilin actin binding protein 1	5756	123,080303	-0,213050178	0,186299	-1,143595059	0,252791612	0,708754466
ENSG00000198728	LDB1	LIM domain binding 1	8861	479,0512116	-0,245137628	0,086859	-2,822238806	0,004768965	0,096039621
ENSG00000122482	ZNF644	zinc finger protein 644	84146	199,4734541	0,047043593	0,148569	0,316645736	0,751512428	0,94158578
ENSG00000156966	B3GNT7	UDP-GlcNAc:betaGal beta-1,3-N-acetylglucosaminyltransferase 7	93010	104,6606375	-0,050437766	0,17074	-0,295406859	0,767683095	0,944698783
ENSG00000005483	KMT2E	lysine methyltransferase 2E (inactive)	55904	715,4952044	0,325475203	0,084656	3,844670639	0,000120714	0,007584731
ENSG00000088888	MAVS	mitochondrial antiviral signaling protein	57506	878,0128244	-0,235627918	0,056149	-4,196461589	2,71E-05	0,00228963
ENSG00000078142	PIK3C3	phosphatidylinositol 3-kinase catalytic subunit type 3	5289	216,413539	-0,090564915	0,11455	-0,790616034	0,429168087	0,832060923
ENSG00000034677	RNF19A	ring finger protein 19A, RBR E3 ubiquitin protein ligase	25897	283,2225586	-0,075516674	0,105773	-0,71395291	0,475256298	0,853811879
ENSG00000118922	KLF12	KLF transcription factor 12	11278	401,5946895	-0,3090056	0,084851	-3,641730332	0,000270812	0,012885133
ENSG00000155868	MED7	mediator complex subunit 7	9443	57,33831801	0,117717447	0,240876	0,488705216	0,62505041	0,90479929
ENSG00000166295	ANAPC16	anaphase promoting complex subunit 16	119504	267,2691585	-0,178938625	0,115234	-1,55283418	0,120462755	0,554378286
ENSG00000124228	DDX27	DEAD-box helicase 27	55661	220,1817866	-0,020768519	0,11279	-0,184134563	0,853907893	0,968377739
ENSG00000133422	MORC2	MORC family CW-type zinc finger 2	22880	189,5613152	0,069445948	0,125204	0,554661553	0,579126178	0,887537774
ENSG00000171320	ESCO2	establishment of sister chromatid cohesion N-acetyltransferase 2	157570	39,76713117	0,962775892	0,36019	2,672967162	0,007518361	NA
ENSG00000197498	RPF2	ribosome production factor 2 homolog	84154	38,68533754	0,039512561	0,356402	0,110865049	0,911723361	NA
ENSG00000197619	ZNF615	zinc finger protein 615	284370	73,35776625	-0,445644685	0,273296	-1,630629818	0,102968454	0,515621608
ENSG00000109685	NSD2	nuclear receptor binding SET domain protein 2	7468	412,7806649	0,549291809	0,087709	6,262665422	3,78E-10	1,86E-07
ENSG00000080503	SMARCA2	SWI/SNF related, matrix associated, actin dependent regulator of chromatin, subfamily a, member 2	6595	1620,257653	-0,112119439	0,082315	-1,362070371	0,173175678	0,629558603
ENSG00000120875	DUSP4	dual specificity phosphatase 4	1846	1205,875667	0,182223659	0,052324	3,482605346	0,00049656	0,020744578
ENSG00000136161	RCBTB2	RCC1 and BTB domain containing protein 2	1102	1555,92953	-0,027195793	0,061377	-0,443090927	0,657699968	0,919088619
ENSG00000178966	RMI1	RecQ mediated genome instability 1	80010	64,45912371	0,197898259	0,249347	0,793666209	0,427389777	0,832060923
ENSG00000251369	ZNF550	zinc finger protein 550	162972	13,27182753	5,102983202	1,588711	3,212026872	0,00131802	NA
ENSG00000278129	ZNF8	zinc finger protein 8	7554	96,41025263	0,027448409	0,183124	0,149890109	0,880851315	0,976300778
ENSG00000169871	TRIM56	tripartite motif containing 56	81844	780,7862692	-0,178651449	0,063854	-2,797800471	0,005145189	0,100843978
ENSG00000165030	NFIL3	nuclear factor, interleukin 3 regulated	4783	272,6894585	0,088255559	0,108606	0,812618653	0,416436733	0,82446781
ENSG00000164327	RICTOR	RPTOR independent companion of MTOR complex 2	253260	925,1224256	-0,056690417	0,077423	-0,732220885	0,464033759	0,848549742
ENSG00000176658	MYO1D	myosin ID	4642	142,2804025	-0,082032353	0,137657	-0,595918945	0,551229377	0,880819693

ENSG00000163104	SMARCAD1	SWI/SNF-related, matrix-associated actin-dependent regulator of chromatin, subfamily a, containing DEAD/H box 1	56916	152,6087164	-0,506673251	0,181008	-2,799183352	0,005123204	0,100843978
ENSG00000170542	SERPINB9	serpin family B member 9	5272	294,3014163	-0,326713054	0,102985	-3,172426909	0,001511706	0,045665152
ENSG00000134996	OSTF1	osteoclast stimulating factor 1	26578	865,0651365	-0,132953945	0,059289	-2,242467267	0,024931189	0,253311197
ENSG00000065613	SLK	STE20 like kinase	9748	654,0663384	-0,075410386	0,070378	-1,071511926	0,283939311	0,737423617
ENSG00000163191	S100A11	S100 calcium binding protein A11	6282	284,3905115	0,165997505	0,109383	1,517580089	0,12912029	0,56464964
ENSG00000132475	H3-3B	H3,3 histone B	3021	1641,770432	0,182305349	0,210484	0,86612475	0,386421754	0,809653368
ENSG00000198885	ITPRIPL1	ITPRIP like 1	150771	172,6659899	-0,037901538	0,130981	-0,289366533	0,772300902	0,946409165
ENSG00000158321	AUTS2	activator of transcription and developmental regulator AUTS2	26053	553,5736393	-0,242746607	0,10858	-2,23563848	0,025375462	0,25682845
ENSG00000159063	ALG8	ALG8 alpha-1,3-glucosyltransferase	79053	70,46239975	0,462149843	0,221747	2,084130096	0,037148335	0,306351862
ENSG00000179456	ZBTB18	zinc finger and BTB domain containing 18	10472	27,3524874	0,209652113	0,349373	0,600080935	0,548452297	NA
ENSG00000134698	AGO4	argonaute RISC component 4	192670	341,1361283	-0,177780404	0,10019	-1,774433101	0,075991539	0,446400557
ENSG00000179364	PACS2	phosphofurin acidic cluster sorting protein 2	23241	157,5233665	-0,337529766	0,209292	-1,612718268	0,10680573	0,525374026
ENSG00000179941	BBS10	Bardet-Biedl syndrome 10	79738	31,38262883	-0,424661911	0,36434	-1,165565089	0,24379033	NA
ENSG00000215472	RPL17-C18orf32	RPL17-C18orf32 readthrough	100526842	68,44942924	-0,170128839	0,240255	-0,708119216	0,478871227	0,854008769
ENSG00000292343	AKAP17A	A-kinase anchoring protein 17A	8227	371,2785875	-0,237553707	0,184373	-1,288444062	0,197591429	0,65618735
ENSG00000105698	USF2	upstream transcription factor 2, c-fos interacting	7392	331,4973649	-0,332296139	0,114213	-2,909450896	0,003620643	0,080556733
ENSG00000137841	PLCB2	phospholipase C beta 2	5330	630,726521	-0,062086641	0,100856	-0,615596077	0,538161141	0,875096812
ENSG00000105829	BET1	Bet1 golgi vesicular membrane trafficking protein	10282	39,0065684	0,104089485	0,300407	0,346494973	0,728970762	NA
ENSG00000135932	CAB39	calcium binding protein 39	51719	1112,636324	-0,03559162	0,062704	-0,567613522	0,570297424	0,886795904
ENSG00000109971	HSPA8	heat shock protein family A (Hsp70) member 8	3312	3606,121548	0,109261498	0,046476	2,350907666	0,01872768	0,213533226
ENSG00000128965	CHAC1	ChaC glutathione specific gamma-glutamylcyclotransferase 1	79094	17,54047682	-0,224617944	0,436822	-0,514209329	0,607105646	NA
ENSG00000112312	GMNN	geminin DNA replication inhibitor	51053	30,71406683	0,909357525	0,342776	2,652920302	0,007979871	NA
ENSG00000124688	MAD2L1BP	MAD2L1 binding protein	9587	65,13025881	0,252696322	0,234537	1,077425229	0,281290338	0,734459547
ENSG00000152818	UTRN	utrophin	7402	2161,186035	0,016624104	0,042031	0,395520806	0,69245857	0,927787546
ENSG00000198961	PJA2	praja ring finger ubiquitin ligase 2	9867	774,2033407	-0,07364635	0,074656	-0,986472568	0,32390127	0,767603859
ENSG00000197045	GMFB	glia maturation factor beta	2764	207,1741478	-0,412104396	0,122365	-3,367821122	0,000757647	0,028608473
ENSG00000113810	SMC4	structural maintenance of chromosomes 4	10051	692,4377031	0,274487781	0,101127	2,714293661	0,006641726	0,118759094
ENSG00000110057	UNC93B1	unc-93 homolog B1, TLR signaling regulator	81622	235,9320878	-0,240042524	0,115136	-2,084868883	0,037081204	0,306351862
ENSG00000151287	TEX30	testis expressed 30	93081	169,491697	0,342314108	0,147296	2,323982354	0,02012644	0,223582826
ENSG00000040275	SPDL1	spindle apparatus coiled-coil protein 1	54908	52,80903495	0,098135052	0,395682	0,24801496	0,804122832	0,957051178
ENSG00000071051	NCK2	NCK adaptor protein 2	8440	104,1204947	-0,212501232	0,202718	-1,048259657	0,294518991	0,746659832
ENSG00000111875	ASF1A	anti-silencing function 1A histone chaperone	25842	112,1993165	0,253298376	0,164387	1,540862151	0,123350339	0,559349633
ENSG00000141622	ARK2C	arkadia (RNF111) C-terminal like ring finger ubiquitin ligase 2C	494470	25,70204465	0,186979972	0,43905	0,42587373	0,670199858	NA
ENSG00000172794	RAB37	RAB37, member RAS oncogene family	326624	257,0078681	-0,139832023	0,118115	-1,18385976	0,236468581	0,691328984
ENSG00000136925	TSTD2	thiosulfate sulfurtransferase like domain containing 2	158427	84,90558122	0,132159746	0,236034	0,559918369	0,575535119	0,887069818
ENSG00000137492	THAP12	THAP domain containing 12	5612	262,1692096	-0,145233651	0,11809	-1,229853858	0,218751836	0,674549241
ENSG00000186810	CXCR3	C-X-C motif chemokine receptor 3	2833	414,8617902	-0,026924276	0,095241	-0,282697271	0,777408908	0,947109268
ENSG00000106415	GLCCI1	glucocorticoid induced 1	113263	176,150416	-0,281691455	0,159111	-1,770412055	0,076658523	0,448306806
ENSG00000110880	CORO1C	coronin 1C	23603	287,2681622	0,020700355	0,098529	0,210094516	0,833593905	0,963446071
ENSG00000158691	ZSCAN12	zinc finger and SCAN domain containing 12	9753	100,1764787	-0,048596604	0,17536	-0,277125303	0,781683894	0,948894173
ENSG00000117632	STMN1	stathmin 1	3925	408,4183988	0,625449055	0,115208	5,428885481	5,67E-08	1,35E-05
ENSG00000118655	DCLRE1B	DNA cross-link repair 1B	64858	40,09409783	0,738110781	0,288924	2,554692746	0,010628157	NA
ENSG00000167615	LENG8	leukocyte receptor cluster member 8	114823	1903,108762	-0,172801263	0,046205	-3,739918918	0,00018408	0,010326867
ENSG00000178623	GPR35	G protein-coupled receptor 35	2859	232,009076	0,131719818	0,12143	1,08474281	0,278035582	0,731800087
ENSG00000164976	MYORG	myogenesis regulating glycosidase (putative)	57462	86,45069575	0,221676779	0,25267	0,877335902	0,380304217	0,805974027
ENSG00000125449	ARMC7	armadillo repeat containing 7	79637	49,55996653	0,12107998	0,247539	0,489135689	0,624745636	0,90479929

ENSG00000116133	DHCR24	24-dehydrocholesterol reductase	1718	1087,385578	0,210733377	0,050965	4,134891034	3,55E-05	0,00281731
ENSG00000239900	ADSL	adenylosuccinate lyase	158	102,8800329	0,339631066	0,379092	0,895907353	0,370302247	0,799217874
ENSG00000243811	APOBEC3D	apolipoprotein B mRNA editing enzyme catalytic subunit 3D	140564	192,7178339	0,110753431	0,198611	0,557638819	0,577091043	0,887069818
ENSG00000086589	RBM22	RNA binding motif protein 22	55696	313,3850615	-0,010060574	0,104607	-0,096175296	0,923381351	0,984284131
ENSG00000076554	TPD52	tumor protein D52	7163	250,6200507	-0,084006977	0,126812	-0,662454449	0,507680018	0,864738156
ENSG00000076554	TPD52-MRPS28	TPD52-MRPS28 readthrough	124188259	250,6200507	-0,084006977	0,126812	-0,662454449	0,507680018	0,864738156
ENSG00000123975	CKS2	CDC28 protein kinase regulatory subunit 2	1164	56,1254179	1,038459852	0,28384	3,658614465	0,000253582	0,012374745
ENSG00000101421	CHMP4B	charged multivesicular body protein 4B	128866	352,5998314	-0,281985772	0,098815	-2,853668191	0,004321764	0,090819559
ENSG00000122862	SRGN	serglycin	5552	2000,576022	0,141139002	0,052317	2,697749785	0,006980989	0,122385464
ENSG00000137504	CREBZF	CREB/ATF bZIP transcription factor	58487	243,7049143	0,002908895	0,149053	0,019515851	0,984429593	0,996185513
ENSG00000130779	CLIP1	CAP-Gly domain containing linker protein 1	6249	370,9255353	-0,082048536	0,116086	-0,706792999	0,479695126	0,854606339
ENSG00000286185	NA	NA	NA	31,65946029	2,531234621	1,072846	2,359364933	0,018306244	NA
ENSG00000101138	CSTF1	cleavage stimulation factor subunit 1	1477	32,73091969	-0,377915149	0,359976	-1,049835402	0,293793796	NA
ENSG00000172890	NADSYN1	NAD synthetase 1	55191	246,9179387	-0,096500876	0,107863	-0,894660698	0,370968494	0,79977671
ENSG00000140199	SLC12A6	solute carrier family 12 member 6	9990	589,3479018	-0,05666962	0,077228	-0,733800338	0,463070435	0,847577533
ENSG00000292333	P2RY8	P2Y receptor family member 8	286530	180,3645704	0,099566214	0,145318	0,685159197	0,493243469	0,860513824
ENSG00000164011	ZNF691	zinc finger protein 691	51058	15,35657617	-0,583416663	0,547956	-1,064714156	0,287005302	NA
ENSG00000085433	WDR47	WD repeat domain 47	22911	65,2503476	-0,00442409	0,231206	-0,019134845	0,984733535	0,996185513
ENSG00000142961	MOB3C	MOB kinase activator 3C	148932	229,0255633	-0,097387808	0,1121	-0,86875458	0,384981381	0,809109919
ENSG00000180917	CMTR2	cap methyltransferase 2	55783	54,41632371	-0,000887667	0,363908	-0,002439264	0,998053751	0,999300722
ENSG00000112576	CCND3	cyclin D3	896	719,1673207	-0,150462677	0,072227	-2,083189125	0,037233988	0,306351862
ENSG00000167842	MIS12	MIS12 kinetochore complex component	79003	123,4411532	0,08499816	0,160509	0,529553473	0,59642156	0,894229873
ENSG00000167562	ZNF701	zinc finger protein 701	55762	95,09604266	0,228479205	0,191717	1,191750792	0,233358976	0,688764147
ENSG00000171863	RPS7	ribosomal protein S7	6201	1326,368627	0,07827548	0,066355	1,179647435	0,238140468	0,693290718
ENSG00000153989	NUS1	NUS1 dehydrodolichyl diphosphate synthase subunit	116150	368,5330002	0,070001845	0,091615	0,764086944	0,444815432	0,840611262
ENSG00000138092	CENPO	centromere protein O	79172	75,78751336	0,792058564	0,207414	3,818741203	0,000134134	0,008166601
ENSG00000034533	ASTE1	asteroid homolog 1	28990	47,30143426	-0,238744245	0,268878	-0,887926643	0,374580212	0,801403701
ENSG00000145386	CCNA2	cyclin A2	890	116,5161943	0,371401145	0,16054	2,313452545	0,020697771	0,225859999
ENSG00000169228	RAB24	RAB24, member RAS oncogene family	53917	82,13065148	0,009278476	0,189103	0,049065788	0,960866868	0,992235217
ENSG00000182768	NGRN	neugrin, neurite outgrowth associated	51335	34,76853351	0,12360979	0,350698	0,352467919	0,724487374	NA
ENSG00000185825	BCAP31	B cell receptor associated protein 31	10134	252,1208938	-0,267672799	0,105461	-2,538113079	0,011145197	0,164848163
ENSG00000263002	ZNF234	zinc finger protein 234	10780	69,92437579	-0,274038714	0,271797	-1,008248402	0,313335227	0,758611242
ENSG00000198719	DLL1	delta like canonical Notch ligand 1	28514	339,7256001	-0,355862414	0,108281	-3,28645916	0,001014555	0,035572829
ENSG00000290292	HOMEZ	homeobox and leucine zipper encoding	57594	48,75083149	-0,039652489	0,289141	-0,137138771	0,890921107	0,977990819
ENSG00000007392	LUC7L	LUC7 like	55692	48,7239825	-0,428921281	0,370026	-1,159164217	0,246389254	0,703431917
ENSG00000167635	ZNF146	zinc finger protein 146	7705	319,098987	0,115395656	0,095367	1,210020163	0,226271156	0,681621061
ENSG00000100380	ST13	ST13 Hsp70 interacting protein	6767	445,3779174	0,03258549	0,088008	0,370255432	0,711192178	0,930846779
ENSG00000048028	USP28	ubiquitin specific peptidase 28	57646	306,9475724	-0,070681039	0,106338	-0,664682446	0,506253624	0,864630986
ENSG00000104738	MCM4	minichromosome maintenance complex component 4	4173	215,9467448	0,331555565	0,131691	2,517675383	0,011813214	0,170148395
ENSG00000134186	PRPF38B	pre-mRNA processing factor 38B	55119	696,8162925	-0,035845339	0,074497	-0,481167859	0,630397202	0,905960607
ENSG00000165501	LRR1	leucine rich repeat protein 1	122769	31,3386254	0,764856119	0,329797	2,319171027	0,020385765	NA
ENSG00000167208	SNX20	sorting nexin 20	124460	539,7541412	0,099103346	0,075207	1,317747309	0,187588252	0,645758518
ENSG00000188295	ZNF669	zinc finger protein 669	79862	38,19121422	-0,221044214	0,327691	-0,674550597	0,49996133	NA
ENSG00000260828	NA	NA	NA	52,93709171	-0,113070974	0,265018	-0,426654642	0,669630892	0,922641254
ENSG00000171466	ZNF562	zinc finger protein 562	54811	182,8329736	-0,11013438	0,189149	-0,58226185	0,560390318	0,883921037
ENSG00000135404	CD63	CD63 molecule	967	924,0608435	-0,041373177	0,103028	-0,401572538	0,687998644	0,926748702
ENSG00000162909	CAPN2	calpain 2	824	660,4987234	-0,054560272	0,066867	-0,815953222	0,414526891	0,824435097
ENSG00000112851	ERBIN	erbb2 interacting protein	55914	755,4558108	-0,151802537	0,088864	-1,708256829	0,087588698	0,483091035
ENSG00000278384	NA	NA	NA	69,44336139	0,131864548	0,209161	0,630443704	0,528404325	0,872142129
ENSG00000121274	TENT4B	terminal nucleotidyltransferase 4B	64282	113,2327948	-0,300657252	0,204591	-1,469550544	0,141683523	0,585983354
ENSG00000145041	DCAF1	DDB1 and CUL4 associated factor 1	9730	281,8620144	0,013451907	0,112744	0,119313262	0,905027177	0,978460866
ENSG00000204923	FBXO48	F-box protein 48	554251	16,54481231	0,510640852	0,606258	0,842283312	0,39962939	NA
ENSG00000169679	BUB1	BUB1 mitotic checkpoint serine/threonine kinase	699	201,3860113	0,698117709	0,198554	3,516007475	0,000438089	0,018598639

ENSG00000075420	FNDC3B	fibronectin type III domain containing 3B	64778	553,9311451	-0,163418385	0,089738	-1,821065351	0,068596923	0,425225128
ENSG00000106628	POLD2	DNA polymerase delta 2, accessory subunit	5425	61,91250873	0,307679194	0,233484	1,317774041	0,1875793	0,645758518
ENSG00000119541	VPS4B	vacuolar protein sorting 4 homolog B	9525	362,9473787	0,045875713	0,098856	0,464065679	0,64260069	0,912085896
ENSG00000164022	AIMP1	aminoacyl tRNA synthetase complex interacting multifunctional protein 1	9255	193,9949978	-0,120662403	0,129704	-0,930288532	0,352221714	0,786565067
ENSG00000173193	PARP14	poly(ADP-ribose) polymerase family member 14	54625	819,2651455	-0,002249664	0,08906	-0,025260125	0,979847479	0,995712569
ENSG00000126705	AHDC1	AT-hook DNA binding motif containing 1	27245	351,9749611	-0,012509617	0,094947	-0,131754088	0,895178803	0,977990819
ENSG00000177119	ANO6	anoctamin 6	196527	435,4829578	-0,104814328	0,079717	-1,314837201	0,188564622	0,646436727
ENSG00000107854	TNKS2	tankyrase 2	80351	671,3383878	-0,073040635	0,06507	-1,122490562	0,261653918	0,717538364
ENSG00000221978	CCNL2	cyclin L2	81669	141,8581455	-0,105674063	0,165087	-0,640110783	0,522100579	0,870221868
ENSG00000153015	CWC27	CWC27 spliceosome associated cyclophilin	10283	136,4545944	0,054011097	0,155138	0,348148576	0,727728605	0,937287711
ENSG00000079313	REXO1	RNA exonuclease 1 homolog	57455	233,7406717	-0,183491379	0,123897	-1,480995387	0,138607803	0,580290876
ENSG00000110422	HIPK3	homeodomain interacting protein kinase 3	10114	392,0878298	-0,131065764	0,094156	-1,392008824	0,163919726	0,623148851
ENSG00000152443	ZNF776	zinc finger protein 776	284309	81,3326724	-0,17728643	0,27111	-0,653927466	0,513158537	0,868235061
ENSG00000196235	SUPT5H	SPT5 homolog, DSIF elongation factor subunit	6829	671,195671	-0,011109204	0,082033	-0,135422804	0,892277595	0,977990819
ENSG00000102384	CENPI	centromere protein I	2491	41,7294043	0,753078994	0,33671	2,236580577	0,025313766	NA
ENSG00000177191	B3GNT8	UDP-GlcNAc:betaGal beta-1,3-N-acetylglucosaminyltransferase 8	374907	32,25672148	0,15902012	0,3265	0,487045094	0,626226375	NA
ENSG00000100297	MCM5	minichromosome maintenance complex component 5	4174	330,371899	0,196905774	0,108761	1,810436507	0,070228122	0,430243114
ENSG00000141759	TXNL4A	thioredoxin like 4A	10907	128,2692803	0,847708692	0,287568	2,947852651	0,003199895	0,075020833
ENSG00000275700	AATF	apoptosis antagonizing transcription factor	26574	229,9153677	-0,038127119	0,133955	-0,28462552	0,775931057	0,946448613
ENSG00000029725	RABEP1	rabaptin, RAB GTPase binding effector protein 1	9135	289,2160234	-0,055370876	0,106442	-0,520199937	0,602924229	0,897212117
ENSG00000198105	ZNF248	zinc finger protein 248	57209	66,41523902	-0,235080517	0,230577	-1,019532638	0,307950166	0,754562945
ENSG00000106003	LFNG	LFNG O-fucosylpeptide 3-beta-N-acetylglucosaminyltransferase	3955	188,5546219	0,012624165	0,17128	0,073704747	0,941245321	0,988441237
ENSG00000113240	CLK4	CDC like kinase 4	57396	61,77589579	-0,547499624	0,362725	-1,509407034	0,131194797	0,567474728
ENSG00000174652	ZNF266	zinc finger protein 266	10781	303,2300461	0,153275793	0,102592	1,494038881	0,135165457	0,576011666
ENSG00000149485	FADS1	fatty acid desaturase 1	3992	203,6858657	0,271527547	0,115252	2,355945605	0,018475623	0,212696759
ENSG00000174028	NA	NA	NA	117,2477785	-0,250592562	0,261097	-0,95976912	0,337171427	0,77693253
ENSG00000154229	PRKCA	protein kinase C alpha	5578	480,2072958	-0,121701791	0,076081	-1,599626647	0,109681433	0,529522903
ENSG00000120334	CENPL	centromere protein L	91687	36,56994964	0,235386018	0,372326	0,632204505	0,527253254	NA
ENSG00000141556	TBCD	tubulin folding cofactor D	6904	260,5667383	0,025207326	0,114537	0,220080179	0,825808711	0,961917534
ENSG00000263874	LASP1NB	LASP1 neighbor	100505576	45,92161136	-0,095179529	0,257659	-0,369400865	0,711828953	0,930846779
ENSG00000287839	NA	NA	NA	164,680042	0,200644407	0,16485	1,217133202	0,22355354	0,67869714
ENSG00000072786	STK10	serine/threonine kinase 10	6793	1666,970829	-0,125748172	0,052989	-2,373095488	0,017639703	0,20765035
ENSG00000090975	PITPNM2	phosphatidylinositol transfer protein membrane associated 2	57605	375,3239605	-0,529016033	0,095017	-5,567575622	2,58E-08	6,54E-06
ENSG00000241973	PI4KA	phosphatidylinositol 4-kinase alpha	5297	1026,515265	-0,033072339	0,0673	-0,491415024	0,62313294	0,90479929
ENSG00000140534	TICRR	TOPBP1 interacting checkpoint and replication regulator	90381	35,65155474	0,948929066	0,325088	2,918992033	0,003511652	NA
ENSG00000177666	PNPLA2	patatin like phospholipase domain containing 2	57104	142,6945579	-0,218612861	0,143826	-1,519983887	0,128515025	0,563689485
ENSG00000181704	YIPF6	Yip1 domain family member 6	286451	110,4670047	0,095662966	0,169001	0,566051004	0,571359109	0,886795904
ENSG00000157654	PALM2AKAP2	PALM2 and AKAP2 fusion	445815	128,7272561	0,063082945	0,146142	0,431654242	0,665992729	0,922184616
ENSG00000160957	RECQL4	RecQ like helicase 4	9401	45,48089702	0,547925942	0,269717	2,031488291	0,042205486	0,32787526
ENSG00000132842	AP3B1	adaptor related protein complex 3 subunit beta 1	8546	278,9244913	-0,053080844	0,098625	-0,538210125	0,590431991	0,892294181
ENSG00000144674	GOLGA4	golgin A4	2803	507,1390288	0,083180544	0,087639	0,94912403	0,342557534	0,780064992
ENSG00000078596	ITM2A	integral membrane protein 2A	9452	273,8977876	0,056173336	0,099539	0,564334965	0,57252619	0,886795904
ENSG00000275131	NA	NA	NA	191,5835951	0,329277489	0,134121	2,455076975	0,014085442	0,185304965
ENSG00000166946	CCNDBP1	cyclin D1 binding protein 1	23582	47,05984024	-0,424520656	0,279317	-1,519852004	0,128548176	0,563689485
ENSG00000101367	MAPRE1	microtubule associated protein RP/EB family member 1	22919	689,8630377	-0,105201534	0,083688	-1,257067946	0,208729033	0,662366797
ENSG00000115966	ATF2	activating transcription factor 2	1386	207,0047097	-0,226394909	0,119722	-1,890999738	0,058624379	0,39312401

ENSG00000116016	EPAS1	endothelial PAS domain protein 1	2034	27,3725297	-0,119815158	0,336844	-0,35569976	0,722065425	NA
ENSG00000121864	ZNF639	zinc finger protein 639	51193	102,2300735	-0,382441366	0,175365	-2,180825982	0,02919629	0,275445021
ENSG00000275395	FCGBP	Fc gamma binding protein	8857	126,4643141	0,521636223	0,252038	2,069675046	0,038482785	0,31159154
ENSG00000129353	SLC44A2	solute carrier family 44 member 2	57153	520,0596231	-0,231577578	0,101875	-2,273146784	0,023017336	0,241682027
ENSG00000186716	BCR	BCR activator of RhoGEF and GTPase	613	207,6715299	-0,170308275	0,120829	-1,409497737	0,158688042	0,614461481
ENSG00000157557	ETS2	ETS proto-oncogene 2, transcription factor	2114	82,6570846	0,575336191	0,220121	2,613732309	0,008955918	0,14473205
ENSG00000144554	FANCD2	FA complementation group D2	2177	126,8374261	0,567933355	0,178066	3,189449196	0,001425442	0,043903611
ENSG00000160218	TRAPPC10	trafficking protein particle complex subunit 10	7109	1036,420962	-0,026761321	0,073915	-0,362054342	0,717311422	0,932279316
ENSG00000101290	CDS2	CDP-diacylglycerol synthase 2	8760	328,5452981	-0,242955627	0,098915	-2,456198104	0,014041573	0,185037772
ENSG00000088992	TESC	tescalcin	54997	154,9901116	0,003358617	0,141092	0,023804435	0,981008602	0,995752963
ENSG00000115902	SLC1A4	solute carrier family 1 member 4	6509	995,6853956	0,020678648	0,074942	0,275930187	0,782601688	0,949714718
ENSG00000173559	NABP1	nucleic acid binding protein 1	64859	310,8032457	-0,011504362	0,195452	-0,058860427	0,953063278	0,991212866
ENSG00000052723	SIKE1	suppressor of IKBKE 1	80143	139,4857024	-0,211269303	0,166079	-1,272102521	0,203336696	0,660954946
ENSG00000119402	FBXW2	F-box and WD repeat domain containing 2	26190	488,9348948	0,070859866	0,089727	0,789729342	0,429685851	0,832060923
ENSG00000169016	E2F6	E2F transcription factor 6	1876	39,32285839	-0,384180978	0,285669	-1,344847584	0,17867446	NA
ENSG00000167565	SERTAD3	SERTA domain containing 3	29946	18,40414555	-0,61778252	0,47138	-1,310582189	0,18999896	NA
ENSG00000143878	RHOB	ras homolog family member B	388	62,79153263	-0,269143862	0,27377	-0,983102101	0,32555719	0,768407005
ENSG00000118707	TGIF2	TGFB induced factor homeobox 2	60436	78,73567052	0,058191152	0,200494	0,290238775	0,771633574	0,946052153
ENSG00000112640	PPP2R5D	protein phosphatase 2 regulatory subunit B'delta	5528	168,3707651	0,197231623	0,160621	1,227930507	0,219473056	0,675978948
ENSG00000105771	SMG9	SMG9 nonsense mediated mRNA decay factor	56006	112,8406734	0,475853635	0,16993	2,800297433	0,005105554	0,100843978
ENSG00000198925	ATG9A	autophagy related 9A	79065	191,0798024	0,428715952	0,14549	2,946695267	0,003211896	0,075078059
ENSG00000001084	GCLC	glutamate-cysteine ligase catalytic subunit	2729	323,8083721	-0,002106268	0,09631	-0,021869612	0,982551965	0,996026814
ENSG00000078070	MCCC1	methylcrotonyl-CoA carboxylase subunit 1	56922	93,88425456	-0,176781991	0,20311	-0,870375814	0,384095059	0,808586507
ENSG00000085449	WDFY1	WD repeat and FYVE domain containing 1	57590	378,0478613	-0,047231639	0,11111	-0,425088972	0,670771817	0,922641254
ENSG00000064666	CNN2	calponin 2	1265	1294,737916	-0,203298535	0,060529	-3,35868405	0,000783146	0,029289642
ENSG00000184445	KNTC1	kinetochore associated 1	9735	216,1081129	0,390046324	0,131314	2,970322259	0,002974875	0,071451584
ENSG00000198846	TOX	thymocyte selection associated high mobility group box	9760	318,2448609	-0,016049568	0,095272	-0,16846122	0,86622045	0,971722599
ENSG00000204519	ZNF551	zinc finger protein 551	90233	85,8889005	-0,111146923	0,18968	-0,585972043	0,557894302	0,883921037
ENSG00000107485	GATA3	GATA binding protein 3	2625	478,4742336	0,014221332	0,08973	0,158490103	0,874070619	0,974569239
ENSG00000176087	SLC35A4	solute carrier family 35 member A4	113829	237,7924378	0,086775762	0,121657	0,713279829	0,475672616	0,853811879
ENSG00000076108	BAZ2A	bromodomain adjacent to zinc finger domain 2A	11176	1156,054723	-0,081933628	0,059819	-1,369701693	0,17078004	0,629558603
ENSG00000116863	ADPRS	ADP-ribosylserine hydrolase	54936	38,89944845	0,07975234	0,31811	0,250707144	0,802040537	NA
ENSG00000048405	ZNF800	zinc finger protein 800	168850	326,6913054	-0,040059342	0,093252	-0,429582674	0,667499243	0,922308959
ENSG00000174405	LIG4	DNA ligase 4	3981	114,8263989	-0,024636126	0,182333	-0,135115847	0,892520281	0,977990819
ENSG00000141580	WDR45B	WD repeat domain 45B	56270	205,6169248	-0,042213661	0,128649	-0,328129282	0,742813915	0,939310978
ENSG00000231500	RPS18	ribosomal protein S18	6222	1870,28338	0,173871166	0,076472	2,273646962	0,022987221	0,241682027
ENSG00000198146	ZNF770	zinc finger protein 770	54989	219,9862057	-0,333121367	0,117729	-2,829569741	0,004661064	0,09483937
ENSG00000119820	YIPF4	Yip1 domain family member 4	84272	269,473726	-0,13238843	0,101093	-1,309566647	0,190342478	0,647836333
ENSG00000204394	VARS1	valyl-tRNA synthetase 1	7407	209,7753201	0,523223584	0,23189	2,256340096	0,02404934	0,247679511
ENSG00000093000	NUP50	nucleoporin 50	10762	466,235519	0,007444527	0,085605	0,08696372	0,93070035	0,986491322
ENSG00000048140	TSPAN17	tetraspanin 17	26262	439,4572394	0,050596289	0,099703	0,5074687	0,611825993	0,900006614
ENSG00000138670	RASGEF1B	RasGEF domain family member 1B	153020	167,6365117	0,357004958	0,12916	2,76404349	0,005708993	0,10771718
ENSG00000173542	MOB1B	MOB kinase activator 1B	92597	140,3180503	-0,323459427	0,150162	-2,154065284	0,031235053	0,281657534
ENSG00000197535	MYO5A	myosin VA	4644	379,0101332	-0,039307801	0,108976	-0,360700378	0,718323442	0,932668593
ENSG00000123124	WWP1	WW domain containing E3 ubiquitin protein ligase 1	11059	222,1394543	-0,077266703	0,123898	-0,623632943	0,532868671	0,873191491
ENSG00000179950	PUF60	poly(U) binding splicing factor 60	22827	245,1492347	-0,175744117	0,114656	-1,532789722	0,125327674	0,5624706
ENSG00000145375	AFG2A	AFG2 AAA ATPase homolog A	166378	57,76542095	0,373355496	0,323027	1,15580393	0,247761365	0,706065951
ENSG00000138678	GPAT3	glycerol-3-phosphate acyltransferase 3	84803	12,62789831	0,620611044	0,609216	1,018704952	0,308343061	NA
ENSG00000117586	TNFSF4	TNF superfamily member 4	7292	78,65537251	0,728257938	0,198401	3,670627538	0,000241956	0,01226013
ENSG00000112245	PTP4A1	protein tyrosine phosphatase 4A1	7803	349,0176443	-0,004689893	0,099168	-0,047292407	0,96228018	0,992328441
ENSG00000177602	HASPIN	histone H3 associated protein kinase	83903	20,63537828	0,628618605	0,413963	1,518538049	0,128878815	NA
ENSG00000162222	TTC9C	tetratricopeptide repeat domain 9C	283237	30,46244035	0,218923476	0,353225	0,619784403	0,535399739	NA

ENSG00000130038	CRACR2A	calcium release activated channel regulator 2A	84766	234,0222453	-0,024079711	0,11796	-0,204135151	0,838247886	0,965627959
ENSG00000160352	ZNF714	zinc finger protein 714	148206	63,36563647	0,517049904	0,24868	2,079177388	0,037601049	0,307740511
ENSG00000184110	EIF3C	eukaryotic translation initiation factor 3 subunit C	8663	1269,551505	-0,069588552	0,05023	-1,385394151	0,165931978	0,624933502
ENSG00000148396	SEC16A	SEC16 homolog A, endoplasmic reticulum export factor	9919	930,7989521	0,112978518	0,134233	0,841662455	0,399976919	0,814673014
ENSG00000187866	PABIR1	PP2A Aalpha (PPP2R1A) and B55A (PPP2R2A) interacting phosphatase regulator 1	116224	98,20230013	-0,098474619	0,190464	-0,517024444	0,605139095	0,897347689
ENSG00000048828	FAM120A	family with sequence similarity 120 member A	23196	780,5851212	-0,087379419	0,061709	-1,415998874	0,156775834	0,61428162
ENSG00000140993	TIGD7	tigger transposable element derived 7	91151	31,26503151	-0,044362062	0,362979	-0,122216648	0,902727442	NA
ENSG00000100288	CHKB	choline kinase beta	1120	46,31992541	1,179675983	0,275954	4,274898967	1,91E-05	0,001740504
ENSG00000113593	PPWD1	peptidylprolyl isomerase domain and WD repeat containing 1	23398	176,051829	0,120529136	0,139479	0,8641354	0,387513514	0,809653368
ENSG00000173726	TOMM20	translocase of outer mitochondrial membrane 20	9804	318,7537706	-0,166868222	0,098276	-1,697952648	0,089516699	0,488772026
ENSG00000152061	RABGAP1L	RAB GTPase activating protein 1 like	9910	522,1644767	-0,020934314	0,1051	-0,199183941	0,842118861	0,966959288
ENSG00000107566	ERLIN1	ER lipid raft associated 1	10613	49,44211165	-0,148032098	0,308848	-0,479304689	0,631721887	0,906830052
ENSG00000121390	PSPC1	paraspeckle component 1	55269	116,4321084	0,388327971	0,153685	2,526774423	0,011511541	0,168364333
ENSG00000148832	PAOX	polyamine oxidase	196743	12,03231848	2,151927538	0,696192	3,090998552	0,001994846	NA
ENSG00000186298	PPP1CC	protein phosphatase 1 catalytic subunit gamma	5501	455,4295663	0,018791987	0,099977	0,187963171	0,850905522	0,967902179
ENSG00000147274	RBMX	RNA binding motif protein X-linked	27316	571,2042585	0,027540787	0,080371	0,34266941	0,731847178	0,939050439
ENSG00000170088	TMEM192	transmembrane protein 192	201931	95,95699442	-0,391894227	0,190602	-2,056083357	0,039774482	0,317468275
ENSG00000132603	NIP7	nucleolar pre-rRNA processing protein NIP7	51388	124,5730775	0,033180819	0,153519	0,216135529	0,828882106	0,962723654
ENSG00000115421	PAPOLG	poly(A) polymerase gamma	64895	82,34526248	0,065225626	0,208223	0,31324824	0,754092071	0,942901768
ENSG00000198898	CAPZA2	capping actin protein of muscle Z-line subunit alpha 2	830	138,7576644	-0,497752618	0,261992	-1,899876578	0,057449318	0,38821726
ENSG00000177479	ARIH2	ariadne RBR E3 ubiquitin protein ligase 2	10425	314,5487966	-0,153571118	0,115227	-1,332766084	0,182608579	0,637116189
ENSG00000058063	ATP11B	ATPase phospholipid transporting 11B (putative)	23200	991,084275	-0,019980394	0,058901	-0,339218196	0,734445362	0,939157822
ENSG00000173153	ESRRA	estrogen related receptor alpha	2101	71,20222621	0,00763648	0,226805	0,033669769	0,973140486	0,994534085
ENSG00000172732	MUS81	MUS81 structure-specific endonuclease subunit	80198	148,0483485	0,197047803	0,15965	1,234246921	0,217110908	0,672923864
ENSG00000125630	POLR1B	RNA polymerase I subunit B	84172	179,0660017	0,738722622	0,201745	3,661662498	0,000250584	0,012374745
ENSG00000069329	VPS35	VPS35 retromer complex component	55737	110,7064849	0,251095632	0,316564	0,793191831	0,427666066	0,832060923
ENSG00000010165	METTL13	methyltransferase 13, eEF1A N-terminus and K55	51603	119,807293	0,143608811	0,166025	0,864982575	0,387048353	0,809653368
ENSG00000123329	ARHGAP9	Rho GTPase activating protein 9	64333	1449,624808	0,064548215	0,059044	1,093230181	0,274292749	0,727065559
ENSG00000080819	CPOX	coproporphyrinogen oxidase	1371	101,3534508	0,015618101	0,176991	0,088242098	0,929684259	0,986487751
ENSG00000086061	DNAJA1	DnaJ heat shock protein family (Hsp40) member A1	3301	413,5617356	0,221099048	0,10299	2,146793242	0,031809746	0,284548681
ENSG00000108848	LUC7L3	LUC7 like 3 pre-mRNA splicing factor	51747	842,5843982	-0,085967747	0,076248	-1,127471001	0,259543394	0,715701578
ENSG00000110344	UBE4A	ubiquitination factor E4A	9354	610,213488	-0,04885704	0,087214	-0,560197234	0,575344914	0,887069818
ENSG00000126773	PCNX4	pecanex 4	64430	341,5872167	0,130303631	0,106916	1,218750346	0,222938963	0,678694696
ENSG00000180530	NRIP1	nuclear receptor interacting protein 1	8204	183,9110292	0,125202789	0,127635	0,980946379	0,326619185	0,769687654
ENSG00000187210	GCNT1	glucosaminyl (N-acetyl) transferase 1	2650	527,6705677	0,054782078	0,0742	0,738302244	0,460330803	0,846622987
ENSG00000114738	MAPKAPK3	MAPK activated protein kinase 3	7867	586,0700041	-0,069311439	0,075379	-0,919508078	0,357829882	0,790102866
ENSG00000162630	B3GALT2	beta-1,3-galactosyltransferase 2	8707	13,94787137	-0,179750232	0,714851	-0,251451397	0,801465135	NA
ENSG00000114120	SLC25A36	solute carrier family 25 member 36	55186	362,1504922	-0,492818949	0,108852	-4,527422963	5,97E-06	0,000660481
ENSG00000133246	PRAM1	PML-RARA regulated adaptor molecule 1	84106	39,62303677	-0,312083434	0,277652	-1,124009559	0,261008971	NA
ENSG00000134057	CCNB1	cyclin B1	891	46,63585741	0,706589594	0,321893	2,195103779	0,028156154	0,270084577
ENSG00000204954	UQCC6	ubiquinol-cytochrome c reductase complex assembly factor 6	728568	12,04616121	1,345323444	0,628221	2,141479786	0,032235367	NA
ENSG00000198888	ND1	NADH dehydrogenase subunit 1	4535	10314,83208	0,13962428	0,158118	0,883039889	0,37721472	0,803537947
ENSG00000165322	ARHGAP12	Rho GTPase activating protein 12	94134	52,33187823	0,15045504	0,24476	0,614704999	0,538749558	0,875342836
ENSG00000197958	RPL12	ribosomal protein L12	6136	1138,449621	0,057604779	0,060705	0,948929422	0,342656509	0,780064992
ENSG00000115368	WDR75	WD repeat domain 75	84128	105,022889	0,22712235	0,209826	1,082433449	0,279059972	0,732510365

ENSG00000078140	UBE2K	ubiquitin conjugating enzyme E2 K	3093	243,6468319	-0,120179405	0,147977	-0,812148653	0,416706338	0,82446781
ENSG00000137955	RABGGTB	Rab geranylgeranyltransferase subunit beta	5876	30,90975553	1,239097421	0,404566	3,062782263	0,002192896	NA
ENSG00000134046	MBD2	methyl-CpG binding domain protein 2	8932	295,409139	-0,136437375	0,10203	-1,337224416	0,181149394	0,634628658
ENSG00000164484	TMEM200A	transmembrane protein 200A	114801	470,1108305	0,057769624	0,079238	0,729066095	0,465961233	0,849305994
ENSG00000164169	PRMT9	protein arginine methyltransferase 9	90826	69,90900572	0,225739589	0,245691	0,918794349	0,358203149	0,790482588
ENSG00000170633	RNF34	ring finger protein 34	80196	158,9031915	-0,067917822	0,167892	-0,40453283	0,685820941	0,926450534
ENSG00000163479	SSR2	signal sequence receptor subunit 2	6746	613,9005779	0,023444461	0,072343	0,324074852	0,745881365	0,939843468
ENSG00000108312	UBTF	upstream binding transcription factor	7343	749,4674993	-0,176467811	0,094606	-1,865292217	0,062140455	0,403681669
ENSG00000120280	TASL	TLR adaptor interacting with endolysosomal SLC15A4	80231	17,61106687	0,285945383	0,579808	0,493172441	0,621890749	NA
ENSG00000135164	DMTF1	cyclin D binding myb like transcription factor 1	9988	193,0719783	-0,21965942	0,202626	-1,084063719	0,278336548	0,732101556
ENSG00000137817	PARP6	poly(ADP-ribose) polymerase family member 6	56965	70,93768663	0,000507559	0,217941	0,002328884	0,998141821	0,999300722
ENSG00000198642	KLHL9	kelch like family member 9	55958	231,6542338	-0,149958525	0,116084	-1,291814345	0,196421446	0,655659726
ENSG00000138246	DNAJC13	DnaJ heat shock protein family (Hsp40) member C13	23317	389,8157145	-0,06354103	0,097551	-0,651359881	0,514814202	0,868514045
ENSG00000103249	CLCN7	chloride voltage-gated channel 7	1186	215,0428158	-0,132069356	0,134101	-0,984847535	0,324698968	0,76812822
ENSG00000181847	TIGIT	T cell immunoreceptor with Ig and ITIM domains	201633	534,725059	0,132658743	0,073319	1,809344648	0,070397477	0,430678857
ENSG00000115504	EHBP1	EH domain binding protein 1	23301	92,33181694	0,37264628	0,196136	1,899934629	0,057441699	0,38821726
ENSG00000106052	TAX1BP1	Tax1 binding protein 1	8887	519,7396264	0,052397208	0,09158	0,572149672	0,56722058	0,885785777
ENSG00000115307	AUP1	AUP1 lipid droplet regulating VLDL assembly factor	550	172,3560314	0,209887053	0,184816	1,135654494	0,256101192	0,71225877
ENSG00000005238	ATOSB	atos homolog B	80256	63,4031353	0,16761597	0,223933	0,748510238	0,454152452	0,843234809
ENSG00000071127	WDR1	WD repeat domain 1	9948	2599,173392	-0,061493482	0,049886	-1,232672174	0,217698098	0,673519155
ENSG00000136932	TRMO	tRNA methyltransferase O	51531	41,08541024	0,368023378	0,379148	0,970659461	0,331717885	NA
ENSG00000198093	ZNF649	zinc finger protein 649	65251	34,9546535	0,024735762	0,352451	0,070182147	0,944048684	NA
ENSG00000136541	ERMN	ermin	57471	20,5453555	-0,545026041	0,429429	-1,2691886	0,204373806	NA
ENSG00000141098	GFOD2	Gfo/Idh/MocA-like oxidoreductase domain containing 2	81577	74,00670856	-0,13550422	0,224381	-0,603902602	0,545908399	0,878739902
ENSG00000148175	STOM	stomatin	2040	772,1588559	-0,165286294	0,086228	-1,916842273	0,055257973	0,381367414
ENSG00000003400	CASP10	caspase 10	843	328,7142444	-0,245060138	0,115011	-2,130761555	0,033108791	0,291520679
ENSG00000138778	CENPE	centromere protein E	1062	148,9863186	0,532291844	0,145557	3,656937242	0,000255247	0,012374745
ENSG00000002016	RAD52	RAD52 homolog, DNA repair protein	5893	35,68057765	-0,440355274	0,379417	-1,160610414	0,245800368	NA
ENSG00000254838	GVINP1	GTPase, very large interferon inducible pseudogene 1	387751	885,3050925	0,050338968	0,066875	0,752733293	0,451610199	0,842736177
ENSG00000134460	IL2RA	interleukin 2 receptor subunit alpha	3559	440,4845893	0,262975387	0,106301	2,473874213	0,013365675	0,180367712
ENSG00000187735	TCEA1	transcription elongation factor A1	6917	465,4677821	-0,064658075	0,0869	-0,744053175	0,456844306	0,844645758
ENSG00000186918	ZNF395	zinc finger protein 395	55893	109,7287399	-0,268338372	0,167702	-1,600092845	0,109577988	0,529522903
ENSG00000105063	PPP6R1	protein phosphatase 6 regulatory subunit 1	22870	1034,426866	-0,076958995	0,052525	-1,465186487	0,142870045	0,587795357
ENSG00000077684	JADE1	jade family PHD finger 1	79960	254,0978579	-0,226771471	0,116289	-1,950073999	0,0511673	0,364671481
ENSG00000109917	ZPR1	ZPR1 zinc finger	8882	77,78066265	-0,207731215	0,238854	-0,869700266	0,384464227	0,808672212
ENSG00000174456	C12orf76	chromosome 12 open reading frame 76	400073	15,52909288	0,471326432	0,606875	0,776644783	0,437368372	NA
ENSG00000130592	LSP1	lymphocyte specific protein 1	4046	1623,188195	-0,034440271	0,061564	-0,559420505	0,575874771	0,887069818
ENSG00000158985	CDC42SE2	CDC42 small effector 2	56990	1066,459811	-0,140143357	0,055439	-2,527865779	0,011475821	0,168264867
ENSG00000135898	GPR55	G protein-coupled receptor 55	9290	152,2653287	-0,227583869	0,171924	-1,323744184	0,185588019	0,642967931
ENSG00000113845	TIMMDC1	translocase of inner mitochondrial membrane domain containing 1	51300	58,8014688	0,596031993	0,261549	2,278855477	0,022675658	0,240343612
ENSG00000103657	HERC1	HECT and RLD domain containing E3 ubiquitin protein ligase family member 1	8925	1965,874616	0,172658719	0,049271	3,504277469	0,000457848	0,019333003
ENSG00000158290	CUL4B	cullin 4B	8450	217,4051501	-0,167612761	0,20086	-0,83447646	0,404012547	0,817992511
ENSG00000164587	RPS14	ribosomal protein S14	6208	1403,149341	0,02183187	0,070515	0,309606299	0,756860365	0,942901768
ENSG00000177873	ZNF619	zinc finger protein 619	285267	71,09642937	0,073478843	0,208157	0,352996845	0,724090806	0,934902053
ENSG00000005156	LIG3	DNA ligase 3	3980	166,574719	-0,180631482	0,13169	-1,371645646	0,170173777	0,629558603
ENSG00000138138	ATAD1	ATPase family AAA domain containing 1	84896	198,4645687	0,07760805	0,131486	0,590240078	0,555029707	0,882854594
ENSG00000095261	PSMD5	proteasome 26S subunit, non-ATPase 5	5711	155,1890056	0,361407705	0,148018	2,441654237	0,014620142	0,189796021
ENSG00000104957	YJU2B	YJU2 splicing factor homolog B	81576	47,10131143	-0,36732202	0,325968	-1,126865172	0,259799491	0,715701578

ENSG00000166130	IKBIP	IKBKB interacting protein	121457	56,14336825	0,10058151	0,238861	0,421087425	0,673691244	0,923602509
ENSG00000102699	PARP4	poly(ADP-ribose) polymerase family member 4	143	998,3080606	-0,014441092	0,057826	-0,249732457	0,802794257	0,956339465
ENSG00000119801	YPEL5	yippee like 5	51646	207,6486871	-0,15905276	0,111995	-1,420177185	0,155556104	0,61428162
ENSG00000123146	ADGRE5	adhesion G protein-coupled receptor E5	976	1702,743156	-0,041346079	0,049598	-0,833625479	0,404492063	0,81836184
ENSG00000137166	FOXP4	forkhead box P4	116113	89,94987179	-0,042396863	0,177228	-0,23922274	0,81093287	0,959038743
ENSG00000137449	CPEB2	cytoplasmic polyadenylation element binding protein 2	132864	125,2103572	0,019469136	0,175707	0,11080477	0,911771162	0,97975793
ENSG00000156869	FRRS1	ferric chelate reductase 1	391059	20,642096	0,257423463	0,434149	0,592937729	0,553222827	NA
ENSG00000157600	TMEM164	transmembrane protein 164	84187	391,0428124	-0,117929027	0,091416	-1,290022853	0,197042724	0,655659726
ENSG00000165119	HNRNPK	heterogeneous nuclear ribonucleoprotein K	3190	2389,789375	-0,025906009	0,069723	-0,371553923	0,710225	0,930846779
ENSG00000179899	NA	NA	NA	18,20165149	0,349598977	0,506581	0,690115198	0,490121746	NA
ENSG00000143315	PIGM	phosphatidylinositol glycan anchor biosynthesis class M	93183	97,75209498	0,01220695	0,19409	0,062893188	0,949851559	0,990721666
ENSG00000171634	BPTF	bromodomain PHD finger transcription factor	2186	390,8226547	-0,156575993	0,171726	-0,911775694	0,361886808	0,791409024
ENSG00000106460	TMEM106B	transmembrane protein 106B	54664	427,5704164	-0,354304292	0,111525	-3,17690926	0,001488536	0,045138849
ENSG00000135506	OS9	OS9 endoplasmic reticulum lectin	10956	593,7703182	-0,092060392	0,089558	-1,027939448	0,303978312	0,751870158
ENSG00000134262	AP4B1	adaptor related protein complex 4 subunit beta 1	10717	36,74636317	-0,748804529	0,416056	-1,799768376	0,071897219	NA
ENSG00000071537	SEL1L	SEL1L adaptor subunit of SYVN1 ubiquitin ligase	6400	667,0491617	-0,189911723	0,068744	-2,762583308	0,005734593	0,10771718
ENSG00000115484	CCT4	chaperonin containing TCP1 subunit 4	10575	407,9098071	0,2309651	0,08109	2,848261535	0,004395878	0,091822416
ENSG00000104866	PPP1R37	protein phosphatase 1 regulatory subunit 37	284352	57,91595206	0,081492928	0,25075	0,324997047	0,745183307	0,939745744
ENSG00000110665	C11orf21	chromosome 11 open reading frame 21	29125	305,7390027	-0,215342927	0,09463	-2,27562437	0,022868501	0,241086188
ENSG00000143493	INTS7	integrator complex subunit 7	25896	179,6659993	0,079804252	0,133517	0,597707442	0,550035159	0,88072908
ENSG00000170234	PWWP2A	PWWP domain containing 2A	114825	137,8084247	0,169015307	0,157454	1,073426938	0,283079603	0,736682308
ENSG00000090054	SPTLC1	serine palmitoyltransferase long chain base subunit 1	10558	190,7375639	-0,182734771	0,140217	-1,303232637	0,192495351	0,649982152
ENSG00000116128	BCL9	BCL9 transcription coactivator	607	116,1919819	-0,264065797	0,161937	-1,6306676	0,102960477	0,515621608
ENSG00000179335	CLK3	CDC like kinase 3	1198	83,78984266	-0,173412002	0,198221	-0,874840882	0,381660489	0,806821656
ENSG00000099381	SETD1A	SET domain containing 1A, histone lysine methyltransferase	9739	312,8115698	-0,191329338	0,109582	-1,745992366	0,080812278	0,4605948
ENSG00000166845	C18orf54	chromosome 18 open reading frame 54	162681	37,05067571	0,684709493	0,373843	1,831543116	0,067019518	NA
ENSG00000132432	SEC61G	SEC61 translocon subunit gamma	23480	109,8216683	-0,209121098	0,193744	-1,079368302	0,280423575	0,733410709
ENSG00000067167	TRAM1	translocation associated membrane protein 1	23471	819,5314745	-0,325759816	0,068258	-4,772468706	1,82E-06	0,000234308
ENSG00000145569	OTULINL	OTU deubiquitinase with linear linkage specificity like	54491	319,4492466	-0,027589782	0,105576	-0,261326642	0,793840623	0,952276252
ENSG00000135250	SRPK2	SRSF protein kinase 2	6733	254,1111468	-0,172640687	0,133746	-1,29080961	0,196769705	0,655659726
ENSG00000160185	UBASH3A	ubiquitin associated and SH3 domain containing A	53347	91,63229973	0,464880271	0,197736	2,351017213	0,018722168	0,213533226
ENSG00000065978	YBX1	Y-box binding protein 1	4904	313,9880018	0,242179854	0,121527	1,992805591	0,046282739	0,343577159
ENSG00000128641	MYO1B	myosin IB	4430	28,73862406	1,13562262	0,336308	3,376736869	0,000733512	NA
ENSG00000010292	NCAPD2	non-SMC condensin I complex subunit D2	9918	681,1741274	0,382070433	0,072789	5,249030712	1,53E-07	2,86E-05
ENSG00000115414	FN1	fibronectin 1	2335	337,2794723	0,554474426	0,098446	5,632269745	1,78E-08	5,37E-06
ENSG00000147324	MFHAS1	multifunctional ROCO family signaling regulator 1	9258	174,3706363	0,063049577	0,234547	0,26881448	0,78807245	0,95148885
ENSG00000168411	RFWD3	ring finger and WD repeat domain 3	55159	183,368997	0,021861358	0,154549	0,141452318	0,887512626	0,977990819
ENSG00000163374	YY1AP1	YY1 associated protein 1	55249	256,1527762	0,522103045	0,156964	3,326260923	0,000880195	0,032153725
ENSG00000132792	CTNNBL1	catenin beta like 1	56259	156,3523515	-0,101572385	0,142696	-0,711808827	0,476583166	0,853811879
ENSG00000184619	KRBA2	KRAB-A domain containing 2	124751	89,52182804	0,578543601	0,250629	2,308363884	0,020978906	0,226641441
ENSG00000101782	RIOK3	RIO kinase 3	8780	297,6153256	-0,058759843	0,119503	-0,491701519	0,622930364	0,90479929
ENSG00000196154	S100A4	S100 calcium binding protein A4	6275	334,8631517	0,428637573	0,11563	3,706971835	0,000209752	0,011117984
ENSG00000162676	GFI1	growth factor independent 1 transcriptional repressor	2672	178,6455364	0,121910563	0,151051	0,807083994	0,419618093	0,825985089
ENSG00000138172	CALHM2	calcium homeostasis modulator family member 2	51063	157,9585088	0,005259762	0,150306	0,034993675	0,972084785	0,994108581
ENSG00000183542	KLRC4	killer cell lectin like receptor C4	8302	213,6299497	0,206917786	0,126422	1,636721284	0,10168872	0,514437479
ENSG00000141376	BCAS3	BCAS3 microtubule associated cell migration factor	54828	123,8389939	-0,046510315	0,172059	-0,270316647	0,78691666	0,951039147
ENSG00000004139	SARM1	sterile alpha and TIR motif containing 1	23098	152,3427925	-0,074733693	0,142084	-0,525982592	0,598900302	0,89463553
ENSG00000023171	GRAMD1B	GRAM domain containing 1B	57476	418,1099102	0,043205951	0,106202	0,406827446	0,684134727	0,925586358

ENSG00000100129	EIF3L	eukaryotic translation initiation factor 3 subunit L	51386	259,3834065	1,537044608	0,338819	4,536481812	5,72E-06	0,00065109
ENSG00000108100	CCNY	cyclin Y	219771	267,1905961	-0,077304661	0,110842	-0,697428398	0,485534734	0,858001067
ENSG00000115415	STAT1	signal transducer and activator of transcription 1	6772	2634,455936	0,066783099	0,043914	1,520759789	0,128320129	0,563689485
ENSG00000102575	ACP5	acid phosphatase 5, tartrate resistant	54	197,3665922	-0,120078346	0,123947	-0,968790484	0,332649736	0,773425407
ENSG00000138376	BARD1	BRCA1 associated RING domain 1	580	87,82068997	-0,129667564	0,205518	-0,63093019	0,528086171	0,872142129
ENSG00000126814	TRMT5	tRNA methyltransferase 5	57570	13,11971357	0,332386752	0,537132	0,618817448	0,536036627	NA
ENSG00000172578	KLHL6	kelch like family member 6	89857	338,5385403	-0,505863943	0,179426	-2,819347539	0,004812138	0,096414632
ENSG00000181555	SETD2	SET domain containing 2, histone lysine methyltransferase	29072	605,6145463	0,009807692	0,097021	0,101088437	0,919480257	0,984137086
ENSG00000063177	RPL18	ribosomal protein L18	6141	1136,094901	-0,072848815	0,059464	-1,225094097	0,220539769	0,677095199
ENSG00000143514	TP53BP2	tumor protein p53 binding protein 2	7159	407,6278613	-0,004734812	0,094708	-0,049993686	0,96012742	0,992235217
ENSG00000119392	GLE1	GLE1 RNA export mediator	2733	295,2859043	0,253540505	0,115534	2,194516656	0,028198288	0,270084577
ENSG00000101557	USP14	ubiquitin specific peptidase 14	9097	228,0080948	-0,037290098	0,184851	-0,201730261	0,840127604	0,966505956
ENSG00000082258	CCNT2	cyclin T2	905	147,2789857	0,187428324	0,197086	0,950998071	0,341605355	0,779479506
ENSG00000122257	RBBP6	RB binding protein 6, ubiquitin ligase	5930	596,0079911	-0,081897343	0,081173	-1,008921472	0,313012296	0,758381858
ENSG00000272325	NUDT3	nudix hydrolase 3	11165	390,0171714	-0,116116479	0,083667	-1,387843383	0,165184741	0,624454568
ENSG00000088451	TGDS	TDP-glucose 4,6-dehydratase	23483	62,19212027	0,400952014	0,245854	1,630855959	0,102920717	0,515621608
ENSG00000107201	RIGI	RNA sensor RIG-I	23586	84,18784209	0,454533616	0,202349	2,246286135	0,024685686	0,252449711
ENSG00000092531	SNAP23	synaptosome associated protein 23	8773	297,3722168	-0,11480929	0,168908	-0,679714861	0,496685024	0,861283987
ENSG00000177683	THAP5	THAP domain containing 5	168451	75,383644	-0,001639445	0,258551	-0,006340907	0,994940722	0,998500439
ENSG00000189298	ZKSCAN3	zinc finger with KRAB and SCAN domains 3	80317	35,34655165	0,299987141	0,399538	0,750834777	0,452752097	NA
ENSG00000102471	NDFIP2	Nedd4 family interacting protein 2	54602	278,9580185	0,28056806	0,114079	2,459416098	0,013916322	0,18410007
ENSG00000164430	CGAS	cyclic GMP-AMP synthase	115004	58,22507734	0,274944095	0,329614	0,8341403	0,404201928	0,817992511
ENSG00000126945	HNRNPH2	heterogeneous nuclear ribonucleoprotein H2	3188	390,0890288	-0,069797191	0,088219	-0,791182529	0,428837485	0,832060923
ENSG00000175137	SH3BP5L	SH3 binding domain protein 5 like	80851	116,1153129	-0,138051764	0,16585	-0,832389532	0,405189109	0,818437414
ENSG00000112308	C6orf62	chromosome 6 open reading frame 62	81688	823,3405075	-0,021689249	0,07625	-0,284449715	0,776065764	0,946448613
ENSG00000131171	SH3BGRL	SH3 domain binding glutamate rich protein like	6451	279,2344408	-0,327166675	0,104914	-3,118415013	0,001818266	0,051326425
ENSG00000065675	PRKCQ	protein kinase C theta	5588	341,9174342	0,043051778	0,092789	0,463977261	0,642664038	0,912085896
ENSG00000089280	FUS	FUS RNA binding protein	2521	522,8002417	-0,202107765	0,110251	-1,833166504	0,066777814	0,417162318
ENSG00000164244	PRRC1	proline rich coiled-coil 1	133619	283,0788515	0,040519012	0,125684	0,322387493	0,747159158	0,939899419
ENSG00000067560	RHOA	ras homolog family member A	387	2061,517896	-0,00688107	0,041735	-0,164875908	0,869041656	0,973118501
ENSG00000133302	SLF1	SMC5-SMC6 complex localization factor 1	84250	84,47038904	0,056396432	0,225801	0,249761569	0,802771741	0,956339465
ENSG00000136731	UGGT1	UDP-glucose glycoprotein glucosyltransferase 1	56886	748,9295096	-0,189899204	0,078942	-2,405564688	0,016147493	0,200668369
ENSG00000111371	SLC38A1	solute carrier family 38 member 1	81539	3144,566367	-0,026515459	0,048451	-0,547266846	0,584195418	0,88998582
ENSG00000066230	SLC9A3	solute carrier family 9 member A3	6550	64,55042817	-0,010636406	0,234854	-0,045289499	0,963876558	0,992694268
ENSG00000069424	KCNAB2	potassium voltage-gated channel subfamily A regulatory beta subunit 2	8514	1174,506059	-0,129024221	0,062081	-2,078306889	0,037681102	0,307894574
ENSG00000125148	MT2A	metallothionein 2A	4502	34,80430942	0,514959396	0,307391	1,675260548	0,093883122	NA
ENSG00000188917	TRMT2B	tRNA methyltransferase 2 homolog B	79979	134,5655983	-0,079243673	0,142315	-0,556820609	0,577650002	0,887069818
ENSG00000149308	NPAT	nuclear protein, coactivator of histone transcription	4863	281,0460383	-0,243420051	0,107051	-2,273872487	0,022973654	0,241682027
ENSG00000153443	UBALD1	UBA like domain containing 1	124402	97,34038741	-0,485754333	0,17939	-2,707808406	0,006772911	0,120349418
ENSG00000165494	PCF11	PCF11 cleavage and polyadenylation factor subunit	51585	437,0511795	-0,031937951	0,086123	-0,370841265	0,710755764	0,930846779
ENSG00000186834	HEXIM1	HEXIM P-TEFb complex subunit 1	10614	192,027232	-0,223876135	0,121201	-1,847148931	0,064725559	0,410155553
ENSG00000114353	GNAI2	G protein subunit alpha i2	2771	1504,554508	-0,285996463	0,056867	-5,029186087	4,93E-07	7,74E-05
ENSG00000122042	UBL3	ubiquitin like 3	5412	175,6978501	-0,11205946	0,139739	-0,80192241	0,422597842	0,827843798
ENSG00000146729	NIPSNAP2	nipsnap homolog 2	2631	79,40386996	0,06096884	0,187926	0,324429189	0,745613124	0,939843468
ENSG00000102401	ARMCX3	armadillo repeat containing X-linked 3	51566	122,2355449	-0,049509709	0,162803	-0,304108646	0,76104512	0,942901768
ENSG00000109944	JHY	junctional cadherin complex regulator	79864	51,57153642	0,579614451	0,281512	2,058931899	0,039500763	0,316570399
ENSG00000158773	USF1	upstream transcription factor 1	7391	75,54492486	0,279052187	0,243046	1,148144764	0,250908807	0,707335884
ENSG00000168564	CDKN2AIP	CDKN2A interacting protein	55602	122,6893021	0,12277569	0,178777	0,686754183	0,492237646	0,860513824
ENSG00000122224	LY9	lymphocyte antigen 9	4063	54,47584232	-0,608505788	0,235202	-2,587159159	0,009677088	0,151402093

ENSG00000156239	N6AMT1	N-6 adenine-specific DNA methyltransferase 1	29104	22,8104192	-0,384854291	0,434224	-0,886303533	0,375453986	NA
ENSG00000161298	ZNF382	zinc finger protein 382	84911	37,84452678	0,509835822	0,364964	1,396949648	0,162428713	NA
ENSG00000134283	PPHLN1	periphilin 1	51535	251,9279643	-0,120375225	0,119945	-1,003584962	0,315578706	0,76048697
ENSG00000196981	WDR5B	WD repeat domain 5B	54554	34,39140549	-0,486095131	0,332105	-1,463678953	0,143281689	NA
ENSG00000142945	KIF2C	kinesin family member 2C	11004	76,84015444	0,567946359	0,223269	2,543776299	0,010966129	0,163343491
ENSG00000180694	TMEM64	transmembrane protein 64	169200	136,6210003	0,294217025	0,143836	2,045498119	0,040805774	0,322423091
ENSG00000124570	SERPINB6	serpin family B member 6	5269	93,05292952	-0,141249922	0,211436	-0,668049077	0,504102266	0,863331152
ENSG00000185418	TARS3	threonyl-tRNA synthetase 3	123283	195,7994456	-0,099688538	0,120526	-0,827109506	0,408174997	0,820514158
ENSG00000080802	CNOT4	CCR4-NOT transcription complex subunit 4	4850	129,3570306	-0,069459591	0,159572	-0,435287979	0,663353409	0,920978907
ENSG00000139990	DCAF5	DDB1 and CUL4 associated factor 5	8816	587,052938	-0,004624245	0,082456	-0,056081054	0,955277237	0,991420853
ENSG00000166734	GOLM2	golgi membrane protein 2	113201	373,3998057	-0,211560716	0,094565	-2,237197518	0,025273434	0,256125867
ENSG00000070718	AP3M2	adaptor related protein complex 3 subunit mu 2	10947	71,03316285	0,439118036	0,206667	2,124759317	0,033606701	0,293600701
ENSG00000065135	GNAI3	G protein subunit alpha i3	2773	399,4619589	-0,177354115	0,091324	-1,942028136	0,052133694	0,369238926
ENSG00000133794	BMAL1	basic helix-loop-helix ARNT like 1	406	144,0967874	0,335241137	0,223822	1,497801413	0,134184853	0,574309297
ENSG00000021776	AQR	aquarius intron-binding spliceosomal factor	9716	201,8975988	0,322117016	0,152727	2,109107193	0,03493533	0,298241395
ENSG00000008952	SEC62	SEC62 homolog, preprotein translocation factor	7095	358,356953	0,02906243	0,115827	0,250912183	0,801882005	0,956122858
ENSG00000100601	ALKBH1	alkB homolog 1, histone H2A dioxygenase	8846	27,86559652	0,480659317	0,480067	1,001233543	0,316713914	NA
ENSG00000115020	PIKFYVE	phosphoinositide kinase, FYVE-type zinc finger containing	200576	633,6663766	-0,054765272	0,073412	-0,745998262	0,455668465	0,844061349
ENSG00000116044	NFE2L2	NFE2 like bZIP transcription factor 2	4780	275,4433167	0,048602757	0,121394	0,4003728	0,688881955	0,926748702
ENSG00000069399	BCL3	BCL3 transcription coactivator	602	81,0298956	0,361439683	0,230293	1,569479382	0,11653628	0,5441593
ENSG00000188739	RBM34	RNA binding motif protein 34	23029	83,46709417	0,377022907	0,273052	1,380772991	0,167348771	0,62720477
ENSG00000102921	N4BP1	NEDD4 binding protein 1	9683	417,8551483	-0,08981801	0,087132	-1,030828884	0,302621071	0,751870158
ENSG00000116473	RAP1A	RAP1A, member of RAS oncogene family	5906	519,1376286	-0,150895211	0,075285	-2,004321741	0,045035606	0,340112828
ENSG00000175354	PTPN2	protein tyrosine phosphatase non-receptor type 2	5771	205,2618222	0,134784061	0,124414	1,083351418	0,278652471	0,732196891
ENSG00000137275	RIPK1	receptor interacting serine/threonine kinase 1	8737	193,4438525	-0,215955107	0,177373	-1,217521314	0,223405932	0,67869714
ENSG00000027869	SH2D2A	SH2 domain containing 2A	9047	300,6527433	-0,144698323	0,114169	-1,267404143	0,205010819	0,660954946
ENSG00000152904	GGPS1	geranylgeranyl diphosphate synthase 1	9453	60,74709029	0,44288666	0,230407	1,922192607	0,054581527	0,378695505
ENSG00000163376	KBTBD8	kelch repeat and BTB domain containing 8	84541	48,11058937	-0,214925955	0,28493	-0,754312524	0,450661584	0,84263906
ENSG00000139842	CUL4A	cullin 4A	8451	612,9530413	0,096002594	0,073428	1,307436098	0,191064644	0,649057836
ENSG00000237649	KIFC1	kinesin family member C1	3833	142,9049245	0,479402651	0,184232	2,602171253	0,009263558	0,147356636
ENSG00000172671	ZFAND4	zinc finger AN1-type containing 4	93550	30,94525276	-0,532611483	0,330675	-1,610681415	0,107249178	NA
ENSG00000198363	ASPH	aspartate beta-hydroxylase	444	81,20255442	0,261982654	0,206655	1,26773193	0,204893698	0,660954946
ENSG00000128829	EIF2AK4	eukaryotic translation initiation factor 2 alpha kinase 4	440275	153,0784223	-0,051244295	0,158988	-0,322315971	0,747213335	0,939899419
ENSG00000166261	ZNF202	zinc finger protein 202	7753	70,49004066	0,125727097	0,20819	0,603906794	0,545905612	0,878739902
ENSG00000105287	PRKD2	protein kinase D2	25865	474,7256969	-0,058663147	0,084911	-0,690881344	0,489640111	0,860369726
ENSG00000147649	MTDH	metadherin	92140	1011,849544	-0,0289051	0,066933	-0,431851957	0,665849014	0,922184616
ENSG00000180357	ZNF609	zinc finger protein 609	23060	514,2808623	-0,086888976	0,079712	-1,09003352	0,275698379	0,728333356
ENSG00000138398	PPIG	peptidylprolyl isomerase G	9360	457,5676372	0,03559662	0,100153	0,355422321	0,722273229	0,934860571
ENSG00000141012	GALNS	galactosamine (N-acetyl)-6-sulfatase	2588	61,60415076	0,069077388	0,235636	0,293153371	0,769404923	0,944829104
ENSG00000205339	IPO7	importin 7	10527	435,8486067	0,010382129	0,082492	0,125855843	0,899846036	0,977990819
ENSG00000154642	C21orf91	chromosome 21 open reading frame 91	54149	251,9968343	0,107521598	0,162353	0,662268437	0,507799201	0,864738156
ENSG00000105255	FSD1	fibronectin type III and SPRY domain containing 1	79187	19,67716558	-0,887197692	0,404838	-2,191487017	0,028416568	NA
ENSG00000198915	RASGEF1A	RasGEF domain family member 1A	221002	168,3957004	-0,445012373	0,126426	-3,519933606	0,000431655	0,018525776
ENSG00000123485	HJURP	Holliday junction recognition protein	55355	69,5272343	0,934079644	0,299229	3,121622348	0,001798575	0,051326425
ENSG00000177200	CHD9	chromodomain helicase DNA binding protein 9	80205	625,5271698	-0,184508594	0,089143	-2,069804056	0,038470697	0,31159154
ENSG00000136819	C9orf78	chromosome 9 open reading frame 78	51759	445,2957814	0,092645947	0,08122	1,140676057	0,254004754	0,709921781
ENSG00000111300	NAA25	N-alpha-acetyltransferase 25, NatB auxiliary subunit	80018	87,2485304	0,332835677	0,202818	1,64105621	0,100785748	0,512343859
ENSG00000155629	PIK3AP1	phosphoinositide-3-kinase adaptor protein 1	118788	1841,430596	0,129151502	0,045359	2,847338547	0,004408645	0,091844823
ENSG00000103222	ABCC1	ATP binding cassette subfamily C member 1	4363	385,3698213	0,230296383	0,103358	2,228135475	0,025871482	0,259507819

ENSG00000066557	LRRC40	leucine rich repeat containing 40	55631	85,83801143	0,003931521	0,19427	0,02023739	0,983854001	0,996185513
ENSG00000205268	PDE7A	phosphodiesterase 7A	5150	537,0673189	0,003071518	0,082454	0,037251353	0,970284593	0,993542794
ENSG00000184508	HDDC3	HD domain containing 3	374659	20,3714142	0,579057916	0,427357	1,354973229	0,175426086	NA
ENSG00000113194	FAF2	Fas associated factor family member 2	23197	255,8751601	0,140645836	0,109039	1,289861856	0,197098627	0,655659726
ENSG00000151150	ANK3	ankyrin 3	288	69,58646462	0,903282981	0,296478	3,046710932	0,002313601	0,058616198
ENSG00000182944	EWSR1	EWS RNA binding protein 1	2130	492,5261764	-0,185136396	0,115685	-1,600343793	0,109522337	0,529522903
ENSG00000136156	ITM2B	integral membrane protein 2B	9445	606,884331	-0,20233227	0,088581	-2,284155316	0,022362405	0,238309804
ENSG00000078403	MLLT10	MLLT10 histone lysine methyltransferase DOT1L cofactor	8028	156,6298835	0,009684376	0,200838	0,048219765	0,961541098	0,992328441
ENSG00000102738	MRPS31	mitochondrial ribosomal protein S31	10240	75,90084716	0,536072261	0,197596	2,712971139	0,006668291	0,118759094
ENSG00000135451	TROAP	trophinin associated protein	10024	60,51798771	0,796075284	0,25871	3,077092605	0,002090304	0,05587092
ENSG00000130255	RPL36	ribosomal protein L36	25873	350,2336209	-0,118248933	0,104481	-1,131769569	0,257731325	0,713362633
ENSG00000143333	RGS16	regulator of G protein signaling 16	6004	143,7928619	0,733293177	0,147771	4,962361126	6,96E-07	0,000101289
ENSG00000159131	GART	phosphoribosylglycinamide formyltransferase, phosphoribosylglycinamide synthetase, phosphoribosylaminoimidazole synthetase	2618	239,7623872	-0,035123658	0,115798	-0,303318137	0,761647426	0,942934881
ENSG00000157570	TSPAN18	tetraspanin 18	90139	22,39775324	-0,725840857	0,401314	-1,808660965	0,070503691	NA
ENSG00000095139	ARCN1	archain 1	372	786,8630269	0,041734572	0,065876	0,633528296	0,526388709	0,871470682
ENSG00000158158	CNNM4	cyclin and CBS domain divalent metal cation transport mediator 4	26504	73,05564111	0,214118694	0,222388	0,962815103	0,33564032	0,775557244
ENSG00000137812	KNL1	kinetochore scaffold 1	57082	199,2505379	0,169312472	0,168252	1,006305256	0,314268753	0,759554708
ENSG00000180667	YOD1	YOD1 deubiquitinase	55432	141,8269056	-0,233249034	0,157056	-1,485130696	0,137509206	0,578689141
ENSG00000119673	ACOT2	acyl-CoA thioesterase 2	10965	33,13334374	-0,265517104	0,306408	-0,866548879	0,386189234	NA
ENSG00000144161	ZC3H8	zinc finger CCCH-type containing 8	84524	46,0158417	0,004029698	0,267977	0,01503749	0,988002271	0,996894778
ENSG00000263528	IKBKE	inhibitor of nuclear factor kappa B kinase subunit epsilon	9641	269,5232384	0,180254359	0,134124	1,343937775	0,178968509	0,633385915
ENSG00000112249	ASCC3	activating signal cointegrator 1 complex subunit 3	10973	537,5615544	0,078670411	0,07845	1,002815881	0,315949705	0,76048697
ENSG00000113658	SMAD5	SMAD family member 5	4090	146,6885468	-0,08974813	0,165876	-0,541056686	0,588468512	0,891557039
ENSG00000164663	USP49	ubiquitin specific peptidase 49	25862	79,33791873	0,110401585	0,201516	0,547854809	0,583791601	0,889619565
ENSG00000213079	SCAF8	SR-related CTD associated factor 8	22828	423,5163999	0,023313445	0,094263	0,247324534	0,804657073	0,957328863
ENSG00000146285	SCML4	Scm polycomb group protein like 4	256380	404,5022958	-0,158525525	0,091255	-1,737175975	0,082356114	0,467021604
ENSG00000083799	CYLD	CYLD lysine 63 deubiquitinase	1540	1495,528363	0,139863795	0,056239	2,486955808	0,01288414	0,177810208
ENSG00000109906	ZBTB16	zinc finger and BTB domain containing 16	7704	556,2115803	-0,476926964	0,09807	-4,863106324	1,16E-06	0,000165016
ENSG00000100092	SH3BP1	SH3 domain binding protein 1	23616	676,6766868	-0,078952571	0,071851	-1,09883923	0,271838197	0,725949406
ENSG00000125810	CD93	CD93 molecule	22918	27,07085173	-0,286290652	0,342776	-0,835211541	0,403598613	NA
ENSG00000185324	CDK10	cyclin dependent kinase 10	8558	46,94023537	-0,395836997	0,958629	-0,412920075	0,679665173	0,925586358
ENSG00000188167	TMPPE	transmembrane protein with metallophosphoesterase domain	643853	29,10001708	0,613855983	0,357608	1,716562208	0,086059193	NA
ENSG00000135974	C2orf49	chromosome 2 open reading frame 49	79074	111,4626149	0,410550844	0,217807	1,884926393	0,059439776	0,39462384
ENSG00000069956	MAPK6	mitogen-activated protein kinase 6	5597	215,6738369	-0,144713343	0,116119	-1,246250326	0,212672508	0,667215123
ENSG00000123143	PKN1	protein kinase N1	5585	423,915027	-0,049463151	0,08139	-0,60773068	0,543366111	0,877542071
ENSG00000143476	DTL	denticleless E3 ubiquitin protein ligase homolog	51514	70,63041148	0,664400535	0,270958	2,452046821	0,014204618	0,186248861
ENSG00000171488	LRRC8C	leucine rich repeat containing 8 VRAC subunit C	84230	263,2977986	0,159647507	0,10647	1,499461722	0,133753892	0,573252035
ENSG00000173706	HEG1	heart development protein with EGF like domains 1	57493	1805,497436	0,169264911	0,05316	3,184083455	0,001452131	0,044377588
ENSG00000186575	NF2	NF2, moesin-ezrin-radixin like (MERLIN) tumor suppressor	4771	180,6628944	0,177267097	0,14008	1,265471576	0,205702331	0,660954946
ENSG00000189114	BLOC1S3	biogenesis of lysosomal organelles complex 1 subunit 3	388552	47,73184521	-0,694385539	0,312181	-2,22430109	0,026128193	0,260750739
ENSG00000089505	CMTM1	CKLF like MARVEL transmembrane domain containing 1	113540	20,69578549	-0,182127163	0,459621	-0,396254846	0,691917034	NA
ENSG00000100625	SIX4	SIX homeobox 4	51804	25,42628417	-0,516476029	0,494538	-1,044359805	0,296318953	NA

ENSG00000006652	IFRD1	interferon related developmental regulator 1	3475	61,92068894	0,428309529	0,334348	1,281030854	0,200182829	0,659218424
ENSG00000185220	PGBD2	piggyBac transposable element derived 2	267002	35,26887944	0,173875361	0,327939	0,530206468	0,595968787	NA
ENSG00000213722	DDAH2	DDAH family member 2, ADMA-independent	23564	33,47883102	-0,545884591	0,540637	-1,009706122	0,312636109	NA
ENSG00000121716	PILRB	paired immunoglobin like type 2 receptor beta	29990	528,1048564	-0,22515754	0,093495	-2,408232549	0,016029967	0,19984025
ENSG00000138764	CCNG2	cyclin G2	901	114,6981897	-0,036519537	0,199616	-0,182949018	0,854838021	0,968377739
ENSG00000119285	HEATR1	HEAT repeat containing 1	55127	367,5924764	-0,001498739	0,126938	-0,011806828	0,990579733	0,997437593
ENSG00000108582	CPD	carboxypeptidase D	1362	828,5104185	0,056072062	0,069869	0,802529023	0,422247007	0,827816808
ENSG00000135905	DOCK10	dedicator of cytokinesis 10	55619	2600,230952	-0,003346368	0,038025	-0,088003505	0,929873891	0,986487751
ENSG00000118496	FBXO30	F-box protein 30	84085	86,14432191	-0,085544525	0,201263	-0,425038657	0,670808495	0,922641254
ENSG00000149177	PTPRJ	protein tyrosine phosphatase receptor type J	5795	1395,896295	-0,039475536	0,045723	-0,863366167	0,387936175	0,809686612
ENSG00000165006	UBAP1	ubiquitin associated protein 1	51271	211,7043592	-0,007436416	0,134333	-0,055358191	0,955853103	0,991510463
ENSG00000177156	TALDO1	transaldolase 1	6888	243,4856385	0,336244635	0,107854	3,117602713	0,001823284	0,051326425
ENSG00000213024	NUP62	nucleoporin 62	23636	328,7878661	0,056245197	0,103077	0,545660018	0,58529966	0,890362877
ENSG00000165672	PRDX3	peroxiredoxin 3	10935	203,2812057	0,34810956	0,147605	2,358379401	0,018354923	0,21231158
ENSG00000149257	SERPINH1	serpin family H member 1	871	42,00731791	-0,138257501	0,301215	-0,458999887	0,646234247	NA
ENSG00000019582	CD74	CD74 molecule	972	2174,68496	-0,099363436	0,055668	-1,784914817	0,074275135	0,443476943
ENSG00000088930	XRN2	5'-3' exoribonuclease 2	22803	485,4822715	-0,018213903	0,084752	-0,214907335	0,829839566	0,962996447
ENSG00000138160	KIF11	kinesin family member 11	3832	223,754104	0,56070413	0,123792	4,529387685	5,92E-06	0,000660481
ENSG00000110852	CLEC2B	C-type lectin domain family 2 member B	9976	228,8613748	0,113489455	0,137707	0,824138474	0,409860881	0,82130194
ENSG00000118855	MFSD1	major facilitator superfamily domain containing 1	64747	132,1642652	0,123370553	0,164266	0,751042694	0,452626962	0,843234809
ENSG00000175348	TMEM9B	TMEM9 domain family member B	56674	214,337584	-0,282266504	0,117743	-2,39730725	0,016516068	0,202942572
ENSG00000198089	SFI1	SFI1 centrin binding protein	9814	147,0901418	-0,121721448	0,148632	-0,818945788	0,412817344	0,82225544
ENSG00000100427	MLC1	modulator of VRAC current 1	23209	258,7568696	0,039008845	0,104948	0,371698649	0,710117231	0,930846779
ENSG00000131634	TMEM204	transmembrane protein 204	79652	40,30791159	0,185655684	0,286657	0,647657571	0,517206452	NA
ENSG00000214367	HAUS3	HAUS augmin like complex subunit 3	79441	109,8974738	0,125048439	0,1869	0,669066767	0,503452888	0,863218221
ENSG00000182473	EXOC7	exocyst complex component 7	23265	531,8415656	-0,124974236	0,093547	-1,335956458	0,181563506	0,6349064
ENSG00000164056	SPRY1	sprouty RTK signaling antagonist 1	10252	135,8056692	0,386619498	0,143188	2,700076009	0,006932364	0,121804885
ENSG00000143499	SMYD2	SET and MYND domain containing 2	56950	88,64219803	-0,431750405	0,229354	-1,882461133	0,059773432	0,395835192
ENSG00000119396	RAB14	RAB14, member RAS oncogene family	51552	421,2996509	-0,019382828	0,127062	-0,152546537	0,878755889	0,976237908
ENSG00000070413	DGCR2	DiGeorge syndrome critical region gene 2	9993	165,7037593	0,11503808	0,174969	0,65747791	0,51087367	0,866180216
ENSG00000122390	NAA60	N-alpha-acetyltransferase 60, NatF catalytic subunit	79903	123,1425788	-0,120199163	0,168108	-0,715011874	0,474601706	0,853811879
ENSG00000019995	ZRANB1	zinc finger RANBP2-type containing 1	54764	191,803825	-0,213543985	0,127653	-1,672850533	0,094356724	0,498337741
ENSG00000042493	CAPG	capping actin protein, gelsolin like	822	1023,626895	-0,002504157	0,055991	-0,044724597	0,964326828	0,992728748
ENSG00000146842	TMEM209	transmembrane protein 209	84928	108,0117177	-0,406341917	0,181829	-2,234744397	0,025434134	0,257063603
ENSG00000150457	LATS2	large tumor suppressor kinase 2	26524	208,3948491	-0,164145275	0,115821	-1,417237849	0,156413399	0,61428162
ENSG00000163564	PYHIN1	pyrin and HIN domain family member 1	149628	151,8297726	0,23385102	0,16736	1,397292895	0,162325511	0,619487156
ENSG00000128872	TMOD2	tropomodulin 2	29767	91,21694983	0,134656202	0,218483	0,616324695	0,537680243	0,874923109
ENSG00000076513	ANKRD13A	ankyrin repeat domain 13A	88455	416,648014	-0,100315794	0,092669	-1,082518795	0,279022069	0,732510365
ENSG00000212907	ND4L	NADH dehydrogenase subunit 4L	4539	2004,334932	0,235135742	0,074606	3,151704136	0,001623207	0,047927309
ENSG00000084774	CAD	carbamoyl-phosphate synthetase 2, aspartate transcarbamylase, and dihydroorotase	790	339,5874322	0,092337409	0,101114	0,913199134	0,361137819	0,791408368
ENSG00000135801	TAF5L	TATA-box binding protein associated factor 5 like	27097	173,4838289	-0,025572053	0,150775	-0,16960399	0,865321585	0,971722599
ENSG00000144579	CTDSP1	CTD small phosphatase 1	58190	322,1142098	-0,211560455	0,094892	-2,229494098	0,025781048	0,259252915
ENSG00000177565	TBL1XR1	TBL1X/Y related 1	79718	246,0461318	0,815126544	0,231083	3,527413251	0,000419641	0,018310702
ENSG00000113716	HMGXB3	HMG-box containing 3	22993	219,9897539	0,144370983	0,118017	1,223303743	0,221214994	0,677288877
ENSG00000120029	ARMH3	armadillo like helical domain containing 3	79591	154,7448256	-0,135036598	0,171946	-0,785342206	0,432252956	0,834541474
ENSG00000203747	FCGR3A	Fc gamma receptor IIIa	2214	2965,151207	-0,340753782	0,047452	-7,181075533	6,92E-13	6,47E-10
ENSG00000137486	ARRB1	arrestin beta 1	408	727,0067545	-0,040659669	0,07833	-0,519081172	0,603704137	0,897212117
ENSG00000072571	HMMR	hyaluronan mediated motility receptor	3161	90,62573615	0,505881774	0,200746	2,520008409	0,011735203	0,169426994
ENSG00000167972	ABCA3	ATP binding cassette subfamily A member 3	21	144,6392068	0,336086138	0,221249	1,51903758	0,128753037	0,563689485
ENSG00000034152	MAP2K3	mitogen-activated protein kinase kinase 3	5606	151,5475061	0,265074942	0,189596	1,398106794	0,162081	0,619487156

ENSG00000131165	CHMP1A	charged multivesicular body protein 1A	5119	180,9165445	-0,015705063	0,149503	-0,105048223	0,916337544	0,982006
ENSG00000213516	RBMXL1	RBMX like 1	494115	153,4164871	-0,182418381	0,14293	-1,276277675	0,201857379	0,660119983
ENSG00000159111	MRPL10	mitochondrial ribosomal protein L10	124995	45,56358492	0,178551218	0,331478	0,538651876	0,590127086	0,892294181
ENSG00000115053	NCL	nucleolin	4691	1832,076793	-0,160296384	0,084937	-1,887240278	0,059128015	0,393218823
ENSG00000136888	ATP6V1G1	ATPase H+ transporting V1 subunit G1	9550	221,1625488	-0,006776069	0,117466	-0,057685524	0,953999125	0,991212866
ENSG00000125485	DDX31	DEAD-box helicase 31	64794	45,92166532	0,074882086	0,291831	0,256593744	0,797492405	0,95354564
ENSG00000022567	SLC45A4	solute carrier family 45 member 4	57210	93,95549605	-0,09900174	0,178092	-0,555901399	0,578278262	0,887069818
ENSG00000160746	ANO10	anoctamin 10	55129	82,14232491	-0,188464414	0,200074	-0,941972377	0,346206782	0,782042713
ENSG00000111802	TDP2	tyrosyl-DNA phosphodiesterase 2	51567	165,0495276	-0,030647208	0,134103	-0,228535355	0,819230074	0,961067311
ENSG00000235173	HGH1	HGH1 homolog	51236	30,71241278	1,135364612	0,567712	1,999894629	0,045511643	NA
ENSG00000107036	RIC1	RIC1 homolog, RAB6A GEF complex partner 1	57589	181,4777335	0,09897079	0,127645	0,775357327	0,438128543	0,837388481
ENSG00000163510	CWC22	CWC22 spliceosome associated protein homolog	57703	116,9612193	0,266656392	0,170467	1,564274104	0,117753211	0,546915268
ENSG00000112159	MDN1	midasin AAA ATPase 1	23195	1168,081593	0,18240655	0,076752	2,376584643	0,017473752	0,206510834
ENSG00000167548	KMT2D	lysine methyltransferase 2D	8085	1966,505537	0,045877274	0,076973	0,596015226	0,551165055	0,880819693
ENSG00000181029	TRAPPC5	trafficking protein particle complex subunit 5	126003	30,94065365	-0,528373099	0,323262	-1,634502999	0,102153277	NA
ENSG00000257524	NA	NA	NA	53,74931728	-0,522092647	0,396884	-1,315478199	0,188349239	0,646436727
ENSG00000131979	GCH1	GTP cyclohydrolase 1	2643	79,05611028	0,105093153	0,421196	0,2495111	0,802965456	0,956398346
ENSG00000119906	SLF2	SMC5-SMC6 complex localization factor 2	55719	348,6128888	-0,195653794	0,144661	-1,352493929	0,17621736	0,632039836
ENSG00000213799	ZNF845	zinc finger protein 845	91664	84,94188653	0,235039681	0,193671	1,213601863	0,224899795	0,680155176
ENSG00000179562	GCC1	GRIP and coiled-coil domain containing 1	79571	117,7202852	0,003025688	0,159323	0,01899088	0,984848381	0,996185513
ENSG00000167173	C15orf39	chromosome 15 open reading frame 39	56905	694,957884	-0,04472178	0,067468	-0,662855984	0,507422795	0,864738156
ENSG00000170776	AKAP13	A-kinase anchoring protein 13	11214	2432,342762	-0,023267434	0,057754	-0,402871511	0,68704275	0,926748702
ENSG00000007264	MATK	megakaryocyte-associated tyrosine kinase	4145	1459,458895	-0,064722063	0,048342	-1,338843822	0,180621521	0,634062228
ENSG00000138073	PREB	prolactin regulatory element binding	10113	139,1130131	0,064054041	0,161006	0,397835823	0,690751209	0,927252461
ENSG00000123739	PLA2G12A	phospholipase A2 group XIIA	81579	47,74806731	-0,887185321	0,278003	-3,191278464	0,001416447	0,043903611
ENSG00000166822	TMEM170A	transmembrane protein 170A	124491	126,7547143	0,244854482	0,169728	1,442627644	0,149125392	0,603105472
ENSG00000138750	NUP54	nucleoporin 54	53371	105,4665385	0,153945681	0,183326	0,839737115	0,401055799	0,814798664
ENSG00000177888	ZBTB41	zinc finger and BTB domain containing 41	360023	87,31034861	0,081151402	0,190878	0,425148939	0,670728104	0,922641254
ENSG00000033170	FUT8	fucosyltransferase 8	2530	376,1662602	0,080283086	0,128966	0,622513444	0,533604306	0,873191491
ENSG00000165915	SLC39A13	solute carrier family 39 member 13	91252	92,81720819	-0,00209305	0,224151	-0,00933768	0,992549717	0,998500439
ENSG00000108702	CCL1	C-C motif chemokine ligand 1	6346	31,89970141	0,803194831	0,360156	2,230127678	0,025738968	NA
ENSG00000129696	TTI2	TELO2 interacting protein 2	80185	42,59723811	0,033958386	0,28129	0,120723634	0,903909939	0,978460866
ENSG00000140941	MAP1LC3B	microtubule associated protein 1 light chain 3 beta	81631	488,7294182	0,08021917	0,080997	0,990397495	0,321979869	0,766544375
ENSG00000118507	AKAP7	A-kinase anchoring protein 7	9465	34,47102631	-0,551065988	0,310066	-1,777255732	0,075526174	NA
ENSG00000136936	XPA	XPA, DNA damage recognition and repair factor	7507	49,66099136	-0,230386756	0,27887	-0,826143956	0,408722436	0,820793685
ENSG00000136942	RPL35	ribosomal protein L35	11224	656,594104	-0,006235209	0,066233	-0,094139837	0,924998075	0,984515928
ENSG00000114554	PLXNA1	plexin A1	5361	80,2885035	0,318481608	0,215279	1,479391223	0,139035784	0,580974065
ENSG00000172888	ZNF621	zinc finger protein 621	285268	303,0288485	-0,036075484	0,109649	-0,329008801	0,742149036	0,939193793
ENSG00000104472	CHRAC1	chromatin accessibility complex subunit 1	54108	102,5189488	-0,135062457	0,166395	-0,811697936	0,416964979	0,82446781
ENSG00000103168	TAF1C	TATA-box binding protein associated factor, RNA polymerase I subunit C	9013	61,21107203	0,125002918	0,437344	0,285823016	0,775013679	0,946448613
ENSG00000198162	MAN1A2	mannosidase alpha class 1A member 2	10905	462,7925423	-0,089886128	0,084963	-1,057942233	0,29008178	0,741876358
ENSG00000123240	OPTN	optineurin	10133	769,0437244	-0,058598061	0,069412	-0,844203791	0,398555536	0,814457852
ENSG00000163820	FYCO1	FYVE and coiled-coil domain autophagy adaptor 1	79443	296,0275771	0,052292877	0,140753	0,371523475	0,710247675	0,930846779
ENSG00000196182	STK40	serine/threonine kinase 40	83931	361,8632513	-0,092053588	0,089235	-1,03158476	0,302266683	0,751870158
ENSG00000080986	NDC80	NDC80 kinetochore complex component	10403	70,92672444	0,737401938	0,221921	3,322806343	0,000891168	0,032254517
ENSG00000133997	MED6	mediator complex subunit 6	10001	66,75999378	0,226165241	0,261119	0,866140146	0,386413312	0,809653368
ENSG00000136108	CKAP2	cytoskeleton associated protein 2	26586	182,6333343	0,404382707	0,143839	2,811356037	0,004933316	0,098340776
ENSG00000172932	ANKRD13D	ankyrin repeat domain 13D	338692	88,01914299	0,057911025	0,283575	0,204217497	0,838183539	0,965627959
ENSG00000100644	HIF1A	hypoxia inducible factor 1 subunit alpha	3091	731,8134875	0,137776776	0,075916	1,814869391	0,069543984	0,428367551
ENSG00000131143	COX4I1	cytochrome c oxidase subunit 4I1	1327	460,8797446	0,014959184	0,101056	0,148027964	0,882320699	0,976300778
ENSG00000130517	PGPEP1	pyroglutamyl-peptidase I	54858	329,6965653	0,036474715	0,168392	0,216606109	0,828515324	0,962723654
ENSG00000162244	RPL29	ribosomal protein L29	6159	1112,103551	-0,049458632	0,06529	-0,757525428	0,448735137	0,84263906
ENSG00000116852	KIF21B	kinesin family member 21B	23046	1039,697904	-0,216777186	0,057555	-3,766449515	0,000165586	0,009656023

ENSG00000141219	C17orf80	chromosome 17 open reading frame 80	55028	94,59122798	0,32943391	0,211924	1,554490279	0,120067514	0,553734734
ENSG00000166012	TAF1D	TATA-box binding protein associated factor, RNA polymerase I subunit D	79101	67,98349566	0,188936213	0,243718	0,775225949	0,438206157	0,837388481
ENSG00000101152	DNAJC5	DnaJ heat shock protein family (Hsp40) member C5	80331	233,2789989	0,270855248	0,178001	1,521646528	0,128097672	0,563689485
ENSG00000127445	PIN1	peptidylprolyl cis/trans isomerase, NIMA-interacting 1	5300	99,60537507	-0,229530173	0,180192	-1,273805909	0,20273221	0,660688291
ENSG00000141524	TMC6	transmembrane channel like 6	11322	661,3738445	-0,283764003	0,112729	-2,517219894	0,011828498	0,170148395
ENSG00000180628	PCGF5	polycomb group ring finger 5	84333	439,9334089	0,007192052	0,089686	0,080191858	0,936084665	0,987098279
ENSG00000147416	ATP6V1B2	ATPase H+ transporting V1 subunit B2	526	181,5619389	0,177678773	0,143484	1,238314731	0,215599384	0,672218166
ENSG00000116786	PLEKHM2	pleckstrin homology and RUN domain containing M2	23207	222,0675824	-0,191385574	0,117765	-1,625145326	0,104131597	0,517954124
ENSG00000179912	R3HDM2	R3H domain containing 2	22864	306,9512546	-0,230519757	0,101877	-2,262721511	0,023652862	0,245726955
ENSG00000189067	LITAF	lipopolysaccharide induced TNF factor	9516	338,393191	-0,121914702	0,157006	-0,776498347	0,437454796	0,837249449
ENSG00000147789	ZNF7	zinc finger protein 7	7553	73,54383312	0,080135791	0,217831	0,367880923	0,712962026	0,930846779
ENSG00000128245	YWHAH	tyrosine 3-monooxygenase/tryptophan 5-monooxygenase activation protein eta	7533	255,5762329	0,229723147	0,109422	2,099431616	0,03577887	0,300864289
ENSG00000135919	SERPINE2	serpin family E member 2	5270	132,0469639	-0,171885401	0,16318	-1,053350432	0,292180413	0,74421837
ENSG00000161217	PCYT1A	phosphate cytidylyltransferase 1A, choline	5130	323,6817806	0,065803178	0,09641	0,682536875	0,494899534	0,860513824
ENSG00000056558	TRAF1	TNF receptor associated factor 1	7185	236,5463276	0,12423617	0,114983	1,080477617	0,279929549	0,733157579
ENSG00000134001	EIF2S1	eukaryotic translation initiation factor 2 subunit alpha	1965	238,778965	-0,030671161	0,125272	-0,244837267	0,806582437	0,958091116
ENSG00000186666	BCDIN3D	BCDIN3 domain containing RNA methyltransferase	144233	28,40624253	-0,055558036	0,384681	-0,144426127	0,885163987	NA
ENSG00000197323	TRIM33	tripartite motif containing 33	51592	474,2624254	-0,278082858	0,097561	-2,850355707	0,004367036	0,091463202
ENSG00000135945	REV1	REV1 DNA directed polymerase	51455	108,7015338	-0,003673486	0,199072	-0,018453031	0,985277447	0,996185513
ENSG00000048740	CELF2	CUGBP Elav-like family member 2	10659	1709,301564	0,072269853	0,055511	1,301893861	0,192952671	0,650901454
ENSG00000128595	CALU	calumenin	813	293,6065852	-0,129799401	0,10905	-1,190274967	0,233938337	0,688858646
ENSG00000156299	TIAM1	TIAM Rac1 associated GEF 1	7074	1811,955021	-0,150145533	0,046711	-3,214379088	0,00130727	0,041400384
ENSG00000187801	ZFP69B	ZFP69 zinc finger protein B	65243	16,96947539	-0,369747082	0,511041	-0,723517409	0,469362062	NA
ENSG00000134508	CABLES1	Cdk5 and Abl enzyme substrate 1	91768	55,17489359	-0,323269632	0,219541	-1,472477651	0,140891938	0,584590139
ENSG00000213523	SRA1	steroid receptor RNA activator 1	10011	58,87695011	-0,383386291	0,213765	-1,793492599	0,072894193	0,438705741
ENSG00000117228	GBP1	guanylate binding protein 1	2633	214,2572304	0,304242619	0,221694	1,372354354	0,169953153	0,629558603
ENSG00000123200	ZC3H13	zinc finger CCCH-type containing 13	23091	682,2749288	-0,052250338	0,078719	-0,66375388	0,506847849	0,864738156
ENSG00000171169	NAIF1	nuclear apoptosis inducing factor 1	203245	57,90980131	-0,232069643	0,226284	-1,025570149	0,305094244	0,751870158
ENSG00000215251	FASTKD5	FAST kinase domains 5	60493	100,8881723	0,14888999	0,162468	0,9164241	0,3594445	0,790999468
ENSG00000135446	CDK4	cyclin dependent kinase 4	1019	67,87266238	0,268987838	0,252517	1,065227232	0,286773113	0,738005299
ENSG00000162441	LZIC	leucine zipper and CTNNBIP1 domain containing	84328	145,5279308	0,156215671	0,155244	1,006258536	0,314291221	0,759554708
ENSG00000134602	STK26	serine/threonine kinase 26	51765	286,2938992	-0,206595032	0,104477	-1,977423785	0,047993748	0,350971037
ENSG00000101574	METTL4	methyltransferase 4, N6-adenosine	64863	84,29045675	0,235048191	0,20405	1,151916619	0,249355338	0,707335884
ENSG00000132341	RAN	RAN, member RAS oncogene family	5901	601,0204005	0,33070508	0,081607	4,052429784	5,07E-05	0,003752175
ENSG00000121542	SEC22A	SEC22 homolog A, vesicle trafficking protein	26984	44,35390716	0,480803096	0,318712	1,508582306	0,131405559	0,567689361
ENSG00000115694	STK25	serine/threonine kinase 25	10494	165,8401754	-0,031664807	0,137979	-0,229490225	0,818487917	0,961041127
ENSG00000136240	KDELR2	KDEL endoplasmic reticulum protein retention receptor 2	11014	104,0254881	0,645288256	0,221228	2,91684136	0,003535956	0,0795742
ENSG00000089685	BIRC5	baculoviral IAP repeat containing 5	332	70,03956522	0,526933775	0,221126	2,382951979	0,017174435	0,204685901
ENSG00000105808	RASA4	RAS p21 protein activator 4	10156	16,28980518	-0,330945234	0,485016	-0,682338435	0,495024975	NA
ENSG00000131871	SELENOS	selenoprotein S	55829	277,8338499	0,111614214	0,115198	0,968892589	0,332598783	0,773425407
ENSG00000139722	VPS37B	VPS37B subunit of ESCRT-I	79720	246,5938363	-0,008580886	0,103484	-0,082920186	0,933915003	0,987098279
ENSG00000142102	PGGHG	protein-glucosylgalactosylhydroxylysine glucosidase	80162	431,3442417	-0,250578555	0,113888	-2,200219613	0,027791317	0,269631574
ENSG00000164151	ICE1	interactor of little elongation complex ELL subunit 1	23379	578,3455287	-0,017176551	0,074035	-0,23200691	0,816532647	0,959707099
ENSG00000166794	PPIB	peptidylprolyl isomerase B	5479	817,1130286	0,018804949	0,068565	0,27426429	0,783881525	0,950093441
ENSG00000127603	MACF1	microtubule actin crosslinking factor 1	23499	2400,979948	0,032215727	0,268333	0,120058914	0,904436479	0,978460866

ENSG00000120784	ZFP30	ZFP30 zinc finger protein	22835	64,7786688	0,131811116	0,335546	0,392826064	0,694447953	0,92884779
ENSG00000141429	GALNT1	polypeptide N-acetylgalactosaminyltransferase 1	2589	303,0090454	-0,089321547	0,12354	-0,723015314	0,469670476	0,852363442
ENSG00000182606	TRAK1	trafficking kinesin protein 1	22906	196,84968	0,048110362	0,116989	0,411237535	0,680898368	0,925586358
ENSG00000198727	CYTB	cytochrome b	4519	17575,95662	0,133545751	0,103028	1,29621173	0,194902549	0,653056579
ENSG00000110925	CSRNP2	cysteine and serine rich nuclear protein 2	81566	94,93781411	0,057675543	0,225236	0,256066605	0,797899408	0,95354564
ENSG00000197471	SPN	sialophorin	6693	2895,308425	-0,216344681	0,04744	-4,560405966	5,11E-06	0,000591357
ENSG00000108733	PEX12	peroxisomal biogenesis factor 12	5193	16,12652841	0,44290997	0,490369	0,903217968	0,366410223	NA
ENSG00000048392	RRM2B	ribonucleotide reductase regulatory TP53 inducible subunit M2B	50484	190,8105788	0,248072279	0,134584	1,843250375	0,065292463	0,412224279
ENSG00000090339	ICAM1	intercellular adhesion molecule 1	3383	183,4636534	-0,129055081	0,124066	-1,0402107	0,298242021	0,748846814
ENSG00000082213	C5orf22	chromosome 5 open reading frame 22	55322	82,68623717	0,45717491	0,322223	1,418814164	0,155953202	0,61428162
ENSG00000113621	TXNDC15	thioredoxin domain containing 15	79770	111,6297653	0,083874244	0,167285	0,501385686	0,616099711	0,902090873
ENSG00000153487	ING1	inhibitor of growth family member 1	3621	105,2738216	-0,11455571	0,226295	-0,506223294	0,612699904	0,900033105
ENSG00000204120	GIGYF2	GRB10 interacting GYF protein 2	26058	429,2757727	0,260600157	0,124422	2,094486795	0,036216638	0,302116994
ENSG00000053254	FOXN3	forkhead box N3	1112	1563,136053	0,044268834	0,064614	0,685130394	0,493261643	0,860513824
ENSG00000134321	RSAD2	radical S-adenosyl methionine domain containing 2	91543	123,1588487	0,334766819	0,218163	1,534479162	0,124911816	0,5624706
ENSG00000185236	RAB11B	RAB11B, member RAS oncogene family	9230	303,1064856	-0,179270275	0,115401	-1,553457833	0,120313796	0,554219678
ENSG00000166974	MAPRE2	microtubule associated protein RP/EB family member 2	10982	359,592076	-0,124096863	0,093411	-1,328501293	0,184012557	0,640457926
ENSG00000117226	GBP3	guanylate binding protein 3	2635	104,4515127	-0,074100302	0,212289	-0,349053598	0,727049072	0,936626457
ENSG00000159147	DONSON	DNA replication fork stabilization factor DONSON	29980	39,42337943	0,535572446	0,293948	1,82199902	0,068455134	NA
ENSG00000166913	YWHAB	tyrosine 3-monooxygenase/tryptophan 5-monooxygenase activation protein beta	7529	1596,369768	-0,111697278	0,044	-2,538546712	0,011131394	0,164848163
ENSG00000162368	CMPK1	cytidine/uridine monophosphate kinase 1	51727	477,924717	-0,131800214	0,08389	-1,571111462	0,116156761	0,543356283
ENSG00000108506	INTS2	integrator complex subunit 2	57508	118,4397042	-0,218457552	0,17276	-1,264514178	0,206045535	0,660954946
ENSG00000241839	PLEKHO2	pleckstrin homology domain containing O2	80301	171,267291	-0,150049943	0,150885	-0,994466869	0,319995627	0,764369117
ENSG00000073584	SMARCE1	SWI/SNF related, matrix associated, actin dependent regulator of chromatin, subfamily e, member 1	6605	648,3690913	-0,247897915	0,093356	-2,655417562	0,007921029	0,132558158
ENSG00000180008	SOCS4	suppressor of cytokine signaling 4	122809	233,2798622	-0,021660764	0,127931	-0,169316332	0,86554783	0,971722599
ENSG00000211829	NA	NA	NA	1507,107877	-0,042972233	0,050415	-0,852363245	0,394012514	0,812498986
ENSG00000156504	PABIR2	PABIR family member 2	159090	321,7608532	-0,051376885	0,102586	-0,500819317	0,616498289	0,902090873
ENSG00000175216	CKAP5	cytoskeleton associated protein 5	9793	565,7155971	0,230725089	0,09341	2,470022663	0,013510449	0,18084297
ENSG00000188846	RPL14	ribosomal protein L14	9045	118,0594479	-0,167382246	0,213366	-0,784485082	0,432755532	0,834691048
ENSG00000079616	KIF22	kinesin family member 22	3835	83,32391248	0,328325367	0,42101	0,77985112	0,435478513	0,836566451
ENSG00000103111	MON1B	MON1 homolog B, secretory trafficking associated	22879	361,3489306	0,01511721	0,118904	0,127138028	0,898831154	0,977990819
ENSG00000111731	C2CD5	C2 calcium dependent domain containing 5	9847	252,9878643	0,043224489	0,118905	0,363520063	0,716216429	0,932040329
ENSG00000124172	ATP5F1E	ATP synthase F1 subunit epsilon	514	375,4630852	0,172871453	0,196544	0,879557743	0,379098939	0,804903799
ENSG00000137265	IRF4	interferon regulatory factor 4	3662	56,9660548	1,119903786	0,301301	3,716895947	0,000201685	0,010849572
ENSG00000140153	WDR20	WD repeat domain 20	91833	93,32912087	0,040898796	0,208289	0,196356203	0,844331374	0,967231052
ENSG00000157450	RNF111	ring finger protein 111	54778	165,5963397	0,08029043	0,134307	0,597812013	0,549965374	0,88072908
ENSG00000162702	ZNF281	zinc finger protein 281	23528	111,9781115	0,205615348	0,172859	1,189496909	0,234244186	0,689304549
ENSG00000164985	PSIP1	PC4 and SRSF1 interacting protein 1	11168	452,5000417	-0,042973674	0,081126	-0,529714821	0,59630967	0,894229873
ENSG00000165891	E2F7	E2F transcription factor 7	144455	57,38451756	0,638309538	0,251197	2,54106793	0,011051445	0,164079481
ENSG00000172167	MTBP	MDM2 binding protein	27085	26,13748407	0,599240459	0,408142	1,468216672	0,142045377	NA
ENSG00000181610	MRPS23	mitochondrial ribosomal protein S23	51649	93,44644845	-0,111352038	0,222639	-0,50014517	0,616972863	0,90216458
ENSG00000106682	EIF4H	eukaryotic translation initiation factor 4H	7458	913,891012	-0,080580761	0,057933	-1,390937741	0,164244307	0,623778911
ENSG00000162889	MAPKAPK2	MAPK activated protein kinase 2	9261	734,4915178	0,030256089	0,061733	0,49011151	0,624054994	0,90479929
ENSG00000185619	PCGF3	polycomb group ring finger 3	10336	253,8449337	-0,134244914	0,147799	-0,908296711	0,36372148	0,792858313
ENSG00000197608	ZNF841	zinc finger protein 841	284371	287,6461984	0,027262667	0,115417	0,236209372	0,813270208	0,959207071
ENSG00000183735	TBK1	TANK binding kinase 1	29110	175,2095651	0,077971237	0,151438	0,514871941	0,606642509	0,897481254

ENSG00000189241	TSPYL1	TSPY like 1	7259	269,9532777	-0,14957377	0,107812	-1,387356137	0,165333193	0,624454568
ENSG00000183354	KIAA2026	KIAA2026	158358	458,544483	0,005689881	0,101076	0,056293008	0,955108388	0,991420853
ENSG00000288825	H2AC18	H2A clustered histone 18	8337	43,05664571	0,394953986	0,297119	1,329279682	0,183755715	0,64000771
ENSG00000142676	RPL11	ribosomal protein L11	6135	865,1043811	0,158322867	0,069523	2,277286568	0,022769119	0,240361098
ENSG00000174238	PITPNA	phosphatidylinositol transfer protein alpha	5306	336,2108101	-0,064199356	0,095562	-0,671811932	0,501703432	0,862227298
ENSG00000081307	UBA5	ubiquitin like modifier activating enzyme 5	79876	106,7560484	-0,29764892	0,246087	-1,209526134	0,226460777	0,681621061
ENSG00000240344	PPIL3	peptidylprolyl isomerase like 3	53938	59,58595447	0,840211379	0,250445	3,354872708	0,000794015	0,029416013
ENSG00000113163	CERT1	ceramide transporter 1	10087	196,7438959	-0,046058317	0,149461	-0,308163601	0,757957847	0,942901768
ENSG00000179295	PTPN11	protein tyrosine phosphatase non-receptor type 11	5781	571,1232668	-0,019201981	0,086598	-0,221737303	0,82451839	0,961917534
ENSG00000068878	PSME4	proteasome activator subunit 4	23198	642,7380032	-0,067257732	0,073628	-0,9134841	0,360987992	0,791408368
ENSG00000167394	ZNF668	zinc finger protein 668	79759	93,01216983	0,195856573	0,17895	1,094476837	0,273745901	0,726913977
ENSG00000171045	TSNARE1	t-SNARE domain containing 1	203062	45,1779403	-0,304850067	0,243721	-1,250817966	0,211000899	0,6647417
ENSG00000033011	ALG1	ALG1 chitobiosyldiphosphodolichol beta-mannosyltransferase	56052	65,75803792	0,118074148	0,229603	0,514254242	0,607074249	0,897481254
ENSG00000177225	GATD1	glutamine amidotransferase class 1 domain containing 1	347862	144,0192295	-0,098850683	0,178245	-0,554576101	0,579184639	0,887537774
ENSG00000160691	SHC1	SHC adaptor protein 1	6464	253,5913079	-0,263108582	0,126554	-2,07902186	0,037615341	0,307740511
ENSG00000089060	SLC8B1	solute carrier family 8 member B1	80024	93,24976661	0,18891822	0,212928	0,887239518	0,374949961	0,801976304
ENSG00000171130	ATP6V0E2	ATPase H+ transporting V0 subunit e2	155066	306,5252614	-0,132422414	0,110824	-1,194891169	0,232129554	0,687201475
ENSG00000100991	TRPC4AP	transient receptor potential cation channel subfamily C member 4 associated protein	26133	390,3961408	-0,053355253	0,086727	-0,61520742	0,538417748	0,87533285
ENSG00000147162	OGT	O-linked N-acetylglucosamine (GlcNAc) transferase	8473	1265,541043	-0,145308301	0,11648	-1,24749346	0,212216617	0,666699723
ENSG00000104408	EIF3E	eukaryotic translation initiation factor 3 subunit E	3646	486,6985277	0,060271885	0,157921	0,381659356	0,702714051	0,930846779
ENSG00000136250	AOAH	acyloxyacyl hydrolase	313	1187,370564	-0,005142995	0,058729	-0,087571186	0,930217505	0,986487751
ENSG00000100401	RANGAP1	Ran GTPase activating protein 1	5905	273,6733494	0,180326386	0,112567	1,601942491	0,109168327	0,529264223
ENSG00000152256	PDK1	pyruvate dehydrogenase kinase 1	5163	146,1914843	-0,332534779	0,200246	-1,660635452	0,096786684	0,503085783
ENSG00000213918	DNASE1	deoxyribonuclease 1	1773	279,129631	-0,307213757	0,110775	-2,773306448	0,005548984	0,10681794
ENSG00000117399	CDC20	cell division cycle 20	991	77,17603089	0,857127618	0,219376	3,907111589	9,34E-05	0,006164797
ENSG00000169129	AFAP1L2	actin filament associated protein 1 like 2	84632	1506,09747	0,221352758	0,059388	3,727234228	0,000193593	0,01048604
ENSG00000172216	CEBPB	CCAAT enhancer binding protein beta	1051	108,5536233	0,179399972	0,163729	1,095710306	0,273205571	0,726913977
ENSG00000229117	RPL41	ribosomal protein L41	6171	2451,701074	-0,01584419	0,060006	-0,264044194	0,791745865	0,951719761
ENSG00000133119	RFC3	replication factor C subunit 3	5983	40,82806615	0,293125536	0,275563	1,063733815	0,287449301	NA
ENSG00000084463	WBP11	WW domain binding protein 11	51729	395,4049135	-0,058698686	0,109038	-0,538330508	0,590348893	0,892294181
ENSG00000169905	TOR1AIP2	torsin 1A interacting protein 2	163590	193,7538571	-0,094520827	0,158075	-0,597947809	0,549874758	0,88072908
ENSG00000186185	KIF18B	kinesin family member 18B	146909	79,70210307	0,182490407	0,197397	0,924483971	0,355234393	0,788341988
ENSG00000182199	SHMT2	serine hydroxymethyltransferase 2	6472	456,7537694	0,236240875	0,09211	2,564765558	0,010324554	0,157711213
ENSG00000084092	NOA1	nitric oxide associated 1	84273	102,3683337	0,270261083	0,172409	1,567554614	0,116985111	0,545494102
ENSG00000113838	TBCCD1	TBCC domain containing 1	55171	77,5681876	0,069185338	0,198871	0,347891373	0,727921764	0,937382774
ENSG00000137145	DENND4C	DENN domain containing 4C	55667	476,7997083	-0,104867597	0,093082	-1,126610551	0,259907176	0,715747182
ENSG00000151414	NEK7	NIMA related kinase 7	140609	367,4832415	-0,030369821	0,09658	-0,314451765	0,753177945	0,942402355
ENSG00000198912	C1orf174	chromosome 1 open reading frame 174	339448	40,28494223	-0,005095968	0,279572	-0,018227777	0,985457143	NA
ENSG00000214253	FIS1	fission, mitochondrial 1	51024	137,93373	-0,261730526	0,183819	-1,423847208	0,154490707	0,611886036
ENSG00000119401	TRIM32	tripartite motif containing 32	22954	45,24929592	0,113752328	0,271356	0,419199474	0,675070356	0,923693829
ENSG00000167996	FTH1	ferritin heavy chain 1	2495	889,8535448	0,069952845	0,0801	0,87331796	0,382489794	0,807686858
ENSG00000113141	IK	IK cytokine	3550	410,1468016	-0,004055958	0,094056	-0,043122804	0,965603642	0,992789539
ENSG00000173110	HSPA6	heat shock protein family A (Hsp70) member 6	3310	93,76259932	0,008400374	0,172163	0,048793002	0,961084259	0,992235217
ENSG00000102409	BEX4	brain expressed X-linked 4	56271	57,5405083	-0,305201009	0,220014	-1,38718668	0,165384846	0,624454568
ENSG00000103365	GGA2	golgi associated, gamma adaptin ear containing, ARF binding protein 2	23062	342,7248865	-0,01054077	0,086977	-0,1211907	0,90353999	0,978460866
ENSG00000197057	DTHD1	death domain containing 1	401124	20,19594084	-0,757877062	0,472815	-1,602902898	0,108956094	NA
ENSG00000162073	PAQR4	progestin and adipoQ receptor family member 4	124222	60,3110043	0,281120335	0,249883	1,125009213	0,26058513	0,716404964
ENSG00000100949	RABGGTA	Rab geranylgeranyltransferase subunit alpha	5875	30,14702626	-0,012691857	0,381014	-0,033310747	0,973426784	NA
ENSG00000134897	BIVM	basic, immunoglobulin-like variable motif containing	54841	363,0375578	-0,078916607	0,106391	-0,741757971	0,458233989	0,845558274

ENSG00000166311	SMPD1	sphingomyelin phosphodiesterase 1	6609	135,0483018	-0,290251793	0,161171	-1,80088844	0,071720465	0,435172111
ENSG00000197147	LRRC8B	leucine rich repeat containing 8 VRAC subunit B	23507	49,55264307	0,200377205	0,27147	0,738119149	0,460442048	0,846622987
ENSG00000259075	POC1B-GALNT4	POC1B-GALNT4 readthrough	100528030	39,49821824	-0,295173642	0,638242	-0,462478867	0,643737954	NA
ENSG00000145020	AMT	aminomethyltransferase	275	22,13011867	0,455256429	0,589686	0,772032592	0,440095124	NA
ENSG00000154832	CXXC1	CXXC finger protein 1	30827	186,1303524	-0,221952289	0,166007	-1,337002029	0,181221975	0,634628658
ENSG00000180938	ZNF572	zinc finger protein 572	137209	13,1265117	0,522214257	0,550508	0,948604153	0,342821978	NA
ENSG00000185621	LMLN	leishmanolysin like peptidase	89782	17,93808156	0,702142689	0,483872	1,451092655	0,146754062	NA
ENSG00000097007	ABL1	ABL proto-oncogene 1, non-receptor tyrosine kinase	25	210,2573765	-0,055499215	0,113537	-0,488818634	0,624970104	0,90479929
ENSG00000104228	TRIM35	tripartite motif containing 35	23087	209,6101652	-0,123739171	0,11685	-1,058960413	0,289617809	0,741447348
ENSG00000120742	SERP1	stress associated endoplasmic reticulum protein 1	27230	390,8652546	-0,095954172	0,117183	-0,818837735	0,412878998	0,82225544
ENSG00000127314	RAP1B	RAP1B, member of RAS oncogene family	5908	1133,557754	-0,045343439	0,058236	-0,778613353	0,436207511	0,836566451
ENSG00000142347	MYO1F	myosin IF	4542	1051,93267	-0,108933131	0,065896	-1,653110642	0,098308348	0,507302079
ENSG00000157625	TAB3	TGF-beta activated kinase 1 (MAP3K7) binding protein 3	257397	192,8261581	-0,049039944	0,139436	-0,351701888	0,725061845	0,935847464
ENSG00000166037	CEP57	centrosomal protein 57	9702	207,0157182	0,217576427	0,119453	1,821445276	0,068539198	0,425202892
ENSG00000170515	PA2G4	proliferation-associated 2G4	5036	624,8198556	-0,013851627	0,088013	-0,157381977	0,874943817	0,975139597
ENSG00000112078	KCTD20	potassium channel tetramerization domain containing 20	222658	551,5645915	0,113967313	0,072033	1,582148606	0,11361565	0,539502607
ENSG00000243302	NA	NA	NA	101,0713113	-0,023338765	0,182821	-0,127659344	0,898418566	0,977990819
ENSG00000072518	MARK2	microtubule affinity regulating kinase 2	2011	422,3444864	-0,182665607	0,115211	-1,585484893	0,112856207	0,537847479
ENSG00000185896	LAMP1	lysosomal associated membrane protein 1	3916	926,5849885	-0,091496646	0,07275	-1,257689152	0,208504197	0,662366797
ENSG00000170379	TCAF2	TRPM8 channel associated factor 2	285966	48,54140399	0,100047859	0,261653	0,382369145	0,702187573	0,930846779
ENSG00000087365	SF3B2	splicing factor 3b subunit 2	10992	737,550563	-0,001707791	0,088475	-0,019302457	0,984599824	0,996185513
ENSG00000188725	SMIM15	small integral membrane protein 15	643155	166,4909308	0,229884679	0,210416	1,092526643	0,274601686	0,727393472
ENSG00000184588	PDE4B	phosphodiesterase 4B	5142	53,05345081	0,178298068	0,236049	0,75534267	0,450043402	0,84263906
ENSG00000136404	TM6SF1	transmembrane 6 superfamily member 1	53346	85,38213698	0,118022279	0,210495	0,560689573	0,575009177	0,887069818
ENSG00000127526	SLC35E1	solute carrier family 35 member E1	79939	360,5935697	0,055924621	0,141334	0,395691571	0,692332575	0,927787546
ENSG00000135269	TES	testin LIM domain protein	26136	542,3244116	-0,288973457	0,090637	-3,188239194	0,001431421	0,043915541
ENSG00000128590	DNAJB9	DnaJ heat shock protein family (Hsp40) member B9	4189	128,4515774	-0,430034139	0,172939	-2,48662012	0,012896302	0,177810208
ENSG00000135837	CEP350	centrosomal protein 350	9857	590,8396536	0,029274205	0,10241	0,28585405	0,774989909	0,946448613
ENSG00000083123	BCKDHB	branched chain keto acid dehydrogenase E1 subunit beta	594	53,71052254	-0,386874425	0,257818	-1,500574162	0,133465738	0,572495854
ENSG00000077984	CST7	cystatin F	8530	473,901938	-0,16081293	0,085188	-1,887750459	0,059059459	0,39312401
ENSG00000100461	RBM23	RNA binding motif protein 23	55147	346,0656404	0,186417482	0,104947	1,776295881	0,075684162	0,446229554
ENSG00000166860	ZBTB39	zinc finger and BTB domain containing 39	9880	92,59750743	0,227683363	0,195455	1,164890479	0,244063328	0,700945995
ENSG00000075975	MKRN2	makorin ring finger protein 2	23609	110,3781204	0,295626531	0,176807	1,672032062	0,094518	0,498337741
ENSG00000150527	MIA2	MIA SH3 domain ER export factor 2	4253	363,8181583	-0,103527108	0,093562	-1,10651181	0,268505055	0,723940509
ENSG00000119682	AREL1	apoptosis resistant E3 ubiquitin protein ligase 1	9870	245,7423773	0,188984364	0,129957	1,454201523	0,145890443	0,593998723
ENSG00000100722	ZC3H14	zinc finger CCCH-type containing 14	79882	126,1485329	-0,390154482	0,172588	-2,260608569	0,023783507	0,246757809
ENSG00000059728	MXD1	MAX dimerization protein 1	4084	138,4360388	-0,194364368	0,146413	-1,327508477	0,184340538	0,640907739
ENSG00000152642	GPD1L	glycerol-3-phosphate dehydrogenase 1 like	23171	246,7982532	-0,079128471	0,126209	-0,626962042	0,530684117	0,872577406
ENSG00000031003	FAM13B	family with sequence similarity 13 member B	51306	557,5643808	-0,137681454	0,081293	-1,693651443	0,090331537	0,489623111
ENSG00000079999	KEAP1	kelch like ECH associated protein 1	9817	158,1152941	-0,032017	0,13488	-0,237373343	0,812367168	0,959038743
ENSG00000130402	ACTN4	actinin alpha 4	81	902,3096142	-0,096845755	0,127724	-0,758243878	0,448304998	0,84263906
ENSG00000147536	GINS4	GINS complex subunit 4	84296	39,16402894	0,831290853	0,295749	2,810798858	0,004941867	NA
ENSG00000149091	DGKZ	diacylglycerol kinase zeta	8525	997,6768518	-0,066597378	0,057113	-1,166066613	0,243587514	0,700525938
ENSG00000166508	MCM7	minichromosome maintenance complex component 7	4176	300,0142205	0,187919173	0,104416	1,799718865	0,071905041	0,435172111
ENSG00000169410	PTPN9	protein tyrosine phosphatase non-receptor type 9	5780	288,861406	-0,031765454	0,098284	-0,323201009	0,746543017	0,939843468
ENSG00000172053	QARS1	glutaminyl-tRNA synthetase 1	5859	350,6896924	-0,053887109	0,096812	-0,556617161	0,577789026	0,887069818
ENSG00000118985	ELL2	elongation factor for RNA polymerase II 2	22936	268,3386736	-0,179772629	0,105777	-1,699544251	0,089216684	0,488176025

ENSG00000188486	H2AX	H2A,X variant histone	3014	113,4197931	0,313169427	0,231501	1,352780026	0,176125916	0,632039836
ENSG00000094804	CDC6	cell division cycle 6	990	47,69221562	0,815622851	0,378099	2,157167048	0,030992651	0,281657534
ENSG00000178425	NT5DC1	5'-nucleotidase domain containing 1	221294	64,76385902	-0,044528837	0,208227	-0,213847255	0,830666173	0,963156386
ENSG00000159352	PSMD4	proteasome 26S subunit ubiquitin receptor, non-ATPase 4	5710	275,5438675	-0,081537339	0,10094	-0,807782474	0,419215816	0,825672195
ENSG00000100485	SOS2	SOS Ras/Rho guanine nucleotide exchange factor 2	6655	377,8554083	-0,182511784	0,092794	-1,966855855	0,049199831	0,355815353
ENSG00000175463	TBC1D10C	TBC1 domain family member 10C	374403	350,429435	0,055366128	0,104573	0,52944784	0,596494819	0,894229873
ENSG00000110906	KCTD10	potassium channel tetramerization domain containing 10	83892	198,8579823	-0,367772018	0,123955	-2,966987775	0,003007329	0,071791986
ENSG00000086102	NFX1	nuclear transcription factor, X-box binding 1	4799	260,0418411	0,062658935	0,123337	0,508028813	0,611433137	0,900006614
ENSG00000060491	OGFR	opioid growth factor receptor	11054	292,2407083	-0,274047921	0,098495	-2,782358433	0,00539654	0,10491195
ENSG00000180758	GPR157	G protein-coupled receptor 157	80045	89,74475928	0,456607628	0,207461	2,200931519	0,027740872	0,269631574
ENSG00000099194	SCD	stearoyl-CoA desaturase	6319	180,7897659	-0,247474986	0,131862	-1,876766559	0,060550096	0,398308113
ENSG00000168487	BMP1	bone morphogenetic protein 1	649	42,46498156	0,22675543	0,323381	0,701202594	0,483176591	0,856436233
ENSG00000143575	HAX1	HCLS1 associated protein X-1	10456	113,7791327	0,266064645	0,174271	1,526726289	0,126829088	0,563689485
ENSG00000099949	LZTR1	leucine zipper like post translational regulator 1	8216	188,4264944	-0,059931848	0,13549	-0,442334463	0,658247196	0,919088619
ENSG00000105355	PLIN3	perilipin 3	10226	96,08582463	-0,198263652	0,172613	-1,148599244	0,250721269	0,707335884
ENSG00000107282	APBA1	amyloid beta precursor protein binding family A member 1	320	327,9778276	0,251133888	0,103113	2,43551724	0,014870516	0,1911648
ENSG00000166398	GARRE1	granule associated Rac and RHOG effector 1	9710	180,1631025	-0,096272609	0,134525	-0,71564919	0,474207992	0,853811879
ENSG00000113552	GNPDA1	glucosamine-6-phosphate deaminase 1	10007	77,97789381	0,278091423	0,196417	1,415823542	0,156827175	0,61428162
ENSG00000146247	PHIP	pleckstrin homology domain interacting protein	55023	575,9900381	-0,159169585	0,074003	-2,150847035	0,031488275	0,282669444
ENSG00000171953	ATPAF2	ATP synthase mitochondrial F1 complex assembly factor 2	91647	22,26622759	-0,555593302	0,561758	-0,989026923	0,32264997	NA
ENSG00000172893	DHCR7	7-dehydrocholesterol reductase	1717	109,3445083	0,158028558	0,178474	0,885440629	0,375919027	0,803373958
ENSG00000174485	DENND4A	DENN domain containing 4A	10260	300,3476005	0,149184792	0,117529	1,269339483	0,20432001	0,660954946
ENSG00000051523	CYBA	cytochrome b-245 alpha chain	1535	509,9877434	-0,229950467	0,073363	-3,134417662	0,001721956	0,05038513
ENSG00000120992	LYPLA1	lysophospholipase 1	10434	222,4365516	-0,159130693	0,121155	-1,313445749	0,18903279	0,6473259
ENSG00000165943	MOAP1	modulator of apoptosis 1	64112	187,87143	-0,267885227	0,17384	-1,54098973	0,123319286	0,559349633
ENSG00000196150	ZNF250	zinc finger protein 250	58500	25,09572645	0,71102351	0,428332	1,659983767	0,096917718	NA
ENSG00000196782	MAML3	mastermind like transcriptional coactivator 3	55534	238,3825164	0,001611301	0,113383	0,014211077	0,988661582	0,997032569
ENSG00000065526	SPEN	spen family transcriptional repressor	23013	775,2678217	0,008463133	0,101431	0,083437183	0,933503924	0,987098279
ENSG00000155111	CDK19	cyclin dependent kinase 19	23097	392,0015612	-0,118421667	0,087641	-1,351216781	0,176626001	0,632039836
ENSG00000135114	OASL	2'-5'-oligoadenylate synthetase like	8638	115,7266137	0,309584636	0,173571	1,783617892	0,074485776	0,443526374
ENSG00000119383	PTPA	protein phosphatase 2 phosphatase activator	5524	358,7973908	-0,276674471	0,127709	-2,166449578	0,030276849	0,279077601
ENSG00000153922	CHD1	chromodomain helicase DNA binding protein 1	1105	137,9059242	0,078826229	0,314957	0,250276471	0,802373551	0,956331071
ENSG00000018510	AGPS	alkylglycerone phosphate synthase	8540	199,1637119	0,016456559	0,368872	0,044613191	0,964415628	0,992728748
ENSG00000165410	CFL2	cofilin 2	1073	44,09131312	0,151327753	0,347522	0,435447792	0,663237427	0,920978907
ENSG00000125952	MAX	MYC associated factor X	4149	257,4285666	-0,002557447	0,178172	-0,014353816	0,988547705	0,997032569
ENSG00000138964	PARVG	parvin gamma	64098	236,1438021	-0,140753273	0,107736	-1,306466627	0,191393921	0,649525167
ENSG00000175567	UCP2	uncoupling protein 2	7351	398,5280454	0,315125753	0,101004	3,119946014	0,001808842	0,051326425
ENSG00000120685	PROSER1	proline and serine rich 1	80209	340,0484409	-0,170466874	0,098822	-1,724994863	0,084528398	0,474371941
ENSG00000132256	TRIM5	tripartite motif containing 5	85363	113,865321	0,104606268	0,177455	0,589479732	0,555539504	0,882854594
ENSG00000147677	EIF3H	eukaryotic translation initiation factor 3 subunit H	8667	514,807784	-0,058886351	0,078804	-0,747250686	0,454912254	0,84325722
ENSG00000274750	H3C6	H3 clustered histone 6	8353	16,25055933	0,280226212	0,497643	0,563107038	0,573361999	NA
ENSG00000116062	MSH6	mutS homolog 6	2956	164,1060596	0,33952775	0,182062	1,864901031	0,062195278	0,403703895
ENSG00000126067	PSMB2	proteasome 20S subunit beta 2	5690	226,9265729	-0,058034145	0,121518	-0,477575852	0,632952122	0,907570154
ENSG00000147471	PLPBP	pyridoxal phosphate binding protein	11212	114,8335079	0,024465101	0,167436	0,146116447	0,883829463	0,976751663
ENSG00000274180	NATD1	N-acetyltransferase domain containing 1	256302	64,07956314	-0,012934947	0,24429	-0,052949123	0,957772445	0,991911599
ENSG00000105136	ZNF419	zinc finger protein 419	79744	30,00506636	-0,816384588	0,409153	-1,995304009	0,046009733	NA
ENSG00000258230	NA	NA	NA	15,54914593	-0,302961681	0,47004	-0,6445438	0,519222865	NA
ENSG00000163453	IGFBP7	insulin like growth factor binding protein 7	3490	46,74401929	-0,19412732	0,262383	-0,739861772	0,459383873	0,846574514

ENSG00000124151	NCOA3	nuclear receptor coactivator 3	8202	632,5222321	0,02682576	0,074779	0,35873539	0,719793051	0,933530522
ENSG00000079335	CDC14A	cell division cycle 14A	8556	114,7796561	-0,154668651	0,183468	-0,843028958	0,399212249	0,814478813
ENSG00000172466	ZNF24	zinc finger protein 24	7572	432,3618689	-0,067547952	0,085864	-0,786683843	0,431466965	0,833786615
ENSG00000103248	MTHFSD	methenyltetrahydrofolate synthetase domain containing	64779	57,19661755	-0,081906503	0,243163	-0,336838407	0,736238719	0,939157822
ENSG00000163686	ABHD6	abhydrolase domain containing 6, acylglycerol lipase	57406	27,37171788	0,538850897	0,342876	1,571564078	0,116051683	NA
ENSG00000090470	PDCD7	programmed cell death 7	10081	119,1788563	-0,213041677	0,187166	-1,138248034	0,255016926	0,709997498
ENSG00000171703	TCEA2	transcription elongation factor A2	6919	16,65822563	-0,583998989	0,448567	-1,30192216	0,192942996	NA
ENSG00000173145	NOC3L	NOC3 like DNA replication regulator	64318	88,91123668	-0,096014083	0,187484	-0,512118858	0,608567827	0,897640621
ENSG00000109618	SEPSECS	Sep (O-phosphoserine) tRNA:Sec (selenocysteine) tRNA synthase	51091	18,38105671	1,39067267	1,671594	0,831944247	0,405440415	NA
ENSG00000153406	NMRAL1	NmrA like redox sensor 1	57407	27,1895861	-0,525848681	0,346317	-1,518404164	0,128912543	NA
ENSG00000122223	CD244	CD244 molecule	51744	721,8877717	-0,101231456	0,067926	-1,490312953	0,136141968	0,576527754
ENSG00000115325	DOK1	docking protein 1	1796	68,04425701	0,037652659	0,237787	0,158346343	0,874183892	0,974569239
ENSG00000136504	KAT7	lysine acetyltransferase 7	11143	368,4364061	0,134175935	0,140383	0,955784486	0,339181128	0,777431905
ENSG00000137656	BUD13	BUD13 homolog	84811	78,28122779	0,235664487	0,226537	1,040291976	0,298204271	0,748846814
ENSG00000100815	TRIP11	thyroid hormone receptor interactor 11	9321	172,408528	-0,156750547	0,220374	-0,711292451	0,476903029	0,853811879
ENSG00000197312	DDI2	DNA damage inducible 1 homolog 2	84301	277,9113391	-0,077355497	0,108038	-0,716004761	0,473988409	0,853811879
ENSG00000255154	HTD2	hydroxyacyl-thioester dehydratase type 2	109703458	22,2812021	1,272127747	0,830227	1,532264698	0,125457129	NA
ENSG00000100744	GSKIP	GSK3B interacting protein	51527	98,82171827	-0,094252868	0,171266	-0,550331665	0,58209191	0,888501395
ENSG00000109332	UBE2D3	ubiquitin conjugating enzyme E2 D3	7323	997,6070208	-0,16958306	0,098118	-1,728350632	0,083925381	0,471737097
ENSG00000196177	ACADSB	acyl-CoA dehydrogenase short/branched chain	36	189,050841	-0,225392987	0,131663	-1,711892961	0,086916396	0,481411407
ENSG00000117625	RCOR3	REST corepressor 3	55758	295,7867646	-0,069846461	0,131785	-0,53000289	0,596109927	0,894229873
ENSG00000148634	HERC4	HECT and RLD domain containing E3 ubiquitin protein ligase 4	26091	279,5946388	0,265790764	0,140397	1,893143695	0,058338763	0,3920639
ENSG00000145868	FBXO38	F-box protein 38	81545	194,2861995	-0,029338591	0,14899	-0,196916351	0,843892998	0,967231052
ENSG00000254413	CHKB-CPT1B	CHKB-CPT1B readthrough (NMD candidate)	386593	23,02598346	1,1507241	0,691786	1,663410716	0,09623025	NA
ENSG00000162066	AMDHD2	amidohydrolase domain containing 2	51005	61,40112336	0,380488087	0,23513	1,618202503	0,105618964	0,522649494
ENSG00000129657	SEC14L1	SEC14 like lipid binding 1	6397	962,7892151	0,110758869	0,059601	1,858350363	0,063119276	0,404317327
ENSG00000164400	CSF2	colony stimulating factor 2	1437	46,95186104	0,50393907	0,251245	2,005770078	0,044880784	0,339916761
ENSG00000122490	SLC66A2	solute carrier family 66 member 2	80148	26,5154562	0,789008472	0,452033	1,745468054	0,080903429	NA
ENSG00000176619	LMNB2	lamin B2	84823	228,8643029	0,036678046	0,111153	0,329978763	0,741416009	0,939193793
ENSG00000198887	SMC5	structural maintenance of chromosomes 5	23137	392,3027957	-0,11967872	0,094458	-1,267003556	0,205154018	0,660954946
ENSG00000025434	NR1H3	nuclear receptor subfamily 1 group H member 3	10062	48,07289458	0,607739846	0,380947	1,595337895	0,110636696	0,531462146
ENSG00000124209	RAB22A	RAB22A, member RAS oncogene family	57403	229,8012306	-0,164237663	0,116218	-1,413189405	0,157600041	0,61428162
ENSG00000182196	ARL6IP4	ADP ribosylation factor like GTPase 6 interacting protein 4	51329	168,7170151	-0,028120685	0,156957	-0,179161784	0,85781067	0,96924831
ENSG00000141699	RETREG3	reticulophagy regulator family member 3	162427	257,8660818	-0,332728587	0,113103	-2,941815393	0,003262944	0,075374002
ENSG00000155438	NIFK	nucleolar protein interacting with the FHA domain of MKI67	84365	116,1032678	-0,056045698	0,220476	-0,254203479	0,799338363	0,954455285
ENSG00000165997	ARL5B	ADP ribosylation factor like GTPase 5B	221079	148,0729422	-0,100523225	0,147834	-0,679974882	0,496520365	0,86123475
ENSG00000196646	ZNF136	zinc finger protein 136	7695	61,5134586	0,125618031	0,250276	0,50191787	0,615725294	0,902090873
ENSG00000179119	SPTY2D1	SPT2 chromatin protein domain containing 1	144108	268,8383399	-0,085587837	0,103523	-0,826753831	0,408376603	0,820514158
ENSG00000099849	RASSF7	Ras association domain family member 7	8045	43,29863928	0,324887689	0,289104	1,123774759	0,261108592	0,716543286
ENSG00000113916	BCL6	BCL6 transcription repressor	604	48,40926653	-0,261760857	0,286348	-0,914136301	0,36064523	0,791408368
ENSG00000112695	COX7A2	cytochrome c oxidase subunit 7A2	1347	129,8760091	0,065571283	0,146959	0,446186343	0,655462647	0,91813869
ENSG00000113068	PFDN1	prefoldin subunit 1	5201	123,9767966	-0,295628057	0,220319	-1,341818724	0,17965478	0,633591125
ENSG00000114770	ABCC5	ATP binding cassette subfamily C member 5	10057	255,9378354	0,284647263	0,14179	2,007520212	0,044694301	0,33915849
ENSG00000240857	RDH14	retinol dehydrogenase 14	57665	42,43176274	0,269349939	0,327427	0,822625282	0,410721117	0,82130516
ENSG00000117475	BLZF1	basic leucine zipper nuclear factor 1	8548	80,70140982	0,101603488	0,206545	0,491920154	0,62277579	0,90479929
ENSG00000155850	SLC26A2	solute carrier family 26 member 2	1836	132,2357117	-0,014911571	0,15264	-0,09769099	0,92217767	0,984284131

ENSG00000228716	DHFR	dihydrofolate reductase	1719	251,8737433	0,126767365	0,113	1,121830822	0,261934378	0,717555845
ENSG00000118762	PKD2	polycystin 2, transient receptor potential cation channel	5311	87,65130595	-0,341909612	0,197064	-1,735019725	0,082737312	0,468169196
ENSG00000111640	GAPDH	glyceraldehyde-3-phosphate dehydrogenase	2597	3290,550649	0,256410024	0,039327	6,520019318	7,03E-11	4,25E-08
ENSG00000112079	STK38	serine/threonine kinase 38	11329	624,4804383	-0,045923119	0,084469	-0,543667009	0,586670639	0,891324625
ENSG00000165861	ZFYVE1	zinc finger FYVE-type containing 1	53349	120,6452602	0,273849065	0,191952	1,426657211	0,153678725	0,6111586
ENSG00000176463	SLCO3A1	solute carrier organic anion transporter family member 3A1	28232	74,50671786	-0,60433435	0,260963	-2,31578329	0,020570104	0,225859999
ENSG00000254681	PKD1P5	polycystin 1, transient receptor potential channel interacting pseudogene 5	348156	275,5568592	-0,090428903	0,097854	-0,924122891	0,355422337	0,788341988
ENSG00000101558	VAPA	VAMP associated protein A	9218	295,1629029	-0,080977365	0,143263	-0,565234048	0,571914581	0,886795904
ENSG00000111727	HCFC2	host cell factor C2	29915	62,66834028	0,258157115	0,23829	1,083375016	0,278642001	0,732196891
ENSG00000147854	UHRF2	ubiquitin like with PHD and ring finger domains 2	115426	52,46892604	0,661509168	0,401661	1,64693243	0,099571935	0,509429216
ENSG00000157184	CPT2	carnitine palmitoyltransferase 2	1376	37,79684385	0,439023826	0,300744	1,459793216	0,144346914	NA
ENSG00000164674	SYTL3	synaptotagmin like 3	94120	711,9034344	-0,114387899	0,065238	-1,753399443	0,079533466	0,45762333
ENSG00000174437	ATP2A2	ATPase sarcoplasmic/endoplasmic reticulum Ca2+ transporting 2	488	536,8781941	-0,047207063	0,138491	-0,340866234	0,733204286	0,93912057
ENSG00000186026	ZNF284	zinc finger protein 284	342909	26,23664109	-0,154608335	0,367327	-0,420900729	0,673827573	NA
ENSG00000180787	ZFP3	ZFP3 zinc finger protein	124961	46,57639951	-0,039704675	0,27152	-0,146231052	0,883738993	0,976751663
ENSG00000119638	NEK9	NIMA related kinase 9	91754	53,8804602	1,302526008	0,513978	2,534206116	0,011270242	0,166384359
ENSG00000119471	HSDL2	hydroxysteroid dehydrogenase like 2	84263	188,4684885	-0,103362729	0,119442	-0,865379161	0,386830715	0,809653368
ENSG00000168496	FEN1	flap structure-specific endonuclease 1	2237	145,0351265	0,585823044	0,151821	3,858645309	0,000114017	0,00728042
ENSG00000257218	GATC	glutamyl-tRNA amidotransferase subunit C	283459	146,7852259	-0,021643289	0,154619	-0,139978478	0,888676995	0,977990819
ENSG00000089123	TASP1	taspase 1	55617	15,83063361	-0,476390538	0,662179	-0,719428364	0,471877026	NA
ENSG00000007255	TRAPPC6A	trafficking protein particle complex subunit 6A	79090	47,71943576	-0,125363518	0,243925	-0,513942212	0,607292394	0,897481254
ENSG00000173674	EIF1AX	eukaryotic translation initiation factor 1A X-linked	1964	366,4953623	-0,200905765	0,186251	-1,078685588	0,280727912	0,733722803
ENSG00000173674	LOC124905557	eukaryotic translation initiation factor 1A, X-chromosomal	124905557	366,4953623	-0,200905765	0,186251	-1,078685588	0,280727912	0,733722803
ENSG00000161671	EMC10	ER membrane protein complex subunit 10	284361	205,3773657	-0,005659936	0,211326	-0,026782923	0,978632874	0,995712569
ENSG00000100201	DDX17	DEAD-box helicase 17	10521	2981,226025	-0,262259021	0,045106	-5,814233282	6,09E-09	2,08E-06
ENSG00000149499	EML3	EMAP like 3	256364	305,7533007	-0,186001116	0,127394	-1,460045937	0,144277449	0,59024645
ENSG00000149527	PLCH2	phospholipase C eta 2	9651	16,87649195	-1,262355997	3,002562	-0,420426261	0,674174087	NA
ENSG00000162408	NOL9	nucleolar protein 9	79707	171,8581945	0,123989112	0,144134	0,860232533	0,389660875	0,810747116
ENSG00000138346	DNA2	DNA replication helicase/nuclease 2	1763	45,48668308	0,400672802	0,285609	1,402872252	0,160654937	0,618147537
ENSG00000133835	HSD17B4	hydroxysteroid 17-beta dehydrogenase 4	3295	222,2306086	0,208330081	0,119263	1,746819561	0,080668642	0,4605948
ENSG00000112972	HMGCS1	3-hydroxy-3-methylglutaryl-CoA synthase 1	3157	330,0303868	0,003792207	0,109983	0,034480011	0,972494382	0,994397979
ENSG00000080345	RIF1	replication timing regulatory factor 1	55183	475,5344745	-0,138803832	0,091048	-1,524520225	0,127378809	0,563689485
ENSG00000089154	GCN1	GCN1 activator of EIF2AK4	10985	769,1832087	0,081429742	0,064223	1,267919477	0,204826708	0,660954946
ENSG00000163516	ANKZF1	ankyrin repeat and zinc finger peptidyl tRNA hydrolase 1	55139	79,12031098	0,137946415	0,214786	0,642250851	0,520710321	0,869770068
ENSG00000030110	BAK1	BCL2 antagonist/killer 1	578	123,6382028	-0,034121596	0,158756	-0,21493164	0,829820616	0,962996447
ENSG00000078369	GNB1	G protein subunit beta 1	2782	1298,852531	-0,044818536	0,051217	-0,875078045	0,381531442	0,806821656
ENSG00000147118	ZNF182	zinc finger protein 182	7569	80,61979229	0,031046319	0,202092	0,153624984	0,877905437	0,976223885
ENSG00000147548	NSD3	nuclear receptor binding SET domain protein 3	54904	577,5067069	-0,037445599	0,084307	-0,444159407	0,656927338	0,919088619
ENSG00000010244	ZNF207	zinc finger protein 207	7756	1768,242215	-0,055643264	0,05524	-1,007296302	0,313792407	0,759483994
ENSG00000130270	ATP8B3	ATPase phospholipid transporting 8B3	148229	16,45398375	0,24554169	0,729471	0,336602404	0,736416644	NA
ENSG00000165929	TC2N	tandem C2 domains, nuclear	123036	521,8053016	-0,297814631	0,110979	-2,683525318	0,007285046	0,125896872
ENSG00000180257	ZNF816	zinc finger protein 816	125893	37,41526052	0,513449069	0,501002	1,024843404	0,305437083	NA
ENSG00000115268	RPS15	ribosomal protein S15	6209	660,7308254	-0,176517247	0,081912	-2,154959136	0,031165032	0,281657534
ENSG00000163002	NUP35	nucleoporin 35	129401	20,70342305	0,673452386	0,407085	1,654330052	0,09806047	NA
ENSG00000159140	SON	SON DNA and RNA binding protein	6651	2423,85252	-0,08804188	0,07501	-1,173741394	0,240498625	0,695620773
ENSG00000125817	CENPB	centromere protein B	1059	265,7929208	-0,316158397	0,099992	-3,161826303	0,001567831	0,046820308
ENSG00000155621	C9orf85	chromosome 9 open reading frame 85	138241	38,36017513	0,181799866	0,292137	0,622309739	0,533738219	NA
ENSG00000156970	BUB1B	BUB1 mitotic checkpoint serine/threonine kinase B	701	113,7157621	0,378915652	0,162547	2,331116272	0,019747229	0,220932679

ENSG00000168394	TAP1	transporter 1, ATP binding cassette subfamily B member	6890	466,328657	0,580178352	0,178405	3,25203114	0,001145835	0,03749744
ENSG00000176473	WDR25	WD repeat domain 25	79446	16,77477664	0,896473437	0,456079	1,965609909	0,049343689	NA
ENSG00000144426	NBEAL1	neurobeachin like 1	65065	176,5523139	0,275727432	0,234997	1,173321996	0,240666705	0,695620773
ENSG00000140575	IQGAP1	IQ motif containing GTPase activating protein 1	8826	3441,838538	-0,104895537	0,060028	-1,74743935	0,080561157	0,4605948
ENSG00000185905	C16orf54	chromosome 16 open reading frame 54	283897	254,1557185	-0,098419926	0,114464	-0,85983197	0,389881673	0,810747116
ENSG00000117222	RBBP5	RB binding protein 5, histone lysine methyltransferase complex subunit	5929	149,4951166	-0,090477838	0,147807	-0,612133518	0,540449423	0,876149366
ENSG00000129315	CCNT1	cyclin T1	904	326,0105107	-0,050616884	0,097585	-0,518695697	0,603972963	0,897212117
ENSG00000065802	ASB1	ankyrin repeat and SOCS box containing 1	51665	241,4932518	0,41250069	0,191789	2,150803739	0,031491694	0,282669444
ENSG00000108055	SMC3	structural maintenance of chromosomes 3	9126	475,3857575	0,397508915	0,102498	3,878227559	0,00010522	0,006773771
ENSG00000120910	PPP3CC	protein phosphatase 3 catalytic subunit gamma	5533	165,6417181	0,046622115	0,133134	0,350190694	0,72619559	0,93640838
ENSG00000143889	HNRNPLL	heterogeneous nuclear ribonucleoprotein L like	92906	107,1294372	0,38131447	0,167413	2,277680936	0,022745594	0,240361098
ENSG00000017260	ATP2C1	ATPase secretory pathway Ca2+ transporting 1	27032	239,317404	0,115489079	0,195804	0,589819942	0,555311372	0,882854594
ENSG00000181038	METTL23	methyltransferase 23, arginine	124512	89,87960059	0,117231033	0,19592	0,598360843	0,549599187	0,88072908
ENSG00000070423	RNF126	ring finger protein 126	55658	86,33201718	-0,291397362	0,190048	-1,533285623	0,125205495	0,5624706
ENSG00000025796	SEC63	SEC63 homolog, protein translocation regulator	11231	423,5095087	-0,170208802	0,106803	-1,593669069	0,111010176	0,53211348
ENSG00000167632	TRAPPC9	trafficking protein particle complex subunit 9	83696	70,79854158	0,09157064	0,240039	0,381481736	0,702845821	0,930846779
ENSG00000015171	ZMYND11	zinc finger MYND-type containing 11	10771	404,3974465	-0,074917893	0,10538	-0,710929225	0,477128095	0,853811879
ENSG00000112701	SENP6	SUMO specific peptidase 6	26054	336,9973222	0,135543565	0,108357	1,25089789	0,210971734	0,6647417
ENSG00000100139	MICALL1	MICAL like 1	85377	224,192448	-0,195523336	0,127501	-1,533500203	0,125152657	0,5624706
ENSG00000156875	MFSD14A	major facilitator superfamily domain containing 14A	64645	272,0931529	-0,057475639	0,101072	-0,56866175	0,569585711	0,886263374
ENSG00000029639	TFB1M	transcription factor B1, mitochondrial	51106	28,20746886	-0,083543939	0,361275	-0,231247387	0,817122618	NA
ENSG00000119922	IFIT2	interferon induced protein with tetratricopeptide repeats 2	3433	27,11085766	0,881124781	0,333858	2,639221477	0,008309667	NA
ENSG00000124207	CSE1L	chromosome segregation 1 like	1434	218,6692755	0,164266531	0,124374	1,320745508	0,186586235	0,645571933
ENSG00000140511	HAPLN3	hyaluronan and proteoglycan link protein 3	145864	94,84747971	0,614850237	0,219862	2,796531263	0,005165441	0,100918846
ENSG00000156873	PHKG2	phosphorylase kinase catalytic subunit gamma 2	5261	35,08322875	0,161588989	0,43584	0,370752803	0,710821657	NA
ENSG00000198189	HSD17B11	hydroxysteroid 17-beta dehydrogenase 11	51170	144,2753792	-0,328636887	0,148404	-2,214477698	0,026795935	0,265726358
ENSG00000176953	NFATC2IP	nuclear factor of activated T cells 2 interacting protein	84901	573,8230636	0,135680648	0,100738	1,346867722	0,178022839	0,632379636
ENSG00000102100	SLC35A2	solute carrier family 35 member A2	7355	35,37903665	0,579925944	0,362523	1,599693182	0,109666665	NA
ENSG00000175087	PDIK1L	PDLIM1 interacting kinase 1 like	149420	47,70348302	-0,096689024	0,305044	-0,316967339	0,751268385	0,941512988
ENSG00000140718	FTO	FTO alpha-ketoglutarate dependent dioxygenase	79068	55,42792419	-0,528128566	0,802889	-0,657785556	0,510675937	0,866180216
ENSG00000096384	HSP90AB1	heat shock protein 90 alpha family class B member 1	3326	2043,669158	0,062631325	0,049312	1,270103541	0,204047751	0,660954946
ENSG00000125450	NUP85	nucleoporin 85	79902	78,83133137	0,097975266	0,266219	0,368024987	0,712854603	0,930846779
ENSG00000137770	CTDSPL2	CTD small phosphatase like 2	51496	235,8880614	-0,104977209	0,169446	-0,61953293	0,535565336	0,874894595
ENSG00000139977	NAA30	N-alpha-acetyltransferase 30, NatC catalytic subunit	122830	122,8066258	-0,407913813	0,331085	-1,232051485	0,217929852	0,673519155
ENSG00000115568	ZNF142	zinc finger protein 142	7701	275,4688902	0,136392843	0,119788	1,138614666	0,254863909	0,709964783
ENSG00000172530	BANP	BTG3 associated nuclear protein	54971	56,7596896	0,201712075	0,243648	0,827884194	0,407736087	0,820276442
ENSG00000167553	TUBA1C	tubulin alpha 1c	84790	157,5057291	0,375349013	0,148668	2,524752835	0,011577969	0,169021127
ENSG00000198786	ND5	NADH dehydrogenase subunit 5	4540	17352,45958	0,038428651	0,130103	0,295370072	0,767711193	0,944698783
ENSG00000129173	E2F8	E2F transcription factor 8	79733	22,32993081	0,155284377	0,420841	0,36898553	0,71213851	NA
ENSG00000251247	ZNF345	zinc finger protein 345	25850	40,97524609	-0,299319772	0,339323	-0,88210764	0,377718601	NA
ENSG00000126261	UBA2	ubiquitin like modifier activating enzyme 2	10054	493,6091087	0,157307886	0,087591	1,795932942	0,072505183	0,437369972
ENSG00000081721	DUSP12	dual specificity phosphatase 12	11266	73,70845915	0,105230312	0,209913	0,501303975	0,616157208	0,902090873
ENSG00000055950	MRPL43	mitochondrial ribosomal protein L43	84545	48,16815912	0,393177185	0,28855	1,362594519	0,173010337	0,629558603
ENSG00000102172	SMS	spermine synthase	6611	211,0895505	0,452228768	0,120214	3,761878268	0,000168642	0,009667995
ENSG00000105379	ETFB	electron transfer flavoprotein subunit beta	2109	137,3264259	-0,288004657	0,17715	-1,62576672	0,103999291	0,517786851

ENSG00000168904	LRRC28	leucine rich repeat containing 28	123355	68,65693499	0,0076499	0,378005	0,020237553	0,983853871	0,996185513
ENSG00000184402	SS18L1	SS18L1 subunit of BAF chromatin remodeling complex	26039	85,10612374	-0,008107926	0,186698	-0,043428094	0,965360283	0,992789539
ENSG00000187037	GPR141	G protein-coupled receptor 141	353345	22,07306718	0,94381957	0,392038	2,407468135	0,016063564	NA
ENSG00000005194	CIAPIN1	cytokine induced apoptosis inhibitor 1	57019	106,2249613	-0,097539494	0,18555	-0,525677353	0,599112402	0,89463553
ENSG00000105374	NKG7	natural killer cell granule protein 7	4818	2054,476145	-0,038705853	0,056895	-0,680308762	0,496308978	0,86123475
ENSG00000145545	SRD5A1	steroid 5 alpha-reductase 1	6715	32,42230536	0,397619459	0,342741	1,160118093	0,246000729	NA
ENSG00000185305	ARL15	ADP ribosylation factor like GTPase 15	54622	48,85929482	0,962838081	0,43798	2,198363047	0,027923245	0,269753518
ENSG00000132716	DCAF8	DDB1 and CUL4 associated factor 8	50717	445,6998336	-0,143732595	0,082056	-1,751648664	0,079834239	0,458350463
ENSG00000284946	NA	NA	NA	58,08605584	0,298626473	0,267113	1,117977265	0,263576703	0,719682465
ENSG00000162739	SLAMF6	SLAM family member 6	114836	490,3997927	0,038680111	0,08314	0,4652409	0,641758952	0,912063088
ENSG00000188404	SELL	selectin L	6402	29,32640824	-0,864573311	0,312588	-2,765853952	0,005677396	NA
ENSG00000244754	N4BP2L2	NEDD4 binding protein 2 like 2	10443	261,8375644	0,287356263	0,162376	1,769695094	0,076777948	0,448671131
ENSG00000122565	CBX3	chromobox 3	11335	495,4592253	0,187333007	0,096345	1,94439654	0,051847651	0,368517148
ENSG00000160796	NBEAL2	neurobeachin like 2	23218	1424,287397	0,086804666	0,057006	1,522715862	0,127829808	0,563689485
ENSG00000136810	TXN	thioredoxin	7295	105,581508	0,77586121	0,174509	4,445958491	8,75E-06	0,000881066
ENSG00000149346	SLX4IP	SLX4 interacting protein	128710	85,18102609	-0,123517775	0,198517	-0,622203638	0,533807974	0,873238108
ENSG00000185920	PTCH1	patched 1	5727	95,58643638	-0,616882611	0,202749	-3,042596007	0,00234547	0,059232541
ENSG00000175826	CTDNEP1	CTD nuclear envelope phosphatase 1	23399	314,2976591	-0,082333077	0,120519	-0,683152785	0,494510304	0,860513824
ENSG00000125520	SLC2A4RG	SLC2A4 regulator	56731	37,26373934	-0,093819665	0,323342	-0,290156156	0,771696776	NA
ENSG00000123144	TRIR	telomerase RNA component interacting RNase	79002	341,6324008	-0,292843527	0,106136	-2,759137676	0,005795411	0,108374192
ENSG00000145833	DDX46	DEAD-box helicase 46	9879	563,3342733	0,076907318	0,078761	0,976467172	0,328833008	0,770885747
ENSG00000011451	WIZ	WIZ zinc finger	58525	213,9604508	-0,104539941	0,185924	-0,562272069	0,573930666	0,886894319
ENSG00000108840	HDAC5	histone deacetylase 5	10014	154,2942077	-0,372958728	0,153855	-2,424098345	0,015346449	0,194404855
ENSG00000149269	PAK1	p21 (RAC1) activated kinase 1	5058	50,52245468	0,342674571	0,243336	1,408234178	0,159061739	0,615101377
ENSG00000166938	DIS3L	DIS3 like exosome 3'-5' exoribonuclease	115752	109,9651006	-0,018168784	0,315715	-0,057548037	0,954108641	0,991212866
ENSG00000179981	TSHZ1	teashirt zinc finger homeobox 1	10194	178,3443225	-0,129175312	0,1368	-0,944265567	0,345033957	0,781625815
ENSG00000182580	EPHB3	EPH receptor B3	2049	15,31931442	0,218313767	0,501474	0,435343899	0,663312825	NA
ENSG00000204920	ZNF155	zinc finger protein 155	7711	42,5752834	0,218908353	0,310911	0,704087351	0,481378377	0,855577353
ENSG00000170185	USP38	ubiquitin specific peptidase 38	84640	177,0168892	0,230182348	0,236477	0,973380142	0,330364405	0,771479881
ENSG00000171853	TRAPPC12	trafficking protein particle complex subunit 12	51112	109,8160506	-0,231086431	0,157343	-1,468682727	0,141918865	0,586367972
ENSG00000183458	PKD1P3	polycystin 1, transient receptor potential channel interacting pseudogene 3	339039	132,6342434	-0,481470104	0,290388	-1,658022534	0,097312918	0,504815947
ENSG00000174371	EXO1	exonuclease 1	9156	27,71960455	0,877371691	0,384596	2,281282042	0,022531763	NA
ENSG00000103994	ZNF106	zinc finger protein 106	64397	614,9644332	0,119009545	0,080481	1,478721292	0,139214819	0,580974065
ENSG00000123595	RAB9A	RAB9A, member RAS oncogene family	9367	35,67676881	1,187781226	0,332601	3,57119338	0,000355358	NA
ENSG00000140521	POLG	DNA polymerase gamma, catalytic subunit	5428	379,5340705	-0,109730985	0,135162	-0,811848332	0,416878665	0,82446781
ENSG00000140521	POLGARF	POLG alternative reading frame	125316803	379,5340705	-0,109730985	0,135162	-0,811848332	0,416878665	0,82446781
ENSG00000145349	CAMK2D	calcium/calmodulin dependent protein kinase II delta	817	269,9717642	-0,375122482	0,126006	-2,977026357	0,00291059	0,070337762
ENSG00000173692	PSMD1	proteasome 26S subunit, non-ATPase 1	5707	373,9460654	0,343913378	0,084998	4,046119194	5,21E-05	0,003752175
ENSG00000186272	ZNF17	zinc finger protein 17	7565	41,41486058	0,059641286	0,340327	0,175247146	0,86088544	NA
ENSG00000198938	COX3	cytochrome c oxidase subunit III	4514	14705,96588	0,132126213	0,09723	1,358906655	0,17417617	0,631278097
ENSG00000204568	MRPS18B	mitochondrial ribosomal protein S18B	28973	107,7648095	0,128721638	0,167627	0,767907146	0,442542352	0,838678081
ENSG00000139211	AMIGO2	adhesion molecule with Ig like domain 2	347902	73,04049068	0,094571707	0,21471	0,440461968	0,659602552	0,919368176
ENSG00000179222	MAGED1	MAGE family member D1	9500	154,5766671	-0,121323891	0,152233	-0,796961537	0,42547337	0,830443782
ENSG00000184083	FAM120C	family with sequence similarity 120 member C	54954	74,51786569	0,162638208	0,298276	0,545260226	0,585574555	0,890608551
ENSG00000170445	HARS1	histidyl-tRNA synthetase 1	3035	157,9038601	-0,120399846	0,163815	-0,7349736	0,462355572	0,847058704
ENSG00000136709	WDR33	WD repeat domain 33	55339	389,8748599	0,127319474	0,095516	1,332958782	0,182545331	0,637116189
ENSG00000139687	RB1	RB transcriptional corepressor 1	5925	533,302934	0,016750103	0,075217	0,222689674	0,823777038	0,961917534
ENSG00000146731	CCT6A	chaperonin containing TCP1 subunit 6A	908	457,1790125	0,192208301	0,079135	2,428856536	0,015146524	0,1934322
ENSG00000181472	ZBTB2	zinc finger and BTB domain containing 2	57621	180,6181382	-0,009172935	0,132382	-0,069291422	0,944757653	0,989205143

ENSG00000111859	NEDD9	neural precursor cell expressed, developmentally down-regulated 9	4739	788,0364703	-0,065684219	0,075818	-0,86633967	0,386303918	0,809653368
ENSG00000140545	MFGE8	milk fat globule EGF and factor V/VIII domain containing	4240	132,3211034	-0,108462841	0,154725	-0,701006061	0,483299233	0,856460328
ENSG00000140987	ZSCAN32	zinc finger and SCAN domain containing 32	54925	49,02235603	0,198372097	0,288661	0,687215252	0,491947095	0,860513824
ENSG00000166189	HPS6	HPS6 biogenesis of lysosomal organelles complex 2 subunit 3	79803	62,21148222	-0,274969388	0,226634	-1,213274126	0,225025032	0,680271979
ENSG00000135723	FHOD1	formin homology 2 domain containing 1	29109	202,8157273	-0,209080197	0,139592	-1,497797138	0,134185965	0,574309297
ENSG00000141034	GID4	GID complex subunit 4 homolog	79018	80,30080303	-0,291233601	0,213458	-1,364360809	0,172454036	0,629558603
ENSG00000076248	UNG	uracil DNA glycosylase	7374	74,43843982	0,061307924	0,241948	0,253393008	0,79996453	0,95485128
ENSG00000108604	SMARCD2	SWI/SNF related, matrix associated, actin dependent regulator of chromatin, subfamily d, member 2	6603	235,8792524	-0,114564518	0,119331	-0,960056609	0,337026725	0,77693253
ENSG00000108774	RAB5C	RAB5C, member RAS oncogene family	5878	378,511373	-0,185945865	0,105637	-1,76023808	0,078367448	0,453238539
ENSG00000167325	RRM1	ribonucleotide reductase catalytic subunit M1	6240	208,9433025	0,346217685	0,142091	2,436592411	0,014826381	0,1911648
ENSG00000168439	STIP1	stress induced phosphoprotein 1	10963	529,8279239	0,122975304	0,09467	1,298991286	0,19394692	0,651961247
ENSG00000147140	NONO	non-POU domain containing octamer binding	4841	1173,110609	-0,142868247	0,071461	-1,999244021	0,045581958	0,340953043
ENSG00000165689	ENTR1	endosome associated trafficking regulator 1	10807	148,1106349	0,14648896	0,13891	1,054558683	0,291627208	0,743239565
ENSG00000154945	ANKRD40	ankyrin repeat domain 40	91369	189,7501731	-0,224070939	0,117522	-1,906632618	0,056568178	0,38600041
ENSG00000106554	CHCHD3	coiled-coil-helix-coiled-coil-helix domain containing 3	54927	86,42345028	0,082161128	0,177294	0,463417766	0,643064947	0,912160392
ENSG00000121104	FAM117A	family with sequence similarity 117 member A	81558	206,8627809	-0,011297906	0,123258	-0,091660355	0,926967897	0,985570037
ENSG00000133243	BTBD2	BTB domain containing 2	55643	142,7287227	-0,095904193	0,148771	-0,644643943	0,519157951	0,869025267
ENSG00000163960	UBXN7	UBX domain protein 7	26043	374,6218206	-0,137154329	0,092936	-1,475789222	0,140000485	0,583013685
ENSG00000196214	ZNF766	zinc finger protein 766	90321	63,61236045	0,111008592	0,257405	0,431260376	0,666279058	0,922184616
ENSG00000197362	ZNF786	zinc finger protein 786	136051	43,19342006	-0,230037403	0,324085	-0,709804735	0,477825232	0,854008769
ENSG00000151665	PIGF	phosphatidylinositol glycan anchor biosynthesis class F	5281	18,12068524	0,827340091	0,464494	1,78116423	0,074885625	NA
ENSG00000100941	PNN	pinin, desmosome associated protein	5411	892,4879482	-0,100270826	0,094989	-1,055601595	0,291150272	0,743239565
ENSG00000137642	SORL1	sortilin related receptor 1	6653	1940,146231	-0,098710892	0,054797	-1,801404971	0,071639073	0,435172111
ENSG00000130396	AFDN	afadin, adherens junction formation factor	4301	334,738743	0,101979718	0,145057	0,703031315	0,482036234	0,855766859
ENSG00000189171	S100A13	S100 calcium binding protein A13	6284	12,56363558	-0,016277188	0,572264	-0,028443481	0,977308446	NA
ENSG00000058272	PPP1R12A	protein phosphatase 1 regulatory subunit 12A	4659	585,8706186	-0,057783102	0,101254	-0,570674535	0,568220283	0,88603113
ENSG00000099364	FBXL19	F-box and leucine rich repeat protein 19	54620	48,71110049	0,526081011	0,34497	1,525004427	0,127257993	0,563689485
ENSG00000185127	C6orf120	chromosome 6 open reading frame 120	387263	90,70674725	-0,150622875	0,195028	-0,772314258	0,439928323	0,83848252
ENSG00000197694	SPTAN1	spectrin alpha, non-erythrocytic 1	6709	1928,931316	0,059974286	0,055982	1,07132137	0,284024954	0,737423617
ENSG00000125430	HS3ST3B1	heparan sulfate-glucosamine 3-sulfotransferase 3B1	9953	57,68903051	-0,179773991	0,230993	-0,778265163	0,436412708	0,836608594
ENSG00000118620	ZNF430	zinc finger protein 430	80264	114,7069647	0,070248067	0,1897	0,370312158	0,711149916	0,930846779
ENSG00000133961	NUMB	NUMB endocytic adaptor protein	8650	224,8946791	-0,030028386	0,133569	-0,224814882	0,822123288	0,961917534
ENSG00000116857	TMEM9	transmembrane protein 9	252839	13,21176791	-0,461697437	0,481186	-0,959499513	0,337307165	NA
ENSG00000171448	ZBTB26	zinc finger and BTB domain containing 26	57684	67,70467605	0,108197729	0,266454	0,406064611	0,684695127	0,925735157
ENSG00000126746	ZNF384	zinc finger protein 384	171017	260,0457766	-0,027554234	0,112925	-0,244005075	0,807226889	0,958276902
ENSG00000235750	KIAA0040	KIAA0040	9674	186,6629863	0,107503889	0,150333	0,715103807	0,474544901	0,853811879
ENSG00000107614	TRDMT1	tRNA aspartic acid methyltransferase 1	1787	43,33726663	0,134596362	0,373139	0,360713552	0,718313593	0,932668593
ENSG00000198483	ANKRD35	ankyrin repeat domain 35	148741	25,66841809	0,157479244	0,381734	0,412536073	0,679946546	NA
ENSG00000113391	ARB2A	ARB2 cotranscriptional regulator A	83989	139,3948093	0,076355618	0,162094	0,47105705	0,637599995	0,909767927
ENSG00000114423	CBLB	Cbl proto-oncogene B	868	384,3872138	0,002363642	0,146016	0,016187533	0,987084781	0,996894778
ENSG00000161791	FMNL3	formin like 3	91010	1684,80659	0,068472827	0,051643	1,325887664	0,18487691	0,641636433
ENSG00000139168	ZCRB1	zinc finger CCHC-type and RNA binding motif containing 1	85437	90,72583792	-0,28773144	0,242706	-1,185516166	0,235813427	0,691328984
ENSG00000143457	GOLPH3L	golgi phosphoprotein 3 like	55204	77,79457301	0,020161768	0,227897	0,088468651	0,9295042	0,986487751
ENSG00000089220	PEBP1	phosphatidylethanolamine binding protein 1	5037	233,469088	-0,086098867	0,121292	-0,709847277	0,477798848	0,854008769

ENSG00000243927	MRPS6	mitochondrial ribosomal protein S6	64968	126,1784419	0,515444032	0,168392	3,060972686	0,002206192	0,056998137
ENSG00000169136	ATF5	activating transcription factor 5	22809	40,99164627	-0,360753751	0,293031	-1,231110737	0,21828145	NA
ENSG00000066027	PPP2R5A	protein phosphatase 2 regulatory subunit B'alpha	5525	494,413712	-0,123700756	0,082728	-1,495279646	0,134841475	0,575414344
ENSG00000188933	NA	NA	NA	58,69903995	-0,433213209	0,227788	-1,901824651	0,057194082	0,38821726
ENSG00000172613	RAD9A	RAD9 checkpoint clamp component A	5883	43,27280815	0,634563548	0,452983	1,400856337	0,161257038	0,618965617
ENSG00000138760	SCARB2	scavenger receptor class B member 2	950	76,95095533	-0,181692662	0,298843	-0,607987966	0,543195455	0,877542071
ENSG00000138814	PPP3CA	protein phosphatase 3 catalytic subunit alpha	5530	641,6098167	-0,256672699	0,069109	-3,714029475	0,000203985	0,010898628
ENSG00000179240	GVQW3	GVQW motif containing 3	100506127	26,66966548	-0,353228534	0,453248	-0,779327912	0,435786577	NA
ENSG00000185728	YTHDF3	YTH N6-methyladenosine RNA binding protein F3	253943	381,101539	-0,055429758	0,107139	-0,517365386	0,604901118	0,897347689
ENSG00000206503	HLA-A	major histocompatibility complex, class I, A	3105	3925,456494	-0,142981162	0,041724	-3,426822445	0,000610688	0,023744284
ENSG00000032219	ARID4A	AT-rich interaction domain 4A	5926	299,8015355	-0,258520097	0,108456	-2,383636295	0,017142535	0,204616219
ENSG00000171497	PPID	peptidylprolyl isomerase D	5481	116,1591943	0,118257077	0,170632	0,693053167	0,488276187	0,859508239
ENSG00000007545	CRAMP1	cramped chromatin regulator homolog 1	57585	741,3580305	-0,06962335	0,075047	-0,927728827	0,353548251	0,788228969
ENSG00000076003	MCM6	minichromosome maintenance complex component 6	4175	280,2920975	0,218087389	0,104993	2,07715146	0,037787582	0,308186575
ENSG00000109452	INPP4B	inositol polyphosphate-4-phosphatase type II B	8821	273,6543519	0,006172054	0,115527	0,053425395	0,957392972	0,991911599
ENSG00000149084	HSD17B12	hydroxysteroid 17-beta dehydrogenase 12	51144	135,8615114	0,043523445	0,148205	0,293670694	0,76900955	0,944813709
ENSG00000214029	ZNF891	zinc finger protein 891	101060200	100,0646303	0,299853935	0,34876	0,859771957	0,38991476	0,810747116
ENSG00000100453	GZMB	granzyme B	3002	1015,969894	0,212443706	0,074265	2,860626539	0,004228048	0,089992108
ENSG00000198231	DDX42	DEAD-box helicase 42	11325	488,3963269	-0,025846526	0,082519	-0,313220024	0,754113506	0,942901768
ENSG00000104979	C19orf53	chromosome 19 open reading frame 53	28974	102,7211481	-0,43780864	0,174665	-2,506562182	0,012191161	0,17377383
ENSG00000145743	FBXL17	F-box and leucine rich repeat protein 17	64839	106,6558181	-0,076980313	0,174176	-0,441969696	0,658511135	0,919293718
ENSG00000135720	DYNC1LI2	dynein cytoplasmic 1 light intermediate chain 2	1783	458,6798956	-0,234700093	0,075906	-3,09199282	0,001988177	0,054949576
ENSG00000146833	TRIM4	tripartite motif containing 4	89122	162,9172636	0,124477988	0,136063	0,914854697	0,360267916	0,791408368
ENSG00000205730	ITPRIPL2	ITPRIP like 2	162073	58,32311176	-0,218169137	0,241612	-0,902974278	0,366539547	0,79459056
ENSG00000108561	C1QBP	complement C1q binding protein	708	152,4886264	0,070439339	0,182505	0,38595827	0,699527574	0,930783276
ENSG00000149289	ZC3H12C	zinc finger CCCH-type containing 12C	85463	16,12974717	1,689446053	0,479387	3,524176596	0,000424801	NA
ENSG00000214135	NA	NA	NA	17,35108148	0,527592226	0,424286	1,243482148	0,213690215	NA
ENSG00000032444	PNPLA6	patatin like phospholipase domain containing 6	10908	605,3332737	0,07215814	0,074068	0,974208412	0,329953069	0,771479881
ENSG00000101986	ABCD1	ATP binding cassette subfamily D member 1	215	67,73309813	-0,533347921	0,224277	-2,378075795	0,017403248	0,206472979
ENSG00000114861	FOXP1	forkhead box P1	27086	255,0992488	-0,040608355	0,139158	-0,29181419	0,770428695	0,945219782
ENSG00000131374	TBC1D5	TBC1 domain family member 5	9779	349,5542641	-0,121149161	0,096006	-1,261886874	0,206989496	0,661502129
ENSG00000142252	GEMIN7	gem nuclear organelle associated protein 7	79760	50,20287841	0,141775735	0,253826	0,558554765	0,576465619	0,887069818
ENSG00000171044	XKR6	XK related 6	286046	11,97626058	1,120196151	0,626919	1,786828959	0,073965137	NA
ENSG00000244398	RPL36AP37	ribosomal protein L36a pseudogene 37	729362	293,8583085	-0,066521121	0,11845	-0,561595098	0,574391921	0,886894319
ENSG00000107077	KDM4C	lysine demethylase 4C	23081	286,335284	0,002405764	0,120921	0,019895275	0,984126915	0,996185513
ENSG00000134954	ETS1	ETS proto-oncogene 1, transcription factor	2113	4231,234966	-0,093854017	0,038826	-2,417298468	0,01563619	0,196490617
ENSG00000158092	NCK1	NCK adaptor protein 1	4690	135,6176101	-0,132826103	0,146493	-0,906704128	0,36456328	0,793491925
ENSG00000188229	TUBB4B	tubulin beta 4B class IVb	10383	253,8566784	0,189464544	0,109545	1,729560951	0,083708748	0,471627336
ENSG00000151466	SCLT1	sodium channel and clathrin linker 1	132320	84,64834015	0,293523392	0,206491	1,421484863	0,155175851	0,61398042
ENSG00000198171	DDRGK1	DDRGK domain containing 1	65992	104,3357034	-0,181430952	0,221661	-0,818505364	0,413068681	0,82225544
ENSG00000189042	ZNF567	zinc finger protein 567	163081	49,10499194	0,319708802	0,278028	1,1499153	0,250178759	0,707335884
ENSG00000136146	MED4	mediator complex subunit 4	29079	193,9769909	-0,303556051	0,124728	-2,433748331	0,014943381	0,1911648
ENSG00000158636	EMSY	EMSY transcriptional repressor, BRCA2 interacting	56946	172,2485258	-0,405505006	0,186198	-2,177820099	0,029419433	0,277050631
ENSG00000130816	DNMT1	DNA methyltransferase 1	1786	492,7592893	0,427138221	0,153605	2,780752681	0,005423304	0,104917427
ENSG00000101544	ADNP2	ADNP homeobox 2	22850	162,4730181	0,160632421	0,143247	1,121369544	0,262130594	0,717842988
ENSG00000172667	ZMAT3	zinc finger matrin-type 3	64393	391,3693148	-0,018886262	0,095444	-0,197877085	0,843141233	0,967210584
ENSG00000143110	C1orf162	chromosome 1 open reading frame 162	128346	119,0441009	-0,30067901	0,169653	-1,772320938	0,076341298	0,447360046
ENSG00000205808	PLPP6	phospholipid phosphatase 6	403313	47,83063346	-0,012035311	0,259956	-0,046297419	0,963073197	0,992517634
ENSG00000065000	AP3D1	adaptor related protein complex 3 subunit delta 1	8943	795,4757716	-0,143711629	0,079618	-1,805022571	0,07107115	0,433716249
ENSG00000169951	ZNF764	zinc finger protein 764	92595	73,63184321	0,014327118	0,225443	0,063550915	0,949327817	0,990700279

ENSG00000112486	CCR6	C-C motif chemokine receptor 6	1235	194,8457339	0,140747333	0,117299	1,199902823	0,230177084	0,684730271
ENSG00000152484	USP12	ubiquitin specific peptidase 12	219333	258,0949891	-0,053296091	0,148182	-0,359667284	0,71909596	0,933054629
ENSG00000146834	MEPCE	methylphosphate capping enzyme	56257	146,5047424	0,178026084	0,142082	1,252985123	0,210211121	0,664554962
ENSG00000157637	SLC38A10	solute carrier family 38 member 10	124565	412,0481661	-0,207619191	0,089405	-2,322231834	0,020220456	0,224146577
ENSG00000167074	TEF	TEF transcription factor, PAR bZIP family member	7008	39,58379702	0,176097624	0,322474	0,546082557	0,58500919	NA
ENSG00000103479	RBL2	RB transcriptional corepressor like 2	5934	1432,892808	-0,100734022	0,060542	-1,663881685	0,096136076	0,502134421
ENSG00000119522	DENND1A	DENN domain containing 1A	57706	98,73191683	0,033401512	0,218897	0,152589999	0,878721613	0,976237908
ENSG00000153879	CEBPG	CCAAT enhancer binding protein gamma	1054	122,2895815	0,251514991	0,211338	1,190105449	0,234004949	0,688858646
ENSG00000010539	ZNF200	zinc finger protein 200	7752	34,83321075	-0,071931	0,3389	-0,212248462	0,8319132	NA
ENSG00000088387	DOCK9	dedicator of cytokinesis 9	23348	371,8339061	-0,018009187	0,098908	-0,18208092	0,855519222	0,968439087
ENSG00000100519	PSMC6	proteasome 26S subunit, ATPase 6	5706	157,4175273	0,115205558	0,134506	0,856509166	0,391716205	0,812491435
ENSG00000088986	DYNLL1	dynein light chain LC8-type 1	8655	283,9049279	0,091262427	0,112128	0,813915592	0,415693308	0,82446781
ENSG00000152454	ZNF256	zinc finger protein 256	10172	33,40785647	-0,18097099	0,318199	-0,568735819	0,569535436	NA
ENSG00000290183	NA	NA	NA	12,47015899	-0,478979188	2,888317	-0,165833342	0,868288107	NA
ENSG00000181090	EHMT1	euchromatic histone lysine methyltransferase 1	79813	389,2395075	-0,050584899	0,093403	-0,541578526	0,588108888	0,891557039
ENSG00000134255	CEPT1	choline/ethanolamine phosphotransferase 1	10390	76,81769681	0,093682049	0,237242	0,394879089	0,692932123	0,928002746
ENSG00000135521	LTV1	LTV1 ribosome biogenesis factor	84946	140,2745018	0,023543026	0,138138	0,170431154	0,864671073	0,971561202
ENSG00000148400	NOTCH1	notch receptor 1	4851	1581,778229	-0,067231736	0,073878	-0,910032695	0,362805267	0,791740086
ENSG00000187741	FANCA	FA complementation group A	2175	69,23002258	-0,035747837	0,269833	-0,132481357	0,894603568	0,977990819
ENSG00000112697	TMEM30A	transmembrane protein 30A	55754	672,584281	0,016852445	0,070273	0,239815524	0,810473272	0,959038743
ENSG00000196436	NPIPB15	nuclear pore complex interacting protein family member B15	440348	22,02804088	-0,412971934	0,383047	-1,078122778	0,280978968	NA
ENSG00000196436	LOC124907807	nuclear pore complex-interacting protein family member B15	124907807	22,02804088	-0,412971934	0,383047	-1,078122778	0,280978968	NA
ENSG00000196436	LOC124907808	nuclear pore complex-interacting protein family member B15	124907808	22,02804088	-0,412971934	0,383047	-1,078122778	0,280978968	NA
ENSG00000100342	APOL1	apolipoprotein L1	8542	312,9784611	-0,164507653	0,098895	-1,663461687	0,096220054	0,502134421
ENSG00000039319	ZFYVE16	zinc finger FYVE-type containing 16	9765	109,2442935	0,111803593	0,288293	0,387812451	0,698154831	0,930783276
ENSG00000161847	RAVER1	ribonucleoprotein, PTB binding 1	125950	264,7852177	0,069419006	0,124407	0,558000027	0,576844366	0,887069818
ENSG00000115602	IL1RL1	interleukin 1 receptor like 1	9173	49,0563382	0,177857991	0,248853	0,714711519	0,474787319	0,853811879
ENSG00000131323	TRAF3	TNF receptor associated factor 3	7187	376,748343	0,153356471	0,126874	1,20873161	0,226765974	0,681621061
ENSG00000126804	ZBTB1	zinc finger and BTB domain containing 1	22890	578,784679	-0,003015886	0,077166	-0,039083276	0,968823995	0,993542794
ENSG00000148840	PPRC1	PPARG related coactivator 1	23082	193,454631	0,121807126	0,15688	0,77643325	0,437493219	0,837249449
ENSG00000125868	DSTN	destrin, actin depolymerizing factor	11034	191,0418183	-0,227566597	0,122243	-1,861584512	0,062661678	0,404317327
ENSG00000173409	ARV1	ARV1 homolog, fatty acid homeostasis modulator	64801	43,23932169	0,236655905	0,268691	0,880772357	0,378441044	0,804187219
ENSG00000063245	EPN1	epsin 1	29924	180,9810474	-0,292751651	0,15297	-1,913779851	0,055648291	0,382350454
ENSG00000165458	INPPL1	inositol polyphosphate phosphatase like 1	3636	561,7853121	-0,036280684	0,078534	-0,461974116	0,644099882	0,912530858
ENSG00000065328	MCM10	minichromosome maintenance 10 replication initiation factor	55388	44,79341845	0,470602856	0,392261	1,199718674	0,230248618	0,684730271
ENSG00000115540	MOB4	MOB family member 4, phocein	25843	149,1206619	-0,106160855	0,192597	-0,551207275	0,581491594	0,888501395
ENSG00000125730	C3	complement C3	718	27,47654132	1,540285194	0,742812	2,07358607	0,03811777	NA
ENSG00000109475	RPL34	ribosomal protein L34	6164	723,7374569	0,158375679	0,069716	2,271711112	0,023103965	0,242267741
ENSG00000135655	USP15	ubiquitin specific peptidase 15	9958	473,6452405	0,117937659	0,126408	0,932991173	0,350824526	0,785712602
ENSG00000085999	RAD54L	RAD54 like	8438	27,89453835	0,560849918	0,408742	1,372137554	0,170020621	NA
ENSG00000170846	MRFAP1L2	Morf4 family associated protein 1 like 2	93622	48,4516207	-0,336323135	0,298962	-1,124969141	0,260602111	0,716404964
ENSG00000147905	ZCCHC7	zinc finger CCHC-type containing 7	84186	119,3184552	-0,187914493	0,194341	-0,966932304	0,333577876	0,773674527
ENSG00000148356	LRSAM1	leucine rich repeat and sterile alpha motif containing 1	90678	42,20206799	-0,141380275	0,354422	-0,398904164	0,689963821	NA
ENSG00000136875	PRPF4	pre-mRNA processing factor 4	9128	116,5330252	0,123253251	0,168218	0,7327005	0,463741119	0,848212098
ENSG00000168078	PBK	PDZ binding kinase	55872	25,08466529	1,350702399	0,481756	2,803706036	0,005051894	NA
ENSG00000164603	BMT2	base methyltransferase of 25S rRNA 2 homolog	154743	73,68119596	-0,118818998	0,212131	-0,560119851	0,575397691	0,887069818

ENSG00000125844	RRBP1	ribosome binding protein 1	6238	454,2213181	0,063757193	0,081492	0,78237124	0,43399643	0,835238413
ENSG00000121774	KHDRBS1	KH RNA binding domain containing, signal transduction associated 1	10657	562,4591037	0,085458097	0,06936	1,232087836	0,217916275	0,673519155
ENSG00000282034	NA	NA	NA	509,5486478	0,434827809	0,244833	1,776017681	0,075730003	0,446229554
ENSG00000154781	CCDC174	coiled-coil domain containing 174	51244	79,23640344	-0,430182963	0,201393	-2,136037504	0,032676357	0,288946532
ENSG00000145780	FEM1C	fem-1 homolog C	56929	104,1010804	0,01712288	0,17711	0,09667936	0,922981032	0,984284131
ENSG00000100034	PPM1F	protein phosphatase, Mg2+/Mn2+ dependent 1F	9647	232,8865005	-0,267524382	0,115718	-2,311867908	0,020784964	0,225859999
ENSG00000163608	NEPRO	nucleolus and neural progenitor protein	25871	102,2227657	-0,09181521	0,203762	-0,450599263	0,652278398	0,916482964
ENSG00000049245	VAMP3	vesicle associated membrane protein 3	9341	114,3935383	0,023317106	0,182089	0,128053089	0,89810696	0,977990819
ENSG00000169612	RAMAC	RNA guanine-7 methyltransferase activating subunit	83640	123,4641453	0,032750819	0,161788	0,20243038	0,83958028	0,96627649
ENSG00000162607	USP1	ubiquitin specific peptidase 1	7398	315,6577221	0,21142859	0,09932	2,128765795	0,033273642	0,292316763
ENSG00000100376	FAM118A	family with sequence similarity 118 member A	55007	51,36989342	-0,037146295	0,25132	-0,147804927	0,882496721	0,976300778
ENSG00000259924	NA	NA	NA	12,51668514	0,472206142	0,519334	0,909253748	0,363216199	NA
ENSG00000125898	FAM110A	family with sequence similarity 110 member A	83541	30,17227563	0,552298015	0,364139	1,516721329	0,129337057	NA
ENSG00000142556	ZNF614	zinc finger protein 614	80110	84,79608665	0,146060091	0,202956	0,719662287	0,471732952	0,85368447
ENSG00000168066	SF1	splicing factor 1	7536	858,4913922	-0,165589659	0,068005	-2,434951147	0,0148938	0,1911648
ENSG00000058262	SEC61A1	SEC61 translocon subunit alpha 1	29927	794,9417516	-0,087276744	0,072646	-1,201401866	0,229595353	0,684082663
ENSG00000011243	AKAP8L	A-kinase anchoring protein 8 like	26993	92,95657957	-0,743838346	1,025631	-0,725249254	0,468299132	0,851393838
ENSG00000185760	KCNQ5	potassium voltage-gated channel subfamily Q member 5	56479	96,63285704	-0,168240546	0,259913	-0,647294524	0,517441345	0,868791049
ENSG00000106105	GARS1	glycyl-tRNA synthetase 1	2617	188,1070731	-0,071640801	0,170387	-0,420459435	0,674149857	0,923602509
ENSG00000170854	RIOX2	ribosomal oxygenase 2	84864	73,99538384	-0,099948741	0,253495	-0,394282359	0,693372586	0,928041631
ENSG00000197275	RAD54B	RAD54 homolog B	25788	21,1179604	0,528140593	0,487024	1,084424347	0,278176693	NA
ENSG00000153767	GTF2E1	general transcription factor IIE subunit 1	2960	54,04397919	-0,148056908	0,25841	-0,572953103	0,566676448	0,88521771
ENSG00000035403	VCL	vinculin	7414	171,1476336	-0,337629743	0,141637	-2,383775596	0,017136048	0,204616219
ENSG00000087191	PSMC5	proteasome 26S subunit, ATPase 5	5705	255,746233	-0,095174	0,102715	-0,926585323	0,354141878	0,788228969
ENSG00000167005	NUDT21	nudix hydrolase 21	11051	406,4909804	-0,049487675	0,100497	-0,492431182	0,62241456	0,904764752
ENSG00000105339	DENND3	DENN domain containing 3	22898	673,619012	-0,036335031	0,099874	-0,363810149	0,715999784	0,932040329
ENSG00000140526	ABHD2	abhydrolase domain containing 2, acylglycerol lipase	11057	1251,249578	-0,036286267	0,070637	-0,513699457	0,607462132	0,897481254
ENSG00000159082	SYNJ1	synaptojanin 1	8867	184,5351952	-0,022365261	0,133101	-0,168032367	0,866557816	0,971722599
ENSG00000184277	TM2D3	TM2 domain containing 3	80213	70,5263633	0,288164494	0,230353	1,250970246	0,210945333	0,6647417
ENSG00000198879	SFMBT2	Scm like with four mbt domains 2	57713	1498,526492	0,106035296	0,056735	1,86897351	0,0616265	0,402674318
ENSG00000166598	HSP90B1	heat shock protein 90 beta family member 1	7184	2126,53965	0,156006482	0,041736	3,737977551	0,000185507	0,010333107
ENSG00000099901	RANBP1	RAN binding protein 1	5902	226,346974	0,239947451	0,11448	2,095973891	0,036084507	0,302084897
ENSG00000146574	CCZ1B	CCZ1 homolog B, vacuolar protein trafficking and biogenesis associated	221960	259,7766279	-0,005749552	0,103739	-0,055423418	0,95580114	0,991510463
ENSG00000112339	HBS1L	HBS1 like translational GTPase	10767	135,3493261	0,482436486	0,210116	2,296050303	0,021673005	0,232567042
ENSG00000160285	LSS	lanosterol synthase	4047	197,3587299	0,33689707	0,151664	2,221345698	0,026327556	0,262074302
ENSG00000171552	BCL2L1	BCL2 like 1	598	550,1606645	0,149547698	0,087164	1,715695721	0,086217748	0,47957096
ENSG00000204351	SKIC2	SKI2 subunit of superkiller complex	6499	19,31261575	0,471016033	3,029851	0,155458482	0,876459882	NA
ENSG00000184900	SUMO3	small ubiquitin like modifier 3	6612	251,3274416	-0,06921862	0,108932	-0,635428927	0,525148707	0,871254318
ENSG00000185504	FAAP100	FA core complex associated protein 100	80233	89,30368401	-0,367891943	0,259992	-1,41501093	0,157065291	0,61428162
ENSG00000143258	USP21	ubiquitin specific peptidase 21	27005	78,95280751	0,007042732	0,222775	0,031613645	0,974780161	0,994534085
ENSG00000070831	CDC42	cell division cycle 42	998	700,5047369	-0,137956758	0,213442	-0,646342756	0,518057404	0,868791049
ENSG00000145331	TRMT10A	tRNA methyltransferase 10A	93587	21,58195546	0,468072892	0,401707	1,165209353	0,24393426	NA
ENSG00000100628	ASB2	ankyrin repeat and SOCS box containing 2	51676	66,94850184	-0,280409421	0,254502	-1,101797522	0,270549708	0,725477256
ENSG00000119335	SET	SET nuclear proto-oncogene	6418	1621,060295	-0,066348545	0,070107	-0,946388003	0,343950722	0,781132432
ENSG00000122376	SHLD2	shieldin complex subunit 2	54537	204,9242523	-0,086203204	0,120379	-0,716095708	0,473932254	0,853811879
ENSG00000143799	PARP1	poly(ADP-ribose) polymerase 1	142	749,6312795	0,056546456	0,0603	0,937755658	0,348369994	0,784431746
ENSG00000158467	AHCYL2	adenosylhomocysteinase like 2	23382	100,8734505	-0,192333727	0,174939	-1,099431431	0,271579927	0,725708516
ENSG00000239282	CASTOR1	cytosolic arginine sensor for mTORC1 subunit 1	652968	19,22988708	0,856541239	0,457355	1,872812931	0,061094222	NA

ENSG00000147202	DIAPH2	diaphanous related formin 2	1730	58,92593269	-0,179215542	0,261181	-0,686172881	0,492604097	0,860513824
ENSG00000167397	VKORC1	vitamin K epoxide reductase complex subunit 1	79001	99,597503	0,167645463	0,220291	0,761018628	0,446645941	0,842246632
ENSG00000107929	LARP4B	La ribonucleoprotein 4B	23185	264,6257904	-0,134274876	0,114025	-1,177593252	0,238958801	0,693819751
ENSG00000172469	MANEA	mannosidase endo-alpha	79694	86,06939051	0,203832387	0,196138	1,039230273	0,298697654	0,749216326
ENSG00000128203	ASPHD2	aspartate beta-hydroxylase domain containing 2	57168	30,32321977	0,453769159	0,321049	1,413397268	0,157538948	NA
ENSG00000133250	ZNF414	zinc finger protein 414	84330	46,18888015	-0,938336231	0,373602	-2,511592917	0,012018764	0,172254331
ENSG00000127946	HIP1	huntingtin interacting protein 1	3092	476,2869215	0,003181131	0,088562	0,035919928	0,971346206	0,994000404
ENSG00000166747	AP1G1	adaptor related protein complex 1 subunit gamma 1	164	668,0379985	0,050523926	0,077223	0,654262695	0,512942576	0,86823028
ENSG00000198771	RCSD1	RCSD domain containing 1	92241	1029,648329	-0,075514189	0,055358	-1,364107784	0,172533645	0,629558603
ENSG00000008853	RHOBTB2	Rho related BTB domain containing 2	23221	157,8249596	-0,222751957	0,127918	-1,741362273	0,081620097	0,464231481
ENSG00000105640	RPL18A	ribosomal protein L18a	6142	1333,775873	-0,120439647	0,057791	-2,084073422	0,037153489	0,306351862
ENSG00000143420	ENSA	endosulfine alpha	2029	475,5464234	0,034069879	0,07458	0,456825718	0,647796319	0,9144223
ENSG00000188827	SLX4	SLX4 structure-specific endonuclease subunit	84464	159,5336449	0,223447566	0,142297	1,570293789	0,116346778	0,543917551
ENSG00000213626	LBH	LBH regulator of WNT signaling pathway	81606	108,835476	-0,840691581	0,225551	-3,727284873	0,000193554	0,01048604
ENSG00000067840	PDZD4	PDZ domain containing 4	57595	11,85825541	-0,749748684	0,51349	-1,460104916	0,144261242	NA
ENSG00000135766	EGLN1	egl-9 family hypoxia inducible factor 1	54583	221,4508114	-0,279996115	0,176723	-1,584381934	0,113106831	0,538390853
ENSG00000177885	GRB2	growth factor receptor bound protein 2	2885	545,0169491	0,002122852	0,074596	0,028458139	0,977296755	0,995291586
ENSG00000101413	RPRD1B	regulation of nuclear pre-mRNA domain containing 1B	58490	113,0657691	-0,254581484	0,187831	-1,355377287	0,175297382	0,632039836
ENSG00000006062	MAP3K14	mitogen-activated protein kinase kinase kinase 14	9020	80,44287507	-0,032462613	0,21272	-0,15260717	0,87870807	0,976237908
ENSG00000157350	ST3GAL2	ST3 beta-galactoside alpha-2,3-sialyltransferase 2	6483	297,5890854	0,016247507	0,107328	0,151381979	0,879674404	0,976300778
ENSG00000082996	RNF13	ring finger protein 13	11342	127,0586253	-0,386533364	0,155161	-2,491184055	0,012731815	0,177307233
ENSG00000112343	TRIM38	tripartite motif containing 38	10475	349,5618957	0,0678254	0,106275	0,638207914	0,523338346	0,870221868
ENSG00000157349	DDX19B	DEAD-box helicase 19B	11269	139,3204179	-0,062794664	0,190738	-0,329219896	0,741989486	0,939193793
ENSG00000163872	YEATS2	YEATS domain containing 2	55689	233,8275224	0,068159863	0,115124	0,592057987	0,553811759	0,882816634
ENSG00000170100	ZNF778	zinc finger protein 778	197320	106,2896912	-0,322431242	0,285601	-1,128956086	0,258916359	0,714772965
ENSG00000198000	NOL8	nucleolar protein 8	55035	166,5704931	-0,082573814	0,146112	-0,565142058	0,571977144	0,886795904
ENSG00000139793	MBNL2	muscleblind like splicing regulator 2	10150	108,1166715	0,150680916	0,179573	0,839108291	0,401408544	0,815200159
ENSG00000166881	NEMP1	nuclear envelope integral membrane protein 1	23306	152,936917	0,225874685	0,150269	1,5031396	0,132803049	0,570166586
ENSG00000197818	SLC9A8	solute carrier family 9 member A8	23315	213,764501	0,054665387	0,158928	0,343963637	0,730873633	0,938729652
ENSG00000163939	PBRM1	polybromo 1	55193	611,321362	0,100957147	0,101631	0,993366471	0,320531395	0,764486358
ENSG00000085511	MAP3K4	mitogen-activated protein kinase kinase kinase 4	4216	147,9402206	0,118046131	0,156506	0,754258972	0,450693733	0,84263906
ENSG00000100359	SGSM3	small G protein signaling modulator 3	27352	238,4816141	-0,202826244	0,119576	-1,696209013	0,089846304	0,488772026
ENSG00000111142	METAP2	methionyl aminopeptidase 2	10988	267,0957826	-0,072715484	0,124069	-0,586089627	0,557815287	0,883921037
ENSG00000182979	MTA1	metastasis associated 1	9112	216,5035132	-0,241911067	0,214898	-1,125700119	0,260292474	0,716055022
ENSG00000062598	ELMO2	engulfment and cell motility 2	63916	438,6654481	0,171366358	0,09364	1,830059929	0,067240978	0,418803046
ENSG00000214753	HNRNPUL2	heterogeneous nuclear ribonucleoprotein U like 2	221092	1026,529789	-0,055347374	0,071672	-0,772236278	0,439974499	0,83848252
ENSG00000023516	AKAP11	A-kinase anchoring protein 11	11215	566,5853812	-0,0239039	0,077342	-0,309065907	0,757271392	0,942901768
ENSG00000005007	UPF1	UPF1 RNA helicase and ATPase	5976	387,0973281	-0,176176766	0,13384	-1,316322619	0,188065782	0,646419542
ENSG00000198160	MIER1	MIER1 transcriptional regulator	57708	377,3798034	-0,196369652	0,093675	-2,096283453	0,036057054	0,302084897
ENSG00000154059	IMPACT	impact RWD domain protein	55364	67,93152666	-0,079577642	0,21496	-0,370197622	0,711235248	0,930846779
ENSG00000180488	MIGA1	mitoguardin 1	374986	86,74517978	-0,043579823	0,238172	-0,182976129	0,854816749	0,968377739
ENSG00000186815	TPCN1	two pore segment channel 1	53373	131,6343852	-0,143616915	0,147988	-0,970466447	0,331814042	0,772855126
ENSG00000138380	CARF	calcium responsive transcription factor	79800	92,23017113	0,255569558	0,198272	1,288984436	0,197403498	0,65618735
ENSG00000181852	RNF41	ring finger protein 41	10193	155,4564385	0,292708413	0,13502	2,167887333	0,03016726	0,279077601
ENSG00000115750	TAF1B	TATA-box binding protein associated factor, RNA polymerase I subunit B	9014	57,65863611	-0,178426525	0,312458	-0,571042541	0,567970806	0,88603113
ENSG00000156469	MTERF3	mitochondrial transcription termination factor 3	51001	35,17181346	-0,299820401	0,322713	-0,929063074	0,3528564	NA
ENSG00000162063	CCNF	cyclin F	899	106,3809784	0,085546651	0,246757	0,346684272	0,728828527	0,937916952
ENSG00000070961	ATP2B1	ATPase plasma membrane Ca2+ transporting 1	490	199,7768927	0,185127008	0,153138	1,208888784	0,226705576	0,681621061
ENSG00000077232	DNAJC10	DnaJ heat shock protein family (Hsp40) member C10	54431	281,2706533	-0,21680265	0,144822	-1,497024204	0,13438696	0,574309297
ENSG00000137500	CCDC90B	coiled-coil domain containing 90B	60492	32,96348889	-0,325959567	0,489619	-0,665741077	0,505576613	NA
ENSG00000151092	NGLY1	N-glycanase 1	55768	77,6472163	0,083391119	0,255756	0,326057855	0,744380583	0,939745744

ENSG00000166133	RPUSD2	RNA pseudouridine synthase domain containing 2	27079	36,74864541	-0,024943317	0,293299	-0,085043996	0,932226413	NA
ENSG00000180096	SEPTIN1	septin 1	1731	362,2838299	-0,079317959	0,110873	-0,715392794	0,474366364	0,853811879
ENSG00000198718	TOGARAM1	TOG array regulator of axonemal microtubules 1	23116	51,32223875	0,082012442	0,345766	0,237190818	0,81250876	0,959038743
ENSG00000221890	NPTXR	neuronal pentraxin receptor	23467	38,33239488	-0,39532543	0,312665	-1,264375644	0,20609523	NA
ENSG00000111885	MAN1A1	mannosidase alpha class 1A member 1	4121	190,3795272	-0,251989671	0,124314	-2,027036057	0,042658725	0,328150469
ENSG00000004961	HCCS	holocytochrome c synthase	3052	42,24349674	0,718134953	0,267743	2,682183185	0,00731434	NA
ENSG00000174327	SLC16A13	solute carrier family 16 member 13	201232	38,53172373	0,079763505	0,314065	0,253971453	0,799517612	NA
ENSG00000026508	CD44	CD44 molecule (Indian blood group)	960	1849,750454	-0,054633784	0,076566	-0,713548928	0,475506148	0,853811879
ENSG00000103335	PIEZO1	piezo type mechanosensitive ion channel component 1	9780	306,5806757	0,029684662	0,107855	0,27522829	0,783140854	0,94992869
ENSG00000063854	HAGH	hydroxyacylglutathione hydrolase	3029	69,03779883	0,182167942	0,331311	0,549840223	0,582428968	0,888501395
ENSG00000117543	DPH5	diphthamide biosynthesis 5	51611	39,33693204	0,100858738	0,276154	0,365226404	0,71494241	NA
ENSG00000164951	PDP1	pyruvate dehydrogenase phosphatase catalytic subunit 1	54704	157,2327249	-0,221689809	0,134751	-1,645181204	0,099932448	0,509429216
ENSG00000179218	CALR	calreticulin	811	2396,125934	-0,061542081	0,060792	-1,012346543	0,311372388	0,757361021
ENSG00000184992	BRI3BP	BRI3 binding protein	140707	171,2105607	0,273561198	0,176456	1,550307872	0,121067639	0,555411936
ENSG00000127364	TAS2R4	taste 2 receptor member 4	50832	14,56668343	-0,222551194	0,634343	-0,350837444	0,725710304	NA
ENSG00000204116	CHIC1	cysteine rich hydrophobic domain 1	53344	91,55137356	-0,407003518	0,19368	-2,101427696	0,035603439	0,300111909
ENSG00000116584	ARHGEF2	Rho/Rac guanine nucleotide exchange factor 2	9181	378,1184292	0,017293304	0,133291	0,129740856	0,896771457	0,977990819
ENSG00000062822	POLD1	DNA polymerase delta 1, catalytic subunit	5424	79,20858426	0,46085119	0,246778	1,867473511	0,061835493	0,402854784
ENSG00000087087	SRRT	serrate, RNA effector molecule	51593	384,4262531	0,036508265	0,110458	0,330516882	0,741009439	0,939193793
ENSG00000130589	HELZ2	helicase with zinc finger 2	85441	285,29706	-0,029128862	0,123199	-0,23643843	0,813092479	0,959207071
ENSG00000138134	STAMBPL1	STAM binding protein like 1	57559	55,88163937	0,215320294	0,285344	0,754597894	0,450490287	0,84263906
ENSG00000211696	TRGV8	T cell receptor gamma variable 8	6982	24,48367051	0,497930388	0,388564	1,281464231	0,200030656	NA
ENSG00000139055	ERP27	endoplasmic reticulum protein 27	121506	14,04405183	0,257693042	0,450146	0,572465637	0,567006559	NA
ENSG00000241878	PISD	phosphatidylserine decarboxylase	23761	135,6099188	-0,10529264	0,210678	-0,499779792	0,617230141	0,90216458
ENSG00000167447	SMG8	SMG8 nonsense mediated mRNA decay factor	55181	88,73102035	-0,032355919	0,202315	-0,159928314	0,872937544	0,974533542
ENSG00000138032	PPM1B	protein phosphatase, Mg2+/Mn2+ dependent 1B	5495	301,5766748	0,25384097	0,12151	2,08905951	0,036702367	0,305037446
ENSG00000079805	DNM2	dynamin 2	1785	772,570344	-0,164276567	0,060169	-2,730267729	0,006328291	0,115368797
ENSG00000163029	SMC6	structural maintenance of chromosomes 6	79677	198,072822	-0,103199648	0,130964	-0,788003119	0,430694881	0,833171822
ENSG00000173611	SCAI	suppressor of cancer cell invasion	286205	121,9364194	-0,342910738	0,193954	-1,767998284	0,077061191	0,448990052
ENSG00000174021	GNG5	G protein subunit gamma 5	2787	181,7925351	0,204279646	0,132298	1,544086745	0,12256732	0,558690088
ENSG00000177646	ACAD9	acyl-CoA dehydrogenase family member 9	28976	32,0711085	1,449520902	0,417	3,476070707	0,000508818	NA
ENSG00000117616	RSRP1	arginine and serine rich protein 1	57035	18,08312958	-0,58567571	0,800526	-0,731613331	0,464404609	NA
ENSG00000167526	RPL13	ribosomal protein L13	6137	1421,033785	-0,004653707	0,069271	-0,067181227	0,94643743	0,989789558
ENSG00000122188	LAX1	lymphocyte transmembrane adaptor 1	54900	138,4500381	-0,122791195	0,151592	-0,810012114	0,417933213	0,82446781
ENSG00000184661	CDCA2	cell division cycle associated 2	157313	53,15610066	0,688033087	0,266901	2,577861586	0,009941381	0,153700013
ENSG00000185485	NA	NA	NA	57,60982175	0,209311062	0,248609	0,841927576	0,399828494	0,814673014
ENSG00000249709	ZNF564	zinc finger protein 564	163050	65,6532775	0,101991878	0,296801	0,343637282	0,731119084	0,938729652
ENSG00000238227	TMEM250	transmembrane protein 250	90120	94,06794014	-0,072442382	0,183639	-0,39448178	0,693225377	0,928002746
ENSG00000071859	FAM50A	family with sequence similarity 50 member A	9130	127,2174508	0,105502404	0,182648	0,57762772	0,563515488	0,884816202
ENSG00000177946	CENPBD1P	CENPB DNA-binding domain containing 1, pseudogene	92806	31,20230089	0,175543611	0,376845	0,465824158	0,641341372	NA
ENSG00000181274	FRAT2	FRAT regulator of WNT signaling pathway 2	23401	41,04106347	-0,614778295	0,31443	-1,95521267	0,050557974	NA
ENSG00000165280	VCP	valosin containing protein	7415	764,5504767	0,359024627	0,137778	2,605811371	0,009165692	0,146613742
ENSG00000184162	NR2C2AP	nuclear receptor 2C2 associated protein	126382	22,95744284	0,327899687	0,613657	0,534336842	0,593108507	NA
ENSG00000168785	TSPAN5	tetraspanin 5	10098	257,3235647	0,038979378	0,15813	0,246502092	0,805293585	0,957328863
ENSG00000100439	ABHD4	abhydrolase domain containing 4, N-acyl phospholipase B	63874	126,549032	0,077357568	0,161322	0,479522498	0,631566967	0,906823941
ENSG00000133612	AGAP3	ArfGAP with GTPase domain, ankyrin repeat and PH domain 3	116988	167,2710997	-0,083154072	0,145135	-0,572944769	0,566682091	0,88521771
ENSG00000067064	IDI1	isopentenyl-diphosphate delta isomerase 1	3422	345,9288411	0,187955806	0,096857	1,940541398	0,052313928	0,36949064

ENSG00000071553	ATP6AP1	ATPase H+ transporting accessory protein 1	537	252,3030535	0,081119475	0,109	0,744213689	0,456747208	0,844645758
ENSG00000105849	POLR1F	RNA polymerase I subunit F	221830	114,0496372	-0,332874324	0,16157	-2,060248109	0,039374828	0,316271423
ENSG00000124279	FASTKD3	FAST kinase domains 3	79072	30,86606632	0,3915309	0,474439	0,825250126	0,409229601	NA
ENSG00000147419	CCDC25	coiled-coil domain containing 25	55246	84,35537834	-0,244777377	0,298437	-0,820197466	0,412103546	0,822245236
ENSG00000164919	COX6C	cytochrome c oxidase subunit 6C	1345	15,94507704	0,335340871	0,45876	0,730972697	0,46479583	NA
ENSG00000172007	RAB33B	RAB33B, member RAS oncogene family	83452	72,89570119	-0,08403197	0,207804	-0,404381788	0,685931991	0,926450534
ENSG00000179134	SAMD4B	sterile alpha motif domain containing 4B	55095	177,5249189	-0,21287108	0,125499	-1,696203858	0,08984728	0,488772026
ENSG00000180855	ZNF443	zinc finger protein 443	10224	16,96687524	0,007391122	0,520507	0,01419986	0,988670531	NA
ENSG00000112118	MCM3	minichromosome maintenance complex component 3	4172	372,4868884	0,228583835	0,09885	2,312433643	0,020753798	0,225859999
ENSG00000122591	HYCC1	hyccin PI4KA lipid kinase complex subunit 1	84668	131,5570117	0,052294011	0,467038	0,111969411	0,91084766	0,979595261
ENSG00000068120	COASY	Coenzyme A synthase	80347	158,3756154	-0,110192454	0,138052	-0,798195117	0,424757271	0,830220569
ENSG00000256771	ZNF253	zinc finger protein 253	56242	56,74181448	0,388473578	0,255341	1,521389298	0,128162173	0,563689485
ENSG00000134278	SPIRE1	spire type actin nucleation factor 1	56907	108,8956625	-0,064610119	0,170272	-0,379452683	0,704351735	0,930846779
ENSG00000148180	GSN	gelsolin	2934	581,4357613	-0,014698177	0,076391	-0,192406736	0,847423616	0,967411651
ENSG00000135124	P2RX4	purinergic receptor P2X 4	5025	263,6963662	-0,020315123	0,114331	-0,177687072	0,858968739	0,969719775
ENSG00000143751	SDE2	SDE2 telomere maintenance homolog	163859	176,7016811	0,090919498	0,130471	0,696857314	0,485892094	0,858001067
ENSG00000172638	EFEMP2	EGF containing fibulin extracellular matrix protein 2	30008	14,06667983	-0,715257632	0,53108	-1,346798954	0,178044992	NA
ENSG00000163110	PDLIM5	PDZ and LIM domain 5	10611	97,07482864	0,333835057	0,235861	1,415390925	0,156953909	0,61428162
ENSG00000171453	POLR1C	RNA polymerase I and III subunit C	9533	28,6487704	0,396138829	0,338734	1,169470428	0,242214149	NA
ENSG00000121152	NCAPH	non-SMC condensin I complex subunit H	23397	69,66346125	1,430465695	0,391775	3,651245792	0,000260971	0,012574655
ENSG00000106397	PLOD3	procollagen-lysine,2-oxoglutarate 5-dioxygenase 3	8985	75,60579508	-0,036702028	0,214688	-0,170954851	0,864259266	0,971561202
ENSG00000139370	SLC15A4	solute carrier family 15 member 4	121260	91,52338066	0,425579183	0,281411	1,512304905	0,130456314	0,566497377
ENSG00000164252	AGGF1	angiogenic factor with G-patch and FHA domains 1	55109	204,680784	-0,046663988	0,125608	-0,371504764	0,710261609	0,930846779
ENSG00000182087	TMEM259	transmembrane protein 259	91304	477,5529138	-0,229920892	0,080133	-2,869254931	0,0041144	0,088853402
ENSG00000164168	TMEM184C	transmembrane protein 184C	55751	149,9582779	0,100139565	0,137866	0,72635485	0,467621262	0,851116896
ENSG00000100578	KIAA0586	KIAA0586	9786	97,60447339	-0,049279845	0,317841	-0,155045421	0,876785509	0,975875037
ENSG00000171298	GAA	alpha glucosidase	2548	23,09165719	3,687613656	1,778389	2,073569423	0,038119317	NA
ENSG00000221909	FAM200A	family with sequence similarity 200 member A	221786	14,97936747	-0,289994668	0,494124	-0,586886884	0,557279679	NA
ENSG00000112584	FAM120B	family with sequence similarity 120 member B	84498	251,6202293	0,042801318	0,131015	0,326689472	0,743902764	0,939745744
ENSG00000119969	HELLS	helicase, lymphoid specific	3070	69,09263684	0,60863707	0,378555	1,607790743	0,107881017	0,527254203
ENSG00000154114	TBCEL	tubulin folding cofactor E like	219899	85,2410292	0,330609963	0,204593	1,615937716	0,10610778	0,522853317
ENSG00000160991	ORAI2	ORAI calcium release-activated calcium modulator 2	80228	786,6905349	-0,116855178	0,105913	-1,103316785	0,26988962	0,725477256
ENSG00000136279	DBNL	drebrin like	28988	422,1383401	-0,392274282	0,278209	-1,410000121	0,158539647	0,614461481
ENSG00000114491	UMPS	uridine monophosphate synthetase	7372	76,49457925	0,060703768	0,213514	0,284307647	0,776174627	0,946448613
ENSG00000164576	SAP30L	SAP30 like	79685	134,1908516	-0,095289832	0,165747	-0,574910801	0,565351625	0,885045179
ENSG00000167900	TK1	thymidine kinase 1	7083	59,8411856	0,736544737	0,285116	2,583316961	0,009785538	0,151888562
ENSG00000213782	DDX47	DEAD-box helicase 47	51202	99,16325247	0,344695937	0,210987	1,633730085	0,102315538	0,515119384
ENSG00000163877	SNIP1	Smad nuclear interacting protein 1	79753	54,03362304	0,051841763	0,267874	0,193530163	0,846543786	0,967231052
ENSG00000154727	GABPA	GA binding protein transcription factor subunit alpha	2551	269,8331953	-0,166359748	0,104664	-1,589457509	0,11195714	0,534832035
ENSG00000146859	TMEM140	transmembrane protein 140	55281	64,4243563	0,267608135	0,227382	1,176908469	0,239232041	0,694099909
ENSG00000100147	CCDC134	coiled-coil domain containing 134	79879	125,8059801	0,028469861	0,238355	0,119443191	0,904924245	0,978460866
ENSG00000151247	EIF4E	eukaryotic translation initiation factor 4E	1977	219,4882649	0,291708114	0,148526	1,964016273	0,049528205	0,357023063
ENSG00000158615	PPP1R15B	protein phosphatase 1 regulatory subunit 15B	84919	627,132835	-0,112078028	0,072479	-1,546359158	0,122017852	0,557491687
ENSG00000000938	FGR	FGR proto-oncogene, Src family tyrosine kinase	2268	200,8209738	-0,193287512	0,136158	-1,419586023	0,155728237	0,61428162
ENSG00000101811	CSTF2	cleavage stimulation factor subunit 2	1478	77,56015772	0,302228012	0,197626	1,529296023	0,126191075	0,563689485
ENSG00000198663	C6orf89	chromosome 6 open reading frame 89	221477	552,5159461	0,101841943	0,100824	1,010095535	0,312449521	0,757868604
ENSG00000259660	NA	NA	NA	31,84944972	0,93796935	0,322124	2,911823894	0,003593252	NA
ENSG00000085840	ORC1	origin recognition complex subunit 1	4998	29,56304304	0,904366696	0,354891	2,548297257	0,010825019	NA

ENSG00000144815	NXPE3	neurexophilin and PC-esterase domain family member 3	91775	266,4458949	-0,023415416	0,134985	-0,173466482	0,862284772	0,970951197
ENSG00000234545	FAM133B	family with sequence similarity 133 member B	257415	70,86666264	0,536085394	0,212592	2,52166097	0,011680222	0,169255469
ENSG00000061273	HDAC7	histone deacetylase 7	51564	359,722454	-0,214396801	0,109352	-1,960613372	0,049924141	0,357759312
ENSG00000140829	DHX38	DEAH-box helicase 38	9785	415,7030452	-0,055395551	0,086124	-0,643208722	0,520088675	0,86944974
ENSG00000185477	GPRIN3	GPRIN family member 3	285513	666,5493412	-0,121284491	0,087447	-1,386953924	0,165455813	0,624454568
ENSG00000015153	YAF2	YY1 associated factor 2	10138	88,6862351	0,119690765	0,308599	0,387852643	0,698125086	0,930783276
ENSG00000179115	FARSA	phenylalanyl-tRNA synthetase subunit alpha	2193	161,1967133	0,004707669	0,138506	0,033988984	0,972885935	0,994534085
ENSG00000172123	SLFN12	schlafen family member 12	55106	71,51645241	-0,198994165	0,259238	-0,767613004	0,442717134	0,838678081
ENSG00000166266	CUL5	cullin 5	8065	150,2290004	-0,097452342	0,166935	-0,583772438	0,559373424	0,883921037
ENSG00000099785	MARCHF2	membrane associated ring-CH-type finger 2	51257	70,49111693	-0,248973459	0,198743	-1,252740323	0,210300226	0,664554962
ENSG00000106524	ANKMY2	ankyrin repeat and MYND domain containing 2	57037	80,76730299	-0,406298623	0,258584	-1,57124639	0,116125428	0,543356283
ENSG00000153207	AHCTF1	AT-hook containing transcription factor 1	25909	275,2649051	-0,005718393	0,131749	-0,043403566	0,965379835	0,992789539
ENSG00000163607	GTPBP8	GTP binding protein 8 (putative)	29083	51,39381396	-0,135772402	0,234397	-0,579241468	0,562426254	0,884605624
ENSG00000172432	GTPBP2	GTP binding protein 2	54676	190,5543396	-0,003277369	0,180701	-0,018136927	0,985529619	0,996312219
ENSG00000100056	ESS2	ess-2 splicing factor homolog	8220	28,06155722	-0,2536503	0,485426	-0,522530971	0,601300688	NA
ENSG00000112304	ACOT13	acyl-CoA thioesterase 13	55856	34,11332343	0,302151569	0,318392	0,948991509	0,342624931	NA
ENSG00000135482	ZC3H10	zinc finger CCCH-type containing 10	84872	61,45680119	0,150258213	0,226372	0,663767093	0,50683939	0,864738156
ENSG00000153914	SREK1	splicing regulatory glutamic acid and lysine rich protein 1	140890	180,3039457	0,012693937	0,15133	0,083882546	0,933149817	0,987098279
ENSG00000198815	FOXJ3	forkhead box J3	22887	362,0670241	-0,011039659	0,096703	-0,114160129	0,909110858	0,97930822
ENSG00000067113	PLPP1	phospholipid phosphatase 1	8611	12,06296672	-0,160241478	0,55359	-0,289458825	0,772230284	NA
ENSG00000132589	FLOT2	flotillin 2	2319	426,9887546	-0,145456114	0,078193	-1,860228408	0,062853218	0,404317327
ENSG00000127022	CANX	calnexin	821	894,9495548	-0,279639175	0,098	-2,853449441	0,004324741	0,090819559
ENSG00000161533	ACOX1	acyl-CoA oxidase 1	51	147,6140843	-0,078387368	0,151957	-0,515853408	0,605956797	0,897380562
ENSG00000131462	TUBG1	tubulin gamma 1	7283	49,48913203	-0,054531043	0,259882	-0,209829834	0,833800488	0,963446071
ENSG00000188529	SRSF10	serine and arginine rich splicing factor 10	10772	428,6932061	0,17695298	0,098578	1,795057085	0,072644605	0,437874697
ENSG00000089818	NECAP1	NECAP endocytosis associated 1	25977	46,64087929	2,735768196	1,513125	1,808025278	0,070602566	0,431527281
ENSG00000234616	JRK	Jrk helix-turn-helix protein	8629	239,9006369	-0,203301707	0,130767	-1,554684803	0,120021156	0,553734734
ENSG00000128951	DUT	deoxyuridine triphosphatase	1854	108,5632153	0,264978143	0,191931	1,380592735	0,167404218	0,62720477
ENSG00000136122	BORA	BORA aurora kinase A activator	79866	51,24135512	0,210801767	0,334023	0,631098748	0,527975959	0,872142129
ENSG00000156831	NSMCE2	NSE2 (MMS21) homolog, SMC5-SMC6 complex SUMO ligase	286053	34,29298734	0,510706193	0,337932	1,511270242	0,130719612	NA
ENSG00000183617	MRPL54	mitochondrial ribosomal protein L54	116541	84,59439174	-0,208057743	0,202293	-1,02849672	0,303716233	0,751870158
ENSG00000100360	IFT27	intraflagellar transport 27	11020	24,76778209	-0,455516023	0,347442	-1,311057403	0,189838371	NA
ENSG00000174125	TLR1	toll like receptor 1	7096	39,46764075	-0,100832176	0,310832	-0,324394105	0,745639682	NA
ENSG00000100014	SPECC1L	sperm antigen with calponin homology and coiled-coil domains 1 like	23384	318,8975942	-0,064088805	0,103594	-0,618651	0,536146297	0,874923109
ENSG00000066855	MTFR1	mitochondrial fission regulator 1	9650	22,56285438	1,121972261	0,479793	2,338451933	0,019363816	NA
ENSG00000109814	UGDH	UDP-glucose 6-dehydrogenase	7358	166,4693575	-0,406247923	0,128242	-3,167830598	0,00153581	0,046039123
ENSG00000139132	FGD4	FYVE, RhoGEF and PH domain containing 4	121512	93,80724707	0,333032119	0,200448	1,661437226	0,096625667	0,502914503
ENSG00000168010	ATG16L2	autophagy related 16 like 2	89849	46,3189684	-1,283656264	0,867467	-1,479774847	0,138933343	0,580974065
ENSG00000103035	PSMD7	proteasome 26S subunit, non-ATPase 7	5713	216,010531	0,438503673	0,144247	3,03994991	0,002366175	0,059432797
ENSG00000046647	GEMIN8	gem nuclear organelle associated protein 8	54960	30,86065812	-0,239725904	0,342368	-0,700199566	0,483802682	NA
ENSG00000143771	CNIH4	cornichon family AMPA receptor auxiliary protein 4	29097	69,67012063	-0,179015471	0,281617	-0,635670662	0,524991103	0,871176869
ENSG00000117523	PRRC2C	proline rich coiled-coil 2C	23215	2589,135059	0,052512758	0,041265	1,272560304	0,203174112	0,660954946
ENSG00000155903	RASA2	RAS p21 protein activator 2	5922	992,6700446	-0,063040272	0,067122	-0,939190702	0,347632842	0,784103633
ENSG00000054965	FAM168A	family with sequence similarity 168 member A	23201	330,5162655	-0,05839506	0,097442	-0,599277986	0,548987526	0,880487651
ENSG00000170315	UBB	ubiquitin B	7314	1621,590717	0,048698785	0,049964	0,974675072	0,329721462	0,771479881
ENSG00000167528	ZNF641	zinc finger protein 641	121274	264,295155	0,170062909	0,125018	1,360303595	0,173733871	0,630547978
ENSG00000175395	ZNF25	zinc finger protein 25	219749	60,9868666	0,4707809	0,298355	1,577919539	0,114584096	0,54024053
ENSG00000068097	HEATR6	HEAT repeat containing 6	63897	82,19490747	-0,685001099	0,248003	-2,762067506	0,00574366	0,10771718
ENSG00000163681	SLMAP	sarcolemma associated protein	7871	241,917669	-0,320146591	0,119847	-2,671286674	0,007556108	0,129012338
ENSG00000066777	ARFGEF1	ADP ribosylation factor guanine nucleotide exchange factor 1	10565	467,8925157	0,088027325	0,080672	1,091169468	0,27519832	0,727877072
ENSG00000137337	MDC1	mediator of DNA damage checkpoint 1	9656	671,9776178	0,081075053	0,081315	0,997048158	0,318741135	0,763389521

ENSG00000067829	IDH3G	isocitrate dehydrogenase (NAD(+)) 3 non-catalytic subunit gamma	3421	147,2162004	-0,080781571	0,149267	-0,541187193	0,588378564	0,891557039
ENSG00000178307	TMEM11	transmembrane protein 11	8834	22,79319513	-0,051795431	0,39783	-0,130194951	0,896412189	NA
ENSG00000178921	PFAS	phosphoribosylformylglycinamidine synthase	5198	110,1897709	0,166439839	0,246195	0,676048091	0,499010112	0,86161226
ENSG00000189060	H1-0	H1,0 linker histone	3005	51,37801159	0,482929216	0,234446	2,059869868	0,039410983	0,316271423
ENSG00000178996	SNX18	sorting nexin 18	112574	336,8559475	0,135451998	0,090594	1,495152286	0,134874703	0,575414344
ENSG00000102781	KATNAL1	katanin catalytic subunit A1 like 1	84056	182,2041646	-0,355342509	0,164105	-2,165336335	0,030361938	0,279077601
ENSG00000198276	UCKL1	uridine-cytidine kinase 1 like 1	54963	92,36168041	-0,049315084	0,171288	-0,287907192	0,773417782	0,946448613
ENSG00000270629	NBPF14	NBPF member 14	25832	804,6758759	0,071749808	0,124328	0,577103199	0,563869744	0,885045179
ENSG00000272772	NA	NA	NA	125,7650777	0,051791647	0,313855	0,165017618	0,868930115	0,973118501
ENSG00000099917	MED15	mediator complex subunit 15	51586	169,1914646	0,312349766	0,203347	1,536041999	0,12452808	0,5624706
ENSG00000117280	RAB29	RAB29, member RAS oncogene family	8934	293,756173	0,070179928	0,109063	0,643479656	0,519912912	0,86944974
ENSG00000132199	ENOSF1	enolase superfamily member 1	55556	22,32657797	-0,209393688	0,46267	-0,452576659	0,650853609	NA
ENSG00000122912	SLC25A16	solute carrier family 25 member 16	8034	72,20262753	0,11203791	0,211027	0,530917429	0,595476	0,894229873
ENSG00000129197	RPAIN	RPA interacting protein	84268	39,06934006	0,293454976	0,290524	1,010087777	0,312453238	NA
ENSG00000106771	TMEM245	transmembrane protein 245	23731	283,1661483	0,019464286	0,148734	0,130866557	0,895880871	0,977990819
ENSG00000160007	ARHGAP35	Rho GTPase activating protein 35	2909	614,8822662	0,03215507	0,078221	0,411077145	0,681015968	0,925586358
ENSG00000165819	METTL3	methyltransferase 3, N6-adenosine-methyltransferase complex catalytic subunit	56339	41,93975032	1,503857515	0,999171	1,505105946	0,132296841	NA
ENSG00000170525	PFKFB3	6-phosphofructo-2-kinase/fructose-2,6-biphosphatase 3	5209	153,910602	0,05974713	0,140189	0,426190255	0,669969218	0,922641254
ENSG00000173960	UBXN2A	UBX domain protein 2A	165324	75,89482861	-0,272043924	0,282422	-0,963254819	0,335419661	0,775275461
ENSG00000271121	NA	NA	NA	14,63212958	0,037735028	0,626688	0,060213398	0,951985675	NA
ENSG00000023902	PLEKHO1	pleckstrin homology domain containing O1	51177	329,1068326	-0,075199573	0,116194	-0,647191907	0,517507749	0,868791049
ENSG00000076984	MAP2K7	mitogen-activated protein kinase kinase 7	5609	232,2506827	-0,245926808	0,111636	-2,202931254	0,027599594	0,269631574
ENSG00000100097	LGALS1	galectin 1	3956	492,9006588	0,303728082	0,103224	2,942412137	0,003256662	0,075374002
ENSG00000102763	VWA8	von Willebrand factor A domain containing 8	23078	199,6564072	0,148051838	0,118229	1,252247736	0,210479607	0,664672917
ENSG00000102910	LONP2	lon peptidase 2, peroxisomal	83752	417,7032431	0,129923055	0,11609	1,119155932	0,263073622	0,719422085
ENSG00000108963	DPH1	diphthamide biosynthesis 1	1801	62,06110712	-0,35184398	0,284944	-1,234782601	0,216911425	0,672891335
ENSG00000138759	FRAS1	Fraser extracellular matrix complex subunit 1	80144	49,99275154	-0,428527039	0,25367	-1,68930647	0,091160714	0,490934677
ENSG00000175895	PLEKHF2	pleckstrin homology and FYVE domain containing 2	79666	76,43717305	0,188906409	0,220091	0,858310354	0,390721113	0,811187847
ENSG00000182108	DEXI	Dexi homolog	28955	76,31332578	0,181501442	0,209379	0,866855481	0,386021199	0,809653368
ENSG00000183484	GPR132	G protein-coupled receptor 132	29933	128,5321492	-0,234911911	0,152192	-1,543519082	0,122704881	0,558690088
ENSG00000078808	SDF4	stromal cell derived factor 4	51150	488,4097711	-0,047968862	0,09753	-0,491835032	0,622835969	0,90479929
ENSG00000130024	PHF10	PHD finger protein 10	55274	221,0623215	0,045843682	0,114477	0,400463137	0,68881543	0,926748702
ENSG00000148362	PAXX	PAXX non-homologous end joining factor	286257	48,75181664	0,069686952	0,306314	0,227501954	0,82003345	0,961418528
ENSG00000245680	ZNF585B	zinc finger protein 585B	92285	107,3957288	0,073494214	0,241818	0,303923351	0,761186287	0,942901768
ENSG00000027075	PRKCH	protein kinase C eta	5583	1559,222384	-0,038896906	0,048002	-0,810315729	0,417758737	0,82446781
ENSG00000153140	CETN3	centrin 3	1070	26,01084581	0,14338538	0,365837	0,391938141	0,69510392	NA
ENSG00000293268	NA	NA	NA	171,0342459	0,14007498	0,154281	0,907919412	0,363920801	0,793072689
ENSG00000109381	ELF2	E74 like ETS transcription factor 2	1998	164,1306513	0,039630949	0,13483	0,293932008	0,768809859	0,944802477
ENSG00000115808	STRN	striatin	6801	364,8454164	0,05113184	0,087884	0,581808447	0,560695714	0,884021857
ENSG00000205426	KRT81	keratin 81	3887	306,783933	0,018845567	0,101747	0,185220777	0,853055873	0,968333694
ENSG00000219200	RNASEK	ribonuclease K	440400	188,5864315	-0,017318238	0,121934	-0,142029189	0,88705695	0,977954139
ENSG00000134882	UBAC2	UBA domain containing 2	337867	341,1871095	0,112503113	0,107048	1,050963831	0,293275202	0,744745216
ENSG00000182405	PGBD4	piggyBac transposable element derived 4	161779	33,25555343	0,248317786	0,339633	0,731135694	0,464696274	NA
ENSG00000013288	MAN2B2	mannosidase alpha class 2B member 2	23324	235,0534084	-0,116384845	0,113036	-1,029624075	0,303186509	0,751870158
ENSG00000102898	NUTF2	nuclear transport factor 2	10204	166,5092275	0,46924553	0,160382	2,925805852	0,003435653	0,077987331
ENSG00000135604	STX11	syntaxin 11	8676	130,7688666	0,266233715	0,194921	1,365855811	0,171984225	0,629558603
ENSG00000176476	SGF29	SAGA complex associated factor 29	112869	43,60462015	0,088644851	0,284251	0,311854579	0,755151043	0,942901768
ENSG00000179943	FIZ1	FLT3 interacting zinc finger 1	84922	26,7376165	0,089838341	0,374313	0,240008622	0,810323572	NA
ENSG00000164970	FAM219A	family with sequence similarity 219 member A	203259	61,24499828	-0,416438602	0,276509	-1,506057557	0,132052399	0,568919111
ENSG00000147403	RPL10	ribosomal protein L10	6134	585,4410413	-0,256239952	0,121574	-2,107691368	0,035057694	0,298636803
ENSG00000124783	SSR1	signal sequence receptor subunit 1	6745	865,8174788	0,010301276	0,073417	0,140312727	0,888412909	0,977990819
ENSG00000125871	MGME1	mitochondrial genome maintenance exonuclease 1	92667	140,5006821	0,270576292	0,157179	1,721454905	0,085168308	0,47675331

ENSG00000040487	SLC66A1	solute carrier family 66 member 1	54896	23,65005112	0,437566242	0,411362	1,063700019	0,287464616	NA
ENSG00000128609	NDUFA5	NADH:ubiquinone oxidoreductase subunit A5	4698	111,9099628	0,027705362	0,188847	0,146707785	0,883362674	0,976751663
ENSG00000120539	MASTL	microtubule associated serine/threonine kinase like	84930	88,15901198	0,223976552	0,187614	1,193813176	0,232551057	0,687930696
ENSG00000204642	HLA-F	major histocompatibility complex, class I, F	3134	206,7739395	-0,15121749	0,159982	-0,945216874	0,344548166	0,781366475
ENSG00000206560	ANKRD28	ankyrin repeat domain 28	23243	283,9383869	-0,17093891	0,159549	-1,071384991	0,283996359	0,737423617
ENSG00000154001	PPP2R5E	protein phosphatase 2 regulatory subunit B'epsilon	5529	305,8498803	-0,080913298	0,105685	-0,765610725	0,443907961	0,8399068
ENSG00000176438	SYNE3	spectrin repeat containing nuclear envelope family member 3	161176	384,4955412	0,103270148	0,092648	1,114654747	0,2649984	0,720421404
ENSG00000206127	GOLGA8O	golgin A8 family member O	728047	1046,590194	0,090639301	0,084794	1,068940653	0,285096412	0,737867955
ENSG00000125534	PPDPF	pancreatic progenitor cell differentiation and proliferation factor	79144	185,111415	0,063079434	0,140168	0,450027943	0,652690293	0,916707718
ENSG00000134910	STT3A	STT3 oligosaccharyltransferase complex catalytic subunit A	3703	314,5904702	0,034054318	0,092181	0,369428293	0,711808513	0,930846779
ENSG00000130511	SSBP4	single stranded DNA binding protein 4	170463	196,6772727	-0,126053762	0,118042	-1,067874526	0,285577115	0,737867955
ENSG00000085832	EPS15	epidermal growth factor receptor pathway substrate 15	2060	435,5723312	-0,114150459	0,119749	-0,953250646	0,340463091	0,777431905
ENSG00000143537	ADAM15	ADAM metallopeptidase domain 15	8751	28,97056285	-0,694719624	0,324938	-2,138004733	0,032516359	NA
ENSG00000068885	IFT80	intraflagellar transport 80	57560	136,0346092	-0,297690179	0,155102	-1,919318734	0,054944009	0,379868172
ENSG00000107643	MAPK8	mitogen-activated protein kinase 8	5599	82,87447193	-0,296331444	0,363664	-0,814848992	0,415158754	0,82446781
ENSG00000126351	THRA	thyroid hormone receptor alpha	7067	53,64370424	-0,470602998	0,224617	-2,095138459	0,036158686	0,302116994
ENSG00000161800	RACGAP1	Rac GTPase activating protein 1	29127	201,8177801	0,575912012	0,136034	4,233581356	2,30E-05	0,001985068
ENSG00000172375	C2CD2L	C2CD2 like	9854	80,53284951	-0,347063475	0,24332	-1,426367337	0,153762337	0,6111586
ENSG00000163755	HPS3	HPS3 biogenesis of lysosomal organelles complex 2 subunit 1	84343	279,2901705	0,189483146	0,099897	1,896791239	0,057855496	0,390095203
ENSG00000033800	PIAS1	protein inhibitor of activated STAT 1	8554	287,0176721	-0,030744384	0,134673	-0,228289936	0,819420848	0,961128271
ENSG00000119403	PHF19	PHD finger protein 19	26147	200,985691	0,093318883	0,134657	0,693011657	0,488302236	0,859508239
ENSG00000213689	TREX1	three prime repair exonuclease 1	11277	17,07101547	1,1660111	0,482633	2,415939798	0,015694656	NA
ENSG00000177383	MAGEF1	MAGE family member F1	64110	32,26254181	-0,472290665	0,295104	-1,600418708	0,109505728	NA
ENSG00000015676	NUDCD3	NudC domain containing 3	23386	101,421463	-0,119128276	0,192554	-0,618673406	0,536131533	0,874923109
ENSG00000126226	PCID2	PCI domain containing 2	55795	214,8892774	0,312147364	0,116517	2,678984135	0,007384589	0,126634419
ENSG00000167645	YIF1B	Yip1 interacting factor homolog B, membrane trafficking protein	90522	44,52058389	0,127062561	0,345848	0,367394523	0,713324756	0,930949258
ENSG00000140943	MBTPS1	membrane bound transcription factor peptidase, site 1	8720	390,2324781	-0,054347402	0,109604	-0,495853395	0,61999785	0,904264273
ENSG00000155366	RHOC	ras homolog family member C	389	437,8168277	-0,132335717	0,102171	-1,295239449	0,19523764	0,653343172
ENSG00000173442	EHBP1L1	EH domain binding protein 1 like 1	254102	572,5537299	-0,020635036	0,095509	-0,216053144	0,828946322	0,962723654
ENSG00000165490	DDIAS	DNA damage induced apoptosis suppressor	220042	21,70516124	1,202169098	0,497961	2,414182638	0,015770555	NA
ENSG00000170348	TMED10	transmembrane p24 trafficking protein 10	10972	1120,577517	-0,194158533	0,054809	-3,542438856	0,000396445	0,017591421
ENSG00000183647	ZNF530	zinc finger protein 530	348327	15,32436573	1,169569002	0,530634	2,204096031	0,027517591	NA
ENSG00000169925	BRD3	bromodomain containing 3	8019	315,5959543	-0,208738522	0,099395	-2,10009569	0,035720424	0,300694762
ENSG00000211456	SACM1L	SAC1 like phosphatidylinositide phosphatase	22908	449,2519279	0,005813935	0,277691	0,02093672	0,983296135	0,996185513
ENSG00000205758	CRYZL1	crystallin zeta like 1	9946	50,50472421	-0,408365467	0,279352	-1,461829577	0,143787917	0,59024645
ENSG00000270505	NA	NA	NA	43,12639638	-0,174369564	0,266464	-0,654383979	0,512864453	0,86823028
ENSG00000031823	RANBP3	RAN binding protein 3	8498	202,4136578	-0,213876187	0,125507	-1,704099428	0,088362513	0,486308725
ENSG00000081386	ZNF510	zinc finger protein 510	22869	121,7437724	0,208443634	0,155635	1,339314131	0,18046843	0,634062228
ENSG00000178057	NDUFAF3	NADH:ubiquinone oxidoreductase complex assembly factor 3	25915	39,52792836	0,413206415	0,312012	1,324329957	0,185393486	NA
ENSG00000183281	PLGLB1	plasminogen like B1	5343	20,20037027	0,875011554	0,499413	1,752080386	0,079759986	NA
ENSG00000010404	IDS	iduronate 2-sulfatase	3423	827,7627894	-0,063149401	0,115546	-0,546530337	0,58470144	0,890019742
ENSG00000062650	WAPL	WAPL cohesin release factor	23063	539,0727643	-0,09425046	0,072054	-1,308055307	0,190854551	0,648905473
ENSG00000109390	NDUFC1	NADH:ubiquinone oxidoreductase subunit C1	4717	12,20877801	-0,284564604	0,944301	-0,301349435	0,763148048	NA
ENSG00000146232	NFKBIE	NFKB inhibitor epsilon	4794	39,85877595	0,260431907	0,27247	0,955820155	0,339163104	NA

ENSG00000196365	LONP1	lon peptidase 1, mitochondrial	9361	130,9955702	0,263755222	0,196398	1,342962901	0,179283987	0,633591125
ENSG00000221983	UBA52	ubiquitin A-52 residue ribosomal protein fusion product 1	7311	855,6310951	-0,040955653	0,068644	-0,596640483	0,550747437	0,880819693
ENSG00000126882	FAM78A	family with sequence similarity 78 member A	286336	357,049093	-0,191454117	0,117427	-1,630405857	0,103015748	0,515621608
ENSG00000047621	C12orf4	chromosome 12 open reading frame 4	57102	93,02168085	0,105851435	0,205961	0,51393944	0,607294332	0,897481254
ENSG00000196417	ZNF765	zinc finger protein 765	91661	89,00134849	-0,216392248	0,228502	-0,947003171	0,343637163	0,781132432
ENSG00000075790	BCAP29	B cell receptor associated protein 29	55973	77,13118564	-0,963464959	0,247144	-3,898396059	9,68E-05	0,006285274
ENSG00000127993	RBM48	RNA binding motif protein 48	84060	97,53251448	-0,069460396	0,189958	-0,365662648	0,714616821	0,931861284
ENSG00000129680	MAP7D3	MAP7 domain containing 3	79649	120,5087143	0,058046758	0,176482	0,328910236	0,742223538	0,939193793
ENSG00000197858	GPAA1	glycosylphosphatidylinositol anchor attachment 1	8733	114,985423	0,207067545	0,226121	0,91573856	0,359804035	0,79112567
ENSG00000033867	SLC4A7	solute carrier family 4 member 7	9497	126,4901113	-0,26763125	0,461987	-0,579304212	0,562383924	0,884605624
ENSG00000198853	RUSC2	RUN and SH3 domain containing 2	9853	97,35994804	-0,132648827	0,200243	-0,662440906	0,507688695	0,864738156
ENSG00000131013	PPIL4	peptidylprolyl isomerase like 4	85313	87,23677245	0,313379786	0,19034	1,646420616	0,099677191	0,509429216
ENSG00000082512	TRAF5	TNF receptor associated factor 5	7188	439,7368708	-0,031143222	0,109824	-0,283573731	0,776737071	0,946987419
ENSG00000145901	TNIP1	TNFAIP3 interacting protein 1	10318	694,6027168	-0,060062448	0,074145	-0,810068901	0,417900577	0,82446781
ENSG00000162695	SLC30A7	solute carrier family 30 member 7	148867	223,0017722	-0,100015896	0,139343	-0,717768591	0,472899983	0,853811879
ENSG00000270647	TAF15	TATA-box binding protein associated factor 15	8148	529,1587267	-0,178352908	0,263863	-0,675930172	0,49908498	0,86161226
ENSG00000108559	NUP88	nucleoporin 88	4927	130,65723	1,078746723	0,205062	5,260596865	1,44E-07	2,75E-05
ENSG00000027697	IFNGR1	interferon gamma receptor 1	3459	134,821471	0,221878107	0,149507	1,484063519	0,13779207	0,578980755
ENSG00000104177	MYEF2	myelin expression factor 2	50804	17,90499744	-0,512528505	0,572474	-0,895287614	0,37063336	NA
ENSG00000123933	MXD4	MAX dimerization protein 4	10608	285,5627405	-0,178692012	0,125649	-1,422157748	0,154980461	0,613516404
ENSG00000114388	NPRL2	NPR2 like, GATOR1 complex subunit	10641	47,63337698	0,70806846	0,313683	2,257274337	0,023990938	0,247679511
ENSG00000258890	CEP95	centrosomal protein 95	90799	90,08679435	0,15597357	0,20603	0,757042136	0,449024619	0,84263906
ENSG00000101052	IFT52	intraflagellar transport 52	51098	59,54373653	-0,057787832	0,222165	-0,260112175	0,794777246	0,952742807
ENSG00000136152	COG3	component of oligomeric golgi complex 3	83548	299,2957226	0,042389573	0,112026	0,378389926	0,705140951	0,930846779
ENSG00000139289	PHLDA1	pleckstrin homology like domain family A member 1	22822	220,2969419	0,516126507	0,11419	4,51988095	6,19E-06	0,000665701
ENSG00000146670	CDCA5	cell division cycle associated 5	113130	82,62291762	0,910084057	0,206175	4,414136037	1,01E-05	0,000995634
ENSG00000174953	DHX36	DEAH-box helicase 36	170506	203,4784588	-0,121159923	0,173941	-0,696558292	0,486079265	0,858095426
ENSG00000146426	TIAM2	TIAM Rac1 associated GEF 2	26230	83,06822202	-0,244708832	0,193811	-1,26261494	0,206727596	0,661359894
ENSG00000170160	CCDC144A	coiled-coil domain containing 144A	9720	243,8527652	0,02411383	0,137264	0,175674569	0,860549615	0,97061627
ENSG00000234745	HLA-B	major histocompatibility complex, class I, B	3106	4217,108586	-0,037734473	0,041151	-0,916983921	0,359151066	0,790999468
ENSG00000075785	RAB7A	RAB7A, member RAS oncogene family	7879	728,4288642	0,028123607	0,063274	0,444472549	0,656700971	0,919053711
ENSG00000169991	IFFO2	intermediate filament family orphan 2	126917	180,2296114	-0,075290492	0,138589	-0,543262971	0,586948756	0,891326322
ENSG00000198924	DCLRE1A	DNA cross-link repair 1A	9937	133,2749271	0,09376677	0,157563	0,595104751	0,551773453	0,881150198
ENSG00000145358	DDIT4L	DNA damage inducible transcript 4 like	115265	17,5719187	0,620930041	0,508682	1,220664821	0,222212952	NA
ENSG00000185716	MOSMO	modulator of smoothened	730094	39,87418843	0,557571324	0,328731	1,696133374	0,089860624	NA
ENSG00000197111	PCBP2	poly(rC) binding protein 2	5094	1294,443866	0,078982023	0,055447	1,42445814	0,154313894	0,611607348
ENSG00000141279	NPEPPS	aminopeptidase puromycin sensitive	9520	234,5160482	-0,017511511	0,147909	-0,118393507	0,90575587	0,978460866
ENSG00000065615	CYB5R4	cytochrome b5 reductase 4	51167	133,2029542	-0,387097718	0,310873	-1,245194425	0,213060291	0,667215123
ENSG00000065615	RIPPLY2-CYB5R4	RIPPLY2-CYB5R4 readthrough	123956252	133,2029542	-0,387097718	0,310873	-1,245194425	0,213060291	0,667215123
ENSG00000182287	AP1S2	adaptor related protein complex 1 subunit sigma 2	8905	107,9356336	-0,166817448	0,170241	-0,979888823	0,327141	0,769732001
ENSG00000186517	ARHGAP30	Rho GTPase activating protein 30	257106	1681,861967	-0,139309044	0,047422	-2,93764398	0,003307166	0,075727344
ENSG00000131558	EXOC4	exocyst complex component 4	60412	322,8148103	-0,065929607	0,100205	-0,657944374	0,510573876	0,866180216
ENSG00000170631	ZNF16	zinc finger protein 16	7564	19,03727056	0,777925869	0,424445	1,832805829	0,066831452	NA
ENSG00000146263	MMS22L	MMS22 like, DNA repair protein	253714	82,60713796	0,237976266	0,219146	1,085927966	0,277510863	0,731689696
ENSG00000065183	WDR3	WD repeat domain 3	10885	135,5816774	-0,10035935	0,295999	-0,339053207	0,734569648	0,939157822
ENSG00000124762	CDKN1A	cyclin dependent kinase inhibitor 1A	1026	55,76763391	0,725296536	0,275887	2,628958414	0,008564683	0,141021013
ENSG00000125779	PANK2	pantothenate kinase 2	80025	177,8045693	-0,206756337	0,272067	-0,759947025	0,447286251	0,842562288
ENSG00000126934	MAP2K2	mitogen-activated protein kinase kinase 2	5605	309,0576601	-0,123367724	0,10832	-1,138923744	0,254734961	0,709964783
ENSG00000134825	TMEM258	transmembrane protein 258	746	61,97481423	-0,264984986	0,219409	-1,207723457	0,227153652	0,682242748

ENSG00000137513	NARS2	asparaginyl-tRNA synthetase 2, mitochondrial	79731	29,20058512	0,530657593	0,352737	1,504401518	0,132478014	NA
ENSG00000143156	NME7	NME/NM23 family member 7	29922	19,32931301	-0,730592513	0,400216	-1,82549594	0,067926223	NA
ENSG00000161980	POLR3K	RNA polymerase III subunit K	51728	44,49088594	0,119928369	0,25974	0,461724407	0,644278966	0,912530858
ENSG00000164626	KCNK5	potassium two pore domain channel subfamily K member 5	8645	27,6341	0,507601548	0,389928	1,301782196	0,192990852	NA
ENSG00000175048	ZDHHC14	zinc finger DHHC-type palmitoyltransferase 14	79683	228,4292592	0,362098049	0,141343	2,561836448	0,010412034	0,157997752
ENSG00000204397	CARD16	caspase recruitment domain family member 16	114769	39,04879256	-0,124040483	0,529128	-0,2344243	0,814655595	NA
ENSG00000183785	TUBA8	tubulin alpha 8	51807	58,39331645	-0,160767213	0,374232	-0,429592017	0,667492445	0,922308959
ENSG00000100614	PPM1A	protein phosphatase, Mg2+/Mn2+ dependent 1A	5494	277,1442539	0,275955692	0,12085	2,283457768	0,022403418	0,238423365
ENSG00000072736	NFATC3	nuclear factor of activated T cells 3	4775	1154,444445	-0,061094639	0,060855	-1,003933359	0,315410736	0,760454518
ENSG00000132763	MMACHC	metabolism of cobalamin associated C	25974	17,76297556	0,226444016	0,629979	0,359446719	0,71926093	NA
ENSG00000133657	ATP13A3	ATPase 13A3	79572	326,0177069	-0,083232859	0,145709	-0,571227182	0,567845655	0,88603113
ENSG00000077235	GTF3C1	general transcription factor IIIC subunit 1	2975	557,8117048	-0,008521771	0,068963	-0,12356993	0,901655802	0,978122192
ENSG00000257800	NA	NA	NA	13,53563428	-0,498913046	0,556322	-0,896806776	0,369822031	NA
ENSG00000136159	NUDT15	nudix hydrolase 15	55270	33,37317301	-0,17473854	0,308406	-0,566586701	0,570995013	NA
ENSG00000138031	ADCY3	adenylate cyclase 3	109	478,5826221	0,298310605	0,077751	3,836761435	0,000124667	0,00768001
ENSG00000159885	ZNF222	zinc finger protein 222	7673	14,75512609	0,413503497	0,47621	0,868322413	0,385217856	NA
ENSG00000125812	GZF1	GDNF inducible zinc finger protein 1	64412	84,20026881	-0,184492089	0,201122	-0,917316372	0,35897688	0,790999468
ENSG00000070501	POLB	DNA polymerase beta	5423	25,2205059	0,098842047	0,394941	0,250270686	0,802378024	NA
ENSG00000197798	FAM118B	family with sequence similarity 118 member B	79607	37,90396571	0,199041286	0,320642	0,620759112	0,534758129	NA
ENSG00000131149	GSE1	Gse1 coiled-coil protein	23199	405,4297935	-0,094424785	0,112449	-0,83971001	0,401071	0,814798664
ENSG00000160014	CALM3	calmodulin 3	808	868,9513834	-0,039155775	0,074848	-0,52313586	0,600879712	0,896014669
ENSG00000169299	PGM2	phosphoglucomutase 2	55276	184,8957111	0,331648815	0,161647	2,051686761	0,040200114	0,319566495
ENSG00000187790	FANCM	FA complementation group M	57697	12,97430062	1,192928899	0,594269	2,007389682	0,044708187	NA
ENSG00000160783	PMF1	polyamine modulated factor 1	11243	110,6117012	0,048179567	0,154502	0,311837602	0,755163946	0,942901768
ENSG00000141076	UTP4	UTP4 small subunit processome component	84916	95,48222066	-0,193434558	0,217319	-0,890093343	0,373415767	0,800776142
ENSG00000116791	CRYZ	crystallin zeta	1429	87,36247749	0,03421159	0,189425	0,180607738	0,856675482	0,968618668
ENSG00000173905	GOLIM4	golgi integral membrane protein 4	27333	486,0165359	-0,218183511	0,109071	-2,000378852	0,04545937	0,340953043
ENSG00000257594	GALNT4	polypeptide N-acetylgalactosaminyltransferase 4	8693	237,0633275	0,020820467	0,169067	0,123149572	0,901988657	0,978348144
ENSG00000289346	NA	NA	NA	113,6506247	-0,27346195	0,173395	-1,577100676	0,114772362	0,54024053
ENSG00000196705	ZNF431	zinc finger protein 431	170959	204,9078287	-0,018965175	0,189503	-0,100078455	0,92028204	0,984284131
ENSG00000148153	INIP	INTS3 and NABP interacting protein	58493	163,63888	0,250909863	0,152553	1,644740391	0,10002336	0,5094575
ENSG00000119041	GTF3C3	general transcription factor IIIC subunit 3	9330	127,172669	0,2538889	0,207345	1,22447832	0,22077184	0,677095199
ENSG00000105755	ETHE1	ETHE1 persulfide dioxygenase	23474	48,28243011	0,696759681	0,287725	2,421617573	0,015451602	0,195124944
ENSG00000122218	COPA	COPI coat complex subunit alpha	1314	386,6533072	0,068269023	0,428758	0,159224907	0,873491681	0,974533542
ENSG00000163660	CCNL1	cyclin L1	57018	70,17270195	0,596056574	0,398141	1,497100509	0,134367107	0,574309297
ENSG00000187736	NHEJ1	non-homologous end joining factor 1	79840	55,10080609	0,444515849	0,33261	1,336446803	0,181403277	0,634628658
ENSG00000033627	ATP6V0A1	ATPase H+ transporting V0 subunit a1	535	12,99263506	-0,802623097	0,802728	-0,999869879	0,317373483	NA
ENSG00000095574	IKZF5	IKAROS family zinc finger 5	64376	153,5318633	-0,030190302	0,165104	-0,182856568	0,854910562	0,968377739
ENSG00000144357	UBR3	ubiquitin protein ligase E3 component n-recognin 3	130507	275,776488	-0,048928891	0,110575	-0,442496147	0,658130218	0,919088619
ENSG00000087338	GMCL1	germ cell-less 1, spermatogenesis associated	64395	323,8978341	-0,159548029	0,106121	-1,503460546	0,132720324	0,570166586
ENSG00000136732	GYPC	glycophorin C (Gerbich blood group)	2995	170,749726	-0,131207118	0,139653	-0,93952455	0,347461494	0,783959372
ENSG00000136238	RAC1	Rac family small GTPase 1	5879	444,4814522	-0,046327803	0,092287	-0,501995621	0,615670601	0,902090873
ENSG00000142065	ZFP14	ZFP14 zinc finger protein	57677	47,5412887	0,015575442	0,359925	0,043274179	0,965482974	0,992789539
ENSG00000119977	TCTN3	tectonic family member 3	26123	93,35746914	0,522231882	0,229259	2,2779118	0,022731833	0,240361098
ENSG00000174106	LEMD3	LEM domain containing 3	23592	236,6591964	-0,054275329	0,1171	-0,463493653	0,643010564	0,912160392
ENSG00000175029	CTBP2	C-terminal binding protein 2	1488	127,6868965	0,15963065	0,161835	0,986381217	0,323946079	0,767603859
ENSG00000126214	KLC1	kinesin light chain 1	3831	452,8549834	-0,039702518	0,096744	-0,410388492	0,681520987	0,925586358
ENSG00000160888	IER2	immediate early response 2	9592	422,5525257	-0,108109419	0,093825	-1,15225037	0,249218205	0,707335884
ENSG00000182986	ZNF320	zinc finger protein 320	162967	71,02847764	0,074304525	0,225614	0,329343869	0,74189579	0,939193793
ENSG00000120694	HSPH1	heat shock protein family H (Hsp110) member 1	10808	210,3414644	0,256732094	0,172206	1,490845205	0,13600214	0,576449438
ENSG00000138794	CASP6	caspase 6	839	42,76150192	0,756562921	0,423935	1,784621116	0,074322794	0,443476943
ENSG00000071539	TRIP13	thyroid hormone receptor interactor 13	9319	25,97458147	1,007463798	0,362404	2,779950295	0,005436722	NA

ENSG00000111665	CDCA3	cell division cycle associated 3	83461	28,34772502	0,887811541	0,371892	2,387285997	0,016973281	NA
ENSG00000141985	SH3GL1	SH3 domain containing GRB2 like 1, endophilin A2	6455	145,0158111	0,108386654	0,145447	0,745196277	0,456153073	0,844219046
ENSG00000121578	B4GALT4	beta-1,4-galactosyltransferase 4	8702	91,3130006	0,422294863	0,229959	1,836394419	0,066299344	0,41677041
ENSG00000008382	MPND	MPN domain containing	84954	37,96559986	-0,160268734	0,301967	-0,530749652	0,595592274	NA
ENSG00000141971	MVB12A	multivesicular body subunit 12A	93343	18,46597981	0,71535872	0,927022	0,771673624	0,440307756	NA
ENSG00000160209	PDXK	pyridoxal kinase	8566	333,3436004	0,007712761	0,11475	0,067213575	0,946411678	0,989789558
ENSG00000249839	NA	NA	NA	40,72267435	0,477276012	0,31274	1,526111319	0,126982143	NA
ENSG00000047315	POLR2B	RNA polymerase II subunit B	5431	731,9626177	-0,006293123	0,082554	-0,07623048	0,939235734	0,987813564
ENSG00000160789	LMNA	lamin A/C	4000	41,1379283	0,273777963	0,278919	0,981569189	0,326312133	NA
ENSG00000197016	ZNF470	zinc finger protein 470	388566	36,54426809	0,088747322	0,351276	0,252642494	0,80054449	NA
ENSG00000205423	CNEP1R1	CTD nuclear envelope phosphatase 1 regulatory subunit 1	255919	64,92200572	-0,205222653	0,212899	-0,963942746	0,335074631	0,774934085
ENSG00000034693	PEX3	peroxisomal biogenesis factor 3	8504	36,73499909	0,70653759	0,283982	2,487965771	0,01284761	NA
ENSG00000126106	TMEM53	transmembrane protein 53	79639	12,80631868	0,467775439	0,560779	0,83415347	0,404194508	NA
ENSG00000197142	ACSL5	acyl-CoA synthetase long chain family member 5	51703	404,9650233	-0,081086768	0,087857	-0,922943797	0,356036495	0,788804127
ENSG00000255641	NA	NA	NA	194,0303438	0,000307522	0,139887	0,002198358	0,998245965	0,999300722
ENSG00000181817	LSM10	LSM10, U7 small nuclear RNA associated	84967	68,11717672	-0,215200011	0,220636	-0,975360063	0,329381687	0,771437864
ENSG00000148943	LIN7C	lin-7 homolog C, crumbs cell polarity complex component	55327	77,97753225	0,168330626	0,19689	0,854949384	0,392579169	0,812491435
ENSG00000176485	PLAAT3	phospholipase A and acyltransferase 3	11145	21,65375295	-0,105090871	0,390403	-0,269185872	0,787786651	NA
ENSG00000168818	STX18	syntaxin 18	53407	59,63253478	0,012496776	0,220342	0,056715303	0,954771983	0,991420853
ENSG00000198830	HMGN2	high mobility group nucleosomal binding domain 2	3151	811,4066402	0,253613355	0,080929	3,133781351	0,001725694	0,05038513
ENSG00000137992	DBT	dihydrolipoamide branched chain transacylase E2	1629	114,6621173	-0,110151492	0,245454	-0,44876695	0,653599783	0,917134445
ENSG00000115942	ORC2	origin recognition complex subunit 2	4999	121,5992858	0,172976666	0,175017	0,988340136	0,322986095	0,767603859
ENSG00000241343	RPL36A	ribosomal protein L36a	6173	126,7481863	0,31164983	0,205116	1,519381835	0,128666412	0,563689485
ENSG00000002822	MAD1L1	mitotic arrest deficient 1 like 1	8379	42,4987466	-0,058337184	0,260618	-0,223841809	0,822880396	0,961917534
ENSG00000164253	WDR41	WD repeat domain 41	55255	114,4179097	-0,420907059	0,196921	-2,137444678	0,032561841	0,288320967
ENSG00000172766	NAA16	N-alpha-acetyltransferase 16, NatA auxiliary subunit	79612	89,76174327	-0,467353548	0,212435	-2,199988835	0,027807687	0,269631574
ENSG00000131475	VPS25	vacuolar protein sorting 25 homolog	84313	94,27832674	0,220774624	0,171113	1,290229894	0,19697085	0,655659726
ENSG00000141258	SGSM2	small G protein signaling modulator 2	9905	352,2642128	-0,218511053	0,10707	-2,040825912	0,041268134	0,323534763
ENSG00000151116	UEVLD	UEV and lactate/malate dehyrogenase domains	55293	128,1295983	-0,097037761	0,179258	-0,541328958	0,588280864	0,891557039
ENSG00000160179	ABCG1	ATP binding cassette subfamily G member 1	9619	53,20495868	-0,093130986	0,270976	-0,34368664	0,731081959	0,938729652
ENSG00000198821	CD247	CD247 molecule	919	872,2378289	-0,143832801	0,060291	-2,385649215	0,017049004	0,204616219
ENSG00000110651	CD81	CD81 molecule	975	447,4210401	-0,042139687	0,08597	-0,490169996	0,624013611	0,90479929
ENSG00000100599	RIN3	Ras and Rab interactor 3	79890	1050,308205	-0,031477224	0,06421	-0,490222136	0,623976718	0,90479929
ENSG00000157833	GAREM2	GRB2 associated regulator of MAPK1 subtype 2	150946	16,3492031	-0,600530063	0,488836	-1,228490782	0,219262788	NA
ENSG00000156052	GNAQ	G protein subunit alpha q	2776	61,69874882	0,055894748	0,244453	0,228652601	0,819138937	0,961067311
ENSG00000030419	IKZF2	IKAROS family zinc finger 2	22807	348,9027463	-0,072134613	0,131065	-0,550374716	0,582062387	0,888501395
ENSG00000183207	RUVBL2	RuvB like AAA ATPase 2	10856	96,94205712	-0,15736809	0,170581	-0,922542957	0,356245434	0,78894346
ENSG00000140968	IRF8	interferon regulatory factor 8	3394	437,0834428	-0,175181748	0,138086	-1,268639458	0,204569684	0,660954946
ENSG00000099203	TMED1	transmembrane p24 trafficking protein 1	11018	31,08042973	0,085878009	0,371194	0,231355911	0,817038314	NA
ENSG00000136816	TOR1B	torsin family 1 member B	27348	123,8391645	0,191313049	0,183273	1,043867676	0,296546615	0,74817768
ENSG00000124942	AHNAK	AHNAK nucleoprotein	79026	11560,96172	-0,013615122	0,049821	-0,273279192	0,784638609	0,950277816
ENSG00000068650	ATP11A	ATPase phospholipid transporting 11A	23250	432,213782	-0,193835924	0,089833	-2,157725137	0,030949208	0,281657534
ENSG00000130856	ZNF236	zinc finger protein 236	7776	148,9209899	-0,086737586	0,186596	-0,464842837	0,642044009	0,912063088
ENSG00000131981	LGALS3	galectin 3	3958	52,16022611	-0,132284239	0,279395	-0,473467353	0,635879787	0,909036096
ENSG00000139531	SUOX	sulfite oxidase	6821	142,7677473	0,222694156	0,178109	1,250328302	0,211179644	0,664818718
ENSG00000160799	CCDC12	coiled-coil domain containing 12	151903	108,7154901	-0,254361049	0,164562	-1,54568626	0,122180357	0,557738568
ENSG00000203880	PCMTD2	protein-L-isoaspartate (D-aspartate) O-methyltransferase domain containing 2	55251	194,9068695	-0,294686148	0,144466	-2,039831066	0,041367154	0,323603218
ENSG00000140577	CRTC3	CREB regulated transcription coactivator 3	64784	339,7453737	-0,038146458	0,11087	-0,344064986	0,730797414	0,938729652
ENSG00000109911	ELP4	elongator acetyltransferase complex subunit 4	26610	66,2825007	0,119739079	0,245909	0,486925226	0,626311321	0,90479929

ENSG00000025770	NCAPH2	non-SMC condensin II complex subunit H2	29781	142,12795	0,351748494	0,150608	2,335523918	0,019516068	0,219283544
ENSG00000049089	COL9A2	collagen type IX alpha 2 chain	1298	156,7460138	-0,317299096	0,138712	-2,287467862	0,022168529	0,236886569
ENSG00000110200	ANAPC15	anaphase promoting complex subunit 15	25906	44,07087312	0,082513177	0,289885	0,284641009	0,77591919	0,946448613
ENSG00000128923	MINDY2	MINDY lysine 48 deubiquitinase 2	54629	253,179481	-0,016261004	0,144779	-0,112316399	0,91057254	0,979595261
ENSG00000134287	ARF3	ADP ribosylation factor 3	377	616,1713313	-0,276043598	0,132005	-2,09116363	0,036513398	0,30378838
ENSG00000152784	PRDM8	PR/SET domain 8	56978	93,12196286	-0,026687292	0,207584	-0,128561377	0,897704729	0,977990819
ENSG00000197785	ATAD3A	ATPase family AAA domain containing 3A	55210	84,51868327	-0,273117507	0,190608	-1,432877017	0,151892986	0,608564843
ENSG00000051825	MPHOSPH9	M-phase phosphoprotein 9	10198	238,7255325	-0,033676268	0,124117	-0,271326618	0,786139836	0,950624564
ENSG00000231259	NA	NA	NA	68,33963963	-0,255206099	0,214364	-1,190528993	0,233838542	0,688858646
ENSG00000166483	WEE1	WEE1 G2 checkpoint kinase	7465	68,13026517	-0,136301205	0,210968	-0,646074478	0,518231123	0,868791049
ENSG00000143127	ITGA10	integrin subunit alpha 10	8515	27,79906599	0,369244994	0,375634	0,982992534	0,325611113	NA
ENSG00000123091	RNF11	ring finger protein 11	26994	203,8409445	-0,0886333	0,114681	-0,772868324	0,439600313	0,83848252
ENSG00000131844	MCCC2	methylcrotonyl-CoA carboxylase subunit 2	64087	100,1237933	0,242006428	0,275594	0,878127873	0,379874328	0,805706988
ENSG00000160613	PCSK7	proprotein convertase subtilisin/kexin type 7	9159	884,4907965	-0,037990133	0,069292	-0,54826379	0,583510787	0,889364199
ENSG00000066427	ATXN3	ataxin 3	4287	86,67547683	-0,01642412	0,203415	-0,080741803	0,935647292	0,987098279
ENSG00000135362	PRR5L	proline rich 5 like	79899	43,17866612	-0,168532324	0,362043	-0,465503046	0,641571256	0,912063088
ENSG00000156711	MAPK13	mitogen-activated protein kinase 13	5603	197,8910456	-0,147709461	0,13674	-1,080218455	0,280044912	0,733157579
ENSG00000128731	HERC2	HECT and RLD domain containing E3 ubiquitin protein ligase 2	8924	569,0253594	0,20156312	0,085694	2,352113109	0,018667098	0,213533226
ENSG00000158526	TSR2	TSR2 ribosome maturation factor	90121	141,594003	-0,124382804	0,162079	-0,767420702	0,442831424	0,838678081
ENSG00000139684	ESD	esterase D	2098	152,7041567	0,19999061	0,145835	1,371348629	0,170266303	0,629558603
ENSG00000120451	SNX19	sorting nexin 19	399979	539,3779969	0,069947609	0,134829	0,518789093	0,603907825	0,897212117
ENSG00000152464	RPP38	ribonuclease P/MRP subunit p38	10557	23,97690191	0,3568586	0,59197	0,602831984	0,546620467	NA
ENSG00000026103	FAS	Fas cell surface death receptor	355	77,54725522	0,319394526	0,764887	0,417570716	0,676261011	0,924186156
ENSG00000066583	ISOC1	isochorismatase domain containing 1	51015	78,76922423	0,108719522	0,193615	0,561525427	0,574439401	0,886894319
ENSG00000119661	DNAL1	dynein axonemal light chain 1	83544	40,83375814	0,716573124	0,655379	1,093371397	0,274230767	NA
ENSG00000144224	UBXN4	UBX domain protein 4	23190	726,9705424	0,132357179	0,092492	1,431010246	0,152427279	0,608624223
ENSG00000198586	TLK1	tousled like kinase 1	9874	238,1842218	0,211853274	0,153671	1,378612932	0,168014127	0,627733684
ENSG00000110955	ATP5F1B	ATP synthase F1 subunit beta	506	899,7454149	0,259639538	0,060202	4,312819142	1,61E-05	0,001507089
ENSG00000156384	SFR1	SWI5 dependent homologous recombination repair protein 1	119392	20,63056606	0,255498523	0,437644	0,583804885	0,559351591	NA
ENSG00000173821	RNF213	ring finger protein 213	57674	9579,489631	0,088711929	0,053902	1,645788671	0,099807276	0,509429216
ENSG00000025772	TOMM34	translocase of outer mitochondrial membrane 34	10953	46,27004616	0,094598978	0,250916	0,377014767	0,706162631	0,930846779
ENSG00000104356	POP1	POP1 homolog, ribonuclease P/MRP subunit	10940	43,16168091	0,34841265	0,289532	1,203365264	0,228835004	0,68351286
ENSG00000181754	AMIGO1	adhesion molecule with Ig like domain 1	57463	38,98879519	-0,337913436	0,288185	-1,172556743	0,240973605	NA
ENSG00000167981	ZNF597	zinc finger protein 597	146434	36,87827543	0,392086018	0,297766	1,316757918	0,187919783	NA
ENSG00000181019	NQO1	NAD(P)H quinone dehydrogenase 1	1728	33,57932951	0,32211653	0,322759	0,998008946	0,318275021	NA
ENSG00000175634	RPS6KB2	ribosomal protein S6 kinase B2	6199	120,5053176	-0,260442001	0,185945	-1,400639332	0,161321953	0,618965617
ENSG00000165895	ARHGAP42	Rho GTPase activating protein 42	143872	15,84342643	0,070029412	0,444466	0,157558569	0,874804653	NA
ENSG00000135108	FBXO21	F-box protein 21	23014	136,1168506	-0,16755601	0,210349	-0,796562706	0,425705044	0,830680103
ENSG00000163946	TASOR	transcription activation suppressor	23272	809,6796018	-0,151378365	0,098923	-1,530263304	0,125951568	0,563339187
ENSG00000166888	STAT6	signal transducer and activator of transcription 6	6778	600,6293213	-0,198288055	0,093511	-2,120478115	0,033965745	0,294612488
ENSG00000146109	ABT1	activator of basal transcription 1	29777	75,67386584	-0,202471244	0,273046	-0,741527499	0,458373664	0,845558274
ENSG00000115607	IL18RAP	interleukin 18 receptor accessory protein	8807	194,6840672	0,007223836	0,138437	0,052181494	0,958384078	0,991911599
ENSG00000186010	NDUFA13	NADH:ubiquinone oxidoreductase subunit A13	51079	149,6838306	-0,144465526	0,144815	-0,997589268	0,318478567	0,763136371
ENSG00000015475	BID	BH3 interacting domain death agonist	637	73,61305409	0,00494279	0,217229	0,022753839	0,98184663	0,995974079
ENSG00000150995	ITPR1	inositol 1,4,5-trisphosphate receptor type 1	3708	325,0161118	0,280655658	0,145111	1,934077837	0,053103558	0,372224799
ENSG00000157216	SSBP3	single stranded DNA binding protein 3	23648	211,8310488	-0,136099475	0,146403	-0,92962238	0,352566636	0,787082432
ENSG00000103353	UBFD1	ubiquitin family domain containing 1	56061	168,3326118	0,203688177	0,14932	1,364105214	0,172534454	0,629558603
ENSG00000138448	ITGAV	integrin subunit alpha V	3685	54,90661466	0,053386755	0,237234	0,225037965	0,82194974	0,961917534

ENSG00000177685	CRACR2B	calcium release activated channel regulator 2B	283229	38,95159074	-0,272329803	0,266184	-1,023089774	0,306265402	NA
ENSG00000074706	IPCEF1	interaction protein for cytohesin exchange factors 1	26034	403,3575272	-0,22749536	0,146993	-1,54765641	0,121705042	0,556808172
ENSG00000088812	ATRN	attractin	8455	185,8088738	-0,183127324	0,155055	-1,18104804	0,23758364	0,692643617
ENSG00000136986	DERL1	derlin 1	79139	404,5914354	-0,032161186	0,085343	-0,37684463	0,706289072	0,930846779
ENSG00000183520	UTP11	UTP11 small subunit processome component	51118	57,40201191	-0,070415962	0,296328	-0,237628499	0,812169245	0,959038743
ENSG00000197417	SHPK	sedoheptulokinase	23729	38,93858901	0,212497699	0,284288	0,747473712	0,454777665	NA
ENSG00000204104	TRAF3IP1	TRAF3 interacting protein 1	26146	47,77702176	-0,245726696	0,24408	-1,006747301	0,314056225	0,759554708
ENSG00000215790	NA	NA	NA	35,82639119	-0,093803911	0,313088	-0,299609028	0,764475397	NA
ENSG00000038274	MAT2B	methionine adenosyltransferase 2 non-catalytic beta subunit	27430	484,1153123	0,010276646	0,087467	0,117491396	0,906470662	0,978516824
ENSG00000131370	SH3BP5	SH3 domain binding protein 5	9467	93,99963073	-0,3582695	0,296938	-1,206547999	0,227606263	0,683140794
ENSG00000134109	EDEM1	ER degradation enhancing alpha-mannosidase like protein 1	9695	812,0502592	-0,080358342	0,072664	-1,105890359	0,268773978	0,724168379
ENSG00000156983	BRPF1	bromodomain and PHD finger containing 1	7862	451,2495061	-0,066490493	0,096248	-0,690827822	0,489673749	0,860369726
ENSG00000156374	PCGF6	polycomb group ring finger 6	84108	31,01243097	-0,131447838	0,300915	-0,436827002	0,662236815	NA
ENSG00000256683	ZNF350	zinc finger protein 350	59348	66,08114514	-0,137417592	0,226774	-0,605966615	0,544536922	0,87855238
ENSG00000170540	ARL6IP1	ADP ribosylation factor like GTPase 6 interacting protein 1	23204	703,5255352	0,099915528	0,062785	1,591401918	0,111519153	0,533417434
ENSG00000075711	DLG1	discs large MAGUK scaffold protein 1	1739	220,767241	0,058118096	0,157428	0,369173684	0,71199827	0,930846779
ENSG00000196678	ERI2	ERI1 exoribonuclease family member 2	112479	34,96349851	0,771552285	0,310251	2,486866074	0,01288739	NA
ENSG00000174720	LARP7	La ribonucleoprotein 7, transcriptional regulator	51574	201,9723759	0,43130344	0,132564	3,253549537	0,001139729	0,037467385
ENSG00000106993	CDC37L1	cell division cycle 37 like 1, HSP90 cochaperone	55664	29,07424309	0,070166459	0,378996	0,185137607	0,853121105	NA
ENSG00000169062	UPF3A	UPF3A regulator of nonsense mediated mRNA decay	65110	97,12767649	0,443752526	0,27092	1,637948342	0,10143247	0,514437479
ENSG00000095015	MAP3K1	mitogen-activated protein kinase kinase kinase 1	4214	227,7853603	-0,153450802	0,114163	-1,344139373	0,178903322	0,633385915
ENSG00000102893	PHKB	phosphorylase kinase regulatory subunit beta	5257	128,1624949	0,638619259	0,282829	2,257972683	0,023947363	0,247679511
ENSG00000141456	PELP1	proline, glutamate and leucine rich protein 1	27043	151,618707	-0,16610224	0,15095	-1,10037912	0,271166972	0,725477256
ENSG00000151327	FAM177A1	family with sequence similarity 177 member A1	283635	64,38120718	-0,158102847	0,215045	-0,735209656	0,462211819	0,846992914
ENSG00000151327	LOC101927178	uncharacterized LOC101927178	101927178	64,38120718	-0,158102847	0,215045	-0,735209656	0,462211819	0,846992914
ENSG00000062194	GPBP1	GC-rich promoter binding protein 1	65056	461,4615806	0,111735963	0,079878	1,398834141	0,161862726	0,619487156
ENSG00000153201	RANBP2	RAN binding protein 2	5903	1162,419001	0,04816246	0,055966	0,860563862	0,389478297	0,810747116
ENSG00000159069	FBXW5	F-box and WD repeat domain containing 5	54461	216,6831146	0,122523946	0,162856	0,752345289	0,451843439	0,842919189
ENSG00000160213	CSTB	cystatin B	1476	156,727937	0,244407792	0,150082	1,628495024	0,103419964	0,516898333
ENSG00000239306	RBM14	RNA binding motif protein 14	10432	269,5851724	-0,068590539	0,165668	-0,414024046	0,678856497	0,925294441
ENSG00000265491	RNF115	ring finger protein 115	27246	330,2534701	0,052403032	0,088804	0,590095567	0,555126581	0,882854594
ENSG00000277791	PSMB3	proteasome 20S subunit beta 3	5691	167,8848558	0,215610021	0,173114	1,245482863	0,212954311	0,667215123
ENSG00000186468	RPS23	ribosomal protein S23	6228	1011,442235	0,036610886	0,077589	0,471859375	0,637027167	0,90943397
ENSG00000120899	PTK2B	protein tyrosine kinase 2 beta	2185	1366,372094	-0,066922644	0,052923	-1,26453633	0,206037589	0,660954946
ENSG00000105438	KDELR1	KDEL endoplasmic reticulum protein retention receptor 1	10945	191,6966897	0,136600158	0,125426	1,089087711	0,276115209	0,728944151
ENSG00000065989	PDE4A	phosphodiesterase 4A	5141	520,1460439	-0,155590781	0,079081	-1,967498629	0,049125754	0,355687755
ENSG00000243646	IL10RB	interleukin 10 receptor subunit beta	3588	104,3052858	-0,011275404	0,230755	-0,048863095	0,9610284	0,992235217
ENSG00000249915	PDCD6	programmed cell death 6	10016	158,9406402	-0,040954589	0,13929	-0,294023366	0,768740049	0,944802477
ENSG00000110218	PANX1	pannexin 1	24145	80,87730901	0,108048869	0,186669	0,578827496	0,562705576	0,884605624
ENSG00000112759	SLC29A1	solute carrier family 29 member 1 (Augustine blood group)	2030	18,7651652	0,26243862	0,447081	0,587004773	0,557200501	NA
ENSG00000131100	ATP6V1E1	ATPase H+ transporting V1 subunit E1	529	208,8484283	0,127201001	0,119418	1,065177969	0,286795401	0,738005299
ENSG00000136280	CCM2	CCM2 scaffold protein	83605	179,1930751	-0,275985924	0,157184	-1,755819725	0,079119195	0,456242408
ENSG00000145592	RPL37	ribosomal protein L37	6167	301,6001509	0,057091341	0,205895	0,277284159	0,781561922	0,948892771
ENSG00000151413	NUBPL	NUBP iron-sulfur cluster assembly factor, mitochondrial	80224	18,39113413	-0,19575201	0,453337	-0,431802109	0,665885247	NA
ENSG00000198081	ZBTB14	zinc finger and BTB domain containing 14	7541	126,5715279	0,11092594	0,174892	0,63425478	0,525914563	0,871384275
ENSG00000131018	SYNE1	spectrin repeat containing nuclear envelope protein 1	23345	274,7919275	-0,366445769	0,434271	-0,843818687	0,398770731	0,814457852

ENSG00000149716	LTO1	LTO1 maturation factor of ABCE1	220064	40,02538329	0,126115474	0,34585	0,364654036	0,715369673	NA
ENSG00000184863	RBM33	RNA binding motif protein 33	155435	606,2162744	0,008159086	0,09599	0,08499954	0,932261756	0,987098279
ENSG00000127980	PEX1	peroxisomal biogenesis factor 1	5189	139,7186463	-0,088732118	0,13883	-0,639142379	0,522730313	0,870221868
ENSG00000135596	MICAL1	microtubule associated monooxygenase, calponin and LIM domain containing 1	64780	275,5426266	-0,153254212	0,099796	-1,535676226	0,124617809	0,5624706
ENSG00000100425	BRD1	bromodomain containing 1	23774	258,6741021	0,096367503	0,112037	0,860137831	0,38971307	0,810747116
ENSG00000196628	TCF4	transcription factor 4	6925	15,42935421	4,215720587	1,960504	2,150325353	0,031529488	NA
ENSG00000107341	UBE2R2	ubiquitin conjugating enzyme E2 R2	54926	182,0855113	-0,114272623	0,125935	-0,907395165	0,364197866	0,793456321
ENSG00000119596	YLPM1	YLP motif containing 1	56252	354,4123374	-0,047808694	0,116233	-0,411319132	0,680838543	0,925586358
ENSG00000173041	ZNF680	zinc finger protein 680	340252	39,19640801	-0,031602541	0,298847	-0,105748266	0,915782084	NA
ENSG00000072803	FBXW11	F-box and WD repeat domain containing 11	23291	210,7992871	-0,051832231	0,134256	-0,386069472	0,699445218	0,930783276
ENSG00000198839	ZNF277	zinc finger protein 277	11179	45,96551319	-0,537469927	0,306685	-1,752512768	0,079685676	0,458163472
ENSG00000088833	NSFL1C	NSFL1 cofactor	55968	74,24614662	0,026825368	0,281135	0,095418142	0,923982707	0,984284131
ENSG00000092470	WDR76	WD repeat domain 76	79968	84,77403871	0,312979396	0,215804	1,450294645	0,146976372	0,597490903
ENSG00000142039	CCDC97	coiled-coil domain containing 97	90324	315,6075749	-0,201906003	0,109838	-1,838210993	0,066031321	0,415552878
ENSG00000166164	BRD7	bromodomain containing 7	29117	306,818566	-0,16636179	0,117958	-1,410344515	0,15843798	0,614461481
ENSG00000248333	CDK11B	cyclin dependent kinase 11B	984	235,3357442	-0,267449677	0,186225	-1,436162888	0,150955997	0,607670525
ENSG00000089195	TRMT6	tRNA methyltransferase 6 non-catalytic subunit	51605	66,15487043	-0,12926544	0,284765	-0,453936934	0,649874219	0,914926418
ENSG00000162777	DENND2D	DENN domain containing 2D	79961	435,3396289	-0,159463841	0,088729	-1,797195995	0,07230451	0,436830479
ENSG00000111424	VDR	vitamin D receptor	7421	25,32668647	0,588183319	0,368205	1,597434668	0,11016885	NA
ENSG00000163125	RPRD2	regulation of nuclear pre-mRNA domain containing 2	23248	256,2882225	-0,084429172	0,142485	-0,592548153	0,553483586	0,882816634
ENSG00000143093	STRIP1	striatin interacting protein 1	85369	178,3285911	-0,098195425	0,147941	-0,663748118	0,506851536	0,864738156
ENSG00000136848	DAB2IP	DAB2 interacting protein	153090	18,2396559	-0,461215364	0,474983	-0,97101461	0,331541003	NA
ENSG00000176715	ACSF3	acyl-CoA synthetase family member 3	197322	77,82880126	0,441511804	0,268932	1,641722854	0,100647453	0,51200808
ENSG00000180448	ARHGAP45	Rho GTPase activating protein 45	23526	1639,444837	-0,149812038	0,054941	-2,726803995	0,006395101	0,115569573
ENSG00000157540	DYRK1A	dual specificity tyrosine phosphorylation regulated kinase 1A	1859	527,0719545	-0,208772739	0,088809	-2,35081359	0,018732416	0,213533226
ENSG00000075275	CELSR1	cadherin EGF LAG seven-pass G-type receptor 1	9620	57,64256719	0,048347115	0,266342	0,181522835	0,855957211	0,968439087
ENSG00000198589	LRBA	LPS responsive beige-like anchor protein	987	607,8380769	-0,036955235	0,110715	-0,333786912	0,738540361	0,939193793
ENSG00000242689	CNTF	ciliary neurotrophic factor	1270	13,6712239	0,046200115	0,479629	0,096324763	0,923262644	NA
ENSG00000196821	ILRUN	inflammation and lipid regulator with UBA-like and NBR1-like domains	64771	545,8261685	-0,118178837	0,070828	-1,668539366	0,095208704	0,499178345
ENSG00000247626	MARS2	methionyl-tRNA synthetase 2, mitochondrial	92935	79,60124158	0,012672274	0,211438	0,059933775	0,95220838	0,991212866
ENSG00000144843	ADPRH	ADP-ribosylarginine hydrolase	141	125,2312828	0,239293957	0,151374	1,580807588	0,113922039	0,539617633
ENSG00000155287	SLC25A28	solute carrier family 25 member 28	81894	44,23088522	0,126206001	0,373271	0,338107873	0,735281898	0,939157822
ENSG00000111652	COPS7A	COP9 signalosome subunit 7A	50813	70,05997028	0,424305232	0,205999	2,059742245	0,039423189	0,316271423
ENSG00000114904	NEK4	NIMA related kinase 4	6787	85,09949679	0,543601988	0,2866	1,896729674	0,057863625	0,390095203
ENSG00000151835	SACS	sacsin molecular chaperone	26278	577,3534202	-0,243168041	0,091591	-2,654926842	0,007932561	0,132558158
ENSG00000178202	POGLUT3	protein O-glucosyltransferase 3	143888	111,8487292	-0,027035221	0,198543	-0,136168344	0,8916882	0,977990819
ENSG00000008282	SYPL1	synaptophysin like 1	6856	103,8429138	-0,00133244	0,169356	-0,007867687	0,993722559	0,998500439
ENSG00000182195	LDOC1	LDOC1 regulator of NFKB signaling	23641	74,3335362	-0,455745312	0,197408	-2,30864575	0,020963247	0,226641441
ENSG00000224531	SMIM13	small integral membrane protein 13	221710	54,48845252	-0,018854497	0,260049	-0,072503704	0,942201058	0,988475138
ENSG00000107223	EDF1	endothelial differentiation related factor 1	8721	314,3890734	-0,110747119	0,104451	-1,060278375	0,289017974	0,740602666
ENSG00000198018	ENTPD7	ectonucleoside triphosphate diphosphohydrolase 7	57089	93,22525316	0,253231771	0,180637	1,40187919	0,160951326	0,618147537
ENSG00000158435	CNOT11	CCR4-NOT transcription complex subunit 11	55571	218,0876843	0,090961623	0,117984	0,77096816	0,440725805	0,838678081
ENSG00000254093	PINX1	PIN2 (TERF1) interacting telomerase inhibitor 1	54984	27,55070366	0,593429471	0,36662	1,618651552	0,105522257	NA
ENSG00000198841	KTI12	KTI12 chromatin associated homolog	112970	52,2977462	-0,123088249	0,262839	-0,468302496	0,639568284	0,910841232
ENSG00000160094	ZNF362	zinc finger protein 362	149076	80,3043116	-0,450098289	0,200467	-2,245245416	0,024752382	0,252751889
ENSG00000185201	IFITM2	interferon induced transmembrane protein 2	10581	317,8772364	-0,240225751	0,134514	-1,785882595	0,07411827	0,443476943
ENSG00000074800	ENO1	enolase 1	2023	3331,545811	0,04663277	0,040848	1,141626823	0,25360917	0,709599722
ENSG00000078269	SYNJ2	synaptojanin 2	8871	308,8248159	-0,112422432	0,110801	-1,014634063	0,310280296	0,75688983
ENSG00000100351	GRAP2	GRB2 related adaptor protein 2	9402	116,2017441	0,216365796	0,169196	1,278788759	0,200971454	0,660006147

ENSG00000114439	BBX	BBX high mobility group box domain containing	56987	375,4291347	-0,105296052	0,103593	-1,016440066	0,309419875	0,755889174
ENSG00000174007	CEP19	centrosomal protein 19	84984	26,6202885	0,215383153	0,339004	0,635341425	0,525205761	NA
ENSG00000107874	CUEDC2	CUE domain containing 2	79004	76,25782816	0,006870261	0,195655	0,035114139	0,971988727	0,994108581
ENSG00000114982	KANSL3	KAT8 regulatory NSL complex subunit 3	55683	228,2751202	-0,365122665	0,223811	-1,631385403	0,102809023	0,515621608
ENSG00000182400	TRAPPC6B	trafficking protein particle complex subunit 6B	122553	182,234092	-0,050988178	0,160117	-0,318442354	0,750149415	0,941011581
ENSG00000130204	TOMM40	translocase of outer mitochondrial membrane 40	10452	115,5936014	0,070851987	0,161005	0,440061627	0,659892474	0,919368176
ENSG00000158019	BABAM2	BRISC and BRCA1 A complex member 2	9577	151,8182667	-0,150583409	0,134795	-1,117132379	0,263937728	0,719682465
ENSG00000204525	HLA-C	major histocompatibility complex, class I, C	3107	1987,265412	-0,083706552	0,053501	-1,564588602	0,117679403	0,546895877
ENSG00000064703	DDX20	DEAD-box helicase 20	11218	28,30138084	1,915580307	2,09097	0,91612061	0,359603639	NA
ENSG00000122512	PMS2	PMS1 homolog 2, mismatch repair system component	5395	72,78714397	-0,326684994	0,309306	-1,056186128	0,290883187	0,743239565
ENSG00000135473	PAN2	poly(A) specific ribonuclease subunit PAN2	9924	119,2778211	-0,208278072	0,178763	-1,165109428	0,243974701	0,700945995
ENSG00000175221	MED16	mediator complex subunit 16	10025	93,14622078	-0,502614683	0,188662	-2,664104545	0,007719357	0,130945634
ENSG00000090263	MRPS33	mitochondrial ribosomal protein S33	51650	48,00139975	0,043273996	0,310498	0,139369868	0,889157882	0,977990819
ENSG00000172500	FIBP	FGF1 intracellular binding protein	9158	77,34879382	-0,085280054	0,22707	-0,375566878	0,707238918	0,930846779
ENSG00000163812	ZDHHC3	zinc finger DHHC-type palmitoyltransferase 3	51304	287,6537609	0,086735208	0,111235	0,779746634	0,435540024	0,836566451
ENSG00000149187	CELF1	CUGBP Elav-like family member 1	10658	641,7690757	-0,001339467	0,080111	-0,016720242	0,986659799	0,996813231
ENSG00000259781	NA	NA	NA	157,1585488	0,044446889	0,188496	0,235797372	0,813589907	0,959207071
ENSG00000135077	HAVCR2	hepatitis A virus cellular receptor 2	84868	973,4516809	0,103533542	0,076625	1,351174652	0,176639492	0,632039836
ENSG00000141905	NFIC	nuclear factor I C	4782	96,63429096	-0,328521738	0,232349	-1,413914565	0,157386989	0,61428162
ENSG00000147050	KDM6A	lysine demethylase 6A	7403	195,964809	0,042277232	0,459193	0,092068468	0,926643641	0,985359831
ENSG00000155961	RAB39B	RAB39B, member RAS oncogene family	116442	46,02337453	-0,211890243	0,269276	-0,786889832	0,43134636	0,833786615
ENSG00000166200	COPS2	COP9 signalosome subunit 2	9318	181,916343	0,053599762	0,152186	0,352199461	0,724688681	0,935520201
ENSG00000185917	SETD4	SET domain containing 4	54093	36,25359226	-0,006393739	0,351579	-0,018185758	0,985490664	NA
ENSG00000102931	ARL2BP	ADP ribosylation factor like GTPase 2 binding protein	23568	241,3906421	-0,051610559	0,116402	-0,443382075	0,657489399	0,919088619
ENSG00000152056	AP1S3	adaptor related protein complex 1 subunit sigma 3	130340	47,92554157	0,299432441	0,396715	0,754779698	0,450381177	0,84263906
ENSG00000103152	MPG	N-methylpurine DNA glycosylase	4350	141,5214916	-0,016030487	0,136116	-0,117771094	0,906249034	0,978516824
ENSG00000108826	MRPL27	mitochondrial ribosomal protein L27	51264	57,98059281	0,426047745	0,254224	1,675877026	0,093762283	0,497328138
ENSG00000121897	LIAS	lipoic acid synthetase	11019	11,94189044	0,266902988	0,64843	0,411614359	0,680622106	NA
ENSG00000172071	EIF2AK3	eukaryotic translation initiation factor 2 alpha kinase 3	9451	251,4144087	-0,150700083	0,203693	-0,739840865	0,45939656	0,846574514
ENSG00000127533	F2RL3	F2R like thrombin or trypsin receptor 3	9002	36,74109921	0,101670294	0,29008	0,350489967	0,725971019	NA
ENSG00000169683	LRRC45	leucine rich repeat containing 45	201255	56,6379472	0,131567712	0,225016	0,584703661	0,558746992	0,883921037
ENSG00000184178	SCFD2	sec1 family domain containing 2	152579	64,57537537	-0,028401644	0,239981	-0,118349558	0,905790692	0,978460866
ENSG00000067955	CBFB	core-binding factor subunit beta	865	456,0120509	-0,324539383	0,181683	-1,78629417	0,07405164	0,443476943
ENSG00000136451	VEZF1	vascular endothelial zinc finger 1	7716	346,5108201	-0,088082864	0,089689	-0,982087207	0,326056887	0,76925527
ENSG00000124571	XPO5	exportin 5	57510	246,2589987	0,22478573	0,114139	1,969400214	0,048907151	0,354876255
ENSG00000173914	RBM4B	RNA binding motif protein 4B	83759	50,99477518	-0,299463326	0,285789	-1,047849314	0,294708038	0,746855268
ENSG00000109466	KLHL2	kelch like family member 2	11275	74,22605031	0,083972996	0,252343	0,332773453	0,739305299	0,939193793
ENSG00000132182	NUP210	nucleoporin 210	23225	793,6861402	0,092622255	0,06686	1,385316058	0,165955846	0,624933502
ENSG00000077420	APBB1IP	amyloid beta precursor protein binding family B member 1 interacting protein	54518	307,3730938	-0,190599873	0,100548	-1,895618016	0,058010573	0,390415632
ENSG00000163131	CTSS	cathepsin S	1520	344,1324448	-0,361153484	0,201425	-1,792990034	0,072974518	0,43885288
ENSG00000058673	ZC3H11A	zinc finger CCCH-type containing 11A	9877	612,5990265	0,009460999	0,153675	0,061564882	0,950909344	0,991212866
ENSG00000182899	RPL35A	ribosomal protein L35a	6165	700,2327488	0,176244499	0,072694	2,424463077	0,015331042	0,194404855
ENSG00000221823	PPP3R1	protein phosphatase 3 regulatory subunit B, alpha	5534	271,8132194	-0,085695606	0,106931	-0,801406984	0,422896074	0,828079223
ENSG00000124356	STAMBP	STAM binding protein	10617	94,68146064	0,053769814	0,281734	0,19085321	0,848640599	0,967767008
ENSG00000171456	ASXL1	ASXL transcriptional regulator 1	171023	510,1160853	-0,171371888	0,0796	-2,152923331	0,031324705	0,281814696
ENSG00000111247	RAD51AP1	RAD51 associated protein 1	10635	29,49631729	1,303501429	0,467206	2,789993363	0,005270912	NA
ENSG00000001167	NFYA	nuclear transcription factor Y subunit alpha	4800	219,2315564	-0,16973834	0,148475	-1,143211231	0,252950901	0,708754466
ENSG00000133606	MKRN1	makorin ring finger protein 1	23608	322,875962	-0,101572582	0,108837	-0,933250591	0,3506906	0,785712602

ENSG00000100364	KIAA0930	KIAA0930	23313	526,4472535	-0,291478433	0,170103	-1,713544665	0,086612384	0,481317095
ENSG00000119899	SLC17A5	solute carrier family 17 member 5	26503	47,45993069	-0,076738304	0,309801	-0,24770212	0,804364892	0,957194221
ENSG00000138642	HERC6	HECT and RLD domain containing E3 ubiquitin protein ligase family member 6	55008	63,21738384	0,290883591	0,214725	1,354681284	0,175519123	0,632039836
ENSG00000092820	EZR	ezrin	7430	438,2129459	-0,0664425	0,085347	-0,778499524	0,436274587	0,836566451
ENSG00000129084	PSMA1	proteasome 20S subunit alpha 1	5682	345,3548186	0,375018916	0,094169	3,982384585	6,82E-05	0,004659625
ENSG00000173281	PPP1R3B	protein phosphatase 1 regulatory subunit 3B	79660	52,29906354	0,172194615	0,333249	0,516714817	0,605355251	0,897347689
ENSG00000154760	SLFN13	schlafen family member 13	146857	684,8904039	-0,034010101	0,089427	-0,380309648	0,703715575	0,930846779
ENSG00000088832	FKBP1A	FKBP prolyl isomerase 1A	2280	857,6410013	-0,161975243	0,071009	-2,28106169	0,022544797	0,239603292
ENSG00000066379	POLR1H	RNA polymerase I subunit H	30834	21,58833676	0,727897133	0,527675	1,37944096	0,167758838	NA
ENSG00000107551	RASSF4	Ras association domain family member 4	83937	18,40998974	-0,549218487	1,53052	-0,358844302	0,719711569	NA
ENSG00000118217	ATF6	activating transcription factor 6	22926	297,8604508	-0,093688981	0,140309	-0,667734462	0,504303108	0,863483019
ENSG00000118900	UBN1	ubinuclein 1	29855	434,9642335	0,087906502	0,08217	1,069815069	0,284702558	0,737867955
ENSG00000148688	RPP30	ribonuclease P/MRP subunit p30	10556	34,88016319	0,525249442	0,306294	1,714855482	0,086371726	NA
ENSG00000167257	RNF214	ring finger protein 214	257160	117,2275096	-0,110491797	0,15315	-0,721462546	0,470624977	0,852690218
ENSG00000171241	SHCBP1	SHC binding and spindle associated 1	79801	61,08060699	0,969376298	0,230903	4,198202788	2,69E-05	0,00228963
ENSG00000175467	SART1	spliceosome associated factor 1, recruiter of U4/U6,U5 tri-snRNP	9092	277,7455528	-0,300534106	0,110639	-2,716349782	0,006600614	0,118629802
ENSG00000180979	LRRC57	leucine rich repeat containing 57	255252	110,9898762	-0,269345988	0,165229	-1,630140523	0,103071801	0,515621608
ENSG00000100027	YPEL1	yippee like 1	29799	461,3153063	-0,224798541	0,171431	-1,311304323	0,189754969	0,647690362
ENSG00000146701	MDH2	malate dehydrogenase 2	4191	283,0470134	-0,059762611	0,111816	-0,534471306	0,593015497	0,893619284
ENSG00000100441	KHNYN	KH and NYN domain containing	23351	383,0253431	-0,132040225	0,133508	-0,989005813	0,322660298	0,767466377
ENSG00000167110	GOLGA2	golgin A2	2801	278,0371949	-0,002702589	0,129314	-0,02089941	0,983325897	0,996185513
ENSG00000105514	RAB3D	RAB3D, member RAS oncogene family	9545	71,49536118	0,263453067	0,266771	0,987561314	0,32336754	0,767603859
ENSG00000109756	RAPGEF2	Rap guanine nucleotide exchange factor 2	9693	120,0568726	-0,109678174	0,152722	-0,718157539	0,472660156	0,853811879
ENSG00000166167	BTRC	beta-transducin repeat containing E3 ubiquitin protein ligase	8945	93,25528259	-0,125795274	0,184653	-0,681252487	0,495711742	0,861163463
ENSG00000160570	DEDD2	death effector domain containing 2	162989	60,54237142	0,432679992	0,375757	1,151489183	0,249531043	0,707335884
ENSG00000106785	TRIM14	tripartite motif containing 14	9830	365,5379002	-0,071585261	0,091708	-0,780574756	0,435052644	0,836319408
ENSG00000130021	PUDP	pseudouridine 5'-phosphatase	8226	59,15246631	0,18381918	0,248603	0,739408708	0,459658856	0,846622987
ENSG00000136213	CHST12	carbohydrate sulfotransferase 12	55501	940,2736227	0,03526946	0,068819	0,512497518	0,608302857	0,897640621
ENSG00000143486	EIF2D	eukaryotic translation initiation factor 2D	1939	101,5465161	0,107043699	0,169685	0,630838447	0,528146162	0,872142129
ENSG00000149761	NUDT22	nudix hydrolase 22	84304	41,67437173	0,022710062	0,271723	0,083577847	0,933392081	NA
ENSG00000168275	COA6	cytochrome c oxidase assembly factor 6	388753	20,94837482	0,15400569	0,382338	0,402800011	0,687095353	NA
ENSG00000114867	EIF4G1	eukaryotic translation initiation factor 4 gamma 1	1981	1410,651215	0,025490816	0,052367	0,486768007	0,626422745	0,90479929
ENSG00000157933	SKI	SKI proto-oncogene	6497	393,1180294	-0,115440044	0,111801	-1,032546155	0,301816337	0,751870158
ENSG00000106069	CHN2	chimerin 2	1124	294,3773708	-0,067503005	0,109479	-0,616583086	0,537509754	0,874923109
ENSG00000002919	SNX11	sorting nexin 11	29916	73,0568143	-0,008139909	0,256581	-0,031724551	0,974691716	0,994534085
ENSG00000143569	UBAP2L	ubiquitin associated protein 2 like	9898	506,0860505	0,052360856	0,093088	0,56248477	0,573785777	0,886894319
ENSG00000129219	PLD2	phospholipase D2	5338	46,24600364	-0,055297712	0,313322	-0,176488227	0,859910397	0,970364405
ENSG00000120137	PANK3	pantothenate kinase 3	79646	600,4014247	-0,036971994	0,074342	-0,497323628	0,618960852	0,903255023
ENSG00000142546	NOSIP	nitric oxide synthase interacting protein	51070	77,63270247	-0,103047982	0,241838	-0,426103327	0,670032556	0,922641254
ENSG00000163492	CCDC141	coiled-coil domain containing 141	285025	26,35598572	0,186505451	0,371411	0,502153933	0,615559245	NA
ENSG00000168246	UBTD2	ubiquitin domain containing 2	92181	45,04971603	-0,039304779	0,283643	-0,138571403	0,889788842	0,977990819
ENSG00000114796	KLHL24	kelch like family member 24	54800	233,5118049	-0,084688064	0,118416	-0,715172429	0,474502503	0,853811879
ENSG00000205352	PRR13	proline rich 13	54458	378,6037873	-0,143602084	0,084488	-1,699674089	0,089192246	0,488176025
ENSG00000172809	RPL38	ribosomal protein L38	6169	395,2520684	-0,038939719	0,088906	-0,437987841	0,661395098	0,91978661
ENSG00000107099	DOCK8	dedicator of cytokinesis 8	81704	2791,059652	0,147756628	0,041637	3,548648486	0,000387214	0,017478021
ENSG00000135503	ACVR1B	activin A receptor type 1B	91	332,1287804	-0,071515915	0,10159	-0,703965784	0,481454082	0,855577353
ENSG00000142686	C1orf216	chromosome 1 open reading frame 216	127703	117,371574	0,144948076	0,153062	0,946988349	0,343644716	0,781132432
ENSG00000164074	ABHD18	abhydrolase domain containing 18	80167	77,29765925	-0,088219148	0,26259	-0,335957138	0,736903189	0,939157822
ENSG00000198886	ND4	NADH dehydrogenase subunit 4	4538	25721,80232	0,13253565	0,120767	1,097446772	0,272446137	0,726831508

ENSG00000244687	UBE2V1	ubiquitin conjugating enzyme E2 V1	7335	487,6035533	-0,057060767	0,091344	-0,624682485	0,532179471	0,872967783
ENSG00000111335	OAS2	2'-5'-oligoadenylate synthetase 2	4939	491,1524691	0,029900857	0,098202	0,304483138	0,760759837	0,942901768
ENSG00000188677	PARVB	parvin beta	29780	50,2120539	-0,162669814	0,317389	-0,512524855	0,60828373	0,897640621
ENSG00000127870	RNF6	ring finger protein 6	6049	272,8219185	0,046842578	0,106321	0,440575219	0,659520546	0,919368176
ENSG00000005100	DHX33	DEAH-box helicase 33	56919	93,9683984	0,026153049	0,222873	0,117344898	0,906586747	0,978516824
ENSG00000065243	PKN2	protein kinase N2	5586	226,4945739	-0,168149715	0,118776	-1,415692048	0,156865688	0,61428162
ENSG00000109519	GRPEL1	GrpE like 1, mitochondrial	80273	64,89234614	0,103628078	0,247681	0,418393768	0,675659242	0,924052604
ENSG00000055955	ITIH4	inter-alpha-trypsin inhibitor heavy chain 4	3700	13,94936999	-0,456833217	0,704422	-0,648522211	0,51664725	NA
ENSG00000171604	CXXC5	CXXC finger protein 5	51523	400,6121548	-0,04003947	0,09143	-0,437925019	0,661440639	0,91978661
ENSG00000166971	AKTIP	AKT interacting protein	64400	121,4935473	0,058179579	0,164514	0,353645451	0,723604607	0,934902053
ENSG00000176148	TCP11L1	t-complex 11 like 1	55346	79,34178895	0,138335035	0,192669	0,71799313	0,472761523	0,853811879
ENSG00000242114	MTFP1	mitochondrial fission process 1	51537	30,92626214	0,55277483	0,327235	1,689228968	0,09117556	NA
ENSG00000147439	BIN3	bridging integrator 3	55909	80,58250794	0,147301691	0,200737	0,733803066	0,463068771	0,847577533
ENSG00000112210	RAB23	RAB23, member RAS oncogene family	51715	13,47091032	0,180426107	0,554119	0,325609083	0,744720139	NA
ENSG00000133818	RRAS2	RAS related 2	22800	250,5907177	0,251064344	0,11601	2,164161775	0,030451936	0,279077601
ENSG00000133321	PLAAT4	phospholipase A and acyltransferase 4	5920	104,8082665	0,194361983	0,179174	1,084768389	0,278024249	0,731800087
ENSG00000026751	SLAMF7	SLAM family member 7	57823	2087,367245	-0,020945492	0,043451	-0,482051082	0,629769659	0,905567722
ENSG00000169504	CLIC4	chloride intracellular channel 4	25932	58,67388535	-0,362848877	0,264086	-1,373981705	0,169447362	0,629558603
ENSG00000118804	STBD1	starch binding domain 1	8987	109,0326673	-0,076176664	0,190082	-0,400756573	0,688599354	0,926748702
ENSG00000162616	DNAJB4	DnaJ heat shock protein family (Hsp40) member B4	11080	19,55981122	0,122265331	0,466541	0,262067952	0,793269054	NA
ENSG00000131697	NPHP4	nephrocystin 4	261734	82,27622676	-0,108708167	0,246768	-0,44052801	0,65955473	0,919368176
ENSG00000100294	MCAT	malonyl-CoA-acyl carrier protein transacylase	27349	12,406649	1,014502132	0,53538	1,894918125	0,058103249	NA
ENSG00000162241	SLC25A45	solute carrier family 25 member 45	283130	52,78006114	-0,501171348	0,2552	-1,963839952	0,049548655	0,357023063
ENSG00000156876	SASS6	SAS-6 centriolar assembly protein	163786	42,29501935	0,420623366	0,363874	1,155957937	0,247698363	NA
ENSG00000163535	SGO2	shugoshin 2	151246	100,8874595	0,574813216	0,197356	2,91257359	0,003584637	0,080139791
ENSG00000082701	GSK3B	glycogen synthase kinase 3 beta	2932	373,04093	-0,072916438	0,142015	-0,513442814	0,607641603	0,897481254
ENSG00000104043	ATP8B4	ATPase phospholipid transporting 8B4 (putative)	79895	861,3631077	-0,009479808	0,096687	-0,098046743	0,921895176	0,984284131
ENSG00000153066	TXNDC11	thioredoxin domain containing 11	51061	190,8331485	-0,079433181	0,13843	-0,573815106	0,566092925	0,88521771
ENSG00000176142	TMEM39A	transmembrane protein 39A	55254	72,07603296	0,237086136	1,272045	0,18638192	0,852145269	0,968180755
ENSG00000095319	NUP188	nucleoporin 188	23511	467,2441293	0,097877162	0,091396	1,070910923	0,284209484	0,737423617
ENSG00000164211	STARD4	StAR related lipid transfer domain containing 4	134429	345,1120003	0,219586212	0,135823	1,616711055	0,105940667	0,522649494
ENSG00000089737	DDX24	DEAD-box helicase 24	57062	32,6638932	-0,089143982	0,321703	-0,277100288	0,781703101	NA
ENSG00000124155	PIGT	phosphatidylinositol glycan anchor biosynthesis class T	51604	150,9094169	-0,110694693	0,142556	-0,776497836	0,437455098	0,837249449
ENSG00000124226	RNF114	ring finger protein 114	55905	193,2982216	0,026490794	0,123705	0,214144601	0,830434296	0,963156386
ENSG00000105327	BBC3	BCL2 binding component 3	27113	32,59331868	0,145738695	0,513196	0,283982785	0,776423573	NA
ENSG00000162086	ZNF75A	zinc finger protein 75A	7627	59,59364431	0,175068536	0,242724	0,721264409	0,47074685	0,852690218
ENSG00000205937	RNPS1	RNA binding protein with serine rich domain 1	10921	391,8445033	-0,069136713	0,09311	-0,7425264	0,457768462	0,84552083
ENSG00000119314	PTBP3	polypyrimidine tract binding protein 3	9991	983,6250537	-0,011853747	0,058265	-0,203446399	0,838786137	0,965956938
ENSG00000147364	FBXO25	F-box protein 25	26260	128,1495381	0,026311201	0,18835	0,139693124	0,888902459	0,977990819
ENSG00000167637	ZNF283	zinc finger protein 283	284349	49,81478728	0,31010128	0,330586	0,938035182	0,34822633	0,784333122
ENSG00000184857	TMEM186	transmembrane protein 186	25880	17,6839018	0,025143357	0,444826	0,056523994	0,954924382	NA
ENSG00000011275	RNF216	ring finger protein 216	54476	272,2501814	-0,263797152	0,120081	-2,196827367	0,028032778	0,270084577
ENSG00000111269	CREBL2	cAMP responsive element binding protein like 2	1389	122,4130985	0,04163005	0,153403	0,271376305	0,786101624	0,950624564
ENSG00000169118	CSNK1G1	casein kinase 1 gamma 1	53944	258,1302556	0,215577958	0,118944	1,812439222	0,069918357	0,429815384
ENSG00000147036	LANCL3	LanC like family member 3	347404	17,91026406	-0,808495782	0,589262	-1,372046969	0,170048817	NA
ENSG00000163349	HIPK1	homeodomain interacting protein kinase 1	204851	1027,705508	-0,141319584	0,063531	-2,224425955	0,026119799	0,260750739
ENSG00000131242	RAB11FIP4	RAB11 family interacting protein 4	84440	177,2736325	-0,045260809	0,234186	-0,193268919	0,846748367	0,967231052
ENSG00000137497	NUMA1	nuclear mitotic apparatus protein 1	4926	860,8824391	-0,174317266	0,087168	-1,999796041	0,045522292	0,340953043
ENSG00000122694	GLIPR2	GLI pathogenesis related 2	152007	471,6090177	-0,221071931	0,082575	-2,677221784	0,007423548	0,12702515
ENSG00000138071	ACTR2	actin related protein 2	10097	2965,035205	-0,02573985	0,040574	-0,634395277	0,525822891	0,871384275
ENSG00000196470	SIAH1	siah E3 ubiquitin protein ligase 1	6477	51,36931202	0,082564414	0,244425	0,337790758	0,735520875	0,939157822
ENSG00000085377	PREP	prolyl endopeptidase	5550	413,6059704	0,110199519	0,084844	1,298852422	0,19399458	0,651961247
ENSG00000175334	BANF1	BAF nuclear assembly factor 1	8815	238,7519152	0,098950083	0,106169	0,932006395	0,35133322	0,785712602
ENSG00000061938	TNK2	tyrosine kinase non receptor 2	10188	366,8864007	-0,149296477	0,097925	-1,524595566	0,127360004	0,563689485

ENSG00000129675	ARHGEF6	Rac/Cdc42 guanine nucleotide exchange factor 6	9459	1363,233116	0,060759126	0,048258	1,259042515	0,208014976	0,662274754
ENSG00000142657	PGD	phosphogluconate dehydrogenase	5226	232,7253165	0,318864454	0,117863	2,705378914	0,006822652	0,120959608
ENSG00000149231	CCDC82	coiled-coil domain containing 82	79780	184,9099658	0,185541611	0,170649	1,087267645	0,276918543	0,730699811
ENSG00000164975	SNAPC3	small nuclear RNA activating complex polypeptide 3	6619	24,21822611	-0,641506366	0,604746	-1,060785765	0,288787273	NA
ENSG00000165304	MELK	maternal embryonic leucine zipper kinase	9833	61,91689314	0,664855675	0,266712	2,492784959	0,012674558	0,177128078
ENSG00000167114	SLC27A4	solute carrier family 27 member 4	10999	126,2083478	0,069684942	0,163178	0,427048005	0,669344363	0,922641254
ENSG00000181896	ZNF101	zinc finger protein 101	94039	149,3420978	0,644460216	0,458757	1,404796787	0,160081715	0,617437616
ENSG00000196329	GIMAP5	GTPase, IMAP family member 5	55340	114,7936886	-0,111943512	0,49922	-0,224236847	0,822573014	0,961917534
ENSG00000198804	COX1	cytochrome c oxidase subunit I	4512	60914,57553	0,015535747	0,108822	0,142762981	0,886477374	0,977928921
ENSG00000205903	ZNF316	zinc finger protein 316	100131017	238,9756243	-0,208342227	0,11367	-1,832867683	0,066822251	0,417162318
ENSG00000221968	FADS3	fatty acid desaturase 3	3995	39,10051954	0,42825195	0,355594	1,204330035	0,228462042	NA
ENSG00000293600	NA	NA	NA	12,62053335	-1,615788516	0,831264	-1,943771954	0,051922957	NA
ENSG00000164053	ATRIP	ATR interacting protein	84126	44,35607199	0,088329661	0,298763	0,2956513	0,767496392	0,944698783
ENSG00000164053	ATRIP-TREX1	ATRIP-TREX1 readthrough	111822955	44,35607199	0,088329661	0,298763	0,2956513	0,767496392	0,944698783
ENSG00000183172	SMDT1	single-pass membrane protein with aspartate rich tail 1	91689	45,23731065	-0,225622244	0,26586	-0,848650934	0,396075556	0,812799303
ENSG00000164219	PGGT1B	protein geranylgeranyltransferase type I subunit beta	5229	158,4028433	-0,009983381	0,194278	-0,051387055	0,9590171	0,991911599
ENSG00000256235	SMIM3	small integral membrane protein 3	85027	164,6505994	0,289466946	0,14182	2,041082044	0,041242673	0,323534763
ENSG00000059588	TARBP1	TAR (HIV-1) RNA binding protein 1	6894	111,0292784	-0,257386883	0,200906	-1,281133785	0,200146679	0,659218424
ENSG00000173917	HOXB2	homeobox B2	3212	97,22424339	0,381839122	0,179529	2,126891633	0,033429088	0,293026852
ENSG00000180776	ZDHHC20	zinc finger DHHC-type palmitoyltransferase 20	253832	933,6586085	-0,144214226	0,089284	-1,615236666	0,106259453	0,523236203
ENSG00000094975	SUCO	SUN domain containing ossification factor	51430	271,8291288	-0,059375787	0,121308	-0,489463389	0,624513668	0,90479929
ENSG00000175854	SWI5	SWI5 homologous recombination repair protein	375757	27,84467358	-0,444083383	0,315761	-1,406390662	0,159608152	NA
ENSG00000241489	NA	NA	NA	204,9510564	-0,021798831	0,12599	-0,173020311	0,862635463	0,97107038
ENSG00000129007	CALML4	calmodulin like 4	91860	12,31525733	-1,404332423	0,826143	-1,699866038	0,089156126	NA
ENSG00000008018	PSMB1	proteasome 20S subunit beta 1	5689	277,2118933	0,127375383	0,100948	1,261788975	0,20702473	0,661502129
ENSG00000197714	ZNF460	zinc finger protein 460	10794	104,8954017	0,274019366	0,240318	1,140238342	0,254187018	0,709949543
ENSG00000023909	GCLM	glutamate-cysteine ligase modifier subunit	2730	86,78331567	-0,042635581	0,191494	-0,222647468	0,82380989	0,961917534
ENSG00000162972	MAIP1	matrix AAA peptidase interacting protein 1	79568	19,88192278	-0,056672991	0,45593	-0,12430185	0,901076283	NA
ENSG00000186001	LRCH3	leucine rich repeats and calponin homology domain containing 3	84859	320,5031314	-0,006716552	0,130526	-0,051457491	0,958960974	0,991911599
ENSG00000233476	NA	NA	NA	12,57297857	-1,323626336	0,529789	-2,498400809	0,012475505	NA
ENSG00000068831	RASGRP2	RAS guanyl releasing protein 2	10235	60,91995059	-0,498664229	0,377814	-1,31986544	0,186879948	0,645758518
ENSG00000164307	ERAP1	endoplasmic reticulum aminopeptidase 1	51752	1265,470428	-0,057711675	0,057899	-0,996762267	0,318879918	0,763389521
ENSG00000166181	API5	apoptosis inhibitor 5	8539	653,1197424	-0,012372361	0,072595	-0,170429926	0,864672039	0,971561202
ENSG00000189339	SLC35E2B	solute carrier family 35 member E2B	728661	988,4420329	-0,083991126	0,074037	-1,134440714	0,256609729	0,712699297
ENSG00000173588	CEP83	centrosomal protein 83	51134	54,14197152	0,20425841	0,246352	0,829133852	0,40702867	0,819272982
ENSG00000117091	CD48	CD48 molecule	962	925,1603599	0,133637017	0,063179	2,115213782	0,034411731	0,296348392
ENSG00000177576	C18orf32	chromosome 18 open reading frame 32	497661	57,98896727	0,438961574	0,923156	0,475501216	0,634429766	0,908608932
ENSG00000108443	RPS6KB1	ribosomal protein S6 kinase B1	6198	186,9789463	0,012583852	0,14356	0,087655741	0,930150298	0,986487751
ENSG00000114062	UBE3A	ubiquitin protein ligase E3A	7337	367,2162844	0,135621009	0,097854	1,385952416	0,165761434	0,624933502
ENSG00000103150	MLYCD	malonyl-CoA decarboxylase	23417	106,0753621	0,272257849	0,201803	1,349127429	0,177296039	0,632039836
ENSG00000100321	SYNGR1	synaptogyrin 1	9145	91,22887978	-0,259594558	0,195469	-1,328059946	0,184158304	0,640557715
ENSG00000196968	FUT11	fucosyltransferase 11	170384	86,34438977	-0,093919198	0,221904	-0,423241977	0,672118723	0,923389485
ENSG00000050748	MAPK9	mitogen-activated protein kinase 9	5601	187,6869877	-0,285505941	0,177282	-1,610464933	0,107296395	0,526362202
ENSG00000120860	WASHC3	WASH complex subunit 3	51019	50,69279687	0,393585498	0,263859	1,491650121	0,135790891	0,576449438
ENSG00000153560	UBP1	upstream binding protein 1	7342	420,1482502	-0,145332236	0,096312	-1,508978937	0,131304166	0,56756352
ENSG00000157538	VPS26C	VPS26 endosomal protein sorting factor C	10311	201,5349737	0,06274439	0,163997	0,382595716	0,702019547	0,930846779
ENSG00000167674	HDGFL2	HDGF like 2	84717	133,8279698	-0,261771704	0,1602	-1,634028367	0,102252894	0,515119384
ENSG00000168118	RAB4A	RAB4A, member RAS oncogene family	5867	77,05496125	-0,152652494	0,195277	-0,781724693	0,434376386	0,835560161

ENSG00000169203	NPIPB12	nuclear pore complex interacting protein family member B12	440353	296,479045	-0,31170612	0,188822	-1,650797596	0,098779911	0,509400803
ENSG00000179918	SEPHS2	selenophosphate synthetase 2	22928	340,3067381	0,050646835	0,090613	0,558938027	0,576204016	0,887069818
ENSG00000198466	ZNF587	zinc finger protein 587	84914	230,7530413	-0,211611079	0,21614	-0,979044991	0,32755775	0,769876712
ENSG00000131876	SNRPA1	small nuclear ribonucleoprotein polypeptide A'	6627	57,84500316	-0,060213036	0,226385	-0,265976063	0,790257644	0,95167209
ENSG00000133731	IMPA1	inositol monophosphatase 1	3612	98,14142865	-0,540588878	0,22676	-2,383970318	0,017126984	0,204616219
ENSG00000116663	FBXO6	F-box protein 6	26270	225,1254562	0,227679188	0,136689	1,665675137	0,095778137	0,501511212
ENSG00000122678	POLM	DNA polymerase mu	27434	49,26254844	0,293518349	0,355381	0,825924906	0,408846691	0,820793685
ENSG00000055483	USP36	ubiquitin specific peptidase 36	57602	307,0513673	-0,253543701	0,149908	-1,691330009	0,090773792	0,490665767
ENSG00000105618	PRPF31	pre-mRNA processing factor 31	26121	139,9221972	0,066042219	0,176234	0,374741214	0,707852937	0,930846779
ENSG00000185800	DMWD	DM1 locus, WD repeat containing	1762	100,4004845	-0,349071885	0,186035	-1,876378794	0,060603284	0,398308113
ENSG00000137563	GGH	gamma-glutamyl hydrolase	8836	25,53451107	0,842958938	0,424778	1,984467475	0,04720375	NA
ENSG00000012983	MAP4K5	mitogen-activated protein kinase kinase kinase kinase 5	11183	201,3229303	-0,143326051	0,120995	-1,184562269	0,236190563	0,691328984
ENSG00000132109	TRIM21	tripartite motif containing 21	6737	151,3835662	-0,046186233	0,148407	-0,311213715	0,755638158	0,942901768
ENSG00000103005	USB1	U6 snRNA biogenesis phosphodiesterase 1	79650	264,5291256	-0,025815007	0,147126	-0,175462376	0,860716332	0,97061627
ENSG00000018869	ZNF582	zinc finger protein 582	147948	11,89457461	0,380164724	0,61351	0,619655083	0,535484893	NA
ENSG00000159023	EPB41	erythrocyte membrane protein band 4,1	2035	781,3695274	0,020784883	0,077772	0,267254829	0,789272964	0,95148885
ENSG00000177728	TMEM94	transmembrane protein 94	9772	164,2995434	0,005617939	0,170835	0,032885198	0,973766137	0,994534085
ENSG00000006576	PHTF2	putative homeodomain transcription factor 2	57157	270,0993038	0,234079193	0,110402	2,120248524	0,033985092	0,294612488
ENSG00000117385	P3H1	prolyl 3-hydroxylase 1	64175	89,76271827	-0,000856133	0,238974	-0,00358253	0,99714156	0,999300722
ENSG00000090863	GLG1	golgi glycoprotein 1	2734	958,370257	0,043425698	0,115299	0,376635786	0,706444289	0,930846779
ENSG00000170113	NIPA1	NIPA magnesium transporter 1	123606	145,9760685	-0,117223439	0,169384	-0,69205832	0,488900705	0,860176107
ENSG00000178567	EPM2AIP1	EPM2A interacting protein 1	9852	244,6544596	-0,142718289	0,116649	-1,223486532	0,221145988	0,677288877
ENSG00000076382	SPAG5	sperm associated antigen 5	10615	117,3586879	0,588706777	0,181423	3,244948338	0,001174719	0,037968089
ENSG00000169918	OTUD7A	OTU deubiquitinase 7A	161725	38,43553876	-0,059011739	0,311713	-0,189314377	0,849846427	NA
ENSG00000186187	ZNRF1	zinc and ring finger 1	84937	37,90880686	0,033310849	0,299809	0,111106802	0,911531655	NA
ENSG00000163933	RFT1	RFT1 homolog	91869	71,039153	0,248587124	0,224868	1,10548078	0,268951318	0,724397686
ENSG00000102974	CTCF	CCCTC-binding factor	10664	421,0830014	-0,127836263	0,084542	-1,512107469	0,130506525	0,566497377
ENSG00000130150	MOSPD2	motile sperm domain containing 2	158747	79,99131228	0,145340891	0,235517	0,617113911	0,537159593	0,874923109
ENSG00000271383	NBPF19	NBPF member 19	101060226	1475,378604	0,083068446	0,083564	0,994064567	0,320191434	0,76448423
ENSG00000140368	PSTPIP1	proline-serine-threonine phosphatase interacting protein 1	9051	403,015453	-0,264888386	0,108841	-2,433717882	0,014944638	0,1911648
ENSG00000196453	ZNF777	zinc finger protein 777	27153	68,79555323	-0,250857123	0,254171	-0,986963394	0,323660584	0,767603859
ENSG00000215012	RTL10	retrotransposon Gag like 10	79680	64,88531738	-0,045878037	0,229553	-0,199858247	0,841591445	0,966777711
ENSG00000162231	NXF1	nuclear RNA export factor 1	10482	811,3443336	-0,149780411	0,066023	-2,268612739	0,023291886	0,243912633
ENSG00000178295	GEN1	GEN1 Holliday junction 5' flap endonuclease	348654	89,58145678	0,450805678	0,175392	2,57027515	0,010161777	0,15588007
ENSG00000054983	GALC	galactosylceramidase	2581	187,1816597	-0,169156011	0,13494	-1,253568648	0,209998833	0,664554962
ENSG00000101407	TTI1	TELO2 interacting protein 1	9675	124,4058827	0,070947572	0,170542	0,416012488	0,677400865	0,924466793
ENSG00000127564	PKMYT1	protein kinase, membrane associated tyrosine/threonine 1	9088	21,28824225	1,055578978	0,590063	1,788926748	0,073626613	NA
ENSG00000197808	ZNF461	zinc finger protein 461	92283	16,54094344	-0,481231674	0,643222	-0,748157506	0,454365159	NA
ENSG00000072958	AP1M1	adaptor related protein complex 1 subunit mu 1	8907	267,6034591	0,107005364	0,11018	0,9711906	0,331453374	0,772473234
ENSG00000085185	BCORL1	BCL6 corepressor like 1	63035	39,97394683	-0,134144037	0,334832	-0,400630687	0,688692049	NA
ENSG00000136448	NMT1	N-myristoyltransferase 1	4836	385,1087062	-0,05540742	0,150386	-0,368435009	0,712548897	0,930846779
ENSG00000142751	GPN2	GPN-loop GTPase 2	54707	134,5983453	-0,102815459	0,166163	-0,618761053	0,536073783	0,874923109
ENSG00000153774	CFDP1	craniofacial development protein 1	10428	58,62330842	-0,133354274	0,239845	-0,556002496	0,578209148	0,887069818
ENSG00000167881	SRP68	signal recognition particle 68	6730	224,0949613	0,033780818	0,155815	0,216801089	0,828363363	0,962723654
ENSG00000181404	WASHC1	WASH complex subunit 1	100287171	47,9833755	-0,260174185	0,277953	-0,936037421	0,349253921	0,784537594
ENSG00000181404	LOC124908094	WAS protein family homolog 2-like	124908094	47,9833755	-0,260174185	0,277953	-0,936037421	0,349253921	0,784537594
ENSG00000181404	LOC124908102	WAS protein family homolog 2-like	124908102	47,9833755	-0,260174185	0,277953	-0,936037421	0,349253921	0,784537594
ENSG00000187688	TRPV2	transient receptor potential cation channel subfamily V member 2	51393	325,4670833	-0,038688839	0,089512	-0,432218991	0,665582258	0,922184616
ENSG00000124574	ABCC10	ATP binding cassette subfamily C member 10	89845	146,3401706	-0,041451405	0,152534	-0,271752574	0,785812274	0,950624564
ENSG00000151233	GXYLT1	glucoside xylosyltransferase 1	283464	98,24414814	-0,113294941	0,205616	-0,55100134	0,581632756	0,888501395
ENSG00000168329	CX3CR1	C-X3-C motif chemokine receptor 1	1524	34,81349871	-1,682712439	0,358317	-4,69616121	2,65E-06	NA

ENSG00000169057	MECP2	methyl-CpG binding protein 2	4204	382,7519102	0,17375443	0,113877	1,525813109	0,127056415	0,563689485
ENSG00000226479	TMEM185B	transmembrane protein 185B	79134	250,7707137	0,038412708	0,116565	0,329540129	0,741747468	0,939193793
ENSG00000103274	NUBP1	NUBP iron-sulfur cluster assembly factor 1, cytosolic	4682	67,72289967	0,202436335	0,209943	0,964243404	0,334923908	0,774813659
ENSG00000107672	NSMCE4A	NSE4 homolog A, SMC5-SMC6 complex component	54780	70,65335684	-0,004892687	0,218691	-0,022372613	0,982150726	0,995974079
ENSG00000152620	NADK2	NAD kinase 2, mitochondrial	133686	42,25666176	-0,39950819	0,271691	-1,470450042	0,141439907	NA
ENSG00000185591	SP1	Sp1 transcription factor	6667	637,5177848	-0,167009327	0,069888	-2,389669889	0,016863522	0,204616219
ENSG00000292373	IL9R	interleukin 9 receptor	3581	388,0006662	0,09631193	0,099566	0,967322066	0,333383056	0,773674527
ENSG00000139436	GIT2	GIT ArfGAP 2	9815	569,5915109	-0,113793287	0,100163	-1,136082628	0,255921985	0,71206165
ENSG00000182197	EXT1	exostosin glycosyltransferase 1	2131	65,64633357	-0,081522024	0,220746	-0,369301558	0,711902964	0,930846779
ENSG00000186469	GNG2	G protein subunit gamma 2	54331	333,3657941	-0,032801771	0,103001	-0,318460549	0,750135615	0,941011581
ENSG00000186230	ZNF749	zinc finger protein 749	388567	37,39351996	-0,300504322	0,296101	-1,014872512	0,310166603	NA
ENSG00000168803	ADAL	adenosine deaminase like	161823	43,93853612	0,009158948	0,289329	0,031655822	0,974746526	0,994534085
ENSG00000028528	SNX1	sorting nexin 1	6642	541,7579842	0,044386148	0,150196	0,295521828	0,76759528	0,944698783
ENSG00000112877	CEP72	centrosomal protein 72	55722	21,46126866	-0,134590459	0,406434	-0,33114923	0,740531767	NA
ENSG00000158234	FAIM	Fas apoptotic inhibitory molecule	55179	29,04478431	0,225668689	0,32828	0,687428168	0,491812952	NA
ENSG00000189164	ZNF527	zinc finger protein 527	84503	28,57653163	-0,107153664	0,434029	-0,246881405	0,805000008	NA
ENSG00000225630	MTND2P28	MT-ND2 pseudogene 28	100652939	52,99114745	0,081854844	0,270855	0,302208953	0,76249278	0,943004836
ENSG00000011007	ELOA	elongin A	6924	184,3703229	0,096998763	0,145011	0,668908164	0,503554062	0,863218221
ENSG00000108953	YWHAE	tyrosine 3-monooxygenase/tryptophan 5-monooxygenase activation protein epsilon	7531	303,7529233	0,158212195	0,183132	0,863925839	0,387628631	0,809653368
ENSG00000126698	DNAJC8	DnaJ heat shock protein family (Hsp40) member C8	22826	396,9506097	-0,033795704	0,124627	-0,271175151	0,786256324	0,950624564
ENSG00000155096	AZIN1	antizyme inhibitor 1	51582	451,0283275	0,111873581	0,091779	1,21894188	0,222866252	0,678694696
ENSG00000213186	TRIM59	tripartite motif containing 59	286827	135,7707766	-0,056730927	0,154315	-0,367631858	0,713147756	0,93087294
ENSG00000142544	CTU1	cytosolic thiouridylase subunit 1	90353	11,80924635	0,420393739	0,552219	0,761280848	0,446489337	NA
ENSG00000135441	BLOC1S1	biogenesis of lysosomal organelles complex 1 subunit 1	2647	103,6309867	-0,219814629	0,194191	-1,131949085	0,257655841	0,713362633
ENSG00000124596	OARD1	O-acyl-ADP-ribose deacylase 1	221443	89,56811632	-0,553873957	0,296345	-1,86901979	0,061620061	0,402674318
ENSG00000167513	CDT1	chromatin licensing and DNA replication factor 1	81620	35,45851145	0,40048439	0,316212	1,266506366	0,205331852	NA
ENSG00000137409	MTCH1	mitochondrial carrier 1	23787	115,8706444	-0,272490196	0,192739	-1,413779036	0,157426791	0,61428162
ENSG00000233232	NPIPB7	nuclear pore complex interacting protein family member B7	440350	26,0706214	0,465674929	0,374742	1,242653458	0,213995562	NA
ENSG00000113649	TCERG1	transcription elongation regulator 1	10915	154,7396931	-0,339845847	0,198374	-1,713159863	0,086683133	0,481317095
ENSG00000174744	BRMS1	BRMS1 transcriptional repressor and anoikis regulator	25855	100,733352	-0,072886402	0,195367	-0,373074152	0,709093256	0,930846779
ENSG00000111196	MAGOHB	mago homolog B, exon junction complex subunit	55110	46,69375667	0,582785604	0,351823	1,656472633	0,097626143	0,505442139
ENSG00000152944	MED21	mediator complex subunit 21	9412	60,90429175	-0,072472845	0,233959	-0,309767798	0,756737541	0,942901768
ENSG00000163938	GNL3	G protein nucleolar 3	26354	112,4410488	0,206934191	0,245787	0,84192343	0,399830814	0,814673014
ENSG00000166716	ZNF592	zinc finger protein 592	9640	252,7313921	-0,077221072	0,121805	-0,6339751	0,526097072	0,871384275
ENSG00000161267	BDH1	3-hydroxybutyrate dehydrogenase 1	622	95,31587541	-0,35770202	0,229714	-1,557165091	0,119431294	0,552466951
ENSG00000250644	NA	NA	NA	182,146187	-0,126044643	0,164465	-0,766390355	0,44344407	0,839231259
ENSG00000213366	GSTM2	glutathione S-transferase mu 2	2946	117,0340694	-0,075851779	0,156062	-0,486036913	0,626940995	0,90479929
ENSG00000100150	DEPDC5	DEP domain containing 5, GATOR1 subcomplex subunit	9681	47,66715083	-0,481984944	0,375796	-1,282569864	0,199642814	0,658544586
ENSG00000203326	ZNF525	zinc finger protein 525	170958	56,16728562	-0,047141618	0,340754	-0,138344939	0,889967811	0,977990819
ENSG00000152217	SETBP1	SET binding protein 1	26040	197,6054086	0,271808829	0,124649	2,180588508	0,029213866	0,275445021
ENSG00000111667	USP5	ubiquitin specific peptidase 5	8078	256,9390792	-0,131396924	0,116702	-1,125918785	0,260199897	0,716051154
ENSG00000163823	CCR1	C-C motif chemokine receptor 1	1230	843,9531717	-0,019781432	0,059766	-0,330982523	0,740657687	0,939193793
ENSG00000168036	CTNNB1	catenin beta 1	1499	756,2037	-0,083283199	0,094185	-0,884255676	0,376558209	0,803373958
ENSG00000180884	ZNF792	zinc finger protein 792	126375	128,6612604	-0,056819129	0,150951	-0,376408449	0,706613265	0,930846779
ENSG00000137100	DCTN3	dynactin subunit 3	11258	89,49233099	-0,12126558	0,180438	-0,672060469	0,501545201	0,862227298
ENSG00000204389	HSPA1A	heat shock protein family A (Hsp70) member 1A	3303	259,0075592	0,097911198	0,138573	0,706565335	0,479836638	0,854606339
ENSG00000160551	TAOK1	TAO kinase 1	57551	779,2892938	-0,077863753	0,064419	-1,208713098	0,226773088	0,681621061
ENSG00000082781	ITGB5	integrin subunit beta 5	3693	22,73625454	0,560709838	0,435919	1,2862699	0,198348883	NA
ENSG00000249115	HAUS5	HAUS augmin like complex subunit 5	23354	54,23113377	-0,005820967	0,252351	-0,023066946	0,981596872	0,995974079
ENSG00000099783	HNRNPM	heterogeneous nuclear ribonucleoprotein M	4670	606,2940173	-0,110561323	0,072087	-1,533717943	0,125099058	0,5624706
ENSG00000156642	NPTN	neuroplastin	27020	125,6326968	0,051881709	0,15587	0,332851733	0,739246205	0,939193793

ENSG00000178952	TUFM	Tu translation elongation factor, mitochondrial	7284	294,2694303	-0,083633106	0,100458	-0,832519879	0,405115562	0,818437414
ENSG00000204172	AGAP9	ArfGAP with GTPase domain, ankyrin repeat and PH domain 9	642517	49,72093324	-0,59598238	0,281063	-2,120457063	0,033967519	0,294612488
ENSG00000254858	MPV17L2	MPV17 mitochondrial inner membrane protein like 2	84769	22,5735945	0,013537863	0,381811	0,035457017	0,97171532	NA
ENSG00000144566	RAB5A	RAB5A, member RAS oncogene family	5868	302,0688923	-0,160926351	0,096881	-1,661064174	0,096700559	0,502970986
ENSG00000197128	ZNF772	zinc finger protein 772	400720	69,5191225	-0,147762473	0,242635	-0,608991624	0,542529991	0,877396094
ENSG00000008130	NADK	NAD kinase	65220	209,9390971	-0,257083656	0,130373	-1,971913343	0,0486195	0,354159983
ENSG00000010818	HIVEP2	HIVEP zinc finger 2	3097	374,2700554	-0,09460827	0,123058	-0,768808523	0,442006989	0,838678081
ENSG00000140332	TLE3	TLE family member 3, transcriptional corepressor	7090	254,4631542	-0,108890944	0,158067	-0,688893165	0,490890501	0,860513824
ENSG00000165389	SPTSSA	serine palmitoyltransferase small subunit A	171546	74,79810585	-0,528794475	0,206308	-2,563134127	0,010373197	0,157889703
ENSG00000135272	MDFIC	MyoD family inhibitor domain containing	29969	1143,568331	0,0182703	0,058428	0,312695965	0,754511662	0,942901768
ENSG00000143190	POU2F1	POU class 2 homeobox 1	5451	240,8756234	0,041809712	0,190699	0,219244793	0,826459364	0,961917534
ENSG00000205795	CYS1	cystin 1	192668	33,16594387	-0,095053645	0,347012	-0,27391999	0,784146109	NA
ENSG00000089057	SLC23A2	solute carrier family 23 member 2	9962	303,8501629	-0,129308318	0,114387	-1,130441862	0,258290083	0,714048892
ENSG00000123575	FAM199X	family with sequence similarity 199, X-linked	139231	265,2496972	-0,333454442	0,108035	-3,086531524	0,002025065	0,054949576
ENSG00000176208	ATAD5	ATPase family AAA domain containing 5	79915	70,08623446	0,338682395	0,261043	1,297419883	0,194486755	0,652365446
ENSG00000138166	DUSP5	dual specificity phosphatase 5	1847	279,8102507	0,145749955	0,101652	1,433810356	0,151626389	0,60852001
ENSG00000071655	MBD3	methyl-CpG binding domain protein 3	53615	63,03116435	0,873319683	0,430434	2,028929305	0,042465491	0,328150469
ENSG00000103356	EARS2	glutamyl-tRNA synthetase 2, mitochondrial	124454	52,01045765	-0,468373316	0,290523	-1,612171658	0,106924591	0,525522989
ENSG00000168283	BMI1	BMI1 proto-oncogene, polycomb ring finger	648	169,220178	-0,274751026	0,146264	-1,878461098	0,060318114	0,398308113
ENSG00000100242	SUN2	Sad1 and UNC84 domain containing 2	25777	2329,575044	-0,135595146	0,076949	-1,762145455	0,078044725	0,452159899
ENSG00000138375	SMARCAL1	SWI/SNF related, matrix associated, actin dependent regulator of chromatin, subfamily a like 1	50485	123,4564806	0,223063181	0,194851	1,144790934	0,252295772	0,708754466
ENSG00000160298	C21orf58	chromosome 21 open reading frame 58	54058	35,0609696	0,378573567	0,324739	1,165779651	0,243703547	NA
ENSG00000035499	DEPDC1B	DEP domain containing 1B	55789	36,86096926	0,735634349	0,287747	2,556535757	0,010572021	NA
ENSG00000117697	NSL1	NSL1 component of MIS12 kinetochore complex	25936	142,6784623	0,132366999	0,195095	0,678474677	0,497470775	0,861283987
ENSG00000150753	CCT5	chaperonin containing TCP1 subunit 5	22948	572,4060753	0,124346112	0,08765	1,418667858	0,155995872	0,61428162
ENSG00000168615	ADAM9	ADAM metallopeptidase domain 9	8754	16,3849929	0,385786993	0,790927	0,487765358	0,625716052	NA
ENSG00000108262	GIT1	GIT ArfGAP 1	28964	243,7856998	-0,017581881	0,114376	-0,153720143	0,877830401	0,976223885
ENSG00000278619	MRM1	mitochondrial rRNA methyltransferase 1	79922	14,91905215	0,15203375	0,591526	0,257019515	0,797163709	NA
ENSG00000170442	KRT86	keratin 86	3892	442,2623066	-0,010038382	0,079053	-0,126982906	0,898953928	0,977990819
ENSG00000257727	CNPY2	canopy FGF signaling regulator 2	10330	55,0092613	-0,080086425	0,293845	-0,272546449	0,785201881	0,950624564
ENSG00000005436	GCFC2	GC-rich sequence DNA-binding factor 2	6936	40,04024636	-0,194387847	0,3521	-0,552080622	0,580893118	NA
ENSG00000100368	CSF2RB	colony stimulating factor 2 receptor subunit beta	1439	311,0778815	-0,179156601	0,118494	-1,511951356	0,130546238	0,566497377
ENSG00000168092	PAFAH1B2	platelet activating factor acetylhydrolase 1b catalytic subunit 2	5049	190,9260486	-0,199241825	0,128709	-1,54800263	0,121621663	0,556808172
ENSG00000174606	ANGEL2	angel homolog 2	90806	161,2173764	0,020591074	0,139243	0,147879137	0,882438153	0,976300778
ENSG00000145945	FAM50B	family with sequence similarity 50 member B	26240	23,05025951	0,304431012	0,380615	0,799840559	0,423803181	NA
ENSG00000095370	SH2D3C	SH2 domain containing 3C	10044	421,5320113	-0,078710873	0,096211	-0,818103508	0,413298086	0,822407694
ENSG00000157617	C2CD2	C2 calcium dependent domain containing 2	25966	20,61436532	-1,094896901	0,809161	-1,353125859	0,176015426	NA
ENSG00000168386	FILIP1L	filamin A interacting protein 1 like	11259	12,61940238	0,099207042	0,567641	0,174770767	0,861259759	NA
ENSG00000178980	SELENOW	selenoprotein W	6415	55,92211278	-0,017866492	0,241214	-0,074069065	0,94095543	0,988399618
ENSG00000115998	C2orf42	chromosome 2 open reading frame 42	54980	70,64826964	-0,244786724	0,234866	-1,042238856	0,297300953	0,748371252
ENSG00000137574	TGS1	trimethylguanosine synthase 1	96764	131,8525122	0,32487311	0,210058	1,546589128	0,121962353	0,557491687
ENSG00000154719	MRPL39	mitochondrial ribosomal protein L39	54148	39,81636158	0,249928543	0,270683	0,923324299	0,355838228	NA
ENSG00000168961	LGALS9	galectin 9	3965	23,04393988	-0,411517003	0,529474	-0,777218218	0,437030036	NA
ENSG00000213047	DENND1B	DENN domain containing 1B	163486	350,153536	-0,128271371	0,090948	-1,410378857	0,158427845	0,614461481

ENSG00000122644	ARL4A	ADP ribosylation factor like GTPase 4A	10124	40,64853369	0,374453348	0,37639	0,994853476	0,319807533	NA
ENSG00000165516	KLHDC2	kelch domain containing 2	23588	62,91098285	0,179478242	0,364297	0,492670656	0,622245315	0,904764752
ENSG00000109445	ZNF330	zinc finger protein 330	27309	87,77701126	0,014839013	0,216979	0,068389314	0,945475728	0,98927293
ENSG00000131381	RBSN	rabenosyn, RAB effector	64145	123,5526544	0,403884404	0,179576	2,249099738	0,024506151	0,251596486
ENSG00000046651	OFD1	OFD1 centriole and centriolar satellite protein	8481	119,3012995	0,071826514	0,294362	0,244007602	0,807224933	0,958276902
ENSG00000071575	TRIB2	tribbles pseudokinase 2	28951	466,2058026	0,040975465	0,087005	0,470956834	0,637671561	0,909767927
ENSG00000101307	SIRPB1	signal regulatory protein beta 1	10326	13,60801802	0,462519583	0,602059	0,76822982	0,44235066	NA
ENSG00000121797	CCRL2	C-C motif chemokine receptor like 2	9034	21,75466319	0,595551703	0,442185	1,346838717	0,178032182	NA
ENSG00000171443	ZNF524	zinc finger protein 524	147807	28,67469061	0,176401117	0,413676	0,426423654	0,669799169	NA
ENSG00000215845	TSTD1	thiosulfate sulfurtransferase like domain containing 1	100131187	47,02860115	-0,197060317	0,267682	-0,736174471	0,461624526	0,846765498
ENSG00000102030	NAA10	N-alpha-acetyltransferase 10, NatA catalytic subunit	8260	39,49211239	-0,311505364	0,291044	-1,070304594	0,284482227	NA
ENSG00000072210	ALDH3A2	aldehyde dehydrogenase 3 family member A2	224	121,6215967	-0,183960886	0,241907	-0,760460403	0,44697943	0,842554058
ENSG00000152926	ZNF117	zinc finger protein 117	51351	98,90750496	0,052728143	0,192214	0,274319956	0,78383875	0,950093441
ENSG00000152926	ERV3-1-ZNF117	ERV3-1-ZNF117 readthrough	109504726	98,90750496	0,052728143	0,192214	0,274319956	0,78383875	0,950093441
ENSG00000133874	RNF122	ring finger protein 122	79845	56,99290883	-0,230699106	0,244091	-0,945136712	0,344589085	0,781366475
ENSG00000113328	CCNG1	cyclin G1	900	137,9500302	0,170366165	0,144202	1,181442863	0,237426839	0,692443518
ENSG00000106089	STX1A	syntaxin 1A	6804	41,28573848	-0,425911916	0,332563	-1,280697012	0,20030011	NA
ENSG00000068912	ERLEC1	endoplasmic reticulum lectin 1	27248	194,2142447	0,067347404	0,145975	0,461363569	0,644537784	0,912530858
ENSG00000141298	SSH2	slingshot protein phosphatase 2	85464	1016,526248	-0,220372221	0,085285	-2,583956079	0,009767423	0,151888562
ENSG00000188610	FAM72B	family with sequence similarity 72 member B	653820	29,23597625	-0,022421381	0,405038	-0,055356271	0,955854633	NA
ENSG00000188021	UBQLN2	ubiquilin 2	29978	155,3161234	-0,199319409	0,148806	-1,339455117	0,180422557	0,634062228
ENSG00000105278	ZFR2	zinc finger RNA binding protein 2	23217	38,75662709	0,460672902	0,386518	1,191852684	0,233319014	NA
ENSG00000020922	MRE11	MRE11 homolog, double strand break repair nuclease	4361	233,648865	0,088063656	0,151188	0,58247811	0,560244682	0,883921037
ENSG00000163328	GPR155	G protein-coupled receptor 155	151556	110,8778109	-0,434503988	0,172262	-2,522345723	0,011657507	0,169255469
ENSG00000088448	ANKRD10	ankyrin repeat domain 10	55608	384,6038218	-0,027078519	0,09041	-0,299509305	0,764551473	0,944297417
ENSG00000096654	ZNF184	zinc finger protein 184	7738	60,13366646	0,27811147	0,237477	1,171109911	0,241554601	0,696802579
ENSG00000111252	SH2B3	SH2B adaptor protein 3	10019	740,9655867	-0,042172769	0,068873	-0,612323918	0,540323468	0,876149366
ENSG00000120254	MTHFD1L	methylenetetrahydrofolate dehydrogenase (NADP+ dependent) 1 like	25902	61,53742825	0,553953792	0,278746	1,987309447	0,046888115	0,347086954
ENSG00000135372	NAT10	N-acetyltransferase 10	55226	245,7116231	-0,02846405	0,134307	-0,211933222	0,832159132	0,963446071
ENSG00000146802	TMEM168	transmembrane protein 168	64418	177,0505654	-0,462736456	0,145993	-3,169569893	0,001526647	0,045939802
ENSG00000197756	RPL37A	ribosomal protein L37a	6168	1056,763204	0,059359019	0,065075	0,91216928	0,361679613	0,791408368
ENSG00000077152	UBE2T	ubiquitin conjugating enzyme E2 T	29089	18,9411161	0,668313121	0,569308	1,173904661	0,240433215	NA
ENSG00000111639	MRPL51	mitochondrial ribosomal protein L51	51258	152,3177481	0,255930651	0,142666	1,793911138	0,072827353	0,438639596
ENSG00000116350	SRSF4	serine and arginine rich splicing factor 4	6429	277,297535	-0,267701425	0,135211	-1,979880266	0,047716984	0,350251584
ENSG00000131508	UBE2D2	ubiquitin conjugating enzyme E2 D2	7322	311,6243682	0,058766876	0,140061	0,419580447	0,674791975	0,923602509
ENSG00000140320	BAHD1	bromo adjacent homology domain containing 1	22893	265,2535053	-0,147082859	0,108983	-1,349593148	0,177146523	0,632039836
ENSG00000150477	KIAA1328	KIAA1328	57536	21,30451902	0,087705468	0,411585	0,213092223	0,83125503	NA
ENSG00000011405	PIK3C2A	phosphatidylinositol-4-phosphate 3-kinase catalytic subunit type 2 alpha	5286	277,070716	-0,162779803	0,110283	-1,476019253	0,139938724	0,583013685
ENSG00000240303	ACAD11	acyl-CoA dehydrogenase family member 11	84129	56,73991576	-0,363624181	0,973548	-0,373504183	0,708773233	0,930846779
ENSG00000253729	PRKDC	protein kinase, DNA-activated, catalytic subunit	5591	1892,802626	0,149351484	0,053709	2,78073859	0,005423539	0,104917427
ENSG00000112335	SNX3	sorting nexin 3	8724	280,698482	-0,060228878	0,113405	-0,531094715	0,595353147	0,894229873
ENSG00000182831	HAPSTR1	HUWE1 associated protein modifying stress responses	29035	182,9160462	-0,365911513	0,171333	-2,135673092	0,032706069	0,288946532
ENSG00000006459	KDM7A	lysine demethylase 7A	80853	211,4264385	-0,057134737	0,131999	-0,432843132	0,665128736	0,922184616
ENSG00000012232	EXTL3	exostosin like glycosyltransferase 3	2137	163,8631492	-0,171572632	0,152121	-1,127872228	0,259373884	0,715533012
ENSG00000112561	TFEB	transcription factor EB	7942	64,9816856	0,026460776	0,24217	0,109265162	0,912992178	0,980488351
ENSG00000169714	CNBP	CCHC-type zinc finger nucleic acid binding protein	7555	997,1458306	0,092871798	0,074234	1,251068675	0,210909423	0,6647417
ENSG00000170899	GSTA4	glutathione S-transferase alpha 4	2941	44,04738138	-0,050141265	0,297635	-0,168465745	0,86621689	0,971722599
ENSG00000198198	SZT2	SZT2 subunit of KICSTOR complex	23334	421,973448	0,224194992	0,088697	2,527636529	0,011483317	0,168264867
ENSG00000115282	TTC31	tetratricopeptide repeat domain 31	64427	112,4791313	0,309739126	0,295818	1,047060789	0,295071543	0,746855268

ENSG00000112893	MAN2A1	mannosidase alpha class 2A member 1	4124	215,6439894	-0,159724454	0,125107	-1,276703789	0,201706843	0,660119983
ENSG00000151929	BAG3	BAG cochaperone 3	9531	24,88610944	-0,353031063	0,386734	-0,912853274	0,361319715	NA
ENSG00000108179	PPIF	peptidylprolyl isomerase F	10105	159,3836745	-0,078773678	0,129224	-0,60959192	0,542132165	0,877014629
ENSG00000198816	ZNF358	zinc finger protein 358	140467	23,57729495	-0,100801467	0,377192	-0,267241766	0,78928302	NA
ENSG00000083845	RPS5	ribosomal protein S5	6193	625,2298063	-0,068476253	0,075788	-0,903521897	0,366248972	0,79459056
ENSG00000105223	PLD3	phospholipase D family member 3	23646	235,7405952	-0,171850432	0,106931	-1,60711669	0,108028773	0,527320063
ENSG00000130349	MTRES1	mitochondrial transcription rescue factor 1	51250	22,98271865	0,514872233	0,406134	1,267738283	0,204891429	NA
ENSG00000106443	PHF14	PHD finger protein 14	9678	121,2528705	0,236212208	0,158642	1,488964169	0,136496804	0,577299893
ENSG00000007168	PAFAH1B1	platelet activating factor acetylhydrolase 1b regulatory subunit 1	5048	672,7439934	-0,000304114	0,091068	-0,003339431	0,997335524	0,999300722
ENSG00000113811	SELENOK	selenoprotein K	58515	89,791115	0,033497558	0,183274	0,182773227	0,854975957	0,968377739
ENSG00000240342	RPS2P5	ribosomal protein S2 pseudogene 5	100130562	211,3697638	-0,298101875	0,140143	-2,127122191	0,033409932	0,293026852
ENSG00000117450	PRDX1	peroxiredoxin 1	5052	154,2446918	0,462763832	0,155143	2,982827479	0,002855989	0,069661304
ENSG00000172262	ZNF131	zinc finger protein 131	7690	152,2820177	-0,118306308	0,150102	-0,788170849	0,430596778	0,833171822
ENSG00000134049	IER3IP1	immediate early response 3 interacting protein 1	51124	124,045294	-0,102206836	0,174989	-0,584077259	0,559168334	0,883921037
ENSG00000035115	SH3YL1	SH3 and SYLF domain containing 1	26751	91,44872774	0,036301123	0,208953	0,17372906	0,862078398	0,970863509
ENSG00000271503	CCL5	C-C motif chemokine ligand 5	6352	2362,268863	-0,309660782	0,057947	-5,343816141	9,10E-08	1,93E-05
ENSG00000139645	ANKRD52	ankyrin repeat domain 52	283373	516,92606	-0,063583665	0,081026	-0,784728291	0,432612891	0,834691048
ENSG00000292344	PLCXD1	phosphatidylinositol specific phospholipase C X domain containing 1	55344	114,81281	-0,004864092	0,18285	-0,026601532	0,978777551	0,995712569
ENSG00000204475	NCR3	natural cytotoxicity triggering receptor 3	259197	402,7642849	-0,127571026	0,161095	-0,791898618	0,428419793	0,832060923
ENSG00000196535	MYO18A	myosin XVIIIA	399687	105,2135115	0,733324143	0,273368	2,682557235	0,007306165	0,125896872
ENSG00000129250	KIF1C	kinesin family member 1C	10749	200,4917576	-0,134854687	0,126234	-1,068290221	0,285389618	0,737867955
ENSG00000170989	S1PR1	sphingosine-1-phosphate receptor 1	1901	32,0737172	-0,15749163	0,342456	-0,459889288	0,64559569	NA
ENSG00000138182	KIF20B	kinesin family member 20B	9585	139,8907757	0,343828325	0,159652	2,153607314	0,03127098	0,281657534
ENSG00000109066	TMEM104	transmembrane protein 104	54868	94,49248848	0,081163611	0,197509	0,410935562	0,681119785	0,925586358
ENSG00000175573	C11orf68	chromosome 11 open reading frame 68	83638	81,8650139	0,067584023	0,226974	0,297760507	0,765885959	0,944698783
ENSG00000106636	YKT6	YKT6 v-SNARE homolog	10652	202,0994311	-0,236337162	0,133662	-1,768164208	0,077033456	0,448990052
ENSG00000161016	RPL8	ribosomal protein L8	6132	856,6138256	-0,100262642	0,197866	-0,50671958	0,61235159	0,900006614
ENSG00000099817	POLR2E	RNA polymerase II, I and III subunit E	5434	82,59139682	-0,166151337	0,270644	-0,61391063	0,539274385	0,875342836
ENSG00000112305	SMAP1	small ArfGAP 1	60682	356,3481128	-0,126384438	0,095767	-1,319706173	0,186933138	0,645758518
ENSG00000119431	HDHD3	haloacid dehalogenase like hydrolase domain containing 3	81932	25,03561375	0,420941406	0,420958	0,999961051	0,317329357	NA
ENSG00000163156	SCNM1	sodium channel modifier 1	79005	18,1380972	1,633935416	0,548989	2,976261993	0,002917855	NA
ENSG00000163156	TNFAIP8L2-SCNM1	TNFAIP8L2-SCNM1 readthrough	100534012	18,1380972	1,633935416	0,548989	2,976261993	0,002917855	NA
ENSG00000171161	ZNF672	zinc finger protein 672	79894	71,17438904	-0,014601492	0,230906	-0,063235753	0,949578775	0,990700279
ENSG00000171813	PWWP2B	PWWP domain containing 2B	170394	35,61446362	0,103132968	0,302976	0,340399866	0,73355542	NA
ENSG00000197579	TOPORS	TOP1 binding arginine/serine rich protein, E3 ubiquitin ligase	10210	137,7076753	0,041648578	0,148022	0,281367878	0,778428253	0,947109268
ENSG00000267673	FDX2	ferredoxin 2	112812	13,52635741	0,979343117	0,75981	1,288931931	0,197421752	NA
ENSG00000185651	UBE2L3	ubiquitin conjugating enzyme E2 L3	7332	342,3557543	0,113576117	0,11301	1,005009909	0,314892078	0,760305504
ENSG00000115163	CENPA	centromere protein A	1058	30,32474734	0,66435682	0,341338	1,946331927	0,051614881	NA
ENSG00000198205	ZXDA	zinc finger X-linked duplicated A	7789	24,01669092	-0,31943562	0,42175	-0,757405231	0,448807123	NA
ENSG00000099795	NDUFB7	NADH:ubiquinone oxidoreductase subunit B7	4713	77,61544042	-0,172909834	0,197176	-0,876933162	0,380522943	0,806167487
ENSG00000111145	ELK3	ETS transcription factor ELK3	2004	66,83897559	0,153217452	0,231361	0,662242923	0,50781555	0,864738156
ENSG00000115486	GGCX	gamma-glutamyl carboxylase	2677	90,49050038	0,459752272	0,628743	0,731224137	0,464642259	0,848967535
ENSG00000254634	SMG1P6	SMG1 pseudogene 6	100422558	40,3341663	-0,040262879	0,274539	-0,146656495	0,883403159	NA
ENSG00000107104	KANK1	KN motif and ankyrin repeat domains 1	23189	20,45326573	-0,240755942	0,845534	-0,284738441	0,775844537	NA
ENSG00000173114	LRRN3	leucine rich repeat neuronal 3	54674	12,61900678	-0,125842881	0,524686	-0,239844124	0,810451099	NA
ENSG00000267680	ZNF224	zinc finger protein 224	7767	206,155962	0,019947299	0,134734	0,148048966	0,882304125	0,976300778
ENSG00000102804	TSC22D1	TSC22 domain family member 1	8848	186,6757308	-0,037631831	0,119404	-0,315164548	0,752636722	0,942025309
ENSG00000146918	NCAPG2	non-SMC condensin II complex subunit G2	54892	156,5973749	0,514459508	0,200133	2,570594407	0,010152415	0,15588007
ENSG00000138867	GUCD1	guanylyl cyclase domain containing 1	83606	194,3470582	-0,097427662	0,125805	-0,774433988	0,438674191	0,837388481
ENSG00000116977	LGALS8	galectin 8	3964	681,5323522	-0,048258953	0,071114	-0,678618631	0,497379535	0,861283987
ENSG00000100142	POLR2F	RNA polymerase II, I and III subunit F	5435	51,1776569	-0,389893573	0,239844	-1,625612844	0,104032041	0,517786851
ENSG00000204131	NHSL2	NHS like 2	340527	16,33176559	-1,429643117	0,453386	-3,153258544	0,001614587	NA

ENSG00000077514	POLD3	DNA polymerase delta 3, accessory subunit	10714	73,93058262	0,295969467	0,224358	1,319185348	0,187107156	0,645758518
ENSG00000085117	CD82	CD82 molecule	3732	743,7875844	-0,006022359	0,071858	-0,083809467	0,933207921	0,987098279
ENSG00000141569	TRIM65	tripartite motif containing 65	201292	129,5091921	-0,056817784	0,180676	-0,314472489	0,753162208	0,942402355
ENSG00000157778	PSMG3	proteasome assembly chaperone 3	84262	40,93055504	0,045819839	0,328306	0,139564413	0,889004159	NA
ENSG00000136689	IL1RN	interleukin 1 receptor antagonist	3557	12,4210377	1,032793183	0,557549	1,852382573	0,063970906	NA
ENSG00000185561	TLCD2	TLC domain containing 2	727910	25,80572625	-0,602388723	0,360697	-1,670067092	0,09490609	NA
ENSG00000054116	TRAPPC3	trafficking protein particle complex subunit 3	27095	110,4431528	-0,199798296	0,1814	-1,101425859	0,270711356	0,725477256
ENSG00000116824	CD2	CD2 molecule	914	3038,209795	0,140234281	0,050683	2,766891582	0,005659357	0,10771718
ENSG00000198824	CHAMP1	chromosome alignment maintaining phosphoprotein 1	283489	128,3380185	-0,021768196	0,150768	-0,144381897	0,885198911	0,977414909
ENSG00000089012	SIRPG	signal regulatory protein gamma	55423	117,5695298	-0,162436987	0,169349	-0,959187196	0,337464449	0,77693253
ENSG00000278540	ACACA	acetyl-CoA carboxylase alpha	31	152,5707332	-0,10832254	0,281925	-0,384224841	0,700811808	0,930783276
ENSG00000178951	ZBTB7A	zinc finger and BTB domain containing 7A	51341	460,2821661	-0,100320198	0,13619	-0,736620624	0,46135309	0,846765498
ENSG00000164048	ZNF589	zinc finger protein 589	51385	85,62115077	-0,219262246	0,210349	-1,04237395	0,297238339	0,748371252
ENSG00000040633	PHF23	PHD finger protein 23	79142	112,8086989	-0,285088988	0,191995	-1,484873876	0,137577237	0,578689141
ENSG00000090924	PLEKHG2	pleckstrin homology and RhoGEF domain containing G2	64857	159,4238043	-0,307371075	0,196823	-1,561661537	0,118367744	0,5494446
ENSG00000105402	NAPA	NSF attachment protein alpha	8775	229,4870284	0,019792358	0,148742	0,133065436	0,89414163	0,977990819
ENSG00000171827	ZNF570	zinc finger protein 570	148268	41,26690589	0,21725575	0,291882	0,744327602	0,456678306	NA
ENSG00000105193	RPS16	ribosomal protein S16	6217	936,940138	0,120995459	0,072511	1,668659202	0,095184939	0,499178345
ENSG00000168016	TRANK1	tetratricopeptide repeat and ankyrin repeat containing 1	9881	871,0439568	0,05040719	0,064326	0,783622097	0,433261886	0,835093272
ENSG00000227232	NA	NA	NA	98,48600507	0,053262424	0,17914	0,297322853	0,766220036	0,944698783
ENSG00000102007	PLP2	proteolipid protein 2	5355	335,2798164	0,056285355	0,116535	0,482992649	0,629100956	0,905567722
ENSG00000105993	DNAJB6	DnaJ heat shock protein family (Hsp40) member B6	10049	347,1877158	-0,03905762	0,103862	-0,37605227	0,706878037	0,930846779
ENSG00000117481	NSUN4	NOP2/Sun RNA methyltransferase 4	387338	53,73796034	-0,374628588	0,311196	-1,20383375	0,228653842	0,68351286
ENSG00000165244	ZNF367	zinc finger protein 367	195828	41,4713448	0,267635631	0,293505	0,911860153	0,36184234	NA
ENSG00000172322	CLEC12A	C-type lectin domain family 12 member A	160364	112,9943593	0,104140825	0,158089	0,6587497	0,510056512	0,866180216
ENSG00000172943	PHF8	PHD finger protein 8	23133	308,631919	-0,106384795	0,128831	-0,825771451	0,408933752	0,820793685
ENSG00000185946	RNPC3	RNA binding region (RNP1, RRM) containing 3	55599	128,6191109	0,022818787	0,166508	0,137043177	0,890996667	0,977990819
ENSG00000213145	CRIP1	cysteine rich protein 1	1396	118,8853669	0,099389501	0,191446	0,519150569	0,603655746	0,897212117
ENSG00000224470	ATXN1L	ataxin 1 like	342371	409,6832334	-0,172798836	0,086415	-1,999650381	0,04553803	0,340953043
ENSG00000065518	NDUFB4	NADH:ubiquinone oxidoreductase subunit B4	4710	99,40389486	0,638045684	0,175012	3,645721432	0,000266643	0,012769584
ENSG00000090857	PDPR	pyruvate dehydrogenase phosphatase regulatory subunit	55066	698,1488605	-0,095778498	0,073843	-1,297064045	0,194609151	0,652365446
ENSG00000090857	LOC124907803	pyruvate dehydrogenase phosphatase regulatory subunit, mitochondrial-like	124907803	698,1488605	-0,095778498	0,073843	-1,297064045	0,194609151	0,652365446
ENSG00000092330	TINF2	TERF1 interacting nuclear factor 2	26277	35,81624781	-0,619553547	0,631282	-0,981421267	0,326385043	NA
ENSG00000158828	PINK1	PTEN induced kinase 1	65018	91,19667904	0,07200111	0,190711	0,377539592	0,705772647	0,930846779
ENSG00000189376	C8orf76	chromosome 8 open reading frame 76	84933	33,83619637	0,349431071	0,397839	0,878321863	0,379769074	NA
ENSG00000119725	ZNF410	zinc finger protein 410	57862	142,0720773	0,444788949	0,179376	2,479641981	0,013151436	0,18006752
ENSG00000107758	PPP3CB	protein phosphatase 3 catalytic subunit beta	5532	332,7557102	-0,148244713	0,098458	-1,505663077	0,132153687	0,569043343
ENSG00000064419	TNPO3	transportin 3	23534	326,4430451	0,060104515	0,094131	0,638519109	0,523135819	0,870221868
ENSG00000110063	DCPS	decapping enzyme, scavenger	28960	90,64392601	0,282582664	0,208484	1,355417395	0,17528461	0,632039836
ENSG00000107779	BMPR1A	bone morphogenetic protein receptor type 1A	657	29,69351914	0,59918391	0,376103	1,593136161	0,111129649	NA
ENSG00000122687	MRM2	mitochondrial rRNA methyltransferase 2	29960	41,11640694	0,223145557	0,405009	0,550963889	0,58165843	NA
ENSG00000130338	TULP4	TUB like protein 4	56995	58,95119971	0,240135402	0,218114	1,100960708	0,270913758	0,725477256
ENSG00000170248	PDCD6IP	programmed cell death 6 interacting protein	10015	625,8935473	-0,069767782	0,084037	-0,830200241	0,406425579	0,819098895
ENSG00000171956	FOXB1	forkhead box B1	27023	11,85812195	0,160849113	0,536345	0,299898583	0,764254515	NA
ENSG00000185883	ATP6V0C	ATPase H+ transporting V0 subunit c	527	296,2102032	-0,185846417	0,111245	-1,670610384	0,09479866	0,499027263
ENSG00000221914	PPP2R2A	protein phosphatase 2 regulatory subunit Balpha	5520	104,4181234	0,635979269	0,553192	1,149653288	0,250286701	0,707335884
ENSG00000243696	NA	NA	NA	51,04165659	0,034116515	0,254252	0,13418395	0,893257117	0,977990819
ENSG00000103018	CYB5B	cytochrome b5 type B	80777	210,797201	0,379842489	0,179127	2,120525306	0,03396177	0,294612488
ENSG00000103591	AAGAB	alpha and gamma adaptin binding protein	79719	196,5732292	0,003415687	0,135565	0,025195916	0,979898694	0,995712569

ENSG00000123870	NA	NA	NA	19,40040223	-0,181419532	0,410757	-0,441671356	0,65872704	NA
ENSG00000126561	STAT5A	signal transducer and activator of transcription 5A	6776	216,1542705	-0,115320222	0,114064	-1,011017356	0,312008123	0,757868604
ENSG00000145781	COMMD10	COMM domain containing 10	51397	38,12842027	0,04509009	0,327201	0,137805382	0,89039423	NA
ENSG00000230124	ACBD6	acyl-CoA binding domain containing 6	84320	93,99507486	0,196980088	0,236679	0,832265921	0,405258862	0,818437414
ENSG00000230124	LHX4-AS1	LHX4 antisense RNA 1	100527964	93,99507486	0,196980088	0,236679	0,832265921	0,405258862	0,818437414
ENSG00000236609	ZNF853	zinc finger protein 853	54753	37,71520195	-0,205376526	0,282939	-0,725868704	0,467919263	NA
ENSG00000135829	DHX9	DExH-box helicase 9	1660	903,7173872	-0,002846821	0,068035	-0,041843345	0,966623581	0,992789539
ENSG00000163512	AZI2	5-azacytidine induced 2	64343	71,5055871	0,368938847	0,289373	1,274959085	0,202323724	0,660119983
ENSG00000101182	PSMA7	proteasome 20S subunit alpha 7	5688	255,5690763	0,264960047	0,113627	2,331835475	0,019709348	0,220823423
ENSG00000110801	PSMD9	proteasome 26S subunit, non-ATPase 9	5715	49,87087423	0,025088344	0,353626	0,070946042	0,9434407	0,988630188
ENSG00000205707	ETFRF1	electron transfer flavoprotein regulatory factor 1	144363	23,19560376	-0,125980594	0,44078	-0,285813064	0,775021302	NA
ENSG00000077585	GPR137B	G protein-coupled receptor 137B	7107	38,78919842	0,136918013	0,33915	0,403709567	0,686426304	NA
ENSG00000115604	IL18R1	interleukin 18 receptor 1	8809	263,0941079	-0,235998532	0,137118	-1,721139172	0,085225573	0,47675331
ENSG00000110066	KMT5B	lysine methyltransferase 5B	51111	221,4873292	0,172170518	0,379495	0,453683662	0,650056528	0,914926418
ENSG00000122779	TRIM24	tripartite motif containing 24	8805	165,8793521	-0,181879597	0,156881	-1,159350314	0,246313421	0,703431917
ENSG00000286264	LOC114841035	Uncharacterized protein LOC114841035	114841035	32,72980362	-0,390667746	0,300875	-1,298438319	0,194136759	NA
ENSG00000180773	SLC36A4	solute carrier family 36 member 4	120103	29,96544726	-0,335116494	0,407433	-0,82250755	0,410788092	NA
ENSG00000130119	GNL3L	G protein nucleolar 3 like	54552	363,3355813	-0,441853663	0,12498	-3,535400882	0,000407157	0,017965229
ENSG00000160753	RUSC1	RUN and SH3 domain containing 1	23623	58,94638435	-0,190828042	0,243537	-0,783569105	0,43329299	0,835093272
ENSG00000119655	NPC2	NPC intracellular cholesterol transporter 2	10577	42,59192519	0,390856165	0,290784	1,344145783	0,17890125	0,633385915
ENSG00000092964	DPYSL2	dihydropyrimidinase like 2	1808	247,6082914	-0,250105356	0,11684	-2,140581577	0,032307796	0,287228571
ENSG00000100298	APOBEC3H	apolipoprotein B mRNA editing enzyme catalytic subunit 3H	164668	72,43880178	-0,189800923	0,217581	-0,872323272	0,383032046	0,808474519
ENSG00000078699	CBFA2T2	CBFA2/RUNX1 partner transcriptional co-repressor 2	9139	220,6657211	-0,194318907	0,123223	-1,576968244	0,114802832	0,54024053
ENSG00000144791	LIMD1	LIM domain containing 1	8994	276,3009301	-0,003965108	0,138377	-0,028654323	0,977140287	0,995291586
ENSG00000180964	TCEAL8	transcription elongation factor A like 8	90843	72,07442964	-0,141410748	0,209323	-0,675562753	0,499318296	0,86161226
ENSG00000137501	SYTL2	synaptotagmin like 2	54843	945,4823112	-0,067809542	0,069266	-0,978970414	0,327594598	0,769876712
ENSG00000119321	FKBP15	FKBP prolyl isomerase family member 15	23307	143,8380843	0,109944061	0,264274	0,416022408	0,677393606	0,924466793
ENSG00000138180	CEP55	centrosomal protein 55	55165	87,70799016	0,616376088	0,188349	3,27252372	0,001065919	0,036879868
ENSG00000153317	ASAP1	ArfGAP with SH3 domain, ankyrin repeat and PH domain 1	50807	242,859499	-0,314499996	0,243729	-1,290365991	0,196923614	0,655659726
ENSG00000106829	TLE4	TLE family member 4, transcriptional corepressor	7091	255,0303913	-0,115881276	0,106917	-1,083841742	0,278434974	0,732115261
ENSG00000138785	INTS12	integrator complex subunit 12	57117	55,63127942	0,109352611	0,252008	0,43392557	0,664342491	0,921854961
ENSG00000171302	CANT1	calcium activated nucleotidase 1	124583	195,1002198	-0,101481357	0,141729	-0,716023754	0,473976681	0,853811879
ENSG00000180376	CCDC66	coiled-coil domain containing 66	285331	16,92695058	3,236116578	2,084	1,552838822	0,120461645	NA
ENSG00000198521	ZNF43	zinc finger protein 43	7594	79,30092211	-0,337572706	0,275694	-1,224444964	0,220784416	0,677095199
ENSG00000023608	SNAPC1	small nuclear RNA activating complex polypeptide 1	6617	22,47445351	0,24198389	0,406901	0,594700132	0,552043934	NA
ENSG00000159461	AMFR	autocrine motility factor receptor	267	158,8051274	-0,13669542	0,152937	-0,893799503	0,371429175	0,800231702
ENSG00000160256	SLX9	SLX9 ribosome biogenesis factor	85395	38,77390217	0,090960476	0,272507	0,333791591	0,738536829	NA
ENSG00000196369	SRGAP2B	SLIT-ROBO Rho GTPase activating protein 2B	647135	83,11526833	0,449547419	0,293961	1,529273564	0,12619664	0,563689485
ENSG00000132963	POMP	proteasome maturation protein	51371	146,8631145	0,119631716	0,141013	0,848373847	0,396229803	0,812799303
ENSG00000136193	SCRN1	secernin 1	9805	60,37851745	-0,683282939	0,386905	-1,766021569	0,07739223	0,449917526
ENSG00000231925	TAPBP	TAP binding protein	6892	1278,515662	-0,336856013	0,058335	-5,774523689	7,72E-09	2,53E-06
ENSG00000122557	HERPUD2	HERPUD family member 2	64224	496,433459	-0,101091337	0,084322	-1,198869567	0,230578667	0,685179309
ENSG00000041880	PARP3	poly(ADP-ribose) polymerase family member 3	10039	33,40206525	-0,29582213	0,309545	-0,955666703	0,33924065	NA
ENSG00000090861	AARS1	alanyl-tRNA synthetase 1	16	355,3464322	0,104119307	0,099164	1,049974459	0,293729856	0,745140274
ENSG00000122203	KIAA1191	KIAA1191	57179	208,1724591	-0,119076514	0,163159	-0,729817434	0,465501786	0,84908342
ENSG00000136982	DSCC1	DNA replication and sister chromatid cohesion 1	79075	20,69789694	0,116006962	0,451172	0,257123533	0,797083412	NA
ENSG00000151883	PARP8	poly(ADP-ribose) polymerase family member 8	79668	1212,765281	-0,183667056	0,059539	-3,084818389	0,002036765	0,054971652
ENSG00000196700	ZNF512B	zinc finger protein 512B	57473	155,3704581	-0,239337062	0,181966	-1,315282377	0,188415019	0,646436727
ENSG00000211460	TSN	translin	7247	201,8358563	-0,322757287	0,363424	-0,888101726	0,374486034	0,801403701

ENSG00000177613	CSTF2T	cleavage stimulation factor subunit 2 tau variant	23283	352,7575826	-0,093358014	0,089608	-1,041850692	0,297480908	0,748371252
ENSG00000139433	GLTP	glycolipid transfer protein	51228	152,4536591	-0,078239855	0,132464	-0,590648235	0,554756139	0,882854594
ENSG00000198055	GRK6	G protein-coupled receptor kinase 6	2870	415,6985457	-0,194752481	0,115273	-1,689483725	0,091126768	0,490934677
ENSG00000104964	TLE5	TLE family member 5, transcriptional modulator	166	693,9006242	-0,221009031	0,067931	-3,253445534	0,001140146	0,037467385
ENSG00000138433	CIR1	corepressor interacting with RBPJ, CIR1	9541	160,0120888	0,162097171	0,189613	0,854883558	0,392615614	0,812491435
ENSG00000080608	PUM3	pumilio RNA binding family member 3	9933	102,3438762	0,179872003	0,182141	0,987543304	0,323376365	0,767603859
ENSG00000085788	DDHD2	DDHD domain containing 2	23259	229,438425	-0,239957319	0,121253	-1,978977672	0,04781852	0,350669148
ENSG00000261221	ZNF865	zinc finger protein 865	100507290	100,701152	-0,221035151	0,200421	-1,10285197	0,270091455	0,725477256
ENSG00000065970	FOXJ2	forkhead box J2	55810	198,0625698	0,15451702	0,122358	1,262829507	0,206650458	0,661359894
ENSG00000121671	CRY2	cryptochrome circadian regulator 2	1408	108,3441644	0,107574948	0,182875	0,588242256	0,556369698	0,883586042
ENSG00000144029	MRPS5	mitochondrial ribosomal protein S5	64969	41,07048888	0,518444341	0,383889	1,35050622	0,17685366	NA
ENSG00000086598	TMED2	transmembrane p24 trafficking protein 2	10959	735,4049539	-0,115344796	0,077749	-1,483548873	0,137928642	0,578980755
ENSG00000267796	LIN37	lin-37 DREAM MuvB core complex component	55957	19,9423684	-0,210461762	0,445361	-0,472564043	0,636524241	NA
ENSG00000154803	FLCN	folliculin	201163	122,3559226	-0,167818001	0,177333	-0,946341969	0,343974194	0,781132432
ENSG00000144524	COPS7B	COP9 signalosome subunit 7B	64708	71,77813025	-0,167307631	0,27071	-0,618033515	0,536553247	0,874923109
ENSG00000151135	TMEM263	transmembrane protein 263	90488	106,5888617	0,153804998	0,232441	0,661695106	0,508166642	0,864738156
ENSG00000134108	ARL8B	ADP ribosylation factor like GTPase 8B	55207	238,8955221	-0,443412193	0,120119	-3,691455059	0,000222975	0,011545615
ENSG00000139826	ABHD13	abhydrolase domain containing 13	84945	138,302562	-0,142845512	0,166228	-0,859337239	0,390154485	0,810747116
ENSG00000286140	DERPC	DERPC proline and glycine rich nuclear protein	113455421	71,85827482	2,093897179	1,185477	1,766290453	0,077347132	0,449917526
ENSG00000130811	EIF3G	eukaryotic translation initiation factor 3 subunit G	8666	453,323032	-0,070315896	0,090761	-0,774738928	0,438493943	0,837388481
ENSG00000163444	TMEM183A	transmembrane protein 183A	92703	238,8639465	-0,034617278	0,130902	-0,264451841	0,791431769	0,951705903
ENSG00000167461	RAB8A	RAB8A, member RAS oncogene family	4218	464,6863797	-0,124394241	0,098792	-1,259153906	0,207974747	0,662274754
ENSG00000176248	ANAPC2	anaphase promoting complex subunit 2	29882	113,8263726	-0,048872948	0,205013	-0,23838989	0,811578708	0,959038743
ENSG00000172409	CLP1	cleavage factor polyribonucleotide kinase subunit 1	10978	39,05265861	-0,025565715	0,286225	-0,089320385	0,928827294	NA
ENSG00000198899	ATP6	ATP synthase F0 subunit 6	4508	12784,9256	0,104740247	0,177901	0,588754857	0,556025732	0,88329816
ENSG00000171033	PKIA	cAMP-dependent protein kinase inhibitor alpha	5569	13,32634936	-0,085966638	0,692447	-0,12414903	0,901197278	NA
ENSG00000120800	UTP20	UTP20 small subunit processome component	27340	181,4941713	0,003722036	0,135619	0,027444826	0,978104946	0,995598269
ENSG00000127774	EMC6	ER membrane protein complex subunit 6	83460	33,87554854	-0,099890425	0,318006	-0,31411513	0,753433598	NA
ENSG00000172780	RAB43	RAB43, member RAS oncogene family	339122	222,8838578	0,09207446	0,391404	0,235241354	0,814021411	0,959207071
ENSG00000072858	SIDT1	SID1 transmembrane family member 1	54847	134,9045837	-0,239970611	0,160556	-1,494623639	0,135012693	0,575673014
ENSG00000125484	GTF3C4	general transcription factor IIIC subunit 4	9329	166,5108539	0,01423045	0,159269	0,089348733	0,928804766	0,986487751
ENSG00000126070	AGO3	argonaute RISC catalytic component 3	192669	341,8923953	-0,082780274	0,151694	-0,545704339	0,585269189	0,890362877
ENSG00000145715	RASA1	RAS p21 protein activator 1	5921	398,1509652	-0,254428298	0,092121	-2,761902588	0,005746562	0,10771718
ENSG00000159579	RSPRY1	ring finger and SPRY domain containing 1	89970	106,4458112	-0,179512662	0,167002	-1,074916257	0,282412223	0,736165151
ENSG00000169288	MRPL1	mitochondrial ribosomal protein L1	65008	62,71962114	-0,047547519	0,216604	-0,21951363	0,826249964	0,961917534
ENSG00000269713	NBPF9	NBPF member 9	400818	96,0786281	0,02710955	0,186276	0,145534599	0,8842888	0,977096826
ENSG00000129255	MPDU1	mannose-P-dolichol utilization defect 1	9526	83,19689534	0,030605956	0,195057	0,156908027	0,875317334	0,975344937
ENSG00000120675	DNAJC15	DnaJ heat shock protein family (Hsp40) member C15	29103	78,2070747	-0,316422555	0,234833	-1,347435461	0,177840026	0,632142851
ENSG00000121210	TMEM131L	transmembrane 131 like	23240	348,4756675	-0,126325685	0,11481	-1,100304334	0,271199545	0,725477256
ENSG00000180425	C11orf71	chromosome 11 open reading frame 71	54494	21,26515053	-0,32696663	0,415794	-0,7863664	0,431652862	NA
ENSG00000103642	LACTB	lactamase beta	114294	136,6279437	-0,08731193	0,164436	-0,530978397	0,595433749	0,894229873
ENSG00000169738	DCXR	dicarbonyl and L-xylulose reductase	51181	63,34735485	-0,040214521	0,307568	-0,130750024	0,895973059	0,977990819
ENSG00000130818	ZNF426	zinc finger protein 426	79088	87,14632534	0,193257266	0,195975	0,986134343	0,324067193	0,767603859
ENSG00000292345	ZBED1	zinc finger BED-type containing 1	9189	156,1040814	0,223934365	0,138864	1,612618624	0,10682739	0,525374026
ENSG00000104131	EIF3J	eukaryotic translation initiation factor 3 subunit J	8669	258,6439358	-0,144054695	0,111979	-1,286447702	0,19828686	0,656945827
ENSG00000181830	SLC35C1	solute carrier family 35 member C1	55343	112,1079972	0,207197032	0,174863	1,184911817	0,236052315	0,691328984

ENSG00000144468	RHBDD1	rhomboid domain containing 1	84236	80,51755779	-0,001670401	0,211739	-0,007888956	0,993705589	0,998500439
ENSG00000184207	PGP	phosphoglycolate phosphatase	283871	98,37225417	0,058401886	0,185001	0,315683436	0,752242801	0,941832449
ENSG00000109180	OCIAD1	OCIA domain containing 1	54940	276,5526372	0,045029432	0,10063	0,447474417	0,654532555	0,917490396
ENSG00000173598	NUDT4	nudix hydrolase 4	11163	306,0944504	-0,162796397	0,111444	-1,460788187	0,144073579	0,59024645
ENSG00000128294	TPST2	tyrosylprotein sulfotransferase 2	8459	624,2836113	-0,09171812	0,076222	-1,203300987	0,228859867	0,68351286
ENSG00000139719	VPS33A	VPS33A core subunit of CORVET and HOPS complexes	65082	114,1763287	-0,510106618	0,216466	-2,35652286	0,018446932	0,212696759
ENSG00000106351	AGFG2	ArfGAP with FG repeats 2	3268	92,6307661	-0,153908134	0,230113	-0,668837849	0,50359892	0,863218221
ENSG00000106609	TMEM248	transmembrane protein 248	55069	459,6823717	-0,172839026	0,095106	-1,817334611	0,069165893	0,427066766
ENSG00000165175	MID1IP1	MID1 interacting protein 1	58526	76,38450463	-0,239097562	0,231109	-1,034565306	0,300871963	0,751870158
ENSG00000205810	KLRC3	killer cell lectin like receptor C3	3823	281,0319669	-0,052760378	0,104253	-0,506080736	0,612799973	0,900033105
ENSG00000121281	ADCY7	adenylate cyclase 7	113	1854,398756	0,000814482	0,056937	0,014304869	0,988586755	0,997032569
ENSG00000116906	GNPAT	glyceronephosphate O-acyltransferase	8443	147,7529867	0,29418638	0,143342	2,052338572	0,04013677	0,319386212
ENSG00000186635	ARAP1	ArfGAP with RhoGAP domain, ankyrin repeat and PH domain 1	116985	609,9178328	0,042580043	0,104299	0,408249383	0,683090596	0,925586358
ENSG00000127824	TUBA4A	tubulin alpha 4a	7277	276,1059714	0,312193827	0,106698	2,925956524	0,003433989	0,077987331
ENSG00000170903	MSANTD4	Myb/SANT DNA binding domain containing 4 with coiled-coils	84437	89,97522869	-0,100454348	0,22158	-0,453354823	0,650293262	0,914977299
ENSG00000183208	GDPGP1	GDP-D-glucose phosphorylase 1	390637	19,51070731	-0,466499188	0,434361	-1,073990565	0,28282691	NA
ENSG00000165118	QNG1	Q-nucleotide N-glycosylase 1	84267	59,91287547	0,197947637	0,244027	0,811172209	0,417266783	0,82446781
ENSG00000104442	ARMC1	armadillo repeat containing 1	55156	122,682344	-0,379672922	0,16866	-2,251113062	0,024378377	0,250612272
ENSG00000083097	DOP1A	DOP1 leucine zipper like protein A	23033	182,7393132	0,141441795	0,149425	0,94657513	0,343855322	0,781132432
ENSG00000125870	SNRPB2	small nuclear ribonucleoprotein polypeptide B2	6629	177,6576687	0,256403213	0,12586	2,037205342	0,041629469	0,325007801
ENSG00000139112	GABARAPL1	GABA type A receptor associated protein like 1	23710	232,2561316	0,421470304	0,204277	2,06323145	0,039090643	0,314890167
ENSG00000090889	KIF4A	kinesin family member 4A	24137	67,76586201	0,631574696	0,21258	2,971002651	0,002968292	0,071451584
ENSG00000138668	HNRNPD	heterogeneous nuclear ribonucleoprotein D	3184	636,9101888	-0,021973552	0,089624	-0,245175959	0,80632019	0,958037095
ENSG00000185658	BRWD1	bromodomain and WD repeat domain containing 1	54014	800,953174	-0,214721095	0,112514	-1,908394162	0,056340291	0,385114573
ENSG00000164300	SERINC5	serine incorporator 5	256987	34,99757345	-0,08879455	0,296956	-0,299015538	0,76492819	NA
ENSG00000129351	ILF3	interleukin enhancer binding factor 3	3609	968,7313264	-0,07180758	0,059361	-1,2096755	0,226403434	0,681621061
ENSG00000163930	BAP1	BRCA1 associated protein 1	8314	306,6909325	-0,107542225	0,097183	-1,106590936	0,268470828	0,723940509
ENSG00000198270	TMEM116	transmembrane protein 116	89894	12,40373795	0,330207552	1,086462	0,303929146	0,761181872	NA
ENSG00000087269	NOP14	NOP14 nucleolar protein	8602	254,4807745	0,030649711	0,129951	0,235855761	0,813544597	0,959207071
ENSG00000188064	WNT7B	Wnt family member 7B	7477	19,72659572	0,161344935	0,55716	0,289584501	0,772134125	NA
ENSG00000121644	DESI2	desumoylating isopeptidase 2	51029	180,3042994	0,132952034	0,126256	1,053036805	0,292324124	0,74421837
ENSG00000122783	CYREN	cell cycle regulator of NHEJ	78996	73,48878139	0,240333614	0,213427	1,126067873	0,260136792	0,716051154
ENSG00000163832	ELP6	elongator acetyltransferase complex subunit 6	54859	47,71653674	0,13986154	0,262774	0,532249425	0,594553255	0,894229873
ENSG00000166803	PCLAF	PCNA clamp associated factor	9768	34,40369458	0,405232991	0,381597	1,061938741	0,288263496	NA
ENSG00000117620	SLC35A3	solute carrier family 35 member A3	23443	150,2018697	-0,423425118	0,299754	-1,412577212	0,157780073	0,61428162
ENSG00000114030	KPNA1	karyopherin subunit alpha 1	3836	213,6919732	0,012401774	0,115035	0,107808417	0,914147666	0,981103549
ENSG00000008988	RPS20	ribosomal protein S20	6224	1251,071377	0,025910718	0,072292	0,358419546	0,720029367	0,933530522
ENSG00000028277	POU2F2	POU class 2 homeobox 2	5452	125,9794817	-0,262723718	0,299021	-0,878613539	0,379610852	0,805365649
ENSG00000123473	STIL	STIL centriolar assembly protein	6491	78,55338655	0,555680512	0,274621	2,023447683	0,043027012	0,330336417
ENSG00000178999	AURKB	aurora kinase B	9212	41,91743206	0,587078612	0,308793	1,901205458	0,057275107	NA
ENSG00000253816	NA	NA	NA	45,29324246	0,2218287	0,279634	0,793280977	0,427614138	0,832060923
ENSG00000023572	GLRX2	glutaredoxin 2	51022	12,30042681	-0,083134221	0,559302	-0,148639235	0,881838312	NA
ENSG00000134297	PLEKHA8P1	pleckstrin homology domain containing A8 pseudogene 1	51054	18,69182498	0,416528822	0,468216	0,889609124	0,373675805	NA
ENSG00000108306	FBXL20	F-box and leucine rich repeat protein 20	84961	217,377513	-0,215229254	0,168353	-1,278440051	0,20109431	0,660006147
ENSG00000128165	ADM2	adrenomedullin 2	79924	26,24199041	-0,042924407	0,325497	-0,131873244	0,895084552	NA
ENSG00000173992	CCS	copper chaperone for superoxide dismutase	9973	21,72188868	0,183103683	0,666081	0,274897061	0,783395325	NA
ENSG00000139192	TAPBPL	TAP binding protein like	55080	113,9522857	-0,064547041	0,174247	-0,370435021	0,711058384	0,930846779
ENSG00000183431	SF3A3	splicing factor 3a subunit 3	10946	265,2643633	0,107893095	0,11051	0,976317146	0,328907326	0,770885747
ENSG00000248487	ABHD14A	abhydrolase domain containing 14A	25864	14,08508649	-0,284554624	0,47439	-0,599832591	0,548617811	NA
ENSG00000169895	SYAP1	synapse associated protein 1	94056	178,5309518	-0,131863593	0,125006	-1,054855525	0,291491406	0,743239565
ENSG00000179476	C14orf28	chromosome 14 open reading frame 28	122525	12,14789149	0,42292423	0,749014	0,564641007	0,572317967	NA

ENSG00000005882	PDK2	pyruvate dehydrogenase kinase 2	5164	111,7144482	-0,247503172	0,164324	-1,506188935	0,132018679	0,568919111
ENSG00000196550	FAM72A	family with sequence similarity 72 member A	729533	29,80412493	-0,135698275	0,345869	-0,392340463	0,69480667	NA
ENSG00000134291	TMEM106C	transmembrane protein 106C	79022	109,3674766	0,239813622	0,161313	1,48663863	0,137110279	0,57855532
ENSG00000164051	CCDC51	coiled-coil domain containing 51	79714	18,6103211	0,060126581	0,413271	0,145489617	0,884324313	NA
ENSG00000136243	NUP42	nucleoporin 42	11097	29,66580371	0,614397711	0,513945	1,195454949	0,231909328	NA
ENSG00000091409	ITGA6	integrin subunit alpha 6	3655	20,89920029	-0,765320515	0,381831	-2,004344223	0,0450332	NA
ENSG00000165630	PRPF18	pre-mRNA processing factor 18	8559	110,2553197	-0,138517636	0,16344	-0,847513556	0,396708934	0,812799303
ENSG00000080824	HSP90AA1	heat shock protein 90 alpha family class A member 1	3320	2740,764939	0,112560519	0,047378	2,375786037	0,017511614	0,206510834
ENSG00000144589	STK11IP	serine/threonine kinase 11 interacting protein	114790	109,0104902	-0,192539678	0,190569	-1,010342643	0,312331158	0,757868604
ENSG00000196247	ZNF107	zinc finger protein 107	51427	78,40442608	-0,373126793	0,270643	-1,378668194	0,16799708	0,627733684
ENSG00000102144	PGK1	phosphoglycerate kinase 1	5230	1392,620994	0,155071957	0,066018	2,348950967	0,018826386	0,213983263
ENSG00000100883	SRP54	signal recognition particle 54	6729	185,099125	0,025887609	0,195575	0,132366905	0,89469409	0,977990819
ENSG00000011132	APBA3	amyloid beta precursor protein binding family A member 3	9546	65,89613464	-0,066925162	0,254241	-0,263234616	0,792369752	0,952007348
ENSG00000123358	NR4A1	nuclear receptor subfamily 4 group A member 1	3164	23,35455935	0,06908934	0,364023	0,189793764	0,849470743	NA
ENSG00000146281	PM20D2	peptidase M20 domain containing 2	135293	74,74034789	0,149564519	0,234624	0,637464598	0,523822263	0,870612376
ENSG00000164933	SLC25A32	solute carrier family 25 member 32	81034	91,39391974	-0,15099248	0,229399	-0,658209188	0,510403722	0,866180216
ENSG00000166562	SEC11C	SEC11 homolog C, signal peptidase complex subunit	90701	106,3491159	0,052736783	0,160505	0,328566882	0,742483084	0,939195062
ENSG00000083828	ZNF586	zinc finger protein 586	54807	64,87231684	-0,268577956	0,314929	-0,852821159	0,393758488	0,812498986
ENSG00000105426	PTPRS	protein tyrosine phosphatase receptor type S	5802	34,10604297	-0,626550287	0,309853	-2,022087288	0,043167335	NA
ENSG00000106144	CASP2	caspase 2	835	262,6070001	-0,206439711	0,135659	-1,52175414	0,128070696	0,563689485
ENSG00000106459	NRF1	nuclear respiratory factor 1	4899	98,39620247	-0,050056509	0,175558	-0,285128095	0,775546006	0,946448613
ENSG00000119231	SENP5	SUMO specific peptidase 5	205564	229,3758605	-0,00884926	0,158977	-0,055663879	0,955609575	0,991510463
ENSG00000136270	TBRG4	transforming growth factor beta regulator 4	9238	81,3120652	0,206862313	0,198992	1,039552179	0,298548004	0,74913611
ENSG00000167182	SP2	Sp2 transcription factor	6668	160,5634147	-0,094468435	0,157092	-0,601359051	0,547600866	0,879578516
ENSG00000173933	RBM4	RNA binding motif protein 4	5936	373,4588053	-0,169701415	0,121426	-1,397574761	0,162240802	0,619487156
ENSG00000196305	IARS1	isoleucyl-tRNA synthetase 1	3376	368,4334192	0,190244947	0,10015	1,89959562	0,057486207	0,38821726
ENSG00000196371	FUT4	fucosyltransferase 4	2526	45,05489167	-0,085609168	0,266227	-0,321564679	0,747782505	0,939899419
ENSG00000266338	NBPF15	NBPF member 15	284565	98,48701166	0,248984335	0,188951	1,317717798	0,187598134	0,645758518
ENSG00000115687	PASK	PAS domain containing serine/threonine kinase	23178	71,57219157	0,092329876	0,221435	0,416962195	0,67670606	0,924186156
ENSG00000145675	PIK3R1	phosphoinositide-3-kinase regulatory subunit 1	5295	1475,422286	-0,196099506	0,085782	-2,286009504	0,022253703	0,237473614
ENSG00000162419	GMEB1	glucocorticoid modulatory element binding protein 1	10691	187,9610554	0,157845624	0,258927	0,609615193	0,542116745	0,877014629
ENSG00000065427	KARS1	lysyl-tRNA synthetase 1	3735	483,3666898	-0,015360711	0,079077	-0,194249986	0,845980145	0,967231052
ENSG00000105221	AKT2	AKT serine/threonine kinase 2	208	154,4485147	-0,43663761	0,162593	-2,685468554	0,007242819	0,125574173
ENSG00000123552	USP45	ubiquitin specific peptidase 45	85015	47,69051334	-0,222639223	0,304161	-0,731978886	0,464181455	0,848622241
ENSG00000262814	MRPL12	mitochondrial ribosomal protein L12	6182	65,8938549	0,051466839	0,227107	0,226619578	0,820719567	0,961891299
ENSG00000198554	WDHD1	WD repeat and HMG-box DNA binding protein 1	11169	70,43391209	0,178648011	0,242333	0,737200895	0,461000189	0,846765498
ENSG00000115295	CLIP4	CAP-Gly domain containing linker protein family member 4	79745	27,67649855	0,846913314	1,299824	0,651559801	0,514685187	NA
ENSG00000164362	TERT	telomerase reverse transcriptase	7015	285,3088303	9,770799582	0,837804	11,66239835	1,98E-31	1,56E-27
ENSG00000178904	DPY19L3	dpy-19 like C-mannosyltransferase 3	147991	102,8436458	-0,550209117	0,22226	-2,475520487	0,013304213	0,180367712
ENSG00000246705	H2AJ	H2A,J histone	55766	45,18340673	0,054361606	0,25928	0,209663742	0,833930126	0,963446071
ENSG00000108518	PFN1	profilin 1	5216	3456,999566	0,065680098	0,057529	1,141680704	0,253586764	0,709599722
ENSG00000186501	TMEM222	transmembrane protein 222	84065	68,99013989	-0,072780751	0,208271	-0,349451605	0,726750297	0,936607802
ENSG00000180817	PPA1	inorganic pyrophosphatase 1	5464	227,5771682	0,163474689	0,110217	1,483210325	0,138018539	0,578980755
ENSG00000132952	USPL1	ubiquitin specific peptidase like 1	10208	161,520001	-0,007003112	0,135444	-0,051704705	0,958763989	0,991911599
ENSG00000111860	CEP85L	centrosomal protein 85 like	387119	309,6235857	-0,166142511	0,153022	-1,085740459	0,277593835	0,731689696
ENSG00000107625	DDX50	DExD-box helicase 50	79009	182,7473033	0,164935587	0,134517	1,226133361	0,220148493	0,676903242
ENSG00000005022	SLC25A5	solute carrier family 25 member 5	292	447,360859	0,335982828	0,083013	4,047355214	5,18E-05	0,003752175
ENSG00000108588	CCDC47	coiled-coil domain containing 47	57003	333,4864224	0,108737553	0,10412	1,044343557	0,296326468	0,747862492
ENSG00000164111	ANXA5	annexin A5	308	250,4108379	0,221585345	0,105738	2,09561522	0,036116338	0,302084897
ENSG00000143379	SETDB1	SET domain bifurcated histone lysine methyltransferase 1	9869	183,2322719	0,49771998	0,436086	1,141334432	0,253730779	0,709687156

ENSG00000101189	MRGBP	MRG domain binding protein	55257	90,63920325	0,088230446	0,19294	0,457295291	0,647458814	0,914264928
ENSG00000102390	PBDC1	polysaccharide biosynthesis domain containing 1	51260	82,39953628	-0,065189645	0,192631	-0,338417901	0,735048286	0,939157822
ENSG00000168214	RBPJ	recombination signal binding protein for immunoglobulin kappa J region	3516	713,9797531	-0,071102317	0,109279	-0,650646648	0,51527461	0,868562431
ENSG00000198551	ZNF627	zinc finger protein 627	199692	28,78671531	-0,091375946	0,36662	-0,249238895	0,803175995	NA
ENSG00000197372	ZNF675	zinc finger protein 675	171392	46,83422002	0,295615279	0,309077	0,95644492	0,338847499	0,777431905
ENSG00000141510	TP53	tumor protein p53	7157	338,7463338	-0,076326879	0,107333	-0,711118908	0,477010554	0,853811879
ENSG00000123472	ATPAF1	ATP synthase mitochondrial F1 complex assembly factor 1	64756	88,50406041	-0,316777453	0,28292	-1,119671576	0,262853742	0,719071156
ENSG00000144136	SLC20A1	solute carrier family 20 member 1	6574	459,006274	0,155938435	0,095516	1,632584328	0,10255645	0,515621608
ENSG00000099995	SF3A1	splicing factor 3a subunit 1	10291	788,2799092	-0,056126552	0,083403	-0,672957712	0,500974195	0,861917048
ENSG00000198369	SPRED2	sprouty related EVH1 domain containing 2	200734	94,65623514	-0,059025	0,195194	-0,30239188	0,762353344	0,943004836
ENSG00000074054	CLASP1	cytoplasmic linker associated protein 1	23332	643,1666634	0,092821671	0,080371	1,154913296	0,248125934	0,706134779
ENSG00000168792	ABHD15	abhydrolase domain containing 15	116236	218,5746183	-0,097760829	0,135638	-0,720750611	0,471062968	0,852690218
ENSG00000183137	CEP57L1	centrosomal protein 57 like 1	285753	59,78258005	-0,340525853	0,297736	-1,143718856	0,252740251	0,708754466
ENSG00000183696	UPP1	uridine phosphorylase 1	7378	22,76856838	0,193417114	0,602901	0,32081085	0,748353736	NA
ENSG00000135049	AGTPBP1	ATP/GTP binding carboxypeptidase 1	23287	228,8633256	0,057297111	0,113084	0,506678584	0,612380359	0,900006614
ENSG00000158793	NIT1	nitrilase 1	4817	48,14049185	-0,28687346	0,26442	-1,084914652	0,277959458	0,731800087
ENSG00000124275	MTRR	5-methyltetrahydrofolate-homocysteine methyltransferase reductase	4552	83,27980252	0,259040907	0,601635	0,430561322	0,66678737	0,922184616
ENSG00000021762	OSBPL5	oxysterol binding protein like 5	114879	386,5980809	-0,132732945	0,12903	-1,028700376	0,303620493	0,751870158
ENSG00000048991	R3HDM1	R3H domain containing 1	23518	270,5628926	-0,01964462	0,105515	-0,186178505	0,85230478	0,968180755
ENSG00000151702	FLI1	Fli-1 proto-oncogene, ETS transcription factor	2313	577,8689752	-0,050598128	0,158875	-0,318476888	0,750123224	0,941011581
ENSG00000185787	MORF4L1	mortality factor 4 like 1	10933	578,7889037	-0,019036695	0,094896	-0,200606519	0,84100626	0,966505956
ENSG00000076685	NT5C2	5'-nucleotidase, cytosolic II	22978	241,2836497	-0,192258453	0,167268	-1,149403861	0,250389489	0,707335884
ENSG00000161920	MED11	mediator complex subunit 11	400569	30,3769115	0,440857289	0,333125	1,323397711	0,185703153	NA
ENSG00000123908	AGO2	argonaute RISC catalytic component 2	27161	1440,318041	0,082225849	0,058627	1,402519889	0,160760056	0,618147537
ENSG00000112029	FBXO5	F-box protein 5	26271	85,50261985	0,268559979	0,198427	1,353448055	0,175912534	0,632039836
ENSG00000131873	CHSY1	chondroitin sulfate synthase 1	22856	289,8024433	-0,053851395	0,105491	-0,510481444	0,609714211	0,898865155
ENSG00000143653	SCCPDH	saccharopine dehydrogenase (putative)	51097	87,73870923	-0,159304355	0,180211	-0,883986928	0,376703269	0,803373958
ENSG00000204592	HLA-E	major histocompatibility complex, class I, E	3133	225,1928395	-0,241076413	0,226653	-1,063637673	0,287492869	0,738622504
ENSG00000166669	ATF7IP2	activating transcription factor 7 interacting protein 2	80063	63,89386367	0,807364387	0,496646	1,62563233	0,104027893	0,517786851
ENSG00000109929	SC5D	sterol-C5-desaturase	6309	84,94163505	-0,306112791	0,22678	-1,349823296	0,17707267	0,632039836
ENSG00000149823	VPS51	VPS51 subunit of GARP complex	738	215,254112	-0,127126143	0,16542	-0,768507131	0,442185956	0,838678081
ENSG00000172113	NME6	NME/NM23 nucleoside diphosphate kinase 6	10201	132,2041373	0,167138943	0,206156	0,810741271	0,417514267	0,82446781
ENSG00000186871	ERCC6L	ERCC excision repair 6 like, spindle assembly checkpoint helicase	54821	20,58950173	0,755717245	0,438157	1,724762722	0,084570242	NA
ENSG00000213614	HEXA	hexosaminidase subunit alpha	3073	224,0947835	0,13581143	0,128793	1,054496286	0,29165576	0,743239565
ENSG00000086848	ALG9	ALG9 alpha-1,2-mannosyltransferase	79796	46,90139103	0,121801583	0,319203	0,381579971	0,702772943	0,930846779
ENSG00000186654	PRR5	proline rich 5	55615	38,51643845	0,16341903	0,31435	0,519863281	0,603158869	NA
ENSG00000104517	UBR5	ubiquitin protein ligase E3 component n-recognin 5	51366	738,7350791	-0,138693012	0,091365	-1,518017745	0,129009925	0,564481311
ENSG00000146083	RNF44	ring finger protein 44	22838	575,0280753	-0,222815808	0,095823	-2,325279633	0,020057013	0,223127163
ENSG00000104320	NBN	nibrin	4683	216,9985216	0,057605328	0,173962	0,331137847	0,740540365	0,939193793
ENSG00000124171	PARD6B	par-6 family cell polarity regulator beta	84612	15,55985167	0,05090829	0,449712	0,113201892	0,909870495	NA
ENSG00000152439	ZNF773	zinc finger protein 773	374928	34,41017802	0,187356461	0,311992	0,600516597	0,548162003	NA
ENSG00000154874	NA	NA	NA	99,68143055	0,27716539	0,192788	1,437672479	0,150527005	0,606528499
ENSG00000167083	GNGT2	G protein subunit gamma transducin 2	2793	117,4073875	-0,013320799	0,154067	-0,086460823	0,931100098	0,986491322
ENSG00000011454	RABGAP1	RAB GTPase activating protein 1	23637	138,5150777	0,15536839	0,145747	1,066011026	0,286418658	0,738005299
ENSG00000204604	ZNF468	zinc finger protein 468	90333	109,5706973	-0,223579337	0,174374	-1,282179948	0,199779529	0,6587189
ENSG00000009335	UBE3C	ubiquitin protein ligase E3C	9690	394,3353916	-0,131574274	0,090048	-1,461164013	0,143970436	0,59024645
ENSG00000188994	ZNF292	zinc finger protein 292	23036	264,9599683	-0,199539094	0,340207	-0,586523282	0,557523921	0,883921037
ENSG00000157353	FCSK	fucose kinase	197258	55,20337153	0,305846197	0,331218	0,923399279	0,355799167	0,788500751
ENSG00000139278	GLIPR1	GLI pathogenesis related 1	11010	320,890349	0,095156901	0,118538	0,802756036	0,422115758	0,827816808
ENSG00000143947	RPS27A	ribosomal protein S27a	6233	1126,858296	0,154116778	0,081099	1,900348095	0,057387454	0,38821726
ENSG00000113643	RARS1	arginyl-tRNA synthetase 1	5917	127,1567674	0,362877803	0,189526	1,914659158	0,055535985	0,382157692

ENSG00000083535	PIBF1	progesterone immunomodulatory binding factor 1	10464	68,2821741	-0,149887146	0,207965	-0,720731098	0,471074975	0,852690218
ENSG00000115738	ID2	inhibitor of DNA binding 2	3398	1708,911772	-0,028704245	0,057782	-0,496765248	0,619354605	0,903661725
ENSG00000137075	RNF38	ring finger protein 38	152006	359,1138729	-0,266970017	0,097787	-2,730124801	0,006331035	0,115368797
ENSG00000115594	IL1R1	interleukin 1 receptor type 1	3554	17,45856665	-0,456206306	0,446129	-1,022588482	0,306502458	NA
ENSG00000173276	ZBTB21	zinc finger and BTB domain containing 21	49854	103,8632303	0,144611249	0,169711	0,852104271	0,394156222	0,812498986
ENSG00000131351	HAUS8	HAUS augmin like complex subunit 8	93323	23,22412817	0,015002966	0,44542	0,033682724	0,973130155	NA
ENSG00000164366	CCDC127	coiled-coil domain containing 127	133957	72,78756994	-0,380916477	0,231462	-1,645700159	0,099825506	0,509429216
ENSG00000198707	CEP290	centrosomal protein 290	80184	84,56594274	-0,128069331	0,63872	-0,200509367	0,841082233	0,966505956
ENSG00000173889	PHC3	polyhomeotic homolog 3	80012	751,598674	-0,088248489	0,146394	-0,602813571	0,546632717	0,878739902
ENSG00000186073	CDIN1	CDAN1 interacting nuclease 1	84529	23,43744776	0,791868109	0,51809	1,528435902	0,126404347	NA
ENSG00000167987	VPS37C	VPS37C subunit of ESCRT-I	55048	13,99152632	0,771160604	0,586295	1,315311492	0,188405237	NA
ENSG00000149503	INCENP	inner centromere protein	3619	190,0538372	0,108449653	0,157824	0,687155961	0,491984453	0,860513824
ENSG00000168061	SAC3D1	SAC3 domain containing 1	29901	12,65406523	0,641011131	0,636554	1,007001854	0,313933883	NA
ENSG00000155368	DBI	diazepam binding inhibitor, acyl-CoA binding protein	1622	238,5332705	0,310886485	0,13438	2,313495751	0,020695398	0,225859999
ENSG00000115392	FANCL	FA complementation group L	55120	19,39981783	0,290290482	0,539707	0,537866518	0,590669205	NA
ENSG00000167333	TRIM68	tripartite motif containing 68	55128	50,9294316	0,216491353	0,240256	0,901084628	0,367543325	0,795318634
ENSG00000051620	HEBP2	heme binding protein 2	23593	66,93284714	0,438748668	0,266703	1,645081796	0,099952944	0,509429216
ENSG00000170340	B3GNT2	UDP-GlcNAc:betaGal beta-1,3-N-acetylglucosaminyltransferase 2	10678	323,0866426	-0,10074426	0,098177	-1,026146707	0,304822437	0,751870158
ENSG00000116406	EDEM3	ER degradation enhancing alpha-mannosidase like protein 3	80267	268,5387109	-0,017883317	0,106247	-0,168319104	0,866332245	0,971722599
ENSG00000101843	PSMD10	proteasome 26S subunit, non-ATPase 10	5716	92,44618422	-0,107379061	0,182679	-0,587802126	0,556665117	0,883708056
ENSG00000001561	ENPP4	ectonucleotide pyrophosphatase/phosphodiesterase 4	22875	53,32127678	0,1321329	0,245461	0,538305577	0,590366102	0,892294181
ENSG00000089335	ZNF302	zinc finger protein 302	55900	73,89584223	0,196626913	0,2256	0,871571267	0,383442311	0,808586507
ENSG00000166986	MARS1	methionyl-tRNA synthetase 1	4141	400,6025557	0,054535306	0,110734	0,492487998	0,622374405	0,904764752
ENSG00000179715	PCED1B	PC-esterase domain containing 1B	91523	114,2684887	0,051302458	0,184397	0,278217479	0,780845418	0,948265435
ENSG00000000419	DPM1	dolichyl-phosphate mannosyltransferase subunit 1, catalytic	8813	96,2191894	0,303838192	0,172819	1,758126087	0,078726058	0,454642985
ENSG00000237765	FAM200B	family with sequence similarity 200 member B	285550	22,66247502	-0,291304734	0,53205	-0,547514337	0,584025423	NA
ENSG00000145354	CISD2	CDGSH iron sulfur domain 2	493856	46,08691383	1,509301369	0,89661	1,683341189	0,092309061	0,4945733
ENSG00000101096	NFATC2	nuclear factor of activated T cells 2	4773	820,935714	-0,079856156	0,076329	-1,046213634	0,29546241	0,747362888
ENSG00000248527	MTATP6P1	MT-ATP6 pseudogene 1	106480796	1470,454701	0,221679883	0,170442	1,300615121	0,193390229	0,650901454
ENSG00000102858	MGRN1	mahogunin ring finger 1	23295	414,3716223	-0,214010587	0,098775	-2,166639629	0,030262343	0,279077601
ENSG00000198399	ITSN2	intersectin 2	50618	494,8752919	-0,167587334	0,083249	-2,013091078	0,044105049	0,336311702
ENSG00000135069	PSAT1	phosphoserine aminotransferase 1	29968	176,093023	0,115832597	0,138833	0,83432809	0,404096127	0,817992511
ENSG00000143842	SOX13	SRY-box transcription factor 13	9580	18,77875892	-0,329888557	0,420541	-0,784438379	0,432782926	NA
ENSG00000103495	MAZ	MYC associated zinc finger protein	4150	548,4338894	-0,255176989	0,102109	-2,499069294	0,012451996	0,175323113
ENSG00000289746	TARP	TCR gamma alternate reading frame protein	445347	263,9801992	0,050372815	0,122116	0,412500111	0,6799729	0,925586358
ENSG00000198890	PRMT6	protein arginine methyltransferase 6	55170	73,02978595	-0,034340232	0,241118	-0,142420571	0,886747814	0,977928921
ENSG00000189350	TOGARAM2	TOG array regulator of axonemal microtubules 2	165186	20,13525907	-0,552263491	0,409692	-1,347997446	0,177659204	NA
ENSG00000196510	ANAPC7	anaphase promoting complex subunit 7	51434	126,7840342	0,016580979	0,204291	0,081163668	0,935311793	0,987098279
ENSG00000125304	TM9SF2	transmembrane 9 superfamily member 2	9375	536,2842645	0,073640778	0,071952	1,02347644	0,306082634	0,752888508
ENSG00000166439	RNF169	ring finger protein 169	254225	249,0532187	-0,08178883	0,108962	-0,75061689	0,452883253	0,843234809
ENSG00000111581	NUP107	nucleoporin 107	57122	182,8235356	-0,031587627	0,130077	-0,242838298	0,808130666	0,958576399
ENSG00000138768	USO1	USO1 vesicle transport factor	8615	564,3992534	0,038329184	0,075626	0,506825601	0,612277192	0,900006614
ENSG00000217555	CKLF	chemokine like factor	51192	181,6171728	-0,123084985	0,14143	-0,87028948	0,384142227	0,808586507
ENSG00000089693	MLF2	myeloid leukemia factor 2	8079	202,8729859	-0,177184835	0,131494	-1,347472694	0,177828042	0,632142851
ENSG00000141682	PMAIP1	phorbol-12-myristate-13-acetate-induced protein 1	5366	46,4921774	0,191544812	0,268939	0,712225158	0,476325361	0,853811879
ENSG00000131779	PEX11B	peroxisomal biogenesis factor 11 beta	8799	114,4730278	0,030328705	0,189131	0,160358168	0,87259894	0,974533542
ENSG00000106635	BCL7B	BAF chromatin remodeling complex subunit BCL7B	9275	99,64497675	-0,105861859	0,197505	-0,535996528	0,59196095	0,892834002
ENSG00000171100	MTM1	myotubularin 1	4534	211,3135487	-0,020728209	0,13233	-0,156640523	0,875528164	0,975344937
ENSG00000117151	CTBS	chitobiase	1486	34,4300384	0,126572003	0,326804	0,387303002	0,698531903	NA

ENSG00000171208	NETO2	neuropilin and tolloid like 2	81831	16,82374378	0,032200958	0,586149	0,054936475	0,956189073	NA
ENSG00000182541	LIMK2	LIM domain kinase 2	3985	114,6008732	0,378218861	0,206148	1,834693233	0,066551156	0,416786686
ENSG00000110435	PDHX	pyruvate dehydrogenase complex component X	8050	82,23054429	-0,074217012	0,183957	-0,403447073	0,686619363	0,926741447
ENSG00000054118	THRAP3	thyroid hormone receptor associated protein 3	9967	943,0821181	0,021227675	0,082058	0,258692501	0,795872506	0,953436042
ENSG00000092853	CLSPN	claspin	63967	107,7083422	0,318911091	0,182392	1,748491182	0,080379011	0,460464445
ENSG00000184840	TMED9	transmembrane p24 trafficking protein 9	54732	213,6634157	-0,2678234	0,136521	-1,96176998	0,04978927	0,357759312
ENSG00000205189	ZBTB10	zinc finger and BTB domain containing 10	65986	36,69375371	0,802139753	0,396774	2,021654167	0,043212092	NA
ENSG00000160588	MPZL3	myelin protein zero like 3	196264	648,9225589	0,061461703	0,086361	0,711681266	0,476662171	0,853811879
ENSG00000004897	CDC27	cell division cycle 27	996	330,2266726	0,169403214	0,113303	1,495136724	0,134878763	0,575414344
ENSG00000158427	TMSB15B	thymosin beta 15B	286527	18,52564833	0,706567018	0,58746	1,202748406	0,229073696	NA
ENSG00000164284	GRPEL2	GrpE like 2, mitochondrial	134266	54,5785116	0,098828805	0,252456	0,391469355	0,695450336	0,929397132
ENSG00000169026	SLC49A3	solute carrier family 49 member 3	84179	22,40499337	-0,016558235	0,467605	-0,035410769	0,971752198	NA
ENSG00000197043	ANXA6	annexin A6	309	2559,557742	0,011002982	0,041778	0,263370413	0,792265093	0,952007348
ENSG00000215021	PHB2	prohibitin 2	11331	318,5046638	0,022059118	0,103719	0,212682124	0,831574911	0,963444365
ENSG00000092203	TOX4	TOX high mobility group box family member 4	9878	254,5483879	0,018821707	0,120657	0,155993287	0,876038313	0,97552884
ENSG00000101040	ZMYND8	zinc finger MYND-type containing 8	23613	225,1966372	-0,034851996	0,117249	-0,297248309	0,766276943	0,944698783
ENSG00000101191	DIDO1	death inducer-obliterator 1	11083	961,0512288	-0,107368928	0,058045	-1,849755761	0,06434876	0,408565205
ENSG00000134759	ELP2	elongator acetyltransferase complex subunit 2	55250	148,6556387	0,701781097	0,301372	2,328619255	0,019879245	0,221777828
ENSG00000139679	LPAR6	lysophosphatidic acid receptor 6	10161	628,4917797	-0,492579843	0,097741	-5,039628237	4,66E-07	7,63E-05
ENSG00000148308	GTF3C5	general transcription factor IIIC subunit 5	9328	176,8460922	-0,109365416	0,136937	-0,798652965	0,424491667	0,829962049
ENSG00000159596	TMEM69	transmembrane protein 69	51249	42,02044471	0,232983994	0,312459	0,745646254	0,455881134	NA
ENSG00000174442	ZWILCH	zwilch kinetochore protein	55055	89,89744219	0,36542162	0,182625	2,000939083	0,045398955	0,340953043
ENSG00000174695	TMEM167A	transmembrane protein 167A	153339	599,7480572	-0,307407646	0,093047	-3,303783273	0,000953895	0,033747274
ENSG00000204370	SDHD	succinate dehydrogenase complex subunit D	6392	150,5181363	0,188998786	0,142492	1,326384132	0,184712492	0,64134921
ENSG00000157916	RER1	retention in endoplasmic reticulum sorting receptor 1	11079	334,8923974	-0,08584381	0,113964	-0,753256091	0,451296039	0,842718653
ENSG00000075089	ACTR6	actin related protein 6	64431	76,86558526	0,341983897	0,306782	1,114746911	0,264958892	0,720421404
ENSG00000066739	ATG2B	autophagy related 2B	55102	496,7912829	-0,049532639	0,076581	-0,646803894	0,517758872	0,868791049
ENSG00000267534	S1PR2	sphingosine-1-phosphate receptor 2	9294	25,90868375	-0,535105396	0,41119	-1,301357789	0,193136015	NA
ENSG00000122882	ECD	ecdysoneless cell cycle regulator	11319	161,9903965	-0,020509142	0,139091	-0,147450817	0,882776198	0,976387024
ENSG00000124588	NQO2	N-ribosyldihydronicotinamide:quinone dehydrogenase 2	4835	29,3032781	-0,011006924	0,340759	-0,032301234	0,974231825	NA
ENSG00000110583	NAA40	N-alpha-acetyltransferase 40, NatD catalytic subunit	79829	116,2452771	0,91140699	0,198742	4,585875301	4,52E-06	0,000537985
ENSG00000174780	SRP72	signal recognition particle 72	6731	627,3961341	-0,100787531	0,124009	-0,81274323	0,41636529	0,82446781
ENSG00000096060	FKBP5	FKBP prolyl isomerase 5	2289	437,199653	0,206490521	0,080716	2,5582373	0,010520427	0,158898917
ENSG00000196961	AP2A1	adaptor related protein complex 2 subunit alpha 1	160	408,8784529	0,021607161	0,08184	0,264016948	0,79176686	0,951719761
ENSG00000078687	TNRC6C	trinucleotide repeat containing adaptor 6C	57690	518,5040796	-0,205876762	0,125649	-1,638508163	0,101315733	0,514372181
ENSG00000112855	HARS2	histidyl-tRNA synthetase 2, mitochondrial	23438	99,2957321	0,113268698	0,185105	0,611916044	0,540593306	0,876149366
ENSG00000119950	MXI1	MAX interactor 1, dimerization protein	4601	37,85719984	-0,450458617	0,359829	-1,251869242	0,210617514	NA
ENSG00000169230	PRELID1	PRELI domain containing 1	27166	345,3392824	0,176154299	0,120115	1,466544932	0,14249989	0,587503482
ENSG00000117155	SSX2IP	SSX family member 2 interacting protein	117178	26,02680634	0,479580276	0,426917	1,123357184	0,261285826	NA
ENSG00000181804	SLC9A9	solute carrier family 9 member A9	285195	35,22323014	-0,329760733	0,303723	-1,085727884	0,2775994	NA
ENSG00000141068	KSR1	kinase suppressor of ras 1	8844	600,134218	-0,006204407	0,076453	-0,081152738	0,935320485	0,987098279
ENSG00000060749	QSER1	glutamine and serine rich 1	79832	23,30731432	-0,058651769	0,381023	-0,153932217	0,877663181	NA
ENSG00000119632	IFI27L2	interferon alpha inducible protein 27 like 2	83982	48,46885387	0,27546046	0,250246	1,100757113	0,271002382	0,725477256
ENSG00000120802	TMPO	thymopoietin	7112	842,8744183	0,182305055	0,071681	2,543300483	0,010981075	0,163343491
ENSG00000188985	NA	NA	NA	13,53773509	2,202296047	1,035544	2,126703814	0,0334447	NA
ENSG00000169718	DUS1L	dihydrouridine synthase 1 like	64118	105,9163825	0,373450454	0,236089	1,581822418	0,113690116	0,539530012
ENSG00000021355	SERPINB1	serpin family B member 1	1992	278,4498319	-0,148680289	0,113749	-1,307092881	0,191181169	0,649172893
ENSG00000163539	CLASP2	cytoplasmic linker associated protein 2	23122	218,7243613	0,205372008	0,146403	1,402782535	0,160681697	0,618147537
ENSG00000050344	NFE2L3	NFE2 like bZIP transcription factor 3	9603	138,3172383	-0,078329547	0,192067	-0,407824802	0,683402303	0,925586358
ENSG00000144401	METTL21A	methyltransferase 21A, HSPA lysine	151194	24,33377421	0,222307335	0,364155	0,610475147	0,541547102	NA
ENSG00000137040	RANBP6	RAN binding protein 6	26953	267,9333538	0,02709426	0,11081	0,244511904	0,806834384	0,958100582
ENSG00000204713	TRIM27	tripartite motif containing 27	5987	189,7827352	-0,112431366	0,133817	-0,840190299	0,400801697	0,814673014

ENSG00000263001	GTF2I	general transcription factor IIi	2969	1047,192085	0,01846162	0,055174	0,334605448	0,737922735	0,939193793
ENSG00000113300	CNOT6	CCR4-NOT transcription complex subunit 6	57472	259,3766563	0,005892938	0,104154	0,05657893	0,954880619	0,991420853
ENSG00000134590	RTL8C	retrotransposon Gag like 8C	8933	74,45135087	-0,041310672	0,211841	-0,195007735	0,845386892	0,967231052
ENSG00000124145	SDC4	syndecan 4	6385	65,18091789	-0,009614595	0,225299	-0,042674884	0,965960701	0,992789539
ENSG00000113971	NPHP3	nephrocystin 3	27031	60,13351466	-0,493564763	0,309157	-1,596486978	0,110380111	0,530878989
ENSG00000143793	C1orf35	chromosome 1 open reading frame 35	79169	27,88137777	-0,081921184	0,432661	-0,189342514	0,849824377	NA
ENSG00000196290	NIF3L1	NGG1 interacting factor 3 like 1	60491	52,30514306	0,495627249	0,242512	2,043718942	0,040981321	0,322789747
ENSG00000214160	ALG3	ALG3 alpha-1,3-mannosyltransferase	10195	51,06699427	0,200590679	0,236616	0,847747605	0,396578548	0,812799303
ENSG00000108061	SHOC2	SHOC2 leucine rich repeat scaffold protein	8036	294,3603827	-0,02627378	0,095992	-0,273707657	0,784309293	0,95013509
ENSG00000130958	SLC35D2	solute carrier family 35 member D2	11046	90,47954802	0,242204814	0,17916	1,351893156	0,176409497	0,632039836
ENSG00000197586	ENTPD6	ectonucleoside triphosphate diphosphohydrolase 6	955	57,31458047	-0,193232861	0,25569	-0,755731016	0,449810484	0,84263906
ENSG00000175782	SLC35E3	solute carrier family 35 member E3	55508	50,3676045	-0,049664701	0,311765	-0,159301528	0,873431317	0,974533542
ENSG00000205302	SNX2	sorting nexin 2	6643	40,68115606	0,556622971	0,56639	0,982756115	0,325727485	NA
ENSG00000139266	MARCHF9	membrane associated ring-CH-type finger 9	92979	24,43603868	0,197016112	0,354438	0,555854987	0,578309992	NA
ENSG00000179094	PER1	period circadian regulator 1	5187	65,13732366	0,028525307	0,212108	0,134484576	0,893019407	0,977990819
ENSG00000101417	PXMP4	peroxisomal membrane protein 4	11264	56,70396499	0,085772013	0,446155	0,192247107	0,847548648	0,967411651
ENSG00000131473	ACLY	ATP citrate lyase	47	754,7948054	0,032088032	0,061912	0,518281817	0,604261658	0,897212117
ENSG00000159445	THEM4	thioesterase superfamily member 4	117145	103,8054527	-0,33690125	0,270425	-1,245820568	0,212830277	0,667215123
ENSG00000167491	GATAD2A	GATA zinc finger domain containing 2A	54815	721,4195689	-0,163812557	0,101106	-1,6202112	0,105186916	0,52117398
ENSG00000149932	TMEM219	transmembrane protein 219	124446	66,55381069	0,345021297	0,23461	1,470617575	0,141394568	0,585546769
ENSG00000136824	SMC2	structural maintenance of chromosomes 2	10592	353,5964695	0,333583317	0,102658	3,249477812	0,001156171	0,037594481
ENSG00000256514	NA	NA	NA	26,85286262	0,47557146	0,775238	0,613452152	0,539577411	NA
ENSG00000155849	ELMO1	engulfment and cell motility 1	9844	173,7872811	0,154321495	0,151642	1,017668558	0,308835494	0,754797782
ENSG00000182841	NA	NA	NA	13,77475596	0,13776391	0,569862	0,241749513	0,808974261	NA
ENSG00000185009	AP3M1	adaptor related protein complex 3 subunit mu 1	26985	348,8068011	-0,006153085	0,103752	-0,059305645	0,952708665	0,991212866
ENSG00000164465	DCBLD1	discoidin, CUB and LCCL domain containing 1	285761	18,55528607	0,075369228	0,427318	0,176377506	0,859997375	NA
ENSG00000168827	GFM1	G elongation factor mitochondrial 1	85476	206,6683247	-0,112828684	0,164097	-0,687573457	0,491721428	0,860513824
ENSG00000104549	SQLE	squalene epoxidase	6713	327,6510241	0,261726156	0,100982	2,591801202	0,009547492	0,150574309
ENSG00000205593	DENND6B	DENN domain containing 6B	414918	17,06329145	-0,514912773	0,573569	-0,89773454	0,36932709	NA
ENSG00000104897	SF3A2	splicing factor 3a subunit 2	8175	223,5164876	-0,23209422	0,12645	-1,835460373	0,066437505	0,416786686
ENSG00000170860	LSM3	LSM3 homolog, U6 small nuclear RNA and mRNA degradation associated	27258	115,1441591	0,008125685	0,165133	0,049206804	0,960754489	0,992235217
ENSG00000188283	ZNF383	zinc finger protein 383	163087	51,26525832	0,112452837	0,368296	0,305332989	0,760112553	0,942901768
ENSG00000100575	TIMM9	translocase of inner mitochondrial membrane 9	26520	42,56266983	0,253397107	0,378228	0,669957764	0,502884715	0,863089257
ENSG00000132591	ERAL1	Era like 12S mitochondrial rRNA chaperone 1	26284	82,78385287	0,289883957	0,274715	1,055217084	0,291326052	0,743239565
ENSG00000138078	PREPL	prolyl endopeptidase like	9581	223,1897479	-0,021710391	0,178992	-0,121292728	0,90345918	0,978460866
ENSG00000169221	TBC1D10B	TBC1 domain family member 10B	26000	208,880605	0,001709555	0,125256	0,013648445	0,989110455	0,997108653
ENSG00000171865	RNASEH1	ribonuclease H1	246243	250,8438852	-0,013504173	0,105201	-0,128365535	0,897859704	0,977990819
ENSG00000173273	TNKS	tankyrase	8658	12,49224716	0,917531882	0,656132	1,398394884	0,161994519	NA
ENSG00000261247	GOLGA8T	golgin A8 family member T	653075	40,00119056	-0,148387645	0,287993	-0,515246737	0,606380613	NA
ENSG00000261247	GOLGA8K	golgin A8 family member K	653125	40,00119056	-0,148387645	0,287993	-0,515246737	0,606380613	NA
ENSG00000117090	SLAMF1	signaling lymphocytic activation molecule family member 1	6504	13,8973266	0,174304272	0,511196	0,340973661	0,733123411	NA
ENSG00000141424	SLC39A6	solute carrier family 39 member 6	25800	236,6913157	0,015832389	0,119001	0,133044163	0,894158454	0,977990819
ENSG00000112983	BRD8	bromodomain containing 8	10902	145,8947991	-0,130521876	0,539919	-0,241743368	0,808979023	0,958764958
ENSG00000197943	PLCG2	phospholipase C gamma 2	5336	929,3182623	-0,174728936	0,100051	-1,746398841	0,080741671	0,4605948
ENSG00000128944	KNSTRN	kinetochore localized astrin (SPAG5) binding protein	90417	45,34303598	0,102122799	0,464387	0,219908799	0,825942183	0,961917534
ENSG00000171222	SCAND1	SCAN domain containing 1	51282	58,45648232	-0,167520151	0,221833	-0,75516265	0,450151396	0,84263906
ENSG00000185753	CXorf38	chromosome X open reading frame 38	159013	207,7638315	-0,070031544	0,127411	-0,549650064	0,582559413	0,888501395
ENSG00000070669	ASNS	asparagine synthetase (glutamine-hydrolyzing)	440	149,4804363	0,017923648	0,157178	0,114034334	0,909210577	0,97930822
ENSG00000102349	KLF8	KLF transcription factor 8	11279	39,8908474	-0,878611608	0,382142	-2,299175928	0,021494951	NA
ENSG00000140995	DEF8	differentially expressed in FDCP 8 homolog	54849	70,94451427	-0,330063749	0,294555	-1,120551048	0,262479012	0,718296223

ENSG00000169967	MAP3K2	mitogen-activated protein kinase kinase kinase 2	10746	481,5316681	0,081739978	0,105275	0,776441896	0,437488115	0,837249449
ENSG00000168209	DDIT4	DNA damage inducible transcript 4	54541	622,3438269	-0,145275592	0,076455	-1,900147689	0,057413741	0,38821726
ENSG00000149658	YTHDF1	YTH N6-methyladenosine RNA binding protein F1	54915	232,3066855	-0,257935404	0,125924	-2,048334145	0,040527269	0,321200165
ENSG00000174206	KICS2	KICSTOR subunit 2	144577	31,16061111	-0,24031699	0,360089	-0,667382117	0,504528085	NA
ENSG00000172845	SP3	Sp3 transcription factor	6670	425,1129979	-0,087925818	0,151559	-0,580141639	0,561819106	0,884605624
ENSG00000111711	GOLT1B	golgi transport 1B	51026	121,3809776	-0,444352638	0,154531	-2,875491447	0,004033991	0,087764441
ENSG00000267041	ZNF850	zinc finger protein 850	342892	24,61707902	-0,116063436	0,39523	-0,29366021	0,769017562	NA
ENSG00000086200	IPO11	importin 11	51194	57,61895702	0,016759534	0,254734	0,065792361	0,947543138	0,990418394
ENSG00000086200	IPO11-LRRC70	IPO11-LRRC70 readthrough	101180901	57,61895702	0,016759534	0,254734	0,065792361	0,947543138	0,990418394
ENSG00000163344	PMVK	phosphomevalonate kinase	10654	58,38743317	-0,093881922	0,224709	-0,417794246	0,676097558	0,924186156
ENSG00000028203	VEZT	vezatin, adherens junctions transmembrane protein	55591	25,49975405	0,581811444	0,433546	1,341982652	0,179601621	NA
ENSG00000096872	IFT74	intraflagellar transport 74	80173	19,1285042	0,818263322	0,55328	1,478930405	0,139158916	NA
ENSG00000081059	TCF7	transcription factor 7	6932	249,3925984	0,140959135	0,128187	1,099634853	0,27149125	0,725708516
ENSG00000094841	UPRT	uracil phosphoribosyltransferase homolog	139596	46,71627498	0,29223654	0,28181	1,036997574	0,299736988	0,750202136
ENSG00000116237	ICMT	isoprenylcysteine carboxyl methyltransferase	23463	234,4311324	0,067284984	0,139026	0,483974768	0,628403776	0,905567722
ENSG00000126749	EMG1	EMG1 N1-specific pseudouridine methyltransferase	10436	74,90500567	-0,037117706	0,203027	-0,182821207	0,854938309	0,968377739
ENSG00000138744	NAAA	N-acylethanolamine acid amidase	27163	68,91271772	-0,066238428	0,216113	-0,306498861	0,759224847	0,942901768
ENSG00000165609	NUDT5	nudix hydrolase 5	11164	230,5056321	0,68065726	0,220515	3,08666785	0,002024136	0,054949576
ENSG00000108465	CDK5RAP3	CDK5 regulatory subunit associated protein 3	80279	76,92791832	0,361406459	0,259457	1,392934155	0,163639703	0,622687125
ENSG00000164332	UBLCP1	ubiquitin like domain containing CTD phosphatase 1	134510	294,3094557	-0,116607166	0,103762	-1,123789694	0,261102255	0,716543286
ENSG00000074695	LMAN1	lectin, mannose binding 1	3998	430,8461788	-0,148881817	0,094612	-1,573610958	0,115577418	0,541856098
ENSG00000080603	SRCAP	Snf2 related CREBBP activator protein	10847	919,8532524	-0,21201657	0,102258	-2,073356808	0,038139086	0,31021219
ENSG00000130222	GADD45G	growth arrest and DNA damage inducible gamma	10912	22,21377116	-0,149965382	0,397988	-0,376808511	0,706315915	NA
ENSG00000135334	AKIRIN2	akirin 2	55122	218,3529297	-0,043294243	0,113831	-0,380338093	0,703694463	0,930846779
ENSG00000103942	HOMER2	homer scaffold protein 2	9455	13,10187058	-0,51837481	0,497262	-1,042458565	0,297199126	NA
ENSG00000149548	CCDC15	coiled-coil domain containing 15	80071	20,59939047	0,242270024	0,468914	0,516661666	0,60539236	NA
ENSG00000163728	TTC14	tetratricopeptide repeat domain 14	151613	382,5203556	-0,111460515	0,092601	-1,203664258	0,228719372	0,68351286
ENSG00000169696	ASPSCR1	ASPSCR1 tether for SLC2A4, UBX domain containing	79058	55,09920813	0,025894351	0,338216	0,076561674	0,938972249	0,987813564
ENSG00000173599	PC	pyruvate carboxylase	5091	12,16643915	-0,312845315	0,695411	-0,449870821	0,652803588	NA
ENSG00000186591	UBE2H	ubiquitin conjugating enzyme E2 H	7328	254,7993326	-0,084255417	0,120385	-0,699880935	0,484001664	0,85698884
ENSG00000197971	MBP	myelin basic protein	4155	1446,417688	-0,23783569	0,047793	-4,976375539	6,48E-07	9,79E-05
ENSG00000204231	RXRB	retinoid X receptor beta	6257	82,04673383	0,576468353	0,284243	2,028086318	0,042551439	0,328150469
ENSG00000213341	CHUK	component of inhibitor of nuclear factor kappa B kinase complex	1147	126,2033601	0,148527823	0,169669	0,875397708	0,381357547	0,806821656
ENSG00000273154	NA	NA	NA	26,81068415	0,215819626	0,428557	0,503596512	0,614544944	NA
ENSG00000007080	CCDC124	coiled-coil domain containing 124	115098	83,15604281	-0,468209336	0,18942	-2,471811493	0,013443038	0,18084297
ENSG00000047249	ATP6V1H	ATPase H+ transporting V1 subunit H	51606	115,3233613	-0,023535628	0,152206	-0,154629826	0,877113155	0,975885813
ENSG00000187601	MAGEH1	MAGE family member H1	28986	37,38596512	0,278213752	0,273211	1,018309563	0,308530865	NA
ENSG00000204842	ATXN2	ataxin 2	6311	104,8465925	-0,112803152	0,222931	-0,505999833	0,612856767	0,900033105
ENSG00000115539	PDCL3	phosducin like 3	79031	64,75526614	-0,120678404	0,208574	-0,578587249	0,562867711	0,884605624
ENSG00000136518	ACTL6A	actin like 6A	86	102,9650652	0,574867254	0,175804	3,269937471	0,001075713	0,037055468
ENSG00000087263	OGFOD1	2-oxoglutarate and iron dependent oxygenase domain containing 1	55239	74,0001654	0,211439229	0,368023	0,574526546	0,565611543	0,885151063
ENSG00000117305	HMGCL	3-hydroxy-3-methylglutaryl-CoA lyase	3155	33,5644721	0,240907604	0,318163	0,757184086	0,448939583	NA
ENSG00000128268	MGAT3	beta-1,4-mannosyl-glycoprotein 4-beta-N-acetylglucosaminyltransferase	4248	209,8809253	-0,411470118	0,118317	-3,477700188	0,000505735	0,021016109
ENSG00000198130	HIBCH	3-hydroxyisobutyryl-CoA hydrolase	26275	66,01617058	0,464214186	0,230419	2,01465525	0,043940784	0,33571101
ENSG00000136048	DRAM1	DNA damage regulated autophagy modulator 1	55332	17,22704948	0,382821816	0,47351	0,8084764	0,418816387	NA
ENSG00000109736	MFSD10	major facilitator superfamily domain containing 10	10227	44,51227752	0,282433339	0,30996	0,911192808	0,362193793	0,791409024

ENSG00000197170	PSMD12	proteasome 26S subunit, non-ATPase 12	5718	132,8737264	-0,000620114	0,210614	-0,002944315	0,99765078	0,999300722
ENSG00000124587	PEX6	peroxisomal biogenesis factor 6	5190	76,70489403	-0,003188451	0,207693	-0,015351784	0,98775153	0,996894778
ENSG00000203879	GDI1	GDP dissociation inhibitor 1	2664	336,7864742	-0,050507837	0,122698	-0,411644497	0,680600012	0,925586358
ENSG00000177084	POLE	DNA polymerase epsilon, catalytic subunit	5426	281,9311232	0,224843214	0,108904	2,064592227	0,038961599	0,314172896
ENSG00000008277	ADAM22	ADAM metallopeptidase domain 22	53616	27,6222156	0,953914142	0,425524	2,241739239	0,02497823	NA
ENSG00000168395	ING5	inhibitor of growth family member 5	84289	85,86065151	-0,058154337	0,21201	-0,274300217	0,783853918	0,950093441
ENSG00000177932	ZNF354C	zinc finger protein 354C	30832	67,24116174	0,209460639	0,209024	1,002090584	0,316299845	0,760863395
ENSG00000198040	ZNF84	zinc finger protein 84	7637	173,4655718	-0,154422999	0,147468	-1,047164061	0,295023918	0,746855268
ENSG00000148824	MTG1	mitochondrial ribosome associated GTPase 1	92170	80,72299679	-0,3098866	0,204782	-1,513251206	0,130215862	0,566497377
ENSG00000213983	AP1G2	adaptor related protein complex 1 subunit gamma 2	8906	287,1668885	-0,097967911	0,155903	-0,628388445	0,529749505	0,872145761
ENSG00000174165	ZDHHC24	zinc finger DHHC-type containing 24	254359	105,2969432	0,012416298	0,181707	0,068331574	0,945521691	0,98927293
ENSG00000143374	TARS2	threonyl-tRNA synthetase 2, mitochondrial	80222	115,6537328	0,111096667	0,179848	0,617724187	0,536757166	0,874923109
ENSG00000149476	TKFC	triokinase and FMN cyclase	26007	30,44363531	-0,360590474	0,447675	-0,805474006	0,420546201	NA
ENSG00000104231	ZFAND1	zinc finger AN1-type containing 1	79752	59,779697	-0,377129339	0,373896	-1,008647075	0,313143922	0,758381858
ENSG00000134982	APC	APC regulator of WNT signaling pathway	324	163,0119112	0,227226958	0,273882	0,829653721	0,406734594	0,819272982
ENSG00000174243	DDX23	DEAD-box helicase 23	9416	369,0673461	-0,140880378	0,115033	-1,224692841	0,220690972	0,677095199
ENSG00000093009	CDC45	cell division cycle 45	8318	26,51912208	0,791679801	0,371345	2,131926117	0,033012921	NA
ENSG00000167380	ZNF226	zinc finger protein 226	7769	102,1853849	0,553633716	0,211649	2,615816222	0,008901445	0,14473205
ENSG00000197355	UAP1L1	UDP-N-acetylglucosamine pyrophosphorylase 1 like 1	91373	79,08985668	0,135218743	0,200445	0,674591595	0,499935274	0,86161226
ENSG00000123374	CDK2	cyclin dependent kinase 2	1017	131,2010272	0,367486749	0,154106	2,384639212	0,017095878	0,204616219
ENSG00000104884	ERCC2	ERCC excision repair 2, TFIIH core complex helicase subunit	2068	40,88762459	-0,482795745	0,503296	-0,959268046	0,337423729	NA
ENSG00000165209	STRBP	spermatid perinuclear RNA binding protein	55342	280,2187938	0,323704951	0,096446	3,356345214	0,000789799	0,029398496
ENSG00000101846	STS	steroid sulfatase	412	93,60402293	-0,192024191	0,181601	-1,05739764	0,290330148	0,742269851
ENSG00000080839	RBL1	RB transcriptional corepressor like 1	5933	180,8269469	0,20172164	0,164365	1,227278874	0,219717793	0,676295699
ENSG00000130164	LDLR	low density lipoprotein receptor	3949	380,6862012	-0,052079617	0,162677	-0,320142124	0,748860594	0,940446292
ENSG00000165288	BRWD3	bromodomain and WD repeat domain containing 3	254065	243,2151376	-0,190575729	0,117553	-1,62118496	0,104977977	0,52084462
ENSG00000105671	DDX49	DEAD-box helicase 49	54555	80,52394056	-0,281613473	0,268004	-1,050780041	0,293359625	0,744745216
ENSG00000113721	PDGFRB	platelet derived growth factor receptor beta	5159	18,22187162	-0,849340253	0,477912	-1,777189199	0,075537116	NA
ENSG00000146576	INTS15	integrator complex subunit 15	79034	127,3307313	-0,016675117	0,144436	-0,115449915	0,908088517	0,97914981
ENSG00000213402	PTPRCAP	protein tyrosine phosphatase receptor type C associated protein	5790	570,0099392	-0,034701994	0,092766	-0,374080722	0,708344261	0,930846779
ENSG00000163528	CHCHD4	coiled-coil-helix-coiled-coil-helix domain containing 4	131474	21,1790683	0,499142703	0,438923	1,137199992	0,255454691	NA
ENSG00000178585	CTNNBIP1	catenin beta interacting protein 1	56998	126,7726421	-0,27661201	0,148531	-1,862315877	0,062558579	0,404317327
ENSG00000012048	BRCA1	BRCA1 DNA repair associated	672	118,2352051	0,365670752	0,214633	1,703702856	0,088436614	0,486308725
ENSG00000196236	XPNPEP3	X-prolyl aminopeptidase 3	63929	44,78858671	-0,047847055	0,300595	-0,159174408	0,873531466	0,974533542
ENSG00000196152	ZNF79	zinc finger protein 79	7633	54,18214623	0,334667958	0,257972	1,297305138	0,194526217	0,652365446
ENSG00000197013	ZNF429	zinc finger protein 429	353088	19,76040042	-0,554472919	0,518756	-1,068851379	0,285136643	NA
ENSG00000177830	CHID1	chitinase domain containing 1	66005	62,77496667	0,315087075	0,234906	1,341332159	0,179812633	0,633591125
ENSG00000101773	RBBP8	RB binding protein 8, endonuclease	5932	119,0615381	0,424779843	0,182942	2,321939971	0,020236168	0,224146577
ENSG00000243708	PLA2G4B	phospholipase A2 group IVB	100137049	19,28341594	-0,638515115	2,776735	-0,229951794	0,818129229	NA
ENSG00000114346	ECT2	epithelial cell transforming 2	1894	89,22639818	0,166967135	0,20193	0,826857882	0,408317618	0,820514158
ENSG00000105429	MEGF8	multiple EGF like domains 8	1954	163,6465231	-0,059881189	0,127417	-0,469962617	0,638381726	0,909954642
ENSG00000101901	ALG13	ALG13 UDP-N-acetylglucosaminyltransferase subunit	79868	139,8726939	-0,2143257	0,22616	-0,947673983	0,34329545	0,781132432
ENSG00000165102	HGSNAT	heparan-alpha-glucosaminide N-acetyltransferase	138050	137,6877434	-0,389861983	0,207741	-1,876671655	0,06056311	0,398308113
ENSG00000077312	SNRPA	small nuclear ribonucleoprotein polypeptide A	6626	119,793031	-0,027178636	0,17152	-0,158457311	0,874096457	0,974569239
ENSG00000180354	MTURN	maturin, neural progenitor differentiation regulator homolog	222166	20,1526908	0,043084668	0,546017	0,078907226	0,937106416	NA

ENSG00000112081	SRSF3	serine and arginine rich splicing factor 3	6428	474,7758244	0,126240599	0,098262	1,284736645	0,198884328	0,657700006
ENSG00000111452	ADGRD1	adhesion G protein-coupled receptor D1	283383	30,24225712	0,204264019	0,623848	0,327425756	0,743345888	NA
ENSG00000119900	OGFRL1	opioid growth factor receptor like 1	79627	243,254394	-0,095316148	0,124256	-0,767094374	0,443025407	0,838843189
ENSG00000163932	PRKCD	protein kinase C delta	5580	120,1363896	-0,121297983	0,17092	-0,709678159	0,477903739	0,854008769
ENSG00000217128	FNIP1	folliculin interacting protein 1	96459	180,015157	0,065566035	0,148205	0,442400799	0,658199201	0,919088619
ENSG00000070367	EXOC5	exocyst complex component 5	10640	196,5257958	-0,557654826	0,381333	-1,462381207	0,143636776	0,59024645
ENSG00000092108	SCFD1	sec1 family domain containing 1	23256	130,3521263	-0,008882167	0,195961	-0,045326163	0,963847333	0,992694268
ENSG00000118873	RAB3GAP2	RAB3 GTPase activating non-catalytic protein subunit 2	25782	321,3801986	-0,078180458	0,104396	-0,748884148	0,453927036	0,843234809
ENSG00000112685	EXOC2	exocyst complex component 2	55770	364,4301535	0,019520861	0,088429	0,22075157	0,825285875	0,961917534
ENSG00000151576	QTRT2	queuine tRNA-ribosyltransferase accessory subunit 2	79691	83,41973347	0,049507044	0,191661	0,258305427	0,796171198	0,953436042
ENSG00000141552	ANAPC11	anaphase promoting complex subunit 11	51529	45,39797778	0,051345721	0,266281	0,192825446	0,847095675	0,967300004
ENSG00000148384	INPP5E	inositol polyphosphate-5-phosphatase E	56623	99,83111253	-0,276046108	0,17795	-1,551255762	0,120840404	0,555017854
ENSG00000141384	TAF4B	TATA-box binding protein associated factor 4b	6875	53,72427804	-0,045470371	0,383161	-0,118671849	0,90553534	0,978460866
ENSG00000124788	ATXN1	ataxin 1	6310	334,6982952	0,098069138	0,157526	0,622558154	0,533574917	0,873191491
ENSG00000197448	GSTK1	glutathione S-transferase kappa 1	373156	297,8645613	0,003843021	0,097335	0,039482573	0,968505648	0,993542794
ENSG00000075234	TTC38	tetratricopeptide repeat domain 38	55020	175,5039483	-0,215940302	0,133501	-1,6175183	0,105766449	0,522649494
ENSG00000147475	ERLIN2	ER lipid raft associated 2	11160	87,94703199	0,308096158	0,236856	1,300774508	0,19333565	0,650901454
ENSG00000154511	DIPK1A	divergent protein kinase domain 1A	388650	26,06850467	-0,107407759	0,369794	-0,290453218	0,771469536	NA
ENSG00000123159	GIPC1	GIPC PDZ domain containing family member 1	10755	97,67255903	-0,121201571	0,191915	-0,631537503	0,527689136	0,872142129
ENSG00000133275	CSNK1G2	casein kinase 1 gamma 2	1455	411,4361504	-0,064150213	0,095219	-0,673714868	0,500492607	0,861654743
ENSG00000156500	PABIR3	PABIR family member 3	159091	26,55924113	-0,591465564	0,505	-1,171218847	0,241510822	NA
ENSG00000163655	GMPS	guanine monophosphate synthase	8833	230,3582292	0,231165328	0,132145	1,749331191	0,080233787	0,459968005
ENSG00000185112	FAM43A	family with sequence similarity 43 member A	131583	68,1459706	-0,275999047	0,241739	-1,141722239	0,253569494	0,709599722
ENSG00000197077	KIAA1671	KIAA1671	85379	673,3548864	0,004885306	0,071499	0,068326835	0,945525463	0,98927293
ENSG00000063601	MTMR1	myotubularin related protein 1	8776	309,6229996	0,004564907	0,103816	0,043971211	0,964927352	0,992789539
ENSG00000117640	MTFR1L	mitochondrial fission regulator 1 like	56181	134,5676156	0,003479213	0,146918	0,02368129	0,98110683	0,995752963
ENSG00000146556	NA	NA	NA	53,94229344	-0,377923828	0,247933	-1,524301026	0,127433531	0,563689485
ENSG00000235859	NA	NA	NA	32,15909285	-0,007502964	0,325316	-0,023063647	0,981599504	NA
ENSG00000121039	RDH10	retinol dehydrogenase 10	157506	31,44172796	0,2905829	0,458384	0,633929184	0,526127039	NA
ENSG00000143977	SNRPG	small nuclear ribonucleoprotein polypeptide G	6637	103,630192	0,525741822	0,179935	2,921845996	0,003479635	0,078531773
ENSG00000213977	TAX1BP3	Tax1 binding protein 3	30851	37,95314391	0,20449955	0,315806	0,647548526	0,517276999	NA
ENSG00000104047	DTWD1	DTW domain containing 1	56986	33,31875723	1,736179035	1,048846	1,655322399	0,097859118	NA
ENSG00000109118	PHF12	PHD finger protein 12	57649	312,2297507	-0,136144033	0,104037	-1,308608962	0,190666844	0,648548026
ENSG00000163161	ERCC3	ERCC excision repair 3, TFIIH core complex helicase subunit	2071	192,9366993	0,05718501	0,126078	0,453566863	0,650140609	0,914926418
ENSG00000166435	XRRA1	X-ray radiation resistance associated 1	143570	31,22178001	-0,167469806	0,473313	-0,353824732	0,723470237	NA
ENSG00000170653	ATF7	activating transcription factor 7	11016	309,9866848	-0,278802465	0,118291	-2,356914669	0,018427481	0,212696759
ENSG00000198087	CD2AP	CD2 associated protein	23607	180,5368522	-0,059498904	0,146775	-0,405374213	0,685202463	0,925942901
ENSG00000012061	ERCC1	ERCC excision repair 1, endonuclease non-catalytic subunit	2067	118,943107	0,09341358	0,261116	0,357747248	0,720532471	0,933530522
ENSG00000038219	BOD1L1	biorientation of chromosomes in cell division 1 like 1	259282	975,6596659	0,062772566	0,058688	1,069600546	0,284799149	0,737867955
ENSG00000100353	EIF3D	eukaryotic translation initiation factor 3 subunit D	8664	420,1402226	0,036936703	0,111968	0,329886126	0,741486007	0,939193793
ENSG00000101972	STAG2	STAG2 cohesin complex component	10735	807,7673873	0,097506259	0,092114	1,058543772	0,289807606	0,741447348
ENSG00000115866	DARS1	aspartyl-tRNA synthetase 1	1615	297,0027533	0,03584425	0,09884	0,362648274	0,716867644	0,932040329
ENSG00000174353	NA	NA	NA	72,88568023	0,085026692	0,208447	0,407904858	0,683343526	0,925586358
ENSG00000164548	TRA2A	transformer 2 alpha homolog	29896	235,8165618	0,003584082	0,138879	0,025807188	0,979411128	0,995712569
ENSG00000114125	RNF7	ring finger protein 7	9616	111,648921	-0,067621835	0,154523	-0,437617957	0,661663252	0,919853106
ENSG00000179409	GEMIN4	gem nuclear organelle associated protein 4	50628	95,0048804	0,132793441	0,187501	0,708228564	0,47880333	0,854008769

ENSG00000087053	MTMR2	myotubularin related protein 2	8898	114,493572	0,225414633	0,28521	0,790346015	0,429325721	0,832060923
ENSG00000165406	MARCHF8	membrane associated ring-CH-type finger 8	220972	339,0633976	0,039889178	0,118975	0,335274626	0,737417933	0,939193793
ENSG00000076604	TRAF4	TNF receptor associated factor 4	9618	45,79804499	0,431727288	0,257626	1,675789944	0,093779345	0,497328138
ENSG00000117133	RPF1	ribosome production factor 1 homolog	80135	136,5780343	0,192898925	0,147869	1,304526892	0,192053997	0,649982152
ENSG00000143126	CELSR2	cadherin EGF LAG seven-pass G-type receptor 2	1952	85,95390335	-0,138115021	0,179407	-0,76984324	0,441392887	0,838678081
ENSG00000180198	RCC1	regulator of chromosome condensation 1	1104	100,3280486	0,43174011	0,181042	2,384746826	0,017090879	0,204616219
ENSG00000132466	ANKRD17	ankyrin repeat domain 17	26057	429,4214631	0,325592648	0,31672	1,028014347	0,303943079	0,751870158
ENSG00000250312	ZNF718	zinc finger protein 718	255403	39,01566346	-0,039437835	0,335881	-0,117416153	0,906530285	NA
ENSG00000066056	TIE1	tyrosine kinase with immunoglobulin like and EGF like domains 1	7075	27,85466734	1,461044361	0,68899	2,120558557	0,033958969	NA
ENSG00000110696	C11orf58	chromosome 11 open reading frame 58	10944	370,9988804	0,022153969	0,114545	0,193407639	0,846639734	0,967231052
ENSG00000154305	MIA3	MIA SH3 domain ER export factor 3	375056	328,0480508	-0,239181345	0,114124	-2,095807839	0,036099241	0,302084897
ENSG00000174886	NDUFA11	NADH:ubiquinone oxidoreductase subunit A11	126328	34,42083379	0,399881915	0,427037	0,936411357	0,349061433	NA
ENSG00000177951	BET1L	Bet1 golgi vesicular membrane trafficking protein like	51272	201,1605033	0,210157789	0,138351	1,519021097	0,128757186	0,563689485
ENSG00000135926	TMBIM1	transmembrane BAX inhibitor motif containing 1	64114	184,5064628	0,038500565	0,148033	0,26008075	0,794801486	0,952742807
ENSG00000132823	OSER1	oxidative stress responsive serine rich 1	51526	108,2986087	0,025486096	0,175502	0,145218355	0,884538474	0,977108443
ENSG00000167272	POP5	POP5 homolog, ribonuclease P/MRP subunit	51367	14,87452	-1,031218735	0,617762	-1,669282279	0,09506145	NA
ENSG00000075188	NUP37	nucleoporin 37	79023	37,0693136	0,710001037	0,294025	2,414762298	0,015745481	NA
ENSG00000038427	VCAN	versican	1462	17,16701211	-0,276799571	0,5119	-0,540729389	0,588694119	NA
ENSG00000110660	SLC35F2	solute carrier family 35 member F2	54733	76,68786115	-0,088443475	0,222506	-0,39748818	0,691007501	0,927252461
ENSG00000124486	USP9X	ubiquitin specific peptidase 9 X-linked	8239	945,846775	-0,058907563	0,08393	-0,70186787	0,482761568	0,855925045
ENSG00000055130	CUL1	cullin 1	8454	614,0953265	0,105286249	0,078664	1,338427925	0,180756981	0,634062228
ENSG00000196116	TDRD7	tudor domain containing 7	23424	105,1115388	0,01432841	0,161931	0,088484725	0,929491425	0,986487751
ENSG00000204161	TMEM273	transmembrane protein 273	170371	150,2584654	-0,416288516	0,142958	-2,911958865	0,003591699	0,080139791
ENSG00000053900	ANAPC4	anaphase promoting complex subunit 4	29945	149,6078193	-0,160987319	0,14779	-1,089294667	0,276023964	0,728944151
ENSG00000108091	CCDC6	coiled-coil domain containing 6	8030	197,2304931	-0,039393759	0,122656	-0,321173861	0,748078639	0,939899419
ENSG00000234231	ANAPC1P4	ANAPC1 pseudogene 4	730268	21,56405333	0,479521457	0,401803	1,193425067	0,232702943	NA
ENSG00000088325	TPX2	TPX2 microtubule nucleation factor	22974	292,66309	0,632843286	0,122075	5,184066363	2,17E-07	3,88E-05
ENSG00000160058	BSDC1	BSD domain containing 1	55108	248,8857198	0,148438427	0,150958	0,983306973	0,325456379	0,768407005
ENSG00000168813	ZNF507	zinc finger protein 507	22847	199,1820535	0,141528923	0,132414	1,068839078	0,285142187	0,737867955
ENSG00000113384	GOLPH3	golgi phosphoprotein 3	64083	389,4001236	-0,056637179	0,094272	-0,600787289	0,547981671	0,879578516
ENSG00000025293	PHF20	PHD finger protein 20	51230	336,4965469	-0,177734396	0,12311	-1,443705	0,148821974	0,602188452
ENSG00000169926	KLF13	KLF transcription factor 13	51621	523,2529092	0,164057598	0,140196	1,170204331	0,24191875	0,696890628
ENSG00000075142	SRI	sorcin	6717	108,6693638	-0,020693835	0,191539	-0,108039989	0,913963971	0,981103549
ENSG00000138190	EXOC6	exocyst complex component 6	54536	84,67959479	0,194702479	0,206698	0,941963692	0,346211229	0,782042713
ENSG00000196642	RABL6	RAB, member RAS oncogene family like 6	55684	305,7948641	-0,201877527	0,126686	-1,593521321	0,111043289	0,53211348
ENSG00000132330	SCLY	selenocysteine lyase	51540	109,9082784	0,306117496	0,226667	1,350518439	0,176849743	0,632039836
ENSG00000035928	RFC1	replication factor C subunit 1	5981	286,8569262	0,217750695	0,110197	1,976021274	0,048152368	0,351803439
ENSG00000197780	TAF13	TATA-box binding protein associated factor 13	6884	73,73058471	-0,070429844	0,23897	-0,294722886	0,768205579	0,944698783
ENSG00000100804	PSMB5	proteasome 20S subunit beta 5	5693	47,46591524	0,010713759	0,258854	0,041389233	0,966985596	0,99290167
ENSG00000117758	STX12	syntaxin 12	23673	85,8523307	0,072449536	0,202165	0,358367894	0,720068015	0,933530522
ENSG00000163322	ABRAXAS1	abraxas 1, BRCA1 A complex subunit	84142	37,5572619	0,008083496	0,372115	0,021723117	0,982668823	NA
ENSG00000163785	RYK	receptor like tyrosine kinase	6259	81,71499868	0,17344233	0,207762	0,834810881	0,403824198	0,817992511
ENSG00000124523	SIRT5	sirtuin 5	23408	69,42678761	0,315742091	0,324838	0,971997497	0,331051794	0,772473234
ENSG00000153944	MSI2	musashi RNA binding protein 2	124540	312,5003706	0,062418809	0,155588	0,401179142	0,688288236	0,926748702
ENSG00000106780	MEGF9	multiple EGF like domains 9	1955	214,4271032	0,129769985	0,115145	1,127017218	0,259735201	0,715701578
ENSG00000173020	GRK2	G protein-coupled receptor kinase 2	156	1269,630683	-0,086260431	0,051673	-1,669346086	0,095048812	0,499178345
ENSG00000131828	PDHA1	pyruvate dehydrogenase E1 subunit alpha 1	5160	187,7075713	0,379749633	0,148097	2,564200128	0,01034139	0,157711213
ENSG00000102710	SUPT20H	SPT20 homolog, SAGA complex component	55578	131,9111876	0,077867226	0,186789	0,416872505	0,676771666	0,924186156
ENSG00000100612	DHRS7	dehydrogenase/reductase 7	51635	190,2025648	0,170847049	0,144318	1,18382062	0,236484078	0,691328984
ENSG00000103489	XYLT1	xylosyltransferase 1	64131	812,2056131	-0,13465913	0,333169	-0,404176751	0,686082748	0,926454406

ENSG00000163714	U2SURP	U2 snRNP associated SURP domain containing	23350	364,1109731	-0,29356712	0,171572	-1,711045401	0,087072732	0,481598055
ENSG00000100325	ASCC2	activating signal cointegrator 1 complex subunit 2	84164	217,2850519	-0,055201742	0,130811	-0,421997322	0,673026972	0,923602509
ENSG00000143294	PRCC	proline rich mitotic checkpoint control factor	5546	203,0961593	-0,124258364	0,124909	-0,994793368	0,319836773	0,764222091
ENSG00000140416	TPM1	tropomyosin 1	7168	38,52724282	0,004921111	0,365744	0,013455062	0,989264738	NA
ENSG00000165156	ZHX1	zinc fingers and homeoboxes 1	11244	190,5104276	-0,175368794	0,124458	-1,409057962	0,15881803	0,614461481
ENSG00000196923	PDLIM7	PDZ and LIM domain 7	9260	52,86833251	-0,419155285	0,262624	-1,596027962	0,110482551	0,531046482
ENSG00000121073	SLC35B1	solute carrier family 35 member B1	10237	47,71447078	0,347860855	0,418255	0,831694972	0,405581139	0,818464021
ENSG00000266028	SRGAP2	SLIT-ROBO Rho GTPase activating protein 2	23380	292,8659863	0,172545902	0,104625	1,649187108	0,099109308	0,509429216
ENSG00000060339	CCAR1	cell division cycle and apoptosis regulator 1	55749	305,3658508	-0,036722389	0,313088	-0,117291133	0,906629351	0,978516824
ENSG00000101161	PRPF6	pre-mRNA processing factor 6	24148	499,5630533	0,093012228	0,081306	1,143977365	0,252633024	0,708754466
ENSG00000120063	GNA13	G protein subunit alpha 13	10672	874,3752241	0,030732955	0,080892	0,379927352	0,703999343	0,930846779
ENSG00000136492	BRIP1	BRCA1 interacting helicase 1	83990	24,27000699	1,072247233	0,514597	2,083663459	0,03719079	NA
ENSG00000101216	GMEB2	glucocorticoid modulatory element binding protein 2	26205	87,5113396	-0,012736088	0,196343	-0,064866542	0,94828026	0,990608484
ENSG00000115806	GORASP2	golgi reassembly stacking protein 2	26003	330,7500112	-0,402798402	0,131233	-3,06934081	0,002145317	0,055967203
ENSG00000088298	EDEM2	ER degradation enhancing alpha-mannosidase like protein 2	55741	110,0124515	0,075417516	0,167119	0,451279458	0,651788148	0,91625991
ENSG00000088298	MMP24-AS1-EDEM2	MMP24-AS1-EDEM2 readthrough	111089941	110,0124515	0,075417516	0,167119	0,451279458	0,651788148	0,91625991
ENSG00000100354	TNRC6B	trinucleotide repeat containing adaptor 6B	23112	955,1098507	-0,0564364	0,06237	-0,904865002	0,36553691	0,794170647
ENSG00000107371	EXOSC3	exosome component 3	51010	44,10149943	0,152630065	0,294414	0,51842011	0,604165187	0,897212117
ENSG00000136636	KCTD3	potassium channel tetramerization domain containing 3	51133	44,96020154	0,204912254	0,251342	0,815272902	0,414916116	0,82446781
ENSG00000141127	PRPSAP2	phosphoribosyl pyrophosphate synthetase associated protein 2	5636	82,335578	0,184361419	0,187523	0,983138485	0,325539286	0,768407005
ENSG00000162430	SELENON	selenoprotein N	57190	63,72197654	-0,335537889	0,228516	-1,468331654	0,142014157	0,586424389
ENSG00000163874	ZC3H12A	zinc finger CCCH-type containing 12A	80149	161,0759853	0,384493054	0,144622	2,658615475	0,007846246	0,132525621
ENSG00000165097	KDM1B	lysine demethylase 1B	221656	59,80166913	0,142509575	0,290566	0,490455504	0,623811609	0,90479929
ENSG00000169131	ZNF354A	zinc finger protein 354A	6940	88,04125551	-0,092455364	0,187692	-0,492589873	0,622302405	0,904764752
ENSG00000189190	ZNF600	zinc finger protein 600	162966	165,9023118	-0,192433121	0,169539	-1,135040121	0,256358508	0,712469825
ENSG00000143612	C1orf43	chromosome 1 open reading frame 43	25912	279,157937	-0,040982401	0,126051	-0,325125326	0,745086222	0,939745744
ENSG00000155858	LSM11	LSM11, U7 small nuclear RNA associated	134353	33,45548486	-0,038660642	0,305284	-0,126638197	0,899226764	NA
ENSG00000242247	ARFGAP3	ADP ribosylation factor GTPase activating protein 3	26286	356,1543939	-0,05952764	0,091731	-0,648939822	0,516377274	0,868791049
ENSG00000116874	WARS2	tryptophanyl tRNA synthetase 2, mitochondrial	10352	26,35009965	0,166350114	0,37665	0,441656596	0,658737723	NA
ENSG00000074266	EED	embryonic ectoderm development	8726	66,61010001	-0,112708526	0,268188	-0,420259931	0,674295578	0,923602509
ENSG00000101928	MOSPD1	motile sperm domain containing 1	56180	24,89613968	0,327054794	0,364332	0,897683065	0,36935454	NA
ENSG00000101193	GID8	GID complex subunit 8 homolog	54994	311,7615606	-0,105834881	0,095312	-1,110398864	0,266827188	0,722327312
ENSG00000153814	JAZF1	JAZF zinc finger 1	221895	210,4790643	0,090929195	0,118319	0,768506721	0,442186199	0,838678081
ENSG00000166851	PLK1	polo like kinase 1	5347	78,66774195	0,590181338	0,239813	2,461009297	0,013854678	0,18410007
ENSG00000178607	ERN1	endoplasmic reticulum to nucleus signaling 1	2081	835,7612374	-0,138684672	0,352454	-0,393483455	0,693962441	0,9283565
ENSG00000184708	EIF4ENIF1	eukaryotic translation initiation factor 4E nuclear import factor 1	56478	108,4015507	0,244903409	0,160647	1,524478675	0,12738918	0,563689485
ENSG00000197451	HNRNPAB	heterogeneous nuclear ribonucleoprotein A/B	3182	481,2844308	0,195842913	0,0931	2,103566408	0,035416287	0,299740858
ENSG00000114686	MRPL3	mitochondrial ribosomal protein L3	11222	157,7307889	0,184170375	0,137411	1,340291211	0,180150688	0,633917339
ENSG00000128578	STRIP2	striatin interacting protein 2	57464	12,25724139	-0,610975152	0,501528	-1,218227751	0,223137437	NA
ENSG00000104660	LEPROTL1	leptin receptor overlapping transcript like 1	23484	379,8153257	0,171991055	0,086596	1,986136849	0,047018131	0,347721656
ENSG00000115825	PRKD3	protein kinase D3	23683	789,2115631	0,171944114	0,065715	2,616503073	0,008883556	0,14473205
ENSG00000040341	STAU2	staufen double-stranded RNA binding protein 2	27067	404,0828674	0,101858933	0,099635	1,022322099	0,306628477	0,753759018
ENSG00000102753	KPNA3	karyopherin subunit alpha 3	3839	279,1421711	-0,013733532	0,103764	-0,132353812	0,894704446	0,977990819
ENSG00000127666	TICAM1	TIR domain containing adaptor molecule 1	148022	63,16956862	-0,046634038	0,211462	-0,22053113	0,825457531	0,961917534
ENSG00000147174	GCNA	germ cell nuclear acidic peptidase	93953	43,69841933	0,048283555	0,316208	0,152695398	0,87863849	0,976237908

ENSG00000075292	ZNF638	zinc finger protein 638	27332	428,4647828	0,122123393	0,121573	1,00452526	0,315125502	0,760367341
ENSG00000167770	OTUB1	OTU deubiquitinase, ubiquitin aldehyde binding 1	55611	170,0758192	0,021681701	0,144045	0,150520024	0,880354354	0,976300778
ENSG00000105186	ANKRD27	ankyrin repeat domain 27	84079	325,5505436	0,018049845	0,096338	0,187359315	0,85137892	0,967902179
ENSG00000147010	SH3KBP1	SH3 domain containing kinase binding protein 1	30011	193,7318342	0,031768887	0,142708	0,222614491	0,823835557	0,961917534
ENSG00000158882	TOMM40L	translocase of outer mitochondrial membrane 40 like	84134	99,15677772	0,160121234	0,17568	0,911438276	0,362064494	0,791409024
ENSG00000179933	C14orf119	chromosome 14 open reading frame 119	55017	132,5909398	0,418993419	0,271632	1,542505437	0,122950818	0,558690088
ENSG00000106268	NUDT1	nudix hydrolase 1	4521	25,05298669	0,82249246	0,359989	2,284771077	0,022326254	NA
ENSG00000109670	FBXW7	F-box and WD repeat domain containing 7	55294	135,0482906	-0,07769724	0,270408	-0,287333855	0,773856703	0,946448613
ENSG00000124406	ATP8A1	ATPase phospholipid transporting 8A1	10396	812,0444429	-0,055753636	0,076961	-0,724443158	0,468793712	0,851701553
ENSG00000144182	LIPT1	lipoyltransferase 1	51601	12,77780159	-0,288938408	0,531889	-0,543230238	0,58697129	NA
ENSG00000148158	SNX30	sorting nexin family member 30	401548	148,6644879	-0,22495102	0,165131	-1,362256162	0,173117057	0,629558603
ENSG00000155093	PTPRN2	protein tyrosine phosphatase receptor type N2	5799	67,24160279	-0,725514052	0,219539	-3,30471524	0,000950729	0,033747274
ENSG00000166025	AMOTL1	angiomotin like 1	154810	19,45046705	0,293215776	0,416621	0,703795592	0,481560079	NA
ENSG00000167699	GLOD4	glyoxalase domain containing 4	51031	224,6915644	-0,097111722	0,131727	-0,737218201	0,460989667	0,846765498
ENSG00000184860	SDR42E1	short chain dehydrogenase/reductase family 42E, member 1	93517	105,9276714	0,187944403	0,175834	1,068871124	0,285127745	0,737867955
ENSG00000262664	OVCA2	OVCA2 serine hydrolase domain containing	124641	75,98675525	0,228678129	0,194898	1,173319349	0,240667766	0,695620773
ENSG00000159055	MIS18A	MIS18 kinetochore protein A	54069	22,20705884	0,42029081	0,398829	1,05381145	0,29196925	NA
ENSG00000112237	CCNC	cyclin C	892	126,1777748	-0,101008389	0,15023	-0,672357014	0,501356442	0,862227298
ENSG00000133812	SBF2	SET binding factor 2	81846	24,27669775	0,065648825	0,478431	0,137216778	0,89085945	NA
ENSG00000186480	INSIG1	insulin induced gene 1	3638	187,9141602	-0,100214168	0,124526	-0,804766998	0,42095415	0,827182516
ENSG00000168404	MLKL	mixed lineage kinase domain like pseudokinase	197259	70,55162383	0,194037526	0,212574	0,91280129	0,36134706	0,791408368
ENSG00000142784	WDTC1	WD and tetratricopeptide repeats 1	23038	299,869702	-0,10745275	0,145376	-0,739137356	0,459823595	0,846622987
ENSG00000225697	SLC26A6	solute carrier family 26 member 6	65010	53,67241366	0,488198673	0,262802	1,857666355	0,063216409	0,404317327
ENSG00000139116	KIF21A	kinesin family member 21A	55605	170,3833979	0,311353911	0,233766	1,331902514	0,182892225	0,637282846
ENSG00000103932	RPAP1	RNA polymerase II associated protein 1	26015	85,34058605	0,38157191	0,337607	1,130225237	0,258381328	0,714048892
ENSG00000188487	INSC	INSC spindle orientation adaptor protein	387755	50,75272479	-0,648558566	0,260344	-2,491164132	0,012732529	0,177307233
ENSG00000160310	PRMT2	protein arginine methyltransferase 2	3275	471,7675558	-0,106713303	0,086685	-1,231045939	0,218305683	0,673519155
ENSG00000124120	TTPAL	alpha tocopherol transfer protein like	79183	149,0726066	0,123269917	0,180252	0,683874076	0,494054685	0,860513824
ENSG00000015285	WAS	WASP actin nucleation promoting factor	7454	470,7238642	-0,198105928	0,099217	-1,996689748	0,045858897	0,342372408
ENSG00000196531	NACA	nascent polypeptide associated complex subunit alpha	4666	706,2595266	0,267927061	0,068281	3,923897873	8,71E-05	0,005799159
ENSG00000143772	ITPKB	inositol-trisphosphate 3-kinase B	3707	676,2765187	-0,089292186	0,068491	-1,303707191	0,192333437	0,649982152
ENSG00000155506	LARP1	La ribonucleoprotein 1, translational regulator	23367	908,6081864	-0,063890228	0,062579	-1,020951498	0,307277418	0,754087936
ENSG00000164654	MIOS	meiosis regulator for oocyte development	54468	75,64945106	0,099414746	0,197667	0,502941191	0,61500562	0,901934707
ENSG00000173473	SMARCC1	SWI/SNF related, matrix associated, actin dependent regulator of chromatin subfamily c member 1	6599	488,7173671	-0,231868416	0,115129	-2,013993163	0,044010251	0,335914979
ENSG00000174013	FBXO45	F-box protein 45	200933	86,4967411	-0,150608574	0,267264	-0,563519359	0,573081281	0,886892687
ENSG00000197226	TBC1D9B	TBC1 domain family member 9B	23061	443,8347441	0,022213805	0,110021	0,201904868	0,839991096	0,966494297
ENSG00000198356	GET3	guided entry of tail-anchored proteins factor 3, ATPase	439	160,9049426	-0,256080445	0,163486	-1,56637726	0,117260323	0,54624115
ENSG00000001630	CYP51A1	cytochrome P450 family 51 subfamily A member 1	1595	271,9543738	-0,043821819	0,109791	-0,399138745	0,689790975	0,927252461
ENSG00000278053	DDX52	DExD-box helicase 52	11056	120,6258902	-0,176374657	0,226731	-0,777902345	0,436626586	0,836814349
ENSG00000211455	STK38L	serine/threonine kinase 38 like	23012	114,7211191	0,231857489	0,171916	1,348665789	0,177444339	0,632039836
ENSG00000131725	WDR44	WD repeat domain 44	54521	175,7704128	0,162445581	0,134261	1,209927767	0,226306611	0,681621061
ENSG00000176393	RNPEP	arginyl aminopeptidase	6051	150,1262168	0,07527995	0,132357	0,568764943	0,569515668	0,886263374
ENSG00000083750	RRAGB	Ras related GTP binding B	10325	28,49854111	-0,196862118	0,444784	-0,442601671	0,658053876	NA
ENSG00000131732	ZCCHC9	zinc finger CCHC-type containing 9	84240	61,27678145	0,117399446	0,248934	0,471609003	0,6372059	0,90943397

ENSG00000154265	ABCA5	ATP binding cassette subfamily A member 5	23461	145,9368406	-0,233144651	0,171517	-1,359312217	0,174047674	0,631278097
ENSG00000119979	DENND10	DENN domain containing 10	404636	113,6072566	-0,03804547	0,183961	-0,206812496	0,836156297	0,964625669
ENSG00000176853	FAM91A1	family with sequence similarity 91 member A1	157769	363,770455	-0,094993836	0,107247	-0,885745598	0,375754631	0,8032599
ENSG00000075856	SART3	spliceosome associated factor 3, U4/U6 recycling protein	9733	251,5038956	-0,073233901	0,154731	-0,473297157	0,63600119	0,909036096
ENSG00000141027	NCOR1	nuclear receptor corepressor 1	9611	988,6044499	0,026676943	0,097418	0,273840942	0,784206858	0,95013509
ENSG00000186017	ZNF566	zinc finger protein 566	84924	41,01883245	0,270514002	0,311824	0,867520972	0,385656626	NA
ENSG00000042445	RETSAT	retinol saturase	54884	62,3018242	0,040537357	0,250258	0,161982468	0,87131966	0,974150353
ENSG00000072501	SMC1A	structural maintenance of chromosomes 1A	8243	971,921475	0,176411559	0,076266	2,313110767	0,02071655	0,225859999
ENSG00000113088	GZMK	granzyme K	3003	174,8602165	-0,170662164	0,141155	-1,209043835	0,226646005	0,681621061
ENSG00000159363	ATP13A2	ATPase cation transporting 13A2	23400	142,8900599	0,080626225	0,201448	0,400233314	0,688984679	0,926748702
ENSG00000124496	TRERF1	transcriptional regulating factor 1	55809	456,6483847	-0,09069439	0,103657	-0,874951002	0,381600567	0,806821656
ENSG00000101577	LPIN2	lipin 2	9663	344,2151211	0,106897563	0,095271	1,122040657	0,261845154	0,717555845
ENSG00000174227	PIGG	phosphatidylinositol glycan anchor biosynthesis class G	54872	147,899594	-0,146867024	0,167742	-0,875552077	0,381273588	0,806821656
ENSG00000162819	BROX	BRO1 domain and CAAX motif containing	148362	217,0630425	-0,253041149	0,135832	-1,862898176	0,062476593	0,404317327
ENSG00000197329	PELI1	pellino E3 ubiquitin protein ligase 1	57162	27,08754474	-0,226263483	0,352417	-0,642033768	0,520851258	NA
ENSG00000170946	DNAJC24	DnaJ heat shock protein family (Hsp40) member C24	120526	51,79974681	0,060884587	0,289695	0,210167921	0,833536616	0,963446071
ENSG00000179750	APOBEC3B	apolipoprotein B mRNA editing enzyme catalytic subunit 3B	9582	29,24361771	0,778907579	0,338063	2,304028373	0,021221051	NA
ENSG00000063438	AHRR	aryl hydrocarbon receptor repressor	57491	543,911316	0,106273751	0,077832	1,365424871	0,172119551	0,629558603
ENSG00000106992	AK1	adenylate kinase 1	203	75,65064168	-0,719031066	0,278919	-2,577916369	0,009939805	0,153700013
ENSG00000214087	ARL16	ADP ribosylation factor like GTPase 16	339231	20,89485522	-0,502259661	0,46617	-1,077417998	0,281293567	NA
ENSG00000150776	NKAPD1	NKAP domain containing 1	55216	121,2856888	-0,065653894	0,179749	-0,365253174	0,714922429	0,932016096
ENSG00000177051	FBXO46	F-box protein 46	23403	76,32630793	-0,165455626	0,218239	-0,758140163	0,448367079	0,84263906
ENSG00000124782	RREB1	ras responsive element binding protein 1	6239	167,7536708	-0,241866816	0,13094	-1,847161272	0,06472377	0,410155553
ENSG00000013503	POLR3B	RNA polymerase III subunit B	55703	43,17833428	0,070958148	0,289562	0,245053538	0,806414977	0,958037095
ENSG00000226979	LTA	lymphotoxin alpha	4049	17,17865877	0,266654725	0,693856	0,38430842	0,700749868	NA
ENSG00000104814	MAP4K1	mitogen-activated protein kinase kinase kinase kinase 1	11184	494,8549752	-0,106072496	0,096401	-1,100325407	0,271190366	0,725477256
ENSG00000107864	CPEB3	cytoplasmic polyadenylation element binding protein 3	22849	39,99174979	0,000816986	0,295494	0,00276481	0,997794003	NA
ENSG00000114383	TUSC2	tumor suppressor 2, mitochondrial calcium regulator	11334	61,61767535	-0,02302608	0,2146	-0,107297831	0,914552705	0,981270075
ENSG00000137310	TCF19	transcription factor 19	6941	81,69704636	0,193767831	0,308102	0,628908868	0,52940872	0,872145761
ENSG00000142089	IFITM3	interferon induced transmembrane protein 3	10410	15,29609398	-0,357470823	0,477848	-0,748085232	0,454408749	NA
ENSG00000143776	CDC42BPA	CDC42 binding protein kinase alpha	8476	215,0873185	-0,222846213	0,145578	-1,530764565	0,12582759	0,563113596
ENSG00000149196	HIKESHI	heat shock protein nuclear import factor hikeshi	51501	62,86347096	0,18387179	0,231979	0,792621389	0,427998443	0,832060923
ENSG00000159348	CYB5R1	cytochrome b5 reductase 1	51706	51,06666193	-0,173077884	0,256579	-0,674559354	0,499955764	0,86161226
ENSG00000162337	LRP5	LDL receptor related protein 5	4041	67,03466045	0,610902309	0,252849	2,416078775	0,015688667	0,196835126
ENSG00000180182	MED14	mediator complex subunit 14	9282	280,5506807	0,041033904	0,117414	0,349480911	0,726728299	0,936607802
ENSG00000185298	CCDC137	coiled-coil domain containing 137	339230	71,39730694	-0,110930503	0,237671	-0,466740339	0,640685665	0,911523706
ENSG00000109063	MYH3	myosin heavy chain 3	4621	14,87081015	0,28205292	0,513394	0,549388461	0,582738891	NA
ENSG00000135040	NAA35	N-alpha-acetyltransferase 35, NatC auxiliary subunit	60560	184,1820332	0,075407132	0,134681	0,559896047	0,575550345	0,887069818
ENSG00000167112	TRUB2	TruB pseudouridine synthase family member 2	26995	111,1937771	-0,137865479	0,168112	-0,820081705	0,412169531	0,822245236
ENSG00000008710	PKD1	polycystin 1, transient receptor potential channel interacting	5310	331,5880475	-0,092997416	0,095555	-0,973232464	0,330437779	0,771479881
ENSG00000060688	SNRNP40	small nuclear ribonucleoprotein U5 subunit 40	9410	139,8486177	0,029879108	0,153449	0,194717376	0,845614208	0,967231052
ENSG00000130332	LSM7	LSM7 homolog, U6 small nuclear RNA and mRNA degradation associated	51690	56,70991203	-0,164588582	0,248195	-0,663143306	0,507238778	0,864738156
ENSG00000146733	PSPH	phosphoserine phosphatase	5723	19,67637579	0,655533728	0,485503	1,350215178	0,176946971	NA
ENSG00000284770	TBCE	tubulin folding cofactor E	6905	23,79465692	2,922564421	1,355664	2,155817795	0,031097895	NA
ENSG00000072364	AFF4	ALF transcription elongation factor 4	27125	556,4501129	0,050712371	0,08418	0,602425654	0,546890838	0,878739902

ENSG00000153046	CDYL	chromodomain Y like	9425	156,7733741	0,106965129	0,146335	0,730959733	0,464803749	0,848969452
ENSG00000169896	ITGAM	integrin subunit alpha M	3684	1629,000797	0,107075867	0,055379	1,933497252	0,053174971	0,372224799
ENSG00000171843	MLLT3	MLLT3 super elongation complex subunit	4300	124,9789039	-0,017467093	0,207943	-0,083999255	0,933057024	0,987098279
ENSG00000187531	SIRT7	sirtuin 7	51547	48,22027769	-0,038286328	0,400619	-0,095567928	0,923863739	0,984284131
ENSG00000180398	MCFD2	multiple coagulation factor deficiency 2, ER cargo receptor complex subunit	90411	161,274136	-0,234176751	0,14557	-1,608688905	0,107684383	0,527254203
ENSG00000178878	APOLD1	apolipoprotein L domain containing 1	81575	25,03709943	0,259565275	0,349209	0,743295978	0,457302506	NA
ENSG00000109881	CCDC34	coiled-coil domain containing 34	91057	13,10065211	0,58484511	0,641494	0,911692728	0,361930493	NA
ENSG00000137575	SDCBP	syndecan binding protein	6386	284,9613328	-0,046519651	0,106142	-0,438278379	0,661184497	0,91978661
ENSG00000164117	FBXO8	F-box protein 8	26269	28,86465608	0,156873708	0,393595	0,398566451	0,690212686	NA
ENSG00000173930	SLCO4C1	solute carrier organic anion transporter family member 4C1	353189	100,9664879	-0,122313411	0,163321	-0,748915295	0,453908261	0,843234809
ENSG00000120509	PDZD11	PDZ domain containing 11	51248	52,99610314	0,049725136	0,302499	0,164381423	0,869430887	0,973310365
ENSG00000163249	CCNYL1	cyclin Y like 1	151195	48,94517607	0,326931174	0,258416	1,265136549	0,205822383	0,660954946
ENSG00000157978	LDLRAP1	low density lipoprotein receptor adaptor protein 1	26119	65,55037892	-0,16435087	0,257952	-0,637137778	0,524035103	0,870612376
ENSG00000126787	DLGAP5	DLG associated protein 5	9787	108,3692031	0,6186784	0,179799	3,440936699	0,000579704	0,022961201
ENSG00000171067	C11orf24	chromosome 11 open reading frame 24	53838	73,29817068	-0,096230077	0,218888	-0,439632533	0,660203274	0,919368176
ENSG00000185624	P4HB	prolyl 4-hydroxylase subunit beta	5034	1149,438953	-0,021164967	0,062031	-0,341199524	0,732953382	0,93912057
ENSG00000104324	CPQ	carboxypeptidase Q	10404	48,90044403	-0,249767712	0,289797	-0,861871119	0,388758443	0,810326119
ENSG00000139354	GAS2L3	growth arrest specific 2 like 3	283431	14,1535559	-0,189794513	0,526709	-0,360340094	0,718592819	NA
ENSG00000168385	SEPTIN2	septin 2	4735	1863,196885	-0,086278142	0,054651	-1,578713523	0,114401782	0,54024053
ENSG00000105866	SP4	Sp4 transcription factor	6671	166,6751216	-5,72E-05	0,184607	-0,000309636	0,999752946	0,999764435
ENSG00000117408	IPO13	importin 13	9670	137,9015161	-0,014630512	0,167005	-0,087605485	0,930190243	0,986487751
ENSG00000120458	MSANTD2	Myb/SANT DNA binding domain containing 2	79684	51,57943337	-0,103558939	0,387219	-0,267442882	0,789128186	0,95148885
ENSG00000136104	RNASEH2B	ribonuclease H2 subunit B	79621	81,05545941	0,973505448	0,416022	2,340031424	0,019282117	0,217588722
ENSG00000158623	COPG2	COPI coat complex subunit gamma 2	26958	60,92052586	0,122843955	0,227891	0,539047005	0,589854421	0,892279781
ENSG00000160917	CPSF4	cleavage and polyadenylation specific factor 4	10898	55,47188302	0,475645658	0,26482	1,796106194	0,072477629	0,437369972
ENSG00000198911	SREBF2	sterol regulatory element binding transcription factor 2	6721	667,6025599	-0,002047858	0,069126	-0,029625182	0,976365982	0,995074982
ENSG00000196975	ANXA4	annexin A4	307	76,87482502	0,113248026	0,199146	0,568669248	0,569580621	0,886263374
ENSG00000197429	IPP	intracisternal A particle-promoted polypeptide	3652	55,7228892	0,024947166	0,286196	0,087168006	0,93053797	0,986491322
ENSG00000131966	ACTR10	actin related protein 10	55860	60,43923148	-0,236375252	0,46771	-0,505388206	0,613286201	0,900495387
ENSG00000011143	MKS1	MKS transition zone complex subunit 1	54903	15,69167465	0,182877546	0,69788	0,262047149	0,793285092	NA
ENSG00000086015	MAST2	microtubule associated serine/threonine kinase 2	23139	150,7340037	0,204037924	0,13731	1,485966527	0,137287975	0,57855532
ENSG00000090376	IRAK3	interleukin 1 receptor associated kinase 3	11213	66,62326555	-0,159252025	0,231531	-0,687821464	0,491565218	0,860513824
ENSG00000175390	EIF3F	eukaryotic translation initiation factor 3 subunit F	8665	257,9576276	0,178084259	0,172675	1,031324239	0,302388795	0,751870158
ENSG00000185164	NOMO2	NODAL modulator 2	283820	504,1001722	0,177284389	0,100205	1,769209808	0,076858868	0,448810074
ENSG00000185164	LOC102723728	nodal modulator 3-like	102723728	504,1001722	0,177284389	0,100205	1,769209808	0,076858868	0,448810074
ENSG00000229419	RALGAPA1P1	RALGAPA1 pseudogene 1	26134	11,76363194	0,109638798	0,616582	0,17781693	0,858866751	NA
ENSG00000052841	TTC17	tetratricopeptide repeat domain 17	55761	315,1240146	0,042489212	0,103432	0,410791732	0,681225256	0,925586358
ENSG00000101474	APMAP	adipocyte plasma membrane associated protein	57136	661,357034	-0,037880404	0,068554	-0,552560716	0,580564248	0,888049268
ENSG00000105778	AVL9	AVL9 cell migration associated	23080	203,4912172	-0,077142627	0,130142	-0,592758576	0,553342734	0,882785665
ENSG00000112977	DAP	death associated protein	1611	252,6264253	0,026645859	0,116603	0,228516904	0,819244416	0,961067311
ENSG00000158161	EYA3	EYA transcriptional coactivator and phosphatase 3	2140	184,7287395	-0,387954809	0,15774	-2,459464925	0,013914429	0,18410007
ENSG00000111481	COPZ1	COPI coat complex subunit zeta 1	22818	288,8437317	-0,267857133	0,150765	-1,77665737	0,07562463	0,446229554
ENSG00000143801	PSEN2	presenilin 2	5664	55,2920818	-0,300808221	0,357688	-0,840980295	0,400358974	0,814673014
ENSG00000176390	CRLF3	cytokine receptor like factor 3	51379	208,3457779	-0,094867638	0,140739	-0,674069115	0,500267374	0,86161226
ENSG00000185088	RPS27L	ribosomal protein S27 like	51065	154,206632	0,631608196	0,167719	3,765863564	0,000165974	0,009656023
ENSG00000166068	SPRED1	sprouty related EVH1 domain containing 1	161742	14,16422348	0,133905408	0,598959	0,22356345	0,823097007	NA
ENSG00000176083	ZNF683	zinc finger protein 683	257101	693,8438113	0,090771691	0,068923	1,317001109	0,187838253	0,645920156
ENSG00000117877	POLR1G	RNA polymerase I subunit G	10849	24,42133699	0,488759649	0,411254	1,18846184	0,234651506	NA
ENSG00000137804	NUSAP1	nucleolar and spindle associated protein 1	51203	107,6142024	0,734723927	0,256255	2,867158647	0,004141753	0,088853402

ENSG00000141380	SS18	SS18 subunit of BAF chromatin remodeling complex	6760	314,1155416	-0,075705275	0,127503	-0,593753107	0,552677257	0,882068269
ENSG00000111911	HINT3	histidine triad nucleotide binding protein 3	135114	72,07152633	-0,251076874	0,207195	-1,211787646	0,225593679	0,681466443
ENSG00000134375	TIMM17A	translocase of inner mitochondrial membrane 17A	10440	87,38060486	0,390794465	0,192457	2,030559282	0,042299722	0,327927224
ENSG00000145687	SSBP2	single stranded DNA binding protein 2	23635	62,95490381	0,08236328	0,27154	0,303318746	0,761646961	0,942934881
ENSG00000163617	CCDC191	coiled-coil domain containing 191	57577	12,94828196	-0,107961525	0,467951	-0,230711149	0,817539211	NA
ENSG00000175198	PCCA	propionyl-CoA carboxylase subunit alpha	5095	53,15999598	-0,080370806	0,251716	-0,319292174	0,749504966	0,940653884
ENSG00000188171	ZNF626	zinc finger protein 626	199777	22,48308044	-0,070688024	0,398913	-0,177201807	0,859349876	NA
ENSG00000105447	GRWD1	glutamate rich WD repeat containing 1	83743	136,5093738	-0,024177586	0,14932	-0,161917531	0,871370798	0,974150353
ENSG00000144749	LRIG1	leucine rich repeats and immunoglobulin like domains 1	26018	230,1569114	0,168290643	0,133781	1,257960311	0,20840611	0,662366797
ENSG00000117410	ATP6V0B	ATPase H+ transporting V0 subunit b	533	96,89920561	0,30367792	0,174592	1,739359026	0,081971631	0,465718853
ENSG00000105229	PIAS4	protein inhibitor of activated STAT 4	51588	87,09501041	-0,040800461	0,184799	-0,220782774	0,825261577	0,961917534
ENSG00000107957	SH3PXD2A	SH3 and PX domains 2A	9644	188,4224416	-0,200978529	0,122102	-1,645985534	0,099766737	0,509429216
ENSG00000141385	AFG3L2	AFG3 like matrix AAA peptidase subunit 2	10939	198,2680075	0,026611976	0,122465	0,217302193	0,827972848	0,962723654
ENSG00000173275	ZNF449	zinc finger protein 449	203523	41,60403757	-0,008170347	0,28875	-0,028295599	0,977426391	NA
ENSG00000070366	SMG6	SMG6 nonsense mediated mRNA decay factor	23293	413,7329472	-0,050141162	0,146626	-0,341965691	0,732376712	0,93912057
ENSG00000183726	TMEM50A	transmembrane protein 50A	23585	416,2273214	0,132887341	0,079931	1,662519877	0,096408581	0,502784194
ENSG00000196391	ZNF774	zinc finger protein 774	342132	17,69587635	0,310000313	0,443695	0,698679051	0,484752629	NA
ENSG00000068654	POLR1A	RNA polymerase I subunit A	25885	519,8679643	-0,024593112	0,074274	-0,331114237	0,740558198	0,939193793
ENSG00000151651	ADAM8	ADAM metallopeptidase domain 8	101	855,6754349	-0,133260458	0,060903	-2,188079742	0,028663794	0,272623046
ENSG00000188177	ZC3H6	zinc finger CCCH-type containing 6	376940	84,52683079	-0,169888911	0,209784	-0,809827814	0,418039144	0,82446781
ENSG00000128739	SNRPN	small nuclear ribonucleoprotein polypeptide N	6638	157,6340177	-0,053912348	0,136227	-0,395754074	0,69228646	0,927787546
ENSG00000160993	ALKBH4	alkB homolog 4, lysine demethylase	54784	32,57997739	0,490074774	0,37944	1,291574974	0,196504375	NA
ENSG00000136100	VPS36	vacuolar protein sorting 36 homolog	51028	353,5490844	-0,126646282	0,101872	-1,243196001	0,213795616	0,668717948
ENSG00000171262	FAM98B	family with sequence similarity 98 member B	283742	113,7649231	-0,034741352	0,176874	-0,196418601	0,844282538	0,967231052
ENSG00000167930	FAM234A	family with sequence similarity 234 member A	83986	45,20455714	-0,145103403	0,354352	-0,409489108	0,682180758	0,925586358
ENSG00000141458	NPC1	NPC intracellular cholesterol transporter 1	4864	192,7948009	0,064200854	0,124673	0,514953514	0,606585504	0,897481254
ENSG00000158373	H2BC5	H2B clustered histone 5	3017	16,3771626	0,632499078	0,523732	1,207675892	0,227171955	NA
ENSG00000154898	NA	NA	NA	12,38569503	-0,583956716	0,52671	-1,108688053	0,267564777	NA
ENSG00000178127	NDUFV2	NADH:ubiquinone oxidoreductase core subunit V2	4729	135,2132878	0,126419864	0,153681	0,822612798	0,410728218	0,82130516
ENSG00000237065	NA	NA	NA	12,39472376	0,987771846	0,478389	2,06478709	0,03894315	NA
ENSG00000258366	RTEL1	regulator of telomere elongation helicase 1	51750	59,39397631	0,030066894	1,123045	0,026772651	0,978641067	0,995712569
ENSG00000184384	MAML2	mastermind like transcriptional coactivator 2	84441	332,9564519	-0,160646123	0,122164	-1,31500652	0,188507711	0,646436727
ENSG00000070444	MNT	MAX network transcriptional repressor	4335	109,9066808	-0,020607109	0,212534	-0,096959286	0,922758727	0,984284131
ENSG00000123064	DDX54	DEAD-box helicase 54	79039	214,1016413	-0,192178679	0,114724	-1,675140071	0,093906752	0,49738244
ENSG00000125652	ALKBH7	alkB homolog 7	84266	30,28240025	-0,287283651	0,322491	-0,890827231	0,373021864	NA
ENSG00000138468	SENP7	SUMO specific peptidase 7	57337	329,7433949	-0,105356632	0,101504	-1,037951636	0,299292572	0,750045903
ENSG00000166452	AKIP1	A-kinase interacting protein 1	56672	23,52257804	0,342726823	0,384579	0,89117452	0,372835552	NA
ENSG00000167515	TRAPPC2L	trafficking protein particle complex subunit 2L	51693	58,650357	1,374918139	0,455516	3,018377506	0,002541321	0,06316309
ENSG00000185730	ZNF696	zinc finger protein 696	79943	32,55396524	-0,576774175	0,325606	-1,771387724	0,076496249	NA
ENSG00000168883	USP39	ubiquitin specific peptidase 39	10713	196,1255707	0,269661226	0,136014	1,982605131	0,047411554	0,349139303
ENSG00000136059	VILL	villin like	50853	60,3374047	-0,471581529	0,247201	-1,907685429	0,056431886	0,385405247
ENSG00000013441	CLK1	CDC like kinase 1	1195	67,14282888	-0,052233552	0,36625	-0,142617047	0,886592634	0,977928921
ENSG00000142875	PRKACB	protein kinase cAMP-activated catalytic subunit beta	5567	1035,05945	-0,018689031	0,055883	-0,334428659	0,738056117	0,939193793
ENSG00000176058	TPRN	taperin	286262	20,0931958	-0,59965821	0,660586	-0,907767116	0,364001276	NA
ENSG00000198792	TMEM184B	transmembrane protein 184B	25829	176,1127783	-0,078276898	0,126812	-0,617265498	0,53705962	0,874923109
ENSG00000250462	NA	NA	NA	15,41087853	0,337336412	0,485304	0,695103401	0,486990503	NA

ENSG00000102181	CD99L2	CD99 molecule like 2	83692	113,2182442	-0,386036348	0,193391	-1,996142101	0,045918457	0,342491515
ENSG00000118007	STAG1	STAG1 cohesin complex component	10274	254,4717395	0,213051021	0,114084	1,867496267	0,061832318	0,402854784
ENSG00000197050	ZNF420	zinc finger protein 420	147923	37,75682147	-0,1541757	0,304807	-0,505814057	0,61298719	NA
ENSG00000228253	ATP8	ATP synthase F0 subunit 8	4509	1337,526485	0,288856594	0,180438	1,600866155	0,109406567	0,529522903
ENSG00000090273	NUDC	nuclear distribution C, dynein complex regulator	10726	183,6210475	-0,058521699	0,151803	-0,385510624	0,699859137	0,930783276
ENSG00000100151	PICK1	protein interacting with PRKCA 1	9463	39,03898166	-0,949730517	0,305727	-3,10646419	0,001893392	NA
ENSG00000136631	VPS45	vacuolar protein sorting 45 homolog	11311	104,264953	0,407951871	0,626386	0,651278661	0,514866621	0,868514045
ENSG00000138385	SSB	small RNA binding exonuclease protection factor La	6741	284,9844827	0,054601674	0,116418	0,469015983	0,639058212	0,910510986
ENSG00000140367	UBE2Q2	ubiquitin conjugating enzyme E2 Q2	92912	242,9750235	-0,052070413	0,116113	-0,448444125	0,653832703	0,917134445
ENSG00000160211	G6PD	glucose-6-phosphate dehydrogenase	2539	423,1495333	-0,0365326	0,10458	-0,349328169	0,726842953	0,936607802
ENSG00000171603	CLSTN1	calsyntenin 1	22883	314,5984277	-0,23408088	0,156993	-1,491027591	0,135954251	0,576449438
ENSG00000181396	OGFOD3	2-oxoglutarate and iron dependent oxygenase domain containing 3	79701	51,58859291	0,646902718	0,27409	2,360186682	0,018265741	0,211904176
ENSG00000204611	ZNF616	zinc finger protein 616	90317	41,36319899	-0,280631222	0,430203	-0,652322252	0,514193308	NA
ENSG00000233369	GTF2IP4	general transcription factor IIi pseudogene 4	100093631	394,4702457	0,250022398	0,108082	2,313264459	0,020708103	0,225859999
ENSG00000256525	POLG2	DNA polymerase gamma 2, accessory subunit	11232	23,75902386	-0,782680288	0,378484	-2,067934516	0,038646181	NA
ENSG00000145725	PPIP5K2	diphosphoinositol pentakisphosphate kinase 2	23262	360,9672576	-0,098459462	0,13646	-0,721523907	0,470587237	0,852690218
ENSG00000198677	SKIC3	SKI3 subunit of superkiller complex	9652	327,3712863	-0,208292256	0,094678	-2,200003293	0,027806661	0,269631574
ENSG00000221866	PLXNA4	plexin A4	91584	582,5625705	-0,097346917	0,08433	-1,154362049	0,248351767	0,706211
ENSG00000256797	KLRF2	killer cell lectin like receptor F2	100431172	99,23449689	0,270615427	0,175438	1,542509456	0,122949842	0,558690088
ENSG00000058668	ATP2B4	ATPase plasma membrane Ca2+ transporting 4	493	1204,006131	0,028124617	0,062735	0,448311637	0,653928303	0,917134445
ENSG00000129636	ITFG1	integrin alpha FG-GAP repeat containing 1	81533	102,9761116	0,016870612	0,278209	0,060640024	0,951645898	0,991212866
ENSG00000171163	ZNF692	zinc finger protein 692	55657	29,26385754	1,004663052	0,45916	2,188046118	0,028666243	NA
ENSG00000165775	FUNDC2	FUN14 domain containing 2	65991	33,84973732	0,32455731	0,319104	1,017089397	0,309110904	NA
ENSG00000141499	WRAP53	WD repeat containing antisense to TP53	55135	38,22288837	0,801714422	0,41137	1,948890986	0,051308445	NA
ENSG00000187778	MCRS1	microspherule protein 1	10445	261,2697502	-0,247103881	0,131266	-1,882464315	0,059773001	0,395835192
ENSG00000132405	TBC1D14	TBC1 domain family member 14	57533	479,7332925	-0,07857928	0,080395	-0,977413067	0,328364697	0,770763995
ENSG00000142208	AKT1	AKT serine/threonine kinase 1	207	483,2765343	-0,152527875	0,1059	-1,440297951	0,149783121	0,604831173
ENSG00000087299	L2HGDH	L-2-hydroxyglutarate dehydrogenase	79944	34,57084896	0,770124412	0,409708	1,87969114	0,060150184	NA
ENSG00000106012	IQCE	IQ motif containing E	23288	70,17905732	-0,27506027	0,266812	-1,030912983	0,302581628	0,751870158
ENSG00000058799	YIPF1	Yip1 domain family member 1	54432	44,16145416	-0,287397621	0,317325	-0,905689349	0,365100302	0,793880889
ENSG00000104852	SNRNP70	small nuclear ribonucleoprotein U1 subunit 70	6625	241,4222038	-0,032664084	0,155717	-0,209766138	0,833850203	0,963446071
ENSG00000204178	MACO1	macoilin 1	55219	111,8020506	-0,366618231	0,299289	-1,224963431	0,220588999	0,677095199
ENSG00000040531	CTNS	cystinosin, lysosomal cystine transporter	1497	59,42232525	0,125698393	0,219024	0,573901087	0,566034737	0,88521771
ENSG00000065457	ADAT1	adenosine deaminase tRNA specific 1	23536	204,5535974	0,041116166	0,195809	0,209981136	0,833682396	0,963446071
ENSG00000105298	CACTIN	cactin, spliceosome C complex subunit	58509	109,6921641	-0,195009669	0,162148	-1,202664994	0,229105986	0,68351286
ENSG00000136450	SRSF1	serine and arginine rich splicing factor 1	6426	754,4481787	-0,038350315	0,070605	-0,543169537	0,587013079	0,891326322
ENSG00000233757	ZNF892	zinc finger protein 892	344065	42,77241535	-0,229689809	0,263872	-0,870460226	0,384048946	0,808586507
ENSG00000266412	NCOA4	nuclear receptor coactivator 4	8031	899,3429426	-0,015944761	0,056985	-0,279808016	0,779624802	0,947712923
ENSG00000122435	TRMT13	tRNA methyltransferase 13 homolog	54482	83,82281889	-0,342081258	0,263737	-1,297052215	0,194613221	0,652365446
ENSG00000078668	VDAC3	voltage dependent anion channel 3	7419	241,9905145	0,296955885	0,116434	2,550421706	0,010759269	0,161574181
ENSG00000120093	HOXB3	homeobox B3	3213	111,5780377	-0,188692343	0,166651	-1,132259479	0,257525361	0,713362633
ENSG00000058729	RIOK2	RIO kinase 2	55781	85,00151652	0,175717915	0,203107	0,865149446	0,386956769	0,809653368
ENSG00000033178	UBA6	ubiquitin like modifier activating enzyme 6	55236	471,3913246	0,14540107	0,145184	1,001494717	0,316587693	0,761322639
ENSG00000114573	ATP6V1A	ATPase H+ transporting V1 subunit A	523	196,0254273	-0,271809509	0,134086	-2,027125696	0,04264956	0,328150469
ENSG00000123106	CCDC91	coiled-coil domain containing 91	55297	133,1776642	0,289941046	0,160253	1,809270953	0,070408919	0,430678857

ENSG00000157426	AASDH	aminoadipate-semialdehyde dehydrogenase	132949	49,23731947	0,268813945	0,270515	0,993710619	0,320363771	0,76448423
ENSG00000119048	UBE2B	ubiquitin conjugating enzyme E2 B	7320	78,95366763	-0,052649083	0,21714	-0,242466534	0,808418686	0,958576399
ENSG00000186908	ZDHHC17	zinc finger DHHC-type palmitoyltransferase 17	23390	186,0698156	-0,189658681	0,125643	-1,509510403	0,131168399	0,567474728
ENSG00000101224	CDC25B	cell division cycle 25B	994	667,673918	-0,012493924	0,06643	-0,188075893	0,850817158	0,967902179
ENSG00000107819	SFXN3	sideroflexin 3	81855	163,4475017	0,009349278	0,15328	0,06099495	0,951363231	0,991212866
ENSG00000119487	MAPKAP1	MAPK associated protein 1	79109	234,9340037	-0,116578619	0,134447	-0,867094154	0,385890424	0,809653368
ENSG00000171159	BBLN	bublin coiled coil protein	79095	130,1579021	-0,487351441	0,146825	-3,319259368	0,000902566	0,032517201
ENSG00000171502	COL24A1	collagen type XXIV alpha 1 chain	255631	46,58549797	0,242484611	0,271309	0,893756455	0,371452212	0,800231702
ENSG00000273841	TAF9	TATA-box binding protein associated factor 9	6880	110,2829343	-0,180983624	0,176204	-1,027126416	0,304360942	0,751870158
ENSG00000115234	SNX17	sorting nexin 17	9784	259,2050797	-0,214712673	0,182971	-1,17347943	0,240603601	0,695620773
ENSG00000166123	GPT2	glutamic--pyruvic transaminase 2	84706	34,62270157	0,293114077	0,343011	0,854532733	0,392809881	NA
ENSG00000101445	PPP1R16B	protein phosphatase 1 regulatory subunit 16B	26051	1360,628063	0,012712566	0,050569	0,251388768	0,801513551	0,956122858
ENSG00000119737	GPR75	G protein-coupled receptor 75	10936	26,9633481	-0,220696743	0,366954	-0,601428202	0,54755482	NA
ENSG00000164535	DAGLB	diacylglycerol lipase beta	221955	29,18805052	0,991125733	0,381376	2,59881192	0,009354701	NA
ENSG00000257365	FNTB	farnesyltransferase, CAAX box, beta	2342	21,44357595	0,896387346	0,684722	1,309125577	0,190491817	NA
ENSG00000257365	CHURC1-FNTB	CHURC1-FNTB readthrough	100529261	21,44357595	0,896387346	0,684722	1,309125577	0,190491817	NA
ENSG00000119004	CYP20A1	cytochrome P450 family 20 subfamily A member 1	57404	189,6927993	-0,795741123	0,21752	-3,658245056	0,000253948	0,012374745
ENSG00000085415	SEH1L	SEH1 like nucleoporin	81929	75,63627492	-0,011452219	0,203606	-0,056246933	0,955145093	0,991420853
ENSG00000119844	AFTPH	aftiphilin	54812	233,3349383	0,094508645	0,118931	0,794647819	0,426818394	0,83202573
ENSG00000188647	PTAR1	protein prenyltransferase alpha subunit repeat containing 1	375743	426,0794051	-0,410536638	0,085204	-4,818285839	1,45E-06	0,000192752
ENSG00000038210	PI4K2B	phosphatidylinositol 4-kinase type 2 beta	55300	99,76641693	0,321425322	0,187809	1,711444066	0,086999168	0,48153028
ENSG00000111271	ACAD10	acyl-CoA dehydrogenase family member 10	80724	74,27214286	-0,336582941	0,198255	-1,697724882	0,089559699	0,488772026
ENSG00000108799	EZH1	enhancer of zeste 1 polycomb repressive complex 2 subunit	2145	280,7848348	-0,193186941	0,131143	-1,473103479	0,140723136	0,584473563
ENSG00000142230	SAE1	SUMO1 activating enzyme subunit 1	10055	165,3792768	-0,222278262	0,284786	-0,780510346	0,43509054	0,836319408
ENSG00000100055	CYTH4	cytohesin 4	27128	659,7729659	-0,046711851	0,108162	-0,43187056	0,665835493	0,922184616
ENSG00000127527	EPS15L1	epidermal growth factor receptor pathway substrate 15 like 1	58513	212,5825305	0,212272661	0,171814	1,235478782	0,216652368	0,672891335
ENSG00000160233	LRRC3	leucine rich repeat containing 3	81543	21,50323876	0,186180347	0,414775	0,448871041	0,653524688	NA
ENSG00000149573	MPZL2	myelin protein zero like 2	10205	117,0637363	0,278302778	0,189588	1,467934541	0,142122005	0,586525698
ENSG00000072135	PTPN18	protein tyrosine phosphatase non-receptor type 18	26469	306,47767	-0,309362356	0,132307	-2,338215468	0,019376074	0,218022466
ENSG00000102908	NFAT5	nuclear factor of activated T cells 5	10725	370,8417349	-0,296169782	0,184757	-1,603026161	0,108928878	0,52908614
ENSG00000173681	BCLAF3	BCLAF1 and THRAP3 family member 3	256643	94,87825997	0,054299406	0,192082	0,282688096	0,777415942	0,947109268
ENSG00000163154	TNFAIP8L2	TNF alpha induced protein 8 like 2	79626	97,68694039	-0,024677017	0,177229	-0,139238144	0,889261967	0,977990819
ENSG00000205809	KLRC2	killer cell lectin like receptor C2	3822	369,1427621	-0,0938536	0,092616	-1,013357235	0,310889558	0,75688983
ENSG00000143622	RIT1	Ras like without CAAX 1	6016	196,4255815	-0,42031779	0,146589	-2,867325766	0,004139567	0,088853402
ENSG00000173083	HPSE	heparanase	10855	197,3703544	0,153964297	0,148204	1,038867956	0,298866151	0,749216326
ENSG00000104343	UBE2W	ubiquitin conjugating enzyme E2 W	55284	141,1150658	-0,168843057	0,225509	-0,748720768	0,454025523	0,843234809
ENSG00000157404	KIT	KIT proto-oncogene, receptor tyrosine kinase	3815	1994,515959	-0,077800191	0,070125	-1,109457602	0,267232825	0,722449525
ENSG00000108424	KPNB1	karyopherin subunit beta 1	3837	775,865326	0,129121085	0,093603	1,379454062	0,167754801	0,627402954
ENSG00000166595	CIAO2B	cytosolic iron-sulfur assembly component 2B	51647	66,62349848	-0,094842452	0,207875	-0,45624671	0,64821258	0,914533708
ENSG00000173480	ZNF417	zinc finger protein 417	147687	98,80785529	-0,101883259	0,236947	-0,429982967	0,667208032	0,922308959
ENSG00000074071	MRPS34	mitochondrial ribosomal protein S34	65993	83,01746265	0,394522385	0,257199	1,533919996	0,125049336	0,5624706
ENSG00000122359	ANXA11	annexin A11	311	547,9373639	0,014327388	0,110371	0,129811624	0,896715466	0,977990819
ENSG00000070610	GBA2	glucosylceramidase beta 2	57704	264,2444531	-0,065357468	0,103699	-0,630263673	0,528522086	0,872142129
ENSG00000112511	PHF1	PHD finger protein 1	5252	37,07927049	-0,832857601	1,128544	-0,737993107	0,460518638	NA
ENSG00000122026	RPL21	ribosomal protein L21	6144	1194,164045	0,147981877	0,090666	1,632160007	0,102645783	0,515621608
ENSG00000183914	DNAH2	dynein axonemal heavy chain 2	146754	15,06361662	1,446105869	0,54971	2,630672871	0,008521602	NA
ENSG00000116199	FAM20B	FAM20B glycosaminoglycan xylosylkinase	9917	275,6116171	-0,130779966	0,113696	-1,150263427	0,250035389	0,707335884

ENSG00000006555	TTC22	tetratricopeptide repeat domain 22	55001	53,5498818	-0,060514122	0,268473	-0,225401108	0,82166725	0,961898134
ENSG00000102316	MAGED2	MAGE family member D2	10916	185,2981404	-0,112229095	0,128333	-0,87451463	0,381838057	0,806821656
ENSG00000073111	MCM2	minichromosome maintenance complex component 2	4171	137,1783029	0,113223852	0,199103	0,568670312	0,569579899	0,886263374
ENSG00000158411	MITD1	microtubule interacting and trafficking domain containing 1	129531	55,16606352	-0,153137979	0,264626	-0,578696358	0,562794074	0,884605624
ENSG00000105220	GPI	glucose-6-phosphate isomerase	2821	352,0704562	-0,124212453	0,093109	-1,334061079	0,18218384	0,636302884
ENSG00000198700	IPO9	importin 9	55705	368,6951431	0,170718067	0,090436	1,887718853	0,059063704	0,39312401
ENSG00000078043	PIAS2	protein inhibitor of activated STAT 2	9063	15,66712093	-0,137850998	0,500396	-0,275483558	0,782944756	NA
ENSG00000134444	RELCH	RAB11 binding and LisH domain, coiled-coil and HEAT repeat containing	57614	272,3125778	0,061016976	0,129796	0,470100333	0,638283336	0,909954642
ENSG00000243725	TTC4	tetratricopeptide repeat domain 4	7268	74,38227184	-0,051729762	0,216121	-0,239355681	0,810829793	0,959038743
ENSG00000122958	VPS26A	VPS26 retromer complex component A	9559	410,0769542	0,230704468	0,083129	2,775258756	0,00551578	0,106439654
ENSG00000134748	PRPF38A	pre-mRNA processing factor 38A	84950	263,7220133	0,056083362	0,11464	0,489211011	0,624692315	0,90479929
ENSG00000224877	NDUFAF8	NADH:ubiquinone oxidoreductase complex assembly factor 8	284184	29,90655778	0,684162176	0,314411	2,176010107	0,029554505	NA
ENSG00000101109	STK4	serine/threonine kinase 4	6789	1360,028718	-0,095168022	0,056405	-1,687225426	0,091560014	0,492205579
ENSG00000074621	SLC24A1	solute carrier family 24 member 1	9187	15,23440828	0,377143891	0,539526	0,699028114	0,484534461	NA
ENSG00000116221	MRPL37	mitochondrial ribosomal protein L37	51253	127,1506038	0,201057642	0,167893	1,197536452	0,231097528	0,685179309
ENSG00000124767	GLO1	glyoxalase I	2739	518,7410275	-0,032500328	0,075347	-0,431344325	0,666218026	0,922184616
ENSG00000127418	FGFRL1	fibroblast growth factor receptor like 1	53834	88,6587791	-0,03597497	0,197294	-0,182342397	0,855314028	0,968377739
ENSG00000151470	C4orf33	chromosome 4 open reading frame 33	132321	57,01926649	0,260696695	0,249611	1,044413386	0,296294173	0,747862492
ENSG00000198826	ARHGAP11A	Rho GTPase activating protein 11A	9824	217,5539975	0,349012106	0,164746	2,118492243	0,034133401	0,295021466
ENSG00000204406	MBD5	methyl-CpG binding domain protein 5	55777	50,40388111	-0,04742532	0,306032	-0,154968424	0,876846211	0,975875037
ENSG00000215421	ZNF407	zinc finger protein 407	55628	140,6909127	0,212055517	0,154653	1,371168042	0,170322578	0,629558603
ENSG00000023318	ERP44	endoplasmic reticulum protein 44	23071	244,5593252	0,154490384	0,120124	1,286093139	0,198410558	0,656945827
ENSG00000106305	AIMP2	aminoacyl tRNA synthetase complex interacting multifunctional protein 2	7965	42,72525627	-0,03906972	0,303112	-0,128895408	0,89744041	0,977990819
ENSG00000173272	MZT2A	mitotic spindle organizing protein 2A	653784	25,0839296	0,017512524	0,349306	0,050135194	0,960014654	NA
ENSG00000113263	ITK	IL2 inducible T cell kinase	3702	752,8944271	-0,022805347	0,064463	-0,353773998	0,723508262	0,934902053
ENSG00000073605	GSDMB	gasdermin B	55876	22,29880636	0,322228318	0,66001	0,488217378	0,625395877	NA
ENSG00000124160	NCOA5	nuclear receptor coactivator 5	57727	183,9367347	0,127776593	0,133523	0,956963081	0,33858589	0,777431905
ENSG00000256294	ZNF225	zinc finger protein 225	7768	50,841801	-0,189954156	0,298739	-0,63585353	0,524871893	0,871163113
ENSG00000166900	STX3	syntaxin 3	6809	734,2953314	0,189624558	0,078871	2,404224558	0,016206814	0,201087394
ENSG00000198856	OSTC	oligosaccharyltransferase complex non-catalytic subunit	58505	183,5753586	0,151797425	0,129778	1,169669444	0,242134019	0,697111652
ENSG00000135679	MDM2	MDM2 proto-oncogene	4193	582,8926352	0,174245507	0,094896	1,836180837	0,066330916	0,41677041
ENSG00000183741	CBX6	chromobox 6	23466	363,2021011	-0,304064479	0,088392	-3,439970631	0,000581777	0,022961201
ENSG00000057757	PITHD1	PITH domain containing 1	57095	138,1217796	0,067508119	0,15249	0,442704187	0,657979714	0,919088619
ENSG00000149480	MTA2	metastasis associated 1 family member 2	9219	618,4706761	-0,058171664	0,075787	-0,767569352	0,442743076	0,838678081
ENSG00000181036	FCRL6	Fc receptor like 6	343413	51,76945271	0,04342677	0,288564	0,150492873	0,880375774	0,976300778
ENSG00000221869	CEBPD	CCAAT enhancer binding protein delta	1052	49,32956124	-0,10469532	0,25295	-0,413896624	0,678949817	0,925294441
ENSG00000004487	KDM1A	lysine demethylase 1A	23028	120,4604681	-0,019731248	0,172752	-0,11421729	0,909065546	0,97930822
ENSG00000074319	TSG101	tumor susceptibility 101	7251	112,3122733	0,090889225	0,175695	0,517311712	0,60493858	0,897347689
ENSG00000163788	SNRK	SNF related kinase	54861	672,113091	-0,224639414	0,077593	-2,895094333	0,003790446	0,083389813
ENSG00000196428	TSC22D2	TSC22 domain family member 2	9819	46,13272861	-0,039063895	0,298889	-0,130697154	0,896014884	0,977990819
ENSG00000163359	COL6A3	collagen type VI alpha 3 chain	1293	17,40344806	-0,23086165	0,449295	-0,513831154	0,607370045	NA
ENSG00000167536	DHRS13	dehydrogenase/reductase 13	147015	38,25521979	0,413075445	0,289137	1,428649333	0,153105047	NA
ENSG00000164077	MON1A	MON1 homolog A, secretory trafficking associated	84315	20,20715255	0,238221035	0,459334	0,518623143	0,604023567	NA
ENSG00000139514	SLC7A1	solute carrier family 7 member 1	6541	359,384697	-0,061501898	0,094291	-0,652258236	0,514234597	0,868514045
ENSG00000138002	IFT172	intraflagellar transport 172	26160	30,53075551	0,480387357	0,683993	0,702327837	0,482474737	NA
ENSG00000167550	RHEBL1	RHEB like 1	121268	11,66679975	-0,445249092	0,519296	-0,857408497	0,391219165	NA

ENSG00000168924	LETM1	leucine zipper and EF-hand containing transmembrane protein 1	3954	215,201492	-0,077661399	0,132891	-0,584398983	0,558951911	0,883921037
ENSG00000009790	TRAF3IP3	TRAF3 interacting protein 3	80342	483,1674203	-0,054126097	0,09162	-0,590769841	0,554674645	0,882854594
ENSG00000175548	ALG10B	ALG10 alpha-1,2-glucosyltransferase B	144245	75,82258647	0,469862416	0,39247	1,197191914	0,231231761	0,685318585
ENSG00000177042	TMEM80	transmembrane protein 80	283232	78,20523161	0,107330182	0,210354	0,51023478	0,609886989	0,898865155
ENSG00000123411	IKZF4	IKAROS family zinc finger 4	64375	88,73609384	-0,107068748	0,278886	-0,383916454	0,70104037	0,930846779
ENSG00000227500	SCAMP4	secretory carrier membrane protein 4	113178	95,91419631	-0,225083632	0,179399	-1,25465556	0,209603825	0,66406956
ENSG00000196440	ARMCX4	armadillo repeat containing X-linked 4	100131755	19,07044234	-0,300973818	0,398523	-0,755224144	0,450114504	NA
ENSG00000029993	HMGB3	high mobility group box 3	3149	26,66636714	0,152678176	0,329035	0,464017897	0,642634923	NA
ENSG00000082641	NFE2L1	NFE2 like bZIP transcription factor 1	4779	451,7316508	-0,149487243	0,089389	-1,672320581	0,094461123	0,498337741
ENSG00000089351	GRAMD1A	GRAM domain containing 1A	57655	323,7007927	-0,177031349	0,123813	-1,429825876	0,152767	0,609428683
ENSG00000115129	TP53I3	tumor protein p53 inducible protein 3	9540	20,16308487	0,069368773	0,512408	0,135377948	0,892313059	NA
ENSG00000119242	CCDC92	coiled-coil domain containing 92	80212	65,48289795	-1,035280961	0,491052	-2,108291489	0,035005784	0,298518377
ENSG00000119537	KDSR	3-ketodihydrosphingosine reductase	2531	43,26706434	0,039851773	0,351038	0,113525418	0,909614012	0,979548024
ENSG00000120526	NUDCD1	NudC domain containing 1	84955	36,2613522	-0,263500518	0,434351	-0,606653333	0,544080998	NA
ENSG00000126003	PLAGL2	PLAG1 like zinc finger 2	5326	148,7211946	-0,128321814	0,134576	-0,953528053	0,340322589	0,777431905
ENSG00000134152	KATNBL1	katanin regulatory subunit B1 like 1	79768	72,17031452	-0,154872049	0,229806	-0,673926211	0,500358227	0,86161226
ENSG00000134815	DHX34	DExH-box helicase 34	9704	75,0511492	0,019563901	0,20339	0,096188968	0,923370492	0,984284131
ENSG00000147251	DOCK11	dedicator of cytokinesis 11	139818	580,8295791	0,072120633	0,093718	0,769553029	0,441565077	0,838678081
ENSG00000152229	PSTPIP2	proline-serine-threonine phosphatase interacting protein 2	9050	129,7844519	-0,040242953	0,147086	-0,273602375	0,784390209	0,95013509
ENSG00000160075	SSU72	SSU72 homolog, RNA polymerase II CTD phosphatase	29101	309,8497107	0,046918368	0,107522	0,436361661	0,662574351	0,920548196
ENSG00000171444	MCC	MCC regulator of WNT signaling pathway	4163	48,63522464	-0,096746048	0,243397	-0,397482008	0,691012052	0,927252461
ENSG00000171612	SLC25A33	solute carrier family 25 member 33	84275	44,88788511	-0,069502799	0,258485	-0,268885747	0,788017606	0,95148885
ENSG00000204217	BMPR2	bone morphogenetic protein receptor type 2	659	252,1675663	0,067693854	0,116886	0,579146567	0,562490281	0,884605624
ENSG00000160299	PCNT	pericentrin	5116	552,7526709	0,058150987	0,08411	0,691366268	0,489335397	0,860369726
ENSG00000104969	SGTA	small glutamine rich tetratricopeptide repeat co-chaperone alpha	6449	115,9558936	-0,184006858	0,1779	-1,034328381	0,300982673	0,751870158
ENSG00000159921	GNE	glucosamine (UDP-N-acetyl)-2-epimerase/N-acetylmannosamine kinase	10020	84,8564505	0,271871435	0,21316	1,275431369	0,202156602	0,660119983
ENSG00000143179	UCK2	uridine-cytidine kinase 2	7371	126,9642941	-0,318986502	0,257384	-1,239341748	0,215218964	0,671565255
ENSG00000103978	TMEM87A	transmembrane protein 87A	25963	246,7487748	0,063097963	0,116358	0,542276027	0,587628368	0,891487966
ENSG00000116539	ASH1L	ASH1 like histone lysine methyltransferase	55870	584,0821256	-0,059682377	0,146335	-0,407848693	0,683384762	0,925586358
ENSG00000148516	ZEB1	zinc finger E-box binding homeobox 1	6935	262,2157509	-0,139281076	0,187531	-0,742711025	0,457656653	0,84552083
ENSG00000100412	ACO2	aconitase 2	50	266,7547263	-0,098085469	0,115624	-0,848316566	0,396261694	0,812799303
ENSG00000115758	ODC1	ornithine decarboxylase 1	4953	80,37075356	0,238230851	0,207951	1,145608151	0,251957323	0,70851157
ENSG00000110108	TMEM109	transmembrane protein 109	79073	148,9040934	-0,061169823	0,152406	-0,401361833	0,688153746	0,926748702
ENSG00000054793	ATP9A	ATPase phospholipid transporting 9A (putative)	10079	19,0926088	0,514928974	0,450489	1,143044047	0,253020304	NA
ENSG00000073849	ST6GAL1	ST6 beta-galactoside alpha-2,6-sialyltransferase 1	6480	621,0960452	0,011249953	0,082351	0,136610057	0,891339027	0,977990819
ENSG00000181061	HIGD1A	HIG1 hypoxia inducible domain family member 1A	25994	148,1286092	-0,106146103	0,232799	-0,45595577	0,648421784	0,914533708
ENSG00000154217	PITPNC1	phosphatidylinositol transfer protein cytoplasmic 1	26207	319,0825484	-0,108539857	0,106016	-1,023807947	0,305925996	0,752738964
ENSG00000087903	RFX2	regulatory factor X2	5990	34,83592189	0,251765582	0,295108	0,853131456	0,393586409	NA
ENSG00000152782	PANK1	pantothenate kinase 1	53354	24,44571923	0,486199882	0,389704	1,247614855	0,212172136	NA
ENSG00000169972	PUSL1	pseudouridine synthase like 1	126789	18,1634053	0,293618091	0,484805	0,605641244	0,544753008	NA
ENSG00000168288	MMADHC	metabolism of cobalamin associated D	27249	188,1711378	0,275384037	0,142634	1,930701832	0,053519936	0,373308685
ENSG00000168268	NT5DC2	5'-nucleotidase domain containing 2	64943	12,86397213	0,834085193	0,833253	1,000998775	0,316827401	NA
ENSG00000064607	SUGP2	SURP and G-patch domain containing 2	10147	604,7384831	-0,203485754	0,086431	-2,354317901	0,018556733	0,213076877
ENSG00000108384	RAD51C	RAD51 paralog C	5889	18,0168326	-0,377659689	0,804743	-0,469292267	0,638860742	NA
ENSG00000160688	FLAD1	flavin adenine dinucleotide synthetase 1	80308	114,1727849	-0,087270878	0,187887	-0,464486004	0,642299585	0,912063088
ENSG00000197299	BLM	BLM RecQ like helicase	641	93,0122309	-0,78755694	0,578374	-1,361673478	0,173300955	0,629558603
ENSG00000067221	STOML1	stomatin like 1	9399	16,35670167	-0,47568707	0,506728	-0,938742409	0,347863014	NA
ENSG00000145439	CBR4	carbonyl reductase 4	84869	92,72021418	0,043032619	0,209698	0,205211878	0,837406589	0,965359071

ENSG00000100403	ZC3H7B	zinc finger CCCH-type containing 7B	23264	407,9593894	0,082719974	0,090261	0,916449335	0,359431269	0,790999468
ENSG00000102760	RGCC	regulator of cell cycle	28984	16,37712273	0,487161658	0,490891	0,992403238	0,32100086	NA
ENSG00000115137	DNAJC27	DnaJ heat shock protein family (Hsp40) member C27	51277	39,21821561	0,23798243	0,311972	0,762831471	0,44556391	NA
ENSG00000116198	CEP104	centrosomal protein 104	9731	139,2019005	-0,034125101	0,224226	-0,152190444	0,879036731	0,976237908
ENSG00000135821	GLUL	glutamate-ammonia ligase	2752	454,6859484	0,143848532	0,096529	1,490216085	0,136167428	0,576527754
ENSG00000166783	MARF1	meiosis regulator and mRNA stability factor 1	9665	446,651486	0,016844872	0,107623	0,156517918	0,875624797	0,975344937
ENSG00000105821	DNAJC2	DnaJ heat shock protein family (Hsp40) member C2	27000	87,26556874	0,080126727	0,231322	0,346385404	0,729053093	0,937916952
ENSG00000067057	PFKP	phosphofructokinase, platelet	5214	271,2640033	-0,075594767	0,11115	-0,680113554	0,496432563	0,86123475
ENSG00000014164	ZC3H3	zinc finger CCCH-type containing 3	23144	75,69620744	-0,565267652	0,263723	-2,143412269	0,032080009	0,286639805
ENSG00000103429	BFAR	bifunctional apoptosis regulator	51283	116,1193191	-0,034633278	0,231988	-0,149288788	0,881325762	0,976300778
ENSG00000125885	MCM8	minichromosome maintenance 8 homologous recombination repair factor	84515	129,1494174	0,124616828	0,184589	0,675102982	0,499610339	0,86161226
ENSG00000162852	CNST	consortin, connexin sorting protein	163882	130,7768491	-0,030161362	0,233334	-0,129262415	0,89715001	0,977990819
ENSG00000086827	ZW10	zw10 kinetochore protein	9183	81,48286418	0,283261905	0,21941	1,291014233	0,196698742	0,655659726
ENSG00000162572	SCNN1D	sodium channel epithelial 1 subunit delta	6339	12,10546014	-0,864829751	0,578694	-1,494449679	0,135058125	NA
ENSG00000099337	KCNK6	potassium two pore domain channel subfamily K member 6	9424	117,8693729	-0,008734418	0,154512	-0,056528887	0,954920484	0,991420853
ENSG00000145604	SKP2	S-phase kinase associated protein 2	6502	58,189684	-0,153848443	0,357264	-0,430629692	0,666737648	0,922184616
ENSG00000197852	INKA2	inka box actin regulator 2	55924	14,76320029	0,347285305	0,511685	0,678709657	0,497321846	NA
ENSG00000237296	SMG1P1	SMG1 pseudogene 1	641298	471,4456818	-0,06479844	0,15646	-0,414153843	0,678761443	0,925294441
ENSG00000197063	MAFG	MAF bZIP transcription factor G	4097	121,8226743	0,046716671	0,167115	0,279547357	0,7798248	0,947749187
ENSG00000292348	CD99	CD99 molecule (Xg blood group)	4267	759,5213029	-0,153506672	0,076772	-1,999511492	0,04555304	0,340953043
ENSG00000109689	STIM2	stromal interaction molecule 2	57620	233,0966879	-0,23205891	0,133939	-1,732570945	0,083171957	0,469613624
ENSG00000117984	CTSD	cathepsin D	1509	2216,694774	0,033947904	0,066295	0,512074359	0,608598969	0,897640621
ENSG00000155115	GTF3C6	general transcription factor IIIC subunit 6	112495	97,32743771	-0,12112497	0,17914	-0,676148521	0,498946353	0,86161226
ENSG00000161999	JMJD8	jumonji domain containing 8	339123	54,34375621	0,543585208	0,324369	1,675823918	0,093772688	0,497328138
ENSG00000162032	SPSB3	splA/ryanodine receptor domain and SOCS box containing 3	90864	54,89846504	0,116056826	0,345575	0,335836853	0,736993898	0,939157822
ENSG00000163626	COX18	cytochrome c oxidase assembly factor COX18	285521	17,05973748	0,42664289	0,49137	0,86827238	0,385245239	NA
ENSG00000166912	MTMR10	myotubularin related protein 10	54893	232,8553742	-0,120339479	0,201833	-0,596231636	0,551020494	0,880819693
ENSG00000198113	TOR4A	torsin family 4 member A	54863	92,71794791	0,093708157	0,196676	0,47646056	0,633746299	0,908447084
ENSG00000089041	P2RX7	purinergic receptor P2X 7	5027	201,0886956	0,070660347	0,113492	0,622599369	0,533547826	0,873191491
ENSG00000100726	TELO2	telomere maintenance 2	9894	88,95141497	-0,111493968	0,204656	-0,544785884	0,585900788	0,890759735
ENSG00000124802	EEF1E1	eukaryotic translation elongation factor 1 epsilon 1	9521	38,34246013	0,367732957	0,290588	1,265480384	0,205699176	NA
ENSG00000136908	DPM2	dolichyl-phosphate mannosyltransferase subunit 2, regulatory	8818	47,33419534	0,404854426	0,260788	1,552427432	0,120559984	0,554378286
ENSG00000157193	LRP8	LDL receptor related protein 8	7804	195,5952001	0,264924506	0,158209	1,674517763	0,094028886	0,497643446
ENSG00000158109	TPRG1L	tumor protein p63 regulated 1 like	127262	125,6647176	-0,352413986	0,153984	-2,2886431	0,022100098	0,236477065
ENSG00000030066	NUP160	nucleoporin 160	23279	266,3106313	0,135873319	0,123979	1,095938279	0,273105786	0,726913977
ENSG00000113249	HAVCR1	hepatitis A virus cellular receptor 1	26762	75,26924558	-0,408625585	0,294496	-1,387539999	0,165277163	0,624454568
ENSG00000126883	NUP214	nucleoporin 214	8021	284,8439015	-0,193876868	0,138992	-1,394880213	0,163051965	0,621052441
ENSG00000151131	NOPCHAP1	NOP protein chaperone 1	121053	66,08828639	0,492964721	0,685447	0,719186942	0,472025744	0,853811879
ENSG00000164088	PPM1M	protein phosphatase, Mg2+/Mn2+ dependent 1M	132160	248,5059837	0,108231248	0,14693	0,736615995	0,461355906	0,846765498
ENSG00000169246	NPIPB3	nuclear pore complex interacting protein family member B3	23117	276,487	-0,324789219	0,157795	-2,058298797	0,03956146	0,316733643
ENSG00000106479	ZNF862	zinc finger protein 862	643641	213,8318416	-0,114430244	0,13175	-0,868538682	0,385099506	0,809141659
ENSG00000141568	FOXK2	forkhead box K2	3607	224,9859597	-0,056907111	0,108704	-0,523507472	0,600621153	0,896014669
ENSG00000143515	ATP8B2	ATPase phospholipid transporting 8B2	57198	576,1946009	-0,135558648	0,071769	-1,888829609	0,058914664	0,39312401
ENSG00000197566	ZNF624	zinc finger protein 624	57547	38,93607982	0,139929627	0,331657	0,421911154	0,673089869	NA
ENSG00000213551	DNAJC9	DnaJ heat shock protein family (Hsp40) member C9	23234	124,0044542	-0,163579879	0,151225	-1,081698015	0,279386736	0,732760553
ENSG00000099821	POLRMT	RNA polymerase mitochondrial	5442	139,2444352	-0,177157521	0,176479	-1,003847545	0,315452103	0,760454518

ENSG00000107938	EDRF1	erythroid differentiation regulatory factor 1	26098	64,66477216	-0,713002965	0,31939	-2,232392531	0,025589031	0,257992618
ENSG00000157456	CCNB2	cyclin B2	9133	58,39117218	0,673143151	0,256195	2,627464469	0,008602383	0,141050339
ENSG00000100865	CINP	cyclin dependent kinase 2 interacting protein	51550	34,95694288	-0,48659057	0,316672	-1,536575577	0,124397277	NA
ENSG00000116667	C1orf21	chromosome 1 open reading frame 21	81563	19,21863156	0,250194125	0,473523	0,528366994	0,597244641	NA
ENSG00000168229	PTGDR	prostaglandin D2 receptor	5729	326,993153	-0,089090741	0,107013	-0,832522102	0,405114308	0,818437414
ENSG00000197548	ATG7	autophagy related 7	10533	53,11179733	-0,449419508	0,285316	-1,575163144	0,115218792	0,541225115
ENSG00000162654	GBP4	guanylate binding protein 4	115361	650,0365861	0,192853198	0,073541	2,622377876	0,008731856	0,14257795
ENSG00000101365	IDH3B	isocitrate dehydrogenase (NAD(+)) 3 non-catalytic subunit beta	3420	156,5057798	-0,134268588	0,174682	-0,768646686	0,442103082	0,838678081
ENSG00000100994	PYGB	glycogen phosphorylase B	5834	332,5022766	-0,06072553	0,11469	-0,52947627	0,596475102	0,894229873
ENSG00000155097	ATP6V1C1	ATPase H+ transporting V1 subunit C1	528	164,9147655	0,138189715	0,202021	0,684036732	0,493951971	0,860513824
ENSG00000180611	MB21D2	Mab-21 domain containing 2	151963	81,31640821	0,098401588	0,191537	0,513747867	0,607428281	0,897481254
ENSG00000196867	ZFP28	ZFP28 zinc finger protein	140612	26,12149904	-0,065621594	0,334785	-0,196011011	0,844601548	NA
ENSG00000056972	TRAF3IP2	TRAF3 interacting protein 2	10758	41,2069396	0,679336289	0,510765	1,330037521	0,183505909	NA
ENSG00000154767	XPC	XPC complex subunit, DNA damage recognition and repair factor	7508	251,3881491	0,025872242	0,109946	0,235317541	0,813962282	0,959207071
ENSG00000103091	WDR59	WD repeat domain 59	79726	157,4547063	-0,173706089	0,139791	-1,242610299	0,214011473	0,668985134
ENSG00000112742	TTK	TTK protein kinase	7272	57,14693155	1,38766139	0,426487	3,253699895	0,001139125	0,037467385
ENSG00000067248	DHX29	DExH-box helicase 29	54505	133,3497418	0,103271232	0,161316	0,640178545	0,522056529	0,870221868
ENSG00000082068	WDR70	WD repeat domain 70	55100	83,96325142	-0,166097297	0,200288	-0,82929311	0,406938569	0,819272982
ENSG00000180370	PAK2	p21 (RAC1) activated kinase 2	5062	859,0761125	0,010864337	0,06365	0,170689842	0,864467651	0,971561202
ENSG00000140694	PARN	poly(A)-specific ribonuclease	5073	149,1923943	0,00339856	0,194233	0,017497335	0,986039859	0,99657149
ENSG00000165898	ISCA2	iron-sulfur cluster assembly 2	122961	26,43204702	-0,012881127	0,435538	-0,029575223	0,976405826	NA
ENSG00000122741	DCAF10	DDB1 and CUL4 associated factor 10	79269	129,4524197	-0,11635199	0,207994	-0,559401191	0,575887949	0,887069818
ENSG00000227345	PARG	poly(ADP-ribose) glycohydrolase	8505	116,4787014	0,219118922	0,222905	0,98301612	0,325599505	0,768407005
ENSG00000016864	GLT8D1	glycosyltransferase 8 domain containing 1	55830	80,5633005	0,698580653	0,227906	3,065218404	0,002175111	0,056380599
ENSG00000137413	TAF8	TATA-box binding protein associated factor 8	129685	209,6443398	-0,15702264	0,166657	-0,942192442	0,346094123	0,782042713
ENSG00000183475	ASB7	ankyrin repeat and SOCS box containing 7	140460	114,9493676	0,004257409	0,193859	0,021961337	0,982478797	0,996026814
ENSG00000158286	RNF207	ring finger protein 207	388591	29,86988448	-0,698039869	0,522043	-1,337130373	0,181180085	NA
ENSG00000148488	ST8SIA6	ST8 alpha-N-acetyl-neuraminide alpha-2,8-sialyltransferase 6	338596	16,55421606	-0,769990204	0,463404	-1,661597456	0,096593514	NA
ENSG00000181381	DDX60L	DExD/H-box 60 like	91351	231,3197309	-0,181912028	0,154802	-1,175129495	0,239942912	0,695135238
ENSG00000102879	CORO1A	coronin 1A	11151	1879,87106	0,054310555	0,077383	0,701839577	0,482779214	0,855925045
ENSG00000185813	PCYT2	phosphate cytidylyltransferase 2, ethanolamine	5833	132,08618	-0,339881528	0,206454	-1,646286041	0,099704882	0,509429216
ENSG00000047056	WDR37	WD repeat domain 37	22884	173,102801	-0,221977	0,162206	-1,368490153	0,171158702	0,629558603
ENSG00000090581	GNPTG	N-acetylglucosamine-1-phosphate transferase subunit gamma	84572	80,81486439	0,141490107	0,211601	0,668665587	0,503708824	0,863218221
ENSG00000107902	LHPP	phospholysine phosphohistidine inorganic pyrophosphate phosphatase	64077	18,24270759	-0,103723468	0,425086	-0,244005926	0,80722623	NA
ENSG00000124614	RPS10	ribosomal protein S10	6204	748,8263851	0,123652428	0,077855	1,588232934	0,112233676	0,535466371
ENSG00000126247	CAPNS1	calpain small subunit 1	826	607,3562936	-0,149337807	0,079664	-1,874597143	0,060848167	0,399583194
ENSG00000158856	DMTN	dematin actin binding protein	2039	257,421354	-0,051080188	0,105039	-0,486297441	0,626756294	0,90479929
ENSG00000185650	ZFP36L1	ZFP36 ring finger protein like 1	677	2151,587492	0,165314442	0,050144	3,296774221	0,000978021	0,034445632
ENSG00000134070	IRAK2	interleukin 1 receptor associated kinase 2	3656	236,904709	-0,022922118	0,124321	-0,184378761	0,853716331	0,968377739
ENSG00000135045	C9orf40	chromosome 9 open reading frame 40	55071	14,69279692	0,093645839	0,57063	0,164109618	0,86964485	NA
ENSG00000139117	CPNE8	copine 8	144402	23,30846129	0,500763452	0,408191	1,226788411	0,219902128	NA
ENSG00000111642	CHD4	chromodomain helicase DNA binding protein 4	1108	1190,922339	0,070979586	0,089225	0,795511573	0,426315982	0,831459081
ENSG00000205307	SAP25	Sin3A associated protein 25	100316904	36,46850255	-0,413722472	0,319876	-1,293385808	0,195877656	NA
ENSG00000024048	UBR2	ubiquitin protein ligase E3 component n-recognin 2	23304	543,8753096	-0,107690379	0,084637	-1,27237573	0,203239653	0,660954946
ENSG00000186376	ZNF75D	zinc finger protein 75D	7626	363,4263387	0,0187531	0,101656	0,184476976	0,853639288	0,968377739
ENSG00000005059	MCUB	mitochondrial calcium uniporter dominant negative subunit beta	55013	133,3803923	0,176972655	0,16836	1,051156051	0,293186924	0,744745216
ENSG00000115523	GNLY	granulysin	10578	5572,054764	-0,025837027	0,053098	-0,486591013	0,626548194	0,90479929
ENSG00000184678	H2BC21	H2B clustered histone 21	8349	82,02516333	0,171386522	0,196113	0,873917667	0,382163093	0,807291266
ENSG00000102547	CAB39L	calcium binding protein 39 like	81617	20,97522233	0,002070335	0,403649	0,005129045	0,995907632	NA

ENSG00000138496	PARP9	poly(ADP-ribose) polymerase family member 9	83666	398,2065734	0,126333699	0,097901	1,290421456	0,196904366	0,655659726
ENSG00000186350	RXRA	retinoid X receptor alpha	6256	272,3443454	-0,022213641	0,113513	-0,195693239	0,844850276	0,967231052
ENSG00000179627	ZBTB42	zinc finger and BTB domain containing 42	100128927	17,86520993	0,027065169	0,488511	0,055403394	0,955817092	NA
ENSG00000073921	PICALM	phosphatidylinositol binding clathrin assembly protein	8301	573,1079921	-0,16580077	0,102349	-1,619948063	0,105243434	0,52117398
ENSG00000115827	DCAF17	DDB1 and CUL4 associated factor 17	80067	114,9067773	-0,271316734	0,207736	-1,306063089	0,191531104	0,649525167
ENSG00000118965	WDR35	WD repeat domain 35	57539	46,0143461	-0,192882753	0,254708	-0,757271294	0,448887345	0,84263906
ENSG00000128059	PPAT	phosphoribosyl pyrophosphate amidotransferase	5471	72,8923589	-0,18468719	0,21484	-0,859649964	0,389982024	0,810747116
ENSG00000003509	NDUFAF7	NADH:ubiquinone oxidoreductase complex assembly factor 7	55471	19,21882217	1,149993056	0,515881	2,229183821	0,025801677	NA
ENSG00000086544	ITPKC	inositol-trisphosphate 3-kinase C	80271	35,85860632	0,111864268	0,294326	0,380069378	0,703893916	NA
ENSG00000117569	PTBP2	polypyrimidine tract binding protein 2	58155	54,45015169	-0,617589682	0,759783	-0,81285007	0,416304024	0,82446781
ENSG00000148110	MFSD14B	major facilitator superfamily domain containing 14B	84641	285,1684869	-0,135542143	0,105578	-1,2838135	0,199207219	0,658208139
ENSG00000149273	RPS3	ribosomal protein S3	6188	1633,749028	0,102028581	0,071371	1,429557604	0,152844031	0,609428683
ENSG00000165434	PGM2L1	phosphoglucomutase 2 like 1	283209	83,55250267	0,020613056	0,184272	0,111862182	0,910932683	0,979595261
ENSG00000172954	LCLAT1	lysocardiolipin acyltransferase 1	253558	43,46316068	-0,079503967	0,343275	-0,231604281	0,816845382	0,959707099
ENSG00000214655	ZSWIM8	zinc finger SWIM-type containing 8	23053	468,7496522	0,043710228	0,089942	0,485981859	0,626980028	0,90479929
ENSG00000075945	KIFAP3	kinesin associated protein 3	22920	144,6287901	0,034030181	0,138741	0,245279118	0,80624032	0,958037095
ENSG00000094880	CDC23	cell division cycle 23	8697	153,1228365	0,237693433	0,150661	1,577667244	0,114642075	0,54024053
ENSG00000159086	PAXBP1	PAX3 and PAX7 binding protein 1	94104	57,5171294	-0,742044203	0,349138	-2,125359663	0,033556613	0,293547274
ENSG00000197620	EOLA1	endothelium and lymphocyte associated ASCH domain 1	91966	111,3146231	0,147484438	0,157107	0,938753201	0,347857472	0,784103633
ENSG00000100084	HIRA	histone cell cycle regulator	7290	222,739168	0,036731994	0,114176	0,321713476	0,747669768	0,939899419
ENSG00000255423	EBLN2	endogenous Bornavirus like nucleoprotein 2	55096	11,85876847	0,726576717	0,514727	1,411575482	0,158074996	NA
ENSG00000074855	ANO8	anoctamin 8	57719	65,4199797	-0,10513515	0,265806	-0,395533834	0,692448957	0,927787546
ENSG00000128789	PSMG2	proteasome assembly chaperone 2	56984	88,46056881	0,209904171	0,173139	1,212346237	0,225379872	0,681082537
ENSG00000151422	FER	FER tyrosine kinase	2241	38,77181352	-0,315084544	0,333932	-0,943558615	0,345395249	NA
ENSG00000168067	MAP4K2	mitogen-activated protein kinase kinase kinase kinase 2	5871	70,35715746	-0,334774812	0,44394	-0,754099593	0,450789422	0,84263906
ENSG00000139921	TMX1	thioredoxin related transmembrane protein 1	81542	131,5607249	0,189026021	0,149027	1,268404084	0,204653684	0,660954946
ENSG00000176731	RBIS	ribosomal biogenesis factor	401466	53,04012289	-0,055174741	0,245871	-0,224405535	0,822441764	0,961917534
ENSG00000205560	CPT1B	carnitine palmitoyltransferase 1B	1375	48,54318354	-0,490873672	0,351293	-1,397333613	0,162313272	0,619487156
ENSG00000158528	PPP1R9A	protein phosphatase 1 regulatory subunit 9A	55607	13,89891003	0,348363762	0,532392	0,654337111	0,512894642	NA
ENSG00000104081	BMF	Bcl2 modifying factor	90427	462,3663643	0,012693089	0,102218	0,124176365	0,901175635	0,977990819
ENSG00000196074	SYCP2	synaptonemal complex protein 2	10388	45,20246211	-0,135858333	0,314126	-0,432496399	0,665380669	0,922184616
ENSG00000146063	TRIM41	tripartite motif containing 41	90933	93,38061634	-0,496824423	0,549584	-0,904001303	0,365994711	0,794505931
ENSG00000126456	IRF3	interferon regulatory factor 3	3661	134,9884117	-0,102323307	0,162936	-0,6279983	0,530005053	0,872145761
ENSG00000266086	NA	NA	NA	40,57850666	-0,035302523	0,349068	-0,101133643	0,919444372	NA
ENSG00000151176	PLBD2	phospholipase B domain containing 2	196463	153,578248	-0,100933876	0,169123	-0,596805969	0,550636932	0,880819693
ENSG00000123643	SLC36A1	solute carrier family 36 member 1	206358	124,4252153	0,070456373	0,191445	0,368024189	0,712855198	0,930846779
ENSG00000140307	GTF2A2	general transcription factor IIA subunit 2	2958	81,23071405	-0,064558151	0,279968	-0,230591137	0,817632453	0,960422122
ENSG00000152749	GPR180	G protein-coupled receptor 180	160897	80,86811488	-0,116316544	0,224727	-0,517590359	0,60474411	0,897347689
ENSG00000156735	BAG4	BAG cochaperone 4	9530	68,85317741	-0,082318984	0,232463	-0,354115994	0,723251956	0,934902053
ENSG00000171004	HS6ST2	heparan sulfate 6-O-sulfotransferase 2	90161	55,31781217	0,286222507	0,224978	1,272225578	0,203292982	0,660954946
ENSG00000171425	ZNF581	zinc finger protein 581	51545	23,64197902	0,288111007	0,356126	0,809014609	0,418506743	NA
ENSG00000177173	NA	NA	NA	19,68598277	0,397752537	0,424656	0,936646828	0,348940256	NA
ENSG00000278615	C11orf98	chromosome 11 open reading frame 98	102288414	111,1203646	-0,053807874	0,180106	-0,298756254	0,765126031	0,944359403
ENSG00000154310	TNIK	TRAF2 and NCK interacting kinase	23043	391,1242406	0,096563339	0,098747	0,977889137	0,328129158	0,770441378
ENSG00000114988	LMAN2L	lectin, mannose binding 2 like	81562	28,99638638	0,262480948	0,342829	0,765632164	0,443895201	NA
ENSG00000176915	ANKLE2	ankyrin repeat and LEM domain containing 2	23141	348,7512544	0,157619453	0,092231	1,708964542	0,087457518	0,482849084
ENSG00000067992	PDK3	pyruvate dehydrogenase kinase 3	5165	211,8781203	-0,152531874	0,18544	-0,822542427	0,41076825	0,82130516

ENSG00000088766	CRLS1	cardiolipin synthase 1	54675	63,62348329	-0,299983338	0,296285	-1,012482592	0,311307366	0,757361021
ENSG00000103061	SLC7A6OS	solute carrier family 7 member 6 opposite strand	84138	105,1458978	-0,336531341	0,362262	-0,928972435	0,352903372	0,787192015
ENSG00000133065	SLC41A1	solute carrier family 41 member 1	254428	163,5858115	0,032898945	0,159467	0,206306287	0,836551668	0,96494005
ENSG00000137522	RNF121	ring finger protein 121	55298	54,62789277	0,281291003	0,293355	0,958876845	0,337620791	0,77693253
ENSG00000175376	EIF1AD	eukaryotic translation initiation factor 1A domain containing	84285	120,6869665	-0,358067133	0,192337	-1,861661318	0,062650844	0,404317327
ENSG00000041988	THAP3	THAP domain containing 3	90326	24,69008147	0,168666397	0,422715	0,39900759	0,689887612	NA
ENSG00000173451	THAP2	THAP domain containing 2	83591	16,37536735	-0,046661818	0,539411	-0,086505174	0,931064844	NA
ENSG00000117533	VAMP4	vesicle associated membrane protein 4	8674	94,38108764	-0,400041623	0,180598	-2,215091125	0,02675381	0,26564403
ENSG00000159079	CFAP298	cilia and flagella associated protein 298	56683	45,15308139	-0,262604912	0,38363	-0,684525801	0,493643203	0,860513824
ENSG00000182903	ZNF721	zinc finger protein 721	170960	200,6598847	0,006602874	0,13004	0,050775628	0,959504312	0,992094111
ENSG00000182307	C8orf33	chromosome 8 open reading frame 33	65265	29,40248375	2,35089614	1,884368	1,247577738	0,212185735	NA
ENSG00000176014	TUBB6	tubulin beta 6 class V	84617	24,14150695	0,990474075	0,399925	2,476652361	0,013262101	NA
ENSG00000119953	SMNDC1	survival motor neuron domain containing 1	10285	111,5042804	-0,433202445	0,269347	-1,60834098	0,10776052	0,527254203
ENSG00000137842	TMEM62	transmembrane protein 62	80021	47,94922103	0,425268901	0,251742	1,689306087	0,091160787	0,490934677
ENSG00000198742	SMURF1	SMAD specific E3 ubiquitin protein ligase 1	57154	118,4052776	0,027030388	0,169383	0,159581258	0,873210944	0,974533542
ENSG00000246596	NA	NA	NA	18,72670724	-0,201987465	0,449196	-0,44966465	0,652952264	NA
ENSG00000135924	DNAJB2	DnaJ heat shock protein family (Hsp40) member B2	3300	110,8938537	-0,191227417	0,193778	-0,986837973	0,323722076	0,767603859
ENSG00000100982	PCIF1	phosphorylated CTD interacting factor 1	63935	174,6181426	-0,025680831	0,131862	-0,194754855	0,845584866	0,967231052
ENSG00000125731	SH2D3A	SH2 domain containing 3A	10045	31,13700015	0,128557758	0,381672	0,336827954	0,736246599	NA
ENSG00000165312	OTUD1	OTU deubiquitinase 1	220213	33,63809902	-0,02212458	0,324872	-0,068102422	0,945704102	NA
ENSG00000198960	ARMCX6	armadillo repeat containing X-linked 6	54470	40,58162747	-0,226244377	0,29881	-0,757151288	0,44895923	NA
ENSG00000185340	GAS2L1	growth arrest specific 2 like 1	10634	64,9900324	-0,378112515	0,214688	-1,761221582	0,078200907	0,452608637
ENSG00000179841	AKAP5	A-kinase anchoring protein 5	9495	472,4406195	-0,156499414	0,087042	-1,797985935	0,072179237	0,436409334
ENSG00000145740	SLC30A5	solute carrier family 30 member 5	64924	179,7694706	0,127517359	0,130851	0,974521384	0,329797727	0,771479881
ENSG00000185010	F8	coagulation factor VIII	2157	16,40682708	-0,580494799	0,449605	-1,291120333	0,196661955	NA
ENSG00000003402	CFLAR	CASP8 and FADD like apoptosis regulator	8837	1545,671203	-0,054744006	0,093812	-0,58354708	0,559525073	0,883921037
ENSG00000048162	NOP16	NOP16 nucleolar protein	51491	41,58764331	0,152593072	0,265068	0,575676068	0,564834154	NA
ENSG00000115241	PPM1G	protein phosphatase, Mg2+/Mn2+ dependent 1G	5496	395,7319928	0,019992449	0,091195	0,219227486	0,826472845	0,961917534
ENSG00000137824	RMDN3	regulator of microtubule dynamics 3	55177	91,28729681	-0,07403102	0,17744	-0,417216831	0,676519816	0,924186156
ENSG00000145014	TMEM44	transmembrane protein 44	93109	13,02287461	0,103794499	0,592984	0,175037482	0,861050182	NA
ENSG00000150768	DLAT	dihydrolipoamide S-acetyltransferase	1737	103,6608661	0,353513982	0,230941	1,530757114	0,125829432	0,563113596
ENSG00000154127	UBASH3B	ubiquitin associated and SH3 domain containing B	84959	686,3429436	0,023502228	0,066336	0,354288112	0,723122976	0,934902053
ENSG00000187535	IFT140	intraflagellar transport 140	9742	21,75426383	0,594682083	0,393289	1,512074223	0,130514982	NA
ENSG00000196081	ZNF724	zinc finger protein 724	440519	18,38286488	-0,807113466	0,504836	-1,598762812	0,109873315	NA
ENSG00000214176	NA	NA	NA	144,0474882	-0,00083678	0,139854	-0,005983236	0,995226097	0,998659226
ENSG00000113312	TTC1	tetratricopeptide repeat domain 1	7265	174,9335672	-0,010600001	0,139001	-0,076258652	0,939213321	0,987813564
ENSG00000197763	TXNRD3	thioredoxin reductase 3	114112	11,92971013	0,351120965	0,577157	0,608362844	0,542946849	NA
ENSG00000140688	RUSF1	RUS family member 1	64755	99,83574458	-0,248197623	0,192277	-1,290835628	0,196760681	0,655659726
ENSG00000243317	STMP1	short transmembrane mitochondrial protein 1	647087	144,7595535	-0,147587272	0,165866	-0,889800192	0,373573184	0,800776142
ENSG00000103199	ZNF500	zinc finger protein 500	26048	37,33466748	0,783444355	0,322405	2,430000506	0,015098802	NA
ENSG00000160695	VPS11	VPS11 core subunit of CORVET and HOPS complexes	55823	151,6060977	0,128294549	0,144071	0,890497975	0,373198555	0,800776142
ENSG00000065060	BLTP3A	bridge-like lipid transfer protein family member 3A	54887	141,5624758	0,094519999	0,158368	0,596839514	0,550614534	0,880819693
ENSG00000104635	SLC39A14	solute carrier family 39 member 14	23516	115,3166357	0,205255749	0,170713	1,202344564	0,229230058	0,68351286
ENSG00000164086	DUSP7	dual specificity phosphatase 7	1849	122,3513077	-0,109421795	0,155426	-0,704010209	0,481426416	0,855577353
ENSG00000240849	PEDS1	plasmanylethanolamine desaturase 1	387521	117,0646752	-0,276725447	0,621859	-0,444996741	0,656322109	0,918687194
ENSG00000118193	KIF14	kinesin family member 14	9928	105,9561183	0,428161875	0,161211	2,655906786	0,007909547	0,132558158
ENSG00000076321	KLHL20	kelch like family member 20	27252	100,1996373	0,223961873	0,188841	1,185982792	0,235629095	0,691328984
ENSG00000091947	TMEM101	transmembrane protein 101	84336	46,88594648	0,141012839	0,250962	0,561888858	0,574191745	0,886894319
ENSG00000109606	DHX15	DEAH-box helicase 15	1665	520,3884893	0,104093474	0,088391	1,177641651	0,238939497	0,693819751
ENSG00000123268	ATF1	activating transcription factor 1	466	117,5741231	0,048367184	0,179046	0,270137922	0,787054148	0,951039147
ENSG00000131791	PRKAB2	protein kinase AMP-activated non-catalytic subunit beta 2	5565	109,8809945	-0,059515452	0,176322	-0,337537732	0,735711573	0,939157822

ENSG00000197081	IGF2R	insulin like growth factor 2 receptor	3482	1483,442797	0,126367124	0,061843	2,043362433	0,041016573	0,322789747
ENSG00000113615	SEC24A	SEC24 homolog A, COPII coat complex component	10802	218,3152272	-0,058633206	0,117112	-0,500657208	0,616612394	0,902090873
ENSG00000179632	MAF1	MAF1 homolog, negative regulator of RNA polymerase III	84232	213,6546136	-0,227049055	0,13791	-1,646353743	0,099690951	0,509429216
ENSG00000198721	ECI2	enoyl-CoA delta isomerase 2	10455	112,4550356	0,201646124	0,166079	1,214159763	0,224686722	0,680034496
ENSG00000102531	FNDC3A	fibronectin type III domain containing 3A	22862	334,7297729	0,12956745	0,089032	1,455284699	0,145590461	0,593084792
ENSG00000107679	PLEKHA1	pleckstrin homology domain containing A1	59338	348,4531864	0,159483549	0,11111	1,435366776	0,151182608	0,607670525
ENSG00000160336	ZNF761	zinc finger protein 761	388561	77,03402967	-0,684447409	0,264324	-2,589430509	0,009613482	0,151166315
ENSG00000160336	ZNF765-ZNF761	ZNF765-ZNF761 readthrough	110116772	77,03402967	-0,684447409	0,264324	-2,589430509	0,009613482	0,151166315
ENSG00000173638	SLC19A1	solute carrier family 19 member 1	6573	26,36151636	0,910316553	0,373462	2,437508414	0,01478887	NA
ENSG00000075240	GRAMD4	GRAM domain containing 4	23151	305,4724585	-0,176540991	0,099833	-1,768357998	0,077001074	0,448990052
ENSG00000232838	PET117	PET117 cytochrome c oxidase chaperone	100303755	12,07306651	-0,034266291	1,090148	-0,031432698	0,974924465	NA
ENSG00000259956	RBM15B	RNA binding motif protein 15B	29890	378,4584934	0,004188729	0,090026	0,046527798	0,962889578	0,992458628
ENSG00000264538	NA	NA	NA	23,68037109	0,139077502	0,395105	0,352001712	0,724836978	NA
ENSG00000005486	RHBDD2	rhomboid domain containing 2	57414	47,16651067	0,337178441	0,44615	0,755751883	0,44979797	0,84263906
ENSG00000071205	ARHGAP10	Rho GTPase activating protein 10	79658	45,46145007	-0,186458352	0,281678	-0,66195657	0,507999055	0,864738156
ENSG00000089063	TMEM230	transmembrane protein 230	29058	301,794563	0,011436462	0,112665	0,101508992	0,91914642	0,984062493
ENSG00000119812	FAM98A	family with sequence similarity 98 member A	25940	73,21624692	-0,302524308	0,246951	-1,225037152	0,220561223	0,677095199
ENSG00000119812	RASGRP3-AS1	RASGRP3 antisense RNA 1	105374454	73,21624692	-0,302524308	0,246951	-1,225037152	0,220561223	0,677095199
ENSG00000125901	MRPS26	mitochondrial ribosomal protein S26	64949	44,61206381	-0,020266804	0,281168	-0,072080808	0,9425376	0,988475138
ENSG00000167657	DAPK3	death associated protein kinase 3	1613	83,80595957	-0,302403833	0,194253	-1,556753323	0,119529063	0,552466951
ENSG00000184743	ATL3	atlastin GTPase 3	25923	351,5464716	-0,105399034	0,107072	-0,984374604	0,32493136	0,768215203
ENSG00000162664	ZNF326	zinc finger protein 326	284695	177,8753689	-0,059206512	0,151963	-0,38961103	0,696824195	0,930422107
ENSG00000139180	NDUFA9	NADH:ubiquinone oxidoreductase subunit A9	4704	71,62478237	-0,422938301	0,24863	-1,701077538	0,088928428	0,488044705
ENSG00000105058	FAM32A	family with sequence similarity 32 member A	26017	220,4265758	-0,120432517	0,109721	-1,097626309	0,2723677	0,726831508
ENSG00000163684	RPP14	ribonuclease P/MRP subunit p14	11102	77,51461417	0,386978122	0,621714	0,622437724	0,533654081	0,873191491
ENSG00000115275	MOGS	mannosyl-oligosaccharide glucosidase	7841	131,2663551	-0,421811053	0,15972	-2,64094164	0,008267596	0,137280547
ENSG00000145819	ARHGAP26	Rho GTPase activating protein 26	23092	357,9567125	0,08110209	0,175875	0,461134321	0,644702239	0,912530858
ENSG00000136197	C7orf25	chromosome 7 open reading frame 25	79020	22,16008083	0,996728282	1,382264	0,721083988	0,470857842	NA
ENSG00000100258	LMF2	lipase maturation factor 2	91289	233,7323939	0,003461887	0,141059	0,024542192	0,980420129	0,995752963
ENSG00000111737	RAB35	RAB35, member RAS oncogene family	11021	323,2116973	-0,18030507	0,113814	-1,584211166	0,113145673	0,538390853
ENSG00000112773	TENT5A	terminal nucleotidyltransferase 5A	55603	79,70512565	-0,195893527	0,199765	-0,980621411	0,326779472	0,769687654
ENSG00000115841	RMDN2	regulator of microtubule dynamics 2	151393	13,96951699	-0,01708946	0,479235	-0,03565985	0,971553585	NA
ENSG00000137944	KYAT3	kynurenine aminotransferase 3	56267	108,5353243	0,363457373	0,167708	2,167205029	0,030219224	0,279077601
ENSG00000152457	DCLRE1C	DNA cross-link repair 1C	64421	182,7680309	-0,075809747	0,166788	-0,454528909	0,64944819	0,914926418
ENSG00000161513	FDXR	ferredoxin reductase	2232	35,99848842	0,775820247	0,371689	2,087283431	0,036862522	NA
ENSG00000164880	INTS1	integrator complex subunit 1	26173	430,2942842	-0,066842816	0,111937	-0,597148085	0,550408517	0,880819693
ENSG00000153037	SRP19	signal recognition particle 19	6728	123,789453	-0,119747583	0,178145	-0,672191181	0,501461994	0,862227298
ENSG00000185101	ANO9	anoctamin 9	338440	103,6702078	0,174732084	0,243597	0,717298688	0,473189817	0,853811879
ENSG00000176903	PNMA1	PNMA family member 1	9240	81,74639068	-0,009221412	0,189344	-0,048701973	0,961156803	0,992235217
ENSG00000186566	GPATCH8	G-patch domain containing 8	23131	522,9007546	-0,00183928	0,103675	-0,01774077	0,985845656	0,996503447
ENSG00000185024	BRF1	BRF1 RNA polymerase III transcription initiation factor subunit	2972	118,5338247	0,031688913	0,181992	0,17412236	0,861769301	0,970863509
ENSG00000072682	P4HA2	prolyl 4-hydroxylase subunit alpha 2	8974	46,33220266	-0,22824744	0,251913	-0,906055978	0,364906224	0,793678617
ENSG00000092140	G2E3	G2/M-phase specific E3 ubiquitin protein ligase	55632	98,98057598	0,032852247	0,183023	0,179497503	0,857547078	0,969089892
ENSG00000109046	WSB1	WD repeat and SOCS box containing 1	26118	89,23178522	-0,008354513	0,327077	-0,025542969	0,979621875	0,995712569
ENSG00000139131	YARS2	tyrosyl-tRNA synthetase 2	51067	36,61485838	-0,049865303	0,328483	-0,151804821	0,879340879	NA
ENSG00000133858	ZFC3H1	zinc finger C3H1-type containing	196441	289,667482	-0,035020416	0,15597	-0,224533456	0,822342237	0,961917534
ENSG00000173226	IQCB1	IQ motif containing B1	9657	68,76557092	0,024508819	0,219635	0,111589085	0,911149227	0,979595261
ENSG00000171148	TADA3	transcriptional adaptor 3	10474	393,1538726	0,014428566	0,097577	0,147869078	0,882446092	0,976300778

ENSG00000197903	H2BC12	H2B clustered histone 12	85236	71,01215497	0,026867948	0,250981	0,107051671	0,914747988	0,98134554
ENSG00000073578	SDHA	succinate dehydrogenase complex flavoprotein subunit A	6389	272,3331848	-0,072357374	0,136608	-0,529671017	0,596340046	0,894229873
ENSG00000111615	KRR1	KRR1 small subunit processome component homolog	11103	278,7332554	0,315643032	0,17894	1,763963822	0,07773807	0,451064976
ENSG00000131931	THAP1	THAP domain containing 1	55145	60,77071289	-0,1042759	0,232516	-0,448468043	0,653815445	0,917134445
ENSG00000134851	TMEM165	transmembrane protein 165	55858	119,3106678	0,097870946	0,156495	0,625393728	0,531712677	0,872945174
ENSG00000170476	MZB1	marginal zone B and B1 cell specific protein	51237	20,31018926	-0,352097685	0,417402	-0,843546136	0,398923074	NA
ENSG00000148842	CNNM2	cyclin and CBS domain divalent metal cation transport mediator 2	54805	103,0974764	0,406781998	0,209556	1,941164413	0,052238338	0,36949064
ENSG00000187231	SESTD1	SEC14 and spectrin domain containing 1	91404	15,66911737	0,673343946	0,548731	1,227093264	0,219787539	NA
ENSG00000101255	TRIB3	tribbles pseudokinase 3	57761	42,17623908	0,031779819	0,281747	0,112795519	0,910192671	NA
ENSG00000148660	CAMK2G	calcium/calmodulin dependent protein kinase II gamma	818	168,2888264	-0,101853509	0,143114	-0,711694477	0,476653989	0,853811879
ENSG00000166340	TPP1	tripeptidyl peptidase 1	1200	589,8776037	-0,20124185	0,113356	-1,775304919	0,075847554	0,446229554
ENSG00000271361	NA	NA	NA	15,31825269	0,684088605	0,535784	1,276800428	0,201672714	NA
ENSG00000096264	NCR2	natural cytotoxicity triggering receptor 2	9436	444,586587	-0,067198389	0,087152	-0,771049944	0,440677329	0,838678081
ENSG00000165632	TAF3	TATA-box binding protein associated factor 3	83860	144,2947908	0,037260902	0,137216	0,271550094	0,785967977	0,950624564
ENSG00000178761	FAM219B	family with sequence similarity 219 member B	57184	139,6479852	-0,39283093	0,214756	-1,829198427	0,067369888	0,418806958
ENSG00000066933	MYO9A	myosin IXA	4649	279,4712874	-0,023647636	0,112303	-0,21056924	0,833223417	0,963446071
ENSG00000132323	ILKAP	ILK associated serine/threonine phosphatase	80895	57,35846614	0,21919037	0,257289	0,851924122	0,394256207	0,812498986
ENSG00000197969	VPS13A	vacuolar protein sorting 13 homolog A	23230	856,3660406	-0,030852185	0,069735	-0,442420046	0,658185276	0,919088619
ENSG00000014216	CAPN1	calpain 1	823	553,0008479	-0,105352151	0,077348	-1,362056971	0,173179906	0,629558603
ENSG00000196476	C20orf96	chromosome 20 open reading frame 96	140680	13,49902631	-0,332526783	0,490807	-0,677510864	0,498081879	NA
ENSG00000111011	RSRC2	arginine and serine rich coiled-coil 2	65117	50,8870262	0,254992619	0,318965	0,799437515	0,424036765	0,829279072
ENSG00000135900	MRPL44	mitochondrial ribosomal protein L44	65080	86,53958669	-0,030560542	0,209193	-0,146087823	0,883852059	0,976751663
ENSG00000157881	PANK4	pantothenate kinase 4 (inactive)	55229	89,77872056	-0,159062592	0,203258	-0,782564581	0,433882847	0,835238413
ENSG00000090989	EXOC1	exocyst complex component 1	55763	170,4239875	-0,121280304	0,205314	-0,590705225	0,554717947	0,882854594
ENSG00000100916	BRMS1L	BRMS1 like transcriptional repressor	84312	15,04477181	-0,135389418	0,462928	-0,292463155	0,769932527	NA
ENSG00000114166	KAT2B	lysine acetyltransferase 2B	8850	937,0391871	0,062235455	0,064918	0,958681273	0,337719335	0,77693253
ENSG00000135912	TTLL4	tubulin tyrosine ligase like 4	9654	82,39627148	-0,081842831	0,246864	-0,331530111	0,740244101	0,939193793
ENSG00000196498	NCOR2	nuclear receptor corepressor 2	9612	492,556489	-0,153904681	0,114766	-1,341034408	0,179909282	0,633591125
ENSG00000261115	TMEM178B	transmembrane protein 178B	100507421	23,32384494	0,085154684	0,364879	0,233377991	0,815467902	NA
ENSG00000100300	TSPO	translocator protein	706	64,48212517	0,239815655	0,286778	0,836240178	0,403019799	0,817066986
ENSG00000116903	EXOC8	exocyst complex component 8	149371	154,2547191	-0,370991159	0,14829	-2,501786608	0,012356838	0,174865955
ENSG00000130303	BST2	bone marrow stromal cell antigen 2	684	470,2919543	-0,046077273	0,081019	-0,568723058	0,569544097	0,886263374
ENSG00000131236	CAP1	cyclase associated actin cytoskeleton regulatory protein 1	10487	2122,95593	-0,057711885	0,054214	-1,064522822	0,287091921	0,738104868
ENSG00000136560	TANK	TRAF family member associated NFKB activator	10010	361,4715352	0,292965906	0,095329	3,073200666	0,00211776	0,05587092
ENSG00000138386	NAB1	NGFI-A binding protein 1	4664	117,4106654	-0,089053091	0,160244	-0,555733	0,578393394	0,887073172
ENSG00000156515	HK1	hexokinase 1	3098	633,6233065	0,042563617	0,073944	0,575619718	0,56487225	0,885045179
ENSG00000163918	RFC4	replication factor C subunit 4	5984	48,52137231	0,409093683	0,288085	1,420046581	0,155594121	0,61428162
ENSG00000164241	C5orf63	chromosome 5 open reading frame 63	401207	21,49745907	-0,01788252	0,564559	-0,031675177	0,974731091	NA
ENSG00000213619	NDUFS3	NADH:ubiquinone oxidoreductase core subunit S3	4722	97,22364333	0,153929425	0,194828	0,790079305	0,429481455	0,832060923
ENSG00000227124	ZNF717	zinc finger protein 717	100131827	23,56804446	0,285608376	0,393711	0,725425843	0,468190824	NA
ENSG00000285508	MKKS	MKKS centrosomal shuttling protein	8195	13,38035257	-0,169557375	0,528599	-0,320767734	0,748386413	NA
ENSG00000285508	LOC128706666	uncharacterized LOC128706666	128706666	13,38035257	-0,169557375	0,528599	-0,320767734	0,748386413	NA
ENSG00000072274	TFRC	transferrin receptor	7037	615,8737249	0,429591125	0,102882	4,175560379	2,97E-05	0,002431904
ENSG00000141040	ZNF287	zinc finger protein 287	57336	12,18538884	0,315117362	0,550894	0,57201104	0,567314495	NA
ENSG00000111647	BLTP3B	bridge-like lipid transfer protein family member 3B	23074	183,9187041	0,048976356	0,132878	0,36858209	0,712439247	0,930846779

ENSG00000132849	PATJ	PATJ crumbs cell polarity complex component	10207	118,18965	-0,504879848	0,189217	-2,668256265	0,007624608	0,129618332
ENSG00000139620	KANSL2	KAT8 regulatory NSL complex subunit 2	54934	69,84111743	-0,170215193	0,257237	-0,661705496	0,508159982	0,864738156
ENSG00000106803	SEC61B	SEC61 translocon subunit beta	10952	172,9022721	-0,032531269	0,131829	-0,24676821	0,805087615	0,957328863
ENSG00000168002	POLR2G	RNA polymerase II subunit G	5436	117,3350342	0,163543608	0,163814	0,998351078	0,318109147	0,762879158
ENSG00000131174	COX7B	cytochrome c oxidase subunit 7B	1349	123,9122565	0,086191877	0,178333	0,483320424	0,628868241	0,905567722
ENSG00000081019	RSBN1	round spermatid basic protein 1	54665	172,9309981	0,435564003	0,175923	2,475876743	0,013290945	0,180367712
ENSG00000006015	REX1BD	required for excision 1-B domain containing	55049	19,38951908	-0,168365327	0,532752	-0,316029281	0,751980283	NA
ENSG00000108515	ENO3	enolase 3	2027	12,65954347	1,425458593	0,77568	1,837688248	0,066108357	NA
ENSG00000102189	EEA1	early endosome antigen 1	8411	155,9750549	-0,283019055	0,148036	-1,911820095	0,055899273	0,38263345
ENSG00000284691	LOC728743	zinc finger domain-containing protein LOC728743	728743	32,36145183	-0,484636762	0,385354	-1,257639035	0,20852233	NA
ENSG00000178105	DDX10	DEAD-box helicase 10	1662	87,87656593	0,056326699	0,202475	0,278190874	0,78086584	0,948265435
ENSG00000123178	SPRYD7	SPRY domain containing 7	57213	43,08038602	-0,049677441	0,264206	-0,188025648	0,850856546	0,967902179
ENSG00000120437	ACAT2	acetyl-CoA acetyltransferase 2	39	129,5706599	0,348589407	0,15079	2,311748571	0,020791543	0,225859999
ENSG00000170581	STAT2	signal transducer and activator of transcription 2	6773	282,0941275	-0,092884676	0,179957	-0,516148662	0,605750583	0,897380562
ENSG00000100284	TOM1	target of myb1 membrane trafficking protein	10043	111,0758404	0,242732273	0,218406	1,111381254	0,266404278	0,722327312
ENSG00000149016	TUT1	terminal uridylyl transferase 1, U6 snRNA-specific	64852	54,33518421	-0,281161268	0,261407	-1,075569576	0,2821198	0,735655069
ENSG00000183793	NPIPA5	nuclear pore complex interacting protein family member A5	100288332	17,0125345	1,682322941	2,057338	0,817718299	0,413518059	NA
ENSG00000247315	ZCCHC3	zinc finger CCHC-type containing 3	85364	162,1723397	-0,384545049	0,134157	-2,866382512	0,004151922	0,088853402
ENSG00000122068	FYTTD1	forty-two-three domain containing 1	84248	640,4226234	0,36221666	0,117806	3,074693999	0,002107186	0,05587092
ENSG00000147894	C9orf72	C9orf72-SMCR8 complex subunit	203228	18,57800022	0,800444285	0,465968	1,717811129	0,085831071	NA
ENSG00000116954	RRAGC	Ras related GTP binding C	64121	136,0247791	0,045246248	0,143292	0,315761937	0,752183211	0,941832449
ENSG00000164172	MOCS2	molybdenum cofactor synthesis 2	4338	93,16055907	0,007436587	0,239239	0,031084363	0,97520226	0,994594828
ENSG00000164754	RAD21	RAD21 cohesin complex component	5885	960,4777993	0,171143926	0,055446	3,086693488	0,002023962	0,054949576
ENSG00000168286	THAP11	THAP domain containing 11	57215	98,77138191	0,047388735	0,207334	0,228561995	0,819209366	0,961067311
ENSG00000177697	CD151	CD151 molecule (Raph blood group)	977	84,82534107	-0,174923442	0,256925	-0,680835808	0,495975389	0,86123475
ENSG00000211450	SELENOH	selenoprotein H	280636	131,4824056	0,007489447	0,176068	0,042537125	0,966070517	0,992789539
ENSG00000261052	SULT1A3	sulfotransferase family 1A member 3	6818	63,41645731	-0,109252884	0,221596	-0,493027121	0,621993424	0,904764752
ENSG00000261052	LOC124907837	sulfotransferase 1A3	124907837	63,41645731	-0,109252884	0,221596	-0,493027121	0,621993424	0,904764752
ENSG00000151014	NOCT	nocturnin	25819	11,96638044	0,024373717	0,495489	0,049191208	0,960766918	NA
ENSG00000134352	IL6ST	interleukin 6 cytokine family signal transducer	3572	37,64366514	1,1053837	1,468905	0,752522119	0,451737133	NA
ENSG00000102125	TAFAZZIN	tafazzin, phospholipid-lysophospholipid transacylase	6901	56,28747846	-0,291627018	0,315313	-0,924880805	0,355027912	0,788341988
ENSG00000134318	ROCK2	Rho associated coiled-coil containing protein kinase 2	9475	204,523299	-0,043083459	0,119429	-0,360744562	0,718290409	0,932668593
ENSG00000137760	ALKBH8	alkB homolog 8, tRNA methyltransferase	91801	96,13216413	0,263684998	0,183146	1,439751101	0,14993783	0,605144768
ENSG00000140543	DET1	DET1 partner of COP1 E3 ubiquitin ligase	55070	15,68874598	-0,360491633	0,47128	-0,764919996	0,444319186	NA
ENSG00000141446	ESCO1	establishment of sister chromatid cohesion N-acetyltransferase 1	114799	121,1929133	-0,061042653	0,268234	-0,227572463	0,81997863	0,961418528
ENSG00000147996	ZNG1E	Zn regulated GTPase metalloprotein activator 1E	220869	20,49401203	0,386548956	0,510273	0,757534337	0,448729802	NA
ENSG00000149806	FAU	FAU ubiquitin like and ribosomal protein S30 fusion	2197	525,1379505	0,143556554	0,093095	1,542045142	0,123062624	0,558690088
ENSG00000164889	SLC4A2	solute carrier family 4 member 2	6522	224,553703	0,179781972	0,118893	1,512133317	0,130499951	0,566497377
ENSG00000168374	ARF4	ADP ribosylation factor 4	378	203,180966	0,016218664	0,124722	0,130038652	0,896535846	0,977990819
ENSG00000272916	NA	NA	NA	50,51684843	0,646071179	0,312456	2,067720149	0,038666346	0,312755389
ENSG00000158417	EIF5B	eukaryotic translation initiation factor 5B	9669	512,5844845	0,037706195	0,082425	0,457459568	0,647340757	0,914264928
ENSG00000176102	CSTF3	cleavage stimulation factor subunit 3	1479	89,40271611	-0,025789707	0,205914	-0,125244952	0,900329631	0,977990819
ENSG00000122643	NT5C3A	5'-nucleotidase, cytosolic IIIA	51251	57,47432221	-1,355437631	0,416372	-3,255356067	0,001132503	0,037467385
ENSG00000153790	SPMIP4	sperm microtubule inner protein 4	136895	28,3779031	0,507083875	0,349317	1,451641935	0,146601192	NA

ENSG00000166477	LEO1	LEO1 homolog, Paf1/RNA polymerase II complex component	123169	110,5091323	-0,017687626	0,164	-0,10785143	0,914113546	0,981103549
ENSG00000182544	MFSD5	major facilitator superfamily domain containing 5	84975	92,3981453	0,151758371	0,177315	0,855868308	0,392070627	0,812491435
ENSG00000139343	SNRPF	small nuclear ribonucleoprotein polypeptide F	6636	64,25131831	0,161251462	0,203375	0,792879009	0,427848318	0,832060923
ENSG00000128973	CLN6	CLN6 transmembrane ER protein	54982	46,34169195	0,09097417	0,276076	0,329525352	0,741758635	0,939193793
ENSG00000197951	ZNF71	zinc finger protein 71	58491	40,95669182	-0,067030711	0,286543	-0,233929164	0,815039972	NA
ENSG00000157869	RAB28	RAB28, member RAS oncogene family	9364	58,44823049	0,143276338	0,227982	0,628455981	0,529705274	0,872145761
ENSG00000285943	NA	NA	NA	26,04973804	0,050617272	0,367944	0,137567716	0,890582072	NA
ENSG00000163171	CDC42EP3	CDC42 effector protein 3	10602	294,4667532	0,000753167	0,104791	0,007187352	0,994265372	0,998500439
ENSG00000100784	RPS6KA5	ribosomal protein S6 kinase A5	9252	382,749384	-0,015942637	0,097305	-0,163842189	0,869855378	0,973613245
ENSG00000054148	PHPT1	phosphohistidine phosphatase 1	29085	57,91059852	0,166600337	0,2209	0,754187827	0,450736446	0,84263906
ENSG00000169592	INO80E	INO80 complex subunit E	283899	84,50581448	-0,249812241	0,24197	-1,032410576	0,30187982	0,751870158
ENSG00000173757	STAT5B	signal transducer and activator of transcription 5B	6777	209,8452152	0,150392935	0,198934	0,755993382	0,449653164	0,84263906
ENSG00000125247	TMTC4	transmembrane O-mannosyltransferase targeting cadherins 4	84899	94,13458691	-0,05453618	0,166987	-0,326589069	0,743978712	0,939745744
ENSG00000050426	LETMD1	LETM1 domain containing 1	25875	43,55464381	-0,33942508	0,418022	-0,811979307	0,416803505	0,82446781
ENSG00000162604	TM2D1	TM2 domain containing 1	83941	64,08982739	-0,041455578	0,24115	-0,171908047	0,86350982	0,971561202
ENSG00000258790	PRORP	protein only RNase P catalytic subunit	9692	69,08100884	0,655998472	0,31062	2,111897062	0,034695279	0,297161096
ENSG00000186174	BCL9L	BCL9 like	283149	508,2869652	-0,147146052	0,090067	-1,633741423	0,102313157	0,515119384
ENSG00000265808	SEC22B	SEC22 homolog B, vesicle trafficking protein	9554	430,5430431	-0,007107343	0,082187	-0,086478043	0,93108641	0,986491322
ENSG00000189362	NEMP2	nuclear envelope integral membrane protein 2	100131211	101,5080472	0,130160379	0,189262	0,687725555	0,491625624	0,860513824
ENSG00000110031	LPXN	leupaxin	9404	861,9436445	0,106593656	0,060572	1,759772346	0,078446414	0,453361393
ENSG00000040199	PHLPP2	PH domain and leucine rich repeat protein phosphatase 2	23035	90,14008654	-0,428009983	0,223776	-1,912673469	0,055789868	0,382350454
ENSG00000184206	GOLGA6L4	golgin A6 family like 4	643707	61,72047887	-0,383268077	0,234079	-1,63734782	0,101557814	0,514437479
ENSG00000100266	PACSIN2	protein kinase C and casein kinase substrate in neurons 2	11252	415,4180467	0,193817011	0,095572	2,027971739	0,042563132	0,328150469
ENSG00000158805	ZNF276	zinc finger protein 276	92822	305,6866336	0,04732018	0,101699	0,465296502	0,641719139	0,912063088
ENSG00000116688	MFN2	mitofusin 2	9927	261,5967247	-0,248128165	0,126544	-1,960800261	0,049902328	0,357759312
ENSG00000004975	DVL2	dishevelled segment polarity protein 2	1856	91,50428092	0,316306307	0,20015	1,580349454	0,11402686	0,539617633
ENSG00000008256	CYTH3	cytohesin 3	9265	32,44393264	-0,182545094	0,305591	-0,597351384	0,550272804	NA
ENSG00000067606	PRKCZ	protein kinase C zeta	5590	63,27572032	-0,491352556	0,267951	-1,833741689	0,066692347	0,417039566
ENSG00000099940	SNAP29	synaptosome associated protein 29	9342	244,9151256	-0,121830667	0,121251	-1,004779432	0,31500307	0,760305504
ENSG00000102978	POLR2C	RNA polymerase II subunit C	5432	142,8933926	0,056034167	0,155205	0,361034333	0,718073781	0,932668593
ENSG00000103194	USP10	ubiquitin specific peptidase 10	9100	255,5399773	-0,079824986	0,125779	-0,634643341	0,525661054	0,871384275
ENSG00000107537	PHYH	phytanoyl-CoA 2-hydroxylase	5264	30,68056424	0,612691853	0,336773	1,819301894	0,068865385	NA
ENSG00000126903	SLC10A3	solute carrier family 10 member 3	8273	79,068117	-0,260842585	0,193207	-1,350071147	0,176993162	0,632039836
ENSG00000153563	CD8A	CD8 subunit alpha	925	244,5226802	0,254089482	0,132184	1,922241349	0,054575396	0,378695505
ENSG00000165480	SKA3	spindle and kinetochore associated complex subunit 3	221150	31,36875712	0,054361559	0,52097	0,104346807	0,916894134	NA
ENSG00000188938	FAM120AOS	family with sequence similarity 120 member A opposite strand	158293	123,3922831	-0,5819971	0,287048	-2,027526172	0,042608631	0,328150469
ENSG00000292366	VAMP7	vesicle associated membrane protein 7	6845	198,6183944	-0,034185982	0,117923	-0,289900107	0,77189266	0,946183835
ENSG00000129055	ANAPC13	anaphase promoting complex subunit 13	25847	37,45916746	-0,17617992	0,29161	-0,604162362	0,545735701	NA
ENSG00000197321	SVIL	supervillin	6840	15,69066165	-0,535597233	0,434836	-1,231723738	0,218052299	NA
ENSG00000213066	CEP43	centrosomal protein 43	11116	91,68204754	0,281317569	0,184801	1,522273268	0,127940623	0,563689485
ENSG00000114978	MOB1A	MOB kinase activator 1A	55233	1244,11585	-0,186987174	0,054632	-3,422670805	0,000620091	0,023991117
ENSG00000127922	SEM1	SEM1 26S proteasome subunit	7979	80,68493198	0,083149105	0,215095	0,386569718	0,69907478	0,930783276
ENSG00000140030	GPR65	G protein-coupled receptor 65	8477	234,6797411	0,02770342	0,119618	0,231599544	0,816849062	0,959707099
ENSG00000168807	SNTB2	syntrophin beta 2	6645	587,5742433	-0,259941663	0,084234	-3,085961562	0,00202895	0,054949576
ENSG00000105404	RABAC1	Rab acceptor 1	10567	119,1154625	0,091564195	0,151848	0,603000993	0,546508029	0,878739902
ENSG00000149531	NA	NA	NA	54,09160912	-0,088039892	0,263324	-0,334341109	0,738122173	0,939193793
ENSG00000004059	ARF5	ADP ribosylation factor 5	381	173,9438057	-0,07286995	0,128813	-0,565703557	0,571595317	0,886795904
ENSG00000187607	ZNF286A	zinc finger protein 286A	57335	74,55822361	0,007361351	0,360133	0,020440642	0,983691863	0,996185513
ENSG00000128654	MTX2	metaxin 2	10651	29,22132598	0,162190824	0,381533	0,425102562	0,67076191	NA

ENSG00000120889	TNFRSF10B	TNF receptor superfamily member 10b	8795	208,0711077	0,148596751	0,117945	1,259880133	0,207712607	0,662274754
ENSG00000126756	UXT	ubiquitously expressed prefoldin like chaperone	8409	82,32298441	-0,179586895	0,195958	-0,916456881	0,359427313	0,790999468
ENSG00000198918	RPL39	ribosomal protein L39	6170	856,5229382	0,158492443	0,096219	1,64720113	0,099516712	0,509429216
ENSG00000083838	ZNF446	zinc finger protein 446	55663	17,3432973	-0,63086363	0,503767	-1,252291325	0,210463729	NA
ENSG00000124795	DEK	DEK proto-oncogene	7913	383,8108279	0,970964998	0,341162	2,84605275	0,004426486	0,091955749
ENSG00000164109	MAD2L1	mitotic arrest deficient 2 like 1	4085	36,75496264	0,742042856	0,277789	2,671243448	0,007557082	NA
ENSG00000164118	CEP44	centrosomal protein 44	80817	54,57026526	-0,174416297	0,292245	-0,596816193	0,550630105	0,880819693
ENSG00000164885	CDK5	cyclin dependent kinase 5	1020	35,18136712	0,317030475	0,300029	1,056665002	0,290664503	NA
ENSG00000099385	BCL7C	BAF chromatin remodeling complex subunit BCL7C	9274	49,17075952	-0,004943668	0,259075	-0,019082005	0,984775687	0,996185513
ENSG00000145979	TBC1D7	TBC1 domain family member 7	51256	34,87202423	0,157751847	0,328093	0,480814759	0,63064816	NA
ENSG00000145979	TBC1D7-LOC100130357	TBC1D7-LOC100130357 readthrough	107080638	34,87202423	0,157751847	0,328093	0,480814759	0,63064816	NA
ENSG00000169021	UQCRFS1	ubiquinol-cytochrome c reductase, Rieske iron-sulfur polypeptide 1	7386	145,9406727	0,138974686	0,144119	0,964306355	0,334892356	0,774813659
ENSG00000198355	PIM3	Pim-3 proto-oncogene, serine/threonine kinase	415116	54,76841749	0,138397952	0,244782	0,565391594	0,571807441	0,886795904
ENSG00000107651	SEC23IP	SEC23 interacting protein	11196	263,9160325	0,009208908	0,117466	0,078396139	0,937512945	0,987194684
ENSG00000096070	BRPF3	bromodomain and PHD finger containing 3	27154	182,784171	-0,260983471	0,158366	-1,647977828	0,099357221	0,509429216
ENSG00000169583	CLIC3	chloride intracellular channel 3	9022	15,83041301	-0,292164607	0,47897	-0,609985503	0,541871411	NA
ENSG00000100207	TCF20	transcription factor 20	6942	439,502612	0,027417574	0,283429	0,096735362	0,922936557	0,984284131
ENSG00000196267	ZNF836	zinc finger protein 836	162962	52,79057408	0,431977808	0,318969	1,354294004	0,175642598	0,632039836
ENSG00000169756	LIMS1	LIM zinc finger domain containing 1	3987	248,5253382	-0,055342298	0,13194	-0,419451737	0,67488602	0,923602509
ENSG00000110274	CEP164	centrosomal protein 164	22897	94,9938101	-0,114701088	0,176316	-0,650541323	0,515342619	0,868562431
ENSG00000005302	MSL3	MSL complex subunit 3	10943	97,66357572	0,05305437	0,182708	0,290378328	0,771526822	0,946052153
ENSG00000164398	ACSL6	acyl-CoA synthetase long chain family member 6	23305	37,68344439	-0,275712087	0,426126	-0,64701969	0,5176192	NA
ENSG00000205643	CDPF1	cysteine rich DPF motif domain containing 1	150383	21,7959623	-0,399616363	0,450316	-0,887413068	0,374856551	NA
ENSG00000100478	AP4S1	adaptor related protein complex 4 subunit sigma 1	11154	62,02404308	-0,038976354	0,269533	-0,144606756	0,885021362	0,977414909
ENSG00000267554	NA	NA	NA	22,76766007	-0,068753016	0,636663	-0,10798972	0,914003847	NA
ENSG00000188599	NPIPP1	nuclear pore complex interacting protein pseudogene 1	100874381	38,84360117	0,159687594	0,287343	0,555738527	0,578389615	NA
ENSG00000178913	TAF7	TATA-box binding protein associated factor 7	6879	420,1919701	0,003875504	0,104215	0,037187605	0,970335422	0,993542794
ENSG00000013810	TACC3	transforming acidic coiled-coil containing protein 3	10460	259,9223776	0,187007315	0,146143	1,279616857	0,200679918	0,659555076
ENSG00000163743	RCHY1	ring finger and CHY zinc finger domain containing 1	25898	82,8463561	-0,387593103	0,305595	-1,268321365	0,20468321	0,660954946
ENSG00000143614	GATAD2B	GATA zinc finger domain containing 2B	57459	495,4646371	-0,273974057	0,123955	-2,210270824	0,027086372	0,267256743
ENSG00000065357	DGKA	diacylglycerol kinase alpha	1606	191,462487	0,152093005	0,156072	0,974504166	0,329806272	0,771479881
ENSG00000106355	LSM5	LSM5 homolog, U6 small nuclear RNA and mRNA degradation associated	23658	67,65428757	0,58273512	0,223111	2,611859159	0,009005134	0,145228594
ENSG00000180957	PITPNB	phosphatidylinositol transfer protein beta	23760	316,3287753	-0,027948446	0,09956	-0,280719853	0,77892528	0,947321361
ENSG00000135211	TMEM60	transmembrane protein 60	85025	33,00326223	0,438188364	0,343043	1,277355465	0,20147678	NA
ENSG00000143373	ZNF687	zinc finger protein 687	57592	142,3252969	-0,208673054	0,234489	-0,889906797	0,373515934	0,800776142
ENSG00000272655	NA	NA	NA	11,73636256	0,386680086	0,573961	0,673704009	0,500499512	NA
ENSG00000162139	NEU3	neuraminidase 3	10825	108,7530948	0,112404762	0,291315	0,385852718	0,699605749	0,930783276
ENSG00000148700	ADD3	adducin 3	120	688,5365981	-0,061179383	0,070253	-0,870846172	0,383838151	0,808586507
ENSG00000107951	MTPAP	mitochondrial poly(A) polymerase	55149	95,23301804	-0,028649427	0,192654	-0,148709521	0,881782848	0,976300778
ENSG00000114473	IQCG	IQ motif containing G	84223	29,37020131	0,168333403	0,340659	0,494140949	0,62120664	NA
ENSG00000185246	PRPF39	pre-mRNA processing factor 39	55015	28,78401357	-0,336322339	0,498582	-0,674558279	0,499956447	NA
ENSG00000167986	DDB1	damage specific DNA binding protein 1	1642	656,7423829	0,130685607	0,095029	1,375211169	0,169065987	0,628714138
ENSG00000181894	ZNF329	zinc finger protein 329	79673	31,33560027	-0,058079667	0,307022	-0,189171114	0,849958707	NA
ENSG00000009844	VTA1	vesicle trafficking 1	51534	150,8031527	0,003086978	0,199203	0,015496675	0,987635937	0,996894778
ENSG00000243716	NPIPB5	nuclear pore complex interacting protein family member B5	100132247	1442,613856	0,008949344	0,096215	0,093014063	0,925892389	0,985226775
ENSG00000101447	FAM83D	family with sequence similarity 83 member D	81610	29,90453081	0,349940898	0,307628	1,137544217	0,255310851	NA
ENSG00000140470	ADAMTS17	ADAM metallopeptidase with thrombospondin type 1 motif 17	170691	114,3293604	-0,326464253	0,160346	-2,036001647	0,04175019	0,325303563

ENSG00000154473	BUB3	BUB3 mitotic checkpoint protein	9184	687,4301323	0,223025297	0,115199	1,935994929	0,052868322	0,371734827
ENSG00000180900	SCRIB	scribble planar cell polarity protein	23513	279,051267	-0,0941052	0,099904	-0,941958838	0,346213714	0,782042713
ENSG00000135899	SP110	SP110 nuclear body protein	3431	401,9661546	-0,03212578	0,084414	-0,380574928	0,703518688	0,930846779
ENSG00000152382	TADA1	transcriptional adaptor 1	117143	88,14344855	-0,156647051	0,18742	-0,835808703	0,40326253	0,817348106
ENSG00000116668	SWT1	SWT1 RNA endoribonuclease homolog	54823	23,8194121	-0,039016587	0,42011	-0,092872278	0,92600503	NA
ENSG00000117000	RLF	RLF zinc finger	6018	259,7792022	-0,152434665	0,108122	-1,40984538	0,158585344	0,614461481
ENSG00000140319	SRP14	signal recognition particle 14	6727	391,578574	0,24144011	0,105157	2,296007343	0,021675462	0,232567042
ENSG00000104774	MAN2B1	mannosidase alpha class 2B member 1	4125	255,0877559	0,075718376	0,129507	0,584664273	0,558773481	0,883921037
ENSG00000085741	WNT11	Wnt family member 11	7481	176,893357	0,144122167	0,141379	1,019401646	0,308012325	0,754562945
ENSG00000129518	EAPP	E2F associated phosphoprotein	55837	123,2885742	-0,04427416	0,16541	-0,267663238	0,78895855	0,95148885
ENSG00000164081	TEX264	testis expressed 264, ER-phagy receptor	51368	77,70899903	-0,251929553	0,246861	-1,020532178	0,307476136	0,754087936
ENSG00000198843	SELENOT	selenoprotein T	51714	339,1478157	-0,181234474	0,099612	-1,819402026	0,068850118	0,426428338
ENSG00000121057	AKAP1	A-kinase anchoring protein 1	8165	140,5663875	-0,303398938	0,160004	-1,896192635	0,057934577	0,390238564
ENSG00000140350	ANP32A	acidic nuclear phosphoprotein 32 family member A	8125	763,3782266	0,092377724	0,065271	1,415305607	0,156978912	0,61428162
ENSG00000167384	ZNF180	zinc finger protein 180	7733	36,14961837	-0,98761134	0,460354	-2,145330856	0,031926403	NA
ENSG00000008324	SS18L2	SS18 like 2	51188	63,22672661	0,249272719	0,224717	1,109271926	0,267312892	0,722449525
ENSG00000093144	ECHDC1	ethylmalonyl-CoA decarboxylase 1	55862	155,5656192	-0,016195527	0,206695	-0,078354765	0,937545855	0,987194684
ENSG00000100888	CHD8	chromodomain helicase DNA binding protein 8	57680	625,4092623	-0,081719106	0,082029	-0,99622358	0,319141525	0,763489959
ENSG00000112306	RPS12	ribosomal protein S12	6206	916,8832265	0,181878654	0,082942	2,192842653	0,028318718	0,270907684
ENSG00000132718	SYT11	synaptotagmin 11	23208	319,3695898	-0,013739414	0,10154	-0,135310997	0,892365991	0,977990819
ENSG00000135775	COG2	component of oligomeric golgi complex 2	22796	213,8786319	-0,036476465	0,127758	-0,285511322	0,775252432	0,946448613
ENSG00000137312	FLOT1	flotillin 1	10211	336,2969428	0,512769668	0,342538	1,496972677	0,134400368	0,574309297
ENSG00000152778	IFIT5	interferon induced protein with tetratricopeptide repeats 5	24138	65,06316805	0,177616494	0,265348	0,669371405	0,503258588	0,863200032
ENSG00000167487	KLHL26	kelch like family member 26	55295	30,67691469	0,109128512	0,3756	0,290544187	0,771399951	NA
ENSG00000176182	MYPOP	Myb related transcription factor, partner of profilin	339344	44,19518218	0,006697791	0,262681	0,025497769	0,979657928	0,995712569
ENSG00000136856	SLC2A8	solute carrier family 2 member 8	29988	20,58186052	0,200224448	0,471649	0,424520227	0,671186457	NA
ENSG00000127804	METTL16	methyltransferase 16, RNA N6-adenosine	79066	156,6810251	-0,09240212	0,141786	-0,651702317	0,514593227	0,868514045
ENSG00000100220	RTCB	RNA 2',3'-cyclic phosphate and 5'-OH ligase	51493	174,6294429	0,029321568	0,13006	0,225447062	0,821631504	0,961898134
ENSG00000140105	WARS1	tryptophanyl-tRNA synthetase 1	7453	557,5955979	0,142486802	0,06981	2,041070911	0,041243779	0,323534763
ENSG00000010310	GIPR	gastric inhibitory polypeptide receptor	2696	53,90896853	-0,269391202	0,238216	-1,130868066	0,258110626	0,714048892
ENSG00000106617	PRKAG2	protein kinase AMP-activated non-catalytic subunit gamma 2	51422	34,72764654	-0,755530297	0,403528	-1,872311617	0,061163505	NA
ENSG00000162300	ZFPL1	zinc finger protein like 1	7542	31,16290547	-0,26351756	0,330255	-0,797922321	0,424915569	NA
ENSG00000197978	GOLGA6L9	golgin A6 family like 9	440295	49,89034644	-0,092227327	0,489267	-0,188501053	0,85048389	0,967902179
ENSG00000166526	ZNF3	zinc finger protein 3	7551	82,44604695	0,039279123	0,224934	0,17462521	0,861374138	0,970863509
ENSG00000182712	CMC4	C-X9-C motif containing 4	100272147	17,5228486	0,304301018	0,505657	0,601793545	0,547311573	NA
ENSG00000174915	PTDSS2	phosphatidylserine synthase 2	81490	44,00268403	0,141031869	0,561496	0,251171556	0,801681475	0,956122858
ENSG00000036054	TBC1D23	TBC1 domain family member 23	55773	148,0886597	0,06939406	0,165843	0,418432233	0,675631124	0,924052604
ENSG00000044446	PHKA2	phosphorylase kinase regulatory subunit alpha 2	5256	129,0441297	-0,066484003	0,174625	-0,380725075	0,70340726	0,930846779
ENSG00000119640	ACYP1	acylphosphatase 1	97	22,14083546	-0,342408423	0,419955	-0,815344752	0,414874999	NA
ENSG00000125503	PPP1R12C	protein phosphatase 1 regulatory subunit 12C	54776	183,9547933	0,229739362	0,17439	1,317386729	0,187709027	0,645758518
ENSG00000136040	PLXNC1	plexin C1	10154	284,5127657	0,164874061	0,13917	1,184694912	0,236138095	0,691328984
ENSG00000184281	TSSC4	tumor suppressing subtransferable candidate 4	10078	73,49400281	-0,190295019	0,194283	-0,979474591	0,327345537	0,769876712
ENSG00000186532	SMYD4	SET and MYND domain containing 4	114826	101,7160686	0,001244827	0,183695	0,00677658	0,994593113	0,998500439
ENSG00000196268	ZNF493	zinc finger protein 493	284443	57,02567331	-0,135203248	0,276466	-0,489040406	0,624813091	0,90479929
ENSG00000164068	RNF123	ring finger protein 123	63891	121,1496223	0,180312048	0,189191	0,953070329	0,340554438	0,777431905
ENSG00000095564	BTAF1	B-TFIID TATA-box binding protein associated factor 1	9044	526,3274206	-0,001142738	0,080077	-0,014270442	0,988614221	0,997032569
ENSG00000138378	STAT4	signal transducer and activator of transcription 4	6775	642,1620031	0,072839401	0,078819	0,924139766	0,355413551	0,788341988
ENSG00000162413	KLHL21	kelch like family member 21	9903	158,3618042	-0,201022408	0,129615	-1,550918762	0,120921154	0,555064138
ENSG00000164114	MAP9	microtubule associated protein 9	79884	38,42164616	0,196129838	0,321734	0,609601538	0,542125792	NA

ENSG00000104643	MTMR9	myotubularin related protein 9	66036	132,1307819	0,026345992	0,168284	0,15655661	0,875594301	0,975344937
ENSG00000161960	EIF4A1	eukaryotic translation initiation factor 4A1	1973	1176,538188	0,128895382	0,056961	2,262859487	0,023644352	0,245726955
ENSG00000158714	SLAMF8	SLAM family member 8	56833	354,6884051	-0,352625791	0,11897	-2,963981534	0,003036865	0,072162376
ENSG00000166377	ATP9B	ATPase phospholipid transporting 9B (putative)	374868	102,8899665	-0,084457381	0,169378	-0,498631511	0,618039002	0,902245041
ENSG00000117118	SDHB	succinate dehydrogenase complex iron sulfur subunit B	6390	97,37168155	0,262196073	0,163984	1,598908459	0,109840944	0,529583042
ENSG00000134250	NOTCH2	notch receptor 2	4853	1701,891815	0,060726352	0,044396	1,367834196	0,171363981	0,629558603
ENSG00000164180	TMEM161B	transmembrane protein 161B	153396	48,12007792	-0,331429544	0,27556	-1,202751136	0,229072639	0,68351286
ENSG00000090060	PAPOLA	poly(A) polymerase alpha	10914	981,1370442	-0,048122917	0,058423	-0,823700541	0,410109732	0,82130194
ENSG00000177542	SLC25A22	solute carrier family 25 member 22	79751	81,8749865	-0,02859077	0,209722	-0,136327196	0,891562625	0,977990819
ENSG00000175130	MARCKSL1	MARCKS like 1	65108	25,95126349	-0,12369768	0,350673	-0,352743885	0,724280456	NA
ENSG00000163466	ARPC2	actin related protein 2/3 complex subunit 2	10109	1577,036668	-0,004074068	0,051401	-0,079259909	0,936825894	0,987098279
ENSG00000184164	CRELD2	cysteine rich with EGF like domains 2	79174	139,6124959	-0,136530366	0,142579	-0,957577333	0,338275933	0,777431905
ENSG00000196396	PTPN1	protein tyrosine phosphatase non-receptor type 1	5770	512,1436085	0,002334783	0,076583	0,030486865	0,975678769	0,994804758
ENSG00000167780	SOAT2	sterol O-acyltransferase 2	8435	17,00754402	-0,050422298	0,51862	-0,097224017	0,922548496	NA
ENSG00000141551	CSNK1D	casein kinase 1 delta	1453	455,1907703	-0,132737673	0,087341	-1,519770963	0,128568549	0,563689485
ENSG00000123836	PFKFB2	6-phosphofructo-2-kinase/fructose-2,6-biphosphatase 2	5208	172,1377173	-0,016808752	0,150552	-0,111647248	0,911103108	0,979595261
ENSG00000173218	VANGL1	VANGL planar cell polarity protein 1	81839	139,4155679	0,097830379	0,195847	0,49952504	0,617409551	0,90216458
ENSG00000196781	TLE1	TLE family member 1, transcriptional corepressor	7088	226,4113075	-0,084465368	0,124608	-0,677851369	0,497865937	0,861283987
ENSG00000108773	KAT2A	lysine acetyltransferase 2A	2648	76,24200943	-0,505608059	0,242999	-2,080701856	0,037461204	0,307119308
ENSG00000130731	METTL26	methyltransferase like 26	84326	27,77964266	-0,173053377	0,466368	-0,371066128	0,710588279	NA
ENSG00000143195	ILDR2	immunoglobulin like domain containing receptor 2	387597	43,0285188	0,657676169	0,449155	1,464252638	0,143124933	0,588535718
ENSG00000162065	TBC1D24	TBC1 domain family member 24	57465	91,35360156	-0,311109364	0,216431	-1,437452073	0,150589582	0,606528499
ENSG00000166147	FBN1	fibrillin 1	2200	13,64151569	-0,158332125	0,943675	-0,167782557	0,866754346	NA
ENSG00000104331	BPNT2	3'(2'), 5'-bisphosphate nucleotidase 2	54928	380,4802518	-0,070373585	0,104547	-0,673127367	0,500866264	0,861917048
ENSG00000123094	RASSF8	Ras association domain family member 8	11228	191,0072539	-0,0991281	0,121758	-0,814137906	0,415565953	0,82446781
ENSG00000138185	ENTPD1	ectonucleoside triphosphate diphosphohydrolase 1	953	652,5710689	0,17254697	0,214411	0,804750535	0,420963651	0,827182516
ENSG00000169258	GPRIN1	G protein regulated inducer of neurite outgrowth 1	114787	31,82980249	-0,035127391	0,378416	-0,092827467	0,92604063	NA
ENSG00000135452	TSPAN31	tetraspanin 31	6302	64,72082424	1,329485982	0,397138	3,347667117	0,000814948	0,029909366
ENSG00000103043	VAC14	VAC14 component of PIKFYVE complex	55697	95,47436547	-0,057280666	0,170824	-0,335320755	0,737383139	0,939193793
ENSG00000228168	HNRNPA1P21	heterogeneous nuclear ribonucleoprotein A1 pseudogene 21	344697	38,59607289	-0,606341587	0,310311	-1,953978941	0,050703708	NA
ENSG00000277400	MAFIP	MAFF interacting protein	727764	15,12140201	-0,478800219	0,4823	-0,992744497	0,320834484	NA
ENSG00000126012	KDM5C	lysine demethylase 5C	8242	812,6162777	-0,105304316	0,066477	-1,584078432	0,113175872	0,538390853
ENSG00000177707	NECTIN3	nectin cell adhesion molecule 3	25945	99,98996843	0,15492402	0,180975	0,856049704	0,391970287	0,812491435
ENSG00000140807	NKD1	NKD inhibitor of WNT signaling pathway 1	85407	23,75566067	0,07025434	0,355045	0,197874483	0,843143269	NA
ENSG00000078674	PCM1	pericentriolar material 1	5108	864,1085618	-0,003221179	0,062774	-0,051314131	0,959075208	0,991911599
ENSG00000100271	TTLL1	TTL family tubulin polyglutamylase complex subunit L1	25809	26,92315122	-0,996328037	0,692398	-1,438953661	0,150163652	NA
ENSG00000131089	ARHGEF9	Cdc42 guanine nucleotide exchange factor 9	23229	225,3004589	-0,164214189	0,167686	-0,979295337	0,327434074	0,769876712
ENSG00000006432	MAP3K9	mitogen-activated protein kinase kinase kinase 9	4293	26,3968472	-0,176277847	0,471518	-0,373851657	0,708514685	NA
ENSG00000029364	SLC39A9	solute carrier family 39 member 9	55334	230,1977511	-0,072185558	0,12918	-0,558799495	0,576298567	0,887069818
ENSG00000121892	PDS5A	PDS5 cohesin associated factor A	23244	994,9365797	0,004964548	0,082182	0,06040895	0,951829931	0,991212866
ENSG00000142634	EFHD2	EF-hand domain family member D2	79180	358,4276036	-0,267780683	0,099398	-2,694014022	0,007059719	0,123215637
ENSG00000159335	PTMS	parathymosin	5763	20,8517224	-0,1465991	0,4339	-0,337863986	0,735465689	NA
ENSG00000141084	RANBP10	RAN binding protein 10	57610	134,9875463	0,152789582	0,15007	1,018120039	0,308620913	0,754797782
ENSG00000088305	DNMT3B	DNA methyltransferase 3 beta	1789	14,52685044	2,009533518	2,064736	0,973264116	0,330422052	NA
ENSG00000066926	FECH	ferrochelatase	2235	75,01172843	1,52001239	1,006557	1,510111206	0,131015051	0,567474728
ENSG00000145191	EIF2B5	eukaryotic translation initiation factor 2B subunit epsilon	8893	83,19546267	0,372660922	0,337828	1,103107257	0,26998059	0,725477256

ENSG00000254986	DPP3	dipeptidyl peptidase 3	10072	125,9892942	0,19265814	0,174022	1,107091299	0,268254457	0,723761767
ENSG00000074842	MYDGF	myeloid derived growth factor	56005	143,4041404	-0,090256287	0,14894	-0,605991371	0,544520484	0,87855238
ENSG00000163605	PPP4R2	protein phosphatase 4 regulatory subunit 2	151987	277,1343444	0,067498505	0,108282	0,623360936	0,533047363	0,873191491
ENSG00000136169	SETDB2	SET domain bifurcated histone lysine methyltransferase 2	83852	173,5384273	-0,220083041	0,130229	-1,689968136	0,091034051	0,490934677
ENSG00000119929	CUTC	cutC copper transporter	51076	62,79422823	0,054085621	0,243799	0,221844798	0,824434704	0,961917534
ENSG00000063322	MED29	mediator complex subunit 29	55588	205,3451091	-0,102913569	0,142446	-0,722476247	0,470001724	0,852690218
ENSG00000101752	MIB1	MIB E3 ubiquitin protein ligase 1	57534	722,8196909	0,001988635	0,08271	0,024043562	0,980817861	0,995752963
ENSG00000103326	CAPN15	calpain 15	6650	270,0948453	0,153862517	0,158958	0,967945234	0,333071722	0,773674527
ENSG00000112739	PRPF4B	pre-mRNA processing factor 4B	8899	454,7954934	0,004569612	0,142709	0,032020444	0,974455747	0,994534085
ENSG00000149923	PPP4C	protein phosphatase 4 catalytic subunit	5531	198,5326545	0,146858525	0,134665	1,090543141	0,275473961	0,727985359
ENSG00000171867	PRNP	prion protein	5621	302,1770745	-0,163779568	0,113324	-1,445233964	0,148392179	0,601349859
ENSG00000264522	OTUD7B	OTU deubiquitinase 7B	56957	140,8579915	-0,123103188	0,141372	-0,870772217	0,383878538	0,808586507
ENSG00000111605	CPSF6	cleavage and polyadenylation specific factor 6	11052	451,800834	0,027140553	0,079116	0,343048693	0,731561828	0,938991109
ENSG00000113532	ST8SIA4	ST8 alpha-N-acetyl-neuraminide alpha-2,8-sialyltransferase 4	7903	289,6213851	0,067855035	0,119957	0,565659056	0,571625574	0,886795904
ENSG00000260914	NA	NA	NA	36,04724151	0,192251125	0,306172	0,627918005	0,530057656	NA
ENSG00000000460	FIRRM	FIGNL1 interacting regulator of recombination and mitosis	55732	35,60232875	0,734528077	0,298603	2,459883796	0,013898201	NA
ENSG00000002330	BAD	BCL2 associated agonist of cell death	572	24,61585533	0,101727681	0,343447	0,296195837	0,767080528	NA
ENSG00000119685	TTLL5	tubulin tyrosine ligase like 5	23093	87,32385687	0,26438575	0,190441	1,388280412	0,165051674	0,624454568
ENSG00000092439	TRPM7	transient receptor potential cation channel subfamily M member 7	54822	703,28346	-0,030031579	0,079788	-0,376392594	0,706625051	0,930846779
ENSG00000115211	EIF2B4	eukaryotic translation initiation factor 2B subunit delta	8890	68,90094098	0,080879718	0,219442	0,368569366	0,712448732	0,930846779
ENSG00000126777	KTN1	kinectin 1	3895	604,7826303	0,013199783	0,110997	0,118920163	0,905338607	0,978460866
ENSG00000174151	CYB561D1	cytochrome b561 family member D1	284613	257,6684428	0,315896585	0,152947	2,065403544	0,038884834	0,313876138
ENSG00000189007	ADAT2	adenosine deaminase tRNA specific 2	134637	68,4941239	-0,298379015	0,241545	-1,235296182	0,216720294	0,672891335
ENSG00000197302	KRBOX5	KRAB box domain containing 5	124411	89,03009696	-0,138931773	0,214841	-0,646673563	0,517843237	0,868791049
ENSG00000198380	GFPT1	glutamine--fructose-6-phosphate transaminase 1	2673	249,1947637	0,098908515	0,142108	0,696009936	0,486422607	0,858315694
ENSG00000198646	NCOA6	nuclear receptor coactivator 6	23054	442,8009365	0,077932719	0,087251	0,8931969	0,371751738	0,800366818
ENSG00000214013	GANC	glucosidase alpha, neutral C	2595	68,23026404	-0,132182848	0,262868	-0,502849171	0,61507032	0,901934707
ENSG00000028137	TNFRSF1B	TNF receptor superfamily member 1B	7133	966,8969111	-0,03999926	0,094684	-0,422447929	0,6726981	0,923602509
ENSG00000182551	ADI1	acireductone dioxygenase 1	55256	114,0184485	-0,092837577	0,178927	-0,518856177	0,60386104	0,897212117
ENSG00000101444	AHCY	adenosylhomocysteinase	191	91,415211	0,424587397	0,193458	2,19473165	0,028182853	0,270084577
ENSG00000106723	SPIN1	spindlin 1	10927	367,6935676	-0,003192359	0,115773	-0,027574245	0,978001724	0,995598269
ENSG00000113749	HRH2	histamine receptor H2	3274	188,5697409	-0,176936118	0,144322	-1,225983966	0,220204709	0,676903242
ENSG00000132781	MUTYH	mutY DNA glycosylase	4595	28,65365354	0,566279364	0,372158	1,521611144	0,128106543	NA
ENSG00000160190	SLC37A1	solute carrier family 37 member 1	54020	179,8894123	-0,262839541	0,152844	-1,719661905	0,085493916	0,477891497
ENSG00000171135	JAGN1	jagunal homolog 1	84522	73,94965966	0,111553051	0,211161	0,528284006	0,597302231	0,894257182
ENSG00000284842	NA	NA	NA	41,68708887	0,169705487	0,267812	0,633673009	0,526294243	NA
ENSG00000088876	ZNF343	zinc finger protein 343	79175	44,90611884	-0,071591658	0,298197	-0,240081452	0,810267113	0,959038743
ENSG00000184117	NIPSNAP1	nipsnap homolog 1	8508	158,759393	-0,018362563	0,130304	-0,140921434	0,887932009	0,977990819
ENSG00000164187	LMBRD2	LMBR1 domain containing 2	92255	172,3701169	0,040580415	0,13515	0,300262719	0,763976768	0,944181516
ENSG00000138942	RNF185	ring finger protein 185	91445	132,2928583	0,113010979	0,166572	0,678450219	0,497486277	0,861283987
ENSG00000152223	EPG5	ectopic P-granules 5 autophagy tethering factor	57724	315,7753953	-0,236787812	0,397228	-0,596100275	0,551108241	0,880819693
ENSG00000197728	RPS26	ribosomal protein S26	6231	369,0525881	0,215900977	0,097626	2,211520731	0,026999798	0,266737626
ENSG00000008128	CDK11A	cyclin dependent kinase 11A	728642	190,667754	0,221400717	0,133792	1,654808636	0,097963322	0,506186795
ENSG00000070214	SLC44A1	solute carrier family 44 member 1	23446	173,8592529	0,10273221	0,146701	0,70028448	0,483749662	0,85698884
ENSG00000104756	KCTD9	potassium channel tetramerization domain containing 9	54793	256,3455768	-0,059946127	0,104874	-0,57159881	0,567593802	0,88603113
ENSG00000117448	AKR1A1	aldo-keto reductase family 1 member A1	10327	123,829455	0,156122784	0,178229	0,875965595	0,381048738	0,806699466
ENSG00000126249	PDCD2L	programmed cell death 2 like	84306	15,43878584	1,155915065	0,770903	1,499430058	0,133762101	NA
ENSG00000127054	INTS11	integrator complex subunit 11	54973	169,9115367	0,201553466	0,153525	1,312842303	0,189236092	0,6473259
ENSG00000132821	VSTM2L	V-set and transmembrane domain containing 2 like	128434	65,92469392	-0,090528573	0,242622	-0,373125724	0,709054874	0,930846779

ENSG00000148343	MIGA2	mitoguardin 2	84895	75,16257727	-0,06894647	0,315668	-0,218414754	0,827105971	0,962241193
ENSG00000155307	SAMSN1	SAM domain, SH3 domain and nuclear localization signals 1	64092	629,1778665	0,201554901	0,073924	2,726504284	0,006400912	0,115569573
ENSG00000170949	ZNF160	zinc finger protein 160	90338	175,6090521	-0,248805512	0,153349	-1,6224816	0,10470027	0,519795144
ENSG00000182179	UBA7	ubiquitin like modifier activating enzyme 7	7318	346,8425857	-0,151285513	0,094728	-1,597056565	0,110253099	0,530593038
ENSG00000174871	CNIH2	cornichon family AMPA receptor auxiliary protein 2	254263	28,73766341	0,038155998	0,318218	0,11990537	0,90455811	NA
ENSG00000111880	RNGTT	RNA guanylyltransferase and 5'-phosphatase	8732	171,8265913	0,15242107	0,128527	1,1859032	0,235660529	0,691328984
ENSG00000166136	NDUFB8	NADH:ubiquinone oxidoreductase subunit B8	4714	151,3127244	0,075073157	0,148351	0,506050767	0,612821011	0,900033105
ENSG00000054611	TBC1D22A	TBC1 domain family member 22A	25771	100,9152873	-0,141619914	0,328967	-0,430499098	0,666832623	0,922184616
ENSG00000074582	BCS1L	BCS1 homolog, ubiquinol-cytochrome c reductase complex chaperone	617	48,22808539	-0,124953003	0,294062	-0,424920888	0,670894347	0,922641254
ENSG00000101343	CRNKL1	crooked neck pre-mRNA splicing factor 1	51340	131,0458821	-0,118571055	0,202222	-0,586340754	0,55764655	0,883921037
ENSG00000155329	ZCCHC10	zinc finger CCHC-type containing 10	54819	57,32356824	-0,286766387	0,246814	-1,161872793	0,24528714	0,702327815
ENSG00000185049	NELFA	negative elongation factor complex member A	7469	86,71261579	0,111518255	0,199612	0,558675846	0,576382967	0,887069818
ENSG00000155660	PDIA4	protein disulfide isomerase family A member 4	9601	605,0701289	-0,121532008	0,079783	-1,523289597	0,127686269	0,563689485
ENSG00000177606	JUN	Jun proto-oncogene, AP-1 transcription factor subunit	3725	40,06231854	0,055191503	1,852098	0,029799446	0,976227001	NA
ENSG00000171428	NAT1	N-acetyltransferase 1	9	22,25286629	0,399391434	0,456327	0,875230152	0,38144869	NA
ENSG00000136271	DDX56	DEAD-box helicase 56	54606	80,67682846	-0,003824225	0,332799	-0,011491101	0,990831629	0,99756334
ENSG00000170242	USP47	ubiquitin specific peptidase 47	55031	582,3178142	0,039445286	0,085549	0,461083627	0,644738608	0,912530858
ENSG00000175582	RAB6A	RAB6A, member RAS oncogene family	5870	587,1917352	-0,148462539	0,096227	-1,542834765	0,122870872	0,558690088
ENSG00000099814	CEP170B	centrosomal protein 170B	283638	95,62678266	-0,146453667	0,180041	-0,813445668	0,415962585	0,82446781
ENSG00000168958	MFF	mitochondrial fission factor	56947	152,7677605	-0,03699668	0,15368	-0,240738349	0,809757915	0,959038743
ENSG00000172292	CERS6	ceramide synthase 6	253782	393,7176586	-0,06824196	0,107818	-0,632937027	0,526774767	0,871653726
ENSG00000198712	COX2	cytochrome c oxidase subunit II	4513	15051,72574	0,244959532	0,089837	2,726720273	0,006396724	0,115569573
ENSG00000244509	APOBEC3C	apolipoprotein B mRNA editing enzyme catalytic subunit 3C	27350	632,2685434	-0,03280902	0,072842	-0,450411743	0,652413579	0,916482964
ENSG00000123395	ATG101	autophagy related 101	60673	49,48081655	-0,263875075	0,275955	-0,956226112	0,33895801	0,777431905
ENSG00000168769	TET2	tet methylcytosine dioxygenase 2	54790	284,2671836	0,049262159	0,12992	0,379173784	0,704558818	0,930846779
ENSG00000171219	CDC42BPG	CDC42 binding protein kinase gamma	55561	36,77255531	-0,112493708	0,320526	-0,350965857	0,725613963	NA
ENSG00000175213	ZNF408	zinc finger protein 408	79797	45,86292464	-0,165285713	0,300863	-0,549371212	0,582750726	0,888501395
ENSG00000289694	NA	NA	NA	850,6740597	-0,209824524	0,135987	-1,542978619	0,122835964	0,558690088
ENSG00000108946	PRKAR1A	protein kinase cAMP-dependent type I regulatory subunit alpha	5573	1298,115965	-0,199003884	0,084153	-2,364795978	0,018040006	0,210216924
ENSG00000196850	PPTC7	protein phosphatase targeting COQ7	160760	284,5889522	-0,103866535	0,104506	-0,993884113	0,32027929	0,76448423
ENSG00000160714	UBE2Q1	ubiquitin conjugating enzyme E2 Q1	55585	283,0076187	-0,060323311	0,104626	-0,576563625	0,564234277	0,885045179
ENSG00000273820	USP27X	ubiquitin specific peptidase 27 X-linked	389856	15,70644161	0,319744388	0,461496	0,692843275	0,488407911	NA
ENSG00000090372	STRN4	striatin 4	29888	136,9902767	-0,235149174	0,158563	-1,483002621	0,138073714	0,578980755
ENSG00000127580	WDR24	WD repeat domain 24	84219	49,02083621	-0,036810614	0,275833	-0,133452416	0,893835594	0,977990819
ENSG00000129625	REEP5	receptor accessory protein 5	7905	633,3953154	-0,015834874	0,078829	-0,200876636	0,840795037	0,966505956
ENSG00000144827	ABHD10	abhydrolase domain containing 10, depalmitoylase	55347	61,48369965	0,212003176	0,278073	0,762401284	0,44582054	0,841259829
ENSG00000150316	CWC15	CWC15 spliceosome associated protein homolog	51503	80,35906271	0,362006052	0,209958	1,72417992	0,084675368	0,47468975
ENSG00000196576	PLXNB2	plexin B2	23654	16,48693638	0,237046347	0,732696	0,323526097	0,746296847	NA
ENSG00000007237	GAS7	growth arrest specific 7	8522	994,2173494	0,073005275	0,060636	1,203984388	0,228595613	0,68351286
ENSG00000116353	MECR	mitochondrial trans-2-enoyl-CoA reductase	51102	12,14478684	0,941013495	0,718539	1,309621302	0,190323978	NA
ENSG00000074966	TXK	TXK tyrosine kinase	7294	1095,38063	-0,059817173	0,073255	-0,816557304	0,414181463	0,823956741
ENSG00000166987	MBD6	methyl-CpG binding domain protein 6	114785	382,8307575	-0,120884138	0,109512	-1,10384732	0,269659374	0,725477256
ENSG00000168758	SEMA4C	semaphorin 4C	54910	109,888386	0,263036881	1,938139	0,135716188	0,89204565	0,977990819
ENSG00000086300	SNX10	sorting nexin 10	29887	223,8902782	0,197297223	0,14161	1,393238721	0,163547614	0,622638372
ENSG00000162736	NCSTN	nicastrin	23385	224,6905803	0,124198038	0,198114	0,626901983	0,530723487	0,872577406
ENSG00000159618	ADGRG5	adhesion G protein-coupled receptor G5	221188	341,6868652	-0,014919451	0,119172	-0,125192608	0,900371069	0,977990819
ENSG00000134222	PSRC1	proline and serine rich coiled-coil 1	84722	12,05684049	0,698801893	0,878605	0,795354256	0,426407461	NA

ENSG00000204310	AGPAT1	1-acylglycerol-3-phosphate O-acyltransferase 1	10554	75,6237544	0,234691023	0,29124	0,805835015	0,420337986	0,82698761
ENSG00000165678	GHITM	growth hormone inducible transmembrane protein	27069	474,9190834	0,183288255	0,093785	1,954342134	0,050660769	0,362047026
ENSG00000130305	NSUN5	NOP2/Sun RNA methyltransferase 5	55695	48,1368075	-0,301248811	0,374278	-0,804880766	0,420888488	0,827182516
ENSG00000133935	ERG28	ergosterol biosynthesis 28 homolog	11161	86,8107267	0,030605305	0,193987	0,157769941	0,874638086	0,974937202
ENSG00000160307	S100B	S100 calcium binding protein B	6285	31,12979079	-0,397947944	0,318297	-1,250239981	0,211211896	NA
ENSG00000114450	GNB4	G protein subunit beta 4	59345	69,20725766	-0,125597795	0,262103	-0,47919186	0,631802145	0,906830052
ENSG00000100330	MTMR3	myotubularin related protein 3	8897	361,3931788	-0,059473301	0,094038	-0,632436597	0,527101626	0,871653726
ENSG00000169375	SIN3A	SIN3 transcription regulator family member A	25942	567,3811636	0,033097108	0,082202	0,402631605	0,687219256	0,926748702
ENSG00000171475	WIPF2	WAS/WASL interacting protein family member 2	147179	312,1586136	-0,381890157	0,130578	-2,924620704	0,003448763	0,078059322
ENSG00000163006	CCDC138	coiled-coil domain containing 138	165055	11,8194699	-0,203488466	0,514672	-0,395374945	0,692566197	NA
ENSG00000081237	PTPRC	protein tyrosine phosphatase receptor type C	5788	3744,288831	-0,492544098	0,157436	-3,128535879	0,001756796	0,05104416
ENSG00000104375	STK3	serine/threonine kinase 3	6788	12,12248516	0,430527522	0,517037	0,832682568	0,405023778	NA
ENSG00000106991	ENG	endoglin	2022	103,0435337	-0,001093668	0,169272	-0,006461012	0,994844894	0,998500439
ENSG00000148057	IDNK	IDNK gluconokinase	414328	19,67145239	-0,530625632	0,682606	-0,777352566	0,43695079	NA
ENSG00000166965	RCCD1	RCC1 domain containing 1	91433	18,46841116	0,427132574	0,607266	0,703369739	0,48182536	NA
ENSG00000288920	RPSA2	ribosomal protein SA 2	388524	93,43789194	0,021731975	0,210003	0,103484116	0,917578755	0,983173744
ENSG00000241685	ARPC1A	actin related protein 2/3 complex subunit 1A	10552	82,59795426	0,008145621	0,210025	0,038783994	0,969062606	0,993542794
ENSG00000189369	GSPT2	G1 to S phase transition 2	23708	59,3334678	-0,226791373	0,234151	-0,968569454	0,33276005	0,773452925
ENSG00000145723	GIN1	gypsy retrotransposon integrase 1	54826	23,82930141	0,161677189	0,517632	0,312340162	0,75478202	NA
ENSG00000132329	RAMP1	receptor activity modifying protein 1	10267	86,80614071	0,014367148	0,198569	0,072353531	0,942320565	0,988475138
ENSG00000204316	MRPL38	mitochondrial ribosomal protein L38	64978	86,77543074	0,111457587	0,189651	0,587699777	0,556733825	0,883708056
ENSG00000069998	HDHD5	haloacid dehalogenase like hydrolase domain containing 5	27440	48,03176506	-0,377823105	0,262579	-1,438893455	0,150180712	0,605789839
ENSG00000120438	TCP1	t-complex 1	6950	364,6044272	0,121364979	0,109159	1,11182009	0,266215512	0,722230271
ENSG00000165185	KIAA1958	KIAA1958	158405	62,26509308	0,092305037	0,260467	0,354382596	0,723052175	0,934902053
ENSG00000198015	MRPL42	mitochondrial ribosomal protein L42	28977	143,4402692	0,102844004	0,291299	0,353053353	0,724048443	0,934902053
ENSG00000280828	NA	NA	NA	71,14415175	-0,147003057	0,227341	-0,646619202	0,517878427	0,868791049
ENSG00000182324	KCNJ14	potassium inwardly rectifying channel subfamily J member 14	3770	11,83252362	-0,10964511	0,516579	-0,212252559	0,831910004	NA
ENSG00000147099	HDAC8	histone deacetylase 8	55869	43,78060606	0,616022925	0,308851	1,994564089	0,046090444	0,342966833
ENSG00000039123	MTREX	Mtr4 exosome RNA helicase	23517	253,2362211	0,037316095	0,190314	0,196076908	0,844549971	0,967231052
ENSG00000132155	RAF1	Raf-1 proto-oncogene, serine/threonine kinase	5894	158,7816825	-0,209142099	0,196229	-1,065807866	0,286510505	0,738005299
ENSG00000137185	ZSCAN9	zinc finger and SCAN domain containing 9	7746	46,27272213	0,030374864	0,299281	0,101492864	0,919159222	0,984062493
ENSG00000102226	USP11	ubiquitin specific peptidase 11	8237	228,8096802	-0,086411937	0,125852	-0,686615046	0,492325344	0,860513824
ENSG00000125835	SNRPB	small nuclear ribonucleoprotein polypeptides B and B1	6628	233,1486004	-0,095789783	0,157403	-0,608563654	0,542813702	0,877542071
ENSG00000183426	NPIPA1	nuclear pore complex interacting protein family member A1	9284	97,74494747	-0,616677547	0,286148	-2,15509822	0,031154149	0,281657534
ENSG00000183426	NPIPA2	nuclear pore complex interacting protein family member A2	642799	97,74494747	-0,616677547	0,286148	-2,15509822	0,031154149	0,281657534
ENSG00000107372	ZFAND5	zinc finger AN1-type containing 5	7763	284,389711	-0,129903663	0,124961	-1,039556771	0,29854587	0,74913611
ENSG00000175764	TTLL11	tubulin tyrosine ligase like 11	158135	27,85675185	0,473190924	0,71977	0,6574197	0,510911088	NA
ENSG00000284431	NA	NA	NA	29,92855074	-0,816131267	0,580715	-1,405390942	0,159905059	NA
ENSG00000004478	FKBP4	FKBP prolyl isomerase 4	2288	105,7636569	0,045455068	0,166901	0,272347671	0,785354705	0,950624564
ENSG00000196588	MRTFA	myocardin related transcription factor A	57591	265,8625536	-0,072271561	0,133795	-0,540165986	0,589082568	0,891627382
ENSG00000106344	RBM28	RNA binding motif protein 28	55131	282,1353338	-0,330020971	0,149691	-2,204678006	0,027476698	0,269079779
ENSG00000162650	ATXN7L2	ataxin 7 like 2	127002	29,45424752	-0,611639639	0,665754	-0,9187178	0,358243197	NA
ENSG00000203668	CHML	CHM like Rab escort protein	1122	110,9222209	0,081923529	0,164112	0,499192849	0,617643532	0,902181653
ENSG00000203668	OPN3	opsin 3	23596	110,9222209	0,081923529	0,164112	0,499192849	0,617643532	0,902181653
ENSG00000168303	MPLKIP	M-phase specific PLK1 interacting protein	136647	49,41025778	0,118789772	0,290658	0,408692357	0,682765444	0,925586358
ENSG00000099999	RNF215	ring finger protein 215	200312	18,46541029	0,165639668	0,530382	0,312302435	0,754810689	NA
ENSG00000108523	RNF167	ring finger protein 167	26001	392,3683216	-0,037953689	0,105723	-0,358991174	0,719601692	0,933530522
ENSG00000183496	MEX3B	mex-3 RNA binding family member B	84206	11,95435675	0,243389225	0,62763	0,387790869	0,698170803	NA

ENSG00000094916	CBX5	chromobox 5	23468	613,1308311	0,392708499	0,233664	1,680655084	0,09282993	0,494966917
ENSG00000095002	MSH2	mutS homolog 2	4436	174,8551232	0,112558658	0,136383	0,825314725	0,409192934	0,821104064
ENSG00000188243	COMMD6	COMM domain containing 6	170622	90,29967501	0,052548077	0,243182	0,216085506	0,828921097	0,962723654
ENSG00000139218	SCAF11	SR-related CTD associated factor 11	9169	973,5012959	0,073250431	0,066824	1,096172082	0,273003475	0,726913977
ENSG00000091542	ALKBH5	alkB homolog 5, RNA demethylase	54890	289,9725664	-0,189157518	0,103118	-1,834371682	0,06659884	0,416786686
ENSG00000159210	SNF8	SNF8 subunit of ESCRT-II	11267	53,67575947	-0,152420763	0,370303	-0,411610596	0,680624864	0,925586358
ENSG00000069849	ATP1B3	ATPase Na+/K+ transporting subunit beta 3	483	165,693814	0,562352333	0,13849	4,060589648	4,89E-05	0,003696586
ENSG00000169255	B3GALNT1	beta-1,3-N-acetylgalactosaminyltransferase 1 (globoside blood group)	8706	33,74459015	-0,412607729	0,324341	-1,272141541	0,203322834	NA
ENSG00000100350	FOXRED2	FAD dependent oxidoreductase domain containing 2	80020	22,99380444	0,654698834	0,447153	1,464150351	0,143152872	NA
ENSG00000100890	PRORP	protein only RNase P catalytic subunit	9692	147,1858436	-0,005247216	0,203313	-0,025808607	0,979409997	0,995712569
ENSG00000109083	IFT20	intraflagellar transport 20	90410	37,49646452	0,234099941	0,267874	0,873917425	0,382163224	NA
ENSG00000121594	CD80	CD80 molecule	941	15,6598666	0,255587518	0,502727	0,508402047	0,611171418	NA
ENSG00000168310	IRF2	interferon regulatory factor 2	3660	302,6704581	0,011720391	0,124668	0,094012786	0,925099	0,984515928
ENSG00000171596	NMUR1	neuromedin U receptor 1	10316	54,22909853	-0,457783707	0,250784	-1,825409802	0,067939211	0,421813886
ENSG00000174943	KCTD13	potassium channel tetramerization domain containing 13	253980	98,12769388	-0,312785332	0,212528	-1,471738051	0,141091628	0,584767096
ENSG00000174989	FBXW8	F-box and WD repeat domain containing 8	26259	135,213387	-0,050706038	0,153154	-0,331079271	0,740584609	0,939193793
ENSG00000100060	MFNG	MFNG O-fucosylpeptide 3-beta-N-acetylglucosaminyltransferase	4242	401,7367044	0,014220082	0,099196	0,143353503	0,886011003	0,977761756
ENSG00000235194	PPP1R3E	protein phosphatase 1 regulatory subunit 3E	90673	41,12915559	-0,616462001	0,315186	-1,955870215	0,050480445	NA
ENSG00000175416	CLTB	clathrin light chain B	1212	86,6397694	-0,162986839	0,200435	-0,813167229	0,416122185	0,82446781
ENSG00000115514	TXNDC9	thioredoxin domain containing 9	10190	101,2378111	0,017970419	0,179971	0,099851477	0,920462239	0,984284131
ENSG00000100029	PES1	pescadillo ribosomal biogenesis factor 1	23481	147,3724738	-0,209029501	0,141221	-1,480161206	0,138830231	0,580912431
ENSG00000117614	SYF2	SYF2 pre-mRNA splicing factor	25949	191,7371267	-0,037696221	0,142602	-0,264346436	0,791512982	0,951705903
ENSG00000172831	CES2	carboxylesterase 2	8824	127,1699073	0,063080037	0,166442	0,378990738	0,704694742	0,930846779
ENSG00000173207	CKS1B	CDC28 protein kinase regulatory subunit 1B	1163	71,34925154	0,158748049	0,203072	0,781734272	0,434370756	0,835560161
ENSG00000101003	GINS1	GINS complex subunit 1	9837	27,83349682	-0,10267407	0,367754	-0,279191903	0,780097557	NA
ENSG00000132635	PCED1A	PC-esterase domain containing 1A	64773	150,9157635	-0,310486712	0,147535	-2,104488176	0,035335885	0,299505978
ENSG00000136003	ISCU	iron-sulfur cluster assembly enzyme	23479	148,2375353	0,102544028	0,141518	0,724601919	0,468696281	0,851701553
ENSG00000198730	CTR9	CTR9 homolog, Paf1/RNA polymerase II complex component	9646	496,6266491	-0,103657636	0,080149	-1,293319437	0,195900601	0,654724816
ENSG00000101247	NDUFAF5	NADH:ubiquinone oxidoreductase complex assembly factor 5	79133	27,75767729	-0,985983662	0,637365	-1,546968392	0,121870868	NA
ENSG00000170852	KBTBD2	kelch repeat and BTB domain containing 2	25948	229,531511	0,032075799	0,109627	0,292590529	0,769835154	0,945027399
ENSG00000204514	ZNF814	zinc finger protein 814	730051	49,40576447	0,69915538	0,2865	2,440331076	0,014673807	0,190178349
ENSG00000162688	AGL	amylo-alpha-1, 6-glucosidase, 4-alpha-glucanotransferase	178	196,0648581	0,153814053	0,122726	1,253316135	0,210090679	0,664554962
ENSG00000285437	POLR2J3	RNA polymerase II subunit J3	548644	191,1040034	0,28586271	0,465435	0,614184419	0,539093468	0,875342836
ENSG00000122696	SLC25A51	solute carrier family 25 member 51	92014	33,70827567	-0,094716548	0,35607	-0,266005118	0,790235267	NA
ENSG00000130165	ELOF1	elongation factor 1	84337	86,64251118	-0,277586492	0,180903	-1,53444919	0,124919184	0,5624706
ENSG00000211695	TRGV9	T cell receptor gamma variable 9	6983	61,80450941	-0,176379371	0,239779	-0,73559057	0,461979903	0,846765498
ENSG00000151914	DST	dystonin	667	139,7588525	-0,030662811	0,165388	-0,18539917	0,85291596	0,968314823
ENSG00000189306	RRP7A	ribosomal RNA processing 7 homolog A	27341	136,884918	-0,26254699	0,154331	-1,701197838	0,088905844	0,488044705
ENSG00000198746	GPATCH3	G-patch domain containing 3	63906	64,08014568	-0,28961908	0,203025	-1,426521558	0,153717849	0,6111586
ENSG00000095794	CREM	cAMP responsive element modulator	1390	310,3882099	0,243642939	0,101579	2,398556439	0,01645984	0,202942572
ENSG00000164494	PDSS2	decaprenyl diphosphate synthase subunit 2	57107	64,74134185	0,268376133	0,261521	1,026212	0,304791666	0,751870158
ENSG00000183684	ALYREF	Aly/REF export factor	10189	127,6985388	0,168075965	0,155421	1,081427212	0,279507122	0,732760553
ENSG00000146830	GIGYF1	GRB10 interacting GYF protein 1	64599	491,085863	-0,150142436	0,088889	-1,689108778	0,091198586	0,490934677
ENSG00000158769	F11R	F11 receptor	50848	96,62366847	0,398555824	0,295008	1,351000593	0,176695243	0,632039836

ENSG00000151923	TIAL1	TIA1 cytotoxic granule associated RNA binding protein like 1	7073	306,5544484	0,00208312	0,109496	0,019024593	0,984821487	0,996185513
ENSG00000129472	RAB2B	RAB2B, member RAS oncogene family	84932	122,9172093	-0,249812807	0,155056	-1,61111144	0,107155435	0,526328198
ENSG00000168038	ULK4	unc-51 like kinase 4	54986	15,95891866	-0,151362599	0,550992	-0,27470932	0,78353957	NA
ENSG00000105122	RASAL3	RAS protein activator like 3	64926	485,9292003	-0,061922575	0,106047	-0,583917104	0,559276085	0,883921037
ENSG00000129480	DTD2	D-aminoacyl-tRNA deacylase 2	112487	37,55910784	-0,371545484	0,386622	-0,961005156	0,336549575	NA
ENSG00000149089	APIP	APAF1 interacting protein	51074	48,92511992	0,141284381	0,299074	0,472405644	0,636637276	0,90943397
ENSG00000077549	CAPZB	capping actin protein of muscle Z-line subunit beta	832	871,2113052	-0,142597353	0,067519	-2,111970738	0,034688959	0,297161096
ENSG00000105341	DMAC2	distal membrane arm assembly component 2	55101	79,6578587	-0,12993993	0,224781	-0,578072661	0,563215064	0,884816202
ENSG00000112234	FBXL4	F-box and leucine rich repeat protein 4	26235	90,70740527	0,063154151	0,240117	0,263014261	0,792539588	0,952065757
ENSG00000124098	FAM210B	family with sequence similarity 210 member B	116151	120,6245237	-0,138237611	0,147308	-0,938427796	0,348024607	0,784103633
ENSG00000157259	GATAD1	GATA zinc finger domain containing 1	57798	144,5010752	-0,009513637	0,163482	-0,058193959	0,953594132	0,991212866
ENSG00000265241	RBM8A	RNA binding motif protein 8A	9939	524,4613689	-0,008297012	0,128726	-0,064455053	0,948607895	0,990608484
ENSG00000132305	IMMT	inner membrane mitochondrial protein	10989	308,7616778	0,241978116	0,119501	2,024905383	0,042877081	0,329507429
ENSG00000132376	INPP5K	inositol polyphosphate-5-phosphatase K	51763	173,2527308	-0,229518071	0,151543	-1,514544927	0,129887688	0,565894171
ENSG00000106868	SUSD1	sushi domain containing 1	64420	164,7196435	0,038213271	0,163496	0,233726016	0,81519769	0,959616705
ENSG00000141349	G6PC3	glucose-6-phosphatase catalytic subunit 3	92579	30,02155148	0,240430724	1,032283	0,232911613	0,815830041	NA
ENSG00000133872	SARAF	store-operated calcium entry associated regulatory factor	51669	1130,565717	-0,042467052	0,08488	-0,500318968	0,6168505	0,90216458
ENSG00000108984	MAP2K6	mitogen-activated protein kinase kinase 6	5608	119,7003972	-0,702968903	0,583997	-1,203719216	0,228698123	0,68351286
ENSG00000164258	NDUFS4	NADH:ubiquinone oxidoreductase subunit S4	4724	36,70899097	0,223691929	0,325178	0,687906897	0,491511413	NA
ENSG00000112146	FBXO9	F-box protein 9	26268	151,3114636	-0,089909883	0,168328	-0,534134528	0,593248463	0,893798854
ENSG00000115310	RTN4	reticulon 4	57142	317,8490767	-0,014477778	0,096839	-0,149503685	0,881156202	0,976300778
ENSG00000006695	COX10	cytochrome c oxidase assembly factor heme A:farnesyltransferase COX10	1352	53,82811414	0,128578923	0,256613	0,501060662	0,616328431	0,902090873
ENSG00000079819	EPB41L2	erythrocyte membrane protein band 4,1 like 2	2037	69,30707817	0,582471193	0,249474	2,334794531	0,019554157	0,219397642
ENSG00000123136	DDX39A	DExD-box helicase 39A	10212	174,2625473	0,190917871	0,149485	1,277169891	0,201542275	0,660119983
ENSG00000186141	POLR3C	RNA polymerase III subunit C	10623	50,83027508	0,04255243	0,564819	0,075338139	0,939945677	0,987813564
ENSG00000276045	ORAI1	ORAI calcium release-activated calcium modulator 1	84876	90,63113003	-0,276874384	0,305399	-0,906600062	0,364618329	0,793491925
ENSG00000104976	SNAPC2	small nuclear RNA activating complex polypeptide 2	6618	58,95740349	-0,059039108	0,229607	-0,257131413	0,797077328	0,95354564
ENSG00000125977	EIF2S2	eukaryotic translation initiation factor 2 subunit beta	8894	367,5752627	0,205304861	0,096988	2,116807346	0,034276202	0,295829986
ENSG00000163964	PIGX	phosphatidylinositol glycan anchor biosynthesis class X	54965	96,18464198	0,27542884	0,222546	1,237627754	0,215854118	0,672410621
ENSG00000168237	GLYCTK	glycerate kinase	132158	67,38819913	0,365578455	0,279169	1,309522751	0,190357336	0,647836333
ENSG00000169609	C15orf40	chromosome 15 open reading frame 40	123207	68,39209355	-0,92037252	0,907888	-1,013751627	0,310701282	0,75688983
ENSG00000171763	AFG2B	AFG2 AAA ATPase homolog B	79029	15,96752932	0,026675772	0,621692	0,042908343	0,965774598	NA
ENSG00000175387	SMAD2	SMAD family member 2	4087	532,9860514	0,01350234	0,080351	0,168042865	0,866549557	0,971722599
ENSG00000198900	TOP1	DNA topoisomerase I	7150	502,0447799	0,063669981	0,095532	0,666480882	0,50510378	0,864630986
ENSG00000282936	LOC122526780	uncharacterized LOC122526780	122526780	24,51957084	0,048881898	0,37545	0,130195651	0,896411636	NA
ENSG00000108423	TUBD1	tubulin delta 1	51174	17,27905778	-0,085681063	0,451063	-0,189953518	0,849345555	NA
ENSG00000130706	ADRM1	ADRM1 26S proteasome ubiquitin receptor	11047	155,1317483	-0,174169283	0,145551	-1,196617977	0,23145549	0,685722905
ENSG00000164916	FOXK1	forkhead box K1	221937	382,5085861	-0,190801252	0,092319	-2,066767604	0,038756057	0,313158514
ENSG00000068745	IP6K2	inositol hexakisphosphate kinase 2	51447	189,0372422	-0,041395939	0,123108	-0,336257177	0,736676939	0,939157822
ENSG00000169239	CA5B	carbonic anhydrase 5B	11238	114,3061138	-0,276031685	0,176417	-1,564650927	0,117664781	0,546895877
ENSG00000178773	CPNE7	copine 7	27132	40,79265285	0,348226308	0,297641	1,169952202	0,242020205	NA
ENSG00000065809	FAM107B	family with sequence similarity 107 member B	83641	514,167945	0,037725537	0,091183	0,413732671	0,679069897	0,925297532
ENSG00000087502	ERGIC2	ERGIC and golgi 2	51290	97,81238937	-0,039857928	0,185794	-0,214527781	0,830135505	0,963156386
ENSG00000101624	CEP76	centrosomal protein 76	79959	26,35301351	-0,165894409	0,393229	-0,421877151	0,673114688	NA
ENSG00000087008	ACOX3	acyl-CoA oxidase 3, pristanoyl	8310	49,75349676	0,086028356	0,281706	0,305383583	0,760074023	0,942901768
ENSG00000096996	IL12RB1	interleukin 12 receptor subunit beta 1	3594	235,3956134	-0,066034767	0,109048	-0,60555468	0,544810504	0,878589015

ENSG00000175595	ERCC4	ERCC excision repair 4, endonuclease catalytic subunit	2072	54,91749407	-0,337041474	0,255662	-1,318310223	0,18739982	0,645758518
ENSG00000134690	CDCA8	cell division cycle associated 8	55143	86,64423477	0,477622789	0,205385	2,325503127	0,020045073	0,223127163
ENSG00000140326	CDAN1	codanin 1	146059	50,10675354	0,192117141	0,256287	0,749618588	0,453484452	0,843234809
ENSG00000196597	ZNF782	zinc finger protein 782	158431	34,744013	0,150560469	0,378551	0,397728432	0,690830377	NA
ENSG00000135636	DYSF	dysferlin	8291	25,57609219	-0,136777669	0,431458	-0,317012987	0,751233748	NA
ENSG00000105722	ERF	ETS2 repressor factor	2077	39,59487333	0,021312022	0,306963	0,069428573	0,944648485	NA
ENSG00000148411	NACC2	NACC family member 2	138151	70,23674145	-0,069487685	0,216251	-0,321329174	0,74796095	0,939899419
ENSG00000044115	CTNNA1	catenin alpha 1	1495	486,672347	0,104931402	0,094062	1,115554246	0,264612986	0,720266173
ENSG00000076555	ACACB	acetyl-CoA carboxylase beta	32	14,25163798	0,196728706	0,559681	0,351501394	0,725212227	NA
ENSG00000151366	NDUFC2	NADH:ubiquinone oxidoreductase subunit C2	4718	147,7920716	0,198048067	0,159395	1,242502027	0,214051393	0,668985134
ENSG00000134247	PTGFRN	prostaglandin F2 receptor inhibitor	5738	68,97671643	0,129923659	0,208542	0,623009128	0,533278523	0,873191491
ENSG00000156521	TYSND1	trypsin like peroxisomal matrix peptidase 1	219743	20,12396169	-0,543662336	0,419626	-1,295586464	0,195117995	NA
ENSG00000163412	EIF4E3	eukaryotic translation initiation factor 4E family member 3	317649	229,9242323	-0,288582992	0,153753	-1,876925994	0,060528238	0,398308113
ENSG00000167377	ZNF23	zinc finger protein 23	7571	24,52119088	0,748900039	0,760522	0,984718218	0,324762501	NA
ENSG00000128604	IRF5	interferon regulatory factor 5	3663	61,7660269	0,098294978	0,255481	0,384744557	0,70042668	0,930783276
ENSG00000154370	TRIM11	tripartite motif containing 11	81559	234,9124769	-0,041353005	0,117633	-0,351543689	0,725180502	0,935847464
ENSG00000178935	ZNF552	zinc finger protein 552	79818	38,63821253	0,2118138	0,299982	0,706087856	0,480133503	NA
ENSG00000171681	ATF7IP	activating transcription factor 7 interacting protein	55729	569,7873152	-0,115636638	0,076324	-1,515081479	0,129751771	0,565894171
ENSG00000141428	C18orf21	chromosome 18 open reading frame 21	83608	41,17997905	-0,148595117	0,307786	-0,482786575	0,629247284	NA
ENSG00000134744	TUT4	terminal uridylyl transferase 4	23318	217,617071	-0,070076002	0,17804	-0,393598033	0,693877833	0,9283565
ENSG00000174428	GTF2IRD2B	GTF2I repeat domain containing 2B	389524	117,6373376	-0,485678127	0,211241	-2,299162954	0,021495688	0,231270044
ENSG00000171421	MRPL36	mitochondrial ribosomal protein L36	64979	35,49935448	0,316389397	0,303803	1,041428672	0,297676641	NA
ENSG00000165233	CARD19	caspase recruitment domain family member 19	84270	15,89705486	0,137667794	0,660806	0,208333042	0,834968934	NA
ENSG00000102309	PIN4	peptidylprolyl cis/trans isomerase, NIMA-interacting 4	5303	44,03652452	0,081625814	0,264308	0,308828136	0,757452265	0,942901768
ENSG00000105963	ADAP1	ArfGAP with dual PH domains 1	11033	187,7322211	0,10024812	0,197395	0,507855298	0,611554826	0,900006614
ENSG00000157593	SLC35B2	solute carrier family 35 member B2	347734	105,70537	-0,132045446	0,191023	-0,691254355	0,489405711	0,860369726
ENSG00000172239	PAIP1	poly(A) binding protein interacting protein 1	10605	91,4376576	0,100643611	0,184958	0,544142328	0,586343535	0,89126033
ENSG00000185900	POMK	protein O-mannose kinase	84197	168,7893201	-0,386606171	0,198227	-1,950325182	0,051137373	0,364671481
ENSG00000112110	MRPL18	mitochondrial ribosomal protein L18	29074	87,63567348	0,030076213	0,18581	0,161865628	0,871411671	0,974150353
ENSG00000197841	ZNF181	zinc finger protein 181	339318	33,95167624	-0,244212973	0,365125	-0,668847929	0,503592489	NA
ENSG00000138613	APH1B	aph-1 homolog B, gamma-secretase subunit	83464	64,2447437	0,514036935	0,315243	1,630607903	0,102973081	0,515621608
ENSG00000282826	FRG1CP	FSHD region gene 1 family member C, pseudogene	100289097	62,10800423	-0,015365069	0,210729	-0,07291382	0,941874696	0,988475138
ENSG00000089327	FXYD5	FXYD domain containing ion transport regulator 5	53827	566,2532212	-0,027297876	0,079952	-0,341429484	0,732780282	0,93912057
ENSG00000107185	RGP1	RGP1 homolog, RAB6A GEF complex partner 1	9827	296,0545482	0,137264088	0,107995	1,271023282	0,203720366	0,660954946
ENSG00000118308	IRAG2	inositol 1,4,5-triphosphate receptor associated 2	4033	108,124309	0,161578725	0,25873	0,624506934	0,532294718	0,872967783
ENSG00000176407	KCMF1	potassium channel modulatory factor 1	56888	129,2202196	0,172824289	0,15471	1,117088998	0,263956274	0,719682465
ENSG00000151461	UPF2	UPF2 regulator of nonsense mediated mRNA decay	26019	382,0854807	-0,012928353	0,096765	-0,133605836	0,893714269	0,977990819
ENSG00000138795	LEF1	lymphoid enhancer binding factor 1	51176	454,2112355	0,101888633	0,088826	1,147063337	0,251355445	0,70779361
ENSG00000095787	WAC	WW domain containing adaptor with coiled-coil	51322	413,2971496	-0,050483833	0,123551	-0,408605665	0,682829072	0,925586358
ENSG00000022277	RTF2	replication termination factor 2	51507	411,4924053	-0,015539414	0,084945	-0,182934764	0,854849206	0,968377739
ENSG00000102786	INTS6	integrator complex subunit 6	26512	354,2642187	-0,062131057	0,278505	-0,223087502	0,823467405	0,961917534
ENSG00000104687	GSR	glutathione-disulfide reductase	2936	405,9322235	-0,158727259	0,082153	-1,932082254	0,053349354	0,372780987
ENSG00000108622	ICAM2	intercellular adhesion molecule 2	3384	35,00096664	-0,204387993	0,321981	-0,634782174	0,525570491	NA
ENSG00000139899	CBLN3	cerebellin 3 precursor	643866	17,38503549	0,312466226	0,486714	0,641991744	0,520878544	NA
ENSG00000141002	TCF25	transcription factor 25	22980	345,9629697	-0,189073821	0,093523	-2,02167241	0,043210206	0,331418904
ENSG00000171649	ZIK1	zinc finger protein interacting with K protein 1	284307	42,69358207	-0,039399187	0,266341	-0,147927508	0,882399979	0,976300778
ENSG00000179859	RNF227	ring finger protein 227	284023	18,89406393	-0,319377875	0,464208	-0,688005689	0,491449199	NA

ENSG00000182095	TNRC18	trinucleotide repeat containing 18	84629	642,3429103	-0,036847151	0,099057	-0,371980525	0,709907349	0,930846779
ENSG00000185527	PDE6G	phosphodiesterase 6G	5148	26,73788572	-0,564146224	0,386821	-1,458416482	0,14472578	NA
ENSG00000188211	NCR3LG1	natural killer cell cytotoxicity receptor 3 ligand 1	374383	15,54419067	0,877571635	0,473943	1,851640063	0,064077526	NA
ENSG00000255529	POLR2M	RNA polymerase II subunit M	81488	182,657223	-0,673304339	0,516434	-1,303757537	0,192316265	0,649982152
ENSG00000129235	TXNDC17	thioredoxin domain containing 17	84817	27,53735957	0,03033676	0,387333	0,078322156	0,937571794	NA
ENSG00000154930	ACSS1	acyl-CoA synthetase short chain family member 1	84532	216,0552551	-0,168257241	0,119359	-1,409674325	0,158635869	0,614461481
ENSG00000104490	NCALD	neurocalcin delta	83988	506,4398414	-0,439672834	0,078877	-5,574139635	2,49E-08	6,51E-06
ENSG00000144233	AMMECR1L	AMMECR1 like	83607	101,1847957	0,127370183	0,316902	0,401922793	0,687740848	0,926748702
ENSG00000006194	ZNF263	zinc finger protein 263	10127	101,1417512	0,125967187	0,234133	0,538014542	0,590567009	0,892326528
ENSG00000035687	ADSS2	adenylosuccinate synthase 2	159	303,9165154	0,011903481	0,112461	0,105845829	0,915704674	0,981835428
ENSG00000103496	STX4	syntaxin 4	6810	182,2345991	-0,11827439	0,154649	-0,764790116	0,444396535	0,840223973
ENSG00000001461	NIPAL3	NIPA like domain containing 3	57185	287,0398643	0,167335766	0,119952	1,395018374	0,163010299	0,621052441
ENSG00000120256	LRP11	LDL receptor related protein 11	84918	21,45923502	0,211799354	0,396396	0,534313188	0,593124869	NA
ENSG00000166887	VPS39	VPS39 subunit of HOPS complex	23339	399,8169644	-0,017267591	0,092203	-0,187277121	0,85144336	0,967902179
ENSG00000131378	RFTN1	raftlin, lipid raft linker 1	23180	450,5655904	-0,092089212	0,089215	-1,032213357	0,301972179	0,751870158
ENSG00000100591	AHSA1	activator of HSP90 ATPase activity 1	10598	204,2458029	-0,263047089	0,154864	-1,698565414	0,089401098	0,488626459
ENSG00000140264	SERF2	small EDRK-rich factor 2	10169	548,7693207	-0,011599456	0,089702	-0,129310493	0,897111969	0,977990819
ENSG00000168175	MAPK1IP1L	mitogen-activated protein kinase 1 interacting protein 1 like	93487	317,7310831	-0,112234163	0,095235	-1,178501979	0,238596544	0,693537104
ENSG00000153214	TMEM87B	transmembrane protein 87B	84910	187,92606	-0,069760705	0,12636	-0,552077749	0,580895086	0,888209149
ENSG00000172301	COPRS	coordinator of PRMT5 and differentiation stimulator	55352	20,45156962	0,797706238	0,444617	1,794144199	0,072790156	NA
ENSG00000107521	HPS1	HPS1 biogenesis of lysosomal organelles complex 3 subunit 1	3257	17,05712299	-0,334890462	0,68653	-0,487801891	0,625690172	NA
ENSG00000197863	ZNF790	zinc finger protein 790	388536	31,26949436	0,204823169	0,318961	0,642157865	0,520770688	NA
ENSG00000095951	HIVEP1	HIVEP zinc finger 1	3096	106,0381111	0,500294786	0,633072	0,79026543	0,429372771	0,832060923
ENSG00000129355	CDKN2D	cyclin dependent kinase inhibitor 2D	1032	73,80476139	-0,160673624	0,192005	-0,836820765	0,402693324	0,816750249
ENSG00000183621	ZNF438	zinc finger protein 438	220929	25,76904139	0,062548018	0,374032	0,167226334	0,867191964	NA
ENSG00000150459	SAP18	Sin3A associated protein 18	10284	263,1355667	-0,15171837	0,130723	-1,160613996	0,245798911	0,703024271
ENSG00000198258	UBL5	ubiquitin like 5	59286	118,7118289	0,051085352	0,172682	0,295835465	0,767355737	0,944698783
ENSG00000145388	METTL14	methyltransferase 14, N6-adenosine-methyltransferase subunit	57721	112,6756361	0,027566075	0,200108	0,137756067	0,890433206	0,977990819
ENSG00000122952	ZWINT	ZW10 interacting kinetochore protein	11130	55,6529961	0,33699559	0,293447	1,1484039	0,250801865	0,707335884
ENSG00000182154	MRPL41	mitochondrial ribosomal protein L41	64975	61,86680472	-0,370858758	0,235365	-1,575675618	0,115100579	0,541225115
ENSG00000146282	RARS2	arginyl-tRNA synthetase 2, mitochondrial	57038	37,25879688	0,732045496	0,658714	1,111325688	0,266428186	NA
ENSG00000140830	TXNL4B	thioredoxin like 4B	54957	40,30577444	-0,151201906	0,276251	-0,547335195	0,584148469	NA
ENSG00000185722	ANKFY1	ankyrin repeat and FYVE domain containing 1	51479	415,9336568	-0,099536758	0,085866	-1,159208261	0,246371305	0,703431917
ENSG00000187837	H1-2	H1,2 linker histone, cluster member	3006	32,64714393	0,839519644	0,323044	2,598778689	0,009355607	NA
ENSG00000136286	MYO1G	myosin IG	64005	635,8114203	-0,062586564	0,090868	-0,688762571	0,490972693	0,860513824
ENSG00000138688	BLTP1	bridge-like lipid transfer protein family member 1	84162	396,5917801	0,497871334	0,55768	0,892754893	0,371988447	0,800437606
ENSG00000135249	RINT1	RAD50 interactor 1	60561	97,60807243	0,271293371	0,170841	1,587990875	0,112288403	0,535466371
ENSG00000159314	ARHGAP27	Rho GTPase activating protein 27	201176	78,41888148	0,855837217	0,66698	1,283152653	0,1994386	0,658208139
ENSG00000103966	EHD4	EH domain containing 4	30844	60,46458939	0,075125278	0,212652	0,353278635	0,723879561	0,934902053
ENSG00000107745	MICU1	mitochondrial calcium uptake 1	10367	81,80291149	0,092139962	0,202594	0,454801634	0,649251954	0,91482326
ENSG00000163281	GNPDA2	glucosamine-6-phosphate deaminase 2	132789	52,68351967	0,084069638	0,24015	0,35007176	0,726284844	0,93640838
ENSG00000146955	RAB19	RAB19, member RAS oncogene family	401409	13,47547227	-0,377335903	0,606821	-0,621824377	0,534057355	NA
ENSG00000140400	MAN2C1	mannosidase alpha class 2C member 1	4123	177,1959072	-0,260677435	0,13238	-1,969162563	0,048934426	0,354876255
ENSG00000071246	VASH1	vasohibin 1	22846	175,0539	-0,020828196	0,153307	-0,13585938	0,891932447	0,977990819
ENSG00000115084	SLC35F5	solute carrier family 35 member F5	80255	215,585692	-0,566136184	0,153386	-3,690919841	0,000223445	0,011545615
ENSG00000147421	HMBOX1	homeobox containing 1	79618	149,3361781	0,122627005	0,15997	0,766560413	0,44334292	0,839231259
ENSG00000196591	HDAC2	histone deacetylase 2	3066	179,0525753	0,129932619	0,162471	0,799727	0,423868986	0,829157414
ENSG00000270149	NA	NA	NA	13,29704447	1,989437486	2,182129	0,911695703	0,361928927	NA
ENSG00000274070	CASTOR2	cytosolic arginine sensor for mTORC1 subunit 2	729438	73,90256328	0,09366607	0,197274	0,474802468	0,634927774	0,908844904
ENSG00000171492	LRRC8D	leucine rich repeat containing 8 VRAC subunit D	55144	239,3913674	-0,023461599	0,114801	-0,204367805	0,838066087	0,965627959

ENSG00000107954	NEURL1	neuralized E3 ubiquitin protein ligase 1	9148	16,34607039	-0,324706855	0,424656	-0,764635341	0,444488719	NA
ENSG00000125834	STK35	serine/threonine kinase 35	140901	120,1396852	-0,061246961	0,177739	-0,34458904	0,730403347	0,938729652
ENSG00000147601	TERF1	telomeric repeat binding factor 1	7013	102,7916712	-0,221349958	0,216921	-1,020417734	0,307530387	0,754087936
ENSG00000275342	PRAG1	PEAK1 related, kinase-activating pseudokinase 1	157285	100,3779282	-0,027894589	0,162695	-0,171452854	0,863867698	0,971561202
ENSG00000033100	CHPF2	chondroitin polymerizing factor 2	54480	290,1288395	-0,025951314	0,111407	-0,232942369	0,815806157	0,959707099
ENSG00000079950	STX7	syntaxin 7	8417	200,9793747	0,018802518	0,156429	0,120198429	0,904325962	0,978460866
ENSG00000102218	RP2	RP2 activator of ARL3 GTPase	6102	116,3351669	0,178014694	0,180388	0,986841362	0,323720414	0,767603859
ENSG00000107872	FBXL15	F-box and leucine rich repeat protein 15	79176	18,09547311	0,541833655	0,439968	1,231529505	0,218124889	NA
ENSG00000118564	FBXL5	F-box and leucine rich repeat protein 5	26234	249,9797363	-0,049668494	0,149804	-0,331555782	0,740224713	0,939193793
ENSG00000124641	MED20	mediator complex subunit 20	9477	44,6569769	0,15908534	0,373706	0,425696669	0,670328889	0,922641254
ENSG00000139517	LNX2	ligand of numb-protein X 2	222484	65,0661497	-0,073732632	0,216851	-0,340015735	0,733844678	0,939157822
ENSG00000148730	EIF4EBP2	eukaryotic translation initiation factor 4E binding protein 2	1979	940,9496937	-0,096770488	0,065652	-1,473983916	0,140485922	0,583797053
ENSG00000152242	ARK2N	arkadia (RNF111) N-terminal like PKA signaling regulator 2N	147339	193,5133744	-0,127882477	0,145069	-0,881530609	0,378030694	0,804187219
ENSG00000160392	C19orf47	chromosome 19 open reading frame 47	126526	48,51545262	0,340361428	0,253118	1,344677065	0,178729544	0,633385915
ENSG00000164896	FASTK	Fas activated serine/threonine kinase	10922	38,84951575	-0,470825053	0,901961	-0,522001732	0,601669125	NA
ENSG00000166004	CEP295	centrosomal protein 295	85459	136,6159662	0,162285275	0,157435	1,030806407	0,302631613	0,751870158
ENSG00000174796	THAP6	THAP domain containing 6	152815	41,53690062	-0,46245104	0,379971	-1,21706912	0,223577919	NA
ENSG00000196155	PLEKHG4	pleckstrin homology and RhoGEF domain containing G4	25894	55,26537145	0,220026689	0,238231	0,923585031	0,35570241	0,788500751
ENSG00000196937	FAM3C	FAM3 metabolism regulating signaling molecule C	10447	645,3327949	-0,024857239	0,06855	-0,362617137	0,716890907	0,932040329
ENSG00000197343	ZNF655	zinc finger protein 655	79027	155,9572256	0,054936431	0,270643	0,202985161	0,83914663	0,966230411
ENSG00000205236	NA	NA	NA	19,41790965	-0,876468279	0,443532	-1,976109501	0,048142377	NA
ENSG00000251201	TMED7-TICAM2	TMED7-TICAM2 readthrough	100302736	17,03395633	0,41406299	0,468134	0,884496743	0,37642812	NA
ENSG00000288674	NA	NA	NA	26,5901372	-0,17779	1,473305	-0,120674244	0,90394906	NA
ENSG00000162227	TAF6L	TATA-box binding protein associated factor 6 like	10629	26,71606716	0,386683919	0,416846	0,927642512	0,353593038	NA
ENSG00000163428	LRRC58	leucine rich repeat containing 58	116064	217,6715609	-0,08049453	0,15285	-0,52662589	0,598453409	0,894604697
ENSG00000169087	HSPBAP1	HSPB1 associated protein 1	79663	28,25026719	-0,575565587	0,413909	-1,390560353	0,164358786	NA
ENSG00000274602	NA	NA	NA	18,01335653	-0,168654094	0,397163	-0,42464727	0,671093828	NA
ENSG00000143702	CEP170	centrosomal protein 170	9859	65,12381359	0,544725544	1,247305	0,436721957	0,662313004	0,9203479
ENSG00000143702	CEP170P1	centrosomal protein 170 pseudogene 1	645455	65,12381359	0,544725544	1,247305	0,436721957	0,662313004	0,9203479
ENSG00000144043	TEX261	testis expressed 261	113419	267,2441508	0,080180721	0,115627	0,69344113	0,488032758	0,859508239
ENSG00000261739	GOLGA8S	golgin A8 family member S	653061	23,18729879	-0,58653625	0,460275	-1,274318202	0,202550668	NA
ENSG00000027001	MIPEP	mitochondrial intermediate peptidase	4285	20,81752145	-0,148416124	0,509816	-0,291116953	0,770961875	NA
ENSG00000060069	CTDP1	CTD phosphatase subunit 1	9150	214,0222504	-0,01481465	0,180864	-0,081910557	0,934717839	0,987098279
ENSG00000069248	NUP133	nucleoporin 133	55746	213,9587611	0,164439538	0,120626	1,363213418	0,17281526	0,629558603
ENSG00000070061	ELP1	elongator acetyltransferase complex subunit 1	8518	164,5296884	0,638385892	0,262049	2,436134588	0,01484516	0,1911648
ENSG00000086504	MRPL28	mitochondrial ribosomal protein L28	10573	80,35040757	0,250476234	0,268562	0,932657244	0,350996967	0,785712602
ENSG00000089022	MAPKAPK5	MAPK activated protein kinase 5	8550	187,2952239	-0,144866708	0,241105	-0,600845058	0,54794319	0,879578516
ENSG00000090612	ZNF268	zinc finger protein 268	10795	112,9057369	0,087321257	0,247077	0,353417736	0,723775291	0,934902053
ENSG00000100345	MYH9	myosin heavy chain 9	4627	4598,280786	0,039617265	0,053373	0,742267579	0,457925231	0,84552083
ENSG00000105258	POLR2I	RNA polymerase II subunit I	5438	15,49848232	-0,179734619	0,472947	-0,380031322	0,703922164	NA
ENSG00000111641	NOP2	NOP2 nucleolar protein	4839	183,8761436	0,189628369	0,141379	1,341279562	0,179829703	0,633591125
ENSG00000112782	CLIC5	chloride intracellular channel 5	53405	34,05487072	-0,091476373	0,364886	-0,250698487	0,802047231	NA
ENSG00000116918	TSNAX	translin associated factor X	7257	234,1907616	-0,077055781	0,111905	-0,688580184	0,491087495	0,860513824
ENSG00000118482	PHF3	PHD finger protein 3	23469	490,8892994	-0,087690614	0,096983	-0,904187496	0,36589599	0,794505931
ENSG00000124357	NAGK	N-acetylglucosamine kinase	55577	48,23629264	-0,014122936	0,283563	-0,049805242	0,96027759	0,992235217
ENSG00000130313	PGLS	6-phosphogluconolactonase	25796	75,43146299	0,16458456	0,210036	0,783602684	0,433273281	0,835093272
ENSG00000132604	TERF2	telomeric repeat binding factor 2	7014	106,3154443	0,061301842	0,178341	0,343733118	0,731047002	0,938729652
ENSG00000138621	PPCDC	phosphopantothenoylcysteine decarboxylase	60490	70,52598412	0,1547105	0,234867	0,658715908	0,510078215	0,866180216
ENSG00000151881	TMEM267	transmembrane protein 267	64417	43,84634333	-0,356336581	0,312444	-1,140481317	0,254085832	0,709921781
ENSG00000169189	NSMCE1	NSE1 homolog, SMC5-SMC6 complex component	197370	109,4745034	-0,322995714	0,199283	-1,620787201	0,105063284	0,520938785
ENSG00000169519	METTL15	methyltransferase 15, mitochondrial 12S rRNA N4-cytidine	196074	49,49513937	0,02623644	0,321437	0,08162239	0,934946996	0,987098279

ENSG00000169826	CSGALNACT2	chondroitin sulfate N-acetylgalactosaminyltransferase 2	55454	334,5843748	0,059280663	0,12775	0,464035082	0,642622611	0,912085896
ENSG00000172009	THOP1	thimet oligopeptidase 1	7064	56,25577626	0,176723818	0,248286	0,711775803	0,476603619	0,853811879
ENSG00000179958	DCTPP1	dCTP pyrophosphatase 1	79077	50,01722971	0,294083079	0,286363	1,026959635	0,304439472	0,751870158
ENSG00000168781	PPIP5K1	diphosphoinositol pentakisphosphate kinase 1	9677	119,3492438	-0,122142038	0,179269	-0,681333901	0,495660237	0,861163463
ENSG00000168887	C2orf68	chromosome 2 open reading frame 68	388969	157,882513	-0,134860561	0,155211	-0,868882796	0,38491124	0,809109919
ENSG00000132436	FIGNL1	fidgetin like 1	63979	97,28395315	0,063922571	0,176089	0,363013785	0,716594587	0,932040329
ENSG00000237651	C2orf74	chromosome 2 open reading frame 74	339804	36,50455351	0,3785104	0,312174	1,212496386	0,225322425	NA
ENSG00000174373	RALGAPA1	Ral GTPase activating protein catalytic subunit alpha 1	253959	386,3598259	0,026457548	0,098836	0,267690251	0,788937755	0,95148885
ENSG00000068001	HYAL2	hyaluronidase 2	8692	14,07899232	0,47593585	0,474175	1,003714159	0,31551641	NA
ENSG00000155158	TTC39B	tetratricopeptide repeat domain 39B	158219	212,7700609	-0,221831622	0,219321	-1,011445879	0,311803072	0,757868604
ENSG00000163166	IWS1	interacts with SUPT6H, CTD assembly factor 1	55677	225,5170166	0,113598258	0,121192	0,937342286	0,348582518	0,784460486
ENSG00000100997	ABHD12	abhydrolase domain containing 12, lysophospholipase	26090	101,4492951	-0,086711317	0,187807	-0,461704064	0,644293556	0,912530858
ENSG00000186104	CYP2R1	cytochrome P450 family 2 subfamily R member 1	120227	48,11671779	0,210212652	0,333423	0,630467517	0,528388749	0,872142129
ENSG00000100365	NCF4	neutrophil cytosolic factor 4	4689	609,243408	0,235258803	0,084042	2,799303633	0,005121296	0,100843978
ENSG00000032389	EIPR1	EARP complex and GARP complex interacting protein 1	7260	107,6466535	0,114170275	0,169119	0,675090328	0,499618378	0,86161226
ENSG00000059122	FLYWCH1	FLYWCH-type zinc finger 1	84256	114,0601632	-0,14016227	0,179672	-0,78010264	0,435330463	0,836566451
ENSG00000068784	SRBD1	S1 RNA binding domain 1	55133	121,8072795	-0,097784947	0,163384	-0,598498824	0,549507143	0,88072908
ENSG00000166337	TAF10	TATA-box binding protein associated factor 10	6881	93,14282541	0,405358381	0,768683	0,52734154	0,597956432	0,894542823
ENSG00000206527	HACD2	3-hydroxyacyl-CoA dehydratase 2	201562	255,4893408	-0,019117259	0,125549	-0,152269027	0,878974753	0,976237908
ENSG00000143797	MBOAT2	membrane bound O-acyltransferase domain containing 2	129642	14,22461536	0,130326373	0,480275	0,271357711	0,786115924	NA
ENSG00000157741	UBN2	ubinuclein 2	254048	286,3379672	-0,130437007	0,111186	-1,173147485	0,240736667	0,695620773
ENSG00000122140	MRPS2	mitochondrial ribosomal protein S2	51116	61,70045573	-0,181320553	0,231651	-0,782730068	0,433785641	0,835238413
ENSG00000138600	SPPL2A	signal peptide peptidase like 2A	84888	246,4611564	-0,202451313	0,340878	-0,593911659	0,552571201	0,882068269
ENSG00000185304	RGPD2	RANBP2 like and GRIP domain containing 2	729857	35,22706436	0,323880089	0,470095	0,688967752	0,490843561	NA
ENSG00000267648	NA	NA	NA	24,95343374	0,02953818	0,340713	0,086695298	0,930913714	NA
ENSG00000133884	DPF2	double PHD fingers 2	5977	156,1993763	0,246861499	0,242561	1,017730621	0,308805991	0,754797782
ENSG00000141644	MBD1	methyl-CpG binding domain protein 1	4152	343,0272406	0,167595573	0,101143	1,657014378	0,097516568	0,505207868
ENSG00000163527	STT3B	STT3 oligosaccharyltransferase complex catalytic subunit B	201595	914,1663878	-0,045839133	0,066351	-0,690858118	0,489654708	0,860369726
ENSG00000197747	S100A10	S100 calcium binding protein A10	6281	187,2104621	0,239474918	0,132138	1,812302933	0,069939402	0,429815384
ENSG00000011009	LYPLA2	lysophospholipase 2	11313	141,7503608	-0,095534851	0,147307	-0,648541607	0,516634709	0,868791049
ENSG00000106789	CORO2A	coronin 2A	7464	111,436061	-0,314510448	0,177193	-1,774964019	0,075903828	0,446229554
ENSG00000146676	PURB	purine rich element binding protein B	5814	432,6552389	-0,04988341	0,103681	-0,481124797	0,630427805	0,905960607
ENSG00000163590	PPM1L	protein phosphatase, Mg2+/Mn2+ dependent 1L	151742	54,59642408	0,272283524	0,394687	0,689872179	0,490274573	0,860513824
ENSG00000114021	NIT2	nitrilase family member 2	56954	68,3664178	-0,113869193	0,212436	-0,536015766	0,591947654	0,892834002
ENSG00000158480	SPATA2	spermatogenesis associated 2	9825	34,78634272	-0,310756254	0,330433	-0,940452491	0,346985508	NA
ENSG00000163931	TKT	transketolase	7086	220,0447453	0,189842336	0,181991	1,043140697	0,296883134	0,748371252
ENSG00000085872	CHERP	calcium homeostasis endoplasmic reticulum protein	10523	201,4409525	-0,053830481	0,134059	-0,401543532	0,688019995	0,926748702
ENSG00000117751	PPP1R8	protein phosphatase 1 regulatory subunit 8	5511	182,7626466	-0,018269066	0,132973	-0,137389302	0,890723086	0,977990819
ENSG00000164828	SUN1	Sad1 and UNC84 domain containing 1	23353	33,58017462	1,176900473	1,352879	0,869923258	0,384342344	NA
ENSG00000135622	SEMA4F	ssemaphorin 4F	10505	44,14393606	0,422503089	0,451946	0,934852618	0,349864258	0,785095396
ENSG00000154845	PPP4R1	protein phosphatase 4 regulatory subunit 1	9989	168,9627228	-0,090389126	0,131874	-0,685419729	0,493079098	0,860513824
ENSG00000263647	BPTFP1	bromodomain PHD finger transcription factor pseudogene 1	109286551	32,15001829	0,409596633	0,311563	1,314652873	0,188626592	NA
ENSG00000102265	TIMP1	TIMP metallopeptidase inhibitor 1	7076	97,45032652	-0,024463595	0,183648	-0,133208801	0,894028251	0,977990819
ENSG00000140262	TCF12	transcription factor 12	6938	236,1202259	0,009268948	0,126644	0,07318915	0,9416556	0,988474082

ENSG00000122674	CCZ1	CCZ1 homolog, vacuolar protein trafficking and biogenesis associated	51622	94,17131483	0,055152913	0,186942	0,295027632	0,767972771	0,944698783
ENSG00000150593	PDCD4	programmed cell death 4	27250	898,8972982	-0,045525239	0,075736	-0,601104518	0,547770374	0,879578516
ENSG00000170430	MGMT	O-6-methylguanine-DNA methyltransferase	4255	18,78442478	0,118094118	0,461155	0,256083201	0,797886594	NA
ENSG00000172531	PPP1CA	protein phosphatase 1 catalytic subunit alpha	5499	725,6400066	-0,009806383	0,07022	-0,13965163	0,888935245	0,977990819
ENSG00000180822	PSMG4	proteasome assembly chaperone 4	389362	40,52717991	0,436993995	0,294175	1,485490509	0,137413936	NA
ENSG00000075407	ZNF37A	zinc finger protein 37A	7587	244,5792973	0,135196069	0,133097	1,015773069	0,309737463	0,755959614
ENSG00000099804	CDC34	cell division cycle 34, ubiqiutin conjugating enzyme	997	55,14080582	0,020905734	0,253966	0,082317099	0,934394558	0,987098279
ENSG00000124067	SLC12A4	solute carrier family 12 member 4	6560	71,93740283	-0,033063756	0,261115	-0,126625378	0,89923691	0,977990819
ENSG00000170468	RIOX1	ribosomal oxygenase 1	79697	77,15682011	-0,475004778	0,195156	-2,433972136	0,014934145	0,1911648
ENSG00000103544	VPS35L	VPS35 endosomal protein sorting factor like	57020	235,0357114	0,080007655	0,106293	0,752705741	0,451626759	0,842736177
ENSG00000106399	RPA3	replication protein A3	6119	35,10525432	0,091969346	0,374771	0,245401299	0,806145723	NA
ENSG00000135838	NPL	N-acetylneuraminate pyruvate lyase	80896	18,61759486	0,168471918	0,422637	0,398621307	0,69017226	NA
ENSG00000076944	STXBP2	syntaxin binding protein 2	6813	258,815452	-0,018133412	0,117381	-0,154483332	0,877228653	0,975885813
ENSG00000006607	FARP2	FERM, ARH/RhoGEF and pleckstrin domain protein 2	9855	17,50048762	-0,168894134	0,445651	-0,378982595	0,704700789	NA
ENSG00000048544	MRPS10	mitochondrial ribosomal protein S10	55173	110,1464879	0,141275624	0,165353	0,854390285	0,392888777	0,812491435
ENSG00000123415	SMUG1	single-strand-selective monofunctional uracil-DNA glycosylase 1	23583	39,22885366	-0,124316433	0,313942	-0,395985618	0,692115638	NA
ENSG00000165752	STK32C	serine/threonine kinase 32C	282974	69,24889887	-0,080671405	0,210814	-0,382665796	0,701967578	0,930846779
ENSG00000177733	HNRNPA0	heterogeneous nuclear ribonucleoprotein A0	10949	561,7732268	-0,115574229	0,085605	-1,350083595	0,17698917	0,632039836
ENSG00000198373	WWP2	WW domain containing E3 ubiquitin protein ligase 2	11060	287,728436	0,045760392	0,111901	0,408936661	0,682586144	0,925586358
ENSG00000013523	ANGEL1	angel homolog 1	23357	43,67863411	-0,762457358	0,306638	-2,486507192	0,012900396	0,177810208
ENSG00000037042	TUBG2	tubulin gamma 2	27175	34,35181628	-0,107899238	0,361345	-0,298604345	0,76524195	NA
ENSG00000048471	SNX29	sorting nexin 29	92017	125,9293301	0,059763568	0,167008	0,357847591	0,720457373	0,933530522
ENSG00000099985	OSM	oncostatin M	5008	30,23611705	0,013528047	0,391623	0,034543569	0,9724437	NA
ENSG00000105245	NUMBL	NUMB like endocytic adaptor protein	9253	16,20164475	0,258985443	0,54712	0,473361513	0,635955283	NA
ENSG00000123352	SPATS2	spermatogenesis associated serine rich 2	65244	33,15690526	-0,032675469	0,348738	-0,093696424	0,925350311	NA
ENSG00000126254	RBM42	RNA binding motif protein 42	79171	128,1744666	0,037426341	0,1522	0,245902991	0,805757328	0,957690384
ENSG00000131116	ZNF428	zinc finger protein 428	126299	50,57355291	-0,23409202	0,251094	-0,932287779	0,351187822	0,785712602
ENSG00000137801	THBS1	thrombospondin 1	7057	27,10677482	-0,118560944	0,365311	-0,324547855	0,745523298	NA
ENSG00000141452	RMC1	regulator of MON1-CCZ1	29919	91,77211977	0,297014257	0,251883	1,179175304	0,238328378	0,693489081
ENSG00000176155	CCDC57	coiled-coil domain containing 57	284001	53,53528156	0,080135786	0,321509	0,24924859	0,803168496	0,956495127
ENSG00000183291	SELENOF	selenoprotein F	9403	377,5984136	0,102422039	0,089305	1,146878961	0,251431649	0,70779361
ENSG00000185414	MRPL30	mitochondrial ribosomal protein L30	51263	106,0391689	-0,451724919	0,35993	-1,255035032	0,209466043	0,663900848
ENSG00000188735	TMEM120B	transmembrane protein 120B	144404	62,72797664	-0,266594241	0,286829	-0,929454071	0,352653817	0,787082432
ENSG00000189308	LIN54	lin-54 DREAM MuvB core complex component	132660	143,9117312	-0,111836343	0,259156	-0,431540236	0,666075603	0,922184616
ENSG00000125910	S1PR4	sphingosine-1-phosphate receptor 4	8698	94,74454773	-0,342651558	0,203578	-1,683147603	0,092346521	0,4945733
ENSG00000196584	XRCC2	X-ray repair cross complementing 2	7516	13,38256179	2,355483231	1,95597	1,204253286	0,228491696	NA
ENSG00000123104	ITPR2	inositol 1,4,5-trisphosphate receptor type 2	3709	558,7645475	0,025120868	0,086542	0,290273823	0,771606763	0,946052153
ENSG00000145919	BOD1	biorientation of chromosomes in cell division 1	91272	26,09579341	-0,088634628	0,547249	-0,161964036	0,871334175	NA
ENSG00000181904	C5orf24	chromosome 5 open reading frame 24	134553	173,2416469	-0,094347589	0,236087	-0,399629993	0,689429062	0,927028909
ENSG00000174579	MSL2	MSL complex subunit 2	55167	376,9585832	-0,202039454	0,097014	-2,082579358	0,037289582	0,306351862
ENSG00000077713	SLC25A43	solute carrier family 25 member 43	203427	67,57754336	0,037036116	0,21772	0,170108849	0,864924534	0,971694649
ENSG00000205356	TECPR1	tectonin beta-propeller repeat containing 1	25851	163,6326209	0,107400658	0,151145	0,710581869	0,477343382	0,853811879
ENSG00000249471	ZNF324B	zinc finger protein 324B	388569	21,77358423	-0,557246054	0,451998	-1,232850853	0,217631414	NA
ENSG00000116704	SLC35D1	solute carrier family 35 member D1	23169	182,3452713	0,00492926	0,129418	0,03808794	0,969617567	0,993542794
ENSG00000107863	ARHGAP21	Rho GTPase activating protein 21	57584	198,846004	-0,070144458	0,185672	-0,377786922	0,70558889	0,930846779
ENSG00000127124	HIVEP3	HIVEP zinc finger 3	59269	184,0245972	0,301889353	0,12356	2,443265686	0,014555018	0,189263425
ENSG00000136717	BIN1	bridging integrator 1	274	298,4631924	-0,137754258	0,109727	-1,255423673	0,209325	0,663721659
ENSG00000113272	THG1L	tRNA-histidine guanylyltransferase 1 like	54974	47,60917199	0,136957295	0,243477	0,562507227	0,573770481	0,886894319

ENSG00000114416	FXR1	FMR1 autosomal homolog 1	8087	139,1089475	0,05833472	0,451046	0,129332131	0,897094848	0,977990819
ENSG00000139133	ALG10	ALG10 alpha-1,2-glucosyltransferase	84920	36,095714	-0,072486394	0,426799	-0,169837217	0,865138157	NA
ENSG00000156136	DCK	deoxycytidine kinase	1633	230,4660869	0,202280026	0,120557	1,677879234	0,093370677	0,496838278
ENSG00000133789	SWAP70	switching B cell complex subunit SWAP70	23075	84,73414039	0,043283428	0,182145	0,237631258	0,812167105	0,959038743
ENSG00000130590	SAMD10	sterile alpha motif domain containing 10	140700	138,8853356	-0,006898415	0,156567	-0,04406046	0,96485621	0,992789539
ENSG00000111669	TPI1	triosephosphate isomerase 1	7167	884,9502595	0,159162889	0,06286	2,532039627	0,011340117	0,167101843
ENSG00000119718	EIF2B2	eukaryotic translation initiation factor 2B subunit beta	8892	49,17193273	0,405372586	0,251989	1,608689233	0,107684311	0,527254203
ENSG00000165219	GAPVD1	GTPase activating protein and VPS9 domains 1	26130	434,5703267	-0,031094282	0,080642	-0,385582159	0,699806148	0,930783276
ENSG00000101181	MTG2	mitochondrial ribosome associated GTPase 2	26164	59,28986376	-0,295255514	0,342903	-0,861047603	0,389211826	0,810747116
ENSG00000050438	SLC4A8	solute carrier family 4 member 8	9498	95,4869767	-0,122521213	0,245652	-0,498758549	0,617949492	0,902245041
ENSG00000082212	ME2	malic enzyme 2	4200	168,3515573	-0,136348848	0,234118	-0,58239484	0,560300756	0,883921037
ENSG00000115364	MRPL19	mitochondrial ribosomal protein L19	9801	169,6810723	0,345037146	0,25069	1,376348106	0,168713886	0,628297231
ENSG00000115661	STK16	serine/threonine kinase 16	8576	61,68717413	0,013200157	0,339432	0,038888984	0,968978899	0,993542794
ENSG00000119689	DLST	dihydrolipoamide S-succinyltransferase	1743	530,4711017	-0,017263416	0,073731	-0,234139927	0,81487635	0,95938223
ENSG00000162490	DRAXIN	dorsal inhibitory axon guidance protein	374946	660,38735	-0,280383217	0,068107	-4,116825955	3,84E-05	0,002987054
ENSG00000163634	THOC7	THO complex subunit 7	80145	85,46665705	0,049337462	0,201652	0,244665913	0,806715123	0,958100582
ENSG00000178096	BOLA1	bolA family member 1	51027	13,98472934	-0,057620075	0,571182	-0,100878692	0,919646758	NA
ENSG00000196296	ATP2A1	ATPase sarcoplasmic/endoplasmic reticulum Ca2+ transporting 1	487	15,22379854	-0,294152812	0,477947	-0,61545042	0,538257302	NA
ENSG00000276256	LOC389831	uncharacterized LOC389831	389831	24,08555513	0,194944376	0,374741	0,520210517	0,602916856	NA
ENSG00000189077	TMEM120A	transmembrane protein 120A	83862	50,35882644	0,414482724	0,296373	1,39851913	0,161957232	0,619487156
ENSG00000102119	EMD	emerin	2010	51,31728192	-0,405465964	0,373468	-1,08567909	0,277620995	0,731689696
ENSG00000198919	DZIP3	DAZ interacting zinc finger protein 3	9666	204,5987252	0,049589429	0,166919	0,297086571	0,766400417	0,944698783
ENSG00000125743	SNRPD2	small nuclear ribonucleoprotein D2 polypeptide	6633	199,741353	-0,058172401	0,151394	-0,38424443	0,700797291	0,930783276
ENSG00000178719	GRINA	glutamate ionotropic receptor NMDA type subunit associated protein 1	2907	100,5124837	0,049974807	0,181889	0,274754449	0,783504896	0,950082577
ENSG00000120832	MTERF2	mitochondrial transcription termination factor 2	80298	18,66708669	-0,018514989	0,49254	-0,037590827	0,970013922	NA
ENSG00000135052	GOLM1	golgi membrane protein 1	51280	43,96293617	-0,238821944	0,26797	-0,891225286	0,372808322	0,800776142
ENSG00000143458	GABPB2	GA binding protein transcription factor subunit beta 2	126626	153,654028	-0,556496431	0,246423	-2,258296471	0,023927182	0,247679511
ENSG00000008869	HEATR5B	HEAT repeat containing 5B	54497	348,2980751	-0,066970085	0,090668	-0,738630716	0,460131268	0,846622987
ENSG00000111653	ING4	inhibitor of growth family member 4	51147	55,71679003	0,164145043	0,311623	0,526742906	0,598372136	0,894604697
ENSG00000143933	CALM2	calmodulin 2	805	530,4855673	0,005162255	0,089133	0,057916303	0,953815297	0,991212866
ENSG00000198780	FAM169A	family with sequence similarity 169 member A	26049	38,43280231	0,238361439	0,318941	0,747353409	0,454850261	NA
ENSG00000034510	TMSB10	thymosin beta 10	9168	954,1975259	0,000622468	0,072156	0,008626638	0,993117024	0,998500439
ENSG00000165424	ZCCHC24	zinc finger CCHC-type containing 24	219654	44,31857655	0,116318652	0,272103	0,427480206	0,669029599	0,922641254
ENSG00000174891	RSRC1	arginine and serine rich coiled-coil 1	51319	71,29726682	-0,187206515	0,240345	-0,778908905	0,436033378	0,836566451
ENSG00000084072	PPIE	peptidylprolyl isomerase E	10450	94,9217906	-0,074341328	0,187959	-0,395519667	0,69245941	0,927787546
ENSG00000128881	TTBK2	tau tubulin kinase 2	146057	192,9985777	-0,090860851	0,12708	-0,714987929	0,474616502	0,853811879
ENSG00000143774	GUK1	guanylate kinase 1	2987	132,4151097	0,025731876	0,162161	0,158681502	0,873919813	0,974569239
ENSG00000099331	MYO9B	myosin IXB	4650	965,0058491	-0,138978329	0,09695	-1,43350544	0,151713445	0,608558427
ENSG00000100162	CENPM	centromere protein M	79019	18,01903016	0,844067184	0,464083	1,818785887	0,068944102	NA
ENSG00000116678	LEPR	leptin receptor	3953	14,22436265	-0,155204671	0,456877	-0,339707914	0,734076502	NA
ENSG00000139323	POC1B	POC1 centriolar protein B	282809	170,3248522	-0,196485125	0,137248	-1,431603114	0,152257438	0,608564843
ENSG00000146143	PRIM2	DNA primase subunit 2	5558	60,36281184	-0,068960037	0,249642	-0,276235202	0,782367421	0,949577148
ENSG00000164323	CFAP97	cilia and flagella associated protein 97	57587	239,6110048	-0,159406521	0,115008	-1,386044163	0,165733419	0,624933502
ENSG00000116396	KCNC4	potassium voltage-gated channel subfamily C member 4	3749	20,47225792	-0,648445652	0,417793	-1,55207416	0,12064448	NA
ENSG00000288701	PRRC2B	proline rich coiled-coil 2B	84726	3139,284538	-0,030630968	0,051859	-0,590656139	0,554750842	0,882854594
ENSG00000185627	PSMD13	proteasome 26S subunit, non-ATPase 13	5719	323,9441201	0,23284738	0,093003	2,503665929	0,012291403	0,174531515
ENSG00000269858	EGLN2	egl-9 family hypoxia inducible factor 2	112398	135,0841832	-0,096625957	0,149519	-0,646246622	0,518119651	0,868791049
ENSG00000133460	SLC2A11	solute carrier family 2 member 11	66035	44,05283526	0,604324836	0,285159	2,119253598	0,034069041	0,295014605
ENSG00000141503	MINK1	misshapen like kinase 1	50488	362,3060772	-0,249403905	0,117021	-2,131267307	0,033067127	0,291480599

ENSG00000162236	STX5	syntaxin 5	6811	148,4606032	-0,294706184	0,168767	-1,746236137	0,080769928	0,4605948
ENSG00000171517	LPAR3	lysophosphatidic acid receptor 3	23566	49,0282451	-0,291814307	0,263827	-1,106081115	0,268691412	0,724168379
ENSG00000285077	ARHGAP11B	Rho GTPase activating protein 11B	89839	42,15401812	0,719933187	0,477997	1,506144373	0,132030116	NA
ENSG00000084234	APLP2	amyloid beta precursor like protein 2	334	233,9639684	0,157418502	0,116534	1,350842622	0,176745852	0,632039836
ENSG00000089234	BRAP	BRCA1 associated protein	8315	124,7497863	0,024531346	0,173932	0,141040058	0,887838297	0,977990819
ENSG00000110395	CBL	Cbl proto-oncogene	867	948,7823251	-0,056903737	0,056727	-1,003111023	0,315807297	0,76048697
ENSG00000125686	MED1	mediator complex subunit 1	5469	617,3168034	-0,058680324	0,079062	-0,742204346	0,457963536	0,84552083
ENSG00000133106	EPSTI1	epithelial stromal interaction 1	94240	98,31853884	0,137946858	0,196126	0,703358123	0,481832597	0,855598738
ENSG00000166263	STXBP4	syntaxin binding protein 4	252983	47,43236784	0,726136582	0,43627	1,664418809	0,096028764	0,502134421
ENSG00000197024	ZNF398	zinc finger protein 398	57541	118,705806	0,125294853	0,168052	0,745569992	0,455927216	0,844219046
ENSG00000228049	POLR2J2	RNA polymerase II subunit J2	246721	71,84866001	-0,651468405	0,460589	-1,414423588	0,157237568	0,61428162
ENSG00000084754	HADHA	hydroxyacyl-CoA dehydrogenase trifunctional multienzyme complex subunit alpha	3030	447,5262044	0,326787602	0,092938	3,516192504	0,000437783	0,018598639
ENSG00000132793	LPIN3	lipin 3	64900	11,84989361	-0,090512639	0,601227	-0,15054653	0,880333443	NA
ENSG00000162928	PEX13	peroxisomal biogenesis factor 13	5194	85,290537	-0,035048336	0,244465	-0,143367676	0,88599981	0,977761756
ENSG00000171295	ZNF440	zinc finger protein 440	126070	57,35405135	0,1257775	0,250448	0,502210632	0,615519365	0,902090873
ENSG00000197540	GZMM	granzyme M	3004	41,04349895	-0,364155782	0,329307	-1,105826163	0,268801769	NA
ENSG00000205534	NA	NA	NA	159,070169	0,466984063	0,198706	2,350119749	0,018767372	0,213621654
ENSG00000104613	INTS10	integrator complex subunit 10	55174	208,3471179	0,07518328	0,136062	0,552567552	0,580559566	0,888049268
ENSG00000075914	EXOSC7	exosome component 7	23016	23,59221936	-0,368656357	0,418471	-0,880959926	0,378339511	NA
ENSG00000113648	MACROH2A1	macroH2A,1 histone	9555	302,2104818	0,276680607	0,101428	2,727865228	0,006374565	0,115569573
ENSG00000123338	NCKAP1L	NCK associated protein 1 like	3071	979,6866033	0,248908801	0,100745	2,470669234	0,013486049	0,18084297
ENSG00000131115	ZNF227	zinc finger protein 227	7770	104,6143555	-0,246069022	0,178307	-1,380030565	0,167577234	0,62720477
ENSG00000087302	RTRAF	RNA transcription, translation and transport factor	51637	200,2244357	-0,003897739	0,12063	-0,03231154	0,974223607	0,994534085
ENSG00000004766	VPS50	VPS50 subunit of EARP/GARPII complex	55610	59,4270618	0,058233493	0,765574	0,076065159	0,939367259	0,987813564
ENSG00000047932	GOPC	golgi associated PDZ and coiled-coil motif containing	57120	327,7309546	-0,136160351	0,101118	-1,346550295	0,178125112	0,632456884
ENSG00000101745	ANKRD12	ankyrin repeat domain 12	23253	1032,718781	0,041145893	0,069958	0,588149495	0,556431953	0,883586042
ENSG00000140386	SCAPER	S-phase cyclin A associated protein in the ER	49855	58,89688589	-0,068112576	0,282561	-0,241054618	0,809512785	0,959038743
ENSG00000140853	NLRC5	NLR family CARD domain containing 5	84166	1264,868194	0,181511387	0,059068	3,072902189	0,00211988	0,05587092
ENSG00000146386	ABRACL	ABRA C-terminal like	58527	174,4856737	0,364537337	0,140497	2,594633789	0,009469175	0,150244246
ENSG00000164758	MED30	mediator complex subunit 30	90390	30,12937192	0,025797766	0,312059	0,082669534	0,934114311	NA
ENSG00000168884	TNIP2	TNFAIP3 interacting protein 2	79155	76,51542419	-0,405330629	0,194237	-2,086786463	0,036907441	0,306351862
ENSG00000196455	PIK3R4	phosphoinositide-3-kinase regulatory subunit 4	30849	160,473351	-0,091173289	0,16393	-0,556171576	0,578093568	0,887069818
ENSG00000251192	ZNF674	zinc finger protein 674	641339	13,06436641	0,297597158	0,574354	0,518142578	0,604358795	NA
ENSG00000119328	ABITRAM	actin binding transcription modulator	54942	37,08683953	-0,559200286	0,297825	-1,877610653	0,060434447	NA
ENSG00000179091	CYC1	cytochrome c1	1537	96,29157196	0,147330838	0,178715	0,824389181	0,40971846	0,82130194
ENSG00000197894	ADH5	alcohol dehydrogenase 5 (class III), chi polypeptide	128	206,295541	0,046539503	0,16343	0,284768053	0,77582185	0,946448613
ENSG00000117906	RCN2	reticulocalbin 2	5955	154,0177873	0,103477458	0,140955	0,73411573	0,462878207	0,847577533
ENSG00000164961	WASHC5	WASH complex subunit 5	9897	262,421992	0,13693642	0,13039	1,05020979	0,293621669	0,745106491
ENSG00000074935	TUBE1	tubulin epsilon 1	51175	35,39922533	0,475493378	0,330462	1,438876115	0,150185626	NA
ENSG00000134242	PTPN22	protein tyrosine phosphatase non-receptor type 22	26191	804,8435398	0,068957527	0,070612	0,976571608	0,328781281	0,770885747
ENSG00000197965	MPZL1	myelin protein zero like 1	9019	47,76368901	-0,108349778	0,256823	-0,421885821	0,67310836	0,923602509
ENSG00000151876	FBXO4	F-box protein 4	26272	34,40162787	0,482556966	0,316625	1,524066608	0,127492073	NA
ENSG00000054282	SDCCAG8	SHH signaling and ciliogenesis regulator SDCCAG8	10806	24,42115515	-0,923358589	2,183111	-0,422955377	0,672327818	NA
ENSG00000072121	ZFYVE26	zinc finger FYVE-type containing 26	23503	221,5976108	0,064589505	0,122291	0,528162275	0,597386711	0,894257182
ENSG00000078246	TULP3	TUB like protein 3	7289	58,82776561	-0,023245257	0,22799	-0,101957459	0,918790442	0,984062493
ENSG00000100227	POLDIP3	DNA polymerase delta interacting protein 3	84271	335,1097222	0,056077117	0,105537	0,531351243	0,595175401	0,894229873
ENSG00000159459	UBR1	ubiquitin protein ligase E3 component n-recognin 1	197131	403,8289324	0,051630983	0,091197	0,566150033	0,571291794	0,886795904
ENSG00000229018	NA	NA	NA	19,8248506	0,088529724	0,40568	0,21822545	0,827253456	NA
ENSG00000176225	RTTN	rotatin	25914	36,983148	0,687670782	0,39761	1,729511091	0,083717663	NA
ENSG00000141232	TOB1	transducer of ERBB2, 1	10140	20,61495021	-0,075181019	0,411561	-0,182673027	0,855054581	NA
ENSG00000170037	CNTROB	centrobin, centriole duplication and spindle assembly protein	116840	142,6847815	0,013734282	0,174941	0,078508254	0,937423764	0,987194684

ENSG00000151893	CACUL1	CDK2 associated cullin domain 1	143384	206,9851878	-0,219804215	0,134689	-1,631943261	0,102691439	0,515621608
ENSG00000108469	RECQL5	RecQ like helicase 5	9400	92,43337596	-0,125923813	0,241676	-0,521043939	0,602336163	0,896931787
ENSG00000115526	CHST10	carbohydrate sulfotransferase 10	9486	28,21339331	0,225125384	0,322729	0,697567007	0,485448021	NA
ENSG00000117010	ZNF684	zinc finger protein 684	127396	13,39044705	0,29911889	0,488754	0,612003086	0,540535716	NA
ENSG00000141562	NARF	nuclear prelamin A recognition factor	26502	83,28445163	-0,054382324	0,281596	-0,193121533	0,84686379	0,967231052
ENSG00000163159	VPS72	vacuolar protein sorting 72 homolog	6944	90,68643184	0,169977691	0,261157	0,650862815	0,515135047	0,868562431
ENSG00000131368	MRPS25	mitochondrial ribosomal protein S25	64432	33,52447897	0,012047676	1,958353	0,006151944	0,99509149	NA
ENSG00000161677	JOSD2	Josephin domain containing 2	126119	22,57946526	0,067667421	0,426338	0,158717825	0,873891194	NA
ENSG00000151694	ADAM17	ADAM metallopeptidase domain 17	6868	131,2346383	0,00080841	0,171635	0,004710063	0,996241927	0,999295542
ENSG00000159479	MED8	mediator complex subunit 8	112950	75,83860299	-0,131501953	0,203323	-0,646764152	0,517784596	0,868791049
ENSG00000230989	HSBP1	heat shock factor binding protein 1	3281	15,17684715	-2,983940741	0,670364	-4,451225802	8,54E-06	NA
ENSG00000162836	ACP6	acid phosphatase 6, lysophosphatidic	51205	25,21529219	0,139480639	0,552328	0,252532093	0,800629811	NA
ENSG00000163507	CIP2A	cellular inhibitor of PP2A	57650	35,74812363	0,365316368	1,125415	0,324606027	0,745479265	NA
ENSG00000165521	EML5	EMAP like 5	161436	19,91898007	0,591973809	0,404437	1,463697517	0,143276614	NA
ENSG00000067191	CACNB1	calcium voltage-gated channel auxiliary subunit beta 1	782	110,8785085	-0,043872529	0,230463	-0,190367156	0,849021433	0,967810498
ENSG00000137815	RTF1	RTF1 homolog, Paf1/RNA polymerase II complex component	23168	373,0784336	0,027996938	0,093743	0,298655575	0,765202857	0,944359403
ENSG00000145414	NAF1	nuclear assembly factor 1 ribonucleoprotein	92345	30,34321804	0,33246084	0,373357	0,890464679	0,373216426	NA
ENSG00000166128	RAB8B	RAB8B, member RAS oncogene family	51762	892,7727226	-0,226674233	0,065078	-3,48309956	0,000495644	0,020744578
ENSG00000213443	NA	NA	NA	40,02744437	0,337584262	0,31298	1,07861127	0,280761055	NA
ENSG00000153339	TRAPPC8	trafficking protein particle complex subunit 8	22878	289,2836988	0,085215748	0,182141	0,467856688	0,639887079	0,910851161
ENSG00000172731	LRRC20	leucine rich repeat containing 20	55222	36,25780035	-0,316200678	0,405933	-0,778947921	0,436010393	NA
ENSG00000064933	PMS1	PMS1 homolog 1, mismatch repair system component	5378	130,8879362	-0,09646777	0,18805	-0,512990564	0,607957921	0,897640621
ENSG00000257315	ZBED6	zinc finger BED-type containing 6	100381270	352,7622124	0,076874399	0,157649	0,487630352	0,625811693	0,90479929
ENSG00000134265	NAPG	NSF attachment protein gamma	8774	57,45764319	-0,317782902	0,304039	-1,045205711	0,295927904	0,747862492
ENSG00000138593	SECISBP2L	SECIS binding protein 2 like	9728	467,9395399	0,055384247	0,089426	0,619332265	0,535697493	0,874894595
ENSG00000116213	WRAP73	WD repeat containing, antisense to TP73	49856	65,92448487	0,212144467	0,236146	0,898360653	0,368993306	0,797217623
ENSG00000122034	GTF3A	general transcription factor IIIA	2971	251,9424193	0,289810571	0,131987	2,195753168	0,028109615	0,270084577
ENSG00000156671	SAMD8	sterile alpha motif domain containing 8	142891	208,825299	-0,160722177	0,134012	-1,199309823	0,230407497	0,684943406
ENSG00000023734	STRAP	serine/threonine kinase receptor associated protein	11171	291,8471178	-0,062310761	0,115872	-0,537757248	0,59074465	0,892423251
ENSG00000118246	FASTKD2	FAST kinase domains 2	22868	107,149086	-0,235093269	0,209264	-1,123431421	0,261254311	0,716692756
ENSG00000198301	SDAD1	SDA1 domain containing 1	55153	159,7845537	0,119142341	0,169174	0,704259385	0,481271255	0,855577353
ENSG00000117016	RIMS3	regulating synaptic membrane exocytosis 3	9783	207,6720338	-0,055383905	0,142349	-0,389071379	0,697223345	0,930422107
ENSG00000136811	ODF2	outer dense fiber of sperm tails 2	4957	92,54016759	0,459382928	0,333526	1,377354385	0,168402708	0,627733684
ENSG00000143256	PFDN2	prefoldin subunit 2	5202	43,04476805	-0,236690459	0,280453	-0,843958382	0,398692662	0,814457852
ENSG00000165934	CPSF2	cleavage and polyadenylation specific factor 2	53981	334,8898899	0,032396306	0,097808	0,331224317	0,740475054	0,939193793
ENSG00000213719	CLIC1	chloride intracellular channel 1	1192	403,3050145	0,117971747	0,18717	0,630290404	0,5285046	0,872142129
ENSG00000147408	CSGALNACT1	chondroitin sulfate N-acetylgalactosaminyltransferase 1	55790	18,90178497	-0,498497226	0,424114	-1,175385411	0,239840557	NA
ENSG00000151806	GUF1	GTP binding elongation factor GUF1	60558	115,977674	0,250915427	0,273329	0,917996775	0,358620554	0,790915737
ENSG00000185432	TMT1A	thiol methyltransferase 1A	25840	99,3787495	-0,317763002	0,210826	-1,507228149	0,131752187	0,568561363
ENSG00000187792	ZNF70	zinc finger protein 70	7621	51,30479327	0,001227758	0,244919	0,005012917	0,996000288	0,99927023
ENSG00000213246	SUPT4H1	SPT4 homolog, DSIF elongation factor subunit	6827	200,0488886	-0,021166572	0,199272	-0,106219601	0,915408119	0,981785521
ENSG00000069943	PIGB	phosphatidylinositol glycan anchor biosynthesis class B	9488	22,12051035	-0,412709969	0,511526	-0,806820893	0,41976968	NA
ENSG00000106086	PLEKHA8	pleckstrin homology domain containing A8	84725	47,43641469	0,158269279	0,291756	0,542472263	0,58749321	0,891455115
ENSG00000010295	IFFO1	intermediate filament family orphan 1	25900	64,90137797	-0,321875783	0,237128	-1,357392456	0,174656544	0,632039836

ENSG00000138592	USP8	ubiquitin specific peptidase 8	9101	304,7514981	0,056451732	0,117492	0,480474731	0,630889867	0,906183068
ENSG00000261556	SMG1P7	SMG1 pseudogene 7	100506060	20,71347404	-0,429181034	0,405697	-1,05788488	0,290107929	NA
ENSG00000012211	PRICKLE3	prickle planar cell polarity protein 3	4007	30,95628746	0,088021892	0,428576	0,2053823	0,837273448	NA
ENSG00000162496	DHRS3	dehydrogenase/reductase 3	9249	117,4511523	0,222145434	0,195122	1,138492759	0,254914781	0,709964783
ENSG00000213654	GPSM3	G protein signaling modulator 3	63940	249,4452484	0,136145673	0,154748	0,879789348	0,378973436	0,804903799
ENSG00000244187	TMEM141	transmembrane protein 141	85014	30,45345943	-0,399041056	0,360819	-1,105930333	0,268756674	NA
ENSG00000139350	NEDD1	NEDD1 gamma-tubulin ring complex targeting factor	121441	145,2064213	0,096234848	0,145973	0,65926456	0,509725896	0,866180216
ENSG00000130559	CAMSAP1	calmodulin regulated spectrin associated protein 1	157922	188,8602522	0,003463628	0,12448	0,027824739	0,977801935	0,995547887
ENSG00000102103	PQBP1	polyglutamine binding protein 1	10084	99,74263062	0,093432988	0,168561	0,554298827	0,579374353	0,887537774
ENSG00000115762	PLEKHB2	pleckstrin homology domain containing B2	55041	524,816145	-0,007295845	0,086197	-0,084641904	0,932546084	0,987098279
ENSG00000119878	CRIPT	CXXC repeat containing interactor of PDZ3 domain	9419	83,23562351	-0,157801494	0,258566	-0,610293734	0,541667247	0,876623646
ENSG00000120688	WBP4	WW domain binding protein 4	11193	67,82227253	-0,040657716	0,224413	-0,18117334	0,85623152	0,96843928
ENSG00000161551	ZNF577	zinc finger protein 577	84765	35,34610351	-0,123081287	0,325431	-0,378209769	0,705274769	NA
ENSG00000164978	NUDT2	nudix hydrolase 2	318	31,16840131	0,822578899	0,313933	2,620233124	0,008786968	NA
ENSG00000166710	B2M	beta-2-microglobulin	567	14710,52189	0,090668532	0,055855	1,623291478	0,10452711	0,519421475
ENSG00000168488	ATXN2L	ataxin 2 like	11273	757,3486701	-0,033175481	0,06816	-0,486726346	0,626452272	0,90479929
ENSG00000204152	TIMM23B	translocase of inner mitochondrial membrane 23 homolog B	100652748	61,95320405	-0,303675913	0,337994	-0,898464282	0,368938079	0,797217623
ENSG00000213967	ZNF726	zinc finger protein 726	730087	17,0691637	-0,322637123	0,447398	-0,721141408	0,470822516	NA
ENSG00000100280	AP1B1	adaptor related protein complex 1 subunit beta 1	162	355,6683049	0,073521864	0,089798	0,818745694	0,412931521	0,82225544
ENSG00000142082	SIRT3	sirtuin 3	23410	67,14880276	0,059937589	0,341334	0,175597908	0,860609846	0,97061627
ENSG00000139629	GALNT6	polypeptide N-acetylgalactosaminyltransferase 6	11226	306,2117084	-0,067982173	0,124317	-0,546845017	0,584485213	0,88998582
ENSG00000008517	IL32	interleukin 32	9235	1841,37458	-0,139057644	0,044073	-3,155151135	0,00160415	0,047543369
ENSG00000136715	SAP130	Sin3A associated protein 130	79595	158,1518575	0,061999128	0,152259	0,407196209	0,683863886	0,925586358
ENSG00000138381	ASNSD1	asparagine synthetase domain containing 1	54529	129,7225464	-0,396920349	0,183225	-2,166303628	0,030287993	0,279077601
ENSG00000165698	SPACA9	sperm acrosome associated 9	11092	15,43554253	0,717481135	0,478204	1,500365698	0,1335197	NA
ENSG00000163519	TRAT1	T cell receptor associated transmembrane adaptor 1	50852	16,52378788	-0,023139362	0,446239	-0,051854188	0,958644877	NA
ENSG00000135083	CCNJL	cyclin J like	79616	217,8912924	-0,36178799	0,242649	-1,490994219	0,135963013	0,576449438
ENSG00000164902	PHAX	phosphorylated adaptor for RNA export	51808	174,6317072	0,111371786	0,174043	0,639907947	0,522232447	0,870221868
ENSG00000068305	MEF2A	myocyte enhancer factor 2A	4205	587,5164742	0,117761032	0,07659	1,537543546	0,12416026	0,562307116
ENSG00000215440	NPEPL1	aminopeptidase like 1	79716	102,8595327	-0,145099057	0,184309	-0,787257863	0,431130931	0,833786615
ENSG00000215440	LOC124904942	uncharacterized LOC124904942	124904942	102,8595327	-0,145099057	0,184309	-0,787257863	0,431130931	0,833786615
ENSG00000153786	ZDHHC7	zinc finger DHHC-type palmitoyltransferase 7	55625	184,361154	-0,38130612	0,206919	-1,842782809	0,065360728	0,41232382
ENSG00000169231	THBS3	thrombospondin 3	7059	50,81468135	-0,255402156	0,254007	-1,005491182	0,314660393	0,760179246
ENSG00000174718	RESF1	retroelement silencing factor 1	55196	1806,853078	-0,083372439	0,072873	-1,144083418	0,252589044	0,708754466
ENSG00000175224	ATG13	autophagy related 13	9776	398,1238605	-0,124149238	0,117247	-1,058865912	0,289660851	0,741447348
ENSG00000240682	ISY1	ISY1 splicing factor homolog	57461	141,000695	0,506391728	0,268206	1,888072561	0,05901621	0,39312401
ENSG00000248098	BCKDHA	branched chain keto acid dehydrogenase E1 subunit alpha	593	118,6145982	-0,044522733	0,18766	-0,237251777	0,81246147	0,959038743
ENSG00000206190	ATP10A	ATPase phospholipid transporting 10A (putative)	57194	601,3353252	-0,161591648	0,106242	-1,520981731	0,128264422	0,563689485
ENSG00000101166	PRELID3B	PRELI domain containing 3B	51012	226,1068629	0,027737498	0,138692	0,199993996	0,841485277	0,966777711
ENSG00000125089	SH3TC1	SH3 domain and tetratricopeptide repeats 1	54436	24,61355079	0,852693804	1,211035	0,7041031	0,48136857	NA
ENSG00000133816	MICAL2	microtubule associated monooxygenase, calponin and LIM domain containing 2	9645	85,36987993	-0,378065156	0,319362	-1,183814873	0,236486353	0,691328984
ENSG00000148719	DNAJB12	DnaJ heat shock protein family (Hsp40) member B12	54788	220,8260923	0,073631708	0,12587	0,584982027	0,558559802	0,883921037
ENSG00000154122	ANKH	ANKH inorganic pyrophosphate transport regulator	56172	68,62459946	-0,233946523	0,213293	-1,096833898	0,272714009	0,726913977
ENSG00000164038	SLC9B2	solute carrier family 9 member B2	133308	64,22841831	-0,001479874	0,301944	-0,004901153	0,996089461	0,99927023
ENSG00000164039	BDH2	3-hydroxybutyrate dehydrogenase 2	56898	38,86982181	0,004693842	0,309526	0,015164604	0,987900861	NA
ENSG00000164171	ITGA2	integrin subunit alpha 2	3673	110,7513012	0,096551475	0,166708	0,579164817	0,562477968	0,884605624
ENSG00000185252	ZNF74	zinc finger protein 74	7625	37,27442814	-0,214709519	0,310342	-0,691847928	0,489032834	NA
ENSG00000187815	ZFP69	ZFP69 zinc finger protein	339559	30,11631234	0,045003962	0,326912	0,137663756	0,890506165	NA

ENSG00000148848	ADAM12	ADAM metallopeptidase domain 12	8038	19,83541788	-0,703573522	0,432113	-1,628217018	0,103478878	NA
ENSG00000179832	MROH1	maestro heat like repeat family member 1	727957	221,4840639	-0,050569495	0,13941	-0,362738929	0,716799916	0,932040329
ENSG00000184887	BTBD6	BTB domain containing 6	90135	33,74015502	-0,092706544	0,683287	-0,135677409	0,892076308	NA
ENSG00000221944	TIGD1	tigger transposable element derived 1	200765	26,2160658	0,683394819	0,34673	1,970970938	0,0487272	NA
ENSG00000162775	RBM15	RNA binding motif protein 15	64783	167,9528596	-0,095063436	0,140142	-0,678338704	0,497556964	0,861283987
ENSG00000142166	IFNAR1	interferon alpha and beta receptor subunit 1	3454	152,9575472	0,016316966	0,212291	0,076861433	0,938733779	0,987813564
ENSG00000184432	COPB2	COPI coat complex subunit beta 2	9276	479,7227799	0,047555702	0,082781	0,574477587	0,565644663	0,885151063
ENSG00000086758	HUWE1	HECT, UBA and WWE domain containing E3 ubiquitin protein ligase 1	10075	3161,584703	0,131684772	0,04014	3,280625292	0,001035772	0,035995386
ENSG00000144451	SPAG16	sperm associated antigen 16	79582	18,31786373	-0,296302364	0,42402	-0,698793236	0,484681256	NA
ENSG00000148481	MINDY3	MINDY lysine 48 deubiquitinase 3	80013	12,87700233	0,520791111	0,700784	0,74315497	0,457387862	NA
ENSG00000165526	RPUSD4	RNA pseudouridine synthase D4	84881	64,01410175	-0,073895901	0,23427	-0,315430488	0,752434822	0,941922711
ENSG00000129460	NGDN	neuroguidin	25983	70,65532592	-0,13155662	0,210735	-0,624275648	0,532446574	0,873034529
ENSG00000104859	CLASRP	CLK4 associating serine/arginine rich protein	11129	57,30298586	0,075565365	0,890282	0,084877965	0,93235841	0,987098279
ENSG00000076067	RBMS2	RNA binding motif single stranded interacting protein 2	5939	17,26009083	-0,37428835	0,563986	-0,66364812	0,506915552	NA
ENSG00000117500	TMED5	transmembrane p24 trafficking protein 5	50999	215,4963463	-0,354222063	0,165376	-2,141920562	0,032199875	0,287228571
ENSG00000125991	ERGIC3	ERGIC and golgi 3	51614	224,1606922	-0,065359937	0,112631	-0,580299146	0,561712903	0,884605624
ENSG00000168495	POLR3D	RNA polymerase III subunit D	661	100,510425	-0,112854975	0,184284	-0,612396071	0,540275741	0,876149366
ENSG00000082146	STRADB	STE20 related adaptor beta	55437	61,55604653	-0,228919001	0,214597	-1,066737191	0,286090528	0,738005299
ENSG00000085982	USP40	ubiquitin specific peptidase 40	55230	106,6523098	0,064687751	0,198633	0,325665396	0,744677528	0,939745744
ENSG00000101473	ACOT8	acyl-CoA thioesterase 8	10005	22,75872969	0,423208828	0,705902	0,59952934	0,548819951	NA
ENSG00000105879	CBLL1	Cbl proto-oncogene like 1	79872	185,9440335	0,267204458	0,219941	1,214889751	0,224408143	0,679715217
ENSG00000107815	TWNK	twinkle mtDNA helicase	56652	61,88063938	-0,413174123	0,317705	-1,300497222	0,193430608	0,650901454
ENSG00000108591	DRG2	developmentally regulated GTP binding protein 2	1819	79,15287159	-0,039287206	0,219763	-0,178770396	0,858117991	0,969285743
ENSG00000115875	SRSF7	serine and arginine rich splicing factor 7	6432	192,5324512	0,202849393	0,198329	1,022789859	0,306407214	0,753450925
ENSG00000186577	SMIM29	small integral membrane protein 29	221491	35,19619146	0,090577701	0,358239	0,252841375	0,800390793	NA
ENSG00000188706	ZDHHC9	zinc finger DHHC-type palmitoyltransferase 9	51114	21,1878672	-0,376004376	0,400886	-0,937934481	0,348278082	NA
ENSG00000175470	PPP2R2D	protein phosphatase 2 regulatory subunit Bdelta	55844	38,53048588	-0,632933856	1,037882	-0,60983246	0,541972797	NA
ENSG00000205060	SLC35B4	solute carrier family 35 member B4	84912	155,1235536	0,096092643	0,186225	0,516002081	0,605852956	0,897380562
ENSG00000074201	CLNS1A	chloride nucleotide-sensitive channel 1A	1207	174,8764118	0,197701348	0,129028	1,532231567	0,125465301	0,562766692
ENSG00000077097	TOP2B	DNA topoisomerase II beta	7155	896,8662488	0,000680649	0,0861	0,007905313	0,993692538	0,998500439
ENSG00000100490	CDKL1	cyclin dependent kinase like 1	8814	104,5625173	-0,001158259	0,180144	-0,006429633	0,99486993	0,998500439
ENSG00000101337	TM9SF4	transmembrane 9 superfamily member 4	9777	278,7697764	-0,061858099	0,115407	-0,53599875	0,591959414	0,892834002
ENSG00000112996	MRPS30	mitochondrial ribosomal protein S30	10884	73,98757914	-0,019777386	0,224093	-0,088255404	0,929673683	0,986487751
ENSG00000125967	NECAB3	N-terminal EF-hand calcium binding protein 3	63941	42,56063188	-0,306938585	0,432851	-0,709108889	0,478256907	0,854008769
ENSG00000148985	PGAP2	post-GPI attachment to proteins 2	27315	34,26436691	0,16067199	0,353079	0,455059052	0,649066756	NA
ENSG00000170142	UBE2E1	ubiquitin conjugating enzyme E2 E1	7324	54,5781025	-0,289153778	0,271634	-1,064497394	0,287103434	0,738104868
ENSG00000186470	BTN3A2	butyrophilin subfamily 3 member A2	11118	796,3510432	-0,168664301	0,064128	-2,630132727	0,008535154	0,140830038
ENSG00000143158	MPC2	mitochondrial pyruvate carrier 2	25874	82,66104012	0,140850867	0,205927	0,683984062	0,49398523	0,860513824
ENSG00000198954	KIFBP	kinesin family binding protein	26128	31,15685516	0,168220854	1,738855	0,096742297	0,922931049	NA
ENSG00000104218	CSPP1	centrosome and spindle pole associated protein 1	79848	46,0419392	0,063178589	1,182622	0,053422469	0,957395303	0,991911599
ENSG00000106244	PDAP1	PDGFA associated protein 1	11333	136,3603162	-0,190375543	0,15658	-1,215838762	0,22404635	0,6792803
ENSG00000141378	PTRH2	peptidyl-tRNA hydrolase 2	51651	38,43523052	-0,117357071	0,325479	-0,360567398	0,718422864	NA
ENSG00000175106	TVP23C	trans-golgi network vesicle protein 23 homolog C	201158	93,23447649	-0,228858667	0,21383	-1,070284188	0,284491409	0,737805171
ENSG00000213390	ARHGAP19	Rho GTPase activating protein 19	84986	172,0225628	0,167813046	0,157528	1,065291642	0,286743974	0,738005299
ENSG00000251079	NA	NA	NA	16,67181402	0,039545939	0,448875	0,088100148	0,929797079	NA
ENSG00000064201	TSPAN32	tetraspanin 32	10077	22,42170378	-1,023202371	0,463671	-2,206740565	0,02733219	NA

ENSG00000012822	CALCOCO1	calcium binding and coiled-coil domain 1	57658	402,2073608	-0,351410163	0,14191	-2,476280751	0,013275914	0,180367712
ENSG00000188419	CHM	CHM Rab escort protein	1121	136,6072967	0,099427588	0,158555	0,627084721	0,530603702	0,872577406
ENSG00000077721	UBE2A	ubiquitin conjugating enzyme E2 A	7319	191,7491516	0,227514582	0,12744	1,785275031	0,074216717	0,443476943
ENSG00000124562	SNRPC	small nuclear ribonucleoprotein polypeptide C	6631	104,7240976	0,167315199	0,163024	1,026324621	0,304738596	0,751870158
ENSG00000150403	TMCO3	transmembrane and coiled-coil domains 3	55002	110,4490103	0,089955253	0,154036	0,583989097	0,559227648	0,883921037
ENSG00000155463	OXA1L	OXA1L mitochondrial inner membrane protein	5018	234,817258	0,058027117	0,118845	0,488260696	0,625365198	0,90479929
ENSG00000181915	ADO	2-aminoethanethiol dioxygenase	84890	248,4046068	0,043920917	0,128714	0,341228181	0,73293181	0,93912057
ENSG00000256229	ZNF486	zinc finger protein 486	90649	20,09187462	-0,597356377	0,419794	-1,422973676	0,154743787	NA
ENSG00000196199	MPHOSPH8	M-phase phosphoprotein 8	54737	408,666919	-0,155241223	0,099768	-1,556024799	0,119702196	0,552699027
ENSG00000087589	CASS4	Cas scaffold protein family member 4	57091	305,132149	-0,094647432	0,106299	-0,89038684	0,373258206	0,800776142
ENSG00000129187	DCTD	dCMP deaminase	1635	184,030833	-0,170040333	0,161994	-1,049669056	0,293870295	0,745255828
ENSG00000169710	FASN	fatty acid synthase	2194	673,1372909	0,070535336	0,073296	0,962335373	0,335881166	0,775885493
ENSG00000175938	ORAI3	ORAI calcium release-activated calcium modulator 3	93129	91,75692447	0,10416213	0,216619	0,480854886	0,630619639	0,905960607
ENSG00000135046	ANXA1	annexin A1	301	361,7631782	0,216910148	0,111268	1,94943967	0,051242941	0,364879472
ENSG00000002549	LAP3	leucine aminopeptidase 3	51056	174,3079428	0,142114071	0,126647	1,122124101	0,261809678	0,717555845
ENSG00000165264	NDUFB6	NADH:ubiquinone oxidoreductase subunit B6	4712	51,41764518	0,385235548	0,37608	1,024344309	0,305672676	0,752351363
ENSG00000122484	RPAP2	RNA polymerase II associated protein 2	79871	296,3072628	0,165521945	0,099805	1,658456634	0,097225334	0,504697799
ENSG00000112531	QKI	QKI, KH domain containing RNA binding	9444	650,3357294	-0,377901671	0,139865	-2,701893745	0,006894579	0,121412612
ENSG00000175215	CTDSP2	CTD small phosphatase 2	10106	551,452366	-0,057523608	0,088733	-0,648280156	0,516803766	0,868791049
ENSG00000165121	NA	NA	NA	15,5881036	0,651747509	0,468591	1,390867167	0,164265711	NA
ENSG00000170385	SLC30A1	solute carrier family 30 member 1	7779	235,4172519	0,033762962	0,145551	0,231965881	0,816564514	0,959707099
ENSG00000122986	HVCN1	hydrogen voltage gated channel 1	84329	215,2474795	0,095424091	0,117144	0,814587415	0,415308517	0,82446781
ENSG00000060138	YBX3	Y-box binding protein 3	8531	41,06559711	-0,038870605	0,296867	-0,13093596	0,895825968	NA
ENSG00000167106	EEIG1	estrogen-induced osteoclastogenesis regulator 1	399665	640,684221	-0,07930358	0,067264	-1,178986812	0,238403427	0,693489081
ENSG00000115289	PCGF1	polycomb group ring finger 1	84759	25,09940739	0,002570174	0,3676	0,006991764	0,994421425	NA
ENSG00000134153	EMC7	ER membrane protein complex subunit 7	56851	91,29170426	-0,039202951	0,188785	-0,207659452	0,83549488	0,964287552
ENSG00000146535	GNA12	G protein subunit alpha 12	2768	98,31113401	0,124117813	0,533777	0,232527447	0,816128372	0,959707099
ENSG00000177868	SVBP	small vasohibin binding protein	374969	22,74795483	0,022952425	0,394082	0,058242763	0,953555258	NA
ENSG00000140564	FURIN	furin, paired basic amino acid cleaving enzyme	5045	855,4922793	-0,074679995	0,060645	-1,231437306	0,218159351	0,673519155
ENSG00000130487	KLHDC7B	kelch domain containing 7B	113730	18,48350677	0,02901352	0,408995	0,070938626	0,943446601	NA
ENSG00000183513	COA5	cytochrome c oxidase assembly factor 5	493753	49,87319232	-0,362624995	0,244123	-1,485418891	0,137432895	0,578689141
ENSG00000274512	TBC1D3	TBC1 domain family member 3	729873	138,7276101	-0,08758931	0,145091	-0,603686713	0,54605195	0,878739902
ENSG00000274512	TBC1D3L	TBC1 domain family member 3L	101060376	138,7276101	-0,08758931	0,145091	-0,603686713	0,54605195	0,878739902
ENSG00000168405	CMAHP	cytidine monophospho-N-acetylneuraminic acid hydroxylase, pseudogene	8418	120,0086713	0,123162555	0,170766	0,721234092	0,4707655	0,852690218
ENSG00000284773	NA	NA	NA	23,02676487	0,016235663	0,406747	0,039915839	0,968160224	NA
ENSG00000143851	PTPN7	protein tyrosine phosphatase non-receptor type 7	5778	1956,888251	0,026845033	0,050837	0,528064894	0,597454296	0,894257182
ENSG00000189430	NCR1	natural cytotoxicity triggering receptor 1	9437	89,14035841	-0,299848232	0,351047	-0,854154766	0,393019243	0,812491435
ENSG00000196458	ZNF605	zinc finger protein 605	100289635	74,01095869	-0,042336197	0,199829	-0,211861646	0,832214973	0,963446071
ENSG00000106348	IMPDH1	inosine monophosphate dehydrogenase 1	3614	129,3323626	-0,177395904	0,190195	-0,932703826	0,350972909	0,785712602
ENSG00000181744	DIPK2A	divergent protein kinase domain 2A	205428	101,0893548	-0,322907273	0,207168	-1,55867222	0,11907398	0,551744566
ENSG00000108094	CUL2	cullin 2	8453	100,0839336	-0,040831172	0,239203	-0,170696447	0,864462456	0,971561202
ENSG00000161888	SPC24	SPC24 component of NDC80 kinetochore complex	147841	14,68096019	-0,308111028	0,468079	-0,658245633	0,510380307	NA
ENSG00000111670	GNPTAB	N-acetylglucosamine-1-phosphate transferase subunits alpha and beta	79158	5044,514469	0,041241525	0,033733	1,222576693	0,221489621	0,67740634
ENSG00000241506	PSMC1P1	proteasome 26S subunit, ATPase 1 pseudogene 1	151645	41,27574111	0,009936233	1,104406	0,008996901	0,992821608	NA
ENSG00000116985	BMP8B	bone morphogenetic protein 8b	656	19,61654211	-0,217533885	0,405917	-0,535907751	0,592022307	NA

ENSG00000137802	MAPKBP1	mitogen-activated protein kinase binding protein 1	23005	233,4725256	0,305728629	0,144324	2,118355642	0,03414496	0,295021466
ENSG00000092529	CAPN3	calpain 3	825	12,27797935	1,025180391	0,664095	1,543724833	0,122655008	NA
ENSG00000145996	CDKAL1	CDK5 regulatory subunit associated protein 1 like 1	54901	93,97355001	-0,213724727	0,211392	-1,011035195	0,311999585	0,757868604
ENSG00000172725	CORO1B	coronin 1B	57175	94,22841484	-0,352814521	0,210869	-1,673142736	0,0942992	0,498337741
ENSG00000212916	MAP10	microtubule associated protein 10	54627	13,40476888	0,234105074	0,491543	0,476265866	0,63388498	NA
ENSG00000077454	LRCH4	leucine rich repeats and calponin homology domain containing 4	4034	375,7292464	-0,11243444	0,108731	-1,034056253	0,301109866	0,751870158
ENSG00000108039	XPNPEP1	X-prolyl aminopeptidase 1	7511	239,8815673	0,194046122	0,117315	1,654059856	0,098115352	0,506639035
ENSG00000156273	BACH1	BTB domain and CNC homolog 1	571	171,2778321	-0,134019853	0,140508	-0,95382386	0,34017281	0,777431905
ENSG00000156273	GRIK1-AS2	GRIK1 antisense RNA 2	100379661	171,2778321	-0,134019853	0,140508	-0,95382386	0,34017281	0,777431905
ENSG00000163956	LRPAP1	LDL receptor related protein associated protein 1	4043	97,55847858	0,214198501	0,203532	1,052407686	0,292612544	0,744711251
ENSG00000170873	MTSS1	MTSS I-BAR domain containing 1	9788	208,2594244	-0,313045731	0,16624	-1,883092589	0,059687821	0,395835192
ENSG00000171206	TRIM8	tripartite motif containing 8	81603	195,2953402	-0,045702131	0,188338	-0,242659671	0,808269052	0,958576399
ENSG00000213930	GALT	galactose-1-phosphate uridylyltransferase	2592	42,14347216	-0,430150433	0,416885	-1,03182141	0,302155787	NA
ENSG00000137200	CMTR1	cap methyltransferase 1	23070	290,8725623	0,14458071	0,098509	1,467691473	0,142188048	0,586525698
ENSG00000175203	DCTN2	dynactin subunit 2	10540	190,1892587	0,019704067	0,16296	0,120913768	0,903759337	0,978460866
ENSG00000096093	EFHC1	EF-hand domain containing 1	114327	13,26801394	0,574525297	0,6691	0,85865419	0,390531331	NA
ENSG00000114735	HEMK1	HemK methyltransferase family member 1	51409	162,9625088	0,076403725	0,159871	0,477907659	0,63271593	0,907570154
ENSG00000135913	USP37	ubiquitin specific peptidase 37	57695	153,7251721	-0,061374344	0,168335	-0,364596578	0,715412569	0,932040329
ENSG00000145016	RUBCN	rubicon autophagy regulator	9711	369,1164365	-0,10608025	0,115522	-0,918266111	0,358479565	0,790870366
ENSG00000243749	TMEM35B	transmembrane protein 35B	100506144	25,0291128	-0,025969637	0,394418	-0,065842934	0,947502874	NA
ENSG00000177105	RHOG	ras homolog family member G	391	144,7986855	-0,118299624	0,144422	-0,819125226	0,412714971	0,82225544
ENSG00000187583	PLEKHN1	pleckstrin homology domain containing N1	84069	45,1108328	-0,001893375	0,257895	-0,007341659	0,994142256	0,998500439
ENSG00000040933	INPP4A	inositol polyphosphate-4-phosphatase type I A	3631	1679,465391	-0,054129581	0,062599	-0,864708086	0,38719903	0,809653368
ENSG00000077463	SIRT6	sirtuin 6	51548	37,86723672	-0,024225354	0,314772	-0,076961552	0,938654131	NA
ENSG00000078487	ZCWPW1	zinc finger CW-type and PWWP domain containing 1	55063	30,38031661	0,570036675	0,354733	1,60694647	0,108066111	NA
ENSG00000100299	ARSA	arylsulfatase A	410	109,6004784	0,164508861	0,161451	1,018939594	0,308231645	0,754797782
ENSG00000105819	PMPCB	peptidase, mitochondrial processing subunit beta	9512	106,9494571	-0,002205396	0,318912	-0,006915385	0,994482365	0,998500439
ENSG00000116830	TTF2	transcription termination factor 2	8458	242,1659477	0,127971058	0,106145	1,205627847	0,227961017	0,683361002
ENSG00000118369	USP35	ubiquitin specific peptidase 35	57558	92,27733359	0,044455969	0,278022	0,159900858	0,872959173	0,974533542
ENSG00000126522	ASL	argininosuccinate lyase	435	43,95908012	0,594557738	1,357531	0,437969808	0,66140817	0,91978661
ENSG00000132740	IGHMBP2	immunoglobulin mu DNA binding protein 2	3508	73,57847235	-0,041225917	0,224727	-0,183448529	0,854446101	0,968377739
ENSG00000139083	ETV6	ETS variant transcription factor 6	2120	124,3350657	-0,324674177	0,179202	-1,811780382	0,070020138	0,429975106
ENSG00000139697	SBNO1	strawberry notch homolog 1	55206	495,2036655	-0,002555641	0,087405	-0,029239052	0,976673936	0,995173468
ENSG00000148019	CEP78	centrosomal protein 78	84131	217,51808	0,317951918	0,138614	2,293797895	0,02180211	0,233606788
ENSG00000159917	ZNF235	zinc finger protein 235	9310	41,32982599	0,135373446	0,338479	0,399946665	0,6891958	NA
ENSG00000164687	FABP5	fatty acid binding protein 5	2171	38,64564404	0,391733704	0,309101	1,267333364	0,205036116	NA
ENSG00000165525	NEMF	nuclear export mediator factor	9147	133,6201898	0,833323216	0,28363	2,938062512	0,003302704	0,075727344
ENSG00000173141	MRPL57	mitochondrial ribosomal protein L57	78988	65,36412706	0,192031488	0,218378	0,879354034	0,379209347	0,804903799
ENSG00000178896	EXOSC4	exosome component 4	54512	12,16144247	-0,524472023	0,512493	-1,02337473	0,306130703	NA
ENSG00000187325	TAF9B	TATA-box binding protein associated factor 9b	51616	60,00807376	-0,089450385	0,228234	-0,391923818	0,695114504	0,929397132
ENSG00000198793	MTOR	mechanistic target of rapamycin kinase	2475	459,1386807	-0,017431226	0,135207	-0,128922655	0,89741885	0,977990819
ENSG00000111850	SMIM8	small integral membrane protein 8	57150	21,36949684	0,779553289	0,433817	1,796961791	0,072341686	NA
ENSG00000111850	LINC01590	long intergenic non-protein coding RNA 1590	63914	21,36949684	0,779553289	0,433817	1,796961791	0,072341686	NA
ENSG00000125863	MKKS	MKKS centrosomal shuttling protein	8195	62,49034138	0,167848593	0,259846	0,645955148	0,518308403	0,868791049
ENSG00000135316	SYNCRIP	synaptotagmin binding cytoplasmic RNA interacting protein	10492	882,1588908	0,109990388	0,065298	1,684430827	0,092098437	0,494085469
ENSG00000155252	PI4K2A	phosphatidylinositol 4-kinase type 2 alpha	55361	103,3371935	0,234801434	0,176214	1,332482544	0,182701674	0,637116189
ENSG00000159346	ADIPOR1	adiponectin receptor 1	51094	207,108332	-0,005244167	0,140307	-0,037376468	0,970184835	0,993542794
ENSG00000159720	ATP6V0D1	ATPase H+ transporting V0 subunit d1	9114	230,7856382	0,135601852	0,107621	1,259990628	0,207672743	0,662274754

ENSG00000170049	KCNAB3	potassium voltage-gated channel subfamily A regulatory beta subunit 3	9196	17,04968014	0,643717751	0,46046	1,397987478	0,162116828	NA
ENSG00000174444	RPL4	ribosomal protein L4	6124	906,6217786	0,244153956	0,124317	1,963966568	0,049533969	0,357023063
ENSG00000185973	TMLHE	trimethyllysine hydroxylase, epsilon	55217	33,10580084	-0,247846268	0,380556	-0,651273426	0,514869999	NA
ENSG00000100302	RASD2	RASD family member 2	23551	38,11396697	0,03009293	0,275911	0,109067378	0,913149049	NA
ENSG00000068383	INPP5A	inositol polyphosphate-5-phosphatase A	3632	24,84007246	-0,011199829	0,528998	-0,021171766	0,983108636	NA
ENSG00000140284	SLC27A2	solute carrier family 27 member 2	11001	90,38432548	0,337123132	0,201783	1,67072026	0,094776945	0,499027263
ENSG00000156973	PDE6D	phosphodiesterase 6D	5147	67,70949545	0,004984877	0,240552	0,020722624	0,983466922	0,996185513
ENSG00000083444	PLOD1	procollagen-lysine,2-oxoglutarate 5-dioxygenase 1	5351	215,8559139	-0,143846973	0,110594	-1,300680331	0,193367897	0,650901454
ENSG00000168795	ZBTB5	zinc finger and BTB domain containing 5	9925	97,73494244	-0,209494025	0,181776	-1,152487134	0,249120954	0,707335884
ENSG00000263956	NBPF11	NBPF member 11	200030	28,91552554	-0,419195633	0,521272	-0,804177681	0,421294367	NA
ENSG00000136758	YME1L1	YME1 like 1 ATPase	10730	735,6242273	0,033323283	0,061656	0,54047089	0,588872331	0,891627382
ENSG00000172936	MYD88	MYD88 innate immune signal transduction adaptor	4615	172,961466	0,197800229	0,429209	0,460848353	0,644907408	0,912530858
ENSG00000035664	DAPK2	death associated protein kinase 2	23604	81,13011649	-0,485560713	0,210107	-2,311017267	0,020831901	0,225985845
ENSG00000175970	UNC119B	unc-119 lipid binding chaperone B	84747	135,4988514	-0,073081468	0,143779	-0,508291375	0,611249018	0,900006614
ENSG00000049618	ARID1B	AT-rich interaction domain 1B	57492	321,2899651	0,11513815	0,309814	0,371636791	0,710163293	0,930846779
ENSG00000068796	KIF2A	kinesin family member 2A	3796	532,5835068	-0,222796811	0,132243	-1,684757966	0,092035278	0,494084123
ENSG00000090615	GOLGA3	golgin A3	2802	580,6881154	-0,057715415	0,081601	-0,707283831	0,479390112	0,854426127
ENSG00000135074	ADAM19	ADAM metallopeptidase domain 19	8728	933,5981294	-0,084128212	0,057059	-1,474400449	0,140373804	0,583639944
ENSG00000168014	C2CD3	C2 domain containing 3 centriole elongation regulator	26005	90,82703856	0,796195578	0,793811	1,003004417	0,31585873	0,76048697
ENSG00000172785	ZNG1A	Zn regulated GTPase metalloprotein activator 1A	55871	47,12707397	0,026066816	0,268276	0,097164016	0,922596144	0,984284131
ENSG00000188566	NDOR1	NADPH dependent diflavin oxidoreductase 1	27158	141,9344794	-0,07522928	0,166178	-0,452702254	0,650763156	0,915310499
ENSG00000213762	ZNF134	zinc finger protein 134	7693	110,9713337	-0,123831239	0,278872	-0,444043102	0,657011422	0,919088619
ENSG00000172819	RARG	retinoic acid receptor gamma	5916	159,6930017	-0,075372104	0,137796	-0,546981446	0,584391479	0,88998582
ENSG00000108557	RAI1	retinoic acid induced 1	10743	118,705306	-0,418707953	0,165571	-2,528872101	0,011442972	0,168264867
ENSG00000112679	DUSP22	dual specificity phosphatase 22	56940	154,1925144	-0,169592487	0,132647	-1,278520932	0,201065809	0,660006147
ENSG00000112679	LOC124907782	dual specificity protein phosphatase 22	124907782	154,1925144	-0,169592487	0,132647	-1,278520932	0,201065809	0,660006147
ENSG00000186395	KRT10	keratin 10	3858	45,94010052	-0,181612115	0,267991	-0,677680402	0,497974355	0,861283987
ENSG00000198695	ND6	NADH dehydrogenase subunit 6	4541	259,9099808	0,178719016	0,1245	1,435493336	0,151146566	0,607670525
ENSG00000255112	CHMP1B	charged multivesicular body protein 1B	57132	231,4360029	-0,075908824	0,120612	-0,629361845	0,529112191	0,872145761
ENSG00000110911	SLC11A2	solute carrier family 11 member 2	4891	62,01433798	-0,139002526	0,375017	-0,370656344	0,71089351	0,930846779
ENSG00000257923	CUX1	cut like homeobox 1	1523	308,2111011	-0,102684996	0,140738	-0,729619328	0,465622904	0,84908342
ENSG00000107789	MINPP1	multiple inositol-polyphosphate phosphatase 1	9562	43,56170006	-0,125058321	0,292573	-0,427443013	0,669056684	0,922641254
ENSG00000121064	SCPEP1	serine carboxypeptidase 1	59342	468,4758402	0,195322847	0,082798	2,359026347	0,018322955	0,212254406
ENSG00000137818	RPLP1	ribosomal protein lateral stalk subunit P1	6176	1618,093873	0,198891973	0,092267	2,155608272	0,031114266	0,281657534
ENSG00000143363	PRUNE1	prune exopolyphosphatase 1	58497	76,414395	-0,110721069	0,2045	-0,541424286	0,588215171	0,891557039
ENSG00000154518	ATP5MC3	ATP synthase membrane subunit c locus 3	518	173,6513656	0,292498389	0,134699	2,171498574	0,029893505	0,279077601
ENSG00000174574	AKIRIN1	akirin 1	79647	316,6026418	-0,246476518	0,116197	-2,121194928	0,033905402	0,294612488
ENSG00000108468	CBX1	chromobox 1	10951	159,763692	0,01802958	0,137915	0,130729918	0,895988964	0,977990819
ENSG00000104325	DECR1	2,4-dienoyl-CoA reductase 1	1666	31,1407589	0,194206568	0,468318	0,414689357	0,678369325	NA
ENSG00000041357	PSMA4	proteasome 20S subunit alpha 4	5685	187,7987254	0,154124286	0,131569	1,171430334	0,241425845	0,696802579
ENSG00000152127	MGAT5	alpha-1,6-mannosylglycoprotein 6-beta-N-acetylglucosaminyltransferase	4249	1071,872456	0,17231454	0,056338	3,058583937	0,002223858	0,057258849
ENSG00000268043	NBPF12	NBPF member 12	149013	244,8649894	-0,008385438	0,114152	-0,073458258	0,94144146	0,988441237
ENSG00000141337	ARSG	arylsulfatase G	22901	65,77656671	-0,339858453	0,237457	-1,431243903	0,152360325	0,608624223
ENSG00000131153	GINS2	GINS complex subunit 2	51659	24,59188963	0,470226717	0,359334	1,308605946	0,190667866	NA
ENSG00000187118	CMC1	C-X9-C motif containing 1	152100	20,95160157	0,141160708	0,820869	0,171964887	0,863465134	NA
ENSG00000203761	NA	NA	NA	16,80022721	-0,529206489	0,448971	-1,178710063	0,238513647	NA
ENSG00000152520	PAN3	poly(A) specific ribonuclease subunit PAN3	255967	318,731451	-0,117617073	0,094072	-1,250287235	0,21119464	0,664818718

ENSG00000134644	PUM1	pumilio RNA binding family member 1	9698	367,4907885	-0,036395708	0,098782	-0,368444879	0,712541539	0,930846779
ENSG00000005810	MYCBP2	MYC binding protein 2	23077	1096,957964	0,003399625	0,06915	0,04916329	0,960789166	0,992235217
ENSG00000172086	KRCC1	lysine rich coiled-coil 1	51315	126,1513494	-0,015997488	0,160022	-0,099970388	0,920367835	0,984284131
ENSG00000187189	TSPYL4	TSPY like 4	23270	131,8697039	-0,372436499	0,163346	-2,28004415	0,02260507	0,239919217
ENSG00000090316	MAEA	macrophage erythroblast attacher, E3 ubiquitin ligase	10296	156,072417	-0,086186204	0,162117	-0,531630986	0,594981599	0,894229873
ENSG00000250299	NA	NA	NA	21,97364167	0,869074915	0,384539	2,26004213	0,023818636	NA
ENSG00000009780	FAM76A	family with sequence similarity 76 member A	199870	54,56777212	0,130863956	0,290535	0,450423424	0,652405158	0,916482964
ENSG00000109861	CTSC	cathepsin C	1075	951,1734222	0,174165938	0,093694	1,85887425	0,063044965	0,404317327
ENSG00000111786	SRSF9	serine and arginine rich splicing factor 9	8683	235,8187533	-0,014305803	0,110202	-0,129814726	0,896713012	0,977990819
ENSG00000227372	GFOD3P	Gfo/Idh/MocA-like oxidoreductase domain containing 3, pseudogene	57212	13,77687614	-0,214869089	0,478495	-0,449051939	0,65339419	NA
ENSG00000043462	LCP2	lymphocyte cytosolic protein 2	3937	2154,455922	0,101834822	0,077232	1,318563374	0,187315125	0,645758518
ENSG00000169020	ATP5ME	ATP synthase membrane subunit e	521	57,87235564	0,219763906	0,255767	0,859236235	0,390210196	0,810747116
ENSG00000128928	IVD	isovaleryl-CoA dehydrogenase	3712	251,361452	-0,041317862	0,123659	-0,334126508	0,738284098	0,939193793
ENSG00000039650	PNKP	polynucleotide kinase 3'-phosphatase	11284	75,32983645	-0,166578726	0,231062	-0,720926531	0,470954718	0,852690218
ENSG00000053501	USE1	unconventional SNARE in the ER 1	55850	43,09261337	-0,116090968	0,296508	-0,391526647	0,695407996	0,929397132
ENSG00000085365	SCAMP1	secretory carrier membrane protein 1	9522	33,46455029	0,353857001	0,475134	0,744752185	0,456421545	NA
ENSG00000101160	CTSZ	cathepsin Z	1522	173,3087865	-0,498979921	0,208149	-2,39722421	0,016519812	0,202942572
ENSG00000121417	ZNF211	zinc finger protein 211	10520	93,46962099	-0,017727921	0,288672	-0,061411902	0,951031173	0,991212866
ENSG00000131269	ABCB7	ATP binding cassette subfamily B member 7	22	48,58651874	0,941867706	0,508485	1,852301633	0,063982521	0,407557764
ENSG00000132467	UTP3	UTP3 small subunit processome component	57050	188,6378246	-0,052978595	0,120888	-0,438245104	0,661208615	0,91978661
ENSG00000140650	PMM2	phosphomannomutase 2	5373	89,55228619	-0,142768225	0,191799	-0,744363777	0,456656427	0,844645758
ENSG00000140650	LOC100130283	uncharacterized LOC100130283	100130283	89,55228619	-0,142768225	0,191799	-0,744363777	0,456656427	0,844645758
ENSG00000143549	TPM3	tropomyosin 3	7170	1372,505756	-0,070264347	0,07764	-0,904999488	0,365465658	0,794170647
ENSG00000157870	PRXL2B	peroxiredoxin like 2B	127281	57,26586622	0,02726728	1,249114	0,02182929	0,98258413	0,996026814
ENSG00000164733	CTSB	cathepsin B	1508	63,63677934	-0,320191007	1,058419	-0,302518119	0,762257123	0,943004836
ENSG00000167664	TMIGD2	transmembrane and immunoglobulin domain containing 2	126259	17,67151362	-0,166249682	0,431076	-0,385662171	0,699746882	NA
ENSG00000168522	FNTA	farnesyltransferase, CAAX box, alpha	2339	156,5543356	-0,030247077	0,164887	-0,183441392	0,854451701	0,968377739
ENSG00000175564	UCP3	uncoupling protein 3	7352	15,49959278	-0,358284225	0,47782	-0,749831089	0,453356442	NA
ENSG00000178234	GALNT11	polypeptide N-acetylgalactosaminyltransferase 11	63917	197,5060792	-0,056081769	0,132822	-0,422232289	0,672855476	0,923602509
ENSG00000182749	PAQR7	progestin and adipoQ receptor family member 7	164091	15,92098857	0,165976179	0,462879	0,358573503	0,719914172	NA
ENSG00000185875	THNSL1	threonine synthase like 1	79896	21,37634402	-0,058632797	0,38108	-0,153859623	0,877720421	NA
ENSG00000188626	GOLGA8M	golgin A8 family member M	653720	34,94973048	0,666121386	0,291083	2,288427289	0,02211265	NA
ENSG00000137947	GTF2B	general transcription factor IIB	2959	102,9127461	-0,119147474	0,164638	-0,723692515	0,469254529	0,852144526
ENSG00000175040	CHST2	carbohydrate sulfotransferase 2	9435	91,40139711	0,290579102	0,174127	1,668773827	0,095162212	0,499178345
ENSG00000169251	NMD3	NMD3 ribosome export adaptor	51068	186,2971475	0,030626463	0,121869	0,251306473	0,801577171	0,956122858
ENSG00000054267	ARID4B	AT-rich interaction domain 4B	51742	418,5079856	0,006255304	0,120345	0,051977995	0,958546227	0,991911599
ENSG00000183309	ZNF623	zinc finger protein 623	9831	80,88619844	-0,242929045	0,790353	-0,307367963	0,758563311	0,942901768
ENSG00000112624	BICRAL	BICRA like chromatin remodeling complex associated protein	23506	412,4335902	-0,008118169	0,08786	-0,092399333	0,926380769	0,985345959
ENSG00000119787	ATL2	atlastin GTPase 2	64225	100,6539008	0,254690948	0,202815	1,255777875	0,209196515	0,663582162
ENSG00000216490	IFI30	IFI30 lysosomal thiol reductase	10437	31,8734172	0,265308072	0,325118	0,816035453	0,414479859	NA
ENSG00000063587	ZNF275	zinc finger protein 275	10838	94,47921101	-0,065543518	0,213102	-0,307568321	0,758410829	0,942901768
ENSG00000063587	LOC105373378	uncharacterized LOC105373378	105373378	94,47921101	-0,065543518	0,213102	-0,307568321	0,758410829	0,942901768
ENSG00000063978	RNF4	ring finger protein 4	6047	492,0673481	0,005648847	0,081557	0,069262585	0,944780607	0,989205143
ENSG00000162377	COA7	cytochrome c oxidase assembly factor 7	65260	44,70224922	0,274155502	0,281114	0,97524739	0,32943756	0,771437864
ENSG00000180185	FAHD1	fumarylacetoacetate hydrolase domain containing 1	81889	49,66255667	-0,323006164	0,254123	-1,271063587	0,203706028	0,660954946
ENSG00000198393	ZNF26	zinc finger protein 26	7574	106,6135766	0,201783663	0,169547	1,190133146	0,233994065	0,688858646
ENSG00000153113	CAST	calpastatin	831	634,0074801	-0,023424263	0,10958	-0,213764621	0,830730616	0,963156386
ENSG00000240038	AMY2B	amylase alpha 2B	280	36,37167292	-0,206767073	0,312289	-0,662101638	0,507906086	NA

ENSG00000111666	CHPT1	choline phosphotransferase 1	56994	27,55355399	0,086286706	0,360985	0,239031353	0,811081271	NA
ENSG00000124181	PLCG1	phospholipase C gamma 1	5335	855,2936962	0,063388411	0,056079	1,130338717	0,258333526	0,714048892
ENSG00000154165	GPR15	G protein-coupled receptor 15	2838	637,9682789	-0,314804711	0,084118	-3,742409594	0,000182264	0,010298578
ENSG00000006744	ELAC2	elaC ribonuclease Z 2	60528	240,7890997	0,108845166	0,130838	0,831908937	0,405460347	0,818464021
ENSG00000089006	SNX5	sorting nexin 5	27131	123,6289689	0,019125519	0,326478	0,058581341	0,953285573	0,991212866
ENSG00000114784	EIF1B	eukaryotic translation initiation factor 1B	10289	91,15246718	0,234384724	0,17391	1,347739342	0,177742234	0,632142851
ENSG00000147874	HAUS6	HAUS augmin like complex subunit 6	54801	228,1143743	0,009367564	0,130966	0,07152692	0,9429784	0,988624545
ENSG00000167535	CACNB3	calcium voltage-gated channel auxiliary subunit beta 3	784	17,33591199	0,654482558	0,767645	0,852585311	0,393889311	NA
ENSG00000167977	KCTD5	potassium channel tetramerization domain containing 5	54442	241,8347295	0,118422684	0,115588	1,024526351	0,305586731	0,752351363
ENSG00000168476	REEP4	receptor accessory protein 4	80346	48,07170371	0,112007783	0,272828	0,410544173	0,681406807	0,925586358
ENSG00000178409	BEND3	BEN domain containing 3	57673	20,79412603	-0,0854482	0,381432	-0,224019457	0,822742164	NA
ENSG00000182534	MXRA7	matrix remodeling associated 7	439921	198,1658565	0,199378191	0,13542	1,472298832	0,140940199	0,584590139
ENSG00000184574	LPAR5	lysophosphatidic acid receptor 5	57121	15,07146744	-0,511532504	0,612683	-0,834905227	0,403771071	NA
ENSG00000188186	LAMTOR4	late endosomal/lysosomal adaptor, MAPK and MTOR activator 4	389541	78,74594538	-0,008817547	0,206731	-0,042652355	0,96597866	0,992789539
ENSG00000198648	STK39	serine/threonine kinase 39	27347	203,5898223	0,151340368	0,140708	1,075562702	0,282122876	0,735655069
ENSG00000261609	GAN	gigaxonin	8139	98,32558103	-0,166710767	0,196609	-0,847931813	0,396475946	0,812799303
ENSG00000049769	PPP1R3F	protein phosphatase 1 regulatory subunit 3F	89801	42,20300964	0,095607547	0,302474	0,316085319	0,75193775	NA
ENSG00000143401	ANP32E	acidic nuclear phosphoprotein 32 family member E	81611	505,5933264	0,167762638	0,082719	2,028110345	0,042548987	0,328150469
ENSG00000203760	CENPW	centromere protein W	387103	14,10688521	0,702139868	0,457981	1,533119494	0,125246415	NA
ENSG00000143553	SNAPIN	SNAP associated protein	23557	66,58044573	0,272545111	0,225401	1,209154312	0,226603566	0,681621061
ENSG00000130227	XPO7	exportin 7	23039	314,7660239	0,138459953	0,093014	1,48859564	0,13659388	0,577399536
ENSG00000065029	ZNF76	zinc finger protein 76	7629	76,73033667	-0,140619416	0,205456	-0,684425559	0,493706481	0,860513824
ENSG00000178385	PLEKHM3	pleckstrin homology domain containing M3	389072	106,9273085	0,11250388	0,16955	0,66354257	0,506983124	0,864738156
ENSG00000152409	JMY	junction mediating and regulatory protein, p53 cofactor	133746	111,7388665	-0,31537307	0,207086	-1,522907321	0,127781894	0,563689485
ENSG00000167670	CHAF1A	chromatin assembly factor 1 subunit A	10036	92,23024536	0,33922029	0,17242	1,967401859	0,0491369	0,355687755
ENSG00000101442	ACTR5	actin related protein 5	79913	57,43050019	-0,040847269	0,218793	-0,186693999	0,851900561	0,968147715
ENSG00000105227	PRX	periaxin	57716	18,38361915	0,280710851	0,472757	0,593774389	0,552663021	NA
ENSG00000109501	WFS1	wolframin ER transmembrane glycoprotein	7466	32,05909615	-0,286188805	0,430159	-0,665309902	0,505852299	NA
ENSG00000114544	SLC41A3	solute carrier family 41 member 3	54946	68,15201223	0,029512416	0,246785	0,119587682	0,904809778	0,978460866
ENSG00000117682	DHDDS	dehydrodolichyl diphosphate synthase subunit	79947	75,66671951	0,387478146	0,265013	1,462110587	0,143710908	0,59024645
ENSG00000133056	PIK3C2B	phosphatidylinositol-4-phosphate 3-kinase catalytic subunit type 2 beta	5287	346,9140792	-0,12428363	0,097131	-1,279545738	0,200704944	0,659555076
ENSG00000135763	URB2	URB2 ribosome biogenesis homolog	9816	88,00619484	0,011122886	0,186992	0,05948331	0,952567159	0,991212866
ENSG00000143207	COP1	COP1 E3 ubiquitin ligase	64326	71,55332677	-0,108052849	0,340735	-0,317117178	0,751154691	0,941512988
ENSG00000170734	POLH	DNA polymerase eta	5429	267,8070237	-0,006023143	0,142885	-0,042153702	0,96637617	0,992789539
ENSG00000183648	NDUFB1	NADH:ubiquinone oxidoreductase subunit B1	4707	45,47948525	0,141995256	0,254271	0,558439818	0,576544089	0,887069818
ENSG00000186063	AIDA	axin interactor, dorsalization associated	64853	184,4589035	-0,205972919	0,138043	-1,492092792	0,135674821	0,576449438
ENSG00000197056	ZMYM1	zinc finger MYM-type containing 1	79830	37,42673613	-0,135372674	0,514778	-0,262972908	0,792571461	NA
ENSG00000214331	NA	NA	NA	91,46571118	-0,02402	0,205758	-0,116739122	0,907066787	0,978585515
ENSG00000263513	FAM72C	family with sequence similarity 72 member C	554282	18,72138397	0,48270711	0,621442	0,776752969	0,437304529	NA
ENSG00000013561	RNF14	ring finger protein 14	9604	105,6267619	0,136434717	0,177762	0,767512803	0,442776684	0,838678081
ENSG00000092020	PPP2R3C	protein phosphatase 2 regulatory subunit B''gamma	55012	84,46427505	-0,077818037	0,197748	-0,393520989	0,693934725	0,9283565
ENSG00000106299	WASL	WASP like actin nucleation promoting factor	8976	54,85888931	0,041602007	0,244119	0,170417199	0,864682047	0,971561202
ENSG00000181991	MRPS11	mitochondrial ribosomal protein S11	64963	31,26042949	0,548694417	0,337293	1,626760817	0,103787906	NA
ENSG00000197483	ZNF628	zinc finger protein 628	89887	25,38527006	-0,389875112	0,354298	-1,100414509	0,27115156	NA
ENSG00000152465	NMT2	N-myristoyltransferase 2	9397	236,643787	-0,052948331	0,133732	-0,395929243	0,692157228	0,927787546

ENSG00000184076	UQCR10	ubiquinol-cytochrome c reductase, complex III subunit X	29796	72,90861418	0,416232856	0,209962	1,982421456	0,04743209	0,349139303
ENSG00000113460	BRIX1	biogenesis of ribosomes BRX1	55299	82,40262293	-0,081005753	0,188221	-0,430375642	0,666922411	0,922184616
ENSG00000059377	TBXAS1	thromboxane A synthase 1	6916	34,09097129	0,04301665	0,387112	0,11112203	0,911519579	NA
ENSG00000111206	FOXM1	forkhead box M1	2305	139,1983108	0,673180901	0,149042	4,516714715	6,28E-06	0,000666597
ENSG00000116679	IVNS1ABP	influenza virus NS1A binding protein	10625	816,4449922	-0,052702761	0,102185	-0,515758291	0,606023237	0,897380562
ENSG00000167470	MIDN	midnolin	90007	234,0782452	-0,306765297	0,11398	-2,691387352	0,007115552	0,123914742
ENSG00000185475	TMEM179B	transmembrane protein 179B	374395	47,55960961	-0,054439345	0,254706	-0,213734204	0,830754337	0,963156386
ENSG00000186222	BLOC1S4	biogenesis of lysosomal organelles complex 1 subunit 4	55330	43,8893005	-0,0224987	0,267611	-0,084072476	0,932998808	0,987098279
ENSG00000215302	NA	NA	NA	50,5488493	0,152652718	0,265316	0,575361819	0,565046621	0,885045179
ENSG00000110876	SELPLG	selectin P ligand	6404	1015,105514	-0,133670229	0,095542	-1,39908027	0,161788913	0,619487156
ENSG00000143178	TBX19	T-box transcription factor 19	9095	13,52550444	-0,128895565	0,59316	-0,217303335	0,827971958	NA
ENSG00000116809	ZBTB17	zinc finger and BTB domain containing 17	7709	80,12460785	0,014652203	0,186715	0,078473659	0,937451282	0,987194684
ENSG00000114742	WDR48	WD repeat domain 48	57599	192,4404127	0,123968843	0,127313	0,973730328	0,330190455	0,771479881
ENSG00000005020	SKAP2	src kinase associated phosphoprotein 2	8935	247,1632782	0,023356031	0,110716	0,2109538	0,832923323	0,963446071
ENSG00000132563	REEP2	receptor accessory protein 2	51308	17,66130159	-0,018801031	0,413521	-0,045465671	0,963736137	NA
ENSG00000144228	SPOPL	speckle type BTB/POZ protein like	339745	140,3352423	-0,059702652	0,154558	-0,386280001	0,69928931	0,930783276
ENSG00000153391	INO80C	INO80 complex subunit C	125476	16,47239045	0,353762176	0,45843	0,771682209	0,44030267	NA
ENSG00000162194	LBHD1	LBH domain containing 1	79081	118,1183505	0,240177868	0,18756	1,280539959	0,200355301	0,659373545
ENSG00000101150	TPD52L2	TPD52 like 2	7165	191,943765	-0,157583315	0,140153	-1,124363721	0,260858756	0,716543286
ENSG00000142046	TMEM91	transmembrane protein 91	641649	11,71439501	-1,476161543	0,567084	-2,603072789	0,009239234	NA
ENSG00000158201	ABHD3	abhydrolase domain containing 3, phospholipase	171586	151,371422	0,129482624	0,192302	0,673328535	0,500738303	0,861888808
ENSG00000171621	SPSB1	splA/ryanodine receptor domain and SOCS box containing 1	80176	16,95307316	-0,064029639	0,486075	-0,131727809	0,895199589	NA
ENSG00000100336	APOL4	apolipoprotein L4	80832	69,08829802	0,057942306	0,231787	0,249980985	0,802602053	0,956339465
ENSG00000137073	UBAP2	ubiquitin associated protein 2	55833	134,8948061	0,062871691	0,193985	0,324105367	0,745858264	0,939843468
ENSG00000173486	FKBP2	FKBP prolyl isomerase 2	2286	56,15507159	-0,142436159	0,319581	-0,445696436	0,65581654	0,918143156
ENSG00000227097	NA	NA	NA	63,21456649	0,009002148	0,214689	0,041931028	0,966553682	0,992789539
ENSG00000112365	ZBTB24	zinc finger and BTB domain containing 24	9841	137,9005758	-0,260687915	0,2052	-1,270411884	0,203937952	0,660954946
ENSG00000130518	IQCN	IQ motif containing N	80726	29,84283325	0,235858323	0,31876	0,739924902	0,459345564	NA
ENSG00000142892	PIGK	phosphatidylinositol glycan anchor biosynthesis class K	10026	114,8400459	-0,002250109	0,160022	-0,014061281	0,98878109	0,997032569
ENSG00000106477	CEP41	centrosomal protein 41	95681	89,90488275	-0,122529171	0,302	-0,405726057	0,684943895	0,92575277
ENSG00000123353	ORMDL2	ORMDL sphingolipid biosynthesis regulator 2	29095	67,36003752	-0,285970426	0,261525	-1,093470761	0,27418716	0,727065559
ENSG00000129158	SERGEF	secretion regulating guanine nucleotide exchange factor	26297	21,04813481	-0,703216053	0,904719	-0,777275318	0,436996354	NA
ENSG00000159176	CSRP1	cysteine and glycine rich protein 1	1465	160,170701	0,061593341	0,154531	0,398583436	0,690200168	0,927252461
ENSG00000166188	ZNF319	zinc finger protein 319	57567	163,4530743	0,005285444	0,146872	0,035986656	0,971292999	0,994000404
ENSG00000168763	CNNM3	cyclin and CBS domain divalent metal cation transport mediator 3	26505	198,9599806	-0,103734434	0,137381	-0,755087468	0,450196502	0,84263906
ENSG00000198909	MAP3K3	mitogen-activated protein kinase kinase kinase 3	4215	196,2719846	-0,147350919	0,120143	-1,22646172	0,220024972	0,676880584
ENSG00000204138	PHACTR4	phosphatase and actin regulator 4	65979	277,2933851	-0,029189767	0,145193	-0,20104108	0,840666452	0,966505956
ENSG00000163378	EOGT	EGF domain specific O-linked N-acetylglucosamine transferase	285203	86,2448789	-0,067615787	0,253697	-0,266521581	0,789837541	0,951585219
ENSG00000234284	ZNF879	zinc finger protein 879	345462	21,75910483	0,078988628	0,399375	0,197780713	0,843216637	NA
ENSG00000089048	ESF1	ESF1 nucleolar pre-rRNA processing protein homolog	51575	116,8442351	-0,211074041	0,190002	-1,110902236	0,266610433	0,722327312
ENSG00000099797	TECR	trans-2,3-enoyl-CoA reductase	9524	121,9723622	-0,145422917	0,157806	-0,921527687	0,356774993	0,789494009
ENSG00000132912	DCTN4	dynactin subunit 4	51164	189,0642876	-0,000287372	0,144214	-0,001992671	0,998410079	0,999300722
ENSG00000138081	FBXO11	F-box protein 11	80204	204,3698442	-0,058606029	0,126054	-0,464926991	0,641983741	0,912063088
ENSG00000146112	PPP1R18	protein phosphatase 1 regulatory subunit 18	170954	901,2869455	-0,052650996	0,071773	-0,73357251	0,463209321	0,847634204
ENSG00000146112	LOC107987457	phostensin-like	107987457	901,2869455	-0,052650996	0,071773	-0,73357251	0,463209321	0,847634204
ENSG00000149781	FERMT3	FERM domain containing kindlin 3	83706	507,6160753	-0,052086865	0,085341	-0,610336514	0,541638914	0,876623646
ENSG00000181450	ZNF678	zinc finger protein 678	339500	45,69892178	0,120018291	0,390279	0,307518974	0,758448383	0,942901768
ENSG00000183665	TRMT12	tRNA methyltransferase 12 homolog	55039	57,6638248	-0,128900797	0,25075	-0,514060546	0,60720966	0,897481254
ENSG00000196735	HLA-DQA1	major histocompatibility complex, class II, DQ alpha 1	3117	49,19153473	0,234926252	0,25236	0,930918609	0,351895668	0,786165473

ENSG00000198185	ZNF334	zinc finger protein 334	55713	13,76850413	0,114660405	0,598054	0,19172244	0,847959628	NA	
ENSG00000126005	MMP24OS	MMP24 opposite strand	101410538	93,43054617	0,272475351	0,180773	1,507281565	0,131738501	0,568561363	
ENSG00000105699	LSR	lipolysis stimulated lipoprotein receptor	51599	25,99032489	-0,087398381	0,342977	-0,254822555	0,798860153	NA	
ENSG00000155393	HEATR3	HEAT repeat containing 3	55027	26,98591894	-0,003352419	0,741942	-0,004518435	0,996394822	NA	
ENSG00000069974	RAB27A	RAB27A, member RAS oncogene family	5873	388,8839549	0,029173221	0,173508	0,168137775	0,866474892	0,971722599	
ENSG00000086475	SEPHS1	selenophosphate synthetase 1	22929	146,7682569	0,146743363	0,143613	1,021794694	0,306878081	0,754087936	
ENSG00000162385	MAGOH	mago homolog, exon junction complex subunit	4116	99,82049183	0,1453267	0,191122	0,76038661	0,447023524	0,842554058	
ENSG00000173546	CSPG4	chondroitin sulfate proteoglycan 4	1464	45,10757492	-0,411566528	0,260979	-1,577012266	0,114792703	0,54024053	
ENSG00000198855	FICD	FIC domain protein adenylyltransferase	11153	39,84196372	0,075358711	0,335832	0,224393996	0,822450742	NA	
ENSG00000133030	MPRIP	myosin phosphatase Rho interacting protein	23164	1124,161073	0,040319944	0,083364	0,483662225	0,628625606	0,905567722	
ENSG00000143669	LYST	lysosomal trafficking regulator	1130	1286,410654	0,113516895	0,109067	1,040796688	0,297969918	0,748846814	
ENSG00000145348	TBCK	TBC1 domain containing kinase	93627	38,033298	0,436805027	1,005105	0,434586452	0,663862631	NA	
ENSG00000164308	ERAP2	endoplasmic reticulum aminopeptidase 2	64167	166,2292234	-0,317135101	0,221357	-1,432682971	0,151948458	0,608564843	
ENSG00000198060	MARCHF5	membrane associated ring-CH-type finger 5	54708	92,4660233	-0,242827189	0,184911	-1,313211175	0,189111799	0,6473259	
ENSG00000103363	ELOB	elongin B	6923	174,2680697	0,155416686	0,180962	0,858835762	0,390431134	0,81080014	
ENSG00000132002	DNAJB1	DnaJ heat shock protein family (Hsp40) member B1	3337	192,233345	-0,133556205	0,122397	-1,091174012	0,275196321	0,727877072	
ENSG00000139675	HNRNPA1L2	heterogeneous nuclear ribonucleoprotein A1 like 2	144983	45,69137195	-0,152581611	0,310963	-0,490674693	0,623656548	0,90479929	
ENSG00000149262	INTS4	integrator complex subunit 4	92105	99,09054136	0,090535846	0,210311	0,430485407	0,66684258	0,922184616	
ENSG00000198546	ZNF511	zinc finger protein 511	118472	38,47672522	-0,227750524	0,378962	-0,600985615	0,547849567	NA	
ENSG00000168297	PXK	PX domain containing serine/threonine kinase like	54899	229,278696	0,141130314	0,158173	0,892255358	0,372256076	0,800625279	
ENSG00000130684	ZNF337	zinc finger protein 337	26152	131,7604659	0,065470913	0,153106	0,427618001	0,668929258	0,922641254	
ENSG00000111674	ENO2	enolase 2	2026	61,82067794	0,021167215	0,245922	0,086072818	0,931408531	0,986551936	
ENSG00000115970	THADA	THADA armadillo repeat containing	63892	174,7018716	0,137935887	0,141966	0,971612739	0,331243243	0,772473234	
ENSG00000124422	USP22	ubiquitin specific peptidase 22	23326	613,4348331	-0,065059201	0,072222	-0,900817229	0,367685505	0,795318634	
ENSG00000150961	SEC24D	SEC24 homolog D, COPII coat complex component	9871	337,5309405	0,240076165	0,09024	2,660414641	0,007804451	0,132103784	
ENSG00000156414	TDRD9	tudor domain containing 9	122402	44,40685527	-0,053371664	0,324757	-0,164343358	0,869460851	0,973310365	
ENSG00000176749	CDK5R1	cyclin dependent kinase 5 regulatory subunit 1	8851	168,2689342	-0,117326217	0,130218	-0,901000431	0,36758809	0,795318634	
ENSG00000141696	P3H4	prolyl 3-hydroxylase family member 4 (inactive)	10609	33,55283674	0,0039371	0,341366	0,011533371	0,990797905	NA	
ENSG00000103066	PLA2G15	phospholipase A2 group XV	23659	31,20318801	-0,228221245	0,405445	-0,56289141	0,57350883	NA	
ENSG00000143621	ILF2	interleukin enhancer binding factor 2	3608	420,0605438	0,098859642	0,088397	1,118353955	0,263415852	0,719682465	
ENSG00000031698	SARS1	seryl-tRNA synthetase 1	6301	324,7712355	-0,066584832	0,095861	-0,694595957	0,487308547	0,858914122	
ENSG00000071189	SNX13	sorting nexin 13	23161	153,7035548	0,162297106	0,162953	0,995977838	0,319260913	0,763543	
ENSG00000100417	PMM1	phosphomannomutase 1	5372	34,99556573	-0,335585524	0,313837	-1,069299375	0,284934792	NA	
ENSG00000124243	BCAS4	breast carcinoma amplified sequence 4	55653	42,03562524	-0,066292521	0,290108	-0,228509668	0,819250041	NA	
ENSG00000137752	CASP1	caspase 1	834	173,1428466	0,00258115	0,139811	0,018461766	0,985270479	0,996185513	
ENSG00000169955	ZNF747	zinc finger protein 747	65988	68,21163333	-0,211980438	0,259933	-0,815520135	0,414774644	0,82446781	
ENSG00000196586	MYO6	myosin VI	4646	36,65933145	0,75492416	0,516956	1,460325363	0,144200675	NA	
ENSG00000213096	ZNF254	zinc finger protein 254	9534	35,54540722	-0,043802294	0,453839	-0,09651502	0,923111545	NA	
ENSG00000099624	ATP5F1D	ATP synthase F1 subunit delta	513	64,98300616	0,275846005	0,233337	1,182180633	0,237134036	0,692360861	
ENSG00000154174	TOMM70	translocase of outer mitochondrial membrane 70	9868	248,5720701	-0,181435106	0,13307	-1,363455368	0,172739041	0,629558603	
ENSG00000115677	HDLBP	high density lipoprotein binding protein	3069	1681,57817	0,03434011	0,073507	0,467170662	0,640377782	0,911311306	
ENSG00000181104	F2R	coagulation factor II thrombin receptor	2149	268,6313717	-0,175012123	0,103184	-1,696118943	0,089863357	0,488772026	
ENSG00000023287	RB1CC1	RB1 inducible coiled-coil 1	9821	305,4502754	-0,116129389	0,115836	-1,002532237	0,316086605	0,760583393	
ENSG00000165732	DDX21	DExD-box helicase 21	9188	625,996861	0,076187243	0,07677	0,992408015	0,320998531	0,764671659	
ENSG00000047346	ATOSA	atos homolog A	56204	217,4433239	-0,142702883	0,159709	-0,89351892	0,371579345	0,800231702	
ENSG00000038532	CLEC16A	C-type lectin domain containing 16A	23274	292,3399158	-0,011959524	0,098679	-0,121195845	0,903535915	0,978460866	
ENSG00000102780	DGKH	diacylglycerol kinase eta	160851	121,54742	0,454819589	0,154251	2,948565897	0,003192521	0,075020833	
ENSG00000105738	SIPA1L3	signal induced proliferation associated 1 like 3	23094	169,3616915	-0,055632715	0,126027	-0,441436013	0,658897376	0,919368176	
ENSG00000109189	USP46	ubiquitin specific peptidase 46	64854	67,09392724	-0,310972346	0,29314	-1,06083043	0,288766971	0,740200975	
ENSG00000110171	TRIM3	tripartite motif containing 3	10612	29,93363341	-0,114743	0,305474	-0,375622497	0,707197563	NA	
ENSG00000116525	TRIM62	tripartite motif containing 62	55223	93,05658447	-0,306291817	0,171653	-1,784363952	0,074364545	0,443476943	

ENSG00000122971	ACADS	acyl-CoA dehydrogenase short chain	35	38,09883176	-0,080336686	0,27865	-0,288306657	0,773112012	NA
ENSG00000140983	RHOT2	ras homolog family member T2	89941	156,6741399	-0,428025934	0,214725	-1,993369334	0,046221019	0,3434436
ENSG00000169032	MAP2K1	mitogen-activated protein kinase kinase 1	5604	377,3958984	-0,073282964	0,119418	-0,613665763	0,539436217	0,875357861
ENSG00000243147	MRPL33	mitochondrial ribosomal protein L33	9553	72,90391473	0,174774039	0,204997	0,852569891	0,393897865	0,812498986
ENSG00000255274	SMIM35	small integral membrane protein 35	100526771	21,36048422	0,228814662	0,421148	0,543311516	0,586915337	NA
ENSG00000112699	GMDS	GDP-mannose 4,6-dehydratase	2762	59,95175545	0,199594976	0,261464	0,763374413	0,445240135	0,840933482
ENSG00000075539	FRYL	FRY like transcription coactivator	285527	696,4554118	-0,021428444	0,104967	-0,20414465	0,838240463	0,965627959
ENSG00000144895	EIF2A	eukaryotic translation initiation factor 2A	83939	223,0112931	-0,272656969	0,163693	-1,665658952	0,095781362	0,501511212
ENSG00000147155	EBP	EBP cholestenol delta-isomerase	10682	173,6223457	0,315251341	0,135831	2,320916626	0,020291344	0,224146577
ENSG00000148331	ASB6	ankyrin repeat and SOCS box containing 6	140459	130,3806301	-0,084866082	0,160284	-0,529474482	0,596476342	0,894229873
ENSG00000133704	IPO8	importin 8	10526	412,1963421	0,025773465	0,100053	0,257597583	0,796717495	0,95354564
ENSG00000070770	CSNK2A2	casein kinase 2 alpha 2	1459	46,14346249	-0,128633027	0,254841	-0,504758769	0,613728278	0,90080768
ENSG00000112144	CILK1	ciliogenesis associated kinase 1	22858	171,4852502	0,015457203	0,129519	0,119343192	0,905003465	0,978460866
ENSG00000116171	SCP2	sterol carrier protein 2	6342	300,5698326	0,090931413	0,106497	0,853836382	0,393195653	0,812491435
ENSG00000089486	CDIP1	cell death inducing p53 target 1	29965	163,9213343	-0,043783903	0,207517	-0,210989392	0,832895549	0,963446071
ENSG00000119669	IRF2BPL	interferon regulatory factor 2 binding protein like	64207	405,7887457	-0,190683385	0,090745	-2,101318987	0,035612974	0,300111909
ENSG00000118680	MYL12B	myosin light chain 12B	103910	677,1404114	0,169687089	0,076336	2,222892949	0,026223019	0,261364967
ENSG00000121621	KIF18A	kinesin family member 18A	81930	43,10883206	0,406341421	0,273274	1,486939756	0,137030723	0,57855532
ENSG00000168234	TTC39C	tetratricopeptide repeat domain 39C	125488	499,1959906	0,311400036	0,100431	3,100633453	0,001931072	0,053973799
ENSG00000094631	HDAC6	histone deacetylase 6	10013	95,82704795	0,075938367	0,254167	0,298773078	0,765113194	0,944359403
ENSG00000104886	PLEKHJ1	pleckstrin homology domain containing J1	55111	64,47295332	0,061797183	0,230349	0,268276735	0,788486313	0,95148885
ENSG00000064995	TAF11	TATA-box binding protein associated factor 11	6882	60,00808812	-0,481350118	0,248551	-1,93662505	0,052791194	0,371734827
ENSG00000157873	TNFRSF14	TNF receptor superfamily member 14	8764	79,48124954	0,841787151	1,233782	0,682282045	0,495060624	0,860603396
ENSG00000130414	NDUFA10	NADH:ubiquinone oxidoreductase subunit A10	4705	170,7193576	0,169432942	0,230075	0,736426238	0,461471342	0,846765498
ENSG00000141736	ERBB2	erb-b2 receptor tyrosine kinase 2	2064	15,08223197	-0,443684334	0,52329	-0,847874667	0,396507774	NA
ENSG00000160932	LY6E	lymphocyte antigen 6 family member E	4061	97,12389861	-0,809694967	2,621625	-0,308852287	0,757433893	0,942901768
ENSG00000187961	KLHL17	kelch like family member 17	339451	50,65774631	-0,330481854	0,244983	-1,348997059	0,177337911	0,632039836
ENSG00000196459	TRAPPC2	trafficking protein particle complex subunit 2	6399	116,2497347	0,06555994	0,187856	0,348991169	0,727095939	0,936626457
ENSG00000037241	RPL26L1	ribosomal protein L26 like 1	51121	25,47534174	0,557349426	0,398681	1,3979831	0,162118142	NA
ENSG00000140598	EFL1	elongation factor like GTPase 1	79631	123,9951275	-0,065601552	0,222066	-0,295414909	0,767676945	0,944698783
ENSG00000004142	POLDIP2	DNA polymerase delta interacting protein 2	26073	178,11138	0,239319071	0,154548	1,548504666	0,121500839	0,556748886
ENSG00000145703	IQGAP2	IQ motif containing GTPase activating protein 2	10788	1013,702934	-0,346493345	0,253777	-1,365348154	0,172143651	0,629558603
ENSG00000196419	XRCC6	X-ray repair cross complementing 6	2547	790,3652222	0,118526157	0,062852	1,885794997	0,059322585	0,394179007
ENSG00000119772	DNMT3A	DNA methyltransferase 3 alpha	1788	463,100216	0,199577733	0,100318	1,989459303	0,04665053	0,345654018
ENSG00000184465	WDR27	WD repeat domain 27	253769	37,62501418	-0,038474313	0,38813	-0,099127461	0,921037069	NA
ENSG00000177054	ZDHHC13	zinc finger DHHC-type palmitoyltransferase 13	54503	62,74331658	0,15183945	0,237397	0,63960015	0,522432585	0,870221868
ENSG00000119616	FCF1	FCF1 rRNA-processing protein	51077	168,5893725	0,18014277	0,177599	1,01432562	0,310427403	0,75688983
ENSG00000147586	MRPS28	mitochondrial ribosomal protein S28	28957	22,7332448	0,432136804	0,416358	1,037896635	0,299318181	NA
ENSG00000125457	MIF4GD	MIF4G domain containing	57409	65,58419741	-0,171890435	0,207253	-0,829374582	0,40689248	0,819272982
ENSG00000157800	SLC37A3	solute carrier family 37 member 3	84255	51,69564872	-0,132710508	0,238456	-0,556540364	0,577841509	0,887069818
ENSG00000100462	PRMT5	protein arginine methyltransferase 5	10419	95,67533127	0,12535308	0,18541	0,676087669	0,498984985	0,86161226
ENSG00000162734	PEA15	proliferation and apoptosis adaptor protein 15	8682	183,5612905	0,077006829	0,137136	0,561537489	0,574431181	0,886894319
ENSG00000204463	BAG6	BAG cochaperone 6	7917	239,0926795	-0,23734477	0,188579	-1,25859264	0,208177507	0,662274754
ENSG00000102054	RBBP7	RB binding protein 7, chromatin remodeling factor	5931	321,6175464	0,160260178	0,115365	1,389161076	0,164783775	0,624454568
ENSG00000109270	LAMTOR3	late endosomal/lysosomal adaptor, MAPK and MTOR activator 3	8649	98,54292017	0,275516535	0,242524	1,136040408	0,255939653	0,71206165

ENSG00000118518	RNF146	ring finger protein 146	81847	137,1462343	-0,092158289	0,147947	-0,622913496	0,533341369	0,873191491
ENSG00000134504	KCTD1	potassium channel tetramerization domain containing 1	284252	19,17207174	0,3229739	0,401079	0,805262353	0,420668302	NA
ENSG00000143228	NUF2	NUF2 component of NDC80 kinetochore complex	83540	59,8657361	0,505965009	0,248309	2,037641434	0,041585805	0,324989964
ENSG00000162601	MYSM1	Myb like, SWIRM and MPN domains 1	114803	320,9300778	0,262522366	0,312904	0,838987689	0,401476218	0,815200159
ENSG00000049167	ERCC8	ERCC excision repair 8, CSA ubiquitin ligase complex subunit	1161	44,18499311	-1,587445714	0,524134	-3,02870381	0,002456053	0,0612376
ENSG00000105810	CDK6	cyclin dependent kinase 6	1021	711,6282737	-0,223760411	0,073233	-3,055451513	0,002247219	0,057304094
ENSG00000160741	CRTC2	CREB regulated transcription coactivator 2	200186	245,2292299	-0,130903982	0,108554	-1,205886139	0,227861396	0,683323178
ENSG00000173915	ATP5MK	ATP synthase membrane subunit k	84833	75,75332048	0,361071272	0,2233	1,616975958	0,105883471	0,522649494
ENSG00000174177	CTU2	cytosolic thiouridylase subunit 2	348180	32,59539615	0,22199038	0,332192	0,668259054	0,503968246	NA
ENSG00000061936	SFSWAP	splicing factor SWAP	6433	175,0908358	-0,081464	0,186466	-0,43688343	0,662195889	0,9203479
ENSG00000166002	SMCO4	single-pass membrane protein with coiled-coil domains 4	56935	64,994505	0,302891074	0,293607	1,031621477	0,302249475	0,751870158
ENSG00000110330	BIRC2	baculoviral IAP repeat containing 2	329	358,7415226	0,038579002	0,091712	0,420653951	0,674007791	0,923602509
ENSG00000123453	SARDH	sarcosine dehydrogenase	1757	16,39968695	0,994046962	0,554727	1,791958161	0,073139668	NA
ENSG00000128791	TWSG1	twisted gastrulation BMP signaling modulator 1	57045	40,12140946	0,177029157	0,313752	0,564232527	0,572595894	NA
ENSG00000131504	DIAPH1	diaphanous related formin 1	1729	2091,647844	-0,014653371	0,061402	-0,23864689	0,811379402	0,959038743
ENSG00000138658	ZGRF1	zinc finger GRF-type containing 1	55345	50,36512458	1,109522961	0,819556	1,353810629	0,175796802	0,632039836
ENSG00000143543	JTB	jumping translocation breakpoint	10899	218,9993219	0,112500934	0,132345	0,850058055	0,39529281	0,812799303
ENSG00000160593	JAML	junction adhesion molecule like	120425	548,1657322	0,170757474	0,091481	1,866589	0,061959005	0,402854784
ENSG00000149428	HYOU1	hypoxia up-regulated 1	10525	648,659482	-0,113756902	0,428828	-0,265273672	0,790798645	0,951704496
ENSG00000198585	NUDT16	nudix hydrolase 16	131870	164,2865543	-0,099449143	0,20099	-0,494797407	0,620743135	0,90467927
ENSG00000198917	SPOUT1	SPOUT domain containing methyltransferase 1	51490	172,3565487	-0,128127025	0,155423	-0,824377123	0,40972531	0,82130194
ENSG00000203667	COX20	cytochrome c oxidase assembly factor COX20	116228	75,7405136	0,469922562	0,226666	2,073191722	0,03815444	0,31021219
ENSG00000162946	DISC1	DISC1 scaffold protein	27185	79,07976923	-0,128922436	0,205247	-0,628134468	0,529915855	0,872145761
ENSG00000165675	ENOX2	ecto-NOX disulfide-thiol exchanger 2	10495	64,43727025	0,002970578	0,242656	0,012241921	0,990232604	0,99732426
ENSG00000080854	IGSF9B	immunoglobulin superfamily member 9B	22997	257,0640419	-0,042514049	0,126151	-0,337007909	0,736110938	0,939157822
ENSG00000223496	EXOSC6	exosome component 6	118460	183,7822536	-0,162574426	0,127556	-1,274535284	0,202473776	0,660119983
ENSG00000205683	DPF3	double PHD fingers 3	8110	235,0417453	-0,181084114	0,138048	-1,311745078	0,189606161	0,647463823
ENSG00000117724	CENPF	centromere protein F	1063	587,3569862	0,650858759	0,090756	7,17150366	7,42E-13	6,47E-10
ENSG00000185019	UBOX5	U-box domain containing 5	22888	55,82844863	0,019087131	0,274902	0,069432585	0,944645292	0,989205143
ENSG00000215695	RSC1A1	regulator of solute carriers 1	6248	23,53223786	0,003575257	0,799982	0,00446917	0,99643413	NA
ENSG00000006530	AGK	acylglycerol kinase	55750	331,5962045	0,141870536	0,103596	1,36945836	0,170856042	0,629558603
ENSG00000066654	THUMPD1	THUMP domain containing 1	55623	271,8150772	-0,276890648	0,106415	-2,601991277	0,009268421	0,147356636
ENSG00000198668	CALM1	calmodulin 1	801	1684,428918	0,00280754	0,066659	0,042117766	0,966404817	0,992789539
ENSG00000115760	BIRC6	baculoviral IAP repeat containing 6	57448	1772,400324	-0,09318791	0,08865	-1,051191887	0,293170469	0,744745216
ENSG00000173846	PLK3	polo like kinase 3	1263	21,05230939	1,557299993	1,559478	0,998603346	0,317986878	NA
ENSG00000196792	STRN3	striatin 3	29966	76,99765633	0,114268911	0,191634	0,596287521	0,550983166	0,880819693
ENSG00000108587	GOSR1	golgi SNAP receptor complex member 1	9527	194,9363042	0,225383152	0,264032	0,853621856	0,393314545	0,812491435
ENSG00000140382	HMG20A	high mobility group 20A	10363	200,2778872	-0,251925639	0,146058	-1,724829086	0,084558278	0,474371941
ENSG00000157106	SMG1	SMG1 nonsense mediated mRNA decay associated PI3K related kinase	23049	2677,455134	-0,042395635	0,062517	-0,678145563	0,497679404	0,861283987
ENSG00000115274	INO80B	INO80 complex subunit B	83444	39,42379116	-0,29975719	0,352368	-0,850693862	0,394939434	NA
ENSG00000104980	TIMM44	translocase of inner mitochondrial membrane 44	10469	54,00212181	-0,247567574	0,246035	-1,006229532	0,314305169	0,759554708
ENSG00000164934	DCAF13	DDB1 and CUL4 associated factor 13	25879	123,6293132	0,219933894	0,184444	1,192414248	0,233098856	0,688695219
ENSG00000119414	PPP6C	protein phosphatase 6 catalytic subunit	5537	292,2578777	-0,028415242	0,099895	-0,284451318	0,776064536	0,946448613
ENSG00000047410	TPR	translocated promoter region, nuclear basket protein	7175	1277,55709	0,130871166	0,082817	1,580239147	0,11405211	0,539617633
ENSG00000100138	SNU13	small nuclear ribonucleoprotein 13	4809	195,9038508	-0,169917265	0,126084	-1,347652094	0,177770307	0,632142851
ENSG00000170191	NANP	N-acetylneuraminic acid phosphatase	140838	25,39829688	-0,040966139	0,359628	-0,113912647	0,90930704	NA
ENSG00000134574	DDB2	damage specific DNA binding protein 2	1643	108,4719024	0,394110415	0,216845	1,817473374	0,069144662	0,427066766
ENSG00000149115	TNKS1BP1	tankyrase 1 binding protein 1	85456	28,89985323	0,197196145	0,435979	0,452306693	0,651048055	NA

ENSG00000066697	MSANTD3	Myb/SANT DNA binding domain containing 3	91283	45,54002664	0,196233273	0,268394	0,731138425	0,464694606	0,848967535
ENSG00000116489	CAPZA1	capping actin protein of muscle Z-line subunit alpha 1	829	1064,844969	-0,046983362	0,063661	-0,738023192	0,460500356	0,846622987
ENSG00000143398	PIP5K1A	phosphatidylinositol-4-phosphate 5-kinase type 1 alpha	8394	156,3990331	-0,172049564	0,42232	-0,407391644	0,683720363	0,925586358
ENSG00000197857	ZNF44	zinc finger protein 44	51710	72,05668716	0,259154872	0,209348	1,237915742	0,215747305	0,672410621
ENSG00000196689	TRPV1	transient receptor potential cation channel subfamily V member 1	7442	26,10502796	-0,43906019	0,417439	-1,051794437	0,292893872	NA
ENSG00000066044	ELAVL1	ELAV like RNA binding protein 1	1994	343,4489476	0,017509239	0,114897	0,152390718	0,878878777	0,976237908
ENSG00000167081	PBX3	PBX homeobox 3	5090	45,77871239	-0,132107726	0,283836	-0,465437492	0,641618191	0,912063088
ENSG00000031081	ARHGAP31	Rho GTPase activating protein 31	57514	187,8013097	0,592833052	0,133815	4,430253567	9,41E-06	0,000935743
ENSG00000110721	CHKA	choline kinase alpha	1119	70,25893192	0,014568073	0,255779	0,056955775	0,954580424	0,991420853
ENSG00000178700	DHFR2	dihydrofolate reductase 2	200895	67,7973186	0,008590823	0,232456	0,036956713	0,97051952	0,993542794
ENSG00000181722	ZBTB20	zinc finger and BTB domain containing 20	26137	99,30399574	-0,068353111	0,242926	-0,281374348	0,778423291	0,947109268
ENSG00000187742	SECISBP2	SECIS binding protein 2	79048	163,5880247	-0,410114933	0,137569	-2,981152222	0,00287166	0,069826683
ENSG00000007541	PIGQ	phosphatidylinositol glycan anchor biosynthesis class Q	9091	48,00798942	0,567628438	0,373475	1,519856368	0,128547078	0,563689485
ENSG00000113732	ATP6V0E1	ATPase H+ transporting V0 subunit e1	8992	222,9504884	0,228411174	0,149738	1,525404675	0,127158193	0,563689485
ENSG00000176209	SMIM19	small integral membrane protein 19	114926	73,55152834	0,216525669	0,266184	0,813443983	0,415963551	0,82446781
ENSG00000076043	REXO2	RNA exonuclease 2	25996	53,65905672	0,176522461	0,26542	0,665069407	0,506006101	0,864630986
ENSG00000115935	WIPF1	WAS/WASL interacting protein family member 1	7456	1910,719142	-0,196466213	0,081647	-2,406276048	0,016116082	0,200595414
ENSG00000084623	EIF3I	eukaryotic translation initiation factor 3 subunit I	8668	280,9029997	0,098470122	0,14454	0,681263677	0,495704663	0,861163463
ENSG00000159784	FAM131B	family with sequence similarity 131 member B	9715	13,27610299	-0,115779748	0,648581	-0,178512406	0,858320579	NA
ENSG00000108666	C17orf75	chromosome 17 open reading frame 75	64149	53,1163605	0,149764136	0,29972	0,499679874	0,617300506	0,90216458
ENSG00000121775	TMEM39B	transmembrane protein 39B	55116	27,98045912	0,340763639	0,445337	0,76518219	0,444163064	NA
ENSG00000137216	TMEM63B	transmembrane protein 63B	55362	47,74143391	0,610464822	1,257116	0,485607408	0,627245543	0,90479929
ENSG00000128342	LIF	LIF interleukin 6 family cytokine	3976	121,8103637	1,177483174	0,244156	4,822665898	1,42E-06	0,000191817
ENSG00000168936	TMEM129	transmembrane protein 129, E3 ubiquitin ligase	92305	71,99312183	-0,130709542	0,247659	-0,527780583	0,597651636	0,894257182
ENSG00000170632	ARMC10	armadillo repeat containing 10	83787	45,44256228	0,140601552	0,344562	0,408058214	0,683230937	0,925586358
ENSG00000159231	CBR3	carbonyl reductase 3	874	12,83627873	-0,004334311	0,528193	-0,00820593	0,993452689	NA
ENSG00000136141	LRCH1	leucine rich repeats and calponin homology domain containing 1	23143	200,0302898	-0,03794774	0,118778	-0,319484122	0,749359429	0,940621537
ENSG00000158292	GPR153	G protein-coupled receptor 153	387509	27,62298558	-0,550509252	0,349673	-1,574354939	0,115405415	NA
ENSG00000105171	POP4	POP4 homolog, ribonuclease P/MRP subunit	10775	64,07649698	0,034320712	0,241061	0,142373429	0,886785049	0,977928921
ENSG00000198574	SH2D1B	SH2 domain containing 1B	117157	757,494562	-0,156315888	0,068989	-2,265821922	0,023462289	0,245043637
ENSG00000159202	UBE2Z	ubiquitin conjugating enzyme E2 Z	65264	345,4123357	0,048736094	0,098279	0,495894702	0,619968704	0,904264273
ENSG00000101412	E2F1	E2F transcription factor 1	1869	97,71311702	0,069051558	0,177276	0,389515103	0,696895141	0,930422107
ENSG00000148672	GLUD1	glutamate dehydrogenase 1	2746	489,3486051	-0,068681329	0,122236	-0,561875876	0,574200591	0,886894319
ENSG00000254469	XNDC1N	XRCC1 N-terminal domain containing 1, N-terminal like	100133315	22,06045984	0,356969283	0,598321	0,596618555	0,55076208	NA
ENSG00000116497	S100PBP	S100P binding protein	64766	470,159916	-0,081942435	0,08716	-0,940141687	0,347144888	0,783470101
ENSG00000167118	URM1	ubiquitin related modifier 1	81605	155,1783845	0,089306177	0,17502	0,510263967	0,609866543	0,898865155
ENSG00000181666	ZNF875	zinc finger protein 875	284459	98,55096854	-0,247626557	0,189955	-1,303607727	0,192367365	0,649982152
ENSG00000205542	TMSB4X	thymosin beta 4 X-linked	7114	6254,108534	0,114032015	0,058144	1,961187688	0,049857133	0,357759312
ENSG00000110429	FBXO3	F-box protein 3	26273	213,8333941	-0,16394548	0,12107	-1,354136019	0,175692987	0,632039836
ENSG00000276293	PIP4K2B	phosphatidylinositol-5-phosphate 4-kinase type 2 beta	8396	429,7984094	-0,164719824	0,13343	-1,234505126	0,217014738	0,672891335
ENSG00000113638	TTC33	tetratricopeptide repeat domain 33	23548	51,88282546	0,270341806	0,250248	1,080295986	0,280010397	0,733157579
ENSG00000100379	KCTD17	potassium channel tetramerization domain containing 17	79734	98,96170797	0,071803973	0,216152	0,33219216	0,739744164	0,939193793
ENSG00000119326	CTNNAL1	catenin alpha like 1	8727	14,74899354	1,130322412	0,505075	2,237930505	0,025225587	NA
ENSG00000172687	ZNF738	zinc finger protein 738	148203	63,56397173	-0,022989263	0,273791	-0,083966358	0,93308318	0,987098279
ENSG00000185947	ZNF267	zinc finger protein 267	10308	133,2123703	0,125787249	0,168101	0,748284493	0,454288576	0,843234809
ENSG00000175274	TP53I11	tumor protein p53 inducible protein 11	9537	38,48165843	-0,35625068	0,46757	-0,761919637	0,446107969	NA
ENSG00000179934	CCR8	C-C motif chemokine receptor 8	1237	64,75335627	-0,060567162	0,209008	-0,289783434	0,771981922	0,946183835

ENSG00000131943	C19orf12	chromosome 19 open reading frame 12	83636	136,6535664	-0,166233988	0,178543	-0,931058068	0,351823528	0,786165473
ENSG00000148335	NTMT1	N-terminal Xaa-Pro-Lys N-methyltransferase 1	28989	33,35681908	0,03890059	0,363886	0,106903137	0,914865824	NA
ENSG00000101849	TBL1X	transducin beta like 1 X-linked	6907	93,29531784	1,012777527	1,02563	0,987468583	0,323412977	0,767603859
ENSG00000167700	MFSD3	major facilitator superfamily domain containing 3	113655	15,83196306	-0,087192101	0,492451	-0,177057478	0,859463242	NA
ENSG00000049883	PTCD2	pentatricopeptide repeat domain 2	79810	42,5544671	-0,673868098	0,400685	-1,68178959	0,092609648	0,49480012
ENSG00000134779	TPGS2	tubulin polyglutamylase complex subunit 2	25941	195,7467831	-0,087182929	0,133685	-0,652153922	0,514301882	0,868514045
ENSG00000116455	WDR77	WD repeat domain 77	79084	109,3927073	0,207464201	0,174226	1,190778314	0,233740624	0,688858646
ENSG00000108479	GALK1	galactokinase 1	2584	19,27522319	0,29098062	0,513163	0,567032979	0,570691775	NA
ENSG00000064012	CASP8	caspase 8	841	329,73542	0,10093879	0,116537	0,866151777	0,386406934	0,809653368
ENSG00000077348	EXOSC5	exosome component 5	56915	20,3078883	-0,077732416	0,389674	-0,199480763	0,84188669	NA
ENSG00000103275	UBE2I	ubiquitin conjugating enzyme E2 I	7329	333,2312294	0,282301216	0,172468	1,636835723	0,101664799	0,514437479
ENSG00000083223	TUT7	terminal uridylyl transferase 7	79670	409,5279389	-0,084394006	0,103118	-0,818420922	0,413116879	0,82225544
ENSG00000026652	AGPAT4	1-acylglycerol-3-phosphate O-acyltransferase 4	56895	91,96868947	0,345854942	0,337187	1,025705563	0,305030391	0,751870158
ENSG00000063660	GPC1	glypican 1	2817	17,11867479	0,374336655	0,469169	0,797871871	0,424944849	NA
ENSG00000071626	DAZAP1	DAZ associated protein 1	26528	339,6472028	-0,027229847	0,121922	-0,223338988	0,823271685	0,961917534
ENSG00000107443	CCNJ	cyclin J	54619	26,809609	0,059462454	0,361703	0,164395784	0,869419582	NA
ENSG00000110011	DNAJC4	DnaJ heat shock protein family (Hsp40) member C4	3338	79,39956948	-0,658067158	0,249436	-2,638217635	0,008334308	0,137805582
ENSG00000113319	RASGRF2	Ras protein specific guanine nucleotide releasing factor 2	5924	284,4243266	-0,37945753	0,222757	-1,703461769	0,088481687	0,486308725
ENSG00000117505	DR1	down-regulator of transcription 1	1810	492,0894825	0,095706601	0,093298	1,025816732	0,304977978	0,751870158
ENSG00000160221	GATD3	glutamine amidotransferase class 1 domain containing 3	8209	133,4498758	-0,085975089	0,164648	-0,522175587	0,601548082	0,89650069
ENSG00000182957	SPATA13	spermatogenesis associated 13	221178	1200,568869	0,000227195	0,069076	0,003289074	0,997375703	0,999300722
ENSG00000052749	RRP12	ribosomal RNA processing 12 homolog	23223	68,4240582	-0,380964161	0,224488	-1,697034195	0,089690196	0,488772026
ENSG00000120314	WDR55	WD repeat domain 55	54853	164,7402181	-0,066176875	0,134349	-0,492575436	0,622312608	0,904764752
ENSG00000145817	YIPF5	Yip1 domain family member 5	81555	171,6851991	0,095235768	0,141252	0,674224396	0,500168662	0,86161226
ENSG00000151725	CENPU	centromere protein U	79682	25,7268659	0,415447668	0,471866	0,880435818	0,37862326	NA
ENSG00000172869	DMXL1	Dmx like 1	1657	385,1784695	-0,100711099	0,091941	-1,095388285	0,273346564	0,726913977
ENSG00000181924	COA4	cytochrome c oxidase assembly factor 4 homolog	51287	53,19197653	-0,094562065	0,230934	-0,409476469	0,682190032	0,925586358
ENSG00000155755	TMEM237	transmembrane protein 237	65062	14,8616702	0,638015925	0,558475	1,142424394	0,253277656	NA
ENSG00000114026	OGG1	8-oxoguanine DNA glycosylase	4968	64,36499658	-0,299540533	0,228749	-1,309470193	0,190375128	0,647836333
ENSG00000164610	RP9	RP9 pre-mRNA splicing factor	6100	22,4013049	0,506587499	0,398011	1,272796198	0,203090371	NA
ENSG00000168301	KCTD6	potassium channel tetramerization domain containing 6	200845	15,75946014	-0,058406566	0,5748	-0,101611925	0,919064714	NA
ENSG00000059573	ALDH18A1	aldehyde dehydrogenase 18 family member A1	5832	201,1102203	0,026162286	0,12159	0,215168605	0,829635867	0,962996447
ENSG00000198931	APRT	adenine phosphoribosyltransferase	353	135,1123819	-0,12603278	0,158811	-0,793601064	0,427427713	0,832060923
ENSG00000165506	DNAAF2	dynein axonemal assembly factor 2	55172	33,19130103	0,164529505	0,307475	0,535099401	0,592581124	NA
ENSG00000082516	GEMIN5	gem nuclear organelle associated protein 5	25929	112,7722835	0,055209118	0,175803	0,314038849	0,753491533	0,942644552
ENSG00000090020	SLC9A1	solute carrier family 9 member A1	6548	149,6444563	-0,150176872	0,151392	-0,991973357	0,321210523	0,764944646
ENSG00000115761	NOL10	nucleolar protein 10	79954	22,40743354	-2,52389258	2,930551	-0,861234875	0,389108696	NA
ENSG00000186432	KPNA4	karyopherin subunit alpha 4	3840	306,9600892	-0,289816435	0,177986	-1,628312489	0,103458643	0,516898333
ENSG00000196110	ZNF699	zinc finger protein 699	374879	59,14988986	-0,059491482	0,320435	-0,185658606	0,852712492	0,968229875
ENSG00000127080	IPPK	inositol-pentakisphosphate 2-kinase	64768	38,38218208	0,225862005	0,433631	0,520862748	0,602462387	NA
ENSG00000132773	TOE1	target of EGR1, exonuclease	114034	39,16057966	0,221973859	0,324136	0,684817423	0,49345914	NA
ENSG00000144320	LNPK	lunapark, ER junction formation factor	80856	178,2370018	-0,154897443	0,132799	-1,166403072	0,243451517	0,700391287
ENSG00000186866	POFUT2	protein O-fucosyltransferase 2	23275	81,61534592	0,408859612	0,277145	1,475257777	0,140143254	0,58302406
ENSG00000103148	NPRL3	NPR3 like, GATOR1 complex subunit	8131	54,88731225	0,599821759	0,373186	1,607299324	0,107988723	0,527320063
ENSG00000169217	CD2BP2	CD2 cytoplasmic tail binding protein 2	10421	483,036506	0,047729907	0,092169	0,517849529	0,604563259	0,897347689
ENSG00000168264	IRF2BP2	interferon regulatory factor 2 binding protein 2	359948	292,2408954	0,111517271	0,098231	1,135250618	0,256270326	0,712469825
ENSG00000184209	SNRNP35	small nuclear ribonucleoprotein U11/U12 subunit 35	11066	74,07827848	-0,055349849	0,206029	-0,26865042	0,788198709	0,95148885

ENSG00000184988	TMEM106A	transmembrane protein 106A	113277	70,81633587	0,035419872	0,25347	0,139739632	0,888865712	0,977990819
ENSG00000124256	ZBP1	Z-DNA binding protein 1	81030	97,71989244	-0,404205748	0,191971	-2,105554991	0,035243026	0,299241865
ENSG00000166801	FAM111A	FAM111 trypsin like peptidase A	63901	673,9572705	-0,023694184	0,074907	-0,316313407	0,751764636	0,941684123
ENSG00000169359	SLC33A1	solute carrier family 33 member 1	9197	83,03243676	-0,087392184	0,318081	-0,274748252	0,783509658	0,950082577
ENSG00000065923	SLC9A7	solute carrier family 9 member A7	84679	19,95315178	-0,78539668	0,496142	-1,583006593	0,113419962	NA
ENSG00000108578	BLMH	bleomycin hydrolase	642	203,4087945	-0,017543301	0,11812	-0,148521132	0,88193151	0,976300778
ENSG00000114648	KLHL18	kelch like family member 18	23276	110,8887623	0,268603965	0,185085	1,451246501	0,146711233	0,596721917
ENSG00000136021	SCYL2	SCY1 like pseudokinase 2	55681	378,0484369	-0,000335316	0,093689	-0,003579043	0,997144343	0,999300722
ENSG00000136937	NCBP1	nuclear cap binding protein subunit 1	4686	228,2505006	-0,255515133	0,212469	-1,202598051	0,229131902	0,68351286
ENSG00000164543	STK17A	serine/threonine kinase 17a	9263	304,2544779	0,102769257	0,126739	0,810874123	0,417437962	0,82446781
ENSG00000165661	QSOX2	quiescin sulfhydryl oxidase 2	169714	110,6395076	0,362848813	0,286806	1,26513506	0,205822917	0,660954946
ENSG00000219545	UMAD1	UBAP1-MVB12-associated (UMA) domain containing 1	729852	35,77633742	0,07129478	0,291351	0,244704059	0,806685585	NA
ENSG00000259494	MRPL46	mitochondrial ribosomal protein L46	26589	36,75532555	-0,121080965	0,317224	-0,381689576	0,702691633	NA
ENSG00000138443	ABI2	abl interactor 2	10152	208,012052	0,094444061	0,129255	0,730680101	0,464974573	0,84908342
ENSG00000169599	NFU1	NFU1 iron-sulfur cluster scaffold	27247	50,7316193	0,121657118	0,268453	0,453178769	0,650420019	0,91499173
ENSG00000125454	SLC25A19	solute carrier family 25 member 19	60386	23,95445341	0,165857743	0,405355	0,409166907	0,682417178	NA
ENSG00000175294	CATSPER1	cation channel sperm associated 1	117144	13,60700297	-0,384835916	0,513178	-0,749906895	0,453310782	NA
ENSG00000159216	RUNX1	RUNX family transcription factor 1	861	137,8981055	-0,021288502	0,167294	-0,127251774	0,898741129	0,977990819
ENSG00000159216	LOC100506403	uncharacterized LOC100506403	100506403	137,8981055	-0,021288502	0,167294	-0,127251774	0,898741129	0,977990819
ENSG00000161202	DVL3	dishevelled segment polarity protein 3	1857	411,7558668	-0,114762166	0,10883	-1,054506008	0,291651311	0,743239565
ENSG00000197121	PGAP1	post-GPI attachment to proteins inositol deacylase 1	80055	28,05713952	0,3630582	0,563156	0,644685033	0,519131317	NA
ENSG00000145022	TCTA	T cell leukemia translocation altered	6988	183,2549468	-0,13474369	0,124274	-1,084250623	0,278253692	0,732101556
ENSG00000104805	NUCB1	nucleobindin 1	4924	509,3784438	-0,098023158	0,105148	-0,932244254	0,35121031	0,785712602
ENSG00000163541	SUCLG1	succinate-CoA ligase GDP/ADP-forming subunit alpha	8802	109,5227592	-0,053356572	0,157511	-0,338748079	0,734799518	0,939157822
ENSG00000171490	RSL1D1	ribosomal L1 domain containing 1	26156	176,281978	-0,030541552	0,142645	-0,214108457	0,830462481	0,963156386
ENSG00000182858	ALG12	ALG12 alpha-1,6-mannosyltransferase	79087	96,05321376	-0,186899159	0,208174	-0,8978007	0,369291811	0,79747536
ENSG00000106266	SNX8	sorting nexin 8	29886	35,05985667	-0,266913648	0,315213	-0,846771395	0,397122555	NA
ENSG00000154640	BTG3	BTG anti-proliferation factor 3	10950	27,63369997	0,030229353	0,397636	0,076022745	0,939401003	NA
ENSG00000158604	TMED4	transmembrane p24 trafficking protein 4	222068	250,3761766	0,033926414	0,103891	0,326556999	0,744002972	0,939745744
ENSG00000160752	FDPS	farnesyl diphosphate synthase	2224	218,9561267	0,245947671	0,124173	1,980690667	0,047625974	0,349910571
ENSG00000172164	SNTB1	syntrophin beta 1	6641	341,3976304	-0,214472592	0,105111	-2,040435569	0,041306962	0,323534763
ENSG00000178988	MRFAP1L1	Morf4 family associated protein 1 like 1	114932	276,0981693	0,067649228	0,130459	0,518547679	0,604076203	0,897212117
ENSG00000214193	SH3D21	SH3 domain containing 21	79729	142,0812914	-0,132818735	0,18919	-0,70203797	0,482655484	0,855925045
ENSG00000236287	ZBED5	zinc finger BED-type containing 5	58486	191,2430942	-0,179685752	0,126709	-1,418102343	0,156160887	0,61428162
ENSG00000075884	ARHGAP15	Rho GTPase activating protein 15	55843	206,2535912	0,137365041	0,115668	1,187575406	0,235000732	0,690495978
ENSG00000064651	SLC12A2	solute carrier family 12 member 2	6558	134,1486855	-0,137940606	0,172039	-0,801796923	0,422670439	0,827843798
ENSG00000197816	CCDC180	coiled-coil domain containing 180	100499483	17,43097184	0,598777014	0,538635	1,111656712	0,266285779	NA
ENSG00000243943	ZNF512	zinc finger protein 512	84450	180,8284365	-0,090012067	0,128616	-0,699851558	0,484020012	0,85698884
ENSG00000169223	LMAN2	lectin, mannose binding 2	10960	393,5705928	-0,05247907	0,141743	-0,37024167	0,711202431	0,930846779
ENSG00000069493	CLEC2D	C-type lectin domain family 2 member D	29121	327,9004395	-0,051352639	0,120665	-0,425581134	0,67041309	0,922641254
ENSG00000118263	KLF7	KLF transcription factor 7	8609	15,79248661	0,326623026	0,603153	0,541526001	0,588145081	NA
ENSG00000125637	PSD4	pleckstrin and Sec7 domain containing 4	23550	992,9064827	-0,066041594	0,071801	-0,9197858	0,357684705	0,790004408
ENSG00000144647	POMGNT2	protein O-linked mannose N-acetylglucosaminyltransferase 2 (beta 1,4-)	84892	24,29710709	-0,500228479	0,391887	-1,276461393	0,201792466	NA
ENSG00000175110	MRPS22	mitochondrial ribosomal protein S22	56945	66,89206014	-0,018479087	0,234729	-0,078725127	0,937251259	0,987194684
ENSG00000035681	NSMAF	neutral sphingomyelinase activation associated factor	8439	210,9048998	0,033398075	0,126135	0,2647808	0,791178329	0,951705903
ENSG00000051382	PIK3CB	phosphatidylinositol-4,5-bisphosphate 3-kinase catalytic subunit beta	5291	48,17050958	0,078780913	0,267289	0,294740716	0,768191957	0,944698783

ENSG00000106153	CHCHD2	coiled-coil-helix-coiled-coil-helix domain containing 2	51142	260,5421572	0,307172366	0,153803	1,997178004	0,04580585	0,342301753
ENSG00000179526	SHARPIN	SHANK associated RH domain interactor	81858	52,55667383	0,049062734	0,251587	0,19501299	0,845382778	0,967231052
ENSG00000261794	GOLGA8H	golgin A8 family member H	728498	42,31669545	-0,156297446	0,270073	-0,578722345	0,562776536	NA
ENSG00000176022	B3GALT6	beta-1,3-galactosyltransferase 6	126792	109,6406175	0,009669573	0,167607	0,057691935	0,953994018	0,991212866
ENSG00000136867	SLC31A2	solute carrier family 31 member 2	1318	15,34555722	-0,189286084	0,531096	-0,356406625	0,721536068	NA
ENSG00000172922	RNASEH2C	ribonuclease H2 subunit C	84153	60,88197526	0,35599928	0,370366	0,961209481	0,33644685	0,776552413
ENSG00000180329	CCDC43	coiled-coil domain containing 43	124808	82,64914173	0,020865477	0,213021	0,097950362	0,921971709	0,984284131
ENSG00000116871	MAP7D1	MAP7 domain containing 1	55700	462,1691882	-0,144993097	0,094407	-1,535827945	0,124580584	0,5624706
ENSG00000011478	QPCTL	glutaminyl-peptide cyclotransferase like	54814	22,72930565	-0,826625325	0,417364	-1,980585863	0,047637736	NA
ENSG00000107331	ABCA2	ATP binding cassette subfamily A member 2	20	1636,774421	-0,018611108	0,10168	-0,183035833	0,854769903	0,968377739
ENSG00000130717	UCK1	uridine-cytidine kinase 1	83549	85,74508237	-0,004913951	0,188215	-0,026108137	0,979171087	0,995712569
ENSG00000132581	SDF2	stromal cell derived factor 2	6388	55,92698534	0,67045636	0,245032	2,736198213	0,006215358	0,114173394
ENSG00000171862	PTEN	phosphatase and tensin homolog	5728	159,7733451	0,033204794	0,263841	0,125851393	0,89984956	0,977990819
ENSG00000196313	POM121	POM121 transmembrane nucleoporin	9883	612,6211907	0,024288958	0,077911	0,311751146	0,755229655	0,942901768
ENSG00000117335	CD46	CD46 molecule	4179	460,0321773	0,086918493	0,094487	0,919899368	0,357625349	0,790004408
ENSG00000128284	APOL3	apolipoprotein L3	80833	128,0971388	-0,308335949	0,260334	-1,184383984	0,236261097	0,691328984
ENSG00000076356	PLXNA2	plexin A2	5362	63,84213803	-0,108243936	0,222185	-0,487180269	0,626130587	0,90479929
ENSG00000139579	NABP2	nucleic acid binding protein 2	79035	113,4632746	0,068894696	0,171931	0,400711616	0,688632457	0,926748702
ENSG00000116793	PHTF1	putative homeodomain transcription factor 1	10745	99,51285868	0,112363586	0,197406	0,569199936	0,569220464	0,886263374
ENSG00000183048	SLC25A10	solute carrier family 25 member 10	1468	18,61285386	0,736074591	0,438707	1,677825688	0,093381133	NA
ENSG00000158710	TAGLN2	transgelin 2	8407	745,8990021	-0,009484427	0,073387	-0,129239265	0,897168328	0,977990819
ENSG00000187522	HSPA14	heat shock protein family A (Hsp70) member 14	51182	65,10976961	0,184350702	0,263935	0,698468843	0,484884036	0,857621265
ENSG00000169372	CRADD	CASP2 and RIPK1 domain containing adaptor with death domain	8738	16,13282892	0,313563379	0,771548	0,406408295	0,684442626	NA
ENSG00000234444	ZNF736	zinc finger protein 736	728927	69,55784451	-0,048586093	0,291317	-0,166780702	0,867542603	0,972132916
ENSG00000010072	SPRTN	SprT-like N-terminal domain	83932	82,81524665	0,181843547	0,195357	0,930827737	0,351942679	0,786165473
ENSG00000155975	VPS37A	VPS37A subunit of ESCRT-I	137492	100,4968111	0,203236645	0,239489	0,848627754	0,396088458	0,812799303
ENSG00000167283	ATP5MG	ATP synthase membrane subunit g	10632	323,8784113	0,298993795	0,116724	2,561553164	0,010420529	0,157997752
ENSG00000196757	ZNF700	zinc finger protein 700	90592	160,560557	-0,010813636	0,135306	-0,079919844	0,936301007	0,987098279
ENSG00000187840	EIF4EBP1	eukaryotic translation initiation factor 4E binding protein 1	1978	35,97893277	-0,048142231	0,349449	-0,137766031	0,890425332	NA
ENSG00000109762	SNX25	sorting nexin 25	83891	58,75191052	0,092504902	0,222639	0,415493376	0,677780763	0,924663907
ENSG00000165669	FAM204A	family with sequence similarity 204 member A	63877	99,85545605	-0,016475611	0,186377	-0,088399253	0,929559355	0,986487751
ENSG00000079974	RABL2B	RAB, member of RAS oncogene family like 2B	11158	102,8961291	-0,095928738	0,194363	-0,493553518	0,621621535	0,904764752
ENSG00000100100	PIK3IP1	phosphoinositide-3-kinase interacting protein 1	113791	319,9765073	0,07770848	0,104975	0,740257548	0,459143735	0,846574514
ENSG00000204267	TAP2	transporter 2, ATP binding cassette subfamily B member	6891	30,93534918	0,059932111	0,74357	0,080600514	0,935759658	NA
ENSG00000156990	RPUSD3	RNA pseudouridine synthase D3	285367	75,29505531	-0,209071898	0,21638	-0,966225845	0,333931183	0,773674527
ENSG00000057252	SOAT1	sterol O-acyltransferase 1	6646	451,0120476	0,019392038	0,083737	0,231581608	0,816862994	0,959707099
ENSG00000089902	RCOR1	REST corepressor 1	23186	177,2643364	-0,069216449	0,143083	-0,483751918	0,628561943	0,905567722
ENSG00000188234	AGAP4	ArfGAP with GTPase domain, ankyrin repeat and PH domain 4	119016	56,1058293	-0,305846654	0,280064	-1,092059566	0,27480692	0,727446428
ENSG00000063169	BICRA	BRD4 interacting chromatin remodeling complex associated protein	29998	126,6223526	-0,385543286	0,157393	-2,449550599	0,014303461	0,186300802
ENSG00000104907	TRMT1	tRNA methyltransferase 1	55621	72,76290728	-0,075446915	0,205083	-0,367884463	0,712959386	0,930846779
ENSG00000166704	ZNF606	zinc finger protein 606	80095	28,60532719	0,116383142	0,446038	0,260926739	0,794149004	NA
ENSG00000186416	NKRF	NFKB repressing factor	55922	13,41965706	-1,578711818	2,836272	-0,55661501	0,577790496	NA
ENSG00000204859	ZBTB48	zinc finger and BTB domain containing 48	3104	45,13732852	-0,887636037	0,381013	-2,329671923	0,019823498	0,221470484
ENSG00000104679	R3HCC1	R3H domain and coiled-coil containing 1	203069	83,20087882	-0,228655276	0,203424	-1,124033713	0,260998724	0,716543286
ENSG00000118197	DDX59	DEAD-box helicase 59	83479	62,60782944	-0,166868882	0,205988	-0,810091161	0,417887784	0,82446781
ENSG00000120697	ALG5	ALG5 dolichyl-phosphate beta-glucosyltransferase	29880	63,97907518	0,545866287	0,253081	2,15688292	0,031014788	0,281657534
ENSG00000196358	NTNG2	netrin G2	84628	20,67549095	-0,325112053	0,415659	-0,782159946	0,434120581	NA
ENSG00000221829	FANCG	FA complementation group G	2189	34,71505992	0,970829746	1,107448	0,876636999	0,380683836	NA
ENSG00000239883	NA	NA	NA	19,92576644	0,496617882	0,440788	1,126658393	0,25988694	NA
ENSG00000279457	NA	NA	NA	27,64119759	0,245157186	0,408504	0,600134737	0,548416444	NA

ENSG00000133895	MEN1	menin 1	4221	98,17270159	-0,055014967	0,196542	-0,279914933	0,779542771	0,947712923
ENSG00000177943	MAMDC4	MAM domain containing 4	158056	18,82179404	0,373435297	0,488139	0,765018548	0,4442605	NA
ENSG00000081665	ZNF506	zinc finger protein 506	440515	89,82480509	0,163497781	0,219417	0,74514585	0,456183554	0,844219046
ENSG00000105497	ZNF175	zinc finger protein 175	7728	27,65266834	0,558491296	0,377039	1,481257989	0,138537839	NA
ENSG00000106245	BUD31	BUD31 homolog	8896	100,371482	0,574141096	0,221762	2,588998979	0,009625538	0,151166315
ENSG00000146376	ARHGAP18	Rho GTPase activating protein 18	93663	226,8286142	0,121412101	0,115093	1,05490519	0,291468688	0,743239565
ENSG00000173085	COQ2	coenzyme Q2, polyprenyltransferase	27235	36,10838699	0,191579737	0,291997	0,656102841	0,511757957	NA
ENSG00000188322	SBK1	SH3 domain binding kinase 1	388228	26,94832998	-0,599584723	0,337528	-1,776400155	0,075666986	NA
ENSG00000254996	ANKHD1-EIF4EBP3	ANKHD1-EIF4EBP3 readthrough	404734	261,3145183	-0,129743303	0,416283	-0,311671066	0,755290519	0,942901768
ENSG00000273611	ZNHIT3	zinc finger HIT-type containing 3	9326	53,96421748	0,24414316	1,022103	0,238863459	0,811211461	0,959038743
ENSG00000162769	FLVCR1	FLVCR choline and heme transporter 1	28982	73,37394334	0,122070359	0,232244	0,525612478	0,599157485	0,89463553
ENSG00000069345	DNAJA2	DnaJ heat shock protein family (Hsp40) member A2	10294	156,9942491	-0,108740553	0,152015	-0,715328064	0,474406351	0,853811879
ENSG00000081760	AACS	acetoacetyl-CoA synthetase	65985	171,9611391	0,105990314	0,135755	0,780746181	0,434951793	0,836319408
ENSG00000172046	USP19	ubiquitin specific peptidase 19	10869	282,2893017	0,044999917	0,10923	0,411975521	0,680357366	0,925586358
ENSG00000183474	GTF2H2C	GTF2H2 family member C	728340	46,76211619	0,779101785	0,533705	1,459799478	0,144345192	0,59024645
ENSG00000218739	CEBPZOS	CEBPZ opposite strand	100505876	118,240225	0,021950428	0,225553	0,097318084	0,922473795	0,984284131
ENSG00000176531	PHLDB3	pleckstrin homology like domain family B member 3	653583	12,39936915	-0,758690111	0,569475	-1,332262797	0,182773848	NA
ENSG00000170145	SIK2	salt inducible kinase 2	23235	100,7143355	-0,087811967	0,190155	-0,461792722	0,644229971	0,912530858
ENSG00000142409	ZNF787	zinc finger protein 787	126208	65,25159638	-0,021048115	0,206281	-0,10203625	0,918727902	0,984062493
ENSG00000166313	APBB1	amyloid beta precursor protein binding family B member 1	322	127,1764752	-0,064396029	0,151416	-0,425290743	0,670624741	0,922641254
ENSG00000143119	CD53	CD53 molecule	963	1734,040588	0,061176287	0,050302	1,216186417	0,223913917	0,679266088
ENSG00000084090	STARD7	StAR related lipid transfer domain containing 7	56910	570,1066366	0,004130897	0,078565	0,052579573	0,958066893	0,991911599
ENSG00000153093	ACOXL	acyl-CoA oxidase like	55289	18,7674238	-0,068634295	0,415641	-0,16512861	0,868842755	NA
ENSG00000137161	CNPY3	canopy FGF signaling regulator 3	10695	174,2378388	-0,022635199	0,12472	-0,181487761	0,855984739	0,968439087
ENSG00000100241	SBF1	SET binding factor 1	6305	924,468349	-0,090389896	0,110255	-0,819828017	0,412314157	0,82225544
ENSG00000105516	DBP	D-box binding PAR bZIP transcription factor	1628	23,32864753	0,089028987	0,511866	0,173930347	0,861920203	NA
ENSG00000109854	HTATIP2	HIV-1 Tat interactive protein 2	10553	70,31108789	0,06150764	0,210729	0,291879713	0,770378595	0,945219782
ENSG00000140280	LYSMD2	LysM domain containing 2	256586	60,00729029	0,07842134	0,247718	0,316574825	0,751566241	0,94158578
ENSG00000147121	KRBOX4	KRAB box domain containing 4	55634	40,46003818	0,606965049	0,297616	2,039420689	0,041408059	NA
ENSG00000170296	GABARAP	GABA type A receptor-associated protein	11337	316,8312382	0,063694325	0,112544	0,565949144	0,571428353	0,886795904
ENSG00000050405	LIMA1	LIM domain and actin binding 1	51474	12,41296107	0,65850054	0,652058	1,009880385	0,312552601	NA
ENSG00000092199	HNRNPC	heterogeneous nuclear ribonucleoprotein C	3183	1495,462035	0,058652476	0,063742	0,920156501	0,357490982	0,790004408
ENSG00000117394	SLC2A1	solute carrier family 2 member 1	6513	71,09001369	-0,280376986	0,643366	-0,435796729	0,662984217	0,920791873
ENSG00000123505	AMD1	adenosylmethionine decarboxylase 1	262	289,4214111	0,059893194	0,099717	0,600632625	0,548084703	0,879578516
ENSG00000166479	TMX3	thioredoxin related transmembrane protein 3	54495	419,8795231	0,021425402	0,09145	0,234284489	0,814764127	0,95938223
ENSG00000168872	DDX19A	DEAD-box helicase 19A	55308	159,4113952	-0,207293159	0,152691	-1,357594639	0,174592345	0,632039836
ENSG00000203485	INF2	inverted formin 2	64423	17,1144397	0,097788306	0,571588	0,171081901	0,864159367	NA
ENSG00000178950	GAK	cyclin G associated kinase	2580	629,4461975	-0,0906639	0,07997	-1,133730971	0,256907414	0,71283265
ENSG00000196437	ZNF569	zinc finger protein 569	148266	37,88886253	0,270203269	0,310554	0,870068245	0,38426311	NA
ENSG00000149100	EIF3M	eukaryotic translation initiation factor 3 subunit M	10480	184,5887622	0,400056742	0,167829	2,383723245	0,017138486	0,204616219
ENSG00000157500	APPL1	adaptor protein, phosphotyrosine interacting with PH domain and leucine zipper 1	26060	336,9242309	-0,06220272	0,098783	-0,629693464	0,528895159	0,872145761
ENSG00000226232	NPIPB14P	nuclear pore complex interacting protein family member B14, pseudogene	109729168	17,35005319	0,168946038	0,404301	0,417872097	0,676040634	NA
ENSG00000147124	ZNF41	zinc finger protein 41	7592	81,47729644	0,08607196	0,210366	0,409152477	0,682427768	0,925586358
ENSG00000001036	FUCA2	alpha-L-fucosidase 2	2519	93,43620107	-0,057887351	0,210075	-0,275556133	0,782889006	0,949847725
ENSG00000084693	AGBL5	AGBL carboxypeptidase 5	60509	22,26915546	0,429843089	0,81799	0,525487144	0,599244588	NA
ENSG00000105370	LIM2	lens intrinsic membrane protein 2	3982	48,09664127	-0,382216228	0,248627	-1,537310375	0,124217322	0,562307116
ENSG00000120662	MTRF1	mitochondrial translation release factor 1	9617	47,97421084	-0,085815691	0,280298	-0,306159012	0,759483578	0,942901768
ENSG00000131043	AAR2	AAR2 splicing factor	25980	137,7907292	0,008129318	0,150937	0,053859118	0,957047408	0,991911599
ENSG00000138758	SEPTIN11	septin 11	55752	1079,558575	-0,005577766	0,056224	-0,099206058	0,920974665	0,984284131
ENSG00000159322	ADPGK	ADP dependent glucokinase	83440	191,9234299	0,200872857	0,178243	1,126963331	0,259757985	0,715701578

ENSG00000162542	TMCO4	transmembrane and coiled-coil domains 4	255104	66,10788671	0,287277266	0,258902	1,109596906	0,267172765	0,722449525
ENSG00000170836	PPM1D	protein phosphatase, Mg2+/Mn2+ dependent 1D	8493	34,37077915	-0,526979663	0,35418	-1,48788648	0,136780834	NA
ENSG00000178741	COX5A	cytochrome c oxidase subunit 5A	9377	158,7024484	0,101610009	0,147312	0,689762286	0,490343689	0,860513824
ENSG00000183605	SFXN4	sideroflexin 4	119559	16,35267863	-1,040990557	0,959891	-1,084488333	0,278148337	NA
ENSG00000187764	SEMA4D	semaphorin 4D	10507	2282,289495	-0,050541138	0,044018	-1,148198977	0,250886432	0,707335884
ENSG00000196466	ZNF799	zinc finger protein 799	90576	12,31624561	-0,059983059	0,573894	-0,104519475	0,916757114	NA
ENSG00000032742	IFT88	intraflagellar transport 88	8100	41,69752076	-0,242412148	0,312194	-0,776478318	0,437466618	NA
ENSG00000047578	KATNIP	katanin interacting protein	23247	120,6522044	-0,069153131	0,178473	-0,38747114	0,698407446	0,930783276
ENSG00000196418	ZNF124	zinc finger protein 124	7678	31,20675251	-0,079436504	0,337159	-0,235605753	0,813738609	NA
ENSG00000148835	TAF5	TATA-box binding protein associated factor 5	6877	33,63579914	-0,244194284	0,348926	-0,699845032	0,484024088	NA
ENSG00000167323	STIM1	stromal interaction molecule 1	6786	411,9103587	-0,63943168	0,146576	-4,362451483	1,29E-05	0,001231863
ENSG00000196605	ZNF846	zinc finger protein 846	162993	21,93511047	-0,494227603	0,484998	-1,019029334	0,308189041	NA
ENSG00000196605	LOC100505555	uncharacterized LOC100505555	100505555	21,93511047	-0,494227603	0,484998	-1,019029334	0,308189041	NA
ENSG00000168028	RPSA	ribosomal protein SA	3921	1932,527721	-0,061117068	0,080845	-0,755978331	0,449662188	0,84263906
ENSG00000005893	LAMP2	lysosomal associated membrane protein 2	3920	442,8529477	-0,030696785	0,093872	-0,327007602	0,743662136	0,939745744
ENSG00000014824	SLC30A9	solute carrier family 30 member 9	10463	125,2157159	-0,391698257	0,191667	-2,043637117	0,04098941	0,322789747
ENSG00000182872	RBM10	RNA binding motif protein 10	8241	238,8568902	-0,126710574	0,131528	-0,963370892	0,335361429	0,775275461
ENSG00000183943	PRKX	protein kinase cAMP-dependent X-linked catalytic subunit	5613	1205,283512	-0,031635256	0,051717	-0,611704368	0,540733371	0,876195564
ENSG00000156853	ZNF689	zinc finger protein 689	115509	66,49435552	-0,143068287	0,204856	-0,698383957	0,484937107	0,857621265
ENSG00000129667	RHBDF2	rhomboid 5 homolog 2	79651	205,7529202	-0,136837222	0,192573	-0,710575083	0,477347588	0,853811879
ENSG00000173113	TRMT112	tRNA methyltransferase activator subunit 11-2	51504	129,7829749	0,20803713	0,148138	1,404344873	0,160216178	0,617437616
ENSG00000187446	CHP1	calcineurin like EF-hand protein 1	11261	244,0642453	0,110396592	0,126624	0,87184445	0,383293241	0,808586507
ENSG00000070540	WIPI1	WD repeat domain, phosphoinositide interacting 1	55062	114,278283	0,192816918	0,165852	1,162583099	0,244998691	0,701819279
ENSG00000170876	TMEM43	transmembrane protein 43	79188	289,519713	-0,126137573	0,136056	-0,927102029	0,353873563	0,788228969
ENSG00000181045	SLC26A11	solute carrier family 26 member 11	284129	15,77734596	-0,055212171	0,479409	-0,11516711	0,908312668	NA
ENSG00000275074	NUDT18	nudix hydrolase 18	79873	35,10405354	-0,188071698	0,3174	-0,592538245	0,553490218	NA
ENSG00000163754	GYG1	glycogenin 1	2992	290,093727	0,011587903	0,098477	0,11767092	0,906328409	0,978516824
ENSG00000112419	PHACTR2	phosphatase and actin regulator 2	9749	197,3750558	-0,014251113	0,126682	-0,112495271	0,910430719	0,979595261
ENSG00000103145	HCFC1R1	host cell factor C1 regulator 1	54985	29,50693187	-0,16986579	0,40477	-0,419659662	0,674734097	NA
ENSG00000178498	DTX3	deltex E3 ubiquitin ligase 3	196403	53,41315204	-0,058768356	0,260124	-0,225924367	0,821260246	0,961898134
ENSG00000110324	IL10RA	interleukin 10 receptor subunit alpha	3587	922,6358256	0,017184085	0,093054	0,18466843	0,85348911	0,968377739
ENSG00000136878	USP20	ubiquitin specific peptidase 20	10868	604,43282	-0,024248631	0,073445	-0,330162173	0,741277427	0,939193793
ENSG00000171606	ZNF274	zinc finger protein 274	10782	76,14612284	-0,070364514	0,202888	-0,346814175	0,728730928	0,937916952
ENSG00000165733	BMS1	BMS1 ribosome biogenesis factor	9790	285,0604574	0,193586888	0,109243	1,772079778	0,076381315	0,447360046
ENSG00000181513	ACBD4	acyl-CoA binding domain containing 4	79777	27,69179865	-0,225754807	0,392056	-0,575823073	0,564734777	NA
ENSG00000277072	NA	NA	NA	43,40421773	-0,038067827	0,27817	-0,136850997	0,891148573	0,977990819
ENSG00000133805	AMPD3	adenosine monophosphate deaminase 3	272	188,1980344	0,030119747	0,130993	0,229933184	0,81814369	0,960780583
ENSG00000169371	SNUPN	snurportin 1	10073	49,71964896	0,408399808	0,308964	1,321835894	0,186222803	0,644598455
ENSG00000108064	TFAM	transcription factor A, mitochondrial	7019	179,9593076	-0,143385026	0,194056	-0,738884863	0,459976915	0,846622987
ENSG00000132383	RPA1	replication protein A1	6117	836,8433444	0,062567613	0,064756	0,966210286	0,333938967	0,773674527
ENSG00000169682	SPNS1	SPNS lysolipid transporter 1, lysophospholipid	83985	88,77165155	0,332026061	0,260412	1,275002183	0,202308469	0,660119983
ENSG00000131148	EMC8	ER membrane protein complex subunit 8	10328	63,91831982	-0,250487516	0,213356	-1,174034606	0,240381165	0,695620773
ENSG00000135953	MFSD9	major facilitator superfamily domain containing 9	84804	84,91501997	-0,30060435	0,237717	-1,264545089	0,206034448	0,660954946
ENSG00000170325	PRDM10	PR/SET domain 10	56980	107,1861871	0,063736374	0,18448	0,345492589	0,729724081	0,938472725
ENSG00000167604	NFKBID	NFKB inhibitor delta	84807	39,01474363	0,315050065	0,280644	1,122598648	0,26160799	NA
ENSG00000169976	SF3B5	splicing factor 3b subunit 5	83443	120,1714539	0,048636932	0,157008	0,309774244	0,756732639	0,942901768
ENSG00000129810	SGO1	shugoshin 1	151648	34,52455862	0,427539785	0,367154	1,164470772	0,244233281	NA
ENSG00000109171	SLAIN2	SLAIN motif family member 2	57606	268,8469851	0,043043134	0,108567	0,396467884	0,691759896	0,927787546
ENSG00000135185	TMEM243	transmembrane protein 243	79161	76,29397566	-0,134811228	0,200797	-0,671381662	0,501977424	0,862509448
ENSG00000168826	ZBTB49	zinc finger and BTB domain containing 49	166793	24,1351344	0,284063933	0,475434	0,597483326	0,550184736	NA
ENSG00000179041	RRS1	ribosome biogenesis regulator 1 homolog	23212	89,19481609	-0,196269283	0,190476	-1,030412962	0,302816191	0,751870158
ENSG00000204899	MZT1	mitotic spindle organizing protein 1	440145	60,79700155	0,699606063	0,243611	2,871811154	0,004081268	0,088547727

ENSG00000108406	DHX40	DEAH-box helicase 40	79665	157,7668751	-0,084339494	0,144163	-0,58502769	0,558529098	0,883921037
ENSG00000139044	B4GALNT3	beta-1,4-N-acetyl-galactosaminyltransferase 3	283358	18,01949045	-0,973392983	2,246663	-0,433261691	0,664824666	NA
ENSG00000107020	PLGRKT	plasminogen receptor with a C-terminal lysine	55848	30,91716225	-0,055662149	0,338095	-0,164634589	0,869231605	NA
ENSG00000125633	CCDC93	coiled-coil domain containing 93	54520	303,5093107	-0,217991157	0,113489	-1,920807009	0,054756043	0,379236295
ENSG00000113273	ARSB	arylsulfatase B	411	49,67709868	0,161602155	0,286473	0,564108912	0,572680013	0,886795904
ENSG00000100038	TOP3B	DNA topoisomerase III beta	8940	74,08375442	0,183695932	0,314424	0,58422971	0,559065775	0,883921037
ENSG00000119729	RHOQ	ras homolog family member Q	23433	98,31672107	-0,081558154	0,165535	-0,492694598	0,622228395	0,904764752
ENSG00000116337	AMPD2	adenosine monophosphate deaminase 2	271	54,0617235	-0,607240681	0,497221	-1,221269732	0,22198391	0,678334201
ENSG00000125733	TRIP10	thyroid hormone receptor interactor 10	9322	30,64244989	0,124502204	0,32315	0,385276786	0,700032359	NA
ENSG00000130382	MLLT1	MLLT1 super elongation complex subunit	4298	221,0231434	-0,132457588	0,115147	-1,150334825	0,250005993	0,707335884
ENSG00000197961	ZNF121	zinc finger protein 121	7675	203,5306235	-0,09486487	0,126533	-0,749725917	0,453419795	0,843234809
ENSG00000056277	ZNF280C	zinc finger protein 280C	55609	36,87943652	0,185206165	0,290639	0,637238543	0,523969476	NA
ENSG00000183604	NA	NA	NA	118,7384903	-0,034749396	0,256876	-0,135276781	0,892393042	0,977990819
ENSG00000072778	ACADVL	acyl-CoA dehydrogenase very long chain	37	404,6078976	-0,000110218	0,142697	-0,000772387	0,999383724	0,999703675
ENSG00000087470	DNM1L	dynamin 1 like	10059	227,8116518	0,076687148	0,159865	0,479698561	0,631441751	0,906809932
ENSG00000118961	LDAH	lipid droplet associated hydrolase	60526	99,6518247	-0,142843562	0,190897	-0,748275773	0,454293835	0,843234809
ENSG00000125648	SLC25A23	solute carrier family 25 member 23	79085	89,08542339	-0,296809455	0,217845	-1,36247891	0,173046795	0,629558603
ENSG00000131778	CHD1L	chromodomain helicase DNA binding protein 1 like	9557	95,02380143	0,439919028	0,955388	0,460461347	0,64518511	0,912530858
ENSG00000107937	GTPBP4	GTP binding protein 4	23560	191,3067045	-0,068874661	0,130986	-0,525815926	0,599016108	0,89463553
ENSG00000135677	GNS	glucosamine (N-acetyl)-6-sulfatase	2799	224,5999163	-0,079996253	0,12985	-0,616067386	0,537850046	0,874923109
ENSG00000076770	MBNL3	muscleblind like splicing regulator 3	55796	215,1241468	-0,030584656	0,158421	-0,193059795	0,846912139	0,967231052
ENSG00000129493	HEATR5A	HEAT repeat containing 5A	25938	82,20918067	0,599154783	0,719848	0,832334825	0,405219979	0,818437414
ENSG00000130363	RSPH3	radial spoke head 3	83861	15,96605237	0,14238166	0,635034	0,22421121	0,822592961	NA
ENSG00000101187	SLCO4A1	solute carrier organic anion transporter family member 4A1	28231	34,11626307	-0,324089609	0,404163	-0,801879184	0,422622849	NA
ENSG00000178252	WDR6	WD repeat domain 6	11180	462,3542475	-0,029312129	0,087485	-0,335053762	0,737584532	0,939193793
ENSG00000139597	N4BP2L1	NEDD4 binding protein 2 like 1	90634	89,67639479	0,024729781	0,218966	0,11293866	0,910079186	0,979548024
ENSG00000136603	SKIL	SKI like proto-oncogene	6498	153,7343274	0,10306752	0,191145	0,539212305	0,589740371	0,892279113
ENSG00000106692	FKTN	fukutin	2218	88,28795296	-0,139506777	0,216222	-0,645201223	0,518796794	0,868791049
ENSG00000127481	UBR4	ubiquitin protein ligase E3 component n-recognin 4	23352	2251,244879	0,109438176	0,058884	1,85852802	0,063094068	0,404317327
ENSG00000114779	ABHD14B	abhydrolase domain containing 14B	84836	128,2841398	-0,058399251	0,153446	-0,380584876	0,703511305	0,930846779
ENSG00000141469	SLC14A1	solute carrier family 14 member 1 (Kidd blood group)	6563	30,25047819	-0,766187721	0,38798	-1,974810137	0,048289698	NA
ENSG00000168701	TMEM208	transmembrane protein 208	29100	35,25167569	0,105545756	0,279421	0,377730345	0,705630923	NA
ENSG00000170855	TRIAP1	TP53 regulated inhibitor of apoptosis 1	51499	62,46194628	0,135062516	0,215074	0,627980414	0,530016771	0,872145761
ENSG00000183044	ABAT	4-aminobutyrate aminotransferase	18	121,1980018	-0,038444545	0,169639	-0,226625012	0,820715341	0,961891299
ENSG00000184979	USP18	ubiquitin specific peptidase 18	11274	26,20710198	0,13804192	0,379942	0,363323614	0,716363156	NA
ENSG00000256269	HMBS	hydroxymethylbilane synthase	3145	26,67148666	-0,038687655	0,403601	-0,095856181	0,923634797	NA
ENSG00000104412	EMC2	ER membrane protein complex subunit 2	9694	42,28945506	-0,113773618	0,288163	-0,394823965	0,692972807	NA
ENSG00000170322	NFRKB	nuclear factor related to kappaB binding protein	4798	169,9851776	0,031961273	0,148367	0,21542101	0,829439091	0,962958554
ENSG00000187555	USP7	ubiquitin specific peptidase 7	7874	592,8947613	0,055660162	0,241493	0,2304834	0,817716161	0,960422122
ENSG00000143570	SLC39A1	solute carrier family 39 member 1	27173	95,17484481	0,021194914	0,169084	0,125351659	0,900245157	0,977990819
ENSG00000163811	WDR43	WD repeat domain 43	23160	192,5731007	0,044375049	0,124367	0,356807328	0,72123605	0,933829316
ENSG00000066084	DIP2B	disco interacting protein 2 homolog B	57609	239,8806928	-0,09241583	0,109568	-0,843452562	0,398975386	0,814478813
ENSG00000073803	MAP3K13	mitogen-activated protein kinase kinase kinase 13	9175	35,35682422	0,71905559	0,323147	2,225162661	0,02607032	NA
ENSG00000128050	PAICS	phosphoribosylaminoimidazole carboxylase and phosphoribosylaminoimidazole succinocarboxamide synthase	10606	223,2023843	0,119839452	0,111049	1,079162884	0,280515122	0,733410709
ENSG00000002834	LASP1	LIM and SH3 protein 1	3927	2756,811576	0,052826728	0,04465	1,183131562	0,236757011	0,691517131
ENSG00000188352	FOCAD	focadhesin	54914	211,8213747	0,067534701	0,132856	0,508331319	0,61122101	0,900006614

ENSG00000174945	AMZ1	archaelysin family metallopeptidase 1	155185	20,04762638	0,701635852	0,446152	1,572639996	0,115802201	NA
ENSG00000167220	HDHD2	haloacid dehalogenase like hydrolase domain containing 2	84064	79,53462567	-0,104541841	0,310876	-0,336281775	0,736658392	0,939157822
ENSG00000214706	IFRD2	interferon related developmental regulator 2	7866	47,43349499	-0,336173581	0,276771	-1,214627577	0,224508166	0,679756028
ENSG00000103507	BCKDK	branched chain keto acid dehydrogenase kinase	10295	74,96798485	0,125022838	0,197714	0,63234129	0,527163889	0,871653726
ENSG00000117593	DARS2	aspartyl-tRNA synthetase 2, mitochondrial	55157	87,49090196	0,051133949	0,200691	0,25478897	0,798886095	0,954289184
ENSG00000179029	TMEM107	transmembrane protein 107	84314	20,79407481	-0,17750709	0,537743	-0,330096363	0,741327151	NA
ENSG00000091073	DTX2	deltex E3 ubiquitin ligase 2	113878	51,82899914	-0,093748707	0,235227	-0,398545887	0,690227841	0,927252461
ENSG00000152804	HHEX	hematopoietically expressed homeobox	3087	20,34322082	-0,414520285	0,418418	-0,990684721	0,321839554	NA
ENSG00000095485	CWF19L1	CWF19 like cell cycle control factor 1	55280	116,0462787	-0,138386032	0,199197	-0,694718948	0,487231451	0,858914122
ENSG00000107290	SETX	senataxin	23064	1770,675223	-0,078868969	0,053196	-1,482619324	0,13817558	0,579098722
ENSG00000125726	CD70	CD70 molecule	970	16,29910454	1,376292938	0,483518	2,846412352	0,00442149	NA
ENSG00000134901	POGLUT2	protein O-glucosyltransferase 2	79070	107,738282	0,262904192	0,184238	1,426978908	0,153585974	0,6111586
ENSG00000137441	FGFBP2	fibroblast growth factor binding protein 2	83888	44,27182579	-0,769318507	0,270385	-2,845269522	0,004437386	0,091955749
ENSG00000139734	DIAPH3	diaphanous related formin 3	81624	31,95355979	0,369696829	0,40218	0,919232514	0,357973968	NA
ENSG00000160305	DIP2A	disco interacting protein 2 homolog A	23181	695,690775	0,117591339	0,073459	1,60077861	0,109425963	0,529522903
ENSG00000165416	SUGT1	SGT1 homolog, MIS12 kinetochore complex assembly cochaperone	10910	189,4051116	-0,195601837	0,147247	-1,32839373	0,18404807	0,640457926
ENSG00000181026	AEN	apoptosis enhancing nuclease	64782	97,41893708	-0,084964822	0,167474	-0,507331689	0,611922108	0,900006614
ENSG00000175324	LSM1	LSM1 homolog, mRNA degradation associated	27257	91,48045383	0,169668411	0,198669	0,854025434	0,393090897	0,812491435
ENSG00000166579	NDEL1	nudE neurodevelopment protein 1 like 1	81565	145,6705482	-0,216402463	0,17693	-1,223095732	0,22129354	0,677288877
ENSG00000151746	BICD1	BICD cargo adaptor 1	636	42,94609388	-0,561933924	0,300206	-1,871830443	0,061230065	0,401755164
ENSG00000160226	CFAP410	cilia and flagella associated protein 410	755	40,51122593	-0,211941564	0,287962	-0,736004124	0,461728188	NA
ENSG00000164442	CITED2	Cbp/p300 interacting transactivator with Glu/Asp rich carboxy-terminal domain 2	10370	118,1438203	-0,351468722	0,168754	-2,08272763	0,037276057	0,306351862
ENSG00000108883	EFTUD2	elongation factor Tu GTP binding domain containing 2	9343	355,7142052	0,098627047	0,097067	1,016070398	0,309595864	0,755959614
ENSG00000148187	MRRF	mitochondrial ribosome recycling factor	92399	70,49123201	0,108213762	0,335453	0,322590398	0,747005466	0,939899419
ENSG00000152683	SLC30A6	solute carrier family 30 member 6	55676	163,9267504	0,070693624	0,160689	0,439941165	0,65997972	0,919368176
ENSG00000180479	ZNF571	zinc finger protein 571	51276	17,35413024	-0,115759108	0,445284	-0,259967176	0,794889093	NA
ENSG00000187609	EXD3	exonuclease 3'-5' domain containing 3	54932	38,12799491	-0,252430639	0,416439	-0,606164328	0,544405638	NA
ENSG00000223705	NSUN5P1	NSUN5 pseudogene 1	155400	61,37476431	-0,391233071	0,230505	-1,697285571	0,089642684	0,488772026
ENSG00000101966	XIAP	X-linked inhibitor of apoptosis	331	341,8493242	-0,287062985	0,131451	-2,183797583	0,028977125	0,274531173
ENSG00000179909	ZNF154	zinc finger protein 154	7710	32,71273054	-0,376269747	0,349102	-1,077823279	0,281112629	NA
ENSG00000103064	SLC7A6	solute carrier family 7 member 6	9057	921,6104266	0,026116264	0,070062	0,372761857	0,709325693	0,930846779
ENSG00000099899	TRMT2A	tRNA methyltransferase 2 homolog A	27037	112,7631669	-0,098074687	0,176246	-0,5564637	0,577893903	0,887069818
ENSG00000119927	GPAM	glycerol-3-phosphate acyltransferase, mitochondrial	57678	60,92489875	0,230945158	0,213562	1,081398096	0,279520068	0,732760553
ENSG00000138663	COPS4	COP9 signalosome subunit 4	51138	118,2548011	-0,200188329	0,157793	-1,268677658	0,204556054	0,660954946
ENSG00000145246	ATP10D	ATPase phospholipid transporting 10D (putative)	57205	341,873551	0,211320334	0,105959	1,994355778	0,046113188	0,342966833
ENSG00000173039	RELA	RELA proto-oncogene, NF-kB subunit	5970	327,99316	-0,083889256	0,095919	-0,874582966	0,381800859	0,806821656
ENSG00000179532	DNHD1	dynein heavy chain domain 1	144132	88,30152297	0,196335266	0,223788	0,877328769	0,380308091	0,805974027
ENSG00000180304	OAZ2	ornithine decarboxylase antizyme 2	4947	80,03578153	-0,269864477	0,216694	-1,245373306	0,212994561	0,667215123
ENSG00000198237	NA	NA	NA	30,10947595	0,276204653	0,41142	0,671344486	0,502001101	NA
ENSG00000104064	GABPB1	GA binding protein transcription factor subunit beta 1	2553	92,92639563	-0,383178437	0,215758	-1,775967713	0,075738239	0,446229554
ENSG00000139146	SINHCAF	SIN3-HDAC complex associated factor	58516	171,7825079	0,034349299	0,127745	0,268888539	0,788015457	0,95148885
ENSG00000197948	FCHSD1	FCH and double SH3 domains 1	89848	82,1349592	-0,327205486	0,210016	-1,558000844	0,119233048	0,552155873

ENSG00000130520	LSM4	LSM4 homolog, U6 small nuclear RNA and mRNA degradation associated	25804	117,6861016	0,014681411	0,188071	0,078063176	0,937777799	0,987306546
ENSG00000005884	ITGA3	integrin subunit alpha 3	3675	60,02982569	0,413063982	0,369912	1,116656055	0,264141414	0,719682465
ENSG00000111981	ULBP1	UL16 binding protein 1	80329	18,92037418	0,350026728	0,460607	0,759925304	0,447299235	NA
ENSG00000065665	SEC61A2	SEC61 translocon subunit alpha 2	55176	78,16622906	0,095171758	0,226262	0,420627049	0,674027439	0,923602509
ENSG00000122515	ZMIZ2	zinc finger MIZ-type containing 2	83637	238,0336006	-0,151679312	0,156158	-0,971317838	0,331390029	0,772473234
ENSG00000285171	NA	NA	NA	15,29809915	-0,115920093	2,157696	-0,053724025	0,957155042	NA
ENSG00000059758	CDK17	cyclin dependent kinase 17	5128	392,6483128	-0,003758091	0,102701	-0,03659245	0,970809963	0,993710602
ENSG00000106952	TNFSF8	TNF superfamily member 8	944	105,5240457	0,480151857	0,173553	2,766608351	0,005664276	0,10771718
ENSG00000165832	TRUB1	TruB pseudouridine synthase family member 1	142940	57,35980005	-0,2884793	0,241961	-1,192257616	0,233160248	0,688695219
ENSG00000100075	SLC25A1	solute carrier family 25 member 1	6576	106,8061666	0,361866533	0,171443	2,110716527	0,034796685	0,297704968
ENSG00000168710	AHCYL1	adenosylhomocysteinase like 1	10768	338,2540445	0,06700718	0,09227	0,726204631	0,467713334	0,851116896
ENSG00000261236	BOP1	BOP1 ribosomal biogenesis factor	23246	109,7302683	0,006402454	0,168645	0,037964067	0,969716332	0,993542794
ENSG00000001631	KRIT1	KRIT1 ankyrin repeat containing	889	32,26338181	-1,037400515	1,371829	-0,756217036	0,44951908	NA
ENSG00000100099	HPS4	HPS4 biogenesis of lysosomal organelles complex 3 subunit 2	89781	42,95576618	-0,96968741	1,455493	-0,66622624	0,505266503	0,864630986
ENSG00000105127	AKAP8	A-kinase anchoring protein 8	10270	63,45779761	-1,255464202	1,35669	-0,925387967	0,354764136	0,788341988
ENSG00000130749	ZC3H4	zinc finger CCCH-type containing 4	23211	331,0497574	-0,092790399	0,160877	-0,576779038	0,564088732	0,885045179
ENSG00000151491	EPS8	epidermal growth factor receptor pathway substrate 8	2059	14,68723415	0,002139425	0,476216	0,004492551	0,996415475	NA
ENSG00000153898	MCOLN2	mucolipin TRP cation channel 2	255231	145,4731942	0,049501077	0,165156	0,299722329	0,764388965	0,944245192
ENSG00000160216	AGPAT3	1-acylglycerol-3-phosphate O-acyltransferase 3	56894	167,9741035	0,010429269	0,145543	0,071657662	0,94287435	0,988624545
ENSG00000066117	SMARCD1	SWI/SNF related, matrix associated, actin dependent regulator of chromatin, subfamily d, member 1	6602	285,0470743	0,09451847	0,105229	0,898214968	0,369070955	0,797217623
ENSG00000112294	ALDH5A1	aldehyde dehydrogenase 5 family member A1	7915	29,83675259	0,046088657	0,552027	0,083489826	0,933462067	NA
ENSG00000126970	ZC4H2	zinc finger C4H2-type containing	55906	15,89516937	0,195757444	0,440317	0,444583206	0,656620986	NA
ENSG00000164087	POC1A	POC1 centriolar protein A	25886	27,14098183	0,347997067	0,330601	1,05262016	0,292515114	NA
ENSG00000166401	SERPINB8	serpin family B member 8	5271	59,8836035	0,400072344	0,284399	1,406728477	0,159507918	0,615917006
ENSG00000117748	RPA2	replication protein A2	6118	205,669915	-0,114314017	0,125082	-0,913913768	0,360762159	0,791408368
ENSG00000101019	UQCC1	ubiquinol-cytochrome c reductase complex assembly factor 1	55245	100,739861	0,108864477	0,220682	0,49330939	0,621793995	0,904764752
ENSG00000166575	TMEM135	transmembrane protein 135	65084	75,17331295	0,068494352	0,347302	0,19721815	0,843656828	0,967231052
ENSG00000181467	RAP2B	RAP2B, member of RAS oncogene family	5912	151,7730226	-0,530225671	0,144696	-3,664421464	0,000247898	0,012374745
ENSG00000186814	ZSCAN30	zinc finger and SCAN domain containing 30	100101467	87,44952397	0,075134317	0,221997	0,338446852	0,735026472	0,939157822
ENSG00000198937	CCDC167	coiled-coil domain containing 167	154467	24,4320704	-0,186964452	0,369302	-0,506264747	0,612670807	NA
ENSG00000234857	HNRNPUL2-BSCL2	HNRNPUL2-BSCL2 readthrough (NMD candidate)	100534595	233,0579517	-0,073328565	0,182272	-0,402303594	0,68746061	0,926748702
ENSG00000101246	ARFRP1	ADP ribosylation factor related protein 1	10139	70,01971106	-0,025921895	0,229432	-0,112982791	0,910044199	0,979548024
ENSG00000116815	CD58	CD58 molecule	965	127,5669547	-0,16217414	0,147374	-1,100423407	0,271147684	0,725477256
ENSG00000123600	METTL8	methyltransferase 8, tRNA N3-cytidine	79828	47,53329313	0,096064834	0,284972	0,337102768	0,736039431	0,939157822
ENSG00000131061	ZNF341	zinc finger protein 341	84905	24,66826016	0,2055917	0,520681	0,39485152	0,692952471	NA
ENSG00000188522	FAM83G	family with sequence similarity 83 member G	644815	33,12036692	0,042977031	0,302415	0,142112976	0,886990769	NA
ENSG00000188707	NA	NA	NA	22,91466385	0,314696155	0,374344	0,840660797	0,400537989	NA
ENSG00000125445	MRPS7	mitochondrial ribosomal protein S7	51081	88,26327939	0,169915414	0,191121	0,889048261	0,373977143	0,801401633
ENSG00000172586	CHCHD1	coiled-coil-helix-coiled-coil-helix domain containing 1	118487	45,48874358	-0,011265267	0,267403	-0,042128494	0,966396266	0,992789539
ENSG00000117020	AKT3	AKT serine/threonine kinase 3	10000	134,610704	0,18419012	0,285567	0,644997617	0,51892873	0,868826742
ENSG00000173065	FAM222B	family with sequence similarity 222 member B	55731	59,80578506	-0,278666541	0,239985	-1,161184777	0,245566764	0,702616162
ENSG00000177628	GBA1	glucosylceramidase beta 1	2629	190,6049984	0,0901154	0,119632	0,753271347	0,451286873	0,842718653
ENSG00000116984	MTR	5-methyltetrahydrofolate-homocysteine methyltransferase	4548	246,2337119	-0,007650326	0,1563	-0,04894645	0,960961972	0,992235217

ENSG00000156017	CARNMT1	carnosine N-methyltransferase 1	138199	108,2852296	0,057991187	0,179321	0,323393613	0,746397166	0,939843468
ENSG00000214113	LYRM4	LYR motif containing 4	57128	47,43248103	0,065061876	0,245043	0,265512292	0,790614842	0,95167209
ENSG00000117362	APH1A	aph-1 homolog A, gamma-secretase subunit	51107	366,6080042	0,10397041	0,113421	0,916676935	0,359311956	0,790999468
ENSG00000164414	SLC35A1	solute carrier family 35 member A1	10559	100,6890405	-0,125697486	0,173769	-0,723360291	0,469458561	0,85231797
ENSG00000021574	SPAST	spastin	6683	148,8882889	0,010604363	0,153355	0,069148999	0,944871019	0,989205143
ENSG00000053371	AKR7A2	aldo-keto reductase family 7 member A2	8574	55,66470229	-0,227215581	0,235215	-0,965992118	0,334048125	0,773699196
ENSG00000103174	NAGPA	N-acetylglucosamine-1-phosphodiester alpha-N-acetylglucosaminidase	51172	35,20324135	-0,187352834	0,317585	-0,589929813	0,555237705	NA
ENSG00000111328	CDK2AP1	cyclin dependent kinase 2 associated protein 1	8099	39,24237394	0,182324271	0,312327	0,583761753	0,559380614	NA
ENSG00000114626	ABTB1	ankyrin repeat and BTB domain containing 1	80325	94,91812169	-0,283028745	0,185835	-1,523008137	0,12775667	0,563689485
ENSG00000130299	GTPBP3	GTP binding protein 3, mitochondrial	84705	44,50818208	-0,143448948	0,277336	-0,51723915	0,604989226	0,897347689
ENSG00000144036	EXOC6B	exocyst complex component 6B	23233	27,8128907	0,308650819	0,413455	0,74651681	0,455355281	NA
ENSG00000172349	IL16	interleukin 16	3603	1513,762148	-0,15219598	0,067816	-2,244249454	0,024816356	0,252798519
ENSG00000188157	AGRN	agrin	375790	74,74610895	-0,230207458	0,331612	-0,69420801	0,48755177	0,859150012
ENSG00000237693	IRGM	immunity related GTPase M	345611	16,57856763	0,614469718	0,421433	1,458049495	0,144826901	NA
ENSG00000166135	HIF1AN	hypoxia inducible factor 1 subunit alpha inhibitor	55662	383,3000608	-0,193317811	0,088038	-2,195857514	0,028102144	0,270084577
ENSG00000014138	POLA2	DNA polymerase alpha 2, accessory subunit	23649	83,82868449	0,23671288	0,221928	1,066620427	0,286143272	0,738005299
ENSG00000125821	DTD1	D-aminoacyl-tRNA deacylase 1	92675	43,82590989	-0,419755744	0,35454	-1,183946057	0,236434417	0,691328984
ENSG00000213625	LEPROT	leptin receptor overlapping transcript	54741	150,1005519	-0,20393773	0,167438	-1,217987763	0,223228623	0,67869714
ENSG00000119707	RBM25	RNA binding motif protein 25	58517	478,2519717	-0,105311387	0,128493	-0,819590691	0,412449482	0,82225544
ENSG00000101400	SNTA1	syntrophin alpha 1	6640	12,35034911	-0,446538865	0,540089	-0,82678808	0,408357187	NA
ENSG00000141293	SKAP1	src kinase associated phosphoprotein 1	8631	307,295322	-0,034568801	0,102934	-0,335833856	0,736996158	0,939157822
ENSG00000165392	WRN	WRN RecQ like helicase	7486	70,5573615	0,026619418	0,354592	0,075070529	0,940158596	0,987826838
ENSG00000085063	CD59	CD59 molecule (CD59 blood group)	966	652,3844011	0,214470654	0,089546	2,395090323	0,016616271	0,203098821
ENSG00000196187	TMEM63A	transmembrane protein 63A	9725	369,056244	-0,075882721	0,120413	-0,630188921	0,528570987	0,872142129
ENSG00000028310	BRD9	bromodomain containing 9	65980	88,92899295	0,084681243	0,205773	0,411526557	0,680686472	0,925586358
ENSG00000065882	TBC1D1	TBC1 domain family member 1	23216	156,8881227	0,282652064	0,169059	1,671916578	0,094540773	0,498337741
ENSG00000146021	KLHL3	kelch like family member 3	26249	16,99957987	-0,130086483	0,450977	-0,288454549	0,772998818	NA
ENSG00000149577	SIDT2	SID1 transmembrane family member 2	51092	145,3255901	-0,070424158	0,157155	-0,448117714	0,654068244	0,917166933
ENSG00000150782	IL18	interleukin 18	3606	11,75610218	0,332724948	0,564394	0,58952571	0,55550867	NA
ENSG00000163900	TMEM41A	transmembrane protein 41A	90407	42,76133859	-0,329556576	0,291768	-1,129516822	0,258679878	0,714371223
ENSG00000168056	LTBP3	latent transforming growth factor beta binding protein 3	4054	140,0430553	-0,732830237	0,538696	-1,360377629	0,173710454	0,630547978
ENSG00000181220	ZNF746	zinc finger protein 746	155061	171,2557043	-0,158816051	0,125034	-1,27018127	0,204020068	0,660954946
ENSG00000204348	DXO	decapping exoribonuclease	1797	14,11053711	-1,328620158	0,987456	-1,345498714	0,178464236	NA
ENSG00000228672	PROB1	proline rich basic protein 1	389333	29,71130564	-0,336891992	0,335698	-1,003555507	0,31559291	NA
ENSG00000259330	INAFM2	InaF motif containing 2	100505573	55,13166748	0,192925508	0,272604	0,707713201	0,479123378	0,854264474
ENSG00000101665	SMAD7	SMAD family member 7	4092	150,1443251	0,500667267	0,237295	2,109891739	0,034867682	0,297987785
ENSG00000118960	HS1BP3	HCLS1 binding protein 3	64342	47,21396147	-0,542612202	0,278939	-1,945275366	0,051741845	0,368098237
ENSG00000141030	COPS3	COP9 signalosome subunit 3	8533	147,8722127	0,147346794	0,141401	1,042049788	0,297388597	0,748371252
ENSG00000143162	CREG1	cellular repressor of E1A stimulated genes 1	8804	43,26296993	0,198638285	0,264424	0,751211497	0,452525382	0,843234809
ENSG00000173660	UQCRH	ubiquinol-cytochrome c reductase hinge protein	7388	106,5882669	0,1108102	0,191249	0,579402666	0,562317505	0,884605624
ENSG00000055917	PUM2	pumilio RNA binding family member 2	23369	663,5888205	-0,198943818	0,080548	-2,469875182	0,01351602	0,18084297
ENSG00000122705	CLTA	clathrin light chain A	1211	155,284041	0,082396525	0,137633	0,59867014	0,549392873	0,88072908
ENSG00000211751	TRBC1	T cell receptor beta constant 1	28639	165,322197	0,075217173	0,150681	0,499182284	0,617650974	0,902181653
ENSG00000104763	ASAH1	N-acylsphingosine amidohydrolase 1	427	140,3489438	-0,212264929	0,449993	-0,471706852	0,637136046	0,90943397
ENSG00000112592	TBP	TATA-box binding protein	6908	49,44833818	0,036207906	0,27416	0,132068466	0,894930138	0,977990819
ENSG00000103257	SLC7A5	solute carrier family 7 member 5	8140	1214,462542	-0,029838865	0,051762	-0,576459262	0,564304798	0,885045179
ENSG00000114520	SNX4	sorting nexin 4	8723	87,58848267	-0,179760283	0,195433	-0,919807382	0,357673425	0,790004408
ENSG00000167863	ATP5PD	ATP synthase peripheral stalk subunit d	10476	129,939415	0,092402583	0,147756	0,625374645	0,531725199	0,872945174
ENSG00000187024	PTRH1	peptidyl-tRNA hydrolase 1 homolog	138428	12,55491517	-0,310113394	0,553051	-0,560732046	0,574980218	NA
ENSG00000108389	MTMR4	myotubularin related protein 4	9110	322,4160662	0,033777774	0,090933	0,371459108	0,710295608	0,930846779
ENSG00000123219	CENPK	centromere protein K	64105	61,51975048	0,154540586	0,226138	0,68339073	0,494359976	0,860513824

ENSG00000139625	MAP3K12	mitogen-activated protein kinase kinase kinase 12	7786	89,1244142	0,045401293	0,180939	0,250919944	0,801876005	0,956122858
ENSG00000113048	MRPS27	mitochondrial ribosomal protein S27	23107	198,9029162	0,12793111	0,148894	0,859211416	0,390223886	0,810747116
ENSG00000114127	XRN1	5'-3' exoribonuclease 1	54464	674,8904723	-0,109374366	0,086484	-1,264676338	0,205987376	0,660954946
ENSG00000150779	TIMM8B	translocase of inner mitochondrial membrane 8 homolog B	26521	36,34272985	0,152598106	0,30265	0,504206756	0,614116094	NA
ENSG00000115548	KDM3A	lysine demethylase 3A	55818	249,1769723	-0,02066615	0,104982	-0,196853972	0,843941814	0,967231052
ENSG00000119397	CNTRL	centriolin	11064	84,22867304	0,11575634	1,953731	0,059248858	0,952753895	0,991212866
ENSG00000163257	DCAF16	DDB1 and CUL4 associated factor 16	54876	209,4707973	-0,241352964	0,113298	-2,130250767	0,033150916	0,291564717
ENSG00000198959	TGM2	transglutaminase 2	7052	20,25994338	-0,107562611	0,43214	-0,248906674	0,803432973	NA
ENSG00000151690	MFSD6	major facilitator superfamily domain containing 6	54842	345,0930728	0,084046902	0,088463	0,950081455	0,342070865	0,780064992
ENSG00000169692	AGPAT2	1-acylglycerol-3-phosphate O-acyltransferase 2	10555	57,03922125	-0,133239718	0,235646	-0,565423194	0,571785952	0,886795904
ENSG00000116922	AIRIM	AFG2 interacting ribosome maturation factor	54955	29,30992197	-0,152909221	0,406805	-0,375878407	0,707007294	NA
ENSG00000213339	QTRT1	queuine tRNA-ribosyltransferase catalytic subunit 1	81890	22,15630085	-0,04687179	0,445333	-0,105251137	0,916176534	NA
ENSG00000051108	HERPUD1	homocysteine inducible ER protein with ubiquitin like domain 1	9709	259,742027	0,201090547	0,157597	1,275980238	0,201962505	0,660119983
ENSG00000106608	URGCP	upregulator of cell proliferation	55665	84,92901073	0,018749428	0,190823	0,098255846	0,921729138	0,984284131
ENSG00000131037	EPS8L1	EPS8 like 1	54869	18,42337692	0,164505387	0,491532	0,334678884	0,737867332	NA
ENSG00000119280	C1orf198	chromosome 1 open reading frame 198	84886	44,47168164	-0,183414247	0,263871	-0,695090601	0,486998525	0,858914122
ENSG00000172270	BSG	basigin (Ok blood group)	682	517,4815241	-0,125205951	0,073448	-1,704691126	0,088252046	0,486094161
ENSG00000173120	KDM2A	lysine demethylase 2A	22992	867,7198157	-0,073442346	0,068437	-1,073132249	0,283211784	0,736782162
ENSG00000180098	TRNAU1AP	tRNA selenocysteine 1 associated protein 1	54952	33,04226445	-0,074849083	0,357415	-0,20941801	0,834121935	NA
ENSG00000198932	GPRASP1	G protein-coupled receptor associated sorting protein 1	9737	11,84242589	-0,268598748	0,55913	-0,480387331	0,630952001	NA
ENSG00000204070	SYS1	SYS1 golgi trafficking protein	90196	106,6921576	-0,069720758	0,169718	-0,41080439	0,681215974	0,925586358
ENSG00000105085	MED26	mediator complex subunit 26	9441	29,91585769	-0,497666952	0,514161	-0,967920179	0,333084236	NA
ENSG00000166199	ALKBH3	alkB homolog 3, alpha-ketoglutarate dependent dioxygenase	221120	58,34208891	0,058306044	0,243315	0,239631796	0,810615713	0,959038743
ENSG00000101608	MYL12A	myosin light chain 12A	10627	1271,950532	0,04716921	0,055513	0,849696445	0,395493875	0,812799303
ENSG00000135070	ISCA1	iron-sulfur cluster assembly 1	81689	99,94093529	-0,051876404	0,179557	-0,288913245	0,772647767	0,946409165
ENSG00000198464	ZNF480	zinc finger protein 480	147657	80,39259206	0,136393026	0,211345	0,64535585	0,518696607	0,868791049
ENSG00000109534	GAR1	GAR1 ribonucleoprotein	54433	26,19547427	-0,468499009	0,344871	-1,358474197	0,174313265	NA
ENSG00000118418	HMGN3	high mobility group nucleosomal binding domain 3	9324	132,3812604	0,127977736	0,154739	0,827057903	0,408204243	0,820514158
ENSG00000160781	PAQR6	progestin and adipoQ receptor family member 6	79957	35,33189761	-1,554484717	1,333494	-1,165722701	0,243726579	NA
ENSG00000074603	DPP8	dipeptidyl peptidase 8	54878	349,3015221	0,132649134	0,107022	1,239452528	0,215177959	0,671565255
ENSG00000155085	AK9	adenylate kinase 9	221264	32,24291576	-0,080590994	0,315862	-0,255146284	0,798610118	NA
ENSG00000177485	ZBTB33	zinc finger and BTB domain containing 33	10009	222,160704	-0,117537994	0,121157	-0,970131512	0,331980943	0,773014624
ENSG00000136861	CDK5RAP2	CDK5 regulatory subunit associated protein 2	55755	212,4195528	0,235683863	0,134666	1,750138819	0,080094363	0,459504109
ENSG00000175309	PHYKPL	5-phosphohydroxy-L-lysine phospho-lyase	85007	97,74373754	0,219112023	0,198292	1,104998179	0,269160378	0,724712242
ENSG00000268480	NFILZ	NFIL3 like basic leucine zipper	105372267	915,8607318	-0,052155872	0,084627	-0,616306003	0,537692578	0,874923109
ENSG00000081177	EXD2	exonuclease 3'-5' domain containing 2	55218	20,2262603	-1,133932064	0,760006	-1,492003426	0,135698247	NA
ENSG00000105793	GTPBP10	GTP binding protein 10	85865	81,14151556	-0,30568652	0,316206	-0,966730906	0,333678573	0,773674527
ENSG00000105793	LOC101927446	uncharacterized LOC101927446	101927446	81,14151556	-0,30568652	0,316206	-0,966730906	0,333678573	0,773674527
ENSG00000167196	FBXO22	F-box protein 22	26263	148,8998928	-0,738851182	0,240245	-3,075408331	0,002102145	0,05587092
ENSG00000178809	TRIM73	tripartite motif containing 73	375593	22,42858136	0,146635478	0,407753	0,35961844	0,719132492	NA
ENSG00000183718	TRIM52	tripartite motif containing 52	84851	151,1473097	-0,259784343	0,1366	-1,901788203	0,057198849	0,38821726
ENSG00000214021	TTLL3	tubulin tyrosine ligase like 3	26140	54,08225712	-0,011422298	0,296958	-0,038464315	0,969317483	0,993542794
ENSG00000235655	NA	NA	NA	20,2297361	-2,149730723	3,735585	-0,575473647	0,564971008	NA
ENSG00000272886	DCP1A	decapping mRNA 1A	55802	219,8858742	-0,125654882	0,123903	-1,014139645	0,310516123	0,75688983
ENSG00000109062	NHERF1	NHERF family PDZ scaffold protein 1	9368	868,1042492	-0,206847914	0,092144	-2,244821454	0,024779597	0,252751889
ENSG00000138363	ATIC	5-aminoimidazole-4-carboxamide ribonucleotide formyltransferase/IMP cyclohydrolase	471	228,6846592	0,176486943	0,123825	1,425297357	0,154071264	0,611607348
ENSG00000182149	IST1	IST1 factor associated with ESCRT-III	9798	234,6425884	0,019999161	0,381251	0,052456699	0,958164797	0,991911599

ENSG00000100393	EP300	E1A binding protein p300	2033	1141,59431	0,029261888	0,076621	0,381902003	0,702534055	0,930846779
ENSG00000126767	ELK1	ETS transcription factor ELK1	2002	137,6135965	0,105049062	0,170981	0,614390846	0,538957084	0,875342836
ENSG00000173209	AHSA2P	activator of HSP90 ATPase homolog 2, pseudogene	130872	51,66563056	0,062089854	0,24222	0,256337015	0,797690618	0,95354564
ENSG00000090520	DNAJB11	DnaJ heat shock protein family (Hsp40) member B11	51726	253,5043936	0,162953751	0,152967	1,065288161	0,286745549	0,738005299
ENSG00000132334	PTPRE	protein tyrosine phosphatase receptor type E	5791	291,4796667	0,044119022	0,117501	0,375476843	0,707305865	0,930846779
ENSG00000182185	RAD51B	RAD51 paralog B	5890	16,31930382	0,062568186	0,458865	0,136354129	0,891541334	NA
ENSG00000111716	LDHB	lactate dehydrogenase B	3945	673,5387726	0,076365293	0,069754	1,094782567	0,273611906	0,726913977
ENSG00000147459	DOCK5	dedicator of cytokinesis 5	80005	503,4771817	-0,047270081	0,125833	-0,375655832	0,707172778	0,930846779
ENSG00000174547	MRPL11	mitochondrial ribosomal protein L11	65003	47,72915752	0,269534173	0,262945	1,025058392	0,305335636	0,752229011
ENSG00000198064	NPIPB13	nuclear pore complex interacting protein family, member B13	613037	338,4777357	0,196609968	0,129764	1,515134009	0,129738471	0,565894171
ENSG00000126870	DYNC2I1	dynein 2 intermediate chain 1	55112	56,46332303	-0,066486617	0,246156	-0,270099662	0,787083582	0,951039147
ENSG00000131507	NDFIP1	Nedd4 family interacting protein 1	80762	290,5360903	0,164289045	0,10048	1,635034118	0,102041896	0,515062371
ENSG00000139626	ITGB7	integrin subunit beta 7	3695	1956,030173	-0,05559762	0,051873	-1,071807699	0,283806414	0,737423617
ENSG00000157764	BRAF	B-Raf proto-oncogene, serine/threonine kinase	673	190,5571167	-0,250624642	0,185249	-1,352904532	0,176086131	0,632039836
ENSG00000153551	CMTM7	CKLF like MARVEL transmembrane domain containing 7	112616	21,26892171	0,850567216	0,514006	1,654779669	0,0979692	NA
ENSG00000179085	DPM3	dolichyl-phosphate mannosyltransferase subunit 3, regulatory	54344	16,77482609	0,237756403	0,439246	0,541282666	0,588312767	NA
ENSG00000105647	PIK3R2	phosphoinositide-3-kinase regulatory subunit 2	5296	46,78584436	-0,107340999	0,255317	-0,420422009	0,674177193	0,923602509
ENSG00000132780	NASP	nuclear autoantigenic sperm protein	4678	265,6976939	0,190787655	0,108739	1,75454328	0,079337461	0,457165382
ENSG00000058056	USP13	ubiquitin specific peptidase 13	8975	32,6624623	0,359980466	0,341511	1,054082804	0,291845008	NA
ENSG00000111897	SERINC1	serine incorporator 1	57515	834,0983357	-0,000282915	0,073944	-0,003826087	0,996947232	0,999300722
ENSG00000119705	SLIRP	SRA stem-loop interacting RNA binding protein	81892	17,35506386	-0,394225029	0,428825	-0,919314268	0,357931217	NA
ENSG00000130340	SNX9	sorting nexin 9	51429	70,4714375	-0,282128358	0,346067	-0,815241819	0,414933905	0,82446781
ENSG00000157911	PEX10	peroxisomal biogenesis factor 10	5192	36,87743438	-0,048844793	0,336602	-0,145111222	0,884623058	NA
ENSG00000198502	HLA-DRB5	major histocompatibility complex, class II, DR beta 5	3127	12,32006543	-0,254535824	0,512961	-0,496209029	0,619746942	NA
ENSG00000244005	NFS1	NFS1 cysteine desulfurase	9054	25,3699445	0,199237968	0,539259	0,369465929	0,711780464	NA
ENSG00000275052	PPP4R3B	protein phosphatase 4 regulatory subunit 3B	57223	571,0217669	-0,129318767	0,092131	-1,403638699	0,160426465	0,617944806
ENSG00000242485	MRPL20	mitochondrial ribosomal protein L20	55052	92,09670506	0,65410268	0,184622	3,542921516	0,00039572	0,017591421
ENSG00000183688	RFLNB	refilin B	359845	421,5126343	0,030561083	0,303287	0,100766106	0,919736133	0,984142724
ENSG00000132471	WBP2	WW domain binding protein 2	23558	219,1132979	-0,172813032	0,114849	-1,504704192	0,132400145	0,569480142
ENSG00000054523	KIF1B	kinesin family member 1B	23095	183,0805833	0,140251132	0,219226	0,639757008	0,522330587	0,870221868
ENSG00000163297	ANTXR2	ANTXR cell adhesion molecule 2	118429	451,0229359	0,153314908	0,104309	1,469808977	0,141613497	0,585983354
ENSG00000174384	NA	NA	NA	52,34026659	0,026012204	0,261611	0,099430931	0,920796125	0,984284131
ENSG00000100105	PATZ1	POZ/BTB and AT hook containing zinc finger 1	23598	158,5201362	-0,098033024	0,146348	-0,669860659	0,502946621	0,863089257
ENSG00000160961	ZNF333	zinc finger protein 333	84449	52,54629632	-0,284607654	0,274415	-1,037144717	0,299668418	0,750202136
ENSG00000168216	LMBRD1	LMBR1 domain containing 1	55788	43,98405675	-1,885070939	1,370182	-1,375781376	0,168889329	0,628354709
ENSG00000255769	NA	NA	NA	19,16117187	-0,487837579	0,411206	-1,186358241	0,235480855	NA
ENSG00000039523	RIPOR1	RHO family interacting cell polarization regulator 1	79567	33,70477177	-0,419066423	0,350608	-1,195254979	0,231987424	NA
ENSG00000134248	LAMTOR5	late endosomal/lysosomal adaptor, MAPK and MTOR activator 5	10542	107,6035826	-0,131395754	0,16473	-0,797640922	0,425078896	0,830443782
ENSG00000149636	DSN1	DSN1 component of MIS12 kinetochore complex	79980	45,66133857	0,50896167	0,282528	1,801456087	0,071631023	0,435172111
ENSG00000119013	NDUFB3	NADH:ubiquinone oxidoreductase subunit B3	4709	84,38545306	0,16988327	0,20025	0,848353805	0,396240961	0,812799303
ENSG00000171657	GPR82	G protein-coupled receptor 82	27197	87,83593739	-0,655507425	0,193238	-3,392230623	0,000693261	0,026431403
ENSG00000072506	HSD17B10	hydroxysteroid 17-beta dehydrogenase 10	3028	98,54841516	0,174376769	0,190735	0,914237036	0,360592307	0,791408368
ENSG00000133226	SRRM1	serine and arginine repetitive matrix 1	10250	518,1258054	0,068175365	0,08832	0,771913	0,440165957	0,83848252
ENSG00000196367	TRRAP	transformation/transcription domain associated protein	8295	1070,166092	0,154322499	0,08922	1,729688866	0,083685879	0,471627336
ENSG00000172331	BPGM	bisphosphoglycerate mutase	669	48,76474336	0,217177175	0,251429	0,863770327	0,387714072	0,809653368
ENSG00000183401	CCDC159	coiled-coil domain containing 159	126075	19,41676119	-0,266395875	0,477851	-0,557487307	0,577194529	NA

ENSG00000198517	MAFK	MAF bZIP transcription factor K	7975	103,6157095	-0,221244438	0,181723	-1,217482823	0,223420568	0,67869714
ENSG00000020129	NCDN	neurochondrin	23154	139,214735	0,060764323	0,143112	0,424594008	0,671132662	0,922645883
ENSG00000089597	GANAB	glucosidase II alpha subunit	23193	1447,450209	0,040699506	0,060667	0,670871307	0,502302517	0,86280677
ENSG00000100749	VRK1	VRK serine/threonine kinase 1	7443	93,12522091	0,456114021	0,178561	2,554386133	0,010637522	0,160359107
ENSG00000113597	TRAPPC13	trafficking protein particle complex subunit 13	80006	56,97702937	0,326798381	0,250466	1,304763242	0,191973479	0,649982152
ENSG00000123545	NDUFAF4	NADH:ubiquinone oxidoreductase complex assembly factor 4	29078	22,0527284	-0,01228145	0,537271	-0,022858949	0,981762786	NA
ENSG00000135341	MAP3K7	mitogen-activated protein kinase kinase kinase 7	6885	134,5746234	0,052785185	0,326233	0,161802064	0,871461728	0,974150353
ENSG00000147457	CHMP7	charged multivesicular body protein 7	91782	72,21883596	0,198505861	0,4023	0,493427654	0,621710447	0,904764752
ENSG00000162959	MEMO1	mediator of cell motility 1	51072	124,0365332	-0,030837659	0,145141	-0,21246718	0,83174258	0,963446071
ENSG00000183597	TANGO2	transport and golgi organization 2 homolog	128989	138,7401555	-0,044308337	0,153353	-0,288929837	0,77263507	0,946409165
ENSG00000132326	PER2	period circadian regulator 2	8864	100,3595375	-0,144293908	0,180388	-0,79990972	0,423763106	0,829156809
ENSG00000164930	FZD6	frizzled class receptor 6	8323	108,4856073	-0,181263384	0,184225	-0,98392518	0,325152302	0,768275024
ENSG00000185420	SMYD3	SET and MYND domain containing 3	64754	34,41304471	0,057785071	0,311781	0,18533892	0,852963213	NA
ENSG00000157954	WIPI2	WD repeat domain, phosphoinositide interacting 2	26100	221,4015658	-0,187083795	0,130946	-1,428706931	0,153088485	0,609793649
ENSG00000215375	MYL5	myosin light chain 5	4636	15,9104996	-0,258728182	0,592308	-0,436813531	0,662246586	NA
ENSG00000179889	PDXDC1	pyridoxal dependent decarboxylase domain containing 1	23042	141,4234687	-0,064292179	0,174557	-0,368316742	0,712637071	0,930846779
ENSG00000087086	FTL	ferritin light chain	2512	704,7696982	0,133645672	0,071422	1,871219283	0,061314693	0,401974624
ENSG00000137714	FDX1	ferredoxin 1	2230	63,10200085	0,087082579	0,218586	0,398389976	0,690342746	0,927252461
ENSG00000144840	RABL3	RAB, member of RAS oncogene family like 3	285282	27,86754888	0,9526744	1,150148	0,828306118	0,407497159	NA
ENSG00000067704	IARS2	isoleucyl-tRNA synthetase 2, mitochondrial	55699	303,8966917	0,08020126	0,112316	0,714071074	0,475183231	0,853811879
ENSG00000071462	BUD23	BUD23 rRNA methyltransferase and ribosome maturation factor	114049	99,74654427	-0,028312572	0,214862	-0,13177083	0,895165559	0,977990819
ENSG00000113583	C5orf15	chromosome 5 open reading frame 15	56951	156,4747122	0,067724709	0,151912	0,445814272	0,655731411	0,918143156
ENSG00000137509	PRCP	prolylcarboxypeptidase	5547	99,22521312	0,422171957	0,193204	2,18511189	0,028880644	0,273947555
ENSG00000205981	DNAJC19	DnaJ heat shock protein family (Hsp40) member C19	131118	40,48540737	0,288475629	0,278257	1,036721898	0,299865483	NA
ENSG00000053770	AP5M1	adaptor related protein complex 5 subunit mu 1	55745	210,6433881	-0,085854769	0,176815	-0,485563306	0,627276818	0,90479929
ENSG00000130347	RTN4IP1	reticulon 4 interacting protein 1	84816	25,73866204	-0,043657973	0,374657	-0,116527972	0,907234119	NA
ENSG00000132003	ZSWIM4	zinc finger SWIM-type containing 4	65249	73,46024276	0,018627898	0,22775	0,081791061	0,934812865	0,987098279
ENSG00000109065	NAT9	N-acetyltransferase 9 (putative)	26151	26,50432842	-0,782405369	0,353855	-2,211089772	0,027029621	NA
ENSG00000141179	PCTP	phosphatidylcholine transfer protein	58488	71,37951065	-0,020766507	0,211668	-0,098108853	0,921845858	0,984284131
ENSG00000177463	NR2C2	nuclear receptor subfamily 2 group C member 2	7182	450,3565203	-0,176381053	0,091589	-1,925785236	0,054131196	0,376569009
ENSG00000259332	ST20-MTHFS	ST20-MTHFS readthrough	100528021	13,26761535	1,015800489	1,131797	0,897511225	0,369446186	NA
ENSG00000145736	GTF2H2	general transcription factor IIH subunit 2	2966	12,55286032	-0,373604772	1,152672	-0,324120651	0,745846693	NA
ENSG00000145736	GTF2H2C	GTF2H2 family member C	728340	12,55286032	-0,373604772	1,152672	-0,324120651	0,745846693	NA
ENSG00000160602	NEK8	NIMA related kinase 8	284086	37,6298956	-0,512599524	0,299276	-1,712797072	0,086749879	NA
ENSG00000196449	YRDC	yrdC N6-threonylcarbamoyltransferase domain containing	79693	51,83423648	0,330772911	0,258497	1,279598503	0,200686377	0,659555076
ENSG00000216937	CCDC7	coiled-coil domain containing 7	79741	15,78279436	-1,065525196	1,050548	-1,014256316	0,310460463	NA
ENSG00000065268	WDR18	WD repeat domain 18	57418	40,66706186	0,021960882	0,304456	0,07213145	0,942497298	NA
ENSG00000125249	RAP2A	RAP2A, member of RAS oncogene family	5911	172,7670059	-0,17395318	0,132728	-1,310594195	0,189994902	0,647812504
ENSG00000167085	PHB1	prohibitin 1	5245	152,7435254	-0,212610666	0,419462	-0,50686519	0,612249412	0,900006614
ENSG00000126231	PROZ	protein Z, vitamin K dependent plasma glycoprotein	8858	21,08955326	-0,025124129	0,3959	-0,063460797	0,949399575	NA
ENSG00000124198	ARFGEF2	ADP ribosylation factor guanine nucleotide exchange factor 2	10564	310,3735472	-0,165256339	0,144988	-1,139790439	0,254373618	0,709962756
ENSG00000005469	CROT	carnitine O-octanoyltransferase	54677	41,24164561	-0,002021454	0,270546	-0,007471768	0,994038448	NA
ENSG00000086619	ERO1B	endoplasmic reticulum oxidoreductase 1 beta	56605	62,22684302	-0,14083172	0,321875	-0,437535971	0,661722695	0,919853106
ENSG00000129646	QRICH2	glutamine rich 2	84074	25,77610553	-0,062630708	0,422561	-0,148217129	0,882171414	NA

ENSG00000144848	ATG3	autophagy related 3	64422	131,0109192	-0,146129901	0,171731	-0,85092178	0,394812805	0,812498986
ENSG00000152207	CYSLTR2	cysteinyl leukotriene receptor 2	57105	113,5763323	-0,091443992	0,152688	-0,59889444	0,549243279	0,88071799
ENSG00000105372	RPS19	ribosomal protein S19	6223	1592,863475	0,16679659	0,086292	1,932932874	0,053244467	0,372379383
ENSG00000130956	HABP4	hyaluronan binding protein 4	22927	56,42388539	-0,003662215	0,266103	-0,013762422	0,989019522	0,997108653
ENSG00000005812	FBXL3	F-box and leucine rich repeat protein 3	26224	222,6938136	-0,166141515	0,113311	-1,466240027	0,142582908	0,587537333
ENSG00000100429	HDAC10	histone deacetylase 10	83933	75,58399884	0,283758284	0,430647	0,658910954	0,509952951	0,866180216
ENSG00000166387	PPFIBP2	PPFIA binding protein 2	8495	16,45363367	-0,464133033	0,448171	-1,03561659	0,300381049	NA
ENSG00000116641	DOCK7	dedicator of cytokinesis 7	85440	22,75555016	0,281467541	0,669859	0,420189324	0,674347153	NA
ENSG00000135931	ARMC9	armadillo repeat containing 9	80210	24,44629284	0,599129904	0,495976	1,207981605	0,227054338	NA
ENSG00000136933	RABEPK	Rab9 effector protein with kelch motifs	10244	30,01625787	-0,053835222	0,336044	-0,160202996	0,872721169	NA
ENSG00000150990	DHX37	DEAH-box helicase 37	57647	116,4655663	-0,36561116	0,182825	-1,999789498	0,045522999	0,340953043
ENSG00000254999	BRK1	BRICK1 subunit of SCAR/WAVE actin nucleating complex	55845	247,1078178	0,10114093	0,109388	0,924611245	0,355168162	0,788341988
ENSG00000264343	NOTCH2NLA	notch 2 N-terminal like A	388677	234,6066648	0,351413292	0,161741	2,172686115	0,02980395	0,279077601
ENSG00000159658	EFCAB14	EF-hand calcium binding domain 14	9813	708,8631949	0,022576053	0,072836	0,309959207	0,756591978	0,942901768
ENSG00000164938	TP53INP1	tumor protein p53 inducible nuclear protein 1	94241	197,5774051	-0,222562604	0,146857	-1,515508329	0,129643723	0,565894171
ENSG00000112941	TENT4A	terminal nucleotidyltransferase 4A	11044	178,5514323	0,185610087	0,125511	1,478830827	0,139185535	0,580974065
ENSG00000136371	MTHFS	methenyltetrahydrofolate synthetase	10588	22,57534156	-0,729303209	0,76415	-0,954397734	0,339882353	NA
ENSG00000149792	MRPL49	mitochondrial ribosomal protein L49	740	124,890779	0,242625946	0,186449	1,301296233	0,193157076	0,650901454
ENSG00000188428	BLOC1S5	biogenesis of lysosomal organelles complex 1 subunit 5	63915	81,02367583	-0,255061144	0,205152	-1,243280988	0,213764308	0,668717948
ENSG00000214776	NA	NA	NA	49,19049398	0,622204555	0,259163	2,40081961	0,016358398	0,202648045
ENSG00000136247	ZDHHC4	zinc finger DHHC-type palmitoyltransferase 4	55146	42,1081438	-0,369285771	0,460582	-0,801781178	0,422679548	NA
ENSG00000239713	APOBEC3G	apolipoprotein B mRNA editing enzyme catalytic subunit 3G	60489	568,9832772	0,15695189	0,079135	1,983353412	0,047327967	0,349139303
ENSG00000134324	LPIN1	lipin 1	23175	466,2380971	-0,204980778	0,097302	-2,106648239	0,035148082	0,299082379
ENSG00000198783	ZNF830	zinc finger protein 830	91603	82,5314847	-0,023919514	0,250017	-0,095671694	0,923781322	0,984284131
ENSG00000115977	AAK1	AP2 associated kinase 1	22848	823,9196501	0,173883467	0,060127	2,891954843	0,00382853	0,083992381
ENSG00000125351	UPF3B	UPF3B regulator of nonsense mediated mRNA decay	65109	76,61280697	-0,130409069	0,207104	-0,629678092	0,528905219	0,872145761
ENSG00000145332	KLHL8	kelch like family member 8	57563	94,80074119	-0,133949309	0,196084	-0,683120494	0,494530706	0,860513824
ENSG00000110717	NDUFS8	NADH:ubiquinone oxidoreductase core subunit S8	4728	87,22304032	0,252572321	0,205112	1,231388306	0,218177669	0,673519155
ENSG00000144283	PKP4	plakophilin 4	8502	37,94817143	-0,453814007	0,409954	-1,106986414	0,268299802	NA
ENSG00000241360	PDXP	pyridoxal phosphatase	57026	45,49776847	0,119952282	0,327427	0,366347816	0,714105553	0,931512238
ENSG00000101974	ATP11C	ATPase phospholipid transporting 11C	286410	158,8045654	0,235770659	0,137155	1,719007575	0,085612993	0,477891497
ENSG00000149925	ALDOA	aldolase, fructose-bisphosphate A	226	877,3434728	0,111807199	0,061062	1,831031556	0,067095833	0,418230694
ENSG00000196950	SLC39A10	solute carrier family 39 member 10	57181	408,1832543	-0,070722083	0,086026	-0,822099801	0,411020099	0,821394592
ENSG00000276234	TADA2A	transcriptional adaptor 2A	6871	42,67886671	0,519356464	0,611403	0,849450719	0,395630541	0,812799303
ENSG00000119912	IDE	insulin degrading enzyme	3416	23,76179036	0,835495945	0,69964	1,194180503	0,232407368	NA
ENSG00000140553	UNC45A	unc-45 myosin chaperone A	55898	176,2889357	-0,02784219	0,147157	-0,189201207	0,849935122	0,967902179
ENSG00000163950	SLBP	stem-loop histone mRNA binding protein	7884	157,8444957	0,074942742	0,153599	0,48791009	0,625613527	0,90479929
ENSG00000277149	TYW1B	tRNA-yW synthesizing protein 1 homolog B	441250	15,77853056	0,132392436	0,455341	0,290754553	0,771239045	NA
ENSG00000014641	MDH1	malate dehydrogenase 1	4190	217,2392454	0,22102654	0,124525	1,774953212	0,075905613	0,446229554
ENSG00000044459	CNTLN	centlein	54875	13,32689463	0,683746358	0,531649	1,286087104	0,198412664	NA
ENSG00000110536	PTPMT1	protein tyrosine phosphatase mitochondrial 1	114971	54,26174926	0,000719753	0,2427	0,002965604	0,997633794	0,999300722
ENSG00000110844	PRPF40B	pre-mRNA processing factor 40 homolog B	25766	52,63748721	-0,172590737	0,250643	-0,688590627	0,491080921	0,860513824
ENSG00000113456	RAD1	RAD1 checkpoint DNA exonuclease	5810	53,62422162	0,377631066	0,328561	1,149348101	0,250412471	0,707335884
ENSG00000120519	SLC10A7	solute carrier family 10 member 7	84068	81,85011534	-0,273433799	0,19968	-1,369357304	0,170887614	0,629558603
ENSG00000125037	EMC3	ER membrane protein complex subunit 3	55831	58,84974203	-0,21985708	0,237256	-0,926667377	0,354099261	0,788228969
ENSG00000147592	LACTB2	lactamase beta 2	51110	26,18796246	-0,063495193	0,349416	-0,181717831	0,855804172	NA
ENSG00000151553	FHIP2A	FHF complex subunit HOOK interacting protein 2A	57700	192,5033115	0,192469791	0,196305	0,980464161	0,326857052	0,769687654
ENSG00000158545	ZC3H18	zinc finger CCCH-type containing 18	124245	345,3093643	-0,178307739	0,108112	-1,649294564	0,099087303	0,509429216
ENSG00000166446	CDYL2	chromodomain Y like 2	124359	164,8381204	0,256911504	0,131155	1,958836759	0,050131907	0,358920692

ENSG00000168071	CCDC88B	coiled-coil domain containing 88B	283234	514,4365285	0,23974033	0,369559	0,648720325	0,516519164	0,868791049
ENSG00000168675	LDLRAD4	low density lipoprotein receptor class A domain containing 4	753	26,89965299	0,024404681	0,388375	0,062837993	0,949895511	NA
ENSG00000170006	TMEM154	transmembrane protein 154	201799	39,8154733	-0,450015989	0,604209	-0,744801829	0,456391529	NA
ENSG00000176024	ZNF613	zinc finger protein 613	79898	36,93229172	-0,224248945	0,307921	-0,728268545	0,466449213	NA
ENSG00000204435	CSNK2B	casein kinase 2 beta	1460	242,6980853	0,230437817	0,110555	2,084381272	0,0371255	0,306351862
ENSG00000214517	PPME1	protein phosphatase methylesterase 1	51400	56,4007422	-0,377991808	0,26758	-1,412633326	0,157763565	0,61428162
ENSG00000173349	SFT2D3	SFT2 domain containing 3	84826	39,64786746	-0,40431323	0,292387	-1,382799317	0,166726408	NA
ENSG00000172197	MBOAT1	membrane bound O-acyltransferase domain containing 1	154141	15,41913365	0,176051084	0,453822	0,387929766	0,69806801	NA
ENSG00000205250	E2F4	E2F transcription factor 4	1874	229,1798427	0,127220017	0,134758	0,944065693	0,345136079	0,781631709
ENSG00000181638	ZFP41	ZFP41 zinc finger protein	286128	54,43827262	0,213094968	0,251895	0,845966415	0,39757148	0,813581659
ENSG00000255185	PDXDC2P	pyridoxal dependent decarboxylase domain containing 2, pseudogene	109731405	52,74197192	0,011005082	0,282654	0,038934887	0,968942302	0,993542794
ENSG00000140398	NEIL1	nei like DNA glycosylase 1	79661	13,06297408	-1,182007232	0,6034	-1,958910838	0,05012323	NA
ENSG00000182504	CEP97	centrosomal protein 97	79598	45,39050778	0,345271456	1,282331	0,269253086	0,787734932	0,95148885
ENSG00000146457	WTAP	WT1 associated protein	9589	382,1752603	0,075790211	0,115973	0,653513416	0,513425343	0,86831237
ENSG00000115956	PLEK	pleckstrin	5341	257,2209062	-0,108694787	0,105659	-1,028734733	0,303604344	0,751870158
ENSG00000133641	RLIG1	RNA 5'-phosphate and 3'-OH ligase 1	91298	40,68501342	0,65121942	0,389826	1,670538694	0,09481283	NA
ENSG00000147168	IL2RG	interleukin 2 receptor subunit gamma	3561	2465,661198	0,074883042	0,036885	2,030187993	0,042337434	0,327927224
ENSG00000135365	PHF21A	PHD finger protein 21A	51317	217,8551052	0,147227169	0,153937	0,956411806	0,338864223	0,777431905
ENSG00000141506	PIK3R5	phosphoinositide-3-kinase regulatory subunit 5	23533	670,1270616	-0,098740354	0,078763	-1,253645794	0,209970779	0,664554962
ENSG00000135127	BICDL1	BICD family like cargo adaptor 1	92558	60,42541293	0,378307195	0,218856	1,728563486	0,083887249	0,471737097
ENSG00000157601	MX1	MX dynamin like GTPase 1	4599	36,30697611	-0,278384285	0,412049	-0,675610292	0,499288105	NA
ENSG00000136738	STAM	signal transducing adaptor molecule	8027	244,509839	-0,039103102	0,105914	-0,369198114	0,711980062	0,930846779
ENSG00000090061	CCNK	cyclin K	8812	247,3903067	-0,383836313	0,160889	-2,385720522	0,017045699	0,204616219
ENSG00000137807	KIF23	kinesin family member 23	9493	89,58166579	0,56115837	0,224615	2,498317602	0,012478434	0,175323113
ENSG00000151611	MMAA	metabolism of cobalamin associated A	166785	21,3128335	0,21634486	0,624267	0,346558307	0,728923173	NA
ENSG00000151611	LINC02491	long intergenic non-protein coding RNA 2491	105377467	21,3128335	0,21634486	0,624267	0,346558307	0,728923173	NA
ENSG00000084207	GSTP1	glutathione S-transferase pi 1	2950	686,0044319	0,155187083	0,07418	2,092036448	0,036435255	0,303459699
ENSG00000105875	WDR91	WD repeat domain 91	29062	35,47617655	-0,245134407	0,446102	-0,549503615	0,582659885	NA
ENSG00000110442	COMMD9	COMM domain containing 9	29099	72,07727428	0,607113337	0,276523	2,195529063	0,028125668	0,270084577
ENSG00000126368	NR1D1	nuclear receptor subfamily 1 group D member 1	9572	13,79215599	0,122431844	0,495493	0,247091085	0,804837733	NA
ENSG00000131495	NDUFA2	NADH:ubiquinone oxidoreductase subunit A2	4695	46,94571901	0,298465602	0,252266	1,183137514	0,236754652	0,691517131
ENSG00000138175	ARL3	ADP ribosylation factor like GTPase 3	403	25,93738559	0,428628988	0,377627	1,135058523	0,256350799	NA
ENSG00000173875	ZNF791	zinc finger protein 791	163049	265,9791781	0,026430746	0,110268	0,23969496	0,810566742	0,959038743
ENSG00000175581	MRPL48	mitochondrial ribosomal protein L48	51642	45,66510335	0,333646917	0,277058	1,20424917	0,228493287	0,68351286
ENSG00000261796	ISY1-RAB43	ISY1-RAB43 readthrough	100534599	212,4228781	-0,287001875	1,329335	-0,215898755	0,829066667	0,962723654
ENSG00000081154	PCNP	PEST proteolytic signal containing nuclear protein	57092	275,6287267	0,305543872	0,152649	2,001610743	0,045326613	0,340953043
ENSG00000134330	IAH1	isoamyl acetate hydrolyzing esterase 1 (putative)	285148	63,72398651	-0,280405128	0,492785	-0,569021573	0,5693415	0,886263374
ENSG00000160563	MED27	mediator complex subunit 27	9442	62,66238518	0,111402701	0,260634	0,427429665	0,669066404	0,922641254
ENSG00000105397	TYK2	tyrosine kinase 2	7297	87,28008026	0,003016904	2,12651	0,001418711	0,998868033	0,999631693
ENSG00000178028	DMAP1	DNA methyltransferase 1 associated protein 1	55929	58,64334404	-0,812086552	0,299285	-2,713420599	0,006659252	0,118759094
ENSG00000105483	CARD8	caspase recruitment domain family member 8	22900	330,5592152	-0,044770251	0,159005	-0,281564233	0,778277669	0,947109268
ENSG00000057608	GDI2	GDP dissociation inhibitor 2	2665	695,886355	0,062582269	0,064432	0,971286286	0,331405736	0,772473234
ENSG00000152332	UHMK1	U2AF homology motif kinase 1	127933	1021,397428	-0,156414164	0,085266	-1,834415365	0,066592361	0,416786686
ENSG00000100418	DESI1	desumoylating isopeptidase 1	27351	111,9425752	-0,042405057	0,164006	-0,258557659	0,795976555	0,953436042
ENSG00000104129	DNAJC17	DnaJ heat shock protein family (Hsp40) member C17	55192	45,56345109	-0,344325569	0,5076	-0,678340075	0,497556095	0,861283987
ENSG00000138069	RAB1A	RAB1A, member RAS oncogene family	5861	227,6884079	-0,279409573	0,124706	-2,240546785	0,025055447	0,254244807
ENSG00000144485	HES6	hes family bHLH transcription factor 6	55502	12,71592214	-0,004555201	0,497652	-0,00915339	0,992696754	NA
ENSG00000155275	TRMT44	tRNA methyltransferase 44 homolog	152992	41,17815297	-0,138471109	0,274193	-0,505013273	0,613549514	NA
ENSG00000175482	POLD4	DNA polymerase delta 4, accessory subunit	57804	43,69600074	0,198759568	0,419904	0,473345201	0,635966918	0,909036096

ENSG00000257093	DENND11	DENN domain containing 11	57189	14,0881747	3,124641268	3,782425	0,826094724	0,40875036	NA
ENSG00000116095	PLEKHA3	pleckstrin homology domain containing A3	65977	95,80065707	-0,10321636	0,195184	-0,528814533	0,596934115	0,894257182
ENSG00000119408	NEK6	NIMA related kinase 6	10783	434,6733015	0,092612268	0,089135	1,039009465	0,298800335	0,749216326
ENSG00000145782	ATG12	autophagy related 12	9140	146,8784189	0,185792378	0,20569	0,903264004	0,366385796	0,79459056
ENSG00000083812	ZNF324	zinc finger protein 324	25799	58,7152438	0,117915413	0,290589	0,405780464	0,684903914	0,92575277
ENSG00000092148	HECTD1	HECT domain E3 ubiquitin protein ligase 1	25831	546,5386289	-0,062267557	0,099634	-0,624960224	0,531997164	0,872967783
ENSG00000100714	MTHFD1	methylenetetrahydrofolate dehydrogenase, cyclohydrolase and formyltetrahydrofolate synthetase 1	4522	288,2606226	0,201272582	0,112653	1,78665836	0,073992723	0,443476943
ENSG00000102977	ACD	ACD shelterin complex subunit and telomerase recruitment factor	65057	36,68074494	-0,223414842	0,997216	-0,224038659	0,822727222	NA
ENSG00000105835	NAMPT	nicotinamide phosphoribosyltransferase	10135	84,58115663	-0,561776533	0,323248	-1,737913767	0,082226009	0,466620721
ENSG00000109133	TMEM33	transmembrane protein 33	55161	211,5805089	-0,055809988	0,220775	-0,252791145	0,800429611	0,955261231
ENSG00000125651	GTF2F1	general transcription factor IIF subunit 1	2962	253,3368416	-0,020221462	0,120568	-0,167718884	0,86680444	0,971807194
ENSG00000129968	ABHD17A	abhydrolase domain containing 17A, depalmitoylase	81926	250,2725232	-0,101535277	0,114975	-0,883108831	0,377177473	0,803537947
ENSG00000174944	P2RY14	purinergic receptor P2Y14	9934	25,84831366	0,073663453	0,365814	0,201368377	0,840410541	NA
ENSG00000178449	COX14	cytochrome c oxidase assembly factor COX14	84987	32,31738487	0,380874293	0,376814	1,01077628	0,312123519	NA
ENSG00000136628	EPRS1	glutamyl-prolyl-tRNA synthetase 1	2058	814,0557864	0,095412208	0,06275	1,520508843	0,128383138	0,563689485
ENSG00000139641	ESYT1	extended synaptotagmin 1	23344	1425,184383	-0,034056994	0,050518	-0,674161078	0,500208912	0,86161226
ENSG00000017483	SLC38A5	solute carrier family 38 member 5	92745	93,02750926	-0,201063694	0,267306	-0,752185548	0,451939484	0,842919189
ENSG00000122085	MTERF4	mitochondrial transcription termination factor 4	130916	88,1955344	-0,100157717	0,202224	-0,495280893	0,620401856	0,904349699
ENSG00000115946	PNO1	partner of NOB1 homolog	56902	57,76081419	0,289949389	0,222689	1,302037596	0,192903534	0,650901454
ENSG00000163635	ATXN7	ataxin 7	6314	337,441955	0,024848318	0,132597	0,187397308	0,851349133	0,967902179
ENSG00000169629	RGPD8	RANBP2 like and GRIP domain containing 8	727851	171,7088706	0,007373472	0,157402	0,0468448	0,962636923	0,992328441
ENSG00000139613	SMARCC2	SWI/SNF related, matrix associated, actin dependent regulator of chromatin subfamily c member 2	6601	777,785718	-0,037607943	0,073925	-0,508728479	0,610942558	0,899995226
ENSG00000171469	ZNF561	zinc finger protein 561	93134	99,59563088	0,131027634	0,19694	0,66531585	0,505848495	0,864630986
ENSG00000164405	UQCRQ	ubiquinol-cytochrome c reductase complex III subunit VII	27089	59,85039003	0,049316124	0,252323	0,195448261	0,845042038	0,967231052
ENSG00000172336	POP7	POP7 homolog, ribonuclease P/MRP subunit	10248	32,35912011	0,270340754	0,367949	0,734722586	0,462508462	NA
ENSG00000186184	POLR1D	RNA polymerase I and III subunit D	51082	221,0412739	-0,035261855	0,131058	-0,269055012	0,787887349	0,95148885
ENSG00000254206	NPIPB11	nuclear pore complex interacting protein family member B11	728888	82,81748187	-0,089555147	0,2788	-0,321216542	0,748046297	0,939899419
ENSG00000168995	SIGLEC7	sialic acid binding Ig like lectin 7	27036	23,62085677	-0,543210417	0,363153	-1,49581814	0,134701053	NA
ENSG00000019485	PRDM11	PR/SET domain 11	56981	138,1617583	0,07585419	0,171126	0,44326431	0,657574568	0,919088619
ENSG00000095321	CRAT	carnitine O-acetyltransferase	1384	49,57723852	-0,134882316	0,2842	-0,474603002	0,635069966	0,908862885
ENSG00000213160	KLHL23	kelch like family member 23	151230	144,696641	-0,231572823	0,151681	-1,526712154	0,126832604	0,563689485
ENSG00000213160	PHOSPHO2-KLHL23	PHOSPHO2-KLHL23 readthrough	100526832	144,696641	-0,231572823	0,151681	-1,526712154	0,126832604	0,563689485
ENSG00000136938	ANP32B	acidic nuclear phosphoprotein 32 family member B	10541	453,7298295	0,163721644	0,087531	1,870436962	0,061423163	0,402334407
ENSG00000271699	NA	NA	NA	18,05740907	-0,295929172	0,42844	-0,690713876	0,489745367	NA
ENSG00000156398	SFXN2	sideroflexin 2	118980	32,04097188	-0,200470743	0,357612	-0,560581452	0,575082899	NA
ENSG00000161956	SENP3	SUMO specific peptidase 3	26168	153,4770254	0,071962015	0,146747	0,490382555	0,623863219	0,90479929
ENSG00000164080	RAD54L2	RAD54 like 2	23132	310,2535586	-0,015812471	0,094071	-0,168091002	0,866511688	0,971722599
ENSG00000166333	ILK	integrin linked kinase	3611	480,9688377	-0,100721327	0,085348	-1,180130982	0,237948124	0,693191605
ENSG00000149591	TAGLN	transgelin	6876	12,55138537	-1,478231127	0,698586	-2,116034209	0,034341898	NA
ENSG00000127415	IDUA	alpha-L-iduronidase	3425	22,99568245	-1,416345873	1,637057	-0,865178122	0,386941032	NA
ENSG00000151445	VIPAS39	VPS33B interacting protein, apical-basolateral polarity regulator, spe-39 homolog	63894	83,4215471	-0,194945288	0,20449	-0,953325474	0,340425188	0,777431905
ENSG00000156639	ZFAND3	zinc finger AN1-type containing 3	60685	236,9208086	-0,020294197	0,107181	-0,189344473	0,849822841	0,967902179
ENSG00000104626	ERI1	exoribonuclease 1	90459	189,0275398	0,08081837	0,143516	0,56312989	0,57334644	0,886894319
ENSG00000064393	HIPK2	homeodomain interacting protein kinase 2	28996	1165,512467	0,019079348	0,081833	0,233149484	0,815645331	0,959707099
ENSG00000112062	MAPK14	mitogen-activated protein kinase 14	1432	311,76104	0,211355815	0,121045	1,746086163	0,080795981	0,4605948

ENSG00000127184	COX7C	cytochrome c oxidase subunit 7C	1350	226,241751	0,146496259	0,174945	0,837385822	0,402375732	0,816750249
ENSG00000148334	PTGES2	prostaglandin E synthase 2	80142	69,8547685	-0,085716468	0,206775	-0,414539595	0,678478976	0,925294441
ENSG00000197594	ENPP1	ectonucleotide pyrophosphatase/phosphodiesterase 1	5167	28,92343361	-0,183801417	0,319942	-0,574482925	0,565641052	NA
ENSG00000004455	AK2	adenylate kinase 2	204	279,2275969	0,101407277	0,140481	0,721858354	0,470381568	0,852690218
ENSG00000119446	RBM18	RNA binding motif protein 18	92400	116,9971751	0,087198464	0,154247	0,565316961	0,571858195	0,886795904
ENSG00000149679	CABLES2	Cdk5 and Abl enzyme substrate 2	81928	79,51039288	0,033557917	0,207293	0,161886209	0,871395463	0,974150353
ENSG00000028116	VRK2	VRK serine/threonine kinase 2	7444	100,6617194	0,15306719	0,227072	0,674089815	0,500254214	0,86161226
ENSG00000144455	SUMF1	sulfatase modifying factor 1	285362	50,4317701	0,166988404	0,239292	0,69784388	0,485274834	0,857915459
ENSG00000144455	LOC100130207	uncharacterized LOC100130207	100130207	50,4317701	0,166988404	0,239292	0,69784388	0,485274834	0,857915459
ENSG00000143633	C1orf131	chromosome 1 open reading frame 131	128061	56,7250111	0,017494747	0,229172	0,076338938	0,939149448	0,987813564
ENSG00000100196	KDELR3	KDEL endoplasmic reticulum protein retention receptor 3	11015	16,69383056	-0,351423753	0,456657	-0,769556926	0,441562765	NA
ENSG00000169814	BTD	biotinidase	686	107,6775335	0,43535383	0,342682	1,270432426	0,203930639	0,660954946
ENSG00000134014	ELP3	elongator acetyltransferase complex subunit 3	55140	180,718251	-0,08058464	0,261648	-0,307989288	0,758090484	0,942901768
ENSG00000178381	ZFAND2A	zinc finger AN1-type containing 2A	90637	26,99640326	-0,310829234	0,363525	-0,855042063	0,392527862	NA
ENSG00000168393	DTYMK	deoxythymidylate kinase	1841	40,90050772	0,498338914	0,276211	1,804197717	0,071200317	NA
ENSG00000196151	WDSUB1	WD repeat, sterile alpha motif and U-box domain containing 1	151525	92,29852503	0,036359401	0,170458	0,213304424	0,831089523	0,963156386
ENSG00000205629	LCMT1	leucine carboxyl methyltransferase 1	51451	62,55536177	0,291451488	0,240861	1,210037736	0,226264413	0,681621061
ENSG00000240230	COX19	cytochrome c oxidase assembly factor COX19	90639	128,4357621	0,151961221	0,158909	0,95627954	0,338931024	0,777431905
ENSG00000128915	ICE2	interactor of little elongation complex ELL subunit 2	79664	78,65935564	0,543465107	0,3757	1,446541226	0,148025458	0,601032765
ENSG00000248124	NA	NA	NA	65,86217609	-0,071001982	0,227999	-0,311414009	0,755485905	0,942901768
ENSG00000284906	NA	NA	NA	55,77416385	0,32707756	0,230892	1,416580643	0,156605571	0,61428162
ENSG00000126216	TUBGCP3	tubulin gamma complex component 3	10426	140,8438374	0,129517491	0,154783	0,83676902	0,402722414	0,816750249
ENSG00000132879	FBXO44	F-box protein 44	93611	277,7722471	-0,061347517	0,110088	-0,557259253	0,577350311	0,887069818
ENSG00000143748	NVL	nuclear VCP like	4931	132,6579972	-0,029309033	0,183391	-0,159816909	0,873025304	0,974533542
ENSG00000149547	EI24	EI24 autophagy associated transmembrane protein	9538	134,7870181	-0,171859907	0,153912	-1,1166093	0,264161414	0,719682465
ENSG00000160271	RALGDS	ral guanine nucleotide dissociation stimulator	5900	270,6028871	-0,171020146	0,115612	-1,47926368	0,139069856	0,580974065
ENSG00000102606	ARHGEF7	Rho guanine nucleotide exchange factor 7	8874	405,5599358	0,065426149	0,086582	0,755658831	0,449853773	0,84263906
ENSG00000080815	PSEN1	presenilin 1	5663	213,51902	0,080929947	0,214792	0,376783428	0,706334557	0,930846779
ENSG00000168291	PDHB	pyruvate dehydrogenase E1 subunit beta	5162	180,3224483	0,160299166	0,128678	1,245738332	0,212860476	0,667215123
ENSG00000153827	TRIP12	thyroid hormone receptor interactor 12	9320	1349,059488	0,072123823	0,059096	1,220443535	0,222296782	0,678553801
ENSG00000065361	ERBB3	erb-b2 receptor tyrosine kinase 3	2065	14,1472861	-0,159288306	0,848812	-0,18766036	0,851142906	NA
ENSG00000107021	TBC1D13	TBC1 domain family member 13	54662	179,6808566	0,002861427	0,142675	0,020055546	0,983999062	0,996185513
ENSG00000113282	CLINT1	clathrin interactor 1	9685	291,9351592	0,044830528	0,10995	0,407737362	0,683466505	0,925586358
ENSG00000166166	TRMT61A	tRNA methyltransferase 61A	115708	25,70102072	0,021890039	0,390681	0,056030488	0,955317519	NA
ENSG00000101158	NELFCD	negative elongation factor complex member C/D	51497	162,0118452	-0,031930499	0,139411	-0,229038881	0,818838697	0,961067311
ENSG00000137207	YIPF3	Yip1 domain family member 3	25844	137,6688088	0,021644568	0,144729	0,149552833	0,881117423	0,976300778
ENSG00000006025	OSBPL7	oxysterol binding protein like 7	114881	101,1998309	-0,125304466	0,197411	-0,634740319	0,525597793	0,871384275
ENSG00000127616	SMARCA4	SWI/SNF related, matrix associated, actin dependent regulator of chromatin, subfamily a, member 4	6597	328,1926407	-0,064437752	0,129175	-0,498840539	0,617891726	0,902245041
ENSG00000166289	PLEKHF1	pleckstrin homology and FYVE domain containing 1	79156	149,6673749	0,060200986	0,134512	0,447552578	0,654476134	0,917490396
ENSG00000137831	UACA	uveal autoantigen with coiled-coil domains and ankyrin repeats	55075	34,66073062	0,20327262	0,325158	0,625149442	0,531872981	NA
ENSG00000011376	LARS2	leucyl-tRNA synthetase 2, mitochondrial	23395	132,8863232	-0,113599584	0,189397	-0,599795904	0,548642264	0,880168143
ENSG00000099968	BCL2L13	BCL2 like 13	23786	102,7253382	-0,077478631	0,23473	-0,330075596	0,741342842	0,939193793
ENSG00000102743	SLC25A15	solute carrier family 25 member 15	10166	50,86038721	-0,010255627	0,251483	-0,040780547	0,967470848	0,993270071
ENSG00000103351	CLUAP1	clusterin associated protein 1	23059	36,36502104	-0,091147934	0,44942	-0,202812371	0,839281688	NA

ENSG00000111725	PRKAB1	protein kinase AMP-activated non-catalytic subunit beta 1	5564	81,70430302	0,273065446	0,327038	0,834966148	0,403736769	0,817992511
ENSG00000132294	EFR3A	EFR3 homolog A	23167	464,6669227	0,028269746	0,112982	0,250213921	0,80242192	0,956331071
ENSG00000136068	FLNB	filamin B	2317	181,7449502	-0,196040927	0,149697	-1,309582653	0,19033706	0,647836333
ENSG00000141564	RPTOR	regulatory associated protein of MTOR complex 1	57521	237,5434912	-0,249066795	0,113154	-2,201131297	0,02772673	0,269631574
ENSG00000141576	RNF157	ring finger protein 157	114804	620,7450549	0,049084139	0,065644	0,747735424	0,45461976	0,843234809
ENSG00000149532	CPSF7	cleavage and polyadenylation specific factor 7	79869	461,4544549	-0,02433639	0,094961	-0,256276755	0,797737145	0,95354564
ENSG00000149582	TMEM25	transmembrane protein 25	84866	18,05766136	0,348746169	0,437562	0,797021508	0,42543854	NA
ENSG00000160633	SAFB	scaffold attachment factor B	6294	413,7785844	-0,17342719	0,108432	-1,599408672	0,109729827	0,529522903
ENSG00000162517	PEF1	penta-EF-hand domain containing 1	553115	141,7666969	-0,005575873	0,142112	-0,03923585	0,968702352	0,993542794
ENSG00000163468	CCT3	chaperonin containing TCP1 subunit 3	7203	459,6831454	0,176860529	0,081688	2,16506475	0,030382727	0,279077601
ENSG00000169733	RFNG	RFNG O-fucosylpeptide 3-beta-N-acetylglucosaminyltransferase	5986	28,35152724	-0,796163908	0,722095	-1,102574937	0,270211799	NA
ENSG00000173264	GPR137	G protein-coupled receptor 137	56834	43,74806718	-0,062842021	0,313465	-0,200475444	0,841108761	0,966505956
ENSG00000178177	LCORL	ligand dependent nuclear receptor corepressor like	254251	82,26815286	-0,160909094	0,385512	-0,417391047	0,676392402	0,924186156
ENSG00000197119	SLC25A29	solute carrier family 25 member 29	123096	89,69592703	-0,235186109	0,210793	-1,115720479	0,264541802	0,720266173
ENSG00000136111	TBC1D4	TBC1 domain family member 4	9882	280,8866083	0,038709576	0,099998	0,387103164	0,698679835	0,930783276
ENSG00000148444	COMMD3	COMM domain containing 3	23412	73,07931008	0,016504091	0,204376	0,080753508	0,935637983	0,987098279
ENSG00000145908	ZNF300	zinc finger protein 300	91975	14,71446108	-0,295183412	0,519769	-0,567912671	0,570094268	NA
ENSG00000135297	MTO1	mitochondrial tRNA translation optimization 1	25821	60,80514935	0,149747747	0,519825	0,288073493	0,773290483	0,946448613
ENSG00000159259	CHAF1B	chromatin assembly factor 1 subunit B	8208	49,06936605	0,06498858	0,242885	0,267569451	0,789030748	0,95148885
ENSG00000105676	ARMC6	armadillo repeat containing 6	93436	71,91841906	-0,063356913	0,195798	-0,323582321	0,746254274	0,939843468
ENSG00000143157	POGK	pogo transposable element derived with KRAB domain	57645	358,0456004	-0,020467887	0,087167	-0,234813385	0,814353578	0,959207071
ENSG00000173614	NMNAT1	nicotinamide nucleotide adenylyltransferase 1	64802	30,27260016	-0,96895506	0,446476	-2,170229212	0,029989486	NA
ENSG00000005189	REXO5	RNA exonuclease 5	81691	16,76604923	0,317113975	0,721378	0,439594757	0,660230639	NA
ENSG00000064601	CTSA	cathepsin A	5476	531,5785113	0,07380088	0,09846	0,749549806	0,453525891	0,843234809
ENSG00000116731	PRDM2	PR/SET domain 2	7799	619,889834	-0,053710111	0,081531	-0,658772298	0,510041999	0,866180216
ENSG00000155959	VBP1	VHL binding protein 1	7411	119,4198584	-0,060862961	0,160601	-0,378969765	0,704710316	0,930846779
ENSG00000160072	ATAD3B	ATPase family AAA domain containing 3B	83858	27,70038773	-0,073956143	0,461382	-0,160292753	0,872650467	NA
ENSG00000168003	SLC3A2	solute carrier family 3 member 2	6520	317,5483587	-0,017718161	0,123128	-0,143900058	0,88557939	0,977697572
ENSG00000206341	NA	NA	NA	14,36397587	-0,112127144	0,490781	-0,228466846	0,819283327	NA
ENSG00000105677	TMEM147	transmembrane protein 147	10430	58,24463132	0,132462317	0,224701	0,589505683	0,555522101	0,882854594
ENSG00000120690	ELF1	E74 like ETS transcription factor 1	1997	758,1945395	-0,050086969	0,095802	-0,522817948	0,601100948	0,896174421
ENSG00000136527	TRA2B	transformer 2 beta homolog	6434	93,37993954	0,000301717	0,281257	0,001072747	0,999144072	0,99965319
ENSG00000158796	DEDD	death effector domain containing	9191	104,314407	-0,160588975	0,214577	-0,74839748	0,454220442	0,843234809
ENSG00000111224	PARP11	poly(ADP-ribose) polymerase family member 11	57097	74,7910205	0,90697696	1,242884	0,729735662	0,465551778	0,84908342
ENSG00000140992	PDPK1	3-phosphoinositide dependent protein kinase 1	5170	458,9464005	0,142592468	0,092467	1,542083631	0,123053272	0,558690088
ENSG00000120913	PDLIM2	PDZ and LIM domain 2	64236	173,0723175	-0,20096663	0,128751	-1,560888275	0,118550114	0,549966094
ENSG00000129317	PUS7L	pseudouridine synthase 7 like	83448	261,0115831	0,218911253	0,126283	1,733501581	0,083006557	0,469016904
ENSG00000161395	PGAP3	post-GPI attachment to proteins phospholipase 3	93210	45,78435147	-0,02531216	0,334651	-0,075637516	0,939707488	0,987813564
ENSG00000129083	COPB1	COPI coat complex subunit beta 1	1315	613,0111424	-0,057571461	0,069928	-0,823300277	0,410337256	0,82130194
ENSG00000096717	SIRT1	sirtuin 1	23411	108,1286801	-0,096117566	0,182784	-0,525853146	0,598990245	0,89463553
ENSG00000174446	SNAPC5	small nuclear RNA activating complex polypeptide 5	10302	41,38849619	-0,255046222	0,279007	-0,91411973	0,360653936	NA
ENSG00000182150	ERCC6L2	ERCC excision repair 6 like 2	375748	177,7360731	0,106641209	0,142856	0,746495069	0,455368409	0,843704526
ENSG00000189180	ZNF33A	zinc finger protein 33A	7581	285,5344053	-0,096272243	0,100188	-0,960915984	0,336594413	0,776552413
ENSG00000132612	VPS4A	vacuolar protein sorting 4 homolog A	27183	19,38240794	-0,37819239	1,346711	-0,28082673	0,7788433	NA
ENSG00000135823	STX6	syntaxin 6	10228	187,4952931	-0,115027557	0,129249	-0,889969499	0,373482264	0,800776142
ENSG00000137338	PGBD1	piggyBac transposable element derived 1	84547	12,17327968	0,24081919	0,512562	0,46983464	0,638473164	NA
ENSG00000143106	PSMA5	proteasome 20S subunit alpha 5	5686	148,0954126	-0,101355969	0,572849	-0,176933118	0,859560924	0,970109427
ENSG00000110888	CAPRIN2	caprin family member 2	65981	39,09928391	-0,689598348	1,121565	-0,614853835	0,538651253	NA

ENSG00000189043	NDUFA4	NDUFA4 mitochondrial complex associated	4697	158,8763421	0,318060519	0,158081	2,012013739	0,044218489	0,336849671
ENSG00000186260	MRTFB	myocardin related transcription factor B	57496	31,66000536	0,316397275	0,474622	0,666629768	0,50500865	NA
ENSG00000231389	HLA-DPA1	major histocompatibility complex, class II, DP alpha 1	3113	162,8024438	0,234388052	0,178184	1,315423866	0,188367489	0,646436727
ENSG00000137210	TMEM14B	transmembrane protein 14B	81853	96,18540907	0,113736239	0,180401	0,630464519	0,52839071	0,872142129
ENSG00000095383	TBC1D2	TBC1 domain family member 2	55357	24,11658723	-0,034867583	0,362275	-0,096246121	0,923325101	NA
ENSG00000178467	P4HTM	prolyl 4-hydroxylase, transmembrane	54681	19,39921794	0,121994113	0,582826	0,209314913	0,834202411	NA
ENSG00000110711	AIP	aryl hydrocarbon receptor interacting protein	9049	197,0048004	-0,090288785	0,138983	-0,649640893	0,515924212	0,868791049
ENSG00000151726	ACSL1	acyl-CoA synthetase long chain family member 1	2180	122,995848	0,04448856	0,169762	0,262064363	0,793271821	0,952276252
ENSG00000004866	ST7	suppression of tumorigenicity 7	7982	44,69069484	0,348704027	0,257346	1,355002808	0,175416663	0,632039836
ENSG00000004866	ST7-OT3	ST7 overlapping transcript 3	93655	44,69069484	0,348704027	0,257346	1,355002808	0,175416663	0,632039836
ENSG00000048649	RSF1	remodeling and spacing factor 1	51773	339,0468035	0,03380517	0,119499	0,282891613	0,777259924	0,947109268
ENSG00000071242	RPS6KA2	ribosomal protein S6 kinase A2	6196	107,9533914	0,035971159	0,187808	0,1915318	0,848108969	0,967472454
ENSG00000107796	ACTA2	actin alpha 2, smooth muscle	59	19,87571023	0,414085284	0,477364	0,867441429	0,38570019	NA
ENSG00000109775	UFSP2	UFM1 specific peptidase 2	55325	46,64938804	0,112027548	0,303106	0,369598442	0,711681712	0,930846779
ENSG00000123297	TSFM	Ts translation elongation factor, mitochondrial	10102	43,27194275	-0,596896954	0,416515	-1,433073418	0,151836857	0,608564843
ENSG00000127952	STYXL1	serine/threonine/tyrosine interacting like 1	51657	56,88715281	0,119285457	0,283943	0,420103772	0,674409647	0,923602509
ENSG00000132423	COQ3	coenzyme Q3, methyltransferase	51805	12,32332307	0,219482874	0,5081	0,43196825	0,665764489	NA
ENSG00000139746	RBM26	RNA binding motif protein 26	64062	397,37143	-0,096765458	0,105943	-0,91337533	0,361045176	0,791408368
ENSG00000166347	CYB5A	cytochrome b5 type A	1528	17,84498398	0,368513576	0,575567	0,640261712	0,522002468	NA
ENSG00000177548	RABEP2	rabaptin, RAB GTPase binding effector protein 2	79874	47,89595826	0,021301166	0,263808	0,080744874	0,935644849	0,987098279
ENSG00000204822	MRPL53	mitochondrial ribosomal protein L53	116540	49,17208078	0,219870713	0,261518	0,840748567	0,400488807	0,814673014
ENSG00000083817	ZNF416	zinc finger protein 416	55659	27,20195946	0,017609313	0,344833	0,051066182	0,959272784	NA
ENSG00000140740	UQCRC2	ubiquinol-cytochrome c reductase core protein 2	7385	297,3746843	0,274864266	0,10957	2,508578126	0,012121816	0,173414831
ENSG00000108395	TRIM37	tripartite motif containing 37	4591	104,6966838	0,026294049	0,204991	0,128269575	0,897935642	0,977990819
ENSG00000164062	APEH	acylaminoacyl-peptide hydrolase	327	261,8581595	0,008147081	0,112055	0,07270577	0,942040257	0,988475138
ENSG00000079263	SP140	SP140 nuclear body protein	11262	263,8296346	0,05641385	0,116918	0,482509076	0,629444352	0,905567722
ENSG00000099977	DDT	D-dopachrome tautomerase	1652	50,46205872	0,14420537	0,271754	0,530645773	0,59566427	0,894229873
ENSG00000099977	DDTL	D-dopachrome tautomerase like	100037417	50,46205872	0,14420537	0,271754	0,530645773	0,59566427	0,894229873
ENSG00000196967	ZNF585A	zinc finger protein 585A	199704	65,82204367	0,188959917	0,30679	0,615926668	0,53794292	0,874923109
ENSG00000090266	NDUFB2	NADH:ubiquinone oxidoreductase subunit B2	4708	85,19392869	0,079855778	0,200182	0,398915505	0,689955464	0,927252461
ENSG00000116205	TCEANC2	transcription elongation factor A N-terminal and central domain containing 2	127428	118,982515	-0,119391206	0,157463	-0,758216317	0,448321494	0,84263906
ENSG00000156411	ATP5MJ	ATP synthase membrane subunit j	9556	63,84952584	0,092291735	0,209866	0,439764621	0,660107594	0,919368176
ENSG00000205571	SMN1	survival of motor neuron 1, telomeric	6606	56,13472638	0,151263517	1,294023	0,11689402	0,906944037	0,978585515
ENSG00000205571	SMN2	survival of motor neuron 2, centromeric	6607	56,13472638	0,151263517	1,294023	0,11689402	0,906944037	0,978585515
ENSG00000125912	NCLN	nicalin	56926	154,0770149	0,055355809	0,150498	0,367817666	0,713009196	0,930846779
ENSG00000081985	IL12RB2	interleukin 12 receptor subunit beta 2	3595	725,8105263	-0,334043542	0,089588	-3,728677461	0,000192487	0,01048604
ENSG00000115828	QPCT	glutaminyl-peptide cyclotransferase	25797	31,46733967	0,405443012	0,337596	1,200969998	0,229762839	NA
ENSG00000175265	GOLGA8A	golgin A8 family member A	23015	115,8520551	0,193411716	0,685757	0,282041198	0,777911921	0,947109268
ENSG00000083642	PDS5B	PDS5 cohesin associated factor B	23047	215,2585945	0,091649907	0,165295	0,554461964	0,579262729	0,887537774
ENSG00000115464	USP34	ubiquitin specific peptidase 34	9736	1349,441115	0,054259466	0,081679	0,664301668	0,506497254	0,864738156
ENSG00000115128	SF3B6	splicing factor 3b subunit 6	51639	122,1290462	0,415174831	0,167977	2,47161054	0,013450596	0,18084297
ENSG00000123992	DNPEP	aspartyl aminopeptidase	23549	77,51207365	-0,424111173	0,38752	-1,094423336	0,273769354	0,726913977
ENSG00000184056	VPS33B	VPS33B late endosome and lysosome associated	26276	36,60933472	-0,003013984	1,10557	-0,002726181	0,997824825	NA
ENSG00000100104	SRRD	SRR1 domain containing	402055	93,33035398	0,050864957	0,191565	0,265522866	0,790606697	0,95167209
ENSG00000130827	PLXNA3	plexin A3	55558	341,6562956	-0,169091527	0,090934	-1,859490007	0,062957714	0,404317327
ENSG00000177971	IMP3	IMP U3 small nucleolar ribonucleoprotein 3	55272	111,6611227	0,006125468	0,186019	0,032929253	0,973731005	0,994534085
ENSG00000184205	TSPYL2	TSPY like 2	64061	88,46864775	-0,314180868	0,24422	-1,28646408	0,198281147	0,656945827

ENSG00000071073	MGAT4A	alpha-1,3-mannosyl-glycoprotein 4-beta-N-acetylglucosaminyltransferase A	11320	509,5496219	0,269258458	0,113569	2,370870451	0,01774625	0,20802843
ENSG00000137038	DMAC1	distal membrane arm assembly component 1	90871	50,8033677	0,199610104	0,246573	0,809538944	0,418205212	0,82446781
ENSG00000146085	MMUT	methylmalonyl-CoA mutase	4594	87,75440477	0,108885224	0,196257	0,554810694	0,579024151	0,887537774
ENSG00000176401	EID2B	EP300 interacting inhibitor of differentiation 2B	126272	11,69718907	-0,436759181	0,597835	-0,730567594	0,465043312	NA
ENSG00000165275	TRMT10B	tRNA methyltransferase 10B	158234	42,56204529	-0,189815447	0,314306	-0,603919083	0,545897441	0,878739902
ENSG00000184939	ZFP90	ZFP90 zinc finger protein	146198	135,0833284	-0,487762066	0,198339	-2,459230098	0,013923535	0,18410007
ENSG00000120333	MRPS14	mitochondrial ribosomal protein S14	63931	71,99388431	0,029673422	0,246743	0,120260349	0,904276913	0,978460866
ENSG00000130449	ZSWIM6	zinc finger SWIM-type containing 6	57688	63,9669542	-0,004108654	0,240397	-0,017091093	0,986363945	0,996756734
ENSG00000136143	SUCLA2	succinate-CoA ligase ADP-forming subunit beta	8803	86,87903269	0,182782874	0,191265	0,955652582	0,339247787	0,777431905
ENSG00000086730	LAT2	linker for activation of T cells family member 2	7462	531,3299717	0,024923106	0,088453	0,281765511	0,778123318	0,947109268
ENSG00000105612	DNASE2	deoxyribonuclease 2, lysosomal	1777	122,6176364	0,226806325	0,198296	1,14377464	0,25271711	0,708754466
ENSG00000111684	LPCAT3	lysophosphatidylcholine acyltransferase 3	10162	100,3606423	0,098527087	0,174652	0,564133389	0,572663357	0,886795904
ENSG00000129521	EGLN3	egl-9 family hypoxia inducible factor 3	112399	22,95750441	-0,463244524	0,371088	-1,248341008	0,211906203	NA
ENSG00000146909	NOM1	nucleolar protein with MIF4G domain 1	64434	123,2253396	-0,058484484	0,151823	-0,385214337	0,700078622	0,930783276
ENSG00000167797	CDK2AP2	cyclin dependent kinase 2 associated protein 2	10263	176,9173164	0,08485014	0,128047	0,66264814	0,507555931	0,864738156
ENSG00000168282	MGAT2	alpha-1,6-mannosyl-glycoprotein 2-beta-N-acetylglucosaminyltransferase	4247	256,0111762	-0,060837268	0,113519	-0,535923324	0,592011544	0,892834002
ENSG00000180879	SSR4	signal sequence receptor subunit 4	6748	241,3674899	0,164900955	0,115182	1,431651245	0,152243656	0,608564843
ENSG00000183978	COA3	cytochrome c oxidase assembly factor 3	28958	53,87160188	0,048812045	0,240862	0,202655987	0,839403927	0,96627649
ENSG00000186787	SPIN2B	spindlin family member 2B	474343	20,19185634	-0,638649684	0,457893	-1,394757761	0,1630889	NA
ENSG00000189091	SF3B3	splicing factor 3b subunit 3	23450	593,9454441	-0,101341981	0,070048	-1,446751526	0,147966528	0,601032765
ENSG00000197872	CYRIA	CYFIP related Rac1 interactor A	81553	509,0095432	0,077287564	0,109853	0,703552311	0,481711619	0,855577353
ENSG00000198346	ZNF813	zinc finger protein 813	126017	42,66994108	-0,428369956	0,29233	-1,465364612	0,142821467	0,587795357
ENSG00000198604	BAZ1A	bromodomain adjacent to zinc finger domain 1A	11177	442,5540026	0,086760296	0,080772	1,074138016	0,282760828	0,736584923
ENSG00000198756	COLGALT2	collagen beta(1-O)galactosyltransferase 2	23127	185,7864301	-0,063735564	0,133811	-0,476309232	0,63385409	0,908447084
ENSG00000203791	EEF1AKMT2	EEF1A lysine methyltransferase 2	399818	47,03634974	-0,409216364	0,421088	-0,971806364	0,331146889	0,772473234
ENSG00000072609	CHFR	checkpoint with forkhead and ring finger domains	55743	229,4978466	-0,092134364	0,140969	-0,653577165	0,51338426	0,86831237
ENSG00000101347	SAMHD1	SAM and HD domain containing deoxynucleoside triphosphate triphosphohydrolase 1	25939	1390,293375	0,007788612	0,082117	0,094847478	0,924435974	0,984284131
ENSG00000105173	CCNE1	cyclin E1	898	17,29750077	-0,164895668	0,474786	-0,347305325	0,72836195	NA
ENSG00000105393	BABAM1	BRISC and BRCA1 A complex member 1	29086	159,6047478	0,045607601	0,13863	0,328987739	0,742164956	0,939193793
ENSG00000108854	SMURF2	SMAD specific E3 ubiquitin protein ligase 2	64750	189,6009282	0,100562477	0,182997	0,549531485	0,582640763	0,888501395
ENSG00000115286	NDUFS7	NADH:ubiquinone oxidoreductase core subunit S7	374291	48,20643188	0,0674596	0,282256	0,239001498	0,811104421	0,959038743
ENSG00000116752	BCAS2	BCAS2 pre-mRNA processing factor	10286	95,61240004	0,267027955	0,206085	1,295716301	0,195073243	0,653071292
ENSG00000125352	RNF113A	ring finger protein 113A	7737	76,77251976	-0,084860237	0,24175	-0,351024892	0,725569674	0,936042086
ENSG00000127528	KLF2	KLF transcription factor 2	10365	24,36521753	-0,424119082	0,58136	-0,729529474	0,465677845	NA
ENSG00000130775	THEMIS2	thymocyte selection associated family member 2	9473	119,7987729	-0,131584531	0,188836	-0,696818206	0,485916571	0,858001067
ENSG00000134900	TPP2	tripeptidyl peptidase 2	7174	340,0376358	-0,180135289	0,101198	-1,780030907	0,075070903	0,44532241
ENSG00000136950	ARPC5L	actin related protein 2/3 complex subunit 5 like	81873	189,1814094	0,087406225	0,127857	0,683627339	0,494210517	0,860513824
ENSG00000140374	ETFA	electron transfer flavoprotein subunit alpha	2108	102,0646238	0,00673602	0,524576	0,012840877	0,989754744	0,997211687
ENSG00000144867	SRPRB	SRP receptor subunit beta	58477	128,2405356	0,232873327	0,147413	1,57973757	0,114166978	0,539835908
ENSG00000149483	TMEM138	transmembrane protein 138	51524	88,55216364	0,349396324	0,219139	1,594403247	0,110845746	0,53211348
ENSG00000160445	ZER1	zyg-11 related cell cycle regulator	10444	216,1115768	-0,041101446	0,123773	-0,332072239	0,739834712	0,939193793
ENSG00000161091	MFSD12	major facilitator superfamily domain containing 12	126321	53,51865144	0,00606912	0,257183	0,023598424	0,981172929	0,995752963

ENSG00000164162	ANAPC10	anaphase promoting complex subunit 10	10393	21,20180853	0,378570337	0,391084	0,968002205	0,333043268	NA
ENSG00000164715	LMTK2	lemur tyrosine kinase 2	22853	210,6491495	-0,06196133	0,135471	-0,457378273	0,647399178	0,914264928
ENSG00000169379	ARL13B	ADP ribosylation factor like GTPase 13B	200894	16,21028215	0,293075162	0,860198	0,340706564	0,733324497	NA
ENSG00000177239	MAN1B1	mannosidase alpha class 1B member 1	11253	111,8706166	0,174568333	0,252619	0,691032692	0,489544996	0,860369726
ENSG00000177595	PIDD1	p53-induced death domain protein 1	55367	28,65789017	-0,368743912	0,494667	-0,745438742	0,45600653	NA
ENSG00000184208	C22orf46P	CTA-216E10,6	79640	93,59662534	0,040328439	0,182386	0,221115691	0,825002353	0,961917534
ENSG00000184470	TXNRD2	thioredoxin reductase 2	10587	34,85033312	0,228914035	0,317805	0,720297893	0,471341602	NA
ENSG00000188158	NHS	NHS actin remodeling regulator	4810	144,4663837	0,370455813	0,171976	2,154107237	0,031231763	0,281657534
ENSG00000197562	RAB40C	RAB40C, member RAS oncogene family	57799	31,26638874	-0,222496759	0,66434	-0,334913869	0,73769006	NA
ENSG00000228570	NUTM2E	NUT family member 2E	283008	19,12734391	0,234843985	0,431728	0,543962828	0,586467053	NA
ENSG00000125107	CNOT1	CCR4-NOT transcription complex subunit 1	23019	1308,13218	0,105001388	0,05465	1,921330606	0,054690041	0,379113488
ENSG00000155256	ZFYVE27	zinc finger FYVE-type containing 27	118813	121,2325302	-0,102912069	0,160023	-0,643107173	0,52015456	0,86944974
ENSG00000078124	ACER3	alkaline ceramidase 3	55331	76,23007499	-0,719781846	0,404567	-1,779143209	0,075216286	0,445848085
ENSG00000104973	MED25	mediator complex subunit 25	81857	127,877678	-0,004733445	0,177111	-0,026725816	0,978678422	0,995712569
ENSG00000143368	SF3B4	splicing factor 3b subunit 4	10262	282,220731	-0,112759117	0,120381	-0,936682415	0,348921944	0,784537594
ENSG00000153044	CENPH	centromere protein H	64946	24,35755809	0,311155819	0,347573	0,895224654	0,370667008	NA
ENSG00000168060	NAALADL1	N-acetylated alpha-linked acidic dipeptidase like 1	10004	14,44367491	-0,5713653	0,547508	-1,043575149	0,296681995	NA
ENSG00000066294	CD84	CD84 molecule	8832	80,771651	1,177927951	0,284363	4,142333834	3,44E-05	0,002755227
ENSG00000091009	RBM27	RNA binding motif protein 27	54439	281,7478437	0,00573923	0,103808	0,055286758	0,955910011	0,991510463
ENSG00000104695	PPP2CB	protein phosphatase 2 catalytic subunit beta	5516	153,1447256	-0,167134901	0,139249	-1,200261784	0,230037687	0,684730271
ENSG00000135541	AHI1	Abelson helper integration site 1	54806	60,5922094	1,012771768	0,312675	3,239056339	0,001199259	0,038444811
ENSG00000173436	MICOS10	mitochondrial contact site and cristae organizing system subunit 10	440574	38,00292279	-0,537240436	0,413612	-1,298900307	0,193978144	NA
ENSG00000183386	FHL3	four and a half LIM domains 3	2275	12,55844044	0,207258584	0,529896	0,391130403	0,695700849	NA
ENSG00000185808	PIGP	phosphatidylinositol glycan anchor biosynthesis class P	51227	20,93363764	0,022454709	0,386311	0,058125938	0,953648313	NA
ENSG00000196839	ADA	adenosine deaminase	100	61,66341627	0,316386227	0,275265	1,149387883	0,250396074	0,707335884
ENSG00000026025	VIM	vimentin	7431	2313,675882	0,096151547	0,051932	1,851484267	0,064099916	0,407974669
ENSG00000159399	HK2	hexokinase 2	3099	39,70658305	-0,039543301	0,274859	-0,143867452	0,885605138	NA
ENSG00000067596	DHX8	DEAH-box helicase 8	1659	305,1747379	0,09097228	0,099746	0,91204244	0,361746377	0,791408368
ENSG00000112242	E2F3	E2F transcription factor 3	1871	316,5378729	0,118030269	0,107443	1,09854135	0,271968172	0,726049634
ENSG00000164104	HMGB2	high mobility group box 2	3148	474,2747332	0,438619108	0,115179	3,808161676	0,000140004	0,008393811
ENSG00000188313	PLSCR1	phospholipid scramblase 1	5359	65,72319584	-0,027233045	0,259325	-0,105015024	0,916363887	0,982006
ENSG00000258659	TRIM34	tripartite motif containing 34	53840	65,22663794	-0,117150277	0,209231	-0,559909924	0,57554088	0,887069818
ENSG00000112378	PERP	p53 apoptosis effector related to PMP22	64065	33,13850634	-0,00952444	0,328328	-0,029008946	0,976857456	NA
ENSG00000115267	IFIH1	interferon induced with helicase C domain 1	64135	57,95390352	0,39424306	0,234301	1,682638211	0,09244515	0,4945733
ENSG00000116670	MAD2L2	mitotic arrest deficient 2 like 2	10459	105,4374419	-0,008086905	0,169809	-0,047623463	0,962016333	0,992328441
ENSG00000180573	H2AC6	H2A clustered histone 6	8334	25,41423314	0,222276346	0,734602	0,302580837	0,76220932	NA
ENSG00000113522	RAD50	RAD50 double strand break repair protein	10111	87,16890339	0,179963897	1,468239	0,122571274	0,902446603	0,978453541
ENSG00000129911	KLF16	KLF transcription factor 16	83855	42,41687329	-0,356546092	0,266381	-1,338481603	0,180739493	0,634062228
ENSG00000184635	ZNF93	zinc finger protein 93	81931	12,88988047	-0,184756091	0,54167	-0,341086218	0,733038676	NA
ENSG00000197555	SIPA1L1	signal induced proliferation associated 1 like 1	26037	286,3327041	0,123456055	0,12181	1,013511691	0,310815815	0,75688983
ENSG00000105568	PPP2R1A	protein phosphatase 2 scaffold subunit Aalpha	5518	456,622852	0,045292831	0,082396	0,549697479	0,582526886	0,888501395
ENSG00000267303	NA	NA	NA	44,52507306	-2,37540574	2,375266	-1,000058912	0,317281999	0,762292941
ENSG00000064545	TMEM161A	transmembrane protein 161A	54929	12,32685198	0,789292073	2,173001	0,363226822	0,716435453	NA
ENSG00000142864	SERBP1	SERPINE1 mRNA binding protein 1	26135	1268,53219	-0,007932215	0,082951	-0,09562481	0,92381856	0,984284131
ENSG00000100711	ZFYVE21	zinc finger FYVE-type containing 21	79038	23,36013399	-0,445051875	0,391905	-1,135611389	0,25611924	NA
ENSG00000133703	KRAS	KRAS proto-oncogene, GTPase	3845	371,0216686	-0,00304375	0,094555	-0,032190327	0,97432027	0,994534085
ENSG00000146463	ZMYM4	zinc finger MYM-type containing 4	9202	240,5852472	0,087001538	0,127873	0,680375682	0,496266614	0,86123475
ENSG00000182827	ACBD3	acyl-CoA binding domain containing 3	64746	166,357348	-0,083803541	0,152101	-0,550971623	0,581653127	0,888501395
ENSG00000063180	CA11	carbonic anhydrase 11	770	15,6107711	-0,258195722	0,465668	-0,554463422	0,579261732	NA
ENSG00000076242	MLH1	mutL homolog 1	4292	141,4254503	0,356152134	0,191664	1,85821071	0,063139098	0,404317327
ENSG00000162980	ARL5A	ADP ribosylation factor like GTPase 5A	26225	174,2130234	0,119062729	0,225587	0,527791167	0,597644289	0,894257182
ENSG00000170619	COMMD5	COMM domain containing 5	28991	38,01651394	-0,285909387	0,338477	-0,844693903	0,398281764	NA
ENSG00000171365	CLCN5	chloride voltage-gated channel 5	1184	31,06292351	-0,271167701	0,327456	-0,828103126	0,407612099	NA

ENSG00000188321	ZNF559	zinc finger protein 559	84527	75,06358784	0,179563896	0,245938	0,73011859	0,465317699	0,84908342
ENSG00000272047	GTF2H5	general transcription factor IIH subunit 5	404672	30,08652692	0,403843697	0,703156	0,574330451	0,565744207	NA
ENSG00000179163	FUCA1	alpha-L-fucosidase 1	2517	586,3937097	0,075171522	0,073061	1,028884851	0,303533788	0,751870158
ENSG00000132570	PCBD2	pterin-4 alpha-carbinolamine dehydratase 2	84105	63,38562535	0,176110155	0,256408	0,686835372	0,492186477	0,860513824
ENSG00000043143	JADE2	jade family PHD finger 2	23338	497,5712725	-0,023595481	0,105779	-0,223063105	0,823486393	0,961917534
ENSG00000101266	CSNK2A1	casein kinase 2 alpha 1	1457	366,6913628	-0,121141571	0,101134	-1,197827932	0,230984011	0,685179309
ENSG00000101940	WDR13	WD repeat domain 13	64743	31,62287393	0,424098233	0,505192	0,839479395	0,401200347	NA
ENSG00000108010	GLRX3	glutaredoxin 3	10539	135,581995	-0,107298624	0,174137	-0,616172146	0,53778091	0,874923109
ENSG00000111358	GTF2H3	general transcription factor IIH subunit 3	2967	137,4797064	0,203951786	0,215465	0,946566297	0,343859825	0,781132432
ENSG00000144659	SLC25A38	solute carrier family 25 member 38	54977	266,2893866	0,12480485	0,117176	1,06510939	0,28682643	0,738005299
ENSG00000166925	TSC22D4	TSC22 domain family member 4	81628	428,0140967	-0,082498809	0,099888	-0,825915725	0,4088519	0,820793685
ENSG00000179344	HLA-DQB1	major histocompatibility complex, class II, DQ beta 1	3119	149,5655721	0,05382094	0,138301	0,389158167	0,697159147	0,930422107
ENSG00000215114	UBXN2B	UBX domain protein 2B	137886	200,7315758	-0,271279401	0,126747	-2,140321955	0,032328757	0,287228571
ENSG00000242294	NA	NA	NA	14,51917677	-0,059564922	0,510493	-0,116681228	0,907112666	NA
ENSG00000123131	PRDX4	peroxiredoxin 4	10549	29,47032169	-0,01736864	0,359916	-0,048257511	0,961511017	NA
ENSG00000130748	TMEM160	transmembrane protein 160	54958	22,26521175	0,367711664	0,39096	0,940535716	0,346942838	NA
ENSG00000215193	PEX26	peroxisomal biogenesis factor 26	55670	330,3036515	0,350429709	0,374424	0,935916826	0,349316013	0,784537594
ENSG00000164815	ORC5	origin recognition complex subunit 5	5001	37,07719628	0,227400662	0,290052	0,783999786	0,433040236	NA
ENSG00000126246	IGFLR1	IGF like family receptor 1	79713	301,6531556	-0,209269347	0,10441	-2,004312597	0,045036585	0,340112828
ENSG00000198860	TSEN15	tRNA splicing endonuclease subunit 15	116461	188,314554	0,134752442	0,141066	0,955247096	0,339452754	0,777431905
ENSG00000065485	PDIA5	protein disulfide isomerase family A member 5	10954	16,11830775	-1,12121138	0,75472	-1,485598295	0,137385406	NA
ENSG00000213859	KCTD11	potassium channel tetramerization domain containing 11	147040	84,05152746	-0,204853867	0,203993	-1,004220322	0,315272429	0,760454518
ENSG00000165195	PIGA	phosphatidylinositol glycan anchor biosynthesis class A	5277	16,50584141	1,292976189	2,034316	0,635582765	0,525048406	NA
ENSG00000178163	ZNF518B	zinc finger protein 518B	85460	145,3824546	-0,028548898	0,152403	-0,187324777	0,851405998	0,967902179
ENSG00000104450	SPAG1	sperm associated antigen 1	6674	99,53282991	0,249961812	0,207886	1,202396751	0,229209847	0,68351286
ENSG00000143337	TOR1AIP1	torsin 1A interacting protein 1	26092	577,6674748	0,03205817	0,078081	0,410574676	0,681384436	0,925586358
ENSG00000100410	PHF5A	PHD finger protein 5A	84844	67,21302013	0,269125519	0,220784	1,218952748	0,222862127	0,678694696
ENSG00000119723	COQ6	coenzyme Q6, monooxygenase	51004	13,8074808	0,031695514	0,513189	0,061761849	0,950752485	NA
ENSG00000143390	RFX5	regulatory factor X5	5993	364,6355148	0,122818254	0,088382	1,389635241	0,164639669	0,624374678
ENSG00000160703	NLRX1	NLR family member X1	79671	55,95664969	0,139890457	0,269414	0,519240085	0,603593329	0,897212117
ENSG00000185129	PURA	purine rich element binding protein A	5813	211,3327512	-0,235521703	0,213674	-1,102245293	0,270355046	0,725477256
ENSG00000111676	ATN1	atrophin 1	1822	252,7756217	-0,208489759	0,166605	-1,251403636	0,210787251	0,6647417
ENSG00000135722	FBXL8	F-box and leucine rich repeat protein 8	55336	14,66638671	-0,937860742	0,563202	-1,665229755	0,095866927	NA
ENSG00000175455	CCDC14	coiled-coil domain containing 14	64770	283,7650161	-0,342184439	0,1715	-1,995243697	0,046016307	0,342895709
ENSG00000288722	F8A1	coagulation factor VIII associated 1	8263	89,68031229	-0,0296091	0,196761	-0,150482468	0,880383982	0,976300778
ENSG00000125741	OPA3	outer mitochondrial membrane lipid metabolism regulator OPA3	80207	161,8218948	-0,018112068	0,14929	-0,12132177	0,903436177	0,978460866
ENSG00000078804	TP53INP2	tumor protein p53 inducible nuclear protein 2	58476	16,91697841	0,189120608	0,434667	0,435092744	0,66349511	NA
ENSG00000114650	SCAP	SREBF chaperone	22937	232,0445995	-0,040635111	0,122133	-0,332712158	0,739351571	0,939193793
ENSG00000130066	SAT1	spermidine/spermine N1-acetyltransferase 1	6303	62,78266018	0,000510365	0,23982	0,002128117	0,998302009	0,999300722
ENSG00000214765	NA	NA	NA	16,87251462	0,587082859	0,454156	1,292689548	0,196118454	NA
ENSG00000113761	ZNF346	zinc finger protein 346	23567	77,34901512	-0,064548113	0,229086	-0,281763178	0,778125107	0,947109268
ENSG00000118363	SPCS2	signal peptidase complex subunit 2	9789	285,0747786	0,095097454	0,161363	0,589338676	0,555634105	0,882854594
ENSG00000114739	ACVR2B	activin A receptor type 2B	93	64,15797417	-0,185727508	0,230888	-0,804405758	0,421162677	0,827332857
ENSG00000121957	GPSM2	G protein signaling modulator 2	29899	64,14761698	0,486164123	0,361119	1,34626994	0,178215477	0,6324918
ENSG00000196922	NA	NA	NA	36,79232433	-0,074072019	0,294658	-0,251382717	0,801518229	NA
ENSG00000172354	GNB2	G protein subunit beta 2	2783	327,1107114	0,007968148	0,104742	0,07607438	0,939359923	0,987813564
ENSG00000163935	SFMBT1	Scm like with four mbt domains 1	51460	134,2468718	-0,257617671	0,150757	-1,708825099	0,087483352	0,482849084
ENSG00000121851	POLR3GL	RNA polymerase III subunit GL	84265	147,2994628	0,014800714	0,149853	0,098768528	0,921322057	0,984284131
ENSG00000167595	PROSER3	proline and serine rich 3	148137	74,65447613	-0,36946277	0,255464	-1,446239832	0,148109945	0,601032765
ENSG00000100731	PCNX1	pecanex 1	22990	1320,450396	0,076884761	0,08421	0,913014781	0,361234768	0,791408368
ENSG00000163719	MTMR14	myotubularin related protein 14	64419	142,1426081	0,173693259	0,165308	1,050725939	0,293384479	0,744745216

ENSG00000175063	UBE2C	ubiquitin conjugating enzyme E2 C	11065	60,34172861	0,498441665	0,212887	2,341342942	0,019214508	0,217137768
ENSG00000136840	ST6GALNAC4	ST6 N-acetylgalactosaminide alpha-2,6-sialyltransferase 4	27090	102,2436409	0,023341223	0,163727	0,142561894	0,886636194	0,977928921
ENSG00000075399	VPS9D1	VPS9 domain containing 1	9605	64,81826581	0,080077739	0,258964	0,309223352	0,757151631	0,942901768
ENSG00000147443	DOK2	docking protein 2	9046	334,6719059	0,009308026	0,107377	0,086685256	0,930921697	0,986491322
ENSG00000163513	TGFBR2	transforming growth factor beta receptor 2	7048	553,0453359	-0,154666581	0,082788	-1,868214354	0,061732199	0,402854784
ENSG00000099246	RAB18	RAB18, member RAS oncogene family	22931	120,1615412	-0,656848033	0,808658	-0,812269709	0,416636888	0,82446781
ENSG00000160219	GAB3	GRB2 associated binding protein 3	139716	158,2779474	-0,060722884	0,150998	-0,402143452	0,687578456	0,926748702
ENSG00000109107	ALDOC	aldolase, fructose-bisphosphate C	230	105,0266066	-0,064875268	0,161583	-0,401498157	0,688053395	0,926748702
ENSG00000041802	LSG1	large 60S subunit nuclear export GTPase 1	55341	202,0717573	0,13686926	0,125328	1,092090218	0,274793449	0,727446428
ENSG00000048707	VPS13D	vacuolar protein sorting 13 homolog D	55187	807,2965325	0,059072317	0,101466	0,582187005	0,560440726	0,883921037
ENSG00000104894	CD37	CD37 molecule	951	596,8117523	-0,185399654	0,074937	-2,474061897	0,013358655	0,180367712
ENSG00000112787	FBRSL1	fibrosin like 1	57666	174,5348233	-0,193092388	0,128786	-1,499330153	0,133788004	0,573252035
ENSG00000119508	NR4A3	nuclear receptor subfamily 4 group A member 3	8013	14,38326482	-0,263553802	0,528309	-0,498863408	0,617875614	NA
ENSG00000121350	PYROXD1	pyridine nucleotide-disulphide oxidoreductase domain 1	79912	187,2479025	0,018207788	0,129334	0,140781054	0,888042911	0,977990819
ENSG00000123130	ACOT9	acyl-CoA thioesterase 9	23597	91,55765147	0,055835486	0,218719	0,255284181	0,798503617	0,953977397
ENSG00000131373	HACL1	2-hydroxyacyl-CoA lyase 1	26061	82,99285578	0,309387101	0,197394	1,567360283	0,117030502	0,545494102
ENSG00000132361	CLUH	clustered mitochondria homolog	23277	159,298923	-0,012311697	0,166062	-0,074139068	0,940899729	0,988399618
ENSG00000135336	ORC3	origin recognition complex subunit 3	23595	65,07560693	0,009372721	0,251421	0,037278918	0,970262615	0,993542794
ENSG00000138279	ANXA7	annexin A7	310	355,6262225	0,286739833	0,099998	2,867458472	0,004137831	0,088853402
ENSG00000145365	TIFA	TRAF interacting protein with forkhead associated domain	92610	31,95512385	-0,234656911	0,463751	-0,505997701	0,612858264	NA
ENSG00000155313	USP25	ubiquitin specific peptidase 25	29761	391,8301708	-0,102250263	0,106383	-0,961152636	0,336475427	0,776552413
ENSG00000157181	ODR4	odr-4 GPCR localization factor homolog	54953	91,00448941	0,237006737	0,174401	1,358972989	0,174155148	0,631278097
ENSG00000168610	STAT3	signal transducer and activator of transcription 3	6774	983,5513884	0,015707595	0,110485	0,142169078	0,886946455	0,977954139
ENSG00000172508	CARNS1	carnosine synthase 1	57571	17,36963023	0,279024529	0,785034	0,355429812	0,722267618	NA
ENSG00000175073	VCPIP1	valosin containing protein interacting protein 1	80124	473,612483	-0,116036501	0,079833	-1,45349348	0,146086789	0,594489968
ENSG00000198951	NAGA	alpha-N-acetylgalactosaminidase	4668	87,56885395	-0,099883001	0,187885	-0,53161698	0,594991302	0,894229873
ENSG00000213639	PPP1CB	protein phosphatase 1 catalytic subunit beta	5500	650,8416062	0,14509483	0,115034	1,261320269	0,207193481	0,661772104
ENSG00000172081	MOB3A	MOB kinase activator 3A	126308	1309,941745	-0,050800177	0,062754	-0,80951768	0,418217437	0,82446781
ENSG00000188559	RALGAPA2	Ral GTPase activating protein catalytic subunit alpha 2	57186	502,8406984	0,060401154	0,080103	0,754042506	0,450823699	0,84263906
ENSG00000133318	RTN3	reticulon 3	10313	293,3087113	0,100872978	0,13692	0,736729586	0,461286811	0,846765498
ENSG00000167065	DUSP18	dual specificity phosphatase 18	150290	33,47599545	-0,616819484	0,3672	-1,679791354	0,092997918	NA
ENSG00000081923	ATP8B1	ATPase phospholipid transporting 8B1	5205	19,32872272	-1,225462836	1,641623	-0,746494534	0,455368733	NA
ENSG00000132965	ALOX5AP	arachidonate 5-lipoxygenase activating protein	241	197,1639233	0,057738961	0,127095	0,454298296	0,649614142	0,914926418
ENSG00000155729	KCTD18	potassium channel tetramerization domain containing 18	130535	100,1846905	0,27892603	0,169243	1,648079599	0,099336338	0,509429216
ENSG00000090621	PABPC4	poly(A) binding protein cytoplasmic 4	8761	187,3674401	-0,011881488	0,14426	-0,082361356	0,934359365	0,987098279
ENSG00000116138	DNAJC16	DnaJ heat shock protein family (Hsp40) member C16	23341	122,2823685	-0,352813562	0,202865	-1,739153371	0,082007789	0,465718853
ENSG00000178947	SMIM10L2A	small integral membrane protein 10 like 2A	399668	60,55859199	-0,187845645	0,221846	-0,846737145	0,39714165	0,813102153
ENSG00000112297	CRYBG1	crystallin beta-gamma domain containing 1	202	254,4000658	-0,00251815	0,112561	-0,022371426	0,982151673	0,995974079
ENSG00000159228	CBR1	carbonyl reductase 1	873	62,55826305	0,210826553	0,223079	0,945074158	0,344621017	0,781366475
ENSG00000167904	TMEM68	transmembrane protein 68	137695	48,70208648	0,089631532	0,298001	0,300776229	0,763585136	0,943846027
ENSG00000164164	OTUD4	OTU deubiquitinase 4	54726	390,719494	-0,103420696	0,08903	-1,161635717	0,245383468	0,702347578
ENSG00000182810	DDX28	DEAD-box helicase 28	55794	37,75643209	-0,345611893	0,287473	-1,202239372	0,229270799	NA
ENSG00000185361	TNFAIP8L1	TNF alpha induced protein 8 like 1	126282	202,7643026	-0,103391717	0,115883	-0,89221157	0,372279542	0,800625279
ENSG00000244480	NA	NA	NA	19,9792079	-0,049293315	0,413541	-0,119198212	0,905118323	NA
ENSG00000076053	RBM7	RNA binding motif protein 7	10179	86,36820105	-0,928575464	0,439228	-2,114107149	0,034506117	0,296835751
ENSG00000180346	TIGD2	tigger transposable element derived 2	166815	28,95320108	-0,09112611	0,39772	-0,229121286	0,81877465	NA
ENSG00000157796	WDR19	WD repeat domain 19	57728	38,91706507	-0,179438979	0,527664	-0,340062922	0,733809143	NA
ENSG00000186106	ANKRD46	ankyrin repeat domain 46	157567	37,19998276	-0,213902204	0,300303	-0,712288076	0,476286406	NA

ENSG00000069869	NEDD4	NEDD4 E3 ubiquitin protein ligase	4734	18,24885442	0,775766247	1,729404	0,448574271	0,653738798	NA
ENSG00000104689	TNFRSF10A	TNF receptor superfamily member 10a	8797	14,34802112	0,207550631	0,46104	0,450179435	0,652581063	NA
ENSG00000134864	GGACT	gamma-glutamylamine cyclotransferase	87769	24,15974623	-0,261405923	0,400697	-0,652377732	0,514157526	NA
ENSG00000136444	RSAD1	radical S-adenosyl methionine domain containing 1	55316	104,2397928	0,013102425	0,184024	0,07119954	0,943238948	0,988624545
ENSG00000164347	GFM2	GTP dependent ribosome recycling factor mitochondrial 2	84340	118,5509331	0,160516115	0,166094	0,966418337	0,333834892	0,773674527
ENSG00000172493	AFF1	ALF transcription elongation factor 1	4299	499,6381982	0,045782393	0,094248	0,485766214	0,627132931	0,90479929
ENSG00000173456	RNF26	ring finger protein 26	79102	104,6256743	0,097585108	0,18061	0,540307637	0,588984893	0,891627382
ENSG00000187608	ISG15	ISG15 ubiquitin like modifier	9636	51,96199788	0,090333371	0,292037	0,309321899	0,757076674	0,942901768
ENSG00000067365	METTL22	methyltransferase 22, Kin17 lysine	79091	22,63024058	0,720957368	0,395607	1,822408361	0,068393047	NA
ENSG00000120733	KDM3B	lysine demethylase 3B	51780	593,0875438	-0,042852114	0,099815	-0,429316344	0,667693024	0,922308959
ENSG00000148814	LRRC27	leucine rich repeat containing 27	80313	27,1756999	-0,032641165	0,369017	-0,088454429	0,929515503	NA
ENSG00000089157	RPLP0	ribosomal protein lateral stalk subunit P0	6175	2083,715923	0,287931489	0,053226	5,409580027	6,32E-08	1,46E-05
ENSG00000055609	KMT2C	lysine methyltransferase 2C	58508	956,122426	0,295547383	0,18351	1,610523964	0,107283518	0,526362202
ENSG00000172059	KLF11	KLF transcription factor 11	8462	62,52941268	-0,243809582	0,221757	-1,099445597	0,271573751	0,725708516
ENSG00000119684	MLH3	mutL homolog 3	27030	101,4693144	-0,302668053	0,246487	-1,227928834	0,219473684	0,675978948
ENSG00000105519	CAPS	calcyphosine	828	46,61315996	-0,439811156	0,279472	-1,573722597	0,115551595	0,541856098
ENSG00000164091	WDR82	WD repeat domain 82	80335	1131,648823	-0,162374828	0,073557	-2,207457981	0,027282079	0,268177037
ENSG00000111671	SPSB2	splA/ryanodine receptor domain and SOCS box containing 2	84727	17,42452322	0,162928922	0,418258	0,389542079	0,696875189	NA
ENSG00000111671	LOC105369632	uncharacterized LOC105369632	105369632	17,42452322	0,162928922	0,418258	0,389542079	0,696875189	NA
ENSG00000074370	ATP2A3	ATPase sarcoplasmic/endoplasmic reticulum Ca2+ transporting 3	489	781,877609	-0,049993582	0,067907	-0,7362094	0,461603273	0,846765498
ENSG00000152556	PFKM	phosphofructokinase, muscle	5213	19,17457884	0,177694482	0,558375	0,318234831	0,750306814	NA
ENSG00000175054	ATR	ATR serine/threonine kinase	545	116,4951489	0,263036756	0,340981	0,77141264	0,440462385	0,838640381
ENSG00000178685	PARP10	poly(ADP-ribose) polymerase family member 10	84875	230,5894318	-0,168594901	0,116988	-1,441135956	0,149546277	0,604312127
ENSG00000178764	ZHX2	zinc fingers and homeoboxes 2	22882	83,6299281	-0,021561143	0,222925	-0,096719159	0,922949425	0,984284131
ENSG00000108439	PNPO	pyridoxamine 5'-phosphate oxidase	55163	49,1016939	-0,29570543	0,299923	-0,985939078	0,324163009	0,767603859
ENSG00000140455	USP3	ubiquitin specific peptidase 3	9960	132,8879777	-0,244659564	0,289979	-0,843713993	0,398829246	0,814457852
ENSG00000142453	CARM1	coactivator associated arginine methyltransferase 1	10498	108,836381	-0,071513962	0,165281	-0,432680825	0,665246662	0,922184616
ENSG00000151882	CCL28	C-C motif chemokine ligand 28	56477	45,85854552	-0,649937972	0,27262	-2,3840393	0,017123774	0,204616219
ENSG00000168899	VAMP5	vesicle associated membrane protein 5	10791	24,38119769	-0,443570901	0,450959	-0,98361798	0,325303381	NA
ENSG00000189050	RNFT1	ring finger protein, transmembrane 1	51136	63,46583561	-0,278755682	0,402171	-0,69312788	0,488229302	0,859508239
ENSG00000102901	CENPT	centromere protein T	80152	21,50994081	-0,395901591	0,534545	-0,740632711	0,458916169	NA
ENSG00000129562	DAD1	defender against cell death 1	1603	195,2589618	0,031581565	0,143059	0,220758672	0,825280345	0,961917534
ENSG00000159692	CTBP1	C-terminal binding protein 1	1487	545,6316979	-0,073293032	0,076561	-0,957310965	0,338410323	0,777431905
ENSG00000131759	RARA	retinoic acid receptor alpha	5914	147,1767474	-0,00471754	0,139237	-0,033881446	0,972971688	0,994534085
ENSG00000068438	FTSJ1	FtsJ RNA 2'-O-methyltransferase 1	24140	124,9284787	-0,12989044	0,169827	-0,76483924	0,444367278	0,840223973
ENSG00000130254	SAFB2	scaffold attachment factor B2	9667	262,8764431	0,036776138	0,120303	0,305695972	0,759836141	0,942901768
ENSG00000167925	GHDC	GH3 domain containing	84514	82,26652691	0,071181233	0,208878	0,340778614	0,733270251	0,93912057
ENSG00000178074	C2orf69	chromosome 2 open reading frame 69	205327	46,96608844	-0,279230154	0,258683	-1,079431859	0,280395255	0,733410709
ENSG00000103047	TANGO6	transport and golgi organization 6 homolog	79613	88,82858133	0,083704944	0,222293	0,376552311	0,706506333	0,930846779
ENSG00000133114	GPALPP1	GPALPP motifs containing 1	55425	56,61008852	-0,319285418	0,278339	-1,147111478	0,251335551	0,70779361
ENSG00000141968	VAV1	vav guanine nucleotide exchange factor 1	7409	353,0170113	0,165257447	0,093693	1,7638223	0,077761902	0,451064976
ENSG00000247627	MTND4P12	MT-ND4 pseudogene 12	100293090	20,33230413	0,081742521	0,469042	0,17427543	0,861649008	NA
ENSG00000121297	TSHZ3	teashirt zinc finger homeobox 3	57616	14,18126225	-0,212421543	0,511054	-0,415654176	0,677663077	NA
ENSG00000129071	MBD4	methyl-CpG binding domain 4, DNA glycosylase	8930	172,6245126	-0,046251476	0,139367	-0,331869336	0,739987926	0,939193793
ENSG00000130560	UBAC1	UBA domain containing 1	10422	71,66402638	-0,015033271	0,285152	-0,052720277	0,957954783	0,991911599
ENSG00000140950	MEAK7	MTOR associated protein, eak-7 homolog	57707	49,10524375	0,100576768	0,271348	0,370656407	0,710893463	0,930846779

ENSG00000143164	DCAF6	DDB1 and CUL4 associated factor 6	55827	129,703834	0,163591439	0,185711	0,88089291	0,378375785	0,804187219
ENSG00000198455	ZXDB	zinc finger X-linked duplicated B	158586	68,94541214	-0,26515312	0,223181	-1,188063165	0,234808525	0,69018943
ENSG00000181192	DHTKD1	dehydrogenase E1 and transketolase domain containing 1	55526	115,523584	0,097385491	0,153663	0,633759386	0,526237862	0,871404632
ENSG00000106330	MOSPD3	motile sperm domain containing 3	64598	58,20718071	-0,046375888	0,225531	-0,205629417	0,837080397	0,965266398
ENSG00000120053	GOT1	glutamic-oxaloacetic transaminase 1	2805	71,70133088	0,278764627	0,21127	1,319470715	0,187011794	0,645758518
ENSG00000137288	UQCC2	ubiquinol-cytochrome c reductase complex assembly factor 2	84300	15,30211372	0,036377613	0,504355	0,07212704	0,942500807	NA
ENSG00000214194	SMIM30	small integral membrane protein 30	401397	55,9148452	0,253691544	0,228172	1,111842742	0,266205771	0,722230271
ENSG00000091483	FH	fumarate hydratase	2271	100,4068542	0,209717182	0,181258	1,157012379	0,247267303	0,705258787
ENSG00000095209	TMEM38B	transmembrane protein 38B	55151	29,22791543	0,338295009	0,346416	0,9765575	0,328788268	NA
ENSG00000119720	NRDE2	NRDE-2, necessary for RNA interference, domain containing	55051	118,3419014	-0,131026639	0,169187	-0,774450349	0,438664519	0,837388481
ENSG00000103876	FAH	fumarylacetoacetate hydrolase	2184	53,49184227	0,155997713	0,251564	0,620112042	0,535184025	0,874783627
ENSG00000134056	MRPS36	mitochondrial ribosomal protein S36	92259	46,11720249	0,503544144	0,2611	1,928547369	0,053787078	0,374590746
ENSG00000273173	SNURF	SNRPN upstream open reading frame	8926	12,29316551	-0,729001599	0,814445	-0,895090169	0,370738889	NA
ENSG00000118894	EEF2KMT	eukaryotic elongation factor 2 lysine methyltransferase	196483	24,22302428	-0,038393998	0,365102	-0,105159735	0,916249061	NA
ENSG00000147650	LRP12	LDL receptor related protein 12	29967	13,02919939	0,217815408	0,651082	0,33454364	0,737969366	NA
ENSG00000163945	UVSSA	UV stimulated scaffold protein A	57654	280,9272383	-0,226654647	0,152665	-1,484653003	0,137635767	0,578689141
ENSG00000176896	TCEANC	transcription elongation factor A N-terminal and central domain containing	170082	16,82618354	0,101022127	0,446266	0,226371869	0,820912206	NA
ENSG00000198056	PRIM1	DNA primase subunit 1	5557	30,40193876	1,014665184	1,289982	0,786573177	0,431531767	NA
ENSG00000253276	CCDC71L	coiled-coil domain containing 71 like	168455	40,72899806	0,285206553	0,299163	0,953349641	0,340412948	NA
ENSG00000266173	STRADA	STE20 related adaptor alpha	92335	95,13091863	0,107499442	0,200603	0,535882646	0,592039659	0,892834002
ENSG00000198298	ZNF485	zinc finger protein 485	220992	28,66582045	0,161937933	0,345611	0,468555872	0,639387126	NA
ENSG00000176894	PXMP2	peroxisomal membrane protein 2	5827	16,71850378	-0,36885486	0,460103	-0,801678876	0,422738738	NA
ENSG00000183155	RABIF	RAB interacting factor	5877	139,6969078	0,110613854	0,15744	0,702579252	0,482317996	0,855834779
ENSG00000131437	KIF3A	kinesin family member 3A	11127	78,40312266	-0,472792083	0,199327	-2,371944081	0,017694768	0,207734995
ENSG00000168397	ATG4B	autophagy related 4B cysteine peptidase	23192	113,0938183	0,183621546	0,329397	0,557447485	0,57722173	0,887069818
ENSG00000215187	CIMIP2B	ciliary microtubule inner protein 2B	730112	56,16729371	-0,323094111	0,261686	-1,234663319	0,216955833	0,672891335
ENSG00000120837	NFYB	nuclear transcription factor Y subunit beta	4801	89,00975503	0,237647331	0,251198	0,946056113	0,344119966	0,781132432
ENSG00000138430	OLA1	Obg like ATPase 1	29789	151,2415665	0,096763951	0,13233	0,731230643	0,464638286	0,848967535
ENSG00000140391	TSPAN3	tetraspanin 3	10099	61,65595703	0,368970345	0,227318	1,623142864	0,104558868	0,519421475
ENSG00000168612	ZSWIM1	zinc finger SWIM-type containing 1	90204	16,43664347	-0,2911979	0,452525	-0,643495466	0,519902656	NA
ENSG00000176092	CRYBG2	crystallin beta-gamma domain containing 2	55057	46,56919031	0,033364303	0,257295	0,129673181	0,896825002	0,977990819
ENSG00000178397	FAM220A	family with sequence similarity 220 member A	84792	44,18208832	-0,165322919	0,25958	-0,63688608	0,524199051	0,870612376
ENSG00000180011	PTGR3	prostaglandin reductase 3	284273	149,9691764	0,142900354	0,151977	0,940277904	0,34707503	0,783470101
ENSG00000185043	CIB1	calcium and integrin binding 1	10519	140,4582684	0,085541186	0,177369	0,482277587	0,629608767	0,905567722
ENSG00000000971	CFH	complement factor H	3075	92,79298728	0,42994562	0,349832	1,229006755	0,219069273	0,67526298
ENSG00000126215	XRCC3	X-ray repair cross complementing 3	7517	19,96905044	0,078116955	0,525049	0,148780308	0,88172699	NA
ENSG00000112303	VNN2	vanin 2	8875	77,16011748	-0,348394346	0,248792	-1,400341574	0,161411057	0,619005098
ENSG00000116030	SUMO1	small ubiquitin like modifier 1	7341	309,3833521	0,169866379	0,101489	1,673746288	0,094180473	0,498110055
ENSG00000183765	CHEK2	checkpoint kinase 2	11200	45,25604973	-0,365501708	0,284439	-1,28499146	0,198795268	0,657682409
ENSG00000133983	COX16	cytochrome c oxidase assembly factor COX16	51241	56,84959493	-0,004163762	0,31712	-0,013129934	0,989524129	0,997211687
ENSG00000124608	AARS2	alanyl-tRNA synthetase 2, mitochondrial	57505	91,59512952	0,113017007	0,179897	0,628230873	0,529852708	0,872145761
ENSG00000159110	IFNAR2	interferon alpha and beta receptor subunit 2	3455	393,1993719	-0,077059556	0,104735	-0,735754537	0,461880093	0,846765498
ENSG00000123815	COQ8B	coenzyme Q8B	79934	25,73294976	0,030865679	0,440163	0,070123216	0,944095588	NA
ENSG00000188878	FBF1	Fas binding factor 1	85302	22,62241544	-0,285042313	0,424991	-0,670702394	0,502410138	NA
ENSG00000197956	S100A6	S100 calcium binding protein A6	6277	86,64714265	0,110495397	0,180561	0,611955047	0,5405675	0,876149366

ENSG00000143952	VPS54	VPS54 subunit of GARP complex	51542	69,83919891	-0,070385767	0,220023	-0,319901457	0,749043034	0,940489812
ENSG00000055208	TAB2	TGF-beta activated kinase 1 (MAP3K7) binding protein 2	23118	434,3351275	0,052013108	0,090787	0,572915904	0,566701636	0,88521771
ENSG00000108599	AKAP10	A-kinase anchoring protein 10	11216	173,1282201	-0,127963188	0,165057	-0,775266516	0,438182191	0,837388481
ENSG00000115170	ACVR1	activin A receptor type 1	90	45,52476238	0,302990089	0,291207	1,040462132	0,298125248	0,748846814
ENSG00000174600	CMKLR1	chemerin chemokine-like receptor 1	1240	23,64135107	-0,671058462	0,415639	-1,614521161	0,106414431	NA
ENSG00000180423	HARBI1	harbinger transposase derived 1	283254	15,15825104	0,09729373	0,50189	0,193854582	0,846289747	NA
ENSG00000189046	ALKBH2	alkB homolog 2, alpha-ketoglutarate dependent dioxygenase	121642	11,71629712	-0,639645195	0,556834	-1,148717146	0,250672633	NA
ENSG00000291317	TMEM276	transmembrane protein 276	84773	11,74559874	-0,210234031	0,498327	-0,421879488	0,673112983	NA
ENSG00000144747	TMF1	TATA element modulatory factor 1	7110	329,5553785	0,027206999	0,149564	0,181909155	0,855654019	0,968439087
ENSG00000005801	ZNF195	zinc finger protein 195	7748	67,331008	0,143022938	0,252308	0,566859152	0,570809879	0,886795904
ENSG00000037474	NSUN2	NOP2/Sun RNA methyltransferase 2	54888	322,5929005	-0,0222567	0,105609	-0,210745677	0,83308573	0,963446071
ENSG00000091127	PUS7	pseudouridine synthase 7	54517	45,35450711	-0,169321269	0,257579	-0,657356862	0,510951482	0,866180216
ENSG00000127152	BCL11B	BCL11 transcription factor B	64919	180,4477335	-0,136664281	0,1211	-1,128524919	0,259098299	0,715023907
ENSG00000136854	STXBP1	syntaxin binding protein 1	6812	68,10512376	-0,03459316	0,351064	-0,098538134	0,921504993	0,984284131
ENSG00000158042	MRPL17	mitochondrial ribosomal protein L17	63875	36,83577648	0,397922384	0,324702	1,225500085	0,220386858	NA
ENSG00000167578	RAB4B	RAB4B, member RAS oncogene family	53916	90,72007071	-0,106723521	0,200047	-0,533493042	0,593692326	0,894229873
ENSG00000240891	PLCXD2	phosphatidylinositol specific phospholipase C X domain containing 2	257068	231,8011021	-0,22409605	0,119261	-1,879034627	0,060239765	0,398251782
ENSG00000285043	LOC112694756	uncharaterized LOC112694756	112694756	94,1251697	0,181153496	0,214884	0,843027557	0,399213032	0,814478813
ENSG00000177301	KCNA2	potassium voltage-gated channel subfamily A member 2	3737	55,21279105	0,392690092	0,256871	1,528742425	0,12632831	0,563689485
ENSG00000186891	TNFRSF18	TNF receptor superfamily member 18	8784	238,4353609	-0,271477091	0,116964	-2,321036959	0,020284849	0,224146577
ENSG00000267120	NA	NA	NA	61,89108185	0,123532745	0,276754	0,44636236	0,655335517	0,918124358
ENSG00000163584	RPL22L1	ribosomal protein L22 like 1	200916	29,00257259	-1,159491939	1,083348	-1,070285843	0,284490665	NA
ENSG00000189159	JPT1	Jupiter microtubule associated homolog 1	51155	233,102028	-0,072209356	0,247499	-0,291755897	0,770473268	0,945219782
ENSG00000087088	BAX	BCL2 associated X, apoptosis regulator	581	279,1161692	0,023223282	0,12974	0,178998386	0,857938969	0,969253835
ENSG00000143155	TIPRL	TOR signaling pathway regulator	261726	216,2561414	-0,104101738	0,172043	-0,605090367	0,545118955	0,878589015
ENSG00000143641	GALNT2	polypeptide N-acetylgalactosaminyltransferase 2	2590	233,1982658	0,17097909	0,124529	1,373001125	0,169751997	0,629558603
ENSG00000174483	BBS1	Bardet-Biedl syndrome 1	582	23,31049701	-0,292188489	0,43499	-0,671712716	0,501766605	NA
ENSG00000126001	CEP250	centrosomal protein 250	11190	203,2314616	0,220405509	0,144832	1,521805325	0,128057867	0,563689485
ENSG00000214114	MYCBP	MYC binding protein	26292	92,61191381	0,002922715	0,228744	0,012777228	0,989805524	0,997211687
ENSG00000254505	CHMP4A	charged multivesicular body protein 4A	29082	120,9180133	-0,079655863	0,174723	-0,455896718	0,64846425	0,914533708
ENSG00000139998	RAB15	RAB15, member RAS oncogene family	376267	16,1871874	-0,598737125	0,740191	-0,808895198	0,418575431	NA
ENSG00000036549	ZZZ3	zinc finger ZZ-type containing 3	26009	161,4442732	0,200827007	0,244749	0,820543581	0,411906296	0,82213775
ENSG00000260916	CCPG1	cell cycle progression 1	9236	192,5483632	-0,183378396	0,210824	-0,86981845	0,384399627	0,808672212
ENSG00000109790	KLHL5	kelch like family member 5	51088	180,2585492	0,248173356	0,123815	2,004389765	0,045028325	0,340112828
ENSG00000165055	METTL2B	methyltransferase 2B, tRNA N3-cytidine	55798	105,0281891	-0,200908268	0,164219	-1,22341712	0,221172191	0,677288877
ENSG00000172340	SUCLG2	succinate-CoA ligase GDP-forming subunit beta	8801	205,3340203	0,028937505	0,124756	0,231952707	0,816574746	0,959707099
ENSG00000215712	TMEM242	transmembrane protein 242	729515	101,8166557	0,359710386	0,167027	2,1536023	0,031271374	0,281657534
ENSG00000292358	GTPBP6	GTP binding protein 6 (putative)	8225	112,0679083	0,039254834	0,191136	0,205375992	0,837278376	0,965352961
ENSG00000185404	SP140L	SP140 nuclear body protein like	93349	247,6068628	0,031148493	0,108049	0,288281396	0,773131346	0,946448613
ENSG00000117592	PRDX6	peroxiredoxin 6	9588	372,6030537	0,348850029	0,129851	2,686534869	0,007219741	0,12545099
ENSG00000152495	CAMK4	calcium/calmodulin dependent protein kinase IV	814	171,0200809	0,953430163	0,252806	3,771386578	0,000162343	0,009586778
ENSG00000140451	PIF1	PIF1 5'-to-3' DNA helicase	80119	46,79538115	0,102617329	0,242103	0,423858336	0,671669131	0,923060255
ENSG00000143224	PPOX	protoporphyrinogen oxidase	5498	21,67050368	-0,611136541	0,406981	-1,50163486	0,133191435	NA
ENSG00000102393	GLA	galactosidase alpha	2717	14,60257835	-0,963515807	0,74861	-1,287072944	0,198068865	NA
ENSG00000172830	SSH3	slingshot protein phosphatase 3	54961	77,13741379	-0,025701307	0,322469	-0,079701571	0,93647461	0,987098279
ENSG00000146826	TRAPPC14	trafficking protein particle complex subunit 14	55262	83,43489667	-0,268168122	0,198081	-1,353831848	0,17579003	0,632039836
ENSG00000170604	IRF2BP1	interferon regulatory factor 2 binding protein 1	26145	62,39935174	-0,18721266	0,231806	-0,807627726	0,419304921	0,825672195

ENSG00000143319	ISG20L2	interferon stimulated exonuclease gene 20 like 2	81875	185,235625	-0,096998519	0,150293	-0,645394028	0,518671873	0,868791049
ENSG00000180747	SMG1P3	SMG1 pseudogene 3	100271836	863,3084671	0,327746664	0,211185	1,551943912	0,120675645	0,554585438
ENSG00000198715	GLMP	glycosylated lysosomal membrane protein	112770	57,72719488	-0,089070174	0,265027	-0,336079351	0,736811029	0,939157822
ENSG00000104154	SLC30A4	solute carrier family 30 member 4	7782	103,5900184	-0,247883349	0,180753	-1,371395558	0,170251681	0,629558603
ENSG00000090432	MUL1	mitochondrial E3 ubiquitin protein ligase 1	79594	105,4890191	0,036566796	0,217128	0,168411351	0,866259679	0,971722599
ENSG00000116209	TMEM59	transmembrane protein 59	9528	299,8631624	0,249219652	0,135124	1,844377138	0,065128197	0,411797014
ENSG00000075303	SLC25A40	solute carrier family 25 member 40	55972	116,6016171	-0,128906434	0,157338	-0,819296251	0,412617411	0,82225544
ENSG00000156587	UBE2L6	ubiquitin conjugating enzyme E2 L6	9246	305,9110866	0,053974202	0,093992	0,574240993	0,565804733	0,88521771
ENSG00000121691	CAT	catalase	847	202,1437725	-0,015596516	0,127261	-0,122554902	0,902459568	0,978453541
ENSG00000123360	PDE1B	phosphodiesterase 1B	5153	13,37707355	0,476914066	1,655017	0,288162722	0,773222183	NA
ENSG00000124784	RIOK1	RIO kinase 1	83732	138,4315602	0,103185362	0,154962	0,665875835	0,505490468	0,864630986
ENSG00000136783	NIPSNAP3A	nipsnap homolog 3A	25934	56,57911666	0,041320846	0,229634	0,179942211	0,857197936	0,968974178
ENSG00000166411	IDH3A	isocitrate dehydrogenase (NAD(+)) 3 catalytic subunit alpha	3419	16,66811558	1,056181371	0,849778	1,242890932	0,213908028	NA
ENSG00000168575	SLC20A2	solute carrier family 20 member 2	6575	114,3916804	0,031346246	0,160986	0,19471371	0,845617078	0,967231052
ENSG00000173545	ZNF622	zinc finger protein 622	90441	81,01533884	-0,168029765	0,188163	-0,892998991	0,371857713	0,800375577
ENSG00000141741	MIEN1	migration and invasion enhancer 1	84299	41,87894077	0,141635632	0,28207	0,502129344	0,61557654	NA
ENSG00000157693	TMEM268	transmembrane protein 268	203197	66,55887838	-0,286228939	0,212212	-1,348787544	0,177405217	0,632039836
ENSG00000103494	RPGRIP1L	RPGRIP1 like	23322	19,8514878	-0,42032222	0,582007	-0,72219452	0,470174892	NA
ENSG00000104824	HNRNPL	heterogeneous nuclear ribonucleoprotein L	3191	117,3136805	-0,605688474	0,490313	-1,235310351	0,216715023	0,672891335
ENSG00000090447	TFAP4	transcription factor AP-4	7023	14,11647241	0,069765645	0,546516	0,127655313	0,898421756	NA
ENSG00000101596	SMCHD1	structural maintenance of chromosomes flexible hinge domain containing 1	23347	720,6698688	-0,041755928	0,090647	-0,460642345	0,645055226	0,912530858
ENSG00000114956	DGUOK	deoxyguanosine kinase	1716	71,02777256	0,562068039	0,26066	2,156322878	0,031058462	0,281657534
ENSG00000123080	CDKN2C	cyclin dependent kinase inhibitor 2C	1031	96,14990537	0,16371383	0,180574	0,906630114	0,364602431	0,793491925
ENSG00000125826	RBCK1	RANBP2-type and C3HC4-type zinc finger containing 1	10616	172,7127254	0,35493571	0,202408	1,753561969	0,079505592	0,45762333
ENSG00000130844	ZNF331	zinc finger protein 331	55422	41,79337075	-0,17559908	0,314942	-0,557559354	0,577145318	NA
ENSG00000213380	COG8	component of oligomeric golgi complex 8	84342	127,9603758	-0,2504319	0,214885	-1,165424289	0,24384729	0,700945995
ENSG00000121579	NAA50	N-alpha-acetyltransferase 50, NatE catalytic subunit	80218	368,2913545	-0,13240045	0,12711	-1,041622395	0,297586782	0,748397881
ENSG00000163867	ZMYM6	zinc finger MYM-type containing 6	9204	129,6271907	-0,134417653	0,274128	-0,490346383	0,623888811	0,90479929
ENSG00000156931	VPS8	VPS8 subunit of CORVET complex	23355	255,8200932	0,079587535	0,113059	0,703948477	0,481464861	0,855577353
ENSG00000099904	ZDHHC8	zinc finger DHHC-type palmitoyltransferase 8	29801	146,4446266	-0,021920576	0,17507	-0,125210402	0,900356983	0,977990819
ENSG00000135587	SMPD2	sphingomyelin phosphodiesterase 2	6610	31,59196788	0,025594259	0,324367	0,078905327	0,937107926	NA
ENSG00000023892	DEF6	DEF6 guanine nucleotide exchange factor	50619	243,4305028	-0,297457473	0,158464	-1,877127758	0,060500586	0,398308113
ENSG00000048052	HDAC9	histone deacetylase 9	9734	83,51682319	0,2958005	0,203123	1,456262326	0,145320116	0,59259823
ENSG00000051180	RAD51	RAD51 recombinase	5888	27,60376503	0,124716063	0,395722	0,315160818	0,752639554	NA
ENSG00000095066	HOOK2	hook microtubule tethering protein 2	29911	28,0524023	-0,218621355	0,444103	-0,492276722	0,622523734	NA
ENSG00000101194	SLC17A9	solute carrier family 17 member 9	63910	41,22917089	0,351960069	0,460462	0,764362141	0,444651463	NA
ENSG00000119185	ITGB1BP1	integrin subunit beta 1 binding protein 1	9270	113,6799444	-0,022399566	0,178113	-0,125760075	0,899921846	0,977990819
ENSG00000124733	MEA1	male-enhanced antigen 1	4201	100,2810339	0,082165706	0,180445	0,455350329	0,648857224	0,914799182
ENSG00000130159	ECSIT	ECSIT signaling integrator	51295	25,33508992	-0,166357823	0,447348	-0,371875252	0,709985732	NA
ENSG00000130758	MAP3K10	mitogen-activated protein kinase kinase kinase 10	4294	70,33307344	0,159553048	0,206026	0,774430375	0,438676327	0,837388481
ENSG00000134146	DPH6	diphthamine biosynthesis 6	89978	13,16400896	-0,236394511	0,540784	-0,437132744	0,662015082	NA
ENSG00000138029	HADHB	hydroxyacyl-CoA dehydrogenase trifunctional multienzyme complex subunit beta	3032	167,3943507	0,081166501	0,156187	0,519676581	0,603289012	0,897212117
ENSG00000138646	HERC5	HECT and RLD domain containing E3 ubiquitin protein ligase 5	51191	33,76321279	0,675594196	0,352111	1,918699014	0,055022436	NA
ENSG00000155957	TMBIM4	transmembrane BAX inhibitor motif containing 4	51643	192,1293212	-0,131794112	0,149902	-0,879204002	0,379290675	0,804903799
ENSG00000159200	RCAN1	regulator of calcineurin 1	1827	78,75480116	-0,157336982	0,188044	-0,836702922	0,402759577	0,816750249
ENSG00000166225	FRS2	fibroblast growth factor receptor substrate 2	10818	109,7967959	-0,055466679	0,168408	-0,329359092	0,741884284	0,939193793
ENSG00000181544	FANCB	FA complementation group B	2187	18,98169069	-0,100424921	0,530012	-0,189476819	0,849719122	NA

ENSG00000183576	SETD3	SET domain containing 3, actin N3(tau)-histidine methyltransferase	84193	231,2932742	0,143104695	0,11153	1,283100091	0,199457012	0,658208139
ENSG00000183918	SH2D1A	SH2 domain containing 1A	4068	281,0489512	0,129224646	0,115888	1,115079276	0,264816451	0,720421404
ENSG00000185721	DRG1	developmentally regulated GTP binding protein 1	4733	98,89003572	-0,190696524	0,203691	-0,936207213	0,34916651	0,784537594
ENSG00000214389	RPS3AP26	RPS3A pseudogene 26	644972	11,8158494	0,4234989	0,758281	0,558498406	0,576504093	NA
ENSG00000234420	ZNF37BP	zinc finger protein 37B, pseudogene	100129482	84,66644256	-0,106207349	0,191782	-0,553793451	0,579720211	0,887548253
ENSG00000274211	SOCS7	suppressor of cytokine signaling 7	30837	67,43590505	0,384878606	0,291865	1,31868653	0,187273932	0,645758518
ENSG00000131408	NR1H2	nuclear receptor subfamily 1 group H member 2	7376	139,8736658	-0,196322277	0,270792	-0,724991828	0,468457044	0,851483828
ENSG00000105982	RNF32	ring finger protein 32	140545	21,44621897	-0,159761674	0,410278	-0,389399043	0,69698098	NA
ENSG00000163781	TOPBP1	DNA topoisomerase II binding protein 1	11073	341,9349614	0,074337034	0,089691	0,828810176	0,407211829	0,819431644
ENSG00000102897	LYRM1	LYR motif containing 1	57149	43,67191387	0,288868432	0,288965	0,999664667	0,317472816	0,762292941
ENSG00000129925	PGAP6	post-GPI attachment to proteins 6	58986	151,4782131	0,205988045	0,157966	1,30400468	0,192231987	0,649982152
ENSG00000132664	POLR3F	RNA polymerase III subunit F	10621	34,91920975	-0,122532265	0,449813	-0,272407045	0,785309056	NA
ENSG00000135205	CCDC146	coiled-coil domain containing 146	57639	49,94286038	0,146810747	0,315972	0,464632418	0,642194713	0,912063088
ENSG00000136261	BZW2	basic leucine zipper and W2 domains 2	28969	103,295991	0,006027955	0,171125	0,035225462	0,971899959	0,994108581
ENSG00000169446	MMGT1	membrane magnesium transporter 1	93380	166,6393338	-0,329649698	0,127504	-2,585412193	0,009726264	0,151567619
ENSG00000178971	CTC1	CST telomere replication complex component 1	80169	421,4522981	0,00721075	0,11257	0,064055526	0,948926014	0,9906219
ENSG00000189319	FAM53B	family with sequence similarity 53 member B	9679	143,2774016	-0,101108817	0,152131	-0,664618373	0,506294615	0,864630986
ENSG00000196205	NA	NA	NA	24,37303115	0,538662428	0,408175	1,319686053	0,186939859	NA
ENSG00000106799	TGFBR1	transforming growth factor beta receptor 1	7046	426,8799882	-0,142583465	0,445879	-0,319780486	0,749134742	0,940489812
ENSG00000110514	MADD	MAP kinase activating death domain	8567	692,2893041	0,040413545	0,071677	0,563830128	0,572869746	0,886892687
ENSG00000115944	COX7A2L	cytochrome c oxidase subunit 7A2 like	9167	127,0425405	0,033161948	0,157356	0,21074539	0,833085954	0,963446071
ENSG00000163041	H3-3A	H3,3 histone A	3020	890,806774	0,078159367	0,07137	1,095136413	0,273456878	0,726913977
ENSG00000134452	FBH1	F-box DNA helicase 1	84893	249,1867062	-0,123319511	0,106803	-1,154646893	0,248235056	0,706134779
ENSG00000204149	AGAP6	ArfGAP with GTPase domain, ankyrin repeat and PH domain 6	414189	47,53589512	-0,197767542	0,295412	-0,669462345	0,503200593	0,863200032
ENSG00000143578	CREB3L4	cAMP responsive element binding protein 3 like 4	148327	23,63586424	0,032833144	0,371018	0,088494827	0,929483395	NA
ENSG00000240065	PSMB9	proteasome 20S subunit beta 9	5698	33,61145121	0,294178019	0,326483	0,901051656	0,367560855	NA
ENSG00000042088	TDP1	tyrosyl-DNA phosphodiesterase 1	55775	168,0820928	0,176664379	0,161545	1,093595517	0,274132416	0,727065559
ENSG00000107262	BAG1	BAG cochaperone 1	573	159,4900115	0,423017355	0,15844	2,669890714	0,007587594	0,129268898
ENSG00000133739	LRRCC1	leucine rich repeat and coiled-coil centrosomal protein 1	85444	20,55072435	-0,469210297	0,653709	-0,717766356	0,472901361	NA
ENSG00000135749	PCNX2	pecanex 2	80003	293,1662299	0,173107857	0,157016	1,10248235	0,270252028	0,725477256
ENSG00000221923	ZNF880	zinc finger protein 880	400713	34,89676517	0,3496508	0,343405	1,018186704	0,308589237	NA
ENSG00000005175	RPAP3	RNA polymerase II associated protein 3	79657	136,2996596	0,079016785	0,259106	0,304959797	0,760396772	0,942901768
ENSG00000135018	UBQLN1	ubiquilin 1	29979	434,0859375	-0,064495351	0,096155	-0,670745803	0,502382479	0,86280677
ENSG00000137547	MRPL15	mitochondrial ribosomal protein L15	29088	22,87021234	0,0761727	0,553501	0,137619701	0,890540985	NA
ENSG00000161904	LEMD2	LEM domain nuclear envelope protein 2	221496	123,4481923	0,097582285	0,223809	0,436006709	0,662831862	0,920743093
ENSG00000205323	SARNP	SAP domain containing ribonucleoprotein	84324	111,6669696	0,09357734	0,155738	0,600865299	0,547929707	0,879578516
ENSG00000101605	MYOM1	myomesin 1	8736	17,60857164	-0,128597357	0,446831	-0,287798543	0,773500952	NA
ENSG00000144028	SNRNP200	small nuclear ribonucleoprotein U5 subunit 200	23020	975,8022721	0,42494994	0,247683	1,71570295	0,086216424	0,47957096
ENSG00000171943	SRGAP2C	SLIT-ROBO Rho GTPase activating protein 2C	653464	106,7356055	-0,052401771	0,260362	-0,201264849	0,840491487	0,966505956
ENSG00000065548	ZC3H15	zinc finger CCCH-type containing 15	55854	238,9295536	0,055322349	0,129791	0,426243126	0,669930695	0,922641254
ENSG00000156697	UTP14A	UTP14A small subunit processome component	10813	89,71246313	0,177062175	0,201297	0,879605597	0,379073006	0,804903799
ENSG00000215908	NA	NA	NA	40,71347512	-0,242147545	0,321692	-0,752730813	0,45161169	NA
ENSG00000118412	CASP8AP2	caspase 8 associated protein 2	9994	37,23632378	2,250875016	3,25273	0,691995577	0,488940107	NA
ENSG00000196387	ZNF140	zinc finger protein 140	7699	15,56821956	-0,159166617	0,859444	-0,1851973	0,853074287	NA
ENSG00000229180	NA	NA	NA	19,47447714	-0,032646472	0,434372	-0,07515781	0,940089152	NA
ENSG00000242802	AP5Z1	adaptor related protein complex 5 subunit zeta 1	9907	49,79879264	0,48191129	1,17537	0,410008001	0,681800078	0,925586358

ENSG00000099377	HSD3B7	hydroxy-delta-5-steroid dehydrogenase, 3 beta- and steroid delta-isomerase 7	80270	24,73461627	-0,067542019	0,38305	-0,176326838	0,860037179	NA
ENSG00000011566	MAP4K3	mitogen-activated protein kinase kinase kinase kinase 3	8491	41,92849352	0,049744885	0,268004	0,185612687	0,852748504	NA
ENSG00000263465	SRSF8	serine and arginine rich splicing factor 8	10929	206,9177514	0,04816375	0,131473	0,366339255	0,714111941	0,931512238
ENSG00000080371	RAB21	RAB21, member RAS oncogene family	23011	201,8219112	-0,502754352	0,123946	-4,056234884	4,99E-05	0,003730284
ENSG00000103037	SETD6	SET domain containing 6, protein lysine methyltransferase	79918	57,44499405	-0,105967944	0,337796	-0,313704167	0,753745735	0,942812391
ENSG00000119421	NDUFA8	NADH:ubiquinone oxidoreductase subunit A8	4702	45,24452019	-0,080029791	0,277203	-0,288705109	0,772807053	0,946448613
ENSG00000126062	TMEM115	transmembrane protein 115	11070	64,93856188	-0,201452241	0,269131	-0,748528031	0,454141724	0,843234809
ENSG00000164830	OXR1	oxidation resistance 1	55074	161,0399614	0,109369292	0,15913	0,687294883	0,491896923	0,860513824
ENSG00000169567	HINT1	histidine triad nucleotide binding protein 1	3094	373,6816953	0,301081811	0,100891	2,984220527	0,002843018	0,069661304
ENSG00000175792	RUVBL1	RuvB like AAA ATPase 1	8607	70,13829868	0,05437271	0,210159	0,258721186	0,795850372	0,953436042
ENSG00000177842	ZNF620	zinc finger protein 620	253639	19,82099475	0,505364188	0,423655	1,192868301	0,232920955	NA
ENSG00000179588	ZFPM1	zinc finger protein, FOG family member 1	161882	80,82144097	0,190217647	0,237124	0,802185163	0,422445858	0,827816808
ENSG00000101057	MYBL2	MYB proto-oncogene like 2	4605	73,36667421	0,420728638	0,241615	1,741319076	0,081627664	0,464231481
ENSG00000070269	TMEM260	transmembrane protein 260	54916	68,9516572	0,685195245	1,225004	0,559341082	0,575928963	0,887069818
ENSG00000122033	MTIF3	mitochondrial translational initiation factor 3	219402	53,73772507	-0,027281334	0,258338	-0,105603256	0,915897141	0,981907746
ENSG00000130429	ARPC1B	actin related protein 2/3 complex subunit 1B	10095	518,1055013	-0,035086583	0,088229	-0,397676447	0,690868701	0,927252461
ENSG00000131238	PPT1	palmitoyl-protein thioesterase 1	5538	322,4391382	0,196350213	0,103254	1,901614104	0,057221623	0,38821726
ENSG00000159256	MORC3	MORC family CW-type zinc finger 3	23515	270,6428498	-0,042928627	0,100865	-0,425604011	0,670396417	0,922641254
ENSG00000062582	MRPS24	mitochondrial ribosomal protein S24	64951	47,36634663	0,004724715	0,278754	0,016949421	0,986476966	0,996756734
ENSG00000089916	GPATCH2L	G-patch domain containing 2 like	55668	581,3497028	-0,120744065	0,111834	-1,079670976	0,280288723	0,733410709
ENSG00000162976	SLC66A3	solute carrier family 66 member 3	130814	93,8816721	0,081263269	0,214362	0,379094168	0,704617937	0,930846779
ENSG00000167792	NDUFV1	NADH:ubiquinone oxidoreductase core subunit V1	4723	274,6197612	0,001976686	0,127215	0,015538208	0,987602803	0,996894778
ENSG00000182004	SNRPE	small nuclear ribonucleoprotein polypeptide E	6635	74,32177484	-0,160801109	0,684366	-0,234963596	0,814236988	0,959207071
ENSG00000197442	MAP3K5	mitogen-activated protein kinase kinase kinase 5	4217	164,6182072	-0,008928756	0,142943	-0,062463542	0,950193695	0,990946923
ENSG00000198382	UVRAG	UV radiation resistance associated	7405	90,24190419	-0,037038105	0,181447	-0,204126593	0,838254574	0,965627959
ENSG00000225614	ZNF469	zinc finger protein 469	84627	60,62432735	0,041345343	0,25055	0,165018323	0,86892956	0,973118501
ENSG00000108021	TASOR2	transcription activation suppressor family member 2	54906	420,5226474	0,024582489	0,092756	0,265024002	0,790990972	0,951704496
ENSG00000100023	PPIL2	peptidylprolyl isomerase like 2	23759	307,749922	-0,207350821	0,180478	-1,148897865	0,250598098	0,707335884
ENSG00000072310	SREBF1	sterol regulatory element binding transcription factor 1	6720	75,99512214	-0,079776329	0,198572	-0,401749248	0,687868576	0,926748702
ENSG00000090661	CERS4	ceramide synthase 4	79603	105,476731	0,17217289	0,184753	0,931906149	0,35138503	0,785712602
ENSG00000105851	PIK3CG	phosphatidylinositol-4,5-bisphosphate 3-kinase catalytic subunit gamma	5294	765,7794107	-0,00691499	0,078744	-0,087816539	0,930022493	0,986487751
ENSG00000124532	MRS2	magnesium transporter MRS2	57380	80,15261315	0,262633254	0,218178	1,203755307	0,228684169	0,68351286
ENSG00000128563	PRKRIP1	PRKR interacting protein 1	79706	86,2097991	-0,697217153	0,279362	-2,495748973	0,012569151	0,176138115
ENSG00000139437	TCHP	trichoplein keratin filament binding	84260	104,5906289	-0,185357814	0,194413	-0,953421453	0,340376576	0,777431905
ENSG00000147853	AK3	adenylate kinase 3	50808	140,6186556	0,068736652	0,161044	0,426818415	0,669511593	0,922641254
ENSG00000154222	CC2D1B	coiled-coil and C2 domain containing 1B	200014	177,6889568	0,024694311	0,138298	0,17855881	0,858284139	0,969285743
ENSG00000158863	FHIP2B	FHF complex subunit HOOK interacting protein 2B	64760	86,73508737	0,235569195	0,323592	0,727981768	0,466624747	0,850142242
ENSG00000167525	PROCA1	protein interacting with cyclin A1	147011	12,77931906	-0,170677872	0,716153	-0,238325826	0,811628392	NA
ENSG00000188315	C3orf62	chromosome 3 open reading frame 62	375341	55,40663776	0,085657651	0,292076	0,293271816	0,769314395	0,944829104
ENSG00000196704	AMZ2	archaelysin family metallopeptidase 2	51321	246,8402901	0,067965525	0,146216	0,46482843	0,642054326	0,912063088
ENSG00000198252	STYX	serine/threonine/tyrosine interacting protein	6815	142,173945	-0,040240253	0,154058	-0,261201355	0,793937233	0,952276252
ENSG00000253352	TUG1	taurine up-regulated 1	55000	225,8753525	-0,279825119	0,14685	-1,905515517	0,056713092	0,386653322
ENSG00000100528	CNIH1	cornichon family AMPA receptor auxiliary protein 1	10175	100,8656388	0,257034697	0,327638	0,784508822	0,432741607	0,834691048

ENSG00000213672	NCKIPSD	NCK interacting protein with SH3 domain	51517	44,05444228	-0,014346954	0,285321	-0,050283481	0,959896488	0,992235217
ENSG00000120963	ZNF706	zinc finger protein 706	51123	130,2710902	-0,412927947	0,172543	-2,393192207	0,016702489	0,203697743
ENSG00000157036	EXOG	exo/endonuclease G	9941	102,2703708	0,191719001	0,215687	0,88887724	0,374069059	0,801401633
ENSG00000244720	NA	NA	NA	67,76164368	-0,102361735	0,212079	-0,482659168	0,62933776	0,905567722
ENSG00000078747	ITCH	itchy E3 ubiquitin protein ligase	83737	235,6180277	0,315212451	0,473345	0,665925046	0,505459011	0,864630986
ENSG00000144746	ARL6IP5	ADP ribosylation factor like GTPase 6 interacting protein 5	10550	1270,883682	0,193751292	0,052287	3,705562894	0,000210922	0,011117984
ENSG00000171503	ETFDH	electron transfer flavoprotein dehydrogenase	2110	26,51124399	-0,201788168	0,724001	-0,278712384	0,780465558	NA
ENSG00000183963	SMTN	smoothelin	6525	14,43668756	-0,550574072	2,145219	-0,256651724	0,797447642	NA
ENSG00000185869	ZNF829	zinc finger protein 829	374899	33,39388505	0,437082737	0,409481	1,067406049	0,285788518	NA
ENSG00000089775	ZBTB25	zinc finger and BTB domain containing 25	7597	246,6715943	-0,096882192	0,12792	-0,757362961	0,44883244	0,84263906
ENSG00000186153	WWOX	WW domain containing oxidoreductase	51741	23,43706057	1,467531329	0,412934	3,553914753	0,000379542	NA
ENSG00000188747	NOXA1	NADPH oxidase activator 1	10811	60,44880744	0,07564721	0,235637	0,321032467	0,748185787	0,939899419
ENSG00000087152	ATXN7L3	ataxin 7 like 3	56970	304,863808	-0,068647235	0,100311	-0,684342087	0,493759177	0,860513824
ENSG00000105401	CDC37	cell division cycle 37, HSP90 cochaperone	11140	451,4983484	-0,210244231	0,099376	-2,115647775	0,034374775	0,296348392
ENSG00000105643	ARRDC2	arrestin domain containing 2	27106	163,1840373	0,025326439	0,285263	0,088782701	0,929254606	0,986487751
ENSG00000110367	DDX6	DEAD-box helicase 6	1656	1310,53632	-0,16695712	0,062313	-2,679346395	0,007376604	0,126634419
ENSG00000113712	CSNK1A1	casein kinase 1 alpha 1	1452	674,5289337	0,134455599	0,073922	1,818884052	0,068929121	0,426428338
ENSG00000116106	EPHA4	EPH receptor A4	2043	125,8983655	-0,031907464	0,151875	-0,210090714	0,833596873	0,963446071
ENSG00000125257	ABCC4	ATP binding cassette subfamily C member 4	10257	84,95002378	0,173829308	0,226017	0,769099243	0,441834397	0,838678081
ENSG00000130699	TAF4	TATA-box binding protein associated factor 4	6874	109,7437552	0,19168817	0,224336	0,854470252	0,392844485	0,812491435
ENSG00000135637	CCDC142	coiled-coil domain containing 142	84865	62,00574291	-0,039532421	0,266408	-0,148390399	0,882034677	0,976300778
ENSG00000135778	NTPCR	nucleoside-triphosphatase, cancer-related	84284	37,27823403	-0,416952818	0,302331	-1,379126335	0,167855805	NA
ENSG00000138796	HADH	hydroxyacyl-CoA dehydrogenase	3033	59,25513717	0,207611063	0,240678	0,862610812	0,388351484	0,810040214
ENSG00000141959	PFKL	phosphofructokinase, liver type	5211	328,6865192	-0,227548229	0,095328	-2,387006739	0,01698618	0,204616219
ENSG00000144567	RETREG2	reticulophagy regulator family member 2	79137	220,1503236	-0,167839926	0,18328	-0,915756769	0,359794483	0,79112567
ENSG00000145220	LYAR	Ly1 antibody reactive	55646	51,77022615	-0,011249796	0,277065	-0,040603457	0,967612028	0,993285175
ENSG00000153107	ANAPC1	anaphase promoting complex subunit 1	64682	349,0427965	0,184115401	0,134707	1,366787737	0,171691848	0,629558603
ENSG00000156535	CD109	CD109 molecule	135228	133,1022121	0,162236325	0,169833	0,955271174	0,339440581	0,777431905
ENSG00000160050	CCDC28B	coiled-coil domain containing 28B	79140	15,06237182	-0,683560752	0,46476	-1,47078189	0,141350112	NA
ENSG00000165476	REEP3	receptor accessory protein 3	221035	65,30774089	0,079786857	0,236179	0,337824229	0,73549565	0,939157822
ENSG00000169252	ADRB2	adrenoceptor beta 2	154	28,58645635	-0,206410437	0,360475	-0,572607074	0,566910769	NA
ENSG00000171806	METTL18	methyltransferase 18, RPL3 N3(tau)-histidine	92342	32,33903121	0,069772897	0,343737	0,202983508	0,839147922	NA
ENSG00000172057	ORMDL3	ORMDL sphingolipid biosynthesis regulator 3	94103	298,1278958	0,036818279	0,099271	0,370887746	0,710721143	0,930846779
ENSG00000177432	NAP1L5	nucleosome assembly protein 1 like 5	266812	21,61542466	-0,00304711	0,388709	-0,00783906	0,993745399	NA
ENSG00000181409	AATK	apoptosis associated tyrosine kinase	9625	15,35831057	-0,417623481	0,892606	-0,467869804	0,639877698	NA
ENSG00000196227	FAM217B	family with sequence similarity 217 member B	63939	159,2935766	0,002885904	0,14513	0,019884924	0,984135172	0,996185513
ENSG00000215717	TMEM167B	transmembrane protein 167B	56900	170,1932587	-0,155519966	0,125781	-1,23643528	0,216296809	0,672891335
ENSG00000106638	TBL2	transducin beta like 2	26608	16,58218028	-0,696413541	0,804623	-0,865515596	0,38675586	NA
ENSG00000137221	TJAP1	tight junction associated protein 1	93643	105,0181776	-0,131332951	0,174231	-0,753788477	0,450976244	0,842718653
ENSG00000101294	HM13	histocompatibility minor 13	81502	329,9472735	0,09157678	0,096777	0,946262405	0,344014763	0,781132432
ENSG00000134077	THUMPD3	THUMP domain containing 3	25917	144,9761467	0,205420187	0,2279	0,901359179	0,367397378	0,795318634
ENSG00000091140	DLD	dihydrolipoamide dehydrogenase	1738	159,3288758	0,169773488	0,421141	0,403126967	0,686854823	0,926748702
ENSG00000099875	MKNK2	MAPK interacting serine/threonine kinase 2	2872	287,1530699	-0,189526268	0,101001	-1,876481564	0,060589184	0,398308113
ENSG00000111331	OAS3	2'-5'-oligoadenylate synthetase 3	4940	238,0472878	0,41532975	0,424573	0,978228324	0,327961411	0,770277788
ENSG00000131669	NINJ1	ninjurin 1	4814	18,40128187	0,181235326	0,426762	0,424674928	0,671073664	NA
ENSG00000172269	DPAGT1	dolichyl-phosphate N-acetylglucosaminephosphotransferase 1	1798	77,75193764	0,161942375	0,29337	0,552007056	0,580943519	0,888209149
ENSG00000185338	SOCS1	suppressor of cytokine signaling 1	8651	167,5272106	0,030874759	0,129169	0,239026979	0,811084663	0,959038743
ENSG00000144741	SLC25A26	solute carrier family 25 member 26	115286	18,51747131	-0,49495997	0,595059	-0,83178259	0,405531673	NA
ENSG00000138382	METTL5	methyltransferase 5, N6-adenosine	29081	51,64738981	0,182908798	0,286518	0,638384467	0,52322344	0,870221868

ENSG00000174032	SLC25A30	solute carrier family 25 member 30	253512	235,7526191	-0,020494778	0,123931	-0,165372092	0,868651119	0,973097402
ENSG00000183828	NUDT14	nudix hydrolase 14	256281	39,72049125	-0,15747657	0,273428	-0,575934949	0,564659152	NA
ENSG00000115233	PSMD14	proteasome 26S subunit, non-ATPase 14	10213	172,4935747	0,332806495	0,134228	2,479409428	0,013160015	0,18006752
ENSG00000244038	DDOST	dolichyl-diphosphooligosaccharide--protein glycosyltransferase non-catalytic subunit	1650	629,5041104	-0,028105984	0,071039	-0,395640351	0,692370365	0,927787546
ENSG00000024862	CCDC28A	coiled-coil domain containing 28A	25901	61,84259717	-0,312495376	0,228956	-1,364869686	0,172294011	0,629558603
ENSG00000069702	TGFBR3	transforming growth factor beta receptor 3	7049	273,9520257	0,024818874	0,105331	0,235626994	0,813722125	0,959207071
ENSG00000084093	REST	RE1 silencing transcription factor	5978	468,269318	-0,235977269	0,09173	-2,572531454	0,010095778	0,155474984
ENSG00000136381	IREB2	iron responsive element binding protein 2	3658	431,7091321	0,107757237	0,098284	1,096387795	0,272909103	0,726913977
ENSG00000137628	DDX60	DExD/H-box helicase 60	55601	210,1223824	-0,039434577	0,166112	-0,237398065	0,812347991	0,959038743
ENSG00000155508	CNOT8	CCR4-NOT transcription complex subunit 8	9337	307,5757492	-0,074567404	0,105957	-0,703753853	0,481586077	0,855577353
ENSG00000241837	ATP5PO	ATP synthase peripheral stalk subunit OSCP	539	178,4736013	0,293451671	0,135592	2,164231196	0,03044661	0,279077601
ENSG00000242259	C22orf39	chromosome 22 open reading frame 39	128977	119,1714025	-0,235025066	0,170058	-1,382025757	0,166963793	0,626832518
ENSG00000135776	ABCB10	ATP binding cassette subfamily B member 10	23456	154,5291903	0,094676258	0,146663	0,645536675	0,51857946	0,868791049
ENSG00000103502	CDIPT	CDP-diacylglycerol--inositol 3-phosphatidyltransferase	10423	170,7046045	0,097449365	0,138469	0,703762703	0,481580564	0,855577353
ENSG00000168434	COG7	component of oligomeric golgi complex 7	91949	62,68308381	0,106893736	0,229072	0,46663764	0,640759152	0,911523706
ENSG00000083814	ZNF671	zinc finger protein 671	79891	28,73238717	-1,059580389	1,088446	-0,973480149	0,330314721	NA
ENSG00000175931	UBE2O	ubiquitin conjugating enzyme E2 O	63893	168,6114915	-0,313887308	0,15559	-2,017399381	0,043653852	0,333843576
ENSG00000243667	DNAAF10	dynein axonemal assembly factor 10	116143	44,01249862	0,010604723	0,286884	0,036965162	0,970512784	0,993542794
ENSG00000140464	PML	PML nuclear body scaffold	5371	235,0482827	0,024428051	0,122632	0,199198791	0,842107245	0,966959288
ENSG00000155008	APOOL	apolipoprotein O like	139322	72,05524403	-0,284630376	0,224603	-1,267257277	0,205063312	0,660954946
ENSG00000182054	IDH2	isocitrate dehydrogenase (NADP(+)) 2	3418	288,2272508	0,105578143	0,12547	0,841460302	0,400090116	0,814673014
ENSG00000113407	TARS1	threonyl-tRNA synthetase 1	6897	518,4522116	0,068291957	0,093848	0,727688141	0,466804511	0,850251074
ENSG00000150401	DCUN1D2	defective in cullin neddylation 1 domain containing 2	55208	26,08915121	-0,042624973	0,685483	-0,06218239	0,950417586	NA
ENSG00000151689	INPP1	inositol polyphosphate-1-phosphatase	3628	54,51820848	0,293884892	0,238316	1,233174663	0,217510606	0,673519155
ENSG00000228300	FAM174C	family with sequence similarity 174 member C	55009	39,11126542	-0,543327774	0,286947	-1,893476222	0,058294567	NA
ENSG00000104885	DOT1L	DOT1 like histone lysine methyltransferase	84444	375,6575165	-0,165990265	0,108115	-1,535314769	0,124706528	0,5624706
ENSG00000003056	M6PR	mannose-6-phosphate receptor, cation dependent	4074	513,9246434	-0,089998503	0,076535	-1,175913922	0,239629275	0,694482777
ENSG00000109084	TMEM97	transmembrane protein 97	27346	44,60849287	0,399948459	0,341347	1,171675775	0,241327253	0,696802579
ENSG00000160791	CCR5	C-C motif chemokine receptor 5	1234	317,7626078	-0,259664764	0,100416	-2,585882885	0,009712993	0,151567619
ENSG00000136802	LRRC8A	leucine rich repeat containing 8 VRAC subunit A	56262	101,0613072	-0,023957333	0,169713	-0,14116373	0,887740598	0,977990819
ENSG00000044090	CUL7	cullin 7	9820	44,45837891	-0,147940884	0,461184	-0,320785068	0,748373276	0,939984601
ENSG00000128228	SDF2L1	stromal cell derived factor 2 like 1	23753	67,70600894	0,166457016	0,201841	0,824693071	0,409545866	0,82130194
ENSG00000149554	CHEK1	checkpoint kinase 1	1111	53,11536958	0,836497978	0,242404	3,450843091	0,000558838	0,022393452
ENSG00000160208	RRP1B	ribosomal RNA processing 1B	23076	272,8840594	0,057396527	0,14119	0,406520486	0,684360208	0,925601011
ENSG00000175550	DRAP1	DR1 associated protein 1	10589	182,9465356	0,009695591	0,15038	0,064473865	0,948592916	0,990608484
ENSG00000180902	D2HGDH	D-2-hydroxyglutarate dehydrogenase	728294	17,18797374	0,423610469	0,750169	0,564686841	0,572286786	NA
ENSG00000174839	DENND6A	DENN domain containing 6A	201627	128,4142688	0,057669608	0,165968	0,347474785	0,728234659	0,937631969
ENSG00000120709	FAM53C	family with sequence similarity 53 member C	51307	164,2735277	-0,111887968	0,133162	-0,840238196	0,400774846	0,814673014
ENSG00000120709	LOC100128966	uncharacterized LOC100128966	100128966	164,2735277	-0,111887968	0,133162	-0,840238196	0,400774846	0,814673014
ENSG00000013374	NUB1	negative regulator of ubiquitin like proteins 1	51667	318,7665006	-0,049448583	0,109407	-0,451969118	0,65129123	0,915725264
ENSG00000059378	PARP12	poly(ADP-ribose) polymerase family member 12	64761	72,71547272	-0,004164809	0,343998	-0,012107064	0,990340196	0,99732426
ENSG00000076650	GPATCH1	G-patch domain containing 1	55094	55,78263155	-0,077738429	0,297783	-0,261057024	0,794048533	0,952276252
ENSG00000100124	ANKRD54	ankyrin repeat domain 54	129138	36,99066623	-0,054316088	0,299396	-0,181419013	0,856038696	NA
ENSG00000105708	ZNF14	zinc finger protein 14	7561	64,10485521	0,019730406	0,259652	0,075987906	0,93942872	0,987813564
ENSG00000126524	SBDS	SBDS ribosome maturation factor	51119	97,12110143	0,135011893	0,215332	0,626995018	0,530662501	0,872577406

ENSG00000134539	KLRD1	killer cell lectin like receptor D1	3824	2897,995346	-0,19927896	0,099098	-2,010929547	0,044332899	0,33739398
ENSG00000137055	PLAA	phospholipase A2 activating protein	9373	164,5415153	0,042716806	0,150145	0,284503817	0,77602431	0,946448613
ENSG00000147130	ZMYM3	zinc finger MYM-type containing 3	9203	277,1394125	-0,10881347	0,119221	-0,912705735	0,361397327	0,791408368
ENSG00000164941	INTS8	integrator complex subunit 8	55656	57,62198417	-0,060760215	0,355601	-0,170866361	0,864328847	0,971561202
ENSG00000165688	PMPCA	peptidase, mitochondrial processing subunit alpha	23203	118,5798912	-0,106609534	0,165785	-0,643058034	0,520186444	0,86944974
ENSG00000175662	TOM1L2	target of myb1 like 2 membrane trafficking protein	146691	106,0597044	-0,252041115	0,183066	-1,376780126	0,168580237	0,628097335
ENSG00000187097	ENTPD5	ectonucleoside triphosphate diphosphohydrolase 5 (inactive)	957	59,45064858	0,102655073	0,225694	0,454842567	0,649222504	0,91482326
ENSG00000165512	ZNF22	zinc finger protein 22	7570	151,1600884	0,009616999	0,151784	0,063359819	0,949479982	0,990700279
ENSG00000151612	ZNF827	zinc finger protein 827	152485	13,35066219	0,145341962	0,938676	0,154837145	0,876949707	NA
ENSG00000011304	PTBP1	polypyrimidine tract binding protein 1	5725	747,1955617	0,015105624	0,062312	0,242418923	0,808455573	0,958576399
ENSG00000163848	ZNF148	zinc finger protein 148	7707	332,5112151	0,014044828	0,113374	0,123880139	0,901410178	0,977990819
ENSG00000140931	CMTM3	CKLF like MARVEL transmembrane domain containing 3	123920	305,400934	0,081520593	0,093793	0,869158796	0,384760281	0,809078245
ENSG00000257949	TEN1	TEN1 subunit of CST complex	100134934	23,46853718	0,247564936	0,379996	0,651493358	0,514728063	NA
ENSG00000271303	SRXN1	sulfiredoxin 1	140809	80,5703104	-0,074096499	0,205733	-0,360157848	0,718729095	0,932886847
ENSG00000125629	INSIG2	insulin induced gene 2	51141	64,32130479	0,099069881	0,26222	0,377812477	0,705569904	0,930846779
ENSG00000197622	CDC42SE1	CDC42 small effector 1	56882	1206,566435	-0,063455684	0,04965	-1,278071833	0,201224099	0,660119983
ENSG00000087995	METTL2A	methyltransferase 2A, tRNA N3-cytidine	339175	46,86381772	-0,270782814	0,334231	-0,810165724	0,417844934	0,82446781
ENSG00000141956	PRDM15	PR/SET domain 15	63977	88,28195499	-0,352893315	0,182139	-1,93749501	0,052684863	0,371734827
ENSG00000112320	SOBP	sine oculis binding protein homolog	55084	33,33610745	-0,186956368	0,348308	-0,536756123	0,591436084	NA
ENSG00000121058	COIL	coilin	8161	83,90796362	0,045008837	0,201214	0,223686022	0,823001623	0,961917534
ENSG00000176410	DNAJC30	DnaJ heat shock protein family (Hsp40) member C30	84277	37,26690176	-0,029156404	0,342208	-0,085200712	0,932101825	NA
ENSG00000227671	NA	NA	NA	433,1047243	-0,104044495	0,088385	-1,177177401	0,239124707	0,694044881
ENSG00000169018	FEM1B	fem-1 homolog B	10116	306,4904024	-0,367396912	0,104198	-3,52594111	0,000421981	0,018310702
ENSG00000198740	ZNF652	zinc finger protein 652	22834	484,7968296	-0,026549561	0,12127	-0,218929084	0,826705291	0,961917534
ENSG00000078177	N4BP2	NEDD4 binding protein 2	55728	238,0855848	0,591890488	0,421055	1,405733591	0,159803248	0,616754158
ENSG00000124459	ZNF45	zinc finger protein 45	7596	110,1433978	-0,178851997	0,198612	-0,900510852	0,367848453	0,795452024
ENSG00000225921	NOL7	nucleolar protein 7	51406	85,99203513	0,174873638	0,206436	0,847108411	0,396934697	0,81291398
ENSG00000283782	NA	NA	NA	104,7610523	0,078955472	1,449716	0,05446272	0,95656651	0,991798465
ENSG00000100852	ARHGAP5	Rho GTPase activating protein 5	394	157,3508147	0,037643687	0,232866	0,161653901	0,871578409	0,974150353
ENSG00000105726	ATP13A1	ATPase 13A1	57130	157,9246566	-0,093265106	0,16077	-0,580115496	0,561836734	0,884605624
ENSG00000107130	NCS1	neuronal calcium sensor 1	23413	121,238281	0,274787799	0,207288	1,325632807	0,184961354	0,641645969
ENSG00000128699	ORMDL1	ORMDL sphingolipid biosynthesis regulator 1	94101	200,0292653	-0,073125644	0,130175	-0,561750334	0,574286135	0,886894319
ENSG00000153130	SCOC	short coiled-coil protein	60592	70,26369346	-0,203083759	0,213986	-0,949051456	0,342594442	0,780064992
ENSG00000160447	PKN3	protein kinase N3	29941	30,68668348	-0,71512532	0,314853	-2,271302533	0,02312867	NA
ENSG00000164967	RPP25L	ribonuclease P/MRP subunit p25 like	138716	22,95779943	0,11190376	0,388234	0,288237596	0,773164872	NA
ENSG00000183506	PI4KAP2	phosphatidylinositol 4-kinase alpha pseudogene 2	375133	46,28213516	-0,042839848	0,26465	-0,161873596	0,871405396	0,974150353
ENSG00000196083	IL1RAP	interleukin 1 receptor accessory protein	3556	34,51824798	0,382436895	0,377386	1,013382585	0,310877454	NA
ENSG00000284194	SCO2	synthesis of cytochrome C oxidase 2	9997	19,54857151	0,286026567	0,379322	0,754047032	0,450820981	NA
ENSG00000058600	POLR3E	RNA polymerase III subunit E	55718	70,47019074	-0,264890487	0,372244	-0,711604374	0,476709798	0,853811879
ENSG00000099308	MAST3	microtubule associated serine/threonine kinase 3	23031	514,4046774	-0,397027358	0,077916	-5,095611977	3,48E-07	6,07E-05
ENSG00000107263	RAPGEF1	Rap guanine nucleotide exchange factor 1	2889	1118,298793	0,023368885	0,051343	0,455149184	0,649001916	0,914799182
ENSG00000096968	JAK2	Janus kinase 2	3717	197,6435793	0,041471045	0,14494	0,286125644	0,774781891	0,946448613
ENSG00000258186	NA	NA	NA	17,89654835	-0,061086844	0,439438	-0,139011193	0,889441304	NA
ENSG00000161914	ZNF653	zinc finger protein 653	115950	11,64900042	-0,487618077	0,869318	-0,56092002	0,574852061	NA
ENSG00000079387	SENP1	SUMO specific peptidase 1	29843	164,1461632	-0,052584586	0,21666	-0,242705034	0,808233908	0,958576399
ENSG00000132434	LANCL2	LanC like glutathione S-transferase 2	55915	46,29018077	0,158015263	0,272158	0,580601097	0,561509333	0,884605624
ENSG00000135469	COQ10A	coenzyme Q10A	93058	24,07153632	0,08500654	0,388452	0,218833823	0,826779498	NA
ENSG00000132824	SERINC3	serine incorporator 3	10955	670,677136	-0,104950275	0,089239	-1,176057082	0,239572066	0,694482777
ENSG00000166024	R3HCC1L	R3H domain and coiled-coil containing 1 like	27291	115,1095542	0,306916195	0,165855	1,850505336	0,06424075	0,408209424
ENSG00000166170	BAG5	BAG cochaperone 5	9529	214,5308477	-0,098601219	0,126635	-0,778626788	0,436199595	0,836566451
ENSG00000185238	PRMT3	protein arginine methyltransferase 3	10196	48,26494415	-0,596210488	0,370822	-1,607808331	0,107877163	0,527254203
ENSG00000213658	LAT	linker for activation of T cells	27040	164,7345785	-0,130094645	0,151437	-0,859069136	0,390302374	0,810747116
ENSG00000138095	LRPPRC	leucine rich pentatricopeptide repeat containing	10128	123,5091066	-0,354875638	0,475093	-0,746959843	0,455087801	0,843383575

ENSG00000115947	ORC4	origin recognition complex subunit 4	5000	92,00613476	0,005587981	0,195582	0,028571106	0,977206657	0,995291586
ENSG00000119333	DYNC2I2	dynein 2 intermediate chain 2	89891	20,50354931	0,551490315	0,386306	1,427597851	0,153407643	NA
ENSG00000118242	MREG	melanoregulin	55686	101,6760851	0,123532083	0,197532	0,625376748	0,531723819	0,872945174
ENSG00000147535	PLPP5	phospholipid phosphatase 5	84513	65,63368629	-0,374840977	0,274442	-1,365830568	0,17199215	0,629558603
ENSG00000166348	USP54	ubiquitin specific peptidase 54	159195	16,87509488	0,813865732	0,862818	0,943264721	0,345545516	NA
ENSG00000225830	ERCC6	ERCC excision repair 6, chromatin remodeling factor	2074	208,4551766	-0,09748528	0,187033	-0,521218939	0,602214262	0,896931787
ENSG00000109684	CLNK	cytokine dependent hematopoietic cell linker	116449	251,0747966	0,026873657	0,12972	0,20716644	0,835879876	0,964577442
ENSG00000126953	TIMM8A	translocase of inner mitochondrial membrane 8A	1678	15,21393087	-0,085213361	0,47156	-0,180705347	0,856598862	NA
ENSG00000184898	RBM43	RNA binding motif protein 43	375287	44,68267107	-0,139479307	0,30012	-0,464745739	0,642113549	0,912063088
ENSG00000170266	GLB1	galactosidase beta 1	2720	113,3304732	0,035440694	0,173825	0,203887482	0,838441428	0,965701565
ENSG00000197020	ZNF100	zinc finger protein 100	163227	48,53145329	-1,850100275	1,687305	-1,096482404	0,27286772	0,726913977
ENSG00000067334	DNTTIP2	deoxynucleotidyltransferase terminal interacting protein 2	30836	95,93005005	-0,010225685	0,176396	-0,057970065	0,953772473	0,991212866
ENSG00000159267	HLCS	holocarboxylase synthetase	3141	113,4833519	-0,028877745	0,350291	-0,082439217	0,934297452	0,987098279
ENSG00000134285	FKBP11	FKBP prolyl isomerase 11	51303	76,47388266	0,211444755	0,218776	0,966488058	0,33380002	0,773674527
ENSG00000135972	MRPS9	mitochondrial ribosomal protein S9	64965	73,62047235	0,045427435	0,210354	0,215957154	0,829021146	0,962723654
ENSG00000247596	TWF2	twinfilin actin binding protein 2	11344	261,132475	-0,060907694	0,104704	-0,581715317	0,560758453	0,884021857
ENSG00000213780	GTF2H4	general transcription factor IIH subunit 4	2968	45,94634457	-0,143996082	1,154753	-0,124698615	0,900762154	0,977990819
ENSG00000197774	EME2	essential meiotic structure-specific endonuclease subunit 2	197342	56,43966788	0,162732295	0,418713	0,388648633	0,697536086	0,930601056
ENSG00000198874	TYW1	tRNA-yW synthesizing protein 1 homolog	55253	106,3989779	-0,030754108	0,177913	-0,17285992	0,862761537	0,97107038
ENSG00000135698	MPHOSPH6	M-phase phosphoprotein 6	10200	53,84268447	-0,005752653	0,24095	-0,023874913	0,980952385	0,995752963
ENSG00000100983	GSS	glutathione synthetase	2937	85,85729443	0,295902745	0,186925	1,58299916	0,113421656	0,53909841
ENSG00000148843	PDCD11	programmed cell death 11	22984	400,7177635	0,030375309	0,087689	0,346396987	0,729044389	0,937916952
ENSG00000088205	DDX18	DEAD-box helicase 18	8886	406,8775567	0,11695081	0,108485	1,078034843	0,281018207	0,734237191
ENSG00000100319	ZMAT5	zinc finger matrin-type 5	55954	31,25067374	-0,023739189	0,322275	-0,073661343	0,941279859	NA
ENSG00000117597	UTP25	UTP25 small subunit processome component	27042	125,3079966	-0,00493388	0,164767	-0,029944501	0,976111315	0,995074982
ENSG00000118495	PLAGL1	PLAG1 like zinc finger 1	5325	50,33704461	-0,258950984	0,283063	-0,914816116	0,360288173	0,791408368
ENSG00000131023	LATS1	large tumor suppressor kinase 1	9113	322,1185768	-0,022729387	0,137416	-0,165405348	0,868624945	0,973097402
ENSG00000162927	PUS10	pseudouridine synthase 10	150962	37,98300829	-0,184783491	0,300448	-0,61502618	0,538537431	NA
ENSG00000163866	SMIM12	small integral membrane protein 12	113444	185,8991089	0,228111911	0,135866	1,678947761	0,093162225	0,496065163
ENSG00000165813	CCDC186	coiled-coil domain containing 186	55088	309,4273208	-0,133319204	0,139013	-0,959044283	0,337536437	0,77693253
ENSG00000170275	CRTAP	cartilage associated protein	10491	78,37598207	0,003344373	0,26039	0,012843687	0,989752502	0,997211687
ENSG00000197779	ZNF81	zinc finger protein 81	347344	315,3772917	-0,056131025	0,105741	-0,530834063	0,595533774	0,894229873
ENSG00000101079	NDRG3	NDRG family member 3	57446	271,5116694	0,009064448	0,111818	0,081064393	0,935390743	0,987098279
ENSG00000136522	MRPL47	mitochondrial ribosomal protein L47	57129	82,27876927	0,256684582	0,233507	1,09925789	0,271655594	0,725708516
ENSG00000164120	HPGD	15-hydroxyprostaglandin dehydrogenase	3248	91,74362435	-0,156060857	0,256522	-0,608371169	0,542941329	0,877542071
ENSG00000093183	SEC22C	SEC22 homolog C, vesicle trafficking protein	9117	199,7559797	0,047728498	0,155262	0,307405569	0,75853469	0,942901768
ENSG00000070814	TCOF1	treacle ribosome biogenesis factor 1	6949	326,4719701	0,066187574	0,12343	0,536236595	0,591795045	0,892834002
ENSG00000163882	POLR2H	RNA polymerase II, I and III subunit H	5437	60,17657876	0,24838039	0,354953	0,699755704	0,484079882	0,85698884
ENSG00000167315	ACAA2	acetyl-CoA acyltransferase 2	10449	305,1229247	0,139776561	0,099179	1,409337095	0,158735515	0,614461481
ENSG00000213793	ZNF888	zinc finger protein 888	388559	50,71342986	0,155335551	0,245493	0,632749913	0,52689697	0,871653726
ENSG00000170448	NFXL1	nuclear transcription factor, X-box binding like 1	152518	74,48080706	0,26225136	0,244882	1,070930449	0,284200704	0,737423617
ENSG00000242110	AMACR	alpha-methylacyl-CoA racemase	23600	33,92944047	0,147764027	0,433517	0,340849466	0,733216909	NA
ENSG00000061794	MRPS35	mitochondrial ribosomal protein S35	60488	100,3108363	-0,070778884	0,174194	-0,406321341	0,684506507	0,92563948
ENSG00000155100	OTUD6B	OTU deubiquitinase 6B	51633	70,24031116	0,125798117	0,215645	0,583357796	0,559652463	0,883921037
ENSG00000164105	SAP30	Sin3A associated protein 30	8819	16,12809308	0,026046993	0,575509	0,045259042	0,963900834	NA
ENSG00000137814	HAUS2	HAUS augmin like complex subunit 2	55142	96,30336683	0,116336247	0,218482	0,532475487	0,594396715	0,894229873
ENSG00000071994	PDCD2	programmed cell death 2	5134	188,2338764	-0,082286592	0,169235	-0,486226671	0,626806463	0,90479929
ENSG00000128626	MRPS12	mitochondrial ribosomal protein S12	6183	29,75183388	0,079255784	0,342956	0,23109584	0,817240347	NA
ENSG00000185187	SIGIRR	single Ig and TIR domain containing	59307	220,5904924	-0,131102178	0,123045	-1,065482061	0,286657839	0,738005299
ENSG00000241553	ARPC4	actin related protein 2/3 complex subunit 4	10093	581,0436068	-0,265050563	0,086995	-3,046741149	0,002313368	0,058616198

ENSG00000075826	SEC31B	SEC31 homolog B, COPII coat complex component	25956	18,86951739	0,63053853	2,1709	0,29045028	0,771471783	NA
ENSG00000103160	HSDL1	hydroxysteroid dehydrogenase like 1	83693	58,5955726	0,093265171	0,245218	0,380336135	0,703695915	0,930846779
ENSG00000068028	RASSF1	Ras association domain family member 1	11186	317,9281711	-0,008819893	0,137845	-0,063983991	0,948982974	0,9906219
ENSG00000198453	ZNF568	zinc finger protein 568	374900	44,89862315	-0,139622036	0,29462	-0,473906113	0,63556686	0,909036096
ENSG00000262246	CORO7	coronin 7	79585	293,9927176	0,108213197	0,166674	0,649251461	0,516175854	0,868791049
ENSG00000125375	DMAC2L	distal membrane arm assembly component 2 like	27109	63,13322539	0,070936355	0,276237	0,256795578	0,797336584	0,95354564
ENSG00000205670	SMIM11	small integral membrane protein 11	54065	44,62579025	0,370711171	0,290851	1,274574876	0,202459755	0,660119983
ENSG00000205670	LOC102723553	small integral membrane protein 11B	102723553	44,62579025	0,370711171	0,290851	1,274574876	0,202459755	0,660119983
ENSG00000127511	SIN3B	SIN3 transcription regulator family member B	23309	245,4271551	0,077457646	0,104267	0,74287659	0,4575564	0,84552083
ENSG00000110697	PITPNM1	phosphatidylinositol transfer protein membrane associated 1	9600	273,1602811	-0,042906676	0,111071	-0,386299863	0,699274602	0,930783276
ENSG00000107164	FUBP3	far upstream element binding protein 3	8939	174,5618418	0,1615409	0,192172	0,840605678	0,400568877	0,814673014
ENSG00000147164	SNX12	sorting nexin 12	29934	109,4603756	-0,263972319	0,241601	-1,092597907	0,274570382	0,727393472
ENSG00000243156	MICAL3	microtubule associated monooxygenase, calponin and LIM domain containing 3	57553	629,5373826	0,126014349	0,075749	1,663572931	0,096197805	0,502134421
ENSG00000105583	WDR83OS	WD repeat domain 83 opposite strand	51398	153,3528566	0,21499752	0,152758	1,407435501	0,15929829	0,615712978
ENSG00000084731	KIF3C	kinesin family member 3C	3797	90,61593595	0,03996359	0,176482	0,226445367	0,820855047	0,961891299
ENSG00000156162	DPY19L4	dpy-19 like 4	286148	56,80189458	-0,128535368	0,296453	-0,433577472	0,664595297	0,921888283
ENSG00000157227	MMP14	matrix metallopeptidase 14	4323	58,66945854	0,813125487	1,052186	0,772796681	0,439642718	0,83848252
ENSG00000163577	EIF5A2	eukaryotic translation initiation factor 5A2	56648	66,47315805	0,329813363	0,24341	1,354970575	0,175426932	0,632039836
ENSG00000186625	KATNA1	katanin catalytic subunit A1	11104	44,76663354	0,198101998	0,293397	0,675200325	0,4995485	0,86161226
ENSG00000186687	LYRM7	LYR motif containing 7	90624	174,9656765	-0,0847948	0,142521	-0,594962056	0,551868834	0,881150198
ENSG00000198625	MDM4	MDM4 regulator of p53	4194	563,0049971	-0,174048179	0,135041	-1,288856824	0,197447867	0,65618735
ENSG00000273748	NA	NA	NA	19,91879325	-0,475648295	0,556382	-0,854895877	0,392608793	NA
ENSG00000275066	SYNRG	synergin gamma	11276	1259,284234	-0,238726218	0,057899	-4,123122062	3,74E-05	0,002935601
ENSG00000131591	C1orf159	chromosome 1 open reading frame 159	54991	17,83818831	-0,111332696	0,506296	-0,219896307	0,825951912	NA
ENSG00000007047	MARK4	microtubule affinity regulating kinase 4	57787	112,8498975	-0,078920039	0,177068	-0,445704902	0,655810423	0,918143156
ENSG00000180233	ZNRF2	zinc and ring finger 2	223082	73,08511762	-0,044652467	0,227517	-0,196259805	0,844406821	0,967231052
ENSG00000008294	SPAG9	sperm associated antigen 9	9043	461,5036094	-0,035815461	0,085056	-0,421080623	0,673696211	0,923602509
ENSG00000086189	DIMT1	DIM1 rRNA methyltransferase and ribosome maturation factor	27292	50,56223844	-0,212756835	0,288254	-0,738088269	0,460460812	0,846622987
ENSG00000103423	DNAJA3	DnaJ heat shock protein family (Hsp40) member A3	9093	23,46849001	-0,929137924	1,775479	-0,523316725	0,600753864	NA
ENSG00000152422	XRCC4	X-ray repair cross complementing 4	7518	42,14630425	0,005779182	0,407714	0,01417461	0,988690676	NA
ENSG00000160201	U2AF1	U2 small nuclear RNA auxiliary factor 1	7307	202,6074743	-0,06601618	0,124607	-0,529794954	0,596254104	0,894229873
ENSG00000160201	LOC102724594	U2 small nuclear RNA auxiliary factor 1 like 5	102724594	202,6074743	-0,06601618	0,124607	-0,529794954	0,596254104	0,894229873
ENSG00000196072	BLOC1S2	biogenesis of lysosomal organelles complex 1 subunit 2	282991	148,8228693	0,16476952	0,154839	1,064135211	0,287267452	0,738284871
ENSG00000255150	EID3	EP300 interacting inhibitor of differentiation 3	493861	21,73678167	0,116614144	0,4186	0,278581103	0,780566316	NA
ENSG00000116717	GADD45A	growth arrest and DNA damage inducible alpha	1647	36,94087576	0,659989131	0,312722	2,110462852	0,034818508	NA
ENSG00000162402	USP24	ubiquitin specific peptidase 24	23358	678,2249052	0,023443915	0,076465	0,306596445	0,75915056	0,942901768
ENSG00000054654	SYNE2	spectrin repeat containing nuclear envelope protein 2	23224	3532,923189	0,213909989	0,086717	2,466758062	0,013634244	0,181497213
ENSG00000079435	LIPE	lipase E, hormone sensitive type	3991	20,0683242	0,201422881	0,469154	0,429331768	0,667681801	NA
ENSG00000079462	PAFAH1B3	platelet activating factor acetylhydrolase 1b catalytic subunit 3	5050	17,33537333	-0,0062011	0,442795	-0,014004447	0,988826433	NA
ENSG00000186193	SAPCD2	suppressor APC domain containing 2	89958	31,46653869	0,488609281	0,348797	1,400839729	0,161262005	NA
ENSG00000115839	RAB3GAP1	RAB3 GTPase activating protein catalytic subunit 1	22930	107,2786729	0,526716184	0,314067	1,677082353	0,093526379	0,496993357
ENSG00000188895	MSL1	MSL complex subunit 1	339287	338,6977905	-0,10064634	0,116957	-0,860538315	0,389492372	0,810747116
ENSG00000168228	ZCCHC4	zinc finger CCHC-type containing 4	29063	33,65446852	-0,391070322	0,30807	-1,269420286	0,204291205	NA
ENSG00000115685	PPP1R7	protein phosphatase 1 regulatory subunit 7	5510	159,1624351	0,111160708	0,180004	0,617547207	0,536873854	0,874923109

ENSG00000162961	DPY30	dpy-30 histone methyltransferase complex regulatory subunit	84661	47,21309698	-0,295991929	0,242638	-1,219888723	0,222507061	0,678694696
ENSG00000157927	RADIL	Rap associating with DIL domain	55698	15,11413543	0,834113317	2,86176	0,291468696	0,770692882	NA
ENSG00000091640	SPAG7	sperm associated antigen 7	9552	113,9419455	0,099882935	0,151885	0,657621484	0,510781386	0,866180216
ENSG00000104731	KLHDC4	kelch domain containing 4	54758	379,7506133	-0,079504024	0,105879	-0,75089258	0,452717306	0,843234809
ENSG00000183814	LIN9	lin-9 DREAM MuvB core complex component	286826	15,37821542	-0,016465229	0,872534	-0,018870592	0,984944339	NA
ENSG00000152689	RASGRP3	RAS guanyl releasing protein 3	25780	108,605587	0,364829257	0,193098	1,889349387	0,058845028	0,39312401
ENSG00000230521	NA	NA	NA	11,66658292	-0,311381146	0,580842	-0,536085735	0,591899299	NA
ENSG00000043514	TRIT1	tRNA isopentenyltransferase 1	54802	22,63803839	0,012974969	0,810263	0,016013281	0,987223796	NA
ENSG00000104133	SPG11	SPG11 vesicle trafficking associated, spatacsin	80208	613,3695409	-0,058083637	0,156054	-0,372202693	0,709741941	0,930846779
ENSG00000127663	KDM4B	lysine demethylase 4B	23030	251,7653851	-0,113506943	0,128337	-0,884441271	0,376458053	0,803373958
ENSG00000160124	MIX23	mitochondrial matrix import factor 23	131076	14,97706104	-0,306853015	0,484517	-0,633317802	0,526526131	NA
ENSG00000080200	CRYBG3	crystallin beta-gamma domain containing 3	131544	22,0324003	0,149679196	0,385901	0,387869099	0,698112907	NA
ENSG00000138231	DBR1	debranching RNA lariats 1	51163	71,53767679	0,18094379	0,246903	0,73285462	0,463647103	0,848212098
ENSG00000106392	C1GALT1	core 1 synthase, glycoprotein-N-acetylgalactosamine 3-beta-galactosyltransferase 1	56913	170,8460904	-0,156504101	0,178498	-0,876782722	0,380604665	0,806167487
ENSG00000056097	ZFR	zinc finger RNA binding protein	51663	452,9127571	0,096760936	0,084247	1,148539625	0,250745865	0,707335884
ENSG00000073169	SELENOO	selenoprotein O	83642	37,70403826	-0,325607951	0,320477	-1,01600955	0,309624839	NA
ENSG00000167778	SPRYD3	SPRY domain containing 3	84926	141,0422702	-0,11843953	0,147147	-0,804906077	0,420873881	0,827182516
ENSG00000114745	GORASP1	golgi reassembly stacking protein 1	64689	29,07089445	0,268651363	0,650939	0,412713565	0,679816485	NA
ENSG00000151779	NBAS	NBAS subunit of NRZ tethering complex	51594	202,7866821	0,208748699	0,117012	1,783986523	0,074425855	0,443505816
ENSG00000166478	ZNF143	zinc finger protein 143	7702	78,0618109	0,009877766	0,217658	0,045382138	0,963802718	0,992694268
ENSG00000154124	OTULIN	OTU deubiquitinase with linear linkage specificity	90268	325,5993156	0,224412968	0,097582	2,299736498	0,021463153	0,231236768
ENSG00000254901	BORCS8	BLOC-1 related complex subunit 8	729991	27,98476988	0,100592021	0,423381	0,237592387	0,812197256	NA
ENSG00000145284	SCD5	stearoyl-CoA desaturase 5	79966	38,74488712	0,016107357	0,275827	0,058396669	0,953432668	NA
ENSG00000179021	C3orf38	chromosome 3 open reading frame 38	285237	119,4249179	-0,31120825	0,194686	-1,598517447	0,109927866	0,529676968
ENSG00000204356	NELFE	negative elongation factor complex member E	7936	31,51512175	-0,02367279	2,384633	-0,009927224	0,992079352	NA
ENSG00000233927	RPS28	ribosomal protein S28	6234	173,3589046	-0,018580832	0,164128	-0,113209407	0,909864538	0,979548024
ENSG00000133026	MYH10	myosin heavy chain 10	4628	13,47590469	0,716838127	2,187426	0,327708448	0,743132115	NA
ENSG00000047579	DTNBP1	dystrobrevin binding protein 1	84062	54,39158122	-0,352611645	0,319748	-1,102778699	0,27012328	0,725477256
ENSG00000069966	GNB5	G protein subunit beta 5	10681	113,063864	-0,026379817	0,192576	-0,136984028	0,89104342	0,977990819
ENSG00000144713	RPL32	ribosomal protein L32	6161	827,9127902	0,037320194	0,131902	0,282937997	0,777224367	0,947109268
ENSG00000197879	MYO1C	myosin IC	4641	82,58036607	0,031102525	0,200969	0,154762818	0,877008305	0,975885813
ENSG00000092201	SUPT16H	SPT16 homolog, facilitates chromatin remodeling subunit	11198	760,1035199	-0,056928618	0,069922	-0,814177994	0,41554299	0,82446781
ENSG00000135845	PIGC	phosphatidylinositol glycan anchor biosynthesis class C	5279	91,03707279	0,178116757	0,193251	0,921684564	0,356693135	0,789494009
ENSG00000106346	USP42	ubiquitin specific peptidase 42	84132	94,48802098	-0,215525476	0,278405	-0,774144575	0,4388453	0,837388481
ENSG00000135624	CCT7	chaperonin containing TCP1 subunit 7	10574	425,2009308	0,065293767	0,095132	0,686347876	0,492493765	0,860513824
ENSG00000198042	MAK16	MAK16 homolog	84549	106,1200761	-0,007163288	0,168177	-0,042593641	0,966025464	0,992789539
ENSG00000083168	KAT6A	lysine acetyltransferase 6A	7994	681,5197942	0,033557885	0,079336	0,422985045	0,672306172	0,923450975
ENSG00000137364	TPMT	thiopurine S-methyltransferase	7172	70,40954511	-0,03489357	0,194366	-0,179524962	0,857525519	0,969089892
ENSG00000139154	AEBP2	AE binding protein 2	121536	199,8450823	0,161490556	0,189648	0,851527321	0,394476493	0,812498986
ENSG00000167088	SNRPD1	small nuclear ribonucleoprotein D1 polypeptide	6632	147,4200424	0,153162944	0,197593	0,775143168	0,438255066	0,837388481
ENSG00000196814	MVB12B	multivesicular body subunit 12B	89853	122,1292013	-0,097956812	0,157786	-0,620820243	0,534717903	0,874203665
ENSG00000022267	FHL1	four and a half LIM domains 1	2273	32,92783519	0,492162854	0,297365	1,655077431	0,097908792	NA
ENSG00000105552	BCAT2	branched chain amino acid transaminase 2	587	48,32812015	-0,226430973	0,286641	-0,789946381	0,429559083	0,832060923
ENSG00000146007	ZMAT2	zinc finger matrin-type 2	153527	226,7162437	0,010912284	0,135435	0,080572114	0,935782244	0,987098279
ENSG00000132953	XPO4	exportin 4	64328	223,2997009	0,060040313	0,109208	0,549778488	0,582471315	0,888501395
ENSG00000169857	AVEN	apoptosis and caspase activation inhibitor	57099	44,61225297	0,110492365	0,256594	0,430611045	0,666751209	0,922184616
ENSG00000111361	EIF2B1	eukaryotic translation initiation factor 2B subunit alpha	1967	213,1003202	0,101518913	0,127354	0,797139489	0,425370024	0,830443782
ENSG00000140379	BCL2A1	BCL2 related protein A1	597	14,76272953	0,632194469	0,483207	1,308330931	0,190761088	NA

ENSG00000141252	VPS53	VPS53 subunit of GARP complex	55275	78,25497195	-0,230718297	0,328445	-0,70245698	0,482394222	0,855834779
ENSG00000160318	CLDND2	claudin domain containing 2	125875	25,31764099	-0,434181024	0,366924	-1,183299682	0,236690399	NA
ENSG00000163644	PPM1K	protein phosphatase, Mg2+/Mn2+ dependent 1K	152926	170,8538721	-0,404031365	0,204962	-1,971247377	0,048695587	0,354159983
ENSG00000166557	TMED3	transmembrane p24 trafficking protein 3	23423	119,5247751	0,160911597	0,168854	0,952962616	0,340609013	0,777431905
ENSG00000179598	PLD6	phospholipase D family member 6	201164	35,68907532	-0,181618955	0,356573	-0,509345353	0,610510173	NA
ENSG00000104714	ERICH1	glutamate rich 1	157697	92,26521788	-0,185460697	0,183671	-1,009743618	0,312618139	0,75804349
ENSG00000176845	METRNL	meteorin like, glial cell differentiation regulator	284207	48,67949674	-0,242083865	0,339853	-0,712319986	0,476266651	0,853811879
ENSG00000197324	LRP10	LDL receptor related protein 10	26020	695,1952836	0,125318425	0,074152	1,690029397	0,091022331	0,490934677
ENSG00000205581	HMGN1	high mobility group nucleosome binding domain 1	3150	390,3626328	0,013428463	0,108142	0,124174744	0,901176918	0,977990819
ENSG00000140406	TLNRD1	talin rod domain containing 1	59274	139,3849157	0,037646657	0,143631	0,262107571	0,79323851	0,952276252
ENSG00000100764	PSMC1	proteasome 26S subunit, ATPase 1	5700	188,706541	0,239042567	0,158105	1,511926283	0,130552617	0,566497377
ENSG00000127586	CHTF18	chromosome transmission fidelity factor 18	63922	38,87097198	-0,288060459	0,332269	-0,866950414	0,385969179	NA
ENSG00000132661	NXT1	nuclear transport factor 2 like export factor 1	29107	35,96508614	0,330615586	0,310639	1,064307728	0,287189318	NA
ENSG00000250317	SMIM20	small integral membrane protein 20	389203	15,66939849	0,157576444	0,441726	0,356728802	0,721294842	NA
ENSG00000108829	LRRC59	leucine rich repeat containing 59	55379	202,2940091	0,117696052	0,132715	0,886833036	0,375168799	0,802225904
ENSG00000009954	BAZ1B	bromodomain adjacent to zinc finger domain 1B	9031	836,5335315	0,082141478	0,068094	1,206293662	0,227704281	0,683140794
ENSG00000137710	RDX	radixin	5962	136,8828167	-0,106726672	0,140864	-0,757660203	0,448654429	0,84263906
ENSG00000223959	AFG3L1P	AFG3 like matrix AAA peptidase subunit 1, pseudogene	172	29,00226601	-0,130731917	0,32756	-0,399108672	0,689813133	NA
ENSG00000175643	RMI2	RecQ mediated genome instability 2	116028	15,69479146	-0,047003038	0,481642	-0,097589239	0,92225847	NA
ENSG00000196511	TPK1	thiamin pyrophosphokinase 1	27010	24,52311639	0,625963355	0,405658	1,543081288	0,122811054	NA
ENSG00000066136	NFYC	nuclear transcription factor Y subunit gamma	4802	122,8617964	-0,01255724	0,189944	-0,066110101	0,947290169	0,990285769
ENSG00000158470	B4GALT5	beta-1,4-galactosyltransferase 5	9334	481,8966488	0,035918123	0,089816	0,399906932	0,689225067	0,9269133
ENSG00000134987	WDR36	WD repeat domain 36	134430	167,2476789	-0,118624181	0,136842	-0,866867033	0,386014869	0,809653368
ENSG00000204469	PRRC2A	proline rich coiled-coil 2A	7916	409,7101488	0,226946801	0,303629	0,74744863	0,4547928	0,843234809
ENSG00000239779	WBP1	WW domain binding protein 1	23559	53,18479348	0,203061034	0,261831	0,775542796	0,438018987	0,837388481
ENSG00000107968	MAP3K8	mitogen-activated protein kinase kinase kinase 8	1326	332,172668	0,193549381	0,108827	1,778506882	0,075320643	0,446129961
ENSG00000068366	ACSL4	acyl-CoA synthetase long chain family member 4	2182	146,6115657	-0,089072271	0,154796	-0,575416305	0,56500978	0,885045179
ENSG00000147679	UTP23	UTP23 small subunit processome component	84294	91,30506259	0,011089915	0,210167	0,052767242	0,957917363	0,991911599
ENSG00000196352	CD55	CD55 molecule (Cromer blood group)	1604	692,2694088	0,172699976	0,07269	2,375834709	0,017509304	0,206510834
ENSG00000204516	MICB	MHC class I polypeptide-related sequence B	4277	61,05735494	-0,136967567	0,223755	-0,61213242	0,54045015	0,876149366
ENSG00000188002	NA	NA	NA	15,83731564	-0,600501881	0,453431	-1,324350011	0,185386828	NA
ENSG00000172992	DCAKD	dephospho-CoA kinase domain containing	79877	44,0242184	-0,435094476	0,344716	-1,262182614	0,206883083	0,661502129
ENSG00000241852	C8orf58	chromosome 8 open reading frame 58	541565	20,43968919	-0,241844532	0,37222	-0,649736311	0,515862565	NA
ENSG00000162704	ARPC5	actin related protein 2/3 complex subunit 5	10092	563,3656406	0,140355675	0,118121	1,188236135	0,234740391	0,69018943
ENSG00000135002	RFK	riboflavin kinase	55312	71,32704849	-0,136695104	0,217345	-0,628930815	0,529394351	0,872145761
ENSG00000269343	ZNF587B	zinc finger protein 587B	100293516	106,2768138	0,292325308	0,267062	1,094596788	0,273693323	0,726913977
ENSG00000181007	ZFP82	ZFP82 zinc finger protein	284406	22,54605534	-0,014531977	0,470502	-0,030886109	0,975360368	NA
ENSG00000114841	DNAH1	dynein axonemal heavy chain 1	25981	128,2192724	0,862469261	1,37754	0,626093965	0,53125331	0,872900314
ENSG00000182093	GET1	guided entry of tail-anchored proteins factor 1	7485	24,19855986	0,110664572	0,371994	0,297490372	0,766092157	NA
ENSG00000197062	ZSCAN26	zinc finger and SCAN domain containing 26	7741	67,20747262	0,157916165	0,378785	0,416901747	0,676750276	0,924186156
ENSG00000082515	MRPL22	mitochondrial ribosomal protein L22	29093	46,70839252	0,069244516	0,2986	0,231897384	0,816617717	0,959707099
ENSG00000088808	PPP1R13B	protein phosphatase 1 regulatory subunit 13B	23368	41,22549605	0,091581984	1,117643	0,081942061	0,934692787	NA
ENSG00000152270	PDE3B	phosphodiesterase 3B	5140	222,8086382	-0,226437351	0,121768	-1,859586941	0,062943988	0,404317327
ENSG00000156110	ADK	adenosine kinase	132	34,91277088	0,033756399	0,304347	0,110914293	0,911684311	NA
ENSG00000283515	NA	NA	NA	19,23141917	0,206390081	1,639994	0,125848045	0,89985221	NA
ENSG00000138709	LARP1B	La ribonucleoprotein 1B	55132	101,9778852	0,147336498	0,173044	0,851438166	0,394525998	0,812498986

ENSG00000110080	ST3GAL4	ST3 beta-galactoside alpha-2,3-sialyltransferase 4	6484	22,14704218	-0,732171512	0,42133	-1,73776294	0,082252593	NA
ENSG00000164808	SPIDR	scaffold protein involved in DNA repair	23514	60,83551159	-0,025947245	1,370405	-0,018933999	0,984893757	0,996185513
ENSG00000176624	MEX3C	mex-3 RNA binding family member C	51320	750,7857169	-0,173158204	0,077609	-2,231152231	0,025671047	0,258487696
ENSG00000241015	TPM3P9	tropomyosin 3 pseudogene 9	147804	21,38396438	-0,472913471	0,372073	-1,271024612	0,203719893	NA
ENSG00000107404	DVL1	dishevelled segment polarity protein 1	1855	91,40062631	-0,162495119	0,215728	-0,75324089	0,451305172	0,842718653
ENSG00000113758	DBN1	drebrin 1	1627	66,40786108	-0,205135085	0,203356	-1,008748017	0,313095497	0,758381858
ENSG00000131019	ULBP3	UL16 binding protein 3	79465	21,72170011	0,090936282	0,38432	0,236616244	0,812954517	NA
ENSG00000136877	FPGS	folylpolyglutamate synthase	2356	157,9272171	-0,134513638	0,170016	-0,791184165	0,42883653	0,832060923
ENSG00000162378	ZYG11B	zyg-11 family member B, cell cycle regulator	79699	47,45848226	0,222917642	0,26171	0,851774773	0,39433911	0,812498986
ENSG00000171960	PPIH	peptidylprolyl isomerase H	10465	78,72896495	-0,182438412	0,200791	-0,908600496	0,363561044	0,792858313
ENSG00000100285	NEFH	neurofilament heavy chain	4744	31,27642834	0,092282035	0,344543	0,267838741	0,788823448	NA
ENSG00000104889	RNASEH2A	ribonuclease H2 subunit A	10535	35,28866129	0,322104797	0,346463	0,929693583	0,352529758	NA
ENSG00000135930	EIF4E2	eukaryotic translation initiation factor 4E family member 2	9470	289,9757941	-0,126184646	0,113011	-1,116573343	0,264176795	0,719682465
ENSG00000144040	SFXN5	sideroflexin 5	94097	77,54549592	0,18896388	0,209243	0,903081687	0,366482542	0,79459056
ENSG00000149743	TRPT1	tRNA phosphotransferase 1	83707	18,60000173	0,061754543	0,448885	0,137573053	0,890577853	NA
ENSG00000153006	SREK1IP1	SREK1 interacting protein 1	285672	151,9320965	-0,150466013	0,146253	-1,028804218	0,303571684	0,751870158
ENSG00000163636	PSMD6	proteasome 26S subunit, non-ATPase 6	9861	158,2303176	0,178243809	0,151306	1,178034325	0,238782922	0,693819751
ENSG00000180228	PRKRA	protein activator of interferon induced protein kinase EIF2AK2	8575	46,00853308	-0,798657937	0,261201	-3,057641316	0,002230864	0,057258849
ENSG00000226942	NA	NA	NA	22,64179856	-0,054017417	0,405699	-0,133146503	0,894077518	NA
ENSG00000067369	TP53BP1	tumor protein p53 binding protein 1	7158	202,5962964	-0,00809154	0,181038	-0,044695171	0,964350283	0,992728748
ENSG00000068394	GPKOW	G-patch domain and KOW motifs	27238	72,35649	0,240705784	0,211224	1,139578186	0,254462078	0,709962756
ENSG00000170265	ZNF282	zinc finger protein 282	8427	118,9931497	-0,037898008	0,153398	-0,247055946	0,804864927	0,957328863
ENSG00000235316	NA	NA	NA	23,69583691	-0,567231943	0,426505	-1,329954407	0,183533294	NA
ENSG00000091164	TXNL1	thioredoxin like 1	9352	128,7367184	-0,013500674	0,156847	-0,086075215	0,931406626	0,986551936
ENSG00000144677	CTDSPL	CTD small phosphatase like	10217	30,53829902	-0,319679277	0,364445	-0,877167739	0,380395536	NA
ENSG00000162623	TYW3	tRNA-yW synthesizing protein 3 homolog	127253	130,3978273	0,102301779	0,178447	0,573290105	0,566448284	0,88521771
ENSG00000104518	GSDMD	gasdermin D	79792	121,5789312	0,546694328	1,30236	0,419772189	0,674651883	0,923602509
ENSG00000184182	UBE2F	ubiquitin conjugating enzyme E2 F (putative)	140739	437,4195813	-0,21086618	0,100594	-2,09622053	0,036062633	0,302084897
ENSG00000154839	SKA1	spindle and kinetochore associated complex subunit 1	220134	34,03438649	0,312405661	0,301427	1,036422669	0,300004999	NA
ENSG00000089053	ANAPC5	anaphase promoting complex subunit 5	51433	324,1188065	-0,017256738	0,091817	-0,187946693	0,850918439	0,967902179
ENSG00000155906	RMND1	required for meiotic nuclear division 1 homolog	55005	19,37579434	-0,030795785	0,528399	-0,058281346	0,953524526	NA
ENSG00000177556	ATOX1	antioxidant 1 copper chaperone	475	85,22509243	0,033812034	0,192752	0,17541761	0,860751505	0,97061627
ENSG00000275318	LOC100419515	zinc finger protein 41 pseudogene	100419515	12,90442702	-0,278082043	0,613282	-0,453432406	0,650237406	NA
ENSG00000017427	IGF1	insulin like growth factor 1	3479	26,29713389	-0,258694455	0,457408	-0,565566634	0,571688415	NA
ENSG00000151532	VTI1A	vesicle transport through interaction with t-SNAREs 1A	143187	144,7802388	0,018812873	0,1609	0,116922611	0,90692138	0,978585515
ENSG00000105053	VRK3	VRK serine/threonine kinase 3	51231	106,9946876	-0,042148529	0,191534	-0,220057599	0,825826297	0,961917534
ENSG00000107560	RAB11FIP2	RAB11 family interacting protein 2	22841	98,80733677	-0,269772486	0,210691	-1,280418924	0,200397842	0,659373545
ENSG00000107816	LZTS2	leucine zipper tumor suppressor 2	84445	65,23272263	-0,272225825	0,2666	-1,021103552	0,30720538	0,754087936
ENSG00000121022	COPS5	COP9 signalosome subunit 5	10987	126,8598578	0,028490209	0,170274	0,167319627	0,867118562	0,971935091
ENSG00000270231	NBPF8	NBPF member 8	728841	156,9228758	-0,269116397	0,145172	-1,853773794	0,063771529	0,406570577
ENSG00000285976	LOC128125822	uncharacterized LOC128125822	128125822	16,4460952	0,362851448	0,459021	0,790490721	0,429241239	NA
ENSG00000144026	ZNF514	zinc finger protein 514	84874	79,17279672	0,00620442	0,273528	0,022682952	0,981903174	0,995974079
ENSG00000148572	NRBF2	nuclear receptor binding factor 2	29982	85,24685476	-0,077396999	0,212083	-0,36493667	0,715158681	0,932016096
ENSG00000197635	DPP4	dipeptidyl peptidase 4	1803	101,9662776	0,175327196	0,211102	0,830532237	0,406237929	0,819098895
ENSG00000198168	SVIP	small VCP interacting protein	258010	34,63775336	0,136012205	0,294995	0,461066016	0,644751243	NA
ENSG00000074755	ZZEF1	zinc finger ZZ-type and EF-hand domain containing 1	23140	892,6475304	-0,017436813	0,066138	-0,263643262	0,792054819	0,951920207
ENSG00000132541	RIDA	reactive intermediate imine deaminase A homolog	10247	14,98709676	-0,270166517	0,516688	-0,522881067	0,601057021	NA
ENSG00000144959	NCEH1	neutral cholesterol ester hydrolase 1	57552	83,52718488	0,10185725	0,217076	0,469224282	0,638909331	0,910510986
ENSG00000171307	ZDHHC16	zinc finger DHHC-type palmitoyltransferase 16	84287	50,36990225	0,371839136	0,299911	1,239831455	0,215037741	0,671533367
ENSG00000176422	SPRYD4	SPRY domain containing 4	283377	77,36490591	-0,063148503	0,207776	-0,303925967	0,761184295	0,942901768

ENSG00000116691	MIIP	migration and invasion inhibitory protein	60672	57,78259284	-0,15918009	0,247229	-0,64385768	0,519667727	0,86944974
ENSG00000185261	KIAA0825	KIAA0825	285600	53,32270804	-0,18653133	0,380755	-0,48989849	0,624205731	0,90479929
ENSG00000100592	DAAM1	dishevelled associated activator of morphogenesis 1	23002	119,3287556	0,057982817	0,188331	0,307877282	0,758175713	0,942901768
ENSG00000178573	MAF	MAF bZIP transcription factor	4094	135,0815533	0,02351086	0,198739	0,118299924	0,905830018	0,978460866
ENSG00000181788	SIAH2	siah E3 ubiquitin protein ligase 2	6478	90,18823158	-0,221214661	0,189325	-1,168439751	0,242629429	0,698281983
ENSG00000176101	SSNA1	SS nuclear autoantigen 1	8636	58,66008464	0,007775041	0,274921	0,028281009	0,977438027	0,9953064
ENSG00000108651	UTP6	UTP6 small subunit processome component	55813	56,84470252	0,075747364	0,396431	0,191073443	0,848468054	0,967741518
ENSG00000136868	SLC31A1	solute carrier family 31 member 1	1317	72,13839629	0,313271128	0,204192	1,534201169	0,12498017	0,5624706
ENSG00000164331	ANKRA2	ankyrin repeat family A member 2	57763	72,96438578	0,266413112	0,250994	1,061430904	0,288494119	0,740096492
ENSG00000168538	TRAPPC11	trafficking protein particle complex subunit 11	60684	220,28576	-0,055883272	0,128604	-0,434537068	0,663898483	0,921409911
ENSG00000227057	WDR46	WD repeat domain 46	9277	145,009675	0,066875757	0,146925	0,455170584	0,648986521	0,914799182
ENSG00000240445	FOXO3B	forkhead box O3B	2310	35,66441266	-0,023266406	0,379569	-0,061296961	0,951122711	NA
ENSG00000136463	TACO1	translational activator of cytochrome c oxidase I	51204	34,5603347	-0,658648941	0,512496	-1,285179461	0,198729579	NA
ENSG00000105701	FKBP8	FKBP prolyl isomerase 8	23770	430,8794628	-0,113880451	0,08267	-1,377535985	0,168346597	0,627733684
ENSG00000132274	TRIM22	tripartite motif containing 22	10346	285,0380668	0,093121689	0,128076	0,727080946	0,46717637	0,850533892
ENSG00000108932	SLC16A6	solute carrier family 16 member 6	9120	50,37685935	0,102766609	0,273149	0,376228857	0,706746764	0,930846779
ENSG00000154813	DPH3	diphthamide biosynthesis 3	285381	86,77700637	-0,06801969	0,204625	-0,3324114	0,739578632	0,939193793
ENSG00000183527	PSMG1	proteasome assembly chaperone 1	8624	25,38795135	-1,253675852	0,402316	-3,116149587	0,001832293	NA
ENSG00000102543	CDADC1	cytidine and dCMP deaminase domain containing 1	81602	31,71970218	-0,568497803	0,348338	-1,632029824	0,102673204	NA
ENSG00000121940	CLCC1	chloride channel CLIC like 1	23155	46,26136908	-0,13579748	0,428019	-0,317269794	0,751038895	0,941512988
ENSG00000101639	CEP192	centrosomal protein 192	55125	248,2384038	0,066640683	0,162881	0,409138091	0,682438324	0,925586358
ENSG00000013725	CD6	CD6 molecule	923	14,95956539	-1,312248109	0,723152	-1,814623547	0,069581781	NA
ENSG00000105662	CRTC1	CREB regulated transcription coactivator 1	23373	197,8965433	-0,037671388	0,168844	-0,223112949	0,823447601	0,961917534
ENSG00000116459	ATP5PB	ATP synthase peripheral stalk-membrane subunit b	515	238,2169669	1,30702954	0,652774	2,002268382	0,045255875	0,340953043
ENSG00000117419	ERI3	ERI1 exoribonuclease family member 3	79033	58,04579863	0,17754871	0,249373	0,711979746	0,476477318	0,853811879
ENSG00000120896	SORBS3	sorbin and SH3 domain containing 3	10174	14,17841744	-0,012256433	0,538442	-0,022762778	0,981839499	NA
ENSG00000127540	UQCR11	ubiquinol-cytochrome c reductase, complex III subunit XI	10975	116,0921607	0,10180697	0,158591	0,641947669	0,520907161	0,869884979
ENSG00000139726	DENR	density regulated re-initiation and release factor	8562	274,5262254	0,012537893	0,112541	0,111407679	0,911293071	0,979595261
ENSG00000146410	MTFR2	mitochondrial fission regulator 2	113115	22,74595316	0,490628944	0,414875	1,182595988	0,236969303	NA
ENSG00000171574	ZNF584	zinc finger protein 584	201514	13,25531218	-0,485454397	0,626847	-0,774438197	0,438671703	NA
ENSG00000174996	KLC2	kinesin light chain 2	64837	230,1206183	-0,126852603	0,148263	-0,855590367	0,3922244	0,812491435
ENSG00000197006	METTL9	methyltransferase 9, His-X-His N1(pi)-histidine	51108	245,8775359	0,050032828	0,108267	0,462123344	0,643992871	0,912530858
ENSG00000214413	BBIP1	BBSome interacting protein 1	92482	25,55490315	-0,056984397	0,482377	-0,11813244	0,90596272	NA
ENSG00000114107	CEP70	centrosomal protein 70	80321	20,83906177	-0,126069519	0,486713	-0,259022347	0,795617998	NA
ENSG00000134594	RAB33A	RAB33A, member RAS oncogene family	9363	32,70275261	-0,161131937	0,291418	-0,55292408	0,580315398	NA
ENSG00000135956	TMEM127	transmembrane protein 127	55654	343,1889371	-0,158041606	0,156212	-1,011709557	0,311676944	0,757867097
ENSG00000165300	SLITRK5	SLIT and NTRK like family member 5	26050	17,69540521	0,423077855	0,641557	0,65945443	0,509604	NA
ENSG00000018699	TTC27	tetratricopeptide repeat domain 27	55622	57,67695684	0,088028387	0,238032	0,369817636	0,711518374	0,930846779
ENSG00000131652	THOC6	THO complex subunit 6	79228	78,13657694	-0,288104498	0,238839	-1,206269489	0,227713598	0,683140794
ENSG00000142669	SH3BGRL3	SH3 domain binding glutamate rich protein like 3	83442	1818,827655	0,008946574	0,067673	0,132202838	0,894823856	0,977990819
ENSG00000174775	HRAS	HRas proto-oncogene, GTPase	3265	12,49909799	0,622554506	0,9225	0,674856185	0,49976714	NA
ENSG00000125611	CHCHD5	coiled-coil-helix-coiled-coil-helix domain containing 5	84269	20,49883309	-0,149019623	0,418528	-0,356056572	0,7217982	NA
ENSG00000145982	FARS2	phenylalanyl-tRNA synthetase 2, mitochondrial	10667	35,14433557	0,243955708	0,291162	0,837870348	0,402103524	NA
ENSG00000143436	MRPL9	mitochondrial ribosomal protein L9	65005	115,1604125	-0,147588235	0,153552	-0,961163822	0,336469804	0,776552413
ENSG00000119574	ZBTB45	zinc finger and BTB domain containing 45	84878	39,54517044	-0,221157845	0,299883	-0,737479235	0,460830966	NA
ENSG00000138780	GSTCD	glutathione S-transferase C-terminal domain containing	79807	43,62224051	0,183347977	0,268329	0,683295655	0,494420039	0,860513824
ENSG00000167685	ZNF444	zinc finger protein 444	55311	35,48771872	-0,283512803	0,311432	-0,910351186	0,362637332	NA
ENSG00000015133	CCDC88C	coiled-coil domain containing 88C	440193	663,1994324	-0,149288827	0,068236	-2,187840625	0,028681214	0,272623046

ENSG00000106028	SSBP1	single stranded DNA binding protein 1	6742	123,7925422	0,327779567	0,18393	1,782090994	0,074734394	0,444354325
ENSG00000112200	ZNF451	zinc finger protein 451	26036	309,7385413	-0,118151744	0,193389	-0,610953606	0,541230295	0,876458296
ENSG00000115652	UXS1	UDP-glucuronate decarboxylase 1	80146	92,64560817	-0,027270968	0,180412	-0,151159522	0,87984988	0,976300778
ENSG00000121964	GTDC1	glycosyltransferase like domain containing 1	79712	105,50985	-0,068476651	0,178523	-0,383573375	0,701294677	0,930846779
ENSG00000138604	GLCE	glucuronic acid epimerase	26035	13,54072672	0,094466212	0,635526	0,148642511	0,881835727	NA
ENSG00000162910	MRPL55	mitochondrial ribosomal protein L55	128308	23,27909036	-0,175552497	0,448292	-0,391603385	0,695351287	NA
ENSG00000177879	AP3S1	adaptor related protein complex 3 subunit sigma 1	1176	167,8534133	-0,118484074	0,133405	-0,888151887	0,374459055	0,801403701
ENSG00000070759	TESK2	testis associated actin remodelling kinase 2	10420	14,35961918	0,672689889	1,056124	0,636942116	0,524162548	NA
ENSG00000113558	SKP1	S-phase kinase associated protein 1	6500	190,5631656	0,295011477	0,372759	0,791426202	0,428695325	0,832060923
ENSG00000141933	TPGS1	tubulin polyglutamylase complex subunit 1	91978	15,09761545	0,009411818	0,497708	0,018910311	0,984912654	NA
ENSG00000155090	KLF10	KLF transcription factor 10	7071	46,76562138	-0,086777953	0,255754	-0,339301911	0,734382303	0,939157822
ENSG00000128311	TST	thiosulfate sulfurtransferase	7263	52,66239578	0,133078531	0,252629	0,526774111	0,598350463	0,894604697
ENSG00000170522	ELOVL6	ELOVL fatty acid elongase 6	79071	264,1213794	0,107888238	0,102574	1,051804574	0,29288922	0,744745216
ENSG00000239672	NME1	NME/NM23 nucleoside diphosphate kinase 1	4830	74,71779587	0,430978104	0,203147	2,121512975	0,033878657	0,294612488
ENSG00000102871	TRADD	TNFRSF1A associated via death domain	8717	37,1487876	-0,043683076	0,3303	-0,132252697	0,894784421	NA
ENSG00000104221	BRF2	BRF2 RNA polymerase III transcription initiation factor subunit	55290	31,24249331	-0,033678285	0,343003	-0,098186475	0,921784222	NA
ENSG00000127081	ZNF484	zinc finger protein 484	83744	117,4986826	0,182454465	0,163225	1,117808567	0,263648762	0,719682465
ENSG00000134215	VAV3	vav guanine nucleotide exchange factor 3	10451	203,555973	-0,024989157	0,149734	-0,166890658	0,867456084	0,972132916
ENSG00000165271	NOL6	nucleolar protein 6	65083	172,6311088	-0,140407318	0,420805	-0,333663457	0,738633528	0,939193793
ENSG00000172939	OXSR1	oxidative stress responsive kinase 1	9943	272,2492062	-0,010294329	0,101071	-0,1018527	0,918873595	0,984062493
ENSG00000173852	DPY19L1	dpy-19 like C-mannosyltransferase 1	23333	201,1488321	-0,075150899	0,115245	-0,652094678	0,514340097	0,868514045
ENSG00000175820	CCDC168	coiled-coil domain containing 168	643677	15,29534108	0,503639235	0,451312	1,115945488	0,264445469	NA
ENSG00000138286	FAM149B1	family with sequence similarity 149 member B1	317662	23,31350718	0,024265671	0,418042	0,058046037	0,953711958	NA
ENSG00000198863	RUNDC1	RUN domain containing 1	146923	98,65867277	-0,129243228	0,16941	-0,762902926	0,445521291	0,840933482
ENSG00000178691	SUZ12	SUZ12 polycomb repressive complex 2 subunit	23512	371,3964399	0,057530576	0,085791	0,670592705	0,502480031	0,86280677
ENSG00000064115	TM7SF3	transmembrane 7 superfamily member 3	51768	173,7204087	0,034744734	0,13034	0,266570466	0,789799898	0,951585219
ENSG00000088543	C3orf18	chromosome 3 open reading frame 18	51161	73,49052996	-0,475588239	0,229714	-2,070353638	0,03841924	0,31159154
ENSG00000100221	JOSD1	Josephin domain containing 1	9929	249,2733151	-0,09789493	0,124546	-0,786014723	0,431858862	0,834190728
ENSG00000104853	CLPTM1	CLPTM1 regulator of GABA type A receptor forward trafficking	1209	339,0868683	-0,113738067	0,110562	-1,02873083	0,303606178	0,751870158
ENSG00000126243	LRFN3	leucine rich repeat and fibronectin type III domain containing 3	79414	19,48066224	0,042666386	0,501175	0,085132661	0,932155925	NA
ENSG00000130244	FAM98C	family with sequence similarity 98 member C	147965	13,50313335	-0,008220211	0,461678	-0,017805093	0,985794342	NA
ENSG00000139197	PEX5	peroxisomal biogenesis factor 5	5830	42,1988582	-0,20965497	1,186231	-0,17674048	0,859712243	NA
ENSG00000139636	LMBR1L	limb development membrane protein 1 like	55716	18,86574148	1,082311612	2,31108	0,468314188	0,639559924	NA
ENSG00000169727	GPS1	G protein pathway suppressor 1	2873	212,4638016	-0,145481066	0,115175	-1,263134733	0,206540764	0,661359894
ENSG00000198265	HELZ	helicase with zinc finger	9931	1415,608169	0,023714067	0,076617	0,309513052	0,756931285	0,942901768
ENSG00000204271	SPIN3	spindlin family member 3	169981	78,29045876	-0,038299903	0,221556	-0,172867843	0,862755309	0,97107038
ENSG00000013364	MVP	major vault protein	9961	535,5657186	-0,118547752	0,088695	-1,336576508	0,181360911	0,634628658
ENSG00000090565	RAB11FIP3	RAB11 family interacting protein 3	9727	60,69912622	0,388295867	2,00608	0,193559484	0,846520826	0,967231052
ENSG00000114023	FAM162A	family with sequence similarity 162 member A	26355	38,75168335	0,451697517	0,400807	1,126970257	0,259755057	NA
ENSG00000135315	CEP162	centrosomal protein 162	22832	58,45950492	-0,039288496	0,252199	-0,155783846	0,876203404	0,97557436
ENSG00000189144	ZNF573	zinc finger protein 573	126231	16,36030717	-1,063007678	2,153785	-0,493553343	0,621621658	NA
ENSG00000196911	KPNA5	karyopherin subunit alpha 5	3841	90,92726733	-0,397296769	0,190422	-2,086403262	0,03694211	0,306351862
ENSG00000258289	CHURC1	churchill domain containing 1	91612	312,2753953	-0,208392075	0,117991	-1,766173749	0,077366704	0,449917526
ENSG00000111412	SPRING1	SREBF pathway regulator in golgi 1	79794	317,9326797	0,016798113	0,122116	0,137558913	0,890589029	0,977990819
ENSG00000161204	ABCF3	ATP binding cassette subfamily F member 3	55324	108,8839949	0,222850851	0,170629	1,306057474	0,191533013	0,649525167
ENSG00000177144	NUDT4B	nudix hydrolase 4B	440672	42,0970613	-0,277070016	0,300602	-0,921718075	0,35667565	NA

ENSG00000049239	H6PD	hexose-6-phosphate dehydrogenase/glucose 1-dehydrogenase	9563	585,0834575	-0,011752846	0,085323	-0,137745754	0,890441358	0,977990819
ENSG00000073050	XRCC1	X-ray repair cross complementing 1	7515	111,951743	-0,165059865	0,159834	-1,032693176	0,301747508	0,751870158
ENSG00000085760	MTIF2	mitochondrial translational initiation factor 2	4528	127,3976806	0,044022065	0,168241	0,261660436	0,793583245	0,952276252
ENSG00000102034	ELF4	E74 like ETS transcription factor 4	2000	441,0882034	-0,102037036	0,080552	-1,266720646	0,205255195	0,660954946
ENSG00000179271	GADD45GIP1	GADD45G interacting protein 1	90480	37,50744718	-0,116111217	0,279028	-0,41612714	0,677316971	NA
ENSG00000171735	CAMTA1	calmodulin binding transcription activator 1	23261	16,71992659	-0,648782367	0,603543	-1,074955668	0,282394577	NA
ENSG00000011260	UTP18	UTP18 small subunit processome component	51096	114,7948056	-0,054424951	0,160833	-0,338394549	0,735065882	0,939157822
ENSG00000139505	MTMR6	myotubularin related protein 6	9107	197,501206	-0,012311685	0,129722	-0,094908421	0,924387568	0,984284131
ENSG00000135392	DNAJC14	DnaJ heat shock protein family (Hsp40) member C14	85406	236,2290189	-0,12254709	0,115488	-1,061127465	0,288631978	0,740096492
ENSG00000223501	VPS52	VPS52 subunit of GARP complex	6293	68,91789687	-0,118163981	0,487399	-0,242438072	0,808440737	0,958576399
ENSG00000143149	ALDH9A1	aldehyde dehydrogenase 9 family member A1	223	160,5214106	0,096372399	0,158273	0,608898986	0,542591396	0,877396094
ENSG00000166889	PATL1	PAT1 homolog 1, processing body mRNA decay factor	219988	241,0463066	-0,015353619	0,140373	-0,109377147	0,912903359	0,980488351
ENSG00000170085	SIMC1	SUMO interacting motifs containing 1	375484	50,45250807	0,006191745	0,248592	0,024907272	0,980128927	0,995722751
ENSG00000133619	KRBA1	KRAB-A domain containing 1	84626	29,97553354	0,109431781	0,387621	0,28231664	0,777700727	NA
ENSG00000011485	PPP5C	protein phosphatase 5 catalytic subunit	5536	250,2962087	-0,02183701	0,116971	-0,186687204	0,85190589	0,968147715
ENSG00000178974	FBXO34	F-box protein 34	55030	208,7791574	-0,167821776	0,120039	-1,398056426	0,162096124	0,619487156
ENSG00000011295	TTC19	tetratricopeptide repeat domain 19	54902	62,28134999	-0,146401858	0,279507	-0,523786153	0,600427286	0,896014669
ENSG00000173418	NAA20	N-alpha-acetyltransferase 20, NatB catalytic subunit	51126	70,7871527	0,173043201	0,229433	0,754220383	0,4507169	0,84263906
ENSG00000084073	ZMPSTE24	zinc metallopeptidase STE24	10269	244,7941752	0,163649958	0,115894	1,412059758	0,157932367	0,61428162
ENSG00000197776	KLHDC1	kelch domain containing 1	122773	16,42692591	-0,064336749	0,44931	-0,143190001	0,886140126	NA
ENSG00000001497	LAS1L	LAS1 like ribosome biogenesis factor	81887	90,21054735	-0,042752404	0,215589	-0,198305142	0,842806329	0,967210584
ENSG00000166260	COX11	cytochrome c oxidase copper chaperone COX11	1353	48,3823658	-0,709438059	0,412695	-1,719035411	0,085607924	0,477891497
ENSG00000160199	PKNOX1	PBX/knotted 1 homeobox 1	5316	86,65557187	0,076199071	0,177551	0,429167992	0,667800975	0,922308959
ENSG00000196141	SPATS2L	spermatogenesis associated serine rich 2 like	26010	129,8409219	0,081461213	0,171486	0,475031399	0,634764593	0,908844904
ENSG00000006715	VPS41	VPS41 subunit of HOPS complex	27072	258,6607571	-0,203804944	0,111045	-1,835337582	0,066455686	0,416786686
ENSG00000084733	RAB10	RAB10, member RAS oncogene family	10890	351,1350401	-0,05960301	0,111847	-0,532897541	0,594104508	0,894229873
ENSG00000108798	ABI3	ABI family member 3	51225	333,8836253	-0,218287686	0,101633	-2,14781229	0,03172867	0,284147066
ENSG00000117153	KLHL12	kelch like family member 12	59349	143,785222	-0,037093306	0,149123	-0,24874381	0,803558958	0,956670011
ENSG00000126822	PLEKHG3	pleckstrin homology and RhoGEF domain containing G3	26030	61,46207581	-0,768018537	0,737032	-1,042042468	0,29739199	0,748371252
ENSG00000134030	CTIF	cap binding complex dependent translation initiation factor	9811	26,77778246	0,224636977	0,333705	0,673160793	0,500845002	NA
ENSG00000136045	PWP1	PWP1 homolog, endonuclein	11137	177,6942837	0,178491985	0,141809	1,25867973	0,208146036	0,662274754
ENSG00000140743	CDR2	cerebellar degeneration related protein 2	1039	52,99122396	-0,015363044	0,237018	-0,064817972	0,948318932	0,990608484
ENSG00000145734	BDP1	B double prime 1, subunit of RNA polymerase III transcription initiation factor IIIB	55814	315,6099334	0,196666799	1,485794	0,132364824	0,894695736	0,977990819
ENSG00000152684	PELO	pelota mRNA surveillance and ribosome rescue factor	53918	73,88194318	0,145649631	0,195622	0,744545827	0,456546327	0,844645758
ENSG00000169193	CCDC126	coiled-coil domain containing 126	90693	35,4679578	-0,16701853	0,35156	-0,475077753	0,634731555	NA
ENSG00000188603	CLN3	CLN3 lysosomal/endosomal transmembrane protein, battenin	1201	209,7195932	0,242782375	0,192044	1,264204187	0,206156748	0,660954946
ENSG00000198176	TFDP1	transcription factor Dp-1	7027	241,3773616	0,214789868	0,108934	1,971741186	0,048639159	0,354159983
ENSG00000058804	NDC1	NDC1 transmembrane nucleoporin	55706	111,7714783	0,043103454	0,154247	0,279443924	0,779904167	0,947749187
ENSG00000061987	MON2	MON2 homolog, regulator of endosome-to-Golgi trafficking	23041	395,0243081	0,043380831	0,12232	0,354649529	0,722852166	0,934902053
ENSG00000085998	POMGNT1	protein O-linked mannose N-acetylglucosaminyltransferase 1 (beta 1,2-)	55624	77,79642891	-0,336591077	0,396975	-0,847889847	0,396499319	0,812799303

ENSG00000104835	SARS2	seryl-tRNA synthetase 2, mitochondrial	54938	61,63225726	-0,079927201	0,235389	-0,339553649	0,734192689	0,939157822
ENSG00000106367	AP1S1	adaptor related protein complex 1 subunit sigma 1	1174	98,48079352	-0,254456681	0,198278	-1,2833322	0,199375716	0,658208139
ENSG00000138030	KHK	ketohexokinase	3795	16,46811142	0,280331773	0,5917	0,473773561	0,63566139	NA
ENSG00000157823	AP3S2	adaptor related protein complex 3 subunit sigma 2	10239	114,9117533	0,168937614	0,598718	0,282165556	0,777816568	0,947109268
ENSG00000158006	PAFAH2	platelet activating factor acetylhydrolase 2	5051	121,7595287	0,009635501	0,163381	0,058975674	0,952971484	0,991212866
ENSG00000159593	NAE1	NEDD8 activating enzyme E1 subunit 1	8883	112,7708732	-0,009693529	0,166987	-0,058049557	0,953709154	0,991212866
ENSG00000163162	RNF149	ring finger protein 149	284996	123,0007058	-0,346477362	0,158702	-2,183197581	0,029021263	0,27461807
ENSG00000164306	PRIMPOL	primase and DNA directed polymerase	201973	64,43765435	-0,295111024	0,219668	-1,343439882	0,17912958	0,633446071
ENSG00000171617	ENC1	ectodermal-neural cortex 1	8507	14,0961403	-0,139630169	0,64428	-0,21672284	0,828424347	NA
ENSG00000183624	HMCES	5-hydroxymethylcytosine binding, ES cell specific	56941	227,6260931	0,080332756	0,116955	0,68686936	0,492165057	0,860513824
ENSG00000141577	CEP131	centrosomal protein 131	22994	55,41930168	-0,069857993	0,310444	-0,225026095	0,821958974	0,961917534
ENSG00000069275	NUCKS1	nuclear casein kinase and cyclin dependent kinase substrate 1	64710	884,468655	0,031421308	0,058546	0,536694598	0,591478588	0,892834002
ENSG00000105669	COPE	COPI coat complex subunit epsilon	11316	208,4425473	-0,043442502	0,121755	-0,356803808	0,721238685	0,933829316
ENSG00000106683	LIMK1	LIM domain kinase 1	3984	486,5529551	-0,189691908	0,077439	-2,449555762	0,014303256	0,186300802
ENSG00000116212	LRRC42	leucine rich repeat containing 42	115353	36,19898961	0,255612927	0,296557	0,861936562	0,388722428	NA
ENSG00000120727	PAIP2	poly(A) binding protein interacting protein 2	51247	314,2699981	0,076547909	0,10223	0,748778846	0,453990512	0,843234809
ENSG00000137871	ZNF280D	zinc finger protein 280D	54816	115,6642265	-0,149068626	0,172796	-0,862684438	0,388310991	0,810040214
ENSG00000140157	NIPA2	NIPA magnesium transporter 2	81614	236,6849043	0,062902608	0,122528	0,513372147	0,607691025	0,897481254
ENSG00000147123	NDUFB11	NADH:ubiquinone oxidoreductase subunit B11	54539	90,11245805	0,496104567	0,176664	2,808179769	0,004982241	0,099064602
ENSG00000171823	FBXL14	F-box and leucine rich repeat protein 14	144699	36,22289715	0,012078799	0,29246	0,041300621	0,967056238	NA
ENSG00000179922	ZNF784	zinc finger protein 784	147808	14,59961679	-0,061730304	0,457295	-0,134990065	0,89261973	NA
ENSG00000198894	CIPC	CLOCK interacting pacemaker	85457	58,0696742	-0,867552616	0,467847	-1,854352224	0,063688785	0,406570577
ENSG00000213585	VDAC1	voltage dependent anion channel 1	7416	482,8888237	0,06963292	0,080508	0,864915858	0,387084973	0,809653368
ENSG00000063176	SPHK2	sphingosine kinase 2	56848	69,69235415	-0,151943285	0,261362	-0,581352921	0,56100262	0,884229295
ENSG00000107890	ANKRD26	ankyrin repeat domain containing 26	22852	109,5556266	0,0838942	0,202566	0,414156834	0,678759253	0,925294441
ENSG00000179833	SERTAD2	SERTA domain containing 2	9792	316,7307633	0,14043237	0,099466	1,411866015	0,157989416	0,61428162
ENSG00000213397	HAUS7	HAUS augmin like complex subunit 7	55559	18,06881092	0,172092841	0,438827	0,392165471	0,694935955	NA
ENSG00000152700	SAR1B	secretion associated Ras related GTPase 1B	51128	174,8423016	0,247626664	0,16947	1,4611837	0,143965035	0,59024645
ENSG00000111679	PTPN6	protein tyrosine phosphatase non-receptor type 6	5777	1231,040008	-0,155272164	0,079936	-1,942456891	0,052081814	0,369238926
ENSG00000116161	CACYBP	calcyclin binding protein	27101	142,9336653	0,557649172	0,257301	2,167306785	0,030211469	0,279077601
ENSG00000142507	PSMB6	proteasome 20S subunit beta 6	5694	129,7037711	0,136544371	0,160789	0,849215484	0,395761399	0,812799303
ENSG00000143919	CAMKMT	calmodulin-lysine N-methyltransferase	79823	20,78424883	-0,659895507	0,576201	-1,145253098	0,252104329	NA
ENSG00000175606	TMEM70	transmembrane protein 70	54968	50,70979712	0,390562197	0,313649	1,245219205	0,213051185	0,667215123
ENSG00000178537	SLC25A20	solute carrier family 25 member 20	788	163,0193072	-0,048650556	0,141801	-0,343089607	0,73153105	0,938991109
ENSG00000166484	MAPK7	mitogen-activated protein kinase 7	5598	28,55356598	-0,435700228	1,070284	-0,407088242	0,683943179	NA
ENSG00000009413	REV3L	REV3 like, DNA directed polymerase zeta catalytic subunit	5980	278,7497423	0,045923763	0,728193	0,063065355	0,949714462	0,990710238
ENSG00000018610	STEEP1	STING1 ER exit protein 1	63932	76,87754441	-0,021598916	0,259636	-0,083189357	0,933700976	0,987098279
ENSG00000100372	SLC25A17	solute carrier family 25 member 17	10478	46,50905279	0,33855409	0,301222	1,123934812	0,261040682	0,716543286
ENSG00000128185	DGCR6L	DiGeorge syndrome critical region gene 6 like	85359	51,92484071	-0,091722167	0,2732	-0,335732391	0,737072678	0,939157822
ENSG00000130309	COLGALT1	collagen beta(1-O)galactosyltransferase 1	79709	203,9568606	-0,122582102	0,127041	-0,964899776	0,334595014	0,77450906
ENSG00000134058	CDK7	cyclin dependent kinase 7	1022	32,7582481	0,423515584	1,080385	0,392004333	0,695055012	NA
ENSG00000139725	RHOF	ras homolog family member F, filopodia associated	54509	365,0081592	-0,042514132	0,099026	-0,429324029	0,667687432	0,922308959
ENSG00000166847	DCTN5	dynactin subunit 5	84516	387,4907456	0,074468881	0,126941	0,586642159	0,557444062	0,883921037
ENSG00000171097	KYAT1	kynurenine aminotransferase 1	883	14,00578821	0,447157475	0,505683	0,884265117	0,376553114	NA
ENSG00000171659	GPR34	G protein-coupled receptor 34	2857	16,76097927	0,431968847	0,457147	0,944923501	0,344697933	NA
ENSG00000213071	NA	NA	NA	100,8269018	0,100246273	0,169462	0,591555392	0,554148352	0,882854594
ENSG00000108590	MED31	mediator complex subunit 31	51003	17,05651015	0,047362434	0,492612	0,096145611	0,923404927	NA

ENSG00000145860	RNF145	ring finger protein 145	153830	410,4263203	0,064028491	0,113312	0,565064375	0,572029978	0,886795904
ENSG00000203705	TATDN3	TatD DNase domain containing 3	128387	18,99070408	-0,217643393	0,466354	-0,466691589	0,640720549	NA
ENSG00000274523	RCC1L	RCC1 like	81554	112,2805882	0,183171656	0,186886	0,98012554	0,327024153	0,769687654
ENSG00000188785	ZNF548	zinc finger protein 548	147694	143,8499	-0,461764479	0,195113	-2,366648008	0,017949995	0,209790571
ENSG00000100483	VCPKMT	valosin containing protein lysine methyltransferase	79609	16,72210989	0,802696055	0,709197	1,131837128	0,257702915	NA
ENSG00000116127	ALMS1	ALMS1 centrosome and basal body associated protein	7840	195,3683009	0,296050472	0,39312	0,753078451	0,451402772	0,842718653
ENSG00000162923	WDR26	WD repeat domain 26	80232	358,6158845	-0,130720101	0,286155	-0,456816261	0,647803117	0,9144223
ENSG00000101997	CCDC22	coiled-coil domain containing 22	28952	76,88424045	-0,010322037	0,202556	-0,05095888	0,959358287	0,992073731
ENSG00000110315	RNF141	ring finger protein 141	50862	41,5871766	0,176437098	0,302352	0,583547712	0,559524648	NA
ENSG00000126264	HCST	hematopoietic cell signal transducer	10870	285,6378601	0,008302082	0,13738	0,060431434	0,951812023	0,991212866
ENSG00000134686	PHC2	polyhomeotic homolog 2	1912	130,0072969	-0,015388883	0,154062	-0,099887617	0,920433548	0,984284131
ENSG00000167720	SRR	serine racemase	63826	39,42599275	0,131167192	0,2833	0,462998207	0,643365652	NA
ENSG00000287542	HERC3	HECT and RLD domain containing E3 ubiquitin protein ligase 3	8916	48,19079815	0,325157237	0,839634	0,387260563	0,698563318	0,930783276
ENSG00000047188	YTHDC2	YTH N6-methyladenosine RNA binding protein C2	64848	394,4210877	0,160672165	0,100048	1,605956822	0,108283397	0,52823466
ENSG00000101350	KIF3B	kinesin family member 3B	9371	144,7914856	-0,007942955	0,164464	-0,048295998	0,961480344	0,992328441
ENSG00000121895	TMEM156	transmembrane protein 156	80008	38,46612356	-0,238213586	0,313894	-0,758898196	0,447913458	NA
ENSG00000162869	PPP1R21	protein phosphatase 1 regulatory subunit 21	129285	124,1351223	0,394324453	0,237335	1,661464956	0,096620102	0,502914503
ENSG00000167291	TBC1D16	TBC1 domain family member 16	125058	14,11307514	0,022238236	0,560133	0,03970171	0,968330939	NA
ENSG00000003393	ALS2	alsin Rho guanine nucleotide exchange factor ALS2	57679	39,35564922	0,062415238	0,486635	0,128258834	0,897944142	NA
ENSG00000078061	ARAF	A-Raf proto-oncogene, serine/threonine kinase	369	249,4877342	0,248039962	0,112829	2,19836225	0,027923302	0,269753518
ENSG00000125962	ARMCX5	armadillo repeat containing X-linked 5	64860	37,2112081	-0,060339677	0,397211	-0,151908343	0,879259228	NA
ENSG00000184613	NELL2	neural EGFL like 2	4753	54,05105053	0,443288	0,31313	1,415666503	0,15687317	0,61428162
ENSG00000125149	PHAF1	phagosome assembly factor 1	80262	67,7642603	0,036403343	0,233979	0,155584127	0,876360838	0,975611342
ENSG00000087111	PIGS	phosphatidylinositol glycan anchor biosynthesis class S	94005	176,2300368	-0,200382174	0,168651	-1,188150041	0,234774302	0,69018943
ENSG00000108639	SYNGR2	synaptogyrin 2	9144	131,9060321	-0,003931721	0,156473	-0,025127147	0,979953547	0,995712569
ENSG00000108784	NAGLU	N-acetyl-alpha-glucosaminidase	4669	71,78073675	-0,17060907	0,216907	-0,786553153	0,431543492	0,833786615
ENSG00000110931	CAMKK2	calcium/calmodulin dependent protein kinase kinase 2	10645	214,5334721	-0,13114763	0,139744	-0,938481989	0,347996769	0,784103633
ENSG00000115561	CHMP3	charged multivesicular body protein 3	51652	150,1630292	0,113717627	0,18961	0,599744922	0,548676245	0,880168143
ENSG00000122873	CISD1	CDGSH iron sulfur domain 1	55847	28,16307804	0,560426936	0,408883	1,370628565	0,170490774	NA
ENSG00000125971	DYNLRB1	dynein light chain roadblock-type 1	83658	182,5916674	0,125583817	0,126018	0,996557671	0,318979261	0,763389521
ENSG00000243678	NME2	NME/NM23 nucleoside diphosphate kinase 2	4831	335,3556332	0,280637234	0,098746	2,842013256	0,004482963	0,092655762
ENSG00000272779	NA	NA	NA	37,5457456	-0,0844211	0,31163	-0,270901428	0,786466847	NA
ENSG00000119509	INVS	inversin	27130	111,7120031	0,133937294	0,214799	0,623547599	0,532924733	0,873191491
ENSG00000148358	GPR107	G protein-coupled receptor 107	57720	306,2392962	-0,01338281	0,096858	-0,138169674	0,890106322	0,977990819
ENSG00000165071	TMEM71	transmembrane protein 71	137835	48,80305204	0,010115882	0,265286	0,038131917	0,969582504	0,993542794
ENSG00000160131	VMA21	vacuolar ATPase assembly factor VMA21	203547	148,782748	-0,123094358	0,197068	-0,624628406	0,532214972	0,872967783
ENSG00000198482	ZNF808	zinc finger protein 808	388558	102,2058261	0,019143798	0,254311	0,075277203	0,939994159	0,987813564
ENSG00000136830	NIBAN2	niban apoptosis regulator 2	64855	178,7470753	-0,126445518	0,128376	-0,98496417	0,324641671	0,76812822
ENSG00000067836	ROGDI	rogdi atypical leucine zipper	79641	15,74831947	0,805320048	1,62706	0,494954054	0,620632553	NA
ENSG00000105953	OGDH	oxoglutarate dehydrogenase	4967	565,7514837	0,010762809	0,093043	0,11567549	0,907909732	0,979091451
ENSG00000143891	GALM	galactose mutarotase	130589	27,76547981	-0,053014658	0,3631	-0,146005751	0,883916849	NA
ENSG00000185986	NA	NA	NA	11,7399574	0,01869363	0,553498	0,033773607	0,973057682	NA
ENSG00000176108	CHMP6	charged multivesicular body protein 6	79643	86,96519418	-0,200893308	0,183562	-1,094415482	0,273772797	0,726913977
ENSG00000277734	TRAC	T cell receptor alpha constant	28755	62,287903	-0,125059647	0,217186	-0,575818932	0,564737576	0,885045179
ENSG00000140403	DNAJA4	DnaJ heat shock protein family (Hsp40) member A4	55466	45,99778581	-0,042503373	0,284289	-0,149507531	0,881153167	0,976300778
ENSG00000160584	SIK3	SIK family kinase 3	23387	282,7501897	0,005245969	0,109404	0,047950224	0,961755913	0,992328441
ENSG00000133195	SLC39A11	solute carrier family 39 member 11	201266	53,98375953	0,19450753	0,244427	0,795770949	0,42616518	0,831371417
ENSG00000115904	SOS1	SOS Ras/Rac guanine nucleotide exchange factor 1	6654	1182,127969	-0,331988369	0,119748	-2,77239608	0,005564528	0,106855271
ENSG00000182628	SKA2	spindle and kinetochore associated complex subunit 2	348235	160,2130234	0,025419807	0,180641	0,140719843	0,888091269	0,977990819
ENSG00000165272	AQP3	aquaporin 3 (Gill blood group)	360	36,62869196	0,256847347	0,293828	0,874142572	0,382040616	NA

ENSG00000168566	SNRNP48	small nuclear ribonucleoprotein U11/U12 subunit 48	154007	82,26107998	0,011069374	0,190764	0,058026538	0,95372749	0,991212866
ENSG00000114902	SPCS1	signal peptidase complex subunit 1	28972	152,4198229	-0,009775505	0,152829	-0,0639637	0,948999131	0,9906219
ENSG00000196220	SRGAP3	SLIT-ROBO Rho GTPase activating protein 3	9901	499,5802653	0,2751608	0,077603	3,545731781	0,000391524	0,017571619
ENSG00000198026	ZNF335	zinc finger protein 335	63925	337,8066098	0,048426462	0,094278	0,51365589	0,607492596	0,897481254
ENSG00000135605	TEC	tec protein tyrosine kinase	7006	116,1106167	0,196860962	0,152268	1,292855238	0,196061131	0,654982614
ENSG00000163050	COQ8A	coenzyme Q8A	56997	60,42034314	0,410083896	1,114796	0,367855648	0,712980872	0,930846779
ENSG00000106400	ZNHIT1	zinc finger HIT-type containing 1	10467	85,77028363	0,064441707	0,182472	0,353159017	0,72396923	0,934902053
ENSG00000196636	SDHAF3	succinate dehydrogenase complex assembly factor 3	57001	16,53512404	-0,353097011	0,426235	-0,828409142	0,407438831	NA
ENSG00000160087	UBE2J2	ubiquitin conjugating enzyme E2 J2	118424	128,8440884	-0,140260013	0,168983	-0,830024347	0,406525018	0,819098895
ENSG00000205531	NAP1L4	nucleosome assembly protein 1 like 4	4676	609,2496542	0,054167675	0,10791	0,501972971	0,615686534	0,902090873
ENSG00000099219	ERMP1	endoplasmic reticulum metallopeptidase 1	79956	736,5882394	-0,354105542	0,237463	-1,491203262	0,135908137	0,576449438
ENSG00000091490	SEL1L3	SEL1L family member 3	23231	698,9921212	0,07364575	0,077913	0,945234371	0,344539236	0,781366475
ENSG00000112033	PPARD	peroxisome proliferator activated receptor delta	5467	221,2405016	0,022213152	0,114148	0,194599928	0,845706159	0,967231052
ENSG00000114767	RRP9	ribosomal RNA processing 9, U3 small nucleolar RNA binding protein	9136	40,01474923	0,06240301	0,281964	0,221315581	0,824846719	NA
ENSG00000170464	DNAJC18	DnaJ heat shock protein family (Hsp40) member C18	202052	30,64840246	-0,17366837	0,351681	-0,493823455	0,621430867	NA
ENSG00000174851	YIF1A	Yip1 interacting factor homolog A, membrane trafficking protein	10897	47,47223559	-0,363447691	0,27628	-1,315505645	0,188340022	0,646436727
ENSG00000292372	WASH6P	WASP family homolog 6, pseudogene	653440	35,7572851	0,048152113	0,325854	0,147771893	0,882522792	NA
ENSG00000164346	NSA2	NSA2 ribosome biogenesis factor	10412	134,6025528	0,441599887	0,166788	2,647672355	0,008104803	0,134862554
ENSG00000198945	L3MBTL3	L3MBTL histone methyl-lysine binding protein 3	84456	65,13718736	-0,098271815	0,259764	-0,378312062	0,705198786	0,930846779
ENSG00000011523	CEP68	centrosomal protein 68	23177	64,68605308	-0,192454962	0,268715	-0,716205424	0,473864514	0,853811879
ENSG00000049249	TNFRSF9	TNF receptor superfamily member 9	3604	23,64411742	0,59240204	0,411407	1,43994033	0,149884282	NA
ENSG00000072864	NDE1	nudE neurodevelopment protein 1	54820	47,76965705	1,018506614	1,864164	0,546361096	0,584817747	0,890019742
ENSG00000114395	CYB561D2	cytochrome b561 family member D2	11068	61,75043202	0,187521266	0,346886	0,540584146	0,588794248	0,891627382
ENSG00000292327	PPP2R3B	protein phosphatase 2 regulatory subunit B''beta	28227	19,29658762	0,158546722	0,49131	0,322702104	0,746920859	NA
ENSG00000103653	CSK	C-terminal Src kinase	1445	725,5247811	0,1169656	0,092106	1,269905739	0,204118209	0,660954946
ENSG00000257267	ZNF271P	zinc finger protein 271, pseudogene	10778	70,15837332	0,051984945	0,204555	0,254136999	0,799389721	0,954455285
ENSG00000181027	FKRP	fukutin related protein	79147	56,22404047	-0,141001572	0,240348	-0,586654914	0,557435494	0,883921037
ENSG00000101190	TCFL5	transcription factor like 5	10732	50,6635875	-0,27177608	0,242485	-1,120793971	0,262375571	0,718263414
ENSG00000105270	CLIP3	CAP-Gly domain containing linker protein 3	25999	14,73887975	-0,2414569	0,497849	-0,485000386	0,627676072	NA
ENSG00000069509	FUNDC1	FUN14 domain containing 1	139341	25,94275271	-0,05455821	0,377316	-0,144595389	0,885030338	NA
ENSG00000130311	DDA1	DET1 and DDB1 associated 1	79016	135,695299	-0,056807856	0,163768	-0,346879461	0,728681878	0,937916952
ENSG00000163703	CRELD1	cysteine rich with EGF like domains 1	78987	49,02989199	-0,326684653	0,396533	-0,823853061	0,410023054	0,82130194
ENSG00000167721	TSR1	TSR1 ribosome maturation factor	55720	197,7243634	0,030253519	0,120781	0,250483363	0,802213569	0,956331071
ENSG00000100442	FKBP3	FKBP prolyl isomerase 3	2287	104,7495785	0,292554304	0,17778	1,645597651	0,099846623	0,509429216
ENSG00000105926	PALS2	protein associated with LIN7 2, MAGUK p55 family member	51678	52,21001202	0,332393675	0,381955	0,870243687	0,384167246	0,808586507
ENSG00000137106	GRHPR	glyoxylate and hydroxypyruvate reductase	9380	99,55611651	0,13897207	0,171585	0,809932863	0,417978763	0,82446781
ENSG00000120318	ARAP3	ArfGAP with RhoGAP domain, ankyrin repeat and PH domain 3	64411	12,67454095	0,033741959	0,576451	0,058533954	0,953323317	NA
ENSG00000139618	BRCA2	BRCA2 DNA repair associated	675	65,58541657	-1,16807601	2,572062	-0,454139825	0,649728191	0,914926418
ENSG00000163382	NAXE	NAD(P)HX epimerase	128240	71,83610433	-0,043076865	0,190922	-0,22562537	0,821492807	0,961898134
ENSG00000171840	NINJ2	ninjurin 2	4815	24,4284697	0,151527362	0,430283	0,352157293	0,724720304	NA
ENSG00000100216	TOMM22	translocase of outer mitochondrial membrane 22	56993	110,9030071	0,238934798	0,187247	1,27604211	0,201940634	0,660119983
ENSG00000166707	ZCCHC18	zinc finger CCHC-type containing 18	644353	20,92310189	-0,423782246	0,388372	-1,091175007	0,275195883	NA
ENSG00000167716	WDR81	WD repeat domain 81	124997	231,8975522	0,034180632	0,122034	0,280091579	0,779407245	0,947712923
ENSG00000182742	HOXB4	homeobox B4	3214	57,03985994	-0,368753954	0,230577	-1,599268075	0,10976105	0,529522903
ENSG00000230202	NA	NA	NA	39,73549309	0,226235185	0,287514	0,786866152	0,431360224	NA
ENSG00000242028	HYPK	huntingtin interacting protein K	25764	58,99713968	-0,636437644	0,50561	-1,258752853	0,208119615	0,662274754

ENSG00000134809	TIMM10	translocase of inner mitochondrial membrane 10	26519	24,68980962	0,317197265	0,392871	0,807382995	0,419445861	NA
ENSG00000064490	RFXANK	regulatory factor X associated ankyrin containing protein	8625	45,982121	-0,34237306	0,270746	-1,264555055	0,206030873	0,660954946
ENSG00000100335	MIEF1	mitochondrial elongation factor 1	54471	231,3570988	-0,27498413	0,124871	-2,202144295	0,027655117	0,269631574
ENSG00000188811	NHLRC3	NHL repeat containing 3	387921	128,1202626	-0,072601859	0,148462	-0,489027561	0,624822184	0,90479929
ENSG00000256594	NA	NA	NA	14,33170473	0,063620936	0,458651	0,138713216	0,889676774	NA
ENSG00000273213	H3-7	H3,7 histone (putative)	440686	12,59126055	-0,746300964	0,569826	-1,309700875	0,190297047	NA
ENSG00000178149	DALRD3	DALR anticodon binding domain containing 3	55152	28,69605722	0,370123267	0,464096	0,797515015	0,425151986	NA
ENSG00000133265	HSPBP1	HSPA (Hsp70) binding protein 1	23640	37,35607553	0,042962166	0,272633	0,157582688	0,874785646	NA
ENSG00000165359	INTS6L	integrator complex subunit 6 like	203522	135,3795776	0,069312823	0,151094	0,458740909	0,646420233	0,913619671
ENSG00000196663	TECPR2	tectonin beta-propeller repeat containing 2	9895	69,38055824	-0,118777538	0,223121	-0,532344997	0,594487073	0,894229873
ENSG00000099992	TBC1D10A	TBC1 domain family member 10A	83874	106,9814728	-0,285114844	0,187419	-1,52126627	0,128193031	0,563689485
ENSG00000165983	PTER	phosphotriesterase related	9317	61,52294247	-0,192407304	0,225374	-0,853724729	0,393257529	0,812491435
ENSG00000166619	BLCAP	BLCAP apoptosis inducing factor	10904	181,0005253	-0,142566179	0,144239	-0,988400066	0,322956755	0,767603859
ENSG00000105576	TNPO2	transportin 2	30000	241,4389286	-0,181312902	0,129289	-1,402382732	0,160800987	0,618147537
ENSG00000137168	PPIL1	peptidylprolyl isomerase like 1	51645	67,16230703	0,324650251	0,218173	1,4880416	0,136739923	0,577705947
ENSG00000177853	ZNF518A	zinc finger protein 518A	9849	164,6193099	-0,153144394	0,185221	-0,826821462	0,408338263	0,820514158
ENSG00000116649	SRM	spermidine synthase	6723	158,4781318	0,153011942	0,138017	1,108643236	0,267584119	0,722449525
ENSG00000172171	TEFM	transcription elongation factor, mitochondrial	79736	19,03335904	0,351495389	0,483778	0,726562913	0,467493754	NA
ENSG00000173950	XXYLT1	xyloside xylosyltransferase 1	152002	57,28932525	0,051423256	0,245538	0,209430972	0,834111817	0,963446071
ENSG00000197724	PHF2	PHD finger protein 2	5253	257,302772	-0,12778646	0,111495	-1,146114978	0,251747581	0,708175323
ENSG00000271425	NBPF10	NBPF member 10	100132406	379,0381407	0,100410727	0,094283	1,064998136	0,286876773	0,738005299
ENSG00000171860	C3AR1	complement C3a receptor 1	719	63,03351757	0,199430978	0,225701	0,883607064	0,376908365	0,803373958
ENSG00000100219	XBP1	X-box binding protein 1	7494	1450,296718	0,034989975	0,049158	0,711788828	0,476595552	0,853811879
ENSG00000167004	PDIA3	protein disulfide isomerase family A member 3	2923	355,7874359	0,393714801	0,702109	0,560760505	0,574960814	0,887069818
ENSG00000188636	RTL6	retrotransposon Gag like 6	84247	82,63110247	-0,047235177	0,203761	-0,231816589	0,816680472	0,959707099
ENSG00000085382	HACE1	HECT domain and ankyrin repeat containing E3 ubiquitin protein ligase 1	57531	101,3344079	-0,080354751	0,179929	-0,446591938	0,655169717	0,918055836
ENSG00000100226	GTPBP1	GTP binding protein 1	9567	232,7614071	-0,074863342	0,11749	-0,637187555	0,524002683	0,870612376
ENSG00000143811	PYCR2	pyrroline-5-carboxylate reductase 2	29920	157,2899181	-0,049043092	0,149166	-0,328781055	0,742321184	0,939193793
ENSG00000146433	TMEM181	transmembrane protein 181	57583	333,5431865	0,097048912	0,102228	0,949334177	0,342450676	0,780064992
ENSG00000155636	RBM45	RNA binding motif protein 45	129831	25,30464767	0,204189074	0,749195	0,272544704	0,785203223	NA
ENSG00000185278	ZBTB37	zinc finger and BTB domain containing 37	84614	335,4417266	0,101644406	0,310771	0,327071371	0,743613904	0,939745744
ENSG00000175756	AURKAIP1	aurora kinase A interacting protein 1	54998	67,39134922	0,104018695	0,245369	0,423927379	0,671618776	0,923060255
ENSG00000124659	TBCC	tubulin folding cofactor C	6903	116,2869546	-0,04919479	0,151942	-0,323772418	0,746110339	0,939843468
ENSG00000009307	CSDE1	cold shock domain containing E1	7812	1346,695615	0,085548981	0,065186	1,312381457	0,18939146	0,647463823
ENSG00000134575	ACP2	acid phosphatase 2, lysosomal	53	100,1377817	0,089305159	0,172034	0,51911263	0,603682201	0,897212117
ENSG00000164209	SLC25A46	solute carrier family 25 member 46	91137	247,3479525	-0,126443355	0,116306	-1,087159554	0,276966302	0,730699811
ENSG00000140854	KATNB1	katanin regulatory subunit B1	10300	82,32522478	-0,194120144	0,203384	-0,954452416	0,339854685	0,777431905
ENSG00000142599	RERE	arginine-glutamic acid dipeptide repeats	473	94,55268059	0,16972675	0,173414	0,97873716	0,327709866	0,769917226
ENSG00000005448	WDR54	WD repeat domain 54	84058	20,18405362	0,565538916	0,427544	1,322762281	0,185914445	NA
ENSG00000113369	ARRDC3	arrestin domain containing 3	57561	170,0221361	1,33772601	0,161289	8,293945616	1,10E-16	1,43E-13
ENSG00000124508	BTN2A2	butyrophilin subfamily 2 member A2	10385	35,5403863	-0,304567163	0,326377	-0,933176652	0,350728768	NA
ENSG00000134480	CCNH	cyclin H	902	75,24967601	0,420194983	0,195412	2,150299188	0,031531557	0,282704161
ENSG00000076351	SLC46A1	solute carrier family 46 member 1	113235	17,22799253	-0,800299797	0,446758	-1,791351401	0,073236923	NA
ENSG00000115204	MPV17	mitochondrial inner membrane protein MPV17	4358	83,96227437	-0,141473309	0,214531	-0,659452694	0,509605114	0,866180216
ENSG00000089682	RBM41	RNA binding motif protein 41	55285	151,4262384	-0,073099514	0,258979	-0,282259858	0,777744263	0,947109268
ENSG00000164649	CDCA7L	cell division cycle associated 7 like	55536	15,19693297	0,420605599	0,512822	0,820178207	0,412114524	NA
ENSG00000137309	HMGA1	high mobility group AT-hook 1	3159	84,14268855	0,154785643	0,196249	0,788720658	0,430275291	0,832770364
ENSG00000110958	PTGES3	prostaglandin E synthase 3	10728	487,0658362	0,128074326	0,080149	1,5979612	0,110051612	0,529948108
ENSG00000004779	NDUFAB1	NADH:ubiquinone oxidoreductase subunit AB1	4706	64,07812321	0,427068597	0,236526	1,80558849	0,070982642	0,433512962
ENSG00000100902	PSMA6	proteasome 20S subunit alpha 6	5687	221,649745	0,297239452	0,137264	2,165452952	0,030353015	0,279077601

ENSG00000012963	UBR7	ubiquitin protein ligase E3 component n-recognin 7	55148	215,8440763	-0,046616984	0,128	-0,364195177	0,715712268	0,932040329
ENSG00000090238	YPEL3	yippee like 3	83719	247,7861844	-0,403967602	0,122126	-3,307794722	0,000940337	0,033570034
ENSG00000104671	DCTN6	dynactin subunit 6	10671	44,88035097	0,413154258	0,256768	1,609054781	0,107604364	0,527254203
ENSG00000123154	WDR83	WD repeat domain 83	84292	28,14815778	-0,413037488	0,331386	-1,246393083	0,21262012	NA
ENSG00000128394	APOBEC3F	apolipoprotein B mRNA editing enzyme catalytic subunit 3F	200316	157,743381	-0,375830059	0,183647	-2,046480862	0,040709083	0,322307603
ENSG00000142856	ITGB3BP	integrin subunit beta 3 binding protein	23421	18,16715243	-0,22917969	0,4391	-0,521930882	0,601718455	NA
ENSG00000153975	ZUP1	zinc finger containing ubiquitin peptidase 1	221302	54,72753668	0,069446206	0,290931	0,238703633	0,8113354	0,959038743
ENSG00000166503	HDGFL3	HDGF like 3	50810	258,9860197	0,100958116	0,119811	0,842646167	0,399426363	0,814618191
ENSG00000256053	COA8	cytochrome c oxidase assembly factor 8	84334	43,57363235	0,14653006	0,274918	0,532994809	0,594037173	0,894229873
ENSG00000143742	SRP9	signal recognition particle 9	6726	354,7654551	-0,134101083	0,12391	-1,082242399	0,279144833	0,732510365
ENSG00000177169	ULK1	unc-51 like autophagy activating kinase 1	8408	131,674969	-0,311364841	0,149451	-2,083391818	0,037215523	0,306351862
ENSG00000214425	NA	NA	NA	284,6329874	-0,105154435	0,148445	-0,708373057	0,47871362	0,854008769
ENSG00000065150	IPO5	importin 5	3843	283,3683889	0,407964208	0,171441	2,379615034	0,017330733	0,205923714
ENSG00000092841	MYL6	myosin light chain 6	4637	1343,850411	0,05435218	0,046845	1,160243397	0,245949723	0,703199535
ENSG00000100605	ITPK1	inositol-tetrakisphosphate 1-kinase	3705	341,7732126	-0,034537008	0,113354	-0,304681703	0,760608586	0,942901768
ENSG00000133773	CCDC59	coiled-coil domain containing 59	29080	12,83419889	1,439329774	0,79517	1,810091765	0,070281558	NA
ENSG00000136485	DCAF7	DDB1 and CUL4 associated factor 7	10238	881,5154878	0,111618421	0,112431	0,992774058	0,320820075	0,764513157
ENSG00000136813	ECPAS	Ecm29 proteasome adaptor and scaffold	23392	440,9815704	0,035698819	0,12379	0,288382329	0,773054093	0,946448613
ENSG00000154723	ATP5PF	ATP synthase peripheral stalk subunit F6	522	95,90485797	-0,04227504	0,19046	-0,22196236	0,824343185	0,961917534
ENSG00000155304	HSPA13	heat shock protein family A (Hsp70) member 13	6782	296,6073406	-0,11385517	0,118601	-0,959984413	0,33706306	0,77693253
ENSG00000169740	ZNF32	zinc finger protein 32	7580	83,69333937	-0,043189867	0,186343	-0,231776695	0,816711459	0,959707099
ENSG00000173198	CYSLTR1	cysteinyl leukotriene receptor 1	10800	43,43985018	-0,017623313	0,256937	-0,068590081	0,945315915	0,98927293
ENSG00000176340	COX8A	cytochrome c oxidase subunit 8A	1351	162,7960122	0,504246399	0,163654	3,081170176	0,002061888	0,05545913
ENSG00000180992	MRPL14	mitochondrial ribosomal protein L14	64928	51,7607159	0,214636622	0,238957	0,898224139	0,369066067	0,797217623
ENSG00000183336	BOLA2	bolA family member 2	552900	17,16546551	-0,176501378	1,441001	-0,122485261	0,902514718	NA
ENSG00000185798	WDR53	WD repeat domain 53	348793	28,30385382	0,077891204	0,451245	0,172613963	0,862954876	NA
ENSG00000205155	PSENEN	presenilin enhancer, gamma-secretase subunit	55851	64,04449961	-0,420738129	0,23187	-1,814539363	0,069594728	0,428367551
ENSG00000101199	ARFGAP1	ADP ribosylation factor GTPase activating protein 1	55738	151,161887	0,178774614	0,189231	0,944743857	0,344789661	0,781523231
ENSG00000096092	TMEM14A	transmembrane protein 14A	28978	45,46369349	0,278160393	0,278849	0,997528809	0,318507897	0,763136371
ENSG00000101457	DNTTIP1	deoxynucleotidyltransferase terminal interacting protein 1	116092	42,86647576	0,080345561	0,316369	0,253961515	0,79952529	0,954472052
ENSG00000130479	MAP1S	microtubule associated protein 1S	55201	103,8528547	-0,23624083	0,182028	-1,297825222	0,1943474	0,652365446
ENSG00000171723	GPHN	gephyrin	10243	29,67719776	0,336756397	0,400279	0,841304189	0,400177545	NA
ENSG00000099956	SMARCB1	SWI/SNF related, matrix associated, actin dependent regulator of chromatin, subfamily b, member 1	6598	15,02777094	-0,588920304	0,87489	-0,673136427	0,500860501	NA
ENSG00000147533	GOLGA7	golgin A7	51125	198,891283	-0,10816097	0,172665	-0,626419956	0,531039524	0,872770879
ENSG00000169554	ZEB2	zinc finger E-box binding homeobox 2	9839	28,39428356	1,233261479	1,17825	1,046688875	0,295243096	NA
ENSG00000137404	NRM	nurim	11270	31,52078624	-0,042515969	0,358095	-0,118728175	0,905490714	NA
ENSG00000054219	LY75	lymphocyte antigen 75	4065	334,6037704	0,062949877	0,108134	0,582144412	0,560469412	0,883921037
ENSG00000145050	MANF	mesencephalic astrocyte derived neurotrophic factor	7873	153,1941521	-0,021842068	0,139876	-0,156153574	0,875911971	0,975526463
ENSG00000182158	CREB3L2	cAMP responsive element binding protein 3 like 2	64764	604,6488947	0,042993411	0,085489	0,502914036	0,615024712	0,901934707
ENSG00000063244	U2AF2	U2 small nuclear RNA auxiliary factor 2	11338	600,3302222	-0,103506345	0,080401	-1,287381948	0,197961193	0,656945827
ENSG00000140443	IGF1R	insulin like growth factor 1 receptor	3480	27,01178733	0,023333583	0,343802	0,067869228	0,945889735	NA
ENSG00000069020	MAST4	microtubule associated serine/threonine kinase family member 4	375449	41,36445314	0,472904231	0,328631	1,439010682	0,150147496	NA
ENSG00000078319	NA	NA	NA	28,92538842	0,041805791	0,332927	0,125570605	0,900071832	NA
ENSG00000104904	OAZ1	ornithine decarboxylase antizyme 1	4946	93,38713441	0,151955564	0,330706	0,459488752	0,645883228	0,913189356
ENSG00000144597	EAF1	ELL associated factor 1	85403	255,9018738	0,010267146	0,108317	0,094787808	0,924483371	0,984284131
ENSG00000186951	PPARA	peroxisome proliferator activated receptor alpha	5465	285,7026259	-0,007650882	0,120379	-0,063556663	0,949323239	0,990700279
ENSG00000213064	SFT2D2	SFT2 domain containing 2	375035	963,5746305	-0,151040683	0,110585	-1,365836943	0,171990148	0,629558603

ENSG00000104381	GDAP1	ganglioside induced differentiation associated protein 1	54332	16,73444893	-0,877366158	0,628241	-1,396543693	0,162550833	NA
ENSG00000096401	CDC5L	cell division cycle 5 like	988	228,5933072	-0,010946696	0,12174	-0,089918547	0,928351942	0,986487751
ENSG00000100246	DNAL4	dynein axonemal light chain 4	10126	18,28357126	-0,23303119	0,428746	-0,543518433	0,586772903	NA
ENSG00000104164	BLOC1S6	biogenesis of lysosomal organelles complex 1 subunit 6	26258	205,5867796	-0,165112784	0,11859	-1,392302578	0,163830791	0,623112365
ENSG00000112667	DNPH1	2'-deoxynucleoside 5'-phosphate N-hydrolase 1	10591	19,5614728	0,298568717	0,417733	0,714734991	0,474772812	NA
ENSG00000125356	NDUFA1	NADH:ubiquinone oxidoreductase subunit A1	4694	97,24465557	0,228915502	0,183157	1,249834792	0,211359904	0,66507239
ENSG00000143314	MRPL24	mitochondrial ribosomal protein L24	79590	59,61700975	0,01771026	0,249051	0,071110967	0,94330944	0,988624545
ENSG00000162076	FLYWCH2	FLYWCH family member 2	114984	31,18646936	-0,239831294	0,348094	-0,688983468	0,490833671	NA
ENSG00000269743	SLC25A53	solute carrier family 25 member 53	401612	89,82545078	-0,290344546	0,212218	-1,368142485	0,17126748	0,629558603
ENSG00000140563	MCTP2	multiple C2 and transmembrane domain containing 2	55784	768,7745552	0,109024464	0,1085	1,004829157	0,314979122	0,760305504
ENSG00000006757	PNPLA4	patatin like phospholipase domain containing 4	8228	12,74759947	-0,582659226	0,513799	-1,13402163	0,256785475	NA
ENSG00000107554	DNMBP	dynamin binding protein	23268	34,8261596	-0,092493806	0,295307	-0,313211872	0,754119699	NA
ENSG00000109572	CLCN3	chloride voltage-gated channel 3	1182	275,3218383	0,091110053	0,119543	0,762153408	0,44596845	0,841259829
ENSG00000169019	COMMD8	COMM domain containing 8	54951	84,5558529	0,12644803	0,198168	0,63808595	0,523417732	0,870221868
ENSG00000171476	HOPX	HOP homeobox	84525	1925,056294	0,015599643	0,074919	0,20822054	0,835056771	0,964065248
ENSG00000214826	NA	NA	NA	48,07613611	0,184831159	0,27063	0,682967252	0,494627536	0,860513824
ENSG00000163069	SGCB	sarcoglycan beta	6443	20,06196826	0,301497819	0,441418	0,683020725	0,494593747	NA
ENSG00000066322	ELOVL1	ELOVL fatty acid elongase 1	64834	147,9090622	-0,187893853	0,162637	-1,155297007	0,247968821	0,706134779
ENSG00000100058	NA	NA	NA	12,35957672	0,334478611	0,520074	0,64313676	0,520135363	NA
ENSG00000184481	FOXO4	forkhead box O4	4303	92,28756307	0,089501868	0,232758	0,384527004	0,700587885	0,930783276
ENSG00000151151	IPMK	inositol polyphosphate multikinase	253430	49,51097129	-0,213459787	0,239519	-0,89120339	0,372820066	0,800776142
ENSG00000165724	ZMYND19	zinc finger MYND-type containing 19	116225	39,96989346	-0,345397768	0,291862	-1,183427153	0,236639901	NA
ENSG00000152128	TMEM163	transmembrane protein 163	81615	57,29161845	0,095594782	0,261999	0,364867638	0,715210213	0,932016096
ENSG00000171109	MFN1	mitofusin 1	55669	107,8446362	0,021172722	0,258943	0,081765867	0,934832899	0,987098279
ENSG00000159423	ALDH4A1	aldehyde dehydrogenase 4 family member A1	8659	18,13505503	0,204724312	0,409786	0,499588799	0,617364646	NA
ENSG00000130584	ZBTB46	zinc finger and BTB domain containing 46	140685	48,97942397	-0,324375406	0,290444	-1,116826522	0,264068506	0,719682465
ENSG00000169689	CENPX	centromere protein X	201254	26,55199099	-0,339366023	0,34718	-0,977493213	0,328325036	NA
ENSG00000128309	MPST	mercaptopyruvate sulfurtransferase	4357	98,61802595	0,070439426	0,186184	0,378331708	0,705184193	0,930846779
ENSG00000156795	NTAQ1	N-terminal glutamine amidase 1	55093	35,42762472	0,220923749	0,299597	0,737404092	0,460876648	NA
ENSG00000159199	ATP5MC1	ATP synthase membrane subunit c locus 1	516	56,41925087	0,241652248	0,258177	0,935992743	0,349276924	0,784537594
ENSG00000198964	SGMS1	sphingomyelin synthase 1	259230	107,0778285	0,02028697	0,161417	0,125680426	0,899984897	0,977990819
ENSG00000204482	LST1	leukocyte specific transcript 1	7940	25,96437859	0,789789468	0,553245	1,427558582	0,153418952	NA
ENSG00000104872	PIH1D1	PIH1 domain containing 1	55011	72,2929689	0,274972249	0,213842	1,28586844	0,19848898	0,656945827
ENSG00000127838	PNKD	PNKD metallo-beta-lactamase domain containing	25953	35,90101924	-0,293557512	0,288002	-1,019290855	0,308064904	NA
ENSG00000114054	PCCB	propionyl-CoA carboxylase subunit beta	5096	66,6595347	0,205397897	0,212361	0,967209877	0,333439126	0,773674527
ENSG00000182552	RWDD4	RWD domain containing 4	201965	65,94285733	-0,02967201	0,248174	-0,119561243	0,904830723	0,978460866
ENSG00000269226	TMSB15B	thymosin beta 15B	286527	12,92961081	-0,267495366	0,627248	-0,426458706	0,669773632	NA
ENSG00000269226	TMSB15C	thymosin beta 15C	122394733	12,92961081	-0,267495366	0,627248	-0,426458706	0,669773632	NA
ENSG00000160803	UBQLN4	ubiquilin 4	56893	169,9552294	-0,165806836	0,130534	-1,270219391	0,204006492	0,660954946
ENSG00000175893	ZDHHC21	zinc finger DHHC-type palmitoyltransferase 21	340481	258,661884	0,105316265	0,130949	0,804253062	0,42125084	0,827332857
ENSG00000150756	ATPSCKMT	ATP synthase c subunit lysine N-methyltransferase	134145	56,06381635	-0,012771624	0,271309	-0,047074084	0,962454183	0,992328441
ENSG00000065413	ANKRD44	ankyrin repeat domain 44	91526	1117,091839	-0,083667291	0,065054	-1,286127373	0,198398613	0,656945827
ENSG00000073756	PTGS2	prostaglandin-endoperoxide synthase 2	5743	12,53096177	1,417820439	2,123357	0,667725966	0,504308532	NA
ENSG00000101265	RASSF2	Ras association domain family member 2	9770	253,8865449	-0,15431654	0,109161	-1,41365797	0,157462351	0,61428162
ENSG00000103707	MTFMT	mitochondrial methionyl-tRNA formyltransferase	123263	14,60046031	-0,604464128	0,699903	-0,86363991	0,387785733	NA
ENSG00000122335	SERAC1	serine active site containing 1	84947	28,68001508	-0,850246281	0,466302	-1,823381547	0,068245623	NA
ENSG00000137776	SLTM	SAFB like transcription modulator	79811	507,3686791	-0,033074927	0,088868	-0,372179039	0,709759551	0,930846779
ENSG00000141504	SAT2	spermidine/spermine N1-acetyltransferase family member 2	112483	14,95216769	0,88288134	0,765712	1,15301967	0,248902311	NA

ENSG00000144909	OSBPL11	oxysterol binding protein like 11	114885	116,7466476	0,207871388	0,160649	1,293945143	0,19568437	0,654519463
ENSG00000156860	FBRS	fibrosin	64319	711,0785187	-0,081546651	0,073319	-1,112222778	0,266042376	0,722230271
ENSG00000162129	CLPB	ClpB family mitochondrial disaggregase	81570	121,337494	0,417672371	0,490865	0,850890176	0,394830363	0,812498986
ENSG00000165637	VDAC2	voltage dependent anion channel 2	7417	176,7027504	0,069683854	0,195121	0,357131923	0,720993047	0,933829316
ENSG00000184216	IRAK1	interleukin 1 receptor associated kinase 1	3654	270,214435	0,11318239	0,146019	0,775118901	0,438269404	0,837388481
ENSG00000198522	GPN1	GPN-loop GTPase 1	11321	146,1162779	0,133493461	0,146583	0,910702717	0,362452032	0,791409024
ENSG00000205746	PKD1P4	polycystin 1, transient receptor potential channel interacting pseudogene 4	353512	200,9201153	0,049116272	0,151446	0,324316242	0,745698624	0,939843468
ENSG00000171824	EXOSC10	exosome component 10	5394	307,1052213	0,143093051	0,101344	1,411949924	0,157964706	0,61428162
ENSG00000093010	COMT	catechol-O-methyltransferase	1312	102,9906873	0,155426449	0,233621	0,665292902	0,505863169	0,864630986
ENSG00000120071	KANSL1	KAT8 regulatory NSL complex subunit 1	284058	110,1114225	-0,008023285	0,51692	-0,015521332	0,987616266	0,996894778
ENSG00000125818	PSMF1	proteasome inhibitor subunit 1	9491	295,3757259	0,092884551	0,109612	0,8473946	0,396775214	0,812799303
ENSG00000214530	STARD10	StAR related lipid transfer domain containing 10	10809	43,39999601	0,335037947	0,437888	0,76512237	0,44419868	0,840223973
ENSG00000140612	SEC11A	SEC11 homolog A, signal peptidase complex subunit	23478	312,285865	0,055044533	0,10342	0,532242333	0,594558167	0,894229873
ENSG00000158169	FANCC	FA complementation group C	2176	45,07297582	0,545311095	0,333212	1,636527821	0,101729168	0,514437479
ENSG00000158169	LOC105376156	uncharacterized LOC105376156	105376156	45,07297582	0,545311095	0,333212	1,636527821	0,101729168	0,514437479
ENSG00000169621	APLF	aprataxin and PNKP like factor	200558	12,03273481	-0,314277263	0,614721	-0,51125207	0,609174562	NA
ENSG00000160193	WDR4	WD repeat domain 4	10785	33,00845267	0,240660655	0,357392	0,673379335	0,500705992	NA
ENSG00000198125	MB	myoglobin	4151	104,2712008	0,088996744	0,225599	0,394490221	0,693219146	0,928002746
ENSG00000103266	STUB1	STIP1 homology and U-box containing protein 1	10273	100,8220042	-0,246389571	0,188417	-1,307683575	0,190980657	0,649053257
ENSG00000110047	EHD1	EH domain containing 1	10938	826,5900514	-0,048635503	0,068493	-0,710077297	0,477656204	0,854008769
ENSG00000120725	SIL1	SIL1 nucleotide exchange factor	64374	45,46643655	-0,16622785	0,279011	-0,595775235	0,55132539	0,880819693
ENSG00000132286	TIMM10B	translocase of inner mitochondrial membrane 10B	26515	177,6600776	0,003492026	0,139242	0,02507878	0,979992126	0,995712569
ENSG00000172673	THEMIS	thymocyte selection associated	387357	19,40526509	0,034003469	1,677201	0,020273937	0,983824847	NA
ENSG00000103404	USP31	ubiquitin specific peptidase 31	57478	54,99155953	-0,394903423	0,259027	-1,524563251	0,127368069	0,563689485
ENSG00000109919	MTCH2	mitochondrial carrier 2	23788	18,4496187	0,768121474	1,637925	0,468959967	0,639098252	NA
ENSG00000110107	PRPF19	pre-mRNA processing factor 19	27339	270,6672142	0,025912079	0,124728	0,207749512	0,835424556	0,964287552
ENSG00000149474	KAT14	lysine acetyltransferase 14	57325	87,42641131	0,015648806	0,188636	0,08295769	0,933885182	0,987098279
ENSG00000173200	PARP15	poly(ADP-ribose) polymerase family member 15	165631	310,1319086	-0,255698767	0,11778	-2,170983081	0,029932451	0,279077601
ENSG00000134824	FADS2	fatty acid desaturase 2	9415	560,6929735	-0,01225037	0,083671	-0,146411313	0,883596697	0,976751663
ENSG00000089248	ERP29	endoplasmic reticulum protein 29	10961	268,7506747	0,229528118	0,105439	2,176880148	0,029489511	0,277377987
ENSG00000179151	EDC3	enhancer of mRNA decapping 3	80153	179,8234174	-0,025206522	0,144088	-0,174938956	0,8611276	0,970761615
ENSG00000174749	FAM241A	family with sequence similarity 241 member A	132720	25,28753879	-0,050194045	0,348478	-0,144038079	0,885470401	NA
ENSG00000100603	SNW1	SNW domain containing 1	22938	277,5609842	0,061719552	0,101019	0,610972421	0,541217838	0,876458296
ENSG00000114993	RTKN	rhotekin	6242	17,78911295	0,357499438	0,404633	0,883514211	0,376958508	NA
ENSG00000131788	PIAS3	protein inhibitor of activated STAT 3	10401	96,46856224	0,249031666	0,235098	1,059265465	0,289478898	0,741447348
ENSG00000171566	PLRG1	pleiotropic regulator 1	5356	155,2800919	0,206841165	0,154737	1,336730156	0,181310735	0,634628658
ENSG00000119599	DCAF4	DDB1 and CUL4 associated factor 4	26094	73,82212083	0,021313415	0,222697	0,095706086	0,923754007	0,984284131
ENSG00000145495	MARCHF6	membrane associated ring-CH-type finger 6	10299	511,154346	-0,138209794	0,159643	-0,865743861	0,386630641	0,809653368
ENSG00000162600	OMA1	OMA1 zinc metallopeptidase	115209	63,58685628	-0,03635824	0,274497	-0,132453912	0,894625275	0,977990819
ENSG00000102882	MAPK3	mitogen-activated protein kinase 3	5595	113,0858024	-0,289702014	0,160832	-1,801266711	0,071660852	0,435172111
ENSG00000108106	UBE2S	ubiquitin conjugating enzyme E2 S	27338	90,6367317	0,367868609	0,378972	0,970701651	0,331696869	0,772811395
ENSG00000136643	RPS6KC1	ribosomal protein S6 kinase C1	26750	159,1183311	0,207805146	0,134584	1,544051461	0,122575866	0,558690088
ENSG00000167965	MLST8	MTOR associated protein, LST8 homolog	64223	31,24508699	-0,407910554	0,553205	-0,737358563	0,460904328	NA
ENSG00000180901	KCTD2	potassium channel tetramerization domain containing 2	23510	106,9507258	0,146502458	2,026473	0,072294297	0,942367703	0,988475138
ENSG00000196123	MATCAP1	microtubule associated tyrosine carboxypeptidase 1	653319	37,1507556	-0,156599067	0,295572	-0,529817628	0,596238382	NA

ENSG00000197982	C1orf122	chromosome 1 open reading frame 122	127687	19,10262221	0,328319887	0,408637	0,803450714	0,421714273	NA
ENSG00000055332	EIF2AK2	eukaryotic translation initiation factor 2 alpha kinase 2	5610	364,575422	0,375432239	0,126981	2,956596435	0,003110549	0,07336413
ENSG00000144021	CIAO1	cytosolic iron-sulfur assembly component 1	9391	455,2670092	-0,089045803	0,085017	-1,047389845	0,294919813	0,746855268
ENSG00000232112	TMA7	translation machinery associated 7 homolog	51372	220,8391459	-0,059036073	0,124148	-0,475528471	0,634410345	0,908608932
ENSG00000131653	TRAF7	TNF receptor associated factor 7	84231	200,4469231	0,12809773	0,14799	0,865585323	0,386717607	0,809653368
ENSG00000105705	SUGP1	SURP and G-patch domain containing 1	57794	45,56228078	-0,962190284	1,171759	-0,821150638	0,411560469	0,82165631
ENSG00000141425	RPRD1A	regulation of nuclear pre-mRNA domain containing 1A	55197	53,27657423	-0,374517348	0,328517	-1,140023394	0,254276555	0,709949543
ENSG00000148218	ALAD	aminolevulinate dehydratase	210	144,4462923	0,125191356	0,148085	0,845403893	0,397885372	0,813948101
ENSG00000162642	C1orf52	chromosome 1 open reading frame 52	148423	41,13862023	-0,237035802	0,450068	-0,526666844	0,598424964	NA
ENSG00000175283	DOLK	dolichol kinase	22845	32,0904408	-0,207263082	0,40378	-0,513306578	0,607736883	NA
ENSG00000186767	SPIN4	spindlin family member 4	139886	113,3303086	-0,179979427	0,167262	-1,076032597	0,281912678	0,735595407
ENSG00000004864	SLC25A13	solute carrier family 25 member 13	10165	33,75648802	-0,058031421	0,321387	-0,180565528	0,856708616	NA
ENSG00000015532	XYLT2	xylosyltransferase 2	64132	74,21828519	-0,274616069	0,211933	-1,295771181	0,19505433	0,653071292
ENSG00000055044	NOP58	NOP58 ribonucleoprotein	51602	252,7475184	0,19312648	0,111709	1,728843152	0,083837171	0,471737097
ENSG00000070010	UFD1	ubiquitin recognition factor in ER associated degradation 1	7353	122,6537373	-0,120033489	0,200736	-0,597966038	0,549862594	0,88072908
ENSG00000100106	TRIOBP	TRIO and F-actin binding protein	11078	196,7841744	-0,242451721	0,133598	-1,814785562	0,06955687	0,428367551
ENSG00000100324	TAB1	TGF-beta activated kinase 1 (MAP3K7) binding protein 1	10454	241,4070482	-0,131376735	0,120436	-1,09084678	0,275340308	0,727877072
ENSG00000100554	ATP6V1D	ATPase H+ transporting V1 subunit D	51382	86,90487171	0,01855063	0,200559	0,09249471	0,926304994	0,985345959
ENSG00000105854	PON2	paraoxonase 2	5445	44,70159084	-0,145075126	0,253607	-0,57204592	0,567290865	0,885785777
ENSG00000117411	B4GALT2	beta-1,4-galactosyltransferase 2	8704	25,27777207	0,324266871	0,357373	0,90736156	0,36421563	NA
ENSG00000126464	PRR12	proline rich 12	57479	366,6489767	-0,219767357	0,092247	-2,382389888	0,017200675	0,204688035
ENSG00000136295	TTYH3	tweety family member 3	80727	255,4402864	-0,047163159	0,115146	-0,409592689	0,682104761	0,925586358
ENSG00000143418	CERS2	ceramide synthase 2	29956	291,3812285	0,143612711	0,136584	1,051461448	0,293046707	0,744745216
ENSG00000146872	TLK2	tousled like kinase 2	11011	143,6306185	-0,019712889	0,185187	-0,106448721	0,915226339	0,98172462
ENSG00000148690	FRA10AC1	FRA10A associated CGG repeat 1	118924	40,84185189	-0,258859406	0,305067	-0,848533073	0,396141162	NA
ENSG00000152240	HAUS1	HAUS augmin like complex subunit 1	115106	50,19162886	0,161808809	0,283552	0,570648865	0,568237688	0,88603113
ENSG00000155561	NUP205	nucleoporin 205	23165	384,6960756	0,120616281	0,083488	1,444714261	0,148538162	0,601349859
ENSG00000158716	DUSP23	dual specificity phosphatase 23	54935	37,0988956	0,027678285	0,296366	0,09339213	0,925592042	NA
ENSG00000164818	DNAAF5	dynein axonemal assembly factor 5	54919	47,06828983	0,289945838	0,310906	0,93258395	0,351034824	0,785712602
ENSG00000165792	METTL17	methyltransferase like 17	64745	68,0582399	-0,038344553	0,267386	-0,14340499	0,885970342	0,977761756
ENSG00000166046	TCP11L2	t-complex 11 like 2	255394	47,02191804	-0,355140702	0,260801	-1,361729543	0,173283254	0,629558603
ENSG00000166529	ZSCAN21	zinc finger and SCAN domain containing 21	7589	29,5955487	-0,250800774	0,340083	-0,737469446	0,460836917	NA
ENSG00000167113	COQ4	coenzyme Q4	51117	63,49869222	-0,157133465	0,255906	-0,614028592	0,539196433	0,875342836
ENSG00000168944	CEP120	centrosomal protein 120	153241	136,0204854	-0,325752425	0,253204	-1,286520524	0,198261461	0,656945827
ENSG00000171530	TBCA	tubulin folding cofactor A	6902	110,3356204	0,357316087	0,175928	2,031035855	0,042251357	0,327907274
ENSG00000174306	ZHX3	zinc fingers and homeoboxes 3	23051	126,3833244	-0,030915704	0,260822	-0,118531743	0,905646345	0,978460866
ENSG00000177674	AGTRAP	angiotensin II receptor associated protein	57085	259,0667274	-0,048986714	0,144046	-0,340076516	0,733798907	0,939157822
ENSG00000197217	ENTPD4	ectonucleoside triphosphate diphosphohydrolase 4	9583	367,4956054	-0,031830527	0,095363	-0,333781197	0,738544673	0,939193793
ENSG00000166471	TMEM41B	transmembrane protein 41B	440026	122,6475846	-0,206010144	0,174644	-1,179601639	0,238158691	0,693290718
ENSG00000221838	AP4M1	adaptor related protein complex 4 subunit mu 1	9179	17,58711813	-1,084307216	0,652522	-1,661717923	0,096569346	NA
ENSG00000122884	P4HA1	prolyl 4-hydroxylase subunit alpha 1	5033	65,12462327	0,158556687	0,213836	0,741488873	0,458397076	0,845558274
ENSG00000128513	POT1	protection of telomeres 1	25913	76,32435724	0,113747833	0,295716	0,384652714	0,700494733	0,930783276
ENSG00000126602	TRAP1	TNF receptor associated protein 1	10131	99,47673445	-0,158685882	0,1861	-0,852692727	0,393829725	0,812498986
ENSG00000168646	AXIN2	axin 2	8313	43,9993005	0,186279904	0,284787	0,65410344	0,513045165	0,86823028
ENSG00000172534	HCFC1	host cell factor C1	3054	817,4092323	-0,042208407	0,068643	-0,614895453	0,538623766	0,875342836
ENSG00000215883	CYB5RL	cytochrome b5 reductase like	606495	34,16127201	0,067122833	0,390869	0,171727232	0,863651976	NA
ENSG00000042753	AP2S1	adaptor related protein complex 2 subunit sigma 1	1175	98,12091751	0,007783998	0,218481	0,035627878	0,971579079	0,994108581
ENSG00000054967	RELT	RELT TNF receptor	84957	46,33685283	-0,281183619	0,338163	-0,831504165	0,405688876	0,818464021
ENSG00000082458	DLG3	discs large MAGUK scaffold protein 3	1741	125,2489018	-0,229411068	0,155509	-1,475227308	0,140151442	0,58302406

ENSG00000124786	SLC35B3	solute carrier family 35 member B3	51000	69,42448381	0,302781646	0,218087	1,38835294	0,165029599	0,624454568
ENSG00000237973	MTCO1P12	MT-CO1 pseudogene 12	107075141	46,88004814	0,220606357	0,242216	0,910782834	0,362409809	0,791409024
ENSG00000263142	LRRC37A17P	leucine rich repeat containing 37 member A17, pseudogene	644397	13,63882978	-0,503656152	0,4984	-1,010545448	0,312234037	NA
ENSG00000136828	RALGPS1	Ral GEF with PH domain and SH3 binding motif 1	9649	22,51775136	0,424936302	0,397005	1,07035451	0,284459767	NA
ENSG00000144362	PHOSPHO2	phosphatase, orphan 2	493911	27,23619188	-0,824199753	0,401809	-2,051221474	0,040245384	NA
ENSG00000141965	FEM1A	fem-1 homolog A	55527	186,7534725	-0,298990646	0,168341	-1,77610036	0,075716377	0,446229554
ENSG00000153179	RASSF3	Ras association domain family member 3	283349	148,4152251	-0,337278229	0,142502	-2,366835221	0,017940919	0,209790571
ENSG00000174010	KLHL15	kelch like family member 15	80311	141,6752161	0,102861544	0,204279	0,503534595	0,614588464	0,901765987
ENSG00000100347	SAMM50	SAMM50 sorting and assembly machinery component	25813	130,5726848	-0,053620747	0,151624	-0,353643138	0,723606341	0,934902053
ENSG00000171316	CHD7	chromodomain helicase DNA binding protein 7	55636	36,29289061	-0,68358989	1,537157	-0,444710442	0,656529022	NA
ENSG00000156127	BATF	basic leucine zipper ATF-like transcription factor	10538	32,32695289	-0,146279727	0,325317	-0,449653562	0,65296026	NA
ENSG00000103550	KNOP1	lysine rich nucleolar protein 1	400506	59,68938754	-0,304509359	0,423819	-0,718489726	0,47245538	0,853811879
ENSG00000096080	MRPS18A	mitochondrial ribosomal protein S18A	55168	51,48547994	0,320093435	0,234608	1,364375874	0,172449297	0,629558603
ENSG00000100934	SEC23A	SEC23 homolog A, COPII coat complex component	10484	215,6418637	0,036465152	0,122057	0,298754493	0,765127375	0,944359403
ENSG00000152404	CWF19L2	CWF19 like cell cycle control factor 2	143884	131,3923048	-0,002218925	0,146612	-0,015134657	0,987924752	0,996894778
ENSG00000156804	FBXO32	F-box protein 32	114907	75,30802547	0,069522028	0,281589	0,246892248	0,804991616	0,957328863
ENSG00000125166	GOT2	glutamic-oxaloacetic transaminase 2	2806	181,3625027	-0,068001346	0,125632	-0,5412746	0,588318325	0,891557039
ENSG00000102158	MAGT1	magnesium transporter 1	84061	399,0146225	-0,066491714	0,101303	-0,656367751	0,511587535	0,866510351
ENSG00000071082	RPL31	ribosomal protein L31	6160	328,3488914	0,07356751	0,102611	0,716954653	0,473402078	0,853811879
ENSG00000110090	CPT1A	carnitine palmitoyltransferase 1A	1374	892,2203451	-0,029258462	0,068375	-0,42790887	0,668717469	0,922641254
ENSG00000111726	CMAS	cytidine monophosphate N-acetylneuraminic acid synthetase	55907	84,54649713	0,057087301	0,262767	0,217254224	0,828010229	0,962723654
ENSG00000125746	EML2	EMAP like 2	24139	153,7748705	-0,024687276	0,161056	-0,153283734	0,878174527	0,976237908
ENSG00000144635	DYNC1LI1	dynein cytoplasmic 1 light intermediate chain 1	51143	139,9417493	-0,202767937	0,149753	-1,354017846	0,175730684	0,632039836
ENSG00000147044	CASK	calcium/calmodulin dependent serine protein kinase	8573	242,4680301	-0,000958818	0,141403	-0,006780743	0,994589791	0,998500439
ENSG00000152969	JAKMIP1	janus kinase and microtubule interacting protein 1	152789	69,92022683	-0,147074312	0,206401	-0,712565743	0,476114516	0,853811879
ENSG00000180104	EXOC3	exocyst complex component 3	11336	501,819189	-0,060451042	0,077644	-0,778570227	0,436232923	0,836566451
ENSG00000182134	TDRKH	tudor and KH domain containing	11022	46,85467171	0,220422299	0,255619	0,862307836	0,388518142	0,810040214
ENSG00000060642	PIGV	phosphatidylinositol glycan anchor biosynthesis class V	55650	56,8745616	-0,000150406	0,217839	-0,000690447	0,999449103	0,999703675
ENSG00000114126	TFDP2	transcription factor Dp-2	7029	222,6367086	0,22473783	0,17296	1,299365868	0,193818399	0,65192707
ENSG00000132530	XAF1	XIAP associated factor 1	54739	472,0152189	-0,018359302	0,092537	-0,198400029	0,842732095	0,967210584
ENSG00000138614	INTS14	integrator complex subunit 14	81556	112,3230054	0,262937749	0,178605	1,472171764	0,1409745	0,584590139
ENSG00000177600	RPLP2	ribosomal protein lateral stalk subunit P2	6181	755,6150068	0,084390939	0,079945	1,055612352	0,291145355	0,743239565
ENSG00000177854	TMEM187	transmembrane protein 187	8269	14,53385744	-0,392951017	0,551218	-0,712877678	0,475921453	NA
ENSG00000275835	TUBGCP5	tubulin gamma complex component 5	114791	78,18446292	0,132899662	0,265798	0,500002476	0,617073334	0,90216458
ENSG00000109674	NEIL3	nei like DNA glycosylase 3	55247	16,28294029	0,174973674	0,433654	0,403486992	0,686590002	NA
ENSG00000162384	CZIB	CXXC motif containing zinc binding protein	54987	46,43008846	-0,033207737	0,294807	-0,112642308	0,910314141	0,979595261
ENSG00000100567	PSMA3	proteasome 20S subunit alpha 3	5684	144,7302162	0,149675954	0,182193	0,821525285	0,411347128	0,821439192
ENSG00000105619	TFPT	TCF3 fusion partner	29844	27,22280123	-0,707972204	1,843477	-0,384041685	0,700947552	NA
ENSG00000140548	ZNF710	zinc finger protein 710	374655	67,85282342	-0,120888408	0,214913	-0,562498834	0,573776198	0,886894319
ENSG00000184924	PTRHD1	peptidyl-tRNA hydrolase domain containing 1	391356	36,00148341	0,143292196	0,34932	0,410203617	0,681656588	NA
ENSG00000205138	SDHAF1	succinate dehydrogenase complex assembly factor 1	644096	26,8075256	-0,396300143	0,340177	-1,164981043	0,244026666	NA
ENSG00000273559	CWC25	CWC25 spliceosome associated protein homolog	54883	146,4650293	0,190457316	0,178725	1,065641731	0,286585627	0,738005299
ENSG00000204136	GGTA1	glycoprotein alpha-galactosyltransferase 1 (inactive)	2681	64,66006328	0,214846683	0,217964	0,985696955	0,324281844	0,767603859
ENSG00000093167	LRRFIP2	LRR binding FLII interacting protein 2	9209	116,7949774	0,22653335	0,183065	1,237446023	0,215921542	0,672410621
ENSG00000141480	ARRB2	arrestin beta 2	409	626,2512728	0,08927466	0,076298	1,170080641	0,241968518	0,696890628

ENSG00000163611	SPICE1	spindle and centriole associated protein 1	152185	36,5847172	-0,071366922	0,363012	-0,196596852	0,844143034	NA
ENSG00000164136	IL15	interleukin 15	3600	43,0502911	0,143349652	0,262392	0,546317874	0,584847452	0,890019742
ENSG00000130703	OSBPL2	oxysterol binding protein like 2	9885	234,4070077	0,109108686	0,14354	0,760126695	0,447178857	0,842562288
ENSG00000130703	LOC105369209	uncharacterized LOC105369209	105369209	234,4070077	0,109108686	0,14354	0,760126695	0,447178857	0,842562288
ENSG00000250021	ARPIN-AP3S2	ARPIN-AP3S2 readthrough	100526783	12,66180317	0,615509114	3,043419	0,20224267	0,839727017	NA
ENSG00000181827	RFX7	regulatory factor X7	64864	336,3742787	0,315432272	0,409731	0,769852243	0,441387545	0,838678081
ENSG00000135148	TRAFD1	TRAF-type zinc finger domain containing 1	10906	273,0217803	-0,043367511	0,109934	-0,394486207	0,693222109	0,928002746
ENSG00000156304	SCAF4	SR-related CTD associated factor 4	57466	205,3115959	-0,000999732	0,131681	-0,007592049	0,99394248	0,998500439
ENSG00000107175	CREB3	cAMP responsive element binding protein 3	10488	57,45034487	0,122136122	0,299422	0,407906804	0,683342097	0,925586358
ENSG00000171150	SOCS5	suppressor of cytokine signaling 5	9655	92,70782834	-0,215611256	0,185463	-1,162554389	0,245010345	0,701819279
ENSG00000115159	GPD2	glycerol-3-phosphate dehydrogenase 2	2820	122,3428213	-0,334456215	0,154191	-2,169106485	0,0300746	0,279077601
ENSG00000198933	TBKBP1	TBK1 binding protein 1	9755	76,64360736	0,014825718	0,21217	0,069876757	0,944291753	0,989126091
ENSG00000107742	SPOCK2	SPARC (osteonectin), cwcv and kazal like domains proteoglycan 2	9806	169,6622767	-0,283819851	0,143796	-1,973770345	0,048407862	0,353341404
ENSG00000122692	SMU1	SMU1 DNA replication regulator and spliceosomal factor	55234	294,1271514	-0,042162417	0,101399	-0,415808785	0,67754993	0,924509582
ENSG00000124380	SNRNP27	small nuclear ribonucleoprotein U4/U6,U5 subunit 27	11017	102,2786229	0,175360629	0,202299	0,866840827	0,386029229	0,809653368
ENSG00000127220	ABHD8	abhydrolase domain containing 8	79575	25,21553795	0,474996555	0,338326	1,40396177	0,160330234	NA
ENSG00000165060	FXN	frataxin	2395	74,22632429	0,298010025	0,29607	1,006552158	0,314150035	0,759554708
ENSG00000185480	PARPBP	PARP1 binding protein	55010	13,7696919	-0,272767305	0,803977	-0,339272425	0,734404513	NA
ENSG00000225217	HSPA7	heat shock protein family A (Hsp70) member 7 (pseudogene)	3311	25,45664855	-0,431409664	0,353118	-1,221714607	0,221815571	NA
ENSG00000291306	NA	NA	NA	41,22437185	-0,202649644	0,402344	-0,503672636	0,614491441	NA
ENSG00000168259	DNAJC7	DnaJ heat shock protein family (Hsp40) member C7	7266	218,583592	0,116317921	0,13188	0,88200109	0,377776218	0,804079787
ENSG00000169641	LUZP1	leucine zipper protein 1	7798	82,54763431	-0,122248944	0,384934	-0,317583939	0,750800559	0,941512988
ENSG00000196712	NF1	neurofibromin 1	4763	217,7356319	-0,117992957	0,205037	-0,575472074	0,564972072	0,885045179
ENSG00000276023	DUSP14	dual specificity phosphatase 14	11072	18,64571189	-0,483841467	2,69366	-0,179622315	0,857449084	NA
ENSG00000102225	CDK16	cyclin dependent kinase 16	5127	121,1129377	-0,08499105	0,176009	-0,48288004	0,629180915	0,905567722
ENSG00000125814	NAPB	NSF attachment protein beta	63908	104,8506462	-0,074016905	0,163564	-0,452524766	0,650890984	0,915326371
ENSG00000142197	DOP1B	DOP1 leucine zipper like protein B	9980	194,5190332	-0,167638817	0,122116	-1,372778752	0,169821138	0,629558603
ENSG00000154978	VOPP1	VOPP1 WW domain binding protein	81552	486,752282	0,082892365	0,075822	1,093245829	0,27428588	0,727065559
ENSG00000164031	DNAJB14	DnaJ heat shock protein family (Hsp40) member B14	79982	407,7402316	-1,030890821	0,273945	-3,763136981	0,000167795	0,009667995
ENSG00000167207	NOD2	nucleotide binding oligomerization domain containing 2	64127	136,1859494	-0,002281906	0,267022	-0,008545754	0,993181558	0,998500439
ENSG00000198324	PHETA1	PH domain containing endocytic trafficking adaptor 1	144717	19,54893299	-0,045872489	0,390288	-0,117534849	0,90643623	NA
ENSG00000198920	KIAA0753	KIAA0753	9851	51,1798362	-0,213486556	0,249756	-0,854781123	0,39267233	0,812491435
ENSG00000078237	TIGAR	TP53 induced glycolysis regulatory phosphatase	57103	108,5980474	0,148000848	0,177959	0,831656895	0,405602638	0,818464021
ENSG00000136521	NDUFB5	NADH:ubiquinone oxidoreductase subunit B5	4711	48,87935884	-0,186583459	0,382679	-0,48757134	0,6258535	0,90479929
ENSG00000142186	SCYL1	SCY1 like pseudokinase 1	57410	216,8157894	-0,046811252	0,151174	-0,309651899	0,756825685	0,942901768
ENSG00000160229	ZNF66	zinc finger protein 66	7617	56,4119723	0,217627471	0,338724	0,642492442	0,520553495	0,869693076
ENSG00000161558	TMEM143	transmembrane protein 143	55260	19,7638385	0,266863928	0,403673	0,661090006	0,508554594	NA
ENSG00000213923	CSNK1E	casein kinase 1 epsilon	1454	151,4424978	-4,88E-05	0,165188	-0,000295237	0,999764435	0,999764435
ENSG00000147383	NSDHL	NAD(P) dependent steroid dehydrogenase-like	50814	41,6588116	0,379462066	0,311178	1,219439106	0,222677575	NA
ENSG00000179010	MRFAP1	Morf4 family associated protein 1	93621	366,3590925	-0,067931533	0,094194	-0,721187339	0,470794261	0,852690218
ENSG00000106404	CLDN15	claudin 15	24146	53,06769257	-0,473093495	0,253786	-1,86414263	0,062301677	0,40406059
ENSG00000168101	NUDT16L1	nudix hydrolase 16 like 1	84309	86,36534042	-0,365571626	0,22084	-1,655369598	0,097849549	0,50593177
ENSG00000134970	TMED7	transmembrane p24 trafficking protein 7	51014	266,793901	-0,315381539	0,123923	-2,54497647	0,01092851	0,163343491
ENSG00000087301	TXNDC16	thioredoxin domain containing 16	57544	59,22378682	-0,065584655	0,220145	-0,297915172	0,765767908	0,944698783
ENSG00000103227	LMF1	lipase maturation factor 1	64788	26,08440916	0,086923611	0,363364	0,23921884	0,810935894	NA
ENSG00000125386	FAM193A	family with sequence similarity 193 member A	8603	206,3978411	0,028475622	0,156671	0,18175372	0,855776006	0,968439087

ENSG00000100239	PPP6R2	protein phosphatase 6 regulatory subunit 2	9701	310,5108871	-0,040366327	0,12149	-0,33226161	0,739691726	0,939193793
ENSG00000169764	UGP2	UDP-glucose pyrophosphorylase 2	7360	336,1066275	0,010285662	0,092405	0,111310247	0,91137033	0,979595261
ENSG00000110046	ATG2A	autophagy related 2A	23130	143,4262964	0,150962062	0,203063	0,743426181	0,457223698	0,845148253
ENSG00000205744	DENND1C	DENN domain containing 1C	79958	322,7383443	0,207535939	0,115175	1,801915546	0,071558694	0,435172111
ENSG00000110060	PUS3	pseudouridine synthase 3	83480	26,21979014	0,047710379	0,347515	0,137290121	0,890801479	NA
ENSG00000134545	KLRC1	killer cell lectin like receptor C1	3821	1581,192847	0,123457482	0,056344	2,191128444	0,028442499	0,271466056
ENSG00000176871	WSB2	WD repeat and SOCS box containing 2	55884	119,311395	-0,055345064	0,161836	-0,341982963	0,732363714	0,93912057
ENSG00000223459	TCAF1P1	TRPM8 channel associated factor 1 pseudogene 1	653199	16,28967802	-0,100248141	0,495558	-0,202293269	0,839687462	NA
ENSG00000121481	RNF2	ring finger protein 2	6045	84,83510616	-0,139205113	0,219936	-0,632935849	0,526775536	0,871653726
ENSG00000124702	KLHDC3	kelch domain containing 3	116138	169,982116	0,052941498	0,138296	0,382812912	0,701858487	0,930846779
ENSG00000132313	MRPL35	mitochondrial ribosomal protein L35	51318	116,8475692	-0,109306527	0,222887	-0,49041135	0,623842846	0,90479929
ENSG00000144118	RALB	RAS like proto-oncogene B	5899	139,6731453	-0,282901341	0,154649	-1,829315375	0,067352377	0,418806958
ENSG00000168438	CDC40	cell division cycle 40	51362	169,478353	-0,041067365	0,129263	-0,317703391	0,750709939	0,941512988
ENSG00000175787	ZNF169	zinc finger protein 169	169841	37,60864678	-0,607715769	0,304088	-1,998485656	0,045664033	NA
ENSG00000116661	FBXO2	F-box protein 2	26232	74,08328904	0,019348613	0,198285	0,097579679	0,922266062	0,984284131
ENSG00000174840	PDE12	phosphodiesterase 12	201626	195,1861925	-0,166802336	0,122015	-1,367067924	0,171604016	0,629558603
ENSG00000117862	TXNDC12	thioredoxin domain containing 12	51060	169,2844637	-0,017111159	0,169734	-0,100811484	0,919700111	0,984142724
ENSG00000182359	KBTBD3	kelch repeat and BTB domain containing 3	143879	14,87954596	-0,294045541	0,535325	-0,549283967	0,582810588	NA
ENSG00000247077	PGAM5	PGAM family member 5, mitochondrial serine/threonine protein phosphatase	192111	117,7773101	-0,222163933	0,154938	-1,43389371	0,151602597	0,60852001
ENSG00000073417	PDE8A	phosphodiesterase 8A	5151	49,24792661	0,312096769	0,33121	0,94229133	0,346043506	0,782042713
ENSG00000088035	ALG6	ALG6 alpha-1,3-glucosyltransferase	29929	25,43967492	-0,133265329	0,421557	-0,316126478	0,75190651	NA
ENSG00000124222	STX16	syntaxin 16	8675	824,0774662	-0,170346524	0,068032	-2,503927904	0,012282306	0,174531515
ENSG00000125703	ATG4C	autophagy related 4C cysteine peptidase	84938	35,33923988	0,009380863	0,310116	0,030249487	0,975868082	NA
ENSG00000142168	SOD1	superoxide dismutase 1	6647	151,8910989	0,306075226	0,152445	2,007772066	0,044667518	0,33915849
ENSG00000169813	HNRNPF	heterogeneous nuclear ribonucleoprotein F	3185	1168,753197	0,10173345	0,065352	1,556698787	0,119542017	0,552466951
ENSG00000078618	NRDC	nardilysin convertase	4898	684,9222228	-0,026117126	0,08352	-0,312703778	0,754505725	0,942901768
ENSG00000148341	SH3GLB2	SH3 domain containing GRB2 like, endophilin B2	56904	125,5548898	0,051022509	0,170178	0,299819173	0,76431509	0,944245192
ENSG00000271741	NA	NA	NA	50,88120437	-0,322504847	0,297148	-1,08533397	0,277773765	0,731800087
ENSG00000213445	SIPA1	signal-induced proliferation-associated 1	6494	443,1834337	-0,144987957	0,082152	-1,764879896	0,077583953	0,450698497
ENSG00000082269	FAM135A	family with sequence similarity 135 member A	57579	13,55703124	-0,037258022	1,656321	-0,022494444	0,982053544	NA
ENSG00000204946	ZNF783	zinc finger protein 783	100289678	94,2636384	-0,067456935	0,166553	-0,405018538	0,685463884	0,926136822
ENSG00000220205	VAMP2	vesicle associated membrane protein 2	6844	417,3997174	-0,130048236	0,089096	-1,459645887	0,144387421	0,59024645
ENSG00000274615	NPEPPSP1	NPEPPS pseudogene 1	440434	33,79998372	0,699980997	1,070462	0,653905781	0,513172509	NA
ENSG00000233276	GPX1	glutathione peroxidase 1	2876	69,12369561	-0,258066019	0,220016	-1,172942018	0,240819058	0,695620773
ENSG00000121289	CEP89	centrosomal protein 89	84902	43,17784602	-0,209380044	0,344611	-0,60758343	0,543463794	0,877542071
ENSG00000167543	TP53I13	tumor protein p53 inducible protein 13	90313	89,57256942	-0,357228362	0,189033	-1,889768171	0,058788972	0,39312401
ENSG00000126432	PRDX5	peroxiredoxin 5	25824	239,0135066	-0,058876275	0,125122	-0,470552139	0,637960592	0,909829037
ENSG00000137996	RTCA	RNA 3'-terminal phosphate cyclase	8634	212,2074519	0,113298901	0,118697	0,95452367	0,339818634	0,777431905
ENSG00000007520	TSR3	TSR3 ribosome maturation factor	115939	71,35818437	-0,150848271	0,219364	-0,687661726	0,491665828	0,860513824
ENSG00000153064	BANK1	B cell scaffold protein with ankyrin repeats 1	55024	14,13617805	0,647860683	0,619451	1,045861998	0,295624752	NA
ENSG00000101361	NOP56	NOP56 ribonucleoprotein	10528	224,8833644	-0,126103257	0,127628	-0,988050998	0,323127673	0,767603859
ENSG00000110619	CARS1	cysteinyl-tRNA synthetase 1	833	194,218754	0,155864838	0,182207	0,855429132	0,392313622	0,812491435
ENSG00000117308	GALE	UDP-galactose-4-epimerase	2582	32,50516079	0,386501269	0,402211	0,960942336	0,336581162	NA
ENSG00000128340	RAC2	Rac family small GTPase 2	5880	2376,905216	-0,022682646	0,047023	-0,482372267	0,629541519	0,905567722
ENSG00000131467	PSME3	proteasome activator subunit 3	10197	443,0530162	-0,045844012	0,097148	-0,471900543	0,63699778	0,90943397
ENSG00000145390	USP53	ubiquitin specific peptidase 53	54532	50,43912701	0,378718712	0,542757	0,697768594	0,485321923	0,857915459
ENSG00000155666	KDM8	lysine demethylase 8	79831	25,51220875	0,238855831	0,363129	0,657772063	0,510684609	NA
ENSG00000171700	RGS19	regulator of G protein signaling 19	10287	108,6695142	-0,125425006	0,175071	-0,716422587	0,473730452	0,853811879
ENSG00000204673	AKT1S1	AKT1 substrate 1	84335	78,19230812	-0,090167484	0,213831	-0,421675952	0,67326156	0,923602509
ENSG00000130638	ATXN10	ataxin 10	25814	276,2673442	0,126081408	0,124518	1,012559255	0,31127073	0,757361021
ENSG00000126267	COX6B1	cytochrome c oxidase subunit 6B1	1340	192,0666481	0,13921352	0,120562	1,154709066	0,248209586	0,706134779
ENSG00000139372	TDG	thymine DNA glycosylase	6996	214,5075298	0,093971533	0,146549	0,64122794	0,521374599	0,870221868
ENSG00000104856	RELB	RELB proto-oncogene, NF-kB subunit	5971	102,4956426	0,095223143	0,167168	0,56962628	0,5689312	0,886263374

ENSG00000160959	LRRC14	leucine rich repeat containing 14	9684	114,9875757	0,03325872	0,174539	0,190551687	0,848876844	0,967786142
ENSG00000170584	NUDCD2	NudC domain containing 2	134492	59,65457238	-0,234922578	0,769494	-0,30529492	0,760141545	0,942901768
ENSG00000175166	PSMD2	proteasome 26S subunit ubiquitin receptor, non-ATPase 2	5708	460,2344253	0,214985242	0,097109	2,213860033	0,026838409	0,265811933
ENSG00000163399	ATP1A1	ATPase Na+/K+ transporting subunit alpha 1	476	831,3245252	0,254243064	0,068127	3,731882003	0,000190055	0,01048604
ENSG00000257529	RPL36A-HNRNPH2	RPL36A-HNRNPH2 readthrough	100529097	37,96455376	0,52250801	1,137788	0,459231507	0,646067927	NA
ENSG00000049449	RCN1	reticulocalbin 1	5954	29,86697329	-0,033910913	0,36148	-0,093811442	0,925258943	NA
ENSG00000164032	H2AZ1	H2A,Z variant histone 1	3015	354,3343112	0,385779728	0,096723	3,988492598	6,65E-05	0,00458112
ENSG00000177917	ARL6IP6	ADP ribosylation factor like GTPase 6 interacting protein 6	151188	63,87440502	0,276192668	0,292877	0,94303377	0,34566363	0,782042713
ENSG00000124203	ZNF831	zinc finger protein 831	128611	130,371066	-0,171023122	0,158006	-1,082386904	0,279080645	0,732510365
ENSG00000151292	CSNK1G3	casein kinase 1 gamma 3	1456	154,6666835	-0,193169641	0,134911	-1,431830231	0,152192413	0,608564843
ENSG00000157020	SEC13	SEC13 homolog, nuclear pore and COPII coat complex component	6396	185,960829	0,15219405	0,137889	1,103740818	0,269705584	0,725477256
ENSG00000174842	GLMN	glomulin, FKBP associated protein	11146	51,1316656	0,095434193	0,306477	0,311391323	0,75550315	0,942901768
ENSG00000160953	PWWP3A	PWWP domain containing 3A, DNA repair factor	84939	189,7921289	-0,313234939	0,130564	-2,399089699	0,016435888	0,202942572
ENSG00000183011	NAA38	N-alpha-acetyltransferase 38, NatC auxiliary subunit	84316	38,99711612	0,10801866	0,322487	0,334954871	0,73765913	NA
ENSG00000111877	MCM9	minichromosome maintenance 9 homologous recombination repair factor	254394	113,279786	-0,077243621	0,199297	-0,387580839	0,698326251	0,930783276
ENSG00000111913	RIPOR2	RHO family interacting cell polarization regulator 2	9750	306,0836944	-0,279415961	0,124577	-2,242920586	0,024901936	0,253311197
ENSG00000127720	METTL25	methyltransferase like 25	84190	21,11831307	-0,527047635	0,437378	-1,205015787	0,228197208	NA
ENSG00000128908	INO80	INO80 complex ATPase subunit	54617	266,497339	0,003114761	0,122525	0,025421518	0,979718748	0,995712569
ENSG00000145730	PAM	peptidylglycine alpha-amidating monooxygenase	5066	71,50770202	-0,039868455	0,237847	-0,167621923	0,866880723	0,971807194
ENSG00000167815	PRDX2	peroxiredoxin 2	7001	171,2437752	0,13337779	0,133567	0,998586452	0,317995066	0,762879158
ENSG00000181929	PRKAG1	protein kinase AMP-activated non-catalytic subunit gamma 1	5571	164,1303282	0,050039477	0,170431	0,293605212	0,769059593	0,944813709
ENSG00000005889	ZFX	zinc finger protein X-linked	7543	454,8026872	-0,108894147	0,095074	-1,145359615	0,25206022	0,708547233
ENSG00000037757	MRI1	methylthioribose-1-phosphate isomerase 1	84245	136,0885442	-0,196963202	0,142462	-1,382565199	0,166798226	0,62651041
ENSG00000068489	PRR11	proline rich 11	55771	145,1461248	0,105252346	0,144223	0,729788459	0,4655195	0,84908342
ENSG00000088247	KHSRP	KH-type splicing regulatory protein	8570	412,788889	-0,118482512	0,096129	-1,232532623	0,217750188	0,673519155
ENSG00000096063	SRPK1	SRSF protein kinase 1	6732	285,4939364	-0,078566175	0,101776	-0,771953138	0,440142183	0,83848252
ENSG00000108256	NUFIP2	nuclear FMR1 interacting protein 2	57532	664,6551896	-0,413327436	0,128673	-3,212238326	0,001317051	0,041542635
ENSG00000115816	CEBPZ	CCAAT enhancer binding protein zeta	10153	240,3066548	-0,027565776	0,114725	-0,240277629	0,810115036	0,959038743
ENSG00000120942	UBIAD1	UbiA prenyltransferase domain containing 1	29914	104,316125	-0,061793191	0,160572	-0,384830637	0,700362899	0,930783276
ENSG00000014123	UFL1	UFM1 specific ligase 1	23376	245,2499701	0,03911552	0,120361	0,324985809	0,745191812	0,939745744
ENSG00000087206	UIMC1	ubiquitin interaction motif containing 1	51720	105,9587101	0,323970887	0,56764	0,570733485	0,568180317	0,88603113
ENSG00000114446	IFT57	intraflagellar transport 57	55081	14,11635822	-0,467835712	0,613291	-0,762828412	0,445565734	NA
ENSG00000198825	INPP5F	inositol polyphosphate-5-phosphatase F	22876	222,3095441	0,108959243	0,178341	0,610959231	0,541226571	0,876458296
ENSG00000135870	RC3H1	ring finger and CCCH-type domains 1	149041	423,4397218	-0,04458337	0,108273	-0,411770005	0,680508009	0,925586358
ENSG00000050130	JKAMP	JNK1/MAPK8 associated membrane protein	51528	100,5158213	0,307718615	0,271259	1,134410102	0,256622563	0,712699297
ENSG00000157483	MYO1E	myosin IE	4643	608,2047784	0,063515976	0,079168	0,802297213	0,422381054	0,827816808
ENSG00000162521	RBBP4	RB binding protein 4, chromatin remodeling factor	5928	692,6296812	0,17300115	0,093287	1,85451421	0,063665629	0,406570577
ENSG00000166685	COG1	component of oligomeric golgi complex 1	9382	67,60281702	-0,178451558	0,252003	-0,708132312	0,478863096	0,854008769
ENSG00000173848	NET1	neuroepithelial cell transforming 1	10276	59,04983169	0,029197398	0,256104	0,114006118	0,909232944	0,97930822
ENSG00000250479	CHCHD10	coiled-coil-helix-coiled-coil-helix domain containing 10	400916	15,27706981	-0,27604036	0,482176	-0,572489033	0,566990713	NA
ENSG00000053372	MRTO4	MRT4 homolog, ribosome maturation factor	51154	79,98531784	0,237881264	0,187363	1,269628775	0,204216895	0,660954946
ENSG00000139428	MMAB	metabolism of cobalamin associated B	326625	29,53164083	-0,369807114	0,339537	-1,089152088	0,276086823	NA
ENSG00000101856	PGRMC1	progesterone receptor membrane component 1	10857	67,17117234	0,179064319	0,242627	0,738022437	0,460500815	0,846622987

ENSG00000115446	UNC50	unc-50 inner nuclear membrane RNA binding protein	25972	122,2699724	-0,2640112	0,148664	-1,77588865	0,075751272	0,446229554
ENSG00000099910	KLHL22	kelch like family member 22	84861	75,334332	-0,222256969	0,224548	-0,989795996	0,322273842	0,76684605
ENSG00000109586	GALNT7	polypeptide N-acetylgalactosaminyltransferase 7	51809	89,27980903	-0,097541796	0,196854	-0,495504316	0,620244176	0,904287685
ENSG00000137713	PPP2R1B	protein phosphatase 2 scaffold subunit Abeta	5519	115,9286147	0,079707326	0,206682	0,385651728	0,699754618	0,930783276
ENSG00000183077	AFMID	arylformamidase	125061	15,76700061	0,2841981	0,764574	0,371707881	0,710110356	NA
ENSG00000011638	LDAF1	lipid droplet assembly factor 1	57146	15,66422742	0,245879786	0,536637	0,458186372	0,64681855	NA
ENSG00000023041	ZDHHC6	zinc finger DHHC-type palmitoyltransferase 6	64429	87,8143014	0,702427161	0,915138	0,767564099	0,442746198	0,838678081
ENSG00000132600	PRMT7	protein arginine methyltransferase 7	54496	18,61554473	-0,853210917	1,823944	-0,467783477	0,639939437	NA
ENSG00000196504	PRPF40A	pre-mRNA processing factor 40 homolog A	55660	576,0045506	0,072358154	0,081453	0,888338591	0,374358647	0,801403701
ENSG00000060237	WNK1	WNK lysine deficient protein kinase 1	65125	1343,482534	-0,055351384	0,073823	-0,749780935	0,453386653	0,843234809
ENSG00000029153	BMAL2	basic helix-loop-helix ARNT like 2	56938	72,56079264	0,45678062	0,255037	1,791034544	0,073287752	0,440062693
ENSG00000182934	SRPRA	SRP receptor subunit alpha	6734	501,687616	-0,079779465	0,083436	-0,956179611	0,338981499	0,777431905
ENSG00000130669	PAK4	p21 (RAC1) activated kinase 4	10298	81,20925769	-0,21165809	0,190498	-1,111078787	0,266534438	0,722327312
ENSG00000164190	NIPBL	NIPBL cohesin loading factor	25836	937,2099729	0,021438199	0,074184	0,2889849	0,772592933	0,946409165
ENSG00000272602	ZNF595	zinc finger protein 595	152687	28,96360971	-0,624166172	0,350784	-1,779345481	0,075183139	NA
ENSG00000022840	RNF10	ring finger protein 10	9921	422,3135607	-0,040022074	0,095765	-0,417919125	0,676006249	0,924186156
ENSG00000033050	ABCF2	ATP binding cassette subfamily F member 2	10061	126,4383243	0,045460989	0,184461	0,246453404	0,80533127	0,957328863
ENSG00000070495	JMJD6	jumonji domain containing 6, arginine demethylase and lysine hydroxylase	23210	117,0130539	-0,111805459	0,193454	-0,577942629	0,563302854	0,884816202
ENSG00000078967	UBE2D4	ubiquitin conjugating enzyme E2 D4 (putative)	51619	26,68689987	0,249671339	0,503451	0,495919593	0,619951142	NA
ENSG00000079432	CIC	capicua transcriptional repressor	23152	386,1746179	-0,105346304	0,091422	-1,152313648	0,249192211	0,707335884
ENSG00000105185	PDCD5	programmed cell death 5	9141	83,82200751	0,186690838	0,218499	0,854424586	0,392869778	0,812491435
ENSG00000106605	BLVRA	biliverdin reductase A	644	13,79368091	-0,133885077	0,474774	-0,281997337	0,777945552	NA
ENSG00000109220	CHIC2	cysteine rich hydrophobic domain 2	26511	56,79545828	0,329188521	0,225331	1,460911062	0,144039851	0,59024645
ENSG00000110871	COQ5	coenzyme Q5, methyltransferase	84274	57,56358988	0,17193324	0,223924	0,767818604	0,44259496	0,838678081
ENSG00000127948	POR	cytochrome p450 oxidoreductase	5447	154,9242425	0,029656722	0,13898	0,213388767	0,831023741	0,963156386
ENSG00000129003	VPS13C	vacuolar protein sorting 13 homolog C	54832	1666,973612	-0,078559537	0,077172	-1,017983757	0,308685676	0,754797782
ENSG00000129473	BCL2L2	BCL2 like 2	599	100,7800008	-0,077685173	0,205729	-0,37760999	0,705720342	0,930846779
ENSG00000135426	TESPA1	thymocyte expressed, positive selection associated 1	9840	399,3942412	-0,00068362	0,09899	-0,006905942	0,9944899	0,998500439
ENSG00000142731	PLK4	polo like kinase 4	10733	75,6001304	0,149771794	0,229972	0,651260047	0,514878634	0,868514045
ENSG00000152234	ATP5F1A	ATP synthase F1 subunit alpha	498	970,0005986	0,179789408	0,070455	2,551847853	0,01071533	0,161222609
ENSG00000165475	CRYL1	crystallin lambda 1	51084	26,03461509	-0,328532511	0,360489	-0,911352198	0,362109832	NA
ENSG00000166855	CLPX	caseinolytic mitochondrial matrix peptidase chaperone subunit X	10845	176,8551557	0,087267666	0,137191	0,636101492	0,524710273	0,871078945
ENSG00000167702	KIFC2	kinesin family member C2	90990	59,59590157	0,661497578	1,276499	0,518212273	0,604310173	0,897212117
ENSG00000168591	TMUB2	transmembrane and ubiquitin like domain containing 2	79089	141,0270143	-0,089492587	0,169134	-0,529121229	0,596721355	0,894257182
ENSG00000168906	MAT2A	methionine adenosyltransferase 2A	4144	772,4609906	-0,080126946	0,063733	-1,257234874	0,208668599	0,662366797
ENSG00000172765	TMCC1	transmembrane and coiled-coil domain family 1	23023	65,71289297	0,189014275	0,239031	0,790752987	0,429088149	0,832060923
ENSG00000177981	ASB8	ankyrin repeat and SOCS box containing 8	140461	180,9611106	0,080189812	0,174954	0,458347924	0,646702499	0,913689769
ENSG00000214654	B3GALT9	beta-1,3-galactosyltransferase 9	100288842	21,77235893	-0,111070633	0,383988	-0,289255681	0,772385725	NA
ENSG00000258315	C17orf49	chromosome 17 open reading frame 49	124944	218,4103291	0,03445958	0,150758	0,228575653	0,81919875	0,961067311
ENSG00000266472	MRPS21	mitochondrial ribosomal protein S21	54460	110,87032	-0,043711519	0,161127	-0,271286458	0,786170721	0,950624564
ENSG00000161048	NAPEPLD	N-acyl phosphatidylethanolamine phospholipase D	222236	13,01288657	0,485985401	2,172209	0,223728703	0,82296841	NA
ENSG00000163319	MRPS18C	mitochondrial ribosomal protein S18C	51023	46,37108998	-0,248245301	0,296503	-0,837242749	0,402456133	0,816750249
ENSG00000171314	PGAM1	phosphoglycerate mutase 1	5223	636,7978411	0,024526416	0,067306	0,364401931	0,715557894	0,932040329

ENSG00000204272	NBDY	negative regulator of P-body association	550643	53,41513517	-0,031514111	0,270827	-0,116362587	0,907365185	0,978772993
ENSG00000214736	TOMM6	translocase of outer mitochondrial membrane 6	100188893	113,6924419	0,102232678	0,160603	0,63655386	0,524415488	0,870773623
ENSG00000129103	SUMF2	sulfatase modifying factor 2	25870	228,5644155	-0,016038839	0,12819	-0,125118179	0,900429992	0,977990819
ENSG00000177700	POLR2L	RNA polymerase II, I and III subunit L	5441	48,70000585	-0,062419247	0,296443	-0,210560501	0,833230237	0,963446071
ENSG00000172590	MRPL52	mitochondrial ribosomal protein L52	122704	52,80139295	0,076699541	0,297777	0,257573894	0,79673578	0,95354564
ENSG00000154358	OBSCN	obscurin, cytoskeletal calmodulin and titin-interacting RhoGEF	84033	71,48630014	-0,205027944	0,21735	-0,943309833	0,345522448	0,782042713
ENSG00000150977	RILPL2	Rab interacting lysosomal protein like 2	196383	65,22653846	0,11974082	0,22149	0,540614852	0,588773079	0,891627382
ENSG00000138085	ATRAID	all-trans retinoic acid induced differentiation factor	51374	118,5178991	0,066755489	0,162115	0,411778754	0,680501595	0,925586358
ENSG00000008838	MED24	mediator complex subunit 24	9862	224,5235488	0,057291048	0,122343	0,468281111	0,639583575	0,910841232
ENSG00000129292	PHF20L1	PHD finger protein 20 like 1	51105	207,4835464	0,125373219	0,125973	0,995236994	0,319621013	0,764171518
ENSG00000157107	FCHO2	FCH and mu domain containing endocytic adaptor 2	115548	18,87700371	-1,026174885	0,544979	-1,882961895	0,059705532	NA
ENSG00000096746	HNRNPH3	heterogeneous nuclear ribonucleoprotein H3	3189	283,52666	0,116802639	0,110904	1,053185772	0,292255858	0,74421837
ENSG00000138686	BBS7	Bardet-Biedl syndrome 7	55212	52,53351738	0,079545958	0,230092	0,345713884	0,729557749	0,938412473
ENSG00000148090	AUH	AU RNA binding methylglutaconyl-CoA hydratase	549	58,57255187	0,064708252	0,230525	0,28069957	0,778940839	0,947321361
ENSG00000278311	GGNBP2	gametogenetin binding protein 2	79893	259,3743217	0,134414418	1,373699	0,097848518	0,92205258	0,984284131
ENSG00000079739	PGM1	phosphoglucomutase 1	5236	158,738911	-0,045620641	0,15564	-0,29311661	0,769433021	0,944829104
ENSG00000103024	NME3	NME/NM23 nucleoside diphosphate kinase 3	4832	29,61205014	-0,162932912	0,373716	-0,435980411	0,662850942	NA
ENSG00000139651	ZNF740	zinc finger protein 740	283337	225,9159515	-0,192628581	0,133896	-1,4386422	0,150251923	0,605789839
ENSG00000140323	DISP2	dispatched RND transporter family member 2	85455	12,32645278	0,054020573	0,576177	0,093756948	0,925302231	NA
ENSG00000146409	SLC18B1	solute carrier family 18 member B1	116843	40,09569261	0,416204817	0,311884	1,334484192	0,182045224	NA
ENSG00000161638	ITGA5	integrin subunit alpha 5	3678	228,7053691	0,024891628	0,132511	0,187845919	0,850997438	0,967902179
ENSG00000185838	GNB1L	G protein subunit beta 1 like	54584	16,13700192	-0,068645993	0,559531	-0,122684948	0,902356583	NA
ENSG00000197070	ARRDC1	arrestin domain containing 1	92714	52,88541198	-0,045649587	0,23543	-0,193898846	0,846255087	0,967231052
ENSG00000101310	SEC23B	SEC23 homolog B, COPII coat complex component	10483	284,5869636	0,114434711	0,115029	0,994836612	0,319815736	0,764222091
ENSG00000111832	RWDD1	RWD domain containing 1	51389	140,1874945	0,05480855	0,161741	0,338865117	0,734711344	0,939157822
ENSG00000056050	HPF1	histone PARylation factor 1	54969	36,16398859	0,240291211	0,320768	0,749111184	0,453790195	NA
ENSG00000205336	ADGRG1	adhesion G protein-coupled receptor G1	9289	96,52210117	-0,427299882	0,197161	-2,167259612	0,030215064	0,279077601
ENSG00000138674	SEC31A	SEC31 homolog A, COPII coat complex component	22872	673,4571169	0,114674997	0,078643	1,458170629	0,144793517	0,591371963
ENSG00000170017	ALCAM	activated leukocyte cell adhesion molecule	214	13,30870026	0,218139864	0,610996	0,357023368	0,721074312	NA
ENSG00000106462	EZH2	enhancer of zeste 2 polycomb repressive complex 2 subunit	2146	83,75601332	0,204346507	0,329882	0,619453394	0,535617716	0,874894595
ENSG00000111445	RFC5	replication factor C subunit 5	5985	53,15187694	0,160165412	0,232255	0,689610759	0,490439	0,860513824
ENSG00000130640	TUBGCP2	tubulin gamma complex component 2	10844	119,2768195	-0,204391197	0,261197	-0,782516047	0,433911359	0,835238413
ENSG00000213563	C8orf82	chromosome 8 open reading frame 82	414919	34,31190763	0,272609565	0,339198	0,803689508	0,421576315	NA
ENSG00000075239	ACAT1	acetyl-CoA acetyltransferase 1	38	80,74747859	0,240190238	0,200463	1,198174499	0,230849091	0,685179309
ENSG00000075651	PLD1	phospholipase D1	5337	33,8422972	-0,362218646	0,321533	-1,126534775	0,25993923	NA
ENSG00000080298	RFX3	regulatory factor X3	5991	51,59301553	0,147173168	0,242062	0,607998914	0,543188194	0,877542071
ENSG00000082898	XPO1	exportin 1	7514	622,5957404	-0,274444658	0,096139	-2,854668477	0,004308177	0,090819559
ENSG00000108349	CASC3	CASC3 exon junction complex subunit	22794	380,8328313	-0,071865932	0,106137	-0,677102508	0,498340918	0,86161226
ENSG00000110497	AMBRA1	autophagy and beclin 1 regulator 1	55626	245,9699106	-0,059343131	0,106683	-0,556258251	0,578034323	0,887069818
ENSG00000145029	NICN1	nicolin 1, tubulin polyglutamylase complex subunit	84276	32,48587831	0,355752995	1,153665	0,308367558	0,757802665	NA
ENSG00000146476	ARMT1	acidic residue methyltransferase 1	79624	86,10426642	-0,199250688	0,195808	-1,017581223	0,308877015	0,754797782
ENSG00000147224	PRPS1	phosphoribosyl pyrophosphate synthetase 1	5631	143,5006484	0,355905395	0,194592	1,828986851	0,067401578	0,418806958
ENSG00000148803	FUOM	fucose mutarotase	282969	14,55711882	-0,056876049	0,444144	-0,128057817	0,898103218	NA
ENSG00000175575	PAAF1	proteasomal ATPase associated factor 1	80227	45,8266187	0,208434422	0,322084	0,64714312	0,51753932	0,868791049
ENSG00000255561	FDXACB1	ferredoxin-fold anticodon binding domain containing 1	91893	18,04531346	-0,358251355	0,422127	-0,848682218	0,396058143	NA

ENSG00000136144	RCBTB1	RCC1 and BTB domain containing protein 1	55213	117,3882712	-0,338731096	0,163384	-2,073221924	0,038151631	0,31021219
ENSG00000164054	SHISA5	shisa family member 5	51246	411,2595007	-0,114737611	0,100382	-1,143005389	0,253036354	0,708754466
ENSG00000066455	GOLGA5	golgin A5	9950	134,5394626	0,05470321	0,145969	0,374758954	0,707839743	0,930846779
ENSG00000068024	HDAC4	histone deacetylase 4	9759	121,1131992	-0,555969808	0,189364	-2,935991805	0,003324831	0,075910532
ENSG00000143643	TTC13	tetratricopeptide repeat domain 13	79573	351,8770564	0,056421964	0,105044	0,537125157	0,591181165	0,892834002
ENSG00000145391	SETD7	SET domain containing 7, histone lysine methyltransferase	80854	11,66567311	0,224742256	0,559478	0,401699604	0,687905116	NA
ENSG00000145391	LOC105377622	uncharacterized LOC105377622	105377622	11,66567311	0,224742256	0,559478	0,401699604	0,687905116	NA
ENSG00000169499	PLEKHA2	pleckstrin homology domain containing A2	59339	402,7414528	-0,216505962	0,087076	-2,486394456	0,012904484	0,177810208
ENSG00000197937	ZNF347	zinc finger protein 347	84671	21,50416226	0,142391793	0,432798	0,329002883	0,742153509	NA
ENSG00000214078	CPNE1	copine 1	8904	475,4212551	0,058801884	0,110967	0,52990271	0,596179387	0,894229873
ENSG00000250251	PKD1P6	polycystin 1, transient receptor potential channel interacting pseudogene 6	353511	38,79095326	0,165011846	0,315176	0,523555293	0,600587884	NA
ENSG00000130348	QRSL1	glutaminyl-tRNA amidotransferase subunit QRSL1	55278	71,2567193	-0,184559189	0,231493	-0,797255939	0,425302404	0,830443782
ENSG00000143167	GPA33	glycoprotein A33	10223	86,83450182	0,143148483	0,182768	0,783223792	0,433495706	0,835093272
ENSG00000064999	ANKS1A	ankyrin repeat and sterile alpha motif domain containing 1A	23294	89,95834061	0,009158345	0,186823	0,049021424	0,960902222	0,992235217
ENSG00000105135	ILVBL	ilvB acetolactate synthase like	10994	47,29654981	-0,122653537	0,253639	-0,483574896	0,628687594	0,905567722
ENSG00000141873	SLC39A3	solute carrier family 39 member 3	29985	68,9280561	-0,045220038	0,216134	-0,209221779	0,834275112	0,963446071
ENSG00000176809	LRRC37A3	leucine rich repeat containing 37 member A3	374819	27,07589595	0,044636473	0,391568	0,113994081	0,909242485	NA
ENSG00000228474	OST4	oligosaccharyltransferase complex subunit 4, non-catalytic	100128731	221,0761984	0,016893788	0,116343	0,145206122	0,884548132	0,977108443
ENSG00000292338	DHRSX	dehydrogenase/reductase X-linked	207063	31,00828637	0,473697317	0,344079	1,37670917	0,168602182	NA
ENSG00000166233	ARIH1	ariadne RBR E3 ubiquitin protein ligase 1	25820	192,0603825	0,12175477	0,148141	0,821881639	0,411144264	0,821394592
ENSG00000074356	NCBP3	nuclear cap binding subunit 3	55421	270,9475794	-0,084039679	0,122046	-0,688587977	0,491082589	0,860513824
ENSG00000111961	SASH1	SAM and SH3 domain containing 1	23328	11,79726002	0,146536927	0,695205	0,210782299	0,833057152	NA
ENSG00000121067	SPOP	speckle type BTB/POZ protein	8405	140,3890588	-0,410713546	0,165253	-2,485361526	0,012941993	0,178014729
ENSG00000134256	CD101	CD101 molecule	9398	138,362292	-0,113425591	0,14594	-0,777208609	0,437035704	0,837249449
ENSG00000136718	IMP4	IMP U3 small nucleolar ribonucleoprotein 4	92856	34,44043022	0,127227025	0,603468	0,210826589	0,83302259	NA
ENSG00000150433	TMEM218	transmembrane protein 218	219854	25,93908533	-0,258039421	0,587689	-0,439074439	0,6606076	NA
ENSG00000153395	LPCAT1	lysophosphatidylcholine acyltransferase 1	79888	309,4425842	0,432624523	0,326605	1,324611116	0,185300167	0,642537533
ENSG00000155893	PXYLP1	2-phosphoxylose phosphatase 1	92370	39,55799612	-0,399177986	0,292602	-1,364237574	0,172492806	NA
ENSG00000167613	LAIR1	leukocyte associated immunoglobulin like receptor 1	3903	50,90934925	0,092735464	0,306429	0,302632771	0,762169738	0,943004836
ENSG00000170677	SOCS6	suppressor of cytokine signaling 6	9306	19,9499241	-0,027617699	0,530737	-0,05203653	0,958499586	NA
ENSG00000229809	ZNF688	zinc finger protein 688	146542	22,58803981	0,117527529	0,46113	0,254868396	0,798824747	NA
ENSG00000277053	NA	NA	NA	107,9383813	0,361559331	0,287233	1,258764933	0,20811525	0,662274754
ENSG00000087157	PGS1	phosphatidylglycerophosphate synthase 1	9489	37,2559343	-0,074700625	1,193385	-0,062595572	0,950088555	NA
ENSG00000173327	MAP3K11	mitogen-activated protein kinase kinase kinase 11	4296	180,3755322	-0,043331493	0,142641	-0,303780993	0,761294748	0,942901768
ENSG00000169442	CD52	CD52 molecule	1043	1714,989486	0,042257888	0,057417	0,735978903	0,461743537	0,846765498
ENSG00000270170	NCBP2AS2	NCBP2 antisense 2 (head to head)	152217	34,11677646	0,214498402	0,373017	0,575037191	0,565266145	NA
ENSG00000188921	HACD4	3-hydroxyacyl-CoA dehydratase 4	401494	240,1591019	-0,083808038	0,107629	-0,778677233	0,436169871	0,836566451
ENSG00000105518	TMEM205	transmembrane protein 205	374882	35,19246599	-0,831921813	0,34328	-2,423447262	0,015373985	NA
ENSG00000169139	UBE2V2	ubiquitin conjugating enzyme E2 V2	7336	118,2067347	0,205659121	0,176588	1,164629864	0,24416885	0,700945995
ENSG00000103490	PYCARD	PYD and CARD domain containing	29108	62,54734709	0,156779168	0,274401	0,571350706	0,567761936	0,88603113
ENSG00000105443	CYTH2	cytohesin 2	9266	67,69908463	0,25342424	0,256052	0,989738687	0,32230186	0,76684605
ENSG00000121680	PEX16	peroxisomal biogenesis factor 16	9409	53,11147049	0,093601296	0,271865	0,344293104	0,73062587	0,938729652
ENSG00000126107	HECTD3	HECT domain E3 ubiquitin protein ligase 3	79654	117,5683208	-0,251912181	0,168653	-1,493673886	0,135260877	0,576105711
ENSG00000165629	ATP5F1C	ATP synthase F1 subunit gamma	509	310,4708329	0,117012509	0,147713	0,792162384	0,428265999	0,832060923

ENSG00000189233	NUGGC	nuclear GTPase, germinal center associated	389643	92,70475561	-0,10645918	0,188625	-0,564395184	0,572485216	0,886795904
ENSG00000276141	NA	NA	NA	19,554672	0,236988883	0,411056	0,57653614	0,564252849	NA
ENSG00000129691	ASH2L	ASH2 like, histone lysine methyltransferase complex subunit	9070	195,9767052	-0,228516448	0,137348	-1,663773124	0,096157777	0,502134421
ENSG00000164611	PTTG1	PTTG1 regulator of sister chromatid separation, securin	9232	39,46409786	0,491149957	0,270354	1,816694502	0,069263904	NA
ENSG00000010803	SCMH1	Scm polycomb group protein homolog 1	22955	28,16899318	-0,178299773	0,370261	-0,481551787	0,630124383	NA
ENSG00000091157	WDR7	WD repeat domain 7	23335	409,3777465	0,026971357	0,091474	0,29485151	0,768107315	0,944698783
ENSG00000111752	PHC1	polyhomeotic homolog 1	1911	145,1503159	-0,108738618	0,142361	-0,763822897	0,44497279	0,840706349
ENSG00000143321	HDGF	heparin binding growth factor	3068	520,7167205	0,266221753	0,077943	3,415589572	0,000636441	0,024502976
ENSG00000168090	COPS6	COP9 signalosome subunit 6	10980	130,0617627	0,129825013	0,160337	0,809699889	0,418112682	0,82446781
ENSG00000213221	DNLZ	DNL-type zinc finger	728489	18,96626147	0,255895132	0,409445	0,624980346	0,531983958	NA
ENSG00000114857	NKTR	natural killer cell triggering receptor	4820	784,058934	-0,189917147	0,303944	-0,624841645	0,532074995	0,872967783
ENSG00000183826	BTBD9	BTB domain containing 9	114781	86,29186089	-0,292187588	0,211748	-1,379885336	0,167621952	0,62720477
ENSG00000163013	FBXO41	F-box protein 41	150726	221,2721055	-0,164311239	0,110153	-1,491668178	0,135786155	0,576449438
ENSG00000116685	KIAA2013	KIAA2013	90231	168,1178597	0,016674838	0,138799	0,120136743	0,904374826	0,978460866
ENSG00000120008	WDR11	WD repeat domain 11	55717	312,848514	0,201939479	0,118071	1,710316341	0,087207392	0,482003415
ENSG00000186132	C2orf76	chromosome 2 open reading frame 76	130355	16,02717805	-0,022748507	0,475174	-0,047874041	0,961816628	NA
ENSG00000052795	FNIP2	folliculin interacting protein 2	57600	89,68609317	0,202738022	0,183878	1,102569835	0,270214016	0,725477256
ENSG00000083635	NUFIP1	nuclear FMR1 interacting protein 1	26747	34,32742151	0,014619743	0,324685	0,045027534	0,964085362	NA
ENSG00000086065	CHMP5	charged multivesicular body protein 5	51510	124,2693107	0,119793219	0,1627	0,736284223	0,461557745	0,846765498
ENSG00000108961	RANGRF	RAN guanine nucleotide release factor	29098	65,97740509	-0,272479591	0,234964	-1,159666609	0,24618457	0,703431917
ENSG00000115216	NRBP1	nuclear receptor binding protein 1	29959	259,1556098	-0,099240326	0,11913	-0,833041668	0,40482123	0,818437414
ENSG00000117479	SLC19A2	solute carrier family 19 member 2	10560	21,87775126	0,048143345	0,468734	0,102709309	0,918193686	NA
ENSG00000127337	YEATS4	YEATS domain containing 4	8089	55,49143238	0,262034262	0,230067	1,1389462	0,254725594	0,709964783
ENSG00000132768	DPH2	diphthamide biosynthesis 2	1802	64,58802936	-0,562681757	1,28233	-0,43879633	0,66080912	0,91978661
ENSG00000137076	TLN1	talin 1	7094	4913,924969	-0,014700135	0,032697	-0,449582363	0,653011607	0,916753963
ENSG00000162341	TPCN2	two pore segment channel 2	219931	279,9723657	-0,31608965	0,106693	-2,962611955	0,003050409	0,072162376
ENSG00000177465	ACOT4	acyl-CoA thioesterase 4	122970	16,77333848	0,057913861	0,446518	0,129701182	0,896802848	NA
ENSG00000186704	NA	NA	NA	21,53353783	-0,489757709	0,466588	-1,049657272	0,293875715	NA
ENSG00000188807	TMEM201	transmembrane protein 201	199953	54,59277663	-0,02437998	0,248274	-0,098197958	0,921775104	0,984284131
ENSG00000198133	TMEM229B	transmembrane protein 229B	161145	211,3854484	-0,161894736	0,237823	-0,680735995	0,496038556	0,86123475
ENSG00000204392	LSM2	LSM2 homolog, U6 small nuclear RNA and mRNA degradation associated	57819	62,15838855	-0,220434293	0,235353	-0,936612115	0,348958118	0,784537594
ENSG00000204439	C6orf47	chromosome 6 open reading frame 47	57827	88,42272019	-0,371172972	0,32529	-1,141054144	0,253847392	0,709760562
ENSG00000264364	DYNLL2	dynein light chain LC8-type 2	140735	382,4199578	-0,170048137	0,092312	-1,842093739	0,065461439	0,412627725
ENSG00000160439	RDH13	retinol dehydrogenase 13	112724	24,0530977	0,292668707	2,402319	0,121827598	0,903035557	NA
ENSG00000114744	COMMD2	COMM domain containing 2	51122	128,6333345	-0,085495155	0,162422	-0,526375212	0,598627535	0,89463553
ENSG00000156603	MED19	mediator complex subunit 19	219541	23,55152267	0,182196737	0,400608	0,454800263	0,649252941	NA
ENSG00000161654	LSM12	LSM12 homolog	124801	148,2880198	-0,03644484	0,142112	-0,256452177	0,797601703	0,95354564
ENSG00000113580	NR3C1	nuclear receptor subfamily 3 group C member 1	2908	540,9911926	-0,024414613	0,080699	-0,302538577	0,76224153	0,943004836
ENSG00000147684	NDUFB9	NADH:ubiquinone oxidoreductase subunit B9	4715	135,560895	0,10346724	0,161213	0,641803727	0,521000629	0,869884979
ENSG00000160949	TONSL	tonsoku like, DNA repair protein	4796	50,19335165	0,122664156	0,28802	0,42588698	0,670190203	0,922641254
ENSG00000167984	NLRC3	NLR family CARD domain containing 3	197358	1325,003938	-0,07203213	0,055678	-1,293738329	0,195755821	0,654519463
ENSG00000134490	TMEM241	transmembrane protein 241	85019	16,0419332	-2,238127082	2,104766	-1,063361619	0,287617991	NA
ENSG00000141642	ELAC1	elaC ribonuclease Z 1	55520	36,02896854	0,180060129	0,350946	0,513070208	0,60790221	NA
ENSG00000167747	C19orf48P	multidrug resistance-related protein	84798	19,7894216	0,465774254	0,462733	1,006572739	0,314140141	NA
ENSG00000173818	ENDOV	endonuclease V	284131	22,14333526	-0,036654055	0,431606	-0,08492478	0,932321191	NA
ENSG00000101384	JAG1	jagged canonical Notch ligand 1	182	27,36298699	0,660478676	0,328076	2,013188131	0,044094841	NA
ENSG00000100802	C14orf93	chromosome 14 open reading frame 93	60686	52,27552368	0,001908056	0,250718	0,007610368	0,993927863	0,998500439
ENSG00000162585	FAAP20	FA core complex associated protein 20	199990	22,18938547	-0,026924716	0,535272	-0,050301013	0,959882517	NA
ENSG00000242616	GNG10	G protein subunit gamma 10	2790	53,40230518	0,288841409	0,283329	1,01945659	0,307986252	0,754562945
ENSG00000130734	ATG4D	autophagy related 4D cysteine peptidase	84971	31,09075467	-0,35745512	1,877327	-0,190406429	0,84899066	NA
ENSG00000204308	RNF5	ring finger protein 5	6048	99,54593091	-0,001590573	0,162105	-0,00981199	0,99217129	0,998500439
ENSG00000053524	MCF2L2	MCF,2 cell line derived transforming sequence-like 2	23101	16,97069574	-0,301114122	0,486902	-0,61842885	0,536292685	NA

ENSG00000177030	DEAF1	DEAF1 transcription factor	10522	41,97298749	0,279538447	0,603507	0,463190184	0,643228051	NA
ENSG00000167566	NCKAP5L	NCK associated protein 5 like	57701	16,78732614	-0,457161393	0,519811	-0,879475556	0,379143481	NA
ENSG00000125434	SLC25A35	solute carrier family 25 member 35	399512	51,48472571	-0,056726454	0,282057	-0,201116736	0,840607296	0,966505956
ENSG00000143971	ETAA1	ETAA1 activator of ATR kinase	54465	67,64942313	-0,067079785	0,219344	-0,305819588	0,759742013	0,942901768
ENSG00000156858	PRR14	proline rich 14	78994	174,9352797	0,132736715	0,153568	0,864349056	0,387396169	0,809653368
ENSG00000128159	TUBGCP6	tubulin gamma complex component 6	85378	376,2857791	0,141602754	0,1224	1,156883368	0,247320015	0,705258787
ENSG00000038002	AGA	aspartylglucosaminidase	175	70,39882681	-0,110721422	0,203582	-0,54386677	0,586533158	0,891324625
ENSG00000156475	PPP2R2B	protein phosphatase 2 regulatory subunit Bbeta	5521	99,40112628	-0,040021941	0,669824	-0,059749907	0,952354822	0,991212866
ENSG00000213413	PVRIG	PVR related immunoglobulin domain containing	79037	196,5952944	-0,150288291	0,125479	-1,19771302	0,231028759	0,685179309
ENSG00000166788	SAAL1	serum amyloid A like 1	113174	34,31307824	0,099193155	0,335342	0,295797178	0,767384978	NA
ENSG00000101911	PRPS2	phosphoribosyl pyrophosphate synthetase 2	5634	169,2028704	-0,065957052	0,136644	-0,482691861	0,629314543	0,905567722
ENSG00000196976	LAGE3	L antigen family member 3	8270	19,92414495	0,056166168	0,458151	0,122593253	0,902429197	NA
ENSG00000125656	CLPP	caseinolytic mitochondrial matrix peptidase proteolytic subunit	8192	97,15052466	-0,028606799	0,208844	-0,136977127	0,891048874	0,977990819
ENSG00000185499	MUC1	mucin 1, cell surface associated	4582	13,90327741	0,05441749	0,571515	0,095216128	0,92414316	NA
ENSG00000141627	DYM	dymeclin	54808	193,7515082	-0,051150084	0,149998	-0,341005678	0,733099307	0,93912057
ENSG00000147955	SIGMAR1	sigma non-opioid intracellular receptor 1	10280	82,57659269	0,174660004	0,413501	0,422393231	0,672738018	0,923602509
ENSG00000099326	MZF1	myeloid zinc finger 1	7593	17,35625671	-0,232380854	0,432818	-0,536901881	0,591335392	NA
ENSG00000133103	COG6	component of oligomeric golgi complex 6	57511	102,8517268	0,204986326	0,223774	0,916041963	0,359644886	0,79112567
ENSG00000137274	BPHL	biphenyl hydrolase like	670	12,30535809	-0,184487715	0,663438	-0,278078325	0,780952234	NA
ENSG00000163565	IFI16	interferon gamma inducible protein 16	3428	1033,664614	0,046936968	0,080327	0,584325517	0,559001328	0,883921037
ENSG00000166272	WBP1L	WW domain binding protein 1 like	54838	363,0664637	-0,08929473	0,095819	-0,931913318	0,351381324	0,785712602
ENSG00000166272	CYP17A1-AS1	CYP17A1 antisense RNA 1	102724307	363,0664637	-0,08929473	0,095819	-0,931913318	0,351381324	0,785712602
ENSG00000068079	IFI35	interferon induced protein 35	3430	71,30356113	-0,15682494	0,227064	-0,690662642	0,489777571	0,860369726
ENSG00000134531	EMP1	epithelial membrane protein 1	2012	23,99490922	0,981519337	0,472769	2,076106398	0,037884112	NA
ENSG00000166436	TRIM66	tripartite motif containing 66	9866	279,6792248	-0,304883463	0,117627	-2,591958822	0,009543119	0,150574309
ENSG00000169635	HIC2	HIC ZBTB transcriptional repressor 2	23119	36,67247796	-0,137792138	0,297763	-0,462757602	0,643538125	NA
ENSG00000198498	TMA16	translation machinery associated 16 homolog	55319	35,19686791	0,015863729	0,291451	0,054430136	0,956592469	NA
ENSG00000104219	ZDHHC2	zinc finger DHHC-type palmitoyltransferase 2	51201	169,6387058	-0,076200139	0,130695	-0,583039891	0,559866447	0,883921037
ENSG00000185386	MAPK11	mitogen-activated protein kinase 11	5600	29,46395709	-0,213620895	0,400645	-0,533192122	0,593900594	NA
ENSG00000196544	BORCS6	BLOC-1 related complex subunit 6	54785	61,89015947	-0,393323259	0,235716	-1,668632737	0,095190187	0,499178345
ENSG00000286019	NOTCH2NLB	notch 2 N-terminal like B	100996763	70,28705407	0,57693435	0,323317	1,784423085	0,074354943	0,443476943
ENSG00000117262	GPR89A	G protein-coupled receptor 89A	653519	32,61235924	0,270116426	0,512005	0,527566108	0,597800522	NA
ENSG00000118260	CREB1	cAMP responsive element binding protein 1	1385	353,8628566	-0,265323423	0,16122	-1,645723704	0,099820657	0,509429216
ENSG00000120647	CCDC77	coiled-coil domain containing 77	84318	27,33804572	0,348574326	0,497989	0,699964451	0,483949505	NA
ENSG00000156313	RPGR	retinitis pigmentosa GTPase regulator	6103	43,70144693	-0,155272193	0,319343	-0,486223811	0,626808491	0,90479929
ENSG00000254995	STX16-NPEPL1	STX16-NPEPL1 readthrough (NMD candidate)	100534593	19,18502071	0,606941602	0,657592	0,922976209	0,356019603	NA
ENSG00000130724	CHMP2A	charged multivesicular body protein 2A	27243	137,901546	-0,002554539	0,169985	-0,015028056	0,988009798	0,996894778
ENSG00000109458	GAB1	GRB2 associated binding protein 1	2549	22,71405191	0,564802742	0,593562	0,951547934	0,341326298	NA
ENSG00000153933	DGKE	diacylglycerol kinase epsilon	8526	158,3696986	0,092114675	0,134097	0,686926842	0,492128831	0,860513824
ENSG00000004700	RECQL	RecQ like helicase	5965	608,9131501	0,220849079	0,093386	2,364908371	0,018034532	0,210216924
ENSG00000103769	RAB11A	RAB11A, member RAS oncogene family	8766	215,0694052	0,039919786	0,168597	0,236776372	0,812830283	0,959129964
ENSG00000120533	ENY2	ENY2 transcription and export complex 2 subunit	56943	73,6058812	0,180428843	0,233555	0,772531177	0,439799889	0,83848252
ENSG00000183010	PYCR1	pyrroline-5-carboxylate reductase 1	5831	25,94281351	0,020043711	0,347302	0,057712588	0,953977566	NA
ENSG00000011021	CLCN6	chloride voltage-gated channel 6	1185	129,5707714	0,269904207	0,15763	1,712269107	0,086847087	0,481411407
ENSG00000184517	ZFP1	ZFP1 zinc finger protein	162239	34,99057869	0,143175636	0,372807	0,384047383	0,700943329	NA
ENSG00000110025	SNX15	sorting nexin 15	29907	55,60821945	-0,084063858	0,234947	-0,357799525	0,720493346	0,933530522
ENSG00000130826	DKC1	dyskerin pseudouridine synthase 1	1736	123,1624161	0,587045648	0,504074	1,164602795	0,244179812	0,700945995

ENSG00000008086	CDKL5	cyclin dependent kinase like 5	6792	14,07982566	-0,254205437	0,516254	-0,492403586	0,622434065	NA
ENSG00000139631	CSAD	cysteine sulfinic acid decarboxylase	51380	21,23541929	-0,352312702	0,902235	-0,390488744	0,696175177	NA
ENSG00000112651	MRPL2	mitochondrial ribosomal protein L2	51069	20,40950924	-0,819849401	0,422134	-1,942154114	0,052118446	NA
ENSG00000158864	NDUFS2	NADH:ubiquinone oxidoreductase core subunit S2	4720	182,7194528	0,136389625	0,164164	0,830815699	0,406077752	0,819038179
ENSG00000064547	LPAR2	lysophosphatidic acid receptor 2	9170	54,31076399	-0,360782005	0,247555	-1,457378487	0,145011932	0,591897348
ENSG00000089094	KDM2B	lysine demethylase 2B	84678	276,5744026	-0,101796622	0,108178	-0,941014461	0,346697448	0,782910224
ENSG00000158850	B4GALT3	beta-1,4-galactosyltransferase 3	8703	83,72844109	-0,070467553	0,217972	-0,32328763	0,746477421	0,939843468
ENSG00000175066	GK5	glycerol kinase 5	256356	140,3533098	-0,12929892	0,162242	-0,796953222	0,425478199	0,830443782
ENSG00000197771	MCMBP	minichromosome maintenance complex binding protein	79892	377,3869171	0,269542284	0,095182	2,831862575	0,004627773	0,094406576
ENSG00000119608	PROX2	prospero homeobox 2	283571	44,39260617	-0,499399135	0,295851	-1,688011065	0,091409105	0,491730898
ENSG00000008300	CELSR3	cadherin EGF LAG seven-pass G-type receptor 3	1951	36,45240499	0,133527285	0,303593	0,43982327	0,660065112	NA
ENSG00000101452	DHX35	DEAH-box helicase 35	60625	40,32412613	-0,095878904	0,610463	-0,157059218	0,875198178	NA
ENSG00000118276	B4GALT6	beta-1,4-galactosyltransferase 6	9331	33,7727647	0,276103528	0,333416	0,828104322	0,407611422	NA
ENSG00000125875	TBC1D20	TBC1 domain family member 20	128637	344,4208391	0,100995316	0,149347	0,67624647	0,498884173	0,86161226
ENSG00000127419	TMEM175	transmembrane protein 175	84286	20,71659089	0,416846335	2,404879	0,173333591	0,862389222	NA
ENSG00000143324	XPR1	xenotropic and polytropic retrovirus receptor 1	9213	172,4414503	0,087391189	0,136012	0,642526116	0,520531638	0,869693076
ENSG00000151079	KCNA6	potassium voltage-gated channel subfamily A member 6	3742	27,60032834	-0,391773945	0,36082	-1,085786714	0,277573366	NA
ENSG00000160404	TOR2A	torsin family 2 member A	27433	48,70147776	0,043972589	0,295785	0,148664036	0,881818741	0,976300778
ENSG00000162825	NBPF20	NBPF member 20	100288142	105,2384132	0,135229936	0,248774	0,543586149	0,586726294	0,891324625
ENSG00000165169	DYNLT3	dynein light chain Tctex-type 3	6990	89,1822649	-0,233666713	0,206478	-1,131678341	0,257769691	0,713362633
ENSG00000196812	ZSCAN16	zinc finger and SCAN domain containing 16	80345	22,33378729	-0,042682411	0,371189	-0,11498833	0,908454372	NA
ENSG00000244045	TMEM199	transmembrane protein 199	147007	100,7410487	0,342812045	0,16473	2,081058012	0,037428596	0,307119308
ENSG00000269335	IKBKG	inhibitor of nuclear factor kappa B kinase regulatory subunit gamma	8517	158,0308164	-0,01290431	0,143532	-0,089905268	0,928362495	0,986487751
ENSG00000006451	RALA	RAS like proto-oncogene A	5898	138,9461907	-0,160752538	0,144791	-1,110241284	0,266895067	0,722327312
ENSG00000006756	ARSD	arylsulfatase D	414	140,4477038	-0,069565541	0,161013	-0,432048685	0,66570603	0,922184616
ENSG00000060762	MPC1	mitochondrial pyruvate carrier 1	51660	55,61610892	-0,059774178	0,231465	-0,258243373	0,796219086	0,953436042
ENSG00000077458	FAM76B	family with sequence similarity 76 member B	143684	87,00417138	0,26703213	0,270861	0,985864059	0,324199826	0,767603859
ENSG00000095059	DHPS	deoxyhypusine synthase	1725	53,49799531	-0,186929086	0,232813	-0,802915732	0,422023442	0,827816808
ENSG00000108528	SLC25A11	solute carrier family 25 member 11	8402	129,7851585	-0,072530105	0,14463	-0,501487152	0,616028317	0,902090873
ENSG00000109103	UNC119	unc-119 lipid binding chaperone	9094	110,7073893	-0,155394095	0,18868	-0,823586956	0,41017429	0,82130194
ENSG00000112208	BAG2	BAG cochaperone 2	9532	25,36002163	0,215543763	0,350983	0,614115093	0,539139275	NA
ENSG00000117395	EBNA1BP2	EBNA1 binding protein 2	10969	77,33911384	0,099559697	0,23525	0,423207554	0,672143836	0,923389485
ENSG00000119760	SUPT7L	SPT7 like, STAGA complex subunit gamma	9913	366,6152694	-0,090810038	0,087805	-1,034220478	0,301033103	0,751870158
ENSG00000130939	UBE4B	ubiquitination factor E4B	10277	259,4320566	0,075733099	0,10657	0,710644065	0,47730483	0,853811879
ENSG00000133059	DSTYK	dual serine/threonine and tyrosine protein kinase	25778	135,265325	-0,091524823	0,154969	-0,590599529	0,554788781	0,882854594
ENSG00000135709	KIAA0513	KIAA0513	9764	141,5720565	-0,155258673	0,163443	-0,949926974	0,34214936	0,780064992
ENSG00000137767	SQOR	sulfide quinone oxidoreductase	58472	154,7793445	0,295939378	0,134105	2,206768521	0,027330235	0,268314586
ENSG00000156502	SUPV3L1	Suv3 like RNA helicase	6832	155,6975147	0,028770685	0,145452	0,197802291	0,843199754	0,967210584
ENSG00000160049	DFFA	DNA fragmentation factor subunit alpha	1676	210,8169875	0,070028564	0,12374	0,565932379	0,571439749	0,886795904
ENSG00000162851	TFB2M	transcription factor B2, mitochondrial	64216	48,66917215	0,312640078	0,271534	1,151384389	0,249574133	0,707335884
ENSG00000165240	ATP7A	ATPase copper transporting alpha	538	102,0908788	0,278597263	0,260015	1,071465773	0,283960053	0,737423617
ENSG00000167766	ZNF83	zinc finger protein 83	55769	75,09552193	-0,849922734	0,2998	-2,834965913	0,004583058	0,093982595
ENSG00000173540	GMPPB	GDP-mannose pyrophosphorylase B	29925	114,69945	-0,237846621	0,15536	-1,53094124	0,125783916	0,563113596
ENSG00000173786	CNP	2',3'-cyclic nucleotide 3' phosphodiesterase	1267	155,8220841	-0,021135217	0,184447	-0,114587087	0,908772416	0,97930822
ENSG00000198858	R3HDM4	R3H domain containing 4	91300	135,9407259	-0,425683704	0,150823	-2,822411869	0,004766392	0,096039621
ENSG00000205659	LIN52	lin-52 DREAM MuvB core complex component	91750	47,54999838	-0,01529457	0,259853	-0,058858658	0,953064687	0,991212866
ENSG00000176105	YES1	YES proto-oncogene 1, Src family tyrosine kinase	7525	105,8003469	-0,054894873	0,214698	-0,255683633	0,798195134	0,95375393

ENSG00000275740	LOC127814297	RBM27-POU4F3	127814297	19,31428212	-0,854914673	1,59896	-0,534669312	0,592878546	NA
ENSG00000196743	GM2A	ganglioside GM2 activator	2760	97,05098223	0,156269164	0,172283	0,907050616	0,364380031	0,793491925
ENSG00000127125	PPCS	phosphopantothenoylcysteine synthetase	79717	84,94930865	0,304064271	0,265001	1,147406336	0,251213724	0,70779361
ENSG00000172172	MRPL13	mitochondrial ribosomal protein L13	28998	61,27315888	0,274670014	0,234641	1,170597736	0,241760508	0,696802579
ENSG00000077238	IL4R	interleukin 4 receptor	3566	810,4886054	0,033828609	0,061507	0,54999947	0,582319737	0,888501395
ENSG00000105700	KXD1	KxDL motif containing 1	79036	186,7332712	-0,074389833	0,128152	-0,580479386	0,561591384	0,884605624
ENSG00000108592	FTSJ3	FtsJ RNA 2'-O-methyltransferase 3	117246	197,3138483	0,010983036	0,134986	0,081364504	0,935152077	0,987098279
ENSG00000147669	POLR2K	RNA polymerase II, I and III subunit K	5440	115,3701667	0,26843656	0,163746	1,639347397	0,10114093	0,51381686
ENSG00000010256	UQCRC1	ubiquinol-cytochrome c reductase core protein 1	7384	144,5883302	0,070686025	0,149419	0,473072097	0,636161743	0,909100133
ENSG00000081692	JMJD4	jumonji domain containing 4	65094	62,61872607	0,079749144	0,222681	0,358131252	0,720245093	0,933530522
ENSG00000113360	DROSHA	drosha ribonuclease III	29102	250,8673504	-0,035481598	0,117502	-0,301966245	0,762677795	0,943021317
ENSG00000139718	SETD1B	SET domain containing 1B, histone lysine methyltransferase	23067	380,7143075	-0,126684816	0,091712	-1,381333017	0,167176591	0,62703197
ENSG00000261740	BOLA2-SMG1P6	BOLA2-SMG1P6 readthrough	107282092	33,10993896	-0,60371685	0,371227	-1,626275761	0,103891006	NA
ENSG00000025156	HSF2	heat shock transcription factor 2	3298	53,50827443	0,112869785	0,229094	0,492679788	0,622238862	0,904764752
ENSG00000106603	COA1	cytochrome c oxidase assembly factor 1	55744	56,4554559	0,026966919	0,224614	0,120058678	0,904436666	0,978460866
ENSG00000072756	TRNT1	tRNA nucleotidyl transferase 1	51095	63,78025074	-0,183887211	0,357934	-0,513746513	0,607429228	0,897481254
ENSG00000137133	HINT2	histidine triad nucleotide binding protein 2	84681	21,6190152	0,040918098	0,367685	0,111285757	0,91138975	NA
ENSG00000141570	CBX8	chromobox 8	57332	18,43691367	-0,041088472	0,417058	-0,098519733	0,921519604	NA
ENSG00000154144	TBRG1	transforming growth factor beta regulator 1	84897	63,05336036	-0,214415385	0,379072	-0,565632922	0,571643343	0,886795904
ENSG00000145779	TNFAIP8	TNF alpha induced protein 8	25816	408,8662494	-0,026801463	0,110686	-0,242140131	0,808671583	0,958687791
ENSG00000008405	CRY1	cryptochrome circadian regulator 1	1407	57,37028117	0,146689443	0,249999	0,586761036	0,557364209	0,883921037
ENSG00000050393	MCUR1	mitochondrial calcium uniporter regulator 1	63933	73,74199864	0,026482114	0,195392	0,135533526	0,892190059	0,977990819
ENSG00000104299	INTS9	integrator complex subunit 9	55756	64,6013458	-0,014115165	0,29356	-0,048082719	0,961650318	0,992328441
ENSG00000182518	VCF2	VCP nuclear cofactor family member 2	90736	15,81381125	-0,217121411	0,67994	-0,319324319	0,749480593	NA
ENSG00000133561	GIMAP6	GTPase, IMAP family member 6	474344	1123,324858	-0,245700602	0,067525	-3,638662719	0,000274057	0,012885133
ENSG00000168970	JMJD7-PLA2G4B	JMJD7-PLA2G4B readthrough	8681	40,19407373	-0,37570107	0,550421	-0,682570884	0,494878038	NA
ENSG00000130023	ERMARD	ER membrane associated RNA degradation	55780	56,74800466	-0,019496805	1,237502	-0,015754974	0,987429869	0,996894778
ENSG00000143252	SDHC	succinate dehydrogenase complex subunit C	6391	173,1702845	0,141864595	0,136358	1,040386596	0,298160327	0,748846814
ENSG00000146350	TBC1D32	TBC1 domain family member 32	221322	16,30478892	-0,24851639	0,42733	-0,581556284	0,560865596	NA
ENSG00000170779	CDCA4	cell division cycle associated 4	55038	76,4015349	0,035842219	0,208888	0,171586017	0,863763001	0,971561202
ENSG00000104529	EEF1D	eukaryotic translation elongation factor 1 delta	1936	86,38693159	0,703611265	0,382606	1,838999095	0,065915319	0,415155507
ENSG00000136770	DNAJC1	DnaJ heat shock protein family (Hsp40) member C1	64215	77,9533602	-0,014251748	0,189406	-0,075244485	0,94002019	0,987813564
ENSG00000169220	RGS14	regulator of G protein signaling 14	10636	85,78416436	0,063353004	0,195312	0,324368228	0,745659271	0,939843468
ENSG00000166507	NDST2	N-deacetylase and N-sulfotransferase 2	8509	193,0282398	-0,046849516	0,13666	-0,342818335	0,731735132	0,939050439
ENSG00000090487	SPG21	SPG21 abhydrolase domain containing, maspardin	51324	151,1832915	-0,077634523	0,134927	-0,575382352	0,565032737	0,885045179
ENSG00000177889	UBE2N	ubiquitin conjugating enzyme E2 N	7334	285,822519	0,022478016	0,120879	0,185955047	0,852480014	0,968229875
ENSG00000225648	NA	NA	NA	28,72921722	0,490809584	0,309512	1,585754361	0,112795042	NA
ENSG00000103671	TRIP4	thyroid hormone receptor interactor 4	9325	139,3104497	0,053779141	0,135168	0,397870109	0,690725934	0,927252461
ENSG00000127957	NA	NA	NA	25,07935535	0,10311991	0,331059	0,311484662	0,755432201	NA
ENSG00000143303	METTL25B	methyltransferase like 25B	51093	53,99996561	0,188268314	0,269275	0,699167338	0,48444746	0,857141327
ENSG00000102967	DHODH	dihydroorotate dehydrogenase (quinone)	1723	23,7784914	-0,190826603	0,352791	-0,540904773	0,588573221	NA
ENSG00000104142	VPS18	VPS18 core subunit of CORVET and HOPS complexes	57617	193,7610405	-0,136395313	0,139701	-0,976334777	0,328898591	0,770885747
ENSG00000107611	CUBN	cubilin	8029	20,53300429	-0,00533676	0,482793	-0,011053935	0,991180416	NA
ENSG00000111203	ITFG2	integrin alpha FG-GAP repeat containing 2	55846	91,13705619	-0,0492751	0,184167	-0,267557151	0,789040217	0,95148885
ENSG00000130810	PPAN	peter pan homolog	56342	131,4151722	-0,145104951	0,159082	-0,912141759	0,361694098	0,791408368

ENSG00000134326	CMPK2	cytidine/uridine monophosphate kinase 2	129607	41,21674763	-1,017652808	0,437007	-2,328687922	0,019875604 NA
ENSG00000138802	SEC24B	SEC24 homolog B, COPII coat complex component	10427	216,477479	-0,124519648	0,116355	-1,070166393	0,284544418	0,737805171
ENSG00000164002	EXO5	exonuclease 5	64789	27,42862624	-0,048094607	0,436808	-0,110104708	0,912326334	NA
ENSG00000065057	NTHL1	nth like DNA glycosylase 1	4913	18,44833586	-0,361503733	0,446571	-0,809510103	0,418221794	NA
ENSG00000105968	H2AZ2	H2A,Z variant histone 2	94239	492,1403111	0,284428046	0,127852	2,224663681	0,026103824	0,260750739
ENSG00000170638	TRABD	TraB domain containing	80305	219,0611635	-0,068100577	0,166075	-0,410058515	0,681763024	0,925586358
ENSG00000186792	HYAL3	hyaluronidase 3	8372	12,67574846	-0,651101975	0,656722	-0,991441563	0,321470014	NA
ENSG00000070761	CFAP20	cilia and flagella associated protein 20	29105	56,34657772	0,056988065	0,283747	0,200841212	0,840822737	0,966505956
ENSG00000229474	PATL2	PAT1 homolog 2	197135	56,44715279	-0,163235468	0,233378	-0,699446972	0,484272742	0,857025262
ENSG00000105723	GSK3A	glycogen synthase kinase 3 alpha	2931	266,2452379	-0,139620829	0,103213	-1,352743356	0,176137634	0,632039836
ENSG00000005075	POLR2J	RNA polymerase II subunit J	5439	90,11138247	0,245392383	0,188661	1,300707666	0,193358537	0,650901454
ENSG00000126453	BCL2L12	BCL2 like 12	83596	31,64201874	0,0779292	0,331373	0,235170397	0,814076482	NA
ENSG00000126709	IFI6	interferon alpha inducible protein 6	2537	58,96549584	0,027050687	0,226973	0,119180012	0,905132742	0,978460866
ENSG00000130803	ZNF317	zinc finger protein 317	57693	191,23529	-0,032684686	0,147613	-0,221420733	0,82476485	0,961917534
ENSG00000131503	ANKHD1	ankyrin repeat and KH domain containing 1	54882	169,1358171	0,768064891	1,027486	0,747518593	0,454750584	0,843234809
ENSG00000132300	PTCD3	pentatricopeptide repeat domain 3	55037	307,5852909	-0,367305536	0,997835	-0,368102502	0,712796805	0,930846779
ENSG00000141026	MED9	mediator complex subunit 9	55090	33,2173354	-0,412539339	0,325031	-1,269230018	0,204359038	NA
ENSG00000145241	CENPC	centromere protein C	1060	84,33188151	0,690794751	0,249536	2,768321832	0,005634578	0,107673915
ENSG00000169957	ZNF768	zinc finger protein 768	79724	33,81341001	-0,401840439	0,306326	-1,311806169	0,189585543	NA
ENSG00000174917	MICOS13	mitochondrial contact site and cristae organizing system subunit 13	125988	40,1450074	0,033650587	0,265424	0,126780522	0,899114112	NA
ENSG00000175193	PARL	presenilin associated rhomboid like	55486	55,77122193	-0,002892306	0,367819	-0,007863388	0,993725988	0,998500439
ENSG00000198331	HYLS1	HYLS1 centriolar and ciliogenesis associated	219844	26,04629805	-0,146147097	0,360235	-0,405698781	0,684963938	NA
ENSG00000073350	LLGL2	LLGL scribble cell polarity complex component 2	3993	104,014584	0,09042878	0,212103	0,426342721	0,669858132	0,922641254
ENSG00000093217	XYLB	xylulokinase	9942	21,02840967	0,090603786	0,761175	0,119031504	0,905250397	NA
ENSG00000115520	COQ10B	coenzyme Q10B	80219	122,5174908	-0,072919862	0,192378	-0,379044471	0,704654841	0,930846779
ENSG00000120158	RCL1	RNA terminal phosphate cyclase like 1	10171	42,11091986	0,31560905	0,344773	0,915411371	0,359975711	NA
ENSG00000124383	MPHOSPH10	M-phase phosphoprotein 10	10199	155,4475622	0,103932278	0,135309	0,768109502	0,442422132	0,838678081
ENSG00000164609	SLU7	SLU7 homolog, splicing factor	10569	271,1946081	0,053597501	0,116761	0,459037257	0,646207412	0,913483263
ENSG00000172273	HINFP	histone H4 transcription factor	25988	47,81713484	0,32075572	0,350907	0,914077302	0,360676228	0,791408368
ENSG00000171311	EXOSC1	exosome component 1	51013	47,39824595	-0,14291178	0,299296	-0,477492984	0,633011116	0,907570154
ENSG00000145214	DGKQ	diacylglycerol kinase theta	1609	131,1789817	-0,375926393	0,226254	-1,661522631	0,096608528	0,502914503
ENSG00000173171	MTX1	metaxin 1	4580	35,84798787	-0,343091536	0,335461	-1,02274727	0,306427355	NA
ENSG00000133193	VCF1	VCP nuclear cofactor family member 1	84923	177,4389289	0,108329771	0,138015	0,784911936	0,432505203	0,834691048
ENSG00000137764	MAP2K5	mitogen-activated protein kinase kinase 5	5607	58,58713488	0,222995133	0,262029	0,851032181	0,394751477	0,812498986
ENSG00000099800	TIMM13	translocase of inner mitochondrial membrane 13	26517	62,59344446	-0,307663912	0,333985	-0,921190439	0,356951009	0,789494009
ENSG00000106948	AKNA	AT-hook transcription factor	80709	3613,181113	-0,217821161	0,038771	-5,618102874	1,93E-08	5,62E-06
ENSG00000130940	CASZ1	castor zinc finger 1	54897	48,45919078	-0,280685543	0,29088	-0,964952362	0,334568673	0,77450906
ENSG00000137502	RAB30	RAB30, member RAS oncogene family	27314	67,6490679	0,243272394	0,318795	0,763100664	0,445403364	0,840933482
ENSG00000141994	DUS3L	dihydrouridine synthase 3 like	56931	52,29211087	-0,006738724	0,293348	-0,022971778	0,981672785	0,995974079
ENSG00000169564	PCBP1	poly(rC) binding protein 1	5093	1233,547795	0,02166107	0,054769	0,395499928	0,692473974	0,927787546
ENSG00000172428	COPS9	COP9 signalosome subunit 9	150678	45,06032203	0,049070313	0,255821	0,191815192	0,84788697	0,967411651
ENSG00000196262	PPIA	peptidylprolyl isomerase A	5478	1394,440669	0,246100253	0,080205	3,068406505	0,002152037	0,055967203
ENSG00000258311	NA	NA	NA	44,56118845	0,062940191	0,264962	0,237544401	0,812234477	0,959038743
ENSG00000015568	RGPD5	RANBP2 like and GRIP domain containing 5	84220	18,3301311	-6,783399536	3,911519	-1,73421113	0,082880629	NA
ENSG00000235162	C12orf75	chromosome 12 open reading frame 75	387882	128,462497	0,219615904	0,204546	1,073676504	0,282967695	0,736635161
ENSG00000243477	NAA80	N-alpha-acetyltransferase 80, NatH catalytic subunit	24142	35,71783658	0,11163452	0,329776	0,338516591	0,734973927	NA
ENSG00000101084	RAB5IF	RAB5 interacting factor	55969	119,4832546	0,251663812	0,211222	1,191464337	0,233471349	0,688836955
ENSG00000166035	LIPC	lipase C, hepatic type	3990	14,70996667	0,084168841	0,460606	0,182734934	0,855006004	NA
ENSG00000248483	POU5F2	POU domain class 5, transcription factor 2	134187	16,15813789	0,304795446	0,444178	0,686200879	0,492586444	NA
ENSG00000178338	ZNF354B	zinc finger protein 354B	117608	34,7289054	0,117921635	0,306388	0,384876748	0,700328734	NA
ENSG00000001629	ANKIB1	ankyrin repeat and IBR domain containing 1	54467	342,4091921	-0,157232243	0,091506	-1,718274382	0,08574658	0,477965676
ENSG00000089289	IGBP1	immunoglobulin binding protein 1	3476	175,8778525	0,158870176	0,13299	1,194600753	0,232243055	0,68727843
ENSG00000106546	AHR	aryl hydrocarbon receptor	196	259,8145841	-0,189385994	0,148477	-1,275527834	0,202122479	0,660119983

ENSG00000115145	STAM2	signal transducing adaptor molecule 2	10254	120,6538986	-0,153184846	0,165278	-0,926828835	0,354015411	0,788228969
ENSG00000116898	MRPS15	mitochondrial ribosomal protein S15	64960	89,485968	-0,14447892	0,187479	-0,770641888	0,440919226	0,838678081
ENSG00000136710	CCDC115	coiled-coil domain containing 115	84317	97,58914234	-0,000830933	0,201314	-0,004127554	0,996706698	0,999300722
ENSG00000138617	PARP16	poly(ADP-ribose) polymerase family member 16	54956	50,79988023	-0,145823999	0,241972	-0,602648612	0,546742474	0,878739902
ENSG00000145494	NDUFS6	NADH:ubiquinone oxidoreductase subunit S6	4726	62,71025305	0,054434939	0,2108	0,258229794	0,796229565	0,953436042
ENSG00000164574	GALNT10	polypeptide N-acetylgalactosaminyltransferase 10	55568	360,8504978	0,100362598	0,095818	1,047427226	0,29490258	0,746855268
ENSG00000172115	CYCS	cytochrome c, somatic	54205	247,991853	0,140836317	0,124233	1,133646496	0,256942861	0,71283265
ENSG00000181826	RELL1	RELT like 1	768211	140,3931208	-0,254395663	0,166128	-1,531325427	0,125688984	0,563113596
ENSG00000205476	CCDC85C	coiled-coil domain containing 85C	317762	60,43263304	0,075965117	0,355995	0,213388152	0,831024221	0,963156386
ENSG00000241058	NSUN6	NOP2/Sun RNA methyltransferase 6	221078	53,63229232	-0,382761955	0,258001	-1,483568311	0,137923482	0,578980755
ENSG00000151503	NCAPD3	non-SMC condensin II complex subunit D3	23310	140,8040444	-0,038874598	0,24345	-0,1596819	0,87313166	0,974533542
ENSG00000118596	SLC16A7	solute carrier family 16 member 7	9194	376,2576629	-0,074905429	0,099803	-0,750532693	0,452933941	0,843234809
ENSG00000231550	NA	NA	NA	14,67789044	0,481341944	0,49908	0,964458149	0,334816281	NA
ENSG00000158122	PRXL2C	peroxiredoxin like 2C	195827	34,45519217	0,278293052	0,405109	0,686958424	0,492108928	NA
ENSG00000188542	DUSP28	dual specificity phosphatase 28	285193	28,04251127	-0,305499689	0,378982	-0,806106605	0,420181383	NA
ENSG00000024526	DEPDC1	DEP domain containing 1	55635	22,07569549	0,473824759	0,454847	1,041724509	0,297539423	NA
ENSG00000060971	ACAA1	acetyl-CoA acyltransferase 1	30	127,9868506	0,007251437	0,182347	0,039767155	0,968278762	0,993542794
ENSG00000158517	NCF1	neutrophil cytosolic factor 1	653361	18,28748178	0,109218746	0,435877	0,250572645	0,802144533	NA
ENSG00000163596	ICA1L	islet cell autoantigen 1 like	130026	35,7561278	-0,085119238	0,324035	-0,262685057	0,792793335	NA
ENSG00000164024	METAP1	methionyl aminopeptidase 1	23173	206,8769099	0,084971138	0,116525	0,729210735	0,465872765	0,849305994
ENSG00000175711	B3GNTL1	UDP-GlcNAc:betaGal beta-1,3-N-acetylglucosaminyltransferase like 1	146712	18,20266132	0,497517172	0,540446	0,920568564	0,357275721	NA
ENSG00000178104	PDE4DIP	phosphodiesterase 4D interacting protein	9659	220,9196462	0,364252098	0,112558	3,236122151	0,001211655	0,038684309
ENSG00000178104	LOC124904395	uncharacterized LOC124904395	124904395	220,9196462	0,364252098	0,112558	3,236122151	0,001211655	0,038684309
ENSG00000237238	BMS1P10	BMS1 pseudogene 10	728611	12,88642325	-0,280132236	0,487237	-0,574940856	0,565331298	NA
ENSG00000013563	DNASE1L1	deoxyribonuclease 1 like 1	1774	12,35481466	-0,394255164	1,121808	-0,351446098	0,725253704	NA
ENSG00000038358	EDC4	enhancer of mRNA decapping 4	23644	199,6740685	-0,177078982	0,164904	-1,073831116	0,282898381	0,736635161
ENSG00000042286	AIFM2	apoptosis inducing factor mitochondria associated 2	84883	83,36209423	0,08817225	0,182713	0,482572573	0,629399257	0,905567722
ENSG00000068697	LAPTM4A	lysosomal protein transmembrane 4 alpha	9741	118,7843045	-0,13754528	0,19407	-0,708739411	0,478486203	0,854008769
ENSG00000100292	HMOX1	heme oxygenase 1	3162	51,63420453	-0,02390998	0,297955	-0,080246973	0,936040831	0,987098279
ENSG00000101220	ADISSP	adipose secreted signaling protein	54976	28,19532146	0,041035134	0,312038	0,13150698	0,895374265	NA
ENSG00000101546	RBFA	ribosome binding factor A	79863	39,56199416	-0,309927229	0,311756	-0,994133784	0,32015774	NA
ENSG00000106080	FKBP14	FKBP prolyl isomerase 14	55033	24,17890277	0,575099406	0,37123	1,549171502	0,121340498	NA
ENSG00000107771	CCSER2	coiled-coil serine rich protein 2	54462	448,5059967	0,159750945	0,105634	1,512304678	0,130456372	0,566497377
ENSG00000117713	ARID1A	AT-rich interaction domain 1A	8289	525,8881378	-0,395376438	0,136806	-2,890054672	0,003851748	0,084266382
ENSG00000120805	ARL1	ADP ribosylation factor like GTPase 1	400	162,1637199	0,269801264	0,14517	1,858514269	0,063096019	0,404317327
ENSG00000125459	MSTO1	misato mitochondrial distribution and morphology regulator 1	55154	65,20190108	-0,314444886	0,229267	-1,37152474	0,170211437	0,629558603
ENSG00000125843	AP5S1	adaptor related protein complex 5 subunit sigma 1	55317	64,4441984	-0,104209994	0,260069	-0,400701951	0,688639574	0,926748702
ENSG00000132623	ANKEF1	ankyrin repeat and EF-hand domain containing 1	63926	13,18037961	-0,200396499	0,738558	-0,271334986	0,786133401	NA
ENSG00000133466	C1QTNF6	C1q and TNF related 6	114904	27,19442428	-2,09507627	2,206683	-0,949423093	0,34240547	NA
ENSG00000138442	WDR12	WD repeat domain 12	55759	93,8066102	0,006179047	0,189617	0,032586993	0,974003943	0,994534085
ENSG00000144306	SCRN3	secernin 3	79634	50,32499578	-0,034079719	0,31427	-0,108440962	0,91364591	0,980967187
ENSG00000146414	SHPRH	SNF2 histone linker PHD RING helicase	257218	198,4419381	-0,144407306	0,117673	-1,227196505	0,219748743	0,676295699
ENSG00000154079	SDHAF4	succinate dehydrogenase complex assembly factor 4	135154	18,62216323	-0,238586768	0,406006	-0,58764366	0,556771499	NA
ENSG00000163558	PRKCI	protein kinase C iota	5584	72,68810008	0,114257367	0,240652	0,474782954	0,634941684	0,908844904
ENSG00000167130	DOLPP1	dolichyldiphosphatase 1	57171	53,20451764	0,00772053	0,247654	0,031174658	0,97513025	0,994594828
ENSG00000167967	E4F1	E4F transcription factor 1	1877	84,13738795	-0,266398933	0,19903	-1,338485245	0,180738307	0,634062228
ENSG00000170606	HSPA4	heat shock protein family A (Hsp70) member 4	3308	494,0130197	0,077973712	0,075509	1,03263754	0,301773554	0,751870158
ENSG00000178502	KLHL11	kelch like family member 11	55175	94,59395784	0,008080793	0,169443	0,047690229	0,961963121	0,992328441

ENSG00000182568	SATB1	SATB homeobox 1	6304	289,2183407	-0,179317115	0,196823	-0,911058168	0,362264726	0,791409024
ENSG00000183955	KMT5A	lysine methyltransferase 5A	387893	115,2877388	0,022062495	0,272034	0,081101972	0,935360858	0,987098279
ENSG00000187109	NAP1L1	nucleosome assembly protein 1 like 1	4673	890,4360767	0,099532776	0,072212	1,378338613	0,168098767	0,627733684
ENSG00000198818	SFT2D1	SFT2 domain containing 1	113402	36,21292131	0,309797118	0,285352	1,085666698	0,277626479	NA
ENSG00000205269	TMEM170B	transmembrane protein 170B	100113407	14,1661473	-0,226365893	0,507418	-0,446113461	0,65551529	NA
ENSG00000213699	SLC35F6	solute carrier family 35 member F6	54978	144,1810005	-0,040627978	0,216843	-0,18736111	0,851377512	0,967902179
ENSG00000226210	WASH8P	WAS protein family homolog 8, pseudogene	100288778	23,05616904	-0,463946163	0,391264	-1,185763782	0,235715598	NA
ENSG00000169684	CHRNA5	cholinergic receptor nicotinic alpha 5 subunit	1138	12,0090217	0,272859171	0,578818	0,471407942	0,637349446	NA
ENSG00000149311	ATM	ATM serine/threonine kinase	472	1949,663045	-0,348996977	0,271384	-1,285989679	0,198446664	0,656945827
ENSG00000163904	SENP2	SUMO specific peptidase 2	59343	83,90659661	-0,057408821	0,244196	-0,235092943	0,814136596	0,959207071
ENSG00000212127	TAS2R14	taste 2 receptor member 14	50840	11,89287186	0,075673451	0,572575	0,132163427	0,894855028	NA
ENSG00000212127	PRH1-TAS2R14	PRH1-TAS2R14 readthrough	106707243	11,89287186	0,075673451	0,572575	0,132163427	0,894855028	NA
ENSG00000090554	FLT3LG	fms related receptor tyrosine kinase 3 ligand	2323	97,22849518	-0,058851126	0,172326	-0,341509722	0,732719887	0,93912057
ENSG00000159884	CCDC107	coiled-coil domain containing 107	203260	77,17461675	0,119770585	0,214001	0,55967208	0,57570313	0,887069818
ENSG00000112367	FIG4	FIG4 phosphoinositide 5-phosphatase	9896	59,29547087	0,318930956	0,46067	0,692319643	0,488736617	0,860080079
ENSG00000068971	PPP2R5B	protein phosphatase 2 regulatory subunit B'beta	5526	94,90106396	-0,029158758	0,216847	-0,134467216	0,893033133	0,977990819
ENSG00000105321	CCDC9	coiled-coil domain containing 9	26093	66,30723057	-0,088641747	0,229723	-0,385863494	0,699597768	0,930783276
ENSG00000111801	BTN3A3	butyrophilin subfamily 3 member A3	10384	254,6053441	0,025735129	0,105617	0,243665721	0,807489724	0,958444052
ENSG00000126391	FRMD8	FERM domain containing 8	83786	138,2791526	-0,250046577	0,165106	-1,514459878	0,129909243	0,565894171
ENSG00000147872	PLIN2	perilipin 2	123	68,12092263	0,255079349	0,210884	1,209569512	0,226444123	0,681621061
ENSG00000241945	PWP2	PWP2 small subunit processome component	5822	172,8810185	-0,0331368	0,128968	-0,256938321	0,797226387	0,95354564
ENSG00000083844	ZNF264	zinc finger protein 264	9422	224,3870182	0,287939545	0,364447	0,790072311	0,429485539	0,832060923
ENSG00000115425	PECR	peroxisomal trans-2-enoyl-CoA reductase	55825	28,8920201	-0,158019069	0,395965	-0,399073427	0,689839101	NA
ENSG00000244257	NA	NA	NA	67,12064998	-0,944625801	0,806906	-1,170677093	0,241728596	0,696802579
ENSG00000059145	UNKL	unk like zinc finger	64718	79,07527927	-0,412749861	0,295049	-1,398919849	0,16183702	0,619487156
ENSG00000101935	AMMECR1	AMMECR nuclear protein 1	9949	56,52236761	0,189382206	0,26504	0,714541211	0,474892583	0,853811879
ENSG00000135241	PNPLA8	patatin like phospholipase domain containing 8	50640	151,3974355	-0,02213715	0,186576	-0,118649473	0,905553069	0,978460866
ENSG00000151773	CCDC122	coiled-coil domain containing 122	160857	14,73316632	0,359221276	0,470685	0,763187818	0,445351393	NA
ENSG00000168116	KIAA1586	KIAA1586	57691	39,82828131	-0,315581606	0,278561	-1,132899037	0,257256654	NA
ENSG00000273749	CYFIP1	cytoplasmic FMR1 interacting protein 1	23191	61,49336422	-0,239036936	0,456734	-0,523361514	0,600722701	0,896014669
ENSG00000107833	NPM3	nucleophosmin/nucleoplasmin 3	10360	26,29906768	0,298022867	0,403622	0,738371753	0,460288575	NA
ENSG00000111450	STX2	syntaxin 2	2054	92,39379229	0,100028257	0,311098	0,32153294	0,747806554	0,939899419
ENSG00000155016	CYP2U1	cytochrome P450 family 2 subfamily U member 1	113612	17,34434463	0,192386269	0,47557	0,404538071	0,685817088	NA
ENSG00000185158	LRRC37B	leucine rich repeat containing 37B	114659	158,9567774	-0,056078412	0,154259	-0,363534201	0,716205869	0,932040329
ENSG00000198467	TPM2	tropomyosin 2	7169	29,15918311	-0,105450806	0,352745	-0,298943854	0,764982886	NA
ENSG00000007341	ST7L	suppression of tumorigenicity 7 like	54879	29,27672704	-0,30999933	0,363408	-0,853033391	0,393640788	NA
ENSG00000013016	EHD3	EH domain containing 3	30845	16,71041128	-0,177395419	0,411841	-0,430737241	0,666659437	NA
ENSG00000026297	RNASET2	ribonuclease T2	8635	52,41466738	0,085798923	0,266613	0,321810152	0,747596523	0,939899419
ENSG00000077254	USP33	ubiquitin specific peptidase 33	23032	331,0210673	0,00687845	0,113208	0,060759502	0,951550743	0,991212866
ENSG00000107959	PITRM1	pitrilysin metallopeptidase 1	10531	185,6905179	0,043398493	0,228504	0,189924582	0,849368229	0,967902179
ENSG00000116251	RPL22	ribosomal protein L22	6146	584,999471	0,244358484	0,155507	1,571370911	0,116096519	0,543356283
ENSG00000124214	STAU1	staufen double-stranded RNA binding protein 1	6780	263,6361463	0,070841163	0,117012	0,605419276	0,544900446	0,878589015
ENSG00000127452	FBXL12	F-box and leucine rich repeat protein 12	54850	54,93613377	0,23361165	0,256573	0,910507514	0,362554921	0,79141366
ENSG00000139291	TMEM19	transmembrane protein 19	55266	146,5460866	-0,24915898	0,144634	-1,722687062	0,084945131	0,475862383
ENSG00000143727	ACP1	acid phosphatase 1	52	234,1832999	0,080772247	0,116235	0,694907435	0,487113313	0,858914122
ENSG00000145912	NHP2	NHP2 ribonucleoprotein	55651	79,05071109	0,349814853	0,252833	1,383581805	0,166486543	0,625938395
ENSG00000155363	MOV10	Mov10 RISC complex RNA helicase	4343	98,30120979	0,285316789	0,290802	0,981137607	0,326524887	0,769687654
ENSG00000169570	DTWD2	DTW domain containing 2	285605	25,21802056	-0,435568668	0,382807	-1,137829584	0,255191649	NA
ENSG00000173402	DAG1	dystroglycan 1	1605	175,0383553	0,140191745	0,140091	1,000718359	0,316962989	0,761991833
ENSG00000205413	SAMD9	sterile alpha motif domain containing 9	54809	754,0740717	0,006884892	0,072656	0,094760295	0,924505225	0,984284131
ENSG00000110917	MLEC	malectin	9761	947,8219279	0,002057249	0,069058	0,029789951	0,976234573	0,995074982
ENSG00000124535	WRNIP1	WRN helicase interacting protein 1	56897	176,3198901	0,009859173	0,136523	0,072216073	0,942429954	0,988475138
ENSG00000127483	HP1BP3	heterochromatin protein 1 binding protein 3	50809	1086,155213	-0,138310285	0,061287	-2,256778373	0,024021927	0,247679511

ENSG00000170190	SLC16A5	solute carrier family 16 member 5	9121	15,86064898	0,244570637	0,452019	0,541063072	0,58846411	NA
ENSG00000205220	PSMB10	proteasome 20S subunit beta 10	5699	208,3087942	0,156483575	0,141125	1,108826051	0,26750523	0,722449525
ENSG00000243679	NA	NA	NA	37,96373279	0,012495562	0,316701	0,039455341	0,968527358	NA
ENSG00000259431	THTPA	thiamine triphosphatase	79178	44,08009041	-0,337676696	0,26823	-1,258907794	0,208063638	0,662274754
ENSG00000187240	DYNC2H1	dynein cytoplasmic 2 heavy chain 1	79659	81,20583456	-0,334181434	0,273098	-1,223669592	0,221076895	0,677288877
ENSG00000104765	BNIP3L	BCL2 interacting protein 3 like	665	120,0471169	0,294966209	0,32589	0,905110237	0,365406989	0,794170647
ENSG00000111696	NT5DC3	5'-nucleotidase domain containing 3	51559	26,8231273	-0,166608344	0,403554	-0,412852279	0,679714846	NA
ENSG00000198492	YTHDF2	YTH N6-methyladenosine RNA binding protein F2	51441	305,5005609	-0,064389084	0,123602	-0,520940316	0,602408349	0,896931787
ENSG00000072415	PALS1	protein associated with LIN7 1, MAGUK p55 family member	64398	17,72726866	-0,040992693	0,763486	-0,053691512	0,957180946	NA
ENSG00000131446	MGAT1	alpha-1,3-mannosyl-glycoprotein 2-beta-N-acetylglucosaminyltransferase	4245	640,7517388	-0,041630002	0,088234	-0,471815387	0,637058567	0,90943397
ENSG00000171103	TRMT61B	tRNA methyltransferase 61B	55006	31,69022127	-0,690376387	0,482581	-1,430593187	0,152546841	NA
ENSG00000171720	HDAC3	histone deacetylase 3	8841	108,2630131	0,259279279	0,19432	1,334293571	0,182107664	0,636302884
ENSG00000169919	GUSB	glucuronidase beta	2990	163,9974639	0,178331536	0,130306	1,368556176	0,171138051	0,629558603
ENSG00000127463	EMC1	ER membrane protein complex subunit 1	23065	208,4778619	-0,095832103	0,176477	-0,543030119	0,587109065	0,891326322
ENSG00000144535	DIS3L2	DIS3 like 3'-5' exoribonuclease 2	129563	104,7427327	-0,056314222	0,21302	-0,26436099	0,791501768	0,951705903
ENSG00000169744	LDB2	LIM domain binding 2	9079	28,37158228	-0,074591956	0,331065	-0,225308816	0,821739042	NA
ENSG00000185697	MYBL1	MYB proto-oncogene like 1	4603	331,8369279	-0,220368204	0,092739	-2,376230288	0,017490543	0,206510834
ENSG00000147133	TAF1	TATA-box binding protein associated factor 1	6872	427,6108131	-0,042004313	0,087916	-0,477776167	0,632809526	0,907570154
ENSG00000204574	ABCF1	ATP binding cassette subfamily F member 1	23	45,58540459	0,928175821	1,92512	0,482139321	0,629706978	0,905567722
ENSG00000285292	ABCF2-H2BK1	ABCF2-H2BK1 readthrough	114483834	72,29813641	0,052468078	0,27509	0,190730373	0,848736841	0,967767008
ENSG00000149218	ENDOD1	endonuclease domain containing 1	23052	111,0083378	-0,126430642	0,16443	-0,768901097	0,441952026	0,838678081
ENSG00000164506	STXBP5	syntaxin binding protein 5	134957	61,66485291	-0,174200099	1,355606	-0,128503485	0,897750541	0,977990819
ENSG00000145293	ENOPH1	enolase-phosphatase 1	58478	138,6853682	0,192087678	0,140341	1,368717598	0,171087567	0,629558603
ENSG00000089177	KIF16B	kinesin family member 16B	55614	33,1107872	0,322982281	0,337936	0,955750335	0,339198385	NA
ENSG00000198934	MAGEE1	MAGE family member E1	57692	27,57575645	-0,190365518	0,33824	-0,562812555	0,57356253	NA
ENSG00000064313	TAF2	TATA-box binding protein associated factor 2	6873	89,66440104	0,2081626	0,95047	0,219010151	0,82664214	0,961917534
ENSG00000108828	VAT1	vesicle amine transport 1	10493	151,671064	-0,081806227	0,138457	-0,590840993	0,554626965	0,882854594
ENSG00000117298	ECE1	endothelin converting enzyme 1	1889	112,5314014	0,177869846	0,193889	0,917381826	0,358942593	0,790999468
ENSG00000188070	ZFTA	zinc finger translocation associated	65998	37,99797494	0,187929938	0,317153	0,59255258	0,553480623	NA
ENSG00000114480	GBE1	1,4-alpha-glucan branching enzyme 1	2632	65,29837064	0,168366525	0,21014	0,801210175	0,423009982	0,828095812
ENSG00000118640	VAMP8	vesicle associated membrane protein 8	8673	197,0107515	-0,049795002	0,123238	-0,404057038	0,686170777	0,926454406
ENSG00000169241	SLC50A1	solute carrier family 50 member 1	55974	46,14682027	-0,251787127	0,352633	-0,714019588	0,475215067	0,853811879
ENSG00000171858	RPS21	ribosomal protein S21	6227	302,3105547	0,039716253	0,127436	0,311656778	0,755301379	0,942901768
ENSG00000259024	TVP23C-CDRT4	TVP23C-CDRT4 readthrough	100533496	21,75608246	-0,73958323	2,461677	-0,300438726	0,763842528	NA
ENSG00000167775	CD320	CD320 molecule	51293	69,03781561	0,226353897	0,213051	1,062440269	0,288035861	0,73953372
ENSG00000105639	JAK3	Janus kinase 3	3718	352,9834782	0,288898396	0,360118	0,802233065	0,422418153	0,827816808
ENSG00000063241	ISOC2	isochorismatase domain containing 2	79763	31,53976194	-0,187924047	0,306325	-0,6134803	0,539558805	NA
ENSG00000086062	B4GALT1	beta-1,4-galactosyltransferase 1	2683	572,5015021	-0,175085174	0,073048	-2,39683852	0,01653721	0,202942572
ENSG00000196715	VKORC1L1	vitamin K epoxide reductase complex subunit 1 like 1	154807	123,0797187	0,040685424	0,185059	0,219850518	0,825987574	0,961917534
ENSG00000234127	TRIM26	tripartite motif containing 26	7726	38,59082395	-0,614929727	3,411152	-0,180270397	0,856940294	NA
ENSG00000107362	ABHD17B	abhydrolase domain containing 17B, depalmitoylase	51104	154,5771992	-0,122060511	0,144495	-0,844735884	0,39825832	0,814350649
ENSG00000175182	FAM131A	family with sequence similarity 131 member A	131408	21,88343886	-0,413121473	0,432045	-0,95619977	0,338971316	NA
ENSG00000135968	GCC2	GRIP and coiled-coil domain containing 2	9648	306,3192058	-0,054857999	0,167911	-0,326708048	0,743888713	0,939745744
ENSG00000006712	PAF1	PAF1 homolog, Paf1/RNA polymerase II complex component	54623	149,542051	0,110989132	0,156506	0,709170274	0,478218818	0,854008769
ENSG00000088356	PDRG1	p53 and DNA damage regulated 1	81572	36,1039274	-0,362952647	0,294103	-1,234100876	0,217165317	NA
ENSG00000088682	COQ9	coenzyme Q9	57017	54,6813438	-0,009601867	0,257741	-0,037253947	0,970282525	0,993542794

ENSG00000104497	SNX16	sorting nexin 16	64089	17,87764073	-0,168884158	0,416723	-0,405267401	0,685280966	NA
ENSG00000107882	SUFU	SUFU negative regulator of hedgehog signaling	51684	80,84532736	0,017930173	0,194751	0,092067096	0,926644731	0,985359831
ENSG00000111144	LTA4H	leukotriene A4 hydrolase	4048	228,6365794	0,256323108	0,115812	2,21327197	0,026878901	0,265877692
ENSG00000119943	PYROXD2	pyridine nucleotide-disulphide oxidoreductase domain 2	84795	32,72032127	-1,079008493	0,563724	-1,91407221	0,05561093	NA
ENSG00000125505	MBOAT7	membrane bound O-acyltransferase domain containing 7	79143	54,65113211	-0,506778595	0,281489	-1,800351982	0,071805078	0,435172111
ENSG00000127837	AAMP	angio associated migratory cell protein	14	114,6288783	0,034544672	0,204759	0,168708846	0,86602566	0,971722599
ENSG00000139508	SLC46A3	solute carrier family 46 member 3	283537	59,7736848	-0,275055985	0,242946	-1,132168879	0,257563441	0,713362633
ENSG00000148484	RSU1	Ras suppressor protein 1	6251	334,89436	-0,062976467	0,094742	-0,664715811	0,506232279	0,864630986
ENSG00000151692	RNF144A	ring finger protein 144A	9781	961,1457793	-0,079621148	0,07734	-1,029494761	0,30324724	0,751870158
ENSG00000184675	AMER1	APC membrane recruitment protein 1	139285	66,80210627	-0,280845025	0,229963	-1,221259718	0,2219877	0,678334201
ENSG00000188986	NELFB	negative elongation factor complex member B	25920	199,4046869	0,019293683	0,120836	0,159667763	0,873142796	0,974533542
ENSG00000204628	RACK1	receptor for activated C kinase 1	10399	2215,404152	0,159760404	0,079146	2,018556867	0,043533298	0,333571241
ENSG00000273899	NOL12	nucleolar protein 12	79159	67,99636332	0,30823391	0,468197	0,658342089	0,510318338	0,866180216
ENSG00000280789	PAGR1	PAXIP1 associated glutamate rich protein 1	79447	107,8636006	0,151057235	0,215045	0,702445471	0,482401396	0,855834779
ENSG00000292339	ASMTL	acetylserotonin O-methyltransferase like	8623	99,45823912	-0,106889892	0,162797	-0,656583078	0,511449032	0,866462619
ENSG00000171202	TMEM126A	transmembrane protein 126A	84233	37,78087495	0,061940345	0,299191	0,207026385	0,835989253	NA
ENSG00000118096	IFT46	intraflagellar transport 46	56912	27,05782545	0,483974764	0,366098	1,321981376	0,186174353	NA
ENSG00000156599	ZDHHC5	zinc finger DHHC-type palmitoyltransferase 5	25921	507,3743902	0,042571423	0,096531	0,441015025	0,65920212	0,919368176
ENSG00000133313	CNDP2	carnosine dipeptidase 2	55748	304,861504	-0,046015121	0,094198	-0,488496072	0,625198507	0,90479929
ENSG00000091436	MAP3K20	mitogen-activated protein kinase kinase kinase 20	51776	112,4093655	0,341268514	0,17569	1,94245157	0,052082457	0,369238926
ENSG00000104205	SGK3	serum/glucocorticoid regulated kinase family member 3	23678	56,99927215	-0,051351196	0,347656	-0,147707117	0,882573914	0,976300778
ENSG00000136940	PDCL	phosducin like	5082	89,42691927	-0,154334188	0,180797	-0,853633155	0,393308282	0,812491435
ENSG00000143753	DEGS1	delta 4-desaturase, sphingolipid 1	8560	424,1117702	0,208293203	0,094647	2,20073588	0,027754727	0,269631574
ENSG00000060982	BCAT1	branched chain amino acid transaminase 1	586	62,12067162	-0,41846598	0,329971	-1,26819012	0,204730064	0,660954946
ENSG00000185359	HGS	hepatocyte growth factor-regulated tyrosine kinase substrate	9146	128,3386233	0,105519084	0,530951	0,19873588	0,842469356	0,967210584
ENSG00000031691	CENPQ	centromere protein Q	55166	27,81175969	0,393184314	0,315949	1,244453547	0,213332686	NA
ENSG00000085662	AKR1B1	aldo-keto reductase family 1 member B	231	155,5725178	0,099344037	0,144694	0,68657936	0,492347839	0,860513824
ENSG00000119965	C10orf88	chromosome 10 open reading frame 88	80007	38,6929744	0,289948233	0,347214	0,835070245	0,403678159	NA
ENSG00000124813	RUNX2	RUNX family transcription factor 2	860	557,1231762	-0,133763554	0,081873	-1,633788967	0,10230317	0,515119384
ENSG00000135736	CCDC102A	coiled-coil domain containing 102A	92922	28,5041145	0,047552637	0,379778	0,125211636	0,900356006	NA
ENSG00000186994	KANK3	KN motif and ankyrin repeat domains 3	256949	24,62046423	-0,175823149	0,351844	-0,499718345	0,617273413	NA
ENSG00000271601	LIX1L	limb and CNS expressed 1 like	128077	268,0509226	-0,49365759	0,115365	-4,279084028	1,88E-05	0,001734015
ENSG00000284671	NA	NA	NA	57,67948911	0,025870261	0,26116	0,099059003	0,921091423	0,984284131
ENSG00000134371	CDC73	cell division cycle 73	79577	202,9559809	0,043321359	0,162321	0,266886895	0,789556247	0,951553231
ENSG00000141367	CLTC	clathrin heavy chain	1213	1353,210625	0,101945963	0,064045	1,591792584	0,111431317	0,533322099
ENSG00000013583	HEBP1	heme binding protein 1	50865	18,92950892	0,169414576	0,563494	0,300650365	0,763681122	NA
ENSG00000240972	MIF	macrophage migration inhibitory factor	4282	151,9336382	0,182883641	0,158689	1,152462274	0,249131164	0,707335884
ENSG00000112039	FANCE	FA complementation group E	2178	21,22929854	-0,564692228	0,460713	-1,225692985	0,220314231	NA
ENSG00000198612	COPS8	COP9 signalosome subunit 8	10920	131,3058933	-0,155990975	0,196797	-0,792648583	0,427982595	0,832060923
ENSG00000221926	TRIM16	tripartite motif containing 16	10626	18,56789318	-0,572188241	0,480988	-1,189609112	0,234200063	NA
ENSG00000136044	APPL2	adaptor protein, phosphotyrosine interacting with PH domain and leucine zipper 2	55198	120,3779534	-0,144173292	0,249222	-0,578493697	0,562930852	0,884605624
ENSG00000138111	MFSD13A	major facilitator superfamily domain containing 13A	79847	21,2349299	-0,209741394	0,397099	-0,528184218	0,597371482	NA
ENSG00000138641	HERC3	HECT and RLD domain containing E3 ubiquitin protein ligase 3	8916	339,9560677	0,155650308	0,122067	1,275125669	0,202264765	0,660119983
ENSG00000139644	TMBIM6	transmembrane BAX inhibitor motif containing 6	7009	1872,804135	0,006968049	0,052234	0,133401666	0,893875728	0,977990819
ENSG00000160214	RRP1	ribosomal RNA processing 1	8568	75,12982889	-0,108327774	0,230284	-0,470410278	0,638061922	0,909829037
ENSG00000183255	PTTG1IP	PTTG1 interacting protein	754	249,3782324	0,024578437	0,119434	0,205791681	0,83695364	0,965261951

ENSG00000176261	ZBTB8OS	zinc finger and BTB domain containing 8 opposite strand	339487	44,45010033	0,018643858	0,318197	0,058592252	0,953276882	0,991212866
ENSG00000163481	RNF25	ring finger protein 25	64320	46,46257009	-0,241907064	0,307518	-0,786644481	0,431490013	0,833786615
ENSG00000138821	SLC39A8	solute carrier family 39 member 8	64116	154,6963029	0,041579243	0,256451	0,162133348	0,871200846	0,974150353
ENSG00000197102	DYNC1H1	dynein cytoplasmic 1 heavy chain 1	1778	558,4080966	0,275776337	0,438452	0,62897765	0,529363689	0,872145761
ENSG00000206562	METTL6	methyltransferase 6, tRNA N3-cytidine	131965	58,31263442	0,366784263	0,225626	1,625629831	0,104028425	0,517786851
ENSG00000168672	LRATD2	LRAT domain containing 2	157638	47,60697666	0,057237181	0,270572	0,211541388	0,83246484	0,963446071
ENSG00000065883	CDK13	cyclin dependent kinase 13	8621	326,2307868	-0,032129622	0,103567	-0,310231527	0,756384897	0,942901768
ENSG00000128463	EMC4	ER membrane protein complex subunit 4	51234	109,8427467	0,159959759	0,187611	0,852614017	0,393873387	0,812498986
ENSG00000167740	CYB5D2	cytochrome b5 domain containing 2	124936	16,91729276	0,468866361	0,508739	0,92162482	0,356724307	NA
ENSG00000170917	NUDT6	nudix hydrolase 6	11162	13,17228132	-0,478415178	0,540009	-0,885940025	0,375649846	NA
ENSG00000176444	CLK2	CDC like kinase 2	1196	55,0420225	-1,19654168	0,91743	-1,304232093	0,192154461	0,649982152
ENSG00000083720	OXCT1	3-oxoacid CoA-transferase 1	5019	244,1431728	-0,120750086	0,115495	-1,04549982	0,295792024	0,747862492
ENSG00000121879	PIK3CA	phosphatidylinositol-4,5-bisphosphate 3-kinase catalytic subunit alpha	5290	212,3657833	0,141907079	0,370306	0,383215224	0,701560191	0,930846779
ENSG00000123609	NMI	N-myc and STAT interactor	9111	128,0408934	0,030676932	0,161352	0,190124183	0,84921182	0,967887046
ENSG00000126858	RHOT1	ras homolog family member T1	55288	114,0925023	-0,589687957	0,228932	-2,575817496	0,010000341	0,154307823
ENSG00000167508	MVD	mevalonate diphosphate decarboxylase	4597	82,84497522	0,008111149	0,216277	0,0375035	0,970083549	0,993542794
ENSG00000167523	SPATA33	spermatogenesis associated 33	124045	11,82302142	-0,180883257	0,71066	-0,254528481	0,799087303	NA
ENSG00000000457	SCYL3	SCY1 like pseudokinase 3	57147	136,3255898	-0,116153001	0,152865	-0,759840844	0,447349725	0,842562288
ENSG00000113734	BNIP1	BCL2 interacting protein 1	662	22,32549052	0,240131666	0,392486	0,611821639	0,540655771	NA
ENSG00000136052	SLC41A2	solute carrier family 41 member 2	84102	28,75088306	-0,007631431	0,354602	-0,021521103	0,982829969	NA
ENSG00000139405	RITA1	RBPJ interacting and tubulin associated 1	84934	75,9422956	-0,053848887	0,193212	-0,278703314	0,780472519	0,948156406
ENSG00000160294	MCM3AP	minichromosome maintenance complex component 3 associated protein	8888	532,6516626	0,267713435	0,083027	3,224402737	0,001262357	0,040139896
ENSG00000158747	NBL1	NBL1, DAN family BMP antagonist	4681	154,4850975	-0,07772341	0,147069	-0,528484159	0,597163339	0,894257182
ENSG00000158747	MICOS10-NBL1	MICOS10-NBL1 readthrough	100532736	154,4850975	-0,07772341	0,147069	-0,528484159	0,597163339	0,894257182
ENSG00000113966	ARL6	ADP ribosylation factor like GTPase 6	84100	12,59303886	-0,947776758	0,576693	-1,643469333	0,100285865	NA
ENSG00000176978	DPP7	dipeptidyl peptidase 7	29952	120,1464997	-0,149229099	0,225932	-0,660504676	0,508930019	0,865743203
ENSG00000241468	ATP5MF	ATP synthase membrane subunit f	9551	117,4833245	0,112877243	0,221666	0,509221749	0,6105968	0,899742452
ENSG00000108861	DUSP3	dual specificity phosphatase 3	1845	61,46689191	0,007007909	0,237403	0,029519102	0,976450584	0,995074982
ENSG00000131389	SLC6A6	solute carrier family 6 member 6	6533	764,8362691	0,124087201	0,116867	1,061785213	0,288333204	0,740055225
ENSG00000145911	N4BP3	NEDD4 binding protein 3	23138	22,96629301	0,390198275	0,410069	0,951543989	0,341328299	NA
ENSG00000147526	TACC1	transforming acidic coiled-coil containing protein 1	6867	1113,739303	0,057739291	0,058144	0,993036947	0,32069195	0,764513157
ENSG00000164073	MFSD8	major facilitator superfamily domain containing 8	256471	34,04033718	-0,172681234	0,491044	-0,351661074	0,725092456	NA
ENSG00000074696	HACD3	3-hydroxyacyl-CoA dehydratase 3	51495	139,8969467	0,100679329	0,157553	0,63901932	0,522810363	0,870221868
ENSG00000083937	CHMP2B	charged multivesicular body protein 2B	25978	101,7445173	0,294765897	0,17161	1,717645149	0,08586136	0,478266043
ENSG00000092929	UNC13D	unc-13 homolog D	201294	540,7622954	0,033271881	0,088101	0,377657537	0,705685017	0,930846779
ENSG00000100629	CEP128	centrosomal protein 128	145508	26,73481566	0,265349495	0,357179	0,742904223	0,457539669	NA
ENSG00000111788	NA	NA	NA	80,62444956	-0,006222033	0,200948	-0,030963455	0,975298684	0,994594828
ENSG00000130726	TRIM28	tripartite motif containing 28	10155	445,5000036	0,070956654	0,114514	0,619634546	0,535498417	0,874894595
ENSG00000151012	SLC7A11	solute carrier family 7 member 11	23657	13,13076753	0,349666844	0,496927	0,703657898	0,481645846	NA
ENSG00000154429	CCSAP	centriole, cilia and spindle associated protein	126731	39,50772642	-0,359995199	0,349744	-1,029309121	0,303334439	NA
ENSG00000157837	SPPL3	signal peptide peptidase like 3	121665	144,117209	0,075135822	0,150807	0,498226028	0,618324739	0,902494425
ENSG00000177374	HIC1	HIC ZBTB transcriptional repressor 1	3090	237,0643559	0,006724549	0,125193	0,053713474	0,957163448	0,991911599
ENSG00000177380	PPFIA3	PTPRF interacting protein alpha 3	8541	20,52572672	0,220854874	0,448211	0,492747786	0,622190809	NA
ENSG00000179195	ZNF664	zinc finger protein 664	144348	456,2002051	0,00943859	0,089601	0,105340228	0,916105843	0,981997447
ENSG00000237190	CDKN2AIPNL	CDKN2A interacting protein N-terminal like	91368	35,9133077	0,047355523	0,349053	0,135668761	0,892083145	NA
ENSG00000139737	SLAIN1	SLAIN motif family member 1	122060	59,06866047	-0,00332873	0,265416	-0,012541571	0,989993537	0,997230889

ENSG00000137806	NDUFAF1	NADH:ubiquinone oxidoreductase complex assembly factor 1	51103	34,17539322	-0,013210518	0,400547	-0,03298118	0,973689596	NA
ENSG00000160446	ZDHHC12	zinc finger DHHC-type palmitoyltransferase 12	84885	42,86407348	0,305300995	0,321661	0,949137844	0,342550509	0,780064992
ENSG00000124444	ZNF576	zinc finger protein 576	79177	29,65358749	-0,434534824	0,336555	-1,291125088	0,196660306	NA
ENSG00000278662	GOLGA6L4	golgin A6 family like 4	643707	15,58323452	0,530790995	0,604605	0,877914349	0,379990202	NA
ENSG00000278662	GOLGA6L10	golgin A6 family like 10	647042	15,58323452	0,530790995	0,604605	0,877914349	0,379990202	NA
ENSG00000278662	LOC102724117	golgin subfamily A member 6-like protein 4	102724117	15,58323452	0,530790995	0,604605	0,877914349	0,379990202	NA
ENSG00000059691	GATB	glutamyl-tRNA amidotransferase subunit B	5188	28,6835209	-0,153115828	0,34088	-0,449177494	0,653303621	NA
ENSG00000100068	NA	NA	NA	27,19227416	-0,529357112	0,366181	-1,445617478	0,148284522	NA
ENSG00000111229	ARPC3	actin related protein 2/3 complex subunit 3	10094	710,6936748	0,216755022	0,068228	3,176914937	0,001488507	0,045138849
ENSG00000122729	ACO1	aconitase 1	48	66,58588381	0,088834847	0,225109	0,394631004	0,693115229	0,928002746
ENSG00000130733	YIPF2	Yip1 domain family member 2	78992	58,26962759	-0,43826685	0,232062	-1,888573472	0,058949004	0,39312401
ENSG00000132388	UBE2G1	ubiquitin conjugating enzyme E2 G1	7326	296,8824521	-0,283590953	0,109563	-2,588382959	0,009642771	0,151166315
ENSG00000164163	ABCE1	ATP binding cassette subfamily E member 1	6059	278,3799267	-0,020516584	0,121027	-0,169520481	0,865387264	0,971722599
ENSG00000164466	SFXN1	sideroflexin 1	94081	1027,77057	-0,115081847	0,06459	-1,781722151	0,074794553	0,444354325
ENSG00000198001	IRAK4	interleukin 1 receptor associated kinase 4	51135	119,5396278	-0,242261517	0,663958	-0,364874996	0,71520472	0,932016096
ENSG00000100077	GRK3	G protein-coupled receptor kinase 3	157	115,5677982	0,130099143	0,175463	0,7414602	0,458414455	0,845558274
ENSG00000101888	NXT2	nuclear transport factor 2 like export factor 2	55916	55,62538724	0,150326806	0,253871	0,592139338	0,553757286	0,882816634
ENSG00000119777	TMEM214	transmembrane protein 214	54867	274,3820858	0,063298455	0,102576	0,617088536	0,53717633	0,874923109
ENSG00000051596	THOC3	THO complex subunit 3	84321	136,3225785	-0,081381516	0,156489	-0,52004493	0,60303226	0,897212117
ENSG00000068308	OTUD5	OTU deubiquitinase 5	55593	329,6224512	0,027787192	0,13036	0,213158014	0,831203716	0,963156386
ENSG00000115459	ELMOD3	ELMO domain containing 3	84173	28,5750325	-0,560735604	0,429808	-1,304618148	0,192022906	NA
ENSG00000116521	SCAMP3	secretory carrier membrane protein 3	10067	101,7121479	-0,216420638	0,200322	-1,080362396	0,279980834	0,733157579
ENSG00000122008	POLK	DNA polymerase kappa	51426	107,5424913	-0,686267768	0,714284	-0,960777124	0,336664243	0,776552413
ENSG00000129534	MIS18BP1	MIS18 binding protein 1	55320	275,1941209	-0,064393672	0,106706	-0,603467977	0,546197412	0,878739902
ENSG00000141295	SCRN2	secernin 2	90507	36,1653068	-0,217956596	0,343623	-0,634289208	0,525892098	NA
ENSG00000142687	KIAA0319L	KIAA0319 like	79932	193,0832119	-0,21590689	0,156442	-1,380109919	0,167552804	0,62720477
ENSG00000142694	EVA1B	eva-1 homolog B	55194	39,88851523	-0,142795126	0,273391	-0,522310306	0,601454295	NA
ENSG00000147889	CDKN2A	cyclin dependent kinase inhibitor 2A	1029	42,25244014	-0,097030956	0,349	-0,278025875	0,780992496	NA
ENSG00000163482	STK36	serine/threonine kinase 36	27148	79,23896465	-0,288862051	0,237715	-1,215159811	0,224305145	0,679665359
ENSG00000171861	MRM3	mitochondrial rRNA methyltransferase 3	55178	44,78087248	0,073735397	0,303645	0,242834201	0,808133839	0,958576399
ENSG00000177311	ZBTB38	zinc finger and BTB domain containing 38	253461	416,278883	0,054555145	0,083631	0,652334159	0,514185628	0,868514045
ENSG00000188554	NBR1	NBR1 autophagy cargo receptor	4077	659,311198	-0,023598431	0,076955	-0,306654227	0,759106574	0,942901768
ENSG00000221955	SLC12A8	solute carrier family 12 member 8	84561	66,1640617	0,36204012	0,297799	1,215720064	0,224091579	0,6792803
ENSG00000072849	DERL2	derlin 2	51009	118,6981424	0,112852357	0,587817	0,1919855	0,847753563	0,967411651
ENSG00000125458	NT5C	5', 3'-nucleotidase, cytosolic	30833	40,2890771	0,227556181	0,324687	0,700848834	0,483397358	NA
ENSG00000006007	GDE1	glycerophosphodiester phosphodiesterase 1	51573	122,6209511	-0,25882138	0,161276	-1,604832015	0,108530778	0,528784574
ENSG00000101391	CDK5RAP1	CDK5 regulatory subunit associated protein 1	51654	69,56519516	0,247725901	0,221656	1,117614169	0,263731815	0,719682465
ENSG00000108474	PIGL	phosphatidylinositol glycan anchor biosynthesis class L	9487	17,74315566	-0,158092821	0,420272	-0,37616793	0,706792055	NA
ENSG00000126088	UROD	uroporphyrinogen decarboxylase	7389	59,89179458	0,517568331	0,244984	2,112664743	0,034629473	0,297161096
ENSG00000147604	RPL7	ribosomal protein L7	6129	1397,439193	0,122267903	0,054418	2,246834167	0,024650627	0,252419853
ENSG00000261371	PECAM1	platelet and endothelial cell adhesion molecule 1	5175	1943,927654	0,246600205	0,059009	4,178999014	2,93E-05	0,002420644
ENSG00000122550	KLHL7	kelch like family member 7	55975	17,60058373	-0,486292757	0,531545	-0,914866215	0,360261868	NA
ENSG00000100522	GNPNAT1	glucosamine-phosphate N-acetyltransferase 1	64841	63,63930269	0,00888276	0,272648	0,032579574	0,974009859	0,994534085
ENSG00000168653	NDUFS5	NADH:ubiquinone oxidoreductase subunit S5	4725	86,4190761	-0,047455417	0,234574	-0,20230439	0,839678768	0,96627649
ENSG00000079277	MKNK1	MAPK interacting serine/threonine kinase 1	8569	326,7854243	0,202058433	0,148994	1,356155644	0,175049652	0,632039836
ENSG00000113441	LNPEP	leucyl and cystinyl aminopeptidase	4012	1633,970742	-0,214499529	0,059909	-3,580414663	0,000343049	0,015664592
ENSG00000126581	BECN1	beclin 1	8678	228,532395	0,009395821	0,118236	0,079466858	0,936661292	0,987098279
ENSG00000058453	CROCC	ciliary rootlet coiled-coil, rootletin	9696	79,80414876	-0,142369638	0,196244	-0,725474313	0,468161098	0,851339954
ENSG00000187953	PMS2CL	PMS2 C-terminal like (pseudogene)	441194	26,06204788	0,050608451	0,374014	0,135311751	0,892365395	NA
ENSG00000120656	TAF12	TATA-box binding protein associated factor 12	6883	72,57787555	-0,153278536	0,199425	-0,768602895	0,442129086	0,838678081

ENSG00000130312	MRPL34	mitochondrial ribosomal protein L34	64981	59,0617519	0,315182399	0,274962	1,146274302	0,251681672	0,708175323
ENSG00000138756	BMP2K	BMP2 inducible kinase	55589	61,15645697	0,60919721	0,26282	2,317925688	0,02045336	0,224986961
ENSG00000147813	NAPRT	nicotinate phosphoribosyltransferase	93100	20,12204013	-0,698421758	0,804378	-0,868275482	0,385243541	NA
ENSG00000170473	PYM1	PYM homolog 1, exon junction complex associated factor	84305	64,08891488	-0,189009258	0,23561	-0,802214054	0,422429148	0,827816808
ENSG00000085719	CPNE3	copine 3	8895	472,7108363	-0,046967369	0,078908	-0,595216374	0,551698847	0,881150198
ENSG00000092931	MFSD11	major facilitator superfamily domain containing 11	79157	90,57508237	-0,080581269	0,211706	-0,380628357	0,703479037	0,930846779
ENSG00000135632	SMYD5	SMYD family member 5	10322	53,03399665	-0,06404349	0,241233	-0,265483956	0,790636668	0,95167209
ENSG00000184381	PLA2G6	phospholipase A2 group VI	8398	260,7754705	-0,280783395	0,183961	-1,526317502	0,126930812	0,563689485
ENSG00000030582	GRN	granulin precursor	2896	84,20944629	0,224399876	0,177441	1,264647403	0,205997752	0,660954946
ENSG00000092036	HAUS4	HAUS augmin like complex subunit 4	54930	44,64133033	0,277231947	0,446518	0,620875022	0,534681857	0,874203665
ENSG00000100938	GMPR2	guanosine monophosphate reductase 2	51292	22,45102839	-0,212180499	2,418249	-0,087741382	0,930082228	NA
ENSG00000110719	TCIRG1	T cell immune regulator 1, ATPase H+ transporting V0 subunit a3	10312	831,6191039	0,141086708	0,089557	1,575380788	0,115168576	0,541225115
ENSG00000149179	CSTPP1	centriolar satellite-associated tubulin polyglutamylase complex regulator 1	79096	13,28536667	-0,779226956	0,83806	-0,929798678	0,352475331	NA
ENSG00000165283	STOML2	stomatin like 2	30968	107,1562898	0,19041772	0,160742	1,18461846	0,236168335	0,691328984
ENSG00000087266	SH3BP2	SH3 domain binding protein 2	6452	499,1371125	0,045756849	0,126095	0,362877409	0,716696463	0,932040329
ENSG00000138801	PAPSS1	3'-phosphoadenosine 5'-phosphosulfate synthase 1	9061	148,5167161	-0,022875051	0,143893	-0,158973053	0,873690105	0,974569239
ENSG00000278259	MYO19	myosin XIX	80179	46,92492285	-1,310025234	1,843145	-0,710755233	0,477235926	0,853811879
ENSG00000135597	REPS1	RALBP1 associated Eps domain containing 1	85021	151,3175252	0,045273601	0,153322	0,295285405	0,767775865	0,944698783
ENSG00000137414	FAM8A1	family with sequence similarity 8 member A1	51439	220,753702	-0,08567611	0,179327	-0,477764619	0,632817746	0,907570154
ENSG00000072042	RDH11	retinol dehydrogenase 11	51109	213,8484949	0,079085249	0,133534	0,592246157	0,553685765	0,882816634
ENSG00000027847	B4GALT7	beta-1,4-galactosyltransferase 7	11285	26,78432939	-0,125929922	0,386765	-0,325597686	0,744728763	NA
ENSG00000149182	ARFGAP2	ADP ribosylation factor GTPase activating protein 2	84364	315,9783273	0,03047307	0,103616	0,29409646	0,768684196	0,944802477
ENSG00000132510	KDM6B	lysine demethylase 6B	23135	109,0938552	0,011337492	0,166053	0,068276467	0,945565557	0,98927293
ENSG00000179304	FAM156B	family with sequence similarity 156 member B	727866	94,57842584	-0,045304787	1,465993	-0,030903825	0,975346239	0,994594828
ENSG00000254470	AP5B1	adaptor related protein complex 5 subunit beta 1	91056	205,5699358	0,018866748	0,11581	0,162911275	0,870588293	0,974150353
ENSG00000095906	NUBP2	NUBP iron-sulfur cluster assembly factor 2, cytosolic	10101	54,01351745	0,087277308	0,271474	0,321494089	0,747835991	0,939899419
ENSG00000116747	RO60	Ro60, Y RNA binding protein	6738	259,4080927	-0,291206574	0,160116	-1,818721647	0,068953907	0,426428338
ENSG00000156709	AIFM1	apoptosis inducing factor mitochondria associated 1	9131	100,5330905	0,185545824	0,185818	0,998533381	0,318020786	0,762879158
ENSG00000101236	RNF24	ring finger protein 24	11237	102,3912543	-0,239843652	0,231135	-1,037677495	0,299420226	0,750126461
ENSG00000161036	LRWD1	leucine rich repeats and WD repeat domain containing 1	222229	65,05048116	-0,150710375	0,247944	-0,607841416	0,543292658	0,877542071
ENSG00000173653	RCE1	Ras converting CAAX endopeptidase 1	9986	22,95429809	0,031431848	0,431983	0,072761749	0,94199571	NA
ENSG00000177352	CCDC71	coiled-coil domain containing 71	64925	54,0126826	-0,089561371	0,297719	-0,300825378	0,763547655	0,943846027
ENSG00000182173	TSEN54	tRNA splicing endonuclease subunit 54	283989	76,97076752	0,207365306	0,936679	0,221383546	0,824793803	0,961917534
ENSG00000130985	UBA1	ubiquitin like modifier activating enzyme 1	7317	930,6508308	-0,025731463	0,058133	-0,442630124	0,658033292	0,919088619
ENSG00000153048	CARHSP1	calcium regulated heat stable protein 1	23589	193,8935905	0,195887946	0,187521	1,044619283	0,296198964	0,747862492
ENSG00000089050	RBBP9	RB binding protein 9, serine hydrolase	10741	62,90529594	-0,076877669	0,222729	-0,345162818	0,72997197	0,938637828
ENSG00000164096	C4orf3	chromosome 4 open reading frame 3	401152	275,440387	-0,074907538	0,107669	-0,69571779	0,486605582	0,858445697
ENSG00000197296	FITM2	fat storage inducing transmembrane protein 2	128486	56,88227083	-0,457519941	0,230743	-1,982809415	0,047388722	0,349139303
ENSG00000128335	APOL2	apolipoprotein L2	23780	217,2405583	-0,313632156	0,144892	-2,164595323	0,03041869	0,279077601
ENSG00000100568	VTI1B	vesicle transport through interaction with t-SNAREs 1B	10490	174,4894043	-0,200084992	0,132546	-1,509556618	0,131156598	0,567474728
ENSG00000088836	SLC4A11	solute carrier family 4 member 11	83959	11,70924309	-0,257704232	0,530318	-0,485942673	0,627007812	NA
ENSG00000151304	SRFBP1	serum response factor binding protein 1	153443	50,37857372	-0,158489891	0,238255	-0,665211969	0,505914926	0,864630986
ENSG00000197563	PIGN	phosphatidylinositol glycan anchor biosynthesis class N	23556	126,1751574	-0,158389405	0,153767	-1,030063634	0,302980135	0,751870158
ENSG00000110841	PPFIBP1	PPFIA binding protein 1	8496	99,67792951	0,973151588	0,229814	4,234509846	2,29E-05	0,001985068

ENSG00000138767	CNOT6L	CCR4-NOT transcription complex subunit 6 like	246175	734,0226925	-0,18363296	0,080971	-2,267899028	0,023335362	0,244042517
ENSG00000010278	CD9	CD9 molecule	928	118,1016357	0,114147131	0,15082	0,75684539	0,449142496	0,84263906
ENSG00000088038	CNOT3	CCR4-NOT transcription complex subunit 3	4849	136,4509571	-0,251643097	0,23595	-1,06650871	0,286193743	0,738005299
ENSG00000116005	PCYOX1	prenylcysteine oxidase 1	51449	165,2693748	0,070149609	0,129552	0,541477138	0,588178751	0,891557039
ENSG00000182732	RGS6	regulator of G protein signaling 6	9628	13,76032096	-0,739303253	0,580408	-1,273765456	0,202746551	NA
ENSG00000104880	ARHGEF18	Rho/Rac guanine nucleotide exchange factor 18	23370	195,5063086	-0,197250507	0,123058	-1,60290107	0,108956497	0,52908614
ENSG00000130741	EIF2S3	eukaryotic translation initiation factor 2 subunit gamma	1968	761,6010552	-0,087031285	0,067023	-1,298523833	0,194107392	0,652061359
ENSG00000132356	PRKAA1	protein kinase AMP-activated catalytic subunit alpha 1	5562	513,0769459	0,032663447	0,0784	0,416623023	0,676954166	0,924186156
ENSG00000115239	ASB3	ankyrin repeat and SOCS box containing 3	51130	81,29324763	-0,048296698	0,194374	-0,248472538	0,803768816	0,956774822
ENSG00000115239	GPR75-ASB3	GPR75-ASB3 readthrough	100302652	81,29324763	-0,048296698	0,194374	-0,248472538	0,803768816	0,956774822
ENSG00000174233	ADCY6	adenylate cyclase 6	112	11,70574058	-0,41149031	0,661179	-0,622358147	0,533706395	NA
ENSG00000145337	PYURF	PIGY upstream open reading frame	100996939	169,6123458	0,049523383	0,145362	0,340690354	0,733336701	0,93912057
ENSG00000241258	CRCP	CGRP receptor component	27297	152,5454953	-0,349685045	0,170069	-2,056138889	0,039769131	0,317468275
ENSG00000146066	HIGD2A	HIG1 hypoxia inducible domain family member 2A	192286	132,7588393	0,122576698	0,161831	0,757435222	0,448789161	0,84263906
ENSG00000117139	KDM5B	lysine demethylase 5B	10765	297,2418136	0,344394946	0,38482	0,894951158	0,370813198	0,79977671
ENSG00000224578	HNRNPA1L3	heterogeneous nuclear ribonucleoprotein A1 like 3	642659	11,64471151	0,413783913	0,532601	0,77691137	0,437211063	NA
ENSG00000049656	CLPTM1L	CLPTM1 like	81037	265,2151321	0,039503719	0,323019	0,122295229	0,90266521	0,978460866
ENSG00000082805	ERC1	ELKS/RAB6-interacting/CAST family member 1	23085	98,25624463	-0,025544152	0,268817	-0,095024487	0,924295377	0,984284131
ENSG00000070476	ZXDC	ZXD family zinc finger C	79364	117,7635718	-0,137382324	0,19158	-0,717100218	0,473312261	0,853811879
ENSG00000162735	PEX19	peroxisomal biogenesis factor 19	5824	132,4335672	-0,243288903	0,236681	-1,027917802	0,303988495	0,751870158
ENSG00000090905	TNRC6A	trinucleotide repeat containing adaptor 6A	27327	232,0068194	-0,204012409	0,113976	-1,789954004	0,073461306	0,440767836
ENSG00000274847	MAFIP	MAFF interacting protein	727764	22,5465457	-0,417362505	0,386289	-1,080441379	0,279945678	NA
ENSG00000103051	COG4	component of oligomeric golgi complex 4	25839	107,3401767	-0,301260185	0,533404	-0,564788507	0,572217625	0,886795904
ENSG00000164751	PEX2	peroxisomal biogenesis factor 2	5828	149,1860713	0,077389217	0,213003	0,363325255	0,71636193	0,932040329
ENSG00000285589	NA	NA	NA	54,96537901	0,090180571	0,225198	0,40044927	0,688825641	0,926748702
ENSG00000143322	ABL2	ABL proto-oncogene 2, non-receptor tyrosine kinase	27	87,54199117	0,240704175	0,189285	1,271647373	0,203498437	0,660954946
ENSG00000169598	DFFB	DNA fragmentation factor subunit beta	1677	20,17641791	-0,51532161	1,798226	-0,286572223	0,774439885	NA
ENSG00000214941	ZSWIM7	zinc finger SWIM-type containing 7	125150	17,03089965	0,171939876	0,764746	0,224832684	0,822109439	NA
ENSG00000276231	PIK3R6	phosphoinositide-3-kinase regulatory subunit 6	146850	112,224978	-0,191262262	0,209246	-0,914052864	0,360689069	0,791408368
ENSG00000011258	MBTD1	mbt domain containing 1	54799	146,9860304	-0,497469975	0,152268	-3,267068818	0,001086673	0,037269549
ENSG00000117174	ZNHIT6	zinc finger HIT-type containing 6	54680	166,0859009	0,060719978	0,146695	0,413919278	0,678933225	0,925294441
ENSG00000127989	MTERF1	mitochondrial transcription termination factor 1	7978	59,55429617	-0,243936141	0,374125	-0,652017074	0,514390158	0,868514045
ENSG00000163138	PACRGL	parkin coregulated like	133015	25,7641157	-0,535032373	0,367493	-1,455896209	0,145421314	NA
ENSG00000189079	ARID2	AT-rich interaction domain 2	196528	306,575552	-0,060097679	0,19715	-0,304832364	0,760493831	0,942901768
ENSG00000149150	SLC43A1	solute carrier family 43 member 1	8501	53,58871628	0,060534416	0,258285	0,234370209	0,814697585	0,95938223
ENSG00000256667	NA	NA	NA	128,7613123	0,126639679	0,147632	0,857805607	0,390999813	0,811337525
ENSG00000149782	PLCB3	phospholipase C beta 3	5331	88,47545487	-0,076554038	0,178397	-0,429122116	0,667834358	0,922308959
ENSG00000051009	FHIP1B	FHF complex subunit HOOK interacting protein 1B	84067	137,8389115	-0,086570358	0,187949	-0,460604693	0,645082244	0,912530858
ENSG00000158717	RNF166	ring finger protein 166	115992	254,5438751	-0,037539783	0,1105	-0,339726936	0,734062175	0,939157822
ENSG00000155380	SLC16A1	solute carrier family 16 member 1	6566	13,0290842	1,442879299	3,74177	0,3856141	0,699782489	NA
ENSG00000125734	GPR108	G protein-coupled receptor 108	56927	241,123029	0,172758898	0,118665	1,455857589	0,145431992	0,592746686
ENSG00000223509	NA	NA	NA	111,6258352	-0,074451381	0,177229	-0,420086617	0,674422179	0,923602509
ENSG00000111906	HDDC2	HD domain containing 2	51020	43,91172099	-0,048985897	0,253142	-0,19351183	0,846558142	0,967231052
ENSG00000091592	NLRP1	NLR family pyrin domain containing 1	22861	388,037291	0,142781561	0,123382	1,157228453	0,247179036	0,705258787
ENSG00000150907	FOXO1	forkhead box O1	2308	94,1177851	0,266280444	0,19477	1,367155995	0,171576415	0,629558603
ENSG00000153574	RPIA	ribose 5-phosphate isomerase A	22934	85,70048047	0,121600763	0,225239	0,53987368	0,589284151	0,891760639
ENSG00000164983	TMEM65	transmembrane protein 65	157378	134,6353165	-0,257847323	0,251599	-1,024836472	0,305440354	0,752251032
ENSG00000196683	TOMM7	translocase of outer mitochondrial membrane 7	54543	193,9095026	0,093592986	0,152337	0,614379342	0,538964684	0,875342836

ENSG00000196683	LOC107986346	mitochondrial import receptor subunit TOM7 homolog	107986346	193,9095026	0,093592986	0,152337	0,614379342	0,538964684	0,875342836
ENSG00000235106	BRD3OS	BRD3 opposite strand	266655	50,60319865	0,367666708	0,240656	1,527766723	0,126570467	0,563689485
ENSG00000033327	GAB2	GRB2 associated binding protein 2	9846	71,00229365	-0,20382659	0,225604	-0,903471444	0,366275737	0,79459056
ENSG00000132676	DAP3	death associated protein 3	7818	195,3612464	-0,012473535	0,151021	-0,082594661	0,934173846	0,987098279
ENSG00000156232	WHAMM	WASP homolog associated with actin, golgi membranes and microtubules	123720	124,0114323	-0,097081798	0,160894	-0,603391065	0,546248565	0,878739902
ENSG00000116514	RNF19B	ring finger protein 19B	127544	125,0727253	0,151782756	0,200963	0,755275943	0,45008343	0,84263906
ENSG00000125995	ROMO1	reactive oxygen species modulator 1	140823	27,3002703	0,227251694	0,330143	0,688344094	0,49123612	NA
ENSG00000131196	NFATC1	nuclear factor of activated T cells 1	4772	41,99581718	-0,195378093	0,343239	-0,569218941	0,569207568	NA
ENSG00000164713	BRI3	brain protein I3	25798	28,07525045	0,154483289	0,342851	0,450583997	0,652289403	NA
ENSG00000173457	PPP1R14B	protein phosphatase 1 regulatory inhibitor subunit 14B	26472	67,75618926	-0,203570123	0,22096	-0,921299439	0,356894114	0,789494009
ENSG00000174173	TRMT10C	tRNA methyltransferase 10C, mitochondrial RNase P subunit	54931	55,87667617	-0,254843172	0,250401	-1,017739319	0,308801856	0,754797782
ENSG00000187713	TMEM203	transmembrane protein 203	94107	71,49596471	0,176851516	0,210191	0,84138642	0,400131491	0,814673014
ENSG00000004777	ARHGAP33	Rho GTPase activating protein 33	115703	108,1615182	-0,090672652	0,17963	-0,504774018	0,613717567	0,90080768
ENSG00000136891	TEX10	testis expressed 10	54881	76,83143249	0,272893442	0,212909	1,281736799	0,199934991	0,658954856
ENSG00000150455	TIRAP	TIR domain containing adaptor protein	114609	48,86705023	-0,106756167	0,484757	-0,220226133	0,825695045	0,961917534
ENSG00000185453	ZSWIM9	zinc finger SWIM-type containing 9	374920	37,18313603	-0,103521801	0,278697	-0,371449805	0,710302536	NA
ENSG00000103549	RNF40	ring finger protein 40	9810	319,2946575	-0,160271753	0,095555	-1,677271575	0,093489388	0,496993357
ENSG00000120699	EXOSC8	exosome component 8	11340	60,03377514	0,595229757	0,248547	2,394842191	0,01662752	0,203098821
ENSG00000173511	VEGFB	vascular endothelial growth factor B	7423	39,65968377	-0,166869618	0,352902	-0,472850226	0,636320038	NA
ENSG00000198440	ZNF583	zinc finger protein 583	147949	26,99138369	-0,159167396	0,379702	-0,419190022	0,675077264	NA
ENSG00000083544	TDRD3	tudor domain containing 3	81550	89,99442193	0,063708672	0,176883	0,360174213	0,718716857	0,932886847
ENSG00000163795	ZNF513	zinc finger protein 513	130557	79,90428358	-0,349765517	0,223341	-1,5660588	0,117334852	0,546264333
ENSG00000100445	SDR39U1	short chain dehydrogenase/reductase family 39U member 1	56948	16,48698752	-1,015144357	1,0045	-1,01059636	0,312209659	NA
ENSG00000097033	SH3GLB1	SH3 domain containing GRB2 like, endophilin B1	51100	444,7713461	0,064282682	0,092197	0,697235392	0,485655494	0,858001067
ENSG00000104419	NDRG1	N-myc downstream regulated 1	10397	102,9278512	0,024812832	0,16475	0,150609359	0,880283878	0,976300778
ENSG00000110104	CCDC86	coiled-coil domain containing 86	79080	44,24346223	-0,188797833	0,260176	-0,725655522	0,468049975	0,851339954
ENSG00000153561	RMND5A	required for meiotic nuclear division 5 homolog A	64795	246,8725837	-0,049111836	0,126255	-0,388990074	0,697283489	0,930422107
ENSG00000167862	MRPL58	mitochondrial ribosomal protein L58	3396	39,30643623	0,01050793	0,270502	0,038846061	0,969013121	NA
ENSG00000020256	ZFP64	ZFP64 zinc finger protein	55734	50,23313341	-0,147421751	0,261575	-0,563592227	0,573031678	0,886892687
ENSG00000105887	MTPN	myotrophin	136319	903,9686129	-0,009045357	0,071735	-0,126093837	0,899657646	0,977990819
ENSG00000121406	ZNF549	zinc finger protein 549	256051	78,97670558	-0,070064524	0,182968	-0,382934181	0,701768567	0,930846779
ENSG00000080947	CROCCP3	CROCC pseudogene 3	114819	13,25181499	0,188308282	0,483588	0,389397897	0,696981827	NA
ENSG00000109184	DCUN1D4	defective in cullin neddylation 1 domain containing 4	23142	39,06797485	-0,215334628	0,371298	-0,579951227	0,561947509	NA
ENSG00000111364	DDX55	DEAD-box helicase 55	57696	82,32301495	0,000650084	0,215443	0,003017435	0,997592439	0,999300722
ENSG00000122707	RECK	reversion inducing cysteine rich protein with kazal motifs	8434	70,87677044	-0,095270099	0,201491	-0,472826137	0,636337225	0,909185477
ENSG00000075336	TIMM21	translocase of inner mitochondrial membrane 21	29090	18,96000784	0,252771208	0,529147	0,477695601	0,632866875	NA
ENSG00000086232	EIF2AK1	eukaryotic translation initiation factor 2 alpha kinase 1	27102	482,0699745	0,087619283	0,110952	0,789702021	0,429701811	0,832060923
ENSG00000107829	FBXW4	F-box and WD repeat domain containing 4	6468	95,32023679	-0,000862658	0,22219	-0,003882528	0,996902199	0,999300722
ENSG00000125846	ZNF133	zinc finger protein 133	7692	42,48122271	-0,396544523	0,304253	-1,303337998	0,192459394	0,649982152
ENSG00000138303	ASCC1	activating signal cointegrator 1 complex subunit 1	51008	80,85294434	0,174762301	0,284699	0,613848407	0,539315506	0,875342836
ENSG00000159377	PSMB4	proteasome 20S subunit beta 4	5692	283,5729313	0,080094277	0,113363	0,706529081	0,479859174	0,854606339
ENSG00000163462	TRIM46	tripartite motif containing 46	80128	22,59419483	0,018403504	0,465314	0,039550713	0,968451322	NA
ENSG00000164040	PGRMC2	progesterone receptor membrane component 2	10424	120,8146472	-0,111206013	0,247723	-0,448913361	0,653494158	0,917134445
ENSG00000171793	CTPS1	CTP synthase 1	1503	83,91727379	-0,262356291	0,376453	-0,696915908	0,485855422	0,858001067
ENSG00000174799	CEP135	centrosomal protein 135	9662	99,64551486	0,062802999	0,174587	0,35972354	0,719053887	0,933054629
ENSG00000181873	IBA57	iron-sulfur cluster assembly factor IBA57	200205	65,36218359	-0,073641327	0,250099	-0,294448683	0,768415071	0,944760797

ENSG00000188976	NOC2L	NOC2 like nucleolar associated transcriptional repressor	26155	141,232022	-0,107229218	0,167758	-0,639190526	0,522698994	0,870221868
ENSG00000071564	TCF3	transcription factor 3	6929	148,928288	-0,231492044	0,149097	-1,552625473	0,120512636	0,554378286
ENSG00000079257	LXN	latexin	56925	15,97111137	0,05531343	0,456649	0,121128953	0,903588897	NA
ENSG00000134440	NARS1	asparaginyl-tRNA synthetase 1	4677	400,9022786	0,054735292	0,120634	0,453728761	0,650024063	0,914926418
ENSG00000144560	VGLL4	vestigial like family member 4	9686	129,1045412	-0,02820529	0,149876	-0,188190428	0,850727376	0,967902179
ENSG00000267312	NA	NA	NA	16,10954312	-0,272858764	0,507309	-0,537854964	0,590677183	NA
ENSG00000162813	BPNT1	3'(2'), 5'-bisphosphate nucleotidase 1	10380	85,86812958	0,215183756	0,228335	0,942405436	0,345985105	0,782042713
ENSG00000117036	ETV3	ETS variant transcription factor 3	2117	305,7799707	-0,037621555	0,103548	-0,363323894	0,716362946	0,932040329
ENSG00000185104	FAF1	Fas associated factor 1	11124	108,3860656	0,229881358	0,195935	1,17325589	0,240693205	0,695620773
ENSG00000076662	ICAM3	intercellular adhesion molecule 3	3385	587,0933416	-0,028097206	0,106984	-0,26262984	0,792835898	0,95213045
ENSG00000170270	GON7	GON7 subunit of KEOPS complex	84520	17,17157622	-1,456630202	2,257779	-0,64516068	0,518823064	NA
ENSG00000171310	CHST11	carbohydrate sulfotransferase 11	50515	667,4898871	-0,023912096	0,085979	-0,278114486	0,780924476	0,948265435
ENSG00000158792	SPATA2L	spermatogenesis associated 2 like	124044	18,34589031	-0,13635335	0,415414	-0,328234606	0,742734284	NA
ENSG00000198561	CTNND1	catenin delta 1	1500	30,75054829	-0,57158304	0,574232	-0,995386791	0,31954818	NA
ENSG00000185090	MANEAL	mannosidase endo-alpha like	149175	15,14470316	-0,178454778	0,506702	-0,352188969	0,724696549	NA
ENSG00000104915	STX10	syntaxin 10	8677	74,77205112	-0,103800267	0,215077	-0,482618684	0,62936651	0,905567722
ENSG00000113013	HSPA9	heat shock protein family A (Hsp70) member 9	3313	506,0801405	0,089428365	0,085811	1,042154096	0,297340241	0,748371252
ENSG00000128524	ATP6V1F	ATPase H+ transporting V1 subunit F	9296	112,3257768	0,011160605	0,172852	0,064567383	0,948518455	0,990608484
ENSG00000155744	HYCC2	hyccin PI4KA lipid kinase complex subunit 2	285172	357,1749406	-0,102271026	0,100162	-1,021057555	0,307227171	0,754087936
ENSG00000152942	RAD17	RAD17 checkpoint clamp loader component	5884	46,10584053	0,189582956	1,175003	0,161346721	0,871820327	0,974150353
ENSG00000107949	BCCIP	BRCA2 and CDKN1A interacting protein	56647	131,8870158	0,186504619	0,153661	1,213738249	0,224847693	0,680155176
ENSG00000081014	AP4E1	adaptor related protein complex 4 subunit epsilon 1	23431	196,0999448	-0,040472986	0,124238	-0,325770533	0,744597975	0,939745744
ENSG00000133597	ADCK2	aarF domain containing kinase 2	90956	37,12400408	0,294548651	0,341431	0,862687876	0,3883091	NA
ENSG00000175691	ZNF77	zinc finger protein 77	58492	17,30362183	0,319288577	0,452146	0,706163167	0,480086673	NA
ENSG00000160194	NDUFV3	NADH:ubiquinone oxidoreductase subunit V3	4731	95,5933543	-0,253267534	0,181382	-1,396324461	0,162616811	0,620297442
ENSG00000181523	SGSH	N-sulfoglucosamine sulfohydrolase	6448	20,95865255	0,311166456	2,423913	0,12837359	0,89785333	NA
ENSG00000118454	ANKRD13C	ankyrin repeat domain 13C	81573	252,4870117	0,049851942	0,121019	0,411934619	0,680387345	0,925586358
ENSG00000114503	NCBP2	nuclear cap binding protein subunit 2	22916	184,6892236	0,007064885	0,134994	0,052334921	0,958261829	0,991911599
ENSG00000204427	ABHD16A	abhydrolase domain containing 16A, phospholipase	7920	64,57400655	0,116467367	0,328724	0,354300892	0,723113399	0,934902053
ENSG00000179262	RAD23A	RAD23 homolog A, nucleotide excision repair protein	5886	192,6515906	-0,121850546	0,144887	-0,841002955	0,400346279	0,814673014
ENSG00000136051	WASHC4	WASH complex subunit 4	23325	406,7395147	-0,234966164	0,118367	-1,985059845	0,047137814	0,348278822
ENSG00000144231	POLR2D	RNA polymerase II subunit D	5433	174,181114	-0,009815096	0,164102	-0,059810969	0,952306189	0,991212866
ENSG00000172183	ISG20	interferon stimulated exonuclease gene 20	3669	83,83962568	-0,126131078	0,195342	-0,645694494	0,518477227	0,868791049
ENSG00000018189	RUFY3	RUN and FYVE domain containing 3	22902	110,4914982	0,214445691	0,161594	1,32706771	0,184486285	0,641130658
ENSG00000066651	TRMT11	tRNA methyltransferase 11 homolog	60487	26,63917212	0,212563036	0,735183	0,289129379	0,772482372	NA
ENSG00000142327	RNPEPL1	arginyl aminopeptidase like 1	57140	186,3911474	-0,063988492	0,147063	-0,435108492	0,66348368	0,920996964
ENSG00000161547	SRSF2	serine and arginine rich splicing factor 2	6427	586,0818617	0,038459747	0,095928	0,400921495	0,688477924	0,926748702
ENSG00000135686	KLHL36	kelch like family member 36	79786	291,0213247	-0,074479825	0,115146	-0,646831442	0,51774104	0,868791049
ENSG00000138463	SLC49A4	solute carrier family 49 member 4	84925	12,9634029	0,079552137	0,49865	0,159534988	0,873247395	NA
ENSG00000081870	IFT25	intraflagellar transport 25	51668	72,08505279	-0,039425755	0,198927	-0,19819165	0,84289512	0,967210584
ENSG00000175220	ARHGAP1	Rho GTPase activating protein 1	392	438,8503847	-0,060768461	0,085792	-0,708323223	0,478744559	0,854008769
ENSG00000103540	CCP110	centriolar coiled-coil protein 110	9738	126,5169814	0,004150343	0,170743	0,024307585	0,980607263	0,995752963
ENSG00000132406	TMEM128	transmembrane protein 128	85013	47,44364325	-0,066668533	0,275785	-0,241741046	0,808980822	0,958764958
ENSG00000132485	ZRANB2	zinc finger RANBP2-type containing 2	9406	338,2256088	-0,030006787	0,112265	-0,267285079	0,789249674	0,95148885
ENSG00000164597	COG5	component of oligomeric golgi complex 5	10466	102,1512042	-0,696032885	0,233745	-2,977742485	0,002903798	0,070337762

ENSG00000197345	MRPL21	mitochondrial ribosomal protein L21	219927	33,49196812	0,088353148	0,307326	0,287490051	0,773737118	NA
ENSG00000256664	NA	NA	NA	13,68976693	-0,959695766	0,569059	-1,686459752	0,091707281	NA
ENSG00000198865	CCDC152	coiled-coil domain containing 152	100129792	19,42649722	-0,130028835	0,445706	-0,291736604	0,77048802	NA
ENSG00000182511	FES	FES proto-oncogene, tyrosine kinase	2242	93,94341256	-0,367401746	0,182931	-2,00842251	0,044598413	0,339086093
ENSG00000006125	AP2B1	adaptor related protein complex 2 subunit beta 1	163	1087,717053	0,006393189	0,054378	0,117570229	0,906408195	0,978516824
ENSG00000026950	BTN3A1	butyrophilin subfamily 3 member A1	11119	431,8863636	-0,160172649	0,097956	-1,635147552	0,10201812	0,515062371
ENSG00000125877	ITPA	inosine triphosphatase	3704	89,57078757	0,16955607	0,192164	0,882350541	0,377587273	0,804050464
ENSG00000151229	SLC2A13	solute carrier family 2 member 13	114134	66,66483025	0,16334119	0,239267	0,682672369	0,494813893	0,860513824
ENSG00000153147	SMARCA5	SWI/SNF related, matrix associated, actin dependent regulator of chromatin, subfamily a, member 5	8467	659,4953157	0,080145757	0,065108	1,230975492	0,218332031	0,673519155
ENSG00000162398	CIMAP2	ciliary microtubule associated protein 2	163747	21,99609856	-0,173120124	0,406701	-0,425669099	0,670348982	NA
ENSG00000167969	ECI1	enoyl-CoA delta isomerase 1	1632	26,10644393	0,001031849	0,502342	0,002054078	0,998361084	NA
ENSG00000168765	GSTM4	glutathione S-transferase mu 4	2948	36,94957873	-0,147380768	0,327363	-0,450205579	0,652562214	NA
ENSG00000173214	MFSD4B	major facilitator superfamily domain containing 4B	91749	154,4775879	0,01954478	0,458897	0,042590786	0,96602774	0,992789539
ENSG00000187049	TMEM216	transmembrane protein 216	51259	42,8873173	-0,27806197	0,286907	-0,969171945	0,332459406	0,773425407
ENSG00000197601	FAR1	fatty acyl-CoA reductase 1	84188	177,5990013	-0,300214166	0,282882	-1,06126891	0,28856771	0,740096492
ENSG00000198242	RPL23A	ribosomal protein L23a	6147	382,9145234	-0,202476319	0,120875	-1,6750917	0,093916241	0,49738244
ENSG00000014919	COX15	cytochrome c oxidase assembly homolog COX15	1355	130,931313	-0,413704281	0,193182	-2,141520632	0,032232077	0,287228571
ENSG00000124541	RRP36	ribosomal RNA processing 36	88745	110,0988586	0,143404598	0,164229	0,8731964	0,382556037	0,807686858
ENSG00000101945	SUV39H1	SUV39H1 histone lysine methyltransferase	6839	73,35152043	0,039889683	0,201576	0,197888881	0,843132004	0,967210584
ENSG00000104365	IKBKB	inhibitor of nuclear factor kappa B kinase subunit beta	3551	148,3995256	-0,309543799	0,469307	-0,659576513	0,509525631	0,866180216
ENSG00000142002	DPP9	dipeptidyl peptidase 9	91039	642,5135074	0,146387857	0,103878	1,40922844	0,158767631	0,614461481
ENSG00000197381	ADARB1	adenosine deaminase RNA specific B1	104	687,1660263	0,031593204	0,099525	0,317438394	0,750910979	0,941512988
ENSG00000242338	NA	NA	NA	23,01313469	-0,349311149	0,391018	-0,893337894	0,371676251	NA
ENSG00000049246	PER3	period circadian regulator 3	8863	27,2038874	0,319437121	0,438073	0,729187444	0,465887011	NA
ENSG00000107798	LIPA	lipase A, lysosomal acid type	3988	238,2936943	0,04709907	0,10617	0,443620137	0,657317244	0,919088619
ENSG00000184752	NDUFA12	NADH:ubiquinone oxidoreductase subunit A12	55967	168,7987816	0,185650534	0,133032	1,395535608	0,162854385	0,620902105
ENSG00000256043	CTSO	cathepsin O	1519	63,14861835	-0,942632788	0,702121	-1,34254945	0,179417908	0,633591125
ENSG00000023697	DERA	deoxyribose-phosphate aldolase	51071	71,70980769	0,246632444	0,202605	1,217306043	0,223487796	0,67869714
ENSG00000129347	KRI1	KRI1 homolog	65095	267,2135681	0,011830906	0,134861	0,087726984	0,930093673	0,986487751
ENSG00000138018	SELENOI	selenoprotein I	85465	142,8581196	0,080075879	0,136452	0,586843163	0,557309045	0,883921037
ENSG00000143033	MTF2	metal response element binding transcription factor 2	22823	183,2466787	0,300174509	0,145628	2,061247282	0,039279455	0,316086925
ENSG00000161057	PSMC2	proteasome 26S subunit, ATPase 2	5701	206,959482	0,281105664	0,16949	1,658537759	0,097208973	0,504697799
ENSG00000164402	SEPTIN8	septin 8	23176	59,56705905	0,14027103	0,225496	0,622056161	0,53390494	0,873238108
ENSG00000185608	MRPL40	mitochondrial ribosomal protein L40	64976	28,64961229	-0,018917305	0,320613	-0,05900364	0,952949209	NA
ENSG00000034713	GABARAPL2	GABA type A receptor associated protein like 2	11345	114,711426	0,161218558	0,17198	0,93742646	0,348539235	0,784460486
ENSG00000049860	HEXB	hexosaminidase subunit beta	3074	106,1707072	-0,28076226	0,191169	-1,468657038	0,141925836	0,586367972
ENSG00000116017	ARID3A	AT-rich interaction domain 3A	1820	63,8805998	-0,171297239	0,263089	-0,651099649	0,514982163	0,868514045
ENSG00000127914	AKAP9	A-kinase anchoring protein 9	10142	601,8789129	0,243315411	0,131457	1,85090701	0,064182932	0,408172266
ENSG00000141664	ZCCHC2	zinc finger CCHC-type containing 2	54877	215,9586661	-0,041528215	0,136755	-0,303668351	0,761380572	0,942901768
ENSG00000163001	CFAP36	cilia and flagella associated protein 36	112942	16,25375885	0,382410898	0,523846	0,730006016	0,465386508	NA
ENSG00000165879	FRAT1	FRAT regulator of WNT signaling pathway 1	10023	29,21414388	0,151225535	0,366851	0,412225595	0,680174078	NA
ENSG00000166169	POLL	DNA polymerase lambda	27343	46,19453681	-0,198045376	0,373465	-0,530291262	0,595910004	0,894229873
ENSG00000166902	MRPL16	mitochondrial ribosomal protein L16	54948	102,8970111	0,229093353	0,181225	1,264138718	0,206180242	0,660954946
ENSG00000169762	TAPT1	transmembrane anterior posterior transformation 1	202018	80,58297975	-0,241515729	0,21205	-1,138957581	0,254720847	0,709964783
ENSG00000173531	MST1	macrophage stimulating 1	4485	14,07489126	-0,15127978	0,570024	-0,265391896	0,790707579	NA
ENSG00000176973	FAM89B	family with sequence similarity 89 member B	23625	112,8618321	0,187103573	0,155926	1,199954249	0,230157109	0,684730271
ENSG00000203965	EFCAB7	EF-hand calcium binding domain 7	84455	17,9526509	-0,324544675	0,452223	-0,717665726	0,472963421	NA
ENSG00000213463	SYNJ2BP	synaptojanin 2 binding protein	55333	103,4199862	-0,054078002	0,190022	-0,284588069	0,775959753	0,946448613

ENSG00000213676	ATF6B	activating transcription factor 6 beta	1388	37,6273082	-0,421644567	0,398842	-1,057171299	0,290433416	NA	
ENSG00000267645	NA	NA	NA	60,41901084	0,058615541	0,421135	0,139184715	0,889304187	0,977990819	
ENSG00000272410	NA	NA	NA	54,75422774	-0,0993781	0,816571	-0,121701716	0,903135254	0,978460866	
ENSG00000163126	ANKRD23	ankyrin repeat domain 23	200539	18,67790587	-0,882974014	0,813435	-1,085488719	0,277705257	NA	
ENSG00000105325	FZR1	fizzy and cell division cycle 20 related 1	51343	205,8040035	-0,232403189	0,160847	-1,444874336	0,148493186	0,601349859	
ENSG00000167985	SDHAF2	succinate dehydrogenase complex assembly factor 2	54949	87,43386857	0,417880455	0,20372	2,051245068	0,040243087	0,319584636	
ENSG00000138439	FAM117B	family with sequence similarity 117 member B	150864	28,49704848	-0,260426502	0,37451	-0,695380174	0,486817082	NA	
ENSG00000288558	DUS4L-BCAP29	DUS4L-BCAP29 readthrough	115253422	35,0233672	1,238992402	1,768465	0,700603485	0,483550503	NA	
ENSG00000116260	QSOX1	quiescin sulfhydryl oxidase 1	5768	367,9076136	0,14505833	0,09822	1,476867802	0,139711077	0,582426115	
ENSG00000135424	ITGA7	integrin subunit alpha 7	3679	25,18576727	-1,353314362	1,200877	-1,126937951	0,259768716	NA	
ENSG00000141646	SMAD4	SMAD family member 4	4089	124,7387141	-0,757541636	0,53739	-1,409667407	0,158637913	0,614461481	
ENSG00000148399	DPH7	diphthamide biosynthesis 7	92715	45,59775384	0,053554943	0,350672	0,152721001	0,878618298	0,976237908	
ENSG00000159733	ZFYVE28	zinc finger FYVE-type containing 28	57732	135,2557202	0,115905557	0,151925	0,762914947	0,445514121	0,840933482	
ENSG00000168137	SETD5	SET domain containing 5	55209	485,4709801	-0,264347947	0,101437	-2,60602009	0,009160109	0,146613742	
ENSG00000137074	APTX	aprataxin	54840	34,42591753	-0,186961284	0,391779	-0,477210778	0,633212037	NA	
ENSG00000155827	RNF20	ring finger protein 20	56254	239,9157256	-0,041651453	0,110963	-0,375363002	0,707390516	0,930846779	
ENSG00000173221	GLRX	glutaredoxin	2745	127,6297201	0,160720241	0,163912	0,9805259	0,326826592	0,769687654	
ENSG00000177963	RIC8A	RIC8 guanine nucleotide exchange factor A	60626	262,2329922	0,117200804	0,118057	0,992744151	0,320834653	0,764513157	
ENSG00000211689	TRGC1	T cell receptor gamma constant 1	6966	162,4805555	-0,207713439	0,150953	-1,376018098	0,16881603	0,628354709	
ENSG00000211689	TARP	TCR gamma alternate reading frame protein	445347	162,4805555	-0,207713439	0,150953	-1,376018098	0,16881603	0,628354709	
ENSG00000167671	UBXN6	UBX domain protein 6	80700	144,7410427	0,031756164	0,142404	0,222999807	0,823535657	0,961917534	
ENSG00000013573	DDX11	DEAD/H-box helicase 11	1663	146,9463283	0,212261319	0,18009	1,178639388	0,2385418	0,693537104	
ENSG00000162302	RPS6KA4	ribosomal protein S6 kinase A4	8986	132,3125814	-0,042448101	0,190147	-0,223238162	0,823350152	0,961917534	
ENSG00000182621	PLCB1	phospholipase C beta 1	23236	29,18025258	0,144685189	0,391886	0,369202086	0,711977101	NA	
ENSG00000182621	LOC105372522	uncharacterized LOC105372522	105372522	29,18025258	0,144685189	0,391886	0,369202086	0,711977101	NA	
ENSG00000138107	ACTR1A	actin related protein 1A	10121	316,0147953	0,059733528	0,103779	0,575584256	0,564896225	0,885045179	
ENSG00000140396	NCOA2	nuclear receptor coactivator 2	10499	698,6081518	-0,135970107	0,082427	-1,649585233	0,099027797	0,509429216	
ENSG00000151093	OXSM	3-oxoacyl-ACP synthase, mitochondrial	54995	15,84538788	0,184589901	0,601349	0,306959884	0,758873907	NA	
ENSG00000151348	EXT2	exostosin glycosyltransferase 2	2132	131,6080669	0,024958782	0,203225	0,122813625	0,902254685	0,978453541	
ENSG00000167468	GPX4	glutathione peroxidase 4	2879	200,8499852	-0,040992675	0,125118	-0,32763293	0,74318922	0,939634278	
ENSG00000181191	PJA1	praja ring finger ubiquitin ligase 1	64219	84,6058732	0,078211544	0,201011	0,389091045	0,697208797	0,930422107	
ENSG00000186638	KIF24	kinesin family member 24	347240	27,62578119	0,293981703	0,32313	0,909792733	0,362931828	NA	
ENSG00000232434	AJM1	apical junction component 1 homolog	389813	56,81952498	-0,322210101	0,233226	-1,381536907	0,167113938	0,62703197	
ENSG00000239857	GET4	guided entry of tail-anchored proteins factor 4	51608	90,70046933	-0,091652099	0,211968	-0,432385671	0,665461131	0,922184616	
ENSG00000198406	NA	NA	NA	79,03231934	0,686851152	0,37926	1,811028339	0,070136465	0,430243114	
ENSG00000006625	GGCT	gamma-glutamylcyclotransferase	79017	58,41674083	0,114847529	0,224269	0,512096346	0,608583582	0,897640621	
ENSG00000103254	ANTKMT	adenine nucleotide translocase lysine methyltransferase	65990	16,35210703	0,190252343	0,522128	0,364378869	0,715575113	NA	
ENSG00000256660	CLEC12B	C-type lectin domain family 12 member B	387837	35,86289182	0,315548645	0,298191	1,058209681	0,289959858	NA	
ENSG00000131584	ACAP3	ArfGAP with coiled-coil, ankyrin repeat and PH domains 3	116983	70,90442885	-0,055105596	0,276418	-0,199356328	0,84198402	0,966959288	
ENSG00000189266	PNRC2	proline rich nuclear receptor coactivator 2	55629	469,8957725	-0,240259595	0,084041	-2,858844257	0,004251875	0,090254661	
ENSG00000182919	C11orf54	chromosome 11 open reading frame 54	28970	81,01784385	0,083374359	0,223228	0,373494119	0,708780722	0,930846779	
ENSG00000243989	ACY1	aminoacylase 1	95	11,89592223	-0,225025049	2,172147	-0,103595667	0,917490227	NA	
ENSG00000265354	TIMM23	translocase of inner mitochondrial membrane 23	100287932	99,80135584	0,151349919	0,175199	0,863874501	0,387656836	0,809653368	
ENSG00000085871	MGST2	microsomal glutathione S-transferase 2	4258	21,08366246	-0,323840283	0,390336	-0,829644721	0,406739684	NA	
ENSG00000101654	RNMT	RNA guanine-7 methyltransferase	8731	227,8767437	-0,165587689	0,124288	-1,332294979	0,182763276	0,637116189	
ENSG00000122481	RWDD3	RWD domain containing 3	25950	19,93366173	-0,247205648	0,422057	-0,585715911	0,55806644	NA	
ENSG00000070882	OSBPL3	oxysterol binding protein like 3	26031	640,225298	-0,016382252	0,116678	-0,140405605	0,888339529	0,977990819	
ENSG00000100526	CDKN3	cyclin dependent kinase inhibitor 3	1033	18,61748259	0,279839591	0,426251	0,656513165	0,511493999	NA	
ENSG00000125657	TNFSF9	TNF superfamily member 9	8744	15,08265277	0,616486956	0,500616	1,231456227	0,218152279	NA	
ENSG00000100307	CBX7	chromobox 7	23492	84,24707618	-0,314651744	0,190685	-1,650109476	0,098920547	0,509429216	

ENSG00000011600	TYROBP	transmembrane immune signaling adaptor TYROBP	7305	342,3359804	-0,007084821	0,106501	-0,066523406	0,946961123	0,990073571
ENSG00000112984	KIF20A	kinesin family member 20A	10112	65,7862281	0,517750268	0,238134	2,174201709	0,02968999	0,278929644
ENSG00000113070	HBEGF	heparin binding EGF like growth factor	1839	13,08617287	0,500547737	0,465961	1,074227395	0,282720777	NA
ENSG00000143013	LMO4	LIM domain only 4	8543	309,9114579	0,174182525	0,100326	1,736163869	0,082534864	0,467697562
ENSG00000152133	GPATCH11	G-patch domain containing 11	253635	105,326642	-0,062768487	0,16802	-0,373577097	0,708718976	0,930846779
ENSG00000165282	PIGO	phosphatidylinositol glycan anchor biosynthesis class O	84720	26,45658517	-0,623690214	0,746172	-0,835853044	0,403237581	NA
ENSG00000170471	RALGAPB	Ral GTPase activating protein non-catalytic subunit beta	57148	539,202404	0,094175554	0,101832	0,924811314	0,355064064	0,788341988
ENSG00000175602	CCDC85B	coiled-coil domain containing 85B	11007	58,22790953	-0,00166949	0,256898	-0,006498647	0,994814867	0,998500439
ENSG00000172650	AGAP5	ArfGAP with GTPase domain, ankyrin repeat and PH domain 5	729092	50,88942581	-0,229559617	0,239935	-0,956757922	0,338689455	0,777431905
ENSG00000112149	CD83	CD83 molecule	9308	59,75187632	0,511638364	0,246221	2,077965064	0,037712577	0,307894574
ENSG00000121749	TBC1D15	TBC1 domain family member 15	64786	172,6625409	0,27193254	0,19023	1,429496653	0,152861536	0,609428683
ENSG00000163814	CDCP1	CUB domain containing protein 1	64866	363,9996419	0,059483757	0,123682	0,480942679	0,630557239	0,905960607
ENSG00000126457	PRMT1	protein arginine methyltransferase 1	3276	268,3830111	0,003893327	0,136726	0,028475428	0,977282966	0,995291586
ENSG00000128607	KLHDC10	kelch domain containing 10	23008	136,9689776	-0,12864651	0,145428	-0,884603795	0,376370359	0,803373958
ENSG00000188493	ACTMAP	actin maturation protease	284325	61,11241294	0,077225309	0,237209	0,325557904	0,744758866	0,939745744
ENSG00000168005	SPINDOC	spindlin interactor and repressor of chromatin binding	144097	61,26336555	-0,455157804	0,24665	-1,845355368	0,064985861	0,411280381
ENSG00000272391	POM121C	POM121 transmembrane nucleoporin C	100101267	531,804953	-0,031755666	0,110774	-0,286669999	0,774365011	0,946448613
ENSG00000170502	NUDT9	nudix hydrolase 9	53343	36,39840459	0,331793605	0,320132	1,036427032	0,300002965	NA
ENSG00000198689	SLC9A6	solute carrier family 9 member A6	10479	98,28598356	-0,067956635	0,259811	-0,261561674	0,793659396	0,952276252
ENSG00000134905	CARS2	cysteinyl-tRNA synthetase 2, mitochondrial	79587	80,72125487	0,266423813	0,198411	1,342790076	0,179339958	0,633591125
ENSG00000160877	NACC1	nucleus accumbens associated 1	112939	135,9553904	-0,438780031	1,424614	-0,307999314	0,758082854	0,942901768
ENSG00000187676	B3GLCT	beta 3-glucosyltransferase	145173	46,94608395	0,127122033	0,271753	0,467784837	0,639938465	0,910851161
ENSG00000197114	ZGPAT	zinc finger CCCH-type and G-patch domain containing	84619	52,90171426	-0,236796841	0,230246	-1,028452129	0,303737198	0,751870158
ENSG00000065911	MTHFD2	methylenetetrahydrofolate dehydrogenase (NADP+ dependent) 2, methenyltetrahydrofolate cyclohydrolase	10797	227,1668299	-0,343229723	0,498116	-0,689055977	0,490788042	0,860513824
ENSG00000086712	TXLNG	taxilin gamma	55787	131,393501	0,06224304	0,146544	0,424738341	0,67102743	0,922645883
ENSG00000132382	MYBBP1A	MYB binding protein 1a	10514	190,1829633	-0,237573025	0,141167	-1,68291931	0,092390713	0,4945733
ENSG00000010270	STARD3NL	STARD3 N-terminal like	83930	120,5646412	0,086069441	0,18172	0,473636509	0,635759136	0,909036096
ENSG00000104812	GYS1	glycogen synthase 1	2997	212,109105	0,015191997	0,139171	0,109160618	0,913075096	0,980488351
ENSG00000119688	ABCD4	ATP binding cassette subfamily D member 4	5826	21,22274516	-2,548992513	0,650302	-3,919704717	8,87E-05	NA
ENSG00000127399	LRRC61	leucine rich repeat containing 61	65999	21,26541637	0,330791193	0,417993	0,791380514	0,428721977	NA
ENSG00000145476	CYP4V2	cytochrome P450 family 4 subfamily V member 2	285440	131,3991013	-0,077483737	0,144719	-0,535407539	0,592368078	0,893157782
ENSG00000149639	MTCL2	microtubule crosslinking factor 2	140710	25,17776069	0,308239301	0,345149	0,89306253	0,371823688	NA
ENSG00000156471	PTDSS1	phosphatidylserine synthase 1	9791	91,81112374	-0,023370508	0,317374	-0,073637049	0,94129919	0,988441237
ENSG00000182923	CEP63	centrosomal protein 63	80254	45,86623314	0,862543691	0,533141	1,617852295	0,105694433	0,522649494
ENSG00000183891	TTC32	tetratricopeptide repeat domain 32	130502	12,62435966	0,279786384	0,581853	0,480853732	0,630620458	NA
ENSG00000064932	SBNO2	strawberry notch homolog 2	22904	197,4992798	-0,325939603	0,136555	-2,386873804	0,016992323	0,204616219
ENSG00000128534	LSM8	LSM8 homolog, U6 small nuclear RNA associated	51691	256,8418251	0,161377077	0,105164	1,534534478	0,124898218	0,5624706
ENSG00000141560	FN3KRP	fructosamine 3 kinase related protein	79672	95,43264801	-0,038039246	0,17624	-0,215837799	0,829114184	0,962723654
ENSG00000079482	OPHN1	oligophrenin 1	4983	89,63666183	0,381709377	0,241157	1,582822669	0,113461888	0,53909841
ENSG00000125388	GRK4	G protein-coupled receptor kinase 4	2868	13,71617801	0,050429665	0,59101	0,085327889	0,93200072	NA
ENSG00000144648	ACKR2	atypical chemokine receptor 2	1238	19,13206785	-0,634843506	0,414151	-1,532880204	0,125305374	NA
ENSG00000266714	MYO15B	myosin XVB	80022	65,95765268	-0,795686424	1,34632	-0,591008469	0,554514747	0,882854594
ENSG00000100296	THOC5	THO complex subunit 5	8563	145,4496935	-0,010040514	0,154482	-0,064994667	0,948178247	0,990608484
ENSG00000116698	SMG7	SMG7 nonsense mediated mRNA decay factor	9887	455,2685254	-0,038030002	0,086486	-0,439724416	0,660136717	0,919368176
ENSG00000131051	RBM39	RNA binding motif protein 39	9584	280,2807067	-0,036474569	0,322805	-0,112992439	0,910036549	0,979548024
ENSG00000138835	RGS3	regulator of G protein signaling 3	5998	270,0555215	-0,009501487	0,107252	-0,088590153	0,929407634	0,986487751

ENSG00000198131	ZNF544	zinc finger protein 544	27300	70,25126453	0,10168621	0,342751	0,296676425	0,766713557	0,944698783
ENSG00000269001	NA	NA	NA	31,18894674	-0,311593519	0,318569	-0,978104279	0,328022752	NA
ENSG00000111790	FGFR1OP2	FGFR1 oncogene partner 2	26127	431,6009067	0,150386249	0,085349	1,762020678	0,078065804	0,452159899
ENSG00000079246	XRCC5	X-ray repair cross complementing 5	7520	1154,291925	0,130579028	0,059149	2,207635458	0,027269695	0,268177037
ENSG00000103485	QPRT	quinolinate phosphoribosyltransferase	23475	14,37403968	0,170956099	0,4974	0,343699431	0,731072339	NA
ENSG00000133678	TMEM254	transmembrane protein 254	80195	32,20112551	-0,663254245	1,059403	-0,626064387	0,53127271	NA
ENSG00000142684	ZNF593	zinc finger protein 593	51042	19,68098029	-0,183005778	0,438071	-0,417754036	0,67612696	NA
ENSG00000156171	DRAM2	DNA damage regulated autophagy modulator 2	128338	90,31618546	0,012573489	0,229255	0,054845058	0,956261903	0,991744485
ENSG00000239704	CDRT4	CMT1A duplicated region transcript 4	284040	21,93022816	0,129473367	3,199418	0,040467789	0,967720187	NA
ENSG00000135317	SNX14	sorting nexin 14	57231	147,5002544	0,290019675	0,491628	0,589917041	0,555246269	0,882854594
ENSG00000253710	ALG11	ALG11 alpha-1,2-mannosyltransferase	440138	149,5779418	-1,138685209	0,298717	-3,811925185	0,000137889	0,008330594
ENSG00000142396	ERVK3-1	endogenous retrovirus group K3 member 1	105372481	56,73286693	-0,313074872	0,354155	-0,884004257	0,376693915	0,803373958
ENSG00000111596	CNOT2	CCR4-NOT transcription complex subunit 2	4848	312,8540134	-0,056139404	0,097912	-0,573367992	0,566395558	0,88521771
ENSG00000133706	LARS1	leucyl-tRNA synthetase 1	51520	340,1712613	0,346956507	0,160056	2,167719304	0,03018005	0,279077601
ENSG00000151332	MBIP	MAP3K12 binding inhibitory protein 1	51562	53,09247089	0,094066026	0,278888	0,337289164	0,735898927	0,939157822
ENSG00000167261	DPEP2	dipeptidase 2	64174	11,76660376	-0,86895024	0,60387	-1,438969455	0,150159177	NA
ENSG00000139546	TARBP2	TARBP2 subunit of RISC loading complex	6895	22,6414964	0,226334461	0,397815	0,568944525	0,569393788	NA
ENSG00000149541	B3GAT3	beta-1,3-glucuronyltransferase 3	26229	98,97405026	-0,01572254	0,16625	-0,09457146	0,92465522	0,984310395
ENSG00000020633	RUNX3	RUNX family transcription factor 3	864	1655,997385	0,027958905	0,051125	0,546870115	0,584467968	0,88998582
ENSG00000081913	PHLPP1	PH domain and leucine rich repeat protein phosphatase 1	23239	30,89782996	0,359595047	0,31782	1,131442703	0,257868806	NA
ENSG00000168802	CHTF8	chromosome transmission fidelity factor 8	54921	128,3379889	-0,195311825	0,717369	-0,272261303	0,785421108	0,950624564
ENSG00000100030	MAPK1	mitogen-activated protein kinase 1	5594	1934,288191	0,118028221	0,04811	2,453319855	0,014154442	0,185901312
ENSG00000080189	SLC35C2	solute carrier family 35 member C2	51006	101,0382336	-0,026242844	0,206539	-0,127059971	0,898892933	0,977990819
ENSG00000089127	OAS1	2'-5'-oligoadenylate synthetase 1	4938	25,92159612	-0,013348658	0,495421	-0,026944069	0,978504344	NA
ENSG00000143951	WDPCP	WD repeat containing planar cell polarity effector	51057	16,2246378	0,237388869	0,4825	0,491997276	0,622721269	NA
ENSG00000122507	BBS9	Bardet-Biedl syndrome 9	27241	29,18129417	1,144516304	1,317402	0,868767468	0,384974331	NA
ENSG00000134072	CAMK1	calcium/calmodulin dependent protein kinase I	8536	253,9043261	0,100611934	0,112446	0,894759848	0,370915478	0,79977671
ENSG00000176055	MBLAC2	metallo-beta-lactamase domain containing 2	153364	44,78460884	-0,414850544	0,303426	-1,367221594	0,171555859	0,629558603
ENSG00000181588	MEX3D	mex-3 RNA binding family member D	399664	17,0397675	-0,410514408	0,510373	-0,804342314	0,421199307	NA
ENSG00000070190	DAPP1	dual adaptor of phosphotyrosine and 3-phosphoinositides 1	27071	68,91066466	0,127464128	0,251257	0,507306237	0,611939963	0,900006614
ENSG00000065559	MAP2K4	mitogen-activated protein kinase kinase 4	6416	172,9858728	-0,01688038	0,125309	-0,134709675	0,892841423	0,977990819
ENSG00000101493	ZNF516	zinc finger protein 516	9658	21,67484122	-0,42902735	0,389296	-1,102059024	0,270436012	NA
ENSG00000112659	CUL9	cullin 9	23113	141,4485065	0,529437399	0,72057	0,734748068	0,46249294	0,847112768
ENSG00000123179	EBPL	EBP like	84650	16,12879665	-0,567246029	0,509031	-1,114363969	0,265123074	NA
ENSG00000135093	USP30	ubiquitin specific peptidase 30	84749	28,6407908	0,268144018	1,15665	0,231828071	0,816671554	NA
ENSG00000164615	CAMLG	calcium modulating ligand	819	79,44949542	0,266783822	0,253783	1,051228521	0,293153647	0,744745216
ENSG00000174231	PRPF8	pre-mRNA processing factor 8	10594	1882,749863	-0,136248451	0,22953	-0,5935984	0,552780751	0,882068269
ENSG00000078900	TP73	tumor protein p73	7161	23,92808068	0,322160077	0,401958	0,801477631	0,422855189	NA
ENSG00000103264	FBXO31	F-box protein 31	79791	85,8652326	-0,277526431	0,277622	-0,999657116	0,317476472	0,762292941
ENSG00000137094	DNAJB5	DnaJ heat shock protein family (Hsp40) member B5	25822	23,86878957	0,147472629	0,485496	0,303756396	0,761313489	NA
ENSG00000103522	IL21R	interleukin 21 receptor	50615	160,502427	-0,187082026	0,150998	-1,238972722	0,2153556	0,671724735
ENSG00000106701	FSD1L	fibronectin type III and SPRY domain containing 1 like	83856	45,4332829	0,287956144	0,300061	0,959657501	0,337227619	0,77693253
ENSG00000133316	WDR74	WD repeat domain 74	54663	45,09443948	-0,428967171	0,306657	-1,398848462	0,16185843	0,619487156
ENSG00000162298	SYVN1	synoviolin 1	84447	209,5198	-0,039782674	0,120379	-0,330477827	0,741038944	0,939193793
ENSG00000168894	RNF181	ring finger protein 181	51255	54,08710467	-0,027246159	0,2573	-0,105892586	0,915667576	0,981835428
ENSG00000112812	PRSS16	serine protease 16	10279	36,24402504	-0,259521953	1,542559	-0,168241216	0,866393517	NA
ENSG00000255639	NA	NA	NA	11,81999679	-2,056467406	2,092322	-0,982863848	0,325674453	NA
ENSG00000143393	PI4KB	phosphatidylinositol 4-kinase beta	5298	343,096953	0,041169789	0,101014	0,407566394	0,683592041	0,925586358
ENSG00000248919	ATP5MF-PTCD1	ATP5MF-PTCD1 readthrough	100526740	22,60530582	-0,159134131	0,493664	-0,322353247	0,747185099	NA

ENSG00000006282	SPATA20	spermatogenesis associated 20	64847	39,96363455	-2,056439	1,707104	-1,204635977	0,228343862	NA
ENSG00000253251	SHLD3	shieldin complex subunit 3	112441434	20,11222428	0,172270512	0,451623	0,381447669	0,702871095	NA
ENSG00000105176	URI1	URI1 prefoldin like chaperone	8725	88,53391289	0,234840767	0,329118	0,713545297	0,475508394	0,853811879
ENSG00000105364	MRPL4	mitochondrial ribosomal protein L4	51073	83,8791272	-0,217943048	0,179142	-1,216594997	0,223758347	0,679056437
ENSG00000148337	CIZ1	CDKN1A interacting zinc finger protein 1	25792	376,2704757	0,15477067	0,107403	1,441024934	0,149577639	0,604312127
ENSG00000111321	LTBR	lymphotoxin beta receptor	4055	28,22191263	-0,1042994	0,342375	-0,304634684	0,760644401	NA
ENSG00000114541	FRMD4B	FERM domain containing 4B	23150	18,66254433	-0,09322877	0,451089	-0,206675114	0,836263594	NA
ENSG00000115091	ACTR3	actin related protein 3	10096	2072,721108	-0,046355339	0,060651	-0,764292703	0,444692833	0,840581832
ENSG00000182718	ANXA2	annexin A2	302	806,5759654	0,085494612	0,06511	1,313080981	0,189155662	0,6473259
ENSG00000127951	FGL2	fibrinogen like 2	10875	41,69943865	-0,361824009	0,287627	-1,257961443	0,208405701	NA
ENSG00000253719	ATXN7L3B	ataxin 7 like 3B	552889	458,041566	0,015816808	0,086736	0,182356352	0,855303077	0,968377739
ENSG00000197044	ZNF441	zinc finger protein 441	126068	34,20076343	0,365401327	0,363649	1,004817719	0,31498463	NA
ENSG00000085274	MYNN	myoneurin	55892	126,2573658	0,071545364	0,284085	0,251844875	0,80116097	0,955988796
ENSG00000112514	CUTA	cutA divalent cation tolerance homolog	51596	142,4776135	0,158579064	0,209522	0,756862639	0,449132161	0,84263906
ENSG00000164483	SAMD3	sterile alpha motif domain containing 3	154075	438,9297858	-0,025143091	0,088066	-0,285502124	0,775259477	0,946448613
ENSG00000075413	MARK3	microtubule affinity regulating kinase 3	4140	171,1657538	0,13566211	0,145506	0,932345662	0,351157917	0,785712602
ENSG00000129933	MAU2	MAU2 sister chromatid cohesion factor	23383	262,4250927	0,019628675	0,122385	0,160385285	0,87257758	0,974533542
ENSG00000131626	PPFIA1	PTPRF interacting protein alpha 1	8500	15,36466333	1,326194659	3,05388	0,434265448	0,664095691	NA
ENSG00000197008	ZNF138	zinc finger protein 138	7697	24,94092297	-0,031124279	0,421561	-0,073830945	0,941144903	NA
ENSG00000080845	DLGAP4	DLG associated protein 4	22839	217,9976828	-0,181985235	0,149275	-1,21912812	0,222795569	0,678694696
ENSG00000142444	TIMM29	translocase of inner mitochondrial membrane 29	90580	40,13973882	-0,49668309	0,272476	-1,822851457	0,068325892	NA
ENSG00000062725	APPBP2	amyloid beta precursor protein binding protein 2	10513	160,7104898	-0,396298124	0,15081	-2,627805075	0,008593774	0,141050339
ENSG00000138074	SLC5A6	solute carrier family 5 member 6	8884	73,97375961	0,022019054	0,237223	0,092820149	0,926046444	0,985257216
ENSG00000151552	QDPR	quinoid dihydropteridine reductase	5860	31,91724971	0,102422462	0,449204	0,228008953	0,81963928	NA
ENSG00000197892	KIF13B	kinesin family member 13B	23303	212,48483	-0,103426922	0,13639	-0,758319858	0,448259522	0,84263906
ENSG00000130177	CDC16	cell division cycle 16	8881	246,742373	-0,06176798	0,115906	-0,532912525	0,594094134	0,894229873
ENSG00000140365	COMMD4	COMM domain containing 4	54939	60,6746945	0,218129368	0,30501	0,71515397	0,474513908	0,853811879
ENSG00000173208	ABCD2	ATP binding cassette subfamily D member 2	225	102,1982945	-0,266239211	0,187825	-1,417483191	0,156341705	0,61428162
ENSG00000167552	TUBA1A	tubulin alpha 1a	7846	143,963412	-0,043407982	0,166181	-0,261208951	0,793931376	0,952276252
ENSG00000182362	YBEY	ybeY metalloendoribonuclease	54059	23,79604548	0,068749515	0,402055	0,170995491	0,864227311	NA
ENSG00000196547	MAN2A2	mannosidase alpha class 2A member 2	4122	224,2824702	-0,038314839	0,183121	-0,209231934	0,834267185	0,963446071
ENSG00000176946	THAP4	THAP domain containing 4	51078	69,14930146	-0,140010677	0,220468	-0,635062244	0,52538782	0,871384275
ENSG00000090686	USP48	ubiquitin specific peptidase 48	84196	355,0225749	-0,092614883	0,091689	-1,010098513	0,312448095	0,757868604
ENSG00000111231	GPN3	GPN-loop GTPase 3	51184	23,01574696	-0,679408553	0,388766	-1,747601114	0,080533122	NA
ENSG00000188305	PEAK3	PEAK family member 3	374872	34,69556613	-0,241056806	0,332287	-0,725447549	0,468177512	NA
ENSG00000206418	RAB12	RAB12, member RAS oncogene family	201475	58,01905534	0,081944611	0,23819	0,344030466	0,730823375	0,938729652
ENSG00000114999	TTL	tubulin tyrosine ligase	150465	59,37414067	-0,065058586	0,245525	-0,264977086	0,791027113	0,951704496
ENSG00000033030	ZCCHC8	zinc finger CCHC-type containing 8	55596	113,7382937	0,384383105	0,163814	2,346463264	0,018952534	0,214721666
ENSG00000097021	ACOT7	acyl-CoA thioesterase 7	11332	156,6639695	0,121474416	0,1594	0,762074529	0,446015524	0,841259829
ENSG00000136754	ABI1	abl interactor 1	10006	350,6286281	-0,045382726	0,108174	-0,419536431	0,674824135	0,923602509
ENSG00000204209	DAXX	death domain associated protein	1616	290,4815442	0,572679852	2,227704	0,257071754	0,797123382	0,95354564
ENSG00000204209	SMIM40	small integral membrane protein 40	113523636	290,4815442	0,572679852	2,227704	0,257071754	0,797123382	0,95354564
ENSG00000162191	UBXN1	UBX domain protein 1	51035	128,2001136	-0,031586835	0,245905	-0,128451168	0,897791941	0,977990819
ENSG00000166343	MSS51	MSS51 mitochondrial translational activator	118490	27,15505502	-0,348608007	0,371715	-0,937837695	0,348327826	NA
ENSG00000169180	XPO6	exportin 6	23214	558,3505393	-0,079400756	0,070282	-1,129751423	0,258580982	0,714349292
ENSG00000187051	RPS19BP1	ribosomal protein S19 binding protein 1	91582	75,78961275	0,169307604	0,196327	0,862373376	0,388482087	0,810040214
ENSG00000047644	WWC3	WWC family member 3	55841	517,6755663	-0,070564387	0,072478	-0,973590756	0,330259778	0,771479881
ENSG00000163697	APBB2	amyloid beta precursor protein binding family B member 2	323	42,39300182	0,414593831	0,539449	0,768550981	0,442159915	0,838678081
ENSG00000179104	TMTC2	transmembrane O-mannosyltransferase targeting cadherins 2	160335	11,70211702	0,142217426	0,597529	0,238009363	0,811873832	NA
ENSG00000092978	GPATCH2	G-patch domain containing 2	55105	73,15707569	-0,049505916	0,227264	-0,217834758	0,827557862	0,962624326
ENSG00000133247	KMT5C	lysine methyltransferase 5C	84787	14,22502638	-0,975385947	0,773338	-1,261266924	0,207212694	NA
ENSG00000156958	GALK2	galactokinase 2	2585	53,8529446	-0,40965751	0,324346	-1,263024864	0,206580244	0,661359894

ENSG00000176623	RMDN1	regulator of microtubule dynamics 1	51115	121,0932499	0,357785731	0,190256	1,880545811	0,060033729	0,397224017
ENSG00000120949	TNFRSF8	TNF receptor superfamily member 8	943	39,3925782	-0,016980429	0,31182	-0,054455917	0,956571929	NA
ENSG00000160408	ST6GALNAC6	ST6 N-acetylgalactosaminide alpha-2,6-sialyltransferase 6	30815	90,26724816	-0,770341754	0,231807	-3,323200871	0,000889908	0,032254517
ENSG00000136827	TOR1A	torsin family 1 member A	1861	225,7547868	0,151212443	0,112081	1,349139987	0,177292007	0,632039836
ENSG00000117360	PRPF3	pre-mRNA processing factor 3	9129	166,5660398	-0,08018515	0,132301	-0,606081948	0,544460338	0,87855238
ENSG00000137124	ALDH1B1	aldehyde dehydrogenase 1 family member B1	219	50,82397583	-0,161441451	0,252374	-0,639691216	0,522373367	0,870221868
ENSG00000010322	NISCH	nischarin	11188	397,6717006	-0,155071036	0,091668	-1,691660565	0,090710711	0,490662485
ENSG00000070950	RAD18	RAD18 E3 ubiquitin protein ligase	56852	78,5536998	-0,141938105	0,320063	-0,443468882	0,657426622	0,919088619
ENSG00000242372	EIF6	eukaryotic translation initiation factor 6	3692	155,0685659	0,111877242	0,141369	0,791385466	0,428719088	0,832060923
ENSG00000105520	PLPPR2	phospholipid phosphatase related 2	64748	23,26355979	-0,366957146	0,359677	-1,020239775	0,307614758	NA
ENSG00000119523	ALG2	ALG2 alpha-1,3/1,6-mannosyltransferase	85365	113,6220176	-0,414659088	0,178724	-2,320107558	0,02033506	0,224313986
ENSG00000171792	RHNO1	RAD9-HUS1-RAD1 interacting nuclear orphan 1	83695	93,56633061	0,31011117	0,190733	1,62588958	0,103973147	0,517786851
ENSG00000166997	CNPY4	canopy FGF signaling regulator 4	245812	36,32354442	0,072206854	0,321499	0,224594464	0,822294772	NA
ENSG00000186283	TOR3A	torsin family 3 member A	64222	100,3528835	0,217172865	0,188798	1,150293057	0,25002319	0,707335884
ENSG00000173465	ZNRD2	zinc ribbon domain containing 2	10534	32,56146984	-0,250432067	0,307371	-0,814754121	0,415213067	NA
ENSG00000162694	EXTL2	exostosin like glycosyltransferase 2	2135	27,25804504	0,201354578	0,386047	0,521580372	0,601962533	NA
ENSG00000162971	TYW5	tRNA-yW synthesizing protein 5	129450	30,72203623	-0,292832657	0,421995	-0,693924462	0,487729582	NA
ENSG00000028839	TBPL1	TATA-box binding protein like 1	9519	60,04296748	0,252402086	0,27963	0,902628476	0,366723108	0,79476912
ENSG00000132964	CDK8	cyclin dependent kinase 8	1024	49,31538239	-0,93907325	1,110969	-0,845274099	0,397957819	0,813948101
ENSG00000132478	UNK	unk zinc finger	85451	111,6556145	-0,001657123	0,180957	-0,009157532	0,992693448	0,998500439
ENSG00000150712	MTMR12	myotubularin related protein 12	54545	207,9670305	-0,138987469	0,137008	-1,014444331	0,31037078	0,75688983
ENSG00000165660	ABRAXAS2	abraxas 2, BRISC complex subunit	23172	84,44730534	0,130242716	0,208176	0,62563872	0,531551936	0,872945174
ENSG00000123892	RAB38	RAB38, member RAS oncogene family	23682	49,03371684	0,126103737	0,301426	0,418357816	0,675685524	0,924052604
ENSG00000243789	JMJD7	jumonji domain containing 7	100137047	37,4414564	-0,532533772	0,367322	-1,449771823	0,14712216	NA
ENSG00000130830	MPP1	MAGUK p55 scaffold protein 1	4354	78,6193354	-0,063010722	0,207335	-0,303907792	0,761198142	0,942901768
ENSG00000177426	TGIF1	TGFB induced factor homeobox 1	7050	16,25226324	0,55065013	0,582136	0,945913894	0,344192506	NA
ENSG00000204130	RUFY2	RUN and FYVE domain containing 2	55680	44,74572592	-0,62109916	0,389988	-1,592609552	0,111247809	0,532768473
ENSG00000213965	NUDT19	nudix hydrolase 19	390916	77,06108252	-0,29584357	0,188597	-1,568656147	0,116728082	0,544731049
ENSG00000073536	NLE1	notchless homolog 1	54475	42,41639282	-0,422492147	0,296585	-1,424524515	0,154294694	0,611607348
ENSG00000116750	UCHL5	ubiquitin C-terminal hydrolase L5	51377	143,2684829	-0,264529731	0,177971	-1,486368056	0,137181794	0,57855532
ENSG00000017797	RALBP1	ralA binding protein 1	10928	358,3213981	0,003648585	0,096869	0,037664998	0,969954784	0,993542794
ENSG00000141526	SLC16A3	solute carrier family 16 member 3	9123	156,408581	-0,415242685	0,450158	-0,922437749	0,356300287	0,78894346
ENSG00000112031	MTRF1L	mitochondrial translation release factor 1 like	54516	43,85461016	-0,740691098	0,56466	-1,31174758	0,189605317	0,647463823
ENSG00000036257	CUL3	cullin 3	8452	360,8568365	0,013595639	0,091401	0,148746936	0,881753323	0,976300778
ENSG00000041353	RAB27B	RAB27B, member RAS oncogene family	5874	158,8309508	-0,24716788	0,146006	-1,692864892	0,090481187	0,490096031
ENSG00000100348	TXN2	thioredoxin 2	25828	133,1488319	-0,058048535	0,17676	-0,328402958	0,742607007	0,939200553
ENSG00000112578	BYSL	bystin like	705	38,16145151	0,027020006	0,340131	0,079440003	0,936682652	NA
ENSG00000115317	HTRA2	HtrA serine peptidase 2	27429	46,49713947	-0,000402728	0,353818	-0,001138235	0,99909182	0,99965319
ENSG00000120925	RNF170	ring finger protein 170	81790	97,3875175	-0,24833834	0,227115	-1,093449273	0,27419659	0,727065559
ENSG00000137474	MYO7A	myosin VIIA	4647	112,9043174	0,44993554	0,165687	2,715571351	0,006616152	0,118637572
ENSG00000155657	TTN	titin	7273	267,6436538	0,712242831	0,479339	1,485885975	0,137309284	0,57855532
ENSG00000156531	PHF6	PHD finger protein 6	84295	246,3714628	0,037006624	0,13127	0,281912832	0,77801035	0,947109268
ENSG00000168569	TMEM223	transmembrane protein 223	79064	23,16412628	-0,379387361	0,38544	-0,984295681	0,324970153	NA
ENSG00000211584	SLC48A1	solute carrier family 48 member 1	55652	16,16721829	-0,468765344	0,458842	-1,021625993	0,30695795	NA
ENSG00000230715	NA	NA	NA	12,82118731	0,771654763	0,598246	1,289862628	0,197098358	NA
ENSG00000101126	ADNP	activity dependent neuroprotector homeobox	23394	648,6397328	0,036243872	0,070237	0,516024749	0,605837124	0,897380562
ENSG00000101868	POLA1	DNA polymerase alpha 1, catalytic subunit	5422	117,612193	0,054853659	0,166548	0,329355652	0,741886884	0,939193793
ENSG00000119559	C19orf25	chromosome 19 open reading frame 25	148223	42,75932706	0,024395308	0,273214	0,08929002	0,928851425	0,986487751
ENSG00000111554	MDM1	Mdm1 nuclear protein	56890	96,03803087	0,159233001	0,180492	0,882215898	0,377660066	0,804050464
ENSG00000105254	TBCB	tubulin folding cofactor B	1155	144,4279462	0,007233187	0,153984	0,046973485	0,96253436	0,992328441
ENSG00000108771	DHX58	DExH-box helicase 58	79132	38,00719986	-0,168368737	0,325323	-0,517542946	0,604777198	NA

ENSG00000177721	ANXA2R	annexin A2 receptor	389289	52,68447721	-0,021380873	0,238709	-0,089568628	0,928630015	0,986487751
ENSG00000232388	SMIM26	small integral membrane protein 26	388789	42,37319602	-0,163891959	0,284183	-0,576712736	0,564133527	0,885045179
ENSG00000158669	GPAT4	glycerol-3-phosphate acyltransferase 4	137964	185,2979748	0,083075715	0,130195	0,638084925	0,523418399	0,870221868
ENSG00000132967	HMGB1P5	high mobility group box 1 pseudogene 5	10354	14,73071743	0,205117352	0,696027	0,29469731	0,768225118	NA
ENSG00000159753	CARMIL2	capping protein regulator and myosin 1 linker 2	146206	329,3126606	-0,048446377	0,127187	-0,380907002	0,703272257	0,930846779
ENSG00000022976	ZNF839	zinc finger protein 839	55778	42,59898836	-0,98969039	1,120088	-0,883582808	0,376921463	0,803373958
ENSG00000060558	GNA15	G protein subunit alpha 15	2769	51,105925	0,013624977	0,262872	0,051831283	0,958663129	0,991911599
ENSG00000077157	PPP1R12B	protein phosphatase 1 regulatory subunit 12B	4660	198,6948882	0,103402436	0,19211	0,538247325	0,590406312	0,892294181
ENSG00000081791	DELE1	DAP3 binding cell death enhancer 1	9812	173,5373607	-0,266426515	0,234936	-1,134037694	0,256778737	0,71283265
ENSG00000087095	NLK	nemo like kinase	51701	89,82020864	-0,315732953	0,187856	-1,680716696	0,092817956	0,494966917
ENSG00000088854	DNAAF9	dynein axonemal assembly factor 9	25943	26,78964313	-0,013956766	0,345084	-0,040444549	0,967738715	NA
ENSG00000100422	CERK	ceramide kinase	64781	242,0493672	0,010027746	0,130572	0,076798708	0,938783678	0,987813564
ENSG00000100479	POLE2	DNA polymerase epsilon 2, accessory subunit	5427	13,41747372	0,708228218	0,54746	1,293662966	0,195781863	NA
ENSG00000101882	NKAP	NFKB activating protein	79576	45,44040457	0,265579819	0,260251	1,020476301	0,307502623	0,754087936
ENSG00000102081	FMR1	fragile X messenger ribonucleoprotein 1	2332	109,7324081	-0,823076409	0,275749	-2,984877902	0,002836915	0,069661304
ENSG00000106009	BRAT1	BRCA1 associated ATM activator 1	221927	61,7528245	0,075861686	0,223476	0,339462209	0,734261562	0,939157822
ENSG00000108344	PSMD3	proteasome 26S subunit, non-ATPase 3	5709	237,8163541	0,07879955	0,111795	0,704859157	0,480897889	0,855577353
ENSG00000113812	ACTR8	actin related protein 8	93973	185,9081036	0,079394375	0,14227	0,558053265	0,576808012	0,887069818
ENSG00000128040	SPINK2	serine peptidase inhibitor Kazal type 2	6691	205,5544336	0,028564958	0,126196	0,226353383	0,820926582	0,961891299
ENSG00000132275	RRP8	ribosomal RNA processing 8	23378	106,4670212	-0,359240083	0,524192	-0,685322114	0,49314068	0,860513824
ENSG00000134899	ERCC5	ERCC excision repair 5, endonuclease	2073	236,5702459	0,008451825	0,448262	0,018854634	0,98495707	0,996185513
ENSG00000137822	TUBGCP4	tubulin gamma complex component 4	27229	136,5385079	-0,11289554	0,202529	-0,557429785	0,57723382	0,887069818
ENSG00000138660	AP1AR	adaptor related protein complex 1 associated regulatory protein	55435	67,55842411	-0,249544441	0,246214	-1,013527724	0,310808161	0,75688983
ENSG00000149600	COMMD7	COMM domain containing 7	149951	239,1166109	0,167390654	0,118369	1,414145509	0,157319183	0,61428162
ENSG00000160972	PPP1R16A	protein phosphatase 1 regulatory subunit 16A	84988	27,18419451	0,00088137	0,402613	0,002189125	0,998253333	NA
ENSG00000163683	SMIM14	small integral membrane protein 14	201895	87,59544296	-0,27375772	0,307255	-0,890977493	0,372941245	0,800776142
ENSG00000165699	TSC1	TSC complex subunit 1	7248	94,39540126	0,199928209	0,571206	0,350010906	0,726330513	0,93640838
ENSG00000172062	SMN1	survival of motor neuron 1, telomeric	6606	61,94281442	-0,004055084	0,906747	-0,004472125	0,996431772	0,999300722
ENSG00000175104	TRAF6	TNF receptor associated factor 6	7189	132,8438019	-0,229427007	0,156021	-1,470490879	0,141428854	0,585546769
ENSG00000179361	ARID3B	AT-rich interaction domain 3B	10620	525,525207	-0,041265908	0,080724	-0,511200399	0,60921074	0,89837423
ENSG00000180867	NA	NA	NA	11,80353168	-0,004652358	0,523515	-0,008886766	0,99290948	NA
ENSG00000181135	ZNF707	zinc finger protein 707	286075	12,8239871	0,243042534	0,844403	0,287827635	0,773478683	NA
ENSG00000181135	LOC101928160	uncharacterized LOC101928160	101928160	12,8239871	0,243042534	0,844403	0,287827635	0,773478683	NA
ENSG00000181215	C4orf50	chromosome 4 open reading frame 50	389197	13,06585177	-0,208207214	0,538751	-0,386463066	0,699153751	NA
ENSG00000182885	ADGRG3	adhesion G protein-coupled receptor G3	222487	18,66657462	0,078269749	0,424059	0,184572657	0,853564234	NA
ENSG00000183742	MACC1	MET transcriptional regulator MACC1	346389	34,48234447	0,0617545	0,405814	0,152174569	0,879049252	NA
ENSG00000183763	TRAIP	TRAF interacting protein	10293	14,73648862	0,432590768	0,568557	0,760857457	0,446742212	NA
ENSG00000184575	XPOT	exportin for tRNA	11260	381,4532456	0,089331164	0,088418	1,01033073	0,312336863	0,757868604
ENSG00000184990	SIVA1	SIVA1 apoptosis inducing factor	10572	42,02124002	0,170440769	0,342513	0,4976181	0,618753244	NA
ENSG00000197265	GTF2E2	general transcription factor IIE subunit 2	2961	92,2314027	0,187859301	0,183163	1,025639162	0,305061701	0,751870158
ENSG00000213337	ANKRD39	ankyrin repeat domain 39	51239	26,77959927	-0,175662427	0,422486	-0,41578287	0,677568895	NA
ENSG00000263563	NA	NA	NA	14,49873631	-0,346230984	0,457594	-0,756632925	0,44926981	NA
ENSG00000277161	PIGW	phosphatidylinositol glycan anchor biosynthesis class W	284098	30,19653655	0,327127756	1,849843	0,176840847	0,859633403	NA
ENSG00000183337	BCOR	BCL6 corepressor	54880	349,5289202	-0,112275181	0,108971	-1,030317035	0,302861205	0,751870158
ENSG00000070785	EIF2B3	eukaryotic translation initiation factor 2B subunit gamma	8891	48,20364088	0,105388238	0,283756	0,371403963	0,710336675	0,930846779
ENSG00000086666	ZFAND6	zinc finger AN1-type containing 6	54469	160,661112	0,030494138	0,170844	0,17849092	0,858337451	0,969285743
ENSG00000105197	TIMM50	translocase of inner mitochondrial membrane 50	92609	57,42505103	-0,472891452	0,836166	-0,565547588	0,571701366	0,886795904

ENSG00000118939	UCHL3	ubiquitin C-terminal hydrolase L3	7347	40,10063196	0,455459754	0,359871	1,265617798	0,205649951	NA
ENSG00000139324	TMTC3	transmembrane O-mannosyltransferase targeting cadherins 3	160418	47,48634868	-0,268051556	0,30778	-0,870918084	0,383798882	0,808586507
ENSG00000154582	ELOC	elongin C	6921	77,97014555	0,108993319	0,206815	0,527007594	0,598188315	0,894604697
ENSG00000158552	ZFAND2B	zinc finger AN1-type containing 2B	130617	126,4525086	0,095159284	0,17997	0,528751397	0,596977918	0,894257182
ENSG00000197037	ZSCAN25	zinc finger and SCAN domain containing 25	221785	80,79802428	-0,180339996	0,213598	-0,844296082	0,398503975	0,814457852
ENSG00000198759	EGFL6	EGF like domain multiple 6	25975	13,2488307	0,040526802	0,499707	0,081101174	0,935361492	NA
ENSG00000239521	NA	NA	NA	24,98163357	-0,425487143	0,368326	-1,155192931	0,248011429	NA
ENSG00000266820	NA	NA	NA	47,80597349	0,334773081	0,249481	1,341876767	0,179635956	0,633591125
ENSG00000100532	CGRRF1	cell growth regulator with ring finger domain 1	10668	21,68023359	-0,378840423	0,820462	-0,461740362	0,644267523	NA
ENSG00000141101	NOB1	NIN1 (RPN12) binding protein 1 homolog	28987	113,8754828	0,225175231	0,165253	1,362610211	0,173005389	0,629558603
ENSG00000087448	KLHL42	kelch like family member 42	57542	124,3469749	-0,211734404	0,156295	-1,354713999	0,175508696	0,632039836
ENSG00000113119	TMCO6	transmembrane and coiled-coil domains 6	55374	12,60190762	0,229980646	0,803562	0,286201616	0,774723706	NA
ENSG00000124224	NA	NA	NA	20,06208855	-0,379379034	0,433156	-0,875849394	0,381111914	NA
ENSG00000152117	SMPD4BP	sphingomyelin phosphodiesterase 4B, pseudogene	150776	69,23865282	-0,080114905	0,205624	-0,389618657	0,696818554	0,930422107
ENSG00000171055	FEZ2	fasciculation and elongation protein zeta 2	9637	55,96819463	-0,070426263	0,226773	-0,310557933	0,756136711	0,942901768
ENSG00000180921	FAM83H	family with sequence similarity 83 member H	286077	35,44071312	0,570725633	1,728928	0,330103829	0,74132151	NA
ENSG00000182372	CLN8	CLN8 transmembrane ER and ERGIC protein	2055	104,0783204	0,210193812	0,247669	0,848688736	0,396054515	0,812799303
ENSG00000204524	ZNF805	zinc finger protein 805	390980	123,1621522	0,025238549	0,203568	0,12398102	0,901330303	0,977990819
ENSG00000096433	ITPR3	inositol 1,4,5-trisphosphate receptor type 3	3710	782,9808246	0,027564096	0,063545	0,433772159	0,664453901	0,921854961
ENSG00000100083	GGA1	golgi associated, gamma adaptin ear containing, ARF binding protein 1	26088	175,1394617	0,126334826	0,12972	0,973904725	0,330103848	0,771479881
ENSG00000125245	GPR18	G protein-coupled receptor 18	2841	139,0133766	0,049701006	0,183013	0,271570272	0,78595246	0,950624564
ENSG00000140025	EFCAB11	EF-hand calcium binding domain 11	90141	14,20423122	0,295722192	0,630382	0,469116164	0,638986606	NA
ENSG00000146425	DYNLT1	dynein light chain Tctex-type 1	6993	47,15316677	0,43757506	0,242782	1,802334334	0,07149282	0,435172111
ENSG00000164823	OSGIN2	oxidative stress induced growth inhibitor family member 2	734	104,5369408	0,175290899	0,308285	0,568599597	0,569627898	0,886263374
ENSG00000178035	IMPDH2	inosine monophosphate dehydrogenase 2	3615	119,5524813	0,175773188	0,160377	1,095996613	0,273080257	0,726913977
ENSG00000198952	SMG5	SMG5 nonsense mediated mRNA decay factor	23381	490,9190218	0,070130038	0,086529	0,810483985	0,417662066	0,82446781
ENSG00000164944	VIRMA	vir like m6A methyltransferase associated	25962	383,4868852	0,226780786	0,082099	2,76227394	0,00574003	0,10771718
ENSG00000225190	PLEKHM1	pleckstrin homology and RUN domain containing M1	9842	71,52044802	0,284672618	0,930619	0,305895839	0,759683954	0,942901768
ENSG00000213741	RPS29	ribosomal protein S29	6235	459,9080738	0,022130681	0,127391	0,173722843	0,862083284	0,970863509
ENSG00000163463	KRTCAP2	keratinocyte associated protein 2	200185	72,44166015	-0,083452033	0,227691	-0,366513816	0,713981705	0,931512238
ENSG00000130193	THEM6	thioesterase superfamily member 6	51337	48,5675538	0,113111952	0,237653	0,475954009	0,634107143	0,908608932
ENSG00000101844	ATG4A	autophagy related 4A cysteine peptidase	115201	61,88725324	0,156854794	0,245391	0,639204426	0,522689953	0,870221868
ENSG00000100387	RBX1	ring-box 1	9978	106,5563094	0,233878626	0,173587	1,347324658	0,177875694	0,632142851
ENSG00000145907	G3BP1	G3BP stress granule assembly factor 1	10146	581,9060384	-0,034554468	0,106291	-0,325092883	0,745110775	0,939745744
ENSG00000158941	CCAR2	cell cycle and apoptosis regulator 2	57805	442,6631374	0,430714178	0,317654	1,355924037	0,175123339	0,632039836
ENSG00000110628	SLC22A18	solute carrier family 22 member 18	5002	19,31192502	0,259386418	0,436272	0,594551425	0,552143358	NA
ENSG00000172775	PSME3IP1	proteasome activator subunit 3 interacting protein 1	80011	217,414712	-0,081202648	0,180652	-0,449496694	0,653073392	0,916753963
ENSG00000146587	RBAK	RB associated KRAB zinc finger	57786	148,4465522	-0,378571165	0,141131	-2,682403649	0,00730952	0,125896872
ENSG00000188786	MTF1	metal regulatory transcription factor 1	4520	137,1498073	-0,127264816	0,147803	-0,861041763	0,389215042	0,810747116
ENSG00000204147	ASAH2B	N-acylsphingosine amidohydrolase 2B	653308	17,18663129	-0,514002066	1,006962	-0,510448237	0,60973747	NA
ENSG00000123636	BAZ2B	bromodomain adjacent to zinc finger domain 2B	29994	106,4694015	-0,086527066	0,196378	-0,440615826	0,659491144	0,919368176

ENSG00000132017	DCAF15	DDB1 and CUL4 associated factor 15	90379	81,02817156	0,258861239	2,009181	0,128839162	0,897484917	0,977990819
ENSG00000133943	DGLUCY	D-glutamate cyclase	80017	176,5380892	0,10672023	0,192488	0,554424141	0,579288608	0,887537774
ENSG00000115207	GTF3C2	general transcription factor IIIC subunit 2	2976	243,6325223	0,026487857	0,110558	0,239582462	0,810653962	0,959038743
ENSG00000115365	LANCL1	LanC like glutathione S-transferase 1	10314	191,8564412	0,176831998	0,14961	1,181953786	0,237224038	0,692366257
ENSG00000148908	RGS10	regulator of G protein signaling 10	6001	242,0076674	0,061953711	0,130737	0,473879831	0,635585603	0,909036096
ENSG00000164008	C1orf50	chromosome 1 open reading frame 50	79078	52,26892791	0,119895718	0,22945	0,522535573	0,601297485	0,896297295
ENSG00000164050	PLXNB1	plexin B1	5364	19,72239797	0,835092141	0,406056	2,056592969	0,039725395	NA
ENSG00000166908	PIP4K2C	phosphatidylinositol-5-phosphate 4-kinase type 2 gamma	79837	144,4915464	0,098740787	0,140294	0,70381403	0,481548595	0,855577353
ENSG00000047230	CTPS2	CTP synthase 2	56474	45,63129661	0,028221374	0,262526	0,107499247	0,914392923	0,981232684
ENSG00000177150	FAM210A	family with sequence similarity 210 member A	125228	39,27959875	-0,343393884	0,351682	-0,976432378	0,328850243	NA
ENSG00000184220	CMSS1	cms1 ribosomal small subunit homolog	84319	35,17155771	0,399106684	0,609962	0,654313687	0,51290973	NA
ENSG00000187954	ZFTRAF1	zinc finger TRAF-type containing 1	50626	54,99915499	-0,042982496	0,23804	-0,180568647	0,856706167	0,968618668
ENSG00000025708	TYMP	thymidine phosphorylase	1890	25,85067964	0,213882222	0,488669	0,437683012	0,661616086	NA
ENSG00000100564	PIGH	phosphatidylinositol glycan anchor biosynthesis class H	5283	42,51689068	0,081271101	0,295024	0,27547261	0,782953166	0,949847725
ENSG00000100632	ERH	ERH mRNA splicing and mitosis factor	2079	178,2681612	0,239426272	0,141503	1,692028513	0,090640537	0,4906208
ENSG00000132254	ARFIP2	ADP ribosylation factor interacting protein 2	23647	75,38578793	0,289627184	0,216007	1,340824896	0,179977311	0,633591125
ENSG00000135387	CAPRIN1	cell cycle associated protein 1	4076	614,2525138	0,091917419	0,080382	1,143513575	0,252825422	0,708754466
ENSG00000160255	ITGB2	integrin subunit beta 2	3689	2806,825776	0,017434991	0,053954	0,323144762	0,746585612	0,939843468
ENSG00000213203	GIMAP1	GTPase, IMAP family member 1	170575	183,6024701	-0,451940019	0,125686	-3,59578874	0,00032341	0,014854173
ENSG00000123989	CHPF	chondroitin polymerizing factor	79586	70,57055198	0,130542096	0,202572	0,644421751	0,519301983	0,869081137
ENSG00000138434	ITPRID2	ITPR interacting domain containing 2	6744	138,8802877	0,103576602	0,193543	0,535160366	0,59253897	0,893243967
ENSG00000198873	GRK5	G protein-coupled receptor kinase 5	2869	77,33716646	-0,270688914	0,198604	-1,362956204	0,172896314	0,629558603
ENSG00000171954	CYP4F22	cytochrome P450 family 4 subfamily F member 22	126410	36,52639646	-0,305466519	0,298171	-1,024468563	0,305614012	NA
ENSG00000156026	MCU	mitochondrial calcium uniporter	90550	32,04386294	0,055051093	0,501046	0,10987233	0,912510627	NA
ENSG00000111707	SUDS3	SDS3 homolog, SIN3A corepressor complex component	64426	231,3269814	-0,135643875	0,113684	-1,19317114	0,232802355	0,6881557
ENSG00000161021	MAML1	mastermind like transcriptional coactivator 1	9794	124,7187997	-0,234486713	0,883088	-0,26553042	0,790600879	0,95167209
ENSG00000137269	LRRC1	leucine rich repeat containing 1	55227	32,20787081	-0,02054161	0,330356	-0,062180207	0,950419325	NA
ENSG00000065600	PACC1	proton activated chloride channel 1	55248	35,79098884	-0,102568589	0,313144	-0,327544759	0,743255895	NA
ENSG00000073008	PVR	PVR cell adhesion molecule	5817	48,02956123	0,532256014	0,295742	1,79972958	0,071903348	0,435172111
ENSG00000127311	HELB	DNA helicase B	92797	58,08206853	-0,225257558	1,174656	-0,191764722	0,847926506	0,967411651
ENSG00000267368	UPK3BL1	uroplakin 3B like 1	100134938	104,2287887	-0,230140605	0,162293	-1,418052567	0,156175418	0,61428162
ENSG00000286219	NOTCH2NLC	notch 2 N-terminal like C	100996717	425,8640076	0,289387904	0,180555	1,602771986	0,108985004	0,52908614
ENSG00000100523	DDHD1	DDHD domain containing 1	80821	471,3381181	-0,154401579	0,088147	-1,751644728	0,079834916	0,458350463
ENSG00000132128	LRRC41	leucine rich repeat containing 41	10489	144,4378532	0,103074231	0,143793	0,716823891	0,473482768	0,853811879
ENSG00000151498	ACAD8	acyl-CoA dehydrogenase family member 8	27034	58,65056458	0,446525163	0,242141	1,844074	0,065172357	0,411797014
ENSG00000100416	TRMU	tRNA mitochondrial 2-thiouridylase	55687	14,00363259	-2,337202364	1,743931	-1,340191991	0,180182935	NA
ENSG00000106246	PTCD1	pentatricopeptide repeat domain 1	26024	52,10059938	0,308813342	0,299635	1,030633013	0,302712949	0,751870158
ENSG00000111775	COX6A1	cytochrome c oxidase subunit 6A1	1337	172,5347648	0,277116013	0,152605	1,815903129	0,069385233	0,428084542
ENSG00000130714	POMT1	protein O-mannosyltransferase 1	10585	14,3217379	-0,491770148	1,690173	-0,290958433	0,77108311	NA
ENSG00000142655	PEX14	peroxisomal biogenesis factor 14	5195	58,38124629	-0,067721771	0,231636	-0,292362847	0,770009211	0,94509335
ENSG00000163312	HELQ	helicase, POLQ like	113510	76,3522682	-0,107654197	0,207653	-0,518433971	0,604155518	0,897212117
ENSG00000164329	TENT2	terminal nucleotidyltransferase 2	167153	281,5327538	-0,076661828	0,105061	-0,729687008	0,465581524	0,84908342
ENSG00000185829	ARL17A	ADP ribosylation factor like GTPase 17A	51326	415,1406813	-0,077770797	0,083852	-0,927477299	0,353678773	0,788228969
ENSG00000185829	ARL17B	ADP ribosylation factor like GTPase 17B	100506084	415,1406813	-0,077770797	0,083852	-0,927477299	0,353678773	0,788228969

ENSG00000197912	SPG7	SPG7 matrix AAA peptidase subunit, paraplegin	6687	42,79170237	-0,185062539	0,380779	-0,486010701	0,626959579	0,90479929
ENSG00000197912	LOC101930112	uncharacterized LOC101930112	101930112	42,79170237	-0,185062539	0,380779	-0,486010701	0,626959579	0,90479929
ENSG00000277957	NA	NA	NA	11,92120124	0,029708411	1,184404	0,025082995	0,979988764	NA
ENSG00000155330	C16orf87	chromosome 16 open reading frame 87	388272	34,60226293	-0,008706304	0,33763	-0,025786513	0,979427619	NA
ENSG00000162144	CYB561A3	cytochrome b561 family member A3	220002	115,2353188	0,058349479	0,174071	0,335206005	0,737469692	0,939193793
ENSG00000147316	MCPH1	microcephalin 1	79648	130,6382241	0,31854734	0,238792	1,333993747	0,182205906	0,636302884
ENSG00000169188	APEX2	apurinic/apyrimidinic endodeoxyribonuclease 2	27301	95,19119331	-0,372946454	0,182325	-2,045509141	0,040804689	0,322423091
ENSG00000104691	UBXN8	UBX domain protein 8	7993	23,83489776	-0,007026122	0,370444	-0,018966755	0,984867627	NA
ENSG00000185515	BRCC3	BRCA1/BRCA2-containing complex subunit 3	79184	144,5503675	0,184790005	0,158854	1,163267302	0,244721068	0,701819279
ENSG00000140525	FANCI	FA complementation group I	55215	219,5482383	0,597505035	0,180394	3,312231466	0,000925549	0,033192989
ENSG00000171204	TMEM126B	transmembrane protein 126B	55863	49,51893947	-0,947102073	0,321902	-2,942208499	0,003258804	0,075374002
ENSG00000013306	SLC25A39	solute carrier family 25 member 39	51629	180,3211721	0,20987493	0,130759	1,605055151	0,108481668	0,528784574
ENSG00000100281	HMGXB4	HMG-box containing 4	10042	69,03759545	0,375124337	1,250224	0,300045626	0,764142354	0,944237578
ENSG00000105656	ELL	elongation factor for RNA polymerase II	8178	188,0396705	0,075224553	0,128481	0,585491877	0,558217027	0,883921037
ENSG00000108219	TSPAN14	tetraspanin 14	81619	1167,377888	-0,0193219	0,114277	-0,169079137	0,865734395	0,971722599
ENSG00000108433	GOSR2	golgi SNAP receptor complex member 2	9570	221,1699583	0,114769512	0,109852	1,044760171	0,296133827	0,747862492
ENSG00000110074	FOXRED1	FAD dependent oxidoreductase domain containing 1	55572	33,2705506	-0,043892401	0,603664	-0,072709944	0,942036935	NA
ENSG00000116288	PARK7	Parkinsonism associated deglycase	11315	205,0161978	0,376179265	0,117736	3,195117922	0,001397737	0,043562809
ENSG00000117054	ACADM	acyl-CoA dehydrogenase medium chain	34	157,7597147	-0,072049788	0,163139	-0,441647061	0,658744624	0,919368176
ENSG00000117859	OSBPL9	oxysterol binding protein like 9	114883	114,0546163	-0,166510702	0,262394	-0,634582307	0,52570087	0,871384275
ENSG00000124212	PTGIS	prostaglandin I2 synthase	5740	1188,54274	-0,297998509	0,0663	-4,494696902	6,97E-06	0,000719975
ENSG00000144591	GMPPA	GDP-mannose pyrophosphorylase A	29926	83,47278814	-0,145479546	0,206182	-0,705587073	0,480444966	0,855455625
ENSG00000170035	UBE2E3	ubiquitin conjugating enzyme E2 E3	10477	91,47386224	0,123652361	0,214977	0,575187702	0,565164359	0,885045179
ENSG00000184677	ZBTB40	zinc finger and BTB domain containing 40	9923	390,2406991	0,062746287	0,092834	0,675901234	0,499103354	0,86161226
ENSG00000213347	MXD3	MAX dimerization protein 3	83463	23,06441172	0,283520257	0,514115	0,551472436	0,581309857	NA
ENSG00000228594	FNDC10	fibronectin type III domain containing 10	643988	19,73435377	-0,242875748	0,405066	-0,599596206	0,548775376	NA
ENSG00000136720	HS6ST1	heparan sulfate 6-O-sulfotransferase 1	9394	26,67450452	0,232406257	0,364217	0,638097595	0,523410152	NA
ENSG00000166548	TK2	thymidine kinase 2	7084	83,70661105	0,060033847	0,195681	0,306794936	0,758999463	0,942901768
ENSG00000169914	OTUD3	OTU deubiquitinase 3	23252	91,27537138	-0,259350046	0,192126	-1,349897309	0,177048925	0,632039836
ENSG00000161981	SNRNP25	small nuclear ribonucleoprotein U11/U12 subunit 25	79622	56,29541067	0,197577155	0,326337	0,605439797	0,544886815	0,878589015
ENSG00000102900	NUP93	nucleoporin 93	9688	121,0304252	0,163259073	0,165827	0,984512269	0,324863702	0,768215203
ENSG00000115350	POLE4	DNA polymerase epsilon 4, accessory subunit	56655	16,44891381	-0,109690076	0,463284	-0,236766245	0,812838139	NA
ENSG00000125676	THOC2	THO complex subunit 2	57187	418,0682896	-0,02680963	0,136242	-0,196779129	0,844000384	0,967231052
ENSG00000178188	SH2B1	SH2B adaptor protein 1	25970	252,2716847	-0,072699607	0,113414	-0,641012762	0,521514391	0,870221868
ENSG00000185033	SEMA4B	semaphorin 4B	10509	38,65183303	-0,029598992	0,285982	-0,103499367	0,917566652	NA
ENSG00000104972	LILRB1	leukocyte immunoglobulin like receptor B1	10859	106,8098318	-0,252291483	0,193357	-1,30479792	0,191961668	0,649982152
ENSG00000143198	MGST3	microsomal glutathione S-transferase 3	4259	39,11973149	0,058439923	0,282618	0,20678076	0,836181083	NA
ENSG00000140265	ZSCAN29	zinc finger and SCAN domain containing 29	146050	219,7343321	-0,088667159	0,130393	-0,679998235	0,496505578	0,86123475
ENSG00000168556	ING2	inhibitor of growth family member 2	3622	16,06458152	0,3315726	0,448183	0,739815729	0,459411814	NA
ENSG00000187193	MT1X	metallothionein 1X	4501	15,18920217	0,399237772	0,447234	0,892682876	0,372027023	NA
ENSG00000204160	ZDHHC18	zinc finger DHHC-type palmitoyltransferase 18	84243	301,2315973	0,113311747	0,101332	1,118219856	0,263473106	0,719682465
ENSG00000259205	NA	NA	NA	13,57789992	-0,236577436	0,553656	-0,427300323	0,669160598	NA
ENSG00000129932	DOHH	deoxyhypusine hydroxylase	83475	19,84233046	0,216308948	0,462322	0,467874803	0,639874123	NA
ENSG00000145247	OCIAD2	OCIA domain containing 2	132299	63,86309336	-0,216100773	0,277592	-0,778483195	0,43628421	0,836566451
ENSG00000185710	SMG1P4	SMG1 pseudogene 4	100507526	141,5442343	0,105096678	0,147772	0,711206957	0,476955998	0,853811879
ENSG00000197822	OCLN	occludin	100506658	24,40968308	-0,038520131	0,419986	-0,091717755	0,92692229	NA
ENSG00000105607	GCDH	glutaryl-CoA dehydrogenase	2639	72,10991265	-0,546515611	0,315842	-1,730343766	0,083568874	0,471515762
ENSG00000166228	PCBD1	pterin-4 alpha-carbinolamine dehydratase 1	5092	26,21769548	-0,22729096	0,335981	-0,676498488	0,498724206	NA
ENSG00000101146	RAE1	ribonucleic acid export 1	8480	90,52315031	0,176923896	0,267419	0,661597676	0,508229097	0,864738156
ENSG00000108671	PSMD11	proteasome 26S subunit, non-ATPase 11	5717	255,432545	0,035014551	0,122352	0,286178192	0,774741646	0,946448613

ENSG00000139668	WDFY2	WD repeat and FYVE domain containing 2	115825	616,7346171	0,176237756	0,073466	2,398918438	0,016443577	0,202942572
ENSG00000163214	DHX57	DExH-box helicase 57	90957	76,03182391	-0,232602112	0,339291	-0,685553745	0,492994558	0,860513824
ENSG00000103342	GSPT1	G1 to S phase transition 1	2935	445,5114644	0,089473308	0,101927	0,87781839	0,380042283	0,805845596
ENSG00000103343	ZNF174	zinc finger protein 174	7727	46,85874318	0,032678101	0,298713	0,109396351	0,912888128	0,980488351
ENSG00000106133	NA	NA	NA	27,07689323	0,081614914	0,377627	0,216125512	0,828889914	NA
ENSG00000106591	MRPL32	mitochondrial ribosomal protein L32	64983	62,33213727	0,061611928	0,225198	0,273589913	0,784399786	0,95013509
ENSG00000137054	POLR1E	RNA polymerase I subunit E	64425	72,37288438	0,115926995	0,20651	0,561362665	0,57455033	0,886894319
ENSG00000140948	ZCCHC14	zinc finger CCHC-type containing 14	23174	55,0615633	-0,59252652	0,309965	-1,911591605	0,055928597	0,38263345
ENSG00000143409	MINDY1	MINDY lysine 48 deubiquitinase 1	55793	61,56751897	-0,491455475	0,226769	-2,167205535	0,030219186	0,279077601
ENSG00000144559	TAMM41	TAM41 mitochondrial translocator assembly and maintenance homolog	132001	18,72043374	0,244185962	0,47547	0,513567698	0,607554268	NA
ENSG00000239697	TNFSF12	TNF superfamily member 12	8742	157,144601	-0,105068546	0,267977	-0,392080204	0,694998954	0,929397132
ENSG00000256223	ZNF10	zinc finger protein 10	7556	59,27903359	-0,075462876	0,246721	-0,305863495	0,759708581	0,942901768
ENSG00000117143	UAP1	UDP-N-acetylglucosamine pyrophosphorylase 1	6675	80,58120598	-0,095643859	0,183376	-0,521573449	0,601967354	0,896785204
ENSG00000276345	MRPL23	mitochondrial ribosomal protein L23	6150	18,36162047	0,388824378	0,483402	0,80434972	0,42119503	NA
ENSG00000165806	CASP7	caspase 7	840	109,8523896	0,239220977	0,182495	1,310838849	0,189912214	0,647812504
ENSG00000106615	RHEB	Ras homolog, mTORC1 binding	6009	138,8639666	0,196032971	0,157966	1,240983218	0,214611948	0,670470264
ENSG00000103042	SLC38A7	solute carrier family 38 member 7	55238	65,45661356	-0,037622418	0,296555	-0,126864905	0,899047324	0,977990819
ENSG00000106290	TAF6	TATA-box binding protein associated factor 6	6878	120,9087921	-0,370472475	0,223485	-1,657706884	0,097376644	0,504815947
ENSG00000115649	CNPPD1	cyclin Pas1/PHO80 domain containing 1	27013	227,9639611	-0,110897939	0,127447	-0,870151574	0,384217576	0,808586507
ENSG00000116120	FARSB	phenylalanyl-tRNA synthetase subunit beta	10056	126,1770425	0,100024665	0,179843	0,556176655	0,578090097	0,887069818
ENSG00000135476	ESPL1	extra spindle pole bodies like 1, separase	9700	85,4780083	0,886841405	0,643743	1,377632147	0,16831689	0,627733684
ENSG00000143862	ARL8A	ADP ribosylation factor like GTPase 8A	127829	76,17576113	-0,247745285	0,197752	-1,252808241	0,210275502	0,664554962
ENSG00000150093	ITGB1	integrin subunit beta 1	3688	1326,736023	0,056789572	0,062675	0,906098101	0,36488393	0,793678617
ENSG00000151148	UBE3B	ubiquitin protein ligase E3B	89910	210,0922054	-0,043065998	0,139041	-0,309736309	0,756761489	0,942901768
ENSG00000179387	ELMOD2	ELMO domain containing 2	255520	116,2600954	0,070999759	0,202131	0,351257011	0,725395543	0,935971184
ENSG00000043093	DCUN1D1	defective in cullin neddylation 1 domain containing 1	54165	347,5796299	-0,137479934	0,093821	-1,465339402	0,142828341	0,587795357
ENSG00000095380	NANS	N-acetylneuraminate synthase	54187	44,28546379	-0,352312201	0,302923	-1,163043929	0,24481168	0,701819279
ENSG00000123737	EXOSC9	exosome component 9	5393	72,65884738	0,365884974	0,296005	1,236077651	0,216429701	0,672891335
ENSG00000090006	LTBP4	latent transforming growth factor beta binding protein 4	8425	25,2422962	0,016663451	0,424784	0,039228014	0,968708599	NA
ENSG00000100580	TMED8	transmembrane p24 trafficking protein family member 8	283578	121,6336642	0,04716853	0,157836	0,298845795	0,765057707	0,944359403
ENSG00000130764	LRRC47	leucine rich repeat containing 47	57470	163,7353711	-0,099444413	0,13439	-0,739966751	0,45932017	0,846574514
ENSG00000136147	PHF11	PHD finger protein 11	51131	101,7238845	0,098426873	0,178008	0,552934473	0,580308281	0,888049268
ENSG00000138101	DTNB	dystrobrevin beta	1838	29,80819826	-0,460133553	0,479752	-0,959106911	0,337504889	NA
ENSG00000139624	CERS5	ceramide synthase 5	91012	63,21940429	-0,661700799	1,291166	-0,512483095	0,608312949	0,897640621
ENSG00000154734	ADAMTS1	ADAM metallopeptidase with thrombospondin type 1 motif 1	9510	292,4976292	-0,133772541	0,373701	-0,357966604	0,720368306	0,933530522
ENSG00000163870	TPRA1	transmembrane protein adipocyte associated 1	131601	25,37958463	-0,258168265	0,347353	-0,743244194	0,457333852	NA
ENSG00000170043	TRAPPC1	trafficking protein particle complex subunit 1	58485	268,1443199	-0,166843745	0,110541	-1,509342141	0,131211371	0,567474728
ENSG00000173548	SNX33	sorting nexin 33	257364	55,60992093	-0,276771907	0,240871	-1,149044978	0,250537435	0,707335884
ENSG00000177000	MTHFR	methylenetetrahydrofolate reductase	4524	232,7585109	-0,158475564	0,119875	-1,322004297	0,18616672	0,644598455
ENSG00000177034	MTX3	metaxin 3	345778	156,4591736	0,283252509	0,14805	1,913219365	0,055719975	0,382350454
ENSG00000177192	PUS1	pseudouridine synthase 1	80324	48,95318209	-0,269878465	0,32773	-0,823478151	0,410236137	0,82130194
ENSG00000197603	CPLANE1	ciliogenesis and planar polarity effector complex subunit 1	65250	123,199746	-0,443752599	0,177025	-2,506727962	0,012185445	0,17377383
ENSG00000213020	ZNF611	zinc finger protein 611	81856	114,3523477	0,161860287	0,279688	0,57871732	0,562779928	0,884605624
ENSG00000262919	CCNQ	cyclin Q	92002	28,45190197	0,618382179	0,431647	1,432610446	0,151969195	NA
ENSG00000277406	NA	NA	NA	12,1811986	-0,862849441	0,552828	-1,56079307	0,118572583	NA
ENSG00000153187	HNRNPU	heterogeneous nuclear ribonucleoprotein U	3192	1328,141484	0,215923147	0,109013	1,980707033	0,047624138	0,349910571
ENSG00000007376	RPUSD1	RNA pseudouridine synthase domain containing 1	113000	60,2568786	-0,122650231	0,238201	-0,514902072	0,606621453	0,897481254
ENSG00000061337	LZTS1	leucine zipper tumor suppressor 1	11178	308,3416584	0,099428916	0,095864	1,037186126	0,299649123	0,750202136

ENSG00000076201	PTPN23	protein tyrosine phosphatase non-receptor type 23	25930	201,16259	0,010329758	0,159744	0,064664647	0,948441011	0,990608484
ENSG00000124177	CHD6	chromodomain helicase DNA binding protein 6	84181	502,5246119	-0,025691701	0,078813	-0,325984823	0,744435838	0,939745744
ENSG00000155545	MIER3	MIER family member 3	166968	114,3311604	-0,292788134	0,20247	-1,446083672	0,148153735	0,601032765
ENSG00000178802	MPI	mannose phosphate isomerase	4351	65,9991853	0,132539802	0,261541	0,506765136	0,612319621	0,900006614
ENSG00000188342	GTF2F2	general transcription factor IIF subunit 2	2963	48,07463943	0,299189271	0,259989	1,150776933	0,249824017	0,707335884
ENSG00000213465	ARL2	ADP ribosylation factor like GTPase 2	402	20,29973608	-0,221879601	0,454309	-0,488389119	0,625274248	NA
ENSG00000151465	CDC123	cell division cycle 123	8872	264,1062327	0,032310906	0,108757	0,297094006	0,766394741	0,944698783
ENSG00000173258	ZNF483	zinc finger protein 483	158399	14,87923421	-0,661875331	0,858742	-0,770750146	0,440855042	NA
ENSG00000178927	CYBC1	cytochrome b-245 chaperone 1	79415	90,99136434	0,256419002	0,296971	0,863449127	0,387890578	0,809686612
ENSG00000149657	LSM14B	LSM family member 14B	149986	140,7679441	0,027765446	0,141926	0,195633878	0,844896742	0,967231052
ENSG00000163389	POGLUT1	protein O-glucosyltransferase 1	56983	144,5469973	-0,236645346	0,153782	-1,538836233	0,123844282	0,561265429
ENSG00000175105	ZNF654	zinc finger protein 654	55279	155,0839957	-0,017952817	0,156615	-0,114629959	0,908738433	0,97930822
ENSG00000111678	C12orf57	chromosome 12 open reading frame 57	113246	119,0410994	0,198704468	0,178887	1,110779614	0,266663223	0,722327312
ENSG00000170832	USP32	ubiquitin specific peptidase 32	84669	228,403837	-0,006027365	0,145124	-0,041532517	0,96687137	0,99290167
ENSG00000226259	NA	NA	NA	30,07128376	-0,13326355	0,374511	-0,355833646	0,72196515	NA
ENSG00000251634	NA	NA	NA	29,2413014	0,516663805	2,457996	0,210197156	0,833513799	NA
ENSG00000256537	SMIM10L1	small integral membrane protein 10 like 1	100129361	35,83501204	0,022633819	0,402098	0,056289358	0,955111297	NA
ENSG00000055211	GINM1	glycosylated integral membrane protein 1	116254	40,76408261	-0,16531214	0,298949	-0,552978029	0,580278455	NA
ENSG00000118046	STK11	serine/threonine kinase 11	6794	161,1297642	-0,087651944	0,177424	-0,494025096	0,621288456	0,904764752
ENSG00000129515	SNX6	sorting nexin 6	58533	280,4504705	0,05155994	0,112459	0,458479243	0,646608171	0,913689769
ENSG00000144744	UBA3	ubiquitin like modifier activating enzyme 3	9039	322,6021726	0,035717077	0,100233	0,356341625	0,72158474	0,934123381
ENSG00000154710	RABGEF1	RAB guanine nucleotide exchange factor 1	27342	123,616737	0,030977503	0,191998	0,161342521	0,871823635	0,974150353
ENSG00000168876	ANKRD49	ankyrin repeat domain 49	54851	102,2605234	-0,644877289	0,206677	-3,120224086	0,001807135	0,051326425
ENSG00000133111	RFXAP	regulatory factor X associated protein	5994	46,14456152	-0,365087817	0,272264	-1,34093217	0,179942476	0,633591125
ENSG00000143437	ARNT	aryl hydrocarbon receptor nuclear translocator	405	384,5661903	-0,078995716	0,099668	-0,792585156	0,42801956	0,832060923
ENSG00000065491	TBC1D22B	TBC1 domain family member 22B	55633	104,8337535	-0,155094678	0,188467	-0,822927623	0,410549153	0,82130516
ENSG00000136490	LIMD2	LIM domain containing 2	80774	474,0394805	-0,106035302	0,122732	-0,863961045	0,38760929	0,809653368
ENSG00000165704	HPRT1	hypoxanthine phosphoribosyltransferase 1	3251	117,8800512	0,019315713	0,155408	0,124290319	0,901085412	0,977990819
ENSG00000165916	PSMC3	proteasome 26S subunit, ATPase 3	5702	210,7872484	0,231050558	0,122368	1,888167506	0,059003467	0,39312401
ENSG00000187091	PLCD1	phospholipase C delta 1	5333	28,38264292	0,024770569	0,338806	0,073111309	0,941717543	NA
ENSG00000213949	ITGA1	integrin subunit alpha 1	3672	238,8230685	0,587587931	0,183496	3,202191957	0,001363861	0,042847051
ENSG00000239264	TXNDC5	thioredoxin domain containing 5	81567	234,0411068	0,08997251	0,128596	0,699654583	0,484143046	0,85698884
ENSG00000166140	ZFYVE19	zinc finger FYVE-type containing 19	84936	41,26751971	-0,259620395	0,338869	-0,766136978	0,443594802	NA
ENSG00000261732	NA	NA	NA	25,12920186	0,32551946	0,372211	0,874555975	0,381815551	NA
ENSG00000116266	STXBP3	syntaxin binding protein 3	6814	189,2578189	-0,058756013	0,153182	-0,383569797	0,701297329	0,930846779
ENSG00000136153	LMO7	LIM domain 7	4008	54,85265707	0,599508442	0,345798	1,73369498	0,082972218	0,469016904
ENSG00000138399	FASTKD1	FAST kinase domains 1	79675	91,24627803	-0,037302996	0,201951	-0,184712935	0,8534542	0,968377739
ENSG00000166226	CCT2	chaperonin containing TCP1 subunit 2	10576	294,1586037	0,013224003	0,106068	0,124674656	0,900781122	0,977990819
ENSG00000049541	RFC2	replication factor C subunit 2	5982	91,74417714	0,171838091	0,179592	0,956827367	0,338654396	0,777431905
ENSG00000165115	KIF27	kinesin family member 27	55582	31,29993341	0,358584624	0,340231	1,053944339	0,291908402	NA
ENSG00000115339	GALNT3	polypeptide N-acetylgalactosaminyltransferase 3	2591	44,70381958	0,227425912	0,304255	0,747485001	0,454770853	0,843234809
ENSG00000158711	ELK4	ETS transcription factor ELK4	2005	681,7989404	-0,046949415	0,074664	-0,628807162	0,529475311	0,872145761
ENSG00000204227	RING1	ring finger protein 1	6015	85,29106047	0,847459712	1,935005	0,437962532	0,661413444	0,91978661
ENSG00000270800	RPS10-NUDT3	RPS10-NUDT3 readthrough	100529239	23,7910052	-0,066759343	2,553614	-0,02614308	0,979143216	NA
ENSG00000020426	MNAT1	MNAT1 component of CDK activating kinase	4331	81,4138725	-0,076346834	0,194936	-0,391650945	0,69531614	0,929397132
ENSG00000055147	FAM114A2	family with sequence similarity 114 member A2	10827	102,9517568	0,158937928	0,179849	0,883731224	0,376841321	0,803373958
ENSG00000099341	PSMD8	proteasome 26S subunit, non-ATPase 8	5714	233,5129041	0,289550099	0,151247	1,914415314	0,05556711	0,382157692
ENSG00000099810	MTAP	methylthioadenosine phosphorylase	4507	98,16150565	-0,082992786	0,321618	-0,258047354	0,796370362	0,953459271
ENSG00000100413	POLR3H	RNA polymerase III subunit H	171568	108,1272967	-0,103760579	0,171674	-0,604406054	0,545573712	0,878739902
ENSG00000106049	HIBADH	3-hydroxyisobutyrate dehydrogenase	11112	51,99922844	-0,007849593	0,243437	-0,032244824	0,974276811	0,994534085

ENSG00000114853	ZBTB47	zinc finger and BTB domain containing 47	92999	24,97161412	-0,393135447	0,366097	-1,073856542	0,282886984	NA
ENSG00000116586	LAMTOR2	late endosomal/lysosomal adaptor, MAPK and MTOR activator 2	28956	58,85338966	-0,147837242	0,231086	-0,63974871	0,522335982	0,870221868
ENSG00000118690	ARMC2	armadillo repeat containing 2	84071	33,75794894	-0,309718256	0,312543	-0,990960956	0,321704646	NA
ENSG00000125618	PAX8	paired box 8	7849	16,03876308	-0,110616852	0,641644	-0,172396079	0,863126154	NA
ENSG00000125753	VASP	vasodilator stimulated phosphoprotein	7408	549,5371441	0,086970823	0,101941	0,853151539	0,393575274	0,812498986
ENSG00000126458	RRAS	RAS related	6237	16,29294066	-0,867748783	0,48364	-1,79420508	0,072780442	NA
ENSG00000126775	ATG14	autophagy related 14	22863	106,7461286	-0,455008971	0,212523	-2,140984762	0,032275267	0,287228571
ENSG00000133104	SPART	spartin	23111	28,64341951	0,203346699	0,325126	0,62544025	0,531682152	NA
ENSG00000145882	PCYOX1L	prenylcysteine oxidase 1 like	78991	19,19951914	-0,123427258	1,747928	-0,070613453	0,943705403	NA
ENSG00000162636	EEIG2	EEIG family member 2	284611	265,8213378	-0,081019387	0,131127	-0,617871574	0,536659998	0,874923109
ENSG00000162729	IGSF8	immunoglobulin superfamily member 8	93185	80,23409067	-0,234614095	0,198703	-1,180724916	0,23771202	0,692760744
ENSG00000183444	OR7E38P	olfactory receptor family 7 subfamily E member 38 pseudogene	389537	25,17737252	-0,030662782	0,399147	-0,076820684	0,938766196	NA
ENSG00000244462	RBM12	RNA binding motif protein 12	10137	449,8462043	0,004980624	0,09344	0,053302815	0,957490638	0,991911599
ENSG00000250575	NA	NA	NA	15,9377627	-0,477065879	0,509488	-0,936363587	0,349086019	NA
ENSG00000120798	NR2C1	nuclear receptor subfamily 2 group C member 1	7181	80,42160573	0,293919034	0,244177	1,203710664	0,228701429	0,68351286
ENSG00000146540	C7orf50	chromosome 7 open reading frame 50	84310	16,80162837	-0,579565928	0,496191	-1,168030741	0,242794365	NA
ENSG00000198315	ZKSCAN8	zinc finger with KRAB and SCAN domains 8	7745	362,6050192	-0,159429924	0,110291	-1,445538099	0,1483068	0,60134311
ENSG00000204599	TRIM39	tripartite motif containing 39	56658	23,61635438	-7,632006321	3,308167	-2,307019652	0,021053725	NA
ENSG00000114354	TFG	trafficking from ER to golgi regulator	10342	99,22385269	-0,093467432	0,241014	-0,387809477	0,698157031	0,930783276
ENSG00000092847	AGO1	argonaute RISC component 1	26523	283,3519956	-0,027045396	0,38622	-0,070025873	0,944173067	0,989126091
ENSG00000100811	YY1	YY1 transcription factor	7528	603,8262217	0,29286815	0,086764	3,375464881	0,000736911	0,027959897
ENSG00000104960	PTOV1	PTOV1 extended AT-hook containing adaptor protein	53635	64,03069201	-0,085030915	0,318622	-0,266870749	0,789568679	0,951553231
ENSG00000112167	SAYSD1	SAYSVFN motif domain containing 1	55776	15,82318304	-0,233953776	0,531164	-0,440455126	0,659607506	NA
ENSG00000120159	CAAP1	caspase activity and apoptosis inhibitor 1	79886	68,92830535	0,219876538	0,22582	0,97368001	0,330215446	0,771479881
ENSG00000121807	CCR2	C-C motif chemokine receptor 2	729230	35,7654578	-0,035524426	0,316976	-0,112072973	0,910765547	NA
ENSG00000132549	VPS13B	vacuolar protein sorting 13 homolog B	157680	525,6428485	0,014202005	0,260649	0,054487113	0,956547075	0,991798465
ENSG00000136273	HUS1	HUS1 checkpoint clamp component	3364	56,50810453	-0,230390353	0,235925	-0,976539403	0,328797232	0,770885747
ENSG00000139190	VAMP1	vesicle associated membrane protein 1	6843	269,7571802	-0,004515922	0,100216	-0,045062101	0,96405781	0,992728748
ENSG00000170310	STX8	syntaxin 8	9482	58,82543359	-0,040925844	0,223045	-0,18348723	0,854415737	0,968377739
ENSG00000170634	ACYP2	acylphosphatase 2	98	23,92643423	0,443737506	0,357385	1,241622458	0,214375892	NA
ENSG00000181035	SLC25A42	solute carrier family 25 member 42	284439	103,467058	-0,212147887	0,198572	-1,068366342	0,285355294	0,737867955
ENSG00000181938	GINS3	GINS complex subunit 3	64785	24,94188802	0,533491504	0,429155	1,243120506	0,21382343	NA
ENSG00000269825	LOC122539214	Zinc finger protein LOC122539214	122539214	118,8177692	-0,102147261	0,168202	-0,607290327	0,543658257	0,877675632
ENSG00000215630	GUSBP9	GUSB pseudogene 9	118126072	26,68781871	0,286788483	0,37964	0,755421858	0,449995902	NA
ENSG00000220517	ASS1P1	argininosuccinate synthetase 1 pseudogene 1	442167	15,65153965	0,732360576	0,537479	1,362583686	0,173013753	NA
ENSG00000196505	GDAP2	ganglioside induced differentiation associated protein 2	54834	110,48455	0,081071818	0,190112	0,426441738	0,669785994	0,922641254
ENSG00000130725	UBE2M	ubiquitin conjugating enzyme E2 M	9040	91,7011724	0,204130099	0,189989	1,074429806	0,28263009	0,736488628
ENSG00000165487	MICU2	mitochondrial calcium uptake 2	221154	162,7212124	0,021204499	0,160023	0,132509442	0,894581355	0,977990819
ENSG00000188641	DPYD	dihydropyrimidine dehydrogenase	1806	284,0313429	0,021899394	0,105782	0,207023278	0,835991679	0,964577442
ENSG00000083290	ULK2	unc-51 like autophagy activating kinase 2	9706	90,43111724	-0,014017623	0,227556	-0,061600644	0,950880864	0,991212866
ENSG00000138459	SLC35A5	solute carrier family 35 member A5	55032	62,78136818	-0,417656938	0,27369	-1,52602044	0,127004774	0,563689485
ENSG00000131375	CAPN7	calpain 7	23473	202,1860597	0,038529596	0,126736	0,304014689	0,7611167	0,942901768
ENSG00000147437	GNRH1	gonadotropin releasing hormone 1	2796	20,12557431	-0,449705041	0,510876	-0,880262499	0,378717123	NA
ENSG00000134243	SORT1	sortilin 1	6272	307,9195763	0,117504236	0,105801	1,110619701	0,266732079	0,722327312
ENSG00000113318	MSH3	mutS homolog 3	4437	181,272462	0,086715007	0,143286	0,605189801	0,545052892	0,878589015
ENSG00000134453	RBM17	RNA binding motif protein 17	84991	197,4361236	0,208575988	0,12497	1,669006499	0,095116092	0,499178345
ENSG00000143442	POGZ	pogo transposable element derived with ZNF domain	23126	422,5321432	-0,132245485	0,105624	-1,252038261	0,210555923	0,664672917
ENSG00000267855	NDUFA7	NADH:ubiquinone oxidoreductase subunit A7	4701	23,51268098	-0,065394228	0,469565	-0,139265549	0,889240313	NA
ENSG00000269556	TMEM185A	transmembrane protein 185A	84548	104,9269357	-0,037301145	0,16517	-0,225835518	0,821329351	0,961898134

ENSG00000123562	MORF4L2	mortality factor 4 like 2	9643	179,3623856	-0,244443481	0,152817	-1,599584017	0,109690896	0,529522903
ENSG00000111785	RIC8B	RIC8 guanine nucleotide exchange factor B	55188	46,87027867	-0,122805443	0,272587	-0,450518488	0,652336626	0,916482964
ENSG00000205784	ARRDC5	arrestin domain containing 5	645432	24,13088496	-0,01622202	0,423505	-0,038304213	0,969445132	NA
ENSG00000159842	ABR	ABR activator of RhoGEF and GTPase	29	136,8526305	0,3807865	0,76832	0,495609096	0,620170234	0,904287685
ENSG00000156787	TBC1D31	TBC1 domain family member 31	93594	126,9885222	0,058092288	0,222988	0,260518134	0,794464129	0,952629202
ENSG00000136478	TEX2	testis expressed 2	55852	171,2816574	0,012922053	0,127346	0,101472173	0,919175646	0,984062493
ENSG00000119711	ALDH6A1	aldehyde dehydrogenase 6 family member A1	4329	78,91163686	-0,192599439	0,31556	-0,610341388	0,541635686	0,876623646
ENSG00000183323	CCDC125	coiled-coil domain containing 125	202243	62,22309174	0,466757284	0,341656	1,366159988	0,171888753	0,629558603
ENSG00000106263	EIF3B	eukaryotic translation initiation factor 3 subunit B	8662	481,7961558	0,089827377	0,080739	1,112560697	0,265897148	0,722114869
ENSG00000161011	SQSTM1	sequestosome 1	8878	296,213862	-0,150392318	0,125473	-1,198601687	0,230682862	0,685179309
ENSG00000035141	FAM136A	family with sequence similarity 136 member A	84908	107,0528637	0,146444945	0,173864	0,842296408	0,399622061	0,814673014
ENSG00000090674	MCOLN1	mucolipin TRP cation channel 1	57192	29,56350147	0,114073854	0,389236	0,29307132	0,769467638	NA
ENSG00000140497	SCAMP2	secretory carrier membrane protein 2	10066	317,730853	0,043523779	0,096775	0,4497418	0,652896627	0,916753963
ENSG00000158156	XKR8	XK related 8	55113	20,45034383	0,621931509	1,672276	0,371907285	0,709961881	NA
ENSG00000159433	STARD9	StAR related lipid transfer domain containing 9	57519	1066,718277	0,163655123	0,083435	1,961476387	0,049823477	0,357759312
ENSG00000164167	LSM6	LSM6 homolog, U6 small nuclear RNA and mRNA degradation associated	11157	18,18800828	0,112510605	0,440328	0,255515591	0,798324903	NA
ENSG00000176386	CDC26	cell division cycle 26	246184	65,09161323	-0,036631578	0,209044	-0,175233688	0,860896014	0,970639865
ENSG00000188690	UROS	uroporphyrinogen III synthase	7390	99,3583471	0,213727522	0,173391	1,232636182	0,217711532	0,673519155
ENSG00000196465	MYL6B	myosin light chain 6B	140465	21,79826691	0,4429803	0,360338	1,22934721	0,218941654	NA
ENSG00000197019	SERTAD1	SERTA domain containing 1	29950	30,14730292	0,084484011	0,306804	0,27536772	0,783033742	NA
ENSG00000204922	UQCC3	ubiquinol-cytochrome c reductase complex assembly factor 3	790955	11,71628525	-0,595210935	0,529739	-1,123593122	0,261185675	NA
ENSG00000206530	CFAP44	cilia and flagella associated protein 44	55779	26,65928736	0,374993344	0,438693	0,854797165	0,392663448	NA
ENSG00000107669	ATE1	arginyltransferase 1	11101	176,5273389	0,189825562	0,202989	0,935151221	0,349710373	0,784974355
ENSG00000113569	NUP155	nucleoporin 155	9631	230,5232342	-0,119706172	0,127831	-0,93643948	0,349046959	0,784537594
ENSG00000118689	FOXO3	forkhead box O3	2309	408,5760795	-0,048313951	0,105032	-0,459990803	0,645522822	0,91284412
ENSG00000132207	SLX1A	SLX1 homolog A, structure-specific endonuclease subunit	548593	50,29540913	0,378099746	0,310357	1,218271898	0,223120665	0,678694696
ENSG00000132207	LOC124907837	sulfotransferase 1A3	124907837	50,29540913	0,378099746	0,310357	1,218271898	0,223120665	0,678694696
ENSG00000214226	C17orf67	chromosome 17 open reading frame 67	339210	13,36458195	0,007369165	0,525021	0,014035951	0,988801299	NA
ENSG00000229833	PET100	PET100 cytochrome c oxidase chaperone	100131801	46,44078004	0,377576855	0,282165	1,338140421	0,180850666	0,634107649
ENSG00000068354	TBC1D25	TBC1 domain family member 25	4943	119,2366924	-0,123044183	0,154179	-0,798060637	0,424835303	0,830220569
ENSG00000063127	SLC6A16	solute carrier family 6 member 16	28968	17,62007569	-0,003216496	0,421709	-0,007627282	0,993914369	NA
ENSG00000175727	MLXIP	MLX interacting protein	22877	291,4000547	0,129270685	0,101182	1,277606698	0,201388138	0,660119983
ENSG00000185379	RAD51D	RAD51 paralog D	5892	146,3530609	-0,08880457	0,202411	-0,438733539	0,660854622	0,91978661
ENSG00000131899	LLGL1	LLGL scribble cell polarity complex component 1	3996	114,6524029	-0,29652952	0,170278	-1,741446716	0,081605306	0,464231481
ENSG00000012174	MBTPS2	membrane bound transcription factor peptidase, site 2	51360	67,56138278	0,263245848	0,213003	1,235875809	0,21650473	0,672891335
ENSG00000067533	RRP15	ribosomal RNA processing 15 homolog	51018	74,64945917	-0,037198209	0,196403	-0,189397449	0,849781324	0,967902179
ENSG00000073614	KDM5A	lysine demethylase 5A	5927	981,760145	0,066273549	0,073263	0,904601099	0,365676753	0,794254762
ENSG00000123983	ACSL3	acyl-CoA synthetase long chain family member 3	2181	122,6509561	0,20081548	0,198919	1,009532608	0,312719271	0,758054678
ENSG00000124299	PEPD	peptidase D	5184	92,5876348	0,298672276	0,177677	1,680985143	0,0927658	0,494966917
ENSG00000149136	SSRP1	structure specific recognition protein 1	6749	396,7269835	0,105482377	0,084462	1,24887768	0,211709816	0,665906647
ENSG00000169105	CHST14	carbohydrate sulfotransferase 14	113189	71,18250732	0,045618984	0,216768	0,210450951	0,83331573	0,963446071
ENSG00000182180	MRPS16	mitochondrial ribosomal protein S16	51021	167,4681622	-0,129608339	0,147134	-0,880888203	0,378378333	0,804187219
ENSG00000182220	ATP6AP2	ATPase H+ transporting accessory protein 2	10159	291,9998614	0,176678474	0,143226	1,233565217	0,217364961	0,673445523
ENSG00000266967	AARSD1	alanyl-tRNA synthetase domain containing 1	80755	28,95219239	0,325296054	0,401188	0,810831437	0,417462478	NA
ENSG00000114850	SSR3	signal sequence receptor subunit 3	6747	458,91771	-0,046072457	0,136872	-0,336610604	0,736410462	0,939157822
ENSG00000128271	ADORA2A	adenosine A2a receptor	135	18,76435399	-1,781439438	1,529296	-1,164875415	0,244069426	NA

ENSG00000132950	ZMYM5	zinc finger MYM-type containing 5	9205	89,66954597	-0,373246521	0,219643	-1,699332628	0,089256528	0,488176025
ENSG00000196873	ZNG1C	Zn regulated GTPase metalloprotein activator 1C	445571	26,56073423	-0,394307723	0,58511	-0,673903215	0,500372848	NA
ENSG00000106588	PSMA2	proteasome 20S subunit alpha 2	5683	232,3170733	0,331109555	0,206617	1,602531132	0,109038209	0,52908614
ENSG00000113575	PPP2CA	protein phosphatase 2 catalytic subunit alpha	5515	479,5608624	0,028905992	0,077112	0,374857744	0,707766267	0,930846779
ENSG00000137601	NEK1	NIMA related kinase 1	4750	75,06993007	0,322738087	0,892603	0,36156974	0,71767358	0,932595682
ENSG00000142528	ZNF473	zinc finger protein 473	25888	44,96981825	0,072014842	0,275567	0,261333504	0,793835331	0,952276252
ENSG00000143870	PDIA6	protein disulfide isomerase family A member 6	10130	801,9104949	0,056710805	0,06896	0,822378129	0,410861723	0,82130516
ENSG00000144504	ANKMY1	ankyrin repeat and MYND domain containing 1	51281	36,74402575	-0,377975815	0,49191	-0,768384389	0,442258852	NA
ENSG00000171984	SHLD1	shieldin complex subunit 1	149840	15,96755681	-0,323480538	0,501174	-0,645446133	0,518638115	NA
ENSG00000203950	RTL8A	retrotransposon Gag like 8A	26071	21,03661211	-0,329134484	0,489981	-0,67172901	0,50175623	NA
ENSG00000130813	SHFL	shiftless antiviral inhibitor of ribosomal frameshifting	55337	80,38990283	0,184635727	0,238467	0,774262285	0,438775702	0,837388481
ENSG00000261208	NA	NA	NA	47,8451318	-0,40223274	0,270056	-1,489439837	0,136371585	0,57708105
ENSG00000268350	FAM156A	family with sequence similarity 156 member A	29057	25,08313511	-2,102516992	2,286242	-0,91963892	0,357761481	NA
ENSG00000137496	IL18BP	interleukin 18 binding protein	10068	44,06805775	0,220241117	0,2913	0,756063613	0,449611057	0,84263906
ENSG00000062716	VMP1	vacuole membrane protein 1	81671	152,9407571	0,267068613	0,150793	1,771096251	0,076544697	0,447974704
ENSG00000147400	CETN2	centrin 2	1069	42,30360223	0,001598638	0,262106	0,006099199	0,995133573	NA
ENSG00000183060	LYSMD4	LysM domain containing 4	145748	31,62204432	0,163986247	0,470898	0,348241757	0,72765863	NA
ENSG00000224051	CPTP	ceramide-1-phosphate transfer protein	80772	59,32636039	-0,195734435	0,217039	-0,901841932	0,367140841	0,795235567
ENSG00000005206	SPPL2B	signal peptide peptidase like 2B	56928	110,9065438	0,027753022	0,192017	0,144533913	0,885078879	0,977414909
ENSG00000071889	FAM3A	FAM3 metabolism regulating signaling molecule A	60343	42,86939896	-0,196586292	0,282328	-0,69630416	0,486238368	0,858183403
ENSG00000073910	FRY	FRY microtubule binding protein	10129	35,30130281	-0,333215823	0,320883	-1,038432497	0,299068747	NA
ENSG00000089639	GMIP	GEM interacting protein	51291	518,1949412	-0,05024138	0,085872	-0,585074666	0,558497512	0,883921037
ENSG00000100109	TFIP11	tuftelin interacting protein 11	24144	208,1682178	-0,056354502	0,127803	-0,440948791	0,65925007	0,919368176
ENSG00000103187	COTL1	coactosin like F-actin binding protein 1	23406	1577,460476	0,055511371	0,059916	0,926481846	0,354195627	0,788228969
ENSG00000106100	NOD1	nucleotide binding oligomerization domain containing 1	10392	50,43102044	0,096563116	0,289673	0,333351797	0,738868745	0,939193793
ENSG00000110921	MVK	mevalonate kinase	4598	29,47508367	0,346466349	0,669763	0,517296707	0,604949052	NA
ENSG00000112992	NNT	nicotinamide nucleotide transhydrogenase	23530	322,8618815	0,157249434	0,119272	1,318411148	0,187366051	0,645758518
ENSG00000114209	PDCD10	programmed cell death 10	11235	178,7940311	-0,03817936	0,173351	-0,220243626	0,825681422	0,961917534
ENSG00000117528	ABCD3	ATP binding cassette subfamily D member 3	5825	136,3636301	0,20936999	0,151426	1,382656187	0,166770312	0,62651041
ENSG00000121766	ZCCHC17	zinc finger CCHC-type containing 17	51538	99,17566231	-0,032771836	0,181561	-0,180499932	0,856760108	0,968618668
ENSG00000126016	AMOT	angiomotin	154796	52,70487592	-0,191305974	0,241929	-0,79075165	0,429088929	0,832060923
ENSG00000130475	FCHO1	FCH and mu domain containing endocytic adaptor 1	23149	125,4847655	-0,179019552	0,177124	-1,010701861	0,312159146	0,757868604
ENSG00000131748	STARD3	StAR related lipid transfer domain containing 3	10948	123,9495561	0,054781302	0,185449	0,295398467	0,767689505	0,944698783
ENSG00000134597	RBMX2	RNA binding motif protein X-linked 2	51634	68,73070673	0,097767879	0,262273	0,372771422	0,709318574	0,930846779
ENSG00000134802	SLC43A3	solute carrier family 43 member 3	29015	59,77116595	0,023376386	0,230686	0,101334071	0,91928527	0,984062493
ENSG00000137494	ANKRD42	ankyrin repeat domain 42	338699	47,39426582	0,190555285	0,252348	0,755127659	0,450172389	0,84263906
ENSG00000139880	CDH24	cadherin 24	64403	15,86738456	-0,203474473	0,4684	-0,434403219	0,66399566	NA
ENSG00000147231	RADX	RPA1 related single stranded DNA binding protein, X-linked	55086	12,55873797	0,057086404	0,761723	0,074943761	0,940259458	NA
ENSG00000148834	GSTO1	glutathione S-transferase omega 1	9446	120,4303364	0,179357891	0,194086	0,924115077	0,355426404	0,788341988
ENSG00000162639	HENMT1	HEN methyltransferase 1	113802	201,7312046	0,017147197	0,115448	0,148527611	0,881926397	0,976300778
ENSG00000163393	SLC22A15	solute carrier family 22 member 15	55356	15,06149342	0,373413889	0,463225	0,806117806	0,420174926	NA
ENSG00000167107	ACSF2	acyl-CoA synthetase family member 2	80221	14,74487597	-0,160710606	0,620366	-0,259057906	0,795590562	NA
ENSG00000167258	CDK12	cyclin dependent kinase 12	51755	385,9032115	0,014380392	0,129397	0,111133459	0,911510516	0,979611877
ENSG00000167280	ENGASE	endo-beta-N-acetylglucosaminidase	64772	122,2721313	0,151746044	0,233491	0,649900244	0,515756661	0,868791049
ENSG00000169860	P2RY1	purinergic receptor P2Y1	5028	67,14531246	-0,304627447	0,202447	-1,504729649	0,132393597	0,569480142
ENSG00000170791	CHCHD7	coiled-coil-helix-coiled-coil-helix domain containing 7	79145	108,1699197	0,174338901	0,18837	0,925514696	0,354698243	0,788341988
ENSG00000170962	PDGFD	platelet derived growth factor D	80310	85,13383773	-0,1403309	0,244085	-0,574926588	0,565340947	0,885045179

ENSG00000172977	KAT5	lysine acetyltransferase 5	10524	141,3582268	0,118454577	0,153552	0,771429708	0,440452272	0,838640381
ENSG00000173715	TOP6BL	TOP6B like initiator of meiotic double strand breaks	79703	23,27088685	0,445781474	0,398926	1,11745337	0,263800527	NA
ENSG00000182512	GLRX5	glutaredoxin 5	51218	68,1113023	0,073058504	0,206958	0,353010668	0,724080443	0,934902053
ENSG00000184047	DIABLO	diablo IAP-binding mitochondrial protein	56616	40,6443478	-0,746918859	0,563953	-1,324434155	0,185358898	NA
ENSG00000186111	PIP5K1C	phosphatidylinositol-4-phosphate 5-kinase type 1 gamma	23396	317,9449592	-0,048778537	0,133224	-0,366138463	0,714261758	0,931552947
ENSG00000196652	ZKSCAN5	zinc finger with KRAB and SCAN domains 5	23660	50,72038427	0,656737272	0,417403	1,573387657	0,115629083	0,541856098
ENSG00000239382	ALKBH6	alkB homolog 6	84964	23,73046187	0,349277428	0,403981	0,864588617	0,387264623	NA
ENSG00000291237	SOD2	superoxide dismutase 2	6648	44,06864789	0,174821034	0,273083	0,640175391	0,52205858	0,870221868
ENSG00000138698	RAP1GDS1	Rap1 GTPase-GDP dissociation stimulator 1	5910	330,3881853	0,063997329	0,103624	0,617594708	0,536842534	0,874923109
ENSG00000175768	TOMM5	translocase of outer mitochondrial membrane 5	401505	68,48368978	0,215973333	0,223111	0,96800719	0,333040779	0,773674527
ENSG00000196284	SUPT3H	SPT3 homolog, SAGA and STAGA complex component	8464	28,01865695	-0,290242218	0,354957	-0,817683831	0,413537746	NA
ENSG00000168256	NKIRAS2	NFKB inhibitor interacting Ras like 2	28511	94,8424577	0,237793073	0,176869	1,344461186	0,1787993	0,633385915
ENSG00000142459	EVI5L	ecotropic viral integration site 5 like	115704	83,78473504	0,029376172	0,195596	0,150187773	0,880616472	0,976300778
ENSG00000083520	DIS3	DIS3 homolog, exosome endoribonuclease and 3'-5' exoribonuclease	22894	293,2150513	-0,201634457	0,14998	-1,344406474	0,178816982	0,633385915
ENSG00000117899	MESD	mesoderm development LRP chaperone	23184	164,5075784	-0,067644144	0,196556	-0,344146962	0,730735767	0,938729652
ENSG00000173163	COMMD1	copper metabolism domain containing 1	150684	33,56070366	0,276229851	0,370135	0,746293902	0,455489894	NA
ENSG00000100450	GZMH	granzyme H	2999	31,61648041	0,236937679	0,326915	0,724767927	0,468594415	NA
ENSG00000149357	LAMTOR1	late endosomal/lysosomal adaptor, MAPK and MTOR activator 1	55004	142,6226679	-0,035207078	0,14703	-0,239454696	0,810753022	0,959038743
ENSG00000214046	SMIM7	small integral membrane protein 7	79086	39,01283065	0,455947676	0,486093	0,937985346	0,348251941	NA
ENSG00000163320	CGGBP1	CGG triplet repeat binding protein 1	8545	696,4890385	0,102087867	0,095551	1,068407122	0,285336906	0,737867955
ENSG00000075702	WDR62	WD repeat domain 62	284403	30,6514228	0,424355461	0,518007	0,819208301	0,41266758	NA
ENSG00000155592	ZKSCAN2	zinc finger with KRAB and SCAN domains 2	342357	27,83461321	-0,156271634	0,358988	-0,435311147	0,663336595	NA
ENSG00000119139	TJP2	tight junction protein 2	9414	18,77834816	-0,057210911	0,769346	-0,074363054	0,940721506	NA
ENSG00000120265	PCMT1	protein-L-isoaspartate (D-aspartate) O-methyltransferase	5110	192,9522256	0,119683012	0,127518	0,938554174	0,34795969	0,784103633
ENSG00000148606	POLR3A	RNA polymerase III subunit A	11128	56,64166139	-2,386584515	2,558915	-0,932655044	0,350998104	0,785712602
ENSG00000152359	POC5	POC5 centriolar protein	134359	57,53716852	0,018501632	0,277541	0,066662768	0,946850175	0,990073571
ENSG00000178440	TIMM23B-AGAP6	TIMM23B-AGAP6 readthrough (NMD candidate)	113218477	12,07916755	0,025614452	2,241668	0,011426517	0,990883157	NA
ENSG00000197150	ABCB8	ATP binding cassette subfamily B member 8	11194	73,16739945	-0,085414665	0,218745	-0,390475346	0,696185083	0,930220762
ENSG00000160767	ENTREP3	endosomal transmembrane epsin interactor 3	10712	36,74060315	0,258747388	0,301079	0,859400147	0,390119789	NA
ENSG00000101464	PIGU	phosphatidylinositol glycan anchor biosynthesis class U	128869	38,9654603	-0,05458942	0,271887	-0,200779994	0,840870607	NA
ENSG00000269955	FMC1-LUC7L2	FMC1-LUC7L2 readthrough	100996928	43,22270175	-0,253061378	2,027467	-0,124816525	0,900668805	0,977990819
ENSG00000165684	SNAPC4	small nuclear RNA activating complex polypeptide 4	6621	102,3257663	-0,043598805	0,224606	-0,194112412	0,846087863	0,967231052
ENSG00000138594	TMOD3	tropomodulin 3	29766	665,3763232	-0,171639655	0,0681	-2,52041693	0,01172159	0,169426994
ENSG00000167136	ENDOG	endonuclease G	2021	18,68101162	-0,160174857	0,441209	-0,36303585	0,716578104	NA
ENSG00000185344	ATP6V0A2	ATPase H+ transporting V0 subunit a2	23545	145,0754924	0,071495378	0,232926	0,306944461	0,758885646	0,942901768
ENSG00000117650	NEK2	NIMA related kinase 2	4751	41,27072124	0,500238213	0,270693	1,847991761	0,064603535	NA
ENSG00000126461	SCAF1	SR-related CTD associated factor 1	58506	228,4137303	-0,254414613	0,11717	-2,171333317	0,029905986	0,279077601
ENSG00000130304	SLC27A1	solute carrier family 27 member 1	376497	48,09077533	-0,445526263	0,34923	-1,275739913	0,202047474	0,660119983
ENSG00000136930	PSMB7	proteasome 20S subunit beta 7	5695	164,9819728	0,133936238	0,159363	0,840449296	0,400656519	0,814673014
ENSG00000155876	RRAGA	Ras related GTP binding A	10670	115,6625647	-0,151023479	0,165457	-0,912767616	0,361364774	0,791408368
ENSG00000161618	ALDH16A1	aldehyde dehydrogenase 16 family member A1	126133	83,60217971	0,107021364	0,194849	0,549253382	0,582831575	0,888501395
ENSG00000168300	PCMTD1	protein-L-isoaspartate (D-aspartate) O-methyltransferase domain containing 1	115294	267,7049521	-0,173597059	0,119134	-1,457157608	0,145072879	0,591897348
ENSG00000135414	GDF11	growth differentiation factor 11	10220	134,1365892	0,003911223	0,161167	0,024268117	0,980638744	0,995752963

ENSG00000091317	CMTM6	CKLF like MARVEL transmembrane domain containing 6	54918	790,0913543	0,045898306	0,061114	0,751032451	0,452633126	0,843234809
ENSG00000204438	GPANK1	G-patch domain and ankyrin repeats 1	7918	37,63468484	-0,236034662	0,346881	-0,680447918	0,496220889	NA
ENSG00000142330	CAPN10	calpain 10	11132	89,12908942	-0,321531783	0,237665	-1,352880694	0,176093748	0,632039836
ENSG00000151657	KIN	Kin17 DNA and RNA binding protein	22944	60,29752936	-0,078633795	0,216945	-0,36245925	0,71700887	0,932040329
ENSG00000188612	SUMO2	small ubiquitin like modifier 2	6613	608,5900001	0,267829839	0,113703	2,355525091	0,018496548	0,212696759
ENSG00000105983	LMBR1	limb development membrane protein 1	64327	46,21803639	0,214987737	0,389125	0,55248982	0,580612807	0,888049268
ENSG00000062485	CS	citrate synthase	1431	498,5053269	-0,037514955	0,081437	-0,460660802	0,645041982	0,912530858
ENSG00000112130	RNF8	ring finger protein 8	9025	27,93840726	-0,374409317	1,096117	-0,341577885	0,732668583	NA
ENSG00000114316	USP4	ubiquitin specific peptidase 4	7375	427,4310308	-0,08755474	0,081746	-1,071059961	0,28414247	0,737423617
ENSG00000121060	TRIM25	tripartite motif containing 25	7706	320,9762602	0,182915082	0,102647	1,781981558	0,074752239	0,444354325
ENSG00000122965	RBM19	RNA binding motif protein 19	9904	197,2858034	-0,019538754	0,129779	-0,150553771	0,880327731	0,976300778
ENSG00000140463	BBS4	Bardet-Biedl syndrome 4	585	51,37642133	0,018126453	0,266515	0,068012905	0,945775362	0,989360641
ENSG00000143222	UFC1	ubiquitin-fold modifier conjugating enzyme 1	51506	217,1470967	0,09139152	0,125952	0,725606795	0,468079854	0,851339954
ENSG00000149212	SESN3	sestrin 3	143686	19,80390119	0,008736501	0,566564	0,015420144	0,987696993	NA
ENSG00000160710	ADAR	adenosine deaminase RNA specific	103	1614,183496	-0,007318467	0,058709	-0,124656868	0,900795205	0,977990819
ENSG00000173230	GOLGB1	golgin B1	2804	619,9834448	0,147055105	0,149442	0,984025133	0,325103155	0,768275024
ENSG00000176396	EID2	EP300 interacting inhibitor of differentiation 2	163126	44,12836015	-0,145658615	0,255931	-0,569132606	0,569266152	0,886263374
ENSG00000225973	PIGBOS1	PIGB opposite strand 1	101928527	27,21860975	-0,356179915	0,360398	-0,988296728	0,323007347	NA
ENSG00000127995	CASD1	CAS1 domain containing 1	64921	198,9132942	-0,10244241	0,144853	-0,707217026	0,47943162	0,854426127
ENSG00000136935	GOLGA1	golgin A1	2800	143,3579563	-0,308322623	0,162099	-1,902065994	0,057162528	0,38821726
ENSG00000174004	NRROS	negative regulator of reactive oxygen species	375387	24,99939452	-0,204602896	0,340357	-0,601141873	0,547745496	NA
ENSG00000280987	MATR3	matrin 3	9782	107,4431573	-0,197159387	1,543748	-0,127714776	0,898374696	0,977990819
ENSG00000225720	NA	NA	NA	16,49777056	0,186931677	0,460329	0,406082674	0,684681856	NA
ENSG00000078304	PPP2R5C	protein phosphatase 2 regulatory subunit B'gamma	5527	636,6344078	0,073249681	0,082141	0,891750427	0,372526718	0,800776142
ENSG00000104983	CCDC61	coiled-coil domain containing 61	729440	32,66807923	-0,514110456	0,306302	-1,678441944	0,093260855	NA
ENSG00000135617	PRADC1	protease associated domain containing 1	84279	21,51668647	0,023882888	0,416867	0,057291453	0,954313029	NA
ENSG00000136682	ZNG1B	Zn regulated GTPase metalloprotein activator 1B	150472	20,45322013	1,327713487	1,564354	0,848729361	0,396031904	NA
ENSG00000144736	SHQ1	SHQ1, H/ACA ribonucleoprotein assembly factor	55164	72,33393164	-0,184217306	0,24714	-0,745396579	0,456032012	0,844219046
ENSG00000173621	LRFN4	leucine rich repeat and fibronectin type III domain containing 4	78999	30,72771139	-0,197899754	0,323216	-0,612283531	0,540350184	NA
ENSG00000167555	ZNF528	zinc finger protein 528	84436	23,64654511	-0,36567792	0,603856	-0,605571467	0,544799354	NA
ENSG00000170260	ZNF212	zinc finger protein 212	7988	49,83723596	0,010928149	0,247322	0,04418595	0,964756181	0,992789539
ENSG00000239789	MRPS17	mitochondrial ribosomal protein S17	51373	39,63042037	-0,210939228	0,277855	-0,759170075	0,447750825	NA
ENSG00000133028	SCO1	synthesis of cytochrome C oxidase 1	6341	156,3128306	-0,059053976	0,168466	-0,350538458	0,725934633	0,936359108
ENSG00000106665	CLIP2	CAP-Gly domain containing linker protein 2	7461	58,6647182	-0,028317412	0,214536	-0,13199402	0,894989021	0,977990819
ENSG00000157895	C12orf43	chromosome 12 open reading frame 43	64897	85,7328326	0,239764174	0,247318	0,969458172	0,33231664	0,773425407
ENSG00000188822	CNR2	cannabinoid receptor 2	1269	576,4786896	-0,105213957	0,08617	-1,221001077	0,222085612	0,678334201
ENSG00000072401	UBE2D1	ubiquitin conjugating enzyme E2 D1	7321	61,16938723	0,180523277	0,264353	0,682886716	0,494678429	0,860513824
ENSG00000136874	STX17	syntaxin 17	55014	243,5300453	-0,146060478	0,146395	-0,997716368	0,318416914	0,763136371
ENSG00000169627	BOLA2	bolA family member 2	552900	16,56501731	1,438366003	1,511937	0,951340169	0,341431722	NA
ENSG00000169627	BOLA2B	bolA family member 2B	654483	16,56501731	1,438366003	1,511937	0,951340169	0,341431722	NA
ENSG00000169627	LOC124907841	bolA-like protein 2	124907841	16,56501731	1,438366003	1,511937	0,951340169	0,341431722	NA
ENSG00000177425	PAWR	pro-apoptotic WT1 regulator	5074	24,24112187	-0,097497806	0,352979	-0,276214509	0,782383314	NA
ENSG00000142227	EMP3	epithelial membrane protein 3	2014	127,6044664	0,054340322	0,179851	0,302141235	0,762544399	0,943004836
ENSG00000146963	LUC7L2	LUC7 like 2, pre-mRNA splicing factor	51631	279,6444013	0,112078657	0,216944	0,51662479	0,605418107	0,897347689
ENSG00000116741	RGS2	regulator of G protein signaling 2	5997	18,57053132	1,138125893	0,419002	2,71627612	0,006602083	NA
ENSG00000120868	APAF1	apoptotic peptidase activating factor 1	317	288,3179994	-0,255039012	0,102203	-2,495406411	0,012581294	0,176138115
ENSG00000122417	ODF2L	outer dense fiber of sperm tails 2 like	57489	98,70853594	0,173232682	0,312667	0,554048373	0,579545741	0,887548253
ENSG00000135457	TFCP2	transcription factor CP2	7024	228,5242801	0,13672674	0,141458	0,966553149	0,333767465	0,773674527
ENSG00000134684	YARS1	tyrosyl-tRNA synthetase 1	8565	662,7703383	-0,021788501	0,076342	-0,285405586	0,775333428	0,946448613
ENSG00000134697	GNL2	G protein nucleolar 2	29889	152,7551568	0,103992568	0,144099	0,721676605	0,470493329	0,852690218

ENSG00000138336	TET1	tet methylcytosine dioxygenase 1	80312	34,2831778	-0,429012291	0,322047	-1,33214207	0,182813508	NA
ENSG00000141858	SAMD1	sterile alpha motif domain containing 1	90378	100,8893318	-0,188185314	0,183121	-1,027654077	0,304112577	0,751870158
ENSG00000148339	SLC25A25	solute carrier family 25 member 25	114789	90,251807	-0,15051634	0,186388	-0,807542483	0,419354009	0,825672195
ENSG00000220008	LINGO3	leucine rich repeat and Ig domain containing 3	645191	11,77043835	-0,34556606	0,536842	-0,643701451	0,519769049	NA
ENSG00000100395	L3MBTL2	L3MBTL histone methyl-lysine binding protein 2	83746	156,6421536	0,042182017	0,142122	0,296801676	0,766617926	0,944698783
ENSG00000149929	HIRIP3	HIRA interacting protein 3	8479	85,03212297	0,02924571	0,222006	0,131734073	0,895194634	0,977990819
ENSG00000166797	CIAO2A	cytosolic iron-sulfur assembly component 2A	84191	113,9336089	0,293773078	0,163165	1,80046506	0,071787236	0,435172111
ENSG00000167302	TEPSIN	TEPSIN adaptor related protein complex 4 accessory protein	146705	38,88773677	-0,022138702	0,310758	-0,071241026	0,94320593	NA
ENSG00000175155	YPEL2	yippee like 2	388403	85,13131124	-0,362726628	0,188092	-1,92845039	0,053799129	0,374590746
ENSG00000089876	DHX32	DEAH-box helicase 32 (putative)	55760	54,76613577	0,223526832	0,233999	0,955248688	0,339451949	0,777431905
ENSG00000083093	PALB2	partner and localizer of BRCA2	79728	70,72641462	-0,185146277	0,335064	-0,55256926	0,580558396	0,888049268
ENSG00000108352	RAPGEFL1	Rap guanine nucleotide exchange factor like 1	51195	12,10700581	-0,342900174	0,58557	-0,58558375	0,558155271	NA
ENSG00000113790	EHHADH	enoyl-CoA hydratase and 3-hydroxyacyl CoA dehydrogenase	1962	22,39648573	0,309367825	0,435708	0,710034495	0,477682745	NA
ENSG00000135390	ATP5MC2	ATP synthase membrane subunit c locus 2	517	242,8198986	0,156910289	0,128524	1,220861476	0,222138473	0,678334201
ENSG00000182224	CYB5D1	cytochrome b5 domain containing 1	124637	40,95757756	-0,320993615	0,268133	-1,197143099	0,231250784	NA
ENSG00000103415	HMOX2	heme oxygenase 2	3163	108,534143	-0,000762131	0,299207	-0,002547173	0,997967652	0,999300722
ENSG00000283149	NA	NA	NA	12,18439529	-1,179526998	2,227173	-0,529607317	0,59638422	NA
ENSG00000066827	ZFAT	zinc finger and AT-hook domain containing	57623	51,36852656	-0,012829027	0,282514	-0,045410245	0,963780315	0,992694268
ENSG00000108960	MMD	monocyte to macrophage differentiation associated	23531	232,1630561	-0,076478066	0,117813	-0,649145357	0,516244427	0,868791049
ENSG00000121741	ZMYM2	zinc finger MYM-type containing 2	7750	256,0769109	-0,000787342	0,113382	-0,006944181	0,994459389	0,998500439
ENSG00000180035	ZNF48	zinc finger protein 48	197407	37,48675465	-0,430146986	0,284898	-1,509826035	0,13108782	NA
ENSG00000180549	FUT7	fucosyltransferase 7	2529	96,2620455	-0,250145356	0,188539	-1,326754618	0,184589867	0,641206907
ENSG00000103510	KAT8	lysine acetyltransferase 8	84148	67,01053169	0,128463641	0,273951	0,468929793	0,63911982	0,910510986
ENSG00000162645	GBP2	guanylate binding protein 2	2634	460,0350885	0,395548787	0,109447	3,614074354	0,000301423	0,014008123
ENSG00000164144	ARFIP1	ADP ribosylation factor interacting protein 1	27236	133,1330352	-0,169596567	0,18896	-0,897526562	0,369438006	0,797571771
ENSG00000085644	ZNF213	zinc finger protein 213	7760	65,78833125	-0,246768995	0,221232	-1,115430047	0,26466618	0,720266173
ENSG00000011198	ABHD5	abhydrolase domain containing 5, lysophosphatidic acid acyltransferase	51099	41,20181317	0,006454706	0,523276	0,012335178	0,990158201	NA
ENSG00000065154	OAT	ornithine aminotransferase	4942	143,3695514	0,065314628	0,143199	0,456110169	0,648310758	0,914533708
ENSG00000128585	MKLN1	muskelin 1	4289	259,3503246	-0,10152708	0,172012	-0,590232986	0,55503446	0,882854594
ENSG00000130787	HIP1R	huntingtin interacting protein 1 related	9026	208,9142636	0,068347339	0,121355	0,563199888	0,573298779	0,886894319
ENSG00000154920	EME1	essential meiotic structure-specific endonuclease 1	146956	11,79770627	0,084820488	0,602943	0,140677374	0,88812482	NA
ENSG00000181789	COPG1	COPI coat complex subunit gamma 1	22820	429,146439	-0,021593634	0,110374	-0,195640013	0,84489194	0,967231052
ENSG00000228409	NA	NA	NA	12,81764809	0,097679774	0,554434	0,176179371	0,860153027	NA
ENSG00000215305	VPS16	VPS16 core subunit of CORVET and HOPS complexes	64601	206,151819	0,099556095	0,123929	0,803331793	0,421782986	0,827816808
ENSG00000105556	MIER2	MIER family member 2	54531	46,7547816	0,025623732	0,270137	0,094854546	0,924430361	0,984284131
ENSG00000107897	ACBD5	acyl-CoA binding domain containing 5	91452	243,1996817	0,113215313	0,14332	0,789949434	0,4295573	0,832060923
ENSG00000161929	SCIMP	SLP adaptor and CSK interacting membrane protein	388325	14,64414807	-0,230361621	0,579729	-0,397360712	0,691101483	NA
ENSG00000090776	EFNB1	ephrin B1	1947	13,48498779	-0,512536214	0,56523	-0,906774421	0,364526099	NA
ENSG00000127884	ECHS1	enoyl-CoA hydratase, short chain 1	1892	132,0936597	0,051354387	0,165749	0,309833116	0,756687867	0,942901768
ENSG00000164010	ERMAP	erythroblast membrane associated protein (Scianna blood group)	114625	47,24620588	-0,256384413	0,245942	-1,042460197	0,29719837	0,748371252
ENSG00000114098	ARMC8	armadillo repeat containing 8	25852	162,2785667	0,24205217	0,248155	0,975406357	0,329358732	0,771437864
ENSG00000012660	ELOVL5	ELOVL fatty acid elongase 5	60481	509,2240354	0,124284333	0,082693	1,502957044	0,132850121	0,570166586
ENSG00000037897	METTL1	methyltransferase 1, tRNA methylguanosine	4234	12,78604743	0,176150057	0,527992	0,333622284	0,7386646	NA
ENSG00000064961	HMG20B	high mobility group 20B	10362	70,57737709	-0,037160508	0,213643	-0,173937172	0,861914839	0,970863509
ENSG00000072110	ACTN1	actinin alpha 1	87	18,22597445	1,259273417	0,860693	1,463092873	0,143441968	NA

ENSG00000075643	MOCOS	molybdenum cofactor sulfurase	55034	15,10658535	-0,204616103	0,534862	-0,382558437	0,702047192	NA
ENSG00000092208	GEMIN2	gem nuclear organelle associated protein 2	8487	12,88768096	-0,721471038	0,572223	-1,260822471	0,207372816	NA
ENSG00000100304	TTLL12	tubulin tyrosine ligase like 12	23170	86,11367395	-0,226868886	0,247176	-0,917842106	0,358701535	0,790915737
ENSG00000104522	GFUS	GDP-L-fucose synthase	7264	117,1314077	-0,196119697	0,179784	-1,090861014	0,275334044	0,727877072
ENSG00000108509	CAMTA2	calmodulin binding transcription activator 2	23125	212,2816307	0,025317699	0,355117	0,071293888	0,94316386	0,988624545
ENSG00000110002	VWA5A	von Willebrand factor A domain containing 5A	4013	48,39365576	0,123524033	0,253036	0,488166939	0,6254316	0,90479929
ENSG00000110851	PRDM4	PR/SET domain 4	11108	141,5006572	-0,2007124	0,14141	-1,419365031	0,155792622	0,61428162
ENSG00000111237	VPS29	VPS29 retromer complex component	51699	131,9788264	0,045680121	0,156113	0,292610062	0,769820222	0,945027399
ENSG00000115541	HSPE1	heat shock protein family E (Hsp10) member 1	3336	89,40171282	0,02100936	0,182697	0,114995742	0,908448497	0,97930822
ENSG00000115756	HPCAL1	hippocalcin like 1	3241	160,0189898	0,094971755	0,140029	0,678227131	0,497627693	0,861283987
ENSG00000119203	CPSF3	cleavage and polyadenylation specific factor 3	51692	158,5109471	-0,073269891	0,146332	-0,500709298	0,616575728	0,902090873
ENSG00000122299	ZC3H7A	zinc finger CCCH-type containing 7A	29066	322,2004621	0,05920889	0,101558	0,583003368	0,559891033	0,883921037
ENSG00000123607	TTC21B	tetratricopeptide repeat domain 21B	79809	75,78527944	-0,420333311	0,298795	-1,406759552	0,1594987	0,615917006
ENSG00000130544	ZNF557	zinc finger protein 557	79230	98,30761975	-0,005547889	0,168444	-0,032936145	0,973725509	0,994534085
ENSG00000130713	EXOSC2	exosome component 2	23404	29,07550106	-0,458746932	1,762026	-0,260351992	0,794592271	NA
ENSG00000137171	KLC4	kinesin light chain 4	89953	48,31227568	-0,05554422	0,25655	-0,216504402	0,828594595	0,962723654
ENSG00000137720	CFAP68	cilia and flagella associated protein 68	64776	14,82279602	0,852072306	0,52997	1,607775937	0,10788426	NA
ENSG00000140395	SKIC8	SKI8 subunit of superkiller complex	80349	60,14840529	0,379877182	0,243655	1,559078245	0,118977862	0,551624633
ENSG00000140632	GLYR1	glyoxylate reductase 1 homolog	84656	426,7943325	-0,049901827	0,122601	-0,407025011	0,683989619	0,925586358
ENSG00000142185	TRPM2	transient receptor potential cation channel subfamily M member 2	7226	410,4149913	-0,036616145	0,113675	-0,322113941	0,747366377	0,939899419
ENSG00000143756	FBXO28	F-box protein 28	23219	171,3773938	-0,323004289	0,190579	-1,694862157	0,090101573	0,489389871
ENSG00000144580	CNOT9	CCR4-NOT transcription complex subunit 9	9125	369,0146884	-0,087276371	0,109767	-0,795103649	0,426553212	0,831715225
ENSG00000144893	MED12L	mediator complex subunit 12L	116931	29,02653237	0,386400987	0,373583	1,034310035	0,300991247	NA
ENSG00000154917	RAB6B	RAB6B, member RAS oncogene family	51560	12,48004357	0,164362066	0,679498	0,241887316	0,808867479	NA
ENSG00000155229	MMS19	MMS19 homolog, cytosolic iron-sulfur assembly component	64210	168,1612567	0,140260377	0,137816	1,017737927	0,308802518	0,754797782
ENSG00000157014	TATDN2	TatD DNase domain containing 2	9797	504,2238833	0,125710701	0,088892	1,414202182	0,157302548	0,61428162
ENSG00000160679	CHTOP	chromatin target of PRMT1	26097	278,9605082	-0,116900502	0,127186	-0,91913201	0,358026528	0,790314883
ENSG00000162104	ADCY9	adenylate cyclase 9	115	136,2338377	0,087154092	0,183784	0,474219927	0,635343085	0,909036096
ENSG00000164338	UTP15	UTP15 small subunit processome component	84135	65,98505145	-0,964363349	0,378767	-2,546060595	0,010894627	0,163294665
ENSG00000174501	ANKRD36C	ankyrin repeat domain 36C	400986	245,6954993	0,208896578	0,338787	0,616602198	0,537497144	0,874923109
ENSG00000182446	NPLOC4	NPL4 homolog, ubiquitin recognition factor	55666	520,8056774	0,200450864	0,144102	1,391033237	0,164215348	0,623778911
ENSG00000196363	WDR5	WD repeat domain 5	11091	155,4645327	-0,200456545	0,13961	-1,435829578	0,151050841	0,607670525
ENSG00000196526	AFAP1	actin filament associated protein 1	60312	27,54107159	0,564199977	0,335796	1,680185365	0,092921256	NA
ENSG00000197860	SGTB	small glutamine rich tetratricopeptide repeat co-chaperone beta	54557	64,52603922	-0,29129522	0,21458	-1,357514145	0,174617902	0,632039836
ENSG00000198218	QRICH1	glutamine rich 1	54870	368,2727084	-0,011718574	0,131269	-0,089271376	0,928866242	0,986487751
ENSG00000198420	TCAF1	TRPM8 channel associated factor 1	9747	202,5900848	0,140926893	0,117267	1,201759389	0,229456764	0,683929193
ENSG00000213533	STIMATE	STIM activating enhancer	375346	155,6868749	0,198853608	0,158003	1,258546759	0,208194088	0,662274754
ENSG00000100243	CYB5R3	cytochrome b5 reductase 3	1727	141,5847631	-0,057721825	0,216921	-0,266096016	0,790165263	0,95167209
ENSG00000154016	GRAP	GRB2 related adaptor protein	10750	38,90532634	-0,52196889	0,311041	-1,678135715	0,093320608	NA
ENSG00000157212	PAXIP1	PAX interacting protein 1	22976	71,85151329	0,015495051	0,258657	0,059905773	0,952230682	0,991212866
ENSG00000160685	ZBTB7B	zinc finger and BTB domain containing 7B	51043	240,7043194	-0,103512588	0,115653	-0,895023622	0,370774461	0,79977671
ENSG00000167186	COQ7	coenzyme Q7, hydroxylase	10229	34,9141967	0,332796295	0,300261	1,108357281	0,267707546	NA
ENSG00000186020	ZNF529	zinc finger protein 529	57711	88,35991138	-0,373206473	0,194774	-1,916100793	0,055352267	0,381682799
ENSG00000269378	NA	NA	NA	17,61054141	-0,35487965	2,286226	-0,155225106	0,876643856	NA
ENSG00000103245	CIAO3	cytosolic iron-sulfur assembly component 3	64428	53,82928399	0,070295115	0,270965	0,259425447	0,795306998	0,95320329
ENSG00000138495	COX17	cytochrome c oxidase copper chaperone COX17	10063	45,70577016	-0,252456898	0,245598	-1,027926768	0,303984277	0,751870158
ENSG00000143183	TMCO1	transmembrane and coiled-coil domains 1	54499	261,2863402	0,254300287	0,149179	1,704664716	0,088256974	0,486094161
ENSG00000198814	GK	glycerol kinase	2710	27,53469683	0,768139168	0,465402	1,650485568	0,098843663	NA

ENSG00000185347	TEDC1	tubulin epsilon and delta complex 1	283643	13,66590166	0,102591323	0,581452	0,176439748	0,85994848	NA
ENSG00000108510	MED13	mediator complex subunit 13	9969	774,1416658	0,009415091	0,067572	0,139334973	0,889185455	0,977990819
ENSG00000213593	TMX2	thioredoxin related transmembrane protein 2	51075	145,9534608	0,075983607	0,151793	0,500575421	0,616669964	0,902090873
ENSG00000107862	GBF1	golgi brefeldin A resistant guanine nucleotide exchange factor 1	8729	153,491852	0,046020024	0,314527	0,146315049	0,883672686	0,976751663
ENSG00000110700	RPS13	ribosomal protein S13	6207	572,1865191	0,101591394	0,085244	1,191778457	0,233348125	0,688764147
ENSG00000126790	L3HYPDH	trans-L-3-hydroxyproline dehydratase	112849	19,48098383	0,197389541	0,405448	0,486842569	0,626369901	NA
ENSG00000163798	SLC4A1AP	solute carrier family 4 member 1 adaptor protein	22950	149,9715511	-0,098577627	0,159437	-0,618285583	0,536387104	0,874923109
ENSG00000188191	PRKAR1B	protein kinase cAMP-dependent type I regulatory subunit beta	5575	65,65921854	0,083091966	0,316269	0,262725257	0,792762348	0,95213045
ENSG00000205045	SLFN12L	schlafen family member 12 like	100506736	2536,960995	0,030761964	0,063758	0,482480645	0,629464545	0,905567722
ENSG00000125482	TTF1	transcription termination factor 1	7270	117,9639245	0,106159537	0,195533	0,542925121	0,587181359	0,891326322
ENSG00000166197	NOLC1	nucleolar and coiled-body phosphoprotein 1	9221	357,6403931	0,123328097	0,123527	0,998389748	0,318090403	0,762879158
ENSG00000183495	EP400	E1A binding protein p400	57634	653,6605732	0,157024984	0,077278	2,031936793	0,042160055	0,327846607
ENSG00000183808	RBM12B	RNA binding motif protein 12B	389677	169,4621841	0,023037938	0,411179	0,056029015	0,955318693	0,991420853
ENSG00000267059	NA	NA	NA	16,6000332	0,221669769	1,65381	0,134035843	0,893374231	NA
ENSG00000164291	ARSK	arylsulfatase family member K	153642	26,96501229	-0,281606237	0,338882	-0,830986887	0,405981037	NA
ENSG00000105486	LIG1	DNA ligase 1	3978	69,94424372	0,889531094	0,27332	3,254536604	0,001135775	0,037467385
ENSG00000108797	CNTNAP1	contactin associated protein 1	8506	15,33277641	-0,282571809	0,572472	-0,493599145	0,621589304	NA
ENSG00000124164	VAPB	VAMP associated protein B and C	9217	105,0209112	0,363006359	0,352393	1,030117816	0,302954703	0,751870158
ENSG00000125755	SYMPK	symplekin scaffold protein	8189	278,4589879	0,058447661	0,143604	0,407005635	0,68400385	0,925586358
ENSG00000141867	BRD4	bromodomain containing 4	23476	628,6339166	-0,066771068	0,09995	-0,668045592	0,50410449	0,863331152
ENSG00000154957	ZNF18	zinc finger protein 18	7566	35,4184212	0,069748058	0,297195	0,234688227	0,814450726	NA
ENSG00000213015	ZNF580	zinc finger protein 580	51157	53,68391889	-0,466378236	0,286847	-1,625879915	0,103975204	0,517786851
ENSG00000079785	DDX1	DEAD-box helicase 1	1653	220,7915245	0,210777046	0,136377	1,545547399	0,122213913	0,557738568
ENSG00000104946	TBC1D17	TBC1 domain family member 17	79735	90,12524156	-0,313128432	0,222947	-1,404495726	0,160171284	0,617437616
ENSG00000109943	CRTAM	cytotoxic and regulatory T cell molecule	56253	342,3320513	0,277821042	0,098347	2,824900759	0,004729526	0,095983718
ENSG00000156467	UQCRB	ubiquinol-cytochrome c reductase binding protein	7381	130,408723	-0,330879191	0,309853	-1,067858986	0,285584126	0,737867955
ENSG00000162613	FUBP1	far upstream element binding protein 1	8880	479,2301541	-0,089931424	0,083537	-1,076545642	0,2816833	0,735241156
ENSG00000164182	NDUFAF2	NADH:ubiquinone oxidoreductase complex assembly factor 2	91942	15,11949682	-0,018296943	0,474531	-0,038557946	0,969242832	NA
ENSG00000166224	SGPL1	sphingosine-1-phosphate lyase 1	8879	403,1698923	-0,003954125	0,112099	-0,035273512	0,971861645	0,994108581
ENSG00000167264	DUS2	dihydrouridine synthase 2	54920	47,15065818	-0,387345455	0,271941	-1,424375488	0,154337806	0,611607348
ENSG00000178694	NSUN3	NOP2/Sun RNA methyltransferase 3	63899	72,48684721	-0,282166749	0,199516	-1,414256903	0,157286486	0,61428162
ENSG00000183258	DDX41	DEAD-box helicase 41	51428	133,313733	-0,374659784	0,237467	-1,577734659	0,11462658	0,54024053
ENSG00000196378	ZNF34	zinc finger protein 34	80778	28,09141921	0,223165899	0,312451	0,714243324	0,475076731	NA
ENSG00000198720	ANKRD13B	ankyrin repeat domain 13B	124930	21,93569057	0,054995667	2,334883	0,023553927	0,981208423	NA
ENSG00000205208	C4orf46	chromosome 4 open reading frame 46	201725	36,7489932	0,366531936	0,298103	1,229547954	0,21886643	NA
ENSG00000254685	FPGT	fucose-1-phosphate guanylyltransferase	8790	30,84822022	-0,194220778	0,368934	-0,52643792	0,598583974	NA
ENSG00000120833	SOCS2	suppressor of cytokine signaling 2	8835	160,5374538	0,034380155	0,146161	0,235221886	0,81403652	0,959207071
ENSG00000102312	PORCN	porcupine O-acyltransferase	64840	47,15345488	0,082183973	0,266554	0,308320362	0,757838574	0,942901768
ENSG00000105088	OLFM2	olfactomedin 2	93145	19,12468051	-0,591449029	0,425387	-1,390377675	0,164414222	NA
ENSG00000128694	OSGEPL1	O-sialoglycoprotein endopeptidase like 1	64172	20,86899314	0,04635208	0,517245	0,089613336	0,928594487	NA
ENSG00000128891	CCDC32	coiled-coil domain containing 32	90416	38,05009492	-0,05866155	0,426089	-0,137674458	0,890497707	NA
ENSG00000158195	WASF2	WASP family member 2	10163	841,6264803	-0,024053878	0,096568	-0,249087305	0,803293249	0,956498663
ENSG00000160293	VAV2	vav guanine nucleotide exchange factor 2	7410	57,70551438	0,039190274	0,225015	0,174167308	0,861733977	0,970863509
ENSG00000166275	BORCS7	BLOC-1 related complex subunit 7	119032	85,10375659	0,041378996	0,215789	0,191757079	0,847932493	0,967411651
ENSG00000198556	ZNF789	zinc finger protein 789	285989	17,84088359	-0,079031995	0,470952	-0,167813249	0,866730199	NA
ENSG00000160410	SHKBP1	SH3KBP1 binding protein 1	92799	280,0265928	0,071165851	0,112258	0,633948211	0,526114621	0,871384275
ENSG00000100596	SPTLC2	serine palmitoyltransferase long chain base subunit 2	9517	886,1965332	-0,003119178	0,059815	-0,05214686	0,958411675	0,991911599
ENSG00000151353	TMEM18	transmembrane protein 18	129787	72,2760582	-0,778439208	0,63886	-1,218481421	0,223041081	0,678694696
ENSG00000151500	THYN1	thymocyte nuclear protein 1	29087	74,83928688	0,03807909	0,210006	0,181323495	0,856113665	0,96843928

ENSG00000164897	TMUB1	transmembrane and ubiquitin like domain containing 1	83590	75,21907401	-0,310265328	0,224022	-1,384974958	0,166060125	0,624933502
ENSG00000179630	LACC1	laccase domain containing 1	144811	88,45001405	0,079067357	0,185031	0,427319733	0,669146462	0,922641254
ENSG00000237441	RGL2	ral guanine nucleotide dissociation stimulator like 2	5863	35,50400414	0,426519972	3,367445	0,126659825	0,899209644	NA
ENSG00000161542	PRPSAP1	phosphoribosyl pyrophosphate synthetase associated protein 1	5635	132,5496761	0,205955657	0,15033	1,370024858	0,170679142	0,629558603
ENSG00000197021	EOLA2	endothelium and lymphocyte associated ASCH domain 2	541578	143,5687699	0,195083661	0,140565	1,387850322	0,165182628	0,624454568
ENSG00000000003	TSPAN6	tetraspanin 6	7105	0,10488184	-1,076443944	4,080456	-0,263804824	0,791930317	NA
ENSG00000000005	TNMD	tenomodulin	64102	0	NA	NA	NA	NA	NA
ENSG00000001460	STPG1	sperm tail PG-rich repeat containing 1	90529	3,266903193	-0,711588219	1,473045	-0,483073087	0,629043842	NA
ENSG00000001617	SEMA3F	semaphorin 3F	6405	0	NA	NA	NA	NA	NA
ENSG00000001626	CFTR	CF transmembrane conductance regulator	1080	0	NA	NA	NA	NA	NA
ENSG00000002079	MYH16	myosin heavy chain 16 (pseudogene)	84176	0	NA	NA	NA	NA	NA
ENSG00000002587	HS3ST1	heparan sulfate-glucosamine 3-sulfotransferase 1	9957	0,209430041	-1,076238159	4,080456	-0,263754392	0,79196918	NA
ENSG00000002726	AOC1	amine oxidase copper containing 1	26	0,119673877	0,847101401	4,080456	0,207599697	0,835541541	NA
ENSG00000002745	WNT16	Wnt family member 16	51384	0	NA	NA	NA	NA	NA
ENSG00000002746	HECW1	HECT, C2 and WW domain containing E3 ubiquitin protein ligase 1	23072	0	NA	NA	NA	NA	NA
ENSG00000002933	TMEM176A	transmembrane protein 176A	55365	0	NA	NA	NA	NA	NA
ENSG00000003096	KLHL13	kelch like family member 13	90293	1,525370073	1,454347276	1,438695	1,010879633	0,312074043	NA
ENSG00000003137	CYP26B1	cytochrome P450 family 26 subfamily B member 1	56603	0	NA	NA	NA	NA	NA
ENSG00000003147	ICA1	islet cell autoantigen 1	3382	3,060532928	1,44539548	1,643162	0,879642822	0,379052833	NA
ENSG00000003249	DBNDD1	dysbindin domain containing 1	79007	0,988160962	-2,994201855	2,262005	-1,323693614	0,18560482	NA
ENSG00000003436	TFPI	tissue factor pathway inhibitor	7035	0	NA	NA	NA	NA	NA
ENSG00000003987	MTMR7	myotubularin related protein 7	9108	0	NA	NA	NA	NA	NA
ENSG00000003989	SLC7A2	solute carrier family 7 member 2	6542	0	NA	NA	NA	NA	NA
ENSG00000004660	CAMKK1	calcium/calmodulin dependent protein kinase kinase 1	84254	2,658842857	-1,607218395	1,232603	-1,303921843	0,192260232	NA
ENSG00000004776	HSPB6	heat shock protein family B (small) member 6	126393	0	NA	NA	NA	NA	NA
ENSG00000004799	PDK4	pyruvate dehydrogenase kinase 4	5166	2,329687608	1,553037818	2,039547	0,761462247	0,44638102	NA
ENSG00000004809	SLC22A16	solute carrier family 22 member 16	85413	0	NA	NA	NA	NA	NA
ENSG00000004838	ZMYND10	zinc finger MYND-type containing 10	51364	1,249336824	-1,258726408	2,515555	-0,500377129	0,616809555	NA
ENSG00000004846	ABCB5	ATP binding cassette subfamily B member 5	340273	0,653154698	-0,885946654	3,846697	-0,230313617	0,81784808	NA
ENSG00000004848	ARX	aristaless related homeobox	170302	0,435462436	0,844111406	4,080456	0,206866937	0,836113779	NA
ENSG00000004939	SLC4A1	solute carrier family 4 member 1 (Diego blood group)	6521	0,117497108	0,847101401	4,080456	0,207599697	0,835541541	NA
ENSG00000004948	CALCR	calcitonin receptor	799	0	NA	NA	NA	NA	NA
ENSG00000005001	PRSS22	serine protease 22	64063	0	NA	NA	NA	NA	NA
ENSG00000005073	HOXA11	homeobox A11	3207	0,311994459	-2,498367527	3,998294	-0,62485841	0,532063991	NA
ENSG00000005102	MEOX1	mesenchyme homeobox 1	4222	0	NA	NA	NA	NA	NA
ENSG00000005108	THSD7A	thrombospondin type 1 domain containing 7A	221981	0	NA	NA	NA	NA	NA
ENSG00000005187	ACSM3	acyl-CoA synthetase medium chain family member 3	6296	1,114244933	-3,703418045	2,855522	-1,296931864	0,194654632	NA
ENSG00000005243	COPZ2	COPI coat complex subunit zeta 2	51226	5,617406214	-0,310719745	0,787092	-0,394769289	0,693013161	NA
ENSG00000005249	PRKAR2B	protein kinase cAMP-dependent type II regulatory subunit beta	5577	4,080286853	-1,976338226	1,736218	-1,138300984	0,254994823	NA
ENSG00000005381	MPO	myeloperoxidase	4353	0	NA	NA	NA	NA	NA
ENSG00000005421	PON1	paraoxonase 1	5444	0	NA	NA	NA	NA	NA
ENSG00000005471	ABCB4	ATP binding cassette subfamily B member 4	5244	0,458516007	-0,992784056	3,977755	-0,24958401	0,802909066	NA
ENSG00000005513	SOX8	SRY-box transcription factor 8	30812	0,715903024	-3,026907649	2,629515	-1,151127717	0,249679697	NA
ENSG00000005961	ITGA2B	integrin subunit alpha 2b	3674	0,423760506	-1,071623461	4,080456	-0,262623465	0,792840812	NA
ENSG00000005981	ASB4	ankyrin repeat and SOCS box containing 4	51666	0	NA	NA	NA	NA	NA

ENSG00000006016	CRLF1	cytokine receptor like factor 1	9244	0	NA	NA	NA	NA	NA
ENSG00000006042	TMEM98	transmembrane protein 98	26022	0	NA	NA	NA	NA	NA
ENSG00000006071	ABCC8	ATP binding cassette subfamily C member 8	6833	0	NA	NA	NA	NA	NA
ENSG00000006116	CACNG3	calcium voltage-gated channel auxiliary subunit gamma 3	10368	0	NA	NA	NA	NA	NA
ENSG00000006118	TMEM132A	transmembrane protein 132A	54972	0	NA	NA	NA	NA	NA
ENSG00000006128	TAC1	tachykinin precursor 1	6863	0	NA	NA	NA	NA	NA
ENSG00000006210	CX3CL1	C-X3-C motif chemokine ligand 1	6376	0	NA	NA	NA	NA	NA
ENSG00000006283	CACNA1G	calcium voltage-gated channel subunit alpha1 G	8913	0,453185756	0,570624838	3,989253	0,143040523	0,886258178	NA
ENSG00000006327	TNFRSF12A	TNF receptor superfamily member 12A	51330	4,761645524	5,425198586	1,430895	3,79147124	0,000149757	NA
ENSG00000006377	DLX6	distal-less homeobox 6	1750	0	NA	NA	NA	NA	NA
ENSG00000006453	BAIAP2L1	BAR/IMD domain containing adaptor protein 2 like 1	55971	0	NA	NA	NA	NA	NA
ENSG00000006468	ETV1	ETS variant transcription factor 1	2115	2,976320003	0,588050104	1,081529	0,543721318	0,58663326	NA
ENSG00000006534	ALDH3B1	aldehyde dehydrogenase 3 family member B1	221	3,852366776	0,435572206	1,10452	0,394354409	0,693319398	NA
ENSG00000006606	CCL26	C-C motif chemokine ligand 26	10344	0	NA	NA	NA	NA	NA
ENSG00000006611	USH1C	USH1 protein network component harmonin	10083	0,117497108	0,847101401	4,080456	0,207599697	0,835541541	NA
ENSG00000006634	DBF4	DBF4 zinc finger	10926	5,883663429	-2,502290485	1,441953	-1,735348427	0,082679109	NA
ENSG00000006638	TBXA2R	thromboxane A2 receptor	6915	0,78807721	0,454535104	2,297659	0,197825347	0,843181714	NA
ENSG00000006659	LGALS14	galectin 14	56891	0	NA	NA	NA	NA	NA
ENSG00000006704	GTF2IRD1	GTF2I repeat domain containing 1	9569	0	NA	NA	NA	NA	NA
ENSG00000006740	ARHGAP44	Rho GTPase activating protein 44	9912	0	NA	NA	NA	NA	NA
ENSG00000006747	SCIN	scinderin	85477	0,118286652	0	4,080456	0	1	NA
ENSG00000006788	MYH13	myosin heavy chain 13	8735	0	NA	NA	NA	NA	NA
ENSG00000006837	CDKL3	cyclin dependent kinase like 3	51265	2,020601195	-1,175677072	1,773862	-0,662778372	0,507472508	NA
ENSG00000007001	UPP2	uridine phosphorylase 2	151531	0	NA	NA	NA	NA	NA
ENSG00000007038	PRSS21	serine protease 21	10942	0,89054716	-2,15941233	2,326057	-0,92835759	0,35322211	NA
ENSG00000007062	PROM1	prominin 1	8842	0	NA	NA	NA	NA	NA
ENSG00000007129	CEACAM21	CEA cell adhesion molecule 21	90273	0	NA	NA	NA	NA	NA
ENSG00000007171	NOS2	nitric oxide synthase 2	4843	0	NA	NA	NA	NA	NA
ENSG00000007174	DNAH9	dynein axonemal heavy chain 9	1770	0	NA	NA	NA	NA	NA
ENSG00000007216	SLC13A2	solute carrier family 13 member 2	9058	0	NA	NA	NA	NA	NA
ENSG00000007306	CEACAM7	CEA cell adhesion molecule 7	1087	0	NA	NA	NA	NA	NA
ENSG00000007312	CD79B	CD79b molecule	974	1,310930684	-1,901627031	2,532857	-0,75078346	0,452782985	NA
ENSG00000007314	SCN4A	sodium voltage-gated channel alpha subunit 4	6329	0,426487559	-0,110630717	3,607175	-0,030669627	0,975533014	NA
ENSG00000007350	TKTL1	transketolase like 1	8277	0,827934224	-0,925342674	2,334797	-0,396326894	0,69186389	NA
ENSG00000007372	PAX6	paired box 6	5080	0,207996306	-1,916073151	4,043021	-0,473921178	0,635556117	NA
ENSG00000007384	RHBDF1	rhomboid 5 homolog 1	64285	0	NA	NA	NA	NA	NA
ENSG00000007402	CACNA2D2	calcium voltage-gated channel auxiliary subunit alpha2delta 2	9254	1,214093065	-1,54871123	1,799777	-0,860501559	0,389512625	NA
ENSG00000007516	BAIAP3	BAI1 associated protein 3	8938	2,750216645	-1,631175401	1,749786	-0,93221429	0,351225792	NA
ENSG00000007866	TEAD3	TEA domain transcription factor 3	7005	0,999109746	-2,603962055	2,915873	-0,893029948	0,371841135	NA
ENSG00000007908	SELE	selectin E	6401	0	NA	NA	NA	NA	NA
ENSG00000007933	FMO3	flavin containing dimethylaniline monoxygenase 3	2328	0	NA	NA	NA	NA	NA
ENSG00000007952	NOX1	NADPH oxidase 1	27035	0	NA	NA	NA	NA	NA
ENSG00000008056	SYN1	synapsin I	6853	0	NA	NA	NA	NA	NA
ENSG00000008118	CAMK1G	calcium/calmodulin dependent protein kinase IG	57172	0	NA	NA	NA	NA	NA
ENSG00000008196	TFAP2B	transcription factor AP-2 beta	7021	0	NA	NA	NA	NA	NA
ENSG00000008197	TFAP2D	transcription factor AP-2 delta	83741	0	NA	NA	NA	NA	NA
ENSG00000008226	DLEC1	DLEC1 cilia and flagella associated protein	9940	2,156205151	1,327822894	1,937046	0,685488604	0,493035649	NA
ENSG00000008283	CYB561	cytochrome b561	1534	6,068025843	-0,431943559	0,716418	-0,602920742	0,546561417	NA
ENSG00000008311	AASS	aminoadipate-semialdehyde synthase	10157	0,704982649	3,544948029	3,945665	0,898441188	0,368950386	NA
ENSG00000008323	PLEKHG6	pleckstrin homology and RhoGEF domain containing G6	55200	0,431040354	-0,059659212	3,585274	-0,016640071	0,986723757	NA

ENSG00000008394	MGST1	microsomal glutathione S-transferase 1	4257	0,356896058	1,039057448	4,021286	0,258389337	0,796106445	NA
ENSG00000008438	PGLYRP1	peptidoglycan recognition protein 1	8993	0	NA	NA	NA	NA	NA
ENSG00000008441	NFIX	nuclear factor I X	4784	4,403246889	-0,855062195	0,911529	-0,938052173	0,348217599	NA
ENSG00000008735	MAPK8IP2	mitogen-activated protein kinase 8 interacting protein 2	23542	0,336891943	2,504732496	3,986153	0,628358366	0,529769204	NA
ENSG00000009694	TENM1	teneurin transmembrane protein 1	10178	0,349659572	1,687543832	3,707837	0,455128872	0,649016528	NA
ENSG00000009709	PAX7	paired box 7	5081	0	NA	NA	NA	NA	NA
ENSG00000009724	MASP2	MBL associated serine protease 2	10747	0	NA	NA	NA	NA	NA
ENSG00000009765	IYD	iodotyrosine deiodinase	389434	0	NA	NA	NA	NA	NA
ENSG00000009830	POMT2	protein O-mannosyltransferase 2	29954	8,665381578	0,725578298	1,420934	0,510634643	0,609606913	NA
ENSG00000009950	MLXIPL	MLX interacting protein like	51085	0	NA	NA	NA	NA	NA
ENSG00000010030	ETV7	ETS variant transcription factor 7	51513	2,65626472	0,665632183	1,062276	0,626609688	0,530915116	NA
ENSG00000010219	DYRK4	dual specificity tyrosine phosphorylation regulated kinase 4	8798	7,389486422	0,248408666	0,708274	0,350723865	0,725795519	NA
ENSG00000010282	HHATL	hedgehog acyltransferase like	57467	0	NA	NA	NA	NA	NA
ENSG00000010318	PHF7	PHD finger protein 7	51533	6,277306227	-0,17054382	0,823927	-0,206989021	0,836018433	NA
ENSG00000010319	SEMA3G	semaphorin 3G	56920	0,10543123	0	4,080456	0	1	NA
ENSG00000010327	STAB1	stabilin 1	23166	0	NA	NA	NA	NA	NA
ENSG00000010361	FUZ	fuzzy planar cell polarity protein	80199	9,857290595	-1,534075887	0,954347	-1,607461225	0,107953229	NA
ENSG00000010379	SLC6A13	solute carrier family 6 member 13	6540	0,788916861	-0,7631592	2,400383	-0,317932204	0,750536364	NA
ENSG00000010438	PRSS3	serine protease 3	5646	0	NA	NA	NA	NA	NA
ENSG00000010610	CD4	CD4 molecule	920	0	NA	NA	NA	NA	NA
ENSG00000010626	LRRC23	leucine rich repeat containing 23	10233	2,555285048	-0,933452696	1,271981	-0,733857605	0,463035528	NA
ENSG00000010671	BTK	Bruton tyrosine kinase	695	0	NA	NA	NA	NA	NA
ENSG00000010704	HFE	homeostatic iron regulator	3077	3,490772536	1,120867935	1,392167	0,80512466	0,420747747	NA
ENSG00000010932	FMO1	flavin containing dimethylaniline monoxygenase 1	2326	0	NA	NA	NA	NA	NA
ENSG00000011028	MRC2	mannose receptor C type 2	9902	9,762184739	-0,001000084	0,849704	-0,001176979	0,999060907	NA
ENSG00000011052	NME1-NME2	NME1-NME2 readthrough	654364	0,675844884	-0,395171338	3,281246	-0,120433329	0,904139891	NA
ENSG00000011083	SLC6A7	solute carrier family 6 member 7	6534	0,190011793	-0,109711063	4,080456	-0,026886962	0,978549892	NA
ENSG00000011105	TSPAN9	tetraspanin 9	10867	0,10543123	0	4,080456	0	1	NA
ENSG00000011114	BTBD7	BTB domain containing 7	55727	0	NA	NA	NA	NA	NA
ENSG00000011201	ANOS1	anosmin 1	3730	0	NA	NA	NA	NA	NA
ENSG00000011332	DPF1	double PHD fingers 1	8193	0,263579756	-1,066200025	4,080456	-0,26129434	0,793865531	NA
ENSG00000011347	SYT7	synaptotagmin 7	9066	0,196100329	1,133369804	4,080456	0,277755682	0,781199913	NA
ENSG00000011422	PLAUR	plasminogen activator, urokinase receptor	5329	4,847980617	-0,092563136	0,800963	-0,115564756	0,907997496	NA
ENSG00000011465	DCN	decorin	1634	0,119673877	0,847101401	4,080456	0,207599697	0,835541541	NA
ENSG00000011590	ZBTB32	zinc finger and BTB domain containing 32	27033	0	NA	NA	NA	NA	NA
ENSG00000011677	GABRA3	gamma-aminobutyric acid type A receptor subunit alpha3	2556	0	NA	NA	NA	NA	NA
ENSG00000012124	CD22	CD22 molecule	933	0	NA	NA	NA	NA	NA
ENSG00000012171	SEMA3B	semaphorin 3B	7869	0,223009549	-1,082865718	4,080456	-0,265378613	0,79071781	NA
ENSG00000012223	LTF	lactotransferrin	4057	0,315145491	2,382605808	3,993695	0,596591803	0,550779945	NA
ENSG00000012504	NR1H4	nuclear receptor subfamily 1 group H member 4	9971	0	NA	NA	NA	NA	NA
ENSG00000012779	ALOX5	arachidonate 5-lipoxygenase	240	0	NA	NA	NA	NA	NA
ENSG00000012817	KDM5D	lysine demethylase 5D	8284	0	NA	NA	NA	NA	NA
ENSG00000013293	SLC7A14	solute carrier family 7 member 14	57709	0	NA	NA	NA	NA	NA
ENSG00000013297	CLDN11	claudin 11	5010	0	NA	NA	NA	NA	NA
ENSG00000013375	PGM3	phosphoglucomutase 3	5238	11,63283673	0,952947527	1,20191	0,792861177	0,427858709	NA
ENSG00000013392	RWDD2A	RWD domain containing 2A	112611	9,989192657	-0,327153024	0,643892	-0,508087133	0,611392238	NA
ENSG00000013588	GPRC5A	G protein-coupled receptor class C group 5 member A	9052	0,103998153	-1,076443944	4,080456	-0,263804824	0,791930317	NA
ENSG00000013619	MAMLD1	mastermind like domain containing 1	10046	6,441290235	0,952555115	0,877637	1,085363548	0,27776067	NA
ENSG00000014257	ACP3	acid phosphatase 3	55	0	NA	NA	NA	NA	NA
ENSG00000014914	MTMR11	myotubularin related protein 11	10903	0,775975522	-2,405661023	2,557449	-0,940648743	0,346884894	NA
ENSG00000015413	DPEP1	dipeptidase 1	1800	0	NA	NA	NA	NA	NA
ENSG00000015520	NPC1L1	NPC1 like intracellular cholesterol transporter 1	29881	0,176973071	-0,1124679	4,080456	-0,027562582	0,978011025	NA
ENSG00000015592	STMN4	stathmin 4	81551	0,326307745	-1,91644297	4,042998	-0,474015276	0,635489014	NA

ENSG00000016082	ISL1	ISL LIM homeobox 1	3670	0,117497108	0,847101401	4,080456	0,207599697	0,835541541	NA
ENSG00000016391	CHDH	choline dehydrogenase	55349	0,22145722	-0,117439386	4,080456	-0,028780948	0,977039296	NA
ENSG00000016402	IL20RA	interleukin 20 receptor subunit alpha	53832	0	NA	NA	NA	NA	NA
ENSG00000016490	CLCA1	chloride channel accessory 1	1179	0	NA	NA	NA	NA	NA
ENSG00000016602	CLCA4	chloride channel accessory 4	22802	0	NA	NA	NA	NA	NA
ENSG00000018236	CNTN1	contactin 1	1272	0	NA	NA	NA	NA	NA
ENSG00000018280	SLC11A1	solute carrier family 11 member 1	6556	1,179892081	2,177836094	1,980379	1,099706807	0,271459888	NA
ENSG00000018408	WWTR1	WW domain containing transcription regulator 1	25937	0,238025742	0,858027715	4,080155	0,210292945	0,833439041	NA
ENSG00000018607	NA	NA	NA	0	NA	NA	NA	NA	NA
ENSG00000018625	ATP1A2	ATPase Na+/K+ transporting subunit alpha 2	477	0	NA	NA	NA	NA	NA
ENSG00000019102	VSIG2	V-set and immunoglobulin domain containing 2	23584	0,10488184	-1,076443944	4,080456	-0,263804824	0,791930317	NA
ENSG00000019144	PHLDB1	pleckstrin homology like domain family B member 1	23187	0,710436443	2,310218324	3,796827	0,608460215	0,542882285	NA
ENSG00000019169	MARCO	macrophage receptor with collagenous structure	8685	0	NA	NA	NA	NA	NA
ENSG00000019186	CYP24A1	cytochrome P450 family 24 subfamily A member 1	1591	0	NA	NA	NA	NA	NA
ENSG00000019505	SYT13	synaptotagmin 13	57586	0	NA	NA	NA	NA	NA
ENSG00000019549	SNAI2	snail family transcriptional repressor 2	6591	0	NA	NA	NA	NA	NA
ENSG00000019991	HGF	hepatocyte growth factor	3082	0	NA	NA	NA	NA	NA
ENSG00000020181	ADGRA2	adhesion G protein-coupled receptor A2	25960	1,41828548	0,904118738	1,663931	0,543363225	0,586879741	NA
ENSG00000020219	CCT8L1P	chaperonin containing TCP1 subunit 8 like 1, pseudogene	155100	0,381787944	-1,072044548	3,691431	-0,290414366	0,771499255	NA
ENSG00000020577	SAMD4A	sterile alpha motif domain containing 4A	23034	0,207996306	-1,916073151	4,043021	-0,473921178	0,635556117	NA
ENSG00000021300	PLEKHB1	pleckstrin homology domain containing B1	58473	3,124916057	0,056502766	1,129825	0,050010197	0,960114262	NA
ENSG00000021461	CYP3A43	cytochrome P450 family 3 subfamily A member 43	64816	0	NA	NA	NA	NA	NA
ENSG00000021488	SLC7A9	solute carrier family 7 member 9	11136	0,190111146	-0,111827678	4,080456	-0,027405682	0,978136166	NA
ENSG00000021645	NRXN3	neurexin 3	9369	0,258085196	-1,151182322	4,080456	-0,282121008	0,777850725	NA
ENSG00000021826	CPS1	carbamoyl-phosphate synthase 1	1373	0,10488184	-1,076443944	4,080456	-0,263804824	0,791930317	NA
ENSG00000021852	C8B	complement C8 beta chain	732	0	NA	NA	NA	NA	NA
ENSG00000022355	GABRA1	gamma-aminobutyric acid type A receptor subunit alpha1	2554	0	NA	NA	NA	NA	NA
ENSG00000022556	NLRP2	NLR family pyrin domain containing 2	55655	6,492878074	0	4,080456	0	1	NA
ENSG00000023191	RNH1	ribonuclease/angiogenin inhibitor 1	6050	0,945386862	2,924574317	3,628515	0,80599749	0,420244296	NA
ENSG00000023839	ABCC2	ATP binding cassette subfamily C member 2	1244	5,029892283	-0,568597998	0,814987	-0,697677468	0,485378922	NA
ENSG00000024422	EHD2	EH domain containing 2	30846	0,117497108	0,847101401	4,080456	0,207599697	0,835541541	NA
ENSG00000025039	RRAGD	Ras related GTP binding D	58528	0,484135093	0,446713043	3,477393	0,128462063	0,897783319	NA
ENSG00000025423	HSD17B6	hydroxysteroid 17-beta dehydrogenase 6	8630	2,074684937	0,206193101	1,556502	0,13247209	0,894610897	NA
ENSG00000026036	RTEL1-TNFRSF6B	RTEL1-TNFRSF6B readthrough (NMD candidate)	100533107	2,047345722	-2,850870037	2,131507	-1,337490587	0,181062553	NA
ENSG00000026559	KCNG1	potassium voltage-gated channel modifier subfamily G member 1	3755	0	NA	NA	NA	NA	NA
ENSG00000027644	INSRR	insulin receptor related receptor	3645	0,119673877	0,847101401	4,080456	0,207599697	0,835541541	NA
ENSG00000029534	ANK1	ankyrin 1	286	0,41284887	1,803314723	3,514605	0,513091675	0,607887194	NA
ENSG00000029559	IBSP	integrin binding sialoprotein	3381	0	NA	NA	NA	NA	NA
ENSG00000030304	MUSK	muscle associated receptor tyrosine kinase	4593	0	NA	NA	NA	NA	NA
ENSG00000033122	LRRC7	leucine rich repeat containing 7	57554	0,263750917	2,148691403	4,008239	0,536068746	0,59191104	NA
ENSG00000034053	APBA2	amyloid beta precursor protein binding family A member 2	321	7,044162852	4,448322122	1,37083	3,244985622	0,001174566	NA
ENSG00000034239	CLXN	calaxin	79645	0	NA	NA	NA	NA	NA
ENSG00000034971	MYOC	myocilin	4653	0	NA	NA	NA	NA	NA
ENSG00000035862	TIMP2	TIMP metallopeptidase inhibitor 2	7077	2,270659677	-2,233017161	1,899314	-1,175696832	0,239716045	NA
ENSG00000036448	MYOM2	myomesin 2	9172	0	NA	NA	NA	NA	NA
ENSG00000036473	OTC	ornithine transcarbamylase	5009	0	NA	NA	NA	NA	NA
ENSG00000036530	CYP46A1	cytochrome P450 family 46 subfamily A member 1	10858	0	NA	NA	NA	NA	NA

ENSG00000036565	SLC18A1	solute carrier family 18 member A1	6570	0	NA	NA	NA	NA	NA
ENSG00000036672	USP2	ubiquitin specific peptidase 2	9099	1,059522146	0,507764729	1,751799	0,289853302	0,771928469	NA
ENSG00000036828	CASR	calcium sensing receptor	846	0,10488184	-1,076443944	4,080456	-0,263804824	0,791930317	NA
ENSG00000037280	FLT4	fms related receptor tyrosine kinase 4	2324	0	NA	NA	NA	NA	NA
ENSG00000037965	HOXC8	homeobox C8	3224	0	NA	NA	NA	NA	NA
ENSG00000038295	TLL1	tolloid like 1	7092	0	NA	NA	NA	NA	NA
ENSG00000038945	MSR1	macrophage scavenger receptor 1	4481	0	NA	NA	NA	NA	NA
ENSG00000039068	CDH1	cadherin 1	999	0	NA	NA	NA	NA	NA
ENSG00000039139	DNAH5	dynein axonemal heavy chain 5	1767	0,118286652	0	4,080456	0	1	NA
ENSG00000039537	C6	complement C6	729	0	NA	NA	NA	NA	NA
ENSG00000039560	RAI14	retinoic acid induced 14	26064	0	NA	NA	NA	NA	NA
ENSG00000039600	SOX30	SRY-box transcription factor 30	11063	0,671013346	1,519914279	2,671889	0,568853897	0,569455294	NA
ENSG00000039987	BEST2	bestrophin 2	54831	0	NA	NA	NA	NA	NA
ENSG00000040608	RTN4R	reticulon 4 receptor	65078	0	NA	NA	NA	NA	NA
ENSG00000040731	CDH10	cadherin 10	1008	0	NA	NA	NA	NA	NA
ENSG00000041515	MYO16	myosin XVI	23026	0	NA	NA	NA	NA	NA
ENSG00000041982	TNC	tenascin C	3371	0	NA	NA	NA	NA	NA
ENSG00000042062	RIPOR3	RIPOR family member 3	140876	0	NA	NA	NA	NA	NA
ENSG00000042304	NA	NA	NA	0	NA	NA	NA	NA	NA
ENSG00000042317	SPATA7	spermatogenesis associated 7	55812	4,910912869	-0,313669521	0,931248	-0,336827029	0,736247296	NA
ENSG00000042429	MED17	mediator complex subunit 17	9440	6,536904311	0,281703124	0,798086	0,352973569	0,724108256	NA
ENSG00000042781	USH2A	usherin	7399	0	NA	NA	NA	NA	NA
ENSG00000042813	ZPBP	zona pellucida binding protein	11055	0	NA	NA	NA	NA	NA
ENSG00000042832	TG	thyroglobulin	7038	1,088423155	0,796694886	2,275327	0,350145171	0,726229753	NA
ENSG00000042980	ADAM28	ADAM metallopeptidase domain 28	10863	5,54367497	0,059405604	0,999833	0,0594155	0,952621168	NA
ENSG00000043039	BARX2	BARX homeobox 2	8538	0	NA	NA	NA	NA	NA
ENSG00000043355	ZIC2	Zic family member 2	7546	0	NA	NA	NA	NA	NA
ENSG00000043591	ADRB1	adrenoceptor beta 1	153	1,227922504	0,234386944	2,039642	0,114915732	0,908511916	NA
ENSG00000044012	GUCA2B	guanylate cyclase activator 2B	2981	0	NA	NA	NA	NA	NA
ENSG00000044524	EPHA3	EPH receptor A3	2042	0	NA	NA	NA	NA	NA
ENSG00000046604	DSG2	desmoglein 2	1829	0,11808024	-1,076443944	4,080456	-0,263804824	0,791930317	NA
ENSG00000046653	GPM6B	glycoprotein M6B	2824	0,118286652	0	4,080456	0	1	NA
ENSG00000046774	MAGEC2	MAGE family member C2	51438	0	NA	NA	NA	NA	NA
ENSG00000046889	PREX2	phosphatidylinositol-3,4,5-trisphosphate dependent Rac exchange factor 2	80243	0	NA	NA	NA	NA	NA
ENSG00000047457	CP	ceruloplasmin	1356	0	NA	NA	NA	NA	NA
ENSG00000047597	XK	X-linked Kx blood group antigen, Kell and VPS13A binding protein	7504	1,198277749	1,27800111	1,768585	0,722612016	0,469918284	NA
ENSG00000047617	ANO2	anoctamin 2	57101	0	NA	NA	NA	NA	NA
ENSG00000047634	SCML1	Scm polycomb group protein like 1	6322	4,654298634	1,724819424	1,466214	1,176376333	0,239444525	NA
ENSG00000047648	ARHGAP6	Rho GTPase activating protein 6	395	0	NA	NA	NA	NA	NA
ENSG00000047662	FAM184B	family with sequence similarity 184 member B	27146	1,295222839	0,705233995	1,707624	0,412991418	0,679612902	NA
ENSG00000047936	ROS1	ROS proto-oncogene 1, receptor tyrosine kinase	6098	0,117497108	0,847101401	4,080456	0,207599697	0,835541541	NA
ENSG00000048342	CC2D2A	coiled-coil and C2 domain containing 2A	57545	0	NA	NA	NA	NA	NA
ENSG00000048462	TNFRSF17	TNF receptor superfamily member 17	608	0	NA	NA	NA	NA	NA
ENSG00000048540	LMO3	LIM domain only 3	55885	0	NA	NA	NA	NA	NA
ENSG00000048545	GUCA1A	guanylate cyclase activator 1A	2978	0	NA	NA	NA	NA	NA
ENSG00000049130	KITLG	KIT ligand	4254	0	NA	NA	NA	NA	NA
ENSG00000049192	ADAMTS6	ADAM metallopeptidase with thrombospondin type 1 motif 6	11174	0	NA	NA	NA	NA	NA
ENSG00000049247	UTS2	urotensin 2	10911	0,10543123	0	4,080456	0	1	NA
ENSG00000049283	EPN3	epsin 3	55040	0	NA	NA	NA	NA	NA
ENSG00000049323	LTBP1	latent transforming growth factor beta binding protein 1	4052	0,209426181	-1,081680378	4,080456	-0,265088121	0,790941579	NA
ENSG00000049540	ELN	elastin	2006	0	NA	NA	NA	NA	NA
ENSG00000049759	NEDD4L	NEDD4 like E3 ubiquitin protein ligase	23327	4,074973566	3,356765686	1,541687	2,177333334	0,029455706	NA
ENSG00000049768	FOXP3	forkhead box P3	50943	1,460929271	-2,520033941	1,777066	-1,418087071	0,156165346	NA
ENSG00000050030	NEXMIF	neurite extension and migration factor	340533	0	NA	NA	NA	NA	NA

ENSG00000050165	DKK3	dickkopf WNT signaling pathway inhibitor 3	27122	0	NA	NA	NA	NA	NA
ENSG00000050327	ARHGEF5	Rho guanine nucleotide exchange factor 5	7984	0	NA	NA	NA	NA	NA
ENSG00000050555	LAMC3	laminin subunit gamma 3	10319	0	NA	NA	NA	NA	NA
ENSG00000050628	PTGER3	prostaglandin E receptor 3	5733	3,574907191	-0,893416285	1,134671	-0,787379363	0,431059824	NA
ENSG00000050730	TNIP3	TNFAIP3 interacting protein 3	79931	5,045796757	-0,559071725	0,896538	-0,623589851	0,532896978	NA
ENSG00000050767	COL23A1	collagen type XXIII alpha 1 chain	91522	0	NA	NA	NA	NA	NA
ENSG00000050820	BCAR1	BCAR1 scaffold protein, Cas family member	9564	0,807858272	-0,164635256	2,233525	-0,07371094	0,941240393	NA
ENSG00000051128	HOMER3	homer scaffold protein 3	9454	0	NA	NA	NA	NA	NA
ENSG00000052126	PLEKHA5	pleckstrin homology domain containing A5	54477	8,171881911	-0,461549313	1,339117	-0,344666912	0,730344796	NA
ENSG00000052344	PRSS8	serine protease 8	5652	0	NA	NA	NA	NA	NA
ENSG00000052850	ALX4	ALX homeobox 4	60529	0	NA	NA	NA	NA	NA
ENSG00000053108	FSTL4	follistatin like 4	23105	0	NA	NA	NA	NA	NA
ENSG00000053328	METTL24	methyltransferase like 24	728464	2,408251643	1,315969805	1,253517	1,049821895	0,293800007	NA
ENSG00000053438	NNAT	neuronatin	4826	2,069366379	1,919718129	1,482539	1,294885009	0,1953599	NA
ENSG00000053702	NRIP2	nuclear receptor interacting protein 2	83714	0,458620519	-0,121632961	3,541779	-0,034342331	0,97260417	NA
ENSG00000053747	LAMA3	laminin subunit alpha 3	3909	0	NA	NA	NA	NA	NA
ENSG00000053918	KCNQ1	potassium voltage-gated channel subfamily Q member 1	3784	1,697032255	0,774172271	2,009639	0,385229596	0,700067318	NA
ENSG00000054179	ENTPD2	ectonucleoside triphosphate diphosphohydrolase 2	954	0,118286652	0	4,080456	0	1	NA
ENSG00000054356	PTPRN	protein tyrosine phosphatase receptor type N	5798	0	NA	NA	NA	NA	NA
ENSG00000054598	FOXC1	forkhead box C1	2296	3,667970661	0,049546186	1,058806	0,0467944	0,962677092	NA
ENSG00000054690	PLEKHH1	pleckstrin homology, MyTH4 and FERM domain containing H1	57475	0,234994216	1,944111989	4,02324	0,483220515	0,62893917	NA
ENSG00000054796	SPO11	SPO11 initiator of meiotic double strand breaks	23626	0	NA	NA	NA	NA	NA
ENSG00000054803	CBLN4	cerebellin 4 precursor	140689	0	NA	NA	NA	NA	NA
ENSG00000054938	CHRDL2	chordin like 2	25884	0	NA	NA	NA	NA	NA
ENSG00000055118	KCNH2	potassium voltage-gated channel subfamily H member 2	3757	0,072165449	0,847101401	4,080456	0,207599697	0,835541541	NA
ENSG00000055732	MCOLN3	mucolipin TRP cation channel 3	55283	0,23616048	-2,075648354	4,028973	-0,515180498	0,606426895	NA
ENSG00000055813	CCDC85A	coiled-coil domain containing 85A	114800	0,239347754	1,967469188	4,021404	0,489249276	0,624665227	NA
ENSG00000055957	ITIH1	inter-alpha-trypsin inhibitor heavy chain 1	3697	0	NA	NA	NA	NA	NA
ENSG00000056291	NPFFR2	neuropeptide FF receptor 2	10886	0	NA	NA	NA	NA	NA
ENSG00000056487	PHF21B	PHD finger protein 21B	112885	0	NA	NA	NA	NA	NA
ENSG00000056736	IL17RB	interleukin 17 receptor B	55540	0	NA	NA	NA	NA	NA
ENSG00000056998	GYG2	glycogenin 2	8908	0	NA	NA	NA	NA	NA
ENSG00000057019	DCBLD2	discoidin, CUB and LCCL domain containing 2	131566	1,086373616	-0,007991899	2,12767	-0,003756174	0,997003014	NA
ENSG00000057149	SERPINB3	serpin family B member 3	6317	0	NA	NA	NA	NA	NA
ENSG00000057294	PKP2	plakophilin 2	5318	0	NA	NA	NA	NA	NA
ENSG00000057468	MSH4	mutS homolog 4	4438	1,017504213	0,238683739	1,947496	0,122559326	0,902456065	NA
ENSG00000057593	F7	coagulation factor VII	2155	0,263785749	-1,071702764	4,080456	-0,2626429	0,792825831	NA
ENSG00000057704	TMCC3	transmembrane and coiled-coil domain family 3	57458	5,334130361	-0,365541814	0,922012	-0,396460809	0,691765114	NA
ENSG00000057935	MTA3	metastasis associated 1 family member 3	57504	10,94190324	-0,229926837	0,723033	-0,318003059	0,750482616	NA
ENSG00000058085	LAMC2	laminin subunit gamma 2	3918	0,15836288	0	4,080456	0	1	NA
ENSG00000058091	CDK14	cyclin dependent kinase 14	5218	0	NA	NA	NA	NA	NA
ENSG00000058335	RASGRF1	Ras protein specific guanine nucleotide releasing factor 1	5923	0	NA	NA	NA	NA	NA
ENSG00000058404	CAMK2B	calcium/calmodulin dependent protein kinase II beta	816	0,200732474	-0,541973213	4,069999	-0,133162991	0,894064479	NA
ENSG00000058866	DGKG	diacylglycerol kinase gamma	1608	0,507544977	0,948585289	2,848261	0,333040102	0,739104013	NA
ENSG00000059769	DNAJC25	DnaJ heat shock protein family (Hsp40) member C25	548645	9,500587952	0,816531729	2,067415	0,394952927	0,692877629	NA
ENSG00000059915	PSD	pleckstrin and Sec7 domain containing	5662	3,715750336	0,242447136	1,175224	0,206298588	0,836557682	NA
ENSG00000060303	RPS17P5	ribosomal protein S17 pseudogene 5	442216	0	NA	NA	NA	NA	NA
ENSG00000060566	CREB3L3	cAMP responsive element binding protein 3 like 3	84699	4,067551446	0,538602104	1,062955	0,506702863	0,612363322	NA
ENSG00000060656	PTPRU	protein tyrosine phosphatase receptor type U	10076	0,15836288	0	4,080456	0	1	NA
ENSG00000060709	RIMBP2	RIMS binding protein 2	23504	0,117497108	0,847101401	4,080456	0,207599697	0,835541541	NA
ENSG00000060718	COL11A1	collagen type XI alpha 1 chain	1301	0	NA	NA	NA	NA	NA

ENSG00000061455	PRDM6	PR/SET domain 6	93166	0	NA	NA	NA	NA	NA
ENSG00000061492	WNT8A	Wnt family member 8A	7478	0	NA	NA	NA	NA	NA
ENSG00000061656	SPAG4	sperm associated antigen 4	6676	1,760097742	0,421137689	1,556717	0,270529412	0,786752993	NA
ENSG00000061676	NCKAP1	NCK associated protein 1	10787	2,116070215	-0,287356771	1,57588	-0,182346819	0,855310558	NA
ENSG00000061918	GUCY1B1	guanylate cyclase 1 soluble subunit beta 1	2983	1,420934105	-0,754680676	2,130845	-0,354169708	0,723211704	NA
ENSG00000062038	CDH3	cadherin 3	1001	0	NA	NA	NA	NA	NA
ENSG00000062096	ARSF	arylsulfatase F	416	0	NA	NA	NA	NA	NA
ENSG00000062282	DGAT2	diacylglycerol O-acyltransferase 2	84649	0	NA	NA	NA	NA	NA
ENSG00000062370	ZNF112	zinc finger protein 112	7771	0	NA	NA	NA	NA	NA
ENSG00000062524	LTK	leukocyte receptor tyrosine kinase	4058	5,336846144	-0,3243934	0,961584	-0,337353319	0,73585057	NA
ENSG00000063015	SEZ6	seizure related 6 homolog	124925	0	NA	NA	NA	NA	NA
ENSG00000063515	GSC2	goosecoid homeobox 2	2928	0	NA	NA	NA	NA	NA
ENSG00000063761	ADCK1	aarF domain containing kinase 1	57143	9,811503213	0,249693032	0,685195	0,364411671	0,715550621	NA
ENSG00000064042	LIMCH1	LIM and calponin homology domains 1	22998	0	NA	NA	NA	NA	NA
ENSG00000064195	DLX3	distal-less homeobox 3	1747	0	NA	NA	NA	NA	NA
ENSG00000064199	SPA17	sperm autoantigenic protein 17	53340	2,825134242	0,762222623	1,109374	0,687074236	0,492035949	NA
ENSG00000064205	CCN5	cellular communication network factor 5	8839	0	NA	NA	NA	NA	NA
ENSG00000064218	DMRT3	doublesex and mab-3 related transcription factor 3	58524	0	NA	NA	NA	NA	NA
ENSG00000064225	ST3GAL6	ST3 beta-galactoside alpha-2,3-sialyltransferase 6	10402	0	NA	NA	NA	NA	NA
ENSG00000064270	ATP2C2	ATPase secretory pathway Ca2+ transporting 2	9914	0	NA	NA	NA	NA	NA
ENSG00000064300	NGFR	nerve growth factor receptor	4804	0,356316315	0,400010048	3,711272	0,107782462	0,914168255	NA
ENSG00000064309	CDON	cell adhesion associated, oncogene regulated	50937	0,598646235	3,02302539	3,16507	0,955121092	0,339516464	NA
ENSG00000064489	NA	NA	NA	3,129366756	-4,8099871	1,99758	-2,407907425	0,016044249	NA
ENSG00000064652	SNX24	sorting nexin 24	28966	4,893233409	0,389998093	1,047438	0,372335173	0,709643314	NA
ENSG00000064655	EYA2	EYA transcriptional coactivator and phosphatase 2	2139	0	NA	NA	NA	NA	NA
ENSG00000064692	SNCAIP	synuclein alpha interacting protein	9627	0	NA	NA	NA	NA	NA
ENSG00000064763	FAR2	fatty acyl-CoA reductase 2	55711	3,817718218	-0,300077625	1,86509	-0,160891729	0,872178677	NA
ENSG00000064787	BCAS1	brain enriched myelin associated protein 1	8537	0,235788134	0,84536095	4,080456	0,207173163	0,835874625	NA
ENSG00000064835	POU1F1	POU class 1 homeobox 1	5449	0	NA	NA	NA	NA	NA
ENSG00000064886	CHI3L2	chitinase 3 like 2	1117	8,898146792	-0,408315224	0,721765	-0,565717437	0,57158588	NA
ENSG00000064989	CALCRL	calcitonin receptor like receptor	10203	0,45907169	-0,793742707	2,987288	-0,265706809	0,790465019	NA
ENSG00000065054	NHERF2	NHERF family PDZ scaffold protein 2	9351	6,996565107	-1,163758085	0,799721	-1,455204916	0,14561254	NA
ENSG00000065320	NTN1	netrin 1	9423	0,10543123	0	4,080456	0	1	NA
ENSG00000065325	GLP2R	glucagon like peptide 2 receptor	9340	0	NA	NA	NA	NA	NA
ENSG00000065371	ROPN1	rhophilin associated tail protein 1	54763	0	NA	NA	NA	NA	NA
ENSG00000065534	MYLK	myosin light chain kinase	4638	0,11808024	-1,076443944	4,080456	-0,263804824	0,791930317	NA
ENSG00000065609	SNAP91	synaptosome associated protein 91	9892	0	NA	NA	NA	NA	NA
ENSG00000065618	COL17A1	collagen type XVII alpha 1 chain	1308	0	NA	NA	NA	NA	NA
ENSG00000065621	GSTO2	glutathione S-transferase omega 2	119391	1,002717762	1,327725858	2,621019	0,506568601	0,612457544	NA
ENSG00000065717	TLE2	TLE family member 2, transcriptional corepressor	7089	1,660347763	-0,716458415	2,828498	-0,253299994	0,800036401	NA
ENSG00000065833	ME1	malic enzyme 1	4199	1,951423574	0,245825731	1,403655	0,1751326	0,860975443	NA
ENSG00000066032	CTNNA2	catenin alpha 2	1496	0,118286652	0	4,080456	0	1	NA
ENSG00000066185	ZMYND12	zinc finger MYND-type containing 12	84217	0	NA	NA	NA	NA	NA
ENSG00000066248	NGEF	neuronal guanine nucleotide exchange factor	25791	0	NA	NA	NA	NA	NA
ENSG00000066336	SPI1	Spi-1 proto-oncogene	6688	0,15836288	0	4,080456	0	1	NA
ENSG00000066382	MPPED2	metallophosphoesterase domain containing 2	744	0	NA	NA	NA	NA	NA
ENSG00000066405	CLDN18	claudin 18	51208	0	NA	NA	NA	NA	NA
ENSG00000066468	FGFR2	fibroblast growth factor receptor 2	2263	0	NA	NA	NA	NA	NA
ENSG00000066629	EML1	EMAP like 1	2009	0	NA	NA	NA	NA	NA
ENSG00000066735	KIF26A	kinesin family member 26A	26153	0	NA	NA	NA	NA	NA
ENSG00000066813	ACSM2B	acyl-CoA synthetase medium chain family member 2B	348158	0	NA	NA	NA	NA	NA

ENSG00000066923	STAG3	STAG3 cohesin complex component	10734	10,62744216	0,689938195	0,79091	0,872334621	0,383025856	NA
ENSG00000067048	DDX3Y	DEAD-box helicase 3 Y-linked	8653	0	NA	NA	NA	NA	NA
ENSG00000067141	NEO1	neogenin 1	4756	0,838901024	0,426181354	2,06277	0,206606306	0,836317335	NA
ENSG00000067177	PHKA1	phosphorylase kinase regulatory subunit alpha 1	5255	0	NA	NA	NA	NA	NA
ENSG00000067208	EVI5	ecotropic viral integration site 5	7813	1,71823002	1,532015781	1,719746	0,890838466	0,373015836	NA
ENSG00000067445	TRO	trophinin	7216	2,735446642	0,459988259	1,104759	0,416369851	0,677139386	NA
ENSG00000067601	NA	NA	NA	3,332314583	-0,832941747	1,035011	-0,804766187	0,420954617	NA
ENSG00000067646	ZFY	zinc finger protein Y-linked	7544	0	NA	NA	NA	NA	NA
ENSG00000067715	SYT1	synaptotagmin 1	6857	0	NA	NA	NA	NA	NA
ENSG00000067798	NAV3	neuron navigator 3	89795	0	NA	NA	NA	NA	NA
ENSG00000067842	ATP2B3	ATPase plasma membrane Ca2+ transporting 3	492	0	NA	NA	NA	NA	NA
ENSG00000068078	FGFR3	fibroblast growth factor receptor 3	2261	0,11808024	-1,076443944	4,080456	-0,263804824	0,791930317	NA
ENSG00000068137	PLEKHH3	pleckstrin homology, MyTH4 and FERM domain containing H3	79990	0,117497108	0,847101401	4,080456	0,207599697	0,835541541	NA
ENSG00000068615	REEP1	receptor accessory protein 1	65055	0	NA	NA	NA	NA	NA
ENSG00000068781	STON1-GTF2A1L	STON1-GTF2A1L readthrough	286749	0,554089539	0	3,97302	0	1	NA
ENSG00000068976	PYGM	glycogen phosphorylase, muscle associated	5837	5,973252184	0,1341675	0,915503	0,146550656	0,883486704	NA
ENSG00000068985	PAGE1	PAGE family member 1	8712	0	NA	NA	NA	NA	NA
ENSG00000069011	PITX1	paired like homeodomain 1	5307	0	NA	NA	NA	NA	NA
ENSG00000069018	TRPC7	transient receptor potential cation channel subfamily C member 7	57113	0	NA	NA	NA	NA	NA
ENSG00000069122	ADGRF5	adhesion G protein-coupled receptor F5	221395	0	NA	NA	NA	NA	NA
ENSG00000069188	SDK2	sidekick cell adhesion molecule 2	54549	2,038087972	-4,180055127	1,810297	-2,309044075	0,020941136	NA
ENSG00000069206	ADAM7	ADAM metallopeptidase domain 7	8756	0	NA	NA	NA	NA	NA
ENSG00000069431	ABCC9	ATP binding cassette subfamily C member 9	10060	7,288801522	0,988004137	0,769924	1,283249722	0,199404601	NA
ENSG00000069482	GAL	galanin and GMAP prepropeptide	51083	0	NA	NA	NA	NA	NA
ENSG00000069535	MAOB	monoamine oxidase B	4129	3,806401496	-0,561503409	0,955235	-0,587817269	0,556654952	NA
ENSG00000069696	DRD4	dopamine receptor D4	1815	0,644367136	-2,551006053	3,234732	-0,788629732	0,430328448	NA
ENSG00000069764	PLA2G10	phospholipase A2 group X	8399	0	NA	NA	NA	NA	NA
ENSG00000069812	HES2	hes family bHLH transcription factor 2	54626	0,15836288	0	4,080456	0	1	NA
ENSG00000070019	GUCY2C	guanylate cyclase 2C	2984	0	NA	NA	NA	NA	NA
ENSG00000070031	SCT	secretin	6343	0,072165449	0,847101401	4,080456	0,207599697	0,835541541	NA
ENSG00000070047	PHRF1	PHD and ring finger domains 1	57661	11,0456507	-0,773162301	3,740604	-0,206694481	0,836248468	NA
ENSG00000070087	PFN2	profilin 2	5217	4,406756627	-1,24489759	1,180218	-1,054802927	0,291515466	NA
ENSG00000070159	PTPN3	protein tyrosine phosphatase non-receptor type 3	5774	4,906776622	-0,535628923	0,98059	-0,546231143	0,584907061	NA
ENSG00000070182	SPTB	spectrin beta, erythrocytic	6710	0	NA	NA	NA	NA	NA
ENSG00000070193	FGF10	fibroblast growth factor 10	2255	0	NA	NA	NA	NA	NA
ENSG00000070371	CLTCL1	clathrin heavy chain like 1	8218	6,87135833	-2,837121101	1,378952	-2,057448045	0,039643148	NA
ENSG00000070388	FGF22	fibroblast growth factor 22	27006	0,912908362	0,436450853	2,194027	0,198926868	0,842319952	NA
ENSG00000070404	FSTL3	follistatin like 3	10272	5,557096135	-0,652105905	0,74642	-0,8736445	0,382311884	NA
ENSG00000070526	ST6GALNAC1	ST6 N-acetylgalactosaminide alpha-2,6-sialyltransferase 1	55808	0	NA	NA	NA	NA	NA
ENSG00000070601	FRMPD1	FERM and PDZ domain containing 1	22844	0,10543123	0	4,080456	0	1	NA
ENSG00000070614	NDST1	N-deacetylase and N-sulfotransferase 1	3340	0	NA	NA	NA	NA	NA
ENSG00000070729	CNGB1	cyclic nucleotide gated channel subunit beta 1	1258	0	NA	NA	NA	NA	NA
ENSG00000070731	ST6GALNAC2	ST6 N-acetylgalactosaminide alpha-2,6-sialyltransferase 2	10610	0,435961387	0,841893351	4,080456	0,206323356	0,836538335	NA
ENSG00000070748	CHAT	choline O-acetyltransferase	1103	0	NA	NA	NA	NA	NA
ENSG00000070778	PTPN21	protein tyrosine phosphatase non-receptor type 21	11099	0,353716295	0,848523043	4,080456	0,207948099	0,835269492	NA
ENSG00000070808	CAMK2A	calcium/calmodulin dependent protein kinase II alpha	815	0,210862461	0	4,080456	0	1	NA
ENSG00000070886	EPHA8	EPH receptor A8	2046	0	NA	NA	NA	NA	NA
ENSG00000070915	SLC12A3	solute carrier family 12 member 3	6559	0	NA	NA	NA	NA	NA

ENSG00000070985	TRPM5	transient receptor potential cation channel subfamily M member 5	29850	0	NA	NA	NA	NA	NA
ENSG00000071203	MS4A12	membrane spanning 4-domains A12	54860	0	NA	NA	NA	NA	NA
ENSG00000071282	LMCD1	LIM and cysteine rich domains 1	29995	0,072165449	0,847101401	4,080456	0,207599697	0,835541541	NA
ENSG00000071677	PRLH	prolactin releasing hormone	51052	0	NA	NA	NA	NA	NA
ENSG00000071909	MYO3B	myosin IIIB	140469	0	NA	NA	NA	NA	NA
ENSG00000071967	CYBRD1	cytochrome b reductase 1	79901	0,430454526	-2,432293812	3,542928	-0,686520874	0,492384706	NA
ENSG00000071991	CDH19	cadherin 19	28513	0	NA	NA	NA	NA	NA
ENSG00000072041	SLC6A15	solute carrier family 6 member 15	55117	0	NA	NA	NA	NA	NA
ENSG00000072062	PRKACA	protein kinase cAMP-activated catalytic subunit alpha	5566	5,982044961	0	3,341887	0	1	NA
ENSG00000072080	SPP2	secreted phosphoprotein 2	6694	0	NA	NA	NA	NA	NA
ENSG00000072133	RPS6KA6	ribosomal protein S6 kinase A6	27330	0,207996306	-1,916073151	4,043021	-0,473921178	0,635556117	NA
ENSG00000072134	EPN2	epsin 2	22905	7,411569807	-0,466547161	0,650124	-0,717628258	0,472986529	NA
ENSG00000072163	LIMS2	LIM zinc finger domain containing 2	55679	0	NA	NA	NA	NA	NA
ENSG00000072182	ASIC4	acid sensing ion channel subunit family member 4	55515	0,433027771	-1,555835085	3,997357	-0,38921598	0,697116383	NA
ENSG00000072195	SPEG	striated muscle enriched protein kinase	10290	0	NA	NA	NA	NA	NA
ENSG00000072201	LNX1	ligand of numb-protein X 1	84708	0	NA	NA	NA	NA	NA
ENSG00000072315	TRPC5	transient receptor potential cation channel subfamily C member 5	7224	0	NA	NA	NA	NA	NA
ENSG00000072422	RHOBTB1	Rho related BTB domain containing 1	9886	0	NA	NA	NA	NA	NA
ENSG00000072657	TRHDE	thyrotropin releasing hormone degrading enzyme	29953	0	NA	NA	NA	NA	NA
ENSG00000072694	FCGR2B	Fc gamma receptor IIb	2213	4,209629695	0,74126054	1,083383	0,684209074	0,493843153	NA
ENSG00000072832	CRMP1	collapsin response mediator protein 1	1400	0	NA	NA	NA	NA	NA
ENSG00000072840	EVC	EvC ciliary complex subunit 1	2121	3,396583731	0,394269479	1,079775	0,365140477	0,715006547	NA
ENSG00000072952	IRAG1	inositol 1,4,5-triphosphate receptor associated 1	10335	0	NA	NA	NA	NA	NA
ENSG00000072954	TMEM38A	transmembrane protein 38A	79041	0,664085829	0,127091318	3,136501	0,040520091	0,96767849	NA
ENSG00000073060	SCARB1	scavenger receptor class B member 1	949	2,453329635	4,946811967	2,434429	2,032021468	0,042151483	NA
ENSG00000073067	CYP2W1	cytochrome P450 family 2 subfamily W member 1	54905	0	NA	NA	NA	NA	NA
ENSG00000073146	MOV10L1	Mov10 like RISC complex RNA helicase 1	54456	0,883425987	1,135013725	2,353171	0,482333744	0,62956888	NA
ENSG00000073150	PANX2	pannexin 2	56666	0,302805691	1,412719375	4,061131	0,347863555	0,727942657	NA
ENSG00000073282	TP63	tumor protein p63	8626	0	NA	NA	NA	NA	NA
ENSG00000073331	ALPK1	alpha kinase 1	80216	0,34959868	-2,646533008	3,989744	-0,663334082	0,507116613	NA
ENSG00000073464	CLCN4	chloride voltage-gated channel 4	1183	2,306835946	-1,310122828	1,731584	-0,756603581	0,449287395	NA
ENSG00000073598	FNDC8	fibronectin type III domain containing 8	54752	0,11808024	-1,076443944	4,080456	-0,263804824	0,791930317	NA
ENSG00000073670	ADAM11	ADAM metallopeptidase domain 11	4185	0,549282698	-0,594594869	3,366035	-0,176645475	0,859786872	NA
ENSG00000073711	PPP2R3A	protein phosphatase 2 regulatory subunit B''alpha	5523	0,411517282	0,636798588	3,612024	0,176299679	0,860058514	NA
ENSG00000073712	FERMT2	FERM domain containing kindlin 2	10979	0,103998153	-1,076443944	4,080456	-0,263804824	0,791930317	NA
ENSG00000073734	ABCB11	ATP binding cassette subfamily B member 11	8647	0	NA	NA	NA	NA	NA
ENSG00000073737	DHRS9	dehydrogenase/reductase 9	10170	0	NA	NA	NA	NA	NA
ENSG00000073754	CD5L	CD5 molecule like	922	0	NA	NA	NA	NA	NA
ENSG00000073792	IGF2BP2	insulin like growth factor 2 mRNA binding protein 2	10644	0	NA	NA	NA	NA	NA
ENSG00000073905	NA	NA	NA	0,11808024	-1,076443944	4,080456	-0,263804824	0,791930317	NA
ENSG00000073969	NSF	N-ethylmaleimide sensitive factor, vesicle fusing ATPase	4905	11,26096475	0,633636272	1,007586	0,628865567	0,52943707	NA
ENSG00000074047	GLI2	GLI family zinc finger 2	2736	0,15836288	0	4,080456	0	1	NA
ENSG00000074181	NOTCH3	notch receptor 3	4854	0	NA	NA	NA	NA	NA
ENSG00000074211	PPP2R2C	protein phosphatase 2 regulatory subunit Bgamma	5522	0	NA	NA	NA	NA	NA
ENSG00000074219	TEAD2	TEA domain transcription factor 2	8463	0	NA	NA	NA	NA	NA
ENSG00000074276	CDHR2	cadherin related family member 2	54825	2,310834604	0,117788028	1,366377	0,086204608	0,931303768	NA
ENSG00000074317	SNCB	synuclein beta	6620	0,457469659	-1,641534326	3,432841	-0,478185301	0,632518323	NA
ENSG00000074410	CA12	carbonic anhydrase 12	771	0	NA	NA	NA	NA	NA
ENSG00000074416	MGLL	monoglyceride lipase	11343	2,97199758	-0,156468281	1,151075	-0,135932311	0,891874792	NA

ENSG00000074527	NTN4	netrin 4	59277	0	NA	NA	NA	NA	NA
ENSG00000074590	NUAK1	NUAK family kinase 1	9891	2,209468822	0,337211293	1,45562	0,231661612	0,81680085	NA
ENSG00000074657	ZNF532	zinc finger protein 532	55205	6,500940345	-0,761057563	0,766398	-0,993031945	0,320694387	NA
ENSG00000074660	SCARF1	scavenger receptor class F member 1	8578	3,258349528	4,660817387	3,924751	1,187544758	0,235012813	NA
ENSG00000074771	NOX3	NADPH oxidase 3	50508	0	NA	NA	NA	NA	NA
ENSG00000074803	SLC12A1	solute carrier family 12 member 1	6557	0	NA	NA	NA	NA	NA
ENSG00000074964	ARHGEF10L	Rho guanine nucleotide exchange factor 10 like	55160	0	NA	NA	NA	NA	NA
ENSG00000075035	WSCD2	WSC domain containing 2	9671	0,31672576	0	4,080456	0	1	NA
ENSG00000075043	KCNQ2	potassium voltage-gated channel subfamily Q member 2	3785	0	NA	NA	NA	NA	NA
ENSG00000075073	TACR2	tachykinin receptor 2	6865	0,15836288	0	4,080456	0	1	NA
ENSG00000075213	SEMA3A	semaphorin 3A	10371	2,675397105	2,015536508	1,314915	1,532826033	0,125318725	NA
ENSG00000075223	SEMA3C	semaphorin 3C	10512	0	NA	NA	NA	NA	NA
ENSG00000075290	WNT8B	Wnt family member 8B	7479	0	NA	NA	NA	NA	NA
ENSG00000075340	ADD2	adducin 2	119	0	NA	NA	NA	NA	NA
ENSG00000075388	FGF4	fibroblast growth factor 4	2249	0	NA	NA	NA	NA	NA
ENSG00000075391	RASAL2	RAS protein activator like 2	9462	0	NA	NA	NA	NA	NA
ENSG00000075429	CACNG5	calcium voltage-gated channel auxiliary subunit gamma 5	27091	0,117497108	0,847101401	4,080456	0,207599697	0,835541541	NA
ENSG00000075461	CACNG4	calcium voltage-gated channel auxiliary subunit gamma 4	27092	0	NA	NA	NA	NA	NA
ENSG00000075618	FSCN1	fascin actin-bundling protein 1	6624	0,694793128	0,295849308	2,365336	0,125077088	0,900462522	NA
ENSG00000075673	ATP12A	ATPase H+/K+ transporting non-gastric alpha2 subunit	479	0,103998153	-1,076443944	4,080456	-0,263804824	0,791930317	NA
ENSG00000075886	TUBA3D	tubulin alpha 3d	113457	0	NA	NA	NA	NA	NA
ENSG00000075891	PAX2	paired box 2	5076	0	NA	NA	NA	NA	NA
ENSG00000076258	FMO4	flavin containing dimethylaniline monoxygenase 4	2329	1,215629608	0,567999111	1,757313	0,323220158	0,746528516	NA
ENSG00000076344	RGS11	regulator of G protein signaling 11	8786	0	NA	NA	NA	NA	NA
ENSG00000076706	MCAM	melanoma cell adhesion molecule	4162	0,17614548	-0,114114859	4,080456	-0,027966203	0,977689106	NA
ENSG00000076716	GPC4	glypican 4	2239	0	NA	NA	NA	NA	NA
ENSG00000076826	CAMSAP3	calmodulin regulated spectrin associated protein family member 3	57662	1,745044764	0,860600953	1,794065	0,479693349	0,631445458	NA
ENSG00000076864	RAP1GAP	RAP1 GTPase activating protein	5909	0,103998153	-1,076443944	4,080456	-0,263804824	0,791930317	NA
ENSG00000077009	NMRK2	nicotinamide riboside kinase 2	27231	0	NA	NA	NA	NA	NA
ENSG00000077063	CTTNBP2	cortactin binding protein 2	83992	0	NA	NA	NA	NA	NA
ENSG00000077080	ACTL6B	actin like 6B	51412	0	NA	NA	NA	NA	NA
ENSG00000077092	RARB	retinoic acid receptor beta	5915	0,307961314	-0,100013405	3,889764	-0,025711949	0,979487093	NA
ENSG00000077264	PAK3	p21 (RAC1) activated kinase 3	5063	0	NA	NA	NA	NA	NA
ENSG00000077274	CAPN6	calpain 6	827	0	NA	NA	NA	NA	NA
ENSG00000077279	DCX	doublecortin	1641	0	NA	NA	NA	NA	NA
ENSG00000077327	SPAG6	sperm associated antigen 6	9576	0	NA	NA	NA	NA	NA
ENSG00000077498	TYR	tyrosinase	7299	0	NA	NA	NA	NA	NA
ENSG00000077522	ACTN2	actinin alpha 2	88	0	NA	NA	NA	NA	NA
ENSG00000077782	FGFR1	fibroblast growth factor receptor 1	2260	6,125012772	-2,399376437	2,683021	-0,894281597	0,371171244	NA
ENSG00000077800	FKBP6	FKBP prolyl isomerase family member 6 (inactive)	8468	0	NA	NA	NA	NA	NA
ENSG00000077935	SMC1B	structural maintenance of chromosomes 1B	27127	1,027401123	2,19019686	3,005943	0,728622123	0,466232842	NA
ENSG00000077942	FBLN1	fibulin 1	2192	0	NA	NA	NA	NA	NA
ENSG00000077943	ITGA8	integrin subunit alpha 8	8516	0	NA	NA	NA	NA	NA
ENSG00000078018	MAP2	microtubule associated protein 2	4133	0	NA	NA	NA	NA	NA
ENSG00000078053	AMPH	amphiphysin	273	0,645129795	0,831717273	3,117162	0,26681875	0,789608717	NA
ENSG00000078081	LAMP3	lysosomal associated membrane protein 3	27074	3,857329843	0,842308566	0,975055	0,86385753	0,38766616	NA
ENSG00000078098	FAP	fibroblast activation protein alpha	2191	0	NA	NA	NA	NA	NA
ENSG00000078114	NEBL	nebulette	10529	0	NA	NA	NA	NA	NA
ENSG00000078295	ADCY2	adenylate cyclase 2	108	0	NA	NA	NA	NA	NA
ENSG00000078328	RBFOX1	RNA binding fox-1 homolog 1	54715	0	NA	NA	NA	NA	NA
ENSG00000078399	HOXA9	homeobox A9	3205	3,177371594	0,431924812	1,54072	0,28033966	0,779216926	NA
ENSG00000078401	EDN1	endothelin 1	1906	0,780440703	-0,738435108	1,98038	-0,372875488	0,709241116	NA
ENSG00000078549	ADCYAP1R1	ADCYAP receptor type I	117	0	NA	NA	NA	NA	NA
ENSG00000078579	FGF20	fibroblast growth factor 20	26281	0	NA	NA	NA	NA	NA

ENSG00000078725	BRINP1	BMP/retinoic acid inducible neural specific 1	1620	0	NA	NA	NA	NA	NA
ENSG00000078795	PKD2L2	polycystin 2 like 2, transient receptor potential cation channel	27039	0	NA	NA	NA	NA	NA
ENSG00000078814	MYH7B	myosin heavy chain 7B	57644	0,781552941	-2,896716619	3,094349	-0,936131163	0,34920566	NA
ENSG00000078898	BPIFB2	BPI fold containing family B member 2	80341	0	NA	NA	NA	NA	NA
ENSG00000079101	CLUL1	clusterin like 1	27098	0	NA	NA	NA	NA	NA
ENSG00000079102	RUNX1T1	RUNX1 partner transcriptional co-repressor 1	862	0,11808024	-1,076443944	4,080456	-0,263804824	0,791930317	NA
ENSG00000079150	FKBP7	FKBP prolyl isomerase 7	51661	4,647742788	-0,8397819	0,977245	-0,859336106	0,39015511	NA
ENSG00000079156	OSBPL6	oxysterol binding protein like 6	114880	0,621086775	0,45313307	3,99554	0,113409733	0,909705724	NA
ENSG00000079215	SLC1A3	solute carrier family 1 member 3	6507	0	NA	NA	NA	NA	NA
ENSG00000079308	TNS1	tensin 1	7145	0	NA	NA	NA	NA	NA
ENSG00000079337	RAPGEF3	Rap guanine nucleotide exchange factor 3	10411	0,15836288	0	4,080456	0	1	NA
ENSG00000079385	CEACAM1	CEA cell adhesion molecule 1	634	8,371705069	1,519633836	0,712098	2,13402389	0,032840825	NA
ENSG00000079393	DUSP13B	dual specificity phosphatase 13B	51207	0	NA	NA	NA	NA	NA
ENSG00000079393	DUSP13A	dual specificity phosphatase 13A	128854680	0	NA	NA	NA	NA	NA
ENSG00000079459	FDFT1	farnesyl-diphosphate farnesyltransferase 1	2222	4,929820445	0,528216706	1,113989	0,474166921	0,63538088	NA
ENSG00000079557	AFM	afamin	173	0	NA	NA	NA	NA	NA
ENSG00000079689	SCGN	secretagogin, EF-hand calcium binding protein	10590	0	NA	NA	NA	NA	NA
ENSG00000079691	CARMIL1	capping protein regulator and myosin 1 linker 1	55604	0,15836288	0	4,080456	0	1	NA
ENSG00000079841	RIMS1	regulating synaptic membrane exocytosis 1	22999	0	NA	NA	NA	NA	NA
ENSG00000079931	MOXD1	monooxygenase DBH like 1	26002	1,181023975	0,282662525	1,655773	0,170713376	0,864449145	NA
ENSG00000080007	DDX43	DEAD-box helicase 43	55510	10,14475659	-0,287477423	0,618185	-0,465034413	0,641906813	NA
ENSG00000080031	PTPRH	protein tyrosine phosphatase receptor type H	5794	0	NA	NA	NA	NA	NA
ENSG00000080166	DCT	dopachrome tautomerase	1638	0,117497108	0,847101401	4,080456	0,207599697	0,835541541	NA
ENSG00000080224	EPHA6	EPH receptor A6	285220	0,209571606	-1,061718373	4,080456	-0,260196019	0,794712575	NA
ENSG00000080293	SCTR	secretin receptor	6344	0	NA	NA	NA	NA	NA
ENSG00000080493	SLC4A4	solute carrier family 4 member 4	8671	5,107406989	-0,311095517	0,948228	-0,328080846	0,742850536	NA
ENSG00000080511	RDH8	retinol dehydrogenase 8	50700	0	NA	NA	NA	NA	NA
ENSG00000080561	MID2	midline 2	11043	0,70007825	1,492491785	2,753214	0,542090733	0,587756003	NA
ENSG00000080572	DNAAF6	dynein axonemal assembly factor 6	139212	0	NA	NA	NA	NA	NA
ENSG00000080573	COL5A3	collagen type V alpha 3 chain	50509	0	NA	NA	NA	NA	NA
ENSG00000080618	CPB2	carboxypeptidase B2	1361	0	NA	NA	NA	NA	NA
ENSG00000080644	CHRNA3	cholinergic receptor nicotinic alpha 3 subunit	1136	0	NA	NA	NA	NA	NA
ENSG00000080709	KCNN2	potassium calcium-activated channel subfamily N member 2	3781	0	NA	NA	NA	NA	NA
ENSG00000080823	MOK	MOK protein kinase	5891	2,001431054	-2,543697862	1,705344	-1,491603918	0,135803011	NA
ENSG00000081026	MAGI3	membrane associated guanylate kinase, WW and PDZ domain containing 3	260425	1,539157021	2,785058069	1,916908	1,452890717	0,146254099	NA
ENSG00000081041	CXCL2	C-X-C motif chemokine ligand 2	2920	0	NA	NA	NA	NA	NA
ENSG00000081051	AFP	alpha fetoprotein	174	0	NA	NA	NA	NA	NA
ENSG00000081052	COL4A4	collagen type IV alpha 4 chain	1286	1,037895662	-1,099245778	1,997983	-0,550177637	0,58219754	NA
ENSG00000081087	OSTM1	osteoclastogenesis associated transmembrane protein 1	28962	8,531009336	-6,683056156	3,911853	-1,708411848	NA	NA
ENSG00000081138	CDH7	cadherin 7	1005	0,117497108	0,847101401	4,080456	0,207599697	0,835541541	NA
ENSG00000081148	IMPG2	interphotoreceptor matrix proteoglycan 2	50939	8,536512869	0,30683545	0,737088	0,416280658	0,677204645	NA
ENSG00000081181	ARG2	arginase 2	384	1,040936408	0,075726931	2,647155	0,028606914	0,977178098	NA
ENSG00000081189	MEF2C	myocyte enhancer factor 2C	4208	0,567126138	-0,098002834	2,598403	-0,037716571	0,969913664	NA
ENSG00000081248	CACNA1S	calcium voltage-gated channel subunit alpha1 S	779	0	NA	NA	NA	NA	NA
ENSG00000081277	PKP1	plakophilin 1	5317	0	NA	NA	NA	NA	NA
ENSG00000081377	CDC14B	cell division cycle 14B	8555	0,744265057	1,779003105	2,653824	0,670354675	0,502631721	NA
ENSG00000081479	LRP2	LDL receptor related protein 2	4036	0	NA	NA	NA	NA	NA
ENSG00000081800	SLC13A1	solute carrier family 13 member 1	6561	0	NA	NA	NA	NA	NA
ENSG00000081803	CADPS2	calcium dependent secretion activator 2	93664	0	NA	NA	NA	NA	NA

ENSG00000081853	PCDHGA2	protocadherin gamma subfamily A, 2	56113	0,118286652	0	4,080456	0	1	NA
ENSG00000082014	SMARCD3	SWI/SNF related, matrix associated, actin dependent regulator of chromatin, subfamily d, member 3	6604	0,392082898	1,920882505	4,024349	0,477315044	0,6331378	NA
ENSG00000082126	MPP4	MAGUK p55 scaffold protein 4	58538	0,10543123	0	4,080456	0	1	NA
ENSG00000082175	PGR	progesterone receptor	5241	0	NA	NA	NA	NA	NA
ENSG00000082196	C1QTNF3	C1q and TNF related 3	114899	4,819983634	3,645516819	1,332688	2,735461106	0,006229295	NA
ENSG00000082293	COL19A1	collagen type XIX alpha 1 chain	1310	0	NA	NA	NA	NA	NA
ENSG00000082397	EPB41L3	erythrocyte membrane protein band 4,1 like 3	23136	0	NA	NA	NA	NA	NA
ENSG00000082438	COBLL1	cordon-bleu WH2 repeat protein like 1	22837	0,117497108	0,847101401	4,080456	0,207599697	0,835541541	NA
ENSG00000082482	KCNK2	potassium two pore domain channel subfamily K member 2	3776	0	NA	NA	NA	NA	NA
ENSG00000082556	OPRK1	opioid receptor kappa 1	4986	0	NA	NA	NA	NA	NA
ENSG00000082684	SEMA5B	semaphorin 5B	54437	1,055326758	-3,535179485	3,472866	-1,017942895	0,308705095	NA
ENSG00000083067	TRPM3	transient receptor potential cation channel subfamily M member 3	80036	0	NA	NA	NA	NA	NA
ENSG00000083307	GRHL2	grainyhead like transcription factor 2	79977	0,103998153	-1,076443944	4,080456	-0,263804824	0,791930317	NA
ENSG00000083454	P2RX5	purinergic receptor P2X 5	5026	1,730545685	-1,996006284	2,316834	-0,861523353	0,388949865	NA
ENSG00000083457	ITGAE	integrin subunit alpha E	3682	4,679350676	-0,429358057	1,015497	-0,422806	0,67243681	NA
ENSG00000083782	EPYC	epiphycan	1833	0	NA	NA	NA	NA	NA
ENSG00000083807	SLC27A5	solute carrier family 27 member 5	10998	4,824118861	1,546600695	1,04	1,487116404	0,13698407	NA
ENSG00000083857	FAT1	FAT atypical cadherin 1	2195	0,10543123	0	4,080456	0	1	NA
ENSG00000083896	YTHDC1	YTH N6-methyladenosine RNA binding protein C1	91746	0	NA	NA	NA	NA	NA
ENSG00000084110	HAL	histidine ammonia-lyase	3034	1,943903615	-0,234964045	1,67892	-0,139949536	0,888699862	NA
ENSG00000084444	FAM234B	family with sequence similarity 234 member B	57613	7,633265262	0,454981291	0,993083	0,458150491	0,646844327	NA
ENSG00000084453	SLCO1A2	solute carrier organic anion transporter family member 1A2	6579	0	NA	NA	NA	NA	NA
ENSG00000084628	NKAIN1	sodium/potassium transporting ATPase interacting 1	79570	0	NA	NA	NA	NA	NA
ENSG00000084636	COL16A1	collagen type XVI alpha 1 chain	1307	1,787462264	-1,30251459	2,079724	-0,626291992	0,531123438	NA
ENSG00000084674	APOB	apolipoprotein B	338	0,210312395	-1,077404975	4,080456	-0,264040345	0,791748831	NA
ENSG00000084710	EFR3B	EFR3 homolog B	22979	0,664804697	1,725623053	3,282462	0,525710057	0,599089675	NA
ENSG00000084734	GCKR	glucokinase regulator	2646	0	NA	NA	NA	NA	NA
ENSG00000084764	MAPRE3	microtubule associated protein RP/EB family member 3	22924	4,533994386	-0,084873128	0,95477	-0,088893799	0,929166312	NA
ENSG00000085231	AK6	adenylate kinase 6	102157402	8,796519355	0,455295078	1,222389	0,372463234	0,709547981	NA
ENSG00000085265	FCN1	ficolin 1	2219	0	NA	NA	NA	NA	NA
ENSG00000085276	MECOM	MDS1 and EVI1 complex locus	2122	0	NA	NA	NA	NA	NA
ENSG00000085465	OVGP1	oviductal glycoprotein 1	5016	3,358670645	0,865190575	0,921911	0,938475547	0,348000078	NA
ENSG00000085514	PILRA	paired immunoglobin like type 2 receptor alpha	29992	2,554666854	-0,081419018	1,434468	-0,056759053	0,954737132	NA
ENSG00000085552	IGSF9	immunoglobulin superfamily member 9	57549	0	NA	NA	NA	NA	NA
ENSG00000085721	RRN3	RRN3 homolog, RNA polymerase I transcription factor	54700	116,0901473	-1,729170241	3,337549	-0,518095898	NA	NA
ENSG00000085831	TTC39A	tetratricopeptide repeat domain 39A	22996	0,11808024	-1,076443944	4,080456	-0,263804824	0,791930317	NA
ENSG00000086159	AQP6	aquaporin 6	363	0	NA	NA	NA	NA	NA
ENSG00000086205	FOLH1	folate hydrolase 1	2346	0	NA	NA	NA	NA	NA
ENSG00000086288	NME8	NME/NM23 family member 8	51314	1,356495825	2,00062447	2,036751	0,982262473	0,325970557	NA
ENSG00000086289	EPDR1	ependymin related 1	54749	1,034102882	-1,088036331	2,153007	-0,505356612	0,613308387	NA
ENSG00000086506	HBQ1	hemoglobin subunit theta 1	3049	0	NA	NA	NA	NA	NA
ENSG00000086548	CEACAM6	CEA cell adhesion molecule 6	4680	0	NA	NA	NA	NA	NA
ENSG00000086570	FAT2	FAT atypical cadherin 2	2196	0	NA	NA	NA	NA	NA
ENSG00000086696	HSD17B2	hydroxysteroid 17-beta dehydrogenase 2	3294	0	NA	NA	NA	NA	NA
ENSG00000086717	PPEF1	protein phosphatase with EF-hand domain 1	5475	0	NA	NA	NA	NA	NA
ENSG00000086967	MYBPC2	myosin binding protein C2	4606	0	NA	NA	NA	NA	NA
ENSG00000086991	NOX4	NADPH oxidase 4	50507	0,117497108	0,847101401	4,080456	0,207599697	0,835541541	NA
ENSG00000087076	HSD17B14	hydroxysteroid 17-beta dehydrogenase 14	51171	0,10488184	-1,076443944	4,080456	-0,263804824	0,791930317	NA

ENSG00000087077	TRIP6	thyroid hormone receptor interactor 6	7205	1,986190113	-0,425469841	1,600532	-0,265830212	0,790369974	NA
ENSG00000087085	ACHE	acetylcholinesterase (Cartwright blood group)	43	0	NA	NA	NA	NA	NA
ENSG00000087116	ADAMTS2	ADAM metallopeptidase with thrombospondin type 1 motif 2	9509	0	NA	NA	NA	NA	NA
ENSG00000087128	TMPRSS11E	transmembrane serine protease 11E	28983	0,15836288	0	4,080456	0	1	NA
ENSG00000087237	CETP	cholesteryl ester transfer protein	1071	0	NA	NA	NA	NA	NA
ENSG00000087245	MMP2	matrix metallopeptidase 2	4313	0	NA	NA	NA	NA	NA
ENSG00000087250	MT3	metallothionein 3	4504	0	NA	NA	NA	NA	NA
ENSG00000087253	LPCAT2	lysophosphatidylcholine acyltransferase 2	54947	0,381734609	0,860631313	4,080456	0,210915481	0,832953224	NA
ENSG00000087258	GNAO1	G protein subunit alpha o1	2775	0,44593598	-0,889044751	3,00308	-0,296044297	0,767196253	NA
ENSG00000087303	NID2	nidogen 2	22795	10,57942318	1,151054272	0,644728	1,785333194	0,074207288	NA
ENSG00000087494	PTHLH	parathyroid hormone like hormone	5744	0	NA	NA	NA	NA	NA
ENSG00000087495	PHACTR3	phosphatase and actin regulator 3	116154	0	NA	NA	NA	NA	NA
ENSG00000087510	TFAP2C	transcription factor AP-2 gamma	7022	0	NA	NA	NA	NA	NA
ENSG00000087842	PIR	pirin	8544	2,008294143	1,50585966	1,530457	0,98392841	0,325150714	NA
ENSG00000087884	AAMDC	adipogenesis associated Mth938 domain containing	28971	5,535529469	-0,320221336	0,906965	-0,353069029	0,724036691	NA
ENSG00000088002	SULT2B1	sulfotransferase family 2B member 1	6820	0,119673877	0,847101401	4,080456	0,207599697	0,835541541	NA
ENSG00000088256	GNA11	G protein subunit alpha 11	2767	1,57866929	-0,917335371	1,587304	-0,577920559	0,563317754	NA
ENSG00000088280	ASAP3	ArfGAP with SH3 domain, ankyrin repeat and PH domain 3	55616	0	NA	NA	NA	NA	NA
ENSG00000088320	REM1	RRAD and GEM like GTPase 1	28954	0	NA	NA	NA	NA	NA
ENSG00000088340	FER1L4	fer-1 like family member 4 (pseudogene)	80307	0,329803851	0	4,080456	0	1	NA
ENSG00000088367	EPB41L1	erythrocyte membrane protein band 4,1 like 1	2036	0,498469472	-2,799915242	3,343609	-0,837393046	0,402371674	NA
ENSG00000088386	SLC15A1	solute carrier family 15 member 1	6564	0	NA	NA	NA	NA	NA
ENSG00000088538	DOCK3	dedicator of cytokinesis 3	1795	6,72391896	0,34432457	0,673103	0,511547961	0,608967414	NA
ENSG00000088726	TMEM40	transmembrane protein 40	55287	0	NA	NA	NA	NA	NA
ENSG00000088727	KIF9	kinesin family member 9	64147	5,220863153	-0,546490394	0,840285	-0,650363249	0,515457611	NA
ENSG00000088756	ARHGAP28	Rho GTPase activating protein 28	79822	0	NA	NA	NA	NA	NA
ENSG00000088782	DEFB127	defensin beta 127	140850	0	NA	NA	NA	NA	NA
ENSG00000088826	SMOX	spermine oxidase	54498	0,556190537	1,958143098	4,021837	0,486877805	0,626344929	NA
ENSG00000088827	SIGLEC1	sialic acid binding Ig like lectin 1	6614	0	NA	NA	NA	NA	NA
ENSG00000088881	EBF4	EBF family member 4	57593	0	NA	NA	NA	NA	NA
ENSG00000088882	CPXM1	carboxypeptidase X, M14 family member 1	56265	0,523800541	-2,49772722	3,321238	-0,752047104	0,452022733	NA
ENSG00000088899	LZTS3	leucine zipper tumor suppressor family member 3	9762	3,991763777	-0,420940991	1,038403	-0,405373295	0,685203138	NA
ENSG00000088926	F11	coagulation factor XI	2160	0,239347754	1,967469188	4,021404	0,489249276	0,624665227	NA
ENSG00000089091	DZANK1	double zinc ribbon and ankyrin repeat domains 1	55184	5,014142702	-0,572649353	1,492635	-0,383650053	0,701237837	NA
ENSG00000089101	CFAP61	cilia and flagella associated protein 61	26074	0,118286652	0	4,080456	0	1	NA
ENSG00000089116	LHX5	LIM homeobox 5	64211	0	NA	NA	NA	NA	NA
ENSG00000089163	SIRT4	sirtuin 4	23409	2,666977685	-0,289086174	1,295147	-0,2232072	0,823374248	NA
ENSG00000089169	RPH3A	rabphilin 3A	22895	0	NA	NA	NA	NA	NA
ENSG00000089199	CHGB	chromogranin B	1114	0,11808024	-1,076443944	4,080456	-0,263804824	0,791930317	NA
ENSG00000089225	TBX5	T-box transcription factor 5	6910	0	NA	NA	NA	NA	NA
ENSG00000089250	NOS1	nitric oxide synthase 1	4842	0	NA	NA	NA	NA	NA
ENSG00000089356	FXYD3	FXYD domain containing ion transport regulator 3	5349	0	NA	NA	NA	NA	NA
ENSG00000089472	HEPH	hephaestin	9843	0	NA	NA	NA	NA	NA
ENSG00000089558	KCNH4	potassium voltage-gated channel subfamily H member 4	23415	0,434898695	-0,970794996	3,556473	-0,272965687	0,784879592	NA
ENSG00000089692	LAG3	lymphocyte activating 3	3902	3,420255434	0,029495489	1,061448	0,027787979	0,977831254	NA
ENSG00000089723	OTUB2	OTU deubiquitinase, ubiquitin aldehyde binding 2	78990	10,80227454	0,150339503	1,118559	0,134404655	0,893082601	NA
ENSG00000089847	ANKRD24	ankyrin repeat domain 24	170961	0,738225701	-1,885106293	3,710557	-0,508038568	0,611426296	NA
ENSG00000090013	BLVRB	biliverdin reductase B	645	8,908557277	-0,788781521	0,616084	-1,280314869	0,20043442	NA
ENSG00000090097	PCBP4	poly(rC) binding protein 4	57060	7,953415767	-2,073668805	1,458109	-1,42216319	0,154978882	NA
ENSG00000090382	LYZ	lysozyme	4069	0	NA	NA	NA	NA	NA
ENSG00000090402	SI	sucrase-isomaltase	6476	0	NA	NA	NA	NA	NA
ENSG00000090512	FETUB	fetuin B	26998	0	NA	NA	NA	NA	NA
ENSG00000090530	P3H2	prolyl 3-hydroxylase 2	55214	0	NA	NA	NA	NA	NA

ENSG00000090534	THPO	thrombopoietin	7066	0	NA	NA	NA	NA	NA
ENSG00000090539	CHRD	chordin	8646	0	NA	NA	NA	NA	NA
ENSG00000090659	CD209	CD209 molecule	30835	0	NA	NA	NA	NA	NA
ENSG00000090932	DLL3	delta like canonical Notch ligand 3	10683	0,413731827	-1,135190674	4,080456	-0,278201924	0,780857358	NA
ENSG00000090971	NAT14	N-acetyltransferase 14 (putative)	57106	6,381910211	-0,133377431	0,759791	-0,175544834	0,860651545	NA
ENSG00000091010	POU4F3	POU class 4 homeobox 3	5459	0,10488184	-1,076443944	4,080456	-0,263804824	0,791930317	NA
ENSG00000091106	NLRC4	NLR family CARD domain containing 4	58484	0	NA	NA	NA	NA	NA
ENSG00000091128	LAMB4	laminin subunit beta 4	22798	0	NA	NA	NA	NA	NA
ENSG00000091129	NRCAM	neuronal cell adhesion molecule	4897	0,728830199	-1,159990299	2,273289	-0,510269602	0,609862596	NA
ENSG00000091136	LAMB1	laminin subunit beta 1	3912	0	NA	NA	NA	NA	NA
ENSG00000091137	SLC26A4	solute carrier family 26 member 4	5172	0	NA	NA	NA	NA	NA
ENSG00000091138	SLC26A3	solute carrier family 26 member 3	1811	0	NA	NA	NA	NA	NA
ENSG00000091181	IL5RA	interleukin 5 receptor subunit alpha	3568	0	NA	NA	NA	NA	NA
ENSG00000091262	ABCC6	ATP binding cassette subfamily C member 6	368	0	NA	NA	NA	NA	NA
ENSG00000091428	RAPGEF4	Rap guanine nucleotide exchange factor 4	11069	0	NA	NA	NA	NA	NA
ENSG00000091482	SMPX	small muscle protein X-linked	23676	0	NA	NA	NA	NA	NA
ENSG00000091513	TF	transferrin	7018	1,607794551	0,584780119	1,540235	0,379669519	0,704190749	NA
ENSG00000091536	MYO15A	myosin XVA	51168	0	NA	NA	NA	NA	NA
ENSG00000091583	APOH	apolipoprotein H	350	0	NA	NA	NA	NA	NA
ENSG00000091622	PITPNM3	PITPNM family member 3	83394	2,257804043	-2,173297897	1,612481	-1,347797401	0,177723554	NA
ENSG00000091651	ORC6	origin recognition complex subunit 6	23594	6,728769518	5,075059051	1,575781	3,220663204	0,001278943	NA
ENSG00000091656	ZFHX4	zinc finger homeobox 4	79776	0	NA	NA	NA	NA	NA
ENSG00000091664	SLC17A6	solute carrier family 17 member 6	57084	0	NA	NA	NA	NA	NA
ENSG00000091704	CPA1	carboxypeptidase A1	1357	0	NA	NA	NA	NA	NA
ENSG00000091831	ESR1	estrogen receptor 1	2099	1,224152627	0,643281436	1,873647	0,34333109	0,731349394	NA
ENSG00000091844	RGS17	regulator of G protein signaling 17	26575	7,609989021	0,198481622	0,654784	0,303125338	0,761794345	NA
ENSG00000091879	ANGPT2	angiopoietin 2	285	0	NA	NA	NA	NA	NA
ENSG00000091972	CD200	CD200 molecule	4345	0	NA	NA	NA	NA	NA
ENSG00000091986	CCDC80	coiled-coil domain containing 80	151887	1,113355762	0,411680384	2,036454	0,202155527	0,839795139	NA
ENSG00000092009	CMA1	chymase 1	1215	0	NA	NA	NA	NA	NA
ENSG00000092051	JPH4	junctophilin 4	84502	0	NA	NA	NA	NA	NA
ENSG00000092054	MYH7	myosin heavy chain 7	4625	0	NA	NA	NA	NA	NA
ENSG00000092067	CEBPE	CCAAT enhancer binding protein epsilon	1053	0	NA	NA	NA	NA	NA
ENSG00000092068	SLC7A8	solute carrier family 7 member 8	23428	0	NA	NA	NA	NA	NA
ENSG00000092096	SLC22A17	solute carrier family 22 member 17	51310	0	NA	NA	NA	NA	NA
ENSG00000092098	RNF31	ring finger protein 31	55072	3,123017451	-5,815038177	3,91603	-1,484931809	0,137561889	NA
ENSG00000092200	RPGRIP1	RPGR interacting protein 1	57096	0,117497108	0,847101401	4,080456	0,207599697	0,835541541	NA
ENSG00000092345	DAZL	deleted in azoospermia like	1618	0,359021632	2,56113643	3,98322	0,642981427	0,520236152	NA
ENSG00000092377	TBL1Y	transducin beta like 1 Y-linked	90665	0	NA	NA	NA	NA	NA
ENSG00000092421	SEMA6A	semaphorin 6A	57556	0	NA	NA	NA	NA	NA
ENSG00000092445	TYRO3	TYRO3 protein tyrosine kinase	7301	0	NA	NA	NA	NA	NA
ENSG00000092607	TBX15	T-box transcription factor 15	6913	0	NA	NA	NA	NA	NA
ENSG00000092621	PHGDH	phosphoglycerate dehydrogenase	26227	10,24333702	-1,679850434	2,768071	-0,60686674	0,543939352	NA
ENSG00000092758	COL9A3	collagen type IX alpha 3 chain	1299	0	NA	NA	NA	NA	NA
ENSG00000092850	TEKT2	tektin 2	27285	0,10488184	-1,076443944	4,080456	-0,263804824	0,791930317	NA
ENSG00000092969	TGFB2	transforming growth factor beta 2	7042	0,117497108	0,847101401	4,080456	0,207599697	0,835541541	NA
ENSG00000093134	VNN3P	vanin 3, pseudogene	55350	1,210023667	1,36120424	2,034034	0,669214133	0,503358892	NA
ENSG00000094661	OR1I1	olfactory receptor family 1 subfamily I member 1	126370	0,103998153	-1,076443944	4,080456	-0,263804824	0,791930317	NA
ENSG00000094755	GABRP	gamma-aminobutyric acid type A receptor subunit pi	2568	0	NA	NA	NA	NA	NA
ENSG00000094963	FMO2	flavin containing dimethylaniline monoxygenase 2	2327	0	NA	NA	NA	NA	NA
ENSG00000095110	NXPE1	neurexophilin and PC-esterase domain family member 1	120400	0	NA	NA	NA	NA	NA
ENSG00000095203	EPB41L4B	erythrocyte membrane protein band 4,1 like 4B	54566	0	NA	NA	NA	NA	NA

ENSG00000095303	PTGS1	prostaglandin-endoperoxide synthase 1	5742	7,575028825	0,40497843	0,957228	0,423074266	0,672241077	NA
ENSG00000095397	WHRN	whirlin	25861	0,845663946	-1,534581733	2,480671	-0,618615685	0,536169566	NA
ENSG00000095464	PDE6C	phosphodiesterase 6C	5146	0	NA	NA	NA	NA	NA
ENSG00000095539	SEMA4G	semaphorin 4G	57715	0,573478528	-3,066319242	3,968485	-0,772667536	0,439719164	NA
ENSG00000095585	BLNK	B cell linker	29760	0	NA	NA	NA	NA	NA
ENSG00000095587	TLL2	tolloid like 2	7093	0,072165449	0,847101401	4,080456	0,207599697	0,835541541	NA
ENSG00000095596	CYP26A1	cytochrome P450 family 26 subfamily A member 1	1592	0	NA	NA	NA	NA	NA
ENSG00000095627	TDRD1	tudor domain containing 1	56165	0,10488184	-1,076443944	4,080456	-0,263804824	0,791930317	NA
ENSG00000095637	SORBS1	sorbin and SH3 domain containing 1	10580	4,773564732	1,558513088	0,966745	1,612124448	0,106934862	NA
ENSG00000095713	CRTAC1	cartilage acidic protein 1	55118	0	NA	NA	NA	NA	NA
ENSG00000095739	BAMBI	BMP and activin membrane bound inhibitor	25805	0,207996306	-1,916073151	4,043021	-0,473921178	0,635556117	NA
ENSG00000095752	IL11	interleukin 11	3589	0,804009335	1,981801863	3,651595	0,542722188	0,587321095	NA
ENSG00000095777	MYO3A	myosin IIIA	53904	0,118286652	0	4,080456	0	1	NA
ENSG00000095917	TPSD1	tryptase delta 1	23430	0	NA	NA	NA	NA	NA
ENSG00000095932	SMIM24	small integral membrane protein 24	284422	9,076626845	-0,370185768	0,597471	-0,619587415	0,535529454	NA
ENSG00000095970	TREM2	triggering receptor expressed on myeloid cells 2	54209	0	NA	NA	NA	NA	NA
ENSG00000095981	KCNK16	potassium two pore domain channel subfamily K member 16	83795	0	NA	NA	NA	NA	NA
ENSG00000096006	CRISP3	cysteine rich secretory protein 3	10321	0	NA	NA	NA	NA	NA
ENSG00000096088	PGC	progastricsin	5225	0	NA	NA	NA	NA	NA
ENSG00000096395	MLN	motilin	4295	0	NA	NA	NA	NA	NA
ENSG00000096696	DSP	desmoplakin	1832	0,119673877	0,847101401	4,080456	0,207599697	0,835541541	NA
ENSG00000097096	SYDE2	synapse defective Rho GTPase homolog 2	84144	0,144330898	1,418786819	4,061262	0,349346267	0,726829368	NA
ENSG00000099139	PCSK5	proprotein convertase subtilisin/kexin type 5	5125	1,235977832	-2,764688055	2,18193	-1,267083905	0,20512529	NA
ENSG00000099250	NRP1	neuropilin 1	8829	2,128107044	-0,758958339	1,375882	-0,551615977	0,581211487	NA
ENSG00000099251	NA	NA	NA	6,46666288	0,435990599	0,76202	0,57215142	0,567219396	NA
ENSG00000099256	PRTFDC1	phosphoribosyl transferase domain containing 1	56952	6,296230854	-1,625200635	0,800409	-2,030462773	0,042309522	NA
ENSG00000099260	PALMD	palmdelphin	54873	0	NA	NA	NA	NA	NA
ENSG00000099282	TSPAN15	tetraspanin 15	23555	8,905336783	0,189953405	0,635675	0,298821509	0,765076239	NA
ENSG00000099284	MACROH2A2	macroH2A,2 histone	55506	0,649489042	3,454692166	3,94816	0,875013242	0,381566701	NA
ENSG00000099330	OCEL1	occludin/ELL domain containing 1	79629	11,10763877	0,836832459	1,17051	0,714929521	0,474652594	NA
ENSG00000099338	CATSPERG	cation channel sperm associated auxiliary subunit gamma	57828	0	NA	NA	NA	NA	NA
ENSG00000099365	STX1B	syntaxin 1B	112755	8,695883641	0,648470455	0,804786	0,805767584	0,420376873	NA
ENSG00000099399	MAGEB2	MAGE family member B2	4113	0	NA	NA	NA	NA	NA
ENSG00000099617	EFNA2	ephrin A2	1943	0	NA	NA	NA	NA	NA
ENSG00000099625	CBARP	CACN subunit beta associated regulatory protein	255057	0	NA	NA	NA	NA	NA
ENSG00000099715	PCDH11Y	protocadherin 11 Y-linked	83259	0	NA	NA	NA	NA	NA
ENSG00000099721	AMELY	amelogenin Y-linked	266	0	NA	NA	NA	NA	NA
ENSG00000099725	PRKY	protein kinase Y-linked (pseudogene)	5616	0	NA	NA	NA	NA	NA
ENSG00000099769	IGFALS	insulin like growth factor binding protein acid labile subunit	3483	0,73702399	-0,669638087	2,398606	-0,279178041	0,780108195	NA
ENSG00000099812	MISP	mitotic spindle positioning	126353	0	NA	NA	NA	NA	NA
ENSG00000099822	HCN2	hyperpolarization activated cyclic nucleotide gated potassium and sodium channel 2	610	7,737722455	-0,000884154	0,722678	-0,001223442	0,999023835	NA
ENSG00000099834	CDHR5	cadherin related family member 5	53841	0	NA	NA	NA	NA	NA
ENSG00000099840	IZUMO4	IZUMO family member 4	113177	1,791244022	1,254828771	1,796158	0,698618367	0,484790562	NA
ENSG00000099864	PALM	paralemmin	5064	0	NA	NA	NA	NA	NA
ENSG00000099866	MADCAM1	mucosal vascular addressin cell adhesion molecule 1	8174	0,884706902	0,742464767	3,116616	0,2382279	0,811704339	NA
ENSG00000099889	ARVCF	ARVCF delta catenin family member	421	2,830880095	-1,031215194	1,604131	-0,642849617	0,520321684	NA
ENSG00000099937	SERPIND1	serpin family D member 1	3053	3,512732256	-0,308348503	1,039348	-0,296675065	0,766714596	NA
ENSG00000099953	MMP11	matrix metallopeptidase 11	4320	2,122157685	-0,369601282	2,189825	-0,168781215	0,865968735	NA
ENSG00000099954	CECR2	CECR2 histone acetyl-lysine reader	27443	0	NA	NA	NA	NA	NA
ENSG00000099957	P2RX6	purinergic receptor P2X 6	9127	0,424716708	-1,067598233	4,080456	-0,261637	0,793601316	NA
ENSG00000099958	DERL3	derlin 3	91319	10,55702087	0,702416252	1,249186	0,562299395	0,573912051	NA
ENSG00000099960	SLC7A4	solute carrier family 7 member 4	6545	0	NA	NA	NA	NA	NA

ENSG00000099974	DDTL	D-dopachrome tautomerase like	100037417	0	NA	NA	NA	NA	NA
ENSG00000099984	GSTT2	glutathione S-transferase theta 2 (gene/pseudogene)	2953	0	NA	NA	NA	NA	NA
ENSG00000099994	SUSD2	sushi domain containing 2	56241	1,981641396	1,713281968	1,963566	0,872536151	0,382915955	NA
ENSG00000099998	GGT5	gamma-glutamyltransferase 5	2687	0	NA	NA	NA	NA	NA
ENSG00000100003	SEC14L2	SEC14 like lipid binding 2	23541	10,07703952	0,792146321	0,736058	1,076201332	0,281837224	NA
ENSG00000100012	SEC14L3	SEC14 like lipid binding 3	266629	0,11808024	-1,076443944	4,080456	-0,263804824	0,791930317	NA
ENSG00000100024	UPB1	beta-ureidopropionase 1	51733	0,10488184	-1,076443944	4,080456	-0,263804824	0,791930317	NA
ENSG00000100031	GGT1	gamma-glutamyltransferase 1	2678	5,768326897	2,395755267	1,615574	1,48291257	0,138097641	NA
ENSG00000100033	PRODH	proline dehydrogenase 1	5625	0	NA	NA	NA	NA	NA
ENSG00000100036	SLC35E4	solute carrier family 35 member E4	339665	7,489671923	0,282102714	0,672017	0,419785319	0,674642291	NA
ENSG00000100053	CRYBB3	crystallin beta B3	1417	0,664275115	-1,490279409	2,447686	-0,608852486	0,542622221	NA
ENSG00000100065	CARD10	caspase recruitment domain family member 10	29775	6,454765637	-0,33480551	0,831948	-0,402435442	0,687363591	NA
ENSG00000100078	PLA2G3	phospholipase A2 group III	50487	0	NA	NA	NA	NA	NA
ENSG00000100079	LGALS2	galectin 2	3957	0	NA	NA	NA	NA	NA
ENSG00000100095	SEZ6L	seizure related 6 homolog like	23544	0	NA	NA	NA	NA	NA
ENSG00000100101	NA	NA	NA	0	NA	NA	NA	NA	NA
ENSG00000100116	GCAT	glycine C-acetyltransferase	23464	10,63004247	-0,48513966	0,540357	-0,897813073	0,369285214	NA
ENSG00000100121	GGTLC2	gamma-glutamyltransferase light chain 2	91227	0	NA	NA	NA	NA	NA
ENSG00000100122	CRYBB1	crystallin beta B1	1414	0,103998153	-1,076443944	4,080456	-0,263804824	0,791930317	NA
ENSG00000100146	SOX10	SRY-box transcription factor 10	6663	0	NA	NA	NA	NA	NA
ENSG00000100154	TTC28	tetratricopeptide repeat domain 28	23331	1,287391452	-0,962668266	1,595181	-0,603485131	0,546186004	NA
ENSG00000100156	SLC16A8	solute carrier family 16 member 8	23539	0	NA	NA	NA	NA	NA
ENSG00000100167	SEPTIN3	septin 3	55964	0,10488184	-1,076443944	4,080456	-0,263804824	0,791930317	NA
ENSG00000100170	SLC5A1	solute carrier family 5 member 1	6523	0,103998153	-1,076443944	4,080456	-0,263804824	0,791930317	NA
ENSG00000100181	NA	NA	NA	0	NA	NA	NA	NA	NA
ENSG00000100191	SLC5A4	solute carrier family 5 member 4	6527	0	NA	NA	NA	NA	NA
ENSG00000100197	CYP2D6	cytochrome P450 family 2 subfamily D member 6	1565	0,119673877	0,847101401	4,080456	0,207599697	0,835541541	NA
ENSG00000100206	DMC1	DNA meiotic recombinase 1	11144	1,551624553	1,313719283	1,722542	0,762663058	0,445664367	NA
ENSG00000100209	HSCB	HscB mitochondrial iron-sulfur cluster cochaperone	150274	10,82073476	0,881892747	0,683461	1,290333236	0,196934982	NA
ENSG00000100211	CBY1	chibby 1, beta catenin antagonist	25776	4,887989791	-5,143358758	1,610575	-3,193491374	0,001405635	NA
ENSG00000100218	RSPH14	radial spoke head 14 homolog	27156	0,072165449	0,847101401	4,080456	0,207599697	0,835541541	NA
ENSG00000100228	RAB36	RAB36, member RAS oncogene family	9609	3,463364679	-0,498407764	1,072711	-0,464624401	0,642200455	NA
ENSG00000100234	TIMP3	TIMP metallopeptidase inhibitor 3	7078	0	NA	NA	NA	NA	NA
ENSG00000100249	C22orf31	chromosome 22 open reading frame 31	25770	0	NA	NA	NA	NA	NA
ENSG00000100253	MIOX	myo-inositol oxygenase	55586	0,072165449	0,847101401	4,080456	0,207599697	0,835541541	NA
ENSG00000100263	RHBDD3	rhomboid domain containing 3	25807	10,69738482	0,920917512	0,768964	1,197608769	0,231069361	NA
ENSG00000100276	RASL10A	RAS like family 10 member A	10633	0	NA	NA	NA	NA	NA
ENSG00000100290	BIK	BCL2 interacting killer	638	1,323320043	0,994211574	1,700597	0,584624912	0,558799953	NA
ENSG00000100311	PDGFB	platelet derived growth factor subunit B	5155	0	NA	NA	NA	NA	NA
ENSG00000100314	CABP7	calcium binding protein 7	164633	1,462228454	0,915649218	1,584518	0,577872504	0,5633502	NA
ENSG00000100320	RBFOX2	RNA binding fox-1 homolog 2	23543	1,206003324	0,420690041	1,957828	0,214875847	0,829864116	NA
ENSG00000100341	PNPLA5	patatin like phospholipase domain containing 5	150379	0	NA	NA	NA	NA	NA
ENSG00000100344	PNPLA3	patatin like phospholipase domain containing 3	80339	0	NA	NA	NA	NA	NA
ENSG00000100346	CACNA1I	calcium voltage-gated channel subunit alpha1 I	8911	0	NA	NA	NA	NA	NA
ENSG00000100362	PVALB	parvalbumin	5816	0	NA	NA	NA	NA	NA
ENSG00000100373	UPK3A	uroplakin 3A	7380	0	NA	NA	NA	NA	NA
ENSG00000100399	CHADL	chondroadherin like	150356	0	NA	NA	NA	NA	NA
ENSG00000100433	KCNK10	potassium two pore domain channel subfamily K member 10	54207	0,072165449	0,847101401	4,080456	0,207599697	0,835541541	NA
ENSG00000100448	CTSG	cathepsin G	1511	0	NA	NA	NA	NA	NA
ENSG00000100473	COCH	cochlin	1690	0,715730303	-1,047911629	3,766623	-0,278209884	0,780851248	NA
ENSG00000100504	PYGL	glycogen phosphorylase L	5836	0	NA	NA	NA	NA	NA
ENSG00000100505	TRIM9	tripartite motif containing 9	114088	0,378699958	0,832002901	3,688444	0,225570162	0,82153575	NA

ENSG00000100557	CCDC198	coiled-coil domain containing 198	55195	0	NA	NA	NA	NA	NA
ENSG00000100558	PLEK2	pleckstrin 2	26499	0	NA	NA	NA	NA	NA
ENSG00000100565	LRRC74A	leucine rich repeat containing 74A	145497	0	NA	NA	NA	NA	NA
ENSG00000100577	GSTZ1	glutathione S-transferase zeta 1	2954	10,15666763	1,036385422	0,697616	1,485609549	0,137382428	NA
ENSG00000100583	SAMD15	sterile alpha motif domain containing 15	161394	1,347360903	2,032000797	2,346492	0,865973866	0,386504494	NA
ENSG00000100593	ISM2	isthmin 2	145501	0	NA	NA	NA	NA	NA
ENSG00000100600	LGMN	legumain	5641	0	NA	NA	NA	NA	NA
ENSG00000100604	CHGA	chromogranin A	1113	0	NA	NA	NA	NA	NA
ENSG00000100626	GALNT16	polypeptide N-acetylgalactosaminyltransferase 16	57452	0	NA	NA	NA	NA	NA
ENSG00000100652	SLC10A1	solute carrier family 10 member 1	6554	0	NA	NA	NA	NA	NA
ENSG00000100665	SERPINA4	serpin family A member 4	5267	0	NA	NA	NA	NA	NA
ENSG00000100678	SLC8A3	solute carrier family 8 member A3	6547	0	NA	NA	NA	NA	NA
ENSG00000100721	TCL1A	TCL1 family AKT coactivator A	8115	0	NA	NA	NA	NA	NA
ENSG00000100739	BDKRB1	bradykinin receptor B1	623	0	NA	NA	NA	NA	NA
ENSG00000100767	PAPLN	papilin, proteoglycan like sulfated glycoprotein	89932	2,307459606	5,172268935	1,690413	3,0597668	0,002215094	NA
ENSG00000100842	EFS	embryonal Fyn-associated substrate	10278	0	NA	NA	NA	NA	NA
ENSG00000100867	DHRS2	dehydrogenase/reductase 2	10202	0	NA	NA	NA	NA	NA
ENSG00000100884	CPNE6	copine 6	9362	0	NA	NA	NA	NA	NA
ENSG00000100897	DCAF11	DDB1 and CUL4 associated factor 11	80344	0	NA	NA	NA	NA	NA
ENSG00000100979	PLTP	phospholipid transfer protein	5360	7,904213441	0,328423965	0,644117	0,509882192	0,610133999	NA
ENSG00000100985	MMP9	matrix metallopeptidase 9	4318	0,484018559	0,477475747	3,48773	0,136901566	0,891108601	NA
ENSG00000100987	VSX1	visual system homeobox 1	30813	0	NA	NA	NA	NA	NA
ENSG00000101000	PROCR	protein C receptor	10544	0,264136491	2,151285343	4,007854	0,536767441	0,591428264	NA
ENSG00000101004	NINL	ninein like	22981	0,813949436	0,437936495	2,471716	0,17717914	0,85936768	NA
ENSG00000101017	CD40	CD40 molecule	958	0	NA	NA	NA	NA	NA
ENSG00000101049	SGK2	serum/glucocorticoid regulated kinase 2	10110	0,444386376	-0,972734067	3,005954	-0,323602425	0,746239052	NA
ENSG00000101074	R3HDML	R3H domain containing like	140902	0	NA	NA	NA	NA	NA
ENSG00000101076	HNF4A	hepatocyte nuclear factor 4 alpha	3172	0,10543123	0	4,080456	0	1	NA
ENSG00000101098	RIMS4	regulating synaptic membrane exocytosis 4	140730	0	NA	NA	NA	NA	NA
ENSG00000101104	PABPC1L	poly(A) binding protein cytoplasmic 1 like	80336	10,96359949	0,685159051	0,908484	0,754178463	0,450742068	NA
ENSG00000101115	SALL4	spalt like transcription factor 4	57167	4,961809016	-0,277665268	1,158658	-0,23964381	0,810606399	NA
ENSG00000101132	PFDN4	prefoldin subunit 4	5203	11,33494486	0,626051828	0,944974	0,662506725	0,507646526	NA
ENSG00000101134	DOK5	docking protein 5	55816	0	NA	NA	NA	NA	NA
ENSG00000101144	BMP7	bone morphogenetic protein 7	655	6,046945998	-0,881506559	0,877188	-1,00492373	0,314933577	NA
ENSG00000101162	TUBB1	tubulin beta 1 class VI	81027	0,11808024	-1,076443944	4,080456	-0,263804824	0,791930317	NA
ENSG00000101180	HRH3	histamine receptor H3	11255	0	NA	NA	NA	NA	NA
ENSG00000101188	NTSR1	neurotensin receptor 1	4923	0	NA	NA	NA	NA	NA
ENSG00000101197	BIRC7	baculoviral IAP repeat containing 7	79444	0	NA	NA	NA	NA	NA
ENSG00000101198	NKAIN4	sodium/potassium transporting ATPase interacting 4	128414	0	NA	NA	NA	NA	NA
ENSG00000101200	AVP	arginine vasopressin	551	0	NA	NA	NA	NA	NA
ENSG00000101203	COL20A1	collagen type XX alpha 1 chain	57642	0,117497108	0,847101401	4,080456	0,207599697	0,835541541	NA
ENSG00000101204	CHRNA4	cholinergic receptor nicotinic alpha 4 subunit	1137	0	NA	NA	NA	NA	NA
ENSG00000101210	EEF1A2	eukaryotic translation elongation factor 1 alpha 2	1917	0	NA	NA	NA	NA	NA
ENSG00000101213	PTK6	protein tyrosine kinase 6	5753	2,519476476	0,527111712	1,140441	0,462199723	0,643938102	NA
ENSG00000101222	SPEF1	sperm flagellar 1	25876	0,234994216	1,944111989	4,02324	0,483220515	0,62893917	NA
ENSG00000101230	ISM1	isthmin 1	140862	2,235270856	-0,173731603	1,348929	-0,128792288	0,897522008	NA
ENSG00000101276	SLC52A3	solute carrier family 52 member 3	113278	0	NA	NA	NA	NA	NA
ENSG00000101278	RPS10P5	ribosomal protein S10 pseudogene 5	93144	0	NA	NA	NA	NA	NA
ENSG00000101280	ANGPT4	angiopoietin 4	51378	0	NA	NA	NA	NA	NA
ENSG00000101282	RSPO4	R-spondin 4	343637	0	NA	NA	NA	NA	NA
ENSG00000101292	PROKR2	prokineticin receptor 2	128674	0,119673877	0,847101401	4,080456	0,207599697	0,835541541	NA
ENSG00000101298	SNPH	syntaphilin	9751	7,402397227	0,508117176	0,646076	0,786466624	0,431594165	NA
ENSG00000101306	MYLK2	myosin light chain kinase 2	85366	1,182210444	3,191741522	2,181808	1,462888654	0,143497849	NA

ENSG00000101311	FERMT1	FERM domain containing kindlin 1	55612	0	NA	NA	NA	NA	NA
ENSG00000101323	HAO1	hydroxyacid oxidase 1	54363	0	NA	NA	NA	NA	NA
ENSG00000101327	PDYN	prodynorphin	5173	0	NA	NA	NA	NA	NA
ENSG00000101331	CCM2L	CCM2 like scaffold protein	140706	0	NA	NA	NA	NA	NA
ENSG00000101333	PLCB4	phospholipase C beta 4	5332	0	NA	NA	NA	NA	NA
ENSG00000101335	MYL9	myosin light chain 9	10398	0,389135496	2,573267385	3,575646	0,719664983	0,471731291	NA
ENSG00000101336	HCK	HCK proto-oncogene, Src family tyrosine kinase	3055	0	NA	NA	NA	NA	NA
ENSG00000101342	TLDC2	TBC/LysM-associated domain containing 2	140711	0	NA	NA	NA	NA	NA
ENSG00000101349	PAK5	p21 (RAC1) activated kinase 5	57144	0	NA	NA	NA	NA	NA
ENSG00000101353	MROH8	maestro heat like repeat family member 8	140699	4,431963769	-0,568447632	0,960187	-0,592017691	0,553838742	NA
ENSG00000101405	OXT	oxytocin/neurophysin I prepropeptide	5020	0	NA	NA	NA	NA	NA
ENSG00000101425	BPI	bactericidal permeability increasing protein	671	0	NA	NA	NA	NA	NA
ENSG00000101435	CST9L	cystatin 9 like	128821	0	NA	NA	NA	NA	NA
ENSG00000101438	SLC32A1	solute carrier family 32 member 1	140679	0	NA	NA	NA	NA	NA
ENSG00000101439	CST3	cystatin C	1471	5,685532146	-0,15025738	0,755545	-0,198872821	0,84236223	NA
ENSG00000101440	ASIP	agouti signaling protein	434	0	NA	NA	NA	NA	NA
ENSG00000101441	CST4	cystatin S	1472	0	NA	NA	NA	NA	NA
ENSG00000101443	WFDC2	WAP four-disulfide core domain 2	10406	0	NA	NA	NA	NA	NA
ENSG00000101446	SPINT3	serine peptidase inhibitor, Kunitz type 3	10816	0	NA	NA	NA	NA	NA
ENSG00000101448	EPPIN	epididymal peptidase inhibitor	57119	0	NA	NA	NA	NA	NA
ENSG00000101460	MAP1LC3A	microtubule associated protein 1 light chain 3 alpha	84557	6,176141606	0,783038216	0,888555	0,881249012	0,378183057	NA
ENSG00000101463	SYNDIG1	synapse differentiation inducing 1	79953	0	NA	NA	NA	NA	NA
ENSG00000101470	TNNC2	troponin C2, fast skeletal type	7125	0,10488184	-1,076443944	4,080456	-0,263804824	0,791930317	NA
ENSG00000101489	CELF4	CUGBP Elav-like family member 4	56853	0	NA	NA	NA	NA	NA
ENSG00000101542	CDH20	cadherin 20	28316	0,637189237	0,06866901	2,05653	0,033390718	0,973363012	NA
ENSG00000101638	ST8SIA5	ST8 alpha-N-acetyl-neuraminide alpha-2,8-sialyltransferase 5	29906	0	NA	NA	NA	NA	NA
ENSG00000101670	LIPG	lipase G, endothelial type	9388	0,177033521	-0,114258055	4,080456	-0,028001297	0,977661117	NA
ENSG00000101680	LAMA1	laminin subunit alpha 1	284217	0	NA	NA	NA	NA	NA
ENSG00000101746	NOL4	nucleolar protein 4	8715	0	NA	NA	NA	NA	NA
ENSG00000101812	H2BW2	H2B,W histone 2	286436	0	NA	NA	NA	NA	NA
ENSG00000101825	MXRA5	matrix remodeling associated 5	25878	0	NA	NA	NA	NA	NA
ENSG00000101842	VSIG1	V-set and immunoglobulin domain containing 1	340547	0,341739295	1,007887409	4,022353	0,250571569	0,802145365	NA
ENSG00000101850	GPR143	G protein-coupled receptor 143	4935	0	NA	NA	NA	NA	NA
ENSG00000101871	MID1	midline 1	4281	0	NA	NA	NA	NA	NA
ENSG00000101883	RHOXF1	Rhox homeobox family member 1	158800	0,777238955	0	3,554815	0	1	NA
ENSG00000101883	LINC01402	long intergenic non-protein coding RNA 1402	104797536	0,777238955	0	3,554815	0	1	NA
ENSG00000101890	GUCY2F	guanylate cyclase 2F, retinal	2986	0	NA	NA	NA	NA	NA
ENSG00000101892	ATP1B4	ATPase Na+/K+ transporting family member beta 4	23439	0	NA	NA	NA	NA	NA
ENSG00000101898	MCTS2	MCTS family member 2	100101490	4,617868233	-1,857407329	1,319037	-1,408153705	0,159085561	NA
ENSG00000101916	TLR8	toll like receptor 8	51311	0	NA	NA	NA	NA	NA
ENSG00000101938	CHRDL1	chordin like 1	91851	0	NA	NA	NA	NA	NA
ENSG00000101951	PAGE4	PAGE family member 4	9506	0	NA	NA	NA	NA	NA
ENSG00000101955	SRPX	sushi repeat containing protein X-linked	8406	0	NA	NA	NA	NA	NA
ENSG00000101958	GLRA2	glycine receptor alpha 2	2742	0	NA	NA	NA	NA	NA
ENSG00000101977	MCF2	MCF,2 cell line derived transforming sequence	4168	3,201781401	2,188547869	1,778073	1,230853447	0,218377681	NA
ENSG00000101981	F9	coagulation factor IX	2158	0	NA	NA	NA	NA	NA
ENSG00000102001	CACNA1F	calcium voltage-gated channel subunit alpha1 F	778	0,485470567	-0,966494966	2,93311	-0,329512051	0,741768687	NA
ENSG00000102003	SYP	synaptophysin	6855	0,832515672	-0,203286268	2,599299	-0,078208101	0,937662518	NA
ENSG00000102010	BMX	BMX non-receptor tyrosine kinase	660	0	NA	NA	NA	NA	NA
ENSG00000102021	LUZP4	leucine zipper protein 4	51213	0	NA	NA	NA	NA	NA
ENSG00000102024	PLS3	plastin 3	5358	0,572631045	-2,965398545	2,678293	-1,107197347	0,268208614	NA
ENSG00000102032	RENBP	renin binding protein	5973	6,724194576	-1,00478645	1,153478	-0,871092735	0,38370352	NA

ENSG00000102038	SMARCA1	SWI/SNF related, matrix associated, actin dependent regulator of chromatin, subfamily a, member 1	6594	0	NA	NA	NA	NA	NA
ENSG00000102043	MTMR8	myotubularin related protein 8	55613	9,41673918	0,881234974	0,696915	1,264479966	0,206057807	NA
ENSG00000102048	ASB9	ankyrin repeat and SOCS box containing 9	140462	0,118286652	0	4,080456	0	1	NA
ENSG00000102053	ZC3H12B	zinc finger CCCH-type containing 12B	340554	7,498802513	0,018047128	0,758361	0,023797533	0,981014108	NA
ENSG00000102055	PPP1R2C	PPP1R2C family member C	80316	0,119673877	0,847101401	4,080456	0,207599697	0,835541541	NA
ENSG00000102057	KCND1	potassium voltage-gated channel subfamily D member 1	3750	6,649627357	-0,364916348	0,867637	-0,420586543	0,674057022	NA
ENSG00000102076	OPN1LW	opsin 1, long wave sensitive	5956	0	NA	NA	NA	NA	NA
ENSG00000102078	SLC25A14	solute carrier family 25 member 14	9016	9,498798122	-0,440958609	1,145924	-0,384806173	0,700381025	NA
ENSG00000102098	SCML2	Scm polycomb group protein like 2	10389	0,649529994	-0,257730862	2,832818	-0,090980367	0,927508191	NA
ENSG00000102104	RS1	retinoschisin 1	6247	0	NA	NA	NA	NA	NA
ENSG00000102109	PCSK1N	proprotein convertase subtilisin/kexin type 1 inhibitor	27344	0	NA	NA	NA	NA	NA
ENSG00000102128	RAB40AL	RAB40A like	282808	0,693338635	1,376627833	2,216766	0,621007271	0,534594839	NA
ENSG00000102145	GATA1	GATA binding protein 1	2623	0	NA	NA	NA	NA	NA
ENSG00000102174	PHEX	phosphate regulating endopeptidase X-linked	5251	1,758618732	-0,732151678	1,774483	-0,412600022	0,679899685	NA
ENSG00000102195	GPR50	G protein-coupled receptor 50	9248	0	NA	NA	NA	NA	NA
ENSG00000102221	JADE3	jade family PHD finger 3	9767	0,314311041	-1,92331071	3,823864	-0,502975658	0,614981387	NA
ENSG00000102230	PCYT1B	phosphate cytidylyltransferase 1B, choline	9468	0	NA	NA	NA	NA	NA
ENSG00000102239	BRS3	bombesin receptor subtype 3	680	0	NA	NA	NA	NA	NA
ENSG00000102243	VGLL1	vestigial like family member 1	51442	0	NA	NA	NA	NA	NA
ENSG00000102245	CD40LG	CD40 ligand	959	10,189171	-0,769216564	0,777266	-0,989644393	0,322347963	NA
ENSG00000102271	KLHL4	kelch like family member 4	56062	0,239347754	1,967469188	4,021404	0,489249276	0,624665227	NA
ENSG00000102287	GABRE	gamma-aminobutyric acid type A receptor subunit epsilon	2564	0	NA	NA	NA	NA	NA
ENSG00000102290	PCDH11X	protocadherin 11 X-linked	27328	0	NA	NA	NA	NA	NA
ENSG00000102302	FGD1	FYVE, RhoGEF and PH domain containing 1	2245	0	NA	NA	NA	NA	NA
ENSG00000102313	ITIH6	inter-alpha-trypsin inhibitor heavy chain family member 6	347365	0	NA	NA	NA	NA	NA
ENSG00000102359	SRPX2	sushi repeat containing protein X-linked 2	27286	0	NA	NA	NA	NA	NA
ENSG00000102362	SYTL4	synaptotagmin like 4	94121	0	NA	NA	NA	NA	NA
ENSG00000102383	ZDHHC15	zinc finger DHHC-type palmitoyltransferase 15	158866	0	NA	NA	NA	NA	NA
ENSG00000102385	DRP2	dystrophin related protein 2	1821	1,565982474	0,80162571	1,673099	0,479126195	0,631848855	NA
ENSG00000102387	TAF7L	TATA-box binding protein associated factor 7 like	54457	0,118286652	0	4,080456	0	1	NA
ENSG00000102445	RUBCNL	rubicon like autophagy enhancer	80183	0,10488184	-1,076443944	4,080456	-0,263804824	0,791930317	NA
ENSG00000102452	NALCN	sodium leak channel, non-selective	259232	0	NA	NA	NA	NA	NA
ENSG00000102466	FGF14	fibroblast growth factor 14	2259	0,22356181	-1,078302778	4,080456	-0,26426037	0,791579295	NA
ENSG00000102468	HTR2A	5-hydroxytryptamine receptor 2A	3356	0	NA	NA	NA	NA	NA
ENSG00000102524	TNFSF13B	TNF superfamily member 13b	10673	6,888525674	0,79445336	0,840432	0,945292043	0,3445098	NA
ENSG00000102539	MLNR	motilin receptor	2862	0	NA	NA	NA	NA	NA
ENSG00000102554	KLF5	KLF transcription factor 5	688	11,41376252	0,091721898	0,531869	0,172452064	0,863082144	NA
ENSG00000102595	UGGT2	UDP-glucose glycoprotein glucosyltransferase 2	55757	6,516942965	0,136364754	0,767002	0,1777893	0,858888451	NA
ENSG00000102678	FGF9	fibroblast growth factor 9	2254	0,210862461	0	4,080456	0	1	NA
ENSG00000102683	SGCG	sarcoglycan gamma	6445	0	NA	NA	NA	NA	NA
ENSG00000102755	FLT1	fms related receptor tyrosine kinase 1	2321	0,10543123	0	4,080456	0	1	NA
ENSG00000102794	ACOD1	aconitate decarboxylase 1	730249	0	NA	NA	NA	NA	NA
ENSG00000102796	DHRS12	dehydrogenase/reductase 12	79758	9,426511754	-1,441600221	0,883895	-1,630962344	0,102898265	NA
ENSG00000102802	MEDAG	mesenteric estrogen dependent adipogenesis	84935	0	NA	NA	NA	NA	NA
ENSG00000102805	CLN5	CLN5 intracellular trafficking protein	1203	7,716692231	0,644281726	1,191798	0,540596359	0,588785828	NA
ENSG00000102837	OLFM4	olfactomedin 4	10562	0	NA	NA	NA	NA	NA
ENSG00000102854	MSLN	mesothelin	10232	0,207996306	-1,916073151	4,043021	-0,473921178	0,635556117	NA
ENSG00000102870	ZNF629	zinc finger protein 629	23361	4,581520066	-0,171727989	0,850809	-0,201840795	0,840041188	NA

ENSG00000102878	HSF4	heat shock transcription factor 4	3299	0,632587382	0	4,080456	0	1	NA
ENSG00000102886	GDPD3	glycerophosphodiester phosphodiesterase domain containing 3	79153	4,13630632	0,114670252	1,916489	0,059833514	0,952288233	NA
ENSG00000102890	ELMO3	engulfment and cell motility 3	79767	10,57313747	-0,114374806	0,647848	-0,176545848	0,859865133	NA
ENSG00000102891	MT4	metallothionein 4	84560	0	NA	NA	NA	NA	NA
ENSG00000102904	TSNAXIP1	translin associated factor X interacting protein 1	55815	0	NA	NA	NA	NA	NA
ENSG00000102924	CBLN1	cerebellin 1 precursor	869	0	NA	NA	NA	NA	NA
ENSG00000102934	PLLP	plasmolipin	51090	0	NA	NA	NA	NA	NA
ENSG00000102935	ZNF423	zinc finger protein 423	23090	0	NA	NA	NA	NA	NA
ENSG00000102962	CCL22	C-C motif chemokine ligand 22	6367	0,119673877	0,847101401	4,080456	0,207599697	0,835541541	NA
ENSG00000102970	CCL17	C-C motif chemokine ligand 17	6361	0	NA	NA	NA	NA	NA
ENSG00000102981	PARD6A	par-6 family cell polarity regulator alpha	50855	7,153384209	0,980150361	0,887746	1,104088638	0,269554689	NA
ENSG00000102984	ZNF821	zinc finger protein 821	55565	7,316251424	-0,587675038	0,869059	-0,676220108	0,498900908	NA
ENSG00000102996	MMP15	matrix metallopeptidase 15	4324	2,762054991	-0,632993034	1,364433	-0,46392375	0,642702376	NA
ENSG00000103021	CCDC113	coiled-coil domain containing 113	29070	0,278363346	0,843150198	4,080456	0,206631373	0,836297757	NA
ENSG00000103023	PRSS54	serine protease 54	221191	0	NA	NA	NA	NA	NA
ENSG00000103034	NDRG4	NDRG family member 4	65009	0	NA	NA	NA	NA	NA
ENSG00000103044	HAS3	hyaluronan synthase 3	3038	3,914392481	-0,043383507	0,921053	-0,047102064	0,962431882	NA
ENSG00000103056	SMPD3	sphingomyelin phosphodiesterase 3	55512	10,9823585	-1,129013885	0,717456	-1,573633985	0,115572092	NA
ENSG00000103067	ESRP2	epithelial splicing regulatory protein 2	80004	0	NA	NA	NA	NA	NA
ENSG00000103089	FA2H	fatty acid 2-hydroxylase	79152	0	NA	NA	NA	NA	NA
ENSG00000103154	NECAB2	N-terminal EF-hand calcium binding protein 2	54550	0	NA	NA	NA	NA	NA
ENSG00000103175	WFDC1	WAP four-disulfide core domain 1	58189	0	NA	NA	NA	NA	NA
ENSG00000103184	SEC14L5	SEC14 like lipid binding 5	9717	0	NA	NA	NA	NA	NA
ENSG00000103196	CRISPLD2	cysteine rich secretory protein LCCL domain containing 2	83716	0,119673877	0,847101401	4,080456	0,207599697	0,835541541	NA
ENSG00000103200	NA	NA	NA	0,072165449	0,847101401	4,080456	0,207599697	0,835541541	NA
ENSG00000103202	NME4	NME/NM23 nucleoside diphosphate kinase 4	4833	1,064399215	-2,562585326	2,003268	-1,279202468	0,200825767	NA
ENSG00000103226	NOMO3	NODAL modulator 3	408050	1,99080787	1,11987392	2,740926	0,408575059	0,682851537	NA
ENSG00000103241	FOXF1	forkhead box F1	2294	0,421724922	0	4,080456	0	1	NA
ENSG00000103253	HAGHL	hydroxyacylglutathione hydrolase like	84264	10,84431522	-2,117559137	0,867355	-2,441398883	0,014630485	NA
ENSG00000103260	METRN	meteorin, glial cell differentiation regulator	79006	6,738395747	-0,780547185	0,95064	-0,821075523	0,411603251	NA
ENSG00000103269	RHBDL1	rhomboid like 1	9028	0,88435324	-3,818158488	2,442952	-1,562927862	0,118069563	NA
ENSG00000103310	ZP2	zona pellucida glycoprotein 2	7783	0	NA	NA	NA	NA	NA
ENSG00000103313	MEFV	MEFV innate immunity regulator, pyrin	4210	0	NA	NA	NA	NA	NA
ENSG00000103316	CRYM	crystallin mu	1428	0	NA	NA	NA	NA	NA
ENSG00000103319	EEF2K	eukaryotic elongation factor 2 kinase	29904	0	NA	NA	NA	NA	NA
ENSG00000103355	PRSS33	serine protease 33	260429	0,103998153	-1,076443944	4,080456	-0,263804824	0,791930317	NA
ENSG00000103375	AQP8	aquaporin 8	343	0	NA	NA	NA	NA	NA
ENSG00000103381	CPPED1	calcineurin like phosphoesterase domain containing 1	55313	6,657125966	0,594449444	0,820452	0,724539375	0,468734663	NA
ENSG00000103449	SALL1	spalt like transcription factor 1	6299	0	NA	NA	NA	NA	NA
ENSG00000103460	TOX3	TOX high mobility group box family member 3	27324	0	NA	NA	NA	NA	NA
ENSG00000103512	NOMO1	NODAL modulator 1	23420	0	NA	NA	NA	NA	NA
ENSG00000103528	SYT17	synaptotagmin 17	51760	0	NA	NA	NA	NA	NA
ENSG00000103534	TMC5	transmembrane channel like 5	79838	0	NA	NA	NA	NA	NA
ENSG00000103546	SLC6A2	solute carrier family 6 member 2	6530	0	NA	NA	NA	NA	NA
ENSG00000103569	AQP9	aquaporin 9	366	0	NA	NA	NA	NA	NA
ENSG00000103599	IQCH	IQ motif containing H	64799	0,812910048	1,569274183	2,235457	0,701992712	0,482683709	NA
ENSG00000103647	CORO2B	coronin 2B	10391	0	NA	NA	NA	NA	NA
ENSG00000103710	RASL12	RAS like family 12	51285	0	NA	NA	NA	NA	NA
ENSG00000103723	AP3B2	adaptor related protein complex 3 subunit beta 2	8120	0	NA	NA	NA	NA	NA
ENSG00000103740	ACSBG1	acyl-CoA synthetase bubblegum family member 1	23205	0,103998153	-1,076443944	4,080456	-0,263804824	0,791930317	NA
ENSG00000103742	IGDCC4	immunoglobulin superfamily DCC subclass member 4	57722	0,843119074	-1,92874424	3,017694	-0,639145166	0,5227285	NA

ENSG00000103811	CTSH	cathepsin H	1512	9,796154318	-0,259078329	1,007618	-0,257119624	0,797086429	NA
ENSG00000103832	NA	NA	NA	0,23637925	-1,071936542	4,080456	-0,262700192	0,792781669	NA
ENSG00000103852	TTC23	tetratricopeptide repeat domain 23	64927	1,324795753	-0,976795863	1,616918	-0,604109726	0,545770694	NA
ENSG00000103855	CD276	CD276 molecule	80381	10,89953292	0,199832568	0,814433	0,245364129	0,806174501	NA
ENSG00000103888	CEMIP	cell migration inducing hyaluronidase 1	57214	0,434179436	-1,998092412	3,532104	-0,565694652	0,571601372	NA
ENSG00000104044	OCA2	OCA2 melanosomal transmembrane protein	4948	0	NA	NA	NA	NA	NA
ENSG00000104055	TGM5	transglutaminase 5	9333	1,394925512	0,52946759	1,834202	0,288663675	0,772838763	NA
ENSG00000104059	ENTREP2	endosomal transmembrane epsin interactor 2	23359	0	NA	NA	NA	NA	NA
ENSG00000104067	TJP1	tight junction protein 1	7082	3,305392792	1,690678324	1,082956	1,561170055	0,118483632	NA
ENSG00000104093	DMXL2	Dmx like 2	23312	3,075946549	1,643497651	1,145741	1,434441155	0,15144641	NA
ENSG00000104112	SCG3	secretogranin III	29106	0	NA	NA	NA	NA	NA
ENSG00000104140	RHOV	ras homolog family member V	171177	3,244292146	0,804302381	1,029789	0,781036109	0,434781257	NA
ENSG00000104147	OIP5	Opa interacting protein 5	11339	10,41801554	0,073655842	0,655619	0,112345422	0,910549528	NA
ENSG00000104213	PDGFRL	platelet derived growth factor receptor like	5157	0	NA	NA	NA	NA	NA
ENSG00000104237	RP1	RP1 axonemal microtubule associated	6101	0	NA	NA	NA	NA	NA
ENSG00000104267	CA2	carbonic anhydrase 2	760	0,119673877	0,847101401	4,080456	0,207599697	0,835541541	NA
ENSG00000104290	FZD3	frizzled class receptor 3	7976	2,856919565	-0,653200938	1,083994	-0,602587382	0,546783216	NA
ENSG00000104312	RIPK2	receptor interacting serine/threonine kinase 2	8767	1,6596137	-2,448883417	2,084135	-1,175011886	0,239989961	NA
ENSG00000104313	EYA1	EYA transcriptional coactivator and phosphatase 1	2138	0	NA	NA	NA	NA	NA
ENSG00000104321	TRPA1	transient receptor potential cation channel subfamily A member 1	8989	0	NA	NA	NA	NA	NA
ENSG00000104327	CALB1	calbindin 1	793	0	NA	NA	NA	NA	NA
ENSG00000104332	SFRP1	secreted frizzled related protein 1	6422	0	NA	NA	NA	NA	NA
ENSG00000104341	LAPTM4B	lysosomal protein transmembrane 4 beta	55353	6,50384753	0,50822506	0,710176	0,71563271	0,47421817	NA
ENSG00000104361	NIPAL2	NIPA like domain containing 2	79815	1,444756177	-0,947477392	1,776013	-0,53348547	0,593697567	NA
ENSG00000104368	PLAT	plasminogen activator, tissue type	5327	0,333003114	-0,36809022	3,803153	-0,096785551	0,922896698	NA
ENSG00000104369	JPH1	junctophilin 1	56704	0,189411655	-0,096481839	4,080456	-0,023644868	0,981135883	NA
ENSG00000104371	DKK4	dickkopf WNT signaling pathway inhibitor 4	27121	0,117497108	0,847101401	4,080456	0,207599697	0,835541541	NA
ENSG00000104413	ESRP1	epithelial splicing regulatory protein 1	54845	0	NA	NA	NA	NA	NA
ENSG00000104415	CCN4	cellular communication network factor 4	8840	0	NA	NA	NA	NA	NA
ENSG00000104427	ZC2HC1A	zinc finger C2HC-type containing 1A	51101	0	NA	NA	NA	NA	NA
ENSG00000104432	IL7	interleukin 7	3574	0	NA	NA	NA	NA	NA
ENSG00000104435	STMN2	stathmin 2	11075	0	NA	NA	NA	NA	NA
ENSG00000104447	TRPS1	transcriptional repressor GATA binding 1	7227	5,428369573	0,401579511	0,948333	0,42345816	0,671961019	NA
ENSG00000104499	GML	glycosylphosphatidylinositol anchored molecule like	2765	0,653455061	-1,980365805	2,704659	-0,732205322	0,464043257	NA
ENSG00000104524	PYCR3	pyrroline-5-carboxylate reductase 3	65263	6,950466015	0,288035353	2,400522	0,119988631	0,904492154	NA
ENSG00000104537	ANXA13	annexin A13	312	0	NA	NA	NA	NA	NA
ENSG00000104611	SH2D4A	SH2 domain containing 4A	63898	2,029679811	0,816965701	1,351479	0,60449741	0,545512991	NA
ENSG00000104722	NEFM	neurofilament medium chain	4741	0	NA	NA	NA	NA	NA
ENSG00000104723	TUSC3	tumor suppressor candidate 3	7991	0	NA	NA	NA	NA	NA
ENSG00000104728	ARHGEF10	Rho guanine nucleotide exchange factor 10	9639	0	NA	NA	NA	NA	NA
ENSG00000104755	ADAM2	ADAM metallopeptidase domain 2	2515	0	NA	NA	NA	NA	NA
ENSG00000104760	FGL1	fibrinogen like 1	2267	0	NA	NA	NA	NA	NA
ENSG00000104783	KCNN4	potassium calcium-activated channel subfamily N member 4	3783	6,203837765	2,642242912	1,062128	2,4876871	0,01285768	NA
ENSG00000104804	TULP2	TUB like protein 2	7288	0	NA	NA	NA	NA	NA
ENSG00000104808	DHDH	dihydrodiol dehydrogenase	27294	0,10543123	0	4,080456	0	1	NA
ENSG00000104818	CGB2	chorionic gonadotropin subunit beta 2	114336	0	NA	NA	NA	NA	NA
ENSG00000104826	LHB	luteinizing hormone subunit beta	3972	0	NA	NA	NA	NA	NA
ENSG00000104827	CGB3	chorionic gonadotropin subunit beta 3	1082	0	NA	NA	NA	NA	NA
ENSG00000104833	TUBB4A	tubulin beta 4A class IVa	10382	0,48363575	1,593350181	3,481272	0,457692008	0,64717373	NA

ENSG00000104848	KCNA7	potassium voltage-gated channel subfamily A member 7	3743	0,117497108	0,847101401	4,080456	0,207599697	0,835541541	NA	
ENSG00000104863	LIN7B	lin-7 homolog B, crumbs cell polarity complex component	64130	1,160549851	4,265101995	2,361925	1,80577394	0,070953658	NA	
ENSG00000104870	FCGRT	Fc gamma receptor and transporter	2217	1,089409843	-4,157182084	2,676894	-1,552986931	0,120426257	NA	
ENSG00000104879	CKM	creatine kinase, M-type	1158	0	NA	NA	NA	NA	NA	
ENSG00000104881	PPP1R13L	protein phosphatase 1 regulatory subunit 13 like	10848	6,638624228	-0,112898817	1,455827	-0,077549625	0,938186315	NA	
ENSG00000104883	PEX11G	peroxisomal biogenesis factor 11 gamma	92960	1,314654975	0,030707952	2,158766	0,014224772	0,988650657	NA	
ENSG00000104888	SLC17A7	solute carrier family 17 member 7	57030	1,246938238	3,534073392	2,871994	1,230529706	0,21849881	NA	
ENSG00000104892	KLC3	kinesin light chain 3	147700	0	NA	NA	NA	NA	NA	
ENSG00000104899	AMH	anti-Mullerian hormone	268	0	NA	NA	NA	NA	NA	
ENSG00000104901	DKKL1	dickkopf like acrosomal protein 1	27120	0	NA	NA	NA	NA	NA	
ENSG00000104903	LYL1	LYL1 basic helix-loop-helix family member	4066	0,966557288	-0,049234679	1,890667	-0,026040905	0,979224712	NA	
ENSG00000104918	RETN	resistin	56729	0	NA	NA	NA	NA	NA	
ENSG00000104921	FCER2	Fc epsilon receptor II	2208	0	NA	NA	NA	NA	NA	
ENSG00000104936	DMPK	DM1 protein kinase	1760	4,973606631	-4,756206954	1,936487	-2,456100496	0,014045387	NA	
ENSG00000104938	CLEC4M	C-type lectin domain family 4 member M	10332	0	NA	NA	NA	NA	NA	
ENSG00000104941	RSPH6A	radial spoke head 6 homolog A	81492	0,556963003	2,031295901	2,833228	0,716954731	0,47340203	NA	
ENSG00000104951	IL4I1	interleukin 4 induced 1	259307	1,489740077	-1,499464362	1,708674	-0,877560347	0,380182356	NA	
ENSG00000104953	TLE6	TLE family member 6, subcortical maternal complex member	79816	2,14340312	0,957402265	1,635144	0,585515551	0,558201113	NA	
ENSG00000104967	NOVA2	NOVA alternative splicing regulator 2	4858	0	NA	NA	NA	NA	NA	
ENSG00000104970	NA	NA	NA	0	NA	NA	NA	NA	NA	
ENSG00000104974	LILRA1	leukocyte immunoglobulin like receptor A1	11024	0	NA	NA	NA	NA	NA	
ENSG00000105048	TNNT1	troponin T1, slow skeletal type	7138	0	NA	NA	NA	NA	NA	
ENSG00000105072	C19orf44	chromosome 19 open reading frame 44	84167	5,083958262	3,392090319	1,8312	1,852386569	0,063970333	NA	
ENSG00000105131	EPHX3	epoxide hydrolase 3	79852	0,698189923	2,651038003	3,126222	0,84800051	0,396437686	NA	
ENSG00000105137	SYDE1	synapse defective Rho GTPase homolog 1	85360	0	NA	NA	NA	NA	NA	
ENSG00000105141	CASP14	caspase 14	23581	0	NA	NA	NA	NA	NA	
ENSG00000105143	SLC1A6	solute carrier family 1 member 6	6511	0	NA	NA	NA	NA	NA	
ENSG00000105146	AURKC	aurora kinase C	6795	0,565626436	0	3,951243	0	1	NA	
ENSG00000105198	LGALS13	galectin 13	29124	0	NA	NA	NA	NA	NA	
ENSG00000105205	CLC	Charcot-Leyden crystal galectin	1178	0	NA	NA	NA	NA	NA	
ENSG00000105219	CCNP	cyclin P	79935	0,288661797	2,306206831	3,997774	0,57687273	0,564025434	NA	
ENSG00000105246	EBI3	Epstein-Barr virus induced 3	10148	0,689858447	2,294282034	2,500087	0,917681013	0,358785891	NA	
ENSG00000105251	SHD	Src homology 2 domain containing transforming protein D	56961	0	NA	NA	NA	NA	NA	
ENSG00000105261	OVOL3	ovo like zinc finger 3	728361	0	NA	NA	NA	NA	NA	
ENSG00000105289	TJP3	tight junction protein 3	27134	6,411064215	-0,345436708	0,737832	-0,468178285	0,6396571	NA	
ENSG00000105290	APLP1	amyloid beta precursor like protein 1	333	0	NA	NA	NA	NA	NA	
ENSG00000105352	CEACAM4	CEA cell adhesion molecule 4	1089	0	NA	NA	NA	NA	NA	
ENSG00000105357	MYH14	myosin heavy chain 14	79784	0	NA	NA	NA	NA	NA	
ENSG00000105366	SIGLEC8	sialic acid binding Ig like lectin 8	27181	0	NA	NA	NA	NA	NA	
ENSG00000105369	CD79A	CD79a molecule	973	0,314354155	-1,913317757	3,823407	-0,500422236	0,6167778	NA	
ENSG00000105371	ICAM4	intercellular adhesion molecule 4 (Landsteiner-Wiener blood group)	3386	0,432066812	-1,540372088	3,998178	-0,385268469	0,70003852	NA	
ENSG00000105376	ICAM5	intercellular adhesion molecule 5	7087	0,855199304	-0,057898313	2,023024	-0,028619687	0,97716791	NA	
ENSG00000105383	CD33	CD33 molecule	945	0,534158492	-0,431920091	2,52251	-0,171226308	0,864045822	NA	
ENSG00000105388	CEACAM5	CEA cell adhesion molecule 5	1048	0	NA	NA	NA	NA	NA	
ENSG00000105392	CRX	cone-rod homeobox	1406	0,119673877	0,847101401	4,080456	0,207599697	0,835541541	NA	
ENSG00000105398	SULT2A1	sulfotransferase family 2A member 1	6822	0	NA	NA	NA	NA	NA	
ENSG00000105409	ATP1A3	ATPase Na+/K+ transporting subunit alpha 3	478	5,31275262	-0,398622573	1,348048	-0,295703554	0,767456483	NA	
ENSG00000105419	MEIS3	Meis homeobox 3	56917	0	NA	NA	NA	NA	NA	
ENSG00000105427	CNFN	cornifelin	84518	0	NA	NA	NA	NA	NA	
ENSG00000105428	ZNRF4	zinc and ring finger 4	148066	0	NA	NA	NA	NA	NA	

ENSG00000105464	GRIN2D	glutamate ionotropic receptor NMDA type subunit 2D	2906	1,566884366	1,554546205	1,621586	0,958658016	0,337731055	NA
ENSG00000105467	SYNGR4	synaptogyrin 4	23546	0	NA	NA	NA	NA	NA
ENSG00000105472	CLEC11A	C-type lectin domain containing 11A	6320	0,707145637	2,728460982	3,205154	0,851272942	0,394617752	NA
ENSG00000105479	ODAD1	outer dynein arm docking complex subunit 1	93233	0,607296512	0,898461985	3,152126	0,285033625	0,775618381	NA
ENSG00000105492	SIGLEC6	sialic acid binding Ig like lectin 6	946	2,646875193	-1,591296757	1,285471	-1,237909059	0,215749783	NA
ENSG00000105499	PLA2G4C	phospholipase A2 group IVC	8605	5,707261862	-0,056681375	0,862999	-0,065679519	0,947632978	NA
ENSG00000105507	CABP5	calcium binding protein 5	56344	0	NA	NA	NA	NA	NA
ENSG00000105509	HAS1	hyaluronan synthase 1	3036	0	NA	NA	NA	NA	NA
ENSG00000105523	FAM83E	family with sequence similarity 83 member E	54854	0,967130673	0,596648814	2,259284	0,264087562	0,791712448	NA
ENSG00000105538	RASIP1	Ras interacting protein 1	54922	0,448664543	-2,626060022	3,446816	-0,761880053	0,446131596	NA
ENSG00000105549	SPMAP2	sperm microtubule associated protein 2	51298	0	NA	NA	NA	NA	NA
ENSG00000105550	FGF21	fibroblast growth factor 21	26291	0	NA	NA	NA	NA	NA
ENSG00000105559	PLEKHA4	pleckstrin homology domain containing A4	57664	0,82656168	-3,893126895	3,941823	-0,987646176	0,323325963	NA
ENSG00000105605	CACNG7	calcium voltage-gated channel auxiliary subunit gamma 7	59284	0	NA	NA	NA	NA	NA
ENSG00000105609	LILRB5	leukocyte immunoglobulin like receptor B5	10990	0	NA	NA	NA	NA	NA
ENSG00000105610	KLF1	KLF transcription factor 1	10661	1,710159129	1,755957935	1,648278	1,065328506	0,286727297	NA
ENSG00000105613	MAST1	microtubule associated serine/threonine kinase 1	22983	1,079377392	-1,712330537	1,96226	-0,872631789	0,382863808	NA
ENSG00000105617	LENG1	leukocyte receptor cluster member 1	79165	0,984739271	-3,579640917	2,826655	-1,266387429	0,205374409	NA
ENSG00000105641	SLC5A5	solute carrier family 5 member 5	6528	0,10543123	0	4,080456	0	1	NA
ENSG00000105642	KCNN1	potassium calcium-activated channel subfamily N member 1	3780	0	NA	NA	NA	NA	NA
ENSG00000105649	RAB3A	RAB3A, member RAS oncogene family	5864	4,064411031	0,665886627	0,953824	0,698123249	0,48510012	NA
ENSG00000105650	PDE4C	phosphodiesterase 4C	5143	0,10543123	0	4,080456	0	1	NA
ENSG00000105655	ISYNA1	inositol-3-phosphate synthase 1	51477	8,37366608	0,341951924	0,937184	0,364871852	0,715207067	NA
ENSG00000105664	COMP	cartilage oligomeric matrix protein	1311	0,117497108	0,847101401	4,080456	0,207599697	0,835541541	NA
ENSG00000105668	UPK1A	uroplakin 1A	11045	0	NA	NA	NA	NA	NA
ENSG00000105672	ETV2	ETS variant transcription factor 2	2116	1,945116833	-0,598238404	1,473928	-0,405880382	0,684830493	NA
ENSG00000105675	ATP4A	ATPase H+/K+ transporting subunit alpha	495	0	NA	NA	NA	NA	NA
ENSG00000105679	GAPDHS	glyceraldehyde-3-phosphate dehydrogenase, spermatogenic	26330	0,103998153	-1,076443944	4,080456	-0,263804824	0,791930317	NA
ENSG00000105694	NA	NA	NA	0	NA	NA	NA	NA	NA
ENSG00000105695	MAG	myelin associated glycoprotein	4099	0	NA	NA	NA	NA	NA
ENSG00000105696	TMEM59L	transmembrane protein 59 like	25789	0	NA	NA	NA	NA	NA
ENSG00000105697	HAMP	hepcidin antimicrobial peptide	57817	0,454795258	1,051926717	4,080456	0,257796371	0,796564065	NA
ENSG00000105707	HPN	hepsin	3249	0,117497108	0,847101401	4,080456	0,207599697	0,835541541	NA
ENSG00000105711	SCN1B	sodium voltage-gated channel beta subunit 1	6324	3,917625219	1,547141916	0,856881	1,805550508	0,070988579	NA
ENSG00000105717	PBX4	PBX homeobox 4	80714	4,886550674	-1,139020348	1,57285	-0,724176043	0,468957664	NA
ENSG00000105737	GRIK5	glutamate ionotropic receptor kainate type subunit 5	2901	0,382222216	0	3,632223	0	1	NA
ENSG00000105750	ZNF85	zinc finger protein 85	7639	7,640467534	-0,116289733	0,880915	-0,132010152	0,894976262	NA
ENSG00000105767	CADM4	cell adhesion molecule 4	199731	0,176009189	-0,109933579	4,080456	-0,026941494	0,978506398	NA
ENSG00000105784	RUNDC3B	RUN domain containing 3B	154661	0,710215925	-1,04561049	3,173667	-0,329464441	0,741804667	NA
ENSG00000105792	CFAP69	cilia and flagella associated protein 69	79846	0	NA	NA	NA	NA	NA
ENSG00000105825	TFPI2	tissue factor pathway inhibitor 2	7980	0	NA	NA	NA	NA	NA
ENSG00000105852	PON3	paraoxonase 3	5446	0	NA	NA	NA	NA	NA
ENSG00000105855	ITGB8	integrin subunit beta 8	3696	0	NA	NA	NA	NA	NA
ENSG00000105877	DNAH11	dynein axonemal heavy chain 11	8701	0,36866518	-1,078695606	3,713819	-0,290454578	0,771468495	NA
ENSG00000105880	DLX5	distal-less homeobox 5	1749	0	NA	NA	NA	NA	NA
ENSG00000105889	STEAP1B	STEAP family member 1B	256227	0	NA	NA	NA	NA	NA
ENSG00000105889	LOC401312	uncharacterized LOC401312	401312	0	NA	NA	NA	NA	NA
ENSG00000105894	PTN	pleiotrophin	5764	0	NA	NA	NA	NA	NA

ENSG00000105928	GSDME	gasdermin E	1687	0,118286652	0	4,080456	0	1	NA
ENSG00000105929	ATP6V0A4	ATPase H+ transporting V0 subunit a4	50617	0	NA	NA	NA	NA	NA
ENSG00000105948	IFT56	intraflagellar transport 56	79989	8,925163105	-1,408211968	0,83619	-1,684080445	0,092166123	NA
ENSG00000105954	NPVF	neuropeptide VF precursor	64111	0	NA	NA	NA	NA	NA
ENSG00000105967	TFEC	transcription factor EC	22797	0	NA	NA	NA	NA	NA
ENSG00000105971	CAV2	caveolin 2	858	0	NA	NA	NA	NA	NA
ENSG00000105974	CAV1	caveolin 1	857	0,072165449	0,847101401	4,080456	0,207599697	0,835541541	NA
ENSG00000105976	MET	MET proto-oncogene, receptor tyrosine kinase	4233	0	NA	NA	NA	NA	NA
ENSG00000105988	NA	NA	NA	0	NA	NA	NA	NA	NA
ENSG00000105989	WNT2	Wnt family member 2	7472	0	NA	NA	NA	NA	NA
ENSG00000105991	HOXA1	homeobox A1	3198	3,676046547	0,84668943	1,075743	0,787074152	0,43123846	NA
ENSG00000105996	HOXA2	homeobox A2	3199	0,118286652	0	4,080456	0	1	NA
ENSG00000105997	HOXA3	homeobox A3	3200	2,239020901	-0,999326938	1,196579	-0,835153679	0,403631187	NA
ENSG00000106004	HOXA5	homeobox A5	3202	2,516031826	0,302795647	1,272079	0,238032104	0,811856195	NA
ENSG00000106006	HOXA6	homeobox A6	3203	0,51853909	0,539444268	2,806382	0,192220526	0,847569468	NA
ENSG00000106013	ANKRD7	ankyrin repeat domain 7	56311	0,117497108	0,847101401	4,080456	0,207599697	0,835541541	NA
ENSG00000106018	VIPR2	vasoactive intestinal peptide receptor 2	7434	0	NA	NA	NA	NA	NA
ENSG00000106025	TSPAN12	tetraspanin 12	23554	0,117497108	0,847101401	4,080456	0,207599697	0,835541541	NA
ENSG00000106031	HOXA13	homeobox A13	3209	0	NA	NA	NA	NA	NA
ENSG00000106034	CPED1	cadherin like and PC-esterase domain containing 1	79974	0,15836288	0	4,080456	0	1	NA
ENSG00000106038	EVX1	even-skipped homeobox 1	2128	0	NA	NA	NA	NA	NA
ENSG00000106066	CPVL	carboxypeptidase vitellogenic like	54504	0,341711868	0,970823065	4,022602	0,24134209	0,809289991	NA
ENSG00000106070	GRB10	growth factor receptor bound protein 10	2887	1,828498887	0,389914867	1,673731	0,232961421	0,815791363	NA
ENSG00000106077	ABHD11	abhydrolase domain containing 11	83451	11,07998377	-0,212603542	0,670695	-0,316989731	0,751251394	NA
ENSG00000106078	COBL	cordon-bleu WH2 repeat protein	23242	0	NA	NA	NA	NA	NA
ENSG00000106113	CRHR2	corticotropin releasing hormone receptor 2	1395	0	NA	NA	NA	NA	NA
ENSG00000106123	EPHB6	EPH receptor B6	2051	0,527156152	0	4,080456	0	1	NA
ENSG00000106125	MINDY4	MINDY lysine 48 deubiquitinase 4	84182	0,248953381	0,448279127	4,061143	0,110382516	0,912106019	NA
ENSG00000106128	GHRHR	growth hormone releasing hormone receptor	2692	0	NA	NA	NA	NA	NA
ENSG00000106178	CCL24	C-C motif chemokine ligand 24	6369	0	NA	NA	NA	NA	NA
ENSG00000106211	HSPB1	heat shock protein family B (small) member 1	3315	5,295685845	-0,103911838	1,126707	-0,092226104	0,926518399	NA
ENSG00000106236	NPTX2	neuronal pentraxin 2	4885	0	NA	NA	NA	NA	NA
ENSG00000106258	CYP3A5	cytochrome P450 family 3 subfamily A member 5	1577	0	NA	NA	NA	NA	NA
ENSG00000106278	PTPRZ1	protein tyrosine phosphatase receptor type Z1	5803	0	NA	NA	NA	NA	NA
ENSG00000106302	HYAL4	hyaluronidase 4	23553	0	NA	NA	NA	NA	NA
ENSG00000106304	SPAM1	sperm adhesion molecule 1	6677	0	NA	NA	NA	NA	NA
ENSG00000106327	TFR2	transferrin receptor 2	7036	0,890284946	-4,003202721	3,294353	-1,215171112	0,224300836	NA
ENSG00000106328	FSCN3	fascin actin-bundling protein 3	29999	0,118286652	0	4,080456	0	1	NA
ENSG00000106331	PAX4	paired box 4	5078	0	NA	NA	NA	NA	NA
ENSG00000106333	PCOLCE	procollagen C-endopeptidase enhancer	5118	2,723506271	2,054180363	1,523654	1,348193525	0,177596147	NA
ENSG00000106336	FBXO24	F-box protein 24	26261	0,15836288	0	4,080456	0	1	NA
ENSG00000106341	PPP1R17	protein phosphatase 1 regulatory subunit 17	10842	0	NA	NA	NA	NA	NA
ENSG00000106366	SERPINE1	serpin family E member 1	5054	1,915102395	1,055712334	1,424195	0,741269462	0,458530074	NA
ENSG00000106384	MOGAT3	monoacylglycerol O-acyltransferase 3	346606	0,10543123	0	4,080456	0	1	NA
ENSG00000106410	NOBOX	NOBOX oogenesis homeobox	135935	0	NA	NA	NA	NA	NA
ENSG00000106436	MYL10	myosin light chain 10	93408	0	NA	NA	NA	NA	NA
ENSG00000106483	SFRP4	secreted frizzled related protein 4	6424	0	NA	NA	NA	NA	NA
ENSG00000106484	MEST	mesoderm specific transcript	4232	4,026354288	0,423452889	0,829593	0,510434261	0,609747259	NA
ENSG00000106511	MEOX2	mesenchyme homeobox 2	4223	0	NA	NA	NA	NA	NA
ENSG00000106526	ACTR3C	actin related protein 3C	653857	5,866085483	-0,065818374	0,873479	-0,075352	0,939934649	NA
ENSG00000106536	POU6F2	POU class 6 homeobox 2	11281	0	NA	NA	NA	NA	NA
ENSG00000106537	TSPAN13	tetraspanin 13	27075	0,295142228	1,689167759	3,896503	0,433508657	0,664645278	NA
ENSG00000106538	RARRES2	retinoic acid receptor responder 2	5919	1,764225287	0,686839933	1,441492	0,476478546	0,633733488	NA
ENSG00000106540	NA	NA	NA	0	NA	NA	NA	NA	NA
ENSG00000106541	AGR2	anterior gradient 2, protein disulphide isomerase family member	10551	0	NA	NA	NA	NA	NA

ENSG00000106565	TMEM176B	transmembrane protein 176B	28959	0	NA	NA	NA	NA	NA
ENSG00000106571	GLI3	GLI family zinc finger 3	2737	0	NA	NA	NA	NA	NA
ENSG00000106610	NA	NA	NA	9,694452547	-0,211031987	0,607267	-0,347510946	0,728207497	NA
ENSG00000106624	AEBP1	AE binding protein 1	165	0,210314523	-1,075523623	4,080456	-0,26357928	0,792104126	NA
ENSG00000106631	MYL7	myosin light chain 7	58498	0	NA	NA	NA	NA	NA
ENSG00000106633	GCK	glucokinase	2645	0	NA	NA	NA	NA	NA
ENSG00000106648	GALNTL5	polypeptide N-acetylgalactosaminyltransferase like 5	168391	0	NA	NA	NA	NA	NA
ENSG00000106686	SPATA6L	spermatogenesis associated 6 like	55064	2,517502769	-0,270904399	1,099822	-0,246316529	0,805437216	NA
ENSG00000106688	SLC1A1	solute carrier family 1 member 1	6505	0	NA	NA	NA	NA	NA
ENSG00000106689	LHX2	LIM homeobox 2	9355	0	NA	NA	NA	NA	NA
ENSG00000106714	CNTNAP3	contactin associated protein family member 3	79937	0,15836288	0	4,080456	0	1	NA
ENSG00000106772	PRUNE2	prune homolog 2 with BCH domain	158471	0,190648629	0,844884878	4,080431	0,207057764	0,835964747	NA
ENSG00000106804	C5	complement C5	727	10,75501417	-0,424039739	0,658158	-0,644283032	0,519391916	NA
ENSG00000106809	OGN	osteoglycin	4969	0,263034206	0	4,080456	0	1	NA
ENSG00000106819	ASPN	asporin	54829	0	NA	NA	NA	NA	NA
ENSG00000106852	LHX6	LIM homeobox 6	26468	0	NA	NA	NA	NA	NA
ENSG00000106853	PTGR1	prostaglandin reductase 1	22949	0,118286652	0	4,080456	0	1	NA
ENSG00000106927	AMBP	alpha-1-microglobulin/bikunin precursor	259	0	NA	NA	NA	NA	NA
ENSG00000106976	DNM1	dynamin 1	1759	0,932451882	0,218523963	2,520255	0,086707099	0,930904333	NA
ENSG00000107014	RLN2	relaxin 2	6019	1,16160717	-3,910857886	2,850612	-1,37193637	0,170083247	NA
ENSG00000107018	RLN1	relaxin 1	6013	0,745595875	0,908344679	2,606771	0,348455862	0,727497856	NA
ENSG00000107105	ELAVL2	ELAV like RNA binding protein 2	1993	0,214453617	-1,172982082	4,080456	-0,287463489	0,773757454	NA
ENSG00000107147	KCNT1	potassium sodium-activated channel subfamily T member 1	57582	0,480797059	-1,045736749	2,950604	-0,354414431	0,723028321	NA
ENSG00000107159	CA9	carbonic anhydrase 9	768	0	NA	NA	NA	NA	NA
ENSG00000107165	TYRP1	tyrosinase related protein 1	7306	0	NA	NA	NA	NA	NA
ENSG00000107186	MPDZ	multiple PDZ domain crumbs cell polarity complex component	8777	0	NA	NA	NA	NA	NA
ENSG00000107187	LHX3	LIM homeobox 3	8022	0,38369611	-0,116332467	3,661242	-0,031774044	0,974652246	NA
ENSG00000107242	PIP5K1B	phosphatidylinositol-4-phosphate 5-kinase type 1 beta	8395	0,15836288	0	4,080456	0	1	NA
ENSG00000107249	GLIS3	GLIS family zinc finger 3	169792	0	NA	NA	NA	NA	NA
ENSG00000107281	NPDC1	neural proliferation, differentiation and control 1	56654	5,571064332	0,118898235	0,807091	0,147317057	0,88288177	NA
ENSG00000107295	SH3GL2	SH3 domain containing GRB2 like 2, endophilin A1	6456	0	NA	NA	NA	NA	NA
ENSG00000107317	PTGDS	prostaglandin D2 synthase	5730	0,550377308	-0,474765183	3,939264	-0,120521305	0,904070204	NA
ENSG00000107338	SHB	SH2 domain containing adaptor protein B	6461	0,385706507	-1,54056128	3,586151	-0,429586321	0,667496589	NA
ENSG00000107438	PDLIM1	PDZ and LIM domain 1	9124	6,365619873	-0,339922594	0,910306	-0,373415704	0,708839074	NA
ENSG00000107447	DNTT	DNA nucleotidylexotransferase	1791	0	NA	NA	NA	NA	NA
ENSG00000107518	ATRNL1	attractin like 1	26033	0	NA	NA	NA	NA	NA
ENSG00000107562	CXCL12	C-X-C motif chemokine ligand 12	6387	0	NA	NA	NA	NA	NA
ENSG00000107593	PKD2L1	polycystin 2 like 1, transient receptor potential cation channel	9033	0	NA	NA	NA	NA	NA
ENSG00000107731	UNC5B	unc-5 netrin receptor B	219699	0,119673877	0,847101401	4,080456	0,207599697	0,835541541	NA
ENSG00000107736	CDH23	cadherin related 23	64072	7,436479174	-0,1894307	0,697654	-0,271525382	0,785986981	NA
ENSG00000107807	TLX1	T cell leukemia homeobox 1	3195	0	NA	NA	NA	NA	NA
ENSG00000107821	KAZALD1	Kazal type serine peptidase inhibitor domain 1	81621	0,231192803	0,84253533	4,08033	0,206487049	0,83641048	NA
ENSG00000107831	FGF8	fibroblast growth factor 8	2253	0	NA	NA	NA	NA	NA
ENSG00000107859	PITX3	paired like homeodomain 3	5309	0	NA	NA	NA	NA	NA
ENSG00000107984	DKK1	dickkopf WNT signaling pathway inhibitor 1	22943	0	NA	NA	NA	NA	NA
ENSG00000108001	EBF3	EBF transcription factor 3	253738	0	NA	NA	NA	NA	NA
ENSG00000108018	SORCS1	sortilin related VPS10 domain containing receptor 1	114815	0,288661797	2,306206831	3,997774	0,57687273	0,564025434	NA
ENSG00000108176	DNAJC12	DnaJ heat shock protein family (Hsp40) member C12	56521	0	NA	NA	NA	NA	NA
ENSG00000108187	PBLD	phenazine biosynthesis like protein domain containing	64081	7,537458458	0,055582778	0,702704	0,079098379	0,936954373	NA
ENSG00000108231	LGI1	leucine rich glioma inactivated 1	9211	0	NA	NA	NA	NA	NA
ENSG00000108239	TBC1D12	TBC1 domain family member 12	23232	8,063797103	0,446823631	0,643832	0,694007038	0,487677795	NA

ENSG00000108242	CYP2C18	cytochrome P450 family 2 subfamily C member 18	1562	0,072165449	0,847101401	4,080456	0,207599697	0,835541541	NA	
ENSG00000108244	KRT23	keratin 23	25984	0	NA	NA	NA	NA	NA	
ENSG00000108255	CRYBA1	crystallin beta A1	1411	0	NA	NA	NA	NA	NA	
ENSG00000108309	RUNDC3A	RUN domain containing 3A	10900	0,072165449	0,847101401	4,080456	0,207599697	0,835541541	NA	
ENSG00000108342	CSF3	colony stimulating factor 3	1440	0	NA	NA	NA	NA	NA	
ENSG00000108375	RNF43	ring finger protein 43	54894	0,603114591	-2,991904282	3,129835	-0,955930427	0,339107385	NA	
ENSG00000108379	WNT3	Wnt family member 3	7473	0,103998153	-1,076443944	4,080456	-0,263804824	0,791930317	NA	
ENSG00000108379	LOC101929777	uncharacterized LOC101929777	101929777	0,103998153	-1,076443944	4,080456	-0,263804824	0,791930317	NA	
ENSG00000108381	ASPA	aspartoacylase	443	0	NA	NA	NA	NA	NA	
ENSG00000108387	SEPTIN4	septin 4	5414	0,45593274	0,04201047	3,491924	0,01203075	0,990401082	NA	
ENSG00000108405	P2RX1	purinergic receptor P2X 1	5023	0,10488184	-1,076443944	4,080456	-0,263804824	0,791930317	NA	
ENSG00000108442	NA	NA	NA	0	NA	NA	NA	NA	NA	
ENSG00000108448	NA	NA	NA	3,49716846	-0,230165289	0,986066	-0,233417825	0,815436973	NA	
ENSG00000108452	ZNF29P	zinc finger protein 29, pseudogene	7577	0	NA	NA	NA	NA	NA	
ENSG00000108511	HOXB6	homeobox B6	3216	3,671312048	0,203957063	1,044212	0,195321472	0,845141289	NA	
ENSG00000108551	RASD1	ras related dexamethasone induced 1	51655	1,084409819	0,326713784	1,800527	0,1814545	0,856010844	NA	
ENSG00000108556	CHRNE	cholinergic receptor nicotinic epsilon subunit	1145	0,914301431	2,486724132	2,477835	1,00358729	0,315577584	NA	
ENSG00000108576	SLC6A4	solute carrier family 6 member 4	6532	0,10488184	-1,076443944	4,080456	-0,263804824	0,791930317	NA	
ENSG00000108602	ALDH3A1	aldehyde dehydrogenase 3 family member A1	218	0	NA	NA	NA	NA	NA	
ENSG00000108641	B9D1	B9 domain containing 1	27077	0,368826705	0	4,080456	0	1	NA	
ENSG00000108679	LGALS3BP	galectin 3 binding protein	3959	2,451296919	-0,425660553	1,578989	-0,26957785	0,787485042	NA	
ENSG00000108684	ASIC2	acid sensing ion channel subunit 2	40	0	NA	NA	NA	NA	NA	
ENSG00000108688	CCL7	C-C motif chemokine ligand 7	6354	0	NA	NA	NA	NA	NA	
ENSG00000108691	CCL2	C-C motif chemokine ligand 2	6347	0	NA	NA	NA	NA	NA	
ENSG00000108700	CCL8	C-C motif chemokine ligand 8	6355	0	NA	NA	NA	NA	NA	
ENSG00000108759	KRT32	keratin 32	3882	0	NA	NA	NA	NA	NA	
ENSG00000108785	NA	NA	NA	0,210318549	-1,074016958	4,080456	-0,263210041	0,792388692	NA	
ENSG00000108786	HSD17B1	hydroxysteroid 17-beta dehydrogenase 1	3292	0,94650947	2,636946596	2,474901	1,065475788	0,286660676	NA	
ENSG00000108813	DLX4	distal-less homeobox 4	1748	0,815087649	0,716962978	2,082394	0,344297453	0,730622599	NA	
ENSG00000108821	COL1A1	collagen type I alpha 1 chain	1277	0	NA	NA	NA	NA	NA	
ENSG00000108823	SGCA	sarcoglycan alpha	6442	0	NA	NA	NA	NA	NA	
ENSG00000108825	PTGES3L-AARSD1	PTGES3L-AARSD1 readthrough	100885850	0	NA	NA	NA	NA	NA	
ENSG00000108830	RND2	Rho family GTPase 2	8153	0,24899568	0,449730402	4,061162	0,110739331	0,911823056	NA	
ENSG00000108839	ALOX12	arachidonate 12-lipoxygenase, 12S type	239	0	NA	NA	NA	NA	NA	
ENSG00000108846	ABCC3	ATP binding cassette subfamily C member 3	8714	0	NA	NA	NA	NA	NA	
ENSG00000108849	PPY	pancreatic polypeptide	5539	0	NA	NA	NA	NA	NA	
ENSG00000108852	MPP2	MAGUK p55 scaffold protein 2	4355	0,072165449	0,847101401	4,080456	0,207599697	0,835541541	NA	
ENSG00000108878	CACNG1	calcium voltage-gated channel auxiliary subunit gamma 1	786	0	NA	NA	NA	NA	NA	
ENSG00000108924	HLF	HLF transcription factor, PAR bZIP family member	3131	0,072165449	0,847101401	4,080456	0,207599697	0,835541541	NA	
ENSG00000108947	EFNB3	ephrin B3	1949	0	NA	NA	NA	NA	NA	
ENSG00000108950	FAM20A	FAM20A golgi associated secretory pathway pseudokinase	54757	0	NA	NA	NA	NA	NA	
ENSG00000108958	NA	NA	NA	0	NA	NA	NA	NA	NA	
ENSG00000109016	DHRS7B	dehydrogenase/reductase 7B	25979	10,44447158	-0,240963111	1,095888	-0,219879284	0,82596517	NA	
ENSG00000109047	RCVRN	recoverin	5957	3,327151728	-0,47664355	1,013807	-0,470152206	0,638246278	NA	
ENSG00000109061	MYH1	myosin heavy chain 1	4619	0	NA	NA	NA	NA	NA	
ENSG00000109072	VTN	vitronectin	7448	0,396621722	1,931035639	3,606993	0,535358784	0,592401785	NA	
ENSG00000109089	CDR2L	cerebellar degeneration related protein 2 like	30850	0,118286652	0	4,080456	0	1	NA	
ENSG00000109099	PMP22	peripheral myelin protein 22	5376	0	NA	NA	NA	NA	NA	
ENSG00000109101	FOXN1	forkhead box N1	8456	0	NA	NA	NA	NA	NA	
ENSG00000109113	RAB34	RAB34, member RAS oncogene family	83871	10,08950239	-0,918179131	0,684415	-1,341553232	0,179740899	NA	
ENSG00000109132	PHOX2B	paired like homeobox 2B	8929	0	NA	NA	NA	NA	NA	
ENSG00000109158	GABRA4	gamma-aminobutyric acid type A receptor subunit alpha4	2557	0	NA	NA	NA	NA	NA	
ENSG00000109163	GNRHR	gonadotropin releasing hormone receptor	2798	0,117497108	0,847101401	4,080456	0,207599697	0,835541541	NA	
ENSG00000109181	UGT2B10	UDP glucuronosyltransferase family 2 member B10	7365	0	NA	NA	NA	NA	NA	
ENSG00000109182	CWH43	cell wall biogenesis 43 C-terminal homolog	80157	0	NA	NA	NA	NA	NA	

ENSG00000109193	SULT1E1	sulfotransferase family 1E member 1	6783	0	NA	NA	NA	NA	NA
ENSG00000109205	ODAM	odontogenic, ameloblast associated	54959	0	NA	NA	NA	NA	NA
ENSG00000109208	SMR3A	submaxillary gland androgen regulated protein 3A	26952	0	NA	NA	NA	NA	NA
ENSG00000109255	NMU	neuromedin U	10874	0	NA	NA	NA	NA	NA
ENSG00000109265	CRACD	capping protein inhibiting regulator of actin dynamics	57482	0,47508864	0	4,080456	0	1	NA
ENSG00000109272	PF4V1	platelet factor 4 variant 1	5197	0	NA	NA	NA	NA	NA
ENSG00000109323	MANBA	mannosidase beta	4126	8,55445237	-0,024843436	2,74691	-0,00904414	0,992783919	NA
ENSG00000109339	MAPK10	mitogen-activated protein kinase 10	5602	0	NA	NA	NA	NA	NA
ENSG00000109424	UCP1	uncoupling protein 1	7350	0	NA	NA	NA	NA	NA
ENSG00000109436	TBC1D9	TBC1 domain family member 9	23158	0,234994216	1,944111989	4,02324	0,483220515	0,62893917	NA
ENSG00000109471	IL2	interleukin 2	3558	0,208896628	-1,921447128	4,042528	-0,475308318	0,634567231	NA
ENSG00000109472	CPE	carboxypeptidase E	1363	0	NA	NA	NA	NA	NA
ENSG00000109511	ANXA10	annexin A10	11199	0	NA	NA	NA	NA	NA
ENSG00000109576	AADAT	aminoadipate aminotransferase	51166	0	NA	NA	NA	NA	NA
ENSG00000109610	SOD3	superoxide dismutase 3	6649	0	NA	NA	NA	NA	NA
ENSG00000109625	CPZ	carboxypeptidase Z	8532	0	NA	NA	NA	NA	NA
ENSG00000109654	TRIM2	tripartite motif containing 2	23321	0	NA	NA	NA	NA	NA
ENSG00000109667	SLC2A9	solute carrier family 2 member 9	56606	1,453007114	-0,025472758	2,039301	-0,012490928	0,99003394	NA
ENSG00000109680	TBC1D19	TBC1 domain family member 19	55296	11,51225229	0,026909458	0,741103	0,036309992	0,971035183	NA
ENSG00000109705	NKX3-2	NK3 homeobox 2	579	0,103998153	-1,076443944	4,080456	-0,263804824	0,791930317	NA
ENSG00000109738	GLRB	glycine receptor beta	2743	0	NA	NA	NA	NA	NA
ENSG00000109743	BST1	bone marrow stromal cell antigen 1	683	0,191917532	1,678978273	4,04623	0,41494878	0,678179399	NA
ENSG00000109758	HGFAC	HGF activator	3083	0	NA	NA	NA	NA	NA
ENSG00000109771	LRP2BP	LRP2 binding protein	55805	3,960239021	-0,213039315	1,041775	-0,20449655	0,837965488	NA
ENSG00000109787	KLF3	KLF transcription factor 3	51274	4,991107936	-1,217644727	0,826916	-1,472513737	0,1408822	NA
ENSG00000109794	FAM149A	family with sequence similarity 149 member A	25854	0	NA	NA	NA	NA	NA
ENSG00000109819	PPARGC1A	PPARG coactivator 1 alpha	10891	0	NA	NA	NA	NA	NA
ENSG00000109832	DDX25	DEAD-box helicase 25	29118	0,342123144	0,995752892	3,763168	0,26460497	0,791313791	NA
ENSG00000109846	CRYAB	crystallin alpha B	1410	0	NA	NA	NA	NA	NA
ENSG00000109851	DBX1	developing brain homeobox 1	120237	0	NA	NA	NA	NA	NA
ENSG00000109927	TECTA	tectorin alpha	7007	1,303397409	3,443833128	2,12397	1,621413178	0,104929056	NA
ENSG00000109927	TBCEL-TECTA	TBCEL-TECTA readthrough	116804918	1,303397409	3,443833128	2,12397	1,621413178	0,104929056	NA
ENSG00000109956	B3GAT1	beta-1,3-glucuronyltransferase 1	27087	3,016838277	-1,180163971	1,40839	-0,83795264	0,402057303	NA
ENSG00000109991	P2RX3	purinergic receptor P2X 3	5024	0	NA	NA	NA	NA	NA
ENSG00000110013	SIAE	sialic acid acetylesterase	54414	7,492295005	0,009291192	0,755172	0,012303407	0,990183549	NA
ENSG00000110042	DTX4	deltex E3 ubiquitin ligase 4	23220	1,809651376	1,009342622	1,509448	0,668683054	0,50369768	NA
ENSG00000110076	NRXN2	neurexin 2	9379	0,175927639	-0,107381535	4,080456	-0,026316063	0,979005243	NA
ENSG00000110077	MS4A6A	membrane spanning 4-domains A6A	64231	0	NA	NA	NA	NA	NA
ENSG00000110079	MS4A4A	membrane spanning 4-domains A4A	51338	0	NA	NA	NA	NA	NA
ENSG00000110092	CCND1	cyclin D1	595	3,894950751	0,641304556	0,91178	0,703354311	0,481834972	NA
ENSG00000110148	CCKBR	cholecystokinin B receptor	887	0	NA	NA	NA	NA	NA
ENSG00000110169	HPX	hemopexin	3263	0,501727442	-1,022712389	2,918507	-0,350423188	0,726021127	NA
ENSG00000110195	FOLR1	folate receptor alpha	2348	0	NA	NA	NA	NA	NA
ENSG00000110203	FOLR3	folate receptor gamma	2352	0	NA	NA	NA	NA	NA
ENSG00000110237	ARHGEF17	Rho guanine nucleotide exchange factor 17	9828	0,236573303	0	4,080456	0	1	NA
ENSG00000110243	APOA5	apolipoprotein A5	116519	0	NA	NA	NA	NA	NA
ENSG00000110244	APOA4	apolipoprotein A4	337	0	NA	NA	NA	NA	NA
ENSG00000110245	APOC3	apolipoprotein C3	345	0	NA	NA	NA	NA	NA
ENSG00000110318	CEP126	centrosomal protein 126	57562	7,924498147	0,181579651	0,75618	0,240127428	0,810231472	NA
ENSG00000110328	GALNT18	polypeptide N-acetylgalactosaminyltransferase 18	374378	0	NA	NA	NA	NA	NA
ENSG00000110375	UPK2	uroplakin 2	7379	0,210322678	-1,072943209	4,080456	-0,262946897	0,792591509	NA
ENSG00000110400	NECTIN1	nectin cell adhesion molecule 1	5818	10,86000443	-0,443524275	0,936493	-0,473601358	0,635784207	NA
ENSG00000110427	KIAA1549L	KIAA1549 like	25758	0,144330898	1,418786819	4,061262	0,349346267	0,726829368	NA
ENSG00000110436	SLC1A2	solute carrier family 1 member 2	6506	0,234994216	1,944111989	4,02324	0,483220515	0,62893917	NA
ENSG00000110446	SLC15A3	solute carrier family 15 member 3	51296	0	NA	NA	NA	NA	NA
ENSG00000110448	CD5	CD5 molecule	921	0,779509507	-1,228509058	3,008367	-0,408364056	0,683006418	NA

ENSG00000110455	ACCS	1-aminocyclopropane-1-carboxylate synthase homolog (inactive)	84680	11,24329735	0,179005875	0,589856	0,303473732	0,761528863	NA
ENSG00000110484	SCGB2A2	secretoglobin family 2A member 2	4250	0	NA	NA	NA	NA	NA
ENSG00000110492	MDK	midkine	4192	2,481807002	0,255737849	1,434867	0,178231075	0,858541506	NA
ENSG00000110675	ELMOD1	ELMO domain containing 1	55531	0	NA	NA	NA	NA	NA
ENSG00000110680	CALCA	calcitonin related polypeptide alpha	796	0	NA	NA	NA	NA	NA
ENSG00000110693	SOX6	SRY-box transcription factor 6	55553	0	NA	NA	NA	NA	NA
ENSG00000110723	EXPH5	exophilin 5	23086	2,774234327	0,978097293	1,100618	0,888679896	0,374175138	NA
ENSG00000110777	POU2AF1	POU class 2 homeobox associating factor 1	5450	0,342517221	1,94712247	3,761546	0,51763883	0,604710284	NA
ENSG00000110786	PTPN5	protein tyrosine phosphatase non-receptor type 5	84867	0	NA	NA	NA	NA	NA
ENSG00000110799	VWF	von Willebrand factor	7450	1,855787567	-1,480270898	1,626157	-0,910287952	0,362670671	NA
ENSG00000110811	P3H3	prolyl 3-hydroxylase 3	10536	0	NA	NA	NA	NA	NA
ENSG00000110881	ASIC1	acid sensing ion channel subunit 1	41	0,118286652	0	4,080456	0	1	NA
ENSG00000110887	DAO	D-amino acid oxidase	1610	0	NA	NA	NA	NA	NA
ENSG00000110900	TSPAN11	tetraspanin 11	441631	0	NA	NA	NA	NA	NA
ENSG00000110944	IL23A	interleukin 23 subunit alpha	51561	1,895403961	1,205502454	1,694698	0,711337776	0,476874948	NA
ENSG00000110975	SYT10	synaptotagmin 10	341359	0	NA	NA	NA	NA	NA
ENSG00000110987	BCL7A	BAF chromatin remodeling complex subunit BCL7A	605	0,48395036	-0,956121859	3,448865	-0,277227992	0,781605047	NA
ENSG00000111012	CYP27B1	cytochrome P450 family 27 subfamily B member 1	1594	1,061477785	1,680284596	2,478316	0,677994475	0,497775197	NA
ENSG00000111046	MYF6	myogenic factor 6	4618	0	NA	NA	NA	NA	NA
ENSG00000111049	MYF5	myogenic factor 5	4617	0	NA	NA	NA	NA	NA
ENSG00000111052	LIN7A	lin-7 homolog A, crumbs cell polarity complex component	8825	0	NA	NA	NA	NA	NA
ENSG00000111057	KRT18	keratin 18	3875	0,5633938	-1,993064468	2,8439	-0,700820904	0,483414791	NA
ENSG00000111058	ACSS3	acyl-CoA synthetase short chain family member 3	79611	0	NA	NA	NA	NA	NA
ENSG00000111077	TNS2	tensin 2	23371	4,909730494	1,114834379	0,959363	1,162056344	0,245212579	NA
ENSG00000111087	GLI1	GLI family zinc finger 1	2735	0,555498178	0,908726899	3,308168	0,274691883	0,783552968	NA
ENSG00000111110	PPM1H	protein phosphatase, Mg2+/Mn2+ dependent 1H	57460	0	NA	NA	NA	NA	NA
ENSG00000111181	SLC6A12	solute carrier family 6 member 12	6539	0	NA	NA	NA	NA	NA
ENSG00000111186	WNT5B	Wnt family member 5B	81029	0,11808024	-1,076443944	4,080456	-0,263804824	0,791930317	NA
ENSG00000111199	TRPV4	transient receptor potential cation channel subfamily V member 4	59341	0,144330898	1,418786819	4,061262	0,349346267	0,726829368	NA
ENSG00000111218	PRMT8	protein arginine methyltransferase 8	56341	0	NA	NA	NA	NA	NA
ENSG00000111241	FGF6	fibroblast growth factor 6	2251	0	NA	NA	NA	NA	NA
ENSG00000111245	MYL2	myosin light chain 2	4633	0	NA	NA	NA	NA	NA
ENSG00000111249	CUX2	cut like homeobox 2	23316	0	NA	NA	NA	NA	NA
ENSG00000111254	AKAP3	A-kinase anchoring protein 3	10566	6,069541064	-0,925850726	0,829994	-1,11549107	0,264640043	NA
ENSG00000111262	KCNA1	potassium voltage-gated channel subfamily A member 1	3736	0,103998153	-1,076443944	4,080456	-0,263804824	0,791930317	NA
ENSG00000111275	ALDH2	aldehyde dehydrogenase 2 family member	217	0,927021422	1,634822616	2,43967	0,670099784	0,502794182	NA
ENSG00000111291	GPRC5D	G protein-coupled receptor class C group 5 member D	55507	0	NA	NA	NA	NA	NA
ENSG00000111305	GSG1	germ cell associated 1	83445	0	NA	NA	NA	NA	NA
ENSG00000111319	SCNN1A	sodium channel epithelial 1 subunit alpha	6337	0,326093339	-2,555635698	3,994837	-0,639734616	0,522345147	NA
ENSG00000111325	OGFOD2	2-oxoglutarate and iron dependent oxygenase domain containing 2	79676	10,76492251	0,791399343	0,651688	1,214383231	0,224601416	NA
ENSG00000111339	ART4	ADP-ribosyltransferase 4 (inactive) (Dombrock blood group)	420	0	NA	NA	NA	NA	NA
ENSG00000111341	MGP	matrix Gla protein	4256	0	NA	NA	NA	NA	NA
ENSG00000111344	RASAL1	RAS protein activator like 1	8437	0	NA	NA	NA	NA	NA
ENSG00000111404	RERGL	RERG like	79785	0	NA	NA	NA	NA	NA
ENSG00000111405	ENDOU	endonuclease, poly(U) specific	8909	0	NA	NA	NA	NA	NA
ENSG00000111432	FZD10	frizzled class receptor 10	11211	0	NA	NA	NA	NA	NA
ENSG00000111490	TBC1D30	TBC1 domain family member 30	23329	1,072431318	0,6244164	2,181202	0,286271652	0,774670068	NA
ENSG00000111536	IL26	interleukin 26	55801	0,15836288	0	4,080456	0	1	NA
ENSG00000111644	ACRBP	acrosin binding protein	84519	1,07461773	-1,019178295	2,439632	-0,417759097	0,676123259	NA
ENSG00000111664	GNB3	G protein subunit beta 3	2784	0	NA	NA	NA	NA	NA

ENSG00000111700	SLCO1B3	solute carrier organic anion transporter family member 1B3	28234	0	NA	NA	NA	NA	NA
ENSG00000111701	APOBEC1	apolipoprotein B mRNA editing enzyme catalytic subunit 1	339	0	NA	NA	NA	NA	NA
ENSG00000111704	NANOG	Nanog homeobox	79923	0	NA	NA	NA	NA	NA
ENSG00000111713	GYS2	glycogen synthase 2	2998	0	NA	NA	NA	NA	NA
ENSG00000111728	ST8SIA1	ST8 alpha-N-acetyl-neuraminide alpha-2,8-sialyltransferase 1	6489	0,795149177	1,009075224	2,714938	0,371675226	0,710134672	NA
ENSG00000111729	CLEC4A	C-type lectin domain family 4 member A	50856	3,011332302	1,688285666	1,359225	1,242094415	0,214201729	NA
ENSG00000111732	AICDA	activation induced cytidine deaminase	57379	0	NA	NA	NA	NA	NA
ENSG00000111780	NA	NA	NA	0	NA	NA	NA	NA	NA
ENSG00000111783	RFX4	regulatory factor X4	5992	0	NA	NA	NA	NA	NA
ENSG00000111799	COL12A1	collagen type XII alpha 1 chain	1303	0,10488184	-1,076443944	4,080456	-0,263804824	0,791930317	NA
ENSG00000111816	FRK	fyn related Src family tyrosine kinase	2444	0,310388424	1,692592085	3,84046	0,440726352	0,659411116	NA
ENSG00000111817	DSE	dermatan sulfate epimerase	29940	4,989047106	0,16215709	1,08817	0,149018221	0,881539255	NA
ENSG00000111834	RSPH4A	radial spoke head component 4A	345895	3,644464939	0,243971837	0,965009	0,252818243	0,80040867	NA
ENSG00000111837	MAK	male germ cell associated kinase	4117	0	NA	NA	NA	NA	NA
ENSG00000111846	GCNT2	glucosaminyl (N-acetyl) transferase 2 (I blood group)	2651	0	NA	NA	NA	NA	NA
ENSG00000111863	ADTRP	androgen dependent TFPI regulating protein	84830	1,062710439	-4,022498304	2,679829	-1,501028205	0,133348267	NA
ENSG00000111879	FAM184A	family with sequence similarity 184 member A	79632	0	NA	NA	NA	NA	NA
ENSG00000111886	GABRR2	gamma-aminobutyric acid type A receptor subunit rho2	2570	4,611314837	0,452462556	0,883576	0,512080814	0,608594451	NA
ENSG00000111907	TPD52L1	TPD52 like 1	7164	0	NA	NA	NA	NA	NA
ENSG00000111962	UST	uronyl 2-sulfotransferase	10090	0,379872083	1,17679583	3,585404	0,328218507	0,742746456	NA
ENSG00000112038	OPRM1	opioid receptor mu 1	4988	0,221927529	-0,085712031	4,079945	-0,021008134	0,983239167	NA
ENSG00000112041	TULP1	TUB like protein 1	7287	0	NA	NA	NA	NA	NA
ENSG00000112053	SLC26A8	solute carrier family 26 member 8	116369	0	NA	NA	NA	NA	NA
ENSG00000112077	RHAG	Rh associated glycoprotein	6005	0	NA	NA	NA	NA	NA
ENSG00000112115	IL17A	interleukin 17A	3605	0	NA	NA	NA	NA	NA
ENSG00000112116	IL17F	interleukin 17F	112744	0	NA	NA	NA	NA	NA
ENSG00000112137	PHACTR1	phosphatase and actin regulator 1	221692	0,772908265	0,332750502	3,229392	0,103038141	0,9179327	NA
ENSG00000112139	MDGA1	MAM domain containing glycosylphosphatidylinositol anchor 1	266727	0	NA	NA	NA	NA	NA
ENSG00000112164	GLP1R	glucagon like peptide 1 receptor	2740	0,262573501	1,417378639	4,061232	0,349002144	0,7270877	NA
ENSG00000112175	BMP5	bone morphogenetic protein 5	653	0	NA	NA	NA	NA	NA
ENSG00000112183	RBM24	RNA binding motif protein 24	221662	0	NA	NA	NA	NA	NA
ENSG00000112186	CAP2	cyclase associated actin cytoskeleton regulatory protein 2	10486	0	NA	NA	NA	NA	NA
ENSG00000112195	TREML2	triggering receptor expressed on myeloid cells like 2	79865	3,333606221	-0,791698122	1,033107	-0,766327349	0,443481549	NA
ENSG00000112212	TSPO2	translocator protein 2	222642	0	NA	NA	NA	NA	NA
ENSG00000112214	FHL5	four and a half LIM domains 5	9457	0	NA	NA	NA	NA	NA
ENSG00000112218	GPR63	G protein-coupled receptor 63	81491	6,041230723	0,776623865	0,945774	0,821151891	0,411559756	NA
ENSG00000112232	KHDRBS2	KH RNA binding domain containing, signal transduction associated 2	202559	0	NA	NA	NA	NA	NA
ENSG00000112238	PRDM13	PR/SET domain 13	59336	1,458172431	0,685103578	1,56043	0,439048063	0,66062671	NA
ENSG00000112246	SIM1	SIM bHLH transcription factor 1	6492	0	NA	NA	NA	NA	NA
ENSG00000112273	HDGFL1	HDGF like 1	154150	0	NA	NA	NA	NA	NA
ENSG00000112276	BVES	blood vessel epicardial substance	11149	0,401855828	-2,340089147	3,603853	-0,649329777	0,516125243	NA
ENSG00000112280	COL9A1	collagen type IX alpha 1 chain	1297	0	NA	NA	NA	NA	NA
ENSG00000112290	WASF1	WASP family member 1	8936	4,853620505	0,183755444	0,861091	0,213398438	0,831016199	NA
ENSG00000112293	GPLD1	glycosylphosphatidylinositol specific phospholipase D1	2822	1,583551905	-2,160604912	1,647081	-1,311778463	0,189594894	NA
ENSG00000112299	VNN1	vanin 1	8876	0,189777822	-0,104646967	4,080456	-0,025645901	0,979539775	NA
ENSG00000112309	B3GAT2	beta-1,3-glucuronyltransferase 2	135152	0,072165449	0,847101401	4,080456	0,207599697	0,835541541	NA

ENSG00000112319	EYA4	EYA transcriptional coactivator and phosphatase 4	2070	0	NA	NA	NA	NA	NA
ENSG00000112333	NR2E1	nuclear receptor subfamily 2 group E member 1	7101	0	NA	NA	NA	NA	NA
ENSG00000112337	SLC17A2	solute carrier family 17 member 2	10246	0	NA	NA	NA	NA	NA
ENSG00000112357	PEX7	peroxisomal biogenesis factor 7	5191	10,03177367	0,7611416	0,630417	1,207361346	0,227293015	NA
ENSG00000112379	ARFGEF3	ARFGEF family member 3	57221	0,229624587	0,852208006	4,080456	0,208851176	0,83456442	NA
ENSG00000112394	SLC16A10	solute carrier family 16 member 10	117247	1,856727495	-1,10243458	1,265531	-0,87112436	0,383686254	NA
ENSG00000112414	ADGRG6	adhesion G protein-coupled receptor G6	57211	0,680017461	1,175460568	3,114659	0,377396233	0,705879166	NA
ENSG00000112425	EPM2A	EPM2A glucan phosphatase, laforin	7957	3,24859903	0,120209107	2,257825	0,05324111	0,957539801	NA
ENSG00000112462	OR12D3	olfactory receptor family 12 subfamily D member 3	81797	0	NA	NA	NA	NA	NA
ENSG00000112473	SLC39A7	solute carrier family 39 member 7	7922	16,81454965	21,59185819	3,908374	5,524512128	NA	NA
ENSG00000112494	UNC93A	unc-93 homolog A	54346	0	NA	NA	NA	NA	NA
ENSG00000112499	SLC22A2	solute carrier family 22 member 2	6582	0	NA	NA	NA	NA	NA
ENSG00000112530	PACRG	parkin coregulated	135138	0	NA	NA	NA	NA	NA
ENSG00000112539	C6orf118	chromosome 6 open reading frame 118	168090	0	NA	NA	NA	NA	NA
ENSG00000112541	PDE10A	phosphodiesterase 10A	10846	0	NA	NA	NA	NA	NA
ENSG00000112541	LINC00473	long intergenic non-protein coding RNA 473	90632	0	NA	NA	NA	NA	NA
ENSG00000112559	MDFI	MyoD family inhibitor	4188	0	NA	NA	NA	NA	NA
ENSG00000112562	SMOC2	SPARC related modular calcium binding 2	64094	0	NA	NA	NA	NA	NA
ENSG00000112599	GUCA1B	guanylate cyclase activator 1B	2979	1,409076909	0,741793272	2,158629	0,34364097	0,73111631	NA
ENSG00000112619	PRPH2	peripherin 2	5961	0,072165449	0,847101401	4,080456	0,207599697	0,835541541	NA
ENSG00000112655	PTK7	protein tyrosine kinase 7 (inactive)	5754	0,10543123	0	4,080456	0	1	NA
ENSG00000112706	IMPG1	interphotoreceptor matrix proteoglycan 1	3617	0	NA	NA	NA	NA	NA
ENSG00000112715	VEGFA	vascular endothelial growth factor A	7422	3,316940528	-1,243600273	2,050731	-0,606418183	0,544237097	NA
ENSG00000112761	CCN6	cellular communication network factor 6	8838	0	NA	NA	NA	NA	NA
ENSG00000112769	LAMA4	laminin subunit alpha 4	3910	0	NA	NA	NA	NA	NA
ENSG00000112796	ENPP5	ectonucleotide pyrophosphatase/phosphodiesterase family member 5	59084	0,256675851	-1,181624138	4,080456	-0,289581403	0,772136496	NA
ENSG00000112799	LY86	lymphocyte antigen 86	9450	0,224863354	0,837381319	4,080456	0,20521759	0,837402127	NA
ENSG00000112818	MEP1A	meprin A subunit alpha	4224	0	NA	NA	NA	NA	NA
ENSG00000112837	TBX18	T-box transcription factor 18	9096	0	NA	NA	NA	NA	NA
ENSG00000112874	NUDT12	nudix hydrolase 12	83594	0,223983395	0	4,080456	0	1	NA
ENSG00000112902	SEMA5A	semaphorin 5A	9037	0	NA	NA	NA	NA	NA
ENSG00000112936	C7	complement C7	730	0	NA	NA	NA	NA	NA
ENSG00000112964	GHR	growth hormone receptor	2690	0	NA	NA	NA	NA	NA
ENSG00000112981	NME5	NME/NM23 family member 5	8382	0	NA	NA	NA	NA	NA
ENSG00000113073	SLC4A9	solute carrier family 4 member 9	83697	0,117497108	0,847101401	4,080456	0,207599697	0,835541541	NA
ENSG00000113083	LOX	lysyl oxidase	4015	0	NA	NA	NA	NA	NA
ENSG00000113100	CDH9	cadherin 9	1007	0	NA	NA	NA	NA	NA
ENSG00000113108	APBB3	amyloid beta precursor protein binding family B member 3	10307	5,146324618	-0,686361625	1,201792	-0,571115035	0,567921668	NA
ENSG00000113140	SPARC	secreted protein acidic and cysteine rich	6678	0	NA	NA	NA	NA	NA
ENSG00000113196	HAND1	heart and neural crest derivatives expressed 1	9421	0	NA	NA	NA	NA	NA
ENSG00000113205	PCDHB3	protocadherin beta 3	56132	0	NA	NA	NA	NA	NA
ENSG00000113231	PDE8B	phosphodiesterase 8B	8622	0	NA	NA	NA	NA	NA
ENSG00000113262	GRM6	glutamate metabotropic receptor 6	2916	0	NA	NA	NA	NA	NA
ENSG00000113296	THBS4	thrombospondin 4	7060	0,072165449	0,847101401	4,080456	0,207599697	0,835541541	NA
ENSG00000113302	IL12B	interleukin 12B	3593	0,956417103	1,877171212	2,525805	0,743197243	0,457362272	NA
ENSG00000113303	BTNL8	butyrophilin like 8	79908	0	NA	NA	NA	NA	NA
ENSG00000113327	GABRG2	gamma-aminobutyric acid type A receptor subunit gamma2	2566	0	NA	NA	NA	NA	NA
ENSG00000113356	POLR3G	RNA polymerase III subunit G	10622	4,770572221	-0,095754023	1,602193	-0,059764334	0,952343332	NA
ENSG00000113361	CDH6	cadherin 6	1004	0,10488184	-1,076443944	4,080456	-0,263804824	0,791930317	NA
ENSG00000113389	NPR3	natriuretic peptide receptor 3	4883	0	NA	NA	NA	NA	NA

ENSG00000113396	SLC27A6	solute carrier family 27 member 6	28965	0	NA	NA	NA	NA	NA
ENSG00000113430	IRX4	iroquois homeobox 4	50805	0	NA	NA	NA	NA	NA
ENSG00000113492	AGXT2	alanine--glyoxylate aminotransferase 2	64902	0	NA	NA	NA	NA	NA
ENSG00000113494	PRLR	prolactin receptor	5618	0	NA	NA	NA	NA	NA
ENSG00000113504	SLC12A7	solute carrier family 12 member 7	10723	2,912574322	-0,865660316	1,792012	-0,483066169	0,629048755	NA
ENSG00000113520	IL4	interleukin 4	3565	0	NA	NA	NA	NA	NA
ENSG00000113525	IL5	interleukin 5	3567	0	NA	NA	NA	NA	NA
ENSG00000113555	PCDH12	protocadherin 12	51294	0,222267135	-1,077052454	4,080456	-0,263953952	0,791815401	NA
ENSG00000113578	FGF1	fibroblast growth factor 1	2246	0	NA	NA	NA	NA	NA
ENSG00000113594	LIFR	LIF receptor subunit alpha	3977	0	NA	NA	NA	NA	NA
ENSG00000113600	C9	complement C9	735	0	NA	NA	NA	NA	NA
ENSG00000113645	WWC1	WW and C2 domain containing 1	23286	0	NA	NA	NA	NA	NA
ENSG00000113657	DPYSL3	dihydropyrimidinase like 3	1809	0,144330898	1,418786819	4,061262	0,349346267	0,726829368	NA
ENSG00000113722	CDX1	caudal type homeobox 1	1044	0	NA	NA	NA	NA	NA
ENSG00000113739	STC2	stanniocalcin 2	8614	0	NA	NA	NA	NA	NA
ENSG00000113763	UNC5A	unc-5 netrin receptor A	90249	0	NA	NA	NA	NA	NA
ENSG00000113805	CNTN3	contactin 3	5067	0,072165449	0,847101401	4,080456	0,207599697	0,835541541	NA
ENSG00000113889	KNG1	kininogen 1	3827	0	NA	NA	NA	NA	NA
ENSG00000113905	HRG	histidine rich glycoprotein	3273	0	NA	NA	NA	NA	NA
ENSG00000113924	HGD	homogentisate 1,2-dioxygenase	3081	0	NA	NA	NA	NA	NA
ENSG00000113946	CLDN16	claudin 16	10686	0	NA	NA	NA	NA	NA
ENSG00000114013	CD86	CD86 molecule	942	0,449338472	0,373808463	2,985116	0,125224101	0,900346137	NA
ENSG00000114019	AMOTL2	angiomotin like 2	51421	0	NA	NA	NA	NA	NA
ENSG00000114113	RBP2	retinol binding protein 2	5948	0	NA	NA	NA	NA	NA
ENSG00000114115	RBP1	retinol binding protein 1	5947	0	NA	NA	NA	NA	NA
ENSG00000114124	GRK7	G protein-coupled receptor kinase 7	131890	1,768481906	-0,456941014	1,729245	-0,264243147	0,791592565	NA
ENSG00000114200	BCHE	butyrylcholinesterase	590	0	NA	NA	NA	NA	NA
ENSG00000114204	SERPINI2	serpin family I member 2	5276	0,117497108	0,847101401	4,080456	0,207599697	0,835541541	NA
ENSG00000114248	LRRC31	leucine rich repeat containing 31	79782	0	NA	NA	NA	NA	NA
ENSG00000114251	WNT5A	Wnt family member 5A	7474	0	NA	NA	NA	NA	NA
ENSG00000114268	PFKFB4	6-phosphofructo-2-kinase/fructose-2,6-biphosphatase 4	5210	9,366980386	-0,199800966	0,615314	-0,324713945	0,74539758	NA
ENSG00000114270	COL7A1	collagen type VII alpha 1 chain	1294	1,15846507	2,997597286	2,409997	1,243817671	0,213566675	NA
ENSG00000114279	FGF12	fibroblast growth factor 12	2257	0	NA	NA	NA	NA	NA
ENSG00000114315	HES1	hes family bHLH transcription factor 1	3280	0	NA	NA	NA	NA	NA
ENSG00000114349	GNAT1	G protein subunit alpha transducin 1	2779	0	NA	NA	NA	NA	NA
ENSG00000114374	USP9Y	ubiquitin specific peptidase 9 Y-linked	8287	0	NA	NA	NA	NA	NA
ENSG00000114374	TTTY15	testis expressed transcript, Y-linked 15	64595	0	NA	NA	NA	NA	NA
ENSG00000114378	HYAL1	hyaluronidase 1	3373	0,235782768	0,847898644	4,080456	0,207795077	0,835388976	NA
ENSG00000114405	CEP15	centrosomal protein 15	57415	6,701178128	-0,418437786	0,719458	-0,581601121	0,560835388	NA
ENSG00000114455	HHLA2	HHLA2 member of B7 family	11148	0	NA	NA	NA	NA	NA
ENSG00000114487	MORC1	MORC family CW-type zinc finger 1	27136	0	NA	NA	NA	NA	NA
ENSG00000114529	C3orf52	chromosome 3 open reading frame 52	79669	0	NA	NA	NA	NA	NA
ENSG00000114547	ROPN1B	rhophilin associated tail protein 1B	152015	0	NA	NA	NA	NA	NA
ENSG00000114631	PODXL2	podocalyxin like 2	50512	2,166103545	-0,879679325	1,375736	-0,639424719	0,522546672	NA
ENSG00000114638	UPK1B	uroplakin 1B	7348	0	NA	NA	NA	NA	NA
ENSG00000114646	CSPG5	chondroitin sulfate proteoglycan 5	10675	1,113999911	-0,30201717	2,021845	-0,149376992	0,881256166	NA
ENSG00000114654	EFCC1	EF-hand and coiled-coil domain containing 1	79825	0,330314307	-0,982019848	4,0413	-0,242996012	0,808008487	NA
ENSG00000114656	CFAP92	cilia and flagella associated protein 92 (putative)	57501	0	NA	NA	NA	NA	NA
ENSG00000114670	NEK11	NIMA related kinase 11	79858	1,005242122	2,629176603	2,938563	0,894715073	0,370939419	NA
ENSG00000114698	PLSCR4	phospholipid scramblase 4	57088	0	NA	NA	NA	NA	NA
ENSG00000114757	PEX5L	peroxisomal biogenesis factor 5 like	51555	0	NA	NA	NA	NA	NA
ENSG00000114771	AADAC	arylacetamide deacetylase	13	0	NA	NA	NA	NA	NA
ENSG00000114786	ABHD14A-ACY1	ABHD14A-ACY1 readthrough	100526760	0	NA	NA	NA	NA	NA
ENSG00000114790	ARHGEF26	Rho guanine nucleotide exchange factor 26	26084	0	NA	NA	NA	NA	NA
ENSG00000114805	PLCH1	phospholipase C eta 1	23007	2,410425171	0,942176759	1,372707	0,686364094	0,492483541	NA
ENSG00000114812	VIPR1	vasoactive intestinal peptide receptor 1	7433	0	NA	NA	NA	NA	NA

ENSG00000114854	TNNC1	troponin C1, slow skeletal and cardiac type	7134	0,446794723	-1,04915269	4,080456	-0,257116539	0,797088811	NA
ENSG00000114859	CLCN2	chloride voltage-gated channel 2	1181	7,454500815	-2,121290663	0,956745	-2,217195705	0,026609721	NA
ENSG00000114923	SLC4A3	solute carrier family 4 member 3	6508	0	NA	NA	NA	NA	NA
ENSG00000114948	ADAM23	ADAM metallopeptidase domain 23	8745	0	NA	NA	NA	NA	NA
ENSG00000115008	IL1A	interleukin 1 alpha	3552	1,276513496	2,662778336	1,958557	1,359561339	0,173968779	NA
ENSG00000115009	CCL20	C-C motif chemokine ligand 20	6364	0	NA	NA	NA	NA	NA
ENSG00000115041	KCNIP3	potassium voltage-gated channel interacting protein 3	30818	0	NA	NA	NA	NA	NA
ENSG00000115107	STEAP3	STEAP3 metalloreductase	55240	4,32343985	0,666648998	0,8376	0,795904015	0,426087827	NA
ENSG00000115109	EPB41L5	erythrocyte membrane protein band 4,1 like 5	57669	8,431273609	-0,801629827	0,813803	-0,985042175	0,324603355	NA
ENSG00000115112	TFCP2L1	transcription factor CP2 like 1	29842	0,072165449	0,847101401	4,080456	0,207599697	0,835541541	NA
ENSG00000115138	POMC	proopiomelanocortin	5443	3,512748279	0,507530134	0,958598	0,529450327	0,596493094	NA
ENSG00000115155	OTOF	otoferlin	9381	7,299101858	-0,55626986	0,656784	-0,846959704	0,397017582	NA
ENSG00000115183	TANC1	tetratricopeptide repeat, ankyrin repeat and coiled-coil containing 1	85461	0,288661797	2,306206831	3,997774	0,57687273	0,564025434	NA
ENSG00000115194	SLC30A3	solute carrier family 30 member 3	7781	0,10488184	-1,076443944	4,080456	-0,263804824	0,791930317	NA
ENSG00000115221	ITGB6	integrin subunit beta 6	3694	0	NA	NA	NA	NA	NA
ENSG00000115226	FNDC4	fibronectin type III domain containing 4	64838	0,223434687	-1,073356892	4,080456	-0,263048278	0,792513368	NA
ENSG00000115252	PDE1A	phosphodiesterase 1A	5136	0	NA	NA	NA	NA	NA
ENSG00000115255	REEP6	receptor accessory protein 6	92840	10,1314364	-1,30530071	0,705917	-1,84908642	0,064445335	NA
ENSG00000115257	PCSK4	proprotein convertase subtilisin/kexin type 4	54760	1,639272154	0,333530843	2,789441	0,119569063	0,904824528	NA
ENSG00000115263	GCG	glucagon	2641	0	NA	NA	NA	NA	NA
ENSG00000115266	APC2	APC regulator of WNT signaling pathway 2	10297	0,763843553	-0,058046126	2,262973	-0,025650384	0,979536198	NA
ENSG00000115271	GCA	grancalcin	25801	1,9502608	-0,247761704	1,464173	-0,169216127	0,865626646	NA
ENSG00000115290	GRB14	growth factor receptor bound protein 14	2888	0	NA	NA	NA	NA	NA
ENSG00000115297	TLX2	T cell leukemia homeobox 2	3196	0,118286652	0	4,080456	0	1	NA
ENSG00000115318	LOXL3	lysyl oxidase like 3	84695	0,888542262	-0,296901503	2,202337	-0,134811998	0,892760519	NA
ENSG00000115353	TACR1	tachykinin receptor 1	6869	0	NA	NA	NA	NA	NA
ENSG00000115355	CCDC88A	coiled-coil domain containing 88A	55704	4,69123081	2,018148695	1,112905	1,813405585	0,069769289	NA
ENSG00000115361	ACADL	acyl-CoA dehydrogenase long chain	33	0	NA	NA	NA	NA	NA
ENSG00000115363	EVA1A	eva-1 homolog A, regulator of programmed cell death	84141	8,779486533	-0,163362889	0,676482	-0,241488734	0,809176347	NA
ENSG00000115380	EFEMP1	EGF containing fibulin extracellular matrix protein 1	2202	0	NA	NA	NA	NA	NA
ENSG00000115386	REG1A	regenerating family member 1 alpha	5967	0	NA	NA	NA	NA	NA
ENSG00000115423	DNAH6	dynein axonemal heavy chain 6	1768	2,923225136	0,339901544	1,274372	0,266720891	0,789684068	NA
ENSG00000115457	IGFBP2	insulin like growth factor binding protein 2	3485	0,242852145	-0,32003206	4,080456	-0,078430467	0,937485639	NA
ENSG00000115461	IGFBP5	insulin like growth factor binding protein 5	3488	4,074029648	0,108175125	1,010002	0,107103864	0,914706582	NA
ENSG00000115468	EFHD1	EF-hand domain family member D1	80303	0	NA	NA	NA	NA	NA
ENSG00000115474	KCNJ13	potassium inwardly rectifying channel subfamily J member 13	3769	0	NA	NA	NA	NA	NA
ENSG00000115488	NEU2	neuraminidase 2	4759	0	NA	NA	NA	NA	NA
ENSG00000115507	OTX1	orthodenticle homeobox 1	5013	0	NA	NA	NA	NA	NA
ENSG00000115525	ST3GAL5	ST3 beta-galactoside alpha-2,3-sialyltransferase 5	8869	8,499046569	-0,838389656	3,747397	-0,223725886	0,822970603	NA
ENSG00000115556	PLCD4	phospholipase C delta 4	84812	0,23616048	-2,075648354	4,028973	-0,515180498	0,606426895	NA
ENSG00000115590	IL1R2	interleukin 1 receptor type 2	7850	0	NA	NA	NA	NA	NA
ENSG00000115592	PRKAG3	protein kinase AMP-activated non-catalytic subunit gamma 3	53632	0	NA	NA	NA	NA	NA
ENSG00000115593	SMYD1	SET and MYND domain containing 1	150572	0	NA	NA	NA	NA	NA
ENSG00000115596	WNT6	Wnt family member 6	7475	0	NA	NA	NA	NA	NA
ENSG00000115598	IL1RL2	interleukin 1 receptor like 2	8808	0	NA	NA	NA	NA	NA
ENSG00000115616	SLC9A2	solute carrier family 9 member A2	6549	0	NA	NA	NA	NA	NA
ENSG00000115641	FHL2	four and a half LIM domains 2	2274	0,189707075	1,665286192	4,047518	0,411433885	0,680754412	NA
ENSG00000115648	MLPH	melanophilin	79083	0	NA	NA	NA	NA	NA

ENSG00000115657	ABCB6	ATP binding cassette subfamily B member 6 (Langereis blood group)	10058	7,794268769	-0,498570282	1,077135	-0,462867229	0,643459539	NA
ENSG00000115665	SLC5A7	solute carrier family 5 member 7	60482	0	NA	NA	NA	NA	NA
ENSG00000115705	TPO	thyroid peroxidase	7173	0	NA	NA	NA	NA	NA
ENSG00000115718	PROC	protein C, inactivator of coagulation factors Va and VIIIa	5624	0	NA	NA	NA	NA	NA
ENSG00000115840	SLC25A12	solute carrier family 25 member 12	8604	0	NA	NA	NA	NA	NA
ENSG00000115844	DLX2	distal-less homeobox 2	1746	0,119673877	0,847101401	4,080456	0,207599697	0,835541541	NA
ENSG00000115850	LCT	lactase	3938	0,545406795	1,664528166	3,398875	0,489729127	0,624325588	NA
ENSG00000115884	SDC1	syndecan 1	6382	0	NA	NA	NA	NA	NA
ENSG00000115896	PLCL1	phospholipase C like 1 (inactive)	5334	0	NA	NA	NA	NA	NA
ENSG00000115919	KYNU	kynureninase	8942	2,25109559	1,006968795	2,287863	0,440135147	0,659839227	NA
ENSG00000115963	RND3	Rho family GTPase 3	390	0	NA	NA	NA	NA	NA
ENSG00000116014	KISS1R	KISS1 receptor	84634	0	NA	NA	NA	NA	NA
ENSG00000116031	CD207	CD207 molecule	50489	0	NA	NA	NA	NA	NA
ENSG00000116032	GRIN3B	glutamate ionotropic receptor NMDA type subunit 3B	116444	0,10543123	0	4,080456	0	1	NA
ENSG00000116035	VAX2	ventral anterior homeobox 2	25806	0	NA	NA	NA	NA	NA
ENSG00000116039	ATP6V1B1	ATPase H+ transporting V1 subunit B1	525	0	NA	NA	NA	NA	NA
ENSG00000116096	SPR	sepiapterin reductase	6697	0,565395831	-1,402013385	3,955101	-0,354482358	0,722977423	NA
ENSG00000116117	PARD3B	par-3 family cell polarity regulator beta	117583	0,233964918	0,539651521	4,067496	0,132674135	0,8944511	NA
ENSG00000116132	PRRX1	paired related homeobox 1	5396	0	NA	NA	NA	NA	NA
ENSG00000116141	MARK1	microtubule affinity regulating kinase 1	4139	0,072165449	0,847101401	4,080456	0,207599697	0,835541541	NA
ENSG00000116147	TNR	tenascin R	7143	3,229049468	0,962812739	1,362363	0,706722656	0,479738848	NA
ENSG00000116151	MORN1	MORN repeat containing 1	79906	2,40896148	0,626259576	1,308291	0,478685262	0,632162552	NA
ENSG00000116157	GPX7	glutathione peroxidase 7	2882	8,689474476	-0,548366097	0,641069	-0,855393688	0,392333237	NA
ENSG00000116176	TPSG1	tryptase gamma 1	25823	0	NA	NA	NA	NA	NA
ENSG00000116183	PAPPA2	pappalysin 2	60676	0,117497108	0,847101401	4,080456	0,207599697	0,835541541	NA
ENSG00000116191	RALGPS2	Ral GEF with PH domain and SH3 binding motif 2	55103	0,743568246	1,458340834	2,387755	0,610758127	0,541359718	NA
ENSG00000116194	ANGPTL1	angiopoietin like 1	9068	0	NA	NA	NA	NA	NA
ENSG00000116218	NPHS2	NPHS2 stomatin family member, podocin	7827	0	NA	NA	NA	NA	NA
ENSG00000116254	CHD5	chromodomain helicase DNA binding protein 5	26038	2,310964887	-1,955025137	1,681348	-1,162772152	0,244921959	NA
ENSG00000116285	ERRFI1	ERBB receptor feedback inhibitor 1	54206	0,676103706	-1,639990235	2,445269	-0,670678891	0,502425113	NA
ENSG00000116299	ELAPOR1	endosome-lysosome associated apoptosis and autophagy regulator 1	57535	8,841479525	0,576351479	2,099255	0,274550472	0,783661622	NA
ENSG00000116329	OPRD1	opioid receptor delta 1	4985	0,118286652	0	4,080456	0	1	NA
ENSG00000116544	DLGAP3	DLG associated protein 3	58512	1,271326489	0,856586408	1,554776	0,55093864	0,581675738	NA
ENSG00000116675	DNAJC6	DnaJ heat shock protein family (Hsp40) member C6	9829	3,537316318	-0,911619051	0,964389	-0,945281215	0,344515326	NA
ENSG00000116690	PRG4	proteoglycan 4	10216	0,263007986	-1,070885018	4,080456	-0,262442495	0,792980314	NA
ENSG00000116701	NCF2	neutrophil cytosolic factor 2	4688	0	NA	NA	NA	NA	NA
ENSG00000116703	PDC	phosducin	5132	0	NA	NA	NA	NA	NA
ENSG00000116711	PLA2G4A	phospholipase A2 group IVA	5321	4,73226171	0,111550566	0,855252	0,130430047	0,896226195	NA
ENSG00000116721	PRAMEF1	PRAME family member 1	65121	0	NA	NA	NA	NA	NA
ENSG00000116726	PRAMEF12	PRAME family member 12	390999	0	NA	NA	NA	NA	NA
ENSG00000116729	WLS	Wnt ligand secretion mediator	79971	0	NA	NA	NA	NA	NA
ENSG00000116745	RPE65	retinoid isomerohydrolase RPE65	6121	0	NA	NA	NA	NA	NA
ENSG00000116748	AMPD1	adenosine monophosphate deaminase 1	270	0	NA	NA	NA	NA	NA
ENSG00000116761	CTH	cystathionine gamma-lyase	1491	2,74588898	-0,056597083	1,1279	-0,050179168	0,959979612	NA
ENSG00000116771	AGMAT	agmatinase	79814	11,55982901	-0,68988797	0,570635	-1,208982168	0,226669696	NA
ENSG00000116774	OLFML3	olfactomedin like 3	56944	0,334594153	-1,091655271	4,036688	-0,270433396	0,78682685	NA
ENSG00000116783	TNNI3K	TNNI3 interacting kinase	51086	0	NA	NA	NA	NA	NA
ENSG00000116785	CFHR3	complement factor H related 3	10878	0	NA	NA	NA	NA	NA
ENSG00000116819	TFAP2E	transcription factor AP-2 epsilon	339488	3,314984537	-0,341104233	1,779088	-0,191729874	0,847953804	NA
ENSG00000116882	HAO2	hydroxyacid oxidase 2	51179	0	NA	NA	NA	NA	NA
ENSG00000116885	OSCP1	organic solute carrier partner 1	127700	0,435108979	2,010606016	3,538806	0,568159434	0,569926714	NA
ENSG00000116962	NID1	nidogen 1	4811	0	NA	NA	NA	NA	NA
ENSG00000116981	NT5C1A	5'-nucleotidase, cytosolic IA	84618	0	NA	NA	NA	NA	NA
ENSG00000116983	HPCAL4	hippocalcin like 4	51440	0	NA	NA	NA	NA	NA

ENSG00000116990	MYCL	MYCL proto-oncogene, bHLH transcription factor	4610	0	NA	NA	NA	NA	NA
ENSG00000116991	SIPA1L2	signal induced proliferation associated 1 like 2	57568	4,293004316	1,153497049	1,08727	1,06091115	0,288730282	NA
ENSG00000116996	ZP4	zona pellucida glycoprotein 4	57829	0	NA	NA	NA	NA	NA
ENSG00000117009	KMO	kynurenine 3-monooxygenase	8564	0	NA	NA	NA	NA	NA
ENSG00000117013	KCNQ4	potassium voltage-gated channel subfamily Q member 4	9132	1,718965478	-0,914724127	1,919146	-0,47663082	0,633625033	NA
ENSG00000117069	ST6GALNAC5	ST6 N-acetylgalactosaminide alpha-2,6-sialyltransferase 5	81849	0	NA	NA	NA	NA	NA
ENSG00000117114	ADGRL2	adhesion G protein-coupled receptor L2	23266	7,574919902	0,761864796	0,689007	1,105742805	0,268837857	NA
ENSG00000117122	MFAP2	microfibril associated protein 2	4237	0	NA	NA	NA	NA	NA
ENSG00000117148	ACTL8	actin like 8	81569	0	NA	NA	NA	NA	NA
ENSG00000117152	RGS4	regulator of G protein signaling 4	5999	0	NA	NA	NA	NA	NA
ENSG00000117154	IGSF21	immunoglobin superfamily member 21	84966	0	NA	NA	NA	NA	NA
ENSG00000117215	PLA2G2D	phospholipase A2 group IID	26279	0	NA	NA	NA	NA	NA
ENSG00000117245	KIF17	kinesin family member 17	57576	0	NA	NA	NA	NA	NA
ENSG00000117266	CDK18	cyclin dependent kinase 18	5129	5,309997034	-0,32616786	1,163749	-0,280273414	0,779267746	NA
ENSG00000117322	CR2	complement C3d receptor 2	1380	0	NA	NA	NA	NA	NA
ENSG00000117400	MPL	MPL proto-oncogene, thrombopoietin receptor	4352	0	NA	NA	NA	NA	NA
ENSG00000117407	ARTN	artemin	9048	0,361648653	0,733194132	4,080456	0,179684367	0,857400367	NA
ENSG00000117425	PTCH2	patched 2	8643	2,877491459	-0,181401134	1,048237	-0,17305361	0,862609288	NA
ENSG00000117472	TSPAN1	tetraspanin 1	10103	0	NA	NA	NA	NA	NA
ENSG00000117477	CCDC181	coiled-coil domain containing 181	57821	0	NA	NA	NA	NA	NA
ENSG00000117480	FAAH	fatty acid amide hydrolase	2166	5,246348233	-0,370382482	0,813256	-0,455431335	0,648798957	NA
ENSG00000117501	MROH9	maestro heat like repeat family member 9	80133	0	NA	NA	NA	NA	NA
ENSG00000117507	FMO6P	flavin containing dimethylaniline monoxygenase 6, pseudogene	388714	0	NA	NA	NA	NA	NA
ENSG00000117519	CNN3	calponin 3	1266	0	NA	NA	NA	NA	NA
ENSG00000117525	F3	coagulation factor III, tissue factor	2152	0	NA	NA	NA	NA	NA
ENSG00000117594	HSD11B1	hydroxysteroid 11-beta dehydrogenase 1	3290	0	NA	NA	NA	NA	NA
ENSG00000117595	IRF6	interferon regulatory factor 6	3664	1,675332832	1,609958439	1,52545	1,055398866	0,291242941	NA
ENSG00000117598	PLPPR5	phospholipid phosphatase related 5	163404	0	NA	NA	NA	NA	NA
ENSG00000117600	PLPPR4	phospholipid phosphatase related 4	9890	0	NA	NA	NA	NA	NA
ENSG00000117601	SERPINC1	serpin family C member 1	462	0	NA	NA	NA	NA	NA
ENSG00000117691	NENF	neudesin neurotrophic factor	29937	7,666855176	0,309203631	1,112818	0,277856503	0,781122515	NA
ENSG00000117707	PROX1	prospero homeobox 1	5629	0,118286652	0	4,080456	0	1	NA
ENSG00000117791	MTARC2	mitochondrial amidoxime reducing component 2	54996	0	NA	NA	NA	NA	NA
ENSG00000117834	SLC5A9	solute carrier family 5 member 9	200010	0	NA	NA	NA	NA	NA
ENSG00000117971	CHRNB4	cholinergic receptor nicotinic beta 4 subunit	1143	0,15836288	0	4,080456	0	1	NA
ENSG00000117983	MUC5B	mucin 5B, oligomeric mucus/gel-forming	727897	0,23616048	-2,075648354	4,028973	-0,515180498	0,606426895	NA
ENSG00000118004	COLEC11	collectin subfamily member 11	78989	0,118286652	0	4,080456	0	1	NA
ENSG00000118017	A4GNT	alpha-1,4-N-acetylglucosaminyltransferase	51146	0	NA	NA	NA	NA	NA
ENSG00000118094	TREH	trehalase	11181	0	NA	NA	NA	NA	NA
ENSG00000118113	MMP8	matrix metallopeptidase 8	4317	0	NA	NA	NA	NA	NA
ENSG00000118137	APOA1	apolipoprotein A1	335	0,222202577	-1,079355666	4,080456	-0,264518402	0,791380487	NA
ENSG00000118156	ZNF541	zinc finger protein 541	84215	0	NA	NA	NA	NA	NA
ENSG00000118160	SLC8A2	solute carrier family 8 member A2	6543	0,072165449	0,847101401	4,080456	0,207599697	0,835541541	NA
ENSG00000118162	KPTN	kaptin, actin binding protein	11133	8,632674935	0,70558032	0,803674	0,877943698	0,379974274	NA
ENSG00000118194	TNNT2	troponin T2, cardiac type	7139	0	NA	NA	NA	NA	NA
ENSG00000118200	CAMSAP2	calmodulin regulated spectrin associated protein family member 2	23271	0,221314523	-0,129389968	4,080456	-0,031709685	0,974703572	NA
ENSG00000118245	TNP1	transition protein 1	7141	0,818538705	-0,19898409	2,065308	-0,096345941	0,923245825	NA
ENSG00000118257	NRP2	neuropilin 2	8828	0	NA	NA	NA	NA	NA
ENSG00000118271	TTR	transthyretin	7276	0	NA	NA	NA	NA	NA

ENSG00000118292	C1orf54	chromosome 1 open reading frame 54	79630	2,650827913	-0,992911006	1,157784	-0,857596053	0,391115555	NA
ENSG00000118298	CA14	carbonic anhydrase 14	23632	0	NA	NA	NA	NA	NA
ENSG00000118307	DNAI7	dynein axonemal intermediate chain 7	55259	0,234994216	1,944111989	4,02324	0,483220515	0,62893917	NA
ENSG00000118322	ATP10B	ATPase phospholipid transporting 10B (putative)	23120	0	NA	NA	NA	NA	NA
ENSG00000118402	ELOVL4	ELOVL fatty acid elongase 4	6785	0,385188166	-0,070853512	3,689911	-0,019201955	0,984679998	NA
ENSG00000118407	FILIP1	filamin A interacting protein 1	27145	0	NA	NA	NA	NA	NA
ENSG00000118420	UBE3D	ubiquitin protein ligase E3D	90025	3,405918941	-1,372848722	1,923273	-0,713808424	0,47534565	NA
ENSG00000118432	CNR1	cannabinoid receptor 1	1268	0	NA	NA	NA	NA	NA
ENSG00000118434	SPACA1	sperm acrosome associated 1	81833	0	NA	NA	NA	NA	NA
ENSG00000118473	SGIP1	SH3GL interacting endocytic adaptor 1	84251	0,10488184	-1,076443944	4,080456	-0,263804824	0,791930317	NA
ENSG00000118491	ZC2HC1B	zinc finger C2HC-type containing 1B	153918	0	NA	NA	NA	NA	NA
ENSG00000118492	ADGB	androglobin	79747	0	NA	NA	NA	NA	NA
ENSG00000118508	RAB32	RAB32, member RAS oncogene family	10981	6,460323148	-0,479670114	0,824495	-0,58177435	0,560718683	NA
ENSG00000118513	MYB	MYB proto-oncogene, transcription factor	4602	1,074670221	-3,514503767	2,496312	-1,407878571	0,159167029	NA
ENSG00000118514	ALDH8A1	aldehyde dehydrogenase 8 family member A1	64577	1,568636329	-0,158536422	1,380036	-0,114878449	0,908541467	NA
ENSG00000118520	ARG1	arginase 1	383	0	NA	NA	NA	NA	NA
ENSG00000118523	CCN2	cellular communication network factor 2	1490	0	NA	NA	NA	NA	NA
ENSG00000118526	TCF21	transcription factor 21	6943	0	NA	NA	NA	NA	NA
ENSG00000118557	PMFBP1	polyamine modulated factor 1 binding protein 1	83449	1,589452411	4,047733092	2,444783	1,65566167	0,097790354	NA
ENSG00000118600	RXYLT1	ribitol xylosyltransferase 1	10329	4,30825958	6,163297235	3,913117	1,575035312	0,115248294	NA
ENSG00000118729	CASQ2	calsequestrin 2	845	0	NA	NA	NA	NA	NA
ENSG00000118733	OLFM3	olfactomedin 3	118427	0	NA	NA	NA	NA	NA
ENSG00000118777	ABCG2	ATP binding cassette subfamily G member 2 (Junior blood group)	9429	0,418968383	1,399320976	2,960323	0,472692044	0,636432904	NA
ENSG00000118785	SPP1	secreted phosphoprotein 1	6696	0	NA	NA	NA	NA	NA
ENSG00000118849	RARRES1	retinoic acid receptor responder 1	5918	0	NA	NA	NA	NA	NA
ENSG00000118898	PPL	periplakin	5493	0	NA	NA	NA	NA	NA
ENSG00000118903	NA	NA	NA	0	NA	NA	NA	NA	NA
ENSG00000118946	PCDH17	protocadherin 17	27253	0	NA	NA	NA	NA	NA
ENSG00000118972	FGF23	fibroblast growth factor 23	8074	0	NA	NA	NA	NA	NA
ENSG00000118976	NA	NA	NA	0,22282282	0,842478885	4,080456	0,206466854	0,836426254	NA
ENSG00000118990	NA	NA	NA	0	NA	NA	NA	NA	NA
ENSG00000118997	DNAH7	dynein axonemal heavy chain 7	56171	0,359021632	2,56113643	3,98322	0,642981427	0,520236152	NA
ENSG00000119042	SATB2	SATB homeobox 2	23314	2,525054556	-0,833793169	1,225163	-0,680557149	0,496151749	NA
ENSG00000119121	TRPM6	transient receptor potential cation channel subfamily M member 6	140803	0,10543123	0	4,080456	0	1	NA
ENSG00000119125	GDA	guanine deaminase	9615	0	NA	NA	NA	NA	NA
ENSG00000119147	ECRG4	ECRG4 augurin precursor	84417	0	NA	NA	NA	NA	NA
ENSG00000119227	PIGZ	phosphatidylinositol glycan anchor biosynthesis class Z	80235	4,270196081	-0,590804629	1,230415	-0,480166775	0,63110881	NA
ENSG00000119283	TRIM67	tripartite motif containing 67	440730	0,117497108	0,847101401	4,080456	0,207599697	0,835541541	NA
ENSG00000119411	BSPRY	B-box and SPRY domain containing	54836	9,306769818	0,178902384	0,665361	0,268880168	0,788021899	NA
ENSG00000119457	SLC46A2	solute carrier family 46 member 2	57864	0	NA	NA	NA	NA	NA
ENSG00000119514	GALNT12	polypeptide N-acetylgalactosaminyltransferase 12	79695	0,666988775	1,145416297	2,568177	0,446003602	0,655594644	NA
ENSG00000119535	CSF3R	colony stimulating factor 3 receptor	1441	0	NA	NA	NA	NA	NA
ENSG00000119547	ONECUT2	one cut homeobox 2	9480	0	NA	NA	NA	NA	NA
ENSG00000119614	VSX2	visual system homeobox 2	338917	0	NA	NA	NA	NA	NA
ENSG00000119630	PGF	placental growth factor	5228	0,103998153	-1,076443944	4,080456	-0,263804824	0,791930317	NA
ENSG00000119636	BBOF1	basal body orientation factor 1	80127	4,194701057	-0,167647201	0,959093	-0,174797587	0,861238684	NA
ENSG00000119650	IFT43	intraflagellar transport 43	112752	7,300389656	-0,379503499	0,679108	-0,55882678	0,576279944	NA
ENSG00000119660	NA	NA	NA	0,072165449	0,847101401	4,080456	0,207599697	0,835541541	NA
ENSG00000119681	LTBP2	latent transforming growth factor beta binding protein 2	4053	0,230890527	-2,037605746	4,034639	-0,505028067	0,613539123	NA
ENSG00000119686	FLVCR2	FLVCR choline and putative heme transporter 2	55640	5,076062004	-0,683902297	0,981764	-0,696605522	0,486049699	NA
ENSG00000119698	PPP4R4	protein phosphatase 4 regulatory subunit 4	57718	0	NA	NA	NA	NA	NA
ENSG00000119699	TGFB3	transforming growth factor beta 3	7043	7,875047949	0,292191425	0,805047	0,362949696	0,716642462	NA

ENSG00000119703	ZC2HC1C	zinc finger C2HC-type containing 1C	79696	3,612665122	0,977209989	1,502694	0,65030542	0,515494957	NA
ENSG00000119715	ESRRB	estrogen related receptor beta	2103	0	NA	NA	NA	NA	NA
ENSG00000119771	KLHL29	kelch like family member 29	114818	0	NA	NA	NA	NA	NA
ENSG00000119782	FKBP1B	FKBP prolyl isomerase 1B	2281	5,539242085	0,975342158	1,228704	0,793797506	0,427313325	NA
ENSG00000119862	LGALSL	galectin like	29094	0	NA	NA	NA	NA	NA
ENSG00000119865	CNRIP1	cannabinoid receptor interacting protein 1	25927	0	NA	NA	NA	NA	NA
ENSG00000119866	BCL11A	BCL11 transcription factor A	53335	0,687856261	-2,441924437	2,694179	-0,906370659	0,364739698	NA
ENSG00000119888	EPCAM	epithelial cell adhesion molecule	4072	0	NA	NA	NA	NA	NA
ENSG00000119913	TECTB	tectorin beta	6975	0	NA	NA	NA	NA	NA
ENSG00000119915	ELOVL3	ELOVL fatty acid elongase 3	83401	0	NA	NA	NA	NA	NA
ENSG00000119919	NKX2-3	NK2 homeobox 3	159296	0	NA	NA	NA	NA	NA
ENSG00000119938	PPP1R3C	protein phosphatase 1 regulatory subunit 3C	5507	0	NA	NA	NA	NA	NA
ENSG00000119946	CNNM1	cyclin and CBS domain divalent metal cation transport mediator 1	26507	0	NA	NA	NA	NA	NA
ENSG00000119973	PRLHR	prolactin releasing hormone receptor	2834	0	NA	NA	NA	NA	NA
ENSG00000119986	AVPI1	arginine vasopressin induced 1	60370	0,737899607	-1,990962997	2,683226	-0,742003579	0,458085167	NA
ENSG00000120049	KCNIP2	potassium voltage-gated channel interacting protein 2	30819	6,480572882	1,095852966	0,739013	1,482859536	0,138111734	NA
ENSG00000120051	CFAP58	cilia and flagella associated protein 58	159686	0,841782182	-0,124434247	2,432552	-0,051153793	0,959202971	NA
ENSG00000120054	CPN1	carboxypeptidase N subunit 1	1369	0	NA	NA	NA	NA	NA
ENSG00000120055	C10orf95	chromosome 10 open reading frame 95	79946	0,56334964	-1,047575398	2,551546	-0,410564963	0,681391559	NA
ENSG00000120057	SFRP5	secreted frizzled related protein 5	6425	0	NA	NA	NA	NA	NA
ENSG00000120068	HOXB8	homeobox B8	3218	0	NA	NA	NA	NA	NA
ENSG00000120075	HOXB5	homeobox B5	3215	0,294456692	0,695823494	3,897978	0,178508844	0,858323377	NA
ENSG00000120088	CRHR1	corticotropin releasing hormone receptor 1	1394	0	NA	NA	NA	NA	NA
ENSG00000120088	LINC02210-CRHR1	LINC02210-CRHR1 readthrough	104909134	0	NA	NA	NA	NA	NA
ENSG00000120094	HOXB1	homeobox B1	3211	0	NA	NA	NA	NA	NA
ENSG00000120149	MSX2	msh homeobox 2	4488	0	NA	NA	NA	NA	NA
ENSG00000120156	TEK	TEK receptor tyrosine kinase	7010	0,072165449	0,847101401	4,080456	0,207599697	0,835541541	NA
ENSG00000120160	EQTN	equatorin	54586	0	NA	NA	NA	NA	NA
ENSG00000120162	MOB3B	MOB kinase activator 3B	79817	0,207996306	-1,916073151	4,043021	-0,473921178	0,635556117	NA
ENSG00000120210	INSL6	insulin like 6	11172	0	NA	NA	NA	NA	NA
ENSG00000120211	INSL4	insulin like 4	3641	0	NA	NA	NA	NA	NA
ENSG00000120215	MLANA	melan-A	2315	0,117497108	0,847101401	4,080456	0,207599697	0,835541541	NA
ENSG00000120217	CD274	CD274 molecule	29126	7,189068742	0,656682781	0,657445	0,998840066	0,317872174	NA
ENSG00000120235	IFNA6	interferon alpha 6	3443	0	NA	NA	NA	NA	NA
ENSG00000120242	IFNA8	interferon alpha 8	3445	0	NA	NA	NA	NA	NA
ENSG00000120251	GRIA2	glutamate ionotropic receptor AMPA type subunit 2	2891	0	NA	NA	NA	NA	NA
ENSG00000120262	CCDC170	coiled-coil domain containing 170	80129	0	NA	NA	NA	NA	NA
ENSG00000120278	PLEKHG1	pleckstrin homology and RhoGEF domain containing G1	57480	0	NA	NA	NA	NA	NA
ENSG00000120279	MYCT1	MYC target 1	80177	0	NA	NA	NA	NA	NA
ENSG00000120289	MAGEB4	MAGE family member B4	4115	0	NA	NA	NA	NA	NA
ENSG00000120306	CYSTM1	cysteine rich transmembrane module containing 1	84418	8,954985376	0,149512826	0,615697	0,242834975	0,80813324	NA
ENSG00000120329	SLC25A2	solute carrier family 25 member 2	83884	1,878636962	1,369595905	1,581931	0,865775	0,386613562	NA
ENSG00000120332	TNN	tenascin N	63923	0	NA	NA	NA	NA	NA
ENSG00000120337	TNFSF18	TNF superfamily member 18	8995	0,309897119	2,360499491	3,771279	0,625914789	0,531370834	NA
ENSG00000120341	SEC16B	SEC16 homolog B, endoplasmic reticulum export factor	89866	0,072165449	0,847101401	4,080456	0,207599697	0,835541541	NA
ENSG00000120341	CRYZL2P-SEC16B	CRYZL2P-SEC16B readthrough	111240474	0,072165449	0,847101401	4,080456	0,207599697	0,835541541	NA
ENSG00000120370	GORAB	golgin, RAB6 interacting	92344	4,122272238	0,274702521	1,355133	0,202712547	0,839359715	NA
ENSG00000120436	GPR31	G protein-coupled receptor 31	2853	0	NA	NA	NA	NA	NA
ENSG00000120440	TTLL2	tubulin tyrosine ligase like 2	83887	0	NA	NA	NA	NA	NA
ENSG00000120457	KCNJ5	potassium inwardly rectifying channel subfamily J member 5	3762	0	NA	NA	NA	NA	NA

ENSG00000120471	TP53AIP1	tumor protein p53 regulated apoptosis inducing protein 1	63970	0	NA	NA	NA	NA	NA
ENSG00000120498	TEX11	testis expressed 11	56159	0	NA	NA	NA	NA	NA
ENSG00000120500	ARR3	arrestin 3	407	0	NA	NA	NA	NA	NA
ENSG00000120549	KIAA1217	KIAA1217	56243	0,234994216	1,944111989	4,02324	0,483220515	0,62893917	NA
ENSG00000120563	LYZL1	lysozyme like 1	84569	0	NA	NA	NA	NA	NA
ENSG00000120594	PLXDC2	plexin domain containing 2	84898	4,817642835	0,568900496	0,80183	0,709502505	0,478012698	NA
ENSG00000120645	IQSEC3	IQ motif and Sec7 domain ArfGEF 3	440073	0	NA	NA	NA	NA	NA
ENSG00000120658	ENOX1	ecto-NOX disulfide-thiol exchanger 1	55068	0,177336852	-0,124083225	4,0803	-0,03041032	0,975739814	NA
ENSG00000120659	TNFSF11	TNF superfamily member 11	8600	10,55233479	0,28307955	0,581168	0,487086993	0,626196683	NA
ENSG00000120669	SOHLH2	spermatogenesis and oogenesis specific basic helix-loop-helix 2	54937	0	NA	NA	NA	NA	NA
ENSG00000120693	SMAD9	SMAD family member 9	4093	0	NA	NA	NA	NA	NA
ENSG00000120708	TGFBI	transforming growth factor beta induced	7045	0	NA	NA	NA	NA	NA
ENSG00000120729	MYOT	myotilin	9499	0	NA	NA	NA	NA	NA
ENSG00000120756	PLS1	plastin 1	5357	3,715125537	-0,048280065	0,979516	-0,049289713	0,960688417	NA
ENSG00000120820	GLT8D2	glycosyltransferase 8 domain containing 2	83468	0,848565185	0,078622565	2,239349	0,035109556	0,971992382	NA
ENSG00000120885	CLU	clusterin	1191	1,346440764	-2,50177244	2,745507	-0,911224293	0,362177207	NA
ENSG00000120903	CHRNA2	cholinergic receptor nicotinic alpha 2 subunit	1135	0	NA	NA	NA	NA	NA
ENSG00000120907	ADRA1A	adrenoceptor alpha 1A	148	0	NA	NA	NA	NA	NA
ENSG00000120915	EPHX2	epoxide hydrolase 2	2053	0,235578703	-0,116320864	4,080456	-0,028506831	0,97725792	NA
ENSG00000120937	NPPB	natriuretic peptide B	4879	0	NA	NA	NA	NA	NA
ENSG00000121005	CRISPLD1	cysteine rich secretory protein LCCL domain containing 1	83690	9,889057496	0,896995556	0,62176	1,442671469	0,14911304	NA
ENSG00000121053	EPX	eosinophil peroxidase	8288	0	NA	NA	NA	NA	NA
ENSG00000121068	TBX2	T-box transcription factor 2	6909	0	NA	NA	NA	NA	NA
ENSG00000121075	TBX4	T-box transcription factor 4	9496	0	NA	NA	NA	NA	NA
ENSG00000121089	NACA3P	NACA family member 3, pseudogene	389240	0,35296819	1,940513194	4,02341	0,482305569	0,629588892	NA
ENSG00000121101	TEX14	testis expressed 14, intercellular bridge forming factor	56155	0,624865194	-0,181381649	2,482465	-0,073065138	0,941754283	NA
ENSG00000121207	LRAT	lecithin retinol acyltransferase	9227	0,190107389	1,667455255	4,047448	0,411976938	0,680356327	NA
ENSG00000121211	MND1	meiotic nuclear divisions 1	84057	7,808361395	0,847398523	0,659134	1,285624808	0,198574035	NA
ENSG00000121236	TRIM6	tripartite motif containing 6	117854	0,282291042	-0,127414214	3,989059	-0,031940918	0,974519167	NA
ENSG00000121270	ABCC11	ATP binding cassette subfamily C member 11	85320	0,118286652	0	4,080456	0	1	NA
ENSG00000121310	ECHDC2	enoyl-CoA hydratase domain containing 2	55268	5,75978223	-1,330646543	0,953253	-1,395900324	0,162744513	NA
ENSG00000121314	TAS2R8	taste 2 receptor member 8	50836	0	NA	NA	NA	NA	NA
ENSG00000121316	PLBD1	phospholipase B domain containing 1	79887	0	NA	NA	NA	NA	NA
ENSG00000121318	TAS2R10	taste 2 receptor member 10	50839	0	NA	NA	NA	NA	NA
ENSG00000121335	PRB2	proline rich protein BstNI subfamily 2	653247	0	NA	NA	NA	NA	NA
ENSG00000121351	IAPP	islet amyloid polypeptide	3375	0	NA	NA	NA	NA	NA
ENSG00000121361	KCNJ8	potassium inwardly rectifying channel subfamily J member 8	3764	0	NA	NA	NA	NA	NA
ENSG00000121381	TAS2R9	taste 2 receptor member 9	50835	0	NA	NA	NA	NA	NA
ENSG00000121388	NA	NA	NA	0	NA	NA	NA	NA	NA
ENSG00000121410	A1BG	alpha-1-B glycoprotein	1	0,47508864	0	4,080456	0	1	NA
ENSG00000121413	ZSCAN18	zinc finger and SCAN domain containing 18	65982	0,279627912	0	4,080456	0	1	NA
ENSG00000121440	PDZRN3	PDZ domain containing ring finger 3	23024	0	NA	NA	NA	NA	NA
ENSG00000121446	RGSL1	regulator of G protein signaling like 1	353299	0	NA	NA	NA	NA	NA
ENSG00000121454	LHX4	LIM homeobox 4	89884	10,2565114	0,247104013	0,59454	0,415622254	0,67768644	NA
ENSG00000121552	CSTA	cystatin A	1475	0	NA	NA	NA	NA	NA
ENSG00000121570	DPPA4	developmental pluripotency associated 4	55211	0	NA	NA	NA	NA	NA
ENSG00000121577	POPDC2	popeye domain containing 2	64091	7,167527445	0,250250819	0,842172	0,297149149	0,766352643	NA
ENSG00000121634	GJA8	gap junction protein alpha 8	2703	0	NA	NA	NA	NA	NA
ENSG00000121653	MAPK8IP1	mitogen-activated protein kinase 8 interacting protein 1	9479	5,289460509	-0,321856818	0,780887	-0,412168388	0,680216006	NA
ENSG00000121690	DEPDC7	DEP domain containing 7	91614	5,876640515	0,183448819	0,76413	0,240075536	0,810271699	NA
ENSG00000121742	GJB6	gap junction protein beta 6	10804	2,016840781	-0,209323797	1,454433	-0,143921217	0,885562681	NA
ENSG00000121743	GJA3	gap junction protein alpha 3	2700	0	NA	NA	NA	NA	NA
ENSG00000121753	ADGRB2	adhesion G protein-coupled receptor B2	576	1,012715269	-3,394378863	2,977377	-1,140056927	0,254262585	NA
ENSG00000121764	HCRTR1	hypocretin receptor 1	3061	0	NA	NA	NA	NA	NA

ENSG00000121769	FABP3	fatty acid binding protein 3	2170	0	NA	NA	NA	NA	NA
ENSG00000121853	GHSR	growth hormone secretagogue receptor	2693	0,992095236	-0,639853775	1,727281	-0,370439782	0,711054837	NA
ENSG00000121871	SLITRK3	SLIT and NTRK like family member 3	22865	0,24825249	0,454533991	4,061228	0,11192033	0,910886577	NA
ENSG00000121898	CPXM2	carboxypeptidase X, M14 family member 2	119587	0	NA	NA	NA	NA	NA
ENSG00000121900	TMEM54	transmembrane protein 54	113452	0	NA	NA	NA	NA	NA
ENSG00000121903	ZSCAN20	zinc finger and SCAN domain containing 20	7579	6,199725187	-0,023859149	0,868458	-0,027473002	0,978082473	NA
ENSG00000121904	CSMD2	CUB and Sushi multiple domains 2	114784	0	NA	NA	NA	NA	NA
ENSG00000121905	HPCA	hippocalcin	3208	0	NA	NA	NA	NA	NA
ENSG00000121933	TMIGD3	transmembrane and immunoglobulin domain containing 3	57413	0	NA	NA	NA	NA	NA
ENSG00000121988	ZRANB3	zinc finger RANBP2-type containing 3	84083	10,91228501	0,149901835	0,605132	0,247717539	0,80435296	NA
ENSG00000122012	SV2C	synaptic vesicle glycoprotein 2C	22987	0,514730807	1,919919993	3,373505	0,569117258	0,569276567	NA
ENSG00000122025	FLT3	fms related receptor tyrosine kinase 3	2322	0	NA	NA	NA	NA	NA
ENSG00000122035	RASL11A	RAS like family 11 member A	387496	1,082075506	1,370588126	2,237846	0,612458548	0,540234416	NA
ENSG00000122121	XPNPEP2	X-prolyl aminopeptidase 2	7512	1,658070011	0,874940386	1,559814	0,560926096	0,574847919	NA
ENSG00000122126	OCRL	OCRL inositol polyphosphate-5-phosphatase	4952	0,072165449	0,847101401	4,080456	0,207599697	0,835541541	NA
ENSG00000122133	PAEP	progestagen associated endometrial protein	5047	0	NA	NA	NA	NA	NA
ENSG00000122136	OBP2A	odorant binding protein 2A	29991	0	NA	NA	NA	NA	NA
ENSG00000122145	TBX22	T-box transcription factor 22	50945	0	NA	NA	NA	NA	NA
ENSG00000122176	FMOD	fibromodulin	2331	0,118286652	0	4,080456	0	1	NA
ENSG00000122180	MYOG	myogenin	4656	0	NA	NA	NA	NA	NA
ENSG00000122194	PLG	plasminogen	5340	0,118286652	0	4,080456	0	1	NA
ENSG00000122254	HS3ST2	heparan sulfate-glucosamine 3-sulfotransferase 2	9956	0	NA	NA	NA	NA	NA
ENSG00000122304	PRM2	protamine 2	5620	0	NA	NA	NA	NA	NA
ENSG00000122367	LDB3	LIM domain binding 3	11155	0,537804961	-2,558611261	2,807276	-0,911421297	0,362073437	NA
ENSG00000122375	OPN4	opsin 4	94233	0	NA	NA	NA	NA	NA
ENSG00000122378	PRXL2A	peroxiredoxin like 2A	84293	3,073450997	1,719282134	1,222851	1,405962653	0,159735215	NA
ENSG00000122386	ZNF205	zinc finger protein 205	7755	0	NA	NA	NA	NA	NA
ENSG00000122420	PTGFR	prostaglandin F receptor	5737	0	NA	NA	NA	NA	NA
ENSG00000122432	SPATA1	spermatogenesis associated 1	100505741	0,499464131	0,527862065	3,958789	0,13333928	0,893925064	NA
ENSG00000122477	LRRC39	leucine rich repeat containing 39	127495	3,185971818	0,341679165	1,212273	0,281850039	0,7780585	NA
ENSG00000122543	OCM	oncomodulin	654231	0,436905265	0,359992761	3,523432	0,102171042	0,918620913	NA
ENSG00000122547	EEPD1	endonuclease/exonuclease/phosphatase family domain containing 1	80820	0,293519253	0,704171709	3,901812	0,180473011	0,85678124	NA
ENSG00000122574	WIPF3	WAS/WASL interacting protein family member 3	644150	0,654528845	-1,563604237	2,427132	-0,644218941	0,51943347	NA
ENSG00000122584	NXPH1	neurexophilin 1	30010	0	NA	NA	NA	NA	NA
ENSG00000122585	NPY	neuropeptide Y	4852	0	NA	NA	NA	NA	NA
ENSG00000122592	HOXA7	homeobox A7	3204	1,997361714	0,271708578	1,620987	0,167619189	0,866882874	NA
ENSG00000122641	INHBA	inhibin subunit beta A	3624	0,072165449	0,847101401	4,080456	0,207599697	0,835541541	NA
ENSG00000122642	FKBP9	FKBP prolyl isomerase 9	11328	8,406309279	-0,222811582	0,649398	-0,343104874	0,731519564	NA
ENSG00000122679	RAMP3	receptor activity modifying protein 3	10268	0	NA	NA	NA	NA	NA
ENSG00000122691	TWIST1	twist family bHLH transcription factor 1	7291	0,103998153	-1,076443944	4,080456	-0,263804824	0,791930317	NA
ENSG00000122711	SPINK4	serine peptidase inhibitor Kazal type 4	27290	0	NA	NA	NA	NA	NA
ENSG00000122728	TAF1L	TATA-box binding protein associated factor 1 like	138474	0	NA	NA	NA	NA	NA
ENSG00000122733	PHF24	PHD finger protein 24	23349	0	NA	NA	NA	NA	NA
ENSG00000122735	DNAI1	dynein axonemal intermediate chain 1	27019	0	NA	NA	NA	NA	NA
ENSG00000122756	CNTFR	ciliary neurotrophic factor receptor	1271	0	NA	NA	NA	NA	NA
ENSG00000122778	KIAA1549	KIAA1549	57670	1,693740868	0,555723039	1,526698	0,364003345	0,715855512	NA
ENSG00000122786	CALD1	caldesmon 1	800	0,685005818	-1,595703375	2,772171	-0,575615021	0,564875426	NA
ENSG00000122787	AKR1D1	aldo-keto reductase family 1 member D1	6718	0	NA	NA	NA	NA	NA
ENSG00000122824	NUDT10	nudix hydrolase 10	170685	0,117497108	0,847101401	4,080456	0,207599697	0,835541541	NA
ENSG00000122852	SFTPA1	surfactant protein A1	653509	0	NA	NA	NA	NA	NA
ENSG00000122859	NEUROG3	neurogenin 3	50674	0	NA	NA	NA	NA	NA
ENSG00000122861	PLAU	plasminogen activator, urokinase	5328	8,099316555	0,391376263	0,659747	0,593221651	0,553032825	NA
ENSG00000122863	CHST3	carbohydrate sulfotransferase 3	9469	0	NA	NA	NA	NA	NA

ENSG00000122870	BICC1	BicC family RNA binding protein 1	80114	0,384333101	-0,939378357	3,673306	-0,255731042	0,798158524	NA
ENSG00000122872	NA	NA	NA	0	NA	NA	NA	NA	NA
ENSG00000122970	IFT81	intraflagellar transport 81	28981	0,352491325	2,536807152	3,984503	0,636668335	0,524340903	NA
ENSG00000123009	NA	NA	NA	0	NA	NA	NA	NA	NA
ENSG00000123095	BHLHE41	basic helix-loop-helix family member e41	79365	0	NA	NA	NA	NA	NA
ENSG00000123096	SSPN	sarcospan	8082	0	NA	NA	NA	NA	NA
ENSG00000123119	NECAB1	N-terminal EF-hand calcium binding protein 1	64168	0	NA	NA	NA	NA	NA
ENSG00000123165	ACTRT1	actin related protein T1	139741	0	NA	NA	NA	NA	NA
ENSG00000123171	CCDC70	coiled-coil domain containing 70	83446	0	NA	NA	NA	NA	NA
ENSG00000123191	ATP7B	ATPase copper transporting beta	540	1,302977914	0,08739704	2,207313	0,039594306	0,968416567	NA
ENSG00000123201	GUCY1B2	guanylate cyclase 1 soluble subunit beta 2 (pseudogene)	2974	0,992408719	-0,644575633	1,794087	-0,359277727	0,719387335	NA
ENSG00000123213	NLN	neurolysin	57486	8,544667945	0,158773392	0,711567	0,223132169	0,823432642	NA
ENSG00000123243	ITIH5	inter-alpha-trypsin inhibitor heavy chain 5	80760	0	NA	NA	NA	NA	NA
ENSG00000123307	NEUROD4	neuronal differentiation 4	58158	0	NA	NA	NA	NA	NA
ENSG00000123342	MMP19	matrix metallopeptidase 19	4327	0	NA	NA	NA	NA	NA
ENSG00000123364	HOXC13	homeobox C13	3229	0	NA	NA	NA	NA	NA
ENSG00000123384	LRP1	LDL receptor related protein 1	4035	1,694727044	0,251937598	1,563215	0,161166276	0,871962441	NA
ENSG00000123388	HOXC11	homeobox C11	3227	0	NA	NA	NA	NA	NA
ENSG00000123405	NFE2	nuclear factor, erythroid 2	4778	0,10543123	0	4,080456	0	1	NA
ENSG00000123407	HOXC12	homeobox C12	3228	0	NA	NA	NA	NA	NA
ENSG00000123427	EEF1AKMT3	EEF1A lysine methyltransferase 3	25895	5,910907775	0,502039237	0,797053	0,629869437	0,528780011	NA
ENSG00000123447	NA	NA	NA	0	NA	NA	NA	NA	NA
ENSG00000123454	DBH	dopamine beta-hydroxylase	1621	0	NA	NA	NA	NA	NA
ENSG00000123496	IL13RA2	interleukin 13 receptor subunit alpha 2	3598	0	NA	NA	NA	NA	NA
ENSG00000123500	COL10A1	collagen type X alpha 1 chain	1300	0	NA	NA	NA	NA	NA
ENSG00000123560	PLP1	proteolipid protein 1	5354	0	NA	NA	NA	NA	NA
ENSG00000123561	SERPINA7	serpin family A member 7	6906	0	NA	NA	NA	NA	NA
ENSG00000123569	H2BW1	H2B,W histone 1	158983	0	NA	NA	NA	NA	NA
ENSG00000123570	RAB9B	RAB9B, member RAS oncogene family	51209	1,641550087	-1,184162179	1,50767	-0,785425517	0,432204125	NA
ENSG00000123572	NRK	Nik related kinase	203447	0	NA	NA	NA	NA	NA
ENSG00000123576	ESX1	ESX homeobox 1	80712	0	NA	NA	NA	NA	NA
ENSG00000123584	MAGEA9	MAGE family member A9	4108	0	NA	NA	NA	NA	NA
ENSG00000123594	ATXN3L	ataxin 3 like	92552	0	NA	NA	NA	NA	NA
ENSG00000123610	TNFAIP6	TNF alpha induced protein 6	7130	0	NA	NA	NA	NA	NA
ENSG00000123612	ACVR1C	activin A receptor type 1C	130399	0,920057176	-0,311904951	2,312098	-0,134901292	0,892689918	NA
ENSG00000123685	BATF3	basic leucine zipper ATF-like transcription factor 3	55509	6,673067261	-1,48727614	0,840103	-1,770348725	0,076669066	NA
ENSG00000123689	G0S2	G0/G1 switch 2	50486	0,222845427	-2,002032575	4,035234	-0,496137927	0,619797102	NA
ENSG00000123700	KCNJ2	potassium inwardly rectifying channel subfamily J member 2	3759	0	NA	NA	NA	NA	NA
ENSG00000123810	B9D2	B9 domain containing 2	80776	4,875362813	-0,96305135	1,009314	-0,954164179	0,340000544	NA
ENSG00000123838	C4BPA	complement component 4 binding protein alpha	722	0	NA	NA	NA	NA	NA
ENSG00000123843	C4BPB	complement component 4 binding protein beta	725	0,223551938	0	4,080456	0	1	NA
ENSG00000123901	GPR83	G protein-coupled receptor 83	10888	0,919231046	-1,629623729	2,3032	-0,707547694	0,479226185	NA
ENSG00000123965	NA	NA	NA	1,7822692	2,597752565	1,718846	1,511334975	0,130703127	NA
ENSG00000123977	DAW1	dynein assembly factor with WD repeats 1	164781	0	NA	NA	NA	NA	NA
ENSG00000123999	INHA	inhibin subunit alpha	3623	0	NA	NA	NA	NA	NA
ENSG00000124003	MOGAT1	monoacylglycerol O-acyltransferase 1	116255	0	NA	NA	NA	NA	NA
ENSG00000124006	OBSL1	obscurin like cytoskeletal adaptor 1	23363	0,278758929	0,838650104	4,080456	0,205528532	0,837159209	NA
ENSG00000124019	FAM124B	family with sequence similarity 124 member B	79843	5,36955845	-0,015419419	0,7998	-0,019279097	0,984618459	NA
ENSG00000124074	ENKD1	enkurin domain containing 1	84080	4,880234864	-0,038290904	1,000982	-0,038253349	0,969485686	NA
ENSG00000124089	MC3R	melanocortin 3 receptor	4159	0	NA	NA	NA	NA	NA
ENSG00000124091	GCNT7	glucosaminyl (N-acetyl) transferase family member 7	140687	0,10543123	0	4,080456	0	1	NA
ENSG00000124092	CTCFL	CCCTC-binding factor like	140690	0	NA	NA	NA	NA	NA
ENSG00000124097	NA	NA	NA	0,11808024	-1,076443944	4,080456	-0,263804824	0,791930317	NA
ENSG00000124102	PI3	peptidase inhibitor 3	5266	0	NA	NA	NA	NA	NA
ENSG00000124103	FAM209A	family with sequence similarity 209 member A	200232	4,281283571	-0,679610016	1,148561	-0,591705674	0,554047696	NA
ENSG00000124104	SNX21	sorting nexin family member 21	90203	2,698479223	1,391278308	1,67587	0,830182909	0,406435377	NA

ENSG00000124107	SLPI	secretory leukocyte peptidase inhibitor	6590	0	NA	NA	NA	NA	NA
ENSG00000124116	WFDC3	WAP four-disulfide core domain 3	140686	0	NA	NA	NA	NA	NA
ENSG00000124134	KCNS1	potassium voltage-gated channel modifier subfamily S member 1	3787	0	NA	NA	NA	NA	NA
ENSG00000124140	SLC12A5	solute carrier family 12 member 5	57468	1,954954696	1,526486186	1,66507	0,916769704	0,359263331	NA
ENSG00000124143	ARHGAP40	Rho GTPase activating protein 40	343578	0	NA	NA	NA	NA	NA
ENSG00000124157	SEMG2	semenogelin 2	6407	0	NA	NA	NA	NA	NA
ENSG00000124159	MATN4	matrilin 4	8785	0	NA	NA	NA	NA	NA
ENSG00000124191	TOX2	TOX high mobility group box family member 2	84969	7,19876305	-1,069182746	1,188327	-0,899738011	0,36825969	NA
ENSG00000124194	GDAP1L1	ganglioside induced differentiation associated protein 1 like 1	78997	0	NA	NA	NA	NA	NA
ENSG00000124196	GTSF1L	gametocyte specific factor 1 like	149699	0	NA	NA	NA	NA	NA
ENSG00000124205	EDN3	endothelin 3	1908	0,209763681	-1,926644696	4,042043	-0,476651224	0,633610501	NA
ENSG00000124208	PEDS1-UBE2V1	PEDS1-UBE2V1 readthrough	387522	2,943753794	-0,380394592	3,789569	-0,100379388	0,920043133	NA
ENSG00000124215	CDH26	cadherin 26	60437	2,964469982	-0,589762491	1,255055	-0,469909766	0,638419487	NA
ENSG00000124216	SNAI1	snail family transcriptional repressor 1	6615	2,501827258	2,151047688	1,342476	1,602298267	0,109089669	NA
ENSG00000124225	PMEPA1	prostate transmembrane protein, androgen induced 1	56937	1,163226458	1,253874339	2,186953	0,573343156	0,566412371	NA
ENSG00000124227	ANKRD60	ankyrin repeat domain 60	140731	0	NA	NA	NA	NA	NA
ENSG00000124232	RBPJL	recombination signal binding protein for immunoglobulin kappa J region like	11317	0	NA	NA	NA	NA	NA
ENSG00000124233	SEMG1	semenogelin 1	6406	0	NA	NA	NA	NA	NA
ENSG00000124237	CIMIP1	ciliary microtubule inner protein 1	128602	0	NA	NA	NA	NA	NA
ENSG00000124249	KCNK15	potassium two pore domain channel subfamily K member 15	60598	0	NA	NA	NA	NA	NA
ENSG00000124251	TP53TG5	TP53 target 5	27296	0	NA	NA	NA	NA	NA
ENSG00000124253	PCK1	phosphoenolpyruvate carboxykinase 1	5105	0	NA	NA	NA	NA	NA
ENSG00000124257	NEURL2	neuralized E3 ubiquitin protein ligase 2	140825	2,431806088	-1,951326371	1,535441	-1,270857634	0,203779301	NA
ENSG00000124260	MAGEA10	MAGE family member A10	4109	0	NA	NA	NA	NA	NA
ENSG00000124302	CHST8	carbohydrate sulfotransferase 8	64377	0	NA	NA	NA	NA	NA
ENSG00000124313	IQSEC2	IQ motif and Sec7 domain ArfGEF 2	23096	7,73892602	0,900190022	0,694926	1,295374647	0,195191019	NA
ENSG00000124343	XG	Xg glycoprotein (Xg blood group)	7499	0,683418617	-2,839850161	2,50938	-1,13169387	0,257763159	NA
ENSG00000124370	MCEE	methylmalonyl-CoA epimerase	84693	10,75145476	-0,305063436	0,59716	-0,51085753	0,609450822	NA
ENSG00000124374	PAIP2B	poly(A) binding protein interacting protein 2B	400961	6,911965799	-0,591895854	0,762019	-0,776746898	0,437308112	NA
ENSG00000124391	IL17C	interleukin 17C	27189	0,714793466	-0,596116706	3,177157	-0,187625844	0,851169965	NA
ENSG00000124399	NDUFB4P12	NADH:ubiquinone oxidoreductase subunit B4 pseudogene 12	402175	0	NA	NA	NA	NA	NA
ENSG00000124429	POF1B	POF1B actin binding protein	79983	0	NA	NA	NA	NA	NA
ENSG00000124440	HIF3A	hypoxia inducible factor 3 subunit alpha	64344	0,11808024	-1,076443944	4,080456	-0,263804824	0,791930317	NA
ENSG00000124449	IRGC	immunity related GTPase cinema	56269	0	NA	NA	NA	NA	NA
ENSG00000124466	LYPD3	LY6/PLAUR domain containing 3	27076	0,208890305	-1,921416331	4,042529	-0,475300592	0,634572737	NA
ENSG00000124467	PSG8	pregnancy specific beta-1-glycoprotein 8	440533	0	NA	NA	NA	NA	NA
ENSG00000124469	CEACAM8	CEA cell adhesion molecule 8	1088	0	NA	NA	NA	NA	NA
ENSG00000124479	NDP	norrin cystine knot growth factor NDP	4693	0	NA	NA	NA	NA	NA
ENSG00000124490	CRISP2	cysteine rich secretory protein 2	7180	0	NA	NA	NA	NA	NA
ENSG00000124491	F13A1	coagulation factor XIII A chain	2162	0	NA	NA	NA	NA	NA
ENSG00000124493	GRM4	glutamate metabotropic receptor 4	2914	0	NA	NA	NA	NA	NA
ENSG00000124507	PACSIN1	protein kinase C and casein kinase substrate in neurons 1	29993	6,895771864	-2,624182611	0,987427	-2,657596135	0,007870014	NA
ENSG00000124549	NA	NA	NA	3,693707497	0,169793619	0,996823	0,170334797	0,864746847	NA

ENSG00000124564	SLC17A3	solute carrier family 17 member 3	10786	0	NA	NA	NA	NA	NA
ENSG00000124568	SLC17A1	solute carrier family 17 member 1	6568	0	NA	NA	NA	NA	NA
ENSG00000124575	H1-3	H1,3 linker histone, cluster member	3007	2,857783774	1,946101852	1,367335	1,423281402	0,154654596	NA
ENSG00000124593	NA	NA	NA	6,356857329	-0,214054234	1,395875	-0,153347708	0,87812408	NA
ENSG00000124602	UNC5CL	unc-5 family C-terminal like	222643	3,862709506	1,946732085	1,483416	1,312330334	0,189408701	NA
ENSG00000124610	H1-1	H1,1 linker histone, cluster member	3024	0	NA	NA	NA	NA	NA
ENSG00000124613	ZNF391	zinc finger protein 391	346157	1,036186078	3,455461855	2,136961	1,616998147	0,105878681	NA
ENSG00000124615	MOCS1	molybdenum cofactor synthesis 1	4337	0	NA	NA	NA	NA	NA
ENSG00000124635	H2BC11	H2B clustered histone 11	8970	1,832602463	-0,369261513	1,219366	-0,302830758	0,762018842	NA
ENSG00000124657	OR2B6	olfactory receptor family 2 subfamily B member 6	26212	0,560451095	-1,069490749	3,371135	-0,317249423	0,751054351	NA
ENSG00000124664	SPDEF	SAM pointed domain containing ETS transcription factor	25803	0,343057078	1,0018143	4,021536	0,249112327	0,803273894	NA
ENSG00000124678	TCP11	t-complex 11	6954	0	NA	NA	NA	NA	NA
ENSG00000124701	APOBEC2	apolipoprotein B mRNA editing enzyme catalytic subunit 2	10930	0	NA	NA	NA	NA	NA
ENSG00000124713	GNMT	glycine N-methyltransferase	27232	2,870883941	0,072131918	1,416722	0,050914669	0,959393517	NA
ENSG00000124713	CNPY3-GNMT	CNPY3-GNMT readthrough	107080644	2,870883941	0,072131918	1,416722	0,050914669	0,959393517	NA
ENSG00000124721	DNAH8	dynein axonemal heavy chain 8	1769	4,013951004	-1,065772835	1,185215	-0,899223267	0,36853375	NA
ENSG00000124731	TREM1	triggering receptor expressed on myeloid cells 1	54210	0,191703879	1,677831184	4,046267	0,414661554	0,678389681	NA
ENSG00000124743	KLHL31	kelch like family member 31	401265	2,604934735	0,120891147	1,188101	0,101751554	0,91895388	NA
ENSG00000124749	COL21A1	collagen type XXI alpha 1 chain	81578	0	NA	NA	NA	NA	NA
ENSG00000124766	SOX4	SRY-box transcription factor 4	6659	7,502495285	0,34048409	0,674405	0,504866198	0,613652817	NA
ENSG00000124772	CPNE5	copine 5	57699	2,305890995	-0,679961531	1,352818	-0,502625976	0,615227263	NA
ENSG00000124780	KCNK17	potassium two pore domain channel subfamily K member 17	89822	0,119673877	0,847101401	4,080456	0,207599697	0,835541541	NA
ENSG00000124785	NRN1	neuritin 1	51299	0,937628064	2,587534324	2,968275	0,871729907	0,383355741	NA
ENSG00000124787	RPP40	ribonuclease P/MRP subunit p40	10799	7,732965122	-0,318720903	0,851979	-0,374094795	0,708333791	NA
ENSG00000124812	CRISP1	cysteine rich secretory protein 1	167	0	NA	NA	NA	NA	NA
ENSG00000124818	OPN5	opsin 5	221391	0	NA	NA	NA	NA	NA
ENSG00000124827	GCM2	glial cells missing transcription factor 2	9247	0	NA	NA	NA	NA	NA
ENSG00000124839	RAB17	RAB17, member RAS oncogene family	64284	0	NA	NA	NA	NA	NA
ENSG00000124875	CXCL6	C-X-C motif chemokine ligand 6	6372	0	NA	NA	NA	NA	NA
ENSG00000124882	EREG	epiregulin	2069	0,072165449	0,847101401	4,080456	0,207599697	0,835541541	NA
ENSG00000124900	TRIM51	tripartite motif-containing 51	84767	0	NA	NA	NA	NA	NA
ENSG00000124920	MYRF	myelin regulatory factor	745	0	NA	NA	NA	NA	NA
ENSG00000124935	SCGB1D2	secretoglobin family 1D member 2	10647	0	NA	NA	NA	NA	NA
ENSG00000124939	SCGB2A1	secretoglobin family 2A member 1	4246	0	NA	NA	NA	NA	NA
ENSG00000125046	SSUH2	ssu-2 homolog	51066	0	NA	NA	NA	NA	NA
ENSG00000125084	WNT1	Wnt family member 1	7471	1,305040298	-0,036173535	1,452671	-0,024901389	0,980133619	NA
ENSG00000125122	FBXL9P	F-box and leucine rich repeat protein, pseudogene	26231	2,033112666	0,741454511	1,360643	0,544929477	0,585802022	NA
ENSG00000125124	BBS2	Bardet-Biedl syndrome 2	583	0	NA	NA	NA	NA	NA
ENSG00000125144	MT1G	metallothionein 1G	4495	0	NA	NA	NA	NA	NA
ENSG00000125170	DOK4	docking protein 4	55715	7,547028972	-0,015426927	0,650347	-0,023721065	0,981075103	NA
ENSG00000125207	PIWIL1	piwi like RNA-mediated gene silencing 1	9271	0,471122206	-1,910455021	3,487615	-0,547782611	0,58384118	NA
ENSG00000125246	CLYBL	citramalyl-CoA lyase	171425	8,674048892	-1,186206963	0,773204	-1,534144445	0,124994121	NA
ENSG00000125255	SLC10A2	solute carrier family 10 member 2	6555	0	NA	NA	NA	NA	NA
ENSG00000125266	EFNB2	ephrin B2	1948	1,572736511	-4,090528238	2,621499	-1,560377659	0,118670661	NA
ENSG00000125285	SOX21	SRY-box transcription factor 21	11166	0	NA	NA	NA	NA	NA
ENSG00000125319	HROB	homologous recombination factor with OB-fold	78995	9,130047617	0,445153556	0,592006	0,751941398	0,452086301	NA
ENSG00000125337	KIF25	kinesin family member 25	3834	0	NA	NA	NA	NA	NA
ENSG00000125355	TMEM255A	transmembrane protein 255A	55026	0	NA	NA	NA	NA	NA
ENSG00000125363	AMELX	amelogenin X-linked	265	0	NA	NA	NA	NA	NA
ENSG00000125378	BMP4	bone morphogenetic protein 4	652	0	NA	NA	NA	NA	NA
ENSG00000125385	NA	NA	NA	0	NA	NA	NA	NA	NA

ENSG00000125398	SOX9	SRY-box transcription factor 9	6662	0	NA	NA	NA	NA	NA
ENSG00000125409	TEKT3	tektin 3	64518	0	NA	NA	NA	NA	NA
ENSG00000125414	MYH2	myosin heavy chain 2	4620	0	NA	NA	NA	NA	NA
ENSG00000125492	BARHL1	BarH like homeobox 1	56751	0	NA	NA	NA	NA	NA
ENSG00000125498	KIR2DL1	killer cell immunoglobulin like receptor, two Ig domains and long cytoplasmic tail 1	3802	0	NA	NA	NA	NA	NA
ENSG00000125508	SRMS	src-related kinase lacking C-terminal regulatory tyrosine and N-terminal myristylation sites	6725	0	NA	NA	NA	NA	NA
ENSG00000125510	OPRL1	opioid related nociceptin receptor 1	4987	1,646973241	2,018133059	1,664385	1,212539968	0,225305753	NA
ENSG00000125522	NPBWR2	neuropeptides B and W receptor 2	2832	0	NA	NA	NA	NA	NA
ENSG00000125531	FNDC11	fibronectin type III domain containing 11	79025	1,691515084	-1,108339721	1,45353	-0,76251564	0,445752312	NA
ENSG00000125533	BHLHE23	basic helix-loop-helix family member e23	128408	0	NA	NA	NA	NA	NA
ENSG00000125538	IL1B	interleukin 1 beta	3553	0	NA	NA	NA	NA	NA
ENSG00000125551	PLGLB2	plasminogen like B2	5342	6,959442082	-0,028944963	1,014649	-0,028527069	0,977241779	NA
ENSG00000125571	IL37	interleukin 37	27178	0,990551958	-0,364809402	2,550983	-0,143007396	0,88628434	NA
ENSG00000125631	HTR5BP	5-hydroxytryptamine receptor 5B, pseudogene	645694	0	NA	NA	NA	NA	NA
ENSG00000125650	PSPN	persephin	5623	8,059439104	0,159454879	0,731659	0,217936071	0,827478922	NA
ENSG00000125675	GRIA3	glutamate ionotropic receptor AMPA type subunit 3	2892	0	NA	NA	NA	NA	NA
ENSG00000125695	NA	NA	NA	0	NA	NA	NA	NA	NA
ENSG00000125744	RTN2	reticulon 2	6253	3,568373428	0,269782812	1,193812	0,225984266	0,821213658	NA
ENSG00000125775	SDCBP2	syndecan binding protein 2	27111	3,311280286	-0,237139688	0,991063	-0,23927822	0,810889853	NA
ENSG00000125780	TGM3	transglutaminase 3	7053	0	NA	NA	NA	NA	NA
ENSG00000125787	GNRH2	gonadotropin releasing hormone 2	2797	1,460554215	1,225396958	1,708891	0,717071547	0,473329951	NA
ENSG00000125788	DEFB126	defensin beta 126	81623	0	NA	NA	NA	NA	NA
ENSG00000125798	FOXA2	forkhead box A2	3170	0	NA	NA	NA	NA	NA
ENSG00000125813	PAX1	paired box 1	5075	0	NA	NA	NA	NA	NA
ENSG00000125815	CST8	cystatin 8	10047	0	NA	NA	NA	NA	NA
ENSG00000125816	NKX2-4	NK2 homeobox 4	644524	0	NA	NA	NA	NA	NA
ENSG00000125820	NKX2-2	NK2 homeobox 2	4821	0,103998153	-1,076443944	4,080456	-0,263804824	0,791930317	NA
ENSG00000125823	CSTL1	cystatin like 1	128817	0	NA	NA	NA	NA	NA
ENSG00000125831	CST11	cystatin 11	140880	0	NA	NA	NA	NA	NA
ENSG00000125841	NRSN2	neurensin 2	80023	8,04395599	-0,722610391	0,688693	-1,049248444	0,294063788	NA
ENSG00000125845	BMP2	bone morphogenetic protein 2	650	2,858750784	1,937004576	1,286044	1,50617292	0,132022789	NA
ENSG00000125848	FLRT3	fibronectin leucine rich transmembrane protein 3	23767	0	NA	NA	NA	NA	NA
ENSG00000125850	OVOL2	ovo like zinc finger 2	58495	0	NA	NA	NA	NA	NA
ENSG00000125851	PCSK2	proprotein convertase subtilisin/kexin type 2	5126	0	NA	NA	NA	NA	NA
ENSG00000125861	GFRA4	GDNF family receptor alpha 4	64096	0	NA	NA	NA	NA	NA
ENSG00000125864	BFSP1	beaded filament structural protein 1	631	8,158924046	-0,704775997	0,688388	-1,023806188	0,305926827	NA
ENSG00000125869	LAMP5	lysosomal associated membrane protein family member 5	24141	0	NA	NA	NA	NA	NA
ENSG00000125872	LRRN4	leucine rich repeat neuronal 4	164312	0	NA	NA	NA	NA	NA
ENSG00000125878	TCF15	transcription factor 15	6939	0,11808024	-1,076443944	4,080456	-0,263804824	0,791930317	NA
ENSG00000125879	OTOR	otoraplin	56914	0	NA	NA	NA	NA	NA
ENSG00000125888	BANF2	BANF family member 2	140836	0	NA	NA	NA	NA	NA
ENSG00000125895	TMEM74B	transmembrane protein 74B	55321	0,328413044	-0,079504081	3,827612	-0,020771195	0,983428176	NA
ENSG00000125900	SIRPD	signal regulatory protein delta	128646	0	NA	NA	NA	NA	NA
ENSG00000125903	DEFB129	defensin beta 129	140881	0	NA	NA	NA	NA	NA
ENSG00000125931	CITED1	Cbp/p300 interacting transactivator with Glu/Asp rich carboxy-terminal domain 1	4435	0	NA	NA	NA	NA	NA
ENSG00000125954	CHURC1-FNTB	CHURC1-FNTB readthrough	100529261	0	NA	NA	NA	NA	NA
ENSG00000125965	GDF5	growth differentiation factor 5	8200	0	NA	NA	NA	NA	NA
ENSG00000125966	MMP24	matrix metallopeptidase 24	10893	8,761220292	-0,012051512	0,625168	-0,019277227	0,984619951	NA
ENSG00000125968	ID1	inhibitor of DNA binding 1	3397	0,117497108	0,847101401	4,080456	0,207599697	0,835541541	NA
ENSG00000125975	C20orf173	chromosome 20 open reading frame 173	140873	0	NA	NA	NA	NA	NA
ENSG00000125997	BPIFB9P	BPI fold containing family B member 9, pseudogene	402016	0	NA	NA	NA	NA	NA

ENSG00000125998	FAM83C	family with sequence similarity 83 member C	128876	0	NA	NA	NA	NA	NA
ENSG00000125999	BPIFB1	BPI fold containing family B member 1	92747	0	NA	NA	NA	NA	NA
ENSG00000126010	GRPR	gastrin releasing peptide receptor	2925	0,939517234	2,320109802	2,256751	1,028075441	0,303914342	NA
ENSG00000126091	ST3GAL3	ST3 beta-galactoside alpha-2,3-sialyltransferase 3	6487	11,31066123	0,119904299	0,742462	0,161495554	0,871703113	NA
ENSG00000126217	MCF2L	MCF,2 cell line derived transforming sequence like	23263	5,813589412	-0,023058721	0,797601	-0,028910081	0,976936305	NA
ENSG00000126218	F10	coagulation factor X	2159	0	NA	NA	NA	NA	NA
ENSG00000126233	SLURP1	secreted LY6/PLAUR domain containing 1	57152	0	NA	NA	NA	NA	NA
ENSG00000126251	GPR42	G protein-coupled receptor 42	2866	0	NA	NA	NA	NA	NA
ENSG00000126259	KIRREL2	kirre like nephrin family adhesion molecule 2	84063	0,346225899	0,910306656	3,759533	0,242132915	0,808677175	NA
ENSG00000126262	FFAR2	free fatty acid receptor 2	2867	0	NA	NA	NA	NA	NA
ENSG00000126266	FFAR1	free fatty acid receptor 1	2864	0	NA	NA	NA	NA	NA
ENSG00000126337	KRT36	keratin 36	8689	0	NA	NA	NA	NA	NA
ENSG00000126353	CCR7	C-C motif chemokine receptor 7	1236	1,466467436	0,771686808	1,7842	0,432511345	0,665369808	NA
ENSG00000126460	PRRG2	proline rich and Gla domain 2	5639	0,815646679	-3,274447456	2,264463	-1,446015174	0,148172946	NA
ENSG00000126467	TSKS	testis specific serine kinase substrate	60385	0	NA	NA	NA	NA	NA
ENSG00000126500	FLRT1	fibronectin leucine rich transmembrane protein 1	23769	0,10488184	-1,076443944	4,080456	-0,263804824	0,791930317	NA
ENSG00000126545	CSN1S1	casein alpha s1	1446	0	NA	NA	NA	NA	NA
ENSG00000126549	STATH	statherin	6779	0	NA	NA	NA	NA	NA
ENSG00000126550	HTN1	histatin 1	3346	0	NA	NA	NA	NA	NA
ENSG00000126562	WNK4	WNK lysine deficient protein kinase 4	65266	0	NA	NA	NA	NA	NA
ENSG00000126583	PRKCG	protein kinase C gamma	5582	0	NA	NA	NA	NA	NA
ENSG00000126603	GLIS2	GLIS family zinc finger 2	84662	0,103998153	-1,076443944	4,080456	-0,263804824	0,791930317	NA
ENSG00000126733	DACH2	dachshund family transcription factor 2	117154	0	NA	NA	NA	NA	NA
ENSG00000126752	SSX1	SSX family member 1	6756	0	NA	NA	NA	NA	NA
ENSG00000126759	CFP	complement factor properdin	5199	0,38047439	-1,597646357	4,018086	-0,397613798	0,690914888	NA
ENSG00000126778	SIX1	SIX homeobox 1	6495	0,250037874	0,482670071	4,061614	0,118837022	0,905404477	NA
ENSG00000126785	RHOJ	ras homolog family member J	57381	0	NA	NA	NA	NA	NA
ENSG00000126803	HSPA2	heat shock protein family A (Hsp70) member 2	3306	5,796630485	0,290455115	0,745229	0,389752962	0,69671923	NA
ENSG00000126838	PZP	PZP alpha-2-macroglobulin like	5858	0	NA	NA	NA	NA	NA
ENSG00000126856	PRDM7	PR/SET domain 7	11105	0	NA	NA	NA	NA	NA
ENSG00000126878	AIF1L	allograft inflammatory factor 1 like	83543	0	NA	NA	NA	NA	NA
ENSG00000126890	CTAG2	cancer/testis antigen 2	30848	0	NA	NA	NA	NA	NA
ENSG00000126895	AVPR2	arginine vasopressin receptor 2	554	0	NA	NA	NA	NA	NA
ENSG00000126947	ARMCX1	armadillo repeat containing X-linked 1	51309	0	NA	NA	NA	NA	NA
ENSG00000126950	TMEM35A	transmembrane protein 35A	59353	0	NA	NA	NA	NA	NA
ENSG00000126952	NA	NA	NA	0	NA	NA	NA	NA	NA
ENSG00000127074	RGS13	regulator of G protein signaling 13	6003	0,23616048	-2,075648354	4,028973	-0,515180498	0,606426895	NA
ENSG00000127083	OMD	osteomodulin	4958	0	NA	NA	NA	NA	NA
ENSG00000127129	EDN2	endothelin 2	1907	0	NA	NA	NA	NA	NA
ENSG00000127249	ATP13A4	ATPase 13A4	84239	0,681059099	0,087423587	3,238552	0,026994654	0,978463998	NA
ENSG00000127252	PLAAT1	phospholipase A and acyltransferase 1	57110	0	NA	NA	NA	NA	NA
ENSG00000127318	IL22	interleukin 22	50616	0	NA	NA	NA	NA	NA
ENSG00000127324	TSPAN8	tetraspanin 8	7103	0	NA	NA	NA	NA	NA
ENSG00000127325	BEST3	bestrophin 3	144453	0	NA	NA	NA	NA	NA
ENSG00000127329	PTPRB	protein tyrosine phosphatase receptor type B	5787	0	NA	NA	NA	NA	NA
ENSG00000127362	TAS2R3	taste 2 receptor member 3	50831	2,239092668	0,711031967	1,705899	0,416807798	0,676818998	NA
ENSG00000127366	TAS2R5	taste 2 receptor member 5	54429	4,859396783	0,754728084	1,007761	0,74891545	0,453908167	NA
ENSG00000127377	CRYGN	crystallin gamma N	155051	0,10488184	-1,076443944	4,080456	-0,263804824	0,791930317	NA
ENSG00000127412	TRPV5	transient receptor potential cation channel subfamily V member 5	56302	0	NA	NA	NA	NA	NA
ENSG00000127423	AUNIP	aurora kinase A and ninein interacting protein	79000	6,359648319	0,22226665	0,796796	0,278950655	0,780282694	NA
ENSG00000127472	PLA2G5	phospholipase A2 group V	5322	0,395437644	-1,072438596	3,654907	-0,293424295	0,769197857	NA
ENSG00000127507	ADGRE2	adhesion G protein-coupled receptor E2	30817	3,338294769	-0,466116722	0,989727	-0,470954975	0,637672888	NA

ENSG00000127515	OR7A10	olfactory receptor family 7 subfamily A member 10	390892	0	NA	NA	NA	NA	NA
ENSG00000127529	OR7C2	olfactory receptor family 7 subfamily C member 2	26658	0	NA	NA	NA	NA	NA
ENSG00000127530	OR7C1	olfactory receptor family 7 subfamily C member 1	26664	0,22213543	-1,997752277	4,035659	-0,495025056	0,620582434	NA
ENSG00000127578	WFIKKN1	WAP, follistatin/kazal, immunoglobulin, kunitz and netrin domain containing 1	117166	0,36550293	-1,891815294	4,044485	-0,467751887	0,639962031	NA
ENSG00000127585	FBXL16	F-box and leucine rich repeat protein 16	146330	3,120386015	1,018772411	1,201438	0,847960798	0,396459803	NA
ENSG00000127588	GNG13	G protein subunit gamma 13	51764	0,103998153	-1,076443944	4,080456	-0,263804824	0,791930317	NA
ENSG00000127589	TUBBP1	tubulin beta pseudogene 1	92755	0,210862461	0	4,080456	0	1	NA
ENSG00000127743	IL17B	interleukin 17B	27190	0	NA	NA	NA	NA	NA
ENSG00000127780	OR1E2	olfactory receptor family 1 subfamily E member 2	8388	0	NA	NA	NA	NA	NA
ENSG00000127831	VIL1	villin 1	7429	0,75892794	-0,096652379	2,492604	-0,038775658	0,969069252	NA
ENSG00000127863	TNFRSF19	TNF receptor superfamily member 19	55504	5,166167474	-0,094306487	0,87207	-0,108140957	0,91388388	NA
ENSG00000127903	ZNF835	zinc finger protein 835	90485	0	NA	NA	NA	NA	NA
ENSG00000127920	GNG11	G protein subunit gamma 11	2791	0,863023129	3,622821396	2,397623	1,511005488	0,130787053	NA
ENSG00000127928	GNGT1	G protein subunit gamma transducin 1	2792	0	NA	NA	NA	NA	NA
ENSG00000127954	STEAP4	STEAP4 metalloreductase	79689	0,103998153	-1,076443944	4,080456	-0,263804824	0,791930317	NA
ENSG00000127955	GNAI1	G protein subunit alpha i1	2770	2,472594606	-0,314552469	1,770877	-0,17762529	0,859017262	NA
ENSG00000127990	SGCE	sarcoglycan epsilon	8910	0,118286652	0	4,080456	0	1	NA
ENSG00000128039	SRD5A3	steroid 5 alpha-reductase 3	79644	9,700992795	-2,022413929	0,873549	-2,315168314	0,020603723	NA
ENSG00000128045	RASL11B	RAS like family 11 member B	65997	0	NA	NA	NA	NA	NA
ENSG00000128052	KDR	kinase insert domain receptor	3791	0	NA	NA	NA	NA	NA
ENSG00000128191	DGCR8	DGCR8 microprocessor complex subunit	54487	159,2809839	0,125215418	1,514667	0,08266863	NA	NA
ENSG00000128218	VPREB3	V-set pre-B cell surrogate light chain 3	29802	0	NA	NA	NA	NA	NA
ENSG00000128242	GAL3ST1	galactose-3-O-sulfotransferase 1	9514	0,23616048	-2,075648354	4,028973	-0,515180498	0,606426895	NA
ENSG00000128250	RFPL1	ret finger protein like 1	5988	0	NA	NA	NA	NA	NA
ENSG00000128253	RFPL2	ret finger protein like 2	10739	0,409213165	0,945419772	4,076814	0,231901641	0,81661441	NA
ENSG00000128262	NA	NA	NA	0	NA	NA	NA	NA	NA
ENSG00000128266	GNAZ	G protein subunit alpha z	2781	1,552973335	-0,937166988	1,846644	-0,507497294	0,611805935	NA
ENSG00000128274	A4GALT	alpha 1,4-galactosyltransferase (P blood group)	53947	0	NA	NA	NA	NA	NA
ENSG00000128276	RFPL3	ret finger protein like 3	10738	0	NA	NA	NA	NA	NA
ENSG00000128283	CDC42EP1	CDC42 effector protein 1	11135	6,984290371	0,799339492	0,731843	1,092228405	0,27473272	NA
ENSG00000128285	MCHR1	melanin concentrating hormone receptor 1	2847	0	NA	NA	NA	NA	NA
ENSG00000128298	BAIAP2L2	BAR/IMD domain containing adaptor protein 2 like 2	80115	5,846178428	-1,611811389	0,951931	-1,69320232	0,090416963	NA
ENSG00000128310	GALR3	galanin receptor 3	8484	0,188801589	0,865800929	4,080456	0,212182402	0,831964735	NA
ENSG00000128313	APOL5	apolipoprotein L5	80831	0	NA	NA	NA	NA	NA
ENSG00000128322	IGLL1	immunoglobulin lambda like polypeptide 1	3543	0	NA	NA	NA	NA	NA
ENSG00000128346	C22orf23	chromosome 22 open reading frame 23	84645	0,117497108	0,847101401	4,080456	0,207599697	0,835541541	NA
ENSG00000128383	APOBEC3A	apolipoprotein B mRNA editing enzyme catalytic subunit 3A	200315	0	NA	NA	NA	NA	NA
ENSG00000128383	APOBEC3A_B	APOBEC3A and APOBEC3B deletion hybrid	100913187	0	NA	NA	NA	NA	NA
ENSG00000128408	RIBC2	RIB43A domain with coiled-coils 2	26150	1,583482797	0,533575596	2,178447	0,244933889	0,806507621	NA
ENSG00000128422	KRT17	keratin 17	3872	0	NA	NA	NA	NA	NA
ENSG00000128438	NA	NA	NA	0	NA	NA	NA	NA	NA
ENSG00000128482	RNF112	ring finger protein 112	7732	0,536776574	2,650499247	3,222315	0,82254515	0,410766701	NA
ENSG00000128510	CPA4	carboxypeptidase A4	51200	0	NA	NA	NA	NA	NA
ENSG00000128512	DOCK4	dedicator of cytokinesis 4	9732	0	NA	NA	NA	NA	NA
ENSG00000128519	TAS2R16	taste 2 receptor member 16	50833	0	NA	NA	NA	NA	NA
ENSG00000128536	CDHR3	cadherin related family member 3	222256	0,103998153	-1,076443944	4,080456	-0,263804824	0,791930317	NA
ENSG00000128564	VGF	VGF nerve growth factor inducible	7425	1,595313341	-0,676095656	1,666055	-0,405806395	0,684884859	NA
ENSG00000128567	PODXL	podocalyxin like	5420	2,718343021	2,204938034	1,46348	1,506640619	0,1319028	NA
ENSG00000128573	FOXP2	forkhead box P2	93986	0	NA	NA	NA	NA	NA
ENSG00000128581	IFT22	intraflagellar transport 22	64792	4,10618022	-2,631491354	1,354754	-1,94241251	0,052087182	NA
ENSG00000128591	FLNC	filamin C	2318	0	NA	NA	NA	NA	NA
ENSG00000128594	LRRC4	leucine rich repeat containing 4	64101	6,609683213	0,246730531	0,731561	0,337266047	0,735916352	NA
ENSG00000128596	CCDC136	coiled-coil domain containing 136	64753	11,21591368	-0,324514164	0,637394	-0,509126812	0,610663339	NA

ENSG00000128602	SMO	smoothened, frizzled class receptor	6608	0	NA	NA	NA	NA	NA
ENSG00000128606	LRRC17	leucine rich repeat containing 17	10234	0,118286652	0	4,080456	0	1	NA
ENSG00000128610	FEZF1	FEZ family zinc finger 1	389549	0	NA	NA	NA	NA	NA
ENSG00000128617	OPN1SW	opsin 1, short wave sensitive	611	0	NA	NA	NA	NA	NA
ENSG00000128645	HOXD1	homeobox D1	3231	0	NA	NA	NA	NA	NA
ENSG00000128652	HOXD3	homeobox D3	3232	0	NA	NA	NA	NA	NA
ENSG00000128655	PDE11A	phosphodiesterase 11A	50940	0	NA	NA	NA	NA	NA
ENSG00000128656	CHN1	chimerin 1	1123	0,118286652	0	4,080456	0	1	NA
ENSG00000128683	GAD1	glutamate decarboxylase 1	2571	0	NA	NA	NA	NA	NA
ENSG00000128692	NA	NA	NA	0,294130307	0,699214485	3,899836	0,179293295	0,857707412	NA
ENSG00000128708	HAT1	histone acetyltransferase 1	8520	0	NA	NA	NA	NA	NA
ENSG00000128709	HOXD9	homeobox D9	3235	0	NA	NA	NA	NA	NA
ENSG00000128710	HOXD10	homeobox D10	3236	0	NA	NA	NA	NA	NA
ENSG00000128713	HOXD11	homeobox D11	3237	0	NA	NA	NA	NA	NA
ENSG00000128714	HOXD13	homeobox D13	3239	0	NA	NA	NA	NA	NA
ENSG00000128805	ARHGAP22	Rho GTPase activating protein 22	58504	0,103998153	-1,076443944	4,080456	-0,263804824	0,791930317	NA
ENSG00000128815	WDFY4	WDFY family member 4	57705	0	NA	NA	NA	NA	NA
ENSG00000128833	MYO5C	myosin VC	55930	0,814883462	2,660886614	2,553281	1,042144013	0,297344916	NA
ENSG00000128849	CGNL1	cingulin like 1	84952	0	NA	NA	NA	NA	NA
ENSG00000128886	ELL3	elongation factor for RNA polymerase II 3	80237	1,039657064	-3,322826614	2,901528	-1,145198748	0,252126837	NA
ENSG00000128917	DLL4	delta like canonical Notch ligand 4	54567	0	NA	NA	NA	NA	NA
ENSG00000128918	ALDH1A2	aldehyde dehydrogenase 1 family member A2	8854	0	NA	NA	NA	NA	NA
ENSG00000129028	THAP10	THAP domain containing 10	56906	0	NA	NA	NA	NA	NA
ENSG00000129038	LOXL1	lysyl oxidase like 1	4016	0,10488184	-1,076443944	4,080456	-0,263804824	0,791930317	NA
ENSG00000129048	ACKR4	atypical chemokine receptor 4	51554	0,117497108	0,847101401	4,080456	0,207599697	0,835541541	NA
ENSG00000129116	PALLD	palladin, cytoskeletal associated protein	23022	0,923908439	-1,993652629	2,335368	-0,853678055	0,393283397	NA
ENSG00000129151	BBOX1	gamma-butyrobetaine hydroxylase 1	8424	0	NA	NA	NA	NA	NA
ENSG00000129152	MYOD1	myogenic differentiation 1	4654	0	NA	NA	NA	NA	NA
ENSG00000129159	KCNC1	potassium voltage-gated channel subfamily C member 1	3746	0	NA	NA	NA	NA	NA
ENSG00000129167	TPH1	tryptophan hydroxylase 1	7166	4,698606597	1,215902665	0,830912	1,463334376	0,143375906	NA
ENSG00000129170	CSRP3	cysteine and glycine rich protein 3	8048	0	NA	NA	NA	NA	NA
ENSG00000129194	SOX15	SRY-box transcription factor 15	6665	0,793078271	0,869914704	2,288334	0,380152035	0,703832561	NA
ENSG00000129195	PIMREG	PICALM interacting mitotic regulator	54478	8,079659273	0,028695026	0,675114	0,042503958	0,966096957	NA
ENSG00000129204	USP6	ubiquitin specific peptidase 6	9098	0,118286652	0	4,080456	0	1	NA
ENSG00000129214	SHBG	sex hormone binding globulin	6462	0,10543123	0	4,080456	0	1	NA
ENSG00000129221	AIPL1	aryl hydrocarbon receptor interacting protein like 1	23746	0	NA	NA	NA	NA	NA
ENSG00000129226	CD68	CD68 molecule	968	5,063394486	-0,451469758	0,814145	-0,554532476	0,579214487	NA
ENSG00000129244	ATP1B2	ATPase Na+/K+ transporting subunit beta 2	482	0,10543123	0	4,080456	0	1	NA
ENSG00000129295	DNAAF11	dynein axonemal assembly factor 11	23639	0,363600094	-2,252308704	3,675152	-0,61284773	0,539977028	NA
ENSG00000129354	AP1M2	adaptor related protein complex 1 subunit mu 2	10053	0	NA	NA	NA	NA	NA
ENSG00000129422	MTUS1	microtubule associated scaffold protein 1	57509	0	NA	NA	NA	NA	NA
ENSG00000129437	KLK14	kallikrein related peptidase 14	43847	0,944412032	-0,467389586	2,676215	-0,174645723	0,861358019	NA
ENSG00000129450	SIGLEC9	sialic acid binding Ig like lectin 9	27180	6,102047599	-0,705872836	0,916368	-0,770293991	0,44112552	NA
ENSG00000129451	KLK10	kallikrein related peptidase 10	5655	0	NA	NA	NA	NA	NA
ENSG00000129455	KLK8	kallikrein related peptidase 8	11202	0	NA	NA	NA	NA	NA
ENSG00000129467	ADCY4	adenylate cyclase 4	196883	0,632587382	0	4,080456	0	1	NA
ENSG00000129474	AJUBA	ajuba LIM protein	84962	1,181827608	0,604236705	1,536378	0,393286569	0,694107837	NA
ENSG00000129514	FOXA1	forkhead box A1	3169	0	NA	NA	NA	NA	NA
ENSG00000129535	NRL	neural retina leucine zipper	4901	4,535712378	-0,651833641	0,909056	-0,717044386	0,473346709	NA
ENSG00000129538	RNASE1	ribonuclease A family member 1, pancreatic	6035	0	NA	NA	NA	NA	NA
ENSG00000129595	EPB41L4A	erythrocyte membrane protein band 4,1 like 4A	64097	2,467680517	-0,670513401	1,167028	-0,574547863	0,565597121	NA
ENSG00000129596	CDO1	cysteine dioxygenase type 1	1036	0	NA	NA	NA	NA	NA
ENSG00000129654	FOXJ1	forkhead box J1	2302	0,726494087	2,101522641	2,598598	0,808713987	0,418679681	NA

ENSG00000129673	AANAT	aralkylamine N-acetyltransferase	15	0,10488184	-1,076443944	4,080456	-0,263804824	0,791930317	NA
ENSG00000129682	FGF13	fibroblast growth factor 13	2258	0,117497108	0,847101401	4,080456	0,207599697	0,835541541	NA
ENSG00000129744	ART1	ADP-ribosyltransferase 1	417	0	NA	NA	NA	NA	NA
ENSG00000129749	CHRNA10	cholinergic receptor nicotinic alpha 10 subunit	57053	3,682788728	0,416331893	0,94743	0,439432914	0,660347882	NA
ENSG00000129757	CDKN1C	cyclin dependent kinase inhibitor 1C	1028	0	NA	NA	NA	NA	NA
ENSG00000129824	RPS4Y1	ribosomal protein S4 Y-linked 1	6192	0	NA	NA	NA	NA	NA
ENSG00000129862	VCY1B	variable charge Y-linked 1B	353513	0	NA	NA	NA	NA	NA
ENSG00000129864	VCY	variable charge Y-linked	9084	0	NA	NA	NA	NA	NA
ENSG00000129873	CDY2B	chromodomain Y-linked 2B	203611	0	NA	NA	NA	NA	NA
ENSG00000129910	CDH15	cadherin 15	1013	0	NA	NA	NA	NA	NA
ENSG00000129946	SHC2	SHC adaptor protein 2	25759	0	NA	NA	NA	NA	NA
ENSG00000129951	PLPPR3	phospholipid phosphatase related 3	79948	0	NA	NA	NA	NA	NA
ENSG00000129965	INS-IGF2	INS-IGF2 readthrough	723961	0	NA	NA	NA	NA	NA
ENSG00000129988	LBP	lipopolysaccharide binding protein	3929	0	NA	NA	NA	NA	NA
ENSG00000129990	SYT5	synaptotagmin 5	6861	0	NA	NA	NA	NA	NA
ENSG00000129991	TNNI3	troponin I3, cardiac type	7137	0	NA	NA	NA	NA	NA
ENSG00000129993	CBFA2T3	CBFA2/RUNX1 partner transcriptional co-repressor 3	863	3,168315299	0,973128935	1,298001	0,749713376	0,45342735	NA
ENSG00000130005	GAMT	guanidinoacetate N-methyltransferase	2593	2,056503577	0,287062152	1,186247	0,241991798	0,808786518	NA
ENSG00000130032	PRRG3	proline rich and Gla domain 3	79057	0,812394675	0,349290644	2,105269	0,165912607	0,868225726	NA
ENSG00000130035	GALNT8	polypeptide N-acetylgalactosaminyltransferase 8	26290	0,342407094	-1,057075416	3,745452	-0,282229061	0,777767876	NA
ENSG00000130037	KCNA5	potassium voltage-gated channel subfamily A member 5	3741	0	NA	NA	NA	NA	NA
ENSG00000130052	STARD8	StAR related lipid transfer domain containing 8	9754	0,938972046	1,975217821	2,233819	0,884233531	0,376570161	NA
ENSG00000130054	NALF2	NALCN channel auxiliary factor 2	27112	0	NA	NA	NA	NA	NA
ENSG00000130055	GDPD2	glycerophosphodiester phosphodiesterase domain containing 2	54857	0,119673877	0,847101401	4,080456	0,207599697	0,835541541	NA
ENSG00000130147	SH3BP4	SH3 domain binding protein 4	23677	0	NA	NA	NA	NA	NA
ENSG00000130158	DOCK6	dedicator of cytokinesis 6	57572	0,646686547	-0,49669528	2,681065	-0,185260439	0,853024766	NA
ENSG00000130167	TSPAN16	tetraspanin 16	26526	0	NA	NA	NA	NA	NA
ENSG00000130173	ANGPTL8	angiopoietin like 8	55908	0	NA	NA	NA	NA	NA
ENSG00000130176	CNN1	calponin 1	1264	0	NA	NA	NA	NA	NA
ENSG00000130182	ZSCAN10	zinc finger and SCAN domain containing 10	84891	0,072165449	0,847101401	4,080456	0,207599697	0,835541541	NA
ENSG00000130202	NECTIN2	nectin cell adhesion molecule 2	5819	0	NA	NA	NA	NA	NA
ENSG00000130203	APOE	apolipoprotein E	348	0	NA	NA	NA	NA	NA
ENSG00000130208	APOC1	apolipoprotein C1	341	0,119673877	0,847101401	4,080456	0,207599697	0,835541541	NA
ENSG00000130224	LRCH2	leucine rich repeats and calponin homology domain containing 2	57631	0	NA	NA	NA	NA	NA
ENSG00000130226	DPP6	dipeptidyl peptidase like 6	1804	0,578857192	2,672476203	2,789835	0,957933533	0,338096276	NA
ENSG00000130234	ACE2	angiotensin converting enzyme 2	59272	0	NA	NA	NA	NA	NA
ENSG00000130283	GDF1	growth differentiation factor 1	2657	0,118286652	0	4,080456	0	1	NA
ENSG00000130287	NCAN	neurocan	1463	0	NA	NA	NA	NA	NA
ENSG00000130294	KIF1A	kinesin family member 1A	547	0	NA	NA	NA	NA	NA
ENSG00000130300	PLVAP	plasmalemma vesicle associated protein	83483	0	NA	NA	NA	NA	NA
ENSG00000130307	USHBP1	USH1 protein network component harmonin binding protein 1	83878	0	NA	NA	NA	NA	NA
ENSG00000130368	MAS1	MAS1 proto-oncogene, G protein-coupled receptor	4142	0	NA	NA	NA	NA	NA
ENSG00000130377	ACSBG2	acyl-CoA synthetase bubblegum family member 2	81616	0	NA	NA	NA	NA	NA
ENSG00000130383	FUT5	fucosyltransferase 5	2527	0	NA	NA	NA	NA	NA
ENSG00000130385	BMP15	bone morphogenetic protein 15	9210	0	NA	NA	NA	NA	NA
ENSG00000130413	STK33	serine/threonine kinase 33	65975	0,10488184	-1,076443944	4,080456	-0,263804824	0,791930317	NA
ENSG00000130427	EPO	erythropoietin	2056	0,491839781	-0,961217192	2,958424	-0,324908498	0,745250325	NA
ENSG00000130433	CACNG6	calcium voltage-gated channel auxiliary subunit gamma 6	59285	0	NA	NA	NA	NA	NA
ENSG00000130477	UNC13A	unc-13 homolog A	23025	0,189584282	1,664620314	4,04754	0,411267176	0,680876635	NA

ENSG00000130508	PXDN	peroxidasin	7837	10,11090186	0,547662522	0,635128	0,862286528	0,388529864	NA
ENSG00000130513	GDF15	growth differentiation factor 15	9518	0,103998153	-1,076443944	4,080456	-0,263804824	0,791930317	NA
ENSG00000130528	HRC	histidine rich calcium binding protein	3270	0	NA	NA	NA	NA	NA
ENSG00000130529	TRPM4	transient receptor potential cation channel subfamily M member 4	54795	0	NA	NA	NA	NA	NA
ENSG00000130538	OR11H1	olfactory receptor family 11 subfamily H member 1	81061	0	NA	NA	NA	NA	NA
ENSG00000130540	SULT4A1	sulfotransferase family 4A member 1	25830	0	NA	NA	NA	NA	NA
ENSG00000130545	CRB3	crumbs cell polarity complex component 3	92359	0,468520906	-0,552273959	3,51563	-0,157091029	0,875173107	NA
ENSG00000130558	OLFM1	olfactomedin 1	10439	0	NA	NA	NA	NA	NA
ENSG00000130595	TNNT3	troponin T3, fast skeletal type	7140	0	NA	NA	NA	NA	NA
ENSG00000130612	NA	NA	NA	0	NA	NA	NA	NA	NA
ENSG00000130635	COL5A1	collagen type V alpha 1 chain	1289	0,23616048	-2,075648354	4,028973	-0,515180498	0,606426895	NA
ENSG00000130643	CALY	calcyon neuron specific vesicular protein	50632	0	NA	NA	NA	NA	NA
ENSG00000130649	CYP2E1	cytochrome P450 family 2 subfamily E member 1	1571	0,368866608	0	4,080456	0	1	NA
ENSG00000130656	HBZ	hemoglobin subunit zeta	3050	0	NA	NA	NA	NA	NA
ENSG00000130675	MNX1	motor neuron and pancreas homeobox 1	3110	0	NA	NA	NA	NA	NA
ENSG00000130700	GATA5	GATA binding protein 5	140628	0	NA	NA	NA	NA	NA
ENSG00000130701	RBBP8NL	RBBP8 N-terminal like	140893	0	NA	NA	NA	NA	NA
ENSG00000130702	LAMA5	laminin subunit alpha 5	3911	3,357807291	-0,300385718	1,193135	-0,251761646	0,801225305	NA
ENSG00000130707	ASS1	argininosuccinate synthase 1	445	0	NA	NA	NA	NA	NA
ENSG00000130711	PRDM12	PR/SET domain 12	59335	0,15836288	0	4,080456	0	1	NA
ENSG00000130720	FIBCD1	fibrinogen C domain containing 1	84929	0	NA	NA	NA	NA	NA
ENSG00000130751	NPAS1	neuronal PAS domain protein 1	4861	0,144330898	1,418786819	4,061262	0,349346267	0,726829368	NA
ENSG00000130762	ARHGEF16	Rho guanine nucleotide exchange factor 16	27237	0	NA	NA	NA	NA	NA
ENSG00000130768	SMPDL3B	sphingomyelin phosphodiesterase acid like 3B	27293	0,15836288	0	4,080456	0	1	NA
ENSG00000130783	CCDC62	coiled-coil domain containing 62	84660	0,697507893	1,783908893	2,615655	0,682012209	0,495231231	NA
ENSG00000130812	ANGPTL6	angiopoietin like 6	83854	3,868732948	-0,161842143	1,10575	-0,146364134	0,883633939	NA
ENSG00000130821	SLC6A8	solute carrier family 6 member 8	6535	2,603098052	0,062516787	1,278995	0,04887962	0,961015231	NA
ENSG00000130822	PNCK	pregnancy up-regulated nonubiquitous CaM kinase	139728	0	NA	NA	NA	NA	NA
ENSG00000130829	DUSP9	dual specificity phosphatase 9	1852	0	NA	NA	NA	NA	NA
ENSG00000130876	SLC7A10	solute carrier family 7 member 10	56301	0	NA	NA	NA	NA	NA
ENSG00000130881	LRP3	LDL receptor related protein 3	4037	0	NA	NA	NA	NA	NA
ENSG00000130943	PKDREJ	polycystin family receptor for egg jelly	10343	0,399211588	0,69317007	3,072472	0,225606602	0,821507405	NA
ENSG00000130948	HSD17B3	hydroxysteroid 17-beta dehydrogenase 3	3293	0	NA	NA	NA	NA	NA
ENSG00000130950	NUTM2F	NUT family member 2F	54754	0	NA	NA	NA	NA	NA
ENSG00000130957	FBP2	fructose-bisphosphatase 2	8789	0	NA	NA	NA	NA	NA
ENSG00000130962	PRRG1	proline rich and Gla domain 1	5638	0	NA	NA	NA	NA	NA
ENSG00000130988	RGN	regucalcin	9104	0	NA	NA	NA	NA	NA
ENSG00000130997	POLN	DNA polymerase nu	353497	0,469988433	2,955860566	3,965096	0,745470098	0,455987581	NA
ENSG00000131002	NA	NA	NA	0	NA	NA	NA	NA	NA
ENSG00000131015	ULBP2	UL16 binding protein 2	80328	11,35247906	-0,045689116	0,582429	-0,078445746	0,937473485	NA
ENSG00000131016	AKAP12	A-kinase anchoring protein 12	9590	1,34007409	0,714479859	1,677157	0,426006525	0,670103091	NA
ENSG00000131042	LILRB2	leukocyte immunoglobulin like receptor B2	10288	0	NA	NA	NA	NA	NA
ENSG00000131044	TTLL9	tubulin tyrosine ligase like 9	164395	0	NA	NA	NA	NA	NA
ENSG00000131050	BPIFA2	BPI fold containing family A member 2	140683	0,10488184	-1,076443944	4,080456	-0,263804824	0,791930317	NA
ENSG00000131055	COX4I2	cytochrome c oxidase subunit 4I2	84701	0	NA	NA	NA	NA	NA
ENSG00000131059	BPIFA3	BPI fold containing family A member 3	128861	0	NA	NA	NA	NA	NA
ENSG00000131067	GGT7	gamma-glutamyltransferase 7	2686	10,88296079	-0,504649154	0,617516	-0,817224506	0,41380014	NA
ENSG00000131068	DEFB118	defensin beta 118	117285	0	NA	NA	NA	NA	NA
ENSG00000131069	ACSS2	acyl-CoA synthetase short chain family member 2	55902	2,567339588	4,406587371	2,500487	1,76229183	0,078020004	NA

ENSG00000131080	EDA2R	ectodysplasin A2 receptor	60401	7,350859915	0,348133053	0,821782	0,423631683	0,671834445	NA
ENSG00000131094	C1QL1	complement C1q like 1	10882	0,295197408	-1,038309062	3,904667	-0,265914898	0,790304751	NA
ENSG00000131095	GFAP	glial fibrillary acidic protein	2670	0	NA	NA	NA	NA	NA
ENSG00000131096	PYY	peptide YY	5697	0	NA	NA	NA	NA	NA
ENSG00000131097	HIGD1B	HIG1 hypoxia inducible domain family member 1B	51751	0	NA	NA	NA	NA	NA
ENSG00000131126	TEX101	testis expressed 101	83639	0	NA	NA	NA	NA	NA
ENSG00000131142	CCL25	C-C motif chemokine ligand 25	6370	0,298659388	1,625457359	3,882894	0,418620093	0,675493802	NA
ENSG00000131152	NA	NA	NA	0,810528676	2,567465439	2,997717	0,856473627	0,391735855	NA
ENSG00000131183	SLC34A1	solute carrier family 34 member 1	6569	0,540598674	2,74142314	3,970714	0,69041071	0,489935944	NA
ENSG00000131187	F12	coagulation factor XII	2161	0,10543123	0	4,080456	0	1	NA
ENSG00000131188	PRR7	proline rich 7, synaptic	80758	1,927920869	-0,682155247	1,379039	-0,49465985	0,620840247	NA
ENSG00000131203	IDO1	indoleamine 2,3-dioxygenase 1	3620	0	NA	NA	NA	NA	NA
ENSG00000131233	GJA9	gap junction protein alpha 9	81025	0	NA	NA	NA	NA	NA
ENSG00000131264	CDX4	caudal type homeobox 4	1046	0	NA	NA	NA	NA	NA
ENSG00000131355	ADGRE3	adhesion G protein-coupled receptor E3	84658	0	NA	NA	NA	NA	NA
ENSG00000131379	C3orf20	chromosome 3 open reading frame 20	84077	0,485105028	-1,030281945	4,080456	-0,252491873	0,800660895	NA
ENSG00000131386	GALNT15	polypeptide N-acetylgalactosaminyltransferase 15	117248	0	NA	NA	NA	NA	NA
ENSG00000131398	KCNC3	potassium voltage-gated channel subfamily C member 3	3748	0,192687195	1,68310446	4,046099	0,415981983	0,677423187	NA
ENSG00000131400	NAPSA	napsin A aspartic peptidase	9476	0,119673877	0,847101401	4,080456	0,207599697	0,835541541	NA
ENSG00000131401	NA	NA	NA	0	NA	NA	NA	NA	NA
ENSG00000131409	LRRC4B	leucine rich repeat containing 4B	94030	0	NA	NA	NA	NA	NA
ENSG00000131435	PDLIM4	PDZ and LIM domain 4	8572	0,659383822	3,414964776	3,050852	1,119347765	0,262991806	NA
ENSG00000131459	GFPT2	glutamine-fructose-6-phosphate transaminase 2	9945	0,571400325	-0,11924723	2,923202	-0,040793353	0,967460638	NA
ENSG00000131470	PSMC3IP	PSMC3 interacting protein	29893	9,49348985	-0,19195047	0,63802	-0,300853517	0,763526197	NA
ENSG00000131471	AOC3	amine oxidase copper containing 3	8639	0,66336023	-1,003967241	2,715637	-0,369698586	0,711607085	NA
ENSG00000131477	RAMP2	receptor activity modifying protein 2	10266	0	NA	NA	NA	NA	NA
ENSG00000131480	AOC2	amine oxidase copper containing 2	314	3,196044435	0,230277672	1,069299	0,21535389	0,829491417	NA
ENSG00000131482	G6PC1	glucose-6-phosphatase catalytic subunit 1	2538	0	NA	NA	NA	NA	NA
ENSG00000131620	ANO1	anoctamin 1	55107	1,869207921	-0,683113146	2,266195	-0,301436211	0,763081885	NA
ENSG00000131650	KREMEN2	kringle containing transmembrane protein 2	79412	0	NA	NA	NA	NA	NA
ENSG00000131668	BARX1	BARX homeobox 1	56033	0	NA	NA	NA	NA	NA
ENSG00000131686	CA6	carbonic anhydrase 6	765	9,265694203	0,290086903	0,614751	0,471877151	0,637014478	NA
ENSG00000131711	MAP1B	microtubule associated protein 1B	4131	6,376365596	0,673074086	0,777079	0,866159614	0,386402637	NA
ENSG00000131721	RHOXF2	Rhox homeobox family member 2	84528	0	NA	NA	NA	NA	NA
ENSG00000131724	IL13RA1	interleukin 13 receptor subunit alpha 1	3597	0,15836288	0	4,080456	0	1	NA
ENSG00000131730	CKMT2	creatine kinase, mitochondrial 2	1160	0,457983195	-0,125337968	3,54259	-0,035380317	0,97177648	NA
ENSG00000131746	TNS4	tensin 4	84951	1,077606754	3,042449705	2,188557	1,390162601	0,164479507	NA
ENSG00000131771	PPP1R1B	protein phosphatase 1 regulatory inhibitor subunit 1B	84152	0	NA	NA	NA	NA	NA
ENSG00000131773	KHDRBS3	KH RNA binding domain containing, signal transduction associated 3	10656	0,119673877	0,847101401	4,080456	0,207599697	0,835541541	NA
ENSG00000131781	FMO5	flavin containing dimethylaniline monoxygenase 5	2330	5,975113641	1,24541939	0,810733	1,536163734	0,124498228	NA
ENSG00000131797	NA	NA	NA	1,168260168	-0,575953063	1,715203	-0,335792985	0,73702698	NA
ENSG00000131808	FSHB	follicle stimulating hormone subunit beta	2488	0	NA	NA	NA	NA	NA
ENSG00000131831	RAI2	retinoic acid induced 2	10742	0,144330898	1,418786819	4,061262	0,349346267	0,726829368	NA
ENSG00000131848	ZSCAN5A	zinc finger and SCAN domain containing 5A	79149	6,559506665	-0,402697306	0,740053	-0,544146658	0,586340555	NA
ENSG00000131849	ZNF132	zinc finger protein 132	7691	7,697911808	-0,415087519	0,849529	-0,488609083	0,625118481	NA
ENSG00000131864	USP29	ubiquitin specific peptidase 29	57663	0	NA	NA	NA	NA	NA
ENSG00000131885	NA	NA	NA	0	NA	NA	NA	NA	NA
ENSG00000131910	NR0B2	nuclear receptor subfamily 0 group B member 2	8431	0	NA	NA	NA	NA	NA
ENSG00000131914	LIN28A	lin-28 homolog A	79727	0	NA	NA	NA	NA	NA

ENSG00000131941	RHPN2	rhophilin Rho GTPase binding protein 2	85415	0	NA	NA	NA	NA	NA
ENSG00000131944	FAAP24	FA core complex associated protein 24	91442	6,43468371	-1,251635207	1,227793	-1,01941881	0,30800418	NA
ENSG00000131951	LRRC9	leucine rich repeat containing 9	341883	0	NA	NA	NA	NA	NA
ENSG00000131969	ABHD12B	abhydrolase domain containing 12B	145447	0	NA	NA	NA	NA	NA
ENSG00000131982	NA	NA	NA	0	NA	NA	NA	NA	NA
ENSG00000132000	PODNL1	podocan like 1	79883	0	NA	NA	NA	NA	NA
ENSG00000132004	FBXW9	F-box and WD repeat domain containing 9	84261	7,93741242	0,328509578	0,613177	0,535750373	0,592131085	NA
ENSG00000132010	ZNF20	zinc finger protein 20	7568	7,573517978	6,388392979	2,839266	2,250015764	0,024447945	NA
ENSG00000132016	BRME1	break repair meiotic recombinase recruitment factor 1	79173	3,314919435	0,739742954	1,667012	0,443753748	0,657220632	NA
ENSG00000132026	RTBDN	retbindin	83546	0	NA	NA	NA	NA	NA
ENSG00000132031	MATN3	matrilin 3	4148	0	NA	NA	NA	NA	NA
ENSG00000132122	SPATA6	spermatogenesis associated 6	54558	0	NA	NA	NA	NA	NA
ENSG00000132141	CCT6B	chaperonin containing TCP1 subunit 6B	10693	2,567535931	-0,050286798	1,558308	-0,032270138	0,974256623	NA
ENSG00000132164	SLC6A11	solute carrier family 6 member 11	6538	0	NA	NA	NA	NA	NA
ENSG00000132170	PPARG	peroxisome proliferator activated receptor gamma	5468	0	NA	NA	NA	NA	NA
ENSG00000132185	FCRLA	Fc receptor like A	84824	0	NA	NA	NA	NA	NA
ENSG00000132205	EMILIN2	elastin microfibril interfacer 2	84034	5,563631959	-1,210173651	0,987743	-1,225191183	0,220503196	NA
ENSG00000132259	CNGA4	cyclic nucleotide gated channel subunit alpha 4	1262	1,398895733	-1,737556581	2,17754	-0,79794485	0,424902495	NA
ENSG00000132297	HHLA1	HHLA1 neighbor of OC90	10086	0	NA	NA	NA	NA	NA
ENSG00000132321	IQCA1	IQ motif containing with AAA domain 1	79781	2,036011139	-4,406929639	2,173987	-2,027119118	0,042650232	NA
ENSG00000132359	RAP1GAP2	RAP1 GTPase activating protein 2	23108	5,357544885	-1,092802692	0,854749	-1,278507654	0,201070488	NA
ENSG00000132386	SERPINF1	serpin family F member 1	5176	0	NA	NA	NA	NA	NA
ENSG00000132394	EEFSEC	eukaryotic elongation factor, selenocysteine-tRNA specific	60678	1,75375924	0,369687552	1,82658	0,202393272	0,839609288	NA
ENSG00000132429	POPDC3	popeye domain containing 3	64208	0	NA	NA	NA	NA	NA
ENSG00000132437	DDC	dopa decarboxylase	1644	0	NA	NA	NA	NA	NA
ENSG00000132446	FTHL17	ferritin heavy chain like 17	53940	0	NA	NA	NA	NA	NA
ENSG00000132463	GRSF1	G-rich RNA sequence binding factor 1	2926	87,24695773	0,237812185	1,327069	0,179201088	NA	NA
ENSG00000132464	ENAM	enamelin	10117	0,328315563	0,847314335	4,080456	0,20765188	0,835500792	NA
ENSG00000132465	JCHAIN	joining chain of multimeric IgA and IgM	3512	0	NA	NA	NA	NA	NA
ENSG00000132470	ITGB4	integrin subunit beta 4	3691	1,952251835	-2,022345638	1,769042	-1,143187138	0,252960901	NA
ENSG00000132481	TRIM47	tripartite motif containing 47	91107	1,383275261	-0,54873813	2,648086	-0,207220634	0,835837554	NA
ENSG00000132514	CLEC10A	C-type lectin domain containing 10A	10462	0	NA	NA	NA	NA	NA
ENSG00000132517	SLC52A1	solute carrier family 52 member 1	55065	0,117497108	0,847101401	4,080456	0,207599697	0,835541541	NA
ENSG00000132518	GUCY2D	guanylate cyclase 2D, retinal	3000	0,730548379	-1,133272209	2,690781	-0,421168427	0,673632098	NA
ENSG00000132535	DLG4	discs large MAGUK scaffold protein 4	1742	6,753271647	-0,516713986	0,830036	-0,622519995	0,5336	NA
ENSG00000132554	RGS22	regulator of G protein signaling 22	26166	0	NA	NA	NA	NA	NA
ENSG00000132561	MATN2	matrilin 2	4147	0	NA	NA	NA	NA	NA
ENSG00000132622	HSPA12B	heat shock protein family A (Hsp70) member 12B	116835	0	NA	NA	NA	NA	NA
ENSG00000132631	SCP2D1	SCP2 sterol binding domain containing 1	140856	0	NA	NA	NA	NA	NA
ENSG00000132639	SNAP25	synaptosome associated protein 25	6616	0,117497108	0,847101401	4,080456	0,207599697	0,835541541	NA
ENSG00000132640	BTBD3	BTB domain containing 3	22903	4,416039448	0,404427101	1,010517	0,400218192	0,688995817	NA
ENSG00000132669	RIN2	Ras and Rab interactor 2	54453	0	NA	NA	NA	NA	NA
ENSG00000132671	SSTR4	somatostatin receptor 4	6754	0	NA	NA	NA	NA	NA
ENSG00000132677	RHBG	Rh family B glycoprotein	57127	0	NA	NA	NA	NA	NA
ENSG00000132681	ATP1A4	ATPase Na+/K+ transporting subunit alpha 4	480	0	NA	NA	NA	NA	NA
ENSG00000132688	NES	nestin	10763	0	NA	NA	NA	NA	NA
ENSG00000132692	BCAN	brevican	63827	0	NA	NA	NA	NA	NA
ENSG00000132693	CRP	C-reactive protein	1401	0	NA	NA	NA	NA	NA
ENSG00000132694	ARHGEF11	Rho guanine nucleotide exchange factor 11	9826	0,353690896	0,991846912	4,022909	0,246549706	0,805256732	NA
ENSG00000132698	RAB25	RAB25, member RAS oncogene family	57111	0	NA	NA	NA	NA	NA
ENSG00000132703	APCS	amyloid P component, serum	325	0	NA	NA	NA	NA	NA
ENSG00000132704	FCRL2	Fc receptor like 2	79368	0	NA	NA	NA	NA	NA

ENSG00000132744	ACY3	aminoacylase 3	91703	2,420560564	0,039235307	1,139223	0,034440416	0,972525955	NA
ENSG00000132746	ALDH3B2	aldehyde dehydrogenase 3 family member B2	222	0	NA	NA	NA	NA	NA
ENSG00000132749	TESMIN	testis expressed metallothionein like protein	9633	1,171488414	-1,545026408	1,946505	-0,793743753	0,427344623	NA
ENSG00000132837	DMGDH	dimethylglycine dehydrogenase	29958	0	NA	NA	NA	NA	NA
ENSG00000132840	BHMT2	betaine--homocysteine S-methyltransferase 2	23743	0	NA	NA	NA	NA	NA
ENSG00000132846	ZBED3	zinc finger BED-type containing 3	84327	7,441773459	0,222314705	0,635194	0,349995126	0,726342356	NA
ENSG00000132854	KANK4	KN motif and ankyrin repeat domains 4	163782	0	NA	NA	NA	NA	NA
ENSG00000132855	ANGPTL3	angiopoietin like 3	27329	0	NA	NA	NA	NA	NA
ENSG00000132872	SYT4	synaptotagmin 4	6860	0	NA	NA	NA	NA	NA
ENSG00000132874	SLC14A2	solute carrier family 14 member 2	8170	1,136582564	-0,031387288	1,977699	-0,015870606	0,98733762	NA
ENSG00000132911	NMUR2	neuromedin U receptor 2	56923	0	NA	NA	NA	NA	NA
ENSG00000132915	PDE6A	phosphodiesterase 6A	5145	0	NA	NA	NA	NA	NA
ENSG00000132932	ATP8A2	ATPase phospholipid transporting 8A2	51761	0,118286652	0	4,080456	0	1	NA
ENSG00000132938	MTUS2	microtubule associated scaffold protein 2	23281	0	NA	NA	NA	NA	NA
ENSG00000132958	TPTE2	transmembrane phosphoinositide 3-phosphatase and tensin homolog 2	93492	0,119673877	0,847101401	4,080456	0,207599697	0,835541541	NA
ENSG00000132970	WASF3	WASP family member 3	10810	0	NA	NA	NA	NA	NA
ENSG00000132972	RNF17	ring finger protein 17	56163	3,119909295	0,083110006	1,209322	0,068724444	0,945208961	NA
ENSG00000132975	GPR12	G protein-coupled receptor 12	2835	0	NA	NA	NA	NA	NA
ENSG00000133019	CHRM3	cholinergic receptor muscarinic 3	1131	0	NA	NA	NA	NA	NA
ENSG00000133020	MYH8	myosin heavy chain 8	4626	0	NA	NA	NA	NA	NA
ENSG00000133027	PEMT	phosphatidylethanolamine N-methyltransferase	10400	10,57988133	0,301117904	0,999054	0,301403022	0,76310719	NA
ENSG00000133048	CHI3L1	chitinase 3 like 1	1116	0	NA	NA	NA	NA	NA
ENSG00000133055	MYBPH	myosin binding protein H	4608	0	NA	NA	NA	NA	NA
ENSG00000133063	CHIT1	chitinase 1	1118	0,118286652	0	4,080456	0	1	NA
ENSG00000133067	LGR6	leucine rich repeat containing G protein-coupled receptor 6	59352	0	NA	NA	NA	NA	NA
ENSG00000133069	TMCC2	transmembrane and coiled-coil domain family 2	9911	0,656212574	-0,258903741	3,231758	-0,080112356	0,936147895	NA
ENSG00000133083	DCLK1	doublecortin like kinase 1	9201	0	NA	NA	NA	NA	NA
ENSG00000133101	CCNA1	cyclin A1	8900	0,119673877	0,847101401	4,080456	0,207599697	0,835541541	NA
ENSG00000133105	RXFP2	relaxin family peptide receptor 2	122042	0	NA	NA	NA	NA	NA
ENSG00000133107	TRPC4	transient receptor potential cation channel subfamily C member 4	7223	0	NA	NA	NA	NA	NA
ENSG00000133110	POSTN	periostin	10631	0	NA	NA	NA	NA	NA
ENSG00000133115	STOML3	stomatin like 3	161003	0	NA	NA	NA	NA	NA
ENSG00000133116	KL	klotho	9365	0,210862461	0	4,080456	0	1	NA
ENSG00000133121	STARD13	StAR related lipid transfer domain containing 13	90627	0	NA	NA	NA	NA	NA
ENSG00000133124	IRS4	insulin receptor substrate 4	8471	0	NA	NA	NA	NA	NA
ENSG00000133131	MORC4	MORC family CW-type zinc finger 4	79710	5,448319941	0,351323727	0,79047	0,444449187	0,656717859	NA
ENSG00000133134	BEX2	brain expressed X-linked 2	84707	1,392420812	2,023095881	1,947604	1,038761218	0,298915802	NA
ENSG00000133135	RNF128	ring finger protein 128	79589	0	NA	NA	NA	NA	NA
ENSG00000133136	GNG5B	G protein subunit gamma 5B	347687	0	NA	NA	NA	NA	NA
ENSG00000133138	TBC1D8B	TBC1 domain family member 8B	54885	2,4784797	3,661901962	1,562261	2,343975448	0,019079427	NA
ENSG00000133142	TCEAL4	transcription elongation factor A like 4	79921	0,185395892	0,034091427	4,080456	0,008354808	0,993333905	NA
ENSG00000133169	BEX1	brain expressed X-linked 1	55859	0	NA	NA	NA	NA	NA
ENSG00000133216	EPHB2	EPH receptor B2	2048	0	NA	NA	NA	NA	NA
ENSG00000133256	PDE6B	phosphodiesterase 6B	5158	6,254798831	0,185514428	0,675906	0,27446759	0,783725307	NA
ENSG00000133315	MACROD1	mono-ADP ribosylhydrolase 1	28992	5,358219621	-0,199974541	0,998492	-0,200276498	0,841264342	NA
ENSG00000133317	LGALS12	galectin 12	85329	0	NA	NA	NA	NA	NA
ENSG00000133328	PLAAT2	phospholipase A and acyltransferase 2	54979	0,19040219	0,847614965	4,080456	0,207725556	0,835443262	NA
ENSG00000133392	MYH11	myosin heavy chain 11	4629	0,117497108	0,847101401	4,080456	0,207599697	0,835541541	NA
ENSG00000133393	CEP20	centrosomal protein 20	123811	0	NA	NA	NA	NA	NA
ENSG00000133401	PDZD2	PDZ domain containing 2	23037	0	NA	NA	NA	NA	NA
ENSG00000133424	LARGE1	LARGE xylosyl- and glucuronyltransferase 1	9215	0,259158049	0,549076338	4,080456	0,134562499	0,892957794	NA

ENSG00000133433	GSTT2B	glutathione S-transferase theta 2B	653689	0,072165449	0,847101401	4,080456	0,207599697	0,835541541	NA
ENSG00000133454	MYO18B	myosin XVIIIB	84700	7,23005627	-0,163477491	1,476853	-0,110693137	0,911859688	NA
ENSG00000133475	NA	NA	NA	0	NA	NA	NA	NA	NA
ENSG00000133477	FAM83F	family with sequence similarity 83 member F	113828	0	NA	NA	NA	NA	NA
ENSG00000133488	SEC14L4	SEC14 like lipid binding 4	284904	0	NA	NA	NA	NA	NA
ENSG00000133624	NA	NA	NA	11,39686279	-0,274453691	0,574498	-0,477727435	0,632844215	NA
ENSG00000133627	ACTR3B	actin related protein 3B	57180	9,895994654	-0,005052962	0,591538	-0,008542071	0,993184496	NA
ENSG00000133636	NTS	neurotensin	4922	0	NA	NA	NA	NA	NA
ENSG00000133640	LRRIQ1	leucine rich repeats and IQ motif containing 1	84125	0	NA	NA	NA	NA	NA
ENSG00000133661	SFTPD	surfactant protein D	6441	0	NA	NA	NA	NA	NA
ENSG00000133665	DYDC2	DPY30 domain containing 2	84332	0	NA	NA	NA	NA	NA
ENSG00000133687	TMTC1	transmembrane O-mannosyltransferase targeting cadherins 1	83857	0	NA	NA	NA	NA	NA
ENSG00000133710	SPINK5	serine peptidase inhibitor Kazal type 5	11005	0	NA	NA	NA	NA	NA
ENSG00000133740	E2F5	E2F transcription factor 5	1875	4,398566766	0,712620056	1,007668	0,707197273	0,479443893	NA
ENSG00000133742	CA1	carbonic anhydrase 1	759	0	NA	NA	NA	NA	NA
ENSG00000133800	LYVE1	lymphatic vessel endothelial hyaluronan receptor 1	10894	0	NA	NA	NA	NA	NA
ENSG00000133863	TEX15	testis expressed 15, meiosis and synapsis associated	56154	0	NA	NA	NA	NA	NA
ENSG00000133878	DUSP26	dual specificity phosphatase 26	78986	0	NA	NA	NA	NA	NA
ENSG00000133937	GSC	goosecoid homeobox	145258	0	NA	NA	NA	NA	NA
ENSG00000133958	UNC79	unc-79 homolog, NALCN channel complex subunit	57578	0	NA	NA	NA	NA	NA
ENSG00000133962	CATSPERB	cation channel sperm associated auxiliary subunit beta	79820	0,419527361	-2,923289033	3,975145	-0,735391765	0,462100936	NA
ENSG00000133980	VRTN	vertebrae development associated	55237	0	NA	NA	NA	NA	NA
ENSG00000133985	TTC9	tetratricopeptide repeat domain 9	23508	9,000811358	-0,553921859	0,605794	-0,914372563	0,360521114	NA
ENSG00000134007	ADAM20	ADAM metallopeptidase domain 20	8748	1,420759112	2,155964541	1,782461	1,209544005	0,226453916	NA
ENSG00000134013	LOXL2	lysyl oxidase like 2	4017	5,69784239	-0,450967678	1,126628	-0,400281061	0,688949516	NA
ENSG00000134020	PEBP4	phosphatidylethanolamine binding protein 4	157310	0	NA	NA	NA	NA	NA
ENSG00000134028	ADAMDEC1	ADAM like decysin 1	27299	0,47508864	0	4,080456	0	1	NA
ENSG00000134042	MRO	maestro	83876	0,103998153	-1,076443944	4,080456	-0,263804824	0,791930317	NA
ENSG00000134061	CD180	CD180 molecule	4064	0	NA	NA	NA	NA	NA
ENSG00000134115	CNTN6	contactin 6	27255	0	NA	NA	NA	NA	NA
ENSG00000134121	CHL1	cell adhesion molecule L1 like	10752	0	NA	NA	NA	NA	NA
ENSG00000134138	MEIS2	Meis homeobox 2	4212	0	NA	NA	NA	NA	NA
ENSG00000134160	TRPM1	transient receptor potential cation channel subfamily M member 1	4308	0	NA	NA	NA	NA	NA
ENSG00000134183	GNAT2	G protein subunit alpha transducin 2	2780	0	NA	NA	NA	NA	NA
ENSG00000134184	GSTM1	glutathione S-transferase mu 1	2944	0	NA	NA	NA	NA	NA
ENSG00000134193	REG4	regenerating family member 4	83998	0,640188681	-3,316052227	2,607296	-1,271835868	0,203431442	NA
ENSG00000134198	TSPAN2	tetraspanin 2	10100	6,994150901	0,146775659	0,741715	0,197886894	0,843133559	NA
ENSG00000134200	TSHB	thyroid stimulating hormone subunit beta	7252	0	NA	NA	NA	NA	NA
ENSG00000134201	GSTM5	glutathione S-transferase mu 5	2949	0	NA	NA	NA	NA	NA
ENSG00000134202	GSTM3	glutathione S-transferase mu 3	2947	3,749184537	0,229330385	1,092619	0,209890448	0,833753177	NA
ENSG00000134207	SYT6	synaptotagmin 6	148281	0	NA	NA	NA	NA	NA
ENSG00000134216	CHIA	chitinase acidic	27159	0	NA	NA	NA	NA	NA
ENSG00000134240	HMGCS2	3-hydroxy-3-methylglutaryl-CoA synthase 2	3158	0	NA	NA	NA	NA	NA
ENSG00000134245	WNT2B	Wnt family member 2B	7482	0,356762128	0,556810532	3,733946	0,149121225	0,881457978	NA
ENSG00000134249	ADAM30	ADAM metallopeptidase domain 30	11085	0,072165449	0,847101401	4,080456	0,207599697	0,835541541	NA
ENSG00000134253	TRIM45	tripartite motif containing 45	80263	1,157703378	-1,166827299	2,024773	-0,576275637	0,564428888	NA
ENSG00000134258	VTCN1	V-set domain containing T cell activation inhibitor 1	79679	0	NA	NA	NA	NA	NA
ENSG00000134259	NGF	nerve growth factor	4803	0	NA	NA	NA	NA	NA
ENSG00000134317	GRHL1	grainyhead like transcription factor 1	29841	6,680375168	0,006768459	1,288522	0,005252886	0,995808823	NA
ENSG00000134323	MYCN	MYCN proto-oncogene, bHLH transcription factor	4613	0	NA	NA	NA	NA	NA

ENSG00000134339	SAA2	serum amyloid A2	6289	0,11808024	-1,076443944	4,080456	-0,263804824	0,791930317	NA
ENSG00000134343	ANO3	anoctamin 3	63982	0	NA	NA	NA	NA	NA
ENSG00000134363	FST	follistatin	10468	0	NA	NA	NA	NA	NA
ENSG00000134365	CFHR4	complement factor H related 4	10877	0	NA	NA	NA	NA	NA
ENSG00000134369	NAV1	neuron navigator 1	89796	4,836843876	-0,275972753	0,930042	-0,29673153	0,766671483	NA
ENSG00000134376	CRB1	crumbs cell polarity complex component 1	23418	0,117497108	0,847101401	4,080456	0,207599697	0,835541541	NA
ENSG00000134389	CFHR5	complement factor H related 5	81494	0	NA	NA	NA	NA	NA
ENSG00000134398	ERN2	endoplasmic reticulum to nucleus signaling 2	10595	0	NA	NA	NA	NA	NA
ENSG00000134438	RAX	retina and anterior neural fold homeobox	30062	0	NA	NA	NA	NA	NA
ENSG00000134443	GRP	gastrin releasing peptide	2922	0	NA	NA	NA	NA	NA
ENSG00000134461	ANKRD16	ankyrin repeat domain 16	54522	7,677572713	0,074423143	0,706407	0,10535441	0,91609459	NA
ENSG00000134463	ECHDC3	enoyl-CoA hydratase domain containing 3	79746	1,913362324	0,352081224	1,5648	0,225000786	0,821978663	NA
ENSG00000134470	IL15RA	interleukin 15 receptor subunit alpha	3601	9,357983397	-0,472637827	0,936327	-0,504778692	0,613714283	NA
ENSG00000134489	HRH4	histamine receptor H4	59340	0,144330898	1,418786819	4,061262	0,349346267	0,726829368	NA
ENSG00000134532	SOX5	SRY-box transcription factor 5	6660	0,103998153	-1,076443944	4,080456	-0,263804824	0,791930317	NA
ENSG00000134533	RERG	RAS like estrogen regulated growth inhibitor	85004	0,103998153	-1,076443944	4,080456	-0,263804824	0,791930317	NA
ENSG00000134538	SLCO1B1	solute carrier organic anion transporter family member 1B1	10599	0	NA	NA	NA	NA	NA
ENSG00000134548	SPX	spexin hormone	80763	0	NA	NA	NA	NA	NA
ENSG00000134551	PRH2	proline rich protein HaeIII subfamily 2	5555	0	NA	NA	NA	NA	NA
ENSG00000134569	LRP4	LDL receptor related protein 4	4038	0	NA	NA	NA	NA	NA
ENSG00000134571	MYBPC3	myosin binding protein C3	4607	0	NA	NA	NA	NA	NA
ENSG00000134588	USP26	ubiquitin specific peptidase 26	83844	0	NA	NA	NA	NA	NA
ENSG00000134595	SOX3	SRY-box transcription factor 3	6658	0	NA	NA	NA	NA	NA
ENSG00000134612	NA	NA	NA	0	NA	NA	NA	NA	NA
ENSG00000134627	PIWIL4	piwi like RNA-mediated gene silencing 4	143689	2,86394353	-0,157118305	1,733496	-0,090636676	0,927781288	NA
ENSG00000134640	MTNR1B	melatonin receptor 1B	4544	0	NA	NA	NA	NA	NA
ENSG00000134668	SPOCD1	SPOC domain containing 1	90853	0	NA	NA	NA	NA	NA
ENSG00000134709	HOOK1	hook microtubule tethering protein 1	51361	1,700611593	4,147486358	2,152543	1,926784305	0,054006516	NA
ENSG00000134716	CYP2J2	cytochrome P450 family 2 subfamily J member 2	1573	0,11808024	-1,076443944	4,080456	-0,263804824	0,791930317	NA
ENSG00000134755	DSC2	desmocollin 2	1824	0	NA	NA	NA	NA	NA
ENSG00000134757	DSG3	desmoglein 3	1830	0,10543123	0	4,080456	0	1	NA
ENSG00000134760	DSG1	desmoglein 1	1828	0	NA	NA	NA	NA	NA
ENSG00000134762	DSC3	desmocollin 3	1825	0,072165449	0,847101401	4,080456	0,207599697	0,835541541	NA
ENSG00000134765	DSC1	desmocollin 1	1823	0,118286652	0	4,080456	0	1	NA
ENSG00000134769	DTNA	dystrobrevin alpha	1837	0	NA	NA	NA	NA	NA
ENSG00000134775	FHOD3	formin homology 2 domain containing 3	80206	0	NA	NA	NA	NA	NA
ENSG00000134780	DAGLA	diacylglycerol lipase alpha	747	0	NA	NA	NA	NA	NA
ENSG00000134812	CBLIF	cobalamin binding intrinsic factor	2694	0	NA	NA	NA	NA	NA
ENSG00000134817	APLNR	apelin receptor	187	0	NA	NA	NA	NA	NA
ENSG00000134827	TCN1	transcobalamin 1	6947	0	NA	NA	NA	NA	NA
ENSG00000134830	C5AR2	complement C5a receptor 2	27202	0,10543123	0	4,080456	0	1	NA
ENSG00000134853	PDGFRA	platelet derived growth factor receptor alpha	5156	0	NA	NA	NA	NA	NA
ENSG00000134871	COL4A2	collagen type IV alpha 2 chain	1284	0	NA	NA	NA	NA	NA
ENSG00000134873	CLDN10	claudin 10	9071	0	NA	NA	NA	NA	NA
ENSG00000134874	DZIP1	DAZ interacting zinc finger protein 1	22873	0	NA	NA	NA	NA	NA
ENSG00000134909	ARHGAP32	Rho GTPase activating protein 32	9743	0,528939062	-2,845980375	3,243671	-0,877394939	0,380272161	NA
ENSG00000134917	ADAMTS8	ADAM metallopeptidase with thrombospondin type 1 motif 8	11095	0	NA	NA	NA	NA	NA
ENSG00000134940	ACRV1	acrosomal vesicle protein 1	56	0	NA	NA	NA	NA	NA
ENSG00000134955	SLC37A2	solute carrier family 37 member 2	219855	0,708807843	1,92050801	2,658557	0,722387243	0,470056428	NA
ENSG00000134962	KLB	klotho beta	152831	0,781405094	0,517486755	2,358585	0,219405573	0,826334129	NA
ENSG00000134986	NREP	neuronal regeneration related protein	9315	6,279903081	-0,017828811	0,69383	-0,025696231	0,97949963	NA
ENSG00000134986	LOC124906525	neuronal regeneration-related protein	124906525	6,279903081	-0,017828811	0,69383	-0,025696231	0,97949963	NA
ENSG00000135047	CTSL	cathepsin L	1514	0	NA	NA	NA	NA	NA

ENSG00000135063	ENTREP1	endosomal transmembrane epsin interactor 1	9413	0	NA	NA	NA	NA	NA
ENSG00000135094	SDS	serine dehydratase	10993	0	NA	NA	NA	NA	NA
ENSG00000135097	MSI1	musashi RNA binding protein 1	4440	0	NA	NA	NA	NA	NA
ENSG00000135100	HNF1A	HNF1 homeobox A	6927	0	NA	NA	NA	NA	NA
ENSG00000135111	TBX3	T-box transcription factor 3	6926	0	NA	NA	NA	NA	NA
ENSG00000135116	HRK	harakiri, BCL2 interacting protein	8739	0	NA	NA	NA	NA	NA
ENSG00000135119	RNFT2	ring finger protein, transmembrane 2	84900	10,62955935	-0,306767894	0,612746	-0,500644115	0,616621609	NA
ENSG00000135144	DTX1	deltex E3 ubiquitin ligase 1	1840	0	NA	NA	NA	NA	NA
ENSG00000135175	OCM2	oncomodulin 2	4951	0	NA	NA	NA	NA	NA
ENSG00000135218	CD36	CD36 molecule	948	0	NA	NA	NA	NA	NA
ENSG00000135220	UGT2A3	UDP glucuronosyltransferase family 2 member A3	79799	0	NA	NA	NA	NA	NA
ENSG00000135222	CSN2	casein beta	1447	0	NA	NA	NA	NA	NA
ENSG00000135226	UGT2B28	UDP glucuronosyltransferase family 2 member B28	54490	0	NA	NA	NA	NA	NA
ENSG00000135245	HILPDA	hypoxia inducible lipid droplet associated	29923	7,482278537	-0,020597692	0,729589	-0,028231926	0,977477174	NA
ENSG00000135248	GARIN1B	golgi associated RAB2 interactor 1B	84691	0,072165449	0,847101401	4,080456	0,207599697	0,835541541	NA
ENSG00000135253	KCP	kielin cysteine rich BMP regulator	375616	0	NA	NA	NA	NA	NA
ENSG00000135298	ADGRB3	adhesion G protein-coupled receptor B3	577	0	NA	NA	NA	NA	NA
ENSG00000135299	ANKRD6	ankyrin repeat domain 6	22881	3,41336059	0,870976096	1,19063	0,731525633	0,464458153	NA
ENSG00000135312	HTR1B	5-hydroxytryptamine receptor 1B	3351	0	NA	NA	NA	NA	NA
ENSG00000135314	KHDC1	KH domain containing 1	80759	2,817081922	1,556744152	1,271048	1,224772581	0,220660919	NA
ENSG00000135318	NT5E	5'-nucleotidase ecto	4907	1,894597996	0,396048108	1,68365	0,23523182	0,81402881	NA
ENSG00000135324	MRAP2	melanocortin 2 receptor accessory protein 2	112609	0	NA	NA	NA	NA	NA
ENSG00000135333	EPHA7	EPH receptor A7	2045	0	NA	NA	NA	NA	NA
ENSG00000135338	LCA5	lebercilin LCA5	167691	0	NA	NA	NA	NA	NA
ENSG00000135346	CGA	glycoprotein hormones, alpha polypeptide	1081	0	NA	NA	NA	NA	NA
ENSG00000135363	LMO2	LIM domain only 2	4005	0	NA	NA	NA	NA	NA
ENSG00000135373	EHF	ETS homologous factor	26298	0	NA	NA	NA	NA	NA
ENSG00000135374	ELF5	E74 like ETS transcription factor 5	2001	0	NA	NA	NA	NA	NA
ENSG00000135378	PRRG4	proline rich and Gla domain 4	79056	5,913491813	0,313591526	0,741239	0,423064089	0,672248502	NA
ENSG00000135406	PRPH	peripherin	5630	0	NA	NA	NA	NA	NA
ENSG00000135407	AVIL	advillin	10677	1,263001637	0	3,180908	0	1	NA
ENSG00000135409	AMHR2	anti-Mullerian hormone receptor type 2	269	0	NA	NA	NA	NA	NA
ENSG00000135413	LACRT	lacritin	90070	0	NA	NA	NA	NA	NA
ENSG00000135423	GLS2	glutaminase 2	27165	0,864382355	-0,347952905	2,892567	-0,120292085	0,904251774	NA
ENSG00000135436	FAM186B	family with sequence similarity 186 member B	84070	5,320622273	1,817318741	0,922535	1,969919	0,048847655	NA
ENSG00000135437	RDH5	retinol dehydrogenase 5	5959	1,063796784	-3,07973263	3,509279	-0,877597036	0,380162438	NA
ENSG00000135443	KRT85	keratin 85	3891	0	NA	NA	NA	NA	NA
ENSG00000135447	PPP1R1A	protein phosphatase 1 regulatory inhibitor subunit 1A	5502	0	NA	NA	NA	NA	NA
ENSG00000135454	B4GALNT1	beta-1,4-N-acetyl-galactosaminyltransferase 1	2583	0,422078075	-2,157534729	3,98783	-0,541029716	0,588487101	NA
ENSG00000135472	FAIM2	Fas apoptotic inhibitory molecule 2	23017	1,683384372	0,250365079	1,989037	0,125872487	0,899832862	NA
ENSG00000135477	KRT87P	keratin 87, pseudogene	85349	1,804392185	-0,551403274	1,694241	-0,325457488	0,744834852	NA
ENSG00000135480	KRT7	keratin 7	3855	0,191359004	1,675978021	4,046325	0,414197535	0,678729447	NA
ENSG00000135502	SLC26A10P	solute carrier family 26 member 10, pseudogene	65012	0	NA	NA	NA	NA	NA
ENSG00000135517	MIP	major intrinsic protein of lens fiber	4284	0	NA	NA	NA	NA	NA
ENSG00000135519	KCNH3	potassium voltage-gated channel subfamily H member 3	23416	0	NA	NA	NA	NA	NA
ENSG00000135525	MAP7	microtubule associated protein 7	9053	4,970487258	0,104359061	0,849662	0,122824171	0,902246334	NA
ENSG00000135537	AFG1L	AFG1 like ATPase	246269	8,966213331	1,225510385	0,726585	1,686671348	0,091666564	NA
ENSG00000135540	NHSL1	NHS like 1	57224	0,863496019	1,20367038	2,223083	0,54144183	0,588203082	NA
ENSG00000135547	HEY2	hes related family bHLH transcription factor with YRPW motif 2	23493	0	NA	NA	NA	NA	NA
ENSG00000135549	PKIB	cAMP-dependent protein kinase inhibitor beta	5570	0,209763681	-1,926644696	4,042043	-0,476651224	0,633610501	NA
ENSG00000135569	TAAR5	trace amine associated receptor 5	9038	0	NA	NA	NA	NA	NA

ENSG00000135577	NMBR	neuromedin B receptor	4829	0	NA	NA	NA	NA	NA
ENSG00000135625	EGR4	early growth response 4	1961	0,448711411	0,156874304	3,503824	0,04477231	0,964288796	NA
ENSG00000135631	RAB11FIP5	RAB11 family interacting protein 5	26056	1,25715788	0,997195929	1,616229	0,616989274	0,537241801	NA
ENSG00000135638	EMX1	empty spiracles homeobox 1	2016	0,119673877	0,847101401	4,080456	0,207599697	0,835541541	NA
ENSG00000135643	KCNMB4	potassium calcium-activated channel subfamily M regulatory beta subunit 4	27345	4,187929211	-0,691941764	0,897691	-0,770801719	0,440824468	NA
ENSG00000135678	CPM	carboxypeptidase M	1368	0	NA	NA	NA	NA	NA
ENSG00000135697	BCO1	beta-carotene oxygenase 1	53630	0	NA	NA	NA	NA	NA
ENSG00000135702	CHST5	carbohydrate sulfotransferase 5	23563	0,10488184	-1,076443944	4,080456	-0,263804824	0,791930317	NA
ENSG00000135740	SLC9A5	solute carrier family 9 member A5	6553	3,183438537	0,328426526	3,775787	0,086982272	0,930685604	NA
ENSG00000135744	AGT	angiotensinogen	183	0,117497108	0,847101401	4,080456	0,207599697	0,835541541	NA
ENSG00000135747	ZNF670-ZNF695	ZNF670-ZNF695 readthrough (NMD candidate)	100533111	0	NA	NA	NA	NA	NA
ENSG00000135750	KCNK1	potassium two pore domain channel subfamily K member 1	3775	0	NA	NA	NA	NA	NA
ENSG00000135773	CAPN9	calpain 9	10753	0,175291154	-0,052047764	4,080456	-0,01275538	0,989822955	NA
ENSG00000135824	RGS8	regulator of G protein signaling 8	85397	0	NA	NA	NA	NA	NA
ENSG00000135835	KIAA1614	KIAA1614	57710	1,547406164	0,795824887	2,064397	0,38549991	0,699867073	NA
ENSG00000135862	LAMC1	laminin subunit gamma 1	3915	0	NA	NA	NA	NA	NA
ENSG00000135902	CHRND	cholinergic receptor nicotinic delta subunit	1144	0	NA	NA	NA	NA	NA
ENSG00000135903	PAX3	paired box 3	5077	0	NA	NA	NA	NA	NA
ENSG00000135914	HTR2B	5-hydroxytryptamine receptor 2B	3357	0,117497108	0,847101401	4,080456	0,207599697	0,835541541	NA
ENSG00000135917	SLC19A3	solute carrier family 19 member 3	80704	0	NA	NA	NA	NA	NA
ENSG00000135925	WNT10A	Wnt family member 10A	80326	1,501767862	0,892662633	1,559442	0,572424428	0,56703447	NA
ENSG00000135929	CYP27A1	cytochrome P450 family 27 subfamily A member 1	1593	0,15836288	0	4,080456	0	1	NA
ENSG00000135951	TSGA10	testis specific 10	80705	1,489068031	0,23965024	1,889429	0,126837399	0,899069095	NA
ENSG00000135960	EDAR	ectodysplasin A receptor	10913	0	NA	NA	NA	NA	NA
ENSG00000135973	GPR45	G protein-coupled receptor 45	11250	0	NA	NA	NA	NA	NA
ENSG00000136002	ARHGEF4	Rho guanine nucleotide exchange factor 4	50649	0,277446075	-1,065358252	4,080456	-0,261088046	0,79402461	NA
ENSG00000136010	ALDH1L2	aldehyde dehydrogenase 1 family member L2	160428	0,23616048	-2,075648354	4,028973	-0,515180498	0,606426895	NA
ENSG00000136011	STAB2	stabilin 2	55576	0	NA	NA	NA	NA	NA
ENSG00000136014	USP44	ubiquitin specific peptidase 44	84101	0,764012764	1,754318928	2,696993	0,650472088	0,515387326	NA
ENSG00000136026	CKAP4	cytoskeleton associated protein 4	10970	0,278535952	0,841152405	4,080456	0,206141772	0,83668017	NA
ENSG00000136098	NEK3	NIMA related kinase 3	4752	0,530033804	-0,52224897	3,943636	-0,132428294	0,894645536	NA
ENSG00000136099	PCDH8	protocadherin 8	5100	0	NA	NA	NA	NA	NA
ENSG00000136110	CNMD	chondromodulin	11061	0	NA	NA	NA	NA	NA
ENSG00000136114	THSD1	thrombospondin type 1 domain containing 1	55901	3,007514816	1,22485576	1,164211	1,052090774	0,292757905	NA
ENSG00000136149	RPL13AP25	ribosomal protein L13a pseudogene 25	100287887	0,800787506	1,094753869	2,307885	0,47435368	0,635247718	NA
ENSG00000136155	SCEL	sciellin	8796	0	NA	NA	NA	NA	NA
ENSG00000136160	EDNRB	endothelin receptor type B	1910	0	NA	NA	NA	NA	NA
ENSG00000136205	TNS3	tensin 3	64759	0,444527721	-2,556085985	3,446329	-0,741683646	0,45827903	NA
ENSG00000136206	SPDYE1	speedy/RINGO cell cycle regulator family member E1	285955	0,236573303	0	4,080456	0	1	NA
ENSG00000136231	IGF2BP3	insulin like growth factor 2 mRNA binding protein 3	10643	0	NA	NA	NA	NA	NA
ENSG00000136235	GPNMB	glycoprotein nmb	10457	0	NA	NA	NA	NA	NA
ENSG00000136237	RAPGEF5	Rap guanine nucleotide exchange factor 5	9771	6,534562198	0,343509094	0,742975	0,462343	0,643835368	NA
ENSG00000136244	IL6	interleukin 6	3569	0	NA	NA	NA	NA	NA
ENSG00000136267	DGKB	diacylglycerol kinase beta	1607	0	NA	NA	NA	NA	NA
ENSG00000136274	NACAD	NAC alpha domain containing	23148	0,072165449	0,847101401	4,080456	0,207599697	0,835541541	NA
ENSG00000136297	MMD2	monocyte to macrophage differentiation associated 2	221938	0	NA	NA	NA	NA	NA
ENSG00000136305	CIDEB	cell death inducing DFFA like effector b	27141	16,2747805	-0,913828718	3,083712	-0,296340498	NA	NA
ENSG00000136327	NKX2-8	NK2 homeobox 8	26257	0	NA	NA	NA	NA	NA
ENSG00000136352	NKX2-1	NK2 homeobox 1	7080	0	NA	NA	NA	NA	NA
ENSG00000136367	ZFHX2	zinc finger homeobox 2	85446	8,399149635	0,925797088	0,680917	1,359632041	0,173946393	NA
ENSG00000136378	ADAMTS7	ADAM metallopeptidase with thrombospondin type 1 motif 7	11173	0,119673877	0,847101401	4,080456	0,207599697	0,835541541	NA

ENSG00000136379	ABHD17C	abhydrolase domain containing 17C, depalmitoylase	58489	2,254114326	-0,859347664	1,422878	-0,603950536	0,545876529	NA
ENSG00000136383	ALPK3	alpha kinase 3	57538	1,148415943	-0,609484806	1,696295	-0,359303617	0,719367969	NA
ENSG00000136425	CIB2	calcium and integrin binding family member 2	10518	1,318317416	-2,555945458	1,970472	-1,297123391	0,194588735	NA
ENSG00000136449	MYCBPAP	MYCBP associated protein	84073	0	NA	NA	NA	NA	NA
ENSG00000136457	CHAD	chondroadherin	1101	0	NA	NA	NA	NA	NA
ENSG00000136487	GH2	growth hormone 2	2689	0	NA	NA	NA	NA	NA
ENSG00000136488	CSH1	chorionic somatomammotropin hormone 1	1442	0	NA	NA	NA	NA	NA
ENSG00000136531	SCN2A	sodium voltage-gated channel alpha subunit 2	6326	0	NA	NA	NA	NA	NA
ENSG00000136535	TBR1	T-box brain transcription factor 1	10716	0,340844921	0,995741036	4,022773	0,247526007	0,804501167	NA
ENSG00000136542	GALNT5	polypeptide N-acetylgalactosaminyltransferase 5	11227	0	NA	NA	NA	NA	NA
ENSG00000136546	SCN7A	sodium voltage-gated channel alpha subunit 7	6332	0	NA	NA	NA	NA	NA
ENSG00000136573	BLK	BLK proto-oncogene, Src family tyrosine kinase	640	0	NA	NA	NA	NA	NA
ENSG00000136574	GATA4	GATA binding protein 4	2626	0	NA	NA	NA	NA	NA
ENSG00000136630	HLX	H2,0 like homeobox	3142	0,676169091	2,529434265	3,227668	0,783672368	0,43323238	NA
ENSG00000136634	IL10	interleukin 10	3586	0,241472809	0,258344755	4,072543	0,063435727	0,949419538	NA
ENSG00000136688	IL36G	interleukin 36 gamma	56300	0	NA	NA	NA	NA	NA
ENSG00000136694	IL36A	interleukin 36 alpha	27179	0	NA	NA	NA	NA	NA
ENSG00000136695	IL36RN	interleukin 36 receptor antagonist	26525	0	NA	NA	NA	NA	NA
ENSG00000136696	IL36B	interleukin 36 beta	27177	0	NA	NA	NA	NA	NA
ENSG00000136697	IL1F10	interleukin 1 family member 10	84639	0	NA	NA	NA	NA	NA
ENSG00000136698	CFC1	cryptic, EGF-CFC family member 1	55997	0	NA	NA	NA	NA	NA
ENSG00000136750	GAD2	glutamate decarboxylase 2	2572	0	NA	NA	NA	NA	NA
ENSG00000136826	KLF4	KLF transcription factor 4	9314	0,582662	2,014928878	3,241245	0,621652721	0,534170246	NA
ENSG00000136834	OR1J1	olfactory receptor family 1 subfamily J member 1	347168	0	NA	NA	NA	NA	NA
ENSG00000136839	OR13C9	olfactory receptor family 13 subfamily C member 9	286362	0	NA	NA	NA	NA	NA
ENSG00000136842	TMOD1	tropomodulin 1	7111	7,260305893	0,131489102	0,717333	0,183302717	0,854560503	NA
ENSG00000136859	ANGPTL2	angiopoietin like 2	23452	0	NA	NA	NA	NA	NA
ENSG00000136866	ZFP37	ZFP37 zinc finger protein	7539	7,232901672	0,313135065	0,798991	0,391912947	0,695122537	NA
ENSG00000136869	TLR4	toll like receptor 4	7099	5,201125211	0,423638656	0,814697	0,519995645	0,603066611	NA
ENSG00000136872	ALDOB	aldolase, fructose-bisphosphate B	229	0	NA	NA	NA	NA	NA
ENSG00000136881	BAAT	bile acid-CoA:amino acid N-acyltransferase	570	0	NA	NA	NA	NA	NA
ENSG00000136883	KIF12	kinesin family member 12	113220	0	NA	NA	NA	NA	NA
ENSG00000136895	GARNL3	GTPase activating Rap/RanGAP domain like 3	84253	0,119673877	0,847101401	4,080456	0,207599697	0,835541541	NA
ENSG00000136918	WDR38	WD repeat domain 38	401551	0	NA	NA	NA	NA	NA
ENSG00000136928	GABBR2	gamma-aminobutyric acid type B receptor subunit 2	9568	0,15836288	0	4,080456	0	1	NA
ENSG00000136929	HEMGN	hemogen	55363	4,083414594	-1,09010224	1,02973	-1,058629224	0,289768672	NA
ENSG00000136931	NR5A1	nuclear receptor subfamily 5 group A member 1	2516	0	NA	NA	NA	NA	NA
ENSG00000136939	OR1L4	olfactory receptor family 1 subfamily L member 4	254973	0	NA	NA	NA	NA	NA
ENSG00000136943	CTSV	cathepsin V	1515	0	NA	NA	NA	NA	NA
ENSG00000136944	LMX1B	LIM homeobox transcription factor 1 beta	4010	0,725828106	-2,336028957	2,255123	-1,035876392	0,300259813	NA
ENSG00000136960	ENPP2	ectonucleotide pyrophosphatase/phosphodiesterase 2	5168	0	NA	NA	NA	NA	NA
ENSG00000136999	CCN3	cellular communication network factor 3	4856	0	NA	NA	NA	NA	NA
ENSG00000137033	IL33	interleukin 33	90865	0	NA	NA	NA	NA	NA
ENSG00000137070	IL11RA	interleukin 11 receptor subunit alpha	3590	0,696372985	0,021846079	2,762017	0,007909467	0,993689224	NA
ENSG00000137077	CCL21	C-C motif chemokine ligand 21	6366	0	NA	NA	NA	NA	NA
ENSG00000137078	SIT1	signaling threshold regulating transmembrane adaptor 1	27240	0,072165449	0,847101401	4,080456	0,207599697	0,835541541	NA
ENSG00000137080	IFNA21	interferon alpha 21	3452	0	NA	NA	NA	NA	NA
ENSG00000137090	DMRT1	doublesex and mab-3 related transcription factor 1	1761	0,635372412	-1,672649705	2,433881	-0,687235718	0,491934199	NA
ENSG00000137098	SPAG8	sperm associated antigen 8	26206	0,209763681	-1,926644696	4,042043	-0,476651224	0,633610501	NA

ENSG00000137101	CD72	CD72 molecule	971	9,560751691	-0,486961494	1,247675	-0,390295058	0,696318377	NA
ENSG00000137103	TMEM8B	transmembrane protein 8B	51754	11,61981307	-0,783315452	0,816962	-0,958814932	0,337651986	NA
ENSG00000137135	ARHGEF39	Rho guanine nucleotide exchange factor 39	84904	11,33899738	0,434474304	0,685163	0,634118605	0,526003422	NA
ENSG00000137142	IGFBPL1	insulin like growth factor binding protein like 1	347252	0	NA	NA	NA	NA	NA
ENSG00000137177	KIF13A	kinesin family member 13A	63971	0	NA	NA	NA	NA	NA
ENSG00000137198	GMPR	guanosine monophosphate reductase	2766	10,81579569	0,290927114	0,548663	0,530247638	0,595940246	NA
ENSG00000137203	TFAP2A	transcription factor AP-2 alpha	7020	0,103998153	-1,076443944	4,080456	-0,263804824	0,791930317	NA
ENSG00000137204	SLC22A7	solute carrier family 22 member 7	10864	0	NA	NA	NA	NA	NA
ENSG00000137218	FRS3	fibroblast growth factor receptor substrate 3	10817	11,32485516	0,263921772	0,598729	0,440803221	0,659355461	NA
ENSG00000137225	CAPN11	calpain 11	11131	0	NA	NA	NA	NA	NA
ENSG00000137251	TINAG	tubulointerstitial nephritis antigen	27283	0	NA	NA	NA	NA	NA
ENSG00000137252	HCRTR2	hypocretin receptor 2	3062	0	NA	NA	NA	NA	NA
ENSG00000137261	KIAA0319	KIAA0319	9856	0	NA	NA	NA	NA	NA
ENSG00000137266	SLC22A23	solute carrier family 22 member 23	63027	0,682590748	3,451163175	3,749952	0,920321903	0,357404567	NA
ENSG00000137267	TUBB2A	tubulin beta 2A class IIa	7280	2,231018561	3,238154375	1,588447	2,038566234	0,041493338	NA
ENSG00000137270	GCM1	glial cells missing transcription factor 1	8521	0,10488184	-1,076443944	4,080456	-0,263804824	0,791930317	NA
ENSG00000137273	FOXF2	forkhead box F2	2295	0	NA	NA	NA	NA	NA
ENSG00000137285	TUBB2B	tubulin beta 2B class IIb	347733	0,209449485	-1,072311654	4,080456	-0,262792121	0,792710808	NA
ENSG00000137331	IER3	immediate early response 3	8870	0	NA	NA	NA	NA	NA
ENSG00000137343	ATAT1	alpha tubulin acetyltransferase 1	79969	3,222931535	-0,737225518	1,908089	-0,386368548	0,69922374	NA
ENSG00000137392	CLPS	colipase	1208	0	NA	NA	NA	NA	NA
ENSG00000137393	RNF144B	ring finger protein 144B	255488	0,795776488	-0,923557812	2,256741	-0,409244128	0,682360514	NA
ENSG00000137411	VARS2	valyl-tRNA synthetase 2, mitochondrial	57176	1,975611386	-0,673756978	1,617884	-0,416443434	0,677085551	NA
ENSG00000137434	C6orf52	chromosome 6 open reading frame 52	347744	0	NA	NA	NA	NA	NA
ENSG00000137440	FGFBP1	fibroblast growth factor binding protein 1	9982	0	NA	NA	NA	NA	NA
ENSG00000137460	FHDC1	FH2 domain containing 1	85462	4,052527085	-0,119262605	0,963979	-0,123719148	0,90153765	NA
ENSG00000137462	TLR2	toll like receptor 2	7097	0,340717413	-2,613400568	3,991367	-0,654763252	0,512620194	NA
ENSG00000137463	MGARP	mitochondria localized glutamic acid rich protein	84709	0,072165449	0,847101401	4,080456	0,207599697	0,835541541	NA
ENSG00000137473	TTC29	tetratricopeptide repeat domain 29	83894	0	NA	NA	NA	NA	NA
ENSG00000137491	SLCO2B1	solute carrier organic anion transporter family member 2B1	11309	0	NA	NA	NA	NA	NA
ENSG00000137507	LRRC32	leucine rich repeat containing 32	2615	0,10543123	0	4,080456	0	1	NA
ENSG00000137558	PI15	peptidase inhibitor 15	51050	0	NA	NA	NA	NA	NA
ENSG00000137561	TTPA	alpha tocopherol transfer protein	7274	0	NA	NA	NA	NA	NA
ENSG00000137571	SLCO5A1	solute carrier organic anion transporter family member 5A1	81796	0	NA	NA	NA	NA	NA
ENSG00000137573	SULF1	sulfatase 1	23213	0	NA	NA	NA	NA	NA
ENSG00000137634	NXPE4	neurexophilin and PC-esterase domain family member 4	54827	0	NA	NA	NA	NA	NA
ENSG00000137648	TMPRSS4	transmembrane serine protease 4	56649	0	NA	NA	NA	NA	NA
ENSG00000137672	TRPC6	transient receptor potential cation channel subfamily C member 6	7225	0	NA	NA	NA	NA	NA
ENSG00000137673	MMP7	matrix metallopeptidase 7	4316	0	NA	NA	NA	NA	NA
ENSG00000137674	MMP20	matrix metallopeptidase 20	9313	0	NA	NA	NA	NA	NA
ENSG00000137675	MMP27	matrix metallopeptidase 27	64066	0	NA	NA	NA	NA	NA
ENSG00000137691	CFAP300	cilia and flagella associated protein 300	85016	0	NA	NA	NA	NA	NA
ENSG00000137693	YAP1	Yes1 associated transcriptional regulator	10413	0	NA	NA	NA	NA	NA
ENSG00000137699	TRIM29	tripartite motif containing 29	23650	0	NA	NA	NA	NA	NA
ENSG00000137700	SLC37A4	solute carrier family 37 member 4	2542	1,082481737	4,184152341	3,931794	1,064184012	0,287245348	NA
ENSG00000137707	BTG4	BTG anti-proliferation factor 4	54766	0	NA	NA	NA	NA	NA
ENSG00000137709	POU2F3	POU class 2 homeobox 3	25833	0	NA	NA	NA	NA	NA
ENSG00000137726	FXYD6	FXYD domain containing ion transport regulator 6	53826	0	NA	NA	NA	NA	NA

ENSG00000137727	ARHGAP20	Rho GTPase activating protein 20	57569	0,221456361	-0,117503672	4,080456	-0,028796702	0,977026731	NA
ENSG00000137731	FXYD2	FXYD domain containing ion transport regulator 2	486	0,804093228	0,849275085	3,055345	0,2779637	0,781040224	NA
ENSG00000137745	MMP13	matrix metallopeptidase 13	4322	0,11808024	-1,076443944	4,080456	-0,263804824	0,791930317	NA
ENSG00000137747	TMPRSS13	transmembrane serine protease 13	84000	8,477966299	0,19374403	0,642388	0,301599569	0,762957336	NA
ENSG00000137757	CASP5	caspase 5	838	0,15836288	0	4,080456	0	1	NA
ENSG00000137766	UNC13C	unc-13 homolog C	440279	0,103998153	-1,076443944	4,080456	-0,263804824	0,791930317	NA
ENSG00000137809	ITGA11	integrin subunit alpha 11	22801	0	NA	NA	NA	NA	NA
ENSG00000137819	PAQR5	progestin and adipoQ receptor family member 5	54852	0	NA	NA	NA	NA	NA
ENSG00000137821	LRRC49	leucine rich repeat containing 49	54839	0	NA	NA	NA	NA	NA
ENSG00000137825	ITPKA	inositol-trisphosphate 3-kinase A	3706	0,119673877	0,847101401	4,080456	0,207599697	0,835541541	NA
ENSG00000137834	SMAD6	SMAD family member 6	4091	0,118286652	0	4,080456	0	1	NA
ENSG00000137843	PAK6	p21 (RAC1) activated kinase 6	56924	6,738898223	0,119475094	0,805535	0,148317616	0,882092114	NA
ENSG00000137843	BUB1B-PAK6	BUB1B-PAK6 readthrough	106821730	6,738898223	0,119475094	0,805535	0,148317616	0,882092114	NA
ENSG00000137857	DUOX1	dual oxidase 1	53905	0	NA	NA	NA	NA	NA
ENSG00000137860	SLC28A2	solute carrier family 28 member 2	9153	0	NA	NA	NA	NA	NA
ENSG00000137869	CYP19A1	cytochrome P450 family 19 subfamily A member 1	1588	0	NA	NA	NA	NA	NA
ENSG00000137872	SEMA6D	semaphorin 6D	80031	0	NA	NA	NA	NA	NA
ENSG00000137875	BCL2L10	BCL2 like 10	10017	0,353414941	2,570993666	3,981686	0,645704696	0,518470619	NA
ENSG00000137877	SPTBN5	spectrin beta, non-erythrocytic 5	51332	3,7089089	-0,381682346	1,052915	-0,362500604	0,716977972	NA
ENSG00000137878	GCOM1	GCOM1, MYZAP-POLR2M combined locus	145781	0	NA	NA	NA	NA	NA
ENSG00000137880	GCHFR	GTP cyclohydrolase I feedback regulator	2644	8,653796073	-0,522296666	0,700048	-0,746086634	0,455615083	NA
ENSG00000137936	BCAR3	BCAR3 adaptor protein, NSP family member	8412	0,175821551	-0,104001947	4,080456	-0,025487825	0,979665859	NA
ENSG00000137941	TTLL7	tubulin tyrosine ligase like 7	79739	1,756073667	-1,082782348	2,704669	-0,400338279	0,688907377	NA
ENSG00000137942	FNBP1L	formin binding protein 1 like	54874	2,84010387	1,1395977	1,051452	1,083832059	0,278439268	NA
ENSG00000137948	BRDT	bromodomain testis associated	676	0	NA	NA	NA	NA	NA
ENSG00000137959	IFI44L	interferon induced protein 44 like	10964	2,91687066	2,420175198	1,532652	1,579077254	0,114318338	NA
ENSG00000137960	GIPC2	GIPC PDZ domain containing family member 2	54810	0	NA	NA	NA	NA	NA
ENSG00000137962	ARHGAP29	Rho GTPase activating protein 29	9411	0	NA	NA	NA	NA	NA
ENSG00000137962	LOC105378858	uncharacterized LOC105378858	105378858	0	NA	NA	NA	NA	NA
ENSG00000137965	IFI44	interferon induced protein 44	10561	8,720598025	1,173213645	2,11082	0,555809364	0,578341184	NA
ENSG00000137968	SLC44A5	solute carrier family 44 member 5	204962	0,958344733	1,658553453	2,283854	0,726208193	0,46771115	NA
ENSG00000137970	RPL7P9	ribosomal protein L7 pseudogene 9	653702	1,020390319	0,463305343	1,981733	0,233787915	0,815149632	NA
ENSG00000137975	CLCA2	chloride channel accessory 2	9635	0,15836288	0	4,080456	0	1	NA
ENSG00000137976	DNASE2B	deoxyribonuclease 2 beta	58511	0	NA	NA	NA	NA	NA
ENSG00000138028	CGREF1	cell growth regulator with EF-hand domain 1	10669	0	NA	NA	NA	NA	NA
ENSG00000138036	DYNC2LI1	dynein cytoplasmic 2 light intermediate chain 1	51626	9,824519411	0,633102211	0,643268	0,984196851	0,325018734	NA
ENSG00000138039	LHCGR	luteinizing hormone/choriogonadotropin receptor	3973	0	NA	NA	NA	NA	NA
ENSG00000138050	THUMPD2	THUMP domain containing 2	80745	10,6060711	0,556568409	0,859719	0,647384225	0,517383303	NA
ENSG00000138068	SULT6B1	sulfotransferase family 6B member 1	391365	0	NA	NA	NA	NA	NA
ENSG00000138075	ABCG5	ATP binding cassette subfamily G member 5	64240	0	NA	NA	NA	NA	NA
ENSG00000138079	SLC3A1	solute carrier family 3 member 1	6519	0,648923799	-3,178420877	3,128811	-1,015855863	0,30969803	NA
ENSG00000138080	EMILIN1	elastin microfibril interfacer 1	11117	11,43398471	-0,049124236	0,57793	-0,085000313	0,932261142	NA
ENSG00000138083	SIX3	SIX homeobox 3	6496	0	NA	NA	NA	NA	NA
ENSG00000138100	TRIM54	tripartite motif containing 54	57159	0,739409752	-0,72488024	2,358666	-0,307326424	0,758594925	NA
ENSG00000138109	CYP2C9	cytochrome P450 family 2 subfamily C member 9	1559	0	NA	NA	NA	NA	NA
ENSG00000138115	CYP2C8	cytochrome P450 family 2 subfamily C member 8	1558	0	NA	NA	NA	NA	NA
ENSG00000138119	MYOF	myoferlin	26509	0	NA	NA	NA	NA	NA
ENSG00000138131	LOXL4	lysyl oxidase like 4	84171	0,336851945	2,50456325	3,986166	0,628313819	0,529798381	NA
ENSG00000138135	CH25H	cholesterol 25-hydroxylase	9023	0	NA	NA	NA	NA	NA

ENSG00000138136	LBX1	ladybird homeobox 1	10660	0	NA	NA	NA	NA	NA
ENSG00000138152	BTBD16	BTB domain containing 16	118663	0,144330898	1,418786819	4,061262	0,349346267	0,726829368	NA
ENSG00000138161	CUZD1	CUB and zona pellucida like domains 1	50624	1,555466967	0,842445696	3,28577	0,256392169	0,797648034	NA
ENSG00000138162	TACC2	transforming acidic coiled-coil containing protein 2	10579	0	NA	NA	NA	NA	NA
ENSG00000138193	PLCE1	phospholipase C epsilon 1	51196	0,263959327	2,150115196	4,008023	0,536452841	0,591645621	NA
ENSG00000138207	RBP4	retinol binding protein 4	5950	0	NA	NA	NA	NA	NA
ENSG00000138271	GPR87	G protein-coupled receptor 87	53836	0	NA	NA	NA	NA	NA
ENSG00000138308	PLA2G12B	phospholipase A2 group XIIB	84647	0	NA	NA	NA	NA	NA
ENSG00000138311	ZNF365	zinc finger protein 365	22891	0	NA	NA	NA	NA	NA
ENSG00000138315	OIT3	oncoprotein induced transcript 3	170392	0	NA	NA	NA	NA	NA
ENSG00000138316	ADAMTS14	ADAM metallopeptidase with thrombospondin type 1 motif 14	140766	7,993342494	-2,104591902	0,739572	-2,845687713	0,004431563	NA
ENSG00000138347	MYPN	myopalladin	84665	0	NA	NA	NA	NA	NA
ENSG00000138356	AOX1	aldehyde oxidase 1	316	0	NA	NA	NA	NA	NA
ENSG00000138379	MSTN	myostatin	2660	0,117497108	0,847101401	4,080456	0,207599697	0,835541541	NA
ENSG00000138395	CDK15	cyclin dependent kinase 15	65061	0,072165449	0,847101401	4,080456	0,207599697	0,835541541	NA
ENSG00000138400	MDH1B	malate dehydrogenase 1B	130752	0,103998153	-1,076443944	4,080456	-0,263804824	0,791930317	NA
ENSG00000138411	HECW2	HECT, C2 and WW domain containing E3 ubiquitin protein ligase 2	57520	0,676269733	0,058694207	2,739919	0,021421876	0,982909123	NA
ENSG00000138435	CHRNA1	cholinergic receptor nicotinic alpha 1 subunit	1134	0	NA	NA	NA	NA	NA
ENSG00000138449	SLC40A1	solute carrier family 40 member 1	30061	0	NA	NA	NA	NA	NA
ENSG00000138472	GUCA1C	guanylate cyclase activator 1C	9626	0	NA	NA	NA	NA	NA
ENSG00000138483	CCDC54	coiled-coil domain containing 54	84692	0	NA	NA	NA	NA	NA
ENSG00000138587	MNS1	meiosis specific nuclear structural 1	55329	0	NA	NA	NA	NA	NA
ENSG00000138606	SHF	Src homology 2 domain containing F	90525	6,785679248	0,174662592	0,688079	0,25384073	0,799618606	NA
ENSG00000138615	CILP	cartilage intermediate layer protein	8483	3,648895648	-0,949239548	1,051196	-0,903008781	0,366521235	NA
ENSG00000138622	HCN4	hyperpolarization activated cyclic nucleotide gated potassium channel 4	10021	0	NA	NA	NA	NA	NA
ENSG00000138639	ARHGAP24	Rho GTPase activating protein 24	83478	0	NA	NA	NA	NA	NA
ENSG00000138640	FAM13A	family with sequence similarity 13 member A	10144	5,639281799	0,123077691	0,923752	0,133236773	0,894006129	NA
ENSG00000138650	PCDH10	protocadherin 10	57575	0	NA	NA	NA	NA	NA
ENSG00000138653	NDST4	N-deacetylase and N-sulfotransferase 4	64579	0	NA	NA	NA	NA	NA
ENSG00000138669	PRKG2	protein kinase cGMP-dependent 2	5593	0	NA	NA	NA	NA	NA
ENSG00000138675	FGF5	fibroblast growth factor 5	2250	0	NA	NA	NA	NA	NA
ENSG00000138684	IL21	interleukin 21	59067	0	NA	NA	NA	NA	NA
ENSG00000138685	FGF2	fibroblast growth factor 2	2247	2,745969895	-0,784603231	1,396299	-0,561916222	0,5741731	NA
ENSG00000138696	BMPR1B	bone morphogenetic protein receptor type 1B	658	0,103998153	-1,076443944	4,080456	-0,263804824	0,791930317	NA
ENSG00000138722	MMRN1	multimerin 1	22915	9,562808268	-0,233443271	0,702299	-0,332398733	0,739588195	NA
ENSG00000138735	PDE5A	phosphodiesterase 5A	8654	0,356810772	-2,204641482	3,709138	-0,594381153	0,552257211	NA
ENSG00000138738	PRDM5	PR/SET domain 5	11107	0,300514718	-2,447447894	4,002175	-0,611529379	0,540849175	NA
ENSG00000138741	TRPC3	transient receptor potential cation channel subfamily C member 3	7222	0,348928569	1,077171864	4,019008	0,268019368	0,788684411	NA
ENSG00000138755	CXCL9	C-X-C motif chemokine ligand 9	4283	0	NA	NA	NA	NA	NA
ENSG00000138769	CDKL2	cyclin dependent kinase like 2	8999	0,357031816	2,553755314	3,983608	0,641065949	0,521479836	NA
ENSG00000138771	SHROOM3	shroom family member 3	57619	6,249909509	-0,074177529	0,839993	-0,088307266	0,929632464	NA
ENSG00000138772	ANXA3	annexin A3	306	0	NA	NA	NA	NA	NA
ENSG00000138777	PPA2	inorganic pyrophosphatase 2	27068	10,56775864	0,710504369	0,911944	0,779109887	0,435914987	NA
ENSG00000138792	ENPEP	glutamyl aminopeptidase	2028	2,182522944	0,461601036	1,568931	0,294213792	0,768594542	NA
ENSG00000138798	EGF	epidermal growth factor	1950	0	NA	NA	NA	NA	NA
ENSG00000138813	C4orf17	chromosome 4 open reading frame 17	84103	0	NA	NA	NA	NA	NA
ENSG00000138823	MTTP	microsomal triglyceride transfer protein	4547	0,35424072	-2,665130536	3,988406	-0,668219491	0,503993495	NA
ENSG00000138829	FBN2	fibrillin 2	2201	0	NA	NA	NA	NA	NA
ENSG00000138892	TTLL8	tubulin tyrosine ligase like 8	164714	0	NA	NA	NA	NA	NA
ENSG00000138944	SHISAL1	shisa like 1	85352	0,190553964	0,846036095	4,080456	0,207338621	0,835745414	NA
ENSG00000139053	PDE6H	phosphodiesterase 6H	5149	0,207996306	-1,916073151	4,043021	-0,473921178	0,635556117	NA

ENSG00000139144	PIK3C2G	phosphatidylinositol-4-phosphate 3-kinase catalytic subunit type 2 gamma	5288	0	NA	NA	NA	NA	NA
ENSG00000139151	PLCZ1	phospholipase C zeta 1	89869	0	NA	NA	NA	NA	NA
ENSG00000139155	SLCO1C1	solute carrier organic anion transporter family member 1C1	53919	0	NA	NA	NA	NA	NA
ENSG00000139160	ETFBKMT	electron transfer flavoprotein subunit beta lysine methyltransferase	254013	10,30721546	1,110557922	0,672053	1,652484969	0,098435727	NA
ENSG00000139173	TMEM117	transmembrane protein 117	84216	0	NA	NA	NA	NA	NA
ENSG00000139174	PRICKLE1	prickle planar cell polarity protein 1	144165	0	NA	NA	NA	NA	NA
ENSG00000139187	KLRG1	killer cell lectin like receptor G1	10219	8,216193932	-0,100301707	0,616132	-0,162792604	0,870681732	NA
ENSG00000139193	CD27	CD27 molecule	939	1,473550976	0,198855758	1,899655	0,104679922	0,916629795	NA
ENSG00000139200	PIANP	PILR alpha associated neural protein	196500	0,072165449	0,847101401	4,080456	0,207599697	0,835541541	NA
ENSG00000139209	SLC38A4	solute carrier family 38 member 4	55089	0	NA	NA	NA	NA	NA
ENSG00000139219	COL2A1	collagen type II alpha 1 chain	1280	0	NA	NA	NA	NA	NA
ENSG00000139220	PPFIA2	PTPRF interacting protein alpha 2	8499	0	NA	NA	NA	NA	NA
ENSG00000139223	ANP32D	acidic nuclear phosphoprotein 32 family member D	23519	0	NA	NA	NA	NA	NA
ENSG00000139239	RPL14P1	ribosomal protein L14 pseudogene 1	144581	0,913353799	0,43796161	1,949407	0,224663963	0,822240701	NA
ENSG00000139263	LRIG3	leucine rich repeats and immunoglobulin like domains 3	121227	0	NA	NA	NA	NA	NA
ENSG00000139269	INHBE	inhibin subunit beta E	83729	0	NA	NA	NA	NA	NA
ENSG00000139287	TPH2	tryptophan hydroxylase 2	121278	0,15836288	0	4,080456	0	1	NA
ENSG00000139292	LGR5	leucine rich repeat containing G protein-coupled receptor 5	8549	0	NA	NA	NA	NA	NA
ENSG00000139304	PTPRQ	protein tyrosine phosphatase receptor type Q	374462	0	NA	NA	NA	NA	NA
ENSG00000139329	LUM	lumican	4060	0	NA	NA	NA	NA	NA
ENSG00000139330	KERA	keratocan	11081	0	NA	NA	NA	NA	NA
ENSG00000139344	AMDHD1	amidohydrolase domain containing 1	144193	1,789363415	-2,062074566	1,664589	-1,238788744	0,215423744	NA
ENSG00000139351	SYCP3	synaptonemal complex protein 3	50511	4,673140525	-0,766227431	1,048169	-0,731015239	0,464769845	NA
ENSG00000139352	ASCL1	achaete-scute family bHLH transcription factor 1	429	0	NA	NA	NA	NA	NA
ENSG00000139364	TMEM132B	transmembrane protein 132B	114795	0	NA	NA	NA	NA	NA
ENSG00000139410	SDSL	serine dehydratase like	113675	2,649947128	-1,472704741	1,299129	-1,13360947	0,256958399	NA
ENSG00000139438	FAM222A	family with sequence similarity 222 member A	84915	2,796690902	0,557632502	1,051531	0,530305328	0,595900253	NA
ENSG00000139445	FOXN4	forkhead box N4	121643	0	NA	NA	NA	NA	NA
ENSG00000139515	PDX1	pancreatic and duodenal homeobox 1	3651	0	NA	NA	NA	NA	NA
ENSG00000139537	CCDC65	coiled-coil domain containing 65	85478	0,31672576	0	4,080456	0	1	NA
ENSG00000139540	SLC39A5	solute carrier family 39 member 5	283375	0	NA	NA	NA	NA	NA
ENSG00000139547	RDH16	retinol dehydrogenase 16	8608	1,853914311	-0,924572022	2,072557	-0,44610201	0,655523561	NA
ENSG00000139549	DHH	desert hedgehog signaling molecule	50846	0,223550151	-1,077884772	4,080456	-0,264157929	0,791658228	NA
ENSG00000139567	ACVRL1	activin A receptor like type 1	94	0,11808024	-1,076443944	4,080456	-0,263804824	0,791930317	NA
ENSG00000139572	GPR84	G protein-coupled receptor 84	53831	3,805877532	1,722450753	0,932362	1,847404745	0,064688502	NA
ENSG00000139574	NPFF	neuropeptide FF-amide peptide precursor	8620	0,223681252	-0,114099815	4,080456	-0,027962517	0,977692047	NA
ENSG00000139610	CELA1	chymotrypsin like elastase 1	1990	0	NA	NA	NA	NA	NA
ENSG00000139637	MYG1	MYG1 exonuclease	60314	0	NA	NA	NA	NA	NA
ENSG00000139648	KRT71	keratin 71	112802	0	NA	NA	NA	NA	NA
ENSG00000139656	SMIM2	small integral membrane protein 2	79024	0	NA	NA	NA	NA	NA
ENSG00000139714	MORN3	MORN repeat containing 3	283385	2,12384341	0,430368446	1,190234	0,361583145	0,717663562	NA
ENSG00000139767	SRRM4	serine/arginine repetitive matrix 4	84530	0	NA	NA	NA	NA	NA
ENSG00000139780	METTL21C	methyltransferase 21C, AARS1 lysine	196541	0,072165449	0,847101401	4,080456	0,207599697	0,835541541	NA
ENSG00000139797	RNF113B	ring finger protein 113B	140432	0	NA	NA	NA	NA	NA
ENSG00000139800	ZIC5	Zic family member 5	85416	0,119673877	0,847101401	4,080456	0,207599697	0,835541541	NA
ENSG00000139832	RAB20	RAB20, member RAS oncogene family	55647	1,958653962	0,910591125	1,599416	0,569327182	0,569134124	NA

ENSG00000139835	GRTP1	growth hormone regulated TBC protein 1	79774	0	NA	NA	NA	NA	NA
ENSG00000139865	TTC6	tetratricopeptide repeat domain 6	319089	0	NA	NA	NA	NA	NA
ENSG00000139874	SSTR1	somatostatin receptor 1	6751	0	NA	NA	NA	NA	NA
ENSG00000139890	REM2	RRAD and GEM like GTPase 2	161253	3,379303758	0,826223118	1,033218	0,79966028	0,423907652	NA
ENSG00000139910	NOVA1	NOVA alternative splicing regulator 1	4857	0,207996306	-1,916073151	4,043021	-0,473921178	0,635556117	NA
ENSG00000139914	FITM1	fat storage inducing transmembrane protein 1	161247	3,697791723	-2,750402188	1,314073	-2,093035612	0,036345976	NA
ENSG00000139915	MDGA2	MAM domain containing glycosylphosphatidylinositol anchor 2	161357	0	NA	NA	NA	NA	NA
ENSG00000139926	FRMD6	FERM domain containing 6	122786	1,091288535	3,210502456	3,514606	0,913474356	0,360993115	NA
ENSG00000139946	PELI2	pellino E3 ubiquitin protein ligase family member 2	57161	2,056299367	1,204882413	1,297213	0,928823893	0,35298036	NA
ENSG00000139970	RTN1	reticulon 1	6252	0	NA	NA	NA	NA	NA
ENSG00000139971	ARMH4	armadillo like helical domain containing 4	145407	0	NA	NA	NA	NA	NA
ENSG00000139973	SYT16	synaptotagmin 16	83851	0	NA	NA	NA	NA	NA
ENSG00000139974	SLC38A6	solute carrier family 38 member 6	145389	0,486336886	0,824067294	4,080456	0,201954713	0,839952128	NA
ENSG00000139985	ADAM21	ADAM metallopeptidase domain 21	8747	0,117497108	0,847101401	4,080456	0,207599697	0,835541541	NA
ENSG00000139988	RDH12	retinol dehydrogenase 12	145226	0	NA	NA	NA	NA	NA
ENSG00000140009	ESR2	estrogen receptor 2	2100	1,102512507	0,527243455	2,326532	0,22662203	0,82071766	NA
ENSG00000140015	KCNH5	potassium voltage-gated channel subfamily H member 5	27133	0	NA	NA	NA	NA	NA
ENSG00000140022	STON2	stonin 2	85439	0,222124529	-1,997702137	4,03566	-0,495012473	0,620591316	NA
ENSG00000140043	PTGR2	prostaglandin reductase 2	145482	6,526578825	1,4449709	0,847246	1,705491945	0,088102713	NA
ENSG00000140044	JDP2	Jun dimerization protein 2	122953	0,118286652	0	4,080456	0	1	NA
ENSG00000140057	AK7	adenylate kinase 7	122481	0,119673877	0,847101401	4,080456	0,207599697	0,835541541	NA
ENSG00000140067	FAM181A	family with sequence similarity 181 member A	90050	0	NA	NA	NA	NA	NA
ENSG00000140090	SLC24A4	solute carrier family 24 member 4	123041	1,133585335	2,809931257	2,933522	0,957869601	0,338128517	NA
ENSG00000140092	FBLN5	fibulin 5	10516	1,876066427	-3,698442081	2,642878	-1,399399309	0,161693274	NA
ENSG00000140093	SERPINA10	serpin family A member 10	51156	0	NA	NA	NA	NA	NA
ENSG00000140104	CLBA1	clathrin binding box of aftiphilin containing 1	122616	0,671574158	0,860863901	3,253575	0,26459015	0,791325209	NA
ENSG00000140107	SLC25A47	solute carrier family 25 member 47	283600	0	NA	NA	NA	NA	NA
ENSG00000140254	DUOXA1	dual oxidase maturation factor 1	90527	0,10543123	0	4,080456	0	1	NA
ENSG00000140263	SORD	sorbitol dehydrogenase	6652	8,42951711	1,03695074	1,073793	0,965690016	0,334199317	NA
ENSG00000140274	DUOXA2	dual oxidase maturation factor 2	405753	0	NA	NA	NA	NA	NA
ENSG00000140279	DUOX2	dual oxidase 2	50506	0,444537652	-1,917566896	4,042931	-0,474301189	0,635285143	NA
ENSG00000140285	FGF7	fibroblast growth factor 7	2252	0,209763681	-1,926644696	4,042043	-0,476651224	0,633610501	NA
ENSG00000140287	HDC	histidine decarboxylase	3067	0	NA	NA	NA	NA	NA
ENSG00000140297	GCNT3	glucosaminyl (N-acetyl) transferase 3, mucin type	9245	0	NA	NA	NA	NA	NA
ENSG00000140450	ARRDC4	arrestin domain containing 4	91947	1,54546656	1,942401782	1,989355	0,976397656	0,328867443	NA
ENSG00000140465	CYP1A1	cytochrome P450 family 1 subfamily A member 1	1543	2,351937175	0,085696639	1,209713	0,070840461	0,94352473	NA
ENSG00000140478	GOLGA6D	golgin A6 family member D	653643	0	NA	NA	NA	NA	NA
ENSG00000140488	CELF6	CUGBP Elav-like family member 6	60677	2,994437439	0,420365703	1,70361	0,246750014	0,805101697	NA
ENSG00000140505	CYP1A2	cytochrome P450 family 1 subfamily A member 2	1544	0	NA	NA	NA	NA	NA
ENSG00000140506	LMAN1L	lectin, mannose binding 1 like	79748	0,119673877	0,847101401	4,080456	0,207599697	0,835541541	NA
ENSG00000140519	RHCG	Rh family C glycoprotein	51458	0	NA	NA	NA	NA	NA
ENSG00000140522	RLBP1	retinaldehyde binding protein 1	6017	0	NA	NA	NA	NA	NA
ENSG00000140527	WDR93	WD repeat domain 93	56964	0,10543123	0	4,080456	0	1	NA
ENSG00000140538	NTRK3	neurotrophic receptor tyrosine kinase 3	4916	0	NA	NA	NA	NA	NA
ENSG00000140557	ST8SIA2	ST8 alpha-N-acetyl-neuraminide alpha-2,8-sialyltransferase 2	8128	0	NA	NA	NA	NA	NA
ENSG00000140623	SEPTIN12	septin 12	124404	0	NA	NA	NA	NA	NA
ENSG00000140675	SLC5A2	solute carrier family 5 member 2	6524	0,10543123	0	4,080456	0	1	NA
ENSG00000140682	TGFB1I1	transforming growth factor beta 1 induced transcript 1	7041	0	NA	NA	NA	NA	NA
ENSG00000140749	IGSF6	immunoglobulin superfamily member 6	10261	0	NA	NA	NA	NA	NA
ENSG00000140795	MYLK3	myosin light chain kinase 3	91807	0,072165449	0,847101401	4,080456	0,207599697	0,835541541	NA

ENSG00000140798	ABCC12	ATP binding cassette subfamily C member 12	94160	0	NA	NA	NA	NA	NA
ENSG00000140832	MARVELD3	MARVEL domain containing 3	91862	0	NA	NA	NA	NA	NA
ENSG00000140835	CHST4	carbohydrate sulfotransferase 4	10164	0	NA	NA	NA	NA	NA
ENSG00000140839	CLEC18B	C-type lectin domain family 18 member B	497190	0,568231799	2,545497262	3,983713	0,638976008	0,52283854	NA
ENSG00000140848	CPNE2	copine 2	221184	0,397361653	0,840106945	3,657527	0,229692644	0,818330611	NA
ENSG00000140859	KIFC3	kinesin family member C3	3801	3,858194569	-0,65430494	1,011494	-0,646869984	0,517716094	NA
ENSG00000140873	ADAMTS18	ADAM metallopeptidase with thrombospondin type 1 motif 18	170692	0	NA	NA	NA	NA	NA
ENSG00000140876	NUDT7	nudix hydrolase 7	283927	6,562037721	-0,491451228	0,922421	-0,53278399	0,594183118	NA
ENSG00000140932	CMTM2	CKLF like MARVEL transmembrane domain containing 2	146225	2,425083393	-1,563409645	1,364532	-1,145748075	0,251899406	NA
ENSG00000140937	CDH11	cadherin 11	1009	0	NA	NA	NA	NA	NA
ENSG00000140939	NOL3	nucleolar protein 3	8996	0,359021632	2,56113643	3,98322	0,642981427	0,520236152	NA
ENSG00000140945	CDH13	cadherin 13	1012	0	NA	NA	NA	NA	NA
ENSG00000140955	ADAD2	adenosine deaminase domain containing 2	161931	0,234994216	1,944111989	4,02324	0,483220515	0,62893917	NA
ENSG00000140961	OSGIN1	oxidative stress induced growth inhibitor 1	29948	0,652765704	0,410939003	2,367505	0,173574681	0,862199731	NA
ENSG00000141013	GAS8	growth arrest specific 8	2622	2,730150012	4,44596007	2,028764	2,191462921	0,02841831	NA
ENSG00000141028	NA	NA	NA	0	NA	NA	NA	NA	NA
ENSG00000141052	MYOCD	myocardin	93649	0	NA	NA	NA	NA	NA
ENSG00000141086	CTRL	chymotrypsin like	1506	3,758997461	-5,49224284	1,749778	-3,138823545	0,001696276	NA
ENSG00000141096	DPEP3	dipeptidase 3	64180	0,247920512	0,443402903	4,061076	0,109183607	0,913056862	NA
ENSG00000141161	UNC45B	unc-45 myosin chaperone B	146862	1,263699273	1,197499449	2,026117	0,591031634	0,554499225	NA
ENSG00000141194	OR4D1	olfactory receptor family 4 subfamily D member 1	26689	3,334924417	-0,494903259	1,055321	-0,468960017	0,639098216	NA
ENSG00000141198	TOM1L1	target of myb1 like 1 membrane trafficking protein	10040	0,11808024	-1,076443944	4,080456	-0,263804824	0,791930317	NA
ENSG00000141200	KIF2B	kinesin family member 2B	84643	0	NA	NA	NA	NA	NA
ENSG00000141255	SPATA22	spermatogenesis associated 22	84690	0	NA	NA	NA	NA	NA
ENSG00000141294	LRRC46	leucine rich repeat containing 46	90506	0,563417498	0,102495814	3,349443	0,030600858	0,975587858	NA
ENSG00000141314	RHBDL3	rhomboid like 3	162494	1,213737324	-2,246604465	2,413663	-0,930786431	0,35196405	NA
ENSG00000141316	SPACA3	sperm acrosome associated 3	124912	0	NA	NA	NA	NA	NA
ENSG00000141338	ABCA8	ATP binding cassette subfamily A member 8	10351	0	NA	NA	NA	NA	NA
ENSG00000141371	CHCT1	CHD1 helical C-terminal domain containing 1	124773	0	NA	NA	NA	NA	NA
ENSG00000141391	PRELID3A	PRELI domain containing 3A	10650	5,018088245	0,148516768	1,000098	0,148502268	0,881946396	NA
ENSG00000141401	IMPA2	inositol monophosphatase 2	3613	3,312591225	-0,307564889	1,489106	-0,206543283	0,836366559	NA
ENSG00000141404	GNAL	G protein subunit alpha L	2774	0,118286652	0	4,080456	0	1	NA
ENSG00000141431	ASXL3	ASXL transcriptional regulator 3	80816	0	NA	NA	NA	NA	NA
ENSG00000141433	ADCYAP1	adenylate cyclase activating polypeptide 1	116	0	NA	NA	NA	NA	NA
ENSG00000141434	MEP1B	meprin A subunit beta	4225	0,239347754	1,967469188	4,021404	0,489249276	0,624665227	NA
ENSG00000141437	SLC25A52	solute carrier family 25 member 52	147407	0,341035801	-2,011896212	3,742643	-0,537560244	0,590880683	NA
ENSG00000141441	GAREM1	GRB2 associated regulator of MAPK1 subtype 1	64762	0	NA	NA	NA	NA	NA
ENSG00000141447	OSBPL1A	oxysterol binding protein like 1A	114876	0	NA	NA	NA	NA	NA
ENSG00000141448	GATA6	GATA binding protein 6	2627	1,414818095	2,641678617	2,062343	1,280911363	0,200224801	NA
ENSG00000141449	GREB1L	GREB1 like retinoic acid receptor coactivator	80000	0	NA	NA	NA	NA	NA
ENSG00000141485	SLC13A5	solute carrier family 13 member 5	284111	0,11808024	-1,076443944	4,080456	-0,263804824	0,791930317	NA
ENSG00000141497	ZMYND15	zinc finger MYND-type containing 15	84225	0,118286652	0	4,080456	0	1	NA
ENSG00000141505	ASGR1	asialoglycoprotein receptor 1	432	0	NA	NA	NA	NA	NA
ENSG00000141527	CARD14	caspase recruitment domain family member 14	79092	0	NA	NA	NA	NA	NA
ENSG00000141540	TTYH2	tweety family member 2	94015	4,419142201	-0,482349815	0,919682	-0,524474353	0,599948653	NA
ENSG00000141542	RAB40B	RAB40B, member RAS oncogene family	10966	7,450772622	-0,334332003	0,92258	-0,362388054	0,717062065	NA
ENSG00000141574	SECTM1	secreted and transmembrane 1	6398	0	NA	NA	NA	NA	NA
ENSG00000141579	ZNF750	zinc finger protein 750	79755	0	NA	NA	NA	NA	NA
ENSG00000141639	MAPK4	mitogen-activated protein kinase 4	5596	0	NA	NA	NA	NA	NA
ENSG00000141655	TNFRSF11A	TNF receptor superfamily member 11a	8792	2,986745722	-0,253749953	1,085868	-0,23368403	0,815230287	NA
ENSG00000141665	FBXO15	F-box protein 15	201456	0	NA	NA	NA	NA	NA

ENSG00000141668	CBLN2	cerebellin 2 precursor	147381	0	NA	NA	NA	NA	NA
ENSG00000141698	NT5C3B	5'-nucleotidase, cytosolic IIIB	115024	0,950177279	0	4,080456	0	1	NA
ENSG00000141738	GRB7	growth factor receptor bound protein 7	2886	0,118286652	0	4,080456	0	1	NA
ENSG00000141744	PNMT	phenylethanolamine N-methyltransferase	5409	0	NA	NA	NA	NA	NA
ENSG00000141748	ARL5C	ADP ribosylation factor like GTPase 5C	390790	0	NA	NA	NA	NA	NA
ENSG00000141750	STAC2	SH3 and cysteine rich domain 2	342667	0	NA	NA	NA	NA	NA
ENSG00000141753	IGFBP4	insulin like growth factor binding protein 4	3487	0,310366852	2,363466546	3,994208	0,591723511	0,55403575	NA
ENSG00000141756	FKBP10	FKBP prolyl isomerase 10	60681	7,963051883	0,117577272	1,452253	0,080961991	0,93547218	NA
ENSG00000141837	CACNA1A	calcium voltage-gated channel subunit alpha1 A	773	2,062174537	-1,700381068	2,245755	-0,757153431	0,448957946	NA
ENSG00000141854	MISP3	MISP family member 3	113230	0,35424072	-2,665130536	3,988406	-0,668219491	0,503993495	NA
ENSG00000141934	PLPP2	phospholipid phosphatase 2	8612	0	NA	NA	NA	NA	NA
ENSG00000141946	ZIM3	zinc finger imprinted 3	114026	0	NA	NA	NA	NA	NA
ENSG00000141977	CIB3	calcium and integrin binding family member 3	117286	0,422162123	0,67475143	4,080456	0,165361776	0,868659238	NA
ENSG00000141979	NA	NA	NA	0	NA	NA	NA	NA	NA
ENSG00000142025	DMRTC2	DMRT like family C2	63946	0	NA	NA	NA	NA	NA
ENSG00000142149	HUNK	hormonally up-regulated Neu-associated kinase	30811	0	NA	NA	NA	NA	NA
ENSG00000142156	COL6A1	collagen type VI alpha 1 chain	1291	0,639960906	-2,278124129	3,133823	-0,726947266	0,467258261	NA
ENSG00000142163	OR1E3	olfactory receptor family 1 subfamily E member 3 (gene/pseudogene)	8389	0	NA	NA	NA	NA	NA
ENSG00000142173	COL6A2	collagen type VI alpha 2 chain	1292	2,08477345	-1,06074779	1,335612	-0,794203373	0,427077045	NA
ENSG00000142178	SIK1	salt inducible kinase 1	150094	5,224386346	-0,290340935	0,832045	-0,348948443	0,727128016	NA
ENSG00000142182	DNMT3L	DNA methyltransferase 3 like	29947	0	NA	NA	NA	NA	NA
ENSG00000142192	APP	amyloid beta precursor protein	351	3,396303915	1,503660242	1,22667	1,225806445	0,220271521	NA
ENSG00000142224	IL19	interleukin 19	29949	0	NA	NA	NA	NA	NA
ENSG00000142233	NTN5	netrin 5	126147	0,995271396	1,252406542	2,248411	0,557018453	0,577514822	NA
ENSG00000142235	LMTK3	lemur tyrosine kinase 3	114783	1,783724676	2,936203672	2,156305	1,361682665	0,173298054	NA
ENSG00000142273	CBLC	Cbl proto-oncogene C	23624	0	NA	NA	NA	NA	NA
ENSG00000142279	WTIP	WT1 interacting protein	126374	3,544554885	0,42447271	0,926858	0,457969699	0,646974211	NA
ENSG00000142319	SLC6A3	solute carrier family 6 member 3	6531	0	NA	NA	NA	NA	NA
ENSG00000142405	NLRP12	NLR family pyrin domain containing 12	91662	0	NA	NA	NA	NA	NA
ENSG00000142408	CACNG8	calcium voltage-gated channel auxiliary subunit gamma 8	59283	0	NA	NA	NA	NA	NA
ENSG00000142449	FBN3	fibrillin 3	84467	0,315072348	-1,925107942	4,042134	-0,476260316	0,633888933	NA
ENSG00000142484	TM4SF5	transmembrane 4 L six family member 5	9032	0,10543123	0	4,080456	0	1	NA
ENSG00000142494	SLC47A1	solute carrier family 47 member 1	55244	0	NA	NA	NA	NA	NA
ENSG00000142511	GPR32	G protein-coupled receptor 32	2854	0	NA	NA	NA	NA	NA
ENSG00000142512	SIGLEC10	sialic acid binding Ig like lectin 10	89790	1,055707027	-3,131894863	2,201725	-1,422472914	0,154889009	NA
ENSG00000142513	ACP4	acid phosphatase 4	93650	0	NA	NA	NA	NA	NA
ENSG00000142515	KLK3	kallikrein related peptidase 3	354	0	NA	NA	NA	NA	NA
ENSG00000142530	GARIN5A	golgi associated RAB2 interactor 5A	112703	0,117497108	0,847101401	4,080456	0,207599697	0,835541541	NA
ENSG00000142538	PTH2	parathyroid hormone 2	113091	0	NA	NA	NA	NA	NA
ENSG00000142539	NA	NA	NA	0	NA	NA	NA	NA	NA
ENSG00000142549	IGLON5	IgLON family member 5	402665	0	NA	NA	NA	NA	NA
ENSG00000142552	RCN3	reticulocalbin 3	57333	0,516730388	-0,752270475	2,803807	-0,268303239	0,788465913	NA
ENSG00000142583	SLC2A5	solute carrier family 2 member 5	6518	0,15836288	0	4,080456	0	1	NA
ENSG00000142606	MMEL1	membrane metalloendopeptidase like 1	79258	0	NA	NA	NA	NA	NA
ENSG00000142609	CFAP74	cilia and flagella associated protein 74	85452	0	NA	NA	NA	NA	NA
ENSG00000142611	PRDM16	PR/SET domain 16	63976	0	NA	NA	NA	NA	NA
ENSG00000142615	CELA2A	chymotrypsin like elastase 2A	63036	0	NA	NA	NA	NA	NA
ENSG00000142621	FHAD1	forkhead associated phosphopeptide binding domain 1	114827	0,94679116	3,437578133	3,584677	0,958964569	0,337576595	NA
ENSG00000142627	EPHA2	EPH receptor A2	1969	0	NA	NA	NA	NA	NA
ENSG00000142661	MYOM3	myomesin 3	127294	0,10488184	-1,076443944	4,080456	-0,263804824	0,791930317	NA
ENSG00000142675	CNKSR1	connector enhancer of kinase suppressor of Ras 1	10256	0	NA	NA	NA	NA	NA

ENSG00000142677	IL22RA1	interleukin 22 receptor subunit alpha 1	58985	0	NA	NA	NA	NA	NA
ENSG00000142698	C1orf94	chromosome 1 open reading frame 94	84970	0	NA	NA	NA	NA	NA
ENSG00000142700	DMRTA2	DMRT like family A2	63950	0	NA	NA	NA	NA	NA
ENSG00000142733	MAP3K6	mitogen-activated protein kinase kinase kinase 6	9064	0,649489042	3,454692166	3,94816	0,875013242	0,381566701	NA
ENSG00000142748	FCN3	ficolin 3	8547	0,10488184	-1,076443944	4,080456	-0,263804824	0,791930317	NA
ENSG00000142789	CELA3A	chymotrypsin like elastase 3A	10136	0	NA	NA	NA	NA	NA
ENSG00000142794	NBPF3	NBPF member 3	84224	8,881366497	-0,198458985	2,227437	-0,089097465	0,929004452	NA
ENSG00000142798	HSPG2	heparan sulfate proteoglycan 2	3339	2,186033193	-0,293616931	1,375944	-0,213393034	0,831020414	NA
ENSG00000142871	CCN1	cellular communication network factor 1	3491	0	NA	NA	NA	NA	NA
ENSG00000142910	TINAGL1	tubulointerstitial nephritis antigen like 1	64129	0	NA	NA	NA	NA	NA
ENSG00000142920	AZIN2	antizyme inhibitor 2	113451	4,906828973	0,818366453	1,160326	0,705290142	0,480629694	NA
ENSG00000142949	PTPRF	protein tyrosine phosphatase receptor type F	5792	0,23616048	-2,075648354	4,028973	-0,515180498	0,606426895	NA
ENSG00000142959	BEST4	bestrophin 4	266675	1,941549944	0,016627572	1,786823	0,009305662	0,992575263	NA
ENSG00000142973	CYP4B1	cytochrome P450 family 4 subfamily B member 1	1580	0	NA	NA	NA	NA	NA
ENSG00000143001	TMEM61	transmembrane protein 61	199964	0	NA	NA	NA	NA	NA
ENSG00000143006	DMRTB1	DMRT like family B with proline rich C-terminal 1	63948	0	NA	NA	NA	NA	NA
ENSG00000143028	SYPL2	synaptophysin like 2	284612	0,306903602	2,348992666	3,995137	0,587963051	0,556557094	NA
ENSG00000143032	BARHL2	BarH like homeobox 2	343472	0	NA	NA	NA	NA	NA
ENSG00000143036	SLC44A3	solute carrier family 44 member 3	126969	0	NA	NA	NA	NA	NA
ENSG00000143061	IGSF3	immunoglobulin superfamily member 3	3321	3,034194666	0,43600528	1,245074	0,350184258	0,72620042	NA
ENSG00000143067	ZNF697	zinc finger protein 697	90874	0,367780563	-1,078470196	3,716361	-0,290195222	0,771666891	NA
ENSG00000143079	CTTNBP2NL	CTTNBP2 N-terminal like	55917	1,147673396	3,784263528	2,781079	1,360717683	0,173602924	NA
ENSG00000143105	KCNA10	potassium voltage-gated channel subfamily A member 10	3744	0	NA	NA	NA	NA	NA
ENSG00000143107	FNDC7	fibronectin type III domain containing 7	163479	0,412055931	0,693896128	3,048376	0,22762812	0,819935357	NA
ENSG00000143125	PROK1	prokineticin 1	84432	0,237164997	1,955812406	4,022315	0,486240462	0,626796687	NA
ENSG00000143147	GPR161	G protein-coupled receptor 161	23432	0	NA	NA	NA	NA	NA
ENSG00000143171	RXRG	retinoid X receptor gamma	6258	0	NA	NA	NA	NA	NA
ENSG00000143194	MAEL	maelstrom spermatogenic transposon silencer	84944	0,494701444	1,930828194	3,434623	0,562165911	0,574002985	NA
ENSG00000143196	DPT	dermatopontin	1805	0	NA	NA	NA	NA	NA
ENSG00000143199	ADCY10	adenylate cyclase 10	55811	0,551470736	-1,054517077	2,890169	-0,364863491	0,715213308	NA
ENSG00000143217	NECTIN4	nectin cell adhesion molecule 4	81607	0,308531421	-0,116433377	3,887687	-0,029949268	0,976107513	NA
ENSG00000143226	FCGR2A	Fc gamma receptor IIa	2212	10,73951995	-1,029254328	0,674117	-1,526818517	0,126806146	NA
ENSG00000143248	RGS5	regulator of G protein signaling 5	8490	2,136954012	2,007256518	1,921454	1,044654794	0,296182545	NA
ENSG00000143257	NR1I3	nuclear receptor subfamily 1 group I member 3	9970	0	NA	NA	NA	NA	NA
ENSG00000143297	FCRL5	Fc receptor like 5	83416	0	NA	NA	NA	NA	NA
ENSG00000143318	CASQ1	calsequestrin 1	844	0	NA	NA	NA	NA	NA
ENSG00000143320	CRABP2	cellular retinoic acid binding protein 2	1382	2,004923981	-0,881673569	1,662025	-0,530481648	0,59577803	NA
ENSG00000143340	FAM163A	family with sequence similarity 163 member A	148753	0	NA	NA	NA	NA	NA
ENSG00000143341	HMCN1	hemicentin 1	83872	5,345527977	-0,52075201	0,913054	-0,570340957	0,568446467	NA
ENSG00000143344	RGL1	ral guanine nucleotide dissociation stimulator like 1	23179	5,477983497	0,193436051	0,741529	0,26086096	0,794199732	NA
ENSG00000143353	LYPLAL1	lysophospholipase like 1	127018	6,445072481	1,130877858	0,827194	1,367125464	0,171585983	NA
ENSG00000143355	LHX9	LIM homeobox 9	56956	0,674589384	2,383386853	2,607962	0,913888767	0,360775297	NA
ENSG00000143365	RORC	RAR related orphan receptor C	6097	0,735521806	-2,913753106	3,789728	-0,768855552	0,441979066	NA
ENSG00000143367	TUFT1	tuftelin 1	7286	2,595399248	-0,972337651	1,387692	-0,700686856	0,48349846	NA
ENSG00000143369	ECM1	extracellular matrix protein 1	1893	1,857595684	-0,825964575	2,004117	-0,412133906	0,680241278	NA
ENSG00000143375	CGN	cingulin	57530	0,76597909	1,371134749	2,73017	0,502215951	0,615515624	NA
ENSG00000143382	ADAMTSL4	ADAMTS like 4	54507	0,306685724	0,658819361	4,039489	0,16309473	0,870443849	NA
ENSG00000143412	ANXA9	annexin A9	8416	4,172907207	-1,258062121	1,00054	-1,257382763	0,208615068	NA
ENSG00000143416	SELENBP1	selenium binding protein 1	8991	0	NA	NA	NA	NA	NA
ENSG00000143429	LSP1P4	LSP1 pseudogene 4	654342	2,417409741	0,373662765	1,195679	0,312510974	0,754652224	NA
ENSG00000143434	SEMA6C	semaphorin 6C	10500	3,841809572	-0,299631816	3,422939	-0,08753642	0,930245137	NA
ENSG00000143443	C1orf56	chromosome 1 open reading frame 56	54964	6,128313639	-1,121838401	0,776759	-1,444255757	0,148667046	NA
ENSG00000143450	OAZ3	ornithine decarboxylase antizyme 3	51686	1,028674262	0,32825377	2,09192	0,156915058	0,875311792	NA
ENSG00000143452	HORMAD1	HORMA domain containing 1	84072	0	NA	NA	NA	NA	NA

ENSG00000143469	SYT14	synaptotagmin 14	255928	0	NA	NA	NA	NA	NA
ENSG00000143473	KCNH1	potassium voltage-gated channel subfamily H member 1	3756	0	NA	NA	NA	NA	NA
ENSG00000143479	DYRK3	dual specificity tyrosine phosphorylation regulated kinase 3	8444	5,420616968	0,759921052	0,874025	0,869449598	0,384601265	NA
ENSG00000143494	VASH2	vasohibin 2	79805	0,072165449	0,847101401	4,080456	0,207599697	0,835541541	NA
ENSG00000143498	TAF1A	TATA-box binding protein associated factor, RNA polymerase I subunit A	9015	10,97369295	-0,094295808	0,707343	-0,133309869	0,893948323	NA
ENSG00000143502	SUSD4	sushi domain containing 4	55061	0	NA	NA	NA	NA	NA
ENSG00000143512	HHIPL2	HHIP like 2	79802	0	NA	NA	NA	NA	NA
ENSG00000143520	FLG2	filaggrin 2	388698	0	NA	NA	NA	NA	NA
ENSG00000143536	CRNN	cornulin	49860	0	NA	NA	NA	NA	NA
ENSG00000143545	RAB13	RAB13, member RAS oncogene family	5872	5,606194415	-0,007774374	0,807825	-0,009623835	0,992321409	NA
ENSG00000143546	S100A8	S100 calcium binding protein A8	6279	0	NA	NA	NA	NA	NA
ENSG00000143552	NUP210L	nucleoporin 210 like	91181	0,658581394	3,156907237	3,12994	1,008615831	0,313158912	NA
ENSG00000143554	SLC27A3	solute carrier family 27 member 3	11000	11,49200411	0	4,080456	0	NA	NA
ENSG00000143556	S100A7	S100 calcium binding protein A7	6278	0	NA	NA	NA	NA	NA
ENSG00000143590	EFNA3	ephrin A3	1944	5,752575042	-0,01718709	0,850321	-0,020212462	0,983873887	NA
ENSG00000143595	AQP10	aquaporin 10	89872	0	NA	NA	NA	NA	NA
ENSG00000143603	KCNN3	potassium calcium-activated channel subfamily N member 3	3782	0,359021632	2,56113643	3,98322	0,642981427	0,520236152	NA
ENSG00000143624	INTS3	integrator complex subunit 3	65123	0	NA	NA	NA	NA	NA
ENSG00000143627	PKLR	pyruvate kinase L/R	5313	0,224961871	0,84179608	4,080456	0,206299518	0,836556955	NA
ENSG00000143630	HCN3	hyperpolarization activated cyclic nucleotide gated potassium channel 3	57657	2,422927485	-0,097881382	2,895993	-0,033798906	0,973037509	NA
ENSG00000143631	FLG	filaggrin	2312	0	NA	NA	NA	NA	NA
ENSG00000143632	ACTA1	actin alpha 1, skeletal muscle	58	0,103998153	-1,076443944	4,080456	-0,263804824	0,791930317	NA
ENSG00000143674	MAP3K21	mitogen-activated protein kinase kinase kinase 21	84451	2,716717276	-1,591202344	1,092971	-1,455850102	0,145434062	NA
ENSG00000143768	LEFTY2	left-right determination factor 2	7044	0	NA	NA	NA	NA	NA
ENSG00000143786	CNIH3	cornichon family AMPA receptor auxiliary protein 3	149111	1,138297101	0,460159697	1,947221	0,236316133	0,813187369	NA
ENSG00000143816	WNT9A	Wnt family member 9A	7483	7,201642618	-0,116288325	0,736384	-0,157918055	0,874521371	NA
ENSG00000143819	EPHX1	epoxide hydrolase 1	2052	1,871050594	1,24827518	1,472824	0,847538427	0,396695078	NA
ENSG00000143839	REN	renin	5972	0	NA	NA	NA	NA	NA
ENSG00000143845	ETNK2	ethanolamine kinase 2	55224	0,427471889	2,82171989	3,402977	0,82919162	0,406995986	NA
ENSG00000143847	PPFIA4	PTPRF interacting protein alpha 4	8497	0,587302522	0,883055876	2,795782	0,3158529	0,752114163	NA
ENSG00000143850	PLEKHA6	pleckstrin homology domain containing A6	22874	0,880009691	-0,910407243	2,095685	-0,434419935	0,663983524	NA
ENSG00000143858	SYT2	synaptotagmin 2	127833	8,003085443	0,391195338	0,615068	0,636020033	0,524763365	NA
ENSG00000143867	OSR1	odd-skipped related transcription factor 1	130497	0	NA	NA	NA	NA	NA
ENSG00000143869	GDF7	growth differentiation factor 7	151449	0,103998153	-1,076443944	4,080456	-0,263804824	0,791930317	NA
ENSG00000143882	ATP6V1C2	ATPase H+ transporting V1 subunit C2	245973	0	NA	NA	NA	NA	NA
ENSG00000143921	ABCG8	ATP binding cassette subfamily G member 8	64241	0	NA	NA	NA	NA	NA
ENSG00000143942	CHAC2	ChaC glutathione specific gamma-glutamylcyclotransferase 2	494143	11,58659194	-0,341671407	0,563773	-0,606044731	0,544485051	NA
ENSG00000143954	REG3G	regenerating family member 3 gamma	130120	0	NA	NA	NA	NA	NA
ENSG00000143994	ABHD1	abhydrolase domain containing 1	84696	0	NA	NA	NA	NA	NA
ENSG00000143995	MEIS1	Meis homeobox 1	4211	10,56586291	-0,370027739	0,891445	-0,41508768	0,678077718	NA
ENSG00000144010	TRIM43B	tripartite motif containing 43B	653192	0	NA	NA	NA	NA	NA
ENSG00000144031	ANKRD53	ankyrin repeat domain 53	79998	0,10543123	0	4,080456	0	1	NA
ENSG00000144045	DQX1	DEAQ-box RNA dependent ATPase 1	165545	0	NA	NA	NA	NA	NA
ENSG00000144057	ST6GAL2	ST6 beta-galactoside alpha-2,6-sialyltransferase 2	84620	0	NA	NA	NA	NA	NA
ENSG00000144061	NPHP1	nephrocystin 1	4867	0,905238985	-2,370366013	2,985875	-0,793859734	0,427277093	NA
ENSG00000144063	MALL	mal, T cell differentiation protein like	7851	0	NA	NA	NA	NA	NA
ENSG00000144115	THNSL2	threonine synthase like 2	55258	0,117497108	0,847101401	4,080456	0,207599697	0,835541541	NA
ENSG00000144119	C1QL2	complement C1q like 2	165257	0	NA	NA	NA	NA	NA
ENSG00000144120	TMEM177	transmembrane protein 177	80775	8,36180672	-0,913125457	0,65957	-1,38442475	0,166228435	NA

ENSG00000144130	NT5DC4	5'-nucleotidase domain containing 4	284958	0	NA	NA	NA	NA	NA
ENSG00000144134	RABL2A	RAB, member of RAS oncogene family like 2A	11159	9,140697404	-0,582056875	1,002252	-0,580749054	0,561409596	NA
ENSG00000144152	FBLN7	fibulin 7	129804	0	NA	NA	NA	NA	NA
ENSG00000144158	NA	NA	NA	0,072165449	0,847101401	4,080456	0,207599697	0,835541541	NA
ENSG00000144191	CNGA3	cyclic nucleotide gated channel subunit alpha 3	1261	0,072165449	0,847101401	4,080456	0,207599697	0,835541541	NA
ENSG00000144214	LYG1	lysozyme g1	129530	2,933624593	0,092448716	1,12446	0,082216065	0,934474899	NA
ENSG00000144218	AFF3	ALF transcription elongation factor 3	3899	0	NA	NA	NA	NA	NA
ENSG00000144227	NXPH2	neurexophilin 2	11249	0	NA	NA	NA	NA	NA
ENSG00000144229	THSD7B	thrombospondin type 1 domain containing 7B	80731	0	NA	NA	NA	NA	NA
ENSG00000144230	GPR17	G protein-coupled receptor 17	2840	0	NA	NA	NA	NA	NA
ENSG00000144278	GALNT13	polypeptide N-acetylgalactosaminyltransferase 13	114805	0	NA	NA	NA	NA	NA
ENSG00000144285	SCN1A	sodium voltage-gated channel alpha subunit 1	6323	0	NA	NA	NA	NA	NA
ENSG00000144290	SLC4A10	solute carrier family 4 member 10	57282	7,31117482	0,269081892	0,648453	0,414959484	0,678171563	NA
ENSG00000144331	ZNF385B	zinc finger protein 385B	151126	0	NA	NA	NA	NA	NA
ENSG00000144339	TMEFF2	transmembrane protein with EGF like and two follistatin like domains 2	23671	0	NA	NA	NA	NA	NA
ENSG00000144354	CDCA7	cell division cycle associated 7	83879	7,229839249	0,739613599	1,131305	0,653770378	0,513259753	NA
ENSG00000144366	GULP1	GULP PTB domain containing engulfment adaptor 1	51454	0	NA	NA	NA	NA	NA
ENSG00000144369	FAM171B	family with sequence similarity 171 member B	165215	5,22588287	-0,218282884	0,926353	-0,235636859	0,813714469	NA
ENSG00000144395	CCDC150	coiled-coil domain containing 150	284992	1,538015607	1,371681508	2,462663	0,556991117	0,577533498	NA
ENSG00000144406	UNC80	unc-80 homolog, NALCN channel complex subunit	285175	0	NA	NA	NA	NA	NA
ENSG00000144407	PTH2R	parathyroid hormone 2 receptor	5746	0,927675939	-0,857621985	2,134441	-0,40180161	0,687830037	NA
ENSG00000144410	CPO	carboxypeptidase O	130749	0,072165449	0,847101401	4,080456	0,207599697	0,835541541	NA
ENSG00000144445	KANSL1L	KAT8 regulatory NSL complex subunit 1 like	151050	4,337354191	0,318132713	1,309581	0,242927038	0,80806192	NA
ENSG00000144452	ABCA12	ATP binding cassette subfamily A member 12	26154	0	NA	NA	NA	NA	NA
ENSG00000144460	NYAP2	neuronal tyrosine-phosphorylated phosphoinositide-3-kinase adaptor 2	57624	0,454642586	2,515445141	3,403842	0,739001819	0,459905893	NA
ENSG00000144476	ACKR3	atypical chemokine receptor 3	57007	6,239932174	-0,075145803	0,750071	-0,10018486	0,920197565	NA
ENSG00000144481	TRPM8	transient receptor potential cation channel subfamily M member 8	79054	7,152301811	0,042077279	0,810498	0,05191534	0,958596151	NA
ENSG00000144488	ESPNL	espin like	339768	0,709921793	-1,054234885	3,773256	-0,279396572	0,779940501	NA
ENSG00000144550	CPNE9	copine family member 9	151835	0,226845572	0,896625805	4,07866	0,219833434	0,82600088	NA
ENSG00000144583	MARCHF4	membrane associated ring-CH-type finger 4	57574	0,103998153	-1,076443944	4,080456	-0,263804824	0,791930317	NA
ENSG00000144596	GRIP2	glutamate receptor interacting protein 2	80852	0	NA	NA	NA	NA	NA
ENSG00000144619	CNTN4	contactin 4	152330	0	NA	NA	NA	NA	NA
ENSG00000144642	RBMS3	RNA binding motif single stranded interacting protein 3	27303	0,144330898	1,418786819	4,061262	0,349346267	0,726829368	NA
ENSG00000144644	GADL1	glutamate decarboxylase like 1	339896	0	NA	NA	NA	NA	NA
ENSG00000144645	OSBPL10	oxysterol binding protein like 10	114884	0	NA	NA	NA	NA	NA
ENSG00000144649	GASK1A	golgi associated kinase 1A	729085	0	NA	NA	NA	NA	NA
ENSG00000144671	SLC22A14	solute carrier family 22 member 14	9389	0	NA	NA	NA	NA	NA
ENSG00000144681	STAC	SH3 and cysteine rich domain	6769	2,980937486	-1,226088634	1,322289	-0,927247407	0,353798094	NA
ENSG00000144712	CAND2	cullin associated and neddylation dissociated 2 (putative)	23066	0	NA	NA	NA	NA	NA
ENSG00000144724	PTPRG	protein tyrosine phosphatase receptor type G	5793	8,169268727	0,35923694	0,78889	0,455369916	0,648843135	NA
ENSG00000144730	IL17RD	interleukin 17 receptor D	54756	1,352760801	-0,504119148	1,859003	-0,271177166	0,786254774	NA
ENSG00000144771	LRTM1	leucine rich repeats and transmembrane domains 1	57408	0	NA	NA	NA	NA	NA
ENSG00000144785	NA	NA	NA	2,821600551	-0,250088678	2,317078	-0,107932793	0,914049004	NA

ENSG00000144810	COL8A1	collagen type VIII alpha 1 chain	1295	0	NA	NA	NA	NA	NA
ENSG00000144820	ADGRG7	adhesion G protein-coupled receptor G7	84873	0	NA	NA	NA	NA	NA
ENSG00000144821	MYH15	myosin heavy chain 15	22989	0,262361852	1,410283587	4,061078	0,347268296	0,728389767	NA
ENSG00000144824	PHLDB2	pleckstrin homology like domain family B member 2	90102	7,040338319	-0,902951522	0,912457	-0,989582339	0,322378306	NA
ENSG00000144834	TAGLN3	transgelin 3	29114	0,354310989	-1,281527926	4,006514	-0,319861083	0,749073641	NA
ENSG00000144837	PLA1A	phospholipase A1 member A	51365	0	NA	NA	NA	NA	NA
ENSG00000144847	IGSF11	immunoglobulin superfamily member 11	152404	1,201742094	2,10090122	2,070529	1,014668892	0,310263688	NA
ENSG00000144852	NR1I2	nuclear receptor subfamily 1 group I member 2	8856	0	NA	NA	NA	NA	NA
ENSG00000144857	BOC	BOC cell adhesion associated, oncogene regulated	91653	0	NA	NA	NA	NA	NA
ENSG00000144868	TMEM108	transmembrane protein 108	66000	0	NA	NA	NA	NA	NA
ENSG00000144891	AGTR1	angiotensin II receptor type 1	185	0	NA	NA	NA	NA	NA
ENSG00000144908	ALDH1L1	aldehyde dehydrogenase 1 family member L1	10840	0	NA	NA	NA	NA	NA
ENSG00000144935	TRPC1	transient receptor potential cation channel subfamily C member 1	7220	0,176385574	-0,094012413	4,080456	-0,023039684	0,981618618	NA
ENSG00000144962	SPATA16	spermatogenesis associated 16	83893	0	NA	NA	NA	NA	NA
ENSG00000145002	FAM86B2	family with sequence similarity 86 member B2	653333	0	NA	NA	NA	NA	NA
ENSG00000145040	UCN2	urocortin 2	90226	0	NA	NA	NA	NA	NA
ENSG00000145087	STXBP5L	syntaxin binding protein 5L	9515	0	NA	NA	NA	NA	NA
ENSG00000145088	EAF2	ELL associated factor 2	55840	5,102621356	0,501126347	0,870734	0,575521549	0,564938621	NA
ENSG00000145103	ILDR1	immunoglobulin like domain containing receptor 1	286676	0	NA	NA	NA	NA	NA
ENSG00000145107	TM4SF19	transmembrane 4 L six family member 19	116211	0	NA	NA	NA	NA	NA
ENSG00000145113	MUC4	mucin 4, cell surface associated	4585	0	NA	NA	NA	NA	NA
ENSG00000145147	SLIT2	slit guidance ligand 2	9353	0,119673877	0,847101401	4,080456	0,207599697	0,835541541	NA
ENSG00000145192	AHSG	alpha 2-HS glycoprotein	197	0	NA	NA	NA	NA	NA
ENSG00000145194	ECE2	endothelin converting enzyme 2	9718	0	NA	NA	NA	NA	NA
ENSG00000145198	VWA5B2	von Willebrand factor A domain containing 5B2	90113	0	NA	NA	NA	NA	NA
ENSG00000145217	SLC26A1	solute carrier family 26 member 1	10861	0,48836734	-0,136373328	2,99367	-0,045553902	0,963665812	NA
ENSG00000145242	EPHA5	EPH receptor A5	2044	0,119673877	0,847101401	4,080456	0,207599697	0,835541541	NA
ENSG00000145244	CORIN	corin, serine peptidase	10699	0	NA	NA	NA	NA	NA
ENSG00000145248	SLC10A4	solute carrier family 10 member 4	201780	0	NA	NA	NA	NA	NA
ENSG00000145283	SLC10A6	solute carrier family 10 member 6	345274	0	NA	NA	NA	NA	NA
ENSG00000145309	CABS1	calcium binding protein, spermatid associated 1	85438	0	NA	NA	NA	NA	NA
ENSG00000145321	GC	GC vitamin D binding protein	2638	0	NA	NA	NA	NA	NA
ENSG00000145335	SNCA	synuclein alpha	6622	0,897604642	2,595964961	3,064467	0,847118009	0,396929348	NA
ENSG00000145362	ANK2	ankyrin 2	287	0	NA	NA	NA	NA	NA
ENSG00000145384	FABP2	fatty acid binding protein 2	2169	0,103998153	-1,076443944	4,080456	-0,263804824	0,791930317	NA
ENSG00000145416	MARCHF1	membrane associated ring-CH-type finger 1	55016	0,10488184	-1,076443944	4,080456	-0,263804824	0,791930317	NA
ENSG00000145423	SFRP2	secreted frizzled related protein 2	6423	0	NA	NA	NA	NA	NA
ENSG00000145428	RNF175	ring finger protein 175	285533	0	NA	NA	NA	NA	NA
ENSG00000145431	PDGFC	platelet derived growth factor C	56034	0	NA	NA	NA	NA	NA
ENSG00000145451	GLRA3	glycine receptor alpha 3	8001	0	NA	NA	NA	NA	NA
ENSG00000145491	ROPN1L	rhophilin associated tail protein 1 like	83853	0,794677744	-0,870113978	2,419385	-0,359642644	0,719114389	NA
ENSG00000145506	NKD2	NKD inhibitor of WNT signaling pathway 2	85409	0	NA	NA	NA	NA	NA
ENSG00000145526	CDH18	cadherin 18	1016	0	NA	NA	NA	NA	NA
ENSG00000145536	ADAMTS16	ADAM metallopeptidase with thrombospondin type 1 motif 16	170690	0	NA	NA	NA	NA	NA
ENSG00000145555	MYO10	myosin X	4651	0,670408108	1,925878253	3,255566	0,59156488	0,554141997	NA
ENSG00000145623	OSMR	oncostatin M receptor	9180	0,117497108	0,847101401	4,080456	0,207599697	0,835541541	NA
ENSG00000145626	UGT3A1	UDP glycosyltransferase family 3 member A1	133688	0	NA	NA	NA	NA	NA
ENSG00000145632	PLK2	polo like kinase 2	10769	2,61314363	5,31649459	1,635355	3,250972519	0,00115011	NA
ENSG00000145642	SHISAL2B	shisa like 2B	100132916	0	NA	NA	NA	NA	NA
ENSG00000145681	HAPLN1	hyaluronan and proteoglycan link protein 1	1404	0	NA	NA	NA	NA	NA

ENSG00000145685	LHFPL2	LHFPL tetraspan subfamily member 2	10184	0,97900373	-0,551984721	1,667407	-0,331043782	0,740611415	NA
ENSG00000145692	BHMT	betaine--homocysteine S-methyltransferase	635	0	NA	NA	NA	NA	NA
ENSG00000145700	ANKRD31	ankyrin repeat domain 31	256006	0	NA	NA	NA	NA	NA
ENSG00000145708	CRHBP	corticotropin releasing hormone binding protein	1393	0	NA	NA	NA	NA	NA
ENSG00000145721	LIX1	limb and CNS expressed 1	167410	0	NA	NA	NA	NA	NA
ENSG00000145757	SPATA9	spermatogenesis associated 9	83890	0	NA	NA	NA	NA	NA
ENSG00000145777	TSLP	thymic stromal lymphopoietin	85480	0	NA	NA	NA	NA	NA
ENSG00000145794	MEGF10	multiple EGF like domains 10	84466	0	NA	NA	NA	NA	NA
ENSG00000145808	ADAMTS19	ADAM metallopeptidase with thrombospondin type 1 motif 19	171019	0	NA	NA	NA	NA	NA
ENSG00000145824	CXCL14	C-X-C motif chemokine ligand 14	9547	0,118286652	0	4,080456	0	1	NA
ENSG00000145826	LECT2	leukocyte cell derived chemotaxin 2	3950	0	NA	NA	NA	NA	NA
ENSG00000145832	SLC25A48	solute carrier family 25 member 48	153328	0	NA	NA	NA	NA	NA
ENSG00000145835	NA	NA	NA	0	NA	NA	NA	NA	NA
ENSG00000145839	IL9	interleukin 9	3578	0	NA	NA	NA	NA	NA
ENSG00000145850	TIMD4	T cell immunoglobulin and mucin domain containing 4	91937	10,91661463	-0,440518465	0,604874	-0,728281921	0,466441027	NA
ENSG00000145861	C1QTNF2	C1q and TNF related 2	114898	0	NA	NA	NA	NA	NA
ENSG00000145863	GABRA6	gamma-aminobutyric acid type A receptor subunit alpha6	2559	0	NA	NA	NA	NA	NA
ENSG00000145864	GABRB2	gamma-aminobutyric acid type A receptor subunit beta2	2561	0	NA	NA	NA	NA	NA
ENSG00000145879	SPINK7	serine peptidase inhibitor Kazal type 7	84651	0	NA	NA	NA	NA	NA
ENSG00000145888	GLRA1	glycine receptor alpha 1	2741	0	NA	NA	NA	NA	NA
ENSG00000145916	RMND5B	required for meiotic nuclear division 5 homolog B	64777	6,095250006	0,013221968	0,840384	0,015733251	0,9874472	NA
ENSG00000145920	CPLX2	complexin 2	10814	0	NA	NA	NA	NA	NA
ENSG00000145934	TENM2	teneurin transmembrane protein 2	57451	0	NA	NA	NA	NA	NA
ENSG00000145936	KCNMB1	potassium calcium-activated channel subfamily M regulatory beta subunit 1	3779	0	NA	NA	NA	NA	NA
ENSG00000145949	MYLK4	myosin light chain kinase family member 4	340156	8,886003303	-0,34061652	0,775573	-0,439180509	0,660530747	NA
ENSG00000145975	FAM217A	family with sequence similarity 217 member A	222826	0	NA	NA	NA	NA	NA
ENSG00000146005	PSD2	pleckstrin and Sec7 domain containing 2	84249	1,701485032	-0,942223273	1,772715	-0,531514184	0,595062515	NA
ENSG00000146006	LRRTM2	leucine rich repeat transmembrane neuronal 2	26045	1,039882709	-2,444170315	2,253803	-1,084465053	0,278158654	NA
ENSG00000146013	GFRA3	GDNF family receptor alpha 3	2676	0,119673877	0,847101401	4,080456	0,207599697	0,835541541	NA
ENSG00000146038	DCDC2	doublecortin domain containing 2	51473	0	NA	NA	NA	NA	NA
ENSG00000146039	SLC17A4	solute carrier family 17 member 4	10050	0	NA	NA	NA	NA	NA
ENSG00000146047	H2BC1	H2B clustered histone 1	255626	0	NA	NA	NA	NA	NA
ENSG00000146054	TRIM7	tripartite motif containing 7	81786	1,197212808	-3,609687281	2,376148	-1,519134087	0,128728748	NA
ENSG00000146070	PLA2G7	phospholipase A2 group VII	7941	0,072165449	0,847101401	4,080456	0,207599697	0,835541541	NA
ENSG00000146072	TNFRSF21	TNF receptor superfamily member 21	27242	0	NA	NA	NA	NA	NA
ENSG00000146090	RASGEF1C	RasGEF domain family member 1C	255426	0	NA	NA	NA	NA	NA
ENSG00000146094	DOK3	docking protein 3	79930	10,65631514	-0,088477105	0,6113	-0,144735966	0,884919342	NA
ENSG00000146122	DAAM2	dishevelled associated activator of morphogenesis 2	23500	0	NA	NA	NA	NA	NA
ENSG00000146147	MLIP	muscular LMNA interacting protein	90523	0	NA	NA	NA	NA	NA
ENSG00000146147	MLIP-IT1	MLIP intronic transcript 1	100874282	0	NA	NA	NA	NA	NA
ENSG00000146147	LOC101927189	uncharacterized LOC101927189	101927189	0	NA	NA	NA	NA	NA
ENSG00000146151	HMGCLL1	3-hydroxy-3-methylglutaryl-CoA lyase like 1	54511	0	NA	NA	NA	NA	NA
ENSG00000146166	LGSN	lengsin, lens protein with glutamine synthetase domain	51557	0,314893766	-1,922847908	4,042268	-0,475685396	0,634298527	NA
ENSG00000146197	SCUBE3	signal peptide, CUB domain and EGF like domain containing 3	222663	0,119673877	0,847101401	4,080456	0,207599697	0,835541541	NA

ENSG00000146205	ANO7	anoctamin 7	50636	1,994169373	-1,456761588	1,33416	-1,091894437	0,274879503	NA
ENSG00000146215	CRIP3	cysteine rich protein 3	401262	0	NA	NA	NA	NA	NA
ENSG00000146216	TTBK1	tau tubulin kinase 1	84630	0,508829506	-2,711004383	3,339293	-0,811849904	0,416877763	NA
ENSG00000146221	TCTE1	t-complex-associated-testis-expressed 1	202500	0,549491846	-0,444820034	3,325548	-0,133758417	0,89359361	NA
ENSG00000146233	CYP39A1	cytochrome P450 family 39 subfamily A member 1	51302	0	NA	NA	NA	NA	NA
ENSG00000146242	TPBG	trophoblast glycoprotein	7162	0	NA	NA	NA	NA	NA
ENSG00000146243	IRAK1BP1	interleukin 1 receptor associated kinase 1 binding protein 1	134728	10,69598361	0,632570648	0,558117	1,13340247	0,257045277	NA
ENSG00000146250	PRSS35	serine protease 35	167681	0,207996306	-1,916073151	4,043021	-0,473921178	0,635556117	NA
ENSG00000146267	FAXC	failed axon connections homolog, metaxin like GST domain containing	84553	0	NA	NA	NA	NA	NA
ENSG00000146276	GABRR1	gamma-aminobutyric acid type A receptor subunit rho1	2569	0	NA	NA	NA	NA	NA
ENSG00000146352	CLVS2	clavesin 2	134829	0	NA	NA	NA	NA	NA
ENSG00000146360	GPR6	G protein-coupled receptor 6	2830	0	NA	NA	NA	NA	NA
ENSG00000146373	RNF217	ring finger protein 217	154214	2,862273942	0,175860671	1,518333	0,115824854	0,907791353	NA
ENSG00000146374	RSPO3	R-spondin 3	84870	0	NA	NA	NA	NA	NA
ENSG00000146378	TAAR2	trace amine associated receptor 2	9287	0	NA	NA	NA	NA	NA
ENSG00000146383	TAAR6	trace amine associated receptor 6	319100	0	NA	NA	NA	NA	NA
ENSG00000146385	TAAR8	trace amine associated receptor 8	83551	0	NA	NA	NA	NA	NA
ENSG00000146399	TAAR1	trace amine associated receptor 1	134864	0	NA	NA	NA	NA	NA
ENSG00000146411	SLC2A12	solute carrier family 2 member 12	154091	1,429387154	2,686738608	1,892324	1,419808891	0,155663326	NA
ENSG00000146416	AIG1	androgen induced 1	51390	5,049114394	-0,329959644	0,823625	-0,400618899	0,688700729	NA
ENSG00000146453	PNLDC1	PARN like ribonuclease domain containing exonuclease 1	154197	0,484210139	-1,0753962	3,374358	-0,318696574	0,749956613	NA
ENSG00000146469	VIP	vasoactive intestinal peptide	7432	0	NA	NA	NA	NA	NA
ENSG00000146477	SLC22A3	solute carrier family 22 member 3	6581	0,917222578	-3,775398436	2,271778	-1,661869373	0,096538969	NA
ENSG00000146530	VWDE	von Willebrand factor D and EGF domains	221806	0	NA	NA	NA	NA	NA
ENSG00000146555	SDK1	sidekick cell adhesion molecule 1	221935	0	NA	NA	NA	NA	NA
ENSG00000146592	CREB5	cAMP responsive element binding protein 5	9586	0	NA	NA	NA	NA	NA
ENSG00000146618	FERD3L	Fer3 like bHLH transcription factor	222894	0,119673877	0,847101401	4,080456	0,207599697	0,835541541	NA
ENSG00000146648	EGFR	epidermal growth factor receptor	1956	0	NA	NA	NA	NA	NA
ENSG00000146674	IGFBP3	insulin like growth factor binding protein 3	3486	0	NA	NA	NA	NA	NA
ENSG00000146677	RPL32P18	ribosomal protein L32 pseudogene 18	644907	0,10488184	-1,076443944	4,080456	-0,263804824	0,791930317	NA
ENSG00000146678	IGFBP1	insulin like growth factor binding protein 1	3484	0	NA	NA	NA	NA	NA
ENSG00000146700	SSC4D	scavenger receptor cysteine rich family member with 4 domains	136853	0	NA	NA	NA	NA	NA
ENSG00000146707	POMZP3	POM121 and ZP3 fusion	22932	4,494892762	0	4,080456	0	1	NA
ENSG00000146722	NA	NA	NA	0	NA	NA	NA	NA	NA
ENSG00000146755	TRIM50	tripartite motif containing 50	135892	0	NA	NA	NA	NA	NA
ENSG00000146776	ATXN7L1	ataxin 7 like 1	222255	11,00829369	0,338243227	0,641455	0,52730616	0,597980997	NA
ENSG00000146809	ASB15	ankyrin repeat and SOCS box containing 15	142685	0	NA	NA	NA	NA	NA
ENSG00000146839	ZAN	zonadhesin	7455	0	NA	NA	NA	NA	NA
ENSG00000146856	AGBL3	AGBL carboxypeptidase 3	340351	0,503937522	3,066673511	3,960842	0,774247849	0,438784237	NA
ENSG00000146857	STRA8	stimulated by retinoic acid 8	346673	0	NA	NA	NA	NA	NA
ENSG00000146858	ZC3HAV1L	zinc finger CCCH-type containing, antiviral 1 like	92092	5,399182548	-0,663427663	0,918225	-0,722511389	0,469980126	NA
ENSG00000146910	CNPY1	canopy FGF signaling regulator 1	285888	0	NA	NA	NA	NA	NA
ENSG00000146926	ASB10	ankyrin repeat and SOCS box containing 10	136371	0	NA	NA	NA	NA	NA
ENSG00000146938	NLGN4X	neuroligin 4 X-linked	57502	0	NA	NA	NA	NA	NA
ENSG00000146950	SHROOM2	shroom family member 2	357	0,444615789	0,116411446	3,984185	0,029218383	0,97669042	NA
ENSG00000146966	DENND2A	DENN domain containing 2A	27147	0	NA	NA	NA	NA	NA
ENSG00000147003	CLTRN	collectrin, amino acid transport regulator	57393	0,314645521	-2,509429468	3,997603	-0,627733573	0,530178488	NA
ENSG00000147027	TMEM47	transmembrane protein 47	83604	0	NA	NA	NA	NA	NA
ENSG00000147041	SYTL5	synaptotagmin like 5	94122	0,221426773	-0,119764867	4,080456	-0,029350855	0,976584768	NA
ENSG00000147059	SPIN2A	spindlin family member 2A	54466	3,654661751	1,658947345	1,09648	1,512974996	0,13028601	NA

ENSG00000147081	AKAP4	A-kinase anchoring protein 4	8852	0	NA	NA	NA	NA	NA
ENSG00000147082	CCNB3	cyclin B3	85417	1,484406719	0,980239592	1,515952	0,646616537	0,517880152	NA
ENSG00000147100	SLC16A2	solute carrier family 16 member 2	6567	0	NA	NA	NA	NA	NA
ENSG00000147113	DIPK2B	divergent protein kinase domain 2B	79742	0	NA	NA	NA	NA	NA
ENSG00000147117	ZNF157	zinc finger protein 157	7712	0,592895667	-1,703463054	3,304862	-0,51544154	0,606244511	NA
ENSG00000147119	CHST7	carbohydrate sulfotransferase 7	56548	4,166820823	-0,967863797	0,944683	-1,024537831	0,305581312	NA
ENSG00000147127	RAB41	RAB41, member RAS oncogene family	347517	0	NA	NA	NA	NA	NA
ENSG00000147144	CCDC120	coiled-coil domain containing 120	90060	4,096284106	5,13389212	3,919748	1,309750396	0,190280289	NA
ENSG00000147145	LPAR4	lysophosphatidic acid receptor 4	2846	0,493734846	2,049257718	4,010462	0,51097799	0,609366469	NA
ENSG00000147160	AWAT2	acyl-CoA wax alcohol acyltransferase 2	158835	0	NA	NA	NA	NA	NA
ENSG00000147166	ITGB1BP2	integrin subunit beta 1 binding protein 2	26548	3,022222365	0,104317668	1,093805	0,095371354	0,924019868	NA
ENSG00000147180	ZNF711	zinc finger protein 711	7552	0,638035268	1,232616129	2,457583	0,501556221	0,615979721	NA
ENSG00000147183	CPXCR1	CPX chromosome region candidate 1	53336	0	NA	NA	NA	NA	NA
ENSG00000147206	NXF3	nuclear RNA export factor 3	56000	0	NA	NA	NA	NA	NA
ENSG00000147223	RIPPLY1	ripply transcriptional repressor 1	92129	0	NA	NA	NA	NA	NA
ENSG00000147234	FRMPD3	FERM and PDZ domain containing 3	84443	1,337680191	0,08112941	1,762048	0,046042667	0,963276242	NA
ENSG00000147246	HTR2C	5-hydroxytryptamine receptor 2C	3358	0	NA	NA	NA	NA	NA
ENSG00000147255	IGSF1	immunoglobulin superfamily member 1	3547	0	NA	NA	NA	NA	NA
ENSG00000147256	ARHGAP36	Rho GTPase activating protein 36	158763	0	NA	NA	NA	NA	NA
ENSG00000147257	GPC3	glypican 3	2719	0,118286652	0	4,080456	0	1	NA
ENSG00000147262	GPR119	G protein-coupled receptor 119	139760	0	NA	NA	NA	NA	NA
ENSG00000147378	FATE1	fetal and adult testis expressed 1	89885	0	NA	NA	NA	NA	NA
ENSG00000147381	MAGEA4	MAGE family member A4	4103	0	NA	NA	NA	NA	NA
ENSG00000147432	CHRNB3	cholinergic receptor nicotinic beta 3 subunit	1142	0	NA	NA	NA	NA	NA
ENSG00000147434	CHRNA6	cholinergic receptor nicotinic alpha 6 subunit	8973	0	NA	NA	NA	NA	NA
ENSG00000147454	SLC25A37	solute carrier family 25 member 37	51312	5,605792658	0,380732942	1,066647	0,35694361	0,721134021	NA
ENSG00000147465	STAR	steroidogenic acute regulatory protein	6770	0,072165449	0,847101401	4,080456	0,207599697	0,835541541	NA
ENSG00000147481	SNTG1	syntrophin gamma 1	54212	0	NA	NA	NA	NA	NA
ENSG00000147485	PXDNL	peroxidasin like	137902	0,86055137	0,767627892	2,450894	0,313203175	0,754126307	NA
ENSG00000147488	ST18	ST18 C2H2C-type zinc finger transcription factor	9705	0	NA	NA	NA	NA	NA
ENSG00000147509	RGS20	regulator of G protein signaling 20	8601	0	NA	NA	NA	NA	NA
ENSG00000147570	DNAJC5B	DnaJ heat shock protein family (Hsp40) member C5 beta	85479	0,072165449	0,847101401	4,080456	0,207599697	0,835541541	NA
ENSG00000147571	CRH	corticotropin releasing hormone	1392	0	NA	NA	NA	NA	NA
ENSG00000147573	TRIM55	tripartite motif containing 55	84675	0	NA	NA	NA	NA	NA
ENSG00000147576	ADHFE1	alcohol dehydrogenase iron containing 1	137872	3,463333055	-0,829245478	2,034746	-0,407542485	0,683609597	NA
ENSG00000147588	PMP2	peripheral myelin protein 2	5375	0	NA	NA	NA	NA	NA
ENSG00000147596	PRDM14	PR/SET domain 14	63978	0	NA	NA	NA	NA	NA
ENSG00000147606	SLC26A7	solute carrier family 26 member 7	115111	0	NA	NA	NA	NA	NA
ENSG00000147613	PSKH2	protein serine kinase H2	85481	0	NA	NA	NA	NA	NA
ENSG00000147614	ATP6V0D2	ATPase H+ transporting V0 subunit d2	245972	0	NA	NA	NA	NA	NA
ENSG00000147642	SYBU	syntabulin	55638	0	NA	NA	NA	NA	NA
ENSG00000147647	DPYS	dihydropyrimidinase	1807	0	NA	NA	NA	NA	NA
ENSG00000147655	RSPO2	R-spondin 2	340419	0	NA	NA	NA	NA	NA
ENSG00000147676	MAL2	mal, T cell differentiation protein 2	114569	0	NA	NA	NA	NA	NA
ENSG00000147689	FAM83A	family with sequence similarity 83 member A	84985	0,118286652	0	4,080456	0	1	NA
ENSG00000147697	GSDMC	gasdermin C	56169	0	NA	NA	NA	NA	NA
ENSG00000147724	FAM135B	family with sequence similarity 135 member B	51059	0	NA	NA	NA	NA	NA
ENSG00000147799	ARHGAP39	Rho GTPase activating protein 39	80728	0,10543123	0	4,080456	0	1	NA

ENSG00000147804	SLC39A4	solute carrier family 39 member 4	55630	0	NA	NA	NA	NA	NA
ENSG00000147852	VLDLR	very low density lipoprotein receptor	7436	0	NA	NA	NA	NA	NA
ENSG00000147862	NFIB	nuclear factor I B	4781	0	NA	NA	NA	NA	NA
ENSG00000147869	CER1	cerberus 1, DAN family BMP antagonist	9350	0	NA	NA	NA	NA	NA
ENSG00000147873	IFNA5	interferon alpha 5	3442	0	NA	NA	NA	NA	NA
ENSG00000147885	IFNA16	interferon alpha 16	3449	0	NA	NA	NA	NA	NA
ENSG00000147896	IFNK	interferon kappa	56832	0	NA	NA	NA	NA	NA
ENSG00000147912	FBXO10	F-box protein 10	26267	11,12622286	-0,670792538	0,556396	-1,205603359	0,227970463	NA
ENSG00000148053	NTRK2	neurotrophic receptor tyrosine kinase 2	4915	2,554812585	1,059455954	1,200657	0,882396753	0,377562291	NA
ENSG00000148082	SHC3	SHC adaptor protein 3	53358	0	NA	NA	NA	NA	NA
ENSG00000148120	AOPEP	aminopeptidase O (putative)	84909	7,349061773	-0,211927732	0,737535	-0,287345927	0,77384746	NA
ENSG00000148123	PLPPR1	phospholipid phosphatase related 1	54886	0	NA	NA	NA	NA	NA
ENSG00000148136	OR13C4	olfactory receptor family 13 subfamily C member 4	138804	0	NA	NA	NA	NA	NA
ENSG00000148143	ZNF462	zinc finger protein 462	58499	0,440225414	0,047962663	3,477269	0,013793198	0,988994969	NA
ENSG00000148156	ACTL7B	actin like 7B	10880	0,15836288	0	4,080456	0	1	NA
ENSG00000148200	NR6A1	nuclear receptor subfamily 6 group A member 1	2649	3,401865119	1,329773042	1,225498	1,085088068	0,277882652	NA
ENSG00000148204	CRB2	crumbs cell polarity complex component 2	286204	0,11808024	-1,076443944	4,080456	-0,263804824	0,791930317	NA
ENSG00000148215	OR5C1	olfactory receptor family 5 subfamily C member 1	392391	0	NA	NA	NA	NA	NA
ENSG00000148219	ASTN2	astrotactin 2	23245	6,082081114	0,545801106	0,848858	0,64298274	0,520235299	NA
ENSG00000148225	WDR31	WD repeat domain 31	114987	0	NA	NA	NA	NA	NA
ENSG00000148288	GBGT1	globoside alpha-1,3-N-acetylgalactosaminyltransferase 1 (FORS blood group)	26301	3,629988936	-0,461269631	1,063185	-0,43385642	0,664392708	NA
ENSG00000148344	PTGES	prostaglandin E synthase	9536	0	NA	NA	NA	NA	NA
ENSG00000148346	LCN2	lipocalin 2	3934	0	NA	NA	NA	NA	NA
ENSG00000148357	HMCN2	hemicentin 2	256158	0	NA	NA	NA	NA	NA
ENSG00000148377	IDI2	isopentenyl-diphosphate delta isomerase 2	91734	0,117497108	0,847101401	4,080456	0,207599697	0,835541541	NA
ENSG00000148386	LCN9	lipocalin 9	392399	0	NA	NA	NA	NA	NA
ENSG00000148408	CACNA1B	calcium voltage-gated channel subunit alpha1 B	774	0,277254918	0	4,080456	0	1	NA
ENSG00000148426	PROSER2	proline and serine rich 2	254427	0,20888193	-1,921375531	4,04253	-0,475290357	0,634580032	NA
ENSG00000148429	USP6NL	USP6 N-terminal like	9712	0,261376721	-0,211883044	4,052312	-0,052286956	0,958300047	NA
ENSG00000148450	MSRB2	methionine sulfoxide reductase B2	22921	0,072165449	0,847101401	4,080456	0,207599697	0,835541541	NA
ENSG00000148459	PDSS1	decaprenyl diphosphate synthase subunit 1	23590	11,0304672	0,751408836	0,551389	1,362755758	0,172959499	NA
ENSG00000148468	FAM171A1	family with sequence similarity 171 member A1	221061	0,10488184	-1,076443944	4,080456	-0,263804824	0,791930317	NA
ENSG00000148482	SLC39A12	solute carrier family 39 member 12	221074	0,10543123	0	4,080456	0	1	NA
ENSG00000148483	TMEM236	transmembrane protein 236	653567	4,503104474	0,09719996	0,950892	0,102219764	0,918582241	NA
ENSG00000148498	PARD3	par-3 family cell polarity regulator	56288	1,340119099	-1,151972018	2,074368	-0,55533639	0,578664593	NA
ENSG00000148513	ANKRD30A	ankyrin repeat domain 30A	91074	0	NA	NA	NA	NA	NA
ENSG00000148541	FAM13C	family with sequence similarity 13 member C	220965	0	NA	NA	NA	NA	NA
ENSG00000148584	A1CF	APOBEC1 complementation factor	29974	0	NA	NA	NA	NA	NA
ENSG00000148602	LRIT1	leucine rich repeat, Ig-like and transmembrane domains 1	26103	0	NA	NA	NA	NA	NA
ENSG00000148604	RGR	retinal G protein coupled receptor	5995	0	NA	NA	NA	NA	NA
ENSG00000148655	LRMDA	leucine rich melanocyte differentiation associated	83938	0	NA	NA	NA	NA	NA
ENSG00000148671	ADIRF	adipogenesis regulatory factor	10974	0	NA	NA	NA	NA	NA
ENSG00000148677	ANKRD1	ankyrin repeat domain 1	27063	0	NA	NA	NA	NA	NA
ENSG00000148680	HTR7	5-hydroxytryptamine receptor 7	3363	0	NA	NA	NA	NA	NA
ENSG00000148702	HABP2	hyaluronan binding protein 2	3026	0	NA	NA	NA	NA	NA
ENSG00000148704	VAX1	ventral anterior homeobox 1	11023	0	NA	NA	NA	NA	NA
ENSG00000148734	NPFFR1	neuropeptide FF receptor 1	64106	0,312934741	-2,502299275	3,998048	-0,625880325	0,53139344	NA
ENSG00000148735	PLEKHS1	pleckstrin homology domain containing S1	79949	0,207996306	-1,916073151	4,043021	-0,473921178	0,635556117	NA
ENSG00000148737	TCF7L2	transcription factor 7 like 2	6934	0,664295148	-1,363883959	2,670426	-0,510736417	0,609535637	NA
ENSG00000148795	CYP17A1	cytochrome P450 family 17 subfamily A member 1	1586	0	NA	NA	NA	NA	NA

ENSG00000148798	INA	internexin neuronal intermediate filament protein alpha	9118	0,10543123	0	4,080456	0	1	NA
ENSG00000148826	NKX6-2	NK6 homeobox 2	84504	0	NA	NA	NA	NA	NA
ENSG00000148926	ADM	adrenomedullin	133	0,119673877	0,847101401	4,080456	0,207599697	0,835541541	NA
ENSG00000148935	GAS2	growth arrest specific 2	2620	0	NA	NA	NA	NA	NA
ENSG00000148942	SLC5A12	solute carrier family 5 member 12	159963	0	NA	NA	NA	NA	NA
ENSG00000148948	LRRC4C	leucine rich repeat containing 4C	57689	0	NA	NA	NA	NA	NA
ENSG00000148950	IMMP1L	inner mitochondrial membrane peptidase subunit 1	196294	8,849161416	0,295108267	0,638566	0,462142085	0,643979432	NA
ENSG00000148965	SAA4	serum amyloid A4, constitutive	6291	0	NA	NA	NA	NA	NA
ENSG00000149021	SCGB1A1	secretoglobin family 1A member 1	7356	0	NA	NA	NA	NA	NA
ENSG00000149043	SYT8	synaptotagmin 8	90019	0	NA	NA	NA	NA	NA
ENSG00000149050	ZNF214	zinc finger protein 214	7761	0	NA	NA	NA	NA	NA
ENSG00000149054	ZNF215	zinc finger protein 215	7762	2,646445153	0,372063029	1,183484	0,314379492	0,75323283	NA
ENSG00000149090	PAMR1	peptidase domain containing associated with muscle regeneration 1	25891	0	NA	NA	NA	NA	NA
ENSG00000149124	GLYAT	glycine-N-acyltransferase	10249	0	NA	NA	NA	NA	NA
ENSG00000149131	SERPING1	serpin family G member 1	710	0	NA	NA	NA	NA	NA
ENSG00000149133	OR5F1	olfactory receptor family 5 subfamily F member 1	338674	0	NA	NA	NA	NA	NA
ENSG00000149201	CCDC81	coiled-coil domain containing 81	60494	1,795479399	-0,602087265	1,447269	-0,416016085	0,677398233	NA
ENSG00000149243	KLHL35	kelch like family member 35	283212	0,351702411	-0,361086518	4,024629	-0,089719211	0,92851035	NA
ENSG00000149256	TENM4	teneurin transmembrane protein 4	26011	0	NA	NA	NA	NA	NA
ENSG00000149260	CAPN5	calpain 5	726	0,612316921	-1,133993668	3,295399	-0,344114257	0,730760361	NA
ENSG00000149292	TTC12	tetratricopeptide repeat domain 12	54970	10,60049816	0,141073967	0,949338	0,148602388	0,881867388	NA
ENSG00000149295	DRD2	dopamine receptor D2	1813	0	NA	NA	NA	NA	NA
ENSG00000149300	C11orf52	chromosome 11 open reading frame 52	91894	0	NA	NA	NA	NA	NA
ENSG00000149305	HTR3B	5-hydroxytryptamine receptor 3B	9177	0	NA	NA	NA	NA	NA
ENSG00000149328	GLB1L2	galactosidase beta 1 like 2	89944	0,340974805	-1,07822114	4,080456	-0,264240363	0,791594711	NA
ENSG00000149380	P4HA3	prolyl 4-hydroxylase subunit alpha 3	283208	0	NA	NA	NA	NA	NA
ENSG00000149403	GRIK4	glutamate ionotropic receptor kainate type subunit 4	2900	1,418237368	1,164917047	1,836115	0,634446648	0,525789374	NA
ENSG00000149418	ST14	ST14 transmembrane serine protease matriptase	6768	0	NA	NA	NA	NA	NA
ENSG00000149435	GGTLC1	gamma-glutamyltransferase light chain 1	92086	0	NA	NA	NA	NA	NA
ENSG00000149451	ADAM33	ADAM metallopeptidase domain 33	80332	0	NA	NA	NA	NA	NA
ENSG00000149452	SLC22A8	solute carrier family 22 member 8	9376	0	NA	NA	NA	NA	NA
ENSG00000149488	TMC2	transmembrane channel like 2	117532	0,517559288	-1,604343851	2,505737	-0,640268321	0,521998172	NA
ENSG00000149489	ROM1	retinal outer segment membrane protein 1	6094	2,851537874	-0,221318845	1,03533	-0,213766399	0,830729229	NA
ENSG00000149506	ZP1	zona pellucida glycoprotein 1	22917	0	NA	NA	NA	NA	NA
ENSG00000149507	OOSP2	oocyte secreted protein 2	219990	0	NA	NA	NA	NA	NA
ENSG00000149516	MS4A3	membrane spanning 4-domains A3	932	0	NA	NA	NA	NA	NA
ENSG00000149534	MS4A2	membrane spanning 4-domains A2	2206	0,117497108	0,847101401	4,080456	0,207599697	0,835541541	NA
ENSG00000149557	FEZ1	fasciculation and elongation protein zeta 1	9638	1,433812948	1,676898459	2,012211	0,833361109	0,404641102	NA
ENSG00000149557	STT3A-AS1	STT3A antisense RNA 1	105369550	1,433812948	1,676898459	2,012211	0,833361109	0,404641102	NA
ENSG00000149564	ESAM	endothelial cell adhesion molecule	90952	0,49833027	-1,072978835	3,447894	-0,311198361	0,755649829	NA
ENSG00000149571	KIRREL3	kirre like nephrin family adhesion molecule 3	84623	0	NA	NA	NA	NA	NA
ENSG00000149575	SCN2B	sodium voltage-gated channel beta subunit 2	6327	0	NA	NA	NA	NA	NA
ENSG00000149596	JPH2	junctophilin 2	57158	0	NA	NA	NA	NA	NA
ENSG00000149599	DUSP15	dual specificity phosphatase 15	128853	0	NA	NA	NA	NA	NA
ENSG00000149609	C20orf144	chromosome 20 open reading frame 144	128864	0	NA	NA	NA	NA	NA
ENSG00000149633	KIAA1755	KIAA1755	85449	0	NA	NA	NA	NA	NA

ENSG00000149634	SPATA25	spermatogenesis associated 25	128497	0,911616737	-0,791576801	1,952657	-0,405384455	0,685194936	NA
ENSG00000149635	OCSTAMP	osteoclast stimulatory transmembrane protein	128506	0,117497108	0,847101401	4,080456	0,207599697	0,835541541	NA
ENSG00000149646	CNBD2	cyclic nucleotide binding domain containing 2	140894	0,556378837	-1,688197367	2,472954	-0,682664418	0,494818919	NA
ENSG00000149651	SPINT4	serine peptidase inhibitor, Kunitz type 4	391253	0	NA	NA	NA	NA	NA
ENSG00000149654	CDH22	cadherin 22	64405	0	NA	NA	NA	NA	NA
ENSG00000149656	NA	NA	NA	0,83117866	0,958668674	2,601653	0,368484409	0,712512069	NA
ENSG00000149735	GPHA2	glycoprotein hormone subunit alpha 2	170589	0	NA	NA	NA	NA	NA
ENSG00000149742	SLC22A9	solute carrier family 22 member 9	114571	0	NA	NA	NA	NA	NA
ENSG00000149798	CDC42EP2	CDC42 effector protein 2	10435	0	NA	NA	NA	NA	NA
ENSG00000149809	TM7SF2	transmembrane 7 superfamily member 2	7108	9,705952575	-1,606429645	2,070501	-0,775865192	0,437828587	NA
ENSG00000149922	TBX6	T-box transcription factor 6	6911	2,233489062	-2,150740108	1,42695	-1,507229161	0,131751928	NA
ENSG00000149926	TLCD3B	TLC domain containing 3B	83723	1,677669182	2,96477107	1,927813	1,537893547	0,124074645	NA
ENSG00000149927	DOC2A	double C2 domain alpha	8448	0	NA	NA	NA	NA	NA
ENSG00000149948	HMGA2	high mobility group AT-hook 2	8091	0,239347754	1,967469188	4,021404	0,489249276	0,624665227	NA
ENSG00000149968	MMP3	matrix metallopeptidase 3	4314	0	NA	NA	NA	NA	NA
ENSG00000149970	CNKSR2	connector enhancer of kinase suppressor of Ras 2	22866	1,504565336	-1,418770397	2,151561	-0,659414473	0,509629651	NA
ENSG00000149972	CNTN5	contactin 5	53942	0	NA	NA	NA	NA	NA
ENSG00000150048	CLEC1A	C-type lectin domain family 1 member A	51267	0	NA	NA	NA	NA	NA
ENSG00000150051	MKX	mohawk homeobox	283078	0	NA	NA	NA	NA	NA
ENSG00000150054	MPP7	MAGUK p55 scaffold protein 7	143098	7,688814973	-0,932456043	1,075641	-0,866884415	0,386005344	NA
ENSG00000150175	FRMPD2B	FERM and PDZ domain containing 2B (pseudogene)	728798	0,10543123	0	4,080456	0	1	NA
ENSG00000150201	FXYD4	FXYD domain containing ion transport regulator 4	53828	0	NA	NA	NA	NA	NA
ENSG00000150244	TRIM48	tripartite motif containing 48	79097	0	NA	NA	NA	NA	NA
ENSG00000150275	PCDH15	protocadherin related 15	65217	0	NA	NA	NA	NA	NA
ENSG00000150276	PPIAP26	peptidylprolyl isomerase A pseudogene 26	121981	0	NA	NA	NA	NA	NA
ENSG00000150281	CTF1	cardiotrophin 1	1489	0,841984599	0,949971418	2,26074	0,420203816	0,674336567	NA
ENSG00000150337	FCGR1A	Fc gamma receptor Ia	2209	0	NA	NA	NA	NA	NA
ENSG00000150361	KLHL1	kelch like family member 1	57626	0	NA	NA	NA	NA	NA
ENSG00000150394	CDH8	cadherin 8	1006	0,118286652	0	4,080456	0	1	NA
ENSG00000150456	EEF1AKMT1	EEF1A lysine methyltransferase 1	221143	10,89954628	-0,469535424	0,570633	-0,822831895	0,410603596	NA
ENSG00000150471	ADGRL3	adhesion G protein-coupled receptor L3	23284	0	NA	NA	NA	NA	NA
ENSG00000150510	FAM124A	family with sequence similarity 124 member A	220108	0	NA	NA	NA	NA	NA
ENSG00000150540	HNMT	histamine N-methyltransferase	3176	0	NA	NA	NA	NA	NA
ENSG00000150551	LYPD1	LY6/PLAUR domain containing 1	116372	0	NA	NA	NA	NA	NA
ENSG00000150556	LYPD6B	LY6/PLAUR domain containing 6B	130576	0	NA	NA	NA	NA	NA
ENSG00000150594	ADRA2A	adrenoceptor alpha 2A	150	0,10543123	0	4,080456	0	1	NA
ENSG00000150625	GPM6A	glycoprotein M6A	2823	0	NA	NA	NA	NA	NA
ENSG00000150627	WDR17	WD repeat domain 17	116966	0,103998153	-1,076443944	4,080456	-0,263804824	0,791930317	NA
ENSG00000150628	SPATA4	spermatogenesis associated 4	132851	0	NA	NA	NA	NA	NA
ENSG00000150630	VEGFC	vascular endothelial growth factor C	7424	0	NA	NA	NA	NA	NA
ENSG00000150636	CCDC102B	coiled-coil domain containing 102B	79839	0,358844176	1,391891512	4,008947	0,347196314	0,72844384	NA
ENSG00000150656	CNDP1	carnosine dipeptidase 1	84735	0,368234516	-1,929388565	4,04188	-0,47734929	0,633113418	NA
ENSG00000150667	FSIP1	fibrous sheath interacting protein 1	161835	2,502700996	-0,300847469	1,331599	-0,225929456	0,821256287	NA
ENSG00000150672	DLG2	discs large MAGUK scaffold protein 2	1740	3,724083208	-1,231286554	1,204833	-1,021955827	0,306801808	NA
ENSG00000150676	CCDC83	coiled-coil domain containing 83	220047	0	NA	NA	NA	NA	NA
ENSG00000150681	RGS18	regulator of G protein signaling 18	64407	4,966254876	1,202460372	0,902242	1,332747795	0,182614583	NA
ENSG00000150687	PRSS23	serine protease 23	11098	0,348032559	-2,640923009	3,989966	-0,661891072	0,508041034	NA
ENSG00000150722	PPP1R1C	protein phosphatase 1 regulatory inhibitor subunit 1C	151242	0	NA	NA	NA	NA	NA
ENSG00000150732	NA	NA	NA	0	NA	NA	NA	NA	NA
ENSG00000150750	POU2AF2	POU class 2 homeobox associating factor 2	341032	0	NA	NA	NA	NA	NA

ENSG00000150760	DOCK1	dedicator of cytokinesis 1	1793	2,637075659	0,548955898	1,300285	0,422181313	0,67289268	NA
ENSG00000150764	DIXDC1	DIX domain containing 1	85458	0,444172694	-0,72094792	3,987096	-0,180820293	0,856508634	NA
ENSG00000150773	PIH1D2	PIH1 domain containing 2	120379	0,279523518	0,859567177	4,080456	0,210654693	0,833156731	NA
ENSG00000150783	TEX12	testis expressed 12	56158	0,31535374	-2,512344685	3,99743	-0,628490041	0,529682969	NA
ENSG00000150787	PTS	6-pyruvoyltetrahydropterin synthase	5805	3,813242294	-1,424116113	1,066004	-1,335939333	0,181569103	NA
ENSG00000150873	C2orf50	chromosome 2 open reading frame 50	130813	1,250810637	-0,714876262	2,084416	-0,342962387	0,731626757	NA
ENSG00000150893	FREM2	FRAS1 related extracellular matrix 2	341640	0	NA	NA	NA	NA	NA
ENSG00000150967	ABCB9	ATP binding cassette subfamily B member 9	23457	6,158970579	1,323479173	0,893827	1,480687503	0,138689866	NA
ENSG00000151005	TKTL2	transketolase like 2	84076	0	NA	NA	NA	NA	NA
ENSG00000151006	PRSS53	serine protease 53	339105	5,616320622	-0,268581766	1,722343	-0,155939749	0,876080513	NA
ENSG00000151023	ENKUR	enkurin, TRPC channel interacting protein	219670	0	NA	NA	NA	NA	NA
ENSG00000151025	GPR158	G protein-coupled receptor 158	57512	0	NA	NA	NA	NA	NA
ENSG00000151033	LYZL2	lysozyme like 2	119180	0	NA	NA	NA	NA	NA
ENSG00000151090	THRB	thyroid hormone receptor beta	7068	0	NA	NA	NA	NA	NA
ENSG00000151117	TMEM86A	transmembrane protein 86A	144110	4,6034651	0,091865072	0,89043	0,103169309	0,917828598	NA
ENSG00000151136	ABTB3	ankyrin repeat and BTB domain containing 3	121551	2,226075806	1,02022253	1,506023	0,67742837	0,498134203	NA
ENSG00000151164	RAD9B	RAD9 checkpoint clamp component B	144715	2,328319191	-0,137783156	1,121441	-0,122862598	0,902215904	NA
ENSG00000151208	DLG5	discs large MAGUK scaffold protein 5	9231	5,522364088	6,522005196	3,911715	1,667300862	0,095454595	NA
ENSG00000151224	MAT1A	methionine adenosyltransferase 1A	4143	0	NA	NA	NA	NA	NA
ENSG00000151240	DIP2C	disco interacting protein 2 homolog C	22982	7,562888939	-0,617782179	0,783223	-0,788768783	0,430247158	NA
ENSG00000151276	MAGI1	membrane associated guanylate kinase, WW and PDZ domain containing 1	9223	0	NA	NA	NA	NA	NA
ENSG00000151320	AKAP6	A-kinase anchoring protein 6	9472	0	NA	NA	NA	NA	NA
ENSG00000151322	NPAS3	neuronal PAS domain protein 3	64067	0	NA	NA	NA	NA	NA
ENSG00000151338	MIPOL1	mirror-image polydactyly 1	145282	0	NA	NA	NA	NA	NA
ENSG00000151360	ALLC	allantoicase	55821	0	NA	NA	NA	NA	NA
ENSG00000151364	KCTD14	potassium channel tetramerization domain containing 14	65987	0,719684551	1,940384406	2,339762	0,829308581	0,406929817	NA
ENSG00000151365	THRSP	thyroid hormone responsive	7069	0	NA	NA	NA	NA	NA
ENSG00000151376	ME3	malic enzyme 3	10873	0,15836288	0	4,080456	0	1	NA
ENSG00000151379	MSGN1	mesogenin 1	343930	0	NA	NA	NA	NA	NA
ENSG00000151388	ADAMTS12	ADAM metallopeptidase with thrombospondin type 1 motif 12	81792	0	NA	NA	NA	NA	NA
ENSG00000151418	ATP6V1G3	ATPase H+ transporting V1 subunit G3	127124	0	NA	NA	NA	NA	NA
ENSG00000151458	ANKRD50	ankyrin repeat domain containing 50	57182	3,009304509	-0,511655736	1,144731	-0,446965961	0,654899637	NA
ENSG00000151468	CCDC3	coiled-coil domain containing 3	83643	0,695726623	-2,547751313	2,69077	-0,946848353	0,343716059	NA
ENSG00000151474	FRMD4A	FERM domain containing 4A	55691	0,223132954	-1,077756258	4,080456	-0,264126434	0,791682496	NA
ENSG00000151475	SLC25A31	solute carrier family 25 member 31	83447	0	NA	NA	NA	NA	NA
ENSG00000151490	PTPRO	protein tyrosine phosphatase receptor type O	5800	0,10488184	-1,076443944	4,080456	-0,263804824	0,791930317	NA
ENSG00000151572	ANO4	anoctamin 4	121601	0	NA	NA	NA	NA	NA
ENSG00000151575	TEX9	testis expressed 9	374618	0,221825887	-1,106121665	4,080456	-0,271077963	0,78633107	NA
ENSG00000151577	DRD3	dopamine receptor D3	1814	0,103998153	-1,076443944	4,080456	-0,263804824	0,791930317	NA
ENSG00000151615	POU4F2	POU class 4 homeobox 2	5458	0	NA	NA	NA	NA	NA
ENSG00000151617	EDNRA	endothelin receptor type A	1909	0	NA	NA	NA	NA	NA
ENSG00000151623	NR3C2	nuclear receptor subfamily 3 group C member 2	4306	0,323113839	-0,127338178	3,836269	-0,033193234	0,973520494	NA
ENSG00000151631	NA	NA	NA	0	NA	NA	NA	NA	NA
ENSG00000151632	AKR1C2	aldo-keto reductase family 1 member C2	1646	0	NA	NA	NA	NA	NA
ENSG00000151640	DPYSL4	dihydropyrimidinase like 4	10570	3,936206322	-0,815635973	0,985799	-0,827385461	0,408018619	NA
ENSG00000151650	VENTX	VENT homeobox	27287	0,786722061	-3,822147062	2,786325	-1,371751952	0,170140669	NA
ENSG00000151655	ITIH2	inter-alpha-trypsin inhibitor heavy chain 2	3698	0	NA	NA	NA	NA	NA
ENSG00000151687	ANKAR	ankyrin and armadillo repeat containing	150709	4,142199731	-1,402160108	1,8404	-0,761877799	0,446132941	NA
ENSG00000151693	ASAP2	ArfGAP with SH3 domain, ankyrin repeat and PH domain 2	8853	0,144330898	1,418786819	4,061262	0,349346267	0,726829368	NA

ENSG00000151704	KCNJ1	potassium inwardly rectifying channel subfamily J member 1	3758	0,265537393	0,427264742	4,069188	0,104999994	0,916375813	NA
ENSG00000151715	TMEM45B	transmembrane protein 45B	120224	0	NA	NA	NA	NA	NA
ENSG00000151718	WWC2	WW and C2 domain containing 2	80014	2,301711286	0,676421195	1,382421	0,489301877	0,624627992	NA
ENSG00000151729	SLC25A4	solute carrier family 25 member 4	291	1,16459454	3,421346963	2,441592	1,40127735	0,161131152	NA
ENSG00000151743	AMN1	antagonist of mitotic exit network 1 homolog	196394	10,65440866	-0,095625645	0,725205	-0,131860239	0,895094839	NA
ENSG00000151748	SAV1	salvador family WW domain containing protein 1	60485	7,383708615	0,537183165	0,7119	0,75457678	0,450502961	NA
ENSG00000151778	SERP2	stress associated endoplasmic reticulum protein family member 2	387923	0,417804446	-2,917764618	3,975409	-0,733953268	0,46297722	NA
ENSG00000151789	ZNF385D	zinc finger protein 385D	79750	0,229461317	-1,281388063	4,080456	-0,314030614	0,753497787	NA
ENSG00000151790	TDO2	tryptophan 2,3-dioxygenase	6999	0	NA	NA	NA	NA	NA
ENSG00000151812	SLC35F4	solute carrier family 35 member F4	341880	0	NA	NA	NA	NA	NA
ENSG00000151834	GABRA2	gamma-aminobutyric acid type A receptor subunit alpha2	2555	0	NA	NA	NA	NA	NA
ENSG00000151838	CCDC175	coiled-coil domain containing 175	729665	0	NA	NA	NA	NA	NA
ENSG00000151846	PABPC3	poly(A) binding protein cytoplasmic 3	5042	0,263708297	0	4,080456	0	1	NA
ENSG00000151892	GFRA1	GDNF family receptor alpha 1	2674	0,072165449	0,847101401	4,080456	0,207599697	0,835541541	NA
ENSG00000151917	BEND6	BEN domain containing 6	221336	0,072165449	0,847101401	4,080456	0,207599697	0,835541541	NA
ENSG00000151948	GLT1D1	glycosyltransferase 1 domain containing 1	144423	0	NA	NA	NA	NA	NA
ENSG00000151952	TMEM132D	transmembrane protein 132D	121256	0	NA	NA	NA	NA	NA
ENSG00000151962	RBM46	RNA binding motif protein 46	166863	0	NA	NA	NA	NA	NA
ENSG00000151963	ZNF37CP	zinc finger protein 37C, pseudogene	79232	0	NA	NA	NA	NA	NA
ENSG00000151967	SCHIP1	schwannomin interacting protein 1	29970	0,22301909	0,850545367	4,080448	0,208444122	0,834882209	NA
ENSG00000152034	MCHR2	melanin concentrating hormone receptor 2	84539	0	NA	NA	NA	NA	NA
ENSG00000152049	KCNE4	potassium voltage-gated channel subfamily E regulatory subunit 4	23704	0	NA	NA	NA	NA	NA
ENSG00000152076	CCDC74B	coiled-coil domain containing 74B	91409	0	NA	NA	NA	NA	NA
ENSG00000152078	TLCD4	TLC domain containing 4	148534	0,119673877	0,847101401	4,080456	0,207599697	0,835541541	NA
ENSG00000152086	TUBA3E	tubulin alpha 3e	112714	0	NA	NA	NA	NA	NA
ENSG00000152092	ASTN1	astrotactin 1	460	0	NA	NA	NA	NA	NA
ENSG00000152093	CFC1B	cryptic, EGF-CFC family member 1B	653275	0	NA	NA	NA	NA	NA
ENSG00000152104	PTPN14	protein tyrosine phosphatase non-receptor type 14	5784	6,572393917	1,403601559	0,763845	1,837546546	0,066129252	NA
ENSG00000152137	HSPB8	heat shock protein family B (small) member 8	26353	0	NA	NA	NA	NA	NA
ENSG00000152154	TMEM178A	transmembrane protein 178A	130733	0,11808024	-1,076443944	4,080456	-0,263804824	0,791930317	NA
ENSG00000152192	POU4F1	POU class 4 homeobox 1	5457	4,051995864	1,097844618	0,971926	1,129555831	0,258663431	NA
ENSG00000152208	GRID2	glutamate ionotropic receptor delta type subunit 2	2895	0	NA	NA	NA	NA	NA
ENSG00000152213	ARL11	ADP ribosylation factor like GTPase 11	115761	1,301249657	0,010295355	1,693644	0,006078818	0,995149835	NA
ENSG00000152214	RIT2	Ras like without CAAX 2	6014	0	NA	NA	NA	NA	NA
ENSG00000152253	SPC25	SPC25 component of NDC80 kinetochore complex	57405	5,026280771	3,595257836	3,177817	1,131360908	0,257903218	NA
ENSG00000152254	G6PC2	glucose-6-phosphatase catalytic subunit 2	57818	0	NA	NA	NA	NA	NA
ENSG00000152266	PTH	parathyroid hormone	5741	0	NA	NA	NA	NA	NA
ENSG00000152284	TCF7L1	transcription factor 7 like 1	83439	0	NA	NA	NA	NA	NA
ENSG00000152292	SH2D6	SH2 domain containing 6	284948	0	NA	NA	NA	NA	NA
ENSG00000152315	KCNK13	potassium two pore domain channel subfamily K member 13	56659	0	NA	NA	NA	NA	NA
ENSG00000152348	ATG10	autophagy related 10	83734	10,11281198	-0,719829637	0,61691	-1,166830998	0,243278626	NA
ENSG00000152377	SPOCK1	SPARC (osteonectin), cwcv and kazal like domains proteoglycan 1	6695	0	NA	NA	NA	NA	NA
ENSG00000152380	FAM151B	family with sequence similarity 151 member B	167555	0,103998153	-1,076443944	4,080456	-0,263804824	0,791930317	NA
ENSG00000152402	GUCY1A2	guanylate cyclase 1 soluble subunit alpha 2	2977	0	NA	NA	NA	NA	NA
ENSG00000152413	HOMER1	homer scaffold protein 1	9456	9,092800349	0,595423915	0,662874	0,898246478	0,369054159	NA

ENSG00000152430	BOLL	boule homolog, RNA binding protein	66037	0	NA	NA	NA	NA	NA
ENSG00000152433	ZNF547	zinc finger protein 547	284306	6,487374188	0,149264139	0,840297	0,17763263	0,859011497	NA
ENSG00000152455	SUV39H2	SUV39H2 histone lysine methyltransferase	79723	8,674407939	0,721716954	3,743691	0,192782185	NA	NA
ENSG00000152463	OLAH	oleoyl-ACP hydrolase	55301	0	NA	NA	NA	NA	NA
ENSG00000152467	ZSCAN1	zinc finger and SCAN domain containing 1	284312	0	NA	NA	NA	NA	NA
ENSG00000152475	ZNF837	zinc finger protein 837	116412	1,739002405	2,212487198	1,523052	1,452667336	0,14631614	NA
ENSG00000152503	TRIM36	tripartite motif containing 36	55521	0,448713386	0,702076827	3,476798	0,201932018	0,839969871	NA
ENSG00000152527	PLEKHH2	pleckstrin homology, MyTH4 and FERM domain containing H2	130271	0	NA	NA	NA	NA	NA
ENSG00000152578	GRIA4	glutamate ionotropic receptor AMPA type subunit 4	2893	0	NA	NA	NA	NA	NA
ENSG00000152580	IGSF10	immunoglobulin superfamily member 10	285313	0	NA	NA	NA	NA	NA
ENSG00000152582	SPEF2	sperm flagellar 2	79925	9,338198873	0,556560904	0,670079	0,830589545	0,406205543	NA
ENSG00000152583	SPARCL1	SPARC like 1	8404	0	NA	NA	NA	NA	NA
ENSG00000152591	DSPP	dentin sialophosphoprotein	1834	0	NA	NA	NA	NA	NA
ENSG00000152592	DMP1	dentin matrix acidic phosphoprotein 1	1758	0	NA	NA	NA	NA	NA
ENSG00000152595	MEPE	matrix extracellular phosphoglycoprotein	56955	0	NA	NA	NA	NA	NA
ENSG00000152611	CAPSL	calcyphosine like	133690	0	NA	NA	NA	NA	NA
ENSG00000152661	GJA1	gap junction protein alpha 1	2697	0	NA	NA	NA	NA	NA
ENSG00000152669	CCNO	cyclin O	10309	0	NA	NA	NA	NA	NA
ENSG00000152670	DDX4	DEAD-box helicase 4	54514	0,288661797	2,306206831	3,997774	0,57687273	0,564025434	NA
ENSG00000152672	CLEC4F	C-type lectin domain family 4 member F	165530	0	NA	NA	NA	NA	NA
ENSG00000152705	CATSPER3	cation channel sperm associated 3	347732	0,438522238	0,937180332	3,518297	0,26637325	0,789951765	NA
ENSG00000152760	DYNLT5	dynein light chain Tctex-type family member 5	200132	0	NA	NA	NA	NA	NA
ENSG00000152763	DNAI4	dynein axonemal intermediate chain 4	79819	0,595226755	2,98257476	3,93184	0,758569796	0,448109947	NA
ENSG00000152766	ANKRD22	ankyrin repeat domain 22	118932	0,118286652	0	4,080456	0	1	NA
ENSG00000152767	FARP1	FERM, ARH/RhoGEF and pleckstrin domain protein 1	10160	0,262739954	-1,073154812	4,080456	-0,262998755	0,792551539	NA
ENSG00000152779	SLC16A12	solute carrier family 16 member 12	387700	0	NA	NA	NA	NA	NA
ENSG00000152785	BMP3	bone morphogenetic protein 3	651	0	NA	NA	NA	NA	NA
ENSG00000152822	GRM1	glutamate metabotropic receptor 1	2911	0,524647374	0,184749298	2,442835	0,075629063	0,939714214	NA
ENSG00000152894	PTPRK	protein tyrosine phosphatase receptor type K	5796	0	NA	NA	NA	NA	NA
ENSG00000152910	CNTNAP4	contactin associated protein family member 4	85445	0	NA	NA	NA	NA	NA
ENSG00000152932	RAB3C	RAB3C, member RAS oncogene family	115827	0	NA	NA	NA	NA	NA
ENSG00000152936	LMNTD1	lamin tail domain containing 1	160492	0,103998153	-1,076443944	4,080456	-0,263804824	0,791930317	NA
ENSG00000152939	MARVELD2	MARVEL domain containing 2	153562	0,826726647	-0,815292793	2,334373	-0,349255623	0,726897411	NA
ENSG00000152952	PLOD2	procollagen-lysine,2-oxoglutarate 5-dioxygenase 2	5352	0	NA	NA	NA	NA	NA
ENSG00000152953	STK32B	serine/threonine kinase 32B	55351	0	NA	NA	NA	NA	NA
ENSG00000152954	NRSN1	neurensin 1	140767	0,10488184	-1,076443944	4,080456	-0,263804824	0,791930317	NA
ENSG00000152977	ZIC1	Zic family member 1	7545	0	NA	NA	NA	NA	NA
ENSG00000152990	ADGRA3	adhesion G protein-coupled receptor A3	166647	3,674569454	-0,845128966	1,551194	-0,544824945	0,58587392	NA
ENSG00000153002	CPB1	carboxypeptidase B1	1360	0	NA	NA	NA	NA	NA
ENSG00000153012	LGI2	leucine rich repeat LGI family member 2	55203	0	NA	NA	NA	NA	NA
ENSG00000153060	TEKT5	tektin 5	146279	0	NA	NA	NA	NA	NA
ENSG00000153071	DAB2	DAB adaptor protein 2	1601	1,162775724	0,795972808	1,751268	0,454512237	0,649460187	NA
ENSG00000153086	ACMSD	aminocarboxymuconate semialdehyde decarboxylase	130013	0,118286652	0	4,080456	0	1	NA
ENSG00000153132	CLGN	calmegin	1047	0	NA	NA	NA	NA	NA
ENSG00000153157	SYCP2L	synaptonemal complex protein 2 like	221711	0	NA	NA	NA	NA	NA
ENSG00000153162	BMP6	bone morphogenetic protein 6	654	0	NA	NA	NA	NA	NA
ENSG00000153165	RGPD3	RANBP2 like and GRIP domain containing 3	653489	6,787654366	0,02320454	0,807799	0,028725649	0,9770834	NA
ENSG00000153208	MERTK	MER proto-oncogene, tyrosine kinase	10461	0,10488184	-1,076443944	4,080456	-0,263804824	0,791930317	NA

ENSG00000153230	OR14K1	olfactory receptor family 14 subfamily K member 1	343170	0	NA	NA	NA	NA	NA
ENSG00000153233	PTPRR	protein tyrosine phosphatase receptor type R	5801	0	NA	NA	NA	NA	NA
ENSG00000153237	CCDC148	coiled-coil domain containing 148	130940	0	NA	NA	NA	NA	NA
ENSG00000153246	PLA2R1	phospholipase A2 receptor 1	22925	0,5489058	0,273262893	3,416689	0,079978852	0,936254076	NA
ENSG00000153253	SCN3A	sodium voltage-gated channel alpha subunit 3	6328	0	NA	NA	NA	NA	NA
ENSG00000153266	FEZF2	FEZ family zinc finger 2	55079	0,118286652	0	4,080456	0	1	NA
ENSG00000153291	SLC25A27	solute carrier family 25 member 27	9481	0	NA	NA	NA	NA	NA
ENSG00000153292	ADGRF1	adhesion G protein-coupled receptor F1	266977	0	NA	NA	NA	NA	NA
ENSG00000153294	ADGRF4	adhesion G protein-coupled receptor F4	221393	0	NA	NA	NA	NA	NA
ENSG00000153303	FRMD1	FERM domain containing 1	79981	0	NA	NA	NA	NA	NA
ENSG00000153347	FAM81B	family with sequence similarity 81 member B	153643	0	NA	NA	NA	NA	NA
ENSG00000153404	PLEKHG4B	pleckstrin homology and RhoGEF domain containing G4B	153478	0	NA	NA	NA	NA	NA
ENSG00000153446	C16orf89	chromosome 16 open reading frame 89	146556	0	NA	NA	NA	NA	NA
ENSG00000153485	LYSET	lysosomal enzyme trafficking factor	26175	2,366990051	0,580248795	1,470701	0,394538839	0,693183259	NA
ENSG00000153495	TEX29	testis expressed 29	121793	2,884662665	2,085625731	1,398194	1,491656438	0,135789235	NA
ENSG00000153498	SPACA7	sperm acrosome associated 7	122258	0	NA	NA	NA	NA	NA
ENSG00000153531	ADPRHL1	ADP-ribosylhydrolase like 1	113622	4,133890019	0,725451627	1,102289	0,658131962	0,51045334	NA
ENSG00000153558	FBXL2	F-box and leucine rich repeat protein 2	25827	0	NA	NA	NA	NA	NA
ENSG00000153684	GOLGA8F	golgin A8 family member F	100132565	0,590765296	0,622749511	3,932212	0,158371298	0,874164229	NA
ENSG00000153707	PTPRD	protein tyrosine phosphatase receptor type D	5789	0	NA	NA	NA	NA	NA
ENSG00000153714	LURAP1L	leucine rich adaptor protein 1 like	286343	0	NA	NA	NA	NA	NA
ENSG00000153721	CNKSR3	CNKSR family member 3	154043	0,575785728	2,157329961	3,311074	0,651549857	0,514691604	NA
ENSG00000153779	TGIF2LX	TGFB induced factor homeobox 2 like X-linked	90316	0	NA	NA	NA	NA	NA
ENSG00000153789	CIBAR2	CBY1 interacting BAR domain containing 2	339145	0	NA	NA	NA	NA	NA
ENSG00000153802	TMPRSS11D	transmembrane serine protease 11D	9407	0	NA	NA	NA	NA	NA
ENSG00000153820	SPHKAP	SPHK1 interactor, AKAP domain containing	80309	0	NA	NA	NA	NA	NA
ENSG00000153822	KCNJ16	potassium inwardly rectifying channel subfamily J member 16	3773	0	NA	NA	NA	NA	NA
ENSG00000153823	PID1	phosphotyrosine interaction domain containing 1	55022	0,11808024	-1,076443944	4,080456	-0,263804824	0,791930317	NA
ENSG00000153832	FBXO36	F-box protein 36	130888	4,359840993	-0,320117354	0,866476	-0,369447516	0,711794187	NA
ENSG00000153885	KCTD15	potassium channel tetramerization domain containing 15	79047	0	NA	NA	NA	NA	NA
ENSG00000153896	ZNF599	zinc finger protein 599	148103	9,20959755	1,058292337	0,919248	1,151258225	0,249626018	NA
ENSG00000153902	LGI4	leucine rich repeat LGI family member 4	163175	0	NA	NA	NA	NA	NA
ENSG00000153904	DDAH1	dimethylarginine dimethylaminohydrolase 1	23576	0	NA	NA	NA	NA	NA
ENSG00000153923	NA	NA	NA	0	NA	NA	NA	NA	NA
ENSG00000153930	ANKFN1	ankyrin repeat and fibronectin type III domain containing 1	162282	0	NA	NA	NA	NA	NA
ENSG00000153956	CACNA2D1	calcium voltage-gated channel auxiliary subunit alpha2delta 1	781	0	NA	NA	NA	NA	NA
ENSG00000153976	HS3ST3A1	heparan sulfate-glucosamine 3-sulfotransferase 3A1	9955	0,263106742	1,43297508	4,061571	0,352813038	0,724228609	NA
ENSG00000153982	GDPD1	glycerophosphodiester phosphodiesterase domain containing 1	284161	9,259844789	0,39384064	0,723008	0,544724866	0,58594276	NA
ENSG00000153993	SEMA3D	semaphorin 3D	223117	0	NA	NA	NA	NA	NA
ENSG00000154007	ASB17	ankyrin repeat and SOCS box containing 17	127247	0	NA	NA	NA	NA	NA
ENSG00000154025	SLC5A10	solute carrier family 5 member 10	125206	0,10543123	0	4,080456	0	1	NA
ENSG00000154027	AK5	adenylate kinase 5	26289	0,479525674	0,956881614	3,430436	0,278938796	0,780291795	NA
ENSG00000154040	CABYR	calcium binding tyrosine phosphorylation regulated	26256	2,042537519	0,164766241	1,729117	0,09528926	0,924085073	NA
ENSG00000154065	ANKRD29	ankyrin repeat domain 29	147463	0	NA	NA	NA	NA	NA

ENSG00000154080	CHST9	carbohydrate sulfotransferase 9	83539	0	NA	NA	NA	NA	NA
ENSG00000154096	THY1	Thy-1 cell surface antigen	7070	0	NA	NA	NA	NA	NA
ENSG00000154099	DNAAF1	dynein axonemal assembly factor 1	123872	0	NA	NA	NA	NA	NA
ENSG00000154102	C16orf74	chromosome 16 open reading frame 74	404550	2,068823586	-0,542469168	1,484582	-0,365401848	0,714811462	NA
ENSG00000154118	JPH3	junctophilin 3	57338	0	NA	NA	NA	NA	NA
ENSG00000154133	ROBO4	roundabout guidance receptor 4	54538	0	NA	NA	NA	NA	NA
ENSG00000154134	ROBO3	roundabout guidance receptor 3	64221	0,837717141	3,794059551	3,939528	0,963074739	0,335510018	NA
ENSG00000154143	PANX3	pannexin 3	116337	0	NA	NA	NA	NA	NA
ENSG00000154146	NRGN	neurogranin	4900	0,896084818	3,231953553	2,450901	1,318679574	0,187276258	NA
ENSG00000154153	RETREG1	reticulophagy regulator 1	54463	1,753497856	0,703576177	1,808984	0,388934449	0,697324638	NA
ENSG00000154162	CDH12	cadherin 12	1010	0	NA	NA	NA	NA	NA
ENSG00000154175	ABI3BP	ABI family member 3 binding protein	25890	0	NA	NA	NA	NA	NA
ENSG00000154188	ANGPT1	angiopoietin 1	284	0	NA	NA	NA	NA	NA
ENSG00000154198	NA	NA	NA	0	NA	NA	NA	NA	NA
ENSG00000154227	CERS3	ceramide synthase 3	204219	0,207996306	-1,916073151	4,043021	-0,473921178	0,635556117	NA
ENSG00000154237	LRRK1	leucine rich repeat kinase 1	79705	0	NA	NA	NA	NA	NA
ENSG00000154240	CEP112	centrosomal protein 112	201134	2,74886475	-0,121345194	2,660918	-0,045602757	0,963626872	NA
ENSG00000154252	GAL3ST2	galactose-3-O-sulfotransferase 2	64090	0	NA	NA	NA	NA	NA
ENSG00000154258	ABCA9	ATP binding cassette subfamily A member 9	10350	0,588329392	3,217350307	3,85573	0,834433502	0,404036745	NA
ENSG00000154262	ABCA6	ATP binding cassette subfamily A member 6	23460	0,268535477	1,754029256	4,029201	0,435329308	0,663323414	NA
ENSG00000154263	ABCA10	ATP binding cassette subfamily A member 10	10349	2,942720923	-1,905258611	1,59672	-1,19323312	0,232778087	NA
ENSG00000154269	ENPP3	ectonucleotide pyrophosphatase/phosphodiesterase 3	5169	10,43381118	0,375989838	0,594489	0,632459008	0,527086986	NA
ENSG00000154274	C4orf19	chromosome 4 open reading frame 19	55286	0,234994216	1,944111989	4,02324	0,483220515	0,62893917	NA
ENSG00000154277	UCHL1	ubiquitin C-terminal hydrolase L1	7345	0,210862461	0	4,080456	0	1	NA
ENSG00000154309	DISP1	dispatched RND transporter family member 1	84976	4,758336321	-0,717024495	3,597724	-0,199299465	0,842028498	NA
ENSG00000154319	FAM167A	family with sequence similarity 167 member A	83648	0,216496347	1,902771968	4,026559	0,472555327	0,63653046	NA
ENSG00000154328	NEIL2	nei like DNA glycosylase 2	252969	10,57354351	0,363067581	0,839562	0,432448661	0,665415358	NA
ENSG00000154330	PGM5	phosphoglucomutase 5	5239	0	NA	NA	NA	NA	NA
ENSG00000154342	WNT3A	Wnt family member 3A	89780	0	NA	NA	NA	NA	NA
ENSG00000154359	LONRF1	LON peptidase N-terminal domain and ring finger 1	91694	5,011193026	4,942737102	1,495985	3,304001989	0,000953152	NA
ENSG00000154380	ENAH	ENAH actin regulator	55740	0,117497108	0,847101401	4,080456	0,207599697	0,835541541	NA
ENSG00000154415	PPP1R3A	protein phosphatase 1 regulatory subunit 3A	5506	0	NA	NA	NA	NA	NA
ENSG00000154438	ASZ1	ankyrin repeat, SAM and basic leucine zipper domain containing 1	136991	0	NA	NA	NA	NA	NA
ENSG00000154447	SH3RF1	SH3 domain containing ring finger 1	57630	1,524658994	-1,530895821	2,102531	-0,728120619	0,466539753	NA
ENSG00000154478	GPR26	G protein-coupled receptor 26	2849	0	NA	NA	NA	NA	NA
ENSG00000154479	CFAP210	cilia and flagella associated protein 210	129881	0,216496347	1,902771968	4,026559	0,472555327	0,63653046	NA
ENSG00000154485	MMP21	matrix metallopeptidase 21	118856	1,029792781	-1,975018659	2,287512	-0,863391703	0,38792214	NA
ENSG00000154493	C10orf90	chromosome 10 open reading frame 90	118611	0,420190318	-0,902900634	3,652085	-0,247228814	0,804731147	NA
ENSG00000154529	CNTNAP3B	contactin associated protein family member 3B	728577	0	NA	NA	NA	NA	NA
ENSG00000154545	MAGED4	MAGE family member D4	728239	0	NA	NA	NA	NA	NA
ENSG00000154548	SRSF12	serine and arginine rich splicing factor 12	135295	0	NA	NA	NA	NA	NA
ENSG00000154553	PDLIM3	PDZ and LIM domain 3	27295	0	NA	NA	NA	NA	NA
ENSG00000154556	SORBS2	sorbin and SH3 domain containing 2	8470	0,117497108	0,847101401	4,080456	0,207599697	0,835541541	NA
ENSG00000154589	LY96	lymphocyte antigen 96	23643	0,454666753	2,53109073	3,403906	0,743584309	0,457127999	NA
ENSG00000154608	NA	NA	NA	1,272689359	-2,894668956	2,428657	-1,191880667	0,23330804	NA
ENSG00000154611	PSMA8	proteasome 20S subunit alpha 8	143471	2,708445628	0,292379625	1,172062	0,249457482	0,803006926	NA
ENSG00000154620	TMSB4Y	thymosin beta 4 Y-linked	9087	0	NA	NA	NA	NA	NA
ENSG00000154639	CXADR	CXADR Ig-like cell adhesion molecule	1525	0,072165449	0,847101401	4,080456	0,207599697	0,835541541	NA
ENSG00000154645	CHODL	chondrolectin	140578	0,680081052	-0,185294235	2,24225	-0,082637619	0,934139688	NA
ENSG00000154646	TMPRSS15	transmembrane serine protease 15	5651	0	NA	NA	NA	NA	NA

ENSG00000154654	NCAM2	neural cell adhesion molecule 2	4685	0	NA	NA	NA	NA	NA
ENSG00000154655	L3MBTL4	L3MBTL histone methyl-lysine binding protein 4	91133	8,157932445	-0,053283893	0,725176	-0,073477224	0,941426368	NA
ENSG00000154678	PDE1C	phosphodiesterase 1C	5137	1,728706521	2,295571336	1,824138	1,258441897	0,208231988	NA
ENSG00000154721	JAM2	junctional adhesion molecule 2	58494	0	NA	NA	NA	NA	NA
ENSG00000154736	ADAMTS5	ADAM metallopeptidase with thrombospondin type 1 motif 5	11096	0	NA	NA	NA	NA	NA
ENSG00000154743	TSEN2	tRNA splicing endonuclease subunit 2	80746	4,836437933	-1,255624817	1,200498	-1,045919664	0,295598125	NA
ENSG00000154764	WNT7A	Wnt family member 7A	7476	0	NA	NA	NA	NA	NA
ENSG00000154783	FGD5	FYVE, RhoGEF and PH domain containing 5	152273	0	NA	NA	NA	NA	NA
ENSG00000154856	APCDD1	APC down-regulated 1	147495	0	NA	NA	NA	NA	NA
ENSG00000154864	PIEZO2	piezo type mechanosensitive ion channel component 2	63895	0	NA	NA	NA	NA	NA
ENSG00000154914	USP43	ubiquitin specific peptidase 43	124739	6,635439111	1,12309216	2,718725	0,413095148	0,679536904	NA
ENSG00000154928	EPHB1	EPH receptor B1	2047	0,15836288	0	4,080456	0	1	NA
ENSG00000154975	CA10	carbonic anhydrase 10	56934	0	NA	NA	NA	NA	NA
ENSG00000154997	SEPTIN14	septin 14	346288	0	NA	NA	NA	NA	NA
ENSG00000155011	DKK2	dickkopf WNT signaling pathway inhibitor 2	27123	0	NA	NA	NA	NA	NA
ENSG00000155026	RSPH10B	radial spoke head 10 homolog B	222967	0,661669234	-1,064362152	3,164026	-0,336394836	0,736573143	NA
ENSG00000155052	CNTNAP5	contactin associated protein family member 5	129684	0	NA	NA	NA	NA	NA
ENSG00000155070	NA	NA	NA	0	NA	NA	NA	NA	NA
ENSG00000155087	ODF1	outer dense fiber of sperm tails 1	4956	0	NA	NA	NA	NA	NA
ENSG00000155099	PIP4P2	phosphatidylinositol-4,5-bisphosphate 4-phosphatase 2	55529	0,378957686	-1,412073693	3,602826	-0,391935009	0,695106235	NA
ENSG00000155254	MARVELD1	MARVEL domain containing 1	83742	1,889584818	-1,042089798	1,600088	-0,65127031	0,51487201	NA
ENSG00000155265	GOLGA7B	golgin A7 family member B	401647	2,387167701	0,436346768	1,296794	0,336481079	0,736508117	NA
ENSG00000155269	GPR78	G protein-coupled receptor 78	27201	0	NA	NA	NA	NA	NA
ENSG00000155324	GRAMD2B	GRAM domain containing 2B	65983	7,478981983	0,263812564	0,846564	0,311627531	0,755323609	NA
ENSG00000155367	PPM1J	protein phosphatase, Mg2+/Mn2+ dependent 1J	333926	0	NA	NA	NA	NA	NA
ENSG00000155428	TRIM74	tripartite motif containing 74	378108	0,264067816	-1,069312187	4,080456	-0,26205704	0,793277467	NA
ENSG00000155465	SLC7A7	solute carrier family 7 member 7	9056	0,208889805	-1,921413894	4,042529	-0,47529998	0,634573173	NA
ENSG00000155495	MAGEC1	MAGE family member C1	9947	0	NA	NA	NA	NA	NA
ENSG00000155511	GRIA1	glutamate ionotropic receptor AMPA type subunit 1	2890	0	NA	NA	NA	NA	NA
ENSG00000155530	LRGUK	leucine rich repeats and guanylate kinase domain containing	136332	2,365672305	-0,045030303	1,3162	-0,03421236	0,97270781	NA
ENSG00000155542	SETD9	SET domain containing 9	133383	5,316634885	1,806229935	1,464447	1,233387322	0,217431293	NA
ENSG00000155622	XAGE2	X antigen family member 2	9502	0	NA	NA	NA	NA	NA
ENSG00000155659	VSIG4	V-set and immunoglobulin domain containing 4	11326	0	NA	NA	NA	NA	NA
ENSG00000155714	PDZD9	PDZ domain containing 9	255762	0	NA	NA	NA	NA	NA
ENSG00000155719	OTOA	otoancorin	146183	0	NA	NA	NA	NA	NA
ENSG00000155749	FLACC1	flagellum associated containing coiled-coil domains 1	130540	8,343251011	0,605487491	0,646007	0,937277501	0,348615833	NA
ENSG00000155754	C2CD6	C2 calcium dependent domain containing 6	151254	0	NA	NA	NA	NA	NA
ENSG00000155760	FZD7	frizzled class receptor 7	8324	3,937619861	0,40092479	1,273328	0,314863651	0,752865181	NA
ENSG00000155761	SPAG17	sperm associated antigen 17	200162	0	NA	NA	NA	NA	NA
ENSG00000155792	DEPTOR	DEP domain containing MTOR interacting protein	64798	1,057817507	0,961601842	1,996485	0,48164734	0,63005649	NA
ENSG00000155816	FMN2	formin 2	56776	0	NA	NA	NA	NA	NA
ENSG00000155833	CYLC2	cylicin 2	1539	0	NA	NA	NA	NA	NA
ENSG00000155846	PPARGC1B	PPARG coactivator 1 beta	133522	9,223774415	0,555817841	0,624411	0,890147707	0,373386579	NA
ENSG00000155875	SAXO1	stabilizer of axonemal microtubules 1	158297	0,10543123	0	4,080456	0	1	NA
ENSG00000155886	SLC24A2	solute carrier family 24 member 2	25769	0,119673877	0,847101401	4,080456	0,207599697	0,835541541	NA
ENSG00000155890	TRIM42	tripartite motif containing 42	287015	0	NA	NA	NA	NA	NA
ENSG00000155897	ADCY8	adenylate cyclase 8	114	0	NA	NA	NA	NA	NA
ENSG00000155918	RAET1L	retinoic acid early transcript 1L	154064	0	NA	NA	NA	NA	NA
ENSG00000155962	CLIC2	chloride intracellular channel 2	1193	0,17509563	-0,078678846	4,080456	-0,019281876	0,984616242	NA

ENSG00000155966	AFF2	ALF transcription elongation factor 2	2334	0	NA	NA	NA	NA	NA
ENSG00000155970	MICU3	mitochondrial calcium uptake family member 3	286097	2,080182091	1,356531034	1,341241	1,011399747	0,311825142	NA
ENSG00000155974	GRIP1	glutamate receptor interacting protein 1	23426	0,072165449	0,847101401	4,080456	0,207599697	0,835541541	NA
ENSG00000155980	KIF5A	kinesin family member 5A	3798	7,332206457	-0,197756205	0,628945	-0,314425031	0,753198248	NA
ENSG00000156006	NAT2	N-acetyltransferase 2	10	0	NA	NA	NA	NA	NA
ENSG00000156009	MAGEA8	MAGE family member A8	4107	0	NA	NA	NA	NA	NA
ENSG00000156011	PSD3	pleckstrin and Sec7 domain containing 3	23362	0,718128586	-3,458068679	2,472847	-1,398415987	0,161988185	NA
ENSG00000156042	CFAP70	cilia and flagella associated protein 70	118491	1,405125611	-1,024019113	2,434236	-0,420673666	0,673993393	NA
ENSG00000156049	GNA14	G protein subunit alpha 14	9630	0	NA	NA	NA	NA	NA
ENSG00000156050	FAM161B	FAM161 centrosomal protein B	145483	7,769569136	-0,188552384	0,68292	-0,276097283	0,782473348	NA
ENSG00000156076	WIF1	WNT inhibitory factor 1	11197	0	NA	NA	NA	NA	NA
ENSG00000156096	UGT2B4	UDP glucuronosyltransferase family 2 member B4	7363	0	NA	NA	NA	NA	NA
ENSG00000156097	GPR61	G protein-coupled receptor 61	83873	0,629345376	-1,954167807	3,095662	-0,631260108	0,527870466	NA
ENSG00000156103	MMP16	matrix metallopeptidase 16	4325	0,072165449	0,847101401	4,080456	0,207599697	0,835541541	NA
ENSG00000156113	KCNMA1	potassium calcium-activated channel subfamily M alpha 1	3778	0	NA	NA	NA	NA	NA
ENSG00000156140	ADAMTS3	ADAM metallopeptidase with thrombospondin type 1 motif 3	9508	0	NA	NA	NA	NA	NA
ENSG00000156150	ALX3	ALX homeobox 3	257	0	NA	NA	NA	NA	NA
ENSG00000156170	NDUFAF6	NADH:ubiquinone oxidoreductase complex assembly factor 6	137682	10,27068985	1,181602158	0,725292	1,629138941	0,103283609	NA
ENSG00000156172	CFAP418	cilia and flagella associated protein 418	157657	7,044570175	0,52877742	0,684647	0,772335454	0,439915772	NA
ENSG00000156194	PPEF2	protein phosphatase with EF-hand domain 2	5470	0,072165449	0,847101401	4,080456	0,207599697	0,835541541	NA
ENSG00000156206	CFAP161	cilia and flagella associated protein 161	161502	0,177776417	0,844124108	4,080398	0,206872973	0,836109064	NA
ENSG00000156219	ART3	ADP-ribosyltransferase 3 (inactive)	419	0	NA	NA	NA	NA	NA
ENSG00000156222	SLC28A1	solute carrier family 28 member 1	9154	0	NA	NA	NA	NA	NA
ENSG00000156234	CXCL13	C-X-C motif chemokine ligand 13	10563	0	NA	NA	NA	NA	NA
ENSG00000156269	NAA11	N-alpha-acetyltransferase 11, NatA catalytic subunit	84779	0	NA	NA	NA	NA	NA
ENSG00000156282	CLDN17	claudin 17	26285	0	NA	NA	NA	NA	NA
ENSG00000156284	CLDN8	claudin 8	9073	0	NA	NA	NA	NA	NA
ENSG00000156298	TSPAN7	tetraspanin 7	7102	0	NA	NA	NA	NA	NA
ENSG00000156345	CDK20	cyclin dependent kinase 20	23552	0,811241313	-0,48018413	2,645804	-0,181488896	0,855983848	NA
ENSG00000156381	ANKRD9	ankyrin repeat domain 9	122416	9,956157199	0,081689473	0,674085	0,121185678	0,903543968	NA
ENSG00000156395	SORCS3	sortilin related VPS10 domain containing receptor 3	22986	0	NA	NA	NA	NA	NA
ENSG00000156413	FUT6	fucosyltransferase 6	2528	0,15836288	0	4,080456	0	1	NA
ENSG00000156427	FGF18	fibroblast growth factor 18	8817	0,328930741	0,844176535	4,080456	0,206882898	0,836101313	NA
ENSG00000156453	PCDH1	protocadherin 1	5097	0	NA	NA	NA	NA	NA
ENSG00000156463	SH3RF2	SH3 domain containing ring finger 2	153769	0	NA	NA	NA	NA	NA
ENSG00000156466	GDF6	growth differentiation factor 6	392255	0,103998153	-1,076443944	4,080456	-0,263804824	0,791930317	NA
ENSG00000156486	KCNS2	potassium voltage-gated channel modifier subfamily S member 2	3788	0	NA	NA	NA	NA	NA
ENSG00000156509	FBXO43	F-box protein 43	286151	1,233330224	-4,472868605	2,977718	-1,502113074	0,133067909	NA
ENSG00000156510	HKDC1	hexokinase domain containing 1	80201	9,080922516	-0,603873614	0,614125	-0,98330687	0,32545643	NA
ENSG00000156564	LRFN2	leucine rich repeat and fibronectin type III domain containing 2	57497	0	NA	NA	NA	NA	NA
ENSG00000156574	NODAL	nodal growth differentiation factor	4838	0	NA	NA	NA	NA	NA
ENSG00000156575	PRG3	proteoglycan 3, pro eosinophil major basic protein 2	10394	0	NA	NA	NA	NA	NA
ENSG00000156687	UNC5D	unc-5 netrin receptor D	137970	0	NA	NA	NA	NA	NA
ENSG00000156689	GLYATL2	glycine-N-acyltransferase like 2	219970	0	NA	NA	NA	NA	NA
ENSG00000156738	MS4A1	membrane spanning 4-domains A1	931	0,342650506	1,03181899	3,762459	0,274240573	0,78389975	NA

ENSG00000156755	IGKV1OR-2	immunoglobulin kappa variable 1/OR-2 (pseudogene)	3532	0	NA	NA	NA	NA	NA
ENSG00000156885	COX6A2	cytochrome c oxidase subunit 6A2	1339	3,943621662	-1,13842149	0,981194	-1,160240744	0,245950803	NA
ENSG00000156920	ADGRG4	adhesion G protein-coupled receptor G4	139378	0	NA	NA	NA	NA	NA
ENSG00000156925	ZIC3	Zic family member 3	7547	0	NA	NA	NA	NA	NA
ENSG00000156928	MALSU1	mitochondrial assembly of ribosomal large subunit 1	115416	9,100340361	-0,171324599	0,579057	-0,295868281	0,767330675	NA
ENSG00000156959	LHFPL4	LHFPL tetraspan subfamily member 4	375323	0	NA	NA	NA	NA	NA
ENSG00000156968	MPV17L	MPV17 mitochondrial inner membrane protein like	255027	0	NA	NA	NA	NA	NA
ENSG00000157005	SST	somatostatin	6750	0	NA	NA	NA	NA	NA
ENSG00000157017	GHRL	ghrelin and obestatin prepropeptide	51738	1,717360568	-0,196807893	1,594554	-0,123425009	0,901770554	NA
ENSG00000157021	NA	NA	NA	0	NA	NA	NA	NA	NA
ENSG00000157045	NTAN1	N-terminal asparagine amidase	123803	5,121880304	6,426110105	1,516278	4,238082913	2,25E-05	NA
ENSG00000157060	SHCBP1L	SHC binding and spindle associated 1 like	81626	0	NA	NA	NA	NA	NA
ENSG00000157064	NMNAT2	nicotinamide nucleotide adenylyltransferase 2	23057	0,119673877	0,847101401	4,080456	0,207599697	0,835541541	NA
ENSG00000157077	ZFYVE9	zinc finger FYVE-type containing 9	9372	0	NA	NA	NA	NA	NA
ENSG00000157087	ATP2B2	ATPase plasma membrane Ca2+ transporting 2	491	0	NA	NA	NA	NA	NA
ENSG00000157093	LYZL4	lysozyme like 4	131375	0	NA	NA	NA	NA	NA
ENSG00000157103	SLC6A1	solute carrier family 6 member 1	6529	0	NA	NA	NA	NA	NA
ENSG00000157110	RBPMS	RNA binding protein, mRNA processing factor	11030	0	NA	NA	NA	NA	NA
ENSG00000157111	TMEM171	transmembrane protein 171	134285	0	NA	NA	NA	NA	NA
ENSG00000157119	KLHL40	kelch like family member 40	131377	0	NA	NA	NA	NA	NA
ENSG00000157131	C8A	complement C8 alpha chain	731	0	NA	NA	NA	NA	NA
ENSG00000157150	TIMP4	TIMP metallopeptidase inhibitor 4	7079	0	NA	NA	NA	NA	NA
ENSG00000157152	SYN2	synapsin II	6854	0	NA	NA	NA	NA	NA
ENSG00000157168	NRG1	neuregulin 1	3084	0,254221738	1,740742744	4,03085	0,431855021	0,665846787	NA
ENSG00000157211	CDCP2	CUB domain containing protein 2	200008	0,3521638	-0,377100387	4,023898	-0,093715197	0,925335398	NA
ENSG00000157214	STEAP2	STEAP2 metalloreductase	261729	0	NA	NA	NA	NA	NA
ENSG00000157219	HTR5A	5-hydroxytryptamine receptor 5A	3361	0	NA	NA	NA	NA	NA
ENSG00000157224	CLDN12	claudin 12	9069	1,997030842	-0,903834122	1,594353	-0,566897109	0,570784089	NA
ENSG00000157240	FZD1	frizzled class receptor 1	8321	0,103998153	-1,076443944	4,080456	-0,263804824	0,791930317	NA
ENSG00000157303	SUSD3	sushi domain containing 3	203328	7,071926302	-0,352991213	1,01782	-0,346810996	0,728733315	NA
ENSG00000157315	TMED6	transmembrane p24 trafficking protein 6	146456	1,229553219	3,566512345	1,852891	1,924836837	0,054249774	NA
ENSG00000157322	CLEC18A	C-type lectin domain family 18 member A	348174	1,175003485	3,35144916	2,385354	1,405011267	0,160017928	NA
ENSG00000157326	DHRS4	dehydrogenase/reductase 4	10901	2,532616046	4,757001338	2,174026	2,188106441	0,02866185	NA
ENSG00000157330	CFAP107	cilia and flagella associated protein 107	93190	0	NA	NA	NA	NA	NA
ENSG00000157335	CLEC18C	C-type lectin domain family 18 member C	283971	0,10543123	0	4,080456	0	1	NA
ENSG00000157335	LOC124907806	C-type lectin domain family 18 member C-like	124907806	0,10543123	0	4,080456	0	1	NA
ENSG00000157343	ARMC12	armadillo repeat containing 12	221481	0,680842773	0,644237495	2,291324	0,281163841	0,778584737	NA
ENSG00000157368	IL34	interleukin 34	146433	0	NA	NA	NA	NA	NA
ENSG00000157388	CACNA1D	calcium voltage-gated channel subunit alpha1 D	776	0	NA	NA	NA	NA	NA
ENSG00000157399	ARSL	arylsulfatase L	415	0	NA	NA	NA	NA	NA
ENSG00000157423	HYDIN	HYDIN axonemal central pair apparatus protein	54768	0	NA	NA	NA	NA	NA
ENSG00000157429	ZNF19	zinc finger protein 19	7567	3,640377338	-0,704684501	1,282446	-0,549484906	0,58267272	NA
ENSG00000157429	LOC124903710	uncharacterized LOC124903710	124903710	3,640377338	-0,704684501	1,282446	-0,549484906	0,58267272	NA
ENSG00000157445	CACNA2D3	calcium voltage-gated channel auxiliary subunit alpha2delta 3	55799	0	NA	NA	NA	NA	NA
ENSG00000157470	FAM81A	family with sequence similarity 81 member A	145773	1,650274899	0,636846938	1,472613	0,432460516	0,665406744	NA
ENSG00000157502	PWWP3B	PWWP domain containing 3B	139221	0,103998153	-1,076443944	4,080456	-0,263804824	0,791930317	NA
ENSG00000157510	AFAP1L1	actin filament associated protein 1 like 1	134265	0,209763681	-1,926644696	4,042043	-0,476651224	0,633610501	NA

ENSG00000157542	KCNJ6	potassium inwardly rectifying channel subfamily J member 6	3763	0,368212692	0,472268508	3,702304	0,127560719	0,898496619	NA
ENSG00000157551	KCNJ15	potassium inwardly rectifying channel subfamily J member 15	3772	0,192301809	1,681039576	4,046165	0,415464929	0,677801584	NA
ENSG00000157554	ERG	ETS transcription factor ERG	2078	0	NA	NA	NA	NA	NA
ENSG00000157578	LCA5L	lebercilin LCA5 like	150082	0	NA	NA	NA	NA	NA
ENSG00000157613	CREB3L1	cAMP responsive element binding protein 3 like 1	90993	0	NA	NA	NA	NA	NA
ENSG00000157653	C9orf43	chromosome 9 open reading frame 43	257169	0,209763681	-1,926644696	4,042043	-0,476651224	0,633610501	NA
ENSG00000157657	ZNF618	zinc finger protein 618	114991	2,486202251	0,803183738	1,185778	0,677347571	0,498185455	NA
ENSG00000157680	DGKI	diacylglycerol kinase iota	9162	0	NA	NA	NA	NA	NA
ENSG00000157703	SVOPL	SVOP like	136306	0	NA	NA	NA	NA	NA
ENSG00000157734	SNX22	sorting nexin 22	79856	0	NA	NA	NA	NA	NA
ENSG00000157765	SLC34A2	solute carrier family 34 member 2	10568	0	NA	NA	NA	NA	NA
ENSG00000157766	ACAN	aggrecan	176	0	NA	NA	NA	NA	NA
ENSG00000157782	CABP1	calcium binding protein 1	9478	0	NA	NA	NA	NA	NA
ENSG00000157827	FMNL2	formin like 2	114793	11,58244294	0,255452574	0,522153	0,489229071	0,62467953	NA
ENSG00000157851	DPYSL5	dihydropyrimidinase like 5	56896	0	NA	NA	NA	NA	NA
ENSG00000157856	DRC1	dynein regulatory complex subunit 1	92749	0	NA	NA	NA	NA	NA
ENSG00000157884	CIB4	calcium and integrin binding family member 4	130106	0	NA	NA	NA	NA	NA
ENSG00000157890	MEGF11	multiple EGF like domains 11	84465	0	NA	NA	NA	NA	NA
ENSG00000157965	NA	NA	NA	0	NA	NA	NA	NA	NA
ENSG00000157985	AGAP1	ArfGAP with GTPase domain, ankyrin repeat and PH domain 1	116987	1,036617644	-2,050736605	2,681733	-0,764705702	0,44444681	NA
ENSG00000157992	KRTCAP3	keratinocyte associated protein 3	200634	0,327613358	0	4,080456	0	1	NA
ENSG00000157999	ANKRD61	ankyrin repeat domain 61	100310846	0,457708943	-1,037936601	2,981887	-0,348080437	0,727779775	NA
ENSG00000158008	EXTL1	exostosin like glycosyltransferase 1	2134	0	NA	NA	NA	NA	NA
ENSG00000158014	SLC30A2	solute carrier family 30 member 2	7780	0	NA	NA	NA	NA	NA
ENSG00000158022	TRIM63	tripartite motif containing 63	84676	0	NA	NA	NA	NA	NA
ENSG00000158023	CFAP251	cilia and flagella associated protein 251	144406	0,209432085	-1,07564442	4,080456	-0,263608884	0,792081312	NA
ENSG00000158055	GRHL3	grainyhead like transcription factor 3	57822	0	NA	NA	NA	NA	NA
ENSG00000158062	UBXN11	UBX domain protein 11	91544	9,716894242	0,081923676	0,727639	0,112588371	0,910356905	NA
ENSG00000158077	NLRP14	NLR family pyrin domain containing 14	338323	0	NA	NA	NA	NA	NA
ENSG00000158079	PTPDC1	protein tyrosine phosphatase domain containing 1	138639	6,606793093	0,170991725	0,720464	0,23733557	0,81239647	NA
ENSG00000158089	GALNT14	polypeptide N-acetylgalactosaminyltransferase 14	79623	0	NA	NA	NA	NA	NA
ENSG00000158104	HPD	4-hydroxyphenylpyruvate dioxygenase	3242	0	NA	NA	NA	NA	NA
ENSG00000158106	RHPN1	rhophilin Rho GTPase binding protein 1	114822	1,44353346	3,021044102	2,076713	1,45472369	0,145745772	NA
ENSG00000158113	LRRC43	leucine rich repeat containing 43	254050	0,787076573	-2,498127588	3,093535	-0,807531826	0,419360146	NA
ENSG00000158125	XDH	xanthine dehydrogenase	7498	0	NA	NA	NA	NA	NA
ENSG00000158163	DZIP1L	DAZ interacting zinc finger protein 1 like	199221	0,103998153	-1,076443944	4,080456	-0,263804824	0,791930317	NA
ENSG00000158164	TMSB15A	thymosin beta 15A	11013	0,575476767	0,010473572	2,847115	0,003678661	0,99706486	NA
ENSG00000158186	MRAS	muscle RAS oncogene homolog	22808	1,371200717	3,578388875	2,147991	1,665923487	0,095728655	NA
ENSG00000158220	ESYT3	extended synaptotagmin 3	83850	0	NA	NA	NA	NA	NA
ENSG00000158246	TENT5B	terminal nucleotidyltransferase 5B	115572	0	NA	NA	NA	NA	NA
ENSG00000158258	CLSTN2	calsyntenin 2	64084	0	NA	NA	NA	NA	NA
ENSG00000158270	COLEC12	collectin subfamily member 12	81035	0,103998153	-1,076443944	4,080456	-0,263804824	0,791930317	NA
ENSG00000158296	SLC13A3	solute carrier family 13 member 3	64849	3,224287148	0,096587989	1,210342	0,079802207	0,936394569	NA
ENSG00000158301	GPRASP2	G protein-coupled receptor associated sorting protein 2	114928	3,827984243	-0,486608875	1,029508	-0,472661568	0,636454649	NA
ENSG00000158301	ARMCX5-GPRASP2	ARMCX5-GPRASP2 readthrough	100528062	3,827984243	-0,486608875	1,029508	-0,472661568	0,636454649	NA
ENSG00000158315	RHBDL2	rhomboid like 2	54933	0	NA	NA	NA	NA	NA
ENSG00000158352	SHROOM4	shroom family member 4	57477	0	NA	NA	NA	NA	NA
ENSG00000158402	CDC25C	cell division cycle 25C	995	11,33556619	0,703246293	0,640478	1,098001652	0,272203768	NA
ENSG00000158406	H4C8	H4 clustered histone 8	8365	0	NA	NA	NA	NA	NA

ENSG00000158423	RIBC1	RIB43A domain with coiled-coils 1	158787	2,012037273	-0,157794221	1,536203	-0,10271704	0,91818755	NA	
ENSG00000158428	CATIP	ciliogenesis associated TTC17 interacting protein	375307	0	NA	NA	NA	NA	NA	
ENSG00000158445	KCNB1	potassium voltage-gated channel subfamily B member 1	3745	0,072165449	0,847101401	4,080456	0,207599697	0,835541541	NA	
ENSG00000158457	TSPAN33	tetraspanin 33	340348	0	NA	NA	NA	NA	NA	
ENSG00000158458	NRG2	neuregulin 2	9542	0,119673877	0,847101401	4,080456	0,207599697	0,835541541	NA	
ENSG00000158473	CD1D	CD1d molecule	912	0	NA	NA	NA	NA	NA	
ENSG00000158477	CD1A	CD1a molecule	909	0,578679567	-0,029156576	2,850069	-0,010230131	0,991837679	NA	
ENSG00000158481	CD1C	CD1c molecule	911	0	NA	NA	NA	NA	NA	
ENSG00000158482	NA	NA	NA	0,329681734	-2,570343643	3,993843	-0,643576557	0,519850056	NA	
ENSG00000158483	FAM86C1P	family with sequence similarity 86 member C1, pseudogene	55199	0,739282591	0,511421692	2,13619	0,239408339	0,810788965	NA	
ENSG00000158485	CD1B	CD1b molecule	910	0	NA	NA	NA	NA	NA	
ENSG00000158486	DNAH3	dynein axonemal heavy chain 3	55567	0	NA	NA	NA	NA	NA	
ENSG00000158488	CD1E	CD1e molecule	913	0	NA	NA	NA	NA	NA	
ENSG00000158497	HMHB1	histocompatibility minor HB-1	57824	1,327565069	0,840982583	1,517716	0,554110596	0,579503159	NA	
ENSG00000158516	CPA2	carboxypeptidase A2	1358	0	NA	NA	NA	NA	NA	
ENSG00000158525	CPA5	carboxypeptidase A5	93979	0	NA	NA	NA	NA	NA	
ENSG00000158553	POM121L2	POM121 transmembrane nucleoporin like 2	94026	0,461687771	0,985267993	3,521529	0,279784137	0,779643123	NA	
ENSG00000158555	GDPD5	glycerophosphodiester phosphodiesterase domain containing 5	81544	1,780262859	-1,743584996	1,580743	-1,10301616	0,270020147	NA	
ENSG00000158560	DYNC1I1	dynein cytoplasmic 1 intermediate chain 1	1780	0	NA	NA	NA	NA	NA	
ENSG00000158571	PFKFB1	6-phosphofructo-2-kinase/fructose-2,6-biphosphatase 1	5207	1,864761949	1,067982149	1,350851	0,790599517	0,429177729	NA	
ENSG00000158578	ALAS2	5'-aminolevulinate synthase 2	212	0,119673877	0,847101401	4,080456	0,207599697	0,835541541	NA	
ENSG00000158639	PAGE5	PAGE family member 5	90737	0	NA	NA	NA	NA	NA	
ENSG00000158683	PKD1L1	polycystin 1 like 1, transient receptor potential channel interacting	168507	0,369297554	2,148190028	3,617031	0,593909737	0,552572486	NA	
ENSG00000158715	SLC45A3	solute carrier family 45 member 3	85414	0,501961441	3,061503704	3,960966	0,772918436	0,439570653	NA	
ENSG00000158731	OR10J6P	olfactory receptor family 10 subfamily J member 6 pseudogene	401973	0	NA	NA	NA	NA	NA	
ENSG00000158748	HTR6	5-hydroxytryptamine receptor 6	3362	1,381406142	0,627345034	1,923093	0,32621663	0,74426046	NA	
ENSG00000158764	ITLN2	intelectin 2	142683	0	NA	NA	NA	NA	NA	
ENSG00000158786	PLA2G2F	phospholipase A2 group IIF	64600	0	NA	NA	NA	NA	NA	
ENSG00000158806	NPM2	nucleophosmin/nucleoplasmin 2	10361	1,667712387	0,736098552	1,974821	0,372741852	0,709340584	NA	
ENSG00000158813	EDA	ectodysplasin A	1896	0	NA	NA	NA	NA	NA	
ENSG00000158815	FGF17	fibroblast growth factor 17	8822	0,339333775	0,974738185	4,023505	0,242260962	0,808577961	NA	
ENSG00000158816	VWA5B1	von Willebrand factor A domain containing 5B1	127731	0	NA	NA	NA	NA	NA	
ENSG00000158825	CDA	cytidine deaminase	978	0,207996306	-1,916073151	4,043021	-0,473921178	0,635556117	NA	
ENSG00000158859	ADAMTS4	ADAM metallopeptidase with thrombospondin type 1 motif 4	9507	0,683381186	-2,385995392	3,032852	-0,786716776	0,431447681	NA	
ENSG00000158865	SLC5A11	solute carrier family 5 member 11	115584	0	NA	NA	NA	NA	NA	
ENSG00000158874	APOA2	apolipoprotein A2	336	0	NA	NA	NA	NA	NA	
ENSG00000158887	MPZ	myelin protein zero	4359	5,114557862	-0,16846202	0,941408	-0,178946796	0,857979478	NA	
ENSG00000158901	WFDC8	WAP four-disulfide core domain 8	90199	0	NA	NA	NA	NA	NA	
ENSG00000158955	WNT9B	Wnt family member 9B	7484	0	NA	NA	NA	NA	NA	
ENSG00000158966	CACHD1	cache domain containing 1	57685	0	NA	NA	NA	NA	NA	
ENSG00000159128	IFNGR2	interferon gamma receptor 2	3460	0	NA	NA	NA	NA	NA	
ENSG00000159164	SV2A	synaptic vesicle glycoprotein 2A	9900	2,825850202	-1,029807145	1,17039	-0,879884064	0,378922118	NA	
ENSG00000159166	LAD1	ladinin 1	3898	0	NA	NA	NA	NA	NA	
ENSG00000159167	STC1	stanniocalcin 1	6781	0	NA	NA	NA	NA	NA	
ENSG00000159173	TNNI1	troponin I1, slow skeletal type	7135	0	NA	NA	NA	NA	NA	
ENSG00000159182	PRAC1	PRAC1 small nuclear protein	84366	0	NA	NA	NA	NA	NA	
ENSG00000159184	HOXB13	homeobox B13	10481	0	NA	NA	NA	NA	NA	
ENSG00000159186	ATP5POP1	ATP5PO pseudogene 1	100132849	0	NA	NA	NA	NA	NA	
ENSG00000159189	C1QC	complement C1q C chain	714	0	NA	NA	NA	NA	NA	

ENSG00000159197	KCNE2	potassium voltage-gated channel subfamily E regulatory subunit 2	9992	0	NA	NA	NA	NA	NA
ENSG00000159208	CIART	circadian associated repressor of transcription	148523	0	NA	NA	NA	NA	NA
ENSG00000159212	CLIC6	chloride intracellular channel 6	54102	0	NA	NA	NA	NA	NA
ENSG00000159214	CCDC24	coiled-coil domain containing 24	149473	0,684866864	2,148230754	3,182015	0,675116383	0,499601826	NA
ENSG00000159217	IGF2BP1	insulin like growth factor 2 mRNA binding protein 1	10642	0,103998153	-1,076443944	4,080456	-0,263804824	0,791930317	NA
ENSG00000159224	GIP	gastric inhibitory polypeptide	2695	0	NA	NA	NA	NA	NA
ENSG00000159239	NA	NA	NA	0	NA	NA	NA	NA	NA
ENSG00000159247	NA	NA	NA	0	NA	NA	NA	NA	NA
ENSG00000159248	GJD2	gap junction protein delta 2	57369	0	NA	NA	NA	NA	NA
ENSG00000159251	ACTC1	actin alpha cardiac muscle 1	70	0	NA	NA	NA	NA	NA
ENSG00000159261	CLDN14	claudin 14	23562	0	NA	NA	NA	NA	NA
ENSG00000159263	SIM2	SIM bHLH transcription factor 2	6493	0	NA	NA	NA	NA	NA
ENSG00000159289	GOLGA6A	golgin A6 family member A	342096	0,307199368	2,350214075	3,995082	0,588276733	0,55634656	NA
ENSG00000159289	LOC124907523	golgin subfamily A member 6A-like	124907523	0,307199368	2,350214075	3,995082	0,588276733	0,55634656	NA
ENSG00000159307	SCUBE1	signal peptide, CUB domain and EGF like domain containing 1	80274	0,763981631	-1,034523314	2,790718	-0,370701509	0,710859866	NA
ENSG00000159337	PLA2G4D	phospholipase A2 group IVD	283748	0	NA	NA	NA	NA	NA
ENSG00000159374	M1AP	meiosis 1 associated protein	130951	0	NA	NA	NA	NA	NA
ENSG00000159387	IRX6	iroquois homeobox 6	79190	0	NA	NA	NA	NA	NA
ENSG00000159398	CES5A	carboxylesterase 5A	221223	0	NA	NA	NA	NA	NA
ENSG00000159409	CELF3	CUGBP Elav-like family member 3	11189	0	NA	NA	NA	NA	NA
ENSG00000159450	TCHH	trichohyalin	7062	0	NA	NA	NA	NA	NA
ENSG00000159455	LCE2B	late cornified envelope 2B	26239	0	NA	NA	NA	NA	NA
ENSG00000159495	TGM7	transglutaminase 7	116179	0	NA	NA	NA	NA	NA
ENSG00000159496	RGL4	ral guanine nucleotide dissociation stimulator like 4	266747	0,11808024	-1,076443944	4,080456	-0,263804824	0,791930317	NA
ENSG00000159516	SPRR2G	small proline rich protein 2G	6706	0	NA	NA	NA	NA	NA
ENSG00000159527	PGLYRP3	peptidoglycan recognition protein 3	114771	0	NA	NA	NA	NA	NA
ENSG00000159556	ISL2	ISL LIM homeobox 2	64843	0,263246434	-1,068906236	4,080456	-0,261957553	0,793354167	NA
ENSG00000159588	CCDC17	coiled-coil domain containing 17	149483	0	NA	NA	NA	NA	NA
ENSG00000159625	DRC7	dynein regulatory complex subunit 7	84229	0	NA	NA	NA	NA	NA
ENSG00000159640	ACE	angiotensin I converting enzyme	1636	0	NA	NA	NA	NA	NA
ENSG00000159648	SPMIP8	sperm microtubule inner protein 8	374739	0,863475802	-1,241331559	2,200631	-0,564079806	0,572699821	NA
ENSG00000159650	UROC1	urocanate hydratase 1	131669	0	NA	NA	NA	NA	NA
ENSG00000159674	SPON2	spondin 2	10417	0,645942992	-1,55705312	3,248257	-0,479350381	0,631689386	NA
ENSG00000159685	CHCHD6	coiled-coil-helix-coiled-coil-helix domain containing 6	84303	3,767949663	-0,040565912	1,528963	-0,026531651	0,978833289	NA
ENSG00000159708	LRRC36	leucine rich repeat containing 36	55282	0	NA	NA	NA	NA	NA
ENSG00000159712	NA	NA	NA	2,39816455	-0,063960427	1,468826	-0,043545273	0,965266876	NA
ENSG00000159713	TPPP3	tubulin polymerization promoting protein family member 3	51673	0	NA	NA	NA	NA	NA
ENSG00000159714	ZDHHC1	zinc finger DHHC-type containing 1	29800	1,60089885	0,017905619	1,781002	0,010053675	0,991978463	NA
ENSG00000159723	AGRP	agouti related neuropeptide	181	0	NA	NA	NA	NA	NA
ENSG00000159761	C16orf86	chromosome 16 open reading frame 86	388284	0,407422303	2,779863969	3,972521	0,699773276	0,484068906	NA
ENSG00000159763	PIP	prolactin induced protein	5304	0	NA	NA	NA	NA	NA
ENSG00000159788	RGS12	regulator of G protein signaling 12	6002	6,485112573	0,112224636	0,660264	0,169969423	0,865034184	NA
ENSG00000159860	NA	NA	NA	0	NA	NA	NA	NA	NA
ENSG00000159871	LYPD5	LY6/PLAUR domain containing 5	284348	0,562132011	2,379121425	3,386481	0,702535084	0,48234553	NA
ENSG00000159899	NPR2	natriuretic peptide receptor 2	4882	2,73947995	0,874652935	1,971527	0,443642486	0,657301084	NA
ENSG00000159904	NA	NA	NA	0,415352526	2,350221467	3,52288	0,667130751	0,504688619	NA
ENSG00000159905	ZNF221	zinc finger protein 221	7638	10,49983082	-0,393991134	0,641148	-0,614509323	0,538878814	NA
ENSG00000159915	ZNF233	zinc finger protein 233	353355	2,219926337	1,849787746	1,434627	1,289385486	0,197264105	NA
ENSG00000159958	TNFRSF13C	TNF receptor superfamily member 13C	115650	0,808936376	-0,799839227	2,13685	-0,374307666	0,708175429	NA
ENSG00000159961	OR3A3	olfactory receptor family 3 subfamily A member 3	8392	0	NA	NA	NA	NA	NA
ENSG00000160013	PTGIR	prostaglandin I2 receptor	5739	0,103998153	-1,076443944	4,080456	-0,263804824	0,791930317	NA

ENSG00000160051	IQCC	IQ motif containing C	55721	3,767956182	-0,598332638	1,464724	-0,408495073	0,682910247	NA	
ENSG00000160055	TMEM234	transmembrane protein 234	56063	7,247112625	0,362395071	1,250624	0,289771339	0,771991176	NA	
ENSG00000160062	ZBTB8A	zinc finger and BTB domain containing 8A	653121	0,743212483	0,452502618	2,491245	0,181637169	0,855867478	NA	
ENSG00000160097	FNDC5	fibronectin type III domain containing 5	252995	0	NA	NA	NA	NA	NA	
ENSG00000160111	CPAMD8	C3 and PZP like alpha-2-macroglobulin domain containing 8	27151	0	NA	NA	NA	NA	NA	
ENSG00000160113	NR2F6	nuclear receptor subfamily 2 group F member 6	2063	2,174160619	2,468789219	1,527849	1,615859193	0,10612476	NA	
ENSG00000160117	ANKLE1	ankyrin repeat and LEM domain containing 1	126549	1,236134678	-3,212297661	2,2974	-1,398231504	0,162043559	NA	
ENSG00000160145	KALRN	kalirin RhoGEF kinase	8997	1,694356315	0	2,886148	0	1	NA	
ENSG00000160161	CILP2	cartilage intermediate layer protein 2	148113	0,410923438	1,3826326	3,531617	0,391501296	0,695426731	NA	
ENSG00000160172	NA	NA	NA	0,22224662	-1,077764921	4,080456	-0,264128557	0,79168086	NA	
ENSG00000160180	TFF3	trefoil factor 3	7033	0	NA	NA	NA	NA	NA	
ENSG00000160181	TFF2	trefoil factor 2	7032	0	NA	NA	NA	NA	NA	
ENSG00000160182	TFF1	trefoil factor 1	7031	0	NA	NA	NA	NA	NA	
ENSG00000160183	TMPRSS3	transmembrane serine protease 3	64699	0,11808024	-1,076443944	4,080456	-0,263804824	0,791930317	NA	
ENSG00000160188	RSPH1	radial spoke head component 1	89765	0,241404598	-1,17597993	4,080456	-0,288198174	0,773195047	NA	
ENSG00000160191	PDE9A	phosphodiesterase 9A	5152	1,977941061	-3,632964275	1,735425	-2,093414775	0,036312145	NA	
ENSG00000160200	CBS	cystathionine beta-synthase	875	5,027450493	0,121660876	1,060942	0,114672526	0,908704691	NA	
ENSG00000160200	LOC102724560	cystathionine beta-synthase like	102724560	5,027450493	0,121660876	1,060942	0,114672526	0,908704691	NA	
ENSG00000160202	CRYAA	crystallin alpha A	1409	0	NA	NA	NA	NA	NA	
ENSG00000160202	LOC102724652	crystallin alpha A2	102724652	0	NA	NA	NA	NA	NA	
ENSG00000160207	HSF2BP	heat shock transcription factor 2 binding protein	11077	0,072165449	0,847101401	4,080456	0,207599697	0,835541541	NA	
ENSG00000160223	ICOSLG	inducible T cell costimulator ligand	23308	6,320876366	-0,925600981	0,75609	-1,224193496	0,220879242	NA	
ENSG00000160223	LOC102723996	ICOS ligand	102723996	6,320876366	-0,925600981	0,75609	-1,224193496	0,220879242	NA	
ENSG00000160224	AIRE	autoimmune regulator	326	0	NA	NA	NA	NA	NA	
ENSG00000160282	FTCD	formimidoyltransferase cyclodeaminase	10841	0	NA	NA	NA	NA	NA	
ENSG00000160284	SPATC1L	spermatogenesis and centriole associated 1 like	84221	2,06218765	-0,367181749	1,288711	-0,284921643	0,775704174	NA	
ENSG00000160321	ZNF208	zinc finger protein 208	7757	1,254027415	2,848496431	2,565576	1,110275418	0,266880362	NA	
ENSG00000160323	ADAMTS13	ADAM metallopeptidase with thrombospondin type 1 motif 13	11093	0	NA	NA	NA	NA	NA	
ENSG00000160325	CACFD1	calcium channel flower domain containing 1	11094	0	NA	NA	NA	NA	NA	
ENSG00000160339	FCN2	ficolin 2	2220	0	NA	NA	NA	NA	NA	
ENSG00000160345	PIERCE1	piercer of microtubule wall 1	138162	0,604584414	0,461302605	2,55241	0,180732187	0,856577794	NA	
ENSG00000160349	LCN1	lipocalin 1	3933	0	NA	NA	NA	NA	NA	
ENSG00000160360	GPSM1	G protein signaling modulator 1	26086	3,483912127	-0,666874086	1,081163	-0,616811781	0,53735888	NA	
ENSG00000160396	HIPK4	homeodomain interacting protein kinase 4	147746	0,411251592	-1,63432896	3,533798	-0,462485108	0,643733479	NA	
ENSG00000160401	CFAP157	cilia and flagella associated protein 157	286207	0,118286652	0	4,080456	0	1	NA	
ENSG00000160460	SPTBN4	spectrin beta, non-erythrocytic 4	57731	0,207996306	-1,916073151	4,043021	-0,473921178	0,635556117	NA	
ENSG00000160469	BRSK1	BR serine/threonine kinase 1	84446	9,033108608	0,100221727	0,630527	0,158949122	0,873708959	NA	
ENSG00000160471	COX6B2	cytochrome c oxidase subunit 6B2	125965	0,10488184	-1,076443944	4,080456	-0,263804824	0,791930317	NA	
ENSG00000160472	TMEM190	transmembrane protein 190	147744	0	NA	NA	NA	NA	NA	
ENSG00000160505	NLRP4	NLR family pyrin domain containing 4	147945	0	NA	NA	NA	NA	NA	
ENSG00000160539	PLPP7	phospholipid phosphatase 7 (inactive)	84814	0,325565549	-0,955718666	4,042958	-0,236390974	0,813129299	NA	
ENSG00000160606	TLCD1	TLC domain containing 1	116238	0,22488007	-0,989424113	4,080456	-0,242478817	0,808409169	NA	
ENSG00000160654	CD3G	CD3 gamma subunit of T-cell receptor complex	917	5,577770478	-5,781271278	1,354913	-4,266894435	1,98E-05	NA	
ENSG00000160678	S100A1	S100 calcium binding protein A1	6271	0	NA	NA	NA	NA	NA	
ENSG00000160683	CXCR5	C-X-C motif chemokine receptor 5	643	2,116955754	1,135546374	1,395817	0,813535329	0,415911199	NA	
ENSG00000160712	IL6R	interleukin 6 receptor	3570	1,309048169	-0,592152535	2,075288	-0,285335134	0,775387398	NA	
ENSG00000160716	CHRNB2	cholinergic receptor nicotinic beta 2 subunit	1141	0,191134342	1,091635687	4,080456	0,267527875	0,789062755	NA	
ENSG00000160766	NA	NA	NA	6,885483107	1,083979951	0,698772	1,551264461	0,12083832	NA	
ENSG00000160801	PTH1R	parathyroid hormone 1 receptor	5745	0	NA	NA	NA	NA	NA	

ENSG00000160808	MYL3	myosin light chain 3	4634	0,10543123	0	4,080456	0	1	NA
ENSG00000160813	PPP1R35	protein phosphatase 1 regulatory subunit 35	221908	11,26111996	5,936135657	3,103138	1,912946017	0,055754964	NA
ENSG00000160818	GPATCH4	G-patch domain containing 4 (gene/pseudogene)	54865	0	NA	NA	NA	NA	NA
ENSG00000160838	LRRC71	leucine rich repeat containing 71	149499	0	NA	NA	NA	NA	NA
ENSG00000160856	FCRL3	Fc receptor like 3	115352	0,460355384	1,420055733	4,06129	0,349656302	0,726596652	NA
ENSG00000160862	AZGP1	alpha-2-glycoprotein 1, zinc-binding	563	0	NA	NA	NA	NA	NA
ENSG00000160867	FGFR4	fibroblast growth factor receptor 4	2264	0,418606307	-1,790409312	3,98787	-0,44896378	0,653457785	NA
ENSG00000160868	CYP3A4	cytochrome P450 family 3 subfamily A member 4	1576	0	NA	NA	NA	NA	NA
ENSG00000160870	CYP3A7	cytochrome P450 family 3 subfamily A member 7	1551	0	NA	NA	NA	NA	NA
ENSG00000160882	CYP11B1	cytochrome P450 family 11 subfamily B member 1	1584	0	NA	NA	NA	NA	NA
ENSG00000160883	HK3	hexokinase 3	3101	0	NA	NA	NA	NA	NA
ENSG00000160886	LY6K	lymphocyte antigen 6 family member K	54742	0,11808024	-1,076443944	4,080456	-0,263804824	0,791930317	NA
ENSG00000160948	VPS28	VPS28 subunit of ESCRT-I	51160	0	NA	NA	NA	NA	NA
ENSG00000160951	PTGER1	prostaglandin E receptor 1	5731	0,368369863	-1,90978883	3,65105	-0,523079398	0,600919001	NA
ENSG00000160963	COL26A1	collagen type XXVI alpha 1 chain	136227	0	NA	NA	NA	NA	NA
ENSG00000160973	FOXH1	forkhead box H1	8928	0,295526854	0,849817615	3,91993	0,21679407	0,828368833	NA
ENSG00000160994	TEKTL1	tektin like 1	126402	0	NA	NA	NA	NA	NA
ENSG00000160999	SH2B2	SH2B adaptor protein 2	10603	0,57747143	-0,335177154	2,519596	-0,133028112	0,894171148	NA
ENSG00000161031	PGLYRP2	peptidoglycan recognition protein 2	114770	0	NA	NA	NA	NA	NA
ENSG00000161040	FBXL13	F-box and leucine rich repeat protein 13	222235	0,231436919	0,840629235	4,080248	0,206024029	0,836772141	NA
ENSG00000161055	SCGB3A1	secretoglobin family 3A member 1	92304	0	NA	NA	NA	NA	NA
ENSG00000161082	CELF5	CUGBP Elav-like family member 5	60680	0,473736483	-2,672088686	3,988163	-0,670004801	0,50285473	NA
ENSG00000161103	POM121L15P	POM121 transmembrane nucleoporin like 15, pseudogene	102725072	0	NA	NA	NA	NA	NA
ENSG00000161132	PRODHLP	proline dehydrogenase like, pseudogene	440792	0	NA	NA	NA	NA	NA
ENSG00000161133	USP41P	ubiquitin specific peptidase 41, pseudogene	373856	0	NA	NA	NA	NA	NA
ENSG00000161179	YDJC	YdjC chitooligosaccharide deacetylase homolog	150223	4,389252861	-3,870852445	1,846933	-2,095827257	0,036097517	NA
ENSG00000161180	CCDC116	coiled-coil domain containing 116	164592	4,192873685	-0,519377558	0,964893	-0,538274591	0,590387491	NA
ENSG00000161243	FBXO27	F-box protein 27	126433	0,343087497	-2,612102383	3,661833	-0,713331937	0,475640379	NA
ENSG00000161249	DMKN	dermokine	93099	0	NA	NA	NA	NA	NA
ENSG00000161265	U2AF1L4	U2 small nuclear RNA auxiliary factor 1 like 4	199746	3,198756731	-0,184842026	1,181141	-0,156494425	0,875643313	NA
ENSG00000161270	NPHS1	NPHS1 adhesion molecule, nephrin	4868	0,15836288	0	4,080456	0	1	NA
ENSG00000161277	THAP8	THAP domain containing 8	199745	10,65601763	0,137287125	0,568749	0,241384409	0,809257196	NA
ENSG00000161281	COX7A1	cytochrome c oxidase subunit 7A1	1346	0	NA	NA	NA	NA	NA
ENSG00000161328	LRRC56	leucine rich repeat containing 56	115399	0,948881073	0	4,080456	0	1	NA
ENSG00000161381	PLXDC1	plexin domain containing 1	57125	7,213205162	-1,291914607	1,070496	-1,206836908	0,227494959	NA
ENSG00000161509	GRIN2C	glutamate ionotropic receptor NMDA type subunit 2C	2905	0	NA	NA	NA	NA	NA
ENSG00000161544	CYGB	cytoglobin	114757	1,523451459	1,4451342	1,64494	0,878533114	0,379654475	NA
ENSG00000161594	KLHL10	kelch like family member 10	317719	0	NA	NA	NA	NA	NA
ENSG00000161609	KASH5	KASH domain containing 5	147872	0	NA	NA	NA	NA	NA
ENSG00000161610	HCRT	hypocretin neuropeptide precursor	3060	0	NA	NA	NA	NA	NA
ENSG00000161634	DCD	dermcidin	117159	0	NA	NA	NA	NA	NA
ENSG00000161640	SIGLEC11	sialic acid binding Ig like lectin 11	114132	0	NA	NA	NA	NA	NA
ENSG00000161642	ZNF385A	zinc finger protein 385A	25946	1,984822906	-0,481848625	1,317103	-0,365839872	0,714484565	NA
ENSG00000161643	SIGLEC16	sialic acid binding Ig like lectin 16	400709	0,10488184	-1,076443944	4,080456	-0,263804824	0,791930317	NA
ENSG00000161647	MPP3	MAGUK p55 scaffold protein 3	4356	0	NA	NA	NA	NA	NA
ENSG00000161649	CD300LG	CD300 molecule like family member g	146894	0,15836288	0	4,080456	0	1	NA
ENSG00000161652	IZUMO2	IZUMO family member 2	126123	0	NA	NA	NA	NA	NA
ENSG00000161653	NAGS	N-acetylglutamate synthase	162417	0	NA	NA	NA	NA	NA

ENSG00000161664	ASB16	ankyrin repeat and SOCS box containing 16	92591	0	NA	NA	NA	NA	NA
ENSG00000161681	SHANK1	SH3 and multiple ankyrin repeat domains 1	50944	0	NA	NA	NA	NA	NA
ENSG00000161682	FAM171A2	family with sequence similarity 171 member A2	284069	0,295433134	-1,043629804	3,903913	-0,267329147	0,789215747	NA
ENSG00000161692	DBF4B	DBF4 zinc finger B	80174	10,03937652	0,515287013	0,572462	0,900124145	0,368054188	NA
ENSG00000161714	PLCD3	phospholipase C delta 3	113026	6,188998284	0,244584374	0,745821	0,327939585	0,742957342	NA
ENSG00000161798	AQP5	aquaporin 5	362	0	NA	NA	NA	NA	NA
ENSG00000161807	OR7G1	olfactory receptor family 7 subfamily G member 1	125962	0	NA	NA	NA	NA	NA
ENSG00000161835	TAMALIN	trafficking regulator and scaffold protein tamalin	160622	1,278337235	-0,320358772	2,156737	-0,148538605	0,881917721	NA
ENSG00000161849	KRT84	keratin 84	3890	0	NA	NA	NA	NA	NA
ENSG00000161850	KRT82	keratin 82	3888	0	NA	NA	NA	NA	NA
ENSG00000161860	SYCE2	synaptonemal complex central element protein 2	256126	1,759063751	-1,719854996	1,935222	-0,888712059	0,374157848	NA
ENSG00000161896	IP6K3	inositol hexakisphosphate kinase 3	117283	0	NA	NA	NA	NA	NA
ENSG00000161905	ALOX15	arachidonate 15-lipoxygenase	246	0,119673877	0,847101401	4,080456	0,207599697	0,835541541	NA
ENSG00000161911	TREML1	triggering receptor expressed on myeloid cells like 1	340205	0	NA	NA	NA	NA	NA
ENSG00000161912	NA	NA	NA	6,273297538	-0,19393519	0,71124	-0,272671835	0,785105487	NA
ENSG00000161921	CXCL16	C-X-C motif chemokine ligand 16	58191	2,259028361	1,736546745	1,345654	1,29048575	0,196882056	NA
ENSG00000161939	RNASEK-C17orf49	RNASEK-C17orf49 readthrough	100529209	0,354859955	0	4,080456	0	1	NA
ENSG00000161940	BCL6B	BCL6B transcription repressor	255877	2,22806018	0,392954198	1,181832	0,332495871	0,739514857	NA
ENSG00000161944	ASGR2	asialoglycoprotein receptor 2	433	0,234994216	1,944111989	4,02324	0,483220515	0,62893917	NA
ENSG00000161955	TNFSF13	TNF superfamily member 13	8741	7,118044923	-1,205333784	0,773707	-1,557867765	0,119264598	NA
ENSG00000161973	CCDC42	coiled-coil domain containing 42	146849	0,238153703	0,056554852	4,07679	0,013872398	0,988931783	NA
ENSG00000161992	PRR35	proline rich 35	146325	0,222338348	-0,117851777	4,080456	-0,028882012	0,976958692	NA
ENSG00000161996	WDR90	WD repeat domain 90	197335	9,255375746	-0,278285393	1,210079	-0,229972905	0,818112824	NA
ENSG00000162004	CCDC78	coiled-coil domain containing 78	124093	0,642355909	-0,21884167	3,831907	-0,057110386	0,954457263	NA
ENSG00000162006	MSLNL	mesothelin like	401827	0	NA	NA	NA	NA	NA
ENSG00000162009	SSTR5	somatostatin receptor 5	6755	0	NA	NA	NA	NA	NA
ENSG00000162039	MEIOB	meiosis specific with OB-fold	254528	0	NA	NA	NA	NA	NA
ENSG00000162040	HS3ST6	heparan sulfate-glucosamine 3-sulfotransferase 6	64711	0	NA	NA	NA	NA	NA
ENSG00000162062	TEDC2	tubulin epsilon and delta complex 2	80178	2,854650653	2,37367572	2,147947	1,105090679	0,269120299	NA
ENSG00000162068	NTN3	netrin 3	4917	0	NA	NA	NA	NA	NA
ENSG00000162069	BICDL2	BICD family like cargo adaptor 2	146439	5,370000152	0,08912066	1,153372	0,077269687	0,938409005	NA
ENSG00000162105	SHANK2	SH3 and multiple ankyrin repeat domains 2	22941	0	NA	NA	NA	NA	NA
ENSG00000162148	SAXO4	stabilizer of axonemal microtubules 4	220004	6,952731016	-0,38424592	0,658302	-0,583692356	0,559427311	NA
ENSG00000162174	ASRGL1	asparaginase and isoaspartyl peptidase 1	80150	6,454511357	-0,329247895	0,785104	-0,419368518	0,674946829	NA
ENSG00000162188	GNG3	G protein subunit gamma 3	2785	0	NA	NA	NA	NA	NA
ENSG00000162267	ITIH3	inter-alpha-trypsin inhibitor heavy chain 3	3699	0	NA	NA	NA	NA	NA
ENSG00000162344	FGF19	fibroblast growth factor 19	9965	0	NA	NA	NA	NA	NA
ENSG00000162365	CYP4A22	cytochrome P450 family 4 subfamily A member 22	284541	0	NA	NA	NA	NA	NA
ENSG00000162366	PDZK1IP1	PDZK1 interacting protein 1	10158	0	NA	NA	NA	NA	NA
ENSG00000162367	TAL1	TAL bHLH transcription factor 1, erythroid differentiation factor	6886	0,177227025	-0,120298031	4,080395	-0,029481954	0,976480211	NA
ENSG00000162373	BEND5	BEN domain containing 5	79656	0	NA	NA	NA	NA	NA
ENSG00000162374	ELAVL4	ELAV like RNA binding protein 4	1996	0	NA	NA	NA	NA	NA
ENSG00000162383	SLC1A7	solute carrier family 1 member 7	6512	0	NA	NA	NA	NA	NA
ENSG00000162390	ACOT11	acyl-CoA thioesterase 11	26027	2,604680068	2,75025349	1,822326	1,509199161	0,131247895	NA
ENSG00000162391	FAM151A	family with sequence similarity 151 member A	338094	0	NA	NA	NA	NA	NA
ENSG00000162396	PARS2	prolyl-tRNA synthetase 2, mitochondrial	25973	8,49015524	-0,011414948	0,760139	-0,015016931	0,988018673	NA
ENSG00000162399	BSND	barttin CLCNK type accessory subunit beta	7809	0,907090649	1,279603648	1,949407	0,656406648	0,511562514	NA
ENSG00000162407	PLPP3	phospholipid phosphatase 3	8613	0	NA	NA	NA	NA	NA
ENSG00000162409	PRKAA2	protein kinase AMP-activated catalytic subunit alpha 2	5563	0	NA	NA	NA	NA	NA

ENSG00000162415	ZSWIM5	zinc finger SWIM-type containing 5	57643	0,591351825	1,98733097	3,182702	0,624416378	0,532354172	NA
ENSG00000162426	SLC45A1	solute carrier family 45 member 1	50651	0	NA	NA	NA	NA	NA
ENSG00000162433	AK4	adenylate kinase 4	205	3,618923508	-1,820754995	1,136499	-1,602073273	0,109139407	NA
ENSG00000162437	RAVER2	ribonucleoprotein, PTB binding 2	55225	0,666345747	1,404420518	2,752131	0,510302924	0,609839255	NA
ENSG00000162438	CTRC	chymotrypsin C	11330	1,186136685	-0,257666818	1,909109	-0,134967048	0,892637928	NA
ENSG00000162444	RBP7	retinol binding protein 7	116362	0	NA	NA	NA	NA	NA
ENSG00000162456	KNCN	kinocilin	148930	0	NA	NA	NA	NA	NA
ENSG00000162458	FBLIM1	filamin binding LIM protein 1	54751	0	NA	NA	NA	NA	NA
ENSG00000162460	TMEM82	transmembrane protein 82	388595	0	NA	NA	NA	NA	NA
ENSG00000162461	SLC25A34	solute carrier family 25 member 34	284723	7,680408219	-0,685683473	0,683015	-1,00390632	0,31542377	NA
ENSG00000162482	AKR7A3	aldo-keto reductase family 7 member A3	22977	1,381909322	0,44511255	1,865632	0,238585426	0,811427067	NA
ENSG00000162493	PDPN	podoplanin	10630	0	NA	NA	NA	NA	NA
ENSG00000162494	LRRC38	leucine rich repeat containing 38	126755	0	NA	NA	NA	NA	NA
ENSG00000162510	MATN1	matrilin 1	4146	0,691481523	0,262105148	2,515168	0,104209807	0,917002852	NA
ENSG00000162512	SDC3	syndecan 3	9672	0,411797749	2,344231912	3,529641	0,664155905	0,506590532	NA
ENSG00000162520	SYNC	syncoilin, intermediate filament protein	81493	0,11808024	-1,076443944	4,080456	-0,263804824	0,791930317	NA
ENSG00000162522	NHSL3	NHS like 3	57648	0,517326842	2,345765586	3,345905	0,701085456	0,483249686	NA
ENSG00000162526	TSSK3	testis specific serine kinase 3	81629	0	NA	NA	NA	NA	NA
ENSG00000162545	CAMK2N1	calcium/calmodulin dependent protein kinase II inhibitor 1	55450	1,195942278	0,204837442	2,137638	0,095824182	0,923660211	NA
ENSG00000162551	ALPL	alkaline phosphatase, biomineralization associated	249	0	NA	NA	NA	NA	NA
ENSG00000162552	WNT4	Wnt family member 4	54361	0,222327376	-0,118748019	4,080456	-0,029101655	0,976783516	NA
ENSG00000162571	TTLL10	tubulin tyrosine ligase like 10	254173	0,68567878	1,820083785	2,336475	0,778986895	0,435987435	NA
ENSG00000162576	MXRA8	matrix remodeling associated 8	54587	0,234994216	1,944111989	4,02324	0,483220515	0,62893917	NA
ENSG00000162591	MEGF6	multiple EGF like domains 6	1953	3,388390652	-0,618549676	1,000672	-0,618134556	0,536486645	NA
ENSG00000162592	CCDC27	coiled-coil domain containing 27	148870	0	NA	NA	NA	NA	NA
ENSG00000162594	IL23R	interleukin 23 receptor	149233	0,587485541	3,280248886	3,95344	0,829720116	0,406697045	NA
ENSG00000162595	DIRAS3	DIRAS family GTPase 3	9077	0	NA	NA	NA	NA	NA
ENSG00000162598	C1orf87	chromosome 1 open reading frame 87	127795	0	NA	NA	NA	NA	NA
ENSG00000162599	NFIA	nuclear factor I A	4774	3,065622382	0,072573164	1,136034	0,063882919	0,949063453	NA
ENSG00000162614	NEXN	nexilin F-actin binding protein	91624	0,944286416	-3,218174153	3,565234	-0,902654435	0,366709326	NA
ENSG00000162618	ADGRL4	adhesion G protein-coupled receptor L4	64123	0	NA	NA	NA	NA	NA
ENSG00000162620	LRRIQ3	leucine rich repeats and IQ motif containing 3	127255	1,575816811	0,189705914	1,561082	0,121522061	0,903277542	NA
ENSG00000162621	LRRC53	leucine rich repeat containing 53	105378803	0	NA	NA	NA	NA	NA
ENSG00000162624	LHX8	LIM homeobox 8	431707	0	NA	NA	NA	NA	NA
ENSG00000162627	SNX7	sorting nexin 7	51375	0	NA	NA	NA	NA	NA
ENSG00000162631	NTNG1	netrin G1	22854	0	NA	NA	NA	NA	NA
ENSG00000162641	AKNAD1	AKNA domain containing 1	254268	0	NA	NA	NA	NA	NA
ENSG00000162643	DNAI3	dynein axonemal intermediate chain 3	126820	4,606697393	-0,367334888	1,020943	-0,359799689	0,718996936	NA
ENSG00000162669	HFM1	helicase for meiosis 1	164045	0	NA	NA	NA	NA	NA
ENSG00000162670	BRINP3	BMP/retinoic acid inducible neural specific 3	339479	0	NA	NA	NA	NA	NA
ENSG00000162685	NA	NA	NA	0	NA	NA	NA	NA	NA
ENSG00000162692	VCAM1	vascular cell adhesion molecule 1	7412	1,500750657	2,85773352	1,845353	1,548610574	0,121475363	NA
ENSG00000162699	NA	NA	NA	0	NA	NA	NA	NA	NA
ENSG00000162706	CADM3	cell adhesion molecule 3	57863	0	NA	NA	NA	NA	NA
ENSG00000162711	NLRP3	NLR family pyrin domain containing 3	114548	0,825468694	-0,145454538	2,449153	-0,059389724	0,952641697	NA
ENSG00000162722	TRIM58	tripartite motif containing 58	25893	10,5282248	-0,654969913	0,56271	-1,163955937	0,244441869	NA
ENSG00000162723	SLAMF9	SLAM family member 9	89886	0	NA	NA	NA	NA	NA
ENSG00000162727	OR2M5	olfactory receptor family 2 subfamily M member 5	127059	0	NA	NA	NA	NA	NA
ENSG00000162728	KCNJ9	potassium inwardly rectifying channel subfamily J member 9	3765	0	NA	NA	NA	NA	NA
ENSG00000162733	DDR2	discoidin domain receptor tyrosine kinase 2	4921	3,141233711	-0,507307294	1,061635	-0,477854734	0,632753601	NA
ENSG00000162738	VANGL2	VANGL planar cell polarity protein 2	57216	0	NA	NA	NA	NA	NA
ENSG00000162745	OLFML2B	olfactomedin like 2B	25903	0	NA	NA	NA	NA	NA
ENSG00000162746	FCRLB	Fc receptor like B	127943	0,415992612	-2,911962988	3,975679	-0,732444112	0,463897543	NA
ENSG00000162747	FCGR3B	Fc gamma receptor IIIb	2215	0,223494813	-0,126591461	4,080456	-0,031023852	0,975250517	NA

ENSG00000162747	LOC124905743	low affinity immunoglobulin gamma Fc region receptor III-B	124905743	0,223494813	-0,126591461	4,080456	-0,031023852	0,975250517	NA	
ENSG00000162753	SLC9C2	solute carrier family 9 member C2 (putative)	284525	0	NA	NA	NA	NA	NA	
ENSG00000162755	KLHDC9	kelch domain containing 9	126823	0	NA	NA	NA	NA	NA	
ENSG00000162757	C1orf74	chromosome 1 open reading frame 74	148304	7,684898731	1,316612994	1,144292	1,150591659	0,249900266	NA	
ENSG00000162761	LMX1A	LIM homeobox transcription factor 1 alpha	4009	0	NA	NA	NA	NA	NA	
ENSG00000162763	LRRC52	leucine rich repeat containing 52	440699	0	NA	NA	NA	NA	NA	
ENSG00000162771	GARIN4	golgi associated RAB2 interactor family member 4	149647	0	NA	NA	NA	NA	NA	
ENSG00000162772	ATF3	activating transcription factor 3	467	9,134112001	0,065290769	0,636984	0,102499897	0,918359895	NA	
ENSG00000162779	AXDND1	axonemal dynein light chain domain containing 1	126859	0	NA	NA	NA	NA	NA	
ENSG00000162782	TDRD5	tudor domain containing 5	163589	0	NA	NA	NA	NA	NA	
ENSG00000162804	SNED1	sushi, nidogen and EGF like domains 1	25992	0	NA	NA	NA	NA	NA	
ENSG00000162814	SPATA17	spermatogenesis associated 17	128153	0	NA	NA	NA	NA	NA	
ENSG00000162817	C1orf115	chromosome 1 open reading frame 115	79762	0,10488184	-1,076443944	4,080456	-0,263804824	0,791930317	NA	
ENSG00000162843	WDR64	WD repeat domain 64	128025	0	NA	NA	NA	NA	NA	
ENSG00000162849	KIF26B	kinesin family member 26B	55083	0,144330898	1,418786819	4,061262	0,349346267	0,726829368	NA	
ENSG00000162873	KLHDC8A	kelch domain containing 8A	55220	0	NA	NA	NA	NA	NA	
ENSG00000162877	PM20D1	peptidase M20 domain containing 1	148811	0,328753512	-1,936607922	4,041453	-0,479185998	0,631806314	NA	
ENSG00000162878	PKDCC	protein kinase domain containing, cytoplasmic	91461	0	NA	NA	NA	NA	NA	
ENSG00000162881	OXER1	oxoeicosanoid receptor 1	165140	0	NA	NA	NA	NA	NA	
ENSG00000162882	HAAO	3-hydroxyanthranilate 3,4-dioxygenase	23498	0	NA	NA	NA	NA	NA	
ENSG00000162891	IL20	interleukin 20	50604	0	NA	NA	NA	NA	NA	
ENSG00000162892	IL24	interleukin 24	11009	0,224907488	0,839464896	4,080456	0,205728213	0,837003219	NA	
ENSG00000162896	PIGR	polymeric immunoglobulin receptor	5284	0,52376874	3,13347473	3,896308	0,804216451	0,42127198	NA	
ENSG00000162897	FCAMR	Fc alpha and mu receptor	83953	0	NA	NA	NA	NA	NA	
ENSG00000162929	SANBR	SANT and BTB domain regulator of CSR	84542	9,952034631	-0,693724502	0,88611	-0,782887589	0,433693126	NA	
ENSG00000162931	TRIM17	tripartite motif containing 17	51127	1,998226135	1,56856045	1,68006	0,933633735	0,350492857	NA	
ENSG00000162944	RFTN2	raftlin family member 2	130132	0	NA	NA	NA	NA	NA	
ENSG00000162949	CAPN13	calpain 13	92291	0	NA	NA	NA	NA	NA	
ENSG00000162951	LRRTM1	leucine rich repeat transmembrane neuronal 1	347730	0	NA	NA	NA	NA	NA	
ENSG00000162975	KCNF1	potassium voltage-gated channel modifier subfamily F member 1	3754	0	NA	NA	NA	NA	NA	
ENSG00000162981	LRATD1	LRAT domain containing 1	151354	0,10543123	0	4,080456	0	1	NA	
ENSG00000162989	KCNJ3	potassium inwardly rectifying channel subfamily J member 3	3760	0	NA	NA	NA	NA	NA	
ENSG00000162992	NEUROD1	neuronal differentiation 1	4760	0	NA	NA	NA	NA	NA	
ENSG00000162994	CLHC1	clathrin heavy chain linker domain containing 1	130162	9,756643278	0,026543732	0,675424	0,039299362	0,968651715	NA	
ENSG00000162997	PRORSD1P	prolyl-tRNA synthetase associated domain containing 1, pseudogene	344405	3,071048938	-1,502302487	1,212954	-1,238548835	0,215512626	NA	
ENSG00000162998	FRZB	frizzled related protein	2487	0,119673877	0,847101401	4,080456	0,207599697	0,835541541	NA	
ENSG00000162999	DUSP19	dual specificity phosphatase 19	142679	0,440850606	-0,80846923	3,558696	-0,227181341	0,820282736	NA	
ENSG00000163012	ZSWIM2	zinc finger SWIM-type containing 2	151112	0	NA	NA	NA	NA	NA	
ENSG00000163017	ACTG2	actin gamma 2, smooth muscle	72	0	NA	NA	NA	NA	NA	
ENSG00000163032	VSNL1	visinin like 1	7447	0	NA	NA	NA	NA	NA	
ENSG00000163040	CCDC74A	coiled-coil domain containing 74A	90557	2,146484988	0,132836409	1,927669	0,068910396	0,945060943	NA	
ENSG00000163046	ANKRD30BL	ankyrin repeat domain 30B like	554226	0	NA	NA	NA	NA	NA	
ENSG00000163053	SLC16A14	solute carrier family 16 member 14	151473	1,154100343	-0,279600366	2,440362	-0,114573325	0,908783324	NA	
ENSG00000163060	TEKT4	tektin 4	150483	0,10488184	-1,076443944	4,080456	-0,263804824	0,791930317	NA	
ENSG00000163064	EN1	engrailed homeobox 1	2019	0	NA	NA	NA	NA	NA	
ENSG00000163071	SPATA18	spermatogenesis associated 18	132671	0	NA	NA	NA	NA	NA	
ENSG00000163072	NOSTRIN	nitric oxide synthase trafficking	115677	0,207996306	-1,916073151	4,043021	-0,473921178	0,635556117	NA	

ENSG00000163075	CFAP221	cilia and flagella associated protein 221	200373	0	NA	NA	NA	NA	NA
ENSG00000163082	SGPP2	sphingosine-1-phosphate phosphatase 2	130367	0,565669628	0,940978302	2,560273	0,367530478	0,713223362	NA
ENSG00000163083	INHBB	inhibin subunit beta B	3625	0,119673877	0,847101401	4,080456	0,207599697	0,835541541	NA
ENSG00000163092	XIRP2	xin actin binding repeat containing 2	129446	0	NA	NA	NA	NA	NA
ENSG00000163093	BBS5	Bardet-Biedl syndrome 5	129880	6,029549492	-0,09529925	0,946979	-0,100635044	0,919840176	NA
ENSG00000163098	BIRC8	baculoviral IAP repeat containing 8	112401	0	NA	NA	NA	NA	NA
ENSG00000163106	HPGDS	hematopoietic prostaglandin D synthase	27306	0	NA	NA	NA	NA	NA
ENSG00000163114	PDHA2	pyruvate dehydrogenase E1 subunit alpha 2	5161	0	NA	NA	NA	NA	NA
ENSG00000163116	STPG2	sperm tail PG-rich repeat containing 2	285555	0,295716189	-0,115132404	3,935669	-0,029253577	0,976662351	NA
ENSG00000163121	NEURL3	neuralized E3 ubiquitin protein ligase 3	93082	0	NA	NA	NA	NA	NA
ENSG00000163132	MSX1	msh homeobox 1	4487	0,210312757	-1,078650175	4,080456	-0,264345507	0,791513698	NA
ENSG00000163141	BNIPL	BCL2 interacting protein like	149428	2,334564031	0,62258308	1,481872	0,420132755	0,674388476	NA
ENSG00000163145	C1QTNF7	C1q and TNF related 7	114905	0	NA	NA	NA	NA	NA
ENSG00000163155	LYSMD1	LysM domain containing 1	388695	7,833312258	-0,721644335	0,751662	-0,96006477	0,337022618	NA
ENSG00000163157	TMOD4	tropomodulin 4	29765	0	NA	NA	NA	NA	NA
ENSG00000163170	BOLA3	bolA family member 3	388962	8,674904343	0,24700811	0,754499	0,327380472	0,743380135	NA
ENSG00000163202	LCE3D	late cornified envelope 3D	84648	0	NA	NA	NA	NA	NA
ENSG00000163206	SMCP	sperm mitochondria associated cysteine rich protein	4184	0	NA	NA	NA	NA	NA
ENSG00000163207	IVL	involucrin	3713	0	NA	NA	NA	NA	NA
ENSG00000163209	SPRR3	small proline rich protein 3	6707	0	NA	NA	NA	NA	NA
ENSG00000163216	SPRR2D	small proline rich protein 2D	6703	0	NA	NA	NA	NA	NA
ENSG00000163217	BMP10	bone morphogenetic protein 10	27302	0	NA	NA	NA	NA	NA
ENSG00000163218	PGLYRP4	peptidoglycan recognition protein 4	57115	0	NA	NA	NA	NA	NA
ENSG00000163220	S100A9	S100 calcium binding protein A9	6280	0	NA	NA	NA	NA	NA
ENSG00000163221	S100A12	S100 calcium binding protein A12	6283	0	NA	NA	NA	NA	NA
ENSG00000163235	TGFA	transforming growth factor alpha	7039	0,118286652	0	4,080456	0	1	NA
ENSG00000163239	TDRD10	tudor domain containing 10	126668	0	NA	NA	NA	NA	NA
ENSG00000163251	FZD5	frizzled class receptor 5	7855	1,858404531	2,864823184	1,663566	1,722097727	0,085051817	NA
ENSG00000163263	CFAP141	cilia and flagella associated protein 141	388701	0	NA	NA	NA	NA	NA
ENSG00000163273	NPPC	natriuretic peptide C	4880	0,238060938	0,861692617	4,080011	0,211198586	0,832732314	NA
ENSG00000163283	ALPP	alkaline phosphatase, placental	250	0	NA	NA	NA	NA	NA
ENSG00000163285	GABRG1	gamma-aminobutyric acid type A receptor subunit gamma1	2565	0	NA	NA	NA	NA	NA
ENSG00000163286	ALPG	alkaline phosphatase, germ cell	251	0	NA	NA	NA	NA	NA
ENSG00000163288	GABRB1	gamma-aminobutyric acid type A receptor subunit beta1	2560	0	NA	NA	NA	NA	NA
ENSG00000163291	PAQR3	progestin and adipoQ receptor family member 3	152559	5,252350097	0,315275204	0,991468	0,317988327	0,750493792	NA
ENSG00000163293	NIPAL1	NIPA like domain containing 1	152519	1,331744907	-0,673414467	2,380341	-0,282906768	0,777248307	NA
ENSG00000163295	ALPI	alkaline phosphatase, intestinal	248	0	NA	NA	NA	NA	NA
ENSG00000163331	DAPL1	death associated protein like 1	92196	0	NA	NA	NA	NA	NA
ENSG00000163347	CLDN1	claudin 1	9076	0	NA	NA	NA	NA	NA
ENSG00000163352	LENEP	lens epithelial protein	55891	0,233629135	0,824134891	4,079542	0,202016508	0,839903818	NA
ENSG00000163354	DCST2	DC-STAMP domain containing 2	127579	0,223614196	0	4,080456	0	1	NA
ENSG00000163357	DCST1	DC-STAMP domain containing 1	149095	0	NA	NA	NA	NA	NA
ENSG00000163362	INAVA	innate immunity activator	55765	0,103998153	-1,076443944	4,080456	-0,263804824	0,791930317	NA
ENSG00000163377	TAFA4	TAFA chemokine like family member 4	151647	0	NA	NA	NA	NA	NA
ENSG00000163380	LMOD3	leiomodin 3	56203	0,10488184	-1,076443944	4,080456	-0,263804824	0,791930317	NA
ENSG00000163394	CCKAR	cholecystokinin A receptor	886	0,11808024	-1,076443944	4,080456	-0,263804824	0,791930317	NA
ENSG00000163395	IGFN1	immunoglobulin like and fibronectin type III domain containing 1	91156	0,190595992	0,845602619	4,080456	0,207232389	0,835828373	NA
ENSG00000163406	SLC15A2	solute carrier family 15 member 2	6565	7,439735692	0,710320828	0,858199	0,827688138	0,407847139	NA

ENSG00000163421	PROK2	prokineticin 2	60675	0,262937145	1,428300867	4,061469	0,351670991	0,725085018	NA
ENSG00000163424	TEX55	testis expressed 55	152405	0	NA	NA	NA	NA	NA
ENSG00000163430	FSTL1	follistatin like 1	11167	0	NA	NA	NA	NA	NA
ENSG00000163431	LMOD1	leiomodin 1	25802	0,119673877	0,847101401	4,080456	0,207599697	0,835541541	NA
ENSG00000163435	ELF3	E74 like ETS transcription factor 3	1999	4,940976146	-0,170608262	0,789067	-0,216215197	0,828820008	NA
ENSG00000163440	PDCL2	phosducin like 2	132954	0	NA	NA	NA	NA	NA
ENSG00000163449	TMEM169	transmembrane protein 169	92691	2,529407508	-0,839836092	1,16938	-0,71818903	0,472640741	NA
ENSG00000163464	CXCR1	C-X-C motif chemokine receptor 1	3577	0,63965398	-3,267879846	2,611405	-1,251387539	0,210793121	NA
ENSG00000163467	TSACC	TSSK6 activating cochaperone	128229	0,210862461	0	4,080456	0	1	NA
ENSG00000163472	TMEM79	transmembrane protein 79	84283	11,59219755	1,576061527	0,84702	1,860713029	0,062784713	NA
ENSG00000163485	ADORA1	adenosine A1 receptor	134	0	NA	NA	NA	NA	NA
ENSG00000163491	NEK10	NIMA related kinase 10	152110	2,822380667	0,140818332	1,224744	0,114977722	0,90846278	NA
ENSG00000163497	FEV	FEV transcription factor, ETS family member	54738	0	NA	NA	NA	NA	NA
ENSG00000163499	CRYBA2	crystallin beta A2	1412	0	NA	NA	NA	NA	NA
ENSG00000163501	IHH	Indian hedgehog signaling molecule	3549	0,234994216	1,944111989	4,02324	0,483220515	0,62893917	NA
ENSG00000163515	RETNLB	resistin like beta	84666	0	NA	NA	NA	NA	NA
ENSG00000163517	HDAC11	histone deacetylase 11	79885	5,092723023	-2,02811478	1,003935	-2,020165028	0,043366272	NA
ENSG00000163518	FCRL4	Fc receptor like 4	83417	0,22258626	-1,067580192	4,080456	-0,261632579	0,793604725	NA
ENSG00000163520	FBLN2	fibulin 2	2199	0	NA	NA	NA	NA	NA
ENSG00000163521	GLB1L	galactosidase beta 1 like	79411	7,293451446	0,112267638	0,796215	0,141001687	0,88786861	NA
ENSG00000163530	DPPA2	developmental pluripotency associated 2	151871	0	NA	NA	NA	NA	NA
ENSG00000163531	NFASC	neurofascin	23114	0	NA	NA	NA	NA	NA
ENSG00000163534	FCRL1	Fc receptor like 1	115350	0	NA	NA	NA	NA	NA
ENSG00000163536	SERPINI1	serpin family I member 1	5274	9,403781547	0,329723508	0,724148	0,455326423	0,648874419	NA
ENSG00000163545	NUAK2	NUAK family kinase 2	81788	1,968094432	-0,694846119	1,316688	-0,52772265	0,59769185	NA
ENSG00000163554	SPTA1	spectrin alpha, erythrocytic 1	6708	0	NA	NA	NA	NA	NA
ENSG00000163563	MNDA	myeloid cell nuclear differentiation antigen	4332	0	NA	NA	NA	NA	NA
ENSG00000163568	AIM2	absent in melanoma 2	9447	1,685634339	-0,435834738	2,072281	-0,210316458	0,833420691	NA
ENSG00000163576	EFHB	EF-hand domain family member B	151651	2,12574552	-1,014570284	1,521452	-0,666843423	0,504872153	NA
ENSG00000163581	SLC2A2	solute carrier family 2 member 2	6514	0	NA	NA	NA	NA	NA
ENSG00000163586	FABP1	fatty acid binding protein 1	2168	0	NA	NA	NA	NA	NA
ENSG00000163599	CTLA4	cytotoxic T-lymphocyte associated protein 4	1493	4,731779758	1,359990197	0,99461	1,367360356	0,171512382	NA
ENSG00000163602	RYBP	RING1 and YY1 binding protein	23429	4,103527672	-0,865885637	1,584724	-0,546395103	0,584794375	NA
ENSG00000163606	CD200R1	CD200 receptor 1	131450	2,187733179	-0,702669515	1,253188	-0,560705588	0,574998257	NA
ENSG00000163612	FAM86KP	family with sequence similarity 86 member K, pseudogene	100287013	0	NA	NA	NA	NA	NA
ENSG00000163618	CADPS	calcium dependent secretion activator	8618	0	NA	NA	NA	NA	NA
ENSG00000163623	NKX6-1	NK6 homeobox 1	4825	0	NA	NA	NA	NA	NA
ENSG00000163624	CDS1	CDP-diacylglycerol synthase 1	1040	0	NA	NA	NA	NA	NA
ENSG00000163625	WDFY3	WD repeat and FYVE domain containing 3	23001	0	NA	NA	NA	NA	NA
ENSG00000163629	PTPN13	protein tyrosine phosphatase non-receptor type 13	5783	0	NA	NA	NA	NA	NA
ENSG00000163630	SYNPR	synaptoporin	132204	0	NA	NA	NA	NA	NA
ENSG00000163631	ALB	albumin	213	0	NA	NA	NA	NA	NA
ENSG00000163632	C3orf49	chromosome 3 open reading frame 49	132200	0	NA	NA	NA	NA	NA
ENSG00000163633	C4orf36	chromosome 4 open reading frame 36	132989	0	NA	NA	NA	NA	NA
ENSG00000163637	PRICKLE2	prickle planar cell polarity protein 2	166336	0	NA	NA	NA	NA	NA
ENSG00000163638	ADAMTS9	ADAM metallopeptidase with thrombospondin type 1 motif 9	56999	0	NA	NA	NA	NA	NA
ENSG00000163645	ERICH6	glutamate rich 6	131831	0,239347754	1,967469188	4,021404	0,489249276	0,624665227	NA
ENSG00000163646	CLRN1	clarin 1	7401	0	NA	NA	NA	NA	NA
ENSG00000163661	PTX3	pentraxin 3	5806	0	NA	NA	NA	NA	NA
ENSG00000163666	HESX1	HESX homeobox 1	8820	0,297001175	0,758587173	3,890997	0,194959599	0,845424576	NA
ENSG00000163673	DCLK3	doublecortin like kinase 3	85443	0	NA	NA	NA	NA	NA
ENSG00000163687	DNASE1L3	deoxyribonuclease 1 like 3	1776	0	NA	NA	NA	NA	NA
ENSG00000163689	CFAP20DC	CFAP20 domain containing	200844	4,253815908	-0,666981494	0,973369	-0,68523011	0,493198726	NA
ENSG00000163694	RBM47	RNA binding motif protein 47	54502	0,410538812	-0,924583772	4,080456	-0,226588352	0,82074385	NA
ENSG00000163701	IL17RE	interleukin 17 receptor E	132014	0,341062715	0	4,080456	0	1	NA
ENSG00000163702	IL17RC	interleukin 17 receptor C	84818	0,11808024	-1,076443944	4,080456	-0,263804824	0,791930317	NA

ENSG00000163704	PRRT3	proline rich transmembrane protein 3	285368	7,466134365	0,516783879	0,852097	0,606484503	0,54419307	NA
ENSG00000163705	FANCD2OS	FANCD2 opposite strand	115795	0	NA	NA	NA	NA	NA
ENSG00000163710	PCOLCE2	procollagen C-endopeptidase enhancer 2	26577	0	NA	NA	NA	NA	NA
ENSG00000163734	CXCL3	C-X-C motif chemokine ligand 3	2921	0	NA	NA	NA	NA	NA
ENSG00000163735	CXCL5	C-X-C motif chemokine ligand 5	6374	0	NA	NA	NA	NA	NA
ENSG00000163736	PPBP	pro-platelet basic protein	5473	0	NA	NA	NA	NA	NA
ENSG00000163737	PF4	platelet factor 4	5196	0	NA	NA	NA	NA	NA
ENSG00000163738	MTHFD2L	methylenetetrahydrofolate dehydrogenase (NADP+ dependent) 2 like	441024	1,904566073	-2,336487856	2,066083	-1,130877988	0,258106449	NA
ENSG00000163739	CXCL1	C-X-C motif chemokine ligand 1	2919	0	NA	NA	NA	NA	NA
ENSG00000163746	PLSCR2	phospholipid scramblase 2	57047	0,186984793	-0,031583923	4,080456	-0,007740293	0,993824202	NA
ENSG00000163749	CCDC158	coiled-coil domain containing 158	339965	0,103998153	-1,076443944	4,080456	-0,263804824	0,791930317	NA
ENSG00000163751	CPA3	carboxypeptidase A3	1359	0	NA	NA	NA	NA	NA
ENSG00000163762	TM4SF18	transmembrane 4 L six family member 18	116441	0	NA	NA	NA	NA	NA
ENSG00000163792	TCF23	transcription factor 23	150921	0,10488184	-1,076443944	4,080456	-0,263804824	0,791930317	NA
ENSG00000163793	DNAJC5G	DnaJ heat shock protein family (Hsp40) member C5 gamma	285126	0	NA	NA	NA	NA	NA
ENSG00000163794	UCN	urocortin	7349	0,340965167	-0,128270321	3,782872	-0,033908184	0,972950367	NA
ENSG00000163803	PLB1	phospholipase B1	151056	5,972754853	-1,231974522	1,117299	-1,102636361	0,270185113	NA
ENSG00000163806	SPDYA	speedy/RINGO cell cycle regulator family member A	245711	0,15836288	0	4,080456	0	1	NA
ENSG00000163810	TGM4	transglutaminase 4	7047	0	NA	NA	NA	NA	NA
ENSG00000163815	CLEC3B	C-type lectin domain family 3 member B	7123	0,675102066	0,204726432	3,772322	0,054270673	0,956719515	NA
ENSG00000163817	SLC6A20	solute carrier family 6 member 20	54716	0	NA	NA	NA	NA	NA
ENSG00000163818	LZTFL1	leucine zipper transcription factor like 1	54585	7,527539243	5,858873736	1,522532	3,848111723	0,000119032	NA
ENSG00000163825	RTP3	receptor transporter protein 3	83597	0,209444426	-1,073105693	4,080456	-0,262986717	0,792560817	NA
ENSG00000163827	LRRC2	leucine rich repeat containing 2	79442	1,700527773	0,216726706	1,849562	0,117177304	0,906719552	NA
ENSG00000163833	FBXO40	F-box protein 40	51725	0	NA	NA	NA	NA	NA
ENSG00000163864	NMNAT3	nicotinamide nucleotide adenylyltransferase 3	349565	1,643128655	2,582980715	2,12407	1,216052566	0,223964898	NA
ENSG00000163873	GRIK3	glutamate ionotropic receptor kainate type subunit 3	2899	0	NA	NA	NA	NA	NA
ENSG00000163879	DNALI1	dynein axonemal light intermediate chain 1	7802	0	NA	NA	NA	NA	NA
ENSG00000163884	KLF15	KLF transcription factor 15	28999	0	NA	NA	NA	NA	NA
ENSG00000163885	CFAP100	cilia and flagella associated protein 100	348807	0	NA	NA	NA	NA	NA
ENSG00000163888	CAMK2N2	calcium/calmodulin dependent protein kinase II inhibitor 2	94032	0	NA	NA	NA	NA	NA
ENSG00000163898	LIPH	lipase H	200879	0,117497108	0,847101401	4,080456	0,207599697	0,835541541	NA
ENSG00000163909	HEYL	hes related family bHLH transcription factor with YRPW motif like	26508	0	NA	NA	NA	NA	NA
ENSG00000163913	IFT122	intraflagellar transport 122	55764	8,52324313	-1,908917198	1,374636	-1,388670614	0,164932934	NA
ENSG00000163914	RHO	rhodopsin	6010	0	NA	NA	NA	NA	NA
ENSG00000163923	RPL39L	ribosomal protein L39 like	116832	5,115197264	0,051220757	0,813603	0,062955445	0,949801984	NA
ENSG00000163958	ZDHHC19	zinc finger DHHC-type palmitoyltransferase 19	131540	0	NA	NA	NA	NA	NA
ENSG00000163959	SLC51A	solute carrier family 51 subunit alpha	200931	0	NA	NA	NA	NA	NA
ENSG00000163975	MELTF	melanotransferrin	4241	2,142647521	-0,303322848	1,389135	-0,218353743	0,827153504	NA
ENSG00000163982	OTOP1	otopetrin 1	133060	0	NA	NA	NA	NA	NA
ENSG00000163993	S100P	S100 calcium binding protein P	6286	0	NA	NA	NA	NA	NA
ENSG00000163995	ABLIM2	actin binding LIM protein family member 2	84448	1,005724147	-1,684292534	2,270393	-0,741850656	0,458177825	NA
ENSG00000164007	CLDN19	claudin 19	149461	0	NA	NA	NA	NA	NA
ENSG00000164023	SGMS2	sphingomyelin synthase 2	166929	5,772209335	0,289344667	0,77553	0,37309264	0,709079496	NA
ENSG00000164035	EMCN	endomucin	51705	0	NA	NA	NA	NA	NA
ENSG00000164037	SLC9B1	solute carrier family 9 member B1	150159	0,633451519	0	4,080456	0	1	NA
ENSG00000164047	CAMP	cathelicidin antimicrobial peptide	820	0	NA	NA	NA	NA	NA

ENSG00000164049	FBXW12	F-box and WD repeat domain containing 12	285231	0	NA	NA	NA	NA	NA
ENSG00000164061	BSN	bassoon presynaptic cytomatrix protein	8927	0,949447155	-0,799474754	2,152377	-0,371438083	0,710311265	NA
ENSG00000164066	INTU	inturned planar cell polarity protein	27152	0	NA	NA	NA	NA	NA
ENSG00000164070	HSPA4L	heat shock protein family A (Hsp70) member 4 like	22824	0,10488184	-1,076443944	4,080456	-0,263804824	0,791930317	NA
ENSG00000164076	CAMKV	CaM kinase like vesicle associated	79012	0	NA	NA	NA	NA	NA
ENSG00000164078	MST1R	macrophage stimulating 1 receptor	4486	0,236441977	-1,088999706	4,080456	-0,266881873	0,789560114	NA
ENSG00000164082	GRM2	glutamate metabotropic receptor 2	2912	1,734098955	0,708591219	3,071213	0,230720325	0,817532082	NA
ENSG00000164089	ETNPPL	ethanolamine-phosphate phospho-lyase	64850	0	NA	NA	NA	NA	NA
ENSG00000164093	PITX2	paired like homeodomain 2	5308	0	NA	NA	NA	NA	NA
ENSG00000164099	PRSS12	serine protease 12	8492	0	NA	NA	NA	NA	NA
ENSG00000164100	NDST3	N-deacetylase and N-sulfotransferase 3	9348	0	NA	NA	NA	NA	NA
ENSG00000164106	SCRG1	stimulator of chondrogenesis 1	11341	0,117497108	0,847101401	4,080456	0,207599697	0,835541541	NA
ENSG00000164107	HAND2	heart and neural crest derivatives expressed 2	9464	0	NA	NA	NA	NA	NA
ENSG00000164112	SMIM43	small integral membrane protein 43	132332	0	NA	NA	NA	NA	NA
ENSG00000164113	ADAD1	adenosine deaminase domain containing 1	132612	0	NA	NA	NA	NA	NA
ENSG00000164116	GUCY1A1	guanylate cyclase 1 soluble subunit alpha 1	2982	0,10543123	0	4,080456	0	1	NA
ENSG00000164122	ASB5	ankyrin repeat and SOCS box containing 5	140458	0,103998153	-1,076443944	4,080456	-0,263804824	0,791930317	NA
ENSG00000164123	SPMIP2	sperm microtubule inner protein 2	152940	0	NA	NA	NA	NA	NA
ENSG00000164124	TMEM144	transmembrane protein 144	55314	0	NA	NA	NA	NA	NA
ENSG00000164125	GASK1B	golgi associated kinase 1B	51313	0	NA	NA	NA	NA	NA
ENSG00000164128	NPY1R	neuropeptide Y receptor Y1	4886	0	NA	NA	NA	NA	NA
ENSG00000164129	NPY5R	neuropeptide Y receptor Y5	4889	0	NA	NA	NA	NA	NA
ENSG00000164142	FHIP1A	FHF complex subunit HOOK interacting protein 1A	729830	0,11808024	-1,076443944	4,080456	-0,263804824	0,791930317	NA
ENSG00000164161	HHIP	hedgehog interacting protein	64399	0	NA	NA	NA	NA	NA
ENSG00000164175	SLC45A2	solute carrier family 45 member 2	51151	0	NA	NA	NA	NA	NA
ENSG00000164176	EDIL3	EGF like repeats and discoidin domains 3	10085	0	NA	NA	NA	NA	NA
ENSG00000164181	ELOVL7	ELOVL fatty acid elongase 7	79993	0	NA	NA	NA	NA	NA
ENSG00000164185	ZNF474	zinc finger protein 474	133923	0	NA	NA	NA	NA	NA
ENSG00000164188	RANBP3L	RAN binding protein 3 like	202151	0	NA	NA	NA	NA	NA
ENSG00000164197	RNF180	ring finger protein 180	285671	0	NA	NA	NA	NA	NA
ENSG00000164199	ADGRV1	adhesion G protein-coupled receptor V1	84059	0	NA	NA	NA	NA	NA
ENSG00000164220	F2RL2	coagulation factor II thrombin receptor like 2	2151	0	NA	NA	NA	NA	NA
ENSG00000164221	CCDC112	coiled-coil domain containing 112	153733	3,853893714	-0,175284237	1,029068	-0,170333021	0,864748244	NA
ENSG00000164236	ANKRD33B	ankyrin repeat domain 33B	651746	9,71110429	-0,665464774	0,569732	-1,16803198	0,242793865	NA
ENSG00000164237	CMBL	carboxymethylenebutenolidase homolog	134147	0,938398658	3,355356817	2,462892	1,362364512	0,173082877	NA
ENSG00000164251	F2RL1	F2R like trypsin receptor 1	2150	0,474523826	0	4,080456	0	1	NA
ENSG00000164256	PRDM9	PR/SET domain 9	56979	0	NA	NA	NA	NA	NA
ENSG00000164265	SCGB3A2	secretoglobin family 3A member 2	117156	0	NA	NA	NA	NA	NA
ENSG00000164266	SPINK1	serine peptidase inhibitor Kazal type 1	6690	0	NA	NA	NA	NA	NA
ENSG00000164270	HTR4	5-hydroxytryptamine receptor 4	3360	0	NA	NA	NA	NA	NA
ENSG00000164283	ESM1	endothelial cell specific molecule 1	11082	3,32760927	-0,623866616	1,060345	-0,588361891	0,556289411	NA
ENSG00000164287	CDC20B	cell division cycle 20B	166979	0,355122085	-1,074568492	4,080456	-0,263345206	0,79228452	NA
ENSG00000164292	RHOBTB3	Rho related BTB domain containing 3	22836	1,522541566	-0,806080789	1,752594	-0,459935869	0,645562253	NA
ENSG00000164294	GPX8	glutathione peroxidase 8 (putative)	493869	0	NA	NA	NA	NA	NA
ENSG00000164296	TIGD6	tigger transposable element derived 6	81789	5,960431582	-0,021517352	0,800785	-0,026870322	0,978563165	NA
ENSG00000164299	SPZ1	spermatogenic leucine zipper 1	84654	0	NA	NA	NA	NA	NA
ENSG00000164303	ENPP6	ectonucleotide pyrophosphatase/phosphodiesterase 6	133121	3,63423412	-0,731931926	1,264454	-0,578852227	0,562688887	NA

ENSG00000164304	CAGE1	cancer antigen 1	285782	0,236367703	-1,078200251	4,080456	-0,264235244	0,791598655	NA
ENSG00000164309	CMYA5	cardiomyopathy associated 5	202333	2,42228418	0,234507894	1,426499	0,164394	0,869420987	NA
ENSG00000164318	EGFLAM	EGF like, fibronectin type III and laminin G domains	133584	0	NA	NA	NA	NA	NA
ENSG00000164325	TMEM174	transmembrane protein 174	134288	0	NA	NA	NA	NA	NA
ENSG00000164326	CARTPT	CART prepropeptide	9607	0	NA	NA	NA	NA	NA
ENSG00000164330	EBF1	EBF transcription factor 1	1879	0	NA	NA	NA	NA	NA
ENSG00000164334	FAM170A	family with sequence similarity 170 member A	340069	0	NA	NA	NA	NA	NA
ENSG00000164342	TLR3	toll like receptor 3	7098	2,053706441	-0,201648904	1,596319	-0,126321183	0,899477689	NA
ENSG00000164344	KLKB1	kallikrein B1	3818	0,10488184	-1,076443944	4,080456	-0,263804824	0,791930317	NA
ENSG00000164363	SLC6A18	solute carrier family 6 member 18	348932	0	NA	NA	NA	NA	NA
ENSG00000164379	FOXQ1	forkhead box Q1	94234	0	NA	NA	NA	NA	NA
ENSG00000164393	ADGRF2	adhesion G protein-coupled receptor F2	222611	0	NA	NA	NA	NA	NA
ENSG00000164399	IL3	interleukin 3	3562	0,189712639	1,665316361	4,047517	0,411441438	0,680748874	NA
ENSG00000164403	SHROOM1	shroom family member 1	134549	11,32835334	-0,47135011	0,578039	-0,815430262	0,414826068	NA
ENSG00000164404	GDF9	growth differentiation factor 9	2661	0,743722749	0,138620544	2,282386	0,060734926	0,951570316	NA
ENSG00000164406	LEAP2	liver enriched antimicrobial peptide 2	116842	1,649849019	-0,59608468	1,934434	-0,308144208	0,757972604	NA
ENSG00000164411	GJB7	gap junction protein beta 7	375519	0	NA	NA	NA	NA	NA
ENSG00000164418	GRIK2	glutamate ionotropic receptor kainate type subunit 2	2898	0	NA	NA	NA	NA	NA
ENSG00000164434	FABP7	fatty acid binding protein 7	2173	0	NA	NA	NA	NA	NA
ENSG00000164438	TLX3	T cell leukemia homeobox 3	30012	0	NA	NA	NA	NA	NA
ENSG00000164440	TXLNB	taxilin beta	167838	6,946404881	0,338950054	0,742499	0,456499152	0,648031081	NA
ENSG00000164451	CALHM4	calcium homeostasis modulator family member 4	221301	0	NA	NA	NA	NA	NA
ENSG00000164458	TBXT	T-box transcription factor T	6862	0,472161394	-0,386268391	2,95249	-0,130828029	0,89591135	NA
ENSG00000164485	IL22RA2	interleukin 22 receptor subunit alpha 2	116379	0	NA	NA	NA	NA	NA
ENSG00000164488	DACT2	dishevelled binding antagonist of beta catenin 2	168002	0	NA	NA	NA	NA	NA
ENSG00000164500	SPMIP7	sperm microtubule inner protein 7	100130988	0	NA	NA	NA	NA	NA
ENSG00000164508	H2AC1	H2A clustered histone 1	221613	0	NA	NA	NA	NA	NA
ENSG00000164509	IL31RA	interleukin 31 receptor A	133396	0,590756606	-1,705292946	3,306417	-0,515752533	0,606027259	NA
ENSG00000164512	ANKRD55	ankyrin repeat domain 55	79722	0	NA	NA	NA	NA	NA
ENSG00000164520	RAET1E	retinoic acid early transcript 1E	135250	0	NA	NA	NA	NA	NA
ENSG00000164530	PI16	peptidase inhibitor 16	221476	0,494211524	-1,070339683	2,931439	-0,36512429	0,71501863	NA
ENSG00000164532	TBX20	T-box transcription factor 20	57057	0	NA	NA	NA	NA	NA
ENSG00000164542	MATCAP2	microtubule associated tyrosine carboxypeptidase 2	23366	0,103998153	-1,076443944	4,080456	-0,263804824	0,791930317	NA
ENSG00000164556	FAM183BP	acyloxyacyl hydrolase (neutrophil)	340286	0,815358866	-2,955904473	2,082762	-1,419223617	0,155833833	NA
ENSG00000164588	HCN1	hyperpolarization activated cyclic nucleotide gated potassium channel 1	348980	0	NA	NA	NA	NA	NA
ENSG00000164591	MYOZ3	myozenin 3	91977	0	NA	NA	NA	NA	NA
ENSG00000164600	NEUROD6	neuronal differentiation 6	63974	0	NA	NA	NA	NA	NA
ENSG00000164604	GPR85	G protein-coupled receptor 85	54329	0,072165449	0,847101401	4,080456	0,207599697	0,835541541	NA
ENSG00000164616	NA	NA	NA	0	NA	NA	NA	NA	NA
ENSG00000164619	BMPER	BMP binding endothelial regulator	168667	0	NA	NA	NA	NA	NA
ENSG00000164620	RELL2	RELT like 2	285613	10,33190304	1,61198844	0,815595	1,976457403	0,048102996	NA
ENSG00000164627	KIF6	kinesin family member 6	221458	0	NA	NA	NA	NA	NA
ENSG00000164638	SLC29A4	solute carrier family 29 member 4	222962	4,561154897	-0,982355168	0,966574	-1,016326744	0,309473817	NA
ENSG00000164645	TEX47	testis expressed 47	219557	0	NA	NA	NA	NA	NA
ENSG00000164647	STEAP1	STEAP family member 1	26872	0	NA	NA	NA	NA	NA
ENSG00000164651	SP8	Sp8 transcription factor	221833	0	NA	NA	NA	NA	NA
ENSG00000164659	ELAPOR2	endosome-lysosome associated apoptosis and autophagy regulator family member 2	222223	2,434409887	-2,597112837	1,663575	-1,561163515	0,118485175	NA
ENSG00000164669	NA	NA	NA	0	NA	NA	NA	NA	NA
ENSG00000164675	IQUB	IQ motif and ubiquitin domain containing	154865	0	NA	NA	NA	NA	NA
ENSG00000164683	HEY1	hes related family bHLH transcription factor with YRPW motif 1	23462	0	NA	NA	NA	NA	NA
ENSG00000164684	ZNF704	zinc finger protein 704	619279	0,763055817	-2,296404755	2,266961	-1,012988007	0,31106589	NA
ENSG00000164690	SHH	sonic hedgehog signaling molecule	6469	0,221767362	-0,095712977	4,080172	-0,023458074	0,981284881	NA
ENSG00000164692	COL1A2	collagen type I alpha 2 chain	1278	0	NA	NA	NA	NA	NA

ENSG00000164694	FNDC1	fibronectin type III domain containing 1	84624	0	NA	NA	NA	NA	NA
ENSG00000164695	CHMP4C	charged multivesicular body protein 4C	92421	3,952373092	-0,419942544	0,999711	-0,420063996	0,674438704	NA
ENSG00000164707	SLC13A4	solute carrier family 13 member 4	26266	0	NA	NA	NA	NA	NA
ENSG00000164708	PGAM2	phosphoglycerate mutase 2	5224	0,351667494	-0,359845814	4,024686	-0,089409672	0,928756337	NA
ENSG00000164729	SLC35G3	solute carrier family 35 member G3	146861	0	NA	NA	NA	NA	NA
ENSG00000164736	SOX17	SRY-box transcription factor 17	64321	0	NA	NA	NA	NA	NA
ENSG00000164740	SEPTIN7P5	septin 7 pseudogene 5	100418718	0	NA	NA	NA	NA	NA
ENSG00000164741	DLC1	DLC1 Rho GTPase activating protein	10395	0	NA	NA	NA	NA	NA
ENSG00000164742	ADCY1	adenylate cyclase 1	107	0	NA	NA	NA	NA	NA
ENSG00000164743	C8orf48	chromosome 8 open reading frame 48	157773	0	NA	NA	NA	NA	NA
ENSG00000164744	SUN3	Sad1 and UNC84 domain containing 3	256979	0,072165449	0,847101401	4,080456	0,207599697	0,835541541	NA
ENSG00000164746	C7orf57	chromosome 7 open reading frame 57	136288	0,850881106	-1,128412626	2,040631	-0,552972311	0,58028237	NA
ENSG00000164749	HNF4G	hepatocyte nuclear factor 4 gamma	3174	0	NA	NA	NA	NA	NA
ENSG00000164756	SLC30A8	solute carrier family 30 member 8	169026	0	NA	NA	NA	NA	NA
ENSG00000164761	TNFRSF11B	TNF receptor superfamily member 11b	4982	0	NA	NA	NA	NA	NA
ENSG00000164764	SBSPON	somatomedin B and thrombospondin type 1 domain containing	157869	0	NA	NA	NA	NA	NA
ENSG00000164776	PHKG1	phosphorylase kinase catalytic subunit gamma 1	5260	2,85462823	-0,907339854	1,205959	-0,752380132	0,451822491	NA
ENSG00000164778	EN2	engrailed homeobox 2	2020	0,42724731	2,364168865	2,939896	0,804167479	0,421300258	NA
ENSG00000164794	KCNV1	potassium voltage-gated channel modifier subfamily V member 1	27012	0	NA	NA	NA	NA	NA
ENSG00000164796	CSMD3	CUB and Sushi multiple domains 3	114788	0	NA	NA	NA	NA	NA
ENSG00000164825	DEFB1	defensin beta 1	1672	0	NA	NA	NA	NA	NA
ENSG00000164841	TMEM74	transmembrane protein 74	157753	0	NA	NA	NA	NA	NA
ENSG00000164845	NA	NA	NA	0,10488184	-1,076443944	4,080456	-0,263804824	0,791930317	NA
ENSG00000164849	GPR146	G protein-coupled receptor 146	115330	0,117497108	0,847101401	4,080456	0,207599697	0,835541541	NA
ENSG00000164850	GPER1	G protein-coupled estrogen receptor 1	2852	0,15836288	0	4,080456	0	1	NA
ENSG00000164853	UNCX	UNC homeobox	340260	0	NA	NA	NA	NA	NA
ENSG00000164855	TMEM184A	transmembrane protein 184A	202915	0	NA	NA	NA	NA	NA
ENSG00000164867	NOS3	nitric oxide synthase 3	4846	0,333444644	-0,50565726	4,044967	-0,125009002	0,900516424	NA
ENSG00000164871	SPAG11B	sperm associated antigen 11B	10407	0	NA	NA	NA	NA	NA
ENSG00000164871	LOC124906741	sperm-associated antigen 11B-like	124906741	0	NA	NA	NA	NA	NA
ENSG00000164877	MICALL2	MICAL like 2	79778	4,794246296	-3,775017306	1,545425	-2,442705478	0,014577628	NA
ENSG00000164879	CA3	carbonic anhydrase 3	761	0,117497108	0,847101401	4,080456	0,207599697	0,835541541	NA
ENSG00000164893	SLC7A13	solute carrier family 7 member 13	157724	0	NA	NA	NA	NA	NA
ENSG00000164898	FMC1	formation of mitochondrial complex V assembly factor 1 homolog	154791	9,577371256	0,805667682	0,745254	1,08106453	0,27966841	NA
ENSG00000164900	GBX1	gastrulation brain homeobox 1	2636	0	NA	NA	NA	NA	NA
ENSG00000164904	ALDH7A1	aldehyde dehydrogenase 7 family member A1	501	0	NA	NA	NA	NA	NA
ENSG00000164920	OSR2	odd-skipped related transciption factor 2	116039	1,122939825	3,163347598	2,107642	1,500894188	0,133382932	NA
ENSG00000164929	BAALC	BAALC binder of MAP3K1 and KLF4	79870	0	NA	NA	NA	NA	NA
ENSG00000164932	CTHRC1	collagen triple helix repeat containing 1	115908	1,42985902	0,541187604	2,036723	0,265714832	0,790458839	NA
ENSG00000164935	DCSTAMP	dendrocyte expressed seven transmembrane protein	81501	0	NA	NA	NA	NA	NA
ENSG00000164946	FREM1	FRAS1 related extracellular matrix 1	158326	0	NA	NA	NA	NA	NA
ENSG00000164953	TMEM67	transmembrane protein 67	91147	0,15836288	0	4,080456	0	1	NA
ENSG00000164972	SPMIP6	sperm microtubule inner protein 6	84688	0,559347652	3,184941327	3,941732	0,808005509	0,419087411	NA
ENSG00000164989	CCDC171	coiled-coil domain containing 171	203238	10,89302707	-0,343412576	0,589157	-0,582887567	0,559968991	NA
ENSG00000165023	DIRAS2	DIRAS family GTPase 2	54769	0	NA	NA	NA	NA	NA
ENSG00000165028	NIPSNAP3B	nipsnap homolog 3B	55335	5,068693701	0,338754881	0,885146	0,382710944	0,701934099	NA

ENSG00000165029	ABCA1	ATP binding cassette subfamily A member 1	19	0	NA	NA	NA	NA	NA
ENSG00000165046	LETM2	leucine zipper and EF-hand containing transmembrane protein 2	137994	5,485686002	-0,513547451	1,012784	-0,507065177	0,612109088	NA
ENSG00000165059	PRKACG	protein kinase cAMP-activated catalytic subunit gamma	5568	0	NA	NA	NA	NA	NA
ENSG00000165061	ZMAT4	zinc finger matrin-type 4	79698	3,242030368	0,050194476	1,198952	0,041865288	0,966606088	NA
ENSG00000165066	NKX6-3	NK6 homeobox 3	157848	0	NA	NA	NA	NA	NA
ENSG00000165072	MAMDC2	MAM domain containing 2	256691	0	NA	NA	NA	NA	NA
ENSG00000165076	PRSS37	serine protease 37	136242	0	NA	NA	NA	NA	NA
ENSG00000165078	CPA6	carboxypeptidase A6	57094	0	NA	NA	NA	NA	NA
ENSG00000165084	C8orf34	chromosome 8 open reading frame 34	116328	0	NA	NA	NA	NA	NA
ENSG00000165091	TMC1	transmembrane channel like 1	117531	0	NA	NA	NA	NA	NA
ENSG00000165092	ALDH1A1	aldehyde dehydrogenase 1 family member A1	216	0	NA	NA	NA	NA	NA
ENSG00000165105	RASEF	RAS and EF-hand domain containing	158158	0,532451417	0,970971927	3,373365	0,287834822	0,773473181	NA
ENSG00000165113	GKAP1	G kinase anchoring protein 1	80318	5,086730563	-1,120839109	0,942657	-1,189021401	0,234431246	NA
ENSG00000165120	SSMEM1	serine rich single-pass membrane protein 1	136263	0	NA	NA	NA	NA	NA
ENSG00000165124	SVEP1	sushi, von Willebrand factor type A, EGF and pentraxin domain containing 1	79987	0,119673877	0,847101401	4,080456	0,207599697	0,835541541	NA
ENSG00000165125	TRPV6	transient receptor potential cation channel subfamily V member 6	55503	0	NA	NA	NA	NA	NA
ENSG00000165131	LLCFC1	LLLL and CFNLAS motif containing 1	135927	0	NA	NA	NA	NA	NA
ENSG00000165138	ANKS6	ankyrin repeat and sterile alpha motif domain containing 6	203286	1,842634725	1,252135241	1,53215	0,817240417	0,413791049	NA
ENSG00000165140	FBP1	fructose-bisphosphatase 1	2203	1,917578217	0,631491096	1,781628	0,354446078	0,723004608	NA
ENSG00000165152	PGAP4	post-GPI attachment to proteins GalNAc transferase 4	84302	0,118286652	0	4,080456	0	1	NA
ENSG00000165164	CFAP47	cilia and flagella associated protein 47	286464	0	NA	NA	NA	NA	NA
ENSG00000165168	CYBB	cytochrome b-245 beta chain	1536	0,262824103	-1,072436847	4,080456	-0,262822802	0,792687159	NA
ENSG00000165171	METTL27	methyltransferase like 27	155368	2,514313498	-0,113079584	1,106818	-0,102166418	0,918624583	NA
ENSG00000165178	NCF1C	neutrophil cytosolic factor 1C (pseudogene)	654817	10,54453947	0,724722889	0,54828	1,321811861	0,186230808	NA
ENSG00000165181	SHOC1	shortage in chiasmata 1	158401	0,10543123	0	4,080456	0	1	NA
ENSG00000165182	CXorf58	chromosome X open reading frame 58	254158	0,10543123	0	4,080456	0	1	NA
ENSG00000165186	PTCHD1	patched domain containing 1	139411	0,119673877	0,847101401	4,080456	0,207599697	0,835541541	NA
ENSG00000165188	RNF183	ring finger protein 183	138065	0	NA	NA	NA	NA	NA
ENSG00000165192	ASB11	ankyrin repeat and SOCS box containing 11	140456	0	NA	NA	NA	NA	NA
ENSG00000165194	PCDH19	protocadherin 19	57526	0,10488184	-1,076443944	4,080456	-0,263804824	0,791930317	NA
ENSG00000165197	VEGFD	vascular endothelial growth factor D	2277	0	NA	NA	NA	NA	NA
ENSG00000165202	OR1Q1	olfactory receptor family 1 subfamily Q member 1	158131	0	NA	NA	NA	NA	NA
ENSG00000165204	OR1K1	olfactory receptor family 1 subfamily K member 1	392392	0	NA	NA	NA	NA	NA
ENSG00000165215	CLDN3	claudin 3	1365	0	NA	NA	NA	NA	NA
ENSG00000165238	WNK2	WNK lysine deficient protein kinase 2	65268	0	NA	NA	NA	NA	NA
ENSG00000165246	NLGN4Y	neuroligin 4 Y-linked	22829	0	NA	NA	NA	NA	NA
ENSG00000165259	HDX	highly divergent homeobox	139324	0,997168406	1,730272468	2,501719	0,691633394	0,489167585	NA
ENSG00000165269	AQP7	aquaporin 7	364	0	NA	NA	NA	NA	NA
ENSG00000165309	ARMC3	armadillo repeat containing 3	219681	0	NA	NA	NA	NA	NA
ENSG00000165323	FAT3	FAT atypical cadherin 3	120114	0	NA	NA	NA	NA	NA
ENSG00000165325	DEUP1	deuterosome assembly protein 1	159989	0	NA	NA	NA	NA	NA
ENSG00000165338	HECTD2	HECT domain E3 ubiquitin protein ligase 2	143279	4,12435058	0,183975566	1,086617	0,169310467	0,865552443	NA
ENSG00000165349	SLC7A3	solute carrier family 7 member 3	84889	0	NA	NA	NA	NA	NA
ENSG00000165370	GPR101	G protein-coupled receptor 101	83550	0	NA	NA	NA	NA	NA
ENSG00000165376	CLDN2	claudin 2	9075	0	NA	NA	NA	NA	NA
ENSG00000165379	LRFN5	leucine rich repeat and fibronectin type III domain containing 5	145581	0,10543123	0	4,080456	0	1	NA

ENSG00000165383	LRRC18	leucine rich repeat containing 18	474354	0	NA	NA	NA	NA	NA
ENSG00000165409	TSHR	thyroid stimulating hormone receptor	7253	0	NA	NA	NA	NA	NA
ENSG00000165443	PHYHIPL	phytanoyl-CoA 2-hydroxylase interacting protein like	84457	0	NA	NA	NA	NA	NA
ENSG00000165449	SLC16A9	solute carrier family 16 member 9	220963	0,117497108	0,847101401	4,080456	0,207599697	0,835541541	NA
ENSG00000165457	FOLR2	folate receptor beta	2350	1,791855904	-1,194132855	1,535408	-0,777729856	0,436728288	NA
ENSG00000165462	PHOX2A	paired like homeobox 2A	401	0	NA	NA	NA	NA	NA
ENSG00000165471	MBL2	mannose binding lectin 2	4153	0	NA	NA	NA	NA	NA
ENSG00000165474	GJB2	gap junction protein beta 2	2706	0,577213178	1,566076838	2,814662	0,556399649	0,577937679	NA
ENSG00000165478	HEPACAM	hepatic and glial cell adhesion molecule	220296	0,11808024	-1,076443944	4,080456	-0,263804824	0,791930317	NA
ENSG00000165495	PKNOX2	PBX/knotted 1 homeobox 2	63876	0	NA	NA	NA	NA	NA
ENSG00000165496	RPL10L	ribosomal protein L10 like	140801	0	NA	NA	NA	NA	NA
ENSG00000165507	DEPP1	DEPP autophagy regulator 1	11067	0	NA	NA	NA	NA	NA
ENSG00000165509	MAGEC3	MAGE family member C3	139081	0	NA	NA	NA	NA	NA
ENSG00000165533	TTC8	tetratricopeptide repeat domain 8	123016	3,458631076	0,276077186	1,627723	0,169609408	0,865317323	NA
ENSG00000165548	TMEM63C	transmembrane protein 63C	57156	0,3343144	2,494848778	3,986735	0,62578745	0,531454365	NA
ENSG00000165553	NGB	neuroglobin	58157	0	NA	NA	NA	NA	NA
ENSG00000165555	NOXRED1	NADP dependent oxidoreductase domain containing 1	122945	1,905309097	2,345765743	1,558607	1,505039759	0,132313855	NA
ENSG00000165556	CDX2	caudal type homeobox 2	1045	0	NA	NA	NA	NA	NA
ENSG00000165566	AMER2	APC membrane recruitment protein 2	219287	0	NA	NA	NA	NA	NA
ENSG00000165568	AKR1E2	aldo-keto reductase family 1 member E2	83592	0,072165449	0,847101401	4,080456	0,207599697	0,835541541	NA
ENSG00000165583	SSX5	SSX family member 5	6758	0	NA	NA	NA	NA	NA
ENSG00000165584	SSX3	SSX family member 3	10214	0	NA	NA	NA	NA	NA
ENSG00000165588	OTX2	orthodenticle homeobox 2	5015	0	NA	NA	NA	NA	NA
ENSG00000165591	FAAH2	fatty acid amide hydrolase 2	158584	0,552453092	1,925817393	4,024112	0,478569559	0,632244878	NA
ENSG00000165606	DRGX	dorsal root ganglia homeobox	644168	0	NA	NA	NA	NA	NA
ENSG00000165617	DACT1	dishevelled binding antagonist of beta catenin 1	51339	0	NA	NA	NA	NA	NA
ENSG00000165621	OXGR1	oxoglutarate receptor 1	27199	0	NA	NA	NA	NA	NA
ENSG00000165623	UCMA	upper zone of growth plate and cartilage matrix associated	221044	0	NA	NA	NA	NA	NA
ENSG00000165626	BEND7	BEN domain containing 7	222389	0	NA	NA	NA	NA	NA
ENSG00000165633	VSTM4	V-set and transmembrane domain containing 4	196740	1,882676627	-0,065154297	1,553424	-0,041942373	0,966544637	NA
ENSG00000165643	SOHLH1	spermatogenesis and oogenesis specific basic helix-loop-helix 1	402381	0	NA	NA	NA	NA	NA
ENSG00000165644	COMTD1	catechol-O-methyltransferase domain containing 1	118881	11,38486762	-0,418206923	0,681286	-0,613849148	0,539315016	NA
ENSG00000165646	SLC18A2	solute carrier family 18 member A2	6571	0	NA	NA	NA	NA	NA
ENSG00000165655	ZNF503	zinc finger protein 503	84858	0	NA	NA	NA	NA	NA
ENSG00000165682	CLEC1B	C-type lectin domain family 1 member B	51266	0,144330898	1,418786819	4,061262	0,349346267	0,726829368	NA
ENSG00000165685	TMEM52B	transmembrane protein 52B	120939	0	NA	NA	NA	NA	NA
ENSG00000165694	FRMD7	FERM domain containing 7	90167	0	NA	NA	NA	NA	NA
ENSG00000165695	AK8	adenylate kinase 8	158067	1,224568709	-3,026086694	1,854386	-1,631853315	0,102710391	NA
ENSG00000165702	GFI1B	growth factor independent 1B transcriptional repressor	8328	5,247593392	-1,222037461	0,817329	-1,495159792	0,134872744	NA
ENSG00000165716	DIPK1B	divergent protein kinase domain 1B	138311	0	NA	NA	NA	NA	NA
ENSG00000165730	STOX1	storkhead box 1	219736	0,10488184	-1,076443944	4,080456	-0,263804824	0,791930317	NA
ENSG00000165731	RET	ret proto-oncogene	5979	0,314645521	-2,509429468	3,997603	-0,627733573	0,530178488	NA
ENSG00000165757	JCAD	junctional cadherin 5 associated	57608	2,203402817	0,033450095	1,348701	0,024801719	0,98021312	NA
ENSG00000165794	SLC39A2	solute carrier family 39 member 2	29986	0,71930069	-0,707379016	2,360988	-0,2996114	0,764473588	NA
ENSG00000165795	NDRG2	NDRG family member 2	57447	0,365404816	0,996951429	4,010387	0,248592331	0,803676141	NA
ENSG00000165799	RNASE7	ribonuclease A family member 7	84659	0	NA	NA	NA	NA	NA
ENSG00000165801	ARHGEF40	Rho guanine nucleotide exchange factor 40	55701	0	NA	NA	NA	NA	NA
ENSG00000165804	ZNF219	zinc finger protein 219	51222	3,498525107	-0,517225386	1,222093	-0,423229085	0,672128128	NA
ENSG00000165805	C12orf50	chromosome 12 open reading frame 50	160419	0,11808024	-1,076443944	4,080456	-0,263804824	0,791930317	NA
ENSG00000165807	PPP1R36	protein phosphatase 1 regulatory subunit 36	145376	0,264026547	2,150563003	4,007957	0,536573341	0,591562363	NA

ENSG00000165810	BTNL9	butyrophilin like 9	153579	0,144330898	1,418786819	4,061262	0,349346267	0,726829368	NA
ENSG00000165816	VWA2	von Willebrand factor A domain containing 2	340706	0,235784017	0,850510453	4,080449	0,208435495	0,834888944	NA
ENSG00000165821	SALL2	spalt like transcription factor 2	6297	8,205441983	-0,198881938	0,715418	-0,277994063	0,781016916	NA
ENSG00000165828	PRAP1	proline rich acidic protein 1	118471	0	NA	NA	NA	NA	NA
ENSG00000165837	ERICH6B	glutamate rich 6B	220081	0	NA	NA	NA	NA	NA
ENSG00000165841	CYP2C19	cytochrome P450 family 2 subfamily C member 19	1557	0	NA	NA	NA	NA	NA
ENSG00000165868	HSPA12A	heat shock protein family A (Hsp70) member 12A	259217	0,943936562	-4,091095689	3,937345	-1,039049463	0,298781733	NA
ENSG00000165874	SHLD2P1	shieldin complex subunit 2 pseudogene 1	414241	0,366183428	-0,4699538	3,671672	-0,127994496	0,898153329	NA
ENSG00000165886	UBTD1	ubiquitin domain containing 1	80019	1,457774141	-0,44341822	1,728408	-0,256547152	0,797528376	NA
ENSG00000165887	ANKRD2	ankyrin repeat domain 2	26287	0	NA	NA	NA	NA	NA
ENSG00000165899	OTOGL	otogelin like	283310	0,445771398	0,853393079	3,513587	0,242883708	0,808095487	NA
ENSG00000165905	LARGE2	LARGE xylosyl- and glucuronyltransferase 2	120071	0,262980127	1,429507564	4,061495	0,351965828	0,72486389	NA
ENSG00000165912	PACSIN3	protein kinase C and casein kinase substrate in neurons 3	29763	0,435034609	-2,523742874	3,997022	-0,631405829	0,527775205	NA
ENSG00000165914	TTC7B	tetratricopeptide repeat domain 7B	145567	0,973321041	0,806382653	1,869089	0,43143092	0,666155071	NA
ENSG00000165917	RAPSN	receptor associated protein of the synapse	5913	2,121744985	0,043123551	1,802121	0,023929326	0,980908982	NA
ENSG00000165935	SMCO2	single-pass membrane protein with coiled-coil domains 2	341346	0,072165449	0,847101401	4,080456	0,207599697	0,835541541	NA
ENSG00000165948	IFI27L1	interferon alpha inducible protein 27 like 1	122509	7,043250498	0,11045379	1,016675	0,10864219	0,913486296	NA
ENSG00000165949	IFI27	interferon alpha inducible protein 27	3429	0	NA	NA	NA	NA	NA
ENSG00000165953	SERPINA12	serpin family A member 12	145264	0	NA	NA	NA	NA	NA
ENSG00000165959	CLMN	calmin	79789	0,280925803	-0,964782912	4,042515	-0,238659083	0,811369947	NA
ENSG00000165966	PDZRN4	PDZ domain containing ring finger 4	29951	0	NA	NA	NA	NA	NA
ENSG00000165970	SLC6A5	solute carrier family 6 member 5	9152	0	NA	NA	NA	NA	NA
ENSG00000165972	CCDC38	coiled-coil domain containing 38	120935	0,119673877	0,847101401	4,080456	0,207599697	0,835541541	NA
ENSG00000165973	NELL1	neural EGFL like 1	4745	0	NA	NA	NA	NA	NA
ENSG00000165985	C1QL3	complement C1q like 3	389941	0,688873296	1,850003376	2,335895	0,791988946	0,428367122	NA
ENSG00000165995	CACNB2	calcium voltage-gated channel auxiliary subunit beta 2	783	0,10543123	0	4,080456	0	1	NA
ENSG00000165996	HACD1	3-hydroxyacyl-CoA dehydratase 1	9200	9,981074895	-0,37293112	0,663084	-0,562419132	0,573830487	NA
ENSG00000166006	KCNC2	potassium voltage-gated channel subfamily C member 2	3747	0	NA	NA	NA	NA	NA
ENSG00000166007	NA	NA	NA	0	NA	NA	NA	NA	NA
ENSG00000166013	NA	NA	NA	0	NA	NA	NA	NA	NA
ENSG00000166016	ABTB2	ankyrin repeat and BTB domain containing 2	25841	0,795654335	1,061876084	2,002417	0,530297107	0,595905952	NA
ENSG00000166033	HTRA1	HtrA serine peptidase 1	5654	9,220479425	-0,1669296	0,779883	-0,214044399	0,830512434	NA
ENSG00000166049	PASD1	PAS domain containing repressor 1	139135	0	NA	NA	NA	NA	NA
ENSG00000166069	TMCO5A	transmembrane and coiled-coil domains 5A	145942	0	NA	NA	NA	NA	NA
ENSG00000166073	GPR176	G protein-coupled receptor 176	11245	0	NA	NA	NA	NA	NA
ENSG00000166086	JAM3	junctional adhesion molecule 3	83700	0,47508864	0	4,080456	0	1	NA
ENSG00000166090	IL25	interleukin 25	64806	0	NA	NA	NA	NA	NA
ENSG00000166091	CMTM5	CKLF like MARVEL transmembrane domain containing 5	116173	0	NA	NA	NA	NA	NA
ENSG00000166104	NA	NA	NA	0	NA	NA	NA	NA	NA
ENSG00000166105	GLB1L3	galactosidase beta 1 like 3	112937	0	NA	NA	NA	NA	NA
ENSG00000166106	ADAMTS15	ADAM metallopeptidase with thrombospondin type 1 motif 15	170689	0,276347057	0,84157555	4,080456	0,206245473	0,836599169	NA
ENSG00000166111	SVOP	SV2 related protein	55530	0	NA	NA	NA	NA	NA
ENSG00000166118	SPATA19	spermatogenesis associated 19	219938	0	NA	NA	NA	NA	NA
ENSG00000166126	AMN	amnion associated transmembrane protein	81693	0,570463502	-3,355935702	3,120464	-1,075460615	0,282168556	NA
ENSG00000166143	PPP1R14D	protein phosphatase 1 regulatory inhibitor subunit 14D	54866	0	NA	NA	NA	NA	NA

ENSG00000166145	SPINT1	serine peptidase inhibitor, Kunitz type 1	6692	3,700314099	-0,842354144	1,216063	-0,692689465	0,488504452	NA
ENSG00000166148	AVPR1A	arginine vasopressin receptor 1A	552	0	NA	NA	NA	NA	NA
ENSG00000166152	C16orf78	chromosome 16 open reading frame 78	123970	0	NA	NA	NA	NA	NA
ENSG00000166153	DEPDC4	DEP domain containing 4	120863	0,544923219	0,742301427	2,907525	0,25530357	0,798488643	NA
ENSG00000166160	OPN1MW2	opsin 1, medium wave sensitive 2	728458	0	NA	NA	NA	NA	NA
ENSG00000166165	CKB	creatine kinase B	1152	0	NA	NA	NA	NA	NA
ENSG00000166171	DPCD	deleted in primary ciliary dyskinesia homolog (mouse)	25911	0,540675174	0,035025929	2,435214	0,0143831	0,988524342	NA
ENSG00000166173	LARP6	La ribonucleoprotein 6, translational regulator	55323	0	NA	NA	NA	NA	NA
ENSG00000166183	ASPG	asparaginase	374569	0,10488184	-1,076443944	4,080456	-0,263804824	0,791930317	NA
ENSG00000166192	SENP8	SUMO peptidase family member, NEDD8 specific	123228	4,892819636	1,462247869	1,199393	1,219156693	0,222784725	NA
ENSG00000166206	GABRB3	gamma-aminobutyric acid type A receptor subunit beta3	2562	0	NA	NA	NA	NA	NA
ENSG00000166211	SPIC	Spi-C transcription factor	121599	0	NA	NA	NA	NA	NA
ENSG00000166220	TBATA	thymus, brain and testes associated	219793	0	NA	NA	NA	NA	NA
ENSG00000166246	DNAAF8	dynein axonemal assembly factor 8	146562	0	NA	NA	NA	NA	NA
ENSG00000166250	CLMP	CXADR like membrane protein	79827	0	NA	NA	NA	NA	NA
ENSG00000166257	SCN3B	sodium voltage-gated channel beta subunit 3	55800	0,144330898	1,418786819	4,061262	0,349346267	0,726829368	NA
ENSG00000166262	FAM227B	family with sequence similarity 227 member B	196951	2,443032606	1,303299047	1,356684	0,960650669	0,336727843	NA
ENSG00000166265	CYYR1	cysteine and tyrosine rich 1	116159	0	NA	NA	NA	NA	NA
ENSG00000166268	MYRFL	myelin regulatory factor like	196446	0	NA	NA	NA	NA	NA
ENSG00000166278	C2	complement C2	717	1,853188033	-0,275710541	2,039415	-0,135191013	0,892460853	NA
ENSG00000166292	TMEM100	transmembrane protein 100	55273	0	NA	NA	NA	NA	NA
ENSG00000166317	SYNPO2L	synaptopodin 2 like	79933	0,397781277	-0,142199693	3,592848	-0,039578545	0,968429133	NA
ENSG00000166321	NUDT13	nudix hydrolase 13	25961	6,647993749	0,512122212	0,713699	0,717560512	0,473028313	NA
ENSG00000166323	C11orf65	chromosome 11 open reading frame 65	160140	0,246738432	1,280162498	4,05826	0,315446139	0,75242294	NA
ENSG00000166329	CCDC182	coiled-coil domain containing 182	101927581	0	NA	NA	NA	NA	NA
ENSG00000166341	DCHS1	dachsous cadherin-related 1	8642	1,640194453	0,241502763	1,65233	0,146158956	0,883795906	NA
ENSG00000166342	NETO1	neuropilin and tolloid like 1	81832	0	NA	NA	NA	NA	NA
ENSG00000166349	RAG1	recombination activating 1	5896	0,730552433	-2,477968296	2,966261	-0,835384456	0,403501279	NA
ENSG00000166351	POTED	POTE ankyrin domain family member D	317754	0	NA	NA	NA	NA	NA
ENSG00000166352	IFTAP	intraflagellar transport associated protein	119710	0,234994216	1,944111989	4,02324	0,483220515	0,62893917	NA
ENSG00000166359	WDR88	WD repeat domain 88	126248	0,103998153	-1,076443944	4,080456	-0,263804824	0,791930317	NA
ENSG00000166363	OR10A5	olfactory receptor family 10 subfamily A member 5	144124	0	NA	NA	NA	NA	NA
ENSG00000166368	OR2D2	olfactory receptor family 2 subfamily D member 2	120776	0	NA	NA	NA	NA	NA
ENSG00000166391	MOGAT2	monoacylglycerol O-acyltransferase 2	80168	0	NA	NA	NA	NA	NA
ENSG00000166394	CYB5R2	cytochrome b5 reductase 2	51700	0,687734187	-2,018505606	3,273126	-0,616690548	0,537438857	NA
ENSG00000166396	SERPINB7	serpin family B member 7	8710	0	NA	NA	NA	NA	NA
ENSG00000166402	TUB	TUB bipartite transcription factor	7275	0,10543123	0	4,080456	0	1	NA
ENSG00000166405	RIC3	RIC3 acetylcholine receptor chaperone	79608	0,530920805	-0,173309676	2,847287	-0,060868358	0,951464049	NA
ENSG00000166407	LMO1	LIM domain only 1	4004	0	NA	NA	NA	NA	NA
ENSG00000166408	OR5P1P	olfactory receptor family 5 subfamily P member 1 pseudogene	79283	0	NA	NA	NA	NA	NA
ENSG00000166415	WDR72	WD repeat domain 72	256764	0	NA	NA	NA	NA	NA
ENSG00000166426	CRABP1	cellular retinoic acid binding protein 1	1381	0	NA	NA	NA	NA	NA
ENSG00000166428	PLD4	phospholipase D family member 4	122618	0	NA	NA	NA	NA	NA
ENSG00000166432	ZMAT1	zinc finger matrin-type 1	84460	0,951128725	-1,906335371	3,585273	-0,53171279	0,594924932	NA
ENSG00000166444	DENND2B	DENN domain containing 2B	6764	0	NA	NA	NA	NA	NA
ENSG00000166448	TMEM130	transmembrane protein 130	222865	0	NA	NA	NA	NA	NA
ENSG00000166450	PRTG	protogenin	283659	0	NA	NA	NA	NA	NA
ENSG00000166473	PKD1L2	polycystin 1 like 2 (gene/pseudogene)	114780	0	NA	NA	NA	NA	NA
ENSG00000166482	MFAP4	microfibril associated protein 4	4239	0	NA	NA	NA	NA	NA

ENSG00000166492	FAM86GP	family with sequence similarity 86, member A pseudogene	387745	0	NA	NA	NA	NA	NA
ENSG00000166509	CLEC3A	C-type lectin domain family 3 member A	10143	0	NA	NA	NA	NA	NA
ENSG00000166510	CCDC68	coiled-coil domain containing 68	80323	0	NA	NA	NA	NA	NA
ENSG00000166523	CLEC4E	C-type lectin domain family 4 member E	26253	0	NA	NA	NA	NA	NA
ENSG00000166527	CLEC4D	C-type lectin domain family 4 member D	338339	0	NA	NA	NA	NA	NA
ENSG00000166530	NA	NA	NA	0	NA	NA	NA	NA	NA
ENSG00000166532	RIMKLB	ribosomal modification protein rimK like family member B	57494	1,232856303	-0,152387539	2,034943	-0,074885424	0,940305874	NA
ENSG00000166535	A2ML1	alpha-2-macroglobulin like 1	144568	0	NA	NA	NA	NA	NA
ENSG00000166546	BEAN1	brain expressed associated with NEDD4 1	146227	0	NA	NA	NA	NA	NA
ENSG00000166558	SLC38A8	solute carrier family 38 member 8	146167	0	NA	NA	NA	NA	NA
ENSG00000166569	CPLX4	complexin 4	339302	0,10543123	0	4,080456	0	1	NA
ENSG00000166573	GALR1	galanin receptor 1	2587	0	NA	NA	NA	NA	NA
ENSG00000166578	IQCD	IQ motif containing D	115811	1,088035375	0,343847447	1,760477	0,195314887	0,845146444	NA
ENSG00000166582	CENPV	centromere protein V	201161	2,879886207	-1,024431836	1,443233	-0,709817191	0,477817507	NA
ENSG00000166589	CDH16	cadherin 16	1014	0	NA	NA	NA	NA	NA
ENSG00000166592	RRAD	RRAD, Ras related glycolysis inhibitor and calcium channel regulator	6236	1,896119989	-2,297306827	1,775293	-1,294043933	0,195650246	NA
ENSG00000166596	CFAP52	cilia and flagella associated protein 52	146845	0,222783006	0,840454589	4,080456	0,205970758	0,836813753	NA
ENSG00000166603	MC4R	melanocortin 4 receptor	4160	0	NA	NA	NA	NA	NA
ENSG00000166634	SERPINB12	serpin family B member 12	89777	0	NA	NA	NA	NA	NA
ENSG00000166664	CHRFAM7A	CHRNA7 (exons 5-10) and FAM7A (exons A-E) fusion	89832	3,753645065	-0,683094956	1,247754	-0,547459443	0,584063127	NA
ENSG00000166670	MMP10	matrix metallopeptidase 10	4319	0	NA	NA	NA	NA	NA
ENSG00000166676	TVP23A	trans-golgi network vesicle protein 23 homolog A	780776	0	NA	NA	NA	NA	NA
ENSG00000166681	BEX3	brain expressed X-linked 3	27018	0,10543123	0	4,080456	0	1	NA
ENSG00000166682	TMPRSS5	transmembrane serine protease 5	80975	1,13788626	-0,338764548	1,868449	-0,181307872	0,856125927	NA
ENSG00000166689	PLEKHA7	pleckstrin homology domain containing A7	144100	0	NA	NA	NA	NA	NA
ENSG00000166693	NA	NA	NA	0	NA	NA	NA	NA	NA
ENSG00000166736	HTR3A	5-hydroxytryptamine receptor 3A	3359	0	NA	NA	NA	NA	NA
ENSG00000166741	NNMT	nicotinamide N-methyltransferase	4837	0,207996306	-1,916073151	4,043021	-0,473921178	0,635556117	NA
ENSG00000166743	ACSM1	acyl-CoA synthetase medium chain family member 1	116285	0	NA	NA	NA	NA	NA
ENSG00000166762	CATSPER2	cation channel sperm associated 2	117155	6,789190285	0,331706079	0,855235	0,387853757	0,698124261	NA
ENSG00000166763	NA	NA	NA	1,407430128	-0,602698652	2,459616	-0,245037667	0,806427266	NA
ENSG00000166780	BMERB1	bMERB domain containing 1	89927	0,234994216	1,944111989	4,02324	0,483220515	0,62893917	NA
ENSG00000166787	NA	NA	NA	0	NA	NA	NA	NA	NA
ENSG00000166793	YPEL4	yippee like 4	219539	0	NA	NA	NA	NA	NA
ENSG00000166796	LDHC	lactate dehydrogenase C	3948	0	NA	NA	NA	NA	NA
ENSG00000166800	LDHAL6A	lactate dehydrogenase A like 6A	160287	0,583107432	-1,582220463	3,359241	-0,471005362	0,637636906	NA
ENSG00000166813	KIF7	kinesin family member 7	374654	3,949554708	-0,353434603	1,135044	-0,311383978	0,755508732	NA
ENSG00000166816	LDHD	lactate dehydrogenase D	197257	0,579416548	0,342973747	2,766779	0,123961361	0,901345869	NA
ENSG00000166819	PLIN1	perilipin 1	5346	0,440530838	-0,11230623	3,070955	-0,036570454	0,970827502	NA
ENSG00000166821	PEX11A	peroxisomal biogenesis factor 11 alpha	8800	9,989396758	0,227473295	0,879884	0,258526482	0,796000614	NA
ENSG00000166823	MESP1	mesoderm posterior bHLH transcription factor 1	55897	0,799765772	1,974094838	2,271553	0,869050716	0,384819391	NA
ENSG00000166825	ANPEP	alanyl aminopeptidase, membrane	290	0	NA	NA	NA	NA	NA
ENSG00000166828	SCNN1G	sodium channel epithelial 1 subunit gamma	6340	0	NA	NA	NA	NA	NA
ENSG00000166831	RBPMS2	RNA binding protein, mRNA processing factor 2	348093	0	NA	NA	NA	NA	NA
ENSG00000166833	NAV2	neuron navigator 2	89797	3,167125028	-1,538932745	1,135865	-1,354854852	0,175463806	NA
ENSG00000166839	ANKDD1A	ankyrin repeat and death domain containing 1A	348094	2,813178188	-0,600019168	1,641718	-0,365482401	0,714751341	NA
ENSG00000166840	GLYATL1	glycine-N-acyltransferase like 1	92292	0	NA	NA	NA	NA	NA
ENSG00000166856	GPR182	G protein-coupled receptor 182	11318	0	NA	NA	NA	NA	NA

ENSG00000166862	CACNG2	calcium voltage-gated channel auxiliary subunit gamma 2	10369	0	NA	NA	NA	NA	NA
ENSG00000166863	TAC3	tachykinin precursor 3	6866	0,119673877	0,847101401	4,080456	0,207599697	0,835541541	NA
ENSG00000166866	MYO1A	myosin IA	4640	0,10488184	-1,076443944	4,080456	-0,263804824	0,791930317	NA
ENSG00000166869	CHP2	calcineurin like EF-hand protein 2	63928	0	NA	NA	NA	NA	NA
ENSG00000166884	OR4D6	olfactory receptor family 4 subfamily D member 6	219983	0	NA	NA	NA	NA	NA
ENSG00000166896	ATP23	ATP23 metallopeptidase and ATP synthase assembly factor homolog	91419	0,531447696	1,873841945	3,30169	0,567540218	0,57034721	NA
ENSG00000166897	ELFN2	extracellular leucine rich repeat and fibronectin type III domain containing 2	114794	0,399545415	-1,026323355	3,077678	-0,333473261	0,73877707	NA
ENSG00000166920	C15orf48	chromosome 15 open reading frame 48	84419	5,666144814	-0,21441267	0,904426	-0,237070418	0,812602161	NA
ENSG00000166922	SCG5	secretogranin V	6447	0,319658092	0,912659733	4,027787	0,226590847	0,82074191	NA
ENSG00000166924	NYAP1	neuronal tyrosine phosphorylated phosphoinositide-3-kinase adaptor 1	222950	0,479404219	-3,122097492	3,966817	-0,78705354	0,431250525	NA
ENSG00000166926	MS4A6E	membrane spanning 4-domains A6E	245802	0	NA	NA	NA	NA	NA
ENSG00000166927	MS4A7	membrane spanning 4-domains A7	58475	0	NA	NA	NA	NA	NA
ENSG00000166928	MS4A14	membrane spanning 4-domains A14	84689	0	NA	NA	NA	NA	NA
ENSG00000166930	MS4A5	membrane spanning 4-domains A5	64232	0	NA	NA	NA	NA	NA
ENSG00000166947	EPB42	erythrocyte membrane protein band 4,2	2038	0	NA	NA	NA	NA	NA
ENSG00000166948	TGM6	transglutaminase 6	343641	0	NA	NA	NA	NA	NA
ENSG00000166959	MS4A8	membrane spanning 4-domains A8	83661	0	NA	NA	NA	NA	NA
ENSG00000166960	CCDC178	coiled-coil domain containing 178	374864	0	NA	NA	NA	NA	NA
ENSG00000166961	MS4A15	membrane spanning 4-domains A15	219995	0	NA	NA	NA	NA	NA
ENSG00000166963	MAP1A	microtubule associated protein 1A	4130	2,06039475	1,373184981	1,501218	0,914713972	0,360341808	NA
ENSG00000166979	EVA1C	eva-1 homolog C	59271	0,453838075	-0,033259314	3,49522	-0,009515658	0,992407718	NA
ENSG00000166984	NA	NA	NA	0	NA	NA	NA	NA	NA
ENSG00000167011	NAT16	N-acetyltransferase 16 (putative)	375607	0	NA	NA	NA	NA	NA
ENSG00000167014	TERB2	telomere repeat binding bouquet formation protein 2	145645	0	NA	NA	NA	NA	NA
ENSG00000167034	NKX3-1	NK3 homeobox 1	4824	1,376958042	-1,81835451	2,089852	-0,87008771	0,384252473	NA
ENSG00000167037	SGSM1	small G protein signaling modulator 1	129049	2,41092871	-2,055757084	1,497005	-1,373246584	0,169675702	NA
ENSG00000167077	MEI1	meiotic double-stranded break formation protein 1	150365	0,854290116	2,463863684	2,472877	0,996355153	0,319077615	NA
ENSG00000167080	B4GALNT2	beta-1,4-N-acetyl-galactosaminyltransferase 2	124872	0,23616048	-2,075648354	4,028973	-0,515180498	0,606426895	NA
ENSG00000167094	TTC16	tetratricopeptide repeat domain 16	158248	0,072165449	0,847101401	4,080456	0,207599697	0,835541541	NA
ENSG00000167098	SUN5	Sad1 and UNC84 domain containing 5	140732	0	NA	NA	NA	NA	NA
ENSG00000167100	SAMD14	sterile alpha motif domain containing 14	201191	9,074023279	-2,224891818	1,392342	-1,597949103	0,110054305	NA
ENSG00000167103	PIP5KL1	phosphatidylinositol-4-phosphate 5-kinase like 1	138429	0,356362368	-0,558286937	3,734144	-0,149508661	0,881152276	NA
ENSG00000167104	BPIFB6	BPI fold containing family B member 6	128859	0	NA	NA	NA	NA	NA
ENSG00000167105	TMEM92	transmembrane protein 92	162461	0	NA	NA	NA	NA	NA
ENSG00000167117	ANKRD40CL	ANKRD40 C-terminal like	55018	0	NA	NA	NA	NA	NA
ENSG00000167123	CERCAM	cerebral endothelial cell adhesion molecule	51148	9,398118974	-1,29250405	0,890637	-1,45121289	0,146720589	NA
ENSG00000167131	CCDC103	coiled-coil domain containing 103	388389	0,320549828	0,941890489	4,02652	0,233921694	0,815045771	NA
ENSG00000167139	TBC1D21	TBC1 domain family member 21	161514	0	NA	NA	NA	NA	NA
ENSG00000167157	PRRX2	paired related homeobox 2	51450	0	NA	NA	NA	NA	NA
ENSG00000167165	UGT1A6	UDP glucuronosyltransferase family 1 member A6	54578	0	NA	NA	NA	NA	NA
ENSG00000167178	ISLR2	immunoglobulin superfamily containing leucine rich repeat 2	57611	0	NA	NA	NA	NA	NA
ENSG00000167183	PRR15L	proline rich 15 like	79170	0,10488184	-1,076443944	4,080456	-0,263804824	0,791930317	NA

ENSG00000167191	GPRC5B	G protein-coupled receptor class C group 5 member B	51704	0	NA	NA	NA	NA	NA
ENSG00000167194	C16orf92	chromosome 16 open reading frame 92	146378	0	NA	NA	NA	NA	NA
ENSG00000167195	GOLGA6C	golgin A6 family member C	653641	0,223436953	-1,073453894	4,080456	-0,263072051	0,792495046	NA
ENSG00000167195	LOC124907524	golgin subfamily A member 6B	124907524	0,223436953	-1,073453894	4,080456	-0,263072051	0,792495046	NA
ENSG00000167210	LOXHD1	lipoxygenase homology PLAT domains 1	125336	0	NA	NA	NA	NA	NA
ENSG00000167216	KATNAL2	katanin catalytic subunit A1 like 2	83473	0	NA	NA	NA	NA	NA
ENSG00000167244	IGF2	insulin like growth factor 2	3481	0	NA	NA	NA	NA	NA
ENSG00000167281	RBFOX3	RNA binding fox-1 homolog 3	146713	0	NA	NA	NA	NA	NA
ENSG00000167286	CD3D	CD3 delta subunit of T-cell receptor complex	915	7,771509885	-2,187004492	0,860583	-2,541304769	0,01104396	NA
ENSG00000167306	MYO5B	myosin VB	4645	0	NA	NA	NA	NA	NA
ENSG00000167311	ART5	ADP-ribosyltransferase 5	116969	0	NA	NA	NA	NA	NA
ENSG00000167332	OR51E2	olfactory receptor family 51 subfamily E member 2	81285	0,118286652	0	4,080456	0	1	NA
ENSG00000167346	MMP26	matrix metallopeptidase 26	56547	0	NA	NA	NA	NA	NA
ENSG00000167355	OR51B5	olfactory receptor family 51 subfamily B member 5	282763	0	NA	NA	NA	NA	NA
ENSG00000167359	OR51I1	olfactory receptor family 51 subfamily I member 1	390063	0	NA	NA	NA	NA	NA
ENSG00000167360	OR51Q1	olfactory receptor family 51 subfamily Q member 1	390061	0	NA	NA	NA	NA	NA
ENSG00000167363	FN3K	fructosamine 3 kinase	64122	2,454656968	-2,239574452	1,499172	-1,493874236	0,135208494	NA
ENSG00000167371	PRRT2	proline rich transmembrane protein 2	112476	0,947008769	-1,780211598	1,987254	-0,895814917	0,370351621	NA
ENSG00000167390	POM121L3P	POM121 transmembrane nucleoporin like 3, pseudogene	651452	0	NA	NA	NA	NA	NA
ENSG00000167414	GNG8	G protein subunit gamma 8	94235	0,118286652	0	4,080456	0	1	NA
ENSG00000167419	LPO	lactoperoxidase	4025	0	NA	NA	NA	NA	NA
ENSG00000167434	CA4	carbonic anhydrase 4	762	0	NA	NA	NA	NA	NA
ENSG00000167476	JSRP1	junctional sarcoplasmic reticulum protein 1	126306	0	NA	NA	NA	NA	NA
ENSG00000167483	NIBAN3	niban apoptosis regulator 3	199786	0,669814895	3,474308279	3,601519	0,964678677	0,334705779	NA
ENSG00000167524	RSKR	ribosomal protein S6 kinase related	124923	6,946300986	-0,027191011	1,197033	-0,022715345	0,981877335	NA
ENSG00000167531	LALBA	lactalbumin alpha	3906	0	NA	NA	NA	NA	NA
ENSG00000167549	CORO6	coronin 6	84940	0,31672576	0	4,080456	0	1	NA
ENSG00000167554	ZNF610	zinc finger protein 610	162963	0,294464567	1,812856222	3,900371	0,464790661	0,642081377	NA
ENSG00000167580	AQP2	aquaporin 2	359	0	NA	NA	NA	NA	NA
ENSG00000167588	GPD1	glycerol-3-phosphate dehydrogenase 1	2819	0	NA	NA	NA	NA	NA
ENSG00000167600	CYP2S1	cytochrome P450 family 2 subfamily S member 1	29785	0	NA	NA	NA	NA	NA
ENSG00000167601	AXL	AXL receptor tyrosine kinase	558	0	NA	NA	NA	NA	NA
ENSG00000167608	TMC4	transmembrane channel like 4	147798	0	NA	NA	NA	NA	NA
ENSG00000167612	ANKRD33	ankyrin repeat domain 33	341405	0	NA	NA	NA	NA	NA
ENSG00000167614	TTYH1	tweety family member 1	57348	0,288661797	2,306206831	3,997774	0,57687273	0,564025434	NA
ENSG00000167617	CDC42EP5	CDC42 effector protein 5	148170	0	NA	NA	NA	NA	NA
ENSG00000167618	LAIR2	leukocyte associated immunoglobulin like receptor 2	3904	3,664693719	-0,959913406	1,152497	-0,832899116	0,404901628	NA
ENSG00000167619	TMEM145	transmembrane protein 145	284339	0,424552111	0,117344536	3,988626	0,029419787	0,976529792	NA
ENSG00000167634	NLRP7	NLR family pyrin domain containing 7	199713	0	NA	NA	NA	NA	NA
ENSG00000167641	PPP1R14A	protein phosphatase 1 regulatory inhibitor subunit 14A	94274	0	NA	NA	NA	NA	NA
ENSG00000167642	SPINT2	serine peptidase inhibitor, Kunitz type 2	10653	0,693774736	-1,072649546	3,194457	-0,335784597	0,737033306	NA
ENSG00000167644	C19orf33	chromosome 19 open reading frame 33	64073	0	NA	NA	NA	NA	NA
ENSG00000167646	DNAAF3	dynein axonemal assembly factor 3	352909	0	NA	NA	NA	NA	NA
ENSG00000167653	PSCA	prostate stem cell antigen	8000	0	NA	NA	NA	NA	NA
ENSG00000167654	ATCAY	ATCAY kinesin light chain interacting caytaxin	85300	0	NA	NA	NA	NA	NA
ENSG00000167656	LY6D	lymphocyte antigen 6 family member D	8581	0	NA	NA	NA	NA	NA
ENSG00000167676	PLIN4	perilipin 4	729359	0	NA	NA	NA	NA	NA
ENSG00000167680	SEMA6B	semaphorin 6B	10501	0	NA	NA	NA	NA	NA
ENSG00000167693	NXN	nucleoredoxin	64359	1,057678441	-1,476853322	1,914662	-0,771338737	0,440506177	NA
ENSG00000167695	TLCD3A	TLC domain containing 3A	79850	9,180317326	-0,145842633	0,806395	-0,18085764	0,856479319	NA
ENSG00000167701	GPT	glutamic--pyruvic transaminase	2875	0	NA	NA	NA	NA	NA

ENSG00000167703	SLC43A2	solute carrier family 43 member 2	124935	0,587485541	3,280248886	3,95344	0,829720116	0,406697045	NA
ENSG00000167705	RILP	Rab interacting lysosomal protein	83547	8,858501624	1,429271145	2,736246	0,522347492	0,601428408	NA
ENSG00000167711	SERPINF2	serpin family F member 2	5345	0,763078682	-1,162074866	2,674745	-0,434461857	0,663953087	NA
ENSG00000167723	TRPV3	transient receptor potential cation channel subfamily V member 3	162514	1,196738772	4,311091181	3,929692	1,097055628	0,272617076	NA
ENSG00000167733	HSD11B1L	hydroxysteroid 11-beta dehydrogenase 1 like	374875	2,70022392	1,143174795	1,361313	0,839759045	0,4010435	NA
ENSG00000167741	GGT6	gamma-glutamyltransferase 6	124975	0	NA	NA	NA	NA	NA
ENSG00000167748	KLK1	kallikrein 1	3816	0	NA	NA	NA	NA	NA
ENSG00000167749	KLK4	kallikrein related peptidase 4	9622	0	NA	NA	NA	NA	NA
ENSG00000167751	KLK2	kallikrein related peptidase 2	3817	0	NA	NA	NA	NA	NA
ENSG00000167754	KLK5	kallikrein related peptidase 5	25818	0	NA	NA	NA	NA	NA
ENSG00000167755	KLK6	kallikrein related peptidase 6	5653	0	NA	NA	NA	NA	NA
ENSG00000167757	KLK11	kallikrein related peptidase 11	11012	0	NA	NA	NA	NA	NA
ENSG00000167759	KLK13	kallikrein related peptidase 13	26085	0	NA	NA	NA	NA	NA
ENSG00000167767	KRT80	keratin 80	144501	0	NA	NA	NA	NA	NA
ENSG00000167768	KRT1	keratin 1	3848	0	NA	NA	NA	NA	NA
ENSG00000167769	ACER1	alkaline ceramidase 1	125981	2,493740774	-0,699495184	1,271962	-0,549934124	0,582364558	NA
ENSG00000167771	RCOR2	REST corepressor 2	283248	0	NA	NA	NA	NA	NA
ENSG00000167772	ANGPTL4	angiopoietin like 4	51129	0	NA	NA	NA	NA	NA
ENSG00000167774	NA	NA	NA	3,530107178	0,404390111	3,773959	0,107152745	0,914667804	NA
ENSG00000167779	IGFBP6	insulin like growth factor binding protein 6	3489	3,446207498	1,139058971	1,010036	1,127740768	0,259429415	NA
ENSG00000167791	CABP2	calcium binding protein 2	51475	0	NA	NA	NA	NA	NA
ENSG00000167798	NA	NA	NA	0	NA	NA	NA	NA	NA
ENSG00000167799	NUDT8	nudix hydrolase 8	254552	10,87753967	0,201792994	0,54939	0,36730367	0,713392517	NA
ENSG00000167800	TBX10	T-box transcription factor 10	347853	0	NA	NA	NA	NA	NA
ENSG00000167807	NA	NA	NA	4,585460974	-1,082863395	0,869799	-1,244958406	0,21314704	NA
ENSG00000167822	OR8J3	olfactory receptor family 8 subfamily J member 3	81168	0	NA	NA	NA	NA	NA
ENSG00000167825	OR5I1	olfactory receptor family 5 subfamily I member 1	10798	0	NA	NA	NA	NA	NA
ENSG00000167840	ZNF232	zinc finger protein 232	7775	4,333410864	4,941546765	1,711864	2,886647027	0,003893708	NA
ENSG00000167858	TEKT1	tektin 1	83659	0	NA	NA	NA	NA	NA
ENSG00000167861	HID1	HID1 domain containing	283987	0	NA	NA	NA	NA	NA
ENSG00000167874	TMEM88	transmembrane protein 88	92162	1,274496389	1,932576295	1,744964	1,107516397	0,268070727	NA
ENSG00000167880	EVPL	envoplakin	2125	0	NA	NA	NA	NA	NA
ENSG00000167889	MGAT5B	alpha-1,6-mannosylglycoprotein 6-beta-N-acetylglucosaminyltransferase B	146664	0,548245812	0,610153081	2,791643	0,218564116	0,826989609	NA
ENSG00000167910	CYP7A1	cytochrome P450 family 7 subfamily A member 1	1581	0	NA	NA	NA	NA	NA
ENSG00000167914	GSDMA	gasdermin A	284110	0,15836288	0	4,080456	0	1	NA
ENSG00000167916	KRT24	keratin 24	192666	0	NA	NA	NA	NA	NA
ENSG00000167941	SOST	sclerostin	50964	0	NA	NA	NA	NA	NA
ENSG00000167962	ZNF598	zinc finger protein 598, E3 ubiquitin ligase	90850	0	NA	NA	NA	NA	NA
ENSG00000167964	RAB26	RAB26, member RAS oncogene family	25837	0,207996306	-1,916073151	4,043021	-0,473921178	0,635556117	NA
ENSG00000167968	DNASE1L2	deoxyribonuclease 1 like 2	1775	1,823401091	0,972329427	1,595739	0,609328797	0,542306523	NA
ENSG00000167971	CASKIN1	CASK interacting protein 1	57524	0,278468972	0,841921074	4,080456	0,206330151	0,836533028	NA
ENSG00000167992	VWCE	von Willebrand factor C and EGF domains	220001	0,459816412	0,267789656	3,52834	0,075896788	0,939501212	NA
ENSG00000167994	RAB3IL1	RAB3A interacting protein like 1	5866	0	NA	NA	NA	NA	NA
ENSG00000167995	BEST1	bestrophin 1	7439	0,189623321	1,664832041	4,047533	0,411320184	0,680837771	NA
ENSG00000168000	BSCL2	BSCL2 lipid droplet biogenesis associated, seipin	26580	7,788856203	-1,81860822	0,996543	-1,82491762	0,068013462	NA
ENSG00000168004	PLAAT5	phospholipase A and acyltransferase 5	117245	0	NA	NA	NA	NA	NA
ENSG00000168026	TTC21A	tetratricopeptide repeat domain 21A	199223	1,474165985	-0,003516674	2,178015	-0,001614623	0,998711718	NA
ENSG00000168032	ENTPD3	ectonucleoside triphosphate diphosphohydrolase 3	956	0	NA	NA	NA	NA	NA
ENSG00000168062	BATF2	basic leucine zipper ATF-like transcription factor 2	116071	0,144330898	1,418786819	4,061262	0,349346267	0,726829368	NA
ENSG00000168065	SLC22A11	solute carrier family 22 member 11	55867	0	NA	NA	NA	NA	NA
ENSG00000168070	MAJIN	membrane anchored junction protein	283129	0	NA	NA	NA	NA	NA
ENSG00000168077	SCARA3	scavenger receptor class A member 3	51435	0	NA	NA	NA	NA	NA

ENSG00000168079	SCARA5	scavenger receptor class A member 5	286133	0,228311956	0,031396737	4,077338	0,007700303	0,993856108	NA
ENSG00000168081	PNOC	prepronociceptin	5368	0	NA	NA	NA	NA	NA
ENSG00000168096	ANKS3	ankyrin repeat and sterile alpha motif domain containing 3	124401	4,535969426	-0,381094615	1,570446	-0,242666434	0,808263812	NA
ENSG00000168122	NA	NA	NA	0	NA	NA	NA	NA	NA
ENSG00000168124	OR1F1	olfactory receptor family 1 subfamily F member 1	4992	0	NA	NA	NA	NA	NA
ENSG00000168126	OR2W6P	olfactory receptor family 2 subfamily W member 6 pseudogene	81406	0,144330898	1,418786819	4,061262	0,349346267	0,726829368	NA
ENSG00000168129	NA	NA	NA	0	NA	NA	NA	NA	NA
ENSG00000168131	OR2B2	olfactory receptor family 2 subfamily B member 2	81697	0	NA	NA	NA	NA	NA
ENSG00000168135	KCNJ4	potassium inwardly rectifying channel subfamily J member 4	3761	0	NA	NA	NA	NA	NA
ENSG00000168140	VASN	vasorin	114990	0	NA	NA	NA	NA	NA
ENSG00000168143	FAM83B	family with sequence similarity 83 member B	222584	0,103998153	-1,076443944	4,080456	-0,263804824	0,791930317	NA
ENSG00000168148	H3-4	H3,4 histone, cluster member	8290	0	NA	NA	NA	NA	NA
ENSG00000168152	THAP9	THAP domain containing 9	79725	10,08147378	0,196050475	0,54205	0,361683572	0,717588505	NA
ENSG00000168158	OR2C1	olfactory receptor family 2 subfamily C member 1	4993	0,119673877	0,847101401	4,080456	0,207599697	0,835541541	NA
ENSG00000168243	GNG4	G protein subunit gamma 4	2786	0,10543123	0	4,080456	0	1	NA
ENSG00000168255	POLR2J3-UPK3BL2	POLR2J3-UPK3BL2 readthrough	123258399	11,22843283	-0,492338221	0,585216	-0,841293825	0,400183349	NA
ENSG00000168263	KCNV2	potassium voltage-gated channel modifier subfamily V member 2	169522	0	NA	NA	NA	NA	NA
ENSG00000168267	PTF1A	pancreas associated transcription factor 1a	256297	0	NA	NA	NA	NA	NA
ENSG00000168269	FOXI1	forkhead box I1	2299	1,066221097	0,540587075	1,96482	0,275133185	0,783213917	NA
ENSG00000168273	UQCC5	ubiquinol-cytochrome c reductase complex assembly factor 5	440957	11,48895918	1,326007194	0,70286	1,886586706	0,059215936	NA
ENSG00000168280	KIF5C	kinesin family member 5C	3800	0,711495962	3,555452054	3,57691	0,994001057	0,320222353	NA
ENSG00000168298	H1-4	H1,4 linker histone, cluster member	3008	7,983733889	1,821741601	0,866647	2,102056429	0,035548333	NA
ENSG00000168306	ACOX2	acyl-CoA oxidase 2	8309	0	NA	NA	NA	NA	NA
ENSG00000168309	FAM107A	family with sequence similarity 107 member A	11170	0	NA	NA	NA	NA	NA
ENSG00000168314	MOBP	myelin associated oligodendrocyte basic protein	4336	0,236367903	-1,075404137	4,080456	-0,263549998	0,792126692	NA
ENSG00000168333	PPDPFL	pancreatic progenitor cell differentiation and proliferation factor like	492307	0	NA	NA	NA	NA	NA
ENSG00000168334	XIRP1	xin actin binding repeat containing 1	165904	1,399226241	-1,654812419	2,461603	-0,672249996	0,501424558	NA
ENSG00000168348	INSM2	INSM transcriptional repressor 2	84684	0,119673877	0,847101401	4,080456	0,207599697	0,835541541	NA
ENSG00000168350	DEGS2	delta 4-desaturase, sphingolipid 2	123099	0,15836288	0	4,080456	0	1	NA
ENSG00000168356	SCN11A	sodium voltage-gated channel alpha subunit 11	11280	0,446288901	0,897550558	3,014796	0,297715172	0,765920562	NA
ENSG00000168389	MFSD2A	MFSD2 lysolipid transporter A, lysophospholipid	84879	1,697763426	2,689280525	1,796212	1,497195395	0,134342423	NA
ENSG00000168398	BDKRB2	bradykinin receptor B2	624	0	NA	NA	NA	NA	NA
ENSG00000168412	MTNR1A	melatonin receptor 1A	4543	0	NA	NA	NA	NA	NA
ENSG00000168418	KCNG4	potassium voltage-gated channel modifier subfamily G member 4	93107	0,11808024	-1,076443944	4,080456	-0,263804824	0,791930317	NA
ENSG00000168427	KLHL30	kelch like family member 30	377007	0	NA	NA	NA	NA	NA
ENSG00000168447	SCNN1B	sodium channel epithelial 1 subunit beta	6338	0	NA	NA	NA	NA	NA
ENSG00000168453	HR	HR lysine demethylase and nuclear receptor corepressor	55806	0	NA	NA	NA	NA	NA
ENSG00000168454	TXNDC2	thioredoxin domain containing 2	84203	0	NA	NA	NA	NA	NA
ENSG00000168461	RAB31	RAB31, member RAS oncogene family	11031	0,103998153	-1,076443944	4,080456	-0,263804824	0,791930317	NA
ENSG00000168477	TNXB	tenascin XB	7148	0,10488184	-1,076443944	4,080456	-0,263804824	0,791930317	NA
ENSG00000168481	LGI3	leucine rich repeat LGI family member 3	203190	0	NA	NA	NA	NA	NA
ENSG00000168484	SFTPC	surfactant protein C	6440	0	NA	NA	NA	NA	NA

ENSG00000168490	PHYHIP	phytanoyl-CoA 2-hydroxylase interacting protein	9796	0,10543123	0	4,080456	0	1	NA
ENSG00000168491	CCDC110	coiled-coil domain containing 110	256309	0	NA	NA	NA	NA	NA
ENSG00000168497	CAVIN2	caveolae associated protein 2	8436	0	NA	NA	NA	NA	NA
ENSG00000168502	MTCL1	microtubule crosslinking factor 1	23255	0,23616048	-2,075648354	4,028973	-0,515180498	0,606426895	NA
ENSG00000168505	GBX2	gastrulation brain homeobox 2	2637	0	NA	NA	NA	NA	NA
ENSG00000168509	HJV	hemojuvelin BMP co-receptor	148738	0	NA	NA	NA	NA	NA
ENSG00000168515	SCGB1D1	secretoglobin family 1D member 1	10648	0	NA	NA	NA	NA	NA
ENSG00000168517	HEXIM2	HEXIM P-TEFb complex subunit 2	124790	6,56164047	0,273578996	0,868097	0,315147882	0,752649375	NA
ENSG00000168528	SERINC2	serine incorporator 2	347735	0,722266927	0,844323958	2,669345	0,316303854	0,751771886	NA
ENSG00000168530	MYL1	myosin light chain 1	4632	0	NA	NA	NA	NA	NA
ENSG00000168539	CHRM1	cholinergic receptor muscarinic 1	1128	0	NA	NA	NA	NA	NA
ENSG00000168542	COL3A1	collagen type III alpha 1 chain	1281	0	NA	NA	NA	NA	NA
ENSG00000168546	GFRA2	GDNF family receptor alpha 2	2675	0	NA	NA	NA	NA	NA
ENSG00000168582	CRYGA	crystallin gamma A	1418	0	NA	NA	NA	NA	NA
ENSG00000168589	DYNLRB2	dynein light chain roadblock-type 2	83657	0	NA	NA	NA	NA	NA
ENSG00000168594	ADAM29	ADAM metallopeptidase domain 29	11086	0	NA	NA	NA	NA	NA
ENSG00000168619	ADAM18	ADAM metallopeptidase domain 18	8749	0	NA	NA	NA	NA	NA
ENSG00000168621	GDNF	glial cell derived neurotrophic factor	2668	0	NA	NA	NA	NA	NA
ENSG00000168630	SPINT5P	serine peptidase inhibitor, Kunitz type 5, pseudogene	140863	0	NA	NA	NA	NA	NA
ENSG00000168631	MUCL3	mucin like 3	135656	0	NA	NA	NA	NA	NA
ENSG00000168634	WFDC13	WAP four-disulfide core domain 13	164237	0	NA	NA	NA	NA	NA
ENSG00000168658	VWA3B	von Willebrand factor A domain containing 3B	200403	0	NA	NA	NA	NA	NA
ENSG00000168661	ZNF30	zinc finger protein 30	90075	3,936510603	-0,723766085	1,082034	-0,668894071	0,503563053	NA
ENSG00000168671	UGT3A2	UDP glycosyltransferase family 3 member A2	167127	0	NA	NA	NA	NA	NA
ENSG00000168676	KCTD19	potassium channel tetramerization domain containing 19	146212	0,072165449	0,847101401	4,080456	0,207599697	0,835541541	NA
ENSG00000168679	SLC16A4	solute carrier family 16 member 4	9122	0	NA	NA	NA	NA	NA
ENSG00000168702	LRP1B	LDL receptor related protein 1B	53353	0	NA	NA	NA	NA	NA
ENSG00000168703	WFDC12	WAP four-disulfide core domain 12	128488	0	NA	NA	NA	NA	NA
ENSG00000168734	PKIG	cAMP-dependent protein kinase inhibitor gamma	11142	0	NA	NA	NA	NA	NA
ENSG00000168743	NPNT	nephronectin	255743	0,10488184	-1,076443944	4,080456	-0,263804824	0,791930317	NA
ENSG00000168748	CA7	carbonic anhydrase 7	766	0	NA	NA	NA	NA	NA
ENSG00000168754	FAM178B	family with sequence similarity 178 member B	51252	0,276485966	0,840073247	4,080456	0,205877302	0,836886756	NA
ENSG00000168757	TSPY2	testis specific protein Y-linked 2	64591	0	NA	NA	NA	NA	NA
ENSG00000168772	CXXC4	CXXC finger protein 4	80319	0	NA	NA	NA	NA	NA
ENSG00000168778	TCTN2	tectonic family member 2	79867	9,692039659	0,450339933	0,593509	0,758774975	0,447987178	NA
ENSG00000168779	SHOX2	SHOX homeobox 2	6474	0	NA	NA	NA	NA	NA
ENSG00000168811	IL12A	interleukin 12A	3592	0	NA	NA	NA	NA	NA
ENSG00000168824	NSG1	neuronal vesicle trafficking associated 1	27065	0,246586995	-1,217779329	4,080456	-0,29844198	0,765365853	NA
ENSG00000168828	OR13J1	olfactory receptor family 13 subfamily J member 1	392309	0	NA	NA	NA	NA	NA
ENSG00000168830	HTR1E	5-hydroxytryptamine receptor 1E	3354	0	NA	NA	NA	NA	NA
ENSG00000168843	FSTL5	follistatin like 5	56884	0	NA	NA	NA	NA	NA
ENSG00000168852	NA	NA	NA	0,340092862	-0,970820105	3,753539	-0,258641274	0,795912034	NA
ENSG00000168874	ATOH8	atonal bHLH transcription factor 8	84913	0,118286652	0	4,080456	0	1	NA
ENSG00000168875	SOX14	SRY-box transcription factor 14	8403	0	NA	NA	NA	NA	NA
ENSG00000168878	SFTPB	surfactant protein B	6439	0,832228723	1,882766227	2,370494	0,794250493	0,427049619	NA
ENSG00000168890	TMEM150A	transmembrane protein 150A	129303	11,24876841	-1,470713303	0,773595	-1,901142007	0,057283415	NA
ENSG00000168903	BTNL3	butyrophilin like 3	10917	0	NA	NA	NA	NA	NA
ENSG00000168907	PLA2G4F	phospholipase A2 group IVF	255189	0	NA	NA	NA	NA	NA

ENSG00000168913	ENHO	energy homeostasis associated	375704	0	NA	NA	NA	NA	NA
ENSG00000168916	ZNF608	zinc finger protein 608	57507	10,65454996	-0,121593491	0,535084	-0,22724181	0,820235718	NA
ENSG00000168917	SLC35G2	solute carrier family 35 member G2	80723	0,283767938	-0,99056451	4,040728	-0,245145073	0,806344104	NA
ENSG00000168925	CTRB1	chymotrypsinogen B1	1504	0	NA	NA	NA	NA	NA
ENSG00000168928	CTRB2	chymotrypsinogen B2	440387	0	NA	NA	NA	NA	NA
ENSG00000168930	TRIM49	tripartite motif containing 49	57093	0	NA	NA	NA	NA	NA
ENSG00000168938	PPIC	peptidylprolyl isomerase C	5480	0	NA	NA	NA	NA	NA
ENSG00000168939	SPRY3	sprouty RTK signaling antagonist 3	10251	8,986902179	-0,392305287	0,618894	-0,633881419	0,526158213	NA
ENSG00000168952	STXBP6	syntaxin binding protein 6	29091	0	NA	NA	NA	NA	NA
ENSG00000168955	TM4SF20	transmembrane 4 L six family member 20	79853	0,119673877	0,847101401	4,080456	0,207599697	0,835541541	NA
ENSG00000168959	GRM5	glutamate metabotropic receptor 5	2915	0	NA	NA	NA	NA	NA
ENSG00000168967	NA	NA	NA	0	NA	NA	NA	NA	NA
ENSG00000168992	OR7E102P	olfactory receptor family 7 subfamily E member 102 pseudogene	400984	0	NA	NA	NA	NA	NA
ENSG00000168993	CPLX1	complexin 1	10815	0,207996306	-1,916073151	4,043021	-0,473921178	0,635556117	NA
ENSG00000168994	PXDC1	PX domain containing 1	221749	0	NA	NA	NA	NA	NA
ENSG00000169006	NTSR2	neurotensin receptor 2	23620	0	NA	NA	NA	NA	NA
ENSG00000169031	COL4A3	collagen type IV alpha 3 chain	1285	0,189779886	1,66568091	4,047506	0,411532707	0,680681963	NA
ENSG00000169035	KLK7	kallikrein related peptidase 7	5650	0	NA	NA	NA	NA	NA
ENSG00000169047	IRS1	insulin receptor substrate 1	3667	3,836133997	0,915146195	0,961374	0,951914839	0,341140173	NA
ENSG00000169059	VCX3A	variable charge X-linked 3A	51481	0	NA	NA	NA	NA	NA
ENSG00000169064	ZBBX	zinc finger B-box domain containing	79740	0	NA	NA	NA	NA	NA
ENSG00000169067	ACTBL2	actin beta like 2	345651	0	NA	NA	NA	NA	NA
ENSG00000169071	ROR2	receptor tyrosine kinase like orphan receptor 2	4920	0	NA	NA	NA	NA	NA
ENSG00000169075	NA	NA	NA	0	NA	NA	NA	NA	NA
ENSG00000169083	AR	androgen receptor	367	0	NA	NA	NA	NA	NA
ENSG00000169085	VXN	vexin	254778	1,830448605	-0,053310186	1,921978	-0,027737143	0,9778718	NA
ENSG00000169116	PARM1	prostate androgen-regulated mucin-like protein 1	25849	0	NA	NA	NA	NA	NA
ENSG00000169122	FAM110B	family with sequence similarity 110 member B	90362	0,223658091	0	4,080456	0	1	NA
ENSG00000169126	ODAD2	outer dynein arm docking complex subunit 2	55130	0	NA	NA	NA	NA	NA
ENSG00000169154	GOT1L1	glutamic-oxaloacetic transaminase 1 like 1	137362	0	NA	NA	NA	NA	NA
ENSG00000169164	XAGE-4	XAGE-4 protein	139629	0	NA	NA	NA	NA	NA
ENSG00000169169	CPT1C	carnitine palmitoyltransferase 1C	126129	0	NA	NA	NA	NA	NA
ENSG00000169174	PCSK9	proprotein convertase subtilisin/kexin type 9	255738	0	NA	NA	NA	NA	NA
ENSG00000169181	GSG1L	GSG1 like	146395	0	NA	NA	NA	NA	NA
ENSG00000169184	MN1	MN1 proto-oncogene, transcriptional regulator	4330	1,665153144	-1,043462632	2,090861	-0,499058751	0,617737996	NA
ENSG00000169194	IL13	interleukin 13	3596	0	NA	NA	NA	NA	NA
ENSG00000169208	OR10G3	olfactory receptor family 10 subfamily G member 3	26533	0	NA	NA	NA	NA	NA
ENSG00000169213	RAB3B	RAB3B, member RAS oncogene family	5865	0,072165449	0,847101401	4,080456	0,207599697	0,835541541	NA
ENSG00000169214	OR6F1	olfactory receptor family 6 subfamily F member 1	343169	0	NA	NA	NA	NA	NA
ENSG00000169218	RSPO1	R-spondin 1	284654	0	NA	NA	NA	NA	NA
ENSG00000169224	GCSAML	germinal center associated signaling and motility like	148823	0,236573303	0	4,080456	0	1	NA
ENSG00000169242	EFNA1	ephrin A1	1942	0	NA	NA	NA	NA	NA
ENSG00000169245	CXCL10	C-X-C motif chemokine ligand 10	3627	0	NA	NA	NA	NA	NA
ENSG00000169247	SH3TC2	SH3 domain and tetratricopeptide repeats 2	79628	0,10543123	0	4,080456	0	1	NA
ENSG00000169248	CXCL11	C-X-C motif chemokine ligand 11	6373	0	NA	NA	NA	NA	NA
ENSG00000169253	NA	NA	NA	0	NA	NA	NA	NA	NA
ENSG00000169271	HSPB3	heat shock protein family B (small) member 3	8988	0	NA	NA	NA	NA	NA
ENSG00000169282	KCNAB1	potassium voltage-gated channel subfamily A regulatory beta subunit 1	7881	0	NA	NA	NA	NA	NA
ENSG00000169291	SHE	Src homology 2 domain containing E	126669	0	NA	NA	NA	NA	NA
ENSG00000169297	NR0B1	nuclear receptor subfamily 0 group B member 1	190	0	NA	NA	NA	NA	NA
ENSG00000169302	STK32A	serine/threonine kinase 32A	202374	0	NA	NA	NA	NA	NA

ENSG00000169306	IL1RAPL1	interleukin 1 receptor accessory protein like 1	11141	0	NA	NA	NA	NA	NA
ENSG00000169313	P2RY12	purinergic receptor P2Y12	64805	0	NA	NA	NA	NA	NA
ENSG00000169314	C22orf15	chromosome 22 open reading frame 15	150248	0	NA	NA	NA	NA	NA
ENSG00000169325	GUSBP12	GUSB pseudogene 12	642007	0	NA	NA	NA	NA	NA
ENSG00000169327	OR5AU1	olfactory receptor family 5 subfamily AU member 1	390445	0	NA	NA	NA	NA	NA
ENSG00000169330	MINAR1	membrane integral NOTCH2 associated receptor 1	23251	4,503342836	-4,244212338	3,934188	-1,078802531	0,280675766	NA
ENSG00000169340	PDILT	protein disulfide isomerase like, testis expressed	204474	0	NA	NA	NA	NA	NA
ENSG00000169344	UMOD	uromodulin	7369	0	NA	NA	NA	NA	NA
ENSG00000169347	GP2	glycoprotein 2	2813	0	NA	NA	NA	NA	NA
ENSG00000169385	RNASE2	ribonuclease A family member 2	6036	0	NA	NA	NA	NA	NA
ENSG00000169393	ELSPBP1	epididymal sperm binding protein 1	64100	0	NA	NA	NA	NA	NA
ENSG00000169397	RNASE3	ribonuclease A family member 3	6037	0	NA	NA	NA	NA	NA
ENSG00000169398	PTK2	protein tyrosine kinase 2	5747	0,657857685	3,458926198	3,617841	0,956074605	0,339034544	NA
ENSG00000169402	RSPH10B2	radial spoke head 10 homolog B2	728194	0,11808024	-1,076443944	4,080456	-0,263804824	0,791930317	NA
ENSG00000169403	PTAFR	platelet activating factor receptor	5724	5,275705308	0,515520639	0,915482	0,563114185	0,573357133	NA
ENSG00000169413	RNASE6	ribonuclease A family member k6	6039	0,673820085	0,987518911	2,754922	0,358456276	0,720001884	NA
ENSG00000169418	NPR1	natriuretic peptide receptor 1	4881	0	NA	NA	NA	NA	NA
ENSG00000169427	KCNK9	potassium two pore domain channel subfamily K member 9	51305	0,103998153	-1,076443944	4,080456	-0,263804824	0,791930317	NA
ENSG00000169429	CXCL8	C-X-C motif chemokine ligand 8	3576	0	NA	NA	NA	NA	NA
ENSG00000169432	SCN9A	sodium voltage-gated channel alpha subunit 9	6335	0,577733087	0,49757672	3,376895	0,147347427	0,8828578	NA
ENSG00000169435	RASSF6	Ras association domain family member 6	166824	0	NA	NA	NA	NA	NA
ENSG00000169436	COL22A1	collagen type XXII alpha 1 chain	169044	0	NA	NA	NA	NA	NA
ENSG00000169439	SDC2	syndecan 2	6383	0	NA	NA	NA	NA	NA
ENSG00000169469	SPRR1B	small proline rich protein 1B	6699	0	NA	NA	NA	NA	NA
ENSG00000169474	SPRR1A	small proline rich protein 1A	6698	0	NA	NA	NA	NA	NA
ENSG00000169495	HTRA4	HtrA serine peptidase 4	203100	0,85810612	-0,045673935	2,397783	-0,019048401	0,984802494	NA
ENSG00000169507	SLC38A11	solute carrier family 38 member 11	151258	0,31672576	0	4,080456	0	1	NA
ENSG00000169508	GPR183	G protein-coupled receptor 183	1880	1,214627258	1,749147747	2,34602	0,745580954	0,455920592	NA
ENSG00000169509	CRCT1	cysteine rich C-terminal 1	54544	0	NA	NA	NA	NA	NA
ENSG00000169515	CCDC8	coiled-coil domain containing 8	83987	0	NA	NA	NA	NA	NA
ENSG00000169548	ZNF280A	zinc finger protein 280A	129025	0	NA	NA	NA	NA	NA
ENSG00000169550	MUC15	mucin 15, cell surface associated	143662	0,236573303	0	4,080456	0	1	NA
ENSG00000169551	CT55	cancer/testis antigen 55	54967	0	NA	NA	NA	NA	NA
ENSG00000169562	GJB1	gap junction protein beta 1	2705	0,337415223	0,59413108	4,080456	0,145604094	0,884233936	NA
ENSG00000169575	VPREB1	V-set pre-B cell surrogate light chain 1	7441	0	NA	NA	NA	NA	NA
ENSG00000169594	BNC1	basonuclin zinc finger protein 1	646	0	NA	NA	NA	NA	NA
ENSG00000169604	ANTXR1	ANTXR cell adhesion molecule 1	84168	0	NA	NA	NA	NA	NA
ENSG00000169605	GKN1	gastrokine 1	56287	0	NA	NA	NA	NA	NA
ENSG00000169618	PROKR1	prokineticin receptor 1	10887	2,396249097	0,151715252	1,193291	0,1271402	0,898829434	NA
ENSG00000169662	NA	NA	NA	0	NA	NA	NA	NA	NA
ENSG00000169668	NA	NA	NA	0	NA	NA	NA	NA	NA
ENSG00000169676	DRD5	dopamine receptor D5	1816	0	NA	NA	NA	NA	NA
ENSG00000169688	MT1B	metallothionein 1B	4490	0	NA	NA	NA	NA	NA
ENSG00000169704	GP9	glycoprotein IX platelet	2815	0	NA	NA	NA	NA	NA
ENSG00000169715	MT1E	metallothionein 1E	4493	5,71487599	-0,071146731	0,824996	-0,086238924	0,931276489	NA
ENSG00000169717	ACTRT2	actin related protein T2	140625	0	NA	NA	NA	NA	NA
ENSG00000169750	RAC3	Rac family small GTPase 3	5881	1,878318022	1,108516901	1,894926	0,584992188	0,55855297	NA
ENSG00000169752	NRG4	neuregulin 4	145957	0	NA	NA	NA	NA	NA
ENSG00000169758	TMEM266	transmembrane protein 266	123591	0	NA	NA	NA	NA	NA
ENSG00000169760	NLGN1	neuroligin 1	22871	0	NA	NA	NA	NA	NA
ENSG00000169777	TAS2R1	taste 2 receptor member 1	50834	0	NA	NA	NA	NA	NA
ENSG00000169783	LINGO1	leucine rich repeat and Ig domain containing 1	84894	0	NA	NA	NA	NA	NA
ENSG00000169789	PRY	PTPN13 like Y-linked	9081	0	NA	NA	NA	NA	NA

ENSG00000169800	RBMY1F	RNA binding motif protein Y-linked family 1 member F	159163	0	NA	NA	NA	NA	NA
ENSG00000169807	PRY2	PTPN13 like Y-linked 2	442862	0	NA	NA	NA	NA	NA
ENSG00000169811	RBMY1HP	RNA binding motif protein Y-linked family 1 member H, pseudogene	5944	0	NA	NA	NA	NA	NA
ENSG00000169836	TACR3	tachykinin receptor 3	6870	0	NA	NA	NA	NA	NA
ENSG00000169840	GSX1	GS homeobox 1	219409	0	NA	NA	NA	NA	NA
ENSG00000169849	TSPY14P	testis specific protein Y-linked 14, pseudogene	378013	0	NA	NA	NA	NA	NA
ENSG00000169851	PCDH7	protocadherin 7	5099	0	NA	NA	NA	NA	NA
ENSG00000169855	ROBO1	roundabout guidance receptor 1	6091	0,195138267	0,788783202	4,078032	0,193422528	0,846628074	NA
ENSG00000169856	ONECUT1	one cut homeobox 1	3175	0	NA	NA	NA	NA	NA
ENSG00000169862	CTNND2	catenin delta 2	1501	0	NA	NA	NA	NA	NA
ENSG00000169876	MUC17	mucin 17, cell surface associated	140453	0	NA	NA	NA	NA	NA
ENSG00000169877	AHSP	alpha hemoglobin stabilizing protein	51327	0	NA	NA	NA	NA	NA
ENSG00000169884	WNT10B	Wnt family member 10B	7480	3,626527158	-0,468139575	1,117996	-0,41873081	0,675412876	NA
ENSG00000169885	CALML6	calmodulin like 6	163688	1,408045442	1,024059262	2,477549	0,413335542	0,679360793	NA
ENSG00000169891	REPS2	RALBP1 associated Eps domain containing 2	9185	0,342936577	1,967039308	3,761118	0,522993244	0,600978955	NA
ENSG00000169894	MUC3A	mucin 3A, cell surface associated	4584	0	NA	NA	NA	NA	NA
ENSG00000169900	PYDC1	pyrin domain containing 1	260434	0	NA	NA	NA	NA	NA
ENSG00000169902	TPST1	tyrosylprotein sulfotransferase 1	8460	1,113837808	0,434342254	2,117877	0,20508377	0,837506677	NA
ENSG00000169903	TM4SF4	transmembrane 4 L six family member 4	7104	0	NA	NA	NA	NA	NA
ENSG00000169906	S100G	S100 calcium binding protein G	795	0	NA	NA	NA	NA	NA
ENSG00000169908	TM4SF1	transmembrane 4 L six family member 1	4071	0	NA	NA	NA	NA	NA
ENSG00000169933	FRMPD4	FERM and PDZ domain containing 4	9758	0	NA	NA	NA	NA	NA
ENSG00000169946	ZFPM2	zinc finger protein, FOG family member 2	23414	0	NA	NA	NA	NA	NA
ENSG00000169953	HSFY2	heat shock transcription factor Y-linked 2	159119	0	NA	NA	NA	NA	NA
ENSG00000169962	TAS1R3	taste 1 receptor member 3	83756	8,984239302	0,163129024	0,624575	0,261183946	0,793950658	NA
ENSG00000169989	TIGD4	tigger transposable element derived 4	201798	1,138763572	0,390184133	1,579514	0,247028019	0,80488654	NA
ENSG00000169994	MYO7B	myosin VIIB	4648	0	NA	NA	NA	NA	NA
ENSG00000170011	MYRIP	myosin VIIA and Rab interacting protein	25924	0	NA	NA	NA	NA	NA
ENSG00000170044	ZPLD1	zona pellucida like domain containing 1	131368	0,11808024	-1,076443944	4,080456	-0,263804824	0,791930317	NA
ENSG00000170054	SERPINA9	serpin family A member 9	327657	0	NA	NA	NA	NA	NA
ENSG00000170074	FAM153A	family with sequence similarity 153 member A	285596	0	NA	NA	NA	NA	NA
ENSG00000170075	GPR37L1	G protein-coupled receptor 37 like 1	9283	0	NA	NA	NA	NA	NA
ENSG00000170091	NSG2	neuronal vesicle trafficking associated 2	51617	0	NA	NA	NA	NA	NA
ENSG00000170092	SPDYE5	speedy/RINGO cell cycle regulator family member E5	442590	0	NA	NA	NA	NA	NA
ENSG00000170099	SERPINA6	serpin family A member 6	866	0	NA	NA	NA	NA	NA
ENSG00000170122	FOXD4	forkhead box D4	2298	0,693267171	2,813816965	2,703307	1,04087951	0,297931473	NA
ENSG00000170128	GPR25	G protein-coupled receptor 25	2848	0	NA	NA	NA	NA	NA
ENSG00000170153	RNF150	ring finger protein 150	57484	0	NA	NA	NA	NA	NA
ENSG00000170162	VGLL2	vestigial like family member 2	245806	0	NA	NA	NA	NA	NA
ENSG00000170166	HOXD4	homeobox D4	3233	0	NA	NA	NA	NA	NA
ENSG00000170178	HOXD12	homeobox D12	3238	0	NA	NA	NA	NA	NA
ENSG00000170180	GYPA	glycophorin A (MNS blood group)	2993	0	NA	NA	NA	NA	NA
ENSG00000170209	ANKK1	ankyrin repeat and kinase domain containing 1	255239	0,118286652	0	4,080456	0	1	NA
ENSG00000170214	ADRA1B	adrenoceptor alpha 1B	147	0	NA	NA	NA	NA	NA
ENSG00000170222	ADPRM	ADP-ribose/CDP-alcohol diphosphatase, manganese dependent	56985	7,628031672	-0,249839821	1,260059	-0,198276273	0,842828914	NA
ENSG00000170231	FABP6	fatty acid binding protein 6	2172	0	NA	NA	NA	NA	NA
ENSG00000170236	USP50	ubiquitin specific peptidase 50	373509	0	NA	NA	NA	NA	NA
ENSG00000170255	MRGPRX1	MAS related GPR family member X1	259249	0	NA	NA	NA	NA	NA

ENSG00000170255	LOC124906949	mas-related G-protein coupled receptor member X1	124906949	0	NA	NA	NA	NA	NA
ENSG00000170262	MRAP	melanocortin 2 receptor accessory protein	56246	0	NA	NA	NA	NA	NA
ENSG00000170264	FAM161A	FAM161 centrosomal protein A	84140	4,354962729	0,638748622	1,784922	0,357858064	0,720449535	NA
ENSG00000170271	FAXDC2	fatty acid hydroxylase domain containing 2	10826	0,795102569	2,285899023	2,190797	1,043409884	0,296758498	NA
ENSG00000170276	HSPB2	heat shock protein family B (small) member 2	3316	0	NA	NA	NA	NA	NA
ENSG00000170279	C7orf33	chromosome 7 open reading frame 33	202865	0	NA	NA	NA	NA	NA
ENSG00000170289	CNGB3	cyclic nucleotide gated channel subunit beta 3	54714	0	NA	NA	NA	NA	NA
ENSG00000170290	SLN	sarcolipin	6588	0	NA	NA	NA	NA	NA
ENSG00000170291	ELP5	elongator acetyltransferase complex subunit 5	23587	7,994522723	5,295177963	1,725124	3,069448297	0,002144545	NA
ENSG00000170293	CMTM8	CKLF like MARVEL transmembrane domain containing 8	152189	0	NA	NA	NA	NA	NA
ENSG00000170298	LGALS9B	galectin 9B	284194	10,08314588	-0,322837256	0,624141	-0,517250114	0,604981573	NA
ENSG00000170323	FABP4	fatty acid binding protein 4	2167	0	NA	NA	NA	NA	NA
ENSG00000170324	FRMPD2	FERM and PDZ domain containing 2	143162	0	NA	NA	NA	NA	NA
ENSG00000170356	OR2A20P	olfactory receptor family 2 subfamily A member 20 pseudogene	401428	0	NA	NA	NA	NA	NA
ENSG00000170364	SETMAR	SET domain and mariner transposase fusion gene	6419	11,52847243	0,074192814	0,703972	0,105391688	0,916065011	NA
ENSG00000170365	SMAD1	SMAD family member 1	4086	0,598100948	-1,871480036	3,361259	-0,556779466	0,577678115	NA
ENSG00000170367	CST5	cystatin D	1473	0	NA	NA	NA	NA	NA
ENSG00000170369	CST2	cystatin SA	1470	0	NA	NA	NA	NA	NA
ENSG00000170370	EMX2	empty spiracles homeobox 2	2018	0,262646074	-1,073961775	4,080456	-0,263196517	0,792399115	NA
ENSG00000170373	CST1	cystatin SN	1469	0	NA	NA	NA	NA	NA
ENSG00000170381	SEMA3E	semaphorin 3E	9723	0	NA	NA	NA	NA	NA
ENSG00000170382	LRRN2	leucine rich repeat neuronal 2	10446	0,48687302	-1,600669523	3,354447	-0,477178342	0,633235132	NA
ENSG00000170390	DCLK2	doublecortin like kinase 2	166614	0	NA	NA	NA	NA	NA
ENSG00000170396	ZNF804A	zinc finger protein 804A	91752	0	NA	NA	NA	NA	NA
ENSG00000170409	NA	NA	NA	0	NA	NA	NA	NA	NA
ENSG00000170417	TMEM182	transmembrane protein 182	130827	6,463598344	0,496985069	1,115695	0,445449037	0,655995281	NA
ENSG00000170419	VSTM2A	V-set and transmembrane domain containing 2A	222008	0	NA	NA	NA	NA	NA
ENSG00000170421	KRT8	keratin 8	3856	0,190559127	0,845982752	4,080456	0,207325549	0,835755623	NA
ENSG00000170423	KRT78	keratin 78	196374	0	NA	NA	NA	NA	NA
ENSG00000170425	ADORA2B	adenosine A2b receptor	136	0,072165449	0,847101401	4,080456	0,207599697	0,835541541	NA
ENSG00000170426	SDR9C7	short chain dehydrogenase/reductase family 9C member 7	121214	0	NA	NA	NA	NA	NA
ENSG00000170439	TMT1B	thiol methyltransferase 1B	196410	0,555839688	-2,016529796	3,2531	-0,619879471	0,535337142	NA
ENSG00000170454	KRT75	keratin 75	9119	0	NA	NA	NA	NA	NA
ENSG00000170456	DENND5B	DENN domain containing 5B	160518	0,345720691	-2,620552662	3,655323	-0,716914094	0,473427105	NA
ENSG00000170458	CD14	CD14 molecule	929	0,328243497	-1,995333859	3,78423	-0,527276005	0,598001935	NA
ENSG00000170465	KRT6C	keratin 6C	286887	0	NA	NA	NA	NA	NA
ENSG00000170469	SPATA24	spermatogenesis associated 24	202051	3,694939386	0,086661333	1,542668	0,056176252	0,955201399	NA
ENSG00000170477	KRT4	keratin 4	3851	0	NA	NA	NA	NA	NA
ENSG00000170482	SLC23A1	solute carrier family 23 member 1	9963	2,082701971	-0,530005849	1,490661	-0,355550782	0,722177008	NA
ENSG00000170484	KRT74	keratin 74	121391	0	NA	NA	NA	NA	NA
ENSG00000170485	NPAS2	neuronal PAS domain protein 2	4862	0	NA	NA	NA	NA	NA
ENSG00000170486	KRT72	keratin 72	140807	0,208888447	-1,921407277	4,042529	-0,475298321	0,634574356	NA
ENSG00000170498	KISS1	KiSS-1 metastasis suppressor	3814	0	NA	NA	NA	NA	NA
ENSG00000170500	LONRF2	LON peptidase N-terminal domain and ring finger 2	164832	0	NA	NA	NA	NA	NA
ENSG00000170509	HSD17B13	hydroxysteroid 17-beta dehydrogenase 13	345275	0,94959503	-0,043215829	2,026604	-0,021324257	0,982986994	NA
ENSG00000170516	COX7B2	cytochrome c oxidase subunit 7B2	170712	0	NA	NA	NA	NA	NA
ENSG00000170523	KRT83	keratin 83	3889	0,974836826	0,97688276	2,014248	0,484986452	0,627685956	NA
ENSG00000170537	TMC7	transmembrane channel like 7	79905	0	NA	NA	NA	NA	NA
ENSG00000170545	SMAGP	small cell adhesion glycoprotein	57228	2,870394796	0,967565381	1,588736	0,609015768	0,542513988	NA
ENSG00000170549	IRX1	iroquois homeobox 1	79192	0	NA	NA	NA	NA	NA
ENSG00000170558	CDH2	cadherin 2	1000	8,582495595	0,516913165	1,174845	0,439984242	0,65994852	NA
ENSG00000170561	IRX2	iroquois homeobox 2	153572	0	NA	NA	NA	NA	NA
ENSG00000170577	SIX2	SIX homeobox 2	10736	0	NA	NA	NA	NA	NA
ENSG00000170579	DLGAP1	DLG associated protein 1	9229	0	NA	NA	NA	NA	NA

ENSG00000170605	OR9K2	olfactory receptor family 9 subfamily K member 2	441639	0	NA	NA	NA	NA	NA
ENSG00000170608	FOXA3	forkhead box A3	3171	0	NA	NA	NA	NA	NA
ENSG00000170613	GARIN3	golgi associated RAB2B interactor family member 3	153745	0	NA	NA	NA	NA	NA
ENSG00000170615	SLC26A5	solute carrier family 26 member 5	375611	0,118286652	0	4,080456	0	1	NA
ENSG00000170624	SGCD	sarcoglycan delta	6444	0,57269538	-1,393146705	3,449314	-0,403890926	0,686292929	NA
ENSG00000170627	GTSF1	gametocyte specific factor 1	121355	6,378227754	1,300176454	0,745208	1,744715044	0,081034483	NA
ENSG00000170629	NA	NA	NA	1,596245969	2,246771361	1,630761	1,377743705	0,168282432	NA
ENSG00000170667	RASA4	RAS p21 protein activator 4	10156	8,974473444	-0,476498929	1,119896	-0,425485151	0,670483044	NA
ENSG00000170667	RASA4B	RAS p21 protein activator 4B	100271927	8,974473444	-0,476498929	1,119896	-0,425485151	0,670483044	NA
ENSG00000170681	CAVIN4	caveolae associated protein 4	347273	2,870129671	0,399626677	1,081349	0,369563181	0,711707989	NA
ENSG00000170689	HOXB9	homeobox B9	3219	1,178339068	-2,34862386	2,185279	-1,074747659	0,28248772	NA
ENSG00000170703	TTLL6	tubulin tyrosine ligase like 6	284076	0	NA	NA	NA	NA	NA
ENSG00000170743	SYT9	synaptotagmin 9	143425	0	NA	NA	NA	NA	NA
ENSG00000170745	KCNS3	potassium voltage-gated channel modifier subfamily S member 3	3790	0	NA	NA	NA	NA	NA
ENSG00000170748	RBMXL2	RBMX like 2	27288	0	NA	NA	NA	NA	NA
ENSG00000170775	GPR37	G protein-coupled receptor 37	2861	0	NA	NA	NA	NA	NA
ENSG00000170777	TPD52L3	TPD52 like 3	89882	0	NA	NA	NA	NA	NA
ENSG00000170782	OR10A4	olfactory receptor family 10 subfamily A member 4	283297	0	NA	NA	NA	NA	NA
ENSG00000170786	SDR16C5	short chain dehydrogenase/reductase family 16C member 5	195814	0	NA	NA	NA	NA	NA
ENSG00000170788	DYDC1	DPY30 domain containing 1	143241	0	NA	NA	NA	NA	NA
ENSG00000170790	OR10A2	olfactory receptor family 10 subfamily A member 2	341276	0	NA	NA	NA	NA	NA
ENSG00000170801	HTRA3	HtrA serine peptidase 3	94031	0	NA	NA	NA	NA	NA
ENSG00000170807	LMOD2	leiomodin 2	442721	0	NA	NA	NA	NA	NA
ENSG00000170819	BFSP2	beaded filament structural protein 2	8419	0	NA	NA	NA	NA	NA
ENSG00000170820	FSHR	follicle stimulating hormone receptor	2492	0	NA	NA	NA	NA	NA
ENSG00000170827	CELP	carboxyl ester lipase pseudogene	1057	0	NA	NA	NA	NA	NA
ENSG00000170835	CEL	carboxyl ester lipase	1056	4,432114457	0,553607748	0,874076	0,633363213	0,526496482	NA
ENSG00000170837	GPR27	G protein-coupled receptor 27	2850	7,995872022	-0,809121553	0,740755	-1,092293646	0,274704052	NA
ENSG00000170848	PSG6	pregnancy specific beta-1-glycoprotein 6	5675	0	NA	NA	NA	NA	NA
ENSG00000170889	RPS9	ribosomal protein S9	6203	70,42806189	0	4,080456	0	NA	NA
ENSG00000170890	PLA2G1B	phospholipase A2 group IB	5319	0	NA	NA	NA	NA	NA
ENSG00000170891	CYTL1	cytokine like 1	54360	2,134072508	-0,847733075	1,267482	-0,668832573	0,503602286	NA
ENSG00000170892	TSEN34	tRNA splicing endonuclease subunit 34	79042	1,7979131	-3,840116875	2,217518	-1,731718166	0,083323755	NA
ENSG00000170893	TRH	thyrotropin releasing hormone	7200	0	NA	NA	NA	NA	NA
ENSG00000170906	NDUFA3	NADH:ubiquinone oxidoreductase subunit A3	4696	6,125910763	-0,981017348	1,094671	-0,896175624	0,370158972	NA
ENSG00000170909	OSCAR	osteoclast associated Ig-like receptor	126014	0,386471413	-1,494444177	3,641206	-0,410425639	0,681493742	NA
ENSG00000170920	OR7G3	olfactory receptor family 7 subfamily G member 3	390883	0	NA	NA	NA	NA	NA
ENSG00000170921	TANC2	tetratricopeptide repeat, ankyrin repeat and coiled-coil containing 2	26115	3,153288801	1,180533887	1,111991	1,061639652	0,288399306	NA
ENSG00000170923	OR7G2	olfactory receptor family 7 subfamily G member 2	390882	0	NA	NA	NA	NA	NA
ENSG00000170925	TEX13B	testis expressed 13B	56156	0	NA	NA	NA	NA	NA
ENSG00000170927	PKHD1	PKHD1 ciliary IPT domain containing fibrocystin/polyductin	5314	0	NA	NA	NA	NA	NA
ENSG00000170929	OR1M1	olfactory receptor family 1 subfamily M member 1	125963	0	NA	NA	NA	NA	NA
ENSG00000170935	NCBP2L	nuclear cap binding protein subunit 2 like	392517	0	NA	NA	NA	NA	NA
ENSG00000170950	PGK2	phosphoglycerate kinase 2	5232	0	NA	NA	NA	NA	NA
ENSG00000170953	OR8B12	olfactory receptor family 8 subfamily B member 12	219858	0	NA	NA	NA	NA	NA
ENSG00000170954	ZNF415	zinc finger protein 415	55786	0	NA	NA	NA	NA	NA
ENSG00000170955	CAVIN3	caveolae associated protein 3	112464	0	NA	NA	NA	NA	NA
ENSG00000170956	CEACAM3	CEA cell adhesion molecule 3	1084	0	NA	NA	NA	NA	NA
ENSG00000170959	DCDC1	doublecortin domain containing 1	341019	0	NA	NA	NA	NA	NA

ENSG00000170961	HAS2	hyaluronan synthase 2	3037	0	NA	NA	NA	NA	NA	
ENSG00000170965	PLAC1	placenta enriched 1	10761	0	NA	NA	NA	NA	NA	
ENSG00000170967	DDI1	DNA damage inducible 1 homolog 1	414301	0	NA	NA	NA	NA	NA	
ENSG00000171014	OR4D5	olfactory receptor family 4 subfamily D member 5	219875	0	NA	NA	NA	NA	NA	
ENSG00000171016	PYGO1	pygopus family PHD finger 1	26108	0,355656668	1,980900908	3,722302	0,532170937	0,59460761	NA	
ENSG00000171017	LRRC8E	leucine rich repeat containing 8 VRAC subunit E	80131	0	NA	NA	NA	NA	NA	
ENSG00000171049	FPR2	formyl peptide receptor 2	2358	0	NA	NA	NA	NA	NA	
ENSG00000171051	FPR1	formyl peptide receptor 1	2357	0	NA	NA	NA	NA	NA	
ENSG00000171053	PATE1	prostate and testis expressed 1	160065	0	NA	NA	NA	NA	NA	
ENSG00000171054	OR13H1	olfactory receptor family 13 subfamily H member 1	347468	0	NA	NA	NA	NA	NA	
ENSG00000171056	SOX7	SRY-box transcription factor 7	83595	0,791814399	0	4,080456	0	1	NA	
ENSG00000171060	C8orf74	chromosome 8 open reading frame 74	203076	0	NA	NA	NA	NA	NA	
ENSG00000171084	NA	NA	NA	0	NA	NA	NA	NA	NA	
ENSG00000171094	ALK	ALK receptor tyrosine kinase	238	0	NA	NA	NA	NA	NA	
ENSG00000171102	OBP2B	odorant binding protein 2B	29989	0	NA	NA	NA	NA	NA	
ENSG00000171105	INSR	insulin receptor	3643	6,050002387	0,168996517	0,798856	0,211548186	0,832459536	NA	
ENSG00000171116	HSFX1	heat shock transcription factor family, X-linked 1	100506164	5,728001139	-0,755254825	0,888551	-0,849984466	0,395333723	NA	
ENSG00000171119	NRTN	neurturin	4902	0	NA	NA	NA	NA	NA	
ENSG00000171121	KCNMB3	potassium calcium-activated channel subfamily M regulatory beta subunit 3	27094	6,918741951	0,812936758	1,010254	0,804685629	0,421001115	NA	
ENSG00000171124	FUT3	fucosyltransferase 3 (Lewis blood group)	2525	0	NA	NA	NA	NA	NA	
ENSG00000171126	KCNG3	potassium voltage-gated channel modifier subfamily G member 3	170850	0	NA	NA	NA	NA	NA	
ENSG00000171132	PRKCE	protein kinase C epsilon	5581	10,65030699	-0,817639166	0,649451	-1,258969393	0,208041387	NA	
ENSG00000171133	OR2K2	olfactory receptor family 2 subfamily K member 2	26248	0,072165449	0,847101401	4,080456	0,207599697	0,835541541	NA	
ENSG00000171160	MORN4	MORN repeat containing 4	118812	0	NA	NA	NA	NA	NA	
ENSG00000171174	RBKS	ribokinase	64080	3,5925194	-0,217887208	1,730223	-0,125930131	0,899787231	NA	
ENSG00000171180	OR2M4	olfactory receptor family 2 subfamily M member 4	26245	0	NA	NA	NA	NA	NA	
ENSG00000171189	GRIK1	glutamate ionotropic receptor kainate type subunit 1	2897	0	NA	NA	NA	NA	NA	
ENSG00000171195	MUC7	mucin 7, secreted	4589	0	NA	NA	NA	NA	NA	
ENSG00000171199	OPRPN	opiorphin prepropeptide	58503	0	NA	NA	NA	NA	NA	
ENSG00000171201	SMR3B	submaxillary gland androgen regulated protein 3B	10879	0	NA	NA	NA	NA	NA	
ENSG00000171209	CSN3	casein kappa	1448	0	NA	NA	NA	NA	NA	
ENSG00000171217	CLDN20	claudin 20	49861	0,119673877	0,847101401	4,080456	0,207599697	0,835541541	NA	
ENSG00000171224	FAM241B	family with sequence similarity 241 member B	219738	2,956743588	-1,677044458	1,180143	-1,421052189	0,155301588	NA	
ENSG00000171227	TMEM37	transmembrane protein 37	140738	0	NA	NA	NA	NA	NA	
ENSG00000171234	UGT2B7	UDP glucuronosyltransferase family 2 member B7	7364	0	NA	NA	NA	NA	NA	
ENSG00000171236	LRG1	leucine rich alpha-2-glycoprotein 1	116844	3,018093688	-0,327884702	1,113056	-0,294580568	0,768314308	NA	
ENSG00000171243	SOSTDC1	sclerostin domain containing 1	25928	0	NA	NA	NA	NA	NA	
ENSG00000171246	NPTX1	neuronal pentraxin 1	4884	0,80185512	-3,406950926	3,652739	-0,932711351	0,350969023	NA	
ENSG00000171291	ZNF439	zinc finger protein 439	90594	1,3256859	0,467150143	1,728747	0,270224702	0,78698739	NA	
ENSG00000171303	KCNK3	potassium two pore domain channel subfamily K member 3	3777	0	NA	NA	NA	NA	NA	
ENSG00000171345	KRT19	keratin 19	3880	0	NA	NA	NA	NA	NA	
ENSG00000171346	KRT15	keratin 15	3866	0	NA	NA	NA	NA	NA	
ENSG00000171357	LURAP1	leucine rich adaptor protein 1	541468	0	NA	NA	NA	NA	NA	
ENSG00000171360	KRT38	keratin 38	8687	0	NA	NA	NA	NA	NA	
ENSG00000171368	TPPP	tubulin polymerization promoting protein	11076	1,223146806	1,093963375	2,019731	0,541638171	0,58806779	NA	
ENSG00000171385	KCND3	potassium voltage-gated channel subfamily D member 3	3752	0	NA	NA	NA	NA	NA	
ENSG00000171388	APLN	apelin	8862	0,792835074	0,995974963	2,24983	0,442688921	0,657990757	NA	
ENSG00000171401	KRT13	keratin 13	3860	0	NA	NA	NA	NA	NA	
ENSG00000171402	XAGE3	X antigen family member 3	170626	0	NA	NA	NA	NA	NA	
ENSG00000171403	KRT9	keratin 9	3857	0,119673877	0,847101401	4,080456	0,207599697	0,835541541	NA	
ENSG00000171405	XAGE5	X antigen family member 5	170627	0	NA	NA	NA	NA	NA	
ENSG00000171408	PDE7B	phosphodiesterase 7B	27115	9,561302689	-0,757334453	0,607052	-1,247560158	0,212192177	NA	
ENSG00000171431	KRT20	keratin 20	54474	0	NA	NA	NA	NA	NA	

ENSG00000171433	GLOD5	glyoxalase domain containing 5	392465	0	NA	NA	NA	NA	NA
ENSG00000171435	KSR2	kinase suppressor of ras 2	283455	0	NA	NA	NA	NA	NA
ENSG00000171446	KRT27	keratin 27	342574	0	NA	NA	NA	NA	NA
ENSG00000171450	CDK5R2	cyclin dependent kinase 5 regulatory subunit 2	8941	0	NA	NA	NA	NA	NA
ENSG00000171451	DSEL	dermatan sulfate epimerase like	92126	0,499293832	0,211728856	3,347211	0,063255303	0,949563208	NA
ENSG00000171459	OR1L6	olfactory receptor family 1 subfamily L member 6	392390	0	NA	NA	NA	NA	NA
ENSG00000171462	DLK2	delta like non-canonical Notch ligand 2	65989	0,10543123	0	4,080456	0	1	NA
ENSG00000171478	SPACA5B	sperm acrosome associated 5B	729201	0,119673877	0,847101401	4,080456	0,207599697	0,835541541	NA
ENSG00000171481	OR1L3	olfactory receptor family 1 subfamily L member 3	26735	0	NA	NA	NA	NA	NA
ENSG00000171483	NA	NA	NA	0	NA	NA	NA	NA	NA
ENSG00000171487	NLRP5	NLR family pyrin domain containing 5	126206	0	NA	NA	NA	NA	NA
ENSG00000171489	SPACA5	sperm acrosome associated 5	389852	0	NA	NA	NA	NA	NA
ENSG00000171495	MROH2B	maestro heat like repeat family member 2B	133558	0	NA	NA	NA	NA	NA
ENSG00000171496	OR1L8	olfactory receptor family 1 subfamily L member 8	138881	0,15836288	0	4,080456	0	1	NA
ENSG00000171501	OR1N2	olfactory receptor family 1 subfamily N member 2	138882	0	NA	NA	NA	NA	NA
ENSG00000171505	OR1N1	olfactory receptor family 1 subfamily N member 1	138883	0	NA	NA	NA	NA	NA
ENSG00000171509	RXFP1	relaxin family peptide receptor 1	59350	0	NA	NA	NA	NA	NA
ENSG00000171532	NEUROD2	neuronal differentiation 2	4761	0	NA	NA	NA	NA	NA
ENSG00000171533	MAP6	microtubule associated protein 6	4135	0	NA	NA	NA	NA	NA
ENSG00000171540	OTP	orthopedia homeobox	23440	0	NA	NA	NA	NA	NA
ENSG00000171551	ECEL1	endothelin converting enzyme like 1	9427	0	NA	NA	NA	NA	NA
ENSG00000171557	FGG	fibrinogen gamma chain	2266	0	NA	NA	NA	NA	NA
ENSG00000171560	FGA	fibrinogen alpha chain	2243	0	NA	NA	NA	NA	NA
ENSG00000171561	OR2AT4	olfactory receptor family 2 subfamily AT member 4	341152	0	NA	NA	NA	NA	NA
ENSG00000171564	FGB	fibrinogen beta chain	2244	0	NA	NA	NA	NA	NA
ENSG00000171570	NA	NA	NA	0	NA	NA	NA	NA	NA
ENSG00000171587	DSCAM	DS cell adhesion molecule	1826	0	NA	NA	NA	NA	NA
ENSG00000171595	DNAI2	dynein axonemal intermediate chain 2	64446	0,11808024	-1,076443944	4,080456	-0,263804824	0,791930317	NA
ENSG00000171611	PTCRA	pre T cell antigen receptor alpha	171558	0	NA	NA	NA	NA	NA
ENSG00000171631	P2RY6	pyrimidinergic receptor P2Y6	5031	0,117497108	0,847101401	4,080456	0,207599697	0,835541541	NA
ENSG00000171643	S100Z	S100 calcium binding protein Z	170591	0,539861465	-0,429191375	3,374589	-0,127183299	0,898795323	NA
ENSG00000171658	NA	NA	NA	0	NA	NA	NA	NA	NA
ENSG00000171680	PLEKHG5	pleckstrin homology and RhoGEF domain containing G5	57449	0,935150975	0,819728458	2,27062	0,361015275	0,718088029	NA
ENSG00000171695	LKAAEAR1	LKAAEAR motif containing 1	198437	0	NA	NA	NA	NA	NA
ENSG00000171714	ANO5	anoctamin 5	203859	0,117497108	0,847101401	4,080456	0,207599697	0,835541541	NA
ENSG00000171714	LOC102723370	uncharacterized LOC102723370	102723370	0,117497108	0,847101401	4,080456	0,207599697	0,835541541	NA
ENSG00000171722	SPATA46	spermatogenesis associated 46	284680	0,191687874	1,677745228	4,046269	0,414640031	0,678405439	NA
ENSG00000171724	VAT1L	vesicle amine transport 1 like	57687	0	NA	NA	NA	NA	NA
ENSG00000171729	TMEM51	transmembrane protein 51	55092	0	NA	NA	NA	NA	NA
ENSG00000171757	LRRC34	leucine rich repeat containing 34	151827	0	NA	NA	NA	NA	NA
ENSG00000171759	PAH	phenylalanine hydroxylase	5053	0	NA	NA	NA	NA	NA
ENSG00000171766	GATM	glycine amidinotransferase	2628	0,239347754	1,967469188	4,021404	0,489249276	0,624665227	NA
ENSG00000171772	SYCE1	synaptonemal complex central element protein 1	93426	0	NA	NA	NA	NA	NA
ENSG00000171773	NXNL1	nucleoredoxin like 1	115861	0	NA	NA	NA	NA	NA
ENSG00000171777	RASGRP4	RAS guanyl releasing protein 4	115727	0	NA	NA	NA	NA	NA
ENSG00000171786	NHLH1	nescient helix-loop-helix 1	4807	1,07548678	-0,389275442	1,939699	-0,200688591	0,840942081	NA
ENSG00000171790	SLFNL1	schlafen like 1	200172	0	NA	NA	NA	NA	NA
ENSG00000171794	UTF1	undifferentiated embryonic cell transcription factor 1	8433	0	NA	NA	NA	NA	NA
ENSG00000171798	KNDC1	kinase non-catalytic C-lobe domain containing 1	85442	1,203770924	0,48731053	2,157518	0,225866279	0,821305425	NA
ENSG00000171804	WDR87	WD repeat domain 87	83889	0	NA	NA	NA	NA	NA
ENSG00000171811	CFAP46	cilia and flagella associated protein 46	54777	0	NA	NA	NA	NA	NA

ENSG00000171812	COL8A2	collagen type VIII alpha 2 chain	1296	0,223051705	-2,002977641	4,03521	-0,496375108	0,619629784	NA
ENSG00000171817	ZNF540	zinc finger protein 540	163255	6,142519422	0,378450716	1,213756	0,311801338	0,755191507	NA
ENSG00000171819	ANGPTL7	angiopoietin like 7	10218	0	NA	NA	NA	NA	NA
ENSG00000171847	FAM90A1	family with sequence similarity 90 member A1	55138	0,209763681	-1,926644696	4,042043	-0,476651224	0,633610501	NA
ENSG00000171848	RRM2	ribonucleotide reductase regulatory subunit M2	6241	162,5159568	0,768175306	1,374238	0,558982652	NA	NA
ENSG00000171855	IFNB1	interferon beta 1	3456	0	NA	NA	NA	NA	NA
ENSG00000171864	PRND	prion like protein doppel	23627	0,119673877	0,847101401	4,080456	0,207599697	0,835541541	NA
ENSG00000171872	KLF17	KLF transcription factor 17	128209	0	NA	NA	NA	NA	NA
ENSG00000171873	ADRA1D	adrenoceptor alpha 1D	146	0	NA	NA	NA	NA	NA
ENSG00000171877	FRMD5	FERM domain containing 5	84978	0	NA	NA	NA	NA	NA
ENSG00000171885	AQP4	aquaporin 4	361	0	NA	NA	NA	NA	NA
ENSG00000171903	CYP4F11	cytochrome P450 family 4 subfamily F member 11	57834	0	NA	NA	NA	NA	NA
ENSG00000171914	TLN2	talin 2	83660	5,222703905	-0,263054748	0,953511	-0,275880106	0,782640154	NA
ENSG00000171931	FBXW10	F-box and WD repeat domain containing 10	10517	0	NA	NA	NA	NA	NA
ENSG00000171936	OR10H3	olfactory receptor family 10 subfamily H member 3	26532	0	NA	NA	NA	NA	NA
ENSG00000171942	OR10H2	olfactory receptor family 10 subfamily H member 2	26538	0	NA	NA	NA	NA	NA
ENSG00000171944	OR52A5	olfactory receptor family 52 subfamily A member 5	390054	0	NA	NA	NA	NA	NA
ENSG00000171951	SCG2	secretogranin II	7857	0,453989242	-0,124758579	3,061491	-0,04075092	0,967494467	NA
ENSG00000171962	DRC3	dynein regulatory complex subunit 3	83450	0	NA	NA	NA	NA	NA
ENSG00000171970	ZNF57	zinc finger protein 57	126295	8,081418524	0,576538327	0,650717	0,886005097	0,37561478	NA
ENSG00000171989	LDHAL6B	lactate dehydrogenase A like 6B	92483	0	NA	NA	NA	NA	NA
ENSG00000171992	SYNPO	synaptopodin	11346	0	NA	NA	NA	NA	NA
ENSG00000171999	OR52P2P	olfactory receptor family 52 subfamily P member 2 pseudogene	119775	0	NA	NA	NA	NA	NA
ENSG00000172000	ZNF556	zinc finger protein 556	80032	0,32077736	0,945512566	4,026365	0,234830339	0,814340419	NA
ENSG00000172006	ZNF554	zinc finger protein 554	115196	7,898567609	-0,660078007	1,503987	-0,438885304	0,660744645	NA
ENSG00000172014	NA	NA	NA	10,07612605	-0,339578892	0,543434	-0,624876475	0,532052134	NA
ENSG00000172016	REG3A	regenerating family member 3 alpha	5068	0	NA	NA	NA	NA	NA
ENSG00000172020	GAP43	growth associated protein 43	2596	0	NA	NA	NA	NA	NA
ENSG00000172023	REG1B	regenerating family member 1 beta	5968	0	NA	NA	NA	NA	NA
ENSG00000172031	EPHX4	epoxide hydrolase 4	253152	0,103998153	-1,076443944	4,080456	-0,263804824	0,791930317	NA
ENSG00000172037	LAMB2	laminin subunit beta 2	3913	0,237958766	0,846395164	4,080456	0,207426619	0,835676696	NA
ENSG00000172058	SERF1A	small EDRK-rich factor 1A	8293	11,21496061	-0,78165656	0,76895	-1,016524405	0,309379732	NA
ENSG00000172061	LRRC15	leucine rich repeat containing 15	131578	0	NA	NA	NA	NA	NA
ENSG00000172073	SPMIP9	sperm microtubule inner protein 9	200523	0	NA	NA	NA	NA	NA
ENSG00000172116	CD8B	CD8 subunit beta	926	0,903394141	0,678986819	3,622714	0,187424901	0,8513275	NA
ENSG00000172139	SLC9C1	solute carrier family 9 member C1	285335	0	NA	NA	NA	NA	NA
ENSG00000172146	OR1A1	olfactory receptor family 1 subfamily A member 1	8383	0	NA	NA	NA	NA	NA
ENSG00000172148	NA	NA	NA	0	NA	NA	NA	NA	NA
ENSG00000172150	OR1A2	olfactory receptor family 1 subfamily A member 2	26189	0	NA	NA	NA	NA	NA
ENSG00000172154	OR8I2	olfactory receptor family 8 subfamily I member 2	120586	0	NA	NA	NA	NA	NA
ENSG00000172155	LCE1D	late cornified envelope 1D	353134	0	NA	NA	NA	NA	NA
ENSG00000172156	CCL11	C-C motif chemokine ligand 11	6356	0	NA	NA	NA	NA	NA
ENSG00000172159	FRMD3	FERM domain containing 3	257019	1,04597788	0,467744428	2,594243	0,180300946	0,856916313	NA
ENSG00000172179	PRL	prolactin	5617	0	NA	NA	NA	NA	NA
ENSG00000172186	NA	NA	NA	0	NA	NA	NA	NA	NA
ENSG00000172188	OR4C11	olfactory receptor family 4 subfamily C member 11	219429	0	NA	NA	NA	NA	NA
ENSG00000172201	ID4	inhibitor of DNA binding 4	3400	0	NA	NA	NA	NA	NA
ENSG00000172208	OR4X2	olfactory receptor family 4 subfamily X member 2	119764	0	NA	NA	NA	NA	NA
ENSG00000172232	AZU1	azurocidin 1	566	0,15836288	0	4,080456	0	1	NA
ENSG00000172236	TPSAB1	tryptase alpha/beta 1	7177	0	NA	NA	NA	NA	NA
ENSG00000172238	ATOH1	atonal bHLH transcription factor 1	474	0	NA	NA	NA	NA	NA
ENSG00000172243	CLEC7A	C-type lectin domain containing 7A	64581	0,322339277	1,907622291	4,026274	0,473793476	0,635647187	NA
ENSG00000172244	C5orf34	chromosome 5 open reading frame 34	375444	8,627744496	-0,034889447	0,726788	-0,048004988	0,961712267	NA

ENSG00000172250	SERHL	serine hydrolase like (pseudogene)	94009	0	NA	NA	NA	NA	NA
ENSG00000172260	NEGR1	neuronal growth regulator 1	257194	0,15836288	0	4,080456	0	1	NA
ENSG00000172264	MACROD2	mono-ADP ribosylhydrolase 2	140733	0	NA	NA	NA	NA	NA
ENSG00000172283	PRYP4	PTPN13 like Y-linked pseudogene 4	442866	0	NA	NA	NA	NA	NA
ENSG00000172288	CDY1	chromodomain Y-linked 1	9085	0	NA	NA	NA	NA	NA
ENSG00000172289	OR10V1	olfactory receptor family 10 subfamily V member 1	390201	0	NA	NA	NA	NA	NA
ENSG00000172294	NA	NA	NA	0	NA	NA	NA	NA	NA
ENSG00000172296	SPTLC3	serine palmitoyltransferase long chain base subunit 3	55304	0	NA	NA	NA	NA	NA
ENSG00000172297	NA	NA	NA	0	NA	NA	NA	NA	NA
ENSG00000172318	B3GALT1	beta-1,3-galactosyltransferase 1	8708	0,354348058	1,42958936	4,061497	0,351985803	0,72484891	NA
ENSG00000172320	OR5A1	olfactory receptor family 5 subfamily A member 1	219982	0	NA	NA	NA	NA	NA
ENSG00000172324	OR5A2	olfactory receptor family 5 subfamily A member 2	219981	0	NA	NA	NA	NA	NA
ENSG00000172339	ALG14	ALG14 UDP-N-acetylglucosaminyltransferase subunit	199857	7,722365539	0,415343397	0,907176	0,457842344	0,647065712	NA
ENSG00000172342	NA	NA	NA	0	NA	NA	NA	NA	NA
ENSG00000172345	STARD5	StAR related lipid transfer domain containing 5	80765	3,534818451	1,969641409	1,011233	1,947762442	0,051443395	NA
ENSG00000172346	CSDC2	cold shock domain containing C2	27254	0,117497108	0,847101401	4,080456	0,207599697	0,835541541	NA
ENSG00000172348	RCAN2	regulator of calcineurin 2	10231	0	NA	NA	NA	NA	NA
ENSG00000172350	ABCG4	ATP binding cassette subfamily G member 4	64137	0	NA	NA	NA	NA	NA
ENSG00000172352	CDY1B	chromodomain Y-linked 1B	253175	0	NA	NA	NA	NA	NA
ENSG00000172361	CFAP53	cilia and flagella associated protein 53	220136	0,543029536	0,835532481	3,393295	0,24623045	0,805503845	NA
ENSG00000172362	OR5B12	olfactory receptor family 5 subfamily B member 12	390191	0,072165449	0,847101401	4,080456	0,207599697	0,835541541	NA
ENSG00000172365	OR5B2	olfactory receptor family 5 subfamily B member 2	390190	0,256595619	0,369155811	4,070322	0,09069449	0,927735349	NA
ENSG00000172366	MCRIP2	MAPK regulated corepressor interacting protein 2	84331	4,470296352	0,029537791	1,043191	0,02831485	0,977411036	NA
ENSG00000172367	NHERF4	NHERF family PDZ scaffold protein 4	79849	0,439741614	0,839075462	4,080456	0,205632774	0,837077774	NA
ENSG00000172377	OR9I1	olfactory receptor family 9 subfamily I member 1	219954	0	NA	NA	NA	NA	NA
ENSG00000172379	ARNT2	aryl hydrocarbon receptor nuclear translocator 2	9915	0,118286652	0	4,080456	0	1	NA
ENSG00000172380	GNG12	G protein subunit gamma 12	55970	0,144330898	1,418786819	4,061262	0,349346267	0,726829368	NA
ENSG00000172382	PRSS27	serine protease 27	83886	0,770243831	-3,794869219	3,451004	-1,099641997	0,271488136	NA
ENSG00000172399	MYOZ2	myozenin 2	51778	0	NA	NA	NA	NA	NA
ENSG00000172403	SYNPO2	synaptopodin 2	171024	5,8340197	0,455431167	0,708933	0,642417885	0,52060189	NA
ENSG00000172404	DNAJB7	DnaJ heat shock protein family (Hsp40) member B7	150353	0,103998153	-1,076443944	4,080456	-0,263804824	0,791930317	NA
ENSG00000172410	INSL5	insulin like 5	10022	0	NA	NA	NA	NA	NA
ENSG00000172421	EFCAB3	EF-hand calcium binding domain 3	146779	0	NA	NA	NA	NA	NA
ENSG00000172425	TTC36	tetratricopeptide repeat domain 36	143941	0,119673877	0,847101401	4,080456	0,207599697	0,835541541	NA
ENSG00000172426	RSPH9	radial spoke head component 9	221421	0,072165449	0,847101401	4,080456	0,207599697	0,835541541	NA
ENSG00000172456	FGGY	FGGY carbohydrate kinase domain containing	55277	7,776486661	-0,846032069	0,939861	-0,900167005	0,368031382	NA
ENSG00000172457	OR9G4	olfactory receptor family 9 subfamily G member 4	283189	0	NA	NA	NA	NA	NA
ENSG00000172458	IL17D	interleukin 17D	53342	3,753074485	-0,238725723	0,935594	-0,255159523	0,798599892	NA
ENSG00000172459	OR5AR1	olfactory receptor family 5 subfamily AR member 1	219493	0	NA	NA	NA	NA	NA
ENSG00000172460	PRSS30P	serine protease 30, pseudogene	124221	7,885894289	0,401108917	0,733785	0,546629992	0,584632959	NA
ENSG00000172461	FUT9	fucosyltransferase 9	10690	0	NA	NA	NA	NA	NA
ENSG00000172464	OR5AP2	olfactory receptor family 5 subfamily AP member 2	338675	0	NA	NA	NA	NA	NA
ENSG00000172465	TCEAL1	transcription elongation factor A like 1	9338	6,719500175	-0,070059932	0,775589	-0,090331205	0,928024023	NA
ENSG00000172468	HSFY1	heat shock transcription factor Y-linked 1	86614	0	NA	NA	NA	NA	NA
ENSG00000172476	RAB40A	RAB40A, member RAS oncogene family	142684	0,210862461	0	4,080456	0	1	NA
ENSG00000172478	MAB21L4	mab-21 like 4	79919	0	NA	NA	NA	NA	NA
ENSG00000172482	AGXT	alanine--glyoxylate aminotransferase	189	0	NA	NA	NA	NA	NA

ENSG00000172497	ACOT12	acyl-CoA thioesterase 12	134526	0	NA	NA	NA	NA	NA
ENSG00000172519	OR10H5	olfactory receptor family 10 subfamily H member 5	284433	0,11808024	-1,076443944	4,080456	-0,263804824	0,791930317	NA
ENSG00000172538	FAM170B	family with sequence similarity 170 member B	170370	0	NA	NA	NA	NA	NA
ENSG00000172548	NIPAL4	NIPA like domain containing 4	348938	0,119673877	0,847101401	4,080456	0,207599697	0,835541541	NA
ENSG00000172551	MUCL1	mucin like 1	118430	0,518931308	1,769272806	3,289453	0,537862302	0,590672116	NA
ENSG00000172554	SNTG2	syntrophin gamma 2	54221	0	NA	NA	NA	NA	NA
ENSG00000172568	FNDC9	fibronectin type III domain containing 9	408263	0,144330898	1,418786819	4,061262	0,349346267	0,726829368	NA
ENSG00000172572	PDE3A	phosphodiesterase 3A	5139	0,18998621	1,666798939	4,047469	0,411812619	0,680476771	NA
ENSG00000172594	SMPDL3A	sphingomyelin phosphodiesterase acid like 3A	10924	0	NA	NA	NA	NA	NA
ENSG00000172602	RND1	Rho family GTPase 1	27289	1,019229284	0,236129208	2,069415	0,11410431	0,909155106	NA
ENSG00000172640	OR10AD1	olfactory receptor family 10 subfamily AD member 1	121275	0	NA	NA	NA	NA	NA
ENSG00000172663	TMEM134	transmembrane protein 134	80194	5,791310257	0,209160491	0,825772	0,253290913	0,800043418	NA
ENSG00000172680	MOS	MOS proto-oncogene, serine/threonine kinase	4342	0	NA	NA	NA	NA	NA
ENSG00000172689	MS4A10	membrane spanning 4-domains A10	341116	0	NA	NA	NA	NA	NA
ENSG00000172717	GARIN2	golgi associated RAB2 interactor family member 2	161142	0,10543123	0	4,080456	0	1	NA
ENSG00000172724	CCL19	C-C motif chemokine ligand 19	6363	0	NA	NA	NA	NA	NA
ENSG00000172728	FUT10	fucosyltransferase 10	84750	10,04040421	0,456794865	0,566631	0,80615981	0,420150709	NA
ENSG00000172733	PURG	purine rich element binding protein G	29942	0,119673877	0,847101401	4,080456	0,207599697	0,835541541	NA
ENSG00000172738	TMEM217	transmembrane protein 217	221468	6,102689438	0,801069141	0,91708	0,873499497	0,38239088	NA
ENSG00000172742	OR4D9	olfactory receptor family 4 subfamily D member 9	390199	0	NA	NA	NA	NA	NA
ENSG00000172746	NA	NA	NA	0	NA	NA	NA	NA	NA
ENSG00000172748	ZNF596	zinc finger protein 596	169270	6,360516719	0,882186918	0,944675	0,933852235	0,350380121	NA
ENSG00000172752	COL6A5	collagen type VI alpha 5 chain	256076	0	NA	NA	NA	NA	NA
ENSG00000172769	OR5B3	olfactory receptor family 5 subfamily B member 3	441608	0	NA	NA	NA	NA	NA
ENSG00000172771	EFCAB12	EF-hand calcium binding domain 12	90288	5,089231544	-1,071030764	0,817357	-1,310358441	0,190074606	NA
ENSG00000172772	OR10W1	olfactory receptor family 10 subfamily W member 1	81341	0	NA	NA	NA	NA	NA
ENSG00000172782	FADS6	fatty acid desaturase 6	283985	0	NA	NA	NA	NA	NA
ENSG00000172789	HOXC5	homeobox C5	3222	0	NA	NA	NA	NA	NA
ENSG00000172799	NA	NA	NA	0	NA	NA	NA	NA	NA
ENSG00000172803	SNX32	sorting nexin 32	254122	1,57351034	1,554888931	1,709202	0,909716449	0,362972067	NA
ENSG00000172817	CYP7B1	cytochrome P450 family 7 subfamily B member 1	9420	0	NA	NA	NA	NA	NA
ENSG00000172818	OVOL1	ovo like transcriptional repressor 1	5017	0,10543123	0	4,080456	0	1	NA
ENSG00000172824	CES4A	carboxylesterase 4A	283848	9,614320058	0,055772485	0,573347	0,097275221	0,922507834	NA
ENSG00000172828	CES3	carboxylesterase 3	23491	0,118286652	0	4,080456	0	1	NA
ENSG00000172867	KRT2	keratin 2	3849	0,144330898	1,418786819	4,061262	0,349346267	0,726829368	NA
ENSG00000172889	EGFL7	EGF like domain multiple 7	51162	0,10543123	0	4,080456	0	1	NA
ENSG00000172900	ACTE1P	actin epsilon 1, pseudogene	399923	0	NA	NA	NA	NA	NA
ENSG00000172900	LOC124902707	actin, aortic smooth muscle-like	124902707	0	NA	NA	NA	NA	NA
ENSG00000172901	LVRN	laeverin	206338	0	NA	NA	NA	NA	NA
ENSG00000172912	NA	NA	NA	0	NA	NA	NA	NA	NA
ENSG00000172915	NBEA	neurobeachin	26960	0,300361865	0,799433064	4,047969	0,197489909	0,843444178	NA
ENSG00000172927	MYEOV	myeloma overexpressed	26579	0	NA	NA	NA	NA	NA
ENSG00000172935	MRGPRF	MAS related GPR family member F	116535	0,70965039	-1,601255518	2,420846	-0,661444591	0,508327238	NA
ENSG00000172938	MRGPRD	MAS related GPR family member D	116512	0	NA	NA	NA	NA	NA
ENSG00000172940	SLC22A13	solute carrier family 22 member 13	9390	0,393918771	0	4,080456	0	1	NA
ENSG00000172955	ADH6	alcohol dehydrogenase 6 (class V)	130	0	NA	NA	NA	NA	NA
ENSG00000172967	XKR3	XK related 3	150165	0,777792403	0,060892659	2,405822	0,02531054	0,979807267	NA
ENSG00000172969	FRG2C	FSHD region gene 2 family member C	100288801	0	NA	NA	NA	NA	NA
ENSG00000172971	NA	NA	NA	0	NA	NA	NA	NA	NA
ENSG00000172974	NA	NA	NA	0,549328012	-0,133849449	3,452433	-0,038769599	0,969074083	NA
ENSG00000172985	SH3RF3	SH3 domain containing ring finger 3	344558	0	NA	NA	NA	NA	NA
ENSG00000172986	GXYLT2	glucoside xylosyltransferase 2	727936	0	NA	NA	NA	NA	NA
ENSG00000172987	HPSE2	heparanase 2 (inactive)	60495	0	NA	NA	NA	NA	NA

ENSG00000172995	ARPP21	cAMP regulated phosphoprotein 21	10777	0	NA	NA	NA	NA	NA
ENSG00000173013	CCDC96	coiled-coil domain containing 96	257236	2,302483882	-0,574186914	1,282847	-0,447588023	0,654450549	NA
ENSG00000173040	EVC2	EvC ciliary complex subunit 2	132884	0,144330898	1,418786819	4,061262	0,349346267	0,726829368	NA
ENSG00000173068	BNC2	basonuclin zinc finger protein 2	54796	2,493453288	2,279234746	1,870852	1,218287133	0,223114878	NA
ENSG00000173080	RXFP4	relaxin family peptide/INSL5 receptor 4	339403	0,119673877	0,847101401	4,080456	0,207599697	0,835541541	NA
ENSG00000173093	CCDC63	coiled-coil domain containing 63	160762	0	NA	NA	NA	NA	NA
ENSG00000173124	ACSM6	acyl-CoA synthetase medium chain family member 6	142827	0	NA	NA	NA	NA	NA
ENSG00000173156	RHOD	ras homolog family member D	29984	0	NA	NA	NA	NA	NA
ENSG00000173157	ADAMTS20	ADAM metallopeptidase with thrombospondin type 1 motif 20	80070	0	NA	NA	NA	NA	NA
ENSG00000173166	RAPH1	Ras association (RalGDS/AF-6) and pleckstrin homology domains 1	65059	0	NA	NA	NA	NA	NA
ENSG00000173175	ADCY5	adenylate cyclase 5	111	0,10488184	-1,076443944	4,080456	-0,263804824	0,791930317	NA
ENSG00000173210	ABLIM3	actin binding LIM protein family member 3	22885	0	NA	NA	NA	NA	NA
ENSG00000173212	MAB21L3	mab-21 like 3	126868	1,98511112	0,161435605	1,666749	0,096856553	0,922840313	NA
ENSG00000173213	TUBB8B	tubulin beta 8B	260334	0	NA	NA	NA	NA	NA
ENSG00000173213	LOC124908013	tubulin beta 8B	124908013	0	NA	NA	NA	NA	NA
ENSG00000173213	LOC124908015	tubulin beta 8B	124908015	0	NA	NA	NA	NA	NA
ENSG00000173213	LOC124908041	tubulin beta 8B	124908041	0	NA	NA	NA	NA	NA
ENSG00000173227	SYT12	synaptotagmin 12	91683	0	NA	NA	NA	NA	NA
ENSG00000173231	NA	NA	NA	0	NA	NA	NA	NA	NA
ENSG00000173237	C11orf86	chromosome 11 open reading frame 86	254439	0	NA	NA	NA	NA	NA
ENSG00000173239	LIPM	lipase family member M	340654	0	NA	NA	NA	NA	NA
ENSG00000173250	GPR151	G protein-coupled receptor 151	134391	0	NA	NA	NA	NA	NA
ENSG00000173253	DMRT2	doublesex and mab-3 related transcription factor 2	10655	0	NA	NA	NA	NA	NA
ENSG00000173261	PLAC8L1	PLAC8 like 1	153770	0,780906765	-0,779709704	2,136824	-0,36489195	0,715192064	NA
ENSG00000173262	SLC2A14	solute carrier family 2 member 14	144195	0,144330898	1,418786819	4,061262	0,349346267	0,726829368	NA
ENSG00000173267	SNCG	synuclein gamma	6623	0	NA	NA	NA	NA	NA
ENSG00000173269	MMRN2	multimerin 2	79812	0	NA	NA	NA	NA	NA
ENSG00000173285	OR10K1	olfactory receptor family 10 subfamily K member 1	391109	0	NA	NA	NA	NA	NA
ENSG00000173295	FAM86B3P	family with sequence similarity 86, member A pseudogene	286042	0,644402642	0,410118819	3,207849	0,127848555	0,898268824	NA
ENSG00000173302	GPR148	G protein-coupled receptor 148	344561	0	NA	NA	NA	NA	NA
ENSG00000173320	STOX2	storkhead box 2	56977	0,118286652	0	4,080456	0	1	NA
ENSG00000173334	TRIB1	tribbles pseudokinase 1	10221	5,946533501	0,078553208	0,834494	0,094132731	0,92500372	NA
ENSG00000173335	CST9	cystatin 9	128822	0	NA	NA	NA	NA	NA
ENSG00000173338	KCNK7	potassium two pore domain channel subfamily K member 7	10089	1,647701314	-1,127418636	1,488733	-0,757300986	0,448869561	NA
ENSG00000173357	RBMY2MP	RNA binding motif protein Y-linked family 2 member M, pseudogene	140101	0	NA	NA	NA	NA	NA
ENSG00000173366	NA	NA	NA	2,617149375	-0,332069781	1,434836	-0,231433904	0,816977728	NA
ENSG00000173369	C1QB	complement C1q B chain	713	0	NA	NA	NA	NA	NA
ENSG00000173372	C1QA	complement C1q A chain	712	0	NA	NA	NA	NA	NA
ENSG00000173376	NDNF	neuron derived neurotrophic factor	79625	0,072165449	0,847101401	4,080456	0,207599697	0,835541541	NA
ENSG00000173389	IQCF1	IQ motif containing F1	132141	0	NA	NA	NA	NA	NA
ENSG00000173391	OLR1	oxidized low density lipoprotein receptor 1	4973	0,072165449	0,847101401	4,080456	0,207599697	0,835541541	NA
ENSG00000173401	GLIPR1L1	GLIPR1 like 1	256710	0	NA	NA	NA	NA	NA
ENSG00000173404	INSM1	INSM transcriptional repressor 1	3642	0	NA	NA	NA	NA	NA
ENSG00000173406	DAB1	DAB adaptor protein 1	1600	0,210862461	0	4,080456	0	1	NA
ENSG00000173421	IHO1	interactor of HORMAD1 1	339834	0,15836288	0	4,080456	0	1	NA
ENSG00000173431	RNASE8	ribonuclease A family member 8	122665	0	NA	NA	NA	NA	NA
ENSG00000173432	SAA1	serum amyloid A1	6288	0	NA	NA	NA	NA	NA
ENSG00000173452	TMEM196	transmembrane protein 196	256130	0	NA	NA	NA	NA	NA

ENSG00000173464	RNASE11	ribonuclease A family member 11 (inactive)	122651	0	NA	NA	NA	NA	NA
ENSG00000173467	AGR3	anterior gradient 3, protein disulphide isomerase family member	155465	0	NA	NA	NA	NA	NA
ENSG00000173482	PTPRM	protein tyrosine phosphatase receptor type M	5797	0	NA	NA	NA	NA	NA
ENSG00000173530	TNFRSF10D	TNF receptor superfamily member 10d	8793	10,01743079	0,522941874	0,560119	0,9336263	0,350496694	NA
ENSG00000173535	TNFRSF10C	TNF receptor superfamily member 10c	8794	0	NA	NA	NA	NA	NA
ENSG00000173557	CIMIP2C	ciliary microtubule inner protein 2C	339778	0	NA	NA	NA	NA	NA
ENSG00000173567	ADGRF3	adhesion G protein-coupled receptor F3	165082	2,889255449	2,337381498	1,569424	1,489324297	0,136401993	NA
ENSG00000173572	NLRP13	NLR family pyrin domain containing 13	126204	0	NA	NA	NA	NA	NA
ENSG00000173578	XCR1	X-C motif chemokine receptor 1	2829	0,190047249	1,667129561	4,047459	0,411895395	0,680416096	NA
ENSG00000173581	CCDC106	coiled-coil domain containing 106	29903	6,686685529	0,35525709	0,736492	0,482363875	0,629547479	NA
ENSG00000173585	CCR9	C-C motif chemokine receptor 9	10803	0	NA	NA	NA	NA	NA
ENSG00000173597	SULT1B1	sulfotransferase family 1B member 1	27284	5,095143377	-0,563156825	0,859035	-0,655569007	0,512101472	NA
ENSG00000173610	UGT2A1	UDP glucuronosyltransferase family 2 member A1 complex locus	10941	0	NA	NA	NA	NA	NA
ENSG00000173612	GPRC6A	G protein-coupled receptor class C group 6 member A	222545	0,119673877	0,847101401	4,080456	0,207599697	0,835541541	NA
ENSG00000173626	TRAPPC3L	trafficking protein particle complex subunit 3L	100128327	0	NA	NA	NA	NA	NA
ENSG00000173627	APOBEC4	apolipoprotein B mRNA editing enzyme catalytic polypeptide like 4	403314	0	NA	NA	NA	NA	NA
ENSG00000173641	HSPB7	heat shock protein family B (small) member 7	27129	0	NA	NA	NA	NA	NA
ENSG00000173662	TAS1R1	taste 1 receptor member 1	80835	0	NA	NA	NA	NA	NA
ENSG00000173673	HES3	hes family bHLH transcription factor 3	390992	0	NA	NA	NA	NA	NA
ENSG00000173678	SPDYE2B	speedy/RINGO cell cycle regulator family member E2B	100310812	0,176102187	-0,084289105	4,080456	-0,020656786	0,983519441	NA
ENSG00000173679	OR1L1	olfactory receptor family 1 subfamily L member 1	26737	0	NA	NA	NA	NA	NA
ENSG00000173698	ADGRG2	adhesion G protein-coupled receptor G2	10149	0	NA	NA	NA	NA	NA
ENSG00000173699	SPATA3	spermatogenesis associated 3	130560	0	NA	NA	NA	NA	NA
ENSG00000173702	MUC13	mucin 13, cell surface associated	56667	0	NA	NA	NA	NA	NA
ENSG00000173705	SUSD5	sushi domain containing 5	26032	0	NA	NA	NA	NA	NA
ENSG00000173714	WFIKKN2	WAP, follistatin/kazal, immunoglobulin, kunitz and netrin domain containing 2	124857	0	NA	NA	NA	NA	NA
ENSG00000173727	NA	NA	NA	0,355062322	1,964038142	4,0223	0,488287352	0,625346319	NA
ENSG00000173728	SPMIP3	sperm microtubule inner protein 3	200159	0	NA	NA	NA	NA	NA
ENSG00000173769	TOPAZ1	testis and ovary specific TOPAZ 1	375337	0	NA	NA	NA	NA	NA
ENSG00000173801	JUP	junction plakoglobin	3728	0	NA	NA	NA	NA	NA
ENSG00000173805	HAP1	huntingtin associated protein 1	9001	1,139717032	4,071943133	2,240517	1,81741228	0,069154009	NA
ENSG00000173809	TDRD12	tudor domain containing 12	91646	1,881800899	0,505681855	1,899488	0,266220141	0,790069673	NA
ENSG00000173810	NA	NA	NA	0	NA	NA	NA	NA	NA
ENSG00000173825	TIGD3	tigger transposable element derived 3	220359	0,928657928	1,834811019	2,126539	0,862815617	0,388238851	NA
ENSG00000173826	KCNH6	potassium voltage-gated channel subfamily H member 6	81033	0	NA	NA	NA	NA	NA
ENSG00000173838	MARCHF10	membrane associated ring-CH-type finger 10	162333	0	NA	NA	NA	NA	NA
ENSG00000173867	NA	NA	NA	3,055718861	1,395328628	1,797016	0,776469605	0,43747176	NA
ENSG00000173868	PHOSPHO1	phosphoethanolamine/phosphocholine phosphatase 1	162466	0,416409229	-0,600297709	3,590627	-0,16718466	0,867224754	NA
ENSG00000173890	GPR160	G protein-coupled receptor 160	26996	3,958540209	0,296944901	1,174399	0,25284849	0,800385295	NA
ENSG00000173894	CBX2	chromobox 2	84733	5,983513902	-1,023074595	0,866903	-1,180148429	0,237941186	NA
ENSG00000173898	SPTBN2	spectrin beta, non-erythrocytic 2	6712	0,875281353	1,049240861	2,71475	0,386496266	0,699129167	NA
ENSG00000173908	KRT28	keratin 28	162605	0	NA	NA	NA	NA	NA

ENSG00000173918	C1QTNF1	C1q and TNF related 1	114897	0,34115109	-2,004928366	3,742062	-0,535781715	0,592109421	NA
ENSG00000173926	MARCHF3	membrane associated ring-CH-type finger 3	115123	6,592654163	0,236642942	0,8625	0,274368774	0,783801237	NA
ENSG00000173928	SWSAP1	SWIM-type zinc finger 7 associated protein 1	126074	10,98555536	-0,173196686	0,604	-0,286749535	0,774304106	NA
ENSG00000173947	CIMAP3	ciliary microtubule associated protein 3	128344	0	NA	NA	NA	NA	NA
ENSG00000173954	NA	NA	NA	0	NA	NA	NA	NA	NA
ENSG00000173966	COMMD5P1	COMMD5 pseudogene 1	101928879	0	NA	NA	NA	NA	NA
ENSG00000173976	RAX2	retina and anterior neural fold homeobox 2	84839	0,490249257	-0,946095503	3,471904	-0,272500469	0,785237231	NA
ENSG00000173988	LRRC63	leucine rich repeat containing 63	220416	3,840084661	-1,21265782	1,089753	-1,112782113	0,265802019	NA
ENSG00000173991	TCAP	titin-cap	8557	1,008024985	-0,339995968	2,365724	-0,143717511	0,885723543	NA
ENSG00000174015	CBY2	chibby family member 2	220082	0	NA	NA	NA	NA	NA
ENSG00000174016	TENT5D	terminal nucleotidyltransferase 5D	169966	0	NA	NA	NA	NA	NA
ENSG00000174038	SPATA31G1	SPATA31 subfamily G member 1	138724	0	NA	NA	NA	NA	NA
ENSG00000174059	CD34	CD34 molecule	947	0	NA	NA	NA	NA	NA
ENSG00000174080	CTSF	cathepsin F	8722	1,815518981	-4,441024405	3,149794	-1,409941178	0,158557052	NA
ENSG00000174099	MSRB3	methionine sulfoxide reductase B3	253827	0	NA	NA	NA	NA	NA
ENSG00000174109	UQCC4	ubiquinol-cytochrome c reductase complex assembly factor 4	283951	9,270302507	0,927749919	1,058795	0,876231627	0,380904126	NA
ENSG00000174123	TLR10	toll like receptor 10	81793	0,317507192	-1,867811974	3,812687	-0,489893902	0,624208978	NA
ENSG00000174130	TLR6	toll like receptor 6	10333	3,663581828	0,64017317	1,028274	0,622570454	0,533566832	NA
ENSG00000174132	FAM174A	family with sequence similarity 174 member A	345757	8,49268376	-0,246097127	0,655926	-0,375190509	0,707518788	NA
ENSG00000174136	RGMB	repulsive guidance molecule BMP co-receptor b	285704	0	NA	NA	NA	NA	NA
ENSG00000174137	FAM53A	family with sequence similarity 53 member A	152877	1,83167323	0,549246353	1,5869	0,346112835	0,729257918	NA
ENSG00000174145	NWD2	NACHT and WD repeat domain containing 2	57495	0	NA	NA	NA	NA	NA
ENSG00000174156	GSTA3	glutathione S-transferase alpha 3	2940	0	NA	NA	NA	NA	NA
ENSG00000174175	SELP	selectin P	6403	0	NA	NA	NA	NA	NA
ENSG00000174225	ARL13A	ADP ribosylation factor like GTPase 13A	392509	0	NA	NA	NA	NA	NA
ENSG00000174226	SNX31	sorting nexin 31	169166	0	NA	NA	NA	NA	NA
ENSG00000174236	REP15	RAB15 effector protein	387849	0,690320753	1,824444319	2,33664	0,780798282	0,434921145	NA
ENSG00000174255	ZNF80	zinc finger protein 80	7634	2,592231626	0,745263226	2,233005	0,333748958	0,738569003	NA
ENSG00000174276	ZNHIT2	zinc finger HIT-type containing 2	741	8,332626318	-0,33928129	0,661365	-0,513001729	0,607950111	NA
ENSG00000174279	EVX2	even-skipped homeobox 2	344191	0	NA	NA	NA	NA	NA
ENSG00000174292	TNK1	tyrosine kinase non receptor 1	8711	1,759715954	-0,725853972	1,695635	-0,428072143	0,668598598	NA
ENSG00000174307	PHLDA3	pleckstrin homology like domain family A member 3	23612	10,78328838	-1,354066901	0,856769	-1,580433705	0,114007578	NA
ENSG00000174326	SLC16A11	solute carrier family 16 member 11	162515	8,153499554	0,44973908	0,661268	0,68011625	0,496430856	NA
ENSG00000174332	GLIS1	GLIS family zinc finger 1	148979	0	NA	NA	NA	NA	NA
ENSG00000174339	OR2Y1	olfactory receptor family 2 subfamily Y member 1	134083	0	NA	NA	NA	NA	NA
ENSG00000174343	CHRNA9	cholinergic receptor nicotinic alpha 9 subunit	55584	0,435144887	0	4,080456	0	1	NA
ENSG00000174348	PODN	podocan	127435	0,10543123	0	4,080456	0	1	NA
ENSG00000174358	SLC6A19	solute carrier family 6 member 19	340024	0	NA	NA	NA	NA	NA
ENSG00000174408	NA	NA	NA	0	NA	NA	NA	NA	NA
ENSG00000174417	TRHR	thyrotropin releasing hormone receptor	7201	0	NA	NA	NA	NA	NA
ENSG00000174418	RPL35P9	ribosomal protein L35 pseudogene 9	100873796	0	NA	NA	NA	NA	NA
ENSG00000174429	ABRA	actin binding Rho activating protein	137735	0	NA	NA	NA	NA	NA
ENSG00000174448	STARD6	StAR related lipid transfer domain containing 6	147323	0,11808024	-1,076443944	4,080456	-0,263804824	0,791930317	NA
ENSG00000174450	GOLGA6L2	golgin A6 family like 2	283685	0	NA	NA	NA	NA	NA
ENSG00000174453	VWC2L	von Willebrand factor C domain containing 2 like	402117	0	NA	NA	NA	NA	NA
ENSG00000174460	ZCCHC12	zinc finger CCHC-type containing 12	170261	0	NA	NA	NA	NA	NA
ENSG00000174469	CNTNAP2	contactin associated protein 2	26047	0	NA	NA	NA	NA	NA
ENSG00000174473	GALNTL6	polypeptide N-acetylgalactosaminyltransferase like 6	442117	0,223753975	0	4,080456	0	1	NA

ENSG00000174482	LINGO2	leucine rich repeat and Ig domain containing 2	158038	0	NA	NA	NA	NA	NA
ENSG00000174495	NA	NA	NA	0	NA	NA	NA	NA	NA
ENSG00000174498	IGDCC3	immunoglobulin superfamily DCC subclass member 3	9543	0	NA	NA	NA	NA	NA
ENSG00000174502	SLC26A9	solute carrier family 26 member 9	115019	0	NA	NA	NA	NA	NA
ENSG00000174514	MFSD4A	major facilitator superfamily domain containing 4A	148808	0	NA	NA	NA	NA	NA
ENSG00000174516	PELI3	pellino E3 ubiquitin protein ligase family member 3	246330	4,081959919	0,942880968	1,605955	0,587115367	0,557126227	NA
ENSG00000174521	TTC9B	tetratricopeptide repeat domain 9B	148014	0	NA	NA	NA	NA	NA
ENSG00000174527	MYO1H	myosin IH	283446	0	NA	NA	NA	NA	NA
ENSG00000174529	TMEM81	transmembrane protein 81	388730	8,161921346	0,230381443	0,668923	0,344406572	0,730540547	NA
ENSG00000174562	KLK15	kallikrein related peptidase 15	55554	0	NA	NA	NA	NA	NA
ENSG00000174564	IL20RB	interleukin 20 receptor subunit beta	53833	0	NA	NA	NA	NA	NA
ENSG00000174567	GOLT1A	golgi transport 1A	127845	0	NA	NA	NA	NA	NA
ENSG00000174572	RPL10P2	ribosomal protein L10 pseudogene 2	442171	0,264643344	0	4,080456	0	1	NA
ENSG00000174576	NPAS4	neuronal PAS domain protein 4	266743	0	NA	NA	NA	NA	NA
ENSG00000174586	ZNF497	zinc finger protein 497	162968	2,550067207	-0,165700656	1,292603	-0,128191448	0,897997467	NA
ENSG00000174599	TRAM1L1	translocation associated membrane protein 1 like 1	133022	0	NA	NA	NA	NA	NA
ENSG00000174607	UGT8	UDP glycosyltransferase 8	7368	0,118286652	0	4,080456	0	1	NA
ENSG00000174611	KY	kyphoscoliosis peptidase	339855	0	NA	NA	NA	NA	NA
ENSG00000174628	IQCK	IQ motif containing K	124152	0	NA	NA	NA	NA	NA
ENSG00000174640	SLCO2A1	solute carrier organic anion transporter family member 2A1	6578	0	NA	NA	NA	NA	NA
ENSG00000174667	OR7D4	olfactory receptor family 7 subfamily D member 4	125958	0,117497108	0,847101401	4,080456	0,207599697	0,835541541	NA
ENSG00000174669	SLC29A2	solute carrier family 29 member 2	3177	10,11534751	-0,007677715	2,905785	-0,002642217	0,997891818	NA
ENSG00000174677	VN1R6P	vomeronasal 1 receptor 6, pseudogene	653753	0	NA	NA	NA	NA	NA
ENSG00000174678	NA	NA	NA	0,118286652	0	4,080456	0	1	NA
ENSG00000174684	B4GAT1	beta-1,4-glucuronyltransferase 1	11041	7,466982823	-0,269670849	0,679068	-0,397118793	0,691279862	NA
ENSG00000174697	LEP	leptin	3952	0	NA	NA	NA	NA	NA
ENSG00000174705	SH3PXD2B	SH3 and PX domains 2B	285590	0,507835589	0,194209798	3,433928	0,056556161	0,954898757	NA
ENSG00000174715	NA	NA	NA	0,406082007	1,33734887	3,998012	0,334503483	0,737999663	NA
ENSG00000174721	FGFBP3	fibroblast growth factor binding protein 3	143282	6,258365083	-0,187691029	0,704369	-0,266467043	0,789879538	NA
ENSG00000174740	PABPC5	poly(A) binding protein cytoplasmic 5	140886	0,15836288	0	4,080456	0	1	NA
ENSG00000174776	WDR49	WD repeat domain 49	151790	0	NA	NA	NA	NA	NA
ENSG00000174788	PCP2	Purkinje cell protein 2	126006	4,607738722	-1,158434294	1,009167	-1,147910962	0,251005322	NA
ENSG00000174791	RIN1	Ras and Rab interactor 1	9610	2,393348871	-4,521739379	2,242774	-2,016137168	0,043785634	NA
ENSG00000174792	ODAPH	odontogenesis associated phosphoprotein	152816	0,327794323	-2,562662296	3,99435	-0,641571766	0,52115127	NA
ENSG00000174804	FZD4	frizzled class receptor 4	8322	0,239347754	1,967469188	4,021404	0,489249276	0,624665227	NA
ENSG00000174807	CD248	CD248 molecule	57124	0	NA	NA	NA	NA	NA
ENSG00000174808	BTC	betacellulin	685	0	NA	NA	NA	NA	NA
ENSG00000174827	PDZK1	PDZ domain containing 1	5174	6,854497518	-1,020927314	1,020593	-1,000327111	0,317152231	NA
ENSG00000174837	ADGRE1	adhesion G protein-coupled receptor E1	2015	0	NA	NA	NA	NA	NA
ENSG00000174844	DNAH12	dynein axonemal heavy chain 12	201625	0,702455745	0,005705145	2,797691	0,002039233	0,998372928	NA
ENSG00000174876	AMY1B	amylase alpha 1B	277	0	NA	NA	NA	NA	NA
ENSG00000174876	LOC124905666	alpha-amylase 1B	124905666	0	NA	NA	NA	NA	NA
ENSG00000174876	LOC124905668	alpha-amylase 1B	124905668	0	NA	NA	NA	NA	NA
ENSG00000174885	NLRP6	NLR family pyrin domain containing 6	171389	11,13707462	-0,003041983	0,564128	-0,005392366	0,995697535	NA
ENSG00000174898	CATSPERD	cation channel sperm associated auxiliary subunit delta	257062	0	NA	NA	NA	NA	NA
ENSG00000174899	SLC66A1L	solute carrier family 66 member 1 like (pseudogene)	152078	0	NA	NA	NA	NA	NA
ENSG00000174912	NA	NA	NA	3,342542111	1,361646308	1,392757	0,977662224	0,328241411	NA
ENSG00000174914	OR9G1	olfactory receptor family 9 subfamily G member 1	390174	0	NA	NA	NA	NA	NA
ENSG00000174928	C3orf33	chromosome 3 open reading frame 33	285315	6,493901383	-0,806185421	0,806306	-0,999850246	0,317382986	NA
ENSG00000174930	NA	NA	NA	0	NA	NA	NA	NA	NA

ENSG00000174938	SEZ6L2	seizure related 6 homolog like 2	26470	0	NA	NA	NA	NA	NA
ENSG00000174939	ASPHD1	aspartate beta-hydroxylase domain containing 1	253982	0	NA	NA	NA	NA	NA
ENSG00000174948	GPR149	G protein-coupled receptor 149	344758	0	NA	NA	NA	NA	NA
ENSG00000174950	CD164L2	CD164 molecule like 2	388611	0	NA	NA	NA	NA	NA
ENSG00000174951	FUT1	fucosyltransferase 1 (H blood group)	2523	1,006503802	-0,096758687	1,736221	-0,05572947	0,955557323	NA
ENSG00000174957	OR5J2	olfactory receptor family 5 subfamily J member 2	282775	0	NA	NA	NA	NA	NA
ENSG00000174963	ZIC4	Zic family member 4	84107	0	NA	NA	NA	NA	NA
ENSG00000174970	OR10AG1	olfactory receptor family 10 subfamily AG member 1	282770	0	NA	NA	NA	NA	NA
ENSG00000174977	NA	NA	NA	0,231978377	0,836453602	4,08007	0,205009639	0,837564594	NA
ENSG00000174982	OR4S2	olfactory receptor family 4 subfamily S member 2	219431	0	NA	NA	NA	NA	NA
ENSG00000174990	CA5A	carbonic anhydrase 5A	763	0	NA	NA	NA	NA	NA
ENSG00000174992	ZG16	zymogen granule protein 16	653808	0,10488184	-1,076443944	4,080456	-0,263804824	0,791930317	NA
ENSG00000175003	SLC22A1	solute carrier family 22 member 1	6580	1,64600205	-0,261838675	1,888952	-0,138615812	0,889753748	NA
ENSG00000175018	TEX36	testis expressed 36	387718	0	NA	NA	NA	NA	NA
ENSG00000175065	DSG4	desmoglein 4	147409	0	NA	NA	NA	NA	NA
ENSG00000175084	DES	desmin	1674	0	NA	NA	NA	NA	NA
ENSG00000175093	SPSB4	splA/ryanodine receptor domain and SOCS box containing 4	92369	0	NA	NA	NA	NA	NA
ENSG00000175097	RAG2	recombination activating 2	5897	0	NA	NA	NA	NA	NA
ENSG00000175121	WFDC5	WAP four-disulfide core domain 5	149708	0	NA	NA	NA	NA	NA
ENSG00000175161	CADM2	cell adhesion molecule 2	253559	0,177627407	-0,133797117	4,080055	-0,03279297	0,973839684	NA
ENSG00000175164	ABO	ABO, alpha 1-3-N-acetylgalactosaminyltransferase and alpha 1-3-galactosyltransferase	28	0	NA	NA	NA	NA	NA
ENSG00000175175	PPM1E	protein phosphatase, Mg2+/Mn2+ dependent 1E	22843	0	NA	NA	NA	NA	NA
ENSG00000175183	CSRP2	cysteine and glycine rich protein 2	1466	0,117497108	0,847101401	4,080456	0,207599697	0,835541541	NA
ENSG00000175189	INHBC	inhibin subunit beta C	3626	0,558311711	0,639865995	2,729198	0,234451984	0,814634106	NA
ENSG00000175202	HIGD2B	HIG1 hypoxia inducible domain family member 2B	123346	0	NA	NA	NA	NA	NA
ENSG00000175206	NPPA	natriuretic peptide A	4878	0	NA	NA	NA	NA	NA
ENSG00000175229	GAL3ST3	galactose-3-O-sulfotransferase 3	89792	0	NA	NA	NA	NA	NA
ENSG00000175262	C1orf127	chromosome 1 open reading frame 127	148345	0,144330898	1,418786819	4,061262	0,349346267	0,726829368	NA
ENSG00000175264	CHST1	carbohydrate sulfotransferase 1	8534	0	NA	NA	NA	NA	NA
ENSG00000175267	VWA3A	von Willebrand factor A domain containing 3A	146177	0,562606679	-1,477051079	3,321716	-0,444665069	0,656561816	NA
ENSG00000175279	CENPS	centromere protein S	378708	4,847422402	-0,031829647	0,911297	-0,03492785	0,972137273	NA
ENSG00000175287	PHYHD1	phytanoyl-CoA dioxygenase domain containing 1	254295	0	NA	NA	NA	NA	NA
ENSG00000175302	ANKRD30BP1	ankyrin repeat domain 30B pseudogene 1	642460	0	NA	NA	NA	NA	NA
ENSG00000175315	CST6	cystatin E/M	1474	10,62621283	0,777686449	0,570784	1,362487667	0,173044034	NA
ENSG00000175318	GRAMD2A	GRAM domain containing 2A	196996	0	NA	NA	NA	NA	NA
ENSG00000175319	NA	NA	NA	0	NA	NA	NA	NA	NA
ENSG00000175322	ZNF519	zinc finger protein 519	162655	5,581070783	1,515217509	1,119745	1,353181249	0,175997734	NA
ENSG00000175325	PROP1	PROP paired-like homeobox 1	5626	0	NA	NA	NA	NA	NA
ENSG00000175329	ISX	intestine specific homeobox	91464	0	NA	NA	NA	NA	NA
ENSG00000175336	APOF	apolipoprotein F	319	0	NA	NA	NA	NA	NA
ENSG00000175344	CHRNA7	cholinergic receptor nicotinic alpha 7 subunit	1139	0,103998153	-1,076443944	4,080456	-0,263804824	0,791930317	NA
ENSG00000175352	NRIP3	nuclear receptor interacting protein 3	56675	0,222253801	-0,125226729	4,080456	-0,030689397	0,975517247	NA
ENSG00000175356	SCUBE2	signal peptide, CUB domain and EGF like domain containing 2	57758	0	NA	NA	NA	NA	NA
ENSG00000175398	OR10P1	olfactory receptor family 10 subfamily P member 1	121130	0	NA	NA	NA	NA	NA
ENSG00000175426	PCSK1	proprotein convertase subtilisin/kexin type 1	5122	0	NA	NA	NA	NA	NA
ENSG00000175445	LPL	lipoprotein lipase	4023	0	NA	NA	NA	NA	NA
ENSG00000175449	RFESD	Rieske Fe-S domain containing	317671	5,519190093	-0,633385652	0,992655	-0,638072208	0,523426677	NA

ENSG00000175471	MCTP1	multiple C2 and transmembrane domain containing 1	79772	0	NA	NA	NA	NA	NA
ENSG00000175485	OR52W1	olfactory receptor family 52 subfamily W member 1	120787	0	NA	NA	NA	NA	NA
ENSG00000175489	LRRC25	leucine rich repeat containing 25	126364	0,352491325	2,536807152	3,984503	0,636668335	0,524340903	NA
ENSG00000175497	DPP10	dipeptidyl peptidase like 10	57628	0	NA	NA	NA	NA	NA
ENSG00000175505	CLCF1	cardiotrophin like cytokine factor 1	23529	0,251807506	0,311240198	4,071474	0,076444116	0,939065773	NA
ENSG00000175509	ACRP1	ACR pseudogene 1	645529	0	NA	NA	NA	NA	NA
ENSG00000175513	TSGA10IP	testis specific 10 interacting protein	254187	0	NA	NA	NA	NA	NA
ENSG00000175514	GPR152	G protein-coupled receptor 152	390212	0,117497108	0,847101401	4,080456	0,207599697	0,835541541	NA
ENSG00000175518	UBQLNL	ubiquilin like	143630	0,985509242	1,05828913	1,964536	0,538696858	0,590096042	NA
ENSG00000175520	UBQLN3	ubiquilin 3	50613	0	NA	NA	NA	NA	NA
ENSG00000175535	PNLIP	pancreatic lipase	5406	0	NA	NA	NA	NA	NA
ENSG00000175536	LIPT2	lipoyl(octanoyl) transferase 2	387787	1,206770485	-0,026823354	1,872008	-0,014328653	0,98856778	NA
ENSG00000175538	KCNE3	potassium voltage-gated channel subfamily E regulatory subunit 3	10008	0	NA	NA	NA	NA	NA
ENSG00000175544	CABP4	calcium binding protein 4	57010	2,542596997	-0,297949919	1,57522	-0,189148135	0,849976716	NA
ENSG00000175556	LONRF3	LON peptidase N-terminal domain and ring finger 3	79836	0	NA	NA	NA	NA	NA
ENSG00000175591	P2RY2	purinergic receptor P2Y2	5029	0,072165449	0,847101401	4,080456	0,207599697	0,835541541	NA
ENSG00000175592	FOSL1	FOS like 1, AP-1 transcription factor subunit	8061	2,334406403	1,020454573	1,333898	0,765016821	0,444261528	NA
ENSG00000175600	SUGCT	succinyl-CoA:glutarate-CoA transferase	79783	0,072165449	0,847101401	4,080456	0,207599697	0,835541541	NA
ENSG00000175619	OR4B1	olfactory receptor family 4 subfamily B member 1	119765	0,072165449	0,847101401	4,080456	0,207599697	0,835541541	NA
ENSG00000175646	PRM1	protamine 1	5619	0	NA	NA	NA	NA	NA
ENSG00000175658	NA	NA	NA	0	NA	NA	NA	NA	NA
ENSG00000175664	TEX26	testis expressed 26	122046	0	NA	NA	NA	NA	NA
ENSG00000175697	GPR156	G protein-coupled receptor 156	165829	0	NA	NA	NA	NA	NA
ENSG00000175699	CCDC197	coiled-coil domain containing 197	256369	0	NA	NA	NA	NA	NA
ENSG00000175701	MTLN	mitoregulin	205251	11,56611982	0,091759948	0,580234	0,15814305	0,874344078	NA
ENSG00000175707	KDF1	keratinocyte differentiation factor 1	126695	0	NA	NA	NA	NA	NA
ENSG00000175718	RBMXL3	RBMX like 3	139804	0	NA	NA	NA	NA	NA
ENSG00000175730	NA	NA	NA	1,019511875	2,677152014	2,066368	1,2955833	0,195119086	NA
ENSG00000175741	NA	NA	NA	0	NA	NA	NA	NA	NA
ENSG00000175745	NR2F1	nuclear receptor subfamily 2 group F member 1	7025	0	NA	NA	NA	NA	NA
ENSG00000175749	EIF3KP1	eukaryotic translation initiation factor 3 subunit K pseudogene 1	134505	0,295737801	-0,103216348	3,935388	-0,026227743	0,979075688	NA
ENSG00000175766	EIF4E1B	eukaryotic translation initiation factor 4E family member 1B	253314	0	NA	NA	NA	NA	NA
ENSG00000175785	PRIMA1	proline rich membrane anchor 1	145270	0	NA	NA	NA	NA	NA
ENSG00000175793	SFN	stratifin	2810	0,474295205	0,335550907	3,382199	0,099210861	0,920970852	NA
ENSG00000175800	OR52B3P	olfactory receptor family 52 subfamily B member 3 pseudogene	387747	0	NA	NA	NA	NA	NA
ENSG00000175806	MSRA	methionine sulfoxide reductase A	4482	0,15836288	0	4,080456	0	1	NA
ENSG00000175809	CBLL2	Cbl proto-oncogene like 2	158506	0	NA	NA	NA	NA	NA
ENSG00000175832	ETV4	ETS variant transcription factor 4	2118	0,383884657	1,568569755	3,665335	0,427947178	0,668689578	NA
ENSG00000175841	NA	NA	NA	0	NA	NA	NA	NA	NA
ENSG00000175857	GAPT	GRB2 binding adaptor protein, transmembrane	202309	0	NA	NA	NA	NA	NA
ENSG00000175866	BAIAP2	BAR/IMD domain containing adaptor protein 2	10458	6,832487375	-0,096092548	0,791197	-0,121452132	0,903332927	NA
ENSG00000175868	CALCB	calcitonin related polypeptide beta	797	2,516416131	1,585671879	1,218676	1,301143642	0,193209292	NA
ENSG00000175874	CREG2	cellular repressor of E1A stimulated genes 2	200407	0	NA	NA	NA	NA	NA
ENSG00000175877	TMEM270	transmembrane protein 270	135886	0	NA	NA	NA	NA	NA
ENSG00000175879	HOXD8	homeobox D8	3234	0	NA	NA	NA	NA	NA
ENSG00000175886	RPL7AP66	ribosomal protein L7a pseudogene 66	388474	0	NA	NA	NA	NA	NA
ENSG00000175894	TSPEAR	thrombospondin type laminin G domain and EAR repeats	54084	0	NA	NA	NA	NA	NA

ENSG00000175899	A2M	alpha-2-macroglobulin	2	0	NA	NA	NA	NA	NA
ENSG00000175906	ARL4D	ADP ribosylation factor like GTPase 4D	379	4,077215724	-0,626052998	0,878359	-0,712753226	0,475998474	NA
ENSG00000175920	DOK7	docking protein 7	285489	6,264092985	-0,742727963	0,77263	-0,961298669	0,336402017	NA
ENSG00000175928	LRRN1	leucine rich repeat neuronal 1	57633	0	NA	NA	NA	NA	NA
ENSG00000175946	KLHL38	kelch like family member 38	340359	0,072165449	0,847101401	4,080456	0,207599697	0,835541541	NA
ENSG00000175984	DENND2C	DENN domain containing 2C	163259	1,061883452	3,061490458	2,152227	1,422475771	0,15488818	NA
ENSG00000175985	PLEKHD1	pleckstrin homology and coiled-coil domain containing D1	400224	1,498079476	1,331634454	1,778634	0,748683792	0,454047815	NA
ENSG00000176007	NA	NA	NA	0	NA	NA	NA	NA	NA
ENSG00000176009	ASCL3	achaete-scute family bHLH transcription factor 3	56676	0	NA	NA	NA	NA	NA
ENSG00000176020	AMIGO3	adhesion molecule with Ig like domain 3	386724	7,098900328	0,80627036	3,743037	0,21540542	0,829451244	NA
ENSG00000176029	C11orf16	chromosome 11 open reading frame 16	56673	0	NA	NA	NA	NA	NA
ENSG00000176040	TMPRSS7	transmembrane serine protease 7	344805	0	NA	NA	NA	NA	NA
ENSG00000176043	NA	NA	NA	0	NA	NA	NA	NA	NA
ENSG00000176046	NUPR1	nuclear protein 1, transcriptional regulator	26471	0	NA	NA	NA	NA	NA
ENSG00000176076	KCNE5	potassium voltage-gated channel subfamily E regulatory subunit 5	23630	0,393065875	0	4,080456	0	1	NA
ENSG00000176115	NA	NA	NA	0,103998153	-1,076443944	4,080456	-0,263804824	0,791930317	NA
ENSG00000176125	UFSP1	UFM1 specific peptidase 1 (inactive)	402682	1,434888888	-1,205216298	1,72894	-0,697084031	0,485750207	NA
ENSG00000176134	NA	NA	NA	0	NA	NA	NA	NA	NA
ENSG00000176136	MC5R	melanocortin 5 receptor	4161	1,752418111	-0,149960024	1,453902	-0,103143158	0,917849353	NA
ENSG00000176153	GPX2	glutathione peroxidase 2	2877	0	NA	NA	NA	NA	NA
ENSG00000176160	HSF5	heat shock transcription factor 5	124535	1,233449936	-0,18197123	1,76935	-0,102846358	0,918084912	NA
ENSG00000176165	FOXG1	forkhead box G1	2290	0	NA	NA	NA	NA	NA
ENSG00000176170	SPHK1	sphingosine kinase 1	8877	0,918247208	-0,589915511	1,971624	-0,29920288	0,764785252	NA
ENSG00000176171	BNIP3	BCL2 interacting protein 3	664	9,551359141	0,089049553	0,647555	0,137516526	0,890622531	NA
ENSG00000176177	ENTHD1	ENTH domain containing 1	150350	0	NA	NA	NA	NA	NA
ENSG00000176183	NA	NA	NA	0	NA	NA	NA	NA	NA
ENSG00000176194	CIDEA	cell death inducing DFFA like effector a	1149	0	NA	NA	NA	NA	NA
ENSG00000176200	OR4D11	olfactory receptor family 4 subfamily D member 11	219986	0	NA	NA	NA	NA	NA
ENSG00000176204	LRRTM4	leucine rich repeat transmembrane neuronal 4	80059	0,119673877	0,847101401	4,080456	0,207599697	0,835541541	NA
ENSG00000176222	ZNF404	zinc finger protein 404	342908	7,027136791	0,322370861	0,819897	0,393184723	0,694183052	NA
ENSG00000176231	OR10H4	olfactory receptor family 10 subfamily H member 4	126541	0	NA	NA	NA	NA	NA
ENSG00000176232	NA	NA	NA	0	NA	NA	NA	NA	NA
ENSG00000176239	OR51B6	olfactory receptor family 51 subfamily B member 6	390058	0	NA	NA	NA	NA	NA
ENSG00000176243	NA	NA	NA	0	NA	NA	NA	NA	NA
ENSG00000176244	ACBD7	acyl-CoA binding domain containing 7	414149	2,056483435	1,734792147	1,519312	1,141827636	0,253525673	NA
ENSG00000176256	HMGB4	high mobility group box 4	127540	0	NA	NA	NA	NA	NA
ENSG00000176268	NA	NA	NA	0,967149078	-0,371459924	2,060842	-0,180246716	0,856958885	NA
ENSG00000176269	OR4F21	olfactory receptor family 4 subfamily F member 21	441308	0	NA	NA	NA	NA	NA
ENSG00000176273	SLC35G1	solute carrier family 35 member G1	159371	5,239870321	-0,505139794	0,762186	-0,662751585	0,507489666	NA
ENSG00000176289	NA	NA	NA	0	NA	NA	NA	NA	NA
ENSG00000176293	ZNF135	zinc finger protein 135	7694	0,176709267	0,860992396	4,080456	0,211003972	0,832884172	NA
ENSG00000176294	OR4N2	olfactory receptor family 4 subfamily N member 2	390429	0	NA	NA	NA	NA	NA
ENSG00000176299	OR4M1	olfactory receptor family 4 subfamily M member 1	441670	0	NA	NA	NA	NA	NA
ENSG00000176302	FOXR1	forkhead box R1	283150	0	NA	NA	NA	NA	NA
ENSG00000176312	OR4H12P	olfactory receptor family 4 subfamily H member 12 pseudogene	161271	0	NA	NA	NA	NA	NA
ENSG00000176318	FOXN3P1	forkhead box N3 pseudogene 1	643976	0	NA	NA	NA	NA	NA
ENSG00000176343	NA	NA	NA	0	NA	NA	NA	NA	NA
ENSG00000176358	TAC4	tachykinin precursor 4	255061	0	NA	NA	NA	NA	NA
ENSG00000176378	NA	NA	NA	0	NA	NA	NA	NA	NA
ENSG00000176381	PRR18	proline rich 18	285800	0	NA	NA	NA	NA	NA
ENSG00000176383	B3GNT4	UDP-GlcNAc:betaGal beta-1,3-N-acetylglucosaminyltransferase 4	79369	0	NA	NA	NA	NA	NA

ENSG00000176387	HSD11B2	hydroxysteroid 11-beta dehydrogenase 2	3291	0	NA	NA	NA	NA	NA
ENSG00000176399	DMRTA1	DMRT like family A1	63951	0	NA	NA	NA	NA	NA
ENSG00000176402	GJC3	gap junction protein gamma 3	349149	0	NA	NA	NA	NA	NA
ENSG00000176406	RIMS2	regulating synaptic membrane exocytosis 2	9699	0,072165449	0,847101401	4,080456	0,207599697	0,835541541	NA
ENSG00000176428	VPS37D	VPS37D subunit of ESCRT-I	155382	0,223624566	-1,080456797	4,080456	-0,264788257	0,791172584	NA
ENSG00000176435	CLEC14A	C-type lectin domain containing 14A	161198	0	NA	NA	NA	NA	NA
ENSG00000176454	LPCAT4	lysophosphatidylcholine acyltransferase 4	254531	11,82866516	0	4,080456	0	NA	NA
ENSG00000176472	ZNF575	zinc finger protein 575	284346	7,091335452	0,261520839	0,777903	0,336186976	0,736729873	NA
ENSG00000176490	DIRAS1	DIRAS family GTPase 1	148252	0,239347754	1,967469188	4,021404	0,489249276	0,624665227	NA
ENSG00000176495	OR5AN1	olfactory receptor family 5 subfamily AN member 1	390195	0	NA	NA	NA	NA	NA
ENSG00000176532	PRR15	proline rich 15	222171	0	NA	NA	NA	NA	NA
ENSG00000176533	GNG7	G protein subunit gamma 7	2788	0,58728912	1,387343953	2,778431	0,499326348	0,617549496	NA
ENSG00000176540	OR4C5	olfactory receptor family 4 subfamily C member 5	79346	0	NA	NA	NA	NA	NA
ENSG00000176547	OR4C3	olfactory receptor family 4 subfamily C member 3	256144	0	NA	NA	NA	NA	NA
ENSG00000176555	OR4S1	olfactory receptor family 4 subfamily S member 1	256148	0	NA	NA	NA	NA	NA
ENSG00000176563	CNTD1	cyclin N-terminal domain containing 1	124817	0	NA	NA	NA	NA	NA
ENSG00000176566	DCAF4L2	DDB1 and CUL4 associated factor 4 like 2	138009	0	NA	NA	NA	NA	NA
ENSG00000176567	OR4X1	olfactory receptor family 4 subfamily X member 1	390113	0	NA	NA	NA	NA	NA
ENSG00000176571	CNBD1	cyclic nucleotide binding domain containing 1	168975	0	NA	NA	NA	NA	NA
ENSG00000176584	DMBT1L1	deleted in malignant brain tumors 1 like 1 (pseudogene)	375940	0	NA	NA	NA	NA	NA
ENSG00000176595	KBTBD11	kelch repeat and BTB domain containing 11	9920	0	NA	NA	NA	NA	NA
ENSG00000176597	B3GNT5	UDP-GlcNAc:betaGal beta-1,3-N-acetylglucosaminyltransferase 5	84002	3,914865733	0,541632561	0,964272	0,561701166	0,57431964	NA
ENSG00000176601	MAP3K19	mitogen-activated protein kinase kinase kinase 19	80122	0	NA	NA	NA	NA	NA
ENSG00000176635	HORMAD2	HORMA domain containing 2	150280	0	NA	NA	NA	NA	NA
ENSG00000176641	RNF152	ring finger protein 152	220441	3,901957278	1,904146153	1,023766	1,859943621	0,062893503	NA
ENSG00000176654	NA	NA	NA	0	NA	NA	NA	NA	NA
ENSG00000176678	FOXL1	forkhead box L1	2300	0	NA	NA	NA	NA	NA
ENSG00000176679	TGIF2LY	TGFB induced factor homeobox 2 like Y-linked	90655	0	NA	NA	NA	NA	NA
ENSG00000176681	LRRC37A	leucine rich repeat containing 37A	9884	0,426488868	-1,703889727	3,988392	-0,427212254	0,669224737	NA
ENSG00000176692	FOXC2	forkhead box C2	2303	0	NA	NA	NA	NA	NA
ENSG00000176695	OR4F17	olfactory receptor family 4 subfamily F member 17	81099	0	NA	NA	NA	NA	NA
ENSG00000176697	BDNF	brain derived neurotrophic factor	627	0	NA	NA	NA	NA	NA
ENSG00000176714	CCDC121	coiled-coil domain containing 121	79635	6,46899625	-0,556198977	0,83659	-0,664840399	0,50615258	NA
ENSG00000176716	OR10AB1P	olfactory receptor family 10 subfamily AB member 1 pseudogene	390091	0	NA	NA	NA	NA	NA
ENSG00000176720	BOK	BCL2 family apoptosis regulator BOK	666	1,308582466	-0,349381315	1,789525	-0,195236897	0,845207495	NA
ENSG00000176723	ZNF843	zinc finger protein 843	283933	7,57319707	-0,90076222	0,76553	-1,176652294	0,239334316	NA
ENSG00000176732	PFN4	profilin family member 4	375189	0,10488184	-1,076443944	4,080456	-0,263804824	0,791930317	NA
ENSG00000176742	OR51V1	olfactory receptor family 51 subfamily V member 1	283111	0	NA	NA	NA	NA	NA
ENSG00000176746	MAGEB6	MAGE family member B6	158809	0	NA	NA	NA	NA	NA
ENSG00000176748	OR52Z1P	olfactory receptor family 52 subfamily Z member 1 pseudogene	283110	0	NA	NA	NA	NA	NA
ENSG00000176752	NA	NA	NA	0	NA	NA	NA	NA	NA
ENSG00000176761	NA	NA	NA	0	NA	NA	NA	NA	NA
ENSG00000176769	TCERG1L	transcription elongation regulator 1 like	256536	0	NA	NA	NA	NA	NA
ENSG00000176771	NCKAP5	NCK associated protein 5	344148	0	NA	NA	NA	NA	NA
ENSG00000176774	MAGEB18	MAGE family member B18	286514	0	NA	NA	NA	NA	NA
ENSG00000176783	RUFY1	RUN and FYVE domain containing 1	80230	0	NA	NA	NA	NA	NA
ENSG00000176788	BASP1	brain abundant membrane attached signal protein 1	10409	0	NA	NA	NA	NA	NA

ENSG00000176798	OR51L1	olfactory receptor family 51 subfamily L member 1	119682	0	NA	NA	NA	NA	NA
ENSG00000176812	NA	NA	NA	0	NA	NA	NA	NA	NA
ENSG00000176826	NA	NA	NA	0	NA	NA	NA	NA	NA
ENSG00000176834	VSIG10	V-set and immunoglobulin domain containing 10	54621	0,263227981	-1,076602582	4,080456	-0,263843702	0,791900358	NA
ENSG00000176842	IRX5	iroquois homeobox 5	10265	0,11808024	-1,076443944	4,080456	-0,263804824	0,791930317	NA
ENSG00000176855	KRT18P28	keratin 18 pseudogene 28	343326	0	NA	NA	NA	NA	NA
ENSG00000176857	NA	NA	NA	0	NA	NA	NA	NA	NA
ENSG00000176882	NA	NA	NA	0	NA	NA	NA	NA	NA
ENSG00000176884	GRIN1	glutamate ionotropic receptor NMDA type subunit 1	2902	0,22523445	-0,074591666	4,079694	-0,018283645	0,985412575	NA
ENSG00000176887	SOX11	SRY-box transcription factor 11	6664	0,176063251	-0,082902843	4,080456	-0,020317054	0,983790451	NA
ENSG00000176893	OR51G2	olfactory receptor family 51 subfamily G member 2	81282	0	NA	NA	NA	NA	NA
ENSG00000176895	OR51A7	olfactory receptor family 51 subfamily A member 7	119687	0	NA	NA	NA	NA	NA
ENSG00000176900	OR51T1	olfactory receptor family 51 subfamily T member 1	401665	0	NA	NA	NA	NA	NA
ENSG00000176904	NA	NA	NA	0	NA	NA	NA	NA	NA
ENSG00000176907	TCIM	transcriptional and immune response regulator	56892	0	NA	NA	NA	NA	NA
ENSG00000176909	MAMSTR	MEF2 activating motif and SAP domain containing transcriptional regulator	284358	3,901745655	0,783437419	1,051619	0,744982216	0,456282471	NA
ENSG00000176919	C8G	complement C8 gamma chain	733	0	NA	NA	NA	NA	NA
ENSG00000176920	FUT2	fucosyltransferase 2	2524	0	NA	NA	NA	NA	NA
ENSG00000176922	OR51S1	olfactory receptor family 51 subfamily S member 1	119692	0	NA	NA	NA	NA	NA
ENSG00000176923	NA	NA	NA	0,10543123	0	4,080456	0	1	NA
ENSG00000176925	OR51F2	olfactory receptor family 51 subfamily F member 2	119694	0	NA	NA	NA	NA	NA
ENSG00000176927	EFCAB5	EF-hand calcium binding domain 5	374786	0,288661797	2,306206831	3,997774	0,57687273	0,564025434	NA
ENSG00000176928	GCNT4	glucosaminyl (N-acetyl) transferase 4	51301	0,263043241	-1,078280674	4,080456	-0,264254953	0,791583469	NA
ENSG00000176933	NA	NA	NA	0,400424307	-1,026017792	3,075989	-0,333557036	0,738713843	NA
ENSG00000176945	MUC20	mucin 20, cell surface associated	200958	0,434818771	0,84564972	4,080456	0,207243932	0,835819359	NA
ENSG00000176951	OR51N1P	olfactory receptor family 51 subfamily N member 1 pseudogene	119696	0	NA	NA	NA	NA	NA
ENSG00000176956	LY6H	lymphocyte antigen 6 family member H	4062	0	NA	NA	NA	NA	NA
ENSG00000176970	NA	NA	NA	0	NA	NA	NA	NA	NA
ENSG00000176971	FIBIN	fin bud initiation factor homolog	387758	0	NA	NA	NA	NA	NA
ENSG00000176974	SHMT1	serine hydroxymethyltransferase 1	6470	0	NA	NA	NA	NA	NA
ENSG00000176979	TRIM60	tripartite motif containing 60	166655	0	NA	NA	NA	NA	NA
ENSG00000176988	FMR1NB	FMR1 neighbor	158521	0,10488184	-1,076443944	4,080456	-0,263804824	0,791930317	NA
ENSG00000176998	NA	NA	NA	0,590667673	0,859241901	2,321564	0,370113407	0,711297993	NA
ENSG00000177025	C19orf18	chromosome 19 open reading frame 18	147685	1,028636223	-0,1191206	2,572787	-0,046300211	0,963070971	NA
ENSG00000177045	SIX5	SIX homeobox 5	147912	4,380852815	0,514575115	1,253067	0,410652385	0,681327446	NA
ENSG00000177047	IFNW1	interferon omega 1	3467	0	NA	NA	NA	NA	NA
ENSG00000177076	ACER2	alkaline ceramidase 2	340485	4,531864731	1,584643165	1,065655	1,487012858	0,137011415	NA
ENSG00000177096	PHETA2	PH domain containing endocytic trafficking adaptor 2	150368	8,73134313	-0,309870855	0,654418	-0,473505808	0,635852358	NA
ENSG00000177098	SCN4B	sodium voltage-gated channel beta subunit 4	6330	0	NA	NA	NA	NA	NA
ENSG00000177103	DSCAML1	DS cell adhesion molecule like 1	57453	0,117497108	0,847101401	4,080456	0,207599697	0,835541541	NA
ENSG00000177106	EPS8L2	EPS8 like 2	64787	1,944394903	0,082023422	1,560452	0,052563882	0,958079395	NA
ENSG00000177108	ZDHHC22	zinc finger DHHC-type palmitoyltransferase 22	283576	0	NA	NA	NA	NA	NA
ENSG00000177138	FAM9B	family with sequence similarity 9 member B	171483	0	NA	NA	NA	NA	NA
ENSG00000177143	CETN1	centrin 1	1068	0	NA	NA	NA	NA	NA
ENSG00000177151	OR2T35	olfactory receptor family 2 subfamily T member 35	403244	0	NA	NA	NA	NA	NA
ENSG00000177174	OR14C36	olfactory receptor family 14 subfamily C member 36	127066	0	NA	NA	NA	NA	NA
ENSG00000177181	RIMKLA	ribosomal modification protein rimK like family member A	284716	0,49865195	-1,99743291	2,94551	-0,678128084	0,497690486	NA
ENSG00000177182	CLVS1	clavesin 1	157807	0	NA	NA	NA	NA	NA

ENSG00000177186	OR2M7	olfactory receptor family 2 subfamily M member 7	391196	0	NA	NA	NA	NA	NA
ENSG00000177197	NA	NA	NA	0,237959321	0,84663748	4,080456	0,207486003	0,835630323	NA
ENSG00000177201	OR2T12	olfactory receptor family 2 subfamily T member 12	127064	0,10543123	0	4,080456	0	1	NA
ENSG00000177202	SPACA4	sperm acrosome associated 4	171169	0	NA	NA	NA	NA	NA
ENSG00000177212	OR2T33	olfactory receptor family 2 subfamily T member 33	391195	0	NA	NA	NA	NA	NA
ENSG00000177233	OR2M1P	olfactory receptor family 2 subfamily M member 1 pseudogene	388762	0	NA	NA	NA	NA	NA
ENSG00000177238	TRIM72	tripartite motif containing 72	493829	0,76849319	0,915762042	2,052231	0,446227534	0,655432896	NA
ENSG00000177257	DEFB4A	defensin beta 4A	1673	0	NA	NA	NA	NA	NA
ENSG00000177257	DEFB4B	defensin beta 4B	100289462	0	NA	NA	NA	NA	NA
ENSG00000177257	LOC124906748	beta-defensin 4A	124906748	0	NA	NA	NA	NA	NA
ENSG00000177261	NA	NA	NA	0	NA	NA	NA	NA	NA
ENSG00000177272	KCNA3	potassium voltage-gated channel subfamily A member 3	3738	0	NA	NA	NA	NA	NA
ENSG00000177275	OR2AJ1	olfactory receptor family 2 subfamily AJ member 1	127608	0	NA	NA	NA	NA	NA
ENSG00000177283	FZD8	frizzled class receptor 8	8325	0,191493897	1,676703086	4,046302	0,414379085	0,678596503	NA
ENSG00000177291	GJD4	gap junction protein delta 4	219770	0	NA	NA	NA	NA	NA
ENSG00000177294	FBXO39	F-box protein 39	162517	0,784058136	-0,181361497	2,209208	-0,082093461	0,934572393	NA
ENSG00000177300	CLDN22	claudin 22	53842	0	NA	NA	NA	NA	NA
ENSG00000177303	CASKIN2	CASK interacting protein 2	57513	6,558735675	0,432009041	0,748541	0,577134424	0,563848652	NA
ENSG00000177306	OR7E125P	olfactory receptor family 7 subfamily E member 125 pseudogene	389616	0	NA	NA	NA	NA	NA
ENSG00000177324	BEND2	BEN domain containing 2	139105	0	NA	NA	NA	NA	NA
ENSG00000177350	RPL13AP3	ribosomal protein L13a pseudogene 3	645683	0,117497108	0,847101401	4,080456	0,207599697	0,835541541	NA
ENSG00000177354	C10orf71	chromosome 10 open reading frame 71	118461	0	NA	NA	NA	NA	NA
ENSG00000177359	OVOS2	alpha-2-macroglobulin like 1 pseudogene	144203	3,004309536	-1,029973405	1,633122	-0,630677491	0,52825142	NA
ENSG00000177363	LRRN4CL	LRRN4 C-terminal like	221091	0	NA	NA	NA	NA	NA
ENSG00000177370	TIMM22	translocase of inner mitochondrial membrane 22	29928	35,20843041	-0,974964319	2,457555	-0,39672134	NA	NA
ENSG00000177398	UMODL1	uromodulin like 1	89766	0	NA	NA	NA	NA	NA
ENSG00000177414	UBE2U	ubiquitin conjugating enzyme E2 U	148581	0	NA	NA	NA	NA	NA
ENSG00000177418	NA	NA	NA	0	NA	NA	NA	NA	NA
ENSG00000177447	NA	NA	NA	0	NA	NA	NA	NA	NA
ENSG00000177452	RPL19P3	ribosomal protein L19 pseudogene 3	100131759	0	NA	NA	NA	NA	NA
ENSG00000177453	NIM1K	NIM1 serine/threonine protein kinase	167359	0,715597756	-1,564066503	2,394904	-0,65308122	0,513703918	NA
ENSG00000177455	CD19	CD19 molecule	930	0,11808024	-1,076443944	4,080456	-0,263804824	0,791930317	NA
ENSG00000177459	ERICH5	glutamate rich 5	203111	0	NA	NA	NA	NA	NA
ENSG00000177462	OR2T8	olfactory receptor family 2 subfamily T member 8	343172	0,118286652	0	4,080456	0	1	NA
ENSG00000177464	GPR4	G protein-coupled receptor 4	2828	0	NA	NA	NA	NA	NA
ENSG00000177468	OLIG3	oligodendrocyte transcription factor 3	167826	6,540390232	0,164599468	0,718178	0,229190235	0,818721062	NA
ENSG00000177469	CAVIN1	caveolae associated protein 1	284119	0	NA	NA	NA	NA	NA
ENSG00000177476	OR2G3	olfactory receptor family 2 subfamily G member 3	81469	0	NA	NA	NA	NA	NA
ENSG00000177483	RBM44	RNA binding motif protein 44	375316	2,129390141	0,295267939	2,343931	0,125971234	0,899754695	NA
ENSG00000177489	OR2G2	olfactory receptor family 2 subfamily G member 2	81470	0	NA	NA	NA	NA	NA
ENSG00000177494	ZBED2	zinc finger BED-type containing 2	79413	9,027678	1,115098674	0,740555	1,505761553	0,132128396	NA
ENSG00000177504	VCX2	variable charge X-linked 2	51480	0	NA	NA	NA	NA	NA
ENSG00000177508	IRX3	iroquois homeobox 3	79191	0,117497108	0,847101401	4,080456	0,207599697	0,835541541	NA
ENSG00000177511	ST8SIA3	ST8 alpha-N-acetyl-neuraminide alpha-2,8-sialyltransferase 3	51046	0	NA	NA	NA	NA	NA
ENSG00000177519	RPRM	reprimo, TP53 dependent G2 arrest mediator homolog	56475	0	NA	NA	NA	NA	NA
ENSG00000177535	OR2B11	olfactory receptor family 2 subfamily B member 11	127623	0	NA	NA	NA	NA	NA
ENSG00000177551	NHLH2	nescient helix-loop-helix 2	4808	0	NA	NA	NA	NA	NA
ENSG00000177558	FAM187B	family with sequence similarity 187 member B	148109	0	NA	NA	NA	NA	NA
ENSG00000177570	SAMD12	sterile alpha motif domain containing 12	401474	0,4374016	-0,113596137	3,582913	-0,031704968	0,974707333	NA
ENSG00000177575	CD163	CD163 molecule	9332	0,384004782	0,834654021	3,678813	0,226881346	0,820516007	NA

ENSG00000177586	OR7E149P	olfactory receptor family 7 subfamily E member 149 pseudogene	282756	0	NA	NA	NA	NA	NA
ENSG00000177590	GIMAP3P	GTPase, IMAP family member 3 pseudogene	474345	0	NA	NA	NA	NA	NA
ENSG00000177599	ZNF491	zinc finger protein 491	126069	0,308049492	1,662070002	3,84651	0,432098178	0,665670059	NA
ENSG00000177614	PGBD5	piggyBac transposable element derived 5	79605	0,598730312	2,618864822	2,80993	0,93200361	0,35133466	NA
ENSG00000177627	C12orf54	chromosome 12 open reading frame 54	121273	0	NA	NA	NA	NA	NA
ENSG00000177669	MBOAT4	membrane bound O-acyltransferase domain containing 4	619373	0	NA	NA	NA	NA	NA
ENSG00000177673	TEX44	testis expressed 44	165100	0	NA	NA	NA	NA	NA
ENSG00000177675	CD163L1	CD163 molecule like 1	283316	0,10488184	-1,076443944	4,080456	-0,263804824	0,791930317	NA
ENSG00000177679	SRRM3	serine/arginine repetitive matrix 3	222183	0	NA	NA	NA	NA	NA
ENSG00000177684	DEFB114	defensin beta 114	245928	0	NA	NA	NA	NA	NA
ENSG00000177688	SUMO4	small ubiquitin like modifier 4	387082	2,81419396	0,606439731	1,34311	0,451519127	0,651615443	NA
ENSG00000177689	MAGEB10	MAGE family member B10	139422	0	NA	NA	NA	NA	NA
ENSG00000177692	DNAJC28	DnaJ heat shock protein family (Hsp40) member C28	54943	2,468400324	-0,172434729	2,732936	-0,063095048	0,949690818	NA
ENSG00000177694	NAALADL2	N-acetylated alpha-linked acidic dipeptidase like 2	254827	0,117497108	0,847101401	4,080456	0,207599697	0,835541541	NA
ENSG00000177710	SLC35G5	solute carrier family 35 member G5	83650	0,463266168	1,408433999	4,061038	0,346816263	0,728729358	NA
ENSG00000177731	FLII	FLII actin remodeling protein	2314	0	NA	NA	NA	NA	NA
ENSG00000177732	SOX12	SRY-box transcription factor 12	6666	10,96301312	-0,07072703	0,532577	-0,132801466	0,894350395	NA
ENSG00000177736	FXNP2	frataxin pseudogene 2	100418706	0,652973045	-0,961606531	2,82049	-0,340935932	0,733151814	NA
ENSG00000177752	YIPF7	Yip1 domain family member 7	285525	0	NA	NA	NA	NA	NA
ENSG00000177770	NA	NA	NA	0	NA	NA	NA	NA	NA
ENSG00000177776	NA	NA	NA	0	NA	NA	NA	NA	NA
ENSG00000177791	MYOZ1	myozenin 1	58529	0	NA	NA	NA	NA	NA
ENSG00000177803	NA	NA	NA	0	NA	NA	NA	NA	NA
ENSG00000177807	KCNJ10	potassium inwardly rectifying channel subfamily J member 10	3766	0	NA	NA	NA	NA	NA
ENSG00000177855	NA	NA	NA	0	NA	NA	NA	NA	NA
ENSG00000177875	CCDC184	coiled-coil domain containing 184	387856	0,65266251	-0,88496618	3,092719	-0,286145069	0,774767014	NA
ENSG00000177910	SPATA31C2	SPATA31 subfamily C member 2	645961	0	NA	NA	NA	NA	NA
ENSG00000177938	CAPZA3	capping actin protein of muscle Z-line subunit alpha 3	93661	0	NA	NA	NA	NA	NA
ENSG00000177947	CIMAP1A	ciliary microtubule associated protein 1A	113746	0	NA	NA	NA	NA	NA
ENSG00000177984	LCN15	lipocalin 15	389812	0	NA	NA	NA	NA	NA
ENSG00000177989	CIMAP1B	ciliary microtubule associated protein 1B	440836	0,559454047	-0,286971378	2,895302	-0,099116205	0,921046006	NA
ENSG00000177990	DPY19L2	dpy-19 like 2	283417	0,144330898	1,418786819	4,061262	0,349346267	0,726829368	NA
ENSG00000177992	SPATA31E1	SPATA31 subfamily E member 1	286234	0	NA	NA	NA	NA	NA
ENSG00000177994	C2orf73	chromosome 2 open reading frame 73	129852	0	NA	NA	NA	NA	NA
ENSG00000178015	GPR150	G protein-coupled receptor 150	285601	0,408798142	-0,111111279	3,64695	-0,030466901	0,97569469	NA
ENSG00000178021	TSPYL6	TSPY like 6	388951	0	NA	NA	NA	NA	NA
ENSG00000178026	LRRC75B	leucine rich repeat containing 75B	388886	0	NA	NA	NA	NA	NA
ENSG00000178031	ADAMTSL1	ADAMTS like 1	92949	0	NA	NA	NA	NA	NA
ENSG00000178033	CALHM5	calcium homeostasis modulator family member 5	254228	0	NA	NA	NA	NA	NA
ENSG00000178038	ALS2CL	ALS2 C-terminal like	259173	0	NA	NA	NA	NA	NA
ENSG00000178053	MLF1	myeloid leukemia factor 1	4291	3,165553695	-0,738522035	1,180223	-0,625747903	0,531480307	NA
ENSG00000178055	PRSS42P	serine protease 42, pseudogene	339906	0	NA	NA	NA	NA	NA
ENSG00000178075	GRAMD1C	GRAM domain containing 1C	54762	0,103998153	-1,076443944	4,080456	-0,263804824	0,791930317	NA
ENSG00000178078	STAP2	signal transducing adaptor family member 2	55620	1,564297526	-0,791304412	1,956203	-0,404510448	0,685837396	NA
ENSG00000178082	NA	NA	NA	0,281824483	-0,990805094	3,949148	-0,25089087	0,801898484	NA
ENSG00000178084	HTR3C	5-hydroxytryptamine receptor 3C	170572	0	NA	NA	NA	NA	NA
ENSG00000178093	TSSK6	testis specific serine kinase 6	83983	2,65982909	-0,144779994	1,376843	-0,105153631	0,916253904	NA
ENSG00000178125	PPP1R42	protein phosphatase 1 regulatory subunit 42	286187	0	NA	NA	NA	NA	NA
ENSG00000178146	NA	NA	NA	0	NA	NA	NA	NA	NA
ENSG00000178150	ZNF114	zinc finger protein 114	163071	1,365081366	1,954575133	2,295769	0,851381394	0,394557524	NA

ENSG00000178162	NA	NA	NA	0	NA	NA	NA	NA	NA
ENSG00000178171	AMER3	APC membrane recruitment protein 3	205147	0	NA	NA	NA	NA	NA
ENSG00000178172	SPINK6	serine peptidase inhibitor Kazal type 6	404203	0	NA	NA	NA	NA	NA
ENSG00000178175	ZNF366	zinc finger protein 366	167465	0	NA	NA	NA	NA	NA
ENSG00000178184	PARD6G	par-6 family cell polarity regulator gamma	84552	0	NA	NA	NA	NA	NA
ENSG00000178187	ZNF454	zinc finger protein 454	285676	0,11808024	-1,076443944	4,080456	-0,263804824	0,791930317	NA
ENSG00000178199	ZC3H12D	zinc finger CCCH-type containing 12D	340152	0,45374308	1,665974373	2,996105	0,556046714	0,578178921	NA
ENSG00000178201	VN1R1	vomeronasal 1 receptor 1	57191	1,824975465	-0,297554329	1,954049	-0,152275777	0,878969429	NA
ENSG00000178217	SH2D4B	SH2 domain containing 4B	387694	0,38666373	-0,973089593	3,095897	-0,314315836	0,753281172	NA
ENSG00000178222	RNF212	ring finger protein 212	285498	1,299565338	-2,434607534	1,880155	-1,294897208	0,195355692	NA
ENSG00000178226	PRSS36	serine protease 36	146547	0,961574994	1,452030385	2,621221	0,553951869	0,579611786	NA
ENSG00000178233	TMEM151B	transmembrane protein 151B	441151	0,15836288	0	4,080456	0	1	NA
ENSG00000178235	SLITRK1	SLIT and NTRK like family member 1	114798	0,10543123	0	4,080456	0	1	NA
ENSG00000178257	PRM3	protamine 3	58531	0	NA	NA	NA	NA	NA
ENSG00000178279	TNP2	transition protein 2	7142	0	NA	NA	NA	NA	NA
ENSG00000178287	SPAG11A	sperm associated antigen 11A	653423	0	NA	NA	NA	NA	NA
ENSG00000178297	TMPRSS9	transmembrane serine protease 9	360200	0	NA	NA	NA	NA	NA
ENSG00000178301	AQP11	aquaporin 11	282679	0,896811965	-1,932391286	3,031853	-0,637363082	0,52388837	NA
ENSG00000178342	KCNG2	potassium voltage-gated channel modifier subfamily G member 2	26251	0	NA	NA	NA	NA	NA
ENSG00000178343	SHISA3	shisa family member 3	152573	0,207996306	-1,916073151	4,043021	-0,473921178	0,635556117	NA
ENSG00000178358	OR2D3	olfactory receptor family 2 subfamily D member 3	120775	0	NA	NA	NA	NA	NA
ENSG00000178363	CALML3	calmodulin like 3	810	0	NA	NA	NA	NA	NA
ENSG00000178372	CALML5	calmodulin like 5	51806	0	NA	NA	NA	NA	NA
ENSG00000178386	ZNF223	zinc finger protein 223	7766	9,252175946	-0,597265037	1,084414	-0,550772342	0,581789746	NA
ENSG00000178394	HTR1A	5-hydroxytryptamine receptor 1A	3350	0,11808024	-1,076443944	4,080456	-0,263804824	0,791930317	NA
ENSG00000178395	CCDC185	coiled-coil domain containing 185	164127	0	NA	NA	NA	NA	NA
ENSG00000178401	DNAJC22	DnaJ heat shock protein family (Hsp40) member C22	79962	0	NA	NA	NA	NA	NA
ENSG00000178403	NEUROG2	neurogenin 2	63973	0	NA	NA	NA	NA	NA
ENSG00000178404	CEP295NL	CEP295 N-terminal like	100653515	0,117497108	0,847101401	4,080456	0,207599697	0,835541541	NA
ENSG00000178429	RPS3AP5	RPS3A pseudogene 5	439992	1,141498352	-2,606973292	1,9508	-1,336361056	0,181431289	NA
ENSG00000178445	GLDC	glycine decarboxylase	2731	0,11808024	-1,076443944	4,080456	-0,263804824	0,791930317	NA
ENSG00000178458	NA	NA	NA	0,222131921	-0,138859223	4,080456	-0,034030321	0,972852972	NA
ENSG00000178460	MCMDC2	minichromosome maintenance domain containing 2	157777	0,696451978	-1,916511907	2,480519	-0,772625473	0,439744064	NA
ENSG00000178462	TUBAL3	tubulin alpha like 3	79861	0	NA	NA	NA	NA	NA
ENSG00000178464	RPL10P16	ribosomal protein L10 pseudogene 16	284393	6,40234007	0,354801174	0,750923	0,472486685	0,636579444	NA
ENSG00000178473	UCN3	urocortin 3	114131	0	NA	NA	NA	NA	NA
ENSG00000178503	NA	NA	NA	0	NA	NA	NA	NA	NA
ENSG00000178522	AMBN	ameloblastin	258	0	NA	NA	NA	NA	NA
ENSG00000178531	CTXN1	cortexin 1	404217	0,103998153	-1,076443944	4,080456	-0,263804824	0,791930317	NA
ENSG00000178538	CA8	carbonic anhydrase 8	767	0	NA	NA	NA	NA	NA
ENSG00000178556	NA	NA	NA	0	NA	NA	NA	NA	NA
ENSG00000178562	CD28	CD28 molecule	940	0	NA	NA	NA	NA	NA
ENSG00000178568	ERBB4	erb-b2 receptor tyrosine kinase 4	2066	0	NA	NA	NA	NA	NA
ENSG00000178586	OR6B3	olfactory receptor family 6 subfamily B member 3	150681	0	NA	NA	NA	NA	NA
ENSG00000178591	DEFB125	defensin beta 125	245938	0	NA	NA	NA	NA	NA
ENSG00000178596	NA	NA	NA	0	NA	NA	NA	NA	NA
ENSG00000178597	PSAPL1	prosaposin like 1	768239	0,117497108	0,847101401	4,080456	0,207599697	0,835541541	NA
ENSG00000178602	OTOS	otospiralin	150677	0	NA	NA	NA	NA	NA
ENSG00000178631	NA	NA	NA	1,317911018	-0,69050186	1,735427	-0,397885956	0,690714252	NA
ENSG00000178636	NA	NA	NA	0	NA	NA	NA	NA	NA
ENSG00000178645	C10orf53	chromosome 10 open reading frame 53	282966	0,10543123	0	4,080456	0	1	NA
ENSG00000178654	NA	NA	NA	0	NA	NA	NA	NA	NA
ENSG00000178660	NA	NA	NA	0	NA	NA	NA	NA	NA
ENSG00000178662	CSRNP3	cysteine and serine rich nuclear protein 3	80034	0	NA	NA	NA	NA	NA
ENSG00000178665	ZNF713	zinc finger protein 713	349075	2,305107149	-1,602749697	1,556796	-1,029518112	0,303236273	NA
ENSG00000178690	DYNAP	dynactin associated protein	284254	0	NA	NA	NA	NA	NA
ENSG00000178695	KCTD12	potassium channel tetramerization domain containing 12	115207	0,804438946	2,740842578	2,590595	1,057997399	0,290056628	NA

ENSG00000178715	RPS16P1	ribosomal protein S16 pseudogene 1	441876	0	NA	NA	NA	NA	NA
ENSG00000178718	RPP25	ribonuclease P and MRP subunit p25	54913	6,68764838	-0,17238341	0,740524	-0,232785795	0,815927744	NA
ENSG00000178723	GLULP4	glutamate-ammonia ligase pseudogene 4	392305	0	NA	NA	NA	NA	NA
ENSG00000178726	THBD	thrombomodulin	7056	0	NA	NA	NA	NA	NA
ENSG00000178732	GP5	glycoprotein V platelet	2814	0,552103088	-0,980828939	2,558144	-0,383414215	0,701412665	NA
ENSG00000178750	STX19	syntaxin 19	415117	0	NA	NA	NA	NA	NA
ENSG00000178752	ERFE	erythroferrone	151176	0	NA	NA	NA	NA	NA
ENSG00000178762	H2BC2P	H2B clustered histone 2, pseudogene	100288742	0	NA	NA	NA	NA	NA
ENSG00000178772	CPN2	carboxypeptidase N subunit 2	1370	0	NA	NA	NA	NA	NA
ENSG00000178776	C5orf46	chromosome 5 open reading frame 46	389336	0	NA	NA	NA	NA	NA
ENSG00000178795	GDPD4	glycerophosphodiester phosphodiesterase domain containing 4	220032	0	NA	NA	NA	NA	NA
ENSG00000178796	RIIAD1	regulatory subunit of type II PKA R-subunit domain containing 1	284485	0	NA	NA	NA	NA	NA
ENSG00000178804	H1-8	H1,8 linker histone	132243	0	NA	NA	NA	NA	NA
ENSG00000178814	OPLAH	5-oxoprolinase, ATP-hydrolysing	26873	7,100050593	-0,120521806	0,715063	-0,16854718	0,866152831	NA
ENSG00000178821	TMEM52	transmembrane protein 52	339456	0	NA	NA	NA	NA	NA
ENSG00000178826	TMEM139	transmembrane protein 139	135932	0	NA	NA	NA	NA	NA
ENSG00000178828	RNF186	ring finger protein 186	54546	0	NA	NA	NA	NA	NA
ENSG00000178836	LOC100286922	DnaJ heat shock protein family (Hsp40) member B3 pseudogene	100286922	0	NA	NA	NA	NA	NA
ENSG00000178852	EFCAB13	EF-hand calcium binding domain 13	124989	4,108052661	0,379254441	1,098252	0,345325447	0,729849718	NA
ENSG00000178860	MSC	musculin	9242	3,139016618	0,921271036	1,030522	0,893984708	0,371330073	NA
ENSG00000178882	RFLNA	refilin A	144347	0	NA	NA	NA	NA	NA
ENSG00000178882	ZNF664-RFLNA	ZNF664-RFLNA readthrough	100533183	0	NA	NA	NA	NA	NA
ENSG00000178919	FOXE1	forkhead box E1	2304	0,103998153	-1,076443944	4,080456	-0,263804824	0,791930317	NA
ENSG00000178922	HYI	hydroxypyruvate isomerase (putative)	81888	4,074564651	-0,579984679	1,042953	-0,556098451	0,578143554	NA
ENSG00000178928	TPRX1	tetrapeptide repeat homeobox 1	284355	0	NA	NA	NA	NA	NA
ENSG00000178934	LGALS7	galectin 7	3963	0	NA	NA	NA	NA	NA
ENSG00000178934	LGALS7B	galectin 7B	653499	0	NA	NA	NA	NA	NA
ENSG00000178965	ERICH3	glutamate rich 3	127254	0,359021632	2,56113643	3,98322	0,642981427	0,520236152	NA
ENSG00000178997	EXD1	exonuclease 3'-5' domain containing 1	161829	0,117497108	0,847101401	4,080456	0,207599697	0,835541541	NA
ENSG00000179002	TAS1R2	taste 1 receptor member 2	80834	0,11808024	-1,076443944	4,080456	-0,263804824	0,791930317	NA
ENSG00000179008	C14orf39	chromosome 14 open reading frame 39	317761	0	NA	NA	NA	NA	NA
ENSG00000179023	KLHDC7A	kelch domain containing 7A	127707	0	NA	NA	NA	NA	NA
ENSG00000179031	NA	NA	NA	0	NA	NA	NA	NA	NA
ENSG00000179038	NA	NA	NA	0	NA	NA	NA	NA	NA
ENSG00000179044	EXOC3L1	exocyst complex component 3 like 1	283849	0	NA	NA	NA	NA	NA
ENSG00000179046	TRIML2	tripartite motif family like 2	205860	0	NA	NA	NA	NA	NA
ENSG00000179055	OR13D1	olfactory receptor family 13 subfamily D member 1	286365	0	NA	NA	NA	NA	NA
ENSG00000179057	IGSF22	immunoglobulin superfamily member 22	283284	0	NA	NA	NA	NA	NA
ENSG00000179058	C9orf50	chromosome 9 open reading frame 50	375759	0	NA	NA	NA	NA	NA
ENSG00000179059	ZFP42	ZFP42 zinc finger protein	132625	0	NA	NA	NA	NA	NA
ENSG00000179071	CCDC89	coiled-coil domain containing 89	220388	4,406723483	0,81095768	1,103311	0,735022009	0,462326091	NA
ENSG00000179073	NA	NA	NA	0	NA	NA	NA	NA	NA
ENSG00000179083	FAM133A	family with sequence similarity 133 member A	286499	0	NA	NA	NA	NA	NA
ENSG00000179088	C12orf42	chromosome 12 open reading frame 42	374470	0	NA	NA	NA	NA	NA
ENSG00000179097	HTR1F	5-hydroxytryptamine receptor 1F	3355	0	NA	NA	NA	NA	NA
ENSG00000179101	NA	NA	NA	0,178747705	0,825852766	4,079616	0,202433956	0,839577485	NA
ENSG00000179111	HES7	hes family bHLH transcription factor 7	84667	0,655894002	0,729564703	2,844555	0,256477624	0,797582056	NA
ENSG00000179131	RPL31P38	ribosomal protein L31 pseudogene 38	442326	0	NA	NA	NA	NA	NA
ENSG00000179133	C10orf67	chromosome 10 open reading frame 67	256815	0	NA	NA	NA	NA	NA

ENSG00000179142	CYP11B2	cytochrome P450 family 11 subfamily B member 2	1585	0	NA	NA	NA	NA	NA
ENSG00000179148	ALOXE3	arachidonate lipoxygenase 3	59344	0,072165449	0,847101401	4,080456	0,207599697	0,835541541	NA
ENSG00000179157	RPS2P28	ribosomal protein S2 pseudogene 28	100132673	0	NA	NA	NA	NA	NA
ENSG00000179165	PXT1	peroxisomal testis enriched protein 1	222659	0,144330898	1,418786819	4,061262	0,349346267	0,726829368	NA
ENSG00000179168	GGN	gametogenetin	199720	0	NA	NA	NA	NA	NA
ENSG00000179170	OR7E97P	olfactory receptor family 7 subfamily E member 97 pseudogene	79492	0	NA	NA	NA	NA	NA
ENSG00000179172	HNRNPCL1	heterogeneous nuclear ribonucleoprotein C like 1	343069	0	NA	NA	NA	NA	NA
ENSG00000179178	TMEM125	transmembrane protein 125	128218	0	NA	NA	NA	NA	NA
ENSG00000179213	SIGLECL1	SIGLEC family like 1	284369	0	NA	NA	NA	NA	NA
ENSG00000179241	LDLRAD3	low density lipoprotein receptor class A domain containing 3	143458	1,227403548	0,713593904	2,105716	0,338884266	0,734696918	NA
ENSG00000179242	CDH4	cadherin 4	1002	0	NA	NA	NA	NA	NA
ENSG00000179256	SMCO3	single-pass membrane protein with coiled-coil domains 3	440087	0	NA	NA	NA	NA	NA
ENSG00000179270	PCARE	photoreceptor cilium actin regulator	388939	0	NA	NA	NA	NA	NA
ENSG00000179277	NA	NA	NA	0	NA	NA	NA	NA	NA
ENSG00000179284	DAND5	DAN domain BMP antagonist family member 5	199699	0,10488184	-1,076443944	4,080456	-0,263804824	0,791930317	NA
ENSG00000179292	TMEM151A	transmembrane protein 151A	256472	0,144330898	1,418786819	4,061262	0,349346267	0,726829368	NA
ENSG00000179299	NSUN7	NOP2/Sun RNA methyltransferase family member 7	79730	0	NA	NA	NA	NA	NA
ENSG00000179300	RTL3	retrotransposon Gag like 3	203430	2,838902884	1,867839247	1,412245	1,32260247	0,185967612	NA
ENSG00000179314	WSCD1	WSC domain containing 1	23302	0	NA	NA	NA	NA	NA
ENSG00000179331	RAB39A	RAB39A, member RAS oncogene family	54734	0	NA	NA	NA	NA	NA
ENSG00000179342	NA	NA	NA	0,222236773	-1,078113081	4,080456	-0,264213881	0,791615116	NA
ENSG00000179348	GATA2	GATA binding protein 2	2624	0	NA	NA	NA	NA	NA
ENSG00000179362	NA	NA	NA	0,190557889	0,845995542	4,080456	0,207328683	0,835753175	NA
ENSG00000179363	TMEM31	transmembrane protein 31	203562	0	NA	NA	NA	NA	NA
ENSG00000179381	OR4K11P	olfactory receptor family 4 subfamily K member 11 pseudogene	81079	0	NA	NA	NA	NA	NA
ENSG00000179397	CATSPERE	catsper channel auxiliary subunit epsilon	257044	0,598369386	3,305404915	3,952639	0,83625258	0,403012824	NA
ENSG00000179399	GPC5	glypican 5	2262	0,119673877	0,847101401	4,080456	0,207599697	0,835541541	NA
ENSG00000179403	VWA1	von Willebrand factor A domain containing 1	64856	0,176951641	-0,111828874	4,080456	-0,027405975	0,978135932	NA
ENSG00000179407	DNAJB8	DnaJ heat shock protein family (Hsp40) member B8	165721	0	NA	NA	NA	NA	NA
ENSG00000179431	FJX1	four-jointed box kinase 1	24147	0,10543123	0	4,080456	0	1	NA
ENSG00000179443	OR13C6P	olfactory receptor family 13 subfamily C member 6 pseudogene	392311	0	NA	NA	NA	NA	NA
ENSG00000179455	MKRN3	makorin ring finger protein 3	7681	1,234450195	-0,775353013	1,751358	-0,442715413	0,657971592	NA
ENSG00000179460	NA	NA	NA	0	NA	NA	NA	NA	NA
ENSG00000179468	OR9A2	olfactory receptor family 9 subfamily A member 2	135924	0	NA	NA	NA	NA	NA
ENSG00000179477	ALOX12B	arachidonate 12-lipoxygenase, 12R type	242	0	NA	NA	NA	NA	NA
ENSG00000179520	SLC17A8	solute carrier family 17 member 8	246213	0,224058083	-0,09140451	4,080074	-0,022402659	0,982126759	NA
ENSG00000179528	LBX2	ladybird homeobox 2	85474	1,851912564	-0,471541366	1,468309	-0,32114587	0,74809985	NA
ENSG00000179542	SLITRK4	SLIT and NTRK like family member 4	139065	0,15836288	0	4,080456	0	1	NA
ENSG00000179546	HTR1D	5-hydroxytryptamine receptor 1D	3352	0	NA	NA	NA	NA	NA
ENSG00000179564	LSMEM2	leucine rich single-pass membrane protein 2	132228	0	NA	NA	NA	NA	NA
ENSG00000179571	NBPF17P	NBPF member 17, pseudogene	401967	0	NA	NA	NA	NA	NA
ENSG00000179593	ALOX15B	arachidonate 15-lipoxygenase type B	247	0,508497991	-1,085259468	3,403363	-0,318878513	0,749818639	NA
ENSG00000179600	GPHB5	glycoprotein hormone subunit beta 5	122876	0	NA	NA	NA	NA	NA
ENSG00000179603	GRM8	glutamate metabotropic receptor 8	2918	0	NA	NA	NA	NA	NA
ENSG00000179604	CDC42EP4	CDC42 effector protein 4	23580	1,219100728	-0,379037139	1,997545	-0,189751487	0,849503872	NA
ENSG00000179611	DGKZP1	diacylglycerol kinase zeta pseudogene 1	400126	0,235782488	0,84901625	4,080456	0,20806897	0,835175115	NA

ENSG00000179615	OR2AP1	olfactory receptor family 2 subfamily AP member 1	121129	0	NA	NA	NA	NA	NA
ENSG00000179626	OR6C4	olfactory receptor family 6 subfamily C member 4	341418	0	NA	NA	NA	NA	NA
ENSG00000179636	TPPP2	tubulin polymerization promoting protein family member 2	122664	0	NA	NA	NA	NA	NA
ENSG00000179673	RPRML	reprimo like	388394	0,103998153	-1,076443944	4,080456	-0,263804824	0,791930317	NA
ENSG00000179674	ARL14	ADP ribosylation factor like GTPase 14	80117	0	NA	NA	NA	NA	NA
ENSG00000179695	OR6C2	olfactory receptor family 6 subfamily C member 2	341416	0	NA	NA	NA	NA	NA
ENSG00000179698	WDR97	WD repeat domain 97	340390	2,192100269	1,469920893	1,754897	0,837611026	0,402249199	NA
ENSG00000179709	NLRP8	NLR family pyrin domain containing 8	126205	0	NA	NA	NA	NA	NA
ENSG00000179751	SYCN	syncollin	342898	0	NA	NA	NA	NA	NA
ENSG00000179761	PIPOX	pipecolic acid and sarcosine oxidase	51268	0,237164664	1,955810963	4,022315	0,4862401	0,626796943	NA
ENSG00000179766	NA	NA	NA	0	NA	NA	NA	NA	NA
ENSG00000179772	FOXS1	forkhead box S1	2307	0	NA	NA	NA	NA	NA
ENSG00000179774	ATOH7	atonal bHLH transcription factor 7	220202	0	NA	NA	NA	NA	NA
ENSG00000179776	CDH5	cadherin 5	1003	0	NA	NA	NA	NA	NA
ENSG00000179796	LRRC3B	leucine rich repeat containing 3B	116135	0	NA	NA	NA	NA	NA
ENSG00000179799	OR7E22P	olfactory receptor family 7 subfamily E member 22 pseudogene	9431	0	NA	NA	NA	NA	NA
ENSG00000179813	FAM216B	family with sequence similarity 216 member B	144809	0	NA	NA	NA	NA	NA
ENSG00000179817	MRGPRX4	MAS related GPR family member X4	117196	0	NA	NA	NA	NA	NA
ENSG00000179826	MRGPRX3	MAS related GPR family member X3	117195	0	NA	NA	NA	NA	NA
ENSG00000179846	NKPD1	NTPase KAP family P-loop domain containing 1	284353	4,19979931	0,13667705	1,136968	0,120211886	0,904315302	NA
ENSG00000179855	GIPC3	GIPC PDZ domain containing family member 3	126326	0,429773557	-1,539237249	3,998213	-0,384981315	0,700251259	NA
ENSG00000179862	CITED4	Cbp/p300 interacting transactivator with Glu/Asp rich carboxy-terminal domain 4	163732	0,973389915	-1,832056742	2,137376	-0,857152141	0,391360808	NA
ENSG00000179869	ABCA13	ATP binding cassette subfamily A member 13	154664	0,10543123	0	4,080456	0	1	NA
ENSG00000179873	NLRP11	NLR family pyrin domain containing 11	204801	0	NA	NA	NA	NA	NA
ENSG00000179886	TIGD5	tigger transposable element derived 5	84948	0	NA	NA	NA	NA	NA
ENSG00000179902	CFAP276	cilia and flagella associated protein 276	127003	0	NA	NA	NA	NA	NA
ENSG00000179913	B3GNT3	UDP-GlcNAc:betaGal beta-1,3-N-acetylglucosaminyltransferase 3	10331	0,17616426	-0,114683148	4,080456	-0,028105474	0,977578028	NA
ENSG00000179914	ITLN1	intelectin 1	55600	0	NA	NA	NA	NA	NA
ENSG00000179915	NRXN1	neurexin 1	9378	0,10543123	0	4,080456	0	1	NA
ENSG00000179919	OR10A7	olfactory receptor family 10 subfamily A member 7	121364	0	NA	NA	NA	NA	NA
ENSG00000179921	GPBAR1	G protein-coupled bile acid receptor 1	151306	3,538227771	0,094736502	1,033264	0,091686605	0,92694704	NA
ENSG00000179930	ZNF648	zinc finger protein 648	127665	0	NA	NA	NA	NA	NA
ENSG00000179938	GOLGA8J	golgin A8 family member J	653073	4,571396516	-0,44980191	1,072323	-0,41946499	0,674876336	NA
ENSG00000179954	SSC5D	scavenger receptor cysteine rich family member with 5 domains	284297	0,425470622	-1,272988938	3,53615	-0,359992901	0,718852442	NA
ENSG00000179965	ZNF771	zinc finger protein 771	51333	5,007225556	0,066951523	0,80464	0,083206801	0,933687105	NA
ENSG00000179967	NA	NA	NA	0,072165449	0,847101401	4,080456	0,207599697	0,835541541	NA
ENSG00000179988	PSTK	phosphoseryl-tRNA kinase	118672	8,529877239	-0,139972285	0,852378	-0,164213891	0,869562766	NA
ENSG00000179994	NA	NA	NA	0	NA	NA	NA	NA	NA
ENSG00000179997	NA	NA	NA	0	NA	NA	NA	NA	NA
ENSG00000180015	NA	NA	NA	0,954666027	0,784056939	2,775381	0,282504242	0,777556895	NA
ENSG00000180016	OR1E1	olfactory receptor family 1 subfamily E member 1	8387	0	NA	NA	NA	NA	NA
ENSG00000180019	NA	NA	NA	0	NA	NA	NA	NA	NA
ENSG00000180042	OR1R1P	olfactory receptor family 1 subfamily R member 1 pseudogene	9596	0	NA	NA	NA	NA	NA
ENSG00000180043	GARIN5B	golgi associated RAB2 interactor family member 5B	284418	0	NA	NA	NA	NA	NA

ENSG00000180044	C3orf80	chromosome 3 open reading frame 80	401097	0,118286652	0	4,080456	0	1	NA
ENSG00000180053	NKX2-6	NK2 homeobox 6	137814	0	NA	NA	NA	NA	NA
ENSG00000180061	TMEM150B	transmembrane protein 150B	284417	0	NA	NA	NA	NA	NA
ENSG00000180068	NA	NA	NA	0	NA	NA	NA	NA	NA
ENSG00000180071	ANKRD18A	ankyrin repeat domain 18A	253650	0	NA	NA	NA	NA	NA
ENSG00000180071	FAM95C	family with sequence similarity 95 member C	100289137	0	NA	NA	NA	NA	NA
ENSG00000180083	WFDC11	WAP four-disulfide core domain 11	259239	0	NA	NA	NA	NA	NA
ENSG00000180089	TMEM86B	transmembrane protein 86B	255043	5,734095347	-5,241576245	1,933283	-2,711230408	0,006703403	NA
ENSG00000180090	OR3A1	olfactory receptor family 3 subfamily A member 1	4994	0	NA	NA	NA	NA	NA
ENSG00000180113	TDRD6	tudor domain containing 6	221400	0	NA	NA	NA	NA	NA
ENSG00000180116	C12orf40	chromosome 12 open reading frame 40	283461	0,917213879	-0,288804894	2,195909	-0,131519494	0,895364367	NA
ENSG00000180138	CSNK1A1L	casein kinase 1 alpha 1 like	122011	0	NA	NA	NA	NA	NA
ENSG00000180150	NA	NA	NA	0	NA	NA	NA	NA	NA
ENSG00000180152	XIAPP3	X-linked inhibitor of apoptosis pseudogene 3	100128413	0,365912303	1,151615652	3,62507	0,317680949	0,750726964	NA
ENSG00000180155	LYNX1	Ly6/neurotoxin 1	66004	6,913562295	0,394345765	0,787459	0,500782846	0,616523959	NA
ENSG00000180172	NA	NA	NA	0	NA	NA	NA	NA	NA
ENSG00000180176	TH	tyrosine hydroxylase	7054	0	NA	NA	NA	NA	NA
ENSG00000180178	NA	NA	NA	0	NA	NA	NA	NA	NA
ENSG00000180189	NA	NA	NA	0	NA	NA	NA	NA	NA
ENSG00000180190	TDRP	testis development related protein	157695	0	NA	NA	NA	NA	NA
ENSG00000180205	WFDC9	WAP four-disulfide core domain 9	259240	0	NA	NA	NA	NA	NA
ENSG00000180209	MYL11	myosin light chain 11	29895	0	NA	NA	NA	NA	NA
ENSG00000180210	F2	coagulation factor II, thrombin	2147	0	NA	NA	NA	NA	NA
ENSG00000180211	RPL23P6	ribosomal protein L23 pseudogene 6	646949	0	NA	NA	NA	NA	NA
ENSG00000180219	GARIN6	golgi associated RAB2 interactor family member 6	196472	0	NA	NA	NA	NA	NA
ENSG00000180221	NA	NA	NA	1,188698125	-1,351089293	1,757292	-0,768847371	0,441983923	NA
ENSG00000180230	NACAP2	NACA pseudogene 2	651144	0	NA	NA	NA	NA	NA
ENSG00000180245	RRH	retinal pigment epithelium-derived rhodopsin homolog	10692	0,603320886	1,204830225	2,791129	0,431664101	0,665985562	NA
ENSG00000180251	SLC9A4	solute carrier family 9 member A4	389015	0	NA	NA	NA	NA	NA
ENSG00000180263	FGD6	FYVE, RhoGEF and PH domain containing 6	55785	1,584149743	1,13856923	1,678249	0,678426951	0,497501026	NA
ENSG00000180264	ADGRD2	adhesion G protein-coupled receptor D2	347088	0	NA	NA	NA	NA	NA
ENSG00000180269	GPR139	G protein-coupled receptor 139	124274	0	NA	NA	NA	NA	NA
ENSG00000180279	LINC01869	long intergenic non-protein coding RNA 1869	284365	0,15836288	0	4,080456	0	1	NA
ENSG00000180284	CNEP1R1P1	CNEP1R1 pseudogene 1	100130176	0	NA	NA	NA	NA	NA
ENSG00000180287	PLD5	phospholipase D family member 5	200150	0,175794376	-0,103124943	4,080456	-0,025272898	0,979837292	NA
ENSG00000180305	WFDC10A	WAP four-disulfide core domain 10A	140832	0	NA	NA	NA	NA	NA
ENSG00000180316	PNPLA1	patatin like phospholipase domain containing 1	285848	0,31672576	0	4,080456	0	1	NA
ENSG00000180318	ALX1	ALX homeobox 1	8092	0	NA	NA	NA	NA	NA
ENSG00000180332	KCTD4	potassium channel tetramerization domain containing 4	386618	0	NA	NA	NA	NA	NA
ENSG00000180336	MEIOC	meiosis specific with coiled-coil domain	284071	0,15836288	0	4,080456	0	1	NA
ENSG00000180340	FZD2	frizzled class receptor 2	2535	0,445057399	-1,039777536	3,002643	-0,346287437	0,729126709	NA
ENSG00000180347	ITPRID1	ITPR interacting domain containing 1	223075	0	NA	NA	NA	NA	NA
ENSG00000180383	DEFB124	defensin beta 124	245937	0	NA	NA	NA	NA	NA
ENSG00000180385	NA	NA	NA	0,103998153	-1,076443944	4,080456	-0,263804824	0,791930317	NA
ENSG00000180386	KRTAP9-7	keratin associated protein 9-7	100505724	0	NA	NA	NA	NA	NA
ENSG00000180389	NA	NA	NA	0	NA	NA	NA	NA	NA
ENSG00000180409	OR10AA1P	olfactory receptor family 10 subfamily AA member 1 pseudogene	81487	0	NA	NA	NA	NA	NA
ENSG00000180424	DEFB123	defensin beta 123	245936	0	NA	NA	NA	NA	NA
ENSG00000180432	CYP8B1	cytochrome P450 family 8 subfamily B member 1	1582	0	NA	NA	NA	NA	NA
ENSG00000180433	OR6K6	olfactory receptor family 6 subfamily K member 6	128371	0	NA	NA	NA	NA	NA

ENSG00000180437	OR6K4P	olfactory receptor family 6 subfamily K member 4 pseudogene	128370	0	NA	NA	NA	NA	NA
ENSG00000180438	TPRXL	tetrapeptide repeat homeobox like (pseudogene)	348825	0	NA	NA	NA	NA	NA
ENSG00000180440	SERTM1	serine rich and transmembrane domain containing 1	400120	0	NA	NA	NA	NA	NA
ENSG00000180447	GAS1	growth arrest specific 1	2619	3,768835119	0,961869634	1,098981	0,875237897	0,381444477	NA
ENSG00000180475	OR10Q1	olfactory receptor family 10 subfamily Q member 1	219960	0	NA	NA	NA	NA	NA
ENSG00000180481	GLIPR1L2	GLIPR1 like 2	144321	2,088829473	1,188069271	1,592519	0,746031467	0,455648407	NA
ENSG00000180483	DEFB119	defensin beta 119	245932	0	NA	NA	NA	NA	NA
ENSG00000180509	KCNE1	potassium voltage-gated channel subfamily E regulatory subunit 1	3753	0,322343514	1,407780659	4,061579	0,346609244	0,7288849	NA
ENSG00000180532	ZSCAN4	zinc finger and SCAN domain containing 4	201516	0	NA	NA	NA	NA	NA
ENSG00000180535	BHLHA15	basic helix-loop-helix family member a15	168620	0,812646101	-0,881712724	2,583033	-0,341347792	0,732841773	NA
ENSG00000180537	RNF182	ring finger protein 182	221687	0	NA	NA	NA	NA	NA
ENSG00000180543	TSPYL5	TSPY like 5	85453	2,043278271	-0,597387806	1,172157	-0,509648367	0,610297832	NA
ENSG00000180574	EIF2S3B	eukaryotic translation initiation factor 2 subunit gamma B	255308	0,340829443	-0,232704721	3,777872	-0,061596766	0,950883952	NA
ENSG00000180581	NA	NA	NA	0	NA	NA	NA	NA	NA
ENSG00000180592	SKIDA1	SKI/DACH domain containing 1	387640	0,19156092	1,677063237	4,046291	0,414469264	0,678530472	NA
ENSG00000180596	H2BC4	H2B clustered histone 4	8347	4,14438123	0,365105702	0,941843	0,387650167	0,698274939	NA
ENSG00000180610	NA	NA	NA	0	NA	NA	NA	NA	NA
ENSG00000180613	GSX2	GS homeobox 2	170825	0	NA	NA	NA	NA	NA
ENSG00000180616	SSTR2	somatostatin receptor 2	6752	0,118286652	0	4,080456	0	1	NA
ENSG00000180638	SLC47A2	solute carrier family 47 member 2	146802	0	NA	NA	NA	NA	NA
ENSG00000180658	OR2A4	olfactory receptor family 2 subfamily A member 4	79541	0	NA	NA	NA	NA	NA
ENSG00000180660	MAB21L1	mab-21 like 1	4081	0	NA	NA	NA	NA	NA
ENSG00000180662	RPL21P8	ribosomal protein L21 pseudogene 8	319145	0	NA	NA	NA	NA	NA
ENSG00000180663	NA	NA	NA	0	NA	NA	NA	NA	NA
ENSG00000180673	EXOC5P1	exocyst complex component 5 pseudogene 1	644548	0	NA	NA	NA	NA	NA
ENSG00000180697	C3orf22	chromosome 3 open reading frame 22	152065	0	NA	NA	NA	NA	NA
ENSG00000180708	OR10K2	olfactory receptor family 10 subfamily K member 2	391107	0	NA	NA	NA	NA	NA
ENSG00000180714	OR5AZ1P	olfactory receptor family 5 subfamily AZ member 1 pseudogene	283193	0	NA	NA	NA	NA	NA
ENSG00000180720	CHRM4	cholinergic receptor muscarinic 4	1132	8,709926721	0,633744898	0,674163	0,940047666	0,347193111	NA
ENSG00000180723	OR51A9P	olfactory receptor family 51 subfamily A member 9 pseudogene	390039	0	NA	NA	NA	NA	NA
ENSG00000180730	SHISA2	shisa family member 2	387914	0,10543123	0	4,080456	0	1	NA
ENSG00000180739	S1PR5	sphingosine-1-phosphate receptor 5	53637	3,063820701	-0,345395544	1,171374	-0,294863465	0,768098182	NA
ENSG00000180745	CLRN3	clarin 3	119467	0	NA	NA	NA	NA	NA
ENSG00000180764	PIPSL	PIP5K1A and PSMD4 like (pseudogene)	266971	0	NA	NA	NA	NA	NA
ENSG00000180767	CHST13	carbohydrate sulfotransferase 13	166012	0,224541536	-0,115592152	4,080456	-0,028328245	0,977400354	NA
ENSG00000180770	OR7E129P	olfactory receptor family 7 subfamily E member 129 pseudogene	391576	0	NA	NA	NA	NA	NA
ENSG00000180772	AGTR2	angiotensin II receptor type 2	186	0	NA	NA	NA	NA	NA
ENSG00000180777	ANKRD30B	ankyrin repeat domain 30B	374860	0	NA	NA	NA	NA	NA
ENSG00000180785	OR51E1	olfactory receptor family 51 subfamily E member 1	143503	0	NA	NA	NA	NA	NA
ENSG00000180801	ARSJ	arylsulfatase family member J	79642	0,103998153	-1,076443944	4,080456	-0,263804824	0,791930317	NA
ENSG00000180803	NA	NA	NA	0	NA	NA	NA	NA	NA
ENSG00000180806	HOXC9	homeobox C9	3225	0,103998153	-1,076443944	4,080456	-0,263804824	0,791930317	NA
ENSG00000180815	MAP3K15	mitogen-activated protein kinase kinase kinase 15	389840	0	NA	NA	NA	NA	NA
ENSG00000180818	HOXC10	homeobox C10	3226	0	NA	NA	NA	NA	NA
ENSG00000180828	BHLHE22	basic helix-loop-helix family member e22	27319	0	NA	NA	NA	NA	NA
ENSG00000180834	MAP6D1	MAP6 domain containing 1	79929	3,195368384	-2,049464704	2,130166	-0,962114974	0,335991853	NA

ENSG00000180871	CXCR2	C-X-C motif chemokine receptor 2	3579	0	NA	NA	NA	NA	NA
ENSG00000180872	DEFB112	defensin beta 112	245915	0	NA	NA	NA	NA	NA
ENSG00000180875	GREM2	gremlin 2, DAN family BMP antagonist	64388	0,15836288	0	4,080456	0	1	NA
ENSG00000180878	C11orf42	chromosome 11 open reading frame 42	160298	0	NA	NA	NA	NA	NA
ENSG00000180881	CAPS2	calcyphosine 2	84698	4,532423587	-0,567588914	1,226166	-0,462897449	0,643437876	NA
ENSG00000180891	CUEDC1	CUE domain containing 1	404093	0,190162076	-0,392305272	4,07363	-0,096303601	0,923279451	NA
ENSG00000180909	OR52B1P	olfactory receptor family 52 subfamily B member 1 pseudogene	81274	0,119673877	0,847101401	4,080456	0,207599697	0,835541541	NA
ENSG00000180910	NA	NA	NA	0	NA	NA	NA	NA	NA
ENSG00000180913	OR56B3P	olfactory receptor family 56 subfamily B member 3 pseudogene	401675	0	NA	NA	NA	NA	NA
ENSG00000180914	OXTR	oxytocin receptor	5021	1,559291497	0,245640375	1,512823	0,162372182	0,871012776	NA
ENSG00000180919	OR56B4	olfactory receptor family 56 subfamily B member 4	196335	0	NA	NA	NA	NA	NA
ENSG00000180926	OR7E18P	olfactory receptor family 7 subfamily E member 18 pseudogene	390885	0	NA	NA	NA	NA	NA
ENSG00000180929	GPR62	G protein-coupled receptor 62	118442	0	NA	NA	NA	NA	NA
ENSG00000180934	OR56A1	olfactory receptor family 56 subfamily A member 1	120796	0	NA	NA	NA	NA	NA
ENSG00000180974	OR52E4	olfactory receptor family 52 subfamily E member 4	390081	0	NA	NA	NA	NA	NA
ENSG00000180988	OR52N2	olfactory receptor family 52 subfamily N member 2	390077	0	NA	NA	NA	NA	NA
ENSG00000180998	GPR137C	G protein-coupled receptor 137C	283554	0,327779171	1,036874194	4,022514	0,257767699	0,796586194	NA
ENSG00000180999	C1orf105	chromosome 1 open reading frame 105	92346	0	NA	NA	NA	NA	NA
ENSG00000181001	OR52N1	olfactory receptor family 52 subfamily N member 1	79473	0	NA	NA	NA	NA	NA
ENSG00000181009	OR52N5	olfactory receptor family 52 subfamily N member 5	390075	0	NA	NA	NA	NA	NA
ENSG00000181016	LSMEM1	leucine rich single-pass membrane protein 1	286006	0,469988433	2,955860566	3,965096	0,745470098	0,455987581	NA
ENSG00000181017	OR56B2P	olfactory receptor family 56 subfamily B member 2 pseudogene	390073	0,11808024	-1,076443944	4,080456	-0,263804824	0,791930317	NA
ENSG00000181023	OR56B1	olfactory receptor family 56 subfamily B member 1	387748	0	NA	NA	NA	NA	NA
ENSG00000181031	RPH3AL	rabphilin 3A like (without C2 domains)	9501	1,607737149	-2,993181394	2,682346	-1,115881951	0,264472669	NA
ENSG00000181072	CHRM2	cholinergic receptor muscarinic 2	1129	0,11808024	-1,076443944	4,080456	-0,263804824	0,791930317	NA
ENSG00000181074	OR52N4	olfactory receptor family 52 subfamily N member 4	390072	0,354176625	0,846658144	4,080456	0,207491067	0,835626368	NA
ENSG00000181085	MAPK15	mitogen-activated protein kinase 15	225689	0	NA	NA	NA	NA	NA
ENSG00000181092	ADIPOQ	adiponectin, C1Q and collagen domain containing	9370	0	NA	NA	NA	NA	NA
ENSG00000181101	NA	NA	NA	0	NA	NA	NA	NA	NA
ENSG00000181109	OR52P1	olfactory receptor family 52 subfamily P member 1 (gene/pseudogene)	81248	0	NA	NA	NA	NA	NA
ENSG00000181143	MUC16	mucin 16, cell surface associated	94025	0,117497108	0,847101401	4,080456	0,207599697	0,835541541	NA
ENSG00000181195	PENK	proenkephalin	5179	0	NA	NA	NA	NA	NA
ENSG00000181201	H2BC27P	H2B clustered histone 27, pseudogene	337872	0,34140702	1,007795109	3,765777	0,267619461	0,78899225	NA
ENSG00000181214	OR8G2P	olfactory receptor family 8 subfamily G member 2 pseudogene	26492	0	NA	NA	NA	NA	NA
ENSG00000181218	H2AC25	H2A clustered histone 25	92815	0	NA	NA	NA	NA	NA
ENSG00000181222	POLR2A	RNA polymerase II subunit A	5430	0	NA	NA	NA	NA	NA
ENSG00000181227	NA	NA	NA	0,072165449	0,847101401	4,080456	0,207599697	0,835541541	NA
ENSG00000181234	TMEM132C	transmembrane protein 132C	92293	0,103998153	-1,076443944	4,080456	-0,263804824	0,791930317	NA
ENSG00000181240	SLC25A41	solute carrier family 25 member 41	284427	0	NA	NA	NA	NA	NA
ENSG00000181260	MTHFD2P7	methylenetetrahydrofolate dehydrogenase (NADP+ dependent) 2, methenyltetrahydrofolate cyclohydrolase pseudogene 7	442098	0	NA	NA	NA	NA	NA
ENSG00000181264	TLCD5	TLC domain containing 5	219902	2,158052172	2,239916966	1,738118	1,288702675	0,197501472	NA

ENSG00000181273	OR5AK2	olfactory receptor family 5 subfamily AK member 2	390181	0	NA	NA	NA	NA	NA
ENSG00000181282	OR5AK3P	olfactory receptor family 5 subfamily AK member 3 pseudogene	81228	0	NA	NA	NA	NA	NA
ENSG00000181291	TMEM132E	transmembrane protein 132E	124842	1,601711108	0,033324913	1,715141	0,019429841	0,984498205	NA
ENSG00000181322	NME9	NME/NM23 family member 9	347736	0,354859955	0	4,080456	0	1	NA
ENSG00000181333	HEPHL1	hephaestin like 1	341208	0	NA	NA	NA	NA	NA
ENSG00000181350	LRRC75A	leucine rich repeat containing 75A	388341	10,43064342	0,134354261	1,125744	0,119347055	0,905000406	NA
ENSG00000181355	NA	NA	NA	0	NA	NA	NA	NA	NA
ENSG00000181358	CTAGE10P	CTAGE family member 10, pseudogene	220429	0	NA	NA	NA	NA	NA
ENSG00000181359	HSP90AA6P	heat shock protein 90 alpha family class A member 6, pseudogene	441051	0	NA	NA	NA	NA	NA
ENSG00000181374	CCL13	C-C motif chemokine ligand 13	6357	0	NA	NA	NA	NA	NA
ENSG00000181378	CFAP65	cilia and flagella associated protein 65	255101	0,10543123	0	4,080456	0	1	NA
ENSG00000181392	SYNE4	spectrin repeat containing nuclear envelope family member 4	163183	0,118286652	0	4,080456	0	1	NA
ENSG00000181408	UTS2R	urotensin 2 receptor	2837	0,504578	-0,121203944	2,910097	-0,041649456	0,966778147	NA
ENSG00000181418	DDN	dendrin	23109	0,551592289	-0,980712704	2,894162	-0,338858964	0,73471598	NA
ENSG00000181433	SAGE1	sarcoma antigen 1	55511	0	NA	NA	NA	NA	NA
ENSG00000181444	ZNF467	zinc finger protein 467	168544	0,118286652	0	4,080456	0	1	NA
ENSG00000181449	SOX2	SRY-box transcription factor 2	6657	0	NA	NA	NA	NA	NA
ENSG00000181458	TMEM45A	transmembrane protein 45A	55076	0	NA	NA	NA	NA	NA
ENSG00000181499	OR6T1	olfactory receptor family 6 subfamily T member 1	219874	0	NA	NA	NA	NA	NA
ENSG00000181514	LOC643067	HORMA domain containing 1 pseudogene	643067	0	NA	NA	NA	NA	NA
ENSG00000181518	OR8D4	olfactory receptor family 8 subfamily D member 4	338662	0	NA	NA	NA	NA	NA
ENSG00000181524	RPL24P4	RPL24 pseudogene 4	377381	0,366456732	0,457208744	3,707189	0,123330288	0,901845557	NA
ENSG00000181541	MAB21L2	mab-21 like 2	10586	0	NA	NA	NA	NA	NA
ENSG00000181552	EDDM3B	epididymal protein 3B	64184	0	NA	NA	NA	NA	NA
ENSG00000181562	EDDM3A	epididymal protein 3A	10876	0	NA	NA	NA	NA	NA
ENSG00000181585	TMIE	transmembrane inner ear	259236	0	NA	NA	NA	NA	NA
ENSG00000181609	OR52D1	olfactory receptor family 52 subfamily D member 1	390066	0	NA	NA	NA	NA	NA
ENSG00000181616	OR52H1	olfactory receptor family 52 subfamily H member 1	390067	0,355249323	0,987791904	3,722116	0,265384502	0,790713274	NA
ENSG00000181617	FDCSP	follicular dendritic cell secreted protein	260436	0	NA	NA	NA	NA	NA
ENSG00000181619	GPR135	G protein-coupled receptor 135	64582	8,353081879	0,187579475	0,641936	0,292208979	0,770126847	NA
ENSG00000181626	ANKRD62	ankyrin repeat domain 62	342850	0	NA	NA	NA	NA	NA
ENSG00000181631	P2RY13	purinergic receptor P2Y13	53829	0,10488184	-1,076443944	4,080456	-0,263804824	0,791930317	NA
ENSG00000181634	TNFSF15	TNF superfamily member 15	9966	2,210000852	-1,755789009	1,496738	-1,173076899	0,240764969	NA
ENSG00000181649	PHLDA2	pleckstrin homology like domain family A member 2	7262	0	NA	NA	NA	NA	NA
ENSG00000181652	ATG9B	autophagy related 9B	285973	0	NA	NA	NA	NA	NA
ENSG00000181656	GPR88	G protein-coupled receptor 88	54112	2,351751621	1,545993385	1,257845	1,229081083	0,219041407	NA
ENSG00000181690	PLAG1	PLAG1 zinc finger	5324	10,38726086	0,377927627	0,66779	0,565938019	0,571435915	NA
ENSG00000181705	NA	NA	NA	0	NA	NA	NA	NA	NA
ENSG00000181733	OR2Z1	olfactory receptor family 2 subfamily Z member 1	284383	0,955635404	-0,570953911	2,058843	-0,277317797	0,781536095	NA
ENSG00000181741	NA	NA	NA	0	NA	NA	NA	NA	NA
ENSG00000181752	OR8K5	olfactory receptor family 8 subfamily K member 5	219453	0	NA	NA	NA	NA	NA
ENSG00000181761	OR8H3	olfactory receptor family 8 subfamily H member 3	390152	0	NA	NA	NA	NA	NA
ENSG00000181767	OR8H2	olfactory receptor family 8 subfamily H member 2	390151	0	NA	NA	NA	NA	NA
ENSG00000181773	GPR3	G protein-coupled receptor 3	2827	0,468477094	2,344402466	2,872259	0,816222418	0,414372938	NA
ENSG00000181778	TMEM252	transmembrane protein 252	169693	0	NA	NA	NA	NA	NA
ENSG00000181780	OR5J1P	olfactory receptor family 5 subfamily J member 1 pseudogene	401687	0	NA	NA	NA	NA	NA
ENSG00000181781	CIMAP1D	CIMAP1 family member D	284451	0	NA	NA	NA	NA	NA
ENSG00000181785	OR5AS1	olfactory receptor family 5 subfamily AS member 1	219447	0	NA	NA	NA	NA	NA
ENSG00000181786	ACTL9	actin like 9	284382	0	NA	NA	NA	NA	NA
ENSG00000181790	ADGRB1	adhesion G protein-coupled receptor B1	575	0	NA	NA	NA	NA	NA
ENSG00000181803	OR6S1	olfactory receptor family 6 subfamily S member 1	341799	0	NA	NA	NA	NA	NA

ENSG00000181819	NA	NA	NA	0	NA	NA	NA	NA	NA
ENSG00000181837	OR5D17P	olfactory receptor family 5 subfamily D member 17 pseudogene	81196	0	NA	NA	NA	NA	NA
ENSG00000181867	FTMT	ferritin mitochondrial	94033	0	NA	NA	NA	NA	NA
ENSG00000181903	OR4C6	olfactory receptor family 4 subfamily C member 6	219432	0	NA	NA	NA	NA	NA
ENSG00000181927	OR4P4	olfactory receptor family 4 subfamily P member 4	81300	0	NA	NA	NA	NA	NA
ENSG00000181939	OR4C15	olfactory receptor family 4 subfamily C member 15	81309	0	NA	NA	NA	NA	NA
ENSG00000181943	OR4A21P	olfactory receptor family 4 subfamily A member 21 pseudogene	81322	0	NA	NA	NA	NA	NA
ENSG00000181950	OR4A13P	olfactory receptor family 4 subfamily A member 13 pseudogene	81330	0	NA	NA	NA	NA	NA
ENSG00000181958	OR4A15	olfactory receptor family 4 subfamily A member 15	81328	0	NA	NA	NA	NA	NA
ENSG00000181961	OR4A16	olfactory receptor family 4 subfamily A member 16	81327	0	NA	NA	NA	NA	NA
ENSG00000181963	OR52K2	olfactory receptor family 52 subfamily K member 2	119774	0	NA	NA	NA	NA	NA
ENSG00000181965	NEUROG1	neurogenin 1	4762	0	NA	NA	NA	NA	NA
ENSG00000181977	NA	NA	NA	0	NA	NA	NA	NA	NA
ENSG00000181982	CCDC149	coiled-coil domain containing 149	91050	0	NA	NA	NA	NA	NA
ENSG00000181984	GOLGA8CP	golgin A8 family member C, pseudogene	729786	0	NA	NA	NA	NA	NA
ENSG00000181995	LINC00301	long intergenic non-protein coding RNA 301	283197	0	NA	NA	NA	NA	NA
ENSG00000181997	AQP7P2	aquaporin 7 pseudogene 2	389756	0	NA	NA	NA	NA	NA
ENSG00000182000	NA	NA	NA	0	NA	NA	NA	NA	NA
ENSG00000182010	RTKN2	rhotekin 2	219790	5,570596522	-1,562215926	0,931091	-1,67783364	0,09337958	NA
ENSG00000182013	PNMA8A	PNMA family member 8A	55228	0,103998153	-1,076443944	4,080456	-0,263804824	0,791930317	NA
ENSG00000182022	CHST15	carbohydrate sulfotransferase 15	51363	0	NA	NA	NA	NA	NA
ENSG00000182035	ADIG	adipogenin	149685	0,072165449	0,847101401	4,080456	0,207599697	0,835541541	NA
ENSG00000182040	USH1G	USH1 protein network component sans	124590	0	NA	NA	NA	NA	NA
ENSG00000182048	NA	NA	NA	0	NA	NA	NA	NA	NA
ENSG00000182050	MGAT4C	MGAT4 family member C	25834	0	NA	NA	NA	NA	NA
ENSG00000182053	TRIM49B	tripartite motif containing 49B	283116	0	NA	NA	NA	NA	NA
ENSG00000182070	OR52A1	olfactory receptor family 52 subfamily A member 1	23538	0	NA	NA	NA	NA	NA
ENSG00000182083	OR6B2	olfactory receptor family 6 subfamily B member 2	389090	0	NA	NA	NA	NA	NA
ENSG00000182103	FAM181B	family with sequence similarity 181 member B	220382	0	NA	NA	NA	NA	NA
ENSG00000182107	TMEM30B	transmembrane protein 30B	161291	9,540656139	-0,008686197	0,546	-0,015908778	0,987307167	NA
ENSG00000182111	ZNF716	zinc finger protein 716	441234	0	NA	NA	NA	NA	NA
ENSG00000182118	FAM89A	family with sequence similarity 89 member A	375061	5,670562682	-0,359765684	0,732677	-0,49102873	0,623406128	NA
ENSG00000182132	KCNIP1	potassium voltage-gated channel interacting protein 1	30820	0	NA	NA	NA	NA	NA
ENSG00000182156	ENPP7	ectonucleotide pyrophosphatase/phosphodiest erase 7	339221	0	NA	NA	NA	NA	NA
ENSG00000182168	UNC5C	unc-5 netrin receptor C	8633	0	NA	NA	NA	NA	NA
ENSG00000182170	MRGPRG	MAS related GPR family member G	386746	0	NA	NA	NA	NA	NA
ENSG00000182175	RGMA	repulsive guidance molecule BMP co-receptor a	56963	0,072165449	0,847101401	4,080456	0,207599697	0,835541541	NA
ENSG00000182177	ASB18	ankyrin repeat and SOCS box containing 18	401036	0	NA	NA	NA	NA	NA
ENSG00000182183	SHISAL2A	shisa like 2A	348378	1,800393125	-2,831663826	1,848349	-1,531996024	0,125523416	NA
ENSG00000182187	CRYGB	crystallin gamma B	1419	0	NA	NA	NA	NA	NA
ENSG00000182218	HHIPL1	HHIP like 1	84439	0	NA	NA	NA	NA	NA
ENSG00000182223	ZAR1	zygote arrest 1	326340	0	NA	NA	NA	NA	NA
ENSG00000182240	BACE2	beta-secretase 2	25825	0,10488184	-1,076443944	4,080456	-0,263804824	0,791930317	NA
ENSG00000182247	UBE2E2	ubiquitin conjugating enzyme E2 E2	7325	0,189549789	1,664433219	4,047546	0,411220335	0,680910978	NA
ENSG00000182253	SYNM	synemin	23336	2,036186744	0,746410381	1,356776	0,550135467	0,582226462	NA
ENSG00000182255	KCNA4	potassium voltage-gated channel subfamily A member 4	3739	0,119673877	0,847101401	4,080456	0,207599697	0,835541541	NA
ENSG00000182256	GABRG3	gamma-aminobutyric acid type A receptor subunit gamma3	2567	0,117497108	0,847101401	4,080456	0,207599697	0,835541541	NA

ENSG00000182261	NLRP10	NLR family pyrin domain containing 10	338322	0	NA	NA	NA	NA	NA
ENSG00000182263	FIGN	fidgetin, microtubule severing factor	55137	0,311994459	-2,498367527	3,998294	-0,62485841	0,532063991	NA
ENSG00000182264	IZUMO1	izumo sperm-oocyte fusion 1	284359	0,10543123	0	4,080456	0	1	NA
ENSG00000182271	TMIGD1	transmembrane and immunoglobulin domain containing 1	388364	0	NA	NA	NA	NA	NA
ENSG00000182272	B4GALNT4	beta-1,4-N-acetyl-galactosaminyltransferase 4	338707	0,10488184	-1,076443944	4,080456	-0,263804824	0,791930317	NA
ENSG00000182308	DCAF4L1	DDB1 and CUL4 associated factor 4 like 1	285429	2,587761265	-1,58838477	1,526867	-1,040290103	0,298205141	NA
ENSG00000182310	SPACA6	sperm acrosome associated 6	147650	0	NA	NA	NA	NA	NA
ENSG00000182315	MBD3L3	methyl-CpG binding domain protein 3 like 3	653657	0	NA	NA	NA	NA	NA
ENSG00000182326	C1S	complement C1s	716	0,393503516	-2,05764242	4,029902	-0,510593689	0,609635596	NA
ENSG00000182327	GLTPD2	glycolipid transfer protein domain containing 2	388323	0	NA	NA	NA	NA	NA
ENSG00000182329	KIAA2012	KIAA2012	100652824	0	NA	NA	NA	NA	NA
ENSG00000182333	LIPF	lipase F, gastric type	8513	0	NA	NA	NA	NA	NA
ENSG00000182334	OR5P3	olfactory receptor family 5 subfamily P member 3	120066	0	NA	NA	NA	NA	NA
ENSG00000182346	DAOA	D-amino acid oxidase activator	267012	0	NA	NA	NA	NA	NA
ENSG00000182347	NA	NA	NA	0	NA	NA	NA	NA	NA
ENSG00000182348	ZNF804B	zinc finger protein 804B	219578	0	NA	NA	NA	NA	NA
ENSG00000182351	NA	NA	NA	0	NA	NA	NA	NA	NA
ENSG00000182365	OR5F2P	olfactory receptor family 5 subfamily F member 2 pseudogene	81194	0	NA	NA	NA	NA	NA
ENSG00000182379	NXPH4	neurexophilin 4	11247	0,968465704	2,525219758	2,488663	1,014689188	0,31025401	NA
ENSG00000182383	RPL27AP5	ribosomal protein L27a pseudogene 5	100271300	0	NA	NA	NA	NA	NA
ENSG00000182389	CACNB4	calcium voltage-gated channel auxiliary subunit beta 4	785	0,516835582	1,356125312	4,059904	0,334028939	0,738357721	NA
ENSG00000182397	NA	NA	NA	0,806359988	1,680350625	2,651959	0,633626195	0,526324801	NA
ENSG00000182415	CDY2A	chromodomain Y-linked 2A	9426	0	NA	NA	NA	NA	NA
ENSG00000182447	OTOL1	otolin 1	131149	0	NA	NA	NA	NA	NA
ENSG00000182450	KCNK4	potassium two pore domain channel subfamily K member 4	50801	0	NA	NA	NA	NA	NA
ENSG00000182459	TEX19	testis expressed 19	400629	0	NA	NA	NA	NA	NA
ENSG00000182463	TSHZ2	teashirt zinc finger homeobox 2	128553	0	NA	NA	NA	NA	NA
ENSG00000182472	CAPN12	calpain 12	147968	0	NA	NA	NA	NA	NA
ENSG00000182477	OR2B8P	olfactory receptor family 2 subfamily B member 8 pseudogene	65944	0,10543123	0	4,080456	0	1	NA
ENSG00000182487	NA	NA	NA	0,698276784	1,066338323	2,647011	0,402846171	0,687061393	NA
ENSG00000182489	XKRX	XK related X-linked	402415	0	NA	NA	NA	NA	NA
ENSG00000182492	BGN	biglycan	633	0	NA	NA	NA	NA	NA
ENSG00000182508	LHFPL1	LHFPL tetraspan subfamily member 1	340596	0,30996248	0,709054911	3,840876	0,184607582	0,853536838	NA
ENSG00000182521	TBPL2	TATA-box binding protein like 2	387332	0	NA	NA	NA	NA	NA
ENSG00000182531	NA	NA	NA	0	NA	NA	NA	NA	NA
ENSG00000182533	CAV3	caveolin 3	859	0	NA	NA	NA	NA	NA
ENSG00000182557	SPNS3	SPNS lysolipid transporter 3, sphingosine-1-phosphate (putative)	201305	0	NA	NA	NA	NA	NA
ENSG00000182565	NA	NA	NA	0	NA	NA	NA	NA	NA
ENSG00000182566	CLEC4G	C-type lectin domain family 4 member G	339390	0	NA	NA	NA	NA	NA
ENSG00000182574	CSGALNACT2P1	CSGALNACT2 pseudogene 1	286453	0	NA	NA	NA	NA	NA
ENSG00000182575	NXPH3	neurexophilin 3	11248	0	NA	NA	NA	NA	NA
ENSG00000182578	CSF1R	colony stimulating factor 1 receptor	1436	0	NA	NA	NA	NA	NA
ENSG00000182583	VCX	variable charge X-linked	26609	0	NA	NA	NA	NA	NA
ENSG00000182585	EPGN	epithelial mitogen	255324	0,119673877	0,847101401	4,080456	0,207599697	0,835541541	NA
ENSG00000182591	KRTAP11-1	keratin associated protein 11-1	337880	0	NA	NA	NA	NA	NA
ENSG00000182600	SNORC	secondary ossification center associated regulator of chondrocyte maturation	389084	1,118910194	3,658215151	2,831041	1,292180389	0,196294681	NA
ENSG00000182601	HS3ST4	heparan sulfate-glucosamine 3-sulfotransferase 4	9951	0	NA	NA	NA	NA	NA
ENSG00000182612	TSPAN10	tetraspanin 10	83882	0,706264509	1,91233504	3,193261	0,598865888	0,549262321	NA

ENSG00000182613	OR2V2	olfactory receptor family 2 subfamily V member 2	285659	0	NA	NA	NA	NA	NA
ENSG00000182625	NA	NA	NA	0	NA	NA	NA	NA	NA
ENSG00000182631	RXFP3	relaxin family peptide receptor 3	51289	0	NA	NA	NA	NA	NA
ENSG00000182632	NA	NA	NA	0	NA	NA	NA	NA	NA
ENSG00000182634	OR10G7	olfactory receptor family 10 subfamily G member 7	390265	0	NA	NA	NA	NA	NA
ENSG00000182636	NDN	necdin, MAGE family member	4692	0	NA	NA	NA	NA	NA
ENSG00000182645	CCDC172	coiled-coil domain containing 172	374355	0	NA	NA	NA	NA	NA
ENSG00000182652	OR4Q3	olfactory receptor family 4 subfamily Q member 3	441669	0	NA	NA	NA	NA	NA
ENSG00000182667	NTM	neurotrimin	50863	0	NA	NA	NA	NA	NA
ENSG00000182674	KCNB2	potassium voltage-gated channel subfamily B member 2	9312	0	NA	NA	NA	NA	NA
ENSG00000182685	BRICD5	BRICHOS domain containing 5	283870	7,15380328	-0,455730061	0,781773	-0,582944459	0,55993069	NA
ENSG00000182687	GALR2	galanin receptor 2	8811	0,343286354	-0,123272277	3,77811	-0,032628028	0,973971219	NA
ENSG00000182698	RESP18	regulated endocrine specific protein 18	389075	0	NA	NA	NA	NA	NA
ENSG00000182704	TSKU	tsukushi, small leucine rich proteoglycan	25987	0	NA	NA	NA	NA	NA
ENSG00000182707	NA	NA	NA	0	NA	NA	NA	NA	NA
ENSG00000182722	NA	NA	NA	0	NA	NA	NA	NA	NA
ENSG00000182747	SLC35D3	solute carrier family 35 member D3	340146	0	NA	NA	NA	NA	NA
ENSG00000182752	PAPPA	pappalysin 1	5069	0	NA	NA	NA	NA	NA
ENSG00000182759	MAFA	MAF bZIP transcription factor A	389692	0	NA	NA	NA	NA	NA
ENSG00000182771	GRID1	glutamate ionotropic receptor delta type subunit 1	2894	0	NA	NA	NA	NA	NA
ENSG00000182782	HCAR2	hydroxycarboxylic acid receptor 2	338442	0	NA	NA	NA	NA	NA
ENSG00000182783	OR2T29	olfactory receptor family 2 subfamily T member 29	343563	0	NA	NA	NA	NA	NA
ENSG00000182783	OR2T5	olfactory receptor family 2 subfamily T member 5	401993	0	NA	NA	NA	NA	NA
ENSG00000182791	CCDC87	coiled-coil domain containing 87	55231	0	NA	NA	NA	NA	NA
ENSG00000182793	GSTA5	glutathione S-transferase alpha 5	221357	0	NA	NA	NA	NA	NA
ENSG00000182795	C1orf116	chromosome 1 open reading frame 116	79098	0	NA	NA	NA	NA	NA
ENSG00000182796	TMEM198B	transmembrane protein 198B (pseudogene)	440104	5,742001932	0,562460053	0,808078	0,696046327	0,486399818	NA
ENSG00000182798	MAGEB17	MAGE family member B17	645864	0,072165449	0,847101401	4,080456	0,207599697	0,835541541	NA
ENSG00000182809	CRIP2	cysteine rich protein 2	1397	0	NA	NA	NA	NA	NA
ENSG00000182814	NA	NA	NA	0	NA	NA	NA	NA	NA
ENSG00000182816	KRTAP13-2	keratin associated protein 13-2	337959	0	NA	NA	NA	NA	NA
ENSG00000182836	PLCXD3	phosphatidylinositol specific phospholipase C X domain containing 3	345557	0,118286652	0	4,080456	0	1	NA
ENSG00000182853	VMO1	vitelline membrane outer layer 1 homolog	284013	0,697098553	1,967972635	2,661581	0,739399957	0,459664168	NA
ENSG00000182854	OR4F15	olfactory receptor family 4 subfamily F member 15	390649	0	NA	NA	NA	NA	NA
ENSG00000182871	COL18A1	collagen type XVIII alpha 1 chain	80781	0	NA	NA	NA	NA	NA
ENSG00000182888	RPS27AP20	RPS27A pseudogene 20	100128355	0,10543123	0	4,080456	0	1	NA
ENSG00000182896	TMEM95	transmembrane protein 95	339168	0	NA	NA	NA	NA	NA
ENSG00000182898	TCHHL1	trichohyalin like 1	126637	0	NA	NA	NA	NA	NA
ENSG00000182901	RGS7	regulator of G protein signaling 7	6000	0	NA	NA	NA	NA	NA
ENSG00000182902	SLC25A18	solute carrier family 25 member 18	83733	0	NA	NA	NA	NA	NA
ENSG00000182916	TCEAL7	transcription elongation factor A like 7	56849	0	NA	NA	NA	NA	NA
ENSG00000182921	NA	NA	NA	0	NA	NA	NA	NA	NA
ENSG00000182931	WFDC10B	WAP four-disulfide core domain 10B	280664	0	NA	NA	NA	NA	NA
ENSG00000182938	OTOP3	otopetrin 3	347741	0	NA	NA	NA	NA	NA
ENSG00000182950	CIMAP1C	ciliary microtubule associated protein 1C	161753	0,237779291	-0,10522336	4,080388	-0,025787584	0,979426765	NA
ENSG00000182963	GJC1	gap junction protein gamma 1	10052	0,687163089	-0,586873224	2,483368	-0,236321512	0,813183195	NA
ENSG00000182965	NA	NA	NA	0	NA	NA	NA	NA	NA

ENSG00000182968	SOX1	SRY-box transcription factor 1	6656	0	NA	NA	NA	NA	NA
ENSG00000182983	ZNF662	zinc finger protein 662	389114	0,276688933	0	4,080456	0	1	NA
ENSG00000182985	CADM1	cell adhesion molecule 1	23705	1,185818013	-0,582310853	1,821854	-0,319625404	0,749252314	NA
ENSG00000182993	C12orf60	chromosome 12 open reading frame 60	144608	1,540985218	0,940193958	1,497854	0,627694163	0,53020431	NA
ENSG00000183018	SPNS2	SPNS lysolipid transporter 2, sphingosine-1-phosphate	124976	9,511042801	0,417409247	0,649027	0,643131034	0,520139079	NA
ENSG00000183019	MCEMP1	mast cell expressed membrane protein 1	199675	0,119673877	0,847101401	4,080456	0,207599697	0,835541541	NA
ENSG00000183020	AP2A2	adaptor related protein complex 2 subunit alpha 2	161	12,11688285	1,144241005	3,777363	0,302920607	NA	NA
ENSG00000183022	NA	NA	NA	0	NA	NA	NA	NA	NA
ENSG00000183023	SLC8A1	solute carrier family 8 member A1	6546	0,10543123	0	4,080456	0	1	NA
ENSG00000183024	OR1G1	olfactory receptor family 1 subfamily G member 1	8390	0	NA	NA	NA	NA	NA
ENSG00000183032	SLC25A21	solute carrier family 25 member 21	89874	0	NA	NA	NA	NA	NA
ENSG00000183034	OTOP2	otopetrin 2	92736	0	NA	NA	NA	NA	NA
ENSG00000183035	CYLC1	cylicin 1	1538	0	NA	NA	NA	NA	NA
ENSG00000183036	PCP4	Purkinje cell protein 4	5121	0	NA	NA	NA	NA	NA
ENSG00000183054	RGPD6	RANBP2 like and GRIP domain containing 6	729540	1,436086527	4,574530269	3,735788	1,224515545	0,220757806	NA
ENSG00000183055	NA	NA	NA	0	NA	NA	NA	NA	NA
ENSG00000183066	WBP2NL	WBP2 N-terminal like	164684	4,05379215	0,205765909	1,068524	0,192570179	0,847295602	NA
ENSG00000183067	IGSF5	immunoglobulin superfamily member 5	150084	0	NA	NA	NA	NA	NA
ENSG00000183072	NKX2-5	NK2 homeobox 5	1482	0	NA	NA	NA	NA	NA
ENSG00000183087	GAS6	growth arrest specific 6	2621	0	NA	NA	NA	NA	NA
ENSG00000183090	FREM3	FRAS1 related extracellular matrix 3	166752	0	NA	NA	NA	NA	NA
ENSG00000183091	NEB	nebulin	4703	8,664004697	0,321652761	0,631461	0,50937895	0,610486629	NA
ENSG00000183092	BEGAIN	brain enriched guanylate kinase associated	57596	0	NA	NA	NA	NA	NA
ENSG00000183098	GPC6	glypican 6	10082	0	NA	NA	NA	NA	NA
ENSG00000183111	ARHGEF37	Rho guanine nucleotide exchange factor 37	389337	0,763356439	1,203411703	2,396904	0,502069205	0,615618841	NA
ENSG00000183114	FAM43B	family with sequence similarity 43 member B	163933	0	NA	NA	NA	NA	NA
ENSG00000183117	CSMD1	CUB and Sushi multiple domains 1	64478	0	NA	NA	NA	NA	NA
ENSG00000183122	NA	NA	NA	0	NA	NA	NA	NA	NA
ENSG00000183128	CALHM3	calcium homeostasis modulator 3	119395	0,117497108	0,847101401	4,080456	0,207599697	0,835541541	NA
ENSG00000183134	PTGDR2	prostaglandin D2 receptor 2	11251	0,326334988	-0,96507467	3,7885	-0,254737957	0,798925498	NA
ENSG00000183145	RIPPLY3	ripply transcriptional repressor 3	53820	0	NA	NA	NA	NA	NA
ENSG00000183148	NA	NA	NA	0,332950572	2,489020609	3,987196	0,624253316	0,532461237	NA
ENSG00000183150	GPR19	G protein-coupled receptor 19	2842	2,160846843	2,38249506	1,44454	1,649310984	0,099083941	NA
ENSG00000183153	GJD3	gap junction protein delta 3	125111	1,239776413	-0,667362348	1,736417	-0,384332888	0,700731735	NA
ENSG00000183160	TMEM119	transmembrane protein 119	338773	1,084986132	1,167679915	2,254995	0,51781939	0,604584288	NA
ENSG00000183166	CALN1	calneuron 1	83698	0	NA	NA	NA	NA	NA
ENSG00000183169	NA	NA	NA	0	NA	NA	NA	NA	NA
ENSG00000183171	LOC643015	nucleolar protein 11 pseudogene	643015	0	NA	NA	NA	NA	NA
ENSG00000183185	GABRR3	gamma-aminobutyric acid type A receptor subunit rho3	200959	0	NA	NA	NA	NA	NA
ENSG00000183186	C2CD4C	C2 calcium dependent domain containing 4C	126567	0	NA	NA	NA	NA	NA
ENSG00000183196	CHST6	carbohydrate sulfotransferase 6	4166	0,103998153	-1,076443944	4,080456	-0,263804824	0,791930317	NA
ENSG00000183199	HSP90AB3P	heat shock protein 90 alpha family class B member 3, pseudogene	3327	0,474241808	-0,02447184	3,463959	-0,007064703	0,994363229	NA
ENSG00000183206	POTEC	POTE ankyrin domain family member C	388468	0	NA	NA	NA	NA	NA
ENSG00000183230	CTNNA3	catenin alpha 3	29119	0,10488184	-1,076443944	4,080456	-0,263804824	0,791930317	NA
ENSG00000183239	NA	NA	NA	0,10543123	0	4,080456	0	1	NA
ENSG00000183246	RIMBP3C	RIMS binding protein 3C	150221	0	NA	NA	NA	NA	NA
ENSG00000183248	PRR36	proline rich 36	80164	0	NA	NA	NA	NA	NA
ENSG00000183249	NA	NA	NA	0	NA	NA	NA	NA	NA
ENSG00000183251	OR51B4	olfactory receptor family 51 subfamily B member 4	79339	0	NA	NA	NA	NA	NA
ENSG00000183260	ABHD16B	abhydrolase domain containing 16B	140701	0,10543123	0	4,080456	0	1	NA
ENSG00000183269	OR52E8	olfactory receptor family 52 subfamily E member 8	390079	0	NA	NA	NA	NA	NA
ENSG00000183273	CCDC60	coiled-coil domain containing 60	160777	0	NA	NA	NA	NA	NA

ENSG00000183287	CCBE1	collagen and calcium binding EGF domains 1	147372	0,176905722	-0,110451667	4,080456	-0,027068462	0,978405129	NA
ENSG00000183292	NA	NA	NA	0	NA	NA	NA	NA	NA
ENSG00000183298	RPSAP19	ribosomal protein SA pseudogene 19	730029	0,615243528	-2,578628555	2,777193	-0,928501815	0,353147327	NA
ENSG00000183303	OR5P2	olfactory receptor family 5 subfamily P member 2	120065	0	NA	NA	NA	NA	NA
ENSG00000183304	FAM9A	family with sequence similarity 9 member A	171482	0	NA	NA	NA	NA	NA
ENSG00000183305	MAGEA2	MAGE family member A2	4101	0	NA	NA	NA	NA	NA
ENSG00000183305	MAGEA2B	MAGE family member A2B	266740	0	NA	NA	NA	NA	NA
ENSG00000183307	TMEM121B	transmembrane protein 121B	27439	1,051678178	1,513604532	1,775395	0,852545403	0,393911451	NA
ENSG00000183310	OR2T34	olfactory receptor family 2 subfamily T member 34	127068	0	NA	NA	NA	NA	NA
ENSG00000183313	OR52L1	olfactory receptor family 52 subfamily L member 1	338751	0	NA	NA	NA	NA	NA
ENSG00000183317	EPHA10	EPH receptor A10	284656	0,183813657	1,095083291	4,080456	0,268372781	0,788412389	NA
ENSG00000183318	SPDYE4	speedy/RINGO cell cycle regulator family member E4	388333	0,448806146	1,046758379	3,01483	0,347203153	0,728438702	NA
ENSG00000183324	REC114	REC114 meiotic recombination protein	283677	0	NA	NA	NA	NA	NA
ENSG00000183346	CABCOCO1	ciliary associated calcium binding coiled-coil 1	219621	0	NA	NA	NA	NA	NA
ENSG00000183347	GBP6	guanylate binding protein family member 6	163351	0,90966307	1,244367334	2,235031	0,556756248	0,577693981	NA
ENSG00000183378	OVCH2	ovochymase 2	341277	0	NA	NA	NA	NA	NA
ENSG00000183379	SYNDIG1L	synapse differentiation inducing 1 like	646658	0	NA	NA	NA	NA	NA
ENSG00000183389	OR56A4	olfactory receptor family 56 subfamily A member 4	120793	0	NA	NA	NA	NA	NA
ENSG00000183395	PMCH	pro-melanin concentrating hormone	5367	0,439478554	1,536097969	3,437313	0,446889181	0,654955076	NA
ENSG00000183396	TMEM89	transmembrane protein 89	440955	0,237164083	1,955808445	4,022315	0,486239468	0,626797391	NA
ENSG00000183397	TEKTIP1	tektin bundle interacting protein 1	100128569	1,989933169	1,468916183	1,280185	1,147424829	0,251206085	NA
ENSG00000183421	RIPK4	receptor interacting serine/threonine kinase 4	54101	0	NA	NA	NA	NA	NA
ENSG00000183423	LRIT3	leucine rich repeat, Ig-like and transmembrane domains 3	345193	2,159333929	0,686459054	1,357398	0,505716911	0,613055395	NA
ENSG00000183432	NA	NA	NA	0	NA	NA	NA	NA	NA
ENSG00000183434	TFDP3	transcription factor Dp family member 3	51270	0	NA	NA	NA	NA	NA
ENSG00000183439	TRIM61	tripartite motif containing 61	391712	5,528758597	-0,104569704	0,880162	-0,118807404	0,905427942	NA
ENSG00000183454	GRIN2A	glutamate ionotropic receptor NMDA type subunit 2A	2903	0	NA	NA	NA	NA	NA
ENSG00000183463	URAD	ureidoimidazoline (2-oxo-4-hydroxy-4-carboxy-5-) decarboxylase	646625	0	NA	NA	NA	NA	NA
ENSG00000183476	SH2D7	SH2 domain containing 7	646892	0,625245643	1,810802641	2,710099	0,668168464	0,504026063	NA
ENSG00000183479	TREX2	three prime repair exonuclease 2	11219	5,732699826	0,221069746	0,983583	0,224759703	0,822166216	NA
ENSG00000183486	MX2	MX dynamin like GTPase 2	4600	1,900641517	2,13642146	2,373429	0,900141281	0,36804507	NA
ENSG00000183514	NA	NA	NA	0	NA	NA	NA	NA	NA
ENSG00000183549	ACSM5	acyl-CoA synthetase medium chain family member 5	54988	0	NA	NA	NA	NA	NA
ENSG00000183559	C10orf120	chromosome 10 open reading frame 120	399814	0	NA	NA	NA	NA	NA
ENSG00000183560	IZUMO1R	IZUMO1 receptor, JUNO	390243	0	NA	NA	NA	NA	NA
ENSG00000183566	NA	NA	NA	0	NA	NA	NA	NA	NA
ENSG00000183569	SERHL2	serine hydrolase like 2	253190	0,979905512	0,007391057	2,605826	0,002836358	0,997736916	NA
ENSG00000183570	PCBP3	poly(rC) binding protein 3	54039	0,539543894	1,190633623	3,429989	0,347124645	0,728497679	NA
ENSG00000183571	PGPEP1L	pyroglutamyl-peptidase I like	145814	0	NA	NA	NA	NA	NA
ENSG00000183578	TNFAIP8L3	TNF alpha induced protein 8 like 3	388121	0	NA	NA	NA	NA	NA
ENSG00000183579	ZNRF3	zinc and ring finger 3	84133	3,195968877	-0,447983902	1,00868	-0,444129033	0,656949297	NA
ENSG00000183580	FBXL7	F-box and leucine rich repeat protein 7	23194	0	NA	NA	NA	NA	NA
ENSG00000183586	NA	NA	NA	0	NA	NA	NA	NA	NA
ENSG00000183598	H3C13	H3 clustered histone 13	653604	1,353615017	2,605846905	2,405756	1,083171941	0,278732112	NA
ENSG00000183607	GKN2	gastrokine 2	200504	0	NA	NA	NA	NA	NA
ENSG00000183615	FAM167B	family with sequence similarity 167 member B	84734	2,422649877	-1,449587228	1,469651	-0,986348047	0,32396235	NA
ENSG00000183625	CCR3	C-C motif chemokine receptor 3	1232	0,786912483	0,066051505	2,72238	0,024262415	0,980643293	NA
ENSG00000183628	DGCR6	DiGeorge syndrome critical region gene 6	8214	1,843204043	-4,443031985	2,096933	-2,118823688	0,03410537	NA
ENSG00000183629	GOLGA8G	golgin A8 family member G	283768	0	NA	NA	NA	NA	NA

ENSG00000183631	PRR32	proline rich 32	100130613	0	NA	NA	NA	NA	NA
ENSG00000183632	TP53TG3	TP53 target 3	24150	0	NA	NA	NA	NA	NA
ENSG00000183632	LOC102723655	TP53-target gene 3 protein	102723655	0	NA	NA	NA	NA	NA
ENSG00000183632	LOC102723713	TP53-target gene 3 protein	102723713	0	NA	NA	NA	NA	NA
ENSG00000183638	RP1L1	RP1 like 1	94137	0	NA	NA	NA	NA	NA
ENSG00000183640	KRTAP8-1	keratin associated protein 8-1	337879	0	NA	NA	NA	NA	NA
ENSG00000183644	HOATZ	HOATZ cilia and flagella associated protein	399949	0	NA	NA	NA	NA	NA
ENSG00000183654	MARCHF11	membrane associated ring-CH-type finger 11	441061	0	NA	NA	NA	NA	NA
ENSG00000183662	TAFA1	TAFA chemokine like family member 1	407738	3,740351962	0,27610995	0,873444	0,316116454	0,751914118	NA
ENSG00000183663	NA	NA	NA	0	NA	NA	NA	NA	NA
ENSG00000183666	NA	NA	NA	3,847483344	-0,716043124	1,834351	-0,390352373	0,696276001	NA
ENSG00000183668	PSG9	pregnancy specific beta-1-glycoprotein 9	5678	0	NA	NA	NA	NA	NA
ENSG00000183671	CMKLR2	chemerin chemokine-like receptor 2	2825	0	NA	NA	NA	NA	NA
ENSG00000183682	BMP8A	bone morphogenetic protein 8a	353500	0,93948093	1,374456561	2,15824	0,636841308	0,524228216	NA
ENSG00000183690	EFHC2	EF-hand domain containing 2	80258	2,047570612	-2,44780513	1,562256	-1,566839835	0,117152134	NA
ENSG00000183691	NOG	noggin	9241	0	NA	NA	NA	NA	NA
ENSG00000183695	MRGPRX2	MAS related GPR family member X2	117194	0,103998153	-1,076443944	4,080456	-0,263804824	0,791930317	NA
ENSG00000183704	SLC9B1P1	solute carrier family 9 member B1 pseudogene 1	100128190	0	NA	NA	NA	NA	NA
ENSG00000183706	OR4N4	olfactory receptor family 4 subfamily N member 4	283694	0	NA	NA	NA	NA	NA
ENSG00000183715	OPCML	opioid binding protein/cell adhesion molecule like	4978	0	NA	NA	NA	NA	NA
ENSG00000183722	LHFPL6	LHFPL tetraspan subfamily member 6	10186	0,276855823	0	4,080456	0	1	NA
ENSG00000183723	CMTM4	CKLF like MARVEL transmembrane domain containing 4	146223	2,02746137	-5,182424389	2,594646	-1,997353155	0,045786833	NA
ENSG00000183733	FIGLA	folliculogenesis specific bHLH transcription factor	344018	0	NA	NA	NA	NA	NA
ENSG00000183734	ASCL2	achaete-scute family bHLH transcription factor 2	430	1,982507945	0,227501893	1,281409	0,177540486	0,859083867	NA
ENSG00000183747	ACSM2A	acyl-CoA synthetase medium chain family member 2A	123876	0	NA	NA	NA	NA	NA
ENSG00000183753	BPY2	basic charge Y-linked 2	9083	0	NA	NA	NA	NA	NA
ENSG00000183760	ACP7	acid phosphatase 7, tartrate resistant (putative)	390928	0	NA	NA	NA	NA	NA
ENSG00000183762	KREMEN1	kringle containing transmembrane protein 1	83999	2,323800779	1,034910095	1,336881	0,774122683	0,438858245	NA
ENSG00000183770	FOXL2	forkhead box L2	668	0	NA	NA	NA	NA	NA
ENSG00000183773	AIFM3	apoptosis inducing factor mitochondria associated 3	150209	1,145652534	-1,347251636	2,943613	-0,457686458	0,647177719	NA
ENSG00000183775	KCTD16	potassium channel tetramerization domain containing 16	57528	0	NA	NA	NA	NA	NA
ENSG00000183778	B3GALT5	beta-1,3-galactosyltransferase 5	10317	0,339418315	0,975929807	4,023463	0,24255964	0,80834655	NA
ENSG00000183779	ZNF703	zinc finger protein 703	80139	0,103998153	-1,076443944	4,080456	-0,263804824	0,791930317	NA
ENSG00000183780	SLC35F3	solute carrier family 35 member F3	148641	0	NA	NA	NA	NA	NA
ENSG00000183783	KCTD8	potassium channel tetramerization domain containing 8	386617	0	NA	NA	NA	NA	NA
ENSG00000183798	EMILIN3	elastin microfibril interfacer 3	90187	0,103998153	-1,076443944	4,080456	-0,263804824	0,791930317	NA
ENSG00000183801	OLFML1	olfactomedin like 1	283298	0	NA	NA	NA	NA	NA
ENSG00000183807	FAM162B	family with sequence similarity 162 member B	221303	0	NA	NA	NA	NA	NA
ENSG00000183813	CCR4	C-C motif chemokine receptor 4	1233	0,118286652	0	4,080456	0	1	NA
ENSG00000183831	ANKRD45	ankyrin repeat domain 45	339416	0	NA	NA	NA	NA	NA
ENSG00000183833	CFAP91	cilia and flagella associated protein 91	89876	0	NA	NA	NA	NA	NA
ENSG00000183837	PNMA3	PNMA family member 3	29944	5,582371203	0,713862874	1,236192	0,577469118	0,563622595	NA
ENSG00000183840	GPR39	G protein-coupled receptor 39	2863	0	NA	NA	NA	NA	NA
ENSG00000183844	FAM3B	FAM3 metabolism regulating signaling molecule B	54097	0	NA	NA	NA	NA	NA
ENSG00000183850	ZNF730	zinc finger protein 730	100129543	0,708050387	0,797711627	2,50287	0,318718767	0,749939782	NA

ENSG00000183853	KIRREL1	kirre like nephrin family adhesion molecule 1	55243	0	NA	NA	NA	NA	NA
ENSG00000183862	CNGA2	cyclic nucleotide gated channel subunit alpha 2	1260	0	NA	NA	NA	NA	NA
ENSG00000183873	SCN5A	sodium voltage-gated channel alpha subunit 5	6331	0	NA	NA	NA	NA	NA
ENSG00000183876	ARSI	arylsulfatase family member I	340075	0,072165449	0,847101401	4,080456	0,207599697	0,835541541	NA
ENSG00000183878	UTY	ubiquitously transcribed tetratricopeptide repeat containing, Y-linked	7404	0	NA	NA	NA	NA	NA
ENSG00000183888	SRARP	steroid receptor associated and regulated protein	149563	0	NA	NA	NA	NA	NA
ENSG00000183889	NPIPA9	nuclear pore complex interacting protein family, member A9	105376752	7,688632356	0	4,080456	0	NA	NA
ENSG00000183908	LRRC55	leucine rich repeat containing 55	219527	0	NA	NA	NA	NA	NA
ENSG00000183911	NA	NA	NA	0	NA	NA	NA	NA	NA
ENSG00000183921	SDR42E2	short chain dehydrogenase/reductase family 42E, member 2	100288072	0	NA	NA	NA	NA	NA
ENSG00000183935	HTR7P1	5-hydroxytryptamine receptor 7 pseudogene 1	93164	0,744071635	-0,814503184	2,352138	-0,346282029	0,729130773	NA
ENSG00000183938	TRBV21OR9-2	T cell receptor beta variable 21/OR9-2 (pseudogene)	6959	0	NA	NA	NA	NA	NA
ENSG00000183960	KCNH8	potassium voltage-gated channel subfamily H member 8	131096	1,009548565	-1,566948239	2,320153	-0,675364103	0,499444466	NA
ENSG00000183977	PP2D1	protein phosphatase 2C like domain containing 1	151649	0	NA	NA	NA	NA	NA
ENSG00000183979	NPB	neuropeptide B	256933	0,560591913	1,560675893	3,363612	0,463988131	0,64265625	NA
ENSG00000183981	NA	NA	NA	0	NA	NA	NA	NA	NA
ENSG00000184005	ST6GALNAC3	ST6 N-acetylgalactosaminide alpha-2,6-sialyltransferase 3	256435	0	NA	NA	NA	NA	NA
ENSG00000184012	TMPRSS2	transmembrane serine protease 2	7113	0	NA	NA	NA	NA	NA
ENSG00000184012	LOC124905021	uncharacterized LOC124905021	124905021	0	NA	NA	NA	NA	NA
ENSG00000184022	OR2T10	olfactory receptor family 2 subfamily T member 10	127069	0	NA	NA	NA	NA	NA
ENSG00000184032	KRTAP20-2	keratin associated protein 20-2	337976	0	NA	NA	NA	NA	NA
ENSG00000184033	CTAG1B	cancer/testis antigen 1B	1485	0,072165449	0,847101401	4,080456	0,207599697	0,835541541	NA
ENSG00000184055	NA	NA	NA	0	NA	NA	NA	NA	NA
ENSG00000184058	TBX1	T-box transcription factor 1	6899	0,359654512	0,833643798	4,080456	0,204301633	0,838117794	NA
ENSG00000184060	ADAP2	ArfGAP with dual PH domains 2	55803	1,907543267	-0,316305061	1,557397	-0,203098564	0,839057994	NA
ENSG00000184084	NA	NA	NA	0	NA	NA	NA	NA	NA
ENSG00000184100	BRD7P2	bromodomain containing 7 pseudogene 2	647076	0,103998153	-1,076443944	4,080456	-0,263804824	0,791930317	NA
ENSG00000184106	NA	NA	NA	0	NA	NA	NA	NA	NA
ENSG00000184108	TRIML1	tripartite motif family like 1	339976	0	NA	NA	NA	NA	NA
ENSG00000184111	RPL11P4	ribosomal protein L11 pseudogene 4	100271272	0	NA	NA	NA	NA	NA
ENSG00000184113	CLDN5	claudin 5	7122	0,326933813	-1,93604168	3,780163	-0,512158287	0,608540234	NA
ENSG00000184115	NA	NA	NA	0	NA	NA	NA	NA	NA
ENSG00000184139	RPL7AP28	ribosomal protein L7a pseudogene 28	100271209	0	NA	NA	NA	NA	NA
ENSG00000184140	OR4F6	olfactory receptor family 4 subfamily F member 6	390648	0	NA	NA	NA	NA	NA
ENSG00000184144	CNTN2	contactin 2	6900	0	NA	NA	NA	NA	NA
ENSG00000184148	SPRR4	small proline rich protein 4	163778	0	NA	NA	NA	NA	NA
ENSG00000184154	LRRC51	leucine rich repeat containing 51	120356739	5,16177179	0,301868352	0,879475	0,343237093	0,731420102	NA
ENSG00000184155	OR10J5	olfactory receptor family 10 subfamily J member 5	127385	0	NA	NA	NA	NA	NA
ENSG00000184156	KCNQ3	potassium voltage-gated channel subfamily Q member 3	3786	0	NA	NA	NA	NA	NA
ENSG00000184160	ADRA2C	adrenoceptor alpha 2C	152	0	NA	NA	NA	NA	NA
ENSG00000184163	C1QTNF12	C1q and TNF related 12	388581	0	NA	NA	NA	NA	NA
ENSG00000184166	OR1D2	olfactory receptor family 1 subfamily D member 2	4991	0	NA	NA	NA	NA	NA
ENSG00000184185	KCNJ12	potassium inwardly rectifying channel subfamily J member 12	3768	0	NA	NA	NA	NA	NA
ENSG00000184188	NA	NA	NA	0	NA	NA	NA	NA	NA
ENSG00000184194	GPR173	G protein-coupled receptor 173	54328	0	NA	NA	NA	NA	NA

ENSG00000184210	DGAT2L6	diacylglycerol O-acyltransferase 2 like 6	347516	0	NA	NA	NA	NA	NA
ENSG00000184221	OLIG1	oligodendrocyte transcription factor 1	116448	0	NA	NA	NA	NA	NA
ENSG00000184226	PCDH9	protocadherin 9	5101	4,044737836	1,498599027	0,93327	1,605750493	0,108328742	NA
ENSG00000184227	ACOT1	acyl-CoA thioesterase 1	641371	7,920070547	0,924085963	0,640501	1,442754058	0,149089765	NA
ENSG00000184232	OAF	out at first homolog	220323	8,738129391	-0,058073222	0,628288	-0,092430948	0,926355652	NA
ENSG00000184254	ALDH1A3	aldehyde dehydrogenase 1 family member A3	220	0,486337552	1,713161741	2,794951	0,612948859	0,539910156	NA
ENSG00000184260	H2AC20	H2A clustered histone 20	8338	1,996737584	-1,16399041	1,563151	-0,74464373	0,456487124	NA
ENSG00000184261	KCNK12	potassium two pore domain channel subfamily K member 12	56660	0,897809358	2,285380708	1,990266	1,14827915	0,250853344	NA
ENSG00000184270	H2AC21	H2A clustered histone 21	317772	0,307826837	2,352801453	3,994968	0,588941185	0,555900729	NA
ENSG00000184271	POU6F1	POU class 6 homeobox 1	5463	0,675409768	-2,78132774	3,797952	-0,732322998	0,463971445	NA
ENSG00000184276	DEFB108B	defensin beta 108B	245911	0	NA	NA	NA	NA	NA
ENSG00000184292	TACSTD2	tumor associated calcium signal transducer 2	4070	0	NA	NA	NA	NA	NA
ENSG00000184293	CLECL1P	C-type lectin like 1, pseudogene	160365	0,118286652	0	4,080456	0	1	NA
ENSG00000184302	SIX6	SIX homeobox 6	4990	0	NA	NA	NA	NA	NA
ENSG00000184303	NA	NA	NA	0	NA	NA	NA	NA	NA
ENSG00000184304	PRKD1	protein kinase D1	5587	0	NA	NA	NA	NA	NA
ENSG00000184305	CCSER1	coiled-coil serine rich protein 1	401145	0	NA	NA	NA	NA	NA
ENSG00000184307	ZDHHC23	zinc finger DHHC-type palmitoyltransferase 23	254887	4,365218933	-0,296306655	1,752013	-0,169123508	0,865699494	NA
ENSG00000184313	MROH7	maestro heat like repeat family member 7	374977	0	NA	NA	NA	NA	NA
ENSG00000184319	NA	NA	NA	5,340125114	0,102818413	0,760982	0,135112864	0,89252264	NA
ENSG00000184321	OR51J1	olfactory receptor family 51 subfamily J member 1	79470	0	NA	NA	NA	NA	NA
ENSG00000184330	S100A7A	S100 calcium binding protein A7A	338324	0	NA	NA	NA	NA	NA
ENSG00000184343	SRPK3	SRSF protein kinase 3	26576	0	NA	NA	NA	NA	NA
ENSG00000184344	GDF3	growth differentiation factor 3	9573	0	NA	NA	NA	NA	NA
ENSG00000184345	IQCF2	IQ motif containing F2	389123	0	NA	NA	NA	NA	NA
ENSG00000184347	SLIT3	slit guidance ligand 3	6586	4,054412852	0,01116563	0,980157	0,01139168	0,990910951	NA
ENSG00000184349	EFNA5	ephrin A5	1946	6,735217384	-0,821735658	0,970538	-0,846680949	0,39717298	NA
ENSG00000184350	MRGPRE	MAS related GPR family member E	116534	0	NA	NA	NA	NA	NA
ENSG00000184351	KRTAP19-1	keratin associated protein 19-1	337882	0	NA	NA	NA	NA	NA
ENSG00000184357	H1-5	H1,5 linker histone, cluster member	3009	0,751942309	1,684048213	2,134271	0,789050755	0,430082342	NA
ENSG00000184361	SPATA32	spermatogenesis associated 32	124783	0,326570703	-1,920263129	4,04277	-0,47498701	0,634796232	NA
ENSG00000184363	PKP3	plakophilin 3	11187	0	NA	NA	NA	NA	NA
ENSG00000184368	MAP7D2	MAP7 domain containing 2	256714	1,054744351	-2,306640082	1,873813	-1,230987513	0,218327534	NA
ENSG00000184374	COLEC10	collectin subfamily member 10	10584	0	NA	NA	NA	NA	NA
ENSG00000184378	ACTRT3	actin related protein T3	84517	1,676646617	-0,520168735	1,486104	-0,350021772	0,726322359	NA
ENSG00000184388	PABPC1L2B	poly(A) binding protein cytoplasmic 1 like 2B	645974	0	NA	NA	NA	NA	NA
ENSG00000184389	A3GALT2	alpha 1,3-galactosyltransferase 2	127550	0	NA	NA	NA	NA	NA
ENSG00000184408	KCND2	potassium voltage-gated channel subfamily D member 2	3751	0	NA	NA	NA	NA	NA
ENSG00000184414	NA	NA	NA	1,557221154	1,249160452	1,396301	0,894621043	0,370989699	NA
ENSG00000184423	NA	NA	NA	0	NA	NA	NA	NA	NA
ENSG00000184428	TOP1MT	DNA topoisomerase I mitochondrial	116447	6,453210819	-0,787976178	1,138637	-0,692034841	0,488915449	NA
ENSG00000184434	LRRC19	leucine rich repeat containing 19	64922	0	NA	NA	NA	NA	NA
ENSG00000184436	THAP7	THAP domain containing 7	80764	10,44203636	1,821154091	0,826814	2,202615597	0,027621854	NA
ENSG00000184451	CCR10	C-C motif chemokine receptor 10	2826	0,103998153	-1,076443944	4,080456	-0,263804824	0,791930317	NA
ENSG00000184454	NCMAP	non-compact myelin associated protein	400746	0,11808024	-1,076443944	4,080456	-0,263804824	0,791930317	NA
ENSG00000184459	BPIFC	BPI fold containing family C	254240	0	NA	NA	NA	NA	NA
ENSG00000184471	C1QTNF8	C1q and TNF related 8	390664	0	NA	NA	NA	NA	NA
ENSG00000184478	OR56A3	olfactory receptor family 56 subfamily A member 3	390083	0	NA	NA	NA	NA	NA
ENSG00000184486	POU3F2	POU class 3 homeobox 2	5454	0	NA	NA	NA	NA	NA
ENSG00000184492	FOXD4L1	forkhead box D4 like 1	200350	0,920690312	2,51397639	2,582645	0,973411508	0,330348822	NA
ENSG00000184497	TMEM255B	transmembrane protein 255B	348013	0,598089181	1,678634709	3,263348	0,514390337	0,606979114	NA
ENSG00000184500	PROS1	protein S	5627	0,415131544	-2,513341553	3,506594	-0,716747262	0,473530058	NA
ENSG00000184502	GAST	gastrin	2520	0	NA	NA	NA	NA	NA

ENSG00000184507	NUTM1	NUT midline carcinoma family member 1	256646	0	NA	NA	NA	NA	NA
ENSG00000184523	NA	NA	NA	0	NA	NA	NA	NA	NA
ENSG00000184524	CEND1	cell cycle exit and neuronal differentiation 1	51286	0,870315366	-0,303122886	2,153661	-0,140747744	0,888069226	NA
ENSG00000184530	C6orf58	chromosome 6 open reading frame 58	352999	0	NA	NA	NA	NA	NA
ENSG00000184544	DHRS7C	dehydrogenase/reductase 7C	201140	0	NA	NA	NA	NA	NA
ENSG00000184545	DUSP8	dual specificity phosphatase 8	1850	0	NA	NA	NA	NA	NA
ENSG00000184557	SOCS3	suppressor of cytokine signaling 3	9021	5,576827345	-0,266528785	0,764137	-0,348797266	0,727241516	NA
ENSG00000184564	SLITRK6	SLIT and NTRK like family member 6	84189	0	NA	NA	NA	NA	NA
ENSG00000184571	PIWIL3	piwi like RNA-mediated gene silencing 3	440822	0	NA	NA	NA	NA	NA
ENSG00000184599	TAFA3	TAFA chemokine like family member 3	284467	0	NA	NA	NA	NA	NA
ENSG00000184601	C14orf180	chromosome 14 open reading frame 180	400258	0	NA	NA	NA	NA	NA
ENSG00000184602	SNN	stannin	8303	1,784310108	1,900672134	1,606661	1,182995371	0,236810982	NA
ENSG00000184611	KCNH7	potassium voltage-gated channel subfamily H member 7	90134	0	NA	NA	NA	NA	NA
ENSG00000184612	NA	NA	NA	0	NA	NA	NA	NA	NA
ENSG00000184616	SPDYE12	speedy/RINGO cell cycle regulator family member E12	100101268	0	NA	NA	NA	NA	NA
ENSG00000184617	NA	NA	NA	0	NA	NA	NA	NA	NA
ENSG00000184624	NA	NA	NA	0	NA	NA	NA	NA	NA
ENSG00000184647	PRSS55	serine protease 55	203074	0	NA	NA	NA	NA	NA
ENSG00000184650	ODF4	outer dense fiber of sperm tails 4	146852	0	NA	NA	NA	NA	NA
ENSG00000184659	FOXD4L4	forkhead box D4 like 4	349334	0,278990652	0,836134996	4,080456	0,204912153	0,83764076	NA
ENSG00000184669	OR7E14P	olfactory receptor family 7 subfamily E member 14 pseudogene	10819	0	NA	NA	NA	NA	NA
ENSG00000184672	RALYL	RALY RNA binding protein like	138046	0	NA	NA	NA	NA	NA
ENSG00000184697	CLDN6	claudin 6	9074	0	NA	NA	NA	NA	NA
ENSG00000184698	OR51M1	olfactory receptor family 51 subfamily M member 1	390059	0	NA	NA	NA	NA	NA
ENSG00000184702	SEPTIN5	septin 5	5413	0,118286652	0	4,080456	0	1	NA
ENSG00000184709	LRRC26	leucine rich repeat containing 26	389816	0	NA	NA	NA	NA	NA
ENSG00000184716	SERINC4	serine incorporator 4	619189	0,15836288	0	4,080456	0	1	NA
ENSG00000184719	RNLS	renalase, FAD dependent amine oxidase	55328	2,735096875	-0,92950796	1,611199	-0,576904615	0,564003893	NA
ENSG00000184724	KRTAP6-1	keratin associated protein 6-1	337966	0	NA	NA	NA	NA	NA
ENSG00000184731	FAM110C	family with sequence similarity 110 member C	642273	5,089674277	0,065704973	0,917302	0,071628508	0,942897552	NA
ENSG00000184735	DDX53	DEAD-box helicase 53	168400	0	NA	NA	NA	NA	NA
ENSG00000184741	OR5AQ1P	olfactory receptor family 5 subfamily AQ member 1 pseudogene	81221	0	NA	NA	NA	NA	NA
ENSG00000184761	NA	NA	NA	0	NA	NA	NA	NA	NA
ENSG00000184774	MGAT4EP	MGAT4 family member E, pseudogene	641515	0,222239433	-0,126606705	4,080456	-0,031027588	0,975247538	NA
ENSG00000184785	SMIM10	small integral membrane protein 10	644538	6,625663561	-0,050319784	0,77455	-0,064966478	0,94820069	NA
ENSG00000184786	DYNLT2	dynein light chain Tctex-type 2	6991	0	NA	NA	NA	NA	NA
ENSG00000184788	SATL1	spermidine/spermine N1-acetyl transferase like 1	340562	0	NA	NA	NA	NA	NA
ENSG00000184789	OR4C10P	olfactory receptor family 4 subfamily C member 10 pseudogene	79526	0	NA	NA	NA	NA	NA
ENSG00000184792	OSBP2	oxysterol binding protein 2	23762	0,072165449	0,847101401	4,080456	0,207599697	0,835541541	NA
ENSG00000184795	NA	NA	NA	0	NA	NA	NA	NA	NA
ENSG00000184811	TRARG1	trafficking regulator of GLUT4 (SLC2A4) 1	286753	0,65978764	-0,855988792	3,838561	-0,222997336	0,82353758	NA
ENSG00000184814	PRR23B	proline rich 23B	389151	0	NA	NA	NA	NA	NA
ENSG00000184828	ZBTB7C	zinc finger and BTB domain containing 7C	201501	0,103998153	-1,076443944	4,080456	-0,263804824	0,791930317	NA
ENSG00000184831	APOO	apolipoprotein O	79135	9,007547919	0,199501914	0,667138	0,299041455	0,764908416	NA
ENSG00000184838	PRR16	proline rich 16	51334	0	NA	NA	NA	NA	NA
ENSG00000184844	NA	NA	NA	0	NA	NA	NA	NA	NA
ENSG00000184845	DRD1	dopamine receptor D1	1812	0	NA	NA	NA	NA	NA
ENSG00000184867	ARMCX2	armadillo repeat containing X-linked 2	9823	0,770912234	2,349901784	2,365442	0,993430351	0,320500277	NA

ENSG00000184895	SRY	sex determining region Y	6736	0	NA	NA	NA	NA	NA
ENSG00000184903	IMMP2L	inner mitochondrial membrane peptidase subunit 2	83943	7,14105222	0,815588597	0,854141	0,954864591	0,339646178	NA
ENSG00000184906	NA	NA	NA	0	NA	NA	NA	NA	NA
ENSG00000184908	CLCNKB	chloride voltage-gated channel Kb	1188	0	NA	NA	NA	NA	NA
ENSG00000184911	DMRTC1B	DMRT like family C1B	728656	0,176269367	-0,11782992	4,080456	-0,028876656	0,976962964	NA
ENSG00000184923	NUTM2A	NUT family member 2A	728118	6,023806451	0,509598998	0,739744	0,688885599	0,490895263	NA
ENSG00000184925	LCN12	lipocalin 12	286256	0,343085439	1,002197185	4,021523	0,249208345	0,803199624	NA
ENSG00000184933	OR6A2	olfactory receptor family 6 subfamily A member 2	8590	0	NA	NA	NA	NA	NA
ENSG00000184937	WT1	WT1 transcription factor	7490	0	NA	NA	NA	NA	NA
ENSG00000184945	AQP12A	aquaporin 12A	375318	0	NA	NA	NA	NA	NA
ENSG00000184949	FAM227A	family with sequence similarity 227 member A	646851	0	NA	NA	NA	NA	NA
ENSG00000184954	OR6C70	olfactory receptor family 6 subfamily C member 70	390327	0	NA	NA	NA	NA	NA
ENSG00000184956	MUC6	mucin 6, oligomeric mucus/gel-forming	4588	0,307245471	0,714803201	3,849534	0,185685656	0,852691277	NA
ENSG00000184961	RPL7AP49	ribosomal protein L7a pseudogene 49	100271214	0,368215337	0	4,080456	0	1	NA
ENSG00000184983	NDUFA6	NADH:ubiquinone oxidoreductase subunit A6	4700	0	NA	NA	NA	NA	NA
ENSG00000184984	CHRM5	cholinergic receptor muscarinic 5	1133	0,48102951	0,534865415	2,92722	0,18272132	0,855016686	NA
ENSG00000184985	SORCS2	sortilin related VPS10 domain containing receptor 2	57537	11,39446064	-0,47900938	0,567037	-0,844759316	0,398245234	NA
ENSG00000184986	TMEM121	transmembrane protein 121	80757	3,468597083	-1,19931228	0,980283	-1,223434432	0,221165655	NA
ENSG00000184995	IFNE	interferon epsilon	338376	2,448814998	2,360976736	1,653973	1,427458182	0,153447871	NA
ENSG00000184999	SLC22A10	solute carrier family 22 member 10	387775	0	NA	NA	NA	NA	NA
ENSG00000185002	RFX6	regulatory factor X6	222546	0	NA	NA	NA	NA	NA
ENSG00000185008	ROBO2	roundabout guidance receptor 2	6092	0	NA	NA	NA	NA	NA
ENSG00000185013	NT5C1B	5'-nucleotidase, cytosolic IB	93034	0	NA	NA	NA	NA	NA
ENSG00000185015	CA13	carbonic anhydrase 13	377677	5,653637127	-0,221173278	0,89086	-0,248269531	0,803925872	NA
ENSG00000185028	LRRC14B	leucine rich repeat containing 14B	389257	0	NA	NA	NA	NA	NA
ENSG00000185031	NA	NA	NA	0,572767589	-0,231853838	2,488907	-0,093154873	0,925780524	NA
ENSG00000185037	NA	NA	NA	0	NA	NA	NA	NA	NA
ENSG00000185038	MROH2A	maestro heat like repeat family member 2A	339766	0	NA	NA	NA	NA	NA
ENSG00000185040	SPDYE16	speedy/RINGO cell cycle regulator family member E16	102723555	0,11808024	-1,076443944	4,080456	-0,263804824	0,791930317	NA
ENSG00000185046	ANKS1B	ankyrin repeat and sterile alpha motif domain containing 1B	56899	0	NA	NA	NA	NA	NA
ENSG00000185052	SLC24A3	solute carrier family 24 member 3	57419	8,815260626	-0,844896648	0,622448	-1,357377176	0,174661396	NA
ENSG00000185053	SGCZ	sarcoglycan zeta	137868	0	NA	NA	NA	NA	NA
ENSG00000185055	EFCAB10	EF-hand calcium binding domain 10	100130771	0	NA	NA	NA	NA	NA
ENSG00000185056	C5orf47	chromosome 5 open reading frame 47	133491	0	NA	NA	NA	NA	NA
ENSG00000185069	KRT76	keratin 76	51350	0	NA	NA	NA	NA	NA
ENSG00000185070	FLRT2	fibronectin leucine rich transmembrane protein 2	23768	0,479442268	-1,320278357	3,469734	-0,380512796	0,7035648	NA
ENSG00000185070	LOC124907389	leucine-rich repeat transmembrane protein FLRT2	124907389	0,479442268	-1,320278357	3,469734	-0,380512796	0,7035648	NA
ENSG00000185074	NA	NA	NA	0	NA	NA	NA	NA	NA
ENSG00000185100	ADSS1	adenylosuccinate synthase 1	122622	3,340232787	-1,870046821	1,234527	-1,514788162	0,129826059	NA
ENSG00000185105	MYADML2	myeloid associated differentiation marker like 2	255275	0	NA	NA	NA	NA	NA
ENSG00000185115	NSMCE3	NSE3 homolog, SMC5-SMC6 complex component	56160	1,792855574	0,386115443	1,568461	0,246174669	0,805547023	NA
ENSG00000185130	H2BC13	H2B clustered histone 13	8340	0,366487215	1,169140176	3,623819	0,322626568	0,746978071	NA
ENSG00000185133	INPP5J	inositol polyphosphate-5-phosphatase J	27124	0	NA	NA	NA	NA	NA
ENSG00000185149	NPY2R	neuropeptide Y receptor Y2	4887	0	NA	NA	NA	NA	NA
ENSG00000185155	MIXL1	Mix paired-like homeobox	83881	0	NA	NA	NA	NA	NA
ENSG00000185156	MFSD6L	major facilitator superfamily domain containing 6 like	162387	10,19881177	-0,285532198	0,606256	-0,470976121	0,637657787	NA
ENSG00000185176	AQP12B	aquaporin 12B	653437	1,207494175	-1,306502253	1,74857	-0,747183428	0,454952846	NA
ENSG00000185177	ZNF479	zinc finger protein 479	90827	0	NA	NA	NA	NA	NA
ENSG00000185182	GOLGA8EP	golgin A8 family member E, pseudogene	390535	0,10543123	0	4,080456	0	1	NA
ENSG00000185189	NRBP2	nuclear receptor binding protein 2	340371	33,38355399	1,014125525	1,792823	0,565658395	NA	NA

ENSG00000185198	PRSS57	serine protease 57	400668	11,62305863	-0,182743748	0,503694	-0,362807372	0,716748784	NA
ENSG00000185215	TNFAIP2	TNF alpha induced protein 2	7127	0	NA	NA	NA	NA	NA
ENSG00000185221	NA	NA	NA	0	NA	NA	NA	NA	NA
ENSG00000185222	TCEAL9	transcription elongation factor A like 9	51186	0	NA	NA	NA	NA	NA
ENSG00000185231	MC2R	melanocortin 2 receptor	4158	0,117497108	0,847101401	4,080456	0,207599697	0,835541541	NA
ENSG00000185245	GP1BA	glycoprotein Ib platelet subunit alpha	2811	0	NA	NA	NA	NA	NA
ENSG00000185247	MAGEA11	MAGE family member A11	4110	0	NA	NA	NA	NA	NA
ENSG00000185250	PPIL6	peptidylprolyl isomerase like 6	285755	0,10543123	0	4,080456	0	1	NA
ENSG00000185264	CIMIP4	ciliary microtubule inner protein 4	339669	0	NA	NA	NA	NA	NA
ENSG00000185267	CDNF	cerebral dopamine neurotrophic factor	441549	1,947170711	1,985611916	1,896465	1,047006953	0,295096371	NA
ENSG00000185269	NOTUM	notum, palmitoleoyl-protein carboxylesterase	147111	0,15836288	0	4,080456	0	1	NA
ENSG00000185272	RBM11	RNA binding motif protein 11	54033	0	NA	NA	NA	NA	NA
ENSG00000185274	GALNT17	polypeptide N-acetylgalactosaminyltransferase 17	64409	0	NA	NA	NA	NA	NA
ENSG00000185275	NA	NA	NA	0	NA	NA	NA	NA	NA
ENSG00000185290	NUPR2	nuclear protein 2, transcriptional regulator	389493	0	NA	NA	NA	NA	NA
ENSG00000185294	SPPL2C	signal peptide peptidase like 2C	162540	0	NA	NA	NA	NA	NA
ENSG00000185296	LOC646012	aurora kinase A interacting protein 1 pseudogene	646012	0	NA	NA	NA	NA	NA
ENSG00000185303	SFTPA2	surfactant protein A2	729238	0	NA	NA	NA	NA	NA
ENSG00000185306	C12orf56	chromosome 12 open reading frame 56	115749	0	NA	NA	NA	NA	NA
ENSG00000185313	SCN10A	sodium voltage-gated channel alpha subunit 10	6336	0	NA	NA	NA	NA	NA
ENSG00000185319	NA	NA	NA	0	NA	NA	NA	NA	NA
ENSG00000185339	TCN2	transcobalamin 2	6948	2,460074126	-0,026377254	1,269984	-0,020769749	0,98342933	NA
ENSG00000185345	PRKN	parkin RBR E3 ubiquitin protein ligase	5071	0	NA	NA	NA	NA	NA
ENSG00000185352	HS6ST3	heparan sulfate 6-O-sulfotransferase 3	266722	0	NA	NA	NA	NA	NA
ENSG00000185372	OR2V1	olfactory receptor family 2 subfamily V member 1	26693	0	NA	NA	NA	NA	NA
ENSG00000185385	OR7A17	olfactory receptor family 7 subfamily A member 17	26333	0,118286652	0	4,080456	0	1	NA
ENSG00000185390	NA	NA	NA	0	NA	NA	NA	NA	NA
ENSG00000185436	IFNLR1	interferon lambda receptor 1	163702	0	NA	NA	NA	NA	NA
ENSG00000185437	SH3BGR	SH3 domain binding glutamate rich protein	6450	0,32853889	0,846166949	4,080456	0,20737069	0,835720371	NA
ENSG00000185442	FAM174B	family with sequence similarity 174 member B	400451	0	NA	NA	NA	NA	NA
ENSG00000185448	FAM47A	family with sequence similarity 47 member A	158724	0	NA	NA	NA	NA	NA
ENSG00000185467	KPNA7	karyopherin subunit alpha 7	402569	0	NA	NA	NA	NA	NA
ENSG00000185479	KRT6B	keratin 6B	3854	0	NA	NA	NA	NA	NA
ENSG00000185482	STAC3	SH3 and cysteine rich domain 3	246329	3,594788888	-0,468565575	0,976653	-0,479766826	0,631393204	NA
ENSG00000185483	ROR1	receptor tyrosine kinase like orphan receptor 1	4919	0	NA	NA	NA	NA	NA
ENSG00000185495	NA	NA	NA	0,352191475	0,856603133	4,08021	0,209940941	0,833713768	NA
ENSG00000185507	IRF7	interferon regulatory factor 7	3665	0	NA	NA	NA	NA	NA
ENSG00000185513	L3MBTL1	L3MBTL histone methyl-lysine binding protein 1	26013	1,863936192	4,631049031	2,218064	2,08787864	0,036808783	NA
ENSG00000185518	SV2B	synaptic vesicle glycoprotein 2B	9899	0	NA	NA	NA	NA	NA
ENSG00000185519	FAM131C	family with sequence similarity 131 member C	348487	1,761005249	-2,908930203	1,86108	-1,563033442	0,118044729	NA
ENSG00000185522	LMNTD2	lamin tail domain containing 2	256329	0,311994459	-2,498367527	3,998294	-0,62485841	0,532063991	NA
ENSG00000185523	SPATA45	spermatogenesis associated 45	149643	0,365328454	1,162227258	3,627061	0,320432203	0,748640717	NA
ENSG00000185532	PRKG1	protein kinase cGMP-dependent 1	5592	0	NA	NA	NA	NA	NA
ENSG00000185551	NR2F2	nuclear receptor subfamily 2 group F member 2	7026	0,224561887	1,708505083	4,036854	0,423226848	0,67212976	NA
ENSG00000185559	DLK1	delta like non-canonical Notch ligand 1	8788	0	NA	NA	NA	NA	NA
ENSG00000185565	LSAMP	limbic system associated membrane protein	4045	0	NA	NA	NA	NA	NA
ENSG00000185567	AHNAK2	AHNAK nucleoprotein 2	113146	0,117497108	0,847101401	4,080456	0,207599697	0,835541541	NA
ENSG00000185585	OLFML2A	olfactomedin like 2A	169611	0,210313358	-1,076221255	4,080456	-0,26375025	0,791972372	NA
ENSG00000185607	NA	NA	NA	0	NA	NA	NA	NA	NA

ENSG00000185610	DBX2	developing brain homeobox 2	440097	0	NA	NA	NA	NA	NA
ENSG00000185614	INKA1	inka box actin regulator 1	389119	0,177802412	0,843600846	4,080376	0,20674587	0,836208332	NA
ENSG00000185615	PDIA2	protein disulfide isomerase family A member 2	64714	0	NA	NA	NA	NA	NA
ENSG00000185630	PBX1	PBX homeobox 1	5087	0	NA	NA	NA	NA	NA
ENSG00000185631	RPL14P6	RPL14 pseudogene 6	100288533	0,600068671	-2,545378001	2,781642	-0,915063165	0,360158471	NA
ENSG00000185633	NDUFA4L2	NDUFA4 mitochondrial complex associated like 2	56901	0,199986365	-0,298573268	4,075938	-0,073252651	0,94160507	NA
ENSG00000185634	SHC4	SHC adaptor protein 4	399694	1,237111796	-0,468616244	1,763472	-0,265735043	0,790443273	NA
ENSG00000185638	NA	NA	NA	0	NA	NA	NA	NA	NA
ENSG00000185640	KRT79	keratin 79	338785	5,812697001	-0,717284354	0,923265	-0,776899675	0,437217963	NA
ENSG00000185641	NA	NA	NA	0,176609061	-0,12974713	4,080157	-0,031799544	0,97463191	NA
ENSG00000185652	NTF3	neurotrophin 3	4908	0	NA	NA	NA	NA	NA
ENSG00000185662	SMIM23	small integral membrane protein 23	644994	0	NA	NA	NA	NA	NA
ENSG00000185664	PMEL	premelanosome protein	6490	0,359021632	2,56113643	3,98322	0,642981427	0,520236152	NA
ENSG00000185666	SYN3	synapsin III	8224	0,223094566	-1,079238326	4,080456	-0,264489645	0,791402643	NA
ENSG00000185668	POU3F1	POU class 3 homeobox 1	5453	0	NA	NA	NA	NA	NA
ENSG00000185669	SNAI3	snail family transcriptional repressor 3	333929	6,751832458	-0,395838834	0,730125	-0,542152188	0,587713669	NA
ENSG00000185674	LYG2	lysozyme g2	254773	0,10543123	0	4,080456	0	1	NA
ENSG00000185681	MORN5	MORN repeat containing 5	254956	0	NA	NA	NA	NA	NA
ENSG00000185686	PRAME	PRAME nuclear receptor transcriptional regulator	23532	0	NA	NA	NA	NA	NA
ENSG00000185689	TEX56P	testis expressed 56, pseudogene	404220	0	NA	NA	NA	NA	NA
ENSG00000185736	ADARB2	adenosine deaminase RNA specific B2 (inactive)	105	4,932327847	-2,020111312	1,047423	-1,928648565	0,053774506	NA
ENSG00000185737	NRG3	neuregulin 3	10718	0	NA	NA	NA	NA	NA
ENSG00000185739	SRL	sarcalumenin	6345	0	NA	NA	NA	NA	NA
ENSG00000185742	C11orf87	chromosome 11 open reading frame 87	399947	0	NA	NA	NA	NA	NA
ENSG00000185745	IFIT1	interferon induced protein with tetratricopeptide repeats 1	3434	0,870257687	2,177138527	2,038735	1,067886708	0,285571619	NA
ENSG00000185758	CLDN24	claudin 24	100132463	0	NA	NA	NA	NA	NA
ENSG00000185761	ADAMTSL5	ADAMTS like 5	339366	4,455997426	-0,462564326	1,134776	-0,407626058	0,68354823	NA
ENSG00000185774	KCNIP4	potassium voltage-gated channel interacting protein 4	80333	0	NA	NA	NA	NA	NA
ENSG00000185775	SPATA31A6	SPATA31 subfamily A member 6	389730	0	NA	NA	NA	NA	NA
ENSG00000185792	NLRP9	NLR family pyrin domain containing 9	338321	0	NA	NA	NA	NA	NA
ENSG00000185818	NAT8L	N-acetyltransferase 8 like	339983	0,315507891	-1,077534132	4,080456	-0,264071997	0,791724441	NA
ENSG00000185821	OR6C76	olfactory receptor family 6 subfamily C member 76	390326	0	NA	NA	NA	NA	NA
ENSG00000185823	NPAP1	nuclear pore associated protein 1	23742	0,10488184	-1,076443944	4,080456	-0,263804824	0,791930317	NA
ENSG00000185834	NA	NA	NA	0	NA	NA	NA	NA	NA
ENSG00000185839	AK2P1	adenylate kinase 2 pseudogene 1	266920	0,10543123	0	4,080456	0	1	NA
ENSG00000185842	DNAH14	dynein axonemal heavy chain 14	127602	0,209763681	-1,926644696	4,042043	-0,476651224	0,633610501	NA
ENSG00000185860	CCDC190	coiled-coil domain containing 190	339512	0	NA	NA	NA	NA	NA
ENSG00000185863	TMEM210	transmembrane protein 210	100505993	0	NA	NA	NA	NA	NA
ENSG00000185864	NPIPB4	nuclear pore complex interacting protein family member B4	440345	2,40829079	4,752045806	2,526548	1,88084535	0,059992959	NA
ENSG00000185873	TMPRSS11B	transmembrane serine protease 11B	132724	0	NA	NA	NA	NA	NA
ENSG00000185880	TRIM69	tripartite motif containing 69	140691	0	NA	NA	NA	NA	NA
ENSG00000185888	PRSS38	serine protease 38	339501	0	NA	NA	NA	NA	NA
ENSG00000185897	FFAR3	free fatty acid receptor 3	2865	0	NA	NA	NA	NA	NA
ENSG00000185903	OR11N1P	olfactory receptor family 11 subfamily N member 1 pseudogene	392540	0	NA	NA	NA	NA	NA
ENSG00000185909	KLHDC8B	kelch domain containing 8B	200942	2,453085918	0,801413821	1,587042	0,504973178	0,613577675	NA
ENSG00000185915	KLHL34	kelch like family member 34	257240	0,119673877	0,847101401	4,080456	0,207599697	0,835541541	NA
ENSG00000185924	RTN4RL1	reticulon 4 receptor like 1	146760	1,80314353	-2,312891716	1,696356	-1,363447224	0,172741607	NA
ENSG00000185926	OR4C46	olfactory receptor family 4 subfamily C member 46	119749	0	NA	NA	NA	NA	NA
ENSG00000185933	CALHM1	calcium homeostasis modulator 1	255022	8,984446092	-0,319102276	0,614911	-0,518940632	0,603802142	NA
ENSG00000185942	NKAIN3	sodium/potassium transporting ATPase interacting 3	286183	0	NA	NA	NA	NA	NA
ENSG00000185950	IRS2	insulin receptor substrate 2	8660	9,332189956	0,30362962	0,643422	0,471898422	0,636999294	NA

ENSG00000185955	SPACDR	sperm acrosome developmental regulator	402573	0,747236583	-1,960696928	2,658123	-0,737624617	0,460742592	NA
ENSG00000185958	FAM186A	family with sequence similarity 186 member A	121006	0,11808024	-1,076443944	4,080456	-0,263804824	0,791930317	NA
ENSG00000185962	LCE3A	late cornified envelope 3A	353142	0	NA	NA	NA	NA	NA
ENSG00000185966	LCE3E	late cornified envelope 3E	353145	0	NA	NA	NA	NA	NA
ENSG00000185972	CCIN	calicin	881	1,486675413	0,959227356	1,371815	0,699239542	0,484402343	NA
ENSG00000185982	DEFB128	defensin beta 128	245939	0	NA	NA	NA	NA	NA
ENSG00000185985	SLITRK2	SLIT and NTRK like family member 2	84631	0,382477255	-1,986240344	3,620578	-0,548597548	0,583281669	NA
ENSG00000185988	PLK5	polo like kinase 5 (inactive)	126520	0	NA	NA	NA	NA	NA
ENSG00000186007	LEMD1	LEM domain containing 1	93273	0,10543123	0	4,080456	0	1	NA
ENSG00000186008	RPS4XP21	ribosomal protein S4X pseudogene 21	126235	0	NA	NA	NA	NA	NA
ENSG00000186009	ATP4B	ATPase H+/K+ transporting subunit beta	496	0	NA	NA	NA	NA	NA
ENSG00000186038	HTR3E	5-hydroxytryptamine receptor 3E	285242	0	NA	NA	NA	NA	NA
ENSG00000186047	DLEU7	deleted in lymphocytic leukemia 7	220107	0	NA	NA	NA	NA	NA
ENSG00000186047	DLEU1-AS1	DLEU1 antisense RNA 1	103689915	0	NA	NA	NA	NA	NA
ENSG00000186049	KRT73	keratin 73	319101	0,47353176	0,965573518	2,973198	0,324759226	0,745363306	NA
ENSG00000186051	TAL2	TAL bHLH transcription factor 2	6887	0,072165449	0,847101401	4,080456	0,207599697	0,835541541	NA
ENSG00000186074	CD300LF	CD300 molecule like family member f	146722	0	NA	NA	NA	NA	NA
ENSG00000186075	ZPBP2	zona pellucida binding protein 2	124626	0	NA	NA	NA	NA	NA
ENSG00000186076	NA	NA	NA	0,234994216	1,944111989	4,02324	0,483220515	0,62893917	NA
ENSG00000186081	KRT5	keratin 5	3852	0	NA	NA	NA	NA	NA
ENSG00000186082	KRT18P14	keratin 18 pseudogene 14	119722	0	NA	NA	NA	NA	NA
ENSG00000186086	NBPF6	NBPF member 6	653149	0,35424072	-2,665130536	3,988406	-0,668219491	0,503993495	NA
ENSG00000186090	HTR3D	5-hydroxytryptamine receptor 3D	200909	0	NA	NA	NA	NA	NA
ENSG00000186092	OR4F5	olfactory receptor family 4 subfamily F member 5	79501	0	NA	NA	NA	NA	NA
ENSG00000186094	AGBL4	AGBL carboxypeptidase 4	84871	0	NA	NA	NA	NA	NA
ENSG00000186103	ARGFX	arginine-fifty homeobox	503582	0	NA	NA	NA	NA	NA
ENSG00000186105	LRRC70	leucine rich repeat containing 70	100130733	8,526689748	0,420714024	0,590857	0,712040336	0,476439799	NA
ENSG00000186113	OR5D14	olfactory receptor family 5 subfamily D member 14	219436	0	NA	NA	NA	NA	NA
ENSG00000186115	CYP4F2	cytochrome P450 family 4 subfamily F member 2	8529	0	NA	NA	NA	NA	NA
ENSG00000186118	TEX38	testis expressed 38	374973	0,236573303	0	4,080456	0	1	NA
ENSG00000186119	OR5D18	olfactory receptor family 5 subfamily D member 18	219438	0	NA	NA	NA	NA	NA
ENSG00000186124	OR9M1P	olfactory receptor family 9 subfamily M member 1, pseudogene	81153	0	NA	NA	NA	NA	NA
ENSG00000186143	PRR30	proline rich 30	339779	0	NA	NA	NA	NA	NA
ENSG00000186146	DEFB131A	defensin beta 131A	644414	0	NA	NA	NA	NA	NA
ENSG00000186148	NA	NA	NA	0	NA	NA	NA	NA	NA
ENSG00000186150	UBL4B	ubiquitin like 4B	164153	0	NA	NA	NA	NA	NA
ENSG00000186152	NA	NA	NA	0	NA	NA	NA	NA	NA
ENSG00000186160	CYP4Z1	cytochrome P450 family 4 subfamily Z member 1	199974	0	NA	NA	NA	NA	NA
ENSG00000186162	CIDECP1	CIDEC pseudogene 1	152302	9,108615616	0,329444726	0,627761	0,524793028	0,599727078	NA
ENSG00000186163	PRSS59P	serine protease 59, pseudogene	207147	0	NA	NA	NA	NA	NA
ENSG00000186188	FFAR4	free fatty acid receptor 4	338557	0	NA	NA	NA	NA	NA
ENSG00000186190	BPIFB3	BPI fold containing family B member 3	359710	0	NA	NA	NA	NA	NA
ENSG00000186191	BPIFB4	BPI fold containing family B member 4	149954	0	NA	NA	NA	NA	NA
ENSG00000186197	EDARADD	EDAR associated via death domain	128178	11,29700192	-0,242324951	0,596851	-0,406006094	0,684738123	NA
ENSG00000186198	SLC51B	solute carrier family 51 subunit beta	123264	0	NA	NA	NA	NA	NA
ENSG00000186204	CYP4F12	cytochrome P450 family 4 subfamily F member 12	66002	0	NA	NA	NA	NA	NA
ENSG00000186205	MTARC1	mitochondrial amidoxime reducing component 1	64757	0	NA	NA	NA	NA	NA
ENSG00000186207	LCE5A	late cornified envelope 5A	254910	0	NA	NA	NA	NA	NA
ENSG00000186212	SOWAHB	sosondowah ankyrin repeat domain family member B	345079	0	NA	NA	NA	NA	NA
ENSG00000186226	LCE1E	late cornified envelope 1E	353135	0	NA	NA	NA	NA	NA
ENSG00000186231	KLHL32	kelch like family member 32	114792	0,677633762	1,544091228	3,248036	0,475392274	0,634507401	NA

ENSG00000186234	FAM86MP	family with sequence similarity 86, member A pseudogene	644517	0	NA	NA	NA	NA	NA
ENSG00000186244	RPL21P124	ribosomal protein L21 pseudogene 124	100271450	0,119673877	0,847101401	4,080456	0,207599697	0,835541541	NA
ENSG00000186265	BTLA	B and T lymphocyte associated	151888	0	NA	NA	NA	NA	NA
ENSG00000186268	NA	NA	NA	0	NA	NA	NA	NA	NA
ENSG00000186280	KDM4D	lysine demethylase 4D	55693	1,840629752	0,492426181	1,437274	0,342611155	0,731891009	NA
ENSG00000186281	GPAT2	glycerol-3-phosphate acyltransferase 2, mitochondrial	150763	11,41744796	-0,51161291	0,565594	-0,904557823	0,365699688	NA
ENSG00000186288	PABPC1L2A	poly(A) binding protein cytoplasmic 1 like 2A	340529	0	NA	NA	NA	NA	NA
ENSG00000186297	GABRA5	gamma-aminobutyric acid type A receptor subunit alpha5	2558	0	NA	NA	NA	NA	NA
ENSG00000186300	ZNF555	zinc finger protein 555	148254	4,886446784	0,112529373	1,035491	0,108672487	0,913462265	NA
ENSG00000186301	NA	NA	NA	1,03344987	1,345420825	1,900373	0,707977056	0,478959506	NA
ENSG00000186306	OR10T2	olfactory receptor family 10 subfamily T member 2	128360	0	NA	NA	NA	NA	NA
ENSG00000186310	NAP1L3	nucleosome assembly protein 1 like 3	4675	2,923893419	-0,674531072	1,165614	-0,57869186	0,562797109	NA
ENSG00000186312	NA	NA	NA	10,67214884	0,528810526	0,592501	0,892505022	0,372122302	NA
ENSG00000186314	PRELID2	PRELI domain containing 2	153768	5,478767859	-1,256510259	1,217705	-1,031867769	0,302134067	NA
ENSG00000186318	BACE1	beta-secretase 1	23621	8,358094886	0,86056611	0,938725	0,916739011	0,359279419	NA
ENSG00000186326	RGS9BP	regulator of G protein signaling 9 binding protein	388531	9,054216981	0,11415259	0,621266	0,18374178	0,854216031	NA
ENSG00000186328	LOC112267975	V-type proton ATPase subunit F-like	112267975	0	NA	NA	NA	NA	NA
ENSG00000186329	TMEM212	transmembrane protein 212	389177	0	NA	NA	NA	NA	NA
ENSG00000186334	SLC36A3	solute carrier family 36 member 3	285641	0	NA	NA	NA	NA	NA
ENSG00000186335	SLC36A2	solute carrier family 36 member 2	153201	0	NA	NA	NA	NA	NA
ENSG00000186340	THBS2	thrombospondin 2	7058	0	NA	NA	NA	NA	NA
ENSG00000186352	ANKRD37	ankyrin repeat domain 37	353322	2,20891395	-0,050858155	1,540779	-0,033008082	0,973668143	NA
ENSG00000186364	NUDT17	nudix hydrolase 17	200035	6,521852521	-0,482679717	0,819632	-0,588897869	0,555929787	NA
ENSG00000186367	MINAR2	membrane integral NOTCH2 associated receptor 2	100127206	0	NA	NA	NA	NA	NA
ENSG00000186369	NA	NA	NA	0	NA	NA	NA	NA	NA
ENSG00000186377	CYP4X1	cytochrome P450 family 4 subfamily X member 1	260293	0	NA	NA	NA	NA	NA
ENSG00000186393	KRT26	keratin 26	353288	0	NA	NA	NA	NA	NA
ENSG00000186407	CD300E	CD300e molecule	342510	0,10543123	0	4,080456	0	1	NA
ENSG00000186409	CCDC30	coiled-coil domain containing 30	728621	1,852942992	3,710988536	2,016006	1,84076306	0,065656288	NA
ENSG00000186417	GLDN	gliomedin	342035	0,15836288	0	4,080456	0	1	NA
ENSG00000186439	TRDN	triadin	10345	0	NA	NA	NA	NA	NA
ENSG00000186440	OR6P1	olfactory receptor family 6 subfamily P member 1	128366	0	NA	NA	NA	NA	NA
ENSG00000186442	KRT3	keratin 3	3850	0	NA	NA	NA	NA	NA
ENSG00000186451	SPATA12	spermatogenesis associated 12	353324	0	NA	NA	NA	NA	NA
ENSG00000186452	TMPRSS12	transmembrane serine protease 12	283471	0	NA	NA	NA	NA	NA
ENSG00000186453	FAM228A	family with sequence similarity 228 member A	653140	0,210862461	0	4,080456	0	1	NA
ENSG00000186458	DEFB132	defensin beta 132	400830	0	NA	NA	NA	NA	NA
ENSG00000186462	NAP1L2	nucleosome assembly protein 1 like 2	4674	3,689461025	0,129145501	0,926311	0,139419194	0,889118906	NA
ENSG00000186466	NA	NA	NA	0,072165449	0,847101401	4,080456	0,207599697	0,835541541	NA
ENSG00000186471	AKAP14	A-kinase anchoring protein 14	158798	0	NA	NA	NA	NA	NA
ENSG00000186472	PCLO	piccolo presynaptic cytomatrix protein	27445	0,329063166	-0,210975506	4,042199	-0,052193254	0,958374708	NA
ENSG00000186474	KLK12	kallikrein related peptidase 12	43849	0	NA	NA	NA	NA	NA
ENSG00000186479	RGS7BP	regulator of G protein signaling 7 binding protein	401190	0	NA	NA	NA	NA	NA
ENSG00000186481	NA	NA	NA	9,45003803	-0,695579315	0,654459	-1,06283061	0,287858775	NA
ENSG00000186487	MYT1L	myelin transcription factor 1 like	23040	0	NA	NA	NA	NA	NA
ENSG00000186496	ZNF396	zinc finger protein 396	252884	2,514105913	-1,581697515	1,365619	-1,158227605	0,246771166	NA
ENSG00000186508	NA	NA	NA	0	NA	NA	NA	NA	NA
ENSG00000186509	OR9Q1	olfactory receptor family 9 subfamily Q member 1	219956	0	NA	NA	NA	NA	NA
ENSG00000186510	CLCNKA	chloride voltage-gated channel Ka	1187	0	NA	NA	NA	NA	NA

ENSG00000186513	OR9Q2	olfactory receptor family 9 subfamily Q member 2	219957	0	NA	NA	NA	NA	NA
ENSG00000186522	SEPTIN10	septin 10	151011	0,222178114	-1,080279543	4,080456	-0,264744817	0,79120605	NA
ENSG00000186523	FAM86B1	family with sequence similarity 86 member B1	85002	0	NA	NA	NA	NA	NA
ENSG00000186526	CYP4F8	cytochrome P450 family 4 subfamily F member 8	11283	0	NA	NA	NA	NA	NA
ENSG00000186529	CYP4F3	cytochrome P450 family 4 subfamily F member 3	4051	0,10543123	0	4,080456	0	1	NA
ENSG00000186543	NA	NA	NA	0	NA	NA	NA	NA	NA
ENSG00000186564	FOXD2	forkhead box D2	2306	1,85618891	0,17634181	1,519257	0,116071112	0,907596183	NA
ENSG00000186567	CEACAM19	CEA cell adhesion molecule 19	56971	1,227644485	-0,092352195	2,541233	-0,036341485	0,971010071	NA
ENSG00000186583	SPATC1	spermatogenesis and centriole associated 1	375686	0,550683521	2,810827828	2,733454	1,028306387	0,303805728	NA
ENSG00000186603	HPDL	4-hydroxyphenylpyruvate dioxygenase like	84842	2,646458728	0,982135619	1,129707	0,869372218	0,384643574	NA
ENSG00000186628	FSD2	fibronectin type III and SPRY domain containing 2	123722	0,119673877	0,847101401	4,080456	0,207599697	0,835541541	NA
ENSG00000186642	PDE2A	phosphodiesterase 2A	5138	0,144330898	1,418786819	4,061262	0,349346267	0,726829368	NA
ENSG00000186645	SPDYE17	speedy/RINGO cell cycle regulator family member E17	102723849	0,15836288	0	4,080456	0	1	NA
ENSG00000186645	LOC124906603	putative speedy protein E9	124906603	0,15836288	0	4,080456	0	1	NA
ENSG00000186652	PRG2	proteoglycan 2, pro eosinophil major basic protein	5553	0	NA	NA	NA	NA	NA
ENSG00000186665	C17orf58	chromosome 17 open reading frame 58	284018	8,521265893	-0,863387356	0,864993	-0,998143386	0,318209834	NA
ENSG00000186675	MAGEE2	MAGE family member E2	139599	0	NA	NA	NA	NA	NA
ENSG00000186676	NA	NA	NA	0	NA	NA	NA	NA	NA
ENSG00000186678	LOC101060042	X antigen family member 3-like	101060042	0	NA	NA	NA	NA	NA
ENSG00000186684	CYP27C1	cytochrome P450 family 27 subfamily C member 1	339761	0	NA	NA	NA	NA	NA
ENSG00000186710	CFAP73	cilia and flagella associated protein 73	387885	0	NA	NA	NA	NA	NA
ENSG00000186714	CCDC73	coiled-coil domain containing 73	493860	0,189614167	0,856184649	4,080456	0,209825734	0,833803687	NA
ENSG00000186715	MST1L	macrophage stimulating 1 like (pseudogene)	11223	0,380116742	1,185824217	3,584957	0,330777815	0,74081232	NA
ENSG00000186723	OR10H1	olfactory receptor family 10 subfamily H member 1	26539	0	NA	NA	NA	NA	NA
ENSG00000186732	MPPED1	metallophosphoesterase domain containing 1	758	0	NA	NA	NA	NA	NA
ENSG00000186743	NA	NA	NA	0	NA	NA	NA	NA	NA
ENSG00000186765	FSCN2	fascin actin-bundling protein 2, retinal	25794	0,210862461	0	4,080456	0	1	NA
ENSG00000186766	FOXI2	forkhead box I2	399823	0	NA	NA	NA	NA	NA
ENSG00000186777	ZNF732	zinc finger protein 732	654254	0	NA	NA	NA	NA	NA
ENSG00000186788	SPATA31D3	SPATA31 subfamily D member 3	389762	0	NA	NA	NA	NA	NA
ENSG00000186790	FOXE3	forkhead box E3	2301	0,119673877	0,847101401	4,080456	0,207599697	0,835541541	NA
ENSG00000186795	KCNK18	potassium two pore domain channel subfamily K member 18	338567	0	NA	NA	NA	NA	NA
ENSG00000186803	IFNA10	interferon alpha 10	3446	0	NA	NA	NA	NA	NA
ENSG00000186806	VSIG10L	V-set and immunoglobulin domain containing 10 like	147645	4,270490398	-0,679897293	1,442685	-0,471272319	0,63744628	NA
ENSG00000186818	LILRB4	leukocyte immunoglobulin like receptor B4	11006	0	NA	NA	NA	NA	NA
ENSG00000186825	CDRT15P3	CDRT15 pseudogene 3	408029	0,10488184	-1,076443944	4,080456	-0,263804824	0,791930317	NA
ENSG00000186827	TNFRSF4	TNF receptor superfamily member 4	7293	10,60386026	0,784784514	0,829255	0,946372596	0,343958578	NA
ENSG00000186832	KRT16	keratin 16	3868	0	NA	NA	NA	NA	NA
ENSG00000186838	SELENOV	selenoprotein V	348303	0	NA	NA	NA	NA	NA
ENSG00000186844	LCE1A	late cornified envelope 1A	353131	0	NA	NA	NA	NA	NA
ENSG00000186847	KRT14	keratin 14	3861	0	NA	NA	NA	NA	NA
ENSG00000186854	TRABD2A	TraB domain containing 2A	129293	2,314166849	0,892128858	1,561	0,571511221	0,567653156	NA
ENSG00000186854	LOC105374836	uncharacterized LOC105374836	105374836	2,314166849	0,892128858	1,561	0,571511221	0,567653156	NA
ENSG00000186862	PDZD7	PDZ domain containing 7	79955	1,253088245	0,002977638	2,190192	0,001359533	0,99891525	NA
ENSG00000186867	QRFPR	pyroglutamylated RFamide peptide receptor	84109	0	NA	NA	NA	NA	NA
ENSG00000186868	MAPT	microtubule associated protein tau	4137	0,311994459	-2,498367527	3,998294	-0,62485841	0,532063991	NA
ENSG00000186881	OR13F1	olfactory receptor family 13 subfamily F member 1	138805	0	NA	NA	NA	NA	NA

ENSG00000186886	OR5D3P	olfactory receptor family 5 subfamily D member 3 pseudogene	8594	0	NA	NA	NA	NA	NA
ENSG00000186889	TMEM17	transmembrane protein 17	200728	0	NA	NA	NA	NA	NA
ENSG00000186895	FGF3	fibroblast growth factor 3	2248	0	NA	NA	NA	NA	NA
ENSG00000186897	C1QL4	complement C1q like 4	338761	0	NA	NA	NA	NA	NA
ENSG00000186907	RTN4RL2	reticulon 4 receptor like 2	349667	4,938073059	0,882133058	0,80069	1,101715579	0,270585342	NA
ENSG00000186910	SERPINA11	serpin family A member 11	256394	0	NA	NA	NA	NA	NA
ENSG00000186912	P2RY4	pyrimidinergic receptor P2Y4	5030	1,581306075	-0,868103995	1,409609	-0,615847332	0,537995285	NA
ENSG00000186919	ZACN	zinc activated ion channel	353174	0,10488184	-1,076443944	4,080456	-0,263804824	0,791930317	NA
ENSG00000186924	KRTAP22-1	keratin associated protein 22-1	337979	0	NA	NA	NA	NA	NA
ENSG00000186925	KRTAP19-6	keratin associated protein 19-6	337973	0	NA	NA	NA	NA	NA
ENSG00000186930	KRTAP6-2	keratin associated protein 6-2	337967	0	NA	NA	NA	NA	NA
ENSG00000186940	NA	NA	NA	0,10543123	0	4,080456	0	1	NA
ENSG00000186943	OR13C8	olfactory receptor family 13 subfamily C member 8	138802	0	NA	NA	NA	NA	NA
ENSG00000186952	TMEM232	transmembrane protein 232	642987	0	NA	NA	NA	NA	NA
ENSG00000186965	KRTAP19-2	keratin associated protein 19-2	337969	0	NA	NA	NA	NA	NA
ENSG00000186967	KRTAP19-4	keratin associated protein 19-4	337971	0	NA	NA	NA	NA	NA
ENSG00000186970	KRTAP15-1	keratin associated protein 15-1	254950	0	NA	NA	NA	NA	NA
ENSG00000186971	KRTAP13-4	keratin associated protein 13-4	284827	0	NA	NA	NA	NA	NA
ENSG00000186973	CFAP144	cilia and flagella associated protein 144	440585	0	NA	NA	NA	NA	NA
ENSG00000186976	EFCAB6	EF-hand calcium binding domain 6	64800	0	NA	NA	NA	NA	NA
ENSG00000186977	KRTAP19-5	keratin associated protein 19-5	337972	0	NA	NA	NA	NA	NA
ENSG00000186980	KRTAP23-1	keratin associated protein 23-1	337963	0	NA	NA	NA	NA	NA
ENSG00000186998	EMID1	EMI domain containing 1	129080	3,695014948	-1,293495071	1,172483	-1,103210343	0,269935831	NA
ENSG00000187003	ACTL7A	actin like 7A	10881	0,117497108	0,847101401	4,080456	0,207599697	0,835541541	NA
ENSG00000187005	KRTAP21-1	keratin associated protein 21-1	337977	0	NA	NA	NA	NA	NA
ENSG00000187010	RHD	Rh blood group D antigen	6007	0	NA	NA	NA	NA	NA
ENSG00000187017	ESPN	espin	83715	0,412677675	-2,899582801	3,976815	-0,729121849	0,46592713	NA
ENSG00000187026	KRTAP21-2	keratin associated protein 21-2	337978	0	NA	NA	NA	NA	NA
ENSG00000187033	SAMD7	sterile alpha motif domain containing 7	344658	0	NA	NA	NA	NA	NA
ENSG00000187045	TMPRSS6	transmembrane serine protease 6	164656	2,851679814	0,571781476	1,382985	0,41344017	0,679284149	NA
ENSG00000187048	CYP4A11	cytochrome P450 family 4 subfamily A member 11	1579	0	NA	NA	NA	NA	NA
ENSG00000187054	TMPRSS11A	transmembrane serine protease 11A	339967	0	NA	NA	NA	NA	NA
ENSG00000187066	TMEM262	transmembrane protein 262	100130348	0	NA	NA	NA	NA	NA
ENSG00000187068	C3orf70	chromosome 3 open reading frame 70	285382	0,34453295	1,021477543	4,020868	0,25404402	0,79946155	NA
ENSG00000187079	TEAD1	TEA domain transcription factor 1	7003	0,117497108	0,847101401	4,080456	0,207599697	0,835541541	NA
ENSG00000187080	OR2AK2	olfactory receptor family 2 subfamily AK member 2	391191	0,209440094	-1,073870591	4,080456	-0,263174171	0,792416338	NA
ENSG00000187094	CCK	cholecystokinin	885	0	NA	NA	NA	NA	NA
ENSG00000187098	MITF	melanocyte inducing transcription factor	4286	2,680982881	1,329039495	1,549073	0,857958079	0,390915612	NA
ENSG00000187103	NA	NA	NA	0	NA	NA	NA	NA	NA
ENSG00000187105	HEATR4	HEAT repeat containing 4	399671	0,945750112	-0,208444101	2,150262	-0,096938926	0,922774896	NA
ENSG00000187116	LILRA5	leukocyte immunoglobulin like receptor A5	353514	0	NA	NA	NA	NA	NA
ENSG00000187122	SLIT1	slit guidance ligand 1	6585	3,845096446	1,073941147	1,250822	0,858588459	0,390567608	NA
ENSG00000187123	LYPD6	LY6/PLAUR domain containing 6	130574	0	NA	NA	NA	NA	NA
ENSG00000187134	AKR1C1	aldo-keto reductase family 1 member C1	1645	0	NA	NA	NA	NA	NA
ENSG00000187135	VSTM2B	V-set and transmembrane domain containing 2B	342865	0	NA	NA	NA	NA	NA
ENSG00000187140	FOXD3	forkhead box D3	27022	0	NA	NA	NA	NA	NA
ENSG00000187147	RNF220	ring finger protein 220	55182	0,880414313	-0,361382686	3,120897	-0,115794478	0,907815427	NA
ENSG00000187151	ANGPTL5	angiopoietin like 5	253935	0	NA	NA	NA	NA	NA
ENSG00000187164	SHTN1	shootin 1	57698	0	NA	NA	NA	NA	NA
ENSG00000187166	H1-7	H1,7 linker histone	341567	0	NA	NA	NA	NA	NA
ENSG00000187170	LCE4A	late cornified envelope 4A	199834	0	NA	NA	NA	NA	NA
ENSG00000187172	NA	NA	NA	0,103998153	-1,076443944	4,080456	-0,263804824	0,791930317	NA

ENSG00000187173	LCE2A	late cornified envelope 2A	353139	0	NA	NA	NA	NA	NA
ENSG00000187175	KRTAP12-1	keratin associated protein 12-1	353332	0	NA	NA	NA	NA	NA
ENSG00000187180	LCE2C	late cornified envelope 2C	353140	0	NA	NA	NA	NA	NA
ENSG00000187186	LOC730098	uncharacterized LOC730098	730098	2,115844408	-1,599192936	1,992232	-0,802714028	0,422140043	NA
ENSG00000187191	DAZ3	deleted in azoospermia 3	57054	0	NA	NA	NA	NA	NA
ENSG00000187223	LCE2D	late cornified envelope 2D	353141	0	NA	NA	NA	NA	NA
ENSG00000187238	LCE3B	late cornified envelope 3B	353143	0	NA	NA	NA	NA	NA
ENSG00000187242	KRT12	keratin 12	3859	0	NA	NA	NA	NA	NA
ENSG00000187243	MAGED4B	MAGE family member D4B	81557	0	NA	NA	NA	NA	NA
ENSG00000187244	BCAM	basal cell adhesion molecule (Lutheran blood group)	4059	0	NA	NA	NA	NA	NA
ENSG00000187258	NPSR1	neuropeptide S receptor 1	387129	0	NA	NA	NA	NA	NA
ENSG00000187260	WDR86	WD repeat domain 86	349136	0,260823604	2,133024369	4,009847	0,531946552	0,594763014	NA
ENSG00000187266	EPOR	erythropoietin receptor	2057	7,118344008	-0,460441726	0,732723	-0,628397671	0,529743462	NA
ENSG00000187268	FAM9C	family with sequence similarity 9 member C	171484	2,034102955	0,54530535	1,470997	0,370704551	0,7108576	NA
ENSG00000187272	KRTAP9-8	keratin associated protein 9-8	83901	0	NA	NA	NA	NA	NA
ENSG00000187288	CIDEC	cell death inducing DFFA like effector c	63924	0,118286652	0	4,080456	0	1	NA
ENSG00000187323	DCC	DCC netrin 1 receptor	1630	0	NA	NA	NA	NA	NA
ENSG00000187391	MAGI2	membrane associated guanylate kinase, WW and PDZ domain containing 2	9863	2,326583148	1,076020991	1,333397	0,806977398	0,419679505	NA
ENSG00000187398	LUZP2	leucine zipper protein 2	338645	0,118286652	0	4,080456	0	1	NA
ENSG00000187416	LHFPL3	LHFPL tetraspan subfamily member 3	375612	0	NA	NA	NA	NA	NA
ENSG00000187472	NA	NA	NA	0	NA	NA	NA	NA	NA
ENSG00000187474	FPR3	formyl peptide receptor 3	2359	0	NA	NA	NA	NA	NA
ENSG00000187475	H1-6	H1,6 linker histone, cluster member	3010	0	NA	NA	NA	NA	NA
ENSG00000187479	C11orf96	chromosome 11 open reading frame 96	387763	0,118286652	0	4,080456	0	1	NA
ENSG00000187481	NA	NA	NA	0	NA	NA	NA	NA	NA
ENSG00000187483	NA	NA	NA	0	NA	NA	NA	NA	NA
ENSG00000187486	KCNJ11	potassium inwardly rectifying channel subfamily J member 11	3767	10,03741702	-0,528010768	0,559881	-0,943076058	0,345642001	NA
ENSG00000187492	CDHR4	cadherin related family member 4	389118	0	NA	NA	NA	NA	NA
ENSG00000187498	COL4A1	collagen type IV alpha 1 chain	1282	0	NA	NA	NA	NA	NA
ENSG00000187504	RPL7P48	ribosomal protein L7 pseudogene 48	388401	0	NA	NA	NA	NA	NA
ENSG00000187510	PLEKHG7	pleckstrin homology and RhoGEF domain containing G7	440107	0	NA	NA	NA	NA	NA
ENSG00000187513	GJA4	gap junction protein alpha 4	2701	0	NA	NA	NA	NA	NA
ENSG00000187516	H2AP	H2A,P histone	25763	0	NA	NA	NA	NA	NA
ENSG00000187527	ATP13A5	ATPase 13A5	344905	0	NA	NA	NA	NA	NA
ENSG00000187533	PRR27	proline rich 27	401137	0	NA	NA	NA	NA	NA
ENSG00000187536	NA	NA	NA	0,566368324	-0,112425436	3,428309	-0,032793263	0,973839451	NA
ENSG00000187537	POTEG	POTE ankyrin domain family member G	404785	0	NA	NA	NA	NA	NA
ENSG00000187546	AGMO	alkylglycerol monooxygenase	392636	0	NA	NA	NA	NA	NA
ENSG00000187550	SBK2	SH3 domain binding kinase family member 2	646643	0	NA	NA	NA	NA	NA
ENSG00000187553	CYP26C1	cytochrome P450 family 26 subfamily C member 1	340665	0,221486287	-0,115306178	4,080456	-0,028258161	0,97745625	NA
ENSG00000187554	TLR5	toll like receptor 5	7100	0,342645365	-0,126424723	3,779784	-0,033447601	0,973317651	NA
ENSG00000187556	NANOS3	nanos C2HC-type zinc finger 3	342977	0	NA	NA	NA	NA	NA
ENSG00000187559	FOXD4L3	forkhead box D4 like 3	286380	0,234994216	1,944111989	4,02324	0,483220515	0,62893917	NA
ENSG00000187566	NHLRC1	NHL repeat containing E3 ubiquitin protein ligase 1	378884	3,195662627	1,367350565	1,195145	1,144087551	0,25258733	NA
ENSG00000187569	DPPA3	developmental pluripotency associated 3	359787	0	NA	NA	NA	NA	NA
ENSG00000187581	COX8C	cytochrome c oxidase subunit 8C	341947	0	NA	NA	NA	NA	NA
ENSG00000187589	NA	NA	NA	0	NA	NA	NA	NA	NA
ENSG00000187595	ZNF385C	zinc finger protein 385C	201181	0	NA	NA	NA	NA	NA
ENSG00000187612	OR5W2	olfactory receptor family 5 subfamily W member 2	390148	0	NA	NA	NA	NA	NA
ENSG00000187616	MYMK	myomaker, myoblast fusion factor	389827	0	NA	NA	NA	NA	NA
ENSG00000187627	RGPD1	RANBP2 like and GRIP domain containing 1	400966	0	NA	NA	NA	NA	NA

ENSG00000187630	DHRS4L2	dehydrogenase/reductase 4 like 2	317749	2,661253788	-2,404606065	1,304825	-1,842857086	0,065349879	NA
ENSG00000187634	SAMD11	sterile alpha motif domain containing 11	148398	0	NA	NA	NA	NA	NA
ENSG00000187642	PERM1	PPARGC1 and ESRR induced regulator, muscle 1	84808	0	NA	NA	NA	NA	NA
ENSG00000187653	NA	NA	NA	0	NA	NA	NA	NA	NA
ENSG00000187657	TSPY13P	testis specific protein Y-linked 13, pseudogene	378001	0	NA	NA	NA	NA	NA
ENSG00000187658	C5orf52	chromosome 5 open reading frame 52	100190949	0	NA	NA	NA	NA	NA
ENSG00000187664	HAPLN4	hyaluronan and proteoglycan link protein 4	404037	0	NA	NA	NA	NA	NA
ENSG00000187672	ERC2	ELKS/RAB6-interacting/CAST family member 2	26059	0	NA	NA	NA	NA	NA
ENSG00000187678	SPRY4	sprouty RTK signaling antagonist 4	81848	7,95740718	0,096560764	0,667639	0,144630303	0,885002771	NA
ENSG00000187682	ERAS	ES cell expressed Ras	3266	0,11808024	-1,076443944	4,080456	-0,263804824	0,791930317	NA
ENSG00000187686	NA	NA	NA	0	NA	NA	NA	NA	NA
ENSG00000187689	AMTN	amelotin	401138	0	NA	NA	NA	NA	NA
ENSG00000187690	EZHIP	EZH inhibitory protein	340602	0	NA	NA	NA	NA	NA
ENSG00000187699	C2orf88	chromosome 2 open reading frame 88	84281	0	NA	NA	NA	NA	NA
ENSG00000187701	OR2T27	olfactory receptor family 2 subfamily T member 27	403239	0	NA	NA	NA	NA	NA
ENSG00000187714	SLC18A3	solute carrier family 18 member A3	6572	0	NA	NA	NA	NA	NA
ENSG00000187715	KBTBD12	kelch repeat and BTB domain containing 12	166348	0	NA	NA	NA	NA	NA
ENSG00000187720	THSD4	thrombospondin type 1 domain containing 4	79875	0	NA	NA	NA	NA	NA
ENSG00000187721	GTF2IP3	general transcription factor IIi pseudogene 3	645686	0	NA	NA	NA	NA	NA
ENSG00000187726	DNAJB13	DnaJ heat shock protein family (Hsp40) member B13	374407	0	NA	NA	NA	NA	NA
ENSG00000187730	GABRD	gamma-aminobutyric acid type A receptor subunit delta	2563	0	NA	NA	NA	NA	NA
ENSG00000187733	AMY1C	amylase alpha 1C	278	0	NA	NA	NA	NA	NA
ENSG00000187733	LOC124905666	alpha-amylase 1B	124905666	0	NA	NA	NA	NA	NA
ENSG00000187733	LOC124905668	alpha-amylase 1B	124905668	0	NA	NA	NA	NA	NA
ENSG00000187747	OR52B6	olfactory receptor family 52 subfamily B member 6	340980	0	NA	NA	NA	NA	NA
ENSG00000187753	C9orf153	chromosome 9 open reading frame 153	389766	1,058264075	-1,365656072	3,013841	-0,453128035	0,650456549	NA
ENSG00000187754	SSX7	SSX family member 7	280658	0	NA	NA	NA	NA	NA
ENSG00000187758	ADH1A	alcohol dehydrogenase 1A (class I), alpha polypeptide	124	0	NA	NA	NA	NA	NA
ENSG00000187762	NA	NA	NA	0,914160826	0,175651847	2,00597	0,087564552	0,930222777	NA
ENSG00000187763	OR2B7P	olfactory receptor family 2 subfamily B member 7 pseudogene	81695	0	NA	NA	NA	NA	NA
ENSG00000187766	KRTAP10-8	keratin associated protein 10-8	386681	0	NA	NA	NA	NA	NA
ENSG00000187772	LIN28B	lin-28 homolog B	389421	0	NA	NA	NA	NA	NA
ENSG00000187773	DIPK1C	divergent protein kinase domain 1C	125704	0	NA	NA	NA	NA	NA
ENSG00000187775	DNAH17	dynein axonemal heavy chain 17	8632	0	NA	NA	NA	NA	NA
ENSG00000187783	TMEM72	transmembrane protein 72	643236	0	NA	NA	NA	NA	NA
ENSG00000187791	SPATA31F3	SPATA31 subfamily F member 3	100129969	0	NA	NA	NA	NA	NA
ENSG00000187796	CARD9	caspase recruitment domain family member 9	64170	7,703422136	0,127865953	1,50201	0,085129878	0,932158137	NA
ENSG00000187800	PEAR1	platelet endothelial aggregation receptor 1	375033	0	NA	NA	NA	NA	NA
ENSG00000187806	TMEM202	transmembrane protein 202	338949	0	NA	NA	NA	NA	NA
ENSG00000187808	SOWAHD	sosondowah ankyrin repeat domain family member D	347454	1,573483525	-1,071923336	1,853233	-0,57840727	0,562989187	NA
ENSG00000187812	NA	NA	NA	0	NA	NA	NA	NA	NA
ENSG00000187821	HELT	helt bHLH transcription factor	391723	0	NA	NA	NA	NA	NA
ENSG00000187823	RTL4	retrotransposon Gag like 4	340595	0	NA	NA	NA	NA	NA
ENSG00000187824	TMEM220	transmembrane protein 220	388335	1,791455003	-0,350010768	1,629544	-0,214790587	0,829930592	NA
ENSG00000187847	OR7E25P	olfactory receptor family 7 subfamily E member 25 pseudogene	26647	0	NA	NA	NA	NA	NA
ENSG00000187848	P2RX2	purinergic receptor P2X 2	22953	0	NA	NA	NA	NA	NA
ENSG00000187855	ASCL4	achaete-scute family bHLH transcription factor 4	121549	0	NA	NA	NA	NA	NA

ENSG00000187857	OR6C75	olfactory receptor family 6 subfamily C member 75	390323	0	NA	NA	NA	NA	NA
ENSG00000187860	CCDC157	coiled-coil domain containing 157	550631	7,090381979	-0,577660471	0,665242	-0,868346246	0,385204812	NA
ENSG00000187870	NA	NA	NA	0	NA	NA	NA	NA	NA
ENSG00000187871	GFRAL	GDNF family receptor alpha like	389400	0	NA	NA	NA	NA	NA
ENSG00000187889	FYB2	FYN binding protein 2	199920	0	NA	NA	NA	NA	NA
ENSG00000187893	NA	NA	NA	0	NA	NA	NA	NA	NA
ENSG00000187900	OR5H7P	olfactory receptor family 5 subfamily H member 7 pseudogene	79291	0	NA	NA	NA	NA	NA
ENSG00000187902	SHISA7	shisa family member 7	729956	0	NA	NA	NA	NA	NA
ENSG00000187905	LRRC74B	leucine rich repeat containing 74B	400891	0	NA	NA	NA	NA	NA
ENSG00000187908	DMBT1	deleted in malignant brain tumors 1	1755	0	NA	NA	NA	NA	NA
ENSG00000187912	CLEC17A	C-type lectin domain containing 17A	388512	0	NA	NA	NA	NA	NA
ENSG00000187918	OR51I2	olfactory receptor family 51 subfamily I member 2	390064	0	NA	NA	NA	NA	NA
ENSG00000187922	LCN10	lipocalin 10	414332	0	NA	NA	NA	NA	NA
ENSG00000187942	LDLRAD2	low density lipoprotein receptor class A domain containing 2	401944	9,234074093	-0,555163161	0,621186	-0,893715207	0,371474287	NA
ENSG00000187944	C2orf66	chromosome 2 open reading frame 66	401027	0	NA	NA	NA	NA	NA
ENSG00000187950	OVCH1	ovochymase 1	341350	0	NA	NA	NA	NA	NA
ENSG00000187952	NA	NA	NA	0	NA	NA	NA	NA	NA
ENSG00000187955	COL14A1	collagen type XIV alpha 1 chain	7373	0	NA	NA	NA	NA	NA
ENSG00000187957	DNER	delta/notch like EGF repeat containing	92737	0	NA	NA	NA	NA	NA
ENSG00000187959	CPSF4L	cleavage and polyadenylation specific factor 4 like	642843	0	NA	NA	NA	NA	NA
ENSG00000187969	ZCCHC13	zinc finger CCHC-type containing 13	389874	0	NA	NA	NA	NA	NA
ENSG00000187980	PLA2G2C	phospholipase A2 group IIC	391013	0,765271511	0,08330585	2,139139	0,038943639	0,968935324	NA
ENSG00000187984	NA	NA	NA	0	NA	NA	NA	NA	NA
ENSG00000187987	ZSCAN23	zinc finger and SCAN domain containing 23	222696	3,498457032	-0,813039557	1,256973	-0,646823331	0,517746291	NA
ENSG00000187988	NA	NA	NA	0	NA	NA	NA	NA	NA
ENSG00000187997	C17orf99	chromosome 17 open reading frame 99	100141515	0	NA	NA	NA	NA	NA
ENSG00000187999	NA	NA	NA	0,11808024	-1,076443944	4,080456	-0,263804824	0,791930317	NA
ENSG00000188000	OR7D2	olfactory receptor family 7 subfamily D member 2	162998	9,211456153	-0,251226577	0,630534	-0,398434878	0,690309653	NA
ENSG00000188001	TPRG1	tumor protein p63 regulated 1	285386	0,376212802	1,371240341	3,683462	0,372269461	0,709692234	NA
ENSG00000188010	MORN2	MORN repeat containing 2	729967	0,999333807	0,632295095	2,186463	0,289186271	0,772438837	NA
ENSG00000188013	NA	NA	NA	0,103998153	-1,076443944	4,080456	-0,263804824	0,791930317	NA
ENSG00000188015	S100A3	S100 calcium binding protein A3	6274	0	NA	NA	NA	NA	NA
ENSG00000188026	RILPL1	Rab interacting lysosomal protein like 1	353116	4,87973155	-1,14182607	0,814976	-1,401055473	0,161197485	NA
ENSG00000188029	NA	NA	NA	0	NA	NA	NA	NA	NA
ENSG00000188037	CLCN1	chloride voltage-gated channel 1	1180	0	NA	NA	NA	NA	NA
ENSG00000188038	NRN1L	neuritin 1 like	123904	1,653251522	-1,234983633	1,36417	-0,905300066	0,365306442	NA
ENSG00000188039	NWD1	NACHT and WD repeat domain containing 1	284434	0	NA	NA	NA	NA	NA
ENSG00000188050	RNF133	ring finger protein 133	168433	0	NA	NA	NA	NA	NA
ENSG00000188051	TMEM221	transmembrane protein 221	100130519	0	NA	NA	NA	NA	NA
ENSG00000188056	TREML4	triggering receptor expressed on myeloid cells like 4	285852	0	NA	NA	NA	NA	NA
ENSG00000188060	RAB42	RAB42, member RAS oncogene family	115273	0,10543123	0	4,080456	0	1	NA
ENSG00000188076	SCGB1C1	secretoglobin family 1C member 1	147199	0	NA	NA	NA	NA	NA
ENSG00000188078	LOC100500719	HPS1, biogenesis of lysosomal organelles complex 3 subunit 1 pseudogene	100500719	0	NA	NA	NA	NA	NA
ENSG00000188086	NA	NA	NA	0,103998153	-1,076443944	4,080456	-0,263804824	0,791930317	NA
ENSG00000188089	PLA2G4E	phospholipase A2 group IVE	123745	0	NA	NA	NA	NA	NA
ENSG00000188092	GPR89B	G protein-coupled receptor 89B	51463	2,376752741	3,982613113	3,083804	1,291461247	0,196543785	NA

ENSG00000188095	MESP2	mesoderm posterior bHLH transcription factor 2	145873	0	NA	NA	NA	NA	NA
ENSG00000188100	FAM25A	family with sequence similarity 25 member A	643161	0	NA	NA	NA	NA	NA
ENSG00000188101	ALOX15P2	arachidonate 15-lipoxygenase pseudogene 2	642959	0	NA	NA	NA	NA	NA
ENSG00000188107	EYS	eyes shut homolog	346007	0,072165449	0,847101401	4,080456	0,207599697	0,835541541	NA
ENSG00000188112	C6orf132	chromosome 6 open reading frame 132	647024	0,49596583	3,045403514	3,961588	0,768733037	0,442051808	NA
ENSG00000188120	DAZ1	deleted in azoospermia 1	1617	0	NA	NA	NA	NA	NA
ENSG00000188124	OR2AG2	olfactory receptor family 2 subfamily AG member 2	338755	0,10488184	-1,076443944	4,080456	-0,263804824	0,791930317	NA
ENSG00000188130	MAPK12	mitogen-activated protein kinase 12	6300	0,10488184	-1,076443944	4,080456	-0,263804824	0,791930317	NA
ENSG00000188133	TMEM215	transmembrane protein 215	401498	0	NA	NA	NA	NA	NA
ENSG00000188152	NUTM2G	NUT family member 2G	441457	0	NA	NA	NA	NA	NA
ENSG00000188153	COL4A5	collagen type IV alpha 5 chain	1287	0	NA	NA	NA	NA	NA
ENSG00000188155	KRTAP10-6	keratin associated protein 10-6	386674	0	NA	NA	NA	NA	NA
ENSG00000188162	OTOG	otogelin	340990	2,570505524	4,562090481	1,555783	2,932343032	0,003364149	NA
ENSG00000188163	CIMIP2A	ciliary microtubule inner protein 2A	401565	0	NA	NA	NA	NA	NA
ENSG00000188175	HEPACAM2	HEPACAM family member 2	253012	0	NA	NA	NA	NA	NA
ENSG00000188176	SMTNL2	smoothelin like 2	342527	0	NA	NA	NA	NA	NA
ENSG00000188199	NUTM2B	NUT family member 2B	729262	3,864413978	-0,808671924	1,033114	-0,782751522	0,43377304	NA
ENSG00000188219	POTEE	POTE ankyrin domain family member E	445582	0	NA	NA	NA	NA	NA
ENSG00000188223	NA	NA	NA	0	NA	NA	NA	NA	NA
ENSG00000188227	ZNF793	zinc finger protein 793	390927	8,177511706	0,695902265	0,694249	1,002381202	0,316159518	NA
ENSG00000188257	PLA2G2A	phospholipase A2 group IIA	5320	0	NA	NA	NA	NA	NA
ENSG00000188263	IL17REL	interleukin 17 receptor E like	400935	0	NA	NA	NA	NA	NA
ENSG00000188266	HYKK	hydroxylysine kinase	123688	0,332769857	0	4,080456	0	1	NA
ENSG00000188269	OR7A5	olfactory receptor family 7 subfamily A member 5	26659	0	NA	NA	NA	NA	NA
ENSG00000188277	C15orf62	chromosome 15 open reading frame 62	643338	1,366547495	-2,816395038	1,949153	-1,444932852	0,148476748	NA
ENSG00000188282	RUFY4	RUN and FYVE domain containing 4	285180	0	NA	NA	NA	NA	NA
ENSG00000188290	HES4	hes family bHLH transcription factor 4	57801	0,416236074	0,850191804	3,045357	0,279176405	0,78010945	NA
ENSG00000188293	IGFL1	IGF like family member 1	374918	0	NA	NA	NA	NA	NA
ENSG00000188306	LRRIQ4	leucine rich repeats and IQ motif containing 4	344657	0	NA	NA	NA	NA	NA
ENSG00000188314	NA	NA	NA	0	NA	NA	NA	NA	NA
ENSG00000188316	ENO4	enolase 4	387712	0	NA	NA	NA	NA	NA
ENSG00000188324	OR6C6	olfactory receptor family 6 subfamily C member 6	283365	0	NA	NA	NA	NA	NA
ENSG00000188334	BSPH1	binder of sperm protein homolog 1	100131137	0	NA	NA	NA	NA	NA
ENSG00000188338	SLC38A3	solute carrier family 38 member 3	10991	0	NA	NA	NA	NA	NA
ENSG00000188340	OR6N2	olfactory receptor family 6 subfamily N member 2	81442	0	NA	NA	NA	NA	NA
ENSG00000188343	CIBAR1	CBY1 interacting BAR domain containing 1	137392	0,20489287	-0,586147887	4,06894	-0,144054205	0,885457667	NA
ENSG00000188368	PRR19	proline rich 19	284338	0,80473714	1,933414768	2,664244	0,725689854	0,468028923	NA
ENSG00000188372	ZP3	zona pellucida glycoprotein 3	7784	0,31061662	2,364487303	3,994163	0,591985654	0,553860195	NA
ENSG00000188373	GPR15LG	G protein-coupled receptor 15 ligand	387695	0	NA	NA	NA	NA	NA
ENSG00000188375	H3-5	H3,5 histone	440093	0,189859871	1,666114421	4,047491	0,411641242	0,680602398	NA
ENSG00000188379	IFNA2	interferon alpha 2	3440	0	NA	NA	NA	NA	NA
ENSG00000188385	JAKMIP3	Janus kinase and microtubule interacting protein 3	282973	0,471717977	0,120062875	3,971404	0,030231847	0,97588215	NA
ENSG00000188386	PPP3R2	protein phosphatase 3 regulatory subunit B, beta	5535	0	NA	NA	NA	NA	NA
ENSG00000188388	GOLGA6L3P	golgin A6 family like 3, pseudogene	100133220	3,121280296	-2,078010768	1,174545	-1,769204474	0,076859758	NA
ENSG00000188389	PDCD1	programmed cell death 1	5133	3,03757118	-4,506313536	1,73442	-2,598167464	0,009372277	NA
ENSG00000188393	CLEC2A	C-type lectin domain family 2 member A	387836	0,237750918	-0,117027512	4,080456	-0,028680009	0,9771198	NA
ENSG00000188394	GPR21	G protein-coupled receptor 21	2844	1,189488467	-1,287362706	2,019422	-0,637490774	0,523805217	NA
ENSG00000188396	DYNLT4	dynein light chain Tctex-type 4	343521	0,072165449	0,847101401	4,080456	0,207599697	0,835541541	NA
ENSG00000188399	NA	NA	NA	0	NA	NA	NA	NA	NA
ENSG00000188403	IGHV1OR15-9	immunoglobulin heavy variable 1/OR15-9 (non-functional)	390531	0	NA	NA	NA	NA	NA

ENSG00000188408	MAGEB5	MAGE family member B5	347541	0	NA	NA	NA	NA	NA
ENSG00000188425	NANOS2	nanos C2HC-type zinc finger 2	339345	0	NA	NA	NA	NA	NA
ENSG00000188438	DEFB108F	defensin beta 108F (pseudogene)	100133128	0	NA	NA	NA	NA	NA
ENSG00000188439	OR4P1P	olfactory receptor family 4 subfamily P member 1 pseudogene	79308	0	NA	NA	NA	NA	NA
ENSG00000188451	SRP72P2	signal recognition particle 72 pseudogene 2	153932	0	NA	NA	NA	NA	NA
ENSG00000188452	CERKL	ceramide kinase like	375298	0	NA	NA	NA	NA	NA
ENSG00000188459	NA	NA	NA	0	NA	NA	NA	NA	NA
ENSG00000188460	NA	NA	NA	0	NA	NA	NA	NA	NA
ENSG00000188467	SLC24A5	solute carrier family 24 member 5	283652	0	NA	NA	NA	NA	NA
ENSG00000188488	SERPINA5	serpin family A member 5	5104	0	NA	NA	NA	NA	NA
ENSG00000188501	LCTL	lactase like	197021	0,235782817	0,849566257	4,080456	0,208203761	0,835069872	NA
ENSG00000188505	NCCRP1	NCCRP1, F-box associated domain containing	342897	0	NA	NA	NA	NA	NA
ENSG00000188508	KRTDAP	keratinocyte differentiation associated protein	388533	0	NA	NA	NA	NA	NA
ENSG00000188512	CYCSP3	CYCS pseudogene 3	349198	0	NA	NA	NA	NA	NA
ENSG00000188517	COL25A1	collagen type XXV alpha 1 chain	84570	0	NA	NA	NA	NA	NA
ENSG00000188523	CFAP77	cilia and flagella associated protein 77	389799	0	NA	NA	NA	NA	NA
ENSG00000188536	HBA2	hemoglobin subunit alpha 2	3040	0,117497108	0,847101401	4,080456	0,207599697	0,835541541	NA
ENSG00000188549	CCDC9B	coiled-coil domain containing 9B	388115	5,377048574	-1,015304979	3,666813	-0,276890333	0,781864315	NA
ENSG00000188558	OR2G6	olfactory receptor family 2 subfamily G member 6	391211	0	NA	NA	NA	NA	NA
ENSG00000188573	FBLL1	fibrillarin like 1	345630	0,859514223	0,988684695	2,042439	0,484070611	0,628335757	NA
ENSG00000188580	NKAIN2	sodium/potassium transporting ATPase interacting 2	154215	0	NA	NA	NA	NA	NA
ENSG00000188581	KRTAP1-1	keratin associated protein 1-1	81851	0	NA	NA	NA	NA	NA
ENSG00000188582	PAQR9	progestin and adipoQ receptor family member 9	344838	0	NA	NA	NA	NA	NA
ENSG00000188585	CLEC20A	C-type lectin domain containing 20A	400797	0	NA	NA	NA	NA	NA
ENSG00000188596	CFAP54	cilia and flagella associated protein 54	144535	0	NA	NA	NA	NA	NA
ENSG00000188611	ASAH2	N-acylsphingosine amidohydrolase 2	56624	7,259394753	-0,103403912	0,671569	-0,153973688	0,877630482	NA
ENSG00000188613	NANOS1	nanos C2HC-type zinc finger 1	340719	0,10488184	-1,076443944	4,080456	-0,263804824	0,791930317	NA
ENSG00000188620	HMX3	H6 family homeobox 3	340784	0,072165449	0,847101401	4,080456	0,207599697	0,835541541	NA
ENSG00000188624	IGFL3	IGF like family member 3	388555	0	NA	NA	NA	NA	NA
ENSG00000188629	ZNF177	zinc finger protein 177	7730	2,955552924	-0,052849029	1,147877	-0,046040659	0,963277843	NA
ENSG00000188629	ZNF559-ZNF177	ZNF559-ZNF177 readthrough	100529215	2,955552924	-0,052849029	1,147877	-0,046040659	0,963277843	NA
ENSG00000188643	S100A16	S100 calcium binding protein A16	140576	0	NA	NA	NA	NA	NA
ENSG00000188646	NA	NA	NA	0	NA	NA	NA	NA	NA
ENSG00000188649	CC2D2B	coiled-coil and C2 domain containing 2B	387707	2,832236343	0,026005126	1,201713	0,021640052	0,982735084	NA
ENSG00000188655	RNASE9	ribonuclease A family member 9 (inactive)	390443	0	NA	NA	NA	NA	NA
ENSG00000188656	TSPY7P	testis specific protein Y-linked 7, pseudogene	653288	0	NA	NA	NA	NA	NA
ENSG00000188659	SAXO2	stabilizer of axonemal microtubules 2	283726	0,77580811	0,749901279	3,133489	0,23931834	0,810858746	NA
ENSG00000188662	NA	NA	NA	0	NA	NA	NA	NA	NA
ENSG00000188668	OR7E90P	olfactory receptor family 7 subfamily E member 90 pseudogene	79537	0	NA	NA	NA	NA	NA
ENSG00000188672	RHCE	Rh blood group CcEe antigens	6006	0	NA	NA	NA	NA	NA
ENSG00000188674	C2orf80	chromosome 2 open reading frame 80	389073	0	NA	NA	NA	NA	NA
ENSG00000188676	IDO2	indoleamine 2,3-dioxygenase 2	169355	0	NA	NA	NA	NA	NA
ENSG00000188681	NA	NA	NA	0,594034819	-1,063142522	3,299509	-0,322212372	0,747291813	NA
ENSG00000188687	SLC4A5	solute carrier family 4 member 5	57835	4,439182092	0,128300854	1,253105	0,102386391	0,918449986	NA
ENSG00000188691	OR56A5	olfactory receptor family 56 subfamily A member 5	390084	0	NA	NA	NA	NA	NA
ENSG00000188694	KRTAP24-1	keratin associated protein 24-1	643803	0	NA	NA	NA	NA	NA

ENSG00000188710	QRFP	pyroglutamylated RFamide peptide	347148	0,119673877	0,847101401	4,080456	0,207599697	0,835541541	NA
ENSG00000188712	OR13D3P	olfactory receptor family 13 subfamily D member 3 pseudogene	402374	0	NA	NA	NA	NA	NA
ENSG00000188716	DUSP29	dual specificity phosphatase 29	338599	0,103998153	-1,076443944	4,080456	-0,263804824	0,791930317	NA
ENSG00000188729	OSTN	osteocrin	344901	0	NA	NA	NA	NA	NA
ENSG00000188730	VWC2	von Willebrand factor C domain containing 2	375567	0,10543123	0	4,080456	0	1	NA
ENSG00000188732	FAM221A	family with sequence similarity 221 member A	340277	9,003497646	0,051786777	0,941934	0,054979225	0,956155015	NA
ENSG00000188738	FSIP2	fibrous sheath interacting protein 2	401024	0,15836288	0	4,080456	0	1	NA
ENSG00000188755	NA	NA	NA	0	NA	NA	NA	NA	NA
ENSG00000188760	TMEM198	transmembrane protein 198	130612	10,04208574	0,179296365	0,541301	0,331232268	0,740469049	NA
ENSG00000188761	BCL2L15	BCL2 like 15	440603	4,790675795	-0,642325934	1,821964	-0,352545833	0,724428952	NA
ENSG00000188763	FZD9	frizzled class receptor 9	8326	0,10543123	0	4,080456	0	1	NA
ENSG00000188765	NA	NA	NA	0	NA	NA	NA	NA	NA
ENSG00000188766	SPRED3	sprouty related EVH1 domain containing 3	399473	1,299286168	-1,941681512	2,204723	-0,880691838	0,378484636	NA
ENSG00000188770	OPTC	opticin	26254	0	NA	NA	NA	NA	NA
ENSG00000188771	PLET1	placenta expressed transcript 1	349633	0	NA	NA	NA	NA	NA
ENSG00000188778	ADRB3	adrenoceptor beta 3	155	0	NA	NA	NA	NA	NA
ENSG00000188779	SKOR1	SKI family transcriptional corepressor 1	390598	1,599969641	-0,01501068	1,528755	-0,009818891	0,992165785	NA
ENSG00000188782	CATSPER4	cation channel sperm associated 4	378807	0	NA	NA	NA	NA	NA
ENSG00000188783	PRELP	proline and arginine rich end leucine rich repeat protein	5549	0,10488184	-1,076443944	4,080456	-0,263804824	0,791930317	NA
ENSG00000188784	PLA2G2E	phospholipase A2 group IIE	30814	0	NA	NA	NA	NA	NA
ENSG00000188800	TMCO2	transmembrane and coiled-coil domains 2	127391	0	NA	NA	NA	NA	NA
ENSG00000188801	NA	NA	NA	1,241569983	1,561594771	3,23904	0,482116617	0,629723106	NA
ENSG00000188803	SHISA6	shisa family member 6	388336	0	NA	NA	NA	NA	NA
ENSG00000188816	HMX2	H6 family homeobox 2	3167	0,119673877	0,847101401	4,080456	0,207599697	0,835541541	NA
ENSG00000188817	SNTN	sentan, cilia apical structure protein	132203	0,11808024	-1,076443944	4,080456	-0,263804824	0,791930317	NA
ENSG00000188818	ZDHHC11	zinc finger DHHC-type containing 11	79844	8,703136465	0,253178597	0,724827	0,349295262	0,726867655	NA
ENSG00000188820	CALHM6	calcium homeostasis modulator family member 6	441168	0,43041787	-1,556145346	3,477241	-0,447523013	0,654497476	NA
ENSG00000188828	NA	NA	NA	0	NA	NA	NA	NA	NA
ENSG00000188831	NA	NA	NA	0	NA	NA	NA	NA	NA
ENSG00000188833	ENTPD8	ectonucleoside triphosphate diphosphohydrolase 8	377841	7,376071984	-0,860141508	1,187048	-0,724605655	0,468693989	NA
ENSG00000188848	BEND4	BEN domain containing 4	389206	0,429769366	-1,517696549	3,998764	-0,37954142	0,704285852	NA
ENSG00000188850	NA	NA	NA	0	NA	NA	NA	NA	NA
ENSG00000188856	RPSAP47	ribosomal protein SA pseudogene 47	389672	0,144330898	1,418786819	4,061262	0,349346267	0,726829368	NA
ENSG00000188859	FAM78B	family with sequence similarity 78 member B	149297	0	NA	NA	NA	NA	NA
ENSG00000188868	ZNF563	zinc finger protein 563	147837	0,339807918	1,937702006	4,023544	0,481590848	0,630096629	NA
ENSG00000188869	TMC3	transmembrane channel like 3	342125	0	NA	NA	NA	NA	NA
ENSG00000188873	RPL10AP2	ribosomal protein L10a pseudogene 2	253986	0	NA	NA	NA	NA	NA
ENSG00000188877	POTEA	POTE ankyrin domain family member A	340441	0	NA	NA	NA	NA	NA
ENSG00000188883	KLRG2	killer cell lectin like receptor G2	346689	0	NA	NA	NA	NA	NA
ENSG00000188886	ASTL	astacin like metalloendopeptidase	431705	4,152956965	-0,327242595	0,973306	-0,336217748	0,736706671	NA
ENSG00000188897	LOC400499	putative uncharacterized protein LOC400499	400499	5,662888459	0,014041139	1,065814	0,013174098	0,989488894	NA
ENSG00000188906	LRRK2	leucine rich repeat kinase 2	120892	0	NA	NA	NA	NA	NA
ENSG00000188909	BSX	brain specific homeobox	390259	0	NA	NA	NA	NA	NA
ENSG00000188910	GJB3	gap junction protein beta 3	2707	0	NA	NA	NA	NA	NA
ENSG00000188916	INSYN2A	inhibitory synaptic factor 2A	642938	0	NA	NA	NA	NA	NA
ENSG00000188931	CFAP126	cilia and flagella associated protein 126	257177	0	NA	NA	NA	NA	NA
ENSG00000188937	NYX	nyctalopin	60506	0	NA	NA	NA	NA	NA
ENSG00000188958	UTS2B	urotensin 2B	257313	0,103998153	-1,076443944	4,080456	-0,263804824	0,791930317	NA
ENSG00000188959	C9orf152	chromosome 9 open reading frame 152	401546	0,15836288	0	4,080456	0	1	NA
ENSG00000188981	MSANTD1	Myb/SANT DNA binding domain containing 1	345222	3,667420883	-0,639304161	1,088414	-0,587372104	0,556953825	NA
ENSG00000188984	AADACL3	arylacetamide deacetylase like 3	126767	0	NA	NA	NA	NA	NA

ENSG00000188991	SLC15A5	solute carrier family 15 member 5	729025	0	NA	NA	NA	NA	NA
ENSG00000188992	LIPI	lipase I	149998	0	NA	NA	NA	NA	NA
ENSG00000188993	LRRC66	leucine rich repeat containing 66	339977	0,474168701	1,425350855	3,452738	0,412817525	0,67974031	NA
ENSG00000188996	HUS1B	HUS1 checkpoint clamp component B	135458	1,325429644	0,096121117	1,552468	0,061915023	0,950630504	NA
ENSG00000189001	SBSN	suprabasin	374897	0,714991926	-1,044536366	3,748648	-0,278643489	0,780518435	NA
ENSG00000189002	NA	NA	NA	0	NA	NA	NA	NA	NA
ENSG00000189013	KIR2DL4	killer cell immunoglobulin like receptor, two Ig domains and long cytoplasmic tail 4	3805	0	NA	NA	NA	NA	NA
ENSG00000189014	NA	NA	NA	0	NA	NA	NA	NA	NA
ENSG00000189023	MAGEB16	MAGE family member B16	139604	0	NA	NA	NA	NA	NA
ENSG00000189030	VHLL	VHL like	391104	0	NA	NA	NA	NA	NA
ENSG00000189037	DUSP21	dual specificity phosphatase 21	63904	0	NA	NA	NA	NA	NA
ENSG00000189045	ANKDD1B	ankyrin repeat and death domain containing 1B	728780	0	NA	NA	NA	NA	NA
ENSG00000189051	RNF222	ring finger protein 222	643904	0,503775695	-0,962347763	2,950372	-0,326178396	0,744289386	NA
ENSG00000189052	CGB5	chorionic gonadotropin subunit beta 5	93659	0	NA	NA	NA	NA	NA
ENSG00000189056	RELN	reelin	5649	0	NA	NA	NA	NA	NA
ENSG00000189058	APOD	apolipoprotein D	347	0,282309676	-0,126717329	3,988475	-0,031770869	0,974654778	NA
ENSG00000189064	GAGE2E	G antigen 2E	26749	0	NA	NA	NA	NA	NA
ENSG00000189064	GAGE2B	G antigen 2B	645037	0	NA	NA	NA	NA	NA
ENSG00000189064	GAGE2D	G antigen 2D	729408	0	NA	NA	NA	NA	NA
ENSG00000189064	GAGE2A	G antigen 2A	729447	0	NA	NA	NA	NA	NA
ENSG00000189064	GAGE8	G antigen 8	100101629	0	NA	NA	NA	NA	NA
ENSG00000189068	VSTM1	V-set and transmembrane domain containing 1	284415	0	NA	NA	NA	NA	NA
ENSG00000189089	NA	NA	NA	0	NA	NA	NA	NA	NA
ENSG00000189099	PRSS48	serine protease 48	345062	0	NA	NA	NA	NA	NA
ENSG00000189108	IL1RAPL2	interleukin 1 receptor accessory protein like 2	26280	0	NA	NA	NA	NA	NA
ENSG00000189120	SP6	Sp6 transcription factor	80320	0	NA	NA	NA	NA	NA
ENSG00000189127	ANKRD34B	ankyrin repeat domain 34B	340120	0	NA	NA	NA	NA	NA
ENSG00000189129	PLAC9	placenta associated 9	219348	0	NA	NA	NA	NA	NA
ENSG00000189132	FAM47B	family with sequence similarity 47 member B	170062	0	NA	NA	NA	NA	NA
ENSG00000189134	NKAPL	NFKB activating protein like	222698	1,117906244	-0,340278376	1,939646	-0,17543324	0,860739224	NA
ENSG00000189139	FSCB	fibrous sheath CABYR binding protein	84075	0	NA	NA	NA	NA	NA
ENSG00000189143	CLDN4	claudin 4	1364	0,072165449	0,847101401	4,080456	0,207599697	0,835541541	NA
ENSG00000189145	NA	NA	NA	0	NA	NA	NA	NA	NA
ENSG00000189149	NA	NA	NA	0	NA	NA	NA	NA	NA
ENSG00000189152	GRAPL	GRB2 related adaptor protein like	400581	6,169809503	-0,256809125	0,935757	-0,274439959	0,783746539	NA
ENSG00000189157	FAM47E	family with sequence similarity 47 member E	100129583	0,118286652	0	4,080456	0	1	NA
ENSG00000189166	NA	NA	NA	0	NA	NA	NA	NA	NA
ENSG00000189167	ZAR1L	zygote arrest 1 like	646799	0	NA	NA	NA	NA	NA
ENSG00000189169	KRTAP10-12	keratin associated protein 10-12	386685	0	NA	NA	NA	NA	NA
ENSG00000189181	OR14I1	olfactory receptor family 14 subfamily I member 1	401994	0	NA	NA	NA	NA	NA
ENSG00000189182	KRT77	keratin 77	374454	0	NA	NA	NA	NA	NA
ENSG00000189184	PCDH18	protocadherin 18	54510	0	NA	NA	NA	NA	NA
ENSG00000189186	DCAF8L2	DDB1 and CUL4 associated factor 8 like 2	347442	0	NA	NA	NA	NA	NA
ENSG00000189195	BTBD8	BTB domain containing 8	284697	4,286468341	1,775913206	1,536147	1,156082827	0,24764728	NA
ENSG00000189212	NA	NA	NA	0	NA	NA	NA	NA	NA
ENSG00000189221	MAOA	monoamine oxidase A	4128	0	NA	NA	NA	NA	NA
ENSG00000189227	C15orf61	chromosome 15 open reading frame 61	145853	9,812168498	-0,094288755	0,886817	-0,106322679	0,915326338	NA
ENSG00000189252	SPANXN3	SPANX family member N3	139067	0	NA	NA	NA	NA	NA
ENSG00000189253	TRIM64B	tripartite motif containing 64B	642446	0	NA	NA	NA	NA	NA
ENSG00000189269	DRICH1	aspartate rich 1	51233	2,587153198	-0,85061048	1,484394	-0,57303535	0,566620759	NA
ENSG00000189280	GJB5	gap junction protein beta 5	2709	0	NA	NA	NA	NA	NA
ENSG00000189283	FHIT	fragile histidine triad diadenosine triphosphatase	2272	1,416420477	0,538486298	1,941236	0,27739359	0,781477903	NA
ENSG00000189292	ALKAL2	ALK and LTK ligand 2	285016	0,576447813	-0,780874419	3,332829	-0,234297777	0,814753811	NA
ENSG00000189299	FOXR2	forkhead box R2	139628	0	NA	NA	NA	NA	NA
ENSG00000189320	FAM180A	family with sequence similarity 180 member A	389558	0	NA	NA	NA	NA	NA
ENSG00000189325	BNIP5	BCL2 interacting protein 5	389384	0	NA	NA	NA	NA	NA
ENSG00000189326	SPANXN4	SPANX family member N4	441525	0	NA	NA	NA	NA	NA
ENSG00000189332	NA	NA	NA	0,118286652	0	4,080456	0	1	NA

ENSG00000189334	S100A14	S100 calcium binding protein A14	57402	0	NA	NA	NA	NA	NA
ENSG00000189337	KAZN	kazrin, periplakin interacting protein	23254	3,164738571	0,120580374	1,052425	0,114573789	0,908782956	NA
ENSG00000189343	RPS2P46	ribosomal protein S2 pseudogene 46	125208	0,666840986	0,156632795	3,134248	0,049974599	0,96014263	NA
ENSG00000189348	NA	NA	NA	0	NA	NA	NA	NA	NA
ENSG00000189357	SPATA31D4	SPATA31 subfamily D member 4	389761	0	NA	NA	NA	NA	NA
ENSG00000189366	NA	NA	NA	0,489415678	-1,057395575	3,447697	-0,306696158	0,759074654	NA
ENSG00000189367	KIAA0408	KIAA0408	9729	0	NA	NA	NA	NA	NA
ENSG00000189372	NA	NA	NA	0	NA	NA	NA	NA	NA
ENSG00000189375	TBC1D28	TBC1 domain family member 28	254272	0	NA	NA	NA	NA	NA
ENSG00000189377	CXCL17	C-X-C motif chemokine ligand 17	284340	0	NA	NA	NA	NA	NA
ENSG00000189398	OR7E12P	olfactory receptor family 7 subfamily E member 12 pseudogene	10821	0,072165449	0,847101401	4,080456	0,207599697	0,835541541	NA
ENSG00000189401	OTUD6A	OTU deubiquitinase 6A	139562	0	NA	NA	NA	NA	NA
ENSG00000189409	MMP23B	matrix metallopeptidase 23B	8510	0,144330898	1,418786819	4,061262	0,349346267	0,726829368	NA
ENSG00000189410	SH2D5	SH2 domain containing 5	400745	0	NA	NA	NA	NA	NA
ENSG00000189420	ZFP92	ZFP92 zinc finger protein	139735	8,888292562	0,254545052	0,617037	0,412527981	0,679952476	NA
ENSG00000189423	NA	NA	NA	6,511154426	0,254306116	0,777422	0,327114813	0,743581049	NA
ENSG00000189431	RASSF10	Ras association domain family member 10	644943	0	NA	NA	NA	NA	NA
ENSG00000189433	GJB4	gap junction protein beta 4	127534	0	NA	NA	NA	NA	NA
ENSG00000196071	OR2L13	olfactory receptor family 2 subfamily L member 13	284521	0,103998153	-1,076443944	4,080456	-0,263804824	0,791930317	NA
ENSG00000196085	NA	NA	NA	0	NA	NA	NA	NA	NA
ENSG00000196090	PTPRT	protein tyrosine phosphatase receptor type T	11122	0	NA	NA	NA	NA	NA
ENSG00000196091	MYBPC1	myosin binding protein C1	4604	0	NA	NA	NA	NA	NA
ENSG00000196092	PAX5	paired box 5	5079	4,693229263	-0,960570928	1,38018	-0,69597488	0,486444561	NA
ENSG00000196098	OR5K4	olfactory receptor family 5 subfamily K member 4	403278	0	NA	NA	NA	NA	NA
ENSG00000196099	OR6M1	olfactory receptor family 6 subfamily M member 1	390261	0	NA	NA	NA	NA	NA
ENSG00000196104	SPOCK3	SPARC (osteonectin), cwcv and kazal like domains proteoglycan 3	50859	0	NA	NA	NA	NA	NA
ENSG00000196109	ZNF676	zinc finger protein 676	163223	0	NA	NA	NA	NA	NA
ENSG00000196114	NA	NA	NA	0,507943792	-0,141397904	3,520787	-0,040160884	0,967964863	NA
ENSG00000196115	ADAM5	ADAM metallopeptidase domain 5 (pseudogene)	255926	0	NA	NA	NA	NA	NA
ENSG00000196118	CFAP119	cilia and flagella associated protein 119	90835	1,172496029	3,697233398	2,352434	1,571662984	0,116028731	NA
ENSG00000196119	OR8A1	olfactory receptor family 8 subfamily A member 1	390275	0	NA	NA	NA	NA	NA
ENSG00000196126	HLA-DRB1	major histocompatibility complex, class II, DR beta 1	3123	1,421074476	-0,338087208	2,280501	-0,148251277	0,882144465	NA
ENSG00000196131	VN1R2	vomeronasal 1 receptor 2	317701	0	NA	NA	NA	NA	NA
ENSG00000196132	MYT1	myelin transcription factor 1	4661	0	NA	NA	NA	NA	NA
ENSG00000196136	SERPINA3	serpin family A member 3	12	0	NA	NA	NA	NA	NA
ENSG00000196139	AKR1C3	aldo-keto reductase family 1 member C3	8644	1,337996478	-2,948400531	2,055376	-1,434482185	0,151434709	NA
ENSG00000196143	OR11H13P	olfactory receptor family 11 subfamily H member 13 pseudogene	440159	0	NA	NA	NA	NA	NA
ENSG00000196156	KRTAP4-3	keratin associated protein 4-3	85290	0	NA	NA	NA	NA	NA
ENSG00000196159	FAT4	FAT atypical cadherin 4	79633	1,409999644	1,782771023	2,106987	0,846123415	0,3974839	NA
ENSG00000196169	KIF19	kinesin family member 19	124602	0	NA	NA	NA	NA	NA
ENSG00000196171	OR6K2	olfactory receptor family 6 subfamily K member 2	81448	0	NA	NA	NA	NA	NA
ENSG00000196172	ZNF681	zinc finger protein 681	148213	11,15622198	0,030317984	0,622988	0,048665429	0,961185927	NA
ENSG00000196183	RPS2P4	ribosomal protein S2 pseudogene 4	92427	0	NA	NA	NA	NA	NA
ENSG00000196188	CTSE	cathepsin E	1510	0	NA	NA	NA	NA	NA
ENSG00000196196	HRCT1	histidine rich carboxyl terminus 1	646962	0	NA	NA	NA	NA	NA
ENSG00000196208	GREB1	growth regulating estrogen receptor binding 1	9687	2,588942853	1,14049724	1,337649	0,852613336	0,393873765	NA
ENSG00000196209	SIRPB2	signal regulatory protein beta 2	284759	0	NA	NA	NA	NA	NA
ENSG00000196218	RYR1	ryanodine receptor 1	6261	6,95855546	1,233266068	0,748907	1,646755541	0,099608303	NA
ENSG00000196228	SULT1C3	sulfotransferase family 1C member 3	442038	0	NA	NA	NA	NA	NA
ENSG00000196233	LCOR	ligand dependent nuclear receptor corepressor	84458	949,3475621	1,164181364	1,088513	1,069515614	NA	NA

ENSG00000196240	OR2T2	olfactory receptor family 2 subfamily T member 2	401992	0	NA	NA	NA	NA	NA
ENSG00000196242	OR2C3	olfactory receptor family 2 subfamily C member 3	81472	0,072165449	0,847101401	4,080456	0,207599697	0,835541541	NA
ENSG00000196242	GCSAML-AS1	GCSAML antisense RNA 1	148824	0,072165449	0,847101401	4,080456	0,207599697	0,835541541	NA
ENSG00000196248	OR10S1	olfactory receptor family 10 subfamily S member 1	219873	0	NA	NA	NA	NA	NA
ENSG00000196260	SFTA2	surfactant associated 2	389376	0	NA	NA	NA	NA	NA
ENSG00000196263	ZNF471	zinc finger protein 471	57573	1,495841653	1,027364723	2,205025	0,465919689	0,641272988	NA
ENSG00000196266	NA	NA	NA	0	NA	NA	NA	NA	NA
ENSG00000196275	GTF2IRD2	GTF2I repeat domain containing 2	84163	3,779479038	0,966849325	1,539005	0,62823007	0,529853235	NA
ENSG00000196277	GRM7	glutamate metabotropic receptor 7	2917	0	NA	NA	NA	NA	NA
ENSG00000196289	BECN2	beclin 2	441925	0	NA	NA	NA	NA	NA
ENSG00000196301	NA	NA	NA	0	NA	NA	NA	NA	NA
ENSG00000196302	NA	NA	NA	8,954861626	-0,880981837	1,200263	-0,733990599	0,462954468	NA
ENSG00000196312	NA	NA	NA	5,122163761	-1,310337915	1,098052	-1,193330032	0,232740145	NA
ENSG00000196335	STK31	serine/threonine kinase 31	56164	1,320885386	-0,274642761	1,77952	-0,154335292	0,877345372	NA
ENSG00000196337	CGB7	chorionic gonadotropin subunit beta 7	94027	0	NA	NA	NA	NA	NA
ENSG00000196338	NLGN3	neuroligin 3	54413	2,694612631	-0,298863985	1,431362	-0,208796937	0,834606763	NA
ENSG00000196341	OR8D1	olfactory receptor family 8 subfamily D member 1	283159	0	NA	NA	NA	NA	NA
ENSG00000196344	ADH7	alcohol dehydrogenase 7 (class IV), mu or sigma polypeptide	131	0	NA	NA	NA	NA	NA
ENSG00000196350	ZNF729	zinc finger protein 729	100287226	0	NA	NA	NA	NA	NA
ENSG00000196353	CPNE4	copine 4	131034	0	NA	NA	NA	NA	NA
ENSG00000196357	ZNF565	zinc finger protein 565	147929	7,504935799	-0,077420494	0,786199	-0,098474398	0,9215556	NA
ENSG00000196361	ELAVL3	ELAV like RNA binding protein 3	1995	0	NA	NA	NA	NA	NA
ENSG00000196364	NA	NA	NA	0	NA	NA	NA	NA	NA
ENSG00000196368	NUDT11	nudix hydrolase 11	55190	1,020319105	-0,042612804	1,734558	-0,02456695	0,980400381	NA
ENSG00000196372	ASB13	ankyrin repeat and SOCS box containing 13	79754	4,521800323	2,358404422	1,632453	1,444699421	0,148542333	NA
ENSG00000196376	SLC35F1	solute carrier family 35 member F1	222553	0,103998153	-1,076443944	4,080456	-0,263804824	0,791930317	NA
ENSG00000196381	NA	NA	NA	1,278436588	0,114518513	1,713967	0,066814901	0,946729061	NA
ENSG00000196383	NA	NA	NA	0	NA	NA	NA	NA	NA
ENSG00000196388	INCA1	inhibitor of CDK, cyclin A1 interacting protein 1	388324	0,294753267	-0,101285832	3,939388	-0,025711056	0,979487805	NA
ENSG00000196390	NA	NA	NA	0	NA	NA	NA	NA	NA
ENSG00000196395	NA	NA	NA	0	NA	NA	NA	NA	NA
ENSG00000196403	OR10D1P	olfactory receptor family 10 subfamily D member 1 pseudogene	26541	0	NA	NA	NA	NA	NA
ENSG00000196406	SPANXD	SPANX family member D	64648	0	NA	NA	NA	NA	NA
ENSG00000196407	THEM5	thioesterase superfamily member 5	284486	0	NA	NA	NA	NA	NA
ENSG00000196411	EPHB4	EPH receptor B4	2050	1,867223653	-0,212388028	1,502937	-0,141315359	0,887620816	NA
ENSG00000196415	PRTN3	proteinase 3	5657	0	NA	NA	NA	NA	NA
ENSG00000196420	S100A5	S100 calcium binding protein A5	6276	0,263397257	2,146099957	4,008667	0,535364964	0,592397512	NA
ENSG00000196421	C20orf204	chromosome 20 open reading frame 204	284739	1,458126632	0,059622714	2,379783	0,02505384	0,980012019	NA
ENSG00000196422	PPP1R26	protein phosphatase 1 regulatory subunit 26	9858	5,447190032	-1,043442892	0,802072	-1,300933743	0,193281135	NA
ENSG00000196427	NBPF4	NBPF member 4	148545	1,930180879	0,770743025	2,769739	0,278272826	0,780802934	NA
ENSG00000196431	CRYBA4	crystallin beta A4	1413	0,354859955	0	4,080456	0	1	NA
ENSG00000196456	ZNF775	zinc finger protein 775	285971	8,947671173	-0,148285608	0,601412	-0,246562325	0,805246965	NA
ENSG00000196460	RFX8	regulatory factor X8	731220	5,165350188	-0,553939313	1,114855	-0,496871215	0,619279871	NA
ENSG00000196468	FGF16	fibroblast growth factor 16	8823	0	NA	NA	NA	NA	NA
ENSG00000196472	LOC642474	UDP glucuronosyltransferase family 2 member A3 pseudogene	642474	0	NA	NA	NA	NA	NA
ENSG00000196475	GK2	glycerol kinase 2	2712	0	NA	NA	NA	NA	NA
ENSG00000196482	ESRRG	estrogen related receptor gamma	2104	0	NA	NA	NA	NA	NA
ENSG00000196502	SULT1A1	sulfotransferase family 1A member 1	6817	8,640388325	0,177495713	0,642419	0,276292887	0,782323119	NA
ENSG00000196503	ARL9	ADP ribosylation factor like GTPase 9	132946	1,418190753	0,141105289	1,395173	0,101138226	0,919440733	NA
ENSG00000196507	TCEAL3	transcription elongation factor A like 3	85012	1,723117131	0,546751223	1,254217	0,435930238	0,662887346	NA
ENSG00000196517	SLC6A9	solute carrier family 6 member 9	6536	3,765797844	1,401244124	1,059966	1,321970793	0,186177877	NA
ENSG00000196534	NA	NA	NA	0	NA	NA	NA	NA	NA

ENSG00000196549	MME	membrane metalloendopeptidase	4311	0	NA	NA	NA	NA	NA
ENSG00000196553	CCDC196	coiled-coil domain containing 196	440184	0	NA	NA	NA	NA	NA
ENSG00000196557	CACNA1H	calcium voltage-gated channel subunit alpha1 H	8912	0,119673877	0,847101401	4,080456	0,207599697	0,835541541	NA
ENSG00000196562	SULF2	sulfatase 2	55959	0,10488184	-1,076443944	4,080456	-0,263804824	0,791930317	NA
ENSG00000196564	NA	NA	NA	0,11808024	-1,076443944	4,080456	-0,263804824	0,791930317	NA
ENSG00000196565	HBG2	hemoglobin subunit gamma 2	3048	0	NA	NA	NA	NA	NA
ENSG00000196569	LAMA2	laminin subunit alpha 2	3908	3,393233889	0,490396554	1,183159	0,414480543	0,678522214	NA
ENSG00000196570	PFN3	profilin 3	345456	0	NA	NA	NA	NA	NA
ENSG00000196578	OR5AC2	olfactory receptor family 5 subfamily AC member 2	81050	0	NA	NA	NA	NA	NA
ENSG00000196581	AJAP1	adherens junctions associated protein 1	55966	0	NA	NA	NA	NA	NA
ENSG00000196589	MBD3L2B	methyl-CpG binding domain protein 3 like 2B	729458	0	NA	NA	NA	NA	NA
ENSG00000196593	NA	NA	NA	0	NA	NA	NA	NA	NA
ENSG00000196600	SLC22A25	solute carrier family 22 member 25	387601	0	NA	NA	NA	NA	NA
ENSG00000196604	POTEF	POTE ankyrin domain family member F	728378	0	NA	NA	NA	NA	NA
ENSG00000196611	MMP1	matrix metallopeptidase 1	4312	0	NA	NA	NA	NA	NA
ENSG00000196616	ADH1B	alcohol dehydrogenase 1B (class I), beta polypeptide	125	0	NA	NA	NA	NA	NA
ENSG00000196620	UGT2B15	UDP glucuronosyltransferase family 2 member B15	7366	0	NA	NA	NA	NA	NA
ENSG00000196632	WNK3	WNK lysine deficient protein kinase 3	65267	0	NA	NA	NA	NA	NA
ENSG00000196639	HRH1	histamine receptor H1	3269	0	NA	NA	NA	NA	NA
ENSG00000196659	IFT70B	intraflagellar transport 70B	150737	8,371454887	0,155432197	0,664726	0,233828898	0,815117814	NA
ENSG00000196660	SLC30A10	solute carrier family 30 member 10	55532	0	NA	NA	NA	NA	NA
ENSG00000196664	TLR7	toll like receptor 7	51284	0	NA	NA	NA	NA	NA
ENSG00000196666	FAM180B	family with sequence similarity 180 member B	399888	0	NA	NA	NA	NA	NA
ENSG00000196711	ALKAL1	ALK and LTK ligand 1	389658	0,192011017	1,679479956	4,046214	0,4150744	0,678087439	NA
ENSG00000196724	ZNF418	zinc finger protein 418	147686	4,665731076	0,269090432	0,890603	0,302144247	0,762542104	NA
ENSG00000196730	DAPK1	death associated protein kinase 1	1612	0	NA	NA	NA	NA	NA
ENSG00000196734	LCE1B	late cornified envelope 1B	353132	0	NA	NA	NA	NA	NA
ENSG00000196739	COL27A1	collagen type XXVII alpha 1 chain	85301	0	NA	NA	NA	NA	NA
ENSG00000196747	H2AC13	H2A clustered histone 13	8329	0,823051651	0,838346491	2,258952	0,371121885	0,710546751	NA
ENSG00000196748	CLPSL2	colipase like 2	389383	0	NA	NA	NA	NA	NA
ENSG00000196754	S100A2	S100 calcium binding protein A2	6273	0,207996306	-1,916073151	4,043021	-0,473921178	0,635556117	NA
ENSG00000196767	POU3F4	POU class 3 homeobox 4	5456	0	NA	NA	NA	NA	NA
ENSG00000196772	OR14A16	olfactory receptor family 14 subfamily A member 16	284532	0	NA	NA	NA	NA	NA
ENSG00000196778	OR52K1	olfactory receptor family 52 subfamily K member 1	390036	0	NA	NA	NA	NA	NA
ENSG00000196787	H2AC11	H2A clustered histone 11	8969	4,519130139	0,371394997	0,940609	0,394845383	0,692957	NA
ENSG00000196793	ZNF239	zinc finger protein 239	8187	3,997152595	0,058665151	1,043751	0,056206067	0,955177648	NA
ENSG00000196796	NA	NA	NA	0,496120257	0,936385314	3,4488	0,27151051	0,785998418	NA
ENSG00000196800	SPINK14	serine peptidase inhibitor Kazal type 14 (putative)	408187	0	NA	NA	NA	NA	NA
ENSG00000196805	SPRR2B	small proline rich protein 2B	6701	0	NA	NA	NA	NA	NA
ENSG00000196811	CHRNG	cholinergic receptor nicotinic gamma subunit	1146	0	NA	NA	NA	NA	NA
ENSG00000196826	NA	NA	NA	5,228354629	-2,40103208	3,370641	-0,712336855	0,476256207	NA
ENSG00000196834	POTEI	POTE ankyrin domain family member I	653269	0	NA	NA	NA	NA	NA
ENSG00000196844	PATE2	prostate and testis expressed 2	399967	0,11808024	-1,076443944	4,080456	-0,263804824	0,791930317	NA
ENSG00000196859	KRT39	keratin 39	390792	0	NA	NA	NA	NA	NA
ENSG00000196860	TOMM20L	translocase of outer mitochondrial membrane 20 like	387990	0,072165449	0,847101401	4,080456	0,207599697	0,835541541	NA
ENSG00000196862	RGPD4	RANBP2 like and GRIP domain containing 4	285190	8,705616315	-0,355881966	0,691685	-0,514514611	0,606892248	NA
ENSG00000196866	H2AC7	H2A clustered histone 7	3013	2,656288167	0,087490181	1,454214	0,06016319	0,952025663	NA
ENSG00000196872	CRACDL	CRACD like	343990	5,144189453	0,037716123	0,848752	0,04443714	0,964555957	NA
ENSG00000196876	SCN8A	sodium voltage-gated channel alpha subunit 8	6334	2,256976927	-0,155587182	1,335897	-0,116466463	0,907282864	NA
ENSG00000196878	LAMB3	laminin subunit beta 3	3914	2,307913654	-0,43991414	1,154932	-0,380900583	0,70327702	NA
ENSG00000196890	H2BC26	H2B clustered histone 26	128312	8,588763007	0,345603116	1,183174	0,292098205	0,770211539	NA
ENSG00000196900	SPMIP10	sperm microtubule inner protein 10	389320	0	NA	NA	NA	NA	NA

ENSG00000196917	HCAR1	hydroxycarboxylic acid receptor 1	27198	0,295443621	-0,121164	3,93668	-0,030778217	0,975446412	NA
ENSG00000196932	TMEM26	transmembrane protein 26	219623	0	NA	NA	NA	NA	NA
ENSG00000196933	NA	NA	NA	0	NA	NA	NA	NA	NA
ENSG00000196935	SRGAP1	SLIT-ROBO Rho GTPase activating protein 1	57522	0,563168848	-0,116264581	2,683465	-0,043326289	0,965441435	NA
ENSG00000196946	ZNF705A	zinc finger protein 705A	440077	0	NA	NA	NA	NA	NA
ENSG00000196970	NA	NA	NA	0	NA	NA	NA	NA	NA
ENSG00000196972	SMIM10L2B	small integral membrane protein 10 like 2B	644596	0,311994459	-2,498367527	3,998294	-0,62485841	0,532063991	NA
ENSG00000196990	FAM163B	family with sequence similarity 163 member B	642968	0,118286652	0	4,080456	0	1	NA
ENSG00000196993	NPIPB9	nuclear pore complex interacting protein family member B9	100507607	8,755222362	-1,130083654	1,11629	-1,012357115	0,311367335	NA
ENSG00000197023	OR51A6P	olfactory receptor family 51 subfamily A member 6 pseudogene	390046	0	NA	NA	NA	NA	NA
ENSG00000197046	SIGLEC15	sialic acid binding Ig like lectin 15	284266	0	NA	NA	NA	NA	NA
ENSG00000197054	ZNF763	zinc finger protein 763	284390	7,361418066	0,618358222	0,847553	0,72958027	0,465646786	NA
ENSG00000197061	H4C3	H4 clustered histone 3	8364	0,560359961	0	3,920511	0	1	NA
ENSG00000197067	OR2T32P	olfactory receptor family 2 subfamily T member 32 pseudogene	403241	0	NA	NA	NA	NA	NA
ENSG00000197079	KRT35	keratin 35	3886	0	NA	NA	NA	NA	NA
ENSG00000197083	NA	NA	NA	0	NA	NA	NA	NA	NA
ENSG00000197084	LCE1C	late cornified envelope 1C	353133	0	NA	NA	NA	NA	NA
ENSG00000197092	NA	NA	NA	0	NA	NA	NA	NA	NA
ENSG00000197093	GAL3ST4	galactose-3-O-sulfotransferase 4	79690	0,209763681	-1,926644696	4,042043	-0,476651224	0,633610501	NA
ENSG00000197106	SLC6A17	solute carrier family 6 member 17	388662	0	NA	NA	NA	NA	NA
ENSG00000197123	ZNF679	zinc finger protein 679	168417	0	NA	NA	NA	NA	NA
ENSG00000197124	ZNF682	zinc finger protein 682	91120	5,811263146	-0,508210548	1,172432	-0,433466823	0,664675664	NA
ENSG00000197125	OR8B8	olfactory receptor family 8 subfamily B member 8	26493	0	NA	NA	NA	NA	NA
ENSG00000197134	ZNF257	zinc finger protein 257	113835	5,713918302	-2,118383754	1,013991	-2,089154159	0,036693848	NA
ENSG00000197140	ADAM32	ADAM metallopeptidase domain 32	203102	0	NA	NA	NA	NA	NA
ENSG00000197149	RPS2P40	ribosomal protein S2 pseudogene 40	91561	0,630498334	-0,442848587	3,211157	-0,137909358	0,890312055	NA
ENSG00000197153	H3C12	H3 clustered histone 12	8356	0,235794762	0,854882045	4,080278	0,209515652	0,834045718	NA
ENSG00000197161	NA	NA	NA	0	NA	NA	NA	NA	NA
ENSG00000197165	SULT1A2	sulfotransferase family 1A member 2	6799	0,474670837	0	4,080456	0	1	NA
ENSG00000197168	NEK5	NIMA related kinase 5	341676	0,103998153	-1,076443944	4,080456	-0,263804824	0,791930317	NA
ENSG00000197171	OR2B4P	olfactory receptor family 2 subfamily B member 4 pseudogene	442190	0	NA	NA	NA	NA	NA
ENSG00000197172	MAGEA6	MAGE family member A6	4105	0	NA	NA	NA	NA	NA
ENSG00000197177	ADGRA1	adhesion G protein-coupled receptor A1	84435	0	NA	NA	NA	NA	NA
ENSG00000197181	PIWIL2	piwi like RNA-mediated gene silencing 2	55124	1,711733408	0,778013593	1,712075	0,454427183	0,649521391	NA
ENSG00000197185	NA	NA	NA	0	NA	NA	NA	NA	NA
ENSG00000197191	CYSRT1	cysteine rich tail 1	375791	0,328229347	-0,98335507	4,041243	-0,243329869	0,807749867	NA
ENSG00000197208	SLC22A4	solute carrier family 22 member 4	6583	0	NA	NA	NA	NA	NA
ENSG00000197213	ZSCAN5B	zinc finger and SCAN domain containing 5B	342933	0	NA	NA	NA	NA	NA
ENSG00000197214	NA	NA	NA	0	NA	NA	NA	NA	NA
ENSG00000197233	OR1J2	olfactory receptor family 1 subfamily J member 2	26740	0	NA	NA	NA	NA	NA
ENSG00000197238	H4C11	H4 clustered histone 11	8363	0,524409201	-3,24447917	3,961591	-0,818983792	0,412795661	NA
ENSG00000197241	SLC2A7	solute carrier family 2 member 7	155184	0,604734454	-2,553140792	2,45307	-1,040794247	0,297971051	NA
ENSG00000197245	FAM110D	family with sequence similarity 110 member D	79927	0	NA	NA	NA	NA	NA
ENSG00000197249	SERPINA1	serpin family A member 1	5265	0	NA	NA	NA	NA	NA
ENSG00000197253	TPSB2	tryptase beta 2	64499	0	NA	NA	NA	NA	NA
ENSG00000197254	NA	NA	NA	0	NA	NA	NA	NA	NA
ENSG00000197256	KANK2	KN motif and ankyrin repeat domains 2	25959	5,059597851	-0,255287369	1,101634	-0,231735216	0,816743677	NA
ENSG00000197258	NA	NA	NA	1,323995195	1,347261107	1,976589	0,681609015	0,495486213	NA
ENSG00000197261	C6orf141	chromosome 6 open reading frame 141	135398	0	NA	NA	NA	NA	NA
ENSG00000197272	IL27	interleukin 27	246778	0	NA	NA	NA	NA	NA
ENSG00000197273	GUCA2A	guanylate cyclase activator 2A	2980	0	NA	NA	NA	NA	NA

ENSG00000197279	ZNF165	zinc finger protein 165	7718	1,100368005	1,963549624	2,011073	0,976369336	0,328881472	NA	
ENSG00000197309	OR10D3	olfactory receptor family 10 subfamily D member 3	26497	0,118286652	0	4,080456	0	1	NA	
ENSG00000197320	LOC340268	C3 and PZP like alpha-2-macroglobulin domain containing 8 pseudogene	340268	0	NA	NA	NA	NA	NA	
ENSG00000197353	LYPD2	LY6/PLAUR domain containing 2	137797	0	NA	NA	NA	NA	NA	
ENSG00000197358	BNIP3P1	BCL2 interacting protein 3 pseudogene 1	319138	0	NA	NA	NA	NA	NA	
ENSG00000197360	ZNF98	zinc finger protein 98	148198	0	NA	NA	NA	NA	NA	
ENSG00000197361	FBXL22	F-box and leucine rich repeat protein 22	283807	1,756918738	0,488853518	1,348944	0,36239707	0,717055328	NA	
ENSG00000197363	ZNF517	zinc finger protein 517	340385	9,293799232	-0,356516076	0,749594	-0,475612431	0,634350518	NA	
ENSG00000197364	NA	NA	NA	0	NA	NA	NA	NA	NA	
ENSG00000197375	SLC22A5	solute carrier family 22 member 5	6584	3,637537055	0,276253396	2,059557	0,134132407	0,893297874	NA	
ENSG00000197376	NA	NA	NA	0	NA	NA	NA	NA	NA	
ENSG00000197380	DACT3	dishevelled binding antagonist of beta catenin 3	147906	1,321811285	1,373913145	1,775783	0,773694436	0,439111511	NA	
ENSG00000197385	ZNF860	zinc finger protein 860	344787	0	NA	NA	NA	NA	NA	
ENSG00000197403	OR6N1	olfactory receptor family 6 subfamily N member 1	128372	0	NA	NA	NA	NA	NA	
ENSG00000197405	C5AR1	complement C5a receptor 1	728	0,80479118	1,548948021	2,372607	0,65284636	0,513855333	NA	
ENSG00000197406	DIO3	iodothyronine deiodinase 3	1735	0	NA	NA	NA	NA	NA	
ENSG00000197408	CYP2B6	cytochrome P450 family 2 subfamily B member 6	1555	0,10543123	0	4,080456	0	1	NA	
ENSG00000197409	H3C4	H3 clustered histone 4	8351	1,504513935	1,433985984	2,251256	0,636971536	0,524143384	NA	
ENSG00000197410	DCHS2	dachsous cadherin-related 2	54798	0	NA	NA	NA	NA	NA	
ENSG00000197415	VEPH1	ventricular zone expressed PH domain containing 1	79674	0	NA	NA	NA	NA	NA	
ENSG00000197416	FABP12	fatty acid binding protein 12	646486	0	NA	NA	NA	NA	NA	
ENSG00000197421	GGT3P	gamma-glutamyltransferase 3 pseudogene	2679	0	NA	NA	NA	NA	NA	
ENSG00000197428	OR51D1	olfactory receptor family 51 subfamily D member 1	390038	0	NA	NA	NA	NA	NA	
ENSG00000197430	OPALIN	oligodendrocytic myelin paranodal and inner loop protein	93377	0	NA	NA	NA	NA	NA	
ENSG00000197437	OR13G1	olfactory receptor family 13 subfamily G member 1	441933	0	NA	NA	NA	NA	NA	
ENSG00000197444	OGDHL	oxoglutarate dehydrogenase L	55753	0	NA	NA	NA	NA	NA	
ENSG00000197446	CYP2F1	cytochrome P450 family 2 subfamily F member 1	1572	0	NA	NA	NA	NA	NA	
ENSG00000197454	OR2L5	olfactory receptor family 2 subfamily L member 5	81466	0,177208424	0,852892602	4,080456	0,20901895	0,834433445	NA	
ENSG00000197457	STMN3	stathmin 3	50861	9,179603596	0,353481803	0,689865	0,512392677	0,608376215	NA	
ENSG00000197461	PDGFA	platelet derived growth factor subunit A	5154	10,14659764	-0,335454324	0,593364	-0,565343563	0,571840104	NA	
ENSG00000197465	GYPE	glycophorin E (MNS blood group)	2996	8,634929068	1,757782444	1,020197	1,722982773	0,08489164	NA	
ENSG00000197467	COL13A1	collagen type XIII alpha 1 chain	1305	0	NA	NA	NA	NA	NA	
ENSG00000197468	OR7E85BP	olfactory receptor family 7 subfamily E member 85B pseudogene	650293	0	NA	NA	NA	NA	NA	
ENSG00000197472	ZNF695	zinc finger protein 695	57116	0,072165449	0,847101401	4,080456	0,207599697	0,835541541	NA	
ENSG00000197475	ADAM3A	ADAM metallopeptidase domain 3A (pseudogene)	1587	0	NA	NA	NA	NA	NA	
ENSG00000197476	NA	NA	NA	0	NA	NA	NA	NA	NA	
ENSG00000197487	GALP	galanin like peptide	85569	0	NA	NA	NA	NA	NA	
ENSG00000197496	SLC2A10	solute carrier family 2 member 10	81031	0,458725777	-1,102923277	3,51869	-0,313447099	0,753941006	NA	
ENSG00000197497	ZNF665	zinc finger protein 665	79788	1,115591489	1,603026506	1,935525	0,82821268	0,407550064	NA	
ENSG00000197506	SLC28A3	solute carrier family 28 member 3	64078	0,10543123	0	4,080456	0	1	NA	
ENSG00000197520	FAM177B	family with sequence similarity 177 member B	400823	0,177644342	0,846406996	4,080456	0,207429518	0,835674432	NA	
ENSG00000197530	MIB2	MIB E3 ubiquitin protein ligase 2	142678	6,139982573	0,786166203	2,778056	0,282991455	0,777183387	NA	
ENSG00000197532	OR6Y1	olfactory receptor family 6 subfamily Y member 1	391112	0	NA	NA	NA	NA	NA	
ENSG00000197549	NA	NA	NA	0	NA	NA	NA	NA	NA	
ENSG00000197550	NA	NA	NA	0,882792736	-0,268446854	2,109192	-0,127274731	0,89872296	NA	
ENSG00000197557	IFT70A	intraflagellar transport 70A	92104	4,167108173	0,067427129	0,907761	0,074278485	0,940788796	NA	
ENSG00000197558	SSPOP	SCO-spondin, pseudogene	23145	0,11808024	-1,076443944	4,080456	-0,263804824	0,791930317	NA	
ENSG00000197561	ELANE	elastase, neutrophil expressed	1991	0	NA	NA	NA	NA	NA	

ENSG00000197565	COL4A6	collagen type IV alpha 6 chain	1288	0	NA	NA	NA	NA	NA
ENSG00000197575	RPS17P2	ribosomal protein S17 pseudogene 2	6219	0	NA	NA	NA	NA	NA
ENSG00000197576	HOXA4	homeobox A4	3201	0,660181586	-0,930773226	3,041022	-0,306072539	0,759549416	NA
ENSG00000197580	BCO2	beta-carotene oxygenase 2	83875	0,706737901	1,130029983	2,641746	0,427758714	0,668826798	NA
ENSG00000197582	NA	NA	NA	0,223238175	-1,062474004	4,080456	-0,260381202	0,794569742	NA
ENSG00000197584	KCNMB2	potassium calcium-activated channel subfamily M regulatory beta subunit 2	10242	0	NA	NA	NA	NA	NA
ENSG00000197587	DMBX1	diencephalon/mesencephalon homeobox 1	127343	0	NA	NA	NA	NA	NA
ENSG00000197588	NA	NA	NA	0	NA	NA	NA	NA	NA
ENSG00000197591	OR11L1	olfactory receptor family 11 subfamily L member 1	391189	0	NA	NA	NA	NA	NA
ENSG00000197599	CCDC154	coiled-coil domain containing 154	645811	3,968134003	-0,861703049	1,217479	-0,70777677	0,479083894	NA
ENSG00000197614	MFAP5	microfibril associated protein 5	8076	0	NA	NA	NA	NA	NA
ENSG00000197616	MYH6	myosin heavy chain 6	4624	0	NA	NA	NA	NA	NA
ENSG00000197617	VN1R5	vomeronasal 1 receptor 5 (gene/pseudogene)	317705	0	NA	NA	NA	NA	NA
ENSG00000197629	MPEG1	macrophage expressed 1	219972	0,103998153	-1,076443944	4,080456	-0,263804824	0,791930317	NA
ENSG00000197632	SERPINB2	serpin family B member 2	5055	0	NA	NA	NA	NA	NA
ENSG00000197641	SERPINB13	serpin family B member 13	5275	0	NA	NA	NA	NA	NA
ENSG00000197646	PDCD1LG2	programmed cell death 1 ligand 2	80380	0,461333292	1,417454656	4,061234	0,349020701	0,727073768	NA
ENSG00000197647	ZNF433	zinc finger protein 433	163059	0	NA	NA	NA	NA	NA
ENSG00000197651	CCER1	coiled-coil glutamate rich protein 1	196477	0	NA	NA	NA	NA	NA
ENSG00000197653	DNAH10	dynein axonemal heavy chain 10	196385	0,072165449	0,847101401	4,080456	0,207599697	0,835541541	NA
ENSG00000197658	SLC22A24	solute carrier family 22 member 24	283238	0	NA	NA	NA	NA	NA
ENSG00000197674	OR51C1P	olfactory receptor family 51 subfamily C member 1 pseudogene	401661	0	NA	NA	NA	NA	NA
ENSG00000197683	KRTAP26-1	keratin associated protein 26-1	388818	0	NA	NA	NA	NA	NA
ENSG00000197692	NA	NA	NA	0	NA	NA	NA	NA	NA
ENSG00000197702	PARVA	parvin alpha	55742	0,103998153	-1,076443944	4,080456	-0,263804824	0,791930317	NA
ENSG00000197705	KLHL14	kelch like family member 14	57565	0	NA	NA	NA	NA	NA
ENSG00000197706	OR6C74	olfactory receptor family 6 subfamily C member 74	254783	0	NA	NA	NA	NA	NA
ENSG00000197712	FAM114A1	family with sequence similarity 114 member A1	92689	0,10543123	0	4,080456	0	1	NA
ENSG00000197721	CR1L	complement C3b/C4b receptor 1 like	1379	0	NA	NA	NA	NA	NA
ENSG00000197744	NA	NA	NA	0,191766453	1,678167216	4,046256	0,414745694	0,678328078	NA
ENSG00000197745	SCGB1D4	secretoglobin family 1D member 4	404552	0	NA	NA	NA	NA	NA
ENSG00000197748	CFAP43	cilia and flagella associated protein 43	80217	0,11808024	-1,076443944	4,080456	-0,263804824	0,791930317	NA
ENSG00000197753	LHFPL5	LHFPL tetraspan subfamily member 5	222662	0,103998153	-1,076443944	4,080456	-0,263804824	0,791930317	NA
ENSG00000197757	HOXC6	homeobox C6	3223	0	NA	NA	NA	NA	NA
ENSG00000197766	CFD	complement factor D	1675	0	NA	NA	NA	NA	NA
ENSG00000197768	STPG3	sperm-tail PG-rich repeat containing 3	441476	0	NA	NA	NA	NA	NA
ENSG00000197769	MAP1LC3C	microtubule associated protein 1 light chain 3 gamma	440738	0	NA	NA	NA	NA	NA
ENSG00000197782	ZNF780A	zinc finger protein 780A	284323	9,316647682	-2,221232648	3,746286	-0,592915926	0,553237419	NA
ENSG00000197786	OR5B17	olfactory receptor family 5 subfamily B member 17	219965	0	NA	NA	NA	NA	NA
ENSG00000197790	OR52M1	olfactory receptor family 52 subfamily M member 1	119772	0	NA	NA	NA	NA	NA
ENSG00000197826	CFAP299	cilia and flagella associated protein 299	255119	0	NA	NA	NA	NA	NA
ENSG00000197837	H4C16	H4 histone 16	121504	0,587485541	3,280248886	3,95344	0,829720116	0,406697045	NA
ENSG00000197838	CYP2A13	cytochrome P450 family 2 subfamily A member 13	1553	0	NA	NA	NA	NA	NA
ENSG00000197847	SLC22A20P	solute carrier family 22 member 20, pseudogene	440044	2,435558397	-0,465361285	1,23421	-0,377051987	0,706134971	NA
ENSG00000197849	OR8G1	olfactory receptor family 8 subfamily G member 1	26494	0	NA	NA	NA	NA	NA
ENSG00000197859	ADAMTSL2	ADAMTS like 2	9719	2,053868083	0,56462718	1,600255	0,352835657	0,724211651	NA
ENSG00000197866	OR5M12P	olfactory receptor family 5 subfamily M member 12 pseudogene	81188	0	NA	NA	NA	NA	NA

ENSG00000197870	PRB3	proline rich protein BstNI subfamily 3	5544	0	NA	NA	NA	NA	NA
ENSG00000197882	NA	NA	NA	0	NA	NA	NA	NA	NA
ENSG00000197885	NKIRAS1	NFKB inhibitor interacting Ras like 1	28512	11,34082219	-0,838638445	0,574639	-1,459418082	0,144450072	NA
ENSG00000197887	OR1S2	olfactory receptor family 1 subfamily S member 2	219958	0	NA	NA	NA	NA	NA
ENSG00000197888	UGT2B17	UDP glucuronosyltransferase family 2 member B17	7367	0	NA	NA	NA	NA	NA
ENSG00000197889	MEIG1	meiosis/spermiogenesis associated 1	644890	0,117497108	0,847101401	4,080456	0,207599697	0,835541541	NA
ENSG00000197891	SLC22A12	solute carrier family 22 member 12	116085	0	NA	NA	NA	NA	NA
ENSG00000197893	NRAP	nebulin related anchoring protein	4892	0	NA	NA	NA	NA	NA
ENSG00000197901	SLC22A6	solute carrier family 22 member 6	9356	0	NA	NA	NA	NA	NA
ENSG00000197905	TEAD4	TEA domain transcription factor 4	7004	0	NA	NA	NA	NA	NA
ENSG00000197915	HRNR	hornerin	388697	0	NA	NA	NA	NA	NA
ENSG00000197919	IFNA1	interferon alpha 1	3439	0	NA	NA	NA	NA	NA
ENSG00000197921	HES5	hes family bHLH transcription factor 5	388585	0	NA	NA	NA	NA	NA
ENSG00000197927	NBEAP2	neurobeachin pseudogene 2	100418881	0	NA	NA	NA	NA	NA
ENSG00000197928	ZNF677	zinc finger protein 677	342926	4,050838253	-0,117520102	0,953253	-0,123283186	0,901882855	NA
ENSG00000197933	ZNF823	zinc finger protein 823	55552	11,0341313	0,274613664	0,681153	0,40315974	0,686830715	NA
ENSG00000197935	ZNF311	zinc finger protein 311	282890	0,78558168	1,410910583	3,776318	0,373620736	0,708686505	NA
ENSG00000197938	OR5H2	olfactory receptor family 5 subfamily H member 2	79310	0	NA	NA	NA	NA	NA
ENSG00000197953	AADACL2	arylacetamide deacetylase like 2	344752	0	NA	NA	NA	NA	NA
ENSG00000197959	DNM3	dynamin 3	26052	5,173019436	1,404058739	1,356021	1,03542557	0,300470209	NA
ENSG00000197977	ELOVL2	ELOVL fatty acid elongase 2	54898	0	NA	NA	NA	NA	NA
ENSG00000197980	LEKR1	leucine, glutamate and lysine rich 1	389170	1,732436873	-0,41203491	1,398229	-0,294683451	0,768235706	NA
ENSG00000197984	OR51A8P	olfactory receptor family 51 subfamily A member 8 pseudogene	401662	0	NA	NA	NA	NA	NA
ENSG00000197990	NA	NA	NA	0	NA	NA	NA	NA	NA
ENSG00000197991	NA	NA	NA	0	NA	NA	NA	NA	NA
ENSG00000197992	CLEC9A	C-type lectin domain containing 9A	283420	0	NA	NA	NA	NA	NA
ENSG00000197993	KEL	Kell metallo-endopeptidase (Kell blood group)	3792	0	NA	NA	NA	NA	NA
ENSG00000198003	ODAD3	outer dynein arm docking complex subunit 3	115948	0,119673877	0,847101401	4,080456	0,207599697	0,835541541	NA
ENSG00000198010	DLGAP2	DLG associated protein 2	9228	0	NA	NA	NA	NA	NA
ENSG00000198019	NA	NA	NA	0	NA	NA	NA	NA	NA
ENSG00000198021	SPANXA1	sperm protein associated with the nucleus, X-linked, family member A1	30014	0	NA	NA	NA	NA	NA
ENSG00000198022	NA	NA	NA	0	NA	NA	NA	NA	NA
ENSG00000198028	ZNF560	zinc finger protein 560	147741	0,072165449	0,847101401	4,080456	0,207599697	0,835541541	NA
ENSG00000198033	TUBA3C	tubulin alpha 3c	7278	0	NA	NA	NA	NA	NA
ENSG00000198039	ZNF273	zinc finger protein 273	10793	7,485766487	-0,119461916	2,764344	-0,043215282	0,965529923	NA
ENSG00000198046	ZNF667	zinc finger protein 667	63934	0,261706024	2,139455619	4,008794	0,53369053	0,593555662	NA
ENSG00000198049	AVPR1B	arginine vasopressin receptor 1B	553	0	NA	NA	NA	NA	NA
ENSG00000198053	SIRPA	signal regulatory protein alpha	140885	0,11808024	-1,076443944	4,080456	-0,263804824	0,791930317	NA
ENSG00000198062	POTEH	POTE ankyrin domain family member H	23784	0	NA	NA	NA	NA	NA
ENSG00000198074	AKR1B10	aldo-keto reductase family 1 member B10	57016	0	NA	NA	NA	NA	NA
ENSG00000198075	SULT1C4	sulfotransferase family 1C member 4	27233	0	NA	NA	NA	NA	NA
ENSG00000198077	CYP2A7	cytochrome P450 family 2 subfamily A member 7	1549	0	NA	NA	NA	NA	NA
ENSG00000198083	KRTAP9-9	keratin associated protein 9-9	81870	0	NA	NA	NA	NA	NA
ENSG00000198088	NUP62CL	nucleoporin 62 C-terminal like	54830	1,188293114	1,259188665	2,263441	0,55631615	0,577994749	NA
ENSG00000198092	TMPRSS11F	transmembrane serine protease 11F	389208	0	NA	NA	NA	NA	NA
ENSG00000198099	ADH4	alcohol dehydrogenase 4 (class II), pi polypeptide	127	0	NA	NA	NA	NA	NA
ENSG00000198108	CHSY3	chondroitin sulfate synthase 3	337876	0	NA	NA	NA	NA	NA
ENSG00000198121	LPAR1	lysophosphatidic acid receptor 1	1902	0,557777232	-2,481638241	2,46009	-1,008759103	0,313090179	NA

ENSG00000198128	OR2L3	olfactory receptor family 2 subfamily L member 3	391192	0,263290333	0	4,080456	0	1	NA	
ENSG00000198142	SOWAHC	sosondowah ankyrin repeat domain family member C	65124	0,441705466	-0,879771472	3,538719	-0,248612949	0,803660191	NA	
ENSG00000198153	ZNF849P	zinc finger protein 849, pseudogene	100130108	0	NA	NA	NA	NA	NA	
ENSG00000198155	NA	NA	NA	10,0326111	-0,133747281	0,680152	-0,196643165	0,844106789	NA	
ENSG00000198156	NPIPB6	nuclear pore complex interacting protein family member B6	728741	4,611838241	1,769856823	1,333566	1,327161187	0,184455368	NA	
ENSG00000198157	HMGN5	high mobility group nucleosome binding domain 5	79366	3,695462298	0,322292535	0,969982	0,332266674	0,739687902	NA	
ENSG00000198169	ZNF251	zinc finger protein 251	90987	45,87899517	-0,003386145	1,627411	-0,002080695	NA	NA	
ENSG00000198173	FAM47C	family with sequence similarity 47 member C	442444	0	NA	NA	NA	NA	NA	
ENSG00000198178	CLEC4C	C-type lectin domain family 4 member C	170482	0	NA	NA	NA	NA	NA	
ENSG00000198182	ZNF607	zinc finger protein 607	84775	9,404233014	0,351502631	0,737341	0,476716401	0,633564083	NA	
ENSG00000198183	BPIFA1	BPI fold containing family A member 1	51297	0	NA	NA	NA	NA	NA	
ENSG00000198203	SULT1C2	sulfotransferase family 1C member 2	6819	0	NA	NA	NA	NA	NA	
ENSG00000198208	RPS6KL1	ribosomal protein S6 kinase like 1	83694	0,443112496	0,115963968	3,512891	0,033010974	0,973665836	NA	
ENSG00000198211	NA	NA	NA	0	NA	NA	NA	NA	NA	
ENSG00000198216	CACNA1E	calcium voltage-gated channel subunit alpha1 E	777	0	NA	NA	NA	NA	NA	
ENSG00000198217	OR51H2P	olfactory receptor family 51 subfamily H member 2 pseudogene	401664	0	NA	NA	NA	NA	NA	
ENSG00000198225	FKBP1C	FKBP prolyl isomerase family member 1C	642489	1,066230565	-1,096398629	1,853846	-0,591418413	0,554240106	NA	
ENSG00000198246	SLC29A3	solute carrier family 29 member 3	55315	11,03194832	0,356229995	2,286553	0,155793433	0,876195847	NA	
ENSG00000198251	NA	NA	NA	0	NA	NA	NA	NA	NA	
ENSG00000198261	OR5BB1P	olfactory receptor family 5 subfamily BB member 1 pseudogene	81209	0	NA	NA	NA	NA	NA	
ENSG00000198277	NA	NA	NA	0	NA	NA	NA	NA	NA	
ENSG00000198283	OR5B21	olfactory receptor family 5 subfamily B member 21	219968	2,131362311	-0,612770351	1,280326	-0,47860498	0,632219675	NA	
ENSG00000198284	NUP210P1	nucleoporin 210 pseudogene 1	255330	0	NA	NA	NA	NA	NA	
ENSG00000198300	PEG3	paternally expressed 3	5178	0	NA	NA	NA	NA	NA	
ENSG00000198312	BMS1P9	BMS1 pseudogene 9	653458	0,144330898	1,418786819	4,061262	0,349346267	0,726829368	NA	
ENSG00000198326	TMEM239	transmembrane protein 239	100288797	0	NA	NA	NA	NA	NA	
ENSG00000198336	MYL4	myosin light chain 4	4635	0,118286652	0	4,080456	0	1	NA	
ENSG00000198342	ZNF442	zinc finger protein 442	79973	0	NA	NA	NA	NA	NA	
ENSG00000198353	HOXC4	homeobox C4	3221	3,232758778	-0,800781607	1,051889	-0,761279735	0,446490002	NA	
ENSG00000198354	DCAF12L2	DDB1 and CUL4 associated factor 12 like 2	340578	0	NA	NA	NA	NA	NA	
ENSG00000198367	OR7A11P	olfactory receptor family 7 subfamily A member 11 pseudogene	26669	0	NA	NA	NA	NA	NA	
ENSG00000198390	KRTAP13-1	keratin associated protein 13-1	140258	0	NA	NA	NA	NA	NA	
ENSG00000198398	TMEM207	transmembrane protein 207	131920	0	NA	NA	NA	NA	NA	
ENSG00000198400	NTRK1	neurotrophic receptor tyrosine kinase 1	4914	10,92232018	0,040318099	0,646601	0,062353953	0,950280964	NA	
ENSG00000198414	TATDN2P1	TatD DNase domain containing 2 pseudogene 1	100131103	0	NA	NA	NA	NA	NA	
ENSG00000198416	NA	NA	NA	3,219279992	-0,085514082	1,330934	-0,064251201	0,948770209	NA	
ENSG00000198417	MT1F	metallothionein 1F	4494	4,899806472	0,474523486	0,859335	0,552198281	0,580812512	NA	
ENSG00000198429	ZNF69	zinc finger protein 69	7620	0,978240972	-1,493607466	2,086909	-0,715703257	0,474174599	NA	
ENSG00000198435	NRARP	NOTCH regulated ankyrin repeat protein	441478	9,766293338	-0,857010408	0,598898	-1,430978435	0,152436396	NA	
ENSG00000198443	KRTAP4-1	keratin associated protein 4-1	85285	0	NA	NA	NA	NA	NA	
ENSG00000198445	CCT8L2	chaperonin containing TCP1 subunit 8 like 2	150160	0	NA	NA	NA	NA	NA	
ENSG00000198452	OR14L1	olfactory receptor family 14 subfamily L member 1	127617	0	NA	NA	NA	NA	NA	
ENSG00000198471	RTP2	receptor transporter protein 2	344892	0	NA	NA	NA	NA	NA	
ENSG00000198478	SH3BGRL2	SH3 domain binding glutamate rich protein like 2	83699	0	NA	NA	NA	NA	NA	

ENSG00000198488	B3GNT6	UDP-GlcNAc:betaGal beta-1,3-N-acetylglucosaminyltransferase 6	192134	0	NA	NA	NA	NA	NA
ENSG00000198513	ATL1	atlastin GTPase 1	51062	0,482435914	-3,101748556	3,289555	-0,942908133	0,345727895	NA
ENSG00000198515	CNGA1	cyclic nucleotide gated channel subunit alpha 1	1259	0	NA	NA	NA	NA	NA
ENSG00000198520	ARMH1	armadillo like helical domain containing 1	339541	1,31944632	-0,746020612	2,274459	-0,327999088	0,742912352	NA
ENSG00000198523	PLN	phospholamban	5350	0	NA	NA	NA	NA	NA
ENSG00000198526	NA	NA	NA	0	NA	NA	NA	NA	NA
ENSG00000198535	C2CD4A	C2 calcium dependent domain containing 4A	145741	0	NA	NA	NA	NA	NA
ENSG00000198542	ITGBL1	integrin subunit beta like 1	9358	0,10488184	-1,076443944	4,080456	-0,263804824	0,791930317	NA
ENSG00000198547	C20orf203	chromosome 20 open reading frame 203	284805	5,575786808	0,081600879	1,113337	0,073293964	0,941572195	NA
ENSG00000198553	KCNRG	potassium channel regulator	283518	2,16776151	-2,66697101	1,617082	-1,649249114	0,09909661	NA
ENSG00000198555	NA	NA	NA	0	NA	NA	NA	NA	NA
ENSG00000198569	SLC34A3	solute carrier family 34 member 3	142680	0	NA	NA	NA	NA	NA
ENSG00000198570	RD3	RD3 regulator of GUCY2D	343035	0	NA	NA	NA	NA	NA
ENSG00000198573	SPANXC	SPANX family member C	64663	0	NA	NA	NA	NA	NA
ENSG00000198576	ARC	activity regulated cytoskeleton associated protein	23237	0	NA	NA	NA	NA	NA
ENSG00000198580	NA	NA	NA	1,203674518	0,331603903	1,937015	0,171193273	0,864071797	NA
ENSG00000198597	ZNF536	zinc finger protein 536	9745	0	NA	NA	NA	NA	NA
ENSG00000198598	MMP17	matrix metallopeptidase 17	4326	0,829734747	-1,058541405	2,402725	-0,440558726	0,659532488	NA
ENSG00000198601	OR2M2	olfactory receptor family 2 subfamily M member 2	391194	0	NA	NA	NA	NA	NA
ENSG00000198610	AKR1C4	aldo-keto reductase family 1 member C4	1109	0	NA	NA	NA	NA	NA
ENSG00000198618	NA	NA	NA	1,51600841	-0,90193252	1,603491	-0,562480556	0,573788648	NA
ENSG00000198626	RYR2	ryanodine receptor 2	6262	0,144330898	1,418786819	4,061262	0,349346267	0,726829368	NA
ENSG00000198633	ZNF534	zinc finger protein 534	147658	0,381911477	2,67165625	3,977473	0,671696929	0,501776657	NA
ENSG00000198643	FAM3D	FAM3 metabolism regulating signaling molecule D	131177	0	NA	NA	NA	NA	NA
ENSG00000198650	TAT	tyrosine aminotransferase	6898	0,209431118	-1,075914009	4,080456	-0,263674953	0,792030398	NA
ENSG00000198658	NA	NA	NA	0,223464851	0	4,080456	0	1	NA
ENSG00000198670	LPA	lipoprotein(a)	4018	0	NA	NA	NA	NA	NA
ENSG00000198673	TAFA2	TAFA chemokine like family member 2	338811	0	NA	NA	NA	NA	NA
ENSG00000198674	OR10G6	olfactory receptor family 10 subfamily G member 6	79490	0	NA	NA	NA	NA	NA
ENSG00000198678	OR5BS1P	olfactory receptor family 5 subfamily BS member 1 pseudogene	390313	0	NA	NA	NA	NA	NA
ENSG00000198680	TUSC1	tumor suppressor candidate 1	286319	0,10488184	-1,076443944	4,080456	-0,263804824	0,791930317	NA
ENSG00000198681	MAGEA1	MAGE family member A1	4100	0	NA	NA	NA	NA	NA
ENSG00000198682	PAPSS2	3'-phosphoadenosine 5'-phosphosulfate synthase 2	9060	0	NA	NA	NA	NA	NA
ENSG00000198690	FAN1	FANCD2 and FANCI associated nuclease 1	22909	10,17548366	-0,441955632	0,803247	-0,550211592	0,582174253	NA
ENSG00000198691	ABCA4	ATP binding cassette subfamily A member 4	24	0	NA	NA	NA	NA	NA
ENSG00000198692	EIF1AY	eukaryotic translation initiation factor 1A Y-linked	9086	0	NA	NA	NA	NA	NA
ENSG00000198703	OR10R3P	olfactory receptor family 10 subfamily R member 3 pseudogene	391110	0	NA	NA	NA	NA	NA
ENSG00000198704	GPX6	glutathione peroxidase 6	257202	0	NA	NA	NA	NA	NA
ENSG00000198722	UNC13B	unc-13 homolog B	10497	0,118286652	0	4,080456	0	1	NA
ENSG00000198723	SAXO5	stabilizer of axonemal microtubules 5	374877	0	NA	NA	NA	NA	NA
ENSG00000198729	PPP1R14C	protein phosphatase 1 regulatory inhibitor subunit 14C	81706	5,830070584	-0,5316854	0,89827	-0,591899561	0,553917848	NA
ENSG00000198732	SMOC1	SPARC related modular calcium binding 1	64093	0,118286652	0	4,080456	0	1	NA
ENSG00000198734	F5	coagulation factor V	2153	0,11808024	-1,076443944	4,080456	-0,263804824	0,791930317	NA
ENSG00000198738	NA	NA	NA	0	NA	NA	NA	NA	NA
ENSG00000198739	LRRTM3	leucine rich repeat transmembrane neuronal 3	347731	0,30838133	2,355083577	3,994868	0,589527207	0,555507667	NA
ENSG00000198744	MTCO3P12	MT-CO3 pseudogene 12	107075270	0,619473758	-3,215607425	3,153004	-1,019855036	0,307797217	NA
ENSG00000198752	CDC42BPB	CDC42 binding protein kinase beta	9578	2,767663967	0,296473287	1,126153	0,263261937	0,792348695	NA
ENSG00000198753	PLXNB3	plexin B3	5365	0,11808024	-1,076443944	4,080456	-0,263804824	0,791930317	NA
ENSG00000198754	OXCT2	3-oxoacid CoA-transferase 2	64064	1,863434657	-1,013938473	1,752538	-0,578554183	0,562890028	NA
ENSG00000198758	EPS8L3	EPS8 like 3	79574	0,310277825	-1,136912283	4,027923	-0,282257712	0,777745909	NA

ENSG00000198765	SYCP1	synaptonemal complex protein 1	6847	0	NA	NA	NA	NA	NA
ENSG00000198768	APCDD1L	APC down-regulated 1 like	164284	0	NA	NA	NA	NA	NA
ENSG00000198774	RASSF9	Ras association domain family member 9	9182	0	NA	NA	NA	NA	NA
ENSG00000198785	GRIN3A	glutamate ionotropic receptor NMDA type subunit 3A	116443	0,177731807	0,845026239	4,080437	0,207092099	0,835937933	NA
ENSG00000198787	OR7E103P	olfactory receptor family 7 subfamily E member 103 pseudogene	282754	0	NA	NA	NA	NA	NA
ENSG00000198788	MUC2	mucin 2, oligomeric mucus/gel-forming	4583	0	NA	NA	NA	NA	NA
ENSG00000198794	SCAMP5	secretory carrier membrane protein 5	192683	0,794331633	-0,15129677	2,420724	-0,062500636	0,950164155	NA
ENSG00000198795	ZNF521	zinc finger protein 521	25925	0	NA	NA	NA	NA	NA
ENSG00000198796	ALPK2	alpha kinase 2	115701	0	NA	NA	NA	NA	NA
ENSG00000198797	BRINP2	BMP/retinoic acid inducible neural specific 2	57795	0	NA	NA	NA	NA	NA
ENSG00000198798	MAGEB3	MAGE family member B3	4114	0	NA	NA	NA	NA	NA
ENSG00000198807	PAX9	paired box 9	5083	0	NA	NA	NA	NA	NA
ENSG00000198812	LRRC10	leucine rich repeat containing 10	376132	0	NA	NA	NA	NA	NA
ENSG00000198822	GRM3	glutamate metabotropic receptor 3	2913	0	NA	NA	NA	NA	NA
ENSG00000198829	SUCNR1	succinate receptor 1	56670	0	NA	NA	NA	NA	NA
ENSG00000198832	SELENOM	selenoprotein M	140606	5,98207738	-0,273417806	0,844155	-0,323895093	0,746017459	NA
ENSG00000198835	GJC2	gap junction protein gamma 2	57165	1,348039582	-0,377486296	1,975892	-0,191046055	0,848489511	NA
ENSG00000198838	RYR3	ryanodine receptor 3	6263	0	NA	NA	NA	NA	NA
ENSG00000198842	STYXL2	serine/threonine/tyrosine interacting like 2	92235	0	NA	NA	NA	NA	NA
ENSG00000198844	ARHGEF15	Rho guanine nucleotide exchange factor 15	22899	0	NA	NA	NA	NA	NA
ENSG00000198848	CES1	carboxylesterase 1	1066	0	NA	NA	NA	NA	NA
ENSG00000198854	KPLCE	KPRP N-terminal and LCE C-terminal like protein	100129271	0	NA	NA	NA	NA	NA
ENSG00000198857	NA	NA	NA	0	NA	NA	NA	NA	NA
ENSG00000198868	MTND4LP30	MT-ND4L pseudogene 30	107075244	0	NA	NA	NA	NA	NA
ENSG00000198881	ASB12	ankyrin repeat and SOCS box containing 12	142689	0,191648357	1,677532973	4,046276	0,414586883	0,678444352	NA
ENSG00000198883	PNMA5	PNMA family member 5	114824	0	NA	NA	NA	NA	NA
ENSG00000198889	DCAF12L1	DDB1 and CUL4 associated factor 12 like 1	139170	0	NA	NA	NA	NA	NA
ENSG00000198892	SHISA4	shisa family member 4	149345	0	NA	NA	NA	NA	NA
ENSG00000198908	GPRASP3	G protein-coupled receptor associated sorting protein family member 3	80823	3,631923727	-1,010100867	1,145349	-0,88191511	0,377822715	NA
ENSG00000198910	L1CAM	L1 cell adhesion molecule	3897	9,124634425	0,582192691	0,716839	0,812166435	0,416696136	NA
ENSG00000198914	POU3F3	POU class 3 homeobox 3	5455	0	NA	NA	NA	NA	NA
ENSG00000198923	NA	NA	NA	0	NA	NA	NA	NA	NA
ENSG00000198929	NOS1AP	nitric oxide synthase 1 adaptor protein	9722	2,03158575	-0,384200217	1,81636	-0,211522061	0,832479919	NA
ENSG00000198930	CSAG1	chondrosarcoma associated gene 1	158511	0	NA	NA	NA	NA	NA
ENSG00000198939	ZFP2	ZFP2 zinc finger protein	80108	0,773813145	0,025222019	2,747347	0,009180501	0,992675123	NA
ENSG00000198944	SOWAHA	sosondowah ankyrin repeat domain family member A	134548	0,311994459	-2,498367527	3,998294	-0,62485841	0,532063991	NA
ENSG00000198947	DMD	dystrophin	1756	0,672281981	-1,456108977	2,754656	-0,528599144	0,597083554	NA
ENSG00000198948	MFAP3L	microfibril associated protein 3 like	9848	0,869789735	0,183975019	2,352293	0,078210912	0,937660282	NA
ENSG00000198948	LOC101928198	uncharacterized LOC101928198	101928198	0,869789735	0,183975019	2,352293	0,078210912	0,937660282	NA
ENSG00000198963	RORB	RAR related orphan receptor B	6096	2,920906821	-0,370049764	1,043531	-0,354613137	0,722879433	NA
ENSG00000198965	OR10R2	olfactory receptor family 10 subfamily R member 2	343406	0	NA	NA	NA	NA	NA
ENSG00000198967	OR10Z1	olfactory receptor family 10 subfamily Z member 1	128368	0	NA	NA	NA	NA	NA
ENSG00000199202	RNA5SP289	RNA, 5S ribosomal pseudogene 289	100873542	0	NA	NA	NA	NA	NA
ENSG00000199240	RNA5SP46	RNA, 5S ribosomal pseudogene 46	100873285	0	NA	NA	NA	NA	NA
ENSG00000199276	RNA5SP26	RNA, 5S ribosomal pseudogene 26	100873358	0	NA	NA	NA	NA	NA
ENSG00000199299	RNA5SP430	RNA, 5S ribosomal pseudogene 430	100873680	0	NA	NA	NA	NA	NA
ENSG00000199315	RNA5SP347	RNA, 5S ribosomal pseudogene 347	100873609	0	NA	NA	NA	NA	NA

ENSG00000199318	RNA5SP52	RNA, 5S ribosomal pseudogene 52	100873290	0	NA	NA	NA	NA	NA
ENSG00000199322	RNA5SP183	RNA, 5S ribosomal pseudogene 183	100873445	0	NA	NA	NA	NA	NA
ENSG00000199350	RNA5SP432	RNA, 5S ribosomal pseudogene 432	100873682	0	NA	NA	NA	NA	NA
ENSG00000199354	RNA5SP273	RNA, 5S ribosomal pseudogene 273	100873526	0	NA	NA	NA	NA	NA
ENSG00000199364	RNA5SP327	RNA, 5S ribosomal pseudogene 327	100873593	0	NA	NA	NA	NA	NA
ENSG00000199373	RNA5SP453	RNA, 5S ribosomal pseudogene 453	100873698	0	NA	NA	NA	NA	NA
ENSG00000199395	RNA5SP93	RNA, 5S ribosomal pseudogene 93	100873326	0	NA	NA	NA	NA	NA
ENSG00000199402	RNA5SP308	RNA, 5S ribosomal pseudogene 308	100873582	0	NA	NA	NA	NA	NA
ENSG00000199407	RNA5SP301	RNA, 5S ribosomal pseudogene 301	100873576	0	NA	NA	NA	NA	NA
ENSG00000199415	NA	NA	NA	0	NA	NA	NA	NA	NA
ENSG00000199450	RNA5SP276	RNA, 5S ribosomal pseudogene 276	100873529	0	NA	NA	NA	NA	NA
ENSG00000199454	RNA5SP388	RNA, 5S ribosomal pseudogene 388	100873642	0	NA	NA	NA	NA	NA
ENSG00000199455	RNA5SP191	RNA, 5S ribosomal pseudogene 191	100873453	0	NA	NA	NA	NA	NA
ENSG00000199480	NA	NA	NA	0	NA	NA	NA	NA	NA
ENSG00000199508	RNA5SP110	RNA, 5S ribosomal pseudogene 110	100873380	0	NA	NA	NA	NA	NA
ENSG00000199509	RNA5SP477	RNA, 5S ribosomal pseudogene 477	100873719	0	NA	NA	NA	NA	NA
ENSG00000199523	NA	NA	NA	0	NA	NA	NA	NA	NA
ENSG00000199525	RNA5SP295	RNA, 5S ribosomal pseudogene 295	100873548	0	NA	NA	NA	NA	NA
ENSG00000199535	RNA5SP305	RNA, 5S ribosomal pseudogene 305	100873579	0	NA	NA	NA	NA	NA
ENSG00000199545	RNA5SP195	RNA, 5S ribosomal pseudogene 195	100873457	0	NA	NA	NA	NA	NA
ENSG00000199556	RNA5SP361	RNA, 5S ribosomal pseudogene 361	100873620	0	NA	NA	NA	NA	NA
ENSG00000199564	RNA5SP502	RNA, 5S ribosomal pseudogene 502	100873553	0	NA	NA	NA	NA	NA
ENSG00000199572	RNA5SP174	RNA, 5S ribosomal pseudogene 174	106478998	0	NA	NA	NA	NA	NA
ENSG00000199585	RNA5SP119	RNA, 5S ribosomal pseudogene 119	100873389	0	NA	NA	NA	NA	NA
ENSG00000199592	RNA5SP321	RNA, 5S ribosomal pseudogene 321	100873587	0	NA	NA	NA	NA	NA
ENSG00000199609	RNA5SP124	RNA, 5S ribosomal pseudogene 124	100873394	0	NA	NA	NA	NA	NA
ENSG00000199620	RNA5SP258	RNA, 5S ribosomal pseudogene 258	100873512	0	NA	NA	NA	NA	NA
ENSG00000199638	RNA5SP319	RNA, 5S ribosomal pseudogene 319	100873585	0	NA	NA	NA	NA	NA
ENSG00000199733	RNA5SP307	RNA, 5S ribosomal pseudogene 307	100873581	0	NA	NA	NA	NA	NA
ENSG00000199771	RNA5SP426	RNA, 5S ribosomal pseudogene 426	100873677	0	NA	NA	NA	NA	NA
ENSG00000199786	NA	NA	NA	0	NA	NA	NA	NA	NA
ENSG00000199806	RNA5SP491	RNA, 5S ribosomal pseudogene 491	100873733	0	NA	NA	NA	NA	NA
ENSG00000199809	RNA5SP374	RNA, 5S ribosomal pseudogene 374	100873632	0	NA	NA	NA	NA	NA
ENSG00000199843	RNA5SP358	RNA, 5S ribosomal pseudogene 358	100873617	0	NA	NA	NA	NA	NA
ENSG00000199845	RNA5SP375	RNA, 5S ribosomal pseudogene 375	100873633	0	NA	NA	NA	NA	NA
ENSG00000199874	RNA5SP452	RNA, 5S ribosomal pseudogene 452	100873697	0	NA	NA	NA	NA	NA
ENSG00000199929	RNA5SP405	RNA, 5S ribosomal pseudogene 405	100873657	0	NA	NA	NA	NA	NA
ENSG00000199985	RNA5SP264	RNA, 5S ribosomal pseudogene 264	100873517	0	NA	NA	NA	NA	NA
ENSG00000199994	RNA5SP145	RNA, 5S ribosomal pseudogene 145	100873412	0	NA	NA	NA	NA	NA
ENSG00000200021	RNA5SP448	RNA, 5S ribosomal pseudogene 448	106479015	0	NA	NA	NA	NA	NA
ENSG00000200028	RNA5SP98	RNA, 5S ribosomal pseudogene 98	100873331	0	NA	NA	NA	NA	NA
ENSG00000200036	RNA5SP65	RNA, 5S ribosomal pseudogene 65	100873300	0	NA	NA	NA	NA	NA

ENSG00000200058	RNA5SP218	RNA, 5S ribosomal pseudogene 218	100873478	0	NA	NA	NA	NA	NA
ENSG00000200079	RNA5SP326	RNA, 5S ribosomal pseudogene 326	100873592	0	NA	NA	NA	NA	NA
ENSG00000200114	RNA5SP123	RNA, 5S ribosomal pseudogene 123	100873393	0	NA	NA	NA	NA	NA
ENSG00000200168	RNA5SP350	RNA, 5S ribosomal pseudogene 350	106481794	0	NA	NA	NA	NA	NA
ENSG00000200227	RNA5SP197	RNA, 5S ribosomal pseudogene 197	100873459	0	NA	NA	NA	NA	NA
ENSG00000200238	RNA5SP133	RNA, 5S ribosomal pseudogene 133	100873402	0	NA	NA	NA	NA	NA
ENSG00000200246	RNA5SP263	RNA, 5S ribosomal pseudogene 263	106479002	0	NA	NA	NA	NA	NA
ENSG00000200248	RNA5SP214	RNA, 5S ribosomal pseudogene 214	100873474	0	NA	NA	NA	NA	NA
ENSG00000200275	RNA5SP199	RNA, 5S ribosomal pseudogene 199	106480438	0	NA	NA	NA	NA	NA
ENSG00000200278	RNA5SP352	RNA, 5S ribosomal pseudogene 352	100873612	0	NA	NA	NA	NA	NA
ENSG00000200293	RNA5SP393	RNA, 5S ribosomal pseudogene 393	100873646	0	NA	NA	NA	NA	NA
ENSG00000200301	RNA5SP483	RNA, 5S ribosomal pseudogene 483	100873725	0	NA	NA	NA	NA	NA
ENSG00000200310	RNA5SP215	RNA, 5S ribosomal pseudogene 215	100873475	0	NA	NA	NA	NA	NA
ENSG00000200313	RNA5-8SP7	RNA, 5,8S ribosomal pseudogene 7	100873573	0	NA	NA	NA	NA	NA
ENSG00000200326	RNA5SP391	RNA, 5S ribosomal pseudogene 391	100873644	0	NA	NA	NA	NA	NA
ENSG00000200327	RNA5SP62	RNA, 5S ribosomal pseudogene 62	100873297	0	NA	NA	NA	NA	NA
ENSG00000200408	RNA5SP74	RNA, 5S ribosomal pseudogene 74	100873308	0	NA	NA	NA	NA	NA
ENSG00000200411	RNA5SP346	RNA, 5S ribosomal pseudogene 346	100873608	0	NA	NA	NA	NA	NA
ENSG00000200434	RNA5-8SP2	RNA, 5,8S ribosomal pseudogene 2	100873571	0	NA	NA	NA	NA	NA
ENSG00000200434	LOC124907486	5,8S ribosomal RNA	124907486	0	NA	NA	NA	NA	NA
ENSG00000200434	LOC124908528	5,8S ribosomal RNA	124908528	0	NA	NA	NA	NA	NA
ENSG00000200468	RNA5SP403	RNA, 5S ribosomal pseudogene 403	106479012	0	NA	NA	NA	NA	NA
ENSG00000200473	RNA5SP507	RNA, 5S ribosomal pseudogene 507	100873558	0	NA	NA	NA	NA	NA
ENSG00000200487	RNA5SP322	RNA, 5S ribosomal pseudogene 322	100873588	0	NA	NA	NA	NA	NA
ENSG00000200516	RNA5SP378	RNA, 5S ribosomal pseudogene 378	100873634	0	NA	NA	NA	NA	NA
ENSG00000200527	RNA5SP457	RNA, 5S ribosomal pseudogene 457	100873702	0	NA	NA	NA	NA	NA
ENSG00000200558	RNA5SP429	RNA, 5S ribosomal pseudogene 429	106480767	0	NA	NA	NA	NA	NA
ENSG00000200587	RNA5SP514	RNA, 5S ribosomal pseudogene 514	100873565	0	NA	NA	NA	NA	NA
ENSG00000200601	RNA5SP50	RNA, 5S ribosomal pseudogene 50	106478992	0	NA	NA	NA	NA	NA
ENSG00000200613	RNA5SP349	RNA, 5S ribosomal pseudogene 349	100873611	0	NA	NA	NA	NA	NA
ENSG00000200619	RNA5SP447	RNA, 5S ribosomal pseudogene 447	100873693	0	NA	NA	NA	NA	NA
ENSG00000200626	RNA5SP325	RNA, 5S ribosomal pseudogene 325	100873591	0	NA	NA	NA	NA	NA
ENSG00000200650	RNA5SP114	RNA, 5S ribosomal pseudogene 114	100873384	0	NA	NA	NA	NA	NA
ENSG00000200687	RNA5SP335	RNA, 5S ribosomal pseudogene 335	106479007	0	NA	NA	NA	NA	NA
ENSG00000200709	RNA5SP27	RNA, 5S ribosomal pseudogene 27	100873359	0	NA	NA	NA	NA	NA
ENSG00000200711	RNA5SP28	RNA, 5S ribosomal pseudogene 28	100873360	0	NA	NA	NA	NA	NA
ENSG00000200719	RNA5SP260	RNA, 5S ribosomal pseudogene 260	100873514	0	NA	NA	NA	NA	NA
ENSG00000200738	RNA5SP472	RNA, 5S ribosomal pseudogene 472	100873715	0	NA	NA	NA	NA	NA
ENSG00000200741	NA	NA	NA	0	NA	NA	NA	NA	NA
ENSG00000200755	RNA5SP68	RNA, 5S ribosomal pseudogene 68	100873303	0	NA	NA	NA	NA	NA

ENSG00000200786	RNA5SP234	RNA, 5S ribosomal pseudogene 234	100873492	0	NA	NA	NA	NA	NA
ENSG00000200806	RNA5SP275	RNA, 5S ribosomal pseudogene 275	100873528	0	NA	NA	NA	NA	NA
ENSG00000200839	RNA5SP48	RNA, 5S ribosomal pseudogene 48	100873287	0	NA	NA	NA	NA	NA
ENSG00000200852	RNA5SP499	RNA, 5S ribosomal pseudogene 499	100873550	0	NA	NA	NA	NA	NA
ENSG00000200872	RNA5SP456	RNA, 5S ribosomal pseudogene 456	100873701	0	NA	NA	NA	NA	NA
ENSG00000200873	RNA5SP399	RNA, 5S ribosomal pseudogene 399	100873652	0	NA	NA	NA	NA	NA
ENSG00000200890	NA	NA	NA	0	NA	NA	NA	NA	NA
ENSG00000200914	RNA5SP435	RNA, 5S ribosomal pseudogene 435	106479013	0	NA	NA	NA	NA	NA
ENSG00000200985	RNA5SP493	RNA, 5S ribosomal pseudogene 493	100873735	0	NA	NA	NA	NA	NA
ENSG00000201000	RNA5SP516	RNA, 5S ribosomal pseudogene 516	100873567	0	NA	NA	NA	NA	NA
ENSG00000201014	RNA5SP232	RNA, 5S ribosomal pseudogene 232	100873490	0	NA	NA	NA	NA	NA
ENSG00000201035	RNA5SP469	RNA, 5S ribosomal pseudogene 469	100873712	0	NA	NA	NA	NA	NA
ENSG00000201041	RNA5SP242	RNA, 5S ribosomal pseudogene 242	100873500	0	NA	NA	NA	NA	NA
ENSG00000201059	RNA5SP336	RNA, 5S ribosomal pseudogene 336	100873599	0	NA	NA	NA	NA	NA
ENSG00000201086	RNA5SP394	RNA, 5S ribosomal pseudogene 394	100873647	0	NA	NA	NA	NA	NA
ENSG00000201096	RNA5SP387	RNA, 5S ribosomal pseudogene 387	107133508	0	NA	NA	NA	NA	NA
ENSG00000201109	RNA5SP245	RNA, 5S ribosomal pseudogene 245	100873501	0	NA	NA	NA	NA	NA
ENSG00000201148	RNA5SP42	RNA, 5S ribosomal pseudogene 42	100873281	0	NA	NA	NA	NA	NA
ENSG00000201168	RNA5SP368	RNA, 5S ribosomal pseudogene 368	100873627	0	NA	NA	NA	NA	NA
ENSG00000201185	RNA5SP202	RNA, 5S ribosomal pseudogene 202	100873463	0	NA	NA	NA	NA	NA
ENSG00000201210	RNA5SP139	RNA, 5S ribosomal pseudogene 139	100873407	0	NA	NA	NA	NA	NA
ENSG00000201274	RNA5SP192	RNA, 5S ribosomal pseudogene 192	100873454	0	NA	NA	NA	NA	NA
ENSG00000201301	RNA5SP130	RNA, 5S ribosomal pseudogene 130	106478995	0	NA	NA	NA	NA	NA
ENSG00000201312	RNA5SP72	RNA, 5S ribosomal pseudogene 72	100873306	0	NA	NA	NA	NA	NA
ENSG00000201325	RNA5SP142	RNA, 5S ribosomal pseudogene 142	100873409	0	NA	NA	NA	NA	NA
ENSG00000201347	RNA5SP69	RNA, 5S ribosomal pseudogene 69	100873304	0	NA	NA	NA	NA	NA
ENSG00000201356	RNA5SP500	RNA, 5S ribosomal pseudogene 500	100873551	0	NA	NA	NA	NA	NA
ENSG00000201361	RNA5SP433	RNA, 5S ribosomal pseudogene 433	100873683	0	NA	NA	NA	NA	NA
ENSG00000201394	RNA5SP140	RNA, 5S ribosomal pseudogene 140	106478996	0	NA	NA	NA	NA	NA
ENSG00000201413	RNA5SP141	RNA, 5S ribosomal pseudogene 141	100873408	0	NA	NA	NA	NA	NA
ENSG00000201415	RNA5SP204	RNA, 5S ribosomal pseudogene 204	100873465	0	NA	NA	NA	NA	NA
ENSG00000201420	RNA5SP512	RNA, 5S ribosomal pseudogene 512	100873563	0	NA	NA	NA	NA	NA
ENSG00000201440	RNA5SP515	RNA, 5S ribosomal pseudogene 515	100873566	0	NA	NA	NA	NA	NA
ENSG00000201447	RNA5SP509	RNA, 5S ribosomal pseudogene 509	100873560	0	NA	NA	NA	NA	NA
ENSG00000201469	RNA5SP379	RNA, 5S ribosomal pseudogene 379	100873635	0	NA	NA	NA	NA	NA
ENSG00000201476	RNA5SP353	RNA, 5S ribosomal pseudogene 353	100873613	0	NA	NA	NA	NA	NA
ENSG00000201492	RNA5SP78	RNA, 5S ribosomal pseudogene 78	100873312	0	NA	NA	NA	NA	NA
ENSG00000201518	RNA5SP513	RNA, 5S ribosomal pseudogene 513	100873564	0	NA	NA	NA	NA	NA
ENSG00000201523	RNA5SP205	RNA, 5S ribosomal pseudogene 205	100873466	0	NA	NA	NA	NA	NA
ENSG00000201527	RNA5SP478	RNA, 5S ribosomal pseudogene 478	100873720	0	NA	NA	NA	NA	NA

ENSG00000201567	RNA5SP510	RNA, 5S ribosomal pseudogene 510	100873561	0	NA	NA	NA	NA	NA
ENSG00000201594	RNA5SP517	RNA, 5S ribosomal pseudogene 517	100873568	0	NA	NA	NA	NA	NA
ENSG00000201595	RNA5SP132	RNA, 5S ribosomal pseudogene 132	100873401	0	NA	NA	NA	NA	NA
ENSG00000201610	RNA5SP84	RNA, 5S ribosomal pseudogene 84	100873317	0	NA	NA	NA	NA	NA
ENSG00000201618	RNA5SP505	RNA, 5S ribosomal pseudogene 505	100873556	0	NA	NA	NA	NA	NA
ENSG00000201620	RNA5SP51	RNA, 5S ribosomal pseudogene 51	100873289	0	NA	NA	NA	NA	NA
ENSG00000201671	RNA5SP90	RNA, 5S ribosomal pseudogene 90	100873323	0	NA	NA	NA	NA	NA
ENSG00000201704	RNA5SP396	RNA, 5S ribosomal pseudogene 396	100873649	0	NA	NA	NA	NA	NA
ENSG00000201708	RNA5SP359	RNA, 5S ribosomal pseudogene 359	100873618	0	NA	NA	NA	NA	NA
ENSG00000201713	RNA5SP125	RNA, 5S ribosomal pseudogene 125	100873395	0	NA	NA	NA	NA	NA
ENSG00000201727	RNA5SP173	RNA, 5S ribosomal pseudogene 173	100873437	0	NA	NA	NA	NA	NA
ENSG00000201728	RNA5SP479	RNA, 5S ribosomal pseudogene 479	100873721	0	NA	NA	NA	NA	NA
ENSG00000201736	RNA5SP160	RNA, 5S ribosomal pseudogene 160	100873426	0	NA	NA	NA	NA	NA
ENSG00000201763	RNA5SP267	RNA, 5S ribosomal pseudogene 267	100873520	0	NA	NA	NA	NA	NA
ENSG00000201766	RNA5SP302	RNA, 5S ribosomal pseudogene 302	106479004	0	NA	NA	NA	NA	NA
ENSG00000201790	RNA5SP189	RNA, 5S ribosomal pseudogene 189	100873451	0	NA	NA	NA	NA	NA
ENSG00000201812	RNA5SP488	RNA, 5S ribosomal pseudogene 488	100873730	0	NA	NA	NA	NA	NA
ENSG00000201822	RNA5SP149	RNA, 5S ribosomal pseudogene 149	106480437	0	NA	NA	NA	NA	NA
ENSG00000201846	RNA5SP166	RNA, 5S ribosomal pseudogene 166	100873431	0	NA	NA	NA	NA	NA
ENSG00000201856	RNA5SP331	RNA, 5S ribosomal pseudogene 331	100873597	0	NA	NA	NA	NA	NA
ENSG00000201861	RNA5SP298	RNA, 5S ribosomal pseudogene 298	106480761	0	NA	NA	NA	NA	NA
ENSG00000201876	RNA5SP97	RNA, 5S ribosomal pseudogene 97	100873330	0	NA	NA	NA	NA	NA
ENSG00000201920	RNA5SP442	RNA, 5S ribosomal pseudogene 442	106480768	0	NA	NA	NA	NA	NA
ENSG00000201923	RNA5SP485	RNA, 5S ribosomal pseudogene 485	100873727	0	NA	NA	NA	NA	NA
ENSG00000201939	RNA5SP224	RNA, 5S ribosomal pseudogene 224	100873483	0	NA	NA	NA	NA	NA
ENSG00000201942	RNA5SP363	RNA, 5S ribosomal pseudogene 363	100873622	0	NA	NA	NA	NA	NA
ENSG00000201962	RNA5SP126	RNA, 5S ribosomal pseudogene 126	100873396	0	NA	NA	NA	NA	NA
ENSG00000201966	RNA5-8SP4	RNA, 5,8S ribosomal pseudogene 4	100873572	0	NA	NA	NA	NA	NA
ENSG00000201968	RNA5SP138	RNA, 5S ribosomal pseudogene 138	100873406	0	NA	NA	NA	NA	NA
ENSG00000201999	RNA5SP428	RNA, 5S ribosomal pseudogene 428	100873679	0	NA	NA	NA	NA	NA
ENSG00000202044	RNA5SP223	RNA, 5S ribosomal pseudogene 223	100873482	0	NA	NA	NA	NA	NA
ENSG00000202047	RNA5SP324	RNA, 5S ribosomal pseudogene 324	100873590	0	NA	NA	NA	NA	NA
ENSG00000202054	RNA5SP152	RNA, 5S ribosomal pseudogene 152	100873418	0	NA	NA	NA	NA	NA
ENSG00000202056	RNA5SP19	RNA, 5S ribosomal pseudogene 19	100873274	0	NA	NA	NA	NA	NA
ENSG00000202060	RNA5SP455	RNA, 5S ribosomal pseudogene 455	100873700	0	NA	NA	NA	NA	NA
ENSG00000202063	RNA5SP79	RNA, 5S ribosomal pseudogene 79	100873313	0	NA	NA	NA	NA	NA
ENSG00000202092	RNA5SP190	RNA, 5S ribosomal pseudogene 190	100873452	0	NA	NA	NA	NA	NA
ENSG00000202147	RNA5SP329	RNA, 5S ribosomal pseudogene 329	100873595	0	NA	NA	NA	NA	NA
ENSG00000202164	RNA5SP117	RNA, 5S ribosomal pseudogene 117	100873387	0	NA	NA	NA	NA	NA
ENSG00000202174	RNA5-8SP5	RNA, 5,8S ribosomal pseudogene 5	100873335	0	NA	NA	NA	NA	NA

ENSG00000202175	RNA5SP128	RNA, 5S ribosomal pseudogene 128	100873398	0	NA	NA	NA	NA	NA
ENSG00000202187	NA	NA	NA	0	NA	NA	NA	NA	NA
ENSG00000202193	RNA5SP427	RNA, 5S ribosomal pseudogene 427	100873678	0	NA	NA	NA	NA	NA
ENSG00000202225	RNA5SP240	RNA, 5S ribosomal pseudogene 240	100873498	0	NA	NA	NA	NA	NA
ENSG00000202263	RNA5SP22	RNA, 5S ribosomal pseudogene 22	100873277	0	NA	NA	NA	NA	NA
ENSG00000202264	RNA5SP77	RNA, 5S ribosomal pseudogene 77	100873311	0	NA	NA	NA	NA	NA
ENSG00000202281	RNA5SP235	RNA, 5S ribosomal pseudogene 235	100873493	0	NA	NA	NA	NA	NA
ENSG00000202290	RNA5SP37	RNA, 5S ribosomal pseudogene 37	100873369	0	NA	NA	NA	NA	NA
ENSG00000202322	RNA5SP131	RNA, 5S ribosomal pseudogene 131	100873400	0	NA	NA	NA	NA	NA
ENSG00000202324	RNA5SP366	RNA, 5S ribosomal pseudogene 366	100873625	0	NA	NA	NA	NA	NA
ENSG00000202331	RNA5SP167	RNA, 5S ribosomal pseudogene 167	100873432	0	NA	NA	NA	NA	NA
ENSG00000202334	RNA5SP238	RNA, 5S ribosomal pseudogene 238	100873496	0	NA	NA	NA	NA	NA
ENSG00000202356	RNA5SP277	RNA, 5S ribosomal pseudogene 277	100873530	0	NA	NA	NA	NA	NA
ENSG00000202383	RNA5SP279	RNA, 5S ribosomal pseudogene 279	100873532	0	NA	NA	NA	NA	NA
ENSG00000202386	RNA5SP211	RNA, 5S ribosomal pseudogene 211	100873471	0	NA	NA	NA	NA	NA
ENSG00000202411	RNA5SP259	RNA, 5S ribosomal pseudogene 259	100873513	0	NA	NA	NA	NA	NA
ENSG00000202422	RNA5SP30	RNA, 5S ribosomal pseudogene 30	100873362	0	NA	NA	NA	NA	NA
ENSG00000202430	RNA5SP88	RNA, 5S ribosomal pseudogene 88	100873321	0	NA	NA	NA	NA	NA
ENSG00000202472	RNA5SP248	RNA, 5S ribosomal pseudogene 248	100873504	0	NA	NA	NA	NA	NA
ENSG00000202474	RNA5SP283	RNA, 5S ribosomal pseudogene 283	100873536	0,072165449	0,847101401	4,080456	0,207599697	0,835541541	NA
ENSG00000202502	RNA5SP151	RNA, 5S ribosomal pseudogene 151	100873417	0	NA	NA	NA	NA	NA
ENSG00000203262	NA	NA	NA	0	NA	NA	NA	NA	NA
ENSG00000203363	NA	NA	NA	0	NA	NA	NA	NA	NA
ENSG00000203387	NA	NA	NA	0	NA	NA	NA	NA	NA
ENSG00000203396	LOC100128906	WDR45-like pseudogene	100128906	0,276137183	0,843902929	4,080456	0,206815845	0,836153681	NA
ENSG00000203397	NA	NA	NA	0	NA	NA	NA	NA	NA
ENSG00000203408	OR6C71P	olfactory receptor family 6 subfamily C member 71 pseudogene	390324	0	NA	NA	NA	NA	NA
ENSG00000203413	NA	NA	NA	0	NA	NA	NA	NA	NA
ENSG00000203414	NA	NA	NA	2,57662447	0,345022534	1,265484	0,272640791	0,785129352	NA
ENSG00000203416	NA	NA	NA	0	NA	NA	NA	NA	NA
ENSG00000203435	NA	NA	NA	0,207996306	-1,916073151	4,043021	-0,473921178	0,635556117	NA
ENSG00000203437	LOC100422622	TAF10 RNA polymerase II, TATA box binding protein (TBP)-associated factor, 30kDa pseudogene	100422622	0	NA	NA	NA	NA	NA
ENSG00000203462	NA	NA	NA	0	NA	NA	NA	NA	NA
ENSG00000203489	HMGB1P39	high mobility group box 1 pseudogene 39	100419972	0	NA	NA	NA	NA	NA
ENSG00000203492	OR2N1P	olfactory receptor family 2 subfamily N member 1 pseudogene	26701	0	NA	NA	NA	NA	NA
ENSG00000203499	IQANK1	IQ motif and ankyrin repeat containing 1	642574	0,339525853	1,934751114	4,023685	0,480840629	0,630629772	NA
ENSG00000203523	TAS2R2P	taste 2 receptor member 2, pseudogene	338396	0	NA	NA	NA	NA	NA
ENSG00000203531	NA	NA	NA	0	NA	NA	NA	NA	NA
ENSG00000203546	NA	NA	NA	0	NA	NA	NA	NA	NA
ENSG00000203581	OR1F2P	olfactory receptor family 1 subfamily F member 2 pseudogene	26184	0,144330898	1,418786819	4,061262	0,349346267	0,726829368	NA
ENSG00000203616	NA	NA	NA	0	NA	NA	NA	NA	NA
ENSG00000203618	GP1BB	glycoprotein Ib platelet subunit beta	2812	0,072165449	0,847101401	4,080456	0,207599697	0,835541541	NA
ENSG00000203647	NA	NA	NA	0	NA	NA	NA	NA	NA
ENSG00000203661	OR2T29	olfactory receptor family 2 subfamily T member 29	343563	0	NA	NA	NA	NA	NA

ENSG00000203661	OR2T5	olfactory receptor family 2 subfamily T member 5	401993	0	NA	NA	NA	NA	NA
ENSG00000203663	OR2L2	olfactory receptor family 2 subfamily L member 2	26246	0,978386486	0,325011502	2,105427	0,154368438	0,877319238	NA
ENSG00000203664	NA	NA	NA	0	NA	NA	NA	NA	NA
ENSG00000203666	EFCAB2	EF-hand calcium binding domain 2	84288	1,443902888	-1,767439592	3,298053	-0,535903933	0,592024946	NA
ENSG00000203685	STUM	stum, mechanosensory transduction mediator homolog	375057	0,610166996	0,431757855	3,184286	0,135590153	0,89214529	NA
ENSG00000203690	NA	NA	NA	0	NA	NA	NA	NA	NA
ENSG00000203697	CAPN8	calpain 8	388743	0,10543123	0	4,080456	0	1	NA
ENSG00000203710	CR1	complement C3b/C4b receptor 1 (Knops blood group)	1378	0	NA	NA	NA	NA	NA
ENSG00000203722	RAET1G	retinoic acid early transcript 1G	353091	0	NA	NA	NA	NA	NA
ENSG00000203724	C1orf53	chromosome 1 open reading frame 53	388722	2,056258886	0,907689276	1,721299	0,527328113	0,597965755	NA
ENSG00000203727	SAMD5	sterile alpha motif domain containing 5	389432	0,10488184	-1,076443944	4,080456	-0,263804824	0,791930317	NA
ENSG00000203730	TEDDM1	transmembrane epididymal protein 1	127670	0	NA	NA	NA	NA	NA
ENSG00000203733	GJE1	gap junction protein epsilon 1	100126572	0	NA	NA	NA	NA	NA
ENSG00000203734	ECT2L	epithelial cell transforming 2 like	345930	0	NA	NA	NA	NA	NA
ENSG00000203737	GPR52	G protein-coupled receptor 52	9293	10,31964568	0,571143897	0,523549	1,090908691	0,275313063	NA
ENSG00000203740	NTMT2	N-terminal Xaa-Pro-Lys N-methyltransferase 2	149281	0	NA	NA	NA	NA	NA
ENSG00000203756	TMEM244	transmembrane protein 244	253582	0	NA	NA	NA	NA	NA
ENSG00000203757	OR6K3	olfactory receptor family 6 subfamily K member 3	391114	0	NA	NA	NA	NA	NA
ENSG00000203758	OR10T1P	olfactory receptor family 10 subfamily T member 1 pseudogene	79514	0	NA	NA	NA	NA	NA
ENSG00000203772	SPRN	shadow of prion protein	503542	0,239347754	1,967469188	4,021404	0,489249276	0,624665227	NA
ENSG00000203778	FAM229B	family with sequence similarity 229 member B	619208	1,524600421	0,896837415	1,783755	0,502780483	0,615118617	NA
ENSG00000203780	FANK1	fibronectin type III and ankyrin repeat domains 1	92565	0	NA	NA	NA	NA	NA
ENSG00000203781	S100A7P1	S100 calcium binding protein A7 pseudogene 1	127481	0	NA	NA	NA	NA	NA
ENSG00000203782	LORICRIN	loricrin cornified envelope precursor protein	4014	0	NA	NA	NA	NA	NA
ENSG00000203783	PRR9	proline rich 9	574414	0,10488184	-1,076443944	4,080456	-0,263804824	0,791930317	NA
ENSG00000203784	LELP1	late cornified envelope like proline rich 1	149018	0	NA	NA	NA	NA	NA
ENSG00000203785	SPRR2E	small proline rich protein 2E	6704	0	NA	NA	NA	NA	NA
ENSG00000203786	KPRP	keratinocyte proline rich protein	448834	0	NA	NA	NA	NA	NA
ENSG00000203795	FAM24A	family with sequence similarity 24 member A	118670	0	NA	NA	NA	NA	NA
ENSG00000203797	DDO	D-aspartate oxidase	8528	0	NA	NA	NA	NA	NA
ENSG00000203799	CCDC162P	coiled-coil domain containing 162, pseudogene	221262	0,343005427	-0,120927057	3,77828	-0,03200585	0,974467386	NA
ENSG00000203805	PLPP4	phospholipid phosphatase 4	196051	0	NA	NA	NA	NA	NA
ENSG00000203811	H3C14	H3 clustered histone 14	126961	0	NA	NA	NA	NA	NA
ENSG00000203814	H2BC18	H2B clustered histone 18	440689	2,650048126	-0,043292248	1,244278	-0,034793072	0,972244745	NA
ENSG00000203825	NA	NA	NA	0	NA	NA	NA	NA	NA
ENSG00000203837	PNLIPRP3	pancreatic lipase related protein 3	119548	0	NA	NA	NA	NA	NA
ENSG00000203852	H3C15	H3 clustered histone 15	333932	0,72263443	1,531420556	2,435109	0,62889206	0,529419724	NA
ENSG00000203855	NA	NA	NA	0	NA	NA	NA	NA	NA
ENSG00000203857	HSD3B1	hydroxy-delta-5-steroid dehydrogenase, 3 beta- and steroid delta-isomerase 1	3283	0	NA	NA	NA	NA	NA
ENSG00000203858	HSD3BP2	hydroxy-delta-5-steroid dehydrogenase, 3 beta, pseudogene 2	440606	0	NA	NA	NA	NA	NA
ENSG00000203859	HSD3B2	hydroxy-delta-5-steroid dehydrogenase, 3 beta- and steroid delta-isomerase 2	3284	0	NA	NA	NA	NA	NA
ENSG00000203867	RBM20	RNA binding motif protein 20	282996	0	NA	NA	NA	NA	NA
ENSG00000203870	SMIM9	small integral membrane protein 9	100132963	0	NA	NA	NA	NA	NA
ENSG00000203872	C6orf163	chromosome 6 open reading frame 163	206412	5,384742073	-0,176876718	0,916965	-0,192893558	0,84704233	NA

ENSG00000203877	RIPPLY2	ripply transcriptional repressor 2	134701	0	NA	NA	NA	NA	NA
ENSG00000203878	NA	NA	NA	0	NA	NA	NA	NA	NA
ENSG00000203883	SOX18	SRY-box transcription factor 18	54345	0,207996306	-1,916073151	4,043021	-0,473921178	0,635556117	NA
ENSG00000203896	LIME1	Lck interacting transmembrane adaptor 1	54923	8,217883737	0,023720439	0,676403	0,035068477	0,972025138	NA
ENSG00000203907	OOEP	oocyte expressed protein	441161	0	NA	NA	NA	NA	NA
ENSG00000203908	KHDC3L	KH domain containing 3 like, subcortical maternal complex member	154288	0	NA	NA	NA	NA	NA
ENSG00000203909	DPPA5	developmental pluripotency associated 5	340168	0	NA	NA	NA	NA	NA
ENSG00000203910	C1orf146	chromosome 1 open reading frame 146	388649	0	NA	NA	NA	NA	NA
ENSG00000203914	HSP90B3P	heat shock protein 90 beta family member 3, pseudogene	343477	0	NA	NA	NA	NA	NA
ENSG00000203923	SPANXN1	SPANX family member N1	494118	0	NA	NA	NA	NA	NA
ENSG00000203926	SPANXA2	SPANX family member A2	728712	0	NA	NA	NA	NA	NA
ENSG00000203933	CXorf66	chromosome X open reading frame 66	347487	0	NA	NA	NA	NA	NA
ENSG00000203942	C10orf62	chromosome 10 open reading frame 62	414157	0	NA	NA	NA	NA	NA
ENSG00000203943	SAMD13	sterile alpha motif domain containing 13	148418	0	NA	NA	NA	NA	NA
ENSG00000203952	CCDC160	coiled-coil domain containing 160	347475	0,540843898	1,935219462	3,288419	0,588495383	0,556199832	NA
ENSG00000203963	C1orf141	chromosome 1 open reading frame 141	400757	0	NA	NA	NA	NA	NA
ENSG00000203970	DEFB110	defensin beta 110	245913	0	NA	NA	NA	NA	NA
ENSG00000203972	GLYATL3	glycine-N-acyltransferase like 3	389396	0	NA	NA	NA	NA	NA
ENSG00000203985	LDLRAD1	low density lipoprotein receptor class A domain containing 1	388633	0	NA	NA	NA	NA	NA
ENSG00000203989	RHOXF2B	Rhox homeobox family member 2B	727940	0,072165449	0,847101401	4,080456	0,207599697	0,835541541	NA
ENSG00000203995	ZYG11A	zyg-11 family member A, cell cycle regulator	440590	0,207996306	-1,916073151	4,043021	-0,473921178	0,635556117	NA
ENSG00000204001	LCN8	lipocalin 8	138307	0	NA	NA	NA	NA	NA
ENSG00000204003	NA	NA	NA	0,236573303	0	4,080456	0	1	NA
ENSG00000204006	C1orf185	chromosome 1 open reading frame 185	284546	0	NA	NA	NA	NA	NA
ENSG00000204007	GLT6D1	glycosyltransferase 6 domain containing 1	360203	0	NA	NA	NA	NA	NA
ENSG00000204010	IFIT1B	interferon induced protein with tetratricopeptide repeats 1B	439996	0	NA	NA	NA	NA	NA
ENSG00000204019	CT83	cancer/testis antigen 83	203413	0	NA	NA	NA	NA	NA
ENSG00000204020	LIPN	lipase family member N	643418	0	NA	NA	NA	NA	NA
ENSG00000204021	LIPK	lipase family member K	643414	0	NA	NA	NA	NA	NA
ENSG00000204022	LIPJ	lipase family member J	142910	0	NA	NA	NA	NA	NA
ENSG00000204025	TRPC5OS	TRPC5 opposite strand	100329135	0	NA	NA	NA	NA	NA
ENSG00000204033	LRIT2	leucine rich repeat, Ig-like and transmembrane domains 2	340745	0	NA	NA	NA	NA	NA
ENSG00000204052	LRRC73	leucine rich repeat containing 73	221424	0	NA	NA	NA	NA	NA
ENSG00000204053	NA	NA	NA	0	NA	NA	NA	NA	NA
ENSG00000204060	FOXO6	forkhead box O6	100132074	0	NA	NA	NA	NA	NA
ENSG00000204065	TCEAL5	transcription elongation factor A like 5	340543	0	NA	NA	NA	NA	NA
ENSG00000204071	TCEAL6	transcription elongation factor A like 6	158931	0	NA	NA	NA	NA	NA
ENSG00000204072	NA	NA	NA	0,478695509	2,980701557	3,96411	0,751922061	0,45209793	NA
ENSG00000204086	RPA4	replication protein A4	29935	1,517272624	-0,767112595	1,936984	-0,396034452	0,692079613	NA
ENSG00000204099	NEU4	neuraminidase 4	129807	0	NA	NA	NA	NA	NA
ENSG00000204103	MAFB	MAF bZIP transcription factor B	9935	0	NA	NA	NA	NA	NA
ENSG00000204113	BMP2KL	BMP2 inducible kinase like (pseudogene)	347359	0	NA	NA	NA	NA	NA
ENSG00000204118	NAP1L6P	nucleosome assembly protein 1 like 6, pseudogene	645996	0	NA	NA	NA	NA	NA
ENSG00000204121	NA	NA	NA	0	NA	NA	NA	NA	NA
ENSG00000204128	C2orf72	chromosome 2 open reading frame 72	257407	0,072165449	0,847101401	4,080456	0,207599697	0,835541541	NA
ENSG00000204140	CLPSL1	colipase like 1	340204	0	NA	NA	NA	NA	NA
ENSG00000204165	CXorf65	chromosome X open reading frame 65	158830	0	NA	NA	NA	NA	NA

ENSG00000204173	NA	NA	NA	0	NA	NA	NA	NA	NA
ENSG00000204174	NPY4R	neuropeptide Y receptor Y4	5540	0	NA	NA	NA	NA	NA
ENSG00000204175	GPRIN2	G protein regulated inducer of neurite outgrowth 2	9721	0	NA	NA	NA	NA	NA
ENSG00000204176	SYT15	synaptotagmin 15	83849	1,85482033	2,205579271	2,270183	0,971542369	0,331278265	NA
ENSG00000204177	NA	NA	NA	7,547428698	-0,78210866	0,702399	-1,113481959	0,265501493	NA
ENSG00000204179	PTPN20	protein tyrosine phosphatase non-receptor type 20	26095	0	NA	NA	NA	NA	NA
ENSG00000204188	GGNBP1	gametogenetin binding protein 1 (pseudogene)	449520	0	NA	NA	NA	NA	NA
ENSG00000204193	TXNDC8	thioredoxin domain containing 8	255220	0	NA	NA	NA	NA	NA
ENSG00000204195	AWAT1	acyl-CoA wax alcohol acyltransferase 1	158833	0	NA	NA	NA	NA	NA
ENSG00000204196	NA	NA	NA	0,366163764	-0,459508619	3,671499	-0,1251556	0,900400367	NA
ENSG00000204220	PFDN6	prefoldin subunit 6	10471	10,38130487	1,247312572	1,172345	1,06394643	0,287352968	NA
ENSG00000204228	HSD17B8	hydroxysteroid 17-beta dehydrogenase 8	7923	7,022225181	-0,892804945	1,005778	-0,887676053	0,374715031	NA
ENSG00000204237	OXLD1	oxidoreductase like domain containing 1	339229	6,347023163	-0,196808374	0,995068	-0,197783746	0,843214264	NA
ENSG00000204246	OR13C3	olfactory receptor family 13 subfamily C member 3	138803	0	NA	NA	NA	NA	NA
ENSG00000204248	COL11A2	collagen type XI alpha 2 chain	1302	0,519990765	-3,232988235	3,962027	-0,815993526	0,414503838	NA
ENSG00000204252	HLA-DOA	major histocompatibility complex, class II, DO alpha	3111	4,3423642	2,243104703	1,126354	1,991474072	0,046428793	NA
ENSG00000204253	NA	NA	NA	0,369206492	-1,064806328	3,71403	-0,286698392	0,774343269	NA
ENSG00000204256	BRD2	bromodomain containing 2	6046	11,47955266	0,243522682	0,678744	0,358784085	0,71975662	NA
ENSG00000204257	HLA-DMA	major histocompatibility complex, class II, DM alpha	3108	0	NA	NA	NA	NA	NA
ENSG00000204262	COL5A2	collagen type V alpha 2 chain	1290	1,044633507	0,971084856	2,047236	0,474339586	0,635257767	NA
ENSG00000204264	PSMB8	proteasome 20S subunit beta 8	5696	2,670121619	5,482836162	3,149705	1,74074581	0,081728145	NA
ENSG00000204278	TMEM235	transmembrane protein 235	283999	0	NA	NA	NA	NA	NA
ENSG00000204279	PAGE3	PAGE family member 3	139793	0	NA	NA	NA	NA	NA
ENSG00000204287	HLA-DRA	major histocompatibility complex, class II, DR alpha	3122	0	NA	NA	NA	NA	NA
ENSG00000204290	BTNL2	butyrophilin like 2	56244	0	NA	NA	NA	NA	NA
ENSG00000204291	COL15A1	collagen type XV alpha 1 chain	1306	3,241848714	-1,309278154	1,005483	-1,302138323	0,192869105	NA
ENSG00000204296	TSBP1	testis expressed basic protein 1	10665	0	NA	NA	NA	NA	NA
ENSG00000204300	TMEM225	transmembrane protein 225	338661	0	NA	NA	NA	NA	NA
ENSG00000204301	NOTCH4	notch receptor 4	4855	0	NA	NA	NA	NA	NA
ENSG00000204304	PBX2	PBX homeobox 2	5089	10,00325821	2,376074691	2,784505	0,853320339	0,393481684	NA
ENSG00000204305	AGER	advanced glycosylation end-product specific receptor	177	1,559142369	-1,423668043	2,130268	-0,668304704	0,503939111	NA
ENSG00000204311	PJVK	pejvakin	494513	0,550763419	1,732488315	4,037106	0,429141168	0,667820494	NA
ENSG00000204314	PRRT1	proline rich transmembrane protein 1	80863	0	NA	NA	NA	NA	NA
ENSG00000204314	LOC100507547	uncharacterized LOC100507547	100507547	0	NA	NA	NA	NA	NA
ENSG00000204315	FKBPL	FKBP prolyl isomerase like	63943	7,509017805	0,167142773	0,761936	0,219365915	0,826365019	NA
ENSG00000204323	SMIM5	small integral membrane protein 5	643008	1,32789714	1,132403298	1,693723	0,668588433	0,503758053	NA
ENSG00000204334	ERICH2	glutamate rich 2	285141	0	NA	NA	NA	NA	NA
ENSG00000204335	SP5	Sp5 transcription factor	389058	0	NA	NA	NA	NA	NA
ENSG00000204338	NA	NA	NA	0	NA	NA	NA	NA	NA
ENSG00000204344	STK19	serine/threonine kinase 19	8859	9,760395664	1,203685351	2,133195	0,564264164	0,572574366	NA
ENSG00000204347	BTBD17	BTB domain containing 17	388419	1,027596109	-0,984547512	1,935417	-0,508700369	0,610962263	NA
ENSG00000204352	NA	NA	NA	0	NA	NA	NA	NA	NA
ENSG00000204361	NXPE2	neurexophilin and PC-esterase domain family member 2	120406	0	NA	NA	NA	NA	NA
ENSG00000204363	SPANXN5	SPANX family member N5	494197	0	NA	NA	NA	NA	NA
ENSG00000204366	ZBTB12	zinc finger and BTB domain containing 12	221527	0	NA	NA	NA	NA	NA
ENSG00000204368	NA	NA	NA	0	NA	NA	NA	NA	NA
ENSG00000204371	EHMT2	euchromatic histone lysine methyltransferase 2	10919	12,10220215	21,06155554	3,909011	5,387949785	NA	NA
ENSG00000204379	XAGE1A	X antigen family member 1A	653220	0	NA	NA	NA	NA	NA
ENSG00000204379	LOC124908558	X antigen family member 1-like	124908558	0	NA	NA	NA	NA	NA
ENSG00000204381	LAYN	layilin	143903	1,011194164	0,311050906	2,18244	0,142524397	0,88666581	NA
ENSG00000204382	XAGE1B	X antigen family member 1B	653067	0	NA	NA	NA	NA	NA
ENSG00000204382	LOC124908558	X antigen family member 1-like	124908558	0	NA	NA	NA	NA	NA
ENSG00000204385	SLC44A4	solute carrier family 44 member 4	80736	0	NA	NA	NA	NA	NA
ENSG00000204386	NEU1	neuraminidase 1	4758	1,536451831	-4,256169116	3,237113	-1,314803951	0,188575799	NA

ENSG00000204388	HSPA1B	heat shock protein family A (Hsp70) member 1B	3304	0	NA	NA	NA	NA	NA
ENSG00000204390	HSPA1L	heat shock protein family A (Hsp70) member 1 like	3305	0	NA	NA	NA	NA	NA
ENSG00000204396	VWA7	von Willebrand factor A domain containing 7	80737	6,52190138	-5,774381614	3,916297	-1,474449481	0,14036061	NA
ENSG00000204399	NA	NA	NA	0	NA	NA	NA	NA	NA
ENSG00000204403	CASP12	caspase 12 (gene/pseudogene)	100506742	0,103998153	-1,076443944	4,080456	-0,263804824	0,791930317	NA
ENSG00000204410	MSH5	mutS homolog 5	4439	0	NA	NA	NA	NA	NA
ENSG00000204414	CSHL1	chorionic somatomammotropin hormone like 1	1444	0	NA	NA	NA	NA	NA
ENSG00000204420	MPIG6B	megakaryocyte and platelet inhibitory receptor G6b	80739	0,119673877	0,847101401	4,080456	0,207599697	0,835541541	NA
ENSG00000204421	LY6G6C	lymphocyte antigen 6 family member G6C	80740	0,103998153	-1,076443944	4,080456	-0,263804824	0,791930317	NA
ENSG00000204424	LY6G6F	lymphocyte antigen 6 family member G6F	259215	0	NA	NA	NA	NA	NA
ENSG00000204428	LY6G5C	lymphocyte antigen 6 family member G5C	80741	2,086986771	-0,710269632	1,484206	-0,478551942	0,632257414	NA
ENSG00000204429	NA	NA	NA	0	NA	NA	NA	NA	NA
ENSG00000204434	NA	NA	NA	0	NA	NA	NA	NA	NA
ENSG00000204437	NA	NA	NA	0	NA	NA	NA	NA	NA
ENSG00000204442	NALF1	NALCN channel auxiliary factor 1	728215	0	NA	NA	NA	NA	NA
ENSG00000204444	APOM	apolipoprotein M	55937	3,304361056	-1,007620764	1,248987	-0,806750235	0,419810396	NA
ENSG00000204449	TRIM49C	tripartite motif containing 49C	642612	0	NA	NA	NA	NA	NA
ENSG00000204450	TRIM64	tripartite motif containing 64	120146	0	NA	NA	NA	NA	NA
ENSG00000204455	TRIM51BP	tripartite motif-containing 51B, pseudogene	399937	0	NA	NA	NA	NA	NA
ENSG00000204456	NA	NA	NA	0	NA	NA	NA	NA	NA
ENSG00000204472	AIF1	allograft inflammatory factor 1	199	0	NA	NA	NA	NA	NA
ENSG00000204478	PRAMEF20	PRAME family member 20	645425	0	NA	NA	NA	NA	NA
ENSG00000204479	PRAMEF17	PRAME family member 17	391004	0	NA	NA	NA	NA	NA
ENSG00000204480	PRAMEF19	PRAME family member 19	645414	0	NA	NA	NA	NA	NA
ENSG00000204481	PRAMEF14	PRAME family member 14	729528	0	NA	NA	NA	NA	NA
ENSG00000204498	NFKBIL1	NFKB inhibitor like 1	4795	0	NA	NA	NA	NA	NA
ENSG00000204501	PRAMEF15	PRAME family member 15	653619	0	NA	NA	NA	NA	NA
ENSG00000204505	PRAMEF9	PRAME family member 9	343070	0	NA	NA	NA	NA	NA
ENSG00000204510	PRAMEF7	PRAME family member 7	441871	0	NA	NA	NA	NA	NA
ENSG00000204511	MCCD1	mitochondrial coiled-coil domain 1	401250	0	NA	NA	NA	NA	NA
ENSG00000204518	AADACL4	arylacetamide deacetylase like 4	343066	0	NA	NA	NA	NA	NA
ENSG00000204520	MICA	MHC class I polypeptide-related sequence A	100507436	4,464024839	-0,294747418	1,143922	-0,257664011	0,796666224	NA
ENSG00000204529	NA	NA	NA	0	NA	NA	NA	NA	NA
ENSG00000204531	POU5F1	POU class 5 homeobox 1	5460	0,10543123	0	4,080456	0	1	NA
ENSG00000204532	ZSCAN5C	zinc finger and SCAN domain containing 5C	649137	0	NA	NA	NA	NA	NA
ENSG00000204538	PSORS1C2	psoriasis susceptibility 1 candidate 2	170680	0	NA	NA	NA	NA	NA
ENSG00000204539	CDSN	corneodesmosin	1041	0	NA	NA	NA	NA	NA
ENSG00000204540	PSORS1C1	psoriasis susceptibility 1 candidate 1	170679	0	NA	NA	NA	NA	NA
ENSG00000204542	C6orf15	chromosome 6 open reading frame 15	29113	0	NA	NA	NA	NA	NA
ENSG00000204544	MUC21	mucin 21, cell surface associated	394263	0	NA	NA	NA	NA	NA
ENSG00000204547	NA	NA	NA	0	NA	NA	NA	NA	NA
ENSG00000204548	DEFB121	defensin beta 121	245934	0	NA	NA	NA	NA	NA
ENSG00000204555	CFTRP3	CFTR pseudogene 3	106481718	0	NA	NA	NA	NA	NA
ENSG00000204556	NA	NA	NA	1,496565289	-0,531603187	1,562114	-0,340310109	0,733623005	NA
ENSG00000204564	C6orf136	chromosome 6 open reading frame 136	221545	3,387755925	1,034468459	1,853543	0,558103176	0,576773932	NA
ENSG00000204569	PPP1R10	protein phosphatase 1 regulatory subunit 10	5514	165,5448363	0,149154566	2,089088	0,071396992	NA	NA
ENSG00000204571	KRTAP5-11	keratin associated protein 5-11	440051	0	NA	NA	NA	NA	NA
ENSG00000204572	KRTAP5-10	keratin associated protein 5-10	387273	0	NA	NA	NA	NA	NA
ENSG00000204576	PRR3	proline rich 3	80742	0	NA	NA	NA	NA	NA
ENSG00000204577	LILRB3	leukocyte immunoglobulin like receptor B3	11025	0,103998153	-1,076443944	4,080456	-0,263804824	0,791930317	NA
ENSG00000204577	LOC102725035	leukocyte immunoglobulin-like receptor subfamily B member 3	102725035	0,103998153	-1,076443944	4,080456	-0,263804824	0,791930317	NA

ENSG00000204577	LOC107987425	leukocyte immunoglobulin-like receptor subfamily B member 3	107987425	0,103998153	-1,076443944	4,080456	-0,263804824	0,791930317	NA
ENSG00000204577	LOC107987462	leukocyte immunoglobulin-like receptor subfamily B member 3	107987462	0,103998153	-1,076443944	4,080456	-0,263804824	0,791930317	NA
ENSG00000204580	DDR1	discoidin domain receptor tyrosine kinase 1	780	2,306249769	0,085212845	2,256616	0,037761342	0,969877967	NA
ENSG00000204583	LRCOL1	leucine rich colipase like 1	100507055	0	NA	NA	NA	NA	NA
ENSG00000204590	GNL1	G protein nucleolar 1 (putative)	2794	0	NA	NA	NA	NA	NA
ENSG00000204595	DPRX	divergent-paired related homeobox	503834	0	NA	NA	NA	NA	NA
ENSG00000204610	TRIM15	tripartite motif containing 15	89870	0	NA	NA	NA	NA	NA
ENSG00000204612	FOXB2	forkhead box B2	442425	0	NA	NA	NA	NA	NA
ENSG00000204613	TRIM10	tripartite motif containing 10	10107	0	NA	NA	NA	NA	NA
ENSG00000204614	TRIM40	tripartite motif containing 40	135644	0	NA	NA	NA	NA	NA
ENSG00000204616	TRIM31	tripartite motif containing 31	11074	0	NA	NA	NA	NA	NA
ENSG00000204618	RNF39	ring finger protein 39	80352	0	NA	NA	NA	NA	NA
ENSG00000204619	PPP1R11	protein phosphatase 1 regulatory inhibitor subunit 11	6992	6,135891026	-6,790067058	3,91148	-1,735933127	NA	NA
ENSG00000204623	POLR1HASP	POLR1H antisense, pseudogene	80862	5,910911767	0,614785149	0,739841	0,830969201	0,405991028	NA
ENSG00000204624	DISP3	dispatched RND transporter family member 3	57540	0,861094706	-0,767356308	2,45423	-0,312666887	0,754533755	NA
ENSG00000204632	HLA-G	major histocompatibility complex, class I, G	3135	0	NA	NA	NA	NA	NA
ENSG00000204634	TBC1D8	TBC1 domain family member 8	11138	1,039404853	1,004307434	1,768493	0,567888743	0,570110516	NA
ENSG00000204637	NA	NA	NA	0	NA	NA	NA	NA	NA
ENSG00000204640	NMS	neuromedin S	129521	0	NA	NA	NA	NA	NA
ENSG00000204644	ZFP57	ZFP57 zinc finger protein	346171	3,685694493	-5,851917573	1,697919	-3,446522625	0,000567851	NA
ENSG00000204648	NA	NA	NA	0	NA	NA	NA	NA	NA
ENSG00000204650	LINC02210	long intergenic non-protein coding RNA 2210	147081	1,552651057	1,262867942	1,930101	0,65430133	0,512917689	NA
ENSG00000204653	ASPDH	aspartate dehydrogenase domain containing	554235	0	NA	NA	NA	NA	NA
ENSG00000204655	MOG	myelin oligodendrocyte glycoprotein	4340	0	NA	NA	NA	NA	NA
ENSG00000204657	OR2H2	olfactory receptor family 2 subfamily H member 2	7932	0	NA	NA	NA	NA	NA
ENSG00000204658	CST9LP2	cystatin 9-like pseudogene 2	106480788	0	NA	NA	NA	NA	NA
ENSG00000204661	C5orf60	chromosome 5 open reading frame 60	285679	0	NA	NA	NA	NA	NA
ENSG00000204662	NA	NA	NA	0	NA	NA	NA	NA	NA
ENSG00000204663	NA	NA	NA	0	NA	NA	NA	NA	NA
ENSG00000204669	C9orf57	chromosome 9 open reading frame 57	138240	0	NA	NA	NA	NA	NA
ENSG00000204671	IL31	interleukin 31	386653	0	NA	NA	NA	NA	NA
ENSG00000204677	FAM153CP	protein FAM153C	653316	0	NA	NA	NA	NA	NA
ENSG00000204681	GABBR1	gamma-aminobutyric acid type B receptor subunit 1	2550	0,62718261	-3,066568091	3,86512	-0,793395269	0,427547566	NA
ENSG00000204687	MAS1L	MAS1 proto-oncogene like, G protein-coupled receptor	116511	0	NA	NA	NA	NA	NA
ENSG00000204688	OR2H1	olfactory receptor family 2 subfamily H member 1	26716	0	NA	NA	NA	NA	NA
ENSG00000204694	OR11A1	olfactory receptor family 11 subfamily A member 1	26531	0	NA	NA	NA	NA	NA
ENSG00000204695	OR14J1	olfactory receptor family 14 subfamily J member 1	442191	0,10488184	-1,076443944	4,080456	-0,263804824	0,791930317	NA
ENSG00000204697	OR2U1P	olfactory receptor family 2 subfamily U member 1 pseudogene	26695	0	NA	NA	NA	NA	NA
ENSG00000204700	OR2J2	olfactory receptor family 2 subfamily J member 2	26707	0	NA	NA	NA	NA	NA
ENSG00000204701	OR2J3	olfactory receptor family 2 subfamily J member 3	442186	0	NA	NA	NA	NA	NA
ENSG00000204702	OR2J1	olfactory receptor family 2 subfamily J member 1	442185	0	NA	NA	NA	NA	NA
ENSG00000204703	OR2B3	olfactory receptor family 2 subfamily B member 3	442184	0	NA	NA	NA	NA	NA
ENSG00000204704	OR2W1	olfactory receptor family 2 subfamily W member 1	26692	0	NA	NA	NA	NA	NA
ENSG00000204710	SPDYC	speedy/RINGO cell cycle regulator family member C	387778	0	NA	NA	NA	NA	NA
ENSG00000204711	CFAP95	cilia and flagella associated protein 95	138255	0	NA	NA	NA	NA	NA
ENSG00000204718	CNN2P12	calponin 2 pseudogene 12	100131550	0	NA	NA	NA	NA	NA

ENSG00000204740	MALRD1	MAM and LDL receptor class A domain containing 1	340895	0	NA	NA	NA	NA	NA
ENSG00000204745	LOC102724642	anaphase-promoting complex subunit 1-like	102724642	3,659879875	0,609341607	1,395077	0,436780022	0,662270889	NA
ENSG00000204764	RANBP17	RAN binding protein 17	64901	0,209763681	-1,926644696	4,042043	-0,476651224	0,633610501	NA
ENSG00000204767	INSYN2B	inhibitory synaptic factor family member 2B	100131897	0	NA	NA	NA	NA	NA
ENSG00000204776	NA	NA	NA	0	NA	NA	NA	NA	NA
ENSG00000204778	ZNG1DP	Zn regulated GTPase metalloprotein activator 1D, pseudogene	653510	0	NA	NA	NA	NA	NA
ENSG00000204779	FOXD4L5	forkhead box D4 like 5	653427	0,322361195	1,907879538	4,026259	0,473859143	0,635600356	NA
ENSG00000204780	NA	NA	NA	0	NA	NA	NA	NA	NA
ENSG00000204787	NA	NA	NA	0	NA	NA	NA	NA	NA
ENSG00000204789	NA	NA	NA	2,497917593	1,854548499	1,316184	1,409034416	0,158824992	NA
ENSG00000204790	LOC728877	COBW domain containing 3 pseudogene	728877	0	NA	NA	NA	NA	NA
ENSG00000204791	NA	NA	NA	0	NA	NA	NA	NA	NA
ENSG00000204801	NA	NA	NA	0	NA	NA	NA	NA	NA
ENSG00000204815	ODAD4	outer dynein arm docking complex subunit 4	83538	0,117497108	0,847101401	4,080456	0,207599697	0,835541541	NA
ENSG00000204816	NA	NA	NA	0,696648178	1,045845021	2,648316	0,3949094	0,692909753	NA
ENSG00000204818	NA	NA	NA	0	NA	NA	NA	NA	NA
ENSG00000204837	NA	NA	NA	0,547985936	0,391319284	2,782183	0,140651909	0,888144938	NA
ENSG00000204839	MROH6	maestro heat like repeat family member 6	642475	4,08797101	-1,017763466	1,1977	-0,849765147	0,39545567	NA
ENSG00000204849	SPATA31A1	SPATA31 subfamily A member 1	647060	0	NA	NA	NA	NA	NA
ENSG00000204851	PNMA8B	PNMA family member 8B	57469	0	NA	NA	NA	NA	NA
ENSG00000204852	TCTN1	tectonic family member 1	79600	2,709811534	-0,035618752	1,674284	-0,021274016	0,983027072	NA
ENSG00000204856	FAM216A	family with sequence similarity 216 member A	29902	9,280505294	0,27980131	1,091458	0,256355619	0,797676254	NA
ENSG00000204866	IGFL2	IGF like family member 2	147920	0	NA	NA	NA	NA	NA
ENSG00000204869	IGFL4	IGF like family member 4	444882	0	NA	NA	NA	NA	NA
ENSG00000204873	KRTAP9-3	keratin associated protein 9-3	83900	0	NA	NA	NA	NA	NA
ENSG00000204887	KRTAP1-4	keratin associated protein 1-4	728255	0	NA	NA	NA	NA	NA
ENSG00000204889	KRT40	keratin 40	125115	0	NA	NA	NA	NA	NA
ENSG00000204894	NA	NA	NA	0	NA	NA	NA	NA	NA
ENSG00000204897	KRT25	keratin 25	147183	0	NA	NA	NA	NA	NA
ENSG00000204909	SPINK9	serine peptidase inhibitor Kazal type 9	643394	0	NA	NA	NA	NA	NA
ENSG00000204913	LRRC3C	leucine rich repeat containing 3C	100505591	0	NA	NA	NA	NA	NA
ENSG00000204915	NA	NA	NA	0	NA	NA	NA	NA	NA
ENSG00000204918	PRR20B	proline rich 20B	729233	0	NA	NA	NA	NA	NA
ENSG00000204928	GRXCR2	glutaredoxin and cysteine rich domain containing 2	643226	0	NA	NA	NA	NA	NA
ENSG00000204930	FAM221B	family with sequence similarity 221 member B	392307	0	NA	NA	NA	NA	NA
ENSG00000204933	NA	NA	NA	0	NA	NA	NA	NA	NA
ENSG00000204936	CD177	CD177 molecule	57126	0	NA	NA	NA	NA	NA
ENSG00000204941	PSG5	pregnancy specific beta-1-glycoprotein 5	5673	0	NA	NA	NA	NA	NA
ENSG00000204947	ZNF425	zinc finger protein 425	155054	9,023345724	-0,377814605	0,611698	-0,617649034	0,536806715	NA
ENSG00000204950	LRRC10B	leucine rich repeat containing 10B	390205	0	NA	NA	NA	NA	NA
ENSG00000204952	FBXO47	F-box protein 47	494188	0,223490528	-1,075643766	4,080456	-0,263608724	0,792081435	NA
ENSG00000204956	PCDHGA1	protocadherin gamma subfamily A, 1	56114	0	NA	NA	NA	NA	NA
ENSG00000204959	NA	NA	NA	0	NA	NA	NA	NA	NA
ENSG00000204978	ERICH4	glutamate rich 4	100170765	0,221406068	-0,121405841	4,080456	-0,029753009	0,976264035	NA
ENSG00000204979	MS4A13	membrane spanning 4-domains A13	503497	0	NA	NA	NA	NA	NA
ENSG00000204982	PRSS3P4	PRSS3 pseudogene 4	106480774	0	NA	NA	NA	NA	NA
ENSG00000204983	PRSS1	serine protease 1	5644	0	NA	NA	NA	NA	NA
ENSG00000204989	OR4D8P	olfactory receptor family 4 subfamily D member 8 pseudogene	401696	0	NA	NA	NA	NA	NA
ENSG00000204991	SPIRE2	spire type actin nucleation factor 2	84501	0,10543123	0	4,080456	0	1	NA
ENSG00000204993	CRTC1P1	CRTC1 pseudogene 1	129656	0	NA	NA	NA	NA	NA
ENSG00000205002	AARD	alanine and arginine rich domain containing protein	441376	0,236369981	-1,074377851	4,080456	-0,263298486	0,792320527	NA
ENSG00000205022	PABPN1L	PABPN1 like, cytoplasmic	390748	0	NA	NA	NA	NA	NA
ENSG00000205029	OR5D16	olfactory receptor family 5 subfamily D member 16	390144	0	NA	NA	NA	NA	NA
ENSG00000205030	OR5L2	olfactory receptor family 5 subfamily L member 2	26338	0	NA	NA	NA	NA	NA

ENSG00000205038	PKHD1L1	PKHD1 like 1	93035	0,33401821	-0,112768315	3,799829	-0,029677211	0,976324487	NA
ENSG00000205044	NA	NA	NA	0	NA	NA	NA	NA	NA
ENSG00000205076	LGALS7	galectin 7	3963	0	NA	NA	NA	NA	NA
ENSG00000205076	LGALS7B	galectin 7B	653499	0	NA	NA	NA	NA	NA
ENSG00000205078	SYCE1L	synaptonemal complex central element protein 1 like	100130958	1,105026405	0,912904374	2,32978	0,391841384	0,695175415	NA
ENSG00000205084	TMEM231	transmembrane protein 231	79583	2,509994325	0,325725754	1,547756	0,210450261	0,833316269	NA
ENSG00000205085	GARIN1A	golgi associated RAB2 interactor 1A	346653	8,446912838	-0,235400507	0,681031	-0,345653095	0,729603438	NA
ENSG00000205089	CCNI2	cyclin I family member 2	645121	0,556598863	0,06467857	2,818953	0,022944182	0,981694798	NA
ENSG00000205090	TMEM240	transmembrane protein 240	339453	0	NA	NA	NA	NA	NA
ENSG00000205097	FRG2	FSHD region gene 2	448831	0	NA	NA	NA	NA	NA
ENSG00000205105	NA	NA	NA	0	NA	NA	NA	NA	NA
ENSG00000205108	SPATA31F1	SPATA31 subfamily F member 1	259308	0	NA	NA	NA	NA	NA
ENSG00000205111	CDKL4	cyclin dependent kinase like 4	344387	2,661176741	0,166854901	1,362811	0,122434401	0,902554996	NA
ENSG00000205116	TMEM88B	transmembrane protein 88B	643965	0	NA	NA	NA	NA	NA
ENSG00000205126	ACCSL	1-aminocyclopropane-1-carboxylate synthase homolog (inactive) like	390110	0	NA	NA	NA	NA	NA
ENSG00000205129	CFAP96	cilia and flagella associated protein 96	441054	0	NA	NA	NA	NA	NA
ENSG00000205133	TRIQK	triple QxxK/R motif containing	286144	4,623568863	2,042003631	1,221611	1,671565999	0,094609934	NA
ENSG00000205143	ARID3C	AT-rich interaction domain 3C	138715	0,312505521	-2,500501748	3,998161	-0,625412948	0,531700067	NA
ENSG00000205176	NA	NA	NA	0	NA	NA	NA	NA	NA
ENSG00000205177	C11orf91	chromosome 11 open reading frame 91	100131378	0,329046182	-0,11525624	3,824271	-0,030138095	0,975956919	NA
ENSG00000205184	SLC10A5P1	SLC10A5 pseudogene 1	100631269	0	NA	NA	NA	NA	NA
ENSG00000205186	FABP9	fatty acid binding protein 9	646480	0	NA	NA	NA	NA	NA
ENSG00000205209	SCGB2B2	secretoglobin family 2B member 2	284402	2,425129171	0,063061819	1,218066	0,051772097	0,958710289	NA
ENSG00000205212	NA	NA	NA	0	NA	NA	NA	NA	NA
ENSG00000205213	LGR4	leucine rich repeat containing G protein-coupled receptor 4	55366	0,539336754	1,451405982	3,379987	0,42941169	0,667623648	NA
ENSG00000205215	NA	NA	NA	0	NA	NA	NA	NA	NA
ENSG00000205221	VIT	vitrin	5212	0	NA	NA	NA	NA	NA
ENSG00000205238	SPDYE2	speedy/RINGO cell cycle regulator family member E2	441273	0	NA	NA	NA	NA	NA
ENSG00000205240	OR7E36P	olfactory receptor family 7 subfamily E member 36 pseudogene	26637	0	NA	NA	NA	NA	NA
ENSG00000205267	DGAT2L7P	diacylglycerol O-acyltransferase 2 like 7, pseudogene	646409	0	NA	NA	NA	NA	NA
ENSG00000205274	TRBV20OR9-2	T cell receptor beta variable 20/OR9-2 (non-functional)	6962	0	NA	NA	NA	NA	NA
ENSG00000205277	MUC12	mucin 12, cell surface associated	10071	0	NA	NA	NA	NA	NA
ENSG00000205279	CTXN3	cortexin 3	613212	0	NA	NA	NA	NA	NA
ENSG00000205301	MGAT4D	MGAT4 family member D	152586	0	NA	NA	NA	NA	NA
ENSG00000205309	NT5M	5',3'-nucleotidase, mitochondrial	56953	4,03971525	0,788361811	1,484968	0,530894785	0,595491692	NA
ENSG00000205312	NA	NA	NA	0	NA	NA	NA	NA	NA
ENSG00000205327	OR6C68	olfactory receptor family 6 subfamily C member 68	403284	0	NA	NA	NA	NA	NA
ENSG00000205328	OR6C65	olfactory receptor family 6 subfamily C member 65	403282	0	NA	NA	NA	NA	NA
ENSG00000205329	OR6C3	olfactory receptor family 6 subfamily C member 3	254786	0	NA	NA	NA	NA	NA
ENSG00000205330	OR6C1	olfactory receptor family 6 subfamily C member 1	390321	0	NA	NA	NA	NA	NA
ENSG00000205331	OR6C72P	olfactory receptor family 6 subfamily C member 72 pseudogene	403288	0	NA	NA	NA	NA	NA
ENSG00000205333	GOLGA2P1	GOLGA2 pseudogene 1	100418751	0	NA	NA	NA	NA	NA
ENSG00000205358	MT1H	metallothionein 1H	4496	0	NA	NA	NA	NA	NA
ENSG00000205359	SLCO6A1	solute carrier organic anion transporter family member 6A1	133482	0	NA	NA	NA	NA	NA
ENSG00000205360	NA	NA	NA	0	NA	NA	NA	NA	NA
ENSG00000205361	NA	NA	NA	0	NA	NA	NA	NA	NA
ENSG00000205362	MT1A	metallothionein 1A	4489	0	NA	NA	NA	NA	NA
ENSG00000205363	INSYN1	inhibitory synaptic factor 1	388135	0	NA	NA	NA	NA	NA
ENSG00000205364	MT1M	metallothionein 1M	4499	0	NA	NA	NA	NA	NA
ENSG00000205403	CFI	complement factor I	3426	0	NA	NA	NA	NA	NA

ENSG00000205409	OR52E6	olfactory receptor family 52 subfamily E member 6	390078	0	NA	NA	NA	NA	NA
ENSG00000205412	NA	NA	NA	0	NA	NA	NA	NA	NA
ENSG00000205420	KRT6A	keratin 6A	3853	0	NA	NA	NA	NA	NA
ENSG00000205433	NA	NA	NA	0	NA	NA	NA	NA	NA
ENSG00000205436	EXOC3L4	exocyst complex component 3 like 4	91828	0	NA	NA	NA	NA	NA
ENSG00000205439	KRTAP12-3	keratin associated protein 12-3	386683	0	NA	NA	NA	NA	NA
ENSG00000205442	IZUMO3	IZUMO family member 3	100129669	0	NA	NA	NA	NA	NA
ENSG00000205445	KRTAP10-2	keratin associated protein 10-2	386679	0	NA	NA	NA	NA	NA
ENSG00000205449	NA	NA	NA	0	NA	NA	NA	NA	NA
ENSG00000205456	TP53TG3D	TP53 target 3D	729264	0	NA	NA	NA	NA	NA
ENSG00000205456	LOC102723655	TP53-target gene 3 protein	102723655	0	NA	NA	NA	NA	NA
ENSG00000205456	LOC102723713	TP53-target gene 3 protein	102723713	0	NA	NA	NA	NA	NA
ENSG00000205457	TP53TG3C	TP53 target 3C	653550	0	NA	NA	NA	NA	NA
ENSG00000205457	LOC102723655	TP53-target gene 3 protein	102723655	0	NA	NA	NA	NA	NA
ENSG00000205457	LOC102723713	TP53-target gene 3 protein	102723713	0	NA	NA	NA	NA	NA
ENSG00000205464	NA	NA	NA	2,99996895	-0,452656334	1,007553	-0,449263149	0,653241838	NA
ENSG00000205482	SPDYE18	speedy/RINGO cell cycle regulator family member E18	100505767	9,197192523	-0,101675836	0,683074	-0,148850501	0,8816716	NA
ENSG00000205496	OR51A2	olfactory receptor family 51 subfamily A member 2	401667	0	NA	NA	NA	NA	NA
ENSG00000205497	OR51A4	olfactory receptor family 51 subfamily A member 4	401666	0	NA	NA	NA	NA	NA
ENSG00000205502	C2CD4B	C2 calcium dependent domain containing 4B	388125	0	NA	NA	NA	NA	NA
ENSG00000205517	RGL3	ral guanine nucleotide dissociation stimulator like 3	57139	0	NA	NA	NA	NA	NA
ENSG00000205572	SERF1A	small EDRK-rich factor 1A	8293	11,49174976	-0,320419622	2,872373	-0,111552227	0,911178453	NA
ENSG00000205572	SERF1B	small EDRK-rich factor 1B	728492	11,49174976	-0,320419622	2,872373	-0,111552227	0,911178453	NA
ENSG00000205578	NA	NA	NA	2,37811119	-0,797330056	1,462655	-0,545125338	0,585667317	NA
ENSG00000205579	NA	NA	NA	0	NA	NA	NA	NA	NA
ENSG00000205583	NA	NA	NA	0	NA	NA	NA	NA	NA
ENSG00000205584	NA	NA	NA	0	NA	NA	NA	NA	NA
ENSG00000205592	MUC19	mucin 19, oligomeric	283463	0,478695509	2,980701557	3,96411	0,751922061	0,45209793	NA
ENSG00000205596	NA	NA	NA	0	NA	NA	NA	NA	NA
ENSG00000205609	EIF3CL	eukaryotic translation initiation factor 3 subunit C like	728689	11,28729828	7,54547312	1,407022	5,362726564	8,20E-08	NA
ENSG00000205625	NA	NA	NA	0	NA	NA	NA	NA	NA
ENSG00000205639	MFSD2B	MFSD2 lysolipid transporter B, sphingolipid	388931	0	NA	NA	NA	NA	NA
ENSG00000205642	VCX3B	variable charge X-linked 3B	425054	0	NA	NA	NA	NA	NA
ENSG00000205644	RPS2P23	ribosomal protein S2 pseudogene 23	100270910	0	NA	NA	NA	NA	NA
ENSG00000205649	HTN3	histatin 3	3347	0	NA	NA	NA	NA	NA
ENSG00000205667	ARSH	arylsulfatase family member H	347527	0	NA	NA	NA	NA	NA
ENSG00000205669	ACOT6	acyl-CoA thioesterase 6	641372	0	NA	NA	NA	NA	NA
ENSG00000205678	TECRL	trans-2,3-enoyl-CoA reductase like	253017	0	NA	NA	NA	NA	NA
ENSG00000205693	MANSC4	MANSC domain containing 4	100287284	0,284243993	-0,997285946	4,040369	-0,246830395	0,805039487	NA
ENSG00000205695	LOC100129554	thyroid hormone receptor interactor 4 pseudogene	100129554	0	NA	NA	NA	NA	NA
ENSG00000205702	NA	NA	NA	0	NA	NA	NA	NA	NA
ENSG00000205704	SMIM45	small integral membrane protein 45	339674	0,072165449	0,847101401	4,080456	0,207599697	0,835541541	NA
ENSG00000205710	C17orf107	chromosome 17 open reading frame 107	100130311	1,846394086	-1,204392914	1,502341	-0,801677535	0,422739514	NA
ENSG00000205716	NA	NA	NA	0,339569492	-1,01476975	3,75402	-0,270315521	0,786917525	NA
ENSG00000205718	MBD3L4	methyl-CpG binding domain protein 3 like 4	653656	0	NA	NA	NA	NA	NA
ENSG00000205726	ITSN1	intersectin 1	6453	7,779442433	0,74175153	0,627254	1,182538241	0,236992201	NA
ENSG00000205754	NA	NA	NA	0	NA	NA	NA	NA	NA
ENSG00000205763	NA	NA	NA	5,497740184	-0,431362216	0,817196	-0,527856729	0,597598779	NA
ENSG00000205767	RPL31P5	ribosomal protein L31 pseudogene 5	100129928	0	NA	NA	NA	NA	NA
ENSG00000205771	NA	NA	NA	0,562121406	-0,124886482	2,916091	-0,04282667	0,965839704	NA
ENSG00000205777	GAGE1	G antigen 1	2543	0	NA	NA	NA	NA	NA
ENSG00000205794	LOC344967	acyl-CoA thioesterase 7 pseudogene	344967	0	NA	NA	NA	NA	NA
ENSG00000205822	NA	NA	NA	0	NA	NA	NA	NA	NA
ENSG00000205832	C16orf96	chromosome 16 open reading frame 96	342346	4,769906178	-0,50111415	0,776178	-0,645617865	0,518526865	NA

ENSG00000205835	GMNC	geminin coiled-coil domain containing	647309	0	NA	NA	NA	NA	NA
ENSG00000205838	TTC23L	tetratricopeptide repeat domain 23 like	153657	0	NA	NA	NA	NA	NA
ENSG00000205846	CLEC6A	C-type lectin domain containing 6A	93978	0	NA	NA	NA	NA	NA
ENSG00000205847	NA	NA	NA	0	NA	NA	NA	NA	NA
ENSG00000205856	C22orf42	chromosome 22 open reading frame 42	150297	0	NA	NA	NA	NA	NA
ENSG00000205857	NANOGNB	NANOG neighbor homeobox	360030	0	NA	NA	NA	NA	NA
ENSG00000205858	LRRC72	leucine rich repeat containing 72	100506049	0	NA	NA	NA	NA	NA
ENSG00000205863	C1QTNF9B	C1q and TNF related 9B	387911	0,117497108	0,847101401	4,080456	0,207599697	0,835541541	NA
ENSG00000205871	RPS3AP47	RPS3A pseudogene 47	146053	2,004310163	-0,821522336	1,292524	-0,635595577	0,525040053	NA
ENSG00000205879	NA	NA	NA	0,117497108	0,847101401	4,080456	0,207599697	0,835541541	NA
ENSG00000205882	DEFB134	defensin beta 134	613211	0	NA	NA	NA	NA	NA
ENSG00000205897	OR7E136P	olfactory receptor family 7 subfamily E member 136 pseudogene	155340	0	NA	NA	NA	NA	NA
ENSG00000205898	LOC100420864	chromosome 11 open reading frame 58 pseudogene	100420864	0	NA	NA	NA	NA	NA
ENSG00000205899	BHLHA9	basic helix-loop-helix family member a9	727857	0	NA	NA	NA	NA	NA
ENSG00000205916	DAZ4	deleted in azoospermia 4	57135	0	NA	NA	NA	NA	NA
ENSG00000205918	PDPK2P	3-phosphoinositide dependent protein kinase 2, pseudogene	653650	2,022149346	3,782466888	3,156181	1,198431427	0,230749104	NA
ENSG00000205922	ONECUT3	one cut homeobox 3	390874	0,117497108	0,847101401	4,080456	0,207599697	0,835541541	NA
ENSG00000205923	CEMP1	cementum protein 1	752014	0,437883123	0	4,080456	0	1	NA
ENSG00000205927	OLIG2	oligodendrocyte transcription factor 2	10215	0	NA	NA	NA	NA	NA
ENSG00000205929	C21orf62	chromosome 21 open reading frame 62	56245	0	NA	NA	NA	NA	NA
ENSG00000205936	PPP1R12BP2	protein phosphatase 1 regulatory subunit 12B pseudogene 2	360020	0	NA	NA	NA	NA	NA
ENSG00000205940	NA	NA	NA	2,430275376	-0,212269917	1,158009	-0,183305911	0,854557997	NA
ENSG00000205944	DAZ2	deleted in azoospermia 2	57055	0	NA	NA	NA	NA	NA
ENSG00000205946	USP17L6P	ubiquitin specific peptidase 17 like family member 6, pseudogene	391622	0	NA	NA	NA	NA	NA
ENSG00000205955	NA	NA	NA	0	NA	NA	NA	NA	NA
ENSG00000205976	NA	NA	NA	0	NA	NA	NA	NA	NA
ENSG00000205978	NYNRIN	NYN domain and retroviral integrase containing	57523	0,11808024	-1,076443944	4,080456	-0,263804824	0,791930317	NA
ENSG00000205989	DEFB109C	defensin beta 109C	100286963	0	NA	NA	NA	NA	NA
ENSG00000206013	IFITM5	interferon induced transmembrane protein 5	387733	0	NA	NA	NA	NA	NA
ENSG00000206014	OR7E161P	olfactory receptor family 7 subfamily E member 161 pseudogene	389626	0	NA	NA	NA	NA	NA
ENSG00000206026	SMIM21	small integral membrane protein 21	284274	0	NA	NA	NA	NA	NA
ENSG00000206042	DEFA7P	defensin alpha 7, pseudogene	724067	0	NA	NA	NA	NA	NA
ENSG00000206043	C18orf63	chromosome 18 open reading frame 63	644041	0	NA	NA	NA	NA	NA
ENSG00000206047	DEFA1	defensin alpha 1	1667	0	NA	NA	NA	NA	NA
ENSG00000206052	DOK6	docking protein 6	220164	0,225167282	-0,078007461	4,079771	-0,019120551	0,984744937	NA
ENSG00000206066	NA	NA	NA	0	NA	NA	NA	NA	NA
ENSG00000206069	LHFPL7	LHFPL tetraspan subfamily member 7	255349	0	NA	NA	NA	NA	NA
ENSG00000206072	SERPINB11	serpin family B member 11	89778	0	NA	NA	NA	NA	NA
ENSG00000206073	SERPINB4	serpin family B member 4	6318	0	NA	NA	NA	NA	NA
ENSG00000206075	SERPINB5	serpin family B member 5	5268	0	NA	NA	NA	NA	NA
ENSG00000206077	ZDHHC11B	zinc finger DHHC-type containing 11B	653082	0	NA	NA	NA	NA	NA
ENSG00000206090	LOC100652871	glutathione S-transferase theta-4-like	100652871	0	NA	NA	NA	NA	NA
ENSG00000206102	KRTAP19-8	keratin associated protein 19-8	728299	0	NA	NA	NA	NA	NA
ENSG00000206104	KRTAP20-3	keratin associated protein 20-3	337985	0	NA	NA	NA	NA	NA
ENSG00000206105	KRTAP20-4	keratin associated protein 20-4	100151643	0	NA	NA	NA	NA	NA
ENSG00000206106	KRTAP22-2	keratin associated protein 22-2	100288287	0	NA	NA	NA	NA	NA
ENSG00000206107	KRTAP27-1	keratin associated protein 27-1	643812	0	NA	NA	NA	NA	NA

ENSG00000206113	CFAP99	cilia and flagella associated protein 99	402160	0	NA	NA	NA	NA	NA
ENSG00000206120	NA	NA	NA	0	NA	NA	NA	NA	NA
ENSG00000206140	TMEM191C	transmembrane protein 191C	645426	0	NA	NA	NA	NA	NA
ENSG00000206144	NA	NA	NA	0	NA	NA	NA	NA	NA
ENSG00000206145	NA	NA	NA	0	NA	NA	NA	NA	NA
ENSG00000206147	RPL23AP57	ribosomal protein L23a pseudogene 57	648294	0	NA	NA	NA	NA	NA
ENSG00000206150	RNASE13	ribonuclease A family member 13 (inactive)	440163	0,119673877	0,847101401	4,080456	0,207599697	0,835541541	NA
ENSG00000206159	NA	NA	NA	0	NA	NA	NA	NA	NA
ENSG00000206168	NA	NA	NA	0,119673877	0,847101401	4,080456	0,207599697	0,835541541	NA
ENSG00000206172	HBA1	hemoglobin subunit alpha 1	3039	0,338901385	-0,122034657	3,788036	-0,032215814	0,974299945	NA
ENSG00000206177	HBM	hemoglobin subunit mu	3042	0	NA	NA	NA	NA	NA
ENSG00000206178	HBZP1	hemoglobin subunit zeta pseudogene 1	3051	0	NA	NA	NA	NA	NA
ENSG00000206181	ELOA2	elongin A2	51224	0	NA	NA	NA	NA	NA
ENSG00000206192	NA	NA	NA	0,565068074	-2,528382899	3,281918	-0,770397902	0,441063897	NA
ENSG00000206199	ANKUB1	ankyrin repeat and ubiquitin domain containing 1	389161	0	NA	NA	NA	NA	NA
ENSG00000206203	TSSK2	testis specific serine kinase 2	23617	0,11808024	-1,076443944	4,080456	-0,263804824	0,791930317	NA
ENSG00000206228	NA	NA	NA	0,103998153	-1,076443944	4,080456	-0,263804824	0,791930317	NA
ENSG00000206260	PRR23A	proline rich 23A	729627	0	NA	NA	NA	NA	NA
ENSG00000206262	FOXL2NB	FOXL2 neighbor	401089	0	NA	NA	NA	NA	NA
ENSG00000206356	LOC100652833	putative POM121-like protein 1-like	100652833	0	NA	NA	NA	NA	NA
ENSG00000206384	COL6A6	collagen type VI alpha 6 chain	131873	0	NA	NA	NA	NA	NA
ENSG00000206422	LRRC30	leucine rich repeat containing 30	339291	0	NA	NA	NA	NA	NA
ENSG00000206432	TMEM200C	transmembrane protein 200C	645369	0	NA	NA	NA	NA	NA
ENSG00000206448	PPIAP30	peptidylprolyl isomerase A pseudogene 30	100192204	0,485895295	1,69634266	3,320618	0,510851556	0,609455005	NA
ENSG00000206474	OR10C1	olfactory receptor family 10 subfamily C member 1	442194	0	NA	NA	NA	NA	NA
ENSG00000206531	CD200R1L	CD200 receptor 1 like	344807	0	NA	NA	NA	NA	NA
ENSG00000206535	LNP1	leukemia NUP98 fusion partner 1	348801	2,027493996	0,239049429	2,029093	0,117810967	0,90621744	NA
ENSG00000206536	OR5K3	olfactory receptor family 5 subfamily K member 3	403277	0	NA	NA	NA	NA	NA
ENSG00000206538	VGLL3	vestigial like family member 3	389136	0	NA	NA	NA	NA	NA
ENSG00000206549	NA	NA	NA	0	NA	NA	NA	NA	NA
ENSG00000206559	ZCWPW2	zinc finger CW-type and PWWP domain containing 2	152098	1,850737362	-0,341465966	2,060098	-0,165752319	0,868351871	NA
ENSG00000206561	COLQ	collagen like tail subunit of asymmetric acetylcholinesterase	8292	6,884302865	-0,371679042	1,230394	-0,302081331	0,762590064	NA
ENSG00000206579	XKR4	XK related 4	114786	0,23616048	-2,075648354	4,028973	-0,515180498	0,606426895	NA
ENSG00000206882	RNA5SP87	RNA, 5S ribosomal pseudogene 87	100873320	0	NA	NA	NA	NA	NA
ENSG00000207129	RNA5SP187	RNA, 5S ribosomal pseudogene 187	100873449	0	NA	NA	NA	NA	NA
ENSG00000207186	RNA5SP122	RNA, 5S ribosomal pseudogene 122	100873392	0	NA	NA	NA	NA	NA
ENSG00000207277	RNA5SP284	RNA, 5S ribosomal pseudogene 284	100873537	0	NA	NA	NA	NA	NA
ENSG00000207341	RNA5SP64	RNA, 5S ribosomal pseudogene 64	100873299	0	NA	NA	NA	NA	NA
ENSG00000211445	GPX3	glutathione peroxidase 3	2878	0	NA	NA	NA	NA	NA
ENSG00000211448	DIO2	iodothyronine deiodinase 2	1734	0,11808024	-1,076443944	4,080456	-0,263804824	0,791930317	NA
ENSG00000211451	GNRHR2	gonadotropin releasing hormone receptor 2 (pseudogene)	114814	1,065654636	0,026391159	1,844063	0,014311421	0,988581528	NA
ENSG00000211452	DIO1	iodothyronine deiodinase 1	1733	0	NA	NA	NA	NA	NA
ENSG00000211454	AKR7L	aldo-keto reductase family 7 like (gene/pseudogene)	246181	0,177050765	-0,114765409	4,080456	-0,028125634	0,977561949	NA
ENSG00000211592	IGKC	immunoglobulin kappa constant	3514	0	NA	NA	NA	NA	NA
ENSG00000211593	IGKJ5	immunoglobulin kappa joining 5	28946	0	NA	NA	NA	NA	NA
ENSG00000211594	IGKJ4	immunoglobulin kappa joining 4	28947	0	NA	NA	NA	NA	NA
ENSG00000211595	IGKJ3	immunoglobulin kappa joining 3	28948	0	NA	NA	NA	NA	NA
ENSG00000211596	IGKJ2	immunoglobulin kappa joining 2	28949	0	NA	NA	NA	NA	NA
ENSG00000211597	IGKJ1	immunoglobulin kappa joining 1	28950	0	NA	NA	NA	NA	NA

ENSG00000211598	IGKV4-1	immunoglobulin kappa variable 4-1	28908	0	NA	NA	NA	NA	NA
ENSG00000211623	IGKV2D-26	immunoglobulin kappa variable 2D-26	28884	0	NA	NA	NA	NA	NA
ENSG00000211625	IGKV3D-20	immunoglobulin kappa variable 3D-20	28874	0	NA	NA	NA	NA	NA
ENSG00000211626	IGKV6D-41	immunoglobulin kappa variable 6D-41 (non-functional)	28869	0	NA	NA	NA	NA	NA
ENSG00000211632	IGKV3D-11	immunoglobulin kappa variable 3D-11	28876	0	NA	NA	NA	NA	NA
ENSG00000211633	IGKV1D-42	immunoglobulin kappa variable 1D-42 (non-functional)	28892	0	NA	NA	NA	NA	NA
ENSG00000211637	IGLV4-69	immunoglobulin lambda variable 4-69	28784	0	NA	NA	NA	NA	NA
ENSG00000211638	IGLV8-61	immunoglobulin lambda variable 8-61	28774	0	NA	NA	NA	NA	NA
ENSG00000211639	IGLV4-60	immunoglobulin lambda variable 4-60	28785	0	NA	NA	NA	NA	NA
ENSG00000211640	IGLV6-57	immunoglobulin lambda variable 6-57	28778	0	NA	NA	NA	NA	NA
ENSG00000211641	IGLV11-55	immunoglobulin lambda variable 11-55 (non-functional)	28770	0	NA	NA	NA	NA	NA
ENSG00000211642	IGLV10-54	immunoglobulin lambda variable 10-54	28772	0	NA	NA	NA	NA	NA
ENSG00000211643	NA	NA	NA	4,543426855	-0,839196274	1,016309	-0,825729849	0,408957356	NA
ENSG00000211644	IGLV1-51	immunoglobulin lambda variable 1-51	28820	0,848499758	-0,1129963	2,769567	-0,040799265	0,967455925	NA
ENSG00000211645	IGLV1-50	immunoglobulin lambda variable 1-50 (non-functional)	28821	0	NA	NA	NA	NA	NA
ENSG00000211647	IGLV5-48	immunoglobulin lambda variable 5-48 (non-functional)	28780	0	NA	NA	NA	NA	NA
ENSG00000211648	IGLV1-47	immunoglobulin lambda variable 1-47	28822	0	NA	NA	NA	NA	NA
ENSG00000211649	IGLV7-46	immunoglobulin lambda variable 7-46	28775	0	NA	NA	NA	NA	NA
ENSG00000211650	IGLV5-45	immunoglobulin lambda variable 5-45	28781	0	NA	NA	NA	NA	NA
ENSG00000211651	IGLV1-44	immunoglobulin lambda variable 1-44	28823	0	NA	NA	NA	NA	NA
ENSG00000211652	IGLV7-43	immunoglobulin lambda variable 7-43	28776	0	NA	NA	NA	NA	NA
ENSG00000211653	IGLV1-40	immunoglobulin lambda variable 1-40	28825	0	NA	NA	NA	NA	NA
ENSG00000211654	IGLV5-37	immunoglobulin lambda variable 5-37	28783	0	NA	NA	NA	NA	NA
ENSG00000211655	IGLV1-36	immunoglobulin lambda variable 1-36	28826	0	NA	NA	NA	NA	NA
ENSG00000211656	IGLV2-33	immunoglobulin lambda variable 2-33 (non-functional)	28811	0	NA	NA	NA	NA	NA
ENSG00000211657	IGLV3-32	immunoglobulin lambda variable 3-32 (non-functional)	28787	0	NA	NA	NA	NA	NA
ENSG00000211658	IGLV3-27	immunoglobulin lambda variable 3-27	28791	0	NA	NA	NA	NA	NA
ENSG00000211659	IGLV3-25	immunoglobulin lambda variable 3-25	28793	0	NA	NA	NA	NA	NA
ENSG00000211660	IGLV2-23	immunoglobulin lambda variable 2-23	28813	0	NA	NA	NA	NA	NA
ENSG00000211661	IGLV3-22	immunoglobulin lambda variable 3-22	28795	0	NA	NA	NA	NA	NA
ENSG00000211662	IGLV3-21	immunoglobulin lambda variable 3-21	28796	0	NA	NA	NA	NA	NA
ENSG00000211663	IGLV3-19	immunoglobulin lambda variable 3-19	28797	0	NA	NA	NA	NA	NA
ENSG00000211664	IGLV2-18	immunoglobulin lambda variable 2-18	28814	0	NA	NA	NA	NA	NA
ENSG00000211665	IGLV3-16	immunoglobulin lambda variable 3-16	28799	0	NA	NA	NA	NA	NA
ENSG00000211666	IGLV2-14	immunoglobulin lambda variable 2-14	28815	0,614246853	-0,126694339	2,877902	-0,044023162	0,964885941	NA
ENSG00000211667	IGLV3-12	immunoglobulin lambda variable 3-12	28802	0	NA	NA	NA	NA	NA
ENSG00000211668	IGLV2-11	immunoglobulin lambda variable 2-11	28816	0	NA	NA	NA	NA	NA

ENSG00000211669	IGLV3-10	immunoglobulin lambda variable 3-10	28803	0	NA	NA	NA	NA	NA
ENSG00000211670	IGLV3-9	immunoglobulin lambda variable 3-9	28804	0,82484125	-3,699709815	2,871403	-1,28846751	0,197583272	NA
ENSG00000211672	IGLV4-3	immunoglobulin lambda variable 4-3	28786	0	NA	NA	NA	NA	NA
ENSG00000211673	NA	NA	NA	0	NA	NA	NA	NA	NA
ENSG00000211674	NA	NA	NA	0	NA	NA	NA	NA	NA
ENSG00000211675	IGLC1	immunoglobulin lambda constant 1	3537	0	NA	NA	NA	NA	NA
ENSG00000211676	NA	NA	NA	0	NA	NA	NA	NA	NA
ENSG00000211677	IGLC2	immunoglobulin lambda constant 2	3538	0	NA	NA	NA	NA	NA
ENSG00000211678	NA	NA	NA	0	NA	NA	NA	NA	NA
ENSG00000211679	IGLC3	immunoglobulin lambda constant 3 (Kern-Oz+ marker)	3539	0	NA	NA	NA	NA	NA
ENSG00000211680	IGLJ4	immunoglobulin lambda joining 4 (non-functional)	28830	0	NA	NA	NA	NA	NA
ENSG00000211681	NA	NA	NA	0	NA	NA	NA	NA	NA
ENSG00000211682	NA	NA	NA	0	NA	NA	NA	NA	NA
ENSG00000211684	IGLJ7	immunoglobulin lambda joining 7	28827	0	NA	NA	NA	NA	NA
ENSG00000211685	IGLC7	immunoglobulin lambda constant 7	28834	0	NA	NA	NA	NA	NA
ENSG00000211687	TRGJ2	T cell receptor gamma joining 2	6969	0	NA	NA	NA	NA	NA
ENSG00000211688	TRGJP2	T cell receptor gamma joining P2	6972	0	NA	NA	NA	NA	NA
ENSG00000211691	TRGJP	T cell receptor gamma joining P	6970	0	NA	NA	NA	NA	NA
ENSG00000211692	TRGJP1	T cell receptor gamma joining P1	6971	0	NA	NA	NA	NA	NA
ENSG00000211693	NA	NA	NA	0,15836288	0	4,080456	0	1	NA
ENSG00000211694	NA	NA	NA	8,684238468	0,3263442	0,597516	0,546167682	0,584950679	NA
ENSG00000211697	TRGV5	T cell receptor gamma variable 5	6978	9,663206786	0,055000351	0,640646	0,085851344	0,93158459	NA
ENSG00000211698	TRGV4	T cell receptor gamma variable 4	6977	4,534099803	-4,268361021	1,403487	-3,041254045	0,00235595	NA
ENSG00000211699	TRGV3	T cell receptor gamma variable 3	6976	8,091567006	0,949367205	0,698817	1,358534969	0,174293994	NA
ENSG00000211701	TRGV1	T cell receptor gamma variable 1 (non-functional)	6973	0,119673877	0,847101401	4,080456	0,207599697	0,835541541	NA
ENSG00000211706	TRBV6-1	T cell receptor beta variable 6-1	28606	0	NA	NA	NA	NA	NA
ENSG00000211707	TRBV7-1	T cell receptor beta variable 7-1 (non-functional)	28597	0	NA	NA	NA	NA	NA
ENSG00000211710	TRBV4-1	T cell receptor beta variable 4-1	28617	0	NA	NA	NA	NA	NA
ENSG00000211713	TRBV6-4	T cell receptor beta variable 6-4	28603	0	NA	NA	NA	NA	NA
ENSG00000211714	TRBV7-3	T cell receptor beta variable 7-3	28595	0	NA	NA	NA	NA	NA
ENSG00000211715	TRBV5-3	T cell receptor beta variable 5-3 (non-functional)	28612	0	NA	NA	NA	NA	NA
ENSG00000211716	TRBV9	T cell receptor beta variable 9	28586	0	NA	NA	NA	NA	NA
ENSG00000211724	TRBV6-6	T cell receptor beta variable 6-6	28601	0	NA	NA	NA	NA	NA
ENSG00000211725	TRBV5-5	T cell receptor beta variable 5-5	28610	0	NA	NA	NA	NA	NA
ENSG00000211727	TRBV7-6	T cell receptor beta variable 7-6	28592	0	NA	NA	NA	NA	NA
ENSG00000211734	TRBV5-1	T cell receptor beta variable 5-1	28614	0	NA	NA	NA	NA	NA
ENSG00000211739	TRBV12-2	T cell receptor beta variable 12-2 (pseudogene)	28578	0	NA	NA	NA	NA	NA
ENSG00000211745	TRBV4-2	T cell receptor beta variable 4-2	28616	0	NA	NA	NA	NA	NA
ENSG00000211746	TRBV19	T cell receptor beta variable 19	28568	0	NA	NA	NA	NA	NA
ENSG00000211747	TRBV20-1	T cell receptor beta variable 20-1	28567	0	NA	NA	NA	NA	NA
ENSG00000211750	TRBV24-1	T cell receptor beta variable 24-1	28563	0	NA	NA	NA	NA	NA
ENSG00000211771	TRBJ2-7	T cell receptor beta joining 2-7	28622	0	NA	NA	NA	NA	NA
ENSG00000211772	TRBC2	T cell receptor beta constant 2	28638	1,232638359	0	3,124265	0	1	NA
ENSG00000211776	TRAV2	T cell receptor alpha variable 2	28691	0	NA	NA	NA	NA	NA

ENSG00000211777	TRAV3	T cell receptor alpha variable 3	28690	0	NA	NA	NA	NA	NA
ENSG00000211778	TRAV4	T cell receptor alpha variable 4	28689	0	NA	NA	NA	NA	NA
ENSG00000211779	TRAV5	T cell receptor alpha variable 5	28688	0	NA	NA	NA	NA	NA
ENSG00000211780	TRAV6	T cell receptor alpha variable 6	6956	0	NA	NA	NA	NA	NA
ENSG00000211781	TRAV7	T cell receptor alpha variable 7	28686	0	NA	NA	NA	NA	NA
ENSG00000211782	TRAV8-1	T cell receptor alpha variable 8-1	28685	0	NA	NA	NA	NA	NA
ENSG00000211783	TRAV9-1	T cell receptor alpha variable 9-1	28678	0	NA	NA	NA	NA	NA
ENSG00000211784	TRAV10	T cell receptor alpha variable 10	28676	0	NA	NA	NA	NA	NA
ENSG00000211785	TRAV12-1	T cell receptor alpha variable 12-1	28674	0	NA	NA	NA	NA	NA
ENSG00000211786	TRAV8-2	T cell receptor alpha variable 8-2	28684	0	NA	NA	NA	NA	NA
ENSG00000211787	TRAV8-3	T cell receptor alpha variable 8-3	28683	0	NA	NA	NA	NA	NA
ENSG00000211788	TRAV13-1	T cell receptor alpha variable 13-1	28671	0,072165449	0,847101401	4,080456	0,207599697	0,835541541	NA
ENSG00000211789	TRAV12-2	T cell receptor alpha variable 12-2	28673	0	NA	NA	NA	NA	NA
ENSG00000211790	TRAV8-4	T cell receptor alpha variable 8-4	28682	0,189612497	1,664773339	4,047535	0,411305487	0,680848547	NA
ENSG00000211791	TRAV13-2	T cell receptor alpha variable 13-2	28670	0	NA	NA	NA	NA	NA
ENSG00000211792	TRAV14DV4	T cell receptor alpha variable 14/delta variable 4	28669	0,384891628	-1,064763392	3,685761	-0,288885592	0,77266893	NA
ENSG00000211793	TRAV9-2	T cell receptor alpha variable 9-2	28677	0	NA	NA	NA	NA	NA
ENSG00000211794	TRAV12-3	T cell receptor alpha variable 12-3	28672	0	NA	NA	NA	NA	NA
ENSG00000211795	TRAV8-6	T cell receptor alpha variable 8-6	28680	0	NA	NA	NA	NA	NA
ENSG00000211796	TRAV16	T cell receptor alpha variable 16	28667	0	NA	NA	NA	NA	NA
ENSG00000211797	TRAV17	T cell receptor alpha variable 17	28666	0	NA	NA	NA	NA	NA
ENSG00000211798	TRAV18	T cell receptor alpha variable 18	28665	0	NA	NA	NA	NA	NA
ENSG00000211799	TRAV19	T cell receptor alpha variable 19	28664	0,15836288	0	4,080456	0	1	NA
ENSG00000211800	TRAV20	T cell receptor alpha variable 20	28663	0	NA	NA	NA	NA	NA
ENSG00000211801	TRAV21	T cell receptor alpha variable 21	28662	0	NA	NA	NA	NA	NA
ENSG00000211802	TRAV22	T cell receptor alpha variable 22	28661	2,820134281	-2,202572281	1,482247	-1,485968878	0,137287353	NA
ENSG00000211803	TRAV23DV6	T cell receptor alpha variable 23/delta variable 6	28660	0	NA	NA	NA	NA	NA
ENSG00000211804	TRDV1	T cell receptor delta variable 1	28518	1,967098846	-2,94044903	1,67005	-1,760695437	0,078289966	NA
ENSG00000211805	TRAV24	T cell receptor alpha variable 24	28659	0	NA	NA	NA	NA	NA
ENSG00000211806	TRAV25	T cell receptor alpha variable 25	28658	0	NA	NA	NA	NA	NA
ENSG00000211807	TRAV26-1	T cell receptor alpha variable 26-1	28657	0	NA	NA	NA	NA	NA
ENSG00000211808	NA	NA	NA	0	NA	NA	NA	NA	NA
ENSG00000211809	TRAV27	T cell receptor alpha variable 27	28655	0	NA	NA	NA	NA	NA
ENSG00000211810	TRAV29DV5	T cell receptor alpha variable 29/delta variable 5	28653	0	NA	NA	NA	NA	NA
ENSG00000211812	TRAV26-2	T cell receptor alpha variable 26-2	28656	0	NA	NA	NA	NA	NA
ENSG00000211813	TRAV34	T cell receptor alpha variable 34	28648	0	NA	NA	NA	NA	NA
ENSG00000211814	TRAV35	T cell receptor alpha variable 35	28647	0	NA	NA	NA	NA	NA
ENSG00000211815	TRAV36DV7	T cell receptor alpha variable 36/delta variable 7	28646	0	NA	NA	NA	NA	NA
ENSG00000211816	TRAV38-1	T cell receptor alpha variable 38-1	28644	0	NA	NA	NA	NA	NA
ENSG00000211817	TRAV38-2DV8	T cell receptor alpha variable 38-2/delta variable 8	28643	0	NA	NA	NA	NA	NA

ENSG00000211818	TRAV39	T cell receptor alpha variable 39	28642	0	NA	NA	NA	NA	NA
ENSG00000211819	TRAV40	T cell receptor alpha variable 40	28641	0	NA	NA	NA	NA	NA
ENSG00000211820	TRAV41	T cell receptor alpha variable 41	28640	0	NA	NA	NA	NA	NA
ENSG00000211821	TRDV2	T cell receptor delta variable 2	28517	1,919900453	-4,27435081	1,692436	-2,525561994	0,01155134	NA
ENSG00000211825	TRDJ1	T cell receptor delta joining 1	28522	0	NA	NA	NA	NA	NA
ENSG00000211826	TRDJ4	T cell receptor delta joining 4	28519	0	NA	NA	NA	NA	NA
ENSG00000211827	TRDJ2	T cell receptor delta joining 2	28521	0	NA	NA	NA	NA	NA
ENSG00000211828	TRDJ3	T cell receptor delta joining 3	28520	0	NA	NA	NA	NA	NA
ENSG00000211831	TRAJ61	T cell receptor alpha joining 61 (non-functional)	28694	0	NA	NA	NA	NA	NA
ENSG00000211832	TRAJ59	T cell receptor alpha joining 59 (non-functional)	28696	0	NA	NA	NA	NA	NA
ENSG00000211833	TRAJ58	T cell receptor alpha joining 58 (non-functional)	28697	0	NA	NA	NA	NA	NA
ENSG00000211834	TRAJ57	T cell receptor alpha joining 57	28698	0,10488184	-1,076443944	4,080456	-0,263804824	0,791930317	NA
ENSG00000211835	TRAJ56	T cell receptor alpha joining 56	28699	0	NA	NA	NA	NA	NA
ENSG00000211836	TRAJ54	T cell receptor alpha joining 54	28701	0	NA	NA	NA	NA	NA
ENSG00000211837	TRAJ53	T cell receptor alpha joining 53	28702	0	NA	NA	NA	NA	NA
ENSG00000211838	TRAJ52	T cell receptor alpha joining 52	28703	0	NA	NA	NA	NA	NA
ENSG00000211839	TRAJ50	T cell receptor alpha joining 50	28705	0	NA	NA	NA	NA	NA
ENSG00000211840	TRAJ49	T cell receptor alpha joining 49	28706	0	NA	NA	NA	NA	NA
ENSG00000211841	TRAJ48	T cell receptor alpha joining 48	28707	0	NA	NA	NA	NA	NA
ENSG00000211842	TRAJ47	T cell receptor alpha joining 47	28708	0	NA	NA	NA	NA	NA
ENSG00000211843	TRAJ46	T cell receptor alpha joining 46	28709	0	NA	NA	NA	NA	NA
ENSG00000211844	TRAJ45	T cell receptor alpha joining 45	28710	0	NA	NA	NA	NA	NA
ENSG00000211845	TRAJ44	T cell receptor alpha joining 44	28711	0	NA	NA	NA	NA	NA
ENSG00000211846	TRAJ43	T cell receptor alpha joining 43	28712	0	NA	NA	NA	NA	NA
ENSG00000211847	TRAJ42	T cell receptor alpha joining 42	28713	0	NA	NA	NA	NA	NA
ENSG00000211848	TRAJ41	T cell receptor alpha joining 41	28714	0	NA	NA	NA	NA	NA
ENSG00000211849	TRAJ40	T cell receptor alpha joining 40	28715	0	NA	NA	NA	NA	NA
ENSG00000211850	TRAJ39	T cell receptor alpha joining 39	28716	0	NA	NA	NA	NA	NA
ENSG00000211851	TRAJ38	T cell receptor alpha joining 38	28717	0	NA	NA	NA	NA	NA
ENSG00000211854	TRAJ35	T cell receptor alpha joining 35 (non-functional)	28720	0	NA	NA	NA	NA	NA
ENSG00000211855	TRAJ34	T cell receptor alpha joining 34	28721	0	NA	NA	NA	NA	NA
ENSG00000211856	TRAJ33	T cell receptor alpha joining 33	28722	0	NA	NA	NA	NA	NA
ENSG00000211857	TRAJ32	T cell receptor alpha joining 32	28723	0	NA	NA	NA	NA	NA
ENSG00000211858	TRAJ31	T cell receptor alpha joining 31	28724	0	NA	NA	NA	NA	NA
ENSG00000211859	TRAJ30	T cell receptor alpha joining 30	28725	0	NA	NA	NA	NA	NA
ENSG00000211860	TRAJ29	T cell receptor alpha joining 29	28726	0	NA	NA	NA	NA	NA
ENSG00000211861	TRAJ28	T cell receptor alpha joining 28	28727	0	NA	NA	NA	NA	NA
ENSG00000211862	TRAJ27	T cell receptor alpha joining 27	28728	0	NA	NA	NA	NA	NA
ENSG00000211863	TRAJ26	T cell receptor alpha joining 26	28729	0	NA	NA	NA	NA	NA
ENSG00000211864	TRAJ25	T cell receptor alpha joining 25 (non-functional)	28730	0	NA	NA	NA	NA	NA
ENSG00000211865	TRAJ24	T cell receptor alpha joining 24	28731	0	NA	NA	NA	NA	NA
ENSG00000211866	TRAJ23	T cell receptor alpha joining 23	28732	0	NA	NA	NA	NA	NA

ENSG00000211867	TRAJ22	T cell receptor alpha joining 22	28733	0	NA	NA	NA	NA	NA
ENSG00000211868	TRAJ21	T cell receptor alpha joining 21	28734	0	NA	NA	NA	NA	NA
ENSG00000211869	TRAJ20	T cell receptor alpha joining 20	28735	0	NA	NA	NA	NA	NA
ENSG00000211870	TRAJ19	T cell receptor alpha joining 19 (non-functional)	28736	0	NA	NA	NA	NA	NA
ENSG00000211871	TRAJ18	T cell receptor alpha joining 18	28737	0	NA	NA	NA	NA	NA
ENSG00000211872	TRAJ17	T cell receptor alpha joining 17	28738	0	NA	NA	NA	NA	NA
ENSG00000211873	TRAJ16	T cell receptor alpha joining 16	28739	0	NA	NA	NA	NA	NA
ENSG00000211875	TRAJ14	T cell receptor alpha joining 14	28741	0	NA	NA	NA	NA	NA
ENSG00000211876	TRAJ13	T cell receptor alpha joining 13	28742	0	NA	NA	NA	NA	NA
ENSG00000211877	TRAJ12	T cell receptor alpha joining 12	28743	0	NA	NA	NA	NA	NA
ENSG00000211878	TRAJ11	T cell receptor alpha joining 11	28744	0	NA	NA	NA	NA	NA
ENSG00000211879	TRAJ10	T cell receptor alpha joining 10	28745	0	NA	NA	NA	NA	NA
ENSG00000211880	TRAJ9	T cell receptor alpha joining 9	28746	0	NA	NA	NA	NA	NA
ENSG00000211881	TRAJ8	T cell receptor alpha joining 8	28747	0	NA	NA	NA	NA	NA
ENSG00000211882	TRAJ7	T cell receptor alpha joining 7	28748	0	NA	NA	NA	NA	NA
ENSG00000211883	TRAJ6	T cell receptor alpha joining 6	28749	0	NA	NA	NA	NA	NA
ENSG00000211884	TRAJ5	T cell receptor alpha joining 5	28750	0	NA	NA	NA	NA	NA
ENSG00000211885	TRAJ4	T cell receptor alpha joining 4	28751	0	NA	NA	NA	NA	NA
ENSG00000211886	TRAJ3	T cell receptor alpha joining 3	28752	0	NA	NA	NA	NA	NA
ENSG00000211887	TRAJ2	T cell receptor alpha joining 2 (non-functional)	28753	0	NA	NA	NA	NA	NA
ENSG00000211888	TRAJ1	T cell receptor alpha joining 1 (non-functional)	28754	0	NA	NA	NA	NA	NA
ENSG00000211890	IGHA2	immunoglobulin heavy constant alpha 2 (A2m marker)	3494	0	NA	NA	NA	NA	NA
ENSG00000211891	IGHE	immunoglobulin heavy constant epsilon	3497	0	NA	NA	NA	NA	NA
ENSG00000211892	IGHG4	immunoglobulin heavy constant gamma 4 (G4m marker)	3503	0	NA	NA	NA	NA	NA
ENSG00000211893	IGHG2	immunoglobulin heavy constant gamma 2 (G2m marker)	3501	0	NA	NA	NA	NA	NA
ENSG00000211895	IGHA1	immunoglobulin heavy constant alpha 1	3493	0	NA	NA	NA	NA	NA
ENSG00000211897	IGHG3	immunoglobulin heavy constant gamma 3 (G3m marker)	3502	0	NA	NA	NA	NA	NA
ENSG00000211899	IGHM	immunoglobulin heavy constant mu	3507	1,09538405	4,18330136	3,16874	1,320178267	0,186775506	NA
ENSG00000211900	IGHJ6	immunoglobulin heavy joining 6	28475	0	NA	NA	NA	NA	NA
ENSG00000211904	IGHJ2	immunoglobulin heavy joining 2	28481	0	NA	NA	NA	NA	NA
ENSG00000211933	IGHV6-1	immunoglobulin heavy variable 6-1	28385	0	NA	NA	NA	NA	NA
ENSG00000211938	IGHV3-7	immunoglobulin heavy variable 3-7	28452	0	NA	NA	NA	NA	NA
ENSG00000211941	IGHV3-11	immunoglobulin heavy variable 3-11	28450	0	NA	NA	NA	NA	NA
ENSG00000211942	IGHV3-13	immunoglobulin heavy variable 3-13	28449	0	NA	NA	NA	NA	NA
ENSG00000211943	IGHV3-15	immunoglobulin heavy variable 3-15	28448	0	NA	NA	NA	NA	NA
ENSG00000211944	IGHV3-16	immunoglobulin heavy variable 3-16 (non-functional)	28447	0	NA	NA	NA	NA	NA
ENSG00000211945	IGHV1-18	immunoglobulin heavy variable 1-18	28468	0,10543123	0	4,080456	0	1	NA
ENSG00000211946	IGHV3-20	immunoglobulin heavy variable 3-20	28445	0	NA	NA	NA	NA	NA
ENSG00000211947	IGHV3-21	immunoglobulin heavy variable 3-21	28444	0	NA	NA	NA	NA	NA
ENSG00000211949	IGHV3-23	immunoglobulin heavy variable 3-23	28442	0	NA	NA	NA	NA	NA
ENSG00000211950	IGHV1-24	immunoglobulin heavy variable 1-24	28467	0	NA	NA	NA	NA	NA

ENSG00000211951	IGHV2-26	immunoglobulin heavy variable 2-26	28455	0	NA	NA	NA	NA	NA
ENSG00000211952	IGHV4-28	immunoglobulin heavy variable 4-28	28400	0	NA	NA	NA	NA	NA
ENSG00000211955	IGHV3-33	immunoglobulin heavy variable 3-33	28434	0	NA	NA	NA	NA	NA
ENSG00000211956	IGHV4-34	immunoglobulin heavy variable 4-34	28395	0	NA	NA	NA	NA	NA
ENSG00000211957	IGHV3-35	immunoglobulin heavy variable 3-35 (non-functional)	28432	0	NA	NA	NA	NA	NA
ENSG00000211958	IGHV3-38	immunoglobulin heavy variable 3-38 (non-functional)	28429	0	NA	NA	NA	NA	NA
ENSG00000211959	IGHV4-39	immunoglobulin heavy variable 4-39	28394	0	NA	NA	NA	NA	NA
ENSG00000211962	IGHV1-46	immunoglobulin heavy variable 1-46	28465	0	NA	NA	NA	NA	NA
ENSG00000211966	IGHV5-51	immunoglobulin heavy variable 5-51	28388	0	NA	NA	NA	NA	NA
ENSG00000211972	IGHV3-66	immunoglobulin heavy variable 3-66	28412	0	NA	NA	NA	NA	NA
ENSG00000211973	IGHV1-69	immunoglobulin heavy variable 1-69	28461	0	NA	NA	NA	NA	NA
ENSG00000211976	IGHV3-73	immunoglobulin heavy variable 3-73	28409	0	NA	NA	NA	NA	NA
ENSG00000211979	IGHV7-81	immunoglobulin heavy variable 7-81 (non-functional)	28378	0	NA	NA	NA	NA	NA
ENSG00000212122	TSSK1B	testis specific serine kinase 1B	83942	0,414125008	1,405990335	2,963697	0,474404216	0,635211687	NA
ENSG00000212123	PRR22	proline rich 22	163154	3,602332598	-0,476857925	1,493149	-0,319363912	0,749450573	NA
ENSG00000212138	RNA5SP372	RNA, 5S ribosomal pseudogene 372	100873630	0	NA	NA	NA	NA	NA
ENSG00000212171	RNA5SP86	RNA, 5S ribosomal pseudogene 86	100873319	0	NA	NA	NA	NA	NA
ENSG00000212176	RNA5SP207	RNA, 5S ribosomal pseudogene 207	100873467	0	NA	NA	NA	NA	NA
ENSG00000212204	RNA5SP91	RNA, 5S ribosomal pseudogene 91	100873324	0	NA	NA	NA	NA	NA
ENSG00000212237	RNA5SP18	RNA, 5S ribosomal pseudogene 18	100873273	0	NA	NA	NA	NA	NA
ENSG00000212238	RNA5SP244	RNA, 5S ribosomal pseudogene 244	100885864	0	NA	NA	NA	NA	NA
ENSG00000212242	RNA5SP219	RNA, 5S ribosomal pseudogene 219	100873479	0	NA	NA	NA	NA	NA
ENSG00000212251	RNA5SP376	RNA, 5S ribosomal pseudogene 376	106479010	0	NA	NA	NA	NA	NA
ENSG00000212258	RNA5SP176	RNA, 5S ribosomal pseudogene 176	100873438	0	NA	NA	NA	NA	NA
ENSG00000212265	RNA5SP185	RNA, 5S ribosomal pseudogene 185	100873447	0	NA	NA	NA	NA	NA
ENSG00000212276	RNA5SP292	RNA, 5S ribosomal pseudogene 292	100873545	0	NA	NA	NA	NA	NA
ENSG00000212280	RNA5SP256	RNA, 5S ribosomal pseudogene 256	100873510	0	NA	NA	NA	NA	NA
ENSG00000212289	RNA5SP339	RNA, 5S ribosomal pseudogene 339	100873602	0	NA	NA	NA	NA	NA
ENSG00000212308	RNA5SP23	RNA, 5S ribosomal pseudogene 23	100873278	0	NA	NA	NA	NA	NA
ENSG00000212312	RNA5SP109	RNA, 5S ribosomal pseudogene 109	100873379	0	NA	NA	NA	NA	NA
ENSG00000212331	RNA5SP297	RNA, 5S ribosomal pseudogene 297	106479003	0	NA	NA	NA	NA	NA
ENSG00000212333	RNA5SP213	RNA, 5S ribosomal pseudogene 213	100873473	0	NA	NA	NA	NA	NA
ENSG00000212336	RNA5SP210	RNA, 5S ribosomal pseudogene 210	100873470	0	NA	NA	NA	NA	NA
ENSG00000212365	RNA5SP332	RNA, 5S ribosomal pseudogene 332	100873598	0	NA	NA	NA	NA	NA
ENSG00000212373	RNA5SP171	RNA, 5S ribosomal pseudogene 171	100873436	0	NA	NA	NA	NA	NA
ENSG00000212396	RNA5SP323	RNA, 5S ribosomal pseudogene 323	100873589	0	NA	NA	NA	NA	NA
ENSG00000212425	RNA5SP105	RNA, 5S ribosomal pseudogene 105	100873376	0	NA	NA	NA	NA	NA
ENSG00000212454	RNA5SP286	RNA, 5S ribosomal pseudogene 286	100873539	0	NA	NA	NA	NA	NA
ENSG00000212497	RNA5SP465	RNA, 5S ribosomal pseudogene 465	100873709	0	NA	NA	NA	NA	NA

ENSG00000212499	RNA5SP300	RNA, 5S ribosomal pseudogene 300	100873575	0	NA	NA	NA	NA	NA
ENSG00000212505	RNA5SP299	RNA, 5S ribosomal pseudogene 299	100873574	0	NA	NA	NA	NA	NA
ENSG00000212525	RNA5SP212	RNA, 5S ribosomal pseudogene 212	100873472	0	NA	NA	NA	NA	NA
ENSG00000212527	RNA5SP63	RNA, 5S ribosomal pseudogene 63	100873298	0	NA	NA	NA	NA	NA
ENSG00000212536	RNA5SP474	RNA, 5S ribosomal pseudogene 474	100873716	0	NA	NA	NA	NA	NA
ENSG00000212542	RNA5SP496	RNA, 5S ribosomal pseudogene 496	100873738	0	NA	NA	NA	NA	NA
ENSG00000212549	RNA5SP354	RNA, 5S ribosomal pseudogene 354	100873614	0	NA	NA	NA	NA	NA
ENSG00000212559	RNA5SP35	RNA, 5S ribosomal pseudogene 35	100873367	0	NA	NA	NA	NA	NA
ENSG00000212571	RNA5SP482	RNA, 5S ribosomal pseudogene 482	100873724	0	NA	NA	NA	NA	NA
ENSG00000212576	RNA5SP467	RNA, 5S ribosomal pseudogene 467	100873711	0	NA	NA	NA	NA	NA
ENSG00000212601	RNA5SP53	RNA, 5S ribosomal pseudogene 53	100873291	0	NA	NA	NA	NA	NA
ENSG00000212625	RNA5SP397	RNA, 5S ribosomal pseudogene 397	100873650	0	NA	NA	NA	NA	NA
ENSG00000212628	RNA5SP241	RNA, 5S ribosomal pseudogene 241	100873499	0	NA	NA	NA	NA	NA
ENSG00000212643	ZRSR2P1	ZRSR2 pseudogene 1	7310	2,972453591	1,223453854	2,138353	0,572147744	0,567221886	NA
ENSG00000212659	KRTAP9-6	keratin associated protein 9-6	100507608	0	NA	NA	NA	NA	NA
ENSG00000212664	RPL17P39	ribosomal protein L17 pseudogene 39	645296	0,575344176	-0,208932052	2,489154	-0,083936985	0,933106533	NA
ENSG00000212695	RPL18AP11	ribosomal protein L18a pseudogene 11	100127999	0	NA	NA	NA	NA	NA
ENSG00000212710	CTAGE1	cutaneous T cell lymphoma-associated antigen 1	64693	0	NA	NA	NA	NA	NA
ENSG00000212712	LOC107985122	kinesin-like protein KIF1C	107985122	0	NA	NA	NA	NA	NA
ENSG00000212717	NA	NA	NA	0	NA	NA	NA	NA	NA
ENSG00000212747	RTL8B	retrotransposon Gag like 8B	441518	0	NA	NA	NA	NA	NA
ENSG00000212769	NA	NA	NA	0,11808024	-1,076443944	4,080456	-0,263804824	0,791930317	NA
ENSG00000212802	RPL15P3	ribosomal protein L15 pseudogene 3	653232	1,903960204	-0,777695034	1,48864	-0,522419882	0,601378016	NA
ENSG00000212807	OR2A42	olfactory receptor family 2 subfamily A member 42	402317	0	NA	NA	NA	NA	NA
ENSG00000212829	NA	NA	NA	0,072165449	0,847101401	4,080456	0,207599697	0,835541541	NA
ENSG00000212864	RNF208	ring finger protein 208	727800	4,797194879	0,166801617	0,885322	0,188407901	0,850556906	NA
ENSG00000212899	KRTAP3-3	keratin associated protein 3-3	85293	0	NA	NA	NA	NA	NA
ENSG00000212900	KRTAP3-2	keratin associated protein 3-2	83897	0	NA	NA	NA	NA	NA
ENSG00000212901	KRTAP3-1	keratin associated protein 3-1	83896	0	NA	NA	NA	NA	NA
ENSG00000212932	RPL23AP4	ribosomal protein L23a pseudogene 4	54029	0	NA	NA	NA	NA	NA
ENSG00000212933	KRTAP12-4	keratin associated protein 12-4	386684	0,117497108	0,847101401	4,080456	0,207599697	0,835541541	NA
ENSG00000212935	KRTAP10-3	keratin associated protein 10-3	386682	0	NA	NA	NA	NA	NA
ENSG00000212938	KRTAP6-3	keratin associated protein 6-3	337968	0	NA	NA	NA	NA	NA
ENSG00000212952	NA	NA	NA	0,103998153	-1,076443944	4,080456	-0,263804824	0,791930317	NA
ENSG00000212961	NA	NA	NA	0,382807046	-1,062286525	3,676233	-0,288960622	0,772611512	NA
ENSG00000212989	LOC100129527	ESF1, nucleolar pre-rRNA processing protein, homolog (S, cerevisiae) pseudogene	100129527	0	NA	NA	NA	NA	NA
ENSG00000212993	POU5F1B	POU class 5 homeobox 1B	5462	0,234994216	1,944111989	4,02324	0,483220515	0,62893917	NA
ENSG00000212994	NA	NA	NA	0,341125508	-0,119274987	3,783006	-0,031529156	0,974847541	NA
ENSG00000213003	NA	NA	NA	0	NA	NA	NA	NA	NA
ENSG00000213005	PTTG3P	pituitary tumor-transforming 3, pseudogene	26255	0	NA	NA	NA	NA	NA
ENSG00000213013	RPS15AP36	ribosomal protein S15a pseudogene 36	100271650	0,118286652	0	4,080456	0	1	NA
ENSG00000213014	NA	NA	NA	0	NA	NA	NA	NA	NA
ENSG00000213016	NA	NA	NA	0	NA	NA	NA	NA	NA
ENSG00000213018	NA	NA	NA	0	NA	NA	NA	NA	NA
ENSG00000213022	KLK9	kallikrein related peptidase 9	284366	0	NA	NA	NA	NA	NA
ENSG00000213023	SYT3	synaptotagmin 3	84258	0	NA	NA	NA	NA	NA
ENSG00000213025	NA	NA	NA	0	NA	NA	NA	NA	NA
ENSG00000213026	NA	NA	NA	0	NA	NA	NA	NA	NA
ENSG00000213028	NA	NA	NA	0	NA	NA	NA	NA	NA

ENSG00000213030	CGB8	chorionic gonadotropin subunit beta 8	94115	0,072165449	0,847101401	4,080456	0,207599697	0,835541541	NA	
ENSG00000213032	NA	NA	NA	0	NA	NA	NA	NA	NA	
ENSG00000213033	NA	NA	NA	3,397065734	-1,317530983	1,582493	-0,832566955	0,405089002	NA	
ENSG00000213035	RPL23AP80	ribosomal protein L23a pseudogene 80	390949	0	NA	NA	NA	NA	NA	
ENSG00000213036	NA	NA	NA	0	NA	NA	NA	NA	NA	
ENSG00000213041	RPL17P8	ribosomal protein L17 pseudogene 8	730255	0	NA	NA	NA	NA	NA	
ENSG00000213045	NA	NA	NA	0	NA	NA	NA	NA	NA	
ENSG00000213046	NA	NA	NA	0,117497108	0,847101401	4,080456	0,207599697	0,835541541	NA	
ENSG00000213048	OR5S1P	olfactory receptor family 5 subfamily S member 1 pseudogene	391496	0	NA	NA	NA	NA	NA	
ENSG00000213049	NA	NA	NA	0	NA	NA	NA	NA	NA	
ENSG00000213050	NA	NA	NA	0	NA	NA	NA	NA	NA	
ENSG00000213051	RPL5P5	ribosomal protein L5 pseudogene 5	730213	0	NA	NA	NA	NA	NA	
ENSG00000213055	EEF1B2P7	eukaryotic translation elongation factor 1 beta 2 pseudogene 7	100421756	0	NA	NA	NA	NA	NA	
ENSG00000213058	NA	NA	NA	0	NA	NA	NA	NA	NA	
ENSG00000213060	NA	NA	NA	0	NA	NA	NA	NA	NA	
ENSG00000213061	NA	NA	NA	0,117497108	0,847101401	4,080456	0,207599697	0,835541541	NA	
ENSG00000213063	NA	NA	NA	0	NA	NA	NA	NA	NA	
ENSG00000213065	LOC100422263	serine palmitoyltransferase long chain base subunit 2 pseudogene	100422263	0	NA	NA	NA	NA	NA	
ENSG00000213067	NA	NA	NA	0	NA	NA	NA	NA	NA	
ENSG00000213068	RPS17P6	ribosomal protein S17 pseudogene 6	391130	0	NA	NA	NA	NA	NA	
ENSG00000213069	NA	NA	NA	0	NA	NA	NA	NA	NA	
ENSG00000213070	HMGB3P6	high mobility group box 3 pseudogene 6	729952	0	NA	NA	NA	NA	NA	
ENSG00000213073	NA	NA	NA	1,18353767	4,291503586	3,078212	1,394154826	0,163270856	NA	
ENSG00000213075	RPL31P11	ribosomal protein L31 pseudogene 11	641311	0	NA	NA	NA	NA	NA	
ENSG00000213076	NA	NA	NA	0	NA	NA	NA	NA	NA	
ENSG00000213078	NA	NA	NA	0,216496347	1,902771968	4,026559	0,472555327	0,63653046	NA	
ENSG00000213080	NA	NA	NA	0,264094357	2,151009916	4,007893	0,53669347	0,591479368	NA	
ENSG00000213081	ARPC1BP1	ARPC1B pseudogene 1	100288663	0	NA	NA	NA	NA	NA	
ENSG00000213082	NA	NA	NA	0	NA	NA	NA	NA	NA	
ENSG00000213083	VPS26CP1	VPS26C pseudogene 1	100420893	0	NA	NA	NA	NA	NA	
ENSG00000213085	CFAP45	cilia and flagella associated protein 45	25790	1,073292261	4,149342664	3,149793	1,317338034	0,187725342	NA	
ENSG00000213087	NA	NA	NA	0	NA	NA	NA	NA	NA	
ENSG00000213088	ACKR1	atypical chemokine receptor 1 (Duffy blood group)	2532	0	NA	NA	NA	NA	NA	
ENSG00000213089	NA	NA	NA	0	NA	NA	NA	NA	NA	
ENSG00000213090	SCYL2P1	SCYL2 pseudogene 1	100420110	5,953239839	0,061134273	0,813832	0,075119026	0,94012001	NA	
ENSG00000213091	NA	NA	NA	0	NA	NA	NA	NA	NA	
ENSG00000213100	KRT18P68	keratin 18 pseudogene 68	139060	0	NA	NA	NA	NA	NA	
ENSG00000213104	NA	NA	NA	0,144330898	1,418786819	4,061262	0,349346267	0,726829368	NA	
ENSG00000213107	AHCYP5	adenosylhomocysteinase pseudogene 5	391470	0	NA	NA	NA	NA	NA	
ENSG00000213108	NA	NA	NA	0	NA	NA	NA	NA	NA	
ENSG00000213109	RPL7AP37	ribosomal protein L7a pseudogene 37	645257	0	NA	NA	NA	NA	NA	
ENSG00000213110	NA	NA	NA	0	NA	NA	NA	NA	NA	
ENSG00000213111	COX5BP2	cytochrome c oxidase subunit 5B pseudogene 2	1331	0	NA	NA	NA	NA	NA	
ENSG00000213113	TUBB8P8	tubulin beta 8 class VIII pseudogene 8	102723626	0	NA	NA	NA	NA	NA	
ENSG00000213115	LOC729141	chromosome 18 open reading frame 25 pseudogene	729141	0	NA	NA	NA	NA	NA	
ENSG00000213117	NA	NA	NA	0	NA	NA	NA	NA	NA	
ENSG00000213118	NA	NA	NA	0	NA	NA	NA	NA	NA	
ENSG00000213120	NA	NA	NA	0	NA	NA	NA	NA	NA	
ENSG00000213122	NA	NA	NA	0	NA	NA	NA	NA	NA	
ENSG00000213123	DYNLT2B	dynein light chain Tctex-type 2B	255758	6,22382535	-0,789342571	0,885893	-0,891013706	0,372921817	NA	
ENSG00000213126	NA	NA	NA	0	NA	NA	NA	NA	NA	
ENSG00000213128	NA	NA	NA	0	NA	NA	NA	NA	NA	
ENSG00000213130	EEF1DP5	eukaryotic translation elongation factor 1 delta pseudogene 5	442258	0	NA	NA	NA	NA	NA	
ENSG00000213131	NA	NA	NA	0,6221962	-1,054603692	2,540934	-0,415045668	0,678108472	NA	
ENSG00000213133	NA	NA	NA	0	NA	NA	NA	NA	NA	
ENSG00000213137	NA	NA	NA	1,25030097	0,028868764	1,816907	0,015888961	0,987322977	NA	

ENSG00000213139	CRYGS	crystallin gamma S	1427	1,811882976	-0,465348393	2,068013	-0,225021984	0,821962172	NA
ENSG00000213140	NA	NA	NA	0	NA	NA	NA	NA	NA
ENSG00000213144	NA	NA	NA	0	NA	NA	NA	NA	NA
ENSG00000213147	RPL23AP60	ribosomal protein L23a pseudogene 60	728933	0,473845236	-1,07463259	4,080456	-0,263360914	0,792272413	NA
ENSG00000213148	NA	NA	NA	0	NA	NA	NA	NA	NA
ENSG00000213149	NA	NA	NA	4,875521291	0,353313729	1,006331	0,351091034	0,725520054	NA
ENSG00000213150	NA	NA	NA	0	NA	NA	NA	NA	NA
ENSG00000213152	RPL7AP60	ribosomal protein L7a pseudogene 60	100271538	6,357258084	0,60059786	0,742972	0,808372287	0,4188763	NA
ENSG00000213153	NA	NA	NA	0	NA	NA	NA	NA	NA
ENSG00000213155	NA	NA	NA	0	NA	NA	NA	NA	NA
ENSG00000213157	NA	NA	NA	0	NA	NA	NA	NA	NA
ENSG00000213158	GAPDHP36	glyceraldehyde 3 phosphate dehydrogenase pseudogene 36	647249	0	NA	NA	NA	NA	NA
ENSG00000213159	NA	NA	NA	0	NA	NA	NA	NA	NA
ENSG00000213169	RPL8P4	ribosomal protein L8 pseudogene 4	402150	0	NA	NA	NA	NA	NA
ENSG00000213170	NA	NA	NA	0	NA	NA	NA	NA	NA
ENSG00000213171	LINGO4	leucine rich repeat and Ig domain containing 4	339398	0,176015471	-0,110128575	4,080456	-0,026989282	0,978468282	NA
ENSG00000213172	NA	NA	NA	0	NA	NA	NA	NA	NA
ENSG00000213174	NA	NA	NA	0	NA	NA	NA	NA	NA
ENSG00000213176	RPL13P6	ribosomal protein L13 pseudogene 6	100129176	0	NA	NA	NA	NA	NA
ENSG00000213177	NA	NA	NA	0	NA	NA	NA	NA	NA
ENSG00000213178	NA	NA	NA	10,59999208	-1,356092262	1,235758	-1,097376639	0,272476781	NA
ENSG00000213179	RPL17P41	ribosomal protein L17 pseudogene 41	100132645	0	NA	NA	NA	NA	NA
ENSG00000213180	RPL36AP48	ribosomal protein L36a pseudogene 48	646175	0	NA	NA	NA	NA	NA
ENSG00000213181	OR10N1P	olfactory receptor family 10 subfamily N member 1 pseudogene	390267	0	NA	NA	NA	NA	NA
ENSG00000213182	OR10D5P	olfactory receptor family 10 subfamily D member 5 pseudogene	79488	0	NA	NA	NA	NA	NA
ENSG00000213183	RPS7P8	ribosomal protein S7 pseudogene 8	442237	0	NA	NA	NA	NA	NA
ENSG00000213184	NA	NA	NA	0	NA	NA	NA	NA	NA
ENSG00000213185	FAM24B	family with sequence similarity 24 member B	196792	3,496846638	1,138863914	1,11115	1,024941238	0,305390915	NA
ENSG00000213187	NA	NA	NA	0	NA	NA	NA	NA	NA
ENSG00000213188	YBX1P4	Y-box binding protein 1 pseudogene 4	100131012	2,16112144	-1,349423103	1,483252	-0,909773276	0,362942091	NA
ENSG00000213189	NA	NA	NA	2,919147905	1,536137213	1,658113	0,926436848	0,354219002	NA
ENSG00000213190	MLLT11	MLLT11 transcription factor 7 cofactor	10962	8,599205044	-0,241911539	0,639365	-0,378362404	0,705161393	NA
ENSG00000213194	NA	NA	NA	0	NA	NA	NA	NA	NA
ENSG00000213197	NA	NA	NA	0	NA	NA	NA	NA	NA
ENSG00000213199	ASIC3	acid sensing ion channel subunit 3	9311	3,566279763	-0,512533731	1,40636	-0,364439994	0,715529475	NA
ENSG00000213201	NA	NA	NA	0	NA	NA	NA	NA	NA
ENSG00000213204	NA	NA	NA	0,427424714	2,821534344	3,403247	0,829071254	0,407064089	NA
ENSG00000213205	NA	NA	NA	0,11808024	-1,076443944	4,080456	-0,263804824	0,791930317	NA
ENSG00000213209	NA	NA	NA	0	NA	NA	NA	NA	NA
ENSG00000213210	NA	NA	NA	0	NA	NA	NA	NA	NA
ENSG00000213211	KRT18P64	keratin 18 pseudogene 64	107133513	0	NA	NA	NA	NA	NA
ENSG00000213212	NA	NA	NA	0,072165449	0,847101401	4,080456	0,207599697	0,835541541	NA
ENSG00000213213	CCDC183	coiled-coil domain containing 183	84960	0,144330898	1,418786819	4,061262	0,349346267	0,726829368	NA
ENSG00000213214	ARHGEF35	Rho guanine nucleotide exchange factor 35	445328	0,352491325	2,536807152	3,984503	0,636668335	0,524340903	NA
ENSG00000213216	NA	NA	NA	0	NA	NA	NA	NA	NA
ENSG00000213218	CSH2	chorionic somatomammotropin hormone 2	1443	0	NA	NA	NA	NA	NA
ENSG00000213222	TOMM40P4	TOMM40 pseudogene 4	100422473	0	NA	NA	NA	NA	NA
ENSG00000213226	LOC100130018	prostaglandin reductase 1 pseudogene	100130018	0	NA	NA	NA	NA	NA
ENSG00000213228	NA	NA	NA	0,440665611	0,919837208	3,561784	0,258251795	0,796212586	NA
ENSG00000213231	TCL1B	TCL1 family AKT coactivator B	9623	0	NA	NA	NA	NA	NA
ENSG00000213232	NA	NA	NA	0	NA	NA	NA	NA	NA
ENSG00000213233	RPL23AP62	ribosomal protein L23a pseudogene 62	100271617	0	NA	NA	NA	NA	NA
ENSG00000213234	NA	NA	NA	0	NA	NA	NA	NA	NA
ENSG00000213235	EEF1A1P16	eukaryotic translation elongation factor 1 alpha 1 pseudogene 16	387845	0	NA	NA	NA	NA	NA

ENSG00000213236	NA	NA	NA	0,117497108	0,847101401	4,080456	0,207599697	0,835541541	NA
ENSG00000213237	NA	NA	NA	0	NA	NA	NA	NA	NA
ENSG00000213238	RPS17P12	ribosomal protein S17 pseudogene 12	442726	0	NA	NA	NA	NA	NA
ENSG00000213239	NA	NA	NA	0	NA	NA	NA	NA	NA
ENSG00000213244	NA	NA	NA	0	NA	NA	NA	NA	NA
ENSG00000213247	NA	NA	NA	0	NA	NA	NA	NA	NA
ENSG00000213250	NA	NA	NA	0,15836288	0	4,080456	0	1	NA
ENSG00000213252	LOC100422300	pyridine nucleotide-disulphide oxidoreductase domain 1 pseudogene	100422300	0	NA	NA	NA	NA	NA
ENSG00000213253	NA	NA	NA	0,103998153	-1,076443944	4,080456	-0,263804824	0,791930317	NA
ENSG00000213260	NA	NA	NA	0,072165449	0,847101401	4,080456	0,207599697	0,835541541	NA
ENSG00000213261	EEF1B2P6	eukaryotic translation elongation factor 1 beta 2 pseudogene 6	647030	0,103998153	-1,076443944	4,080456	-0,263804824	0,791930317	NA
ENSG00000213262	NA	NA	NA	0	NA	NA	NA	NA	NA
ENSG00000213264	NA	NA	NA	0	NA	NA	NA	NA	NA
ENSG00000213265	TSGA13	testis specific 13	114960	0,10488184	-1,076443944	4,080456	-0,263804824	0,791930317	NA
ENSG00000213269	RPS6P26	ribosomal protein S6 pseudogene 26	392497	0	NA	NA	NA	NA	NA
ENSG00000213270	RPL6P25	ribosomal protein L6 pseudogene 25	401725	0	NA	NA	NA	NA	NA
ENSG00000213272	RPL7AP9	ribosomal protein L7a pseudogene 9	441642	0,11808024	-1,076443944	4,080456	-0,263804824	0,791930317	NA
ENSG00000213275	IFITM9P	interferon induced transmembrane protein 9 pseudogene	390218	0	NA	NA	NA	NA	NA
ENSG00000213277	NA	NA	NA	0,10488184	-1,076443944	4,080456	-0,263804824	0,791930317	NA
ENSG00000213280	RPS10P15	ribosomal protein S10 pseudogene 15	654029	0	NA	NA	NA	NA	NA
ENSG00000213287	LOC101929104	mitochondrial import receptor subunit TOM22 homolog	101929104	0	NA	NA	NA	NA	NA
ENSG00000213290	PGK1P2	phosphoglycerate kinase 1, pseudogene 2	5233	0,3282516	-0,98893682	3,782263	-0,261466979	0,793732411	NA
ENSG00000213291	RPL31P39	ribosomal protein L31 pseudogene 39	641790	0	NA	NA	NA	NA	NA
ENSG00000213293	RPL28P5	ribosomal protein L28 pseudogene 5	729279	0	NA	NA	NA	NA	NA
ENSG00000213295	NA	NA	NA	0	NA	NA	NA	NA	NA
ENSG00000213296	RPS2P31	ribosomal protein S2 pseudogene 31	392781	0	NA	NA	NA	NA	NA
ENSG00000213297	NA	NA	NA	11,00824314	-1,346671997	2,130755	-0,632016239	0,527376266	NA
ENSG00000213300	NA	NA	NA	0,877214657	-3,253529249	2,935487	-1,108344064	0,267713252	NA
ENSG00000213301	NA	NA	NA	0	NA	NA	NA	NA	NA
ENSG00000213302	RPL31P37	ribosomal protein L31 pseudogene 37	646787	0	NA	NA	NA	NA	NA
ENSG00000213303	NA	NA	NA	0	NA	NA	NA	NA	NA
ENSG00000213304	RPL18AP13	ribosomal protein L18a pseudogene 13	646189	0	NA	NA	NA	NA	NA
ENSG00000213305	HNRNPCP6	heterogeneous nuclear ribonucleoprotein C pseudogene 6	100421794	0,117497108	0,847101401	4,080456	0,207599697	0,835541541	NA
ENSG00000213307	RPL18P11	ribosomal protein L18 pseudogene 11	390612	0	NA	NA	NA	NA	NA
ENSG00000213309	RPL9P18	ribosomal protein L9 pseudogene 18	389404	0	NA	NA	NA	NA	NA
ENSG00000213310	NA	NA	NA	0	NA	NA	NA	NA	NA
ENSG00000213312	RPL7AP34	ribosomal protein L7a pseudogene 34	441154	0	NA	NA	NA	NA	NA
ENSG00000213315	RPS18P2	ribosomal protein S18 pseudogene 2	326320	0	NA	NA	NA	NA	NA
ENSG00000213318	RPS4Y1P1	RPS4Y1 pseudogene 1	441774	0	NA	NA	NA	NA	NA
ENSG00000213326	RPS7P11	ribosomal protein S7 pseudogene 11	644315	0,072165449	0,847101401	4,080456	0,207599697	0,835541541	NA
ENSG00000213328	NA	NA	NA	0	NA	NA	NA	NA	NA
ENSG00000213331	NA	NA	NA	0	NA	NA	NA	NA	NA
ENSG00000213332	NA	NA	NA	0	NA	NA	NA	NA	NA
ENSG00000213333	NPM1P50	nucleophosmin 1 pseudogene 50	100129795	0	NA	NA	NA	NA	NA
ENSG00000213335	CLNS1AP1	chloride nucleotide-sensitive channel 1A pseudogene 1	1204	0	NA	NA	NA	NA	NA
ENSG00000213343	RPL21P18	ribosomal protein L21 pseudogene 18	729484	0	NA	NA	NA	NA	NA
ENSG00000213344	NA	NA	NA	0	NA	NA	NA	NA	NA
ENSG00000213352	NA	NA	NA	0	NA	NA	NA	NA	NA
ENSG00000213355	NA	NA	NA	0	NA	NA	NA	NA	NA
ENSG00000213358	NAA50P2	NAA50 pseudogene 2	127566427	0	NA	NA	NA	NA	NA

ENSG00000213361	RPL36P12	ribosomal protein L36 pseudogene 12	100271012	0	NA	NA	NA	NA	NA
ENSG00000213362	NA	NA	NA	0	NA	NA	NA	NA	NA
ENSG00000213363	RPS3P6	ribosomal protein S3 pseudogene 6	645000	0	NA	NA	NA	NA	NA
ENSG00000213365	LOC401703	U2 small nuclear RNA auxiliary factor 1 like 4 pseudogene	401703	0	NA	NA	NA	NA	NA
ENSG00000213368	NA	NA	NA	0	NA	NA	NA	NA	NA
ENSG00000213370	NA	NA	NA	0	NA	NA	NA	NA	NA
ENSG00000213371	NAP1L1P3	nucleosome assembly protein 1 like 1 pseudogene 3	100130335	0	NA	NA	NA	NA	NA
ENSG00000213376	NA	NA	NA	0	NA	NA	NA	NA	NA
ENSG00000213383	NA	NA	NA	0	NA	NA	NA	NA	NA
ENSG00000213384	EIF4E2P1	eukaryotic translation initiation factor 4E family member 2 pseudogene 1	128770	0	NA	NA	NA	NA	NA
ENSG00000213385	RPL7AP39	ribosomal protein L7a pseudogene 39	646476	1,038439071	-1,570108805	2,051677	-0,765280576	0,444104488	NA
ENSG00000213386	NA	NA	NA	0	NA	NA	NA	NA	NA
ENSG00000213393	NA	NA	NA	0	NA	NA	NA	NA	NA
ENSG00000213394	RPSAP46	ribosomal protein SA pseudogene 46	652624	0	NA	NA	NA	NA	NA
ENSG00000213398	LCAT	lecithin-cholesterol acyltransferase	3931	5,56363934	-0,057622135	1,385787	-0,0415808	0,96683288	NA
ENSG00000213399	RPS2P17	ribosomal protein S2 pseudogene 17	400963	0,103998153	-1,076443944	4,080456	-0,263804824	0,791930317	NA
ENSG00000213400	NA	NA	NA	0	NA	NA	NA	NA	NA
ENSG00000213401	MAGEA12	MAGE family member A12	4111	0	NA	NA	NA	NA	NA
ENSG00000213403	NA	NA	NA	0	NA	NA	NA	NA	NA
ENSG00000213406	NA	NA	NA	0	NA	NA	NA	NA	NA
ENSG00000213409	NA	NA	NA	0	NA	NA	NA	NA	NA
ENSG00000213411	NA	NA	NA	1,318101696	-1,001753575	1,980803	-0,505731013	0,613045494	NA
ENSG00000213412	HNRNPA1P33	heterogeneous nuclear ribonucleoprotein A1 pseudogene 33	728643	0	NA	NA	NA	NA	NA
ENSG00000213414	LOC345471	cell division cycle 37, HSP90 cochaperone pseudogene	345471	0	NA	NA	NA	NA	NA
ENSG00000213420	GPC2	glypican 2	221914	2,400756373	0,828311218	1,330862	0,622386889	0,5336875	NA
ENSG00000213421	RPL7AP17	ribosomal protein L7a pseudogene 17	100270897	0	NA	NA	NA	NA	NA
ENSG00000213423	NA	NA	NA	0	NA	NA	NA	NA	NA
ENSG00000213424	KRT222	keratin 222	125113	0	NA	NA	NA	NA	NA
ENSG00000213430	NA	NA	NA	0	NA	NA	NA	NA	NA
ENSG00000213431	ABRAXAS1P1	ABRAXAS1 pseudogene 1	100421580	0	NA	NA	NA	NA	NA
ENSG00000213432	RPL17P34	ribosomal protein L17 pseudogene 34	643863	0	NA	NA	NA	NA	NA
ENSG00000213433	NA	NA	NA	0,730877336	2,142339578	2,270872	0,943399328	0,345476687	NA
ENSG00000213434	NA	NA	NA	0	NA	NA	NA	NA	NA
ENSG00000213435	NA	NA	NA	0,117497108	0,847101401	4,080456	0,207599697	0,835541541	NA
ENSG00000213438	NA	NA	NA	0	NA	NA	NA	NA	NA
ENSG00000213439	OR5AC1	olfactory receptor family 5 subfamily AC member 1 (gene/pseudogene)	81431	0	NA	NA	NA	NA	NA
ENSG00000213440	NA	NA	NA	0	NA	NA	NA	NA	NA
ENSG00000213442	RPL18AP3	ribosomal protein L18a pseudogene 3	390354	0	NA	NA	NA	NA	NA
ENSG00000213448	RPS23P2	ribosomal protein S23 pseudogene 2	391701	0,10488184	-1,076443944	4,080456	-0,263804824	0,791930317	NA
ENSG00000213449	NA	NA	NA	0	NA	NA	NA	NA	NA
ENSG00000213450	NA	NA	NA	0	NA	NA	NA	NA	NA
ENSG00000213451	OR6C69P	olfactory receptor family 6 subfamily C member 69 pseudogene	403285	0	NA	NA	NA	NA	NA
ENSG00000213452	NA	NA	NA	0	NA	NA	NA	NA	NA
ENSG00000213453	NA	NA	NA	1,569366602	-0,6647644	1,599326	-0,415652737	0,67766413	NA
ENSG00000213455	NA	NA	NA	0	NA	NA	NA	NA	NA
ENSG00000213461	NA	NA	NA	0	NA	NA	NA	NA	NA
ENSG00000213462	ERV3-1	endogenous retrovirus group 3 member 1, envelope	2086	9,518530817	-1,955861162	2,774082	-0,705048079	0,480780316	NA
ENSG00000213467	NA	NA	NA	0	NA	NA	NA	NA	NA
ENSG00000213470	RPL31P51	ribosomal protein L31 pseudogene 51	100271475	0	NA	NA	NA	NA	NA
ENSG00000213471	TTLL13	tubulin tyrosine ligase like 13	440307	0	NA	NA	NA	NA	NA
ENSG00000213478	NA	NA	NA	0	NA	NA	NA	NA	NA
ENSG00000213480	KIAA1191P1	KIAA1191 pseudogene 1	100418710	0	NA	NA	NA	NA	NA
ENSG00000213483	NA	NA	NA	0	NA	NA	NA	NA	NA
ENSG00000213484	NA	NA	NA	0	NA	NA	NA	NA	NA

ENSG00000213486	NA	NA	NA	0	NA	NA	NA	NA	NA
ENSG00000213487	ASS1P4	argininosuccinate synthetase 1 pseudogene 4	449	0	NA	NA	NA	NA	NA
ENSG00000213488	NA	NA	NA	0	NA	NA	NA	NA	NA
ENSG00000213489	NA	NA	NA	0	NA	NA	NA	NA	NA
ENSG00000213492	NT5C3AP1	NT5C3A pseudogene 1	90859	0	NA	NA	NA	NA	NA
ENSG00000213493	ACTN4P1	actinin alpha 4 pseudogene 1	344978	0	NA	NA	NA	NA	NA
ENSG00000213495	RPL26P13	ribosomal protein L26 pseudogene 13	130865	0	NA	NA	NA	NA	NA
ENSG00000213498	NA	NA	NA	0	NA	NA	NA	NA	NA
ENSG00000213500	LAP3P2	leucine aminopeptidase 3 pseudogene 2	389386	0	NA	NA	NA	NA	NA
ENSG00000213509	NA	NA	NA	0,189644771	1,664948365	4,047529	0,411349307	0,68081642	NA
ENSG00000213512	GBP7	guanylate binding protein 7	388646	0,434077754	-1,070161243	3,533651	-0,302848574	0,762005264	NA
ENSG00000213514	NA	NA	NA	0	NA	NA	NA	NA	NA
ENSG00000213519	RPL36P7	ribosomal protein L36 pseudogene 7	100271010	0,326825874	-2,558772358	3,703927	-0,690826933	0,489674308	NA
ENSG00000213522	NA	NA	NA	0	NA	NA	NA	NA	NA
ENSG00000213525	NA	NA	NA	0	NA	NA	NA	NA	NA
ENSG00000213526	NA	NA	NA	0,10543123	0	4,080456	0	1	NA
ENSG00000213527	RPS12P25	ribosomal protein S12 pseudogene 25	646985	0	NA	NA	NA	NA	NA
ENSG00000213529	RPL32P22	ribosomal protein L32 pseudogene 22	644996	0	NA	NA	NA	NA	NA
ENSG00000213530	NA	NA	NA	0	NA	NA	NA	NA	NA
ENSG00000213536	NA	NA	NA	0	NA	NA	NA	NA	NA
ENSG00000213538	KRT8P41	keratin 8 pseudogene 41	283102	0	NA	NA	NA	NA	NA
ENSG00000213539	NA	NA	NA	0	NA	NA	NA	NA	NA
ENSG00000213540	NA	NA	NA	0,393391056	1,883287883	4,025407	0,467850284	0,639891659	NA
ENSG00000213542	RPL7AP43	ribosomal protein L7a pseudogene 43	644236	1,129296345	2,84917599	2,084403	1,366902443	0,171655886	NA
ENSG00000213543	NA	NA	NA	0	NA	NA	NA	NA	NA
ENSG00000213547	VN1R64P	vomeronasal 1 receptor 64 pseudogene	730196	0	NA	NA	NA	NA	NA
ENSG00000213548	NA	NA	NA	0	NA	NA	NA	NA	NA
ENSG00000213549	NA	NA	NA	0	NA	NA	NA	NA	NA
ENSG00000213553	RPLP0P6	ribosomal protein lateral stalk subunit P0 pseudogene 6	220717	0,32551759	-0,954770674	4,042999	-0,236154068	0,81331312	NA
ENSG00000213556	LOC100288416	ATP synthase membrane subunit f pseudogene	100288416	0	NA	NA	NA	NA	NA
ENSG00000213557	RPL31P43	ribosomal protein L31 pseudogene 43	392382	0	NA	NA	NA	NA	NA
ENSG00000213558	NA	NA	NA	0	NA	NA	NA	NA	NA
ENSG00000213559	HNRNPA1P64	heterogeneous nuclear ribonucleoprotein A1 pseudogene 64	100129325	0	NA	NA	NA	NA	NA
ENSG00000213560	NA	NA	NA	0	NA	NA	NA	NA	NA
ENSG00000213561	NA	NA	NA	0	NA	NA	NA	NA	NA
ENSG00000213568	NA	NA	NA	0	NA	NA	NA	NA	NA
ENSG00000213569	NA	NA	NA	0	NA	NA	NA	NA	NA
ENSG00000213574	NA	NA	NA	0	NA	NA	NA	NA	NA
ENSG00000213578	CPLX3	complexin 3	594855	1,771041346	0,894541515	1,650737	0,541904344	0,587884404	NA
ENSG00000213579	NA	NA	NA	0	NA	NA	NA	NA	NA
ENSG00000213587	NA	NA	NA	0	NA	NA	NA	NA	NA
ENSG00000213588	ZBTB9	zinc finger and BTB domain containing 9	221504	4,963494174	-0,027884911	3,748745	-0,007438465	0,994065018	NA
ENSG00000213590	NA	NA	NA	1,944056123	-0,259807875	1,563015	-0,166222259	0,867982043	NA
ENSG00000213592	NA	NA	NA	0	NA	NA	NA	NA	NA
ENSG00000213594	NA	NA	NA	0	NA	NA	NA	NA	NA
ENSG00000213598	RPL31P4	ribosomal protein L31 pseudogene 4	729646	0	NA	NA	NA	NA	NA
ENSG00000213601	NA	NA	NA	0	NA	NA	NA	NA	NA
ENSG00000213605	NA	NA	NA	0	NA	NA	NA	NA	NA
ENSG00000213606	NA	NA	NA	0	NA	NA	NA	NA	NA
ENSG00000213607	OR4A45P	olfactory receptor family 4 subfamily A member 45 pseudogene	403251	0	NA	NA	NA	NA	NA
ENSG00000213608	NA	NA	NA	0,286070576	0,833003022	4,079922	0,204171304	0,838219635	NA
ENSG00000213609	RPL7AP50	ribosomal protein L7a pseudogene 50	87688	0	NA	NA	NA	NA	NA
ENSG00000213612	LOC124902222	putative protein FAM220BP	124902222	0	NA	NA	NA	NA	NA
ENSG00000213620	NA	NA	NA	0	NA	NA	NA	NA	NA
ENSG00000213621	RPSAP54	ribosomal protein SA pseudogene 54	642641	0,355450234	1,948054177	3,721407	0,523472504	0,600645481	NA
ENSG00000213630	NA	NA	NA	0	NA	NA	NA	NA	NA
ENSG00000213638	ADAT3	adenosine deaminase tRNA specific 3	113179	8,005556563	0,083135142	0,662386	0,125508645	0,900120881	NA

ENSG00000213640	EEF1DP4	eukaryotic translation elongation factor 1 delta pseudogene 4	442325	0,072165449	0,847101401	4,080456	0,207599697	0,835541541	NA	
ENSG00000213641	RPL7AP4	ribosomal protein L7a pseudogene 4	652985	0	NA	NA	NA	NA	NA	
ENSG00000213642	NA	NA	NA	0	NA	NA	NA	NA	NA	
ENSG00000213643	VN1R37P	vomeronasal 1 receptor 37 pseudogene	402273	0	NA	NA	NA	NA	NA	
ENSG00000213644	NA	NA	NA	0	NA	NA	NA	NA	NA	
ENSG00000213645	NA	NA	NA	0	NA	NA	NA	NA	NA	
ENSG00000213648	SULT1A4	sulfotransferase family 1A member 4	445329	2,886693704	-0,836738153	1,632232	-0,512634368	0,608207108	NA	
ENSG00000213648	LOC124907837	sulfotransferase 1A3	124907837	2,886693704	-0,836738153	1,632232	-0,512634368	0,608207108	NA	
ENSG00000213650	NA	NA	NA	0	NA	NA	NA	NA	NA	
ENSG00000213652	HMGB3P30	high mobility group box 3 pseudogene 30	203510	0	NA	NA	NA	NA	NA	
ENSG00000213653	NA	NA	NA	0	NA	NA	NA	NA	NA	
ENSG00000213655	NA	NA	NA	0	NA	NA	NA	NA	NA	
ENSG00000213657	RPL31P44	ribosomal protein L31 pseudogene 44	644522	0	NA	NA	NA	NA	NA	
ENSG00000213659	RSU1P3	Ras suppressor protein 1 pseudogene 3	100419964	0	NA	NA	NA	NA	NA	
ENSG00000213661	RPL18P3	ribosomal protein L18 pseudogene 3	100129374	0	NA	NA	NA	NA	NA	
ENSG00000213663	RPL21P58	ribosomal protein L21 pseudogene 58	100271173	0	NA	NA	NA	NA	NA	
ENSG00000213664	RPS16P8	ribosomal protein S16 pseudogene 8	654170	0	NA	NA	NA	NA	NA	
ENSG00000213667	NA	NA	NA	0	NA	NA	NA	NA	NA	
ENSG00000213669	RPL21P86	ribosomal protein L21 pseudogene 86	100271190	0,286317351	-1,026044167	4,038843	-0,254044071	0,799461511	NA	
ENSG00000213671	OLA1P2	OLA1 pseudogene 2	106479059	0,10543123	0	4,080456	0	1	NA	
ENSG00000213673	NA	NA	NA	0	NA	NA	NA	NA	NA	
ENSG00000213684	NA	NA	NA	0	NA	NA	NA	NA	NA	
ENSG00000213690	NA	NA	NA	0	NA	NA	NA	NA	NA	
ENSG00000213693	SEC14L1P1	SEC14 like 1 pseudogene 1	729799	8,3740787	0,890770581	0,778055	1,144868172	0,25226377	NA	
ENSG00000213694	S1PR3	sphingosine-1-phosphate receptor 3	1903	0	NA	NA	NA	NA	NA	
ENSG00000213695	RPS7P14	ribosomal protein S7 pseudogene 14	646108	0	NA	NA	NA	NA	NA	
ENSG00000213697	NA	NA	NA	0	NA	NA	NA	NA	NA	
ENSG00000213698	CAPZA1P3	CAPZA1 pseudogene 3	645938	0	NA	NA	NA	NA	NA	
ENSG00000213700	RPL17P50	ribosomal protein L17 pseudogene 50	729046	0,628891271	-1,041327454	2,893811	-0,359846358	0,718962033	NA	
ENSG00000213701	NA	NA	NA	0	NA	NA	NA	NA	NA	
ENSG00000213703	NA	NA	NA	0	NA	NA	NA	NA	NA	
ENSG00000213704	NA	NA	NA	0	NA	NA	NA	NA	NA	
ENSG00000213706	RHOG2P	RHOG family member 2, pseudogene	392489	0,3268275	-0,115555043	3,828985	-0,030179026	0,975924275	NA	
ENSG00000213707	NA	NA	NA	0,15836288	0	4,080456	0	1	NA	
ENSG00000213708	SLC16A14P1	solute carrier family 16 member 14 pseudogene 1	100128685	0	NA	NA	NA	NA	NA	
ENSG00000213711	NA	NA	NA	0	NA	NA	NA	NA	NA	
ENSG00000213713	NA	NA	NA	0,237167733	1,955824269	4,022315	0,486243437	0,626794577	NA	
ENSG00000213714	FAM209B	family with sequence similarity 209 member B	388799	2,294046576	0,172015592	1,18032	0,145736463	0,884129436	NA	
ENSG00000213716	FABP5P5	fatty acid binding protein 5 pseudogene 5	100288964	0	NA	NA	NA	NA	NA	
ENSG00000213717	RPL18AP10	ribosomal protein L18a pseudogene 10	100271285	0	NA	NA	NA	NA	NA	
ENSG00000213721	NA	NA	NA	0	NA	NA	NA	NA	NA	
ENSG00000213724	NA	NA	NA	0	NA	NA	NA	NA	NA	
ENSG00000213726	RPS2P52	ribosomal protein S2 pseudogene 52	100271250	0	NA	NA	NA	NA	NA	
ENSG00000213727	LOC100132723	single stranded DNA binding protein 4 pseudogene	100132723	0	NA	NA	NA	NA	NA	
ENSG00000213729	RPS16P2	ribosomal protein S16 pseudogene 2	402069	0	NA	NA	NA	NA	NA	
ENSG00000213730	NA	NA	NA	0	NA	NA	NA	NA	NA	
ENSG00000213731	NA	NA	NA	0	NA	NA	NA	NA	NA	
ENSG00000213735	NA	NA	NA	0	NA	NA	NA	NA	NA	
ENSG00000213736	BRI3P2	brain protein I3 pseudogene 2	441630	0	NA	NA	NA	NA	NA	
ENSG00000213740	NA	NA	NA	0	NA	NA	NA	NA	NA	
ENSG00000213744	RPS10P14	ribosomal protein S10 pseudogene 14	646942	0	NA	NA	NA	NA	NA	
ENSG00000213747	NA	NA	NA	0	NA	NA	NA	NA	NA	
ENSG00000213750	RPS2P33	ribosomal protein S2 pseudogene 33	286150	0	NA	NA	NA	NA	NA	

ENSG00000213752	RPS10P31	ribosomal protein S10 pseudogene 31	100129939	0	NA	NA	NA	NA	NA
ENSG00000213753	CENPBD2P	CENPB DNA-binding domains containing 2, pseudogene	65996	1,362543428	1,905428692	2,706149	0,70411072	0,481363825	NA
ENSG00000213753	RPL23AP79	ribosomal protein L23a pseudogene 79	100271626	1,362543428	1,905428692	2,706149	0,70411072	0,481363825	NA
ENSG00000213754	KPNA4P1	KPNA4 pseudogene 1	100421109	3,113007149	-1,185468885	1,228872	-0,964680824	0,334704703	NA
ENSG00000213755	NA	NA	NA	0	NA	NA	NA	NA	NA
ENSG00000213757	RPS3AP20	RPS3A pseudogene 20	643932	1,486785627	0,378543723	1,632959	0,231814618	0,816682003	NA
ENSG00000213759	UGT2B11	UDP glucuronosyltransferase family 2 member B11	10720	0	NA	NA	NA	NA	NA
ENSG00000213760	ATP6V1G2	ATPase H+ transporting V1 subunit G2	534	0,567914286	0,457234546	3,415554	0,133868322	0,8935067	NA
ENSG00000213761	NA	NA	NA	0	NA	NA	NA	NA	NA
ENSG00000213763	NA	NA	NA	0	NA	NA	NA	NA	NA
ENSG00000213771	KRT8P37	keratin 8 pseudogene 37	100287325	0	NA	NA	NA	NA	NA
ENSG00000213772	NA	NA	NA	0	NA	NA	NA	NA	NA
ENSG00000213774	LOC124905951	cyclin-dependent kinase 2-associated protein 1-like	124905951	0	NA	NA	NA	NA	NA
ENSG00000213777	NA	NA	NA	0,10543123	0	4,080456	0	1	NA
ENSG00000213778	NA	NA	NA	0	NA	NA	NA	NA	NA
ENSG00000213779	RPL29P21	ribosomal protein L29 pseudogene 21	732199	0	NA	NA	NA	NA	NA
ENSG00000213781	PSMC1P2	proteasome 26S subunit, ATPase 1 pseudogene 2	442291	0	NA	NA	NA	NA	NA
ENSG00000213783	NA	NA	NA	0	NA	NA	NA	NA	NA
ENSG00000213785	NA	NA	NA	0	NA	NA	NA	NA	NA
ENSG00000213786	NA	NA	NA	0	NA	NA	NA	NA	NA
ENSG00000213787	RPL7AP38	ribosomal protein L7a pseudogene 38	442660	0	NA	NA	NA	NA	NA
ENSG00000213791	RPS5P5	RPS5 pseudogene 5	340443	0	NA	NA	NA	NA	NA
ENSG00000213798	RPL7AP41	ribosomal protein L7a pseudogene 41	100271213	0	NA	NA	NA	NA	NA
ENSG00000213801	ZNF321P	zinc finger protein 321, pseudogene	399669	1,661634035	-0,715270226	2,32688	-0,30739458	0,758543053	NA
ENSG00000213816	NA	NA	NA	0	NA	NA	NA	NA	NA
ENSG00000213820	RPL13P2	ribosomal protein L13 pseudogene 2	128500	0	NA	NA	NA	NA	NA
ENSG00000213822	CEACAM18	CEA cell adhesion molecule 18	729767	0	NA	NA	NA	NA	NA
ENSG00000213839	NA	NA	NA	0,10543123	0	4,080456	0	1	NA
ENSG00000213849	RPL31P18	ribosomal protein L31 pseudogene 18	285260	0	NA	NA	NA	NA	NA
ENSG00000213851	RPS2P21	ribosomal protein S2 pseudogene 21	441013	0	NA	NA	NA	NA	NA
ENSG00000213853	EMP2	epithelial membrane protein 2	2013	0,117497108	0,847101401	4,080456	0,207599697	0,835541541	NA
ENSG00000213854	CNN2P6	calponin 2 pseudogene 6	339897	0,307501004	2,351458528	3,995028	0,588596321	0,556132102	NA
ENSG00000213856	NA	NA	NA	0	NA	NA	NA	NA	NA
ENSG00000213857	NA	NA	NA	0,223243684	-1,062897583	4,080456	-0,260485009	0,794489678	NA
ENSG00000213860	RPL21P75	ribosomal protein L21 pseudogene 75	728501	0,209763681	-1,926644696	4,042043	-0,476651224	0,633610501	NA
ENSG00000213862	RPL7AP62	ribosomal protein L7a pseudogene 62	644029	0	NA	NA	NA	NA	NA
ENSG00000213864	EEF1B2P2	eukaryotic translation elongation factor 1 beta 2 pseudogene 2	1934	0	NA	NA	NA	NA	NA
ENSG00000213866	NA	NA	NA	0	NA	NA	NA	NA	NA
ENSG00000213867	RPL27P1	ribosomal protein L27 pseudogene 1	326299	0	NA	NA	NA	NA	NA
ENSG00000213871	NA	NA	NA	0	NA	NA	NA	NA	NA
ENSG00000213872	NA	NA	NA	0	NA	NA	NA	NA	NA
ENSG00000213873	PPIAP86	peptidylprolyl isomerase A pseudogene 86	111082970	0	NA	NA	NA	NA	NA
ENSG00000213875	NA	NA	NA	0	NA	NA	NA	NA	NA
ENSG00000213876	RPL7AP64	ribosomal protein L7a pseudogene 64	728486	0	NA	NA	NA	NA	NA
ENSG00000213877	NA	NA	NA	0	NA	NA	NA	NA	NA
ENSG00000213880	RPL7AP7	ribosomal protein L7a pseudogene 7	353013	0	NA	NA	NA	NA	NA
ENSG00000213881	NA	NA	NA	0,103998153	-1,076443944	4,080456	-0,263804824	0,791930317	NA
ENSG00000213882	NA	NA	NA	0	NA	NA	NA	NA	NA
ENSG00000213885	NA	NA	NA	0,741293367	-2,278666298	2,577268	-0,884140143	0,376620566	NA
ENSG00000213886	UBD	ubiquitin D	10537	0	NA	NA	NA	NA	NA
ENSG00000213889	PPM1N	protein phosphatase, Mg2+/Mn2+ dependent 1N (putative)	147699	2,629436623	-1,352778613	1,69029	-0,80032358	0,423523345	NA
ENSG00000213891	RPL3P6	ribosomal protein L3 pseudogene 6	643220	0	NA	NA	NA	NA	NA

ENSG00000213892	CEACAM16	CEA cell adhesion molecule 16, tectorial membrane component	388551	0	NA	NA	NA	NA	NA
ENSG00000213896	RPL31P8	ribosomal protein L31 pseudogene 8	253013	0	NA	NA	NA	NA	NA
ENSG00000213901	SLC23A3	solute carrier family 23 member 3	151295	1,467372237	-1,98662646	1,953706	-1,016850209	0,309224693	NA
ENSG00000213908	CYP2A7P1	cytochrome P450 family 2 subfamily A member 7 pseudogene 1	1550	0	NA	NA	NA	NA	NA
ENSG00000213911	OR2G1P	olfactory receptor family 2 subfamily G member 1 pseudogene	26717	0	NA	NA	NA	NA	NA
ENSG00000213916	RPL13P	ribosomal protein L13 pseudogene	387044	0	NA	NA	NA	NA	NA
ENSG00000213917	RPL5P8	ribosomal protein L5 pseudogene 8	100130523	0	NA	NA	NA	NA	NA
ENSG00000213921	LEUTX	leucine twenty homeobox	342900	0	NA	NA	NA	NA	NA
ENSG00000213924	HNRNPH1P2	heterogeneous nuclear ribonucleoprotein H1 pseudogene 2	100129368	0	NA	NA	NA	NA	NA
ENSG00000213925	NA	NA	NA	0	NA	NA	NA	NA	NA
ENSG00000213927	CCL27	C-C motif chemokine ligand 27	10850	0	NA	NA	NA	NA	NA
ENSG00000213931	HBE1	hemoglobin subunit epsilon 1	3046	0	NA	NA	NA	NA	NA
ENSG00000213934	HBG1	hemoglobin subunit gamma 1	3047	0	NA	NA	NA	NA	NA
ENSG00000213935	RPL22P16	ribosomal protein L22 pseudogene 16	100270959	0,117497108	0,847101401	4,080456	0,207599697	0,835541541	NA
ENSG00000213937	CLDN9	claudin 9	9080	5,453500299	0,108673211	0,793099	0,13702343	0,891012275	NA
ENSG00000213938	SEPHS1P6	selenophosphate synthetase 1 pseudogene 6	729399	0	NA	NA	NA	NA	NA
ENSG00000213939	RPS12P29	ribosomal protein S12 pseudogene 29	728244	0,178270104	-0,153946761	4,079547	-0,037736235	0,969897985	NA
ENSG00000213940	RPL13AP13	ribosomal protein L13a pseudogene 13	402214	0	NA	NA	NA	NA	NA
ENSG00000213942	RPL31P10	ribosomal protein L31 pseudogene 10	390283	0	NA	NA	NA	NA	NA
ENSG00000213943	KRT18P17	keratin 18 pseudogene 17	344866	0	NA	NA	NA	NA	NA
ENSG00000213946	NA	NA	NA	0	NA	NA	NA	NA	NA
ENSG00000213950	RPS10P2	ribosomal protein S10 pseudogene 2	140758	0	NA	NA	NA	NA	NA
ENSG00000213954	ATP5PDP3	ATP synthase peripheral stalk subunit d pseudogene 3	138864	0	NA	NA	NA	NA	NA
ENSG00000213956	NA	NA	NA	0,072165449	0,847101401	4,080456	0,207599697	0,835541541	NA
ENSG00000213958	KRT18P29	keratin 18 pseudogene 29	729050	0	NA	NA	NA	NA	NA
ENSG00000213959	NA	NA	NA	0	NA	NA	NA	NA	NA
ENSG00000213962	NA	NA	NA	0	NA	NA	NA	NA	NA
ENSG00000213964	NA	NA	NA	0	NA	NA	NA	NA	NA
ENSG00000213970	RPS15P7	ribosomal protein S15 pseudogene 7	100271361	0	NA	NA	NA	NA	NA
ENSG00000213972	NA	NA	NA	0	NA	NA	NA	NA	NA
ENSG00000213973	ZNF99	zinc finger protein 99	7652	0	NA	NA	NA	NA	NA
ENSG00000213976	NA	NA	NA	0,314645521	-2,509429468	3,997603	-0,627733573	0,530178488	NA
ENSG00000213985	NA	NA	NA	0	NA	NA	NA	NA	NA
ENSG00000213988	ZNF90	zinc finger protein 90	7643	0,446730493	-1,986507594	2,978745	-0,666894238	0,504839692	NA
ENSG00000213996	TM6SF2	transmembrane 6 superfamily member 2	53345	0	NA	NA	NA	NA	NA
ENSG00000213997	NA	NA	NA	0,103998153	-1,076443944	4,080456	-0,263804824	0,791930317	NA
ENSG00000213999	BORCS8-MEF2B	BORCS8-MEF2B readthrough	4207	4,842397611	-0,218875783	1,010391	-0,21662483	0,828500733	NA
ENSG00000213999	MEF2B	myocyte enhancer factor 2B	100271849	4,842397611	-0,218875783	1,010391	-0,21662483	0,828500733	NA
ENSG00000214003	NA	NA	NA	0	NA	NA	NA	NA	NA
ENSG00000214009	NA	NA	NA	0	NA	NA	NA	NA	NA
ENSG00000214012	KRT18P38	keratin 18 pseudogene 38	441133	0	NA	NA	NA	NA	NA
ENSG00000214015	RPL32P23	ribosomal protein L32 pseudogene 23	644020	0	NA	NA	NA	NA	NA
ENSG00000214016	RPSAP61	ribosomal protein SA pseudogene 61	644464	0,369722193	-1,06876226	3,712121	-0,287911509	0,773414477	NA
ENSG00000214018	NA	NA	NA	0,119673877	0,847101401	4,080456	0,207599697	0,835541541	NA
ENSG00000214019	RBM39P1	RBM39 pseudogene 1	643167	0	NA	NA	NA	NA	NA
ENSG00000214020	NA	NA	NA	0	NA	NA	NA	NA	NA
ENSG00000214024	RPL23AP29	ribosomal protein L23a pseudogene 29	100271458	0	NA	NA	NA	NA	NA
ENSG00000214025	ATP5PBP4	ATP synthase peripheral stalk-membrane subunit b pseudogene 4	100422514	0	NA	NA	NA	NA	NA
ENSG00000214027	NA	NA	NA	0	NA	NA	NA	NA	NA
ENSG00000214031	NA	NA	NA	0	NA	NA	NA	NA	NA

ENSG00000214035	NA	NA	NA	0	NA	NA	NA	NA	NA
ENSG00000214041	NA	NA	NA	0	NA	NA	NA	NA	NA
ENSG00000214042	IFNA7	interferon alpha 7	3444	0	NA	NA	NA	NA	NA
ENSG00000214045	NA	NA	NA	0	NA	NA	NA	NA	NA
ENSG00000214047	RPS11P7	ribosomal protein S11 pseudogene 7	642687	0	NA	NA	NA	NA	NA
ENSG00000214050	FBXO16	F-box protein 16	157574	0	NA	NA	NA	NA	NA
ENSG00000214051	NA	NA	NA	0	NA	NA	NA	NA	NA
ENSG00000214062	RPL7P17	ribosomal protein L7 pseudogene 17	100270851	0	NA	NA	NA	NA	NA
ENSG00000214063	TSPAN4	tetraspanin 4	7106	11,28250535	-0,530300874	0,54125	-0,979770903	0,327199218	NA
ENSG00000214064	RPL6P5	ribosomal protein L6 pseudogene 5	100270844	0	NA	NA	NA	NA	NA
ENSG00000214067	NA	NA	NA	0,15836288	0	4,080456	0	1	NA
ENSG00000214070	MANEALP1	MANEAL pseudogene 1	391448	0,470590306	0,715302533	2,954573	0,242100129	0,808702578	NA
ENSG00000214071	FTH1P29	ferritin heavy chain 1 pseudogene 29	442445	0	NA	NA	NA	NA	NA
ENSG00000214073	RPL21P17	ribosomal protein L21 pseudogene 17	727810	0	NA	NA	NA	NA	NA
ENSG00000214074	RPL23AP39	ribosomal protein L23a pseudogene 39	100271461	0	NA	NA	NA	NA	NA
ENSG00000214076	CPSF1P1	CPSF1 pseudogene 1	129099	1,59749014	-0,092131639	1,50894	-0,061057195	0,951313659	NA
ENSG00000214077	NA	NA	NA	0	NA	NA	NA	NA	NA
ENSG00000214081	NA	NA	NA	0	NA	NA	NA	NA	NA
ENSG00000214089	NA	NA	NA	0	NA	NA	NA	NA	NA
ENSG00000214093	RPS18P14	ribosomal protein S18 pseudogene 14	100128535	0,802495104	0,64920821	2,815316	0,230598713	0,817626568	NA
ENSG00000214097	SMCO1	single-pass membrane protein with coiled-coil domains 1	255798	0,144330898	1,418786819	4,061262	0,349346267	0,726829368	NA
ENSG00000214102	WEE2	WEE2 oocyte meiosis inhibiting kinase	494551	0	NA	NA	NA	NA	NA
ENSG00000214107	MAGEB1	MAGE family member B1	4112	0	NA	NA	NA	NA	NA
ENSG00000214108	TPT1P5	TPT1 pseudogene 5	100421420	0	NA	NA	NA	NA	NA
ENSG00000214110	NA	NA	NA	1,201099585	1,678960261	1,913466	0,87744475	0,380245116	NA
ENSG00000214111	NA	NA	NA	0	NA	NA	NA	NA	NA
ENSG00000214121	NA	NA	NA	0	NA	NA	NA	NA	NA
ENSG00000214124	SNRPEP9	SNRPE pseudogene 9	100874416	0,10543123	0	4,080456	0	1	NA
ENSG00000214125	NA	NA	NA	0	NA	NA	NA	NA	NA
ENSG00000214128	TMEM213	transmembrane protein 213	155006	0	NA	NA	NA	NA	NA
ENSG00000214132	NA	NA	NA	0,11808024	-1,076443944	4,080456	-0,263804824	0,791930317	NA
ENSG00000214140	PRCD	photoreceptor disc component	768206	0	NA	NA	NA	NA	NA
ENSG00000214141	NA	NA	NA	0	NA	NA	NA	NA	NA
ENSG00000214142	RPL7P60	ribosomal protein L7 pseudogene 60	100289264	0	NA	NA	NA	NA	NA
ENSG00000214144	NA	NA	NA	0	NA	NA	NA	NA	NA
ENSG00000214147	ENAHP1	ENAH pseudogene 1	100131685	0	NA	NA	NA	NA	NA
ENSG00000214161	NA	NA	NA	0	NA	NA	NA	NA	NA
ENSG00000214174	NA	NA	NA	10,31754715	0,106217459	0,604551	0,175696461	0,860532416	NA
ENSG00000214178	NA	NA	NA	0	NA	NA	NA	NA	NA
ENSG00000214182	PTMAP5	prothymosin alpha pseudogene 5	150928	0,072165449	0,847101401	4,080456	0,207599697	0,835541541	NA
ENSG00000214185	XPOTP1	exportin for tRNA pseudogene 1	170559	0	NA	NA	NA	NA	NA
ENSG00000214189	NA	NA	NA	0,564293211	0,026680232	2,861034	0,009325381	0,99255953	NA
ENSG00000214190	NA	NA	NA	0,418083389	-2,496438939	3,998134	-0,624400969	0,532364289	NA
ENSG00000214192	NA	NA	NA	0,192209515	1,680544719	4,04618	0,415341016	0,677892279	NA
ENSG00000214195	NA	NA	NA	0	NA	NA	NA	NA	NA
ENSG00000214198	TTC41P	tetratricopeptide repeat domain 41, pseudogene	253724	1,501229414	0,968131903	1,603703	0,603685348	0,546052857	NA
ENSG00000214199	NA	NA	NA	0,837241241	-0,75858777	2,097538	-0,361656326	0,717608868	NA
ENSG00000214200	NA	NA	NA	0	NA	NA	NA	NA	NA
ENSG00000214203	RPS4XP1	ribosomal protein S4X pseudogene 1	400064	0,56513882	0,903360073	2,559613	0,352928343	0,724142161	NA
ENSG00000214204	NA	NA	NA	0	NA	NA	NA	NA	NA
ENSG00000214207	KRT18P10	keratin 18 pseudogene 10	360019	0	NA	NA	NA	NA	NA
ENSG00000214210	NA	NA	NA	0	NA	NA	NA	NA	NA
ENSG00000214211	CTAGE14P	CTAGE family member 14, pseudogene	401018	0	NA	NA	NA	NA	NA
ENSG00000214212	C19orf38	chromosome 19 open reading frame 38	255809	0,672726382	-0,356820663	3,283231	-0,108679738	0,913456514	NA
ENSG00000214216	IQCJ	IQ motif containing J	654502	0	NA	NA	NA	NA	NA
ENSG00000214222	NA	NA	NA	0	NA	NA	NA	NA	NA
ENSG00000214223	NA	NA	NA	1,78311023	-1,83076406	1,592074	-1,149923828	0,250175246	NA
ENSG00000214237	MINDY4B	MINDY family member 4B	646951	0	NA	NA	NA	NA	NA
ENSG00000214244	NA	NA	NA	0	NA	NA	NA	NA	NA
ENSG00000214245	PUDPP3	pseudouridine 5'-phosphatase pseudogene 3	391242	0	NA	NA	NA	NA	NA

ENSG00000214249	CTAGE11P	CTAGE family member 11, pseudogene	647288	0	NA	NA	NA	NA	NA
ENSG00000214252	AZGP1P2	AZGP1 pseudogene 2	401393	0,207996306	-1,916073151	4,043021	-0,473921178	0,635556117	NA
ENSG00000214254	LOC100420466	dual specificity phosphatase 3 pseudogene	100420466	0	NA	NA	NA	NA	NA
ENSG00000214255	RPS2P6	ribosomal protein S2 pseudogene 6	342808	0	NA	NA	NA	NA	NA
ENSG00000214259	RPL10AP12	ribosomal protein L10a pseudogene 12	401863	0	NA	NA	NA	NA	NA
ENSG00000214262	ANKRD36BP1	ankyrin repeat domain 36B pseudogene 1	84832	0	NA	NA	NA	NA	NA
ENSG00000214263	RPSAP53	ribosomal protein SA pseudogene 53	400141	0	NA	NA	NA	NA	NA
ENSG00000214264	NA	NA	NA	0,442650221	0,104938304	3,985051	0,026332992	0,97899174	NA
ENSG00000214265	NA	NA	NA	9,16412183	-0,251652228	0,598766	-0,420284498	0,674277633	NA
ENSG00000214266	NA	NA	NA	0	NA	NA	NA	NA	NA
ENSG00000214268	NA	NA	NA	0	NA	NA	NA	NA	NA
ENSG00000214269	NA	NA	NA	0	NA	NA	NA	NA	NA
ENSG00000214273	NA	NA	NA	2,606075147	0,247477466	1,437484	0,172160145	0,86331163	NA
ENSG00000214274	ANG	angiogenin	283	0,117497108	0,847101401	4,080456	0,207599697	0,835541541	NA
ENSG00000214278	LOC100310782	mitochondrial translational initiation factor 3 pseudogene	100310782	0	NA	NA	NA	NA	NA
ENSG00000214279	SCART1	scavenger receptor family member expressed on T cells 1	619207	9,559840494	-1,065518086	0,685157	-1,555144877	0,119911569	NA
ENSG00000214280	DTWD1P1	DTWD1 pseudogene 1	124296660	0	NA	NA	NA	NA	NA
ENSG00000214281	NA	NA	NA	0	NA	NA	NA	NA	NA
ENSG00000214282	KRT8P14	keratin 8 pseudogene 14	347333	0	NA	NA	NA	NA	NA
ENSG00000214283	NA	NA	NA	0	NA	NA	NA	NA	NA
ENSG00000214285	NPS	neuropeptide S	594857	0	NA	NA	NA	NA	NA
ENSG00000214286	NA	NA	NA	0	NA	NA	NA	NA	NA
ENSG00000214288	NA	NA	NA	0	NA	NA	NA	NA	NA
ENSG00000214289	NA	NA	NA	0	NA	NA	NA	NA	NA
ENSG00000214290	POU2AF3	POU class 2 homeobox associating factor 3	120376	0	NA	NA	NA	NA	NA
ENSG00000214295	FOXO1B	forkhead box O1B (pseudogene)	2311	0	NA	NA	NA	NA	NA
ENSG00000214297	NA	NA	NA	0	NA	NA	NA	NA	NA
ENSG00000214298	NA	NA	NA	0	NA	NA	NA	NA	NA
ENSG00000214300	SPDYE3	speedy/RINGO cell cycle regulator family member E3	441272	0	NA	NA	NA	NA	NA
ENSG00000214301	NA	NA	NA	0	NA	NA	NA	NA	NA
ENSG00000214309	MBLAC1	metallo-beta-lactamase domain containing 1	255374	6,131969238	0,517224848	0,783085	0,660496015	0,508935575	NA
ENSG00000214313	NA	NA	NA	0	NA	NA	NA	NA	NA
ENSG00000214318	NA	NA	NA	0	NA	NA	NA	NA	NA
ENSG00000214319	CXADRP1	CXADR pseudogene 1	653108	0	NA	NA	NA	NA	NA
ENSG00000214321	NA	NA	NA	0	NA	NA	NA	NA	NA
ENSG00000214322	NA	NA	NA	0	NA	NA	NA	NA	NA
ENSG00000214324	PRR23E	PRR23 family member E	285311	0	NA	NA	NA	NA	NA
ENSG00000214326	RPL31P1	ribosomal protein L31 pseudogene 1	54027	0	NA	NA	NA	NA	NA
ENSG00000214329	SLC9B1P2	solute carrier family 9 member B1 pseudogene 2	389000	0	NA	NA	NA	NA	NA
ENSG00000214330	NA	NA	NA	0	NA	NA	NA	NA	NA
ENSG00000214335	CTAGE16P	CTAGE family member 16, pseudogene	341689	0	NA	NA	NA	NA	NA
ENSG00000214336	FOXI3	forkhead box I3	344167	0	NA	NA	NA	NA	NA
ENSG00000214338	MTCL3	MTCL family member 3	387104	0	NA	NA	NA	NA	NA
ENSG00000214342	ATP5PBP2	ATP synthase peripheral stalk-membrane subunit b pseudogene 2	645973	0	NA	NA	NA	NA	NA
ENSG00000214344	NA	NA	NA	0	NA	NA	NA	NA	NA
ENSG00000214347	LOC390877	adenylate kinase isoenzyme 1-like	390877	0	NA	NA	NA	NA	NA
ENSG00000214351	OR1X5P	olfactory receptor family 1 subfamily X member 5 pseudogene	402240	0	NA	NA	NA	NA	NA
ENSG00000214354	NA	NA	NA	0	NA	NA	NA	NA	NA
ENSG00000214357	NEURL1B	neuralized E3 ubiquitin protein ligase 1B	54492	11,45336587	-0,086820491	0,564764	-0,153728879	0,877823513	NA
ENSG00000214359	RPL18P10	ribosomal protein L18 pseudogene 10	100271286	1,208468268	-0,876211164	1,859682	-0,47116171	0,63752526	NA
ENSG00000214360	EFCAB9	EF-hand calcium binding domain 9	285588	0,119673877	0,847101401	4,080456	0,207599697	0,835541541	NA
ENSG00000214362	RPS3AP36	RPS3A pseudogene 36	643981	0	NA	NA	NA	NA	NA
ENSG00000214366	NA	NA	NA	0	NA	NA	NA	NA	NA
ENSG00000214369	NA	NA	NA	0	NA	NA	NA	NA	NA

ENSG00000214374	RPLP2P1	ribosomal protein lateral stalk subunit P2 pseudogene 1	442175	0	NA	NA	NA	NA	NA
ENSG00000214376	VSTM5	V-set and transmembrane domain containing 5	387804	0	NA	NA	NA	NA	NA
ENSG00000214380	NA	NA	NA	0	NA	NA	NA	NA	NA
ENSG00000214402	LCNL1	lipocalin like 1	401562	0	NA	NA	NA	NA	NA
ENSG00000214405	NA	NA	NA	0	NA	NA	NA	NA	NA
ENSG00000214414	TRIM77	tripartite motif containing 77	390231	0	NA	NA	NA	NA	NA
ENSG00000214415	GNAT3	G protein subunit alpha transducin 3	346562	0	NA	NA	NA	NA	NA
ENSG00000214417	KRT18P13	keratin 18 pseudogene 13	392371	0	NA	NA	NA	NA	NA
ENSG00000214424	FAM149B1P1	family with sequence similarity 149, member B2	388181	0	NA	NA	NA	NA	NA
ENSG00000214428	NA	NA	NA	0	NA	NA	NA	NA	NA
ENSG00000214429	NA	NA	NA	0	NA	NA	NA	NA	NA
ENSG00000214433	NA	NA	NA	0	NA	NA	NA	NA	NA
ENSG00000214434	NIFKP1	nucleolar protein interacting with the FHA domain of MKI67 pseudogene 1	401825	0	NA	NA	NA	NA	NA
ENSG00000214435	AS3MT	arsenite methyltransferase	57412	0	NA	NA	NA	NA	NA
ENSG00000214439	NA	NA	NA	4,308247998	-0,045817191	0,979312	-0,046785094	0,96268451	NA
ENSG00000214447	FAM187A	family with sequence similarity 187 member A	100528020	0	NA	NA	NA	NA	NA
ENSG00000214455	NA	NA	NA	5,573845124	0,702400361	0,771264	0,910713198	0,362446508	NA
ENSG00000214456	PLIN5	perilipin 5	440503	0,423657656	-2,442003348	4,000411	-0,610438168	0,541571591	NA
ENSG00000214457	RPL7P29	ribosomal protein L7 pseudogene 29	643971	0	NA	NA	NA	NA	NA
ENSG00000214460	NA	NA	NA	0	NA	NA	NA	NA	NA
ENSG00000214465	NA	NA	NA	2,260026514	0,637389208	1,296735	0,491534046	0,623048778	NA
ENSG00000214485	RPL7P1	ribosomal protein L7 pseudogene 1	6174	0,10488184	-1,076443944	4,080456	-0,263804824	0,791930317	NA
ENSG00000214487	OR7E148P	olfactory receptor family 7 subfamily E member 148 pseudogene	282801	0	NA	NA	NA	NA	NA
ENSG00000214491	SEC14L6	SEC14 like lipid binding 6	730005	0,227665699	0,077214204	4,076343	0,018942028	0,984887352	NA
ENSG00000214510	SPINK13	serine peptidase inhibitor Kazal type 13	153218	0	NA	NA	NA	NA	NA
ENSG00000214511	HIGD1C	HIG1 hypoxia inducible domain family member 1C	613227	0	NA	NA	NA	NA	NA
ENSG00000214513	NOTO	notochord homeobox	344022	0	NA	NA	NA	NA	NA
ENSG00000214514	NA	NA	NA	0	NA	NA	NA	NA	NA
ENSG00000214525	NA	NA	NA	0	NA	NA	NA	NA	NA
ENSG00000214526	RPS10P30	ribosomal protein S10 pseudogene 30	343851	0	NA	NA	NA	NA	NA
ENSG00000214533	NA	NA	NA	0	NA	NA	NA	NA	NA
ENSG00000214534	NA	NA	NA	0	NA	NA	NA	NA	NA
ENSG00000214535	RPS15AP1	ribosomal protein S15a pseudogene 1	92682	0	NA	NA	NA	NA	NA
ENSG00000214541	RPS4XP3	ribosomal protein S4X pseudogene 3	128580	0	NA	NA	NA	NA	NA
ENSG00000214544	GTF2IRD2P1	GTF2I repeat domain containing 2 pseudogene 1	401375	0,264019887	0	4,080456	0	1	NA
ENSG00000214549	NA	NA	NA	0	NA	NA	NA	NA	NA
ENSG00000214552	NA	NA	NA	0,612168531	-0,780085552	2,405253	-0,324325725	0,745691446	NA
ENSG00000214553	NA	NA	NA	0	NA	NA	NA	NA	NA
ENSG00000214558	ZC3H11C	zinc finger CCCH-type containing 11C	441155	2,517998166	-1,066979419	1,308427	-0,815467594	0,414804707	NA
ENSG00000214560	NA	NA	NA	0	NA	NA	NA	NA	NA
ENSG00000214561	RBBP4P4	RBBP4 pseudogene 4	727842	0,15836288	0	4,080456	0	1	NA
ENSG00000214562	NUTM2D	NUT family member 2D	728130	5,223803276	2,985797877	1,035203	2,884263066	0,003923309	NA
ENSG00000214563	GAPDHP15	glyceraldehyde-3-phosphate dehydrogenase pseudogene 15	642317	0	NA	NA	NA	NA	NA
ENSG00000214575	CPEB1	cytoplasmic polyadenylation element binding protein 1	64506	0	NA	NA	NA	NA	NA
ENSG00000214578	NA	NA	NA	0	NA	NA	NA	NA	NA
ENSG00000214581	NA	NA	NA	0	NA	NA	NA	NA	NA
ENSG00000214584	NA	NA	NA	0	NA	NA	NA	NA	NA
ENSG00000214593	NA	NA	NA	0	NA	NA	NA	NA	NA
ENSG00000214595	EML6	EMAP like 6	400954	11,24696788	-1,143011855	1,036615	-1,102638526	0,270184173	NA
ENSG00000214602	NA	NA	NA	0	NA	NA	NA	NA	NA
ENSG00000214604	NPM1P36	nucleophosmin 1 pseudogene 36	652423	0	NA	NA	NA	NA	NA
ENSG00000214607	ADAM24P	ADAM metallopeptidase domain 24, pseudogene	646479	0	NA	NA	NA	NA	NA
ENSG00000214612	RPS19P1	ribosomal protein S19 pseudogene 1	85486	0	NA	NA	NA	NA	NA
ENSG00000214617	NA	NA	NA	0	NA	NA	NA	NA	NA

ENSG00000214626	POLR3DP1	RNA polymerase III subunit D pseudogene 1	100422384	1,072219713	-0,074830251	1,695757	-0,044127928	0,964802431	NA
ENSG00000214628	NDUFB5P2	NADH:ubiquinone oxidoreductase subunit B5 pseudogene 2	100422431	0	NA	NA	NA	NA	NA
ENSG00000214629	RPSAP6	ribosomal protein SA pseudogene 6	414254	1,067873879	-0,524864853	1,799784	-0,291626612	0,770572126	NA
ENSG00000214641	RPS15AP20	ribosomal protein S15a pseudogene 20	647155	0	NA	NA	NA	NA	NA
ENSG00000214642	DEFB113	defensin beta 113	245927	0	NA	NA	NA	NA	NA
ENSG00000214643	DEFB133	defensin beta 133 (pseudogene)	403339	0	NA	NA	NA	NA	NA
ENSG00000214646	NA	NA	NA	0	NA	NA	NA	NA	NA
ENSG00000214651	LOC645482	ATPase H+ transporting V1 subunit B2 pseudogene	645482	0	NA	NA	NA	NA	NA
ENSG00000214652	ZNF727	zinc finger protein 727	442319	0,10488184	-1,076443944	4,080456	-0,263804824	0,791930317	NA
ENSG00000214653	HNRNPA3P3	heterogeneous nuclear ribonucleoprotein A3 pseudogene 3	643689	0,10488184	-1,076443944	4,080456	-0,263804824	0,791930317	NA
ENSG00000214657	NA	NA	NA	0	NA	NA	NA	NA	NA
ENSG00000214659	NA	NA	NA	0	NA	NA	NA	NA	NA
ENSG00000214660	SLC29A4P2	solute carrier family 29 member 4 pseudogene 2	100132308	0	NA	NA	NA	NA	NA
ENSG00000214668	SLC29A4P1	solute carrier family 29 member 4 pseudogene 1	402509	0	NA	NA	NA	NA	NA
ENSG00000214669	NA	NA	NA	0	NA	NA	NA	NA	NA
ENSG00000214671	RPL6P12	ribosomal protein L6 pseudogene 12	100270907	0	NA	NA	NA	NA	NA
ENSG00000214676	RPL9P16	ribosomal protein L9 pseudogene 16	727835	0	NA	NA	NA	NA	NA
ENSG00000214681	IQCF5	IQ motif containing F5	389124	0	NA	NA	NA	NA	NA
ENSG00000214684	NA	NA	NA	0	NA	NA	NA	NA	NA
ENSG00000214686	IQCF6	IQ motif containing F6	440956	0	NA	NA	NA	NA	NA
ENSG00000214688	C10orf105	chromosome 10 open reading frame 105	414152	0,839871663	2,082785789	2,233527	0,932509817	0,351073116	NA
ENSG00000214694	ARHGEF33	Rho guanine nucleotide exchange factor 33	100271715	0,10543123	0	4,080456	0	1	NA
ENSG00000214695	NA	NA	NA	0	NA	NA	NA	NA	NA
ENSG00000214700	C12orf71	chromosome 12 open reading frame 71	728858	0	NA	NA	NA	NA	NA
ENSG00000214702	NA	NA	NA	0	NA	NA	NA	NA	NA
ENSG00000214711	CAPN14	calpain 14	440854	0	NA	NA	NA	NA	NA
ENSG00000214720	KRT18P49	keratin 18 pseudogene 49	392516	0	NA	NA	NA	NA	NA
ENSG00000214725	CDIPTOSP	CDIP transferase opposite strand, pseudogene	440356	0	NA	NA	NA	NA	NA
ENSG00000214727	NA	NA	NA	1,108960045	-1,996563673	2,158796	-0,924850681	0,355043584	NA
ENSG00000214743	NA	NA	NA	0	NA	NA	NA	NA	NA
ENSG00000214745	GOT2P6	GOT2 pseudogene 6	644924	0,713352713	2,659528098	2,303042	1,154789016	0,248176836	NA
ENSG00000214748	NA	NA	NA	0	NA	NA	NA	NA	NA
ENSG00000214754	NA	NA	NA	0	NA	NA	NA	NA	NA
ENSG00000214756	CSKMT	citrate synthase lysine methyltransferase	751071	1,102998554	-2,903977172	2,874027	-1,010420856	0,3122937	NA
ENSG00000214759	NA	NA	NA	0	NA	NA	NA	NA	NA
ENSG00000214760	RPL21P1	ribosomal protein L21 pseudogene 1	6145	0	NA	NA	NA	NA	NA
ENSG00000214761	NA	NA	NA	0	NA	NA	NA	NA	NA
ENSG00000214782	MS4A18	membrane spanning 4-domains A18	728588	0	NA	NA	NA	NA	NA
ENSG00000214784	RPS3AP21	RPS3A pseudogene 21	402287	0,072165449	0,847101401	4,080456	0,207599697	0,835541541	NA
ENSG00000214787	MS4A4E	membrane spanning 4-domains A4E	643680	0,603476097	-0,97271183	2,877182	-0,338078021	0,735304393	NA
ENSG00000214794	KRT18P42	keratin 18 pseudogene 42	391819	0	NA	NA	NA	NA	NA
ENSG00000214796	NA	NA	NA	0,98548262	1,285808037	1,959361	0,656238523	0,511670666	NA
ENSG00000214807	NA	NA	NA	0	NA	NA	NA	NA	NA
ENSG00000214810	NA	NA	NA	0	NA	NA	NA	NA	NA
ENSG00000214812	NA	NA	NA	0	NA	NA	NA	NA	NA
ENSG00000214814	FER1L6	fer-1 like family member 6	654463	0	NA	NA	NA	NA	NA
ENSG00000214815	NA	NA	NA	0	NA	NA	NA	NA	NA
ENSG00000214819	CDRT15L2	CMT1A duplicated region transcript 15 like 2	256223	0	NA	NA	NA	NA	NA
ENSG00000214821	NA	NA	NA	0	NA	NA	NA	NA	NA
ENSG00000214822	NA	NA	NA	0	NA	NA	NA	NA	NA
ENSG00000214823	NA	NA	NA	0	NA	NA	NA	NA	NA
ENSG00000214827	MTCP1	mature T cell proliferation 1	4515	10,94090448	0,157454177	0,555074	0,283663521	0,776668253	NA
ENSG00000214832	NA	NA	NA	0,326790457	-1,92342573	4,042581	-0,475791517	0,634222914	NA
ENSG00000214835	RPL23AP6	ribosomal protein L23a pseudogene 6	128709	0	NA	NA	NA	NA	NA
ENSG00000214837	NA	NA	NA	0	NA	NA	NA	NA	NA
ENSG00000214842	RAD51AP2	RAD51 associated protein 2	729475	0	NA	NA	NA	NA	NA
ENSG00000214843	NA	NA	NA	0	NA	NA	NA	NA	NA

ENSG00000214846	RPL10AP7	ribosomal protein L10a pseudogene 7	100128646	0	NA	NA	NA	NA	NA
ENSG00000214853	NA	NA	NA	0	NA	NA	NA	NA	NA
ENSG00000214855	NA	NA	NA	0	NA	NA	NA	NA	NA
ENSG00000214857	NA	NA	NA	0	NA	NA	NA	NA	NA
ENSG00000214866	DCDC2C	doublecortin domain containing 2C	728597	0	NA	NA	NA	NA	NA
ENSG00000214867	NA	NA	NA	0	NA	NA	NA	NA	NA
ENSG00000214869	NA	NA	NA	0	NA	NA	NA	NA	NA
ENSG00000214872	SMTNL1	smoothelin like 1	219537	0,072165449	0,847101401	4,080456	0,207599697	0,835541541	NA
ENSG00000214875	NA	NA	NA	0,918157648	-0,196000904	1,891708	-0,103610574	0,917478396	NA
ENSG00000214878	RPL5P31	ribosomal protein L5 pseudogene 31	341674	0	NA	NA	NA	NA	NA
ENSG00000214880	NA	NA	NA	0	NA	NA	NA	NA	NA
ENSG00000214881	NA	NA	NA	0	NA	NA	NA	NA	NA
ENSG00000214883	NA	NA	NA	0	NA	NA	NA	NA	NA
ENSG00000214886	NA	NA	NA	0	NA	NA	NA	NA	NA
ENSG00000214888	NA	NA	NA	0	NA	NA	NA	NA	NA
ENSG00000214889	NA	NA	NA	0	NA	NA	NA	NA	NA
ENSG00000214890	NA	NA	NA	0	NA	NA	NA	NA	NA
ENSG00000214891	TRIM64C	tripartite motif containing 64C	646754	0	NA	NA	NA	NA	NA
ENSG00000214892	USP8P1	USP8 pseudogene 1	100287272	0	NA	NA	NA	NA	NA
ENSG00000214896	RPSAP55	ribosomal protein SA pseudogene 55	388122	0	NA	NA	NA	NA	NA
ENSG00000214897	PNMA6E	PNMA family member 6E	649238	0	NA	NA	NA	NA	NA
ENSG00000214903	RPS15AP3	ribosomal protein S15a pseudogene 3	326317	0	NA	NA	NA	NA	NA
ENSG00000214904	NA	NA	NA	0	NA	NA	NA	NA	NA
ENSG00000214908	NA	NA	NA	0	NA	NA	NA	NA	NA
ENSG00000214914	RPL23AP3	ribosomal protein L23a pseudogene 3	8489	0	NA	NA	NA	NA	NA
ENSG00000214917	RPS15AP35	ribosomal protein S15a pseudogene 35	390848	0	NA	NA	NA	NA	NA
ENSG00000214925	RPL36AP52	RPL36A pseudogene 52	207032	0	NA	NA	NA	NA	NA
ENSG00000214929	SPATA31D1	SPATA31 subfamily D member 1	389763	0	NA	NA	NA	NA	NA
ENSG00000214930	KRT8P6	keratin 8 pseudogene 6	392552	0	NA	NA	NA	NA	NA
ENSG00000214940	NPIPA8	nuclear pore complex interacting protein family member A8	101059953	7,978299127	1,342007757	1,08617	1,235541285	0,216629121	NA
ENSG00000214943	GPR33	G protein-coupled receptor 33	2856	0	NA	NA	NA	NA	NA
ENSG00000214944	ARHGEF28	Rho guanine nucleotide exchange factor 28	64283	8,728770083	0,99855393	0,628663	1,588376688	0,112201186	NA
ENSG00000214946	TBC1D26	TBC1 domain family member 26	353149	0	NA	NA	NA	NA	NA
ENSG00000214954	LRRC69	leucine rich repeat containing 69	100130742	1,272308705	1,262444752	2,00446	0,629817993	0,528813672	NA
ENSG00000214960	CRPPA	CDP-L-ribitol pyrophosphorylase A	729920	6,107060414	-1,088176088	1,009177	-1,078280537	0,28090858	NA
ENSG00000214961	NT5DC1P2	NT5DC1 pseudogene 2	402425	0	NA	NA	NA	NA	NA
ENSG00000214967	NPIPA7	nuclear pore complex interacting protein family member A7	101059938	2,202518645	-5,312834979	3,311197	-1,60450593	0,108602578	NA
ENSG00000214973	NA	NA	NA	0	NA	NA	NA	NA	NA
ENSG00000214975	NA	NA	NA	0	NA	NA	NA	NA	NA
ENSG00000214976	NA	NA	NA	0	NA	NA	NA	NA	NA
ENSG00000214978	GSG1L2	GSG1 like 2	644070	0	NA	NA	NA	NA	NA
ENSG00000214980	LOC152845	PLAG1 like zinc finger 2 pseudogene	152845	0	NA	NA	NA	NA	NA
ENSG00000214988	RPL7AP26	ribosomal protein L7a pseudogene 26	728128	0	NA	NA	NA	NA	NA
ENSG00000214992	NA	NA	NA	0	NA	NA	NA	NA	NA
ENSG00000214998	CCNB2P1	cyclin B2 pseudogene 1	100132073	0	NA	NA	NA	NA	NA
ENSG00000215000	GAPDHP77	GAPDH pseudogene 77	118568801	0	NA	NA	NA	NA	NA
ENSG00000215002	NA	NA	NA	0	NA	NA	NA	NA	NA
ENSG00000215004	NA	NA	NA	0	NA	NA	NA	NA	NA
ENSG00000215005	HSPD1P7	heat shock protein family D (Hsp60) member 1 pseudogene 7	54047	0	NA	NA	NA	NA	NA
ENSG00000215007	NA	NA	NA	0	NA	NA	NA	NA	NA
ENSG00000215009	ACSM4	acyl-CoA synthetase medium chain family member 4	341392	0	NA	NA	NA	NA	NA
ENSG00000215016	RPL24P7	RPL24 pseudogene 7	100270963	0	NA	NA	NA	NA	NA
ENSG00000215018	COL28A1	collagen type XXVIII alpha 1 chain	340267	0,785640983	-3,504096937	2,899306	-1,20859862	0,226817087	NA
ENSG00000215029	TCP11X2	t-complex 11 family, X-linked 2	100996648	0	NA	NA	NA	NA	NA

ENSG00000215030	RPL13P12	ribosomal protein L13 pseudogene 12	388344	0,320439357	1,884761769	4,027624	0,467958772	0,639814073	NA
ENSG00000215032	GNL3LP1	G protein nucleolar 3 like pseudogene 1	80060	1,90603322	0,322098955	3,02122	0,106612221	0,915096623	NA
ENSG00000215034	NA	NA	NA	0	NA	NA	NA	NA	NA
ENSG00000215035	FDPSP5	farnesyl diphosphate synthase pseudogene 5	2229	0,11808024	-1,076443944	4,080456	-0,263804824	0,791930317	NA
ENSG00000215037	VENTXP2	VENT homeobox pseudogene 2	100287345	0	NA	NA	NA	NA	NA
ENSG00000215043	GLULP6	glutamate-ammonia ligase pseudogene 6	343981	0	NA	NA	NA	NA	NA
ENSG00000215045	GRID2IP	Grid2 interacting protein	392862	0,58206923	0,817373385	2,668591	0,30629395	0,759380845	NA
ENSG00000215049	NA	NA	NA	0	NA	NA	NA	NA	NA
ENSG00000215054	HNRNPCP10	heterogeneous nuclear ribonucleoprotein C pseudogene 10	100129229	0	NA	NA	NA	NA	NA
ENSG00000215057	NA	NA	NA	0	NA	NA	NA	NA	NA
ENSG00000215063	RPL21P2	ribosomal protein L21 pseudogene 2	140715	0	NA	NA	NA	NA	NA
ENSG00000215065	NA	NA	NA	0	NA	NA	NA	NA	NA
ENSG00000215070	XRCC6P5	X-ray repair cross complementing 6 pseudogene 5	442459	0	NA	NA	NA	NA	NA
ENSG00000215085	GTF3C6P1	GTF3C6 pseudogene 1	100129001	0	NA	NA	NA	NA	NA
ENSG00000215086	NA	NA	NA	0,15836288	0	4,080456	0	1	NA
ENSG00000215088	RPS5P3	RPS5 pseudogene 3	54022	0	NA	NA	NA	NA	NA
ENSG00000215089	KRT18P11	keratin 18 pseudogene 11	3876	0	NA	NA	NA	NA	NA
ENSG00000215093	NA	NA	NA	0	NA	NA	NA	NA	NA
ENSG00000215094	NA	NA	NA	0	NA	NA	NA	NA	NA
ENSG00000215096	NA	NA	NA	0	NA	NA	NA	NA	NA
ENSG00000215097	NA	NA	NA	0	NA	NA	NA	NA	NA
ENSG00000215102	NA	NA	NA	0	NA	NA	NA	NA	NA
ENSG00000215105	NA	NA	NA	0,297616229	1,683725871	3,887935	0,433064296	0,66496806	NA
ENSG00000215110	NA	NA	NA	0	NA	NA	NA	NA	NA
ENSG00000215113	CXorf49B	chromosome X open reading frame 49B	100132994	0	NA	NA	NA	NA	NA
ENSG00000215115	CXorf49	chromosome X open reading frame 49	100130361	0	NA	NA	NA	NA	NA
ENSG00000215120	NA	NA	NA	0	NA	NA	NA	NA	NA
ENSG00000215124	NA	NA	NA	0	NA	NA	NA	NA	NA
ENSG00000215126	ZNG1F	Zn regulated GTPase metalloprotein activator 1F	644019	0,959935521	4,011665633	3,242621	1,237167681	0,216024837	NA
ENSG00000215127	NA	NA	NA	0	NA	NA	NA	NA	NA
ENSG00000215131	C16orf90	chromosome 16 open reading frame 90	646174	0	NA	NA	NA	NA	NA
ENSG00000215146	NA	NA	NA	0,103998153	-1,076443944	4,080456	-0,263804824	0,791930317	NA
ENSG00000215148	PRSS41	serine protease 41	360226	0,11808024	-1,076443944	4,080456	-0,263804824	0,791930317	NA
ENSG00000215149	KRT18P32	keratin 18 pseudogene 32	391179	0	NA	NA	NA	NA	NA
ENSG00000215151	ABCD1P2	ATP binding cassette subfamily D member 1 pseudogene 2	26983	0	NA	NA	NA	NA	NA
ENSG00000215154	NA	NA	NA	1,560191074	0,371887888	1,65764	0,224347812	0,822486676	NA
ENSG00000215156	NA	NA	NA	0,263638619	-1,072972184	4,080456	-0,262953998	0,792586036	NA
ENSG00000215158	NA	NA	NA	8,505347298	-0,216998544	0,612048	-0,354545069	0,722930434	NA
ENSG00000215160	OR7E122P	olfactory receptor family 7 subfamily E member 122 pseudogene	81428	0	NA	NA	NA	NA	NA
ENSG00000215165	NA	NA	NA	0	NA	NA	NA	NA	NA
ENSG00000215168	ATXN7L3P1	ATXN7L3 pseudogene 1	392485	0	NA	NA	NA	NA	NA
ENSG00000215174	NLRP2B	NLR family pyrin domain containing 2B	286430	0	NA	NA	NA	NA	NA
ENSG00000215177	NA	NA	NA	0	NA	NA	NA	NA	NA
ENSG00000215179	NA	NA	NA	0	NA	NA	NA	NA	NA
ENSG00000215182	MUC5AC	mucin 5AC, oligomeric mucus/gel-forming	4586	0	NA	NA	NA	NA	NA
ENSG00000215183	MSMP	microseminoprotein, prostate associated	692094	0	NA	NA	NA	NA	NA
ENSG00000215184	NA	NA	NA	0	NA	NA	NA	NA	NA
ENSG00000215186	GOLGA6B	golgin A6 family member B	55889	0,222732053	-0,089267689	4,080026	-0,021879198	0,982544318	NA
ENSG00000215190	NA	NA	NA	1,661902865	0,534037139	1,540488	0,346667498	0,728841131	NA
ENSG00000215197	NA	NA	NA	0	NA	NA	NA	NA	NA
ENSG00000215198	NA	NA	NA	0	NA	NA	NA	NA	NA
ENSG00000215199	NA	NA	NA	0	NA	NA	NA	NA	NA
ENSG00000215203	GRXCR1	glutaredoxin and cysteine rich domain containing 1	389207	0	NA	NA	NA	NA	NA
ENSG00000215206	TRBV24OR9-2	T cell receptor beta variable 24/OR9-2 (pseudogene)	6961	0	NA	NA	NA	NA	NA
ENSG00000215208	KRT18P60	keratin 18 pseudogene 60	100418829	0	NA	NA	NA	NA	NA
ENSG00000215210	NA	NA	NA	0,10488184	-1,076443944	4,080456	-0,263804824	0,791930317	NA

ENSG00000215217	CFAP90	cilia and flagella associated protein 90	134121	0	NA	NA	NA	NA	NA
ENSG00000215221	NA	NA	NA	2,292895161	0,211933657	1,33466	0,158792246	0,873832558	NA
ENSG00000215223	NA	NA	NA	0	NA	NA	NA	NA	NA
ENSG00000215227	NUTF2P5	nuclear transport factor 2 pseudogene 5	106481706	0	NA	NA	NA	NA	NA
ENSG00000215236	RPL7P33	ribosomal protein L7 pseudogene 33	286348	0	NA	NA	NA	NA	NA
ENSG00000215237	NA	NA	NA	0	NA	NA	NA	NA	NA
ENSG00000215262	KCNU1	potassium calcium-activated channel subfamily U member 1	157855	0	NA	NA	NA	NA	NA
ENSG00000215263	NA	NA	NA	0	NA	NA	NA	NA	NA
ENSG00000215264	RPL10AP3	ribosomal protein L10a pseudogene 3	137107	0	NA	NA	NA	NA	NA
ENSG00000215267	AKR1C7P	aldo-keto reductase family 1 member C7, pseudogene	648947	0,234994216	1,944111989	4,02324	0,483220515	0,62893917	NA
ENSG00000215268	NA	NA	NA	0	NA	NA	NA	NA	NA
ENSG00000215270	NA	NA	NA	0	NA	NA	NA	NA	NA
ENSG00000215274	GAGE10	G antigen 10	102724473	0	NA	NA	NA	NA	NA
ENSG00000215277	RNF212B	ring finger protein 212B	100507650	0	NA	NA	NA	NA	NA
ENSG00000215278	RPS7P6	ribosomal protein S7 pseudogene 6	100270869	0	NA	NA	NA	NA	NA
ENSG00000215283	NA	NA	NA	0	NA	NA	NA	NA	NA
ENSG00000215284	NA	NA	NA	0	NA	NA	NA	NA	NA
ENSG00000215286	NA	NA	NA	0	NA	NA	NA	NA	NA
ENSG00000215288	NA	NA	NA	0	NA	NA	NA	NA	NA
ENSG00000215296	NA	NA	NA	0	NA	NA	NA	NA	NA
ENSG00000215297	NA	NA	NA	0,17891688	0,822901517	4,07949	0,201716786	0,840138139	NA
ENSG00000215310	LOC646506	lysosomal associated membrane protein 1 pseudogene	646506	0	NA	NA	NA	NA	NA
ENSG00000215311	NA	NA	NA	0	NA	NA	NA	NA	NA
ENSG00000215313	RPL6P30	ribosomal protein L6 pseudogene 30	139452	0	NA	NA	NA	NA	NA
ENSG00000215317	NA	NA	NA	0	NA	NA	NA	NA	NA
ENSG00000215319	NA	NA	NA	0	NA	NA	NA	NA	NA
ENSG00000215325	ASS1P10	argininosuccinate synthetase 1 pseudogene 10	455	0	NA	NA	NA	NA	NA
ENSG00000215326	NA	NA	NA	0	NA	NA	NA	NA	NA
ENSG00000215333	KRT18P23	keratin 18 pseudogene 23	642448	0	NA	NA	NA	NA	NA
ENSG00000215339	NA	NA	NA	0	NA	NA	NA	NA	NA
ENSG00000215343	ZNF705D	zinc finger protein 705D	728957	0	NA	NA	NA	NA	NA
ENSG00000215347	NA	NA	NA	0	NA	NA	NA	NA	NA
ENSG00000215349	NA	NA	NA	0	NA	NA	NA	NA	NA
ENSG00000215351	NA	NA	NA	0	NA	NA	NA	NA	NA
ENSG00000215353	NA	NA	NA	0	NA	NA	NA	NA	NA
ENSG00000215354	FAM90A24	family with sequence similarity 90 member A24	441332	0	NA	NA	NA	NA	NA
ENSG00000215356	ZNF705B	zinc finger protein 705B	100132396	0	NA	NA	NA	NA	NA
ENSG00000215357	HSPD1P8	heat shock protein family D (Hsp60) member 1 pseudogene 8	647298	0	NA	NA	NA	NA	NA
ENSG00000215367	TMED11P	transmembrane p24 trafficking protein 11, pseudogene	100379220	0	NA	NA	NA	NA	NA
ENSG00000215368	NA	NA	NA	0	NA	NA	NA	NA	NA
ENSG00000215369	NA	NA	NA	0	NA	NA	NA	NA	NA
ENSG00000215372	ZNF705G	zinc finger protein 705G	100131980	0	NA	NA	NA	NA	NA
ENSG00000215372	ZNF705B	zinc finger protein 705B	100132396	0	NA	NA	NA	NA	NA
ENSG00000215373	NA	NA	NA	0	NA	NA	NA	NA	NA
ENSG00000215378	NA	NA	NA	0	NA	NA	NA	NA	NA
ENSG00000215380	NA	NA	NA	0	NA	NA	NA	NA	NA
ENSG00000215388	NA	NA	NA	6,595429565	-0,678183922	0,717186	-0,945617612	0,344343658	NA
ENSG00000215397	SCRT2	scratch family transcriptional repressor 2	85508	0	NA	NA	NA	NA	NA
ENSG00000215398	NA	NA	NA	0	NA	NA	NA	NA	NA
ENSG00000215399	NA	NA	NA	0	NA	NA	NA	NA	NA
ENSG00000215414	NA	NA	NA	0	NA	NA	NA	NA	NA
ENSG00000215418	NA	NA	NA	0	NA	NA	NA	NA	NA
ENSG00000215441	CTAGE12P	CTAGE family member 12, pseudogene	392298	0	NA	NA	NA	NA	NA
ENSG00000215444	LOC100419570	ribosomal protein L29 pseudogene	100419570	0	NA	NA	NA	NA	NA
ENSG00000215448	NA	NA	NA	0	NA	NA	NA	NA	NA
ENSG00000215450	RPS2P54	ribosomal protein S2 pseudogene 54	100271252	0	NA	NA	NA	NA	NA
ENSG00000215452	NA	NA	NA	0,118286652	0	4,080456	0	1	NA
ENSG00000215454	KRTAP10-4	keratin associated protein 10-4	386672	0	NA	NA	NA	NA	NA

ENSG00000215455	KRTAP10-1	keratin associated protein 10-1	386677	0	NA	NA	NA	NA	NA
ENSG00000215456	BCRP4	BCR pseudogene 4	616	0	NA	NA	NA	NA	NA
ENSG00000215457	RPS8P3	ribosomal protein S8 pseudogene 3	100132995	0	NA	NA	NA	NA	NA
ENSG00000215464	NA	NA	NA	0	NA	NA	NA	NA	NA
ENSG00000215467	NA	NA	NA	0	NA	NA	NA	NA	NA
ENSG00000215474	SKOR2	SKI family transcriptional corepressor 2	652991	0	NA	NA	NA	NA	NA
ENSG00000215475	SIAH3	siah E3 ubiquitin protein ligase family member 3	283514	0,118286652	0	4,080456	0	1	NA
ENSG00000215477	NA	NA	NA	0	NA	NA	NA	NA	NA
ENSG00000215480	NA	NA	NA	0	NA	NA	NA	NA	NA
ENSG00000215482	NA	NA	NA	0,118286652	0	4,080456	0	1	NA
ENSG00000215486	NA	NA	NA	0	NA	NA	NA	NA	NA
ENSG00000215492	NA	NA	NA	0,945462509	3,536895015	2,869485	1,232588772	0,217729228	NA
ENSG00000215493	NA	NA	NA	0,10543123	0	4,080456	0	1	NA
ENSG00000215506	NA	NA	NA	0	NA	NA	NA	NA	NA
ENSG00000215512	NA	NA	NA	0	NA	NA	NA	NA	NA
ENSG00000215515	NA	NA	NA	0,262547291	1,416534092	4,061214	0,348795765	0,727242643	NA
ENSG00000215520	RPS4XP4	ribosomal protein S4X pseudogene 4	441878	0	NA	NA	NA	NA	NA
ENSG00000215529	EFCAB8	EF-hand calcium binding domain 8	388795	6,57566247	0,643300427	0,847505	0,759052373	0,447821228	NA
ENSG00000215537	NA	NA	NA	0	NA	NA	NA	NA	NA
ENSG00000215540	NA	NA	NA	0	NA	NA	NA	NA	NA
ENSG00000215544	BCRP7	BCR pseudogene 7	100133163	0	NA	NA	NA	NA	NA
ENSG00000215545	DEFB116	defensin beta 116	245930	0	NA	NA	NA	NA	NA
ENSG00000215546	NA	NA	NA	0	NA	NA	NA	NA	NA
ENSG00000215547	DEFB115	defensin beta 115	245929	0	NA	NA	NA	NA	NA
ENSG00000215548	NA	NA	NA	6,69476287	0,147206489	0,763905	0,192702712	0,8471918	NA
ENSG00000215553	KRT18P3	keratin 18 pseudogene 3	170527	0	NA	NA	NA	NA	NA
ENSG00000215559	NA	NA	NA	0,10488184	-1,076443944	4,080456	-0,263804824	0,791930317	NA
ENSG00000215562	NA	NA	NA	0	NA	NA	NA	NA	NA
ENSG00000215567	NA	NA	NA	0	NA	NA	NA	NA	NA
ENSG00000215568	GAB4	GRB2 associated binding protein family member 4	128954	0	NA	NA	NA	NA	NA
ENSG00000215571	NA	NA	NA	0,764726588	-0,243168694	2,412437	-0,10079795	0,919710854	NA
ENSG00000215572	NA	NA	NA	0	NA	NA	NA	NA	NA
ENSG00000215580	NA	NA	NA	0	NA	NA	NA	NA	NA
ENSG00000215583	ASS1P6	argininosuccinate synthetase 1 pseudogene 6	451	0	NA	NA	NA	NA	NA
ENSG00000215586	NA	NA	NA	0	NA	NA	NA	NA	NA
ENSG00000215589	NA	NA	NA	0	NA	NA	NA	NA	NA
ENSG00000215595	C20orf202	chromosome 20 open reading frame 202	400831	0,223448181	-1,073928937	4,080456	-0,26318847	0,792405317	NA
ENSG00000215601	TSPY24P	testis specific protein Y-linked 24, pseudogene	100874287	0	NA	NA	NA	NA	NA
ENSG00000215603	ZNF92P1Y	zinc finger protein 92 pseudogene 1, Y-linked	100419554	0	NA	NA	NA	NA	NA
ENSG00000215604	ZNF962P	zinc finger protein 962, pseudogene	729501	0	NA	NA	NA	NA	NA
ENSG00000215606	KRT18P35	keratin 18 pseudogene 35	391584	0	NA	NA	NA	NA	NA
ENSG00000215704	CELA2B	chymotrypsin like elastase 2B	51032	0	NA	NA	NA	NA	NA
ENSG00000215720	NA	NA	NA	0	NA	NA	NA	NA	NA
ENSG00000215734	NA	NA	NA	0	NA	NA	NA	NA	NA
ENSG00000215760	TAF9BP2	TATA-box binding protein associated factor 9b pseudogene 2	100130319	0	NA	NA	NA	NA	NA
ENSG00000215771	LRRC37A14P	leucine rich repeat containing 37 member A14, pseudogene	106479025	0	NA	NA	NA	NA	NA
ENSG00000215784	FAM72D	family with sequence similarity 72 member D	728833	8,35910533	0,341496838	0,756152	0,451624389	0,651539597	NA
ENSG00000215785	NA	NA	NA	0	NA	NA	NA	NA	NA
ENSG00000215788	TNFRSF25	TNF receptor superfamily member 25	8718	0,773025985	-2,80594274	3,733147	-0,751629355	0,452273985	NA
ENSG00000215795	LOC100131465	peroxisomal biogenesis factor 5 pseudogene	100131465	1,606566933	-0,129699782	2,214062	-0,058580002	0,953286639	NA
ENSG00000215796	LOC100420880	DNA primase subunit 1 pseudogene	100420880	0	NA	NA	NA	NA	NA
ENSG00000215800	NA	NA	NA	0	NA	NA	NA	NA	NA
ENSG00000215802	NA	NA	NA	0	NA	NA	NA	NA	NA
ENSG00000215805	LOC128136	3-hydroxy-3-methylglutaryl-Coenzyme A synthase 1 (soluble) pseudogene	128136	0	NA	NA	NA	NA	NA
ENSG00000215807	KRT18P65	keratin 18 pseudogene 65	442249	0	NA	NA	NA	NA	NA
ENSG00000215812	NA	NA	NA	0	NA	NA	NA	NA	NA

ENSG00000215817	ZC3H11B	zinc finger CCCH-type containing 11B	643136	0,234994216	1,944111989	4,02324	0,483220515	0,62893917	NA	
ENSG00000215819	KRT18P12	keratin 18 pseudogene 12	643471	0	NA	NA	NA	NA	NA	
ENSG00000215833	QRSL1P1	QRSL1 pseudogene 1	730016	0	NA	NA	NA	NA	NA	
ENSG00000215834	NA	NA	NA	0	NA	NA	NA	NA	NA	
ENSG00000215835	LOC284685	EWS RNA binding protein 1 pseudogene	284685	0,10488184	-1,076443944	4,080456	-0,263804824	0,791930317	NA	
ENSG00000215838	LOC440700	carbonic anhydrase 14 pseudogene	440700	0	NA	NA	NA	NA	NA	
ENSG00000215840	NA	NA	NA	1,91595148	-1,97032183	1,592117	-1,237548535	0,215883507	NA	
ENSG00000215853	RPTN	repetin	126638	0	NA	NA	NA	NA	NA	
ENSG00000215859	NA	NA	NA	0,207996306	-1,916073151	4,043021	-0,473921178	0,635556117	NA	
ENSG00000215861	NA	NA	NA	0,11808024	-1,076443944	4,080456	-0,263804824	0,791930317	NA	
ENSG00000215864	NA	NA	NA	0	NA	NA	NA	NA	NA	
ENSG00000215867	KRT18P57	keratin 18 pseudogene 57	643329	0	NA	NA	NA	NA	NA	
ENSG00000215869	LOC100129138	THAP domain containing 3 pseudogene	100129138	0	NA	NA	NA	NA	NA	
ENSG00000215871	NA	NA	NA	0	NA	NA	NA	NA	NA	
ENSG00000215873	NA	NA	NA	0	NA	NA	NA	NA	NA	
ENSG00000215874	CAPNS1P1	calpain small subunit 1 pseudogene 1	391053	0	NA	NA	NA	NA	NA	
ENSG00000215875	ST13P20	ST13, Hsp70 interacting protein pseudogene 20	729828	0	NA	NA	NA	NA	NA	
ENSG00000215878	NA	NA	NA	0	NA	NA	NA	NA	NA	
ENSG00000215887	NA	NA	NA	0	NA	NA	NA	NA	NA	
ENSG00000215893	NA	NA	NA	0	NA	NA	NA	NA	NA	
ENSG00000215895	HSPA5P1	HSPA5 pseudogene 1	400750	0,072165449	0,847101401	4,080456	0,207599697	0,835541541	NA	
ENSG00000215899	EEF1A1P46	eukaryotic translation elongation factor 1 alpha 1 pseudogene 46	441880	0	NA	NA	NA	NA	NA	
ENSG00000215900	NA	NA	NA	0	NA	NA	NA	NA	NA	
ENSG00000215905	NA	NA	NA	0	NA	NA	NA	NA	NA	
ENSG00000215906	LACTBL1	lactamase beta like 1	646262	0	NA	NA	NA	NA	NA	
ENSG00000215909	NA	NA	NA	0	NA	NA	NA	NA	NA	
ENSG00000215910	C1orf167	chromosome 1 open reading frame 167	284498	0	NA	NA	NA	NA	NA	
ENSG00000215912	TTC34	tetratricopeptide repeat domain 34	100287898	8,098912218	0,033461903	0,745264	0,044899386	0,964187506	NA	
ENSG00000215914	MMP23A	matrix metallopeptidase 23A (pseudogene)	8511	0,288661797	2,306206831	3,997774	0,57687273	0,564025434	NA	
ENSG00000215915	ATAD3C	ATPase family AAA domain containing 3C	219293	2,084168857	-0,330181717	1,394407	-0,236790033	0,812819684	NA	
ENSG00000216265	FABP12P1	fatty acid binding protein 12 pseudogene 1	106479057	0	NA	NA	NA	NA	NA	
ENSG00000216285	NA	NA	NA	0,10488184	-1,076443944	4,080456	-0,263804824	0,791930317	NA	
ENSG00000216306	KRT19P2	keratin 19 pseudogene 2	160313	0	NA	NA	NA	NA	NA	
ENSG00000216307	NA	NA	NA	0,326690525	-2,558298733	3,704288	-0,69063164	0,489797059	NA	
ENSG00000216316	NA	NA	NA	0,224210008	-0,143868901	4,080456	-0,035258046	0,971873977	NA	
ENSG00000216324	NA	NA	NA	0	NA	NA	NA	NA	NA	
ENSG00000216331	NA	NA	NA	0	NA	NA	NA	NA	NA	
ENSG00000216347	NA	NA	NA	0	NA	NA	NA	NA	NA	
ENSG00000216352	NA	NA	NA	0	NA	NA	NA	NA	NA	
ENSG00000216360	NA	NA	NA	0	NA	NA	NA	NA	NA	
ENSG00000216364	NA	NA	NA	0,11808024	-1,076443944	4,080456	-0,263804824	0,791930317	NA	
ENSG00000216365	RPL37P15	ribosomal protein L37 pseudogene 15	100271020	0	NA	NA	NA	NA	NA	
ENSG00000216368	RPL7AP36	ribosomal protein L7a pseudogene 36	728179	0	NA	NA	NA	NA	NA	
ENSG00000216378	NA	NA	NA	0	NA	NA	NA	NA	NA	
ENSG00000216412	NA	NA	NA	0	NA	NA	NA	NA	NA	
ENSG00000216425	NA	NA	NA	0	NA	NA	NA	NA	NA	
ENSG00000216436	NA	NA	NA	0	NA	NA	NA	NA	NA	
ENSG00000216439	NA	NA	NA	0	NA	NA	NA	NA	NA	
ENSG00000216444	RAET1M	retinoic acid early transcript 1M (pseudogene)	353094	0	NA	NA	NA	NA	NA	
ENSG00000216471	RPSAP43	ribosomal protein SA pseudogene 43	643954	0	NA	NA	NA	NA	NA	
ENSG00000216475	NA	NA	NA	0,072165449	0,847101401	4,080456	0,207599697	0,835541541	NA	
ENSG00000216480	RPL21P69	ribosomal protein L21 pseudogene 69	642738	0,118286652	0	4,080456	0	1	NA	
ENSG00000216516	LOC112267977	mitochondrial import receptor subunit TOM6 homolog	112267977	0	NA	NA	NA	NA	NA	
ENSG00000216518	EEF1GP6	eukaryotic translation elongation factor 1 gamma pseudogene 6	100421733	0	NA	NA	NA	NA	NA	
ENSG00000216519	NA	NA	NA	0	NA	NA	NA	NA	NA	
ENSG00000216523	NA	NA	NA	0	NA	NA	NA	NA	NA	
ENSG00000216548	RPS18P10	ribosomal protein S18 pseudogene 10	729293	0	NA	NA	NA	NA	NA	

ENSG00000216588	IGSF23	immunoglobulin superfamily member 23	147710	0	NA	NA	NA	NA	NA
ENSG00000216613	LOC124901409	phosphopantothenate--cysteine ligase-like	124901409	0,239347754	1,967469188	4,021404	0,489249276	0,624665227	NA
ENSG00000216616	RPL27AP7	ribosomal protein L27a pseudogene 7	100271301	0	NA	NA	NA	NA	NA
ENSG00000216621	LOC105378052	uncharacterized LOC105378052	105378052	0	NA	NA	NA	NA	NA
ENSG00000216624	NA	NA	NA	0	NA	NA	NA	NA	NA
ENSG00000216629	OR2W4P	olfactory receptor family 2 subfamily W member 4 pseudogene	81407	0	NA	NA	NA	NA	NA
ENSG00000216636	RPL7P25	ribosomal protein L7 pseudogene 25	100129061	0	NA	NA	NA	NA	NA
ENSG00000216639	RPL7AP35	ribosomal protein L7a pseudogene 35	100271210	0	NA	NA	NA	NA	NA
ENSG00000216642	RPL31P27	ribosomal protein L31 pseudogene 27	100271198	0	NA	NA	NA	NA	NA
ENSG00000216649	GAGE12E	G antigen 12E	729431	0	NA	NA	NA	NA	NA
ENSG00000216657	NA	NA	NA	0	NA	NA	NA	NA	NA
ENSG00000216663	NA	NA	NA	0	NA	NA	NA	NA	NA
ENSG00000216671	NA	NA	NA	0	NA	NA	NA	NA	NA
ENSG00000216676	NA	NA	NA	0	NA	NA	NA	NA	NA
ENSG00000216687	NA	NA	NA	0	NA	NA	NA	NA	NA
ENSG00000216708	CIR1P3	CIR1 pseudogene 3	106480783	0	NA	NA	NA	NA	NA
ENSG00000216710	NA	NA	NA	0	NA	NA	NA	NA	NA
ENSG00000216713	MTND4P13	MT-ND4 pseudogene 13	100862853	0	NA	NA	NA	NA	NA
ENSG00000216718	RPL21P68	ribosomal protein L21 pseudogene 68	645452	0	NA	NA	NA	NA	NA
ENSG00000216721	NA	NA	NA	0	NA	NA	NA	NA	NA
ENSG00000216723	NA	NA	NA	0	NA	NA	NA	NA	NA
ENSG00000216740	NA	NA	NA	0	NA	NA	NA	NA	NA
ENSG00000216753	NA	NA	NA	0	NA	NA	NA	NA	NA
ENSG00000216754	RPL6P17	ribosomal protein L6 pseudogene 17	100131229	0,210862461	0	4,080456	0	1	NA
ENSG00000216762	VN1R13P	vomeronasal 1 receptor 13 pseudogene	387322	0	NA	NA	NA	NA	NA
ENSG00000216775	NA	NA	NA	1,30209712	3,029651972	1,973655	1,535046257	0,124772466	NA
ENSG00000216777	NA	NA	NA	0	NA	NA	NA	NA	NA
ENSG00000216781	NA	NA	NA	0	NA	NA	NA	NA	NA
ENSG00000216802	NA	NA	NA	0	NA	NA	NA	NA	NA
ENSG00000216809	NA	NA	NA	5,563771853	0,609572722	0,788666	0,772916073	0,439572052	NA
ENSG00000216811	NA	NA	NA	0	NA	NA	NA	NA	NA
ENSG00000216813	NA	NA	NA	0,445466279	-0,14657778	3,032167	-0,048340932	0,961444534	NA
ENSG00000216817	R3HDM2P2	R3H domain containing 2 pseudogene 2	100129694	0	NA	NA	NA	NA	NA
ENSG00000216819	TUBB2BP1	tubulin beta 2B class IIb pseudogene 1	100132153	0	NA	NA	NA	NA	NA
ENSG00000216824	ZNF736P10Y	zinc finger protein 736 pseudogene 10, Y-linked	100419727	0	NA	NA	NA	NA	NA
ENSG00000216829	NA	NA	NA	0	NA	NA	NA	NA	NA
ENSG00000216835	NA	NA	NA	0	NA	NA	NA	NA	NA
ENSG00000216844	NA	NA	NA	0	NA	NA	NA	NA	NA
ENSG00000216853	MTCYBP36	MT-CYB pseudogene 36	109729116	0	NA	NA	NA	NA	NA
ENSG00000216854	RPL17P26	ribosomal protein L17 pseudogene 26	100129784	0	NA	NA	NA	NA	NA
ENSG00000216859	NA	NA	NA	0	NA	NA	NA	NA	NA
ENSG00000216866	RPS2P55	ribosomal protein S2 pseudogene 55	286444	0,11808024	-1,076443944	4,080456	-0,263804824	0,791930317	NA
ENSG00000216867	NA	NA	NA	0	NA	NA	NA	NA	NA
ENSG00000216901	ZNF603P	zinc finger protein 603, pseudogene	493821	0	NA	NA	NA	NA	NA
ENSG00000216902	RPL31P32	ribosomal protein L31 pseudogene 32	643851	0	NA	NA	NA	NA	NA
ENSG00000216904	SOCS5P5	suppressor of cytokine signaling 5 pseudogene 5	643884	0	NA	NA	NA	NA	NA
ENSG00000216906	NA	NA	NA	0,365562777	-1,893633516	4,044374	-0,468214234	0,639631394	NA
ENSG00000216913	NA	NA	NA	0	NA	NA	NA	NA	NA
ENSG00000216915	NA	NA	NA	0,407358564	0,442722279	3,59762	0,123059765	0,902059772	NA
ENSG00000216917	RPL15P9	ribosomal protein L15 pseudogene 9	100270935	0	NA	NA	NA	NA	NA
ENSG00000216921	FAM240C	family with sequence similarity 240 member C	285095	0	NA	NA	NA	NA	NA
ENSG00000216938	NA	NA	NA	0	NA	NA	NA	NA	NA
ENSG00000216966	NA	NA	NA	0	NA	NA	NA	NA	NA
ENSG00000216977	RPL21P65	ribosomal protein L21 pseudogene 65	100271178	0,30771557	0,710453372	3,848053	0,1846267	0,853521843	NA
ENSG00000216990	HSPD1P10	heat shock protein family D (Hsp60) member 1 pseudogene 10	644016	0,223092181	-1,079333199	4,080456	-0,264512896	0,791384729	NA

ENSG00000216998	CYP2AC1P	cytochrome P450 family 2 subfamily AC member 1, pseudogene	106478960	0	NA	NA	NA	NA	NA
ENSG00000217004	NA	NA	NA	0	NA	NA	NA	NA	NA
ENSG00000217026	RPL10P1	ribosomal protein L10 pseudogene 1	100288252	0	NA	NA	NA	NA	NA
ENSG00000217027	NA	NA	NA	0,239347754	1,967469188	4,021404	0,489249276	0,624665227	NA
ENSG00000217030	RPS18P8	ribosomal protein S18 pseudogene 8	643627	0	NA	NA	NA	NA	NA
ENSG00000217041	NA	NA	NA	0	NA	NA	NA	NA	NA
ENSG00000217044	MTCO3P31	MT-CO3 pseudogene 31	107075186	0	NA	NA	NA	NA	NA
ENSG00000217060	LOC100421093	karyopherin subunit alpha 3 pseudogene	100421093	0	NA	NA	NA	NA	NA
ENSG00000217067	NA	NA	NA	0	NA	NA	NA	NA	NA
ENSG00000217078	NA	NA	NA	0	NA	NA	NA	NA	NA
ENSG00000217083	MTCO2P33	MT-CO2 pseudogene 33	107075197	0	NA	NA	NA	NA	NA
ENSG00000217085	NA	NA	NA	0	NA	NA	NA	NA	NA
ENSG00000217089	NA	NA	NA	0	NA	NA	NA	NA	NA
ENSG00000217094	NA	NA	NA	0	NA	NA	NA	NA	NA
ENSG00000217120	NA	NA	NA	0	NA	NA	NA	NA	NA
ENSG00000217130	RPS10P13	ribosomal protein S10 pseudogene 13	646785	0,10488184	-1,076443944	4,080456	-0,263804824	0,791930317	NA
ENSG00000217135	NA	NA	NA	0	NA	NA	NA	NA	NA
ENSG00000217139	LOC100129422	LSM14A, SCD6 homolog A (S, cerevisiae) pseudogene	100129422	0	NA	NA	NA	NA	NA
ENSG00000217159	NA	NA	NA	0,22193874	-1,99684748	4,035682	-0,494797985	0,620742727	NA
ENSG00000217160	MTCO2P31	MT-CO2 pseudogene 31	107075292	0	NA	NA	NA	NA	NA
ENSG00000217165	ANKRD18EP	ankyrin repeat domain 18E, pseudogene	100131031	0	NA	NA	NA	NA	NA
ENSG00000217169	MTHFD2P2	methylenetetrahydrofolate dehydrogenase (NADP+ dependent) 2, methenyltetrahydrofolate cyclohydrolase pseudogene 2	106480237	0	NA	NA	NA	NA	NA
ENSG00000217178	NA	NA	NA	0	NA	NA	NA	NA	NA
ENSG00000217179	MTCYBP2	MT-CYB pseudogene 2	100873168	0	NA	NA	NA	NA	NA
ENSG00000217195	NA	NA	NA	0	NA	NA	NA	NA	NA
ENSG00000217227	TFGP1	TFG pseudogene 1	100128379	0	NA	NA	NA	NA	NA
ENSG00000217228	GSTA12P	glutathione S-transferase alpha 12, pseudogene	647169	0	NA	NA	NA	NA	NA
ENSG00000217231	LOC100131041	FKBP prolyl isomerase 7 pseudogene	100131041	0	NA	NA	NA	NA	NA
ENSG00000217236	SP9	Sp9 transcription factor	100131390	0	NA	NA	NA	NA	NA
ENSG00000217239	RPL34P16	ribosomal protein L34 pseudogene 16	100133294	0	NA	NA	NA	NA	NA
ENSG00000217241	NA	NA	NA	0,23616048	-2,075648354	4,028973	-0,515180498	0,606426895	NA
ENSG00000217261	NA	NA	NA	0	NA	NA	NA	NA	NA
ENSG00000217268	NA	NA	NA	0	NA	NA	NA	NA	NA
ENSG00000217272	NA	NA	NA	0	NA	NA	NA	NA	NA
ENSG00000217275	RPS10P1	ribosomal protein S10 pseudogene 1	394255	0,072165449	0,847101401	4,080456	0,207599697	0,835541541	NA
ENSG00000217314	NA	NA	NA	0	NA	NA	NA	NA	NA
ENSG00000217315	OR2W2P	olfactory receptor family 2 subfamily W member 2 pseudogene	81694	0	NA	NA	NA	NA	NA
ENSG00000217325	PRELID1P1	PRELID1 pseudogene 1	728666	0,11808024	-1,076443944	4,080456	-0,263804824	0,791930317	NA
ENSG00000217327	NA	NA	NA	0	NA	NA	NA	NA	NA
ENSG00000217330	NA	NA	NA	0	NA	NA	NA	NA	NA
ENSG00000217331	NA	NA	NA	0	NA	NA	NA	NA	NA
ENSG00000217334	LOC100127917	heat shock protein 90 alpha family class B member 1 pseudogene	100127917	0,10488184	-1,076443944	4,080456	-0,263804824	0,791930317	NA
ENSG00000217372	NA	NA	NA	0	NA	NA	NA	NA	NA
ENSG00000217377	AK4P5	adenylate kinase 4 pseudogene 5	100526416	0	NA	NA	NA	NA	NA
ENSG00000217379	RPL21P61	ribosomal protein L21 pseudogene 61	645157	0	NA	NA	NA	NA	NA
ENSG00000217385	PSMC1P11	proteasome 26S subunit, ATPase 1 pseudogene 11	442153	0	NA	NA	NA	NA	NA
ENSG00000217404	NA	NA	NA	0	NA	NA	NA	NA	NA
ENSG00000217408	NA	NA	NA	0	NA	NA	NA	NA	NA
ENSG00000217414	DDX18P3	DEAD-box helicase 18 pseudogene 3	442164	0	NA	NA	NA	NA	NA
ENSG00000217416	NA	NA	NA	0	NA	NA	NA	NA	NA
ENSG00000217442	SYCE3	synaptonemal complex central element protein 3	644186	0	NA	NA	NA	NA	NA
ENSG00000217447	NA	NA	NA	0	NA	NA	NA	NA	NA
ENSG00000217477	LOC442225	keratin, type II pseudogene	442225	0	NA	NA	NA	NA	NA

ENSG00000217482	HMGB1P17	high mobility group box 1 pseudogene 17	100419974	0,236352033	0,805014928	4,078725	0,197369267	0,843538578	NA
ENSG00000217483	LOC100422453	DNA polymerase delta 1, catalytic subunit pseudogene	100422453	0	NA	NA	NA	NA	NA
ENSG00000217488	LOC100421091	LSM14A, SCD6 homolog A (S, cerevisiae) pseudogene	100421091	0	NA	NA	NA	NA	NA
ENSG00000217495	NA	NA	NA	0	NA	NA	NA	NA	NA
ENSG00000217512	LOC100419073	nucleoporin 155kDa pseudogene	100419073	1,180868457	-0,00364494	2,117463	-0,001721371	0,998626545	NA
ENSG00000217514	RPL37AP4	ribosomal protein L37a pseudogene 4	100271338	0	NA	NA	NA	NA	NA
ENSG00000217527	NA	NA	NA	1,730108225	2,847573206	1,673146	1,701927542	0,088768952	NA
ENSG00000217539	NA	NA	NA	0	NA	NA	NA	NA	NA
ENSG00000217557	NA	NA	NA	0	NA	NA	NA	NA	NA
ENSG00000217566	NA	NA	NA	0	NA	NA	NA	NA	NA
ENSG00000217585	NA	NA	NA	0	NA	NA	NA	NA	NA
ENSG00000217612	NA	NA	NA	0	NA	NA	NA	NA	NA
ENSG00000217624	NA	NA	NA	3,035745168	0,061986696	0,990761	0,062564704	0,950113136	NA
ENSG00000217631	NA	NA	NA	0	NA	NA	NA	NA	NA
ENSG00000217643	NA	NA	NA	3,339536828	-0,143133501	1,037024	-0,13802332	0,890221988	NA
ENSG00000217644	RPL18AP4	ribosomal protein L18a pseudogene 4	127545	0	NA	NA	NA	NA	NA
ENSG00000217646	H2BC16P	H2B clustered histone 16, pseudogene	10340	0	NA	NA	NA	NA	NA
ENSG00000217648	PARLP2	PARL pseudogene 2	100131471	0,234994216	1,944111989	4,02324	0,483220515	0,62893917	NA
ENSG00000217680	NA	NA	NA	0	NA	NA	NA	NA	NA
ENSG00000217684	RPS3AP24	RPS3A pseudogene 24	100271253	0	NA	NA	NA	NA	NA
ENSG00000217686	NA	NA	NA	0	NA	NA	NA	NA	NA
ENSG00000217707	SERPINB8P1	serpin family B member 8 pseudogene 1	11029	0	NA	NA	NA	NA	NA
ENSG00000217716	RPS10P3	ribosomal protein S10 pseudogene 3	158104	0	NA	NA	NA	NA	NA
ENSG00000217718	NA	NA	NA	0	NA	NA	NA	NA	NA
ENSG00000217733	CCT7P1	chaperonin containing TCP1 subunit 7 pseudogene 1	442267	0,328444355	-1,079111253	3,801664	-0,283852347	0,776523537	NA
ENSG00000217746	NA	NA	NA	0,119673877	0,847101401	4,080456	0,207599697	0,835541541	NA
ENSG00000217767	NDUFAB1P1	NADH:ubiquinone oxidoreductase subunit AB1 pseudogene 1	107075303	0,072165449	0,847101401	4,080456	0,207599697	0,835541541	NA
ENSG00000217769	NA	NA	NA	0	NA	NA	NA	NA	NA
ENSG00000217770	FEM1AP3	fem-1 homolog A pseudogene 3	643859	0	NA	NA	NA	NA	NA
ENSG00000217776	NA	NA	NA	0	NA	NA	NA	NA	NA
ENSG00000217783	NA	NA	NA	1,675408591	1,210434408	1,440776	0,840126811	0,400837289	NA
ENSG00000217786	NA	NA	NA	0	NA	NA	NA	NA	NA
ENSG00000217791	ASS1P9	argininosuccinate synthetase 1 pseudogene 9	454	0	NA	NA	NA	NA	NA
ENSG00000217801	NA	NA	NA	0,763908048	0,920824153	2,311659	0,398339026	0,690380297	NA
ENSG00000217805	NA	NA	NA	0	NA	NA	NA	NA	NA
ENSG00000217809	FAT1P1	FAT atypical cadherin 1 pseudogene 1	170513	0	NA	NA	NA	NA	NA
ENSG00000217811	HNRNPDP2	heterogeneous nuclear ribonucleoprotein D pseudogene 2	106481661	0	NA	NA	NA	NA	NA
ENSG00000217824	NA	NA	NA	0	NA	NA	NA	NA	NA
ENSG00000217835	NA	NA	NA	0,103998153	-1,076443944	4,080456	-0,263804824	0,791930317	NA
ENSG00000217862	NA	NA	NA	0	NA	NA	NA	NA	NA
ENSG00000217874	OR4F7P	olfactory receptor family 4 subfamily F member 7 pseudogene	81390	0	NA	NA	NA	NA	NA
ENSG00000217878	NA	NA	NA	0	NA	NA	NA	NA	NA
ENSG00000217889	KRT18P48	keratin 18 pseudogene 48	340598	0	NA	NA	NA	NA	NA
ENSG00000217896	ZNF839P1	zinc finger protein 839 pseudogene 1	100419733	0	NA	NA	NA	NA	NA
ENSG00000217897	NA	NA	NA	0	NA	NA	NA	NA	NA
ENSG00000217929	NA	NA	NA	6,716619435	-0,557471123	0,818274	-0,681276668	0,495696444	NA
ENSG00000217930	PAM16	presequence translocase associated motor 16	51025	3,871690716	-0,655900593	1,642899	-0,399233626	0,689721068	NA
ENSG00000217950	NA	NA	NA	0	NA	NA	NA	NA	NA
ENSG00000218014	KRT19P1	keratin 19 pseudogene 1	441160	0	NA	NA	NA	NA	NA
ENSG00000218016	ZKSCAN8P2	ZKSCAN8 pseudogene 2	222701	0	NA	NA	NA	NA	NA
ENSG00000218020	THAP12P5	THAP domain containing 12 pseudogene 5	100129074	0	NA	NA	NA	NA	NA
ENSG00000218027	NA	NA	NA	0	NA	NA	NA	NA	NA
ENSG00000218048	NA	NA	NA	0	NA	NA	NA	NA	NA
ENSG00000218049	RPL31P33	ribosomal protein L31 pseudogene 33	442218	0	NA	NA	NA	NA	NA
ENSG00000218052	ADAMTS7P4	ADAMTS7 pseudogene 4	642935	1,083799897	3,60422421	2,816534	1,279666513	0,200662447	NA
ENSG00000218069	NA	NA	NA	0	NA	NA	NA	NA	NA
ENSG00000218073	NA	NA	NA	1,835677061	1,497170933	1,391707	1,075780475	0,282025446	NA

ENSG00000218089	DNAJA1P4	DnaJ heat shock protein family (Hsp40) member A1 pseudogene 4	728614	0	NA	NA	NA	NA	NA
ENSG00000218107	LOC100132242	exosome component 8 pseudogene	100132242	0	NA	NA	NA	NA	NA
ENSG00000218125	NA	NA	NA	0	NA	NA	NA	NA	NA
ENSG00000218143	NA	NA	NA	0	NA	NA	NA	NA	NA
ENSG00000218153	KRT18P22	keratin 18 pseudogene 22	442252	0	NA	NA	NA	NA	NA
ENSG00000218173	NA	NA	NA	0	NA	NA	NA	NA	NA
ENSG00000218175	NA	NA	NA	0,10543123	0	4,080456	0	1	NA
ENSG00000218180	NA	NA	NA	0	NA	NA	NA	NA	NA
ENSG00000218186	KRT8P43	keratin 8 pseudogene 43	100289319	0	NA	NA	NA	NA	NA
ENSG00000218187	NA	NA	NA	0	NA	NA	NA	NA	NA
ENSG00000218189	NA	NA	NA	0	NA	NA	NA	NA	NA
ENSG00000218194	HLFP1	HLF pseudogene 1	100873813	0	NA	NA	NA	NA	NA
ENSG00000218198	NA	NA	NA	0	NA	NA	NA	NA	NA
ENSG00000218208	RPS27AP11	RPS27A pseudogene 11	728590	0	NA	NA	NA	NA	NA
ENSG00000218213	NA	NA	NA	0	NA	NA	NA	NA	NA
ENSG00000218226	NA	NA	NA	0,10543123	0	4,080456	0	1	NA
ENSG00000218227	RPL19P9	ribosomal protein L19 pseudogene 9	653314	0	NA	NA	NA	NA	NA
ENSG00000218233	NA	NA	NA	0	NA	NA	NA	NA	NA
ENSG00000218261	NA	NA	NA	0	NA	NA	NA	NA	NA
ENSG00000218265	RPS4XP7	ribosomal protein S4X pseudogene 7	442162	0	NA	NA	NA	NA	NA
ENSG00000218274	NA	NA	NA	0	NA	NA	NA	NA	NA
ENSG00000218281	H2AC9P	H2A clustered histone 9, pseudogene	387323	0	NA	NA	NA	NA	NA
ENSG00000218283	NA	NA	NA	1,822935207	-0,179508188	1,492114	-0,120304567	0,904241886	NA
ENSG00000218305	CDC14C	cell division cycle 14C	168448	0	NA	NA	NA	NA	NA
ENSG00000218313	NA	NA	NA	0	NA	NA	NA	NA	NA
ENSG00000218336	TENM3	teneurin transmembrane protein 3	55714	0,23578552	0,846245032	4,080456	0,207389826	0,835705428	NA
ENSG00000218337	NA	NA	NA	0	NA	NA	NA	NA	NA
ENSG00000218347	NA	NA	NA	0	NA	NA	NA	NA	NA
ENSG00000218350	NA	NA	NA	0	NA	NA	NA	NA	NA
ENSG00000218351	RPS3AP23	RPS3A pseudogene 23	100271131	0	NA	NA	NA	NA	NA
ENSG00000218358	NA	NA	NA	1,496208824	1,648860354	2,17682	0,757462898	0,448772586	NA
ENSG00000218359	SUMO2P13	SUMO2 pseudogene 13	106480263	0	NA	NA	NA	NA	NA
ENSG00000218363	NA	NA	NA	0	NA	NA	NA	NA	NA
ENSG00000218410	LOC100533723	HERV-FRD provirus ancestral Env polyprotein pseudogene	100533723	0	NA	NA	NA	NA	NA
ENSG00000218418	NA	NA	NA	0,2219925	-1,997094807	4,035676	-0,494860055	0,620698909	NA
ENSG00000218424	NDUFS5P1	NADH:ubiquinone oxidoreductase subunit S5 pseudogene 1	100996944	0	NA	NA	NA	NA	NA
ENSG00000218426	RPL27AP6	ribosomal protein L27a pseudogene 6	389435	0,15836288	0	4,080456	0	1	NA
ENSG00000218428	NA	NA	NA	0	NA	NA	NA	NA	NA
ENSG00000218454	HNRNPA1P19	heterogeneous nuclear ribonucleoprotein A1 pseudogene 19	100874402	0	NA	NA	NA	NA	NA
ENSG00000218459	NA	NA	NA	0	NA	NA	NA	NA	NA
ENSG00000218472	RPS25P7	ribosomal protein S25 pseudogene 7	100271103	0	NA	NA	NA	NA	NA
ENSG00000218475	NA	NA	NA	0	NA	NA	NA	NA	NA
ENSG00000218476	RPL6P18	ribosomal protein L6 pseudogene 18	100131805	0	NA	NA	NA	NA	NA
ENSG00000218483	NA	NA	NA	0	NA	NA	NA	NA	NA
ENSG00000218490	FCF1P10	FCF1 pseudogene 10	106479046	0	NA	NA	NA	NA	NA
ENSG00000218499	NA	NA	NA	0	NA	NA	NA	NA	NA
ENSG00000218502	NA	NA	NA	0,223563162	-0,121711175	4,080456	-0,029827838	0,976204358	NA
ENSG00000218512	NA	NA	NA	0	NA	NA	NA	NA	NA
ENSG00000218520	LOC100128610	putative UPF0607 protein ENSP00000383144 pseudogene	100128610	0	NA	NA	NA	NA	NA
ENSG00000218521	NA	NA	NA	0	NA	NA	NA	NA	NA
ENSG00000218536	NA	NA	NA	0	NA	NA	NA	NA	NA
ENSG00000218549	OR4K12P	olfactory receptor family 4 subfamily K member 12 pseudogene	81078	0	NA	NA	NA	NA	NA
ENSG00000218561	NA	NA	NA	0	NA	NA	NA	NA	NA
ENSG00000218565	LOC100129844	DnaJ heat shock protein family (Hsp40) member C7 pseudogene	100129844	0	NA	NA	NA	NA	NA
ENSG00000218574	NA	NA	NA	0	NA	NA	NA	NA	NA
ENSG00000218577	ANKRD18FP	ankyrin repeat domain 18F, pseudogene	127379688	0	NA	NA	NA	NA	NA
ENSG00000218582	NA	NA	NA	0	NA	NA	NA	NA	NA
ENSG00000218586	NA	NA	NA	0,103998153	-1,076443944	4,080456	-0,263804824	0,791930317	NA

ENSG00000218596	NA	NA	NA	0,475500154	-0,00254487	3,969132	-0,000641165	0,999488424	NA
ENSG00000218617	NA	NA	NA	0	NA	NA	NA	NA	NA
ENSG00000218631	LOC100420839	chromosome 18 open reading frame 21 pseudogene	100420839	1,078831345	-1,187046791	1,862278	-0,637416501	0,523853583	NA
ENSG00000218632	RPL7P28	ribosomal protein L7 pseudogene 28	442242	0	NA	NA	NA	NA	NA
ENSG00000218643	RPL5P20	ribosomal protein L5 pseudogene 20	100131913	0	NA	NA	NA	NA	NA
ENSG00000218676	NA	NA	NA	0	NA	NA	NA	NA	NA
ENSG00000218682	NA	NA	NA	0,235782941	0,849696809	4,080456	0,208235755	0,835044892	NA
ENSG00000218689	RPL5P21	ribosomal protein L5 pseudogene 21	100270838	0,15836288	0	4,080456	0	1	NA
ENSG00000218690	H2AC10P	H2A clustered histone 10, pseudogene	8333	0,118286652	0	4,080456	0	1	NA
ENSG00000218698	NA	NA	NA	0	NA	NA	NA	NA	NA
ENSG00000218713	NA	NA	NA	0,19163895	1,677482439	4,046278	0,41457423	0,678453616	NA
ENSG00000218716	RPL12P23	ribosomal protein L12 pseudogene 23	100270925	0	NA	NA	NA	NA	NA
ENSG00000218725	NA	NA	NA	0	NA	NA	NA	NA	NA
ENSG00000218728	KRT18P44	keratin 18 pseudogene 44	139748	0	NA	NA	NA	NA	NA
ENSG00000218730	NA	NA	NA	0,209763681	-1,926644696	4,042043	-0,476651224	0,633610501	NA
ENSG00000218732	NA	NA	NA	0	NA	NA	NA	NA	NA
ENSG00000218749	NA	NA	NA	0	NA	NA	NA	NA	NA
ENSG00000218754	RPL18AP8	ribosomal protein L18a pseudogene 8	644082	0	NA	NA	NA	NA	NA
ENSG00000218757	NA	NA	NA	0	NA	NA	NA	NA	NA
ENSG00000218766	LOC100133102	Rac family small GTPase 2 pseudogene	100133102	0	NA	NA	NA	NA	NA
ENSG00000218772	NA	NA	NA	0	NA	NA	NA	NA	NA
ENSG00000218776	LOC124901233	ATP synthase subunit a-like	124901233	0	NA	NA	NA	NA	NA
ENSG00000218792	NA	NA	NA	0	NA	NA	NA	NA	NA
ENSG00000218793	NA	NA	NA	0	NA	NA	NA	NA	NA
ENSG00000218803	NA	NA	NA	0	NA	NA	NA	NA	NA
ENSG00000218806	NA	NA	NA	0	NA	NA	NA	NA	NA
ENSG00000218809	NA	NA	NA	0	NA	NA	NA	NA	NA
ENSG00000218813	LOC100422587	platelet activating factor acetylhydrolase 1b regulatory subunit 1 pseudogene	100422587	0	NA	NA	NA	NA	NA
ENSG00000218823	PAPOLB	poly(A) polymerase beta	56903	3,171363354	1,664678904	1,252876	1,328686373	0,183951463	NA
ENSG00000218834	NA	NA	NA	0	NA	NA	NA	NA	NA
ENSG00000218857	LOC100421246	double PHD fingers 2 pseudogene	100421246	0	NA	NA	NA	NA	NA
ENSG00000218868	NA	NA	NA	0	NA	NA	NA	NA	NA
ENSG00000218870	SLC25A6P6	solute carrier family 25 member 6 pseudogene 6	100128312	0	NA	NA	NA	NA	NA
ENSG00000218872	RPS12P13	ribosomal protein S12 pseudogene 13	100271057	0	NA	NA	NA	NA	NA
ENSG00000218890	NA	NA	NA	0	NA	NA	NA	NA	NA
ENSG00000218891	ZNF579	zinc finger protein 579	163033	6,73943995	-0,638242709	0,798967	-0,798834689	0,424386274	NA
ENSG00000218893	SUMO2P12	SUMO2 pseudogene 12	106480262	0	NA	NA	NA	NA	NA
ENSG00000218896	TUBB8P2	tubulin beta 8 class VIII pseudogene 2	106478982	0	NA	NA	NA	NA	NA
ENSG00000218902	NA	NA	NA	0	NA	NA	NA	NA	NA
ENSG00000218965	NA	NA	NA	0	NA	NA	NA	NA	NA
ENSG00000218976	NA	NA	NA	0	NA	NA	NA	NA	NA
ENSG00000218980	NA	NA	NA	0	NA	NA	NA	NA	NA
ENSG00000218986	NA	NA	NA	0	NA	NA	NA	NA	NA
ENSG00000218991	CCNG1P1	cyclin G1 pseudogene 1	100130820	0	NA	NA	NA	NA	NA
ENSG00000218996	NA	NA	NA	0,51602181	1,333220791	2,866162	0,465158847	0,641817707	NA
ENSG00000219023	RPS15AP19	ribosomal protein S15a pseudogene 19	646819	0	NA	NA	NA	NA	NA
ENSG00000219027	NA	NA	NA	0,119673877	0,847101401	4,080456	0,207599697	0,835541541	NA
ENSG00000219039	NA	NA	NA	0	NA	NA	NA	NA	NA
ENSG00000219073	CELA3B	chymotrypsin like elastase 3B	23436	0	NA	NA	NA	NA	NA
ENSG00000219074	SOD1P1	superoxide dismutase 1 pseudogene 1	106479055	0,103998153	-1,076443944	4,080456	-0,263804824	0,791930317	NA
ENSG00000219085	NA	NA	NA	1,056881333	0,504221217	1,745041	0,288945255	0,772623272	NA
ENSG00000219087	MTND4LP19	MT-ND4L pseudogene 19	107075241	0	NA	NA	NA	NA	NA
ENSG00000219088	NA	NA	NA	0	NA	NA	NA	NA	NA
ENSG00000219095	NA	NA	NA	0	NA	NA	NA	NA	NA
ENSG00000219102	HNRNPA3P12	heterogeneous nuclear ribonucleoprotein A3 pseudogene 12	100421395	0,118286652	0	4,080456	0	1	NA
ENSG00000219133	RPL21P19	ribosomal protein L21 pseudogene 19	641293	0,398246341	-0,109885078	3,624721	-0,030315456	0,97581547	NA

ENSG00000219135	RPL23AP48	ribosomal protein L23a pseudogene 48	728567	0	NA	NA	NA	NA	NA
ENSG00000219139	TYMSP1	thymidylate synthetase pseudogene 1	100419610	0	NA	NA	NA	NA	NA
ENSG00000219146	RPS4XP8	ribosomal protein S4X pseudogene 8	646269	0	NA	NA	NA	NA	NA
ENSG00000219149	NA	NA	NA	0	NA	NA	NA	NA	NA
ENSG00000219150	NA	NA	NA	0,072165449	0,847101401	4,080456	0,207599697	0,835541541	NA
ENSG00000219163	NA	NA	NA	0,190211346	-0,113942769	4,080456	-0,027924029	0,977722743	NA
ENSG00000219186	NA	NA	NA	0	NA	NA	NA	NA	NA
ENSG00000219188	CACYBPP3	calcyclin binding protein pseudogene 3	106480809	0	NA	NA	NA	NA	NA
ENSG00000219190	NA	NA	NA	0	NA	NA	NA	NA	NA
ENSG00000219201	NA	NA	NA	0,10543123	0	4,080456	0	1	NA
ENSG00000219222	NA	NA	NA	0	NA	NA	NA	NA	NA
ENSG00000219249	AMZ2P2	AMZ2 pseudogene 2	100421018	0,15836288	0	4,080456	0	1	NA
ENSG00000219253	NA	NA	NA	0	NA	NA	NA	NA	NA
ENSG00000219257	NPM1P38	nucleophosmin 1 pseudogene 38	100128985	0	NA	NA	NA	NA	NA
ENSG00000219262	NA	NA	NA	0	NA	NA	NA	NA	NA
ENSG00000219273	NA	NA	NA	0	NA	NA	NA	NA	NA
ENSG00000219274	NA	NA	NA	0	NA	NA	NA	NA	NA
ENSG00000219280	NA	NA	NA	0	NA	NA	NA	NA	NA
ENSG00000219284	NA	NA	NA	0	NA	NA	NA	NA	NA
ENSG00000219294	NA	NA	NA	0	NA	NA	NA	NA	NA
ENSG00000219297	MRPL42P3	mitochondrial ribosomal protein L42 pseudogene 3	359746	0	NA	NA	NA	NA	NA
ENSG00000219298	NA	NA	NA	0	NA	NA	NA	NA	NA
ENSG00000219302	NA	NA	NA	0	NA	NA	NA	NA	NA
ENSG00000219314	NA	NA	NA	0	NA	NA	NA	NA	NA
ENSG00000219329	NA	NA	NA	0,385363	-1,568821208	3,585494	-0,437546684	0,661714928	NA
ENSG00000219355	RPL31P52	ribosomal protein L31 pseudogene 52	100271476	0,591869784	-1,117625086	3,34093	-0,334525164	0,737983305	NA
ENSG00000219361	RPSAP72	ribosomal protein SA pseudogene 72	107075112	0	NA	NA	NA	NA	NA
ENSG00000219368	NA	NA	NA	0	NA	NA	NA	NA	NA
ENSG00000219375	NA	NA	NA	0	NA	NA	NA	NA	NA
ENSG00000219384	LOC100420052	poly(rC) binding protein 2 pseudogene	100420052	0	NA	NA	NA	NA	NA
ENSG00000219387	NA	NA	NA	0	NA	NA	NA	NA	NA
ENSG00000219391	NA	NA	NA	0	NA	NA	NA	NA	NA
ENSG00000219392	NA	NA	NA	0	NA	NA	NA	NA	NA
ENSG00000219395	HSPA8P15	heat shock protein family A (Hsp70) member 8 pseudogene 15	100420051	2,379210105	1,416754367	1,428662	0,991665144	0,3213609	NA
ENSG00000219404	NA	NA	NA	0	NA	NA	NA	NA	NA
ENSG00000219409	LOC100420214	rhomboid domain containing 1 pseudogene	100420214	0,422523506	1,406568282	3,594915	0,391266091	0,695600561	NA
ENSG00000219430	MBL3P	mannose-binding lectin family member 3, pseudogene	50639	0	NA	NA	NA	NA	NA
ENSG00000219433	NA	NA	NA	0,421724922	0	4,080456	0	1	NA
ENSG00000219435	CATSPERZ	catsper channel auxiliary subunit zeta	25858	0	NA	NA	NA	NA	NA
ENSG00000219438	TAFA5	TAFA chemokine like family member 5	25817	0	NA	NA	NA	NA	NA
ENSG00000219451	RPL23P8	ribosomal protein L23 pseudogene 8	222901	0,10488184	-1,076443944	4,080456	-0,263804824	0,791930317	NA
ENSG00000219453	NA	NA	NA	0	NA	NA	NA	NA	NA
ENSG00000219463	RPSAP42	ribosomal protein SA pseudogene 42	100270919	0	NA	NA	NA	NA	NA
ENSG00000219470	RPS2P29	ribosomal protein S2 pseudogene 29	646294	1,144629469	-0,957233704	2,225366	-0,430146675	0,667088949	NA
ENSG00000219481	NBPF1	NBPF member 1	55672	11,58308205	2,617897732	0,793157	3,300606362	0,000964761	NA
ENSG00000219487	NA	NA	NA	0,502709492	1,890206657	2,950215	0,640701284	0,52171678	NA
ENSG00000219491	NA	NA	NA	0	NA	NA	NA	NA	NA
ENSG00000219492	LOC105378242	zinc finger protein 705A-like	105378242	0	NA	NA	NA	NA	NA
ENSG00000219500	LOC100652960	SUZ RNA binding domain containing 1 pseudogene	100652960	0	NA	NA	NA	NA	NA
ENSG00000219507	NA	NA	NA	0,119673877	0,847101401	4,080456	0,207599697	0,835541541	NA
ENSG00000219529	NA	NA	NA	0,651509875	0,950507625	2,770613	0,343067601	0,731547605	NA
ENSG00000219532	NA	NA	NA	0	NA	NA	NA	NA	NA
ENSG00000219547	RPL17P25	ribosomal protein L17 pseudogene 25	442232	0	NA	NA	NA	NA	NA
ENSG00000219549	NA	NA	NA	0	NA	NA	NA	NA	NA
ENSG00000219553	NA	NA	NA	0,221914149	-1,996734338	4,035685	-0,494769591	0,620762772	NA
ENSG00000219559	RPS19P5	ribosomal protein S19 pseudogene 5	100131286	0	NA	NA	NA	NA	NA
ENSG00000219565	NA	NA	NA	0	NA	NA	NA	NA	NA

ENSG00000219575	NA	NA	NA	0	NA	NA	NA	NA	NA
ENSG00000219582	NA	NA	NA	0	NA	NA	NA	NA	NA
ENSG00000219592	NCSTNP1	nicastrin pseudogene 1	100873743	0	NA	NA	NA	NA	NA
ENSG00000219604	NA	NA	NA	0	NA	NA	NA	NA	NA
ENSG00000219607	PPP1R3G	protein phosphatase 1 regulatory subunit 3G	648791	0	NA	NA	NA	NA	NA
ENSG00000219608	NA	NA	NA	0,118286652	0	4,080456	0	1	NA
ENSG00000219619	NA	NA	NA	0	NA	NA	NA	NA	NA
ENSG00000219622	NA	NA	NA	0	NA	NA	NA	NA	NA
ENSG00000219626	FAM228B	family with sequence similarity 228 member B	375190	7,081413055	-0,505813496	0,718284	-0,704196598	0,481310349	NA
ENSG00000219627	NA	NA	NA	0	NA	NA	NA	NA	NA
ENSG00000219642	BMPR1AP1	bone morphogenetic protein receptor type 1A pseudogene 1	100421515	0	NA	NA	NA	NA	NA
ENSG00000219653	NA	NA	NA	0	NA	NA	NA	NA	NA
ENSG00000219666	NA	NA	NA	0	NA	NA	NA	NA	NA
ENSG00000219669	NA	NA	NA	0	NA	NA	NA	NA	NA
ENSG00000219681	NA	NA	NA	0	NA	NA	NA	NA	NA
ENSG00000219682	NA	NA	NA	0	NA	NA	NA	NA	NA
ENSG00000219693	NA	NA	NA	0	NA	NA	NA	NA	NA
ENSG00000219699	NA	NA	NA	0	NA	NA	NA	NA	NA
ENSG00000219700	NA	NA	NA	0	NA	NA	NA	NA	NA
ENSG00000219702	NA	NA	NA	0	NA	NA	NA	NA	NA
ENSG00000219703	NA	NA	NA	0	NA	NA	NA	NA	NA
ENSG00000219712	MARK2P18	MARK2 pseudogene 18	100421511	2,25748204	3,354197929	1,651746	2,030698826	0,042285556	NA
ENSG00000219722	RPL26P20	ribosomal protein L26 pseudogene 20	100271191	0	NA	NA	NA	NA	NA
ENSG00000219736	NUP50P4	NUP50 pseudogene 4	100127900	0	NA	NA	NA	NA	NA
ENSG00000219738	CD83P1	CD83 molecule pseudogene 1	100128240	0	NA	NA	NA	NA	NA
ENSG00000219747	NA	NA	NA	0,117497108	0,847101401	4,080456	0,207599697	0,835541541	NA
ENSG00000219755	RPS3P5	ribosomal protein S3 pseudogene 5	100131956	0,427252517	1,402285028	2,939943	0,476976958	0,633378529	NA
ENSG00000219757	NA	NA	NA	0	NA	NA	NA	NA	NA
ENSG00000219758	NA	NA	NA	0	NA	NA	NA	NA	NA
ENSG00000219770	VN1R11P	vomeronasal 1 receptor 11, pseudogene	387317	0	NA	NA	NA	NA	NA
ENSG00000219773	RPSAP45	ribosomal protein SA pseudogene 45	728562	0	NA	NA	NA	NA	NA
ENSG00000219776	RPL21P67	ribosomal protein L21 pseudogene 67	100271180	0	NA	NA	NA	NA	NA
ENSG00000219784	NA	NA	NA	0	NA	NA	NA	NA	NA
ENSG00000219790	NA	NA	NA	0	NA	NA	NA	NA	NA
ENSG00000219797	NA	NA	NA	0	NA	NA	NA	NA	NA
ENSG00000219806	NA	NA	NA	0	NA	NA	NA	NA	NA
ENSG00000219807	NA	NA	NA	0	NA	NA	NA	NA	NA
ENSG00000219814	RPL23AP47	ribosomal protein L23a pseudogene 47	729277	0	NA	NA	NA	NA	NA
ENSG00000219863	RPL31P28	ribosomal protein L31 pseudogene 28	442217	0	NA	NA	NA	NA	NA
ENSG00000219867	LOC100129847	ras homolog family member A pseudogene	100129847	0	NA	NA	NA	NA	NA
ENSG00000219870	NA	NA	NA	0	NA	NA	NA	NA	NA
ENSG00000219881	NA	NA	NA	0	NA	NA	NA	NA	NA
ENSG00000219891	NA	NA	NA	0	NA	NA	NA	NA	NA
ENSG00000219902	RPL35P3	ribosomal protein L35 pseudogene 3	728010	1,919087466	2,635863187	1,639401	1,607820985	0,107874391	NA
ENSG00000219926	NA	NA	NA	0	NA	NA	NA	NA	NA
ENSG00000219928	NA	NA	NA	0,223441804	0	4,080456	0	1	NA
ENSG00000219930	NA	NA	NA	0	NA	NA	NA	NA	NA
ENSG00000219932	RPL12P8	ribosomal protein L12 pseudogene 8	645161	0	NA	NA	NA	NA	NA
ENSG00000219940	SPTLC1P3	serine palmitoyltransferase long chain base subunit 1 pseudogene 3	100874499	0	NA	NA	NA	NA	NA
ENSG00000219941	KRT18P50	keratin 18 pseudogene 50	442236	0	NA	NA	NA	NA	NA
ENSG00000219951	NA	NA	NA	0	NA	NA	NA	NA	NA
ENSG00000219986	NA	NA	NA	0	NA	NA	NA	NA	NA
ENSG00000219992	NA	NA	NA	0	NA	NA	NA	NA	NA
ENSG00000219993	NA	NA	NA	0	NA	NA	NA	NA	NA
ENSG00000220069	RPL7P27	ribosomal protein L7 pseudogene 27	643906	0	NA	NA	NA	NA	NA
ENSG00000220076	NA	NA	NA	0	NA	NA	NA	NA	NA
ENSG00000220091	LAP3P1	leucine aminopeptidase 3 pseudogene 1	100873864	0,208976652	-1,921836903	4,042516	-0,475406098	0,634497549	NA
ENSG00000220105	RPS5P6	RPS5 pseudogene 6	392282	0	NA	NA	NA	NA	NA
ENSG00000220110	RPL21P64	ribosomal protein L21 pseudogene 64	100271177	0	NA	NA	NA	NA	NA
ENSG00000220113	MTCYBP4	MT-CYB pseudogene 4	100873169	0	NA	NA	NA	NA	NA

ENSG00000220125	NA	NA	NA	0	NA	NA	NA	NA	NA
ENSG00000220130	NA	NA	NA	0	NA	NA	NA	NA	NA
ENSG00000220131	NA	NA	NA	0	NA	NA	NA	NA	NA
ENSG00000220132	CPSF1P2	CPSF1 pseudogene 2	100422285	0	NA	NA	NA	NA	NA
ENSG00000220139	NA	NA	NA	0	NA	NA	NA	NA	NA
ENSG00000220154	NA	NA	NA	0	NA	NA	NA	NA	NA
ENSG00000220157	NA	NA	NA	0,117497108	0,847101401	4,080456	0,207599697	0,835541541	NA
ENSG00000220181	MTRES1P1	MTRES1 pseudogene 1	100129996	0	NA	NA	NA	NA	NA
ENSG00000220184	HMGB3P18	high mobility group box 3 pseudogene 18	644502	0	NA	NA	NA	NA	NA
ENSG00000220201	ZGLP1	zinc finger GATA like protein 1	100125288	4,286639109	2,631760605	1,433594	1,835777804	0,066390525	NA
ENSG00000220204	NA	NA	NA	0	NA	NA	NA	NA	NA
ENSG00000220212	OR4F1P	olfactory receptor family 4 subfamily F member 1 pseudogene	26685	0	NA	NA	NA	NA	NA
ENSG00000220237	RPS24P12	ribosomal protein S24 pseudogene 12	100271098	0	NA	NA	NA	NA	NA
ENSG00000220240	NA	NA	NA	0	NA	NA	NA	NA	NA
ENSG00000220248	ZNF402P	zinc finger protein 402, pseudogene	266673	0	NA	NA	NA	NA	NA
ENSG00000220267	NA	NA	NA	0	NA	NA	NA	NA	NA
ENSG00000220291	NA	NA	NA	0	NA	NA	NA	NA	NA
ENSG00000220305	HNRNPH1P1	heterogeneous nuclear ribonucleoprotein H1 pseudogene 1	100132803	2,893842872	-0,494421854	1,146945	-0,431077273	0,666412185	NA
ENSG00000220311	RPL35AP18	ribosomal protein L35a pseudogene 18	100271321	0	NA	NA	NA	NA	NA
ENSG00000220323	H2BC19P	H2B clustered histone 19, pseudogene	337874	5,081041015	5,416492095	1,468969	3,687274625	0,000226669	NA
ENSG00000220326	NA	NA	NA	0	NA	NA	NA	NA	NA
ENSG00000220340	MTCO1P56	MT-CO1 pseudogene 56	107075286	0	NA	NA	NA	NA	NA
ENSG00000220343	NA	NA	NA	0	NA	NA	NA	NA	NA
ENSG00000220347	NA	NA	NA	0	NA	NA	NA	NA	NA
ENSG00000220349	GPR166P	G protein-coupled receptor 166 pseudogene	442206	0	NA	NA	NA	NA	NA
ENSG00000220370	NA	NA	NA	0	NA	NA	NA	NA	NA
ENSG00000220377	NA	NA	NA	0	NA	NA	NA	NA	NA
ENSG00000220378	NA	NA	NA	0	NA	NA	NA	NA	NA
ENSG00000220392	FCF1P5	FCF1 pseudogene 5	106479045	1,025142559	2,189673124	1,889147	1,159080171	0,246423508	NA
ENSG00000220412	NA	NA	NA	0,248485161	0,431448163	4,060912	0,106244146	0,915388645	NA
ENSG00000220418	TUBB3P1	tubulin beta 3 class III pseudogene 1	100288513	0	NA	NA	NA	NA	NA
ENSG00000220446	NA	NA	NA	0	NA	NA	NA	NA	NA
ENSG00000220447	RPS15AP21	ribosomal protein S15a pseudogene 21	100271357	0	NA	NA	NA	NA	NA
ENSG00000220472	RPS3P4	ribosomal protein S3 pseudogene 4	100270862	0	NA	NA	NA	NA	NA
ENSG00000220483	NA	NA	NA	0	NA	NA	NA	NA	NA
ENSG00000220494	YAP1P1	YAP1 pseudogene 1	442266	1,041930578	2,361064216	2,157166	1,094521197	0,273726456	NA
ENSG00000220505	NA	NA	NA	0	NA	NA	NA	NA	NA
ENSG00000220506	NA	NA	NA	0	NA	NA	NA	NA	NA
ENSG00000220514	MTND4LP20	MT-ND4L pseudogene 20	107075242	0	NA	NA	NA	NA	NA
ENSG00000220515	NA	NA	NA	1,279336034	1,071627768	1,634143	0,655773751	0,511969708	NA
ENSG00000220522	LOC100287856	cornichon family AMPA receptor auxiliary protein 4 pseudogene	100287856	0	NA	NA	NA	NA	NA
ENSG00000220540	NA	NA	NA	0	NA	NA	NA	NA	NA
ENSG00000220541	NA	NA	NA	0	NA	NA	NA	NA	NA
ENSG00000220553	RPL5P19	ribosomal protein L5 pseudogene 19	100270906	0	NA	NA	NA	NA	NA
ENSG00000220556	LOC100128655	ribosomal protein S2 pseudogene	100128655	0	NA	NA	NA	NA	NA
ENSG00000220557	HMGB1P13	high mobility group box 1 pseudogene 13	100129706	0	NA	NA	NA	NA	NA
ENSG00000220563	PKMP3	pyruvate kinase M1/2 pseudogene 3	100419758	1,588810832	1,79318753	1,686753	1,063100035	0,287736589	NA
ENSG00000220581	VN1R12P	vomeronasal 1 receptor 12, pseudogene	387321	0	NA	NA	NA	NA	NA
ENSG00000220583	NA	NA	NA	0,22319663	-1,05868883	4,080456	-0,259453567	0,795285304	NA
ENSG00000220585	NA	NA	NA	0	NA	NA	NA	NA	NA
ENSG00000220586	TUBBP9	tubulin beta class I pseudogene 9	442210	0	NA	NA	NA	NA	NA
ENSG00000220598	SSR1P1	signal sequence receptor subunit 1 pseudogene 1	100420476	0	NA	NA	NA	NA	NA
ENSG00000220600	NA	NA	NA	0,11808024	-1,076443944	4,080456	-0,263804824	0,791930317	NA
ENSG00000220614	NA	NA	NA	0	NA	NA	NA	NA	NA
ENSG00000220635	NA	NA	NA	0	NA	NA	NA	NA	NA
ENSG00000220643	NA	NA	NA	0	NA	NA	NA	NA	NA
ENSG00000220660	NA	NA	NA	0	NA	NA	NA	NA	NA

ENSG00000220666	NA	NA	NA	0	NA	NA	NA	NA	NA
ENSG00000220685	LOC442155	transcription factor B2, mitochondrial pseudogene	442155	0,284030801	-0,994281388	4,040529	-0,246077006	0,805622622	NA
ENSG00000220694	RPL17P23	ribosomal protein L17 pseudogene 23	100271152	0	NA	NA	NA	NA	NA
ENSG00000220695	NA	NA	NA	0	NA	NA	NA	NA	NA
ENSG00000220721	OR1F12P	olfactory receptor family 1 subfamily F member 12, pseudogene	442179	0	NA	NA	NA	NA	NA
ENSG00000220725	NA	NA	NA	0	NA	NA	NA	NA	NA
ENSG00000220730	NA	NA	NA	0	NA	NA	NA	NA	NA
ENSG00000220733	MTND3P20	MT-ND3 pseudogene 20	107075294	0	NA	NA	NA	NA	NA
ENSG00000220734	NA	NA	NA	0	NA	NA	NA	NA	NA
ENSG00000220739	NA	NA	NA	0	NA	NA	NA	NA	NA
ENSG00000220744	RPL5P18	ribosomal protein L5 pseudogene 18	389428	0,515559535	0,834201932	3,447118	0,241999838	0,808780289	NA
ENSG00000220745	NA	NA	NA	0	NA	NA	NA	NA	NA
ENSG00000220748	NA	NA	NA	0	NA	NA	NA	NA	NA
ENSG00000220749	RPL21P28	ribosomal protein L21 pseudogene 28	100131205	0,934982161	0,608451161	2,09817	0,289991317	0,77182288	NA
ENSG00000220758	NA	NA	NA	0	NA	NA	NA	NA	NA
ENSG00000220771	BOLA2P3	bolA family member 2 pseudogene 3	106480323	0	NA	NA	NA	NA	NA
ENSG00000220773	RPSAP44	ribosomal protein SA pseudogene 44	652411	0	NA	NA	NA	NA	NA
ENSG00000220785	NA	NA	NA	2,985218724	0,497620601	1,111941	0,447524337	0,65449652	NA
ENSG00000220793	RPL21P119	ribosomal protein L21 pseudogene 119	653737	0,15836288	0	4,080456	0	1	NA
ENSG00000220804	NA	NA	NA	6,429021324	0,592568198	0,796299	0,744152978	0,456783932	NA
ENSG00000220831	NA	NA	NA	0	NA	NA	NA	NA	NA
ENSG00000220842	RPL21P16	ribosomal protein L21 pseudogene 16	729402	0	NA	NA	NA	NA	NA
ENSG00000220848	NA	NA	NA	2,017566561	-0,43752719	1,339564	-0,326619217	0,743955907	NA
ENSG00000220867	HSPE1P26	heat shock protein family E (Hsp10) member 1 pseudogene 26	106481677	0,10543123	0	4,080456	0	1	NA
ENSG00000220868	MRPL35P1	mitochondrial ribosomal protein L35 pseudogene 1	359742	0	NA	NA	NA	NA	NA
ENSG00000220871	NA	NA	NA	0	NA	NA	NA	NA	NA
ENSG00000220875	H3C9P	H3 clustered histone 9, pseudogene	100289545	0	NA	NA	NA	NA	NA
ENSG00000220884	NA	NA	NA	0	NA	NA	NA	NA	NA
ENSG00000220913	NA	NA	NA	0	NA	NA	NA	NA	NA
ENSG00000220918	LOC100422671	cell division cycle 73, Paf1/RNA polymerase II complex component, homolog (S, cerevisiae) pseudogene	100422671	0	NA	NA	NA	NA	NA
ENSG00000220920	RPS12P12	ribosomal protein S12 pseudogene 12	100131761	0	NA	NA	NA	NA	NA
ENSG00000220924	NA	NA	NA	0	NA	NA	NA	NA	NA
ENSG00000220937	NA	NA	NA	0	NA	NA	NA	NA	NA
ENSG00000220948	TRIM51G	tripartite motif-containing 51G	120824	0	NA	NA	NA	NA	NA
ENSG00000220949	NA	NA	NA	0	NA	NA	NA	NA	NA
ENSG00000220960	NA	NA	NA	0	NA	NA	NA	NA	NA
ENSG00000221818	EBF2	EBF transcription factor 2	64641	0	NA	NA	NA	NA	NA
ENSG00000221821	C6orf226	chromosome 6 open reading frame 226	441150	7,96032915	0,512889491	0,624911	0,820740031	0,411794364	NA
ENSG00000221826	PSG3	pregnancy specific beta-1-glycoprotein 3	5671	0	NA	NA	NA	NA	NA
ENSG00000221837	KRTAP10-9	keratin associated protein 10-9	386676	0	NA	NA	NA	NA	NA
ENSG00000221840	OR4A5	olfactory receptor family 4 subfamily A member 5	81318	0	NA	NA	NA	NA	NA
ENSG00000221843	SPATA31H1	SPATA31 subfamily H member 1	84226	0	NA	NA	NA	NA	NA
ENSG00000221844	DPP3P2	DPP3 pseudogene 2	138971	0	NA	NA	NA	NA	NA
ENSG00000221849	LOC100631243	zinc finger protein 211 pseudogene	100631243	0	NA	NA	NA	NA	NA
ENSG00000221852	KRTAP1-5	keratin associated protein 1-5	83895	0	NA	NA	NA	NA	NA
ENSG00000221858	OR2A12	olfactory receptor family 2 subfamily A member 12	346525	0	NA	NA	NA	NA	NA
ENSG00000221859	KRTAP10-10	keratin associated protein 10-10	353333	0	NA	NA	NA	NA	NA
ENSG00000221864	KRTAP12-2	keratin associated protein 12-2	353323	0	NA	NA	NA	NA	NA
ENSG00000221867	MAGEA3	MAGE family member A3	4102	0	NA	NA	NA	NA	NA

ENSG00000221874	ZNF816-ZNF321P	ZNF816-ZNF321P readthrough	100529240	10,03738407	0,393271785	0,73142	0,537682538	0,590796237	NA
ENSG00000221878	PSG7	pregnancy specific beta-1-glycoprotein 7	5676	0	NA	NA	NA	NA	NA
ENSG00000221879	NA	NA	NA	0	NA	NA	NA	NA	NA
ENSG00000221880	KRTAP1-3	keratin associated protein 1-3	81850	0	NA	NA	NA	NA	NA
ENSG00000221882	OR3A2	olfactory receptor family 3 subfamily A member 2	4995	0	NA	NA	NA	NA	NA
ENSG00000221886	FAM200C	family with sequence similarity 200 member C	63920	2,635671595	0,865650364	1,63776	0,52855768	0,597112324	NA
ENSG00000221887	HMSD	histocompatibility minor serpin domain containing	284293	0,30606939	-1,088663337	4,029927	-0,27014468	0,78704895	NA
ENSG00000221888	OR1C1	olfactory receptor family 1 subfamily C member 1	26188	0	NA	NA	NA	NA	NA
ENSG00000221891	NA	NA	NA	0	NA	NA	NA	NA	NA
ENSG00000221900	POM121L12	POM121 transmembrane nucleoporin like 12	285877	0	NA	NA	NA	NA	NA
ENSG00000221910	OR2F2	olfactory receptor family 2 subfamily F member 2	135948	0	NA	NA	NA	NA	NA
ENSG00000221916	C19orf73	chromosome 19 open reading frame 73	55150	1,19969861	0,633378252	1,593741	0,397415997	0,691060721	NA
ENSG00000221930	NA	NA	NA	0,11808024	-1,076443944	4,080456	-0,263804824	0,791930317	NA
ENSG00000221931	OR6X1	olfactory receptor family 6 subfamily X member 1	390260	0	NA	NA	NA	NA	NA
ENSG00000221932	HEPN1	hepatocellular carcinoma, down-regulated 1	641654	0	NA	NA	NA	NA	NA
ENSG00000221933	OR2A25	olfactory receptor family 2 subfamily A member 25	392138	0	NA	NA	NA	NA	NA
ENSG00000221937	TAS2R40	taste 2 receptor member 40	259286	0	NA	NA	NA	NA	NA
ENSG00000221938	OR2A14	olfactory receptor family 2 subfamily A member 14	135941	0	NA	NA	NA	NA	NA
ENSG00000221946	FXYD7	FXYD domain containing ion transport regulator 7	53822	2,992627873	0,390337807	1,147044	0,340299014	0,733631359	NA
ENSG00000221947	XKR9	XK related 9	389668	0	NA	NA	NA	NA	NA
ENSG00000221962	TMEM14EP	transmembrane protein 14E, pseudogene	645843	0	NA	NA	NA	NA	NA
ENSG00000221970	OR2A1	olfactory receptor family 2 subfamily A member 1	346528	0	NA	NA	NA	NA	NA
ENSG00000221971	TTC4P1	tetratricopeptide repeat domain 4 pseudogene 1	222052	0	NA	NA	NA	NA	NA
ENSG00000221977	OR4E2	olfactory receptor family 4 subfamily E member 2	26686	0	NA	NA	NA	NA	NA
ENSG00000221986	MYBPHL	myosin binding protein H like	343263	0	NA	NA	NA	NA	NA
ENSG00000221988	PPT2	palmitoyl-protein thioesterase 2	9374	2,424918299	0	3,390547	0	1	NA
ENSG00000221989	OR2A2	olfactory receptor family 2 subfamily A member 2	442361	0	NA	NA	NA	NA	NA
ENSG00000221994	ZNF630	zinc finger protein 630	57232	9,256821978	0,164987971	0,61245	0,269390182	0,787629441	NA
ENSG00000221996	OR52B4	olfactory receptor family 52 subfamily B member 4	143496	0	NA	NA	NA	NA	NA
ENSG00000222009	BTBD19	BTB domain containing 19	149478	1,338824335	0,544506906	2,564145	0,212354206	0,831830709	NA
ENSG00000222011	FAM185A	family with sequence similarity 185 member A	222234	6,059831991	-2,653265034	1,431757	-1,853153541	0,063860355	NA
ENSG00000222018	C21orf140	chromosome 21 open reading frame 140	101928147	0	NA	NA	NA	NA	NA
ENSG00000222018	LOC102723451	family with sequence similarity 243 member B	102723451	0	NA	NA	NA	NA	NA
ENSG00000222019	NA	NA	NA	0	NA	NA	NA	NA	NA
ENSG00000222024	NA	NA	NA	0	NA	NA	NA	NA	NA
ENSG00000222028	PSMB11	proteasome subunit beta 11	122706	0	NA	NA	NA	NA	NA
ENSG00000222036	POTEM	POTE ankyrin domain family member M	641455	0	NA	NA	NA	NA	NA
ENSG00000222037	IGLC6	immunoglobulin lambda constant 6	3542	0	NA	NA	NA	NA	NA
ENSG00000222038	POTEJ	POTE ankyrin domain family member J	653781	0	NA	NA	NA	NA	NA
ENSG00000222046	DCDC2B	doublecortin domain containing 2B	149069	0,223085721	-1,079591872	4,080456	-0,264576289	0,791335888	NA
ENSG00000222054	RNA5SP177	RNA, 5S ribosomal pseudogene 177	100873439	0	NA	NA	NA	NA	NA
ENSG00000222108	RNA5SP317	RNA, 5S ribosomal pseudogene 317	106479006	0	NA	NA	NA	NA	NA
ENSG00000222118	RNA5SP487	RNA, 5S ribosomal pseudogene 487	100873729	0	NA	NA	NA	NA	NA
ENSG00000222123	RNA5SP390	RNA, 5S ribosomal pseudogene 390	100873643	0	NA	NA	NA	NA	NA
ENSG00000222129	RNA5SP36	RNA, 5S ribosomal pseudogene 36	100873368	0	NA	NA	NA	NA	NA

ENSG00000222150	RNA5SP239	RNA, 5S ribosomal pseudogene 239	100873497	0	NA	NA	NA	NA	NA
ENSG00000222178	RNA5SP181	RNA, 5S ribosomal pseudogene 181	100873443	0	NA	NA	NA	NA	NA
ENSG00000222182	RNA5SP156	RNA, 5S ribosomal pseudogene 156	100873422	0	NA	NA	NA	NA	NA
ENSG00000222207	RNA5SP407	RNA, 5S ribosomal pseudogene 407	100873659	0	NA	NA	NA	NA	NA
ENSG00000222208	RNA5SP129	RNA, 5S ribosomal pseudogene 129	100873399	0,207996306	-1,916073151	4,043021	-0,473921178	0,635556117	NA
ENSG00000222209	RNA5SP56	RNA, 5S ribosomal pseudogene 56	100873294	0	NA	NA	NA	NA	NA
ENSG00000222230	RNA5SP158	RNA, 5S ribosomal pseudogene 158	100873424	0	NA	NA	NA	NA	NA
ENSG00000222232	RNA5SP89	RNA, 5S ribosomal pseudogene 89	100873322	0	NA	NA	NA	NA	NA
ENSG00000222236	RNA5SP163	RNA, 5S ribosomal pseudogene 163	100873428	0	NA	NA	NA	NA	NA
ENSG00000222244	RNA5SP431	RNA, 5S ribosomal pseudogene 431	100873681	0	NA	NA	NA	NA	NA
ENSG00000222248	RNA5SP201	RNA, 5S ribosomal pseudogene 201	100873462	0	NA	NA	NA	NA	NA
ENSG00000222251	RNA5SP95	RNA, 5S ribosomal pseudogene 95	100873328	0	NA	NA	NA	NA	NA
ENSG00000222268	RNA5SP425	RNA, 5S ribosomal pseudogene 425	100873676	0	NA	NA	NA	NA	NA
ENSG00000222285	RNA5SP193	RNA, 5S ribosomal pseudogene 193	100873455	0	NA	NA	NA	NA	NA
ENSG00000222302	RNA5SP371	RNA, 5S ribosomal pseudogene 371	100873629	0	NA	NA	NA	NA	NA
ENSG00000222308	RNA5SP198	RNA, 5S ribosomal pseudogene 198	100873460	0	NA	NA	NA	NA	NA
ENSG00000222312	RNA5SP178	RNA, 5S ribosomal pseudogene 178	100873440	0	NA	NA	NA	NA	NA
ENSG00000222317	RNA5SP118	RNA, 5S ribosomal pseudogene 118	100873388	0	NA	NA	NA	NA	NA
ENSG00000222346	RNA5SP237	RNA, 5S ribosomal pseudogene 237	100873495	0	NA	NA	NA	NA	NA
ENSG00000222378	RNA5SP44	RNA, 5S ribosomal pseudogene 44	100873283	0	NA	NA	NA	NA	NA
ENSG00000222383	RNA5SP203	RNA, 5S ribosomal pseudogene 203	100873464	0	NA	NA	NA	NA	NA
ENSG00000222407	RNA5SP80	RNA, 5S ribosomal pseudogene 80	100873314	0	NA	NA	NA	NA	NA
ENSG00000222416	RNA5SP274	RNA, 5S ribosomal pseudogene 274	100873527	0	NA	NA	NA	NA	NA
ENSG00000222418	RNA5SP113	RNA, 5S ribosomal pseudogene 113	100873383	0	NA	NA	NA	NA	NA
ENSG00000222419	RNA5SP511	RNA, 5S ribosomal pseudogene 511	100873562	0	NA	NA	NA	NA	NA
ENSG00000222427	RNA5SP338	RNA, 5S ribosomal pseudogene 338	100873601	0	NA	NA	NA	NA	NA
ENSG00000222428	RNA5SP231	RNA, 5S ribosomal pseudogene 231	100873489	0	NA	NA	NA	NA	NA
ENSG00000222455	RNA5SP296	RNA, 5S ribosomal pseudogene 296	100873549	0	NA	NA	NA	NA	NA
ENSG00000222459	RNA5SP293	RNA, 5S ribosomal pseudogene 293	100873546	0	NA	NA	NA	NA	NA
ENSG00000222468	RNA5SP261	RNA, 5S ribosomal pseudogene 261	100873515	0	NA	NA	NA	NA	NA
ENSG00000222500	RNA5SP475	RNA, 5S ribosomal pseudogene 475	100873717	0	NA	NA	NA	NA	NA
ENSG00000222520	RNA5SP451	RNA, 5S ribosomal pseudogene 451	100873696	0	NA	NA	NA	NA	NA
ENSG00000222546	RNA5SP251	RNA, 5S ribosomal pseudogene 251	100873507	0	NA	NA	NA	NA	NA
ENSG00000222552	RNA5SP60	RNA, 5S ribosomal pseudogene 60	100873295	0	NA	NA	NA	NA	NA
ENSG00000222578	RNA5SP345	RNA, 5S ribosomal pseudogene 345	100873607	0	NA	NA	NA	NA	NA
ENSG00000222585	RNA5SP494	RNA, 5S ribosomal pseudogene 494	100873736	0	NA	NA	NA	NA	NA
ENSG00000222608	RNA5SP504	RNA, 5S ribosomal pseudogene 504	100873555	0	NA	NA	NA	NA	NA
ENSG00000222675	RNA5SP146	RNA, 5S ribosomal pseudogene 146	100873413	0	NA	NA	NA	NA	NA
ENSG00000222682	RNA5SP38	RNA, 5S ribosomal pseudogene 38	100873370	0	NA	NA	NA	NA	NA
ENSG00000222740	RNA5SP328	RNA, 5S ribosomal pseudogene 328	100873594	0	NA	NA	NA	NA	NA

ENSG00000222741	RNA5SP229	RNA, 5S ribosomal pseudogene 229	100873487	0	NA	NA	NA	NA	NA
ENSG00000222747	RNA5SP25	RNA, 5S ribosomal pseudogene 25	100873357	0	NA	NA	NA	NA	NA
ENSG00000222778	RNA5SP144	RNA, 5S ribosomal pseudogene 144	100873411	0	NA	NA	NA	NA	NA
ENSG00000222806	RNA5SP225	RNA, 5S ribosomal pseudogene 225	100873484	0	NA	NA	NA	NA	NA
ENSG00000222832	RNA5SP120	RNA, 5S ribosomal pseudogene 120	100873390	0	NA	NA	NA	NA	NA
ENSG00000222835	RNA5SP100	RNA, 5S ribosomal pseudogene 100	106480755	0	NA	NA	NA	NA	NA
ENSG00000222849	RNA5SP21	RNA, 5S ribosomal pseudogene 21	100873276	0	NA	NA	NA	NA	NA
ENSG00000222920	RNA5SP29	RNA, 5S ribosomal pseudogene 29	100873361	0	NA	NA	NA	NA	NA
ENSG00000222921	RNA5SP104	RNA, 5S ribosomal pseudogene 104	100873375	0	NA	NA	NA	NA	NA
ENSG00000222922	RNA5SP501	RNA, 5S ribosomal pseudogene 501	100873552	0	NA	NA	NA	NA	NA
ENSG00000222952	RNA5SP41	RNA, 5S ribosomal pseudogene 41	100873280	0	NA	NA	NA	NA	NA
ENSG00000222955	RNA5SP265	RNA, 5S ribosomal pseudogene 265	100873518	0	NA	NA	NA	NA	NA
ENSG00000222971	RNA5SP222	RNA, 5S ribosomal pseudogene 222	100873481	0	NA	NA	NA	NA	NA
ENSG00000222983	RNA5SP127	RNA, 5S ribosomal pseudogene 127	100873397	0	NA	NA	NA	NA	NA
ENSG00000223003	RNA5SP184	RNA, 5S ribosomal pseudogene 184	100873446	0	NA	NA	NA	NA	NA
ENSG00000223013	RNA5SP344	RNA, 5S ribosomal pseudogene 344	100873606	0	NA	NA	NA	NA	NA
ENSG00000223019	RNA5SP306	RNA, 5S ribosomal pseudogene 306	100873580	0	NA	NA	NA	NA	NA
ENSG00000223046	RNA5SP369	RNA, 5S ribosomal pseudogene 369	100873628	0	NA	NA	NA	NA	NA
ENSG00000223076	RNA5SP31	RNA, 5S ribosomal pseudogene 31	100873363	0	NA	NA	NA	NA	NA
ENSG00000223086	RNA5SP155	RNA, 5S ribosomal pseudogene 155	100873421	0	NA	NA	NA	NA	NA
ENSG00000223092	RNA5SP115	RNA, 5S ribosomal pseudogene 115	100873385	0	NA	NA	NA	NA	NA
ENSG00000223113	RNA5SP247	RNA, 5S ribosomal pseudogene 247	100873503	0	NA	NA	NA	NA	NA
ENSG00000223131	RNA5SP304	RNA, 5S ribosomal pseudogene 304	100873578	0	NA	NA	NA	NA	NA
ENSG00000223138	RNA5SP450	RNA, 5S ribosomal pseudogene 450	100873695	0	NA	NA	NA	NA	NA
ENSG00000223162	RNA5SP280	RNA, 5S ribosomal pseudogene 280	100873533	0	NA	NA	NA	NA	NA
ENSG00000223169	RNA5SP209	RNA, 5S ribosomal pseudogene 209	100873469	0	NA	NA	NA	NA	NA
ENSG00000223177	RNA5SP39	RNA, 5S ribosomal pseudogene 39	100873371	0	NA	NA	NA	NA	NA
ENSG00000223203	RNA5SP221	RNA, 5S ribosomal pseudogene 221	106480759	0	NA	NA	NA	NA	NA
ENSG00000223238	RNA5SP294	RNA, 5S ribosomal pseudogene 294	100873547	0	NA	NA	NA	NA	NA
ENSG00000223259	RNA5SP508	RNA, 5S ribosomal pseudogene 508	100873559	0	NA	NA	NA	NA	NA
ENSG00000223262	RNA5SP492	RNA, 5S ribosomal pseudogene 492	100873734	0	NA	NA	NA	NA	NA
ENSG00000223290	RNA5SP107	RNA, 5S ribosomal pseudogene 107	100873377	0	NA	NA	NA	NA	NA
ENSG00000223293	RNA5SP270	RNA, 5S ribosomal pseudogene 270	100873523	0	NA	NA	NA	NA	NA
ENSG00000223318	RNA5SP111	RNA, 5S ribosomal pseudogene 111	100873381	0	NA	NA	NA	NA	NA
ENSG00000223345	NA	NA	NA	0,117497108	0,847101401	4,080456	0,207599697	0,835541541	NA
ENSG00000223349	NA	NA	NA	0	NA	NA	NA	NA	NA
ENSG00000223350	IGLV9-49	immunoglobulin lambda variable 9-49	28773	0	NA	NA	NA	NA	NA
ENSG00000223353	NA	NA	NA	0	NA	NA	NA	NA	NA
ENSG00000223361	FTH1P10	ferritin heavy chain 1 pseudogene 10	2502	0,10543123	0	4,080456	0	1	NA
ENSG00000223362	NA	NA	NA	0	NA	NA	NA	NA	NA
ENSG00000223368	LOC100420008	transmembrane protein 126B pseudogene	100420008	0	NA	NA	NA	NA	NA
ENSG00000223375	NA	NA	NA	0,207996306	-1,916073151	4,043021	-0,473921178	0,635556117	NA
ENSG00000223377	NA	NA	NA	0	NA	NA	NA	NA	NA

ENSG00000223383	H2ACP1	H2AC histone family pseudogene 1	100509927	0	NA	NA	NA	NA	NA
ENSG00000223389	SDCBP2P1	syndecan binding protein 2 pseudogene 1	106480801	0,11808024	-1,076443944	4,080456	-0,263804824	0,791930317	NA
ENSG00000223391	NA	NA	NA	0	NA	NA	NA	NA	NA
ENSG00000223394	TRBV29OR9-2	T cell receptor beta variable 29/OR9-2 (non-functional)	6958	0	NA	NA	NA	NA	NA
ENSG00000223395	RBM22P7	RNA binding motif protein 22 pseudogene 7	106478953	0	NA	NA	NA	NA	NA
ENSG00000223396	NA	NA	NA	2,606439643	0,564536415	1,288259	0,438216533	0,661229325	NA
ENSG00000223398	NA	NA	NA	0	NA	NA	NA	NA	NA
ENSG00000223402	NA	NA	NA	0	NA	NA	NA	NA	NA
ENSG00000223407	USP9YP18	USP9Y pseudogene 18	106480424	0	NA	NA	NA	NA	NA
ENSG00000223409	RPL17P17	ribosomal protein L17 pseudogene 17	100270943	0	NA	NA	NA	NA	NA
ENSG00000223416	NA	NA	NA	0,23616048	-2,075648354	4,028973	-0,515180498	0,606426895	NA
ENSG00000223417	TRIM49D1	tripartite motif containing 49D1	399939	0	NA	NA	NA	NA	NA
ENSG00000223418	LIMK2P1	LIMK2 pseudogene 1	139952	0	NA	NA	NA	NA	NA
ENSG00000223419	NA	NA	NA	0	NA	NA	NA	NA	NA
ENSG00000223421	NA	NA	NA	0	NA	NA	NA	NA	NA
ENSG00000223422	NA	NA	NA	0	NA	NA	NA	NA	NA
ENSG00000223427	NA	NA	NA	0	NA	NA	NA	NA	NA
ENSG00000223428	NA	NA	NA	0	NA	NA	NA	NA	NA
ENSG00000223429	NA	NA	NA	0	NA	NA	NA	NA	NA
ENSG00000223431	MTND6P21	MT-ND6 pseudogene 21	100873332	0,580985497	0,987074753	2,879592	0,342782893	0,731761797	NA
ENSG00000223433	NA	NA	NA	0	NA	NA	NA	NA	NA
ENSG00000223437	TMSB4XP3	TMSB4X pseudogene 3	100873877	0	NA	NA	NA	NA	NA
ENSG00000223443	USP17L2	ubiquitin specific peptidase 17 like family member 2	377630	0	NA	NA	NA	NA	NA
ENSG00000223443	USP17L1	ubiquitin specific peptidase 17 like family member 1	401447	0	NA	NA	NA	NA	NA
ENSG00000223443	LOC124906403	ubiquitin carboxyl-terminal hydrolase 17-like protein 22	124906403	0	NA	NA	NA	NA	NA
ENSG00000223443	LOC124906412	ubiquitin carboxyl-terminal hydrolase 17-like protein 22	124906412	0	NA	NA	NA	NA	NA
ENSG00000223443	LOC124906415	ubiquitin carboxyl-terminal hydrolase 17-like protein 20	124906415	0	NA	NA	NA	NA	NA
ENSG00000223443	LOC124906416	ubiquitin carboxyl-terminal hydrolase 17-like protein 22	124906416	0	NA	NA	NA	NA	NA
ENSG00000223445	RPL6P21	ribosomal protein L6 pseudogene 21	100270846	0	NA	NA	NA	NA	NA
ENSG00000223450	RPS24P5	ribosomal protein S24 pseudogene 5	100271094	0	NA	NA	NA	NA	NA
ENSG00000223452	NA	NA	NA	0	NA	NA	NA	NA	NA
ENSG00000223455	NA	NA	NA	0	NA	NA	NA	NA	NA
ENSG00000223460	NA	NA	NA	0	NA	NA	NA	NA	NA
ENSG00000223467	CALM1P1	calmodulin 1 pseudogene 1	802	0	NA	NA	NA	NA	NA
ENSG00000223476	NA	NA	NA	0	NA	NA	NA	NA	NA
ENSG00000223488	NA	NA	NA	0	NA	NA	NA	NA	NA
ENSG00000223489	NEFHP1	neurofilament heavy pseudogene 1	339396	0	NA	NA	NA	NA	NA
ENSG00000223490	CHCHD2P5	coiled-coil-helix-coiled-coil-helix domain containing 2 pseudogene 5	100874508	0	NA	NA	NA	NA	NA
ENSG00000223495	NA	NA	NA	0	NA	NA	NA	NA	NA
ENSG00000223497	H2BL1P	H2B,L histone variant 1, pseudogene	115482689	0	NA	NA	NA	NA	NA
ENSG00000223498	NA	NA	NA	0	NA	NA	NA	NA	NA
ENSG00000223500	RNF14P4	RNF14 pseudogene 4	100419645	0	NA	NA	NA	NA	NA
ENSG00000223503	NA	NA	NA	0	NA	NA	NA	NA	NA
ENSG00000223505	NA	NA	NA	0	NA	NA	NA	NA	NA
ENSG00000223508	NA	NA	NA	3,630304359	0,22976505	1,005363	0,2285394	0,81922693	NA
ENSG00000223510	CDRT15	CMT1A duplicated region transcript 15	146822	0	NA	NA	NA	NA	NA
ENSG00000223513	NA	NA	NA	0	NA	NA	NA	NA	NA
ENSG00000223514	NA	NA	NA	0	NA	NA	NA	NA	NA
ENSG00000223518	NA	NA	NA	0	NA	NA	NA	NA	NA
ENSG00000223519	NA	NA	NA	0	NA	NA	NA	NA	NA
ENSG00000223529	EEF1A1P8	eukaryotic translation elongation factor 1 alpha 1 pseudogene 8	389179	0,382299144	0,831223733	3,681842	0,225763034	0,82138573	NA
ENSG00000223538	NA	NA	NA	0	NA	NA	NA	NA	NA
ENSG00000223539	NA	NA	NA	0	NA	NA	NA	NA	NA
ENSG00000223540	RPS15AP5	ribosomal protein S15a pseudogene 5	414230	0	NA	NA	NA	NA	NA
ENSG00000223543	TCEAL4P1	transcription elongation factor A like 4 pseudogene 1	100874432	0	NA	NA	NA	NA	NA
ENSG00000223544	NA	NA	NA	0	NA	NA	NA	NA	NA
ENSG00000223547	ZNF844	zinc finger protein 844	284391	0,838931368	-1,105592201	2,462389	-0,448991655	0,653437677	NA

ENSG00000223549	MTND5P28	MT-ND5 pseudogene 28	100505755	0,118286652	0	4,080456	0	1	NA
ENSG00000223550	SNRPBP1	small nuclear ribonucleoprotein polypeptides B and B1 pseudogene 1	100499258	0	NA	NA	NA	NA	NA
ENSG00000223551	TMSB4XP4	TMSB4X pseudogene 4	7118	7,605100753	0,817784414	0,711529	1,149334612	0,250418031	NA
ENSG00000223553	SMPD4P1	sphingomyelin phosphodiesterase 4 pseudogene 1	645280	0	NA	NA	NA	NA	NA
ENSG00000223555	USP9YP23	USP9Y pseudogene 23	100500798	0	NA	NA	NA	NA	NA
ENSG00000223558	NA	NA	NA	0	NA	NA	NA	NA	NA
ENSG00000223559	NA	NA	NA	0	NA	NA	NA	NA	NA
ENSG00000223564	CYP4F32P	cytochrome P450 family 4 subfamily F member 32, pseudogene	107133497	0	NA	NA	NA	NA	NA
ENSG00000223565	NA	NA	NA	0	NA	NA	NA	NA	NA
ENSG00000223566	TNRC18P2	trinucleotide repeat containing 18 pseudogene 2	27320	0	NA	NA	NA	NA	NA
ENSG00000223568	RPL3P11	ribosomal protein L3 pseudogene 11	100131893	0	NA	NA	NA	NA	NA
ENSG00000223569	USP17L15	ubiquitin specific peptidase 17 like family member 15	100288520	0	NA	NA	NA	NA	NA
ENSG00000223569	LOC124906401	ubiquitin carboxyl-terminal hydrolase 17-like protein 22	124906401	0	NA	NA	NA	NA	NA
ENSG00000223570	RPL34P7	ribosomal protein L34 pseudogene 7	100127995	0	NA	NA	NA	NA	NA
ENSG00000223572	CKMT1A	creatine kinase, mitochondrial 1A	548596	0	NA	NA	NA	NA	NA
ENSG00000223573	TINCR	TINCR ubiquitin domain containing	257000	2,595463035	-1,989669792	1,355716	-1,467615841	0,142208603	NA
ENSG00000223574	TPMTP4	thiopurine S-methyltransferase pseudogene 4	100129298	0	NA	NA	NA	NA	NA
ENSG00000223575	NA	NA	NA	0,748879977	1,967457723	2,617505	0,751653916	0,452259211	NA
ENSG00000223583	RPL17P9	ribosomal protein L17 pseudogene 9	729301	0,279407855	-1,047189919	4,080456	-0,256635521	0,797460151	NA
ENSG00000223584	TVP23CP1	TVP23C pseudogene 1	100421599	0	NA	NA	NA	NA	NA
ENSG00000223589	NA	NA	NA	0	NA	NA	NA	NA	NA
ENSG00000223592	NA	NA	NA	0	NA	NA	NA	NA	NA
ENSG00000223593	LOC100421787	sulfotransferase family 1C member 2 pseudogene	100421787	0	NA	NA	NA	NA	NA
ENSG00000223595	NA	NA	NA	0	NA	NA	NA	NA	NA
ENSG00000223600	NA	NA	NA	0	NA	NA	NA	NA	NA
ENSG00000223601	EBLN1	endogenous Bornavirus like nucleoprotein 1	340900	0	NA	NA	NA	NA	NA
ENSG00000223602	NA	NA	NA	0	NA	NA	NA	NA	NA
ENSG00000223603	CRPP1	C-reactive protein pseudogene 1	171422	0	NA	NA	NA	NA	NA
ENSG00000223604	RPL17P13	ribosomal protein L17 pseudogene 13	100270939	0	NA	NA	NA	NA	NA
ENSG00000223605	NA	NA	NA	0	NA	NA	NA	NA	NA
ENSG00000223609	HBD	hemoglobin subunit delta	3045	0	NA	NA	NA	NA	NA
ENSG00000223611	SUPT20HL2	SUPT20H like 2	170067	2,791552838	-0,973405806	1,204652	-0,808038718	0,419068294	NA
ENSG00000223612	NA	NA	NA	0,50563183	0,718181637	2,940703	0,244221041	0,807059632	NA
ENSG00000223614	ZNF735	zinc finger protein 735	730291	0	NA	NA	NA	NA	NA
ENSG00000223615	NA	NA	NA	0	NA	NA	NA	NA	NA
ENSG00000223619	MTCO1P17	MT-CO1 pseudogene 17	107075222	0	NA	NA	NA	NA	NA
ENSG00000223620	NA	NA	NA	0	NA	NA	NA	NA	NA
ENSG00000223621	NA	NA	NA	0,996152419	2,357177324	1,95932	1,203058949	0,228953509	NA
ENSG00000223622	GSTA6P	glutathione S-transferase alpha 6, pseudogene	2942	0	NA	NA	NA	NA	NA
ENSG00000223624	NA	NA	NA	0	NA	NA	NA	NA	NA
ENSG00000223625	NA	NA	NA	0	NA	NA	NA	NA	NA
ENSG00000223628	NA	NA	NA	0	NA	NA	NA	NA	NA
ENSG00000223637	NA	NA	NA	0	NA	NA	NA	NA	NA
ENSG00000223638	RFPL4A	ret finger protein like 4A	342931	0	NA	NA	NA	NA	NA
ENSG00000223640	RPL30P3	ribosomal protein L30 pseudogene 3	100270977	0	NA	NA	NA	NA	NA
ENSG00000223648	IGHV3-64	immunoglobulin heavy variable 3-64	28414	0	NA	NA	NA	NA	NA
ENSG00000223650	NA	NA	NA	0	NA	NA	NA	NA	NA
ENSG00000223651	LOC643172	neurofilament heavy chain pseudogene	643172	0	NA	NA	NA	NA	NA
ENSG00000223655	RAB9AP3	RAB9A, member RAS oncogene family pseudogene 3	100287577	0	NA	NA	NA	NA	NA
ENSG00000223656	HMGB3P10	high mobility group box 3 pseudogene 10	100129320	0	NA	NA	NA	NA	NA
ENSG00000223657	NA	NA	NA	0	NA	NA	NA	NA	NA

ENSG00000223658	C1GALT1C1L	C1GALT1 specific chaperone 1 like	728819	0	NA	NA	NA	NA	NA
ENSG00000223663	NDUFB4P8	NADH:ubiquinone oxidoreductase subunit B4 pseudogene 8	107075246	0,117497108	0,847101401	4,080456	0,207599697	0,835541541	NA
ENSG00000223668	EEF1A1P24	eukaryotic translation elongation factor 1 alpha 1 pseudogene 24	645715	1,026170714	1,202003253	2,000583	0,600826484	0,547955562	NA
ENSG00000223671	RPL34P3	ribosomal protein L34 pseudogene 3	54026	0	NA	NA	NA	NA	NA
ENSG00000223672	NA	NA	NA	0	NA	NA	NA	NA	NA
ENSG00000223676	RPL34P26	ribosomal protein L34 pseudogene 26	100130718	0	NA	NA	NA	NA	NA
ENSG00000223677	OR2AD1P	olfactory receptor family 2 subfamily AD member 1 pseudogene	79313	0	NA	NA	NA	NA	NA
ENSG00000223679	NA	NA	NA	0	NA	NA	NA	NA	NA
ENSG00000223683	RPSAP65	ribosomal protein SA pseudogene 65	107075109	0	NA	NA	NA	NA	NA
ENSG00000223684	IFNWP18	interferon omega 1 pseudogene 18	360001	0	NA	NA	NA	NA	NA
ENSG00000223688	NA	NA	NA	0	NA	NA	NA	NA	NA
ENSG00000223691	NA	NA	NA	0	NA	NA	NA	NA	NA
ENSG00000223694	ADH5P3	ADH5 pseudogene 3	100131810	0	NA	NA	NA	NA	NA
ENSG00000223698	NA	NA	NA	0	NA	NA	NA	NA	NA
ENSG00000223702	ZDHHC20P2	zinc finger DHHC-type containing 20 pseudogene 2	100048908	0	NA	NA	NA	NA	NA
ENSG00000223703	IGSF3P2	IGSF3 pseudogene 2	100289350	0	NA	NA	NA	NA	NA
ENSG00000223707	NA	NA	NA	0	NA	NA	NA	NA	NA
ENSG00000223709	TRIM64EP	tripartite motif containing 64E, pseudogene	440061	0	NA	NA	NA	NA	NA
ENSG00000223710	CRYGGP	crystallin gamma G, pseudogene	1424	0	NA	NA	NA	NA	NA
ENSG00000223717	DNAJA1P1	DnaJ heat shock protein family (Hsp40) member A1 pseudogene 1	100129308	0	NA	NA	NA	NA	NA
ENSG00000223718	RPL15P11	ribosomal protein L15 pseudogene 11	402694	0,237164745	1,955811315	4,022315	0,486240188	0,626796881	NA
ENSG00000223719	YWHAQP10	YWHAQ pseudogene	127379682	0	NA	NA	NA	NA	NA
ENSG00000223721	NA	NA	NA	0	NA	NA	NA	NA	NA
ENSG00000223722	NA	NA	NA	0	NA	NA	NA	NA	NA
ENSG00000223723	NA	NA	NA	0,103998153	-1,076443944	4,080456	-0,263804824	0,791930317	NA
ENSG00000223724	NA	NA	NA	0	NA	NA	NA	NA	NA
ENSG00000223728	LOC391092	PRAME family member 17 pseudogene	391092	0	NA	NA	NA	NA	NA
ENSG00000223730	RPL6P28	ribosomal protein L6 pseudogene 28	100271208	0	NA	NA	NA	NA	NA
ENSG00000223731	SUPT20HL1	SUPT20H like 1	100130302	0	NA	NA	NA	NA	NA
ENSG00000223733	RPL18AP14	ribosomal protein L18a pseudogene 14	100271420	0	NA	NA	NA	NA	NA
ENSG00000223735	OR51B3P	olfactory receptor family 51 subfamily B member 3 pseudogene	79342	0	NA	NA	NA	NA	NA
ENSG00000223738	HSP90AB5P	heat shock protein 90 alpha family class B member 5, pseudogene	442083	0	NA	NA	NA	NA	NA
ENSG00000223739	RPS15AP15	ribosomal protein S15a pseudogene 15	100130782	0	NA	NA	NA	NA	NA
ENSG00000223740	LOC100419642	zinc finger protein 85 pseudogene	100419642	0	NA	NA	NA	NA	NA
ENSG00000223741	NA	NA	NA	0	NA	NA	NA	NA	NA
ENSG00000223744	RBMY2GP	RNA binding motif protein Y-linked family 2 member G, pseudogene	378952	0	NA	NA	NA	NA	NA
ENSG00000223746	NA	NA	NA	0	NA	NA	NA	NA	NA
ENSG00000223750	NA	NA	NA	0	NA	NA	NA	NA	NA
ENSG00000223753	LOC100420247	mitochondrial fission factor pseudogene	100420247	0	NA	NA	NA	NA	NA
ENSG00000223756	NA	NA	NA	0	NA	NA	NA	NA	NA
ENSG00000223760	NA	NA	NA	0	NA	NA	NA	NA	NA
ENSG00000223772	GEMIN8P3	gem nuclear organelle associated protein 8 pseudogene 3	100421535	0	NA	NA	NA	NA	NA
ENSG00000223777	NA	NA	NA	0	NA	NA	NA	NA	NA
ENSG00000223779	NA	NA	NA	0	NA	NA	NA	NA	NA
ENSG00000223782	NA	NA	NA	0	NA	NA	NA	NA	NA
ENSG00000223787	NA	NA	NA	0	NA	NA	NA	NA	NA
ENSG00000223788	DIS3L2P1	DIS3 like 3'-5' exoribonuclease 2 pseudogene 1	282697	0	NA	NA	NA	NA	NA

ENSG00000223794	COX5BP8	cytochrome c oxidase subunit 5B pseudogene 8	100859925	0	NA	NA	NA	NA	NA
ENSG00000223802	CERS1	ceramide synthase 1	10715	0,11808024	-1,076443944	4,080456	-0,263804824	0,791930317	NA
ENSG00000223803	NA	NA	NA	0	NA	NA	NA	NA	NA
ENSG00000223804	NA	NA	NA	5,940439015	-0,707755044	0,744357	-0,950828009	0,341691692	NA
ENSG00000223807	BOLA3P4	bolA family member 3 pseudogene 4	106480324	0	NA	NA	NA	NA	NA
ENSG00000223810	KRT8P28	keratin 8 pseudogene 28	391099	0	NA	NA	NA	NA	NA
ENSG00000223816	NA	NA	NA	0	NA	NA	NA	NA	NA
ENSG00000223819	NA	NA	NA	0	NA	NA	NA	NA	NA
ENSG00000223822	NA	NA	NA	0	NA	NA	NA	NA	NA
ENSG00000223824	NA	NA	NA	0	NA	NA	NA	NA	NA
ENSG00000223825	NA	NA	NA	0,118286652	0	4,080456	0	1	NA
ENSG00000223828	NA	NA	NA	0	NA	NA	NA	NA	NA
ENSG00000223836	NA	NA	NA	0	NA	NA	NA	NA	NA
ENSG00000223839	NA	NA	NA	0	NA	NA	NA	NA	NA
ENSG00000223841	NA	NA	NA	0	NA	NA	NA	NA	NA
ENSG00000223845	VN1R40P	vomeronasal 1 receptor 40 pseudogene	100312792	0	NA	NA	NA	NA	NA
ENSG00000223847	NA	NA	NA	0	NA	NA	NA	NA	NA
ENSG00000223849	NA	NA	NA	0	NA	NA	NA	NA	NA
ENSG00000223856	RAB9AP2	RAB9A, member RAS oncogene family pseudogene 2	100287542	0	NA	NA	NA	NA	NA
ENSG00000223861	RPS10P6	ribosomal protein S10 pseudogene 6	729374	0	NA	NA	NA	NA	NA
ENSG00000223864	NA	NA	NA	0	NA	NA	NA	NA	NA
ENSG00000223865	HLA-DPB1	major histocompatibility complex, class II, DP beta 1	3115	6,696008075	0,217386116	0,982553	0,22124616	0,824900769	NA
ENSG00000223866	NA	NA	NA	0	NA	NA	NA	NA	NA
ENSG00000223867	NA	NA	NA	0	NA	NA	NA	NA	NA
ENSG00000223869	NA	NA	NA	0	NA	NA	NA	NA	NA
ENSG00000223873	NA	NA	NA	0	NA	NA	NA	NA	NA
ENSG00000223875	NA	NA	NA	0	NA	NA	NA	NA	NA
ENSG00000223876	RPS27P1	ribosomal protein S27 pseudogene 1	414228	0	NA	NA	NA	NA	NA
ENSG00000223877	RPS8P10	ribosomal protein S8 pseudogene 10	388076	0	NA	NA	NA	NA	NA
ENSG00000223878	NA	NA	NA	0	NA	NA	NA	NA	NA
ENSG00000223885	NA	NA	NA	0	NA	NA	NA	NA	NA
ENSG00000223886	LOC100131785	ring finger protein 181 pseudogene	100131785	0	NA	NA	NA	NA	NA
ENSG00000223889	NA	NA	NA	0	NA	NA	NA	NA	NA
ENSG00000223890	NA	NA	NA	0	NA	NA	NA	NA	NA
ENSG00000223893	GNL2P1	G protein nucleolar 2 pseudogene 1	100270790	0	NA	NA	NA	NA	NA
ENSG00000223896	NA	NA	NA	0	NA	NA	NA	NA	NA
ENSG00000223897	NA	NA	NA	0	NA	NA	NA	NA	NA
ENSG00000223899	NA	NA	NA	0	NA	NA	NA	NA	NA
ENSG00000223903	RPL23P4	ribosomal protein L23 pseudogene 4	100270961	0	NA	NA	NA	NA	NA
ENSG00000223904	HMGB1P12	high mobility group box 1 pseudogene 12	100129723	0	NA	NA	NA	NA	NA
ENSG00000223905	LOC100418965	peroxiredoxin 5 pseudogene	100418965	0	NA	NA	NA	NA	NA
ENSG00000223908	NA	NA	NA	0,10488184	-1,076443944	4,080456	-0,263804824	0,791930317	NA
ENSG00000223912	EEF1A1P36	eukaryotic translation elongation factor 1 alpha 1 pseudogene 36	100421820	0	NA	NA	NA	NA	NA
ENSG00000223915	DPPA2P1	developmental pluripotency associated 2 pseudogene 1	100421137	0	NA	NA	NA	NA	NA
ENSG00000223916	RPL18AP9	ribosomal protein L18a pseudogene 9	731642	0,144330898	1,418786819	4,061262	0,349346267	0,726829368	NA
ENSG00000223917	NA	NA	NA	0	NA	NA	NA	NA	NA
ENSG00000223918	RPS21P5	ribosomal protein S21 pseudogene 5	100271236	0	NA	NA	NA	NA	NA
ENSG00000223920	NA	NA	NA	0	NA	NA	NA	NA	NA
ENSG00000223921	MTND1P27	MT-ND1 pseudogene 27	100288227	0	NA	NA	NA	NA	NA
ENSG00000223922	ASS1P2	argininosuccinate synthetase 1 pseudogene 2	447	1,885459102	-0,564124159	1,422372	-0,396607919	0,691656612	NA
ENSG00000223928	NA	NA	NA	0	NA	NA	NA	NA	NA
ENSG00000223931	NA	NA	NA	0	NA	NA	NA	NA	NA
ENSG00000223945	RPL6P31	ribosomal protein L6 pseudogene 31	441907	0	NA	NA	NA	NA	NA
ENSG00000223948	NA	NA	NA	0	NA	NA	NA	NA	NA
ENSG00000223951	NA	NA	NA	0	NA	NA	NA	NA	NA
ENSG00000223953	C1QTNF5	C1q and TNF related 5	114902	0	NA	NA	NA	NA	NA
ENSG00000223955	MTND6P1	MT-ND6 pseudogene 1	100499469	0	NA	NA	NA	NA	NA
ENSG00000223958	NA	NA	NA	0	NA	NA	NA	NA	NA
ENSG00000223962	NA	NA	NA	0	NA	NA	NA	NA	NA

ENSG00000223963	THAP12P8	THAP domain containing 12 pseudogene 8	645939	0	NA	NA	NA	NA	NA
ENSG00000223965	NA	NA	NA	0	NA	NA	NA	NA	NA
ENSG00000223967	NA	NA	NA	0	NA	NA	NA	NA	NA
ENSG00000223968	NA	NA	NA	0	NA	NA	NA	NA	NA
ENSG00000223970	RRBP1P1	RRBP1 pseudogene 1	392027	0	NA	NA	NA	NA	NA
ENSG00000223972	NA	NA	NA	0	NA	NA	NA	NA	NA
ENSG00000223973	NA	NA	NA	0	NA	NA	NA	NA	NA
ENSG00000223974	BNIP3P42	BCL2 interacting protein 3 pseudogene 42	106481697	0	NA	NA	NA	NA	NA
ENSG00000223976	NA	NA	NA	0	NA	NA	NA	NA	NA
ENSG00000223977	LOC112268410	zinc finger protein 141-like	112268410	0	NA	NA	NA	NA	NA
ENSG00000223978	ZNF736P1Y	zinc finger protein 736 pseudogene 1, Y-linked	442486	0	NA	NA	NA	NA	NA
ENSG00000223982	NA	NA	NA	0	NA	NA	NA	NA	NA
ENSG00000223984	NA	NA	NA	0	NA	NA	NA	NA	NA
ENSG00000223986	NA	NA	NA	0	NA	NA	NA	NA	NA
ENSG00000223987	RPL26P28	ribosomal protein L26 pseudogene 28	100130983	0	NA	NA	NA	NA	NA
ENSG00000223995	RPL32P35	ribosomal protein L32 pseudogene 35	100271393	0	NA	NA	NA	NA	NA
ENSG00000223997	TRDD1	T cell receptor delta diversity 1	28525	0	NA	NA	NA	NA	NA
ENSG00000224001	NA	NA	NA	0	NA	NA	NA	NA	NA
ENSG00000224003	NA	NA	NA	1,837465752	0,252575356	1,377886	0,183306377	0,854557631	NA
ENSG00000224004	ATP5F1CP1	ATP synthase F1 subunit gamma pseudogene 1	645560	0	NA	NA	NA	NA	NA
ENSG00000224007	NA	NA	NA	0	NA	NA	NA	NA	NA
ENSG00000224011	RPL36AP46	ribosomal protein L36a pseudogene 46	729621	0	NA	NA	NA	NA	NA
ENSG00000224012	NA	NA	NA	0	NA	NA	NA	NA	NA
ENSG00000224014	NA	NA	NA	0,454181402	0,839330032	3,499866	0,239817791	0,810471514	NA
ENSG00000224016	NA	NA	NA	0	NA	NA	NA	NA	NA
ENSG00000224019	RPL21P32	ribosomal protein L21 pseudogene 32	100271161	0	NA	NA	NA	NA	NA
ENSG00000224025	LOC347097	dermatan sulfate epimerase pseudogene	347097	0	NA	NA	NA	NA	NA
ENSG00000224033	CDY8P	chromodomain Y-linked 8 pseudogene	386732	0	NA	NA	NA	NA	NA
ENSG00000224035	NA	NA	NA	0	NA	NA	NA	NA	NA
ENSG00000224040	NA	NA	NA	0	NA	NA	NA	NA	NA
ENSG00000224041	IGKV3D-15	immunoglobulin kappa variable 3D-15	28875	0	NA	NA	NA	NA	NA
ENSG00000224042	MTND4P6	MT-ND4 pseudogene 6	100873192	0	NA	NA	NA	NA	NA
ENSG00000224045	PAEPP1	progestagen associated endometrial protein pseudogene 1	138159	0	NA	NA	NA	NA	NA
ENSG00000224054	NA	NA	NA	0	NA	NA	NA	NA	NA
ENSG00000224055	GAPDHP55	glyceraldehyde 3 phosphate dehydrogenase pseudogene 55	100421324	0	NA	NA	NA	NA	NA
ENSG00000224058	RBISP5	RBIS pseudogene 5	127379683	0	NA	NA	NA	NA	NA
ENSG00000224059	NA	NA	NA	0	NA	NA	NA	NA	NA
ENSG00000224060	NA	NA	NA	0	NA	NA	NA	NA	NA
ENSG00000224061	NA	NA	NA	0	NA	NA	NA	NA	NA
ENSG00000224062	TUBB4BP5	TUBB4B pseudogene 5	106480747	0	NA	NA	NA	NA	NA
ENSG00000224064	RPL32P6	ribosomal protein L32 pseudogene 6	100270888	0	NA	NA	NA	NA	NA
ENSG00000224065	NA	NA	NA	0	NA	NA	NA	NA	NA
ENSG00000224067	NA	NA	NA	0	NA	NA	NA	NA	NA
ENSG00000224069	NA	NA	NA	0	NA	NA	NA	NA	NA
ENSG00000224071	NA	NA	NA	0	NA	NA	NA	NA	NA
ENSG00000224072	VEZTP1	VEZT pseudogene 1	100113397	0,354729091	2,545181588	3,984059	0,638841337	0,522926154	NA
ENSG00000224080	NA	NA	NA	1,785842095	1,157026692	1,323271	0,87436883	0,381917427	NA
ENSG00000224081	SLC44A3-AS1	SLC44A3 antisense RNA 1	101928079	0	NA	NA	NA	NA	NA
ENSG00000224082	NA	NA	NA	0	NA	NA	NA	NA	NA
ENSG00000224083	MTCO1P11	MT-CO1 pseudogene 11	107075264	8,038806664	0,449570772	0,667608	0,673405464	0,500689374	NA
ENSG00000224085	RPL23AP41	ribosomal protein L23a pseudogene 41	646685	0	NA	NA	NA	NA	NA
ENSG00000224092	NA	NA	NA	0	NA	NA	NA	NA	NA
ENSG00000224094	NA	NA	NA	0,222642189	0,8302265	4,080456	0,203464154	0,838772261	NA
ENSG00000224097	LOC401127	WD repeat domain 5 pseudogene	401127	0,916127813	-0,524829933	1,953974	-0,268596207	0,788240431	NA
ENSG00000224104	NA	NA	NA	0	NA	NA	NA	NA	NA
ENSG00000224106	NA	NA	NA	0	NA	NA	NA	NA	NA
ENSG00000224107	ETDB	embryonic testis differentiation homolog B	100129515	0	NA	NA	NA	NA	NA
ENSG00000224109	CENPVL3	centromere protein V like 3	347549	0	NA	NA	NA	NA	NA

ENSG00000224110	NA	NA	NA	0	NA	NA	NA	NA	NA
ENSG00000224114	RPS14P1	ribosomal protein S14 pseudogene 1	100271063	0	NA	NA	NA	NA	NA
ENSG00000224117	NA	NA	NA	0	NA	NA	NA	NA	NA
ENSG00000224121	NA	NA	NA	0	NA	NA	NA	NA	NA
ENSG00000224124	NA	NA	NA	0	NA	NA	NA	NA	NA
ENSG00000224126	NA	NA	NA	0	NA	NA	NA	NA	NA
ENSG00000224134	NA	NA	NA	0	NA	NA	NA	NA	NA
ENSG00000224136	NA	NA	NA	0	NA	NA	NA	NA	NA
ENSG00000224144	NA	NA	NA	0	NA	NA	NA	NA	NA
ENSG00000224151	USP9YP28	USP9Y pseudogene 28	107080622	0	NA	NA	NA	NA	NA
ENSG00000224155	NA	NA	NA	0	NA	NA	NA	NA	NA
ENSG00000224159	NA	NA	NA	0	NA	NA	NA	NA	NA
ENSG00000224160	NA	NA	NA	0	NA	NA	NA	NA	NA
ENSG00000224161	NA	NA	NA	0	NA	NA	NA	NA	NA
ENSG00000224162	NA	NA	NA	0	NA	NA	NA	NA	NA
ENSG00000224163	LOC392787	filamin C pseudogene	392787	0	NA	NA	NA	NA	NA
ENSG00000224166	PRYP1	PTPN13 like Y-linked pseudogene 1	442863	0	NA	NA	NA	NA	NA
ENSG00000224169	NA	NA	NA	0	NA	NA	NA	NA	NA
ENSG00000224172	NA	NA	NA	0	NA	NA	NA	NA	NA
ENSG00000224183	NA	NA	NA	0,178379969	0,832486441	4,0799	0,204045808	0,838317702	NA
ENSG00000224185	NA	NA	NA	1,831073401	0,71938687	1,56245	0,460422309	0,645213125	NA
ENSG00000224188	NA	NA	NA	0	NA	NA	NA	NA	NA
ENSG00000224203	RPS23P10	ribosomal protein S23 pseudogene 10	100419471	0	NA	NA	NA	NA	NA
ENSG00000224205	NA	NA	NA	0	NA	NA	NA	NA	NA
ENSG00000224207	NA	NA	NA	0	NA	NA	NA	NA	NA
ENSG00000224208	NA	NA	NA	0	NA	NA	NA	NA	NA
ENSG00000224210	TRIM60P5Y	tripartite motif containing 60 pseudogene 5, Y-linked	100420368	0	NA	NA	NA	NA	NA
ENSG00000224217	NA	NA	NA	0	NA	NA	NA	NA	NA
ENSG00000224219	NA	NA	NA	0	NA	NA	NA	NA	NA
ENSG00000224221	RPS6P8	ribosomal protein S6 pseudogene 8	100128450	0,340103648	-2,003582028	3,744886	-0,53501819	0,592637279	NA
ENSG00000224224	NA	NA	NA	0	NA	NA	NA	NA	NA
ENSG00000224227	OR2L1P	olfactory receptor family 2 subfamily L member 1 pseudogene	26247	0,11808024	-1,076443944	4,080456	-0,263804824	0,791930317	NA
ENSG00000224232	NA	NA	NA	0,10543123	0	4,080456	0	1	NA
ENSG00000224233	OR2J4P	olfactory receptor family 2 subfamily J member 4 pseudogene	442188	0	NA	NA	NA	NA	NA
ENSG00000224236	MRRFP1	mitochondrial ribosome recycling factor pseudogene 1	286423	0	NA	NA	NA	NA	NA
ENSG00000224237	NA	NA	NA	0	NA	NA	NA	NA	NA
ENSG00000224240	NA	NA	NA	0	NA	NA	NA	NA	NA
ENSG00000224244	KRT224P	keratin 224, pseudogene	118732296	0	NA	NA	NA	NA	NA
ENSG00000224245	LOC100421692	leishmanolysin like peptidase pseudogene	100421692	0	NA	NA	NA	NA	NA
ENSG00000224252	NA	NA	NA	0	NA	NA	NA	NA	NA
ENSG00000224254	MTCO1P39	MT-CO1 pseudogene 39	107075282	0	NA	NA	NA	NA	NA
ENSG00000224255	PDCL3P6	PDCL3 pseudogene 6	100419023	0	NA	NA	NA	NA	NA
ENSG00000224256	NA	NA	NA	0	NA	NA	NA	NA	NA
ENSG00000224261	RPSAP18	ribosomal protein SA pseudogene 18	388707	0,325746337	-0,959276377	4,042802	-0,237280092	0,812439505	NA
ENSG00000224263	NA	NA	NA	0	NA	NA	NA	NA	NA
ENSG00000224273	NA	NA	NA	0	NA	NA	NA	NA	NA
ENSG00000224274	ENSAP1	endosulfine alpha pseudogene 1	170511	0	NA	NA	NA	NA	NA
ENSG00000224278	LOC100421343	DEAD-box helicase 19B pseudogene	100421343	0	NA	NA	NA	NA	NA
ENSG00000224279	RPS29P31	ribosomal protein S29 pseudogene 31	109729124	0	NA	NA	NA	NA	NA
ENSG00000224280	LOC317727	ataxin 2 like pseudogene	317727	0,15836288	0	4,080456	0	1	NA
ENSG00000224282	RPL9P17	ribosomal protein L9 pseudogene 17	728523	0	NA	NA	NA	NA	NA
ENSG00000224287	MSL3P1	MSL complex subunit 3 pseudogene 1	151507	7,288156302	0,33100218	0,677011	0,488916827	0,624900582	NA
ENSG00000224288	MTCYBP11	MT-CYB pseudogene 11	107075122	0	NA	NA	NA	NA	NA
ENSG00000224289	NA	NA	NA	0	NA	NA	NA	NA	NA
ENSG00000224291	NA	NA	NA	0	NA	NA	NA	NA	NA
ENSG00000224295	NA	NA	NA	0,10543123	0	4,080456	0	1	NA
ENSG00000224296	MRPL49P1	mitochondrial ribosomal protein L49 pseudogene 1	359748	0	NA	NA	NA	NA	NA
ENSG00000224297	LOC100133316	required for meiotic nuclear division 1 homolog (S, cerevisiae) pseudogene	100133316	0	NA	NA	NA	NA	NA
ENSG00000224299	MTATP6P16	MT-ATP6 pseudogene 16	107075158	0	NA	NA	NA	NA	NA

ENSG00000224302	NA	NA	NA	0	NA	NA	NA	NA	NA
ENSG00000224309	NA	NA	NA	0	NA	NA	NA	NA	NA
ENSG00000224314	TCERG1P1	TCERG1 pseudogene 1	100420957	0	NA	NA	NA	NA	NA
ENSG00000224315	RPL7P7	ribosomal protein L7 pseudogene 7	644896	0,470831238	-1,88966119	3,488342	-0,541707563	0,588019979	NA
ENSG00000224316	GTF2IP5	general transcription factor IIi pseudogene 5	100420546	0,22340896	-0,133409408	4,080456	-0,032694731	0,973918026	NA
ENSG00000224321	NA	NA	NA	0	NA	NA	NA	NA	NA
ENSG00000224323	NA	NA	NA	0	NA	NA	NA	NA	NA
ENSG00000224324	NA	NA	NA	0,223367907	0	4,080456	0	1	NA
ENSG00000224331	NA	NA	NA	0	NA	NA	NA	NA	NA
ENSG00000224333	NA	NA	NA	0	NA	NA	NA	NA	NA
ENSG00000224334	NA	NA	NA	0	NA	NA	NA	NA	NA
ENSG00000224335	NA	NA	NA	0	NA	NA	NA	NA	NA
ENSG00000224336	NA	NA	NA	0	NA	NA	NA	NA	NA
ENSG00000224337	NA	NA	NA	0	NA	NA	NA	NA	NA
ENSG00000224338	MTCYBP45	MT-CYB pseudogene 45	109729120	0	NA	NA	NA	NA	NA
ENSG00000224339	NA	NA	NA	0	NA	NA	NA	NA	NA
ENSG00000224340	RPL21P21	ribosomal protein L21 pseudogene 21	100270877	0	NA	NA	NA	NA	NA
ENSG00000224341	NA	NA	NA	0	NA	NA	NA	NA	NA
ENSG00000224344	NA	NA	NA	0	NA	NA	NA	NA	NA
ENSG00000224346	BICD1P1	BICD cargo adaptor 1 pseudogene 1	391472	0	NA	NA	NA	NA	NA
ENSG00000224348	RPL36P6	ribosomal protein L36 pseudogene 6	100271008	0	NA	NA	NA	NA	NA
ENSG00000224351	CDKN2AIPNLP3	CDKN2A interacting protein N-terminal like pseudogene 3	100874397	0	NA	NA	NA	NA	NA
ENSG00000224352	NA	NA	NA	0	NA	NA	NA	NA	NA
ENSG00000224353	ACE3P	angiotensin I converting enzyme 3, pseudogene	100129123	0	NA	NA	NA	NA	NA
ENSG00000224354	MTND2P5	MT-ND2 pseudogene 5	100873176	0	NA	NA	NA	NA	NA
ENSG00000224357	NA	NA	NA	0	NA	NA	NA	NA	NA
ENSG00000224365	NA	NA	NA	0	NA	NA	NA	NA	NA
ENSG00000224366	NA	NA	NA	0	NA	NA	NA	NA	NA
ENSG00000224367	NA	NA	NA	0	NA	NA	NA	NA	NA
ENSG00000224368	NA	NA	NA	0	NA	NA	NA	NA	NA
ENSG00000224370	NA	NA	NA	0	NA	NA	NA	NA	NA
ENSG00000224383	PRR29	proline rich 29	92340	0	NA	NA	NA	NA	NA
ENSG00000224389	C4B	complement C4B (Chido blood group)	721	0	NA	NA	NA	NA	NA
ENSG00000224396	METTL15P3	methyltransferase like 15 pseudogene 3	729650	0	NA	NA	NA	NA	NA
ENSG00000224401	RPL7P57	ribosomal protein L7 pseudogene 57	643507	0	NA	NA	NA	NA	NA
ENSG00000224402	NA	NA	NA	0	NA	NA	NA	NA	NA
ENSG00000224403	NA	NA	NA	0	NA	NA	NA	NA	NA
ENSG00000224410	NA	NA	NA	0	NA	NA	NA	NA	NA
ENSG00000224411	NA	NA	NA	0,232524743	0,832309599	4,079892	0,204002842	0,838351278	NA
ENSG00000224412	NA	NA	NA	0	NA	NA	NA	NA	NA
ENSG00000224414	LOC100533848	ATPase H+/K+ transporting non-gastric alpha2 subunit pseudogene	100533848	0	NA	NA	NA	NA	NA
ENSG00000224416	NA	NA	NA	0	NA	NA	NA	NA	NA
ENSG00000224419	KRT18P27	keratin 18 pseudogene 27	390418	0	NA	NA	NA	NA	NA
ENSG00000224420	ADM5	adrenomedullin 5 (putative)	199800	0,726835236	-2,31673772	2,248892	-1,030168366	0,302930977	NA
ENSG00000224421	ATP5MFP1	ATP synthase membrane subunit f pseudogene 1	101241877	0	NA	NA	NA	NA	NA
ENSG00000224425	NA	NA	NA	0	NA	NA	NA	NA	NA
ENSG00000224426	NA	NA	NA	0	NA	NA	NA	NA	NA
ENSG00000224427	NA	NA	NA	0	NA	NA	NA	NA	NA
ENSG00000224430	NA	NA	NA	0,190652766	0,844824076	4,080428	0,207042995	0,835976281	NA
ENSG00000224431	NA	NA	NA	0	NA	NA	NA	NA	NA
ENSG00000224435	NA	NA	NA	0	NA	NA	NA	NA	NA
ENSG00000224436	NA	NA	NA	0	NA	NA	NA	NA	NA
ENSG00000224437	PIGUP1	phosphatidylinositol glycan anchor biosynthesis class U pseudogene 1	100422342	0	NA	NA	NA	NA	NA
ENSG00000224438	RPL23AP14	ribosomal protein L23a pseudogene 14	341511	0	NA	NA	NA	NA	NA
ENSG00000224439	RPSAP10	ribosomal protein SA pseudogene 10	401638	0	NA	NA	NA	NA	NA
ENSG00000224440	CXorf51A	chromosome X open reading frame 51A	100129239	0	NA	NA	NA	NA	NA
ENSG00000224442	NA	NA	NA	5,851064164	0,696834763	0,924261	0,753937468	0,450886771	NA
ENSG00000224443	NME2P2	NME2 pseudogene 2	100289315	0	NA	NA	NA	NA	NA
ENSG00000224447	NA	NA	NA	0	NA	NA	NA	NA	NA
ENSG00000224448	NA	NA	NA	0	NA	NA	NA	NA	NA

ENSG00000224451	ATP5PBP1	ATP synthase peripheral stalk-membrane subunit b pseudogene 1	100422523	1,922322745	2,29283761	1,554991	1,47450198	0,140346485	NA
ENSG00000224452	NA	NA	NA	0,117497108	0,847101401	4,080456	0,207599697	0,835541541	NA
ENSG00000224458	NA	NA	NA	0	NA	NA	NA	NA	NA
ENSG00000224462	C4BPAP1	C4BPA pseudogene 1	727859	0	NA	NA	NA	NA	NA
ENSG00000224463	NA	NA	NA	0	NA	NA	NA	NA	NA
ENSG00000224464	PGAM1P6	phosphoglycerate mutase 1 pseudogene 6	100420567	1,020669604	2,305347486	2,073357	1,111891009	0,266185015	NA
ENSG00000224465	NA	NA	NA	0	NA	NA	NA	NA	NA
ENSG00000224466	NA	NA	NA	0	NA	NA	NA	NA	NA
ENSG00000224469	RPL18P5	ribosomal protein L18 pseudogene 5	100270944	0	NA	NA	NA	NA	NA
ENSG00000224471	NA	NA	NA	0	NA	NA	NA	NA	NA
ENSG00000224473	CCND3P1	cyclin D3 pseudogene 1	897	0,15836288	0	4,080456	0	1	NA
ENSG00000224475	RPL7AP40	ribosomal protein L7a pseudogene 40	442712	0	NA	NA	NA	NA	NA
ENSG00000224476	NA	NA	NA	0	NA	NA	NA	NA	NA
ENSG00000224479	NA	NA	NA	0,119673877	0,847101401	4,080456	0,207599697	0,835541541	NA
ENSG00000224482	NA	NA	NA	0	NA	NA	NA	NA	NA
ENSG00000224484	NA	NA	NA	0	NA	NA	NA	NA	NA
ENSG00000224485	NA	NA	NA	0	NA	NA	NA	NA	NA
ENSG00000224488	SAMM50P1	SAMM50 sorting and assembly machinery component pseudogene 1	100271893	0	NA	NA	NA	NA	NA
ENSG00000224493	NA	NA	NA	0	NA	NA	NA	NA	NA
ENSG00000224494	HNRNPA3P14	heterogeneous nuclear ribonucleoprotein A3 pseudogene 14	729817	0	NA	NA	NA	NA	NA
ENSG00000224497	NA	NA	NA	0	NA	NA	NA	NA	NA
ENSG00000224498	MAPKAPK5P1	MAPKAPK5 pseudogene 1	100533849	0	NA	NA	NA	NA	NA
ENSG00000224510	NA	NA	NA	0	NA	NA	NA	NA	NA
ENSG00000224518	NA	NA	NA	0	NA	NA	NA	NA	NA
ENSG00000224519	HMGN1P19	high mobility group nucleosome binding domain 1 pseudogene 19	100874445	0	NA	NA	NA	NA	NA
ENSG00000224520	KRT8P45	keratin 8 pseudogene 45	149501	0	NA	NA	NA	NA	NA
ENSG00000224523	NA	NA	NA	0	NA	NA	NA	NA	NA
ENSG00000224524	CYCSP42	CYCS pseudogene 42	343727	0	NA	NA	NA	NA	NA
ENSG00000224529	LOC100533727	replication factor C subunit 2 pseudogene	100533727	0	NA	NA	NA	NA	NA
ENSG00000224530	PPIAP10	peptidylprolyl isomerase A pseudogene 10	140913	0	NA	NA	NA	NA	NA
ENSG00000224537	NA	NA	NA	0	NA	NA	NA	NA	NA
ENSG00000224539	NA	NA	NA	0	NA	NA	NA	NA	NA
ENSG00000224540	NA	NA	NA	0	NA	NA	NA	NA	NA
ENSG00000224543	NA	NA	NA	0	NA	NA	NA	NA	NA
ENSG00000224545	RPL31P36	ribosomal protein L31 pseudogene 36	100271200	0	NA	NA	NA	NA	NA
ENSG00000224546	EIF4BP3	eukaryotic translation initiation factor 4B pseudogene 3	100128771	0	NA	NA	NA	NA	NA
ENSG00000224547	NA	NA	NA	0	NA	NA	NA	NA	NA
ENSG00000224548	NA	NA	NA	0	NA	NA	NA	NA	NA
ENSG00000224550	NA	NA	NA	0	NA	NA	NA	NA	NA
ENSG00000224551	HMGB3P21	high mobility group box 3 pseudogene 21	100873901	0	NA	NA	NA	NA	NA
ENSG00000224553	NA	NA	NA	0	NA	NA	NA	NA	NA
ENSG00000224555	NA	NA	NA	0	NA	NA	NA	NA	NA
ENSG00000224556	NA	NA	NA	0	NA	NA	NA	NA	NA
ENSG00000224560	LOC100419154	ribosomal protein L4 pseudogene	100419154	0	NA	NA	NA	NA	NA
ENSG00000224566	NA	NA	NA	0	NA	NA	NA	NA	NA
ENSG00000224567	NA	NA	NA	0	NA	NA	NA	NA	NA
ENSG00000224569	C9orf78P2	C9orf78 pseudogene 2	391636	0	NA	NA	NA	NA	NA
ENSG00000224570	NA	NA	NA	0	NA	NA	NA	NA	NA
ENSG00000224571	NA	NA	NA	0	NA	NA	NA	NA	NA
ENSG00000224573	RPL7AP78	ribosomal protein L7a pseudogene 78	122965331	0	NA	NA	NA	NA	NA
ENSG00000224584	NA	NA	NA	0	NA	NA	NA	NA	NA
ENSG00000224585	NA	NA	NA	0	NA	NA	NA	NA	NA
ENSG00000224586	GPX5	glutathione peroxidase 5	2880	0	NA	NA	NA	NA	NA
ENSG00000224589	NA	NA	NA	0	NA	NA	NA	NA	NA
ENSG00000224590	MTND3P16	MT-ND3 pseudogene 16	107075278	0	NA	NA	NA	NA	NA
ENSG00000224593	NA	NA	NA	0	NA	NA	NA	NA	NA
ENSG00000224594	NA	NA	NA	0	NA	NA	NA	NA	NA
ENSG00000224597	NA	NA	NA	0,443804635	-2,567698231	3,44785	-0,744724392	0,45643835	NA
ENSG00000224598	RPS5P2	RPS5 pseudogene 2	100130240	0	NA	NA	NA	NA	NA
ENSG00000224599	NA	NA	NA	3,820344227	1,113689022	1,28507	0,866636855	0,386141013	NA

ENSG00000224600	RPLP1P3	ribosomal protein lateral stalk subunit P1 pseudogene 3	127099	0	NA	NA	NA	NA	NA
ENSG00000224602	NA	NA	NA	0	NA	NA	NA	NA	NA
ENSG00000224603	NA	NA	NA	0	NA	NA	NA	NA	NA
ENSG00000224604	NA	NA	NA	0	NA	NA	NA	NA	NA
ENSG00000224607	NA	NA	NA	0	NA	NA	NA	NA	NA
ENSG00000224611	NA	NA	NA	0	NA	NA	NA	NA	NA
ENSG00000224617	NA	NA	NA	0	NA	NA	NA	NA	NA
ENSG00000224618	KLHL25P1	KLHL25 pseudogene 1	127379729	0	NA	NA	NA	NA	NA
ENSG00000224620	NA	NA	NA	0	NA	NA	NA	NA	NA
ENSG00000224622	OR9R1P	olfactory receptor family 9 subfamily R member 1 pseudogene	81140	0	NA	NA	NA	NA	NA
ENSG00000224625	TUBB8P6	tubulin beta 8 class VIII pseudogene 6	200149	0	NA	NA	NA	NA	NA
ENSG00000224627	RPL37P10	ribosomal protein L37 pseudogene 10	100271015	0	NA	NA	NA	NA	NA
ENSG00000224628	LOC149935	CDK5 regulatory subunit associated protein 3 pseudogene	149935	0	NA	NA	NA	NA	NA
ENSG00000224629	NA	NA	NA	0	NA	NA	NA	NA	NA
ENSG00000224630	NA	NA	NA	0	NA	NA	NA	NA	NA
ENSG00000224631	RPS27AP16	RPS27A pseudogene 16	643358	0,15836288	0	4,080456	0	1	NA
ENSG00000224632	NA	NA	NA	0	NA	NA	NA	NA	NA
ENSG00000224634	ZNF736P6Y	zinc finger protein 736 pseudogene 6, Y-linked	100419732	0	NA	NA	NA	NA	NA
ENSG00000224637	NA	NA	NA	0	NA	NA	NA	NA	NA
ENSG00000224640	NA	NA	NA	0	NA	NA	NA	NA	NA
ENSG00000224646	RPS28P5	ribosomal protein S28 pseudogene 5	100271121	0	NA	NA	NA	NA	NA
ENSG00000224650	IGHV3-74	immunoglobulin heavy variable 3-74	28408	0	NA	NA	NA	NA	NA
ENSG00000224653	NA	NA	NA	0	NA	NA	NA	NA	NA
ENSG00000224656	NA	NA	NA	0	NA	NA	NA	NA	NA
ENSG00000224657	RBMY2BP	RNA binding motif protein Y-linked family 2 member B, pseudogene	375850	0	NA	NA	NA	NA	NA
ENSG00000224659	GAGE12J	G antigen 12J	729396	0	NA	NA	NA	NA	NA
ENSG00000224662	NA	NA	NA	0	NA	NA	NA	NA	NA
ENSG00000224664	NA	NA	NA	0	NA	NA	NA	NA	NA
ENSG00000224667	NA	NA	NA	0	NA	NA	NA	NA	NA
ENSG00000224668	IPO8P1	importin 8 pseudogene 1	100418819	0	NA	NA	NA	NA	NA
ENSG00000224669	NA	NA	NA	0	NA	NA	NA	NA	NA
ENSG00000224670	NA	NA	NA	0	NA	NA	NA	NA	NA
ENSG00000224672	NA	NA	NA	0,19047599	0,846844574	4,080456	0,207536756	0,83559069	NA
ENSG00000224674	EIF3EP2	EIF3E pseudogene 2	100132597	0	NA	NA	NA	NA	NA
ENSG00000224677	NA	NA	NA	0	NA	NA	NA	NA	NA
ENSG00000224678	GAPDHP46	glyceraldehyde 3 phosphate dehydrogenase pseudogene 46	100240711	0	NA	NA	NA	NA	NA
ENSG00000224679	NA	NA	NA	0	NA	NA	NA	NA	NA
ENSG00000224680	NA	NA	NA	0,236373268	-1,080428978	4,080456	-0,264781439	0,791177836	NA
ENSG00000224681	NA	NA	NA	0	NA	NA	NA	NA	NA
ENSG00000224682	SOCS5P2	suppressor of cytokine signaling 5 pseudogene 2	100128299	0	NA	NA	NA	NA	NA
ENSG00000224683	RPL36AP29	ribosomal protein L36a pseudogene 29	100271510	0	NA	NA	NA	NA	NA
ENSG00000224685	NA	NA	NA	0	NA	NA	NA	NA	NA
ENSG00000224686	ELOCP23	elongin C pseudogene 23	100462881	0	NA	NA	NA	NA	NA
ENSG00000224688	NA	NA	NA	0	NA	NA	NA	NA	NA
ENSG00000224689	NA	NA	NA	0	NA	NA	NA	NA	NA
ENSG00000224690	UBE2D3P3	ubiquitin conjugating enzyme E2 D3 pseudogene 3	100874262	0	NA	NA	NA	NA	NA
ENSG00000224692	NA	NA	NA	0	NA	NA	NA	NA	NA
ENSG00000224695	MTND6P7	MT-ND6 pseudogene 7	106478945	0	NA	NA	NA	NA	NA
ENSG00000224697	NEFMP1	neurofilament medium pseudogene 1	100420737	0	NA	NA	NA	NA	NA
ENSG00000224701	NA	NA	NA	0	NA	NA	NA	NA	NA
ENSG00000224706	RPS17P13	ribosomal protein S17 pseudogene 13	100271073	0	NA	NA	NA	NA	NA
ENSG00000224709	OR11M1P	olfactory receptor family 11 subfamily M member 1 pseudogene	121270	0	NA	NA	NA	NA	NA
ENSG00000224712	NPIPA3	nuclear pore complex interacting protein family member A3	642778	11,32478776	0,149790828	1,265553	0,118359955	0,905782454	NA
ENSG00000224722	SRRM1P1	serine/arginine repetitive matrix 1 pseudogene 1	401475	0,177277673	-0,122051843	4,080351	-0,029912093	0,976137161	NA
ENSG00000224723	NA	NA	NA	0	NA	NA	NA	NA	NA

ENSG00000224725	NA	NA	NA	0	NA	NA	NA	NA	NA
ENSG00000224727	NA	NA	NA	0	NA	NA	NA	NA	NA
ENSG00000224728	NA	NA	NA	0,875478551	-0,735284704	2,101323	-0,34991515	0,726402377	NA
ENSG00000224732	NA	NA	NA	0	NA	NA	NA	NA	NA
ENSG00000224735	NA	NA	NA	0	NA	NA	NA	NA	NA
ENSG00000224747	MTCYBP21	MT-CYB pseudogene 21	107075260	0	NA	NA	NA	NA	NA
ENSG00000224751	NA	NA	NA	0	NA	NA	NA	NA	NA
ENSG00000224752	VN1R21P	vomeronasal 1 receptor 21 pseudogene	100128368	0	NA	NA	NA	NA	NA
ENSG00000224755	NA	NA	NA	0	NA	NA	NA	NA	NA
ENSG00000224760	NA	NA	NA	0	NA	NA	NA	NA	NA
ENSG00000224761	NA	NA	NA	0	NA	NA	NA	NA	NA
ENSG00000224762	LOC100419691	zinc finger protein 114 pseudogene	100419691	0	NA	NA	NA	NA	NA
ENSG00000224763	FDPSP8	farnesyl diphosphate synthase pseudogene 8	401981	0,118286652	0	4,080456	0	1	NA
ENSG00000224769	NA	NA	NA	2,59395767	1,264495102	1,326249	0,953437014	0,340368695	NA
ENSG00000224773	HSPA8P7	heat shock protein family A (Hsp70) member 8 pseudogene 7	100420080	0	NA	NA	NA	NA	NA
ENSG00000224775	BRAFP1	BRAF pseudogene 1	286494	0	NA	NA	NA	NA	NA
ENSG00000224776	NA	NA	NA	0	NA	NA	NA	NA	NA
ENSG00000224777	OR4F2P	olfactory receptor family 4 subfamily F member 2 pseudogene	390028	0	NA	NA	NA	NA	NA
ENSG00000224778	NA	NA	NA	0	NA	NA	NA	NA	NA
ENSG00000224781	EIF4A2P4	eukaryotic translation initiation factor 4A2 pseudogene 4	286512	0,072165449	0,847101401	4,080456	0,207599697	0,835541541	NA
ENSG00000224783	NA	NA	NA	0	NA	NA	NA	NA	NA
ENSG00000224784	S100A15A	S100 calcium binding protein A15A (pseudogene)	112488748	0	NA	NA	NA	NA	NA
ENSG00000224785	LOC442517	ATP binding cassette subfamily E member 1 pseudogene	442517	0	NA	NA	NA	NA	NA
ENSG00000224786	NA	NA	NA	0	NA	NA	NA	NA	NA
ENSG00000224791	KRT18P39	keratin 18 pseudogene 39	344462	0	NA	NA	NA	NA	NA
ENSG00000224792	IQCF4P	IQ motif containing F4, pseudogene	100506840	0	NA	NA	NA	NA	NA
ENSG00000224797	RPL37P17	ribosomal protein L37 pseudogene 17	100271202	0	NA	NA	NA	NA	NA
ENSG00000224800	RPS27AP6	RPS27A pseudogene 6	100271144	0	NA	NA	NA	NA	NA
ENSG00000224802	TUBB4BP2	TUBB4B pseudogene 2	100418974	0,561671851	-0,121977719	2,602619	-0,046867305	0,962618986	NA
ENSG00000224806	NA	NA	NA	0	NA	NA	NA	NA	NA
ENSG00000224809	BEND3P2	BEN domain containing 3 pseudogene 2	392368	0	NA	NA	NA	NA	NA
ENSG00000224815	NA	NA	NA	0	NA	NA	NA	NA	NA
ENSG00000224816	NA	NA	NA	0	NA	NA	NA	NA	NA
ENSG00000224820	NA	NA	NA	0	NA	NA	NA	NA	NA
ENSG00000224826	NA	NA	NA	0	NA	NA	NA	NA	NA
ENSG00000224827	NA	NA	NA	0,10543123	0	4,080456	0	1	NA
ENSG00000224829	NA	NA	NA	0	NA	NA	NA	NA	NA
ENSG00000224830	NA	NA	NA	0,119673877	0,847101401	4,080456	0,207599697	0,835541541	NA
ENSG00000224831	TMEM183BP	transmembrane protein 183B, pseudogene	653659	0	NA	NA	NA	NA	NA
ENSG00000224834	NA	NA	NA	0,103998153	-1,076443944	4,080456	-0,263804824	0,791930317	NA
ENSG00000224836	NA	NA	NA	0	NA	NA	NA	NA	NA
ENSG00000224837	NA	NA	NA	1,056537484	0,97356357	3,421184	0,284569213	0,775974201	NA
ENSG00000224852	MTND5P24	MT-ND5 pseudogene 24	100873262	0	NA	NA	NA	NA	NA
ENSG00000224856	NA	NA	NA	0,391706917	-0,154367265	3,674049	-0,042015568	0,966486288	NA
ENSG00000224858	NA	NA	NA	0,966604125	-1,19808312	2,270512	-0,527670902	0,597727772	NA
ENSG00000224860	NA	NA	NA	0	NA	NA	NA	NA	NA
ENSG00000224861	NA	NA	NA	0,699123802	-1,926177362	2,420129	-0,79589878	0,426090871	NA
ENSG00000224864	NA	NA	NA	0,235701784	0,809456844	4,078915	0,198449077	0,842693723	NA
ENSG00000224866	USP9YP25	USP9Y pseudogene 25	106480715	0	NA	NA	NA	NA	NA
ENSG00000224869	NA	NA	NA	0	NA	NA	NA	NA	NA
ENSG00000224880	MTCYBP29	MT-CYB pseudogene 29	107075135	0	NA	NA	NA	NA	NA
ENSG00000224881	NA	NA	NA	0	NA	NA	NA	NA	NA
ENSG00000224886	NA	NA	NA	1,243865216	0,349734281	2,086963	0,167580516	0,8669133	NA
ENSG00000224887	NA	NA	NA	0	NA	NA	NA	NA	NA
ENSG00000224891	ARL14EPP1	ARL14EP pseudogene 1	107985793	0	NA	NA	NA	NA	NA
ENSG00000224892	NA	NA	NA	5,090606143	-1,259870586	0,955395	-1,318690632	0,18727256	NA
ENSG00000224895	VPS26BP1	VPS26B pseudogene 1	100421729	3,001856986	0,530213582	1,198568	0,442372694	0,658219535	NA
ENSG00000224896	NA	NA	NA	0	NA	NA	NA	NA	NA
ENSG00000224902	GAGE12H	G antigen 12H	729442	0	NA	NA	NA	NA	NA
ENSG00000224904	SBF1P2	SBF1 pseudogene 2	390997	0	NA	NA	NA	NA	NA
ENSG00000224906	NA	NA	NA	0	NA	NA	NA	NA	NA
ENSG00000224907	RPL21P91	ribosomal protein L21 pseudogene 91	100271424	0	NA	NA	NA	NA	NA
ENSG00000224908	NA	NA	NA	0	NA	NA	NA	NA	NA

ENSG00000224916	NA	NA	NA	0	NA	NA	NA	NA	NA
ENSG00000224917	NA	NA	NA	0	NA	NA	NA	NA	NA
ENSG00000224920	NA	NA	NA	0	NA	NA	NA	NA	NA
ENSG00000224927	NA	NA	NA	0	NA	NA	NA	NA	NA
ENSG00000224928	NA	NA	NA	0	NA	NA	NA	NA	NA
ENSG00000224931	LOC728307	PWWP domain containing 3B pseudogene	728307	0	NA	NA	NA	NA	NA
ENSG00000224936	NA	NA	NA	0	NA	NA	NA	NA	NA
ENSG00000224940	PRRT4	proline rich transmembrane protein 4	401399	0	NA	NA	NA	NA	NA
ENSG00000224942	EEDP1	EED pseudogene 1	266694	0	NA	NA	NA	NA	NA
ENSG00000224946	NA	NA	NA	0	NA	NA	NA	NA	NA
ENSG00000224947	VN1R25P	vomeronasal 1 receptor 25 pseudogene	100312782	0	NA	NA	NA	NA	NA
ENSG00000224948	NA	NA	NA	0	NA	NA	NA	NA	NA
ENSG00000224949	RPL37P24	ribosomal protein L37 pseudogene 24	100271395	0	NA	NA	NA	NA	NA
ENSG00000224953	SRIP3	sorcin pseudogene 3	100861435	0	NA	NA	NA	NA	NA
ENSG00000224955	KCTD10P1	potassium channel tetramerization domain containing 10 pseudogene 1	100422230	0	NA	NA	NA	NA	NA
ENSG00000224960	PPP4R3C	protein phosphatase 4 regulatory subunit 3C	139420	0	NA	NA	NA	NA	NA
ENSG00000224962	NA	NA	NA	0	NA	NA	NA	NA	NA
ENSG00000224963	NA	NA	NA	0	NA	NA	NA	NA	NA
ENSG00000224964	NA	NA	NA	0	NA	NA	NA	NA	NA
ENSG00000224966	NA	NA	NA	0	NA	NA	NA	NA	NA
ENSG00000224967	NA	NA	NA	0,391500595	-1,026821925	3,656635	-0,280810635	0,778855646	NA
ENSG00000224971	NA	NA	NA	0	NA	NA	NA	NA	NA
ENSG00000224976	PARP4P2	poly(ADP-ribose) polymerase family member 4 pseudogene 2	645771	0,119673877	0,847101401	4,080456	0,207599697	0,835541541	NA
ENSG00000224978	NA	NA	NA	0	NA	NA	NA	NA	NA
ENSG00000224980	RPL23AP85	ribosomal protein L23a pseudogene 85	100419650	0	NA	NA	NA	NA	NA
ENSG00000224981	NA	NA	NA	0	NA	NA	NA	NA	NA
ENSG00000224982	TMEM233	transmembrane protein 233	387890	0	NA	NA	NA	NA	NA
ENSG00000224986	PPP1R8P1	protein phosphatase 1 regulatory subunit 8 pseudogene 1	5512	0	NA	NA	NA	NA	NA
ENSG00000224987	NA	NA	NA	0	NA	NA	NA	NA	NA
ENSG00000224988	NA	NA	NA	0,210317649	-1,074295531	4,080456	-0,263278311	0,792336075	NA
ENSG00000224993	NA	NA	NA	0	NA	NA	NA	NA	NA
ENSG00000224999	VTA1P1	vesicle trafficking 1 pseudogene 1	106481708	0	NA	NA	NA	NA	NA
ENSG00000225003	OR51A5P	olfactory receptor family 51 subfamily A member 5 pseudogene	79525	0	NA	NA	NA	NA	NA
ENSG00000225005	RPL7AP77	ribosomal protein L7a pseudogene 77	122965336	0,278067925	-1,059186629	4,080456	-0,259575563	0,795191188	NA
ENSG00000225008	NA	NA	NA	0,119673877	0,847101401	4,080456	0,207599697	0,835541541	NA
ENSG00000225011	NA	NA	NA	0	NA	NA	NA	NA	NA
ENSG00000225014	NA	NA	NA	3,347448852	0,015197247	1,166165	0,013031819	0,989602407	NA
ENSG00000225016	LINC00328-2P	long intergenic non-protein coding RNA 328-2, pseudogene	100874404	0	NA	NA	NA	NA	NA
ENSG00000225017	LOC100420057	aconitase 2, mitochondrial pseudogene	100420057	0	NA	NA	NA	NA	NA
ENSG00000225018	NA	NA	NA	0	NA	NA	NA	NA	NA
ENSG00000225022	NA	NA	NA	0	NA	NA	NA	NA	NA
ENSG00000225024	RPL37P11	ribosomal protein L37 pseudogene 11	100271016	0	NA	NA	NA	NA	NA
ENSG00000225025	NA	NA	NA	0,545069127	3,171201894	3,174015	0,99911357	0,317739678	NA
ENSG00000225026	NA	NA	NA	0	NA	NA	NA	NA	NA
ENSG00000225027	NA	NA	NA	0	NA	NA	NA	NA	NA
ENSG00000225031	NA	NA	NA	0,294776862	-0,124374528	3,93911	-0,03157427	0,974811563	NA
ENSG00000225036	CDK4P1	cyclin dependent kinase 4 pseudogene 1	359941	0	NA	NA	NA	NA	NA
ENSG00000225043	RPL18AP2	ribosomal protein L18a pseudogene 2	387494	0,327244942	-1,96108207	3,785914	-0,517994308	0,604462241	NA
ENSG00000225045	MTND5P27	MT-ND5 pseudogene 27	100873264	0	NA	NA	NA	NA	NA
ENSG00000225046	NA	NA	NA	0	NA	NA	NA	NA	NA
ENSG00000225051	NA	NA	NA	0,721523639	0,805125013	3,130412	0,25719456	0,797028584	NA
ENSG00000225053	NA	NA	NA	0	NA	NA	NA	NA	NA
ENSG00000225055	NA	NA	NA	0	NA	NA	NA	NA	NA
ENSG00000225066	NA	NA	NA	0	NA	NA	NA	NA	NA
ENSG00000225067	NA	NA	NA	2,161069399	1,351701902	1,321309	1,023002087	0,306306859	NA
ENSG00000225069	NA	NA	NA	0	NA	NA	NA	NA	NA
ENSG00000225071	NA	NA	NA	0,366783935	-0,469277988	3,670146	-0,127863565	0,898256946	NA

ENSG00000225072	OR7E116P	olfactory receptor family 7 subfamily E member 116 pseudogene	81358	0	NA	NA	NA	NA	NA
ENSG00000225078	NA	NA	NA	0	NA	NA	NA	NA	NA
ENSG00000225079	FTH1P22	ferritin heavy chain 1 pseudogene 22	100462772	10,58081198	-0,309022888	0,653472	-0,472894161	0,636288691	NA
ENSG00000225080	NA	NA	NA	0	NA	NA	NA	NA	NA
ENSG00000225082	NA	NA	NA	0,118286652	0	4,080456	0	1	NA
ENSG00000225093	RPL3P7	ribosomal protein L3 pseudogene 7	642741	0,784179601	0,666002013	2,616665	0,254523249	0,799091345	NA
ENSG00000225094	NA	NA	NA	0,455165184	0,842669459	3,007028	0,2802333	0,77929852	NA
ENSG00000225098	NA	NA	NA	0	NA	NA	NA	NA	NA
ENSG00000225099	NA	NA	NA	0	NA	NA	NA	NA	NA
ENSG00000225100	DPY19L2P5	DPY19L2 pseudogene 5	106480421	0	NA	NA	NA	NA	NA
ENSG00000225101	OR52K3P	olfactory receptor family 52 subfamily K member 3 pseudogene	390035	0	NA	NA	NA	NA	NA
ENSG00000225108	NA	NA	NA	0	NA	NA	NA	NA	NA
ENSG00000225110	PNMA6F	PNMA family member 6F	105373377	0	NA	NA	NA	NA	NA
ENSG00000225116	NA	NA	NA	0	NA	NA	NA	NA	NA
ENSG00000225117	NA	NA	NA	0	NA	NA	NA	NA	NA
ENSG00000225118	NA	NA	NA	0	NA	NA	NA	NA	NA
ENSG00000225121	EEF1B2P5	eukaryotic translation elongation factor 1 beta 2 pseudogene 5	442227	0	NA	NA	NA	NA	NA
ENSG00000225122	NA	NA	NA	0	NA	NA	NA	NA	NA
ENSG00000225123	NA	NA	NA	0	NA	NA	NA	NA	NA
ENSG00000225124	RPL23AP36	ribosomal protein L23a pseudogene 36	100271296	0	NA	NA	NA	NA	NA
ENSG00000225125	NA	NA	NA	1,285020604	1,181330109	2,026174	0,583034999	0,55986974	NA
ENSG00000225131	PSME2P2	proteasome activator subunit 2 pseudogene 2	338099	0,295879636	-0,141898583	3,934553	-0,036064725	0,971230749	NA
ENSG00000225133	MORF4L1P4	mortality factor 4 like 1 pseudogene 4	100420030	0	NA	NA	NA	NA	NA
ENSG00000225137	DYNC1I2P1	dynein cytoplasmic 1 intermediate chain 2 pseudogene 1	728532	0	NA	NA	NA	NA	NA
ENSG00000225142	NA	NA	NA	0	NA	NA	NA	NA	NA
ENSG00000225147	RPS12P10	ribosomal protein S12 pseudogene 10	100271056	0	NA	NA	NA	NA	NA
ENSG00000225154	TUBAP9	tubulin alpha pseudogene 9	100533626	0	NA	NA	NA	NA	NA
ENSG00000225155	NA	NA	NA	0	NA	NA	NA	NA	NA
ENSG00000225158	HSPE1P24	heat shock protein family E (Hsp10) member 1 pseudogene 24	106480259	0	NA	NA	NA	NA	NA
ENSG00000225159	NPM1P39	nucleophosmin 1 pseudogene 39	440577	0,574860761	-1,045701449	2,879417	-0,363164341	0,716482123	NA
ENSG00000225165	NA	NA	NA	0	NA	NA	NA	NA	NA
ENSG00000225167	NA	NA	NA	0	NA	NA	NA	NA	NA
ENSG00000225169	NA	NA	NA	0	NA	NA	NA	NA	NA
ENSG00000225170	NA	NA	NA	0	NA	NA	NA	NA	NA
ENSG00000225171	DUTP6	deoxyuridine triphosphatase pseudogene 6	100873912	0,531418731	1,818213967	2,756807	0,659536259	0,50955147	NA
ENSG00000225175	HRGP1	HRG pseudogene 1	100533663	0	NA	NA	NA	NA	NA
ENSG00000225176	NA	NA	NA	0	NA	NA	NA	NA	NA
ENSG00000225180	PVALEF	parvalbumin like EF-hand containing	388428	0	NA	NA	NA	NA	NA
ENSG00000225182	NA	NA	NA	0	NA	NA	NA	NA	NA
ENSG00000225183	NA	NA	NA	0,103998153	-1,076443944	4,080456	-0,263804824	0,791930317	NA
ENSG00000225185	NA	NA	NA	0	NA	NA	NA	NA	NA
ENSG00000225189	NA	NA	NA	0	NA	NA	NA	NA	NA
ENSG00000225191	LOC100131348	adaptor related protein complex 2 subunit beta 1 pseudogene	100131348	0	NA	NA	NA	NA	NA
ENSG00000225192	NA	NA	NA	0,657334604	-0,650674894	2,366438	-0,274959614	0,783347266	NA
ENSG00000225193	NA	NA	NA	0,399895096	0,679346677	3,070924	0,221218996	0,824921919	NA
ENSG00000225196	RPL10P17	ribosomal protein L10 pseudogene 17	390998	0	NA	NA	NA	NA	NA
ENSG00000225198	SSX13P	SSX family member 13, pseudogene	326341	0	NA	NA	NA	NA	NA
ENSG00000225210	DUXAP9	double homeobox A pseudogene 9	503638	0,10488184	-1,076443944	4,080456	-0,263804824	0,791930317	NA
ENSG00000225212	NA	NA	NA	0	NA	NA	NA	NA	NA
ENSG00000225215	SMARCE1P1	SWI/SNF related, matrix associated, actin dependent regulator of chromatin, subfamily e, member 1 pseudogene 1	100271884	0,718199706	0,754497768	2,280178	0,330894188	0,740724412	NA
ENSG00000225221	NA	NA	NA	0	NA	NA	NA	NA	NA

ENSG00000225222	CHCHD4P5	coiled-coil-helix-coiled-coil-helix domain containing 4 pseudogene 5	106479039	0	NA	NA	NA	NA	NA
ENSG00000225224	RPS27AP12	RPS27A pseudogene 12	100130446	2,771806784	0,489201747	1,19364	0,409840186	0,681923185	NA
ENSG00000225225	ARL2BPP10	ADP ribosylation factor like GTPase 2 binding protein pseudogene 10	100874449	0	NA	NA	NA	NA	NA
ENSG00000225236	NA	NA	NA	0	NA	NA	NA	NA	NA
ENSG00000225238	NA	NA	NA	0	NA	NA	NA	NA	NA
ENSG00000225239	RPL21P107	ribosomal protein L21 pseudogene 107	644627	0	NA	NA	NA	NA	NA
ENSG00000225240	NA	NA	NA	0	NA	NA	NA	NA	NA
ENSG00000225242	COX6B1P7	cytochrome c oxidase subunit 6B1 pseudogene 7	100133210	0	NA	NA	NA	NA	NA
ENSG00000225244	NA	NA	NA	0	NA	NA	NA	NA	NA
ENSG00000225246	RPS2P1	ribosomal protein S2 pseudogene 1	140755	0,209500091	-1,066820997	4,080456	-0,261446522	0,793748185	NA
ENSG00000225251	RPL5P25	ribosomal protein L5 pseudogene 25	100271204	0	NA	NA	NA	NA	NA
ENSG00000225253	NA	NA	NA	0	NA	NA	NA	NA	NA
ENSG00000225254	ARMC8P1	armadillo repeat containing 8 pseudogene 1	100130871	0	NA	NA	NA	NA	NA
ENSG00000225256	NA	NA	NA	0	NA	NA	NA	NA	NA
ENSG00000225259	NA	NA	NA	0	NA	NA	NA	NA	NA
ENSG00000225264	NA	NA	NA	0,072165449	0,847101401	4,080456	0,207599697	0,835541541	NA
ENSG00000225267	RPL8P2	ribosomal protein L8 pseudogene 2	100128766	0	NA	NA	NA	NA	NA
ENSG00000225270	CICP12	capicua transcriptional repressor pseudogene 12	100420224	0	NA	NA	NA	NA	NA
ENSG00000225271	NOLC1P1	NOLC1 pseudogene 1	392426	0	NA	NA	NA	NA	NA
ENSG00000225272	RPL21P27	ribosomal protein L21 pseudogene 27	100270952	0	NA	NA	NA	NA	NA
ENSG00000225275	NA	NA	NA	0	NA	NA	NA	NA	NA
ENSG00000225276	MTND1P18	MT-ND1 pseudogene 18	100873338	0	NA	NA	NA	NA	NA
ENSG00000225282	NA	NA	NA	0	NA	NA	NA	NA	NA
ENSG00000225286	RPS2P30	ribosomal protein S2 pseudogene 30	100270911	0	NA	NA	NA	NA	NA
ENSG00000225287	OFD1P13Y	OFD1 pseudogene 13 Y-linked	386697	0	NA	NA	NA	NA	NA
ENSG00000225289	RPL29P29	ribosomal protein L29 pseudogene 29	100271474	0	NA	NA	NA	NA	NA
ENSG00000225294	OSTCP2	oligosaccharyltransferase complex subunit pseudogene 2	646567	0	NA	NA	NA	NA	NA
ENSG00000225295	NA	NA	NA	0	NA	NA	NA	NA	NA
ENSG00000225297	NA	NA	NA	0	NA	NA	NA	NA	NA
ENSG00000225304	LOC100132669	carboxymethylenebutenolidase homolog (Pseudomonas) pseudogene	100132669	0	NA	NA	NA	NA	NA
ENSG00000225308	ASS1P11	argininosuccinate synthetase 1 pseudogene 11	340274	0	NA	NA	NA	NA	NA
ENSG00000225310	DNAJC19P4	DnaJ heat shock protein family (Hsp40) member C19 pseudogene 4	106480691	0	NA	NA	NA	NA	NA
ENSG00000225314	CDCA3P1	CDCA3 pseudogene 1	125775241	0,119673877	0,847101401	4,080456	0,207599697	0,835541541	NA
ENSG00000225323	HBAP1	hemoglobin subunit alpha pseudogene 1	3041	0	NA	NA	NA	NA	NA
ENSG00000225326	NA	NA	NA	0	NA	NA	NA	NA	NA
ENSG00000225327	USP17L3	ubiquitin specific peptidase 17 like family member 3	645836	0	NA	NA	NA	NA	NA
ENSG00000225333	NA	NA	NA	0	NA	NA	NA	NA	NA
ENSG00000225336	NA	NA	NA	0	NA	NA	NA	NA	NA
ENSG00000225338	RPL23AP18	ribosomal protein L23a pseudogene 18	728134	0,10543123	0	4,080456	0	1	NA
ENSG00000225343	RPL21P66	ribosomal protein L21 pseudogene 66	100271179	0	NA	NA	NA	NA	NA
ENSG00000225344	NA	NA	NA	0	NA	NA	NA	NA	NA
ENSG00000225345	NA	NA	NA	0	NA	NA	NA	NA	NA
ENSG00000225347	NA	NA	NA	0	NA	NA	NA	NA	NA
ENSG00000225349	ANKRD49P2	ANKRD49 pseudogene 2	116435289	0	NA	NA	NA	NA	NA
ENSG00000225352	RPL31P53	ribosomal protein L31 pseudogene 53	100271477	0	NA	NA	NA	NA	NA
ENSG00000225354	RPL7AP52	ribosomal protein L7a pseudogene 52	100271534	0	NA	NA	NA	NA	NA
ENSG00000225355	NA	NA	NA	0	NA	NA	NA	NA	NA
ENSG00000225356	NA	NA	NA	0	NA	NA	NA	NA	NA
ENSG00000225357	RPF2P1	ribosome production factor 2 homolog pseudogene 1	729608	0	NA	NA	NA	NA	NA
ENSG00000225358	NA	NA	NA	0,294813666	-1,056471517	3,905445	-0,27051243	0,786766056	NA
ENSG00000225360	NA	NA	NA	0	NA	NA	NA	NA	NA

ENSG00000225364	ATP6V0E1P1	ATPase H+ transporting V0 subunit e1 pseudogene 1	100874502	0	NA	NA	NA	NA	NA
ENSG00000225366	CRIPTO3	cripto, EGF-CFC family member 3	6998	0	NA	NA	NA	NA	NA
ENSG00000225369	NA	NA	NA	0	NA	NA	NA	NA	NA
ENSG00000225371	NA	NA	NA	0	NA	NA	NA	NA	NA
ENSG00000225380	NA	NA	NA	0	NA	NA	NA	NA	NA
ENSG00000225381	RPS5P7	RPS5 pseudogene 7	392520	0	NA	NA	NA	NA	NA
ENSG00000225384	NA	NA	NA	0	NA	NA	NA	NA	NA
ENSG00000225385	NA	NA	NA	0	NA	NA	NA	NA	NA
ENSG00000225392	NA	NA	NA	0	NA	NA	NA	NA	NA
ENSG00000225394	LOC100419812	COBW domain containing 7 pseudogene	100419812	0	NA	NA	NA	NA	NA
ENSG00000225397	NA	NA	NA	0	NA	NA	NA	NA	NA
ENSG00000225398	NA	NA	NA	0	NA	NA	NA	NA	NA
ENSG00000225400	NA	NA	NA	0	NA	NA	NA	NA	NA
ENSG00000225401	NA	NA	NA	0,963218863	-0,224115197	1,84715	-0,121330271	0,903429445	NA
ENSG00000225402	NA	NA	NA	0,144330898	1,418786819	4,061262	0,349346267	0,726829368	NA
ENSG00000225404	RPL26P34	ribosomal protein L26 pseudogene 34	100271470	0	NA	NA	NA	NA	NA
ENSG00000225405	RPS15AP17	ribosomal protein S15a pseudogene 17	391656	0	NA	NA	NA	NA	NA
ENSG00000225406	NA	NA	NA	0	NA	NA	NA	NA	NA
ENSG00000225415	NA	NA	NA	0	NA	NA	NA	NA	NA
ENSG00000225416	NA	NA	NA	0	NA	NA	NA	NA	NA
ENSG00000225418	NA	NA	NA	0	NA	NA	NA	NA	NA
ENSG00000225419	RPL21P3	ribosomal protein L21 pseudogene 3	128760	0,23636736	-1,075826945	4,080456	-0,263653616	0,79204684	NA
ENSG00000225422	NA	NA	NA	0,295752694	-0,135857684	3,935168	-0,034523984	0,972459317	NA
ENSG00000225423	TNPO1P1	transportin 1 pseudogene 1	100418919	3,593855807	0,895771327	1,101056	0,813556518	0,415899056	NA
ENSG00000225428	NDUFB4P7	NADH:ubiquinone oxidoreductase subunit B4 pseudogene 7	107075094	0	NA	NA	NA	NA	NA
ENSG00000225433	MTND1P12	MT-ND1 pseudogene 12	106480744	0	NA	NA	NA	NA	NA
ENSG00000225443	RPL36AP27	ribosomal protein L36a pseudogene 27	100128072	0	NA	NA	NA	NA	NA
ENSG00000225447	NA	NA	NA	2,187068096	-2,805291975	1,595519	-1,758231152	0,078708187	NA
ENSG00000225448	NA	NA	NA	0	NA	NA	NA	NA	NA
ENSG00000225451	NA	NA	NA	0	NA	NA	NA	NA	NA
ENSG00000225455	NA	NA	NA	0	NA	NA	NA	NA	NA
ENSG00000225460	LOC124902324	ubiquitin-conjugating enzyme E2 variant 1-like	124902324	0,15836288	0	4,080456	0	1	NA
ENSG00000225462	FDPSP1	farnesyl diphosphate synthase pseudogene 1	343332	0	NA	NA	NA	NA	NA
ENSG00000225463	NA	NA	NA	0,118286652	0	4,080456	0	1	NA
ENSG00000225466	NA	NA	NA	0	NA	NA	NA	NA	NA
ENSG00000225469	LOC392555	MAGE family member C2 pseudogene	392555	0	NA	NA	NA	NA	NA
ENSG00000225471	LOC121627959	TPT1-like	121627959	0,103998153	-1,076443944	4,080456	-0,263804824	0,791930317	NA
ENSG00000225475	NA	NA	NA	0	NA	NA	NA	NA	NA
ENSG00000225476	MTCO3P5	MT-CO3 pseudogene 5	107075228	0	NA	NA	NA	NA	NA
ENSG00000225477	LOC100631258	isocitrate dehydrogenase (NAD(+)) 3 non-catalytic subunit gamma pseudogene	100631258	0	NA	NA	NA	NA	NA
ENSG00000225478	NA	NA	NA	0	NA	NA	NA	NA	NA
ENSG00000225480	NA	NA	NA	0	NA	NA	NA	NA	NA
ENSG00000225482	RPS6P14	ribosomal protein S6 pseudogene 14	100131396	0	NA	NA	NA	NA	NA
ENSG00000225483	RPL22P4	ribosomal protein L22 pseudogene 4	100129102	0	NA	NA	NA	NA	NA
ENSG00000225486	NA	NA	NA	0	NA	NA	NA	NA	NA
ENSG00000225487	NA	NA	NA	0	NA	NA	NA	NA	NA
ENSG00000225491	UBE2Q2P4Y	UBE2Q2 pseudogene 4, Y-linked	100421716	0	NA	NA	NA	NA	NA
ENSG00000225492	NA	NA	NA	0,400338637	0,674650124	3,069944	0,219759758	0,826058262	NA
ENSG00000225496	NA	NA	NA	0	NA	NA	NA	NA	NA
ENSG00000225499	RPL15P4	ribosomal protein L15 pseudogene 4	100129921	0	NA	NA	NA	NA	NA
ENSG00000225501	SNRPFP3	small nuclear ribonucleoprotein polypeptide F pseudogene 3	100874422	0	NA	NA	NA	NA	NA
ENSG00000225502	GAPDHP16	glyceraldehyde-3-phosphate dehydrogenase pseudogene 16	387491	0	NA	NA	NA	NA	NA
ENSG00000225505	NA	NA	NA	0	NA	NA	NA	NA	NA
ENSG00000225507	NA	NA	NA	0	NA	NA	NA	NA	NA
ENSG00000225508	NA	NA	NA	0	NA	NA	NA	NA	NA
ENSG00000225509	AIFM1P1	AIFM1 pseudogene 1	645120	0	NA	NA	NA	NA	NA
ENSG00000225510	NA	NA	NA	0	NA	NA	NA	NA	NA

ENSG00000225511	NA	NA	NA	0	NA	NA	NA	NA	NA
ENSG00000225513	NA	NA	NA	1,777782809	-1,98073949	1,682257	-1,177429516	0,239024115	NA
ENSG00000225514	MTND1P34	MT-ND1 pseudogene 34	100873219	0	NA	NA	NA	NA	NA
ENSG00000225522	NA	NA	NA	0	NA	NA	NA	NA	NA
ENSG00000225523	IGKV6D-21	immunoglobulin kappa variable 6D-21 (non-functional)	28870	0	NA	NA	NA	NA	NA
ENSG00000225526	MKRN2OS	MKRN2 opposite strand	100129480	0	NA	NA	NA	NA	NA
ENSG00000225528	TMA7B	translation machinery associated 7 homolog B	112268293	5,343957941	0,428952074	0,838048	0,511846649	0,60875834	NA
ENSG00000225530	NA	NA	NA	0	NA	NA	NA	NA	NA
ENSG00000225531	NA	NA	NA	0,15836288	0	4,080456	0	1	NA
ENSG00000225533	PAWRP1	pro-apoptotic WT1 regulator pseudogene 1	100420526	0	NA	NA	NA	NA	NA
ENSG00000225536	NA	NA	NA	0	NA	NA	NA	NA	NA
ENSG00000225538	OR5BE1P	olfactory receptor family 5 subfamily BE member 1 pseudogene	81207	0	NA	NA	NA	NA	NA
ENSG00000225544	NA	NA	NA	0,10488184	-1,076443944	4,080456	-0,263804824	0,791930317	NA
ENSG00000225549	NA	NA	NA	0	NA	NA	NA	NA	NA
ENSG00000225551	NA	NA	NA	0	NA	NA	NA	NA	NA
ENSG00000225556	C2CD4D	C2 calcium dependent domain containing 4D	100191040	0,103998153	-1,076443944	4,080456	-0,263804824	0,791930317	NA
ENSG00000225557	MTCO3P20	MT-CO3 pseudogene 20	107075180	0	NA	NA	NA	NA	NA
ENSG00000225558	NA	NA	NA	0	NA	NA	NA	NA	NA
ENSG00000225568	LOC100421468	ubiquitin like modifier activating enzyme 2 pseudogene	100421468	0,29597281	-1,074210473	3,901332	-0,275344568	0,783051527	NA
ENSG00000225569	CCT4P2	chaperonin containing TCP1 subunit 4 pseudogene 2	100131638	1,278030804	-0,865999652	1,693722	-0,511299818	0,609141132	NA
ENSG00000225573	RPL35P5	ribosomal protein L35 pseudogene 5	441246	0	NA	NA	NA	NA	NA
ENSG00000225574	NA	NA	NA	0	NA	NA	NA	NA	NA
ENSG00000225580	NA	NA	NA	0	NA	NA	NA	NA	NA
ENSG00000225581	TRIM53AP	tripartite motif containing 53A, pseudogene	642569	0	NA	NA	NA	NA	NA
ENSG00000225583	COX6A1P6	COX6A1 pseudogene 6	127566429	0	NA	NA	NA	NA	NA
ENSG00000225585	NA	NA	NA	0	NA	NA	NA	NA	NA
ENSG00000225591	NA	NA	NA	1,119034731	-1,034649358	2,235223	-0,46288421	0,643447366	NA
ENSG00000225594	NA	NA	NA	0	NA	NA	NA	NA	NA
ENSG00000225603	NA	NA	NA	0	NA	NA	NA	NA	NA
ENSG00000225609	CDY20P	chromodomain Y-linked 20 pseudogene	386742	0	NA	NA	NA	NA	NA
ENSG00000225615	NA	NA	NA	0	NA	NA	NA	NA	NA
ENSG00000225616	RPL27P4	ribosomal protein L27 pseudogene 4	646624	0	NA	NA	NA	NA	NA
ENSG00000225622	PPIAP17	peptidylprolyl isomerase A pseudogene 17	170540	0	NA	NA	NA	NA	NA
ENSG00000225624	TBL1YP1	transducin beta like 1 Y-linked pseudogene 1	286555	0	NA	NA	NA	NA	NA
ENSG00000225625	NA	NA	NA	0	NA	NA	NA	NA	NA
ENSG00000225636	HNRNPA3P15	heterogeneous nuclear ribonucleoprotein A3 pseudogene 15	106480234	0	NA	NA	NA	NA	NA
ENSG00000225638	NA	NA	NA	0	NA	NA	NA	NA	NA
ENSG00000225639	NA	NA	NA	0	NA	NA	NA	NA	NA
ENSG00000225642	NA	NA	NA	0	NA	NA	NA	NA	NA
ENSG00000225650	NA	NA	NA	0	NA	NA	NA	NA	NA
ENSG00000225653	NA	NA	NA	0	NA	NA	NA	NA	NA
ENSG00000225658	TAF13P2	TATA-box binding protein associated factor 13 pseudogene 2	100462886	0	NA	NA	NA	NA	NA
ENSG00000225660	NA	NA	NA	0	NA	NA	NA	NA	NA
ENSG00000225662	RPL14P2	ribosomal protein L14 pseudogene 2	100270930	0	NA	NA	NA	NA	NA
ENSG00000225664	NA	NA	NA	0	NA	NA	NA	NA	NA
ENSG00000225665	NA	NA	NA	0	NA	NA	NA	NA	NA
ENSG00000225669	RPL23AP27	ribosomal protein L23a pseudogene 27	100271456	0	NA	NA	NA	NA	NA
ENSG00000225671	NA	NA	NA	0	NA	NA	NA	NA	NA
ENSG00000225672	NA	NA	NA	0	NA	NA	NA	NA	NA
ENSG00000225673	NA	NA	NA	0	NA	NA	NA	NA	NA
ENSG00000225674	NA	NA	NA	0	NA	NA	NA	NA	NA
ENSG00000225678	NA	NA	NA	0	NA	NA	NA	NA	NA
ENSG00000225681	NA	NA	NA	0	NA	NA	NA	NA	NA
ENSG00000225685	NA	NA	NA	0	NA	NA	NA	NA	NA
ENSG00000225690	NA	NA	NA	0	NA	NA	NA	NA	NA
ENSG00000225693	NA	NA	NA	0,338204464	1,920693877	4,024358	0,477267095	0,633171939	NA
ENSG00000225695	NA	NA	NA	0,649791391	-0,165510844	2,289465	-0,072292365	0,942369241	NA

ENSG00000225698	IGHV3-72	immunoglobulin heavy variable 3-72	28410	0	NA	NA	NA	NA	NA
ENSG00000225701	NA	NA	NA	0	NA	NA	NA	NA	NA
ENSG00000225705	NA	NA	NA	0	NA	NA	NA	NA	NA
ENSG00000225710	NA	NA	NA	0	NA	NA	NA	NA	NA
ENSG00000225711	NA	NA	NA	0	NA	NA	NA	NA	NA
ENSG00000225712	NA	NA	NA	0	NA	NA	NA	NA	NA
ENSG00000225713	RPL30P1	ribosomal protein L30 pseudogene 1	100270975	0	NA	NA	NA	NA	NA
ENSG00000225715	NA	NA	NA	0	NA	NA	NA	NA	NA
ENSG00000225716	ELOCP13	elongin C pseudogene 13	100462875	0	NA	NA	NA	NA	NA
ENSG00000225719	NA	NA	NA	0	NA	NA	NA	NA	NA
ENSG00000225722	RPL24P9	RPL24 pseudogene 9	100129487	0	NA	NA	NA	NA	NA
ENSG00000225723	MTND6P15	MT-ND6 pseudogene 15	106478949	0	NA	NA	NA	NA	NA
ENSG00000225724	ASH2LP3	ASH2L pseudogene 3	100736411	0,23616048	-2,075648354	4,028973	-0,515180498	0,606426895	NA
ENSG00000225726	NA	NA	NA	0,794498154	2,913711088	3,109665	0,936985625	0,348765952	NA
ENSG00000225727	LOC124903167	uncharacterized LOC124903167	124903167	0	NA	NA	NA	NA	NA
ENSG00000225728	NA	NA	NA	0	NA	NA	NA	NA	NA
ENSG00000225735	NEK4P1	NIMA-related kinase 4 pseudogene 1	100421718	0	NA	NA	NA	NA	NA
ENSG00000225739	NA	NA	NA	1,036116124	3,014833516	2,138708	1,409652012	0,158642461	NA
ENSG00000225740	ELOCP6	elongin C pseudogene 6	100462867	0	NA	NA	NA	NA	NA
ENSG00000225741	NA	NA	NA	0	NA	NA	NA	NA	NA
ENSG00000225755	NA	NA	NA	0	NA	NA	NA	NA	NA
ENSG00000225758	RPS17P17	ribosomal protein S17 pseudogene 17	100271230	0	NA	NA	NA	NA	NA
ENSG00000225760	NA	NA	NA	0,210862461	0	4,080456	0	1	NA
ENSG00000225769	CROCCP1	CROCC pseudogene 1	392547	0,207996306	-1,916073151	4,043021	-0,473921178	0,635556117	NA
ENSG00000225770	NA	NA	NA	0	NA	NA	NA	NA	NA
ENSG00000225774	NA	NA	NA	0	NA	NA	NA	NA	NA
ENSG00000225777	NA	NA	NA	0	NA	NA	NA	NA	NA
ENSG00000225779	NA	NA	NA	0,189812596	0,853963781	4,080456	0,209281464	0,834228521	NA
ENSG00000225781	OR6V1	olfactory receptor family 6 subfamily V member 1	346517	0	NA	NA	NA	NA	NA
ENSG00000225787	NA	NA	NA	0	NA	NA	NA	NA	NA
ENSG00000225795	NA	NA	NA	0	NA	NA	NA	NA	NA
ENSG00000225796	MTND4P23	MT-ND4 pseudogene 23	100873245	0	NA	NA	NA	NA	NA
ENSG00000225797	NA	NA	NA	0	NA	NA	NA	NA	NA
ENSG00000225801	RABEPKP1	Rab9 effector protein with kelch motifs pseudogene 1	100421226	0	NA	NA	NA	NA	NA
ENSG00000225802	WARS1P1	tryptophanyl-tRNA synthetase 1 pseudogene 1	341112	0	NA	NA	NA	NA	NA
ENSG00000225803	NA	NA	NA	0	NA	NA	NA	NA	NA
ENSG00000225805	DEFB131B	defensin beta 131B	100129216	0	NA	NA	NA	NA	NA
ENSG00000225808	NA	NA	NA	0,103998153	-1,076443944	4,080456	-0,263804824	0,791930317	NA
ENSG00000225809	RBMY2KP	RNA binding motif protein Y-linked family 2 member K, pseudogene	140100	0	NA	NA	NA	NA	NA
ENSG00000225813	NA	NA	NA	0	NA	NA	NA	NA	NA
ENSG00000225814	NA	NA	NA	0	NA	NA	NA	NA	NA
ENSG00000225816	NA	NA	NA	0	NA	NA	NA	NA	NA
ENSG00000225823	RPL7P45	ribosomal protein L7 pseudogene 45	100271225	0	NA	NA	NA	NA	NA
ENSG00000225827	NA	NA	NA	0	NA	NA	NA	NA	NA
ENSG00000225828	FAM229A	family with sequence similarity 229 member A	100128071	1,692179679	-4,329274401	2,224961	-1,945775471	0,051681716	NA
ENSG00000225831	RPS18P1	ribosomal protein S18 pseudogene 1	128676	0	NA	NA	NA	NA	NA
ENSG00000225832	NA	NA	NA	0	NA	NA	NA	NA	NA
ENSG00000225840	NA	NA	NA	0	NA	NA	NA	NA	NA
ENSG00000225842	NA	NA	NA	0	NA	NA	NA	NA	NA
ENSG00000225843	NIPA2P1	NIPA2 pseudogene 1	100132321	0	NA	NA	NA	NA	NA
ENSG00000225846	RPL36P8	ribosomal protein L36 pseudogene 8	643205	0	NA	NA	NA	NA	NA
ENSG00000225849	MKRN7P	makorin ring finger protein 7, pseudogene	7686	0	NA	NA	NA	NA	NA
ENSG00000225851	NA	NA	NA	0	NA	NA	NA	NA	NA
ENSG00000225854	RPL34P4	ribosomal protein L34 pseudogene 4	100270889	0,88649438	-1,517383768	2,240295	-0,677314221	0,49820661	NA
ENSG00000225856	PCNPP2	PEST containing nuclear protein pseudogene 2	100128911	0	NA	NA	NA	NA	NA
ENSG00000225858	NA	NA	NA	0	NA	NA	NA	NA	NA
ENSG00000225864	NA	NA	NA	0	NA	NA	NA	NA	NA
ENSG00000225868	NA	NA	NA	0	NA	NA	NA	NA	NA
ENSG00000225869	NA	NA	NA	0	NA	NA	NA	NA	NA
ENSG00000225871	NA	NA	NA	0,438476043	0	4,080456	0	1	NA
ENSG00000225872	LINC01529	long intergenic non-protein coding RNA 1529	644050	0	NA	NA	NA	NA	NA

ENSG00000225873	C3orf86P	long intergenic non-protein coding RNA 694	102724231	0,440652462	-2,988393936	3,358731	-0,88973894	0,37360608	NA
ENSG00000225876	NA	NA	NA	0	NA	NA	NA	NA	NA
ENSG00000225878	SERBP1P2	SERPINE1 mRNA binding protein 1 pseudogene 2	359996	0	NA	NA	NA	NA	NA
ENSG00000225883	BMS1P11	BMS1 pseudogene 11	644632	4,820178359	-2,120740929	1,537345	-1,379483198	0,167745823	NA
ENSG00000225893	RPS6P11	ribosomal protein S6 pseudogene 11	100270866	0	NA	NA	NA	NA	NA
ENSG00000225895	NA	NA	NA	0	NA	NA	NA	NA	NA
ENSG00000225896	NA	NA	NA	0	NA	NA	NA	NA	NA
ENSG00000225898	NA	NA	NA	0	NA	NA	NA	NA	NA
ENSG00000225899	FRG2B	FSHD region gene 2 family member B	441581	0	NA	NA	NA	NA	NA
ENSG00000225900	NA	NA	NA	5,382591792	0,417559149	0,865383	0,482513856	0,629440958	NA
ENSG00000225901	MTND2P9	MT-ND2 pseudogene 9	100884155	0	NA	NA	NA	NA	NA
ENSG00000225904	NA	NA	NA	0	NA	NA	NA	NA	NA
ENSG00000225906	NA	NA	NA	0	NA	NA	NA	NA	NA
ENSG00000225907	RPS27P16	ribosomal protein S27 pseudogene 16	100271119	0	NA	NA	NA	NA	NA
ENSG00000225911	NA	NA	NA	0	NA	NA	NA	NA	NA
ENSG00000225912	RPL26P36	ribosomal protein L26 pseudogene 36	392501	0	NA	NA	NA	NA	NA
ENSG00000225918	NA	NA	NA	0	NA	NA	NA	NA	NA
ENSG00000225920	NA	NA	NA	0	NA	NA	NA	NA	NA
ENSG00000225922	RPL21P92	ribosomal protein L21 pseudogene 92	100130400	0	NA	NA	NA	NA	NA
ENSG00000225923	LOC101060164	secretory carrier membrane protein 1 pseudogene	101060164	0	NA	NA	NA	NA	NA
ENSG00000225925	NA	NA	NA	0	NA	NA	NA	NA	NA
ENSG00000225928	NA	NA	NA	0	NA	NA	NA	NA	NA
ENSG00000225933	NA	NA	NA	0	NA	NA	NA	NA	NA
ENSG00000225934	FAM133FP	family with sequence similarity 133 member A pseudogene	100421606	0	NA	NA	NA	NA	NA
ENSG00000225935	NA	NA	NA	0	NA	NA	NA	NA	NA
ENSG00000225943	NA	NA	NA	0	NA	NA	NA	NA	NA
ENSG00000225944	RIOK3P1	RIO kinase 3 pseudogene 1	122145	0	NA	NA	NA	NA	NA
ENSG00000225947	NA	NA	NA	0	NA	NA	NA	NA	NA
ENSG00000225949	NA	NA	NA	0	NA	NA	NA	NA	NA
ENSG00000225950	NTF4	neurotrophin 4	4909	0	NA	NA	NA	NA	NA
ENSG00000225957	NA	NA	NA	0	NA	NA	NA	NA	NA
ENSG00000225959	MTND4P3	MT-ND4 pseudogene 3	100873189	0	NA	NA	NA	NA	NA
ENSG00000225965	NA	NA	NA	0	NA	NA	NA	NA	NA
ENSG00000225968	ELFN1	extracellular leucine rich repeat and fibronectin type III domain containing 1	392617	1,056696752	-0,309714477	1,838499	-0,168460504	0,866221013	NA
ENSG00000225969	NA	NA	NA	0,103998153	-1,076443944	4,080456	-0,263804824	0,791930317	NA
ENSG00000225971	NA	NA	NA	0	NA	NA	NA	NA	NA
ENSG00000225972	MTND1P23	MT-ND1 pseudogene 23	100887749	3,435661687	0,884389305	1,002414	0,882259922	0,377636264	NA
ENSG00000225976	NA	NA	NA	0,10543123	0	4,080456	0	1	NA
ENSG00000225980	OR7E19P	olfactory receptor family 7 subfamily E member 19 pseudogene	26651	0	NA	NA	NA	NA	NA
ENSG00000225984	NA	NA	NA	0	NA	NA	NA	NA	NA
ENSG00000225990	NA	NA	NA	0	NA	NA	NA	NA	NA
ENSG00000225991	RPL23AP34	ribosomal protein L23a pseudogene 34	100271294	3,510971333	1,152093721	1,071501	1,075215081	0,282278445	NA
ENSG00000225992	NA	NA	NA	1,100320083	-2,030976014	1,866028	-1,088395291	0,276420639	NA
ENSG00000225997	OR4A8	olfactory receptor family 4 subfamily A member 8 (gene/pseudogene)	81315	0	NA	NA	NA	NA	NA
ENSG00000225999	NDUFA12P1	NADH:ubiquinone oxidoreductase subunit A12 pseudogene 1	100527945	0	NA	NA	NA	NA	NA
ENSG00000226002	GTF2IP14	general transcription factor IIi pseudogene 14	106481735	0,178187569	-0,15145136	4,07961	-0,037123979	0,970386153	NA
ENSG00000226003	PPIAP37	peptidylprolyl isomerase A pseudogene 37	111082976	0	NA	NA	NA	NA	NA
ENSG00000226010	LOC100419786	zinc finger protein 670 pseudogene	100419786	0,395861808	1,705832363	3,615127	0,471859541	0,637027048	NA
ENSG00000226011	OFD1P1Y	OFD1 pseudogene 1 Y-linked	378009	0	NA	NA	NA	NA	NA
ENSG00000226013	NA	NA	NA	0	NA	NA	NA	NA	NA
ENSG00000226014	NA	NA	NA	0	NA	NA	NA	NA	NA
ENSG00000226015	CCT8P1	chaperonin containing TCP1 subunit 8 pseudogene 1	644131	1,969686992	1,093380129	1,639111	0,667056837	0,504735829	NA
ENSG00000226016	NA	NA	NA	0	NA	NA	NA	NA	NA
ENSG00000226020	NA	NA	NA	0	NA	NA	NA	NA	NA
ENSG00000226023	CT47A11	cancer/testis antigen family 47 member A11	255313	0	NA	NA	NA	NA	NA

ENSG00000226023	CT47A6	cancer/testis antigen family 47 member A6	728062	0	NA	NA	NA	NA	NA
ENSG00000226024	NA	NA	NA	0	NA	NA	NA	NA	NA
ENSG00000226025	NA	NA	NA	0	NA	NA	NA	NA	NA
ENSG00000226028	NA	NA	NA	0	NA	NA	NA	NA	NA
ENSG00000226030	NA	NA	NA	0	NA	NA	NA	NA	NA
ENSG00000226034	NA	NA	NA	0	NA	NA	NA	NA	NA
ENSG00000226036	NA	NA	NA	0	NA	NA	NA	NA	NA
ENSG00000226038	NA	NA	NA	0	NA	NA	NA	NA	NA
ENSG00000226040	NA	NA	NA	0	NA	NA	NA	NA	NA
ENSG00000226045	NA	NA	NA	0	NA	NA	NA	NA	NA
ENSG00000226046	NA	NA	NA	0	NA	NA	NA	NA	NA
ENSG00000226048	NA	NA	NA	0	NA	NA	NA	NA	NA
ENSG00000226049	TLK2P1	tousled like kinase 2 pseudogene 1	646202	0	NA	NA	NA	NA	NA
ENSG00000226054	NA	NA	NA	0,458534647	-0,993017169	3,977747	-0,249643109	0,802863357	NA
ENSG00000226055	NA	NA	NA	0,297499969	1,658480314	3,887235	0,426647825	0,669635858	NA
ENSG00000226056	NA	NA	NA	0,11808024	-1,076443944	4,080456	-0,263804824	0,791930317	NA
ENSG00000226057	NA	NA	NA	0	NA	NA	NA	NA	NA
ENSG00000226058	LOC107985789	succinate dehydrogenase [ubiquinone] cytochrome b small subunit, mitochondrial-like	107985789	0	NA	NA	NA	NA	NA
ENSG00000226059	NA	NA	NA	0	NA	NA	NA	NA	NA
ENSG00000226061	NA	NA	NA	0	NA	NA	NA	NA	NA
ENSG00000226064	NA	NA	NA	0	NA	NA	NA	NA	NA
ENSG00000226065	NA	NA	NA	0	NA	NA	NA	NA	NA
ENSG00000226066	NA	NA	NA	0	NA	NA	NA	NA	NA
ENSG00000226068	NA	NA	NA	0	NA	NA	NA	NA	NA
ENSG00000226072	NA	NA	NA	0	NA	NA	NA	NA	NA
ENSG00000226074	NA	NA	NA	0	NA	NA	NA	NA	NA
ENSG00000226075	PHKG1P1	phosphorylase kinase catalytic subunit gamma 1 pseudogene 1	643941	0	NA	NA	NA	NA	NA
ENSG00000226080	RPL6P4	ribosomal protein L6 pseudogene 4	645054	0	NA	NA	NA	NA	NA
ENSG00000226081	NA	NA	NA	0	NA	NA	NA	NA	NA
ENSG00000226082	RPS10P10	ribosomal protein S10 pseudogene 10	389127	0	NA	NA	NA	NA	NA
ENSG00000226084	RPL17P6	ribosomal protein L17 pseudogene 6	645441	0,237166456	1,955818734	4,022315	0,486242049	0,626795562	NA
ENSG00000226085	NA	NA	NA	2,273789195	-0,474217303	1,224648	-0,387227592	0,698587725	NA
ENSG00000226086	EIF3LP3	eukaryotic translation initiation factor 3 subunit L pseudogene 3	340947	0	NA	NA	NA	NA	NA
ENSG00000226093	RPS28P8	ribosomal protein S28 pseudogene 8	100271381	0	NA	NA	NA	NA	NA
ENSG00000226094	RPL7P3	ribosomal protein L7 pseudogene 3	140857	0	NA	NA	NA	NA	NA
ENSG00000226096	NA	NA	NA	0	NA	NA	NA	NA	NA
ENSG00000226098	NA	NA	NA	0	NA	NA	NA	NA	NA
ENSG00000226102	SEPTIN7P3	septin 7 pseudogene 3	646913	3,366723008	-0,971605195	1,111939	-0,87379332	0,382230819	NA
ENSG00000226107	RPL36AP54	ribosomal protein L36a pseudogene 54	100271612	0,436460441	-1,094356066	3,529749	-0,310037894	0,75653214	NA
ENSG00000226108	NA	NA	NA	0	NA	NA	NA	NA	NA
ENSG00000226110	NA	NA	NA	0	NA	NA	NA	NA	NA
ENSG00000226112	FCF1P4	FCF1 pseudogene 4	106480799	0	NA	NA	NA	NA	NA
ENSG00000226113	NA	NA	NA	0	NA	NA	NA	NA	NA
ENSG00000226114	NDUFB4P5	NADH:ubiquinone oxidoreductase subunit B4 pseudogene 5	107075096	0	NA	NA	NA	NA	NA
ENSG00000226116	NA	NA	NA	0	NA	NA	NA	NA	NA
ENSG00000226117	NA	NA	NA	0	NA	NA	NA	NA	NA
ENSG00000226118	MTND3P1	MT-ND3 pseudogene 1	100873208	0	NA	NA	NA	NA	NA
ENSG00000226119	NA	NA	NA	0	NA	NA	NA	NA	NA
ENSG00000226121	NA	NA	NA	0,985901608	2,509032407	2,592485	0,967809857	0,33313934	NA
ENSG00000226124	FTCDNL1	formiminotransferase cyclodeaminase N-terminal like	348751	1,14762671	-0,324944168	1,967609	-0,165146738	0,868828486	NA
ENSG00000226126	PSMC1P12	proteasome 26S subunit, ATPase 1 pseudogene 12	644094	0	NA	NA	NA	NA	NA
ENSG00000226128	RPL26P9	ribosomal protein L26 pseudogene 9	100270885	0	NA	NA	NA	NA	NA
ENSG00000226131	NA	NA	NA	0	NA	NA	NA	NA	NA
ENSG00000226132	RPS3AP46	RPS3A pseudogene 46	100271596	0,117497108	0,847101401	4,080456	0,207599697	0,835541541	NA
ENSG00000226138	NA	NA	NA	0	NA	NA	NA	NA	NA
ENSG00000226141	NA	NA	NA	0	NA	NA	NA	NA	NA
ENSG00000226142	NA	NA	NA	0	NA	NA	NA	NA	NA
ENSG00000226144	RPS27AP3	RPS27A pseudogene 3	140714	0	NA	NA	NA	NA	NA
ENSG00000226145	NA	NA	NA	0	NA	NA	NA	NA	NA

ENSG00000226147	NA	NA	NA	0,216496347	1,902771968	4,026559	0,472555327	0,63653046	NA
ENSG00000226148	NA	NA	NA	0	NA	NA	NA	NA	NA
ENSG00000226153	CHP1P3	CHP1 pseudogene 3	117369447	0	NA	NA	NA	NA	NA
ENSG00000226156	RPL23AP76	ribosomal protein L23a pseudogene 76	441781	0	NA	NA	NA	NA	NA
ENSG00000226158	NA	NA	NA	0,11808024	-1,076443944	4,080456	-0,263804824	0,791930317	NA
ENSG00000226161	NA	NA	NA	0,684500913	0,056458825	3,120335	0,018093832	0,985563998	NA
ENSG00000226164	FGFR3P6	fibroblast growth factor receptor 3 pseudogene 6	100420881	0	NA	NA	NA	NA	NA
ENSG00000226166	NA	NA	NA	0	NA	NA	NA	NA	NA
ENSG00000226168	LOC100421009	POM121 transmembrane nucleoporin pseudogene	100421009	0	NA	NA	NA	NA	NA
ENSG00000226174	TEX22	testis expressed 22	647310	7,065656284	-0,862459097	0,800116	-1,077917036	0,281070782	NA
ENSG00000226183	NA	NA	NA	0	NA	NA	NA	NA	NA
ENSG00000226185	TRIM64FP	tripartite motif containing 64F, pseudogene	390233	0	NA	NA	NA	NA	NA
ENSG00000226186	NA	NA	NA	0	NA	NA	NA	NA	NA
ENSG00000226188	NA	NA	NA	0	NA	NA	NA	NA	NA
ENSG00000226191	NA	NA	NA	0	NA	NA	NA	NA	NA
ENSG00000226196	NA	NA	NA	0	NA	NA	NA	NA	NA
ENSG00000226199	NA	NA	NA	0	NA	NA	NA	NA	NA
ENSG00000226205	NA	NA	NA	0	NA	NA	NA	NA	NA
ENSG00000226209	NA	NA	NA	0	NA	NA	NA	NA	NA
ENSG00000226211	NA	NA	NA	0	NA	NA	NA	NA	NA
ENSG00000226212	NA	NA	NA	0,144330898	1,418786819	4,061262	0,349346267	0,726829368	NA
ENSG00000226213	NA	NA	NA	0	NA	NA	NA	NA	NA
ENSG00000226216	RPS12P5	ribosomal protein S12 pseudogene 5	100271053	0,117497108	0,847101401	4,080456	0,207599697	0,835541541	NA
ENSG00000226217	RPL19P1	ribosomal protein L19 pseudogene 1	140759	0	NA	NA	NA	NA	NA
ENSG00000226220	NA	NA	NA	0	NA	NA	NA	NA	NA
ENSG00000226221	RPL26P19	ribosomal protein L26 pseudogene 19	441073	1,199872273	1,84993769	1,836152	1,00750788	0,313690773	NA
ENSG00000226222	H2BP7	H2B histone pseudogene 7	115482717	0	NA	NA	NA	NA	NA
ENSG00000226226	NA	NA	NA	0	NA	NA	NA	NA	NA
ENSG00000226229	NA	NA	NA	0	NA	NA	NA	NA	NA
ENSG00000226230	NA	NA	NA	0	NA	NA	NA	NA	NA
ENSG00000226233	NA	NA	NA	0	NA	NA	NA	NA	NA
ENSG00000226234	KRT18P24	keratin 18 pseudogene 24	340460	0	NA	NA	NA	NA	NA
ENSG00000226241	LOC100128601	zinc finger protein 770 pseudogene	100128601	0	NA	NA	NA	NA	NA
ENSG00000226242	NA	NA	NA	0	NA	NA	NA	NA	NA
ENSG00000226243	NA	NA	NA	0	NA	NA	NA	NA	NA
ENSG00000226246	KRT18P36	keratin 18 pseudogene 36	442406	0	NA	NA	NA	NA	NA
ENSG00000226247	NA	NA	NA	0	NA	NA	NA	NA	NA
ENSG00000226253	NA	NA	NA	0,176266447	-0,117743254	4,080456	-0,028855417	0,976979903	NA
ENSG00000226254	PTP4A1P3	PTP4A1 pseudogene 3	100861469	0	NA	NA	NA	NA	NA
ENSG00000226255	UBE2V2P1	ubiquitin conjugating enzyme E2 V2 pseudogene 1	100128493	0	NA	NA	NA	NA	NA
ENSG00000226261	NA	NA	NA	0	NA	NA	NA	NA	NA
ENSG00000226262	PHKBP1	phosphorylase kinase regulatory subunit beta pseudogene 1	5258	0	NA	NA	NA	NA	NA
ENSG00000226268	NA	NA	NA	1,256666146	-3,487238802	3,375327	-1,033155766	0,30153101	NA
ENSG00000226270	NA	NA	NA	0	NA	NA	NA	NA	NA
ENSG00000226273	NA	NA	NA	0	NA	NA	NA	NA	NA
ENSG00000226278	PSPHP1	phosphoserine phosphatase pseudogene 1	8781	0	NA	NA	NA	NA	NA
ENSG00000226279	RPL12P10	ribosomal protein L12 pseudogene 10	392872	0	NA	NA	NA	NA	NA
ENSG00000226280	LOC100129144	zinc finger protein 681 pseudogene	100129144	0	NA	NA	NA	NA	NA
ENSG00000226284	NA	NA	NA	0	NA	NA	NA	NA	NA
ENSG00000226285	NA	NA	NA	0	NA	NA	NA	NA	NA
ENSG00000226287	NA	NA	NA	0,755843416	-2,565821831	3,014075	-0,851279892	0,394613892	NA
ENSG00000226288	OR52I2	olfactory receptor family 52 subfamily I member 2	143502	0	NA	NA	NA	NA	NA
ENSG00000226289	CDCA4P3	cell division cycle associated 4 pseudogene 3	100420558	0	NA	NA	NA	NA	NA
ENSG00000226292	RPS3AP10	RPS3A pseudogene 10	401975	0,359021632	2,56113643	3,98322	0,642981427	0,520236152	NA
ENSG00000226297	RPL17P14	ribosomal protein L17 pseudogene 14	100270941	0	NA	NA	NA	NA	NA
ENSG00000226298	RAD23BP3	RAD23B pseudogene 3	54034	0	NA	NA	NA	NA	NA
ENSG00000226299	RPSAP62	ribosomal protein SA pseudogene 62	100131469	0	NA	NA	NA	NA	NA
ENSG00000226301	NA	NA	NA	0	NA	NA	NA	NA	NA
ENSG00000226306	NPY6R	neuropeptide Y receptor Y6 (pseudogene)	4888	0	NA	NA	NA	NA	NA
ENSG00000226307	HNRNPDLP1	HNRNPDL pseudogene 1	100128595	0	NA	NA	NA	NA	NA

ENSG00000226309	SMARCE1P2	SWI/SNF related, matrix associated, actin dependent regulator of chromatin, subfamily e, member 1 pseudogene 2	442233	0	NA	NA	NA	NA	NA
ENSG00000226313	MTCYBP12	MT-CYB pseudogene 12	107075123	0	NA	NA	NA	NA	NA
ENSG00000226314	ZKSCAN8P1	ZKSCAN8 pseudogene 1	651302	3,105136802	0,127773949	1,156449	0,110488174	0,912022228	NA
ENSG00000226318	RPS3AP38	RPS3A pseudogene 38	100271594	4,849481495	-0,154609135	0,859404	-0,179902811	0,857228868	NA
ENSG00000226321	CROCC2	ciliary rootlet coiled-coil, rootletin family member 2	728763	0	NA	NA	NA	NA	NA
ENSG00000226324	NA	NA	NA	0	NA	NA	NA	NA	NA
ENSG00000226327	RPSAP49	ribosomal protein SA pseudogene 49	401537	0	NA	NA	NA	NA	NA
ENSG00000226329	NA	NA	NA	0	NA	NA	NA	NA	NA
ENSG00000226331	RPL23AP28	ribosomal protein L23a pseudogene 28	100271457	0	NA	NA	NA	NA	NA
ENSG00000226336	MRPS16P3	mitochondrial ribosomal protein S16 pseudogene 3	338388	0	NA	NA	NA	NA	NA
ENSG00000226338	NA	NA	NA	0	NA	NA	NA	NA	NA
ENSG00000226339	NA	NA	NA	0	NA	NA	NA	NA	NA
ENSG00000226340	NA	NA	NA	0	NA	NA	NA	NA	NA
ENSG00000226341	MTCYBP39	MT-CYB pseudogene 39	109729152	0	NA	NA	NA	NA	NA
ENSG00000226342	NA	NA	NA	0	NA	NA	NA	NA	NA
ENSG00000226344	CST12P	cystatin 12, pseudogene	106478911	0	NA	NA	NA	NA	NA
ENSG00000226345	ZDHHC1P1	ZDHHC1 pseudogene 1	127566426	0	NA	NA	NA	NA	NA
ENSG00000226348	VN2R10P	vomeronasal 2 receptor 10, pseudogene	100033398	0,119673877	0,847101401	4,080456	0,207599697	0,835541541	NA
ENSG00000226353	NA	NA	NA	0	NA	NA	NA	NA	NA
ENSG00000226356	NA	NA	NA	0	NA	NA	NA	NA	NA
ENSG00000226358	KRT8P38	keratin 8 pseudogene 38	100418799	0	NA	NA	NA	NA	NA
ENSG00000226359	ACTG1P24	actin gamma 1 pseudogene 24	441783	0,70183856	-2,47221498	3,175454	-0,778539134	0,436251246	NA
ENSG00000226360	RPL10AP6	ribosomal protein L10a pseudogene 6	100128936	0,488668768	-1,943966988	2,949718	-0,659034949	0,509873327	NA
ENSG00000226361	NA	NA	NA	0,118286652	0	4,080456	0	1	NA
ENSG00000226366	NA	NA	NA	0	NA	NA	NA	NA	NA
ENSG00000226369	NA	NA	NA	0	NA	NA	NA	NA	NA
ENSG00000226372	DCAF8L1	DDB1 and CUL4 associated factor 8 like 1	139425	0	NA	NA	NA	NA	NA
ENSG00000226388	NA	NA	NA	0	NA	NA	NA	NA	NA
ENSG00000226389	NA	NA	NA	0	NA	NA	NA	NA	NA
ENSG00000226392	MTATP6P21	MT-ATP6 pseudogene 21	107075299	0	NA	NA	NA	NA	NA
ENSG00000226393	IFNA20P	interferon alpha 20, pseudogene	3463	0	NA	NA	NA	NA	NA
ENSG00000226394	NA	NA	NA	0	NA	NA	NA	NA	NA
ENSG00000226395	MRPS21P5	mitochondrial ribosomal protein S21 pseudogene 5	359769	0	NA	NA	NA	NA	NA
ENSG00000226396	NA	NA	NA	3,183592348	0,436871234	1,248981	0,349782023	0,726502292	NA
ENSG00000226400	NA	NA	NA	0	NA	NA	NA	NA	NA
ENSG00000226401	NA	NA	NA	0	NA	NA	NA	NA	NA
ENSG00000226405	NA	NA	NA	0	NA	NA	NA	NA	NA
ENSG00000226406	RBMX2P1	RBMX2 pseudogene 1	100128341	0,224238521	-0,140589142	4,080456	-0,034454273	0,972514905	NA
ENSG00000226407	RPL23P11	ribosomal protein L23 pseudogene 11	100271297	0	NA	NA	NA	NA	NA
ENSG00000226410	NA	NA	NA	0,15836288	0	4,080456	0	1	NA
ENSG00000226411	NA	NA	NA	0	NA	NA	NA	NA	NA
ENSG00000226413	OR8T1P	olfactory receptor family 8 subfamily T member 1 pseudogene	282791	0	NA	NA	NA	NA	NA
ENSG00000226414	MTCYBP38	MT-CYB pseudogene 38	109729117	0	NA	NA	NA	NA	NA
ENSG00000226415	NA	NA	NA	0,222260681	-1,077274767	4,080456	-0,264008435	0,791773419	NA
ENSG00000226420	NA	NA	NA	0	NA	NA	NA	NA	NA
ENSG00000226421	SLC25A5P5	solute carrier family 25 member 5 pseudogene 5	442525	0	NA	NA	NA	NA	NA
ENSG00000226427	NA	NA	NA	0	NA	NA	NA	NA	NA
ENSG00000226428	RPL15P13	ribosomal protein L15 pseudogene 13	100131338	0	NA	NA	NA	NA	NA
ENSG00000226429	NA	NA	NA	0	NA	NA	NA	NA	NA
ENSG00000226430	USP17L7	ubiquitin specific peptidase 17 like family member 7	392197	0	NA	NA	NA	NA	NA
ENSG00000226432	NA	NA	NA	0,103998153	-1,076443944	4,080456	-0,263804824	0,791930317	NA
ENSG00000226436	H3P43	H3 histone pseudogene 43	100506811	0	NA	NA	NA	NA	NA
ENSG00000226439	RPS12P9	ribosomal protein S12 pseudogene 9	727997	0	NA	NA	NA	NA	NA
ENSG00000226443	NA	NA	NA	0	NA	NA	NA	NA	NA
ENSG00000226444	NA	NA	NA	0	NA	NA	NA	NA	NA
ENSG00000226446	NOTCH2P1	notch 2 pseudogene 1	100420260	0	NA	NA	NA	NA	NA
ENSG00000226448	RPL7AP51	ribosomal protein L7a pseudogene 51	100271533	0	NA	NA	NA	NA	NA
ENSG00000226449	NA	NA	NA	0	NA	NA	NA	NA	NA

ENSG00000226450	CYP2D8P	ccytochrome P450 family 2 subfamily D member 8, pseudogene	1568	0	NA	NA	NA	NA	NA
ENSG00000226461	NA	NA	NA	0	NA	NA	NA	NA	NA
ENSG00000226466	NA	NA	NA	0	NA	NA	NA	NA	NA
ENSG00000226468	NA	NA	NA	0	NA	NA	NA	NA	NA
ENSG00000226469	ADAM1B	ADAM metallopeptidase domain 1B (pseudogene)	100420505	1,063411114	-2,559740851	2,493267	-1,026661283	0,304579986	NA
ENSG00000226470	NA	NA	NA	0	NA	NA	NA	NA	NA
ENSG00000226472	NA	NA	NA	0	NA	NA	NA	NA	NA
ENSG00000226473	NA	NA	NA	0	NA	NA	NA	NA	NA
ENSG00000226474	NA	NA	NA	0	NA	NA	NA	NA	NA
ENSG00000226477	NA	NA	NA	0	NA	NA	NA	NA	NA
ENSG00000226478	NA	NA	NA	0	NA	NA	NA	NA	NA
ENSG00000226480	OR7H1P	olfactory receptor family 7 subfamily H member 1 pseudogene	390886	0	NA	NA	NA	NA	NA
ENSG00000226481	NA	NA	NA	0	NA	NA	NA	NA	NA
ENSG00000226485	RBM22P3	RNA binding motif protein 22 pseudogene 3	100128867	0	NA	NA	NA	NA	NA
ENSG00000226489	RPL36AP17	ribosomal protein L36a pseudogene 17	100271326	0	NA	NA	NA	NA	NA
ENSG00000226491	NA	NA	NA	0	NA	NA	NA	NA	NA
ENSG00000226493	RPL26P27	ribosomal protein L26 pseudogene 27	100271471	0	NA	NA	NA	NA	NA
ENSG00000226498	RPSAP21	ribosomal protein SA pseudogene 21	729079	1,175915496	0,044445579	2,050402	0,02167652	0,982705994	NA
ENSG00000226499	NA	NA	NA	0	NA	NA	NA	NA	NA
ENSG00000226500	NA	NA	NA	0	NA	NA	NA	NA	NA
ENSG00000226501	USF1P1	upstream transcription factor 1 pseudogene 1	100151645	0,384487281	-1,073581355	3,685563	-0,291293702	0,770826704	NA
ENSG00000226502	NA	NA	NA	0	NA	NA	NA	NA	NA
ENSG00000226504	NA	NA	NA	0	NA	NA	NA	NA	NA
ENSG00000226507	NA	NA	NA	0	NA	NA	NA	NA	NA
ENSG00000226509	NA	NA	NA	0	NA	NA	NA	NA	NA
ENSG00000226515	NA	NA	NA	0	NA	NA	NA	NA	NA
ENSG00000226521	NA	NA	NA	0	NA	NA	NA	NA	NA
ENSG00000226523	NA	NA	NA	0	NA	NA	NA	NA	NA
ENSG00000226525	RPS7P10	ribosomal protein S7 pseudogene 10	100128060	0	NA	NA	NA	NA	NA
ENSG00000226529	MTND1P1	MT-ND1 pseudogene 1	100499468	0	NA	NA	NA	NA	NA
ENSG00000226532	NA	NA	NA	0	NA	NA	NA	NA	NA
ENSG00000226534	SUSD2P1	sushi domain containing 2 pseudogene 1	729568	0	NA	NA	NA	NA	NA
ENSG00000226535	NA	NA	NA	0	NA	NA	NA	NA	NA
ENSG00000226536	NA	NA	NA	0,10543123	0	4,080456	0	1	NA
ENSG00000226537	NA	NA	NA	0	NA	NA	NA	NA	NA
ENSG00000226539	NA	NA	NA	0	NA	NA	NA	NA	NA
ENSG00000226540	NA	NA	NA	0	NA	NA	NA	NA	NA
ENSG00000226541	RPL36P10	ribosomal protein L36 pseudogene 10	100271011	0	NA	NA	NA	NA	NA
ENSG00000226543	NA	NA	NA	0	NA	NA	NA	NA	NA
ENSG00000226544	RPL7P22	ribosomal protein L7 pseudogene 22	728620	0	NA	NA	NA	NA	NA
ENSG00000226545	RPL27P3	ribosomal protein L27 pseudogene	100128662	0	NA	NA	NA	NA	NA
ENSG00000226547	SSU72P1	SSU72 pseudogene 1	286528	0	NA	NA	NA	NA	NA
ENSG00000226549	NA	NA	NA	0	NA	NA	NA	NA	NA
ENSG00000226552	LOC646804	alkylated DNA repair protein alkB homolog 8-like	646804	0	NA	NA	NA	NA	NA
ENSG00000226553	NA	NA	NA	0	NA	NA	NA	NA	NA
ENSG00000226554	NA	NA	NA	0	NA	NA	NA	NA	NA
ENSG00000226555	AGKP1	AGK pseudogene 1	359793	0	NA	NA	NA	NA	NA
ENSG00000226556	NPM1P49	nucleophosmin 1 pseudogene 49	100422284	0,611348814	1,334471766	2,800497	0,476512503	0,633709302	NA
ENSG00000226557	NA	NA	NA	0	NA	NA	NA	NA	NA
ENSG00000226558	NA	NA	NA	0	NA	NA	NA	NA	NA
ENSG00000226564	NA	NA	NA	0,117497108	0,847101401	4,080456	0,207599697	0,835541541	NA
ENSG00000226565	NA	NA	NA	0	NA	NA	NA	NA	NA
ENSG00000226570	NA	NA	NA	0	NA	NA	NA	NA	NA
ENSG00000226573	RPL36P17	ribosomal protein L36 pseudogene 17	100271336	0	NA	NA	NA	NA	NA
ENSG00000226577	NA	NA	NA	0	NA	NA	NA	NA	NA
ENSG00000226579	MTCYBP31	MT-CYB pseudogene 31	107075136	0	NA	NA	NA	NA	NA
ENSG00000226580	NA	NA	NA	0	NA	NA	NA	NA	NA
ENSG00000226582	UBE2V1P5	UBE2V1 pseudogene 5	100287173	0	NA	NA	NA	NA	NA
ENSG00000226590	UBE2V1P10	UBE2V1 pseudogene 10	102724605	0	NA	NA	NA	NA	NA
ENSG00000226592	NA	NA	NA	0	NA	NA	NA	NA	NA
ENSG00000226595	NA	NA	NA	0	NA	NA	NA	NA	NA

ENSG00000226597	IFNWP9	interferon omega 1 pseudogene 9	100130671	0	NA	NA	NA	NA	NA
ENSG00000226608	NA	NA	NA	0,10543123	0	4,080456	0	1	NA
ENSG00000226611	OFD1P2Y	OFD1 pseudogene 2 Y-linked	378010	0	NA	NA	NA	NA	NA
ENSG00000226616	OR52M2P	olfactory receptor family 52 subfamily M member 2 pseudogene	338637	0	NA	NA	NA	NA	NA
ENSG00000226617	RPL21P110	ribosomal protein L21 pseudogene 110	100271438	0	NA	NA	NA	NA	NA
ENSG00000226621	RPL13AP27	ribosomal protein L13a pseudogene 27	127379736	0	NA	NA	NA	NA	NA
ENSG00000226624	RPL13AP16	ribosomal protein L13a pseudogene 16	100129553	0	NA	NA	NA	NA	NA
ENSG00000226625	RBM17P1	RNA binding motif protein 17 pseudogene 1	100133330	0	NA	NA	NA	NA	NA
ENSG00000226626	NOP56P2	NOP56 ribonucleoprotein pseudogene 2	402360	0	NA	NA	NA	NA	NA
ENSG00000226631	SLC9A3P3	solute carrier family 9 member 3 pseudogene 3	728990	0,117497108	0,847101401	4,080456	0,207599697	0,835541541	NA
ENSG00000226632	NA	NA	NA	0,476515779	0,888165057	3,435767	0,25850558	0,796016743	NA
ENSG00000226633	PPIAP28	peptidylprolyl isomerase A pseudogene 28	390378	0	NA	NA	NA	NA	NA
ENSG00000226636	NA	NA	NA	0	NA	NA	NA	NA	NA
ENSG00000226641	NA	NA	NA	0	NA	NA	NA	NA	NA
ENSG00000226642	ACTG1P12	actin gamma 1 pseudogene 12	100418921	0	NA	NA	NA	NA	NA
ENSG00000226646	RPL7P37	ribosomal protein L7 pseudogene 37	439954	0	NA	NA	NA	NA	NA
ENSG00000226650	KIF4B	kinesin family member 4B	285643	0,264031289	0	4,080456	0	1	NA
ENSG00000226652	NA	NA	NA	0	NA	NA	NA	NA	NA
ENSG00000226653	OR13Z1P	olfactory receptor family 13 subfamily Z member 1 pseudogene	403228	0	NA	NA	NA	NA	NA
ENSG00000226658	NA	NA	NA	0	NA	NA	NA	NA	NA
ENSG00000226660	TRBV2	T cell receptor beta variable 2	28620	0,103998153	-1,076443944	4,080456	-0,263804824	0,791930317	NA
ENSG00000226662	CHMP1AP1	charged multivesicular body protein 1A pseudogene 1	646597	0	NA	NA	NA	NA	NA
ENSG00000226665	SSR1P2	signal sequence receptor subunit 1 pseudogene 2	100131189	0	NA	NA	NA	NA	NA
ENSG00000226666	NA	NA	NA	0	NA	NA	NA	NA	NA
ENSG00000226668	NA	NA	NA	0	NA	NA	NA	NA	NA
ENSG00000226670	NA	NA	NA	0	NA	NA	NA	NA	NA
ENSG00000226671	LOC100420647	voltage dependent anion channel 3 pseudogene	100420647	0	NA	NA	NA	NA	NA
ENSG00000226677	NA	NA	NA	0	NA	NA	NA	NA	NA
ENSG00000226683	NA	NA	NA	1,238899877	-1,036015028	2,002511	-0,517357843	0,604906382	NA
ENSG00000226685	CT47A12	cancer/testis antigen family 47 member A12	100507170	0	NA	NA	NA	NA	NA
ENSG00000226690	LOC102725191	uncharacterized LOC102725191	102725191	0	NA	NA	NA	NA	NA
ENSG00000226693	NA	NA	NA	0	NA	NA	NA	NA	NA
ENSG00000226695	NA	NA	NA	0	NA	NA	NA	NA	NA
ENSG00000226700	MTND4P25	MT-ND4 pseudogene 25	100873246	0	NA	NA	NA	NA	NA
ENSG00000226701	NA	NA	NA	0	NA	NA	NA	NA	NA
ENSG00000226703	NA	NA	NA	0	NA	NA	NA	NA	NA
ENSG00000226705	SDCBPP1	syndecan binding protein pseudogene 1	100128930	0	NA	NA	NA	NA	NA
ENSG00000226718	SNRPGP8	small nuclear ribonucleoprotein polypeptide G pseudogene 8	100874409	0	NA	NA	NA	NA	NA
ENSG00000226721	EEF1DP2	eukaryotic translation elongation factor 1 delta pseudogene 2	442429	6,569254468	-0,51224897	0,731903	-0,699886404	0,483998249	NA
ENSG00000226723	NA	NA	NA	0	NA	NA	NA	NA	NA
ENSG00000226724	HRGP2	HRG pseudogene 2	127525789	0	NA	NA	NA	NA	NA
ENSG00000226726	TMEM256P2	transmembrane protein 256 pseudogene 2	106479054	0	NA	NA	NA	NA	NA
ENSG00000226729	RPL35AP30	ribosomal protein L35a pseudogene 30	100271639	0	NA	NA	NA	NA	NA
ENSG00000226734	SNRPGP12	small nuclear ribonucleoprotein polypeptide G pseudogene 12	100874427	0	NA	NA	NA	NA	NA
ENSG00000226742	HSBP1L1	heat shock factor binding protein 1 like 1	440498	0,548929998	-1,052776794	3,381607	-0,311324439	0,75555399	NA
ENSG00000226744	NA	NA	NA	0	NA	NA	NA	NA	NA
ENSG00000226745	NA	NA	NA	0	NA	NA	NA	NA	NA
ENSG00000226750	NA	NA	NA	0	NA	NA	NA	NA	NA

ENSG00000226752	CUTALP	cutA divalent cation tolerance like, pseudogene	253039	0,103998153	-1,076443944	4,080456	-0,263804824	0,791930317	NA
ENSG00000226753	NA	NA	NA	0	NA	NA	NA	NA	NA
ENSG00000226755	NA	NA	NA	0,222868907	-1,090722524	4,080456	-0,267304085	0,789235042	NA
ENSG00000226763	SRRM5	serine/arginine repetitive matrix 5	100170229	2,056514463	-0,213691842	1,434823	-0,148932505	0,881606892	NA
ENSG00000226765	RPSAP33	ribosomal protein SA pseudogene 33	647158	0	NA	NA	NA	NA	NA
ENSG00000226766	NA	NA	NA	0,19011801	1,667512768	4,047446	0,411991337	0,680345773	NA
ENSG00000226767	NA	NA	NA	0	NA	NA	NA	NA	NA
ENSG00000226769	GAPDHP54	glyceraldehyde 3 phosphate dehydrogenase pseudogene 54	100421319	0	NA	NA	NA	NA	NA
ENSG00000226773	NA	NA	NA	0	NA	NA	NA	NA	NA
ENSG00000226774	NA	NA	NA	0	NA	NA	NA	NA	NA
ENSG00000226781	NA	NA	NA	0	NA	NA	NA	NA	NA
ENSG00000226783	NA	NA	NA	0	NA	NA	NA	NA	NA
ENSG00000226784	PGAM4	phosphoglycerate mutase family member 4	441531	0,10543123	0	4,080456	0	1	NA
ENSG00000226789	NA	NA	NA	0,072165449	0,847101401	4,080456	0,207599697	0,835541541	NA
ENSG00000226790	NA	NA	NA	0	NA	NA	NA	NA	NA
ENSG00000226792	C13orf42	chromosome 13 open reading frame 42	647166	0	NA	NA	NA	NA	NA
ENSG00000226794	MTND1P20	MT-ND1 pseudogene 20	100873339	0	NA	NA	NA	NA	NA
ENSG00000226801	NA	NA	NA	0,10488184	-1,076443944	4,080456	-0,263804824	0,791930317	NA
ENSG00000226802	RUVBL2P1	RUVBL2 pseudogene 1	392538	0	NA	NA	NA	NA	NA
ENSG00000226804	NA	NA	NA	0	NA	NA	NA	NA	NA
ENSG00000226807	MROH5	maestro heat like repeat family member 5 (gene/pseudogene)	389690	0	NA	NA	NA	NA	NA
ENSG00000226810	NA	NA	NA	0	NA	NA	NA	NA	NA
ENSG00000226814	LOC100130137	integrin subunit beta 1 binding protein 1 pseudogene	100130137	0	NA	NA	NA	NA	NA
ENSG00000226818	NA	NA	NA	0	NA	NA	NA	NA	NA
ENSG00000226820	NA	NA	NA	0	NA	NA	NA	NA	NA
ENSG00000226823	NA	NA	NA	0	NA	NA	NA	NA	NA
ENSG00000226827	NA	NA	NA	0	NA	NA	NA	NA	NA
ENSG00000226831	MED15P3	mediator complex subunit 15 pseudogene 3	106481657	0	NA	NA	NA	NA	NA
ENSG00000226836	RPL32P19	ribosomal protein L32 pseudogene 19	646433	0	NA	NA	NA	NA	NA
ENSG00000226837	HMGB1P32	high mobility group box 1 pseudogene 32	100419995	0	NA	NA	NA	NA	NA
ENSG00000226838	NA	NA	NA	0,473029194	-0,115587518	3,036191	-0,038069913	0,96963194	NA
ENSG00000226839	MTND6P11	MT-ND6 pseudogene 11	106480727	0	NA	NA	NA	NA	NA
ENSG00000226840	NA	NA	NA	0	NA	NA	NA	NA	NA
ENSG00000226843	NA	NA	NA	0	NA	NA	NA	NA	NA
ENSG00000226845	EEF1GP3	eukaryotic translation elongation factor 1 gamma pseudogene 3	651628	0	NA	NA	NA	NA	NA
ENSG00000226847	PRPS1P1	phosphoribosyl pyrophosphate synthetase 1 pseudogene 1	100861436	0	NA	NA	NA	NA	NA
ENSG00000226855	RPSAP17	ribosomal protein SA pseudogene 17	100270873	0	NA	NA	NA	NA	NA
ENSG00000226857	DUTP3	deoxyuridine triphosphatase pseudogene 3	100861485	0	NA	NA	NA	NA	NA
ENSG00000226860	NA	NA	NA	0	NA	NA	NA	NA	NA
ENSG00000226863	NA	NA	NA	0	NA	NA	NA	NA	NA
ENSG00000226864	ATE1-AS1	ATE1 antisense RNA 1	100130887	0	NA	NA	NA	NA	NA
ENSG00000226870	NA	NA	NA	0	NA	NA	NA	NA	NA
ENSG00000226874	TRPC6P10	TRPC6 pseudogene 10	123464518	0,118286652	0	4,080456	0	1	NA
ENSG00000226875	NA	NA	NA	0	NA	NA	NA	NA	NA
ENSG00000226878	NA	NA	NA	0	NA	NA	NA	NA	NA
ENSG00000226881	NA	NA	NA	0	NA	NA	NA	NA	NA
ENSG00000226884	RPS29P10	ribosomal protein S29 pseudogene 10	100132037	0	NA	NA	NA	NA	NA
ENSG00000226885	NA	NA	NA	0	NA	NA	NA	NA	NA
ENSG00000226886	NA	NA	NA	0	NA	NA	NA	NA	NA
ENSG00000226887	ERVMER34-1	endogenous retrovirus group MER34 member 1, envelope	100288413	0	NA	NA	NA	NA	NA
ENSG00000226888	RPLP1P10	ribosomal protein lateral stalk subunit P1 pseudogene 10	100271346	0	NA	NA	NA	NA	NA
ENSG00000226902	CHCHD2P1	coiled-coil-helix-coiled-coil-helix domain containing 2 pseudogene 1	100874506	0	NA	NA	NA	NA	NA
ENSG00000226905	NA	NA	NA	0	NA	NA	NA	NA	NA
ENSG00000226908	H2BP6	H2B histone pseudogene 6	100820735	0,23616048	-2,075648354	4,028973	-0,515180498	0,606426895	NA

ENSG00000226912	NA	NA	NA	0,117497108	0,847101401	4,080456	0,207599697	0,835541541	NA
ENSG00000226918	RBMY2SP	RNA binding motif protein Y-linked family 2 member S, pseudogene	379007	0	NA	NA	NA	NA	NA
ENSG00000226926	PDZPH1P	PDZ and pleckstrin homology domains 1, pseudogene	105379197	0	NA	NA	NA	NA	NA
ENSG00000226928	NA	NA	NA	0,735853493	3,054678834	2,537489	1,203819457	0,228659368	NA
ENSG00000226929	CT47A11	cancer/testis antigen family 47 member A11	255313	0	NA	NA	NA	NA	NA
ENSG00000226930	NA	NA	NA	0	NA	NA	NA	NA	NA
ENSG00000226933	NA	NA	NA	0,64458563	0,266112665	2,370198	0,112274431	0,910605815	NA
ENSG00000226937	NA	NA	NA	0	NA	NA	NA	NA	NA
ENSG00000226941	RBMY1J	RNA binding motif protein Y-linked family 1 member J	378951	0	NA	NA	NA	NA	NA
ENSG00000226943	ALG1L5P	ALG1 like 5, pseudogene	647415	0	NA	NA	NA	NA	NA
ENSG00000226945	NA	NA	NA	0	NA	NA	NA	NA	NA
ENSG00000226946	LOC100132762	chromosome 17 open reading frame 80 pseudogene	100132762	0	NA	NA	NA	NA	NA
ENSG00000226947	LCEP4	late cornified envelope pseudogene 4	450210	0	NA	NA	NA	NA	NA
ENSG00000226948	RPS4XP2	ribosomal protein S4X pseudogene 2	92507	0,209472022	-1,069496306	4,080456	-0,262102162	0,79324268	NA
ENSG00000226949	NA	NA	NA	0	NA	NA	NA	NA	NA
ENSG00000226952	NA	NA	NA	0,117497108	0,847101401	4,080456	0,207599697	0,835541541	NA
ENSG00000226955	POLE4P1	POLE4 pseudogene 1	127379694	0	NA	NA	NA	NA	NA
ENSG00000226960	MTCO1P21	MT-CO1 pseudogene 21	107075164	0	NA	NA	NA	NA	NA
ENSG00000226961	NA	NA	NA	0	NA	NA	NA	NA	NA
ENSG00000226962	NA	NA	NA	0	NA	NA	NA	NA	NA
ENSG00000226964	NA	NA	NA	0	NA	NA	NA	NA	NA
ENSG00000226967	NA	NA	NA	0	NA	NA	NA	NA	NA
ENSG00000226970	NA	NA	NA	0,10488184	-1,076443944	4,080456	-0,263804824	0,791930317	NA
ENSG00000226971	NA	NA	NA	0	NA	NA	NA	NA	NA
ENSG00000226972	NA	NA	NA	0	NA	NA	NA	NA	NA
ENSG00000226973	NA	NA	NA	0	NA	NA	NA	NA	NA
ENSG00000226976	NA	NA	NA	0	NA	NA	NA	NA	NA
ENSG00000226977	NA	NA	NA	0	NA	NA	NA	NA	NA
ENSG00000226981	NA	NA	NA	0,961921543	-1,627560409	2,129959	-0,764127655	0,444791173	NA
ENSG00000226982	CENPCP1	centromere protein C pseudogene 1	1061	0,11808024	-1,076443944	4,080456	-0,263804824	0,791930317	NA
ENSG00000226984	RPL26P10	ribosomal protein L26 pseudogene 10	100270886	0	NA	NA	NA	NA	NA
ENSG00000226986	NA	NA	NA	0	NA	NA	NA	NA	NA
ENSG00000226987	NA	NA	NA	0,103998153	-1,076443944	4,080456	-0,263804824	0,791930317	NA
ENSG00000226989	NA	NA	NA	0,944212806	-0,805740409	2,152322	-0,374358743	0,708137434	NA
ENSG00000226991	NA	NA	NA	0	NA	NA	NA	NA	NA
ENSG00000226998	MTATP6P30	MT-ATP6 pseudogene 30	107075302	0	NA	NA	NA	NA	NA
ENSG00000226999	NA	NA	NA	0	NA	NA	NA	NA	NA
ENSG00000227000	HSPD1P14	heat shock protein family D (Hsp60) member 1 pseudogene 14	100287140	0	NA	NA	NA	NA	NA
ENSG00000227001	NA	NA	NA	0,572782297	1,561044312	2,764461	0,564682968	0,572289421	NA
ENSG00000227002	NA	NA	NA	0	NA	NA	NA	NA	NA
ENSG00000227004	NA	NA	NA	0	NA	NA	NA	NA	NA
ENSG00000227008	NA	NA	NA	0	NA	NA	NA	NA	NA
ENSG00000227009	NA	NA	NA	2,594437284	-0,915497851	1,330103	-0,688290922	0,491269597	NA
ENSG00000227013	OR7E96P	olfactory receptor family 7 subfamily E member 96 pseudogene	401450	0	NA	NA	NA	NA	NA
ENSG00000227015	NA	NA	NA	0	NA	NA	NA	NA	NA
ENSG00000227018	NA	NA	NA	0	NA	NA	NA	NA	NA
ENSG00000227019	OR7E101P	olfactory receptor family 7 subfamily E member 101 pseudogene	81138	0	NA	NA	NA	NA	NA
ENSG00000227023	OR51A3P	olfactory receptor family 51 subfamily A member 3 pseudogene	79532	0	NA	NA	NA	NA	NA
ENSG00000227032	RPS2P36	ribosomal protein S2 pseudogene 36	100271243	8,325268401	0,806806281	0,789088	1,022453488	0,306566316	NA
ENSG00000227034	NA	NA	NA	0	NA	NA	NA	NA	NA
ENSG00000227035	MTATP6P18	MT-ATP6 pseudogene 18	107075159	0	NA	NA	NA	NA	NA
ENSG00000227038	NA	NA	NA	0	NA	NA	NA	NA	NA
ENSG00000227040	LOC285453	TXNDC9 pseudogene	285453	0	NA	NA	NA	NA	NA
ENSG00000227048	LOC124904582	peptidyl-prolyl cis-trans isomerase NIMA-interacting 4-like	124904582	0	NA	NA	NA	NA	NA
ENSG00000227051	C14orf132	chromosome 14 open reading frame 132	56967	0	NA	NA	NA	NA	NA
ENSG00000227054	NA	NA	NA	0	NA	NA	NA	NA	NA
ENSG00000227055	NA	NA	NA	0	NA	NA	NA	NA	NA

ENSG00000227056	RPL6P2	ribosomal protein L6 pseudogene 2	343495	0	NA	NA	NA	NA	NA
ENSG00000227058	LOC107985640	X antigen family member 5-like	107985640	0	NA	NA	NA	NA	NA
ENSG00000227059	ANHX	anomalous homeobox	647589	0	NA	NA	NA	NA	NA
ENSG00000227062	NA	NA	NA	0	NA	NA	NA	NA	NA
ENSG00000227063	NA	NA	NA	0	NA	NA	NA	NA	NA
ENSG00000227064	NA	NA	NA	0	NA	NA	NA	NA	NA
ENSG00000227067	NA	NA	NA	0	NA	NA	NA	NA	NA
ENSG00000227069	NA	NA	NA	0	NA	NA	NA	NA	NA
ENSG00000227072	NA	NA	NA	0	NA	NA	NA	NA	NA
ENSG00000227073	NA	NA	NA	0	NA	NA	NA	NA	NA
ENSG00000227077	NA	NA	NA	0	NA	NA	NA	NA	NA
ENSG00000227080	NA	NA	NA	0	NA	NA	NA	NA	NA
ENSG00000227081	NA	NA	NA	0	NA	NA	NA	NA	NA
ENSG00000227087	NA	NA	NA	0	NA	NA	NA	NA	NA
ENSG00000227096	NA	NA	NA	0	NA	NA	NA	NA	NA
ENSG00000227104	PIGQP1	phosphatidylinositol glycan anchor biosynthesis class Q pseudogene 1	106480812	0	NA	NA	NA	NA	NA
ENSG00000227105	PARP1P1	poly(ADP-ribose) polymerase 1 pseudogene 1	144	0	NA	NA	NA	NA	NA
ENSG00000227109	CRIP1P3	cysteine rich protein 1 pseudogene 3	106479040	0	NA	NA	NA	NA	NA
ENSG00000227113	NA	NA	NA	0,072165449	0,847101401	4,080456	0,207599697	0,835541541	NA
ENSG00000227118	NA	NA	NA	0	NA	NA	NA	NA	NA
ENSG00000227123	NA	NA	NA	0	NA	NA	NA	NA	NA
ENSG00000227133	NA	NA	NA	0	NA	NA	NA	NA	NA
ENSG00000227134	OTX2P2	OTX2 pseudogene 2	100419816	0	NA	NA	NA	NA	NA
ENSG00000227138	NA	NA	NA	0	NA	NA	NA	NA	NA
ENSG00000227140	USP17L5	ubiquitin specific peptidase 17 like family member 5	728386	0	NA	NA	NA	NA	NA
ENSG00000227141	NA	NA	NA	0	NA	NA	NA	NA	NA
ENSG00000227149	MTCO1P44	MT-CO1 pseudogene 44	107075226	0	NA	NA	NA	NA	NA
ENSG00000227154	NA	NA	NA	0,11808024	-1,076443944	4,080456	-0,263804824	0,791930317	NA
ENSG00000227158	LOC644909	ligand of numb-protein X 1, E3 ubiquitin protein ligase pseudogene	644909	0	NA	NA	NA	NA	NA
ENSG00000227160	NA	NA	NA	0	NA	NA	NA	NA	NA
ENSG00000227163	NA	NA	NA	0	NA	NA	NA	NA	NA
ENSG00000227166	STSP1	steroid sulfatase (microsomal) pseudogene 1	6803	0	NA	NA	NA	NA	NA
ENSG00000227173	NA	NA	NA	0	NA	NA	NA	NA	NA
ENSG00000227176	NA	NA	NA	0	NA	NA	NA	NA	NA
ENSG00000227177	NA	NA	NA	0	NA	NA	NA	NA	NA
ENSG00000227179	NA	NA	NA	0	NA	NA	NA	NA	NA
ENSG00000227180	NA	NA	NA	0	NA	NA	NA	NA	NA
ENSG00000227182	VN1R28P	vomeronasal 1 receptor 28 pseudogene	100312785	0	NA	NA	NA	NA	NA
ENSG00000227183	NA	NA	NA	0	NA	NA	NA	NA	NA
ENSG00000227187	RPL21P31	ribosomal protein L21 pseudogene 31	100271160	0	NA	NA	NA	NA	NA
ENSG00000227201	NA	NA	NA	0	NA	NA	NA	NA	NA
ENSG00000227204	RBMY2JP	RNA binding motif protein Y-linked family 2 member J, pseudogene	378955	0	NA	NA	NA	NA	NA
ENSG00000227205	NA	NA	NA	0	NA	NA	NA	NA	NA
ENSG00000227209	WARS2P1	tryptophanyl tRNA synthetase 2 pseudogene 1	100421633	0	NA	NA	NA	NA	NA
ENSG00000227211	H3P45	H3 histone pseudogene 45	115482705	0	NA	NA	NA	NA	NA
ENSG00000227212	NA	NA	NA	0,15836288	0	4,080456	0	1	NA
ENSG00000227224	NA	NA	NA	0	NA	NA	NA	NA	NA
ENSG00000227225	MTND1P14	MT-ND1 pseudogene 14	100873337	0	NA	NA	NA	NA	NA
ENSG00000227233	NA	NA	NA	0	NA	NA	NA	NA	NA
ENSG00000227234	SPANXB1	SPANX family member B1	728695	0	NA	NA	NA	NA	NA
ENSG00000227236	NA	NA	NA	0	NA	NA	NA	NA	NA
ENSG00000227238	TTC39DP	tetratricopeptide repeat domain 39D, pseudogene	646640	0	NA	NA	NA	NA	NA
ENSG00000227241	NA	NA	NA	0	NA	NA	NA	NA	NA
ENSG00000227242	NA	NA	NA	0	NA	NA	NA	NA	NA
ENSG00000227243	NA	NA	NA	0	NA	NA	NA	NA	NA
ENSG00000227247	NA	NA	NA	0	NA	NA	NA	NA	NA
ENSG00000227249	LOC100422325	poly(A) binding protein cytoplasmic 4 pseudogene	100422325	0	NA	NA	NA	NA	NA
ENSG00000227251	TRIM60P9Y	tripartite motif containing 60 pseudogene 9, Y-linked	100420374	0	NA	NA	NA	NA	NA
ENSG00000227254	NA	NA	NA	0	NA	NA	NA	NA	NA
ENSG00000227255	NA	NA	NA	0	NA	NA	NA	NA	NA
ENSG00000227257	NA	NA	NA	0	NA	NA	NA	NA	NA
ENSG00000227259	NA	NA	NA	0	NA	NA	NA	NA	NA

ENSG00000227261	NA	NA	NA	0	NA	NA	NA	NA	NA
ENSG00000227262	NA	NA	NA	0,15836288	0	4,080456	0	1	NA
ENSG00000227264	NA	NA	NA	0	NA	NA	NA	NA	NA
ENSG00000227265	GXYLT1P7	GXYLT1 pseudogene 7	100130488	0	NA	NA	NA	NA	NA
ENSG00000227267	DONSONP1	DONSON pseudogene 1	100420050	0	NA	NA	NA	NA	NA
ENSG00000227268	KLLN	killin, p53 regulated DNA replication inhibitor	100144748	3,333782541	-0,160371056	0,934269	-0,171653968	0,863709577	NA
ENSG00000227270	NA	NA	NA	0	NA	NA	NA	NA	NA
ENSG00000227271	NA	NA	NA	0	NA	NA	NA	NA	NA
ENSG00000227275	MTND6P10	MT-ND6 pseudogene 10	106478947	0	NA	NA	NA	NA	NA
ENSG00000227283	RPL35AP12	ribosomal protein L35a pseudogene 12	100271316	0	NA	NA	NA	NA	NA
ENSG00000227284	MARK2P10	MARK2 pseudogene 10	402679	0	NA	NA	NA	NA	NA
ENSG00000227288	NA	NA	NA	0,191440369	1,676415401	4,046312	0,414307051	0,67864925	NA
ENSG00000227289	HSFY3P	heat shock transcription factor Y-linked 3, pseudogene	442479	0	NA	NA	NA	NA	NA
ENSG00000227291	NA	NA	NA	0	NA	NA	NA	NA	NA
ENSG00000227295	NA	NA	NA	0	NA	NA	NA	NA	NA
ENSG00000227298	NA	NA	NA	0	NA	NA	NA	NA	NA
ENSG00000227300	NA	NA	NA	0	NA	NA	NA	NA	NA
ENSG00000227302	NA	NA	NA	0	NA	NA	NA	NA	NA
ENSG00000227304	NA	NA	NA	0,072165449	0,847101401	4,080456	0,207599697	0,835541541	NA
ENSG00000227309	RPL31P19	ribosomal protein L31 pseudogene 19	100270985	0	NA	NA	NA	NA	NA
ENSG00000227311	RPL21P20	ribosomal protein L21 pseudogene 20	388621	0,072165449	0,847101401	4,080456	0,207599697	0,835541541	NA
ENSG00000227312	RPL26P8	ribosomal protein L26 pseudogene 8	100270884	0	NA	NA	NA	NA	NA
ENSG00000227321	MTND4P15	MT-ND4 pseudogene 15	100873240	0	NA	NA	NA	NA	NA
ENSG00000227329	NA	NA	NA	0	NA	NA	NA	NA	NA
ENSG00000227331	RPL7AP22	ribosomal protein L7a pseudogene 22	100271039	0,820441366	-2,534358588	2,706294	-0,936468467	0,349032041	NA
ENSG00000227339	THRAP3P1	THRAP3 pseudogene 1	391524	0	NA	NA	NA	NA	NA
ENSG00000227343	RPL15P1	ribosomal protein L15 pseudogene 1	128756	0	NA	NA	NA	NA	NA
ENSG00000227344	HAUS6P1	HAUS augmin like complex subunit 6 pseudogene 1	653175	0,381896367	0	3,63283	0	1	NA
ENSG00000227347	HNRNPKP2	heterogeneous nuclear ribonucleoprotein K pseudogene 2	389053	0	NA	NA	NA	NA	NA
ENSG00000227348	MTND5P25	MT-ND5 pseudogene 25	100873263	0	NA	NA	NA	NA	NA
ENSG00000227349	CEACAMP7	CEA cell adhesion molecule pseudogene 7	1095	0	NA	NA	NA	NA	NA
ENSG00000227350	PPIAP88	peptidylprolyl isomerase A pseudogene 88	111082971	0	NA	NA	NA	NA	NA
ENSG00000227351	NANOGP6	Nanog homeobox pseudogene 6	414134	0	NA	NA	NA	NA	NA
ENSG00000227353	NA	NA	NA	0	NA	NA	NA	NA	NA
ENSG00000227358	NA	NA	NA	0	NA	NA	NA	NA	NA
ENSG00000227361	NA	NA	NA	0	NA	NA	NA	NA	NA
ENSG00000227368	NA	NA	NA	0	NA	NA	NA	NA	NA
ENSG00000227370	NA	NA	NA	0,209763681	-1,926644696	4,042043	-0,476651224	0,633610501	NA
ENSG00000227371	NA	NA	NA	0	NA	NA	NA	NA	NA
ENSG00000227376	NA	NA	NA	0,996521807	2,90621851	2,265475	1,282829684	0,199551752	NA
ENSG00000227379	NA	NA	NA	0,889007394	0,055265116	1,972094	0,028023566	0,977643356	NA
ENSG00000227382	NA	NA	NA	0,958838643	-0,023705591	1,9996	-0,011855166	0,990541168	NA
ENSG00000227383	NA	NA	NA	0,554672913	-1,606012584	2,810813	-0,571369333	0,567749313	NA
ENSG00000227391	NA	NA	NA	0	NA	NA	NA	NA	NA
ENSG00000227393	NA	NA	NA	0	NA	NA	NA	NA	NA
ENSG00000227394	RPL11P1	ribosomal protein L11 pseudogene 1	100133170	0,786975917	3,107119513	2,255234	1,377736803	0,168284564	NA
ENSG00000227395	EIF2AP4	eukaryotic translation initiation factor 2A pseudogene 4	100874401	0	NA	NA	NA	NA	NA
ENSG00000227397	NA	NA	NA	0	NA	NA	NA	NA	NA
ENSG00000227399	RPL21P82	ribosomal protein L21 pseudogene 82	650059	0	NA	NA	NA	NA	NA
ENSG00000227401	RPL37P1	ribosomal protein L37 pseudogene 1	140696	0	NA	NA	NA	NA	NA
ENSG00000227404	KRT8P20	keratin 8 pseudogene 20	441218	0	NA	NA	NA	NA	NA
ENSG00000227406	NA	NA	NA	0	NA	NA	NA	NA	NA
ENSG00000227408	AMYP1	amylase alpha pseudogene 1	281	0	NA	NA	NA	NA	NA
ENSG00000227411	ACNATP	acyl-CoA:amino acid N-acyltransferase, pseudogene	347275	0	NA	NA	NA	NA	NA
ENSG00000227412	STK33P1	serine/threonine kinase 33 pseudogene 1	553118	0	NA	NA	NA	NA	NA
ENSG00000227413	NA	NA	NA	0	NA	NA	NA	NA	NA
ENSG00000227416	NA	NA	NA	0	NA	NA	NA	NA	NA
ENSG00000227417	NA	NA	NA	0	NA	NA	NA	NA	NA

ENSG00000227423	OR10U1P	olfactory receptor family 10 subfamily U member 1 pseudogene	390318	0	NA	NA	NA	NA	NA
ENSG00000227425	NA	NA	NA	0	NA	NA	NA	NA	NA
ENSG00000227426	VN1R33P	vomeronasal 1 receptor 33 pseudogene	100288022	0	NA	NA	NA	NA	NA
ENSG00000227427	NA	NA	NA	0	NA	NA	NA	NA	NA
ENSG00000227430	H3P9	H3 histone pseudogene 9	115482684	0	NA	NA	NA	NA	NA
ENSG00000227433	RPL31P22	ribosomal protein L31 pseudogene 22	100270989	0	NA	NA	NA	NA	NA
ENSG00000227434	NA	NA	NA	0	NA	NA	NA	NA	NA
ENSG00000227436	NA	NA	NA	0,609020567	-1,037379876	2,540513	-0,408334803	0,683027892	NA
ENSG00000227437	RPS8P4	ribosomal protein S8 pseudogene 4	646119	0	NA	NA	NA	NA	NA
ENSG00000227440	NA	NA	NA	0	NA	NA	NA	NA	NA
ENSG00000227443	H3P30	H3 histone pseudogene 30	115482700	0	NA	NA	NA	NA	NA
ENSG00000227444	NA	NA	NA	0	NA	NA	NA	NA	NA
ENSG00000227445	OR10R1P	olfactory receptor family 10 subfamily R member 1 pseudogene	79513	0	NA	NA	NA	NA	NA
ENSG00000227447	NA	NA	NA	0	NA	NA	NA	NA	NA
ENSG00000227449	NA	NA	NA	0,528560674	0,455514163	2,956516	0,154071248	0,877553559	NA
ENSG00000227453	NA	NA	NA	0	NA	NA	NA	NA	NA
ENSG00000227454	NA	NA	NA	0,119673877	0,847101401	4,080456	0,207599697	0,835541541	NA
ENSG00000227462	PSME2P6	proteasome activator subunit 2 pseudogene 6	338098	0	NA	NA	NA	NA	NA
ENSG00000227465	NA	NA	NA	0	NA	NA	NA	NA	NA
ENSG00000227470	RPL39P16	ribosomal protein L39 pseudogene 16	100271023	0	NA	NA	NA	NA	NA
ENSG00000227471	AKR1B15	aldo-keto reductase family 1 member B15	441282	0	NA	NA	NA	NA	NA
ENSG00000227473	TSSK5P	testis specific serine kinase 5, pseudogene	648630	0	NA	NA	NA	NA	NA
ENSG00000227474	RPL6P24	ribosomal protein L6 pseudogene 24	100271034	0	NA	NA	NA	NA	NA
ENSG00000227491	NA	NA	NA	0	NA	NA	NA	NA	NA
ENSG00000227493	NA	NA	NA	0	NA	NA	NA	NA	NA
ENSG00000227494	USP9YP14	USP9Y pseudogene 14	107133502	0	NA	NA	NA	NA	NA
ENSG00000227497	NA	NA	NA	0	NA	NA	NA	NA	NA
ENSG00000227499	NA	NA	NA	0	NA	NA	NA	NA	NA
ENSG00000227505	NA	NA	NA	0	NA	NA	NA	NA	NA
ENSG00000227511	NA	NA	NA	0	NA	NA	NA	NA	NA
ENSG00000227513	NA	NA	NA	0	NA	NA	NA	NA	NA
ENSG00000227514	NA	NA	NA	0	NA	NA	NA	NA	NA
ENSG00000227515	NA	NA	NA	0	NA	NA	NA	NA	NA
ENSG00000227521	TUBB4BP8	TUBB4B pseudogene 8	643371	0	NA	NA	NA	NA	NA
ENSG00000227523	RPS20P15	ribosomal protein S20 pseudogene 15	100271085	0	NA	NA	NA	NA	NA
ENSG00000227525	RPL7P6	ribosomal protein L7 pseudogene 6	90193	0,237163883	1,955807575	4,022315	0,48623925	0,626797546	NA
ENSG00000227532	NA	NA	NA	0	NA	NA	NA	NA	NA
ENSG00000227534	AP1M2P1	AP1M2 pseudogene 1	645388	0	NA	NA	NA	NA	NA
ENSG00000227536	SOCS5P4	suppressor of cytokine signaling 5 pseudogene 4	100132415	1,86670874	-0,647585849	1,321383	-0,490082009	0,624075868	NA
ENSG00000227537	NA	NA	NA	0	NA	NA	NA	NA	NA
ENSG00000227538	NA	NA	NA	0	NA	NA	NA	NA	NA
ENSG00000227541	NA	NA	NA	0,117497108	0,847101401	4,080456	0,207599697	0,835541541	NA
ENSG00000227545	NA	NA	NA	0	NA	NA	NA	NA	NA
ENSG00000227547	OR4A2P	olfactory receptor family 4 subfamily A member 2 pseudogene	390133	0	NA	NA	NA	NA	NA
ENSG00000227550	TRBV7-5	T cell receptor beta variable 7-5 (pseudogene)	28593	0	NA	NA	NA	NA	NA
ENSG00000227551	USP17L12	ubiquitin specific peptidase 17 like family member 12	100287205	0	NA	NA	NA	NA	NA
ENSG00000227556	NA	NA	NA	0	NA	NA	NA	NA	NA
ENSG00000227557	MTND3P9	MT-ND3 pseudogene 9	100873184	0	NA	NA	NA	NA	NA
ENSG00000227560	RPS15AP30	ribosomal protein S15a pseudogene 30	100271607	0	NA	NA	NA	NA	NA
ENSG00000227563	NA	NA	NA	0	NA	NA	NA	NA	NA
ENSG00000227568	NA	NA	NA	0	NA	NA	NA	NA	NA
ENSG00000227573	NA	NA	NA	0	NA	NA	NA	NA	NA
ENSG00000227578	RPS3AP53	RPS3A pseudogene 53	100287512	0	NA	NA	NA	NA	NA
ENSG00000227581	NA	NA	NA	0	NA	NA	NA	NA	NA
ENSG00000227582	ADGRF5P1	adhesion G protein-coupled receptor F5 pseudogene 1	389740	0	NA	NA	NA	NA	NA
ENSG00000227583	RPS3AP37	RPS3A pseudogene 37	100271593	0,10543123	0	4,080456	0	1	NA
ENSG00000227584	MTND4P5	MT-ND4 pseudogene 5	100873191	0	NA	NA	NA	NA	NA
ENSG00000227585	NA	NA	NA	0	NA	NA	NA	NA	NA
ENSG00000227586	NA	NA	NA	0	NA	NA	NA	NA	NA

ENSG00000227590	NA	NA	NA	0	NA	NA	NA	NA	NA
ENSG00000227592	PIGFP3	phosphatidylinositol glycan anchor biosynthesis class F pseudogene 3	100129049	0	NA	NA	NA	NA	NA
ENSG00000227593	NA	NA	NA	0,10543123	0	4,080456	0	1	NA
ENSG00000227597	WASF1P1	WASF1 pseudogene 1	100131624	0	NA	NA	NA	NA	NA
ENSG00000227604	NA	NA	NA	0	NA	NA	NA	NA	NA
ENSG00000227607	NA	NA	NA	0	NA	NA	NA	NA	NA
ENSG00000227609	TMEM183AP1	TMEM183A pseudogene 1	100048909	0	NA	NA	NA	NA	NA
ENSG00000227613	QRSL1P2	QRSL1 pseudogene 2	100422330	0	NA	NA	NA	NA	NA
ENSG00000227615	NA	NA	NA	0,399789032	-1,012321676	3,070415	-0,329701956	0,741625176	NA
ENSG00000227616	NA	NA	NA	0	NA	NA	NA	NA	NA
ENSG00000227620	ALG1L8P	ALG1 like 8, pseudogene	645015	0	NA	NA	NA	NA	NA
ENSG00000227621	NA	NA	NA	0	NA	NA	NA	NA	NA
ENSG00000227623	NA	NA	NA	0	NA	NA	NA	NA	NA
ENSG00000227624	NA	NA	NA	0	NA	NA	NA	NA	NA
ENSG00000227625	NA	NA	NA	0	NA	NA	NA	NA	NA
ENSG00000227629	NA	NA	NA	0	NA	NA	NA	NA	NA
ENSG00000227633	RBMY2YP	RNA binding motif protein Y-linked family 2 member Y, pseudogene	379024	0	NA	NA	NA	NA	NA
ENSG00000227635	USP9YP21	USP9Y pseudogene 21	106480355	0	NA	NA	NA	NA	NA
ENSG00000227638	NA	NA	NA	0	NA	NA	NA	NA	NA
ENSG00000227644	NA	NA	NA	0	NA	NA	NA	NA	NA
ENSG00000227645	RPL7P54	ribosomal protein L7 pseudogene 54	100271146	0	NA	NA	NA	NA	NA
ENSG00000227649	MTND6P32	MT-ND6 pseudogene 32	107075200	0	NA	NA	NA	NA	NA
ENSG00000227653	NA	NA	NA	0	NA	NA	NA	NA	NA
ENSG00000227657	SERPINA7P1	serpin family A member 7 pseudogene 1	100422644	0	NA	NA	NA	NA	NA
ENSG00000227661	MTCO3P18	MT-CO3 pseudogene 18	107075215	0	NA	NA	NA	NA	NA
ENSG00000227663	NA	NA	NA	0	NA	NA	NA	NA	NA
ENSG00000227666	NA	NA	NA	0	NA	NA	NA	NA	NA
ENSG00000227667	LOC107985222	cytochrome c oxidase subunit 7B, mitochondrial-like	107985222	0	NA	NA	NA	NA	NA
ENSG00000227679	NA	NA	NA	0	NA	NA	NA	NA	NA
ENSG00000227682	ATP5F1AP2	ATP synthase F1 subunit alpha pseudogene 2	504	0	NA	NA	NA	NA	NA
ENSG00000227684	CROCCP4	CROCC pseudogene 4	100421113	0	NA	NA	NA	NA	NA
ENSG00000227688	HNRNPA3P2	heterogeneous nuclear ribonucleoprotein A3 pseudogene 2	170526	0	NA	NA	NA	NA	NA
ENSG00000227689	SRP68P2	signal recognition particle 68 pseudogene 2	252840	0	NA	NA	NA	NA	NA
ENSG00000227692	MED28P3	mediator complex subunit 28 pseudogene 3	100131051	0	NA	NA	NA	NA	NA
ENSG00000227693	GSTM3P1	glutathione S-transferase mu 3 pseudogene 1	170523	0	NA	NA	NA	NA	NA
ENSG00000227694	RPL23AP74	ribosomal protein L23a pseudogene 74	729617	0,340961414	0,972345629	3,766252	0,258173283	0,796273176	NA
ENSG00000227700	NA	NA	NA	0	NA	NA	NA	NA	NA
ENSG00000227707	SDAD1P3	SDA1 domain containing 1 pseudogene 3	100873858	0	NA	NA	NA	NA	NA
ENSG00000227709	NA	NA	NA	0	NA	NA	NA	NA	NA
ENSG00000227710	NA	NA	NA	0	NA	NA	NA	NA	NA
ENSG00000227711	NA	NA	NA	0	NA	NA	NA	NA	NA
ENSG00000227714	MTND6P18	MT-ND6 pseudogene 18	106478951	0	NA	NA	NA	NA	NA
ENSG00000227717	GOLGA6L25	golgin A6 family like 25	100132202	0	NA	NA	NA	NA	NA
ENSG00000227721	RPSAP64	ribosomal protein SA pseudogene 64	100873797	0	NA	NA	NA	NA	NA
ENSG00000227722	NA	NA	NA	0	NA	NA	NA	NA	NA
ENSG00000227725	NA	NA	NA	0	NA	NA	NA	NA	NA
ENSG00000227728	NA	NA	NA	0	NA	NA	NA	NA	NA
ENSG00000227729	RD3L	RD3 like	647286	0	NA	NA	NA	NA	NA
ENSG00000227730	MTND6P5	MT-ND6 pseudogene 5	106480427	2,015910179	1,179775667	1,367828	0,862517695	0,3884027	NA
ENSG00000227735	CYCSP5	CYCS pseudogene 5	360158	0	NA	NA	NA	NA	NA
ENSG00000227736	MTCO3P16	MT-CO3 pseudogene 16	107075155	0	NA	NA	NA	NA	NA
ENSG00000227737	NA	NA	NA	0	NA	NA	NA	NA	NA
ENSG00000227738	NA	NA	NA	0	NA	NA	NA	NA	NA
ENSG00000227742	NA	NA	NA	0	NA	NA	NA	NA	NA
ENSG00000227745	NA	NA	NA	0	NA	NA	NA	NA	NA
ENSG00000227747	NA	NA	NA	0	NA	NA	NA	NA	NA
ENSG00000227753	NA	NA	NA	0	NA	NA	NA	NA	NA
ENSG00000227758	NA	NA	NA	0	NA	NA	NA	NA	NA
ENSG00000227759	VTI1BP4	vesicle transport through interaction with t-SNAREs 1B pseudogene 4	100422540	0	NA	NA	NA	NA	NA

ENSG00000227760	HMGN1P23	high mobility group nucleosome binding domain 1 pseudogene 23	100874461	0	NA	NA	NA	NA	NA
ENSG00000227762	NA	NA	NA	0	NA	NA	NA	NA	NA
ENSG00000227765	NA	NA	NA	0,584215301	1,646065576	2,727963	0,60340472	0,546239483	NA
ENSG00000227766	NA	NA	NA	0,799537838	1,40223502	2,691816	0,52092527	0,602418831	NA
ENSG00000227770	RPL7P12	ribosomal protein L7 pseudogene 12	100131085	0	NA	NA	NA	NA	NA
ENSG00000227775	NA	NA	NA	0	NA	NA	NA	NA	NA
ENSG00000227776	NA	NA	NA	0,610495368	-0,118701446	2,868162	-0,041385892	0,96698826	NA
ENSG00000227777	RPL7AP19	ribosomal protein L7a pseudogene 19	100271036	0,53325309	2,327714008	2,483866	0,937133361	0,348689963	NA
ENSG00000227779	NA	NA	NA	0	NA	NA	NA	NA	NA
ENSG00000227784	NA	NA	NA	0	NA	NA	NA	NA	NA
ENSG00000227785	NA	NA	NA	0	NA	NA	NA	NA	NA
ENSG00000227788	MTCO3P43	MT-CO3 pseudogene 43	107075190	0	NA	NA	NA	NA	NA
ENSG00000227789	MTCYBP7	MT-CYB pseudogene 7	107075257	0	NA	NA	NA	NA	NA
ENSG00000227790	NA	NA	NA	0	NA	NA	NA	NA	NA
ENSG00000227791	RPS10P9	ribosomal protein S10 pseudogene 9	730187	0	NA	NA	NA	NA	NA
ENSG00000227795	MTND4LP9	MT-ND4L pseudogene 9	107075237	0	NA	NA	NA	NA	NA
ENSG00000227799	NA	NA	NA	0,295571354	0,713737695	3,895081	0,183240807	0,854609077	NA
ENSG00000227802	DNAJB3	DnaJ heat shock protein family (Hsp40) member B3	414061	0	NA	NA	NA	NA	NA
ENSG00000227805	RPL21P90	ribosomal protein L21 pseudogene 90	100271423	0,222089633	-1,083923011	4,080456	-0,265637724	0,79051823	NA
ENSG00000227806	OR5W1P	olfactory receptor family 5 subfamily W member 1 pseudogene	81182	0	NA	NA	NA	NA	NA
ENSG00000227813	NA	NA	NA	0	NA	NA	NA	NA	NA
ENSG00000227814	NA	NA	NA	0	NA	NA	NA	NA	NA
ENSG00000227815	LOC100129734	zinc finger AN1-type containing 1 pseudogene	100129734	0,072165449	0,847101401	4,080456	0,207599697	0,835541541	NA
ENSG00000227817	NA	NA	NA	0	NA	NA	NA	NA	NA
ENSG00000227825	SLC9A7P1	solute carrier family 9 member 7 pseudogene 1	121456	0	NA	NA	NA	NA	NA
ENSG00000227827	PKD1P2	polycystin 1, transient receptor potential channel interacting pseudogene 2	283955	8,318042097	-0,557386424	1,241952	-0,448798844	0,653576773	NA
ENSG00000227830	TRIM60P3Y	tripartite motif containing 60 pseudogene 3, Y-linked	100420364	0	NA	NA	NA	NA	NA
ENSG00000227832	NA	NA	NA	0	NA	NA	NA	NA	NA
ENSG00000227834	NA	NA	NA	0	NA	NA	NA	NA	NA
ENSG00000227835	NA	NA	NA	0	NA	NA	NA	NA	NA
ENSG00000227836	NA	NA	NA	0,819201303	0,86089477	2,590853	0,332282347	0,739676068	NA
ENSG00000227837	TRIM60P11Y	tripartite motif containing 60 pseudogene 11, Y-linked	100420377	0	NA	NA	NA	NA	NA
ENSG00000227840	NA	NA	NA	0	NA	NA	NA	NA	NA
ENSG00000227841	MTND5P31	MT-ND5 pseudogene 31	100873267	0,10543123	0	4,080456	0	1	NA
ENSG00000227842	NA	NA	NA	0	NA	NA	NA	NA	NA
ENSG00000227845	NA	NA	NA	0	NA	NA	NA	NA	NA
ENSG00000227846	UGT1A11P	UDP glucuronosyltransferase family 1 member A11, pseudogene	54574	0	NA	NA	NA	NA	NA
ENSG00000227847	PPIAP93	PPIA pseudogene 93	116435287	0	NA	NA	NA	NA	NA
ENSG00000227850	SEPTIN2P1	septin 2 pseudogene 1	100873333	0	NA	NA	NA	NA	NA
ENSG00000227852	NA	NA	NA	0	NA	NA	NA	NA	NA
ENSG00000227854	NA	NA	NA	0	NA	NA	NA	NA	NA
ENSG00000227855	NA	NA	NA	0	NA	NA	NA	NA	NA
ENSG00000227860	MTND5P18	MT-ND5 pseudogene 18	100873271	0	NA	NA	NA	NA	NA
ENSG00000227862	NA	NA	NA	0	NA	NA	NA	NA	NA
ENSG00000227864	NA	NA	NA	0	NA	NA	NA	NA	NA
ENSG00000227867	ELOCP11	elongin C pseudogene 11	100462872	0	NA	NA	NA	NA	NA
ENSG00000227868	TEX46	testis expressed 46	729059	0	NA	NA	NA	NA	NA
ENSG00000227871	NA	NA	NA	0	NA	NA	NA	NA	NA
ENSG00000227873	NA	NA	NA	0	NA	NA	NA	NA	NA
ENSG00000227874	GRAMD4P1	GRAM domain containing 4 pseudogene 1	100631252	0	NA	NA	NA	NA	NA
ENSG00000227875	RPL23AP89	ribosomal protein L23a pseudogene 89	106480336	0,072165449	0,847101401	4,080456	0,207599697	0,835541541	NA
ENSG00000227877	MRLN	myoregulin	100507027	0	NA	NA	NA	NA	NA
ENSG00000227878	LOC100420572	glutathione S-transferase alpha 5 pseudogene	100420572	0	NA	NA	NA	NA	NA
ENSG00000227879	PSPC1P1	paraspeckle component 1 pseudogene 1	642395	0	NA	NA	NA	NA	NA
ENSG00000227881	ASS1P5	argininosuccinate synthetase 1 pseudogene 5	450	0	NA	NA	NA	NA	NA
ENSG00000227882	NA	NA	NA	0	NA	NA	NA	NA	NA
ENSG00000227883	ATP6V0E1P4	ATPase H+ transporting V0 subunit e1 pseudogene 4	100874504	0	NA	NA	NA	NA	NA

ENSG00000227887	NA	NA	NA	0	NA	NA	NA	NA	NA
ENSG00000227890	PSMA2P3	proteasome subunit alpha 2 pseudogene 3	79055	0	NA	NA	NA	NA	NA
ENSG00000227892	OR5P4P	olfactory receptor family 5 subfamily P member 4 pseudogene	81185	0	NA	NA	NA	NA	NA
ENSG00000227905	NA	NA	NA	0	NA	NA	NA	NA	NA
ENSG00000227910	NA	NA	NA	0	NA	NA	NA	NA	NA
ENSG00000227913	KRT8P44	keratin 8 pseudogene 44	100129958	0	NA	NA	NA	NA	NA
ENSG00000227922	SPTLC1P5	serine palmitoyltransferase long chain base subunit 1 pseudogene 5	100874512	0	NA	NA	NA	NA	NA
ENSG00000227923	NA	NA	NA	0	NA	NA	NA	NA	NA
ENSG00000227924	NA	NA	NA	0	NA	NA	NA	NA	NA
ENSG00000227932	NA	NA	NA	0	NA	NA	NA	NA	NA
ENSG00000227934	NA	NA	NA	0	NA	NA	NA	NA	NA
ENSG00000227937	MTND2P30	MT-ND2 pseudogene 30	100873234	0	NA	NA	NA	NA	NA
ENSG00000227939	RPL3P2	ribosomal protein L3 pseudogene 2	116935	0	NA	NA	NA	NA	NA
ENSG00000227941	UQCRBP2	ubiquinol-cytochrome c reductase binding protein pseudogene 2	100130719	0	NA	NA	NA	NA	NA
ENSG00000227942	FRMD8P1	FERM domain containing 8 pseudogene 1	83957	0	NA	NA	NA	NA	NA
ENSG00000227948	LOC100420226	argininosuccinate synthase 1 pseudogene	100420226	0	NA	NA	NA	NA	NA
ENSG00000227949	NA	NA	NA	0	NA	NA	NA	NA	NA
ENSG00000227950	RPL37P9	ribosomal protein L37 pseudogene 9	100270828	0	NA	NA	NA	NA	NA
ENSG00000227952	NA	NA	NA	0	NA	NA	NA	NA	NA
ENSG00000227962	NA	NA	NA	1,190199768	3,579732715	2,431174	1,472429559	0,140904916	NA
ENSG00000227965	LOC100131425	LSM5 homolog, U6 small nuclear RNA associated (S, cerevisiae) pseudogene	100131425	0	NA	NA	NA	NA	NA
ENSG00000227968	NA	NA	NA	0	NA	NA	NA	NA	NA
ENSG00000227969	NA	NA	NA	0	NA	NA	NA	NA	NA
ENSG00000227970	NR1H5P	nuclear receptor subfamily 1 group H member 5, pseudogene	643609	0	NA	NA	NA	NA	NA
ENSG00000227971	NA	NA	NA	0	NA	NA	NA	NA	NA
ENSG00000227972	THAP12P3	THAP domain containing 12 pseudogene 3	399774	0	NA	NA	NA	NA	NA
ENSG00000227973	NA	NA	NA	0	NA	NA	NA	NA	NA
ENSG00000227979	COX6A1P7	COX6A1 pseudogene 7	127566423	0	NA	NA	NA	NA	NA
ENSG00000227982	RPL7P30	ribosomal protein L7 pseudogene 30	100270908	0	NA	NA	NA	NA	NA
ENSG00000227983	BRK1P2	BRK1 pseudogene 2	100874492	0	NA	NA	NA	NA	NA
ENSG00000227986	NA	NA	NA	0	NA	NA	NA	NA	NA
ENSG00000227988	NA	NA	NA	0	NA	NA	NA	NA	NA
ENSG00000227992	NA	NA	NA	0,17888372	0,823475279	4,079514	0,201856217	0,840029131	NA
ENSG00000227994	NA	NA	NA	0	NA	NA	NA	NA	NA
ENSG00000227995	RAB11AP1	RAB11A, member RAS oncogene family pseudogene 1	100129731	0	NA	NA	NA	NA	NA
ENSG00000227999	MTND5P1	MT-ND5 pseudogene 1	100873206	0	NA	NA	NA	NA	NA
ENSG00000228002	NA	NA	NA	0	NA	NA	NA	NA	NA
ENSG00000228004	NA	NA	NA	0	NA	NA	NA	NA	NA
ENSG00000228007	NA	NA	NA	0,118286652	0	4,080456	0	1	NA
ENSG00000228014	NA	NA	NA	0,396944744	1,733535793	3,613216	0,479776371	0,631386416	NA
ENSG00000228015	NA	NA	NA	0	NA	NA	NA	NA	NA
ENSG00000228019	NA	NA	NA	0	NA	NA	NA	NA	NA
ENSG00000228020	NA	NA	NA	0	NA	NA	NA	NA	NA
ENSG00000228024	CYP1D1P	cytochrome P450 family 1 subfamily D member 1, pseudogene	100133307	0	NA	NA	NA	NA	NA
ENSG00000228030	RPL21P76	ribosomal protein L21 pseudogene 76	645373	0	NA	NA	NA	NA	NA
ENSG00000228034	NA	NA	NA	0	NA	NA	NA	NA	NA
ENSG00000228036	NA	NA	NA	0	NA	NA	NA	NA	NA
ENSG00000228038	VN1R51P	vomeronasal 1 receptor 51 pseudogene	100312801	0	NA	NA	NA	NA	NA
ENSG00000228039	NA	NA	NA	0	NA	NA	NA	NA	NA
ENSG00000228040	NA	NA	NA	0	NA	NA	NA	NA	NA
ENSG00000228041	NA	NA	NA	0	NA	NA	NA	NA	NA
ENSG00000228042	NA	NA	NA	0	NA	NA	NA	NA	NA
ENSG00000228045	NA	NA	NA	0	NA	NA	NA	NA	NA
ENSG00000228046	MTCO3P40	MT-CO3 pseudogene 40	107075188	0	NA	NA	NA	NA	NA
ENSG00000228050	NA	NA	NA	0	NA	NA	NA	NA	NA
ENSG00000228051	NA	NA	NA	0	NA	NA	NA	NA	NA

ENSG00000228053	RPL21P87	ribosomal protein L21 pseudogene 87	100131672	0	NA	NA	NA	NA	NA
ENSG00000228054	NA	NA	NA	0	NA	NA	NA	NA	NA
ENSG00000228056	NA	NA	NA	0	NA	NA	NA	NA	NA
ENSG00000228057	SEC63P1	SEC63 homolog, protein translocation regulator pseudogene 1	100420341	0,072165449	0,847101401	4,080456	0,207599697	0,835541541	NA
ENSG00000228069	MTCO3P29	MT-CO3 pseudogene 29	107075185	0,072165449	0,847101401	4,080456	0,207599697	0,835541541	NA
ENSG00000228071	RPL7P47	ribosomal protein L7 pseudogene 47	146110	0	NA	NA	NA	NA	NA
ENSG00000228074	NA	NA	NA	0	NA	NA	NA	NA	NA
ENSG00000228075	BOD1L2	biorientation of chromosomes in cell division 1 like 2	284257	0	NA	NA	NA	NA	NA
ENSG00000228076	NA	NA	NA	0	NA	NA	NA	NA	NA
ENSG00000228078	NA	NA	NA	0	NA	NA	NA	NA	NA
ENSG00000228081	NA	NA	NA	0	NA	NA	NA	NA	NA
ENSG00000228083	IFNA14	interferon alpha 14	3448	0	NA	NA	NA	NA	NA
ENSG00000228085	NA	NA	NA	0	NA	NA	NA	NA	NA
ENSG00000228089	NA	NA	NA	0	NA	NA	NA	NA	NA
ENSG00000228092	COX6CP15	cytochrome c oxidase subunit 6C pseudogene 15	106480268	0	NA	NA	NA	NA	NA
ENSG00000228095	NA	NA	NA	0	NA	NA	NA	NA	NA
ENSG00000228097	MTATP6P11	MT-ATP6 pseudogene 11	107075311	1,683439347	-0,342998859	1,363615	-0,251536503	0,801399344	NA
ENSG00000228098	NA	NA	NA	0	NA	NA	NA	NA	NA
ENSG00000228111	NA	NA	NA	0	NA	NA	NA	NA	NA
ENSG00000228112	NA	NA	NA	0	NA	NA	NA	NA	NA
ENSG00000228121	NA	NA	NA	0	NA	NA	NA	NA	NA
ENSG00000228122	NA	NA	NA	0	NA	NA	NA	NA	NA
ENSG00000228123	LOC442427	putative UPF0607 protein ENSP00000381418	442427	0	NA	NA	NA	NA	NA
ENSG00000228125	NA	NA	NA	0	NA	NA	NA	NA	NA
ENSG00000228132	NA	NA	NA	0	NA	NA	NA	NA	NA
ENSG00000228134	NA	NA	NA	0	NA	NA	NA	NA	NA
ENSG00000228141	LOC338963	epididymal protein pseudogene	338963	0	NA	NA	NA	NA	NA
ENSG00000228143	NA	NA	NA	0	NA	NA	NA	NA	NA
ENSG00000228144	NA	NA	NA	0,474337268	2,968317653	3,964599	0,748705645	0,45403464	NA
ENSG00000228146	CASP16P	caspase 16, pseudogene	197350	0	NA	NA	NA	NA	NA
ENSG00000228149	RPL3P1	ribosomal protein L3 pseudogene 1	8488	0	NA	NA	NA	NA	NA
ENSG00000228154	NA	NA	NA	0	NA	NA	NA	NA	NA
ENSG00000228158	NA	NA	NA	0,414451835	1,36735039	3,524917	0,387909972	0,698082658	NA
ENSG00000228161	NA	NA	NA	0	NA	NA	NA	NA	NA
ENSG00000228166	MTND1P11	MT-ND1 pseudogene 11	100873212	4,160251476	-0,037888507	0,887365	-0,042697774	0,965942454	NA
ENSG00000228167	NA	NA	NA	0	NA	NA	NA	NA	NA
ENSG00000228169	NA	NA	NA	0	NA	NA	NA	NA	NA
ENSG00000228171	C11orf98P3	C11orf98 pseudogene 3	127138860	0	NA	NA	NA	NA	NA
ENSG00000228175	NA	NA	NA	0,224294693	-0,134849514	4,080456	-0,033047659	0,973636582	NA
ENSG00000228181	NA	NA	NA	0	NA	NA	NA	NA	NA
ENSG00000228182	MTND2P25	MT-ND2 pseudogene 25	100873231	0	NA	NA	NA	NA	NA
ENSG00000228184	NA	NA	NA	0	NA	NA	NA	NA	NA
ENSG00000228187	LOC100421400	alanyl-tRNA synthetase domain containing 1 pseudogene	100421400	0	NA	NA	NA	NA	NA
ENSG00000228190	RPL23AP16	ribosomal protein L23a pseudogene 16	647201	0	NA	NA	NA	NA	NA
ENSG00000228191	NA	NA	NA	0	NA	NA	NA	NA	NA
ENSG00000228193	NA	NA	NA	0	NA	NA	NA	NA	NA
ENSG00000228194	NA	NA	NA	0	NA	NA	NA	NA	NA
ENSG00000228196	NA	NA	NA	0	NA	NA	NA	NA	NA
ENSG00000228198	OR2M3	olfactory receptor family 2 subfamily M member 3	127062	0	NA	NA	NA	NA	NA
ENSG00000228205	RPS3P3	ribosomal protein S3 pseudogene 3	440991	0,11808024	-1,076443944	4,080456	-0,263804824	0,791930317	NA
ENSG00000228207	NA	NA	NA	0	NA	NA	NA	NA	NA
ENSG00000228209	NA	NA	NA	0	NA	NA	NA	NA	NA
ENSG00000228210	RPL7AP12	ribosomal protein L7a pseudogene 12	128668	0	NA	NA	NA	NA	NA
ENSG00000228211	NA	NA	NA	0	NA	NA	NA	NA	NA
ENSG00000228212	OFD1P17	OFD1 pseudogene 17	23738	0	NA	NA	NA	NA	NA
ENSG00000228217	NA	NA	NA	0,117497108	0,847101401	4,080456	0,207599697	0,835541541	NA
ENSG00000228218	NA	NA	NA	8,084939352	0,207763635	0,665535	0,31217543	0,754907203	NA
ENSG00000228219	NPM1P30	nucleophosmin 1 pseudogene 30	101928904	0	NA	NA	NA	NA	NA
ENSG00000228224	NACA4P	NACA family member 4, pseudogene	83955	0	NA	NA	NA	NA	NA
ENSG00000228225	NA	NA	NA	0	NA	NA	NA	NA	NA
ENSG00000228232	NA	NA	NA	0	NA	NA	NA	NA	NA
ENSG00000228234	NA	NA	NA	0	NA	NA	NA	NA	NA

ENSG00000228236	NA	NA	NA	0	NA	NA	NA	NA	NA
ENSG00000228238	NA	NA	NA	0,144330898	1,418786819	4,061262	0,349346267	0,726829368	NA
ENSG00000228241	MOB1AP1	MOB kinase activator 1A pseudogene 1	100873874	0	NA	NA	NA	NA	NA
ENSG00000228247	NA	NA	NA	0	NA	NA	NA	NA	NA
ENSG00000228248	NA	NA	NA	0	NA	NA	NA	NA	NA
ENSG00000228252	NA	NA	NA	0,118286652	0	4,080456	0	1	NA
ENSG00000228257	NA	NA	NA	0	NA	NA	NA	NA	NA
ENSG00000228259	NA	NA	NA	0	NA	NA	NA	NA	NA
ENSG00000228264	PSMD8P1	proteasome 26S subunit, non-ATPase, 8 pseudogene 1	100422411	0	NA	NA	NA	NA	NA
ENSG00000228278	ORM2	orosomucoid 2	5005	0	NA	NA	NA	NA	NA
ENSG00000228283	NA	NA	NA	0	NA	NA	NA	NA	NA
ENSG00000228285	NA	NA	NA	0	NA	NA	NA	NA	NA
ENSG00000228286	NA	NA	NA	0,234994216	1,944111989	4,02324	0,483220515	0,62893917	NA
ENSG00000228289	NA	NA	NA	0	NA	NA	NA	NA	NA
ENSG00000228291	NA	NA	NA	0	NA	NA	NA	NA	NA
ENSG00000228292	NA	NA	NA	0	NA	NA	NA	NA	NA
ENSG00000228294	NA	NA	NA	0	NA	NA	NA	NA	NA
ENSG00000228301	RPL7P55	ribosomal protein L7 pseudogene 55	100271147	0	NA	NA	NA	NA	NA
ENSG00000228303	NA	NA	NA	0	NA	NA	NA	NA	NA
ENSG00000228305	NA	NA	NA	0,621717744	-0,072682042	2,643265	-0,027497072	0,978063275	NA
ENSG00000228307	OR2S1P	olfactory receptor family 2 subfamily S member 1 pseudogene	392313	0	NA	NA	NA	NA	NA
ENSG00000228312	NA	NA	NA	0	NA	NA	NA	NA	NA
ENSG00000228314	NA	NA	NA	0	NA	NA	NA	NA	NA
ENSG00000228316	MTATP6P19	MT-ATP6 pseudogene 19	107075275	0	NA	NA	NA	NA	NA
ENSG00000228319	SPATA2P1	spermatogenesis associated 2 pseudogene 1	100873966	0	NA	NA	NA	NA	NA
ENSG00000228327	NA	NA	NA	7,445925498	0,707259483	3,712605	0,190502222	0,848915603	NA
ENSG00000228328	NA	NA	NA	0,341768625	1,936502914	4,022852	0,481375586	0,630249585	NA
ENSG00000228330	NA	NA	NA	0,222946168	-1,086007539	4,080456	-0,266148581	0,790124782	NA
ENSG00000228331	RPL17P43	ribosomal protein L17 pseudogene 43	729259	0	NA	NA	NA	NA	NA
ENSG00000228335	NA	NA	NA	0,707190354	0,178055896	1,956916	0,090988012	0,927502117	NA
ENSG00000228336	OR9H1P	olfactory receptor family 9 subfamily H member 1 pseudogene	81439	0	NA	NA	NA	NA	NA
ENSG00000228337	PPIAP69	peptidylprolyl isomerase A pseudogene 69	111082982	0	NA	NA	NA	NA	NA
ENSG00000228339	AMD1P1	adenosylmethionine decarboxylase 1 pseudogene 1	100133024	0	NA	NA	NA	NA	NA
ENSG00000228341	RPL37AP6	ribosomal protein L37a pseudogene 6	100271339	0	NA	NA	NA	NA	NA
ENSG00000228345	LOC392436	MAGE family member B4 pseudogene	392436	0	NA	NA	NA	NA	NA
ENSG00000228347	NA	NA	NA	0	NA	NA	NA	NA	NA
ENSG00000228348	NA	NA	NA	0	NA	NA	NA	NA	NA
ENSG00000228349	RPS26P4	ribosomal protein S26 pseudogene 4	692146	0	NA	NA	NA	NA	NA
ENSG00000228354	NA	NA	NA	0	NA	NA	NA	NA	NA
ENSG00000228360	NA	NA	NA	0	NA	NA	NA	NA	NA
ENSG00000228364	NA	NA	NA	0	NA	NA	NA	NA	NA
ENSG00000228366	NA	NA	NA	0	NA	NA	NA	NA	NA
ENSG00000228367	LOC100996384	aconitate hydratase, mitochondrial-like	100996384	0	NA	NA	NA	NA	NA
ENSG00000228373	LOC112268032	cytochrome c oxidase subunit NDUFA4-like	112268032	0	NA	NA	NA	NA	NA
ENSG00000228374	RPL9P13	ribosomal protein L9 pseudogene 13	100270856	0	NA	NA	NA	NA	NA
ENSG00000228375	RPS20P25	ribosomal protein S20 pseudogene 25	100271235	0	NA	NA	NA	NA	NA
ENSG00000228376	GAS2L1P2	growth arrest specific 2 like 1 pseudogene 2	340508	0	NA	NA	NA	NA	NA
ENSG00000228376	LOC124902155	uncharacterized LOC124902155	124902155	0	NA	NA	NA	NA	NA
ENSG00000228380	NA	NA	NA	0	NA	NA	NA	NA	NA
ENSG00000228398	NA	NA	NA	0	NA	NA	NA	NA	NA
ENSG00000228399	RPS20P6	ribosomal protein S20 pseudogene 6	100270900	0	NA	NA	NA	NA	NA
ENSG00000228407	NA	NA	NA	0	NA	NA	NA	NA	NA
ENSG00000228411	NA	NA	NA	0	NA	NA	NA	NA	NA
ENSG00000228413	ARL8BP1	ARL8B pseudogene 1	127566425	0	NA	NA	NA	NA	NA
ENSG00000228415	NA	NA	NA	1,422747445	-3,528178838	1,876054	-1,880638354	0,060021131	NA
ENSG00000228429	LOC100419458	ribosomal protein L21 pseudogene	100419458	0	NA	NA	NA	NA	NA
ENSG00000228430	NA	NA	NA	0	NA	NA	NA	NA	NA

ENSG00000228431	ARL5AP3	ARL5A pseudogene 3	106480778	0	NA	NA	NA	NA	NA
ENSG00000228432	NA	NA	NA	0	NA	NA	NA	NA	NA
ENSG00000228433	OR7E23P	olfactory receptor family 7 subfamily E member 23 pseudogene	391279	0	NA	NA	NA	NA	NA
ENSG00000228438	NA	NA	NA	0	NA	NA	NA	NA	NA
ENSG00000228440	MTND5P6	MT-ND5 pseudogene 6	100873196	0	NA	NA	NA	NA	NA
ENSG00000228445	NA	NA	NA	0	NA	NA	NA	NA	NA
ENSG00000228446	NA	NA	NA	2,883140473	0,152323043	1,17391	0,129756972	0,896758706	NA
ENSG00000228450	NA	NA	NA	0	NA	NA	NA	NA	NA
ENSG00000228451	SDAD1P1	SDA1 domain containing 1 pseudogene 1	157489	2,761311259	0,528815815	1,106253	0,478024314	0,6326329	NA
ENSG00000228453	NA	NA	NA	0	NA	NA	NA	NA	NA
ENSG00000228460	NA	NA	NA	0	NA	NA	NA	NA	NA
ENSG00000228462	RPS19P7	ribosomal protein S19 pseudogene 7	728509	0	NA	NA	NA	NA	NA
ENSG00000228463	RPL23AP21	ribosomal protein L23a pseudogene 21	728481	0	NA	NA	NA	NA	NA
ENSG00000228463	LOC127239154	uncharacterized LOC127239154	127239154	0	NA	NA	NA	NA	NA
ENSG00000228464	NA	NA	NA	0	NA	NA	NA	NA	NA
ENSG00000228465	NA	NA	NA	0	NA	NA	NA	NA	NA
ENSG00000228466	NA	NA	NA	0	NA	NA	NA	NA	NA
ENSG00000228473	NA	NA	NA	0	NA	NA	NA	NA	NA
ENSG00000228476	CSTP1	cystatin pseudogene 1	1480	0	NA	NA	NA	NA	NA
ENSG00000228477	NA	NA	NA	0	NA	NA	NA	NA	NA
ENSG00000228480	NA	NA	NA	0	NA	NA	NA	NA	NA
ENSG00000228489	RPL21P50	ribosomal protein L21 pseudogene 50	100271167	0	NA	NA	NA	NA	NA
ENSG00000228492	NA	NA	NA	0,10488184	-1,076443944	4,080456	-0,263804824	0,791930317	NA
ENSG00000228499	NA	NA	NA	0	NA	NA	NA	NA	NA
ENSG00000228501	RPL15P18	ribosomal protein L15 pseudogene 18	728088	0	NA	NA	NA	NA	NA
ENSG00000228502	NA	NA	NA	0,787888594	-0,997504711	2,481244	-0,402017926	0,687670834	NA
ENSG00000228507	NA	NA	NA	0,10543123	0	4,080456	0	1	NA
ENSG00000228510	NA	NA	NA	0,10488184	-1,076443944	4,080456	-0,263804824	0,791930317	NA
ENSG00000228513	LOC100419679	zinc finger protein 670 pseudogene	100419679	0	NA	NA	NA	NA	NA
ENSG00000228517	CT47A7	cancer/testis antigen family 47 member A7	653282	0	NA	NA	NA	NA	NA
ENSG00000228518	NA	NA	NA	0	NA	NA	NA	NA	NA
ENSG00000228519	NA	NA	NA	0	NA	NA	NA	NA	NA
ENSG00000228522	NA	NA	NA	0	NA	NA	NA	NA	NA
ENSG00000228523	DNAJB6P4	DNAJB6 pseudogene 4	100130497	0,190684097	-0,124923611	4,080279	-0,030616441	0,97557543	NA
ENSG00000228525	NA	NA	NA	0	NA	NA	NA	NA	NA
ENSG00000228530	NA	NA	NA	0	NA	NA	NA	NA	NA
ENSG00000228532	NA	NA	NA	0,207996306	-1,916073151	4,043021	-0,473921178	0,635556117	NA
ENSG00000228546	PMS2P12	PMS1 homolog 2, mismatch repair system component pseudogene 12	392713	0,11808024	-1,076443944	4,080456	-0,263804824	0,791930317	NA
ENSG00000228547	NA	NA	NA	0	NA	NA	NA	NA	NA
ENSG00000228550	NA	NA	NA	0,118286652	0	4,080456	0	1	NA
ENSG00000228551	NA	NA	NA	0	NA	NA	NA	NA	NA
ENSG00000228557	HSPA8P17	heat shock protein family A (Hsp70) member 8 pseudogene 17	100533658	0	NA	NA	NA	NA	NA
ENSG00000228567	VN1R4	vomeronasal 1 receptor 4	317703	0	NA	NA	NA	NA	NA
ENSG00000228568	RPS15AP13	ribosomal protein S15a pseudogene 13	100128605	0	NA	NA	NA	NA	NA
ENSG00000228571	HSFY7P	heat shock transcription factor Y-linked 7, pseudogene	106478935	0	NA	NA	NA	NA	NA
ENSG00000228578	NA	NA	NA	0	NA	NA	NA	NA	NA
ENSG00000228585	NA	NA	NA	0	NA	NA	NA	NA	NA
ENSG00000228589	NA	NA	NA	0	NA	NA	NA	NA	NA
ENSG00000228593	NA	NA	NA	0	NA	NA	NA	NA	NA
ENSG00000228595	PAICSP2	phosphoribosylaminoimidazole carboxylase, phosphoribosylaminoimidazole succinocarboxamide synthetase pseudogene 2	780815	0	NA	NA	NA	NA	NA
ENSG00000228597	MTND4P1	MT-ND4 pseudogene 1	100873211	0	NA	NA	NA	NA	NA
ENSG00000228599	RPL7P52	ribosomal protein L7 pseudogene 52	646912	0	NA	NA	NA	NA	NA
ENSG00000228600	NA	NA	NA	0	NA	NA	NA	NA	NA
ENSG00000228601	NA	NA	NA	0	NA	NA	NA	NA	NA
ENSG00000228607	CLDN25	claudin 25	644672	0	NA	NA	NA	NA	NA
ENSG00000228610	PDCL2P1	PDCL2 pseudogene 1	653707	0	NA	NA	NA	NA	NA
ENSG00000228611	NA	NA	NA	0	NA	NA	NA	NA	NA
ENSG00000228612	NA	NA	NA	0	NA	NA	NA	NA	NA

ENSG00000228615	NA	NA	NA	0	NA	NA	NA	NA	NA
ENSG00000228623	ZNF883	zinc finger protein 883	169834	1,134225534	0,279999998	1,772932	0,157930506	0,87451156	NA
ENSG00000228626	NA	NA	NA	0	NA	NA	NA	NA	NA
ENSG00000228638	NA	NA	NA	2,012967154	4,286719002	2,5947	1,652105726	0,098513	NA
ENSG00000228645	NA	NA	NA	0	NA	NA	NA	NA	NA
ENSG00000228646	RPL31P13	ribosomal protein L31 pseudogene 13	100270983	0	NA	NA	NA	NA	NA
ENSG00000228653	HNRNPCP7	heterogeneous nuclear ribonucleoprotein C pseudogene 7	100127907	0	NA	NA	NA	NA	NA
ENSG00000228656	MYO5BP3	myosin VB pseudogene 3	441442	0	NA	NA	NA	NA	NA
ENSG00000228657	NA	NA	NA	0	NA	NA	NA	NA	NA
ENSG00000228660	VN1R35P	vomeronasal 1 receptor 35 pseudogene	100312789	0	NA	NA	NA	NA	NA
ENSG00000228663	NA	NA	NA	3,036415189	-1,342270434	1,051075	-1,277045562	0,201586162	NA
ENSG00000228664	RPL18P2	ribosomal protein L18 pseudogene 2	100129421	0	NA	NA	NA	NA	NA
ENSG00000228665	NA	NA	NA	0	NA	NA	NA	NA	NA
ENSG00000228666	KRT18P1	keratin 18 pseudogene 1	3879	0	NA	NA	NA	NA	NA
ENSG00000228667	NA	NA	NA	0	NA	NA	NA	NA	NA
ENSG00000228668	TRGV5P	T cell receptor gamma variable 5P (pseudogene)	6979	9,777169477	0,266107807	0,589594	0,451340743	0,651743985	NA
ENSG00000228670	NA	NA	NA	0	NA	NA	NA	NA	NA
ENSG00000228674	NA	NA	NA	0	NA	NA	NA	NA	NA
ENSG00000228681	NA	NA	NA	0	NA	NA	NA	NA	NA
ENSG00000228685	NA	NA	NA	0	NA	NA	NA	NA	NA
ENSG00000228687	NA	NA	NA	0	NA	NA	NA	NA	NA
ENSG00000228694	MTND4P18	MT-ND4 pseudogene 18	100873350	0	NA	NA	NA	NA	NA
ENSG00000228695	LOC107987423	liver carboxylesterase 1-like	107987423	0	NA	NA	NA	NA	NA
ENSG00000228700	NA	NA	NA	0	NA	NA	NA	NA	NA
ENSG00000228702	NA	NA	NA	0,237988168	0,852827832	4,080358	0,209008077	0,834441933	NA
ENSG00000228711	NA	NA	NA	0	NA	NA	NA	NA	NA
ENSG00000228717	NAB1P1	NAB1 pseudogene 1	286456	0	NA	NA	NA	NA	NA
ENSG00000228721	NA	NA	NA	0	NA	NA	NA	NA	NA
ENSG00000228725	MTND2P12	MT-ND2 pseudogene 12	100873344	0	NA	NA	NA	NA	NA
ENSG00000228727	SAPCD1	suppressor APC domain containing 1	401251	5,409826647	-0,393895168	2,718599	-0,144889003	0,884798509	NA
ENSG00000228728	PPIAP91	peptidylprolyl isomerase A pseudogene 91	111082972	0	NA	NA	NA	NA	NA
ENSG00000228729	LOC100418874	cathepsin B pseudogene	100418874	0	NA	NA	NA	NA	NA
ENSG00000228744	RPS3AP30	RPS3A pseudogene 30	100271254	0	NA	NA	NA	NA	NA
ENSG00000228751	NA	NA	NA	0	NA	NA	NA	NA	NA
ENSG00000228753	EIF4BP2	eukaryotic translation initiation factor 4B pseudogene 2	100129337	0	NA	NA	NA	NA	NA
ENSG00000228754	RPL13AP19	ribosomal protein L13a pseudogene 19	100132924	0	NA	NA	NA	NA	NA
ENSG00000228755	NA	NA	NA	0	NA	NA	NA	NA	NA
ENSG00000228759	FAF2P1	Fas associated factor family member 2 pseudogene 1	100128043	0	NA	NA	NA	NA	NA
ENSG00000228764	NA	NA	NA	0	NA	NA	NA	NA	NA
ENSG00000228766	RPL7L1P8	RPL7L1 pseudogene 8	402152	0	NA	NA	NA	NA	NA
ENSG00000228767	KRT8P18	keratin 8 pseudogene 18	442078	0	NA	NA	NA	NA	NA
ENSG00000228769	ABCF2P2	ATP binding cassette subfamily F member 2 pseudogene 2	100422059	0	NA	NA	NA	NA	NA
ENSG00000228770	NA	NA	NA	0	NA	NA	NA	NA	NA
ENSG00000228771	LOC107985659	X antigen family member 5-like	107985659	0	NA	NA	NA	NA	NA
ENSG00000228776	NA	NA	NA	0	NA	NA	NA	NA	NA
ENSG00000228780	NA	NA	NA	0	NA	NA	NA	NA	NA
ENSG00000228781	RPL21P84	ribosomal protein L21 pseudogene 84	100271187	0	NA	NA	NA	NA	NA
ENSG00000228782	NA	NA	NA	6,579523644	0,074050325	0,721595	0,102620302	0,918264329	NA
ENSG00000228783	H3P29	H3 histone pseudogene 29	115482721	0	NA	NA	NA	NA	NA
ENSG00000228797	NA	NA	NA	0,117497108	0,847101401	4,080456	0,207599697	0,835541541	NA
ENSG00000228803	MTCYBP42	MT-CYB pseudogene 42	109729153	0	NA	NA	NA	NA	NA
ENSG00000228806	NA	NA	NA	0	NA	NA	NA	NA	NA
ENSG00000228807	NA	NA	NA	0	NA	NA	NA	NA	NA
ENSG00000228808	HMGB3P4	high mobility group box 3 pseudogene 4	100873890	4,837803515	0,020483309	0,801921	0,025542801	0,979622009	NA
ENSG00000228810	NA	NA	NA	0	NA	NA	NA	NA	NA
ENSG00000228814	NA	NA	NA	0	NA	NA	NA	NA	NA
ENSG00000228815	MARK2P13	MARK2 pseudogene 13	100631260	0	NA	NA	NA	NA	NA
ENSG00000228816	NA	NA	NA	0	NA	NA	NA	NA	NA
ENSG00000228818	PSMD2P1	proteasome 26S subunit, non-ATPase, 2 pseudogene 1	266783	0	NA	NA	NA	NA	NA
ENSG00000228819	AGKP2	AGK pseudogene 2	392543	0	NA	NA	NA	NA	NA

ENSG00000228820	RPSAP1	ribosomal protein SA pseudogene 1	170529	0	NA	NA	NA	NA	NA
ENSG00000228823	NA	NA	NA	0	NA	NA	NA	NA	NA
ENSG00000228825	NA	NA	NA	0	NA	NA	NA	NA	NA
ENSG00000228827	NA	NA	NA	0	NA	NA	NA	NA	NA
ENSG00000228828	TLK2P2	tousled like kinase 2 pseudogene 2	553121	1,450738996	-1,330349915	1,630974	-0,81567812	0,414684256	NA
ENSG00000228829	NA	NA	NA	0	NA	NA	NA	NA	NA
ENSG00000228833	NA	NA	NA	0	NA	NA	NA	NA	NA
ENSG00000228834	NA	NA	NA	0	NA	NA	NA	NA	NA
ENSG00000228836	CT45A5	cancer/testis antigen family 45 member A5	441521	0	NA	NA	NA	NA	NA
ENSG00000228837	NA	NA	NA	0	NA	NA	NA	NA	NA
ENSG00000228844	RPS11P2	ribosomal protein S11 pseudogene 2	645916	0	NA	NA	NA	NA	NA
ENSG00000228847	NA	NA	NA	0	NA	NA	NA	NA	NA
ENSG00000228848	NA	NA	NA	0	NA	NA	NA	NA	NA
ENSG00000228850	CDY12P	chromodomain Y-linked 12 pseudogene	386734	0	NA	NA	NA	NA	NA
ENSG00000228851	MTCO3P4	MT-CO3 pseudogene 4	107075216	0	NA	NA	NA	NA	NA
ENSG00000228855	NA	NA	NA	0	NA	NA	NA	NA	NA
ENSG00000228860	NA	NA	NA	0	NA	NA	NA	NA	NA
ENSG00000228861	RPL23AP12	ribosomal protein L23a pseudogene 12	391282	0	NA	NA	NA	NA	NA
ENSG00000228868	MYLKP1	myosin light chain kinase pseudogene 1	9430	0	NA	NA	NA	NA	NA
ENSG00000228869	COX4I1P2	cytochrome c oxidase subunit 4I1 pseudogene 2	652170	0	NA	NA	NA	NA	NA
ENSG00000228872	NA	NA	NA	0	NA	NA	NA	NA	NA
ENSG00000228874	RPS24P10	ribosomal protein S24 pseudogene 10	100271097	0	NA	NA	NA	NA	NA
ENSG00000228879	RPL35AP6	ribosomal protein L35a pseudogene 6	100271312	0	NA	NA	NA	NA	NA
ENSG00000228882	NA	NA	NA	0	NA	NA	NA	NA	NA
ENSG00000228884	RPL23AP26	ribosomal protein L23a pseudogene 26	391490	0,10488184	-1,076443944	4,080456	-0,263804824	0,791930317	NA
ENSG00000228897	NA	NA	NA	0,10543123	0	4,080456	0	1	NA
ENSG00000228898	MTCO1P43	MT-CO1 pseudogene 43	107075173	0	NA	NA	NA	NA	NA
ENSG00000228901	NA	NA	NA	0	NA	NA	NA	NA	NA
ENSG00000228902	ST6GALNAC2P1	ST6 N-acetylgalactosaminide alpha-2,6-sialyltransferase 2 pseudogene 1	100422716	0	NA	NA	NA	NA	NA
ENSG00000228903	NA	NA	NA	6,358015742	0,207313243	0,773688	0,267954545	0,788734307	NA
ENSG00000228914	OR1H1P	olfactory receptor family 1 subfamily H member 1 pseudogene	26742	0	NA	NA	NA	NA	NA
ENSG00000228915	OR7E128P	olfactory receptor family 7 subfamily E member 128 pseudogene	81175	0	NA	NA	NA	NA	NA
ENSG00000228922	LOC650024	cancer/testis antigen 55 pseudogene	650024	0	NA	NA	NA	NA	NA
ENSG00000228927	TSPY3	testis specific protein Y-linked 3	728137	0	NA	NA	NA	NA	NA
ENSG00000228927	LOC124905614	testis-specific Y-encoded protein 3	124905614	0	NA	NA	NA	NA	NA
ENSG00000228927	LOC124905615	testis-specific Y-encoded protein 3	124905615	0	NA	NA	NA	NA	NA
ENSG00000228927	LOC124905616	testis-specific Y-encoded protein 3	124905616	0	NA	NA	NA	NA	NA
ENSG00000228927	LOC124905617	testis-specific Y-encoded protein 3	124905617	0	NA	NA	NA	NA	NA
ENSG00000228927	LOC124905618	testis-specific Y-encoded protein 3-like	124905618	0	NA	NA	NA	NA	NA
ENSG00000228927	LOC124905619	testis-specific Y-encoded protein 3-like	124905619	0	NA	NA	NA	NA	NA
ENSG00000228927	LOC124905620	testis-specific Y-encoded protein 3	124905620	0	NA	NA	NA	NA	NA
ENSG00000228927	LOC124905621	testis-specific Y-encoded protein 3	124905621	0	NA	NA	NA	NA	NA
ENSG00000228927	LOC124905624	testis-specific Y-encoded protein 3	124905624	0	NA	NA	NA	NA	NA
ENSG00000228927	LOC124905625	testis-specific Y-encoded protein 3	124905625	0	NA	NA	NA	NA	NA
ENSG00000228927	LOC124905626	testis-specific Y-encoded protein 2	124905626	0	NA	NA	NA	NA	NA
ENSG00000228927	LOC124905627	testis-specific Y-encoded protein 4-like	124905627	0	NA	NA	NA	NA	NA
ENSG00000228927	LOC124905628	testis-specific Y-encoded protein 3	124905628	0	NA	NA	NA	NA	NA

ENSG00000228927	LOC124905629	testis-specific Y-encoded protein 3-like	124905629	0	NA	NA	NA	NA	NA
ENSG00000228927	LOC124908978	testis-specific Y-encoded protein 3	124908978	0	NA	NA	NA	NA	NA
ENSG00000228927	LOC124908979	testis-specific Y-encoded protein 3	124908979	0	NA	NA	NA	NA	NA
ENSG00000228927	LOC124908980	testis-specific Y-encoded protein 3	124908980	0	NA	NA	NA	NA	NA
ENSG00000228927	LOC124908981	testis-specific Y-encoded protein 3	124908981	0	NA	NA	NA	NA	NA
ENSG00000228927	LOC124909197	testis-specific Y-encoded protein 3-like	124909197	0	NA	NA	NA	NA	NA
ENSG00000228927	LOC124909294	testis-specific Y-encoded protein 3	124909294	0	NA	NA	NA	NA	NA
ENSG00000228927	LOC124909306	testis-specific Y-encoded protein 3-like	124909306	0	NA	NA	NA	NA	NA
ENSG00000228927	LOC124909318	testis-specific Y-encoded protein 3-like	124909318	0	NA	NA	NA	NA	NA
ENSG00000228927	LOC124909320	testis-specific Y-encoded protein 3-like	124909320	0	NA	NA	NA	NA	NA
ENSG00000228927	LOC124909330	testis-specific Y-encoded protein 1-like	124909330	0	NA	NA	NA	NA	NA
ENSG00000228928	NA	NA	NA	0	NA	NA	NA	NA	NA
ENSG00000228929	RPS13P2	ribosomal protein S13 pseudogene 2	729236	0	NA	NA	NA	NA	NA
ENSG00000228930	MTCO1P3	MT-CO1 pseudogene 3	100873205	0	NA	NA	NA	NA	NA
ENSG00000228935	NA	NA	NA	0	NA	NA	NA	NA	NA
ENSG00000228937	MTA3P1	MTA3 pseudogene 1	100130766	0	NA	NA	NA	NA	NA
ENSG00000228938	NA	NA	NA	0	NA	NA	NA	NA	NA
ENSG00000228940	LOC100128362	protein phosphatase 2 catalytic subunit alpha pseudogene	100128362	0	NA	NA	NA	NA	NA
ENSG00000228941	NA	NA	NA	0,11808024	-1,076443944	4,080456	-0,263804824	0,791930317	NA
ENSG00000228943	NA	NA	NA	0	NA	NA	NA	NA	NA
ENSG00000228945	NA	NA	NA	0	NA	NA	NA	NA	NA
ENSG00000228948	NA	NA	NA	0,072165449	0,847101401	4,080456	0,207599697	0,835541541	NA
ENSG00000228949	UGT1A12P	UDP glucuronosyltransferase family 1 member A12, pseudogene	54573	0	NA	NA	NA	NA	NA
ENSG00000228953	NA	NA	NA	0	NA	NA	NA	NA	NA
ENSG00000228955	RPL7AP82	ribosomal protein L7a pseudogene 82	122965338	0	NA	NA	NA	NA	NA
ENSG00000228957	NA	NA	NA	0	NA	NA	NA	NA	NA
ENSG00000228958	NA	NA	NA	0	NA	NA	NA	NA	NA
ENSG00000228963	NA	NA	NA	0	NA	NA	NA	NA	NA
ENSG00000228965	RPSAP60	ribosomal protein SA pseudogene 60	100271149	0	NA	NA	NA	NA	NA
ENSG00000228974	NA	NA	NA	0	NA	NA	NA	NA	NA
ENSG00000228976	NA	NA	NA	0	NA	NA	NA	NA	NA
ENSG00000228979	NA	NA	NA	0	NA	NA	NA	NA	NA
ENSG00000228981	RPS2P20	ribosomal protein S2 pseudogene 20	645018	0	NA	NA	NA	NA	NA
ENSG00000228982	NA	NA	NA	0	NA	NA	NA	NA	NA
ENSG00000228983	NA	NA	NA	0	NA	NA	NA	NA	NA
ENSG00000228984	LOC100130861	zinc finger protein 736 pseudogene	100130861	0	NA	NA	NA	NA	NA
ENSG00000228985	TRDD3	T cell receptor delta diversity 3	28523	0	NA	NA	NA	NA	NA
ENSG00000228986	NA	NA	NA	0	NA	NA	NA	NA	NA
ENSG00000228992	RPL5P32	ribosomal protein L5 pseudogene 32	100271207	0	NA	NA	NA	NA	NA
ENSG00000228995	MTND1P9	MT-ND1 pseudogene 9	100873221	0	NA	NA	NA	NA	NA
ENSG00000228997	ORMDL1P1	ORMDL1 pseudogene 1	124902374	0	NA	NA	NA	NA	NA
ENSG00000228998	NA	NA	NA	1,485477863	-0,040277859	1,667485	-0,024154856	0,980729088	NA
ENSG00000229000	SEPTIN7P8	septin 7 pseudogene 8	100418735	0,072165449	0,847101401	4,080456	0,207599697	0,835541541	NA
ENSG00000229001	ACTBP14	ACTB pseudogene 14	389976	0,118286652	0	4,080456	0	1	NA
ENSG00000229002	NA	NA	NA	0	NA	NA	NA	NA	NA
ENSG00000229007	NA	NA	NA	0	NA	NA	NA	NA	NA
ENSG00000229009	NA	NA	NA	0	NA	NA	NA	NA	NA
ENSG00000229010	NA	NA	NA	0,15836288	0	4,080456	0	1	NA
ENSG00000229013	NA	NA	NA	0	NA	NA	NA	NA	NA
ENSG00000229014	RPL30P13	ribosomal protein L30 pseudogene 13	100271305	0	NA	NA	NA	NA	NA
ENSG00000229015	NA	NA	NA	0	NA	NA	NA	NA	NA
ENSG00000229016	NA	NA	NA	0	NA	NA	NA	NA	NA
ENSG00000229022	RPL6P3	ribosomal protein L6 pseudogene 3	391181	0	NA	NA	NA	NA	NA
ENSG00000229023	NA	NA	NA	9,208430534	0,395314037	0,690964	0,572119516	0,567241008	NA
ENSG00000229027	NA	NA	NA	0	NA	NA	NA	NA	NA
ENSG00000229028	KRT223P	keratin 223, pseudogene	643115	0	NA	NA	NA	NA	NA

ENSG00000229029	CDCA4P1	cell division cycle associated 4 pseudogene 1	100420502	0	NA	NA	NA	NA	NA
ENSG00000229030	NA	NA	NA	0	NA	NA	NA	NA	NA
ENSG00000229031	MTCO1P25	MT-CO1 pseudogene 25	107075167	0	NA	NA	NA	NA	NA
ENSG00000229032	NA	NA	NA	0	NA	NA	NA	NA	NA
ENSG00000229035	NA	NA	NA	0	NA	NA	NA	NA	NA
ENSG00000229036	NA	NA	NA	3,387493363	-0,199595969	1,033152	-0,19319135	0,846809113	NA
ENSG00000229037	NA	NA	NA	0	NA	NA	NA	NA	NA
ENSG00000229046	NA	NA	NA	0	NA	NA	NA	NA	NA
ENSG00000229048	NA	NA	NA	0	NA	NA	NA	NA	NA
ENSG00000229052	NA	NA	NA	0	NA	NA	NA	NA	NA
ENSG00000229054	RPS29P14	ribosomal protein S29 pseudogene 14	100129599	0	NA	NA	NA	NA	NA
ENSG00000229057	NA	NA	NA	0	NA	NA	NA	NA	NA
ENSG00000229060	NA	NA	NA	0	NA	NA	NA	NA	NA
ENSG00000229063	TRBV23OR9-2	T cell receptor beta variable 23/OR9-2 (pseudogene)	28552	0	NA	NA	NA	NA	NA
ENSG00000229064	NA	NA	NA	0	NA	NA	NA	NA	NA
ENSG00000229067	NA	NA	NA	0	NA	NA	NA	NA	NA
ENSG00000229068	NA	NA	NA	0	NA	NA	NA	NA	NA
ENSG00000229072	NA	NA	NA	0	NA	NA	NA	NA	NA
ENSG00000229079	LOC100419693	zinc finger protein 114 pseudogene	100419693	0	NA	NA	NA	NA	NA
ENSG00000229080	MTCO3P30	MT-CO3 pseudogene 30	107075229	0	NA	NA	NA	NA	NA
ENSG00000229082	OR5AW1P	olfactory receptor family 5 subfamily AW member 1 pseudogene	81076	0	NA	NA	NA	NA	NA
ENSG00000229083	NA	NA	NA	0,676101059	2,919149668	2,606203	1,120077579	0,262680704	NA
ENSG00000229087	NA	NA	NA	0,10543123	0	4,080456	0	1	NA
ENSG00000229088	NA	NA	NA	0,210313824	-1,075909609	4,080456	-0,263673874	0,792031229	NA
ENSG00000229089	ANKRD20A8P	ankyrin repeat domain 20 family member A8, pseudogene	729171	0	NA	NA	NA	NA	NA
ENSG00000229090	NA	NA	NA	0	NA	NA	NA	NA	NA
ENSG00000229091	HSPA8P8	heat shock protein family A (Hsp70) member 8 pseudogene 8	100287551	0	NA	NA	NA	NA	NA
ENSG00000229093	NA	NA	NA	0	NA	NA	NA	NA	NA
ENSG00000229097	NA	NA	NA	0,11808024	-1,076443944	4,080456	-0,263804824	0,791930317	NA
ENSG00000229101	NA	NA	NA	0	NA	NA	NA	NA	NA
ENSG00000229104	NA	NA	NA	0	NA	NA	NA	NA	NA
ENSG00000229107	NA	NA	NA	0	NA	NA	NA	NA	NA
ENSG00000229110	LOC100421387	heterogeneous nuclear ribonucleoprotein A3 pseudogene	100421387	0	NA	NA	NA	NA	NA
ENSG00000229112	NA	NA	NA	0	NA	NA	NA	NA	NA
ENSG00000229115	NA	NA	NA	0	NA	NA	NA	NA	NA
ENSG00000229119	NA	NA	NA	0,329696904	-0,131364032	3,820552	-0,034383522	0,972571323	NA
ENSG00000229120	NA	NA	NA	0	NA	NA	NA	NA	NA
ENSG00000229129	NA	NA	NA	0	NA	NA	NA	NA	NA
ENSG00000229132	EIF4A1P10	eukaryotic translation initiation factor 4A1 pseudogene 10	100421533	0,294760666	0,67905486	3,896628	0,174267309	0,86165539	NA
ENSG00000229133	RPS7P4	ribosomal protein S7 pseudogene 4	149224	0	NA	NA	NA	NA	NA
ENSG00000229138	NA	NA	NA	0	NA	NA	NA	NA	NA
ENSG00000229142	NA	NA	NA	0	NA	NA	NA	NA	NA
ENSG00000229143	NA	NA	NA	0	NA	NA	NA	NA	NA
ENSG00000229145	ACTBP1	ACTB pseudogene 1	643309	0	NA	NA	NA	NA	NA
ENSG00000229146	NA	NA	NA	0	NA	NA	NA	NA	NA
ENSG00000229147	SMPD4P2	sphingomyelin phosphodiesterase 4 pseudogene 2	441654	0	NA	NA	NA	NA	NA
ENSG00000229156	NA	NA	NA	0,209763681	-1,926644696	4,042043	-0,476651224	0,633610501	NA
ENSG00000229159	TSPY23P	testis specific protein Y-linked 23, pseudogene	100286964	0	NA	NA	NA	NA	NA
ENSG00000229161	NA	NA	NA	0	NA	NA	NA	NA	NA
ENSG00000229163	NA	NA	NA	0	NA	NA	NA	NA	NA
ENSG00000229165	NA	NA	NA	0	NA	NA	NA	NA	NA
ENSG00000229168	RPL19P20	ribosomal protein L19 pseudogene 20	392451	0	NA	NA	NA	NA	NA
ENSG00000229169	NA	NA	NA	0	NA	NA	NA	NA	NA
ENSG00000229172	NA	NA	NA	0	NA	NA	NA	NA	NA
ENSG00000229173	NA	NA	NA	0	NA	NA	NA	NA	NA
ENSG00000229182	NA	NA	NA	0	NA	NA	NA	NA	NA
ENSG00000229183	PGA4	pepsinogen A4	643847	0	NA	NA	NA	NA	NA
ENSG00000229183	LOC124906959	pepsin A-4-like	124906959	0	NA	NA	NA	NA	NA
ENSG00000229184	ATP5PDP2	ATP synthase peripheral stalk subunit d pseudogene 2	138234	0	NA	NA	NA	NA	NA

ENSG00000229186	NA	NA	NA	0	NA	NA	NA	NA	NA
ENSG00000229197	RPS15AP28	ribosomal protein S15a pseudogene 28	100132400	0	NA	NA	NA	NA	NA
ENSG00000229204	NA	NA	NA	0	NA	NA	NA	NA	NA
ENSG00000229207	NA	NA	NA	0	NA	NA	NA	NA	NA
ENSG00000229208	NA	NA	NA	0	NA	NA	NA	NA	NA
ENSG00000229211	NA	NA	NA	0	NA	NA	NA	NA	NA
ENSG00000229212	NA	NA	NA	7,222384247	-0,358860105	0,750478	-0,478175231	0,63252549	NA
ENSG00000229213	NA	NA	NA	0	NA	NA	NA	NA	NA
ENSG00000229217	NA	NA	NA	0	NA	NA	NA	NA	NA
ENSG00000229220	NA	NA	NA	0	NA	NA	NA	NA	NA
ENSG00000229221	NA	NA	NA	0,118286652	0	4,080456	0	1	NA
ENSG00000229222	KRT18P4	keratin 18 pseudogene 4	391256	1,650806055	-1,577621547	1,641541	-0,961061099	0,336521448	NA
ENSG00000229226	NA	NA	NA	0	NA	NA	NA	NA	NA
ENSG00000229230	MT1P3	metallothionein 1 pseudogene 3	140851	0	NA	NA	NA	NA	NA
ENSG00000229231	FEM1AP1	fem-1 homolog A pseudogene 1	441957	0	NA	NA	NA	NA	NA
ENSG00000229234	RBMY1KP	RNA binding motif protein Y-linked family 1 member K, pseudogene	100874497	0	NA	NA	NA	NA	NA
ENSG00000229235	RPL37P18	ribosomal protein L37 pseudogene 18	100271342	0	NA	NA	NA	NA	NA
ENSG00000229237	NA	NA	NA	0	NA	NA	NA	NA	NA
ENSG00000229238	PPP1R12BP1	protein phosphatase 1 regulatory subunit 12B pseudogene 1	360021	0	NA	NA	NA	NA	NA
ENSG00000229239	NA	NA	NA	0	NA	NA	NA	NA	NA
ENSG00000229241	NA	NA	NA	0,866748118	-1,081638584	3,072421	-0,352047622	0,724802549	NA
ENSG00000229248	WBP2P1	WW domain binding protein 2 pseudogene 1	147468	0	NA	NA	NA	NA	NA
ENSG00000229250	USP9YP31	USP9Y pseudogene 31	106478930	0	NA	NA	NA	NA	NA
ENSG00000229251	NA	NA	NA	0,119673877	0,847101401	4,080456	0,207599697	0,835541541	NA
ENSG00000229254	NA	NA	NA	0	NA	NA	NA	NA	NA
ENSG00000229256	NA	NA	NA	0	NA	NA	NA	NA	NA
ENSG00000229259	NA	NA	NA	0	NA	NA	NA	NA	NA
ENSG00000229266	NA	NA	NA	0	NA	NA	NA	NA	NA
ENSG00000229268	PES1P2	pescadillo ribosomal biogenesis factor 1 pseudogene 2	347193	0	NA	NA	NA	NA	NA
ENSG00000229273	NA	NA	NA	0,779678516	-1,274996525	2,205442	-0,578113922	0,563187208	NA
ENSG00000229280	EEF1DP6	eukaryotic translation elongation factor 1 delta pseudogene 6	644357	0	NA	NA	NA	NA	NA
ENSG00000229281	NA	NA	NA	0	NA	NA	NA	NA	NA
ENSG00000229286	NA	NA	NA	0	NA	NA	NA	NA	NA
ENSG00000229287	NA	NA	NA	0	NA	NA	NA	NA	NA
ENSG00000229288	NA	NA	NA	0	NA	NA	NA	NA	NA
ENSG00000229292	RFPL4AL1	ret finger protein like 4A like 1	729974	0	NA	NA	NA	NA	NA
ENSG00000229298	TUBB8P1	tubulin beta 8 class VIII pseudogene 1	100418937	0	NA	NA	NA	NA	NA
ENSG00000229303	NA	NA	NA	0	NA	NA	NA	NA	NA
ENSG00000229305	LOC100129776	eukaryotic translation initiation factor 2 subunit gamma pseudogene	100129776	0	NA	NA	NA	NA	NA
ENSG00000229310	RPP40P1	ribonuclease P/MRP subunit p40 pseudogene 1	100874225	0	NA	NA	NA	NA	NA
ENSG00000229314	ORM1	orosomucoid 1	5004	0	NA	NA	NA	NA	NA
ENSG00000229316	NA	NA	NA	0	NA	NA	NA	NA	NA
ENSG00000229320	KRT8P12	keratin 8 pseudogene 12	90133	5,519163129	-0,257739256	0,930967	-0,276851037	0,78189449	NA
ENSG00000229322	LOC100420321	MAGE family member A11 pseudogene	100420321	0	NA	NA	NA	NA	NA
ENSG00000229324	NA	NA	NA	0	NA	NA	NA	NA	NA
ENSG00000229326	RPL17P15	ribosomal protein L17 pseudogene 15	100128117	0	NA	NA	NA	NA	NA
ENSG00000229332	NA	NA	NA	0	NA	NA	NA	NA	NA
ENSG00000229336	NA	NA	NA	0	NA	NA	NA	NA	NA
ENSG00000229338	NA	NA	NA	0	NA	NA	NA	NA	NA
ENSG00000229339	RPL7AP83	ribosomal protein L7a pseudogene 83	127379730	0	NA	NA	NA	NA	NA
ENSG00000229343	NA	NA	NA	0	NA	NA	NA	NA	NA
ENSG00000229344	MTCO2P12	MT-CO2 pseudogene 12	107075310	0,411707736	-0,218236616	3,001708	-0,072704147	0,942041548	NA
ENSG00000229347	LOC392440	MAGE family member B18 pseudogene	392440	0	NA	NA	NA	NA	NA
ENSG00000229349	NA	NA	NA	0	NA	NA	NA	NA	NA
ENSG00000229357	NA	NA	NA	0	NA	NA	NA	NA	NA
ENSG00000229358	NA	NA	NA	0,223590531	-1,079307759	4,080456	-0,264506661	0,791389533	NA

ENSG00000229359	PIN1P1	peptidylprolyl cis/trans isomerase, NIMA-interacting 1 pseudogene 1	5301	0	NA	NA	NA	NA	NA
ENSG00000229360	NA	NA	NA	0	NA	NA	NA	NA	NA
ENSG00000229361	UBTFL7	UBTF like 7 (pseudogene)	646759	0	NA	NA	NA	NA	NA
ENSG00000229365	NA	NA	NA	0	NA	NA	NA	NA	NA
ENSG00000229367	NA	NA	NA	0	NA	NA	NA	NA	NA
ENSG00000229369	LOC100419923	density regulated re-initiation and release factor pseudogene	100419923	0	NA	NA	NA	NA	NA
ENSG00000229375	USP24P1	ubiquitin specific peptidase 24 pseudogene 1	100874248	0	NA	NA	NA	NA	NA
ENSG00000229376	NA	NA	NA	0	NA	NA	NA	NA	NA
ENSG00000229384	NA	NA	NA	0	NA	NA	NA	NA	NA
ENSG00000229386	NA	NA	NA	0	NA	NA	NA	NA	NA
ENSG00000229390	NA	NA	NA	0,10488184	-1,076443944	4,080456	-0,263804824	0,791930317	NA
ENSG00000229391	NA	NA	NA	0	NA	NA	NA	NA	NA
ENSG00000229398	LOC100419859	zinc finger MYM-type containing 1 pseudogene	100419859	0	NA	NA	NA	NA	NA
ENSG00000229399	NA	NA	NA	0	NA	NA	NA	NA	NA
ENSG00000229405	LOC100130731	proteasome 20S subunit beta 1 pseudogene	100130731	0	NA	NA	NA	NA	NA
ENSG00000229406	OFD1P4Y	OFD1 pseudogene 4 Y-linked	386688	0	NA	NA	NA	NA	NA
ENSG00000229409	NA	NA	NA	0	NA	NA	NA	NA	NA
ENSG00000229413	NA	NA	NA	0	NA	NA	NA	NA	NA
ENSG00000229417	NPM1P25	nucleophosmin 1 pseudogene 25	399804	0	NA	NA	NA	NA	NA
ENSG00000229421	NA	NA	NA	0	NA	NA	NA	NA	NA
ENSG00000229423	RPL27AP8	ribosomal protein L27a pseudogene 8	100271605	0	NA	NA	NA	NA	NA
ENSG00000229427	ANKRD26P4	ankyrin repeat domain 26 pseudogene 4	100873882	0,327487438	-1,974631881	3,785776	-0,521592312	0,601954217	NA
ENSG00000229435	NA	NA	NA	0	NA	NA	NA	NA	NA
ENSG00000229436	NA	NA	NA	0	NA	NA	NA	NA	NA
ENSG00000229440	NA	NA	NA	0	NA	NA	NA	NA	NA
ENSG00000229442	NA	NA	NA	0	NA	NA	NA	NA	NA
ENSG00000229447	NA	NA	NA	2,075188654	0,146766905	1,540564	0,095268284	0,924101733	NA
ENSG00000229453	SPINK8	serine peptidase inhibitor Kazal type 8 (putative)	646424	0	NA	NA	NA	NA	NA
ENSG00000229455	NA	NA	NA	0	NA	NA	NA	NA	NA
ENSG00000229456	RLIMP1	ring finger protein, LIM domain interacting pseudogene 1	100533695	0	NA	NA	NA	NA	NA
ENSG00000229462	NA	NA	NA	0	NA	NA	NA	NA	NA
ENSG00000229468	NA	NA	NA	0	NA	NA	NA	NA	NA
ENSG00000229473	NA	NA	NA	5,848741391	0,246235262	0,745393	0,330343011	0,741140797	NA
ENSG00000229478	ROBO2P1	roundabout guidance receptor 2 pseudogene 1	106480790	0	NA	NA	NA	NA	NA
ENSG00000229486	NA	NA	NA	0	NA	NA	NA	NA	NA
ENSG00000229492	NA	NA	NA	0	NA	NA	NA	NA	NA
ENSG00000229497	NA	NA	NA	0,118286652	0	4,080456	0	1	NA
ENSG00000229500	RPL17P1	ribosomal protein L17 pseudogene 1	140748	0	NA	NA	NA	NA	NA
ENSG00000229503	RPL21P37	ribosomal protein L21 pseudogene 37	100129141	0,309679571	2,359666018	3,771905	0,625589988	0,531583907	NA
ENSG00000229505	NA	NA	NA	0	NA	NA	NA	NA	NA
ENSG00000229508	NA	NA	NA	0	NA	NA	NA	NA	NA
ENSG00000229509	NA	NA	NA	0	NA	NA	NA	NA	NA
ENSG00000229511	NA	NA	NA	0	NA	NA	NA	NA	NA
ENSG00000229515	FLT1P1	FLT1 pseudogene 1	391533	0	NA	NA	NA	NA	NA
ENSG00000229518	NA	NA	NA	0	NA	NA	NA	NA	NA
ENSG00000229519	NA	NA	NA	0	NA	NA	NA	NA	NA
ENSG00000229530	NA	NA	NA	0	NA	NA	NA	NA	NA
ENSG00000229532	NA	NA	NA	0	NA	NA	NA	NA	NA
ENSG00000229534	NA	NA	NA	0,455457359	0,013216233	3,492612	0,003784055	0,996980769	NA
ENSG00000229541	GAPDHP26	glyceraldehyde 3 phosphate dehydrogenase pseudogene 26	392374	0	NA	NA	NA	NA	NA
ENSG00000229544	NKX1-2	NK1 homeobox 2	390010	0	NA	NA	NA	NA	NA
ENSG00000229547	UBE2DNL	ubiquitin conjugating enzyme E2 D N-terminal like (pseudogene)	100131816	0	NA	NA	NA	NA	NA
ENSG00000229549	TSPY8	testis specific protein Y-linked 8	728403	0	NA	NA	NA	NA	NA
ENSG00000229549	LOC124908988	testis-specific Y-encoded protein 8	124908988	0	NA	NA	NA	NA	NA
ENSG00000229551	NA	NA	NA	0	NA	NA	NA	NA	NA
ENSG00000229553	USP9YP17	USP9Y pseudogene 17	106480714	0	NA	NA	NA	NA	NA
ENSG00000229554	RPL21P24	ribosomal protein L21 pseudogene 24	100270880	0	NA	NA	NA	NA	NA

ENSG00000229559	NA	NA	NA	0	NA	NA	NA	NA	NA
ENSG00000229560	NA	NA	NA	0	NA	NA	NA	NA	NA
ENSG00000229562	ZFYVE9P1	zinc finger FYVE-type containing 9 pseudogene 1	100289259	0	NA	NA	NA	NA	NA
ENSG00000229567	NA	NA	NA	0,117497108	0,847101401	4,080456	0,207599697	0,835541541	NA
ENSG00000229568	SMC4P1	structural maintenance of chromosomes 4 pseudogene 1	100421125	0	NA	NA	NA	NA	NA
ENSG00000229570	GAPDHP58	glyceraldehyde 3 phosphate dehydrogenase pseudogene 58	391077	0	NA	NA	NA	NA	NA
ENSG00000229571	PRAMEF25	PRAME family member 25	441873	0	NA	NA	NA	NA	NA
ENSG00000229571	PRAMEF26	PRAME family member 26	645359	0	NA	NA	NA	NA	NA
ENSG00000229572	EIF4BP4	eukaryotic translation initiation factor 4B pseudogene 4	100130064	0,119673877	0,847101401	4,080456	0,207599697	0,835541541	NA
ENSG00000229585	NA	NA	NA	1,756738104	-0,810291128	1,494751	-0,542090868	0,587755909	NA
ENSG00000229586	TNPO1P3	transportin 1 pseudogene 3	339257	0	NA	NA	NA	NA	NA
ENSG00000229590	NA	NA	NA	2,250913021	-1,019574317	1,856479	-0,549197983	0,582869588	NA
ENSG00000229593	NA	NA	NA	0	NA	NA	NA	NA	NA
ENSG00000229594	NA	NA	NA	0	NA	NA	NA	NA	NA
ENSG00000229595	NA	NA	NA	0	NA	NA	NA	NA	NA
ENSG00000229596	NA	NA	NA	0	NA	NA	NA	NA	NA
ENSG00000229598	NA	NA	NA	0,15836288	0	4,080456	0	1	NA
ENSG00000229601	NA	NA	NA	0	NA	NA	NA	NA	NA
ENSG00000229604	MTATP8P2	MT-ATP8 pseudogene 2	106479042	0	NA	NA	NA	NA	NA
ENSG00000229605	RPL21P93	ribosomal protein L21 pseudogene 93	653665	0,709694745	0,633744997	3,166093	0,200166265	0,84135055	NA
ENSG00000229608	NA	NA	NA	0	NA	NA	NA	NA	NA
ENSG00000229612	NA	NA	NA	0	NA	NA	NA	NA	NA
ENSG00000229616	NA	NA	NA	0	NA	NA	NA	NA	NA
ENSG00000229622	MTND5P2	MT-ND5 pseudogene 2	100873209	1,008461931	0,390667506	2,059868	0,189656605	0,849578227	NA
ENSG00000229623	METTL21AP1	methyltransferase like 21A pseudogene 1	100421629	0,399821065	0,818085681	4,079284	0,200546392	0,841053279	NA
ENSG00000229626	NA	NA	NA	0	NA	NA	NA	NA	NA
ENSG00000229627	BSNDP4	BSND pseudogene 4	106481726	0	NA	NA	NA	NA	NA
ENSG00000229629	NA	NA	NA	0	NA	NA	NA	NA	NA
ENSG00000229635	LOC100421273	F-box and WD repeat domain containing 11 pseudogene	100421273	0	NA	NA	NA	NA	NA
ENSG00000229636	KRT8P21	keratin 8 pseudogene 21	126811	0	NA	NA	NA	NA	NA
ENSG00000229637	PRAC2	PRAC2 small nuclear protein	360205	0	NA	NA	NA	NA	NA
ENSG00000229638	RPL4P4	ribosomal protein L4 pseudogene 4	647276	0,787389857	-0,205854024	2,202788	-0,093451563	0,925544828	NA
ENSG00000229641	CYP2C56P	cytochrome P450 family 2 subfamily C member 56, pseudogene	106478959	0	NA	NA	NA	NA	NA
ENSG00000229644	NAMPTP1	nicotinamide phosphoribosyltransferase pseudogene 1	646309	2,317906475	-2,761228172	1,541966	-1,790719438	0,073338329	NA
ENSG00000229648	RPSAP22	ribosomal protein SA pseudogene 22	100270874	0	NA	NA	NA	NA	NA
ENSG00000229652	NA	NA	NA	0	NA	NA	NA	NA	NA
ENSG00000229657	RPL13AP11	ribosomal protein L13a pseudogene 11	100271276	0	NA	NA	NA	NA	NA
ENSG00000229659	RPL26P6	ribosomal protein L26 pseudogene 6	100131526	2,31935747	-2,014407984	1,308247	-1,539775953	0,123614975	NA
ENSG00000229661	NA	NA	NA	0,10543123	0	4,080456	0	1	NA
ENSG00000229662	LOC100420084	ornithine aminotransferase pseudogene	100420084	0	NA	NA	NA	NA	NA
ENSG00000229663	DPY19L4P1	DPY19L4 pseudogene 1	100131533	0	NA	NA	NA	NA	NA
ENSG00000229667	UBE2V1P9	UBE2V1 pseudogene 9	100131981	0	NA	NA	NA	NA	NA
ENSG00000229668	NA	NA	NA	0,386394709	1,270314046	4,000301	0,317554595	0,750822821	NA
ENSG00000229670	NA	NA	NA	0	NA	NA	NA	NA	NA
ENSG00000229674	H2AL3	H2A,L variant histone 3	115482686	0	NA	NA	NA	NA	NA
ENSG00000229676	ZNF492	zinc finger protein 492	57615	0,53484702	0,586753179	2,790773	0,210247564	0,833474458	NA
ENSG00000229677	RPL17P28	ribosomal protein L17 pseudogene 28	100270940	5,292168914	-0,414115706	0,823962	-0,502590662	0,615252096	NA
ENSG00000229682	NA	NA	NA	0	NA	NA	NA	NA	NA
ENSG00000229687	RPL37P7	ribosomal protein L37 pseudogene 7	100270890	0	NA	NA	NA	NA	NA
ENSG00000229690	MTCO2P1	MT-CO2 pseudogene 1	140909	0	NA	NA	NA	NA	NA
ENSG00000229693	NA	NA	NA	0	NA	NA	NA	NA	NA
ENSG00000229695	LOC100421122	zinc finger protein 106 homolog (mouse) pseudogene	100421122	0	NA	NA	NA	NA	NA
ENSG00000229696	KARS1P1	lysyl-tRNA synthetase 1 pseudogene 1	100419558	0	NA	NA	NA	NA	NA
ENSG00000229700	NA	NA	NA	0	NA	NA	NA	NA	NA
ENSG00000229701	MTND2P20	MT-ND2 pseudogene 20	100873226	0	NA	NA	NA	NA	NA
ENSG00000229703	NA	NA	NA	0	NA	NA	NA	NA	NA

ENSG00000229704	NA	NA	NA	0,072165449	0,847101401	4,080456	0,207599697	0,835541541	NA
ENSG00000229707	NA	NA	NA	0	NA	NA	NA	NA	NA
ENSG00000229708	MARK2P12	MARK2 pseudogene 12	100421565	0	NA	NA	NA	NA	NA
ENSG00000229709	USP9YP36	USP9Y pseudogene 36	106478932	0	NA	NA	NA	NA	NA
ENSG00000229713	LCEP2	late cornified envelope pseudogene 2	450209	0	NA	NA	NA	NA	NA
ENSG00000229715	NA	NA	NA	0,119673877	0,847101401	4,080456	0,207599697	0,835541541	NA
ENSG00000229716	NA	NA	NA	0	NA	NA	NA	NA	NA
ENSG00000229721	RPS8P7	ribosomal protein S8 pseudogene 7	646093	0,631340089	0,305531987	2,377579	0,128505496	0,897748949	NA
ENSG00000229724	OR2AQ1P	olfactory receptor family 2 subfamily AQ member 1 pseudogene	81474	0	NA	NA	NA	NA	NA
ENSG00000229725	NA	NA	NA	0	NA	NA	NA	NA	NA
ENSG00000229730	NA	NA	NA	0	NA	NA	NA	NA	NA
ENSG00000229731	LOC100420323	MAGE family member B18 pseudogene	100420323	0	NA	NA	NA	NA	NA
ENSG00000229733	LOC107985645	cytochrome c oxidase subunit NDUFA4-like	107985645	0	NA	NA	NA	NA	NA
ENSG00000229735	FTLP16	ferritin light chain pseudogene 16	100420158	0	NA	NA	NA	NA	NA
ENSG00000229738	NA	NA	NA	0,103998153	-1,076443944	4,080456	-0,263804824	0,791930317	NA
ENSG00000229742	NA	NA	NA	0	NA	NA	NA	NA	NA
ENSG00000229744	NA	NA	NA	0	NA	NA	NA	NA	NA
ENSG00000229745	NA	NA	NA	0	NA	NA	NA	NA	NA
ENSG00000229747	NA	NA	NA	0	NA	NA	NA	NA	NA
ENSG00000229749	NA	NA	NA	0	NA	NA	NA	NA	NA
ENSG00000229752	RPL7P10	ribosomal protein L7 pseudogene 10	646538	0	NA	NA	NA	NA	NA
ENSG00000229753	RPS27AP15	RPS27A pseudogene 15	100271379	0	NA	NA	NA	NA	NA
ENSG00000229754	NA	NA	NA	0	NA	NA	NA	NA	NA
ENSG00000229756	RPL31P20	ribosomal protein L31 pseudogene 20	100270986	0	NA	NA	NA	NA	NA
ENSG00000229757	RPL7AP21	ribosomal protein L7a pseudogene 21	100271038	0,567068828	1,633258865	2,857346	0,571599999	0,567592996	NA
ENSG00000229758	NA	NA	NA	0,072165449	0,847101401	4,080456	0,207599697	0,835541541	NA
ENSG00000229759	NA	NA	NA	0,072165449	0,847101401	4,080456	0,207599697	0,835541541	NA
ENSG00000229760	MTCO1P52	MT-CO1 pseudogene 52	107075227	0	NA	NA	NA	NA	NA
ENSG00000229761	NA	NA	NA	0	NA	NA	NA	NA	NA
ENSG00000229762	LOC100420540	cell division cycle associated 8 pseudogene	100420540	0	NA	NA	NA	NA	NA
ENSG00000229765	RPL21P62	ribosomal protein L21 pseudogene 62	442160	0	NA	NA	NA	NA	NA
ENSG00000229769	TRBV10-2	T cell receptor beta variable 10-2	28584	0	NA	NA	NA	NA	NA
ENSG00000229781	RRN3P4	RRN3 pseudogene 4	100129955	0	NA	NA	NA	NA	NA
ENSG00000229785	NA	NA	NA	0,189662396	1,665043938	4,047526	0,411373234	0,680798877	NA
ENSG00000229786	NA	NA	NA	0	NA	NA	NA	NA	NA
ENSG00000229794	MTCYBP32	MT-CYB pseudogene 32	107075231	0,382565168	0,859788345	4,080456	0,210708894	0,833114434	NA
ENSG00000229798	KRT18P26	keratin 18 pseudogene 26	100418770	0,453232556	0,968669718	3,486412	0,277841412	0,7811341	NA
ENSG00000229800	ATP8A2P2	ATPase phospholipid transporting 8A2 pseudogene 2	100533851	0	NA	NA	NA	NA	NA
ENSG00000229806	RPS15P5	ribosomal protein S15 pseudogene 5	729789	0	NA	NA	NA	NA	NA
ENSG00000229808	NA	NA	NA	3,347877262	-0,679308532	1,234323	-0,550349128	0,582079934	NA
ENSG00000229814	NA	NA	NA	0	NA	NA	NA	NA	NA
ENSG00000229815	NA	NA	NA	0	NA	NA	NA	NA	NA
ENSG00000229816	NA	NA	NA	7,994500487	0,743942208	0,661371	1,124849085	0,260652991	NA
ENSG00000229817	LOC645266	pre-mRNA processing factor 4B pseudogene	645266	0,623082803	-0,117753118	2,821	-0,041741619	0,966704675	NA
ENSG00000229820	NA	NA	NA	1,124171274	0,665004608	1,716381	0,387445867	0,698426153	NA
ENSG00000229822	RPS15AP39	ribosomal protein S15a pseudogene 39	100129284	0	NA	NA	NA	NA	NA
ENSG00000229824	RPL12P34	ribosomal protein L12 pseudogene 34	100271407	0	NA	NA	NA	NA	NA
ENSG00000229826	NA	NA	NA	0	NA	NA	NA	NA	NA
ENSG00000229827	NA	NA	NA	0,11808024	-1,076443944	4,080456	-0,263804824	0,791930317	NA
ENSG00000229828	NA	NA	NA	0	NA	NA	NA	NA	NA
ENSG00000229829	DUTP4	deoxyuridine triphosphatase pseudogene 4	100873910	0	NA	NA	NA	NA	NA
ENSG00000229830	NA	NA	NA	0	NA	NA	NA	NA	NA
ENSG00000229831	NA	NA	NA	0	NA	NA	NA	NA	NA
ENSG00000229834	NA	NA	NA	0	NA	NA	NA	NA	NA
ENSG00000229835	KHSRPP1	KH-type splicing regulatory protein pseudogene 1	402359	0	NA	NA	NA	NA	NA
ENSG00000229836	NA	NA	NA	0	NA	NA	NA	NA	NA
ENSG00000229840	NA	NA	NA	0	NA	NA	NA	NA	NA
ENSG00000229842	MTATP6P13	MT-ATP6 pseudogene 13	107075272	0	NA	NA	NA	NA	NA
ENSG00000229849	NA	NA	NA	0	NA	NA	NA	NA	NA

ENSG00000229853	NA	NA	NA	0,072165449	0,847101401	4,080456	0,207599697	0,835541541	NA
ENSG00000229857	NA	NA	NA	0	NA	NA	NA	NA	NA
ENSG00000229858	NA	NA	NA	0	NA	NA	NA	NA	NA
ENSG00000229859	PGA3	pepsinogen A3	643834	0	NA	NA	NA	NA	NA
ENSG00000229863	RPL19P2	ribosomal protein L19 pseudogene 2	654382	0,10488184	-1,076443944	4,080456	-0,263804824	0,791930317	NA
ENSG00000229865	RPL31P54	ribosomal protein L31 pseudogene 54	100271483	0	NA	NA	NA	NA	NA
ENSG00000229870	RPL21P89	ribosomal protein L21 pseudogene 89	100271422	0	NA	NA	NA	NA	NA
ENSG00000229871	RPSAP20	ribosomal protein SA pseudogene 20	127406	0	NA	NA	NA	NA	NA
ENSG00000229875	NA	NA	NA	0	NA	NA	NA	NA	NA
ENSG00000229878	NA	NA	NA	0	NA	NA	NA	NA	NA
ENSG00000229880	IMMTP1	inner membrane mitochondrial protein pseudogene 1	54045	0	NA	NA	NA	NA	NA
ENSG00000229885	NA	NA	NA	0	NA	NA	NA	NA	NA
ENSG00000229886	NA	NA	NA	0	NA	NA	NA	NA	NA
ENSG00000229887	NA	NA	NA	0	NA	NA	NA	NA	NA
ENSG00000229890	ACTP1	actin family pseudogene 1	106480335	0	NA	NA	NA	NA	NA
ENSG00000229892	NA	NA	NA	0	NA	NA	NA	NA	NA
ENSG00000229894	GK3	glycerol kinase 3	2713	0,232478121	0,832660548	4,079907	0,20408811	0,838284646	NA
ENSG00000229897	SEPTIN7P7	septin 7 pseudogene 7	100130564	0,072165449	0,847101401	4,080456	0,207599697	0,835541541	NA
ENSG00000229899	NA	NA	NA	0	NA	NA	NA	NA	NA
ENSG00000229900	NA	NA	NA	0	NA	NA	NA	NA	NA
ENSG00000229906	NA	NA	NA	0	NA	NA	NA	NA	NA
ENSG00000229907	NA	NA	NA	0	NA	NA	NA	NA	NA
ENSG00000229909	TRIM60P16	tripartite motif containing 60 pseudogene 16	100420412	0	NA	NA	NA	NA	NA
ENSG00000229910	NA	NA	NA	0	NA	NA	NA	NA	NA
ENSG00000229911	NA	NA	NA	0	NA	NA	NA	NA	NA
ENSG00000229914	RPS10P8	ribosomal protein S10 pseudogene 8	729780	0	NA	NA	NA	NA	NA
ENSG00000229917	NA	NA	NA	1,511379666	0,814951536	1,608093	0,506781443	0,612308179	NA
ENSG00000229919	NA	NA	NA	0	NA	NA	NA	NA	NA
ENSG00000229920	RPS4XP5	ribosomal protein S4X pseudogene 5	100271132	0,235789822	0,844897724	4,080456	0,20705964	0,835963282	NA
ENSG00000229924	FAM90A26	family with sequence similarity 90 member A26	100287045	0	NA	NA	NA	NA	NA
ENSG00000229926	NA	NA	NA	0	NA	NA	NA	NA	NA
ENSG00000229927	NA	NA	NA	0	NA	NA	NA	NA	NA
ENSG00000229930	NA	NA	NA	0	NA	NA	NA	NA	NA
ENSG00000229932	NA	NA	NA	0,072165449	0,847101401	4,080456	0,207599697	0,835541541	NA
ENSG00000229935	MTCYBP9	MT-CYB pseudogene 9	107075124	0	NA	NA	NA	NA	NA
ENSG00000229937	PRPS1L1	phosphoribosyl pyrophosphate synthetase 1 like 1	221823	0	NA	NA	NA	NA	NA
ENSG00000229939	NA	NA	NA	0	NA	NA	NA	NA	NA
ENSG00000229940	NA	NA	NA	0	NA	NA	NA	NA	NA
ENSG00000229944	NA	NA	NA	0,311994459	-2,498367527	3,998294	-0,62485841	0,532063991	NA
ENSG00000229946	NA	NA	NA	0	NA	NA	NA	NA	NA
ENSG00000229948	NA	NA	NA	0	NA	NA	NA	NA	NA
ENSG00000229949	NA	NA	NA	0	NA	NA	NA	NA	NA
ENSG00000229954	MTND2P2	MT-ND2 pseudogene 2	56168	0,11808024	-1,076443944	4,080456	-0,263804824	0,791930317	NA
ENSG00000229961	NA	NA	NA	0	NA	NA	NA	NA	NA
ENSG00000229965	RPS3AP7	RPS3A pseudogene 7	391168	0	NA	NA	NA	NA	NA
ENSG00000229968	LOC100419784	zinc finger protein 484 pseudogene	100419784	0	NA	NA	NA	NA	NA
ENSG00000229972	IQCF3	IQ motif containing F3	401067	0	NA	NA	NA	NA	NA
ENSG00000229975	NA	NA	NA	0	NA	NA	NA	NA	NA
ENSG00000229979	NA	NA	NA	0	NA	NA	NA	NA	NA
ENSG00000229982	NA	NA	NA	0	NA	NA	NA	NA	NA
ENSG00000229985	RPL18AP5	ribosomal protein L18a pseudogene 5	729955	0	NA	NA	NA	NA	NA
ENSG00000229988	NA	NA	NA	0	NA	NA	NA	NA	NA
ENSG00000229991	AKR1B1P1	aldehyde reductase family 1 member B1 pseudogene 1	401982	0	NA	NA	NA	NA	NA
ENSG00000229992	NA	NA	NA	0,119673877	0,847101401	4,080456	0,207599697	0,835541541	NA
ENSG00000229994	RPL5P4	ribosomal protein L5 pseudogene 4	643343	2,273108002	1,008183926	1,44573	0,69735281	0,485582026	NA
ENSG00000230003	NA	NA	NA	0	NA	NA	NA	NA	NA
ENSG00000230006	NA	NA	NA	0	NA	NA	NA	NA	NA
ENSG00000230009	MTCO2P3	MT-CO2 pseudogene 3	100873203	0	NA	NA	NA	NA	NA
ENSG00000230011	NA	NA	NA	0	NA	NA	NA	NA	NA
ENSG00000230018	PPIAP39	peptidylprolyl isomerase A pseudogene 39	111082963	0	NA	NA	NA	NA	NA
ENSG00000230019	NA	NA	NA	0	NA	NA	NA	NA	NA
ENSG00000230021	LOC101928626	uncharacterized LOC101928626	101928626	0	NA	NA	NA	NA	NA

ENSG00000230021	LOC124901156	uncharacterized LOC124901156	124901156	0	NA	NA	NA	NA	NA
ENSG00000230022	NA	NA	NA	0	NA	NA	NA	NA	NA
ENSG00000230025	NA	NA	NA	0	NA	NA	NA	NA	NA
ENSG00000230031	POTEB2	POTE ankyrin domain family member B2	100287399	0	NA	NA	NA	NA	NA
ENSG00000230037	NA	NA	NA	0	NA	NA	NA	NA	NA
ENSG00000230039	NA	NA	NA	0	NA	NA	NA	NA	NA
ENSG00000230042	AK3P3	AK3 pseudogene 3	100419075	2,400424788	-0,022260153	1,315425	-0,016922399	0,986498523	NA
ENSG00000230043	NA	NA	NA	0,402565401	-0,114669383	3,616996	-0,031702932	0,974708957	NA
ENSG00000230044	CDK8P1	cyclin dependent kinase 8 pseudogene 1	100419916	0	NA	NA	NA	NA	NA
ENSG00000230045	NA	NA	NA	0	NA	NA	NA	NA	NA
ENSG00000230047	HIGD2AP1	HIGD2A pseudogene 1	100289360	0	NA	NA	NA	NA	NA
ENSG00000230052	MTND3P2	MT-ND3 pseudogene 2	100873180	0	NA	NA	NA	NA	NA
ENSG00000230053	NA	NA	NA	0	NA	NA	NA	NA	NA
ENSG00000230054	TEX53	testis expressed 53	105376230	0	NA	NA	NA	NA	NA
ENSG00000230062	ANKRD66	ankyrin repeat domain 66	100287718	0	NA	NA	NA	NA	NA
ENSG00000230066	NA	NA	NA	0	NA	NA	NA	NA	NA
ENSG00000230067	NA	NA	NA	0,10488184	-1,076443944	4,080456	-0,263804824	0,791930317	NA
ENSG00000230069	LRRC37A15P	leucine rich repeat containing 37 member A15, pseudogene	106481796	0,884542324	0	3,430055	0	1	NA
ENSG00000230071	RPL4P6	ribosomal protein L4 pseudogene 6	100271033	4,682159926	-0,544108101	1,137562	-0,478310921	0,632428924	NA
ENSG00000230073	NA	NA	NA	0	NA	NA	NA	NA	NA
ENSG00000230076	RPL10P6	ribosomal protein L10 pseudogene 6	285176	0	NA	NA	NA	NA	NA
ENSG00000230077	MTAPP2	methylthioadenosine phosphorylase pseudogene 2	100420621	0	NA	NA	NA	NA	NA
ENSG00000230079	NA	NA	NA	0	NA	NA	NA	NA	NA
ENSG00000230080	NA	NA	NA	0	NA	NA	NA	NA	NA
ENSG00000230081	HSPE1P28	heat shock protein family E (Hsp10) member 1 pseudogene 28	171420	0	NA	NA	NA	NA	NA
ENSG00000230086	VN1R96P	vomeronasal 1 receptor 96 pseudogene	100127969	0,912325278	1,287947177	1,950497	0,660317276	0,509050246	NA
ENSG00000230087	LOC100418885	amphiphysin pseudogene	100418885	0	NA	NA	NA	NA	NA
ENSG00000230088	KRT16P5	keratin 16 pseudogene 5	353196	0	NA	NA	NA	NA	NA
ENSG00000230092	NA	NA	NA	2,167472856	-0,655319021	1,266059	-0,517605403	0,604733611	NA
ENSG00000230097	NA	NA	NA	0	NA	NA	NA	NA	NA
ENSG00000230099	TRBV5-4	T cell receptor beta variable 5-4	28611	0	NA	NA	NA	NA	NA
ENSG00000230101	NA	NA	NA	0	NA	NA	NA	NA	NA
ENSG00000230104	NA	NA	NA	0	NA	NA	NA	NA	NA
ENSG00000230106	SNRPCP15	small nuclear ribonucleoprotein polypeptide C pseudogene 15	100310857	0	NA	NA	NA	NA	NA
ENSG00000230111	H3P11	H3 histone pseudogene 11	115482708	0	NA	NA	NA	NA	NA
ENSG00000230112	NA	NA	NA	0	NA	NA	NA	NA	NA
ENSG00000230116	RPS3AP11	RPS3A pseudogene 11	100270904	0	NA	NA	NA	NA	NA
ENSG00000230118	NA	NA	NA	0	NA	NA	NA	NA	NA
ENSG00000230119	NA	NA	NA	0	NA	NA	NA	NA	NA
ENSG00000230122	NA	NA	NA	0	NA	NA	NA	NA	NA
ENSG00000230123	DLEC1P1	DLEC1 pseudogene 1	100421153	0	NA	NA	NA	NA	NA
ENSG00000230125	EEF1A1P39	eukaryotic translation elongation factor 1 alpha 1 pseudogene 39	100862850	0	NA	NA	NA	NA	NA
ENSG00000230130	MTND2P39	MT-ND2 pseudogene 39	107075312	0	NA	NA	NA	NA	NA
ENSG00000230132	NA	NA	NA	0	NA	NA	NA	NA	NA
ENSG00000230137	NA	NA	NA	0	NA	NA	NA	NA	NA
ENSG00000230146	NA	NA	NA	0	NA	NA	NA	NA	NA
ENSG00000230147	NA	NA	NA	0,117497108	0,847101401	4,080456	0,207599697	0,835541541	NA
ENSG00000230154	NA	NA	NA	0	NA	NA	NA	NA	NA
ENSG00000230158	MTND1P28	MT-ND1 pseudogene 28	100873215	0	NA	NA	NA	NA	NA
ENSG00000230160	NA	NA	NA	0	NA	NA	NA	NA	NA
ENSG00000230161	NA	NA	NA	0	NA	NA	NA	NA	NA
ENSG00000230162	CT45A11P	cancer/testis antigen family 45 member A11, pseudogene	100129813	0,263086274	-1,077887169	4,080456	-0,264158516	0,791657775	NA
ENSG00000230165	NA	NA	NA	0	NA	NA	NA	NA	NA
ENSG00000230166	NA	NA	NA	0	NA	NA	NA	NA	NA
ENSG00000230169	RPL30P16	ribosomal protein L30 pseudogene 16	100289099	0	NA	NA	NA	NA	NA
ENSG00000230170	NA	NA	NA	0	NA	NA	NA	NA	NA
ENSG00000230171	RPL22P18	ribosomal protein L22 pseudogene 18	100271290	0	NA	NA	NA	NA	NA
ENSG00000230175	RPS13P1	ribosomal protein S13 pseudogene 1	100128305	0,210862461	0	4,080456	0	1	NA

ENSG00000230178	OR4F3	olfactory receptor family 4 subfamily F member 3	26683	0	NA	NA	NA	NA	NA
ENSG00000230180	RPL12P49	ribosomal protein L12 pseudogene 49	107075252	0	NA	NA	NA	NA	NA
ENSG00000230182	NA	NA	NA	0	NA	NA	NA	NA	NA
ENSG00000230183	NA	NA	NA	0	NA	NA	NA	NA	NA
ENSG00000230187	NA	NA	NA	0	NA	NA	NA	NA	NA
ENSG00000230188	RPL21P83	ribosomal protein L21 pseudogene 83	100271186	0	NA	NA	NA	NA	NA
ENSG00000230189	RABGEF1P2	RABGEF1 pseudogene 2	122149301	2,928544512	0,437289425	1,389125	0,314794923	0,752917367	NA
ENSG00000230191	NA	NA	NA	0	NA	NA	NA	NA	NA
ENSG00000230192	NA	NA	NA	0	NA	NA	NA	NA	NA
ENSG00000230194	RPL34P35	ribosomal protein L34 pseudogene 35	100271504	0	NA	NA	NA	NA	NA
ENSG00000230196	DDX43P3	DEAD-box helicase 43 pseudogene 3	106481709	0	NA	NA	NA	NA	NA
ENSG00000230198	NA	NA	NA	0	NA	NA	NA	NA	NA
ENSG00000230199	NA	NA	NA	0	NA	NA	NA	NA	NA
ENSG00000230201	NA	NA	NA	0	NA	NA	NA	NA	NA
ENSG00000230203	LOC646216	ribosomal protein S3A pseudogene	646216	0,117497108	0,847101401	4,080456	0,207599697	0,835541541	NA
ENSG00000230204	NA	NA	NA	0,29581837	-1,071467666	3,901872	-0,274603505	0,783620873	NA
ENSG00000230208	IFNNP1	interferon nu 1, pseudogene	654783	0	NA	NA	NA	NA	NA
ENSG00000230213	OR8V1P	olfactory receptor family 8 subfamily V member 1 pseudogene	282776	0	NA	NA	NA	NA	NA
ENSG00000230214	FTLP18	ferritin light chain pseudogene 18	100462786	0	NA	NA	NA	NA	NA
ENSG00000230216	NA	NA	NA	0	NA	NA	NA	NA	NA
ENSG00000230219	CIBAR1P2	CIBAR1 pseudogene 2	403315	0	NA	NA	NA	NA	NA
ENSG00000230221	LOC643342	ATM interactor pseudogene	643342	0	NA	NA	NA	NA	NA
ENSG00000230224	PHB1P9	PHB1 pseudogene 9	100289414	0	NA	NA	NA	NA	NA
ENSG00000230225	MTND5P14	MT-ND5 pseudogene 14	100873258	5,493016897	0,425256895	0,730414	0,582213594	0,560422818	NA
ENSG00000230227	NA	NA	NA	0	NA	NA	NA	NA	NA
ENSG00000230228	H3P1	H3 histone pseudogene 1	115482711	0	NA	NA	NA	NA	NA
ENSG00000230231	FMO7P	flavin containing dimethylaniline monoxygenase 7, pseudogene	100337589	0	NA	NA	NA	NA	NA
ENSG00000230237	NA	NA	NA	0	NA	NA	NA	NA	NA
ENSG00000230239	NA	NA	NA	0	NA	NA	NA	NA	NA
ENSG00000230240	NA	NA	NA	0	NA	NA	NA	NA	NA
ENSG00000230241	NA	NA	NA	0	NA	NA	NA	NA	NA
ENSG00000230243	NA	NA	NA	0	NA	NA	NA	NA	NA
ENSG00000230244	NA	NA	NA	0	NA	NA	NA	NA	NA
ENSG00000230245	NA	NA	NA	0	NA	NA	NA	NA	NA
ENSG00000230246	SPATA31C1	SPATA31 subfamily C member 1	441452	0	NA	NA	NA	NA	NA
ENSG00000230247	HNRNPH3P1	heterogeneous nuclear ribonucleoprotein H3 pseudogene 1	106480233	0	NA	NA	NA	NA	NA
ENSG00000230249	NA	NA	NA	0	NA	NA	NA	NA	NA
ENSG00000230251	PHB1P4	PHB1 pseudogene 4	100418774	0	NA	NA	NA	NA	NA
ENSG00000230252	MTND2P4	MT-ND2 pseudogene 4	100873175	0	NA	NA	NA	NA	NA
ENSG00000230256	NA	NA	NA	0	NA	NA	NA	NA	NA
ENSG00000230267	NA	NA	NA	2,310316067	3,365817754	1,914374	1,758181836	0,078716575	NA
ENSG00000230268	SSU72L6	SSU72 like 6	136157	0	NA	NA	NA	NA	NA
ENSG00000230271	NA	NA	NA	0	NA	NA	NA	NA	NA
ENSG00000230273	NA	NA	NA	0	NA	NA	NA	NA	NA
ENSG00000230274	PGAM1P3	phosphoglycerate mutase 1 pseudogene 3	100873887	0	NA	NA	NA	NA	NA
ENSG00000230275	SERBP1P4	SERPINE1 mRNA binding protein 1 pseudogene 4	100132591	0	NA	NA	NA	NA	NA
ENSG00000230276	NA	NA	NA	0	NA	NA	NA	NA	NA
ENSG00000230280	NA	NA	NA	0	NA	NA	NA	NA	NA
ENSG00000230282	RPL7P61	ribosomal protein L7 pseudogene 61	101929589	0	NA	NA	NA	NA	NA
ENSG00000230283	RPS12P24	ribosomal protein S12 pseudogene 24	100271551	0	NA	NA	NA	NA	NA
ENSG00000230284	NA	NA	NA	0	NA	NA	NA	NA	NA
ENSG00000230287	NA	NA	NA	0	NA	NA	NA	NA	NA
ENSG00000230291	RPL26P32	ribosomal protein L26 pseudogene 32	400055	0	NA	NA	NA	NA	NA
ENSG00000230295	NA	NA	NA	0,470044939	-1,037551463	2,860826	-0,362675489	0,716847312	NA
ENSG00000230301	OR5H6	olfactory receptor family 5 subfamily H member 6	79295	0	NA	NA	NA	NA	NA
ENSG00000230302	MTND3P4	MT-ND3 pseudogene 4	100873181	0	NA	NA	NA	NA	NA
ENSG00000230305	NA	NA	NA	2,232636358	-4,280395546	1,839384	-2,327081439	0,019960931	NA
ENSG00000230306	NA	NA	NA	0,293921408	-0,114722747	3,942614	-0,029098141	0,976786319	NA

ENSG00000230307	OR6C5P	olfactory receptor family 6 subfamily C member 5 pseudogene	390320	0	NA	NA	NA	NA	NA
ENSG00000230311	NA	NA	NA	0	NA	NA	NA	NA	NA
ENSG00000230312	NA	NA	NA	2,932100358	-0,159431047	1,137903	-0,140109487	0,888573485	NA
ENSG00000230318	XRCC6P3	X-ray repair cross complementing 6 pseudogene 3	127086	0	NA	NA	NA	NA	NA
ENSG00000230320	NA	NA	NA	0	NA	NA	NA	NA	NA
ENSG00000230321	MTCO2P27	MT-CO2 pseudogene 27	107075291	0	NA	NA	NA	NA	NA
ENSG00000230326	HMGN1P33	high mobility group nucleosome binding domain 1 pseudogene 33	100874446	0	NA	NA	NA	NA	NA
ENSG00000230327	MTCO1P42	MT-CO1 pseudogene 42	107075283	0	NA	NA	NA	NA	NA
ENSG00000230328	NA	NA	NA	0	NA	NA	NA	NA	NA
ENSG00000230330	NA	NA	NA	0,118286652	0	4,080456	0	1	NA
ENSG00000230331	NA	NA	NA	0	NA	NA	NA	NA	NA
ENSG00000230335	NA	NA	NA	0	NA	NA	NA	NA	NA
ENSG00000230338	NA	NA	NA	0	NA	NA	NA	NA	NA
ENSG00000230339	NA	NA	NA	0	NA	NA	NA	NA	NA
ENSG00000230342	FANCD2P2	FANCD2 pseudogene 2	101929530	0,10488184	-1,076443944	4,080456	-0,263804824	0,791930317	NA
ENSG00000230350	RPL35AP3	ribosomal protein L35a pseudogene 3	387074	0	NA	NA	NA	NA	NA
ENSG00000230353	NA	NA	NA	0	NA	NA	NA	NA	NA
ENSG00000230355	NA	NA	NA	0	NA	NA	NA	NA	NA
ENSG00000230356	NCAPD2P1	non-SMC condensin I complex subunit D2 pseudogene 1	100421175	0	NA	NA	NA	NA	NA
ENSG00000230358	SPDYE21	speedy/RINGO cell cycle regulator family member E21	442572	0,222651478	-0,094620153	4,080147	-0,023190378	0,981498414	NA
ENSG00000230359	TPI1P2	triosephosphate isomerase 1 pseudogene 2	286016	0,807335315	-0,691404154	2,167271	-0,319020567	0,749710917	NA
ENSG00000230360	DDX10P2	DEAD-box helicase 10 pseudogene 2	401533	0	NA	NA	NA	NA	NA
ENSG00000230361	VN1R32P	vomeronasal 1 receptor 32 pseudogene	100312788	0	NA	NA	NA	NA	NA
ENSG00000230362	ACTG1P23	actin gamma 1 pseudogene 23	442097	0	NA	NA	NA	NA	NA
ENSG00000230364	RPL4P3	ribosomal protein L4 pseudogene 3	391135	0	NA	NA	NA	NA	NA
ENSG00000230370	RPL23AP52	ribosomal protein L23a pseudogene 52	100271465	0	NA	NA	NA	NA	NA
ENSG00000230371	KARS1P2	lysyl-tRNA synthetase 1 pseudogene 2	100419560	0	NA	NA	NA	NA	NA
ENSG00000230375	NA	NA	NA	0	NA	NA	NA	NA	NA
ENSG00000230376	MEMO1P4	MEMO1 pseudogene 4	100462785	0	NA	NA	NA	NA	NA
ENSG00000230383	RPL6P19	ribosomal protein L6 pseudogene 19	646483	0,530250345	0,689895389	2,925497	0,235821622	0,813571089	NA
ENSG00000230384	RPSAP25	ribosomal protein SA pseudogene 25	129841	0	NA	NA	NA	NA	NA
ENSG00000230386	LOC402279	glutamate metabotropic receptor 8 pseudogene	402279	0	NA	NA	NA	NA	NA
ENSG00000230391	RPSAP23	ribosomal protein SA pseudogene 23	100270875	0	NA	NA	NA	NA	NA
ENSG00000230395	ANAPC1P3	ANAPC1 pseudogene 3	100420889	0	NA	NA	NA	NA	NA
ENSG00000230397	SPTLC1P1	serine palmitoyltransferase long chain base subunit 1 pseudogene 1	100874511	0,646656466	2,194443581	3,147494	0,697203409	0,485675506	NA
ENSG00000230398	NA	NA	NA	0	NA	NA	NA	NA	NA
ENSG00000230399	RBBP8P1	RBBP8 pseudogene 1	100420552	0	NA	NA	NA	NA	NA
ENSG00000230405	RPS3AP52	RPS3A pseudogene 52	100873818	0	NA	NA	NA	NA	NA
ENSG00000230406	RPL23P5	ribosomal protein L23 pseudogene 5	402120	0	NA	NA	NA	NA	NA
ENSG00000230407	LOC100419513	MIA SH3 domain ER export factor 2 pseudogene	100419513	0	NA	NA	NA	NA	NA
ENSG00000230409	NA	NA	NA	4,722156488	-0,873829126	1,167356	-0,748554035	0,454126045	NA
ENSG00000230411	OR3D1P	olfactory receptor family 3 subfamily D member 1 pseudogene	504175	0	NA	NA	NA	NA	NA
ENSG00000230418	NA	NA	NA	0	NA	NA	NA	NA	NA
ENSG00000230425	NA	NA	NA	0,176490776	-0,125666159	4,08026	-0,030798567	0,975430183	NA
ENSG00000230444	NA	NA	NA	0,10488184	-1,076443944	4,080456	-0,263804824	0,791930317	NA
ENSG00000230445	LRRC37A6P	leucine rich repeat containing 37 member A6, pseudogene	387646	1,354144454	0,799367922	1,997068	0,400270794	0,688957077	NA
ENSG00000230447	CTF2P	cardiotrophin 2, pseudogene	100289419	0	NA	NA	NA	NA	NA
ENSG00000230450	NA	NA	NA	0	NA	NA	NA	NA	NA
ENSG00000230451	RPS29P6	ribosomal protein S29 pseudogene 6	100270903	0	NA	NA	NA	NA	NA
ENSG00000230453	ANKRD18B	ankyrin repeat domain 18B	441459	0,627442964	2,170107327	3,186529	0,681025457	0,495855383	NA

ENSG00000230457	NA	NA	NA	4,914912684	0,024098466	0,970927	0,02482005	0,980198499	NA
ENSG00000230459	NA	NA	NA	0	NA	NA	NA	NA	NA
ENSG00000230465	NA	NA	NA	0	NA	NA	NA	NA	NA
ENSG00000230468	NA	NA	NA	0	NA	NA	NA	NA	NA
ENSG00000230469	RPL5P26	ribosomal protein L5 pseudogene 26	100271206	0	NA	NA	NA	NA	NA
ENSG00000230474	NA	NA	NA	0	NA	NA	NA	NA	NA
ENSG00000230476	OFD1P9Y	OFD1 pseudogene 9 Y-linked	386693	0	NA	NA	NA	NA	NA
ENSG00000230477	NA	NA	NA	0	NA	NA	NA	NA	NA
ENSG00000230478	NA	NA	NA	0	NA	NA	NA	NA	NA
ENSG00000230480	RPL34P11	ribosomal protein L34 pseudogene 11	100130286	0	NA	NA	NA	NA	NA
ENSG00000230481	NA	NA	NA	0	NA	NA	NA	NA	NA
ENSG00000230482	NA	NA	NA	0	NA	NA	NA	NA	NA
ENSG00000230484	OR51A10P	olfactory receptor family 51 subfamily A member 10 pseudogene	81295	0	NA	NA	NA	NA	NA
ENSG00000230485	NA	NA	NA	0	NA	NA	NA	NA	NA
ENSG00000230491	SRD5A2P1	SRD5A2 pseudogene 1	118568808	0	NA	NA	NA	NA	NA
ENSG00000230495	LOC100286962	ras homolog family member G pseudogene	100286962	0,311005654	-2,494181533	3,998569	-0,623768564	0,532779587	NA
ENSG00000230496	MTCO2P7	MT-CO2 pseudogene 7	107075145	0	NA	NA	NA	NA	NA
ENSG00000230497	EEF1B2P8	eukaryotic translation elongation factor 1 beta 2 pseudogene 8	100421774	0	NA	NA	NA	NA	NA
ENSG00000230501	NA	NA	NA	0	NA	NA	NA	NA	NA
ENSG00000230502	NA	NA	NA	0	NA	NA	NA	NA	NA
ENSG00000230507	RPL7AP8	ribosomal protein L7a pseudogene 8	340749	0	NA	NA	NA	NA	NA
ENSG00000230508	RPL19P21	ribosomal protein L19 pseudogene 21	392557	0	NA	NA	NA	NA	NA
ENSG00000230510	NA	NA	NA	0,103998153	-1,076443944	4,080456	-0,263804824	0,791930317	NA
ENSG00000230519	HMGB1P49	high mobility group box 1 pseudogene 49	100420013	0	NA	NA	NA	NA	NA
ENSG00000230520	LOC648442	eukaryotic translation initiation factor 3 subunit I pseudogene	648442	0	NA	NA	NA	NA	NA
ENSG00000230522	MBD3L2	methyl-CpG binding domain protein 3 like 2	125997	0	NA	NA	NA	NA	NA
ENSG00000230524	NA	NA	NA	0,117497108	0,847101401	4,080456	0,207599697	0,835541541	NA
ENSG00000230528	NA	NA	NA	0	NA	NA	NA	NA	NA
ENSG00000230529	CEACAMP9	CEA cell adhesion molecule pseudogene 9	1097	0	NA	NA	NA	NA	NA
ENSG00000230531	MTND5P7	MT-ND5 pseudogene 7	100873197	0	NA	NA	NA	NA	NA
ENSG00000230535	NA	NA	NA	0	NA	NA	NA	NA	NA
ENSG00000230538	NA	NA	NA	0	NA	NA	NA	NA	NA
ENSG00000230543	SNX3P1X	sorting nexin 3 pseudogene 1 X-linked	100873884	0	NA	NA	NA	NA	NA
ENSG00000230546	NA	NA	NA	0	NA	NA	NA	NA	NA
ENSG00000230547	HMGB1P11	high mobility group box 1 pseudogene 11	100419996	0	NA	NA	NA	NA	NA
ENSG00000230548	MTND4P27	MT-ND4 pseudogene 27	100873248	0	NA	NA	NA	NA	NA
ENSG00000230549	USP17L1	ubiquitin specific peptidase 17 like family member 1	401447	0	NA	NA	NA	NA	NA
ENSG00000230556	NA	NA	NA	0	NA	NA	NA	NA	NA
ENSG00000230558	NA	NA	NA	0	NA	NA	NA	NA	NA
ENSG00000230559	RPL17P12	ribosomal protein L17 pseudogene 12	100270938	0	NA	NA	NA	NA	NA
ENSG00000230561	CCDC192	coiled-coil domain containing 192	728586	0	NA	NA	NA	NA	NA
ENSG00000230562	NA	NA	NA	2,858996187	-2,189140173	1,199485	-1,825066292	0,067991026	NA
ENSG00000230564	NA	NA	NA	0	NA	NA	NA	NA	NA
ENSG00000230568	SF3A3P1	splicing factor 3a, subunit 3 pseudogene 1	170548	0	NA	NA	NA	NA	NA
ENSG00000230570	NA	NA	NA	0	NA	NA	NA	NA	NA
ENSG00000230571	NA	NA	NA	0	NA	NA	NA	NA	NA
ENSG00000230572	NA	NA	NA	0	NA	NA	NA	NA	NA
ENSG00000230576	OR6R1P	olfactory receptor family 6 subfamily R member 1 pseudogene	81440	0	NA	NA	NA	NA	NA
ENSG00000230578	NA	NA	NA	0	NA	NA	NA	NA	NA
ENSG00000230580	RPL19P5	ribosomal protein L19 pseudogene 5	646532	0	NA	NA	NA	NA	NA
ENSG00000230581	ACTG1P14	actin gamma 1 pseudogene 14	100418910	2,077967756	-0,003462927	1,272008	-0,002722409	0,997827834	NA
ENSG00000230583	NA	NA	NA	0	NA	NA	NA	NA	NA
ENSG00000230584	CCT5P2	chaperonin containing TCP1 subunit 5 pseudogene 2	390415	0	NA	NA	NA	NA	NA
ENSG00000230585	NA	NA	NA	0	NA	NA	NA	NA	NA
ENSG00000230589	IMP3P1	IMP3 pseudogene 1	646087	0	NA	NA	NA	NA	NA

ENSG00000230592	RPSAP8	ribosomal protein SA pseudogene 8	643617	0,117497108	0,847101401	4,080456	0,207599697	0,835541541	NA	
ENSG00000230593	NA	NA	NA	0	NA	NA	NA	NA	NA	
ENSG00000230595	NA	NA	NA	0	NA	NA	NA	NA	NA	
ENSG00000230596	GPAA1P2	glycosylphosphatidylinositol anchor attachment 1 pseudogene 2	106481722	0	NA	NA	NA	NA	NA	
ENSG00000230598	OR2H4P	olfactory receptor family 2 subfamily H member 4 pseudogene	442189	0	NA	NA	NA	NA	NA	
ENSG00000230601	TEX48	testis expressed 48	100505478	0,366640421	2,079048361	3,622587	0,573912577	0,566026962	NA	
ENSG00000230603	NA	NA	NA	0	NA	NA	NA	NA	NA	
ENSG00000230604	NA	NA	NA	0	NA	NA	NA	NA	NA	
ENSG00000230605	MTCYBP6	MT-CYB pseudogene 6	107075259	0	NA	NA	NA	NA	NA	
ENSG00000230607	NA	NA	NA	0	NA	NA	NA	NA	NA	
ENSG00000230609	COX7CP4	COX7C pseudogene 4	127460873	0	NA	NA	NA	NA	NA	
ENSG00000230611	NA	NA	NA	0	NA	NA	NA	NA	NA	
ENSG00000230614	NA	NA	NA	0	NA	NA	NA	NA	NA	
ENSG00000230617	NA	NA	NA	0	NA	NA	NA	NA	NA	
ENSG00000230618	NA	NA	NA	0	NA	NA	NA	NA	NA	
ENSG00000230621	NA	NA	NA	0	NA	NA	NA	NA	NA	
ENSG00000230622	UQCRHP1	ubiquinol-cytochrome c reductase hinge protein pseudogene 1	100130756	0	NA	NA	NA	NA	NA	
ENSG00000230626	NA	NA	NA	0	NA	NA	NA	NA	NA	
ENSG00000230629	RPS23P8	ribosomal protein S23 pseudogene 8	653658	0,103998153	-1,076443944	4,080456	-0,263804824	0,791930317	NA	
ENSG00000230634	NA	NA	NA	0	NA	NA	NA	NA	NA	
ENSG00000230635	CYP4F60P	cytochrome P450 family 4 subfamily F member 60, pseudogene	107126294	0	NA	NA	NA	NA	NA	
ENSG00000230636	RPL36P1	ribosomal protein L36 pseudogene 1	140697	0	NA	NA	NA	NA	NA	
ENSG00000230637	NA	NA	NA	0	NA	NA	NA	NA	NA	
ENSG00000230638	LOC100418723	vir like m6A methyltransferase associated pseudogene	100418723	0	NA	NA	NA	NA	NA	
ENSG00000230639	NA	NA	NA	0	NA	NA	NA	NA	NA	
ENSG00000230643	NA	NA	NA	0	NA	NA	NA	NA	NA	
ENSG00000230646	KLF2P2	Kruppel like factor 2 pseudogene 2	106480678	0	NA	NA	NA	NA	NA	
ENSG00000230650	LOC442041	zinc finger protein 532 pseudogene	442041	0	NA	NA	NA	NA	NA	
ENSG00000230654	MTCO2P25	MT-CO2 pseudogene 25	107075290	0	NA	NA	NA	NA	NA	
ENSG00000230657	PRB4	proline rich protein BstNI subfamily 4	5545	0	NA	NA	NA	NA	NA	
ENSG00000230659	RPL26P12	ribosomal protein L26 pseudogene 12	391126	0	NA	NA	NA	NA	NA	
ENSG00000230661	NA	NA	NA	0	NA	NA	NA	NA	NA	
ENSG00000230665	NA	NA	NA	0	NA	NA	NA	NA	NA	
ENSG00000230666	NA	NA	NA	0	NA	NA	NA	NA	NA	
ENSG00000230667	SETSIP	SET like protein	646817	0	NA	NA	NA	NA	NA	
ENSG00000230671	NA	NA	NA	0	NA	NA	NA	NA	NA	
ENSG00000230673	NA	NA	NA	0	NA	NA	NA	NA	NA	
ENSG00000230681	NA	NA	NA	0	NA	NA	NA	NA	NA	
ENSG00000230682	NA	NA	NA	0	NA	NA	NA	NA	NA	
ENSG00000230683	DDTP1	D-dopachrome tautomerase pseudogene 1	101060160	0	NA	NA	NA	NA	NA	
ENSG00000230691	MRPL35P4	mitochondrial ribosomal protein L35 pseudogene 4	359745	0	NA	NA	NA	NA	NA	
ENSG00000230697	SAP18P3	SAP18 pseudogene 3	106660616	0	NA	NA	NA	NA	NA	
ENSG00000230701	FBXW4P1	F-box and WD repeat domain containing 4 pseudogene 1	26226	3,239946587	-0,704768758	0,996462	-0,707270861	0,47939817	NA	
ENSG00000230702	RPL31P30	ribosomal protein L31 pseudogene 30	729203	0	NA	NA	NA	NA	NA	
ENSG00000230706	NA	NA	NA	0	NA	NA	NA	NA	NA	
ENSG00000230707	HAPSTR2	HUWE1 associated protein modifying stress responses 2	389895	0	NA	NA	NA	NA	NA	
ENSG00000230711	CTAGE13P	CTAGE family member 13, pseudogene	100421638	0	NA	NA	NA	NA	NA	
ENSG00000230712	GGTLC4P	gamma-glutamyltransferase light chain 4 pseudogene	729838	0	NA	NA	NA	NA	NA	
ENSG00000230716	KRT8P7	keratin 8 pseudogene 7	729131	0	NA	NA	NA	NA	NA	
ENSG00000230718	NA	NA	NA	0	NA	NA	NA	NA	NA	
ENSG00000230721	RPL17P5	ribosomal protein L17 pseudogene 5	388132	0	NA	NA	NA	NA	NA	
ENSG00000230727	RBMY2WP	RNA binding motif protein Y-linked family 2 member W, pseudogene	379011	0	NA	NA	NA	NA	NA	

ENSG00000230734	RPL10P3	ribosomal protein L10 pseudogene 3	619445	0	NA	NA	NA	NA	NA
ENSG00000230739	NA	NA	NA	0	NA	NA	NA	NA	NA
ENSG00000230748	PRELID1P7	PRELID1 pseudogene 7	116435286	0	NA	NA	NA	NA	NA
ENSG00000230750	NA	NA	NA	0	NA	NA	NA	NA	NA
ENSG00000230755	MTATP6P23	MT-ATP6 pseudogene 23	107075300	0	NA	NA	NA	NA	NA
ENSG00000230757	NA	NA	NA	0	NA	NA	NA	NA	NA
ENSG00000230758	SNAP23P1	synaptosome associated protein 23 pseudogene 1	101290500	0	NA	NA	NA	NA	NA
ENSG00000230764	NA	NA	NA	0	NA	NA	NA	NA	NA
ENSG00000230769	NA	NA	NA	2,18711979	-0,097929144	1,373424	-0,071302935	0,943156659	NA
ENSG00000230772	VN1R108P	vomeronasal 1 receptor 108 pseudogene	100312850	0,605325541	0,093268581	3,28506	0,028391744	0,977349709	NA
ENSG00000230777	NA	NA	NA	0	NA	NA	NA	NA	NA
ENSG00000230778	ANKRD63	ankyrin repeat domain 63	100131244	0,282264186	0,849750545	4,080456	0,208248924	0,83503461	NA
ENSG00000230779	RPLP0P4	ribosomal protein lateral stalk subunit P0 pseudogene 4	391102	0	NA	NA	NA	NA	NA
ENSG00000230781	NA	NA	NA	0	NA	NA	NA	NA	NA
ENSG00000230783	NA	NA	NA	0,448637719	0,106082239	3,982131	0,026639566	0,978747216	NA
ENSG00000230785	LOC100421782	heterogeneous nuclear ribonucleoprotein A1 like 2 pseudogene	100421782	0	NA	NA	NA	NA	NA
ENSG00000230787	PSAT1P3	phosphoserine aminotransferase 1 pseudogene 3	729779	0	NA	NA	NA	NA	NA
ENSG00000230788	NA	NA	NA	0	NA	NA	NA	NA	NA
ENSG00000230793	SMARCE1P5	SWI/SNF related, matrix associated, actin dependent regulator of chromatin, subfamily e, member 1 pseudogene 5	400129	0,814017501	-1,060059242	3,058941	-0,346544505	0,728933543	NA
ENSG00000230795	HLA-K	major histocompatibility complex, class I, K (pseudogene)	3138	5,190153784	-1,265666139	0,867175	-1,459527217	0,144420055	NA
ENSG00000230796	NA	NA	NA	0	NA	NA	NA	NA	NA
ENSG00000230797	YY2	YY2 transcription factor	404281	10,09008072	-0,342003215	0,570341	-0,599646885	0,548741594	NA
ENSG00000230799	RPS2P16	ribosomal protein S2 pseudogene 16	100270860	0	NA	NA	NA	NA	NA
ENSG00000230801	NA	NA	NA	0	NA	NA	NA	NA	NA
ENSG00000230804	NA	NA	NA	0	NA	NA	NA	NA	NA
ENSG00000230807	NA	NA	NA	0	NA	NA	NA	NA	NA
ENSG00000230809	RPS6P15	ribosomal protein S6 pseudogene 15	100129732	0	NA	NA	NA	NA	NA
ENSG00000230813	NA	NA	NA	0	NA	NA	NA	NA	NA
ENSG00000230814	USP9YP24	USP9Y pseudogene 24	106478928	0	NA	NA	NA	NA	NA
ENSG00000230818	MTND2P16	MT-ND2 pseudogene 16	100873347	0	NA	NA	NA	NA	NA
ENSG00000230819	NA	NA	NA	0	NA	NA	NA	NA	NA
ENSG00000230820	NA	NA	NA	0	NA	NA	NA	NA	NA
ENSG00000230821	NA	NA	NA	0	NA	NA	NA	NA	NA
ENSG00000230823	NA	NA	NA	0,485960082	1,693435587	3,320593	0,509979798	0,610065615	NA
ENSG00000230828	NA	NA	NA	0	NA	NA	NA	NA	NA
ENSG00000230829	RPL27P10	ribosomal protein L27 pseudogene 10	202122	0	NA	NA	NA	NA	NA
ENSG00000230832	NA	NA	NA	0,223443711	0,867676026	4,079778	0,212677263	0,831578703	NA
ENSG00000230833	NA	NA	NA	0,354929073	2,545930433	3,984019	0,639035749	0,522799676	NA
ENSG00000230835	RPS16P6	ribosomal protein S16 pseudogene 6	100271362	0	NA	NA	NA	NA	NA
ENSG00000230837	RPL31P2	ribosomal protein L31 pseudogene 2	140753	0	NA	NA	NA	NA	NA
ENSG00000230840	NA	NA	NA	0	NA	NA	NA	NA	NA
ENSG00000230845	NA	NA	NA	0	NA	NA	NA	NA	NA
ENSG00000230847	NA	NA	NA	3,755388477	-0,342289092	1,13044	-0,302792771	0,762047793	NA
ENSG00000230849	NA	NA	NA	0,303164946	1,397331797	4,060797	0,344102863	0,73076893	NA
ENSG00000230851	VPS51P5	VPS51 pseudogene 5	127379704	0	NA	NA	NA	NA	NA
ENSG00000230853	RPL10P11	ribosomal protein L10 pseudogene 11	100128030	0	NA	NA	NA	NA	NA
ENSG00000230854	USP9YP20	USP9Y pseudogene 20	106480717	0	NA	NA	NA	NA	NA
ENSG00000230859	YRDCP3	yrdC N(6)-threonylcarbamoyltransferase domain containing pseudogene 3	100861429	0	NA	NA	NA	NA	NA
ENSG00000230860	NA	NA	NA	0	NA	NA	NA	NA	NA
ENSG00000230863	NA	NA	NA	0	NA	NA	NA	NA	NA
ENSG00000230865	NA	NA	NA	0	NA	NA	NA	NA	NA
ENSG00000230867	RPS11P4	ribosomal protein S11 pseudogene 4	100271052	0	NA	NA	NA	NA	NA
ENSG00000230869	NA	NA	NA	0	NA	NA	NA	NA	NA
ENSG00000230870	FBXW11P1	F-box and WD repeat domain containing 11 pseudogene 1	54099	0,801548028	0,32618218	2,315045	0,140896678	0,887951567	NA

ENSG00000230871	RPS6P23	ribosomal protein S6 pseudogene 23	100130313	0	NA	NA	NA	NA	NA
ENSG00000230872	MFSD13B	major facilitator superfamily domain containing 13B (pseudogene)	105371130	0,119673877	0,847101401	4,080456	0,207599697	0,835541541	NA
ENSG00000230873	STMND1	stathmin domain containing 1	401236	0	NA	NA	NA	NA	NA
ENSG00000230879	NA	NA	NA	0	NA	NA	NA	NA	NA
ENSG00000230882	NA	NA	NA	0	NA	NA	NA	NA	NA
ENSG00000230886	NA	NA	NA	0	NA	NA	NA	NA	NA
ENSG00000230889	NA	NA	NA	0	NA	NA	NA	NA	NA
ENSG00000230897	RPS18P12	ribosomal protein S18 pseudogene 12	388339	0	NA	NA	NA	NA	NA
ENSG00000230902	FAM204CP	family with sequence similarity 204 member C, pseudogene	100420916	0	NA	NA	NA	NA	NA
ENSG00000230904	NA	NA	NA	0	NA	NA	NA	NA	NA
ENSG00000230911	NA	NA	NA	0	NA	NA	NA	NA	NA
ENSG00000230913	NA	NA	NA	0	NA	NA	NA	NA	NA
ENSG00000230914	NA	NA	NA	0,192018607	1,679520681	4,046213	0,415084598	0,678079974	NA
ENSG00000230916	MTCO1P53	MT-CO1 pseudogene 53	107075285	0	NA	NA	NA	NA	NA
ENSG00000230926	LOC101060199	acyl-coenzyme A synthetase ACSM6, mitochondrial-like	101060199	0	NA	NA	NA	NA	NA
ENSG00000230927	TMBIM7P	transmembrane BAX inhibitor motif containing 7, pseudogene	105375396	0	NA	NA	NA	NA	NA
ENSG00000230929	NA	NA	NA	0	NA	NA	NA	NA	NA
ENSG00000230931	NA	NA	NA	0	NA	NA	NA	NA	NA
ENSG00000230932	NA	NA	NA	0	NA	NA	NA	NA	NA
ENSG00000230934	NA	NA	NA	0	NA	NA	NA	NA	NA
ENSG00000230936	LOC100129276	RNA exonuclease 1 homolog	100129276	0	NA	NA	NA	NA	NA
ENSG00000230942	HMGN1P5	high mobility group nucleosome binding domain 1 pseudogene 5	100874429	0	NA	NA	NA	NA	NA
ENSG00000230946	NA	NA	NA	0	NA	NA	NA	NA	NA
ENSG00000230948	AURKBP1	aurora kinase B pseudogene 1	644233	0	NA	NA	NA	NA	NA
ENSG00000230950	MTND2P21	MT-ND2 pseudogene 21	100873227	0	NA	NA	NA	NA	NA
ENSG00000230951	GPS2P2	G protein pathway suppressor 2 pseudogene 2	100132058	0,236375728	-1,072692556	4,080456	-0,262885469	0,792638857	NA
ENSG00000230953	NA	NA	NA	0	NA	NA	NA	NA	NA
ENSG00000230956	NA	NA	NA	0	NA	NA	NA	NA	NA
ENSG00000230958	NA	NA	NA	0	NA	NA	NA	NA	NA
ENSG00000230964	NA	NA	NA	0	NA	NA	NA	NA	NA
ENSG00000230965	NA	NA	NA	0,11808024	-1,076443944	4,080456	-0,263804824	0,791930317	NA
ENSG00000230973	LOC100421910	olfactory receptor family 5 subfamily R member 1 (gene/pseudogene) pseudogene	100421910	0	NA	NA	NA	NA	NA
ENSG00000230977	NA	NA	NA	0	NA	NA	NA	NA	NA
ENSG00000230979	RPL18AP6	ribosomal protein L18a pseudogene 6	285053	0,593272425	-2,530347996	2,453406	-1,031361308	0,302371418	NA
ENSG00000230980	NA	NA	NA	0	NA	NA	NA	NA	NA
ENSG00000230982	NA	NA	NA	0,458935727	-1,030088661	2,978003	-0,345899137	0,729418517	NA
ENSG00000230986	DDX3P2	DEAD-box helicase 3 pseudogene 2	100874489	0	NA	NA	NA	NA	NA
ENSG00000230987	LOC647132	DExH-box helicase 29 pseudogene	647132	0,117497108	0,847101401	4,080456	0,207599697	0,835541541	NA
ENSG00000230988	RPL23AP11	ribosomal protein L23a pseudogene 11	326294	0	NA	NA	NA	NA	NA
ENSG00000230992	NA	NA	NA	0	NA	NA	NA	NA	NA
ENSG00000230993	NA	NA	NA	0	NA	NA	NA	NA	NA
ENSG00000230994	NA	NA	NA	0	NA	NA	NA	NA	NA
ENSG00000230997	NA	NA	NA	0	NA	NA	NA	NA	NA
ENSG00000230999	MTND5P8	MT-ND5 pseudogene 8	100873198	0	NA	NA	NA	NA	NA
ENSG00000231001	NA	NA	NA	0	NA	NA	NA	NA	NA
ENSG00000231005	NA	NA	NA	0	NA	NA	NA	NA	NA
ENSG00000231006	RPL7P32	ribosomal protein L7 pseudogene 32	100130892	0	NA	NA	NA	NA	NA
ENSG00000231007	CDC20P1	cell division cycle 20 pseudogene 1	157956	0,637882228	1,384876235	2,762052	0,50139399	0,616093869	NA
ENSG00000231009	CUBNP1	cubilin pseudogene 1	728064	0	NA	NA	NA	NA	NA
ENSG00000231011	NA	NA	NA	0	NA	NA	NA	NA	NA
ENSG00000231017	NA	NA	NA	0	NA	NA	NA	NA	NA
ENSG00000231018	NA	NA	NA	0	NA	NA	NA	NA	NA
ENSG00000231020	NA	NA	NA	0	NA	NA	NA	NA	NA
ENSG00000231022	RPS3AP9	RPS3A pseudogene 9	100271129	0	NA	NA	NA	NA	NA
ENSG00000231026	NA	NA	NA	0	NA	NA	NA	NA	NA

ENSG00000231029	RPS18P4	ribosomal protein S18 pseudogene 4	100271075	0	NA	NA	NA	NA	NA
ENSG00000231032	NA	NA	NA	0	NA	NA	NA	NA	NA
ENSG00000231034	NA	NA	NA	0	NA	NA	NA	NA	NA
ENSG00000231035	RPL7L1P9	RPL7L1 pseudogene 9	100128899	0	NA	NA	NA	NA	NA
ENSG00000231043	LOC644456	IK cytokine, down-regulator of HLA II pseudogene	644456	0,19036645	-0,117179182	4,080456	-0,028717179	0,977090155	NA
ENSG00000231047	GCNT1P3	glucosaminyl (N-acetyl) transferase 1 pseudogene 3	100421581	0,280277762	0,823431952	4,080456	0,201799009	0,840073856	NA
ENSG00000231049	OR52B5P	olfactory receptor family 52 subfamily B member 5 pseudogene	81270	0	NA	NA	NA	NA	NA
ENSG00000231053	TRAPPC2LP1	TRAPPC2L pseudogene 1	100421462	0	NA	NA	NA	NA	NA
ENSG00000231058	MSANTD2P1	Myb/SANT DNA binding domain containing 2 pseudogene 1	100130310	0	NA	NA	NA	NA	NA
ENSG00000231060	NA	NA	NA	0	NA	NA	NA	NA	NA
ENSG00000231063	RPL6P6	ribosomal protein L6 pseudogene 6	100270845	0,103998153	-1,076443944	4,080456	-0,263804824	0,791930317	NA
ENSG00000231066	NA	NA	NA	0,23616048	-2,075648354	4,028973	-0,515180498	0,606426895	NA
ENSG00000231068	KRTAP21-3	keratin associated protein 21-3	100288323	0	NA	NA	NA	NA	NA
ENSG00000231069	NA	NA	NA	0	NA	NA	NA	NA	NA
ENSG00000231070	NA	NA	NA	0	NA	NA	NA	NA	NA
ENSG00000231071	NA	NA	NA	0	NA	NA	NA	NA	NA
ENSG00000231072	NA	NA	NA	0	NA	NA	NA	NA	NA
ENSG00000231084	NA	NA	NA	0	NA	NA	NA	NA	NA
ENSG00000231086	NA	NA	NA	0,144330898	1,418786819	4,061262	0,349346267	0,726829368	NA
ENSG00000231087	FDPSP7	farnesyl diphosphate synthase pseudogene 7	441261	0	NA	NA	NA	NA	NA
ENSG00000231093	TRMT1P1	TRMT1 pseudogene 1	347422	0	NA	NA	NA	NA	NA
ENSG00000231095	NA	NA	NA	0	NA	NA	NA	NA	NA
ENSG00000231096	NDUFB4P3	NADH:ubiquinone oxidoreductase subunit B4 pseudogene 3	100288420	0	NA	NA	NA	NA	NA
ENSG00000231099	NA	NA	NA	0	NA	NA	NA	NA	NA
ENSG00000231100	NA	NA	NA	0	NA	NA	NA	NA	NA
ENSG00000231108	PRELID3BP11	PRELI domain containing 3B pseudogene 11	102723314	0	NA	NA	NA	NA	NA
ENSG00000231112	MTHFD2P3	methylenetetrahydrofolate dehydrogenase (NADP+ dependent) 2, methenyltetrahydrofolate cyclohydrolase pseudogene 3	106481665	0	NA	NA	NA	NA	NA
ENSG00000231120	NA	NA	NA	0	NA	NA	NA	NA	NA
ENSG00000231122	NA	NA	NA	0	NA	NA	NA	NA	NA
ENSG00000231123	NA	NA	NA	0	NA	NA	NA	NA	NA
ENSG00000231124	NA	NA	NA	0	NA	NA	NA	NA	NA
ENSG00000231130	NA	NA	NA	0,425627259	-0,245156321	3,540611	-0,069241256	0,944797584	NA
ENSG00000231136	NA	NA	NA	0	NA	NA	NA	NA	NA
ENSG00000231137	NA	NA	NA	0	NA	NA	NA	NA	NA
ENSG00000231139	NA	NA	NA	0	NA	NA	NA	NA	NA
ENSG00000231144	EEF1A1P40	eukaryotic translation elongation factor 1 alpha 1 pseudogene 40	100874490	0	NA	NA	NA	NA	NA
ENSG00000231148	HMGB1P7	high mobility group box 1 pseudogene 7	100420044	0	NA	NA	NA	NA	NA
ENSG00000231152	MTND2P15	MT-ND2 pseudogene 15	100873346	0	NA	NA	NA	NA	NA
ENSG00000231153	NA	NA	NA	0	NA	NA	NA	NA	NA
ENSG00000231158	NA	NA	NA	0	NA	NA	NA	NA	NA
ENSG00000231159	OFD1P8Y	OFD1 pseudogene 8 Y-linked	386692	0	NA	NA	NA	NA	NA
ENSG00000231162	NA	NA	NA	0	NA	NA	NA	NA	NA
ENSG00000231164	RPL7P56	ribosomal protein L7 pseudogene 56	728962	0	NA	NA	NA	NA	NA
ENSG00000231165	TRBV26OR9-2	T cell receptor beta variable 26/OR9-2 (pseudogene)	246285	0	NA	NA	NA	NA	NA
ENSG00000231166	LOC107987035	tubulin beta chain-like	107987035	0	NA	NA	NA	NA	NA
ENSG00000231167	NA	NA	NA	0	NA	NA	NA	NA	NA
ENSG00000231169	NA	NA	NA	0	NA	NA	NA	NA	NA
ENSG00000231181	RPL9P11	ribosomal protein L9 pseudogene 11	642740	1,97943745	1,28613521	1,400074	0,918619254	0,358294758	NA
ENSG00000231184	NA	NA	NA	0	NA	NA	NA	NA	NA
ENSG00000231192	OR5H1	olfactory receptor family 5 subfamily H member 1	26341	0	NA	NA	NA	NA	NA
ENSG00000231195	IFNA11P	interferon alpha 11, pseudogene	3461	0	NA	NA	NA	NA	NA

ENSG00000231198	LOC100422519	pleckstrin homology domain containing B2 pseudogene	100422519	0	NA	NA	NA	NA	NA
ENSG00000231202	NA	NA	NA	0,577761055	2,520247926	2,799602	0,900216558	0,368005016	NA
ENSG00000231203	KRT8P10	keratin 8 pseudogene 10	344320	0	NA	NA	NA	NA	NA
ENSG00000231205	NA	NA	NA	3,966622318	1,29856727	1,067686	1,216244418	0,223891828	NA
ENSG00000231206	NA	NA	NA	0	NA	NA	NA	NA	NA
ENSG00000231207	NA	NA	NA	0	NA	NA	NA	NA	NA
ENSG00000231211	NA	NA	NA	0	NA	NA	NA	NA	NA
ENSG00000231213	PLSCR5	phospholipid scramblase family member 5	389158	0	NA	NA	NA	NA	NA
ENSG00000231222	NA	NA	NA	0,529305418	-1,894128309	4,04399	-0,468381035	0,639512128	NA
ENSG00000231228	NA	NA	NA	0	NA	NA	NA	NA	NA
ENSG00000231232	NA	NA	NA	0	NA	NA	NA	NA	NA
ENSG00000231234	NA	NA	NA	2,242948795	0,129241677	1,400475	0,092284151	0,92647228	NA
ENSG00000231235	CTNNBL1P1	CTNNBL1 pseudogene 1	392456	0	NA	NA	NA	NA	NA
ENSG00000231237	OR6K1P	olfactory receptor family 6 subfamily K member 1 pseudogene	81449	0	NA	NA	NA	NA	NA
ENSG00000231240	NA	NA	NA	0	NA	NA	NA	NA	NA
ENSG00000231241	RPS3AP3	RPS3A pseudogene 3	140713	0,223838207	-0,104555357	4,080373	-0,025623969	0,979557268	NA
ENSG00000231244	PSMC1P3	proteasome 26S subunit, ATPase 1 pseudogene 3	392100	0	NA	NA	NA	NA	NA
ENSG00000231245	NA	NA	NA	0	NA	NA	NA	NA	NA
ENSG00000231251	NA	NA	NA	0	NA	NA	NA	NA	NA
ENSG00000231254	PCED1CP	PC-esterase domain containing 1C, pseudogene	646249	0	NA	NA	NA	NA	NA
ENSG00000231256	CFAP97D1	CFAP97 domain containing 1	284067	0,11808024	-1,076443944	4,080456	-0,263804824	0,791930317	NA
ENSG00000231258	NA	NA	NA	0	NA	NA	NA	NA	NA
ENSG00000231261	NA	NA	NA	0	NA	NA	NA	NA	NA
ENSG00000231264	NA	NA	NA	0	NA	NA	NA	NA	NA
ENSG00000231270	COBLP1	COBL pseudogene 1	392529	0	NA	NA	NA	NA	NA
ENSG00000231271	NA	NA	NA	0	NA	NA	NA	NA	NA
ENSG00000231274	SBK3	SH3 domain binding kinase family member 3	100130827	0	NA	NA	NA	NA	NA
ENSG00000231276	NA	NA	NA	0	NA	NA	NA	NA	NA
ENSG00000231278	OR9S24P	olfactory receptor family 9 subfamily S member 24 pseudogene	403275	0	NA	NA	NA	NA	NA
ENSG00000231280	NA	NA	NA	0	NA	NA	NA	NA	NA
ENSG00000231292	NA	NA	NA	0	NA	NA	NA	NA	NA
ENSG00000231293	NA	NA	NA	0	NA	NA	NA	NA	NA
ENSG00000231296	NA	NA	NA	0	NA	NA	NA	NA	NA
ENSG00000231299	NA	NA	NA	0	NA	NA	NA	NA	NA
ENSG00000231300	NA	NA	NA	0	NA	NA	NA	NA	NA
ENSG00000231301	RPL13AP	ribosomal protein L13a pseudogene	387043	0	NA	NA	NA	NA	NA
ENSG00000231302	NA	NA	NA	0	NA	NA	NA	NA	NA
ENSG00000231307	RPS3P2	ribosomal protein S3 pseudogene 2	140761	2,991372529	0,741830977	1,057664	0,701386308	0,483061964	NA
ENSG00000231311	PRYP2	PTPN13 like Y-linked pseudogene 2	442864	0	NA	NA	NA	NA	NA
ENSG00000231313	NA	NA	NA	0	NA	NA	NA	NA	NA
ENSG00000231316	RPL31P29	ribosomal protein L31 pseudogene 29	100270990	0	NA	NA	NA	NA	NA
ENSG00000231317	LOC643348	ankyrin repeat domain containing 26 pseudogene	643348	0	NA	NA	NA	NA	NA
ENSG00000231322	RPL13AP17	ribosomal protein L13a pseudogene 17	399670	0	NA	NA	NA	NA	NA
ENSG00000231328	NA	NA	NA	0	NA	NA	NA	NA	NA
ENSG00000231331	NA	NA	NA	0	NA	NA	NA	NA	NA
ENSG00000231333	RPL34P6	ribosomal protein L34 pseudogene 6	100270998	0	NA	NA	NA	NA	NA
ENSG00000231340	NA	NA	NA	0,117497108	0,847101401	4,080456	0,207599697	0,835541541	NA
ENSG00000231341	NA	NA	NA	0	NA	NA	NA	NA	NA
ENSG00000231344	NA	NA	NA	0,190603101	-0,122929675	4,080329	-0,030127393	0,975965454	NA
ENSG00000231345	NA	NA	NA	0,222836196	-2,001990276	4,035235	-0,496127311	0,619804592	NA
ENSG00000231349	RPL7L1P22	RPL7L1 pseudogene 22	100420166	0	NA	NA	NA	NA	NA
ENSG00000231351	NA	NA	NA	0	NA	NA	NA	NA	NA
ENSG00000231353	NA	NA	NA	0	NA	NA	NA	NA	NA
ENSG00000231356	NA	NA	NA	0	NA	NA	NA	NA	NA
ENSG00000231357	LOC100131264	prefoldin subunit 4 pseudogene	100131264	0	NA	NA	NA	NA	NA
ENSG00000231359	RPL31P34	ribosomal protein L31 pseudogene 34	100271193	0	NA	NA	NA	NA	NA
ENSG00000231361	RPS29P23	ribosomal protein S29 pseudogene 23	100271588	0	NA	NA	NA	NA	NA
ENSG00000231362	NA	NA	NA	0	NA	NA	NA	NA	NA
ENSG00000231366	NA	NA	NA	0	NA	NA	NA	NA	NA
ENSG00000231368	RPSAP40	ribosomal protein SA pseudogene 40	100270917	0	NA	NA	NA	NA	NA

ENSG00000231369	NA	NA	NA	0	NA	NA	NA	NA	NA
ENSG00000231371	NA	NA	NA	0	NA	NA	NA	NA	NA
ENSG00000231375	NA	NA	NA	0	NA	NA	NA	NA	NA
ENSG00000231376	NA	NA	NA	0	NA	NA	NA	NA	NA
ENSG00000231378	NA	NA	NA	0	NA	NA	NA	NA	NA
ENSG00000231381	NA	NA	NA	0,117497108	0,847101401	4,080456	0,207599697	0,835541541	NA
ENSG00000231382	NBPF21P	NBPF member 21, pseudogene	205655	0	NA	NA	NA	NA	NA
ENSG00000231384	NAP1L5P1	NAP1L5 pseudogene 1	100420939	0	NA	NA	NA	NA	NA
ENSG00000231386	NA	NA	NA	0	NA	NA	NA	NA	NA
ENSG00000231388	FMN2P1	FMN2 pseudogene 1	100418721	0	NA	NA	NA	NA	NA
ENSG00000231390	NA	NA	NA	0	NA	NA	NA	NA	NA
ENSG00000231392	NA	NA	NA	0	NA	NA	NA	NA	NA
ENSG00000231395	NA	NA	NA	0	NA	NA	NA	NA	NA
ENSG00000231396	USP17L10	ubiquitin specific peptidase 17 like family member 10	100287144	0	NA	NA	NA	NA	NA
ENSG00000231397	NA	NA	NA	0	NA	NA	NA	NA	NA
ENSG00000231398	NA	NA	NA	0,10488184	-1,076443944	4,080456	-0,263804824	0,791930317	NA
ENSG00000231399	SNRPEP8	SNRPE pseudogene 8	100874423	0	NA	NA	NA	NA	NA
ENSG00000231402	WASF5P	WASP family member 5, pseudogene	387122	0	NA	NA	NA	NA	NA
ENSG00000231404	NA	NA	NA	0	NA	NA	NA	NA	NA
ENSG00000231409	NA	NA	NA	0	NA	NA	NA	NA	NA
ENSG00000231411	DUX4L31	double homeobox 4 like 31 (pseudogene)	100887073	0	NA	NA	NA	NA	NA
ENSG00000231414	NA	NA	NA	0,119673877	0,847101401	4,080456	0,207599697	0,835541541	NA
ENSG00000231416	RPL34P5	ribosomal protein L34 pseudogene 5	100270995	0	NA	NA	NA	NA	NA
ENSG00000231417	NA	NA	NA	0	NA	NA	NA	NA	NA
ENSG00000231423	RAB9AP5	RAB9A, member RAS oncogene family pseudogene 5	100287234	0	NA	NA	NA	NA	NA
ENSG00000231431	FAR2P4	fatty acyl-CoA reductase 2 pseudogene 4	100420005	0	NA	NA	NA	NA	NA
ENSG00000231434	NA	NA	NA	0	NA	NA	NA	NA	NA
ENSG00000231442	LARP1BP1	LARP1B pseudogene 1	644578	0	NA	NA	NA	NA	NA
ENSG00000231443	NA	NA	NA	0,207996306	-1,916073151	4,043021	-0,473921178	0,635556117	NA
ENSG00000231445	NA	NA	NA	0	NA	NA	NA	NA	NA
ENSG00000231449	RPS16P4	ribosomal protein S16 pseudogene 4	100271068	0,10543123	0	4,080456	0	1	NA
ENSG00000231454	NA	NA	NA	0	NA	NA	NA	NA	NA
ENSG00000231458	RNASEH1P2	ribonuclease H1 pseudogene 2	390766	0	NA	NA	NA	NA	NA
ENSG00000231461	NA	NA	NA	0	NA	NA	NA	NA	NA
ENSG00000231464	NA	NA	NA	0	NA	NA	NA	NA	NA
ENSG00000231466	NA	NA	NA	4,772676535	1,173170143	0,835793	1,403661692	0,160419615	NA
ENSG00000231468	NA	NA	NA	0	NA	NA	NA	NA	NA
ENSG00000231470	NA	NA	NA	0	NA	NA	NA	NA	NA
ENSG00000231471	NA	NA	NA	0	NA	NA	NA	NA	NA
ENSG00000231475	IGHV4-30-2	immunoglobulin heavy variable 4-30-2	28398	0	NA	NA	NA	NA	NA
ENSG00000231478	FCF1P9	FCF1 pseudogene 9	106480444	0	NA	NA	NA	NA	NA
ENSG00000231480	NA	NA	NA	0	NA	NA	NA	NA	NA
ENSG00000231484	NA	NA	NA	0	NA	NA	NA	NA	NA
ENSG00000231489	NA	NA	NA	0	NA	NA	NA	NA	NA
ENSG00000231490	RPL7L1P2	RPL7L1 pseudogene 2	100420155	0	NA	NA	NA	NA	NA
ENSG00000231491	JKAMPP1	JNK1/MAPK8-associated membrane protein pseudogene 1	100049717	0	NA	NA	NA	NA	NA
ENSG00000231494	RPL21P35	ribosomal protein L21 pseudogene 35	100270955	0,571294161	0,9722781	3,331503	0,291843689	0,770406139	NA
ENSG00000231501	MTND4LP1	MT-ND4L pseudogene 1	106478909	0	NA	NA	NA	NA	NA
ENSG00000231503	NA	NA	NA	0	NA	NA	NA	NA	NA
ENSG00000231504	NA	NA	NA	0	NA	NA	NA	NA	NA
ENSG00000231508	RPL34P20	ribosomal protein L34 pseudogene 20	100271308	0,119673877	0,847101401	4,080456	0,207599697	0,835541541	NA
ENSG00000231512	NA	NA	NA	0	NA	NA	NA	NA	NA
ENSG00000231513	NA	NA	NA	0	NA	NA	NA	NA	NA
ENSG00000231514	NA	NA	NA	0	NA	NA	NA	NA	NA
ENSG00000231516	NA	NA	NA	0	NA	NA	NA	NA	NA
ENSG00000231523	NA	NA	NA	0	NA	NA	NA	NA	NA
ENSG00000231525	NA	NA	NA	0	NA	NA	NA	NA	NA
ENSG00000231531	NA	NA	NA	0	NA	NA	NA	NA	NA
ENSG00000231537	MTCO3P10	MT-CO3 pseudogene 10	107075152	0	NA	NA	NA	NA	NA
ENSG00000231540	NA	NA	NA	0	NA	NA	NA	NA	NA
ENSG00000231541	NA	NA	NA	0	NA	NA	NA	NA	NA
ENSG00000231544	NA	NA	NA	0,210862461	0	4,080456	0	1	NA
ENSG00000231546	NA	NA	NA	0,15836288	0	4,080456	0	1	NA
ENSG00000231547	NA	NA	NA	0	NA	NA	NA	NA	NA
ENSG00000231548	NA	NA	NA	0	NA	NA	NA	NA	NA

ENSG00000231549	NA	NA	NA	0	NA	NA	NA	NA	NA
ENSG00000231551	NA	NA	NA	0,649751291	-1,047334309	2,863747	-0,365721717	0,714572739	NA
ENSG00000231552	IGBP1P3	IGBP1 pseudogene 3	391526	0	NA	NA	NA	NA	NA
ENSG00000231553	MRM3P1	MRM3 pseudogene 1	100420295	0	NA	NA	NA	NA	NA
ENSG00000231556	RSL24D1P3	ribosomal L24 domain containing 1 pseudogene 3	100130721	0	NA	NA	NA	NA	NA
ENSG00000231559	RPL37AP3	ribosomal protein L37a pseudogene 3	100132988	0	NA	NA	NA	NA	NA
ENSG00000231561	NA	NA	NA	0,11808024	-1,076443944	4,080456	-0,263804824	0,791930317	NA
ENSG00000231562	RPL7AP44	ribosomal protein L7a pseudogene 44	100271398	0	NA	NA	NA	NA	NA
ENSG00000231564	NA	NA	NA	0	NA	NA	NA	NA	NA
ENSG00000231565	NA	NA	NA	0	NA	NA	NA	NA	NA
ENSG00000231567	NA	NA	NA	0	NA	NA	NA	NA	NA
ENSG00000231568	NA	NA	NA	0	NA	NA	NA	NA	NA
ENSG00000231569	NA	NA	NA	0	NA	NA	NA	NA	NA
ENSG00000231570	PPIAP63	peptidylprolyl isomerase A pseudogene 63	111082966	0	NA	NA	NA	NA	NA
ENSG00000231576	MTCO2P20	MT-CO2 pseudogene 20	107075230	0,15836288	0	4,080456	0	1	NA
ENSG00000231579	NA	NA	NA	0,216496347	1,902771968	4,026559	0,472555327	0,63653046	NA
ENSG00000231583	NA	NA	NA	0	NA	NA	NA	NA	NA
ENSG00000231584	NA	NA	NA	2,235193762	0,211444503	1,136966	0,185972546	0,852466291	NA
ENSG00000231586	RPS23P9	ribosomal protein S23 pseudogene 9	100419472	0	NA	NA	NA	NA	NA
ENSG00000231588	NA	NA	NA	0	NA	NA	NA	NA	NA
ENSG00000231590	NA	NA	NA	0	NA	NA	NA	NA	NA
ENSG00000231593	NA	NA	NA	0	NA	NA	NA	NA	NA
ENSG00000231600	NA	NA	NA	0	NA	NA	NA	NA	NA
ENSG00000231603	NA	NA	NA	0	NA	NA	NA	NA	NA
ENSG00000231610	PIK3CDP1	phosphatidylinositol-4,5-bisphosphate 3-kinase catalytic subunit delta pseudogene 1	391349	0	NA	NA	NA	NA	NA
ENSG00000231615	LOC646870	centrosomal protein 57kDa pseudogene	646870	0,695704176	0,260648705	2,365421	0,110191276	0,912257681	NA
ENSG00000231619	NA	NA	NA	0	NA	NA	NA	NA	NA
ENSG00000231622	NA	NA	NA	0	NA	NA	NA	NA	NA
ENSG00000231625	NA	NA	NA	0	NA	NA	NA	NA	NA
ENSG00000231635	ATP5F1BP1	ATP synthase F1 subunit beta pseudogene 1	507	0	NA	NA	NA	NA	NA
ENSG00000231643	NA	NA	NA	0	NA	NA	NA	NA	NA
ENSG00000231645	KRT17P6	keratin 17 pseudogene 6	284196	0	NA	NA	NA	NA	NA
ENSG00000231649	SPATA31B1P	SPATA31 subfamily B member 1, pseudogene	404770	0	NA	NA	NA	NA	NA
ENSG00000231650	NA	NA	NA	0	NA	NA	NA	NA	NA
ENSG00000231655	NA	NA	NA	0	NA	NA	NA	NA	NA
ENSG00000231660	RPS8P6	ribosomal protein S8 pseudogene 6	100270871	0	NA	NA	NA	NA	NA
ENSG00000231665	NA	NA	NA	0	NA	NA	NA	NA	NA
ENSG00000231667	OR7E111P	olfactory receptor family 7 subfamily E member 111 pseudogene	81136	0	NA	NA	NA	NA	NA
ENSG00000231668	NA	NA	NA	0	NA	NA	NA	NA	NA
ENSG00000231675	NA	NA	NA	0	NA	NA	NA	NA	NA
ENSG00000231684	NA	NA	NA	0	NA	NA	NA	NA	NA
ENSG00000231686	NA	NA	NA	0,10543123	0	4,080456	0	1	NA
ENSG00000231688	RPL21P43	ribosomal protein L21 pseudogene 43	100271163	0	NA	NA	NA	NA	NA
ENSG00000231694	NA	NA	NA	0	NA	NA	NA	NA	NA
ENSG00000231697	NA	NA	NA	1,985902941	1,562046787	1,496707	1,043655695	0,296644715	NA
ENSG00000231699	NA	NA	NA	0	NA	NA	NA	NA	NA
ENSG00000231700	NA	NA	NA	0	NA	NA	NA	NA	NA
ENSG00000231701	BMS1P13	BMS1 pseudogene 13	644716	0,209763681	-1,926644696	4,042043	-0,476651224	0,633610501	NA
ENSG00000231702	NA	NA	NA	0	NA	NA	NA	NA	NA
ENSG00000231706	NA	NA	NA	0	NA	NA	NA	NA	NA
ENSG00000231707	NA	NA	NA	0	NA	NA	NA	NA	NA
ENSG00000231712	NA	NA	NA	0	NA	NA	NA	NA	NA
ENSG00000231715	NA	NA	NA	0	NA	NA	NA	NA	NA
ENSG00000231716	CDY23P	chromodomain Y-linked 23 pseudogene	386745	0	NA	NA	NA	NA	NA
ENSG00000231722	NA	NA	NA	0	NA	NA	NA	NA	NA
ENSG00000231723	NA	NA	NA	0	NA	NA	NA	NA	NA
ENSG00000231725	VN1R110P	vomeronasal 1 receptor 110 pseudogene	100312803	0	NA	NA	NA	NA	NA
ENSG00000231726	NA	NA	NA	0	NA	NA	NA	NA	NA
ENSG00000231735	RPS2P18	ribosomal protein S2 pseudogene 18	643960	0	NA	NA	NA	NA	NA
ENSG00000231738	TSPAN19	tetraspanin 19	144448	0	NA	NA	NA	NA	NA
ENSG00000231739	NA	NA	NA	0	NA	NA	NA	NA	NA
ENSG00000231741	NA	NA	NA	0	NA	NA	NA	NA	NA

ENSG00000231743	NA	NA	NA	0	NA	NA	NA	NA	NA
ENSG00000231744	NA	NA	NA	0	NA	NA	NA	NA	NA
ENSG00000231747	NA	NA	NA	0	NA	NA	NA	NA	NA
ENSG00000231750	NA	NA	NA	0	NA	NA	NA	NA	NA
ENSG00000231752	NA	NA	NA	4,218146169	-0,286102527	0,902573	-0,316985628	0,751254508	NA
ENSG00000231762	NA	NA	NA	0,10488184	-1,076443944	4,080456	-0,263804824	0,791930317	NA
ENSG00000231763	SLC66A2P1	SLC66A2 pseudogene 1	106479032	0	NA	NA	NA	NA	NA
ENSG00000231765	PPP1R11P2	protein phosphatase 1 regulatory inhibitor subunit 11 pseudogene 2	94298	0	NA	NA	NA	NA	NA
ENSG00000231766	MTCO2P5	MT-CO2 pseudogene 5	107075181	0	NA	NA	NA	NA	NA
ENSG00000231767	RPS27AP5	ribosomal protein S27a pseudogene 5	100271374	0	NA	NA	NA	NA	NA
ENSG00000231777	MTND4P21	MT-ND4 pseudogene 21	100873243	0	NA	NA	NA	NA	NA
ENSG00000231780	RBISP6	RBIS pseudogene 6	127379686	0	NA	NA	NA	NA	NA
ENSG00000231784	DBIL5P	diazepam binding inhibitor-like 5, pseudogene	100131454	0,192175908	1,680364491	4,046186	0,415295887	0,677925312	NA
ENSG00000231787	H2BP9	H2B histone pseudogene 9	115482691	0,22271871	0,83664014	4,080456	0,205035948	0,837544039	NA
ENSG00000231788	RPL31P50	ribosomal protein L31 pseudogene 50	341356	0	NA	NA	NA	NA	NA
ENSG00000231793	NA	NA	NA	6,843109635	-0,716267496	0,720337	-0,994350381	0,320052316	NA
ENSG00000231799	NA	NA	NA	0,236573303	0	4,080456	0	1	NA
ENSG00000231801	NA	NA	NA	0	NA	NA	NA	NA	NA
ENSG00000231802	NA	NA	NA	0	NA	NA	NA	NA	NA
ENSG00000231804	RPL21P85	ribosomal protein L21 pseudogene 85	100271189	0	NA	NA	NA	NA	NA
ENSG00000231809	NA	NA	NA	0	NA	NA	NA	NA	NA
ENSG00000231810	RPL36AP35	ribosomal protein L36a pseudogene 35	100271610	0	NA	NA	NA	NA	NA
ENSG00000231812	SNRPCP9	small nuclear ribonucleoprotein polypeptide C pseudogene 9	100310853	0	NA	NA	NA	NA	NA
ENSG00000231821	NPM1P48	nucleophosmin 1 pseudogene 48	442006	0	NA	NA	NA	NA	NA
ENSG00000231822	NA	NA	NA	0,072165449	0,847101401	4,080456	0,207599697	0,835541541	NA
ENSG00000231824	AKAIN1	A-kinase anchor inhibitor 1	642597	0	NA	NA	NA	NA	NA
ENSG00000231827	NA	NA	NA	0,191388035	1,676134088	4,04632	0,414236613	0,678700831	NA
ENSG00000231831	MTHFD1P1	methylenetetrahydrofolate dehydrogenase (NADP+ dependent) 1 pseudogene 1	4523	0	NA	NA	NA	NA	NA
ENSG00000231837	NA	NA	NA	0	NA	NA	NA	NA	NA
ENSG00000231839	DPY19L4P2	DPY19L4 pseudogene 2	100420073	0	NA	NA	NA	NA	NA
ENSG00000231841	NA	NA	NA	0	NA	NA	NA	NA	NA
ENSG00000231845	HMGB3P14	high mobility group box 3 pseudogene 14	100873899	0	NA	NA	NA	NA	NA
ENSG00000231848	LOC124907760	keratinocyte-associated protein 2-like	124907760	0	NA	NA	NA	NA	NA
ENSG00000231849	UBE2V2P4	ubiquitin conjugating enzyme E2 V2 pseudogene 4	106480802	0	NA	NA	NA	NA	NA
ENSG00000231852	CYP21A2	cytochrome P450 family 21 subfamily A member 2	1589	0	NA	NA	NA	NA	NA
ENSG00000231855	NA	NA	NA	0	NA	NA	NA	NA	NA
ENSG00000231857	MORF4L1P5	mortality factor 4 like 1 pseudogene 5	100129288	0	NA	NA	NA	NA	NA
ENSG00000231859	NA	NA	NA	0	NA	NA	NA	NA	NA
ENSG00000231861	OR5K2	olfactory receptor family 5 subfamily K member 2	402135	0	NA	NA	NA	NA	NA
ENSG00000231866	LOC100652967	ATP synthase membrane subunit j pseudogene	100652967	0	NA	NA	NA	NA	NA
ENSG00000231870	KRT17P3	keratin 17 pseudogene 3	729682	0	NA	NA	NA	NA	NA
ENSG00000231874	TSPY18P	testis specific protein Y-linked 18, pseudogene	642796	0	NA	NA	NA	NA	NA
ENSG00000231875	NA	NA	NA	0,072165449	0,847101401	4,080456	0,207599697	0,835541541	NA
ENSG00000231878	NA	NA	NA	0	NA	NA	NA	NA	NA
ENSG00000231879	TXNL1P1	thioredoxin like 1 pseudogene 1	100419095	0	NA	NA	NA	NA	NA
ENSG00000231884	NA	NA	NA	0	NA	NA	NA	NA	NA
ENSG00000231887	PRH1	proline rich protein HaeIII subfamily 1	5554	0	NA	NA	NA	NA	NA
ENSG00000231888	NA	NA	NA	0,49954487	1,719963389	2,77526	0,61974847	0,535423399	NA
ENSG00000231894	NA	NA	NA	0	NA	NA	NA	NA	NA
ENSG00000231897	MARK2P14	MARK2 pseudogene 14	100631261	0	NA	NA	NA	NA	NA
ENSG00000231900	GOT2P1	GOT2 pseudogene 1	645538	0	NA	NA	NA	NA	NA
ENSG00000231905	NA	NA	NA	0	NA	NA	NA	NA	NA
ENSG00000231906	NA	NA	NA	0	NA	NA	NA	NA	NA
ENSG00000231907	NA	NA	NA	0,10543123	0	4,080456	0	1	NA
ENSG00000231909	MAP1LC3BP1	microtubule associated protein 1 light chain 3 beta pseudogene 1	392288	0	NA	NA	NA	NA	NA

ENSG00000231910	GAMTP2	guanidinoacetate N-methyltransferase pseudogene 2	100129452	0	NA	NA	NA	NA	NA
ENSG00000231911	TPRKBP1	TP53RK binding protein pseudogene 1	101060176	0	NA	NA	NA	NA	NA
ENSG00000231913	RARRES2P3	retinoic acid receptor responder 2 pseudogene 3	106481659	0	NA	NA	NA	NA	NA
ENSG00000231915	SALL4P5	spalt like transcription factor 4 pseudogene 5	100130785	0	NA	NA	NA	NA	NA
ENSG00000231916	NA	NA	NA	0,550339906	-3,309228834	3,09771	-1,068282383	0,285393153	NA
ENSG00000231922	NA	NA	NA	0	NA	NA	NA	NA	NA
ENSG00000231923	NA	NA	NA	0	NA	NA	NA	NA	NA
ENSG00000231924	PSG1	pregnancy specific beta-1-glycoprotein 1	5669	0	NA	NA	NA	NA	NA
ENSG00000231926	NA	NA	NA	0	NA	NA	NA	NA	NA
ENSG00000231929	HMGB3P31	high mobility group box 3 pseudogene 31	392545	0	NA	NA	NA	NA	NA
ENSG00000231931	NA	NA	NA	0	NA	NA	NA	NA	NA
ENSG00000231940	RPS7P3	ribosomal protein S7 pseudogene 3	440732	2,318540891	0,465595664	1,183331	0,393461885	0,69397837	NA
ENSG00000231942	NA	NA	NA	0	NA	NA	NA	NA	NA
ENSG00000231947	NA	NA	NA	0	NA	NA	NA	NA	NA
ENSG00000231952	NA	NA	NA	0	NA	NA	NA	NA	NA
ENSG00000231954	MTND2P22	MT-ND2 pseudogene 22	100873228	0	NA	NA	NA	NA	NA
ENSG00000231956	NA	NA	NA	0	NA	NA	NA	NA	NA
ENSG00000231957	GNAI2P2	G protein subunit alpha i2 pseudogene 2	401646	0	NA	NA	NA	NA	NA
ENSG00000231961	NA	NA	NA	0,239347754	1,967469188	4,021404	0,489249276	0,624665227	NA
ENSG00000231967	NA	NA	NA	0	NA	NA	NA	NA	NA
ENSG00000231976	NA	NA	NA	0,117497108	0,847101401	4,080456	0,207599697	0,835541541	NA
ENSG00000231978	NA	NA	NA	0	NA	NA	NA	NA	NA
ENSG00000231981	RPL7L1P12	RPL7L1 pseudogene 12	100420200	0,216496347	1,902771968	4,026559	0,472555327	0,63653046	NA
ENSG00000231982	MCF2L2P1	MCF2L2 pseudogene 1	127525790	0	NA	NA	NA	NA	NA
ENSG00000231984	LOC107985113	2-iminobutanoate/2-iminopropanoate deaminase-like	107985113	0	NA	NA	NA	NA	NA
ENSG00000231988	NA	NA	NA	0	NA	NA	NA	NA	NA
ENSG00000231989	PPP1R2B	PPP1R2 family member B	153743	0,072165449	0,847101401	4,080456	0,207599697	0,835541541	NA
ENSG00000231991	NA	NA	NA	0,117497108	0,847101401	4,080456	0,207599697	0,835541541	NA
ENSG00000231995	RPL7AP76	ribosomal protein L7a pseudogene 76	643630	0	NA	NA	NA	NA	NA
ENSG00000231996	NA	NA	NA	0	NA	NA	NA	NA	NA
ENSG00000232000	CLCN3P1	chloride voltage-gated channel 3 pseudogene 1	100419056	0	NA	NA	NA	NA	NA
ENSG00000232003	NA	NA	NA	0	NA	NA	NA	NA	NA
ENSG00000232004	CAP1P2	CAP1 pseudogene 2	399748	0	NA	NA	NA	NA	NA
ENSG00000232009	NA	NA	NA	0	NA	NA	NA	NA	NA
ENSG00000232015	NA	NA	NA	0	NA	NA	NA	NA	NA
ENSG00000232022	NA	NA	NA	0	NA	NA	NA	NA	NA
ENSG00000232024	NA	NA	NA	5,424758325	-5,868418588	1,933287	-3,035460962	0,002401683	NA
ENSG00000232027	NA	NA	NA	0	NA	NA	NA	NA	NA
ENSG00000232030	MAGEB6B	MAGE family member B6B	392433	0	NA	NA	NA	NA	NA
ENSG00000232032	NA	NA	NA	0	NA	NA	NA	NA	NA
ENSG00000232036	LOC100129573	tRNA methyltransferase 2 homolog B (S, cerevisiae) pseudogene	100129573	0,15836288	0	4,080456	0	1	NA
ENSG00000232037	RPL21P29	ribosomal protein L21 pseudogene 29	440575	0	NA	NA	NA	NA	NA
ENSG00000232039	NA	NA	NA	0	NA	NA	NA	NA	NA
ENSG00000232040	SCAND3	SCAN domain containing 3	114821	0	NA	NA	NA	NA	NA
ENSG00000232041	PSMD10P3	proteasome 26S subunit, non-ATPase, 10 pseudogene 3	338091	0	NA	NA	NA	NA	NA
ENSG00000232042	LOC107985629	prefoldin subunit 4-like	107985629	0	NA	NA	NA	NA	NA
ENSG00000232048	HNRNPCP9	heterogeneous nuclear ribonucleoprotein C pseudogene 9	100130196	0	NA	NA	NA	NA	NA
ENSG00000232050	NA	NA	NA	0	NA	NA	NA	NA	NA
ENSG00000232054	NA	NA	NA	0	NA	NA	NA	NA	NA
ENSG00000232059	NA	NA	NA	0	NA	NA	NA	NA	NA
ENSG00000232060	NA	NA	NA	0	NA	NA	NA	NA	NA
ENSG00000232064	USP9YP33	USP9Y pseudogene 33	106478931	0	NA	NA	NA	NA	NA
ENSG00000232068	NA	NA	NA	0	NA	NA	NA	NA	NA
ENSG00000232069	RPS29P28	ribosomal protein S29 pseudogene 28	100271402	0	NA	NA	NA	NA	NA
ENSG00000232070	TMEM253	transmembrane protein 253	643382	0	NA	NA	NA	NA	NA
ENSG00000232075	MRPL35P2	mitochondrial ribosomal protein L35 pseudogene 2	359743	0	NA	NA	NA	NA	NA
ENSG00000232083	RPL31P7	ribosomal protein L31 pseudogene 7	654391	0	NA	NA	NA	NA	NA

ENSG00000232087	HCFC2P1	host cell factor C2 pseudogene 1	100419149	0	NA	NA	NA	NA	NA
ENSG00000232089	NA	NA	NA	0	NA	NA	NA	NA	NA
ENSG00000232090	NA	NA	NA	0	NA	NA	NA	NA	NA
ENSG00000232091	NA	NA	NA	0	NA	NA	NA	NA	NA
ENSG00000232096	NA	NA	NA	0	NA	NA	NA	NA	NA
ENSG00000232097	NA	NA	NA	0,072165449	0,847101401	4,080456	0,207599697	0,835541541	NA
ENSG00000232101	NA	NA	NA	0	NA	NA	NA	NA	NA
ENSG00000232102	MTCO3P2	MT-CO3 pseudogene 2	100873207	0	NA	NA	NA	NA	NA
ENSG00000232105	NA	NA	NA	0	NA	NA	NA	NA	NA
ENSG00000232109	VN1R54P	vomeronasal 1 receptor 54 pseudogene	100312807	0,10488184	-1,076443944	4,080456	-0,263804824	0,791930317	NA
ENSG00000232111	NA	NA	NA	0	NA	NA	NA	NA	NA
ENSG00000232113	TEX50	testis expressed 50	730159	0	NA	NA	NA	NA	NA
ENSG00000232114	NA	NA	NA	0	NA	NA	NA	NA	NA
ENSG00000232115	NA	NA	NA	0	NA	NA	NA	NA	NA
ENSG00000232125	DYTN	dystrotelin	391475	0	NA	NA	NA	NA	NA
ENSG00000232128	MTCO3P45	MT-CO3 pseudogene 45	107075218	0	NA	NA	NA	NA	NA
ENSG00000232133	IMPDH1P10	inosine monophosphate dehydrogenase 1 pseudogene 10	100874399	0	NA	NA	NA	NA	NA
ENSG00000232134	RPS15AP12	ribosomal protein S15a pseudogene 12	100129243	0,103998153	-1,076443944	4,080456	-0,263804824	0,791930317	NA
ENSG00000232135	NA	NA	NA	0	NA	NA	NA	NA	NA
ENSG00000232136	NA	NA	NA	0	NA	NA	NA	NA	NA
ENSG00000232138	IFNWP5	interferon omega 1 pseudogene 5	392292	0	NA	NA	NA	NA	NA
ENSG00000232142	NA	NA	NA	0	NA	NA	NA	NA	NA
ENSG00000232144	NA	NA	NA	0	NA	NA	NA	NA	NA
ENSG00000232148	FMO11P	flavin containing dimethylaniline monoxygenase 11, pseudogene	100337590	0	NA	NA	NA	NA	NA
ENSG00000232149	FERP1	FER tyrosine kinase pseudogene 1	553112	0	NA	NA	NA	NA	NA
ENSG00000232150	NA	NA	NA	0	NA	NA	NA	NA	NA
ENSG00000232154	MTCYBP15	MT-CYB pseudogene 15	107075258	0	NA	NA	NA	NA	NA
ENSG00000232155	NA	NA	NA	0	NA	NA	NA	NA	NA
ENSG00000232158	C11orf98P1	C11orf98 pseudogene 1	106480289	0	NA	NA	NA	NA	NA
ENSG00000232159	RAB9BP1	RAB9B, member RAS oncogene family pseudogene 1	9366	0	NA	NA	NA	NA	NA
ENSG00000232161	NA	NA	NA	0	NA	NA	NA	NA	NA
ENSG00000232163	RPLP1P13	ribosomal protein lateral stalk subunit P1 pseudogene 13	100874124	0	NA	NA	NA	NA	NA
ENSG00000232165	VN1R30P	vomeronasal 1 receptor 30 pseudogene	100287919	0	NA	NA	NA	NA	NA
ENSG00000232166	RPL9P10	ribosomal protein L9 pseudogene 10	100270855	0	NA	NA	NA	NA	NA
ENSG00000232167	NA	NA	NA	0	NA	NA	NA	NA	NA
ENSG00000232168	P2RY10BP	P2Y receptor family member 10B, pseudogene	449518	0	NA	NA	NA	NA	NA
ENSG00000232172	RPL19P15	ribosomal protein L19 pseudogene 15	642306	0	NA	NA	NA	NA	NA
ENSG00000232173	NA	NA	NA	0	NA	NA	NA	NA	NA
ENSG00000232174	NA	NA	NA	0,316293691	0	4,080456	0	1	NA
ENSG00000232175	NA	NA	NA	0	NA	NA	NA	NA	NA
ENSG00000232176	RPS6P10	ribosomal protein S6 pseudogene 10	100270865	0,340140402	-1,998362975	3,74457	-0,533669549	0,593570181	NA
ENSG00000232177	MTND4P24	MT-ND4 pseudogene 24	100873254	0,117497108	0,847101401	4,080456	0,207599697	0,835541541	NA
ENSG00000232179	MTATP6P29	MT-ATP6 pseudogene 29	107075315	0	NA	NA	NA	NA	NA
ENSG00000232185	NA	NA	NA	0	NA	NA	NA	NA	NA
ENSG00000232186	TERF1P7	TERF1 pseudogene 7	646127	0	NA	NA	NA	NA	NA
ENSG00000232187	NA	NA	NA	0,458665297	-0,129932743	3,542073	-0,036682679	0,97073802	NA
ENSG00000232195	TOMM22P2	TOMM22 pseudogene 2	100499417	0	NA	NA	NA	NA	NA
ENSG00000232198	MTCO2P34	MT-CO2 pseudogene 34	109729142	0	NA	NA	NA	NA	NA
ENSG00000232199	RPL3P8	ribosomal protein L3 pseudogene 8	646620	0	NA	NA	NA	NA	NA
ENSG00000232202	NA	NA	NA	0,072165449	0,847101401	4,080456	0,207599697	0,835541541	NA
ENSG00000232203	NA	NA	NA	0	NA	NA	NA	NA	NA
ENSG00000232204	NA	NA	NA	0	NA	NA	NA	NA	NA
ENSG00000232205	NA	NA	NA	0	NA	NA	NA	NA	NA
ENSG00000232208	NA	NA	NA	0	NA	NA	NA	NA	NA
ENSG00000232215	OR2L6P	olfactory receptor family 2 subfamily L member 6 pseudogene	81465	0,308561081	-1,116456949	4,028875	-0,277113825	0,781692707	NA
ENSG00000232216	IGHV3-43	immunoglobulin heavy variable 3-43	28426	0	NA	NA	NA	NA	NA

ENSG00000232217	FTLP8	ferritin light chain pseudogene 8	100462790	0	NA	NA	NA	NA	NA
ENSG00000232218	NA	NA	NA	0	NA	NA	NA	NA	NA
ENSG00000232223	CNN2P10	calponin 2 pseudogene 10	646693	0	NA	NA	NA	NA	NA
ENSG00000232226	ARSFP1	arylsulfatase F pseudogene 1	23757	0	NA	NA	NA	NA	NA
ENSG00000232228	NA	NA	NA	0,354719191	2,545144492	3,984061	0,638831706	0,52293242	NA
ENSG00000232230	NA	NA	NA	0	NA	NA	NA	NA	NA
ENSG00000232237	ASCL5	achaete-scute family bHLH transcription factor 5	647219	0	NA	NA	NA	NA	NA
ENSG00000232238	NA	NA	NA	0	NA	NA	NA	NA	NA
ENSG00000232239	NA	NA	NA	0	NA	NA	NA	NA	NA
ENSG00000232240	NA	NA	NA	0	NA	NA	NA	NA	NA
ENSG00000232241	NA	NA	NA	0	NA	NA	NA	NA	NA
ENSG00000232242	NA	NA	NA	0	NA	NA	NA	NA	NA
ENSG00000232249	NA	NA	NA	0	NA	NA	NA	NA	NA
ENSG00000232254	CSF2RBP1	CSF2RB pseudogene 1	23772	0	NA	NA	NA	NA	NA
ENSG00000232258	TMEM114	transmembrane protein 114	283953	0	NA	NA	NA	NA	NA
ENSG00000232260	NA	NA	NA	0	NA	NA	NA	NA	NA
ENSG00000232261	LOC100422548	solute carrier family 5 member 4 pseudogene	100422548	0	NA	NA	NA	NA	NA
ENSG00000232263	KRTAP25-1	keratin associated protein 25-1	100131902	0	NA	NA	NA	NA	NA
ENSG00000232267	ACTR3P2	ACTR3 pseudogene 2	100129013	0	NA	NA	NA	NA	NA
ENSG00000232268	OR52I1	olfactory receptor family 52 subfamily I member 1	390037	0	NA	NA	NA	NA	NA
ENSG00000232273	NA	NA	NA	0	NA	NA	NA	NA	NA
ENSG00000232277	SLC30A6P1	SLC30A6 pseudogene 1	100422091	0	NA	NA	NA	NA	NA
ENSG00000232281	NA	NA	NA	0	NA	NA	NA	NA	NA
ENSG00000232282	MTND1P32	MT-ND1 pseudogene 32	100856881	0,072165449	0,847101401	4,080456	0,207599697	0,835541541	NA
ENSG00000232285	ELOBP3	elongin B pseudogene 3	100856810	0	NA	NA	NA	NA	NA
ENSG00000232292	ALDH7A1P2	aldehyde dehydrogenase 7 family member A1 pseudogene 2	542	0	NA	NA	NA	NA	NA
ENSG00000232293	PAICSP7	phosphoribosylaminoimidazole carboxylase, phosphoribosylaminoimidazole succinocarboxamide synthetase pseudogene 7	780816	0	NA	NA	NA	NA	NA
ENSG00000232296	NA	NA	NA	0	NA	NA	NA	NA	NA
ENSG00000232303	DFFBP1	DNA fragmentation factor subunit beta pseudogene 1	100422581	4,770686651	0,576575597	0,791723	0,728254261	0,466457956	NA
ENSG00000232305	NA	NA	NA	0	NA	NA	NA	NA	NA
ENSG00000232314	NA	NA	NA	0	NA	NA	NA	NA	NA
ENSG00000232320	NA	NA	NA	0	NA	NA	NA	NA	NA
ENSG00000232327	LOC100419739	zinc finger protein 341 pseudogene	100419739	0	NA	NA	NA	NA	NA
ENSG00000232332	NA	NA	NA	0	NA	NA	NA	NA	NA
ENSG00000232333	RPS27AP2	RPS27A pseudogene 2	140752	0,40056643	-1,038806424	3,074102	-0,337921914	0,735422033	NA
ENSG00000232334	NA	NA	NA	0	NA	NA	NA	NA	NA
ENSG00000232337	LOC100131736	DEAD-box helicase 18 pseudogene	100131736	0,190545319	0,846125456	4,080456	0,207360521	0,835728312	NA
ENSG00000232341	RPL4P2	ribosomal protein L4 pseudogene 2	646688	0	NA	NA	NA	NA	NA
ENSG00000232344	LOC100419436	ribosomal protein S2 pseudogene	100419436	0	NA	NA	NA	NA	NA
ENSG00000232346	RPS17P16	ribosomal protein S17 pseudogene 16	402057	0,072165449	0,847101401	4,080456	0,207599697	0,835541541	NA
ENSG00000232349	NA	NA	NA	0	NA	NA	NA	NA	NA
ENSG00000232350	NA	NA	NA	0	NA	NA	NA	NA	NA
ENSG00000232355	NA	NA	NA	0	NA	NA	NA	NA	NA
ENSG00000232361	TRPC6P3	TRPC6 pseudogene 3	100288724	0,30804997	1,672861776	3,847083	0,434839059	0,663679251	NA
ENSG00000232362	NA	NA	NA	0	NA	NA	NA	NA	NA
ENSG00000232366	VDAC1P9	voltage dependent anion channel 1 pseudogene 9	391106	0,072165449	0,847101401	4,080456	0,207599697	0,835541541	NA
ENSG00000232368	NA	NA	NA	0	NA	NA	NA	NA	NA
ENSG00000232369	NA	NA	NA	0	NA	NA	NA	NA	NA
ENSG00000232370	NA	NA	NA	0	NA	NA	NA	NA	NA
ENSG00000232373	MTCYBP3	MT-CYB pseudogene 3	100873204	1,272104004	-0,841994331	1,763934	-0,47733899	0,633120751	NA
ENSG00000232374	GPR79	G protein-coupled receptor 79 (pseudogene)	27200	0	NA	NA	NA	NA	NA
ENSG00000232375	NA	NA	NA	0	NA	NA	NA	NA	NA
ENSG00000232378	NA	NA	NA	0	NA	NA	NA	NA	NA
ENSG00000232379	RPL22P23	ribosomal protein L22 pseudogene 23	100289184	0	NA	NA	NA	NA	NA
ENSG00000232380	NA	NA	NA	0	NA	NA	NA	NA	NA
ENSG00000232381	OR52U1P	olfactory receptor family 52 subfamily U member 1 pseudogene	81243	0	NA	NA	NA	NA	NA
ENSG00000232382	OR5K1	olfactory receptor family 5 subfamily K member 1	26339	0	NA	NA	NA	NA	NA

ENSG00000232383	RPL23P7	ribosomal protein L23 pseudogene 7	442659	0	NA	NA	NA	NA	NA
ENSG00000232385	RPS3AP25	RPS3A pseudogene 25	645968	0,072165449	0,847101401	4,080456	0,207599697	0,835541541	NA
ENSG00000232387	NA	NA	NA	0	NA	NA	NA	NA	NA
ENSG00000232389	NA	NA	NA	0	NA	NA	NA	NA	NA
ENSG00000232390	NA	NA	NA	0	NA	NA	NA	NA	NA
ENSG00000232391	NA	NA	NA	0	NA	NA	NA	NA	NA
ENSG00000232392	GOT2P7	GOT2 pseudogene 7	100422628	0	NA	NA	NA	NA	NA
ENSG00000232393	RPL5P6	ribosomal protein L5 pseudogene 6	646784	0	NA	NA	NA	NA	NA
ENSG00000232398	TMPRSS11CP	transmembrane serine protease 11C, pseudogene	100381178	0	NA	NA	NA	NA	NA
ENSG00000232399	USP17L13	ubiquitin specific peptidase 17 like family member 13	100287238	0	NA	NA	NA	NA	NA
ENSG00000232400	RAD17P1	RAD17 pseudogene 1	9207	0	NA	NA	NA	NA	NA
ENSG00000232403	RPL27P5	ribosomal protein L27 pseudogene 5	100270969	0,118286652	0	4,080456	0	1	NA
ENSG00000232407	NA	NA	NA	0	NA	NA	NA	NA	NA
ENSG00000232409	NA	NA	NA	0	NA	NA	NA	NA	NA
ENSG00000232418	NA	NA	NA	0	NA	NA	NA	NA	NA
ENSG00000232420	NA	NA	NA	0	NA	NA	NA	NA	NA
ENSG00000232422	NA	NA	NA	0,222243674	-0,126194802	4,080456	-0,030926643	0,975328042	NA
ENSG00000232424	USP9YP29	USP9Y pseudogene 29	106480425	0	NA	NA	NA	NA	NA
ENSG00000232429	RPL21P131	ribosomal protein L21 pseudogene 131	388532	0	NA	NA	NA	NA	NA
ENSG00000232430	RPL31P15	ribosomal protein L31 pseudogene 15	100270984	0	NA	NA	NA	NA	NA
ENSG00000232433	NA	NA	NA	0	NA	NA	NA	NA	NA
ENSG00000232437	NA	NA	NA	0,31515285	-1,926122967	4,042074	-0,476518529	0,63370501	NA
ENSG00000232439	RPL18AP7	ribosomal protein L18a pseudogene 7	100133038	0	NA	NA	NA	NA	NA
ENSG00000232446	NA	NA	NA	0	NA	NA	NA	NA	NA
ENSG00000232447	MTND5P3	MT-ND5 pseudogene 3	100873210	0	NA	NA	NA	NA	NA
ENSG00000232450	RPS2P14	ribosomal protein S2 pseudogene 14	100270827	1,671944402	-2,007467185	1,795814	-1,117859483	0,263627012	NA
ENSG00000232454	NA	NA	NA	0,461254722	-0,11403097	3,011416	-0,037866231	0,969794338	NA
ENSG00000232456	FAM131C2P	family with sequence similarity 131 member C pseudogene	440568	0	NA	NA	NA	NA	NA
ENSG00000232457	NA	NA	NA	0,11808024	-1,076443944	4,080456	-0,263804824	0,791930317	NA
ENSG00000232459	OR4F14P	olfactory receptor family 4 subfamily F member 14 pseudogene	390650	0	NA	NA	NA	NA	NA
ENSG00000232460	NA	NA	NA	0	NA	NA	NA	NA	NA
ENSG00000232463	RPL13P7	ribosomal protein L13 pseudogene 7	391140	0	NA	NA	NA	NA	NA
ENSG00000232466	H3P31	H3 histone pseudogene 31	115482710	0,915272754	-0,565278233	1,771049	-0,319177111	0,749592213	NA
ENSG00000232467	NA	NA	NA	0	NA	NA	NA	NA	NA
ENSG00000232471	NA	NA	NA	0	NA	NA	NA	NA	NA
ENSG00000232472	EEF1B2P3	eukaryotic translation elongation factor 1 beta 2 pseudogene 3	644820	0,23527457	0,812416248	4,079041	0,199168438	0,842130987	NA
ENSG00000232475	HSFY5P	heat shock transcription factor Y-linked 5, pseudogene	106478934	0	NA	NA	NA	NA	NA
ENSG00000232479	KRT8P52	keratin 8 pseudogene 52	100887068	1,208367946	1,167324302	1,769825	0,659570386	0,509529564	NA
ENSG00000232486	NA	NA	NA	1,426044279	-1,12396212	1,741496	-0,645400396	0,518667747	NA
ENSG00000232489	NA	NA	NA	0,992935568	-0,714861216	1,981748	-0,360722474	0,718306922	NA
ENSG00000232491	NA	NA	NA	0	NA	NA	NA	NA	NA
ENSG00000232492	NA	NA	NA	0	NA	NA	NA	NA	NA
ENSG00000232493	NA	NA	NA	0	NA	NA	NA	NA	NA
ENSG00000232496	NA	NA	NA	0	NA	NA	NA	NA	NA
ENSG00000232497	NA	NA	NA	0	NA	NA	NA	NA	NA
ENSG00000232499	LOC100421402	heterogeneous nuclear ribonucleoprotein A1 pseudogene	100421402	0,222810181	-2,001871074	4,035238	-0,496097395	0,619825697	NA
ENSG00000232502	NA	NA	NA	0	NA	NA	NA	NA	NA
ENSG00000232503	NA	NA	NA	0	NA	NA	NA	NA	NA
ENSG00000232508	NA	NA	NA	0	NA	NA	NA	NA	NA
ENSG00000232511	OR2AH1P	olfactory receptor family 2 subfamily AH member 1 pseudogene	81336	0	NA	NA	NA	NA	NA
ENSG00000232514	RPL21P25	ribosomal protein L21 pseudogene 25	100270881	0	NA	NA	NA	NA	NA
ENSG00000232515	NA	NA	NA	0	NA	NA	NA	NA	NA
ENSG00000232517	CSPG4BP	chondroitin sulfate proteoglycan family member 4B, pseudogene	727930	0,31672576	0	4,080456	0	1	NA
ENSG00000232522	NA	NA	NA	0	NA	NA	NA	NA	NA
ENSG00000232525	NA	NA	NA	0	NA	NA	NA	NA	NA

ENSG00000232526	VDAC1P13	voltage dependent anion channel 1 pseudogene 13	100420568	0	NA	NA	NA	NA	NA
ENSG00000232528	LOC100289473	cytoskeleton associated protein 2-like pseudogene	100289473	0	NA	NA	NA	NA	NA
ENSG00000232531	KMT5AP2	KMT5A pseudogene 2	100421788	0	NA	NA	NA	NA	NA
ENSG00000232535	NA	NA	NA	0	NA	NA	NA	NA	NA
ENSG00000232540	RPL36P19	ribosomal protein L36 pseudogene 19	100873855	0	NA	NA	NA	NA	NA
ENSG00000232546	NA	NA	NA	0	NA	NA	NA	NA	NA
ENSG00000232549	NA	NA	NA	0	NA	NA	NA	NA	NA
ENSG00000232551	MTCO1P14	MT-CO1 pseudogene 14	107075142	0	NA	NA	NA	NA	NA
ENSG00000232553	NA	NA	NA	0	NA	NA	NA	NA	NA
ENSG00000232554	NA	NA	NA	0	NA	NA	NA	NA	NA
ENSG00000232556	PRCPP1	PRCP pseudogene 1	100421289	0	NA	NA	NA	NA	NA
ENSG00000232562	TPMTP3	thiopurine S-methyltransferase pseudogene 3	100129277	0	NA	NA	NA	NA	NA
ENSG00000232563	CRB3P1	crumbs cell polarity complex component 3 pseudogene 1	106480279	0	NA	NA	NA	NA	NA
ENSG00000232568	NA	NA	NA	0,222654543	0,831530852	4,080456	0,203783812	0,838522444	NA
ENSG00000232573	RPL3P4	ribosomal protein L3 pseudogene 4	326307	1,568920084	-1,284789133	1,995932	-0,643703928	0,519767442	NA
ENSG00000232576	NA	NA	NA	0	NA	NA	NA	NA	NA
ENSG00000232578	LOC100533629	FAST kinase domains 1 pseudogene	100533629	6,515756234	0,429738317	0,900556	0,477192126	0,633225317	NA
ENSG00000232579	MTCO1P27	MT-CO1 pseudogene 27	107075168	0	NA	NA	NA	NA	NA
ENSG00000232582	LOC100419791	zinc finger protein 449 pseudogene	100419791	0	NA	NA	NA	NA	NA
ENSG00000232583	GPR143YP	G protein-coupled receptor 143 Y-linked pseudogene	352896	0	NA	NA	NA	NA	NA
ENSG00000232585	NA	NA	NA	0	NA	NA	NA	NA	NA
ENSG00000232587	EEF1A1P3	eukaryotic translation elongation factor 1 alpha 1 pseudogene 3	100421865	8,458915028	0,409341485	0,610778	0,670196648	0,502732441	NA
ENSG00000232593	KANTR	KANTR integral membrane protein	102723508	4,693548166	-0,552791595	0,898818	-0,61502095	0,538540885	NA
ENSG00000232594	NA	NA	NA	0	NA	NA	NA	NA	NA
ENSG00000232595	NA	NA	NA	0	NA	NA	NA	NA	NA
ENSG00000232599	NA	NA	NA	0	NA	NA	NA	NA	NA
ENSG00000232601	ANAPC15P2	ANAPC15 pseudogene 2	117751731	0	NA	NA	NA	NA	NA
ENSG00000232603	NA	NA	NA	0	NA	NA	NA	NA	NA
ENSG00000232605	NA	NA	NA	0	NA	NA	NA	NA	NA
ENSG00000232608	NA	NA	NA	0	NA	NA	NA	NA	NA
ENSG00000232610	NA	NA	NA	0	NA	NA	NA	NA	NA
ENSG00000232612	RPL31P35	ribosomal protein L31 pseudogene 35	100271199	0	NA	NA	NA	NA	NA
ENSG00000232614	NA	NA	NA	0	NA	NA	NA	NA	NA
ENSG00000232615	NA	NA	NA	0	NA	NA	NA	NA	NA
ENSG00000232617	NA	NA	NA	0	NA	NA	NA	NA	NA
ENSG00000232620	TSPY17P	testis specific protein Y-linked 17, pseudogene	100132124	0	NA	NA	NA	NA	NA
ENSG00000232621	C4BPAP2	C4BPA pseudogene 2	724	0	NA	NA	NA	NA	NA
ENSG00000232622	NA	NA	NA	0	NA	NA	NA	NA	NA
ENSG00000232625	RPL23AP31	ribosomal protein L23a pseudogene 31	100271460	0	NA	NA	NA	NA	NA
ENSG00000232626	LOC100420423	FKBP prolyl isomerase 8 pseudogene	100420423	0,742977815	0,390714308	2,540266	0,153808417	0,877760797	NA
ENSG00000232627	NA	NA	NA	0	NA	NA	NA	NA	NA
ENSG00000232629	HLA-DQB2	major histocompatibility complex, class II, DQ beta 2	3120	0	NA	NA	NA	NA	NA
ENSG00000232630	NA	NA	NA	0	NA	NA	NA	NA	NA
ENSG00000232631	MTND2P23	MT-ND2 pseudogene 23	100873229	0	NA	NA	NA	NA	NA
ENSG00000232634	NA	NA	NA	0	NA	NA	NA	NA	NA
ENSG00000232644	NA	NA	NA	0	NA	NA	NA	NA	NA
ENSG00000232646	NA	NA	NA	0	NA	NA	NA	NA	NA
ENSG00000232647	NA	NA	NA	0	NA	NA	NA	NA	NA
ENSG00000232654	NA	NA	NA	0	NA	NA	NA	NA	NA
ENSG00000232658	NA	NA	NA	0	NA	NA	NA	NA	NA
ENSG00000232662	LDHBP1	lactate dehydrogenase B pseudogene 1	100289475	1,283670173	1,177239039	1,798961	0,65439958	0,512854405	NA
ENSG00000232663	NA	NA	NA	0	NA	NA	NA	NA	NA
ENSG00000232664	NA	NA	NA	0,24922814	0,484299682	4,061636	0,119237589	0,905087128	NA
ENSG00000232665	NA	NA	NA	0	NA	NA	NA	NA	NA
ENSG00000232668	NA	NA	NA	0	NA	NA	NA	NA	NA
ENSG00000232672	ACTG1P21	actin gamma 1 pseudogene 21	100131291	0	NA	NA	NA	NA	NA
ENSG00000232676	ADH5P2	ADH5 pseudogene 2	343296	0	NA	NA	NA	NA	NA
ENSG00000232678	SPTLC1P4	serine palmitoyltransferase long chain base subunit 1 pseudogene 4	100874500	0	NA	NA	NA	NA	NA

ENSG00000232690	NA	NA	NA	0	NA	NA	NA	NA	NA
ENSG00000232695	ELOCP17	elongin C pseudogene 17	100462879	0	NA	NA	NA	NA	NA
ENSG00000232699	NA	NA	NA	0	NA	NA	NA	NA	NA
ENSG00000232701	NA	NA	NA	0	NA	NA	NA	NA	NA
ENSG00000232702	NA	NA	NA	0	NA	NA	NA	NA	NA
ENSG00000232706	NA	NA	NA	0,341126215	-2,003684188	3,74207	-0,53544803	0,592340085	NA
ENSG00000232707	AP1B1P2	AP1B1 pseudogene 2	23781	0	NA	NA	NA	NA	NA
ENSG00000232709	NA	NA	NA	0	NA	NA	NA	NA	NA
ENSG00000232713	NA	NA	NA	2,073559966	-2,16650986	1,370476	-1,580844373	0,113913626	NA
ENSG00000232716	LOC100133252	zinc finger protein 131 pseudogene	100133252	0,117497108	0,847101401	4,080456	0,207599697	0,835541541	NA
ENSG00000232718	NA	NA	NA	0	NA	NA	NA	NA	NA
ENSG00000232722	NA	NA	NA	0	NA	NA	NA	NA	NA
ENSG00000232723	RPS17P7	ribosomal protein S17 pseudogene 7	100271069	0	NA	NA	NA	NA	NA
ENSG00000232724	TRIM80P	tripartite motif containing 80, pseudogene	106480328	0	NA	NA	NA	NA	NA
ENSG00000232727	NA	NA	NA	0	NA	NA	NA	NA	NA
ENSG00000232728	NA	NA	NA	0	NA	NA	NA	NA	NA
ENSG00000232730	NA	NA	NA	0	NA	NA	NA	NA	NA
ENSG00000232734	NA	NA	NA	0	NA	NA	NA	NA	NA
ENSG00000232735	NA	NA	NA	0	NA	NA	NA	NA	NA
ENSG00000232736	NA	NA	NA	0	NA	NA	NA	NA	NA
ENSG00000232742	NA	NA	NA	0	NA	NA	NA	NA	NA
ENSG00000232743	NA	NA	NA	0	NA	NA	NA	NA	NA
ENSG00000232744	USP9YP16	USP9Y pseudogene 16	106478927	0	NA	NA	NA	NA	NA
ENSG00000232747	NA	NA	NA	0	NA	NA	NA	NA	NA
ENSG00000232750	NA	NA	NA	0	NA	NA	NA	NA	NA
ENSG00000232751	RPL26P11	ribosomal protein L26 pseudogene 11	730070	0	NA	NA	NA	NA	NA
ENSG00000232752	MTCO3P21	MT-CO3 pseudogene 21	107075162	0	NA	NA	NA	NA	NA
ENSG00000232757	NA	NA	NA	0	NA	NA	NA	NA	NA
ENSG00000232760	LOC100859919	chromosome 9 open reading frame 86 pseudogene 2	100859919	0	NA	NA	NA	NA	NA
ENSG00000232764	TRIM60P8Y	tripartite motif containing 60 pseudogene 8, Y-linked	100420373	0	NA	NA	NA	NA	NA
ENSG00000232765	NA	NA	NA	0	NA	NA	NA	NA	NA
ENSG00000232766	KIAA1143P2	KIAA1143 pseudogene 2	107986034	0,391565795	-0,158409067	3,674147	-0,043114519	0,965610245	NA
ENSG00000232768	NA	NA	NA	0	NA	NA	NA	NA	NA
ENSG00000232771	NA	NA	NA	0	NA	NA	NA	NA	NA
ENSG00000232772	MTND4P22	MT-ND4 pseudogene 22	100873244	0	NA	NA	NA	NA	NA
ENSG00000232773	DEFB130D	defensin beta 130D (pseudogene)	110806292	0	NA	NA	NA	NA	NA
ENSG00000232775	NA	NA	NA	0	NA	NA	NA	NA	NA
ENSG00000232777	NA	NA	NA	0	NA	NA	NA	NA	NA
ENSG00000232778	RPL23AP50	ribosomal protein L23a pseudogene 50	728106	0,119673877	0,847101401	4,080456	0,207599697	0,835541541	NA
ENSG00000232780	NA	NA	NA	0	NA	NA	NA	NA	NA
ENSG00000232783	NA	NA	NA	0	NA	NA	NA	NA	NA
ENSG00000232786	NA	NA	NA	0	NA	NA	NA	NA	NA
ENSG00000232787	NA	NA	NA	0	NA	NA	NA	NA	NA
ENSG00000232789	NA	NA	NA	0	NA	NA	NA	NA	NA
ENSG00000232791	NA	NA	NA	0	NA	NA	NA	NA	NA
ENSG00000232792	FTH1P25	ferritin heavy chain 1 pseudogene 25	100462799	0	NA	NA	NA	NA	NA
ENSG00000232793	NA	NA	NA	0	NA	NA	NA	NA	NA
ENSG00000232794	NA	NA	NA	0	NA	NA	NA	NA	NA
ENSG00000232795	SCAND3P1	SCAND3 pseudogene 1	100873886	0	NA	NA	NA	NA	NA
ENSG00000232797	NA	NA	NA	0	NA	NA	NA	NA	NA
ENSG00000232798	NA	NA	NA	0	NA	NA	NA	NA	NA
ENSG00000232799	NA	NA	NA	0	NA	NA	NA	NA	NA
ENSG00000232800	SLC7A15P	solute carrier family 7 member 15, pseudogene	100289450	0	NA	NA	NA	NA	NA
ENSG00000232801	SDCBPP3	syndecan binding protein pseudogene 3	100131656	0	NA	NA	NA	NA	NA
ENSG00000232805	NA	NA	NA	0	NA	NA	NA	NA	NA
ENSG00000232809	NA	NA	NA	0	NA	NA	NA	NA	NA
ENSG00000232810	TNF	tumor necrosis factor	7124	49,05884278	0,029197935	2,565733	0,011379958	NA	NA
ENSG00000232813	NA	NA	NA	0	NA	NA	NA	NA	NA
ENSG00000232815	NA	NA	NA	2,101061464	0,751252714	1,721753	0,436330193	0,662597179	NA
ENSG00000232818	RPS2P32	ribosomal protein S2 pseudogene 32	256355	2,273296201	0,199385751	1,374962	0,145011808	0,884701549	NA
ENSG00000232823	NA	NA	NA	0	NA	NA	NA	NA	NA
ENSG00000232824	NA	NA	NA	0	NA	NA	NA	NA	NA
ENSG00000232829	GSTO3P	glutathione S-transferase omega 3, pseudogene	344813	0	NA	NA	NA	NA	NA
ENSG00000232830	NA	NA	NA	0	NA	NA	NA	NA	NA
ENSG00000232834	NA	NA	NA	0	NA	NA	NA	NA	NA

ENSG00000232841	BRWD1P3	bromodomain and WD repeat domain containing 1 pseudogene 3	106480688	0	NA	NA	NA	NA	NA
ENSG00000232842	KAT7P1	KAT7 pseudogene 1	648927	0	NA	NA	NA	NA	NA
ENSG00000232843	SNX18P2	sorting nexin 18 pseudogene 2	642952	0	NA	NA	NA	NA	NA
ENSG00000232845	NA	NA	NA	0	NA	NA	NA	NA	NA
ENSG00000232846	SLC25A6P3	solute carrier family 25 member 6 pseudogene 3	100422686	0	NA	NA	NA	NA	NA
ENSG00000232848	NA	NA	NA	0	NA	NA	NA	NA	NA
ENSG00000232851	NA	NA	NA	0	NA	NA	NA	NA	NA
ENSG00000232852	NA	NA	NA	0	NA	NA	NA	NA	NA
ENSG00000232853	NA	NA	NA	0	NA	NA	NA	NA	NA
ENSG00000232857	NA	NA	NA	0	NA	NA	NA	NA	NA
ENSG00000232858	RPL34P27	ribosomal protein L34 pseudogene 27	651249	0	NA	NA	NA	NA	NA
ENSG00000232859	LYRM9	LYR motif containing 9	201229	8,290584575	0,340388501	0,667341	0,510066769	0,610004685	NA
ENSG00000232862	NA	NA	NA	0	NA	NA	NA	NA	NA
ENSG00000232864	NUCKS1P1	nuclear casein kinase and cyclin dependent kinase substrate 1 pseudogene 1	100130466	0	NA	NA	NA	NA	NA
ENSG00000232867	RPL36AP34	ribosomal protein L36a pseudogene 34	100271513	0	NA	NA	NA	NA	NA
ENSG00000232871	SEC1P	secretory blood group 1, pseudogene	653677	0,27867949	0	4,080456	0	1	NA
ENSG00000232872	CTAGE3P	CTAGE family member 3, pseudogene	220112	0	NA	NA	NA	NA	NA
ENSG00000232873	NA	NA	NA	0	NA	NA	NA	NA	NA
ENSG00000232875	NA	NA	NA	0	NA	NA	NA	NA	NA
ENSG00000232879	GLRX5P2	GLR5 pseudogene 2	118568821	0	NA	NA	NA	NA	NA
ENSG00000232880	NDUFB4P10	NADH:ubiquinone oxidoreductase subunit B4 pseudogene 10	107075105	0	NA	NA	NA	NA	NA
ENSG00000232881	RPS10P21	ribosomal protein S10 pseudogene 21	100271351	0	NA	NA	NA	NA	NA
ENSG00000232882	NA	NA	NA	2,655099845	0,859746991	1,327152	0,647813446	0,517105618	NA
ENSG00000232883	RPLP0P5	ribosomal protein lateral stalk subunit P0 pseudogene 5	643779	0,15836288	0	4,080456	0	1	NA
ENSG00000232887	NA	NA	NA	0	NA	NA	NA	NA	NA
ENSG00000232888	RPS11P5	ribosomal protein S11 pseudogene 5	727826	0,117497108	0,847101401	4,080456	0,207599697	0,835541541	NA
ENSG00000232889	NA	NA	NA	0	NA	NA	NA	NA	NA
ENSG00000232894	NA	NA	NA	0,41109263	-0,151814716	3,641246	-0,041693066	0,966743382	NA
ENSG00000232897	NA	NA	NA	0	NA	NA	NA	NA	NA
ENSG00000232901	NA	NA	NA	0	NA	NA	NA	NA	NA
ENSG00000232905	LOC100132609	programmed cell death 2 pseudogene	100132609	0	NA	NA	NA	NA	NA
ENSG00000232906	LOC100533634	zinc finger protein 737 pseudogene	100533634	0	NA	NA	NA	NA	NA
ENSG00000232908	NA	NA	NA	0	NA	NA	NA	NA	NA
ENSG00000232914	NA	NA	NA	0	NA	NA	NA	NA	NA
ENSG00000232915	NA	NA	NA	0,315475223	-1,077683423	4,080456	-0,264108584	0,79169625	NA
ENSG00000232916	NA	NA	NA	0	NA	NA	NA	NA	NA
ENSG00000232917	NA	NA	NA	0	NA	NA	NA	NA	NA
ENSG00000232918	RPL21P26	ribosomal protein L21 pseudogene 26	100270882	0	NA	NA	NA	NA	NA
ENSG00000232922	MTND6P24	MT-ND6 pseudogene 24	107063611	0	NA	NA	NA	NA	NA
ENSG00000232925	NA	NA	NA	0	NA	NA	NA	NA	NA
ENSG00000232926	RPL8P5	ribosomal protein L8 pseudogene 5	100271044	0	NA	NA	NA	NA	NA
ENSG00000232927	NA	NA	NA	0	NA	NA	NA	NA	NA
ENSG00000232928	NA	NA	NA	0	NA	NA	NA	NA	NA
ENSG00000232943	NA	NA	NA	0	NA	NA	NA	NA	NA
ENSG00000232944	NA	NA	NA	0	NA	NA	NA	NA	NA
ENSG00000232946	NA	NA	NA	0	NA	NA	NA	NA	NA
ENSG00000232950	NA	NA	NA	0	NA	NA	NA	NA	NA
ENSG00000232951	NA	NA	NA	0	NA	NA	NA	NA	NA
ENSG00000232952	LOC401957	matrin 3 pseudogene	401957	0	NA	NA	NA	NA	NA
ENSG00000232953	NA	NA	NA	0,544346187	2,341722817	3,318892	0,705573726	0,480453268	NA
ENSG00000232958	NA	NA	NA	0	NA	NA	NA	NA	NA
ENSG00000232963	NA	NA	NA	0	NA	NA	NA	NA	NA
ENSG00000232965	RPS12P18	ribosomal protein S12 pseudogene 18	100271354	0	NA	NA	NA	NA	NA
ENSG00000232968	EIF4EP4	eukaryotic translation initiation factor 4E pseudogene 4	100421455	0	NA	NA	NA	NA	NA
ENSG00000232970	NA	NA	NA	0	NA	NA	NA	NA	NA
ENSG00000232975	MTCO1P57	MT-CO1 pseudogene 57	109729155	0	NA	NA	NA	NA	NA

ENSG00000232976	TRIM60P10Y	tripartite motif containing 60 pseudogene 10, Y-linked	100420376	0	NA	NA	NA	NA	NA
ENSG00000232979	NA	NA	NA	0	NA	NA	NA	NA	NA
ENSG00000232981	MTCO1P51	MT-CO1 pseudogene 51	100287067	0	NA	NA	NA	NA	NA
ENSG00000232982	MTCO1P45	MT-CO1 pseudogene 45	107075174	0	NA	NA	NA	NA	NA
ENSG00000232983	NA	NA	NA	0	NA	NA	NA	NA	NA
ENSG00000232989	NA	NA	NA	0	NA	NA	NA	NA	NA
ENSG00000232990	MTATP6P7	MT-ATP6 pseudogene 7	106480798	0	NA	NA	NA	NA	NA
ENSG00000232992	AHCYP4	adenosylhomocysteinase pseudogene 4	129560	0	NA	NA	NA	NA	NA
ENSG00000232994	NA	NA	NA	0	NA	NA	NA	NA	NA
ENSG00000232999	NA	NA	NA	0	NA	NA	NA	NA	NA
ENSG00000233003	NA	NA	NA	0	NA	NA	NA	NA	NA
ENSG00000233007	NA	NA	NA	0	NA	NA	NA	NA	NA
ENSG00000233010	NA	NA	NA	1,526116664	1,133248732	1,872837	0,605097401	0,545114281	NA
ENSG00000233011	NA	NA	NA	0	NA	NA	NA	NA	NA
ENSG00000233012	HDAC1P2	histone deacetylase 1 pseudogene 2	100419489	0	NA	NA	NA	NA	NA
ENSG00000233013	NA	NA	NA	0	NA	NA	NA	NA	NA
ENSG00000233015	SLC25A6P1	solute carrier family 25 member 6 pseudogene 1	128790	0	NA	NA	NA	NA	NA
ENSG00000233018	NA	NA	NA	0	NA	NA	NA	NA	NA
ENSG00000233020	NA	NA	NA	0	NA	NA	NA	NA	NA
ENSG00000233026	MTCO1P5	MT-CO1 pseudogene 5	106480318	0	NA	NA	NA	NA	NA
ENSG00000233028	NA	NA	NA	0	NA	NA	NA	NA	NA
ENSG00000233031	LOC100287498	NDUFA4, mitochondrial complex associated pseudogene	100287498	0	NA	NA	NA	NA	NA
ENSG00000233036	NA	NA	NA	0	NA	NA	NA	NA	NA
ENSG00000233039	MTND5P30	MT-ND5 pseudogene 30	100873266	0	NA	NA	NA	NA	NA
ENSG00000233040	NA	NA	NA	1,580747615	1,87135343	1,852629	1,010107004	0,312444027	NA
ENSG00000233041	PHGR1	proline, histidine and glycine rich 1	644844	0	NA	NA	NA	NA	NA
ENSG00000233045	NA	NA	NA	0,23776232	-0,110844017	4,080456	-0,027164616	0,978328438	NA
ENSG00000233055	NA	NA	NA	0	NA	NA	NA	NA	NA
ENSG00000233056	ERVH48-1	endogenous retrovirus group 48 member 1, envelope	90625	0	NA	NA	NA	NA	NA
ENSG00000233057	EEF1A1P14	eukaryotic translation elongation factor 1 alpha 1 pseudogene 14	647167	5,037266213	-1,079285016	0,914029	-1,180799076	0,237682551	NA
ENSG00000233063	NA	NA	NA	0	NA	NA	NA	NA	NA
ENSG00000233071	RPSAP59	ribosomal protein SA pseudogene 59	100271148	0	NA	NA	NA	NA	NA
ENSG00000233072	RPS15AP6	ribosomal protein S15a pseudogene 6	100271141	0,565657308	-2,070484539	3,194683	-0,648103232	0,516918184	NA
ENSG00000233080	LINC01399	long intergenic non-protein coding RNA 1399	104310353	0	NA	NA	NA	NA	NA
ENSG00000233083	FTH1P6	ferritin heavy chain 1 pseudogene 6	106481787	0	NA	NA	NA	NA	NA
ENSG00000233084	RPL23AP25	ribosomal protein L23a pseudogene 25	653789	0,222678072	0,833647208	4,080456	0,204302469	0,838117141	NA
ENSG00000233087	RAB6D	RAB6D, member RAS oncogene family	150786	0	NA	NA	NA	NA	NA
ENSG00000233090	NA	NA	NA	0	NA	NA	NA	NA	NA
ENSG00000233105	NA	NA	NA	0	NA	NA	NA	NA	NA
ENSG00000233106	RPL12P3	ribosomal protein L12 pseudogene 3	128862	0	NA	NA	NA	NA	NA
ENSG00000233107	SUCLA2P2	SUCLA2 pseudogene 2	100129863	0	NA	NA	NA	NA	NA
ENSG00000233109	NA	NA	NA	0	NA	NA	NA	NA	NA
ENSG00000233111	NA	NA	NA	0	NA	NA	NA	NA	NA
ENSG00000233114	COX7BP4	COX7B pseudogene 4	127460871	0	NA	NA	NA	NA	NA
ENSG00000233115	FAM90A11	family with sequence similarity 90 member A11	441331	0	NA	NA	NA	NA	NA
ENSG00000233118	NA	NA	NA	0	NA	NA	NA	NA	NA
ENSG00000233120	USP9YP15	USP9Y pseudogene 15	106480713	0	NA	NA	NA	NA	NA
ENSG00000233121	VN1R20P	vomeronasal 1 receptor 20 pseudogene	100312775	0,10543123	0	4,080456	0	1	NA
ENSG00000233122	CTAGE7P	CTAGE family member 7, pseudogene	119437	0	NA	NA	NA	NA	NA
ENSG00000233125	ACTBP12	ACTB pseudogene 12	100462767	0	NA	NA	NA	NA	NA
ENSG00000233126	NA	NA	NA	0	NA	NA	NA	NA	NA
ENSG00000233129	NA	NA	NA	0	NA	NA	NA	NA	NA
ENSG00000233131	RPS15AP14	ribosomal protein S15a pseudogene 14	100127924	0	NA	NA	NA	NA	NA
ENSG00000233132	LOC124906723	putative protein FAM90A5P	124906723	0	NA	NA	NA	NA	NA
ENSG00000233133	NA	NA	NA	0	NA	NA	NA	NA	NA
ENSG00000233134	MTND3P17	MT-ND3 pseudogene 17	107075279	0	NA	NA	NA	NA	NA
ENSG00000233135	RPS27P18	ribosomal protein S27 pseudogene 18	100271380	0	NA	NA	NA	NA	NA

ENSG00000233136	USP17L11	ubiquitin specific peptidase 17 like family member 11	100287178	0	NA	NA	NA	NA	NA
ENSG00000233139	NA	NA	NA	0,072165449	0,847101401	4,080456	0,207599697	0,835541541	NA
ENSG00000233146	BPIFB5P	BPI fold containing family B member 5, pseudogene	100505383	0	NA	NA	NA	NA	NA
ENSG00000233148	SYF2P2	SYF2 pre-mRNA splicing factor pseudogene 2	100128275	0	NA	NA	NA	NA	NA
ENSG00000233155	NA	NA	NA	0,072165449	0,847101401	4,080456	0,207599697	0,835541541	NA
ENSG00000233157	LOC100419506	ribosomal protein L4 pseudogene	100419506	0	NA	NA	NA	NA	NA
ENSG00000233158	RPS24P6	ribosomal protein S24 pseudogene 6	100216339	0	NA	NA	NA	NA	NA
ENSG00000233159	RPL31P16	ribosomal protein L31 pseudogene 16	100129418	0	NA	NA	NA	NA	NA
ENSG00000233162	NA	NA	NA	0	NA	NA	NA	NA	NA
ENSG00000233163	RPS12P17	ribosomal protein S12 pseudogene 17	100271353	0	NA	NA	NA	NA	NA
ENSG00000233167	EEF1A1P26	eukaryotic translation elongation factor 1 alpha 1 pseudogene 26	100128637	0	NA	NA	NA	NA	NA
ENSG00000233170	NA	NA	NA	0,655565367	-0,52539186	2,718133	-0,193291445	0,846730726	NA
ENSG00000233173	VN1R34P	vomeronasal 1 receptor 34 pseudogene	100129587	0	NA	NA	NA	NA	NA
ENSG00000233186	KLF4P1	KLF4 pseudogene 1	100420113	0	NA	NA	NA	NA	NA
ENSG00000233189	NA	NA	NA	0	NA	NA	NA	NA	NA
ENSG00000233190	NA	NA	NA	0	NA	NA	NA	NA	NA
ENSG00000233196	LOC100131939	parkin coregulated like pseudogene	100131939	0	NA	NA	NA	NA	NA
ENSG00000233197	NA	NA	NA	0	NA	NA	NA	NA	NA
ENSG00000233198	RNF224	ring finger protein 224	643596	0	NA	NA	NA	NA	NA
ENSG00000233204	MAPRE1P3	MAPRE1 pseudogene 3	100422229	0	NA	NA	NA	NA	NA
ENSG00000233205	NA	NA	NA	0	NA	NA	NA	NA	NA
ENSG00000233206	RPS3AP1	RPS3A pseudogene 1	101241875	0	NA	NA	NA	NA	NA
ENSG00000233211	NA	NA	NA	0	NA	NA	NA	NA	NA
ENSG00000233216	NA	NA	NA	0	NA	NA	NA	NA	NA
ENSG00000233217	MROH3P	maestro heat like repeat family member 3, pseudogene	647215	0	NA	NA	NA	NA	NA
ENSG00000233225	NA	NA	NA	0,103998153	-1,076443944	4,080456	-0,263804824	0,791930317	NA
ENSG00000233228	LPCAT2BP	lysophosphatidylcholine acyltransferase 2b, pseudogene	100128094	0	NA	NA	NA	NA	NA
ENSG00000233229	NA	NA	NA	0,10488184	-1,076443944	4,080456	-0,263804824	0,791930317	NA
ENSG00000233231	HNRNPA1P49	heterogeneous nuclear ribonucleoprotein A1 pseudogene 49	106481663	0	NA	NA	NA	NA	NA
ENSG00000233235	NA	NA	NA	0	NA	NA	NA	NA	NA
ENSG00000233243	NA	NA	NA	0	NA	NA	NA	NA	NA
ENSG00000233247	NA	NA	NA	0	NA	NA	NA	NA	NA
ENSG00000233248	MTND6P9	MT-ND6 pseudogene 9	106478946	0	NA	NA	NA	NA	NA
ENSG00000233252	CRIP1P1	cysteine rich protein 1 pseudogene 1	106480793	0	NA	NA	NA	NA	NA
ENSG00000233254	RPL21P134	ribosomal protein L21 pseudogene 134	728782	0	NA	NA	NA	NA	NA
ENSG00000233259	NA	NA	NA	0	NA	NA	NA	NA	NA
ENSG00000233260	RPSAP63	ribosomal protein SA pseudogene 63	100270755	0	NA	NA	NA	NA	NA
ENSG00000233261	NA	NA	NA	1,588726558	-0,257519061	1,478669	-0,174155936	0,861742915	NA
ENSG00000233263	NA	NA	NA	0	NA	NA	NA	NA	NA
ENSG00000233264	NA	NA	NA	6,448297742	-0,439132712	0,734809	-0,597614979	0,550096867	NA
ENSG00000233266	HMGB1P31	high mobility group box 1 pseudogene 31	100873894	0,906526723	1,30633853	2,446306	0,534004577	0,593338368	NA
ENSG00000233268	NA	NA	NA	0	NA	NA	NA	NA	NA
ENSG00000233270	NA	NA	NA	0,072165449	0,847101401	4,080456	0,207599697	0,835541541	NA
ENSG00000233272	NA	NA	NA	0	NA	NA	NA	NA	NA
ENSG00000233273	AMMECR1LP1	AMMECR1 like pseudogene 1	100420921	0	NA	NA	NA	NA	NA
ENSG00000233278	NA	NA	NA	0	NA	NA	NA	NA	NA
ENSG00000233285	MTCYBP10	MT-CYB pseudogene 10	107075121	0	NA	NA	NA	NA	NA
ENSG00000233286	MTND3P10	MT-ND3 pseudogene 10	100873239	0	NA	NA	NA	NA	NA
ENSG00000233287	RPL27P11	ribosomal protein L27 pseudogene 11	442723	0	NA	NA	NA	NA	NA
ENSG00000233291	RPL7AP61	ribosomal protein L7a pseudogene 61	100271539	0	NA	NA	NA	NA	NA
ENSG00000233292	NA	NA	NA	0	NA	NA	NA	NA	NA
ENSG00000233295	NA	NA	NA	0	NA	NA	NA	NA	NA
ENSG00000233297	NA	NA	NA	0	NA	NA	NA	NA	NA
ENSG00000233299	NA	NA	NA	0	NA	NA	NA	NA	NA

ENSG00000233300	EIF4A1P1	eukaryotic translation initiation factor 4A1 pseudogene 1	54052	0	NA	NA	NA	NA	NA
ENSG00000233301	OR4C14P	olfactory receptor family 4 subfamily C member 14 pseudogene	81310	0	NA	NA	NA	NA	NA
ENSG00000233306	TRGV2	T cell receptor gamma variable 2	6974	0,614125642	-0,18058025	2,485222	-0,072661614	0,942075396	NA
ENSG00000233307	NA	NA	NA	0	NA	NA	NA	NA	NA
ENSG00000233309	RPS6P12	ribosomal protein S6 pseudogene 12	100270867	0,11808024	-1,076443944	4,080456	-0,263804824	0,791930317	NA
ENSG00000233311	MTCO1P54	MT-CO1 pseudogene 54	107075178	0,117497108	0,847101401	4,080456	0,207599697	0,835541541	NA
ENSG00000233313	NA	NA	NA	0	NA	NA	NA	NA	NA
ENSG00000233318	NA	NA	NA	0	NA	NA	NA	NA	NA
ENSG00000233319	NA	NA	NA	0	NA	NA	NA	NA	NA
ENSG00000233324	EEF1A1P34	eukaryotic translation elongation factor 1 alpha 1 pseudogene 34	100132358	0	NA	NA	NA	NA	NA
ENSG00000233325	NA	NA	NA	0	NA	NA	NA	NA	NA
ENSG00000233327	NA	NA	NA	0	NA	NA	NA	NA	NA
ENSG00000233328	NA	NA	NA	0,426183343	1,689250863	3,562472	0,474179417	0,63537197	NA
ENSG00000233329	RPS16P3	ribosomal protein S16 pseudogene 3	100271067	0	NA	NA	NA	NA	NA
ENSG00000233333	NA	NA	NA	0	NA	NA	NA	NA	NA
ENSG00000233335	RPL36AP14	ribosomal protein L36a pseudogene 14	100271324	0	NA	NA	NA	NA	NA
ENSG00000233337	UBE2FP3	UBE2F pseudogene 3	106480360	0	NA	NA	NA	NA	NA
ENSG00000233343	ATP6V1G1P4	ATPase H+ transporting V1 subunit G1 pseudogene 4	100462843	0,315774477	-1,076321002	4,080456	-0,263774695	0,791953535	NA
ENSG00000233347	NA	NA	NA	0	NA	NA	NA	NA	NA
ENSG00000233352	NA	NA	NA	0	NA	NA	NA	NA	NA
ENSG00000233353	RPS7P9	ribosomal protein S7 pseudogene 9	100128756	0	NA	NA	NA	NA	NA
ENSG00000233357	NA	NA	NA	0,144330898	1,418786819	4,061262	0,349346267	0,726829368	NA
ENSG00000233366	NA	NA	NA	0	NA	NA	NA	NA	NA
ENSG00000233370	ACTR1AP1	ACTR1A pseudogene 1	100287074	0	NA	NA	NA	NA	NA
ENSG00000233377	NA	NA	NA	0	NA	NA	NA	NA	NA
ENSG00000233378	USP9YP34	USP9Y pseudogene 34	387361	0	NA	NA	NA	NA	NA
ENSG00000233380	RPS2P48	ribosomal protein S2 pseudogene 48	645173	0	NA	NA	NA	NA	NA
ENSG00000233381	NA	NA	NA	0	NA	NA	NA	NA	NA
ENSG00000233382	NKAPP1	NFKB activating protein pseudogene 1	158801	0,072165449	0,847101401	4,080456	0,207599697	0,835541541	NA
ENSG00000233383	NA	NA	NA	0	NA	NA	NA	NA	NA
ENSG00000233385	MTCO3P8	MT-CO3 pseudogene 8	107075151	0	NA	NA	NA	NA	NA
ENSG00000233388	LOC100131536	tRNA methyltransferase 11 homolog (S, cerevisiae) pseudogene	100131536	0	NA	NA	NA	NA	NA
ENSG00000233401	PRKAR1AP1	protein kinase cAMP-dependent type I regulatory subunit alpha pseudogene 1	5574	0,10543123	0	4,080456	0	1	NA
ENSG00000233402	NA	NA	NA	0	NA	NA	NA	NA	NA
ENSG00000233406	RPS2P8	ribosomal protein S2 pseudogene 8	440589	0	NA	NA	NA	NA	NA
ENSG00000233412	OR5H15	olfactory receptor family 5 subfamily H member 15	403274	0	NA	NA	NA	NA	NA
ENSG00000233416	RPS25P3	ribosomal protein S25 pseudogene 3	100271101	0,693151692	2,748633518	2,355281	1,167008595	0,243206899	NA
ENSG00000233419	NA	NA	NA	0	NA	NA	NA	NA	NA
ENSG00000233425	KRT18P67	keratin 18 pseudogene 67	100631239	0	NA	NA	NA	NA	NA
ENSG00000233426	EIF3FP3	eukaryotic translation initiation factor 3 subunit F pseudogene 3	339799	2,141740481	-1,5818718	1,677995	-0,942715535	0,345826425	NA
ENSG00000233428	PPIAP65	peptidylprolyl isomerase A pseudogene 65	111082980	0	NA	NA	NA	NA	NA
ENSG00000233430	NA	NA	NA	0	NA	NA	NA	NA	NA
ENSG00000233432	MTIF2P1	mitochondrial translational initiation factor 2 pseudogene 1	80713	0,103998153	-1,076443944	4,080456	-0,263804824	0,791930317	NA
ENSG00000233433	NA	NA	NA	0	NA	NA	NA	NA	NA
ENSG00000233435	NA	NA	NA	7,576667219	-0,569592022	0,771449	-0,738340162	0,460307767	NA
ENSG00000233436	BTBD18	BTB domain containing 18	643376	0	NA	NA	NA	NA	NA
ENSG00000233437	NA	NA	NA	0	NA	NA	NA	NA	NA
ENSG00000233440	NA	NA	NA	0	NA	NA	NA	NA	NA
ENSG00000233441	CYP2AB1P	cytochrome P450 family 2 subfamily AB member 1, pseudogene	647265	0	NA	NA	NA	NA	NA
ENSG00000233442	NA	NA	NA	0	NA	NA	NA	NA	NA
ENSG00000233444	NA	NA	NA	0	NA	NA	NA	NA	NA

ENSG00000233445	RPL17P11	ribosomal protein L17 pseudogene 11	100270937	0	NA	NA	NA	NA	NA
ENSG00000233446	PDK1P2	PDK1 pseudogene 2	100129843	0	NA	NA	NA	NA	NA
ENSG00000233447	NA	NA	NA	0	NA	NA	NA	NA	NA
ENSG00000233448	NA	NA	NA	2,405181983	1,225368443	1,445666	0,847614903	0,396652472	NA
ENSG00000233449	MTATP6P14	MT-ATP6 pseudogene 14	107075157	0	NA	NA	NA	NA	NA
ENSG00000233454	NA	NA	NA	0	NA	NA	NA	NA	NA
ENSG00000233457	NA	NA	NA	0	NA	NA	NA	NA	NA
ENSG00000233459	LOC729532	zinc finger protein 207 pseudogene	729532	0	NA	NA	NA	NA	NA
ENSG00000233460	NA	NA	NA	0	NA	NA	NA	NA	NA
ENSG00000233467	NA	NA	NA	0	NA	NA	NA	NA	NA
ENSG00000233469	ST6GALNAC4P1	ST6 N-acetylgalactosaminide alpha-2,6-sialyltransferase 4 pseudogene 1	100874524	1,108078639	-0,110779059	2,576119	-0,043002306	0,965699696	NA
ENSG00000233471	KRT18P62	keratin 18 pseudogene 62	100287635	0	NA	NA	NA	NA	NA
ENSG00000233473	NA	NA	NA	0	NA	NA	NA	NA	NA
ENSG00000233477	PPIAP38	peptidylprolyl isomerase A pseudogene 38	111082962	0	NA	NA	NA	NA	NA
ENSG00000233483	EFCAB15P	EF-hand calcium binding domain 15, pseudogene	118568824	1,235558601	-0,467334884	1,943531	-0,24045657	0,809976328	NA
ENSG00000233484	NA	NA	NA	0	NA	NA	NA	NA	NA
ENSG00000233487	NA	NA	NA	0	NA	NA	NA	NA	NA
ENSG00000233489	PAXBP1P1	PAX3 and PAX7 binding protein 1 pseudogene 1	106481704	0	NA	NA	NA	NA	NA
ENSG00000233491	LOC100128317	uncharacterized LOC100128317	100128317	0	NA	NA	NA	NA	NA
ENSG00000233493	TMEM238	transmembrane protein 238	388564	2,147684002	1,879383043	1,60336	1,172152644	0,241135777	NA
ENSG00000233494	NA	NA	NA	0	NA	NA	NA	NA	NA
ENSG00000233497	NA	NA	NA	0	NA	NA	NA	NA	NA
ENSG00000233499	NA	NA	NA	0	NA	NA	NA	NA	NA
ENSG00000233503	HNRNPLP1	heterogeneous nuclear ribonucleoprotein L pseudogene 1	442157	0	NA	NA	NA	NA	NA
ENSG00000233511	NA	NA	NA	0	NA	NA	NA	NA	NA
ENSG00000233514	NA	NA	NA	0	NA	NA	NA	NA	NA
ENSG00000233518	RPL7P35	ribosomal protein L7 pseudogene 35	100271216	0	NA	NA	NA	NA	NA
ENSG00000233523	PHB1P5	PHB1 pseudogene 5	106478939	0	NA	NA	NA	NA	NA
ENSG00000233524	NA	NA	NA	0,369269667	-1,8997662	3,649411	-0,520567835	0,602667861	NA
ENSG00000233526	NA	NA	NA	0	NA	NA	NA	NA	NA
ENSG00000233531	DEFA10P	defensin alpha 10, pseudogene	449493	0	NA	NA	NA	NA	NA
ENSG00000233533	MKNK2P1	MAP kinase interacting serine/threonine kinase 2 pseudogene 1	389948	0	NA	NA	NA	NA	NA
ENSG00000233537	NA	NA	NA	0	NA	NA	NA	NA	NA
ENSG00000233541	NA	NA	NA	0	NA	NA	NA	NA	NA
ENSG00000233543	NA	NA	NA	0	NA	NA	NA	NA	NA
ENSG00000233544	EIF3KP2	eukaryotic translation initiation factor 3 subunit K pseudogene 2	100129045	0	NA	NA	NA	NA	NA
ENSG00000233545	NA	NA	NA	0	NA	NA	NA	NA	NA
ENSG00000233546	NA	NA	NA	0	NA	NA	NA	NA	NA
ENSG00000233548	NA	NA	NA	0	NA	NA	NA	NA	NA
ENSG00000233549	NA	NA	NA	0	NA	NA	NA	NA	NA
ENSG00000233551	NA	NA	NA	0	NA	NA	NA	NA	NA
ENSG00000233555	RPL7AP46	ribosomal protein L7a pseudogene 46	441440	0	NA	NA	NA	NA	NA
ENSG00000233557	NA	NA	NA	0	NA	NA	NA	NA	NA
ENSG00000233558	LOC100287430	ribosomal protein S5 pseudogene	100287430	0	NA	NA	NA	NA	NA
ENSG00000233560	KRT8P39	keratin 8 pseudogene 39	100418781	3,592823641	-0,494292316	1,125933	-0,439007041	0,660656435	NA
ENSG00000233562	RPS3AP8	RPS3A pseudogene 8	100271127	0	NA	NA	NA	NA	NA
ENSG00000233563	OR52E7P	olfactory receptor family 52 subfamily E member 7 pseudogene	81264	0	NA	NA	NA	NA	NA
ENSG00000233565	NA	NA	NA	0	NA	NA	NA	NA	NA
ENSG00000233575	NA	NA	NA	0	NA	NA	NA	NA	NA
ENSG00000233576	NA	NA	NA	0	NA	NA	NA	NA	NA
ENSG00000233578	NA	NA	NA	0	NA	NA	NA	NA	NA
ENSG00000233579	KRT8P15	keratin 8 pseudogene 15	645834	0,849472671	-0,447066479	2,625662	-0,170268111	0,864799288	NA
ENSG00000233583	NA	NA	NA	0,239347754	1,967469188	4,021404	0,489249276	0,624665227	NA
ENSG00000233585	ACAA2P1	ACAA2 pseudogene 1	100420869	0	NA	NA	NA	NA	NA
ENSG00000233586	NA	NA	NA	0,552130449	-1,077107756	3,340773	-0,322412729	0,747140042	NA
ENSG00000233588	NA	NA	NA	0	NA	NA	NA	NA	NA
ENSG00000233594	NA	NA	NA	0	NA	NA	NA	NA	NA
ENSG00000233595	MTND2P29	MT-ND2 pseudogene 29	100873233	0	NA	NA	NA	NA	NA
ENSG00000233597	RPL19P6	ribosomal protein L19 pseudogene 6	100270946	1,114009164	-1,323873619	1,951195	-0,678493722	0,497458703	NA

ENSG00000233601	NA	NA	NA	0	NA	NA	NA	NA	NA
ENSG00000233603	JTBP1	jumping translocation breakpoint pseudogene 1	100874254	0	NA	NA	NA	NA	NA
ENSG00000233606	OR6C66P	olfactory receptor family 6 subfamily C member 66 pseudogene	403283	0	NA	NA	NA	NA	NA
ENSG00000233614	NA	NA	NA	0	NA	NA	NA	NA	NA
ENSG00000233615	HNRNPA1P42	heterogeneous nuclear ribonucleoprotein A1 pseudogene 42	100287266	0	NA	NA	NA	NA	NA
ENSG00000233619	NA	NA	NA	0	NA	NA	NA	NA	NA
ENSG00000233622	CYP2T1P	cytochrome P450 family 2 subfamily T member 1, pseudogene	171523	0	NA	NA	NA	NA	NA
ENSG00000233623	PGAM1P11	phosphoglycerate mutase 1 pseudogene 11	100420660	0,884527068	2,712084042	2,399088	1,130464652	0,258280485	NA
ENSG00000233626	NA	NA	NA	0	NA	NA	NA	NA	NA
ENSG00000233632	NA	NA	NA	0	NA	NA	NA	NA	NA
ENSG00000233634	GOT2P5	GOT2 pseudogene 5	100422625	0	NA	NA	NA	NA	NA
ENSG00000233640	KRTAP13-5P	keratin associated protein 13-5, pseudogene	337964	0	NA	NA	NA	NA	NA
ENSG00000233646	OR52T1P	olfactory receptor family 52 subfamily T member 1 pseudogene	81244	0,514628334	0,97151509	3,417788	0,284252628	0,776216787	NA
ENSG00000233647	NENFP1	neudesin neurotrophic factor pseudogene 1	106480294	0,530603585	0,90405002	3,375362	0,26783792	0,788824081	NA
ENSG00000233648	NA	NA	NA	0	NA	NA	NA	NA	NA
ENSG00000233652	NA	NA	NA	0	NA	NA	NA	NA	NA
ENSG00000233653	NA	NA	NA	0	NA	NA	NA	NA	NA
ENSG00000233659	NA	NA	NA	0	NA	NA	NA	NA	NA
ENSG00000233662	CALM2P4	calmodulin 2 pseudogene 4	643193	0	NA	NA	NA	NA	NA
ENSG00000233663	LOC100420325	MAGE family member B16 pseudogene	100420325	0	NA	NA	NA	NA	NA
ENSG00000233664	NA	NA	NA	0	NA	NA	NA	NA	NA
ENSG00000233668	RPS8P9	ribosomal protein S8 pseudogene 9	100130750	0	NA	NA	NA	NA	NA
ENSG00000233670	PIRT	phosphoinositide interacting regulator of transient receptor potential channels	644139	0	NA	NA	NA	NA	NA
ENSG00000233671	CENPNP1	CENPN pseudogene 1	100132498	0	NA	NA	NA	NA	NA
ENSG00000233673	NA	NA	NA	0	NA	NA	NA	NA	NA
ENSG00000233674	NA	NA	NA	0,389002167	1,852181507	4,027727	0,459857763	0,645618319	NA
ENSG00000233676	FDPSP6	farnesyl diphosphate synthase pseudogene 6	54051	0	NA	NA	NA	NA	NA
ENSG00000233680	HNRNPA1P27	heterogeneous nuclear ribonucleoprotein A1 pseudogene 27	664721	0,119673877	0,847101401	4,080456	0,207599697	0,835541541	NA
ENSG00000233681	CEACAMP1	CEA cell adhesion molecule pseudogene 1	1090	0	NA	NA	NA	NA	NA
ENSG00000233683	NA	NA	NA	0	NA	NA	NA	NA	NA
ENSG00000233685	OR6L1P	olfactory receptor family 6 subfamily L member 1 pseudogene	390012	0	NA	NA	NA	NA	NA
ENSG00000233686	PIEZO1P1	piezo type mechanosensitive ion channel component 1 pseudogene 1	128615	0	NA	NA	NA	NA	NA
ENSG00000233688	ZNF622P1	ZNF622 pseudogene 1	100419792	0	NA	NA	NA	NA	NA
ENSG00000233690	EBAG9P1	EBAG9 pseudogene 1	100422254	0,460422088	0,643007102	2,97168	0,216378288	0,828692889	NA
ENSG00000233691	NA	NA	NA	0	NA	NA	NA	NA	NA
ENSG00000233701	PRR23C	proline rich 23C	389152	0	NA	NA	NA	NA	NA
ENSG00000233702	RPL37P13	ribosomal protein L37 pseudogene 13	100271018	0	NA	NA	NA	NA	NA
ENSG00000233703	NA	NA	NA	0	NA	NA	NA	NA	NA
ENSG00000233707	NA	NA	NA	0	NA	NA	NA	NA	NA
ENSG00000233708	NA	NA	NA	0	NA	NA	NA	NA	NA
ENSG00000233709	MTND4P28	MT-ND4 pseudogene 28	100873249	0	NA	NA	NA	NA	NA
ENSG00000233710	LOC100288853	poly(A) binding protein nuclear 1 pseudogene	100288853	0	NA	NA	NA	NA	NA
ENSG00000233711	NA	NA	NA	0	NA	NA	NA	NA	NA
ENSG00000233712	NA	NA	NA	0	NA	NA	NA	NA	NA
ENSG00000233713	NA	NA	NA	0	NA	NA	NA	NA	NA
ENSG00000233716	NA	NA	NA	0,547691829	-1,503638597	2,818853	-0,533422084	0,593741434	NA
ENSG00000233717	NA	NA	NA	0	NA	NA	NA	NA	NA
ENSG00000233719	GOT2P3	GOT2 pseudogene 3	644138	1,043429615	0,955810197	2,236365	0,427394526	0,669091994	NA
ENSG00000233720	NA	NA	NA	0	NA	NA	NA	NA	NA
ENSG00000233724	LOC100421870	WW domain containing E3 ubiquitin protein ligase 2 pseudogene	100421870	0	NA	NA	NA	NA	NA
ENSG00000233727	NA	NA	NA	0	NA	NA	NA	NA	NA
ENSG00000233732	NA	NA	NA	0	NA	NA	NA	NA	NA

ENSG00000233733	NA	NA	NA	0	NA	NA	NA	NA	NA
ENSG00000233737	NA	NA	NA	0	NA	NA	NA	NA	NA
ENSG00000233740	NA	NA	NA	0	NA	NA	NA	NA	NA
ENSG00000233741	RPL27P7	ribosomal protein L27 pseudogene 7	100129234	0	NA	NA	NA	NA	NA
ENSG00000233747	NA	NA	NA	0	NA	NA	NA	NA	NA
ENSG00000233750	CICP27	capicua transcriptional repressor pseudogene 27	100420257	0,189898792	-0,107279466	4,080456	-0,026291049	0,979025194	NA
ENSG00000233751	NA	NA	NA	0	NA	NA	NA	NA	NA
ENSG00000233752	RPL21P36	ribosomal protein L21 pseudogene 36	100270956	0	NA	NA	NA	NA	NA
ENSG00000233762	RPS15P4	ribosomal protein S15 pseudogene 4	401019	0,144330898	1,418786819	4,061262	0,349346267	0,726829368	NA
ENSG00000233764	MTCO1P20	MT-CO1 pseudogene 20	107075163	0	NA	NA	NA	NA	NA
ENSG00000233765	NA	NA	NA	0	NA	NA	NA	NA	NA
ENSG00000233767	PSMA6P3	proteasome subunit alpha 6 pseudogene 3	100874510	0	NA	NA	NA	NA	NA
ENSG00000233771	NA	NA	NA	0	NA	NA	NA	NA	NA
ENSG00000233774	MED14P1	mediator complex subunit 14 pseudogene 1	359791	0	NA	NA	NA	NA	NA
ENSG00000233778	NA	NA	NA	0	NA	NA	NA	NA	NA
ENSG00000233780	LINC00367	long intergenic non-protein coding RNA 367	101930748	0	NA	NA	NA	NA	NA
ENSG00000233782	NA	NA	NA	0	NA	NA	NA	NA	NA
ENSG00000233786	NA	NA	NA	0	NA	NA	NA	NA	NA
ENSG00000233787	PGBD3P2	piggyBac transposable element derived 3 pseudogene 2	267006	0	NA	NA	NA	NA	NA
ENSG00000233792	NA	NA	NA	0	NA	NA	NA	NA	NA
ENSG00000233802	TRIM49D2	tripartite motif containing 49D2	729384	0	NA	NA	NA	NA	NA
ENSG00000233803	TSPY4	testis specific protein Y-linked 4	728395	0	NA	NA	NA	NA	NA
ENSG00000233803	LOC124905612	testis-specific Y-encoded protein 4	124905612	0	NA	NA	NA	NA	NA
ENSG00000233803	LOC124905613	testis-specific Y-encoded protein 4-like	124905613	0	NA	NA	NA	NA	NA
ENSG00000233803	LOC124905618	testis-specific Y-encoded protein 3-like	124905618	0	NA	NA	NA	NA	NA
ENSG00000233803	LOC124909293	testis-specific Y-encoded protein 4	124909293	0	NA	NA	NA	NA	NA
ENSG00000233803	LOC124909305	testis-specific Y-encoded protein 4-like	124909305	0	NA	NA	NA	NA	NA
ENSG00000233803	LOC124909329	testis-specific Y-encoded protein 4-like	124909329	0	NA	NA	NA	NA	NA
ENSG00000233805	NA	NA	NA	0	NA	NA	NA	NA	NA
ENSG00000233807	NA	NA	NA	0	NA	NA	NA	NA	NA
ENSG00000233810	NA	NA	NA	0	NA	NA	NA	NA	NA
ENSG00000233814	H3P32	H3 histone pseudogene 32	115482701	0,103998153	-1,076443944	4,080456	-0,263804824	0,791930317	NA
ENSG00000233816	IFNA13	interferon alpha 13	3447	0	NA	NA	NA	NA	NA
ENSG00000233819	LCEP1	late cornified envelope pseudogene 1	450208	0	NA	NA	NA	NA	NA
ENSG00000233820	NA	NA	NA	0	NA	NA	NA	NA	NA
ENSG00000233822	H2BC15	H2B clustered histone 15	8341	9,288099141	1,59364805	0,948445	1,680275459	0,092903734	NA
ENSG00000233827	NA	NA	NA	0	NA	NA	NA	NA	NA
ENSG00000233829	NA	NA	NA	0	NA	NA	NA	NA	NA
ENSG00000233830	NA	NA	NA	0,234288424	0,819376111	4,079339	0,200860033	0,84080802	NA
ENSG00000233832	NA	NA	NA	0	NA	NA	NA	NA	NA
ENSG00000233833	NA	NA	NA	0	NA	NA	NA	NA	NA
ENSG00000233836	NA	NA	NA	0	NA	NA	NA	NA	NA
ENSG00000233837	EIF3LP2	eukaryotic translation initiation factor 3 subunit L pseudogene 2	143341	0,119673877	0,847101401	4,080456	0,207599697	0,835541541	NA
ENSG00000233838	NA	NA	NA	0	NA	NA	NA	NA	NA
ENSG00000233839	RPS19P2	ribosomal protein S19 pseudogene 2	85487	0	NA	NA	NA	NA	NA
ENSG00000233843	NA	NA	NA	0	NA	NA	NA	NA	NA
ENSG00000233846	NA	NA	NA	0,11808024	-1,076443944	4,080456	-0,263804824	0,791930317	NA
ENSG00000233859	NA	NA	NA	0	NA	NA	NA	NA	NA
ENSG00000233861	NA	NA	NA	0	NA	NA	NA	NA	NA
ENSG00000233867	SLC9B1P3	solute carrier family 9 member B1 pseudogene 3	100421008	0	NA	NA	NA	NA	NA
ENSG00000233868	NA	NA	NA	0	NA	NA	NA	NA	NA
ENSG00000233869	RPSAP27	ribosomal protein SA pseudogene 27	130429	0	NA	NA	NA	NA	NA
ENSG00000233870	NA	NA	NA	0	NA	NA	NA	NA	NA
ENSG00000233872	NA	NA	NA	0	NA	NA	NA	NA	NA
ENSG00000233873	RPL7P44	ribosomal protein L7 pseudogene 44	390413	0	NA	NA	NA	NA	NA
ENSG00000233874	MFN1P1	MFN1 pseudogene 1	441511	0	NA	NA	NA	NA	NA

ENSG00000233876	NA	NA	NA	0	NA	NA	NA	NA	NA
ENSG00000233877	NA	NA	NA	0	NA	NA	NA	NA	NA
ENSG00000233880	NA	NA	NA	0	NA	NA	NA	NA	NA
ENSG00000233888	MTCO2P17	MT-CO2 pseudogene 17	107075268	0	NA	NA	NA	NA	NA
ENSG00000233892	PAIP1P1	PAIP1 pseudogene 1	100421582	0	NA	NA	NA	NA	NA
ENSG00000233900	RPL17P35	ribosomal protein L17 pseudogene 35	100271284	0	NA	NA	NA	NA	NA
ENSG00000233902	NA	NA	NA	0,530859089	2,347812688	2,831609	0,829144292	0,407022763	NA
ENSG00000233905	NA	NA	NA	0	NA	NA	NA	NA	NA
ENSG00000233908	NA	NA	NA	0	NA	NA	NA	NA	NA
ENSG00000233909	UBE2V1P4	UBE2V1 pseudogene 4	102724816	0	NA	NA	NA	NA	NA
ENSG00000233910	GTF2F2P2	general transcription factor IIF subunit 2 pseudogene 2	728633	0	NA	NA	NA	NA	NA
ENSG00000233913	RPL10P9	ribosomal protein L10 pseudogene 9	389342	0,633382482	1,55741897	2,756438	0,565011478	0,572065957	NA
ENSG00000233915	NA	NA	NA	0	NA	NA	NA	NA	NA
ENSG00000233916	ZDHHC20P1	zinc finger DHHC-type containing 20 pseudogene 1	100048907	5,38631805	0,596875026	1,79439	0,332633895	0,739410654	NA
ENSG00000233917	POTEB	POTE ankyrin domain family member B	100996331	0	NA	NA	NA	NA	NA
ENSG00000233918	NA	NA	NA	0	NA	NA	NA	NA	NA
ENSG00000233921	RPS15AP40	ribosomal protein S15a pseudogene 40	100128083	0	NA	NA	NA	NA	NA
ENSG00000233924	RPSAP13	ribosomal protein SA pseudogene 13	3923	0,444157198	-1,601530445	2,909778	-0,550396037	0,582047766	NA
ENSG00000233932	CTXN2	cortexin 2	399697	0	NA	NA	NA	NA	NA
ENSG00000233933	NA	NA	NA	0	NA	NA	NA	NA	NA
ENSG00000233939	RPL27AP4	ribosomal protein L27a pseudogene 4	100271299	0	NA	NA	NA	NA	NA
ENSG00000233940	RPL12P45	ribosomal protein L12 pseudogene 45	100419477	0	NA	NA	NA	NA	NA
ENSG00000233943	NA	NA	NA	0	NA	NA	NA	NA	NA
ENSG00000233944	NA	NA	NA	0	NA	NA	NA	NA	NA
ENSG00000233946	RPS29P4	ribosomal protein S29 pseudogene 4	100271123	0	NA	NA	NA	NA	NA
ENSG00000233947	ASS1P3	argininosuccinate synthetase 1 pseudogene 3	158452	0	NA	NA	NA	NA	NA
ENSG00000233951	NA	NA	NA	0	NA	NA	NA	NA	NA
ENSG00000233952	NA	NA	NA	0	NA	NA	NA	NA	NA
ENSG00000233954	UQCRHL	ubiquinol-cytochrome c reductase hinge protein like	440567	3,046078381	-0,216767876	1,117775	-0,193927971	0,846232282	NA
ENSG00000233955	AHCYP3	adenosylhomocysteinase pseudogene 3	402093	0	NA	NA	NA	NA	NA
ENSG00000233956	BTF3P6	basic transcription factor 3 pseudogene 6	100499261	0,44710952	0	4,080456	0	1	NA
ENSG00000233962	NA	NA	NA	0	NA	NA	NA	NA	NA
ENSG00000233963	NA	NA	NA	0	NA	NA	NA	NA	NA
ENSG00000233966	NA	NA	NA	0,11808024	-1,076443944	4,080456	-0,263804824	0,791930317	NA
ENSG00000233971	NA	NA	NA	0	NA	NA	NA	NA	NA
ENSG00000233974	NA	NA	NA	0	NA	NA	NA	NA	NA
ENSG00000233979	NA	NA	NA	0	NA	NA	NA	NA	NA
ENSG00000233980	NA	NA	NA	0	NA	NA	NA	NA	NA
ENSG00000233983	LOC124904580	CXXC motif containing zinc binding protein-like	124904580	0	NA	NA	NA	NA	NA
ENSG00000233984	RPSAP14	ribosomal protein SA pseudogene 14	3924	0	NA	NA	NA	NA	NA
ENSG00000233990	NA	NA	NA	0	NA	NA	NA	NA	NA
ENSG00000233994	GDI2P2	GDP dissociation inhibitor 2 pseudogene 2	100420259	0	NA	NA	NA	NA	NA
ENSG00000233995	NA	NA	NA	0	NA	NA	NA	NA	NA
ENSG00000233996	NA	NA	NA	0	NA	NA	NA	NA	NA
ENSG00000233998	NA	NA	NA	0	NA	NA	NA	NA	NA
ENSG00000233999	NA	NA	NA	0	NA	NA	NA	NA	NA
ENSG00000234001	NA	NA	NA	0	NA	NA	NA	NA	NA
ENSG00000234003	MTATP6P27	MT-ATP6 pseudogene 27	107075204	0	NA	NA	NA	NA	NA
ENSG00000234004	NA	NA	NA	0	NA	NA	NA	NA	NA
ENSG00000234005	GAPDHP22	glyceraldehyde 3 phosphate dehydrogenase pseudogene 22	390421	0	NA	NA	NA	NA	NA
ENSG00000234008	NA	NA	NA	0	NA	NA	NA	NA	NA
ENSG00000234009	RPL5P34	ribosomal protein L5 pseudogene 34	388907	1,337013099	-1,834110617	1,844009	-0,994631986	0,319915285	NA
ENSG00000234016	NA	NA	NA	0	NA	NA	NA	NA	NA
ENSG00000234017	NA	NA	NA	0	NA	NA	NA	NA	NA
ENSG00000234020	NA	NA	NA	0	NA	NA	NA	NA	NA
ENSG00000234025	NA	NA	NA	0	NA	NA	NA	NA	NA
ENSG00000234030	TMEM97P1	transmembrane protein 97 pseudogene 1	100873880	0,117497108	0,847101401	4,080456	0,207599697	0,835541541	NA
ENSG00000234031	RPS3AP44	RPS3A pseudogene 44	646527	0	NA	NA	NA	NA	NA

ENSG00000234033	RAC1P8	Rac family small GTPase 1 pseudogene 8	100874398	0	NA	NA	NA	NA	NA
ENSG00000234036	NA	NA	NA	0	NA	NA	NA	NA	NA
ENSG00000234039	NDUFA5P7	NADH:ubiquinone oxidoreductase subunit A5 pseudogene 7	106481784	0	NA	NA	NA	NA	NA
ENSG00000234040	RPL10P12	ribosomal protein L10 pseudogene 12	645422	0	NA	NA	NA	NA	NA
ENSG00000234041	NA	NA	NA	0	NA	NA	NA	NA	NA
ENSG00000234042	NA	NA	NA	0	NA	NA	NA	NA	NA
ENSG00000234043	NA	NA	NA	2,002207493	0,185040254	1,562011	0,118462819	0,905700954	NA
ENSG00000234044	NA	NA	NA	0	NA	NA	NA	NA	NA
ENSG00000234048	CIAO2AP1	CIAO2A pseudogene 1	106480777	0	NA	NA	NA	NA	NA
ENSG00000234059	CASKP1	calcium/calmodulin dependent serine protein kinase pseudogene 1	10029	0	NA	NA	NA	NA	NA
ENSG00000234060	NA	NA	NA	0	NA	NA	NA	NA	NA
ENSG00000234061	NA	NA	NA	0	NA	NA	NA	NA	NA
ENSG00000234062	NA	NA	NA	0,440117845	-0,966786447	3,536576	-0,273367922	0,784570408	NA
ENSG00000234065	MTND4P26	MT-ND4 pseudogene 26	100873247	0	NA	NA	NA	NA	NA
ENSG00000234067	RPL5P10	ribosomal protein L5 pseudogene 10	645807	0	NA	NA	NA	NA	NA
ENSG00000234068	PAGE2	PAGE family member 2	203569	0	NA	NA	NA	NA	NA
ENSG00000234069	GAPDHP53	glyceraldehyde 3 phosphate dehydrogenase pseudogene 53	100421318	0	NA	NA	NA	NA	NA
ENSG00000234071	NA	NA	NA	0	NA	NA	NA	NA	NA
ENSG00000234073	CBX3P10	CBX3 pseudogene 10	107986032	2,569415492	0,273153824	1,436623	0,190136071	0,849202505	NA
ENSG00000234075	RPL35AP	ribosomal protein L35a pseudogene	140716	0	NA	NA	NA	NA	NA
ENSG00000234080	RPS2P11	ribosomal protein S2 pseudogene 11	343184	0,119673877	0,847101401	4,080456	0,207599697	0,835541541	NA
ENSG00000234081	ELOCP10	elongin C pseudogene 10	100462871	0	NA	NA	NA	NA	NA
ENSG00000234085	NA	NA	NA	0,10488184	-1,076443944	4,080456	-0,263804824	0,791930317	NA
ENSG00000234087	NA	NA	NA	0	NA	NA	NA	NA	NA
ENSG00000234088	RPS15AP7	ribosomal protein S15a pseudogene 7	100271142	0	NA	NA	NA	NA	NA
ENSG00000234093	RPS15AP11	ribosomal protein S15a pseudogene 11	644790	0	NA	NA	NA	NA	NA
ENSG00000234099	NA	NA	NA	0	NA	NA	NA	NA	NA
ENSG00000234102	NA	NA	NA	0	NA	NA	NA	NA	NA
ENSG00000234106	SRP14P2	SRP14 pseudogene 2	111644132	0	NA	NA	NA	NA	NA
ENSG00000234107	NA	NA	NA	0	NA	NA	NA	NA	NA
ENSG00000234108	NA	NA	NA	0	NA	NA	NA	NA	NA
ENSG00000234109	RPL7P36	ribosomal protein L7 pseudogene 36	100271218	0	NA	NA	NA	NA	NA
ENSG00000234112	LEFTY3P	left-right determination factor 3, pseudogene	286754	0	NA	NA	NA	NA	NA
ENSG00000234115	NA	NA	NA	0	NA	NA	NA	NA	NA
ENSG00000234118	RPL13AP6	ribosomal protein L13a pseudogene 6	644511	0,118286652	0	4,080456	0	1	NA
ENSG00000234119	NA	NA	NA	0	NA	NA	NA	NA	NA
ENSG00000234120	NA	NA	NA	0	NA	NA	NA	NA	NA
ENSG00000234121	NA	NA	NA	0	NA	NA	NA	NA	NA
ENSG00000234122	NA	NA	NA	0	NA	NA	NA	NA	NA
ENSG00000234123	RHBDF1P1	RHBDF1 pseudogene 1	131973	0	NA	NA	NA	NA	NA
ENSG00000234125	EEF1GP8	eukaryotic translation elongation factor 1 gamma pseudogene 8	391698	0	NA	NA	NA	NA	NA
ENSG00000234130	AP2B1P1	AP2B1 pseudogene 1	401602	0,10543123	0	4,080456	0	1	NA
ENSG00000234131	ELOCP8	elongin C pseudogene 8	100462869	0	NA	NA	NA	NA	NA
ENSG00000234135	RPL23AP83	ribosomal protein L23a pseudogene 83	100131846	0	NA	NA	NA	NA	NA
ENSG00000234143	UGT1A13P	UDP glucuronosyltransferase family 1 member A13, pseudogene	404204	0	NA	NA	NA	NA	NA
ENSG00000234144	COX6CP13	cytochrome c oxidase subunit 6C pseudogene 13	106480267	0	NA	NA	NA	NA	NA
ENSG00000234145	NAP1L4P3	nucleosome assembly protein 1 like 4 pseudogene 3	730174	0	NA	NA	NA	NA	NA
ENSG00000234146	AKAP8P1	A-kinase anchoring protein 8 pseudogene 1	646114	0	NA	NA	NA	NA	NA
ENSG00000234152	ELOBP1	elongin B pseudogene 1	246717	0	NA	NA	NA	NA	NA
ENSG00000234158	RPEP2	ribulose-5-phosphate-3-epimerase pseudogene 2	645065	0	NA	NA	NA	NA	NA
ENSG00000234159	NA	NA	NA	0	NA	NA	NA	NA	NA
ENSG00000234163	MTND4LP11	MT-ND4L pseudogene 11	107075238	0	NA	NA	NA	NA	NA
ENSG00000234167	ELOBP4	elongin B pseudogene 4	100874519	0	NA	NA	NA	NA	NA
ENSG00000234175	NA	NA	NA	0	NA	NA	NA	NA	NA

ENSG00000234176	HSPA8P1	heat shock protein family A (Hsp70) member 8 pseudogene 1	3314	0	NA	NA	NA	NA	NA
ENSG00000234179	MTCYBP1	MT-CYB pseudogene 1	100499418	0	NA	NA	NA	NA	NA
ENSG00000234186	C16orf82	chromosome 16 open reading frame 82	162083	0	NA	NA	NA	NA	NA
ENSG00000234187	AIMP1P1	aminoacyl tRNA synthetase complex interacting multifunctional protein 1 pseudogene 1	170547	0	NA	NA	NA	NA	NA
ENSG00000234189	NA	NA	NA	0	NA	NA	NA	NA	NA
ENSG00000234192	NA	NA	NA	0	NA	NA	NA	NA	NA
ENSG00000234200	NA	NA	NA	0	NA	NA	NA	NA	NA
ENSG00000234201	BMI1P1	BMI1 proto-oncogene, polycomb ring finger pseudogene 1	100127902	0	NA	NA	NA	NA	NA
ENSG00000234202	H3P3	H3 histone pseudogene 3	115482682	0	NA	NA	NA	NA	NA
ENSG00000234204	NA	NA	NA	0	NA	NA	NA	NA	NA
ENSG00000234213	FHP1	fumarate hydratase pseudogene 1	100873790	0	NA	NA	NA	NA	NA
ENSG00000234217	NA	NA	NA	0	NA	NA	NA	NA	NA
ENSG00000234219	NA	NA	NA	0,91277013	2,066794209	2,271124	0,910031505	0,362805895	NA
ENSG00000234224	TMEM229A	transmembrane protein 229A	730130	0	NA	NA	NA	NA	NA
ENSG00000234226	NDUFS5P2	NADH:ubiquinone oxidoreductase subunit S5 pseudogene 2	100286918	0	NA	NA	NA	NA	NA
ENSG00000234227	RPL7L1P1	RPL7L1 pseudogene 1	100129260	0	NA	NA	NA	NA	NA
ENSG00000234228	NA	NA	NA	0	NA	NA	NA	NA	NA
ENSG00000234232	NA	NA	NA	0	NA	NA	NA	NA	NA
ENSG00000234234	MTCO2P8	MT-CO2 pseudogene 8	107075146	0	NA	NA	NA	NA	NA
ENSG00000234239	CFL1P8	cofilin 1 pseudogene 8	100873812	0	NA	NA	NA	NA	NA
ENSG00000234241	LOC149844	synaptotagmin binding cytoplasmic RNA interacting protein pseudogene	149844	0,421724922	0	4,080456	0	1	NA
ENSG00000234247	DNAJB6P6	DNAJB6 pseudogene 6	107985099	0	NA	NA	NA	NA	NA
ENSG00000234253	RPL7P13	ribosomal protein L7 pseudogene 13	130728	0	NA	NA	NA	NA	NA
ENSG00000234256	PTCD2P2	pentatricopeptide repeat domain 2 pseudogene 2	100132487	0	NA	NA	NA	NA	NA
ENSG00000234257	SOD2P1	superoxide dismutase 2 pseudogene 1	100421046	0	NA	NA	NA	NA	NA
ENSG00000234259	NA	NA	NA	0	NA	NA	NA	NA	NA
ENSG00000234262	NA	NA	NA	0	NA	NA	NA	NA	NA
ENSG00000234267	NA	NA	NA	0	NA	NA	NA	NA	NA
ENSG00000234268	NA	NA	NA	1,255662948	1,588901802	2,100262	0,756525434	0,449334229	NA
ENSG00000234269	LOC100421294	phosphoribosyl pyrophosphate synthetase 2 pseudogene	100421294	0	NA	NA	NA	NA	NA
ENSG00000234270	RPL36P20	ribosomal protein L36 pseudogene 20	729032	0	NA	NA	NA	NA	NA
ENSG00000234271	LOC107985380	transmembrane protein 14C-like	107985380	0	NA	NA	NA	NA	NA
ENSG00000234272	NA	NA	NA	0	NA	NA	NA	NA	NA
ENSG00000234274	NA	NA	NA	0	NA	NA	NA	NA	NA
ENSG00000234276	SDR42E1P3	short chain dehydrogenase/reductase family 42E, member 1 pseudogene 3	100422347	0	NA	NA	NA	NA	NA
ENSG00000234282	NA	NA	NA	0	NA	NA	NA	NA	NA
ENSG00000234285	GAPDHP49	glyceraldehyde 3 phosphate dehydrogenase pseudogene 49	100421189	0	NA	NA	NA	NA	NA
ENSG00000234287	NA	NA	NA	0,23616048	-2,075648354	4,028973	-0,515180498	0,606426895	NA
ENSG00000234288	OR1AA1P	olfactory receptor family 1 subfamily AA member 1 pseudogene	79312	0	NA	NA	NA	NA	NA
ENSG00000234289	H2BC12L	H2B clustered histone 12 like	54145	1,700274401	2,713448724	1,728562	1,569771908	0,116468185	NA
ENSG00000234289	LOC102724334	histone H2B type F-S-like	102724334	1,700274401	2,713448724	1,728562	1,569771908	0,116468185	NA
ENSG00000234297	NA	NA	NA	0	NA	NA	NA	NA	NA
ENSG00000234304	NA	NA	NA	0	NA	NA	NA	NA	NA
ENSG00000234309	SLC25A18P1	solute carrier family 25 member 18 pseudogene 1	106480776	0	NA	NA	NA	NA	NA
ENSG00000234312	SAPCD2P4	suppressor APC domain containing 2 pseudogene 4	442318	0	NA	NA	NA	NA	NA
ENSG00000234315	NA	NA	NA	0	NA	NA	NA	NA	NA
ENSG00000234319	RPS12P2	ribosomal protein S12 pseudogene 2	619448	0	NA	NA	NA	NA	NA
ENSG00000234322	NA	NA	NA	0	NA	NA	NA	NA	NA

ENSG00000234324	RPL9P2	ribosomal protein L9 pseudogene 2	729004	0,119673877	0,847101401	4,080456	0,207599697	0,835541541	NA
ENSG00000234325	MRPS10P2	mitochondrial ribosomal protein S10 pseudogene 2	359802	0,118286652	0	4,080456	0	1	NA
ENSG00000234329	RPL7AP16	ribosomal protein L7a pseudogene 16	100270896	7,137632745	-0,18716322	0,676557	-0,276640701	0,782056009	NA
ENSG00000234332	NA	NA	NA	0,209763681	-1,926644696	4,042043	-0,476651224	0,633610501	NA
ENSG00000234333	CYCSP53	CYCS pseudogene 53	360156	0	NA	NA	NA	NA	NA
ENSG00000234335	RPS4XP11	ribosomal protein S4X pseudogene 11	441550	0	NA	NA	NA	NA	NA
ENSG00000234337	NA	NA	NA	0	NA	NA	NA	NA	NA
ENSG00000234338	NA	NA	NA	0	NA	NA	NA	NA	NA
ENSG00000234340	NA	NA	NA	0	NA	NA	NA	NA	NA
ENSG00000234345	NA	NA	NA	4,478475093	-0,038980021	0,859299	-0,045362599	0,963818292	NA
ENSG00000234349	NA	NA	NA	0	NA	NA	NA	NA	NA
ENSG00000234353	NA	NA	NA	9,896341959	-0,507588254	0,653611	-0,776590271	0,437400543	NA
ENSG00000234354	NA	NA	NA	0,511990545	0,712119338	2,935951	0,242551488	0,808352866	NA
ENSG00000234355	NA	NA	NA	0	NA	NA	NA	NA	NA
ENSG00000234356	NA	NA	NA	0	NA	NA	NA	NA	NA
ENSG00000234358	RPL7AP42	ribosomal protein L7a pseudogene 42	100271215	0	NA	NA	NA	NA	NA
ENSG00000234359	ELK1P1	ELK1 pseudogene 1	647102	0	NA	NA	NA	NA	NA
ENSG00000234360	NA	NA	NA	0	NA	NA	NA	NA	NA
ENSG00000234363	PPIAP27	peptidylprolyl isomerase A pseudogene 27	100874405	0	NA	NA	NA	NA	NA
ENSG00000234367	NA	NA	NA	0	NA	NA	NA	NA	NA
ENSG00000234369	TATDN1P1	TatD DNase domain containing 1 pseudogene 1	728737	0	NA	NA	NA	NA	NA
ENSG00000234371	RPSAP31	ribosomal protein SA pseudogene 31	391598	0	NA	NA	NA	NA	NA
ENSG00000234373	NA	NA	NA	0,117497108	0,847101401	4,080456	0,207599697	0,835541541	NA
ENSG00000234376	UBTFL2	UBTF like 2 (pseudogene)	120126	0	NA	NA	NA	NA	NA
ENSG00000234379	NA	NA	NA	0	NA	NA	NA	NA	NA
ENSG00000234381	NA	NA	NA	0	NA	NA	NA	NA	NA
ENSG00000234382	NA	NA	NA	0	NA	NA	NA	NA	NA
ENSG00000234383	NA	NA	NA	0	NA	NA	NA	NA	NA
ENSG00000234385	RCC2P1	regulator of chromosome condensation 2 pseudogene 1	650983	0	NA	NA	NA	NA	NA
ENSG00000234386	OR7E162P	olfactory receptor family 7 subfamily E member 162 pseudogene	442101	0	NA	NA	NA	NA	NA
ENSG00000234388	NA	NA	NA	0	NA	NA	NA	NA	NA
ENSG00000234391	NA	NA	NA	0	NA	NA	NA	NA	NA
ENSG00000234397	NA	NA	NA	0	NA	NA	NA	NA	NA
ENSG00000234399	RBMY2XP	RNA binding motif protein Y-linked family 2 member X, pseudogene	379012	0	NA	NA	NA	NA	NA
ENSG00000234404	SPRYD7P1	SPRY domain containing 7 pseudogene 1	100130843	0	NA	NA	NA	NA	NA
ENSG00000234406	NA	NA	NA	0	NA	NA	NA	NA	NA
ENSG00000234409	CCDC188	coiled-coil domain containing 188	388849	0,435374062	-2,526022821	3,99693	-0,631990791	0,527392895	NA
ENSG00000234413	MPV17L2P1	MPV17L2 pseudogene 1	101060056	0	NA	NA	NA	NA	NA
ENSG00000234414	RBMY1A1	RNA binding motif protein Y-linked family 1 member A1	5940	0	NA	NA	NA	NA	NA
ENSG00000234415	RPL5P7	ribosomal protein L5 pseudogene 7	344178	0,117497108	0,847101401	4,080456	0,207599697	0,835541541	NA
ENSG00000234419	NA	NA	NA	0	NA	NA	NA	NA	NA
ENSG00000234421	NA	NA	NA	0	NA	NA	NA	NA	NA
ENSG00000234422	UBE2WP1	ubiquitin conjugating enzyme E2 W pseudogene 1	619457	0	NA	NA	NA	NA	NA
ENSG00000234424	LOC100419824	kinesin family member 27 pseudogene	100419824	0,223569017	0	4,080456	0	1	NA
ENSG00000234429	ANKRD11P1	ANKRD11 pseudogene 1	100287912	0	NA	NA	NA	NA	NA
ENSG00000234438	KBTBD13	kelch repeat and BTB domain containing 13	390594	0	NA	NA	NA	NA	NA
ENSG00000234439	KRT18P2	keratin 18 pseudogene 2	54044	0	NA	NA	NA	NA	NA
ENSG00000234441	NA	NA	NA	0	NA	NA	NA	NA	NA
ENSG00000234442	NA	NA	NA	0	NA	NA	NA	NA	NA
ENSG00000234448	NA	NA	NA	0	NA	NA	NA	NA	NA
ENSG00000234450	RPL26P23	ribosomal protein L26 pseudogene 23	100129517	0	NA	NA	NA	NA	NA
ENSG00000234451	NA	NA	NA	0	NA	NA	NA	NA	NA
ENSG00000234457	NA	NA	NA	0	NA	NA	NA	NA	NA
ENSG00000234458	OR7E130P	olfactory receptor family 7 subfamily E member 130 pseudogene	389144	0	NA	NA	NA	NA	NA
ENSG00000234460	NA	NA	NA	0	NA	NA	NA	NA	NA

ENSG00000234463	COX6CP12	cytochrome c oxidase subunit 6C pseudogene 12	106481682	0	NA	NA	NA	NA	NA
ENSG00000234465	PINLYP	phospholipase A2 inhibitor and LY6/PLAUR domain containing	390940	0,590298894	-2,756633482	2,799635	-0,984640391	0,324800742	NA
ENSG00000234466	NA	NA	NA	0	NA	NA	NA	NA	NA
ENSG00000234467	NA	NA	NA	0	NA	NA	NA	NA	NA
ENSG00000234469	CLDN34	claudin 34	100288814	0	NA	NA	NA	NA	NA
ENSG00000234473	ZNF101P2	zinc finger protein 101 pseudogene 2	730190	1,192994256	0,254679368	1,838792	0,138503606	0,88984242	NA
ENSG00000234479	NA	NA	NA	0	NA	NA	NA	NA	NA
ENSG00000234480	MTCO2P18	MT-CO2 pseudogene 18	107075149	0	NA	NA	NA	NA	NA
ENSG00000234485	OR7E46P	olfactory receptor family 7 subfamily E member 46 pseudogene	402077	0	NA	NA	NA	NA	NA
ENSG00000234488	RPL27P6	ribosomal protein L27 pseudogene 6	647112	0	NA	NA	NA	NA	NA
ENSG00000234489	NA	NA	NA	0	NA	NA	NA	NA	NA
ENSG00000234493	NA	NA	NA	0,11808024	-1,076443944	4,080456	-0,263804824	0,791930317	NA
ENSG00000234496	NA	NA	NA	0	NA	NA	NA	NA	NA
ENSG00000234498	RPL13AP20	ribosomal protein L13a pseudogene 20	387841	0,103998153	-1,076443944	4,080456	-0,263804824	0,791930317	NA
ENSG00000234500	NA	NA	NA	0	NA	NA	NA	NA	NA
ENSG00000234502	NA	NA	NA	0,338172098	1,920344585	4,024375	0,477178307	0,633235157	NA
ENSG00000234511	C5orf58	chromosome 5 open reading frame 58	133874	0,117497108	0,847101401	4,080456	0,207599697	0,835541541	NA
ENSG00000234512	TLR12P	toll like receptor 12, pseudogene	100131451	0	NA	NA	NA	NA	NA
ENSG00000234513	NA	NA	NA	0	NA	NA	NA	NA	NA
ENSG00000234518	NA	NA	NA	9,907058161	-0,167831204	0,616023	-0,272443114	0,785281325	NA
ENSG00000234521	NA	NA	NA	0	NA	NA	NA	NA	NA
ENSG00000234522	NA	NA	NA	0	NA	NA	NA	NA	NA
ENSG00000234523	NDUFB1P2	NADH:ubiquinone oxidoreductase subunit B1 pseudogene 2	100380272	0	NA	NA	NA	NA	NA
ENSG00000234524	RPL12P43	ribosomal protein L12 pseudogene 43	100271387	0	NA	NA	NA	NA	NA
ENSG00000234529	NA	NA	NA	0	NA	NA	NA	NA	NA
ENSG00000234534	NA	NA	NA	0,484977743	-1,895765	3,473644	-0,545756884	0,585233065	NA
ENSG00000234537	NA	NA	NA	0,15836288	0	4,080456	0	1	NA
ENSG00000234538	NA	NA	NA	0	NA	NA	NA	NA	NA
ENSG00000234541	CHEK2P5	CHEK2 pseudogene 5	106479022	0	NA	NA	NA	NA	NA
ENSG00000234544	PTGES3P5	prostaglandin E synthase 3 pseudogene 5	106480359	0	NA	NA	NA	NA	NA
ENSG00000234557	PPIAP90	peptidylprolyl isomerase A pseudogene 90	111082985	0	NA	NA	NA	NA	NA
ENSG00000234558	NA	NA	NA	0	NA	NA	NA	NA	NA
ENSG00000234560	OR10G8	olfactory receptor family 10 subfamily G member 8	219869	0	NA	NA	NA	NA	NA
ENSG00000234562	NA	NA	NA	0	NA	NA	NA	NA	NA
ENSG00000234564	NA	NA	NA	0	NA	NA	NA	NA	NA
ENSG00000234565	NA	NA	NA	0	NA	NA	NA	NA	NA
ENSG00000234566	RPL7AP71	ribosomal protein L7a pseudogene 71	100271399	0	NA	NA	NA	NA	NA
ENSG00000234568	NA	NA	NA	0	NA	NA	NA	NA	NA
ENSG00000234569	NA	NA	NA	0,11808024	-1,076443944	4,080456	-0,263804824	0,791930317	NA
ENSG00000234570	NA	NA	NA	0	NA	NA	NA	NA	NA
ENSG00000234571	NA	NA	NA	0,426686799	2,369165634	3,494486	0,677972601	0,497789066	NA
ENSG00000234573	RPS21P7	ribosomal protein S21 pseudogene 7	100271366	0	NA	NA	NA	NA	NA
ENSG00000234575	NA	NA	NA	0	NA	NA	NA	NA	NA
ENSG00000234576	MTND1P26	MT-ND1 pseudogene 26	100873214	0	NA	NA	NA	NA	NA
ENSG00000234585	NA	NA	NA	6,669894057	0,729666641	0,718199	1,015966518	0,309645331	NA
ENSG00000234586	LOC100130801	lupus La protein-like	100130801	0	NA	NA	NA	NA	NA
ENSG00000234587	MRPL50P1	mitochondrial ribosomal protein L50 pseudogene 1	351143	0	NA	NA	NA	NA	NA
ENSG00000234588	NA	NA	NA	0	NA	NA	NA	NA	NA
ENSG00000234589	NA	NA	NA	0	NA	NA	NA	NA	NA
ENSG00000234590	NA	NA	NA	0	NA	NA	NA	NA	NA
ENSG00000234592	NA	NA	NA	0	NA	NA	NA	NA	NA
ENSG00000234594	MTCO1P58	MT-CO1 pseudogene 58	127482537	0	NA	NA	NA	NA	NA
ENSG00000234602	MCIDAS	multiciliate differentiation and DNA synthesis associated cell cycle protein	345643	0	NA	NA	NA	NA	NA
ENSG00000234604	NA	NA	NA	0	NA	NA	NA	NA	NA
ENSG00000234607	NA	NA	NA	0,072165449	0,847101401	4,080456	0,207599697	0,835541541	NA
ENSG00000234611	OR2AT2P	olfactory receptor family 2 subfamily AT member 2 pseudogene	390224	0	NA	NA	NA	NA	NA

ENSG00000234612	H2AZP5	H2A,Z histone pseudogene 5	100288330	0	NA	NA	NA	NA	NA
ENSG00000234618	NA	NA	NA	1,876344884	0,623223485	1,252878	0,497433324	0,618883511	NA
ENSG00000234619	RPL7P11	ribosomal protein L7 pseudogene 11	100270823	0	NA	NA	NA	NA	NA
ENSG00000234620	NA	NA	NA	0	NA	NA	NA	NA	NA
ENSG00000234624	NA	NA	NA	0,072165449	0,847101401	4,080456	0,207599697	0,835541541	NA
ENSG00000234627	NA	NA	NA	0	NA	NA	NA	NA	NA
ENSG00000234632	NECAP1P2	NECAP endocytosis associated 1 pseudogene 2	106479026	0	NA	NA	NA	NA	NA
ENSG00000234637	RPS19P6	ribosomal protein S19 pseudogene 6	100271078	0	NA	NA	NA	NA	NA
ENSG00000234639	LOC100128052	protein phosphatase 2 regulatory subunit B', gamma pseudogene	100128052	0	NA	NA	NA	NA	NA
ENSG00000234644	NA	NA	NA	0	NA	NA	NA	NA	NA
ENSG00000234645	NA	NA	NA	0	NA	NA	NA	NA	NA
ENSG00000234648	NA	NA	NA	1,765614797	4,4203274	3,168952	1,394886004	0,163050218	NA
ENSG00000234652	AGPAT5P1	1-acylglycerol-3-phosphate O-acyltransferase 5 pseudogene 1	646231	0	NA	NA	NA	NA	NA
ENSG00000234655	OR7E155P	olfactory receptor family 7 subfamily E member 155 pseudogene	403297	0	NA	NA	NA	NA	NA
ENSG00000234658	NA	NA	NA	0	NA	NA	NA	NA	NA
ENSG00000234667	ACTL11P	actin like 11, pseudogene	389120	0	NA	NA	NA	NA	NA
ENSG00000234670	OR6C64P	olfactory receptor family 6 subfamily C member 64 pseudogene	403281	0	NA	NA	NA	NA	NA
ENSG00000234671	MTCYBP5	MT-CYB pseudogene 5	107075125	0	NA	NA	NA	NA	NA
ENSG00000234680	NA	NA	NA	0	NA	NA	NA	NA	NA
ENSG00000234682	VDAC2P4	VDAC2 pseudogene 4	100420574	0	NA	NA	NA	NA	NA
ENSG00000234683	NA	NA	NA	0	NA	NA	NA	NA	NA
ENSG00000234685	NA	NA	NA	0	NA	NA	NA	NA	NA
ENSG00000234700	MTCO1P8	MT-CO1 pseudogene 8	107075138	0	NA	NA	NA	NA	NA
ENSG00000234701	PRDX3P3	peroxiredoxin 3 pseudogene 3	100128902	0	NA	NA	NA	NA	NA
ENSG00000234702	NA	NA	NA	0	NA	NA	NA	NA	NA
ENSG00000234706	NA	NA	NA	0	NA	NA	NA	NA	NA
ENSG00000234709	NA	NA	NA	2,260139997	0,599392864	1,391989	0,43060185	0,666757896	NA
ENSG00000234711	TUBB8P11	tubulin beta 8 class VIII pseudogene 11	388579	0	NA	NA	NA	NA	NA
ENSG00000234713	NA	NA	NA	0	NA	NA	NA	NA	NA
ENSG00000234716	NA	NA	NA	0	NA	NA	NA	NA	NA
ENSG00000234718	RPL23AP51	ribosomal protein L23a pseudogene 51	100271464	0	NA	NA	NA	NA	NA
ENSG00000234719	NPIPB2	nuclear pore complex interacting protein family member B2	729978	0,326872668	-1,924602038	4,042511	-0,476090756	0,634009722	NA
ENSG00000234720	ATP5F1AP8	ATP synthase F1 subunit alpha pseudogene 8	392322	0	NA	NA	NA	NA	NA
ENSG00000234723	FCRL4P1	FCRL4 pseudogene 1	127379687	0	NA	NA	NA	NA	NA
ENSG00000234724	NA	NA	NA	0	NA	NA	NA	NA	NA
ENSG00000234726	LOC100533679	URI1, prefoldin-like chaperone pseudogene	100533679	0	NA	NA	NA	NA	NA
ENSG00000234731	MTND4LP16	MT-ND4L pseudogene 16	107075210	0	NA	NA	NA	NA	NA
ENSG00000234732	RPEP5	ribulose-5-phosphate-3-epimerase pseudogene 5	106481798	0	NA	NA	NA	NA	NA
ENSG00000234735	RPS25P10	ribosomal protein S25 pseudogene 10	100271372	0	NA	NA	NA	NA	NA
ENSG00000234737	KRT18P15	keratin 18 pseudogene 15	729211	0	NA	NA	NA	NA	NA
ENSG00000234742	RPL17P18	ribosomal protein L17 pseudogene 18	100129657	0,103998153	-1,076443944	4,080456	-0,263804824	0,791930317	NA
ENSG00000234743	NA	NA	NA	0	NA	NA	NA	NA	NA
ENSG00000234744	USP9YP26	USP9Y pseudogene 26	106478929	0	NA	NA	NA	NA	NA
ENSG00000234748	NA	NA	NA	0	NA	NA	NA	NA	NA
ENSG00000234750	RPS2P53	ribosomal protein S2 pseudogene 53	729274	0	NA	NA	NA	NA	NA
ENSG00000234751	RPL15P16	ribosomal protein L15 pseudogene 16	100127949	0	NA	NA	NA	NA	NA
ENSG00000234761	NA	NA	NA	0	NA	NA	NA	NA	NA
ENSG00000234764	NA	NA	NA	0	NA	NA	NA	NA	NA
ENSG00000234765	ELL2P3	elongation factor for RNA polymerase II 2 pseudogene 3	100533713	0	NA	NA	NA	NA	NA
ENSG00000234769	NA	NA	NA	5,153402686	-0,411746742	1,065781	-0,386333425	0,699249749	NA
ENSG00000234770	NA	NA	NA	0	NA	NA	NA	NA	NA
ENSG00000234773	NA	NA	NA	1,371266659	1,963103644	1,906104	1,029903969	0,303055088	NA
ENSG00000234776	FREY1	Frey regulator of sperm-oocyte fusion 1	143678	0	NA	NA	NA	NA	NA

ENSG00000234780	LOC100420083	ornithine aminotransferase pseudogene	100420083	0	NA	NA	NA	NA	NA
ENSG00000234782	NA	NA	NA	0,191854446	1,678639645	4,046241	0,414863989	0,678241473	NA
ENSG00000234784	NA	NA	NA	0	NA	NA	NA	NA	NA
ENSG00000234785	EEF1GP5	eukaryotic translation elongation factor 1 gamma pseudogene 5	642357	0	NA	NA	NA	NA	NA
ENSG00000234788	HSPA8P3	heat shock protein family A (Hsp70) member 8 pseudogene 3	100420143	0,974801026	-0,094925781	1,852045	-0,051254569	0,959122669	NA
ENSG00000234790	NUTF2P4	nuclear transport factor 2 pseudogene 4	128322	0	NA	NA	NA	NA	NA
ENSG00000234792	LOC100420089	ornithine aminotransferase pseudogene	100420089	0	NA	NA	NA	NA	NA
ENSG00000234795	RFTN1P1	raftlin, lipid raft linker 1 pseudogene 1	360015	0	NA	NA	NA	NA	NA
ENSG00000234797	RPS3AP6	RPS3A pseudogene 6	145767	4,057131186	-0,146524549	1,176246	-0,124569697	0,90086422	NA
ENSG00000234800	NA	NA	NA	0	NA	NA	NA	NA	NA
ENSG00000234801	NA	NA	NA	0	NA	NA	NA	NA	NA
ENSG00000234805	FAM151AP1	family with sequence similarity 151 member A pseudogene	285303	0	NA	NA	NA	NA	NA
ENSG00000234806	RPL26P29	ribosomal protein L26 pseudogene 29	100131788	0	NA	NA	NA	NA	NA
ENSG00000234811	NA	NA	NA	0	NA	NA	NA	NA	NA
ENSG00000234812	NA	NA	NA	0	NA	NA	NA	NA	NA
ENSG00000234813	NA	NA	NA	0	NA	NA	NA	NA	NA
ENSG00000234814	NA	NA	NA	0,693035442	-3,032000049	3,118387	-0,972297421	0,330902607	NA
ENSG00000234816	NA	NA	NA	0,15836288	0	4,080456	0	1	NA
ENSG00000234819	NA	NA	NA	0	NA	NA	NA	NA	NA
ENSG00000234821	NA	NA	NA	0	NA	NA	NA	NA	NA
ENSG00000234825	XRCC6P2	X-ray repair cross complementing 6 pseudogene 2	389901	0,10488184	-1,076443944	4,080456	-0,263804824	0,791930317	NA
ENSG00000234828	IQCM	IQ motif containing M	285423	0	NA	NA	NA	NA	NA
ENSG00000234829	IFNA17	interferon alpha 17	3451	0	NA	NA	NA	NA	NA
ENSG00000234830	NA	NA	NA	0	NA	NA	NA	NA	NA
ENSG00000234835	NA	NA	NA	0	NA	NA	NA	NA	NA
ENSG00000234837	LOC100419684	zinc finger protein 114 pseudogene	100419684	0	NA	NA	NA	NA	NA
ENSG00000234838	NA	NA	NA	0	NA	NA	NA	NA	NA
ENSG00000234839	RPS26P30	ribosomal protein S26 pseudogene 30	100271111	0	NA	NA	NA	NA	NA
ENSG00000234841	LOC100422627	TATA-box binding protein associated factor 4b pseudogene	100422627	0	NA	NA	NA	NA	NA
ENSG00000234842	MTCO2P16	MT-CO2 pseudogene 16	107075148	0	NA	NA	NA	NA	NA
ENSG00000234844	NA	NA	NA	0	NA	NA	NA	NA	NA
ENSG00000234850	MTND2P3	MT-ND2 pseudogene 3	100499457	0	NA	NA	NA	NA	NA
ENSG00000234853	NDUFA5P3	NADH:ubiquinone oxidoreductase subunit A5 pseudogene 3	100288035	0	NA	NA	NA	NA	NA
ENSG00000234861	ATP5F1AP1	ATP synthase F1 subunit alpha pseudogene 1	442416	0	NA	NA	NA	NA	NA
ENSG00000234863	NA	NA	NA	0	NA	NA	NA	NA	NA
ENSG00000234865	ADH5P5	ADH5 pseudogene 5	115945146	0,190076746	0,851075151	4,080456	0,208573546	0,834781164	NA
ENSG00000234872	NA	NA	NA	0	NA	NA	NA	NA	NA
ENSG00000234878	HSPE1P1	heat shock protein family E (Hsp10) member 1 pseudogene 1	140895	0	NA	NA	NA	NA	NA
ENSG00000234881	NA	NA	NA	0	NA	NA	NA	NA	NA
ENSG00000234882	EIF3EP1	EIF3E pseudogene 1	3647	9,474976968	0,25994033	0,729402	0,356374638	0,72156002	NA
ENSG00000234886	NA	NA	NA	0,253116234	0,560328782	4,062681	0,13792093	0,890302909	NA
ENSG00000234888	NA	NA	NA	0	NA	NA	NA	NA	NA
ENSG00000234889	KPNB1P1	KPNB1 pseudogene 1	100131072	0	NA	NA	NA	NA	NA
ENSG00000234890	NA	NA	NA	0	NA	NA	NA	NA	NA
ENSG00000234894	NA	NA	NA	0	NA	NA	NA	NA	NA
ENSG00000234895	NA	NA	NA	0,15836288	0	4,080456	0	1	NA
ENSG00000234896	NA	NA	NA	0	NA	NA	NA	NA	NA
ENSG00000234898	NA	NA	NA	0	NA	NA	NA	NA	NA
ENSG00000234901	MTND6P13	MT-ND6 pseudogene 13	106480428	0	NA	NA	NA	NA	NA
ENSG00000234906	APOC2	apolipoprotein C2	344	10,77081418	-0,033932277	0,668608	-0,050750651	0,959524215	NA
ENSG00000234907	NA	NA	NA	0	NA	NA	NA	NA	NA
ENSG00000234910	IL6RP1	interleukin 6 receptor pseudogene 1	157916	0	NA	NA	NA	NA	NA
ENSG00000234911	NA	NA	NA	2,936287629	-0,57070759	1,139605	-0,500794152	0,616516002	NA
ENSG00000234922	NA	NA	NA	0	NA	NA	NA	NA	NA
ENSG00000234925	NA	NA	NA	0,10488184	-1,076443944	4,080456	-0,263804824	0,791930317	NA
ENSG00000234927	NA	NA	NA	0	NA	NA	NA	NA	NA
ENSG00000234931	NA	NA	NA	0	NA	NA	NA	NA	NA

ENSG00000234932	NA	NA	NA	0	NA	NA	NA	NA	NA
ENSG00000234933	CDC42P1	CDC42 pseudogene 1	170503	0	NA	NA	NA	NA	NA
ENSG00000234934	MTND5P29	MT-ND5 pseudogene 29	100873265	0	NA	NA	NA	NA	NA
ENSG00000234937	NA	NA	NA	0	NA	NA	NA	NA	NA
ENSG00000234939	MTND2P18	MT-ND2 pseudogene 18	100873224	0	NA	NA	NA	NA	NA
ENSG00000234941	NA	NA	NA	0	NA	NA	NA	NA	NA
ENSG00000234946	NA	NA	NA	0	NA	NA	NA	NA	NA
ENSG00000234950	RBMY2OP	RNA binding motif protein Y-linked family 2 member O, pseudogene	379005	0	NA	NA	NA	NA	NA
ENSG00000234964	NA	NA	NA	1,1808024	-4,409019753	3,925764	-1,123098489	0,261395668	NA
ENSG00000234965	SHISA8	shisa family member 8	440829	0	NA	NA	NA	NA	NA
ENSG00000234969	AARSD1P1	AARSD1 pseudogene 1	117981788	0,366691663	-0,480215547	3,670561	-0,130828919	0,895910645	NA
ENSG00000234981	RPL7AP20	ribosomal protein L7a pseudogene 20	100271037	1,095824192	2,698153567	2,129813	1,266849876	0,205208974	NA
ENSG00000234982	RPL7AP47	ribosomal protein L7a pseudogene 47	100271211	0	NA	NA	NA	NA	NA
ENSG00000234983	NA	NA	NA	0	NA	NA	NA	NA	NA
ENSG00000234984	NA	NA	NA	0	NA	NA	NA	NA	NA
ENSG00000234985	RPL18P4	ribosomal protein L18 pseudogene 4	392979	0	NA	NA	NA	NA	NA
ENSG00000234993	CUBNP2	cubilin pseudogene 2	100289280	0	NA	NA	NA	NA	NA
ENSG00000234995	RPS3AP28	RPS3A pseudogene 28	100271257	0	NA	NA	NA	NA	NA
ENSG00000234996	ACTG1P25	actin gamma 1 pseudogene 25	148709	1,128980218	0,969865066	2,273635	0,426570208	0,669692401	NA
ENSG00000234999	NA	NA	NA	1,394267976	2,094311789	1,927559	1,086510102	0,277253374	NA
ENSG00000235001	EIF4A1P2	eukaryotic translation initiation factor 4A1 pseudogene 2	359792	0	NA	NA	NA	NA	NA
ENSG00000235002	RPS15AP8	ribosomal protein S15a pseudogene 8	100271143	0	NA	NA	NA	NA	NA
ENSG00000235004	NA	NA	NA	0	NA	NA	NA	NA	NA
ENSG00000235007	NA	NA	NA	4,517004005	0,17536204	1,011194	0,173420805	0,862320672	NA
ENSG00000235008	NA	NA	NA	5,146721699	0,640869028	0,933852	0,686263808	0,492546767	NA
ENSG00000235012	JCADP1	JCAD pseudogene 1	729056	0	NA	NA	NA	NA	NA
ENSG00000235013	NA	NA	NA	0,117497108	0,847101401	4,080456	0,207599697	0,835541541	NA
ENSG00000235014	NA	NA	NA	0	NA	NA	NA	NA	NA
ENSG00000235018	RPL17P48	ribosomal protein L17 pseudogene 48	100271416	1,204469745	-1,016322572	1,926065	-0,527667729	0,597729975	NA
ENSG00000235028	NA	NA	NA	0	NA	NA	NA	NA	NA
ENSG00000235034	C19orf81	chromosome 19 open reading frame 81	342918	0	NA	NA	NA	NA	NA
ENSG00000235036	NA	NA	NA	0	NA	NA	NA	NA	NA
ENSG00000235039	MTND6P12	MT-ND6 pseudogene 12	106481743	0	NA	NA	NA	NA	NA
ENSG00000235040	NA	NA	NA	0	NA	NA	NA	NA	NA
ENSG00000235043	NA	NA	NA	0	NA	NA	NA	NA	NA
ENSG00000235044	NA	NA	NA	0	NA	NA	NA	NA	NA
ENSG00000235045	NA	NA	NA	0	NA	NA	NA	NA	NA
ENSG00000235055	LOC100133029	sirtuin 5 pseudogene	100133029	0	NA	NA	NA	NA	NA
ENSG00000235060	NA	NA	NA	0	NA	NA	NA	NA	NA
ENSG00000235061	UBE2V1P7	UBE2V1 pseudogene 7	106481746	0	NA	NA	NA	NA	NA
ENSG00000235062	NA	NA	NA	0	NA	NA	NA	NA	NA
ENSG00000235064	NA	NA	NA	0	NA	NA	NA	NA	NA
ENSG00000235065	RPL24P2	RPL24 pseudogene 2	140760	0	NA	NA	NA	NA	NA
ENSG00000235071	LOC100132857	heat shock protein 90 alpha family class B member 1 pseudogene	100132857	0	NA	NA	NA	NA	NA
ENSG00000235076	GAPDHP52	glyceraldehyde 3 phosphate dehydrogenase pseudogene 52	100421313	0,307302916	2,350641419	3,995064	0,58838648	0,55627291	NA
ENSG00000235080	MTND5P23	MT-ND5 pseudogene 23	100873261	0	NA	NA	NA	NA	NA
ENSG00000235081	NA	NA	NA	0	NA	NA	NA	NA	NA
ENSG00000235082	NA	NA	NA	0	NA	NA	NA	NA	NA
ENSG00000235083	NA	NA	NA	0	NA	NA	NA	NA	NA
ENSG00000235084	NA	NA	NA	0	NA	NA	NA	NA	NA
ENSG00000235088	NA	NA	NA	0,10543123	0	4,080456	0	1	NA
ENSG00000235089	RPL10AP4	ribosomal protein L10a pseudogene 4	343301	0	NA	NA	NA	NA	NA
ENSG00000235090	NA	NA	NA	1,87704612	0,074564121	1,465671	0,050873707	0,959426157	NA
ENSG00000235094	NA	NA	NA	0	NA	NA	NA	NA	NA
ENSG00000235095	NA	NA	NA	0	NA	NA	NA	NA	NA
ENSG00000235098	ANKRD65	ankyrin repeat domain 65	441869	0	NA	NA	NA	NA	NA
ENSG00000235101	NA	NA	NA	0,103998153	-1,076443944	4,080456	-0,263804824	0,791930317	NA
ENSG00000235102	NA	NA	NA	0	NA	NA	NA	NA	NA
ENSG00000235105	NA	NA	NA	0	NA	NA	NA	NA	NA
ENSG00000235108	NA	NA	NA	0	NA	NA	NA	NA	NA
ENSG00000235109	ZSCAN31	zinc finger and SCAN domain containing 31	64288	2,862847642	0,045316947	1,498896	0,030233549	0,975880793	NA

ENSG00000235110	RPS17P10	ribosomal protein S17 pseudogene 10	100271071	0	NA	NA	NA	NA	NA
ENSG00000235112	HSPE1P27	heat shock protein family E (Hsp10) member 1 pseudogene 27	106480261	0	NA	NA	NA	NA	NA
ENSG00000235113	NA	NA	NA	0	NA	NA	NA	NA	NA
ENSG00000235115	NA	NA	NA	0	NA	NA	NA	NA	NA
ENSG00000235118	FAM237A	family with sequence similarity 237 member A	200726	0	NA	NA	NA	NA	NA
ENSG00000235124	KRT8P17	keratin 8 pseudogene 17	392479	0	NA	NA	NA	NA	NA
ENSG00000235128	NA	NA	NA	2,432981316	0,736546068	1,055716	0,697674507	0,485380774	NA
ENSG00000235129	NA	NA	NA	0	NA	NA	NA	NA	NA
ENSG00000235133	RPL35AP22	ribosomal protein L35a pseudogene 22	100271507	0	NA	NA	NA	NA	NA
ENSG00000235136	PSMA5P1	PSMA5 pseudogene 1	117751738	0	NA	NA	NA	NA	NA
ENSG00000235137	NA	NA	NA	0	NA	NA	NA	NA	NA
ENSG00000235141	NA	NA	NA	2,810266645	1,466031641	1,144848	1,28054693	0,200352851	NA
ENSG00000235145	RPSAP16	ribosomal protein SA pseudogene 16	100270872	0,216496347	1,902771968	4,026559	0,472555327	0,63653046	NA
ENSG00000235147	NA	NA	NA	0	NA	NA	NA	NA	NA
ENSG00000235148	NA	NA	NA	0	NA	NA	NA	NA	NA
ENSG00000235150	NA	NA	NA	0	NA	NA	NA	NA	NA
ENSG00000235156	TMEM30CP	transmembrane protein 30C, pseudogene	644444	0	NA	NA	NA	NA	NA
ENSG00000235163	RPL7AP81	ribosomal protein L7a pseudogene 81	122965333	0	NA	NA	NA	NA	NA
ENSG00000235165	CDK5P1	cyclin dependent kinase 5 pseudogene 1	392265	0	NA	NA	NA	NA	NA
ENSG00000235169	SMIM1	small integral membrane protein 1 (Vel blood group)	388588	0,1909219	0,840915725	4,080261	0,206093623	0,83671778	NA
ENSG00000235170	MTND1P31	MT-ND1 pseudogene 31	100873217	0	NA	NA	NA	NA	NA
ENSG00000235174	NA	NA	NA	0	NA	NA	NA	NA	NA
ENSG00000235175	RPL26P37	ribosomal protein L26 pseudogene 37	441533	0	NA	NA	NA	NA	NA
ENSG00000235182	NA	NA	NA	0	NA	NA	NA	NA	NA
ENSG00000235183	SRP14P3	SRP14 pseudogene 3	111644142	0,853801217	2,635538289	2,334295	1,129050961	0,258876336	NA
ENSG00000235186	NA	NA	NA	0	NA	NA	NA	NA	NA
ENSG00000235190	NA	NA	NA	0	NA	NA	NA	NA	NA
ENSG00000235196	NA	NA	NA	0	NA	NA	NA	NA	NA
ENSG00000235197	ZNF33CP	zinc finger protein 33C, pseudogene	79231	0	NA	NA	NA	NA	NA
ENSG00000235199	CYP2C58P	cytochrome P450 family 2 subfamily C member 58, pseudogene	106480430	0	NA	NA	NA	NA	NA
ENSG00000235203	TBC1D3P3	TBC1 domain family member 3 pseudogene 3	653017	0	NA	NA	NA	NA	NA
ENSG00000235205	NA	NA	NA	0,11808024	-1,076443944	4,080456	-0,263804824	0,791930317	NA
ENSG00000235207	TUBBP6	tubulin beta class I pseudogene 6	442308	0	NA	NA	NA	NA	NA
ENSG00000235208	NA	NA	NA	0	NA	NA	NA	NA	NA
ENSG00000235211	NA	NA	NA	0	NA	NA	NA	NA	NA
ENSG00000235213	OR6E1P	olfactory receptor family 6 subfamily E member 1 pseudogene	79323	0	NA	NA	NA	NA	NA
ENSG00000235217	TSPY26P	testis specific protein Y-linked 26, pseudogene	128854	1,58296628	-0,992354429	1,567004	-0,633281459	0,526549859	NA
ENSG00000235218	NA	NA	NA	0	NA	NA	NA	NA	NA
ENSG00000235219	NA	NA	NA	0	NA	NA	NA	NA	NA
ENSG00000235224	NA	NA	NA	0	NA	NA	NA	NA	NA
ENSG00000235225	RPL31P17	ribosomal protein L31 pseudogene 17	653773	0	NA	NA	NA	NA	NA
ENSG00000235226	RPL27P2	ribosomal protein L27 pseudogene 2	646446	0	NA	NA	NA	NA	NA
ENSG00000235227	VN1R38P	vomeronasal 1 receptor 38 pseudogene	100131065	0	NA	NA	NA	NA	NA
ENSG00000235232	NA	NA	NA	0	NA	NA	NA	NA	NA
ENSG00000235235	IGKV1OR2-1	immunoglobulin kappa variable 1/OR2-1 (pseudogene)	3531	0	NA	NA	NA	NA	NA
ENSG00000235238	NA	NA	NA	0,118286652	0	4,080456	0	1	NA
ENSG00000235239	NA	NA	NA	0	NA	NA	NA	NA	NA
ENSG00000235240	NA	NA	NA	0	NA	NA	NA	NA	NA
ENSG00000235241	LOC107985101	uncharacterized LOC107985101	107985101	0	NA	NA	NA	NA	NA
ENSG00000235243	NA	NA	NA	0	NA	NA	NA	NA	NA
ENSG00000235248	OR13C1P	olfactory receptor family 13 subfamily C member 1 pseudogene	392377	0	NA	NA	NA	NA	NA

ENSG00000235251	LOC100421465	ubiquitin like modifier activating enzyme 2 pseudogene	100421465	0	NA	NA	NA	NA	NA
ENSG00000235253	NA	NA	NA	0	NA	NA	NA	NA	NA
ENSG00000235254	NA	NA	NA	0	NA	NA	NA	NA	NA
ENSG00000235256	FKBP4P7	FKBP prolyl isomerase 4 pseudogene 7	441410	0	NA	NA	NA	NA	NA
ENSG00000235258	NDUFB4P6	NADH:ubiquinone oxidoreductase subunit B4 pseudogene 6	107075097	0	NA	NA	NA	NA	NA
ENSG00000235264	RPL5P28	ribosomal protein L5 pseudogene 28	100131535	0	NA	NA	NA	NA	NA
ENSG00000235266	NA	NA	NA	0	NA	NA	NA	NA	NA
ENSG00000235267	NA	NA	NA	0	NA	NA	NA	NA	NA
ENSG00000235268	KDM4E	lysine demethylase 4E	390245	0	NA	NA	NA	NA	NA
ENSG00000235272	RAMACL	RNA guanine-7 methyltransferase activating subunit like	353267	5,394225621	0,913697606	0,88849	1,028370916	0,303775384	NA
ENSG00000235274	LOC100420320	kinesin family member 2A pseudogene	100420320	0	NA	NA	NA	NA	NA
ENSG00000235275	KRT18P16	keratin 18 pseudogene 16	391827	0	NA	NA	NA	NA	NA
ENSG00000235278	NA	NA	NA	0	NA	NA	NA	NA	NA
ENSG00000235282	NA	NA	NA	0	NA	NA	NA	NA	NA
ENSG00000235286	NA	NA	NA	0	NA	NA	NA	NA	NA
ENSG00000235289	NA	NA	NA	0	NA	NA	NA	NA	NA
ENSG00000235290	NA	NA	NA	0	NA	NA	NA	NA	NA
ENSG00000235294	RPL7AP25	ribosomal protein L7a pseudogene 25	100271042	0	NA	NA	NA	NA	NA
ENSG00000235297	NA	NA	NA	0	NA	NA	NA	NA	NA
ENSG00000235299	MRPL53P1	mitochondrial ribosomal protein L53 pseudogene 1	359752	0	NA	NA	NA	NA	NA
ENSG00000235301	HLA-Z	major histocompatibility complex, class I, Z (pseudogene)	267017	0	NA	NA	NA	NA	NA
ENSG00000235306	GAPDHP34	glyceraldehyde 3 phosphate dehydrogenase pseudogene 34	646877	0	NA	NA	NA	NA	NA
ENSG00000235308	NA	NA	NA	0	NA	NA	NA	NA	NA
ENSG00000235310	GXYLT1P6	GXYLT1 pseudogene 6	100132672	0	NA	NA	NA	NA	NA
ENSG00000235312	NA	NA	NA	0	NA	NA	NA	NA	NA
ENSG00000235315	RPL23AP69	ribosomal protein L23a pseudogene 69	100271623	0	NA	NA	NA	NA	NA
ENSG00000235318	MTND4LP13	MT-ND4L pseudogene 13	107075100	0	NA	NA	NA	NA	NA
ENSG00000235323	NA	NA	NA	0	NA	NA	NA	NA	NA
ENSG00000235325	NA	NA	NA	0	NA	NA	NA	NA	NA
ENSG00000235328	RPL31P62	ribosomal protein L31 pseudogene 62	100271482	0	NA	NA	NA	NA	NA
ENSG00000235329	NA	NA	NA	0	NA	NA	NA	NA	NA
ENSG00000235330	RPL12P25	ribosomal protein L12 pseudogene 25	100270926	1,159806313	-0,122440459	1,744502	-0,070186475	0,944045239	NA
ENSG00000235332	NA	NA	NA	0	NA	NA	NA	NA	NA
ENSG00000235333	NA	NA	NA	0	NA	NA	NA	NA	NA
ENSG00000235334	BTG3P1	BTG anti-proliferation factor 3 pseudogene 1	100419246	0	NA	NA	NA	NA	NA
ENSG00000235338	NA	NA	NA	0	NA	NA	NA	NA	NA
ENSG00000235339	NA	NA	NA	0,072165449	0,847101401	4,080456	0,207599697	0,835541541	NA
ENSG00000235347	NA	NA	NA	0	NA	NA	NA	NA	NA
ENSG00000235349	NA	NA	NA	0	NA	NA	NA	NA	NA
ENSG00000235350	NA	NA	NA	0	NA	NA	NA	NA	NA
ENSG00000235352	NA	NA	NA	0	NA	NA	NA	NA	NA
ENSG00000235354	NA	NA	NA	0	NA	NA	NA	NA	NA
ENSG00000235363	NA	NA	NA	0,10488184	-1,076443944	4,080456	-0,263804824	0,791930317	NA
ENSG00000235368	SAPCD2P2	suppressor APC domain containing 2 pseudogene 2	442317	0	NA	NA	NA	NA	NA
ENSG00000235369	NA	NA	NA	0,119673877	0,847101401	4,080456	0,207599697	0,835541541	NA
ENSG00000235370	NA	NA	NA	0	NA	NA	NA	NA	NA
ENSG00000235371	NA	NA	NA	0	NA	NA	NA	NA	NA
ENSG00000235374	SSR4P1	signal sequence receptor subunit 4 pseudogene 1	728039	2,762053832	-0,81647518	1,256606	-0,649746577	0,515855932	NA
ENSG00000235376	RPEL1	ribulose-5-phosphate-3-epimerase like 1	729020	0,117497108	0,847101401	4,080456	0,207599697	0,835541541	NA
ENSG00000235377	RPS2P34	ribosomal protein S2 pseudogene 34	442426	0	NA	NA	NA	NA	NA
ENSG00000235378	MRPS10P1	mitochondrial ribosomal protein S10 pseudogene 1	359753	0	NA	NA	NA	NA	NA
ENSG00000235379	NA	NA	NA	0	NA	NA	NA	NA	NA
ENSG00000235382	NA	NA	NA	0	NA	NA	NA	NA	NA
ENSG00000235386	NA	NA	NA	0	NA	NA	NA	NA	NA
ENSG00000235387	SPAAR	small regulatory polypeptide of amino acid response	158376	0	NA	NA	NA	NA	NA

ENSG00000235390	NA	NA	NA	0	NA	NA	NA	NA	NA
ENSG00000235400	LOC652549	SNW domain containing 1 pseudogene	652549	0,11808024	-1,076443944	4,080456	-0,263804824	0,791930317	NA
ENSG00000235411	NA	NA	NA	0,399983736	-1,611834988	3,555499	-0,453335856	0,650306917	NA
ENSG00000235413	KRT18P63	keratin 18 pseudogene 63	100288868	0	NA	NA	NA	NA	NA
ENSG00000235414	RPSAP24	ribosomal protein SA pseudogene 24	391462	0	NA	NA	NA	NA	NA
ENSG00000235416	NA	NA	NA	0	NA	NA	NA	NA	NA
ENSG00000235420	NA	NA	NA	0	NA	NA	NA	NA	NA
ENSG00000235421	NA	NA	NA	0,221388396	-0,122848641	4,080456	-0,030106597	0,975982039	NA
ENSG00000235422	NA	NA	NA	0	NA	NA	NA	NA	NA
ENSG00000235424	NA	NA	NA	0	NA	NA	NA	NA	NA
ENSG00000235425	NA	NA	NA	0	NA	NA	NA	NA	NA
ENSG00000235429	RPS15AP23	ribosomal protein S15a pseudogene 23	100271558	0	NA	NA	NA	NA	NA
ENSG00000235430	ZSWIM5P1	zinc finger SWIM-type containing 5 pseudogene 1	100130110	0	NA	NA	NA	NA	NA
ENSG00000235432	NA	NA	NA	0	NA	NA	NA	NA	NA
ENSG00000235433	MTATP6P17	MT-ATP6 pseudogene 17	107075274	0	NA	NA	NA	NA	NA
ENSG00000235436	NA	NA	NA	0	NA	NA	NA	NA	NA
ENSG00000235438	LOC105370105	uncharacterized LOC105370105	105370105	0	NA	NA	NA	NA	NA
ENSG00000235440	RPS2P15	ribosomal protein S2 pseudogene 15	100270858	0	NA	NA	NA	NA	NA
ENSG00000235444	PSMB3P2	proteasome subunit beta 3 pseudogene 2	130700	0	NA	NA	NA	NA	NA
ENSG00000235447	TRAPPC13P1	trafficking protein particle complex 13 pseudogene 1	646193	0	NA	NA	NA	NA	NA
ENSG00000235449	NA	NA	NA	1,260371322	-0,974307075	1,817963	-0,535933351	0,592004614	NA
ENSG00000235451	NA	NA	NA	0	NA	NA	NA	NA	NA
ENSG00000235453	SMIM27	small integral membrane protein 27	100129250	9,741380789	-0,569538673	0,725444	-0,785089669	0,432400996	NA
ENSG00000235454	NA	NA	NA	0,294116842	0,687186575	3,899583	0,176220513	0,860120706	NA
ENSG00000235459	RPS26P31	ribosomal protein S26 pseudogene 31	645979	0,189717951	-0,103332491	4,080456	-0,025323761	0,979796721	NA
ENSG00000235462	TAB3P1	TGF-beta activated kinase 1/MAP3K7 binding protein 3 pseudogene 1	643199	0	NA	NA	NA	NA	NA
ENSG00000235463	NA	NA	NA	0	NA	NA	NA	NA	NA
ENSG00000235467	RPL10AP5	ribosomal protein L10a pseudogene 5	645998	0	NA	NA	NA	NA	NA
ENSG00000235469	NA	NA	NA	0	NA	NA	NA	NA	NA
ENSG00000235472	EIF4A1P7	eukaryotic translation initiation factor 4A1 pseudogene 7	341784	0	NA	NA	NA	NA	NA
ENSG00000235479	NA	NA	NA	0	NA	NA	NA	NA	NA
ENSG00000235485	LOC441996	aconitase 2, mitochondrial pseudogene	441996	0,119673877	0,847101401	4,080456	0,207599697	0,835541541	NA
ENSG00000235486	NA	NA	NA	0	NA	NA	NA	NA	NA
ENSG00000235489	NA	NA	NA	2,829435483	-1,198350394	1,124784	-1,065404538	0,286692904	NA
ENSG00000235500	NA	NA	NA	0,475646076	-0,117134849	3,509613	-0,033375427	0,973375205	NA
ENSG00000235508	RPS2P7	ribosomal protein S2 pseudogene 7	86781	0,758787632	1,391987147	2,173353	0,640479143	0,521861145	NA
ENSG00000235510	NA	NA	NA	0	NA	NA	NA	NA	NA
ENSG00000235511	NA	NA	NA	0	NA	NA	NA	NA	NA
ENSG00000235514	NA	NA	NA	0	NA	NA	NA	NA	NA
ENSG00000235521	USP9YP27	USP9Y pseudogene 27	106480716	0	NA	NA	NA	NA	NA
ENSG00000235524	MTCO1P23	MT-CO1 pseudogene 23	107075281	0	NA	NA	NA	NA	NA
ENSG00000235525	NA	NA	NA	0	NA	NA	NA	NA	NA
ENSG00000235534	NA	NA	NA	0	NA	NA	NA	NA	NA
ENSG00000235539	MTND1P33	MT-ND1 pseudogene 33	100873218	0	NA	NA	NA	NA	NA
ENSG00000235544	RPS11P1	ribosomal protein S11 pseudogene 1	128718	0	NA	NA	NA	NA	NA
ENSG00000235546	NA	NA	NA	0	NA	NA	NA	NA	NA
ENSG00000235547	NDUFB11P1	NADH:ubiquinone oxidoreductase subunit B11 pseudogene 1	390239	0	NA	NA	NA	NA	NA
ENSG00000235548	NA	NA	NA	0	NA	NA	NA	NA	NA
ENSG00000235549	NA	NA	NA	0	NA	NA	NA	NA	NA
ENSG00000235550	NA	NA	NA	0	NA	NA	NA	NA	NA
ENSG00000235552	RPL6P27	ribosomal protein L6 pseudogene 27	645387	2,048205649	0,138037687	1,558991	0,08854299	0,929445117	NA
ENSG00000235554	NA	NA	NA	0	NA	NA	NA	NA	NA
ENSG00000235555	NA	NA	NA	0	NA	NA	NA	NA	NA
ENSG00000235558	NA	NA	NA	2,610939664	4,812800496	2,986552	1,611490473	0,107072862	NA
ENSG00000235559	NA	NA	NA	0	NA	NA	NA	NA	NA
ENSG00000235568	NFAM1	NFAT activating protein with ITAM motif 1	150372	0	NA	NA	NA	NA	NA
ENSG00000235571	NA	NA	NA	0	NA	NA	NA	NA	NA

ENSG00000235573	RPS15AP37	ribosomal protein S15a pseudogene 37	100271651	0	NA	NA	NA	NA	NA
ENSG00000235574	LOC442292	coiled-coil domain containing 86 pseudogene	442292	0,338695656	-2,605782007	3,991754	-0,652791151	0,513890929	NA
ENSG00000235579	NA	NA	NA	0	NA	NA	NA	NA	NA
ENSG00000235581	NA	NA	NA	0	NA	NA	NA	NA	NA
ENSG00000235582	RPL24P5	RPL24 pseudogene 5	100129017	0	NA	NA	NA	NA	NA
ENSG00000235583	NA	NA	NA	0,10488184	-1,076443944	4,080456	-0,263804824	0,791930317	NA
ENSG00000235587	NA	NA	NA	0	NA	NA	NA	NA	NA
ENSG00000235592	NA	NA	NA	0	NA	NA	NA	NA	NA
ENSG00000235594	NA	NA	NA	0	NA	NA	NA	NA	NA
ENSG00000235595	NA	NA	NA	0	NA	NA	NA	NA	NA
ENSG00000235598	RRM2P4	ribonucleotide reductase M2 polypeptide pseudogene 4	6246	0	NA	NA	NA	NA	NA
ENSG00000235602	POU5F1P3	POU class 5 homeobox 1 pseudogene 3	642559	0	NA	NA	NA	NA	NA
ENSG00000235605	RPS15P2	ribosomal protein S15 pseudogene 2	100271066	0	NA	NA	NA	NA	NA
ENSG00000235606	NA	NA	NA	0	NA	NA	NA	NA	NA
ENSG00000235608	NKX1-1	NK1 homeobox 1	54729	0	NA	NA	NA	NA	NA
ENSG00000235613	NSRP1P1	nuclear speckle splicing regulatory protein 1 pseudogene 1	100420557	4,464326925	0,61179798	0,912559	0,670420349	0,502589867	NA
ENSG00000235616	NA	NA	NA	0	NA	NA	NA	NA	NA
ENSG00000235617	NA	NA	NA	0	NA	NA	NA	NA	NA
ENSG00000235618	NA	NA	NA	0,885138308	0,885368046	2,034785	0,435116345	0,66347798	NA
ENSG00000235619	NA	NA	NA	1,372383361	-1,028686672	1,672744	-0,614969587	0,538574805	NA
ENSG00000235623	OR7E110P	olfactory receptor family 7 subfamily E member 110 pseudogene	81354	0	NA	NA	NA	NA	NA
ENSG00000235626	KRT18P66	keratin 18 pseudogene 66	442405	0	NA	NA	NA	NA	NA
ENSG00000235627	SNRPFP4	small nuclear ribonucleoprotein polypeptide F pseudogene 4	100874412	0,10543123	0	4,080456	0	1	NA
ENSG00000235631	RNF148	ring finger protein 148	378925	0	NA	NA	NA	NA	NA
ENSG00000235636	NA	NA	NA	0,11808024	-1,076443944	4,080456	-0,263804824	0,791930317	NA
ENSG00000235639	LOC100419447	lysine acetyltransferase 14 pseudogene	100419447	0	NA	NA	NA	NA	NA
ENSG00000235640	ELOAP1	ELOA pseudogene 1	100422580	0	NA	NA	NA	NA	NA
ENSG00000235642	PTP4A1P5	PTP4A1 pseudogene 5	100289380	0	NA	NA	NA	NA	NA
ENSG00000235644	RPL10P5	ribosomal protein L10 pseudogene 5	644911	0	NA	NA	NA	NA	NA
ENSG00000235646	MTND1P2	MT-ND1 pseudogene 2	100873170	0	NA	NA	NA	NA	NA
ENSG00000235647	NA	NA	NA	0	NA	NA	NA	NA	NA
ENSG00000235649	MXRA5Y	matrix remodeling associated 5 Y-linked (pseudogene)	286544	0	NA	NA	NA	NA	NA
ENSG00000235651	G3BP1P1	G3BP1 pseudogene 1	100422338	0,775437282	3,027778704	2,250327	1,345483713	0,178469077	NA
ENSG00000235653	LOC100419512	zinc finger protein 19 pseudogene	100419512	0	NA	NA	NA	NA	NA
ENSG00000235656	NA	NA	NA	0	NA	NA	NA	NA	NA
ENSG00000235670	RPL21P40	ribosomal protein L21 pseudogene 40	100271158	0	NA	NA	NA	NA	NA
ENSG00000235672	NA	NA	NA	2,440034113	-0,862295881	1,382009	-0,623943573	0,532664643	NA
ENSG00000235673	RPS2P13	ribosomal protein S2 pseudogene 13	100270826	0	NA	NA	NA	NA	NA
ENSG00000235674	NA	NA	NA	0	NA	NA	NA	NA	NA
ENSG00000235677	NA	NA	NA	0	NA	NA	NA	NA	NA
ENSG00000235678	OR5AN2P	olfactory receptor family 5 subfamily AN member 2 pseudogene	81224	0	NA	NA	NA	NA	NA
ENSG00000235679	NA	NA	NA	0	NA	NA	NA	NA	NA
ENSG00000235681	NA	NA	NA	0	NA	NA	NA	NA	NA
ENSG00000235683	NA	NA	NA	1,081181742	1,00773414	1,809836	0,556809622	0,577657509	NA
ENSG00000235685	NA	NA	NA	0	NA	NA	NA	NA	NA
ENSG00000235686	PPIAP20	peptidylprolyl isomerase A pseudogene 20	100500737	0	NA	NA	NA	NA	NA
ENSG00000235687	NA	NA	NA	0	NA	NA	NA	NA	NA
ENSG00000235689	NA	NA	NA	0	NA	NA	NA	NA	NA
ENSG00000235690	CUBNP3	cubilin pseudogene 3	100421634	0	NA	NA	NA	NA	NA
ENSG00000235695	NA	NA	NA	0	NA	NA	NA	NA	NA
ENSG00000235698	NA	NA	NA	0,357490535	0,842052017	4,080456	0,206362241	0,836507964	NA
ENSG00000235699	CXorf51B	chromosome X open reading frame 51B	100133053	0	NA	NA	NA	NA	NA
ENSG00000235700	CYCSP52	CYCS pseudogene 52	360155	0	NA	NA	NA	NA	NA
ENSG00000235701	NA	NA	NA	0,295147633	-1,04686718	3,904657	-0,268107302	0,788616725	NA
ENSG00000235705	LOC729815	uncharacterized LOC729815	729815	0	NA	NA	NA	NA	NA
ENSG00000235707	LOC100129601	cell division cycle associated 7 pseudogene	100129601	0	NA	NA	NA	NA	NA
ENSG00000235711	ANKRD34C	ankyrin repeat domain 34C	390616	0	NA	NA	NA	NA	NA

ENSG00000235713	LOC100128334	gap junction protein gamma 3 pseudogene	100128334	0	NA	NA	NA	NA	NA
ENSG00000235716	KRT18P46	keratin 18 pseudogene 46	391458	0	NA	NA	NA	NA	NA
ENSG00000235718	MFRP	membrane frizzled-related protein	83552	0	NA	NA	NA	NA	NA
ENSG00000235720	NA	NA	NA	0,103998153	-1,076443944	4,080456	-0,263804824	0,791930317	NA
ENSG00000235721	NA	NA	NA	0	NA	NA	NA	NA	NA
ENSG00000235729	NA	NA	NA	0	NA	NA	NA	NA	NA
ENSG00000235730	OR2AF1P	olfactory receptor family 2 subfamily AF member 1 pseudogene	79331	0	NA	NA	NA	NA	NA
ENSG00000235735	RPL7AP72	ribosomal protein L7a pseudogene 72	100270754	0	NA	NA	NA	NA	NA
ENSG00000235737	TRIM60P19	tripartite motif containing 60 pseudogene 19	100533672	0	NA	NA	NA	NA	NA
ENSG00000235738	NA	NA	NA	0	NA	NA	NA	NA	NA
ENSG00000235742	NA	NA	NA	0	NA	NA	NA	NA	NA
ENSG00000235746	NA	NA	NA	0	NA	NA	NA	NA	NA
ENSG00000235748	NA	NA	NA	1,14865359	0,85557043	2,002189	0,42731741	0,669148154	NA
ENSG00000235756	NA	NA	NA	0	NA	NA	NA	NA	NA
ENSG00000235759	NA	NA	NA	0,103998153	-1,076443944	4,080456	-0,263804824	0,791930317	NA
ENSG00000235763	NA	NA	NA	0	NA	NA	NA	NA	NA
ENSG00000235768	BRD7P5	bromodomain containing 7 pseudogene 5	100129259	0	NA	NA	NA	NA	NA
ENSG00000235775	NA	NA	NA	0	NA	NA	NA	NA	NA
ENSG00000235776	RPL7AP70	ribosomal protein L7a pseudogene 70	728139	0,11808024	-1,076443944	4,080456	-0,263804824	0,791930317	NA
ENSG00000235778	LOC402329	ubiquitin carboxyl-terminal hydrolase 17-like protein 2-like	402329	0	NA	NA	NA	NA	NA
ENSG00000235780	USP17L27	ubiquitin specific peptidase 17 like family member 27	728393	0	NA	NA	NA	NA	NA
ENSG00000235784	HNRNPA1P29	heterogeneous nuclear ribonucleoprotein A1 pseudogene 29	100421424	0	NA	NA	NA	NA	NA
ENSG00000235787	NA	NA	NA	0	NA	NA	NA	NA	NA
ENSG00000235794	MTND3P21	MT-ND3 pseudogene 21	107075198	0	NA	NA	NA	NA	NA
ENSG00000235797	NA	NA	NA	0	NA	NA	NA	NA	NA
ENSG00000235803	MTCO2P23	MT-CO2 pseudogene 23	107075192	0	NA	NA	NA	NA	NA
ENSG00000235804	NA	NA	NA	0	NA	NA	NA	NA	NA
ENSG00000235808	NA	NA	NA	0	NA	NA	NA	NA	NA
ENSG00000235810	NA	NA	NA	0	NA	NA	NA	NA	NA
ENSG00000235811	NA	NA	NA	0	NA	NA	NA	NA	NA
ENSG00000235812	ADAM21P1	ADAM metallopeptidase domain 21 pseudogene 1	145241	0	NA	NA	NA	NA	NA
ENSG00000235816	NA	NA	NA	0	NA	NA	NA	NA	NA
ENSG00000235817	RPS24P4	ribosomal protein S24 pseudogene 4	729257	0	NA	NA	NA	NA	NA
ENSG00000235821	IFITM4P	interferon induced transmembrane protein 4 pseudogene	340198	0	NA	NA	NA	NA	NA
ENSG00000235827	TUBB8P9	tubulin beta 8 class VIII pseudogene 9	255208	0	NA	NA	NA	NA	NA
ENSG00000235828	RPL36AP26	ribosomal protein L36a pseudogene 26	729909	0	NA	NA	NA	NA	NA
ENSG00000235829	NA	NA	NA	0	NA	NA	NA	NA	NA
ENSG00000235832	NA	NA	NA	0	NA	NA	NA	NA	NA
ENSG00000235836	KIF3AP1	kinesin family member 3A pseudogene 1	100419880	0	NA	NA	NA	NA	NA
ENSG00000235838	HSP90AB7P	heat shock protein 90 alpha family class B member 7, pseudogene	644495	0	NA	NA	NA	NA	NA
ENSG00000235847	NA	NA	NA	0	NA	NA	NA	NA	NA
ENSG00000235855	OR7E145P	olfactory receptor family 7 subfamily E member 145 pseudogene	390215	0	NA	NA	NA	NA	NA
ENSG00000235857	NA	NA	NA	0	NA	NA	NA	NA	NA
ENSG00000235861	NA	NA	NA	0	NA	NA	NA	NA	NA
ENSG00000235863	B3GALT4	beta-1,3-galactosyltransferase 4	8705	0	NA	NA	NA	NA	NA
ENSG00000235864	HSPA8P6	heat shock protein family A (Hsp70) member 8 pseudogene 6	130195	0	NA	NA	NA	NA	NA
ENSG00000235868	NA	NA	NA	0	NA	NA	NA	NA	NA
ENSG00000235869	RPS26P12	ribosomal protein S26 pseudogene 12	100271104	0	NA	NA	NA	NA	NA
ENSG00000235871	NA	NA	NA	0	NA	NA	NA	NA	NA
ENSG00000235876	NA	NA	NA	0	NA	NA	NA	NA	NA
ENSG00000235879	NA	NA	NA	0	NA	NA	NA	NA	NA
ENSG00000235892	NA	NA	NA	0	NA	NA	NA	NA	NA

ENSG00000235893	NA	NA	NA	0,622301059	-0,134723893	2,255299	-0,059736593	0,952365427	NA
ENSG00000235895	NA	NA	NA	0	NA	NA	NA	NA	NA
ENSG00000235901	NA	NA	NA	0	NA	NA	NA	NA	NA
ENSG00000235907	LOC100422287	proteasome 20S subunit alpha 3 pseudogene	100422287	0	NA	NA	NA	NA	NA
ENSG00000235912	NA	NA	NA	0	NA	NA	NA	NA	NA
ENSG00000235916	MRPS18CP7	MRPS18C pseudogene 7	100288560	0	NA	NA	NA	NA	NA
ENSG00000235917	MTCO2P11	MT-CO2 pseudogene 11	107075214	1,437304052	-1,049945123	1,556148	-0,67470788	0,499861377	NA
ENSG00000235920	THRAP3P3	THRAP3 pseudogene 3	100421595	0	NA	NA	NA	NA	NA
ENSG00000235922	RPL13P14	ribosomal protein L13 pseudogene 14	100130374	0	NA	NA	NA	NA	NA
ENSG00000235924	NA	NA	NA	0	NA	NA	NA	NA	NA
ENSG00000235926	NA	NA	NA	0	NA	NA	NA	NA	NA
ENSG00000235929	NA	NA	NA	0,15836288	0	4,080456	0	1	NA
ENSG00000235930	NA	NA	NA	0	NA	NA	NA	NA	NA
ENSG00000235932	NA	NA	NA	0	NA	NA	NA	NA	NA
ENSG00000235937	NA	NA	NA	0	NA	NA	NA	NA	NA
ENSG00000235940	MTND1P21	MT-ND1 pseudogene 21	100873340	0	NA	NA	NA	NA	NA
ENSG00000235942	LCE6A	late cornified envelope 6A	448835	0	NA	NA	NA	NA	NA
ENSG00000235944	NA	NA	NA	11,48249805	0,500438969	0,54911	0,911363644	0,362103803	NA
ENSG00000235945	NA	NA	NA	0	NA	NA	NA	NA	NA
ENSG00000235946	NA	NA	NA	1,267385743	0,251756558	1,61178	0,156197801	0,87587711	NA
ENSG00000235951	LOC100419685	zinc finger protein 555 pseudogene	100419685	0	NA	NA	NA	NA	NA
ENSG00000235955	NA	NA	NA	0	NA	NA	NA	NA	NA
ENSG00000235957	NA	NA	NA	0	NA	NA	NA	NA	NA
ENSG00000235961	PNMA6A	PNMA family member 6A	84968	0	NA	NA	NA	NA	NA
ENSG00000235962	RPL7AP53	ribosomal protein L7a pseudogene 53	401640	0	NA	NA	NA	NA	NA
ENSG00000235963	MCCD1P1	mitochondrial coiled-coil domain 1 pseudogene 1	100048903	0	NA	NA	NA	NA	NA
ENSG00000235964	NA	NA	NA	0	NA	NA	NA	NA	NA
ENSG00000235967	NA	NA	NA	0	NA	NA	NA	NA	NA
ENSG00000235968	NA	NA	NA	0	NA	NA	NA	NA	NA
ENSG00000235969	CHEK2P4	CHEK2 pseudogene 4	106479021	0	NA	NA	NA	NA	NA
ENSG00000235974	VN2R19P	vomeronasal 2 receptor 19, pseudogene	100033407	0	NA	NA	NA	NA	NA
ENSG00000235975	LOC100130620	tropomyosin 2 pseudogene	100130620	0	NA	NA	NA	NA	NA
ENSG00000235976	H2BP8	H2B histone pseudogene 8	115482725	0	NA	NA	NA	NA	NA
ENSG00000235981	NA	NA	NA	0	NA	NA	NA	NA	NA
ENSG00000235988	NA	NA	NA	0	NA	NA	NA	NA	NA
ENSG00000235990	RPL23AP20	ribosomal protein L23a pseudogene 20	645962	0	NA	NA	NA	NA	NA
ENSG00000235991	NA	NA	NA	0,15836288	0	4,080456	0	1	NA
ENSG00000235992	NA	NA	NA	0	NA	NA	NA	NA	NA
ENSG00000235993	NA	NA	NA	0	NA	NA	NA	NA	NA
ENSG00000235995	UBE2V1P6	UBE2V1 pseudogene 6	100287279	0	NA	NA	NA	NA	NA
ENSG00000235998	TAAR7P	trace amine associated receptor 7, pseudogene	503611	0	NA	NA	NA	NA	NA
ENSG00000236004	RPL9P12	ribosomal protein L9 pseudogene 12	100270825	0,103998153	-1,076443944	4,080456	-0,263804824	0,791930317	NA
ENSG00000236005	NA	NA	NA	0	NA	NA	NA	NA	NA
ENSG00000236007	MTCO1P46	MT-CO1 pseudogene 46	107075175	0	NA	NA	NA	NA	NA
ENSG00000236012	NA	NA	NA	0	NA	NA	NA	NA	NA
ENSG00000236015	NA	NA	NA	0	NA	NA	NA	NA	NA
ENSG00000236018	NA	NA	NA	0	NA	NA	NA	NA	NA
ENSG00000236025	NA	NA	NA	0	NA	NA	NA	NA	NA
ENSG00000236027	PATE3	prostate and testis expressed 3	100169851	0	NA	NA	NA	NA	NA
ENSG00000236032	OR5H14	olfactory receptor family 5 subfamily H member 14	403273	0	NA	NA	NA	NA	NA
ENSG00000236035	NA	NA	NA	0	NA	NA	NA	NA	NA
ENSG00000236040	CHIAP1	chitinase, acidic pseudogene 1	100420342	0	NA	NA	NA	NA	NA
ENSG00000236042	LOC100129947	zinc finger protein 674 pseudogene	100129947	0	NA	NA	NA	NA	NA
ENSG00000236044	NA	NA	NA	0	NA	NA	NA	NA	NA
ENSG00000236047	RPL13AP12	ribosomal protein L13a pseudogene 12	100271277	1,017580132	0,512540984	2,329258	0,220044791	0,825836271	NA
ENSG00000236048	SSBL5P	small RNA binding exonuclease protection factor La like 5, pseudogene	389465	0	NA	NA	NA	NA	NA
ENSG00000236058	RPL17P36	ribosomal protein L17 pseudogene 36	729340	0	NA	NA	NA	NA	NA
ENSG00000236060	NA	NA	NA	0	NA	NA	NA	NA	NA
ENSG00000236062	NA	NA	NA	0	NA	NA	NA	NA	NA
ENSG00000236068	NA	NA	NA	0	NA	NA	NA	NA	NA
ENSG00000236072	SRSF2P1	SRSF2 pseudogene 1	392439	0	NA	NA	NA	NA	NA
ENSG00000236073	NA	NA	NA	0	NA	NA	NA	NA	NA

ENSG00000236077	SLC6A14P1	SLC6A14 pseudogene 1	100422432	0	NA	NA	NA	NA	NA
ENSG00000236079	NA	NA	NA	0	NA	NA	NA	NA	NA
ENSG00000236080	NA	NA	NA	0	NA	NA	NA	NA	NA
ENSG00000236083	OR13E1P	olfactory receptor family 13 subfamily E member 1 pseudogene	392308	0	NA	NA	NA	NA	NA
ENSG00000236085	NA	NA	NA	0,23616048	-2,075648354	4,028973	-0,515180498	0,606426895	NA
ENSG00000236086	NA	NA	NA	0,85401672	-0,107835941	2,756253	-0,039124115	0,968791434	NA
ENSG00000236090	NA	NA	NA	0	NA	NA	NA	NA	NA
ENSG00000236097	BNIP3P2	BCL2 interacting protein 3 pseudogene 2	100421754	0	NA	NA	NA	NA	NA
ENSG00000236099	NA	NA	NA	0	NA	NA	NA	NA	NA
ENSG00000236101	RAC1P7	Rac family small GTPase 1 pseudogene 7	100874390	0	NA	NA	NA	NA	NA
ENSG00000236104	ZBTB22	zinc finger and BTB domain containing 22	9278	1,196738772	4,311091181	3,929692	1,097055628	0,272617076	NA
ENSG00000236105	NA	NA	NA	0	NA	NA	NA	NA	NA
ENSG00000236108	LOC124904585	signal peptidase complex subunit 1-like	124904585	0	NA	NA	NA	NA	NA
ENSG00000236110	OR2AM1P	olfactory receptor family 2 subfamily AM member 1 pseudogene	81366	0	NA	NA	NA	NA	NA
ENSG00000236111	RPL6P1	ribosomal protein L6 pseudogene 1	440587	0	NA	NA	NA	NA	NA
ENSG00000236114	LOC100130881	zinc finger protein 12 pseudogene	100130881	0,948419499	0,050457632	1,864006	0,027069458	0,978404335	NA
ENSG00000236118	NA	NA	NA	0	NA	NA	NA	NA	NA
ENSG00000236121	NA	NA	NA	0	NA	NA	NA	NA	NA
ENSG00000236123	NA	NA	NA	0	NA	NA	NA	NA	NA
ENSG00000236124	NA	NA	NA	0,10488184	-1,076443944	4,080456	-0,263804824	0,791930317	NA
ENSG00000236125	USP17L8	ubiquitin specific peptidase 17 like family member 8	392188	0	NA	NA	NA	NA	NA
ENSG00000236125	USP17L4	ubiquitin specific peptidase 17 like family member 4	645402	0	NA	NA	NA	NA	NA
ENSG00000236131	MED13P1	mediator complex subunit 13 pseudogene 1	100499481	0	NA	NA	NA	NA	NA
ENSG00000236132	NA	NA	NA	0	NA	NA	NA	NA	NA
ENSG00000236136	NA	NA	NA	0	NA	NA	NA	NA	NA
ENSG00000236138	DUX4L26	double homeobox 4 like 26 (pseudogene)	100996350	0	NA	NA	NA	NA	NA
ENSG00000236146	NA	NA	NA	0,103998153	-1,076443944	4,080456	-0,263804824	0,791930317	NA
ENSG00000236148	RPL23AP37	ribosomal protein L23a pseudogene 37	130773	0	NA	NA	NA	NA	NA
ENSG00000236152	NA	NA	NA	0	NA	NA	NA	NA	NA
ENSG00000236155	ZPLD2P	zona pellucida like domain containing 2, pseudogene	101928303	0	NA	NA	NA	NA	NA
ENSG00000236156	NA	NA	NA	0,566679733	0,735811257	3,940022	0,186753091	0,851854228	NA
ENSG00000236158	RFC3P1	replication factor C 3 pseudogene 1	100131711	0	NA	NA	NA	NA	NA
ENSG00000236159	LOC100419562	ribosomal protein L13 pseudogene	100419562	0,39345653	1,884837918	4,025332	0,468244142	0,639610009	NA
ENSG00000236160	NA	NA	NA	0	NA	NA	NA	NA	NA
ENSG00000236165	NA	NA	NA	0	NA	NA	NA	NA	NA
ENSG00000236167	GAPDHP57	glyceraldehyde 3 phosphate dehydrogenase pseudogene 57	100421188	0	NA	NA	NA	NA	NA
ENSG00000236168	NA	NA	NA	0	NA	NA	NA	NA	NA
ENSG00000236171	RPL12P26	ribosomal protein L12 pseudogene 26	100271404	0	NA	NA	NA	NA	NA
ENSG00000236175	SSU72P6	SSU72 pseudogene 6	643263	0	NA	NA	NA	NA	NA
ENSG00000236180	NA	NA	NA	0,10543123	0	4,080456	0	1	NA
ENSG00000236182	NA	NA	NA	0	NA	NA	NA	NA	NA
ENSG00000236184	NA	NA	NA	0,590655374	-1,917759325	2,834639	-0,676544483	0,498695014	NA
ENSG00000236185	HNRNPDLP3	HNRNPDL pseudogene 3	106481662	0	NA	NA	NA	NA	NA
ENSG00000236187	NA	NA	NA	0	NA	NA	NA	NA	NA
ENSG00000236189	RPL18AP15	ribosomal protein L18a pseudogene 15	392522	0	NA	NA	NA	NA	NA
ENSG00000236190	NA	NA	NA	0	NA	NA	NA	NA	NA
ENSG00000236191	RPS15AP25	ribosomal protein S15a pseudogene 25	728774	0	NA	NA	NA	NA	NA
ENSG00000236195	MRM3P2	MRM3 pseudogene 2	100420223	0	NA	NA	NA	NA	NA
ENSG00000236205	LOC100533669	MAGE family member A10 pseudogene	100533669	0	NA	NA	NA	NA	NA
ENSG00000236211	MTCO1P7	MT-CO1 pseudogene 7	107075263	0	NA	NA	NA	NA	NA
ENSG00000236216	NA	NA	NA	0	NA	NA	NA	NA	NA
ENSG00000236217	NA	NA	NA	0,118286652	0	4,080456	0	1	NA
ENSG00000236229	NA	NA	NA	0	NA	NA	NA	NA	NA
ENSG00000236232	MTCYBP24	MT-CYB pseudogene 24	107075132	0	NA	NA	NA	NA	NA
ENSG00000236235	NA	NA	NA	0	NA	NA	NA	NA	NA

ENSG00000236238	RPS14P10	ribosomal protein S14 pseudogene 10	647034	0	NA	NA	NA	NA	NA
ENSG00000236241	DDX5P1	DDX5 pseudogene 1	100421238	0	NA	NA	NA	NA	NA
ENSG00000236243	RPL6P29	ribosomal protein L6 pseudogene 29	392505	0	NA	NA	NA	NA	NA
ENSG00000236246	NA	NA	NA	0	NA	NA	NA	NA	NA
ENSG00000236248	NA	NA	NA	0	NA	NA	NA	NA	NA
ENSG00000236252	LOC101928381	uncharacterized LOC101928381	101928381	0	NA	NA	NA	NA	NA
ENSG00000236253	SLC25A3P1	solute carrier family 25 member 3 pseudogene 1	163742	0	NA	NA	NA	NA	NA
ENSG00000236254	MTND4P14	MT-ND4 pseudogene 14	100653489	5,591777732	0,353072471	0,742754	0,47535591	0,634533315	NA
ENSG00000236257	NA	NA	NA	0	NA	NA	NA	NA	NA
ENSG00000236258	NA	NA	NA	0	NA	NA	NA	NA	NA
ENSG00000236259	PPIAP64	peptidylprolyl isomerase A pseudogene 64	100287361	0	NA	NA	NA	NA	NA
ENSG00000236261	NA	NA	NA	0	NA	NA	NA	NA	NA
ENSG00000236264	NA	NA	NA	0	NA	NA	NA	NA	NA
ENSG00000236274	NA	NA	NA	0	NA	NA	NA	NA	NA
ENSG00000236277	NA	NA	NA	0	NA	NA	NA	NA	NA
ENSG00000236278	NA	NA	NA	0,103998153	-1,076443944	4,080456	-0,263804824	0,791930317	NA
ENSG00000236279	CLEC2L	C-type lectin domain family 2 member L	154790	0	NA	NA	NA	NA	NA
ENSG00000236281	NA	NA	NA	0	NA	NA	NA	NA	NA
ENSG00000236284	VN1R31P	vomeronasal 1 receptor 31 pseudogene	100312787	0	NA	NA	NA	NA	NA
ENSG00000236285	NA	NA	NA	0,10488184	-1,076443944	4,080456	-0,263804824	0,791930317	NA
ENSG00000236290	EEF1GP7	eukaryotic translation elongation factor 1 gamma pseudogene 7	645311	0	NA	NA	NA	NA	NA
ENSG00000236296	GUSBP5	GUSB pseudogene 5	441046	2,093417773	1,236561947	1,469779	0,841324917	0,400165936	NA
ENSG00000236297	EEF1A1P23	eukaryotic translation elongation factor 1 alpha 1 pseudogene 23	100421815	3,017055441	0,765537498	1,051073	0,728338957	0,46640612	NA
ENSG00000236300	MTND4LP12	MT-ND4L pseudogene 12	107075239	0	NA	NA	NA	NA	NA
ENSG00000236307	NA	NA	NA	0	NA	NA	NA	NA	NA
ENSG00000236312	NA	NA	NA	0	NA	NA	NA	NA	NA
ENSG00000236313	VN1R53P	vomeronasal 1 receptor 53 pseudogene	100312806	0	NA	NA	NA	NA	NA
ENSG00000236316	OR7E109P	olfactory receptor family 7 subfamily E member 109 pseudogene	401540	0	NA	NA	NA	NA	NA
ENSG00000236317	RPS28P2	ribosomal protein S28 pseudogene 2	100271120	0,072165449	0,847101401	4,080456	0,207599697	0,835541541	NA
ENSG00000236319	LOC100418888	peptidase D pseudogene	100418888	0	NA	NA	NA	NA	NA
ENSG00000236320	SLFN14	schlafen family member 14	342618	0,117497108	0,847101401	4,080456	0,207599697	0,835541541	NA
ENSG00000236323	NA	NA	NA	0	NA	NA	NA	NA	NA
ENSG00000236325	NA	NA	NA	0,10543123	0	4,080456	0	1	NA
ENSG00000236327	NA	NA	NA	0	NA	NA	NA	NA	NA
ENSG00000236330	RPL5P9	ribosomal protein L5 pseudogene 9	100270832	0,339045525	-1,085476267	4,080456	-0,266018382	0,790225052	NA
ENSG00000236334	PPIAL4G	peptidylprolyl isomerase A like 4G	644591	0	NA	NA	NA	NA	NA
ENSG00000236339	POM121L13P	POM121 transmembrane nucleoporin like 13, pseudogene	100874379	0	NA	NA	NA	NA	NA
ENSG00000236343	NA	NA	NA	0	NA	NA	NA	NA	NA
ENSG00000236348	PSMC1P10	proteasome 26S subunit, ATPase 1 pseudogene 10	388925	0	NA	NA	NA	NA	NA
ENSG00000236349	NA	NA	NA	0	NA	NA	NA	NA	NA
ENSG00000236357	EI24P1	EI24 pseudogene 1	100129423	0	NA	NA	NA	NA	NA
ENSG00000236359	OR51B8P	olfactory receptor family 51 subfamily B member 8 pseudogene	403266	0	NA	NA	NA	NA	NA
ENSG00000236360	NA	NA	NA	0	NA	NA	NA	NA	NA
ENSG00000236362	GAGE2C	G antigen 2C	2574	0	NA	NA	NA	NA	NA
ENSG00000236362	GAGE4	G antigen 4	2576	0	NA	NA	NA	NA	NA
ENSG00000236362	GAGE6	G antigen 6	2578	0	NA	NA	NA	NA	NA
ENSG00000236362	GAGE7	G antigen 7	2579	0	NA	NA	NA	NA	NA
ENSG00000236362	GAGE12I	G antigen 12I	26748	0	NA	NA	NA	NA	NA
ENSG00000236362	GAGE12F	G antigen 12F	100008586	0	NA	NA	NA	NA	NA
ENSG00000236362	LOC124908552	G antigen 4	124908552	0	NA	NA	NA	NA	NA
ENSG00000236362	LOC124908553	G antigen 7-like	124908553	0	NA	NA	NA	NA	NA
ENSG00000236362	LOC124908554	G antigen 7-like	124908554	0	NA	NA	NA	NA	NA
ENSG00000236362	LOC124908555	G antigen 5	124908555	0	NA	NA	NA	NA	NA

ENSG00000236362	LOC124908556	G antigen 7-like	124908556	0	NA	NA	NA	NA	NA
ENSG00000236365	NA	NA	NA	0	NA	NA	NA	NA	NA
ENSG00000236371	CT47A1	cancer/testis antigen family 47 member A1	728096	0	NA	NA	NA	NA	NA
ENSG00000236375	NA	NA	NA	2,692992166	0,086311078	1,710359	0,05046372	0,95975286	NA
ENSG00000236379	NA	NA	NA	0	NA	NA	NA	NA	NA
ENSG00000236380	NA	NA	NA	0	NA	NA	NA	NA	NA
ENSG00000236382	NA	NA	NA	0	NA	NA	NA	NA	NA
ENSG00000236383	CCDC200	coiled-coil domain containing 200	100874261	0	NA	NA	NA	NA	NA
ENSG00000236386	NA	NA	NA	0	NA	NA	NA	NA	NA
ENSG00000236391	NA	NA	NA	0	NA	NA	NA	NA	NA
ENSG00000236395	RPS15AP22	ribosomal protein S15a pseudogene 22	100271557	0	NA	NA	NA	NA	NA
ENSG00000236396	SLC35G4	solute carrier family 35 member G4	646000	0	NA	NA	NA	NA	NA
ENSG00000236397	NA	NA	NA	4,524113256	0,584044921	0,876749	0,666148567	0,505316144	NA
ENSG00000236398	TAS2R39	taste 2 receptor member 39	259285	0	NA	NA	NA	NA	NA
ENSG00000236400	KRTAP21-4P	keratin associated protein 21-4, pseudogene	337986	0	NA	NA	NA	NA	NA
ENSG00000236401	SLC4A1APP2	solute carrier family 4 member 1 adaptor protein pseudogene 2	100422527	0	NA	NA	NA	NA	NA
ENSG00000236407	NA	NA	NA	0	NA	NA	NA	NA	NA
ENSG00000236409	NA	NA	NA	0,470322675	1,645763571	2,98561	0,551231945	0,581474684	NA
ENSG00000236413	LOC107985643	TSC22 domain family protein 3-like	107985643	0	NA	NA	NA	NA	NA
ENSG00000236417	NA	NA	NA	0	NA	NA	NA	NA	NA
ENSG00000236420	NA	NA	NA	0	NA	NA	NA	NA	NA
ENSG00000236424	TSPY10	testis specific protein Y-linked 10	100289087	0	NA	NA	NA	NA	NA
ENSG00000236424	LOC124905614	testis-specific Y-encoded protein 3	124905614	0	NA	NA	NA	NA	NA
ENSG00000236424	LOC124905615	testis-specific Y-encoded protein 3	124905615	0	NA	NA	NA	NA	NA
ENSG00000236424	LOC124905616	testis-specific Y-encoded protein 3	124905616	0	NA	NA	NA	NA	NA
ENSG00000236424	LOC124905617	testis-specific Y-encoded protein 3	124905617	0	NA	NA	NA	NA	NA
ENSG00000236424	LOC124905618	testis-specific Y-encoded protein 3-like	124905618	0	NA	NA	NA	NA	NA
ENSG00000236424	LOC124905619	testis-specific Y-encoded protein 3-like	124905619	0	NA	NA	NA	NA	NA
ENSG00000236424	LOC124905620	testis-specific Y-encoded protein 3	124905620	0	NA	NA	NA	NA	NA
ENSG00000236424	LOC124905621	testis-specific Y-encoded protein 3	124905621	0	NA	NA	NA	NA	NA
ENSG00000236424	LOC124905623	testis-specific Y-encoded protein 1	124905623	0	NA	NA	NA	NA	NA
ENSG00000236424	LOC124905624	testis-specific Y-encoded protein 3	124905624	0	NA	NA	NA	NA	NA
ENSG00000236424	LOC124905625	testis-specific Y-encoded protein 3	124905625	0	NA	NA	NA	NA	NA
ENSG00000236424	LOC124905626	testis-specific Y-encoded protein 2	124905626	0	NA	NA	NA	NA	NA
ENSG00000236424	LOC124905627	testis-specific Y-encoded protein 4-like	124905627	0	NA	NA	NA	NA	NA
ENSG00000236424	LOC124905628	testis-specific Y-encoded protein 3	124905628	0	NA	NA	NA	NA	NA
ENSG00000236424	LOC124905629	testis-specific Y-encoded protein 3-like	124905629	0	NA	NA	NA	NA	NA
ENSG00000236424	LOC124905630	testis-specific Y-encoded protein 3-like	124905630	0	NA	NA	NA	NA	NA
ENSG00000236424	LOC124908978	testis-specific Y-encoded protein 3	124908978	0	NA	NA	NA	NA	NA
ENSG00000236424	LOC124908979	testis-specific Y-encoded protein 3	124908979	0	NA	NA	NA	NA	NA
ENSG00000236424	LOC124908980	testis-specific Y-encoded protein 3	124908980	0	NA	NA	NA	NA	NA
ENSG00000236424	LOC124908981	testis-specific Y-encoded protein 3	124908981	0	NA	NA	NA	NA	NA
ENSG00000236424	LOC124909093	testis-specific Y-encoded protein 3-like	124909093	0	NA	NA	NA	NA	NA
ENSG00000236424	LOC124909197	testis-specific Y-encoded protein 3-like	124909197	0	NA	NA	NA	NA	NA
ENSG00000236424	LOC124909294	testis-specific Y-encoded protein 3	124909294	0	NA	NA	NA	NA	NA
ENSG00000236424	LOC124909306	testis-specific Y-encoded protein 3-like	124909306	0	NA	NA	NA	NA	NA

ENSG00000236424	LOC124909318	testis-specific Y-encoded protein 3-like	124909318	0	NA	NA	NA	NA	NA
ENSG00000236425	RPL31P64	ribosomal protein L31 pseudogene 64	127566430	0	NA	NA	NA	NA	NA
ENSG00000236429	NA	NA	NA	0	NA	NA	NA	NA	NA
ENSG00000236430	NA	NA	NA	0	NA	NA	NA	NA	NA
ENSG00000236433	RPL37P21	ribosomal protein L37 pseudogene 21	100271516	0	NA	NA	NA	NA	NA
ENSG00000236435	TSPY12P	testis specific protein Y-linked 12, pseudogene	377997	0	NA	NA	NA	NA	NA
ENSG00000236438	NA	NA	NA	6,922723732	-0,070059747	2,813216	-0,024903788	0,980131705	NA
ENSG00000236439	NA	NA	NA	0,503619263	-0,147320739	2,994551	-0,049196272	0,960762883	NA
ENSG00000236440	NA	NA	NA	0	NA	NA	NA	NA	NA
ENSG00000236442	NA	NA	NA	0	NA	NA	NA	NA	NA
ENSG00000236444	UBE2L5	ubiquitin conjugating enzyme E2 L5	171222	1,748058295	-3,226810307	2,560701	-1,260127574	0,207623345	NA
ENSG00000236446	CT47B1	cancer/testis antigen family 47 member B1	643311	0	NA	NA	NA	NA	NA
ENSG00000236450	NA	NA	NA	0	NA	NA	NA	NA	NA
ENSG00000236456	NA	NA	NA	0	NA	NA	NA	NA	NA
ENSG00000236459	NA	NA	NA	0	NA	NA	NA	NA	NA
ENSG00000236460	SNX2P2	sorting nexin 2 pseudogene 2	100873785	0	NA	NA	NA	NA	NA
ENSG00000236468	EEF1A1P44	eukaryotic translation elongation factor 1 alpha 1 pseudogene 44	100421840	0	NA	NA	NA	NA	NA
ENSG00000236473	NA	NA	NA	0	NA	NA	NA	NA	NA
ENSG00000236474	NA	NA	NA	0,189802672	1,665804421	4,047502	0,41156363	0,680659294	NA
ENSG00000236476	NA	NA	NA	0	NA	NA	NA	NA	NA
ENSG00000236477	NA	NA	NA	0	NA	NA	NA	NA	NA
ENSG00000236478	NA	NA	NA	0	NA	NA	NA	NA	NA
ENSG00000236480	NA	NA	NA	0	NA	NA	NA	NA	NA
ENSG00000236483	MTND2P40	MT-ND2 pseudogene 40	107075280	0	NA	NA	NA	NA	NA
ENSG00000236484	RRM2P2	ribonucleotide reductase M2 polypeptide pseudogene 2	6244	0	NA	NA	NA	NA	NA
ENSG00000236487	LOC100288233	ribosomal protein L23 pseudogene	100288233	0	NA	NA	NA	NA	NA
ENSG00000236489	RPL34P10	ribosomal protein L34 pseudogene 10	100271004	0,49889932	3,052330952	3,190289	0,956756778	0,338690033	NA
ENSG00000236491	NA	NA	NA	0	NA	NA	NA	NA	NA
ENSG00000236493	NA	NA	NA	2,092532	-0,34881627	1,277566	-0,273031936	0,784828667	NA
ENSG00000236496	GPS2P1	G protein pathway suppressor 2 pseudogene 1	392281	0,11808024	-1,076443944	4,080456	-0,263804824	0,791930317	NA
ENSG00000236500	NA	NA	NA	0	NA	NA	NA	NA	NA
ENSG00000236503	TRPC6P8	TRPC6 pseudogene 8	123464512	0	NA	NA	NA	NA	NA
ENSG00000236504	NA	NA	NA	0	NA	NA	NA	NA	NA
ENSG00000236505	NA	NA	NA	0	NA	NA	NA	NA	NA
ENSG00000236509	RPL21P133	ribosomal protein L21 pseudogene 133	100271391	0	NA	NA	NA	NA	NA
ENSG00000236512	RPL29P1	ribosomal protein L29 pseudogene 1	134756	0,235788685	0,852920862	4,080355	0,209031064	0,834423989	NA
ENSG00000236516	NA	NA	NA	0	NA	NA	NA	NA	NA
ENSG00000236521	NPAP1P4	nuclear pore associated protein 1 pseudogene 4	100533674	0	NA	NA	NA	NA	NA
ENSG00000236523	NPM1P40	nucleophosmin 1 pseudogene 40	100289584	0,326944382	-1,935930596	3,78013	-0,512133375	0,608557668	NA
ENSG00000236527	NA	NA	NA	0	NA	NA	NA	NA	NA
ENSG00000236530	KPNA2P1	karyopherin subunit alpha 2 pseudogene 1	643513	0	NA	NA	NA	NA	NA
ENSG00000236533	NA	NA	NA	0,11808024	-1,076443944	4,080456	-0,263804824	0,791930317	NA
ENSG00000236534	H3P36	H3 histone pseudogene 36	100287087	0,262822372	1,424998606	4,061397	0,350864117	0,725690292	NA
ENSG00000236538	NA	NA	NA	0	NA	NA	NA	NA	NA
ENSG00000236539	NA	NA	NA	0,398517391	-0,12433301	3,019841	-0,041172034	0,967158748	NA
ENSG00000236541	VN2R9P	vomeronasal 2 receptor 9, pseudogene	648315	0	NA	NA	NA	NA	NA
ENSG00000236542	NA	NA	NA	0	NA	NA	NA	NA	NA
ENSG00000236543	LOC102723971	lipocalin 1-like	102723971	0	NA	NA	NA	NA	NA
ENSG00000236549	NA	NA	NA	0	NA	NA	NA	NA	NA
ENSG00000236550	NA	NA	NA	0	NA	NA	NA	NA	NA
ENSG00000236552	RPL13AP5	ribosomal protein L13a pseudogene 5	728658	0,328249897	-1,080366252	3,802203	-0,284142215	0,776301397	NA
ENSG00000236554	ASNSP3	ASNS pseudogene 3	100423061	0	NA	NA	NA	NA	NA
ENSG00000236555	NA	NA	NA	0	NA	NA	NA	NA	NA
ENSG00000236562	NA	NA	NA	0	NA	NA	NA	NA	NA
ENSG00000236564	YWHAQP5	YWHAQ pseudogene 5	100130391	0	NA	NA	NA	NA	NA
ENSG00000236565	HNRNPA3P5	heterogeneous nuclear ribonucleoprotein A3 pseudogene 5	387933	0,391755523	1,91454375	4,024655	0,47570381	0,634285406	NA
ENSG00000236567	NA	NA	NA	3,505801628	0,465603922	0,954029	0,488039774	0,625521669	NA
ENSG00000236569	NA	NA	NA	0	NA	NA	NA	NA	NA

ENSG00000236570	RAD23BP1	RAD23B pseudogene 1	131185	0	NA	NA	NA	NA	NA
ENSG00000236571	NA	NA	NA	0	NA	NA	NA	NA	NA
ENSG00000236574	LOC112267990	translation machinery-associated protein 7-like	112267990	0	NA	NA	NA	NA	NA
ENSG00000236576	LOC100631242	zinc finger protein 852 pseudogene	100631242	4,0088454	0,262517932	0,922366	0,284613586	0,775940201	NA
ENSG00000236577	SNRPGP14	small nuclear ribonucleoprotein polypeptide G pseudogene 14	100874434	0	NA	NA	NA	NA	NA
ENSG00000236580	NA	NA	NA	0	NA	NA	NA	NA	NA
ENSG00000236582	PRPF38AP2	PRP38 domain containing A pseudogene 2	100131947	0,234994216	1,944111989	4,02324	0,483220515	0,62893917	NA
ENSG00000236583	NA	NA	NA	0	NA	NA	NA	NA	NA
ENSG00000236590	MTATP6P4	MT-ATP6 pseudogene 4	106480797	0	NA	NA	NA	NA	NA
ENSG00000236592	NA	NA	NA	0	NA	NA	NA	NA	NA
ENSG00000236594	RPS27AP14	RPS27A pseudogene 14	100271378	0	NA	NA	NA	NA	NA
ENSG00000236596	NA	NA	NA	0	NA	NA	NA	NA	NA
ENSG00000236603	NA	NA	NA	0	NA	NA	NA	NA	NA
ENSG00000236604	NA	NA	NA	0	NA	NA	NA	NA	NA
ENSG00000236606	NA	NA	NA	0	NA	NA	NA	NA	NA
ENSG00000236607	EEF1DP8	eukaryotic translation elongation factor 1 delta pseudogene 8	283236	0	NA	NA	NA	NA	NA
ENSG00000236608	EIF4A1P6	eukaryotic translation initiation factor 4A1 pseudogene 6	390407	0	NA	NA	NA	NA	NA
ENSG00000236610	SOCS5P1	suppressor of cytokine signaling 5 pseudogene 1	100420754	0	NA	NA	NA	NA	NA
ENSG00000236612	NA	NA	NA	0	NA	NA	NA	NA	NA
ENSG00000236615	NA	NA	NA	0	NA	NA	NA	NA	NA
ENSG00000236616	NA	NA	NA	0	NA	NA	NA	NA	NA
ENSG00000236620	NA	NA	NA	0	NA	NA	NA	NA	NA
ENSG00000236626	MTND5P17	MT-ND5 pseudogene 17	100873353	0	NA	NA	NA	NA	NA
ENSG00000236636	NA	NA	NA	0	NA	NA	NA	NA	NA
ENSG00000236637	IFNA4	interferon alpha 4	3441	0	NA	NA	NA	NA	NA
ENSG00000236638	NA	NA	NA	0	NA	NA	NA	NA	NA
ENSG00000236646	NA	NA	NA	0	NA	NA	NA	NA	NA
ENSG00000236647	NA	NA	NA	0	NA	NA	NA	NA	NA
ENSG00000236654	NA	NA	NA	0	NA	NA	NA	NA	NA
ENSG00000236655	SLC6A14P3	SLC6A14 pseudogene 3	100422417	0	NA	NA	NA	NA	NA
ENSG00000236667	RPL27AP3	ribosomal protein L27a pseudogene 3	100271298	0	NA	NA	NA	NA	NA
ENSG00000236670	KRT18P5	keratin 18 pseudogene 5	728300	5,210027686	-0,932438755	1,108787	-0,840954269	0,400373554	NA
ENSG00000236674	LOC441887	DnaJ heat shock protein family (Hsp40) member C7 pseudogene	441887	0	NA	NA	NA	NA	NA
ENSG00000236679	RPL23AP24	ribosomal protein L23a pseudogene 24	728517	1,142192377	-0,698889672	1,890929	-0,369601303	0,71167958	NA
ENSG00000236680	PSMC3P1	proteasome 26S subunit, ATPase 3 pseudogene 1	5703	0	NA	NA	NA	NA	NA
ENSG00000236681	NA	NA	NA	0	NA	NA	NA	NA	NA
ENSG00000236683	NA	NA	NA	0	NA	NA	NA	NA	NA
ENSG00000236686	NA	NA	NA	0	NA	NA	NA	NA	NA
ENSG00000236689	MTCO2P21	MT-CO2 pseudogene 21	107075191	0	NA	NA	NA	NA	NA
ENSG00000236690	NA	NA	NA	0	NA	NA	NA	NA	NA
ENSG00000236691	NA	NA	NA	0	NA	NA	NA	NA	NA
ENSG00000236692	NA	NA	NA	0	NA	NA	NA	NA	NA
ENSG00000236695	NA	NA	NA	0	NA	NA	NA	NA	NA
ENSG00000236698	NA	NA	NA	0	NA	NA	NA	NA	NA
ENSG00000236699	ARHGEF38	Rho guanine nucleotide exchange factor 38	54848	0	NA	NA	NA	NA	NA
ENSG00000236701	LOC100422376	ArfGAP with GTPase domain, ankyrin repeat and PH domain 1 pseudogene	100422376	0	NA	NA	NA	NA	NA
ENSG00000236704	CNOT4P1	CCR4-NOT transcription complex subunit 4 pseudogene 1	100874389	0	NA	NA	NA	NA	NA
ENSG00000236710	NA	NA	NA	2,131891935	0,210148643	1,173465	0,17908384	0,857871871	NA
ENSG00000236712	NA	NA	NA	0	NA	NA	NA	NA	NA
ENSG00000236713	NA	NA	NA	0	NA	NA	NA	NA	NA
ENSG00000236717	NA	NA	NA	0	NA	NA	NA	NA	NA
ENSG00000236718	RBMY2QP	RNA binding motif protein Y-linked family 2 member Q, pseudogene	379006	0	NA	NA	NA	NA	NA
ENSG00000236722	RPL27P9	ribosomal protein L27 pseudogene 9	100270970	0,15836288	0	4,080456	0	1	NA
ENSG00000236731	NA	NA	NA	0	NA	NA	NA	NA	NA
ENSG00000236732	RPL21P137	ribosomal protein L21 pseudogene 137	127379693	0	NA	NA	NA	NA	NA

ENSG00000236735	RPL31P63	ribosomal protein L31 pseudogene 63	100271392	0	NA	NA	NA	NA	NA	
ENSG00000236736	UQCRC2P1	ubiquinol-cytochrome c reductase core protein 2 pseudogene 1	100131327	0	NA	NA	NA	NA	NA	
ENSG00000236737	NA	NA	NA	0	NA	NA	NA	NA	NA	
ENSG00000236739	NA	NA	NA	0	NA	NA	NA	NA	NA	
ENSG00000236741	NA	NA	NA	0	NA	NA	NA	NA	NA	
ENSG00000236745	YRDCP2	yrdC N(6)-threonylcarbamoyltransferase domain containing pseudogene 2	100128237	0	NA	NA	NA	NA	NA	
ENSG00000236748	RPL9P19	ribosomal protein L9 pseudogene 19	100129634	0	NA	NA	NA	NA	NA	
ENSG00000236761	CTAGE9	CTAGE family member 9	643854	0	NA	NA	NA	NA	NA	
ENSG00000236762	NA	NA	NA	0	NA	NA	NA	NA	NA	
ENSG00000236763	NA	NA	NA	0	NA	NA	NA	NA	NA	
ENSG00000236764	NA	NA	NA	0	NA	NA	NA	NA	NA	
ENSG00000236768	RPL17P16	ribosomal protein L17 pseudogene 16	729349	0	NA	NA	NA	NA	NA	
ENSG00000236773	NA	NA	NA	0	NA	NA	NA	NA	NA	
ENSG00000236775	LOC100419806	zinc finger protein 519 pseudogene	100419806	0	NA	NA	NA	NA	NA	
ENSG00000236776	RPL21P23	ribosomal protein L21 pseudogene 23	100270879	0	NA	NA	NA	NA	NA	
ENSG00000236777	NA	NA	NA	0	NA	NA	NA	NA	NA	
ENSG00000236779	NA	NA	NA	0	NA	NA	NA	NA	NA	
ENSG00000236782	ZNF593OS	ZNF593 opposite strand	118568799	0	NA	NA	NA	NA	NA	
ENSG00000236783	RPS15AP27	ribosomal protein S15a pseudogene 27	100271613	0	NA	NA	NA	NA	NA	
ENSG00000236785	SDR42E1P5	short chain dehydrogenase/reductase family 42E, member 1 pseudogene 5	100422339	0,459860188	-1,034720731	2,979502	-0,347279712	0,728381191	NA	
ENSG00000236786	NA	NA	NA	0	NA	NA	NA	NA	NA	
ENSG00000236791	OR2AI1P	olfactory receptor family 2 subfamily AI member 1 pseudogene	134082	0	NA	NA	NA	NA	NA	
ENSG00000236792	NA	NA	NA	0	NA	NA	NA	NA	NA	
ENSG00000236794	BCRP8	BCR pseudogene 8	100133044	0,117497108	0,847101401	4,080456	0,207599697	0,835541541	NA	
ENSG00000236796	NA	NA	NA	0	NA	NA	NA	NA	NA	
ENSG00000236797	NA	NA	NA	0	NA	NA	NA	NA	NA	
ENSG00000236801	RPL24P8	RPL24 pseudogene 8	100270964	0	NA	NA	NA	NA	NA	
ENSG00000236803	SDAD1P4	SDA1 domain containing 1 pseudogene 4	100419955	0	NA	NA	NA	NA	NA	
ENSG00000236804	RPS3AP12	RPS3A pseudogene 12	100270905	0,788851313	0,412166918	2,427705	0,169776335	0,865186039	NA	
ENSG00000236806	RPL7AP15	ribosomal protein L7a pseudogene 15	100270895	0,264176885	-1,061512417	4,080456	-0,260145545	0,794751507	NA	
ENSG00000236807	NA	NA	NA	0	NA	NA	NA	NA	NA	
ENSG00000236809	SNX25P1	sorting nexin 25 pseudogene 1	100419028	1,323122744	0,691668955	1,500816	0,460862044	0,644897585	NA	
ENSG00000236811	NA	NA	NA	0	NA	NA	NA	NA	NA	
ENSG00000236813	NA	NA	NA	0	NA	NA	NA	NA	NA	
ENSG00000236814	NA	NA	NA	0,460466194	-1,670624685	3,426155	-0,487609265	0,625826632	NA	
ENSG00000236816	NA	NA	NA	6,851166034	-0,418485675	0,752204	-0,556346279	0,577974156	NA	
ENSG00000236818	NA	NA	NA	0	NA	NA	NA	NA	NA	
ENSG00000236825	NA	NA	NA	0	NA	NA	NA	NA	NA	
ENSG00000236829	RPL23AP5	ribosomal protein L23a pseudogene 5	729480	0,11808024	-1,076443944	4,080456	-0,263804824	0,791930317	NA	
ENSG00000236829	LOC100134368	uncharacterized LOC100134368	100134368	0,11808024	-1,076443944	4,080456	-0,263804824	0,791930317	NA	
ENSG00000236831	YME1L1P1	YME1L1 pseudogene 1	23749	0	NA	NA	NA	NA	NA	
ENSG00000236832	CBY1P1	CBY1 pseudogene 1	100131562	0	NA	NA	NA	NA	NA	
ENSG00000236844	RPL24P6	RPL24 pseudogene 6	100129808	0,279748476	-1,044366323	4,080456	-0,255943541	0,797994433	NA	
ENSG00000236848	NA	NA	NA	0	NA	NA	NA	NA	NA	
ENSG00000236852	NA	NA	NA	0	NA	NA	NA	NA	NA	
ENSG00000236857	NA	NA	NA	0	NA	NA	NA	NA	NA	
ENSG00000236860	NA	NA	NA	0	NA	NA	NA	NA	NA	
ENSG00000236862	NA	NA	NA	0	NA	NA	NA	NA	NA	
ENSG00000236863	RPL23AP23	ribosomal protein L23a pseudogene 23	100271292	0,072165449	0,847101401	4,080456	0,207599697	0,835541541	NA	
ENSG00000236867	NA	NA	NA	0	NA	NA	NA	NA	NA	
ENSG00000236870	POMPP1	POMP pseudogene 1	101060113	0	NA	NA	NA	NA	NA	
ENSG00000236872	PPIAP36	peptidylprolyl isomerase A pseudogene 36	111082961	0,117497108	0,847101401	4,080456	0,207599697	0,835541541	NA	
ENSG00000236875	NA	NA	NA	0	NA	NA	NA	NA	NA	
ENSG00000236876	NA	NA	NA	0	NA	NA	NA	NA	NA	
ENSG00000236877	NA	NA	NA	0	NA	NA	NA	NA	NA	
ENSG00000236878	MTATP6P26	MT-ATP6 pseudogene 26	107075301	0	NA	NA	NA	NA	NA	

ENSG00000236880	RPS26P57	ribosomal protein S26 pseudogene 57	100271401	0	NA	NA	NA	NA	NA
ENSG00000236887	RPS15P1	ribosomal protein S15 pseudogene 1	728428	0	NA	NA	NA	NA	NA
ENSG00000236888	NA	NA	NA	0	NA	NA	NA	NA	NA
ENSG00000236890	NA	NA	NA	0	NA	NA	NA	NA	NA
ENSG00000236893	ASS1P7	argininosuccinate synthetase 1 pseudogene 7	339845	0	NA	NA	NA	NA	NA
ENSG00000236894	NA	NA	NA	0	NA	NA	NA	NA	NA
ENSG00000236900	NA	NA	NA	0	NA	NA	NA	NA	NA
ENSG00000236905	RPS5P4	RPS5 pseudogene 4	100129090	0,118286652	0	4,080456	0	1	NA
ENSG00000236907	NA	NA	NA	0	NA	NA	NA	NA	NA
ENSG00000236909	OR2P1P	olfactory receptor family 2 subfamily P member 1 pseudogene	442183	0	NA	NA	NA	NA	NA
ENSG00000236913	RPS13P3	ribosomal protein S13 pseudogene 3	100129718	0	NA	NA	NA	NA	NA
ENSG00000236917	DNAJA1P2	DnaJ heat shock protein family (Hsp40) member A1 pseudogene 2	100131207	0	NA	NA	NA	NA	NA
ENSG00000236919	PHKG1P3	phosphorylase kinase catalytic subunit gamma 1 pseudogene 3	5262	0	NA	NA	NA	NA	NA
ENSG00000236928	NA	NA	NA	0,307187724	2,350166012	3,995085	0,588264389	0,556354844	NA
ENSG00000236929	RPL35AP37	ribosomal protein L35a pseudogene 37	100128807	0	NA	NA	NA	NA	NA
ENSG00000236930	NA	NA	NA	0	NA	NA	NA	NA	NA
ENSG00000236932	NA	NA	NA	0	NA	NA	NA	NA	NA
ENSG00000236937	PTGES3P4	prostaglandin E synthase 3 pseudogene 4	106480775	0	NA	NA	NA	NA	NA
ENSG00000236940	NA	NA	NA	0	NA	NA	NA	NA	NA
ENSG00000236941	LOC391711	tripartite motif-containing protein 60-like	391711	0	NA	NA	NA	NA	NA
ENSG00000236942	NA	NA	NA	0	NA	NA	NA	NA	NA
ENSG00000236944	NA	NA	NA	0	NA	NA	NA	NA	NA
ENSG00000236946	HNRNPA1P70	heterogeneous nuclear ribonucleoprotein A1 pseudogene 70	341333	0,381587637	-0,129673679	3,665086	-0,035380804	0,971776092	NA
ENSG00000236956	NF1P8	neurofibromin 1 pseudogene 8	401007	0	NA	NA	NA	NA	NA
ENSG00000236957	NA	NA	NA	0	NA	NA	NA	NA	NA
ENSG00000236959	SULT1D1P	sulfotransferase family 1D member 1, pseudogene	133150	0	NA	NA	NA	NA	NA
ENSG00000236960	NA	NA	NA	0	NA	NA	NA	NA	NA
ENSG00000236965	OR52N3P	olfactory receptor family 52 subfamily N member 3 pseudogene	81251	0	NA	NA	NA	NA	NA
ENSG00000236966	NA	NA	NA	0	NA	NA	NA	NA	NA
ENSG00000236969	NA	NA	NA	0	NA	NA	NA	NA	NA
ENSG00000236972	NA	NA	NA	0	NA	NA	NA	NA	NA
ENSG00000236973	GAPDHP51	glyceraldehyde 3 phosphate dehydrogenase pseudogene 51	100421271	0	NA	NA	NA	NA	NA
ENSG00000236976	RPL6P20	ribosomal protein L6 pseudogene 20	285900	0	NA	NA	NA	NA	NA
ENSG00000236978	LOC100967223	neuroepithelial cell transforming 1 pseudogene	100967223	0	NA	NA	NA	NA	NA
ENSG00000236980	C3orf84	chromosome 3 open reading frame 84	646498	0	NA	NA	NA	NA	NA
ENSG00000236981	OR10G9	olfactory receptor family 10 subfamily G member 9	219870	0	NA	NA	NA	NA	NA
ENSG00000236982	NA	NA	NA	0	NA	NA	NA	NA	NA
ENSG00000236987	NA	NA	NA	0	NA	NA	NA	NA	NA
ENSG00000236988	NA	NA	NA	0	NA	NA	NA	NA	NA
ENSG00000236992	RPL12P12	ribosomal protein L12 pseudogene 12	85824	0	NA	NA	NA	NA	NA
ENSG00000236993	NA	NA	NA	0	NA	NA	NA	NA	NA
ENSG00000236994	YBX1P9	Y-box binding protein 1 pseudogene 9	100861470	0	NA	NA	NA	NA	NA
ENSG00000236998	NA	NA	NA	0	NA	NA	NA	NA	NA
ENSG00000237000	NA	NA	NA	0	NA	NA	NA	NA	NA
ENSG00000237003	RPL36AP11	ribosomal protein L36a pseudogene 11	100129180	0,247839996	0,405324059	4,060556	0,099819854	0,920487345	NA
ENSG00000237004	NA	NA	NA	0,15836288	0	4,080456	0	1	NA
ENSG00000237005	NA	NA	NA	0	NA	NA	NA	NA	NA
ENSG00000237007	KRT18P52	keratin 18 pseudogene 52	100418769	0,855302326	0,320837115	2,107513	0,152234926	0,879001648	NA
ENSG00000237008	NA	NA	NA	0	NA	NA	NA	NA	NA
ENSG00000237014	LRRFIP2P1	LRRFIP2 pseudogene 1	100128738	0	NA	NA	NA	NA	NA

ENSG00000237016	LOC100129456	heat shock protein 90 alpha family class B member 1 pseudogene	100129456	0	NA	NA	NA	NA	NA
ENSG00000237024	RPL21P33	ribosomal protein L21 pseudogene 33	100270953	0	NA	NA	NA	NA	NA
ENSG00000237025	NA	NA	NA	0	NA	NA	NA	NA	NA
ENSG00000237026	NA	NA	NA	0	NA	NA	NA	NA	NA
ENSG00000237032	RPSAP7	ribosomal protein SA pseudogene 7	654506	0	NA	NA	NA	NA	NA
ENSG00000237033	CASP3P1	caspase 3 pseudogene 1	100131616	0	NA	NA	NA	NA	NA
ENSG00000237038	USP17L8	ubiquitin specific peptidase 17 like family member 8	392188	0	NA	NA	NA	NA	NA
ENSG00000237039	NA	NA	NA	0	NA	NA	NA	NA	NA
ENSG00000237049	RPL34P1	ribosomal protein L34 pseudogene 1	26514	0,119673877	0,847101401	4,080456	0,207599697	0,835541541	NA
ENSG00000237053	NA	NA	NA	0,119673877	0,847101401	4,080456	0,207599697	0,835541541	NA
ENSG00000237055	MTCO1P48	MT-CO1 pseudogene 48	107075176	0	NA	NA	NA	NA	NA
ENSG00000237064	EIF3IP1	eukaryotic translation initiation factor 3 subunit I pseudogene 1	442720	0	NA	NA	NA	NA	NA
ENSG00000237068	RPL5P2	ribosomal protein L5 pseudogene 2	140749	0	NA	NA	NA	NA	NA
ENSG00000237072	NA	NA	NA	0	NA	NA	NA	NA	NA
ENSG00000237077	NA	NA	NA	0	NA	NA	NA	NA	NA
ENSG00000237082	NA	NA	NA	0	NA	NA	NA	NA	NA
ENSG00000237083	RPL31P42	ribosomal protein L31 pseudogene 42	100271197	0	NA	NA	NA	NA	NA
ENSG00000237085	NA	NA	NA	0	NA	NA	NA	NA	NA
ENSG00000237088	RPL7AP48	ribosomal protein L7a pseudogene 48	100271212	0	NA	NA	NA	NA	NA
ENSG00000237089	NA	NA	NA	0	NA	NA	NA	NA	NA
ENSG00000237090	NA	NA	NA	0,072165449	0,847101401	4,080456	0,207599697	0,835541541	NA
ENSG00000237094	NA	NA	NA	0	NA	NA	NA	NA	NA
ENSG00000237099	NA	NA	NA	0	NA	NA	NA	NA	NA
ENSG00000237106	NA	NA	NA	0	NA	NA	NA	NA	NA
ENSG00000237107	NA	NA	NA	0	NA	NA	NA	NA	NA
ENSG00000237109	RPL21P111	ribosomal protein L21 pseudogene 111	100271439	0	NA	NA	NA	NA	NA
ENSG00000237110	TAAR9	trace amine associated receptor 9	134860	0	NA	NA	NA	NA	NA
ENSG00000237115	LOC100421775	leucine rich repeat containing 8 VRAC subunit B pseudogene	100421775	0	NA	NA	NA	NA	NA
ENSG00000237118	NA	NA	NA	0,072165449	0,847101401	4,080456	0,207599697	0,835541541	NA
ENSG00000237121	NA	NA	NA	0	NA	NA	NA	NA	NA
ENSG00000237124	MTND2P11	MT-ND2 pseudogene 11	100873222	0	NA	NA	NA	NA	NA
ENSG00000237127	NA	NA	NA	0	NA	NA	NA	NA	NA
ENSG00000237129	MTCYBP34	MT-CYB pseudogene 34	109729115	0	NA	NA	NA	NA	NA
ENSG00000237130	NA	NA	NA	0	NA	NA	NA	NA	NA
ENSG00000237131	LOC107985453	cytochrome c oxidase subunit NDUFA4-like	107985453	0	NA	NA	NA	NA	NA
ENSG00000237135	DDX10P1	DEAD-box helicase 10 pseudogene 1	100421371	0,841234684	0,613350955	2,127683	0,288271778	0,773138709	NA
ENSG00000237136	C4orf51	chromosome 4 open reading frame 51	646603	0	NA	NA	NA	NA	NA
ENSG00000237137	NA	NA	NA	0	NA	NA	NA	NA	NA
ENSG00000237139	NA	NA	NA	0	NA	NA	NA	NA	NA
ENSG00000237140	NA	NA	NA	0	NA	NA	NA	NA	NA
ENSG00000237141	NA	NA	NA	0	NA	NA	NA	NA	NA
ENSG00000237148	NA	NA	NA	0	NA	NA	NA	NA	NA
ENSG00000237154	NA	NA	NA	0	NA	NA	NA	NA	NA
ENSG00000237158	NA	NA	NA	0	NA	NA	NA	NA	NA
ENSG00000237160	NA	NA	NA	0	NA	NA	NA	NA	NA
ENSG00000237161	NA	NA	NA	2,65933049	4,686756084	1,793284	2,613504465	0,008961891	NA
ENSG00000237162	RPS20P9	ribosomal protein S20 pseudogene 9	100271080	0	NA	NA	NA	NA	NA
ENSG00000237163	NA	NA	NA	0	NA	NA	NA	NA	NA
ENSG00000237164	NA	NA	NA	0	NA	NA	NA	NA	NA
ENSG00000237169	NA	NA	NA	0	NA	NA	NA	NA	NA
ENSG00000237170	NA	NA	NA	0	NA	NA	NA	NA	NA
ENSG00000237171	NA	NA	NA	0	NA	NA	NA	NA	NA
ENSG00000237172	B3GNT9	UDP-GlcNAc:betaGal beta-1,3-N-acetylglucosaminyltransferase 9	84752	11,49180754	0,004097785	0,558601	0,007335802	0,994146929	NA
ENSG00000237173	NA	NA	NA	0	NA	NA	NA	NA	NA
ENSG00000237175	NME1P1	NME/NM23 nucleoside diphosphate kinase 1 pseudogene 1	100874501	0	NA	NA	NA	NA	NA

ENSG00000237176	LOC100996696	polyadenylate-binding protein 4-like	100996696	0	NA	NA	NA	NA	NA
ENSG00000237178	H3P7	H3 histone pseudogene 7	115482683	0	NA	NA	NA	NA	NA
ENSG00000237180	NA	NA	NA	0	NA	NA	NA	NA	NA
ENSG00000237182	LOC107987328	cytochrome c oxidase subunit NDUFA4-like	107987328	0	NA	NA	NA	NA	NA
ENSG00000237185	VN1R66P	vomeronasal 1 receptor 66 pseudogene	100129315	0	NA	NA	NA	NA	NA
ENSG00000237186	RPS8P5	ribosomal protein S8 pseudogene 5	100131083	0	NA	NA	NA	NA	NA
ENSG00000237193	NA	NA	NA	0	NA	NA	NA	NA	NA
ENSG00000237194	NA	NA	NA	0	NA	NA	NA	NA	NA
ENSG00000237195	DLGAP5P1	DLGAP5 pseudogene 1	360014	0	NA	NA	NA	NA	NA
ENSG00000237202	BNIP3P43	BNIP3 pseudogene 43	127379721	0	NA	NA	NA	NA	NA
ENSG00000237205	NA	NA	NA	0	NA	NA	NA	NA	NA
ENSG00000237206	NA	NA	NA	0	NA	NA	NA	NA	NA
ENSG00000237207	RBM17P3	RNA binding motif protein 17 pseudogene 3	100132949	0	NA	NA	NA	NA	NA
ENSG00000237210	LOC100130121	solute carrier family 19 member 3 pseudogene	100130121	0	NA	NA	NA	NA	NA
ENSG00000237211	NA	NA	NA	0	NA	NA	NA	NA	NA
ENSG00000237213	RPL23AP22	ribosomal protein L23a pseudogene 22	100271291	0	NA	NA	NA	NA	NA
ENSG00000237214	RPS9P2	ribosomal protein S9 pseudogene 2	267009	0	NA	NA	NA	NA	NA
ENSG00000237215	DEFB131D	defensin beta 131D (pseudogene)	100287066	0	NA	NA	NA	NA	NA
ENSG00000237217	NA	NA	NA	0	NA	NA	NA	NA	NA
ENSG00000237223	NA	NA	NA	0	NA	NA	NA	NA	NA
ENSG00000237225	OR13K1P	olfactory receptor family 13 subfamily K member 1 pseudogene	402424	0	NA	NA	NA	NA	NA
ENSG00000237226	RPS3AP39	RPS3A pseudogene 39	730993	0	NA	NA	NA	NA	NA
ENSG00000237227	NA	NA	NA	0	NA	NA	NA	NA	NA
ENSG00000237235	TRDD2	T cell receptor delta diversity 2	28524	0	NA	NA	NA	NA	NA
ENSG00000237236	NA	NA	NA	0	NA	NA	NA	NA	NA
ENSG00000237242	NA	NA	NA	0	NA	NA	NA	NA	NA
ENSG00000237247	MBD3L5	methyl-CpG binding domain protein 3 like 5	284428	0	NA	NA	NA	NA	NA
ENSG00000237249	NA	NA	NA	0,366579943	-0,44735962	3,67036	-0,121884397	0,902990573	NA
ENSG00000237251	NA	NA	NA	0	NA	NA	NA	NA	NA
ENSG00000237252	NA	NA	NA	0	NA	NA	NA	NA	NA
ENSG00000237254	TRBV30	T cell receptor beta variable 30	28557	0	NA	NA	NA	NA	NA
ENSG00000237256	PGAM3P	phosphoglycerate mutase 3, pseudogene	170535	0	NA	NA	NA	NA	NA
ENSG00000237257	MTND2P8	MT-ND2 pseudogene 8	100873178	0	NA	NA	NA	NA	NA
ENSG00000237260	PPIAP67	peptidylprolyl isomerase A pseudogene 67	111082981	0,207996306	-1,916073151	4,043021	-0,473921178	0,635556117	NA
ENSG00000237263	NA	NA	NA	0	NA	NA	NA	NA	NA
ENSG00000237264	NA	NA	NA	0,237961157	0,847303177	4,080456	0,207649146	0,835502928	NA
ENSG00000237265	LOC100129291	chromosome X open reading frame 49 pseudogene	100129291	0	NA	NA	NA	NA	NA
ENSG00000237268	NA	NA	NA	0,072165449	0,847101401	4,080456	0,207599697	0,835541541	NA
ENSG00000237269	RBMY2TP	RNA binding motif protein Y-linked family 2 member T, pseudogene	379008	0	NA	NA	NA	NA	NA
ENSG00000237272	OR51R1P	olfactory receptor family 51 subfamily R member 1 pseudogene	390034	0	NA	NA	NA	NA	NA
ENSG00000237273	NA	NA	NA	0	NA	NA	NA	NA	NA
ENSG00000237274	NA	NA	NA	0	NA	NA	NA	NA	NA
ENSG00000237275	NA	NA	NA	0	NA	NA	NA	NA	NA
ENSG00000237278	RLIMP2	ring finger protein, LIM domain interacting pseudogene 2	100533731	0	NA	NA	NA	NA	NA
ENSG00000237279	NA	NA	NA	0	NA	NA	NA	NA	NA
ENSG00000237285	NA	NA	NA	0	NA	NA	NA	NA	NA
ENSG00000237289	CKMT1B	creatine kinase, mitochondrial 1B	1159	0	NA	NA	NA	NA	NA
ENSG00000237294	NA	NA	NA	0	NA	NA	NA	NA	NA
ENSG00000237297	NA	NA	NA	0	NA	NA	NA	NA	NA
ENSG00000237299	NA	NA	NA	0	NA	NA	NA	NA	NA
ENSG00000237300	MTCO1P19	MT-CO1 pseudogene 19	107075144	0,119673877	0,847101401	4,080456	0,207599697	0,835541541	NA
ENSG00000237302	OFD1P11Y	OFD1 pseudogene 11 Y-linked	386695	0	NA	NA	NA	NA	NA
ENSG00000237303	HIGD1AP8	HIG1 hypoxia inducible domain family member 1A pseudogene 8	100874451	0,10488184	-1,076443944	4,080456	-0,263804824	0,791930317	NA

ENSG00000237306	HSPE1P22	heat shock protein family E (Hsp10) member 1 pseudogene 22	100289607	0	NA	NA	NA	NA	NA
ENSG00000237307	NA	NA	NA	0	NA	NA	NA	NA	NA
ENSG00000237309	MTND2P6	MT-ND2 pseudogene 6	100873177	0	NA	NA	NA	NA	NA
ENSG00000237314	RPL12P39	ribosomal protein L12 pseudogene 39	100271410	0	NA	NA	NA	NA	NA
ENSG00000237317	NA	NA	NA	0	NA	NA	NA	NA	NA
ENSG00000237319	NA	NA	NA	0	NA	NA	NA	NA	NA
ENSG00000237322	RPL7L1P10	RPL7L1 pseudogene 10	106480729	0	NA	NA	NA	NA	NA
ENSG00000237323	NA	NA	NA	0	NA	NA	NA	NA	NA
ENSG00000237325	NA	NA	NA	0	NA	NA	NA	NA	NA
ENSG00000237329	NA	NA	NA	0	NA	NA	NA	NA	NA
ENSG00000237330	RNF223	ring finger protein 223	401934	0,072165449	0,847101401	4,080456	0,207599697	0,835541541	NA
ENSG00000237345	LOC791091	ornithine aminotransferase pseudogene	791091	0	NA	NA	NA	NA	NA
ENSG00000237347	NA	NA	NA	0	NA	NA	NA	NA	NA
ENSG00000237349	NA	NA	NA	0,239347754	1,967469188	4,021404	0,489249276	0,624665227	NA
ENSG00000237350	NA	NA	NA	0	NA	NA	NA	NA	NA
ENSG00000237351	NA	NA	NA	0	NA	NA	NA	NA	NA
ENSG00000237353	PATE4	prostate and testis expressed 4	399968	0	NA	NA	NA	NA	NA
ENSG00000237358	NA	NA	NA	0	NA	NA	NA	NA	NA
ENSG00000237360	NA	NA	NA	0	NA	NA	NA	NA	NA
ENSG00000237363	NA	NA	NA	0	NA	NA	NA	NA	NA
ENSG00000237378	LOC112267897	keratin-associated protein 21-1-like	112267897	0	NA	NA	NA	NA	NA
ENSG00000237379	NA	NA	NA	0	NA	NA	NA	NA	NA
ENSG00000237381	NT5DC1P1	NT5DC1 pseudogene 1	100131340	0	NA	NA	NA	NA	NA
ENSG00000237383	NA	NA	NA	0	NA	NA	NA	NA	NA
ENSG00000237388	OR4A47	olfactory receptor family 4 subfamily A member 47	403253	0	NA	NA	NA	NA	NA
ENSG00000237398	NA	NA	NA	0	NA	NA	NA	NA	NA
ENSG00000237406	NDUFA9P1	NADH:ubiquinone oxidoreductase subunit A9 pseudogene 1	266625	0,117497108	0,847101401	4,080456	0,207599697	0,835541541	NA
ENSG00000237408	ART2BP	ADP-ribosyltransferase 2B, pseudogene	106481723	0	NA	NA	NA	NA	NA
ENSG00000237409	RPL27AP2	ribosomal protein L27a pseudogene 2	391124	0	NA	NA	NA	NA	NA
ENSG00000237412	PRSS56	serine protease 56	646960	0	NA	NA	NA	NA	NA
ENSG00000237415	NA	NA	NA	0	NA	NA	NA	NA	NA
ENSG00000237417	NA	NA	NA	0	NA	NA	NA	NA	NA
ENSG00000237418	NA	NA	NA	0	NA	NA	NA	NA	NA
ENSG00000237425	RPSAP2	ribosomal protein SA pseudogene 2	442181	0	NA	NA	NA	NA	NA
ENSG00000237426	NA	NA	NA	0	NA	NA	NA	NA	NA
ENSG00000237427	TOMM22P1	TOMM22 pseudogene 1	100499416	0	NA	NA	NA	NA	NA
ENSG00000237428	NA	NA	NA	0	NA	NA	NA	NA	NA
ENSG00000237432	RPS7P12	ribosomal protein S7 pseudogene 12	100270914	0	NA	NA	NA	NA	NA
ENSG00000237433	RPSAP11	ribosomal protein SA pseudogene 11	645326	0,117497108	0,847101401	4,080456	0,207599697	0,835541541	NA
ENSG00000237434	SMNP	survival of motor neuron 1, telomeric pseudogene	64939	0	NA	NA	NA	NA	NA
ENSG00000237437	ASS1P12	argininosuccinate synthetase 1 pseudogene 12	392302	0	NA	NA	NA	NA	NA
ENSG00000237438	NA	NA	NA	0	NA	NA	NA	NA	NA
ENSG00000237439	NA	NA	NA	0	NA	NA	NA	NA	NA
ENSG00000237442	HNRNPA1P57	heterogeneous nuclear ribonucleoprotein A1 pseudogene 57	101060039	0	NA	NA	NA	NA	NA
ENSG00000237443	OR13D2P	olfactory receptor family 13 subfamily D member 2 pseudogene	392375	0	NA	NA	NA	NA	NA
ENSG00000237444	NA	NA	NA	0	NA	NA	NA	NA	NA
ENSG00000237446	NA	NA	NA	0	NA	NA	NA	NA	NA
ENSG00000237447	CDC27P2	cell division cycle 27 pseudogene 2	100130277	0	NA	NA	NA	NA	NA
ENSG00000237449	TAAR4P	trace amine associated receptor 4, pseudogene	503612	0	NA	NA	NA	NA	NA
ENSG00000237450	RPL23AP71	ribosomal protein L23a pseudogene 71	644384	0	NA	NA	NA	NA	NA
ENSG00000237451	NA	NA	NA	0	NA	NA	NA	NA	NA
ENSG00000237452	MEIOSIN	meiosis initiator	388553	0,10488184	-1,076443944	4,080456	-0,263804824	0,791930317	NA
ENSG00000237456	NA	NA	NA	0	NA	NA	NA	NA	NA
ENSG00000237458	NA	NA	NA	0	NA	NA	NA	NA	NA
ENSG00000237466	MTCO3P41	MT-CO3 pseudogene 41	107075288	0	NA	NA	NA	NA	NA
ENSG00000237467	USP9YP35	USP9Y pseudogene 35	106480719	0	NA	NA	NA	NA	NA

ENSG00000237469	TUBB8P10	tubulin beta 8 class VIII pseudogene 10	391171	0	NA	NA	NA	NA	NA
ENSG00000237470	NA	NA	NA	0	NA	NA	NA	NA	NA
ENSG00000237472	LOC100287966	ATP synthase-coupling factor 6, mitochondrial-like	100287966	0	NA	NA	NA	NA	NA
ENSG00000237475	RPL7P53	ribosomal protein L7 pseudogene 53	100271145	0	NA	NA	NA	NA	NA
ENSG00000237478	NA	NA	NA	0	NA	NA	NA	NA	NA
ENSG00000237483	RPS27AP8	RPS27A pseudogene 8	100271375	0	NA	NA	NA	NA	NA
ENSG00000237487	VN1R48P	vomeronasal 1 receptor 48 pseudogene	100312798	0	NA	NA	NA	NA	NA
ENSG00000237489	C10orf143	chromosome 10 open reading frame 143	387723	8,269755248	0,118644908	0,621112	0,191020195	0,848509771	NA
ENSG00000237490	NA	NA	NA	0	NA	NA	NA	NA	NA
ENSG00000237492	OR2L9P	olfactory receptor family 2 subfamily L member 9 pseudogene	81462	0	NA	NA	NA	NA	NA
ENSG00000237493	NA	NA	NA	0	NA	NA	NA	NA	NA
ENSG00000237501	MMACHCP1	MMACHC pseudogene 1	127482538	0	NA	NA	NA	NA	NA
ENSG00000237503	NA	NA	NA	0	NA	NA	NA	NA	NA
ENSG00000237506	RPSAP15	ribosomal protein SA pseudogene 15	220885	0	NA	NA	NA	NA	NA
ENSG00000237510	GPAT2P1	glycerol-3-phosphate acyltransferase 2 pseudogene 1	643219	0	NA	NA	NA	NA	NA
ENSG00000237514	NA	NA	NA	0	NA	NA	NA	NA	NA
ENSG00000237515	SHISA9	shisa family member 9	729993	0,368653519	-1,078219309	3,713863	-0,290322886	0,771569232	NA
ENSG00000237517	NA	NA	NA	0	NA	NA	NA	NA	NA
ENSG00000237521	OR7E24	olfactory receptor family 7 subfamily E member 24	26648	0	NA	NA	NA	NA	NA
ENSG00000237522	NA	NA	NA	7,516108308	-0,136691776	0,619818	-0,220535498	0,825454129	NA
ENSG00000237524	TEX51	testis expressed 51	101929926	0	NA	NA	NA	NA	NA
ENSG00000237528	NA	NA	NA	0	NA	NA	NA	NA	NA
ENSG00000237539	NA	NA	NA	0	NA	NA	NA	NA	NA
ENSG00000237540	NA	NA	NA	0	NA	NA	NA	NA	NA
ENSG00000237541	HLA-DQA1	major histocompatibility complex, class II, DQ alpha 1	3117	0	NA	NA	NA	NA	NA
ENSG00000237541	HLA-DQA2	major histocompatibility complex, class II, DQ alpha 2	3118	0	NA	NA	NA	NA	NA
ENSG00000237542	MTCO3P17	MT-CO3 pseudogene 17	107075271	0	NA	NA	NA	NA	NA
ENSG00000237546	NA	NA	NA	0	NA	NA	NA	NA	NA
ENSG00000237549	RPS6P3	ribosomal protein S6 pseudogene 3	644013	0	NA	NA	NA	NA	NA
ENSG00000237551	RPL21P74	ribosomal protein L21 pseudogene 74	100271184	0	NA	NA	NA	NA	NA
ENSG00000237553	NA	NA	NA	0	NA	NA	NA	NA	NA
ENSG00000237555	NA	NA	NA	0	NA	NA	NA	NA	NA
ENSG00000237557	NA	NA	NA	0	NA	NA	NA	NA	NA
ENSG00000237558	CDY7P	chromodomain Y-linked 7 pseudogene	386731	0	NA	NA	NA	NA	NA
ENSG00000237566	CD24P5	CD24 pseudogene 5	127379728	0	NA	NA	NA	NA	NA
ENSG00000237569	TUBAP1	tubulin alpha pseudogene 1	54016	0	NA	NA	NA	NA	NA
ENSG00000237572	LOC100421386	glutamyl aminopeptidase (aminopeptidase A) pseudogene	100421386	0	NA	NA	NA	NA	NA
ENSG00000237575	NA	NA	NA	0	NA	NA	NA	NA	NA
ENSG00000237583	RPL34P8	ribosomal protein L34 pseudogene 8	100270999	0	NA	NA	NA	NA	NA
ENSG00000237584	NA	NA	NA	0	NA	NA	NA	NA	NA
ENSG00000237586	NA	NA	NA	0	NA	NA	NA	NA	NA
ENSG00000237589	HMGN1P32	high mobility group nucleosome binding domain 1 pseudogene 32	100874465	0	NA	NA	NA	NA	NA
ENSG00000237593	RPL17P24	ribosomal protein L17 pseudogene 24	100271153	0	NA	NA	NA	NA	NA
ENSG00000237601	NA	NA	NA	0	NA	NA	NA	NA	NA
ENSG00000237603	NA	NA	NA	0,103998153	-1,076443944	4,080456	-0,263804824	0,791930317	NA
ENSG00000237606	NA	NA	NA	0	NA	NA	NA	NA	NA
ENSG00000237610	OR4C50P	olfactory receptor family 4 subfamily C member 50 pseudogene	256190	0	NA	NA	NA	NA	NA
ENSG00000237617	NA	NA	NA	0	NA	NA	NA	NA	NA
ENSG00000237618	BTBD7P2	BTB domain containing 7 pseudogene 2	100420392	0	NA	NA	NA	NA	NA
ENSG00000237619	OR3B1P	olfactory receptor family 3 subfamily B member 1 pseudogene	392561	0	NA	NA	NA	NA	NA
ENSG00000237620	NA	NA	NA	0	NA	NA	NA	NA	NA

ENSG00000237621	OR9A1P	olfactory receptor family 9 subfamily A member 1 pseudogene	26495	0	NA	NA	NA	NA	NA
ENSG00000237622	ELOCP18	elongin C pseudogene 18	100130626	0	NA	NA	NA	NA	NA
ENSG00000237623	NA	NA	NA	0	NA	NA	NA	NA	NA
ENSG00000237624	OXCT2P1	3-oxoacid CoA-transferase 2 pseudogene 1	192217	1,126049053	-0,091607569	2,339818	-0,039151575	0,968769542	NA
ENSG00000237628	MTCO2P19	MT-CO2 pseudogene 19	107075223	0,10543123	0	4,080456	0	1	NA
ENSG00000237629	NA	NA	NA	0	NA	NA	NA	NA	NA
ENSG00000237630	NIFKP9	nucleolar protein interacting with the FHA domain of MKI67 pseudogene 9	106480805	0	NA	NA	NA	NA	NA
ENSG00000237631	NA	NA	NA	0,353134462	0,418675371	3,745826	0,111771176	0,911004842	NA
ENSG00000237632	NA	NA	NA	0	NA	NA	NA	NA	NA
ENSG00000237635	DUX4L29	double homeobox 4 like 29 (pseudogene)	106481970	0	NA	NA	NA	NA	NA
ENSG00000237636	ANKRD26P3	ankyrin repeat domain 26 pseudogene 3	100101938	0	NA	NA	NA	NA	NA
ENSG00000237639	NA	NA	NA	0	NA	NA	NA	NA	NA
ENSG00000237641	RPL28P2	ribosomal protein L28 pseudogene 2	100270971	0	NA	NA	NA	NA	NA
ENSG00000237642	NA	NA	NA	0,178392034	-0,157587978	4,079456	-0,038629657	0,969185657	NA
ENSG00000237645	NA	NA	NA	0	NA	NA	NA	NA	NA
ENSG00000237650	OR11Q1P	olfactory receptor family 11 subfamily Q member 1 pseudogene	403227	0	NA	NA	NA	NA	NA
ENSG00000237653	NA	NA	NA	0	NA	NA	NA	NA	NA
ENSG00000237659	NA	NA	NA	0	NA	NA	NA	NA	NA
ENSG00000237661	NA	NA	NA	0	NA	NA	NA	NA	NA
ENSG00000237662	NA	NA	NA	0	NA	NA	NA	NA	NA
ENSG00000237663	NA	NA	NA	0	NA	NA	NA	NA	NA
ENSG00000237666	NA	NA	NA	0	NA	NA	NA	NA	NA
ENSG00000237668	NA	NA	NA	0	NA	NA	NA	NA	NA
ENSG00000237669	NA	NA	NA	4,122485696	-2,273705168	1,806682	-1,258497532	0,208211879	NA
ENSG00000237671	GAGE12C	G antigen 12C	729422	0	NA	NA	NA	NA	NA
ENSG00000237672	NA	NA	NA	0	NA	NA	NA	NA	NA
ENSG00000237674	NA	NA	NA	0	NA	NA	NA	NA	NA
ENSG00000237679	NA	NA	NA	0	NA	NA	NA	NA	NA
ENSG00000237682	NA	NA	NA	0	NA	NA	NA	NA	NA
ENSG00000237684	RPSAP35	ribosomal protein SA pseudogene 35	100270916	0	NA	NA	NA	NA	NA
ENSG00000237691	NA	NA	NA	0	NA	NA	NA	NA	NA
ENSG00000237701	NA	NA	NA	0	NA	NA	NA	NA	NA
ENSG00000237702	TRBV3-1	T cell receptor beta variable 3-1	28619	0	NA	NA	NA	NA	NA
ENSG00000237704	NA	NA	NA	0	NA	NA	NA	NA	NA
ENSG00000237706	NA	NA	NA	0	NA	NA	NA	NA	NA
ENSG00000237708	SPIN2P1	SPIN2 family pseudogene 1	100129997	0	NA	NA	NA	NA	NA
ENSG00000237709	EEF1A1P28	eukaryotic translation elongation factor 1 alpha 1 pseudogene 28	442709	0,119673877	0,847101401	4,080456	0,207599697	0,835541541	NA
ENSG00000237711	MTCO3P11	MT-CO3 pseudogene 11	107075153	2,204194746	0,597861749	1,165192	0,513101288	0,607880471	NA
ENSG00000237719	NA	NA	NA	0,10488184	-1,076443944	4,080456	-0,263804824	0,791930317	NA
ENSG00000237722	NA	NA	NA	0	NA	NA	NA	NA	NA
ENSG00000237729	NA	NA	NA	0,541364289	-1,917064578	3,345156	-0,573086675	0,566586009	NA
ENSG00000237730	RPS5P8	RPS5 pseudogene 8	392424	0	NA	NA	NA	NA	NA
ENSG00000237731	RNGTTP1	RNA guanylyltransferase and 5'-phosphatase pseudogene 1	100421428	0	NA	NA	NA	NA	NA
ENSG00000237732	NA	NA	NA	0	NA	NA	NA	NA	NA
ENSG00000237740	NA	NA	NA	0	NA	NA	NA	NA	NA
ENSG00000237743	NA	NA	NA	0	NA	NA	NA	NA	NA
ENSG00000237745	NA	NA	NA	0	NA	NA	NA	NA	NA
ENSG00000237746	NA	NA	NA	0	NA	NA	NA	NA	NA
ENSG00000237747	NA	NA	NA	0	NA	NA	NA	NA	NA
ENSG00000237748	NA	NA	NA	0	NA	NA	NA	NA	NA
ENSG00000237749	NA	NA	NA	0,221401365	-0,121785891	4,080456	-0,029846148	0,976189754	NA
ENSG00000237752	NCKAP1P1	NCKAP1 pseudogene 1	101060152	0	NA	NA	NA	NA	NA
ENSG00000237754	NA	NA	NA	0	NA	NA	NA	NA	NA
ENSG00000237757	NA	NA	NA	1,122937295	-1,010885916	1,869729	-0,54065894	0,588742684	NA
ENSG00000237758	NA	NA	NA	0	NA	NA	NA	NA	NA
ENSG00000237763	AMY1A	amylase alpha 1A	276	0	NA	NA	NA	NA	NA
ENSG00000237763	LOC124905666	alpha-amylase 1B	124905666	0	NA	NA	NA	NA	NA
ENSG00000237763	LOC124905668	alpha-amylase 1B	124905668	0	NA	NA	NA	NA	NA
ENSG00000237766	GGTA2P	glycoprotein alpha-galactosyltransferase 2, pseudogene	121328	0	NA	NA	NA	NA	NA

ENSG00000237770	SPATA31D2P	SPATA31 subfamily D member 2, pseudogene	100130636	0	NA	NA	NA	NA	NA
ENSG00000237774	RPL14P4	ribosomal protein L14 pseudogene 4	100271282	0	NA	NA	NA	NA	NA
ENSG00000237782	NA	NA	NA	0	NA	NA	NA	NA	NA
ENSG00000237783	RPS2P10	ribosomal protein S2 pseudogene 10	401974	0	NA	NA	NA	NA	NA
ENSG00000237784	NA	NA	NA	0	NA	NA	NA	NA	NA
ENSG00000237788	LOC100421569	glyoxylate reductase 1 homolog (Arabidopsis) pseudogene	100421569	6,35126022	-0,10040153	0,72479	-0,138525056	0,889825469	NA
ENSG00000237793	RPL18AP16	ribosomal protein L18a pseudogene 16	347544	0	NA	NA	NA	NA	NA
ENSG00000237799	NA	NA	NA	0	NA	NA	NA	NA	NA
ENSG00000237804	NA	NA	NA	0,117497108	0,847101401	4,080456	0,207599697	0,835541541	NA
ENSG00000237806	NA	NA	NA	0	NA	NA	NA	NA	NA
ENSG00000237810	NA	NA	NA	0	NA	NA	NA	NA	NA
ENSG00000237816	RPL21P109	ribosomal protein L21 pseudogene 109	100271437	0	NA	NA	NA	NA	NA
ENSG00000237818	RPS3AP29	RPS3A pseudogene 29	730861	0,294347791	0,682609053	3,898767	0,175083328	0,861014158	NA
ENSG00000237821	NA	NA	NA	0	NA	NA	NA	NA	NA
ENSG00000237823	NA	NA	NA	0	NA	NA	NA	NA	NA
ENSG00000237824	RPL23AP33	ribosomal protein L23a pseudogene 33	100271293	0	NA	NA	NA	NA	NA
ENSG00000237827	RPS15AP29	ribosomal protein S15a pseudogene 29	100128863	2,047247555	1,600212889	1,454955	1,099836461	0,271403383	NA
ENSG00000237828	NA	NA	NA	0	NA	NA	NA	NA	NA
ENSG00000237833	NA	NA	NA	0	NA	NA	NA	NA	NA
ENSG00000237835	NA	NA	NA	0	NA	NA	NA	NA	NA
ENSG00000237840	FAM21FP	family with sequence similarity 21 member F, pseudogene	100288690	2,840674523	1,262132795	1,16169	1,086462823	0,27727428	NA
ENSG00000237841	KRTAP19-9P	keratin associated protein 19-9, pseudogene	337982	0	NA	NA	NA	NA	NA
ENSG00000237842	NA	NA	NA	0	NA	NA	NA	NA	NA
ENSG00000237846	NA	NA	NA	0	NA	NA	NA	NA	NA
ENSG00000237848	NA	NA	NA	0	NA	NA	NA	NA	NA
ENSG00000237849	NA	NA	NA	0	NA	NA	NA	NA	NA
ENSG00000237850	GOLGA6L22	golgin A6 family like 22	440243	0	NA	NA	NA	NA	NA
ENSG00000237850	GOLGA6L24	golgin A6 family like 24	645202	0	NA	NA	NA	NA	NA
ENSG00000237854	NA	NA	NA	2,210695203	0,551071205	1,708318	0,322581099	0,74701251	NA
ENSG00000237859	EEF1A1P31	eukaryotic translation elongation factor 1 alpha 1 pseudogene 31	553820	0	NA	NA	NA	NA	NA
ENSG00000237860	NA	NA	NA	0	NA	NA	NA	NA	NA
ENSG00000237861	NA	NA	NA	0	NA	NA	NA	NA	NA
ENSG00000237868	NA	NA	NA	0	NA	NA	NA	NA	NA
ENSG00000237872	POU5F1P4	POU class 5 homeobox 1 pseudogene 4	645682	0	NA	NA	NA	NA	NA
ENSG00000237873	NA	NA	NA	0	NA	NA	NA	NA	NA
ENSG00000237875	NA	NA	NA	0	NA	NA	NA	NA	NA
ENSG00000237876	NA	NA	NA	0	NA	NA	NA	NA	NA
ENSG00000237882	NA	NA	NA	0	NA	NA	NA	NA	NA
ENSG00000237885	NA	NA	NA	0	NA	NA	NA	NA	NA
ENSG00000237887	NA	NA	NA	0	NA	NA	NA	NA	NA
ENSG00000237888	NA	NA	NA	0	NA	NA	NA	NA	NA
ENSG00000237891	NA	NA	NA	0	NA	NA	NA	NA	NA
ENSG00000237897	NA	NA	NA	0	NA	NA	NA	NA	NA
ENSG00000237901	NA	NA	NA	0	NA	NA	NA	NA	NA
ENSG00000237902	NA	NA	NA	0	NA	NA	NA	NA	NA
ENSG00000237904	NA	NA	NA	0	NA	NA	NA	NA	NA
ENSG00000237906	MUPP	major urinary protein, pseudogene	100129193	0	NA	NA	NA	NA	NA
ENSG00000237910	MTCO1P18	MT-CO1 pseudogene 18	107075143	0	NA	NA	NA	NA	NA
ENSG00000237911	SRP68P3	signal recognition particle 68 pseudogene 3	347717	0	NA	NA	NA	NA	NA
ENSG00000237913	FTLP19	ferritin light chain pseudogene 19	100873960	0	NA	NA	NA	NA	NA
ENSG00000237916	RPL37P12	ribosomal protein L37 pseudogene 12	100271017	0	NA	NA	NA	NA	NA
ENSG00000237917	PARP4P1	poly(ADP-ribose) polymerase family member 4 pseudogene 1	347613	0,10543123	0	4,080456	0	1	NA
ENSG00000237920	NA	NA	NA	0	NA	NA	NA	NA	NA
ENSG00000237922	NA	NA	NA	0	NA	NA	NA	NA	NA
ENSG00000237924	RPL21P112	ribosomal protein L21 pseudogene 112	100270753	0	NA	NA	NA	NA	NA
ENSG00000237926	NA	NA	NA	0	NA	NA	NA	NA	NA
ENSG00000237929	NA	NA	NA	0	NA	NA	NA	NA	NA

ENSG00000237930	RPL31P14	ribosomal protein L31 pseudogene 14	100129310	0	NA	NA	NA	NA	NA
ENSG00000237931	NA	NA	NA	0	NA	NA	NA	NA	NA
ENSG00000237936	NA	NA	NA	0	NA	NA	NA	NA	NA
ENSG00000237939	NA	NA	NA	0	NA	NA	NA	NA	NA
ENSG00000237948	MTATP6P20	MT-ATP6 pseudogene 20	107075201	0	NA	NA	NA	NA	NA
ENSG00000237951	NA	NA	NA	0,072165449	0,847101401	4,080456	0,207599697	0,835541541	NA
ENSG00000237952	RPL7AP73	ribosomal protein L7a pseudogene 73	100873817	0	NA	NA	NA	NA	NA
ENSG00000237953	NA	NA	NA	0,072165449	0,847101401	4,080456	0,207599697	0,835541541	NA
ENSG00000237959	NA	NA	NA	0	NA	NA	NA	NA	NA
ENSG00000237963	NA	NA	NA	0	NA	NA	NA	NA	NA
ENSG00000237964	NA	NA	NA	0	NA	NA	NA	NA	NA
ENSG00000237968	NA	NA	NA	0	NA	NA	NA	NA	NA
ENSG00000237970	NA	NA	NA	0	NA	NA	NA	NA	NA
ENSG00000237971	NA	NA	NA	0	NA	NA	NA	NA	NA
ENSG00000237972	TUBG1P	tubulin gamma 1 pseudogene	442673	0	NA	NA	NA	NA	NA
ENSG00000237974	NA	NA	NA	0	NA	NA	NA	NA	NA
ENSG00000237977	NA	NA	NA	0,11808024	-1,076443944	4,080456	-0,263804824	0,791930317	NA
ENSG00000237979	NA	NA	NA	0	NA	NA	NA	NA	NA
ENSG00000237982	NA	NA	NA	0	NA	NA	NA	NA	NA
ENSG00000237984	PTENP1	phosphatase and tensin homolog pseudogene 1	11191	0,384365618	2,679452786	3,977338	0,673679898	0,500514844	NA
ENSG00000237988	OR2I1P	olfactory receptor family 2 subfamily I member 1 pseudogene	442197	0	NA	NA	NA	NA	NA
ENSG00000237991	RPL35P1	ribosomal protein L35 pseudogene 1	440737	0	NA	NA	NA	NA	NA
ENSG00000237997	RCC2P2	regulator of chromosome condensation 2 pseudogene 2	100421075	0	NA	NA	NA	NA	NA
ENSG00000237999	ACTG1P19	actin gamma 1 pseudogene 19	644160	0	NA	NA	NA	NA	NA
ENSG00000238000	PSME2P1	proteasome activator subunit 2 pseudogene 1	257093	0	NA	NA	NA	NA	NA
ENSG00000238001	NA	NA	NA	0	NA	NA	NA	NA	NA
ENSG00000238002	NPAP1P6	nuclear pore associated protein 1 pseudogene 6	100533675	0,223113457	-1,078498764	4,080456	-0,2643084	0,791542288	NA
ENSG00000238003	RPL10P4	ribosomal protein L10 pseudogene 4	391151	0	NA	NA	NA	NA	NA
ENSG00000238008	NA	NA	NA	0	NA	NA	NA	NA	NA
ENSG00000238013	NA	NA	NA	0	NA	NA	NA	NA	NA
ENSG00000238015	RPL23P3	ribosomal protein L23 pseudogene 3	100130630	0	NA	NA	NA	NA	NA
ENSG00000238021	NA	NA	NA	0,117497108	0,847101401	4,080456	0,207599697	0,835541541	NA
ENSG00000238025	ZDHHC4P1	zinc finger DHHC-type containing 4 pseudogene 1	100420920	0	NA	NA	NA	NA	NA
ENSG00000238026	TMEM38BP1	TMEM38B pseudogene 1	100131216	0	NA	NA	NA	NA	NA
ENSG00000238029	NA	NA	NA	0	NA	NA	NA	NA	NA
ENSG00000238032	S100A11P5	S100A11 pseudogene 5	107985628	0	NA	NA	NA	NA	NA
ENSG00000238037	OOSP1P1	oocyte secreted protein 1 pseudogene 1	106480251	0	NA	NA	NA	NA	NA
ENSG00000238038	NA	NA	NA	0	NA	NA	NA	NA	NA
ENSG00000238040	NA	NA	NA	0	NA	NA	NA	NA	NA
ENSG00000238041	NA	NA	NA	0	NA	NA	NA	NA	NA
ENSG00000238046	MTND5P22	MT-ND5 pseudogene 22	100873260	0	NA	NA	NA	NA	NA
ENSG00000238047	MTND1P30	MT-ND1 pseudogene 30	100507157	0	NA	NA	NA	NA	NA
ENSG00000238048	NA	NA	NA	0,117497108	0,847101401	4,080456	0,207599697	0,835541541	NA
ENSG00000238049	NA	NA	NA	0	NA	NA	NA	NA	NA
ENSG00000238051	ISCUP1	iron-sulfur cluster assembly enzyme pseudogene 1	100462833	0	NA	NA	NA	NA	NA
ENSG00000238055	NA	NA	NA	0,247870525	0,406657711	4,060574	0,100147846	0,920226951	NA
ENSG00000238059	NA	NA	NA	0	NA	NA	NA	NA	NA
ENSG00000238061	NA	NA	NA	0,994418109	1,398144495	2,098407	0,66628844	0,505226753	NA
ENSG00000238065	NA	NA	NA	0	NA	NA	NA	NA	NA
ENSG00000238066	NA	NA	NA	0	NA	NA	NA	NA	NA
ENSG00000238069	RPLP0P7	ribosomal protein lateral stalk subunit P0 pseudogene 7	100271047	0	NA	NA	NA	NA	NA
ENSG00000238072	NA	NA	NA	0	NA	NA	NA	NA	NA
ENSG00000238073	NA	NA	NA	0	NA	NA	NA	NA	NA
ENSG00000238074	TSPY9	testis specific protein Y-linked 9	728132	0	NA	NA	NA	NA	NA
ENSG00000238074	LOC124905613	testis-specific Y-encoded protein 4-like	124905613	0	NA	NA	NA	NA	NA
ENSG00000238074	LOC124905614	testis-specific Y-encoded protein 3	124905614	0	NA	NA	NA	NA	NA
ENSG00000238074	LOC124905618	testis-specific Y-encoded protein 3-like	124905618	0	NA	NA	NA	NA	NA

ENSG00000238074	LOC124905627	testis-specific Y-encoded protein 4-like	124905627	0	NA	NA	NA	NA	NA
ENSG00000238074	LOC124909305	testis-specific Y-encoded protein 4-like	124909305	0	NA	NA	NA	NA	NA
ENSG00000238074	LOC124909317	testis-specific Y-encoded protein 4-like	124909317	0	NA	NA	NA	NA	NA
ENSG00000238074	LOC124909329	testis-specific Y-encoded protein 4-like	124909329	0	NA	NA	NA	NA	NA
ENSG00000238074	LOC124909332	testis-specific Y-encoded protein 4-like	124909332	0	NA	NA	NA	NA	NA
ENSG00000238075	RPSAP32	ribosomal protein SA pseudogene 32	402123	0	NA	NA	NA	NA	NA
ENSG00000238076	NA	NA	NA	0	NA	NA	NA	NA	NA
ENSG00000238077	NA	NA	NA	0	NA	NA	NA	NA	NA
ENSG00000238079	LOC100506224	translocase of outer mitochondrial membrane 5 homolog (yeast) pseudogene	100506224	0	NA	NA	NA	NA	NA
ENSG00000238081	NA	NA	NA	0	NA	NA	NA	NA	NA
ENSG00000238082	NA	NA	NA	0,117497108	0,847101401	4,080456	0,207599697	0,835541541	NA
ENSG00000238083	LRRC37A2	leucine rich repeat containing 37 member A2	474170	6,29882095	0,167346663	0,796958	0,209981853	0,833681836	NA
ENSG00000238084	NA	NA	NA	0,119673877	0,847101401	4,080456	0,207599697	0,835541541	NA
ENSG00000238085	NA	NA	NA	0	NA	NA	NA	NA	NA
ENSG00000238086	PPP1R26P1	protein phosphatase 1 regulatory subunit 26 pseudogene 1	100418740	5,222465947	0,130289418	0,879051	0,14821592	0,882172368	NA
ENSG00000238087	FMO8P	flavin containing dimethylaniline monoxygenase 8, pseudogene	100129007	0	NA	NA	NA	NA	NA
ENSG00000238088	OFD1P7Y	OFD1 pseudogene 7 Y-linked	386691	0	NA	NA	NA	NA	NA
ENSG00000238090	LOC100421336	RAB3A interacting protein like 1 pseudogene	100421336	0,222729884	-2,001503162	4,035247	-0,496005063	0,619890839	NA
ENSG00000238092	NA	NA	NA	0	NA	NA	NA	NA	NA
ENSG00000238094	NA	NA	NA	0	NA	NA	NA	NA	NA
ENSG00000238098	ABCA17P	ATP binding cassette subfamily A member 17, pseudogene	650655	0,177798344	0,84368287	4,080379	0,206765795	0,83619277	NA
ENSG00000238103	RPL9P7	ribosomal protein L9 pseudogene 7	6126	2,49611278	-2,516596239	1,964491	-1,281042694	0,200178671	NA
ENSG00000238105	NA	NA	NA	9,576298436	0,113420893	0,579386	0,195760407	0,844797701	NA
ENSG00000238108	NA	NA	NA	0	NA	NA	NA	NA	NA
ENSG00000238109	RNF14P3	RNF14 pseudogene 3	100419643	0,412630713	-0,233316766	3,000453	-0,077760502	0,938018566	NA
ENSG00000238110	NA	NA	NA	0,072165449	0,847101401	4,080456	0,207599697	0,835541541	NA
ENSG00000238111	RPS13P4	ribosomal protein S13 pseudogene 4	100271060	0,234994216	1,944111989	4,02324	0,483220515	0,62893917	NA
ENSG00000238116	RAD21P1	RAD21 pseudogene 1	392508	0	NA	NA	NA	NA	NA
ENSG00000238118	SLC25A24P2	SLC25A24 pseudogene 2	100422608	0	NA	NA	NA	NA	NA
ENSG00000238125	SLC9A3P2	solute carrier family 9 member 3 pseudogene 2	128997	0	NA	NA	NA	NA	NA
ENSG00000238132	NA	NA	NA	0,10488184	-1,076443944	4,080456	-0,263804824	0,791930317	NA
ENSG00000238135	USP9YP10	USP9Y pseudogene 10	100421222	0	NA	NA	NA	NA	NA
ENSG00000238137	NA	NA	NA	0,849469083	1,965146977	2,721104	0,722187292	0,470179335	NA
ENSG00000238138	NA	NA	NA	0	NA	NA	NA	NA	NA
ENSG00000238139	NA	NA	NA	0	NA	NA	NA	NA	NA
ENSG00000238143	NA	NA	NA	0	NA	NA	NA	NA	NA
ENSG00000238145	TUBAP8	tubulin alpha pseudogene 8	100533627	0	NA	NA	NA	NA	NA
ENSG00000238149	RPS15AP31	ribosomal protein S15a pseudogene 31	100271652	0	NA	NA	NA	NA	NA
ENSG00000238150	NA	NA	NA	0	NA	NA	NA	NA	NA
ENSG00000238151	NA	NA	NA	0	NA	NA	NA	NA	NA
ENSG00000238152	OR7E140P	olfactory receptor family 7 subfamily E member 140 pseudogene	344729	0	NA	NA	NA	NA	NA
ENSG00000238153	NA	NA	NA	0	NA	NA	NA	NA	NA
ENSG00000238161	OR7E117P	olfactory receptor family 7 subfamily E member 117 pseudogene	399857	0	NA	NA	NA	NA	NA
ENSG00000238165	NA	NA	NA	0	NA	NA	NA	NA	NA
ENSG00000238166	NA	NA	NA	0	NA	NA	NA	NA	NA
ENSG00000238168	NA	NA	NA	0	NA	NA	NA	NA	NA
ENSG00000238172	RPS2P35	ribosomal protein S2 pseudogene 35	100130980	0	NA	NA	NA	NA	NA
ENSG00000238173	NA	NA	NA	0	NA	NA	NA	NA	NA
ENSG00000238180	RPL21P34	ribosomal protein L21 pseudogene 34	100270954	0	NA	NA	NA	NA	NA
ENSG00000238181	AHCYP2	adenosylhomocysteinase pseudogene 2	392387	0	NA	NA	NA	NA	NA
ENSG00000238183	RPS27P5	ribosomal protein S27 pseudogene 5	100271116	0	NA	NA	NA	NA	NA

ENSG00000238188	NA	NA	NA	0	NA	NA	NA	NA	NA
ENSG00000238190	NA	NA	NA	0	NA	NA	NA	NA	NA
ENSG00000238191	NA	NA	NA	0	NA	NA	NA	NA	NA
ENSG00000238193	HADHBP1	HADHB pseudogene 1	100287928	0	NA	NA	NA	NA	NA
ENSG00000238199	NA	NA	NA	0	NA	NA	NA	NA	NA
ENSG00000238200	NA	NA	NA	0	NA	NA	NA	NA	NA
ENSG00000238205	MPC1L	mitochondrial pyruvate carrier 1 like	347411	0	NA	NA	NA	NA	NA
ENSG00000238210	ETDA	embryonic testis differentiation homolog A	101928677	0	NA	NA	NA	NA	NA
ENSG00000238211	POLR2LP1	RNA polymerase II subunit L pseudogene 1	493825	0	NA	NA	NA	NA	NA
ENSG00000238213	TARDBPP2	TARDBP pseudogene 2	440142	0	NA	NA	NA	NA	NA
ENSG00000238215	NA	NA	NA	0	NA	NA	NA	NA	NA
ENSG00000238220	NA	NA	NA	0	NA	NA	NA	NA	NA
ENSG00000238222	NA	NA	NA	0	NA	NA	NA	NA	NA
ENSG00000238223	NA	NA	NA	0	NA	NA	NA	NA	NA
ENSG00000238225	NA	NA	NA	0	NA	NA	NA	NA	NA
ENSG00000238228	OR7E7P	olfactory receptor family 7 subfamily E member 7 pseudogene	392752	5,820487863	0,896922641	0,769411	1,1657263	0,243725123	NA
ENSG00000238231	NA	NA	NA	0,451362767	0	4,080456	0	1	NA
ENSG00000238235	TSPY11P	testis specific protein Y-linked 11, pseudogene	347584	0	NA	NA	NA	NA	NA
ENSG00000238241	CCR12P	C-C motif chemokine receptor 12, pseudogene	100133168	0	NA	NA	NA	NA	NA
ENSG00000238243	OR2W3	olfactory receptor family 2 subfamily W member 3	343171	0,401166636	-0,979734023	3,63151	-0,269786939	0,787324172	NA
ENSG00000238244	NA	NA	NA	0	NA	NA	NA	NA	NA
ENSG00000238245	MYO5BP2	myosin VB pseudogene 2	392335	0	NA	NA	NA	NA	NA
ENSG00000238247	RDXP3	RDX pseudogene 3	100132076	0	NA	NA	NA	NA	NA
ENSG00000238249	NA	NA	NA	0,072165449	0,847101401	4,080456	0,207599697	0,835541541	NA
ENSG00000238251	NA	NA	NA	0	NA	NA	NA	NA	NA
ENSG00000238254	NA	NA	NA	0	NA	NA	NA	NA	NA
ENSG00000238256	MTND5P20	MT-ND5 pseudogene 20	100873269	0	NA	NA	NA	NA	NA
ENSG00000238257	NA	NA	NA	0	NA	NA	NA	NA	NA
ENSG00000238259	NA	NA	NA	1,059684954	2,866801528	2,476175	1,157754145	0,246964381	NA
ENSG00000238263	RPL21P88	ribosomal protein L21 pseudogene 88	100271421	0	NA	NA	NA	NA	NA
ENSG00000238267	CENPPP1	CENPP pseudogene 1	127379695	0,10488184	-1,076443944	4,080456	-0,263804824	0,791930317	NA
ENSG00000238269	PAGE2B	PAGE family member 2B	389860	0,15836288	0	4,080456	0	1	NA
ENSG00000238271	IFNWP19	interferon omega 1 pseudogene 19	3474	0	NA	NA	NA	NA	NA
ENSG00000238275	NA	NA	NA	0	NA	NA	NA	NA	NA
ENSG00000238278	NA	NA	NA	0	NA	NA	NA	NA	NA
ENSG00000238283	TBC1D3P1	TBC1 domain family member 3 pseudogene 1	400609	0	NA	NA	NA	NA	NA
ENSG00000238285	NA	NA	NA	0	NA	NA	NA	NA	NA
ENSG00000238286	NA	NA	NA	1,947751499	-0,741727419	1,51129	-0,490790852	0,623574381	NA
ENSG00000238288	MTND3P23	MT-ND3 pseudogene 23	107075295	0	NA	NA	NA	NA	NA
ENSG00000238379	RNA5SP103	RNA, 5S ribosomal pseudogene 103	100873374	0	NA	NA	NA	NA	NA
ENSG00000238391	RNA5SP233	RNA, 5S ribosomal pseudogene 233	100873491	0	NA	NA	NA	NA	NA
ENSG00000238627	RNA5SP489	RNA, 5S ribosomal pseudogene 489	100873731	0	NA	NA	NA	NA	NA
ENSG00000238765	RNA5SP57	RNA, 5S ribosomal pseudogene 57	106480754	0	NA	NA	NA	NA	NA
ENSG00000238908	RNA5SP484	RNA, 5S ribosomal pseudogene 484	100873726	0	NA	NA	NA	NA	NA
ENSG00000238965	RNA5SP351	RNA, 5S ribosomal pseudogene 351	106480764	0	NA	NA	NA	NA	NA
ENSG00000239021	RNA5SP246	RNA, 5S ribosomal pseudogene 246	100873502	0	NA	NA	NA	NA	NA
ENSG00000239152	RNA5SP310	RNA, 5S ribosomal pseudogene 310	106480762	0	NA	NA	NA	NA	NA
ENSG00000239184	RNA5SP269	RNA, 5S ribosomal pseudogene 269	100873522	0	NA	NA	NA	NA	NA
ENSG00000239198	RPL5P22	ribosomal protein L5 pseudogene 22	389644	0	NA	NA	NA	NA	NA
ENSG00000239199	RPL21P6	ribosomal protein L21 pseudogene 6	652975	0	NA	NA	NA	NA	NA
ENSG00000239200	RPL22P20	ribosomal protein L22 pseudogene 20	100128664	0	NA	NA	NA	NA	NA
ENSG00000239201	RPL21P106	ribosomal protein L21 pseudogene 106	643264	0,239347754	1,967469188	4,021404	0,489249276	0,624665227	NA
ENSG00000239207	NA	NA	NA	0	NA	NA	NA	NA	NA
ENSG00000239210	NA	NA	NA	0	NA	NA	NA	NA	NA
ENSG00000239212	RPL6P7	ribosomal protein L6 pseudogene 7	100270848	0	NA	NA	NA	NA	NA

ENSG00000239215	RPL27P12	ribosomal protein L27 pseudogene 12	100271302	0	NA	NA	NA	NA	NA
ENSG00000239218	NA	NA	NA	0	NA	NA	NA	NA	NA
ENSG00000239223	RPL34P31	ribosomal protein L34 pseudogene 31	729536	0	NA	NA	NA	NA	NA
ENSG00000239226	MRPS17P3	mitochondrial ribosomal protein S17 pseudogene 3	359757	0	NA	NA	NA	NA	NA
ENSG00000239227	NA	NA	NA	0	NA	NA	NA	NA	NA
ENSG00000239238	NA	NA	NA	0	NA	NA	NA	NA	NA
ENSG00000239246	RPS10P26	ribosomal protein S10 pseudogene 26	100271548	0,326916142	-2,559171989	3,994533	-0,640668708	0,521737949	NA
ENSG00000239253	NA	NA	NA	0	NA	NA	NA	NA	NA
ENSG00000239254	NA	NA	NA	0	NA	NA	NA	NA	NA
ENSG00000239255	RPL29P34	ribosomal protein L29 pseudogene 34	127566424	0	NA	NA	NA	NA	NA
ENSG00000239256	NA	NA	NA	0	NA	NA	NA	NA	NA
ENSG00000239257	RPL23AP1	ribosomal protein L23a pseudogene 1	6148	1,312183454	1,327648828	2,144057	0,619222619	0,535769713	NA
ENSG00000239261	RPL31P41	ribosomal protein L31 pseudogene 41	100271201	0	NA	NA	NA	NA	NA
ENSG00000239263	NA	NA	NA	0	NA	NA	NA	NA	NA
ENSG00000239269	RPSAP4	ribosomal protein SA pseudogene 4	145259	0	NA	NA	NA	NA	NA
ENSG00000239272	RPL21P10	ribosomal protein L21 pseudogene 10	319143	1,682821106	-0,35055562	1,597227	-0,219477585	0,826278039	NA
ENSG00000239280	NA	NA	NA	2,036465886	-0,428210474	1,241616	-0,344881631	0,730183361	NA
ENSG00000239281	NA	NA	NA	0	NA	NA	NA	NA	NA
ENSG00000239288	BRD7P7	BRD7 pseudogene 7	100419877	0	NA	NA	NA	NA	NA
ENSG00000239291	RPS2P47	ribosomal protein S2 pseudogene 47	644388	6,286844628	-0,285747988	0,691003	-0,413526201	0,67922113	NA
ENSG00000239304	NA	NA	NA	0	NA	NA	NA	NA	NA
ENSG00000239315	RPL19P13	ribosomal protein L19 pseudogene 13	392193	0	NA	NA	NA	NA	NA
ENSG00000239317	RPL35AP17	ribosomal protein L35a pseudogene 17	100271319	0	NA	NA	NA	NA	NA
ENSG00000239320	RPS29P26	ribosomal protein S29 pseudogene 26	100271591	0	NA	NA	NA	NA	NA
ENSG00000239323	NA	NA	NA	0	NA	NA	NA	NA	NA
ENSG00000239327	RPS26P9	ribosomal protein S26 pseudogene 9	446209	0	NA	NA	NA	NA	NA
ENSG00000239344	RPS6P9	ribosomal protein S6 pseudogene 9	100131467	0	NA	NA	NA	NA	NA
ENSG00000239345	NA	NA	NA	0,210314854	-1,075363137	4,080456	-0,26353995	0,792134436	NA
ENSG00000239350	CCDC137P2	CCDC137 pseudogene 2	127379757	0	NA	NA	NA	NA	NA
ENSG00000239351	NA	NA	NA	0,10543123	0	4,080456	0	1	NA
ENSG00000239354	RPS17P15	ribosomal protein S17 pseudogene 15	100271363	0	NA	NA	NA	NA	NA
ENSG00000239365	RPS26P49	ribosomal protein S26 pseudogene 49	100271575	0	NA	NA	NA	NA	NA
ENSG00000239374	RPL21P105	ribosomal protein L21 pseudogene 105	728693	0	NA	NA	NA	NA	NA
ENSG00000239383	VPS51P4	VPS51 pseudogene 4	127379744	0	NA	NA	NA	NA	NA
ENSG00000239388	ASB14	ankyrin repeat and SOCS box containing 14	142686	0	NA	NA	NA	NA	NA
ENSG00000239392	NA	NA	NA	0	NA	NA	NA	NA	NA
ENSG00000239393	RPS20P17	ribosomal protein S20 pseudogene 17	100271088	0	NA	NA	NA	NA	NA
ENSG00000239395	NA	NA	NA	0	NA	NA	NA	NA	NA
ENSG00000239397	RPL13AP21	ribosomal protein L13a pseudogene 21	100129799	0	NA	NA	NA	NA	NA
ENSG00000239402	NA	NA	NA	0	NA	NA	NA	NA	NA
ENSG00000239405	NA	NA	NA	0	NA	NA	NA	NA	NA
ENSG00000239408	NA	NA	NA	0	NA	NA	NA	NA	NA
ENSG00000239412	RPL21P71	ribosomal protein L21 pseudogene 71	729962	0	NA	NA	NA	NA	NA
ENSG00000239413	NA	NA	NA	0	NA	NA	NA	NA	NA
ENSG00000239426	OR8F1P	olfactory receptor family 8 subfamily F member 1 pseudogene	390269	0	NA	NA	NA	NA	NA
ENSG00000239428	GM2AP2	GM2A pseudogene 2	100287900	0	NA	NA	NA	NA	NA
ENSG00000239432	NA	NA	NA	0	NA	NA	NA	NA	NA
ENSG00000239435	NA	NA	NA	0,10488184	-1,076443944	4,080456	-0,263804824	0,791930317	NA
ENSG00000239438	RPS26P48	ribosomal protein S26 pseudogene 48	100271574	0	NA	NA	NA	NA	NA
ENSG00000239439	NA	NA	NA	0	NA	NA	NA	NA	NA
ENSG00000239443	NA	NA	NA	0	NA	NA	NA	NA	NA
ENSG00000239455	NA	NA	NA	0	NA	NA	NA	NA	NA
ENSG00000239465	NA	NA	NA	0	NA	NA	NA	NA	NA
ENSG00000239470	RPL21P97	ribosomal protein L21 pseudogene 97	387753	0,326126063	-0,99401497	3,787685	-0,26243337	0,792987348	NA

ENSG00000239473	RPL7P38	ribosomal protein L7 pseudogene 38	120872	0	NA	NA	NA	NA	NA
ENSG00000239474	KLHL41	kelch like family member 41	10324	0,34863706	-0,114492363	3,753585	-0,030502139	0,975666587	NA
ENSG00000239481	RPS3AP41	RPS3A pseudogene 41	100271595	0	NA	NA	NA	NA	NA
ENSG00000239483	RPS15AP16	ribosomal protein S15a pseudogene 16	100271358	0	NA	NA	NA	NA	NA
ENSG00000239486	NA	NA	NA	0	NA	NA	NA	NA	NA
ENSG00000239490	RPS4XP18	ribosomal protein S4X pseudogene 18	646583	0	NA	NA	NA	NA	NA
ENSG00000239503	NA	NA	NA	0	NA	NA	NA	NA	NA
ENSG00000239510	NA	NA	NA	0,209482245	-1,068448311	4,080456	-0,261845329	0,79344069	NA
ENSG00000239511	NA	NA	NA	0	NA	NA	NA	NA	NA
ENSG00000239516	FLYWCH1P1	FLYWCH-type zinc finger 1 pseudogene 1	100130898	0	NA	NA	NA	NA	NA
ENSG00000239517	NA	NA	NA	0	NA	NA	NA	NA	NA
ENSG00000239524	NA	NA	NA	0	NA	NA	NA	NA	NA
ENSG00000239525	RPL30P5	ribosomal protein L30 pseudogene 5	100270978	0	NA	NA	NA	NA	NA
ENSG00000239527	NA	NA	NA	0	NA	NA	NA	NA	NA
ENSG00000239528	NA	NA	NA	0	NA	NA	NA	NA	NA
ENSG00000239532	RPL21P45	ribosomal protein L21 pseudogene 45	727821	0	NA	NA	NA	NA	NA
ENSG00000239533	NA	NA	NA	0	NA	NA	NA	NA	NA
ENSG00000239539	NA	NA	NA	0	NA	NA	NA	NA	NA
ENSG00000239544	NA	NA	NA	0,103998153	-1,076443944	4,080456	-0,263804824	0,791930317	NA
ENSG00000239556	NA	NA	NA	6,183736714	0,60187197	0,699965	0,859860122	0,389866153	NA
ENSG00000239557	PPP2R5CP	protein phosphatase 2 regulatory subunit B'gamma pseudogene	140921	5,995751401	0,752665777	0,751082	1,0021086	0,316291145	NA
ENSG00000239559	NA	NA	NA	0	NA	NA	NA	NA	NA
ENSG00000239570	SETP11	SET pseudogene 11	100419187	0,235802283	0,856713446	4,080206	0,209968186	0,833692504	NA
ENSG00000239571	IGKV2D-30	immunoglobulin kappa variable 2D-30	28881	0	NA	NA	NA	NA	NA
ENSG00000239576	COX6CP14	cytochrome c oxidase subunit 6C pseudogene 14	106481683	0	NA	NA	NA	NA	NA
ENSG00000239586	MTND1P16	MT-ND1 pseudogene 16	100873213	0	NA	NA	NA	NA	NA
ENSG00000239590	OR1J4	olfactory receptor family 1 subfamily J member 4	26219	0	NA	NA	NA	NA	NA
ENSG00000239600	NA	NA	NA	0	NA	NA	NA	NA	NA
ENSG00000239602	NA	NA	NA	0	NA	NA	NA	NA	NA
ENSG00000239605	STPG4	sperm-tail PG-rich repeat containing 4	285051	0,448722877	0,116302913	3,982187	0,029205786	0,976700467	NA
ENSG00000239614	NA	NA	NA	0	NA	NA	NA	NA	NA
ENSG00000239615	RPS13P6	ribosomal protein S13 pseudogene 6	100271062	0	NA	NA	NA	NA	NA
ENSG00000239617	RPL32P27	ribosomal protein L32 pseudogene 27	100271306	0	NA	NA	NA	NA	NA
ENSG00000239620	PRR20G	proline rich 20G	100419008	0	NA	NA	NA	NA	NA
ENSG00000239622	NA	NA	NA	0	NA	NA	NA	NA	NA
ENSG00000239626	NA	NA	NA	4,270117297	0,035363162	0,942415	0,037523989	0,970067213	NA
ENSG00000239627	RPL12P20	ribosomal protein L12 pseudogene 20	100270923	0	NA	NA	NA	NA	NA
ENSG00000239632	MTND4LP3	MT-ND4L pseudogene 3	107075245	0	NA	NA	NA	NA	NA
ENSG00000239642	MEIKIN	meiotic kinetochore factor	728637	0	NA	NA	NA	NA	NA
ENSG00000239648	MTND1P3	MT-ND1 pseudogene 3	100288984	0	NA	NA	NA	NA	NA
ENSG00000239649	MYADML	myeloid associated differentiation marker like (pseudogene)	151325	0	NA	NA	NA	NA	NA
ENSG00000239650	NA	NA	NA	1,922755423	0,415014519	1,398934	0,296664922	0,76672234	NA
ENSG00000239659	NA	NA	NA	0	NA	NA	NA	NA	NA
ENSG00000239670	NA	NA	NA	0	NA	NA	NA	NA	NA
ENSG00000239671	RPL34P30	ribosomal protein L34 pseudogene 30	100271505	0	NA	NA	NA	NA	NA
ENSG00000239683	RPL7AP67	ribosomal protein L7a pseudogene 67	100271542	0	NA	NA	NA	NA	NA
ENSG00000239684	NA	NA	NA	0	NA	NA	NA	NA	NA
ENSG00000239686	RPL34P28	ribosomal protein L34 pseudogene 28	100271500	0	NA	NA	NA	NA	NA
ENSG00000239689	RPL17P46	ribosomal protein L17 pseudogene 46	100271419	0	NA	NA	NA	NA	NA
ENSG00000239694	RPSAP38	ribosomal protein SA pseudogene 38	100130676	0	NA	NA	NA	NA	NA
ENSG00000239696	NA	NA	NA	0	NA	NA	NA	NA	NA
ENSG00000239699	HNRNPA3P8	heterogeneous nuclear ribonucleoprotein A3 pseudogene 8	100129557	0	NA	NA	NA	NA	NA
ENSG00000239701	RPL37P20	ribosomal protein L37 pseudogene 20	100271340	0	NA	NA	NA	NA	NA
ENSG00000239706	NA	NA	NA	0	NA	NA	NA	NA	NA
ENSG00000239719	NA	NA	NA	0	NA	NA	NA	NA	NA

ENSG00000239722	IMP3P2	IMP3 pseudogene 2	106480272	0,072165449	0,847101401	4,080456	0,207599697	0,835541541	NA
ENSG00000239732	TLR9	toll like receptor 9	54106	1,278871906	0,079974508	3,230446	0,024756495	0,980249193	NA
ENSG00000239763	NA	NA	NA	8,506135979	-0,509038306	0,715777	-0,711168574	0,47697978	NA
ENSG00000239780	RPLP0P11	ribosomal protein lateral stalk subunit P0 pseudogene 11	647094	0	NA	NA	NA	NA	NA
ENSG00000239783	NA	NA	NA	0	NA	NA	NA	NA	NA
ENSG00000239792	NA	NA	NA	0	NA	NA	NA	NA	NA
ENSG00000239793	NA	NA	NA	0	NA	NA	NA	NA	NA
ENSG00000239797	RPL21P39	ribosomal protein L21 pseudogene 39	389156	0,10543123	0	4,080456	0	1	NA
ENSG00000239804	NA	NA	NA	0	NA	NA	NA	NA	NA
ENSG00000239805	PSMG3P2	PSMG3 pseudogene 2	127482543	0	NA	NA	NA	NA	NA
ENSG00000239809	NA	NA	NA	0	NA	NA	NA	NA	NA
ENSG00000239819	IGKV1D-8	immunoglobulin kappa variable 1D-8	28904	0	NA	NA	NA	NA	NA
ENSG00000239827	NA	NA	NA	0	NA	NA	NA	NA	NA
ENSG00000239829	KRT8P25	keratin 8 pseudogene 25	402134	0	NA	NA	NA	NA	NA
ENSG00000239830	RPS4XP22	ribosomal protein S4X pseudogene 22	100131614	0,424393115	2,116124489	3,474241	0,609089796	0,542464921	NA
ENSG00000239831	NA	NA	NA	0	NA	NA	NA	NA	NA
ENSG00000239835	PARLP1	PARL pseudogene 1	100129707	0	NA	NA	NA	NA	NA
ENSG00000239839	DEFA3	defensin alpha 3	1668	0	NA	NA	NA	NA	NA
ENSG00000239840	RPL23AP72	ribosomal protein L23a pseudogene 72	100271621	0,224170968	-0,148952283	4,080456	-0,036503834	0,970880622	NA
ENSG00000239861	RPL21P96	ribosomal protein L21 pseudogene 96	100271428	0	NA	NA	NA	NA	NA
ENSG00000239870	RPL21P55	ribosomal protein L21 pseudogene 55	100271170	0	NA	NA	NA	NA	NA
ENSG00000239872	NA	NA	NA	0	NA	NA	NA	NA	NA
ENSG00000239873	NA	NA	NA	0	NA	NA	NA	NA	NA
ENSG00000239880	RALBP1P1	RALBP1 pseudogene 1	131054	0	NA	NA	NA	NA	NA
ENSG00000239881	NA	NA	NA	0	NA	NA	NA	NA	NA
ENSG00000239886	KRTAP9-2	keratin associated protein 9-2	83899	0	NA	NA	NA	NA	NA
ENSG00000239887	C1orf226	chromosome 1 open reading frame 226	400793	3,996326088	-0,296645087	1,073739	-0,276272984	0,782338404	NA
ENSG00000239893	NA	NA	NA	0	NA	NA	NA	NA	NA
ENSG00000239912	NA	NA	NA	0	NA	NA	NA	NA	NA
ENSG00000239917	NA	NA	NA	0	NA	NA	NA	NA	NA
ENSG00000239919	NA	NA	NA	0	NA	NA	NA	NA	NA
ENSG00000239920	NA	NA	NA	0,29732219	1,663863222	3,888019	0,427946303	0,668690215	NA
ENSG00000239924	NA	NA	NA	0	NA	NA	NA	NA	NA
ENSG00000239926	NA	NA	NA	0	NA	NA	NA	NA	NA
ENSG00000239939	RPSAP34	ribosomal protein SA pseudogene 34	100270876	0	NA	NA	NA	NA	NA
ENSG00000239944	NA	NA	NA	0	NA	NA	NA	NA	NA
ENSG00000239961	LILRA4	leukocyte immunoglobulin like receptor A4	23547	0	NA	NA	NA	NA	NA
ENSG00000239967	NA	NA	NA	0	NA	NA	NA	NA	NA
ENSG00000239969	NA	NA	NA	0	NA	NA	NA	NA	NA
ENSG00000239975	IGKV1D-33	immunoglobulin kappa variable 1D-33	28896	0	NA	NA	NA	NA	NA
ENSG00000239978	NA	NA	NA	0	NA	NA	NA	NA	NA
ENSG00000239981	OR2A15P	olfactory receptor family 2 subfamily A member 15 pseudogene	135942	0	NA	NA	NA	NA	NA
ENSG00000239983	RPL31P31	ribosomal protein L31 pseudogene 31	727792	0,072165449	0,847101401	4,080456	0,207599697	0,835541541	NA
ENSG00000239985	NA	NA	NA	0,327428331	-1,082507523	3,805019	-0,284494625	0,776031353	NA
ENSG00000239986	NA	NA	NA	0	NA	NA	NA	NA	NA
ENSG00000239988	RPL31P60	ribosomal protein L31 pseudogene 60	100271486	0	NA	NA	NA	NA	NA
ENSG00000239989	NA	NA	NA	0	NA	NA	NA	NA	NA
ENSG00000239992	TRBVA	T cell receptor beta variable A (pseudogene)	28556	0	NA	NA	NA	NA	NA
ENSG00000239995	NA	NA	NA	0	NA	NA	NA	NA	NA
ENSG00000239997	FCF1P3	FCF1 pseudogene 3	100128733	0	NA	NA	NA	NA	NA
ENSG00000239998	LILRA2	leukocyte immunoglobulin like receptor A2	11027	4,523009905	1,742640348	1,398725	1,245877882	0,212809231	NA
ENSG00000240002	NA	NA	NA	0	NA	NA	NA	NA	NA
ENSG00000240003	RPL7P24	ribosomal protein L7 pseudogene 24	100127893	0	NA	NA	NA	NA	NA
ENSG00000240021	TEX35	testis expressed 35	84066	0	NA	NA	NA	NA	NA
ENSG00000240023	RPLP0P3	ribosomal protein lateral stalk subunit P0 pseudogene 3	122589	0	NA	NA	NA	NA	NA
ENSG00000240024	NA	NA	NA	2,571079358	1,064767138	1,097458	0,970212036	0,331940812	NA
ENSG00000240027	NA	NA	NA	0	NA	NA	NA	NA	NA

ENSG00000240031	OR9A3P	olfactory receptor family 9 subfamily A member 3 pseudogene	392106	0	NA	NA	NA	NA	NA
ENSG00000240034	MTND6P6	MT-ND6 pseudogene 6	106478944	0	NA	NA	NA	NA	NA
ENSG00000240036	NA	NA	NA	0,286798967	-1,032608462	4,038497	-0,255691305	0,79818921	NA
ENSG00000240041	IGHJ4	immunoglobulin heavy joining 4	28477	0	NA	NA	NA	NA	NA
ENSG00000240043	RPS27P26	ribosomal protein S27 pseudogene 26	100271583	0	NA	NA	NA	NA	NA
ENSG00000240045	STRIT1	small transmembrane regulator of ion transport 1	100507537	0	NA	NA	NA	NA	NA
ENSG00000240047	RPS3AP32	RPS3A pseudogene 32	100271258	0	NA	NA	NA	NA	NA
ENSG00000240048	NA	NA	NA	0	NA	NA	NA	NA	NA
ENSG00000240051	RPL23AP10	ribosomal protein L23a pseudogene 10	326293	0	NA	NA	NA	NA	NA
ENSG00000240052	NA	NA	NA	0	NA	NA	NA	NA	NA
ENSG00000240058	NA	NA	NA	0	NA	NA	NA	NA	NA
ENSG00000240068	RPL21P42	ribosomal protein L21 pseudogene 42	100271162	0	NA	NA	NA	NA	NA
ENSG00000240069	GPAA1P1	glycosylphosphatidylinositol anchor attachment 1 pseudogene 1	22945	0	NA	NA	NA	NA	NA
ENSG00000240074	RPL9P30	ribosomal protein L9 pseudogene 30	100128710	0	NA	NA	NA	NA	NA
ENSG00000240083	RPS3AP22	RPS3A pseudogene 22	100130786	0	NA	NA	NA	NA	NA
ENSG00000240084	MTND4LP10	MT-ND4L pseudogene 10	107075099	0	NA	NA	NA	NA	NA
ENSG00000240087	RPSAP12	ribosomal protein SA pseudogene 12	387867	0,189674565	1,665109922	4,047524	0,411389754	0,680786766	NA
ENSG00000240089	BMS1P3	BMS1 pseudogene 3	414217	0	NA	NA	NA	NA	NA
ENSG00000240096	RPL18AP1	ribosomal protein L18a pseudogene 1	326289	0	NA	NA	NA	NA	NA
ENSG00000240097	NA	NA	NA	0	NA	NA	NA	NA	NA
ENSG00000240100	RPL36P15	ribosomal protein L36 pseudogene 15	100271334	0	NA	NA	NA	NA	NA
ENSG00000240103	NA	NA	NA	0	NA	NA	NA	NA	NA
ENSG00000240107	NA	NA	NA	0	NA	NA	NA	NA	NA
ENSG00000240108	NA	NA	NA	0	NA	NA	NA	NA	NA
ENSG00000240121	NA	NA	NA	0	NA	NA	NA	NA	NA
ENSG00000240122	NA	NA	NA	0	NA	NA	NA	NA	NA
ENSG00000240125	RPL23AP75	ribosomal protein L23a pseudogene 75	728207	0	NA	NA	NA	NA	NA
ENSG00000240128	KRT18P43	keratin 18 pseudogene 43	151825	0	NA	NA	NA	NA	NA
ENSG00000240131	NA	NA	NA	0	NA	NA	NA	NA	NA
ENSG00000240132	NA	NA	NA	0,119673877	0,847101401	4,080456	0,207599697	0,835541541	NA
ENSG00000240135	PSMD12P1	PSMD12 pseudogene 1	317753	0	NA	NA	NA	NA	NA
ENSG00000240138	EEF1GP4	eukaryotic translation elongation factor 1 gamma pseudogene 4	100129403	1,77218999	0,203024232	1,317167	0,154137041	0,877501683	NA
ENSG00000240156	NA	NA	NA	0	NA	NA	NA	NA	NA
ENSG00000240159	RPL6P16	ribosomal protein L6 pseudogene 16	728365	0	NA	NA	NA	NA	NA
ENSG00000240163	NA	NA	NA	0	NA	NA	NA	NA	NA
ENSG00000240167	RPS7P7	ribosomal protein S7 pseudogene 7	100270870	0	NA	NA	NA	NA	NA
ENSG00000240174	RPL9P22	ribosomal protein L9 pseudogene 22	100130066	0	NA	NA	NA	NA	NA
ENSG00000240179	RPL26P3	ribosomal protein L26 pseudogene 3	326298	0	NA	NA	NA	NA	NA
ENSG00000240180	NA	NA	NA	0,341855097	0,985397174	4,022099	0,244995764	0,806459711	NA
ENSG00000240184	PCDHGC3	protocadherin gamma subfamily C, 3	5098	0	NA	NA	NA	NA	NA
ENSG00000240194	CYMP	chymosin, pseudogene	643160	0	NA	NA	NA	NA	NA
ENSG00000240201	RPS6P24	ribosomal protein S6 pseudogene 24	100128358	0	NA	NA	NA	NA	NA
ENSG00000240204	SMKR1	small lysine rich protein 1	100287482	6,39953989	-0,027479423	0,710524	-0,038674884	0,969149598	NA
ENSG00000240210	RPL7AP5	ribosomal protein L7a pseudogene 5	326309	0	NA	NA	NA	NA	NA
ENSG00000240215	TRBV25OR9-2	T cell receptor beta variable 25/OR9-2 (pseudogene)	6960	0	NA	NA	NA	NA	NA
ENSG00000240216	NA	NA	NA	1,895722125	1,288204139	1,320511	0,975534618	0,329295138	NA
ENSG00000240225	NA	NA	NA	4,353225476	0,429752426	0,942092	0,456168	0,648269175	NA
ENSG00000240231	NA	NA	NA	0,072165449	0,847101401	4,080456	0,207599697	0,835541541	NA
ENSG00000240236	HNRNPA1P23	heterogeneous nuclear ribonucleoprotein A1 pseudogene 23	100130002	0	NA	NA	NA	NA	NA
ENSG00000240237	RPL21P78	ribosomal protein L21 pseudogene 78	100271185	0	NA	NA	NA	NA	NA
ENSG00000240238	RPL21P117	ribosomal protein L21 pseudogene 117	729241	0	NA	NA	NA	NA	NA
ENSG00000240244	NA	NA	NA	0	NA	NA	NA	NA	NA

ENSG00000240246	NA	NA	NA	0	NA	NA	NA	NA	NA
ENSG00000240253	FAR2P3	fatty acyl-CoA reductase 2 pseudogene 3	100288897	0	NA	NA	NA	NA	NA
ENSG00000240255	PSMC1P6	proteasome 26S subunit, ATPase 1 pseudogene 6	643766	0	NA	NA	NA	NA	NA
ENSG00000240265	NA	NA	NA	0	NA	NA	NA	NA	NA
ENSG00000240268	MOXD2P	monooxygenase DBH like 2, pseudogene	100289017	0	NA	NA	NA	NA	NA
ENSG00000240270	RPL12P37	ribosomal protein L12 pseudogene 37	100129801	0,327474991	-0,138756761	3,825253	-0,036273881	0,971063976	NA
ENSG00000240271	NA	NA	NA	0	NA	NA	NA	NA	NA
ENSG00000240280	TCAM1P	testicular cell adhesion molecule 1, pseudogene	146771	1,438243768	1,178608875	1,873003	0,629261467	0,529177893	NA
ENSG00000240281	NA	NA	NA	0	NA	NA	NA	NA	NA
ENSG00000240286	NA	NA	NA	0	NA	NA	NA	NA	NA
ENSG00000240298	NA	NA	NA	0	NA	NA	NA	NA	NA
ENSG00000240305	RPL6P26	ribosomal protein L6 pseudogene 26	100271035	0	NA	NA	NA	NA	NA
ENSG00000240309	MTCO1P6	MT-CO1 pseudogene 6	107075137	0	NA	NA	NA	NA	NA
ENSG00000240311	RPL18P12	ribosomal protein L18 pseudogene 12	390671	0	NA	NA	NA	NA	NA
ENSG00000240320	RPS29P27	ribosomal protein S29 pseudogene 27	100271592	0	NA	NA	NA	NA	NA
ENSG00000240328	NA	NA	NA	0,15836288	0	4,080456	0	1	NA
ENSG00000240338	LOC100506281	uncharacterized LOC100506281	100506281	0	NA	NA	NA	NA	NA
ENSG00000240356	NA	NA	NA	10,87337544	0,120356355	0,650519	0,18501603	0,853216462	NA
ENSG00000240359	NA	NA	NA	0	NA	NA	NA	NA	NA
ENSG00000240361	OR4G11P	olfactory receptor family 4 subfamily G member 11 pseudogene	403263	0	NA	NA	NA	NA	NA
ENSG00000240364	RPL31P59	ribosomal protein L31 pseudogene 59	100271480	0	NA	NA	NA	NA	NA
ENSG00000240366	RPL36AP45	ribosomal protein L36a pseudogene 45	441727	0,403272484	0,646902046	3,065112	0,211053281	0,832845696	NA
ENSG00000240370	RPL13P5	ribosomal protein L13 pseudogene 5	283345	4,392654997	0,23734203	0,900307	0,263623302	0,792070201	NA
ENSG00000240371	RPS4XP13	ribosomal protein S4X pseudogene 13	390183	0	NA	NA	NA	NA	NA
ENSG00000240375	NA	NA	NA	0	NA	NA	NA	NA	NA
ENSG00000240376	RPS8P8	ribosomal protein S8 pseudogene 8	728553	0,10543123	0	4,080456	0	1	NA
ENSG00000240385	RPS29P20	ribosomal protein S29 pseudogene 20	100271587	0	NA	NA	NA	NA	NA
ENSG00000240386	LCE1F	late cornified envelope 1F	353137	0	NA	NA	NA	NA	NA
ENSG00000240388	RPS18P7	ribosomal protein S18 pseudogene 7	133790	0	NA	NA	NA	NA	NA
ENSG00000240392	RPL9P3	ribosomal protein L9 pseudogene 3	641616	1,148046825	0,857001624	2,002118	0,428047449	0,668616575	NA
ENSG00000240393	DRG1P2	DRG1 pseudogene 2	100421670	0	NA	NA	NA	NA	NA
ENSG00000240395	RPL5P23	ribosomal protein L5 pseudogene 23	100270840	0	NA	NA	NA	NA	NA
ENSG00000240399	NA	NA	NA	0,879887537	0,062216622	2,661228	0,023378918	0,981348022	NA
ENSG00000240403	KIR3DL2	killer cell immunoglobulin like receptor, three Ig domains and long cytoplasmic tail 2	3812	0	NA	NA	NA	NA	NA
ENSG00000240404	NA	NA	NA	0	NA	NA	NA	NA	NA
ENSG00000240409	MTATP8P1	MT-ATP8 pseudogene 1	106480795	0,31672576	0	4,080456	0	1	NA
ENSG00000240411	RPL5P16	ribosomal protein L5 pseudogene 16	100270837	0	NA	NA	NA	NA	NA
ENSG00000240412	NA	NA	NA	0	NA	NA	NA	NA	NA
ENSG00000240416	NA	NA	NA	0	NA	NA	NA	NA	NA
ENSG00000240418	RPS2P51	ribosomal protein S2 pseudogene 51	729679	0	NA	NA	NA	NA	NA
ENSG00000240419	NA	NA	NA	0	NA	NA	NA	NA	NA
ENSG00000240426	RPL36AP18	ribosomal protein L36a pseudogene 18	100271328	0	NA	NA	NA	NA	NA
ENSG00000240427	NA	NA	NA	0	NA	NA	NA	NA	NA
ENSG00000240429	NA	NA	NA	8,621644072	0,560176982	0,591622	0,946849748	0,343715348	NA
ENSG00000240432	KRTAP13-3	keratin associated protein 13-3	337960	0	NA	NA	NA	NA	NA
ENSG00000240435	NA	NA	NA	3,003763632	1,385888208	1,073205	1,291354522	0,196580773	NA
ENSG00000240436	RPL35AP14	ribosomal protein L35a pseudogene 14	100271315	0	NA	NA	NA	NA	NA
ENSG00000240438	OFD1P5Y	OFD1 pseudogene 5 Y-linked	386689	0	NA	NA	NA	NA	NA
ENSG00000240441	RPL17P38	ribosomal protein L17 pseudogene 38	100271417	0,353522477	0,98926595	4,022998	0,245902658	0,805757587	NA
ENSG00000240443	RPS10P20	ribosomal protein S10 pseudogene 20	100271547	0	NA	NA	NA	NA	NA

ENSG00000240452	MTCO1P29	MT-CO1 pseudogene 29	107075225	0	NA	NA	NA	NA	NA
ENSG00000240454	NA	NA	NA	0	NA	NA	NA	NA	NA
ENSG00000240458	NA	NA	NA	0	NA	NA	NA	NA	NA
ENSG00000240459	NA	NA	NA	0	NA	NA	NA	NA	NA
ENSG00000240463	NA	NA	NA	0,859763049	-0,12496235	1,749131	-0,071442527	0,943045564	NA
ENSG00000240471	NA	NA	NA	0	NA	NA	NA	NA	NA
ENSG00000240477	NA	NA	NA	0	NA	NA	NA	NA	NA
ENSG00000240480	RPL29P2	ribosomal protein L29 pseudogene 2	118432	0	NA	NA	NA	NA	NA
ENSG00000240484	RPL31P6	ribosomal protein L31 pseudogene 6	654387	0	NA	NA	NA	NA	NA
ENSG00000240486	RPL23AP54	ribosomal protein L23a pseudogene 54	100271466	0	NA	NA	NA	NA	NA
ENSG00000240487	ATOSBP1	ATOSB pseudogene 1	645180	0,103998153	-1,076443944	4,080456	-0,263804824	0,791930317	NA
ENSG00000240489	NA	NA	NA	0,17844662	0,831260617	4,079847	0,203747974	0,83855045	NA
ENSG00000240494	RPS12P28	ribosomal protein S12 pseudogene 28	100271553	0	NA	NA	NA	NA	NA
ENSG00000240505	TNFRSF13B	TNF receptor superfamily member 13B	23495	0	NA	NA	NA	NA	NA
ENSG00000240509	RPL34P18	ribosomal protein L34 pseudogene 18	100271003	0	NA	NA	NA	NA	NA
ENSG00000240511	MED28P2	mediator complex subunit 28 pseudogene 2	106480445	0	NA	NA	NA	NA	NA
ENSG00000240513	NA	NA	NA	0	NA	NA	NA	NA	NA
ENSG00000240518	RPL36P11	ribosomal protein L36 pseudogene 11	642531	0	NA	NA	NA	NA	NA
ENSG00000240519	RPLP1P9	ribosomal protein lateral stalk subunit P1 pseudogene 9	100271049	0	NA	NA	NA	NA	NA
ENSG00000240520	NA	NA	NA	0	NA	NA	NA	NA	NA
ENSG00000240522	RPL7AP10	ribosomal protein L7a pseudogene 10	161406	0	NA	NA	NA	NA	NA
ENSG00000240531	RPL21P123	ribosomal protein L21 pseudogene 123	100271448	3,404592581	2,047531354	1,35886	1,506800349	0,13186184	NA
ENSG00000240534	RPL34P17	ribosomal protein L34 pseudogene 17	100271001	0	NA	NA	NA	NA	NA
ENSG00000240540	RPL3P9	ribosomal protein L3 pseudogene 9	100128963	0	NA	NA	NA	NA	NA
ENSG00000240542	KRTAP9-1	keratin associated protein 9-1	728318	0	NA	NA	NA	NA	NA
ENSG00000240554	RPL7AP58	ribosomal protein L7a pseudogene 58	645846	0	NA	NA	NA	NA	NA
ENSG00000240563	L1TD1	LINE1 type transposase domain containing 1	54596	0	NA	NA	NA	NA	NA
ENSG00000240566	NA	NA	NA	0	NA	NA	NA	NA	NA
ENSG00000240568	NA	NA	NA	0	NA	NA	NA	NA	NA
ENSG00000240572	ARHGEF28P1	ARHGEF28 pseudogene 1	127379713	0	NA	NA	NA	NA	NA
ENSG00000240579	NA	NA	NA	0	NA	NA	NA	NA	NA
ENSG00000240583	AQP1	aquaporin 1 (Colton blood group)	358	0,117497108	0,847101401	4,080456	0,207599697	0,835541541	NA
ENSG00000240590	RPSAP48	ribosomal protein SA pseudogene 48	392262	0	NA	NA	NA	NA	NA
ENSG00000240601	RPL9P15	ribosomal protein L9 pseudogene 15	100270857	0	NA	NA	NA	NA	NA
ENSG00000240602	NA	NA	NA	0	NA	NA	NA	NA	NA
ENSG00000240611	RPL6P15	ribosomal protein L6 pseudogene 15	100270843	0	NA	NA	NA	NA	NA
ENSG00000240616	RPS6P25	ribosomal protein S6 pseudogene 25	729389	7,706940722	0,189536065	0,693826	0,273175071	0,784718642	NA
ENSG00000240621	NA	NA	NA	0	NA	NA	NA	NA	NA
ENSG00000240622	NA	NA	NA	0	NA	NA	NA	NA	NA
ENSG00000240624	RPL17P4	ribosomal protein L17 pseudogene 4	326288	0	NA	NA	NA	NA	NA
ENSG00000240627	RPS10P12	ribosomal protein S10 pseudogene 12	133569	0	NA	NA	NA	NA	NA
ENSG00000240631	RPL30P12	ribosomal protein L30 pseudogene 12	100271304	0	NA	NA	NA	NA	NA
ENSG00000240632	NA	NA	NA	0,15836288	0	4,080456	0	1	NA
ENSG00000240634	NA	NA	NA	0,262647299	1,419710736	4,061283	0,349572019	0,726659913	NA
ENSG00000240651	SEPTIN7P4	septin 7 pseudogene 4	100418715	0	NA	NA	NA	NA	NA
ENSG00000240652	NA	NA	NA	0	NA	NA	NA	NA	NA
ENSG00000240654	C1QTNF9	C1q and TNF related 9	338872	0,914118762	1,345060736	1,98472	0,677707905	0,497956913	NA
ENSG00000240661	NA	NA	NA	0	NA	NA	NA	NA	NA
ENSG00000240667	NA	NA	NA	0,10543123	0	4,080456	0	1	NA
ENSG00000240668	KRT8P36	keratin 8 pseudogene 36	100418748	0	NA	NA	NA	NA	NA
ENSG00000240669	RPS14P6	ribosomal protein S14 pseudogene 6	646737	0	NA	NA	NA	NA	NA
ENSG00000240673	NA	NA	NA	0	NA	NA	NA	NA	NA

ENSG00000240674	RPL21P51	ribosomal protein L21 pseudogene 51	100271169	0	NA	NA	NA	NA	NA
ENSG00000240677	NA	NA	NA	0,222648025	0,830864307	4,080456	0,203620461	0,838650102	NA
ENSG00000240680	H4P1	H4 histone pseudogene 1	115482692	0	NA	NA	NA	NA	NA
ENSG00000240694	PNMA2	PNMA family member 2	10687	0	NA	NA	NA	NA	NA
ENSG00000240695	NA	NA	NA	2,488182828	-0,751478716	1,226635	-0,612634251	0,540118206	NA
ENSG00000240698	RPS2P39	ribosomal protein S2 pseudogene 39	100271244	0	NA	NA	NA	NA	NA
ENSG00000240704	NA	NA	NA	0	NA	NA	NA	NA	NA
ENSG00000240720	LRRD1	leucine rich repeats and death domain containing 1	401387	0	NA	NA	NA	NA	NA
ENSG00000240721	RPS4XP15	ribosomal protein S4X pseudogene 15	100131055	0	NA	NA	NA	NA	NA
ENSG00000240724	RPS12P15	ribosomal protein S12 pseudogene 15	100271059	0	NA	NA	NA	NA	NA
ENSG00000240729	RPL21P60	ribosomal protein L21 pseudogene 60	100271175	2,621834609	0,438001869	1,143043	0,383189238	0,701579457	NA
ENSG00000240738	RPL6P22	ribosomal protein L6 pseudogene 22	388460	0,10488184	-1,076443944	4,080456	-0,263804824	0,791930317	NA
ENSG00000240739	RPS20P28	ribosomal protein S20 pseudogene 28	100271560	0	NA	NA	NA	NA	NA
ENSG00000240747	KRBOX1	KRAB box domain containing 1	100506243	0,103998153	-1,076443944	4,080456	-0,263804824	0,791930317	NA
ENSG00000240751	NA	NA	NA	0	NA	NA	NA	NA	NA
ENSG00000240752	RPS12P6	ribosomal protein S12 pseudogene 6	100271054	0	NA	NA	NA	NA	NA
ENSG00000240755	NA	NA	NA	0,10488184	-1,076443944	4,080456	-0,263804824	0,791930317	NA
ENSG00000240759	NA	NA	NA	0	NA	NA	NA	NA	NA
ENSG00000240760	RPL31P56	ribosomal protein L31 pseudogene 56	100271478	0	NA	NA	NA	NA	NA
ENSG00000240761	NA	NA	NA	0	NA	NA	NA	NA	NA
ENSG00000240764	PCDHGC5	protocadherin gamma subfamily C, 5	56097	0	NA	NA	NA	NA	NA
ENSG00000240771	ARHGEF25	Rho guanine nucleotide exchange factor 25	115557	0,954334924	-2,492631423	3,038717	-0,820290621	0,412050452	NA
ENSG00000240774	NA	NA	NA	0	NA	NA	NA	NA	NA
ENSG00000240775	RPS26P23	ribosomal protein S26 pseudogene 23	100271107	0	NA	NA	NA	NA	NA
ENSG00000240776	RPS10P4	ribosomal protein S10 pseudogene 4	728791	0	NA	NA	NA	NA	NA
ENSG00000240785	RPL36AP21	ribosomal protein L36a pseudogene 21	100271332	0	NA	NA	NA	NA	NA
ENSG00000240787	NECTIN2P1	NECTIN2 pseudogene 1	127379749	0	NA	NA	NA	NA	NA
ENSG00000240791	MTND4LP7	MT-ND4L pseudogene 7	107075236	0	NA	NA	NA	NA	NA
ENSG00000240793	NA	NA	NA	0,224261181	-0,138174352	4,080456	-0,033862479	0,972986813	NA
ENSG00000240796	MTND2P14	MT-ND2 pseudogene 14	100873223	0	NA	NA	NA	NA	NA
ENSG00000240804	NA	NA	NA	0	NA	NA	NA	NA	NA
ENSG00000240808	RPL34P24	ribosomal protein L34 pseudogene 24	100271309	1,331970757	-0,100373655	1,58524	-0,063317643	0,949513567	NA
ENSG00000240809	CAP1P1	CAP1 pseudogene 1	401076	8,768168701	-0,427246129	0,655265	-0,65202069	0,514387825	NA
ENSG00000240813	NA	NA	NA	0	NA	NA	NA	NA	NA
ENSG00000240821	RPL9P25	ribosomal protein L9 pseudogene 25	654350	0,248637481	1,392377094	4,060689	0,342891809	0,731679854	NA
ENSG00000240824	MTND3P7	MT-ND3 pseudogene 7	100873183	0	NA	NA	NA	NA	NA
ENSG00000240827	RPL19P7	ribosomal protein L19 pseudogene 7	100131527	0,144330898	1,418786819	4,061262	0,349346267	0,726829368	NA
ENSG00000240828	RPL21P4	ribosomal protein L21 pseudogene 4	140660	0	NA	NA	NA	NA	NA
ENSG00000240846	RPS15P9	ribosomal protein S15 pseudogene 9	401895	0	NA	NA	NA	NA	NA
ENSG00000240854	PSMC2P1	PSMC2 pseudogene 1	729674	0	NA	NA	NA	NA	NA
ENSG00000240861	RPS10P23	ribosomal protein S10 pseudogene 23	645713	0	NA	NA	NA	NA	NA
ENSG00000240870	RPL19P14	ribosomal protein L19 pseudogene 14	100270951	0	NA	NA	NA	NA	NA
ENSG00000240873	RPS29P22	ribosomal protein S29 pseudogene 22	100271384	0	NA	NA	NA	NA	NA
ENSG00000240874	RPS13P7	ribosomal protein S13 pseudogene 7	645918	0	NA	NA	NA	NA	NA
ENSG00000240881	RPS2P38	ribosomal protein S2 pseudogene 38	196188	0	NA	NA	NA	NA	NA
ENSG00000240882	PTOV1P1	PTOV1 pseudogene 1	442087	0	NA	NA	NA	NA	NA
ENSG00000240890	G2E3P1	G2E3 pseudogene 1	100130550	0,10488184	-1,076443944	4,080456	-0,263804824	0,791930317	NA
ENSG00000240898	RPL21P125	ribosomal protein L21 pseudogene 125	100129685	0,38654467	0,813345473	3,671557	0,221526066	0,824682843	NA
ENSG00000240902	APOOP2	apolipoprotein O pseudogene 2	100129005	0	NA	NA	NA	NA	NA
ENSG00000240912	H2BP3	H2B histone pseudogene 3	391566	0	NA	NA	NA	NA	NA
ENSG00000240914	NA	NA	NA	0	NA	NA	NA	NA	NA
ENSG00000240919	NA	NA	NA	0	NA	NA	NA	NA	NA

ENSG00000240925	RPS20P31	ribosomal protein S20 pseudogene 31	100271563	0	NA	NA	NA	NA	NA
ENSG00000240934	NA	NA	NA	0	NA	NA	NA	NA	NA
ENSG00000240935	NA	NA	NA	0	NA	NA	NA	NA	NA
ENSG00000240950	RPL8P3	ribosomal protein L8 pseudogene 3	100270854	0	NA	NA	NA	NA	NA
ENSG00000240951	MTCO2P6	MT-CO2 pseudogene 6	107075266	0	NA	NA	NA	NA	NA
ENSG00000240954	RPL4P1	ribosomal protein L4 pseudogene 1	650808	0	NA	NA	NA	NA	NA
ENSG00000240959	NA	NA	NA	0	NA	NA	NA	NA	NA
ENSG00000240960	NA	NA	NA	0	NA	NA	NA	NA	NA
ENSG00000240974	NA	NA	NA	0	NA	NA	NA	NA	NA
ENSG00000240975	RPL18P8	ribosomal protein L18 pseudogene 8	100129670	0	NA	NA	NA	NA	NA
ENSG00000240983	RPS21P6	ribosomal protein S21 pseudogene 6	100271365	0	NA	NA	NA	NA	NA
ENSG00000240991	RPL23AP67	ribosomal protein L23a pseudogene 67	100271622	0	NA	NA	NA	NA	NA
ENSG00000240992	NA	NA	NA	0	NA	NA	NA	NA	NA
ENSG00000240995	NA	NA	NA	0	NA	NA	NA	NA	NA
ENSG00000241002	RPL32P13	ribosomal protein L32 pseudogene 13	100270993	0	NA	NA	NA	NA	NA
ENSG00000241003	RPS15AP26	ribosomal protein S15a pseudogene 26	100271359	0	NA	NA	NA	NA	NA
ENSG00000241007	SEPTIN7P6	septin 7 pseudogene 6	100418720	0	NA	NA	NA	NA	NA
ENSG00000241011	FAUP3	FAU pseudogene 3	100271126	0	NA	NA	NA	NA	NA
ENSG00000241014	GPR199P	G protein-coupled receptor 199, pseudogene	653160	0	NA	NA	NA	NA	NA
ENSG00000241018	RCC2P5	regulator of chromosome condensation 2 pseudogene 5	100131442	0	NA	NA	NA	NA	NA
ENSG00000241020	RPL32P24	ribosomal protein L32 pseudogene 24	100271489	0,570802214	1,9084029	4,013468	0,475499689	0,634430855	NA
ENSG00000241022	NA	NA	NA	0	NA	NA	NA	NA	NA
ENSG00000241026	NA	NA	NA	0	NA	NA	NA	NA	NA
ENSG00000241030	NA	NA	NA	0	NA	NA	NA	NA	NA
ENSG00000241035	NA	NA	NA	0	NA	NA	NA	NA	NA
ENSG00000241045	RPL7P43	ribosomal protein L7 pseudogene 43	100271224	0,10488184	-1,076443944	4,080456	-0,263804824	0,791930317	NA
ENSG00000241052	RPL23AP70	ribosomal protein L23a pseudogene 70	100271619	0	NA	NA	NA	NA	NA
ENSG00000241057	NA	NA	NA	1,151146582	-0,675811415	1,925882	-0,350910077	0,725655811	NA
ENSG00000241061	RPL5P1	ribosomal protein L5 pseudogene 1	647436	0,15836288	0	4,080456	0	1	NA
ENSG00000241069	NA	NA	NA	0	NA	NA	NA	NA	NA
ENSG00000241081	RPL22P2	ribosomal protein L22 pseudogene 2	326292	0,118286652	0	4,080456	0	1	NA
ENSG00000241088	RPL17P44	ribosomal protein L17 pseudogene 44	100129584	0	NA	NA	NA	NA	NA
ENSG00000241095	NA	NA	NA	0,11808024	-1,076443944	4,080456	-0,263804824	0,791930317	NA
ENSG00000241097	RPL36AP38	ribosomal protein L36a pseudogene 38	100271642	0	NA	NA	NA	NA	NA
ENSG00000241102	NA	NA	NA	0	NA	NA	NA	NA	NA
ENSG00000241103	NA	NA	NA	0	NA	NA	NA	NA	NA
ENSG00000241104	LOC284344	uncharacterized LOC284344	284344	0	NA	NA	NA	NA	NA
ENSG00000241105	RPL13P11	ribosomal protein L13 pseudogene 11	100130225	0	NA	NA	NA	NA	NA
ENSG00000241106	HLA-DOB	major histocompatibility complex, class II, DO beta	3112	0,47200832	1,368800945	4,060178	0,337128284	0,736020197	NA
ENSG00000241112	NA	NA	NA	1,715356191	1,993989353	1,537681	1,296751027	0,194716866	NA
ENSG00000241114	NA	NA	NA	0	NA	NA	NA	NA	NA
ENSG00000241119	UGT1A9	UDP glucuronosyltransferase family 1 member A9	54600	0	NA	NA	NA	NA	NA
ENSG00000241120	HMGN1P8	high mobility group nucleosome binding domain 1 pseudogene 8	100874431	1,086175163	-1,665800191	2,659802	-0,626287252	0,531126546	NA
ENSG00000241123	KRTAP10-5	keratin associated protein 10-5	386680	0	NA	NA	NA	NA	NA
ENSG00000241127	YAE1	YAE1 maturation factor of ABCE1	57002	8,145632481	-0,332202586	0,72545	-0,457926138	0,647005508	NA
ENSG00000241128	OR14A2	olfactory receptor family 14 subfamily A member 2	388761	0	NA	NA	NA	NA	NA
ENSG00000241129	RPL22P19	ribosomal protein L22 pseudogene 19	644022	0,117497108	0,847101401	4,080456	0,207599697	0,835541541	NA
ENSG00000241130	RPL32P30	ribosomal protein L32 pseudogene 30	100271492	0	NA	NA	NA	NA	NA
ENSG00000241134	NA	NA	NA	0,560470559	-0,120854862	2,839326	-0,042564633	0,966048589	NA
ENSG00000241136	NA	NA	NA	0	NA	NA	NA	NA	NA
ENSG00000241143	NA	NA	NA	0	NA	NA	NA	NA	NA
ENSG00000241146	NA	NA	NA	0	NA	NA	NA	NA	NA

ENSG00000241149	ZNF722	zinc finger protein 722	100419780	0	NA	NA	NA	NA	NA
ENSG00000241157	RPL32P32	ribosomal protein L32 pseudogene 32	100271488	0,333030889	-1,056712487	4,080456	-0,258969223	0,795658987	NA
ENSG00000241170	NA	NA	NA	3,357756471	0,747282049	1,04038	0,718278036	0,47258587	NA
ENSG00000241179	RPL34P13	ribosomal protein L34 pseudogene 13	100270997	0	NA	NA	NA	NA	NA
ENSG00000241183	RPS10P24	ribosomal protein S10 pseudogene 24	645299	0	NA	NA	NA	NA	NA
ENSG00000241185	NA	NA	NA	0	NA	NA	NA	NA	NA
ENSG00000241186	CRIPTO	cripto, EGF-CFC family member	6997	0	NA	NA	NA	NA	NA
ENSG00000241187	RPL21P57	ribosomal protein L21 pseudogene 57	100271172	0	NA	NA	NA	NA	NA
ENSG00000241200	NA	NA	NA	0	NA	NA	NA	NA	NA
ENSG00000241203	HMGN2P26	high mobility group nucleosomal binding domain 2 pseudogene 26	100874470	0	NA	NA	NA	NA	NA
ENSG00000241204	RPS18P11	ribosomal protein S18 pseudogene 11	100271077	0	NA	NA	NA	NA	NA
ENSG00000241207	SSX18P	SSX family member 18, pseudogene	100420551	0	NA	NA	NA	NA	NA
ENSG00000241216	SNAPC5P1	small nuclear RNA activating complex polypeptide 5 pseudogene 1	100750245	0	NA	NA	NA	NA	NA
ENSG00000241218	CSP2	CS pseudogene 2	649024	0	NA	NA	NA	NA	NA
ENSG00000241221	MTND4LP17	MT-ND4L pseudogene 17	107075240	0	NA	NA	NA	NA	NA
ENSG00000241224	C3orf85	chromosome 3 open reading frame 85	401081	0	NA	NA	NA	NA	NA
ENSG00000241225	NMTRS-TGA3-1	nuclear-encoded mitochondrial tRNA-Ser (TGA) 3-1	100189512	0	NA	NA	NA	NA	NA
ENSG00000241228	NA	NA	NA	0	NA	NA	NA	NA	NA
ENSG00000241233	KRTAP5-8	keratin associated protein 5-8	57830	0,335440025	0,956558825	4,025891	0,23760178	0,81218997	NA
ENSG00000241244	IGKV1D-16	immunoglobulin kappa variable 1D-16	28901	0	NA	NA	NA	NA	NA
ENSG00000241250	RPL17P20	ribosomal protein L17 pseudogene 20	391655	0,10488184	-1,076443944	4,080456	-0,263804824	0,791930317	NA
ENSG00000241251	RPL21P101	ribosomal protein L21 pseudogene 101	100271431	0	NA	NA	NA	NA	NA
ENSG00000241255	NA	NA	NA	0,103998153	-1,076443944	4,080456	-0,263804824	0,791930317	NA
ENSG00000241257	NA	NA	NA	0	NA	NA	NA	NA	NA
ENSG00000241261	RPL17P19	ribosomal protein L17 pseudogene 19	100271150	0	NA	NA	NA	NA	NA
ENSG00000241278	ENPP7P4	ectonucleotide pyrophosphatase/phosphodiesterase 7 pseudogene 4	100421816	0	NA	NA	NA	NA	NA
ENSG00000241281	RPL31P26	ribosomal protein L31 pseudogene 26	100130537	0	NA	NA	NA	NA	NA
ENSG00000241282	NA	NA	NA	0	NA	NA	NA	NA	NA
ENSG00000241286	NA	NA	NA	0	NA	NA	NA	NA	NA
ENSG00000241293	PPATP1	phosphoribosyl pyrophosphate amidotransferase pseudogene 1	100289640	0	NA	NA	NA	NA	NA
ENSG00000241305	NA	NA	NA	0	NA	NA	NA	NA	NA
ENSG00000241307	RPL7AP24	ribosomal protein L7a pseudogene 24	100271041	0	NA	NA	NA	NA	NA
ENSG00000241318	NA	NA	NA	0	NA	NA	NA	NA	NA
ENSG00000241319	SETP6	SET pseudogene 6	100419178	0,178324964	0,833506317	4,079943	0,204293597	0,838124073	NA
ENSG00000241322	FBXW10B	F-box and WD repeat domain containing 10B	374286	0,359021632	2,56113643	3,98322	0,642981427	0,520236152	NA
ENSG00000241334	NA	NA	NA	0	NA	NA	NA	NA	NA
ENSG00000241344	RPL21P47	ribosomal protein L21 pseudogene 47	100271164	0	NA	NA	NA	NA	NA
ENSG00000241346	SNRPCP11	small nuclear ribonucleoprotein polypeptide C pseudogene 11	100310850	0	NA	NA	NA	NA	NA
ENSG00000241350	NA	NA	NA	0	NA	NA	NA	NA	NA
ENSG00000241352	RPS6P1	ribosomal protein S6 pseudogene 1	440086	0,224209638	-0,143913572	4,080456	-0,035268994	0,971865247	NA
ENSG00000241353	NA	NA	NA	0	NA	NA	NA	NA	NA
ENSG00000241354	RPS15AP4	ribosomal protein S15a pseudogene 4	654371	0	NA	NA	NA	NA	NA
ENSG00000241356	OR5G3	olfactory receptor family 5 subfamily G member 3 (gene/pseudogene)	81193	0	NA	NA	NA	NA	NA
ENSG00000241358	NA	NA	NA	0	NA	NA	NA	NA	NA
ENSG00000241361	NA	NA	NA	0	NA	NA	NA	NA	NA

ENSG00000241362	NA	NA	NA	0	NA	NA	NA	NA	NA
ENSG00000241367	NA	NA	NA	0	NA	NA	NA	NA	NA
ENSG00000241370	RPP21	ribonuclease P/MRP subunit p21	79897	0	NA	NA	NA	NA	NA
ENSG00000241370	TRIM39-RPP21	TRIM39-RPP21 readthrough	202658	0	NA	NA	NA	NA	NA
ENSG00000241385	NA	NA	NA	0	NA	NA	NA	NA	NA
ENSG00000241399	CD302	CD302 molecule	9936	1,716951193	1,69724716	1,731765	0,980067705	0,327052699	NA
ENSG00000241400	RPL31P21	ribosomal protein L31 pseudogene 21	100270988	0	NA	NA	NA	NA	NA
ENSG00000241404	EGFL8	EGF like domain multiple 8	80864	0	NA	NA	NA	NA	NA
ENSG00000241411	NA	NA	NA	0	NA	NA	NA	NA	NA
ENSG00000241416	NA	NA	NA	0	NA	NA	NA	NA	NA
ENSG00000241418	NA	NA	NA	0,282477378	-0,972107859	4,041716	-0,240518619	0,809928231	NA
ENSG00000241423	RPL21P103	ribosomal protein L21 pseudogene 103	100271433	0	NA	NA	NA	NA	NA
ENSG00000241429	EEF1A1P25	eukaryotic translation elongation factor 1 alpha 1 pseudogene 25	646612	0	NA	NA	NA	NA	NA
ENSG00000241431	RPL37P6	ribosomal protein L37 pseudogene 6	346950	0	NA	NA	NA	NA	NA
ENSG00000241434	NA	NA	NA	0	NA	NA	NA	NA	NA
ENSG00000241438	NA	NA	NA	0	NA	NA	NA	NA	NA
ENSG00000241439	NA	NA	NA	0	NA	NA	NA	NA	NA
ENSG00000241451	RPS27P22	ribosomal protein S27 pseudogene 22	100131418	0	NA	NA	NA	NA	NA
ENSG00000241458	RPL7P19	ribosomal protein L7 pseudogene 19	100270853	0,119673877	0,847101401	4,080456	0,207599697	0,835541541	NA
ENSG00000241462	NA	NA	NA	0	NA	NA	NA	NA	NA
ENSG00000241464	NA	NA	NA	0	NA	NA	NA	NA	NA
ENSG00000241473	NA	NA	NA	0	NA	NA	NA	NA	NA
ENSG00000241478	NA	NA	NA	0	NA	NA	NA	NA	NA
ENSG00000241484	ARHGAP8	Rho GTPase activating protein 8	23779	0	NA	NA	NA	NA	NA
ENSG00000241493	NA	NA	NA	0	NA	NA	NA	NA	NA
ENSG00000241494	RPL26P4	ribosomal protein L26 pseudogene 4	283603	0	NA	NA	NA	NA	NA
ENSG00000241499	NA	NA	NA	0	NA	NA	NA	NA	NA
ENSG00000241511	RPS15AP24	ribosomal protein S15a pseudogene 24	648729	0	NA	NA	NA	NA	NA
ENSG00000241526	NA	NA	NA	0	NA	NA	NA	NA	NA
ENSG00000241527	CA15P1	CA15 pseudogene 1	100996435	0,177016529	-0,113756666	4,080456	-0,027878421	0,97775912	NA
ENSG00000241532	NA	NA	NA	0	NA	NA	NA	NA	NA
ENSG00000241535	NA	NA	NA	0	NA	NA	NA	NA	NA
ENSG00000241537	U2SURPP1	U2SURP pseudogene 1	124906201	0	NA	NA	NA	NA	NA
ENSG00000241539	NA	NA	NA	0	NA	NA	NA	NA	NA
ENSG00000241546	RFKP3	RFK pseudogene 3	127379691	0	NA	NA	NA	NA	NA
ENSG00000241547	NA	NA	NA	2,513281879	-0,322734682	1,276786	-0,25277109	0,800445109	NA
ENSG00000241549	NA	NA	NA	4,632745346	0,397621117	0,858886	0,462949953	0,64340024	NA
ENSG00000241556	NA	NA	NA	0,117497108	0,847101401	4,080456	0,207599697	0,835541541	NA
ENSG00000241562	RPL7P5	ribosomal protein L7 pseudogene 5	654381	0	NA	NA	NA	NA	NA
ENSG00000241563	CORT	cortistatin	1325	0,221502699	-0,114135743	4,080456	-0,027971321	0,977685024	NA
ENSG00000241566	IGKV2D-24	immunoglobulin kappa variable 2D-24 (non-functional)	28885	0	NA	NA	NA	NA	NA
ENSG00000241571	ATP5F1AP7	ATP synthase F1 subunit alpha pseudogene 7	442415	0	NA	NA	NA	NA	NA
ENSG00000241573	NA	NA	NA	0	NA	NA	NA	NA	NA
ENSG00000241582	RPL23AP8	ribosomal protein L23a pseudogene 8	641614	0	NA	NA	NA	NA	NA
ENSG00000241590	RPL17P37	ribosomal protein L17 pseudogene 37	100271411	0	NA	NA	NA	NA	NA
ENSG00000241592	NA	NA	NA	0	NA	NA	NA	NA	NA
ENSG00000241595	KRTAP9-4	keratin associated protein 9-4	85280	0	NA	NA	NA	NA	NA
ENSG00000241597	RPS9P3	ribosomal protein S9 pseudogene 3	100133054	0,072165449	0,847101401	4,080456	0,207599697	0,835541541	NA
ENSG00000241607	NA	NA	NA	0	NA	NA	NA	NA	NA
ENSG00000241612	RPL21P46	ribosomal protein L21 pseudogene 46	645174	0	NA	NA	NA	NA	NA
ENSG00000241621	NA	NA	NA	0	NA	NA	NA	NA	NA
ENSG00000241622	NA	NA	NA	0	NA	NA	NA	NA	NA
ENSG00000241627	NA	NA	NA	0	NA	NA	NA	NA	NA
ENSG00000241634	RPL13P8	ribosomal protein L13 pseudogene 8	100270928	1,72350469	1,17030626	1,672891	0,699571331	0,484195052	NA
ENSG00000241635	UGT1A1	UDP glucuronosyltransferase family 1 member A1	54658	0	NA	NA	NA	NA	NA
ENSG00000241640	NA	NA	NA	0	NA	NA	NA	NA	NA
ENSG00000241641	NA	NA	NA	0,440999951	0,081793751	3,516321	0,023261173	0,981441943	NA

ENSG00000241644	INMT	indolethylamine N-methyltransferase	11185	0,338380627	0,973816373	4,023537	0,242029914	0,808756984	NA	
ENSG00000241651	NA	NA	NA	0	NA	NA	NA	NA	NA	
ENSG00000241654	RPL19P18	ribosomal protein L19 pseudogene 18	100271288	0	NA	NA	NA	NA	NA	
ENSG00000241656	NA	NA	NA	0	NA	NA	NA	NA	NA	
ENSG00000241661	NA	NA	NA	0	NA	NA	NA	NA	NA	
ENSG00000241668	RPL19P11	ribosomal protein L19 pseudogene 11	100270950	0	NA	NA	NA	NA	NA	
ENSG00000241671	KIAA1328P1	KIAA1328 pseudogene 1	100418712	0	NA	NA	NA	NA	NA	
ENSG00000241673	NA	NA	NA	0	NA	NA	NA	NA	NA	
ENSG00000241678	RPL21P94	ribosomal protein L21 pseudogene 94	100271425	1,428002653	-0,087737128	1,998166	-0,043908824	0,964977082	NA	
ENSG00000241680	RPL31P49	ribosomal protein L31 pseudogene 49	100129882	0	NA	NA	NA	NA	NA	
ENSG00000241690	NA	NA	NA	0	NA	NA	NA	NA	NA	
ENSG00000241695	NA	NA	NA	0	NA	NA	NA	NA	NA	
ENSG00000241697	TMEFF1	transmembrane protein with EGF like and two follistatin like domains 1	8577	0,935865057	1,839147195	3,51319	0,523497784	0,600627893	NA	
ENSG00000241717	VWFP1	von Willebrand factor pseudogene 1	7451	0	NA	NA	NA	NA	NA	
ENSG00000241721	NA	NA	NA	0	NA	NA	NA	NA	NA	
ENSG00000241722	RPL36AP23	ribosomal protein L36a pseudogene 23	729075	0	NA	NA	NA	NA	NA	
ENSG00000241723	NA	NA	NA	0	NA	NA	NA	NA	NA	
ENSG00000241735	FABP5P3	fatty acid binding protein 5 pseudogene 3	220832	0,189682469	1,665152779	4,047523	0,411400484	0,680778899	NA	
ENSG00000241738	NA	NA	NA	0,10488184	-1,076443944	4,080456	-0,263804824	0,791930317	NA	
ENSG00000241739	RPL21P56	ribosomal protein L21 pseudogene 56	100271171	0	NA	NA	NA	NA	NA	
ENSG00000241741	RPL7AP30	ribosomal protein L7a pseudogene 30	441034	0,856696567	-0,254805423	1,874373	-0,135941688	0,891867379	NA	
ENSG00000241746	RPL13AP18	ribosomal protein L13a pseudogene 18	402342	0	NA	NA	NA	NA	NA	
ENSG00000241749	RPSAP52	ribosomal protein SA pseudogene 52	204010	0	NA	NA	NA	NA	NA	
ENSG00000241765	RPS26P45	ribosomal protein S26 pseudogene 45	100271570	0,265937128	0	4,080456	0	1	NA	
ENSG00000241776	TMEM183AP5	TMEM183A pseudogene 5	100130326	0	NA	NA	NA	NA	NA	
ENSG00000241777	NA	NA	NA	0	NA	NA	NA	NA	NA	
ENSG00000241782	RPL21P95	ribosomal protein L21 pseudogene 95	100271427	2,257000412	0,334583702	1,266399	0,264200818	0,791625181	NA	
ENSG00000241787	MTND4P16	MT-ND4 pseudogene 16	100873241	0	NA	NA	NA	NA	NA	
ENSG00000241788	NA	NA	NA	0	NA	NA	NA	NA	NA	
ENSG00000241790	NA	NA	NA	0	NA	NA	NA	NA	NA	
ENSG00000241794	SPRR2A	small proline rich protein 2A	6700	0	NA	NA	NA	NA	NA	
ENSG00000241804	SNRPB2P1	SNRPB2 pseudogene 1	100421672	0	NA	NA	NA	NA	NA	
ENSG00000241808	RPS15AP34	ribosomal protein S15a pseudogene 34	390735	0	NA	NA	NA	NA	NA	
ENSG00000241809	RPL17P21	ribosomal protein L17 pseudogene 21	100271151	0	NA	NA	NA	NA	NA	
ENSG00000241810	NA	NA	NA	0	NA	NA	NA	NA	NA	
ENSG00000241815	NA	NA	NA	0	NA	NA	NA	NA	NA	
ENSG00000241825	NA	NA	NA	0,437694046	-2,54139303	3,996313	-0,635934451	0,524819147	NA	
ENSG00000241828	RPS20P29	ribosomal protein S20 pseudogene 29	100271561	0	NA	NA	NA	NA	NA	
ENSG00000241829	RPL21P54	ribosomal protein L21 pseudogene 54	100271168	0	NA	NA	NA	NA	NA	
ENSG00000241838	NA	NA	NA	0	NA	NA	NA	NA	NA	
ENSG00000241846	NA	NA	NA	0	NA	NA	NA	NA	NA	
ENSG00000241853	RPL31P24	ribosomal protein L31 pseudogene 24	100271194	0	NA	NA	NA	NA	NA	
ENSG00000241859	NA	NA	NA	0	NA	NA	NA	NA	NA	
ENSG00000241861	GAPDHP50	glyceraldehyde 3 phosphate dehydrogenase pseudogene 50	100421236	0	NA	NA	NA	NA	NA	
ENSG00000241870	RPL13P10	ribosomal protein L13 pseudogene 10	100133108	0	NA	NA	NA	NA	NA	
ENSG00000241874	TOMM22P6	TOMM22 pseudogene 6	100287573	0	NA	NA	NA	NA	NA	
ENSG00000241877	RPL19P8	ribosomal protein L19 pseudogene 8	391719	0	NA	NA	NA	NA	NA	
ENSG00000241879	NA	NA	NA	0	NA	NA	NA	NA	NA	
ENSG00000241888	NA	NA	NA	0	NA	NA	NA	NA	NA	
ENSG00000241889	NA	NA	NA	0,10543123	0	4,080456	0	1	NA	
ENSG00000241890	RPL13P4	ribosomal protein L13 pseudogene 4	654383	0	NA	NA	NA	NA	NA	
ENSG00000241891	RPL9P6	ribosomal protein L9 pseudogene 6	326311	0	NA	NA	NA	NA	NA	

ENSG00000241899	TPT1P3	TPT1 pseudogene 3	100421439	0	NA	NA	NA	NA	NA
ENSG00000241905	NDUFS6P1	NADH:ubiquinone oxidoreductase subunit S6 pseudogene 1	107075209	0	NA	NA	NA	NA	NA
ENSG00000241907	NA	NA	NA	0	NA	NA	NA	NA	NA
ENSG00000241911	TRBVB	T cell receptor beta variable B (pseudogene)	28555	0	NA	NA	NA	NA	NA
ENSG00000241913	NA	NA	NA	0	NA	NA	NA	NA	NA
ENSG00000241917	RPL9P24	ribosomal protein L9 pseudogene 24	100271045	0	NA	NA	NA	NA	NA
ENSG00000241923	RPL14P3	ribosomal protein L14 pseudogene 3	730747	0,118286652	0	4,080456	0	1	NA
ENSG00000241926	MTCO1P55	MT-CO1 pseudogene 55	107075179	0	NA	NA	NA	NA	NA
ENSG00000241929	NA	NA	NA	0	NA	NA	NA	NA	NA
ENSG00000241932	NA	NA	NA	0	NA	NA	NA	NA	NA
ENSG00000241935	HOGA1	4-hydroxy-2-oxoglutarate aldolase 1	112817	0	NA	NA	NA	NA	NA
ENSG00000241941	RPL32P26	ribosomal protein L32 pseudogene 26	100271491	0	NA	NA	NA	NA	NA
ENSG00000241942	RPS20P20	ribosomal protein S20 pseudogene 20	100271233	0	NA	NA	NA	NA	NA
ENSG00000241946	NA	NA	NA	0	NA	NA	NA	NA	NA
ENSG00000241947	NA	NA	NA	0,22278444	-2,001753131	4,035241	-0,496067796	0,619846579	NA
ENSG00000241950	NA	NA	NA	0	NA	NA	NA	NA	NA
ENSG00000241961	RPL17P31	ribosomal protein L17 pseudogene 31	100271156	0	NA	NA	NA	NA	NA
ENSG00000241962	NA	NA	NA	1,671044877	-0,66299528	2,375104	-0,279143641	0,780134593	NA
ENSG00000241965	NA	NA	NA	0	NA	NA	NA	NA	NA
ENSG00000241975	NA	NA	NA	6,769199866	0,178321539	0,791803	0,225209412	0,821816367	NA
ENSG00000241981	RPL21P49	ribosomal protein L21 pseudogene 49	100271166	0	NA	NA	NA	NA	NA
ENSG00000241984	NA	NA	NA	0,10543123	0	4,080456	0	1	NA
ENSG00000241991	NA	NA	NA	0	NA	NA	NA	NA	NA
ENSG00000241993	NA	NA	NA	0	NA	NA	NA	NA	NA
ENSG00000242001	NA	NA	NA	0	NA	NA	NA	NA	NA
ENSG00000242017	NA	NA	NA	0	NA	NA	NA	NA	NA
ENSG00000242019	KIR3DL3	killer cell immunoglobulin like receptor, three Ig domains and long cytoplasmic tail 3	115653	0,210862461	0	4,080456	0	1	NA
ENSG00000242034	NA	NA	NA	0	NA	NA	NA	NA	NA
ENSG00000242036	NA	NA	NA	0	NA	NA	NA	NA	NA
ENSG00000242041	NA	NA	NA	0	NA	NA	NA	NA	NA
ENSG00000242052	RPL10P7	ribosomal protein L10 pseudogene 7	442086	0,118286652	0	4,080456	0	1	NA
ENSG00000242058	RPS4XP19	ribosomal protein S4X pseudogene 19	645573	0	NA	NA	NA	NA	NA
ENSG00000242060	RPS3AP49	RPS3A pseudogene 49	400652	0	NA	NA	NA	NA	NA
ENSG00000242061	RPL26P2	ribosomal protein L26 pseudogene 2	326297	0	NA	NA	NA	NA	NA
ENSG00000242062	NA	NA	NA	0	NA	NA	NA	NA	NA
ENSG00000242067	RPL9P28	ribosomal protein L9 pseudogene 28	729146	0	NA	NA	NA	NA	NA
ENSG00000242068	NA	NA	NA	0	NA	NA	NA	NA	NA
ENSG00000242070	NPM1P17	nucleophosmin 1 pseudogene 17	10830	0	NA	NA	NA	NA	NA
ENSG00000242071	RPL7AP6	ribosomal protein L7a pseudogene 6	326310	0,11808024	-1,076443944	4,080456	-0,263804824	0,791930317	NA
ENSG00000242073	NA	NA	NA	6,59162407	0,235511945	0,792433	0,297201022	0,766313042	NA
ENSG00000242080	RPL36AP20	ribosomal protein L36a pseudogene 20	100271329	0	NA	NA	NA	NA	NA
ENSG00000242083	RPL7AP31	ribosomal protein L7a pseudogene 31	728315	0	NA	NA	NA	NA	NA
ENSG00000242085	RPS20P33	ribosomal protein S20 pseudogene 33	100271564	1,281588838	1,05126985	2,228426	0,471754386	0,637102113	NA
ENSG00000242087	RPL36AP41	ribosomal protein L36a pseudogene 41	100271644	0	NA	NA	NA	NA	NA
ENSG00000242088	NA	NA	NA	0,119673877	0,847101401	4,080456	0,207599697	0,835541541	NA
ENSG00000242097	NA	NA	NA	0	NA	NA	NA	NA	NA
ENSG00000242100	RPL9P32	ribosomal protein L9 pseudogene 32	727865	0,430214611	-1,568937214	3,476496	-0,451298393	0,651774503	NA
ENSG00000242103	ARPC1AP1	ARPC1A pseudogene 1	100422044	0	NA	NA	NA	NA	NA
ENSG00000242104	NA	NA	NA	0	NA	NA	NA	NA	NA
ENSG00000242109	NPM1P23	nucleophosmin 1 pseudogene 23	100422256	0	NA	NA	NA	NA	NA
ENSG00000242111	NA	NA	NA	0	NA	NA	NA	NA	NA
ENSG00000242119	NA	NA	NA	0	NA	NA	NA	NA	NA
ENSG00000242120	MDFIC2	MyoD family inhibitor domain containing 2	107986096	0	NA	NA	NA	NA	NA

ENSG00000242123	RPL36AP47	ribosomal protein L36a pseudogene 47	100271647	0	NA	NA	NA	NA	NA
ENSG00000242134	RPL5P13	ribosomal protein L5 pseudogene 13	100270834	0,333893106	0,928368447	4,027104	0,230530012	0,817679945	NA
ENSG00000242135	RPL17P2	ribosomal protein L17 pseudogene 2	652974	0	NA	NA	NA	NA	NA
ENSG00000242137	RPL11P5	ribosomal protein L11 pseudogene 5	643499	0	NA	NA	NA	NA	NA
ENSG00000242140	NA	NA	NA	0	NA	NA	NA	NA	NA
ENSG00000242142	NA	NA	NA	0	NA	NA	NA	NA	NA
ENSG00000242151	NA	NA	NA	0	NA	NA	NA	NA	NA
ENSG00000242152	NA	NA	NA	0	NA	NA	NA	NA	NA
ENSG00000242153	OFD1P6Y	OFD1 pseudogene 6 Y-linked	386690	0	NA	NA	NA	NA	NA
ENSG00000242156	NA	NA	NA	0	NA	NA	NA	NA	NA
ENSG00000242159	ABCF2P1	ATP binding cassette subfamily F member 2 pseudogene 1	344653	0	NA	NA	NA	NA	NA
ENSG00000242162	NA	NA	NA	0	NA	NA	NA	NA	NA
ENSG00000242163	NA	NA	NA	0	NA	NA	NA	NA	NA
ENSG00000242169	RPL7AP27	ribosomal protein L7a pseudogene 27	152663	0	NA	NA	NA	NA	NA
ENSG00000242173	ARHGDIG	Rho GDP dissociation inhibitor gamma	398	0	NA	NA	NA	NA	NA
ENSG00000242176	NA	NA	NA	0	NA	NA	NA	NA	NA
ENSG00000242178	NA	NA	NA	0	NA	NA	NA	NA	NA
ENSG00000242186	NA	NA	NA	0	NA	NA	NA	NA	NA
ENSG00000242193	CRYZL2P	crystallin zeta like 2, pseudogene	730102	0	NA	NA	NA	NA	NA
ENSG00000242195	NA	NA	NA	0	NA	NA	NA	NA	NA
ENSG00000242197	RPL37P14	ribosomal protein L37 pseudogene 14	100271019	0	NA	NA	NA	NA	NA
ENSG00000242198	NA	NA	NA	0	NA	NA	NA	NA	NA
ENSG00000242199	RPS26P22	ribosomal protein S26 pseudogene 22	100271112	0	NA	NA	NA	NA	NA
ENSG00000242206	NA	NA	NA	0	NA	NA	NA	NA	NA
ENSG00000242209	NA	NA	NA	0	NA	NA	NA	NA	NA
ENSG00000242214	MTND3P6	MT-ND3 pseudogene 6	100873182	0	NA	NA	NA	NA	NA
ENSG00000242220	TCP10L	t-complex 10 like	140290	0	NA	NA	NA	NA	NA
ENSG00000242221	PSG2	pregnancy specific beta-1-glycoprotein 2	5670	0	NA	NA	NA	NA	NA
ENSG00000242222	SDHBP1	SDHB pseudogene 1	127460886	0	NA	NA	NA	NA	NA
ENSG00000242229	RPS3AP14	RPS3A pseudogene 14	729150	0	NA	NA	NA	NA	NA
ENSG00000242244	NA	NA	NA	0	NA	NA	NA	NA	NA
ENSG00000242246	NA	NA	NA	0	NA	NA	NA	NA	NA
ENSG00000242248	RPL7AP57	ribosomal protein L7a pseudogene 57	100271537	0	NA	NA	NA	NA	NA
ENSG00000242252	BGLAP	bone gamma-carboxyglutamate protein	632	1,861567875	-0,572740423	1,771618	-0,323286628	0,74647818	NA
ENSG00000242255	NA	NA	NA	0	NA	NA	NA	NA	NA
ENSG00000242261	LOC100418961	aurora kinase A pseudogene	100418961	0	NA	NA	NA	NA	NA
ENSG00000242262	NA	NA	NA	0	NA	NA	NA	NA	NA
ENSG00000242265	PEG10	paternally expressed 10	23089	0,958209288	0,293156776	1,875844	0,156279955	0,875812356	NA
ENSG00000242267	NA	NA	NA	0	NA	NA	NA	NA	NA
ENSG00000242272	NA	NA	NA	0,472716489	0,001164615	3,466293	0,000335983	0,999731924	NA
ENSG00000242276	RPL5P3	ribosomal protein L5 pseudogene 3	400385	0	NA	NA	NA	NA	NA
ENSG00000242278	RPS3AP48	RPS3A pseudogene 48	100127963	0	NA	NA	NA	NA	NA
ENSG00000242279	RPL7AP54	ribosomal protein L7a pseudogene 54	390228	0	NA	NA	NA	NA	NA
ENSG00000242285	NA	NA	NA	0	NA	NA	NA	NA	NA
ENSG00000242291	RPL36AP51	ribosomal protein L36a pseudogene 51	649299	0	NA	NA	NA	NA	NA
ENSG00000242292	NA	NA	NA	0	NA	NA	NA	NA	NA
ENSG00000242293	NA	NA	NA	0	NA	NA	NA	NA	NA
ENSG00000242295	RPL31P55	ribosomal protein L31 pseudogene 55	100128250	0	NA	NA	NA	NA	NA
ENSG00000242296	LOC124905442	beta-defensin 109	124905442	0	NA	NA	NA	NA	NA
ENSG00000242299	RPS18P5	ribosomal protein S18 pseudogene 5	100131863	0	NA	NA	NA	NA	NA
ENSG00000242307	NA	NA	NA	0,15836288	0	4,080456	0	1	NA
ENSG00000242308	NA	NA	NA	0	NA	NA	NA	NA	NA
ENSG00000242314	NA	NA	NA	0	NA	NA	NA	NA	NA
ENSG00000242318	RPL21P48	ribosomal protein L21 pseudogene 48	100271165	0,987183617	0,080708772	2,14534	0,037620502	0,969990261	NA
ENSG00000242320	RPL21P126	ribosomal protein L21 pseudogene 126	100271449	0	NA	NA	NA	NA	NA
ENSG00000242321	RPL23AP40	ribosomal protein L23a pseudogene 40	100271462	0	NA	NA	NA	NA	NA

ENSG00000242324	NA	NA	NA	0,072165449	0,847101401	4,080456	0,207599697	0,835541541	NA
ENSG00000242325	RPS12P31	ribosomal protein S12 pseudogene 31	100130192	0	NA	NA	NA	NA	NA
ENSG00000242326	NA	NA	NA	0	NA	NA	NA	NA	NA
ENSG00000242327	RPS13P8	ribosomal protein S13 pseudogene 8	645630	0	NA	NA	NA	NA	NA
ENSG00000242329	RPL37AP9	ribosomal protein L37a pseudogene 9	100271606	0	NA	NA	NA	NA	NA
ENSG00000242337	NA	NA	NA	0,22276517	0,839475749	4,080456	0,205730873	0,837001141	NA
ENSG00000242352	NA	NA	NA	2,858287829	4,846808751	1,754156	2,763043699	0,00572651	NA
ENSG00000242353	NA	NA	NA	0	NA	NA	NA	NA	NA
ENSG00000242358	NA	NA	NA	0	NA	NA	NA	NA	NA
ENSG00000242364	PRDX5P1	PRDX5 pseudogene 1	111501764	0	NA	NA	NA	NA	NA
ENSG00000242365	NA	NA	NA	0	NA	NA	NA	NA	NA
ENSG00000242366	UGT1A8	UDP glucuronosyltransferase family 1 member A8	54576	0	NA	NA	NA	NA	NA
ENSG00000242387	NA	NA	NA	0	NA	NA	NA	NA	NA
ENSG00000242389	RBMY1E	RNA binding motif protein Y-linked family 1 member E	378950	0	NA	NA	NA	NA	NA
ENSG00000242390	NA	NA	NA	0	NA	NA	NA	NA	NA
ENSG00000242393	NA	NA	NA	0	NA	NA	NA	NA	NA
ENSG00000242399	RPS20P23	ribosomal protein S20 pseudogene 23	100271089	0	NA	NA	NA	NA	NA
ENSG00000242411	NA	NA	NA	0	NA	NA	NA	NA	NA
ENSG00000242412	DBIL5P2	diazepam binding inhibitor-like 5 pseudogene 2	100169989	0	NA	NA	NA	NA	NA
ENSG00000242417	RPL18P1	ribosomal protein L18 pseudogene 1	326291	0	NA	NA	NA	NA	NA
ENSG00000242419	PCDHGC4	protocadherin gamma subfamily C, 4	56098	0	NA	NA	NA	NA	NA
ENSG00000242423	RPL12P36	ribosomal protein L12 pseudogene 36	100271408	0	NA	NA	NA	NA	NA
ENSG00000242431	RPL21P52	ribosomal protein L21 pseudogene 52	100128351	0,072165449	0,847101401	4,080456	0,207599697	0,835541541	NA
ENSG00000242435	UPK3BP1	uroplakin 3B pseudogene 1	107080637	1,550962594	-1,220559577	1,402819	-0,870076538	0,384258579	NA
ENSG00000242439	RPS17P3	ribosomal protein S17 pseudogene 3	646013	0	NA	NA	NA	NA	NA
ENSG00000242441	GTF2A1L	general transcription factor IIA subunit 1 like	11036	0	NA	NA	NA	NA	NA
ENSG00000242444	RPL18P9	ribosomal protein L18 pseudogene 9	100129645	0,119673877	0,847101401	4,080456	0,207599697	0,835541541	NA
ENSG00000242445	RPL7AP11	ribosomal protein L7a pseudogene 11	728992	0,459388967	1,556861893	3,447855	0,451545018	0,651596787	NA
ENSG00000242456	MTCO3P38	MT-CO3 pseudogene 38	107075187	0	NA	NA	NA	NA	NA
ENSG00000242457	RBBP4P2	RBBP4 pseudogene 2	100420527	0	NA	NA	NA	NA	NA
ENSG00000242461	RPS15AP32	ribosomal protein S15a pseudogene 32	100271649	0	NA	NA	NA	NA	NA
ENSG00000242472	IGHJ5	immunoglobulin heavy joining 5	28476	0	NA	NA	NA	NA	NA
ENSG00000242477	RPL10AP9	ribosomal protein L10a pseudogene 9	728979	0,209486148	-1,06807294	4,080456	-0,261753337	0,793511617	NA
ENSG00000242479	EIF2AK1P1	EIF2AK1 pseudogene 1	731755	0	NA	NA	NA	NA	NA
ENSG00000242488	NA	NA	NA	0	NA	NA	NA	NA	NA
ENSG00000242498	ARPIN	actin related protein 2/3 complex inhibitor	348110	0,234994216	1,944111989	4,02324	0,483220515	0,62893917	NA
ENSG00000242507	NA	NA	NA	0	NA	NA	NA	NA	NA
ENSG00000242510	NDUFB4P1	NADH:ubiquinone oxidoreductase subunit B4 pseudogene 1	100288654	0	NA	NA	NA	NA	NA
ENSG00000242515	UGT1A10	UDP glucuronosyltransferase family 1 member A10	54575	0	NA	NA	NA	NA	NA
ENSG00000242520	NA	NA	NA	0	NA	NA	NA	NA	NA
ENSG00000242524	OR2U2P	olfactory receptor family 2 subfamily U member 2 pseudogene	26694	0	NA	NA	NA	NA	NA
ENSG00000242525	OR7E100P	olfactory receptor family 7 subfamily E member 100 pseudogene	81430	0	NA	NA	NA	NA	NA
ENSG00000242527	NA	NA	NA	0	NA	NA	NA	NA	NA
ENSG00000242529	NA	NA	NA	0	NA	NA	NA	NA	NA
ENSG00000242531	NA	NA	NA	0	NA	NA	NA	NA	NA
ENSG00000242534	IGKV2D-28	immunoglobulin kappa variable 2D-28	28883	0	NA	NA	NA	NA	NA
ENSG00000242537	NA	NA	NA	0	NA	NA	NA	NA	NA
ENSG00000242550	SERPINB10	serpin family B member 10	5273	0	NA	NA	NA	NA	NA
ENSG00000242551	POU5F1P6	POU class 5 homeobox 1 pseudogene 6	100009668	0,119673877	0,847101401	4,080456	0,207599697	0,835541541	NA
ENSG00000242552	NA	NA	NA	0	NA	NA	NA	NA	NA
ENSG00000242561	NA	NA	NA	0	NA	NA	NA	NA	NA

ENSG00000242562	DCAF13P1	DDB1 and CUL4 associated factor 13 pseudogene 1	100129009	0	NA	NA	NA	NA	NA
ENSG00000242571	RPL21P11	ribosomal protein L21 pseudogene 11	157206	0	NA	NA	NA	NA	NA
ENSG00000242573	NA	NA	NA	0	NA	NA	NA	NA	NA
ENSG00000242574	HLA-DMB	major histocompatibility complex, class II, DM beta	3109	0	NA	NA	NA	NA	NA
ENSG00000242575	TUBAP13	tubulin alpha pseudogene 13	642635	0	NA	NA	NA	NA	NA
ENSG00000242580	IGKV1D-43	immunoglobulin kappa variable 1D-43	28891	0	NA	NA	NA	NA	NA
ENSG00000242583	KMT5AP3	KMT5A pseudogene 3	100421819	0	NA	NA	NA	NA	NA
ENSG00000242586	HYDINP1	HYDIN pseudogene 1	127379712	0	NA	NA	NA	NA	NA
ENSG00000242595	RPL26P26	ribosomal protein L26 pseudogene 26	100270967	0	NA	NA	NA	NA	NA
ENSG00000242598	NA	NA	NA	0	NA	NA	NA	NA	NA
ENSG00000242599	NA	NA	NA	0	NA	NA	NA	NA	NA
ENSG00000242600	MBL1P	mannose binding lectin 1, pseudogene	8512	0	NA	NA	NA	NA	NA
ENSG00000242602	NA	NA	NA	0	NA	NA	NA	NA	NA
ENSG00000242608	RPS23P5	ribosomal protein S23 pseudogene 5	100271093	0	NA	NA	NA	NA	NA
ENSG00000242609	CCDC137P1	CCDC137 pseudogene 1	642802	0	NA	NA	NA	NA	NA
ENSG00000242610	OR5BH1P	olfactory receptor family 5 subfamily BH member 1 pseudogene	81075	0	NA	NA	NA	NA	NA
ENSG00000242612	DECR2	2,4-dienoyl-CoA reductase 2	26063	5,57239288	0,227620397	0,75399	0,301887681	0,762737687	NA
ENSG00000242613	NA	NA	NA	0	NA	NA	NA	NA	NA
ENSG00000242615	NA	NA	NA	1,311426486	-0,085209207	2,196635	-0,038790798	0,969057181	NA
ENSG00000242634	NA	NA	NA	0	NA	NA	NA	NA	NA
ENSG00000242635	RPS14P7	ribosomal protein S14 pseudogene 7	100271065	0	NA	NA	NA	NA	NA
ENSG00000242636	RPL21P129	ribosomal protein L21 pseudogene 129	100271451	0	NA	NA	NA	NA	NA
ENSG00000242640	NA	NA	NA	0	NA	NA	NA	NA	NA
ENSG00000242654	RPL32P14	ribosomal protein L32 pseudogene 14	100270994	0	NA	NA	NA	NA	NA
ENSG00000242660	NA	NA	NA	0	NA	NA	NA	NA	NA
ENSG00000242661	RPS3AP43	RPS3A pseudogene 43	727970	0	NA	NA	NA	NA	NA
ENSG00000242667	RPS10P22	ribosomal protein S10 pseudogene 22	401817	0	NA	NA	NA	NA	NA
ENSG00000242670	RPL22P13	ribosomal protein L22 pseudogene 13	442108	0	NA	NA	NA	NA	NA
ENSG00000242675	NA	NA	NA	0	NA	NA	NA	NA	NA
ENSG00000242683	NA	NA	NA	0,69625254	1,979263385	2,662326	0,743433867	0,457219047	NA
ENSG00000242692	RPS27AP1	RPS27A pseudogene 1	103911	0	NA	NA	NA	NA	NA
ENSG00000242697	RPL5P12	ribosomal protein L5 pseudogene 12	100270833	0	NA	NA	NA	NA	NA
ENSG00000242703	NA	NA	NA	0	NA	NA	NA	NA	NA
ENSG00000242705	ICE2P2	interactor of little elongation complex ELL subunit 2 pseudogene 2	728432	0	NA	NA	NA	NA	NA
ENSG00000242706	RPS27AP9	RPS27A pseudogene 9	100271376	0	NA	NA	NA	NA	NA
ENSG00000242709	RPL30P9	ribosomal protein L30 pseudogene 9	100270981	0	NA	NA	NA	NA	NA
ENSG00000242715	CCDC169	coiled-coil domain containing 169	728591	0	NA	NA	NA	NA	NA
ENSG00000242727	NA	NA	NA	0	NA	NA	NA	NA	NA
ENSG00000242728	UQCRHP4	ubiquinol-cytochrome c reductase hinge protein pseudogene 4	100128448	0	NA	NA	NA	NA	NA
ENSG00000242729	NA	NA	NA	0	NA	NA	NA	NA	NA
ENSG00000242731	FAM86LP	family with sequence similarity 86, member A pseudogene	641924	0	NA	NA	NA	NA	NA
ENSG00000242732	RTL5	retrotransposon Gag like 5	340526	1,778427749	-1,121868767	2,755342	-0,407161295	0,683889527	NA
ENSG00000242735	NA	NA	NA	1,359293532	0,646949298	2,460004	0,262987073	0,792560542	NA
ENSG00000242736	NA	NA	NA	0	NA	NA	NA	NA	NA
ENSG00000242737	NA	NA	NA	0,812283354	-0,960948479	2,585024	-0,371736842	0,710088792	NA
ENSG00000242747	RPL21P14	ribosomal protein L21 pseudogene 14	729208	0,221369794	-0,12441328	4,080456	-0,030490044	0,975676233	NA
ENSG00000242748	RPL23AP81	ribosomal protein L23a pseudogene 81	100271627	1,913847607	0,943527654	1,60652	0,587311636	0,556994428	NA
ENSG00000242752	NMTRQ-TTG12-1	nuclear-encoded mitochondrial tRNA-Gln (TTG) 12-1	100189478	0	NA	NA	NA	NA	NA
ENSG00000242756	NA	NA	NA	0	NA	NA	NA	NA	NA
ENSG00000242757	RPS18P3	ribosomal protein S18 pseudogene 3	100128258	0	NA	NA	NA	NA	NA
ENSG00000242766	IGKV1D-17	immunoglobulin kappa variable 1D-17	28900	0	NA	NA	NA	NA	NA

ENSG00000242768	RPL31P25	ribosomal protein L31 pseudogene 25	100270987	0	NA	NA	NA	NA	NA
ENSG00000242771	NA	NA	NA	0	NA	NA	NA	NA	NA
ENSG00000242779	ZNF702P	zinc finger protein 702, pseudogene	79986	0,615376513	-1,08337051	3,290158	-0,329276131	0,741946984	NA
ENSG00000242793	RPL9P26	ribosomal protein L9 pseudogene 26	100271227	0	NA	NA	NA	NA	NA
ENSG00000242807	RPL26P31	ribosomal protein L26 pseudogene 31	100271469	0	NA	NA	NA	NA	NA
ENSG00000242810	MRPL42P6	mitochondrial ribosomal protein L42 pseudogene 6	106480794	0	NA	NA	NA	NA	NA
ENSG00000242814	NA	NA	NA	0	NA	NA	NA	NA	NA
ENSG00000242829	RPS26P21	ribosomal protein S26 pseudogene 21	100271105	0,532429376	0,445885774	2,435491	0,183078375	0,854736523	NA
ENSG00000242834	RPL7AP56	ribosomal protein L7a pseudogene 56	100271536	0	NA	NA	NA	NA	NA
ENSG00000242836	MTCO3P35	MT-CO3 pseudogene 35	107075287	0	NA	NA	NA	NA	NA
ENSG00000242837	RPL21P13	ribosomal protein L21 pseudogene 13	319144	0	NA	NA	NA	NA	NA
ENSG00000242841	KRT8P35	keratin 8 pseudogene 35	100418747	0	NA	NA	NA	NA	NA
ENSG00000242849	NA	NA	NA	0	NA	NA	NA	NA	NA
ENSG00000242850	NA	NA	NA	0	NA	NA	NA	NA	NA
ENSG00000242852	ZNF709	zinc finger protein 709	163051	9,603533134	1,401245862	2,720429	0,515082633	0,606495278	NA
ENSG00000242854	NA	NA	NA	0	NA	NA	NA	NA	NA
ENSG00000242856	VN1R105P	vomeronasal 1 receptor 105 pseudogene	100288264	0	NA	NA	NA	NA	NA
ENSG00000242858	RPL13AP14	ribosomal protein L13a pseudogene 14	100271278	0	NA	NA	NA	NA	NA
ENSG00000242866	STRC	stereocilin	161497	0,590903223	0,84672926	4,021965	0,210526273	0,833256948	NA
ENSG00000242875	RBMY1B	RNA binding motif protein Y-linked family 1 member B	378948	0	NA	NA	NA	NA	NA
ENSG00000242882	RPL5P11	ribosomal protein L5 pseudogene 11	402193	0	NA	NA	NA	NA	NA
ENSG00000242887	IGHJ3	immunoglobulin heavy joining 3	28479	0	NA	NA	NA	NA	NA
ENSG00000242888	RPS2P3	ribosomal protein S2 pseudogene 3	326326	0	NA	NA	NA	NA	NA
ENSG00000242899	RPL7P16	ribosomal protein L7 pseudogene 16	729677	0	NA	NA	NA	NA	NA
ENSG00000242915	NA	NA	NA	0	NA	NA	NA	NA	NA
ENSG00000242922	RPL21P104	ribosomal protein L21 pseudogene 104	100271435	0	NA	NA	NA	NA	NA
ENSG00000242931	RPL7P49	ribosomal protein L7 pseudogene 49	100271221	1,50528472	-0,553636078	1,763901	-0,313870227	0,753619604	NA
ENSG00000242936	RPL30P6	ribosomal protein L30 pseudogene 6	100270980	0	NA	NA	NA	NA	NA
ENSG00000242941	RPL12P7	ribosomal protein L12 pseudogene 7	326275	0,216496347	1,902771968	4,026559	0,472555327	0,63653046	NA
ENSG00000242943	NA	NA	NA	0	NA	NA	NA	NA	NA
ENSG00000242945	NA	NA	NA	0	NA	NA	NA	NA	NA
ENSG00000242948	NA	NA	NA	0	NA	NA	NA	NA	NA
ENSG00000242950	ERVW-1	endogenous retrovirus group W member 1, envelope	30816	0	NA	NA	NA	NA	NA
ENSG00000242951	NA	NA	NA	0	NA	NA	NA	NA	NA
ENSG00000242952	RPSAP3	ribosomal protein SA pseudogene 3	158674	0	NA	NA	NA	NA	NA
ENSG00000242958	RPL36AP32	ribosomal protein L36a pseudogene 32	100271512	0,10488184	-1,076443944	4,080456	-0,263804824	0,791930317	NA
ENSG00000242960	NA	NA	NA	0,118286652	0	4,080456	0	1	NA
ENSG00000242963	RPS20P30	ribosomal protein S20 pseudogene 30	100271562	0	NA	NA	NA	NA	NA
ENSG00000242968	SULT1D1P2	sulfotransferase family 1D member 1 pseudogene 2	127482547	0	NA	NA	NA	NA	NA
ENSG00000242970	NA	NA	NA	0,209570285	-1,061799434	4,080456	-0,260215885	0,794697252	NA
ENSG00000242978	PPIAP83	peptidylprolyl isomerase A pseudogene 83	111082969	0	NA	NA	NA	NA	NA
ENSG00000242979	RPS15AP18	ribosomal protein S15a pseudogene 18	100271360	0	NA	NA	NA	NA	NA
ENSG00000242983	CABYRP1	calcium binding tyrosine phosphorylation regulated pseudogene 1	266954	0	NA	NA	NA	NA	NA
ENSG00000242986	RPL21P99	ribosomal protein L21 pseudogene 99	100271429	0	NA	NA	NA	NA	NA
ENSG00000242990	NA	NA	NA	0	NA	NA	NA	NA	NA
ENSG00000242991	RPL7P40	ribosomal protein L7 pseudogene 40	100271220	0	NA	NA	NA	NA	NA
ENSG00000242992	NA	NA	NA	0	NA	NA	NA	NA	NA
ENSG00000242993	RPL32P25	ribosomal protein L32 pseudogene 25	100271490	0	NA	NA	NA	NA	NA

ENSG00000242995	RPL36AP1	ribosomal protein L36a pseudogene 1	100131379	0	NA	NA	NA	NA	NA
ENSG00000243007	NA	NA	NA	0	NA	NA	NA	NA	NA
ENSG00000243014	NA	NA	NA	0	NA	NA	NA	NA	NA
ENSG00000243016	NA	NA	NA	0	NA	NA	NA	NA	NA
ENSG00000243020	RPL7P39	ribosomal protein L7 pseudogene 39	100271217	0	NA	NA	NA	NA	NA
ENSG00000243022	NA	NA	NA	0	NA	NA	NA	NA	NA
ENSG00000243023	NA	NA	NA	0	NA	NA	NA	NA	NA
ENSG00000243024	NA	NA	NA	0	NA	NA	NA	NA	NA
ENSG00000243025	NA	NA	NA	0	NA	NA	NA	NA	NA
ENSG00000243033	GAPDHP47	glyceraldehyde 3 phosphate dehydrogenase pseudogene 47	100240712	0,427145674	1,400144739	2,940015	0,476237226	0,633905382	NA
ENSG00000243038	NA	NA	NA	0	NA	NA	NA	NA	NA
ENSG00000243040	NA	NA	NA	0	NA	NA	NA	NA	NA
ENSG00000243044	NA	NA	NA	0	NA	NA	NA	NA	NA
ENSG00000243048	NA	NA	NA	0	NA	NA	NA	NA	NA
ENSG00000243050	RPL30P10	ribosomal protein L30 pseudogene 10	100270982	0	NA	NA	NA	NA	NA
ENSG00000243053	RPL31P58	ribosomal protein L31 pseudogene 58	100271485	0	NA	NA	NA	NA	NA
ENSG00000243056	EIF4EBP3	eukaryotic translation initiation factor 4E binding protein 3	8637	1,795385959	-0,574551428	1,65554	-0,347047713	0,728555474	NA
ENSG00000243058	NA	NA	NA	0	NA	NA	NA	NA	NA
ENSG00000243064	NA	NA	NA	0	NA	NA	NA	NA	NA
ENSG00000243071	RPL21P98	ribosomal protein L21 pseudogene 98	653079	0,854418146	-2,032969055	2,285644	-0,889451168	0,373760656	NA
ENSG00000243072	YTHDF3P1	YTHDF3 pseudogene 1	127379726	0	NA	NA	NA	NA	NA
ENSG00000243085	NA	NA	NA	0	NA	NA	NA	NA	NA
ENSG00000243094	NA	NA	NA	0,779891084	-1,020423281	2,469505	-0,413209676	0,679453	NA
ENSG00000243095	NA	NA	NA	0	NA	NA	NA	NA	NA
ENSG00000243099	RPL17P27	ribosomal protein L17 pseudogene 27	202789	0	NA	NA	NA	NA	NA
ENSG00000243101	RPS3P7	ribosomal protein S3 pseudogene 7	645957	0,221941749	-1,996861323	4,035682	-0,494801459	0,620740274	NA
ENSG00000243104	NA	NA	NA	0	NA	NA	NA	NA	NA
ENSG00000243116	NA	NA	NA	0	NA	NA	NA	NA	NA
ENSG00000243122	RPS15P8	ribosomal protein S15 pseudogene 8	390576	0	NA	NA	NA	NA	NA
ENSG00000243129	RPS26P46	ribosomal protein S26 pseudogene 46	100271572	0	NA	NA	NA	NA	NA
ENSG00000243130	PSG11	pregnancy specific beta-1-glycoprotein 11	5680	0	NA	NA	NA	NA	NA
ENSG00000243137	PSG4	pregnancy specific beta-1-glycoprotein 4	5672	0	NA	NA	NA	NA	NA
ENSG00000243154	SYPL1P1	synaptophysin like 1 pseudogene 1	100130011	0	NA	NA	NA	NA	NA
ENSG00000243160	RPL7AP32	ribosomal protein L7a pseudogene 32	133748	0	NA	NA	NA	NA	NA
ENSG00000243164	RPL31P48	ribosomal protein L31 pseudogene 48	645654	0	NA	NA	NA	NA	NA
ENSG00000243165	RFKP6	RFK pseudogene 6	127482545	0	NA	NA	NA	NA	NA
ENSG00000243167	NA	NA	NA	0,10488184	-1,076443944	4,080456	-0,263804824	0,791930317	NA
ENSG00000243171	RPL17P32	ribosomal protein L17 pseudogene 32	100270942	0	NA	NA	NA	NA	NA
ENSG00000243175	RPSAP36	ribosomal protein SA pseudogene 36	100128888	0,564390631	-2,55585773	3,279836	-0,779263985	0,435824225	NA
ENSG00000243181	RPL21P80	ribosomal protein L21 pseudogene 80	100133273	0,11808024	-1,076443944	4,080456	-0,263804824	0,791930317	NA
ENSG00000243188	HNRNPA1P17	heterogeneous nuclear ribonucleoprotein A1 pseudogene 17	100421376	0	NA	NA	NA	NA	NA
ENSG00000243195	TUBBP11	tubulin beta class I pseudogene 11	106480745	0	NA	NA	NA	NA	NA
ENSG00000243199	RPL6P10	ribosomal protein L6 pseudogene 10	642828	2,21003443	-0,125465926	1,209331	-0,10374825	0,917369135	NA
ENSG00000243207	PPAN-P2RY11	PPAN-P2RY11 readthrough	692312	4,158688204	0,365587432	3,753971	0,097386844	0,922419192	NA
ENSG00000243225	NA	NA	NA	0	NA	NA	NA	NA	NA
ENSG00000243234	RPL19P19	ribosomal protein L19 pseudogene 19	646820	0	NA	NA	NA	NA	NA
ENSG00000243236	GSTA9P	glutathione S-transferase alpha 9, pseudogene	100174857	0	NA	NA	NA	NA	NA
ENSG00000243244	STON1	stonin 1	11037	1,352602998	-2,981530518	2,049114	-1,455033994	0,145659851	NA
ENSG00000243250	RPS6P16	ribosomal protein S6 pseudogene 16	100128923	0	NA	NA	NA	NA	NA
ENSG00000243256	NA	NA	NA	0	NA	NA	NA	NA	NA
ENSG00000243257	MTCO2P29	MT-CO2 pseudogene 29	107075194	0	NA	NA	NA	NA	NA

ENSG00000243264	IGKV2D-29	immunoglobulin kappa variable 2D-29	28882	0	NA	NA	NA	NA	NA
ENSG00000243265	RPL23AP55	ribosomal protein L23a pseudogene 55	100271467	0	NA	NA	NA	NA	NA
ENSG00000243280	NA	NA	NA	0,072165449	0,847101401	4,080456	0,207599697	0,835541541	NA
ENSG00000243284	VSIG8	V-set and immunoglobulin domain containing 8	391123	0	NA	NA	NA	NA	NA
ENSG00000243287	NA	NA	NA	0	NA	NA	NA	NA	NA
ENSG00000243289	NA	NA	NA	9,694686461	0,187285215	0,546633	0,342615802	0,731887512	NA
ENSG00000243297	RPL31P61	ribosomal protein L31 pseudogene 61	100271487	0	NA	NA	NA	NA	NA
ENSG00000243303	RPL34P32	ribosomal protein L34 pseudogene 32	100271502	0	NA	NA	NA	NA	NA
ENSG00000243304	NA	NA	NA	0	NA	NA	NA	NA	NA
ENSG00000243307	NA	NA	NA	0	NA	NA	NA	NA	NA
ENSG00000243312	RPL6P13	ribosomal protein L6 pseudogene 13	643974	0	NA	NA	NA	NA	NA
ENSG00000243314	RPS12P7	ribosomal protein S12 pseudogene 7	100271055	0,072165449	0,847101401	4,080456	0,207599697	0,835541541	NA
ENSG00000243316	GUCY2GP	guanylate cyclase 2G, pseudogene	390003	0	NA	NA	NA	NA	NA
ENSG00000243323	PTPRVP	protein tyrosine phosphatase receptor type V, pseudogene	148713	4,429579458	-0,948352217	0,835928	-1,134490034	0,256589052	NA
ENSG00000243353	RPS29P19	ribosomal protein S29 pseudogene 19	100271383	0,072165449	0,847101401	4,080456	0,207599697	0,835541541	NA
ENSG00000243355	RPS27P28	ribosomal protein S27 pseudogene 28	100271586	0	NA	NA	NA	NA	NA
ENSG00000243364	EFNA4	ephrin A4	1945	5,692782861	-0,683466833	0,798507	-0,855930651	0,39203614	NA
ENSG00000243378	NA	NA	NA	0	NA	NA	NA	NA	NA
ENSG00000243385	NA	NA	NA	0,10488184	-1,076443944	4,080456	-0,263804824	0,791930317	NA
ENSG00000243388	NA	NA	NA	0	NA	NA	NA	NA	NA
ENSG00000243396	RPL32P7	ribosomal protein L32 pseudogene 7	391560	0	NA	NA	NA	NA	NA
ENSG00000243402	NA	NA	NA	0	NA	NA	NA	NA	NA
ENSG00000243403	NA	NA	NA	0,386798454	-0,110733315	3,656216	-0,030286312	0,975838713	NA
ENSG00000243404	NA	NA	NA	0	NA	NA	NA	NA	NA
ENSG00000243406	NA	NA	NA	7,897966982	0,30020217	0,680419	0,441201906	0,659066833	NA
ENSG00000243414	TICAM2	TIR domain containing adaptor molecule 2	353376	1,591441362	0,035570172	2,767869	0,012851104	0,989746585	NA
ENSG00000243417	RPS3AP18	RPS3A pseudogene 18	391706	0	NA	NA	NA	NA	NA
ENSG00000243422	RPL23AP49	ribosomal protein L23a pseudogene 49	653236	0,224191532	-0,146173599	4,080456	-0,03582286	0,971423605	NA
ENSG00000243423	NA	NA	NA	0	NA	NA	NA	NA	NA
ENSG00000243429	OR7E29P	olfactory receptor family 7 subfamily E member 29 pseudogene	26644	0	NA	NA	NA	NA	NA
ENSG00000243431	RPL5P30	ribosomal protein L5 pseudogene 30	645160	0,263973515	-1,070105282	4,080456	-0,262251404	0,793127625	NA
ENSG00000243438	LARP1BP2	LARP1B pseudogene 2	106479034	0	NA	NA	NA	NA	NA
ENSG00000243445	RPS16P7	ribosomal protein S16 pseudogene 7	441744	1,087353682	-0,082844823	1,979709	-0,041846975	0,966620687	NA
ENSG00000243449	NICOL1	NELL2 interacting cell ontogeny regulator 1	401115	2,639946703	0,542073285	1,19246	0,454583922	0,649408604	NA
ENSG00000243455	RPS18P13	ribosomal protein S18 pseudogene 13	100271364	0	NA	NA	NA	NA	NA
ENSG00000243468	INGX	inhibitor of growth family, X-linked (pseudogene)	27160	0,789220752	0,692707406	2,819718	0,245665527	0,80594116	NA
ENSG00000243469	RPL7P51	ribosomal protein L7 pseudogene 51	100271226	0,117497108	0,847101401	4,080456	0,207599697	0,835541541	NA
ENSG00000243478	AOX2P	aldehyde oxidase 2, pseudogene	344454	0	NA	NA	NA	NA	NA
ENSG00000243480	AMY2A	amylase alpha 2A	279	0	NA	NA	NA	NA	NA
ENSG00000243483	DNAJB1P2	DNAJB1 peudogene 2	127379689	0	NA	NA	NA	NA	NA
ENSG00000243489	KRTAP10-11	keratin associated protein 10-11	386678	0	NA	NA	NA	NA	NA
ENSG00000243494	NA	NA	NA	0,412661176	-1,056164087	3,053441	-0,345893074	0,729423074	NA
ENSG00000243495	NA	NA	NA	0,072165449	0,847101401	4,080456	0,207599697	0,835541541	NA
ENSG00000243498	NA	NA	NA	0	NA	NA	NA	NA	NA
ENSG00000243499	RPS6P21	ribosomal protein S6 pseudogene 21	100271263	0	NA	NA	NA	NA	NA
ENSG00000243501	NA	NA	NA	0	NA	NA	NA	NA	NA
ENSG00000243503	RPL26P25	ribosomal protein L26 pseudogene 25	100270966	0	NA	NA	NA	NA	NA
ENSG00000243504	RPS23P1	ribosomal protein S23 pseudogene 1	93948	0	NA	NA	NA	NA	NA
ENSG00000243507	NA	NA	NA	0	NA	NA	NA	NA	NA
ENSG00000243508	DNAJB6P7	DNAJB6 pseudogene 7	100421437	3,091445435	-0,02874283	1,325603	-0,021682833	0,982700957	NA
ENSG00000243509	TNFRSF6B	TNF receptor superfamily member 6b	8771	0	NA	NA	NA	NA	NA

ENSG00000243514	NA	NA	NA	0	NA	NA	NA	NA	NA
ENSG00000243516	RPL12P40	ribosomal protein L12 pseudogene 40	646898	0	NA	NA	NA	NA	NA
ENSG00000243518	NA	NA	NA	0	NA	NA	NA	NA	NA
ENSG00000243519	IGLCOR22-2	immunoglobulin lambda constant/OR22-2 (pseudogene)	84086	0	NA	NA	NA	NA	NA
ENSG00000243521	RPL5P33	ribosomal protein L5 pseudogene 33	100130929	0	NA	NA	NA	NA	NA
ENSG00000243531	NA	NA	NA	0	NA	NA	NA	NA	NA
ENSG00000243537	RPL32P20	ribosomal protein L32 pseudogene 20	392271	0	NA	NA	NA	NA	NA
ENSG00000243538	NA	NA	NA	0	NA	NA	NA	NA	NA
ENSG00000243543	WFDC6	WAP four-disulfide core domain 6	140870	0	NA	NA	NA	NA	NA
ENSG00000243547	HNRNPKP4	heterogeneous nuclear ribonucleoprotein K pseudogene 4	644063	0,10488184	-1,076443944	4,080456	-0,263804824	0,791930317	NA
ENSG00000243554	CCZ1P1	CCZ1 pseudogene 1	110440226	2,663054881	-0,882242161	1,072327	-0,822736271	0,410657984	NA
ENSG00000243566	UPK3B	uroplakin 3B	105375355	0,535330118	1,531190725	2,494998	0,613704107	0,539410874	NA
ENSG00000243568	NA	NA	NA	0	NA	NA	NA	NA	NA
ENSG00000243581	RPS29P25	ribosomal protein S29 pseudogene 25	100271590	0	NA	NA	NA	NA	NA
ENSG00000243584	NA	NA	NA	0	NA	NA	NA	NA	NA
ENSG00000243592	RPL17P22	ribosomal protein L17 pseudogene 22	727984	0	NA	NA	NA	NA	NA
ENSG00000243596	PLSCR4P1	PLSCR4 pseudogene 1	100419967	0	NA	NA	NA	NA	NA
ENSG00000243601	RPL12P24	ribosomal protein L12 pseudogene 24	100270924	0	NA	NA	NA	NA	NA
ENSG00000243607	RPL35AP26	ribosomal protein L35a pseudogene 26	100271630	0,072165449	0,847101401	4,080456	0,207599697	0,835541541	NA
ENSG00000243609	NA	NA	NA	2,548183844	-0,136267996	1,273783	-0,106978945	0,914805684	NA
ENSG00000243621	NA	NA	NA	0	NA	NA	NA	NA	NA
ENSG00000243627	SMIM34	small integral membrane protein 34	388820	0	NA	NA	NA	NA	NA
ENSG00000243627	LOC107983987	small integral membrane protein 34B	107983987	0	NA	NA	NA	NA	NA
ENSG00000243635	NA	NA	NA	0	NA	NA	NA	NA	NA
ENSG00000243641	OR13C7	olfactory receptor family 13 subfamily C member 7 (gene/pseudogene)	81377	0	NA	NA	NA	NA	NA
ENSG00000243643	NA	NA	NA	0	NA	NA	NA	NA	NA
ENSG00000243648	RPS10P11	ribosomal protein S10 pseudogene 11	391833	0	NA	NA	NA	NA	NA
ENSG00000243649	CFB	complement factor B	629	0	NA	NA	NA	NA	NA
ENSG00000243658	MTND5P16	MT-ND5 pseudogene 16	100862854	0	NA	NA	NA	NA	NA
ENSG00000243659	NA	NA	NA	0,118286652	0	4,080456	0	1	NA
ENSG00000243660	ZNF487	zinc finger protein 487	642819	10,03401974	-0,363873028	0,602015	-0,604424747	0,545561287	NA
ENSG00000243661	WBP1LP1	WBP1L pseudogene 1	106479029	7,151334775	-0,040207992	0,783848	-0,051295625	0,959089955	NA
ENSG00000243664	NA	NA	NA	0	NA	NA	NA	NA	NA
ENSG00000243669	RPL34P23	ribosomal protein L34 pseudogene 23	100271499	0	NA	NA	NA	NA	NA
ENSG00000243672	RPS3AP40	RPS3A pseudogene 40	100129199	0,11808024	-1,076443944	4,080456	-0,263804824	0,791930317	NA
ENSG00000243674	NA	NA	NA	0,10488184	-1,076443944	4,080456	-0,263804824	0,791930317	NA
ENSG00000243675	NA	NA	NA	0	NA	NA	NA	NA	NA
ENSG00000243680	NA	NA	NA	0	NA	NA	NA	NA	NA
ENSG00000243686	NA	NA	NA	0,210862461	0	4,080456	0	1	NA
ENSG00000243687	RPL35AP10	ribosomal protein L35a pseudogene 10	100271313	0	NA	NA	NA	NA	NA
ENSG00000243695	NA	NA	NA	0	NA	NA	NA	NA	NA
ENSG00000243697	RPL7AP63	ribosomal protein L7a pseudogene 63	401865	0,103998153	-1,076443944	4,080456	-0,263804824	0,791930317	NA
ENSG00000243705	NA	NA	NA	0	NA	NA	NA	NA	NA
ENSG00000243709	LEFTY1	left-right determination factor 1	10637	0	NA	NA	NA	NA	NA
ENSG00000243710	CFAP57	cilia and flagella associated protein 57	149465	0	NA	NA	NA	NA	NA
ENSG00000243711	RPL21P116	ribosomal protein L21 pseudogene 116	100271444	0,103998153	-1,076443944	4,080456	-0,263804824	0,791930317	NA
ENSG00000243720	RPL21P127	ribosomal protein L21 pseudogene 127	100271452	0	NA	NA	NA	NA	NA
ENSG00000243721	NA	NA	NA	0	NA	NA	NA	NA	NA
ENSG00000243729	OR5V1	olfactory receptor family 5 subfamily V member 1	81696	0	NA	NA	NA	NA	NA
ENSG00000243730	NA	NA	NA	0	NA	NA	NA	NA	NA
ENSG00000243742	RPLP0P2	ribosomal protein lateral stalk subunit P0 pseudogene 2	113157	0	NA	NA	NA	NA	NA
ENSG00000243744	NA	NA	NA	0	NA	NA	NA	NA	NA

ENSG00000243746	EEF1A1P10	eukaryotic translation elongation factor 1 alpha 1 pseudogene 10	100421829	0,459833132	0,017698206	2,946023	0,006007491	0,995206744	NA
ENSG00000243753	NA	NA	NA	1,020825333	-3,589001962	2,826234	-1,269888322	0,204124413	NA
ENSG00000243758	RPL35AP15	ribosomal protein L35a pseudogene 15	100271317	0	NA	NA	NA	NA	NA
ENSG00000243759	ST13P15	ST13, Hsp70 interacting protein pseudogene 15	100422603	0,455781322	-0,13245292	3,059413	-0,043293579	0,96546751	NA
ENSG00000243761	RPL32P4	ribosomal protein L32 pseudogene 4	100131339	0	NA	NA	NA	NA	NA
ENSG00000243771	RPL21P130	ribosomal protein L21 pseudogene 130	100271453	0	NA	NA	NA	NA	NA
ENSG00000243775	OSTCP1	oligosaccharyltransferase complex subunit pseudogene 1	202459	0	NA	NA	NA	NA	NA
ENSG00000243777	RPS6P17	ribosomal protein S6 pseudogene 17	120318	0	NA	NA	NA	NA	NA
ENSG00000243779	RPL36AP49	ribosomal protein L36a pseudogene 49	284230	0	NA	NA	NA	NA	NA
ENSG00000243780	RPL21P114	ribosomal protein L21 pseudogene 114	100271441	0	NA	NA	NA	NA	NA
ENSG00000243781	NA	NA	NA	0	NA	NA	NA	NA	NA
ENSG00000243792	NA	NA	NA	0	NA	NA	NA	NA	NA
ENSG00000243802	NA	NA	NA	0	NA	NA	NA	NA	NA
ENSG00000243806	RPL7P18	ribosomal protein L7 pseudogene 18	100270852	0	NA	NA	NA	NA	NA
ENSG00000243813	LOC100419755	zinc finger protein 426 pseudogene	100419755	0	NA	NA	NA	NA	NA
ENSG00000243822	NA	NA	NA	0	NA	NA	NA	NA	NA
ENSG00000243824	RPL12P6	ribosomal protein L12 pseudogene 6	440176	0	NA	NA	NA	NA	NA
ENSG00000243828	NA	NA	NA	0	NA	NA	NA	NA	NA
ENSG00000243829	RPS9P4	ribosomal protein S9 pseudogene 4	388556	0	NA	NA	NA	NA	NA
ENSG00000243830	RPL15P17	ribosomal protein L15 pseudogene 17	728002	0	NA	NA	NA	NA	NA
ENSG00000243831	NA	NA	NA	0	NA	NA	NA	NA	NA
ENSG00000243838	PSMC1P7	proteasome 26S subunit, ATPase 1 pseudogene 7	646085	1,798866294	-0,872000762	1,570871	-0,555106517	0,578821805	NA
ENSG00000243844	RPL17P33	ribosomal protein L17 pseudogene 33	646909	0	NA	NA	NA	NA	NA
ENSG00000243853	NA	NA	NA	0	NA	NA	NA	NA	NA
ENSG00000243855	RPL12P5	ribosomal protein L12 pseudogene 5	319142	0	NA	NA	NA	NA	NA
ENSG00000243859	RPL5P17	ribosomal protein L5 pseudogene 17	100270839	0	NA	NA	NA	NA	NA
ENSG00000243864	NA	NA	NA	0	NA	NA	NA	NA	NA
ENSG00000243873	HMGB1P36	high mobility group box 1 pseudogene 36	100419968	0	NA	NA	NA	NA	NA
ENSG00000243877	NA	NA	NA	0	NA	NA	NA	NA	NA
ENSG00000243886	MTCH2P1	MTCH2 pseudogene 1	100289118	0	NA	NA	NA	NA	NA
ENSG00000243889	TRBV8-1	T cell receptor beta variable 8-1 (pseudogene)	28588	0	NA	NA	NA	NA	NA
ENSG00000243894	RPS3AP16	RPS3A pseudogene 16	727951	0	NA	NA	NA	NA	NA
ENSG00000243896	OR2A7	olfactory receptor family 2 subfamily A member 7	401427	0	NA	NA	NA	NA	NA
ENSG00000243904	RPSAP5	ribosomal protein SA pseudogene 5	319129	0	NA	NA	NA	NA	NA
ENSG00000243910	TUBA4B	tubulin alpha 4b	80086	0	NA	NA	NA	NA	NA
ENSG00000243914	RPL5P14	ribosomal protein L5 pseudogene 14	100270835	0	NA	NA	NA	NA	NA
ENSG00000243915	NA	NA	NA	0	NA	NA	NA	NA	NA
ENSG00000243916	RPS2P42	ribosomal protein S2 pseudogene 42	100271247	0	NA	NA	NA	NA	NA
ENSG00000243918	EIF4BP8	eukaryotic translation initiation factor 4B pseudogene 8	339881	0,103998153	-1,076443944	4,080456	-0,263804824	0,791930317	NA
ENSG00000243920	RPS26P24	ribosomal protein S26 pseudogene 24	100271113	0	NA	NA	NA	NA	NA
ENSG00000243925	NA	NA	NA	0	NA	NA	NA	NA	NA
ENSG00000243929	RPL37AP2	ribosomal protein L37a pseudogene 2	100271337	0	NA	NA	NA	NA	NA
ENSG00000243930	NA	NA	NA	0,11808024	-1,076443944	4,080456	-0,263804824	0,791930317	NA
ENSG00000243939	NA	NA	NA	0	NA	NA	NA	NA	NA
ENSG00000243945	ZFAND2AP1	ZFAND2A pseudogene 1	127379714	0	NA	NA	NA	NA	NA
ENSG00000243955	GSTA1	glutathione S-transferase alpha 1	2938	0	NA	NA	NA	NA	NA
ENSG00000243964	RPL23AP65	ribosomal protein L23a pseudogene 65	440027	0,208930911	-1,921614122	4,042523	-0,47535021	0,634537376	NA
ENSG00000243967	NA	NA	NA	0	NA	NA	NA	NA	NA

ENSG00000243970	NA	NA	NA	5,377216035	0,293240273	0,865363	0,338863748	0,734712375	NA
ENSG00000243974	NA	NA	NA	0	NA	NA	NA	NA	NA
ENSG00000243977	H3P13	H3 histone pseudogene 13	115482685	0	NA	NA	NA	NA	NA
ENSG00000243978	RTL9	retrotransposon Gag like 9	57529	0,10543123	0	4,080456	0	1	NA
ENSG00000243979	LOC100128548	ribosomal protein L17 pseudogene	100128548	0	NA	NA	NA	NA	NA
ENSG00000243981	NA	NA	NA	0,144330898	1,418786819	4,061262	0,349346267	0,726829368	NA
ENSG00000243986	ENO1P3	enolase 1 pseudogene 3	100419069	0,232865506	0,829759141	4,079783	0,203383156	0,838835564	NA
ENSG00000243988	RPS24P17	ribosomal protein S24 pseudogene 17	100216341	0	NA	NA	NA	NA	NA
ENSG00000243995	NA	NA	NA	0	NA	NA	NA	NA	NA
ENSG00000244000	NA	NA	NA	0	NA	NA	NA	NA	NA
ENSG00000244002	RPSAP39	ribosomal protein SA pseudogene 39	100131161	0	NA	NA	NA	NA	NA
ENSG00000244004	RPS3AP19	RPS3A pseudogene 19	728700	0,117497108	0,847101401	4,080456	0,207599697	0,835541541	NA
ENSG00000244009	NA	NA	NA	0	NA	NA	NA	NA	NA
ENSG00000244018	RPL35P6	ribosomal protein L35 pseudogene 6	100271007	0	NA	NA	NA	NA	NA
ENSG00000244019	RPS2P24	ribosomal protein S2 pseudogene 24	100270912	0	NA	NA	NA	NA	NA
ENSG00000244020	MT1HL1	metallothionein 1H like 1	645745	0	NA	NA	NA	NA	NA
ENSG00000244021	RPL21P53	ribosomal protein L21 pseudogene 53	653156	0,10543123	0	4,080456	0	1	NA
ENSG00000244024	LARP7P4	LARP7 pseudogene 4	100421509	0	NA	NA	NA	NA	NA
ENSG00000244025	KRTAP19-3	keratin associated protein 19-3	337970	0	NA	NA	NA	NA	NA
ENSG00000244031	NA	NA	NA	0	NA	NA	NA	NA	NA
ENSG00000244039	NA	NA	NA	0	NA	NA	NA	NA	NA
ENSG00000244043	RPS27P27	ribosomal protein S27 pseudogene 27	100271585	0	NA	NA	NA	NA	NA
ENSG00000244048	RPS18P6	ribosomal protein S18 pseudogene 6	100271076	0	NA	NA	NA	NA	NA
ENSG00000244050	NA	NA	NA	0	NA	NA	NA	NA	NA
ENSG00000244052	RPL5P24	ribosomal protein L5 pseudogene 24	100270841	0	NA	NA	NA	NA	NA
ENSG00000244053	RPL13AP2	ribosomal protein L13a pseudogene 2	326278	0	NA	NA	NA	NA	NA
ENSG00000244057	LCE3C	late cornified envelope 3C	353144	0	NA	NA	NA	NA	NA
ENSG00000244060	NA	NA	NA	0,237162043	1,955799597	4,022315	0,486237249	0,626798964	NA
ENSG00000244061	NA	NA	NA	0	NA	NA	NA	NA	NA
ENSG00000244062	NA	NA	NA	0	NA	NA	NA	NA	NA
ENSG00000244065	NA	NA	NA	0	NA	NA	NA	NA	NA
ENSG00000244067	GSTA2	glutathione S-transferase alpha 2	2939	0	NA	NA	NA	NA	NA
ENSG00000244071	RPL9P33	ribosomal protein L9 pseudogene 33	100271229	0	NA	NA	NA	NA	NA
ENSG00000244073	RPS4XP6	ribosomal protein S4X pseudogene 6	391777	0,117497108	0,847101401	4,080456	0,207599697	0,835541541	NA
ENSG00000244076	NA	NA	NA	0	NA	NA	NA	NA	NA
ENSG00000244081	NA	NA	NA	0	NA	NA	NA	NA	NA
ENSG00000244083	RPL34P12	ribosomal protein L34 pseudogene 12	100270996	0	NA	NA	NA	NA	NA
ENSG00000244086	NA	NA	NA	0	NA	NA	NA	NA	NA
ENSG00000244088	NA	NA	NA	0	NA	NA	NA	NA	NA
ENSG00000244089	HMGB1P30	high mobility group box 1 pseudogene 30	100873893	0	NA	NA	NA	NA	NA
ENSG00000244094	SPRR2F	small proline rich protein 2F	6705	0	NA	NA	NA	NA	NA
ENSG00000244099	RPS23P3	ribosomal protein S23 pseudogene 3	100271091	0	NA	NA	NA	NA	NA
ENSG00000244101	HMGN1P10	high mobility group nucleosome binding domain 1 pseudogene 10	100874438	0	NA	NA	NA	NA	NA
ENSG00000244113	RPL9P20	ribosomal protein L9 pseudogene 20	100130392	0	NA	NA	NA	NA	NA
ENSG00000244115	DNAJC25-GNG10	DNAJC25-GNG10 readthrough	552891	8,294174161	-0,951890157	1,171766	-0,812355156	0,41658787	NA
ENSG00000244119	NA	NA	NA	4,055857757	-0,800759496	1,221916	-0,655331173	0,512254554	NA
ENSG00000244122	UGT1A7	UDP glucuronosyltransferase family 1 member A7	54577	0	NA	NA	NA	NA	NA
ENSG00000244130	UBE2Q2P9	UBE2Q2 pseudogene 9	106480276	0	NA	NA	NA	NA	NA
ENSG00000244131	RPSAP51	ribosomal protein SA pseudogene 51	100271267	0	NA	NA	NA	NA	NA
ENSG00000244134	RPS12P20	ribosomal protein S12 pseudogene 20	100271549	0	NA	NA	NA	NA	NA
ENSG00000244142	NA	NA	NA	0,234994216	1,944111989	4,02324	0,483220515	0,62893917	NA
ENSG00000244146	RPL26P16	ribosomal protein L26 pseudogene 16	646966	0	NA	NA	NA	NA	NA
ENSG00000244153	NA	NA	NA	0	NA	NA	NA	NA	NA
ENSG00000244155	NA	NA	NA	0	NA	NA	NA	NA	NA
ENSG00000244157	NA	NA	NA	0	NA	NA	NA	NA	NA

ENSG00000244159	NA	NA	NA	0	NA	NA	NA	NA	NA
ENSG00000244167	NA	NA	NA	0,906281771	1,791086587	2,09634	0,85438727	0,392890447	NA
ENSG00000244171	NA	NA	NA	0,11808024	-1,076443944	4,080456	-0,263804824	0,791930317	NA
ENSG00000244176	RPS2P37	ribosomal protein S2 pseudogene 37	390206	0	NA	NA	NA	NA	NA
ENSG00000244183	NA	NA	NA	0	NA	NA	NA	NA	NA
ENSG00000244192	RPL11P2	ribosomal protein L11 pseudogene 2	133629	0	NA	NA	NA	NA	NA
ENSG00000244193	NA	NA	NA	0	NA	NA	NA	NA	NA
ENSG00000244196	NA	NA	NA	0	NA	NA	NA	NA	NA
ENSG00000244199	NA	NA	NA	0	NA	NA	NA	NA	NA
ENSG00000244213	NA	NA	NA	0	NA	NA	NA	NA	NA
ENSG00000244217	RPS4XP10	ribosomal protein S4X pseudogene 10	100271133	0	NA	NA	NA	NA	NA
ENSG00000244219	TMEM225B	transmembrane protein 225B	100289187	1,791764238	-1,292459046	1,550046	-0,833819756	0,404382561	NA
ENSG00000244222	OR7E121P	olfactory receptor family 7 subfamily E member 121 pseudogene	402133	0	NA	NA	NA	NA	NA
ENSG00000244226	ILF2P1	interleukin enhancer binding factor 2 pseudogene 1	100421506	0	NA	NA	NA	NA	NA
ENSG00000244227	LRRC77P	leucine rich repeat containing 77, pseudogene	646168	0	NA	NA	NA	NA	NA
ENSG00000244229	RPL26P35	ribosomal protein L26 pseudogene 35	100128115	0	NA	NA	NA	NA	NA
ENSG00000244234	GMCL2	germ cell-less 2, spermatogenesis associated	64396	0	NA	NA	NA	NA	NA
ENSG00000244235	RPL17P30	ribosomal protein L17 pseudogene 30	100271155	0	NA	NA	NA	NA	NA
ENSG00000244237	RPL24P3	RPL24 pseudogene 3	145235	0	NA	NA	NA	NA	NA
ENSG00000244242	IFITM10	interferon induced transmembrane protein 10	402778	2,392440504	1,266444626	2,255549	0,561479567	0,574470656	NA
ENSG00000244244	RPS3AP42	RPS3A pseudogene 42	100271597	0	NA	NA	NA	NA	NA
ENSG00000244245	NA	NA	NA	0	NA	NA	NA	NA	NA
ENSG00000244246	NA	NA	NA	0	NA	NA	NA	NA	NA
ENSG00000244249	RPS2P19	ribosomal protein S2 pseudogene 19	100270859	0	NA	NA	NA	NA	NA
ENSG00000244251	RPL9P27	ribosomal protein L9 pseudogene 27	100131246	0	NA	NA	NA	NA	NA
ENSG00000244253	RPS29P24	ribosomal protein S29 pseudogene 24	100271589	0	NA	NA	NA	NA	NA
ENSG00000244255	NA	NA	NA	0	NA	NA	NA	NA	NA
ENSG00000244259	NA	NA	NA	0	NA	NA	NA	NA	NA
ENSG00000244266	RPL26P33	ribosomal protein L26 pseudogene 33	100132547	0,119673877	0,847101401	4,080456	0,207599697	0,835541541	NA
ENSG00000244267	RPL34P22	ribosomal protein L34 pseudogene 22	100271498	3,374816316	-0,622117824	0,972166	-0,639929505	0,522218431	NA
ENSG00000244270	RPL32P29	ribosomal protein L32 pseudogene 29	729215	8,089566748	-0,334544761	0,649537	-0,515051059	0,606517341	NA
ENSG00000244274	DBNDD2	dysbindin domain containing 2	55861	5,105544884	0,673031804	1,053438	0,638890727	0,522894021	NA
ENSG00000244280	NA	NA	NA	0	NA	NA	NA	NA	NA
ENSG00000244281	NA	NA	NA	0	NA	NA	NA	NA	NA
ENSG00000244283	NA	NA	NA	0	NA	NA	NA	NA	NA
ENSG00000244289	RPS3AP35	RPS3A pseudogene 35	100271261	0	NA	NA	NA	NA	NA
ENSG00000244292	NA	NA	NA	0	NA	NA	NA	NA	NA
ENSG00000244295	NA	NA	NA	0	NA	NA	NA	NA	NA
ENSG00000244301	NA	NA	NA	0	NA	NA	NA	NA	NA
ENSG00000244306	DUXAP10	double homeobox A pseudogene 10	503639	0,103998153	-1,076443944	4,080456	-0,263804824	0,791930317	NA
ENSG00000244313	NA	NA	NA	1,788528911	-0,499763159	2,047239	-0,244115742	0,807141181	NA
ENSG00000244329	UBA52P9	ubiquitin A-52 residue ribosomal protein fusion product 1 pseudogene 9	100271344	0	NA	NA	NA	NA	NA
ENSG00000244331	NA	NA	NA	0	NA	NA	NA	NA	NA
ENSG00000244346	NA	NA	NA	0,675505103	-0,97620165	2,834683	-0,344377666	0,730562283	NA
ENSG00000244355	LY6G6D	lymphocyte antigen 6 family member G6D	58530	0	NA	NA	NA	NA	NA
ENSG00000244355	LY6G6F-LY6G6D	LY6G6F-LY6G6D readthrough	110599563	0	NA	NA	NA	NA	NA
ENSG00000244361	NA	NA	NA	0	NA	NA	NA	NA	NA
ENSG00000244362	KRTAP19-7	keratin associated protein 19-7	337974	0	NA	NA	NA	NA	NA
ENSG00000244363	RPL7P23	ribosomal protein L7 pseudogene 23	648000	0,118286652	0	4,080456	0	1	NA
ENSG00000244371	NA	NA	NA	0	NA	NA	NA	NA	NA
ENSG00000244378	NA	NA	NA	0,400564959	-1,028830712	3,075462	-0,334528788	0,737980571	NA
ENSG00000244381	NA	NA	NA	0	NA	NA	NA	NA	NA
ENSG00000244395	RBMY1D	RNA binding motif protein Y-linked family 1 member D	378949	0	NA	NA	NA	NA	NA

ENSG00000244400	RPS4XP12	ribosomal protein S4X pseudogene 12	100271385	0	NA	NA	NA	NA	NA
ENSG00000244405	ETV5	ETS variant transcription factor 5	2119	4,080054795	0,909056974	1,044502	0,870325969	0,384122291	NA
ENSG00000244411	KRTAP5-7	keratin associated protein 5-7	440050	0,191721342	1,677924969	4,046264	0,414685037	0,678372487	NA
ENSG00000244413	RPL23AP56	ribosomal protein L23a pseudogene 56	645809	0	NA	NA	NA	NA	NA
ENSG00000244414	CFHR1	complement factor H related 1	3078	0	NA	NA	NA	NA	NA
ENSG00000244422	NA	NA	NA	0	NA	NA	NA	NA	NA
ENSG00000244427	RPS3AP31	RPS3A pseudogene 31	100271256	0	NA	NA	NA	NA	NA
ENSG00000244429	SSBL6P	small RNA binding exonuclease protection factor La like 6, pseudogene	127482546	0	NA	NA	NA	NA	NA
ENSG00000244432	RPL39P28	ribosomal protein L39 pseudogene 28	100271520	0	NA	NA	NA	NA	NA
ENSG00000244436	RPLP2P4	ribosomal protein lateral stalk subunit P2 pseudogene 4	100271347	0,356800806	1,616295772	3,691771	0,437810454	0,661523692	NA
ENSG00000244441	RPL34P9	ribosomal protein L34 pseudogene 9	100271002	0	NA	NA	NA	NA	NA
ENSG00000244451	RPL34P21	ribosomal protein L34 pseudogene 21	100271495	0	NA	NA	NA	NA	NA
ENSG00000244456	SPECC1P2	SPECC1 pseudogene 2	112488741	0	NA	NA	NA	NA	NA
ENSG00000244457	ENO1P1	enolase 1 pseudogene 1	2025	0,288661797	2,306206831	3,997774	0,57687273	0,564025434	NA
ENSG00000244470	RPL6P14	ribosomal protein L6 pseudogene 14	391679	0	NA	NA	NA	NA	NA
ENSG00000244471	NA	NA	NA	0	NA	NA	NA	NA	NA
ENSG00000244474	UGT1A4	UDP glucuronosyltransferase family 1 member A4	54657	0	NA	NA	NA	NA	NA
ENSG00000244476	ERVFRD-1	endogenous retrovirus group FRD member 1, envelope	405754	0,10488184	-1,076443944	4,080456	-0,263804824	0,791930317	NA
ENSG00000244482	LILRA6	leukocyte immunoglobulin like receptor A6	79168	0	NA	NA	NA	NA	NA
ENSG00000244485	RPL18P13	ribosomal protein L18 pseudogene 13	441775	0	NA	NA	NA	NA	NA
ENSG00000244486	SCARF2	scavenger receptor class F member 2	91179	3,351953969	0,395704042	0,963936	0,41050883	0,681432727	NA
ENSG00000244490	NA	NA	NA	0	NA	NA	NA	NA	NA
ENSG00000244503	EEF1A1P45	eukaryotic translation elongation factor 1 alpha 1 pseudogene 45	100421817	0	NA	NA	NA	NA	NA
ENSG00000244510	NA	NA	NA	0,117497108	0,847101401	4,080456	0,207599697	0,835541541	NA
ENSG00000244515	KRT18P34	keratin 18 pseudogene 34	391589	0	NA	NA	NA	NA	NA
ENSG00000244527	RPL21P128	ribosomal protein L21 pseudogene 128	440487	0	NA	NA	NA	NA	NA
ENSG00000244528	NA	NA	NA	0,286373094	-1,026806104	4,038803	-0,254235258	0,799313814	NA
ENSG00000244535	LOC124902803	60S ribosomal protein L34-like	124902803	0	NA	NA	NA	NA	NA
ENSG00000244537	KRTAP4-2	keratin associated protein 4-2	85291	0	NA	NA	NA	NA	NA
ENSG00000244538	NA	NA	NA	0	NA	NA	NA	NA	NA
ENSG00000244540	RPL35AP29	ribosomal protein L35a pseudogene 29	100271638	0	NA	NA	NA	NA	NA
ENSG00000244551	RPL34P29	ribosomal protein L34 pseudogene 29	100271503	0	NA	NA	NA	NA	NA
ENSG00000244556	NA	NA	NA	0	NA	NA	NA	NA	NA
ENSG00000244559	NA	NA	NA	0	NA	NA	NA	NA	NA
ENSG00000244560	NA	NA	NA	1,721375041	-0,339751099	1,655576	-0,205216308	0,837403129	NA
ENSG00000244561	NA	NA	NA	0	NA	NA	NA	NA	NA
ENSG00000244563	RPS26P19	ribosomal protein S26 pseudogene 19	100271110	0,421724922	0	4,080456	0	1	NA
ENSG00000244565	POLR2DP2	POLR2D pseudogene 2	116435285	0	NA	NA	NA	NA	NA
ENSG00000244571	NA	NA	NA	0	NA	NA	NA	NA	NA
ENSG00000244573	NA	NA	NA	0	NA	NA	NA	NA	NA
ENSG00000244582	RPL21P120	ribosomal protein L21 pseudogene 120	731688	0	NA	NA	NA	NA	NA
ENSG00000244585	NA	NA	NA	0	NA	NA	NA	NA	NA
ENSG00000244588	RAD21L1	RAD21 cohesin complex component like 1	642636	0	NA	NA	NA	NA	NA
ENSG00000244593	RPL10AP11	ribosomal protein L10a pseudogene 11	392206	0	NA	NA	NA	NA	NA
ENSG00000244604	NA	NA	NA	0	NA	NA	NA	NA	NA
ENSG00000244607	CCDC13	coiled-coil domain containing 13	152206	0,524409201	-3,24447917	3,961591	-0,818983792	0,412795661	NA
ENSG00000244615	PSPC1P2	paraspeckle component 1 pseudogene 2	100873875	0	NA	NA	NA	NA	NA
ENSG00000244617	ASPRV1	aspartic peptidase retroviral like 1	151516	1,854357409	0,468997738	1,339561	0,35011304	0,726253865	NA

ENSG00000244621	RPS17P11	ribosomal protein S17 pseudogene 11	100271072	0	NA	NA	NA	NA	NA
ENSG00000244623	OR2AE1	olfactory receptor family 2 subfamily AE member 1	81392	0,617194455	-1,985800509	2,820262	-0,70411916	0,481358569	NA
ENSG00000244624	KRTAP20-1	keratin associated protein 20-1	337975	0	NA	NA	NA	NA	NA
ENSG00000244627	NA	NA	NA	0,45698316	-1,032559492	2,979961	-0,346501063	0,728966185	NA
ENSG00000244630	RPS26P27	ribosomal protein S26 pseudogene 27	100271237	0	NA	NA	NA	NA	NA
ENSG00000244640	RPLP0P8	ribosomal protein lateral stalk subunit P0 pseudogene 8	100131151	0	NA	NA	NA	NA	NA
ENSG00000244641	NA	NA	NA	0	NA	NA	NA	NA	NA
ENSG00000244646	NA	NA	NA	0	NA	NA	NA	NA	NA
ENSG00000244652	NA	NA	NA	0	NA	NA	NA	NA	NA
ENSG00000244653	NA	NA	NA	0	NA	NA	NA	NA	NA
ENSG00000244657	NA	NA	NA	0	NA	NA	NA	NA	NA
ENSG00000244662	RPL21P102	ribosomal protein L21 pseudogene 102	100271432	0	NA	NA	NA	NA	NA
ENSG00000244668	NA	NA	NA	0	NA	NA	NA	NA	NA
ENSG00000244669	RPS6P5	ribosomal protein S6 pseudogene 5	100130347	0	NA	NA	NA	NA	NA
ENSG00000244681	MTHFD2P1	methylenetetrahydrofolate dehydrogenase (NADP+ dependent) 2, methenyltetrahydrofolate cyclohydrolase pseudogene 1	100287639	0	NA	NA	NA	NA	NA
ENSG00000244682	NA	NA	NA	5,723168773	1,11466026	1,344315	0,829165987	0,407010489	NA
ENSG00000244691	RPL10AP1	ribosomal protein L10a pseudogene 1	319135	0	NA	NA	NA	NA	NA
ENSG00000244694	PTCHD4	patched domain containing 4	442213	0	NA	NA	NA	NA	NA
ENSG00000244703	CD46P1	CD46 molecule pseudogene 1	4182	0	NA	NA	NA	NA	NA
ENSG00000244705	RPLP0P10	ribosomal protein lateral stalk subunit P0 pseudogene 10	390578	0	NA	NA	NA	NA	NA
ENSG00000244708	RPL23P10	ribosomal protein L23 pseudogene 10	100270962	0	NA	NA	NA	NA	NA
ENSG00000244712	NA	NA	NA	0	NA	NA	NA	NA	NA
ENSG00000244716	RPL17P7	ribosomal protein L17 pseudogene 7	100132742	1,080325689	-3,7445921	2,139841	-1,749939408	0,08012877	NA
ENSG00000244722	RPSAP29	ribosomal protein SA pseudogene 29	389141	0	NA	NA	NA	NA	NA
ENSG00000244723	NA	NA	NA	0,208924536	-1,921583069	4,042524	-0,47534242	0,634542928	NA
ENSG00000244730	NA	NA	NA	0	NA	NA	NA	NA	NA
ENSG00000244731	C4A	complement C4A (Rodgers blood group)	720	5,87354372	-0,160021839	1,69881	-0,094196424	0,924953126	NA
ENSG00000244731	LOC110384692	complement C4A (Rodgers blood group)-like	110384692	5,87354372	-0,160021839	1,69881	-0,094196424	0,924953126	NA
ENSG00000244734	HBB	hemoglobin subunit beta	3043	0	NA	NA	NA	NA	NA
ENSG00000244740	RPL31P23	ribosomal protein L31 pseudogene 23	391581	0	NA	NA	NA	NA	NA
ENSG00000244743	NA	NA	NA	0	NA	NA	NA	NA	NA
ENSG00000244752	CRYBB2	crystallin beta B2	1415	0,302221886	0,856929089	3,866957	0,221602966	0,824622973	NA
ENSG00000244753	NA	NA	NA	0	NA	NA	NA	NA	NA
ENSG00000244756	RPL37P5	ribosomal protein L37 pseudogene 5	654369	0	NA	NA	NA	NA	NA
ENSG00000244921	MTCYBP18	MT-CYB pseudogene 18	107075130	0	NA	NA	NA	NA	NA
ENSG00000244932	NA	NA	NA	0	NA	NA	NA	NA	NA
ENSG00000245205	EEF1A1P4	eukaryotic translation elongation factor 1 alpha 1 pseudogene 4	100421854	0	NA	NA	NA	NA	NA
ENSG00000245848	CEBPA	CCAAT enhancer binding protein alpha	1050	5,760241521	0,509573912	0,822097	0,619846099	0,535359115	NA
ENSG00000245958	NA	NA	NA	1,350611599	1,025681426	1,964071	0,522222276	0,601515578	NA
ENSG00000246082	NUDT16L2P	nudix hydrolase 16 like 2, pseudogene	152195	2,936852038	-0,557494069	1,178853	-0,472912456	0,636275638	NA
ENSG00000246115	NA	NA	NA	0	NA	NA	NA	NA	NA
ENSG00000246575	NA	NA	NA	0	NA	NA	NA	NA	NA
ENSG00000246922	UBAP1L	ubiquitin associated protein 1 like	390595	4,769454395	0,426512357	0,865898	0,492566253	0,622319098	NA
ENSG00000247570	NA	NA	NA	0	NA	NA	NA	NA	NA
ENSG00000247595	MISFA	mitochondrial sheath formation associated	100506540	1,460628365	0,069921585	2,402444	0,029104352	0,976781365	NA
ENSG00000247746	USP51	ubiquitin specific peptidase 51	158880	3,470089076	-9,79E-06	1,132366	-8,65E-06	0,999993099	NA
ENSG00000247763	TUBAP14	tubulin alpha pseudogene 14	100419036	0	NA	NA	NA	NA	NA
ENSG00000247872	NA	NA	NA	0	NA	NA	NA	NA	NA
ENSG00000248099	INSL3	insulin like 3	3640	1,083953166	-0,017652707	1,748635	-0,010095137	0,991945383	NA

ENSG00000248104	TARS2P1	TARS2 pseudogene 1	100533844	0	NA	NA	NA	NA	NA
ENSG00000248105	NA	NA	NA	0	NA	NA	NA	NA	NA
ENSG00000248106	LOC100421074	ZFP14 zinc finger protein pseudogene	100421074	0	NA	NA	NA	NA	NA
ENSG00000248109	MARCOL	MARCO like	105378220	0	NA	NA	NA	NA	NA
ENSG00000248113	NA	NA	NA	0,10488184	-1,076443944	4,080456	-0,263804824	0,791930317	NA
ENSG00000248114	NA	NA	NA	0	NA	NA	NA	NA	NA
ENSG00000248117	NA	NA	NA	0	NA	NA	NA	NA	NA
ENSG00000248120	NA	NA	NA	0	NA	NA	NA	NA	NA
ENSG00000248121	NA	NA	NA	7,379425249	-0,003000096	0,645782	-0,004645677	0,9962933	NA
ENSG00000248122	NA	NA	NA	0	NA	NA	NA	NA	NA
ENSG00000248126	NA	NA	NA	0	NA	NA	NA	NA	NA
ENSG00000248128	NA	NA	NA	0	NA	NA	NA	NA	NA
ENSG00000248133	MTND4LP22	MT-ND4L pseudogene 22	107075102	0	NA	NA	NA	NA	NA
ENSG00000248134	NA	NA	NA	0	NA	NA	NA	NA	NA
ENSG00000248139	NA	NA	NA	0	NA	NA	NA	NA	NA
ENSG00000248144	ADH1C	alcohol dehydrogenase 1C (class I), gamma polypeptide	126	0	NA	NA	NA	NA	NA
ENSG00000248155	NA	NA	NA	0	NA	NA	NA	NA	NA
ENSG00000248156	NA	NA	NA	0	NA	NA	NA	NA	NA
ENSG00000248159	NA	NA	NA	0	NA	NA	NA	NA	NA
ENSG00000248160	NA	NA	NA	0	NA	NA	NA	NA	NA
ENSG00000248162	NA	NA	NA	0	NA	NA	NA	NA	NA
ENSG00000248167	TRIM39-RPP21	TRIM39-RPP21 readthrough	202658	0	NA	NA	NA	NA	NA
ENSG00000248170	NA	NA	NA	0	NA	NA	NA	NA	NA
ENSG00000248180	NA	NA	NA	0	NA	NA	NA	NA	NA
ENSG00000248185	LOC100420027	dedicator of cytokinesis 9 pseudogene	100420027	0	NA	NA	NA	NA	NA
ENSG00000248188	PFDN1P2	PFDN1 pseudogene 2	100288771	0	NA	NA	NA	NA	NA
ENSG00000248191	PES1P1	pescadillo ribosomal biogenesis factor 1 pseudogene 1	345016	0	NA	NA	NA	NA	NA
ENSG00000248196	LOC100419572	uracil DNA glycosylase pseudogene	100419572	0,224272769	-0,136994217	4,080456	-0,033573263	0,973217443	NA
ENSG00000248200	NA	NA	NA	0	NA	NA	NA	NA	NA
ENSG00000248205	NA	NA	NA	0	NA	NA	NA	NA	NA
ENSG00000248208	NA	NA	NA	0	NA	NA	NA	NA	NA
ENSG00000248209	NA	NA	NA	0	NA	NA	NA	NA	NA
ENSG00000248213	NA	NA	NA	0,117497108	0,847101401	4,080456	0,207599697	0,835541541	NA
ENSG00000248216	NA	NA	NA	0	NA	NA	NA	NA	NA
ENSG00000248229	NA	NA	NA	0,237759234	-0,112088991	4,080456	-0,027469723	0,978085088	NA
ENSG00000248231	NA	NA	NA	0	NA	NA	NA	NA	NA
ENSG00000248234	LOC124901186	60S ribosomal protein L27-like	124901186	0	NA	NA	NA	NA	NA
ENSG00000248235	NA	NA	NA	0	NA	NA	NA	NA	NA
ENSG00000248236	NA	NA	NA	0	NA	NA	NA	NA	NA
ENSG00000248237	LOC100421808	protein phosphatase 2 regulatory subunit B, gamma pseudogene	100421808	0	NA	NA	NA	NA	NA
ENSG00000248249	NA	NA	NA	0	NA	NA	NA	NA	NA
ENSG00000248257	NA	NA	NA	0	NA	NA	NA	NA	NA
ENSG00000248259	MTND4LP31	MT-ND4L pseudogene 31	107075296	0	NA	NA	NA	NA	NA
ENSG00000248271	PGAM1P1	phosphoglycerate mutase 1 pseudogene 1	100130514	0	NA	NA	NA	NA	NA
ENSG00000248278	LOC105371814	uncharacterized LOC105371814	105371814	0	NA	NA	NA	NA	NA
ENSG00000248281	NA	NA	NA	0	NA	NA	NA	NA	NA
ENSG00000248282	MTCYBP44	MT-CYB pseudogene 44	109729154	0	NA	NA	NA	NA	NA
ENSG00000248283	NA	NA	NA	2,809843577	1,394118917	1,142444	1,220295049	0,222353046	NA
ENSG00000248287	SCGB1D5P	secretoglobin family 1D member 5, pseudogene	100874258	0	NA	NA	NA	NA	NA
ENSG00000248288	NA	NA	NA	0	NA	NA	NA	NA	NA
ENSG00000248290	NA	NA	NA	0	NA	NA	NA	NA	NA
ENSG00000248293	NA	NA	NA	0	NA	NA	NA	NA	NA
ENSG00000248295	NA	NA	NA	0	NA	NA	NA	NA	NA
ENSG00000248302	NA	NA	NA	0	NA	NA	NA	NA	NA
ENSG00000248305	NA	NA	NA	0	NA	NA	NA	NA	NA
ENSG00000248308	LOC100419319	UBX domain protein 2A pseudogene	100419319	0	NA	NA	NA	NA	NA
ENSG00000248315	MTND4LP2	MT-ND4L pseudogene 2	107075098	0	NA	NA	NA	NA	NA
ENSG00000248320	NA	NA	NA	0	NA	NA	NA	NA	NA
ENSG00000248327	NOL8P1	nucleolar protein 8 pseudogene 1	402191	0,210862461	0	4,080456	0	1	NA
ENSG00000248328	MTCO3P28	MT-CO3 pseudogene 28	107075184	0	NA	NA	NA	NA	NA
ENSG00000248329	APELA	apelin receptor early endogenous ligand	100506013	0	NA	NA	NA	NA	NA
ENSG00000248336	NA	NA	NA	0	NA	NA	NA	NA	NA
ENSG00000248337	NA	NA	NA	0	NA	NA	NA	NA	NA
ENSG00000248340	NA	NA	NA	0	NA	NA	NA	NA	NA

ENSG00000248347	NA	NA	NA	0	NA	NA	NA	NA	NA
ENSG00000248349	NA	NA	NA	0	NA	NA	NA	NA	NA
ENSG00000248350	NA	NA	NA	0	NA	NA	NA	NA	NA
ENSG00000248351	NA	NA	NA	0	NA	NA	NA	NA	NA
ENSG00000248355	ARL2BPP6	ADP ribosylation factor like GTPase 2 binding protein pseudogene 6	100874421	0	NA	NA	NA	NA	NA
ENSG00000248364	OR7E86P	olfactory receptor family 7 subfamily E member 86 pseudogene	442104	0	NA	NA	NA	NA	NA
ENSG00000248365	NA	NA	NA	0	NA	NA	NA	NA	NA
ENSG00000248366	TRAJ51	T cell receptor alpha joining 51 (pseudogene)	28704	0	NA	NA	NA	NA	NA
ENSG00000248374	NA	NA	NA	0	NA	NA	NA	NA	NA
ENSG00000248375	NA	NA	NA	0	NA	NA	NA	NA	NA
ENSG00000248376	FBLP1	FBL pseudogene 1	100127981	0	NA	NA	NA	NA	NA
ENSG00000248377	HMGN1P9	high mobility group nucleosome binding domain 1 pseudogene	100874410	0	NA	NA	NA	NA	NA
ENSG00000248385	TARM1	T cell-interacting, activating receptor on myeloid cells 1	441864	0	NA	NA	NA	NA	NA
ENSG00000248387	LOC100131038	GC-rich promoter binding protein 1 pseudogene	100131038	0	NA	NA	NA	NA	NA
ENSG00000248394	NA	NA	NA	0,341436881	-1,063926357	3,75923	-0,283017127	0,777163708	NA
ENSG00000248396	TOMM22P4	TOMM22 pseudogene 4	100874517	0	NA	NA	NA	NA	NA
ENSG00000248400	ST13P12	ST13, Hsp70 interacting protein pseudogene 12	100127911	0	NA	NA	NA	NA	NA
ENSG00000248401	LOC391674	aminomethyltransferase pseudogene	391674	0	NA	NA	NA	NA	NA
ENSG00000248403	NACAP6	NACA pseudogene 6	100132778	0	NA	NA	NA	NA	NA
ENSG00000248405	PRR5-ARHGAP8	PRR5-ARHGAP8 readthrough	553158	0,577442622	-0,917317393	2,773671	-0,330723188	0,740853585	NA
ENSG00000248406	NA	NA	NA	1,119818376	-0,388165608	2,082392	-0,186403712	0,852128181	NA
ENSG00000248409	NA	NA	NA	0	NA	NA	NA	NA	NA
ENSG00000248415	NA	NA	NA	0	NA	NA	NA	NA	NA
ENSG00000248418	UBL5P1	ubiquitin like 5 pseudogene 1	100130803	0	NA	NA	NA	NA	NA
ENSG00000248419	LOC100130072	reactive intermediate imine deaminase A homolog pseudogene	100130072	0,10488184	-1,076443944	4,080456	-0,263804824	0,791930317	NA
ENSG00000248420	MTATP6P9	MT-ATP6 pseudogene 9	106479044	0	NA	NA	NA	NA	NA
ENSG00000248422	NA	NA	NA	0	NA	NA	NA	NA	NA
ENSG00000248423	NA	NA	NA	0	NA	NA	NA	NA	NA
ENSG00000248424	OR51K1P	olfactory receptor family 51 subfamily K member 1 pseudogene	81278	0	NA	NA	NA	NA	NA
ENSG00000248426	NA	NA	NA	0	NA	NA	NA	NA	NA
ENSG00000248430	HMGB3P16	high mobility group box 3 pseudogene 16	100873900	0	NA	NA	NA	NA	NA
ENSG00000248439	RPL23AP94	ribosomal protein L23a pseudogene 94	106481971	0	NA	NA	NA	NA	NA
ENSG00000248444	NA	NA	NA	0	NA	NA	NA	NA	NA
ENSG00000248447	LOC100533670	tripartite motif containing 60 pseudogene	100533670	0	NA	NA	NA	NA	NA
ENSG00000248448	NA	NA	NA	0	NA	NA	NA	NA	NA
ENSG00000248449	PCDHGB8P	protocadherin gamma subfamily B, 8 pseudogene	56120	0,11808024	-1,076443944	4,080456	-0,263804824	0,791930317	NA
ENSG00000248452	NA	NA	NA	0	NA	NA	NA	NA	NA
ENSG00000248459	NA	NA	NA	0	NA	NA	NA	NA	NA
ENSG00000248462	NENFP3	neudesin neurotrophic factor pseudogene 3	106481703	0	NA	NA	NA	NA	NA
ENSG00000248466	LOC651644	LIM zinc finger domain containing 2 pseudogene	651644	2,644735088	1,355767164	1,24161	1,091942841	0,274858226	NA
ENSG00000248471	NA	NA	NA	0	NA	NA	NA	NA	NA
ENSG00000248480	NA	NA	NA	0	NA	NA	NA	NA	NA
ENSG00000248485	PCP4L1	Purkinje cell protein 4 like 1	654790	0	NA	NA	NA	NA	NA
ENSG00000248488	PGAM4P2	phosphoglycerate mutase family member 4 pseudogene 2	100131611	0	NA	NA	NA	NA	NA
ENSG00000248493	NA	NA	NA	0	NA	NA	NA	NA	NA
ENSG00000248498	NA	NA	NA	0	NA	NA	NA	NA	NA
ENSG00000248503	NA	NA	NA	0,660448934	0,094057743	2,422169	0,038832027	0,96902431	NA
ENSG00000248506	TUBAP10	tubulin alpha pseudogene 10	645773	0	NA	NA	NA	NA	NA
ENSG00000248507	OR5H4P	olfactory receptor family 5 subfamily H member 4 pseudogene	79299	0	NA	NA	NA	NA	NA
ENSG00000248521	NA	NA	NA	1,074139595	3,5884664	2,097787	1,710596114	0,087155697	NA
ENSG00000248522	SBF1P1	SET binding factor 1 pseudogene 1	100133234	0	NA	NA	NA	NA	NA
ENSG00000248530	BCL2L12P1	BCL2 like 12 pseudogene 1	100289095	0	NA	NA	NA	NA	NA

ENSG00000248531	NA	NA	NA	0	NA	NA	NA	NA	NA
ENSG00000248532	NA	NA	NA	0	NA	NA	NA	NA	NA
ENSG00000248542	LOC391768	TAF11 RNA polymerase II, TATA box binding protein (TBP)-associated factor, 28kDa pseudogene	391768	0,119673877	0,847101401	4,080456	0,207599697	0,835541541	NA
ENSG00000248543	NA	NA	NA	0	NA	NA	NA	NA	NA
ENSG00000248546	NA	NA	NA	0	NA	NA	NA	NA	NA
ENSG00000248547	NA	NA	NA	0	NA	NA	NA	NA	NA
ENSG00000248548	MTND4P8	MT-ND4 pseudogene 8	100873194	0	NA	NA	NA	NA	NA
ENSG00000248552	NA	NA	NA	0	NA	NA	NA	NA	NA
ENSG00000248553	OR52H2P	olfactory receptor family 52 subfamily H member 2 pseudogene	81261	0,176458898	-0,124553098	4,080288	-0,030525565	0,975647905	NA
ENSG00000248557	METTL5P2	METTL5 pseudogene 2	100419878	0	NA	NA	NA	NA	NA
ENSG00000248560	LOC729707	transmembrane O-mannosyltransferase targeting cadherins 4 pseudogene	729707	0	NA	NA	NA	NA	NA
ENSG00000248564	NA	NA	NA	0	NA	NA	NA	NA	NA
ENSG00000248565	TECRP2	trans-2,3-enoyl-CoA reductase pseudogene 2	391696	0	NA	NA	NA	NA	NA
ENSG00000248568	NA	NA	NA	1,449093768	-0,866541014	1,710125	-0,506712223	0,612356753	NA
ENSG00000248569	NA	NA	NA	0,190429627	0,847327961	4,080456	0,20765522	0,835498185	NA
ENSG00000248573	NA	NA	NA	0	NA	NA	NA	NA	NA
ENSG00000248574	NA	NA	NA	0	NA	NA	NA	NA	NA
ENSG00000248577	NA	NA	NA	0	NA	NA	NA	NA	NA
ENSG00000248578	NA	NA	NA	0	NA	NA	NA	NA	NA
ENSG00000248583	NA	NA	NA	0	NA	NA	NA	NA	NA
ENSG00000248585	NA	NA	NA	0	NA	NA	NA	NA	NA
ENSG00000248586	LOC100288510	TSR1, 20S rRNA accumulation, homolog (S, cerevisiae) pseudogene	100288510	0	NA	NA	NA	NA	NA
ENSG00000248590	NA	NA	NA	0	NA	NA	NA	NA	NA
ENSG00000248592	STIMATE-MUSTN1	STIMATE-MUSTN1 readthrough	100526772	5,867332105	-1,484600001	1,278336	-1,161353177	0,245498302	NA
ENSG00000248593	NA	NA	NA	7,612069753	0,952609123	0,743698	1,280909079	0,200225603	NA
ENSG00000248608	LOC645433	inositol-3-phosphate synthase 1 pseudogene	645433	0	NA	NA	NA	NA	NA
ENSG00000248610	HSPA8P4	heat shock protein family A (Hsp70) member 8 pseudogene 4	100420028	0	NA	NA	NA	NA	NA
ENSG00000248611	LOC100422640	5-aminoimidazole-4-carboxamide ribonucleotide formyltransferase/IMP cyclohydrolase pseudogene	100422640	0	NA	NA	NA	NA	NA
ENSG00000248613	NA	NA	NA	0	NA	NA	NA	NA	NA
ENSG00000248616	NA	NA	NA	0	NA	NA	NA	NA	NA
ENSG00000248618	ENPP7P3	ectonucleotide pyrophosphatase/phosphodiesterase 7 pseudogene 3	106481658	0	NA	NA	NA	NA	NA
ENSG00000248621	NA	NA	NA	0	NA	NA	NA	NA	NA
ENSG00000248625	LOC402192	chloride nucleotide-sensitive channel 1A pseudogene	402192	0	NA	NA	NA	NA	NA
ENSG00000248626	NA	NA	NA	0,11808024	-1,076443944	4,080456	-0,263804824	0,791930317	NA
ENSG00000248633	GET1P1	GET1 pseudogene 1	256085	0	NA	NA	NA	NA	NA
ENSG00000248637	NA	NA	NA	0	NA	NA	NA	NA	NA
ENSG00000248639	NA	NA	NA	0	NA	NA	NA	NA	NA
ENSG00000248640	NA	NA	NA	0	NA	NA	NA	NA	NA
ENSG00000248641	NA	NA	NA	0	NA	NA	NA	NA	NA
ENSG00000248643	RBM14-RBM4	RBM14-RBM4 readthrough	100526737	1,867788248	0,735929157	1,805204	0,407670814	0,683515367	NA
ENSG00000248645	NA	NA	NA	0	NA	NA	NA	NA	NA
ENSG00000248648	LOC107986374	cytochrome c oxidase subunit NDUFA4-like	107986374	0	NA	NA	NA	NA	NA
ENSG00000248651	NA	NA	NA	0	NA	NA	NA	NA	NA
ENSG00000248652	NA	NA	NA	0	NA	NA	NA	NA	NA
ENSG00000248654	MTCO3P44	MT-CO3 pseudogene 44	107075289	0,50099132	-1,022259196	2,91876	-0,350237513	0,726160456	NA
ENSG00000248659	CARMIL2P1	CARMIL2 pseudogene 1	127460874	0	NA	NA	NA	NA	NA
ENSG00000248660	NA	NA	NA	0	NA	NA	NA	NA	NA
ENSG00000248669	VPS51P13	VPS51 pseudogene 13	127379746	0	NA	NA	NA	NA	NA
ENSG00000248672	LY75-CD302	LY75-CD302 readthrough	100526664	10,76039167	-0,858377129	2,881478	-0,297894788	0,765783466	NA
ENSG00000248674	NA	NA	NA	0	NA	NA	NA	NA	NA
ENSG00000248684	BIN2P2	bridging integrator 2 pseudogene 2	644601	0	NA	NA	NA	NA	NA
ENSG00000248687	NA	NA	NA	0	NA	NA	NA	NA	NA
ENSG00000248696	NA	NA	NA	0	NA	NA	NA	NA	NA

ENSG00000248697	TOX4P1	TOX high mobility group box family member 4 pseudogene 1	285412	0	NA	NA	NA	NA	NA
ENSG00000248698	LINC01085	long intergenic non-protein coding RNA 1085	152742	0	NA	NA	NA	NA	NA
ENSG00000248699	LOC391741	actin beta like 2 pseudogene	391741	0	NA	NA	NA	NA	NA
ENSG00000248702	NA	NA	NA	0	NA	NA	NA	NA	NA
ENSG00000248704	MTND4P2	MT-ND4 pseudogene 2	100873188	0	NA	NA	NA	NA	NA
ENSG00000248705	NA	NA	NA	0	NA	NA	NA	NA	NA
ENSG00000248710	TRIM59-IFT80	TRIM59-IFT80 readthrough (NMD candidate)	100174949	6,243874047	-0,078230393	1,587619	-0,049275284	0,960699916	NA
ENSG00000248711	NA	NA	NA	0	NA	NA	NA	NA	NA
ENSG00000248712	CCDC153	coiled-coil domain containing 153	283152	0,233088224	-0,906242681	4,080456	-0,222093489	0,824241106	NA
ENSG00000248713	C4orf54	chromosome 4 open reading frame 54	285556	0,10543123	0	4,080456	0	1	NA
ENSG00000248715	NA	NA	NA	0	NA	NA	NA	NA	NA
ENSG00000248716	NA	NA	NA	0	NA	NA	NA	NA	NA
ENSG00000248720	RFPL4AP5	ret finger protein like 4A pseudogene 5	100420058	0	NA	NA	NA	NA	NA
ENSG00000248722	AK3P4	AK3 pseudogene 4	100129959	0	NA	NA	NA	NA	NA
ENSG00000248725	NA	NA	NA	0	NA	NA	NA	NA	NA
ENSG00000248729	MTCO1P30	MT-CO1 pseudogene 30	107075170	0,15836288	0	4,080456	0	1	NA
ENSG00000248735	R3HDM2P1	R3H domain containing 2 pseudogene 1	100419742	0	NA	NA	NA	NA	NA
ENSG00000248739	NA	NA	NA	0	NA	NA	NA	NA	NA
ENSG00000248745	NA	NA	NA	0	NA	NA	NA	NA	NA
ENSG00000248746	ACTN3	actinin alpha 3	89	0,635037017	-0,187472536	2,249017	-0,083357555	0,933567237	NA
ENSG00000248748	MTND5P9	MT-ND5 pseudogene 9	100873199	0	NA	NA	NA	NA	NA
ENSG00000248750	NA	NA	NA	0	NA	NA	NA	NA	NA
ENSG00000248751	NA	NA	NA	0	NA	NA	NA	NA	NA
ENSG00000248755	NA	NA	NA	0	NA	NA	NA	NA	NA
ENSG00000248761	NA	NA	NA	0	NA	NA	NA	NA	NA
ENSG00000248763	NA	NA	NA	0	NA	NA	NA	NA	NA
ENSG00000248765	MTCYBP37	MT-CYB pseudogene 37	109729151	0	NA	NA	NA	NA	NA
ENSG00000248766	NA	NA	NA	0	NA	NA	NA	NA	NA
ENSG00000248767	FOXL3-OT1	FOXL3 overlapping transcript 1	100288524	0	NA	NA	NA	NA	NA
ENSG00000248767	FOXL3	forkhead box L3	116033993	0	NA	NA	NA	NA	NA
ENSG00000248769	NA	NA	NA	0,10543123	0	4,080456	0	1	NA
ENSG00000248770	NA	NA	NA	0	NA	NA	NA	NA	NA
ENSG00000248771	SMIM31	small integral membrane protein 31	100505989	0,117497108	0,847101401	4,080456	0,207599697	0,835541541	NA
ENSG00000248772	NA	NA	NA	0	NA	NA	NA	NA	NA
ENSG00000248775	NA	NA	NA	0	NA	NA	NA	NA	NA
ENSG00000248777	LOC100129344	developmental pluripotency associated 4 pseudogene	100129344	0	NA	NA	NA	NA	NA
ENSG00000248778	NA	NA	NA	0	NA	NA	NA	NA	NA
ENSG00000248780	NA	NA	NA	0	NA	NA	NA	NA	NA
ENSG00000248781	PSMC1P4	proteasome 26S subunit, ATPase 1 pseudogene 4	345645	0	NA	NA	NA	NA	NA
ENSG00000248785	NA	NA	NA	0,22271247	0,836219022	4,080456	0,204932745	0,837624671	NA
ENSG00000248791	LOC100422687	pleckstrin homology domain containing A1 pseudogene	100422687	6,781840773	0,191419916	0,702109	0,272635619	0,785133329	NA
ENSG00000248792	LINC00266-2P	long intergenic non-protein coding RNA 266-2, pseudogene	100287785	0	NA	NA	NA	NA	NA
ENSG00000248794	NA	NA	NA	0,10488184	-1,076443944	4,080456	-0,263804824	0,791930317	NA
ENSG00000248795	NA	NA	NA	0	NA	NA	NA	NA	NA
ENSG00000248796	NA	NA	NA	0	NA	NA	NA	NA	NA
ENSG00000248803	NA	NA	NA	0	NA	NA	NA	NA	NA
ENSG00000248807	NA	NA	NA	0	NA	NA	NA	NA	NA
ENSG00000248817	NA	NA	NA	0	NA	NA	NA	NA	NA
ENSG00000248820	DYNLL1P6	dynein light chain LC8-type 1 pseudogene 6	100288885	0	NA	NA	NA	NA	NA
ENSG00000248822	APOBEC3AP1	apolipoprotein B mRNA editing enzyme catalytic subunit 3A pseudogene 1	105377532	0	NA	NA	NA	NA	NA
ENSG00000248824	NA	NA	NA	0	NA	NA	NA	NA	NA
ENSG00000248826	PCBP2P3	PCBP2 pseudogene 3	100131236	0	NA	NA	NA	NA	NA
ENSG00000248827	LOC345576	ubiquitin specific peptidase 7 (herpes virus-associated) pseudogene	345576	0	NA	NA	NA	NA	NA
ENSG00000248830	NA	NA	NA	0,15836288	0	4,080456	0	1	NA
ENSG00000248831	LOC100421142	developmental pluripotency associated 2 pseudogene	100421142	0	NA	NA	NA	NA	NA
ENSG00000248834	MARK2P5	MARK2 pseudogene 5	100421485	0	NA	NA	NA	NA	NA
ENSG00000248838	PGAM1P13	phosphoglycerate mutase 1 pseudogene 13	100129815	0	NA	NA	NA	NA	NA
ENSG00000248840	NA	NA	NA	0	NA	NA	NA	NA	NA

ENSG00000248843	RPL7AP29	ribosomal protein L7a pseudogene 29	100271043	0	NA	NA	NA	NA	NA
ENSG00000248847	LOC100131441	coiled-coil domain containing 65 pseudogene	100131441	0	NA	NA	NA	NA	NA
ENSG00000248848	NA	NA	NA	0	NA	NA	NA	NA	NA
ENSG00000248850	SF3A2P1	SF3A2 pseudogene 1	645852	0	NA	NA	NA	NA	NA
ENSG00000248851	LOC100421364	fibroblast growth factor binding protein 1 pseudogene	100421364	0	NA	NA	NA	NA	NA
ENSG00000248853	NA	NA	NA	0	NA	NA	NA	NA	NA
ENSG00000248854	NA	NA	NA	0	NA	NA	NA	NA	NA
ENSG00000248863	NA	NA	NA	0,502854802	-1,051085866	2,938116	-0,357741448	0,720536812	NA
ENSG00000248867	NA	NA	NA	0	NA	NA	NA	NA	NA
ENSG00000248871	TNFSF12-TNFSF13	TNFSF12-TNFSF13 readthrough	407977	3,846535788	1,890018409	3,290009	0,574472153	0,565648339	NA
ENSG00000248873	NA	NA	NA	0,10488184	-1,076443944	4,080456	-0,263804824	0,791930317	NA
ENSG00000248877	PGBD4P4	piggyBac transposable element derived 4 pseudogene 4	100421146	0	NA	NA	NA	NA	NA
ENSG00000248878	NA	NA	NA	0	NA	NA	NA	NA	NA
ENSG00000248880	NA	NA	NA	0	NA	NA	NA	NA	NA
ENSG00000248883	B4GALNT2P1	B4GALNT2 pseudogene 1	106481717	0	NA	NA	NA	NA	NA
ENSG00000248885	SEPTIN7P10	septin 7 pseudogene 10	100418707	0	NA	NA	NA	NA	NA
ENSG00000248886	UGT2A3P7	UDP glucuronosyltransferase family 2 member A3 pseudogene 7	100422023	0	NA	NA	NA	NA	NA
ENSG00000248891	LOC105379196	protein mago nashi homolog	105379196	0	NA	NA	NA	NA	NA
ENSG00000248895	NA	NA	NA	0	NA	NA	NA	NA	NA
ENSG00000248903	NA	NA	NA	0	NA	NA	NA	NA	NA
ENSG00000248905	FMN1	formin 1	342184	0	NA	NA	NA	NA	NA
ENSG00000248907	MTND2P33	MT-ND2 pseudogene 33	100873236	0	NA	NA	NA	NA	NA
ENSG00000248909	NA	NA	NA	0	NA	NA	NA	NA	NA
ENSG00000248911	NA	NA	NA	0	NA	NA	NA	NA	NA
ENSG00000248913	PHB1P14	PHB1 pseudogene 14	100873751	0	NA	NA	NA	NA	NA
ENSG00000248915	ACTR6P1	ACTR6 pseudogene 1	100421495	0	NA	NA	NA	NA	NA
ENSG00000248916	NA	NA	NA	0	NA	NA	NA	NA	NA
ENSG00000248920	USP17L19	ubiquitin specific peptidase 17 like family member 19	100287404	0	NA	NA	NA	NA	NA
ENSG00000248921	NA	NA	NA	0	NA	NA	NA	NA	NA
ENSG00000248923	MTND5P11	MT-ND5 pseudogene 11	100506169	0,327719665	-0,120527617	3,826905	-0,031494802	0,974874938	NA
ENSG00000248924	NA	NA	NA	0	NA	NA	NA	NA	NA
ENSG00000248926	NA	NA	NA	0	NA	NA	NA	NA	NA
ENSG00000248928	NA	NA	NA	0	NA	NA	NA	NA	NA
ENSG00000248929	NA	NA	NA	0	NA	NA	NA	NA	NA
ENSG00000248930	NA	NA	NA	0	NA	NA	NA	NA	NA
ENSG00000248931	MTCYBP40	MT-CYB pseudogene 40	109729144	0	NA	NA	NA	NA	NA
ENSG00000248933	USP17L22	ubiquitin specific peptidase 17 like family member 22	100287513	0	NA	NA	NA	NA	NA
ENSG00000248950	LDHBP3	lactate dehydrogenase B pseudogene 3	100129283	0	NA	NA	NA	NA	NA
ENSG00000248955	NA	NA	NA	0	NA	NA	NA	NA	NA
ENSG00000248956	HMGB1P44	high mobility group box 1 pseudogene 44	100130279	0,425802765	2,347612119	3,495902	0,671532674	0,501881252	NA
ENSG00000248958	NA	NA	NA	0	NA	NA	NA	NA	NA
ENSG00000248966	NA	NA	NA	0,10543123	0	4,080456	0	1	NA
ENSG00000248967	LOC124901016	S-phase kinase-associated protein 1-like	124901016	0,103998153	-1,076443944	4,080456	-0,263804824	0,791930317	NA
ENSG00000248971	KRT8P46	keratin 8 pseudogene 46	100418744	2,545552759	0,2761262	1,383157	0,199634756	0,841766244	NA
ENSG00000248977	NA	NA	NA	0	NA	NA	NA	NA	NA
ENSG00000248978	NA	NA	NA	0	NA	NA	NA	NA	NA
ENSG00000248979	NA	NA	NA	0	NA	NA	NA	NA	NA
ENSG00000248987	NA	NA	NA	0	NA	NA	NA	NA	NA
ENSG00000248988	PSME2P3	proteasome activator subunit 2 pseudogene 3	338095	0	NA	NA	NA	NA	NA
ENSG00000248993	NA	NA	NA	0,103998153	-1,076443944	4,080456	-0,263804824	0,791930317	NA
ENSG00000248998	EFL1P2	elongation factor like GTPase 1 pseudogene 2	100422019	0	NA	NA	NA	NA	NA
ENSG00000249002	NA	NA	NA	0	NA	NA	NA	NA	NA
ENSG00000249003	NA	NA	NA	0	NA	NA	NA	NA	NA
ENSG00000249004	NA	NA	NA	0	NA	NA	NA	NA	NA
ENSG00000249005	NA	NA	NA	0	NA	NA	NA	NA	NA
ENSG00000249006	NA	NA	NA	0,558241289	1,983202089	3,178097	0,624021913	0,532613194	NA
ENSG00000249013	NA	NA	NA	0	NA	NA	NA	NA	NA
ENSG00000249014	NA	NA	NA	0	NA	NA	NA	NA	NA
ENSG00000249018	GAPDHP56	glyceraldehyde 3 phosphate dehydrogenase pseudogene 56	100421232	0	NA	NA	NA	NA	NA
ENSG00000249019	NA	NA	NA	0	NA	NA	NA	NA	NA

ENSG00000249025	ZNF519P4	zinc finger protein 519 pseudogene 4	106481981	0	NA	NA	NA	NA	NA
ENSG00000249026	NA	NA	NA	0	NA	NA	NA	NA	NA
ENSG00000249031	NA	NA	NA	0	NA	NA	NA	NA	NA
ENSG00000249038	AARS1P1	alanyl-tRNA synthetase 1 pseudogene 1	106480683	0	NA	NA	NA	NA	NA
ENSG00000249045	NA	NA	NA	0	NA	NA	NA	NA	NA
ENSG00000249047	NA	NA	NA	0	NA	NA	NA	NA	NA
ENSG00000249048	TRAV31	T cell receptor alpha variable 31 (pseudogene)	28651	0	NA	NA	NA	NA	NA
ENSG00000249050	NA	NA	NA	0	NA	NA	NA	NA	NA
ENSG00000249051	NA	NA	NA	0	NA	NA	NA	NA	NA
ENSG00000249053	LOC100420683	EZH inhibitory protein pseudogene	100420683	0	NA	NA	NA	NA	NA
ENSG00000249055	NA	NA	NA	0,470561423	0,45110165	2,988312	0,150955329	0,880010954	NA
ENSG00000249056	NA	NA	NA	0	NA	NA	NA	NA	NA
ENSG00000249064	KRT18P25	keratin 18 pseudogene 25	391647	0	NA	NA	NA	NA	NA
ENSG00000249065	NA	NA	NA	0	NA	NA	NA	NA	NA
ENSG00000249068	NA	NA	NA	0	NA	NA	NA	NA	NA
ENSG00000249072	NA	NA	NA	0,117497108	0,847101401	4,080456	0,207599697	0,835541541	NA
ENSG00000249074	NDUFB2P1	NADH:ubiquinone oxidoreductase subunit B2 pseudogene 2	107075208	0	NA	NA	NA	NA	NA
ENSG00000249077	SPICP5	Spi-C transcription factor pseudogene 5	100421664	0	NA	NA	NA	NA	NA
ENSG00000249079	NA	NA	NA	0	NA	NA	NA	NA	NA
ENSG00000249081	NA	NA	NA	0	NA	NA	NA	NA	NA
ENSG00000249089	AIG1P1	androgen induced 1 pseudogene 1	106479028	0	NA	NA	NA	NA	NA
ENSG00000249092	NA	NA	NA	0,117497108	0,847101401	4,080456	0,207599697	0,835541541	NA
ENSG00000249098	NA	NA	NA	0	NA	NA	NA	NA	NA
ENSG00000249101	NA	NA	NA	0	NA	NA	NA	NA	NA
ENSG00000249104	USP17L17	ubiquitin specific peptidase 17 like family member 17	100287327	0	NA	NA	NA	NA	NA
ENSG00000249105	NA	NA	NA	0	NA	NA	NA	NA	NA
ENSG00000249119	MTND6P4	MT-ND6 pseudogene 4	106478943	1,066130973	3,052027266	2,223602	1,372559845	0,169889222	NA
ENSG00000249127	NA	NA	NA	0	NA	NA	NA	NA	NA
ENSG00000249135	NA	NA	NA	0	NA	NA	NA	NA	NA
ENSG00000249138	SLED1	proteoglycan 3, pro eosinophil major basic protein 2 pseudogene	643036	0,447038289	-1,009107195	3,000211	-0,336345398	0,73661042	NA
ENSG00000249139	EPPIN-WFDC6	EPPIN-WFDC6 readthrough	100526773	0	NA	NA	NA	NA	NA
ENSG00000249141	NA	NA	NA	0,209763681	-1,926644696	4,042043	-0,476651224	0,633610501	NA
ENSG00000249142	TMEM183AP2	TMEM183A pseudogene 2	646616	0	NA	NA	NA	NA	NA
ENSG00000249148	LOC100288683	ribosomal protein L21 pseudogene	100288683	0	NA	NA	NA	NA	NA
ENSG00000249149	NA	NA	NA	0	NA	NA	NA	NA	NA
ENSG00000249156	TAF11L12	TATA-box binding protein associated factor 11 like 12	112488739	0	NA	NA	NA	NA	NA
ENSG00000249157	NA	NA	NA	0	NA	NA	NA	NA	NA
ENSG00000249162	ADAM20P3	ADAM metallopeptidase domain 20 pseudogene 3	644325	0	NA	NA	NA	NA	NA
ENSG00000249170	LOC107986208	probable UDP-sugar transporter protein SLC35A4	107986208	0	NA	NA	NA	NA	NA
ENSG00000249176	NA	NA	NA	0	NA	NA	NA	NA	NA
ENSG00000249177	MTND4P29	MT-ND4 pseudogene 29	100873250	0	NA	NA	NA	NA	NA
ENSG00000249183	NA	NA	NA	0	NA	NA	NA	NA	NA
ENSG00000249188	ENPP7P1	ectonucleotide pyrophosphatase/phosphodiesterase 7 pseudogene 1	100421823	0	NA	NA	NA	NA	NA
ENSG00000249191	UBE2V1P12	UBE2V1 pseudogene 12	106479048	0	NA	NA	NA	NA	NA
ENSG00000249192	MTND3P25	MT-ND3 pseudogene 25	107075297	0	NA	NA	NA	NA	NA
ENSG00000249193	HSPD1P5	heat shock protein family D (Hsp60) member 1 pseudogene 5	345041	0	NA	NA	NA	NA	NA
ENSG00000249200	LOC100419960	kelch domain containing 4 pseudogene	100419960	0	NA	NA	NA	NA	NA
ENSG00000249206	NA	NA	NA	0	NA	NA	NA	NA	NA
ENSG00000249209	NA	NA	NA	0	NA	NA	NA	NA	NA
ENSG00000249210	NA	NA	NA	0,11808024	-1,076443944	4,080456	-0,263804824	0,791930317	NA
ENSG00000249212	NA	NA	NA	1,585647508	0,317493889	1,611383	0,197031967	0,843802522	NA
ENSG00000249213	LOC100129404	matrix metallopeptidase 12 (macrophage elastase) pseudogene	100129404	0	NA	NA	NA	NA	NA
ENSG00000249222	ATP5MGL	ATP synthase membrane subunit g like	267020	0,854217873	0,46645306	2,096498	0,222491515	0,823931278	NA
ENSG00000249226	NA	NA	NA	0	NA	NA	NA	NA	NA

ENSG00000249229	NAMPTP2	nicotinamide phosphoribosyltransferase pseudogene 2	100132712	0	NA	NA	NA	NA	NA
ENSG00000249230	CDH12P2	cadherin 12 pseudogene 2	1011	0	NA	NA	NA	NA	NA
ENSG00000249235	LOC101930041	UDP-glucuronosyltransferase 2B10-like	101930041	0	NA	NA	NA	NA	NA
ENSG00000249237	NA	NA	NA	0	NA	NA	NA	NA	NA
ENSG00000249238	NA	NA	NA	0,11808024	-1,076443944	4,080456	-0,263804824	0,791930317	NA
ENSG00000249239	RACK1P3	RACK1 pseudogene 3	100422562	0	NA	NA	NA	NA	NA
ENSG00000249240	NA	NA	NA	2,221389711	2,591217701	2,89636	0,894646223	0,370976234	NA
ENSG00000249242	TMEM150C	transmembrane protein 150C	441027	0,118286652	0	4,080456	0	1	NA
ENSG00000249244	NA	NA	NA	0	NA	NA	NA	NA	NA
ENSG00000249245	NA	NA	NA	0	NA	NA	NA	NA	NA
ENSG00000249247	MTCYBP17	MT-CYB pseudogene 17	107075213	0	NA	NA	NA	NA	NA
ENSG00000249253	NA	NA	NA	0	NA	NA	NA	NA	NA
ENSG00000249256	ATP5MGP3	ATP synthase membrane subunit g pseudogene 3	53409	0	NA	NA	NA	NA	NA
ENSG00000249257	NA	NA	NA	0	NA	NA	NA	NA	NA
ENSG00000249259	NA	NA	NA	0	NA	NA	NA	NA	NA
ENSG00000249262	NA	NA	NA	0	NA	NA	NA	NA	NA
ENSG00000249263	PARP4P3	poly(ADP-ribose) polymerase family member 4 pseudogene 3	107133524	0	NA	NA	NA	NA	NA
ENSG00000249264	NA	NA	NA	0,53858932	-0,986462129	2,899972	-0,340162586	0,733734092	NA
ENSG00000249266	NA	NA	NA	0	NA	NA	NA	NA	NA
ENSG00000249270	NA	NA	NA	0	NA	NA	NA	NA	NA
ENSG00000249271	NA	NA	NA	0	NA	NA	NA	NA	NA
ENSG00000249272	UNC93B8	unc-93 homolog B8 (pseudogene)	100887748	0	NA	NA	NA	NA	NA
ENSG00000249274	PDLIM1P4	PDZ and LIM domain 1 pseudogene 4	100419185	0	NA	NA	NA	NA	NA
ENSG00000249277	SLIRPP2	SLIRP, pseudogene 2	100913179	0	NA	NA	NA	NA	NA
ENSG00000249278	NA	NA	NA	0	NA	NA	NA	NA	NA
ENSG00000249282	NA	NA	NA	0	NA	NA	NA	NA	NA
ENSG00000249283	NA	NA	NA	0,119673877	0,847101401	4,080456	0,207599697	0,835541541	NA
ENSG00000249284	NA	NA	NA	0	NA	NA	NA	NA	NA
ENSG00000249285	LOC100421308	phosphoribosyl pyrophosphate synthetase 2 pseudogene	100421308	0	NA	NA	NA	NA	NA
ENSG00000249286	NA	NA	NA	1,983309742	-1,300489121	1,648912	-0,788695213	0,430290166	NA
ENSG00000249287	NA	NA	NA	0	NA	NA	NA	NA	NA
ENSG00000249288	NA	NA	NA	0	NA	NA	NA	NA	NA
ENSG00000249289	NA	NA	NA	0	NA	NA	NA	NA	NA
ENSG00000249301	NA	NA	NA	0	NA	NA	NA	NA	NA
ENSG00000249302	NA	NA	NA	0	NA	NA	NA	NA	NA
ENSG00000249305	NA	NA	NA	0	NA	NA	NA	NA	NA
ENSG00000249311	NA	NA	NA	0	NA	NA	NA	NA	NA
ENSG00000249312	NA	NA	NA	0	NA	NA	NA	NA	NA
ENSG00000249319	NA	NA	NA	1,417101077	0,230637513	1,678712	0,137389588	0,89072286	NA
ENSG00000249320	NA	NA	NA	0,118286652	0	4,080456	0	1	NA
ENSG00000249321	OR5H5P	olfactory receptor family 5 subfamily H member 5 pseudogene	79297	0	NA	NA	NA	NA	NA
ENSG00000249333	NA	NA	NA	0	NA	NA	NA	NA	NA
ENSG00000249337	NA	NA	NA	0	NA	NA	NA	NA	NA
ENSG00000249338	NA	NA	NA	0	NA	NA	NA	NA	NA
ENSG00000249347	NA	NA	NA	0	NA	NA	NA	NA	NA
ENSG00000249351	LOC112268475	arf-GAP with GTPase, ANK repeat and PH domain-containing protein 1-like	112268475	0	NA	NA	NA	NA	NA
ENSG00000249353	NA	NA	NA	2,134353564	0,865495653	1,193533	0,72515421	0,468357431	NA
ENSG00000249360	NA	NA	NA	0	NA	NA	NA	NA	NA
ENSG00000249363	NA	NA	NA	0	NA	NA	NA	NA	NA
ENSG00000249367	NA	NA	NA	0	NA	NA	NA	NA	NA
ENSG00000249368	NA	NA	NA	0	NA	NA	NA	NA	NA
ENSG00000249372	NA	NA	NA	0	NA	NA	NA	NA	NA
ENSG00000249380	LOC100132524	UBX domain protein 2A pseudogene	100132524	0	NA	NA	NA	NA	NA
ENSG00000249386	MTCO2P9	MT-CO2 pseudogene 9	107075267	0	NA	NA	NA	NA	NA
ENSG00000249387	NA	NA	NA	0	NA	NA	NA	NA	NA
ENSG00000249400	NA	NA	NA	0	NA	NA	NA	NA	NA
ENSG00000249410	NA	NA	NA	0	NA	NA	NA	NA	NA
ENSG00000249411	LOC100131553	disintegrin and metalloproteinase domain-containing protein 21-like	100131553	0	NA	NA	NA	NA	NA
ENSG00000249416	NA	NA	NA	0	NA	NA	NA	NA	NA
ENSG00000249418	NA	NA	NA	0	NA	NA	NA	NA	NA
ENSG00000249433	NA	NA	NA	0	NA	NA	NA	NA	NA
ENSG00000249435	NA	NA	NA	0	NA	NA	NA	NA	NA
ENSG00000249438	KRT18P45	keratin 18 pseudogene 45	391803	0	NA	NA	NA	NA	NA

ENSG00000249439	HMGN1P14	high mobility group nucleosome binding domain 1 pseudogene 14	100874440	0,072165449	0,847101401	4,080456	0,207599697	0,835541541	NA
ENSG00000249443	VPS51P18	VPS51 pseudogene 18	127379732	0	NA	NA	NA	NA	NA
ENSG00000249444	NA	NA	NA	0	NA	NA	NA	NA	NA
ENSG00000249446	TRAJ60	T cell receptor alpha joining 60 (pseudogene)	28695	0	NA	NA	NA	NA	NA
ENSG00000249448	MTCO1P24	MT-CO1 pseudogene 24	107075166	0	NA	NA	NA	NA	NA
ENSG00000249449	MRPL57P6	mitochondrial ribosomal protein L57 pseudogene 6	134490	0	NA	NA	NA	NA	NA
ENSG00000249452	NA	NA	NA	0	NA	NA	NA	NA	NA
ENSG00000249454	GZMAP1	granzyme A pseudogene 1	106479035	0,10488184	-1,076443944	4,080456	-0,263804824	0,791930317	NA
ENSG00000249459	NA	NA	NA	4,061613362	0,605419881	1,035745	0,58452591	0,558866538	NA
ENSG00000249465	NA	NA	NA	0	NA	NA	NA	NA	NA
ENSG00000249467	NA	NA	NA	0	NA	NA	NA	NA	NA
ENSG00000249472	NA	NA	NA	0	NA	NA	NA	NA	NA
ENSG00000249481	SPATS1	spermatogenesis associated serine rich 1	221409	0	NA	NA	NA	NA	NA
ENSG00000249482	USP17L14P	ubiquitin specific peptidase 17 like family member 14, pseudogene	100287270	0	NA	NA	NA	NA	NA
ENSG00000249485	NA	NA	NA	0,912537865	0,160564461	2,386697	0,067274766	0,946362965	NA
ENSG00000249486	NA	NA	NA	0	NA	NA	NA	NA	NA
ENSG00000249488	NACAP5	NACA pseudogene 5	645108	0	NA	NA	NA	NA	NA
ENSG00000249489	NA	NA	NA	0	NA	NA	NA	NA	NA
ENSG00000249493	ANKRD20A18P	ankyrin repeat domain 20 family member A18, pseudogene	391269	0,222667382	-2,001216831	4,035255	-0,495933205	0,619941538	NA
ENSG00000249495	NA	NA	NA	0	NA	NA	NA	NA	NA
ENSG00000249501	USP9YP2	USP9Y pseudogene 2	387363	0	NA	NA	NA	NA	NA
ENSG00000249503	HMGN1P16	high mobility group nucleosome binding domain 1 pseudogene 16	100874442	0	NA	NA	NA	NA	NA
ENSG00000249505	JMJD4P1	JMJD4 pseudogene 1	100130760	0	NA	NA	NA	NA	NA
ENSG00000249506	ZEB2P1	zinc finger E-box binding homeobox 2 pseudogene 1	100420926	0	NA	NA	NA	NA	NA
ENSG00000249510	RPL6P23	ribosomal protein L6 pseudogene 23	100130524	0,48215655	2,641040179	3,325593	0,794156178	0,427104517	NA
ENSG00000249514	NA	NA	NA	0	NA	NA	NA	NA	NA
ENSG00000249516	NA	NA	NA	0	NA	NA	NA	NA	NA
ENSG00000249518	LOC100288484	formyl peptide receptor 1 pseudogene	100288484	0	NA	NA	NA	NA	NA
ENSG00000249520	NA	NA	NA	0	NA	NA	NA	NA	NA
ENSG00000249522	NA	NA	NA	0	NA	NA	NA	NA	NA
ENSG00000249525	NA	NA	NA	0	NA	NA	NA	NA	NA
ENSG00000249531	LOC100422188	UDP glucuronosyltransferase family 2 member B11 pseudogene	100422188	0	NA	NA	NA	NA	NA
ENSG00000249539	NA	NA	NA	0	NA	NA	NA	NA	NA
ENSG00000249540	NA	NA	NA	0	NA	NA	NA	NA	NA
ENSG00000249541	LOC100130748	3-hydroxyisobutyryl-Coenzyme A hydrolase pseudogene	100130748	0	NA	NA	NA	NA	NA
ENSG00000249542	EEF1A1P21	eukaryotic translation elongation factor 1 alpha 1 pseudogene 21	100421807	0	NA	NA	NA	NA	NA
ENSG00000249555	TMEM248P1	transmembrane protein 248 pseudogene 1	645841	0	NA	NA	NA	NA	NA
ENSG00000249557	NA	NA	NA	0	NA	NA	NA	NA	NA
ENSG00000249558	RCC2P4	regulator of chromosome condensation 2 pseudogene 4	100421090	0	NA	NA	NA	NA	NA
ENSG00000249562	NA	NA	NA	0	NA	NA	NA	NA	NA
ENSG00000249563	EVA1CP1	EVA1C pseudogene 1	100420821	0	NA	NA	NA	NA	NA
ENSG00000249564	NA	NA	NA	0	NA	NA	NA	NA	NA
ENSG00000249565	NA	NA	NA	0,359021632	2,56113643	3,98322	0,642981427	0,520236152	NA
ENSG00000249577	NA	NA	NA	0	NA	NA	NA	NA	NA
ENSG00000249579	NA	NA	NA	0	NA	NA	NA	NA	NA
ENSG00000249580	MTATP6P22	MT-ATP6 pseudogene 22	107075202	0	NA	NA	NA	NA	NA
ENSG00000249581	CLRN2	clarin 2	645104	0	NA	NA	NA	NA	NA
ENSG00000249582	NA	NA	NA	0	NA	NA	NA	NA	NA
ENSG00000249590	NA	NA	NA	0,446626744	-2,050754566	4,03026	-0,50883931	0,610864863	NA
ENSG00000249600	NA	NA	NA	0	NA	NA	NA	NA	NA
ENSG00000249606	NA	NA	NA	0	NA	NA	NA	NA	NA
ENSG00000249607	TRPC6P9	TRPC6 pseudogene 9	123464513	0	NA	NA	NA	NA	NA
ENSG00000249616	CCNB3P1	cyclin B3 pseudogene 1	100131678	0,11808024	-1,076443944	4,080456	-0,263804824	0,791930317	NA
ENSG00000249617	NA	NA	NA	0	NA	NA	NA	NA	NA
ENSG00000249619	HMGN1P13	high mobility group nucleosome binding domain 1 pseudogene 13	100874439	0	NA	NA	NA	NA	NA

ENSG00000249620	H3P18	H3 histone pseudogene 18	391744	0	NA	NA	NA	NA	NA
ENSG00000249624	IFNAR2-IL10RB	IFNAR2-IL10RB readthrough	127882475	1,146199981	1,36234392	3,345916	0,407166247	0,68388589	NA
ENSG00000249626	NA	NA	NA	0,15836288	0	4,080456	0	1	NA
ENSG00000249627	NA	NA	NA	0	NA	NA	NA	NA	NA
ENSG00000249633	OR52V1P	olfactory receptor family 52 subfamily V member 1 pseudogene	81242	0,177211844	-0,119769739	4,080409	-0,029352388	0,976583546	NA
ENSG00000249634	USP9YP5	USP9Y pseudogene 5	100421217	0	NA	NA	NA	NA	NA
ENSG00000249646	OR7E94P	olfactory receptor family 7 subfamily E member 94 pseudogene	79273	0	NA	NA	NA	NA	NA
ENSG00000249649	NA	NA	NA	0	NA	NA	NA	NA	NA
ENSG00000249654	VN1R104P	vomeronasal 1 receptor 104 pseudogene	100288195	0	NA	NA	NA	NA	NA
ENSG00000249661	NA	NA	NA	0	NA	NA	NA	NA	NA
ENSG00000249662	LINC02218	long intergenic non-protein coding RNA 2218	102723526	0	NA	NA	NA	NA	NA
ENSG00000249668	KRT18P56	keratin 18 pseudogene 56	106479027	0	NA	NA	NA	NA	NA
ENSG00000249680	TUBA3GP	tubulin alpha 3g pseudogene	645348	0	NA	NA	NA	NA	NA
ENSG00000249681	KRT19P3	keratin 19 pseudogene 3	442114	0	NA	NA	NA	NA	NA
ENSG00000249686	NA	NA	NA	0	NA	NA	NA	NA	NA
ENSG00000249691	DPPA4P2	DPPA4 pseudogene 2	100421154	0	NA	NA	NA	NA	NA
ENSG00000249692	NA	NA	NA	0	NA	NA	NA	NA	NA
ENSG00000249693	SPMAP2L	sperm microtubule associated protein 2 like	100506564	0	NA	NA	NA	NA	NA
ENSG00000249698	MTCO3P9	MT-CO3 pseudogene 9	107075269	0	NA	NA	NA	NA	NA
ENSG00000249715	FER1L5	fer-1 like family member 5	90342	0	NA	NA	NA	NA	NA
ENSG00000249721	NA	NA	NA	0	NA	NA	NA	NA	NA
ENSG00000249726	NA	NA	NA	0	NA	NA	NA	NA	NA
ENSG00000249729	LOC100419045	tubulin alpha 4a pseudogene	100419045	0	NA	NA	NA	NA	NA
ENSG00000249730	OR10J4	olfactory receptor family 10 subfamily J member 4 (gene/pseudogene)	391121	0	NA	NA	NA	NA	NA
ENSG00000249735	NA	NA	NA	0	NA	NA	NA	NA	NA
ENSG00000249741	NA	NA	NA	9,16440722	0,130071451	0,587251	0,221492249	0,824709171	NA
ENSG00000249744	NA	NA	NA	0	NA	NA	NA	NA	NA
ENSG00000249745	NA	NA	NA	0	NA	NA	NA	NA	NA
ENSG00000249754	NDUFB4P9	NADH:ubiquinone oxidoreductase subunit B4 pseudogene 9	107075211	0	NA	NA	NA	NA	NA
ENSG00000249755	NA	NA	NA	0	NA	NA	NA	NA	NA
ENSG00000249761	NA	NA	NA	0	NA	NA	NA	NA	NA
ENSG00000249763	NA	NA	NA	0	NA	NA	NA	NA	NA
ENSG00000249764	NA	NA	NA	0	NA	NA	NA	NA	NA
ENSG00000249766	NA	NA	NA	0	NA	NA	NA	NA	NA
ENSG00000249767	ENPP7P10	ectonucleotide pyrophosphatase/phosphodiesterase 7 pseudogene 10	100421805	0	NA	NA	NA	NA	NA
ENSG00000249768	NA	NA	NA	0	NA	NA	NA	NA	NA
ENSG00000249770	MTND6P17	MT-ND6 pseudogene 17	106480728	0	NA	NA	NA	NA	NA
ENSG00000249773	NA	NA	NA	1,877179147	0,344642399	2,393492	0,143991429	0,885507238	NA
ENSG00000249774	NA	NA	NA	0	NA	NA	NA	NA	NA
ENSG00000249777	SAP18P1	SAP18 pseudogene 1	106480689	0	NA	NA	NA	NA	NA
ENSG00000249778	TRMT112P2	tRNA methyltransferase subunit 11-2 pseudogene 2	106479033	0	NA	NA	NA	NA	NA
ENSG00000249779	NA	NA	NA	0,262529776	1,415964712	4,061201	0,348656627	0,72734711	NA
ENSG00000249780	NA	NA	NA	0	NA	NA	NA	NA	NA
ENSG00000249795	NA	NA	NA	0	NA	NA	NA	NA	NA
ENSG00000249798	NA	NA	NA	0	NA	NA	NA	NA	NA
ENSG00000249799	NA	NA	NA	0	NA	NA	NA	NA	NA
ENSG00000249811	USP17L21	ubiquitin specific peptidase 17 like family member 21	100287478	0	NA	NA	NA	NA	NA
ENSG00000249819	NA	NA	NA	0	NA	NA	NA	NA	NA
ENSG00000249820	NA	NA	NA	0	NA	NA	NA	NA	NA
ENSG00000249828	NA	NA	NA	0	NA	NA	NA	NA	NA
ENSG00000249829	NA	NA	NA	0	NA	NA	NA	NA	NA
ENSG00000249834	NA	NA	NA	0	NA	NA	NA	NA	NA
ENSG00000249838	NA	NA	NA	0	NA	NA	NA	NA	NA
ENSG00000249840	NA	NA	NA	0,222745319	-2,00157388	4,035246	-0,49602281	0,619878318	NA
ENSG00000249843	NA	NA	NA	0	NA	NA	NA	NA	NA
ENSG00000249844	NA	NA	NA	0	NA	NA	NA	NA	NA
ENSG00000249850	KRT18P31	keratin 18 pseudogene 31	646723	3,195411408	0,205747841	1,072063	0,19191772	0,847806657	NA
ENSG00000249851	RPS27AP18	RPS27A pseudogene 18	100419548	0,117497108	0,847101401	4,080456	0,207599697	0,835541541	NA
ENSG00000249853	HS3ST5	heparan sulfate-glucosamine 3-sulfotransferase 5	222537	0,10488184	-1,076443944	4,080456	-0,263804824	0,791930317	NA
ENSG00000249855	EEF1A1P19	eukaryotic translation elongation factor 1 alpha 1 pseudogene 19	100421796	3,756604214	0,807631911	1,129476	0,715049977	0,474578162	NA

ENSG00000249858	SNX5P1	sorting nexin 5 pseudogene 1	100131940	0	NA	NA	NA	NA	NA
ENSG00000249861	LGALS16	galectin 16	148003	0	NA	NA	NA	NA	NA
ENSG00000249863	NA	NA	NA	8,193853073	-0,75877209	0,647554	-1,171751142	0,241296984	NA
ENSG00000249866	OR7E83P	olfactory receptor family 7 subfamily E member 83 pseudogene	391630	0	NA	NA	NA	NA	NA
ENSG00000249869	VPS51P10	VPS51 pseudogene 10	127379706	0	NA	NA	NA	NA	NA
ENSG00000249876	NA	NA	NA	0,34013258	-1,064515311	3,750691	-0,283818448	0,776549517	NA
ENSG00000249878	LOC643307	actin beta pseudogene	643307	0	NA	NA	NA	NA	NA
ENSG00000249884	RNF103-CHMP3	RNF103-CHMP3 readthrough	100526767	0	NA	NA	NA	NA	NA
ENSG00000249887	NA	NA	NA	0	NA	NA	NA	NA	NA
ENSG00000249888	NA	NA	NA	0	NA	NA	NA	NA	NA
ENSG00000249889	ALG1L11P	ALG1 like 11, pseudogene	106480789	0	NA	NA	NA	NA	NA
ENSG00000249890	LOC100422020	UDP glucuronosyltransferase family 2 member B7 pseudogene	100422020	0	NA	NA	NA	NA	NA
ENSG00000249891	KRT19P6	keratin 19 pseudogene 6	106480807	0	NA	NA	NA	NA	NA
ENSG00000249893	MTND6P16	MT-ND6 pseudogene 16	106478950	0	NA	NA	NA	NA	NA
ENSG00000249896	NA	NA	NA	0	NA	NA	NA	NA	NA
ENSG00000249904	NA	NA	NA	0	NA	NA	NA	NA	NA
ENSG00000249908	BRD9P2	bromodomain containing 9 pseudogene 2	109729114	0	NA	NA	NA	NA	NA
ENSG00000249909	CYCTP	cytochrome c, testis, pseudogene	360163	0	NA	NA	NA	NA	NA
ENSG00000249910	TRIM51CP	tripartite motif-containing 51C, pseudogene	100130105	0	NA	NA	NA	NA	NA
ENSG00000249912	TRBV26	T cell receptor beta variable 26 (pseudogene)	28561	0	NA	NA	NA	NA	NA
ENSG00000249919	FABP5P6	fatty acid binding protein 5 pseudogene 6	100130687	0	NA	NA	NA	NA	NA
ENSG00000249920	NA	NA	NA	0	NA	NA	NA	NA	NA
ENSG00000249921	RACK1P1	RACK1 pseudogene 1	100422561	0	NA	NA	NA	NA	NA
ENSG00000249927	NA	NA	NA	0	NA	NA	NA	NA	NA
ENSG00000249928	UQCRBP3	ubiquinol-cytochrome c reductase binding protein pseudogene 3	100288785	0	NA	NA	NA	NA	NA
ENSG00000249930	NA	NA	NA	0	NA	NA	NA	NA	NA
ENSG00000249931	GOLGA8K	golgin A8 family member K	653125	8,395600518	-0,452618541	0,633489	-0,714485352	0,474927111	NA
ENSG00000249934	NA	NA	NA	0	NA	NA	NA	NA	NA
ENSG00000249936	NA	NA	NA	0	NA	NA	NA	NA	NA
ENSG00000249943	NA	NA	NA	0	NA	NA	NA	NA	NA
ENSG00000249944	LOC112267949	COX assembly mitochondrial protein homolog	112267949	0	NA	NA	NA	NA	NA
ENSG00000249947	NA	NA	NA	1,018032097	0,653340105	2,045343	0,319428203	0,749401827	NA
ENSG00000249948	GBA3	glucosylceramidase beta 3 (gene/pseudogene)	57733	0	NA	NA	NA	NA	NA
ENSG00000249950	NA	NA	NA	0	NA	NA	NA	NA	NA
ENSG00000249956	NA	NA	NA	0	NA	NA	NA	NA	NA
ENSG00000249958	CCT7P2	chaperonin containing TCP1 subunit 7 pseudogene 2	100288772	0	NA	NA	NA	NA	NA
ENSG00000249960	NA	NA	NA	0	NA	NA	NA	NA	NA
ENSG00000249961	TERB1	telomere repeat binding bouquet formation protein 1	283847	0,382246366	2,164308191	3,580685	0,604439683	0,54555136	NA
ENSG00000249963	EEF1A1P20	eukaryotic translation elongation factor 1 alpha 1 pseudogene 20	100421797	0	NA	NA	NA	NA	NA
ENSG00000249965	NA	NA	NA	0,457656501	-0,138879209	3,54274	-0,039201074	0,968730077	NA
ENSG00000249967	NA	NA	NA	0	NA	NA	NA	NA	NA
ENSG00000249970	NA	NA	NA	0	NA	NA	NA	NA	NA
ENSG00000249973	NA	NA	NA	0	NA	NA	NA	NA	NA
ENSG00000249975	OR5H3P	olfactory receptor family 5 subfamily H member 3 pseudogene	79309	0	NA	NA	NA	NA	NA
ENSG00000249976	NA	NA	NA	0	NA	NA	NA	NA	NA
ENSG00000249977	LOC100289037	SNU13 homolog, small nuclear ribonucleoprotein (U4/U6,U5) pseudogene	100289037	0	NA	NA	NA	NA	NA
ENSG00000249985	NA	NA	NA	0	NA	NA	NA	NA	NA
ENSG00000249986	NA	NA	NA	0	NA	NA	NA	NA	NA
ENSG00000249987	RPS4XP20	ribosomal protein S4X pseudogene 20	100271386	0	NA	NA	NA	NA	NA
ENSG00000249990	MTCO2P24	MT-CO2 pseudogene 24	107075193	0	NA	NA	NA	NA	NA
ENSG00000249992	TMEM158	transmembrane protein 158	25907	0	NA	NA	NA	NA	NA
ENSG00000249995	ECM1P2	extracellular matrix protein 1 pseudogene 2	106481698	0	NA	NA	NA	NA	NA
ENSG00000249997	MTCO3P7	MT-CO3 pseudogene 7	107075150	0	NA	NA	NA	NA	NA
ENSG00000250006	LOC100421141	developmental pluripotency associated 2 pseudogene	100421141	0	NA	NA	NA	NA	NA
ENSG00000250011	NA	NA	NA	11,33508399	0,892419785	0,548599	1,626724686	0,103795583	NA

ENSG00000250013	ZP3P1	ZP3 pseudogene 1	100421623	0	NA	NA	NA	NA	NA
ENSG00000250016	NA	NA	NA	0	NA	NA	NA	NA	NA
ENSG00000250017	NA	NA	NA	0	NA	NA	NA	NA	NA
ENSG00000250020	SPICP4	Spi-C transcription factor pseudogene 4	100128803	0	NA	NA	NA	NA	NA
ENSG00000250026	TMPRSS11BNL	TMPRSS11B N-terminal like (pseudogene)	401136	0	NA	NA	NA	NA	NA
ENSG00000250030	NA	NA	NA	0	NA	NA	NA	NA	NA
ENSG00000250031	NA	NA	NA	0	NA	NA	NA	NA	NA
ENSG00000250036	IGKV1D-37	immunoglobulin kappa variable 1D-37 (non-functional)	28894	0	NA	NA	NA	NA	NA
ENSG00000250037	LOC100419318	FAST kinase domains 3 pseudogene	100419318	0	NA	NA	NA	NA	NA
ENSG00000250040	RAF1P1	RAF1 pseudogene 1	348910	0	NA	NA	NA	NA	NA
ENSG00000250045	NA	NA	NA	0	NA	NA	NA	NA	NA
ENSG00000250050	MTND4P9	MT-ND4 pseudogene 9	100873193	1,739081892	0,125725077	1,421053	0,088473184	0,929500597	NA
ENSG00000250067	YJEFN3	YjeF N-terminal domain containing 3	374887	0	NA	NA	NA	NA	NA
ENSG00000250068	NA	NA	NA	0	NA	NA	NA	NA	NA
ENSG00000250071	NA	NA	NA	0	NA	NA	NA	NA	NA
ENSG00000250074	NA	NA	NA	0	NA	NA	NA	NA	NA
ENSG00000250076	NA	NA	NA	0	NA	NA	NA	NA	NA
ENSG00000250079	LOC100533667	T-box transcription factor T pseudogene	100533667	0	NA	NA	NA	NA	NA
ENSG00000250080	NA	NA	NA	0	NA	NA	NA	NA	NA
ENSG00000250082	NA	NA	NA	0	NA	NA	NA	NA	NA
ENSG00000250084	LOC107987327	X antigen family member 5-like	107987327	0	NA	NA	NA	NA	NA
ENSG00000250088	NA	NA	NA	0	NA	NA	NA	NA	NA
ENSG00000250090	NA	NA	NA	0,354859955	0	4,080456	0	1	NA
ENSG00000250099	NA	NA	NA	0	NA	NA	NA	NA	NA
ENSG00000250100	NA	NA	NA	0	NA	NA	NA	NA	NA
ENSG00000250111	LOC101929141	coiled-coil domain-containing protein 144A-like	101929141	6,398247635	-1,533992262	2,692107	-0,569811103	0,568805824	NA
ENSG00000250114	NA	NA	NA	0	NA	NA	NA	NA	NA
ENSG00000250115	NA	NA	NA	0	NA	NA	NA	NA	NA
ENSG00000250118	NA	NA	NA	0	NA	NA	NA	NA	NA
ENSG00000250130	NA	NA	NA	3,557094702	-0,062352667	1,119151	-0,055714281	0,955569422	NA
ENSG00000250138	LOC728488	POM121 membrane glycoprotein (rat) pseudogene	728488	0,222335812	-0,11805748	4,080456	-0,028932424	0,976918486	NA
ENSG00000250140	NA	NA	NA	0	NA	NA	NA	NA	NA
ENSG00000250144	EEF1A1P35	eukaryotic translation elongation factor 1 alpha 1 pseudogene 35	389223	0	NA	NA	NA	NA	NA
ENSG00000250145	NA	NA	NA	0	NA	NA	NA	NA	NA
ENSG00000250147	NA	NA	NA	0	NA	NA	NA	NA	NA
ENSG00000250148	KRT8P31	keratin 8 pseudogene 31	728167	0	NA	NA	NA	NA	NA
ENSG00000250151	ARPC4-TTLL3	ARPC4-TTLL3 readthrough	100526693	1,589137326	1,008331836	2,501424	0,403103123	0,686872363	NA
ENSG00000250161	TRMT112P5	tRNA methyltransferase subunit 11-2 pseudogene 5	106480784	0	NA	NA	NA	NA	NA
ENSG00000250162	NA	NA	NA	0	NA	NA	NA	NA	NA
ENSG00000250169	NA	NA	NA	0	NA	NA	NA	NA	NA
ENSG00000250182	NA	NA	NA	9,47951123	-0,029933007	0,612106	-0,048901683	0,960997648	NA
ENSG00000250183	NA	NA	NA	0	NA	NA	NA	NA	NA
ENSG00000250185	RCC2P8	regulator of chromosome condensation 2 pseudogene 8	100129714	0	NA	NA	NA	NA	NA
ENSG00000250189	NA	NA	NA	0,207996306	-1,916073151	4,043021	-0,473921178	0,635556117	NA
ENSG00000250191	NA	NA	NA	0	NA	NA	NA	NA	NA
ENSG00000250194	NA	NA	NA	0	NA	NA	NA	NA	NA
ENSG00000250197	HMGN1P15	high mobility group nucleosome binding domain 1 pseudogene 15	100874441	0	NA	NA	NA	NA	NA
ENSG00000250200	NA	NA	NA	0	NA	NA	NA	NA	NA
ENSG00000250202	NA	NA	NA	0	NA	NA	NA	NA	NA
ENSG00000250204	NA	NA	NA	0	NA	NA	NA	NA	NA
ENSG00000250205	YWHAEP4	tyrosine 3-monooxygenase/tryptophan 5-monooxygenase activation protein epsilon pseudogene 4	106481785	0	NA	NA	NA	NA	NA
ENSG00000250213	NA	NA	NA	0	NA	NA	NA	NA	NA
ENSG00000250214	NA	NA	NA	0	NA	NA	NA	NA	NA
ENSG00000250215	CIR1P2	CIR1 pseudogene 2	100131828	0	NA	NA	NA	NA	NA
ENSG00000250219	LTV1P1	LTV1 ribosome biogenesis factor pseudogene 1	100289220	0	NA	NA	NA	NA	NA
ENSG00000250221	KRT8P32	keratin 8 pseudogene 32	100289133	0	NA	NA	NA	NA	NA

ENSG00000250227	TRIM60P14	tripartite motif containing 60 pseudogene 14	653794	0,118286652	0	4,080456	0	1	NA
ENSG00000250229	NA	NA	NA	0	NA	NA	NA	NA	NA
ENSG00000250231	NA	NA	NA	0	NA	NA	NA	NA	NA
ENSG00000250238	NA	NA	NA	0	NA	NA	NA	NA	NA
ENSG00000250247	NA	NA	NA	0	NA	NA	NA	NA	NA
ENSG00000250249	NA	NA	NA	0	NA	NA	NA	NA	NA
ENSG00000250253	NA	NA	NA	0	NA	NA	NA	NA	NA
ENSG00000250254	PTTG2	pituitary tumor-transforming 2	10744	0,879659312	0,628636359	2,295516	0,273854009	0,784196817	NA
ENSG00000250257	NA	NA	NA	0	NA	NA	NA	NA	NA
ENSG00000250261	NA	NA	NA	0	NA	NA	NA	NA	NA
ENSG00000250264	NA	NA	NA	0	NA	NA	NA	NA	NA
ENSG00000250268	NA	NA	NA	0	NA	NA	NA	NA	NA
ENSG00000250271	NA	NA	NA	0	NA	NA	NA	NA	NA
ENSG00000250272	NA	NA	NA	0	NA	NA	NA	NA	NA
ENSG00000250273	PSMC1P5	proteasome 26S subunit, ATPase 1 pseudogene 5	106480732	0	NA	NA	NA	NA	NA
ENSG00000250277	NA	NA	NA	0	NA	NA	NA	NA	NA
ENSG00000250289	VWA8P1	von Willebrand factor A domain containing 8 pseudogene 1	106480787	0	NA	NA	NA	NA	NA
ENSG00000250290	NA	NA	NA	6,994530673	0,650580584	0,767834	0,847293296	0,396831662	NA
ENSG00000250292	NA	NA	NA	0	NA	NA	NA	NA	NA
ENSG00000250293	NA	NA	NA	0,118286652	0	4,080456	0	1	NA
ENSG00000250298	GIMD1	GIMAP family P-loop NTPase domain containing 1	100507096	0	NA	NA	NA	NA	NA
ENSG00000250300	NA	NA	NA	0	NA	NA	NA	NA	NA
ENSG00000250304	NA	NA	NA	0,10488184	-1,076443944	4,080456	-0,263804824	0,791930317	NA
ENSG00000250305	TRMT9B	tRNA methyltransferase 9B (putative)	57604	0	NA	NA	NA	NA	NA
ENSG00000250306	NA	NA	NA	0	NA	NA	NA	NA	NA
ENSG00000250307	TUBB8P3	tubulin beta 8 class VIII pseudogene 3	132719	0	NA	NA	NA	NA	NA
ENSG00000250308	SALL4P1	spalt like transcription factor 4 pseudogene 1	100419717	0	NA	NA	NA	NA	NA
ENSG00000250315	NA	NA	NA	0	NA	NA	NA	NA	NA
ENSG00000250316	NA	NA	NA	0	NA	NA	NA	NA	NA
ENSG00000250318	RPL13AP26	ribosomal protein L13a pseudogene 26	729212	4,196696978	-0,495940747	0,84376	-0,587774685	0,556683538	NA
ENSG00000250319	NA	NA	NA	0	NA	NA	NA	NA	NA
ENSG00000250321	NA	NA	NA	0	NA	NA	NA	NA	NA
ENSG00000250322	NA	NA	NA	0	NA	NA	NA	NA	NA
ENSG00000250324	MRPL22P1	mitochondrial ribosomal protein L22 pseudogene 1	359738	0	NA	NA	NA	NA	NA
ENSG00000250325	NA	NA	NA	0	NA	NA	NA	NA	NA
ENSG00000250327	RPSAP70	ribosomal protein SA pseudogene 70	105377595	0	NA	NA	NA	NA	NA
ENSG00000250329	NA	NA	NA	1,031154147	0,058796515	2,242581	0,026218231	0,979083275	NA
ENSG00000250330	NA	NA	NA	0	NA	NA	NA	NA	NA
ENSG00000250332	NA	NA	NA	0	NA	NA	NA	NA	NA
ENSG00000250339	NA	NA	NA	0	NA	NA	NA	NA	NA
ENSG00000250342	SNRPCP16	small nuclear ribonucleoprotein polypeptide C pseudogene 16	106480291	0,072165449	0,847101401	4,080456	0,207599697	0,835541541	NA
ENSG00000250345	LOC100419356	ethanolamine kinase 1 pseudogene	100419356	0	NA	NA	NA	NA	NA
ENSG00000250346	NA	NA	NA	0	NA	NA	NA	NA	NA
ENSG00000250347	NA	NA	NA	0	NA	NA	NA	NA	NA
ENSG00000250349	NA	NA	NA	0	NA	NA	NA	NA	NA
ENSG00000250351	H3P20	H3 histone pseudogene 20	100289141	0	NA	NA	NA	NA	NA
ENSG00000250356	NA	NA	NA	0	NA	NA	NA	NA	NA
ENSG00000250359	NA	NA	NA	0	NA	NA	NA	NA	NA
ENSG00000250361	GYPB	glycophorin B (MNS blood group)	2994	0	NA	NA	NA	NA	NA
ENSG00000250363	KRT18P21	keratin 18 pseudogene 21	132391	0	NA	NA	NA	NA	NA
ENSG00000250366	TUNAR	TCL1 upstream neural differentiation-associated RNA	100507043	0	NA	NA	NA	NA	NA
ENSG00000250372	MARK2P4	MARK2 pseudogene 4	100421496	0	NA	NA	NA	NA	NA
ENSG00000250374	TRIM75	tripartite motif containing 75	391714	0	NA	NA	NA	NA	NA
ENSG00000250376	NA	NA	NA	0	NA	NA	NA	NA	NA
ENSG00000250381	NA	NA	NA	0	NA	NA	NA	NA	NA
ENSG00000250384	NA	NA	NA	0	NA	NA	NA	NA	NA
ENSG00000250386	H3P21	H3 histone pseudogene 21	391765	0	NA	NA	NA	NA	NA
ENSG00000250389	MTND6P2	MT-ND6 pseudogene 2	106478941	0	NA	NA	NA	NA	NA
ENSG00000250391	NA	NA	NA	0	NA	NA	NA	NA	NA
ENSG00000250393	NA	NA	NA	0	NA	NA	NA	NA	NA
ENSG00000250398	NA	NA	NA	0	NA	NA	NA	NA	NA

ENSG00000250403	NA	NA	NA	0	NA	NA	NA	NA	NA
ENSG00000250405	LOC402229	SURP and G-patch domain containing 1 pseudogene	402229	0	NA	NA	NA	NA	NA
ENSG00000250412	KLHL2P1	kelch like family member 2 pseudogene 1	729249	0,072165449	0,847101401	4,080456	0,207599697	0,835541541	NA
ENSG00000250416	SEC63P2	SEC63 homolog, protein translocation regulator pseudogene 2	100420287	0	NA	NA	NA	NA	NA
ENSG00000250420	NA	NA	NA	0	NA	NA	NA	NA	NA
ENSG00000250422	NA	NA	NA	0	NA	NA	NA	NA	NA
ENSG00000250423	KIAA1210	KIAA1210	57481	0	NA	NA	NA	NA	NA
ENSG00000250424	NA	NA	NA	0,15836288	0	4,080456	0	1	NA
ENSG00000250425	OR7E35P	olfactory receptor family 7 subfamily E member 35 pseudogene	391632	0	NA	NA	NA	NA	NA
ENSG00000250426	FTLP10	ferritin light chain pseudogene 10	100130017	0	NA	NA	NA	NA	NA
ENSG00000250428	MRS2P1	MRS2 pseudogene 1	100130448	0	NA	NA	NA	NA	NA
ENSG00000250430	LOC100420385	tripartite motif-containing 75 pseudogene	100420385	0	NA	NA	NA	NA	NA
ENSG00000250441	NA	NA	NA	0	NA	NA	NA	NA	NA
ENSG00000250442	EIF3KP3	eukaryotic translation initiation factor 3 subunit K pseudogene 3	645368	0	NA	NA	NA	NA	NA
ENSG00000250444	CCT5P1	chaperonin containing TCP1 subunit 5 pseudogene 1	402226	0	NA	NA	NA	NA	NA
ENSG00000250455	NA	NA	NA	0	NA	NA	NA	NA	NA
ENSG00000250458	NA	NA	NA	0	NA	NA	NA	NA	NA
ENSG00000250461	NA	NA	NA	1,71937571	-1,441841682	1,770128	-0,814541086	0,415335045	NA
ENSG00000250464	LRRC34P2	leucine rich repeat containing 34 pseudogene 2	100420613	0	NA	NA	NA	NA	NA
ENSG00000250470	NA	NA	NA	0	NA	NA	NA	NA	NA
ENSG00000250471	GMPSP1	guanine monophosphate synthase pseudogene 1	728564	0	NA	NA	NA	NA	NA
ENSG00000250473	DUTP7	deoxyuridine triphosphatase pseudogene 7	100873913	0	NA	NA	NA	NA	NA
ENSG00000250474	NA	NA	NA	1,055951454	-2,605271834	2,168948	-1,201168222	0,229685954	NA
ENSG00000250475	RPL6P11	ribosomal protein L6 pseudogene 11	100132922	0	NA	NA	NA	NA	NA
ENSG00000250476	ENPP7P9	ectonucleotide pyrophosphatase/phosphodiesterase 7 pseudogene 9	100421803	0	NA	NA	NA	NA	NA
ENSG00000250480	RLIG1P1	RLIG1 pseudogene 1	100129574	0	NA	NA	NA	NA	NA
ENSG00000250482	NA	NA	NA	0	NA	NA	NA	NA	NA
ENSG00000250483	PPM1AP1	protein phosphatase, Mg2+/Mn2+ dependent 1A pseudogene 1	137012	0	NA	NA	NA	NA	NA
ENSG00000250484	PPIAP76	peptidylprolyl isomerase A pseudogene 76	111082983	0	NA	NA	NA	NA	NA
ENSG00000250485	NA	NA	NA	0	NA	NA	NA	NA	NA
ENSG00000250492	INTS6P1	integrator complex subunit 6 pseudogene 1	285634	0,11808024	-1,076443944	4,080456	-0,263804824	0,791930317	NA
ENSG00000250494	NA	NA	NA	0	NA	NA	NA	NA	NA
ENSG00000250496	NA	NA	NA	0	NA	NA	NA	NA	NA
ENSG00000250500	NA	NA	NA	0	NA	NA	NA	NA	NA
ENSG00000250504	KRT18P51	keratin 18 pseudogene 51	391703	0	NA	NA	NA	NA	NA
ENSG00000250505	LOC100422637	MX dynamin like GTPase 1 pseudogene	100422637	0	NA	NA	NA	NA	NA
ENSG00000250506	CDK3	cyclin dependent kinase 3	1018	4,068330148	-2,226466606	3,77361	-0,590009742	0,555184118	NA
ENSG00000250507	NA	NA	NA	0	NA	NA	NA	NA	NA
ENSG00000250510	GPR162	G protein-coupled receptor 162	27239	0	NA	NA	NA	NA	NA
ENSG00000250515	NA	NA	NA	0	NA	NA	NA	NA	NA
ENSG00000250516	NA	NA	NA	0,210862461	0	4,080456	0	1	NA
ENSG00000250517	NA	NA	NA	0	NA	NA	NA	NA	NA
ENSG00000250523	NA	NA	NA	0	NA	NA	NA	NA	NA
ENSG00000250526	CCT6P2	chaperonin containing TCP1 subunit 6 pseudogene 2	391739	0	NA	NA	NA	NA	NA
ENSG00000250535	NA	NA	NA	0	NA	NA	NA	NA	NA
ENSG00000250536	NA	NA	NA	0	NA	NA	NA	NA	NA
ENSG00000250539	KRT8P33	keratin 8 pseudogene 33	100418737	7,177928329	-0,641471998	0,710384	-0,902993495	0,366529347	NA
ENSG00000250540	NA	NA	NA	0	NA	NA	NA	NA	NA
ENSG00000250547	LOC133332	mitochondrial ribosomal protein S5 pseudogene	133332	0	NA	NA	NA	NA	NA
ENSG00000250550	NA	NA	NA	0	NA	NA	NA	NA	NA
ENSG00000250556	CCDC34P1	coiled-coil domain containing 34 pseudogene 1	100288584	0	NA	NA	NA	NA	NA
ENSG00000250560	NA	NA	NA	0	NA	NA	NA	NA	NA

ENSG00000250561	OR7E59P	olfactory receptor family 7 subfamily E member 59 pseudogene	402249	0	NA	NA	NA	NA	NA
ENSG00000250562	NA	NA	NA	6,3719592	-0,167851781	0,687983	-0,243976809	0,807248781	NA
ENSG00000250563	NA	NA	NA	0	NA	NA	NA	NA	NA
ENSG00000250565	ATP6V1E2	ATPase H+ transporting V1 subunit E2	90423	5,274167048	-0,36063705	0,807652	-0,446525352	0,655217804	NA
ENSG00000250566	NA	NA	NA	0	NA	NA	NA	NA	NA
ENSG00000250568	NA	NA	NA	0,554000868	-0,030112123	3,325929	-0,009053749	0,992776252	NA
ENSG00000250569	NTAN1P2	N-terminal asparagine amidase pseudogene 2	100420307	1,239847465	-0,129368643	1,7578	-0,073596893	0,941331143	NA
ENSG00000250571	GLI4	GLI family zinc finger 4	2738	7,845532603	0,592888866	0,831628	0,712925911	0,475891604	NA
ENSG00000250573	NA	NA	NA	0	NA	NA	NA	NA	NA
ENSG00000250574	NA	NA	NA	0,617118244	2,477046784	2,788295	0,888373378	0,374339941	NA
ENSG00000250580	SNRPCP8	small nuclear ribonucleoprotein polypeptide C pseudogene 8	100310851	0	NA	NA	NA	NA	NA
ENSG00000250587	NA	NA	NA	0	NA	NA	NA	NA	NA
ENSG00000250591	PRSS3P1	PRSS3 pseudogene 1	168330	0	NA	NA	NA	NA	NA
ENSG00000250594	RNF14P2	RNF14 pseudogene 2	116435284	0	NA	NA	NA	NA	NA
ENSG00000250596	NA	NA	NA	0	NA	NA	NA	NA	NA
ENSG00000250609	NA	NA	NA	0	NA	NA	NA	NA	NA
ENSG00000250611	NA	NA	NA	0	NA	NA	NA	NA	NA
ENSG00000250612	NA	NA	NA	0	NA	NA	NA	NA	NA
ENSG00000250613	NA	NA	NA	0	NA	NA	NA	NA	NA
ENSG00000250618	NA	NA	NA	0	NA	NA	NA	NA	NA
ENSG00000250622	NA	NA	NA	0	NA	NA	NA	NA	NA
ENSG00000250624	MTCYBP43	MT-CYB pseudogene 43	109729119	0	NA	NA	NA	NA	NA
ENSG00000250625	LOC100420228	MAGE family member C3 pseudogene	100420228	0	NA	NA	NA	NA	NA
ENSG00000250627	TTC39CP1	tetratricopeptide repeat domain 39C pseudogene 1	100288825	0	NA	NA	NA	NA	NA
ENSG00000250630	MTCO1P35	MT-CO1 pseudogene 35	107075234	0	NA	NA	NA	NA	NA
ENSG00000250636	NA	NA	NA	0	NA	NA	NA	NA	NA
ENSG00000250637	LOC100420534	Rho GTPase activating protein 20 pseudogene	100420534	0	NA	NA	NA	NA	NA
ENSG00000250640	NA	NA	NA	0	NA	NA	NA	NA	NA
ENSG00000250641	LY6G6F-LY6G6D	LY6G6F-LY6G6D readthrough	110599563	0	NA	NA	NA	NA	NA
ENSG00000250642	NA	NA	NA	0	NA	NA	NA	NA	NA
ENSG00000250645	NA	NA	NA	1,688038609	2,113708653	1,749444	1,208217182	0,226963734	NA
ENSG00000250650	LOC107986375	thymosin beta-15A-like	107986375	0	NA	NA	NA	NA	NA
ENSG00000250651	NA	NA	NA	0,71083437	-0,994395882	2,275279	-0,437043546	0,662079768	NA
ENSG00000250654	NA	NA	NA	0,223874632	-0,102249786	4,080321	-0,025059253	0,980007702	NA
ENSG00000250655	NA	NA	NA	0	NA	NA	NA	NA	NA
ENSG00000250656	NA	NA	NA	0,10488184	-1,076443944	4,080456	-0,263804824	0,791930317	NA
ENSG00000250662	NA	NA	NA	0	NA	NA	NA	NA	NA
ENSG00000250667	NA	NA	NA	0	NA	NA	NA	NA	NA
ENSG00000250669	LOC359819	mitochondrial ribosomal protein L39 pseudogene	359819	0	NA	NA	NA	NA	NA
ENSG00000250672	NA	NA	NA	0	NA	NA	NA	NA	NA
ENSG00000250673	REELD1	reeler domain containing 1	345051	0,119673877	0,847101401	4,080456	0,207599697	0,835541541	NA
ENSG00000250677	NA	NA	NA	0	NA	NA	NA	NA	NA
ENSG00000250678	NA	NA	NA	0	NA	NA	NA	NA	NA
ENSG00000250684	NA	NA	NA	0	NA	NA	NA	NA	NA
ENSG00000250687	NAIPP1	NAIP pseudogene 1	728535	0	NA	NA	NA	NA	NA
ENSG00000250688	TRAJ55	T cell receptor alpha joining 55 (pseudogene)	28700	0	NA	NA	NA	NA	NA
ENSG00000250693	RPF2P2	ribosome production factor 2 homolog pseudogene 2	100128462	0	NA	NA	NA	NA	NA
ENSG00000250703	NA	NA	NA	0	NA	NA	NA	NA	NA
ENSG00000250705	NA	NA	NA	0	NA	NA	NA	NA	NA
ENSG00000250709	CCDC169-SOHLH2	CCDC169-SOHLH2 readthrough	100526761	0	NA	NA	NA	NA	NA
ENSG00000250710	OR7E99P	olfactory receptor family 7 subfamily E member 99 pseudogene	81419	0	NA	NA	NA	NA	NA
ENSG00000250711	NA	NA	NA	0	NA	NA	NA	NA	NA
ENSG00000250712	LOC391713	tripartite motif-containing 75 pseudogene	391713	0	NA	NA	NA	NA	NA
ENSG00000250715	NA	NA	NA	0	NA	NA	NA	NA	NA
ENSG00000250719	P3R3URF	PIK3R3 upstream open reading frame	110117498	0	NA	NA	NA	NA	NA
ENSG00000250721	NA	NA	NA	0	NA	NA	NA	NA	NA
ENSG00000250722	SELENOP	selenoprotein P	6414	0,23616048	-2,075648354	4,028973	-0,515180498	0,606426895	NA
ENSG00000250725	NA	NA	NA	0	NA	NA	NA	NA	NA
ENSG00000250726	NA	NA	NA	0	NA	NA	NA	NA	NA
ENSG00000250727	NA	NA	NA	0	NA	NA	NA	NA	NA
ENSG00000250728	NA	NA	NA	0	NA	NA	NA	NA	NA

ENSG00000250730	HMGB3P3	high mobility group box 3 pseudogene 3	100419949	0	NA	NA	NA	NA	NA
ENSG00000250731	NA	NA	NA	1,157911551	-2,816053883	1,908657	-1,47541111	0,14010205	NA
ENSG00000250732	RPEP1	ribulose-5-phosphate-3-epimerase pseudogene 1	100420512	0	NA	NA	NA	NA	NA
ENSG00000250741	NT5C1B-RDH14	NT5C1B-RDH14 readthrough	100526794	0	NA	NA	NA	NA	NA
ENSG00000250745	USP17L20	ubiquitin specific peptidase 17 like family member 20	100287441	0	NA	NA	NA	NA	NA
ENSG00000250746	LOC100288073	heat shock protein family A (Hsp70) member 5 pseudogene	100288073	0,446165129	0,937845705	3,015873	0,31096994	0,755823474	NA
ENSG00000250747	LOC101060067	40S ribosomal protein S3-like	101060067	0	NA	NA	NA	NA	NA
ENSG00000250749	NA	NA	NA	0	NA	NA	NA	NA	NA
ENSG00000250750	NA	NA	NA	0	NA	NA	NA	NA	NA
ENSG00000250752	NA	NA	NA	0	NA	NA	NA	NA	NA
ENSG00000250753	NA	NA	NA	0	NA	NA	NA	NA	NA
ENSG00000250756	NA	NA	NA	0	NA	NA	NA	NA	NA
ENSG00000250762	NA	NA	NA	0	NA	NA	NA	NA	NA
ENSG00000250765	NA	NA	NA	0,706189256	0,80938629	3,183009	0,254283391	0,79927663	NA
ENSG00000250767	NA	NA	NA	0,236573303	0	4,080456	0	1	NA
ENSG00000250768	NA	NA	NA	0	NA	NA	NA	NA	NA
ENSG00000250769	NA	NA	NA	0	NA	NA	NA	NA	NA
ENSG00000250770	NA	NA	NA	0	NA	NA	NA	NA	NA
ENSG00000250771	NA	NA	NA	0	NA	NA	NA	NA	NA
ENSG00000250778	NA	NA	NA	0	NA	NA	NA	NA	NA
ENSG00000250782	TAF11L14	TATA-box binding protein associated factor 11 like 14	112488740	0	NA	NA	NA	NA	NA
ENSG00000250787	HMGN1P17	high mobility group nucleosome binding domain 1 pseudogene 17	100874443	0	NA	NA	NA	NA	NA
ENSG00000250788	TUBB8P4	tubulin beta 8 class VIII pseudogene 4	260338	0	NA	NA	NA	NA	NA
ENSG00000250794	NA	NA	NA	0	NA	NA	NA	NA	NA
ENSG00000250796	NA	NA	NA	0,473550059	1,598254844	3,983952	0,401173183	0,688292624	NA
ENSG00000250799	PRODH2	proline dehydrogenase 2	58510	0	NA	NA	NA	NA	NA
ENSG00000250803	ZNF475	zinc finger protein 475	100505841	0	NA	NA	NA	NA	NA
ENSG00000250804	OR7E111FP	olfactory receptor family 7 subfamily E member 111F pseudogene	100288392	0	NA	NA	NA	NA	NA
ENSG00000250808	MTCO2P30	MT-CO2 pseudogene 30	107075195	0,119673877	0,847101401	4,080456	0,207599697	0,835541541	NA
ENSG00000250812	METTL5P3	METTL5 pseudogene 3	100128865	0	NA	NA	NA	NA	NA
ENSG00000250813	SERF1AP1	small EDRK-rich factor 1A pseudogene 1	100131921	0	NA	NA	NA	NA	NA
ENSG00000250815	NA	NA	NA	0	NA	NA	NA	NA	NA
ENSG00000250816	NA	NA	NA	0	NA	NA	NA	NA	NA
ENSG00000250821	EXOC1L	exocyst complex component 1 like	644145	0	NA	NA	NA	NA	NA
ENSG00000250825	NA	NA	NA	0	NA	NA	NA	NA	NA
ENSG00000250826	NA	NA	NA	0	NA	NA	NA	NA	NA
ENSG00000250827	MFSD4BP1	major facilitator superfamily domain containing 4B pseudogene 1	100128154	0	NA	NA	NA	NA	NA
ENSG00000250828	NA	NA	NA	0	NA	NA	NA	NA	NA
ENSG00000250830	MRPS35P2	mitochondrial ribosomal protein S35 pseudogene 2	359778	0	NA	NA	NA	NA	NA
ENSG00000250833	NA	NA	NA	0	NA	NA	NA	NA	NA
ENSG00000250834	KRT18P54	keratin 18 pseudogene 54	100418792	0	NA	NA	NA	NA	NA
ENSG00000250835	NA	NA	NA	0	NA	NA	NA	NA	NA
ENSG00000250839	NA	NA	NA	0	NA	NA	NA	NA	NA
ENSG00000250844	USP17L18	ubiquitin specific peptidase 17 like family member 18	100287364	0	NA	NA	NA	NA	NA
ENSG00000250847	NA	NA	NA	0	NA	NA	NA	NA	NA
ENSG00000250848	NA	NA	NA	0,072165449	0,847101401	4,080456	0,207599697	0,835541541	NA
ENSG00000250853	NA	NA	NA	0,457391073	0,700574317	2,975425	0,235453512	0,813856757	NA
ENSG00000250857	NA	NA	NA	0	NA	NA	NA	NA	NA
ENSG00000250858	IGBP1P5	IGBP1 pseudogene 5	391642	0	NA	NA	NA	NA	NA
ENSG00000250859	HNRNPKP1	heterogeneous nuclear ribonucleoprotein K pseudogene 1	389322	0,11808024	-1,076443944	4,080456	-0,263804824	0,791930317	NA
ENSG00000250860	NA	NA	NA	0	NA	NA	NA	NA	NA
ENSG00000250862	NA	NA	NA	0	NA	NA	NA	NA	NA
ENSG00000250868	NA	NA	NA	0	NA	NA	NA	NA	NA
ENSG00000250869	NA	NA	NA	0,117497108	0,847101401	4,080456	0,207599697	0,835541541	NA
ENSG00000250878	METTL21EP	methyltransferase like 21E, pseudogene	121952	0	NA	NA	NA	NA	NA
ENSG00000250884	OR7E85P	olfactory receptor family 7 subfamily E member 85 pseudogene	442105	0	NA	NA	NA	NA	NA

ENSG00000250886	NA	NA	NA	0	NA	NA	NA	NA	NA
ENSG00000250890	NA	NA	NA	0	NA	NA	NA	NA	NA
ENSG00000250892	LOC100288172	WEE1 homolog (S, pombe) pseudogene	100288172	0	NA	NA	NA	NA	NA
ENSG00000250895	TUBAP15	tubulin alpha pseudogene 15	117751739	0	NA	NA	NA	NA	NA
ENSG00000250896	NA	NA	NA	0,10543123	0	4,080456	0	1	NA
ENSG00000250897	HMGB3P15	high mobility group box 3 pseudogene 15	644429	0	NA	NA	NA	NA	NA
ENSG00000250913	NA	NA	NA	0	NA	NA	NA	NA	NA
ENSG00000250914	NA	NA	NA	0	NA	NA	NA	NA	NA
ENSG00000250919	UGT2B26P	UDP glucuronosyltransferase family 2 member B26, pseudogene	54570	0	NA	NA	NA	NA	NA
ENSG00000250922	NA	NA	NA	0	NA	NA	NA	NA	NA
ENSG00000250923	NA	NA	NA	0	NA	NA	NA	NA	NA
ENSG00000250927	MESTP3	mesoderm specific transcript pseudogene 3	645641	0	NA	NA	NA	NA	NA
ENSG00000250928	CATSPER2P2	CATSPER2 pseudogene 2	100127986	0,10543123	0	4,080456	0	1	NA
ENSG00000250933	NA	NA	NA	0	NA	NA	NA	NA	NA
ENSG00000250939	NA	NA	NA	0	NA	NA	NA	NA	NA
ENSG00000250940	NA	NA	NA	0	NA	NA	NA	NA	NA
ENSG00000250942	ENPP7P11	ectonucleotide pyrophosphatase/phosphodiesterase 7 pseudogene 11	100421806	0	NA	NA	NA	NA	NA
ENSG00000250946	NA	NA	NA	0	NA	NA	NA	NA	NA
ENSG00000250951	NA	NA	NA	0	NA	NA	NA	NA	NA
ENSG00000250956	NA	NA	NA	0	NA	NA	NA	NA	NA
ENSG00000250959	NA	NA	NA	0,117497108	0,847101401	4,080456	0,207599697	0,835541541	NA
ENSG00000250962	NA	NA	NA	0	NA	NA	NA	NA	NA
ENSG00000250966	NA	NA	NA	0	NA	NA	NA	NA	NA
ENSG00000250972	NA	NA	NA	0	NA	NA	NA	NA	NA
ENSG00000250973	NA	NA	NA	0	NA	NA	NA	NA	NA
ENSG00000250979	NA	NA	NA	0	NA	NA	NA	NA	NA
ENSG00000250980	NA	NA	NA	0	NA	NA	NA	NA	NA
ENSG00000250982	NA	NA	NA	0,15836288	0	4,080456	0	1	NA
ENSG00000250983	FAM76AP1	FAM76A pseudogene 1	127460885	0	NA	NA	NA	NA	NA
ENSG00000250984	NA	NA	NA	0	NA	NA	NA	NA	NA
ENSG00000250995	NA	NA	NA	0	NA	NA	NA	NA	NA
ENSG00000250997	NA	NA	NA	0	NA	NA	NA	NA	NA
ENSG00000251000	NA	NA	NA	0,144330898	1,418786819	4,061262	0,349346267	0,726829368	NA
ENSG00000251001	NA	NA	NA	0	NA	NA	NA	NA	NA
ENSG00000251005	H3P15	H3 histone pseudogene 15	100419353	0	NA	NA	NA	NA	NA
ENSG00000251008	LTO1P1	LTO1 pseudogene 1	100873907	0	NA	NA	NA	NA	NA
ENSG00000251012	NA	NA	NA	0	NA	NA	NA	NA	NA
ENSG00000251013	GAPDHP62	glyceraldehyde 3 phosphate dehydrogenase pseudogene 62	100128961	1,808224199	4,700144629	2,523153	1,862805967	0,06248957	NA
ENSG00000251014	TMEM183AP6	TMEM183A pseudogene 6	100286958	0	NA	NA	NA	NA	NA
ENSG00000251017	LOC643014	coiled-coil domain containing 90B pseudogene	643014	0	NA	NA	NA	NA	NA
ENSG00000251019	HIGD1AP13	HIG1 hypoxia inducible domain family member 1A pseudogene 13	100874454	0	NA	NA	NA	NA	NA
ENSG00000251025	NDUFB5P1	NADH:ubiquinone oxidoreductase subunit B5 pseudogene 1	100288337	0	NA	NA	NA	NA	NA
ENSG00000251032	NA	NA	NA	0	NA	NA	NA	NA	NA
ENSG00000251035	NA	NA	NA	0	NA	NA	NA	NA	NA
ENSG00000251038	NA	NA	NA	0	NA	NA	NA	NA	NA
ENSG00000251039	IGKV2D-40	immunoglobulin kappa variable 2D-40	28878	0	NA	NA	NA	NA	NA
ENSG00000251046	ZNF969P	zinc finger protein 969, pseudogene	100130995	0,15836288	0	4,080456	0	1	NA
ENSG00000251050	NA	NA	NA	0	NA	NA	NA	NA	NA
ENSG00000251051	ELL2P2	elongation factor for RNA polymerase II 2 pseudogene 2	100421143	0	NA	NA	NA	NA	NA
ENSG00000251056	ANKRD20A17P	ankyrin repeat domain 20 family member A17, pseudogene	100421363	0	NA	NA	NA	NA	NA
ENSG00000251062	NA	NA	NA	0	NA	NA	NA	NA	NA
ENSG00000251066	NA	NA	NA	0	NA	NA	NA	NA	NA
ENSG00000251073	NUDT19P5	NUDT19 pseudogene 5	100422445	0	NA	NA	NA	NA	NA
ENSG00000251074	NA	NA	NA	0	NA	NA	NA	NA	NA
ENSG00000251078	NA	NA	NA	0	NA	NA	NA	NA	NA
ENSG00000251087	ALG1L3P	ALG1 like 3, pseudogene	100132066	0	NA	NA	NA	NA	NA

ENSG00000251090	OR5AC4P	olfactory receptor family 5 subfamily AC member 4 pseudogene	403271	0	NA	NA	NA	NA	NA
ENSG00000251099	NA	NA	NA	0	NA	NA	NA	NA	NA
ENSG00000251101	NA	NA	NA	0	NA	NA	NA	NA	NA
ENSG00000251102	CTBP2P4	CTBP2 pseudogene 4	642909	0,11808024	-1,076443944	4,080456	-0,263804824	0,791930317	NA
ENSG00000251105	NA	NA	NA	0	NA	NA	NA	NA	NA
ENSG00000251106	NA	NA	NA	0	NA	NA	NA	NA	NA
ENSG00000251107	NA	NA	NA	0	NA	NA	NA	NA	NA
ENSG00000251108	NA	NA	NA	0	NA	NA	NA	NA	NA
ENSG00000251111	NA	NA	NA	0	NA	NA	NA	NA	NA
ENSG00000251112	NA	NA	NA	0	NA	NA	NA	NA	NA
ENSG00000251113	LOC100533708	protein inhibitor of activated STAT 2 pseudogene	100533708	0	NA	NA	NA	NA	NA
ENSG00000251118	NA	NA	NA	0	NA	NA	NA	NA	NA
ENSG00000251122	NIFKP2	nucleolar protein interacting with the FHA domain of MKI67 pseudogene 2	645398	0	NA	NA	NA	NA	NA
ENSG00000251123	NA	NA	NA	0	NA	NA	NA	NA	NA
ENSG00000251135	NA	NA	NA	0	NA	NA	NA	NA	NA
ENSG00000251137	NA	NA	NA	0	NA	NA	NA	NA	NA
ENSG00000251142	NA	NA	NA	0	NA	NA	NA	NA	NA
ENSG00000251147	TRPC6P7	TRPC6 pseudogene 7	123464511	0	NA	NA	NA	NA	NA
ENSG00000251149	MTND5P5	MT-ND5 pseudogene 5	100873201	0	NA	NA	NA	NA	NA
ENSG00000251154	PTTG1IP2	PTTG1IP family member 2	102723899	0	NA	NA	NA	NA	NA
ENSG00000251155	NA	NA	NA	0,261644068	2,139045705	4,008853	0,533580421	0,593631857	NA
ENSG00000251158	LOC728506	POM121 membrane glycoprotein (rat) pseudogene	728506	0	NA	NA	NA	NA	NA
ENSG00000251159	NA	NA	NA	0	NA	NA	NA	NA	NA
ENSG00000251162	NA	NA	NA	0	NA	NA	NA	NA	NA
ENSG00000251163	PRELID3BP4	PRELI domain containing 3B pseudogene 4	100420275	0,177525443	-0,130435472	4,08014	-0,031968385	0,974497263	NA
ENSG00000251166	OR7E163P	olfactory receptor family 7 subfamily E member 163 pseudogene	100131415	0	NA	NA	NA	NA	NA
ENSG00000251172	TPTE2P7	TPTE2 pseudogene 7	127379748	0	NA	NA	NA	NA	NA
ENSG00000251174	NA	NA	NA	0	NA	NA	NA	NA	NA
ENSG00000251177	LOC100422024	UDP glucuronosyltransferase family 2 member B7 pseudogene	100422024	0	NA	NA	NA	NA	NA
ENSG00000251178	OR7E21P	olfactory receptor family 7 subfamily E member 21 pseudogene	9432	0	NA	NA	NA	NA	NA
ENSG00000251184	NA	NA	NA	0	NA	NA	NA	NA	NA
ENSG00000251188	NA	NA	NA	0	NA	NA	NA	NA	NA
ENSG00000251193	NA	NA	NA	0	NA	NA	NA	NA	NA
ENSG00000251195	NA	NA	NA	0	NA	NA	NA	NA	NA
ENSG00000251196	NA	NA	NA	0	NA	NA	NA	NA	NA
ENSG00000251203	NA	NA	NA	0	NA	NA	NA	NA	NA
ENSG00000251204	NA	NA	NA	0	NA	NA	NA	NA	NA
ENSG00000251211	LOC645853	NAD(P)HX epimerase pseudogene	645853	0	NA	NA	NA	NA	NA
ENSG00000251212	MTND5P4	MT-ND5 pseudogene 4	100873200	0	NA	NA	NA	NA	NA
ENSG00000251215	NA	NA	NA	2,77498094	-0,424902738	1,36183	-0,312008609	0,755033981	NA
ENSG00000251220	RFPL4AP3	ret finger protein like 4A pseudogene 3	106480810	0	NA	NA	NA	NA	NA
ENSG00000251223	MTCO1P9	MT-CO1 pseudogene 9	107075139	0	NA	NA	NA	NA	NA
ENSG00000251228	LOC107986237	cytochrome b-c1 complex subunit 8-like	107986237	0	NA	NA	NA	NA	NA
ENSG00000251229	NA	NA	NA	0	NA	NA	NA	NA	NA
ENSG00000251234	NA	NA	NA	0	NA	NA	NA	NA	NA
ENSG00000251235	NA	NA	NA	0	NA	NA	NA	NA	NA
ENSG00000251236	LOC100422189	UDP glucuronosyltransferase family 2 member B17 pseudogene	100422189	0	NA	NA	NA	NA	NA
ENSG00000251237	B3GNTL1P1	B3GNTL1 pseudogene 1	106481968	0	NA	NA	NA	NA	NA
ENSG00000251243	LOC101060035	dnaJ homolog subfamily C member 24-like	101060035	0	NA	NA	NA	NA	NA
ENSG00000251246	EFNA4-EFNA3	EFNA4-EFNA3 readthrough	125467750	0,649489042	3,454692166	3,94816	0,875013242	0,381566701	NA
ENSG00000251250	NA	NA	NA	0,281448416	-0,974485138	3,950853	-0,246651859	0,805177666	NA
ENSG00000251252	MTND2P31	MT-ND2 pseudogene 31	100873235	0,210862461	0	4,080456	0	1	NA
ENSG00000251253	NA	NA	NA	0	NA	NA	NA	NA	NA
ENSG00000251254	NA	NA	NA	0	NA	NA	NA	NA	NA
ENSG00000251258	RFPL4B	ret finger protein like 4B	442247	0	NA	NA	NA	NA	NA
ENSG00000251261	NA	NA	NA	0	NA	NA	NA	NA	NA
ENSG00000251271	NA	NA	NA	0	NA	NA	NA	NA	NA
ENSG00000251278	LOC100422638	MX dynamin like GTPase 1 pseudogene	100422638	0	NA	NA	NA	NA	NA

ENSG00000251284	NA	NA	NA	0	NA	NA	NA	NA	NA
ENSG00000251285	NA	NA	NA	0	NA	NA	NA	NA	NA
ENSG00000251286	LOC100419861	metallophosphoesterase 1 pseudogene	100419861	0	NA	NA	NA	NA	NA
ENSG00000251287	ALG1L2	ALG1 chitobiosyldiphosphodolichol beta-mannosyltransferase like 2	644974	0	NA	NA	NA	NA	NA
ENSG00000251288	NA	NA	NA	0	NA	NA	NA	NA	NA
ENSG00000251296	NA	NA	NA	0	NA	NA	NA	NA	NA
ENSG00000251299	NA	NA	NA	0	NA	NA	NA	NA	NA
ENSG00000251300	LOC100287014	LysM domain containing 4 pseudogene	100287014	0	NA	NA	NA	NA	NA
ENSG00000251303	CAB39P1	calcium binding protein 39 pseudogene 1	106481732	0	NA	NA	NA	NA	NA
ENSG00000251306	NDUFB4P2	NADH:ubiquinone oxidoreductase subunit B4 pseudogene 2	100132036	0	NA	NA	NA	NA	NA
ENSG00000251308	NA	NA	NA	0	NA	NA	NA	NA	NA
ENSG00000251312	NA	NA	NA	0	NA	NA	NA	NA	NA
ENSG00000251313	LOC100286946	ribosomal protein S3A pseudogene	100286946	0	NA	NA	NA	NA	NA
ENSG00000251322	SHANK3	SH3 and multiple ankyrin repeat domains 3	85358	0	NA	NA	NA	NA	NA
ENSG00000251332	PSME2P4	proteasome activator subunit 2 pseudogene 4	338096	0	NA	NA	NA	NA	NA
ENSG00000251333	NA	NA	NA	0	NA	NA	NA	NA	NA
ENSG00000251334	NA	NA	NA	0	NA	NA	NA	NA	NA
ENSG00000251338	LOC100422639	MX dynamin like GTPase 1 pseudogene	100422639	0	NA	NA	NA	NA	NA
ENSG00000251340	MTCYBP35	MT-CYB pseudogene 35	109729150	0	NA	NA	NA	NA	NA
ENSG00000251342	NA	NA	NA	0	NA	NA	NA	NA	NA
ENSG00000251345	NA	NA	NA	0	NA	NA	NA	NA	NA
ENSG00000251347	IRF5P1	interferon regulatory factor 5 pseudogene 1	100420406	0	NA	NA	NA	NA	NA
ENSG00000251348	NA	NA	NA	0,117497108	0,847101401	4,080456	0,207599697	0,835541541	NA
ENSG00000251349	MSANTD3-TMEFF1	MSANTD3-TMEFF1 readthrough	100526694	4,890508615	0,051744682	1,543327	0,033528014	0,973253527	NA
ENSG00000251352	LOC100420514	coiled-coil domain containing 86 pseudogene	100420514	0	NA	NA	NA	NA	NA
ENSG00000251353	MTND3P5	MT-ND3 pseudogene 5	100873186	0,499990854	2,686903942	2,774606	0,968391245	0,33284901	NA
ENSG00000251354	LOC100420532	ribosomal protein S4 Y-linked 2 pseudogene	100420532	0	NA	NA	NA	NA	NA
ENSG00000251356	NA	NA	NA	0,23716124	1,955796116	4,022316	0,486236376	0,626799583	NA
ENSG00000251357	NA	NA	NA	0	NA	NA	NA	NA	NA
ENSG00000251360	NA	NA	NA	0	NA	NA	NA	NA	NA
ENSG00000251366	NA	NA	NA	0	NA	NA	NA	NA	NA
ENSG00000251368	NA	NA	NA	0	NA	NA	NA	NA	NA
ENSG00000251373	NA	NA	NA	0	NA	NA	NA	NA	NA
ENSG00000251374	NA	NA	NA	0	NA	NA	NA	NA	NA
ENSG00000251378	NA	NA	NA	0	NA	NA	NA	NA	NA
ENSG00000251380	DCANP1	dendritic cell associated nuclear protein 1	140947	0	NA	NA	NA	NA	NA
ENSG00000251385	MTCYBP16	MT-CYB pseudogene 16	107075126	0	NA	NA	NA	NA	NA
ENSG00000251389	YTHDF1P1	YTH domain family member 1 pseudogene 1	402220	0	NA	NA	NA	NA	NA
ENSG00000251395	FTH1P9	ferritin heavy chain 1 pseudogene 9	106478936	0	NA	NA	NA	NA	NA
ENSG00000251400	ALDH7A1P1	aldehyde dehydrogenase 7 family member A1 pseudogene 1	541	0	NA	NA	NA	NA	NA
ENSG00000251401	NA	NA	NA	0	NA	NA	NA	NA	NA
ENSG00000251402	NA	NA	NA	0	NA	NA	NA	NA	NA
ENSG00000251407	MTND1P22	MT-ND1 pseudogene 22	100287591	0	NA	NA	NA	NA	NA
ENSG00000251411	NA	NA	NA	0,354859955	0	4,080456	0	1	NA
ENSG00000251418	NA	NA	NA	0	NA	NA	NA	NA	NA
ENSG00000251419	LOC391798	karyopherin subunit alpha 7 pseudogene	391798	0	NA	NA	NA	NA	NA
ENSG00000251424	NA	NA	NA	0	NA	NA	NA	NA	NA
ENSG00000251427	NA	NA	NA	0	NA	NA	NA	NA	NA
ENSG00000251429	AIDAP2	AIDA pseudogene 2	646890	0	NA	NA	NA	NA	NA
ENSG00000251430	MTCO3P27	MT-CO3 pseudogene 27	107075183	0	NA	NA	NA	NA	NA
ENSG00000251433	NA	NA	NA	0	NA	NA	NA	NA	NA
ENSG00000251435	NA	NA	NA	0	NA	NA	NA	NA	NA
ENSG00000251436	NA	NA	NA	0	NA	NA	NA	NA	NA
ENSG00000251437	NA	NA	NA	0	NA	NA	NA	NA	NA
ENSG00000251440	NA	NA	NA	0	NA	NA	NA	NA	NA
ENSG00000251441	RTEL1P1	regulator of telomere elongation helicase 1 pseudogene 1	100421429	0	NA	NA	NA	NA	NA

ENSG00000251447	NA	NA	NA	0	NA	NA	NA	NA	NA
ENSG00000251449	MTND1P19	MT-ND1 pseudogene 19	100873220	0	NA	NA	NA	NA	NA
ENSG00000251451	GTF2IP9	general transcription factor IIi pseudogene 9	106481734	0,968381977	0,179768804	1,78826	0,100527196	0,919925793	NA
ENSG00000251452	NA	NA	NA	0,209763681	-1,926644696	4,042043	-0,476651224	0,633610501	NA
ENSG00000251453	NA	NA	NA	0	NA	NA	NA	NA	NA
ENSG00000251463	NA	NA	NA	0,10488184	-1,076443944	4,080456	-0,263804824	0,791930317	NA
ENSG00000251464	NA	NA	NA	0	NA	NA	NA	NA	NA
ENSG00000251467	NA	NA	NA	0,11808024	-1,076443944	4,080456	-0,263804824	0,791930317	NA
ENSG00000251468	LOC100422204	regulator of solute carriers 1 pseudogene	100422204	0	NA	NA	NA	NA	NA
ENSG00000251470	ASNSP4	ASNS pseudogene 4	100419423	0	NA	NA	NA	NA	NA
ENSG00000251473	LOC100288146	nucleoporin Nup43-like	100288146	0	NA	NA	NA	NA	NA
ENSG00000251474	NA	NA	NA	3,034262168	0,018742433	1,133152	0,016540088	0,986803521	NA
ENSG00000251476	MTCO1P31	MT-CO1 pseudogene 31	107075171	0	NA	NA	NA	NA	NA
ENSG00000251477	NA	NA	NA	0	NA	NA	NA	NA	NA
ENSG00000251478	TCP1P2	t-complex 1 pseudogene 2	6952	0	NA	NA	NA	NA	NA
ENSG00000251482	NA	NA	NA	0	NA	NA	NA	NA	NA
ENSG00000251483	NA	NA	NA	0	NA	NA	NA	NA	NA
ENSG00000251484	NA	NA	NA	0	NA	NA	NA	NA	NA
ENSG00000251485	NA	NA	NA	3,739731606	-0,02348928	0,975515	-0,024078844	0,980789718	NA
ENSG00000251489	NA	NA	NA	0	NA	NA	NA	NA	NA
ENSG00000251491	OR7E28P	olfactory receptor family 7 subfamily E member 28 pseudogene	26645	0	NA	NA	NA	NA	NA
ENSG00000251492	NA	NA	NA	0	NA	NA	NA	NA	NA
ENSG00000251493	FOXD1	forkhead box D1	2297	6,63149896	-0,139594115	0,743492	-0,187754778	0,851068887	NA
ENSG00000251495	NA	NA	NA	0	NA	NA	NA	NA	NA
ENSG00000251498	LOC100289568	UDP glucuronosyltransferase family 2 member A1 complex locus pseudogene	100289568	0	NA	NA	NA	NA	NA
ENSG00000251501	PGBD3P4	piggyBac transposable element derived 3 pseudogene 4	267008	0	NA	NA	NA	NA	NA
ENSG00000251503	CENPS-CORT	CENPS-CORT readthrough	100526739	2,191644062	3,152294946	2,266063	1,391089095	0,164198411	NA
ENSG00000251508	NA	NA	NA	0	NA	NA	NA	NA	NA
ENSG00000251515	CFAP53P1	CFAP53 pseudogene 1	100129040	0	NA	NA	NA	NA	NA
ENSG00000251516	NA	NA	NA	0	NA	NA	NA	NA	NA
ENSG00000251520	NA	NA	NA	0	NA	NA	NA	NA	NA
ENSG00000251521	NA	NA	NA	0,11808024	-1,076443944	4,080456	-0,263804824	0,791930317	NA
ENSG00000251525	TCP11X3P	t-complex 11 family, X-linked 3, pseudogene	106481605	0	NA	NA	NA	NA	NA
ENSG00000251529	NA	NA	NA	0	NA	NA	NA	NA	NA
ENSG00000251535	NA	NA	NA	0,31672576	0	4,080456	0	1	NA
ENSG00000251537	NA	NA	NA	0,352491325	2,536807152	3,984503	0,636668335	0,524340903	NA
ENSG00000251539	NA	NA	NA	0	NA	NA	NA	NA	NA
ENSG00000251544	MTND5P12	MT-ND5 pseudogene 12	100873257	0	NA	NA	NA	NA	NA
ENSG00000251545	LOC100289470	chromosome 5 open reading frame 60-like	100289470	0	NA	NA	NA	NA	NA
ENSG00000251546	IGKV1D-39	immunoglobulin kappa variable 1D-39	28893	0	NA	NA	NA	NA	NA
ENSG00000251548	NA	NA	NA	0	NA	NA	NA	NA	NA
ENSG00000251549	NA	NA	NA	0	NA	NA	NA	NA	NA
ENSG00000251550	NA	NA	NA	0	NA	NA	NA	NA	NA
ENSG00000251552	NA	NA	NA	0	NA	NA	NA	NA	NA
ENSG00000251553	NA	NA	NA	0	NA	NA	NA	NA	NA
ENSG00000251557	HNRNPKP3	heterogeneous nuclear ribonucleoprotein K pseudogene 3	399881	0	NA	NA	NA	NA	NA
ENSG00000251563	IARS2P1	IARS2 pseudogene 1	642461	0	NA	NA	NA	NA	NA
ENSG00000251566	HMGB1P35	high mobility group box 1 pseudogene 35	100419950	0	NA	NA	NA	NA	NA
ENSG00000251568	ALG3P1	ALG3 pseudogene 1	442131	0,10488184	-1,076443944	4,080456	-0,263804824	0,791930317	NA
ENSG00000251569	NA	NA	NA	0	NA	NA	NA	NA	NA
ENSG00000251571	NA	NA	NA	0	NA	NA	NA	NA	NA
ENSG00000251572	NA	NA	NA	0	NA	NA	NA	NA	NA
ENSG00000251578	TRBV21-1	T cell receptor beta variable 21-1 (pseudogene)	28566	0	NA	NA	NA	NA	NA
ENSG00000251579	LOC100420298	makorin ring finger protein 1 pseudogene	100420298	0	NA	NA	NA	NA	NA
ENSG00000251583	NA	NA	NA	0	NA	NA	NA	NA	NA
ENSG00000251585	NA	NA	NA	0	NA	NA	NA	NA	NA
ENSG00000251587	NA	NA	NA	0	NA	NA	NA	NA	NA
ENSG00000251588	NA	NA	NA	0	NA	NA	NA	NA	NA
ENSG00000251590	NIFKP7	nucleolar protein interacting with the FHA domain of MKI67 pseudogene 7	106479049	0	NA	NA	NA	NA	NA
ENSG00000251591	NA	NA	NA	0	NA	NA	NA	NA	NA

ENSG00000251593	NA	NA	NA	0,23616048	-2,075648354	4,028973	-0,515180498	0,606426895	NA
ENSG00000251595	ABCA11P	ATP binding cassette subfamily A member 11, pseudogene	79963	7,958699304	0,737842561	0,986022	0,748302674	0,454277612	NA
ENSG00000251596	HADHAP1	HADHA pseudogene 1	3031	0	NA	NA	NA	NA	NA
ENSG00000251597	RPL37P25	ribosomal protein L37 pseudogene 25	106481666	0	NA	NA	NA	NA	NA
ENSG00000251605	NA	NA	NA	0	NA	NA	NA	NA	NA
ENSG00000251608	OR12D1	olfactory receptor family 12 subfamily D member 1 (gene/pseudogene)	26530	0	NA	NA	NA	NA	NA
ENSG00000251609	NA	NA	NA	0	NA	NA	NA	NA	NA
ENSG00000251610	EVA1CP6	EVA1C pseudogene 6	122149299	0	NA	NA	NA	NA	NA
ENSG00000251614	NA	NA	NA	0	NA	NA	NA	NA	NA
ENSG00000251618	NA	NA	NA	0	NA	NA	NA	NA	NA
ENSG00000251623	NA	NA	NA	0	NA	NA	NA	NA	NA
ENSG00000251624	NA	NA	NA	0	NA	NA	NA	NA	NA
ENSG00000251627	NA	NA	NA	0	NA	NA	NA	NA	NA
ENSG00000251630	NA	NA	NA	0	NA	NA	NA	NA	NA
ENSG00000251633	GYG1P1	glycogenin 1 pseudogene 1	100132965	0	NA	NA	NA	NA	NA
ENSG00000251638	NA	NA	NA	0	NA	NA	NA	NA	NA
ENSG00000251639	NA	NA	NA	0,103998153	-1,076443944	4,080456	-0,263804824	0,791930317	NA
ENSG00000251643	COPS3P1	COPS3 pseudogene 1	100652955	0,072165449	0,847101401	4,080456	0,207599697	0,835541541	NA
ENSG00000251644	NA	NA	NA	0	NA	NA	NA	NA	NA
ENSG00000251647	LOC100287427	chromosome 18 open reading frame 21 pseudogene	100287427	0	NA	NA	NA	NA	NA
ENSG00000251648	NA	NA	NA	0	NA	NA	NA	NA	NA
ENSG00000251656	PRELID3BP5	PRELI domain containing 3B pseudogene 5	106480242	0	NA	NA	NA	NA	NA
ENSG00000251663	SUMO2P5	SUMO2 pseudogene 5	100526738	0	NA	NA	NA	NA	NA
ENSG00000251667	BRCC3P1	BRCA1/BRCA2-containing complex subunit 3 pseudogene 1	100421311	1,560153672	0,132567631	1,480957	0,08951485	0,928672753	NA
ENSG00000251668	NA	NA	NA	0	NA	NA	NA	NA	NA
ENSG00000251669	NA	NA	NA	0,10488184	-1,076443944	4,080456	-0,263804824	0,791930317	NA
ENSG00000251682	NA	NA	NA	1,982045978	-0,470200183	1,204566	-0,390348231	0,696279063	NA
ENSG00000251685	NA	NA	NA	0	NA	NA	NA	NA	NA
ENSG00000251689	NA	NA	NA	0	NA	NA	NA	NA	NA
ENSG00000251691	LOC100422022	UDP glucuronosyltransferase family 2 member A3 pseudogene	100422022	0	NA	NA	NA	NA	NA
ENSG00000251692	PTX4	pentraxin 4	390667	0	NA	NA	NA	NA	NA
ENSG00000251694	USP17L9P	ubiquitin specific peptidase 17 like family member 9, pseudogene	391627	0	NA	NA	NA	NA	NA
ENSG00000251698	RNA5SP445	RNA, 5S ribosomal pseudogene 445	100873691	0	NA	NA	NA	NA	NA
ENSG00000251705	RNA5-8SP6	RNA, 5,8S ribosomal pseudogene 6	100873336	0	NA	NA	NA	NA	NA
ENSG00000251705	LOC124907582	5,8S ribosomal RNA	124907582	0	NA	NA	NA	NA	NA
ENSG00000251717	RNA5SP419	RNA, 5S ribosomal pseudogene 419	100873670	0	NA	NA	NA	NA	NA
ENSG00000251729	RNA5SP401	RNA, 5S ribosomal pseudogene 401	100873654	0	NA	NA	NA	NA	NA
ENSG00000251746	RNA5SP112	RNA, 5S ribosomal pseudogene 112	100873382	0	NA	NA	NA	NA	NA
ENSG00000251756	RNA5SP385	RNA, 5S ribosomal pseudogene 385	100873640	0	NA	NA	NA	NA	NA
ENSG00000251760	RNA5SP418	RNA, 5S ribosomal pseudogene 418	100873669	0	NA	NA	NA	NA	NA
ENSG00000251764	RNA5SP446	RNA, 5S ribosomal pseudogene 446	100873692	0	NA	NA	NA	NA	NA
ENSG00000251766	RNA5SP518	RNA, 5S ribosomal pseudogene 518	100873569	0	NA	NA	NA	NA	NA
ENSG00000251768	RNA5SP217	RNA, 5S ribosomal pseudogene 217	100873477	0	NA	NA	NA	NA	NA
ENSG00000251770	RNA5SP486	RNA, 5S ribosomal pseudogene 486	100873728	0	NA	NA	NA	NA	NA
ENSG00000251781	RNA5SP356	RNA, 5S ribosomal pseudogene 356	100873615	0	NA	NA	NA	NA	NA
ENSG00000251785	RNA5SP20	RNA, 5S ribosomal pseudogene 20	100873275	0	NA	NA	NA	NA	NA
ENSG00000251816	RNA5SP157	RNA, 5S ribosomal pseudogene 157	100873423	0	NA	NA	NA	NA	NA
ENSG00000251823	NA	NA	NA	0	NA	NA	NA	NA	NA
ENSG00000251829	RNA5SP436	RNA, 5S ribosomal pseudogene 436	100873685	0	NA	NA	NA	NA	NA

ENSG00000251850	RNA5SP96	RNA, 5S ribosomal pseudogene 96	100873329	0	NA	NA	NA	NA	NA
ENSG00000251857	RNA5SP362	RNA, 5S ribosomal pseudogene 362	100873621	0	NA	NA	NA	NA	NA
ENSG00000251873	RNA5SP182	RNA, 5S ribosomal pseudogene 182	100873444	0	NA	NA	NA	NA	NA
ENSG00000251884	RNA5SP288	RNA, 5S ribosomal pseudogene 288	100873541	0	NA	NA	NA	NA	NA
ENSG00000251890	RNA5SP497	RNA, 5S ribosomal pseudogene 497	100873739	0	NA	NA	NA	NA	NA
ENSG00000251904	RNA5SP272	RNA, 5S ribosomal pseudogene 272	100873525	0	NA	NA	NA	NA	NA
ENSG00000251915	RNA5SP406	RNA, 5S ribosomal pseudogene 406	100873658	0	NA	NA	NA	NA	NA
ENSG00000251920	RNA5SP216	RNA, 5S ribosomal pseudogene 216	100873476	0	NA	NA	NA	NA	NA
ENSG00000251924	RNA5SP408	RNA, 5S ribosomal pseudogene 408	100873660	0	NA	NA	NA	NA	NA
ENSG00000251936	RNA5SP249	RNA, 5S ribosomal pseudogene 249	100873505	0	NA	NA	NA	NA	NA
ENSG00000251965	RNA5SP208	RNA, 5S ribosomal pseudogene 208	100873468	0	NA	NA	NA	NA	NA
ENSG00000251978	RNA5SP236	RNA, 5S ribosomal pseudogene 236	100873494	0	NA	NA	NA	NA	NA
ENSG00000251990	RNA5SP180	RNA, 5S ribosomal pseudogene 180	100873442	0	NA	NA	NA	NA	NA
ENSG00000251993	RNA5SP227	RNA, 5S ribosomal pseudogene 227	100873485	0	NA	NA	NA	NA	NA
ENSG00000251997	RNA5SP458	RNA, 5S ribosomal pseudogene 458	106479016	0	NA	NA	NA	NA	NA
ENSG00000252001	RNA5SP303	RNA, 5S ribosomal pseudogene 303	100873577	0	NA	NA	NA	NA	NA
ENSG00000252002	RNA5SP154	RNA, 5S ribosomal pseudogene 154	100873420	0	NA	NA	NA	NA	NA
ENSG00000252012	RNA5SP521	RNA, 5S ribosomal pseudogene 521	106479018	0	NA	NA	NA	NA	NA
ENSG00000252041	RNA5SP228	RNA, 5S ribosomal pseudogene 228	100873486	0	NA	NA	NA	NA	NA
ENSG00000252060	RNA5SP464	RNA, 5S ribosomal pseudogene 464	100873708	0	NA	NA	NA	NA	NA
ENSG00000252070	RNA5SP341	RNA, 5S ribosomal pseudogene 341	100873604	0	NA	NA	NA	NA	NA
ENSG00000252072	RNA5SP320	RNA, 5S ribosomal pseudogene 320	100873586	0	NA	NA	NA	NA	NA
ENSG00000252086	RNA5SP76	RNA, 5S ribosomal pseudogene 76	100873310	0	NA	NA	NA	NA	NA
ENSG00000252094	RNA5SP410	RNA, 5S ribosomal pseudogene 410	100873662	0	NA	NA	NA	NA	NA
ENSG00000252107	RNA5SP220	RNA, 5S ribosomal pseudogene 220	100873480	0	NA	NA	NA	NA	NA
ENSG00000252161	RNA5SP318	RNA, 5S ribosomal pseudogene 318	100873584	0	NA	NA	NA	NA	NA
ENSG00000252164	RNA5SP282	RNA, 5S ribosomal pseudogene 282	100873535	0	NA	NA	NA	NA	NA
ENSG00000252167	RNA5SP287	RNA, 5S ribosomal pseudogene 287	100873540	0	NA	NA	NA	NA	NA
ENSG00000252169	RNA5SP200	RNA, 5S ribosomal pseudogene 200	100873461	0	NA	NA	NA	NA	NA
ENSG00000252179	RNA5SP255	RNA, 5S ribosomal pseudogene 255	100873509	0	NA	NA	NA	NA	NA
ENSG00000252182	RNA5SP413	RNA, 5S ribosomal pseudogene 413	100873664	0	NA	NA	NA	NA	NA
ENSG00000252207	RNA5SP365	RNA, 5S ribosomal pseudogene 365	100873624	0	NA	NA	NA	NA	NA
ENSG00000252211	NA	NA	NA	0	NA	NA	NA	NA	NA
ENSG00000252231	RNA5SP67	RNA, 5S ribosomal pseudogene 67	100873302	0	NA	NA	NA	NA	NA
ENSG00000252246	RNA5SP92	RNA, 5S ribosomal pseudogene 92	100873325	0	NA	NA	NA	NA	NA
ENSG00000252259	RNA5SP49	RNA, 5S ribosomal pseudogene 49	100873288	0	NA	NA	NA	NA	NA
ENSG00000252260	RNA5SP461	RNA, 5S ribosomal pseudogene 461	100873705	0	NA	NA	NA	NA	NA
ENSG00000252261	RNA5SP262	RNA, 5S ribosomal pseudogene 262	100873516	0	NA	NA	NA	NA	NA
ENSG00000252262	RNA5SP61	RNA, 5S ribosomal pseudogene 61	100873296	0	NA	NA	NA	NA	NA
ENSG00000252267	RNA5SP495	RNA, 5S ribosomal pseudogene 495	100873737	0	NA	NA	NA	NA	NA

ENSG00000252268	RNA5SP417	RNA, 5S ribosomal pseudogene 417	100873668	0	NA	NA	NA	NA	NA
ENSG00000252272	RNA5SP470	RNA, 5S ribosomal pseudogene 470	100873713	0	NA	NA	NA	NA	NA
ENSG00000252287	RNA5SP24	RNA, 5S ribosomal pseudogene 24	100873356	0	NA	NA	NA	NA	NA
ENSG00000252289	RNA5SP519	RNA, 5S ribosomal pseudogene 519	100873570	0	NA	NA	NA	NA	NA
ENSG00000252301	RNA5SP134	RNA, 5S ribosomal pseudogene 134	106480756	0	NA	NA	NA	NA	NA
ENSG00000252302	RNA5SP147	RNA, 5S ribosomal pseudogene 147	100873414	0	NA	NA	NA	NA	NA
ENSG00000252307	RNA5SP153	RNA, 5S ribosomal pseudogene 153	100873419	0	NA	NA	NA	NA	NA
ENSG00000252313	RNA5SP281	RNA, 5S ribosomal pseudogene 281	100873534	0	NA	NA	NA	NA	NA
ENSG00000252315	RNA5SP520	RNA, 5S ribosomal pseudogene 520	106480771	0	NA	NA	NA	NA	NA
ENSG00000252336	RNA5SP148	RNA, 5S ribosomal pseudogene 148	100873415	0	NA	NA	NA	NA	NA
ENSG00000252342	RNA5SP164	RNA, 5S ribosomal pseudogene 164	100873429	0	NA	NA	NA	NA	NA
ENSG00000252346	RNA5SP121	RNA, 5S ribosomal pseudogene 121	100873391	0	NA	NA	NA	NA	NA
ENSG00000252364	RNA5SP360	RNA, 5S ribosomal pseudogene 360	100873619	0	NA	NA	NA	NA	NA
ENSG00000252366	RNA5SP367	RNA, 5S ribosomal pseudogene 367	100873626	0	NA	NA	NA	NA	NA
ENSG00000252368	RNA5SP43	RNA, 5S ribosomal pseudogene 43	100873282	0	NA	NA	NA	NA	NA
ENSG00000252370	RNA5SP438	RNA, 5S ribosomal pseudogene 438	100873687	0	NA	NA	NA	NA	NA
ENSG00000252376	RNA5SP395	RNA, 5S ribosomal pseudogene 395	100873648	0	NA	NA	NA	NA	NA
ENSG00000252422	RNA5SP476	RNA, 5S ribosomal pseudogene 476	100873718	0	NA	NA	NA	NA	NA
ENSG00000252424	RNA5SP384	RNA, 5S ribosomal pseudogene 384	100873639	0	NA	NA	NA	NA	NA
ENSG00000252428	RNA5SP285	RNA, 5S ribosomal pseudogene 285	100873538	0	NA	NA	NA	NA	NA
ENSG00000252437	RNA5SP459	RNA, 5S ribosomal pseudogene 459	100873703	0	NA	NA	NA	NA	NA
ENSG00000252451	RNA5SP168	RNA, 5S ribosomal pseudogene 168	100873433	0	NA	NA	NA	NA	NA
ENSG00000252456	RNA5SP434	RNA, 5S ribosomal pseudogene 434	100873684	0	NA	NA	NA	NA	NA
ENSG00000252479	RNA5SP309	RNA, 5S ribosomal pseudogene 309	100873583	0	NA	NA	NA	NA	NA
ENSG00000252496	RNA5SP33	RNA, 5S ribosomal pseudogene 33	100873365	0	NA	NA	NA	NA	NA
ENSG00000252509	RNA5SP271	RNA, 5S ribosomal pseudogene 271	100873524	0	NA	NA	NA	NA	NA
ENSG00000252510	RNA5SP55	RNA, 5S ribosomal pseudogene 55	100873293	0	NA	NA	NA	NA	NA
ENSG00000252512	RNA5SP206	RNA, 5S ribosomal pseudogene 206	106478999	0	NA	NA	NA	NA	NA
ENSG00000252516	RNA5SP82	RNA, 5S ribosomal pseudogene 82	100873316	0	NA	NA	NA	NA	NA
ENSG00000252539	RNA5SP462	RNA, 5S ribosomal pseudogene 462	100873706	0	NA	NA	NA	NA	NA
ENSG00000252546	RNA5SP466	RNA, 5S ribosomal pseudogene 466	100873710	0	NA	NA	NA	NA	NA
ENSG00000252553	RNA5SP73	RNA, 5S ribosomal pseudogene 73	100873307	0	NA	NA	NA	NA	NA
ENSG00000252563	RNA5SP45	RNA, 5S ribosomal pseudogene 45	100873284	0	NA	NA	NA	NA	NA
ENSG00000252587	RNA5SP422	RNA, 5S ribosomal pseudogene 422	100873673	0	NA	NA	NA	NA	NA
ENSG00000252591	RNA5SP468	RNA, 5S ribosomal pseudogene 468	106480769	0	NA	NA	NA	NA	NA
ENSG00000252598	RNA5SP460	RNA, 5S ribosomal pseudogene 460	100873704	0	NA	NA	NA	NA	NA
ENSG00000252618	RNA5SP441	RNA, 5S ribosomal pseudogene 441	100873689	0	NA	NA	NA	NA	NA
ENSG00000252623	RNA5SP481	RNA, 5S ribosomal pseudogene 481	100873723	0	NA	NA	NA	NA	NA
ENSG00000252624	RNA5SP409	RNA, 5S ribosomal pseudogene 409	100873661	0	NA	NA	NA	NA	NA
ENSG00000252637	RNA5SP268	RNA, 5S ribosomal pseudogene 268	100873521	0	NA	NA	NA	NA	NA

ENSG00000252642	RNA5SP137	RNA, 5S ribosomal pseudogene 137	100873405	0	NA	NA	NA	NA	NA
ENSG00000252647	RNA5SP416	RNA, 5S ribosomal pseudogene 416	100873667	0	NA	NA	NA	NA	NA
ENSG00000252649	RNA5SP480	RNA, 5S ribosomal pseudogene 480	100873722	0	NA	NA	NA	NA	NA
ENSG00000252650	RNA5SP75	RNA, 5S ribosomal pseudogene 75	100873309	0	NA	NA	NA	NA	NA
ENSG00000252653	RNA5SP444	RNA, 5S ribosomal pseudogene 444	100873690	0	NA	NA	NA	NA	NA
ENSG00000252673	RNA5SP421	RNA, 5S ribosomal pseudogene 421	100873672	0	NA	NA	NA	NA	NA
ENSG00000252674	RNA5SP102	RNA, 5S ribosomal pseudogene 102	100873373	0	NA	NA	NA	NA	NA
ENSG00000252680	RNA5SP449	RNA, 5S ribosomal pseudogene 449	100873694	0	NA	NA	NA	NA	NA
ENSG00000252696	RNA5SP34	RNA, 5S ribosomal pseudogene 34	100873366	0	NA	NA	NA	NA	NA
ENSG00000252714	RNA5SP392	RNA, 5S ribosomal pseudogene 392	100873645	0	NA	NA	NA	NA	NA
ENSG00000252726	RNA5SP94	RNA, 5S ribosomal pseudogene 94	100873327	0	NA	NA	NA	NA	NA
ENSG00000252745	RNA5SP143	RNA, 5S ribosomal pseudogene 143	100873410	0	NA	NA	NA	NA	NA
ENSG00000252747	RNA5SP364	RNA, 5S ribosomal pseudogene 364	100873623	0	NA	NA	NA	NA	NA
ENSG00000252760	RNA5SP54	RNA, 5S ribosomal pseudogene 54	100873292	0	NA	NA	NA	NA	NA
ENSG00000252780	RNA5SP471	RNA, 5S ribosomal pseudogene 471	100873714	0	NA	NA	NA	NA	NA
ENSG00000252806	RNA5SP420	RNA, 5S ribosomal pseudogene 420	100873671	0	NA	NA	NA	NA	NA
ENSG00000252816	RNA5SP337	RNA, 5S ribosomal pseudogene 337	100873600	0	NA	NA	NA	NA	NA
ENSG00000252828	RNA5SP135	RNA, 5S ribosomal pseudogene 135	100873403	0	NA	NA	NA	NA	NA
ENSG00000252833	RNA5SP186	RNA, 5S ribosomal pseudogene 186	100873448	0	NA	NA	NA	NA	NA
ENSG00000252845	RNA5SP101	RNA, 5S ribosomal pseudogene 101	100873372	0	NA	NA	NA	NA	NA
ENSG00000252848	RNA5SP230	RNA, 5S ribosomal pseudogene 230	100873488	0	NA	NA	NA	NA	NA
ENSG00000252856	RNA5SP503	RNA, 5S ribosomal pseudogene 503	100873554	0	NA	NA	NA	NA	NA
ENSG00000252864	RNA5SP278	RNA, 5S ribosomal pseudogene 278	100873531	0	NA	NA	NA	NA	NA
ENSG00000252866	RNA5SP243	RNA, 5S ribosomal pseudogene 243	106479001	0	NA	NA	NA	NA	NA
ENSG00000252877	RNA5SP312	RNA, 5S ribosomal pseudogene 312	106480439	0	NA	NA	NA	NA	NA
ENSG00000252905	RNA5SP330	RNA, 5S ribosomal pseudogene 330	100873596	0	NA	NA	NA	NA	NA
ENSG00000252927	RNA5SP404	RNA, 5S ribosomal pseudogene 404	100873656	0	NA	NA	NA	NA	NA
ENSG00000252936	RNA5SP423	RNA, 5S ribosomal pseudogene 423	100873674	0	NA	NA	NA	NA	NA
ENSG00000252942	RNA5SP290	RNA, 5S ribosomal pseudogene 290	100873543	0	NA	NA	NA	NA	NA
ENSG00000252950	RNA5SP490	RNA, 5S ribosomal pseudogene 490	100873732	0	NA	NA	NA	NA	NA
ENSG00000252951	RNA5SP165	RNA, 5S ribosomal pseudogene 165	100873430	0	NA	NA	NA	NA	NA
ENSG00000252956	RNA5SP40	RNA, 5S ribosomal pseudogene 40	100873279	0	NA	NA	NA	NA	NA
ENSG00000252957	RNA5SP402	RNA, 5S ribosomal pseudogene 402	100873655	0	NA	NA	NA	NA	NA
ENSG00000252959	RNA5SP170	RNA, 5S ribosomal pseudogene 170	100873435	0	NA	NA	NA	NA	NA
ENSG00000252964	RNA5SP400	RNA, 5S ribosomal pseudogene 400	100873653	0	NA	NA	NA	NA	NA
ENSG00000252970	RNA5SP159	RNA, 5S ribosomal pseudogene 159	100873425	0	NA	NA	NA	NA	NA
ENSG00000252977	RNA5SP70	RNA, 5S ribosomal pseudogene 70	106480436	0	NA	NA	NA	NA	NA
ENSG00000252987	RNA5SP66	RNA, 5S ribosomal pseudogene 66	100873301	0	NA	NA	NA	NA	NA
ENSG00000253031	RNA5SP291	RNA, 5S ribosomal pseudogene 291	100873544	0	NA	NA	NA	NA	NA
ENSG00000253039	RNA5SP47	RNA, 5S ribosomal pseudogene 47	100873286	0	NA	NA	NA	NA	NA

ENSG00000253040	RNA5SP454	RNA, 5S ribosomal pseudogene 454	100873699	0	NA	NA	NA	NA	NA
ENSG00000253055	RNA5SP415	RNA, 5S ribosomal pseudogene 415	100873666	0	NA	NA	NA	NA	NA
ENSG00000253058	RNA5SP437	RNA, 5S ribosomal pseudogene 437	100873686	0	NA	NA	NA	NA	NA
ENSG00000253077	RNA5SP169	RNA, 5S ribosomal pseudogene 169	100873434	0	NA	NA	NA	NA	NA
ENSG00000253080	RNA5SP373	RNA, 5S ribosomal pseudogene 373	100873631	0	NA	NA	NA	NA	NA
ENSG00000253083	RNA5SP106	RNA, 5S ribosomal pseudogene 106	106478994	0	NA	NA	NA	NA	NA
ENSG00000253093	RNA5SP179	RNA, 5S ribosomal pseudogene 179	100873441	0	NA	NA	NA	NA	NA
ENSG00000253107	LOC100132280	ataxin 3 pseudogene	100132280	0	NA	NA	NA	NA	NA
ENSG00000253109	RPL12P22	ribosomal protein L12 pseudogene 22	100270922	0	NA	NA	NA	NA	NA
ENSG00000253114	NA	NA	NA	0	NA	NA	NA	NA	NA
ENSG00000253117	OC90	otoconin 90	729330	0	NA	NA	NA	NA	NA
ENSG00000253118	NA	NA	NA	0	NA	NA	NA	NA	NA
ENSG00000253119	HNRNPA3P7	heterogeneous nuclear ribonucleoprotein A3 pseudogene 7	100421357	0	NA	NA	NA	NA	NA
ENSG00000253120	NA	NA	NA	0	NA	NA	NA	NA	NA
ENSG00000253124	NA	NA	NA	0	NA	NA	NA	NA	NA
ENSG00000253126	NA	NA	NA	0	NA	NA	NA	NA	NA
ENSG00000253127	IGKV2D-36	immunoglobulin kappa variable 2D-36 (pseudogene)	28880	0	NA	NA	NA	NA	NA
ENSG00000253135	NA	NA	NA	0	NA	NA	NA	NA	NA
ENSG00000253137	PSMC2P2	PSMC2 pseudogene 2	100422271	0	NA	NA	NA	NA	NA
ENSG00000253144	NA	NA	NA	0	NA	NA	NA	NA	NA
ENSG00000253146	CIR1P1	CIR1 pseudogene 1	100131740	0	NA	NA	NA	NA	NA
ENSG00000253148	RGS21	regulator of G protein signaling 21	431704	0	NA	NA	NA	NA	NA
ENSG00000253149	IGHVII-28-1	immunoglobulin heavy variable (II)-28-1 (pseudogene)	28372	0	NA	NA	NA	NA	NA
ENSG00000253152	IGLV3-17	immunoglobulin lambda variable 3-17 (pseudogene)	28798	0	NA	NA	NA	NA	NA
ENSG00000253153	LOC124901995	probable ribosome biogenesis protein RLP24	124901995	0	NA	NA	NA	NA	NA
ENSG00000253155	RPL26P18	ribosomal protein L26 pseudogene 18	100270968	0	NA	NA	NA	NA	NA
ENSG00000253156	H2AZP7	H2A,Z histone pseudogene 7	115482726	0	NA	NA	NA	NA	NA
ENSG00000253159	PCDHGA12	protocadherin gamma subfamily A, 12	26025	0	NA	NA	NA	NA	NA
ENSG00000253160	LOC100420403	lysine acetyltransferase 2B pseudogene	100420403	0	NA	NA	NA	NA	NA
ENSG00000253166	NA	NA	NA	0	NA	NA	NA	NA	NA
ENSG00000253169	IGHVII-65-1	immunoglobulin heavy variable (II)-65-1 (pseudogene)	28359	0	NA	NA	NA	NA	NA
ENSG00000253173	NA	NA	NA	0,384152358	-0,114967229	3,661096	-0,031402404	0,974948624	NA
ENSG00000253175	LOC100286997	ribosomal protein L31 pseudogene	100286997	0	NA	NA	NA	NA	NA
ENSG00000253176	LOC100130612	TAF9 RNA polymerase II, TATA box binding protein (TBP)-associated factor, 32kDa pseudogene	100130612	0,072165449	0,847101401	4,080456	0,207599697	0,835541541	NA
ENSG00000253178	NA	NA	NA	0	NA	NA	NA	NA	NA
ENSG00000253179	CALCP	calcitonin pseudogene	798	0	NA	NA	NA	NA	NA
ENSG00000253180	NA	NA	NA	0,336337822	-1,096188879	4,080456	-0,268643728	0,788203858	NA
ENSG00000253183	ST13P17	ST13, Hsp70 interacting protein pseudogene 17	442730	0	NA	NA	NA	NA	NA
ENSG00000253186	NA	NA	NA	0	NA	NA	NA	NA	NA
ENSG00000253188	TRBV6-7	T cell receptor beta variable 6-7 (non-functional)	28600	0	NA	NA	NA	NA	NA
ENSG00000253191	IGKV1D-32	immunoglobulin kappa variable 1D-32 (pseudogene)	28897	0	NA	NA	NA	NA	NA
ENSG00000253195	NA	NA	NA	0	NA	NA	NA	NA	NA
ENSG00000253198	NA	NA	NA	0	NA	NA	NA	NA	NA
ENSG00000253204	NA	NA	NA	0	NA	NA	NA	NA	NA
ENSG00000253208	PSME2P5	proteasome activator subunit 2 pseudogene 5	338097	0	NA	NA	NA	NA	NA
ENSG00000253213	NA	NA	NA	0,072165449	0,847101401	4,080456	0,207599697	0,835541541	NA
ENSG00000253216	NA	NA	NA	0	NA	NA	NA	NA	NA
ENSG00000253218	NA	NA	NA	0,693498272	0,018891657	2,60664	0,007247513	0,994217372	NA
ENSG00000253219	KRT18P41	keratin 18 pseudogene 41	345430	0	NA	NA	NA	NA	NA

ENSG00000253223	LOC100288001	pre-mRNA processing factor 3 pseudogene	100288001	0	NA	NA	NA	NA	NA
ENSG00000253224	NA	NA	NA	0	NA	NA	NA	NA	NA
ENSG00000253225	NA	NA	NA	0	NA	NA	NA	NA	NA
ENSG00000253226	NA	NA	NA	0	NA	NA	NA	NA	NA
ENSG00000253228	NA	NA	NA	0	NA	NA	NA	NA	NA
ENSG00000253229	HIGD1AP6	HIG1 hypoxia inducible domain family member 1A pseudogene 6	100874436	0	NA	NA	NA	NA	NA
ENSG00000253231	COX6CP8	cytochrome c oxidase subunit 6C pseudogene 8	106481680	0	NA	NA	NA	NA	NA
ENSG00000253232	NA	NA	NA	0	NA	NA	NA	NA	NA
ENSG00000253234	NA	NA	NA	0	NA	NA	NA	NA	NA
ENSG00000253237	NA	NA	NA	0	NA	NA	NA	NA	NA
ENSG00000253239	IGLVI-70	immunoglobulin lambda variable (I)-70 (pseudogene)	28763	0	NA	NA	NA	NA	NA
ENSG00000253240	IGHV3-36	immunoglobulin heavy variable 3-36 (pseudogene)	28431	0	NA	NA	NA	NA	NA
ENSG00000253242	NA	NA	NA	0	NA	NA	NA	NA	NA
ENSG00000253245	MTND1P36	MT-ND1 pseudogene 36	100506721	0	NA	NA	NA	NA	NA
ENSG00000253250	C8orf88	chromosome 8 open reading frame 88	100127983	0,15836288	0	4,080456	0	1	NA
ENSG00000253257	NA	NA	NA	0	NA	NA	NA	NA	NA
ENSG00000253261	NA	NA	NA	0	NA	NA	NA	NA	NA
ENSG00000253262	NA	NA	NA	0	NA	NA	NA	NA	NA
ENSG00000253271	NA	NA	NA	0	NA	NA	NA	NA	NA
ENSG00000253273	NA	NA	NA	0	NA	NA	NA	NA	NA
ENSG00000253283	LOC100422614	solute carrier family 7 member 13 pseudogene	100422614	0	NA	NA	NA	NA	NA
ENSG00000253290	NA	NA	NA	0	NA	NA	NA	NA	NA
ENSG00000253292	MIOXP1	myo-inositol oxygenase pseudogene 1	100419614	0	NA	NA	NA	NA	NA
ENSG00000253293	HOXA10	homeobox A10	3206	5,841479866	-1,942168191	0,859317	-2,260129195	0,023813234	NA
ENSG00000253299	NA	NA	NA	0	NA	NA	NA	NA	NA
ENSG00000253303	IGHVIII-82	immunoglobulin heavy variable (III)-82 (pseudogene)	28338	0	NA	NA	NA	NA	NA
ENSG00000253304	TMEM200B	transmembrane protein 200B	399474	0	NA	NA	NA	NA	NA
ENSG00000253305	PCDHGB6	protocadherin gamma subfamily B, 6	56100	0	NA	NA	NA	NA	NA
ENSG00000253309	SERPINE3	serpin family E member 3	647174	0	NA	NA	NA	NA	NA
ENSG00000253312	NA	NA	NA	0	NA	NA	NA	NA	NA
ENSG00000253313	C1orf210	chromosome 1 open reading frame 210	149466	0	NA	NA	NA	NA	NA
ENSG00000253318	NA	NA	NA	0	NA	NA	NA	NA	NA
ENSG00000253319	NA	NA	NA	0	NA	NA	NA	NA	NA
ENSG00000253325	IGHV7-34-1	immunoglobulin heavy variable 7-34-1 (pseudogene)	28382	0	NA	NA	NA	NA	NA
ENSG00000253326	NA	NA	NA	0,52922181	0	4,017598	0	1	NA
ENSG00000253328	SUMO2P19	SUMO2 pseudogene 19	100422265	0	NA	NA	NA	NA	NA
ENSG00000253329	NA	NA	NA	0	NA	NA	NA	NA	NA
ENSG00000253330	NA	NA	NA	0	NA	NA	NA	NA	NA
ENSG00000253332	RPL7AP80	ribosomal protein L7a pseudogene 80	122965332	0	NA	NA	NA	NA	NA
ENSG00000253333	NA	NA	NA	1,372214496	-0,226817392	2,049692	-0,110659274	0,911886542	NA
ENSG00000253336	LOC392266	hydroxysteroid 17-beta dehydrogenase 4 pseudogene	392266	0,381618008	-1,081927931	3,690685	-0,293150987	0,769406746	NA
ENSG00000253338	NA	NA	NA	0	NA	NA	NA	NA	NA
ENSG00000253340	NA	NA	NA	0	NA	NA	NA	NA	NA
ENSG00000253341	NA	NA	NA	0,11808024	-1,076443944	4,080456	-0,263804824	0,791930317	NA
ENSG00000253345	IGHVII-22-1	immunoglobulin heavy variable (II)-22-1 (pseudogene)	28374	0	NA	NA	NA	NA	NA
ENSG00000253346	NA	NA	NA	0	NA	NA	NA	NA	NA
ENSG00000253349	COX6B1P6	cytochrome c oxidase subunit 6B1 pseudogene 6	100462851	0	NA	NA	NA	NA	NA
ENSG00000253359	IGHV3-37	immunoglobulin heavy variable 3-37 (pseudogene)	28430	0	NA	NA	NA	NA	NA
ENSG00000253362	NA	NA	NA	0	NA	NA	NA	NA	NA
ENSG00000253365	IGKV1D-22	immunoglobulin kappa variable 1D-22 (pseudogene)	28899	0	NA	NA	NA	NA	NA
ENSG00000253366	NA	NA	NA	3,809432885	4,803761397	2,078953	2,310663895	0,020851427	NA
ENSG00000253367	IGHVIII-25-1	immunoglobulin heavy variable (III)-25-1 (pseudogene)	28348	0	NA	NA	NA	NA	NA
ENSG00000253368	TRNP1	TMF1 regulated nuclear protein 1	388610	0	NA	NA	NA	NA	NA
ENSG00000253370	NA	NA	NA	0	NA	NA	NA	NA	NA
ENSG00000253382	NA	NA	NA	0	NA	NA	NA	NA	NA

ENSG00000253383	NA	NA	NA	0	NA	NA	NA	NA	NA
ENSG00000253384	NA	NA	NA	0	NA	NA	NA	NA	NA
ENSG00000253391	NA	NA	NA	0	NA	NA	NA	NA	NA
ENSG00000253393	RANP9	RAN pseudogene 9	100874193	0	NA	NA	NA	NA	NA
ENSG00000253401	NA	NA	NA	0	NA	NA	NA	NA	NA
ENSG00000253409	TRBV7-4	T cell receptor beta variable 7-4	28594	0	NA	NA	NA	NA	NA
ENSG00000253410	NA	NA	NA	0	NA	NA	NA	NA	NA
ENSG00000253412	IGHVIII-13-1	immunoglobulin heavy variable (III)-13-1 (pseudogene)	28351	0	NA	NA	NA	NA	NA
ENSG00000253413	NA	NA	NA	0	NA	NA	NA	NA	NA
ENSG00000253415	NA	NA	NA	0	NA	NA	NA	NA	NA
ENSG00000253418	SNX18P27	sorting nexin 18 pseudogene 27	100873788	0	NA	NA	NA	NA	NA
ENSG00000253421	NA	NA	NA	0,614466205	-0,100861443	2,662937	-0,037876016	0,969786536	NA
ENSG00000253422	NA	NA	NA	0	NA	NA	NA	NA	NA
ENSG00000253423	LOC100533622	sulfite oxidase pseudogene	100533622	0	NA	NA	NA	NA	NA
ENSG00000253425	HSPA8P13	heat shock protein family A (Hsp70) member 8 pseudogene 13	106480803	0	NA	NA	NA	NA	NA
ENSG00000253429	NA	NA	NA	0	NA	NA	NA	NA	NA
ENSG00000253431	SRPK2P	SRSF protein kinase 2 pseudogene	100419416	0	NA	NA	NA	NA	NA
ENSG00000253439	NA	NA	NA	0	NA	NA	NA	NA	NA
ENSG00000253440	IGHV3-33-2	immunoglobulin heavy variable 3-33-2 (pseudogene)	28433	0	NA	NA	NA	NA	NA
ENSG00000253441	IGHV3-25	immunoglobulin heavy variable 3-25 (pseudogene)	28441	0	NA	NA	NA	NA	NA
ENSG00000253448	IGLV3-6	immunoglobulin lambda variable 3-6 (pseudogene)	28806	0	NA	NA	NA	NA	NA
ENSG00000253451	IGLV2-28	immunoglobulin lambda variable 2-28 (pseudogene)	28812	0	NA	NA	NA	NA	NA
ENSG00000253454	NA	NA	NA	0	NA	NA	NA	NA	NA
ENSG00000253457	SMIM18	small integral membrane protein 18	100507341	0	NA	NA	NA	NA	NA
ENSG00000253458	IGHVII-15-1	immunoglobulin heavy variable (II)-15-1 (pseudogene)	28376	0	NA	NA	NA	NA	NA
ENSG00000253460	NA	NA	NA	0	NA	NA	NA	NA	NA
ENSG00000253462	IGHVIII-26-1	immunoglobulin heavy variable (III)-26-1 (pseudogene)	28347	0	NA	NA	NA	NA	NA
ENSG00000253463	HMGB1P19	high mobility group box 1 pseudogene 19	100419979	1,485460081	-0,82602085	1,565588	-0,527610485	0,597769714	NA
ENSG00000253467	NA	NA	NA	0	NA	NA	NA	NA	NA
ENSG00000253468	NA	NA	NA	0	NA	NA	NA	NA	NA
ENSG00000253472	LOC100420127	mediator complex subunit 13 pseudogene	100420127	0	NA	NA	NA	NA	NA
ENSG00000253480	NA	NA	NA	0	NA	NA	NA	NA	NA
ENSG00000253481	NA	NA	NA	0	NA	NA	NA	NA	NA
ENSG00000253482	IGHVII-26-2	immunoglobulin heavy variable (II)-26-2 (pseudogene)	28373	0	NA	NA	NA	NA	NA
ENSG00000253483	NA	NA	NA	0	NA	NA	NA	NA	NA
ENSG00000253485	PCDHGA5	protocadherin gamma subfamily A, 5	56110	0,262874376	-1,07201028	4,080456	-0,262718263	0,792767739	NA
ENSG00000253488	SINHCAFP3	SINHCAF pseudogene 3	646721	0,446890665	0	4,080456	0	1	NA
ENSG00000253491	IGHVII-30-1	immunoglobulin heavy variable (II)-30-1 (pseudogene)	28371	0	NA	NA	NA	NA	NA
ENSG00000253493	NA	NA	NA	0	NA	NA	NA	NA	NA
ENSG00000253495	CYCSP23	CYCS pseudogene 23	360176	0	NA	NA	NA	NA	NA
ENSG00000253499	LOC100420746	NADH:ubiquinone oxidoreductase complex assembly factor 7 pseudogene	100420746	0	NA	NA	NA	NA	NA
ENSG00000253501	NA	NA	NA	0	NA	NA	NA	NA	NA
ENSG00000253502	ATP6V1G1P2	ATPase H+ transporting V1 subunit G1 pseudogene 2	100128541	0	NA	NA	NA	NA	NA
ENSG00000253504	MTCYBP19	MT-CYB pseudogene 19	107075127	0	NA	NA	NA	NA	NA
ENSG00000253506	NACA2	nascent polypeptide associated complex subunit alpha 2	342538	0	NA	NA	NA	NA	NA
ENSG00000253512	NA	NA	NA	0	NA	NA	NA	NA	NA
ENSG00000253516	NA	NA	NA	0	NA	NA	NA	NA	NA
ENSG00000253517	XRCC6P4	X-ray repair cross complementing 6 pseudogene 4	100422546	0	NA	NA	NA	NA	NA

ENSG00000253525	NA	NA	NA	0,45813758	-1,661582475	3,43027	-0,484388295	0,628110324	NA
ENSG00000253528	NA	NA	NA	0	NA	NA	NA	NA	NA
ENSG00000253534	TRBV6-8	T cell receptor beta variable 6-8	28599	0	NA	NA	NA	NA	NA
ENSG00000253537	PCDHGA7	protocadherin gamma subfamily A, 7	56108	0,072165449	0,847101401	4,080456	0,207599697	0,835541541	NA
ENSG00000253540	NA	NA	NA	0,22490703	0,839444259	4,080456	0,205723156	0,83700717	NA
ENSG00000253541	NA	NA	NA	0	NA	NA	NA	NA	NA
ENSG00000253542	SDR16C6P	short chain dehydrogenase/reductase family 16C member 6, pseudogene	442388	0	NA	NA	NA	NA	NA
ENSG00000253543	NA	NA	NA	0	NA	NA	NA	NA	NA
ENSG00000253544	C1GALT1P3	C1GALT1 pseudogene 3	106481730	0	NA	NA	NA	NA	NA
ENSG00000253546	NA	NA	NA	0	NA	NA	NA	NA	NA
ENSG00000253547	TUBAP7	tubulin alpha pseudogene 7	644436	0	NA	NA	NA	NA	NA
ENSG00000253548	PYDC2	pyrin domain containing 2	152138	0	NA	NA	NA	NA	NA
ENSG00000253555	NA	NA	NA	0	NA	NA	NA	NA	NA
ENSG00000253556	MTCO1P4	MT-CO1 pseudogene 4	106480684	0,072165449	0,847101401	4,080456	0,207599697	0,835541541	NA
ENSG00000253558	LOC100422441	heat shock protein 90 alpha family class B member 1 pseudogene	100422441	4,418542473	0,925054435	0,920844	1,004572193	0,315102892	NA
ENSG00000253560	NA	NA	NA	0	NA	NA	NA	NA	NA
ENSG00000253564	NA	NA	NA	0	NA	NA	NA	NA	NA
ENSG00000253568	NA	NA	NA	0	NA	NA	NA	NA	NA
ENSG00000253569	NA	NA	NA	0	NA	NA	NA	NA	NA
ENSG00000253570	NA	NA	NA	0	NA	NA	NA	NA	NA
ENSG00000253571	LOC392180	transcriptional adaptor 1 pseudogene	392180	0	NA	NA	NA	NA	NA
ENSG00000253579	SUMO2P16	SUMO2 pseudogene 16	106480679	0	NA	NA	NA	NA	NA
ENSG00000253580	NA	NA	NA	0	NA	NA	NA	NA	NA
ENSG00000253581	DPPA3P9	DPPA3 pseudogene 9	127460878	0	NA	NA	NA	NA	NA
ENSG00000253587	IGHV3-30-2	immunoglobulin heavy variable 3-30-2 (pseudogene)	28438	0	NA	NA	NA	NA	NA
ENSG00000253590	NA	NA	NA	0	NA	NA	NA	NA	NA
ENSG00000253598	SLC10A5	solute carrier family 10 member 5	347051	0,314645521	-2,509429468	3,997603	-0,627733573	0,530178488	NA
ENSG00000253600	RPS15P6	ribosomal protein S15 pseudogene 6	391845	0,119673877	0,847101401	4,080456	0,207599697	0,835541541	NA
ENSG00000253606	NA	NA	NA	0	NA	NA	NA	NA	NA
ENSG00000253610	NA	NA	NA	0	NA	NA	NA	NA	NA
ENSG00000253611	VN1R46P	vomeronasal 1 receptor 46 pseudogene	100131789	0	NA	NA	NA	NA	NA
ENSG00000253612	NA	NA	NA	0	NA	NA	NA	NA	NA
ENSG00000253615	NA	NA	NA	0	NA	NA	NA	NA	NA
ENSG00000253620	NA	NA	NA	0,593243541	-1,124801356	2,662159	-0,422514774	0,67264932	NA
ENSG00000253621	RPSAP74	ribosomal protein SA pseudogene 74	107075113	0,072165449	0,847101401	4,080456	0,207599697	0,835541541	NA
ENSG00000253623	NA	NA	NA	0,117497108	0,847101401	4,080456	0,207599697	0,835541541	NA
ENSG00000253626	EIF5AL1	eukaryotic translation initiation factor 5A like 1	143244	1,217527611	-0,96285465	2,027946	-0,47479295	0,634934559	NA
ENSG00000253630	NA	NA	NA	0	NA	NA	NA	NA	NA
ENSG00000253631	NA	NA	NA	0	NA	NA	NA	NA	NA
ENSG00000253637	IGLVV-58	immunoglobulin lambda variable (V)-58 (pseudogene)	28758	0	NA	NA	NA	NA	NA
ENSG00000253638	DPPA3P7	DPPA3 pseudogene 7	127460876	0	NA	NA	NA	NA	NA
ENSG00000253639	NA	NA	NA	0	NA	NA	NA	NA	NA
ENSG00000253646	RPL6P32	ribosomal protein L6 pseudogene 32	100131520	0	NA	NA	NA	NA	NA
ENSG00000253649	PRSS51	serine protease 51	346702	0	NA	NA	NA	NA	NA
ENSG00000253650	NA	NA	NA	0	NA	NA	NA	NA	NA
ENSG00000253651	NA	NA	NA	0	NA	NA	NA	NA	NA
ENSG00000253654	NA	NA	NA	0	NA	NA	NA	NA	NA
ENSG00000253655	NA	NA	NA	0	NA	NA	NA	NA	NA
ENSG00000253657	LOC101060000	dnaJ homolog subfamily B member 6-like	101060000	0	NA	NA	NA	NA	NA
ENSG00000253663	NPM1P52	nucleophosmin 1 pseudogene 52	100422272	0	NA	NA	NA	NA	NA
ENSG00000253667	NA	NA	NA	0	NA	NA	NA	NA	NA
ENSG00000253668	LOC100129667	chromosome 5 open reading frame 13 pseudogene	100129667	0	NA	NA	NA	NA	NA
ENSG00000253670	NA	NA	NA	0	NA	NA	NA	NA	NA
ENSG00000253674	IGHVII-78-1	immunoglobulin heavy variable (II)-78-1 (pseudogene)	28356	0	NA	NA	NA	NA	NA
ENSG00000253676	NA	NA	NA	0	NA	NA	NA	NA	NA
ENSG00000253677	NA	NA	NA	0	NA	NA	NA	NA	NA
ENSG00000253680	NA	NA	NA	0	NA	NA	NA	NA	NA
ENSG00000253683	NA	NA	NA	0	NA	NA	NA	NA	NA

ENSG00000253684	BUD31P1	BUD31 homolog pseudogene 1	106480331	0	NA	NA	NA	NA	NA
ENSG00000253685	MTND5P41	MT-ND5 pseudogene 41	107075309	0	NA	NA	NA	NA	NA
ENSG00000253691	NA	NA	NA	0	NA	NA	NA	NA	NA
ENSG00000253692	IGHEP1	immunoglobulin heavy constant epsilon P1 (pseudogene)	3498	0	NA	NA	NA	NA	NA
ENSG00000253697	NA	NA	NA	0	NA	NA	NA	NA	NA
ENSG00000253703	IGHV1-68	immunoglobulin heavy variable 1-68 (pseudogene)	28462	0	NA	NA	NA	NA	NA
ENSG00000253707	NA	NA	NA	0	NA	NA	NA	NA	NA
ENSG00000253709	IGHV1-14	immunoglobulin heavy variable 1-14 (pseudogene)	28470	0	NA	NA	NA	NA	NA
ENSG00000253721	NA	NA	NA	0	NA	NA	NA	NA	NA
ENSG00000253723	NA	NA	NA	0	NA	NA	NA	NA	NA
ENSG00000253725	LOC100129457	coiled-coil domain containing 86 pseudogene	100129457	0	NA	NA	NA	NA	NA
ENSG00000253731	PCDHGA6	protocadherin gamma subfamily A, 6	56109	1,341217136	0,074981299	1,769127	0,042383213	0,966193211	NA
ENSG00000253743	MARK2P11	MARK2 pseudogene 11	100421484	0	NA	NA	NA	NA	NA
ENSG00000253748	CYP4F44P	cytochrome P450 family 4 subfamily F member 44, pseudogene	106480733	0	NA	NA	NA	NA	NA
ENSG00000253750	LOC107986878	cytochrome c oxidase subunit NDUFA4-like	107986878	0	NA	NA	NA	NA	NA
ENSG00000253752	IGLVI-63	immunoglobulin lambda variable (I)-63 (pseudogene)	28765	0	NA	NA	NA	NA	NA
ENSG00000253755	NA	NA	NA	0	NA	NA	NA	NA	NA
ENSG00000253756	CARS1P2	cysteinyl-tRNA synthetase 1 pseudogene 2	106481707	0	NA	NA	NA	NA	NA
ENSG00000253760	NA	NA	NA	0	NA	NA	NA	NA	NA
ENSG00000253765	IGKV2D-19	immunoglobulin kappa variable 2D-19 (pseudogene)	28887	0	NA	NA	NA	NA	NA
ENSG00000253767	PCDHGA8	protocadherin gamma subfamily A, 8	9708	0	NA	NA	NA	NA	NA
ENSG00000253770	HMGB1P23	high mobility group box 1 pseudogene 23	100873891	0	NA	NA	NA	NA	NA
ENSG00000253771	NA	NA	NA	0	NA	NA	NA	NA	NA
ENSG00000253772	NA	NA	NA	0	NA	NA	NA	NA	NA
ENSG00000253777	NA	NA	NA	0	NA	NA	NA	NA	NA
ENSG00000253779	NA	NA	NA	0	NA	NA	NA	NA	NA
ENSG00000253781	ZNF317P1	zinc finger protein 317 pseudogene 1	100288659	0	NA	NA	NA	NA	NA
ENSG00000253785	NA	NA	NA	0,072165449	0,847101401	4,080456	0,207599697	0,835541541	NA
ENSG00000253786	IGLV3-15	immunoglobulin lambda variable 3-15 (pseudogene)	28800	0	NA	NA	NA	NA	NA
ENSG00000253789	NARS1P2	asparaginyl-tRNA synthetase 1 pseudogene 2	100287895	0	NA	NA	NA	NA	NA
ENSG00000253790	LOC100420944	nuclear transcription factor Y subunit beta pseudogene	100420944	0	NA	NA	NA	NA	NA
ENSG00000253793	LPGAT1P1	lysophosphatidylglycerol acyltransferase 1 pseudogene 1	100129398	0	NA	NA	NA	NA	NA
ENSG00000253794	NA	NA	NA	0	NA	NA	NA	NA	NA
ENSG00000253795	MTND1P5	MT-ND1 pseudogene 5	100873174	0	NA	NA	NA	NA	NA
ENSG00000253797	UTP14C	UTP14C small subunit processome component	9724	0	NA	NA	NA	NA	NA
ENSG00000253798	NA	NA	NA	0,103998153	-1,076443944	4,080456	-0,263804824	0,791930317	NA
ENSG00000253800	NA	NA	NA	0	NA	NA	NA	NA	NA
ENSG00000253801	NA	NA	NA	0	NA	NA	NA	NA	NA
ENSG00000253803	LOC728587	zinc finger protein 736 pseudogene	728587	0	NA	NA	NA	NA	NA
ENSG00000253810	NA	NA	NA	0	NA	NA	NA	NA	NA
ENSG00000253813	NA	NA	NA	0	NA	NA	NA	NA	NA
ENSG00000253814	MRPS36P3	mitochondrial ribosomal protein S36 pseudogene 3	347704	0	NA	NA	NA	NA	NA
ENSG00000253817	NA	NA	NA	0	NA	NA	NA	NA	NA
ENSG00000253818	IGLV1-41	immunoglobulin lambda variable 1-41 (pseudogene)	28824	0	NA	NA	NA	NA	NA
ENSG00000253822	NA	NA	NA	0	NA	NA	NA	NA	NA
ENSG00000253823	NA	NA	NA	0	NA	NA	NA	NA	NA
ENSG00000253826	NA	NA	NA	0	NA	NA	NA	NA	NA
ENSG00000253828	MTND2P38	MT-ND2 pseudogene 38	107075276	0	NA	NA	NA	NA	NA
ENSG00000253831	ETV3L	ETS variant transcription factor 3 like	440695	0	NA	NA	NA	NA	NA
ENSG00000253833	DSTNP3	DSTN pseudogene 3	100287016	0	NA	NA	NA	NA	NA
ENSG00000253840	NA	NA	NA	0	NA	NA	NA	NA	NA
ENSG00000253841	NA	NA	NA	0	NA	NA	NA	NA	NA
ENSG00000253845	NA	NA	NA	0	NA	NA	NA	NA	NA

ENSG00000253846	PCDHGA10	protocadherin gamma subfamily A, 10	56106	0,56817448	0,795310028	2,767928	0,287330462	0,7738593	NA	
ENSG00000253861	NA	NA	NA	0	NA	NA	NA	NA	NA	
ENSG00000253866	NA	NA	NA	0	NA	NA	NA	NA	NA	
ENSG00000253869	NA	NA	NA	0	NA	NA	NA	NA	NA	
ENSG00000253873	PCDHGA11	protocadherin gamma subfamily A, 11	56105	0	NA	NA	NA	NA	NA	
ENSG00000253874	NA	NA	NA	0	NA	NA	NA	NA	NA	
ENSG00000253882	LOC154761	family with sequence similarity 115, member C pseudogene	154761	0	NA	NA	NA	NA	NA	
ENSG00000253883	IGHV3-19	immunoglobulin heavy variable 3-19 (pseudogene)	28446	0	NA	NA	NA	NA	NA	
ENSG00000253884	NA	NA	NA	0	NA	NA	NA	NA	NA	
ENSG00000253885	NA	NA	NA	0	NA	NA	NA	NA	NA	
ENSG00000253886	LOC100128833	VPS37A, ESCRT-I subunit pseudogene	100128833	0	NA	NA	NA	NA	NA	
ENSG00000253889	NA	NA	NA	0	NA	NA	NA	NA	NA	
ENSG00000253897	NA	NA	NA	0	NA	NA	NA	NA	NA	
ENSG00000253899	NA	NA	NA	0,10543123	0	4,080456	0	1	NA	
ENSG00000253900	CDH12P4	cadherin 12 pseudogene 4	100093626	0	NA	NA	NA	NA	NA	
ENSG00000253906	NA	NA	NA	0	NA	NA	NA	NA	NA	
ENSG00000253907	NA	NA	NA	0	NA	NA	NA	NA	NA	
ENSG00000253908	NA	NA	NA	0	NA	NA	NA	NA	NA	
ENSG00000253910	PCDHGB2	protocadherin gamma subfamily B, 2	56103	0,458621872	-1,660724766	3,429721	-0,484215685	0,628232807	NA	
ENSG00000253912	NA	NA	NA	0	NA	NA	NA	NA	NA	
ENSG00000253913	NA	NA	NA	0	NA	NA	NA	NA	NA	
ENSG00000253915	NA	NA	NA	0	NA	NA	NA	NA	NA	
ENSG00000253916	MTCO1P49	MT-CO1 pseudogene 49	107075177	0	NA	NA	NA	NA	NA	
ENSG00000253919	NA	NA	NA	0,236573303	0	4,080456	0	1	NA	
ENSG00000253920	NA	NA	NA	0	NA	NA	NA	NA	NA	
ENSG00000253923	NA	NA	NA	0	NA	NA	NA	NA	NA	
ENSG00000253934	MRPL49P2	mitochondrial ribosomal protein L49 pseudogene 2	346711	0	NA	NA	NA	NA	NA	
ENSG00000253935	IGLVIV-53	immunoglobulin lambda variable (IV)-53 (pseudogene)	28762	0	NA	NA	NA	NA	NA	
ENSG00000253937	NA	NA	NA	0	NA	NA	NA	NA	NA	
ENSG00000253940	NA	NA	NA	0	NA	NA	NA	NA	NA	
ENSG00000253942	LOC100419977	myeloid leukemia factor 2 pseudogene	100419977	0	NA	NA	NA	NA	NA	
ENSG00000253943	KRT18P37	keratin 18 pseudogene 37	392214	0,712277344	0,828737941	3,143074	0,263671183	0,792033302	NA	
ENSG00000253945	NA	NA	NA	0	NA	NA	NA	NA	NA	
ENSG00000253946	RPL21P59	ribosomal protein L21 pseudogene 59	100271174	0	NA	NA	NA	NA	NA	
ENSG00000253951	CCT3P1	chaperonin containing TCP1 subunit 3 pseudogene 1	646440	0	NA	NA	NA	NA	NA	
ENSG00000253952	NA	NA	NA	0	NA	NA	NA	NA	NA	
ENSG00000253953	PCDHGB4	protocadherin gamma subfamily B, 4	8641	0,10488184	-1,076443944	4,080456	-0,263804824	0,791930317	NA	
ENSG00000253954	NA	NA	NA	0	NA	NA	NA	NA	NA	
ENSG00000253957	IGHV3-22	immunoglobulin heavy variable 3-22 (pseudogene)	28443	0	NA	NA	NA	NA	NA	
ENSG00000253958	CLDN23	claudin 23	137075	0,119673877	0,847101401	4,080456	0,207599697	0,835541541	NA	
ENSG00000253963	NA	NA	NA	0	NA	NA	NA	NA	NA	
ENSG00000253964	NA	NA	NA	0	NA	NA	NA	NA	NA	
ENSG00000253966	NA	NA	NA	0	NA	NA	NA	NA	NA	
ENSG00000253970	MTND2P7	MT-ND2 pseudogene 7	100873179	0	NA	NA	NA	NA	NA	
ENSG00000253971	NA	NA	NA	0	NA	NA	NA	NA	NA	
ENSG00000253975	NA	NA	NA	0	NA	NA	NA	NA	NA	
ENSG00000253977	MTCO2P4	MT-CO2 pseudogene 4	106480296	0	NA	NA	NA	NA	NA	
ENSG00000253989	IGHVIII-38-1	immunoglobulin heavy variable (III)-38-1 (pseudogene)	28346	0	NA	NA	NA	NA	NA	
ENSG00000253991	NA	NA	NA	0	NA	NA	NA	NA	NA	
ENSG00000253993	NA	NA	NA	0	NA	NA	NA	NA	NA	
ENSG00000253994	NDUFB9P3	NADH:ubiquinone oxidoreductase subunit B9 pseudogene 3	100129370	0	NA	NA	NA	NA	NA	
ENSG00000253995	RPL10P18	ribosomal protein L10 pseudogene 18	100630920	0	NA	NA	NA	NA	NA	
ENSG00000253997	NA	NA	NA	0	NA	NA	NA	NA	NA	
ENSG00000253999	NA	NA	NA	0	NA	NA	NA	NA	NA	
ENSG00000254009	IGKV2D-38	immunoglobulin kappa variable 2D-38 (pseudogene)	28879	0	NA	NA	NA	NA	NA	
ENSG00000254012	RPL10P8	ribosomal protein L10 pseudogene 8	644937	0	NA	NA	NA	NA	NA	
ENSG00000254013	NA	NA	NA	0	NA	NA	NA	NA	NA	
ENSG00000254014	NA	NA	NA	0	NA	NA	NA	NA	NA	

ENSG00000254015	MTND4LP26	MT-ND4L pseudogene 26	107075104	0	NA	NA	NA	NA	NA
ENSG00000254016	ALG1L10P	ALG1 like 10, pseudogene	106479037	0	NA	NA	NA	NA	NA
ENSG00000254017	IGHEP2	immunoglobulin heavy constant epsilon P2 (pseudogene)	3499	2,473055118	0,186704027	1,211023	0,154170512	0,877475292	NA
ENSG00000254023	NA	NA	NA	0	NA	NA	NA	NA	NA
ENSG00000254025	BRIX1P1	BRIX1 pseudogene 1	100422268	0	NA	NA	NA	NA	NA
ENSG00000254029	IGLC4	immunoglobulin lambda constant 4 (pseudogene)	3540	0	NA	NA	NA	NA	NA
ENSG00000254030	IGLC5	immunoglobulin lambda constant 5 (pseudogene)	3541	0	NA	NA	NA	NA	NA
ENSG00000254037	NA	NA	NA	0	NA	NA	NA	NA	NA
ENSG00000254045	NA	NA	NA	0	NA	NA	NA	NA	NA
ENSG00000254046	IGHV1-17	immunoglobulin heavy variable 1-17 (pseudogene)	28469	0,10488184	-1,076443944	4,080456	-0,263804824	0,791930317	NA
ENSG00000254047	LOC100130177	uracil DNA glycosylase pseudogene	100130177	0	NA	NA	NA	NA	NA
ENSG00000254051	NA	NA	NA	0	NA	NA	NA	NA	NA
ENSG00000254056	IGHV3-71	immunoglobulin heavy variable 3-71 (pseudogene)	28411	0	NA	NA	NA	NA	NA
ENSG00000254061	NA	NA	NA	0,189806235	1,665823732	4,047501	0,411568464	0,68065575	NA
ENSG00000254063	NA	NA	NA	0	NA	NA	NA	NA	NA
ENSG00000254065	NA	NA	NA	0	NA	NA	NA	NA	NA
ENSG00000254067	NA	NA	NA	0	NA	NA	NA	NA	NA
ENSG00000254069	NA	NA	NA	0	NA	NA	NA	NA	NA
ENSG00000254070	NA	NA	NA	0	NA	NA	NA	NA	NA
ENSG00000254073	NA	NA	NA	0	NA	NA	NA	NA	NA
ENSG00000254075	IGLVV-66	immunoglobulin lambda variable (V)-66 (pseudogene)	28757	0	NA	NA	NA	NA	NA
ENSG00000254076	LOC100129367	nucleolar and coiled-body phosphoprotein 1 pseudogene	100129367	0	NA	NA	NA	NA	NA
ENSG00000254077	IGLV3-7	immunoglobulin lambda variable 3-7 (pseudogene)	28805	0	NA	NA	NA	NA	NA
ENSG00000254079	NA	NA	NA	0	NA	NA	NA	NA	NA
ENSG00000254088	NA	NA	NA	0	NA	NA	NA	NA	NA
ENSG00000254090	MTND2P32	MT-ND2 pseudogene 32	100873237	0	NA	NA	NA	NA	NA
ENSG00000254091	NA	NA	NA	0	NA	NA	NA	NA	NA
ENSG00000254097	NA	NA	NA	0	NA	NA	NA	NA	NA
ENSG00000254103	NA	NA	NA	0	NA	NA	NA	NA	NA
ENSG00000254104	NA	NA	NA	0	NA	NA	NA	NA	NA
ENSG00000254105	NA	NA	NA	0	NA	NA	NA	NA	NA
ENSG00000254112	NA	NA	NA	0	NA	NA	NA	NA	NA
ENSG00000254114	NA	NA	NA	0	NA	NA	NA	NA	NA
ENSG00000254118	MTCYBP20	MT-CYB pseudogene 20	107075128	0	NA	NA	NA	NA	NA
ENSG00000254122	PCDHGB7	protocadherin gamma subfamily B, 7	56099	0,117497108	0,847101401	4,080456	0,207599697	0,835541541	NA
ENSG00000254124	NA	NA	NA	0	NA	NA	NA	NA	NA
ENSG00000254126	CD8B2	CD8B family member 2	927	0,072165449	0,847101401	4,080456	0,207599697	0,835541541	NA
ENSG00000254127	NA	NA	NA	0	NA	NA	NA	NA	NA
ENSG00000254131	NA	NA	NA	0	NA	NA	NA	NA	NA
ENSG00000254132	MTND6P3	MT-ND6 pseudogene 3	106478942	0	NA	NA	NA	NA	NA
ENSG00000254145	NA	NA	NA	0	NA	NA	NA	NA	NA
ENSG00000254146	HMGB1P46	high mobility group box 1 pseudogene 46	100419978	0	NA	NA	NA	NA	NA
ENSG00000254150	NA	NA	NA	0	NA	NA	NA	NA	NA
ENSG00000254151	NA	NA	NA	0	NA	NA	NA	NA	NA
ENSG00000254152	PPIAP84	peptidylprolyl isomerase A pseudogene 84	111082984	0	NA	NA	NA	NA	NA
ENSG00000254156	MTND6P20	MT-ND6 pseudogene 20	106478952	0	NA	NA	NA	NA	NA
ENSG00000254158	LOC100288803	F-box and WD repeat domain containing 2 pseudogene	100288803	0	NA	NA	NA	NA	NA
ENSG00000254161	IGLVIV-65	immunoglobulin lambda variable (IV)-65 (pseudogene)	28759	0	NA	NA	NA	NA	NA
ENSG00000254167	IGHV8-51-1	immunoglobulin heavy variable IGHV8-51-1 (non-functional)	28343	0	NA	NA	NA	NA	NA
ENSG00000254170	NA	NA	NA	0,15836288	0	4,080456	0	1	NA
ENSG00000254174	IGHV1-12	immunoglobulin heavy variable 1-12 (pseudogene)	28471	0	NA	NA	NA	NA	NA
ENSG00000254175	IGLVI-42	immunoglobulin lambda variable (I)-42 (pseudogene)	28767	0	NA	NA	NA	NA	NA
ENSG00000254181	NA	NA	NA	0	NA	NA	NA	NA	NA
ENSG00000254182	NA	NA	NA	0	NA	NA	NA	NA	NA
ENSG00000254183	NA	NA	NA	0	NA	NA	NA	NA	NA
ENSG00000254185	NA	NA	NA	0	NA	NA	NA	NA	NA
ENSG00000254189	LOC100420845	solute carrier family 25 member 43 pseudogene	100420845	0	NA	NA	NA	NA	NA
ENSG00000254190	NA	NA	NA	0	NA	NA	NA	NA	NA

ENSG00000254193	NA	NA	NA	0	NA	NA	NA	NA	NA
ENSG00000254195	NA	NA	NA	0	NA	NA	NA	NA	NA
ENSG00000254198	NA	NA	NA	0	NA	NA	NA	NA	NA
ENSG00000254200	RPL7AP33	ribosomal protein L7a pseudogene 33	391850	0	NA	NA	NA	NA	NA
ENSG00000254203	IGHVII-33-1	immunoglobulin heavy variable (II)-33-1 (pseudogene)	28369	0	NA	NA	NA	NA	NA
ENSG00000254212	NA	NA	NA	0	NA	NA	NA	NA	NA
ENSG00000254213	NA	NA	NA	0	NA	NA	NA	NA	NA
ENSG00000254215	NA	NA	NA	0	NA	NA	NA	NA	NA
ENSG00000254216	NA	NA	NA	0	NA	NA	NA	NA	NA
ENSG00000254220	IGKV2D-18	immunoglobulin kappa variable 2D-18 (pseudogene)	28888	0	NA	NA	NA	NA	NA
ENSG00000254221	PCDHGB1	protocadherin gamma subfamily B, 1	56104	0,329168796	0,84298109	4,080456	0,206589929	0,836330126	NA
ENSG00000254225	NA	NA	NA	0,103998153	-1,076443944	4,080456	-0,263804824	0,791930317	NA
ENSG00000254228	IGHV3-42	immunoglobulin heavy variable 3-42 (pseudogene)	28427	0	NA	NA	NA	NA	NA
ENSG00000254229	FAM90A12	family with sequence similarity 90 member A12	645879	0	NA	NA	NA	NA	NA
ENSG00000254240	NA	NA	NA	0	NA	NA	NA	NA	NA
ENSG00000254241	MTCO1P47	MT-CO1 pseudogene 47	107075284	0	NA	NA	NA	NA	NA
ENSG00000254244	NA	NA	NA	0	NA	NA	NA	NA	NA
ENSG00000254245	PCDHGA3	protocadherin gamma subfamily A, 3	56112	0,10543123	0	4,080456	0	1	NA
ENSG00000254247	NA	NA	NA	0	NA	NA	NA	NA	NA
ENSG00000254252	RPL7P20	ribosomal protein L7 pseudogene 20	728843	0	NA	NA	NA	NA	NA
ENSG00000254253	NACAP10	NACA pseudogene 10	100132812	0	NA	NA	NA	NA	NA
ENSG00000254255	NA	NA	NA	0	NA	NA	NA	NA	NA
ENSG00000254256	RPSAP71	ribosomal protein SA pseudogene 71	107075249	0,10488184	-1,076443944	4,080456	-0,263804824	0,791930317	NA
ENSG00000254264	IGKV3OR22-2	immunoglobulin kappa variable 3/OR22-2 (pseudogene)	3527	0	NA	NA	NA	NA	NA
ENSG00000254265	NA	NA	NA	0	NA	NA	NA	NA	NA
ENSG00000254268	NA	NA	NA	0	NA	NA	NA	NA	NA
ENSG00000254270	NA	NA	NA	0	NA	NA	NA	NA	NA
ENSG00000254272	NA	NA	NA	1,90416288	0,366560155	1,580477	0,231930073	0,816592326	NA
ENSG00000254273	NA	NA	NA	1,724825681	1,153750918	1,57102	0,734396174	0,462707318	NA
ENSG00000254274	NA	NA	NA	0	NA	NA	NA	NA	NA
ENSG00000254283	NA	NA	NA	0	NA	NA	NA	NA	NA
ENSG00000254285	KRT8P3	keratin 8 pseudogene 3	728638	0	NA	NA	NA	NA	NA
ENSG00000254289	IGHV3-29	immunoglobulin heavy variable 3-29 (pseudogene)	28440	0	NA	NA	NA	NA	NA
ENSG00000254292	NA	NA	NA	0	NA	NA	NA	NA	NA
ENSG00000254294	NA	NA	NA	0,223600412	-0,119210755	4,080456	-0,029215058	0,976693072	NA
ENSG00000254305	NA	NA	NA	0	NA	NA	NA	NA	NA
ENSG00000254308	NA	NA	NA	0	NA	NA	NA	NA	NA
ENSG00000254309	NA	NA	NA	0	NA	NA	NA	NA	NA
ENSG00000254310	NA	NA	NA	0	NA	NA	NA	NA	NA
ENSG00000254312	MRPL57P7	mitochondrial ribosomal protein L57 pseudogene 7	359731	0	NA	NA	NA	NA	NA
ENSG00000254313	NA	NA	NA	0	NA	NA	NA	NA	NA
ENSG00000254316	TAGLN2P2	transgelin 2 pseudogene 2	642319	0	NA	NA	NA	NA	NA
ENSG00000254320	NA	NA	NA	0	NA	NA	NA	NA	NA
ENSG00000254326	IGHV7-27	immunoglobulin heavy variable 7-27 (pseudogene)	28383	0	NA	NA	NA	NA	NA
ENSG00000254328	NA	NA	NA	0	NA	NA	NA	NA	NA
ENSG00000254331	NA	NA	NA	0	NA	NA	NA	NA	NA
ENSG00000254332	NA	NA	NA	0	NA	NA	NA	NA	NA
ENSG00000254336	NA	NA	NA	0	NA	NA	NA	NA	NA
ENSG00000254342	NA	NA	NA	0	NA	NA	NA	NA	NA
ENSG00000254345	NA	NA	NA	0	NA	NA	NA	NA	NA
ENSG00000254346	MTND1P6	MT-ND1 pseudogene 6	100873173	0	NA	NA	NA	NA	NA
ENSG00000254348	NA	NA	NA	0	NA	NA	NA	NA	NA
ENSG00000254351	ARL2BPP5	ADP ribosylation factor like GTPase 2 binding protein pseudogene 5	100874420	0	NA	NA	NA	NA	NA
ENSG00000254352	NA	NA	NA	0	NA	NA	NA	NA	NA
ENSG00000254355	IGLVI-68	immunoglobulin lambda variable (I)-68 (pseudogene)	28764	0	NA	NA	NA	NA	NA
ENSG00000254358	NA	NA	NA	0	NA	NA	NA	NA	NA
ENSG00000254373	NA	NA	NA	0	NA	NA	NA	NA	NA
ENSG00000254376	NA	NA	NA	0	NA	NA	NA	NA	NA
ENSG00000254381	TUBB8P5	tubulin beta 8 class VIII pseudogene 5	121014	0	NA	NA	NA	NA	NA
ENSG00000254384	MTND6P19	MT-ND6 pseudogene 19	106481788	0	NA	NA	NA	NA	NA
ENSG00000254387	NA	NA	NA	0	NA	NA	NA	NA	NA

ENSG00000254388	NA	NA	NA	0	NA	NA	NA	NA	NA
ENSG00000254392	LOC100422267	coagulation factor III, tissue factor pseudogene	100422267	0	NA	NA	NA	NA	NA
ENSG00000254398	LOC100419574	HECT and RLD domain containing E3 ubiquitin protein ligase 2 pseudogene	100419574	0,15836288	0	4,080456	0	1	NA
ENSG00000254399	NA	NA	NA	0	NA	NA	NA	NA	NA
ENSG00000254402	LRRC24	leucine rich repeat containing 24	441381	4,512070942	-0,77554373	0,979775	-0,791553035	0,42862134	NA
ENSG00000254403	OR10Y1P	olfactory receptor family 10 subfamily Y member 1 pseudogene	81340	0	NA	NA	NA	NA	NA
ENSG00000254406	NA	NA	NA	0	NA	NA	NA	NA	NA
ENSG00000254407	PHB1P17	PHB1 pseudogene 17	106480420	0	NA	NA	NA	NA	NA
ENSG00000254408	OR4A1P	olfactory receptor family 4 subfamily A member 1 pseudogene	8596	0	NA	NA	NA	NA	NA
ENSG00000254412	NA	NA	NA	0	NA	NA	NA	NA	NA
ENSG00000254415	SIGLEC14	sialic acid binding Ig like lectin 14	100049587	0,072165449	0,847101401	4,080456	0,207599697	0,835541541	NA
ENSG00000254423	LOC100421094	ubiquitin specific peptidase 17 like family member 2 pseudogene	100421094	0	NA	NA	NA	NA	NA
ENSG00000254424	NA	NA	NA	0	NA	NA	NA	NA	NA
ENSG00000254425	LOC100421673	small nuclear ribonucleoprotein polypeptide B2 pseudogene	100421673	0	NA	NA	NA	NA	NA
ENSG00000254430	OR6M3P	olfactory receptor family 6 subfamily M member 3 pseudogene	79321	0	NA	NA	NA	NA	NA
ENSG00000254437	COX6A1P4	COX6A1 pseudogene 4	127566421	0	NA	NA	NA	NA	NA
ENSG00000254440	PBOV1	prostate and breast cancer overexpressed 1	59351	0	NA	NA	NA	NA	NA
ENSG00000254441	NA	NA	NA	0	NA	NA	NA	NA	NA
ENSG00000254442	NA	NA	NA	0	NA	NA	NA	NA	NA
ENSG00000254445	HSPB2-C11orf52	HSPB2-C11orf52 readthrough (NMD candidate)	100528019	0	NA	NA	NA	NA	NA
ENSG00000254449	NA	NA	NA	0	NA	NA	NA	NA	NA
ENSG00000254454	NA	NA	NA	0,10543123	0	4,080456	0	1	NA
ENSG00000254455	NA	NA	NA	0	NA	NA	NA	NA	NA
ENSG00000254457	NA	NA	NA	0	NA	NA	NA	NA	NA
ENSG00000254463	PPIAP41	peptidylprolyl isomerase A pseudogene 41	111082977	5,596237576	0,579997037	0,722124	0,803182338	0,421869353	NA
ENSG00000254464	OR4A3P	olfactory receptor family 4 subfamily A member 3 pseudogene	81320	0	NA	NA	NA	NA	NA
ENSG00000254465	NA	NA	NA	0	NA	NA	NA	NA	NA
ENSG00000254466	OR4D10	olfactory receptor family 4 subfamily D member 10	390197	0	NA	NA	NA	NA	NA
ENSG00000254471	LOC646112	origin recognition complex subunit 3 pseudogene	646112	0	NA	NA	NA	NA	NA
ENSG00000254472	OR4A49P	olfactory receptor family 4 subfamily A member 49 pseudogene	403255	0	NA	NA	NA	NA	NA
ENSG00000254475	OR2AT1P	olfactory receptor family 2 subfamily AT member 1 pseudogene	390225	0	NA	NA	NA	NA	NA
ENSG00000254478	COX7CP3	COX7C pseudogene 3	127460872	0	NA	NA	NA	NA	NA
ENSG00000254479	NA	NA	NA	0	NA	NA	NA	NA	NA
ENSG00000254481	NA	NA	NA	0	NA	NA	NA	NA	NA
ENSG00000254482	NA	NA	NA	0	NA	NA	NA	NA	NA
ENSG00000254483	NA	NA	NA	0	NA	NA	NA	NA	NA
ENSG00000254491	LOC100421023	nuclear transcription factor Y subunit gamma pseudogene	100421023	0	NA	NA	NA	NA	NA
ENSG00000254492	LOC112268018	ATP synthase subunit ATP5MJ, mitochondrial-like	112268018	0	NA	NA	NA	NA	NA
ENSG00000254496	NA	NA	NA	0	NA	NA	NA	NA	NA
ENSG00000254498	NA	NA	NA	0	NA	NA	NA	NA	NA
ENSG00000254500	NA	NA	NA	0	NA	NA	NA	NA	NA
ENSG00000254502	NA	NA	NA	0	NA	NA	NA	NA	NA
ENSG00000254503	HMGB3P29	high mobility group box 3 pseudogene 29	100420015	0,11808024	-1,076443944	4,080456	-0,263804824	0,791930317	NA
ENSG00000254506	NA	NA	NA	0	NA	NA	NA	NA	NA
ENSG00000254509	NA	NA	NA	0	NA	NA	NA	NA	NA
ENSG00000254512	NA	NA	NA	0	NA	NA	NA	NA	NA
ENSG00000254515	TRPC6P5	TRPC6 pseudogene 5	123464510	0	NA	NA	NA	NA	NA
ENSG00000254517	NA	NA	NA	0	NA	NA	NA	NA	NA
ENSG00000254521	SIGLEC12	sialic acid binding Ig like lectin 12	89858	0,502541674	1,389219838	2,864323	0,485008147	0,627670567	NA
ENSG00000254522	NA	NA	NA	0	NA	NA	NA	NA	NA

ENSG00000254524	OR8I4P	olfactory receptor family 8 subfamily I member 4 pseudogene	403303	0	NA	NA	NA	NA	NA
ENSG00000254527	ENPP7P12	ectonucleotide pyrophosphatase/phosphodiesterase 7 pseudogene 12	106480230	0	NA	NA	NA	NA	NA
ENSG00000254529	MRGPRX7P	MAS related GPR family member X7, pseudogene	441592	0	NA	NA	NA	NA	NA
ENSG00000254534	PLS1P1	PLS1 pseudogene 1	100129471	0	NA	NA	NA	NA	NA
ENSG00000254535	PABPC4L	poly(A) binding protein cytoplasmic 4 like	132430	0	NA	NA	NA	NA	NA
ENSG00000254536	NA	NA	NA	1,298644471	4,426168255	3,041795	1,455116979	0,145636879	NA
ENSG00000254537	NA	NA	NA	0	NA	NA	NA	NA	NA
ENSG00000254541	MRGPRX6P	MAS related GPR family member X6, pseudogene	390098	0	NA	NA	NA	NA	NA
ENSG00000254543	NA	NA	NA	0	NA	NA	NA	NA	NA
ENSG00000254544	PCNAP4	proliferating cell nuclear antigen pseudogene 4	390102	0	NA	NA	NA	NA	NA
ENSG00000254546	MRGPRX13P	MAS related GPR family member X13, pseudogene	645319	0	NA	NA	NA	NA	NA
ENSG00000254547	NA	NA	NA	0	NA	NA	NA	NA	NA
ENSG00000254550	OMP	olfactory marker protein	4975	0	NA	NA	NA	NA	NA
ENSG00000254553	NA	NA	NA	0,103998153	-1,076443944	4,080456	-0,263804824	0,791930317	NA
ENSG00000254555	NA	NA	NA	0	NA	NA	NA	NA	NA
ENSG00000254558	ANKRD33BP8	ANKRD33B pseudogene 8	100420021	0	NA	NA	NA	NA	NA
ENSG00000254564	NA	NA	NA	0	NA	NA	NA	NA	NA
ENSG00000254565	MTND2P26	MT-ND2 pseudogene 26	100873349	0	NA	NA	NA	NA	NA
ENSG00000254567	UBTFL9	UBTF like 9 (pseudogene)	100128427	0	NA	NA	NA	NA	NA
ENSG00000254572	NA	NA	NA	0	NA	NA	NA	NA	NA
ENSG00000254576	OR4C1P	olfactory receptor family 4 subfamily C member 1 pseudogene	26242	0	NA	NA	NA	NA	NA
ENSG00000254579	NA	NA	NA	0	NA	NA	NA	NA	NA
ENSG00000254581	VPS51P19	VPS51 pseudogene 19	127379711	0	NA	NA	NA	NA	NA
ENSG00000254582	NA	NA	NA	0	NA	NA	NA	NA	NA
ENSG00000254583	NA	NA	NA	0	NA	NA	NA	NA	NA
ENSG00000254585	MAGEL2	MAGE family member L2	54551	0	NA	NA	NA	NA	NA
ENSG00000254589	EIF4A2P3	eukaryotic translation initiation factor 4A2 pseudogene 3	100133309	0	NA	NA	NA	NA	NA
ENSG00000254590	NA	NA	NA	0	NA	NA	NA	NA	NA
ENSG00000254591	NA	NA	NA	0	NA	NA	NA	NA	NA
ENSG00000254593	NA	NA	NA	0	NA	NA	NA	NA	NA
ENSG00000254595	LOC644169	voltage dependent anion channel 1 pseudogene	644169	1,030330216	-0,645357212	1,781595	-0,362235729	0,717175882	NA
ENSG00000254596	NA	NA	NA	0	NA	NA	NA	NA	NA
ENSG00000254598	CSNK2A3	casein kinase 2 alpha 3	283106	0	NA	NA	NA	NA	NA
ENSG00000254601	NA	NA	NA	0	NA	NA	NA	NA	NA
ENSG00000254609	NA	NA	NA	0	NA	NA	NA	NA	NA
ENSG00000254610	NA	NA	NA	0	NA	NA	NA	NA	NA
ENSG00000254612	DNAJB6P1	DNAJB6 pseudogene 1	387820	0	NA	NA	NA	NA	NA
ENSG00000254613	OR6M2P	olfactory receptor family 6 subfamily M member 2 pseudogene	79546	0	NA	NA	NA	NA	NA
ENSG00000254616	NA	NA	NA	0	NA	NA	NA	NA	NA
ENSG00000254617	NA	NA	NA	0	NA	NA	NA	NA	NA
ENSG00000254618	NA	NA	NA	0	NA	NA	NA	NA	NA
ENSG00000254621	NA	NA	NA	0,210862461	0	4,080456	0	1	NA
ENSG00000254623	NA	NA	NA	0	NA	NA	NA	NA	NA
ENSG00000254624	OR4R3P	olfactory receptor family 4 subfamily R member 3 pseudogene	81298	0	NA	NA	NA	NA	NA
ENSG00000254629	LOC100420800	suppressor of cytokine signaling 6 pseudogene	100420800	0	NA	NA	NA	NA	NA
ENSG00000254633	NA	NA	NA	7,557148189	0,535981549	0,719739	0,744688661	0,456459955	NA
ENSG00000254636	ARMS2	age-related maculopathy susceptibility 2	387715	0,15836288	0	4,080456	0	1	NA
ENSG00000254637	OR4A18P	olfactory receptor family 4 subfamily A member 18 pseudogene	81325	0	NA	NA	NA	NA	NA
ENSG00000254642	COX6CP5	cytochrome c oxidase subunit 6C pseudogene 5	100862855	0	NA	NA	NA	NA	NA
ENSG00000254644	NA	NA	NA	0	NA	NA	NA	NA	NA
ENSG00000254646	OR8B10P	olfactory receptor family 8 subfamily B member 10 pseudogene	390274	0	NA	NA	NA	NA	NA
ENSG00000254647	INS	insulin	3630	0	NA	NA	NA	NA	NA
ENSG00000254650	MTCYBP41	MT-CYB pseudogene 41	109729118	0	NA	NA	NA	NA	NA
ENSG00000254655	NA	NA	NA	0	NA	NA	NA	NA	NA

ENSG00000254656	RTL1	retrotransposon Gag like 1	388015	0	NA	NA	NA	NA	NA
ENSG00000254663	OR4A11P	olfactory receptor family 4 subfamily A member 11 pseudogene	390136	0	NA	NA	NA	NA	NA
ENSG00000254668	NA	NA	NA	0	NA	NA	NA	NA	NA
ENSG00000254672	WEE2P1	WEE2 pseudogene 1	100420018	0	NA	NA	NA	NA	NA
ENSG00000254673	NA	NA	NA	0	NA	NA	NA	NA	NA
ENSG00000254674	OR4A42P	olfactory receptor family 4 subfamily A member 42 pseudogene	403248	0	NA	NA	NA	NA	NA
ENSG00000254677	NA	NA	NA	0	NA	NA	NA	NA	NA
ENSG00000254678	NA	NA	NA	0	NA	NA	NA	NA	NA
ENSG00000254684	LOC100419092	REST corepressor 1 pseudogene	100419092	0	NA	NA	NA	NA	NA
ENSG00000254697	COPS8P3	COP9 signalosome subunit 8 pseudogene 3	106479058	0	NA	NA	NA	NA	NA
ENSG00000254700	DEFB131C	defensin beta 131C (pseudogene)	100128174	0	NA	NA	NA	NA	NA
ENSG00000254701	NA	NA	NA	10,96211645	0,22259878	0,917553	0,242600329	0,808315026	NA
ENSG00000254702	NA	NA	NA	0	NA	NA	NA	NA	NA
ENSG00000254704	LOC643709	macrophage expressed 1 pseudogene	643709	0	NA	NA	NA	NA	NA
ENSG00000254706	NA	NA	NA	0	NA	NA	NA	NA	NA
ENSG00000254708	NA	NA	NA	0	NA	NA	NA	NA	NA
ENSG00000254709	IGLL5	immunoglobulin lambda like polypeptide 5	100423062	0	NA	NA	NA	NA	NA
ENSG00000254712	NA	NA	NA	0	NA	NA	NA	NA	NA
ENSG00000254713	NA	NA	NA	0	NA	NA	NA	NA	NA
ENSG00000254715	OR7E154P	olfactory receptor family 7 subfamily E member 154 pseudogene	403296	0	NA	NA	NA	NA	NA
ENSG00000254717	GLYATL1P2	glycine-N-acyltransferase like 1 pseudogene 2	100129933	0	NA	NA	NA	NA	NA
ENSG00000254719	NA	NA	NA	0	NA	NA	NA	NA	NA
ENSG00000254720	MRGPRX10P	MAS related GPR family member X10, pseudogene	390101	0	NA	NA	NA	NA	NA
ENSG00000254723	OR4A12P	olfactory receptor family 4 subfamily A member 12 pseudogene	81331	0	NA	NA	NA	NA	NA
ENSG00000254725	NA	NA	NA	0	NA	NA	NA	NA	NA
ENSG00000254726	MEX3A	mex-3 RNA binding family member A	92312	1,471394095	2,297386283	2,212006	1,038598573	0,29899147	NA
ENSG00000254727	NA	NA	NA	0	NA	NA	NA	NA	NA
ENSG00000254728	NA	NA	NA	0	NA	NA	NA	NA	NA
ENSG00000254730	NA	NA	NA	0	NA	NA	NA	NA	NA
ENSG00000254732	NA	NA	NA	0	NA	NA	NA	NA	NA
ENSG00000254735	NA	NA	NA	0	NA	NA	NA	NA	NA
ENSG00000254736	BRD9P1	bromodomain containing 9 pseudogene 1	106481721	0	NA	NA	NA	NA	NA
ENSG00000254737	OR10G4	olfactory receptor family 10 subfamily G member 4	390264	0	NA	NA	NA	NA	NA
ENSG00000254738	MRGPRX11P	MAS related GPR family member X11, pseudogene	645415	0	NA	NA	NA	NA	NA
ENSG00000254743	OR10V3P	olfactory receptor family 10 subfamily V member 3 pseudogene	81342	0	NA	NA	NA	NA	NA
ENSG00000254747	NA	NA	NA	0	NA	NA	NA	NA	NA
ENSG00000254748	HNRNPCP8	heterogeneous nuclear ribonucleoprotein C pseudogene 8	100131842	0	NA	NA	NA	NA	NA
ENSG00000254749	NA	NA	NA	0	NA	NA	NA	NA	NA
ENSG00000254750	NA	NA	NA	0	NA	NA	NA	NA	NA
ENSG00000254751	TRIM64DP	tripartite motif containing 64D, pseudogene	727828	0	NA	NA	NA	NA	NA
ENSG00000254755	NA	NA	NA	0	NA	NA	NA	NA	NA
ENSG00000254758	NA	NA	NA	0	NA	NA	NA	NA	NA
ENSG00000254759	NAP1L1P1	nucleosome assembly protein 1 like 1 pseudogene 1	729561	0	NA	NA	NA	NA	NA
ENSG00000254764	TRIM53CP	tripartite motif containing 53C, pseudogene	340970	0	NA	NA	NA	NA	NA
ENSG00000254765	NA	NA	NA	0,144330898	1,418786819	4,061262	0,349346267	0,726829368	NA
ENSG00000254767	NA	NA	NA	0	NA	NA	NA	NA	NA
ENSG00000254769	OR4A4P	olfactory receptor family 4 subfamily A member 4 pseudogene	390134	0	NA	NA	NA	NA	NA
ENSG00000254770	OR4D7P	olfactory receptor family 4 subfamily D member 7 pseudogene	81304	0	NA	NA	NA	NA	NA
ENSG00000254771	NA	NA	NA	0	NA	NA	NA	NA	NA

ENSG00000254780	NA	NA	NA	0	NA	NA	NA	NA	NA
ENSG00000254781	GVINP2	GTPase, very large interferon inducible pseudogene 2	100128135	0	NA	NA	NA	NA	NA
ENSG00000254783	LOC100289518	immunoglobin superfamily member 21 pseudogene	100289518	4,926130609	0,049065748	0,840718	0,058361717	0,953460508	NA
ENSG00000254784	DNAAF11P1	DNAAF11 pseudogene 1	100420589	0	NA	NA	NA	NA	NA
ENSG00000254785	NA	NA	NA	0	NA	NA	NA	NA	NA
ENSG00000254786	NA	NA	NA	0	NA	NA	NA	NA	NA
ENSG00000254788	CKLF-CMTM1	CKLF-CMTM1 readthrough	100529251	7,743518694	-0,769847554	0,701695	-1,09712626	0,272586202	NA
ENSG00000254792	NA	NA	NA	0	NA	NA	NA	NA	NA
ENSG00000254793	FDPSP4	farnesyl diphosphate synthase pseudogene 4	2228	1,015469013	0,021446155	2,23363	0,009601478	0,992339246	NA
ENSG00000254794	NA	NA	NA	0	NA	NA	NA	NA	NA
ENSG00000254795	OR5AP1P	olfactory receptor family 5 subfamily AP member 1 pseudogene	81223	0	NA	NA	NA	NA	NA
ENSG00000254798	NA	NA	NA	0	NA	NA	NA	NA	NA
ENSG00000254799	NA	NA	NA	0	NA	NA	NA	NA	NA
ENSG00000254800	NA	NA	NA	0	NA	NA	NA	NA	NA
ENSG00000254801	NA	NA	NA	0	NA	NA	NA	NA	NA
ENSG00000254803	NA	NA	NA	0	NA	NA	NA	NA	NA
ENSG00000254805	SNRPCP14	small nuclear ribonucleoprotein polypeptide C pseudogene 14	100310855	0	NA	NA	NA	NA	NA
ENSG00000254806	SYS1-DBNDD2	SYS1-DBNDD2 readthrough (NMD candidate)	767557	0,394628248	1,910729275	4,024079	0,47482397	0,634912446	NA
ENSG00000254807	OR10AK1P	olfactory receptor family 10 subfamily AK member 1 pseudogene	403224	0	NA	NA	NA	NA	NA
ENSG00000254811	NA	NA	NA	0	NA	NA	NA	NA	NA
ENSG00000254816	NA	NA	NA	0	NA	NA	NA	NA	NA
ENSG00000254817	NA	NA	NA	0	NA	NA	NA	NA	NA
ENSG00000254818	NA	NA	NA	0	NA	NA	NA	NA	NA
ENSG00000254824	NA	NA	NA	0	NA	NA	NA	NA	NA
ENSG00000254828	NA	NA	NA	0	NA	NA	NA	NA	NA
ENSG00000254831	KRT18P58	keratin 18 pseudogene 58	644030	0	NA	NA	NA	NA	NA
ENSG00000254832	OR4A40P	olfactory receptor family 4 subfamily A member 40 pseudogene	390116	0	NA	NA	NA	NA	NA
ENSG00000254834	OR5M10	olfactory receptor family 5 subfamily M member 10	390167	0	NA	NA	NA	NA	NA
ENSG00000254840	GTF2IP15	general transcription factor IIi pseudogene 15	107403154	0	NA	NA	NA	NA	NA
ENSG00000254841	OR4V1P	olfactory receptor family 4 subfamily V member 1 pseudogene	79534	0	NA	NA	NA	NA	NA
ENSG00000254843	NA	NA	NA	0	NA	NA	NA	NA	NA
ENSG00000254848	OR5BN1P	olfactory receptor family 5 subfamily BN member 1 pseudogene	81204	0	NA	NA	NA	NA	NA
ENSG00000254850	NA	NA	NA	0	NA	NA	NA	NA	NA
ENSG00000254851	NA	NA	NA	0	NA	NA	NA	NA	NA
ENSG00000254852	NPIPA2	nuclear pore complex interacting protein family member A2	642799	2,360354476	-3,495862173	2,503931	-1,396149349	0,162669526	NA
ENSG00000254853	NA	NA	NA	0	NA	NA	NA	NA	NA
ENSG00000254856	NDUFA3P2	NADH:ubiquinone oxidoreductase subunit A3 pseudogene 2	106479051	0	NA	NA	NA	NA	NA
ENSG00000254857	MRGPRX12P	MAS related GPR family member X12, pseudogene	645297	0	NA	NA	NA	NA	NA
ENSG00000254866	DEFB109D	defensin beta 109D (pseudogene)	503838	0	NA	NA	NA	NA	NA
ENSG00000254867	LOC100420020	phosphatidylinositol-4,5-bisphosphate 4-phosphatase 1 pseudogene	100420020	0,222248489	-0,125731867	4,080456	-0,030813191	0,97541852	NA
ENSG00000254870	ATP6V1G2-DDX39B	ATP6V1G2-DDX39B readthrough (NMD candidate)	100532737	1,779435908	-0,912962378	1,978939	-0,46133928	0,644555208	NA
ENSG00000254871	NA	NA	NA	0	NA	NA	NA	NA	NA
ENSG00000254877	LOC100422398	protein kinase cAMP-dependent type II regulatory subunit beta pseudogene	100422398	0	NA	NA	NA	NA	NA
ENSG00000254883	NA	NA	NA	0	NA	NA	NA	NA	NA
ENSG00000254884	NA	NA	NA	0,92503505	-1,704404496	2,011661	-0,847262186	0,396848999	NA
ENSG00000254888	NA	NA	NA	0	NA	NA	NA	NA	NA
ENSG00000254890	NA	NA	NA	0	NA	NA	NA	NA	NA

ENSG00000254891	OR4A9P	olfactory receptor family 4 subfamily A member 9 pseudogene	390137	0	NA	NA	NA	NA	NA
ENSG00000254892	NA	NA	NA	0	NA	NA	NA	NA	NA
ENSG00000254893	NA	NA	NA	0	NA	NA	NA	NA	NA
ENSG00000254895	MTCO3P15	MT-CO3 pseudogene 15	107075154	0	NA	NA	NA	NA	NA
ENSG00000254897	NA	NA	NA	0	NA	NA	NA	NA	NA
ENSG00000254912	NA	NA	NA	6,135556386	-0,783891333	1,521838	-0,515095122	0,606486551	NA
ENSG00000254914	NA	NA	NA	0	NA	NA	NA	NA	NA
ENSG00000254915	NA	NA	NA	0	NA	NA	NA	NA	NA
ENSG00000254916	NA	NA	NA	0	NA	NA	NA	NA	NA
ENSG00000254917	NA	NA	NA	0	NA	NA	NA	NA	NA
ENSG00000254920	NA	NA	NA	0	NA	NA	NA	NA	NA
ENSG00000254923	LOC392196	ubiquitin specific peptidase 17 like family member 2 pseudogene	392196	0	NA	NA	NA	NA	NA
ENSG00000254924	NA	NA	NA	0	NA	NA	NA	NA	NA
ENSG00000254925	OR4C9P	olfactory receptor family 4 subfamily C member 9 pseudogene	79530	0	NA	NA	NA	NA	NA
ENSG00000254926	LOC100420019	glycine-N-acyltransferase pseudogene	100420019	0	NA	NA	NA	NA	NA
ENSG00000254931	MTATP6P15	MT-ATP6 pseudogene 15	107075273	0	NA	NA	NA	NA	NA
ENSG00000254935	NA	NA	NA	0	NA	NA	NA	NA	NA
ENSG00000254937	LOC100421985	olfactory receptor family 8 subfamily B member 3 pseudogene	100421985	0	NA	NA	NA	NA	NA
ENSG00000254939	LOC643381	zinc finger protein 614 pseudogene	643381	0	NA	NA	NA	NA	NA
ENSG00000254940	OR4A19P	olfactory receptor family 4 subfamily A member 19 pseudogene	81324	0	NA	NA	NA	NA	NA
ENSG00000254941	NA	NA	NA	0	NA	NA	NA	NA	NA
ENSG00000254944	NA	NA	NA	0,314645521	-2,509429468	3,997603	-0,627733573	0,530178488	NA
ENSG00000254947	OR8K4P	olfactory receptor family 8 subfamily K member 4 pseudogene	81166	0	NA	NA	NA	NA	NA
ENSG00000254948	OR7E158P	olfactory receptor family 7 subfamily E member 158 pseudogene	392194	0	NA	NA	NA	NA	NA
ENSG00000254949	NA	NA	NA	0	NA	NA	NA	NA	NA
ENSG00000254954	SLC2A13P1	SLC2A13 pseudogene 1	106480239	0	NA	NA	NA	NA	NA
ENSG00000254957	NA	NA	NA	0	NA	NA	NA	NA	NA
ENSG00000254959	INMT-MINDY4	INMT-MINDY4 readthrough (NMD candidate)	100526825	0	NA	NA	NA	NA	NA
ENSG00000254961	TUBB4BP4	TUBB4B pseudogene 4	106480434	0	NA	NA	NA	NA	NA
ENSG00000254965	C1DP5	C1D nuclear receptor corepressor pseudogene 5	106481729	0,925613411	1,964961977	2,241708	0,876546861	0,380732813	NA
ENSG00000254974	NA	NA	NA	0,10488184	-1,076443944	4,080456	-0,263804824	0,791930317	NA
ENSG00000254976	NA	NA	NA	0	NA	NA	NA	NA	NA
ENSG00000254978	NA	NA	NA	0	NA	NA	NA	NA	NA
ENSG00000254979	NA	NA	NA	0	NA	NA	NA	NA	NA
ENSG00000254984	NA	NA	NA	0	NA	NA	NA	NA	NA
ENSG00000254992	NA	NA	NA	0	NA	NA	NA	NA	NA
ENSG00000254993	TRIM77BP	tripartite motif containing 77B, pseudogene	646770	0	NA	NA	NA	NA	NA
ENSG00000254997	KRTAP5-9	keratin associated protein 5-9	3846	0,119673877	0,847101401	4,080456	0,207599697	0,835541541	NA
ENSG00000255000	NA	NA	NA	0	NA	NA	NA	NA	NA
ENSG00000255001	NA	NA	NA	0	NA	NA	NA	NA	NA
ENSG00000255003	NA	NA	NA	0	NA	NA	NA	NA	NA
ENSG00000255006	ELOCP22	elongin C pseudogene 22	100287579	0	NA	NA	NA	NA	NA
ENSG00000255009	UBTFL1	upstream binding transcription factor like 1	642623	0	NA	NA	NA	NA	NA
ENSG00000255010	NA	NA	NA	0,353873746	-0,107942567	3,745244	-0,028821236	0,977007164	NA
ENSG00000255011	NA	NA	NA	0	NA	NA	NA	NA	NA
ENSG00000255012	OR5M1	olfactory receptor family 5 subfamily M member 1	390168	0	NA	NA	NA	NA	NA
ENSG00000255014	NA	NA	NA	0	NA	NA	NA	NA	NA
ENSG00000255016	NA	NA	NA	0	NA	NA	NA	NA	NA
ENSG00000255018	NA	NA	NA	0	NA	NA	NA	NA	NA
ENSG00000255019	OR5D15P	olfactory receptor family 5 subfamily D member 15 pseudogene	81198	0	NA	NA	NA	NA	NA
ENSG00000255022	NA	NA	NA	0	NA	NA	NA	NA	NA
ENSG00000255025	VPS51P8	VPS51 pseudogene 8	441320	0	NA	NA	NA	NA	NA
ENSG00000255030	OR8B5P	olfactory receptor family 8 subfamily B member 5 pseudogene	26592	0	NA	NA	NA	NA	NA
ENSG00000255033	NA	NA	NA	0	NA	NA	NA	NA	NA

ENSG00000255035	NA	NA	NA	0	NA	NA	NA	NA	NA
ENSG00000255037	PLA2G10EP	phospholipase A2 group XE, pseudogene	127525791	0	NA	NA	NA	NA	NA
ENSG00000255040	NA	NA	NA	0	NA	NA	NA	NA	NA
ENSG00000255042	NA	NA	NA	0	NA	NA	NA	NA	NA
ENSG00000255047	HNRNPRP2	heterogeneous nuclear ribonucleoprotein R pseudogene 2	100421354	0	NA	NA	NA	NA	NA
ENSG00000255048	OR8X1P	olfactory receptor family 8 subfamily X member 1 pseudogene	403305	0	NA	NA	NA	NA	NA
ENSG00000255051	NA	NA	NA	0	NA	NA	NA	NA	NA
ENSG00000255053	OR4A44P	olfactory receptor family 4 subfamily A member 44 pseudogene	403250	0	NA	NA	NA	NA	NA
ENSG00000255054	NA	NA	NA	0	NA	NA	NA	NA	NA
ENSG00000255055	MTND1P35	MT-ND1 pseudogene 35	106480433	0	NA	NA	NA	NA	NA
ENSG00000255057	NA	NA	NA	0,117497108	0,847101401	4,080456	0,207599697	0,835541541	NA
ENSG00000255058	PDCL2P2	PDCL2 pseudogene 2	106480295	0	NA	NA	NA	NA	NA
ENSG00000255059	NA	NA	NA	0	NA	NA	NA	NA	NA
ENSG00000255063	NA	NA	NA	0	NA	NA	NA	NA	NA
ENSG00000255065	NA	NA	NA	0	NA	NA	NA	NA	NA
ENSG00000255070	OSBPL9P3	oxysterol binding protein like 9 pseudogene 3	100420591	0	NA	NA	NA	NA	NA
ENSG00000255071	SAA2-SAA4	SAA2-SAA4 readthrough	100528017	0	NA	NA	NA	NA	NA
ENSG00000255073	NA	NA	NA	0,478695509	2,980701557	3,96411	0,751922061	0,45209793	NA
ENSG00000255074	NA	NA	NA	0	NA	NA	NA	NA	NA
ENSG00000255075	NA	NA	NA	0	NA	NA	NA	NA	NA
ENSG00000255077	OR4X7P	olfactory receptor family 4 subfamily X member 7 pseudogene	403264	0	NA	NA	NA	NA	NA
ENSG00000255078	OR4A6P	olfactory receptor family 4 subfamily A member 6 pseudogene	390131	0	NA	NA	NA	NA	NA
ENSG00000255083	OR5AK1P	olfactory receptor family 5 subfamily AK member 1 pseudogene	81230	0	NA	NA	NA	NA	NA
ENSG00000255086	LOC100533706	NEDD8 activating enzyme E1 subunit 1 pseudogene	100533706	0	NA	NA	NA	NA	NA
ENSG00000255088	NA	NA	NA	0	NA	NA	NA	NA	NA
ENSG00000255093	NA	NA	NA	0	NA	NA	NA	NA	NA
ENSG00000255095	OR1D4	olfactory receptor family 1 subfamily D member 4	653166	0	NA	NA	NA	NA	NA
ENSG00000255096	OR5B10P	olfactory receptor family 5 subfamily B member 10 pseudogene	390186	0	NA	NA	NA	NA	NA
ENSG00000255099	LOC644335	SMG5 nonsense mediated mRNA decay factor pseudogene	644335	0	NA	NA	NA	NA	NA
ENSG00000255104	NA	NA	NA	0	NA	NA	NA	NA	NA
ENSG00000255105	NA	NA	NA	0	NA	NA	NA	NA	NA
ENSG00000255106	RPL7AP79	ribosomal protein L7a pseudogene 79	122965337	0	NA	NA	NA	NA	NA
ENSG00000255107	LOC100288097	BAG cochaperone 4 pseudogene	100288097	0	NA	NA	NA	NA	NA
ENSG00000255110	NA	NA	NA	0	NA	NA	NA	NA	NA
ENSG00000255111	NA	NA	NA	0	NA	NA	NA	NA	NA
ENSG00000255113	NA	NA	NA	0	NA	NA	NA	NA	NA
ENSG00000255115	NA	NA	NA	0,298318456	1,650250349	3,884342	0,424846872	0,670948306	NA
ENSG00000255116	SLC5A4P1	solute carrier family 5 member 4 pseudogene 1	100422550	0	NA	NA	NA	NA	NA
ENSG00000255122	NA	NA	NA	0	NA	NA	NA	NA	NA
ENSG00000255123	NA	NA	NA	0	NA	NA	NA	NA	NA
ENSG00000255127	EEF1A1P49	eukaryotic translation elongation factor 1 alpha 1 pseudogene 49	642791	0	NA	NA	NA	NA	NA
ENSG00000255128	NA	NA	NA	0	NA	NA	NA	NA	NA
ENSG00000255131	OR9L1P	olfactory receptor family 9 subfamily L member 1 pseudogene	79301	0	NA	NA	NA	NA	NA
ENSG00000255138	NA	NA	NA	0	NA	NA	NA	NA	NA
ENSG00000255140	OR5BN2P	olfactory receptor family 5 subfamily BN member 2 pseudogene	504181	0	NA	NA	NA	NA	NA
ENSG00000255141	NA	NA	NA	0	NA	NA	NA	NA	NA
ENSG00000255147	NA	NA	NA	0	NA	NA	NA	NA	NA
ENSG00000255149	NA	NA	NA	0	NA	NA	NA	NA	NA
ENSG00000255151	GLYATL1B	glycine-N-acyltransferase like 1B	100287520	0	NA	NA	NA	NA	NA

ENSG00000255152	MSH5-SAPCD1	MSH5-SAPCD1 readthrough (NMD candidate)	100532732	10,03973058	0,625059057	2,208935	0,282968516	0,777200971	NA
ENSG00000255155	NA	NA	NA	0	NA	NA	NA	NA	NA
ENSG00000255157	NA	NA	NA	0	NA	NA	NA	NA	NA
ENSG00000255161	NA	NA	NA	0	NA	NA	NA	NA	NA
ENSG00000255162	NA	NA	NA	0	NA	NA	NA	NA	NA
ENSG00000255163	NA	NA	NA	6,534802631	1,017934684	0,747775	1,361283883	0,173423994	NA
ENSG00000255166	NA	NA	NA	0	NA	NA	NA	NA	NA
ENSG00000255170	NA	NA	NA	0	NA	NA	NA	NA	NA
ENSG00000255172	OR5AM1P	olfactory receptor family 5 subfamily AM member 1 pseudogene	81226	0	NA	NA	NA	NA	NA
ENSG00000255181	CCDC166	coiled-coil domain containing 166	100130274	0	NA	NA	NA	NA	NA
ENSG00000255184	NA	NA	NA	0	NA	NA	NA	NA	NA
ENSG00000255188	NA	NA	NA	0	NA	NA	NA	NA	NA
ENSG00000255189	GLYATL1P1	glycine-N-acyltransferase like 1 pseudogene 1	100129032	0	NA	NA	NA	NA	NA
ENSG00000255190	NA	NA	NA	0	NA	NA	NA	NA	NA
ENSG00000255192	NANOGP8	Nanog homeobox retrogene P8	388112	0,22343371	-1,073314934	4,080456	-0,263037996	0,792521294	NA
ENSG00000255196	OR4A17P	olfactory receptor family 4 subfamily A member 17 pseudogene	390139	0	NA	NA	NA	NA	NA
ENSG00000255199	GTF2IP11	general transcription factor IIi pseudogene 11	106480346	0	NA	NA	NA	NA	NA
ENSG00000255200	NA	NA	NA	0,223390249	0	4,080456	0	1	NA
ENSG00000255203	OR7E2P	olfactory receptor family 7 subfamily E member 2 pseudogene	8587	0	NA	NA	NA	NA	NA
ENSG00000255204	NA	NA	NA	0	NA	NA	NA	NA	NA
ENSG00000255205	MED28P5	mediator complex subunit 28 pseudogene 5	106479053	0	NA	NA	NA	NA	NA
ENSG00000255207	NA	NA	NA	0	NA	NA	NA	NA	NA
ENSG00000255210	NA	NA	NA	0	NA	NA	NA	NA	NA
ENSG00000255211	LOC392187	ubiquitin carboxyl-terminal hydrolase 17-like protein 2-like	392187	0	NA	NA	NA	NA	NA
ENSG00000255213	NA	NA	NA	0	NA	NA	NA	NA	NA
ENSG00000255214	NA	NA	NA	0	NA	NA	NA	NA	NA
ENSG00000255215	NA	NA	NA	0	NA	NA	NA	NA	NA
ENSG00000255217	OR5J7P	olfactory receptor family 5 subfamily J member 7 pseudogene	403276	0	NA	NA	NA	NA	NA
ENSG00000255218	OR5BC1P	olfactory receptor family 5 subfamily BC member 1 pseudogene	81208	0	NA	NA	NA	NA	NA
ENSG00000255220	NA	NA	NA	0	NA	NA	NA	NA	NA
ENSG00000255221	NA	NA	NA	0,23300754	0,828703606	4,079738	0,203126682	0,839036017	NA
ENSG00000255222	NA	NA	NA	0,497176934	-2,587269696	3,343928	-0,77372168	0,439095397	NA
ENSG00000255223	OR5M11	olfactory receptor family 5 subfamily M member 11	219487	0	NA	NA	NA	NA	NA
ENSG00000255225	OR8B6P	olfactory receptor family 8 subfamily B member 6 pseudogene	26591	0	NA	NA	NA	NA	NA
ENSG00000255230	NA	NA	NA	0	NA	NA	NA	NA	NA
ENSG00000255231	NA	NA	NA	0	NA	NA	NA	NA	NA
ENSG00000255232	NA	NA	NA	0	NA	NA	NA	NA	NA
ENSG00000255235	NA	NA	NA	0	NA	NA	NA	NA	NA
ENSG00000255238	RFPL4AP1	ret finger protein like 4A pseudogene 1	646663	0	NA	NA	NA	NA	NA
ENSG00000255239	NA	NA	NA	0	NA	NA	NA	NA	NA
ENSG00000255245	FXYD6-FXYD2	FXYD6-FXYD2 readthrough	100533181	0	NA	NA	NA	NA	NA
ENSG00000255251	PRR23D1	proline rich 23 domain containing 1	100131608	0	NA	NA	NA	NA	NA
ENSG00000255251	PRR23D2	proline rich 23 domain containing 2	100133251	0	NA	NA	NA	NA	NA
ENSG00000255254	NA	NA	NA	0	NA	NA	NA	NA	NA
ENSG00000255255	PPP1R1AP1	protein phosphatase 1 regulatory inhibitor subunit 1A pseudogene 1	94304	0	NA	NA	NA	NA	NA
ENSG00000255259	ZNF123P	zinc finger protein 123, pseudogene	100188891	0	NA	NA	NA	NA	NA
ENSG00000255261	OR7E4P	olfactory receptor family 7 subfamily E member 4 pseudogene	8585	0	NA	NA	NA	NA	NA
ENSG00000255262	ELOBP2	elongin B pseudogene 2	253665	0	NA	NA	NA	NA	NA
ENSG00000255265	NA	NA	NA	0	NA	NA	NA	NA	NA

ENSG00000255266	LOC107984365	cytochrome c oxidase subunit NDUFA4-like	107984365	0	NA	NA	NA	NA	NA
ENSG00000255273	VPS51P14	VPS51 pseudogene 14	127379708	0	NA	NA	NA	NA	NA
ENSG00000255280	NA	NA	NA	0	NA	NA	NA	NA	NA
ENSG00000255281	LOC100421558	microtubule affinity regulating kinase 2 pseudogene	100421558	0	NA	NA	NA	NA	NA
ENSG00000255282	WTAPP1	Wilms tumor 1 associated protein pseudogene 1	100288077	0	NA	NA	NA	NA	NA
ENSG00000255283	NA	NA	NA	0	NA	NA	NA	NA	NA
ENSG00000255285	NA	NA	NA	0	NA	NA	NA	NA	NA
ENSG00000255286	NA	NA	NA	0	NA	NA	NA	NA	NA
ENSG00000255287	SNRPGP16	small nuclear ribonucleoprotein polypeptide G pseudogene 16	106480270	0	NA	NA	NA	NA	NA
ENSG00000255288	NA	NA	NA	0	NA	NA	NA	NA	NA
ENSG00000255291	NA	NA	NA	0	NA	NA	NA	NA	NA
ENSG00000255292	NA	NA	NA	0,117497108	0,847101401	4,080456	0,207599697	0,835541541	NA
ENSG00000255293	NA	NA	NA	0	NA	NA	NA	NA	NA
ENSG00000255294	NA	NA	NA	0	NA	NA	NA	NA	NA
ENSG00000255295	H3P34	H3 histone pseudogene 34	115482702	0	NA	NA	NA	NA	NA
ENSG00000255296	NA	NA	NA	0	NA	NA	NA	NA	NA
ENSG00000255297	OR4A41P	olfactory receptor family 4 subfamily A member 41 pseudogene	403247	0	NA	NA	NA	NA	NA
ENSG00000255298	OR8G5	olfactory receptor family 8 subfamily G member 5	219865	0	NA	NA	NA	NA	NA
ENSG00000255303	OR5BA1P	olfactory receptor family 5 subfamily BA member 1 pseudogene	81210	0	NA	NA	NA	NA	NA
ENSG00000255304	OR4A46P	olfactory receptor family 4 subfamily A member 46 pseudogene	403252	0	NA	NA	NA	NA	NA
ENSG00000255305	ANKRD33BP9	ANKRD33B pseudogene 9	100420025	0	NA	NA	NA	NA	NA
ENSG00000255307	OR52B2	olfactory receptor family 52 subfamily B member 2	255725	0,189671958	1,665095786	4,047525	0,411386215	0,68078936	NA
ENSG00000255309	NA	NA	NA	0	NA	NA	NA	NA	NA
ENSG00000255312	OR4C7P	olfactory receptor family 4 subfamily C member 7 pseudogene	79294	0	NA	NA	NA	NA	NA
ENSG00000255315	NA	NA	NA	0	NA	NA	NA	NA	NA
ENSG00000255316	MTND6P25	MT-ND6 pseudogene 25	107075262	0	NA	NA	NA	NA	NA
ENSG00000255319	ENPP7P8	ectonucleotide pyrophosphatase/phosphodiesterase 7 pseudogene 8	100421860	0	NA	NA	NA	NA	NA
ENSG00000255330	NA	NA	NA	0	NA	NA	NA	NA	NA
ENSG00000255331	NA	NA	NA	0	NA	NA	NA	NA	NA
ENSG00000255333	LOC100420122	dihydropyrimidinase like 5 pseudogene	100420122	0	NA	NA	NA	NA	NA
ENSG00000255336	NA	NA	NA	0	NA	NA	NA	NA	NA
ENSG00000255338	NA	NA	NA	0	NA	NA	NA	NA	NA
ENSG00000255339	NA	NA	NA	0,880764367	1,68810371	2,81222	0,600274496	0,548323313	NA
ENSG00000255341	OR8Q1P	olfactory receptor family 8 subfamily Q member 1 pseudogene	81163	0	NA	NA	NA	NA	NA
ENSG00000255342	NA	NA	NA	0	NA	NA	NA	NA	NA
ENSG00000255346	NOX5	NADPH oxidase 5	79400	0,072165449	0,847101401	4,080456	0,207599697	0,835541541	NA
ENSG00000255347	NA	NA	NA	0	NA	NA	NA	NA	NA
ENSG00000255349	OR4A7P	olfactory receptor family 4 subfamily A member 7 pseudogene	81316	0	NA	NA	NA	NA	NA
ENSG00000255350	MTCO1P15	MT-CO1 pseudogene 15	107075265	0	NA	NA	NA	NA	NA
ENSG00000255352	PPP1R10P1	protein phosphatase 1 regulatory subunit 10 pseudogene 1	100422381	0	NA	NA	NA	NA	NA
ENSG00000255353	ASS1P13	argininosuccinate synthetase 1 pseudogene 13	341230	0	NA	NA	NA	NA	NA
ENSG00000255356	NA	NA	NA	0,103998153	-1,076443944	4,080456	-0,263804824	0,791930317	NA
ENSG00000255358	NA	NA	NA	0,853051556	-0,751881136	2,364916	-0,317931393	0,750536979	NA
ENSG00000255359	CCDC179	coiled-coil domain containing 179	100500938	0	NA	NA	NA	NA	NA
ENSG00000255360	NA	NA	NA	0	NA	NA	NA	NA	NA
ENSG00000255361	NA	NA	NA	0	NA	NA	NA	NA	NA
ENSG00000255365	NA	NA	NA	0	NA	NA	NA	NA	NA
ENSG00000255368	NA	NA	NA	0	NA	NA	NA	NA	NA
ENSG00000255369	NA	NA	NA	0	NA	NA	NA	NA	NA
ENSG00000255370	NA	NA	NA	0	NA	NA	NA	NA	NA
ENSG00000255374	TAS2R43	taste 2 receptor member 43	259289	0	NA	NA	NA	NA	NA

ENSG00000255374	TAS2R45	taste 2 receptor member 45	259291	0	NA	NA	NA	NA	NA
ENSG00000255376	NA	NA	NA	0	NA	NA	NA	NA	NA
ENSG00000255377	NA	NA	NA	0	NA	NA	NA	NA	NA
ENSG00000255379	NA	NA	NA	0	NA	NA	NA	NA	NA
ENSG00000255380	LOC100289416	zinc finger protein 793 pseudogene	100289416	0	NA	NA	NA	NA	NA
ENSG00000255381	LOC100422399	protein kinase cAMP-dependent type II regulatory subunit beta pseudogene	100422399	0	NA	NA	NA	NA	NA
ENSG00000255385	UBTFL10	UBTF like 10 (pseudogene)	106480293	0	NA	NA	NA	NA	NA
ENSG00000255386	OR5BR1P	olfactory receptor family 5 subfamily BR member 1 pseudogene	81201	0	NA	NA	NA	NA	NA
ENSG00000255387	RDXP1	RDX pseudogene 1	643244	0	NA	NA	NA	NA	NA
ENSG00000255391	HMGB3P25	high mobility group box 3 pseudogene 25	100873908	0	NA	NA	NA	NA	NA
ENSG00000255393	OOSP4B	oocyte secreted protein family member 4B	107987155	0	NA	NA	NA	NA	NA
ENSG00000255396	NA	NA	NA	0	NA	NA	NA	NA	NA
ENSG00000255397	NA	NA	NA	0	NA	NA	NA	NA	NA
ENSG00000255398	HCAR3	hydroxycarboxylic acid receptor 3	8843	0	NA	NA	NA	NA	NA
ENSG00000255401	NA	NA	NA	0	NA	NA	NA	NA	NA
ENSG00000255403	NA	NA	NA	0	NA	NA	NA	NA	NA
ENSG00000255413	LOC100419761	zinc finger protein 654 pseudogene	100419761	0	NA	NA	NA	NA	NA
ENSG00000255415	VPS51P11	VPS51 pseudogene 11	127379745	0,10543123	0	4,080456	0	1	NA
ENSG00000255416	OR10AF1P	olfactory receptor family 10 subfamily AF member 1 pseudogene	282769	0	NA	NA	NA	NA	NA
ENSG00000255417	MTCO2P15	MT-CO2 pseudogene 15	107075147	0	NA	NA	NA	NA	NA
ENSG00000255424	MRGPRX9P	MAS related GPR family member X9, pseudogene	100420121	0	NA	NA	NA	NA	NA
ENSG00000255425	OR8G3P	olfactory receptor family 8 subfamily G member 3 pseudogene	387815	0	NA	NA	NA	NA	NA
ENSG00000255430	NA	NA	NA	0	NA	NA	NA	NA	NA
ENSG00000255431	OR5B19P	olfactory receptor family 5 subfamily B member 19 pseudogene	81211	0	NA	NA	NA	NA	NA
ENSG00000255432	NA	NA	NA	0	NA	NA	NA	NA	NA
ENSG00000255437	LOC100421160	structural maintenance of chromosomes 5 pseudogene	100421160	0	NA	NA	NA	NA	NA
ENSG00000255439	NA	NA	NA	7,486438649	-0,601516508	1,247197	-0,482294741	0,629596583	NA
ENSG00000255442	NA	NA	NA	0	NA	NA	NA	NA	NA
ENSG00000255444	NA	NA	NA	0	NA	NA	NA	NA	NA
ENSG00000255445	NA	NA	NA	0	NA	NA	NA	NA	NA
ENSG00000255450	EEF1A1P47	eukaryotic translation elongation factor 1 alpha 1 pseudogene 47	401677	0	NA	NA	NA	NA	NA
ENSG00000255452	NA	NA	NA	0	NA	NA	NA	NA	NA
ENSG00000255454	LOC107984367	V-type proton ATPase subunit G 1-like	107984367	0	NA	NA	NA	NA	NA
ENSG00000255457	NA	NA	NA	0	NA	NA	NA	NA	NA
ENSG00000255459	NA	NA	NA	0	NA	NA	NA	NA	NA
ENSG00000255460	ZDHHC20P3	zinc finger DHHC-type containing 20 pseudogene 3	100874348	0	NA	NA	NA	NA	NA
ENSG00000255463	NA	NA	NA	0	NA	NA	NA	NA	NA
ENSG00000255464	NA	NA	NA	0	NA	NA	NA	NA	NA
ENSG00000255469	NA	NA	NA	0	NA	NA	NA	NA	NA
ENSG00000255481	OR56A7P	olfactory receptor family 56 subfamily A member 7 pseudogene	81236	0	NA	NA	NA	NA	NA
ENSG00000255483	METTL5P4	METTL5 pseudogene 4	127379692	0	NA	NA	NA	NA	NA
ENSG00000255486	NA	NA	NA	0	NA	NA	NA	NA	NA
ENSG00000255489	NA	NA	NA	0	NA	NA	NA	NA	NA
ENSG00000255490	EIF4A2P5	eukaryotic translation initiation factor 4A2 pseudogene 5	106480446	0	NA	NA	NA	NA	NA
ENSG00000255492	H3P33	H3 histone pseudogene 33	115482722	0	NA	NA	NA	NA	NA
ENSG00000255493	NA	NA	NA	0	NA	NA	NA	NA	NA
ENSG00000255497	NA	NA	NA	0	NA	NA	NA	NA	NA
ENSG00000255499	NA	NA	NA	0	NA	NA	NA	NA	NA
ENSG00000255500	LOC646801	pantothenate kinase 3 pseudogene	646801	0	NA	NA	NA	NA	NA
ENSG00000255501	CARD18	caspase recruitment domain family member 18	59082	0	NA	NA	NA	NA	NA
ENSG00000255504	NA	NA	NA	0	NA	NA	NA	NA	NA
ENSG00000255505	NA	NA	NA	0	NA	NA	NA	NA	NA
ENSG00000255508	NA	NA	NA	9,466533787	0,492487891	0,884021	0,557099478	0,577459464	NA

ENSG00000255510	OR8A2P	olfactory receptor family 8 subfamily A member 2 pseudogene	390273	0,117497108	0,847101401	4,080456	0,207599697	0,835541541	NA
ENSG00000255511	LOC645397	methylenetetrahydrofolate dehydrogenase (NADP+ dependent) 1 like pseudogene	645397	0	NA	NA	NA	NA	NA
ENSG00000255514	OR4B2P	olfactory receptor family 4 subfamily B member 2 pseudogene	26690	0	NA	NA	NA	NA	NA
ENSG00000255515	NA	NA	NA	0	NA	NA	NA	NA	NA
ENSG00000255519	NA	NA	NA	0	NA	NA	NA	NA	NA
ENSG00000255522	NA	NA	NA	0	NA	NA	NA	NA	NA
ENSG00000255524	NPIPB8	nuclear pore complex interacting protein family member B8	728734	0,353380818	0,849895907	4,080456	0,208284548	0,835006796	NA
ENSG00000255527	NA	NA	NA	0	NA	NA	NA	NA	NA
ENSG00000255530	NA	NA	NA	0	NA	NA	NA	NA	NA
ENSG00000255531	NDUFS5P6	NADH:ubiquinone oxidoreductase subunit S5 pseudogene 6	106480785	0	NA	NA	NA	NA	NA
ENSG00000255532	NOX4P1	NOX4 pseudogene 1	729960	0	NA	NA	NA	NA	NA
ENSG00000255533	NA	NA	NA	0,210862461	0	4,080456	0	1	NA
ENSG00000255534	OR4A43P	olfactory receptor family 4 subfamily A member 43 pseudogene	390117	0	NA	NA	NA	NA	NA
ENSG00000255535	NA	NA	NA	0	NA	NA	NA	NA	NA
ENSG00000255536	MRGPRX5P	MAS related GPR family member X5, pseudogene	390099	0	NA	NA	NA	NA	NA
ENSG00000255538	OR10V2P	olfactory receptor family 10 subfamily V member 2 pseudogene	81343	0	NA	NA	NA	NA	NA
ENSG00000255540	LOC100130203	cell adhesion associated, oncogene regulated pseudogene	100130203	0	NA	NA	NA	NA	NA
ENSG00000255541	NA	NA	NA	0,10543123	0	4,080456	0	1	NA
ENSG00000255543	NA	NA	NA	0	NA	NA	NA	NA	NA
ENSG00000255544	NA	NA	NA	0	NA	NA	NA	NA	NA
ENSG00000255547	NA	NA	NA	0	NA	NA	NA	NA	NA
ENSG00000255549	ENPP7P6	ectonucleotide pyrophosphatase/phosphodiesterase 7 pseudogene 6	107133511	0	NA	NA	NA	NA	NA
ENSG00000255550	NA	NA	NA	0	NA	NA	NA	NA	NA
ENSG00000255551	NA	NA	NA	0	NA	NA	NA	NA	NA
ENSG00000255554	OR7E1P	olfactory receptor family 7 subfamily E member 1 pseudogene	341128	0	NA	NA	NA	NA	NA
ENSG00000255556	NA	NA	NA	0	NA	NA	NA	NA	NA
ENSG00000255560	NA	NA	NA	0	NA	NA	NA	NA	NA
ENSG00000255562	UNC93B6	unc-93 homolog B6 (pseudogene)	255620	0	NA	NA	NA	NA	NA
ENSG00000255563	NA	NA	NA	0	NA	NA	NA	NA	NA
ENSG00000255566	LOC100420899	mitochondrial ribosomal protein S25 pseudogene	100420899	0	NA	NA	NA	NA	NA
ENSG00000255569	TRAV1-1	T cell receptor alpha variable 1-1	28693	0	NA	NA	NA	NA	NA
ENSG00000255575	NA	NA	NA	0	NA	NA	NA	NA	NA
ENSG00000255581	NA	NA	NA	0	NA	NA	NA	NA	NA
ENSG00000255582	OR10G2	olfactory receptor family 10 subfamily G member 2	26534	0	NA	NA	NA	NA	NA
ENSG00000255583	NA	NA	NA	0	NA	NA	NA	NA	NA
ENSG00000255585	LOC651337	uncharacterized LOC651337	651337	0	NA	NA	NA	NA	NA
ENSG00000255587	RAB44	RAB44, member RAS oncogene family	401258	4,824132298	0,23072087	0,942935	0,244683806	0,806701268	NA
ENSG00000255624	C10orf88B	C10orf88B (pseudogene)	399815	1,124455778	-1,572662412	2,591588	-0,606833458	0,543961442	NA
ENSG00000255627	NA	NA	NA	0	NA	NA	NA	NA	NA
ENSG00000255642	NA	NA	NA	1,562759022	1,05869198	1,737122	0,609451828	0,542224994	NA
ENSG00000255653	LOC100420801	lysine demethylase 4D pseudogene	100420801	0	NA	NA	NA	NA	NA
ENSG00000255663	NA	NA	NA	1,890574763	-0,755023233	2,55747	-0,295222767	0,767823711	NA
ENSG00000255664	NA	NA	NA	0	NA	NA	NA	NA	NA
ENSG00000255669	NA	NA	NA	0,221479466	-0,115799665	4,080456	-0,0283791	0,977359793	NA
ENSG00000255690	TRIL	TLR4 interactor with leucine rich repeats	9865	0,10543123	0	4,080456	0	1	NA
ENSG00000255700	NA	NA	NA	0	NA	NA	NA	NA	NA
ENSG00000255703	NA	NA	NA	0	NA	NA	NA	NA	NA
ENSG00000255704	NA	NA	NA	0	NA	NA	NA	NA	NA
ENSG00000255710	NA	NA	NA	8,087382405	0,073949698	0,692647	0,106763977	0,914976226	NA
ENSG00000255713	OR4D2	olfactory receptor family 4 subfamily D member 2	124538	0	NA	NA	NA	NA	NA

ENSG00000255725	NA	NA	NA	0,119673877	0,847101401	4,080456	0,207599697	0,835541541	NA
ENSG00000255726	NA	NA	NA	0	NA	NA	NA	NA	NA
ENSG00000255730	NA	NA	NA	0,421724922	0	4,080456	0	1	NA
ENSG00000255734	NA	NA	NA	0	NA	NA	NA	NA	NA
ENSG00000255749	NA	NA	NA	0	NA	NA	NA	NA	NA
ENSG00000255753	NA	NA	NA	0	NA	NA	NA	NA	NA
ENSG00000255757	LOC100419932	FGFR1 oncogene partner 2 pseudogene	100419932	0	NA	NA	NA	NA	NA
ENSG00000255763	NA	NA	NA	0	NA	NA	NA	NA	NA
ENSG00000255776	NA	NA	NA	0,381736451	-0,135071301	3,663671	-0,036867745	0,970590458	NA
ENSG00000255780	NA	NA	NA	0	NA	NA	NA	NA	NA
ENSG00000255786	NA	NA	NA	0	NA	NA	NA	NA	NA
ENSG00000255804	OR6J1	olfactory receptor family 6 subfamily J member 1	79549	0	NA	NA	NA	NA	NA
ENSG00000255807	NA	NA	NA	0	NA	NA	NA	NA	NA
ENSG00000255815	KRT8P11	keratin 8 pseudogene 11	347265	0,368796784	0	4,080456	0	1	NA
ENSG00000255819	NA	NA	NA	7,792715339	-1,360977121	1,476652	-0,921664241	0,356703738	NA
ENSG00000255830	NA	NA	NA	0	NA	NA	NA	NA	NA
ENSG00000255833	TIFAB	TIFA inhibitor	497189	0	NA	NA	NA	NA	NA
ENSG00000255835	NA	NA	NA	0	NA	NA	NA	NA	NA
ENSG00000255836	LOC100419928	M-phase phosphoprotein 8 pseudogene	100419928	0	NA	NA	NA	NA	NA
ENSG00000255837	TAS2R20	taste 2 receptor member 20	259295	9,75862239	0,000849206	0,657353	0,001291857	0,998969247	NA
ENSG00000255838	NA	NA	NA	0	NA	NA	NA	NA	NA
ENSG00000255853	LOC100421201	piggyBac transposable element derived 3 pseudogene	100421201	0	NA	NA	NA	NA	NA
ENSG00000255855	KDM4F	lysine demethylase 4F	100129053	0	NA	NA	NA	NA	NA
ENSG00000255860	FOLR3P1	folate receptor 3 pseudogene 1	100288543	0	NA	NA	NA	NA	NA
ENSG00000255870	LOC390251	SH3 domain containing GRB2 like 1, endophilin A2 pseudogene	390251	0	NA	NA	NA	NA	NA
ENSG00000255872	NA	NA	NA	0,353005927	-1,083290237	4,080456	-0,26548265	0,790637674	NA
ENSG00000255875	NA	NA	NA	0	NA	NA	NA	NA	NA
ENSG00000255883	NA	NA	NA	0,178758119	0,825669343	4,079608	0,202389384	0,839612327	NA
ENSG00000255885	NA	NA	NA	0	NA	NA	NA	NA	NA
ENSG00000255892	NA	NA	NA	0	NA	NA	NA	NA	NA
ENSG00000255899	NA	NA	NA	0	NA	NA	NA	NA	NA
ENSG00000255900	NA	NA	NA	0	NA	NA	NA	NA	NA
ENSG00000255903	RPL12P46	ribosomal protein L12 pseudogene 46	107075251	0	NA	NA	NA	NA	NA
ENSG00000255909	NA	NA	NA	0	NA	NA	NA	NA	NA
ENSG00000255919	NA	NA	NA	0	NA	NA	NA	NA	NA
ENSG00000255923	LOC121296	transmembrane protein 132B pseudogene	121296	0	NA	NA	NA	NA	NA
ENSG00000255951	NA	NA	NA	0	NA	NA	NA	NA	NA
ENSG00000255960	NA	NA	NA	0	NA	NA	NA	NA	NA
ENSG00000255964	HSPD1P12	heat shock protein family D (Hsp60) member 1 pseudogene 12	283320	0	NA	NA	NA	NA	NA
ENSG00000255965	NA	NA	NA	0	NA	NA	NA	NA	NA
ENSG00000255967	NA	NA	NA	0,10488184	-1,076443944	4,080456	-0,263804824	0,791930317	NA
ENSG00000255972	NA	NA	NA	0	NA	NA	NA	NA	NA
ENSG00000255973	NA	NA	NA	0	NA	NA	NA	NA	NA
ENSG00000255974	CYP2A6	cytochrome P450 family 2 subfamily A member 6	1548	0	NA	NA	NA	NA	NA
ENSG00000255976	RAB11AP2	RAB11A, member RAS oncogene family pseudogene 2	106480447	0	NA	NA	NA	NA	NA
ENSG00000255977	NA	NA	NA	0	NA	NA	NA	NA	NA
ENSG00000255982	NXPE2P1	neurexophilin and PC-esterase domain family member 2 pseudogene 1	101926945	0	NA	NA	NA	NA	NA
ENSG00000255986	NA	NA	NA	0	NA	NA	NA	NA	NA
ENSG00000255987	NA	NA	NA	4,170846957	-6,231562148	1,554805	-4,007937575	6,13E-05	NA
ENSG00000255988	TUBB4BP1	TUBB4B pseudogene 1	106478981	0	NA	NA	NA	NA	NA
ENSG00000255991	RPL7AP74	ribosomal protein L7a pseudogene 74	390306	0	NA	NA	NA	NA	NA
ENSG00000255993	PSMC1P9	proteasome 26S subunit, ATPase 1 pseudogene 9	643668	0	NA	NA	NA	NA	NA
ENSG00000255995	NA	NA	NA	0	NA	NA	NA	NA	NA
ENSG00000255996	LOC390282	eukaryotic translation initiation factor 3 subunit F pseudogene	390282	0	NA	NA	NA	NA	NA
ENSG00000256004	NA	NA	NA	0	NA	NA	NA	NA	NA
ENSG00000256021	ELOCP31	elongin C pseudogene 31	100422582	0	NA	NA	NA	NA	NA
ENSG00000256029	NA	NA	NA	0	NA	NA	NA	NA	NA
ENSG00000256030	NA	NA	NA	0	NA	NA	NA	NA	NA
ENSG00000256035	NA	NA	NA	0	NA	NA	NA	NA	NA

ENSG00000256037	MRPL40P1	mitochondrial ribosomal protein L40 pseudogene 1	645328	0,296902737	0,683854529	3,889544	0,175818692	0,860436384	NA
ENSG00000256041	PTTG1P2	PTTG1 pseudogene 2	100127954	0	NA	NA	NA	NA	NA
ENSG00000256044	NA	NA	NA	0	NA	NA	NA	NA	NA
ENSG00000256056	NA	NA	NA	0	NA	NA	NA	NA	NA
ENSG00000256060	TRAPPC2B	trafficking protein particle complex subunit 2B	10597	9,285900164	0,388834803	0,618263	0,628914933	0,529404749	NA
ENSG00000256061	DNAAF4	dynein axonemal assembly factor 4	161582	0	NA	NA	NA	NA	NA
ENSG00000256069	NA	NA	NA	0	NA	NA	NA	NA	NA
ENSG00000256070	ST13P9	ST13, Hsp70 interacting protein pseudogene 9	390301	0	NA	NA	NA	NA	NA
ENSG00000256075	PSMC6P2	proteasome 26S subunit, ATPase, 6 pseudogene 2	160410	0	NA	NA	NA	NA	NA
ENSG00000256079	FAM133GP	family with sequence similarity 133 member A pseudogene	100421617	0	NA	NA	NA	NA	NA
ENSG00000256081	NA	NA	NA	0	NA	NA	NA	NA	NA
ENSG00000256091	MTRF1LP1	mitochondrial translational release factor 1 like pseudogene 1	100132520	0	NA	NA	NA	NA	NA
ENSG00000256093	LOC100131779	translocase of outer mitochondrial membrane 6 homolog (yeast) pseudogene	100131779	0	NA	NA	NA	NA	NA
ENSG00000256098	NA	NA	NA	0	NA	NA	NA	NA	NA
ENSG00000256103	NA	NA	NA	0	NA	NA	NA	NA	NA
ENSG00000256108	NA	NA	NA	0,119673877	0,847101401	4,080456	0,207599697	0,835541541	NA
ENSG00000256125	NA	NA	NA	0	NA	NA	NA	NA	NA
ENSG00000256134	NA	NA	NA	0	NA	NA	NA	NA	NA
ENSG00000256136	VPS51P3	VPS51 pseudogene 3	127379703	0	NA	NA	NA	NA	NA
ENSG00000256138	ZKSCAN7P1	zinc finger with KRAB and SCAN domains 7 pseudogene 1	100419827	0	NA	NA	NA	NA	NA
ENSG00000256148	NA	NA	NA	0,119673877	0,847101401	4,080456	0,207599697	0,835541541	NA
ENSG00000256157	NA	NA	NA	0	NA	NA	NA	NA	NA
ENSG00000256159	NA	NA	NA	0	NA	NA	NA	NA	NA
ENSG00000256162	SMLR1	small leucine rich protein 1	100507203	0	NA	NA	NA	NA	NA
ENSG00000256167	NA	NA	NA	0	NA	NA	NA	NA	NA
ENSG00000256171	NA	NA	NA	0	NA	NA	NA	NA	NA
ENSG00000256181	NA	NA	NA	0	NA	NA	NA	NA	NA
ENSG00000256189	NA	NA	NA	0,10488184	-1,076443944	4,080456	-0,263804824	0,791930317	NA
ENSG00000256192	OOEPP2	OOEP pseudogene 2	117751741	0	NA	NA	NA	NA	NA
ENSG00000256206	NA	NA	NA	1,581753167	3,504566438	1,972433	1,776773033	0,07560559	NA
ENSG00000256210	NA	NA	NA	0	NA	NA	NA	NA	NA
ENSG00000256211	NA	NA	NA	0	NA	NA	NA	NA	NA
ENSG00000256221	NA	NA	NA	0	NA	NA	NA	NA	NA
ENSG00000256238	SUPT16HP1	SPT16 homolog, facilitates chromatin remodeling subunit pseudogene 1	400011	0,342328533	1,934952978	3,761597	0,514396655	0,606974697	NA
ENSG00000256243	RPL7AP3	ribosomal protein L7a pseudogene 3	654370	0	NA	NA	NA	NA	NA
ENSG00000256254	LOC100421658	sacsin molecular chaperone pseudogene	100421658	0	NA	NA	NA	NA	NA
ENSG00000256263	NA	NA	NA	0	NA	NA	NA	NA	NA
ENSG00000256274	TAS2R64P	taste 2 receptor member 64 pseudogene	338412	0	NA	NA	NA	NA	NA
ENSG00000256280	NA	NA	NA	0,189921703	1,666449468	4,047481	0,411725125	0,680540907	NA
ENSG00000256282	LOC112268073	uncharacterized LOC112268073	112268073	0,295706089	0,708564174	3,894553	0,181937236	0,855631982	NA
ENSG00000256283	NA	NA	NA	0	NA	NA	NA	NA	NA
ENSG00000256285	OSBPL9P5	oxysterol binding protein like 9 pseudogene 5	106480232	0	NA	NA	NA	NA	NA
ENSG00000256293	NA	NA	NA	0	NA	NA	NA	NA	NA
ENSG00000256310	NA	NA	NA	0,119673877	0,847101401	4,080456	0,207599697	0,835541541	NA
ENSG00000256331	NIFKP3	nucleolar protein interacting with the FHA domain of MKI67 pseudogene 3	100270648	0,815048919	1,938158769	3,091772	0,626876363	0,530740282	NA
ENSG00000256338	RPL41P2	ribosomal protein L41 pseudogene 2	22970	0	NA	NA	NA	NA	NA
ENSG00000256340	NA	NA	NA	0	NA	NA	NA	NA	NA
ENSG00000256346	NA	NA	NA	0	NA	NA	NA	NA	NA
ENSG00000256347	NA	NA	NA	0	NA	NA	NA	NA	NA
ENSG00000256349	NA	NA	NA	0	NA	NA	NA	NA	NA
ENSG00000256350	NA	NA	NA	0	NA	NA	NA	NA	NA
ENSG00000256351	NA	NA	NA	0,178525239	0,82982842	4,079786	0,203399989	0,838822408	NA
ENSG00000256353	NA	NA	NA	0	NA	NA	NA	NA	NA
ENSG00000256355	NTAN1P3	N-terminal asparagine amidase pseudogene 3	100420356	0	NA	NA	NA	NA	NA
ENSG00000256356	NA	NA	NA	0,118286652	0	4,080456	0	1	NA

ENSG00000256358	RPL31P57	ribosomal protein L31 pseudogene 57	645993	0,072165449	0,847101401	4,080456	0,207599697	0,835541541	NA
ENSG00000256361	NA	NA	NA	2,196531642	-0,140586727	1,343563	-0,10463725	0,916663656	NA
ENSG00000256371	NA	NA	NA	0	NA	NA	NA	NA	NA
ENSG00000256372	NA	NA	NA	0	NA	NA	NA	NA	NA
ENSG00000256378	NA	NA	NA	0	NA	NA	NA	NA	NA
ENSG00000256379	NA	NA	NA	0	NA	NA	NA	NA	NA
ENSG00000256385	NA	NA	NA	0	NA	NA	NA	NA	NA
ENSG00000256394	ASIC5	acid sensing ion channel subunit family member 5	51802	0	NA	NA	NA	NA	NA
ENSG00000256399	LOC100419700	MIA SH3 domain ER export factor 2 pseudogene	100419700	0	NA	NA	NA	NA	NA
ENSG00000256407	NA	NA	NA	0	NA	NA	NA	NA	NA
ENSG00000256420	NA	NA	NA	0	NA	NA	NA	NA	NA
ENSG00000256441	DNAJB6P5	DNAJB6 pseudogene 5	100129779	0	NA	NA	NA	NA	NA
ENSG00000256450	NA	NA	NA	0	NA	NA	NA	NA	NA
ENSG00000256453	DND1	DND microRNA-mediated repression inhibitor 1	373863	3,591608139	0,360702719	1,171855	0,307804792	0,758230875	NA
ENSG00000256463	SALL3	spalt like transcription factor 3	27164	0,118286652	0	4,080456	0	1	NA
ENSG00000256464	NA	NA	NA	2,940988613	-1,199049681	1,063562	-1,1273905	0,259577414	NA
ENSG00000256465	NA	NA	NA	0	NA	NA	NA	NA	NA
ENSG00000256474	TRAV11	T cell receptor alpha variable 11 (pseudogene)	28675	0	NA	NA	NA	NA	NA
ENSG00000256496	LOC100420116	transmembrane protein 132B pseudogene	100420116	0	NA	NA	NA	NA	NA
ENSG00000256500	NA	NA	NA	2,71824474	-4,671972541	3,008608	-1,552868353	0,120454589	NA
ENSG00000256502	NA	NA	NA	0	NA	NA	NA	NA	NA
ENSG00000256518	NA	NA	NA	0	NA	NA	NA	NA	NA
ENSG00000256533	LOC100422382	nocturnin pseudogene	100422382	0,233154829	0,827613629	4,079691	0,202861833	0,839243027	NA
ENSG00000256552	NA	NA	NA	0	NA	NA	NA	NA	NA
ENSG00000256553	TRAV1-2	T cell receptor alpha variable 1-2	28692	0,103998153	-1,076443944	4,080456	-0,263804824	0,791930317	NA
ENSG00000256557	NA	NA	NA	0	NA	NA	NA	NA	NA
ENSG00000256563	NANOGNBP2	NANOGNB pseudogene 2	101060006	0	NA	NA	NA	NA	NA
ENSG00000256566	NA	NA	NA	0,529825426	-0,516860304	3,943587	-0,1310635	0,895725075	NA
ENSG00000256574	OR13A1	olfactory receptor family 13 subfamily A member 1	79290	0	NA	NA	NA	NA	NA
ENSG00000256581	NA	NA	NA	0	NA	NA	NA	NA	NA
ENSG00000256589	NA	NA	NA	0	NA	NA	NA	NA	NA
ENSG00000256590	TRDV3	T cell receptor delta variable 3	28516	2,420615507	-4,775446166	1,614419	-2,957997308	0,003096448	NA
ENSG00000256591	NA	NA	NA	2,582779277	-3,221825988	2,442009	-1,319334006	0,187057474	NA
ENSG00000256612	NA	NA	NA	0	NA	NA	NA	NA	NA
ENSG00000256614	AK6P1	adenylate kinase 6 pseudogene 1	100132086	0	NA	NA	NA	NA	NA
ENSG00000256616	NA	NA	NA	0	NA	NA	NA	NA	NA
ENSG00000256625	LOC124902904	liprin-beta-1-like	124902904	0,382862898	-1,062898873	3,676094	-0,289138126	0,772475679	NA
ENSG00000256626	LOC100421202	C-type lectin domain family 1 member B pseudogene	100421202	0	NA	NA	NA	NA	NA
ENSG00000256627	NA	NA	NA	0	NA	NA	NA	NA	NA
ENSG00000256630	NA	NA	NA	0	NA	NA	NA	NA	NA
ENSG00000256640	NA	NA	NA	0	NA	NA	NA	NA	NA
ENSG00000256646	NA	NA	NA	0	NA	NA	NA	NA	NA
ENSG00000256663	NA	NA	NA	4,033872268	4,767900311	1,669194	2,856409332	0,004284624	NA
ENSG00000256671	LIMS4	LIM zinc finger domain containing 4	100288695	2,394519663	0,119360979	1,782218	0,066973278	0,946602976	NA
ENSG00000256673	NA	NA	NA	0	NA	NA	NA	NA	NA
ENSG00000256674	LOC100132172	histone deacetylase 2 pseudogene	100132172	0	NA	NA	NA	NA	NA
ENSG00000256678	NA	NA	NA	0	NA	NA	NA	NA	NA
ENSG00000256681	NA	NA	NA	0	NA	NA	NA	NA	NA
ENSG00000256704	NA	NA	NA	0	NA	NA	NA	NA	NA
ENSG00000256708	NA	NA	NA	0	NA	NA	NA	NA	NA
ENSG00000256709	NA	NA	NA	0	NA	NA	NA	NA	NA
ENSG00000256713	PGA5	pepsinogen A5	5222	0	NA	NA	NA	NA	NA
ENSG00000256720	NA	NA	NA	0	NA	NA	NA	NA	NA
ENSG00000256723	LOC100421622	family with sequence similarity 91 member A1 pseudogene	100421622	0,210312459	-1,077224851	4,080456	-0,263996202	0,791782846	NA
ENSG00000256734	NA	NA	NA	0	NA	NA	NA	NA	NA
ENSG00000256737	NA	NA	NA	0	NA	NA	NA	NA	NA
ENSG00000256745	NA	NA	NA	0,117497108	0,847101401	4,080456	0,207599697	0,835541541	NA
ENSG00000256748	NA	NA	NA	0	NA	NA	NA	NA	NA
ENSG00000256752	HPRT1P3	hypoxanthine phosphoribosyltransferase 1 pseudogene 3	3255	0	NA	NA	NA	NA	NA

ENSG00000256756	NA	NA	NA	0	NA	NA	NA	NA	NA
ENSG00000256762	STH	saitohin	246744	0	NA	NA	NA	NA	NA
ENSG00000256774	NA	NA	NA	0	NA	NA	NA	NA	NA
ENSG00000256777	PDCL3P7	PDCL3 pseudogene 7	390340	0	NA	NA	NA	NA	NA
ENSG00000256779	NA	NA	NA	0	NA	NA	NA	NA	NA
ENSG00000256799	NA	NA	NA	0,118286652	0	4,080456	0	1	NA
ENSG00000256806	C17orf100	chromosome 17 open reading frame 100	388327	4,617055583	0,667694946	1,049464	0,636224631	0,524630021	NA
ENSG00000256812	CAPNS2	calpain small subunit 2	84290	0	NA	NA	NA	NA	NA
ENSG00000256817	NA	NA	NA	0	NA	NA	NA	NA	NA
ENSG00000256825	NA	NA	NA	0	NA	NA	NA	NA	NA
ENSG00000256826	NA	NA	NA	0	NA	NA	NA	NA	NA
ENSG00000256827	NA	NA	NA	0	NA	NA	NA	NA	NA
ENSG00000256835	NA	NA	NA	0	NA	NA	NA	NA	NA
ENSG00000256843	NA	NA	NA	0	NA	NA	NA	NA	NA
ENSG00000256844	NA	NA	NA	0	NA	NA	NA	NA	NA
ENSG00000256847	NA	NA	NA	0	NA	NA	NA	NA	NA
ENSG00000256849	TCP1P3	t-complex 1 pseudogene 3	400013	0	NA	NA	NA	NA	NA
ENSG00000256851	NA	NA	NA	0	NA	NA	NA	NA	NA
ENSG00000256852	NA	NA	NA	0	NA	NA	NA	NA	NA
ENSG00000256861	NA	NA	NA	0	NA	NA	NA	NA	NA
ENSG00000256870	SLC5A8	solute carrier family 5 member 8	160728	0	NA	NA	NA	NA	NA
ENSG00000256896	PSMC1P8	proteasome 26S subunit, ATPase 1 pseudogene 8	390297	0	NA	NA	NA	NA	NA
ENSG00000256898	NA	NA	NA	0	NA	NA	NA	NA	NA
ENSG00000256902	NA	NA	NA	0	NA	NA	NA	NA	NA
ENSG00000256912	GCNAP1	GCNA pseudogene 1	100419929	0,564076935	-1,604417715	2,46378	-0,651201825	0,514916212	NA
ENSG00000256913	NA	NA	NA	1,909923568	0,13469236	1,387933	0,097045303	0,922690417	NA
ENSG00000256917	NA	NA	NA	0	NA	NA	NA	NA	NA
ENSG00000256937	NA	NA	NA	0,209506194	-1,066309628	4,080456	-0,261321201	0,793844819	NA
ENSG00000256950	NA	NA	NA	0	NA	NA	NA	NA	NA
ENSG00000256963	NA	NA	NA	0	NA	NA	NA	NA	NA
ENSG00000256966	NA	NA	NA	0,117497108	0,847101401	4,080456	0,207599697	0,835541541	NA
ENSG00000256968	NA	NA	NA	0	NA	NA	NA	NA	NA
ENSG00000256975	NA	NA	NA	0,10488184	-1,076443944	4,080456	-0,263804824	0,791930317	NA
ENSG00000256977	LIMS3	LIM zinc finger domain containing 3	96626	0	NA	NA	NA	NA	NA
ENSG00000256980	KHDC1L	KH domain containing 1 like	100129128	0	NA	NA	NA	NA	NA
ENSG00000256984	NA	NA	NA	0	NA	NA	NA	NA	NA
ENSG00000256986	NA	NA	NA	0	NA	NA	NA	NA	NA
ENSG00000256987	NA	NA	NA	0	NA	NA	NA	NA	NA
ENSG00000257005	NA	NA	NA	0	NA	NA	NA	NA	NA
ENSG00000257008	GPR142	G protein-coupled receptor 142	350383	5,082302411	-0,855974137	0,797528	-1,073284647	0,283143422	NA
ENSG00000257012	NA	NA	NA	0	NA	NA	NA	NA	NA
ENSG00000257016	SLC25A39P2	SLC25A39 pseudogene 2	107126289	0	NA	NA	NA	NA	NA
ENSG00000257017	HP	haptoglobin	3240	0	NA	NA	NA	NA	NA
ENSG00000257021	NA	NA	NA	0	NA	NA	NA	NA	NA
ENSG00000257035	LOC100420119	transmembrane protein 132B pseudogene	100420119	0	NA	NA	NA	NA	NA
ENSG00000257037	NA	NA	NA	0	NA	NA	NA	NA	NA
ENSG00000257043	NA	NA	NA	0	NA	NA	NA	NA	NA
ENSG00000257046	SLCO1B3-SLCO1B7	SLCO1B3-SLCO1B7 readthrough	115072896	0	NA	NA	NA	NA	NA
ENSG00000257057	C11orf97	chromosome 11 open reading frame 97	643037	0	NA	NA	NA	NA	NA
ENSG00000257062	NA	NA	NA	0	NA	NA	NA	NA	NA
ENSG00000257065	NA	NA	NA	0	NA	NA	NA	NA	NA
ENSG00000257070	NA	NA	NA	0	NA	NA	NA	NA	NA
ENSG00000257074	NA	NA	NA	0	NA	NA	NA	NA	NA
ENSG00000257075	RPEP6	ribulose-5-phosphate-3-epimerase pseudogene 6	100420590	0	NA	NA	NA	NA	NA
ENSG00000257078	NA	NA	NA	0	NA	NA	NA	NA	NA
ENSG00000257087	NA	NA	NA	0	NA	NA	NA	NA	NA
ENSG00000257094	NA	NA	NA	0,844258823	2,416774909	2,182767	1,107207203	0,268204354	NA
ENSG00000257101	LRRC37A13P	leucine rich repeat containing 37 member A13, pseudogene	106479024	0	NA	NA	NA	NA	NA
ENSG00000257108	NHLRC4	NHL repeat containing 4	283948	0,9136097	1,824018277	1,960369	0,930446224	0,352140095	NA
ENSG00000257109	OR4F28P	olfactory receptor family 4 subfamily F member 28 pseudogene	390652	0	NA	NA	NA	NA	NA
ENSG00000257113	NA	NA	NA	0	NA	NA	NA	NA	NA
ENSG00000257115	OR11H12	olfactory receptor family 11 subfamily H member 12	440153	0	NA	NA	NA	NA	NA
ENSG00000257119	NA	NA	NA	0	NA	NA	NA	NA	NA
ENSG00000257121	LOC100287148	peroxiredoxin 6 pseudogene	100287148	0	NA	NA	NA	NA	NA
ENSG00000257122	NA	NA	NA	7,509281283	-0,572222288	1,455644	-0,393106058	0,69424115	NA

ENSG00000257125	NA	NA	NA	0	NA	NA	NA	NA	NA
ENSG00000257128	LOC100129078	tetratricopeptide repeat domain 5 pseudogene	100129078	0	NA	NA	NA	NA	NA
ENSG00000257129	NA	NA	NA	0	NA	NA	NA	NA	NA
ENSG00000257138	TAS2R38	taste 2 receptor member 38	5726	0	NA	NA	NA	NA	NA
ENSG00000257142	NA	NA	NA	0	NA	NA	NA	NA	NA
ENSG00000257146	NA	NA	NA	0	NA	NA	NA	NA	NA
ENSG00000257150	PGAM1P5	phosphoglycerate mutase 1 pseudogene 5	100132594	0	NA	NA	NA	NA	NA
ENSG00000257157	NA	NA	NA	0	NA	NA	NA	NA	NA
ENSG00000257162	NA	NA	NA	0	NA	NA	NA	NA	NA
ENSG00000257169	NA	NA	NA	3,893710357	0,063173423	0,973707	0,064879267	0,948270129	NA
ENSG00000257171	NA	NA	NA	0	NA	NA	NA	NA	NA
ENSG00000257173	AK6P2	adenylate kinase 6 pseudogene 2	100128817	0	NA	NA	NA	NA	NA
ENSG00000257175	NA	NA	NA	0,117497108	0,847101401	4,080456	0,207599697	0,835541541	NA
ENSG00000257179	NA	NA	NA	0	NA	NA	NA	NA	NA
ENSG00000257184	NA	NA	NA	0	NA	NA	NA	NA	NA
ENSG00000257195	NA	NA	NA	0	NA	NA	NA	NA	NA
ENSG00000257198	NA	NA	NA	0	NA	NA	NA	NA	NA
ENSG00000257199	NA	NA	NA	0	NA	NA	NA	NA	NA
ENSG00000257207	NA	NA	NA	0	NA	NA	NA	NA	NA
ENSG00000257210	NA	NA	NA	0	NA	NA	NA	NA	NA
ENSG00000257220	PRXL2AP1	peroxiredoxin like 2A pseudogene 1	100420900	0	NA	NA	NA	NA	NA
ENSG00000257224	NA	NA	NA	0	NA	NA	NA	NA	NA
ENSG00000257231	NA	NA	NA	0	NA	NA	NA	NA	NA
ENSG00000257237	NA	NA	NA	0	NA	NA	NA	NA	NA
ENSG00000257243	PARK7P1	PARK7 pseudogene 1	100422296	3,231946218	-0,690697292	0,968496	-0,713165038	0,475743638	NA
ENSG00000257246	NA	NA	NA	4,097693322	-0,03087225	0,937676	-0,032924213	0,973735024	NA
ENSG00000257254	NA	NA	NA	0	NA	NA	NA	NA	NA
ENSG00000257281	LOC196469	DNA dC->dU-editing enzyme APOBEC-3G-like	196469	0,11808024	-1,076443944	4,080456	-0,263804824	0,791930317	NA
ENSG00000257287	NA	NA	NA	0	NA	NA	NA	NA	NA
ENSG00000257289	NA	NA	NA	0	NA	NA	NA	NA	NA
ENSG00000257292	NA	NA	NA	0	NA	NA	NA	NA	NA
ENSG00000257296	NA	NA	NA	0	NA	NA	NA	NA	NA
ENSG00000257300	HAUS8P1	HAUS augmin like complex subunit 8 pseudogene 1	100420901	0	NA	NA	NA	NA	NA
ENSG00000257302	NA	NA	NA	0	NA	NA	NA	NA	NA
ENSG00000257307	LOC644584	EWS RNA binding protein 1 pseudogene	644584	0,236573303	0	4,080456	0	1	NA
ENSG00000257308	PGBD3P1	piggyBac transposable element derived 3 pseudogene 1	267005	0	NA	NA	NA	NA	NA
ENSG00000257316	ASS1P14	argininosuccinate synthetase 1 pseudogene 14	459	0	NA	NA	NA	NA	NA
ENSG00000257323	NA	NA	NA	0	NA	NA	NA	NA	NA
ENSG00000257331	NA	NA	NA	0	NA	NA	NA	NA	NA
ENSG00000257332	NA	NA	NA	0	NA	NA	NA	NA	NA
ENSG00000257335	MGAM	maltase-glucoamylase	8972	2,144809845	-2,176484907	2,014038	-1,080657256	0,279849603	NA
ENSG00000257336	NA	NA	NA	0	NA	NA	NA	NA	NA
ENSG00000257341	NA	NA	NA	1,145463894	0,129612653	3,015525	0,042981791	0,965716049	NA
ENSG00000257343	NA	NA	NA	0	NA	NA	NA	NA	NA
ENSG00000257350	NA	NA	NA	0	NA	NA	NA	NA	NA
ENSG00000257355	NA	NA	NA	0	NA	NA	NA	NA	NA
ENSG00000257356	BNIP3P6	BCL2 interacting protein 3 pseudogene 6	100129777	0	NA	NA	NA	NA	NA
ENSG00000257360	NA	NA	NA	0	NA	NA	NA	NA	NA
ENSG00000257364	NA	NA	NA	0	NA	NA	NA	NA	NA
ENSG00000257368	MESDP1	MESD pseudogene 1	100128248	0,307337633	2,350784661	3,995057	0,588423266	0,556248225	NA
ENSG00000257376	NA	NA	NA	0	NA	NA	NA	NA	NA
ENSG00000257384	NA	NA	NA	0	NA	NA	NA	NA	NA
ENSG00000257389	NA	NA	NA	0	NA	NA	NA	NA	NA
ENSG00000257390	NA	NA	NA	0	NA	NA	NA	NA	NA
ENSG00000257392	LOC644746	ret finger protein like 3 pseudogene	644746	0	NA	NA	NA	NA	NA
ENSG00000257402	NA	NA	NA	0	NA	NA	NA	NA	NA
ENSG00000257403	NA	NA	NA	0,11808024	-1,076443944	4,080456	-0,263804824	0,791930317	NA
ENSG00000257404	NA	NA	NA	0	NA	NA	NA	NA	NA
ENSG00000257405	NA	NA	NA	0	NA	NA	NA	NA	NA
ENSG00000257408	NA	NA	NA	0	NA	NA	NA	NA	NA
ENSG00000257411	NA	NA	NA	0	NA	NA	NA	NA	NA
ENSG00000257414	NA	NA	NA	0	NA	NA	NA	NA	NA
ENSG00000257415	NA	NA	NA	0	NA	NA	NA	NA	NA
ENSG00000257426	NA	NA	NA	0	NA	NA	NA	NA	NA
ENSG00000257432	NA	NA	NA	0	NA	NA	NA	NA	NA
ENSG00000257444	NA	NA	NA	0	NA	NA	NA	NA	NA
ENSG00000257446	ZNF878	zinc finger protein 878	729747	0	NA	NA	NA	NA	NA
ENSG00000257464	NA	NA	NA	0	NA	NA	NA	NA	NA

ENSG00000257465	NA	NA	NA	0	NA	NA	NA	NA	NA
ENSG00000257480	MRPL2P1	mitochondrial ribosomal protein L2 pseudogene 1	347894	0	NA	NA	NA	NA	NA
ENSG00000257489	NA	NA	NA	2,179311463	-0,173500295	1,310236	-0,132419086	0,894652819	NA
ENSG00000257493	NA	NA	NA	0	NA	NA	NA	NA	NA
ENSG00000257501	PAFAH1B2P2	PAFAH1B2 pseudogene 2	643711	0	NA	NA	NA	NA	NA
ENSG00000257503	NA	NA	NA	0	NA	NA	NA	NA	NA
ENSG00000257506	PLA2G10HP	phospholipase A2 group XH, pseudogene	127525795	0	NA	NA	NA	NA	NA
ENSG00000257511	LOC100420981	nucleosome assembly protein 1 like 1 pseudogene	100420981	5,501255542	-1,47711454	0,842824	-1,752576995	0,079674643	NA
ENSG00000257512	NA	NA	NA	0	NA	NA	NA	NA	NA
ENSG00000257513	NA	NA	NA	0	NA	NA	NA	NA	NA
ENSG00000257520	SFTA3	surfactant associated 3	253970	0	NA	NA	NA	NA	NA
ENSG00000257528	KRT8P19	keratin 8 pseudogene 19	644998	0	NA	NA	NA	NA	NA
ENSG00000257531	NA	NA	NA	0	NA	NA	NA	NA	NA
ENSG00000257539	NA	NA	NA	0	NA	NA	NA	NA	NA
ENSG00000257542	NA	NA	NA	0	NA	NA	NA	NA	NA
ENSG00000257558	NA	NA	NA	0	NA	NA	NA	NA	NA
ENSG00000257564	NA	NA	NA	0	NA	NA	NA	NA	NA
ENSG00000257569	NA	NA	NA	0	NA	NA	NA	NA	NA
ENSG00000257570	NA	NA	NA	0	NA	NA	NA	NA	NA
ENSG00000257572	SSBL3P	small RNA binding exonuclease protection factor La like 3, pseudogene	100533757	0	NA	NA	NA	NA	NA
ENSG00000257576	NA	NA	NA	1,387920905	-0,20836076	1,683017	-0,123801907	0,901472122	NA
ENSG00000257580	NA	NA	NA	0	NA	NA	NA	NA	NA
ENSG00000257586	LOC100421126	sucrase-isomaltase (alpha-glucosidase) pseudogene	100421126	0	NA	NA	NA	NA	NA
ENSG00000257591	ZNF625	zinc finger protein 625	90589	3,699264267	0,527442095	1,325861	0,397810958	0,690769539	NA
ENSG00000257604	NA	NA	NA	0	NA	NA	NA	NA	NA
ENSG00000257609	VN1R57P	vomeronasal 1 receptor 57 pseudogene	100312809	0	NA	NA	NA	NA	NA
ENSG00000257611	NA	NA	NA	0	NA	NA	NA	NA	NA
ENSG00000257616	LOC400036	DEAD-box helicase 23 pseudogene	400036	0	NA	NA	NA	NA	NA
ENSG00000257624	NA	NA	NA	0	NA	NA	NA	NA	NA
ENSG00000257629	LOC100287355	microtubule associated protein 1 light chain 3 beta pseudogene	100287355	0	NA	NA	NA	NA	NA
ENSG00000257635	NA	NA	NA	0	NA	NA	NA	NA	NA
ENSG00000257639	LOC646828	carbonic anhydrase 5A pseudogene	646828	0	NA	NA	NA	NA	NA
ENSG00000257643	NA	NA	NA	0	NA	NA	NA	NA	NA
ENSG00000257644	NA	NA	NA	0	NA	NA	NA	NA	NA
ENSG00000257645	NA	NA	NA	0	NA	NA	NA	NA	NA
ENSG00000257648	NA	NA	NA	0	NA	NA	NA	NA	NA
ENSG00000257649	NA	NA	NA	0	NA	NA	NA	NA	NA
ENSG00000257658	NA	NA	NA	0	NA	NA	NA	NA	NA
ENSG00000257662	EIF4A1P12	eukaryotic translation initiation factor 4A1 pseudogene 12	100462830	0	NA	NA	NA	NA	NA
ENSG00000257664	NA	NA	NA	0	NA	NA	NA	NA	NA
ENSG00000257666	NA	NA	NA	0	NA	NA	NA	NA	NA
ENSG00000257672	NA	NA	NA	0	NA	NA	NA	NA	NA
ENSG00000257674	NA	NA	NA	0	NA	NA	NA	NA	NA
ENSG00000257675	BTBD10P1	BTB domain containing 10 pseudogene 1	100128678	0	NA	NA	NA	NA	NA
ENSG00000257680	NA	NA	NA	0	NA	NA	NA	NA	NA
ENSG00000257687	NA	NA	NA	0	NA	NA	NA	NA	NA
ENSG00000257691	NA	NA	NA	0	NA	NA	NA	NA	NA
ENSG00000257704	INAFM1	InaF motif containing 1	255783	8,630892517	-0,684099835	0,632979	-1,080762473	0,279802786	NA
ENSG00000257720	NA	NA	NA	0	NA	NA	NA	NA	NA
ENSG00000257721	NA	NA	NA	0	NA	NA	NA	NA	NA
ENSG00000257723	NA	NA	NA	0	NA	NA	NA	NA	NA
ENSG00000257730	NA	NA	NA	0	NA	NA	NA	NA	NA
ENSG00000257731	LOC100132698	single stranded DNA binding protein 4 pseudogene	100132698	0	NA	NA	NA	NA	NA
ENSG00000257738	LOC100419293	carbonyl reductase 1 pseudogene	100419293	0	NA	NA	NA	NA	NA
ENSG00000257743	MGAM2	maltase-glucoamylase 2 (putative)	93432	0	NA	NA	NA	NA	NA
ENSG00000257751	LOC100422528	pleckstrin homology domain containing B2 pseudogene	100422528	0	NA	NA	NA	NA	NA
ENSG00000257752	LOC100420011	transmembrane protein 38A pseudogene	100420011	1,441768107	-0,206113041	1,589108	-0,129703633	0,896800908	NA
ENSG00000257757	OR6C7P	olfactory receptor family 6 subfamily C member 7 pseudogene	390322	0	NA	NA	NA	NA	NA

ENSG00000257758	KRT18P20	keratin 18 pseudogene 20	121054	0	NA	NA	NA	NA	NA
ENSG00000257763	NA	NA	NA	0	NA	NA	NA	NA	NA
ENSG00000257767	NA	NA	NA	0,548320701	-1,06330204	4,080456	-0,260584129	0,79441323	NA
ENSG00000257773	NA	NA	NA	0	NA	NA	NA	NA	NA
ENSG00000257777	NA	NA	NA	0	NA	NA	NA	NA	NA
ENSG00000257779	LOC100506911	chromosome 6 open reading frame 35 pseudogene	100506911	0	NA	NA	NA	NA	NA
ENSG00000257780	GLYCAM1	glycosylation dependent cell adhesion molecule 1 (pseudogene)	644076	0	NA	NA	NA	NA	NA
ENSG00000257786	AKIRIN1P1	akirin 1 pseudogene 1	100418731	0	NA	NA	NA	NA	NA
ENSG00000257790	EIF4A1P4	eukaryotic translation initiation factor 4A1 pseudogene 4	728698	0	NA	NA	NA	NA	NA
ENSG00000257792	OR5BJ1P	olfactory receptor family 5 subfamily BJ member 1 pseudogene	81047	0	NA	NA	NA	NA	NA
ENSG00000257802	NA	NA	NA	0	NA	NA	NA	NA	NA
ENSG00000257803	NA	NA	NA	0,144330898	1,418786819	4,061262	0,349346267	0,726829368	NA
ENSG00000257807	STBD1P1	STBD1 pseudogene 1	100127978	0	NA	NA	NA	NA	NA
ENSG00000257813	LOC400026	THAP domain containing 12 pseudogene	400026	0	NA	NA	NA	NA	NA
ENSG00000257818	NA	NA	NA	1,365130498	-0,730087672	2,29697	-0,317848158	0,750600119	NA
ENSG00000257820	NA	NA	NA	0	NA	NA	NA	NA	NA
ENSG00000257823	NA	NA	NA	0	NA	NA	NA	NA	NA
ENSG00000257825	NA	NA	NA	0	NA	NA	NA	NA	NA
ENSG00000257838	NA	NA	NA	0,330285674	0	4,080456	0	1	NA
ENSG00000257844	NA	NA	NA	0	NA	NA	NA	NA	NA
ENSG00000257846	LOC400174	single stranded DNA binding protein 4 pseudogene	400174	0	NA	NA	NA	NA	NA
ENSG00000257847	NA	NA	NA	0	NA	NA	NA	NA	NA
ENSG00000257848	NA	NA	NA	0	NA	NA	NA	NA	NA
ENSG00000257851	HNRNPA3P10	heterogeneous nuclear ribonucleoprotein A3 pseudogene 10	100421413	0	NA	NA	NA	NA	NA
ENSG00000257852	NA	NA	NA	0,23616048	-2,075648354	4,028973	-0,515180498	0,606426895	NA
ENSG00000257853	NA	NA	NA	0	NA	NA	NA	NA	NA
ENSG00000257855	LOC101059974	p53 and DNA damage-regulated protein 1-like	101059974	0	NA	NA	NA	NA	NA
ENSG00000257858	MTND1P24	MT-ND1 pseudogene 24	100873341	0	NA	NA	NA	NA	NA
ENSG00000257864	LOC100294713	IQ motif containing F1 pseudogene	100294713	0	NA	NA	NA	NA	NA
ENSG00000257865	NA	NA	NA	0	NA	NA	NA	NA	NA
ENSG00000257872	LOC641695	reticulon 4 interacting protein 1 pseudogene	641695	0	NA	NA	NA	NA	NA
ENSG00000257875	NA	NA	NA	0,144330898	1,418786819	4,061262	0,349346267	0,726829368	NA
ENSG00000257884	NA	NA	NA	0	NA	NA	NA	NA	NA
ENSG00000257885	NA	NA	NA	0	NA	NA	NA	NA	NA
ENSG00000257896	NA	NA	NA	0	NA	NA	NA	NA	NA
ENSG00000257897	NA	NA	NA	0	NA	NA	NA	NA	NA
ENSG00000257905	NA	NA	NA	0	NA	NA	NA	NA	NA
ENSG00000257907	EEF1A1P17	eukaryotic translation elongation factor 1 alpha 1 pseudogene 17	401717	0	NA	NA	NA	NA	NA
ENSG00000257915	NA	NA	NA	0	NA	NA	NA	NA	NA
ENSG00000257921	NA	NA	NA	0,314645521	-2,509429468	3,997603	-0,627733573	0,530178488	NA
ENSG00000257927	MRPS36P5	mitochondrial ribosomal protein S36 pseudogene 5	347703	0	NA	NA	NA	NA	NA
ENSG00000257941	NA	NA	NA	0	NA	NA	NA	NA	NA
ENSG00000257943	NA	NA	NA	0	NA	NA	NA	NA	NA
ENSG00000257950	P2RX5-TAX1BP3	P2RX5-TAX1BP3 readthrough (NMD candidate)	100533970	0,39686791	0,842710781	3,658703	0,230330476	0,817834981	NA
ENSG00000257954	NA	NA	NA	0,798064583	-0,979747546	2,333903	-0,419789391	0,674639316	NA
ENSG00000257956	NA	NA	NA	0	NA	NA	NA	NA	NA
ENSG00000257957	QRSL1P3	QRSL1 pseudogene 3	100422374	0	NA	NA	NA	NA	NA
ENSG00000257966	NA	NA	NA	0,675181153	1,05174427	2,241693	0,469174038	0,638945241	NA
ENSG00000257976	NA	NA	NA	0	NA	NA	NA	NA	NA
ENSG00000257979	SNRPGP18	small nuclear ribonucleoprotein polypeptide G pseudogene 18	106480680	0	NA	NA	NA	NA	NA
ENSG00000257987	SPMIP11	sperm microtubule inner protein 11	255411	0	NA	NA	NA	NA	NA
ENSG00000257991	NA	NA	NA	0	NA	NA	NA	NA	NA
ENSG00000257994	NA	NA	NA	0	NA	NA	NA	NA	NA
ENSG00000257998	NA	NA	NA	0	NA	NA	NA	NA	NA
ENSG00000258011	NA	NA	NA	0	NA	NA	NA	NA	NA
ENSG00000258012	NA	NA	NA	0	NA	NA	NA	NA	NA
ENSG00000258013	RPL3P13	ribosomal protein L3 pseudogene 13	106480199	0	NA	NA	NA	NA	NA

ENSG00000258016	NA	NA	NA	0	NA	NA	NA	NA	NA
ENSG00000258021	LOC102724178	filamin-B-like	102724178	0	NA	NA	NA	NA	NA
ENSG00000258024	OR5BT1P	olfactory receptor family 5 subfamily BT member 1 pseudogene	282790	0	NA	NA	NA	NA	NA
ENSG00000258027	NA	NA	NA	0	NA	NA	NA	NA	NA
ENSG00000258033	NA	NA	NA	0	NA	NA	NA	NA	NA
ENSG00000258036	NA	NA	NA	0	NA	NA	NA	NA	NA
ENSG00000258051	NA	NA	NA	0	NA	NA	NA	NA	NA
ENSG00000258052	NA	NA	NA	0,524409201	-3,24447917	3,961591	-0,818983792	0,412795661	NA
ENSG00000258064	NA	NA	NA	0,316293691	0	4,080456	0	1	NA
ENSG00000258065	LOC100420424	Vac14, PIKFYVE complex component pseudogene	100420424	0	NA	NA	NA	NA	NA
ENSG00000258071	NA	NA	NA	0	NA	NA	NA	NA	NA
ENSG00000258072	NA	NA	NA	0,400450098	-1,606927037	3,554939	-0,452026627	0,6512498	NA
ENSG00000258073	NA	NA	NA	0	NA	NA	NA	NA	NA
ENSG00000258074	NA	NA	NA	0	NA	NA	NA	NA	NA
ENSG00000258076	LOC100422529	pleckstrin homology domain containing B2 pseudogene	100422529	0	NA	NA	NA	NA	NA
ENSG00000258080	NA	NA	NA	0	NA	NA	NA	NA	NA
ENSG00000258083	OR9A4	olfactory receptor family 9 subfamily A member 4	130075	0	NA	NA	NA	NA	NA
ENSG00000258090	NA	NA	NA	0	NA	NA	NA	NA	NA
ENSG00000258091	NA	NA	NA	0	NA	NA	NA	NA	NA
ENSG00000258102	MAP1LC3B2	microtubule associated protein 1 light chain 3 beta 2	643246	0,208895441	-1,921441346	4,042528	-0,475306867	0,634568265	NA
ENSG00000258104	NA	NA	NA	0	NA	NA	NA	NA	NA
ENSG00000258111	NA	NA	NA	2,154792561	0,350995953	1,600673	0,219280304	0,826431703	NA
ENSG00000258112	CCNG2P1	cyclin G2 pseudogene 1	100128681	0	NA	NA	NA	NA	NA
ENSG00000258116	PPIAP45	peptidylprolyl isomerase A pseudogene 45	100506099	0	NA	NA	NA	NA	NA
ENSG00000258118	PPIAP44	peptidylprolyl isomerase A pseudogene 44	111082964	0	NA	NA	NA	NA	NA
ENSG00000258120	NA	NA	NA	0	NA	NA	NA	NA	NA
ENSG00000258128	NA	NA	NA	0	NA	NA	NA	NA	NA
ENSG00000258130	NA	NA	NA	0	NA	NA	NA	NA	NA
ENSG00000258133	LOC101060021	ADP-ribosylation factor 1-like	101060021	0	NA	NA	NA	NA	NA
ENSG00000258134	LOC100420186	WW domain binding protein 2 pseudogene	100420186	0	NA	NA	NA	NA	NA
ENSG00000258142	NA	NA	NA	0	NA	NA	NA	NA	NA
ENSG00000258148	NA	NA	NA	0	NA	NA	NA	NA	NA
ENSG00000258150	NA	NA	NA	0	NA	NA	NA	NA	NA
ENSG00000258153	NA	NA	NA	0	NA	NA	NA	NA	NA
ENSG00000258154	NA	NA	NA	0	NA	NA	NA	NA	NA
ENSG00000258159	NA	NA	NA	0	NA	NA	NA	NA	NA
ENSG00000258173	NA	NA	NA	0	NA	NA	NA	NA	NA
ENSG00000258179	NA	NA	NA	0	NA	NA	NA	NA	NA
ENSG00000258184	NA	NA	NA	0	NA	NA	NA	NA	NA
ENSG00000258193	NA	NA	NA	0	NA	NA	NA	NA	NA
ENSG00000258202	NA	NA	NA	0	NA	NA	NA	NA	NA
ENSG00000258204	LOC124902995	probable ribosome biogenesis protein RLP24	124902995	0	NA	NA	NA	NA	NA
ENSG00000258205	NA	NA	NA	0	NA	NA	NA	NA	NA
ENSG00000258206	LOC100129589	TM2 domain containing 3 pseudogene	100129589	0	NA	NA	NA	NA	NA
ENSG00000258212	NA	NA	NA	0	NA	NA	NA	NA	NA
ENSG00000258215	NA	NA	NA	0	NA	NA	NA	NA	NA
ENSG00000258223	PRSS58	serine protease 58	136541	0	NA	NA	NA	NA	NA
ENSG00000258224	NA	NA	NA	0	NA	NA	NA	NA	NA
ENSG00000258227	CLEC5A	C-type lectin domain containing 5A	23601	0	NA	NA	NA	NA	NA
ENSG00000258233	NA	NA	NA	0	NA	NA	NA	NA	NA
ENSG00000258239	MTND2P17	MT-ND2 pseudogene 17	100873348	0	NA	NA	NA	NA	NA
ENSG00000258244	NA	NA	NA	0	NA	NA	NA	NA	NA
ENSG00000258245	RPL10P13	ribosomal protein L10 pseudogene 13	390345	0	NA	NA	NA	NA	NA
ENSG00000258251	NA	NA	NA	0	NA	NA	NA	NA	NA
ENSG00000258262	MTCH2P2	MTCH2 pseudogene 2	100132126	0	NA	NA	NA	NA	NA
ENSG00000258271	NA	NA	NA	0	NA	NA	NA	NA	NA
ENSG00000258273	NA	NA	NA	0	NA	NA	NA	NA	NA
ENSG00000258275	OR7K1P	olfactory receptor family 7 subfamily K member 1 pseudogene	81114	0	NA	NA	NA	NA	NA
ENSG00000258278	NA	NA	NA	0	NA	NA	NA	NA	NA
ENSG00000258282	NA	NA	NA	0	NA	NA	NA	NA	NA
ENSG00000258284	NA	NA	NA	0,26324472	-1,076451951	4,080456	-0,263806786	0,791928805	NA
ENSG00000258288	NA	NA	NA	0	NA	NA	NA	NA	NA
ENSG00000258290	NA	NA	NA	0	NA	NA	NA	NA	NA

ENSG00000258300	NA	NA	NA	0	NA	NA	NA	NA	NA
ENSG00000258304	NA	NA	NA	0	NA	NA	NA	NA	NA
ENSG00000258316	NA	NA	NA	0,232784036	0,830366573	4,079809	0,203530747	0,838720216	NA
ENSG00000258320	LOC100128674	SURP and G-patch domain containing 1 pseudogene	100128674	0	NA	NA	NA	NA	NA
ENSG00000258324	NA	NA	NA	0	NA	NA	NA	NA	NA
ENSG00000258336	NA	NA	NA	0	NA	NA	NA	NA	NA
ENSG00000258350	SYF2P1	SYF2 pre-mRNA splicing factor pseudogene 1	317778	0	NA	NA	NA	NA	NA
ENSG00000258352	NA	NA	NA	3,340303962	0,642711816	1,026453	0,626148512	0,531217535	NA
ENSG00000258357	NA	NA	NA	0	NA	NA	NA	NA	NA
ENSG00000258358	NA	NA	NA	0	NA	NA	NA	NA	NA
ENSG00000258359	NA	NA	NA	2,38094141	0,476419685	1,173827	0,405868653	0,684839112	NA
ENSG00000258360	NF1P12	neurofibromin 1 pseudogene 12	100418983	0	NA	NA	NA	NA	NA
ENSG00000258367	NA	NA	NA	0	NA	NA	NA	NA	NA
ENSG00000258368	NA	NA	NA	0	NA	NA	NA	NA	NA
ENSG00000258369	ADI1P3	acireductone dioxygenase 1 pseudogene 3	100288129	0	NA	NA	NA	NA	NA
ENSG00000258373	NA	NA	NA	0	NA	NA	NA	NA	NA
ENSG00000258375	NA	NA	NA	0	NA	NA	NA	NA	NA
ENSG00000258381	NA	NA	NA	0	NA	NA	NA	NA	NA
ENSG00000258385	LOC100533628	WD repeat domain 75 pseudogene	100533628	0	NA	NA	NA	NA	NA
ENSG00000258387	NA	NA	NA	0	NA	NA	NA	NA	NA
ENSG00000258388	PPT2-EGFL8	PPT2-EGFL8 readthrough (NMD candidate)	100532746	0,118286652	0	4,080456	0	1	NA
ENSG00000258392	NA	NA	NA	0	NA	NA	NA	NA	NA
ENSG00000258397	BCAR1P1	BCAR1 pseudogene 1	100533717	0	NA	NA	NA	NA	NA
ENSG00000258401	NA	NA	NA	0	NA	NA	NA	NA	NA
ENSG00000258405	ZNF578	zinc finger protein 578	147660	3,999812649	0,191636696	0,916804	0,209026795	0,834427321	NA
ENSG00000258406	NA	NA	NA	0	NA	NA	NA	NA	NA
ENSG00000258408	NA	NA	NA	0	NA	NA	NA	NA	NA
ENSG00000258415	NA	NA	NA	0	NA	NA	NA	NA	NA
ENSG00000258417	NA	NA	NA	0	NA	NA	NA	NA	NA
ENSG00000258420	NA	NA	NA	0	NA	NA	NA	NA	NA
ENSG00000258421	NA	NA	NA	0	NA	NA	NA	NA	NA
ENSG00000258423	OR7E105P	olfactory receptor family 7 subfamily E member 105 pseudogene	81116	0	NA	NA	NA	NA	NA
ENSG00000258427	NA	NA	NA	0	NA	NA	NA	NA	NA
ENSG00000258429	PDF	peptide deformylase, mitochondrial	64146	11,3179944	0,286102151	0,889973	0,321472712	0,747852188	NA
ENSG00000258431	TMEM183AP4	TMEM183A pseudogene 4	100287335	0	NA	NA	NA	NA	NA
ENSG00000258436	RNASE12	ribonuclease A family member 12 (inactive)	493901	0	NA	NA	NA	NA	NA
ENSG00000258438	OR11K2P	olfactory receptor family 11 subfamily K member 2 pseudogene	401742	0	NA	NA	NA	NA	NA
ENSG00000258439	LOC100419503	ribosomal protein S2 pseudogene	100419503	0	NA	NA	NA	NA	NA
ENSG00000258440	NA	NA	NA	0	NA	NA	NA	NA	NA
ENSG00000258445	NA	NA	NA	1,3294481	-0,170110499	1,971982	-0,086263728	0,931256772	NA
ENSG00000258446	NA	NA	NA	0	NA	NA	NA	NA	NA
ENSG00000258448	LOC100422493	required for meiotic nuclear division 1 homolog (S, cerevisiae) pseudogene	100422493	0	NA	NA	NA	NA	NA
ENSG00000258452	NA	NA	NA	0	NA	NA	NA	NA	NA
ENSG00000258453	OR11H2	olfactory receptor family 11 subfamily H member 2	79334	0	NA	NA	NA	NA	NA
ENSG00000258456	PTCD2P1	pentatricopeptide repeat domain 2 pseudogene 1	100131302	0	NA	NA	NA	NA	NA
ENSG00000258461	NA	NA	NA	6,613371001	-1,006270323	2,745852	-0,366469248	0,714014955	NA
ENSG00000258462	NA	NA	NA	0	NA	NA	NA	NA	NA
ENSG00000258463	NA	NA	NA	0	NA	NA	NA	NA	NA
ENSG00000258465	NA	NA	NA	0	NA	NA	NA	NA	NA
ENSG00000258466	NA	NA	NA	0	NA	NA	NA	NA	NA
ENSG00000258467	NA	NA	NA	0	NA	NA	NA	NA	NA
ENSG00000258469	NA	NA	NA	3,934002874	-0,435253374	1,077451	-0,403965798	0,68623787	NA
ENSG00000258472	NA	NA	NA	0,119673877	0,847101401	4,080456	0,207599697	0,835541541	NA
ENSG00000258477	NA	NA	NA	0	NA	NA	NA	NA	NA
ENSG00000258482	NA	NA	NA	0	NA	NA	NA	NA	NA
ENSG00000258484	SPESP1	sperm equatorial segment protein 1	246777	1,038756385	0,187304668	2,203406	0,085006889	0,932255914	NA
ENSG00000258485	NA	NA	NA	0	NA	NA	NA	NA	NA
ENSG00000258488	LOC100996379	E3 ubiquitin-protein ligase HERC2	100996379	0	NA	NA	NA	NA	NA
ENSG00000258491	NA	NA	NA	0	NA	NA	NA	NA	NA
ENSG00000258493	NA	NA	NA	0	NA	NA	NA	NA	NA

ENSG00000258494	OR11J5P	olfactory receptor family 11 subfamily J member 5 pseudogene	403226	0	NA	NA	NA	NA	NA
ENSG00000258500	NA	NA	NA	0,237753837	-0,114834583	4,080456	-0,028142587	0,977548428	NA
ENSG00000258501	EIF3LP1	eukaryotic translation initiation factor 3 subunit L pseudogene 1	319117	0	NA	NA	NA	NA	NA
ENSG00000258503	NA	NA	NA	0	NA	NA	NA	NA	NA
ENSG00000258505	LOC100420765	leucine rich pentatricopeptide repeat containing pseudogene	100420765	0	NA	NA	NA	NA	NA
ENSG00000258506	NA	NA	NA	0	NA	NA	NA	NA	NA
ENSG00000258509	NA	NA	NA	0	NA	NA	NA	NA	NA
ENSG00000258510	LOC100422225	ATP binding cassette subfamily G member 2 (Junior blood group) pseudogene	100422225	0	NA	NA	NA	NA	NA
ENSG00000258513	NA	NA	NA	0	NA	NA	NA	NA	NA
ENSG00000258519	NA	NA	NA	0	NA	NA	NA	NA	NA
ENSG00000258524	NA	NA	NA	0	NA	NA	NA	NA	NA
ENSG00000258527	NA	NA	NA	0	NA	NA	NA	NA	NA
ENSG00000258529	NA	NA	NA	0	NA	NA	NA	NA	NA
ENSG00000258531	NA	NA	NA	0	NA	NA	NA	NA	NA
ENSG00000258536	NA	NA	NA	0	NA	NA	NA	NA	NA
ENSG00000258539	NA	NA	NA	2,72624888	0,222977034	1,261784	0,176715638	0,859731757	NA
ENSG00000258540	NA	NA	NA	0	NA	NA	NA	NA	NA
ENSG00000258542	NA	NA	NA	0	NA	NA	NA	NA	NA
ENSG00000258544	NA	NA	NA	0	NA	NA	NA	NA	NA
ENSG00000258546	CENPUP2	centromere protein U pseudogene 2	100419930	0	NA	NA	NA	NA	NA
ENSG00000258549	NA	NA	NA	0	NA	NA	NA	NA	NA
ENSG00000258550	OR7E106P	olfactory receptor family 7 subfamily E member 106 pseudogene	81115	0	NA	NA	NA	NA	NA
ENSG00000258552	NA	NA	NA	0	NA	NA	NA	NA	NA
ENSG00000258555	SPECC1L-ADORA2A	SPECC1L-ADORA2A readthrough (NMD candidate)	101730217	7,167089088	6,748821738	3,910986	1,725606371	0,084418252	NA
ENSG00000258557	NA	NA	NA	0	NA	NA	NA	NA	NA
ENSG00000258562	FTLP20	ferritin light chain pseudogene 20	100652792	0	NA	NA	NA	NA	NA
ENSG00000258563	NA	NA	NA	0	NA	NA	NA	NA	NA
ENSG00000258564	OR4N1P	olfactory receptor family 4 subfamily N member 1 pseudogene	79322	0	NA	NA	NA	NA	NA
ENSG00000258565	NA	NA	NA	0	NA	NA	NA	NA	NA
ENSG00000258566	NA	NA	NA	0	NA	NA	NA	NA	NA
ENSG00000258567	DUX4L16	double homeobox 4 like 16 (pseudogene)	728169	0	NA	NA	NA	NA	NA
ENSG00000258568	NA	NA	NA	2,730622527	1,237075403	1,294407	0,955708189	0,339219684	NA
ENSG00000258570	NA	NA	NA	0	NA	NA	NA	NA	NA
ENSG00000258571	NA	NA	NA	0,117497108	0,847101401	4,080456	0,207599697	0,835541541	NA
ENSG00000258577	NA	NA	NA	0	NA	NA	NA	NA	NA
ENSG00000258578	NA	NA	NA	0	NA	NA	NA	NA	NA
ENSG00000258581	NA	NA	NA	0	NA	NA	NA	NA	NA
ENSG00000258585	NA	NA	NA	0	NA	NA	NA	NA	NA
ENSG00000258587	METTL5P1	METTL5 pseudogene 1	100131950	0	NA	NA	NA	NA	NA
ENSG00000258588	TRIM6-TRIM34	TRIM6-TRIM34 readthrough	445372	0	NA	NA	NA	NA	NA
ENSG00000258590	NA	NA	NA	2,548213205	-1,132783073	1,471819	-0,769648361	0,441508509	NA
ENSG00000258591	NA	NA	NA	0	NA	NA	NA	NA	NA
ENSG00000258597	SERPINA2	serpin family A member 2 (gene/pseudogene)	390502	0	NA	NA	NA	NA	NA
ENSG00000258599	NA	NA	NA	0,590871822	1,584867057	3,317101	0,477786774	0,632801976	NA
ENSG00000258605	NA	NA	NA	0	NA	NA	NA	NA	NA
ENSG00000258608	NA	NA	NA	0	NA	NA	NA	NA	NA
ENSG00000258611	NA	NA	NA	0	NA	NA	NA	NA	NA
ENSG00000258613	RPL21P7	ribosomal protein L21 pseudogene 7	145370	0	NA	NA	NA	NA	NA
ENSG00000258615	NA	NA	NA	0	NA	NA	NA	NA	NA
ENSG00000258618	RPL21P9	ribosomal protein L21 pseudogene 9	283562	0	NA	NA	NA	NA	NA
ENSG00000258619	LOC100533787	HERV-FRD provirus ancestral Env polyprotein pseudogene	100533787	0	NA	NA	NA	NA	NA
ENSG00000258622	NA	NA	NA	0	NA	NA	NA	NA	NA
ENSG00000258624	NA	NA	NA	0	NA	NA	NA	NA	NA
ENSG00000258626	NA	NA	NA	0,10543123	0	4,080456	0	1	NA
ENSG00000258627	NA	NA	NA	0	NA	NA	NA	NA	NA
ENSG00000258628	NA	NA	NA	0	NA	NA	NA	NA	NA
ENSG00000258629	NA	NA	NA	0	NA	NA	NA	NA	NA

ENSG00000258632	RPL26P5	ribosomal protein L26 pseudogene 5	654386	1,123768193	0,316291329	1,662667	0,190231347	0,849127849	NA
ENSG00000258638	NA	NA	NA	0	NA	NA	NA	NA	NA
ENSG00000258639	LOC100418768	ataxin 3 pseudogene	100418768	0	NA	NA	NA	NA	NA
ENSG00000258640	RPL21P5	ribosomal protein L21 pseudogene 5	641613	0,28804161	0,823776373	4,079527	0,201929372	0,83997194	NA
ENSG00000258643	BCL2L2-PABPN1	BCL2L2-PABPN1 readthrough	100529063	5,537301999	-1,151386143	1,09851	-1,048134735	0,294576535	NA
ENSG00000258644	SYNJ2BP-COX16	SYNJ2BP-COX16 readthrough	100529257	0	NA	NA	NA	NA	NA
ENSG00000258645	NA	NA	NA	0	NA	NA	NA	NA	NA
ENSG00000258648	NA	NA	NA	0	NA	NA	NA	NA	NA
ENSG00000258650	NA	NA	NA	0	NA	NA	NA	NA	NA
ENSG00000258653	NA	NA	NA	0,591928173	2,529494015	3,269493	0,773665467	0,439128647	NA
ENSG00000258656	NA	NA	NA	0	NA	NA	NA	NA	NA
ENSG00000258665	NA	NA	NA	0	NA	NA	NA	NA	NA
ENSG00000258668	NA	NA	NA	0	NA	NA	NA	NA	NA
ENSG00000258671	NA	NA	NA	0	NA	NA	NA	NA	NA
ENSG00000258674	NA	NA	NA	3,318009523	0,114730419	1,400675	0,081910816	0,934717634	NA
ENSG00000258677	NA	NA	NA	3,366727443	0	2,000844	0	1	NA
ENSG00000258679	NA	NA	NA	0	NA	NA	NA	NA	NA
ENSG00000258680	NA	NA	NA	0	NA	NA	NA	NA	NA
ENSG00000258681	LOC100128373	AKT interacting protein pseudogene	100128373	0	NA	NA	NA	NA	NA
ENSG00000258684	BMS1P16	BMS1 pseudogene 16	727914	0	NA	NA	NA	NA	NA
ENSG00000258685	NA	NA	NA	0	NA	NA	NA	NA	NA
ENSG00000258691	NA	NA	NA	0	NA	NA	NA	NA	NA
ENSG00000258692	NA	NA	NA	0	NA	NA	NA	NA	NA
ENSG00000258704	NA	NA	NA	1,011134872	-2,044434621	2,062765	-0,991113856	0,321629988	NA
ENSG00000258705	LOC100419912	spalt like transcription factor 4 pseudogene	100419912	0	NA	NA	NA	NA	NA
ENSG00000258706	NA	NA	NA	0	NA	NA	NA	NA	NA
ENSG00000258707	NA	NA	NA	0,366976749	0,461928865	3,705314	0,124666581	0,900787515	NA
ENSG00000258712	NA	NA	NA	0	NA	NA	NA	NA	NA
ENSG00000258713	C20orf141	chromosome 20 open reading frame 141	128653	0	NA	NA	NA	NA	NA
ENSG00000258721	OR11H3P	olfactory receptor family 11 subfamily H member 3 pseudogene	81113	0	NA	NA	NA	NA	NA
ENSG00000258722	NA	NA	NA	0	NA	NA	NA	NA	NA
ENSG00000258724	PINX1	PIN2 (TERF1) interacting telomerase inhibitor 1	54984	0,354859955	0	4,080456	0	1	NA
ENSG00000258726	NA	NA	NA	0	NA	NA	NA	NA	NA
ENSG00000258728	NA	NA	NA	0,144330898	1,418786819	4,061262	0,349346267	0,726829368	NA
ENSG00000258732	NA	NA	NA	0	NA	NA	NA	NA	NA
ENSG00000258734	NA	NA	NA	0	NA	NA	NA	NA	NA
ENSG00000258737	NA	NA	NA	0	NA	NA	NA	NA	NA
ENSG00000258739	NA	NA	NA	0	NA	NA	NA	NA	NA
ENSG00000258741	NA	NA	NA	0,266351894	-1,045867184	4,080456	-0,256311358	0,797710428	NA
ENSG00000258746	NA	NA	NA	0	NA	NA	NA	NA	NA
ENSG00000258750	LOC100422641	STT3A, subunit of the oligosaccharyltransferase complex (catalytic) pseudogene	100422641	0	NA	NA	NA	NA	NA
ENSG00000258758	LOC440180	zinc finger CCHC-type containing 7 pseudogene	440180	0	NA	NA	NA	NA	NA
ENSG00000258759	NA	NA	NA	0	NA	NA	NA	NA	NA
ENSG00000258762	MTND4P33	MT-ND4 pseudogene 33	107075253	0	NA	NA	NA	NA	NA
ENSG00000258764	NA	NA	NA	0	NA	NA	NA	NA	NA
ENSG00000258767	NA	NA	NA	0	NA	NA	NA	NA	NA
ENSG00000258769	NA	NA	NA	0	NA	NA	NA	NA	NA
ENSG00000258771	BCAR1P2	BCAR1 pseudogene 2	100533718	0	NA	NA	NA	NA	NA
ENSG00000258780	BMS1P15	BMS1 pseudogene 15	642311	0,103998153	-1,076443944	4,080456	-0,263804824	0,791930317	NA
ENSG00000258781	NA	NA	NA	0	NA	NA	NA	NA	NA
ENSG00000258783	KRT18P6	keratin 18 pseudogene 6	122592	0	NA	NA	NA	NA	NA
ENSG00000258787	SEPHS1P2	selenophosphate synthetase 1 pseudogene 2	100420292	0	NA	NA	NA	NA	NA
ENSG00000258788	NA	NA	NA	0	NA	NA	NA	NA	NA
ENSG00000258794	DUX4L27	double homeobox 4 like 27 (pseudogene)	100131369	0,103998153	-1,076443944	4,080456	-0,263804824	0,791930317	NA
ENSG00000258796	NA	NA	NA	0,119673877	0,847101401	4,080456	0,207599697	0,835541541	NA
ENSG00000258797	NA	NA	NA	0	NA	NA	NA	NA	NA
ENSG00000258799	NA	NA	NA	0	NA	NA	NA	NA	NA
ENSG00000258802	NA	NA	NA	0	NA	NA	NA	NA	NA
ENSG00000258805	NA	NA	NA	0	NA	NA	NA	NA	NA
ENSG00000258806	OR11H7	olfactory receptor family 11 subfamily H member 7 (gene/pseudogene)	390441	0	NA	NA	NA	NA	NA

ENSG00000258812	TRAV33	T cell receptor alpha variable 33 (pseudogene)	28649	0	NA	NA	NA	NA	NA
ENSG00000258816	NA	NA	NA	0	NA	NA	NA	NA	NA
ENSG00000258817	OR4C13	olfactory receptor family 4 subfamily C member 13	283092	0	NA	NA	NA	NA	NA
ENSG00000258818	RNASE4	ribonuclease A family member 4	6038	0,411896448	1,887862132	3,515817	0,536962516	0,591293506	NA
ENSG00000258823	LOC100421646	von Willebrand factor A domain containing 5B2 pseudogene	100421646	0	NA	NA	NA	NA	NA
ENSG00000258828	NA	NA	NA	0	NA	NA	NA	NA	NA
ENSG00000258830	NA	NA	NA	1,592245888	-4,840742833	2,791756	-1,733941717	0,082928425	NA
ENSG00000258834	DUX4L4	double homeobox 4 like 4 (pseudogene)	441056	0	NA	NA	NA	NA	NA
ENSG00000258835	TRAV28	T cell receptor alpha variable 28 (pseudogene)	28654	0	NA	NA	NA	NA	NA
ENSG00000258836	LOC100420347	polycomb group ring finger 3 pseudogene	100420347	0	NA	NA	NA	NA	NA
ENSG00000258839	MC1R	melanocortin 1 receptor	4157	8,38396833	0,117877383	0,704136	0,167407093	0,867049745	NA
ENSG00000258841	EEF1A1P2	eukaryotic translation elongation factor 1 alpha 1 pseudogene 2	319112	0	NA	NA	NA	NA	NA
ENSG00000258846	NA	NA	NA	0	NA	NA	NA	NA	NA
ENSG00000258848	NA	NA	NA	0	NA	NA	NA	NA	NA
ENSG00000258849	OR7E159P	olfactory receptor family 7 subfamily E member 159 pseudogene	403299	0	NA	NA	NA	NA	NA
ENSG00000258855	OR11J2P	olfactory receptor family 11 subfamily J member 2 pseudogene	81110	0	NA	NA	NA	NA	NA
ENSG00000258856	NA	NA	NA	0	NA	NA	NA	NA	NA
ENSG00000258863	NA	NA	NA	0	NA	NA	NA	NA	NA
ENSG00000258864	NA	NA	NA	0	NA	NA	NA	NA	NA
ENSG00000258870	NA	NA	NA	0	NA	NA	NA	NA	NA
ENSG00000258872	FDPSP3	farnesyl diphosphate synthase pseudogene 3	2227	3,169537892	1,238752513	1,138349	1,088201439	0,276506189	NA
ENSG00000258873	DUXA	double homeobox A	503835	0	NA	NA	NA	NA	NA
ENSG00000258877	ATP5F1AP4	ATP synthase F1 subunit alpha pseudogene 4	100129782	0	NA	NA	NA	NA	NA
ENSG00000258878	LOC100420096	IGF like family receptor 1 pseudogene	100420096	0,117497108	0,847101401	4,080456	0,207599697	0,835541541	NA
ENSG00000258881	NA	NA	NA	0,072165449	0,847101401	4,080456	0,207599697	0,835541541	NA
ENSG00000258883	NBEAP4	neurobeachin pseudogene 4	100418897	0	NA	NA	NA	NA	NA
ENSG00000258886	NA	NA	NA	0	NA	NA	NA	NA	NA
ENSG00000258893	NA	NA	NA	0	NA	NA	NA	NA	NA
ENSG00000258894	NA	NA	NA	0	NA	NA	NA	NA	NA
ENSG00000258895	NA	NA	NA	0	NA	NA	NA	NA	NA
ENSG00000258896	NA	NA	NA	1,096636465	0,368230886	1,773709	0,207604967	0,835537426	NA
ENSG00000258900	NA	NA	NA	2,857813495	-0,129989641	1,703961	-0,076286767	0,939190953	NA
ENSG00000258901	NA	NA	NA	0	NA	NA	NA	NA	NA
ENSG00000258905	TRAV32	T cell receptor alpha variable 32 (pseudogene)	28650	0	NA	NA	NA	NA	NA
ENSG00000258906	LOC100129923	myoneurin pseudogene	100129923	0	NA	NA	NA	NA	NA
ENSG00000258907	PSMB3P1	proteasome subunit beta 3 pseudogene 1	121131	0	NA	NA	NA	NA	NA
ENSG00000258915	NA	NA	NA	0	NA	NA	NA	NA	NA
ENSG00000258916	NA	NA	NA	0	NA	NA	NA	NA	NA
ENSG00000258917	NA	NA	NA	0	NA	NA	NA	NA	NA
ENSG00000258923	NA	NA	NA	0	NA	NA	NA	NA	NA
ENSG00000258925	NA	NA	NA	0	NA	NA	NA	NA	NA
ENSG00000258930	NA	NA	NA	0	NA	NA	NA	NA	NA
ENSG00000258932	MIR3171HG	MIR3171 host gene	728755	0	NA	NA	NA	NA	NA
ENSG00000258934	NA	NA	NA	0	NA	NA	NA	NA	NA
ENSG00000258947	TUBB3	tubulin beta 3 class III	10381	0	NA	NA	NA	NA	NA
ENSG00000258948	NA	NA	NA	0	NA	NA	NA	NA	NA
ENSG00000258951	KRT18P7	keratin 18 pseudogene 7	100418877	0	NA	NA	NA	NA	NA
ENSG00000258956	NA	NA	NA	0	NA	NA	NA	NA	NA
ENSG00000258959	NA	NA	NA	0	NA	NA	NA	NA	NA
ENSG00000258960	NA	NA	NA	0	NA	NA	NA	NA	NA
ENSG00000258963	NA	NA	NA	0	NA	NA	NA	NA	NA
ENSG00000258965	NA	NA	NA	0	NA	NA	NA	NA	NA
ENSG00000258966	GTF3AP2	general transcription factor IIIA pseudogene 2	106480682	0	NA	NA	NA	NA	NA
ENSG00000258967	NA	NA	NA	0	NA	NA	NA	NA	NA
ENSG00000258968	NA	NA	NA	0,117497108	0,847101401	4,080456	0,207599697	0,835541541	NA
ENSG00000258971	NA	NA	NA	0	NA	NA	NA	NA	NA
ENSG00000258972	NDUFB8P1	NADH:ubiquinone oxidoreductase subunit B8 pseudogene 1	326606	0	NA	NA	NA	NA	NA

ENSG00000258978	HIF1AP1	HIF1AP pseudogene 1	100874523	0,118286652	0	4,080456	0	1	NA
ENSG00000258980	EIF1AXP2	EIF1AX pseudogene 2	106479036	0	NA	NA	NA	NA	NA
ENSG00000258981	NA	NA	NA	0	NA	NA	NA	NA	NA
ENSG00000258984	UBE2F-SCLY	UBE2F-SCLY readthrough (NMD candidate)	100533179	0,915559777	-2,677332267	2,553064	-1,048674106	0,294328136	NA
ENSG00000258986	TMEM179	transmembrane protein 179	388021	0	NA	NA	NA	NA	NA
ENSG00000258988	SF3B4P1	splicing factor 3b, subunit 4 pseudogene 1	326276	0,117497108	0,847101401	4,080456	0,207599697	0,835541541	NA
ENSG00000258989	NA	NA	NA	1,351952232	0,707423888	1,878971	0,376495281	0,706548723	NA
ENSG00000258990	NA	NA	NA	0	NA	NA	NA	NA	NA
ENSG00000258991	DUX4L19	double homeobox 4 like 19 (pseudogene)	643034	0	NA	NA	NA	NA	NA
ENSG00000258992	TSPY1	testis specific protein Y-linked 1	7258	0	NA	NA	NA	NA	NA
ENSG00000258992	LOC124905614	testis-specific Y-encoded protein 3	124905614	0	NA	NA	NA	NA	NA
ENSG00000258992	LOC124905615	testis-specific Y-encoded protein 3	124905615	0	NA	NA	NA	NA	NA
ENSG00000258992	LOC124905616	testis-specific Y-encoded protein 3	124905616	0	NA	NA	NA	NA	NA
ENSG00000258992	LOC124905617	testis-specific Y-encoded protein 3	124905617	0	NA	NA	NA	NA	NA
ENSG00000258992	LOC124905618	testis-specific Y-encoded protein 3-like	124905618	0	NA	NA	NA	NA	NA
ENSG00000258992	LOC124905619	testis-specific Y-encoded protein 3-like	124905619	0	NA	NA	NA	NA	NA
ENSG00000258992	LOC124905620	testis-specific Y-encoded protein 3	124905620	0	NA	NA	NA	NA	NA
ENSG00000258992	LOC124905621	testis-specific Y-encoded protein 3	124905621	0	NA	NA	NA	NA	NA
ENSG00000258992	LOC124905623	testis-specific Y-encoded protein 1	124905623	0	NA	NA	NA	NA	NA
ENSG00000258992	LOC124905624	testis-specific Y-encoded protein 3	124905624	0	NA	NA	NA	NA	NA
ENSG00000258992	LOC124905625	testis-specific Y-encoded protein 3	124905625	0	NA	NA	NA	NA	NA
ENSG00000258992	LOC124905626	testis-specific Y-encoded protein 2	124905626	0	NA	NA	NA	NA	NA
ENSG00000258992	LOC124905627	testis-specific Y-encoded protein 4-like	124905627	0	NA	NA	NA	NA	NA
ENSG00000258992	LOC124905628	testis-specific Y-encoded protein 3	124905628	0	NA	NA	NA	NA	NA
ENSG00000258992	LOC124905629	testis-specific Y-encoded protein 3-like	124905629	0	NA	NA	NA	NA	NA
ENSG00000258992	LOC124908978	testis-specific Y-encoded protein 3	124908978	0	NA	NA	NA	NA	NA
ENSG00000258992	LOC124908979	testis-specific Y-encoded protein 3	124908979	0	NA	NA	NA	NA	NA
ENSG00000258992	LOC124908980	testis-specific Y-encoded protein 3	124908980	0	NA	NA	NA	NA	NA
ENSG00000258992	LOC124908981	testis-specific Y-encoded protein 3	124908981	0	NA	NA	NA	NA	NA
ENSG00000258992	LOC124908988	testis-specific Y-encoded protein 8	124908988	0	NA	NA	NA	NA	NA
ENSG00000258992	LOC124909015	testis-specific Y-encoded protein 1	124909015	0	NA	NA	NA	NA	NA
ENSG00000258992	LOC124909084	testis-specific Y-encoded protein 3-like	124909084	0	NA	NA	NA	NA	NA
ENSG00000258992	LOC124909294	testis-specific Y-encoded protein 3	124909294	0	NA	NA	NA	NA	NA
ENSG00000258992	LOC124909306	testis-specific Y-encoded protein 3-like	124909306	0	NA	NA	NA	NA	NA
ENSG00000258992	LOC124909318	testis-specific Y-encoded protein 3-like	124909318	0	NA	NA	NA	NA	NA
ENSG00000258992	LOC124909320	testis-specific Y-encoded protein 3-like	124909320	0	NA	NA	NA	NA	NA
ENSG00000258992	LOC124909330	testis-specific Y-encoded protein 1-like	124909330	0	NA	NA	NA	NA	NA
ENSG00000258993	NA	NA	NA	0	NA	NA	NA	NA	NA
ENSG00000258995	NA	NA	NA	0	NA	NA	NA	NA	NA
ENSG00000258996	NA	NA	NA	0	NA	NA	NA	NA	NA
ENSG00000258997	NA	NA	NA	0	NA	NA	NA	NA	NA
ENSG00000259000	DOCK11P1	dedicator of cytokinesis 11 pseudogene 1	100132537	0	NA	NA	NA	NA	NA
ENSG00000259009	TPRX2	tetrapeptide repeat homeobox 2	503627	0	NA	NA	NA	NA	NA
ENSG00000259010	NA	NA	NA	0,103998153	-1,076443944	4,080456	-0,263804824	0,791930317	NA
ENSG00000259012	MTCYBP27	MT-CYB pseudogene 27	107075133	0	NA	NA	NA	NA	NA
ENSG00000259013	NA	NA	NA	0	NA	NA	NA	NA	NA
ENSG00000259015	NA	NA	NA	0	NA	NA	NA	NA	NA

ENSG00000259019	NA	NA	NA	0	NA	NA	NA	NA	NA
ENSG00000259020	NA	NA	NA	0	NA	NA	NA	NA	NA
ENSG00000259021	NA	NA	NA	0	NA	NA	NA	NA	NA
ENSG00000259022	NA	NA	NA	0	NA	NA	NA	NA	NA
ENSG00000259025	LOC283804	disintegrin and metalloproteinase domain-containing protein 21-like	283804	0	NA	NA	NA	NA	NA
ENSG00000259029	DUX4L18	double homeobox 4 like 18 (pseudogene)	100132421	0	NA	NA	NA	NA	NA
ENSG00000259030	FPGT-TNNI3K	FPGT-TNNI3K readthrough	100526835	1,051074298	0,712289935	1,962172	0,363011007	0,716596662	NA
ENSG00000259032	NA	NA	NA	0	NA	NA	NA	NA	NA
ENSG00000259040	BLOC1S5-TXNDC5	BLOC1S5-TXNDC5 readthrough (NMD candidate)	100526836	0	NA	NA	NA	NA	NA
ENSG00000259045	NA	NA	NA	0	NA	NA	NA	NA	NA
ENSG00000259046	RRAGAP1	Ras related GTP binding A pseudogene 1	100462776	0	NA	NA	NA	NA	NA
ENSG00000259047	NA	NA	NA	0	NA	NA	NA	NA	NA
ENSG00000259050	NA	NA	NA	0	NA	NA	NA	NA	NA
ENSG00000259051	NA	NA	NA	0	NA	NA	NA	NA	NA
ENSG00000259056	DUXAP1	double homeobox A pseudogene 1	503630	0	NA	NA	NA	NA	NA
ENSG00000259057	THRAP3P2	THRAP3 pseudogene 2	100421632	0	NA	NA	NA	NA	NA
ENSG00000259059	NA	NA	NA	0	NA	NA	NA	NA	NA
ENSG00000259060	NA	NA	NA	0	NA	NA	NA	NA	NA
ENSG00000259064	NA	NA	NA	0	NA	NA	NA	NA	NA
ENSG00000259068	NA	NA	NA	0	NA	NA	NA	NA	NA
ENSG00000259078	NA	NA	NA	0	NA	NA	NA	NA	NA
ENSG00000259079	NA	NA	NA	0	NA	NA	NA	NA	NA
ENSG00000259086	NA	NA	NA	0	NA	NA	NA	NA	NA
ENSG00000259089	GRAMD4P5	GRAM domain containing 4 pseudogene 5	100631249	0	NA	NA	NA	NA	NA
ENSG00000259090	SEPTIN7P1	septin 7 pseudogene 1	317774	0	NA	NA	NA	NA	NA
ENSG00000259092	TRAV30	T cell receptor alpha variable 30	28652	0	NA	NA	NA	NA	NA
ENSG00000259096	NA	NA	NA	0	NA	NA	NA	NA	NA
ENSG00000259098	NA	NA	NA	0	NA	NA	NA	NA	NA
ENSG00000259099	NA	NA	NA	0	NA	NA	NA	NA	NA
ENSG00000259100	NA	NA	NA	0,436748839	1,516509891	3,442627	0,44050944	0,659568177	NA
ENSG00000259102	SMARCE1P3	SWI/SNF related, matrix associated, actin dependent regulator of chromatin, subfamily e, member 1 pseudogene 3	401744	0	NA	NA	NA	NA	NA
ENSG00000259105	RPS3AP4	RPS3A pseudogene 4	100132142	0	NA	NA	NA	NA	NA
ENSG00000259109	NA	NA	NA	0,317061319	-1,066839104	4,080456	-0,26145096	0,793744763	NA
ENSG00000259112	NDUFC2-KCTD14	NDUFC2-KCTD14 readthrough	100532726	0,953046409	0,119149648	3,000695	0,039707344	0,968326447	NA
ENSG00000259117	NA	NA	NA	0	NA	NA	NA	NA	NA
ENSG00000259120	SMIM6	small integral membrane protein 6	100130933	0	NA	NA	NA	NA	NA
ENSG00000259121	NA	NA	NA	0	NA	NA	NA	NA	NA
ENSG00000259122	NA	NA	NA	0	NA	NA	NA	NA	NA
ENSG00000259132	NA	NA	NA	2,632825299	5,450269376	3,207114	1,699430919	0,08923802	NA
ENSG00000259136	NA	NA	NA	0	NA	NA	NA	NA	NA
ENSG00000259137	NA	NA	NA	0	NA	NA	NA	NA	NA
ENSG00000259140	NA	NA	NA	0	NA	NA	NA	NA	NA
ENSG00000259144	NA	NA	NA	0	NA	NA	NA	NA	NA
ENSG00000259148	NA	NA	NA	0	NA	NA	NA	NA	NA
ENSG00000259151	CAP2P1	cyclase associated actin cytoskeleton regulatory protein 2 pseudogene 1	353163	6,746910306	0,176737644	0,667835	0,264642565	0,791284826	NA
ENSG00000259154	DUX4L17	double homeobox 4 like 17 (pseudogene)	643001	0	NA	NA	NA	NA	NA
ENSG00000259155	NA	NA	NA	0	NA	NA	NA	NA	NA
ENSG00000259156	NA	NA	NA	0	NA	NA	NA	NA	NA
ENSG00000259157	NA	NA	NA	0	NA	NA	NA	NA	NA
ENSG00000259158	NA	NA	NA	1,855722041	0,885329436	1,492067	0,593357877	0,552941673	NA
ENSG00000259160	NA	NA	NA	0	NA	NA	NA	NA	NA
ENSG00000259161	NA	NA	NA	0	NA	NA	NA	NA	NA
ENSG00000259164	NA	NA	NA	0	NA	NA	NA	NA	NA
ENSG00000259165	DDX18P1	DEAD-box helicase 18 pseudogene 1	319099	0	NA	NA	NA	NA	NA
ENSG00000259167	NA	NA	NA	0	NA	NA	NA	NA	NA
ENSG00000259169	GNRHR2P1	GNRHR2 pseudogene 1	404718	0	NA	NA	NA	NA	NA
ENSG00000259171	NA	NA	NA	0	NA	NA	NA	NA	NA
ENSG00000259173	LOC100289091	ATP synthase membrane subunit g pseudogene	100289091	0	NA	NA	NA	NA	NA
ENSG00000259179	NA	NA	NA	0,389365776	-1,579800214	3,577179	-0,441632953	0,658754834	NA

ENSG00000259183	MED28P6	mediator complex subunit 28 pseudogene 6	106480808	0	NA	NA	NA	NA	NA
ENSG00000259186	MRPS15P1	mitochondrial ribosomal protein S15 pseudogene 1	359755	0,072165449	0,847101401	4,080456	0,207599697	0,835541541	NA
ENSG00000259191	NA	NA	NA	0	NA	NA	NA	NA	NA
ENSG00000259192	NA	NA	NA	0,355260041	1,002709967	3,722479	0,269366154	0,787647929	NA
ENSG00000259195	NA	NA	NA	0	NA	NA	NA	NA	NA
ENSG00000259207	ITGB3	integrin subunit beta 3	3690	0,539071267	-0,530522267	3,361828	-0,157807665	0,874608359	NA
ENSG00000259208	NA	NA	NA	0	NA	NA	NA	NA	NA
ENSG00000259214	NA	NA	NA	0	NA	NA	NA	NA	NA
ENSG00000259216	LOC645405	oxidation resistance 1 pseudogene	645405	0	NA	NA	NA	NA	NA
ENSG00000259217	NA	NA	NA	0	NA	NA	NA	NA	NA
ENSG00000259224	SLC35G6	solute carrier family 35 member G6	643664	0,118286652	0	4,080456	0	1	NA
ENSG00000259227	NA	NA	NA	0	NA	NA	NA	NA	NA
ENSG00000259228	NA	NA	NA	0	NA	NA	NA	NA	NA
ENSG00000259229	NA	NA	NA	0	NA	NA	NA	NA	NA
ENSG00000259231	NA	NA	NA	0	NA	NA	NA	NA	NA
ENSG00000259232	NA	NA	NA	1,171709957	0,926809487	1,663037	0,557299322	0,577322939	NA
ENSG00000259236	NA	NA	NA	0	NA	NA	NA	NA	NA
ENSG00000259239	NA	NA	NA	0	NA	NA	NA	NA	NA
ENSG00000259246	NA	NA	NA	0	NA	NA	NA	NA	NA
ENSG00000259247	NA	NA	NA	0	NA	NA	NA	NA	NA
ENSG00000259257	NA	NA	NA	0,447560629	-0,98281932	3,527957	-0,278580337	0,780566904	NA
ENSG00000259258	NA	NA	NA	0	NA	NA	NA	NA	NA
ENSG00000259259	NA	NA	NA	0	NA	NA	NA	NA	NA
ENSG00000259261	NA	NA	NA	0	NA	NA	NA	NA	NA
ENSG00000259262	NDUFA3P4	NADH:ubiquinone oxidoreductase subunit A3 pseudogene 4	106480806	0	NA	NA	NA	NA	NA
ENSG00000259266	NA	NA	NA	0	NA	NA	NA	NA	NA
ENSG00000259270	NA	NA	NA	0	NA	NA	NA	NA	NA
ENSG00000259271	ANKRD62P1	ankyrin repeat domain 62 pseudogene 1	107080639	0,10488184	-1,076443944	4,080456	-0,263804824	0,791930317	NA
ENSG00000259273	NA	NA	NA	0	NA	NA	NA	NA	NA
ENSG00000259286	NA	NA	NA	0,449271642	0,837435553	3,506369	0,238832685	0,811235325	NA
ENSG00000259288	NA	NA	NA	0	NA	NA	NA	NA	NA
ENSG00000259295	CSPG4P12	chondroitin sulfate proteoglycan 4 pseudogene 12	728121	0	NA	NA	NA	NA	NA
ENSG00000259299	LOC100420707	DDB1 and CUL4 associated factor 6 pseudogene	100420707	0,11808024	-1,076443944	4,080456	-0,263804824	0,791930317	NA
ENSG00000259300	NA	NA	NA	0	NA	NA	NA	NA	NA
ENSG00000259301	NA	NA	NA	0	NA	NA	NA	NA	NA
ENSG00000259303	IGHV2OR16-5	immunoglobulin heavy variable 2/OR16-5 (non-functional)	100129631	0	NA	NA	NA	NA	NA
ENSG00000259304	NA	NA	NA	0	NA	NA	NA	NA	NA
ENSG00000259305	ZHX1-C8orf76	ZHX1-C8orf76 readthrough	100533106	6,280420252	0,434252234	1,069069	0,406196696	0,684598082	NA
ENSG00000259308	NA	NA	NA	0	NA	NA	NA	NA	NA
ENSG00000259315	ACTG1P17	actin gamma 1 pseudogene 17	283693	1,161596199	-0,527725637	2,243482	-0,23522614	0,814033219	NA
ENSG00000259316	NA	NA	NA	2,513151422	5,38306411	3,241852	1,660490399	0,096815838	NA
ENSG00000259317	NA	NA	NA	0	NA	NA	NA	NA	NA
ENSG00000259318	NA	NA	NA	1,783941174	-0,778483761	1,313496	-0,592680539	0,553394968	NA
ENSG00000259324	OR11K1BP	olfactory receptor family 11 subfamily K member 1B pseudogene	118568805	0	NA	NA	NA	NA	NA
ENSG00000259333	NA	NA	NA	0	NA	NA	NA	NA	NA
ENSG00000259335	HNRNPMP1	heterogeneous nuclear ribonucleoprotein M pseudogene 1	645204	0,262564091	1,417076457	4,061225	0,348928301	0,727143138	NA
ENSG00000259337	NA	NA	NA	0	NA	NA	NA	NA	NA
ENSG00000259338	NA	NA	NA	0	NA	NA	NA	NA	NA
ENSG00000259339	NA	NA	NA	0	NA	NA	NA	NA	NA
ENSG00000259340	NA	NA	NA	0	NA	NA	NA	NA	NA
ENSG00000259346	NA	NA	NA	0	NA	NA	NA	NA	NA
ENSG00000259350	NA	NA	NA	0	NA	NA	NA	NA	NA
ENSG00000259358	NA	NA	NA	0,119673877	0,847101401	4,080456	0,207599697	0,835541541	NA
ENSG00000259369	NA	NA	NA	0	NA	NA	NA	NA	NA
ENSG00000259374	NDUFB4P11	NADH:ubiquinone oxidoreductase subunit B4 pseudogene 11	107075247	0	NA	NA	NA	NA	NA
ENSG00000259378	NA	NA	NA	2,604600287	0,337554679	1,292703	0,261123056	0,793997612	NA
ENSG00000259379	MTND5P32	MT-ND5 pseudogene 32	107075254	0	NA	NA	NA	NA	NA
ENSG00000259381	NA	NA	NA	0	NA	NA	NA	NA	NA
ENSG00000259384	GH1	growth hormone 1	2688	0	NA	NA	NA	NA	NA

ENSG00000259388	RLIMP3	ring finger protein, LIM domain interacting pseudogene 3	645453	0	NA	NA	NA	NA	NA
ENSG00000259389	NA	NA	NA	0	NA	NA	NA	NA	NA
ENSG00000259393	NA	NA	NA	0	NA	NA	NA	NA	NA
ENSG00000259394	LOC100287243	Y-box-binding protein 1-like	100287243	0	NA	NA	NA	NA	NA
ENSG00000259397	NA	NA	NA	0	NA	NA	NA	NA	NA
ENSG00000259399	TGIF2-RAB5IF	TGIF2-RAB5IF readthrough	100527943	0	NA	NA	NA	NA	NA
ENSG00000259405	NA	NA	NA	0	NA	NA	NA	NA	NA
ENSG00000259411	HNRNPA1P45	heterogeneous nuclear ribonucleoprotein A1 pseudogene 45	100130660	0	NA	NA	NA	NA	NA
ENSG00000259413	NA	NA	NA	0	NA	NA	NA	NA	NA
ENSG00000259414	NA	NA	NA	0	NA	NA	NA	NA	NA
ENSG00000259417	CTXND1	cortexin domain containing 1	100996492	0	NA	NA	NA	NA	NA
ENSG00000259419	HNRNPCP3	heterogeneous nuclear ribonucleoprotein C pseudogene 3	646956	0	NA	NA	NA	NA	NA
ENSG00000259421	NA	NA	NA	0,248846518	1,397769039	4,060806	0,344209733	0,730688563	NA
ENSG00000259425	NA	NA	NA	0	NA	NA	NA	NA	NA
ENSG00000259427	DPPA5P2	developmental pluripotency associated 5 pseudogene 2	100874380	0	NA	NA	NA	NA	NA
ENSG00000259428	HMGB3P26	high mobility group box 3 pseudogene 26	100130405	0	NA	NA	NA	NA	NA
ENSG00000259433	NA	NA	NA	0	NA	NA	NA	NA	NA
ENSG00000259435	OR4N3P	olfactory receptor family 4 subfamily N member 3 pseudogene	390539	0	NA	NA	NA	NA	NA
ENSG00000259440	NA	NA	NA	0	NA	NA	NA	NA	NA
ENSG00000259441	NA	NA	NA	0	NA	NA	NA	NA	NA
ENSG00000259449	NIFKP8	nucleolar protein interacting with the FHA domain of MKI67 pseudogene 8	642907	0	NA	NA	NA	NA	NA
ENSG00000259452	NA	NA	NA	0	NA	NA	NA	NA	NA
ENSG00000259454	WBP1LP5	WBP1L pseudogene 5	106480780	0	NA	NA	NA	NA	NA
ENSG00000259461	NA	NA	NA	0	NA	NA	NA	NA	NA
ENSG00000259464	NA	NA	NA	0	NA	NA	NA	NA	NA
ENSG00000259465	AHCYP7	adenosylhomocysteinase pseudogene 7	645486	0,237162238	1,955800444	4,022315	0,486237462	0,626798814	NA
ENSG00000259466	NA	NA	NA	0	NA	NA	NA	NA	NA
ENSG00000259467	NA	NA	NA	0	NA	NA	NA	NA	NA
ENSG00000259470	KRT8P9	keratin 8 pseudogene 9	390601	0	NA	NA	NA	NA	NA
ENSG00000259477	NA	NA	NA	0	NA	NA	NA	NA	NA
ENSG00000259479	NA	NA	NA	0	NA	NA	NA	NA	NA
ENSG00000259489	NA	NA	NA	0	NA	NA	NA	NA	NA
ENSG00000259490	NA	NA	NA	0	NA	NA	NA	NA	NA
ENSG00000259493	NA	NA	NA	0,210862461	0	4,080456	0	1	NA
ENSG00000259496	NA	NA	NA	0	NA	NA	NA	NA	NA
ENSG00000259500	NA	NA	NA	0	NA	NA	NA	NA	NA
ENSG00000259502	LOC100419668	protein kinase C eta pseudogene	100419668	0,239347754	1,967469188	4,021404	0,489249276	0,624665227	NA
ENSG00000259505	NA	NA	NA	0	NA	NA	NA	NA	NA
ENSG00000259507	LOC100421087	zinc finger CCHC-type containing 9 pseudogene	100421087	0	NA	NA	NA	NA	NA
ENSG00000259509	LOC112268162	cytochrome b5 type B-like	112268162	0	NA	NA	NA	NA	NA
ENSG00000259512	NA	NA	NA	1,039213533	-0,451036977	2,235512	-0,201760042	0,840104321	NA
ENSG00000259513	NA	NA	NA	0,209510168	-1,065986862	4,080456	-0,2612421	0,793905814	NA
ENSG00000259516	ANP32AP1	acidic nuclear phosphoprotein 32 family member A pseudogene 1	723972	0	NA	NA	NA	NA	NA
ENSG00000259525	GCSHP2	GCSH pseudogene 2	100288298	0	NA	NA	NA	NA	NA
ENSG00000259531	NA	NA	NA	0	NA	NA	NA	NA	NA
ENSG00000259533	NA	NA	NA	0	NA	NA	NA	NA	NA
ENSG00000259535	RPL21P12	ribosomal protein L21 pseudogene 12	122409	0	NA	NA	NA	NA	NA
ENSG00000259538	UBE2Q2P11	UBE2Q2 pseudogene 11	100421767	0	NA	NA	NA	NA	NA
ENSG00000259545	NA	NA	NA	0	NA	NA	NA	NA	NA
ENSG00000259547	CYCSP2	CYCS pseudogene 2	360183	0	NA	NA	NA	NA	NA
ENSG00000259550	NA	NA	NA	0	NA	NA	NA	NA	NA
ENSG00000259556	NA	NA	NA	0,28437345	-0,999105994	4,040272	-0,247286794	0,804686278	NA
ENSG00000259557	NA	NA	NA	0	NA	NA	NA	NA	NA
ENSG00000259558	NA	NA	NA	0	NA	NA	NA	NA	NA
ENSG00000259562	NA	NA	NA	2,929759997	1,467300587	1,258703	1,165723808	0,243726131	NA
ENSG00000259565	NA	NA	NA	0	NA	NA	NA	NA	NA
ENSG00000259569	NA	NA	NA	0	NA	NA	NA	NA	NA
ENSG00000259570	NA	NA	NA	0,265174183	0	4,080456	0	1	NA

ENSG00000259571	BLID	BH3-like motif containing, cell death inducer	414899	0	NA	NA	NA	NA	NA
ENSG00000259573	NA	NA	NA	0	NA	NA	NA	NA	NA
ENSG00000259581	NA	NA	NA	11,49680055	1,045291387	0,681276	1,534315011	0,124952175	NA
ENSG00000259585	NA	NA	NA	0	NA	NA	NA	NA	NA
ENSG00000259586	NA	NA	NA	0	NA	NA	NA	NA	NA
ENSG00000259592	NA	NA	NA	0	NA	NA	NA	NA	NA
ENSG00000259593	NA	NA	NA	0	NA	NA	NA	NA	NA
ENSG00000259600	MTND4LP23	MT-ND4L pseudogene 23	107133487	0	NA	NA	NA	NA	NA
ENSG00000259601	DDX18P2	DEAD-box helicase 18 pseudogene 2	651053	0,10488184	-1,076443944	4,080456	-0,263804824	0,791930317	NA
ENSG00000259608	NA	NA	NA	0	NA	NA	NA	NA	NA
ENSG00000259612	EEF1A1P22	eukaryotic translation elongation factor 1 alpha 1 pseudogene 22	645693	0	NA	NA	NA	NA	NA
ENSG00000259614	TUBAP12	tubulin alpha pseudogene 12	100289303	0	NA	NA	NA	NA	NA
ENSG00000259619	NA	NA	NA	0	NA	NA	NA	NA	NA
ENSG00000259622	LOC100288241	protein (peptidylprolyl cis/trans isomerase) NIMA-interacting, 4 (parvulin) pseudogene	100288241	0	NA	NA	NA	NA	NA
ENSG00000259626	MTND3P12	MT-ND3 pseudogene 12	107075160	0	NA	NA	NA	NA	NA
ENSG00000259630	NA	NA	NA	0,296467223	0,832178217	3,915577	0,212530172	0,831693442	NA
ENSG00000259632	NA	NA	NA	0	NA	NA	NA	NA	NA
ENSG00000259648	NA	NA	NA	0	NA	NA	NA	NA	NA
ENSG00000259651	MTCO3P23	MT-CO3 pseudogene 23	107075182	0	NA	NA	NA	NA	NA
ENSG00000259655	NA	NA	NA	0	NA	NA	NA	NA	NA
ENSG00000259657	NA	NA	NA	0	NA	NA	NA	NA	NA
ENSG00000259658	NA	NA	NA	0	NA	NA	NA	NA	NA
ENSG00000259665	NA	NA	NA	0	NA	NA	NA	NA	NA
ENSG00000259671	MTCYBP23	MT-CYB pseudogene 23	107075131	0	NA	NA	NA	NA	NA
ENSG00000259674	RPL7AP75	ribosomal protein L7a pseudogene 75	100132644	0,796646292	1,417810237	2,575385	0,550523517	0,581960352	NA
ENSG00000259677	NA	NA	NA	0	NA	NA	NA	NA	NA
ENSG00000259680	IGHV3OR16-17	immunoglobulin heavy variable 3/OR16-17 (non-functional)	390714	0	NA	NA	NA	NA	NA
ENSG00000259683	NA	NA	NA	0	NA	NA	NA	NA	NA
ENSG00000259686	NA	NA	NA	0	NA	NA	NA	NA	NA
ENSG00000259688	NA	NA	NA	0	NA	NA	NA	NA	NA
ENSG00000259691	NA	NA	NA	0	NA	NA	NA	NA	NA
ENSG00000259695	NA	NA	NA	0,117497108	0,847101401	4,080456	0,207599697	0,835541541	NA
ENSG00000259696	LOC400347	REX4 homolog, 3'-5' exonuclease pseudogene	400347	0,763780207	0,064109684	2,739257	0,023404042	0,981327981	NA
ENSG00000259698	NA	NA	NA	0	NA	NA	NA	NA	NA
ENSG00000259699	NA	NA	NA	0,072165449	0,847101401	4,080456	0,207599697	0,835541541	NA
ENSG00000259706	HSP90B2P	heat shock protein 90 beta family member 2, pseudogene	7190	3,326285422	0,997735766	0,997719	1,000016327	0,317302606	NA
ENSG00000259707	NA	NA	NA	0	NA	NA	NA	NA	NA
ENSG00000259710	NUTF2P6	nuclear transport factor 2 pseudogene 6	100131623	0	NA	NA	NA	NA	NA
ENSG00000259722	NA	NA	NA	0	NA	NA	NA	NA	NA
ENSG00000259726	NA	NA	NA	0	NA	NA	NA	NA	NA
ENSG00000259738	NA	NA	NA	0	NA	NA	NA	NA	NA
ENSG00000259742	NA	NA	NA	0	NA	NA	NA	NA	NA
ENSG00000259743	LOC100419366	phosphodiesterase 8B pseudogene	100419366	0	NA	NA	NA	NA	NA
ENSG00000259746	NA	NA	NA	0,709112475	0,141942788	2,507989	0,056596256	0,954866817	NA
ENSG00000259749	NCAPGP2	non-SMC condensin I complex subunit G pseudogene 2	100421148	0	NA	NA	NA	NA	NA
ENSG00000259750	NA	NA	NA	0	NA	NA	NA	NA	NA
ENSG00000259751	TUBAP11	tubulin alpha pseudogene 11	100132824	0,872216124	0,314318567	2,203084	0,142672075	0,886549172	NA
ENSG00000259753	NA	NA	NA	0	NA	NA	NA	NA	NA
ENSG00000259769	LOC646071	disintegrin and metalloproteinase domain-containing protein 21-like	646071	0	NA	NA	NA	NA	NA
ENSG00000259770	FDPSP9	farnesyl diphosphate synthase pseudogene 9	401822	0	NA	NA	NA	NA	NA
ENSG00000259784	NA	NA	NA	0	NA	NA	NA	NA	NA
ENSG00000259790	ANP32BP1	acidic nuclear phosphoprotein 32 family member B pseudogene 1	646791	0	NA	NA	NA	NA	NA
ENSG00000259800	LOC107987232	COX assembly mitochondrial protein 2 homolog	107987232	0	NA	NA	NA	NA	NA
ENSG00000259803	SLC22A31	solute carrier family 22 member 31	146429	0	NA	NA	NA	NA	NA
ENSG00000259822	NA	NA	NA	0	NA	NA	NA	NA	NA

ENSG00000259823	LYPD8	LY6/PLAUR domain containing 8	646627	0	NA	NA	NA	NA	NA
ENSG00000259832	NA	NA	NA	0	NA	NA	NA	NA	NA
ENSG00000259836	AGGF1P6	angiogenic factor with G-patch and FHA domains 1 pseudogene 6	106481737	0	NA	NA	NA	NA	NA
ENSG00000259838	NA	NA	NA	0	NA	NA	NA	NA	NA
ENSG00000259842	NA	NA	NA	0	NA	NA	NA	NA	NA
ENSG00000259844	NA	NA	NA	0	NA	NA	NA	NA	NA
ENSG00000259845	NA	NA	NA	0,310830754	2,365361781	3,994125	0,592210224	0,553709824	NA
ENSG00000259848	NA	NA	NA	0	NA	NA	NA	NA	NA
ENSG00000259849	VENTXP1	VENT homeobox pseudogene 1	139538	0	NA	NA	NA	NA	NA
ENSG00000259852	NA	NA	NA	0	NA	NA	NA	NA	NA
ENSG00000259856	NA	NA	NA	3,400462224	-0,709594771	1,663604	-0,42654056	0,669714	NA
ENSG00000259864	NA	NA	NA	0	NA	NA	NA	NA	NA
ENSG00000259866	LOC100420642	Rho GTPase activating protein 20 pseudogene	100420642	0	NA	NA	NA	NA	NA
ENSG00000259873	LOC100419639	ribosomal protein L21 pseudogene	100419639	0	NA	NA	NA	NA	NA
ENSG00000259874	NA	NA	NA	0	NA	NA	NA	NA	NA
ENSG00000259882	NA	NA	NA	0	NA	NA	NA	NA	NA
ENSG00000259892	LOC100422492	amyloid beta precursor protein binding family A member 2 pseudogene	100422492	2,627405817	-0,522573523	1,180819	-0,442551862	0,65808991	NA
ENSG00000259897	NA	NA	NA	0	NA	NA	NA	NA	NA
ENSG00000259900	NA	NA	NA	0	NA	NA	NA	NA	NA
ENSG00000259904	ACTG1P15	actin gamma 1 pseudogene 15	100418951	0	NA	NA	NA	NA	NA
ENSG00000259909	NA	NA	NA	0	NA	NA	NA	NA	NA
ENSG00000259916	AQP7B	aquaporin 7B	100509620	0	NA	NA	NA	NA	NA
ENSG00000259917	NA	NA	NA	0,237757999	-0,112638314	4,080456	-0,027604346	0,977977716	NA
ENSG00000259918	NA	NA	NA	0	NA	NA	NA	NA	NA
ENSG00000259922	CFAP69P1	CFAP69 pseudogene 1	100130044	0	NA	NA	NA	NA	NA
ENSG00000259931	NA	NA	NA	0,222114904	-0,141286059	4,080456	-0,034625067	0,972378713	NA
ENSG00000259934	NA	NA	NA	0	NA	NA	NA	NA	NA
ENSG00000259948	LOC124903574	vesicle-associated membrane protein-associated protein A-like	124903574	0	NA	NA	NA	NA	NA
ENSG00000259950	NA	NA	NA	0	NA	NA	NA	NA	NA
ENSG00000259962	NA	NA	NA	0	NA	NA	NA	NA	NA
ENSG00000259963	NA	NA	NA	0	NA	NA	NA	NA	NA
ENSG00000259966	NA	NA	NA	0	NA	NA	NA	NA	NA
ENSG00000259978	MRPS21P8	mitochondrial ribosomal protein S21 pseudogene 8	350297	0	NA	NA	NA	NA	NA
ENSG00000259979	VN1R68P	vomeronasal 1 receptor 68 pseudogene	100131688	0	NA	NA	NA	NA	NA
ENSG00000259982	NA	NA	NA	0	NA	NA	NA	NA	NA
ENSG00000259983	NA	NA	NA	0	NA	NA	NA	NA	NA
ENSG00000259984	NA	NA	NA	0	NA	NA	NA	NA	NA
ENSG00000259987	NA	NA	NA	0	NA	NA	NA	NA	NA
ENSG00000259990	NA	NA	NA	0	NA	NA	NA	NA	NA
ENSG00000259993	NA	NA	NA	0,239347754	1,967469188	4,021404	0,489249276	0,624665227	NA
ENSG00000259996	NA	NA	NA	0	NA	NA	NA	NA	NA
ENSG00000259997	NA	NA	NA	0	NA	NA	NA	NA	NA
ENSG00000260001	TGFBR3L	transforming growth factor beta receptor 3 like	100507588	0,262956004	2,142051094	4,009493	0,534244857	0,593172138	NA
ENSG00000260007	NA	NA	NA	3,432717231	-4,681409145	3,927102	-1,192077173	0,233230987	NA
ENSG00000260010	NA	NA	NA	0	NA	NA	NA	NA	NA
ENSG00000260012	NA	NA	NA	0,10543123	0	4,080456	0	1	NA
ENSG00000260014	NA	NA	NA	0	NA	NA	NA	NA	NA
ENSG00000260024	MRPS21P7	mitochondrial ribosomal protein S21 pseudogene 7	359771	0	NA	NA	NA	NA	NA
ENSG00000260027	HOXB7	homeobox B7	3217	8,714212991	0,204162216	0,864793	0,236082059	0,813368995	NA
ENSG00000260031	NA	NA	NA	0	NA	NA	NA	NA	NA
ENSG00000260033	NA	NA	NA	0	NA	NA	NA	NA	NA
ENSG00000260036	NA	NA	NA	0	NA	NA	NA	NA	NA
ENSG00000260047	BCAP31P2	B cell receptor associated protein 31 pseudogene 2	100194426	0	NA	NA	NA	NA	NA
ENSG00000260048	NA	NA	NA	0	NA	NA	NA	NA	NA
ENSG00000260053	ABCB10P4	ABCB10 pseudogene 4	100132289	0	NA	NA	NA	NA	NA
ENSG00000260062	NA	NA	NA	0	NA	NA	NA	NA	NA
ENSG00000260067	MTND4LP25	MT-ND4L pseudogene 25	107075243	0	NA	NA	NA	NA	NA
ENSG00000260068	NA	NA	NA	0	NA	NA	NA	NA	NA
ENSG00000260075	LRRC37A2	leucine rich repeat containing 37 member A2	474170	0	NA	NA	NA	NA	NA
ENSG00000260078	NA	NA	NA	0	NA	NA	NA	NA	NA
ENSG00000260081	LOC105373383	uncharacterized LOC105373383	105373383	0	NA	NA	NA	NA	NA
ENSG00000260082	NA	NA	NA	0	NA	NA	NA	NA	NA

ENSG00000260087	NA	NA	NA	0	NA	NA	NA	NA	NA
ENSG00000260089	ADAM3B	ADAM metallopeptidase domain 3B (pseudogene)	1596	0	NA	NA	NA	NA	NA
ENSG00000260092	NA	NA	NA	0	NA	NA	NA	NA	NA
ENSG00000260096	NA	NA	NA	0	NA	NA	NA	NA	NA
ENSG00000260097	SPDYE6	speedy/RINGO cell cycle regulator family member E6	729597	0	NA	NA	NA	NA	NA
ENSG00000260104	NA	NA	NA	0	NA	NA	NA	NA	NA
ENSG00000260105	NA	NA	NA	0	NA	NA	NA	NA	NA
ENSG00000260109	NA	NA	NA	0	NA	NA	NA	NA	NA
ENSG00000260112	NA	NA	NA	0	NA	NA	NA	NA	NA
ENSG00000260113	NA	NA	NA	0	NA	NA	NA	NA	NA
ENSG00000260133	CA5AP1	carbonic anhydrase 5A pseudogene 1	764	0	NA	NA	NA	NA	NA
ENSG00000260134	C1QL1P1	complement C1q like 1 pseudogene 1	106480353	0	NA	NA	NA	NA	NA
ENSG00000260139	CSPG4P13	chondroitin sulfate proteoglycan 4 pseudogene 13	100302666	0	NA	NA	NA	NA	NA
ENSG00000260144	LOC646665	golgin A6 family member A pseudogene	646665	0,235790524	0,844721077	4,080456	0,207016349	0,83599709	NA
ENSG00000260146	LOC107984807	nuclear envelope phosphatase-regulatory subunit 1-like	107984807	0	NA	NA	NA	NA	NA
ENSG00000260147	MTND5P34	MT-ND5 pseudogene 34	107075255	0	NA	NA	NA	NA	NA
ENSG00000260152	NA	NA	NA	0,389601419	1,304379118	3,999129	0,326165793	0,744298921	NA
ENSG00000260153	RARRES2P10	retinoic acid receptor responder 2 pseudogene 10	106481972	0	NA	NA	NA	NA	NA
ENSG00000260157	NA	NA	NA	0	NA	NA	NA	NA	NA
ENSG00000260159	NA	NA	NA	0	NA	NA	NA	NA	NA
ENSG00000260161	MTCYBP28	MT-CYB pseudogene 28	107075134	0,520531164	1,668207286	2,88564	0,578106601	0,563192151	NA
ENSG00000260165	NA	NA	NA	0	NA	NA	NA	NA	NA
ENSG00000260170	NA	NA	NA	0,354859955	0	4,080456	0	1	NA
ENSG00000260174	NA	NA	NA	0	NA	NA	NA	NA	NA
ENSG00000260184	NA	NA	NA	0	NA	NA	NA	NA	NA
ENSG00000260187	NA	NA	NA	0	NA	NA	NA	NA	NA
ENSG00000260207	DUX4L47	double homeobox 4 like 47 (pseudogene)	106481738	0	NA	NA	NA	NA	NA
ENSG00000260211	NA	NA	NA	0	NA	NA	NA	NA	NA
ENSG00000260212	NA	NA	NA	0	NA	NA	NA	NA	NA
ENSG00000260218	NA	NA	NA	0	NA	NA	NA	NA	NA
ENSG00000260220	CCDC187	coiled-coil domain containing 187	399693	0	NA	NA	NA	NA	NA
ENSG00000260224	UBL5P4	ubiquitin like 5 pseudogene 4	106480800	0	NA	NA	NA	NA	NA
ENSG00000260229	PPIAP51	peptidylprolyl isomerase A pseudogene 51	729313	0,072165449	0,847101401	4,080456	0,207599697	0,835541541	NA
ENSG00000260230	FRRS1L	ferric chelate reductase 1 like	23732	0,072165449	0,847101401	4,080456	0,207599697	0,835541541	NA
ENSG00000260234	NA	NA	NA	0	NA	NA	NA	NA	NA
ENSG00000260238	PMF1-BGLAP	PMF1-BGLAP readthrough	100527963	5,337617984	0,034397789	1,428789	0,024074784	0,980792957	NA
ENSG00000260240	NA	NA	NA	0	NA	NA	NA	NA	NA
ENSG00000260247	NA	NA	NA	0	NA	NA	NA	NA	NA
ENSG00000260251	LOC100421259	zinc finger and BTB domain containing 14 pseudogene	100421259	0	NA	NA	NA	NA	NA
ENSG00000260255	NA	NA	NA	0	NA	NA	NA	NA	NA
ENSG00000260258	NA	NA	NA	0	NA	NA	NA	NA	NA
ENSG00000260261	NA	NA	NA	0	NA	NA	NA	NA	NA
ENSG00000260272	NA	NA	NA	1,248949241	-4,496371739	2,962989	-1,517512331	0,129137383	NA
ENSG00000260275	NA	NA	NA	0	NA	NA	NA	NA	NA
ENSG00000260282	NA	NA	NA	0	NA	NA	NA	NA	NA
ENSG00000260284	TPSP2	tryptase pseudogene 2	650474	0	NA	NA	NA	NA	NA
ENSG00000260286	ARMH2	armadillo like helical domain containing 2	101928603	0,847633665	1,817177032	2,648354	0,68615341	0,492616374	NA
ENSG00000260290	NA	NA	NA	0	NA	NA	NA	NA	NA
ENSG00000260291	FRG2GP	FSHD region gene 2 family member G, pseudogene	106480321	0	NA	NA	NA	NA	NA
ENSG00000260298	ACTG1P16	actin gamma 1 pseudogene 16	100418960	0	NA	NA	NA	NA	NA
ENSG00000260307	NA	NA	NA	0	NA	NA	NA	NA	NA
ENSG00000260308	NA	NA	NA	0	NA	NA	NA	NA	NA
ENSG00000260314	MRC1	mannose receptor C-type 1	4360	0,473737727	-1,078463941	3,477445	-0,310131089	0,756461271	NA
ENSG00000260318	NA	NA	NA	0,329096616	-0,999381803	4,040555	-0,247337752	0,804646845	NA
ENSG00000260325	HSPB9	heat shock protein family B (small) member 9	94086	0,117497108	0,847101401	4,080456	0,207599697	0,835541541	NA
ENSG00000260326	NA	NA	NA	0	NA	NA	NA	NA	NA
ENSG00000260327	NA	NA	NA	0	NA	NA	NA	NA	NA
ENSG00000260332	DPPA3P11	DPPA3 pseudogene 11	127460881	0	NA	NA	NA	NA	NA
ENSG00000260335	NA	NA	NA	0	NA	NA	NA	NA	NA

ENSG00000260341	NA	NA	NA	0	NA	NA	NA	NA	NA
ENSG00000260342	NA	NA	NA	0,234994216	1,944111989	4,02324	0,483220515	0,62893917	NA
ENSG00000260344	NA	NA	NA	0	NA	NA	NA	NA	NA
ENSG00000260347	NA	NA	NA	0	NA	NA	NA	NA	NA
ENSG00000260357	NA	NA	NA	0	NA	NA	NA	NA	NA
ENSG00000260371	NA	NA	NA	0,718043263	3,570317912	3,944991	0,90502559	0,36545183	NA
ENSG00000260375	NA	NA	NA	0	NA	NA	NA	NA	NA
ENSG00000260379	LOC100131080	ribosomal protein S14 pseudogene	100131080	0	NA	NA	NA	NA	NA
ENSG00000260382	NA	NA	NA	0	NA	NA	NA	NA	NA
ENSG00000260386	LDC1P	leucine decarboxylase 1, pseudogene	149086	0	NA	NA	NA	NA	NA
ENSG00000260389	WBP11P1	WBP11 pseudogene 1	441818	0	NA	NA	NA	NA	NA
ENSG00000260395	NA	NA	NA	0,10543123	0	4,080456	0	1	NA
ENSG00000260399	NA	NA	NA	0	NA	NA	NA	NA	NA
ENSG00000260404	NA	NA	NA	0	NA	NA	NA	NA	NA
ENSG00000260410	NA	NA	NA	0	NA	NA	NA	NA	NA
ENSG00000260413	NA	NA	NA	4,757285444	1,672849372	0,863744	1,936741755	0,052776919	NA
ENSG00000260414	NA	NA	NA	0	NA	NA	NA	NA	NA
ENSG00000260427	AGGF1P9	angiogenic factor with G-patch and FHA domains 1 pseudogene 9	100127958	0	NA	NA	NA	NA	NA
ENSG00000260428	SCX	scleraxis bHLH transcription factor	642658	5,140882058	-0,194429906	0,777358	-0,250116154	0,802497524	NA
ENSG00000260431	VPS51P1	VPS51 pseudogene 1	127379701	0	NA	NA	NA	NA	NA
ENSG00000260438	MTCH2P3	MTCH2 pseudogene 3	100421594	0	NA	NA	NA	NA	NA
ENSG00000260443	NA	NA	NA	0	NA	NA	NA	NA	NA
ENSG00000260445	NA	NA	NA	0	NA	NA	NA	NA	NA
ENSG00000260451	NA	NA	NA	0	NA	NA	NA	NA	NA
ENSG00000260452	TPRKBP2	TP53RK binding protein pseudogene 2	106480804	2,07660605	-0,20574395	1,548216	-0,132890955	0,89427962	NA
ENSG00000260456	C16orf95	chromosome 16 open reading frame 95	100506581	2,35718717	-1,241403044	1,4415	-0,861188633	0,38913416	NA
ENSG00000260458	KCNJ18	potassium inwardly rectifying channel subfamily J member 18	100134444	0	NA	NA	NA	NA	NA
ENSG00000260459	NA	NA	NA	0	NA	NA	NA	NA	NA
ENSG00000260471	PPIAP49	peptidylprolyl isomerase A pseudogene 49	111082973	0	NA	NA	NA	NA	NA
ENSG00000260472	NA	NA	NA	0	NA	NA	NA	NA	NA
ENSG00000260480	C2orf69P4	chromosome 2 open reading frame 69 pseudogene 4	100420859	0	NA	NA	NA	NA	NA
ENSG00000260483	NA	NA	NA	0	NA	NA	NA	NA	NA
ENSG00000260490	NA	NA	NA	0,368378316	2,117916043	3,618544	0,585295091	0,558349315	NA
ENSG00000260494	RPL7L1P16	RPL7L1 pseudogene 16	100127965	0	NA	NA	NA	NA	NA
ENSG00000260497	NA	NA	NA	0	NA	NA	NA	NA	NA
ENSG00000260514	NA	NA	NA	0	NA	NA	NA	NA	NA
ENSG00000260516	CHEK2P7	CHEK2 pseudogene 7	121847986	0	NA	NA	NA	NA	NA
ENSG00000260518	NA	NA	NA	1,084639151	0,514849016	2,179121	0,23626453	0,813227409	NA
ENSG00000260522	NA	NA	NA	0	NA	NA	NA	NA	NA
ENSG00000260524	CYP3A52P	cytochrome P450 family 3 subfamily A member 52, pseudogene	106478983	0	NA	NA	NA	NA	NA
ENSG00000260525	NA	NA	NA	0	NA	NA	NA	NA	NA
ENSG00000260537	NA	NA	NA	0	NA	NA	NA	NA	NA
ENSG00000260540	NA	NA	NA	0	NA	NA	NA	NA	NA
ENSG00000260549	NA	NA	NA	0,11808024	-1,076443944	4,080456	-0,263804824	0,791930317	NA
ENSG00000260571	NA	NA	NA	0,11808024	-1,076443944	4,080456	-0,263804824	0,791930317	NA
ENSG00000260576	NA	NA	NA	0,222691981	-2,001329511	4,035252	-0,495961483	0,619921586	NA
ENSG00000260582	TPST2P1	tyrosylprotein sulfotransferase 2 pseudogene 1	100420710	0	NA	NA	NA	NA	NA
ENSG00000260584	NA	NA	NA	0	NA	NA	NA	NA	NA
ENSG00000260590	NA	NA	NA	0	NA	NA	NA	NA	NA
ENSG00000260596	DUX4	double homeobox 4	100288687	0,072165449	0,847101401	4,080456	0,207599697	0,835541541	NA
ENSG00000260596	LOC124906452	double homeobox protein 4	124906452	0,072165449	0,847101401	4,080456	0,207599697	0,835541541	NA
ENSG00000260596	LOC124906453	double homeobox protein 4	124906453	0,072165449	0,847101401	4,080456	0,207599697	0,835541541	NA
ENSG00000260596	LOC124906454	double homeobox protein 4	124906454	0,072165449	0,847101401	4,080456	0,207599697	0,835541541	NA
ENSG00000260596	LOC124906456	double homeobox protein 4	124906456	0,072165449	0,847101401	4,080456	0,207599697	0,835541541	NA
ENSG00000260596	LOC124906457	double homeobox protein 4	124906457	0,072165449	0,847101401	4,080456	0,207599697	0,835541541	NA
ENSG00000260596	LOC124906458	double homeobox protein 4	124906458	0,072165449	0,847101401	4,080456	0,207599697	0,835541541	NA
ENSG00000260596	LOC124906459	double homeobox protein 4	124906459	0,072165449	0,847101401	4,080456	0,207599697	0,835541541	NA

ENSG00000260596	LOC124906460	double homeobox protein 4	124906460	0,072165449	0,847101401	4,080456	0,207599697	0,835541541	NA
ENSG00000260596	LOC124906461	double homeobox protein 4	124906461	0,072165449	0,847101401	4,080456	0,207599697	0,835541541	NA
ENSG00000260596	LOC124906462	double homeobox protein 4	124906462	0,072165449	0,847101401	4,080456	0,207599697	0,835541541	NA
ENSG00000260596	LOC124906463	double homeobox protein 4	124906463	0,072165449	0,847101401	4,080456	0,207599697	0,835541541	NA
ENSG00000260596	LOC124906464	double homeobox protein 4	124906464	0,072165449	0,847101401	4,080456	0,207599697	0,835541541	NA
ENSG00000260596	LOC124906465	double homeobox protein 4	124906465	0,072165449	0,847101401	4,080456	0,207599697	0,835541541	NA
ENSG00000260596	LOC124906466	double homeobox protein 4	124906466	0,072165449	0,847101401	4,080456	0,207599697	0,835541541	NA
ENSG00000260598	FRG2IP	FSHD region gene 2 family member I, pseudogene	106480351	0	NA	NA	NA	NA	NA
ENSG00000260602	NA	NA	NA	0	NA	NA	NA	NA	NA
ENSG00000260610	LOC100419016	proline rich 21 pseudogene	100419016	0	NA	NA	NA	NA	NA
ENSG00000260615	NA	NA	NA	0	NA	NA	NA	NA	NA
ENSG00000260622	NA	NA	NA	0	NA	NA	NA	NA	NA
ENSG00000260628	LOC388248	Rho GTPase activating protein 23 pseudogene	388248	0	NA	NA	NA	NA	NA
ENSG00000260631	RBM22P12	RNA binding motif protein 22 pseudogene 12	100131641	0	NA	NA	NA	NA	NA
ENSG00000260644	HERC2P5	HERC2 pseudogene 5	388254	0,447798935	-1,047976003	2,998973	-0,349445011	0,726755247	NA
ENSG00000260649	NA	NA	NA	0	NA	NA	NA	NA	NA
ENSG00000260660	LOC105376731	taste receptor cell protein 1-like	105376731	0	NA	NA	NA	NA	NA
ENSG00000260662	NA	NA	NA	0	NA	NA	NA	NA	NA
ENSG00000260668	NA	NA	NA	0	NA	NA	NA	NA	NA
ENSG00000260674	NA	NA	NA	0	NA	NA	NA	NA	NA
ENSG00000260678	NA	NA	NA	0	NA	NA	NA	NA	NA
ENSG00000260680	NA	NA	NA	0	NA	NA	NA	NA	NA
ENSG00000260685	NA	NA	NA	0	NA	NA	NA	NA	NA
ENSG00000260688	NA	NA	NA	0	NA	NA	NA	NA	NA
ENSG00000260689	HNRNPA3P11	heterogeneous nuclear ribonucleoprotein A3 pseudogene 11	100421431	0	NA	NA	NA	NA	NA
ENSG00000260690	NA	NA	NA	0	NA	NA	NA	NA	NA
ENSG00000260691	ANKRD20A1	ankyrin repeat domain 20 family member A1	84210	0,10488184	-1,076443944	4,080456	-0,263804824	0,791930317	NA
ENSG00000260702	NA	NA	NA	0	NA	NA	NA	NA	NA
ENSG00000260714	NA	NA	NA	0,393493028	-0,100761351	3,634145	-0,027726286	0,977880459	NA
ENSG00000260722	VN1R67P	vomeronasal 1 receptor 67 pseudogene	100131118	0	NA	NA	NA	NA	NA
ENSG00000260724	NA	NA	NA	0	NA	NA	NA	NA	NA
ENSG00000260726	NA	NA	NA	0,15836288	0	4,080456	0	1	NA
ENSG00000260727	SLC7A5P1	solute carrier family 7 member 5 pseudogene 1	81893	7,804149327	0,233644887	0,699823	0,333863049	0,738482904	NA
ENSG00000260729	NA	NA	NA	7,482952799	0,690556781	1,366127	0,505485196	0,613218094	NA
ENSG00000260731	KRT8P22	keratin 8 pseudogene 22	342419	0	NA	NA	NA	NA	NA
ENSG00000260734	TLE7	TLE family member 7	102723796	0	NA	NA	NA	NA	NA
ENSG00000260735	LOC100505915	uncharacterized LOC100505915	100505915	0,51774436	1,35505036	2,864849	0,472991878	0,636218974	NA
ENSG00000260746	NA	NA	NA	0	NA	NA	NA	NA	NA
ENSG00000260747	NA	NA	NA	0,222633166	0,829142251	4,080456	0,203198436	0,838979935	NA
ENSG00000260762	NA	NA	NA	0	NA	NA	NA	NA	NA
ENSG00000260764	NA	NA	NA	0	NA	NA	NA	NA	NA
ENSG00000260765	NA	NA	NA	0	NA	NA	NA	NA	NA
ENSG00000260776	NA	NA	NA	0	NA	NA	NA	NA	NA
ENSG00000260781	ARHGAP23P1	Rho GTPase activating protein 23 pseudogene 1	102577425	0	NA	NA	NA	NA	NA
ENSG00000260782	NA	NA	NA	0	NA	NA	NA	NA	NA
ENSG00000260784	NA	NA	NA	8,033692264	-0,749900659	1,421232	-0,52764112	0,597748447	NA
ENSG00000260795	NA	NA	NA	0	NA	NA	NA	NA	NA
ENSG00000260797	NA	NA	NA	0	NA	NA	NA	NA	NA
ENSG00000260799	KRT8P50	keratin 8 pseudogene 50	102724430	0,707968207	2,075764231	2,265913	0,916082826	0,359623455	NA
ENSG00000260802	SERTM2	serine rich and transmembrane domain containing 2	401613	0	NA	NA	NA	NA	NA
ENSG00000260809	VPS35P1	VPS35 pseudogene 1	100422055	0	NA	NA	NA	NA	NA
ENSG00000260811	OR4C45	olfactory receptor family 4 subfamily C member 45 (gene/pseudogene)	403257	0	NA	NA	NA	NA	NA
ENSG00000260812	NA	NA	NA	0	NA	NA	NA	NA	NA
ENSG00000260815	NA	NA	NA	0	NA	NA	NA	NA	NA
ENSG00000260817	LOC124903639	40S ribosomal protein S24-like	124903639	0	NA	NA	NA	NA	NA
ENSG00000260818	NA	NA	NA	0	NA	NA	NA	NA	NA

ENSG00000260836	NA	NA	NA	0	NA	NA	NA	NA	NA
ENSG00000260846	NA	NA	NA	0	NA	NA	NA	NA	NA
ENSG00000260851	NA	NA	NA	0	NA	NA	NA	NA	NA
ENSG00000260860	NA	NA	NA	0	NA	NA	NA	NA	NA
ENSG00000260861	NA	NA	NA	0	NA	NA	NA	NA	NA
ENSG00000260863	CYP2C60P	cytochrome P450 family 2 subfamily C member 60, pseudogene	106480748	0	NA	NA	NA	NA	NA
ENSG00000260864	NA	NA	NA	0	NA	NA	NA	NA	NA
ENSG00000260865	NA	NA	NA	0,651924784	1,831606728	2,364846	0,774514324	0,438626701	NA
ENSG00000260866	SLC9B1P5	solute carrier family 9 member B1 pseudogene 5	100533705	0	NA	NA	NA	NA	NA
ENSG00000260870	NA	NA	NA	0	NA	NA	NA	NA	NA
ENSG00000260882	NA	NA	NA	0,103998153	-1,076443944	4,080456	-0,263804824	0,791930317	NA
ENSG00000260883	VN1R65P	vomeronasal 1 receptor 65 pseudogene	100128384	0	NA	NA	NA	NA	NA
ENSG00000260889	NA	NA	NA	0	NA	NA	NA	NA	NA
ENSG00000260897	NA	NA	NA	0,15836288	0	4,080456	0	1	NA
ENSG00000260900	NA	NA	NA	0	NA	NA	NA	NA	NA
ENSG00000260903	XKR7	XK related 7	343702	0	NA	NA	NA	NA	NA
ENSG00000260908	NA	NA	NA	0	NA	NA	NA	NA	NA
ENSG00000260909	NA	NA	NA	0	NA	NA	NA	NA	NA
ENSG00000260928	SPCS2P1	signal peptidase complex subunit 2 pseudogene 1	100131250	0	NA	NA	NA	NA	NA
ENSG00000260933	LOC100133137	geminin, DNA replication inhibitor pseudogene 2	100133137	0	NA	NA	NA	NA	NA
ENSG00000260939	MTCH2P4	MTCH2 pseudogene 4	100128523	0	NA	NA	NA	NA	NA
ENSG00000260965	RPL23AP91	ribosomal protein L23a pseudogene 91	106480337	0	NA	NA	NA	NA	NA
ENSG00000260967	RARRES2P5	retinoic acid receptor responder 2 pseudogene 5	106480231	0	NA	NA	NA	NA	NA
ENSG00000260984	NA	NA	NA	0	NA	NA	NA	NA	NA
ENSG00000260991	NA	NA	NA	0	NA	NA	NA	NA	NA
ENSG00000260994	AGGF1P7	angiogenic factor with G-patch and FHA domains 1 pseudogene 7	100132606	0	NA	NA	NA	NA	NA
ENSG00000261007	NA	NA	NA	0	NA	NA	NA	NA	NA
ENSG00000261010	RARRES2P6	retinoic acid receptor responder 2 pseudogene 6	105377820	0	NA	NA	NA	NA	NA
ENSG00000261011	NA	NA	NA	0	NA	NA	NA	NA	NA
ENSG00000261017	NA	NA	NA	0	NA	NA	NA	NA	NA
ENSG00000261024	GS1-279B7,1	microtubule associated protein 1 light chain 3 beta pseudogene	100288079	0	NA	NA	NA	NA	NA
ENSG00000261040	WFDC21P	WAP four-disulfide core domain 21, pseudogene	645638	0	NA	NA	NA	NA	NA
ENSG00000261046	C2orf69P1	chromosome 2 open reading frame 69 pseudogene 1	727964	0	NA	NA	NA	NA	NA
ENSG00000261047	NA	NA	NA	0	NA	NA	NA	NA	NA
ENSG00000261048	VN1R60P	vomeronasal 1 receptor 60 pseudogene	100312812	0	NA	NA	NA	NA	NA
ENSG00000261053	NAMPTP3	nicotinamide phosphoribosyltransferase pseudogene 3	106480319	0	NA	NA	NA	NA	NA
ENSG00000261056	NA	NA	NA	5,910794862	0,344498786	0,741806	0,464405192	0,642357471	NA
ENSG00000261072	NA	NA	NA	2,671705252	1,230494983	1,23186	0,998892021	0,317847002	NA
ENSG00000261077	NA	NA	NA	0	NA	NA	NA	NA	NA
ENSG00000261081	LOC100422491	amyloid beta precursor protein binding family A member 2 pseudogene	100422491	0,382630767	2,673945182	3,977433	0,672279093	0,501406037	NA
ENSG00000261084	NA	NA	NA	0	NA	NA	NA	NA	NA
ENSG00000261089	NA	NA	NA	0	NA	NA	NA	NA	NA
ENSG00000261102	NA	NA	NA	0	NA	NA	NA	NA	NA
ENSG00000261108	NA	NA	NA	0	NA	NA	NA	NA	NA
ENSG00000261111	NA	NA	NA	0	NA	NA	NA	NA	NA
ENSG00000261127	NA	NA	NA	0	NA	NA	NA	NA	NA
ENSG00000261130	MPV17L	MPV17 mitochondrial inner membrane protein like	255027	0	NA	NA	NA	NA	NA
ENSG00000261143	ADAMTS7P3	ADAMTS7 pseudogene 3	400406	0	NA	NA	NA	NA	NA
ENSG00000261144	NA	NA	NA	0	NA	NA	NA	NA	NA
ENSG00000261145	NA	NA	NA	0	NA	NA	NA	NA	NA
ENSG00000261147	NA	NA	NA	1,971834078	-0,499254896	1,618487	-0,308470062	0,757724677	NA
ENSG00000261150	EPPK1	epiplakin 1	83481	0,504538838	-0,120787449	2,910139	-0,041505731	0,966892724	NA
ENSG00000261151	NA	NA	NA	0	NA	NA	NA	NA	NA
ENSG00000261153	LOC342443	ADAM metallopeptidase domain 21 pseudogene	342443	0	NA	NA	NA	NA	NA
ENSG00000261174	HMGB1P33	high mobility group box 1 pseudogene 33	100873905	0	NA	NA	NA	NA	NA
ENSG00000261178	NA	NA	NA	0	NA	NA	NA	NA	NA

ENSG00000261181	PLA2G10FP	phospholipase A2 group XF, pseudogene	127525794	0	NA	NA	NA	NA	NA
ENSG00000261192	RNF126P1	ring finger protein 126 pseudogene 1	376412	0	NA	NA	NA	NA	NA
ENSG00000261196	CYP4A43P	cytochrome P450 family 4 subfamily A member 43, pseudogene	100874495	0	NA	NA	NA	NA	NA
ENSG00000261197	NA	NA	NA	0	NA	NA	NA	NA	NA
ENSG00000261199	NA	NA	NA	0	NA	NA	NA	NA	NA
ENSG00000261203	PLA2G10JP	phospholipase A2 group XJ, pseudogene	127525788	0,072165449	0,847101401	4,080456	0,207599697	0,835541541	NA
ENSG00000261205	NA	NA	NA	0	NA	NA	NA	NA	NA
ENSG00000261209	SULT1C2P2	sulfotransferase family, cytosolic, 1C, member 2 pseudogene 2	106480333	0	NA	NA	NA	NA	NA
ENSG00000261210	CLEC19A	C-type lectin domain containing 19A	728276	0	NA	NA	NA	NA	NA
ENSG00000261217	BCAP31P1	B cell receptor associated protein 31 pseudogene 1	100194425	0	NA	NA	NA	NA	NA
ENSG00000261232	NA	NA	NA	0	NA	NA	NA	NA	NA
ENSG00000261233	NA	NA	NA	0	NA	NA	NA	NA	NA
ENSG00000261234	NA	NA	NA	0	NA	NA	NA	NA	NA
ENSG00000261239	NA	NA	NA	0,11808024	-1,076443944	4,080456	-0,263804824	0,791930317	NA
ENSG00000261245	NA	NA	NA	0	NA	NA	NA	NA	NA
ENSG00000261259	NA	NA	NA	0	NA	NA	NA	NA	NA
ENSG00000261263	NA	NA	NA	0	NA	NA	NA	NA	NA
ENSG00000261272	MUC22	mucin 22	100507679	0	NA	NA	NA	NA	NA
ENSG00000261274	TP53TG3GP	TP53 target 3 family member G, pseudogene	106660619	0	NA	NA	NA	NA	NA
ENSG00000261278	NA	NA	NA	0	NA	NA	NA	NA	NA
ENSG00000261279	NA	NA	NA	0,593633877	0,466277368	3,385846	0,137713678	0,890466709	NA
ENSG00000261281	NA	NA	NA	0	NA	NA	NA	NA	NA
ENSG00000261282	SOD1P2	superoxide dismutase 1 pseudogene 2	100652974	0	NA	NA	NA	NA	NA
ENSG00000261284	RBM22P13	RNA binding motif protein 22 pseudogene 13	100130603	0	NA	NA	NA	NA	NA
ENSG00000261289	NA	NA	NA	0	NA	NA	NA	NA	NA
ENSG00000261290	CYP4A44P	cytochrome P450 family 4 subfamily A member 44, pseudogene	106481791	0	NA	NA	NA	NA	NA
ENSG00000261299	NA	NA	NA	0	NA	NA	NA	NA	NA
ENSG00000261303	NA	NA	NA	0	NA	NA	NA	NA	NA
ENSG00000261308	FIGNL2	fidgetin like 2	401720	6,789359146	0,258756625	0,712645	0,363093154	0,716535298	NA
ENSG00000261315	NA	NA	NA	1,838611375	0,336872931	1,562574	0,215588492	0,829308527	NA
ENSG00000261330	PPIAP50	peptidylprolyl isomerase A pseudogene 50	111082974	0	NA	NA	NA	NA	NA
ENSG00000261333	NA	NA	NA	0	NA	NA	NA	NA	NA
ENSG00000261336	EIF4BP5	eukaryotic translation initiation factor 4B pseudogene 5	100421528	0,119673877	0,847101401	4,080456	0,207599697	0,835541541	NA
ENSG00000261341	SMIM47	small integral membrane protein 47	105372440	0	NA	NA	NA	NA	NA
ENSG00000261349	NA	NA	NA	3,394329648	0,809260109	1,511724	0,535322712	0,592426724	NA
ENSG00000261356	NA	NA	NA	0	NA	NA	NA	NA	NA
ENSG00000261365	NA	NA	NA	0	NA	NA	NA	NA	NA
ENSG00000261375	NA	NA	NA	7,863884646	-0,036127829	1,304972	-0,02768475	0,977913587	NA
ENSG00000261377	NA	NA	NA	0,404757788	-0,183379421	3,579249	-0,051234044	0,959139024	NA
ENSG00000261391	NA	NA	NA	0	NA	NA	NA	NA	NA
ENSG00000261395	NA	NA	NA	0	NA	NA	NA	NA	NA
ENSG00000261405	NA	NA	NA	0	NA	NA	NA	NA	NA
ENSG00000261408	TEN1-CDK3	TEN1-CDK3 readthrough (NMD candidate)	100529145	1,539405905	2,958350215	3,282808	0,901164532	0,367500846	NA
ENSG00000261418	HERC2P6	HERC2 pseudogene 6	8918	0	NA	NA	NA	NA	NA
ENSG00000261419	NA	NA	NA	0	NA	NA	NA	NA	NA
ENSG00000261424	NA	NA	NA	0	NA	NA	NA	NA	NA
ENSG00000261429	NA	NA	NA	0,264463297	0	4,080456	0	1	NA
ENSG00000261440	DUX4L45	double homeobox 4 like 45 (pseudogene)	100887074	0	NA	NA	NA	NA	NA
ENSG00000261444	NA	NA	NA	0	NA	NA	NA	NA	NA
ENSG00000261456	TUBB8	tubulin beta 8 class VIII	347688	0	NA	NA	NA	NA	NA
ENSG00000261457	NA	NA	NA	0	NA	NA	NA	NA	NA
ENSG00000261458	NA	NA	NA	0	NA	NA	NA	NA	NA
ENSG00000261459	NA	NA	NA	0,239347754	1,967469188	4,021404	0,489249276	0,624665227	NA
ENSG00000261461	NA	NA	NA	0	NA	NA	NA	NA	NA
ENSG00000261486	NA	NA	NA	0	NA	NA	NA	NA	NA
ENSG00000261491	NA	NA	NA	0	NA	NA	NA	NA	NA
ENSG00000261497	NA	NA	NA	0	NA	NA	NA	NA	NA
ENSG00000261509	TP53TG3B	TP53 target 3B	729355	0	NA	NA	NA	NA	NA
ENSG00000261509	LOC102723655	TP53-target gene 3 protein	102723655	0	NA	NA	NA	NA	NA

ENSG00000261509	LOC102723713	TP53-target gene 3 protein	102723713	0	NA	NA	NA	NA	NA
ENSG00000261511	CYP3A137P	cytochrome P450 family 3 subfamily A member 137, pseudogene	107063543	0	NA	NA	NA	NA	NA
ENSG00000261515	VN1R70P	vomeronasal 1 receptor 70 pseudogene	100130059	0	NA	NA	NA	NA	NA
ENSG00000261522	NA	NA	NA	0	NA	NA	NA	NA	NA
ENSG00000261524	ABCB10P3	ABCB10 pseudogene 3	728248	0	NA	NA	NA	NA	NA
ENSG00000261527	LOC100420066	transmembrane protein 111 pseudogene	100420066	0,144330898	1,418786819	4,061262	0,349346267	0,726829368	NA
ENSG00000261528	CCNYL7	cyclin Y like 7 (pseudogene)	100419017	0	NA	NA	NA	NA	NA
ENSG00000261536	NA	NA	NA	0	NA	NA	NA	NA	NA
ENSG00000261548	NA	NA	NA	0	NA	NA	NA	NA	NA
ENSG00000261549	NA	NA	NA	0	NA	NA	NA	NA	NA
ENSG00000261554	NA	NA	NA	0	NA	NA	NA	NA	NA
ENSG00000261557	NA	NA	NA	0	NA	NA	NA	NA	NA
ENSG00000261559	NA	NA	NA	0	NA	NA	NA	NA	NA
ENSG00000261564	TPSP1	tryptase pseudogene 1	100129339	0	NA	NA	NA	NA	NA
ENSG00000261580	NA	NA	NA	0	NA	NA	NA	NA	NA
ENSG00000261582	NA	NA	NA	0,340123442	-1,041548656	3,751798	-0,277613193	0,781309303	NA
ENSG00000261587	TMEM249	transmembrane protein 249	340393	0,454099859	0,94953558	3,532286	0,268816156	0,78807116	NA
ENSG00000261588	NA	NA	NA	0	NA	NA	NA	NA	NA
ENSG00000261590	NA	NA	NA	0	NA	NA	NA	NA	NA
ENSG00000261593	CYP4A27P	cytochrome P450 family 4 subfamily A member 27, pseudogene	100422288	0	NA	NA	NA	NA	NA
ENSG00000261594	TPBGL	trophoblast glycoprotein like	100507050	0	NA	NA	NA	NA	NA
ENSG00000261599	HERC2P8	HERC2 pseudogene 8	440366	1,378466579	1,671215242	2,166376	0,77143348	0,440450037	NA
ENSG00000261603	PRSS46P	serine protease 46, pseudogene	100287362	0	NA	NA	NA	NA	NA
ENSG00000261607	NA	NA	NA	0	NA	NA	NA	NA	NA
ENSG00000261608	NA	NA	NA	0	NA	NA	NA	NA	NA
ENSG00000261611	NA	NA	NA	1,275884361	1,918365795	3,347688	0,573041981	0,56661627	NA
ENSG00000261612	NA	NA	NA	0	NA	NA	NA	NA	NA
ENSG00000261614	NA	NA	NA	0	NA	NA	NA	NA	NA
ENSG00000261620	NA	NA	NA	0	NA	NA	NA	NA	NA
ENSG00000261624	NDUFB10P2	NADH:ubiquinone oxidoreductase subunit B10 pseudogene 2	107075304	0	NA	NA	NA	NA	NA
ENSG00000261628	NA	NA	NA	0	NA	NA	NA	NA	NA
ENSG00000261635	NA	NA	NA	0,340329373	-0,14017294	3,784672	-0,037037015	0,970455492	NA
ENSG00000261647	NA	NA	NA	0	NA	NA	NA	NA	NA
ENSG00000261649	GOLGA6L7	golgin A6 family like 7	728310	0,11808024	-1,076443944	4,080456	-0,263804824	0,791930317	NA
ENSG00000261652	PIERCE2	piercer of microtubule wall 2	145788	0,103998153	-1,076443944	4,080456	-0,263804824	0,791930317	NA
ENSG00000261667	LY6L	lymphocyte antigen 6 family member L	101928108	0	NA	NA	NA	NA	NA
ENSG00000261679	ATP5PDP1	ATP synthase peripheral stalk subunit d pseudogene 1	390581	0	NA	NA	NA	NA	NA
ENSG00000261680	NA	NA	NA	0	NA	NA	NA	NA	NA
ENSG00000261689	AGGF1P4	angiogenic factor with G-patch and FHA domains 1 pseudogene 4	100129351	0	NA	NA	NA	NA	NA
ENSG00000261701	HPR	haptoglobin-related protein	3250	0	NA	NA	NA	NA	NA
ENSG00000261702	NA	NA	NA	0	NA	NA	NA	NA	NA
ENSG00000261704	NA	NA	NA	0	NA	NA	NA	NA	NA
ENSG00000261708	NA	NA	NA	0	NA	NA	NA	NA	NA
ENSG00000261711	NA	NA	NA	0	NA	NA	NA	NA	NA
ENSG00000261714	NA	NA	NA	0	NA	NA	NA	NA	NA
ENSG00000261716	H2BC20P	H2B clustered histone 20, pseudogene	337873	5,763726799	-1,084700886	0,98683	-1,09917724	0,271690764	NA
ENSG00000261717	NA	NA	NA	0,223409443	0	4,080456	0	1	NA
ENSG00000261719	NA	NA	NA	0	NA	NA	NA	NA	NA
ENSG00000261725	NA	NA	NA	0	NA	NA	NA	NA	NA
ENSG00000261727	NA	NA	NA	0	NA	NA	NA	NA	NA
ENSG00000261741	NA	NA	NA	0	NA	NA	NA	NA	NA
ENSG00000261748	SPRING1P3	SPRING1 pseudogene 3	441750	0	NA	NA	NA	NA	NA
ENSG00000261752	AGGF1P8	angiogenic factor with G-patch and FHA domains 1 pseudogene 8	100128804	0	NA	NA	NA	NA	NA
ENSG00000261760	NA	NA	NA	0	NA	NA	NA	NA	NA
ENSG00000261764	KRT18P18	keratin 18 pseudogene 18	342374	0	NA	NA	NA	NA	NA
ENSG00000261774	NA	NA	NA	0	NA	NA	NA	NA	NA
ENSG00000261776	NA	NA	NA	0,15836288	0	4,080456	0	1	NA
ENSG00000261778	NA	NA	NA	0	NA	NA	NA	NA	NA
ENSG00000261782	NA	NA	NA	0	NA	NA	NA	NA	NA
ENSG00000261787	TCF24	transcription factor 24	100129654	0	NA	NA	NA	NA	NA
ENSG00000261793	NA	NA	NA	0,103998153	-1,076443944	4,080456	-0,263804824	0,791930317	NA

ENSG00000261809	CYP3A54P	cytochrome P450 family 3 subfamily A member 54, pseudogene	106480749	0	NA	NA	NA	NA	NA
ENSG00000261812	TUBB8P7	tubulin beta 8 class VIII pseudogene 7	197331	0	NA	NA	NA	NA	NA
ENSG00000261813	NA	NA	NA	0,10543123	0	4,080456	0	1	NA
ENSG00000261820	DNM1P49	dynamin 1 pseudogene 49	730058	0	NA	NA	NA	NA	NA
ENSG00000261832	NA	NA	NA	0,234994216	1,944111989	4,02324	0,483220515	0,62893917	NA
ENSG00000261834	NA	NA	NA	0	NA	NA	NA	NA	NA
ENSG00000261835	LOC100130602	regulation of nuclear pre-mRNA domain containing 1A pseudogene	100130602	0	NA	NA	NA	NA	NA
ENSG00000261857	MIA	MIA SH3 domain containing	8190	0,119673877	0,847101401	4,080456	0,207599697	0,835541541	NA
ENSG00000261868	NA	NA	NA	0,072165449	0,847101401	4,080456	0,207599697	0,835541541	NA
ENSG00000261873	SMIM36	small integral membrane protein 36	101927367	0	NA	NA	NA	NA	NA
ENSG00000261882	NA	NA	NA	0	NA	NA	NA	NA	NA
ENSG00000261884	NA	NA	NA	0,618316081	-1,026309394	2,898744	-0,354053164	0,723299042	NA
ENSG00000261904	MTND5P33	MT-ND5 pseudogene 33	107075118	0	NA	NA	NA	NA	NA
ENSG00000261934	PCDHGA9	protocadherin gamma subfamily A, 9	56107	0,339987388	-2,610725896	3,991489	-0,654073222	0,513064633	NA
ENSG00000261939	LARP7P2	LARP7 pseudogene 2	106481699	0	NA	NA	NA	NA	NA
ENSG00000261949	GFY	golgi associated olfactory signaling regulator	100507003	0,103998153	-1,076443944	4,080456	-0,263804824	0,791930317	NA
ENSG00000261965	NA	NA	NA	0	NA	NA	NA	NA	NA
ENSG00000261970	NA	NA	NA	0	NA	NA	NA	NA	NA
ENSG00000261987	NA	NA	NA	0	NA	NA	NA	NA	NA
ENSG00000262000	NA	NA	NA	0	NA	NA	NA	NA	NA
ENSG00000262008	NA	NA	NA	0	NA	NA	NA	NA	NA
ENSG00000262067	NA	NA	NA	0	NA	NA	NA	NA	NA
ENSG00000262081	NA	NA	NA	0	NA	NA	NA	NA	NA
ENSG00000262085	OR1P1	olfactory receptor family 1 subfamily P member 1 (gene/pseudogene)	8391	0	NA	NA	NA	NA	NA
ENSG00000262090	LOC647208	protein phosphatase 2 regulatory subunit B'', beta pseudogene	647208	0	NA	NA	NA	NA	NA
ENSG00000262095	MTATP6P25	MT-ATP6 pseudogene 25	107075203	0	NA	NA	NA	NA	NA
ENSG00000262106	NA	NA	NA	0	NA	NA	NA	NA	NA
ENSG00000262107	MTND6P33	MT-ND6 pseudogene 33	107075298	0	NA	NA	NA	NA	NA
ENSG00000262118	MTCO1P28	MT-CO1 pseudogene 28	107075169	0	NA	NA	NA	NA	NA
ENSG00000262120	NA	NA	NA	0	NA	NA	NA	NA	NA
ENSG00000262135	GARS1P1	glycyl-tRNA synthetase 1 pseudogene 1	100419764	0	NA	NA	NA	NA	NA
ENSG00000262140	NA	NA	NA	1,869818497	0,380424982	1,591655	0,239012148	0,811096163	NA
ENSG00000262141	NA	NA	NA	0	NA	NA	NA	NA	NA
ENSG00000262152	GREP1	glycine rich extracellular protein 1	283875	0	NA	NA	NA	NA	NA
ENSG00000262158	MTCO3P24	MT-CO3 pseudogene 24	107075217	0	NA	NA	NA	NA	NA
ENSG00000262165	C17orf114	chromosome 17 open reading frame 114	119139905	0	NA	NA	NA	NA	NA
ENSG00000262179	MYMX	myomixer, myoblast fusion factor	101929726	0	NA	NA	NA	NA	NA
ENSG00000262181	NA	NA	NA	0	NA	NA	NA	NA	NA
ENSG00000262187	NA	NA	NA	0	NA	NA	NA	NA	NA
ENSG00000262209	PCDHGB3	protocadherin gamma subfamily B, 3	56102	0,493922553	-0,978099319	3,466776	-0,282135137	0,777839892	NA
ENSG00000262235	NA	NA	NA	0	NA	NA	NA	NA	NA
ENSG00000262248	NA	NA	NA	0,335252389	-1,100679997	4,080456	-0,26974437	0,787356923	NA
ENSG00000262259	MTND4LP24	MT-ND4L pseudogene 24	107075103	0	NA	NA	NA	NA	NA
ENSG00000262262	NA	NA	NA	0	NA	NA	NA	NA	NA
ENSG00000262271	NA	NA	NA	0	NA	NA	NA	NA	NA
ENSG00000262304	NA	NA	NA	4,409718643	-0,684294107	3,617087	-0,189183755	0,849948799	NA
ENSG00000262316	NA	NA	NA	0	NA	NA	NA	NA	NA
ENSG00000262322	NA	NA	NA	0	NA	NA	NA	NA	NA
ENSG00000262333	NA	NA	NA	7,618044208	-0,445708004	0,681655	-0,653861503	0,513201038	NA
ENSG00000262366	NDUFA3P6	NADH:ubiquinone oxidoreductase subunit A3 pseudogene 6	106479052	0	NA	NA	NA	NA	NA
ENSG00000262381	MTATP6P24	MT-ATP6 pseudogene 24	107075232	0	NA	NA	NA	NA	NA
ENSG00000262384	NA	NA	NA	0	NA	NA	NA	NA	NA
ENSG00000262400	NA	NA	NA	0	NA	NA	NA	NA	NA
ENSG00000262402	NA	NA	NA	5,06966883	-0,113077758	1,123481	-0,100649477	0,919828719	NA
ENSG00000262406	MMP12	matrix metallopeptidase 12	4321	0	NA	NA	NA	NA	NA
ENSG00000262408	NA	NA	NA	0,117497108	0,847101401	4,080456	0,207599697	0,835541541	NA
ENSG00000262461	SPDYE9	speedy/RINGO cell cycle regulator family member E9	643909	0	NA	NA	NA	NA	NA
ENSG00000262461	LOC124906603	putative speedy protein E9	124906603	0	NA	NA	NA	NA	NA
ENSG00000262470	NA	NA	NA	0	NA	NA	NA	NA	NA

ENSG00000262471	NA	NA	NA	0	NA	NA	NA	NA	NA
ENSG00000262480	SAMD11P1	sterile alpha motif domain containing 11 pseudogene 1	100421331	0	NA	NA	NA	NA	NA
ENSG00000262484	CCER2	coiled-coil glutamate rich protein 2	643669	1,21869529	1,468014608	2,939855	0,499349306	0,617533326	NA
ENSG00000262488	NA	NA	NA	0,118286652	0	4,080456	0	1	NA
ENSG00000262492	NA	NA	NA	0	NA	NA	NA	NA	NA
ENSG00000262495	NA	NA	NA	0	NA	NA	NA	NA	NA
ENSG00000262497	FAM187B2P	family with sequence similarity 187, member B pseudogene	100421621	0	NA	NA	NA	NA	NA
ENSG00000262500	MAPK8IP1P1	mitogen-activated protein kinase 8 interacting protein 1 pseudogene 1	644253	0	NA	NA	NA	NA	NA
ENSG00000262503	SPICP3	Spi-C transcription factor pseudogene 3	100128007	1,291073878	3,085771909	2,05937	1,498406034	0,13402779	NA
ENSG00000262516	DPPA3P6	DPPA3 pseudogene 6	127460882	0	NA	NA	NA	NA	NA
ENSG00000262518	NA	NA	NA	0	NA	NA	NA	NA	NA
ENSG00000262519	NA	NA	NA	0	NA	NA	NA	NA	NA
ENSG00000262526	NA	NA	NA	0	NA	NA	NA	NA	NA
ENSG00000262539	NA	NA	NA	0	NA	NA	NA	NA	NA
ENSG00000262543	SMIM28	small integral membrane protein 28	110806279	0	NA	NA	NA	NA	NA
ENSG00000262560	NA	NA	NA	0	NA	NA	NA	NA	NA
ENSG00000262561	NA	NA	NA	0	NA	NA	NA	NA	NA
ENSG00000262566	NA	NA	NA	0	NA	NA	NA	NA	NA
ENSG00000262576	PCDHGA4	protocadherin gamma subfamily A, 4	56111	0,590286911	2,933972749	3,208643	0,914396676	0,360508448	NA
ENSG00000262583	NA	NA	NA	0	NA	NA	NA	NA	NA
ENSG00000262587	NA	NA	NA	2,328487402	0,795275185	1,293909	0,614629946	0,538799134	NA
ENSG00000262609	BTF3P14	basic transcription factor 3 pseudogene 14	100132641	0	NA	NA	NA	NA	NA
ENSG00000262623	NA	NA	NA	0	NA	NA	NA	NA	NA
ENSG00000262628	OR1D5	olfactory receptor family 1 subfamily D member 5	8386	0	NA	NA	NA	NA	NA
ENSG00000262628	OR1D4	olfactory receptor family 1 subfamily D member 4	653166	0	NA	NA	NA	NA	NA
ENSG00000262633	NA	NA	NA	0	NA	NA	NA	NA	NA
ENSG00000262636	NA	NA	NA	0	NA	NA	NA	NA	NA
ENSG00000262648	PHB1P15	PHB1 pseudogene 15	100873752	0	NA	NA	NA	NA	NA
ENSG00000262651	NA	NA	NA	0	NA	NA	NA	NA	NA
ENSG00000262655	SPON1	spondin 1	10418	0,072165449	0,847101401	4,080456	0,207599697	0,835541541	NA
ENSG00000262660	NA	NA	NA	0	NA	NA	NA	NA	NA
ENSG00000262700	LOC100421173	thyroid hormone receptor interactor 11 pseudogene	100421173	0,103998153	-1,076443944	4,080456	-0,263804824	0,791930317	NA
ENSG00000262730	NA	NA	NA	0	NA	NA	NA	NA	NA
ENSG00000262759	NA	NA	NA	0	NA	NA	NA	NA	NA
ENSG00000262803	NA	NA	NA	1,780919399	0,504788055	1,548325	0,326022037	0,744407682	NA
ENSG00000262818	NA	NA	NA	0	NA	NA	NA	NA	NA
ENSG00000262855	NA	NA	NA	0	NA	NA	NA	NA	NA
ENSG00000262870	NA	NA	NA	0	NA	NA	NA	NA	NA
ENSG00000262874	C19orf84	chromosome 19 open reading frame 84	147646	0,649178328	0,000496462	2,682436	0,000185079	0,999852328	NA
ENSG00000262885	NA	NA	NA	0	NA	NA	NA	NA	NA
ENSG00000262902	MTCO1P40	MT-CO1 pseudogene 40	107075172	0,237754479	-0,114448441	4,080456	-0,028047955	0,977623904	NA
ENSG00000262904	TMPOP2	thymopoietin pseudogene 2	100533619	0,403604424	-1,637518964	3,547506	-0,461597197	0,644370205	NA
ENSG00000262943	NA	NA	NA	6,89033969	0,286705498	0,755935	0,379272652	0,704485406	NA
ENSG00000262953	NA	NA	NA	0	NA	NA	NA	NA	NA
ENSG00000262959	NA	NA	NA	0	NA	NA	NA	NA	NA
ENSG00000262961	NA	NA	NA	0	NA	NA	NA	NA	NA
ENSG00000262962	NA	NA	NA	0	NA	NA	NA	NA	NA
ENSG00000262974	NA	NA	NA	0	NA	NA	NA	NA	NA
ENSG00000262980	OR52L2P	olfactory receptor family 52 subfamily L member 2 pseudogene	79274	0	NA	NA	NA	NA	NA
ENSG00000262990	NA	NA	NA	0	NA	NA	NA	NA	NA
ENSG00000263006	NA	NA	NA	0	NA	NA	NA	NA	NA
ENSG00000263017	NA	NA	NA	0	NA	NA	NA	NA	NA
ENSG00000263020	NA	NA	NA	0,445460278	-3,0035493	3,971436	-0,756287955	0,449476568	NA
ENSG00000263045	NA	NA	NA	0,15836288	0	4,080456	0	1	NA
ENSG00000263070	NA	NA	NA	0	NA	NA	NA	NA	NA
ENSG00000263107	NA	NA	NA	0	NA	NA	NA	NA	NA
ENSG00000263125	NA	NA	NA	0	NA	NA	NA	NA	NA
ENSG00000263155	MYZAP	myocardial zonula adherens protein	100820829	0	NA	NA	NA	NA	NA
ENSG00000263177	MTND1P8	MT-ND1 pseudogene 8	100287461	0,11808024	-1,076443944	4,080456	-0,263804824	0,791930317	NA
ENSG00000263179	NA	NA	NA	0,224231977	-0,141316044	4,080456	-0,034632416	0,972372853	NA
ENSG00000263189	NA	NA	NA	0,10543123	0	4,080456	0	1	NA
ENSG00000263196	MTND3P13	MT-ND3 pseudogene 13	107075277	0	NA	NA	NA	NA	NA
ENSG00000263199	NA	NA	NA	0	NA	NA	NA	NA	NA

ENSG00000263201	DPEP2NB	DPEP2 neighbor	100131303	0	NA	NA	NA	NA	NA
ENSG00000263206	NA	NA	NA	0	NA	NA	NA	NA	NA
ENSG00000263219	NA	NA	NA	0,468160017	1,555816787	3,984964	0,390421827	0,696224651	NA
ENSG00000263232	ATP5F1AP3	ATP synthase F1 subunit alpha pseudogene 3	645443	0,072165449	0,847101401	4,080456	0,207599697	0,835541541	NA
ENSG00000263241	MTCYBP33	MT-CYB pseudogene 33	109729143	0	NA	NA	NA	NA	NA
ENSG00000263252	NA	NA	NA	0	NA	NA	NA	NA	NA
ENSG00000263266	RPS7P1	ribosomal protein S7 pseudogene 1	388363	0,974188058	0,668060778	1,71323	0,389942293	0,696579219	NA
ENSG00000263311	NA	NA	NA	0	NA	NA	NA	NA	NA
ENSG00000263326	NA	NA	NA	0	NA	NA	NA	NA	NA
ENSG00000263343	NA	NA	NA	0	NA	NA	NA	NA	NA
ENSG00000263355	AKR1B10P2	aldo-keto reductase family 1 member B10 pseudogene 2	643582	0	NA	NA	NA	NA	NA
ENSG00000263366	NA	NA	NA	0,10543123	0	4,080456	0	1	NA
ENSG00000263368	NA	NA	NA	0	NA	NA	NA	NA	NA
ENSG00000263369	NA	NA	NA	0	NA	NA	NA	NA	NA
ENSG00000263375	LOC100533653	FERM domain containing kindlin 3 pseudogene	100533653	0	NA	NA	NA	NA	NA
ENSG00000263396	NA	NA	NA	0	NA	NA	NA	NA	NA
ENSG00000263405	NA	NA	NA	0	NA	NA	NA	NA	NA
ENSG00000263429	TMEM238L	transmembrane protein 238 like	100289255	0	NA	NA	NA	NA	NA
ENSG00000263433	NA	NA	NA	0	NA	NA	NA	NA	NA
ENSG00000263460	NA	NA	NA	0	NA	NA	NA	NA	NA
ENSG00000263482	NA	NA	NA	0,342410426	1,940756892	3,761597	0,515939593	0,6058966	NA
ENSG00000263483	MTATP6P3	MT-ATP6 pseudogene 3	106479043	0	NA	NA	NA	NA	NA
ENSG00000263505	NA	NA	NA	0	NA	NA	NA	NA	NA
ENSG00000263535	NA	NA	NA	0	NA	NA	NA	NA	NA
ENSG00000263604	NA	NA	NA	0	NA	NA	NA	NA	NA
ENSG00000263606	NA	NA	NA	3,004646865	0,405506482	1,071764	0,378354348	0,705167377	NA
ENSG00000263620	NA	NA	NA	0,600096006	0,085389743	3,290348	0,025951587	0,979295953	NA
ENSG00000263639	MSMB	microseminoprotein beta	4477	0	NA	NA	NA	NA	NA
ENSG00000263648	NA	NA	NA	0,281976284	-0,979966816	4,041697	-0,242464205	0,80842049	NA
ENSG00000263682	NA	NA	NA	0	NA	NA	NA	NA	NA
ENSG00000263715	CRHR1	corticotropin releasing hormone receptor 1	1394	5,11392387	-0,514017335	0,89036	-0,577314302	0,563727154	NA
ENSG00000263715	LINC02210-CRHR1	LINC02210-CRHR1 readthrough	104909134	5,11392387	-0,514017335	0,89036	-0,577314302	0,563727154	NA
ENSG00000263716	SLC25A51P2	SLC25A51 pseudogene 2	645423	0	NA	NA	NA	NA	NA
ENSG00000263725	FTLP13	ferritin light chain pseudogene 13	100462793	0	NA	NA	NA	NA	NA
ENSG00000263729	NA	NA	NA	0	NA	NA	NA	NA	NA
ENSG00000263733	LOC100132647	signal peptide peptidase like 3 pseudogene	100132647	0	NA	NA	NA	NA	NA
ENSG00000263748	NA	NA	NA	0	NA	NA	NA	NA	NA
ENSG00000263749	NA	NA	NA	0	NA	NA	NA	NA	NA
ENSG00000263761	GDF2	growth differentiation factor 2	2658	0	NA	NA	NA	NA	NA
ENSG00000263772	LOC390846	golgi SNAP receptor complex member 2 pseudogene	390846	0	NA	NA	NA	NA	NA
ENSG00000263781	NA	NA	NA	0	NA	NA	NA	NA	NA
ENSG00000263788	NA	NA	NA	0	NA	NA	NA	NA	NA
ENSG00000263809	NA	NA	NA	2,313047562	-4,632895241	3,056313	-1,515844552	0,129558664	NA
ENSG00000263818	RDM1P5	RDM1 pseudogene 5	100131347	0	NA	NA	NA	NA	NA
ENSG00000263829	NA	NA	NA	0	NA	NA	NA	NA	NA
ENSG00000263846	NA	NA	NA	0	NA	NA	NA	NA	NA
ENSG00000263870	NA	NA	NA	0	NA	NA	NA	NA	NA
ENSG00000263883	EEF1DP7	eukaryotic translation elongation factor 1 delta pseudogene 7	100422656	1,830840063	-1,50761862	1,390776	-1,084012347	0,278359324	NA
ENSG00000263887	NA	NA	NA	0	NA	NA	NA	NA	NA
ENSG00000263914	MTND2P13	MT-ND2 pseudogene 13	100873345	0	NA	NA	NA	NA	NA
ENSG00000263935	TOMM20P3	TOMM20 pseudogene 3	106480301	0	NA	NA	NA	NA	NA
ENSG00000263946	NA	NA	NA	0	NA	NA	NA	NA	NA
ENSG00000263961	RHEX	regulator of hemoglobinization and erythroid cell expansion	440712	0	NA	NA	NA	NA	NA
ENSG00000263976	YWHAEP3	tyrosine 3-monooxygenase/tryptophan 5-monooxygenase activation protein epsilon pseudogene 3	106480273	0	NA	NA	NA	NA	NA
ENSG00000264006	AKR1C8	aldo-keto reductase family 1 member C8	340811	0	NA	NA	NA	NA	NA
ENSG00000264029	NA	NA	NA	0	NA	NA	NA	NA	NA
ENSG00000264040	NA	NA	NA	0	NA	NA	NA	NA	NA
ENSG00000264050	LOC100421028	gap junction protein alpha 4 pseudogene	100421028	0	NA	NA	NA	NA	NA

ENSG00000264054	RPL31P9	ribosomal protein L31 pseudogene 9	619464	0	NA	NA	NA	NA	NA
ENSG00000264057	NA	NA	NA	0,191778309	1,67823088	4,046254	0,414761636	0,678316407	NA
ENSG00000264058	NA	NA	NA	0	NA	NA	NA	NA	NA
ENSG00000264061	NA	NA	NA	0	NA	NA	NA	NA	NA
ENSG00000264093	NA	NA	NA	0	NA	NA	NA	NA	NA
ENSG00000264104	LOC100129135	selenocysteine insertion sequence-binding protein 2-like	100129135	0	NA	NA	NA	NA	NA
ENSG00000264127	SCML2P1	SCML2 pseudogene 1	100421167	0	NA	NA	NA	NA	NA
ENSG00000264138	NA	NA	NA	0	NA	NA	NA	NA	NA
ENSG00000264145	NA	NA	NA	0	NA	NA	NA	NA	NA
ENSG00000264150	NA	NA	NA	0	NA	NA	NA	NA	NA
ENSG00000264164	NA	NA	NA	0	NA	NA	NA	NA	NA
ENSG00000264168	MTND1P15	MT-ND1 pseudogene 15	100288998	0	NA	NA	NA	NA	NA
ENSG00000264172	PDLIM1P3	PDZ and LIM domain 1 pseudogene 3	100419427	0	NA	NA	NA	NA	NA
ENSG00000264176	NA	NA	NA	0	NA	NA	NA	NA	NA
ENSG00000264179	NA	NA	NA	0	NA	NA	NA	NA	NA
ENSG00000264186	NA	NA	NA	0	NA	NA	NA	NA	NA
ENSG00000264187	NA	NA	NA	0,387609674	-1,553668015	3,997422	-0,388667541	0,697522097	NA
ENSG00000264189	NA	NA	NA	0	NA	NA	NA	NA	NA
ENSG00000264193	NA	NA	NA	0	NA	NA	NA	NA	NA
ENSG00000264204	NA	NA	NA	0,072165449	0,847101401	4,080456	0,207599697	0,835541541	NA
ENSG00000264217	NA	NA	NA	0	NA	NA	NA	NA	NA
ENSG00000264230	ANXA8L1	annexin A8 like 1	728113	0,10488184	-1,076443944	4,080456	-0,263804824	0,791930317	NA
ENSG00000264236	LOC100418959	aminoacylase 1 pseudogene	100418959	0	NA	NA	NA	NA	NA
ENSG00000264240	CPDP1	carboxypeptidase D pseudogene 1	109729163	0	NA	NA	NA	NA	NA
ENSG00000264245	NA	NA	NA	0	NA	NA	NA	NA	NA
ENSG00000264246	NA	NA	NA	0	NA	NA	NA	NA	NA
ENSG00000264289	NA	NA	NA	0	NA	NA	NA	NA	NA
ENSG00000264296	NA	NA	NA	0	NA	NA	NA	NA	NA
ENSG00000264300	NA	NA	NA	0,072165449	0,847101401	4,080456	0,207599697	0,835541541	NA
ENSG00000264311	NA	NA	NA	0	NA	NA	NA	NA	NA
ENSG00000264315	NA	NA	NA	0,10488184	-1,076443944	4,080456	-0,263804824	0,791930317	NA
ENSG00000264316	MTCO3P13	MT-CO3 pseudogene 13	107075224	0	NA	NA	NA	NA	NA
ENSG00000264324	NA	NA	NA	3,442939954	0,535802281	1,826378	0,293368822	0,769240254	NA
ENSG00000264350	NA	NA	NA	0	NA	NA	NA	NA	NA
ENSG00000264359	NEK4P2	NIMA-related kinase 4 pseudogene 2	100421734	0	NA	NA	NA	NA	NA
ENSG00000264373	NA	NA	NA	0	NA	NA	NA	NA	NA
ENSG00000264388	NA	NA	NA	0	NA	NA	NA	NA	NA
ENSG00000264424	MYH4	myosin heavy chain 4	4622	0	NA	NA	NA	NA	NA
ENSG00000264431	NA	NA	NA	0	NA	NA	NA	NA	NA
ENSG00000264435	NA	NA	NA	0	NA	NA	NA	NA	NA
ENSG00000264444	NA	NA	NA	0	NA	NA	NA	NA	NA
ENSG00000264497	NA	NA	NA	0	NA	NA	NA	NA	NA
ENSG00000264503	NA	NA	NA	0	NA	NA	NA	NA	NA
ENSG00000264529	DNAJB6P8	DNAJB6 pseudogene 8	100421525	0	NA	NA	NA	NA	NA
ENSG00000264564	NA	NA	NA	0	NA	NA	NA	NA	NA
ENSG00000264570	NA	NA	NA	0	NA	NA	NA	NA	NA
ENSG00000264617	NA	NA	NA	0	NA	NA	NA	NA	NA
ENSG00000264644	NA	NA	NA	0	NA	NA	NA	NA	NA
ENSG00000264655	NA	NA	NA	0,222871603	-1,090532802	4,080456	-0,26725759	0,789270838	NA
ENSG00000264662	NA	NA	NA	0	NA	NA	NA	NA	NA
ENSG00000264663	NA	NA	NA	3,441104634	0,651051461	1,2038	0,540830084	0,588624705	NA
ENSG00000264668	NA	NA	NA	3,5212684	0	2,063856	0	1	NA
ENSG00000264685	PRPF19P1	PRPF19 pseudogene 1	100422496	0	NA	NA	NA	NA	NA
ENSG00000264689	NA	NA	NA	0	NA	NA	NA	NA	NA
ENSG00000264697	NA	NA	NA	0	NA	NA	NA	NA	NA
ENSG00000264714	NA	NA	NA	0	NA	NA	NA	NA	NA
ENSG00000264717	NPY4R2	neuropeptide Y receptor Y4-2	100996758	0	NA	NA	NA	NA	NA
ENSG00000264736	NA	NA	NA	0	NA	NA	NA	NA	NA
ENSG00000264775	NA	NA	NA	0	NA	NA	NA	NA	NA
ENSG00000264801	NA	NA	NA	0,646979851	-0,475330612	2,674048	-0,177756973	0,858913839	NA
ENSG00000264813	NA	NA	NA	0	NA	NA	NA	NA	NA
ENSG00000264837	VN1R71P	vomeronasal 1 receptor 71 pseudogene	100312817	0	NA	NA	NA	NA	NA
ENSG00000264845	LOC105372179	uncharacterized LOC105372179	105372179	0	NA	NA	NA	NA	NA
ENSG00000264874	NA	NA	NA	0	NA	NA	NA	NA	NA
ENSG00000264892	NOS2P4	nitric oxide synthase 2 pseudogene 4	266619	0	NA	NA	NA	NA	NA
ENSG00000264907	NA	NA	NA	0	NA	NA	NA	NA	NA
ENSG00000264911	LOC100419894	zinc finger protein 347 pseudogene	100419894	0	NA	NA	NA	NA	NA
ENSG00000264930	NA	NA	NA	0	NA	NA	NA	NA	NA
ENSG00000264943	NA	NA	NA	4,581287016	-0,397363665	0,940985	-0,42228467	0,672817247	NA

ENSG00000264958	NA	NA	NA	0	NA	NA	NA	NA	NA
ENSG00000264970	NA	NA	NA	0	NA	NA	NA	NA	NA
ENSG00000264971	NA	NA	NA	0	NA	NA	NA	NA	NA
ENSG00000265015	NA	NA	NA	0	NA	NA	NA	NA	NA
ENSG00000265019	NA	NA	NA	0	NA	NA	NA	NA	NA
ENSG00000265038	NA	NA	NA	0	NA	NA	NA	NA	NA
ENSG00000265041	NA	NA	NA	0	NA	NA	NA	NA	NA
ENSG00000265060	NA	NA	NA	0	NA	NA	NA	NA	NA
ENSG00000265095	FTLP12	ferritin light chain pseudogene 12	100462792	0,15836288	0	4,080456	0	1	NA
ENSG00000265097	RBM22P1	RNA binding motif protein 22 pseudogene 1	400645	0	NA	NA	NA	NA	NA
ENSG00000265099	NA	NA	NA	0	NA	NA	NA	NA	NA
ENSG00000265107	GJA5	gap junction protein alpha 5	2702	0	NA	NA	NA	NA	NA
ENSG00000265136	NA	NA	NA	0	NA	NA	NA	NA	NA
ENSG00000265158	NA	NA	NA	0	NA	NA	NA	NA	NA
ENSG00000265182	SRP72P1	signal recognition particle 72 pseudogene 1	100420752	0	NA	NA	NA	NA	NA
ENSG00000265188	LOC100422274	transforming acidic coiled-coil containing protein 3 pseudogene	100422274	0,11808024	-1,076443944	4,080456	-0,263804824	0,791930317	NA
ENSG00000265190	ANXA8	annexin A8	653145	0	NA	NA	NA	NA	NA
ENSG00000265203	RBP3	retinol binding protein 3	5949	0	NA	NA	NA	NA	NA
ENSG00000265233	NA	NA	NA	0	NA	NA	NA	NA	NA
ENSG00000265240	LOC100131011	myotubularin related protein 14 pseudogene	100131011	0	NA	NA	NA	NA	NA
ENSG00000265243	IGLJCOR18	immunoglobulin lambda joining-constant/OR18 (pseudogene)	204777	0	NA	NA	NA	NA	NA
ENSG00000265261	NA	NA	NA	0	NA	NA	NA	NA	NA
ENSG00000265262	NA	NA	NA	0	NA	NA	NA	NA	NA
ENSG00000265273	PGDP1	phosphogluconate dehydrogenase pseudogene 1	342705	0	NA	NA	NA	NA	NA
ENSG00000265279	CLUHP6	clustered mitochondria homolog pseudogene 6	100418754	0	NA	NA	NA	NA	NA
ENSG00000265293	NA	NA	NA	0	NA	NA	NA	NA	NA
ENSG00000265296	FEM1AP2	fem-1 homolog A pseudogene 2	390834	0	NA	NA	NA	NA	NA
ENSG00000265298	NA	NA	NA	0	NA	NA	NA	NA	NA
ENSG00000265303	NA	NA	NA	0	NA	NA	NA	NA	NA
ENSG00000265340	OR4K7P	olfactory receptor family 4 subfamily K member 7 pseudogene	79306	0	NA	NA	NA	NA	NA
ENSG00000265366	NA	NA	NA	0	NA	NA	NA	NA	NA
ENSG00000265402	NA	NA	NA	0	NA	NA	NA	NA	NA
ENSG00000265417	LOC100129774	ATPase family AAA domain containing 1 pseudogene	100129774	0	NA	NA	NA	NA	NA
ENSG00000265428	NA	NA	NA	0	NA	NA	NA	NA	NA
ENSG00000265437	NA	NA	NA	0	NA	NA	NA	NA	NA
ENSG00000265453	NA	NA	NA	0	NA	NA	NA	NA	NA
ENSG00000265460	NA	NA	NA	0	NA	NA	NA	NA	NA
ENSG00000265469	NA	NA	NA	0	NA	NA	NA	NA	NA
ENSG00000265472	NA	NA	NA	0	NA	NA	NA	NA	NA
ENSG00000265480	KRT18P55	keratin 18 pseudogene 55	284085	0	NA	NA	NA	NA	NA
ENSG00000265531	FCGR1CP	Fc gamma receptor Ic, pseudogene	100132417	0	NA	NA	NA	NA	NA
ENSG00000265541	H3P41	H3 histone pseudogene 41	100420410	0	NA	NA	NA	NA	NA
ENSG00000265545	NA	NA	NA	0	NA	NA	NA	NA	NA
ENSG00000265564	PIGPP4	phosphatidylinositol glycan anchor biosynthesis class P pseudogene 4	106480811	0	NA	NA	NA	NA	NA
ENSG00000265574	NA	NA	NA	0	NA	NA	NA	NA	NA
ENSG00000265590	CFAP298-TCP10L	CFAP298-TCP10L readthrough	110091775	8,89277677	0,246279865	0,844305	0,291695433	0,770519501	NA
ENSG00000265593	NA	NA	NA	0	NA	NA	NA	NA	NA
ENSG00000265614	NA	NA	NA	0	NA	NA	NA	NA	NA
ENSG00000265626	NA	NA	NA	0	NA	NA	NA	NA	NA
ENSG00000265630	NA	NA	NA	0,210352599	-1,06830069	4,080456	-0,261809152	0,793468583	NA
ENSG00000265631	BNIP3P3	BCL2 interacting protein 3 pseudogene 3	100421736	0	NA	NA	NA	NA	NA
ENSG00000265639	LOC100421257	lysosomal associated membrane protein 1 pseudogene	100421257	0	NA	NA	NA	NA	NA
ENSG00000265646	TUFMP1	Tu translation elongation factor, mitochondrial pseudogene 1	645668	0	NA	NA	NA	NA	NA
ENSG00000265683	SYPL1P2	synaptophysin like 1 pseudogene 2	100132972	0,297862908	1,661085655	3,886234	0,427428058	0,669067575	NA
ENSG00000265690	NA	NA	NA	0,207996306	-1,916073151	4,043021	-0,473921178	0,635556117	NA

ENSG00000265693	NA	NA	NA	0	NA	NA	NA	NA	NA
ENSG00000265698	NA	NA	NA	0	NA	NA	NA	NA	NA
ENSG00000265712	LOC100422053	intraflagellar transport 57 homolog (Chlamydomonas) pseudogene	100422053	0	NA	NA	NA	NA	NA
ENSG00000265713	LOC100421100	RNA terminal phosphate cyclase like 1 pseudogene	100421100	4,206384747	0,146352568	0,951766	0,153769547	0,877791447	NA
ENSG00000265746	NA	NA	NA	0	NA	NA	NA	NA	NA
ENSG00000265763	ZNF488	zinc finger protein 488	118738	0	NA	NA	NA	NA	NA
ENSG00000265766	CXADRP3	CXADR pseudogene 3	440224	0,210862461	0	4,080456	0	1	NA
ENSG00000265787	NA	NA	NA	0,340268604	-0,1398134	3,785706	-0,036931922	0,970539287	NA
ENSG00000265788	NA	NA	NA	0	NA	NA	NA	NA	NA
ENSG00000265790	RNASEH1P1	ribonuclease H1 pseudogene 1	284203	0	NA	NA	NA	NA	NA
ENSG00000265817	FSBP	fibrinogen silencer binding protein	100861412	5,541692722	-0,359620415	0,841633	-0,427288915	0,669168906	NA
ENSG00000265818	NA	NA	NA	0	NA	NA	NA	NA	NA
ENSG00000265833	NA	NA	NA	0	NA	NA	NA	NA	NA
ENSG00000265836	CCND3P2	cyclin D3 pseudogene 2	106480307	0	NA	NA	NA	NA	NA
ENSG00000265853	NA	NA	NA	0	NA	NA	NA	NA	NA
ENSG00000265863	NA	NA	NA	0,10488184	-1,076443944	4,080456	-0,263804824	0,791930317	NA
ENSG00000265881	PDLIM1P2	PDZ and LIM domain 1 pseudogene 2	100419426	0	NA	NA	NA	NA	NA
ENSG00000265883	NA	NA	NA	0	NA	NA	NA	NA	NA
ENSG00000265916	NA	NA	NA	0,237883245	-0,085026507	4,079929	-0,02084019	0,983373138	NA
ENSG00000265934	NA	NA	NA	0	NA	NA	NA	NA	NA
ENSG00000265936	NA	NA	NA	0	NA	NA	NA	NA	NA
ENSG00000265939	UBE2CP2	ubiquitin conjugating enzyme E2 C pseudogene 2	646595	0,336489068	-2,020979568	4,031826	-0,501256695	0,616190478	NA
ENSG00000265946	NA	NA	NA	0	NA	NA	NA	NA	NA
ENSG00000265973	NA	NA	NA	0	NA	NA	NA	NA	NA
ENSG00000265982	NA	NA	NA	0	NA	NA	NA	NA	NA
ENSG00000266003	NA	NA	NA	0	NA	NA	NA	NA	NA
ENSG00000266009	NA	NA	NA	0	NA	NA	NA	NA	NA
ENSG00000266042	NA	NA	NA	0	NA	NA	NA	NA	NA
ENSG00000266048	NA	NA	NA	0	NA	NA	NA	NA	NA
ENSG00000266050	NA	NA	NA	0	NA	NA	NA	NA	NA
ENSG00000266066	POLRMTP1	RNA polymerase mitochondrial pseudogene 1	284167	0,898890341	0,700874442	2,637177	0,265766908	0,79041873	NA
ENSG00000266074	BAHCC1	BAH domain and coiled-coil containing 1	57597	0	NA	NA	NA	NA	NA
ENSG00000266076	NA	NA	NA	0	NA	NA	NA	NA	NA
ENSG00000266105	LOC100287539	epsin 2 pseudogene	100287539	0	NA	NA	NA	NA	NA
ENSG00000266117	NA	NA	NA	0	NA	NA	NA	NA	NA
ENSG00000266127	ZNF415P1	zinc finger protein 415 pseudogene 1	100419893	0	NA	NA	NA	NA	NA
ENSG00000266129	SRP68P1	signal recognition particle 68 pseudogene 1	252841	0	NA	NA	NA	NA	NA
ENSG00000266145	RHOT1P1	ras homolog family member T1 pseudogene 1	100462813	0,74289423	-0,813526345	1,977569	-0,411377014	0,680796106	NA
ENSG00000266155	NA	NA	NA	0	NA	NA	NA	NA	NA
ENSG00000266179	NA	NA	NA	0	NA	NA	NA	NA	NA
ENSG00000266181	NA	NA	NA	0	NA	NA	NA	NA	NA
ENSG00000266184	NA	NA	NA	0	NA	NA	NA	NA	NA
ENSG00000266190	NA	NA	NA	0	NA	NA	NA	NA	NA
ENSG00000266200	PNLIPRP2	pancreatic lipase related protein 2 (gene/pseudogene)	5408	0	NA	NA	NA	NA	NA
ENSG00000266201	NA	NA	NA	0	NA	NA	NA	NA	NA
ENSG00000266202	NA	NA	NA	0	NA	NA	NA	NA	NA
ENSG00000266217	NA	NA	NA	0	NA	NA	NA	NA	NA
ENSG00000266227	NA	NA	NA	0	NA	NA	NA	NA	NA
ENSG00000266242	GRAMD4P7	GRAM domain containing 4 pseudogene 7	100631251	0,103998153	-1,076443944	4,080456	-0,263804824	0,791930317	NA
ENSG00000266251	NA	NA	NA	0	NA	NA	NA	NA	NA
ENSG00000266265	KLF14	KLF transcription factor 14	136259	0	NA	NA	NA	NA	NA
ENSG00000266282	NA	NA	NA	0	NA	NA	NA	NA	NA
ENSG00000266296	NA	NA	NA	0	NA	NA	NA	NA	NA
ENSG00000266306	NA	NA	NA	1,460342757	-0,231327309	1,796026	-0,128799498	0,897516303	NA
ENSG00000266356	NA	NA	NA	0	NA	NA	NA	NA	NA
ENSG00000266364	NA	NA	NA	0	NA	NA	NA	NA	NA
ENSG00000266365	LOC100996361	ribosomal protein S6 kinase B1 pseudogene	100996361	0	NA	NA	NA	NA	NA
ENSG00000266373	NA	NA	NA	0	NA	NA	NA	NA	NA
ENSG00000266379	NA	NA	NA	0	NA	NA	NA	NA	NA
ENSG00000266405	CBX3P2	CBX3 pseudogene 2	645158	1,675830156	-0,713320744	1,414091	-0,504437677	0,613953848	NA
ENSG00000266416	NA	NA	NA	0	NA	NA	NA	NA	NA
ENSG00000266433	NA	NA	NA	0	NA	NA	NA	NA	NA
ENSG00000266448	NA	NA	NA	0	NA	NA	NA	NA	NA

ENSG00000266470	NA	NA	NA	0	NA	NA	NA	NA	NA
ENSG00000266479	NA	NA	NA	0	NA	NA	NA	NA	NA
ENSG00000266501	NA	NA	NA	0	NA	NA	NA	NA	NA
ENSG00000266504	RDM1P1	RDM1 pseudogene 1	112577465	0,757957464	-0,83037664	2,353376	-0,352844814	0,724204785	NA
ENSG00000266520	NA	NA	NA	0	NA	NA	NA	NA	NA
ENSG00000266522	NA	NA	NA	0	NA	NA	NA	NA	NA
ENSG00000266524	GDF10	growth differentiation factor 10	2662	0	NA	NA	NA	NA	NA
ENSG00000266529	MTCYBP13	MT-CYB pseudogene 13	107075129	0	NA	NA	NA	NA	NA
ENSG00000266537	NA	NA	NA	0	NA	NA	NA	NA	NA
ENSG00000266549	NA	NA	NA	0	NA	NA	NA	NA	NA
ENSG00000266561	NA	NA	NA	0	NA	NA	NA	NA	NA
ENSG00000266563	EIF1P5	eukaryotic translation initiation factor 1 pseudogene 5	106481690	0	NA	NA	NA	NA	NA
ENSG00000266575	NA	NA	NA	0	NA	NA	NA	NA	NA
ENSG00000266605	LONRF2P1	LONRF2 pseudogene 1	100421827	0,11808024	-1,076443944	4,080456	-0,263804824	0,791930317	NA
ENSG00000266613	COP1P1	COP1 pseudogene 1	729391	0	NA	NA	NA	NA	NA
ENSG00000266647	NA	NA	NA	0	NA	NA	NA	NA	NA
ENSG00000266648	NA	NA	NA	0,11808024	-1,076443944	4,080456	-0,263804824	0,791930317	NA
ENSG00000266673	NA	NA	NA	0	NA	NA	NA	NA	NA
ENSG00000266691	LOC100421165	FERM domain containing kindlin 3 pseudogene	100421165	0	NA	NA	NA	NA	NA
ENSG00000266693	OR4K8P	olfactory receptor family 4 subfamily K member 8 pseudogene	390836	0	NA	NA	NA	NA	NA
ENSG00000266707	NA	NA	NA	0	NA	NA	NA	NA	NA
ENSG00000266728	NA	NA	NA	0	NA	NA	NA	NA	NA
ENSG00000266733	NA	NA	NA	0	NA	NA	NA	NA	NA
ENSG00000266736	NA	NA	NA	0	NA	NA	NA	NA	NA
ENSG00000266771	NA	NA	NA	0	NA	NA	NA	NA	NA
ENSG00000266777	NA	NA	NA	10,80288607	-0,047584603	0,573216	-0,083013351	0,933840923	NA
ENSG00000266786	NA	NA	NA	0	NA	NA	NA	NA	NA
ENSG00000266806	NA	NA	NA	0	NA	NA	NA	NA	NA
ENSG00000266809	PPIAP57	peptidylprolyl isomerase A pseudogene 57	111082979	0	NA	NA	NA	NA	NA
ENSG00000266818	NA	NA	NA	0	NA	NA	NA	NA	NA
ENSG00000266826	IGBP1C	IGBP1 family member C	645545	0	NA	NA	NA	NA	NA
ENSG00000266853	ITM2BP1	integral membrane protein 2B pseudogene 1	100420308	0,072165449	0,847101401	4,080456	0,207599697	0,835541541	NA
ENSG00000266858	NA	NA	NA	0	NA	NA	NA	NA	NA
ENSG00000266876	STX18P1	syntaxin 18 pseudogene 1	100418889	0,178503397	0,83022484	4,079803	0,203496309	0,83874713	NA
ENSG00000266885	NA	NA	NA	0	NA	NA	NA	NA	NA
ENSG00000266891	NA	NA	NA	0	NA	NA	NA	NA	NA
ENSG00000266899	NA	NA	NA	0	NA	NA	NA	NA	NA
ENSG00000266906	NA	NA	NA	0	NA	NA	NA	NA	NA
ENSG00000266907	NA	NA	NA	0	NA	NA	NA	NA	NA
ENSG00000266909	SLC25A6P4	solute carrier family 25 member 6 pseudogene 4	100422690	0	NA	NA	NA	NA	NA
ENSG00000266910	NA	NA	NA	4,523277773	0,553317511	0,856557	0,645978567	0,518293236	NA
ENSG00000266915	MRPS5P4	mitochondrial ribosomal protein S5 pseudogene 4	359797	0	NA	NA	NA	NA	NA
ENSG00000266920	NA	NA	NA	0,10543123	0	4,080456	0	1	NA
ENSG00000266931	NA	NA	NA	4,548712791	-0,554475975	0,865699	-0,640494764	0,521850992	NA
ENSG00000266932	NA	NA	NA	0	NA	NA	NA	NA	NA
ENSG00000266938	LOC100419704	zinc finger protein 57 pseudogene	100419704	0	NA	NA	NA	NA	NA
ENSG00000266943	NA	NA	NA	0	NA	NA	NA	NA	NA
ENSG00000266944	NA	NA	NA	0	NA	NA	NA	NA	NA
ENSG00000266946	NA	NA	NA	4,370472015	0,770641284	0,983668	0,78343666	0,433370735	NA
ENSG00000266953	NA	NA	NA	0,306822285	-1,097145236	4,029605	-0,272271192	0,785413505	NA
ENSG00000266959	NA	NA	NA	0	NA	NA	NA	NA	NA
ENSG00000266964	FXYD1	FXYD domain containing ion transport regulator 1	5348	0,747104016	0,720061724	2,466264	0,29196457	0,770313713	NA
ENSG00000266971	OR4F8P	olfactory receptor family 4 subfamily F member 8 pseudogene	390872	0	NA	NA	NA	NA	NA
ENSG00000266984	NA	NA	NA	0	NA	NA	NA	NA	NA
ENSG00000266989	NA	NA	NA	0	NA	NA	NA	NA	NA
ENSG00000266992	DHX40P1	DEAH-box helicase 40 pseudogene 1	107133522	1,7720125	-0,01476654	1,495241	-0,00987569	0,992120468	NA
ENSG00000266995	NA	NA	NA	0	NA	NA	NA	NA	NA
ENSG00000266997	NA	NA	NA	0	NA	NA	NA	NA	NA
ENSG00000267001	NA	NA	NA	0,783406519	-2,514109682	3,738517	-0,672488447	0,501272793	NA
ENSG00000267005	NA	NA	NA	0	NA	NA	NA	NA	NA
ENSG00000267019	NA	NA	NA	0	NA	NA	NA	NA	NA
ENSG00000267022	NA	NA	NA	5,756864779	0,446449763	2,657865	0,167973046	0,866604484	NA
ENSG00000267023	NA	NA	NA	0,637323126	-0,984081823	3,324929	-0,295970747	0,767252421	NA
ENSG00000267032	NA	NA	NA	0	NA	NA	NA	NA	NA
ENSG00000267036	NA	NA	NA	0	NA	NA	NA	NA	NA

ENSG00000267046	NA	NA	NA	2,31452327	-0,576947203	1,249613	-0,46170074	0,644295941	NA	
ENSG00000267056	LOC729654	inositol polyphosphate multikinase pseudogene	729654	0	NA	NA	NA	NA	NA	
ENSG00000267060	PTGES3L	prostaglandin E synthase 3 like	100885848	0	NA	NA	NA	NA	NA	
ENSG00000267066	PLEKHB2P1	PLEKHB2 pseudogene 1	100128022	0	NA	NA	NA	NA	NA	
ENSG00000267076	NA	NA	NA	0	NA	NA	NA	NA	NA	
ENSG00000267083	KRT18P61	keratin 18 pseudogene 61	100418865	0,821454617	-1,158153538	2,250684	-0,51457857	0,606847543	NA	
ENSG00000267084	NA	NA	NA	0	NA	NA	NA	NA	NA	
ENSG00000267085	NA	NA	NA	0	NA	NA	NA	NA	NA	
ENSG00000267088	NA	NA	NA	0,404259628	-0,173850915	3,580259	-0,048558196	0,961271385	NA	
ENSG00000267091	CTBP2P7	CTBP2 pseudogene 7	401914	0,119673877	0,847101401	4,080456	0,207599697	0,835541541	NA	
ENSG00000267099	NA	NA	NA	0	NA	NA	NA	NA	NA	
ENSG00000267102	NA	NA	NA	1,521094606	0,879582479	3,108576	0,282953502	0,777212481	NA	
ENSG00000267110	NA	NA	NA	0	NA	NA	NA	NA	NA	
ENSG00000267113	NA	NA	NA	0	NA	NA	NA	NA	NA	
ENSG00000267116	NA	NA	NA	0	NA	NA	NA	NA	NA	
ENSG00000267119	RPL10P15	ribosomal protein L10 pseudogene 15	100129975	0	NA	NA	NA	NA	NA	
ENSG00000267127	NA	NA	NA	0,118286652	0	4,080456	0	1	NA	
ENSG00000267129	NA	NA	NA	0	NA	NA	NA	NA	NA	
ENSG00000267132	HMGB3P27	high mobility group box 3 pseudogene 27	441795	0	NA	NA	NA	NA	NA	
ENSG00000267135	NA	NA	NA	0,11808024	-1,076443944	4,080456	-0,263804824	0,791930317	NA	
ENSG00000267140	NA	NA	NA	0,224814984	0,834825609	4,080456	0,20459126	0,837891484	NA	
ENSG00000267142	NA	NA	NA	0	NA	NA	NA	NA	NA	
ENSG00000267145	NA	NA	NA	0	NA	NA	NA	NA	NA	
ENSG00000267148	NA	NA	NA	0,264498504	-1,065764006	4,080456	-0,261187485	0,793947929	NA	
ENSG00000267153	NA	NA	NA	0	NA	NA	NA	NA	NA	
ENSG00000267156	NA	NA	NA	0,117497108	0,847101401	4,080456	0,207599697	0,835541541	NA	
ENSG00000267157	NA	NA	NA	0,432992695	2,878653477	3,96827	0,725417712	0,46819581	NA	
ENSG00000267162	NA	NA	NA	0	NA	NA	NA	NA	NA	
ENSG00000267166	NA	NA	NA	0	NA	NA	NA	NA	NA	
ENSG00000267168	NA	NA	NA	0	NA	NA	NA	NA	NA	
ENSG00000267170	NA	NA	NA	0	NA	NA	NA	NA	NA	
ENSG00000267173	NA	NA	NA	0	NA	NA	NA	NA	NA	
ENSG00000267178	NA	NA	NA	0,439020347	1,529202333	3,438623	0,444713533	0,656526789	NA	
ENSG00000267179	NA	NA	NA	1,469932668	-3,915612729	2,694895	-1,452973933	0,146230992	NA	
ENSG00000267180	NA	NA	NA	0	NA	NA	NA	NA	NA	
ENSG00000267182	NA	NA	NA	0	NA	NA	NA	NA	NA	
ENSG00000267184	NA	NA	NA	0	NA	NA	NA	NA	NA	
ENSG00000267185	NA	NA	NA	0,072165449	0,847101401	4,080456	0,207599697	0,835541541	NA	
ENSG00000267187	NA	NA	NA	0	NA	NA	NA	NA	NA	
ENSG00000267189	ATP5MGP7	ATP synthase membrane subunit g pseudogene 7	100287964	0	NA	NA	NA	NA	NA	
ENSG00000267190	TYMSP2	thymidylate synthetase pseudogene 2	109729122	0	NA	NA	NA	NA	NA	
ENSG00000267203	NA	NA	NA	0	NA	NA	NA	NA	NA	
ENSG00000267206	LCN6	lipocalin 6	158062	0	NA	NA	NA	NA	NA	
ENSG00000267218	LOC100421620	RNA polymerase mitochondrial pseudogene	100421620	0	NA	NA	NA	NA	NA	
ENSG00000267220	NA	NA	NA	0	NA	NA	NA	NA	NA	
ENSG00000267221	C17orf113	chromosome 17 open reading frame 113	110806298	0,23716975	1,955833014	4,022315	0,48624563	0,626793023	NA	
ENSG00000267227	NA	NA	NA	0	NA	NA	NA	NA	NA	
ENSG00000267228	NA	NA	NA	1,537726471	0	3,774862	0	1	NA	
ENSG00000267233	HNRNPA3P16	heterogeneous nuclear ribonucleoprotein A3 pseudogene 16	100421451	0	NA	NA	NA	NA	NA	
ENSG00000267235	ZNF861P	zinc finger protein 861, pseudogene	100302737	0	NA	NA	NA	NA	NA	
ENSG00000267241	NA	NA	NA	0	NA	NA	NA	NA	NA	
ENSG00000267245	NA	NA	NA	0	NA	NA	NA	NA	NA	
ENSG00000267253	WHSC1L2P	Wolf-Hirschhorn syndrome candidate 1-like 2, pseudogene	100128016	0,237749424	-0,123355296	4,080456	-0,030230763	0,975883014	NA	
ENSG00000267258	PPIAP58	peptidylprolyl isomerase A pseudogene 58	126170	0	NA	NA	NA	NA	NA	
ENSG00000267261	NA	NA	NA	0	NA	NA	NA	NA	NA	
ENSG00000267264	NA	NA	NA	2,080328377	0,528213381	1,325966	0,398361302	0,690363879	NA	
ENSG00000267271	LOC101060119	RING-box protein 2-like	101060119	0,15836288	0	4,080456	0	1	NA	
ENSG00000267281	ATF7-NPFF	ATF7-NPFF readthrough	114108587	4,316809869	2,581803621	3,344511	0,771952573	0,440142518	NA	
ENSG00000267292	NA	NA	NA	0	NA	NA	NA	NA	NA	
ENSG00000267293	LOC100422497	UTP18, small subunit (SSU) processome component, homolog (yeast) pseudogene	100422497	0,578957889	0,724326183	3,248244	0,222990104	0,823543209	NA	
ENSG00000267295	NA	NA	NA	0	NA	NA	NA	NA	NA	
ENSG00000267301	RPL23AP77	ribosomal protein L23a pseudogene 77	728538	0	NA	NA	NA	NA	NA	

ENSG00000267305	NA	NA	NA	0	NA	NA	NA	NA	NA
ENSG00000267310	OR4G1P	olfactory receptor family 4 subfamily G member 1 pseudogene	26681	0	NA	NA	NA	NA	NA
ENSG00000267315	NA	NA	NA	0	NA	NA	NA	NA	NA
ENSG00000267318	NA	NA	NA	0,210314547	-1,075511843	4,080456	-0,263576394	0,792106351	NA
ENSG00000267319	NA	NA	NA	0	NA	NA	NA	NA	NA
ENSG00000267323	SLC25A1P5	solute carrier family 25 member 1 pseudogene 5	642290	0	NA	NA	NA	NA	NA
ENSG00000267324	NA	NA	NA	0	NA	NA	NA	NA	NA
ENSG00000267333	NA	NA	NA	0	NA	NA	NA	NA	NA
ENSG00000267335	NA	NA	NA	0	NA	NA	NA	NA	NA
ENSG00000267336	NA	NA	NA	0,341086052	0,983315948	3,766149	0,261093245	0,7940206	NA
ENSG00000267340	NA	NA	NA	0,557132558	1,943147982	4,022539	0,483065074	0,629049532	NA
ENSG00000267343	NA	NA	NA	0,539661851	-1,56486482	2,82969	-0,553016421	0,580252166	NA
ENSG00000267346	NA	NA	NA	0,963135733	-0,557490012	1,927724	-0,289195932	0,772431445	NA
ENSG00000267352	NA	NA	NA	0	NA	NA	NA	NA	NA
ENSG00000267355	NA	NA	NA	0	NA	NA	NA	NA	NA
ENSG00000267360	NA	NA	NA	0	NA	NA	NA	NA	NA
ENSG00000267361	SEC24AP1	SEC24 homolog A pseudogene 1	100421166	0	NA	NA	NA	NA	NA
ENSG00000267369	TAF5LP1	TATA-box binding protein associated factor 5 like pseudogene 1	645744	0	NA	NA	NA	NA	NA
ENSG00000267370	NA	NA	NA	0,209763681	-1,926644696	4,042043	-0,476651224	0,633610501	NA
ENSG00000267371	NA	NA	NA	0	NA	NA	NA	NA	NA
ENSG00000267376	NA	NA	NA	0,209429383	-1,076451951	4,080456	-0,263806786	0,791928805	NA
ENSG00000267382	NA	NA	NA	0	NA	NA	NA	NA	NA
ENSG00000267384	NA	NA	NA	0	NA	NA	NA	NA	NA
ENSG00000267385	NA	NA	NA	0	NA	NA	NA	NA	NA
ENSG00000267389	NA	NA	NA	0	NA	NA	NA	NA	NA
ENSG00000267392	LOC124900424	cytochrome P450 4F2-like	124900424	0	NA	NA	NA	NA	NA
ENSG00000267398	NA	NA	NA	0	NA	NA	NA	NA	NA
ENSG00000267400	NA	NA	NA	0	NA	NA	NA	NA	NA
ENSG00000267404	NA	NA	NA	0,103998153	-1,076443944	4,080456	-0,263804824	0,791930317	NA
ENSG00000267408	NA	NA	NA	0	NA	NA	NA	NA	NA
ENSG00000267415	NA	NA	NA	0	NA	NA	NA	NA	NA
ENSG00000267418	NA	NA	NA	0	NA	NA	NA	NA	NA
ENSG00000267419	NA	NA	NA	0,32768476	-0,954587842	4,043007	-0,236108378	0,813348573	NA
ENSG00000267422	NA	NA	NA	0,103998153	-1,076443944	4,080456	-0,263804824	0,791930317	NA
ENSG00000267426	NA	NA	NA	0	NA	NA	NA	NA	NA
ENSG00000267428	DYNAPP2	DYNAP pseudogene 2	125078041	0	NA	NA	NA	NA	NA
ENSG00000267430	LOC100422317	proteasome 26S subunit, ATPase 5 pseudogene	100422317	2,211076283	-0,061364516	1,284469	-0,047774242	0,961896165	NA
ENSG00000267433	NA	NA	NA	0	NA	NA	NA	NA	NA
ENSG00000267444	SMUG1P1	single-strand-selective monofunctional uracil-DNA glycosylase 1 pseudogene 1	100129143	0	NA	NA	NA	NA	NA
ENSG00000267450	OR1AB1P	olfactory receptor family 1 subfamily AB member 1 pseudogene	81090	0	NA	NA	NA	NA	NA
ENSG00000267453	NA	NA	NA	0	NA	NA	NA	NA	NA
ENSG00000267456	NA	NA	NA	0	NA	NA	NA	NA	NA
ENSG00000267463	NA	NA	NA	0	NA	NA	NA	NA	NA
ENSG00000267467	APOC4	apolipoprotein C4	346	0	NA	NA	NA	NA	NA
ENSG00000267471	NA	NA	NA	0	NA	NA	NA	NA	NA
ENSG00000267472	NA	NA	NA	0,10488184	-1,076443944	4,080456	-0,263804824	0,791930317	NA
ENSG00000267477	NA	NA	NA	0	NA	NA	NA	NA	NA
ENSG00000267478	LOC100533852	nuclear factor kappa B subunit 2 pseudogene	100533852	0	NA	NA	NA	NA	NA
ENSG00000267482	RNY4P13	RNY4 pseudogene 13	100379296	0	NA	NA	NA	NA	NA
ENSG00000267486	NA	NA	NA	0	NA	NA	NA	NA	NA
ENSG00000267487	NA	NA	NA	0	NA	NA	NA	NA	NA
ENSG00000267490	NA	NA	NA	0	NA	NA	NA	NA	NA
ENSG00000267497	NA	NA	NA	0	NA	NA	NA	NA	NA
ENSG00000267500	NA	NA	NA	1,676799266	0,045093806	1,37615	0,032768085	0,973859529	NA
ENSG00000267507	NA	NA	NA	0	NA	NA	NA	NA	NA
ENSG00000267508	ZNF285	zinc finger protein 285	26974	3,018736974	-0,06540328	1,291091	-0,050657382	0,959598537	NA
ENSG00000267511	ADAD1P2	adenosine deaminase domain containing 1 pseudogene 2	100422407	0	NA	NA	NA	NA	NA
ENSG00000267513	HMGN1P31	high mobility group nucleosome binding domain 1 pseudogene 31	728111	0	NA	NA	NA	NA	NA
ENSG00000267515	HMGB3P28	high mobility group box 3 pseudogene 28	100131036	0,339301358	0,935690727	4,023827	0,232537516	0,816120552	NA
ENSG00000267518	NA	NA	NA	0	NA	NA	NA	NA	NA
ENSG00000267524	RPSAP57	ribosomal protein SA pseudogene 57	390857	1,151365846	1,871467303	2,102618	0,890065107	0,373430928	NA

ENSG00000267526	NA	NA	NA	1,395536243	0,439023586	1,633301	0,26879524	0,788087257	NA
ENSG00000267533	NA	NA	NA	0,368307947	0	4,080456	0	1	NA
ENSG00000267541	MTCO2P2	MT-CO2 pseudogene 2	100873202	0,119673877	0,847101401	4,080456	0,207599697	0,835541541	NA
ENSG00000267544	HIKESHIP2	HIKESHI pseudogene 2	117751740	0,44174869	-0,950368852	3,534202	-0,268906224	0,788001847	NA
ENSG00000267552	NA	NA	NA	0	NA	NA	NA	NA	NA
ENSG00000267558	NA	NA	NA	0	NA	NA	NA	NA	NA
ENSG00000267561	NA	NA	NA	0	NA	NA	NA	NA	NA
ENSG00000267570	NA	NA	NA	0,26317184	1,434710621	4,061608	0,353237067	0,723910722	NA
ENSG00000267573	KRT8P5	keratin 8 pseudogene 5	342732	0	NA	NA	NA	NA	NA
ENSG00000267585	RPL10AP13	ribosomal protein L10a pseudogene 13	100271602	0	NA	NA	NA	NA	NA
ENSG00000267587	NA	NA	NA	0,117497108	0,847101401	4,080456	0,207599697	0,835541541	NA
ENSG00000267589	NA	NA	NA	0	NA	NA	NA	NA	NA
ENSG00000267590	NA	NA	NA	0	NA	NA	NA	NA	NA
ENSG00000267591	NA	NA	NA	0	NA	NA	NA	NA	NA
ENSG00000267592	NA	NA	NA	0	NA	NA	NA	NA	NA
ENSG00000267594	NA	NA	NA	0	NA	NA	NA	NA	NA
ENSG00000267595	NA	NA	NA	0	NA	NA	NA	NA	NA
ENSG00000267597	NA	NA	NA	0	NA	NA	NA	NA	NA
ENSG00000267609	NA	NA	NA	0	NA	NA	NA	NA	NA
ENSG00000267611	NA	NA	NA	0	NA	NA	NA	NA	NA
ENSG00000267618	NA	NA	NA	0	NA	NA	NA	NA	NA
ENSG00000267620	NA	NA	NA	0	NA	NA	NA	NA	NA
ENSG00000267623	NA	NA	NA	0	NA	NA	NA	NA	NA
ENSG00000267624	NA	NA	NA	0	NA	NA	NA	NA	NA
ENSG00000267630	NA	NA	NA	0	NA	NA	NA	NA	NA
ENSG00000267631	CGB1	chorionic gonadotropin subunit beta 1	114335	0	NA	NA	NA	NA	NA
ENSG00000267634	NA	NA	NA	0	NA	NA	NA	NA	NA
ENSG00000267640	NA	NA	NA	0	NA	NA	NA	NA	NA
ENSG00000267641	BNIP3P16	BCL2 interacting protein 3 pseudogene 16	106480418	0	NA	NA	NA	NA	NA
ENSG00000267647	NA	NA	NA	0	NA	NA	NA	NA	NA
ENSG00000267652	NA	NA	NA	0	NA	NA	NA	NA	NA
ENSG00000267656	NA	NA	NA	0	NA	NA	NA	NA	NA
ENSG00000267661	NA	NA	NA	0	NA	NA	NA	NA	NA
ENSG00000267663	NA	NA	NA	0	NA	NA	NA	NA	NA
ENSG00000267664	RPL17P45	ribosomal protein L17 pseudogene 45	100271414	0	NA	NA	NA	NA	NA
ENSG00000267669	NA	NA	NA	0	NA	NA	NA	NA	NA
ENSG00000267676	NA	NA	NA	0	NA	NA	NA	NA	NA
ENSG00000267678	NA	NA	NA	0	NA	NA	NA	NA	NA
ENSG00000267679	EIF5AP2	eukaryotic translation initiation factor 5A pseudogene 2	1986	0	NA	NA	NA	NA	NA
ENSG00000267681	NA	NA	NA	1,381121537	-1,077970601	1,855957	-0,580816492	0,561364138	NA
ENSG00000267685	NA	NA	NA	0	NA	NA	NA	NA	NA
ENSG00000267687	LOC100421385	testin LIM domain protein pseudogene	100421385	0	NA	NA	NA	NA	NA
ENSG00000267689	NA	NA	NA	0	NA	NA	NA	NA	NA
ENSG00000267691	NA	NA	NA	0	NA	NA	NA	NA	NA
ENSG00000267692	NA	NA	NA	0	NA	NA	NA	NA	NA
ENSG00000267693	NA	NA	NA	0	NA	NA	NA	NA	NA
ENSG00000267699	NA	NA	NA	1,26783522	3,751404777	3,344372	1,12170676	0,261987141	NA
ENSG00000267704	FRG2LP	FSHD region gene 2 family member L, pseudogene	106480352	0	NA	NA	NA	NA	NA
ENSG00000267706	NA	NA	NA	0	NA	NA	NA	NA	NA
ENSG00000267708	NA	NA	NA	0	NA	NA	NA	NA	NA
ENSG00000267710	EDDM13	epididymal protein 13	100506374	0	NA	NA	NA	NA	NA
ENSG00000267710	LOC124900420	uncharacterized LOC124900420	124900420	0	NA	NA	NA	NA	NA
ENSG00000267713	NA	NA	NA	0	NA	NA	NA	NA	NA
ENSG00000267716	NA	NA	NA	0	NA	NA	NA	NA	NA
ENSG00000267717	NA	NA	NA	7,624443428	0,616317178	0,714956	0,862034572	0,388668493	NA
ENSG00000267725	NA	NA	NA	0	NA	NA	NA	NA	NA
ENSG00000267733	NA	NA	NA	0	NA	NA	NA	NA	NA
ENSG00000267736	NA	NA	NA	0	NA	NA	NA	NA	NA
ENSG00000267740	NA	NA	NA	0,279865975	0,827292321	4,080456	0,202745072	0,839334292	NA
ENSG00000267741	NA	NA	NA	0	NA	NA	NA	NA	NA
ENSG00000267742	NA	NA	NA	0	NA	NA	NA	NA	NA
ENSG00000267744	NA	NA	NA	0	NA	NA	NA	NA	NA
ENSG00000267748	NA	NA	NA	0	NA	NA	NA	NA	NA
ENSG00000267752	RPS10P27	ribosomal protein S10 pseudogene 27	100129556	0	NA	NA	NA	NA	NA
ENSG00000267756	NA	NA	NA	0	NA	NA	NA	NA	NA
ENSG00000267768	NA	NA	NA	0	NA	NA	NA	NA	NA
ENSG00000267770	LOC100419624	RAN binding protein 1 pseudogene	100419624	0	NA	NA	NA	NA	NA
ENSG00000267771	NA	NA	NA	0	NA	NA	NA	NA	NA
ENSG00000267773	NA	NA	NA	0	NA	NA	NA	NA	NA

ENSG00000267775	NA	NA	NA	0	NA	NA	NA	NA	NA
ENSG00000267781	SLC25A36P1	SLC25A36 pseudogene 1	106480240	0	NA	NA	NA	NA	NA
ENSG00000267792	NA	NA	NA	0	NA	NA	NA	NA	NA
ENSG00000267793	NA	NA	NA	0	NA	NA	NA	NA	NA
ENSG00000267794	NA	NA	NA	0	NA	NA	NA	NA	NA
ENSG00000267795	SMIM22	small integral membrane protein 22	440335	0	NA	NA	NA	NA	NA
ENSG00000267797	NA	NA	NA	0	NA	NA	NA	NA	NA
ENSG00000267799	NA	NA	NA	0	NA	NA	NA	NA	NA
ENSG00000267800	NA	NA	NA	0	NA	NA	NA	NA	NA
ENSG00000267809	NA	NA	NA	1,982869297	1,599288569	1,651628	0,968310373	0,332889386	NA
ENSG00000267881	NA	NA	NA	0	NA	NA	NA	NA	NA
ENSG00000267895	NA	NA	NA	0,119673877	0,847101401	4,080456	0,207599697	0,835541541	NA
ENSG00000267908	ZSCAN5DP	zinc finger and SCAN domain containing 5D pseudogene	646698	0	NA	NA	NA	NA	NA
ENSG00000267909	CCDC177	coiled-coil domain containing 177	56936	0	NA	NA	NA	NA	NA
ENSG00000267920	NA	NA	NA	0	NA	NA	NA	NA	NA
ENSG00000267938	NA	NA	NA	0	NA	NA	NA	NA	NA
ENSG00000267952	NA	NA	NA	0	NA	NA	NA	NA	NA
ENSG00000267961	OR10B1P	olfactory receptor family 10 subfamily B member 1 pseudogene	401903	0	NA	NA	NA	NA	NA
ENSG00000267978	MAGEA9B	MAGE family member A9B	728269	0	NA	NA	NA	NA	NA
ENSG00000267990	NA	NA	NA	0	NA	NA	NA	NA	NA
ENSG00000268009	SSX4	SSX family member 4	6759	0	NA	NA	NA	NA	NA
ENSG00000268020	OR4G4P	olfactory receptor family 4 subfamily G member 4 pseudogene	79504	0	NA	NA	NA	NA	NA
ENSG00000268041	ERFL	ETS repressor factor like	390937	1,472175133	0,802865236	1,527409	0,525638614	0,599139322	NA
ENSG00000268053	NA	NA	NA	0	NA	NA	NA	NA	NA
ENSG00000268058	BNIP3P40	BCL2 interacting protein 3 pseudogene 40	100421702	0	NA	NA	NA	NA	NA
ENSG00000268062	SRP14P4	SRP14 pseudogene 4	111644147	0	NA	NA	NA	NA	NA
ENSG00000268067	NA	NA	NA	0	NA	NA	NA	NA	NA
ENSG00000268070	NA	NA	NA	0	NA	NA	NA	NA	NA
ENSG00000268074	RPL22P5	ribosomal protein L22 pseudogene 5	100132479	0	NA	NA	NA	NA	NA
ENSG00000268079	BNIP3P30	BCL2 interacting protein 3 pseudogene 30	106481694	0	NA	NA	NA	NA	NA
ENSG00000268080	NA	NA	NA	0	NA	NA	NA	NA	NA
ENSG00000268088	NA	NA	NA	0	NA	NA	NA	NA	NA
ENSG00000268089	GABRQ	gamma-aminobutyric acid type A receptor subunit theta	55879	0	NA	NA	NA	NA	NA
ENSG00000268100	NA	NA	NA	0	NA	NA	NA	NA	NA
ENSG00000268101	CYP2G2P	cytochrome P450 family 2 subfamily G member 2, pseudogene	83757	0	NA	NA	NA	NA	NA
ENSG00000268104	SLC6A14	solute carrier family 6 member 14	11254	0	NA	NA	NA	NA	NA
ENSG00000268105	NA	NA	NA	0,117497108	0,847101401	4,080456	0,207599697	0,835541541	NA
ENSG00000268107	NA	NA	NA	3,515499269	2,754382971	1,564179	1,760913071	0,078253117	NA
ENSG00000268116	NA	NA	NA	0	NA	NA	NA	NA	NA
ENSG00000268117	NA	NA	NA	0	NA	NA	NA	NA	NA
ENSG00000268133	NA	NA	NA	0	NA	NA	NA	NA	NA
ENSG00000268140	NA	NA	NA	0	NA	NA	NA	NA	NA
ENSG00000268144	NA	NA	NA	0,10543123	0	4,080456	0	1	NA
ENSG00000268148	NA	NA	NA	0	NA	NA	NA	NA	NA
ENSG00000268153	NA	NA	NA	0	NA	NA	NA	NA	NA
ENSG00000268163	NA	NA	NA	0,209514313	-1,065658117	4,080456	-0,261161534	0,79396794	NA
ENSG00000268173	NA	NA	NA	2,24829145	-4,556811859	3,079541	-1,479704886	0,138952021	NA
ENSG00000268174	BNIP3P22	BCL2 interacting protein 3 pseudogene 22	100421706	0	NA	NA	NA	NA	NA
ENSG00000268182	SMIM17	small integral membrane protein 17	147670	1,117945327	-1,25287115	1,997389	-0,627254509	0,530492418	NA
ENSG00000268193	NA	NA	NA	0	NA	NA	NA	NA	NA
ENSG00000268209	LOC100422190	UDP glucuronosyltransferase family 2 member B10 pseudogene	100422190	0	NA	NA	NA	NA	NA
ENSG00000268221	OPN1MW	opsin 1, medium wave sensitive	2652	0	NA	NA	NA	NA	NA
ENSG00000268222	NA	NA	NA	0	NA	NA	NA	NA	NA
ENSG00000268223	ARL14EPL	ADP ribosylation factor like GTPase 14 effector protein like	644100	0	NA	NA	NA	NA	NA
ENSG00000268234	FKBP4P6	FKBP prolyl isomerase 4 pseudogene 6	100132268	0	NA	NA	NA	NA	NA
ENSG00000268235	TCP11X1	t-complex 11 family, X-linked 1	100996631	0	NA	NA	NA	NA	NA

ENSG00000268238	IGFL1P2	IGF like family member 1 pseudogene 2	727704	0	NA	NA	NA	NA	NA
ENSG00000268243	NA	NA	NA	0	NA	NA	NA	NA	NA
ENSG00000268267	NA	NA	NA	0	NA	NA	NA	NA	NA
ENSG00000268272	NA	NA	NA	0	NA	NA	NA	NA	NA
ENSG00000268278	NA	NA	NA	0	NA	NA	NA	NA	NA
ENSG00000268279	NA	NA	NA	0,103998153	-1,076443944	4,080456	-0,263804824	0,791930317	NA
ENSG00000268282	NA	NA	NA	0	NA	NA	NA	NA	NA
ENSG00000268293	NA	NA	NA	0	NA	NA	NA	NA	NA
ENSG00000268297	NA	NA	NA	0	NA	NA	NA	NA	NA
ENSG00000268320	SCGB1C2	secretoglobin family 1C member 2	653486	0	NA	NA	NA	NA	NA
ENSG00000268322	BNIP3P25	BCL2 interacting protein 3 pseudogene 25	100421707	0	NA	NA	NA	NA	NA
ENSG00000268335	BNIP3P23	BCL2 interacting protein 3 pseudogene 23	100421697	0	NA	NA	NA	NA	NA
ENSG00000268336	NA	NA	NA	0	NA	NA	NA	NA	NA
ENSG00000268357	NA	NA	NA	0	NA	NA	NA	NA	NA
ENSG00000268361	NA	NA	NA	0,10543123	0	4,080456	0	1	NA
ENSG00000268379	NA	NA	NA	0,119673877	0,847101401	4,080456	0,207599697	0,835541541	NA
ENSG00000268391	MTCO3P42	MT-CO3 pseudogene 42	107075189	0	NA	NA	NA	NA	NA
ENSG00000268400	NA	NA	NA	0	NA	NA	NA	NA	NA
ENSG00000268407	NA	NA	NA	0	NA	NA	NA	NA	NA
ENSG00000268412	NA	NA	NA	0	NA	NA	NA	NA	NA
ENSG00000268433	MTDHP3	metadherin pseudogene 3	100418832	0	NA	NA	NA	NA	NA
ENSG00000268434	NA	NA	NA	0	NA	NA	NA	NA	NA
ENSG00000268438	NA	NA	NA	0	NA	NA	NA	NA	NA
ENSG00000268442	HAVCR1P1	hepatitis A virus cellular receptor 1 pseudogene 1	100101266	0	NA	NA	NA	NA	NA
ENSG00000268447	SSX2B	SSX family member 2B	727837	0	NA	NA	NA	NA	NA
ENSG00000268455	NA	NA	NA	0	NA	NA	NA	NA	NA
ENSG00000268461	NA	NA	NA	0	NA	NA	NA	NA	NA
ENSG00000268465	NA	NA	NA	0	NA	NA	NA	NA	NA
ENSG00000268469	BNIP3P38	BCL2 interacting protein 3 pseudogene 38	100421709	0	NA	NA	NA	NA	NA
ENSG00000268473	NA	NA	NA	0	NA	NA	NA	NA	NA
ENSG00000268483	RPL7AP69	ribosomal protein L7a pseudogene 69	100271545	0	NA	NA	NA	NA	NA
ENSG00000268486	NA	NA	NA	0	NA	NA	NA	NA	NA
ENSG00000268500	SIGLEC5	sialic acid binding Ig like lectin 5	8778	0,866686176	-3,759309745	2,370856	-1,585634085	0,11282234	NA
ENSG00000268521	VN1R83P	vomeronasal 1 receptor 83 pseudogene	100312828	3,781070362	-0,851206856	1,030964	-0,82564141	0,409007537	NA
ENSG00000268529	CYP2T3P	cytochrome P450 family 2 subfamily T member 3, pseudogene	163007	0	NA	NA	NA	NA	NA
ENSG00000268533	NA	NA	NA	0	NA	NA	NA	NA	NA
ENSG00000268535	MTDHP4	metadherin pseudogene 4	100418834	0	NA	NA	NA	NA	NA
ENSG00000268541	NA	NA	NA	0	NA	NA	NA	NA	NA
ENSG00000268545	VN1R107P	vomeronasal 1 receptor 107 pseudogene	100312849	0	NA	NA	NA	NA	NA
ENSG00000268581	NA	NA	NA	0,396536241	0,826780876	4,079656	0,202659461	0,839401211	NA
ENSG00000268589	NA	NA	NA	0	NA	NA	NA	NA	NA
ENSG00000268593	VN1R106P	vomeronasal 1 receptor 106 pseudogene	100312848	0,11808024	-1,076443944	4,080456	-0,263804824	0,791930317	NA
ENSG00000268598	VN1R80P	vomeronasal 1 receptor 80 pseudogene	100312825	0,278315297	0,822467996	4,080456	0,201562772	0,840258552	NA
ENSG00000268606	MAGEA2	MAGE family member A2	4101	0	NA	NA	NA	NA	NA
ENSG00000268614	NA	NA	NA	0	NA	NA	NA	NA	NA
ENSG00000268620	NA	NA	NA	0	NA	NA	NA	NA	NA
ENSG00000268623	LOC646666	NAD(P)HX epimerase pseudogene	646666	0	NA	NA	NA	NA	NA
ENSG00000268629	TEX13A	testis expressed 13A	56157	0	NA	NA	NA	NA	NA
ENSG00000268643	NA	NA	NA	0	NA	NA	NA	NA	NA
ENSG00000268651	CTAG1A	cancer/testis antigen 1A	246100	0	NA	NA	NA	NA	NA
ENSG00000268652	LOC100133225	zinc finger DHHC-type palmitoyltransferase 7 pseudogene	100133225	0	NA	NA	NA	NA	NA
ENSG00000268655	SAXO3	stabilizer of axonemal microtubules 3	101059948	0	NA	NA	NA	NA	NA
ENSG00000268660	NA	NA	NA	0	NA	NA	NA	NA	NA
ENSG00000268663	NA	NA	NA	0	NA	NA	NA	NA	NA
ENSG00000268669	NA	NA	NA	0	NA	NA	NA	NA	NA
ENSG00000268674	LOC124903857	FAM231A/C-like protein LOC102723383	124903857	0	NA	NA	NA	NA	NA
ENSG00000268674	LOC124905556	FAM231A/C-like protein LOC102723383	124905556	0	NA	NA	NA	NA	NA
ENSG00000268674	LOC124905693	FAM231A/C-like protein LOC102723383	124905693	0	NA	NA	NA	NA	NA

ENSG00000268681	COX6CP7	cytochrome c oxidase subunit 6C pseudogene 7	106480265	0	NA	NA	NA	NA	NA
ENSG00000268685	NA	NA	NA	0	NA	NA	NA	NA	NA
ENSG00000268696	ZNF723	zinc finger protein 723	646864	0,103998153	-1,076443944	4,080456	-0,263804824	0,791930317	NA
ENSG00000268705	BNIP3P26	BCL2 interacting protein 3 pseudogene 26	100421699	0	NA	NA	NA	NA	NA
ENSG00000268711	NA	NA	NA	0	NA	NA	NA	NA	NA
ENSG00000268717	BNIP3P33	BCL2 interacting protein 3 pseudogene 33	106481695	0	NA	NA	NA	NA	NA
ENSG00000268723	NA	NA	NA	0	NA	NA	NA	NA	NA
ENSG00000268731	NA	NA	NA	0	NA	NA	NA	NA	NA
ENSG00000268738	HSFX2	heat shock transcription factor family, X-linked 2	100130086	0,119673877	0,847101401	4,080456	0,207599697	0,835541541	NA
ENSG00000268742	NA	NA	NA	0	NA	NA	NA	NA	NA
ENSG00000268747	LOC107985265	cytochrome c oxidase assembly protein COX16 homolog, mitochondrial-like	107985265	0	NA	NA	NA	NA	NA
ENSG00000268750	NA	NA	NA	0	NA	NA	NA	NA	NA
ENSG00000268758	NA	NA	NA	0	NA	NA	NA	NA	NA
ENSG00000268785	RPL7P50	ribosomal protein L7 pseudogene 50	729449	0,15836288	0	4,080456	0	1	NA
ENSG00000268789	VN1R87P	vomeronasal 1 receptor 87 pseudogene	100312832	0	NA	NA	NA	NA	NA
ENSG00000268790	NA	NA	NA	0	NA	NA	NA	NA	NA
ENSG00000268794	VN1R90P	vomeronasal 1 receptor 90 pseudogene	100312835	0	NA	NA	NA	NA	NA
ENSG00000268797	NA	NA	NA	0,117497108	0,847101401	4,080456	0,207599697	0,835541541	NA
ENSG00000268799	H3Y2	H3,Y histone 2	340096	0	NA	NA	NA	NA	NA
ENSG00000268803	NA	NA	NA	0	NA	NA	NA	NA	NA
ENSG00000268830	BNIP3P32	BCL2 interacting protein 3 pseudogene 32	106480283	0	NA	NA	NA	NA	NA
ENSG00000268834	OR7A1P	olfactory receptor family 7 subfamily A member 1 pseudogene	390895	0	NA	NA	NA	NA	NA
ENSG00000268839	NA	NA	NA	0	NA	NA	NA	NA	NA
ENSG00000268847	NA	NA	NA	0	NA	NA	NA	NA	NA
ENSG00000268849	NA	NA	NA	0,27743166	0,830527957	4,080456	0,203538032	0,838714523	NA
ENSG00000268864	NA	NA	NA	0	NA	NA	NA	NA	NA
ENSG00000268866	LOC100419847	zinc finger protein 436 pseudogene	100419847	0	NA	NA	NA	NA	NA
ENSG00000268869	NA	NA	NA	0	NA	NA	NA	NA	NA
ENSG00000268870	NA	NA	NA	0,35737071	1,393979615	4,060724	0,343283502	0,731385191	NA
ENSG00000268879	NA	NA	NA	0	NA	NA	NA	NA	NA
ENSG00000268883	NA	NA	NA	0	NA	NA	NA	NA	NA
ENSG00000268884	NA	NA	NA	0	NA	NA	NA	NA	NA
ENSG00000268892	NA	NA	NA	0	NA	NA	NA	NA	NA
ENSG00000268902	CSAG2	CSAG family member 2	102723547	0	NA	NA	NA	NA	NA
ENSG00000268903	NA	NA	NA	7,254997539	1,314056867	1,084996	1,21111666	0,225850697	NA
ENSG00000268908	VN1R92P	vomeronasal 1 receptor 92 pseudogene	100312837	0	NA	NA	NA	NA	NA
ENSG00000268916	CSAG3	CSAG family member 3	389903	0	NA	NA	NA	NA	NA
ENSG00000268940	CT45A1	cancer/testis antigen family 45 member A1	541466	0	NA	NA	NA	NA	NA
ENSG00000268942	NA	NA	NA	0	NA	NA	NA	NA	NA
ENSG00000268949	NA	NA	NA	0	NA	NA	NA	NA	NA
ENSG00000268951	NA	NA	NA	0	NA	NA	NA	NA	NA
ENSG00000268957	NA	NA	NA	0	NA	NA	NA	NA	NA
ENSG00000268964	ERVV-2	endogenous retrovirus group V member 2, envelope	100271846	0	NA	NA	NA	NA	NA
ENSG00000268967	NA	NA	NA	0	NA	NA	NA	NA	NA
ENSG00000268975	NA	NA	NA	0,574139013	2,340440257	2,812656	0,832110351	0,40534666	NA
ENSG00000268988	SPANXN2	SPANX family member N2	494119	0	NA	NA	NA	NA	NA
ENSG00000268992	NA	NA	NA	0	NA	NA	NA	NA	NA
ENSG00000268993	RPL22P6	ribosomal protein L22 pseudogene 6	100132047	0	NA	NA	NA	NA	NA
ENSG00000268994	FAM236B	family with sequence similarity 236 member B	100132304	0	NA	NA	NA	NA	NA
ENSG00000268995	NA	NA	NA	0,632587382	0	4,080456	0	1	NA
ENSG00000269009	NA	NA	NA	0	NA	NA	NA	NA	NA
ENSG00000269012	NA	NA	NA	0,118286652	0	4,080456	0	1	NA
ENSG00000269014	NA	NA	NA	0	NA	NA	NA	NA	NA
ENSG00000269021	NA	NA	NA	0	NA	NA	NA	NA	NA
ENSG00000269025	NA	NA	NA	0	NA	NA	NA	NA	NA
ENSG00000269026	NA	NA	NA	0	NA	NA	NA	NA	NA
ENSG00000269032	NA	NA	NA	0,648587298	-0,115604863	2,843842	-0,040650946	0,967574169	NA
ENSG00000269035	NA	NA	NA	0	NA	NA	NA	NA	NA
ENSG00000269040	BNIP3P24	BCL2 interacting protein 3 pseudogene 24	100421698	0	NA	NA	NA	NA	NA
ENSG00000269055	BNIP3P18	BCL2 interacting protein 3 pseudogene 18	106481693	0	NA	NA	NA	NA	NA

ENSG00000269058	CALR3	calreticulin 3	125972	0,118286652	0	4,080456	0	1	NA	
ENSG00000269067	ZNF728	zinc finger protein 728	388523	0	NA	NA	NA	NA	NA	
ENSG00000269095	NA	NA	NA	0	NA	NA	NA	NA	NA	
ENSG00000269096	CT45A3	cancer/testis antigen family 45 member A3	441519	0	NA	NA	NA	NA	NA	
ENSG00000269097	TPRG1LP1	tumor protein p63 regulated 1 like pseudogene 1	100130490	0	NA	NA	NA	NA	NA	
ENSG00000269099	LSP1P1	LSP1 pseudogene 1	100463528	0	NA	NA	NA	NA	NA	
ENSG00000269113	TRABD2B	TraB domain containing 2B	388630	0	NA	NA	NA	NA	NA	
ENSG00000269118	NA	NA	NA	0	NA	NA	NA	NA	NA	
ENSG00000269119	NA	NA	NA	0	NA	NA	NA	NA	NA	
ENSG00000269130	NA	NA	NA	0	NA	NA	NA	NA	NA	
ENSG00000269136	CCNYL6	cyclin Y like 6 (pseudogene)	641367	0	NA	NA	NA	NA	NA	
ENSG00000269136	BRI3BPP1	BRI3 binding protein pseudogene 1	100129976	0	NA	NA	NA	NA	NA	
ENSG00000269138	NA	NA	NA	0	NA	NA	NA	NA	NA	
ENSG00000269153	NA	NA	NA	0	NA	NA	NA	NA	NA	
ENSG00000269154	NA	NA	NA	0	NA	NA	NA	NA	NA	
ENSG00000269179	NA	NA	NA	0	NA	NA	NA	NA	NA	
ENSG00000269181	NA	NA	NA	0	NA	NA	NA	NA	NA	
ENSG00000269188	NA	NA	NA	0	NA	NA	NA	NA	NA	
ENSG00000269228	RPS27AP19	RPS27A pseudogene 19	107985274	0	NA	NA	NA	NA	NA	
ENSG00000269236	NA	NA	NA	0	NA	NA	NA	NA	NA	
ENSG00000269237	NA	NA	NA	0,11808024	-1,076443944	4,080456	-0,263804824	0,791930317	NA	
ENSG00000269242	NA	NA	NA	1,44035421	-1,893819048	2,696441	-0,702340127	0,482467074	NA	
ENSG00000269244	RPL23AP78	ribosomal protein L23a pseudogene 78	100271624	0	NA	NA	NA	NA	NA	
ENSG00000269253	NA	NA	NA	0	NA	NA	NA	NA	NA	
ENSG00000269274	NA	NA	NA	0	NA	NA	NA	NA	NA	
ENSG00000269281	NA	NA	NA	0	NA	NA	NA	NA	NA	
ENSG00000269304	NA	NA	NA	0	NA	NA	NA	NA	NA	
ENSG00000269307	NA	NA	NA	0,727987071	-3,717343413	3,946344	-0,94197134	0,346207313	NA	
ENSG00000269316	RAD54L2P1	RAD54L2 pseudogene 1	100420273	0	NA	NA	NA	NA	NA	
ENSG00000269320	VN1R93P	vomeronasal 1 receptor 93 pseudogene	100312838	0	NA	NA	NA	NA	NA	
ENSG00000269332	NA	NA	NA	0	NA	NA	NA	NA	NA	
ENSG00000269345	VN1R85P	vomeronasal 1 receptor 85 pseudogene	100312830	0	NA	NA	NA	NA	NA	
ENSG00000269373	NA	NA	NA	0	NA	NA	NA	NA	NA	
ENSG00000269374	LOC732229	AN1-type zinc finger protein 5-like	732229	1,107721274	0,643406271	1,793152	0,358812924	0,719735044	NA	
ENSG00000269383	NA	NA	NA	0	NA	NA	NA	NA	NA	
ENSG00000269385	NA	NA	NA	0	NA	NA	NA	NA	NA	
ENSG00000269388	NA	NA	NA	0,072165449	0,847101401	4,080456	0,207599697	0,835541541	NA	
ENSG00000269403	NA	NA	NA	4,353804198	-0,581224786	1,022888	-0,568219449	0,569885967	NA	
ENSG00000269404	SPIB	Spi-B transcription factor	6689	0,119673877	0,847101401	4,080456	0,207599697	0,835541541	NA	
ENSG00000269405	NXF2	nuclear RNA export factor 2	56001	0	NA	NA	NA	NA	NA	
ENSG00000269419	NA	NA	NA	0	NA	NA	NA	NA	NA	
ENSG00000269421	NA	NA	NA	0,103998153	-1,076443944	4,080456	-0,263804824	0,791930317	NA	
ENSG00000269431	BNIP3P8	BCL2 interacting protein 3 pseudogene 8	100421710	0	NA	NA	NA	NA	NA	
ENSG00000269433	OPN1MW3	opsin 1, medium wave sensitive 3	101060233	0	NA	NA	NA	NA	NA	
ENSG00000269437	NXF2B	nuclear RNA export factor 2B	728343	0	NA	NA	NA	NA	NA	
ENSG00000269458	NA	NA	NA	0	NA	NA	NA	NA	NA	
ENSG00000269466	H3Y1	H3,Y histone 1	391769	0	NA	NA	NA	NA	NA	
ENSG00000269469	NA	NA	NA	0	NA	NA	NA	NA	NA	
ENSG00000269471	NA	NA	NA	0	NA	NA	NA	NA	NA	
ENSG00000269475	LOC100420250	MAGE family member A4 pseudogene	100420250	0	NA	NA	NA	NA	NA	
ENSG00000269476	NA	NA	NA	0,10488184	-1,076443944	4,080456	-0,263804824	0,791930317	NA	
ENSG00000269483	NA	NA	NA	0	NA	NA	NA	NA	NA	
ENSG00000269502	DMRTC1	DMRT like family C1	63947	0	NA	NA	NA	NA	NA	
ENSG00000269505	NA	NA	NA	0	NA	NA	NA	NA	NA	
ENSG00000269509	BNIP3P34	BCL2 interacting protein 3 pseudogene 34	100128849	0	NA	NA	NA	NA	NA	
ENSG00000269516	NA	NA	NA	0	NA	NA	NA	NA	NA	
ENSG00000269519	NA	NA	NA	0	NA	NA	NA	NA	NA	
ENSG00000269526	ERVV-1	endogenous retrovirus group V member 1, envelope	147664	0	NA	NA	NA	NA	NA	
ENSG00000269533	NA	NA	NA	0	NA	NA	NA	NA	NA	
ENSG00000269540	NA	NA	NA	0	NA	NA	NA	NA	NA	
ENSG00000269546	NA	NA	NA	0	NA	NA	NA	NA	NA	
ENSG00000269552	NA	NA	NA	0	NA	NA	NA	NA	NA	
ENSG00000269565	NA	NA	NA	0	NA	NA	NA	NA	NA	
ENSG00000269575	MTDHP5	metadherin pseudogene 5	100418836	0	NA	NA	NA	NA	NA	
ENSG00000269576	NA	NA	NA	0	NA	NA	NA	NA	NA	
ENSG00000269580	NA	NA	NA	0	NA	NA	NA	NA	NA	
ENSG00000269584	NA	NA	NA	0	NA	NA	NA	NA	NA	

ENSG00000269586	CT45A10	cancer/testis antigen family 45 member A10	102723631	0	NA	NA	NA	NA	NA
ENSG00000269588	NA	NA	NA	0	NA	NA	NA	NA	NA
ENSG00000269590	NA	NA	NA	0	NA	NA	NA	NA	NA
ENSG00000269608	OSTCP3	oligosaccharyltransferase complex subunit pseudogene 3	646557	0	NA	NA	NA	NA	NA
ENSG00000269615	MTDHP2	metadherin pseudogene 2	100418835	0	NA	NA	NA	NA	NA
ENSG00000269622	NA	NA	NA	0	NA	NA	NA	NA	NA
ENSG00000269637	NA	NA	NA	0	NA	NA	NA	NA	NA
ENSG00000269651	LOC100422633	ALG11, alpha-1,2-mannosyltransferase pseudogene	100422633	0	NA	NA	NA	NA	NA
ENSG00000269662	BNIP3P39	BCL2 interacting protein 3 pseudogene 39	100421701	0	NA	NA	NA	NA	NA
ENSG00000269678	NA	NA	NA	0	NA	NA	NA	NA	NA
ENSG00000269681	NA	NA	NA	0	NA	NA	NA	NA	NA
ENSG00000269692	FKBP4P2	FKBP prolyl isomerase 4 pseudogene 2	389727	0	NA	NA	NA	NA	NA
ENSG00000269693	NA	NA	NA	0	NA	NA	NA	NA	NA
ENSG00000269699	ZIM2	zinc finger imprinted 2	23619	0	NA	NA	NA	NA	NA
ENSG00000269706	NA	NA	NA	0	NA	NA	NA	NA	NA
ENSG00000269711	NA	NA	NA	0	NA	NA	NA	NA	NA
ENSG00000269720	CCDC194	coiled-coil domain containing 194	110806280	7,103376398	-0,711199453	0,741758	-0,958802502	0,337658249	NA
ENSG00000269732	NA	NA	NA	0	NA	NA	NA	NA	NA
ENSG00000269737	NA	NA	NA	0,072165449	0,847101401	4,080456	0,207599697	0,835541541	NA
ENSG00000269741	NA	NA	NA	0	NA	NA	NA	NA	NA
ENSG00000269742	BNIP3P29	BCL2 interacting protein 3 pseudogene 29	100421700	0	NA	NA	NA	NA	NA
ENSG00000269755	NA	NA	NA	0	NA	NA	NA	NA	NA
ENSG00000269761	NA	NA	NA	0	NA	NA	NA	NA	NA
ENSG00000269763	NA	NA	NA	0	NA	NA	NA	NA	NA
ENSG00000269765	LOC100422106	staufen double-stranded RNA binding protein 1 pseudogene	100422106	0	NA	NA	NA	NA	NA
ENSG00000269776	NA	NA	NA	0	NA	NA	NA	NA	NA
ENSG00000269779	NA	NA	NA	0	NA	NA	NA	NA	NA
ENSG00000269786	NA	NA	NA	0	NA	NA	NA	NA	NA
ENSG00000269787	NA	NA	NA	0	NA	NA	NA	NA	NA
ENSG00000269791	SSX4B	SSX family member 4B	548313	0	NA	NA	NA	NA	NA
ENSG00000269794	LOC100887072	zinc finger protein 470 pseudogene	100887072	0	NA	NA	NA	NA	NA
ENSG00000269811	NA	NA	NA	0	NA	NA	NA	NA	NA
ENSG00000269833	NA	NA	NA	0	NA	NA	NA	NA	NA
ENSG00000269837	NA	NA	NA	1,058686741	1,49925653	2,432269	0,616402317	0,537629024	NA
ENSG00000269839	NA	NA	NA	0	NA	NA	NA	NA	NA
ENSG00000269848	NA	NA	NA	0	NA	NA	NA	NA	NA
ENSG00000269855	RNF225	ring finger protein 225	646862	0,103998153	-1,076443944	4,080456	-0,263804824	0,791930317	NA
ENSG00000269885	NA	NA	NA	0	NA	NA	NA	NA	NA
ENSG00000269891	ARHGAP19-SLIT1	ARHGAP19-SLIT1 readthrough (NMD candidate)	100533184	0,639487074	-0,94811632	3,824989	-0,247874246	0,804231707	NA
ENSG00000269896	LOC100129534	small nuclear ribonucleoprotein polypeptide N pseudogene	100129534	0	NA	NA	NA	NA	NA
ENSG00000269897	COMMD3-BMI1	COMMD3-BMI1 readthrough	100532731	0	NA	NA	NA	NA	NA
ENSG00000269904	MAP2K4P1	mitogen-activated protein kinase kinase 4 pseudogene 1	139201	1,676557594	0,199826441	2,13982	0,093384694	0,92559795	NA
ENSG00000269907	NA	NA	NA	0	NA	NA	NA	NA	NA
ENSG00000269964	MEI4	meiotic double-stranded break formation protein 4	101928601	0	NA	NA	NA	NA	NA
ENSG00000269981	NA	NA	NA	0,472165202	2,962103962	3,964847	0,747091692	0,455008215	NA
ENSG00000270011	ZNF559-ZNF177	ZNF559-ZNF177 readthrough	100529215	0,558843907	0,657424518	3,944299	0,16667714	0,867624094	NA
ENSG00000270073	TSPY6P	testis specific protein Y-linked 6, pseudogene	653174	0	NA	NA	NA	NA	NA
ENSG00000270098	NA	NA	NA	0	NA	NA	NA	NA	NA
ENSG00000270099	NA	NA	NA	0,599851203	0,243019499	2,752296	0,08829702	0,929640607	NA
ENSG00000270106	TSNAX-DISC1	TSNAX-DISC1 readthrough (NMD candidate)	100303453	2,031000101	1,151400627	1,876194	0,613689537	0,539420504	NA
ENSG00000270136	NA	NA	NA	0	NA	NA	NA	NA	NA
ENSG00000270166	NA	NA	NA	0	NA	NA	NA	NA	NA
ENSG00000270181	BIVM-ERCC5	BIVM-ERCC5 readthrough	100533467	1,315161592	-0,454168988	3,207763	-0,141584343	0,887408335	NA
ENSG00000270191	BNIP3P31	BCL2 interacting protein 3 pseudogene 31	106480282	0	NA	NA	NA	NA	NA
ENSG00000270192	NA	NA	NA	0	NA	NA	NA	NA	NA

ENSG00000270193	NA	NA	NA	0	NA	NA	NA	NA	NA
ENSG00000270196	GTF3C6P3	GTF3C6 pseudogene 3	646745	0	NA	NA	NA	NA	NA
ENSG00000270200	NA	NA	NA	0	NA	NA	NA	NA	NA
ENSG00000270202	NA	NA	NA	0	NA	NA	NA	NA	NA
ENSG00000270204	NA	NA	NA	0	NA	NA	NA	NA	NA
ENSG00000270209	NA	NA	NA	0	NA	NA	NA	NA	NA
ENSG00000270218	NA	NA	NA	0	NA	NA	NA	NA	NA
ENSG00000270222	DUXAP11	double homeobox A pseudogene 11	106481741	0	NA	NA	NA	NA	NA
ENSG00000270225	MTCO2P22	MT-CO2 pseudogene 22	100289404	0	NA	NA	NA	NA	NA
ENSG00000270226	LOC100419811	interleukin 9 receptor pseudogene	100419811	0	NA	NA	NA	NA	NA
ENSG00000270228	NA	NA	NA	0	NA	NA	NA	NA	NA
ENSG00000270230	MTND6P22	MT-ND6 pseudogene 22	107075261	0	NA	NA	NA	NA	NA
ENSG00000270232	MTCYBP22	MT-CYB pseudogene 22	107075221	0	NA	NA	NA	NA	NA
ENSG00000270234	NA	NA	NA	0	NA	NA	NA	NA	NA
ENSG00000270236	NA	NA	NA	0	NA	NA	NA	NA	NA
ENSG00000270241	LOC100420336	ferritin, heavy polypeptide 1 pseudogene	100420336	0	NA	NA	NA	NA	NA
ENSG00000270242	NA	NA	NA	0	NA	NA	NA	NA	NA
ENSG00000270243	NA	NA	NA	0	NA	NA	NA	NA	NA
ENSG00000270244	NA	NA	NA	0	NA	NA	NA	NA	NA
ENSG00000270248	NA	NA	NA	0,210312681	-1,076858662	4,080456	-0,26390646	0,791851998	NA
ENSG00000270249	NA	NA	NA	0,672736449	-3,596792037	2,980243	-1,206878944	0,227478768	NA
ENSG00000270255	NA	NA	NA	0	NA	NA	NA	NA	NA
ENSG00000270257	NA	NA	NA	0,11808024	-1,076443944	4,080456	-0,263804824	0,791930317	NA
ENSG00000270258	NA	NA	NA	0	NA	NA	NA	NA	NA
ENSG00000270259	NA	NA	NA	0	NA	NA	NA	NA	NA
ENSG00000270264	NDUFB8P2	NADH:ubiquinone oxidoreductase subunit B8 pseudogene 2	729565	0	NA	NA	NA	NA	NA
ENSG00000270268	CORO1CP1	CORO1C pseudogene 1	100420760	0	NA	NA	NA	NA	NA
ENSG00000270269	NA	NA	NA	0	NA	NA	NA	NA	NA
ENSG00000270270	BNIP3P10	BCL2 interacting protein 3 pseudogene 10	100421695	0	NA	NA	NA	NA	NA
ENSG00000270273	NA	NA	NA	0	NA	NA	NA	NA	NA
ENSG00000270275	NA	NA	NA	0	NA	NA	NA	NA	NA
ENSG00000270276	H4C15	H4 clustered histone 15	554313	0,519990765	-3,232988235	3,962027	-0,815993526	0,414503838	NA
ENSG00000270280	NA	NA	NA	0	NA	NA	NA	NA	NA
ENSG00000270281	NA	NA	NA	0	NA	NA	NA	NA	NA
ENSG00000270282	NA	NA	NA	0	NA	NA	NA	NA	NA
ENSG00000270285	RPS27P30	ribosomal protein S27 pseudogene 30	127379697	0	NA	NA	NA	NA	NA
ENSG00000270287	NA	NA	NA	0	NA	NA	NA	NA	NA
ENSG00000270289	NA	NA	NA	0	NA	NA	NA	NA	NA
ENSG00000270292	LOC100419046	tubulin alpha 4a pseudogene	100419046	0	NA	NA	NA	NA	NA
ENSG00000270293	DPPA3P8	DPPA3 pseudogene 8	127460877	0	NA	NA	NA	NA	NA
ENSG00000270294	NA	NA	NA	0	NA	NA	NA	NA	NA
ENSG00000270296	NA	NA	NA	0	NA	NA	NA	NA	NA
ENSG00000270299	NA	NA	NA	0	NA	NA	NA	NA	NA
ENSG00000270300	NA	NA	NA	0,391228251	2,28483349	3,998753	0,57138646	0,567737705	NA
ENSG00000270301	NA	NA	NA	0	NA	NA	NA	NA	NA
ENSG00000270304	NA	NA	NA	0	NA	NA	NA	NA	NA
ENSG00000270306	LOC100422449	TIA1 cytotoxic granule associated RNA binding protein like 1 pseudogene	100422449	0	NA	NA	NA	NA	NA
ENSG00000270307	MTATP6P2	MT-ATP6 pseudogene 2	100287745	0	NA	NA	NA	NA	NA
ENSG00000270308	NA	NA	NA	0	NA	NA	NA	NA	NA
ENSG00000270313	NA	NA	NA	0	NA	NA	NA	NA	NA
ENSG00000270314	LOC100533631	5-azacytidine induced 2 pseudogene	100533631	0	NA	NA	NA	NA	NA
ENSG00000270316	NA	NA	NA	1,907661122	0,566148766	1,431183	0,395580929	0,692414208	NA
ENSG00000270317	NA	NA	NA	0	NA	NA	NA	NA	NA
ENSG00000270318	NA	NA	NA	0	NA	NA	NA	NA	NA
ENSG00000270322	NA	NA	NA	0	NA	NA	NA	NA	NA
ENSG00000270323	LOC100419713	MIA SH3 domain ER export factor 2 pseudogene	100419713	0	NA	NA	NA	NA	NA
ENSG00000270324	NA	NA	NA	0	NA	NA	NA	NA	NA
ENSG00000270325	BNIP3P9	BCL2 interacting protein 3 pseudogene 9	106480280	0	NA	NA	NA	NA	NA
ENSG00000270326	SMIM15P2	SMIM15 pseudogene 2	117751734	0	NA	NA	NA	NA	NA
ENSG00000270328	LOC100419616	impact RWD domain protein pseudogene	100419616	0	NA	NA	NA	NA	NA
ENSG00000270330	NA	NA	NA	0	NA	NA	NA	NA	NA
ENSG00000270333	XIAPP1	X-linked inhibitor of apoptosis pseudogene 1	100996851	0	NA	NA	NA	NA	NA
ENSG00000270335	NA	NA	NA	0	NA	NA	NA	NA	NA
ENSG00000270336	NA	NA	NA	0	NA	NA	NA	NA	NA

ENSG00000270339	PPIAL4H	peptidylprolyl isomerase A like 4H	105371242	3,001136832	-1,975364937	1,526511	-1,294039499	0,195651777	NA
ENSG00000270342	NA	NA	NA	0	NA	NA	NA	NA	NA
ENSG00000270343	NA	NA	NA	0	NA	NA	NA	NA	NA
ENSG00000270347	NA	NA	NA	0	NA	NA	NA	NA	NA
ENSG00000270350	MTND4LP5	MT-ND4L pseudogene 5	107075235	0	NA	NA	NA	NA	NA
ENSG00000270352	NA	NA	NA	0	NA	NA	NA	NA	NA
ENSG00000270354	NA	NA	NA	0	NA	NA	NA	NA	NA
ENSG00000270359	NA	NA	NA	0	NA	NA	NA	NA	NA
ENSG00000270367	NA	NA	NA	0	NA	NA	NA	NA	NA
ENSG00000270369	NA	NA	NA	0,103998153	-1,076443944	4,080456	-0,263804824	0,791930317	NA
ENSG00000270377	SMIM11P2	SMIM11 pseudogene 2	127379740	0	NA	NA	NA	NA	NA
ENSG00000270378	NA	NA	NA	0	NA	NA	NA	NA	NA
ENSG00000270379	HEATR9	HEAT repeat containing 9	256957	0,216496347	1,902771968	4,026559	0,472555327	0,63653046	NA
ENSG00000270381	NA	NA	NA	0,072165449	0,847101401	4,080456	0,207599697	0,835541541	NA
ENSG00000270382	NA	NA	NA	0	NA	NA	NA	NA	NA
ENSG00000270385	NA	NA	NA	0	NA	NA	NA	NA	NA
ENSG00000270387	RFPL4AP4	ret finger protein like 4A pseudogene 4	100420036	0	NA	NA	NA	NA	NA
ENSG00000270388	MTCO3P22	MT-CO3 pseudogene 22	100289371	0	NA	NA	NA	NA	NA
ENSG00000270390	SFXN4P1	SFXN4 pseudogene 1	100418906	0	NA	NA	NA	NA	NA
ENSG00000270392	PFN1P2	profilin 1 pseudogene 2	767846	0,705935337	2,151384744	2,675149	0,804211252	0,421274982	NA
ENSG00000270393	NA	NA	NA	0,532244233	1,377041668	2,497366	0,551397581	0,581361158	NA
ENSG00000270397	NA	NA	NA	0	NA	NA	NA	NA	NA
ENSG00000270400	NA	NA	NA	0	NA	NA	NA	NA	NA
ENSG00000270401	NA	NA	NA	0	NA	NA	NA	NA	NA
ENSG00000270402	NA	NA	NA	0	NA	NA	NA	NA	NA
ENSG00000270405	NA	NA	NA	0	NA	NA	NA	NA	NA
ENSG00000270409	NA	NA	NA	0	NA	NA	NA	NA	NA
ENSG00000270411	NA	NA	NA	0	NA	NA	NA	NA	NA
ENSG00000270413	NA	NA	NA	0	NA	NA	NA	NA	NA
ENSG00000270415	DPPA3P3	DPPA3 pseudogene 3	106481963	0	NA	NA	NA	NA	NA
ENSG00000270416	NA	NA	NA	0	NA	NA	NA	NA	NA
ENSG00000270421	NA	NA	NA	0	NA	NA	NA	NA	NA
ENSG00000270422	NA	NA	NA	0	NA	NA	NA	NA	NA
ENSG00000270423	NA	NA	NA	0	NA	NA	NA	NA	NA
ENSG00000270424	PAWRP2	pro-apoptotic WT1 regulator pseudogene 2	100420535	0,144330898	1,418786819	4,061262	0,349346267	0,726829368	NA
ENSG00000270425	LOC100419644	sorbitol dehydrogenase pseudogene	100419644	0	NA	NA	NA	NA	NA
ENSG00000270427	NA	NA	NA	0,262489998	0,46087261	4,061315	0,113478669	0,909651074	NA
ENSG00000270429	KNOP1P2	lysine rich nucleolar protein 1 pseudogene 2	100420898	0	NA	NA	NA	NA	NA
ENSG00000270430	LOC100533677	basal body orientation factor 1 pseudogene	100533677	0	NA	NA	NA	NA	NA
ENSG00000270431	NA	NA	NA	0	NA	NA	NA	NA	NA
ENSG00000270432	OR7E108P	olfactory receptor family 7 subfamily E member 108 pseudogene	81363	0,11808024	-1,076443944	4,080456	-0,263804824	0,791930317	NA
ENSG00000270433	NA	NA	NA	0,279036027	0,835651982	4,080456	0,20479378	0,837733247	NA
ENSG00000270434	NA	NA	NA	0,236573303	0	4,080456	0	1	NA
ENSG00000270435	NA	NA	NA	0	NA	NA	NA	NA	NA
ENSG00000270437	NA	NA	NA	0	NA	NA	NA	NA	NA
ENSG00000270440	NA	NA	NA	0	NA	NA	NA	NA	NA
ENSG00000270441	NA	NA	NA	0,327370886	-1,985375713	3,786451	-0,524336821	0,600044291	NA
ENSG00000270442	PPIGP1	peptidylprolyl isomerase G pseudogene 1	100131033	0	NA	NA	NA	NA	NA
ENSG00000270443	NA	NA	NA	0	NA	NA	NA	NA	NA
ENSG00000270444	AZU1P1	azurocidin 1 pseudogene 1	106480244	0	NA	NA	NA	NA	NA
ENSG00000270445	COPS5P2	COP9 signalosome subunit 5 pseudogene 2	112268476	0	NA	NA	NA	NA	NA
ENSG00000270446	NA	NA	NA	0	NA	NA	NA	NA	NA
ENSG00000270447	NA	NA	NA	0	NA	NA	NA	NA	NA
ENSG00000270449	NA	NA	NA	0	NA	NA	NA	NA	NA
ENSG00000270451	NA	NA	NA	0	NA	NA	NA	NA	NA
ENSG00000270453	CHCHD4P1	coiled-coil-helix-coiled-coil-helix domain containing 4 pseudogene 1	645862	0	NA	NA	NA	NA	NA
ENSG00000270455	NA	NA	NA	0	NA	NA	NA	NA	NA
ENSG00000270456	NA	NA	NA	0	NA	NA	NA	NA	NA
ENSG00000270458	NA	NA	NA	0	NA	NA	NA	NA	NA
ENSG00000270467	NA	NA	NA	0	NA	NA	NA	NA	NA
ENSG00000270469	NA	NA	NA	0	NA	NA	NA	NA	NA
ENSG00000270470	LOC100421651	caseinolytic mitochondrial matrix peptidase chaperone subunit X pseudogene	100421651	0	NA	NA	NA	NA	NA
ENSG00000270472	IGHV3OR16-9	immunoglobulin heavy variable 3/OR16-9 (non-functional)	28307	0	NA	NA	NA	NA	NA

ENSG00000270474	IGHV3-32	immunoglobulin heavy variable 3-32 (pseudogene)	28435	0	NA	NA	NA	NA	NA
ENSG00000270475	NA	NA	NA	0	NA	NA	NA	NA	NA
ENSG00000270477	NA	NA	NA	0	NA	NA	NA	NA	NA
ENSG00000270479	BNIP3P37	BCL2 interacting protein 3 pseudogene 37	106480285	0	NA	NA	NA	NA	NA
ENSG00000270480	SLC66A2P2	SLC66A2 pseudogene 2	100127906	0	NA	NA	NA	NA	NA
ENSG00000270484	NA	NA	NA	0	NA	NA	NA	NA	NA
ENSG00000270488	NA	NA	NA	0	NA	NA	NA	NA	NA
ENSG00000270490	LETM1P1	leucine zipper and EF-hand containing transmembrane protein 1 pseudogene 1	390597	0	NA	NA	NA	NA	NA
ENSG00000270491	NA	NA	NA	0	NA	NA	NA	NA	NA
ENSG00000270492	NA	NA	NA	0	NA	NA	NA	NA	NA
ENSG00000270493	NA	NA	NA	0	NA	NA	NA	NA	NA
ENSG00000270494	NA	NA	NA	0	NA	NA	NA	NA	NA
ENSG00000270495	NA	NA	NA	0	NA	NA	NA	NA	NA
ENSG00000270496	NA	NA	NA	0	NA	NA	NA	NA	NA
ENSG00000270497	NA	NA	NA	0	NA	NA	NA	NA	NA
ENSG00000270499	NA	NA	NA	0	NA	NA	NA	NA	NA
ENSG00000270500	NA	NA	NA	0	NA	NA	NA	NA	NA
ENSG00000270503	NA	NA	NA	0	NA	NA	NA	NA	NA
ENSG00000270506	NA	NA	NA	0	NA	NA	NA	NA	NA
ENSG00000270507	NA	NA	NA	0	NA	NA	NA	NA	NA
ENSG00000270509	LOC100128293	histone-lysine N-methyltransferase SUV39H2-like	100128293	0	NA	NA	NA	NA	NA
ENSG00000270510	LOC100420680	coiled-coil domain containing 90B pseudogene	100420680	0	NA	NA	NA	NA	NA
ENSG00000270512	NA	NA	NA	0	NA	NA	NA	NA	NA
ENSG00000270513	NA	NA	NA	0	NA	NA	NA	NA	NA
ENSG00000270516	NA	NA	NA	0	NA	NA	NA	NA	NA
ENSG00000270518	NA	NA	NA	0	NA	NA	NA	NA	NA
ENSG00000270521	NA	NA	NA	0	NA	NA	NA	NA	NA
ENSG00000270524	QTRT1P1	queuine tRNA-ribosyltransferase catalytic subunit 1 pseudogene 1	100420656	0	NA	NA	NA	NA	NA
ENSG00000270526	SNRPGP19	small nuclear ribonucleoprotein polypeptide G pseudogene 19	106481686	0	NA	NA	NA	NA	NA
ENSG00000270528	FAM133EP	family with sequence similarity 133 member A pseudogene	100505694	0	NA	NA	NA	NA	NA
ENSG00000270531	CSTBP1	CSTB pseudogene 1	127379754	0	NA	NA	NA	NA	NA
ENSG00000270533	NA	NA	NA	0	NA	NA	NA	NA	NA
ENSG00000270538	GCSHP6	GCSH pseudogene 6	127379698	0	NA	NA	NA	NA	NA
ENSG00000270541	NA	NA	NA	0	NA	NA	NA	NA	NA
ENSG00000270542	NA	NA	NA	0	NA	NA	NA	NA	NA
ENSG00000270549	NA	NA	NA	0	NA	NA	NA	NA	NA
ENSG00000270550	IGHV3-30	immunoglobulin heavy variable 3-30	28439	0	NA	NA	NA	NA	NA
ENSG00000270552	NA	NA	NA	0	NA	NA	NA	NA	NA
ENSG00000270553	LOC107984970	splicing factor 3B subunit 6-like	107984970	0	NA	NA	NA	NA	NA
ENSG00000270554	EIF3EP4	EIF3E pseudogene 4	100422200	0	NA	NA	NA	NA	NA
ENSG00000270555	NA	NA	NA	0	NA	NA	NA	NA	NA
ENSG00000270558	NA	NA	NA	0,81450932	1,024889242	2,030755	0,504683918	0,613780858	NA
ENSG00000270560	NA	NA	NA	0	NA	NA	NA	NA	NA
ENSG00000270569	NA	NA	NA	0	NA	NA	NA	NA	NA
ENSG00000270570	NA	NA	NA	0	NA	NA	NA	NA	NA
ENSG00000270575	NA	NA	NA	0	NA	NA	NA	NA	NA
ENSG00000270576	NA	NA	NA	0	NA	NA	NA	NA	NA
ENSG00000270577	NA	NA	NA	0	NA	NA	NA	NA	NA
ENSG00000270578	NA	NA	NA	0	NA	NA	NA	NA	NA
ENSG00000270583	LOC100420872	ribosome binding factor A (putative) pseudogene	100420872	0	NA	NA	NA	NA	NA
ENSG00000270584	NA	NA	NA	0	NA	NA	NA	NA	NA
ENSG00000270585	SNX2P1	sorting nexin 2 pseudogene 1	100418968	0	NA	NA	NA	NA	NA
ENSG00000270587	NA	NA	NA	0	NA	NA	NA	NA	NA
ENSG00000270588	NA	NA	NA	0	NA	NA	NA	NA	NA
ENSG00000270591	NA	NA	NA	0	NA	NA	NA	NA	NA
ENSG00000270593	NA	NA	NA	0	NA	NA	NA	NA	NA
ENSG00000270598	NA	NA	NA	0	NA	NA	NA	NA	NA
ENSG00000270606	PPIAP52	peptidylprolyl isomerase A pseudogene 52	111082965	0	NA	NA	NA	NA	NA
ENSG00000270610	NA	NA	NA	0	NA	NA	NA	NA	NA
ENSG00000270611	NA	NA	NA	0	NA	NA	NA	NA	NA
ENSG00000270612	NA	NA	NA	0	NA	NA	NA	NA	NA
ENSG00000270614	NA	NA	NA	0	NA	NA	NA	NA	NA

ENSG00000270615	SGO1P1	shugoshin 1 pseudogene 1	100533666	0	NA	NA	NA	NA	NA
ENSG00000270617	URGCP-MRPS24	URGCP-MRPS24 readthrough	100534592	0,144330898	1,418786819	4,061262	0,349346267	0,726829368	NA
ENSG00000270619	NA	NA	NA	0	NA	NA	NA	NA	NA
ENSG00000270620	NA	NA	NA	0	NA	NA	NA	NA	NA
ENSG00000270623	NA	NA	NA	0	NA	NA	NA	NA	NA
ENSG00000270624	LOC401913	ABO blood group (transferase A, alpha 1-3-N-acetylgalactosaminyltransferase; transferase B, alpha 1-3-galactosyltransferase) pseudogene	401913	0	NA	NA	NA	NA	NA
ENSG00000270625	NUDT19P6	NUDT19 pseudogene 6	107985644	0	NA	NA	NA	NA	NA
ENSG00000270627	NA	NA	NA	0	NA	NA	NA	NA	NA
ENSG00000270631	LOC100287840	tetraspanin 12 pseudogene	100287840	0	NA	NA	NA	NA	NA
ENSG00000270632	NA	NA	NA	0	NA	NA	NA	NA	NA
ENSG00000270634	NA	NA	NA	0	NA	NA	NA	NA	NA
ENSG00000270639	ECM1P1	extracellular matrix protein 1 pseudogene 1	100420354	0	NA	NA	NA	NA	NA
ENSG00000270648	CYP2C61P	cytochrome P450 family 2 subfamily C member 61, pseudogene	106480734	0	NA	NA	NA	NA	NA
ENSG00000270652	NA	NA	NA	0	NA	NA	NA	NA	NA
ENSG00000270654	NA	NA	NA	0	NA	NA	NA	NA	NA
ENSG00000270655	NA	NA	NA	0	NA	NA	NA	NA	NA
ENSG00000270664	NA	NA	NA	0	NA	NA	NA	NA	NA
ENSG00000270665	NA	NA	NA	0	NA	NA	NA	NA	NA
ENSG00000270666	NA	NA	NA	0	NA	NA	NA	NA	NA
ENSG00000270669	FTLP9	ferritin light chain pseudogene 9	100462780	0	NA	NA	NA	NA	NA
ENSG00000270670	NA	NA	NA	0	NA	NA	NA	NA	NA
ENSG00000270677	RACK1P2	RACK1 pseudogene 2	100422559	0	NA	NA	NA	NA	NA
ENSG00000270678	NA	NA	NA	0	NA	NA	NA	NA	NA
ENSG00000270679	NA	NA	NA	0	NA	NA	NA	NA	NA
ENSG00000270680	NA	NA	NA	0	NA	NA	NA	NA	NA
ENSG00000270682	NA	NA	NA	0	NA	NA	NA	NA	NA
ENSG00000270683	GARIN3P1	GARIN3 pseudogene 1	100421478	0	NA	NA	NA	NA	NA
ENSG00000270685	NA	NA	NA	0	NA	NA	NA	NA	NA
ENSG00000270688	NA	NA	NA	0	NA	NA	NA	NA	NA
ENSG00000270689	NA	NA	NA	0,191659871	1,677594817	4,046274	0,414602369	0,678433014	NA
ENSG00000270690	NA	NA	NA	0	NA	NA	NA	NA	NA
ENSG00000270691	NA	NA	NA	0	NA	NA	NA	NA	NA
ENSG00000270694	NA	NA	NA	0	NA	NA	NA	NA	NA
ENSG00000270695	NA	NA	NA	0	NA	NA	NA	NA	NA
ENSG00000270698	LOC100422319	iron-sulfur cluster assembly 1 homolog (S, cerevisiae) pseudogene	100422319	0	NA	NA	NA	NA	NA
ENSG00000270699	NA	NA	NA	0	NA	NA	NA	NA	NA
ENSG00000270701	NA	NA	NA	0	NA	NA	NA	NA	NA
ENSG00000270702	NA	NA	NA	0	NA	NA	NA	NA	NA
ENSG00000270705	NA	NA	NA	0	NA	NA	NA	NA	NA
ENSG00000270706	NA	NA	NA	0	NA	NA	NA	NA	NA
ENSG00000270708	NA	NA	NA	0	NA	NA	NA	NA	NA
ENSG00000270709	NA	NA	NA	0	NA	NA	NA	NA	NA
ENSG00000270711	NA	NA	NA	0	NA	NA	NA	NA	NA
ENSG00000270712	NA	NA	NA	0	NA	NA	NA	NA	NA
ENSG00000270713	NA	NA	NA	0	NA	NA	NA	NA	NA
ENSG00000270714	NA	NA	NA	0,117497108	0,847101401	4,080456	0,207599697	0,835541541	NA
ENSG00000270716	BNIP3P15	BCL2 interacting protein 3 pseudogene 15	106481692	0	NA	NA	NA	NA	NA
ENSG00000270718	NA	NA	NA	0	NA	NA	NA	NA	NA
ENSG00000270719	NA	NA	NA	0	NA	NA	NA	NA	NA
ENSG00000270723	RPL23AP92	ribosomal protein L23a pseudogene 92	106481731	0	NA	NA	NA	NA	NA
ENSG00000270727	NA	NA	NA	0	NA	NA	NA	NA	NA
ENSG00000270728	NA	NA	NA	0	NA	NA	NA	NA	NA
ENSG00000270733	MRPS6P1	mitochondrial ribosomal protein S6 pseudogene 1	359780	0	NA	NA	NA	NA	NA
ENSG00000270739	NA	NA	NA	0	NA	NA	NA	NA	NA
ENSG00000270741	PRELID3BP8	PRELI domain containing 3B pseudogene 8	106480243	0	NA	NA	NA	NA	NA
ENSG00000270745	NA	NA	NA	0	NA	NA	NA	NA	NA
ENSG00000270747	S100A11P9	S100A11 pseudogene 9	107985655	0	NA	NA	NA	NA	NA
ENSG00000270749	LOC100420545	abhydrolase domain containing 15 pseudogene	100420545	0	NA	NA	NA	NA	NA
ENSG00000270753	NA	NA	NA	0	NA	NA	NA	NA	NA
ENSG00000270754	NA	NA	NA	0	NA	NA	NA	NA	NA
ENSG00000270757	HSPE1-MOB4	HSPE1-MOB4 readthrough	100529241	2,247446381	0	3,456785	0	1	NA

ENSG00000270759	NA	NA	NA	0,15836288	0	4,080456	0	1	NA
ENSG00000270762	NA	NA	NA	0	NA	NA	NA	NA	NA
ENSG00000270763	DDX43P2	DEAD-box helicase 43 pseudogene 2	100533714	0	NA	NA	NA	NA	NA
ENSG00000270764	NA	NA	NA	0	NA	NA	NA	NA	NA
ENSG00000270765	GAS2L2	growth arrest specific 2 like 2	246176	0,119673877	0,847101401	4,080456	0,207599697	0,835541541	NA
ENSG00000270767	NA	NA	NA	0	NA	NA	NA	NA	NA
ENSG00000270772	PDHA1P1	PDHA1 pseudogene 1	100132534	0	NA	NA	NA	NA	NA
ENSG00000270773	MARK2P19	MARK2 pseudogene 19	100421508	0	NA	NA	NA	NA	NA
ENSG00000270775	NA	NA	NA	0	NA	NA	NA	NA	NA
ENSG00000270776	NA	NA	NA	0	NA	NA	NA	NA	NA
ENSG00000270778	NA	NA	NA	0	NA	NA	NA	NA	NA
ENSG00000270779	NA	NA	NA	0	NA	NA	NA	NA	NA
ENSG00000270780	NA	NA	NA	0	NA	NA	NA	NA	NA
ENSG00000270781	NA	NA	NA	0	NA	NA	NA	NA	NA
ENSG00000270782	NA	NA	NA	0	NA	NA	NA	NA	NA
ENSG00000270788	NA	NA	NA	0	NA	NA	NA	NA	NA
ENSG00000270789	NA	NA	NA	0	NA	NA	NA	NA	NA
ENSG00000270790	NA	NA	NA	0	NA	NA	NA	NA	NA
ENSG00000270791	DPRXP6	divergent-paired related homeobox pseudogene 6	503647	0	NA	NA	NA	NA	NA
ENSG00000270793	LLPHP1	LLPH pseudogene 1	106480685	0	NA	NA	NA	NA	NA
ENSG00000270794	LOC100420324	MAGE family member A10 pseudogene	100420324	0	NA	NA	NA	NA	NA
ENSG00000270797	NA	NA	NA	0	NA	NA	NA	NA	NA
ENSG00000270798	NA	NA	NA	0	NA	NA	NA	NA	NA
ENSG00000270799	NA	NA	NA	0	NA	NA	NA	NA	NA
ENSG00000270802	NA	NA	NA	0,430390247	-0,300482154	3,532326	-0,085066376	0,932208622	NA
ENSG00000270804	NA	NA	NA	6,419700468	-0,130088105	0,718011	-0,181178519	0,856227455	NA
ENSG00000270806	C17orf50	chromosome 17 open reading frame 50	146853	0,117497108	0,847101401	4,080456	0,207599697	0,835541541	NA
ENSG00000270807	NA	NA	NA	0	NA	NA	NA	NA	NA
ENSG00000270808	NA	NA	NA	0	NA	NA	NA	NA	NA
ENSG00000270809	CHCHD2P11	coiled-coil-helix-coiled-coil-helix domain containing 2 pseudogene 11	101060040	0	NA	NA	NA	NA	NA
ENSG00000270811	NA	NA	NA	0	NA	NA	NA	NA	NA
ENSG00000270812	NA	NA	NA	0	NA	NA	NA	NA	NA
ENSG00000270813	NA	NA	NA	0,236573303	0	4,080456	0	1	NA
ENSG00000270814	NA	NA	NA	0	NA	NA	NA	NA	NA
ENSG00000270815	NA	NA	NA	0	NA	NA	NA	NA	NA
ENSG00000270818	LOC100129949	protein RCC2-like	100129949	0	NA	NA	NA	NA	NA
ENSG00000270822	NA	NA	NA	0	NA	NA	NA	NA	NA
ENSG00000270826	NA	NA	NA	0	NA	NA	NA	NA	NA
ENSG00000270830	NA	NA	NA	0	NA	NA	NA	NA	NA
ENSG00000270831	NF1P1	neurofibromin 1 pseudogene 1	100419006	0	NA	NA	NA	NA	NA
ENSG00000270832	LOC100130664	cytidine/uridine monophosphate kinase 1 pseudogene	100130664	0	NA	NA	NA	NA	NA
ENSG00000270835	NA	NA	NA	0	NA	NA	NA	NA	NA
ENSG00000270837	NA	NA	NA	0	NA	NA	NA	NA	NA
ENSG00000270838	NA	NA	NA	0	NA	NA	NA	NA	NA
ENSG00000270839	NA	NA	NA	0	NA	NA	NA	NA	NA
ENSG00000270842	NA	NA	NA	0	NA	NA	NA	NA	NA
ENSG00000270846	NA	NA	NA	0	NA	NA	NA	NA	NA
ENSG00000270849	NA	NA	NA	0	NA	NA	NA	NA	NA
ENSG00000270850	NA	NA	NA	0	NA	NA	NA	NA	NA
ENSG00000270852	NA	NA	NA	0	NA	NA	NA	NA	NA
ENSG00000270855	ATP7BP1	ATPase copper transporting beta pseudogene 1	100462825	0	NA	NA	NA	NA	NA
ENSG00000270856	NA	NA	NA	0	NA	NA	NA	NA	NA
ENSG00000270858	NA	NA	NA	0	NA	NA	NA	NA	NA
ENSG00000270859	NA	NA	NA	0	NA	NA	NA	NA	NA
ENSG00000270861	NA	NA	NA	0	NA	NA	NA	NA	NA
ENSG00000270863	DDX55P1	DEAD-box helicase 55 pseudogene 1	100129640	1,140195367	0,046887582	1,981713	0,023660131	0,981123708	NA
ENSG00000270864	NA	NA	NA	0	NA	NA	NA	NA	NA
ENSG00000270865	NA	NA	NA	0	NA	NA	NA	NA	NA
ENSG00000270868	NA	NA	NA	0	NA	NA	NA	NA	NA
ENSG00000270870	NA	NA	NA	0	NA	NA	NA	NA	NA
ENSG00000270872	NA	NA	NA	7,735477324	0,63259534	0,743198	0,851179559	0,394669616	NA
ENSG00000270874	NA	NA	NA	0	NA	NA	NA	NA	NA
ENSG00000270878	GCATP1	glycine C-acetyltransferase pseudogene 1	319140	1,050136531	-0,113408106	2,296867	-0,049375126	0,96062035	NA
ENSG00000270880	ATP5PBP8	ATP5PB pseudogene 8	100127892	0	NA	NA	NA	NA	NA
ENSG00000270885	RASL10B	RAS like family 10 member B	91608	0,528134468	1,846263235	3,30581	0,558490376	0,576509575	NA
ENSG00000270889	NA	NA	NA	0	NA	NA	NA	NA	NA
ENSG00000270890	NA	NA	NA	0	NA	NA	NA	NA	NA

ENSG00000270891	NA	NA	NA	0,144330898	1,418786819	4,061262	0,349346267	0,726829368	NA
ENSG00000270892	NA	NA	NA	0	NA	NA	NA	NA	NA
ENSG00000270893	NA	NA	NA	0	NA	NA	NA	NA	NA
ENSG00000270896	NA	NA	NA	0	NA	NA	NA	NA	NA
ENSG00000270897	NA	NA	NA	0	NA	NA	NA	NA	NA
ENSG00000270900	NUDT19P4	NUDT19 pseudogene 4	100422452	0	NA	NA	NA	NA	NA
ENSG00000270902	NA	NA	NA	0	NA	NA	NA	NA	NA
ENSG00000270903	HNRNPA3P9	heterogeneous nuclear ribonucleoprotein A3 pseudogene 9	100421415	0	NA	NA	NA	NA	NA
ENSG00000270904	ATP6V1G1P5	ATPase H+ transporting V1 subunit G1 pseudogene 5	100462840	0	NA	NA	NA	NA	NA
ENSG00000270906	MTND4P35	MT-ND4 pseudogene 35	107075117	0	NA	NA	NA	NA	NA
ENSG00000270909	NA	NA	NA	0	NA	NA	NA	NA	NA
ENSG00000270911	LOC100419654	MIA SH3 domain ER export factor 2 pseudogene	100419654	0	NA	NA	NA	NA	NA
ENSG00000270914	NA	NA	NA	0	NA	NA	NA	NA	NA
ENSG00000270915	NA	NA	NA	0	NA	NA	NA	NA	NA
ENSG00000270916	NA	NA	NA	0	NA	NA	NA	NA	NA
ENSG00000270917	NA	NA	NA	0,680207724	-1,574519139	2,451081	-0,642377538	0,520628081	NA
ENSG00000270920	MCRIP2P2	MAPK regulated corepressor interacting protein 2 pseudogene 2	106480278	0	NA	NA	NA	NA	NA
ENSG00000270921	NA	NA	NA	0	NA	NA	NA	NA	NA
ENSG00000270923	TAS2R6P	taste 2 receptor member 6 pseudogene	448990	1,425964971	-0,543705347	1,513862	-0,359151221	0,719481965	NA
ENSG00000270924	NA	NA	NA	0	NA	NA	NA	NA	NA
ENSG00000270926	NA	NA	NA	0	NA	NA	NA	NA	NA
ENSG00000270927	NA	NA	NA	0	NA	NA	NA	NA	NA
ENSG00000270929	NA	NA	NA	0,072165449	0,847101401	4,080456	0,207599697	0,835541541	NA
ENSG00000270930	GRAMD4P8	GRAM domain containing 4 pseudogene 8	100631247	0	NA	NA	NA	NA	NA
ENSG00000270934	LOC100420531	abhydrolase domain containing 10, depalmitoylase pseudogene	100420531	0	NA	NA	NA	NA	NA
ENSG00000270935	LOC442172	cell division cycle associated 7 pseudogene	442172	0	NA	NA	NA	NA	NA
ENSG00000270938	RAP2CP1	RAP2C pseudogene 1	106480249	0	NA	NA	NA	NA	NA
ENSG00000270941	AK3P7	AK3 pseudogene 7	127379742	0	NA	NA	NA	NA	NA
ENSG00000270945	NA	NA	NA	0	NA	NA	NA	NA	NA
ENSG00000270946	CT45A9	cancer/testis antigen family 45 member A9	102723680	0	NA	NA	NA	NA	NA
ENSG00000270948	MTDHP1	metadherin pseudogene 1	100418814	0	NA	NA	NA	NA	NA
ENSG00000270954	NA	NA	NA	0	NA	NA	NA	NA	NA
ENSG00000270955	NA	NA	NA	0	NA	NA	NA	NA	NA
ENSG00000270957	NA	NA	NA	0	NA	NA	NA	NA	NA
ENSG00000270960	NA	NA	NA	0	NA	NA	NA	NA	NA
ENSG00000270961	NA	NA	NA	0	NA	NA	NA	NA	NA
ENSG00000270962	NA	NA	NA	8,04613557	-0,040330148	0,733681	-0,054969575	0,956162703	NA
ENSG00000270965	NTAN1P1	N-terminal asparagine amidase pseudogene 1	100420389	0	NA	NA	NA	NA	NA
ENSG00000270969	NA	NA	NA	0	NA	NA	NA	NA	NA
ENSG00000270971	NA	NA	NA	0	NA	NA	NA	NA	NA
ENSG00000270973	NA	NA	NA	0	NA	NA	NA	NA	NA
ENSG00000270975	NA	NA	NA	0	NA	NA	NA	NA	NA
ENSG00000270976	NA	NA	NA	0	NA	NA	NA	NA	NA
ENSG00000270977	NA	NA	NA	4,689575786	1,254400186	1,114211	1,125819742	0,260241826	NA
ENSG00000270978	GLULP1	glutamate-ammonia ligase pseudogene 1	2753	0	NA	NA	NA	NA	NA
ENSG00000270980	NA	NA	NA	0	NA	NA	NA	NA	NA
ENSG00000270981	PCBP2P4	PCBP2 pseudogene 4	100420048	0	NA	NA	NA	NA	NA
ENSG00000270982	NA	NA	NA	0	NA	NA	NA	NA	NA
ENSG00000270983	NA	NA	NA	0	NA	NA	NA	NA	NA
ENSG00000270986	NA	NA	NA	0	NA	NA	NA	NA	NA
ENSG00000270987	NA	NA	NA	0	NA	NA	NA	NA	NA
ENSG00000270989	NA	NA	NA	0	NA	NA	NA	NA	NA
ENSG00000270990	EIF2AP3	eukaryotic translation initiation factor 2A pseudogene 3	100874395	0	NA	NA	NA	NA	NA
ENSG00000270992	NA	NA	NA	0	NA	NA	NA	NA	NA
ENSG00000270993	NA	NA	NA	0	NA	NA	NA	NA	NA
ENSG00000270994	LOC100420254	T-box transcription factor T pseudogene	100420254	0	NA	NA	NA	NA	NA
ENSG00000270995	DPPA3P10	DPPA3 pseudogene 10	127460883	0	NA	NA	NA	NA	NA
ENSG00000270997	NA	NA	NA	0	NA	NA	NA	NA	NA
ENSG00000271002	NA	NA	NA	0,144330898	1,418786819	4,061262	0,349346267	0,726829368	NA
ENSG00000271003	LOC100421548	family with sequence similarity 178 member B pseudogene	100421548	0	NA	NA	NA	NA	NA

ENSG00000271005	CTHRC1P1	collagen triple helix repeat containing 1 pseudogene 1	106480299	0	NA	NA	NA	NA	NA
ENSG00000271010	NA	NA	NA	0	NA	NA	NA	NA	NA
ENSG00000271014	NA	NA	NA	0	NA	NA	NA	NA	NA
ENSG00000271018	NA	NA	NA	0	NA	NA	NA	NA	NA
ENSG00000271021	LOC105373314	UDP-GlcNAc:betaGal beta-1,3-N-acetylglucosaminyltransferase-like protein 1	105373314	0	NA	NA	NA	NA	NA
ENSG00000271022	NA	NA	NA	0,444464563	0,824656805	3,53962	0,23297893	0,815777767	NA
ENSG00000271024	HSPE1P19	heat shock protein family E (Hsp10) member 1 pseudogene 19	106481675	0	NA	NA	NA	NA	NA
ENSG00000271025	NA	NA	NA	0	NA	NA	NA	NA	NA
ENSG00000271026	KATNBL1P1	katanin regulatory subunit B1 like 1 pseudogene 1	100420919	0	NA	NA	NA	NA	NA
ENSG00000271027	NA	NA	NA	0	NA	NA	NA	NA	NA
ENSG00000271028	NA	NA	NA	0	NA	NA	NA	NA	NA
ENSG00000271029	NA	NA	NA	0	NA	NA	NA	NA	NA
ENSG00000271034	RNPC3P1	RNA binding region (RNP1, RRM) containing 3 pseudogene 1	654340	0	NA	NA	NA	NA	NA
ENSG00000271035	DPPA3P12	DPPA3 pseudogene 12	127460880	0	NA	NA	NA	NA	NA
ENSG00000271036	CLPTM1LP1	CLPTM1L pseudogene 1	106480241	0	NA	NA	NA	NA	NA
ENSG00000271042	NA	NA	NA	0	NA	NA	NA	NA	NA
ENSG00000271044	HMGB1P43	high mobility group box 1 pseudogene 43	100420041	0	NA	NA	NA	NA	NA
ENSG00000271047	NA	NA	NA	0	NA	NA	NA	NA	NA
ENSG00000271048	NA	NA	NA	0	NA	NA	NA	NA	NA
ENSG00000271049	NA	NA	NA	0	NA	NA	NA	NA	NA
ENSG00000271052	SRP14P5	SRP14 pseudogene 5	127482536	0	NA	NA	NA	NA	NA
ENSG00000271053	HAVCR1P2	hepatitis A virus cellular receptor 1 pseudogene 2	100421002	0	NA	NA	NA	NA	NA
ENSG00000271056	NA	NA	NA	0	NA	NA	NA	NA	NA
ENSG00000271057	NA	NA	NA	0	NA	NA	NA	NA	NA
ENSG00000271063	NA	NA	NA	0	NA	NA	NA	NA	NA
ENSG00000271064	NA	NA	NA	0	NA	NA	NA	NA	NA
ENSG00000271070	GMCL1P2	GMCL1 pseudogene 2	100420975	0	NA	NA	NA	NA	NA
ENSG00000271071	NA	NA	NA	0,119673877	0,847101401	4,080456	0,207599697	0,835541541	NA
ENSG00000271072	NA	NA	NA	0	NA	NA	NA	NA	NA
ENSG00000271074	LOC100420775	5'-nucleotidase domain containing 2 pseudogene	100420775	0	NA	NA	NA	NA	NA
ENSG00000271075	NA	NA	NA	0	NA	NA	NA	NA	NA
ENSG00000271077	NA	NA	NA	0	NA	NA	NA	NA	NA
ENSG00000271078	NA	NA	NA	0	NA	NA	NA	NA	NA
ENSG00000271081	HIKESHIP3	HIKESHI pseudogene 3	117751737	0,072165449	0,847101401	4,080456	0,207599697	0,835541541	NA
ENSG00000271088	NA	NA	NA	0,15836288	0	4,080456	0	1	NA
ENSG00000271092	TLCD4-RWDD3	TLCD4-RWDD3 readthrough	100527978	3,693011483	-0,76165698	1,160053	-0,65657084	0,511456904	NA
ENSG00000271093	NA	NA	NA	0	NA	NA	NA	NA	NA
ENSG00000271094	NA	NA	NA	0	NA	NA	NA	NA	NA
ENSG00000271095	BNIP3P28	BCL2 interacting protein 3 pseudogene 28	106481966	0	NA	NA	NA	NA	NA
ENSG00000271096	SUMO2P14	SUMO2 pseudogene 14	106480264	0	NA	NA	NA	NA	NA
ENSG00000271097	DBF4P3	DBF4 zinc finger pseudogene 3	112272612	0	NA	NA	NA	NA	NA
ENSG00000271098	IMMP1LP3	inner mitochondrial membrane peptidase subunit 1 pseudogene 3	106481715	0	NA	NA	NA	NA	NA
ENSG00000271099	NA	NA	NA	0	NA	NA	NA	NA	NA
ENSG00000271100	NA	NA	NA	0	NA	NA	NA	NA	NA
ENSG00000271101	NA	NA	NA	0	NA	NA	NA	NA	NA
ENSG00000271105	NA	NA	NA	0,555822133	0,046974572	2,819789	0,016658895	0,986708739	NA
ENSG00000271108	KATNBL1P5	katanin regulatory subunit B1 like 1 pseudogene 5	100420841	0	NA	NA	NA	NA	NA
ENSG00000271111	LOC100420211	SH3 domain binding protein 4 pseudogene	100420211	0	NA	NA	NA	NA	NA
ENSG00000271113	TMX1P2	TMX1 pseudogene 2	100101127	0	NA	NA	NA	NA	NA
ENSG00000271114	NA	NA	NA	0	NA	NA	NA	NA	NA
ENSG00000271115	NA	NA	NA	0	NA	NA	NA	NA	NA
ENSG00000271117	MARK2P7	MARK2 pseudogene 7	100421531	0	NA	NA	NA	NA	NA
ENSG00000271118	NA	NA	NA	0	NA	NA	NA	NA	NA
ENSG00000271127	NA	NA	NA	0	NA	NA	NA	NA	NA
ENSG00000271128	NA	NA	NA	0	NA	NA	NA	NA	NA
ENSG00000271129	NA	NA	NA	0	NA	NA	NA	NA	NA
ENSG00000271130	IGHV3OR16-8	immunoglobulin heavy variable 3/OR16-8 (non-functional)	388255	0	NA	NA	NA	NA	NA
ENSG00000271131	PPIAP75	peptidylprolyl isomerase A pseudogene 75	111082968	0	NA	NA	NA	NA	NA

ENSG00000271134	NA	NA	NA	0	NA	NA	NA	NA	NA	
ENSG00000271136	NA	NA	NA	0	NA	NA	NA	NA	NA	
ENSG00000271137	NA	NA	NA	0	NA	NA	NA	NA	NA	
ENSG00000271138	NA	NA	NA	0	NA	NA	NA	NA	NA	
ENSG00000271140	PRR20FP	proline rich 20F, pseudogene	106480416	0	NA	NA	NA	NA	NA	
ENSG00000271142	YWHAQP7	YWHAQ pseudogene 7	100422674	0,11808024	-1,076443944	4,080456	-0,263804824	0,791930317	NA	
ENSG00000271143	LOC101928952	serine palmitoyltransferase small subunit A-like	101928952	0	NA	NA	NA	NA	NA	
ENSG00000271146	BNIP3P45	BNIP3 pseudogene 45	127379725	0	NA	NA	NA	NA	NA	
ENSG00000271149	NA	NA	NA	0	NA	NA	NA	NA	NA	
ENSG00000271150	NA	NA	NA	0	NA	NA	NA	NA	NA	
ENSG00000271153	RPL23AP88	ribosomal protein L23a pseudogene 88	100289034	0,721654492	3,604856845	3,944092	0,913989001	0,360722625	NA	
ENSG00000271154	NA	NA	NA	0	NA	NA	NA	NA	NA	
ENSG00000271157	NA	NA	NA	0	NA	NA	NA	NA	NA	
ENSG00000271158	WDR4P1	WDR4 pseudogene 1	100130607	0	NA	NA	NA	NA	NA	
ENSG00000271159	NA	NA	NA	0	NA	NA	NA	NA	NA	
ENSG00000271161	BOLA2P2	bolA family member 2 pseudogene 2	106480322	0	NA	NA	NA	NA	NA	
ENSG00000271162	LOC100418956	proline rich 11 pseudogene	100418956	0	NA	NA	NA	NA	NA	
ENSG00000271163	NA	NA	NA	0	NA	NA	NA	NA	NA	
ENSG00000271164	CFAP97P1	CFAP97 pseudogene 1	100418722	0	NA	NA	NA	NA	NA	
ENSG00000271166	LOC100420790	glucosamine-6-phosphate deaminase 2 pseudogene	100420790	0	NA	NA	NA	NA	NA	
ENSG00000271173	NA	NA	NA	0	NA	NA	NA	NA	NA	
ENSG00000271174	NA	NA	NA	0	NA	NA	NA	NA	NA	
ENSG00000271175	NA	NA	NA	0	NA	NA	NA	NA	NA	
ENSG00000271177	NA	NA	NA	0	NA	NA	NA	NA	NA	
ENSG00000271178	NA	NA	NA	0	NA	NA	NA	NA	NA	
ENSG00000271179	BNIP3P46	BNIP3 pseudogene 46	127379734	0	NA	NA	NA	NA	NA	
ENSG00000271180	NA	NA	NA	0	NA	NA	NA	NA	NA	
ENSG00000271181	NA	NA	NA	0	NA	NA	NA	NA	NA	
ENSG00000271182	NA	NA	NA	0	NA	NA	NA	NA	NA	
ENSG00000271184	LOC100419701	CTAGE family member 9 pseudogene	100419701	0	NA	NA	NA	NA	NA	
ENSG00000271187	NA	NA	NA	0	NA	NA	NA	NA	NA	
ENSG00000271190	NA	NA	NA	0	NA	NA	NA	NA	NA	
ENSG00000271194	RNF138P2	ring finger protein 138 pseudogene 2	100419777	0	NA	NA	NA	NA	NA	
ENSG00000271195	NA	NA	NA	0	NA	NA	NA	NA	NA	
ENSG00000271196	NA	NA	NA	0	NA	NA	NA	NA	NA	
ENSG00000271198	NA	NA	NA	0	NA	NA	NA	NA	NA	
ENSG00000271202	NA	NA	NA	0	NA	NA	NA	NA	NA	
ENSG00000271205	NA	NA	NA	0	NA	NA	NA	NA	NA	
ENSG00000271207	MTCO1P22	MT-CO1 pseudogene 22	107075165	0,10488184	-1,076443944	4,080456	-0,263804824	0,791930317	NA	
ENSG00000271209	BRDTP1	bromodomain testis associated pseudogene 1	643486	0	NA	NA	NA	NA	NA	
ENSG00000271214	NA	NA	NA	0	NA	NA	NA	NA	NA	
ENSG00000271215	NA	NA	NA	0	NA	NA	NA	NA	NA	
ENSG00000271219	NA	NA	NA	0	NA	NA	NA	NA	NA	
ENSG00000271220	NA	NA	NA	0	NA	NA	NA	NA	NA	
ENSG00000271222	NA	NA	NA	0	NA	NA	NA	NA	NA	
ENSG00000271225	BNIP3P4	BCL2 interacting protein 3 pseudogene 4	100289028	0	NA	NA	NA	NA	NA	
ENSG00000271227	SREK1IP1P2	SREK1IP1 pseudogene 2	115838655	0	NA	NA	NA	NA	NA	
ENSG00000271230	NA	NA	NA	0	NA	NA	NA	NA	NA	
ENSG00000271231	KRT18P9	keratin 18 pseudogene 9	442205	0	NA	NA	NA	NA	NA	
ENSG00000271232	NA	NA	NA	0	NA	NA	NA	NA	NA	
ENSG00000271235	NA	NA	NA	0	NA	NA	NA	NA	NA	
ENSG00000271236	SUMO2P15	SUMO2 pseudogene 15	106480417	0	NA	NA	NA	NA	NA	
ENSG00000271237	HMGB1P50	high mobility group box 1 pseudogene 50	109729125	0	NA	NA	NA	NA	NA	
ENSG00000271238	NA	NA	NA	0	NA	NA	NA	NA	NA	
ENSG00000271240	NA	NA	NA	0	NA	NA	NA	NA	NA	
ENSG00000271242	PRELID3BP2	PRELI domain containing 3B pseudogene 2	106481667	0	NA	NA	NA	NA	NA	
ENSG00000271243	LOC100422418	proteasome 26S subunit, non-ATPase 12 pseudogene	100422418	0	NA	NA	NA	NA	NA	
ENSG00000271245	NA	NA	NA	0	NA	NA	NA	NA	NA	
ENSG00000271248	NA	NA	NA	0	NA	NA	NA	NA	NA	
ENSG00000271249	DIMT1P1	DIM1 dimethyladenosine transferase 1 homolog pseudogene 1	100422385	0	NA	NA	NA	NA	NA	
ENSG00000271250	NA	NA	NA	0	NA	NA	NA	NA	NA	
ENSG00000271251	NA	NA	NA	0	NA	NA	NA	NA	NA	
ENSG00000271253	NA	NA	NA	0	NA	NA	NA	NA	NA	
ENSG00000271254	LOC102724250	neuroblastoma breakpoint family member 1-like	102724250	5,887099707	0,683073318	1,526027	0,447615591	0,654430649	NA	
ENSG00000271257	NA	NA	NA	0	NA	NA	NA	NA	NA	

ENSG00000271264	NA	NA	NA	0	NA	NA	NA	NA	NA
ENSG00000271266	NA	NA	NA	0	NA	NA	NA	NA	NA
ENSG00000271267	NA	NA	NA	0	NA	NA	NA	NA	NA
ENSG00000271269	NA	NA	NA	0	NA	NA	NA	NA	NA
ENSG00000271271	UGT2A2	UDP glucuronosyltransferase family 2 member A2	574537	0	NA	NA	NA	NA	NA
ENSG00000271272	NA	NA	NA	0	NA	NA	NA	NA	NA
ENSG00000271275	NA	NA	NA	0	NA	NA	NA	NA	NA
ENSG00000271277	NA	NA	NA	0	NA	NA	NA	NA	NA
ENSG00000271278	NA	NA	NA	0	NA	NA	NA	NA	NA
ENSG00000271283	NA	NA	NA	0,15836288	0	4,080456	0	1	NA
ENSG00000271284	NA	NA	NA	0	NA	NA	NA	NA	NA
ENSG00000271286	CYTH1P1	CYTH1 pseudogene 1	100418871	0,10543123	0	4,080456	0	1	NA
ENSG00000271287	BCRP9	BCR pseudogene 9	107126287	0	NA	NA	NA	NA	NA
ENSG00000271288	NA	NA	NA	4,680395702	1,872667164	1,47381	1,270629876	0,203860353	NA
ENSG00000271290	NA	NA	NA	0	NA	NA	NA	NA	NA
ENSG00000271291	NA	NA	NA	0	NA	NA	NA	NA	NA
ENSG00000271296	H3P19	H3 histone pseudogene 19	115482709	0	NA	NA	NA	NA	NA
ENSG00000271298	NA	NA	NA	0	NA	NA	NA	NA	NA
ENSG00000271299	NA	NA	NA	0	NA	NA	NA	NA	NA
ENSG00000271302	NA	NA	NA	0	NA	NA	NA	NA	NA
ENSG00000271304	NA	NA	NA	0	NA	NA	NA	NA	NA
ENSG00000271306	RAC1P9	Rac family small GTPase 1 pseudogene 9	100422658	0	NA	NA	NA	NA	NA
ENSG00000271309	NA	NA	NA	0	NA	NA	NA	NA	NA
ENSG00000271313	NA	NA	NA	0	NA	NA	NA	NA	NA
ENSG00000271318	NA	NA	NA	0	NA	NA	NA	NA	NA
ENSG00000271322	NA	NA	NA	0	NA	NA	NA	NA	NA
ENSG00000271328	B3GNTL1P2	B3GNTL1 pseudogene 2	106480419	0	NA	NA	NA	NA	NA
ENSG00000271329	LOC100130714	metaxin 3 pseudogene	100130714	0,119673877	0,847101401	4,080456	0,207599697	0,835541541	NA
ENSG00000271330	NA	NA	NA	0	NA	NA	NA	NA	NA
ENSG00000271332	NA	NA	NA	0	NA	NA	NA	NA	NA
ENSG00000271333	NA	NA	NA	0	NA	NA	NA	NA	NA
ENSG00000271336	NA	NA	NA	0	NA	NA	NA	NA	NA
ENSG00000271337	NDE1P2	nudE neurodevelopment protein 1 pseudogene 2	106480247	0	NA	NA	NA	NA	NA
ENSG00000271338	NA	NA	NA	0	NA	NA	NA	NA	NA
ENSG00000271339	LOC100422685	solute carrier family 25 member 6 pseudogene	100422685	0	NA	NA	NA	NA	NA
ENSG00000271340	NA	NA	NA	0	NA	NA	NA	NA	NA
ENSG00000271343	NA	NA	NA	0	NA	NA	NA	NA	NA
ENSG00000271349	NA	NA	NA	0	NA	NA	NA	NA	NA
ENSG00000271350	NA	NA	NA	0	NA	NA	NA	NA	NA
ENSG00000271353	NA	NA	NA	0	NA	NA	NA	NA	NA
ENSG00000271355	NA	NA	NA	0	NA	NA	NA	NA	NA
ENSG00000271356	USP21P2	USP21 pseudogene 2	100420428	0	NA	NA	NA	NA	NA
ENSG00000271357	NA	NA	NA	0	NA	NA	NA	NA	NA
ENSG00000271358	NA	NA	NA	0	NA	NA	NA	NA	NA
ENSG00000271362	NA	NA	NA	0	NA	NA	NA	NA	NA
ENSG00000271364	LOC100420205	ribonucleotide reductase regulatory subunit M2 pseudogene	100420205	0	NA	NA	NA	NA	NA
ENSG00000271365	NA	NA	NA	0	NA	NA	NA	NA	NA
ENSG00000271368	LOC100421901	immunoglobulin binding protein 1 pseudogene	100421901	0	NA	NA	NA	NA	NA
ENSG00000271369	NA	NA	NA	0	NA	NA	NA	NA	NA
ENSG00000271373	NANOGP3	Nanog homeobox pseudogene 3	340217	0	NA	NA	NA	NA	NA
ENSG00000271375	IGKV1ORY-1	immunoglobulin kappa variable 1/ORY-1 (pseudogene)	439957	0	NA	NA	NA	NA	NA
ENSG00000271377	NA	NA	NA	0	NA	NA	NA	NA	NA
ENSG00000271378	NA	NA	NA	0	NA	NA	NA	NA	NA
ENSG00000271379	LOC101929308	ATP synthase subunit C lysine N-methyltransferase-like	101929308	0,11808024	-1,076443944	4,080456	-0,263804824	0,791930317	NA
ENSG00000271381	NA	NA	NA	0	NA	NA	NA	NA	NA
ENSG00000271385	NA	NA	NA	0	NA	NA	NA	NA	NA
ENSG00000271386	SRGNP1	serglycin pseudogene 1	106480246	0	NA	NA	NA	NA	NA
ENSG00000271389	OSBPL9P1	oxysterol binding protein like 9 pseudogene 1	100130821	0	NA	NA	NA	NA	NA
ENSG00000271396	NA	NA	NA	0	NA	NA	NA	NA	NA
ENSG00000271397	NA	NA	NA	0	NA	NA	NA	NA	NA
ENSG00000271399	NA	NA	NA	0	NA	NA	NA	NA	NA
ENSG00000271400	NA	NA	NA	0	NA	NA	NA	NA	NA
ENSG00000271404	NA	NA	NA	0	NA	NA	NA	NA	NA
ENSG00000271408	NAPGP1	N-ethylmaleimide-sensitive factor attachment protein, gamma pseudogene 1	100288843	0	NA	NA	NA	NA	NA

ENSG00000271410	LOC100419851	protein serine kinase H1 pseudogene	100419851	0	NA	NA	NA	NA	NA
ENSG00000271412	NA	NA	NA	0	NA	NA	NA	NA	NA
ENSG00000271413	EI24P6	EI24 pseudogene 6	127482540	0	NA	NA	NA	NA	NA
ENSG00000271414	NA	NA	NA	0,295066827	0,67640723	3,895631	0,173632275	0,862154465	NA
ENSG00000271415	RPL23AP90	ribosomal protein L23a pseudogene 90	106480690	0	NA	NA	NA	NA	NA
ENSG00000271418	NA	NA	NA	0	NA	NA	NA	NA	NA
ENSG00000271419	LOC100421116	trafficking kinesin protein 2 pseudogene	100421116	0	NA	NA	NA	NA	NA
ENSG00000271421	NA	NA	NA	0	NA	NA	NA	NA	NA
ENSG00000271423	NA	NA	NA	0	NA	NA	NA	NA	NA
ENSG00000271424	GLRX5P3	GLRX5 pseudogene 3	118568802	0	NA	NA	NA	NA	NA
ENSG00000271426	NA	NA	NA	0	NA	NA	NA	NA	NA
ENSG00000271428	NA	NA	NA	0	NA	NA	NA	NA	NA
ENSG00000271429	NA	NA	NA	0	NA	NA	NA	NA	NA
ENSG00000271433	PPATP2	phosphoribosyl pyrophosphate amidotransferase pseudogene 2	100421601	0	NA	NA	NA	NA	NA
ENSG00000271437	NA	NA	NA	0	NA	NA	NA	NA	NA
ENSG00000271439	NA	NA	NA	0	NA	NA	NA	NA	NA
ENSG00000271440	NA	NA	NA	0	NA	NA	NA	NA	NA
ENSG00000271443	NA	NA	NA	0	NA	NA	NA	NA	NA
ENSG00000271447	MMP28	matrix metallopeptidase 28	79148	0	NA	NA	NA	NA	NA
ENSG00000271449	CT45A2	cancer/testis antigen family 45 member A2	728911	0	NA	NA	NA	NA	NA
ENSG00000271454	NA	NA	NA	0	NA	NA	NA	NA	NA
ENSG00000271455	NA	NA	NA	0	NA	NA	NA	NA	NA
ENSG00000271456	NA	NA	NA	0	NA	NA	NA	NA	NA
ENSG00000271457	NA	NA	NA	0	NA	NA	NA	NA	NA
ENSG00000271459	LOC100422549	solute carrier family 5 member 4 pseudogene	100422549	0	NA	NA	NA	NA	NA
ENSG00000271461	CKAP2LP1	CKAP2L pseudogene 1	100533711	0	NA	NA	NA	NA	NA
ENSG00000271462	NA	NA	NA	0	NA	NA	NA	NA	NA
ENSG00000271464	NA	NA	NA	0	NA	NA	NA	NA	NA
ENSG00000271465	SQSTM1P1	sequestosome 1 pseudogene 1	106480677	0	NA	NA	NA	NA	NA
ENSG00000271466	NA	NA	NA	0	NA	NA	NA	NA	NA
ENSG00000271468	LOC100422334	general transcription factor IIH subunit 3 pseudogene	100422334	0	NA	NA	NA	NA	NA
ENSG00000271475	LOC100421842	eukaryotic translation elongation factor 1 alpha 2 pseudogene	100421842	0	NA	NA	NA	NA	NA
ENSG00000271477	NA	NA	NA	0	NA	NA	NA	NA	NA
ENSG00000271480	MTND3P19	MT-ND3 pseudogene 19	107075161	0	NA	NA	NA	NA	NA
ENSG00000271482	NA	NA	NA	0	NA	NA	NA	NA	NA
ENSG00000271483	NA	NA	NA	0	NA	NA	NA	NA	NA
ENSG00000271484	NA	NA	NA	0,119673877	0,847101401	4,080456	0,207599697	0,835541541	NA
ENSG00000271486	NA	NA	NA	0	NA	NA	NA	NA	NA
ENSG00000271488	RBM11P1	RNA binding motif protein 11 pseudogene 1	100420213	0	NA	NA	NA	NA	NA
ENSG00000271490	NA	NA	NA	0	NA	NA	NA	NA	NA
ENSG00000271491	LOC100131557	programmed cell death 2 like pseudogene	100131557	0	NA	NA	NA	NA	NA
ENSG00000271492	MEMO1P5	MEMO1 pseudogene 5	106481668	0	NA	NA	NA	NA	NA
ENSG00000271494	NA	NA	NA	0	NA	NA	NA	NA	NA
ENSG00000271495	NA	NA	NA	0	NA	NA	NA	NA	NA
ENSG00000271496	SNRPGP20	small nuclear ribonucleoprotein polypeptide G pseudogene 20	106480271	0	NA	NA	NA	NA	NA
ENSG00000271498	NA	NA	NA	0	NA	NA	NA	NA	NA
ENSG00000271499	NA	NA	NA	0	NA	NA	NA	NA	NA
ENSG00000271502	NA	NA	NA	0	NA	NA	NA	NA	NA
ENSG00000271507	NA	NA	NA	0	NA	NA	NA	NA	NA
ENSG00000271508	NA	NA	NA	0	NA	NA	NA	NA	NA
ENSG00000271509	NA	NA	NA	0	NA	NA	NA	NA	NA
ENSG00000271511	NA	NA	NA	0	NA	NA	NA	NA	NA
ENSG00000271519	NA	NA	NA	0,144330898	1,418786819	4,061262	0,349346267	0,726829368	NA
ENSG00000271523	NA	NA	NA	0	NA	NA	NA	NA	NA
ENSG00000271524	BNIP3P17	BCL2 interacting protein 3 pseudogene 17	100421704	0	NA	NA	NA	NA	NA
ENSG00000271525	NA	NA	NA	0	NA	NA	NA	NA	NA
ENSG00000271527	LOC100420658	post-GPI attachment to proteins 2 pseudogene	100420658	0	NA	NA	NA	NA	NA
ENSG00000271530	NA	NA	NA	0	NA	NA	NA	NA	NA
ENSG00000271532	BBIP1P1	BBSome interacting protein 1 pseudogene 1	105373509	0	NA	NA	NA	NA	NA
ENSG00000271536	NA	NA	NA	0	NA	NA	NA	NA	NA

ENSG00000271537	NA	NA	NA	0	NA	NA	NA	NA	NA
ENSG00000271544	NA	NA	NA	0	NA	NA	NA	NA	NA
ENSG00000271546	NA	NA	NA	0	NA	NA	NA	NA	NA
ENSG00000271547	NA	NA	NA	0	NA	NA	NA	NA	NA
ENSG00000271550	BNIP3P11	BCL2 interacting protein 3 pseudogene 11	106480286	0	NA	NA	NA	NA	NA
ENSG00000271558	NA	NA	NA	0	NA	NA	NA	NA	NA
ENSG00000271559	NA	NA	NA	0	NA	NA	NA	NA	NA
ENSG00000271560	NA	NA	NA	0	NA	NA	NA	NA	NA
ENSG00000271563	HIRAP1	histone cell cycle regulator pseudogene 1	100422545	0	NA	NA	NA	NA	NA
ENSG00000271565	NA	NA	NA	0	NA	NA	NA	NA	NA
ENSG00000271567	PPIAL4E	peptidylprolyl isomerase A like 4E	730262	0	NA	NA	NA	NA	NA
ENSG00000271568	EIF2AP2	eukaryotic translation initiation factor 2A pseudogene 2	100874394	0	NA	NA	NA	NA	NA
ENSG00000271573	NA	NA	NA	0	NA	NA	NA	NA	NA
ENSG00000271578	NA	NA	NA	0	NA	NA	NA	NA	NA
ENSG00000271580	GYG2P2	glycogenin 2 pseudogene 2	100418892	0	NA	NA	NA	NA	NA
ENSG00000271581	NA	NA	NA	0,756138649	-0,798202375	2,131118	-0,374546312	0,707997908	NA
ENSG00000271582	EIF2AP1	eukaryotic translation initiation factor 2A pseudogene 1	100874393	0	NA	NA	NA	NA	NA
ENSG00000271583	LOC100420430	nipsnap homolog 2 pseudogene	100420430	0	NA	NA	NA	NA	NA
ENSG00000271585	SELENOTP2	selenoprotein T pseudogene 2	100130316	0	NA	NA	NA	NA	NA
ENSG00000271586	NA	NA	NA	0	NA	NA	NA	NA	NA
ENSG00000271587	NA	NA	NA	0	NA	NA	NA	NA	NA
ENSG00000271588	LARP7P1	LARP7 pseudogene 1	106480287	0	NA	NA	NA	NA	NA
ENSG00000271589	NA	NA	NA	0	NA	NA	NA	NA	NA
ENSG00000271595	ELOCP35	elongin C pseudogene 35	106480250	0	NA	NA	NA	NA	NA
ENSG00000271596	NA	NA	NA	0,103998153	-1,076443944	4,080456	-0,263804824	0,791930317	NA
ENSG00000271597	LOC100421347	golgi reassembly stacking protein 2, 55kDa pseudogene	100421347	0	NA	NA	NA	NA	NA
ENSG00000271598	NA	NA	NA	0	NA	NA	NA	NA	NA
ENSG00000271600	NA	NA	NA	0	NA	NA	NA	NA	NA
ENSG00000271602	KRT8P49	keratin 8 pseudogene 49	643447	0	NA	NA	NA	NA	NA
ENSG00000271605	MILR1	mast cell immunoglobulin like receptor 1	284021	1,719986275	0,231712944	1,315074	0,176197588	0,860138716	NA
ENSG00000271606	NA	NA	NA	0	NA	NA	NA	NA	NA
ENSG00000271607	RBISP4	RBIS pseudogene 4	127379685	0	NA	NA	NA	NA	NA
ENSG00000271608	NA	NA	NA	0,118286652	0	4,080456	0	1	NA
ENSG00000271609	NA	NA	NA	0	NA	NA	NA	NA	NA
ENSG00000271611	NA	NA	NA	0	NA	NA	NA	NA	NA
ENSG00000271612	NA	NA	NA	0	NA	NA	NA	NA	NA
ENSG00000271615	NA	NA	NA	0	NA	NA	NA	NA	NA
ENSG00000271616	NA	NA	NA	0	NA	NA	NA	NA	NA
ENSG00000271618	NA	NA	NA	0	NA	NA	NA	NA	NA
ENSG00000271620	NA	NA	NA	0	NA	NA	NA	NA	NA
ENSG00000271621	NA	NA	NA	0	NA	NA	NA	NA	NA
ENSG00000271623	NA	NA	NA	0	NA	NA	NA	NA	NA
ENSG00000271624	LOC100419058	GPN-loop GTPase 1 pseudogene	100419058	0	NA	NA	NA	NA	NA
ENSG00000271625	NA	NA	NA	0	NA	NA	NA	NA	NA
ENSG00000271626	H3P42	H3 histone pseudogene 42	115482704	0	NA	NA	NA	NA	NA
ENSG00000271627	NA	NA	NA	0	NA	NA	NA	NA	NA
ENSG00000271629	NA	NA	NA	0	NA	NA	NA	NA	NA
ENSG00000271632	LOC100421684	tetratricopeptide repeat domain 34 pseudogene	100421684	0	NA	NA	NA	NA	NA
ENSG00000271635	DBF4P2	DBF4 zinc finger pseudogene 2	100420180	0	NA	NA	NA	NA	NA
ENSG00000271638	BNIP3P19	BCL2 interacting protein 3 pseudogene 19	106480281	0	NA	NA	NA	NA	NA
ENSG00000271639	PPFIA1P1	PPFIA1 pseudogene 1	100422710	0	NA	NA	NA	NA	NA
ENSG00000271642	MARK3P1	microtubule affinity regulating kinase 3 pseudogene 1	100421550	0	NA	NA	NA	NA	NA
ENSG00000271644	NA	NA	NA	0	NA	NA	NA	NA	NA
ENSG00000271647	KRT8P47	keratin 8 pseudogene 47	644743	0	NA	NA	NA	NA	NA
ENSG00000271650	NA	NA	NA	0	NA	NA	NA	NA	NA
ENSG00000271652	NA	NA	NA	0	NA	NA	NA	NA	NA
ENSG00000271654	NA	NA	NA	0	NA	NA	NA	NA	NA
ENSG00000271655	BNIP3P35	BCL2 interacting protein 3 pseudogene 35	106480284	0	NA	NA	NA	NA	NA
ENSG00000271656	NA	NA	NA	0	NA	NA	NA	NA	NA
ENSG00000271657	NA	NA	NA	0,103998153	-1,076443944	4,080456	-0,263804824	0,791930317	NA
ENSG00000271660	NA	NA	NA	0	NA	NA	NA	NA	NA

ENSG00000271661	BNIP3P36	BCL2 interacting protein 3 pseudogene 36	106481696	0	NA	NA	NA	NA	NA
ENSG00000271664	NA	NA	NA	0	NA	NA	NA	NA	NA
ENSG00000271666	NA	NA	NA	0	NA	NA	NA	NA	NA
ENSG00000271667	NA	NA	NA	0	NA	NA	NA	NA	NA
ENSG00000271671	NA	NA	NA	0	NA	NA	NA	NA	NA
ENSG00000271672	NA	NA	NA	0	NA	NA	NA	NA	NA
ENSG00000271676	LOC339966	ubiquitin family domain containing 1 pseudogene	339966	0	NA	NA	NA	NA	NA
ENSG00000271677	NA	NA	NA	0	NA	NA	NA	NA	NA
ENSG00000271679	LSM1P2	LSM1 homolog, mRNA degradation associated pseudogene 2	106480288	0	NA	NA	NA	NA	NA
ENSG00000271680	NA	NA	NA	0	NA	NA	NA	NA	NA
ENSG00000271681	NA	NA	NA	0	NA	NA	NA	NA	NA
ENSG00000271682	TVP23CP3	TVP23C pseudogene 3	127525792	0	NA	NA	NA	NA	NA
ENSG00000271686	TNPO3P1	TNPO3 pseudogene 1	100131029	0	NA	NA	NA	NA	NA
ENSG00000271687	MTND5P10	MT-ND5 pseudogene 10	643031	0	NA	NA	NA	NA	NA
ENSG00000271691	NA	NA	NA	0	NA	NA	NA	NA	NA
ENSG00000271693	NA	NA	NA	0	NA	NA	NA	NA	NA
ENSG00000271696	LOC100420541	abhydrolase domain containing 15 pseudogene	100420541	0	NA	NA	NA	NA	NA
ENSG00000271697	NA	NA	NA	0	NA	NA	NA	NA	NA
ENSG00000271701	NA	NA	NA	0	NA	NA	NA	NA	NA
ENSG00000271702	LOC100421527	DnaJ heat shock protein family (Hsp40) member C8 pseudogene	100421527	0	NA	NA	NA	NA	NA
ENSG00000271704	BNIP3P20	BCL2 interacting protein 3 pseudogene 20	100421705	0	NA	NA	NA	NA	NA
ENSG00000271705	RPL17P3	ribosomal protein L17 pseudogene 3	326286	0	NA	NA	NA	NA	NA
ENSG00000271707	NA	NA	NA	0,10543123	0	4,080456	0	1	NA
ENSG00000271710	NDUFA8P1	NADH:ubiquinone oxidoreductase subunit A8 pseudogene 1	107075207	0	NA	NA	NA	NA	NA
ENSG00000271711	NA	NA	NA	0,11808024	-1,076443944	4,080456	-0,263804824	0,791930317	NA
ENSG00000271712	LOC100421611	golgi associated RAB2B interactor family member 3 pseudogene	100421611	0	NA	NA	NA	NA	NA
ENSG00000271723	MROH7-TTC4	MROH7-TTC4 readthrough (NMD candidate)	100527960	0	NA	NA	NA	NA	NA
ENSG00000271749	NA	NA	NA	0	NA	NA	NA	NA	NA
ENSG00000271779	NA	NA	NA	0	NA	NA	NA	NA	NA
ENSG00000271793	NA	NA	NA	0	NA	NA	NA	NA	NA
ENSG00000271810	NA	NA	NA	0	NA	NA	NA	NA	NA
ENSG00000271824	SMIM32	small integral membrane protein 32	389332	0	NA	NA	NA	NA	NA
ENSG00000271890	NA	NA	NA	0	NA	NA	NA	NA	NA
ENSG00000271900	NA	NA	NA	0	NA	NA	NA	NA	NA
ENSG00000271919	NA	NA	NA	0	NA	NA	NA	NA	NA
ENSG00000271924	RNA5SP108	RNA, 5S ribosomal pseudogene 108	100873378	0	NA	NA	NA	NA	NA
ENSG00000271943	NA	NA	NA	4,227824976	-0,048199251	1,068468	-0,04511062	0,964019136	NA
ENSG00000271949	NA	NA	NA	0,072165449	0,847101401	4,080456	0,207599697	0,835541541	NA
ENSG00000271974	RDM1P4	RDM1 pseudogene 4	112577467	0	NA	NA	NA	NA	NA
ENSG00000271977	NA	NA	NA	0	NA	NA	NA	NA	NA
ENSG00000271985	NA	NA	NA	0	NA	NA	NA	NA	NA
ENSG00000272006	NA	NA	NA	0	NA	NA	NA	NA	NA
ENSG00000272031	ANKRD34A	ankyrin repeat domain 34A	284615	6,036016272	0,89109422	0,799324	1,114809337	0,264932134	NA
ENSG00000272104	NA	NA	NA	0,426653233	1,404287586	3,494637	0,401840788	0,687801202	NA
ENSG00000272134	NA	NA	NA	0	NA	NA	NA	NA	NA
ENSG00000272150	NA	NA	NA	2,558764421	-0,289762221	1,419528	-0,204125735	0,838255244	NA
ENSG00000272162	NA	NA	NA	0	NA	NA	NA	NA	NA
ENSG00000272164	NA	NA	NA	0,118286652	0	4,080456	0	1	NA
ENSG00000272220	NA	NA	NA	0	NA	NA	NA	NA	NA
ENSG00000272253	RNA5SP386	RNA, 5S ribosomal pseudogene 386	100873641	0	NA	NA	NA	NA	NA
ENSG00000272281	NA	NA	NA	0	NA	NA	NA	NA	NA
ENSG00000272297	NA	NA	NA	0	NA	NA	NA	NA	NA
ENSG00000272305	NA	NA	NA	0,072165449	0,847101401	4,080456	0,207599697	0,835541541	NA
ENSG00000272319	NA	NA	NA	0	NA	NA	NA	NA	NA
ENSG00000272346	NA	NA	NA	0	NA	NA	NA	NA	NA
ENSG00000272383	NA	NA	NA	0	NA	NA	NA	NA	NA
ENSG00000272387	NA	NA	NA	0	NA	NA	NA	NA	NA
ENSG00000272395	IFNL4	interferon lambda 4 (gene/pseudogene)	101180976	0	NA	NA	NA	NA	NA
ENSG00000272398	CD24	CD24 molecule	100133941	0,072165449	0,847101401	4,080456	0,207599697	0,835541541	NA
ENSG00000272400	NA	NA	NA	0,103998153	-1,076443944	4,080456	-0,263804824	0,791930317	NA
ENSG00000272407	NA	NA	NA	0,235798457	0,855835977	4,08024	0,209751364	0,833861734	NA

ENSG00000272414	FAM47E-STBD1	FAM47E-STBD1 readthrough	100631383	0	NA	NA	NA	NA	NA
ENSG00000272419	NA	NA	NA	3,852802411	-0,658864409	1,099965	-0,598986943	0,549181591	NA
ENSG00000272435	RNA5SP357	RNA, 5S ribosomal pseudogene 357	100873616	0	NA	NA	NA	NA	NA
ENSG00000272436	NA	NA	NA	0	NA	NA	NA	NA	NA
ENSG00000272442	NA	NA	NA	0	NA	NA	NA	NA	NA
ENSG00000272443	NA	NA	NA	0	NA	NA	NA	NA	NA
ENSG00000272480	OR13Z3P	olfactory receptor family 13 subfamily Z member 3 pseudogene	403230	0	NA	NA	NA	NA	NA
ENSG00000272494	OR1X1P	olfactory receptor family 1 subfamily X member 1 pseudogene	402236	0	NA	NA	NA	NA	NA
ENSG00000272514	CFAP206	cilia and flagella associated protein 206	154313	0	NA	NA	NA	NA	NA
ENSG00000272556	NA	NA	NA	1,93767358	1,809198494	1,651662	1,095380903	0,273349797	NA
ENSG00000272559	OR51F3P	olfactory receptor family 51 subfamily F member 3 pseudogene	390043	0	NA	NA	NA	NA	NA
ENSG00000272569	OR5BL1P	olfactory receptor family 5 subfamily BL member 1 pseudogene	81205	0	NA	NA	NA	NA	NA
ENSG00000272573	MUSTN1	musculoskeletal, embryonic nuclear protein 1	389125	0,625485345	-3,230168499	3,870011	-0,834666549	0,403905481	NA
ENSG00000272578	NA	NA	NA	9,142219086	-0,347456719	0,67418	-0,515376967	0,606289624	NA
ENSG00000272595	OR10AH1P	olfactory receptor family 10 subfamily AH member 1 pseudogene	402246	0	NA	NA	NA	NA	NA
ENSG00000272601	EFCAB3P1	EFCAB3 pseudogene 1	125775236	0,871101496	-1,501293562	2,668941	-0,562505305	0,57377179	NA
ENSG00000272617	NA	NA	NA	4,814200739	1,126853188	3,45797	0,325871338	0,744521702	NA
ENSG00000272634	OR51F5P	olfactory receptor family 51 subfamily F member 5 pseudogene	390040	0	NA	NA	NA	NA	NA
ENSG00000272636	DOC2B	double C2 domain beta	8447	0	NA	NA	NA	NA	NA
ENSG00000272645	NA	NA	NA	0	NA	NA	NA	NA	NA
ENSG00000272647	NA	NA	NA	0,447255242	0,158414288	3,983362	0,039768989	0,9682773	NA
ENSG00000272664	OR51C4P	olfactory receptor family 51 subfamily C member 4 pseudogene	390041	0	NA	NA	NA	NA	NA
ENSG00000272676	OR5AO1P	olfactory receptor family 5 subfamily AO member 1 pseudogene	403272	0	NA	NA	NA	NA	NA
ENSG00000272685	OR9I3P	olfactory receptor family 9 subfamily I member 3 pseudogene	81155	0	NA	NA	NA	NA	NA
ENSG00000272693	NA	NA	NA	0,548896959	0,932878551	3,39466	0,274807673	0,783464003	NA
ENSG00000272710	NA	NA	NA	0	NA	NA	NA	NA	NA
ENSG00000272714	NA	NA	NA	0	NA	NA	NA	NA	NA
ENSG00000272741	NA	NA	NA	0,117497108	0,847101401	4,080456	0,207599697	0,835541541	NA
ENSG00000272804	KRTAP10-7	keratin associated protein 10-7	386675	0	NA	NA	NA	NA	NA
ENSG00000272822	NA	NA	NA	0,23616048	-2,075648354	4,028973	-0,515180498	0,606426895	NA
ENSG00000272837	OR10AE3P	olfactory receptor family 10 subfamily AE member 3 pseudogene	403223	0	NA	NA	NA	NA	NA
ENSG00000272856	NA	NA	NA	0	NA	NA	NA	NA	NA
ENSG00000272882	OR2BH1P	olfactory receptor family 2 subfamily BH member 1 pseudogene	403238	0	NA	NA	NA	NA	NA
ENSG00000272887	NA	NA	NA	0	NA	NA	NA	NA	NA
ENSG00000272896	NA	NA	NA	0,385182688	-0,133808777	3,657019	-0,036589572	0,970812258	NA
ENSG00000272897	NA	NA	NA	0	NA	NA	NA	NA	NA
ENSG00000272899	SPMIP1	sperm microtubule inner protein 1	100130705	4,540449956	0,550201505	0,927014	0,59352006	0,552833162	NA
ENSG00000272900	OR10Q2P	olfactory receptor family 10 subfamily Q member 2 pseudogene	81345	0	NA	NA	NA	NA	NA
ENSG00000272921	NA	NA	NA	1,06272216	-4,256679619	3,934039	-1,082012526	0,279246962	NA
ENSG00000272937	OR6U2P	olfactory receptor family 6 subfamily U member 2 pseudogene	390329	0	NA	NA	NA	NA	NA
ENSG00000272963	OR7A19P	olfactory receptor family 7 subfamily A member 19 pseudogene	387853	0,805671473	0,731945106	2,310529	0,31678677	0,751405403	NA
ENSG00000272968	RBAK-RBAKDN	RBAK-RBAKDN readthrough	100533952	0,11808024	-1,076443944	4,080456	-0,263804824	0,791930317	NA
ENSG00000272980	NA	NA	NA	1,443308983	4,597613364	3,686458	1,24716292	0,212337766	NA

ENSG00000272987	OR5AL1	olfactory receptor family 5 subfamily AL member 1 (gene/pseudogene)	79482	0	NA	NA	NA	NA	NA
ENSG00000273003	ARL2-SNX15	ARL2-SNX15 readthrough (NMD candidate)	100528018	0	NA	NA	NA	NA	NA
ENSG00000273024	INTS4P2	integrator complex subunit 4 pseudogene 2	644619	0	NA	NA	NA	NA	NA
ENSG00000273025	NA	NA	NA	0,23616048	-2,075648354	4,028973	-0,515180498	0,606426895	NA
ENSG00000273036	CYP4F33P	cytochrome P450 family 4 subfamily F member 33, pseudogene	107133499	0	NA	NA	NA	NA	NA
ENSG00000273041	NA	NA	NA	1,214595327	-1,838542451	2,210178	-0,831852789	0,405492043	NA
ENSG00000273045	C2orf15	chromosome 2 open reading frame 15	150590	5,418724288	1,033270568	0,922015	1,120665997	0,262430061	NA
ENSG00000273047	NA	NA	NA	0	NA	NA	NA	NA	NA
ENSG00000273049	NA	NA	NA	0	NA	NA	NA	NA	NA
ENSG00000273051	OR51F4P	olfactory receptor family 51 subfamily F member 4 pseudogene	390044	0	NA	NA	NA	NA	NA
ENSG00000273079	GRIN2B	glutamate ionotropic receptor NMDA type subunit 2B	2904	0	NA	NA	NA	NA	NA
ENSG00000273085	OR52E1	olfactory receptor family 52 subfamily E member 1 (gene/pseudogene)	79296	0	NA	NA	NA	NA	NA
ENSG00000273088	NA	NA	NA	0	NA	NA	NA	NA	NA
ENSG00000273111	LYPD4	LY6/PLAUR domain containing 4	147719	0	NA	NA	NA	NA	NA
ENSG00000273155	NA	NA	NA	0,207996306	-1,916073151	4,043021	-0,473921178	0,635556117	NA
ENSG00000273167	NA	NA	NA	1,074939059	-1,082803201	2,564342	-0,422253888	0,672839712	NA
ENSG00000273171	NA	NA	NA	0	NA	NA	NA	NA	NA
ENSG00000273217	NA	NA	NA	0	NA	NA	NA	NA	NA
ENSG00000273225	NA	NA	NA	0	NA	NA	NA	NA	NA
ENSG00000273228	OR5BQ1P	olfactory receptor family 5 subfamily BQ member 1 pseudogene	81202	0	NA	NA	NA	NA	NA
ENSG00000273234	NA	NA	NA	0	NA	NA	NA	NA	NA
ENSG00000273238	TMEM271	transmembrane protein 271	112441426	0	NA	NA	NA	NA	NA
ENSG00000273252	NA	NA	NA	0	NA	NA	NA	NA	NA
ENSG00000273255	NA	NA	NA	0	NA	NA	NA	NA	NA
ENSG00000273259	NA	NA	NA	0	NA	NA	NA	NA	NA
ENSG00000273269	NA	NA	NA	0	NA	NA	NA	NA	NA
ENSG00000273274	ZBTB8B	zinc finger and BTB domain containing 8B	728116	0	NA	NA	NA	NA	NA
ENSG00000273291	NA	NA	NA	0	NA	NA	NA	NA	NA
ENSG00000273294	C1QTNF3-AMACR	C1QTNF3-AMACR readthrough (NMD candidate)	100534612	2,049673211	0,865873205	2,975183	0,291031881	0,771026937	NA
ENSG00000273327	OR6L2P	olfactory receptor family 6 subfamily L member 2 pseudogene	81356	0	NA	NA	NA	NA	NA
ENSG00000273330	CYP4F36P	cytochrome P450 family 4 subfamily F member 36, pseudogene	100422235	0	NA	NA	NA	NA	NA
ENSG00000273331	NA	NA	NA	0	NA	NA	NA	NA	NA
ENSG00000273336	OR7M1P	olfactory receptor family 7 subfamily M member 1 pseudogene	81352	0,54554658	1,385167482	2,484363	0,557554377	0,577148718	NA
ENSG00000273340	NA	NA	NA	3,156283154	1,297754422	1,20153	1,080085262	0,280104214	NA
ENSG00000273345	NA	NA	NA	0	NA	NA	NA	NA	NA
ENSG00000273398	NA	NA	NA	1,253145297	-0,819988334	2,130938	-0,38480163	0,700384391	NA
ENSG00000273408	OR5B15P	olfactory receptor family 5 subfamily B member 15 pseudogene	81215	0	NA	NA	NA	NA	NA
ENSG00000273423	OR13I1P	olfactory receptor family 13 subfamily I member 1 pseudogene	79538	0	NA	NA	NA	NA	NA
ENSG00000273434	OR8S21P	olfactory receptor family 8 subfamily S member 21 pseudogene	403304	0	NA	NA	NA	NA	NA
ENSG00000273444	NA	NA	NA	0,188774686	-0,081485535	4,080456	-0,019969714	0,984067533	NA
ENSG00000273484	NA	NA	NA	0	NA	NA	NA	NA	NA
ENSG00000273497	NA	NA	NA	0	NA	NA	NA	NA	NA
ENSG00000273509	NA	NA	NA	0,072165449	0,847101401	4,080456	0,207599697	0,835541541	NA
ENSG00000273512	NA	NA	NA	0	NA	NA	NA	NA	NA
ENSG00000273513	TBC1D3K	TBC1 domain family member 3K	101060351	0	NA	NA	NA	NA	NA
ENSG00000273513	TBC1D3E	TBC1 domain family member 3E	102723859	0	NA	NA	NA	NA	NA
ENSG00000273514	FOXD4L6	forkhead box D4 like 6	653404	0	NA	NA	NA	NA	NA

ENSG00000273536	NA	NA	NA	0	NA	NA	NA	NA	NA
ENSG00000273540	AGBL1	AGBL carboxypeptidase 1	123624	0	NA	NA	NA	NA	NA
ENSG00000273542	H4C12	H4 clustered histone 12	8362	2,760670304	0,060364714	1,392069	0,043363309	0,965411925	NA
ENSG00000273554	NA	NA	NA	0	NA	NA	NA	NA	NA
ENSG00000273567	NA	NA	NA	0	NA	NA	NA	NA	NA
ENSG00000273569	LOC100419716	zinc finger protein 709 pseudogene	100419716	0	NA	NA	NA	NA	NA
ENSG00000273580	LOC100419774	zinc finger protein 383 pseudogene	100419774	1,535368439	0,689373382	1,451088	0,475073537	0,63473456	NA
ENSG00000273586	OR10G1P	olfactory receptor family 10 subfamily G member 1 pseudogene	26540	0	NA	NA	NA	NA	NA
ENSG00000273589	NA	NA	NA	0	NA	NA	NA	NA	NA
ENSG00000273595	NA	NA	NA	0	NA	NA	NA	NA	NA
ENSG00000273597	LOC100420114	SPATA31 subfamily D member 1 pseudogene	100420114	0,982902193	2,358464547	1,961791	1,202199747	0,229286147	NA
ENSG00000273598	NA	NA	NA	4,129492171	0,649913391	1,365264	0,476035064	0,634049398	NA
ENSG00000273600	FGFR3P2	fibroblast growth factor receptor 3 pseudogene 2	100462815	0	NA	NA	NA	NA	NA
ENSG00000273604	EPOP	elongin BC and polycomb repressive complex 2 associated protein	100170841	6,884376667	1,053637377	0,818307	1,287582071	0,197891484	NA
ENSG00000273609	NA	NA	NA	0	NA	NA	NA	NA	NA
ENSG00000273628	NA	NA	NA	0,10488184	-1,076443944	4,080456	-0,263804824	0,791930317	NA
ENSG00000273634	NA	NA	NA	0	NA	NA	NA	NA	NA
ENSG00000273640	NA	NA	NA	0	NA	NA	NA	NA	NA
ENSG00000273643	PPP1R26P2	protein phosphatase 1 regulatory subunit 26 pseudogene 2	100133064	0	NA	NA	NA	NA	NA
ENSG00000273644	NA	NA	NA	0	NA	NA	NA	NA	NA
ENSG00000273654	NA	NA	NA	0	NA	NA	NA	NA	NA
ENSG00000273668	NA	NA	NA	0	NA	NA	NA	NA	NA
ENSG00000273675	LOC100419913	zinc finger protein 678 pseudogene	100419913	0	NA	NA	NA	NA	NA
ENSG00000273679	LOC441711	RING finger protein 145-like	441711	0	NA	NA	NA	NA	NA
ENSG00000273682	BLVRBP1	BLVRB pseudogene 1	127379696	0	NA	NA	NA	NA	NA
ENSG00000273692	LONRF2P5	LONRF2 pseudogene 5	115830330	0	NA	NA	NA	NA	NA
ENSG00000273693	NA	NA	NA	0	NA	NA	NA	NA	NA
ENSG00000273696	CT45A7	cancer/testis antigen family 45 member A7	101060211	0	NA	NA	NA	NA	NA
ENSG00000273698	MRPL57P1	mitochondrial ribosomal protein L57 pseudogene 1	126581	0	NA	NA	NA	NA	NA
ENSG00000273703	H2BC14	H2B clustered histone 14	8342	0,189380563	-0,095774166	4,080456	-0,023471438	0,981274222	NA
ENSG00000273704	NA	NA	NA	0	NA	NA	NA	NA	NA
ENSG00000273706	LHX1	LIM homeobox 1	3975	0,725462967	-0,019204414	2,31782	-0,00828555	0,993389164	NA
ENSG00000273712	NA	NA	NA	0	NA	NA	NA	NA	NA
ENSG00000273716	NA	NA	NA	0	NA	NA	NA	NA	NA
ENSG00000273719	NA	NA	NA	0	NA	NA	NA	NA	NA
ENSG00000273720	NA	NA	NA	0	NA	NA	NA	NA	NA
ENSG00000273721	NA	NA	NA	0	NA	NA	NA	NA	NA
ENSG00000273724	NA	NA	NA	0,575197049	-2,473581837	3,322309	-0,744536925	0,456551711	NA
ENSG00000273731	NA	NA	NA	0	NA	NA	NA	NA	NA
ENSG00000273732	NA	NA	NA	0	NA	NA	NA	NA	NA
ENSG00000273734	NA	NA	NA	0,23616048	-2,075648354	4,028973	-0,515180498	0,606426895	NA
ENSG00000273746	LOC100499471	leucine rich repeat containing 14 pseudogene	100499471	0	NA	NA	NA	NA	NA
ENSG00000273756	GOLGA6L26	golgin A6 family like 26	102723623	0	NA	NA	NA	NA	NA
ENSG00000273762	VN1R76P	vomeronasal 1 receptor 76 pseudogene	100312821	0	NA	NA	NA	NA	NA
ENSG00000273763	NA	NA	NA	0	NA	NA	NA	NA	NA
ENSG00000273773	NA	NA	NA	0	NA	NA	NA	NA	NA
ENSG00000273777	CEACAM20	CEA cell adhesion molecule 20	125931	0	NA	NA	NA	NA	NA
ENSG00000273791	BNIP3P47	BNIP3 pseudogene 47	127379681	0	NA	NA	NA	NA	NA
ENSG00000273800	NA	NA	NA	0	NA	NA	NA	NA	NA
ENSG00000273802	H2BC8	H2B clustered histone 8	8339	2,083818043	0,50467206	1,384523	0,364509583	0,715477519	NA
ENSG00000273806	NA	NA	NA	0	NA	NA	NA	NA	NA
ENSG00000273813	NA	NA	NA	0	NA	NA	NA	NA	NA
ENSG00000273819	ENPP7P7	ectonucleotide pyrophosphatase/phosphodiesterase 7 pseudogene 7	100421859	0	NA	NA	NA	NA	NA
ENSG00000273825	NA	NA	NA	0	NA	NA	NA	NA	NA
ENSG00000273843	NA	NA	NA	0	NA	NA	NA	NA	NA
ENSG00000273849	SDR42E1P4	short chain dehydrogenase/reductase family 42E, member 1 pseudogene 4	100422348	0	NA	NA	NA	NA	NA
ENSG00000273851	NA	NA	NA	0	NA	NA	NA	NA	NA
ENSG00000273858	NA	NA	NA	0	NA	NA	NA	NA	NA
ENSG00000273860	NA	NA	NA	0	NA	NA	NA	NA	NA

ENSG00000273863	TRIM60P2Y	tripartite motif containing 60 pseudogene 2, Y-linked	100420363	0	NA	NA	NA	NA	NA
ENSG00000273870	NA	NA	NA	0	NA	NA	NA	NA	NA
ENSG00000273872	NA	NA	NA	0	NA	NA	NA	NA	NA
ENSG00000273876	NA	NA	NA	0	NA	NA	NA	NA	NA
ENSG00000273894	NA	NA	NA	0	NA	NA	NA	NA	NA
ENSG00000273897	NA	NA	NA	3,491476234	1,022032737	1,560458	0,654956969	0,51249546	NA
ENSG00000273900	NA	NA	NA	0	NA	NA	NA	NA	NA
ENSG00000273907	NA	NA	NA	0	NA	NA	NA	NA	NA
ENSG00000273933	NA	NA	NA	0	NA	NA	NA	NA	NA
ENSG00000273957	NA	NA	NA	0	NA	NA	NA	NA	NA
ENSG00000273963	ENPP7P14	ectonucleotide pyrophosphatase/phosphodiesterase 7 pseudogene 14	106481720	0	NA	NA	NA	NA	NA
ENSG00000273976	GOLGA6L1	golgin A6 family like 1	283767	0	NA	NA	NA	NA	NA
ENSG00000273976	LOC124907501	golgin subfamily A member 6-like protein 1	124907501	0	NA	NA	NA	NA	NA
ENSG00000273978	LOC100419762	zinc finger protein 773 pseudogene	100419762	0	NA	NA	NA	NA	NA
ENSG00000273983	H3C8	H3 clustered histone 8	8355	1,164763124	3,587960639	2,29528	1,563190822	0,118007718	NA
ENSG00000273988	NA	NA	NA	0	NA	NA	NA	NA	NA
ENSG00000273994	NA	NA	NA	0	NA	NA	NA	NA	NA
ENSG00000273997	NA	NA	NA	0	NA	NA	NA	NA	NA
ENSG00000273999	RBM17P2	RNA binding motif protein 17 pseudogene 2	642389	0	NA	NA	NA	NA	NA
ENSG00000274010	ZFP91P1	ZFP91 pseudogene 1	100131832	0,992054193	-2,93552571	2,53157	-1,15956726	0,246225037	NA
ENSG00000274019	NA	NA	NA	0	NA	NA	NA	NA	NA
ENSG00000274022	NA	NA	NA	0	NA	NA	NA	NA	NA
ENSG00000274024	NA	NA	NA	0	NA	NA	NA	NA	NA
ENSG00000274033	NA	NA	NA	0	NA	NA	NA	NA	NA
ENSG00000274049	INO80B-WBP1	INO80B-WBP1 readthrough (NMD candidate)	100532735	1,159743534	0	4,048819	0	1	NA
ENSG00000274052	NA	NA	NA	0	NA	NA	NA	NA	NA
ENSG00000274064	NA	NA	NA	0	NA	NA	NA	NA	NA
ENSG00000274067	AQP7P5	aquaporin 7 pseudogene 5	102724655	0,11808024	-1,076443944	4,080456	-0,263804824	0,791930317	NA
ENSG00000274099	NA	NA	NA	0	NA	NA	NA	NA	NA
ENSG00000274102	OR4M2	olfactory receptor family 4 subfamily M member 2	390538	0	NA	NA	NA	NA	NA
ENSG00000274114	NA	NA	NA	0,338719478	0,966017735	4,023811	0,24007534	0,810271851	NA
ENSG00000274116	NA	NA	NA	0	NA	NA	NA	NA	NA
ENSG00000274133	LOC392145	exosome component 6 pseudogene	392145	0	NA	NA	NA	NA	NA
ENSG00000274149	NA	NA	NA	0	NA	NA	NA	NA	NA
ENSG00000274150	NA	NA	NA	0	NA	NA	NA	NA	NA
ENSG00000274154	NA	NA	NA	0	NA	NA	NA	NA	NA
ENSG00000274157	NA	NA	NA	0	NA	NA	NA	NA	NA
ENSG00000274162	NA	NA	NA	0	NA	NA	NA	NA	NA
ENSG00000274164	RNA5SP439	RNA, 5S ribosomal pseudogene 439	106479014	0	NA	NA	NA	NA	NA
ENSG00000274167	NA	NA	NA	0	NA	NA	NA	NA	NA
ENSG00000274175	LOC102723655	TP53-target gene 3 protein	102723655	0	NA	NA	NA	NA	NA
ENSG00000274181	NA	NA	NA	0	NA	NA	NA	NA	NA
ENSG00000274183	H2AB1	H2A,B variant histone 1	474382	0,10488184	-1,076443944	4,080456	-0,263804824	0,791930317	NA
ENSG00000274209	ANTXRL	ANTXR like	195977	0	NA	NA	NA	NA	NA
ENSG00000274212	NA	NA	NA	0	NA	NA	NA	NA	NA
ENSG00000274214	NA	NA	NA	1,361298246	0,209102583	1,605739	0,130221985	0,896390801	NA
ENSG00000274219	LOC100419839	zinc finger protein 300 pseudogene	100419839	0	NA	NA	NA	NA	NA
ENSG00000274226	TBC1D3H	TBC1 domain family member 3H	729877	0	NA	NA	NA	NA	NA
ENSG00000274228	RNA5SP414	RNA, 5S ribosomal pseudogene 414	100873665	0	NA	NA	NA	NA	NA
ENSG00000274231	NA	NA	NA	0	NA	NA	NA	NA	NA
ENSG00000274235	NA	NA	NA	0	NA	NA	NA	NA	NA
ENSG00000274242	NA	NA	NA	0	NA	NA	NA	NA	NA
ENSG00000274252	GGTLC3	gamma-glutamyltransferase light chain family member 3	728226	0	NA	NA	NA	NA	NA
ENSG00000274256	NA	NA	NA	0	NA	NA	NA	NA	NA
ENSG00000274274	GAGE13	G antigen 13	645051	0	NA	NA	NA	NA	NA
ENSG00000274279	NA	NA	NA	0,117497108	0,847101401	4,080456	0,207599697	0,835541541	NA
ENSG00000274282	NA	NA	NA	0	NA	NA	NA	NA	NA
ENSG00000274286	ADRA2B	adrenoceptor alpha 2B	151	0,870355638	-2,60185974	2,575798	-1,010117811	0,31243885	NA
ENSG00000274290	H2BC6	H2B clustered histone 6	8344	3,025200566	1,220105007	2,036373	0,59915607	0,549068814	NA
ENSG00000274295	NA	NA	NA	0	NA	NA	NA	NA	NA
ENSG00000274299	NA	NA	NA	0	NA	NA	NA	NA	NA
ENSG00000274310	NA	NA	NA	0	NA	NA	NA	NA	NA
ENSG00000274316	NA	NA	NA	0	NA	NA	NA	NA	NA
ENSG00000274319	RPSAP73	ribosomal protein SA pseudogene 73	107075250	0	NA	NA	NA	NA	NA

ENSG00000274322	NA	NA	NA	0	NA	NA	NA	NA	NA
ENSG00000274328	NA	NA	NA	0	NA	NA	NA	NA	NA
ENSG00000274330	NA	NA	NA	0	NA	NA	NA	NA	NA
ENSG00000274343	NA	NA	NA	0	NA	NA	NA	NA	NA
ENSG00000274346	NA	NA	NA	0,10543123	0	4,080456	0	1	NA
ENSG00000274347	NA	NA	NA	0	NA	NA	NA	NA	NA
ENSG00000274349	ZNF658	zinc finger protein 658	26149	3,235375717	-0,320479509	1,37115	-0,233730505	0,815194204	NA
ENSG00000274351	THAP12P10	THAP domain containing 12 pseudogene 10	100422715	0	NA	NA	NA	NA	NA
ENSG00000274353	NA	NA	NA	0	NA	NA	NA	NA	NA
ENSG00000274355	NA	NA	NA	0	NA	NA	NA	NA	NA
ENSG00000274356	NA	NA	NA	0	NA	NA	NA	NA	NA
ENSG00000274365	NA	NA	NA	0	NA	NA	NA	NA	NA
ENSG00000274369	GRAMD4P4	GRAM domain containing 4 pseudogene 4	106480248	0	NA	NA	NA	NA	NA
ENSG00000274374	TUBAP6	tubulin alpha pseudogene 6	100129818	0	NA	NA	NA	NA	NA
ENSG00000274376	ADAMTS7P1	ADAMTS7 pseudogene 1	390660	7,864144392	-0,07213545	0,828879	-0,087027696	0,930649498	NA
ENSG00000274378	NA	NA	NA	0	NA	NA	NA	NA	NA
ENSG00000274381	NA	NA	NA	0	NA	NA	NA	NA	NA
ENSG00000274386	TMEM269	transmembrane protein 269	100129924	0,387278436	-0,965156704	3,667173	-0,26318818	0,792405541	NA
ENSG00000274391	TPTE	transmembrane phosphatase with tensin homology	7179	0	NA	NA	NA	NA	NA
ENSG00000274391	LOC105379278	putative tyrosine-protein phosphatase TPTE	105379278	0	NA	NA	NA	NA	NA
ENSG00000274397	NA	NA	NA	0	NA	NA	NA	NA	NA
ENSG00000274398	LOC100533728	endogenous retrovirus group FRD member 1 pseudogene	100533728	0	NA	NA	NA	NA	NA
ENSG00000274419	TBC1D3D	TBC1 domain family member 3D	101060389	0	NA	NA	NA	NA	NA
ENSG00000274423	NA	NA	NA	3,701774029	-0,205622425	0,982853	-0,209209765	0,834284489	NA
ENSG00000274443	C8orf89	chromosome 8 open reading frame 89	100130301	0	NA	NA	NA	NA	NA
ENSG00000274445	NA	NA	NA	0	NA	NA	NA	NA	NA
ENSG00000274446	NA	NA	NA	0	NA	NA	NA	NA	NA
ENSG00000274451	LOC100421330	lysophosphatidylcholine acyltransferase 3 pseudogene	100421330	0	NA	NA	NA	NA	NA
ENSG00000274455	NA	NA	NA	0	NA	NA	NA	NA	NA
ENSG00000274467	RNA5SP257	RNA, 5S ribosomal pseudogene 257	100873511	0	NA	NA	NA	NA	NA
ENSG00000274468	NA	NA	NA	0	NA	NA	NA	NA	NA
ENSG00000274471	NA	NA	NA	3,063308531	0,336800516	1,078132	0,312392758	0,754742053	NA
ENSG00000274481	NA	NA	NA	0	NA	NA	NA	NA	NA
ENSG00000274486	LOC100420802	lysine demethylase 4D pseudogene	100420802	0	NA	NA	NA	NA	NA
ENSG00000274487	NA	NA	NA	1,975964907	-5,156549789	3,402983	-1,51530293	0,129695707	NA
ENSG00000274489	MRPS7P1	mitochondrial ribosomal protein S7 pseudogene 1	359783	0	NA	NA	NA	NA	NA
ENSG00000274493	MRPS17P5	mitochondrial ribosomal protein S17 pseudogene 5	359758	0	NA	NA	NA	NA	NA
ENSG00000274499	NA	NA	NA	0	NA	NA	NA	NA	NA
ENSG00000274502	NA	NA	NA	0	NA	NA	NA	NA	NA
ENSG00000274503	NA	NA	NA	0	NA	NA	NA	NA	NA
ENSG00000274516	FAM74A6	family with sequence similarity 74 member A6	653123	0	NA	NA	NA	NA	NA
ENSG00000274525	NA	NA	NA	0	NA	NA	NA	NA	NA
ENSG00000274529	SEBOX	SEBOX homeobox	645832	0	NA	NA	NA	NA	NA
ENSG00000274541	NA	NA	NA	0	NA	NA	NA	NA	NA
ENSG00000274549	NA	NA	NA	0	NA	NA	NA	NA	NA
ENSG00000274551	NA	NA	NA	0	NA	NA	NA	NA	NA
ENSG00000274553	NA	NA	NA	0	NA	NA	NA	NA	NA
ENSG00000274562	NA	NA	NA	0	NA	NA	NA	NA	NA
ENSG00000274569	NA	NA	NA	0,341044737	-0,151296875	3,780773	-0,040017449	0,968079215	NA
ENSG00000274570	SPDYE10	speedy/RINGO cell cycle regulator family member E10	643862	0	NA	NA	NA	NA	NA
ENSG00000274570	LOC124906603	putative speedy protein E9	124906603	0	NA	NA	NA	NA	NA
ENSG00000274572	ZYXP1	zyxin pseudogene 1	106480342	0	NA	NA	NA	NA	NA
ENSG00000274576	IGHV2-70	immunoglobulin heavy variable 2-70	28454	0	NA	NA	NA	NA	NA
ENSG00000274579	NA	NA	NA	0	NA	NA	NA	NA	NA
ENSG00000274583	FAM74A4	family with sequence similarity 74 member A4	401508	0	NA	NA	NA	NA	NA
ENSG00000274584	NA	NA	NA	0	NA	NA	NA	NA	NA
ENSG00000274588	DGKK	diacylglycerol kinase kappa	139189	0	NA	NA	NA	NA	NA
ENSG00000274594	NA	NA	NA	0	NA	NA	NA	NA	NA
ENSG00000274599	DUX4L24	double homeobox 4 like 24 (pseudogene)	102723449	0	NA	NA	NA	NA	NA
ENSG00000274600	RIMBP3B	RIMS binding protein 3B	440804	1,105780309	3,252115226	2,405139	1,352152662	0,176326484	NA
ENSG00000274601	NA	NA	NA	0	NA	NA	NA	NA	NA

ENSG00000274611	TBC1D3	TBC1 domain family member 3	729873	0	NA	NA	NA	NA	NA
ENSG00000274612	BNIP3P14	BCL2 interacting protein 3 pseudogene 14	100421696	0	NA	NA	NA	NA	NA
ENSG00000274614	SALL4P4	spalt like transcription factor 4 pseudogene 4	100419855	0	NA	NA	NA	NA	NA
ENSG00000274618	H4C6	H4 clustered histone 6	8361	0,288661797	2,306206831	3,997774	0,57687273	0,564025434	NA
ENSG00000274628	NA	NA	NA	0,587833995	0,47367285	3,359654	0,140988572	0,88787897	NA
ENSG00000274631	NA	NA	NA	0	NA	NA	NA	NA	NA
ENSG00000274637	NA	NA	NA	0	NA	NA	NA	NA	NA
ENSG00000274641	H2BC17	H2B clustered histone 17	8348	0,982982643	2,580353915	2,538089	1,016652109	0,309318956	NA
ENSG00000274642	NA	NA	NA	0	NA	NA	NA	NA	NA
ENSG00000274649	NA	NA	NA	0	NA	NA	NA	NA	NA
ENSG00000274658	TBC1D3JP	TBC1 domain family member 3J, pseudogene	100510707	0	NA	NA	NA	NA	NA
ENSG00000274664	NA	NA	NA	0	NA	NA	NA	NA	NA
ENSG00000274666	NA	NA	NA	0	NA	NA	NA	NA	NA
ENSG00000274680	NA	NA	NA	0	NA	NA	NA	NA	NA
ENSG00000274688	NA	NA	NA	0	NA	NA	NA	NA	NA
ENSG00000274691	NA	NA	NA	0	NA	NA	NA	NA	NA
ENSG00000274702	NA	NA	NA	0	NA	NA	NA	NA	NA
ENSG00000274712	NA	NA	NA	3,015566633	-0,198615721	1,792355	-0,110812711	0,911764866	NA
ENSG00000274717	NA	NA	NA	1,359887584	0,413702039	2,088339	0,198100978	0,84296606	NA
ENSG00000274727	IQSEC3P3	IQ motif and Sec7 domain 3 pseudogene 3	728056	0	NA	NA	NA	NA	NA
ENSG00000274730	NA	NA	NA	0	NA	NA	NA	NA	NA
ENSG00000274736	CCL23	C-C motif chemokine ligand 23	6368	0,119673877	0,847101401	4,080456	0,207599697	0,835541541	NA
ENSG00000274748	NA	NA	NA	0	NA	NA	NA	NA	NA
ENSG00000274749	KRTAP7-1	keratin associated protein 7-1	337878	0	NA	NA	NA	NA	NA
ENSG00000274764	PRAMEF27	PRAME family member 27	101929983	0	NA	NA	NA	NA	NA
ENSG00000274775	NA	NA	NA	0	NA	NA	NA	NA	NA
ENSG00000274792	LOC102723532	olfactory receptor 4N4-like	102723532	0	NA	NA	NA	NA	NA
ENSG00000274800	NA	NA	NA	0	NA	NA	NA	NA	NA
ENSG00000274808	TBC1D3B	TBC1 domain family member 3B	414059	0	NA	NA	NA	NA	NA
ENSG00000274808	TBC1D3G	TBC1 domain family member 3G	101060321	0	NA	NA	NA	NA	NA
ENSG00000274810	NPHP3-ACAD11	NPHP3-ACAD11 readthrough (NMD candidate)	100532724	5,503496225	0,836952738	1,809351	0,462570581	0,6436722	NA
ENSG00000274813	NA	NA	NA	0	NA	NA	NA	NA	NA
ENSG00000274820	NA	NA	NA	0	NA	NA	NA	NA	NA
ENSG00000274823	NA	NA	NA	0	NA	NA	NA	NA	NA
ENSG00000274832	NA	NA	NA	0	NA	NA	NA	NA	NA
ENSG00000274837	NA	NA	NA	0	NA	NA	NA	NA	NA
ENSG00000274844	NA	NA	NA	0	NA	NA	NA	NA	NA
ENSG00000274855	NA	NA	NA	0	NA	NA	NA	NA	NA
ENSG00000274867	LOC100421158	piggyBac transposable element derived 4 pseudogene	100421158	0	NA	NA	NA	NA	NA
ENSG00000274879	NA	NA	NA	0	NA	NA	NA	NA	NA
ENSG00000274886	NA	NA	NA	0	NA	NA	NA	NA	NA
ENSG00000274892	NA	NA	NA	0	NA	NA	NA	NA	NA
ENSG00000274899	TRIM60P12Y	tripartite motif containing 60 pseudogene 12, Y-linked	100420378	0	NA	NA	NA	NA	NA
ENSG00000274901	OR4W1P	olfactory receptor family 4 subfamily W member 1 pseudogene	81077	0	NA	NA	NA	NA	NA
ENSG00000274903	NA	NA	NA	0	NA	NA	NA	NA	NA
ENSG00000274919	NA	NA	NA	0	NA	NA	NA	NA	NA
ENSG00000274923	NA	NA	NA	0	NA	NA	NA	NA	NA
ENSG00000274927	KMT2CP3	lysine methyltransferase 2C pseudogene 3	125177372	0	NA	NA	NA	NA	NA
ENSG00000274928	NA	NA	NA	0,512968064	-1,066592772	3,420964	-0,311781368	0,755206685	NA
ENSG00000274930	NA	NA	NA	0	NA	NA	NA	NA	NA
ENSG00000274933	TBC1D3I	TBC1 domain family member 3I	102724862	3,09736459	-0,045968893	1,199751	-0,038315372	0,969436234	NA
ENSG00000274944	NA	NA	NA	0,843449843	0	4,080456	0	1	NA
ENSG00000274966	DNM1P29	dynamin 1 pseudogene 29	100132204	0	NA	NA	NA	NA	NA
ENSG00000274970	LOC100419641	CTAGE family member 5, ER export factor	100419641	0	NA	NA	NA	NA	NA
ENSG00000274973	NA	NA	NA	0	NA	NA	NA	NA	NA
ENSG00000274977	NA	NA	NA	0	NA	NA	NA	NA	NA
ENSG00000274985	NA	NA	NA	0	NA	NA	NA	NA	NA
ENSG00000274997	H2AC12	H2A clustered histone 12	85235	0,490741101	0	3,370548	0	1	NA
ENSG00000275002	ZFAND2AP2	ZFAND2A pseudogene 2	127379733	0	NA	NA	NA	NA	NA
ENSG00000275004	ZNF280B	zinc finger protein 280B	140883	6,339889328	0,11414418	0,896052	0,127385632	0,898635188	NA
ENSG00000275011	NA	NA	NA	0	NA	NA	NA	NA	NA

ENSG00000275012	NA	NA	NA	0	NA	NA	NA	NA	NA
ENSG00000275015	NA	NA	NA	0	NA	NA	NA	NA	NA
ENSG00000275017	NA	NA	NA	0	NA	NA	NA	NA	NA
ENSG00000275026	GXYLT1P4	GXYLT1 pseudogene 4	100132441	0	NA	NA	NA	NA	NA
ENSG00000275034	TP53TG3E	TP53 target 3 family member E	102724101	0	NA	NA	NA	NA	NA
ENSG00000275046	NA	NA	NA	0	NA	NA	NA	NA	NA
ENSG00000275048	BSNDP1	BSND pseudogene 1	106480329	0	NA	NA	NA	NA	NA
ENSG00000275060	NA	NA	NA	0	NA	NA	NA	NA	NA
ENSG00000275061	SEPTIN7P15	septin 7 pseudogene 15	100418714	0	NA	NA	NA	NA	NA
ENSG00000275063	LOC102723407	immunoglobulin heavy variable 4-38-2-like	102723407	0	NA	NA	NA	NA	NA
ENSG00000275064	NA	NA	NA	0	NA	NA	NA	NA	NA
ENSG00000275075	NA	NA	NA	0	NA	NA	NA	NA	NA
ENSG00000275078	NA	NA	NA	0	NA	NA	NA	NA	NA
ENSG00000275094	LOC100420569	ribosomal L24 domain containing 1 pseudogene	100420569	0	NA	NA	NA	NA	NA
ENSG00000275103	NA	NA	NA	0	NA	NA	NA	NA	NA
ENSG00000275113	GAGE12B	G antigen 12B	729428	0	NA	NA	NA	NA	NA
ENSG00000275115	NA	NA	NA	0	NA	NA	NA	NA	NA
ENSG00000275121	NA	NA	NA	0	NA	NA	NA	NA	NA
ENSG00000275126	H4C13	H4 clustered histone 13	8368	0,10543123	0	4,080456	0	1	NA
ENSG00000275129	NA	NA	NA	0	NA	NA	NA	NA	NA
ENSG00000275130	NA	NA	NA	0	NA	NA	NA	NA	NA
ENSG00000275138	NA	NA	NA	0	NA	NA	NA	NA	NA
ENSG00000275140	NA	NA	NA	0	NA	NA	NA	NA	NA
ENSG00000275144	NA	NA	NA	1,037831376	-1,810756617	2,827355	-0,640441825	0,521885399	NA
ENSG00000275152	CCL16	C-C motif chemokine ligand 16	6360	0	NA	NA	NA	NA	NA
ENSG00000275157	NA	NA	NA	0	NA	NA	NA	NA	NA
ENSG00000275158	TRBV12-5	T cell receptor beta variable 12-5	28575	0	NA	NA	NA	NA	NA
ENSG00000275160	RPL7AP45	ribosomal protein L7a pseudogene 45	100132678	0,783434075	-0,970570248	2,257531	-0,429925608	0,667249757	NA
ENSG00000275163	NA	NA	NA	0	NA	NA	NA	NA	NA
ENSG00000275170	FRG2MP	FSHD region gene 2 family member M, pseudogene	106481739	0	NA	NA	NA	NA	NA
ENSG00000275183	LENG9	leukocyte receptor cluster member 9	94059	2,640320717	-4,264409985	3,013437	-1,415131715	0,157029881	NA
ENSG00000275194	NA	NA	NA	0	NA	NA	NA	NA	NA
ENSG00000275201	NA	NA	NA	0	NA	NA	NA	NA	NA
ENSG00000275221	H2AC15	H2A clustered histone 15	8330	0,704032409	-0,249380169	2,470557	-0,100940865	0,919597403	NA
ENSG00000275249	NA	NA	NA	0	NA	NA	NA	NA	NA
ENSG00000275250	NA	NA	NA	0	NA	NA	NA	NA	NA
ENSG00000275256	MRPS31P1	mitochondrial ribosomal protein S31 pseudogene 1	351449	0	NA	NA	NA	NA	NA
ENSG00000275280	NA	NA	NA	0	NA	NA	NA	NA	NA
ENSG00000275295	LOC100419775	zinc finger protein 519 pseudogene	100419775	0	NA	NA	NA	NA	NA
ENSG00000275296	NA	NA	NA	0	NA	NA	NA	NA	NA
ENSG00000275312	NA	NA	NA	0	NA	NA	NA	NA	NA
ENSG00000275314	NA	NA	NA	0	NA	NA	NA	NA	NA
ENSG00000275315	NA	NA	NA	0	NA	NA	NA	NA	NA
ENSG00000275320	NA	NA	NA	0	NA	NA	NA	NA	NA
ENSG00000275325	PDCD6IPP1	PDCD6IP pseudogene 1	100861430	0	NA	NA	NA	NA	NA
ENSG00000275328	NA	NA	NA	0	NA	NA	NA	NA	NA
ENSG00000275339	NA	NA	NA	0	NA	NA	NA	NA	NA
ENSG00000275340	NA	NA	NA	0,15836288	0	4,080456	0	1	NA
ENSG00000275348	NA	NA	NA	0	NA	NA	NA	NA	NA
ENSG00000275350	TUBB6P1	tubulin beta 6 class V pseudogene 1	100418964	0	NA	NA	NA	NA	NA
ENSG00000275352	NA	NA	NA	0	NA	NA	NA	NA	NA
ENSG00000275361	NA	NA	NA	0	NA	NA	NA	NA	NA
ENSG00000275363	NA	NA	NA	0	NA	NA	NA	NA	NA
ENSG00000275374	NA	NA	NA	0	NA	NA	NA	NA	NA
ENSG00000275379	H3C11	H3 clustered histone 11	8354	0,117497108	0,847101401	4,080456	0,207599697	0,835541541	NA
ENSG00000275381	NA	NA	NA	0	NA	NA	NA	NA	NA
ENSG00000275385	CCL18	C-C motif chemokine ligand 18	6362	0	NA	NA	NA	NA	NA
ENSG00000275386	NA	NA	NA	0	NA	NA	NA	NA	NA
ENSG00000275387	NA	NA	NA	0	NA	NA	NA	NA	NA
ENSG00000275400	NA	NA	NA	5,026181867	-1,657334556	0,849108	-1,951853818	0,050955563	NA
ENSG00000275406	NA	NA	NA	0	NA	NA	NA	NA	NA
ENSG00000275410	HNF1B	HNF1 homeobox B	6928	0	NA	NA	NA	NA	NA
ENSG00000275419	NA	NA	NA	0	NA	NA	NA	NA	NA
ENSG00000275423	VN1R18P	vomeronasal 1 receptor 18 pseudogene	100133087	0	NA	NA	NA	NA	NA
ENSG00000275432	NA	NA	NA	0	NA	NA	NA	NA	NA
ENSG00000275448	NA	NA	NA	0	NA	NA	NA	NA	NA
ENSG00000275461	NA	NA	NA	0	NA	NA	NA	NA	NA

ENSG00000275468	NA	NA	NA	2,710262629	3,780654741	1,488863	2,539289843	0,011107776	NA
ENSG00000275480	NA	NA	NA	0	NA	NA	NA	NA	NA
ENSG00000275488	LOC100419830	zinc finger protein 337 pseudogene	100419830	0	NA	NA	NA	NA	NA
ENSG00000275489	SPMAP1	sperm microtubule associated protein 1	388381	0	NA	NA	NA	NA	NA
ENSG00000275493	NA	NA	NA	0	NA	NA	NA	NA	NA
ENSG00000275497	NA	NA	NA	0	NA	NA	NA	NA	NA
ENSG00000275503	NA	NA	NA	0	NA	NA	NA	NA	NA
ENSG00000275506	H3P25	H3 histone pseudogene 25	115482720	0	NA	NA	NA	NA	NA
ENSG00000275508	NA	NA	NA	0	NA	NA	NA	NA	NA
ENSG00000275520	FAM236A	family with sequence similarity 236 member A	100129407	0	NA	NA	NA	NA	NA
ENSG00000275540	NA	NA	NA	0	NA	NA	NA	NA	NA
ENSG00000275547	NA	NA	NA	0	NA	NA	NA	NA	NA
ENSG00000275550	NA	NA	NA	0	NA	NA	NA	NA	NA
ENSG00000275553	NA	NA	NA	0	NA	NA	NA	NA	NA
ENSG00000275562	SEPTIN7P12	septin 7 pseudogene 12	100418753	0	NA	NA	NA	NA	NA
ENSG00000275572	GRIFIN	galectin-related inter-fiber protein	402635	0	NA	NA	NA	NA	NA
ENSG00000275578	NA	NA	NA	0	NA	NA	NA	NA	NA
ENSG00000275585	NA	NA	NA	0,11808024	-1,076443944	4,080456	-0,263804824	0,791930317	NA
ENSG00000275591	XKR5	XK related 5	389610	0	NA	NA	NA	NA	NA
ENSG00000275592	NA	NA	NA	0	NA	NA	NA	NA	NA
ENSG00000275597	ANAPC1P5	ANAPC1 pseudogene 5	100420890	0,11808024	-1,076443944	4,080456	-0,263804824	0,791930317	NA
ENSG00000275598	LOC100533643	zinc finger protein 91 pseudogene	100533643	0	NA	NA	NA	NA	NA
ENSG00000275607	WDR53P1	WDR53 pseudogene 1	127379699	0	NA	NA	NA	NA	NA
ENSG00000275609	NA	NA	NA	0	NA	NA	NA	NA	NA
ENSG00000275610	DUX4L21	double homeobox 4 like 21 (pseudogene)	102723518	0	NA	NA	NA	NA	NA
ENSG00000275628	LOC124902165	uncharacterized LOC124902165	124902165	0	NA	NA	NA	NA	NA
ENSG00000275643	RBM22P6	RNA binding motif protein 22 pseudogene 6	644509	0	NA	NA	NA	NA	NA
ENSG00000275647	NA	NA	NA	0	NA	NA	NA	NA	NA
ENSG00000275654	NA	NA	NA	0	NA	NA	NA	NA	NA
ENSG00000275663	H4C7	H4 clustered histone 7	8369	0	NA	NA	NA	NA	NA
ENSG00000275674	NA	NA	NA	0	NA	NA	NA	NA	NA
ENSG00000275675	NA	NA	NA	0	NA	NA	NA	NA	NA
ENSG00000275681	NA	NA	NA	0	NA	NA	NA	NA	NA
ENSG00000275688	NA	NA	NA	0	NA	NA	NA	NA	NA
ENSG00000275691	NA	NA	NA	0	NA	NA	NA	NA	NA
ENSG00000275695	UBE2Q2P6	UBE2Q2 pseudogene 6	100421769	0	NA	NA	NA	NA	NA
ENSG00000275713	H2BC9	H2B clustered histone 9	8345	1,556956314	0,17681724	1,639482	0,107849439	0,914115125	NA
ENSG00000275714	H3C1	H3 clustered histone 1	8350	1,01895642	1,399300474	2,220179	0,63026464	0,528521454	NA
ENSG00000275716	LOC100420742	tetratricopeptide repeat domain 22 pseudogene	100420742	0	NA	NA	NA	NA	NA
ENSG00000275718	CCL15	C-C motif chemokine ligand 15	6359	0	NA	NA	NA	NA	NA
ENSG00000275722	LYZL6	lysozyme like 6	57151	0	NA	NA	NA	NA	NA
ENSG00000275725	NA	NA	NA	0	NA	NA	NA	NA	NA
ENSG00000275733	NA	NA	NA	0	NA	NA	NA	NA	NA
ENSG00000275747	IGHV3-79	immunoglobulin heavy variable 3-79 (pseudogene)	28405	0	NA	NA	NA	NA	NA
ENSG00000275756	NA	NA	NA	0	NA	NA	NA	NA	NA
ENSG00000275767	NA	NA	NA	0	NA	NA	NA	NA	NA
ENSG00000275768	SLC2AXP1	solute carrier family 2 pseudogene 1	399496	0	NA	NA	NA	NA	NA
ENSG00000275772	NA	NA	NA	0	NA	NA	NA	NA	NA
ENSG00000275778	PRH1-PRR4	PRH1-PRR4 readthrough	100533464	0	NA	NA	NA	NA	NA
ENSG00000275785	NA	NA	NA	0	NA	NA	NA	NA	NA
ENSG00000275787	NA	NA	NA	0	NA	NA	NA	NA	NA
ENSG00000275791	TRBV10-3	T cell receptor beta variable 10-3	28583	0	NA	NA	NA	NA	NA
ENSG00000275793	RIMBP3	RIMS binding protein 3	85376	6,990684539	-1,035689932	0,865354	-1,196839265	0,231369211	NA
ENSG00000275800	NA	NA	NA	0,11808024	-1,076443944	4,080456	-0,263804824	0,791930317	NA
ENSG00000275801	NA	NA	NA	0	NA	NA	NA	NA	NA
ENSG00000275811	NA	NA	NA	0	NA	NA	NA	NA	NA
ENSG00000275826	NA	NA	NA	0	NA	NA	NA	NA	NA
ENSG00000275827	NA	NA	NA	0	NA	NA	NA	NA	NA
ENSG00000275828	NA	NA	NA	0	NA	NA	NA	NA	NA
ENSG00000275832	ARHGAP23	Rho GTPase activating protein 23	57636	2,800807979	0,473745729	1,137742	0,416391004	0,67712391	NA
ENSG00000275833	NA	NA	NA	0	NA	NA	NA	NA	NA
ENSG00000275834	NA	NA	NA	0	NA	NA	NA	NA	NA
ENSG00000275839	NA	NA	NA	0,355243265	0,990021385	3,722189	0,265978269	0,790255945	NA
ENSG00000275860	NA	NA	NA	0	NA	NA	NA	NA	NA

ENSG00000275862	HNRNPMP2	heterogeneous nuclear ribonucleoprotein M pseudogene 2	390948	0	NA	NA	NA	NA	NA
ENSG00000275866	NA	NA	NA	0	NA	NA	NA	NA	NA
ENSG00000275869	LOC102723713	TP53-target gene 3 protein	102723713	0	NA	NA	NA	NA	NA
ENSG00000275875	NA	NA	NA	0	NA	NA	NA	NA	NA
ENSG00000275882	IKBKGP1	inhibitor of nuclear factor kappa B kinase subunit gamma pseudogene 1	246210	0	NA	NA	NA	NA	NA
ENSG00000275896	PRSS2	serine protease 2	5645	0	NA	NA	NA	NA	NA
ENSG00000275905	NA	NA	NA	0	NA	NA	NA	NA	NA
ENSG00000275906	NA	NA	NA	0	NA	NA	NA	NA	NA
ENSG00000275919	NA	NA	NA	0	NA	NA	NA	NA	NA
ENSG00000275928	NA	NA	NA	0	NA	NA	NA	NA	NA
ENSG00000275929	NA	NA	NA	0	NA	NA	NA	NA	NA
ENSG00000275940	MRPL57P3	mitochondrial ribosomal protein L57 pseudogene 3	359730	0	NA	NA	NA	NA	NA
ENSG00000275945	NA	NA	NA	0	NA	NA	NA	NA	NA
ENSG00000275953	YAP1P3	YAP1 pseudogene 3	100419883	0	NA	NA	NA	NA	NA
ENSG00000275956	NA	NA	NA	0	NA	NA	NA	NA	NA
ENSG00000275959	LOC100533736	HERV-FRD provirus ancestral Env polyprotein pseudogene	100533736	0	NA	NA	NA	NA	NA
ENSG00000275969	SPATA31A3	SPATA31 subfamily A member 3	727830	0	NA	NA	NA	NA	NA
ENSG00000275980	NA	NA	NA	0	NA	NA	NA	NA	NA
ENSG00000275989	NA	NA	NA	0	NA	NA	NA	NA	NA
ENSG00000276006	REXO1L12P	REXO1 like 12, pseudogene	100289448	0	NA	NA	NA	NA	NA
ENSG00000276017	NA	NA	NA	0	NA	NA	NA	NA	NA
ENSG00000276036	RNA5SP440	RNA, 5S ribosomal pseudogene 440	100873688	0	NA	NA	NA	NA	NA
ENSG00000276039	NA	NA	NA	0	NA	NA	NA	NA	NA
ENSG00000276040	SPATA31A7	SPATA31 subfamily A member 7	26165	0	NA	NA	NA	NA	NA
ENSG00000276046	DUX4L13	double homeobox 4 like 13 (pseudogene)	100289581	0	NA	NA	NA	NA	NA
ENSG00000276058	NA	NA	NA	0,072165449	0,847101401	4,080456	0,207599697	0,835541541	NA
ENSG00000276064	NA	NA	NA	0	NA	NA	NA	NA	NA
ENSG00000276067	NA	NA	NA	0	NA	NA	NA	NA	NA
ENSG00000276070	CCL4L2	C-C motif chemokine ligand 4 like 2	9560	0	NA	NA	NA	NA	NA
ENSG00000276070	CCL4L1	C-C motif chemokine ligand 4 like 1	388372	0	NA	NA	NA	NA	NA
ENSG00000276087	NA	NA	NA	0	NA	NA	NA	NA	NA
ENSG00000276098	LOC100420990	aurora kinase A interacting protein 1 pseudogene	100420990	0	NA	NA	NA	NA	NA
ENSG00000276109	NA	NA	NA	0	NA	NA	NA	NA	NA
ENSG00000276119	OR13C2	olfactory receptor family 13 subfamily C member 2	392376	0	NA	NA	NA	NA	NA
ENSG00000276127	LOC100132834	coiled-coil domain containing 112 pseudogene	100132834	0	NA	NA	NA	NA	NA
ENSG00000276128	GXYLT1P5	GXYLT1 pseudogene 5	100131760	0,656197529	-3,001415342	2,64186	-1,13609916	0,255915067	NA
ENSG00000276149	NA	NA	NA	0	NA	NA	NA	NA	NA
ENSG00000276156	NA	NA	NA	0	NA	NA	NA	NA	NA
ENSG00000276160	GGTLC5P	gamma-glutamyltransferase light chain 5 pseudogene	653590	0	NA	NA	NA	NA	NA
ENSG00000276180	H4C9	H4 clustered histone 9	8294	0,457791926	-0,117414657	3,058504	-0,038389573	0,969377074	NA
ENSG00000276203	NA	NA	NA	0,755968593	-1,125549471	2,967604	-0,379278884	0,704480778	NA
ENSG00000276204	NA	NA	NA	0	NA	NA	NA	NA	NA
ENSG00000276205	NA	NA	NA	0	NA	NA	NA	NA	NA
ENSG00000276206	NA	NA	NA	0	NA	NA	NA	NA	NA
ENSG00000276208	NA	NA	NA	0	NA	NA	NA	NA	NA
ENSG00000276214	NA	NA	NA	0	NA	NA	NA	NA	NA
ENSG00000276240	OR4E1	olfactory receptor family 4 subfamily E member 1	26687	0	NA	NA	NA	NA	NA
ENSG00000276277	NA	NA	NA	0	NA	NA	NA	NA	NA
ENSG00000276290	RBBP4P3	RBBP4 pseudogene 3	730180	0	NA	NA	NA	NA	NA
ENSG00000276291	NA	NA	NA	5,096720157	-0,245252737	1,082153	-0,226634052	0,820708311	NA
ENSG00000276294	NA	NA	NA	0	NA	NA	NA	NA	NA
ENSG00000276298	NA	NA	NA	0	NA	NA	NA	NA	NA
ENSG00000276302	NA	NA	NA	0,239347754	1,967469188	4,021404	0,489249276	0,624665227	NA
ENSG00000276316	OR8G7P	olfactory receptor family 8 subfamily G member 7 pseudogene	282786	0	NA	NA	NA	NA	NA
ENSG00000276318	NA	NA	NA	0	NA	NA	NA	NA	NA
ENSG00000276332	NA	NA	NA	0	NA	NA	NA	NA	NA
ENSG00000276362	NA	NA	NA	0	NA	NA	NA	NA	NA
ENSG00000276368	H2AC14	H2A clustered histone 14	8331	0,342451626	0,981252268	4,022241	0,243956579	0,807264449	NA
ENSG00000276375	NA	NA	NA	0	NA	NA	NA	NA	NA

ENSG00000276380	UBE2NL	ubiquitin conjugating enzyme E2 N like (gene/pseudogene)	389898	0	NA	NA	NA	NA	NA
ENSG00000276385	NA	NA	NA	0	NA	NA	NA	NA	NA
ENSG00000276386	NA	NA	NA	0	NA	NA	NA	NA	NA
ENSG00000276388	NA	NA	NA	0	NA	NA	NA	NA	NA
ENSG00000276391	NA	NA	NA	0	NA	NA	NA	NA	NA
ENSG00000276393	NR3C1P1	nuclear receptor subfamily 3 group C member 1 pseudogene 1	2910	0	NA	NA	NA	NA	NA
ENSG00000276400	NA	NA	NA	0	NA	NA	NA	NA	NA
ENSG00000276409	CCL14	C-C motif chemokine ligand 14	6358	0	NA	NA	NA	NA	NA
ENSG00000276410	H2BC3	H2B clustered histone 3	3018	0,405466859	0,841176639	3,604625	0,233360377	0,815481578	NA
ENSG00000276413	NA	NA	NA	0	NA	NA	NA	NA	NA
ENSG00000276418	NA	NA	NA	0,859522498	-1,0698358	3,005968	-0,35590395	0,721912498	NA
ENSG00000276427	NA	NA	NA	0	NA	NA	NA	NA	NA
ENSG00000276428	NA	NA	NA	0	NA	NA	NA	NA	NA
ENSG00000276431	NA	NA	NA	0	NA	NA	NA	NA	NA
ENSG00000276457	FKBP4P8	FKBP prolyl isomerase 4 pseudogene 8	643880	0	NA	NA	NA	NA	NA
ENSG00000276460	NA	NA	NA	0	NA	NA	NA	NA	NA
ENSG00000276467	LOC100419783	zinc finger protein 831 pseudogene	100419783	0	NA	NA	NA	NA	NA
ENSG00000276474	LOC100421603	family with sequence similarity 156 member A pseudogene	100421603	0	NA	NA	NA	NA	NA
ENSG00000276475	BNIP3P44	BNIP3 pseudogene 44	127379755	0	NA	NA	NA	NA	NA
ENSG00000276478	LOC100420576	death associated protein kinase 1 pseudogene	100420576	0	NA	NA	NA	NA	NA
ENSG00000276484	PLA2G10IP	phospholipase A2 group XI, pseudogene	127525793	0	NA	NA	NA	NA	NA
ENSG00000276486	NA	NA	NA	0	NA	NA	NA	NA	NA
ENSG00000276490	NA	NA	NA	0	NA	NA	NA	NA	NA
ENSG00000276507	NA	NA	NA	0	NA	NA	NA	NA	NA
ENSG00000276520	NA	NA	NA	0	NA	NA	NA	NA	NA
ENSG00000276521	NA	NA	NA	0	NA	NA	NA	NA	NA
ENSG00000276531	SNX19P4	sorting nexin 19 pseudogene 4	100419047	0	NA	NA	NA	NA	NA
ENSG00000276535	NA	NA	NA	0	NA	NA	NA	NA	NA
ENSG00000276538	NA	NA	NA	0	NA	NA	NA	NA	NA
ENSG00000276540	NA	NA	NA	0	NA	NA	NA	NA	NA
ENSG00000276545	PCDHGB9P	protocadherin gamma subfamily B, 9 pseudogene	84055	0,239347754	1,967469188	4,021404	0,489249276	0,624665227	NA
ENSG00000276547	PCDHGB5	protocadherin gamma subfamily B, 5	56101	0,434348466	-1,08012278	3,532674	-0,305752209	0,759793318	NA
ENSG00000276548	NA	NA	NA	0	NA	NA	NA	NA	NA
ENSG00000276556	NA	NA	NA	0	NA	NA	NA	NA	NA
ENSG00000276557	TRBV18	T cell receptor beta variable 18	28569	0	NA	NA	NA	NA	NA
ENSG00000276563	NA	NA	NA	0	NA	NA	NA	NA	NA
ENSG00000276566	IGKV1D-13	immunoglobulin kappa variable 1D-13	28902	0	NA	NA	NA	NA	NA
ENSG00000276569	NA	NA	NA	0	NA	NA	NA	NA	NA
ENSG00000276576	NA	NA	NA	0	NA	NA	NA	NA	NA
ENSG00000276578	NA	NA	NA	0	NA	NA	NA	NA	NA
ENSG00000276581	SPATA31A5	SPATA31 subfamily A member 5	727905	0	NA	NA	NA	NA	NA
ENSG00000276586	NA	NA	NA	0	NA	NA	NA	NA	NA
ENSG00000276592	PHB1P6	PHB1 pseudogene 6	106478940	0	NA	NA	NA	NA	NA
ENSG00000276599	RNA5SP411	RNA, 5S ribosomal pseudogene 411	100887740	0	NA	NA	NA	NA	NA
ENSG00000276600	RAB7B	RAB7B, member RAS oncogene family	338382	0	NA	NA	NA	NA	NA
ENSG00000276605	LOC100419615	complement C7 pseudogene	100419615	0	NA	NA	NA	NA	NA
ENSG00000276620	NA	NA	NA	0	NA	NA	NA	NA	NA
ENSG00000276623	LOC100420386	tripartite motif-containing 75 pseudogene	100420386	0	NA	NA	NA	NA	NA
ENSG00000276632	NA	NA	NA	0	NA	NA	NA	NA	NA
ENSG00000276637	NA	NA	NA	0	NA	NA	NA	NA	NA
ENSG00000276644	DACH1	dachshund family transcription factor 1	1602	0	NA	NA	NA	NA	NA
ENSG00000276647	KRTAP12-5P	keratin associated protein 12-5, pseudogene	112268487	0	NA	NA	NA	NA	NA
ENSG00000276650	NA	NA	NA	0	NA	NA	NA	NA	NA
ENSG00000276662	DDX20P1	DEAD-box helicase 20 pseudogene 1	100421369	0	NA	NA	NA	NA	NA
ENSG00000276667	NA	NA	NA	0	NA	NA	NA	NA	NA
ENSG00000276674	NA	NA	NA	0,072165449	0,847101401	4,080456	0,207599697	0,835541541	NA

ENSG00000276689	NA	NA	NA	0,117497108	0,847101401	4,080456	0,207599697	0,835541541	NA	
ENSG00000276699	TRAJ36	T cell receptor alpha joining 36	28719	0	NA	NA	NA	NA	NA	
ENSG00000276703	RPS29P32	ribosomal protein S29 pseudogene 32	109729158	0	NA	NA	NA	NA	NA	
ENSG00000276710	CSPG4P10	chondroitin sulfate proteoglycan 4 pseudogene 10	390627	0,558870215	-1,366710093	3,956308	-0,34545086	0,729755447	NA	
ENSG00000276721	NA	NA	NA	0	NA	NA	NA	NA	NA	
ENSG00000276738	NA	NA	NA	0	NA	NA	NA	NA	NA	
ENSG00000276756	NA	NA	NA	0	NA	NA	NA	NA	NA	
ENSG00000276758	NA	NA	NA	0	NA	NA	NA	NA	NA	
ENSG00000276760	LOC102723502	POTE ankyrin domain family member B-like	102723502	0	NA	NA	NA	NA	NA	
ENSG00000276763	NA	NA	NA	0	NA	NA	NA	NA	NA	
ENSG00000276789	MRGPRX8P	MAS related GPR family member X8, pseudogene	100420123	0	NA	NA	NA	NA	NA	
ENSG00000276822	NA	NA	NA	0	NA	NA	NA	NA	NA	
ENSG00000276828	NA	NA	NA	0	NA	NA	NA	NA	NA	
ENSG00000276829	TRIM60P4Y	tripartite motif containing 60 pseudogene 4, Y-linked	100420367	0	NA	NA	NA	NA	NA	
ENSG00000276832	NA	NA	NA	0,221873437	-1,996547026	4,03569	-0,494722583	0,620795958	NA	
ENSG00000276840	NA	NA	NA	3,845331006	0,386081319	1,08645	0,355360281	0,722319701	NA	
ENSG00000276841	NA	NA	NA	0	NA	NA	NA	NA	NA	
ENSG00000276845	NA	NA	NA	0	NA	NA	NA	NA	NA	
ENSG00000276868	NA	NA	NA	0	NA	NA	NA	NA	NA	
ENSG00000276872	NA	NA	NA	0	NA	NA	NA	NA	NA	
ENSG00000276892	NA	NA	NA	0	NA	NA	NA	NA	NA	
ENSG00000276894	NA	NA	NA	0	NA	NA	NA	NA	NA	
ENSG00000276897	NA	NA	NA	0	NA	NA	NA	NA	NA	
ENSG00000276903	H2AC16	H2A clustered histone 16	8332	0,072165449	0,847101401	4,080456	0,207599697	0,835541541	NA	
ENSG00000276904	DUX4L14	double homeobox 4 like 14 (pseudogene)	728022	0	NA	NA	NA	NA	NA	
ENSG00000276915	FGFR3P3	fibroblast growth factor receptor 3 pseudogene 3	100462816	0	NA	NA	NA	NA	NA	
ENSG00000276925	SPMIP3P1	SPMIP3 pseudogene 1	127379741	0	NA	NA	NA	NA	NA	
ENSG00000276928	NA	NA	NA	0	NA	NA	NA	NA	NA	
ENSG00000276929	NA	NA	NA	0	NA	NA	NA	NA	NA	
ENSG00000276940	NA	NA	NA	0	NA	NA	NA	NA	NA	
ENSG00000276945	NA	NA	NA	0,591243875	-3,084912703	3,937268	-0,783516131	0,433324085	NA	
ENSG00000276949	NA	NA	NA	0	NA	NA	NA	NA	NA	
ENSG00000276950	GSTT4	glutathione S-transferase theta 4	25774	0	NA	NA	NA	NA	NA	
ENSG00000276951	NA	NA	NA	0	NA	NA	NA	NA	NA	
ENSG00000276953	TRBV12-4	T cell receptor beta variable 12-4	28576	0	NA	NA	NA	NA	NA	
ENSG00000276958	NA	NA	NA	0	NA	NA	NA	NA	NA	
ENSG00000276960	NA	NA	NA	0	NA	NA	NA	NA	NA	
ENSG00000276963	NA	NA	NA	0	NA	NA	NA	NA	NA	
ENSG00000276966	H4C5	H4 clustered histone 5	8367	0,11808024	-1,076443944	4,080456	-0,263804824	0,791930317	NA	
ENSG00000276975	NA	NA	NA	0,316293691	0	4,080456	0	1	NA	
ENSG00000276992	NA	NA	NA	0	NA	NA	NA	NA	NA	
ENSG00000276998	NA	NA	NA	0	NA	NA	NA	NA	NA	
ENSG00000277000	DUSP12P1	dual specificity phosphatase 12 pseudogene 1	100420473	0	NA	NA	NA	NA	NA	
ENSG00000277003	NA	NA	NA	0	NA	NA	NA	NA	NA	
ENSG00000277004	RNA5SP412	RNA, 5S ribosomal pseudogene 412	100873663	0	NA	NA	NA	NA	NA	
ENSG00000277008	TEX28P2	TEX28 pseudogene 2	653363	0	NA	NA	NA	NA	NA	
ENSG00000277009	TEX28P3	TEX28 pseudogene 3	102030688	0	NA	NA	NA	NA	NA	
ENSG00000277024	NA	NA	NA	0	NA	NA	NA	NA	NA	
ENSG00000277040	NA	NA	NA	0	NA	NA	NA	NA	NA	
ENSG00000277041	NA	NA	NA	0	NA	NA	NA	NA	NA	
ENSG00000277043	EEF1A1P42	eukaryotic translation elongation factor 1 alpha 1 pseudogene 42	442215	0	NA	NA	NA	NA	NA	
ENSG00000277056	NA	NA	NA	0	NA	NA	NA	NA	NA	
ENSG00000277058	HNRNPCL3	heterogeneous nuclear ribonucleoprotein C like 3	649330	0	NA	NA	NA	NA	NA	
ENSG00000277075	H2AC8	H2A clustered histone 8	3012	1,940214905	0,276264742	1,454323	0,189961056	0,849339648	NA	
ENSG00000277083	PRSS3P3	PRSS3 pseudogene 3	136540	0	NA	NA	NA	NA	NA	
ENSG00000277095	NA	NA	NA	0	NA	NA	NA	NA	NA	
ENSG00000277096	NA	NA	NA	0	NA	NA	NA	NA	NA	
ENSG00000277099	NA	NA	NA	0	NA	NA	NA	NA	NA	
ENSG00000277103	NA	NA	NA	0,503607722	-1,565326213	2,870916	-0,545235743	0,585591392	NA	
ENSG00000277109	NA	NA	NA	0	NA	NA	NA	NA	NA	
ENSG00000277112	NA	NA	NA	0,11808024	-1,076443944	4,080456	-0,263804824	0,791930317	NA	
ENSG00000277125	PMS2P14	PMS1 homolog 2, mismatch repair system component pseudogene 14	122149300	1,298979327	0,832864231	3,266107	0,255002135	0,79872145	NA	

ENSG00000277143	NA	NA	NA	0	NA	NA	NA	NA	NA
ENSG00000277146	NA	NA	NA	0	NA	NA	NA	NA	NA
ENSG00000277147	NA	NA	NA	6,1420765	-0,178328402	0,752939	-0,236843082	0,812778527	NA
ENSG00000277150	F8A3	coagulation factor VIII associated 3	474384	8,280448191	-0,134813143	0,618057	-0,218124203	0,82733234	NA
ENSG00000277156	NA	NA	NA	0	NA	NA	NA	NA	NA
ENSG00000277157	H4C4	H4 clustered histone 4	8360	0	NA	NA	NA	NA	NA
ENSG00000277162	NA	NA	NA	0	NA	NA	NA	NA	NA
ENSG00000277171	NA	NA	NA	0	NA	NA	NA	NA	NA
ENSG00000277172	GAPDHP41	glyceraldehyde 3 phosphate dehydrogenase pseudogene 41	100131935	0	NA	NA	NA	NA	NA
ENSG00000277174	NA	NA	NA	0	NA	NA	NA	NA	NA
ENSG00000277191	NA	NA	NA	0	NA	NA	NA	NA	NA
ENSG00000277196	PRODH	proline dehydrogenase 1	5625	0	NA	NA	NA	NA	NA
ENSG00000277200	CCDC92B	coiled-coil domain containing 92B	101928991	0	NA	NA	NA	NA	NA
ENSG00000277206	NA	NA	NA	0	NA	NA	NA	NA	NA
ENSG00000277211	SALL4P3	spalt like transcription factor 4 pseudogene 3	100131046	0	NA	NA	NA	NA	NA
ENSG00000277213	SDR42E1P2	short chain dehydrogenase/reductase family 42E, member 1 pseudogene 2	100422346	0	NA	NA	NA	NA	NA
ENSG00000277224	H2BC7	H2B clustered histone 7	8343	0,771588019	-0,974190445	2,447481	-0,398038052	0,690602137	NA
ENSG00000277227	NA	NA	NA	0	NA	NA	NA	NA	NA
ENSG00000277241	FGFR3P4	fibroblast growth factor receptor 3 pseudogene 4	100420833	0	NA	NA	NA	NA	NA
ENSG00000277252	NA	NA	NA	0	NA	NA	NA	NA	NA
ENSG00000277253	CICP28	capicua transcriptional repressor pseudogene 28	100287136	0	NA	NA	NA	NA	NA
ENSG00000277258	PCGF2	polycomb group ring finger 2	7703	0	NA	NA	NA	NA	NA
ENSG00000277282	IGHV1OR21-1	immunoglobulin heavy variable 1/OR21-1 (non-functional)	390530	0	NA	NA	NA	NA	NA
ENSG00000277289	NA	NA	NA	0	NA	NA	NA	NA	NA
ENSG00000277290	NA	NA	NA	0	NA	NA	NA	NA	NA
ENSG00000277297	NA	NA	NA	0	NA	NA	NA	NA	NA
ENSG00000277304	NA	NA	NA	0	NA	NA	NA	NA	NA
ENSG00000277315	NA	NA	NA	0	NA	NA	NA	NA	NA
ENSG00000277319	NA	NA	NA	0	NA	NA	NA	NA	NA
ENSG00000277322	GOLGA6L6	golgin A6 family like 6	727832	0	NA	NA	NA	NA	NA
ENSG00000277327	NA	NA	NA	0	NA	NA	NA	NA	NA
ENSG00000277340	NA	NA	NA	0	NA	NA	NA	NA	NA
ENSG00000277349	NA	NA	NA	0	NA	NA	NA	NA	NA
ENSG00000277357	NA	NA	NA	0	NA	NA	NA	NA	NA
ENSG00000277358	NA	NA	NA	0	NA	NA	NA	NA	NA
ENSG00000277363	SRCIN1	SRC kinase signaling inhibitor 1	80725	0	NA	NA	NA	NA	NA
ENSG00000277367	NA	NA	NA	0	NA	NA	NA	NA	NA
ENSG00000277397	NA	NA	NA	0	NA	NA	NA	NA	NA
ENSG00000277399	GPR179	G protein-coupled receptor 179	440435	0	NA	NA	NA	NA	NA
ENSG00000277408	NA	NA	NA	0	NA	NA	NA	NA	NA
ENSG00000277410	H2BP5	H2B histone pseudogene 5	115482690	0	NA	NA	NA	NA	NA
ENSG00000277422	REXO1L2P	REXO1 like 2, pseudogene	100288527	0	NA	NA	NA	NA	NA
ENSG00000277426	NA	NA	NA	0	NA	NA	NA	NA	NA
ENSG00000277435	NA	NA	NA	0	NA	NA	NA	NA	NA
ENSG00000277436	NA	NA	NA	0	NA	NA	NA	NA	NA
ENSG00000277438	NA	NA	NA	0	NA	NA	NA	NA	NA
ENSG00000277443	MARCKS	myristoylated alanine rich protein kinase C substrate	4082	0,223653915	-1,062439282	4,080456	-0,260372692	0,794576305	NA
ENSG00000277447	NA	NA	NA	0	NA	NA	NA	NA	NA
ENSG00000277462	ZNF670	zinc finger protein 670	93474	2,302413081	-1,339019029	1,338998	-1,000015811	0,317302856	NA
ENSG00000277466	NA	NA	NA	0	NA	NA	NA	NA	NA
ENSG00000277475	NA	NA	NA	0	NA	NA	NA	NA	NA
ENSG00000277479	NA	NA	NA	0	NA	NA	NA	NA	NA
ENSG00000277481	PKD1L3	polycystin 1 like 3, transient receptor potential channel interacting	342372	0	NA	NA	NA	NA	NA
ENSG00000277486	NA	NA	NA	0	NA	NA	NA	NA	NA
ENSG00000277490	NA	NA	NA	0	NA	NA	NA	NA	NA
ENSG00000277493	NA	NA	NA	0	NA	NA	NA	NA	NA
ENSG00000277494	GPIHBP1	glycosylphosphatidylinositol anchored high density lipoprotein binding protein 1	338328	0	NA	NA	NA	NA	NA
ENSG00000277498	NA	NA	NA	0,15836288	0	4,080456	0	1	NA
ENSG00000277499	LOC100533729	HERV-FRD provirus ancestral Env polyprotein pseudogene	100533729	0	NA	NA	NA	NA	NA
ENSG00000277500	NA	NA	NA	0	NA	NA	NA	NA	NA

ENSG00000277505	NA	NA	NA	0	NA	NA	NA	NA	NA
ENSG00000277508	BSNDP3	BSND pseudogene 3	106481725	0	NA	NA	NA	NA	NA
ENSG00000277513	NA	NA	NA	0	NA	NA	NA	NA	NA
ENSG00000277514	OR52Q1P	olfactory receptor family 52 subfamily Q member 1 pseudogene	81246	0	NA	NA	NA	NA	NA
ENSG00000277516	NA	NA	NA	0	NA	NA	NA	NA	NA
ENSG00000277529	NA	NA	NA	0	NA	NA	NA	NA	NA
ENSG00000277531	PNMA8C	PNMA family member 8C	110806277	5,170140359	0,085378133	0,789396	0,108156249	0,913871749	NA
ENSG00000277532	NA	NA	NA	0	NA	NA	NA	NA	NA
ENSG00000277535	CT47C1	cancer/testis antigen family 47 member C1	727838	0	NA	NA	NA	NA	NA
ENSG00000277544	NA	NA	NA	0	NA	NA	NA	NA	NA
ENSG00000277549	NA	NA	NA	0	NA	NA	NA	NA	NA
ENSG00000277556	OR13C5	olfactory receptor family 13 subfamily C member 5	138799	0	NA	NA	NA	NA	NA
ENSG00000277561	NA	NA	NA	2,952006	-5,732850513	3,916583	-1,463737847	0,14326559	NA
ENSG00000277562	VN1R99P	vomeronasal 1 receptor 99 pseudogene	100312842	0	NA	NA	NA	NA	NA
ENSG00000277575	NA	NA	NA	0	NA	NA	NA	NA	NA
ENSG00000277576	NA	NA	NA	0	NA	NA	NA	NA	NA
ENSG00000277586	NEFL	neurofilament light chain	4747	0	NA	NA	NA	NA	NA
ENSG00000277598	NA	NA	NA	0	NA	NA	NA	NA	NA
ENSG00000277608	LOC100419692	zinc finger protein 791 pseudogene	100419692	0	NA	NA	NA	NA	NA
ENSG00000277611	NA	NA	NA	0	NA	NA	NA	NA	NA
ENSG00000277618	NA	NA	NA	0	NA	NA	NA	NA	NA
ENSG00000277630	LOC100288966	POTE ankyrin domain family member D-like	100288966	0	NA	NA	NA	NA	NA
ENSG00000277639	CHD9NB	CHD9 neighbor	105371267	0,355457858	1,94370868	3,721187	0,522335603	0,601436684	NA
ENSG00000277640	NA	NA	NA	0	NA	NA	NA	NA	NA
ENSG00000277646	PDK1P1	pyruvate dehydrogenase kinase 1 pseudogene 1	100421194	0	NA	NA	NA	NA	NA
ENSG00000277647	NA	NA	NA	0	NA	NA	NA	NA	NA
ENSG00000277654	LOC440311	NOP53 ribosome biogenesis factor pseudogene	440311	0,11808024	-1,076443944	4,080456	-0,263804824	0,791930317	NA
ENSG00000277655	NA	NA	NA	0,325695703	-0,958282455	4,042845	-0,237031693	0,812632204	NA
ENSG00000277670	NA	NA	NA	0	NA	NA	NA	NA	NA
ENSG00000277672	NA	NA	NA	0	NA	NA	NA	NA	NA
ENSG00000277673	NA	NA	NA	0	NA	NA	NA	NA	NA
ENSG00000277690	NA	NA	NA	0,189946245	-0,108303762	4,080456	-0,026542074	0,978824975	NA
ENSG00000277702	NA	NA	NA	0	NA	NA	NA	NA	NA
ENSG00000277704	LOC100420879	chromosome 2 open reading frame 27B pseudogene	100420879	0	NA	NA	NA	NA	NA
ENSG00000277705	NA	NA	NA	0	NA	NA	NA	NA	NA
ENSG00000277708	NA	NA	NA	0	NA	NA	NA	NA	NA
ENSG00000277710	NBEAP5	neurobeachin pseudogene 5	100418903	0	NA	NA	NA	NA	NA
ENSG00000277711	GLRX3P1	glutaredoxin 3 pseudogene 1	100418908	0	NA	NA	NA	NA	NA
ENSG00000277720	H3C5P	H3 clustered histone 5, pseudogene	115482688	0	NA	NA	NA	NA	NA
ENSG00000277737	NA	NA	NA	0	NA	NA	NA	NA	NA
ENSG00000277741	NA	NA	NA	1,485297654	-1,821185056	1,791273	-1,016699049	0,309296618	NA
ENSG00000277743	LOC100419891	zinc finger protein 114 pseudogene	100419891	0	NA	NA	NA	NA	NA
ENSG00000277745	H2AB3	H2A,B variant histone 3	83740	0,119673877	0,847101401	4,080456	0,207599697	0,835541541	NA
ENSG00000277746	NA	NA	NA	0	NA	NA	NA	NA	NA
ENSG00000277748	NA	NA	NA	0	NA	NA	NA	NA	NA
ENSG00000277755	NA	NA	NA	0	NA	NA	NA	NA	NA
ENSG00000277758	SYT15B	synaptotagmin 15B	102724488	2,121251734	-0,339516144	1,815407	-0,187019251	0,851645539	NA
ENSG00000277766	NA	NA	NA	0	NA	NA	NA	NA	NA
ENSG00000277769	RBM22P4	RNA binding motif protein 22 pseudogene 4	100271870	0	NA	NA	NA	NA	NA
ENSG00000277770	PLA2G10GP	phospholipase A2 group XG, pseudogene	127525787	0	NA	NA	NA	NA	NA
ENSG00000277775	H3C7	H3 clustered histone 7	8968	0	NA	NA	NA	NA	NA
ENSG00000277778	NA	NA	NA	0	NA	NA	NA	NA	NA
ENSG00000277803	NA	NA	NA	0	NA	NA	NA	NA	NA
ENSG00000277836	NA	NA	NA	0	NA	NA	NA	NA	NA
ENSG00000277856	NA	NA	NA	0	NA	NA	NA	NA	NA
ENSG00000277865	GOLGA6L22	golgin A6 family like 22	440243	0	NA	NA	NA	NA	NA
ENSG00000277866	LOC100419553	zinc finger protein 35 pseudogene	100419553	0	NA	NA	NA	NA	NA
ENSG00000277867	NA	NA	NA	0,10543123	0	4,080456	0	1	NA
ENSG00000277869	NA	NA	NA	0	NA	NA	NA	NA	NA
ENSG00000277876	NA	NA	NA	0	NA	NA	NA	NA	NA
ENSG00000277878	NA	NA	NA	0	NA	NA	NA	NA	NA
ENSG00000277880	TRBV17	T cell receptor beta variable 17 (non-functional)	28570	0	NA	NA	NA	NA	NA
ENSG00000277881	NA	NA	NA	0	NA	NA	NA	NA	NA

ENSG00000277882	LOC102724602	COMM domain-containing protein 6-like	102724602	0	NA	NA	NA	NA	NA
ENSG00000277883	NA	NA	NA	0	NA	NA	NA	NA	NA
ENSG00000277893	SRD5A2	steroid 5 alpha-reductase 2	6716	0,119673877	0,847101401	4,080456	0,207599697	0,835541541	NA
ENSG00000277899	MLECP1	malectin pseudogene 1	106480317	0	NA	NA	NA	NA	NA
ENSG00000277907	NA	NA	NA	0	NA	NA	NA	NA	NA
ENSG00000277914	NA	NA	NA	0	NA	NA	NA	NA	NA
ENSG00000277920	NA	NA	NA	0	NA	NA	NA	NA	NA
ENSG00000277932	OR52E5	olfactory receptor family 52 subfamily E member 5	390082	0	NA	NA	NA	NA	NA
ENSG00000277952	SNRPCP7	small nuclear ribonucleoprotein polypeptide C pseudogene 7	100310852	0,10543123	0	4,080456	0	1	NA
ENSG00000277971	NA	NA	NA	0,355683489	1,412844225	4,061134	0,347894054	0,727919751	NA
ENSG00000277972	CISD3	CDGSH iron sulfur domain 3	284106	3,320908777	-2,517808377	1,869317	-1,346913398	0,178008126	NA
ENSG00000277973	NA	NA	NA	0	NA	NA	NA	NA	NA
ENSG00000277975	CSTP2	cystatin pseudogene 2	1481	0	NA	NA	NA	NA	NA
ENSG00000277977	NA	NA	NA	0	NA	NA	NA	NA	NA
ENSG00000277981	NA	NA	NA	0	NA	NA	NA	NA	NA
ENSG00000277984	LOC100420803	lysine demethylase 4D pseudogene	100420803	0	NA	NA	NA	NA	NA
ENSG00000277988	NA	NA	NA	0	NA	NA	NA	NA	NA
ENSG00000277998	EIF3EP3	EIF3E pseudogene 3	100462836	0	NA	NA	NA	NA	NA
ENSG00000278001	NA	NA	NA	0	NA	NA	NA	NA	NA
ENSG00000278008	NA	NA	NA	0	NA	NA	NA	NA	NA
ENSG00000278009	NA	NA	NA	0	NA	NA	NA	NA	NA
ENSG00000278023	RDM1	RAD52 motif containing 1	201299	2,609109505	4,381556482	1,773405	2,470703237	0,013484767	NA
ENSG00000278030	TRBV7-9	T cell receptor beta variable 7-9	28589	0	NA	NA	NA	NA	NA
ENSG00000278054	NA	NA	NA	0	NA	NA	NA	NA	NA
ENSG00000278057	TEX28	testis expressed 28	1527	0	NA	NA	NA	NA	NA
ENSG00000278068	NA	NA	NA	0	NA	NA	NA	NA	NA
ENSG00000278077	NA	NA	NA	0	NA	NA	NA	NA	NA
ENSG00000278078	NA	NA	NA	0	NA	NA	NA	NA	NA
ENSG00000278083	NA	NA	NA	0	NA	NA	NA	NA	NA
ENSG00000278085	CT45A8	cancer/testis antigen family 45 member A8	102723737	0	NA	NA	NA	NA	NA
ENSG00000278102	NA	NA	NA	0	NA	NA	NA	NA	NA
ENSG00000278106	NA	NA	NA	0	NA	NA	NA	NA	NA
ENSG00000278110	SUSD2P2	sushi domain containing 2 pseudogene 2	729450	0	NA	NA	NA	NA	NA
ENSG00000278122	NA	NA	NA	0	NA	NA	NA	NA	NA
ENSG00000278128	LOC100129616	putative UPF0607 protein ENSP00000332738-like	100129616	0	NA	NA	NA	NA	NA
ENSG00000278130	NA	NA	NA	0	NA	NA	NA	NA	NA
ENSG00000278131	LOC388996	otopetrin 1 pseudogene	388996	0	NA	NA	NA	NA	NA
ENSG00000278138	NA	NA	NA	0	NA	NA	NA	NA	NA
ENSG00000278139	P3R3URF-PIK3R3	P3R3URF-PIK3R3 readthrough	110117499	0,505158144	3,09728805	3,959697	0,782203361	0,434095069	NA
ENSG00000278143	NA	NA	NA	0	NA	NA	NA	NA	NA
ENSG00000278160	NA	NA	NA	0	NA	NA	NA	NA	NA
ENSG00000278171	NA	NA	NA	0,222693562	-2,001336755	4,035252	-0,495963301	0,619920304	NA
ENSG00000278180	NA	NA	NA	0	NA	NA	NA	NA	NA
ENSG00000278181	NA	NA	NA	0	NA	NA	NA	NA	NA
ENSG00000278182	NA	NA	NA	0	NA	NA	NA	NA	NA
ENSG00000278184	NA	NA	NA	0	NA	NA	NA	NA	NA
ENSG00000278193	NA	NA	NA	0	NA	NA	NA	NA	NA
ENSG00000278195	SSTR3	somatostatin receptor 3	6753	0,342349048	-0,00600348	3,757975	-0,001597531	0,998725356	NA
ENSG00000278196	IGLV2-8	immunoglobulin lambda variable 2-8	28817	0	NA	NA	NA	NA	NA
ENSG00000278197	TRIM60P7Y	tripartite motif containing 60 pseudogene 7, Y-linked	100420370	0	NA	NA	NA	NA	NA
ENSG00000278205	NA	NA	NA	0	NA	NA	NA	NA	NA
ENSG00000278212	NA	NA	NA	0,11808024	-1,076443944	4,080456	-0,263804824	0,791930317	NA
ENSG00000278213	NA	NA	NA	0	NA	NA	NA	NA	NA
ENSG00000278215	NA	NA	NA	0	NA	NA	NA	NA	NA
ENSG00000278224	PRICKLE4	prickle planar cell polarity protein 4	29964	0	NA	NA	NA	NA	NA
ENSG00000278256	NA	NA	NA	0	NA	NA	NA	NA	NA
ENSG00000278263	NA	NA	NA	0	NA	NA	NA	NA	NA
ENSG00000278273	NA	NA	NA	0,10543123	0	4,080456	0	1	NA
ENSG00000278275	NA	NA	NA	0	NA	NA	NA	NA	NA
ENSG00000278283	NA	NA	NA	0	NA	NA	NA	NA	NA
ENSG00000278289	CT45A6	cancer/testis antigen family 45 member A6	541465	0	NA	NA	NA	NA	NA
ENSG00000278292	NA	NA	NA	0	NA	NA	NA	NA	NA
ENSG00000278299	TBC1D3C	TBC1 domain family member 3C	414060	0	NA	NA	NA	NA	NA
ENSG00000278301	GRAMD4P3	GRAM domain containing 4 pseudogene 3	100631248	0	NA	NA	NA	NA	NA

ENSG00000278313	LOC100420930	SWI5 recombination repair homolog (yeast) pseudogene	100420930	0	NA	NA	NA	NA	NA
ENSG00000278318	ZNF229	zinc finger protein 229	7772	2,168606407	1,703546094	1,904867	0,894312564	0,37115468	NA
ENSG00000278319	NA	NA	NA	0	NA	NA	NA	NA	NA
ENSG00000278332	NA	NA	NA	0	NA	NA	NA	NA	NA
ENSG00000278337	NA	NA	NA	0	NA	NA	NA	NA	NA
ENSG00000278346	LOC100421674	kelch repeat and BTB domain containing 2 pseudogene	100421674	0	NA	NA	NA	NA	NA
ENSG00000278358	NA	NA	NA	0	NA	NA	NA	NA	NA
ENSG00000278381	NA	NA	NA	0	NA	NA	NA	NA	NA
ENSG00000278382	NA	NA	NA	0	NA	NA	NA	NA	NA
ENSG00000278388	NA	NA	NA	0	NA	NA	NA	NA	NA
ENSG00000278399	LOC390332	retinol dehydrogenase 16 (all-trans) pseudogene	390332	0,233310439	0,826467116	4,079642	0,20258324	0,839460791	NA
ENSG00000278400	NA	NA	NA	0	NA	NA	NA	NA	NA
ENSG00000278416	NA	NA	NA	7,045176858	-0,105247923	0,733655	-0,143457017	0,885929256	NA
ENSG00000278424	NA	NA	NA	0	NA	NA	NA	NA	NA
ENSG00000278428	NA	NA	NA	0	NA	NA	NA	NA	NA
ENSG00000278441	NA	NA	NA	0	NA	NA	NA	NA	NA
ENSG00000278455	CICP21	capicua transcriptional repressor pseudogene 21	100420263	0	NA	NA	NA	NA	NA
ENSG00000278463	H2AC4	H2A clustered histone 4	8335	0	NA	NA	NA	NA	NA
ENSG00000278473	IGHV3-41	immunoglobulin heavy variable 3-41 (pseudogene)	28428	0	NA	NA	NA	NA	NA
ENSG00000278477	NA	NA	NA	0	NA	NA	NA	NA	NA
ENSG00000278478	NA	NA	NA	0	NA	NA	NA	NA	NA
ENSG00000278480	NA	NA	NA	0	NA	NA	NA	NA	NA
ENSG00000278485	NA	NA	NA	0	NA	NA	NA	NA	NA
ENSG00000278486	YRDCP1	yrdC N(6)-threonylcarbamoyltransferase domain containing pseudogene 1	100420501	0,118286652	0	4,080456	0	1	NA
ENSG00000278487	NA	NA	NA	0	NA	NA	NA	NA	NA
ENSG00000278496	RBBP8P2	RBBP8 pseudogene 2	100420607	0	NA	NA	NA	NA	NA
ENSG00000278499	NA	NA	NA	0	NA	NA	NA	NA	NA
ENSG00000278505	C17orf78	chromosome 17 open reading frame 78	284099	0	NA	NA	NA	NA	NA
ENSG00000278510	NA	NA	NA	0	NA	NA	NA	NA	NA
ENSG00000278513	NA	NA	NA	0	NA	NA	NA	NA	NA
ENSG00000278522	POTEB3	POTE ankyrin domain family member B3	102724631	0	NA	NA	NA	NA	NA
ENSG00000278528	PLA2G10KP	phospholipase A2 group XK, pseudogene	127525796	0	NA	NA	NA	NA	NA
ENSG00000278530	CHMP1B2P	charged multivesicular body protein 1B2, pseudogene	101060146	0	NA	NA	NA	NA	NA
ENSG00000278531	NA	NA	NA	0	NA	NA	NA	NA	NA
ENSG00000278535	DHRS11	dehydrogenase/reductase 11	79154	4,237184892	-1,938837768	1,734146	-1,118036344	0,263551472	NA
ENSG00000278541	NA	NA	NA	0	NA	NA	NA	NA	NA
ENSG00000278543	NA	NA	NA	0	NA	NA	NA	NA	NA
ENSG00000278552	LOC100421667	abhydrolase domain containing 17C, depalmitoylase pseudogene	100421667	0	NA	NA	NA	NA	NA
ENSG00000278558	TMEM191B	transmembrane protein 191B	728229	0,629291042	-3,507005634	3,952522	-0,887283046	0,374926531	NA
ENSG00000278561	NA	NA	NA	0	NA	NA	NA	NA	NA
ENSG00000278570	NR2E3	nuclear receptor subfamily 2 group E member 3	10002	0	NA	NA	NA	NA	NA
ENSG00000278572	NA	NA	NA	0	NA	NA	NA	NA	NA
ENSG00000278573	NA	NA	NA	0	NA	NA	NA	NA	NA
ENSG00000278577	NA	NA	NA	0	NA	NA	NA	NA	NA
ENSG00000278580	NA	NA	NA	0	NA	NA	NA	NA	NA
ENSG00000278582	NA	NA	NA	0	NA	NA	NA	NA	NA
ENSG00000278585	SNX3P1Y	sorting nexin 3 pseudogene 1 Y-linked	100873885	0	NA	NA	NA	NA	NA
ENSG00000278586	NA	NA	NA	0	NA	NA	NA	NA	NA
ENSG00000278588	H2BC10	H2B clustered histone 10	8346	0,59088453	2,547749198	3,269703	0,77919893	0,43586254	NA
ENSG00000278589	NA	NA	NA	0	NA	NA	NA	NA	NA
ENSG00000278593	NA	NA	NA	0,11808024	-1,076443944	4,080456	-0,263804824	0,791930317	NA
ENSG00000278594	NA	NA	NA	0	NA	NA	NA	NA	NA
ENSG00000278599	TBC1D3E	TBC1 domain family member 3E	102723859	0	NA	NA	NA	NA	NA
ENSG00000278602	TRIM60P6Y	tripartite motif containing 60 pseudogene 6, Y-linked	100420369	0	NA	NA	NA	NA	NA
ENSG00000278603	NA	NA	NA	0,072165449	0,847101401	4,080456	0,207599697	0,835541541	NA
ENSG00000278616	NA	NA	NA	0	NA	NA	NA	NA	NA
ENSG00000278631	SOWAHCP5	SOWAHC pseudogene 5	100419815	0	NA	NA	NA	NA	NA
ENSG00000278633	NA	NA	NA	0	NA	NA	NA	NA	NA
ENSG00000278635	NA	NA	NA	0	NA	NA	NA	NA	NA
ENSG00000278637	H4C1	H4 clustered histone 1	8359	0,072165449	0,847101401	4,080456	0,207599697	0,835541541	NA
ENSG00000278641	DUX4L15	double homeobox 4 like 15 (pseudogene)	440017	0	NA	NA	NA	NA	NA

ENSG00000278646	NA	NA	NA	0	NA	NA	NA	NA	NA
ENSG00000278647	NA	NA	NA	0	NA	NA	NA	NA	NA
ENSG00000278650	NA	NA	NA	0	NA	NA	NA	NA	NA
ENSG00000278654	IQSEC3P2	IQ motif and Sec7 domain 3 pseudogene 2	100130073	0,10488184	-1,076443944	4,080456	-0,263804824	0,791930317	NA
ENSG00000278655	NA	NA	NA	0	NA	NA	NA	NA	NA
ENSG00000278661	TRAJ37	T cell receptor alpha joining 37	28718	0	NA	NA	NA	NA	NA
ENSG00000278665	NA	NA	NA	0,296496366	0,705176705	3,891311	0,18121828	0,856196247	NA
ENSG00000278667	NA	NA	NA	0	NA	NA	NA	NA	NA
ENSG00000278673	NA	NA	NA	7,706152372	-1,703000059	0,744124	-2,288597761	0,022102734	NA
ENSG00000278677	H2AC17	H2A clustered histone 17	8336	0,87985081	0,707557658	2,103005	0,336450793	0,736530953	NA
ENSG00000278678	NA	NA	NA	0	NA	NA	NA	NA	NA
ENSG00000278681	NA	NA	NA	0	NA	NA	NA	NA	NA
ENSG00000278683	NA	NA	NA	0	NA	NA	NA	NA	NA
ENSG00000278684	NA	NA	NA	0	NA	NA	NA	NA	NA
ENSG00000278685	IQCA1L	IQ motif containing with AAA domain 1 like	392843	0	NA	NA	NA	NA	NA
ENSG00000278687	NA	NA	NA	0	NA	NA	NA	NA	NA
ENSG00000278694	NA	NA	NA	0	NA	NA	NA	NA	NA
ENSG00000278704	NA	NA	NA	0,144330898	1,418786819	4,061262	0,349346267	0,726829368	NA
ENSG00000278705	H4C2	H4 clustered histone 2	8366	0	NA	NA	NA	NA	NA
ENSG00000278720	LOC100132599	zinc finger protein 114 pseudogene	100132599	0	NA	NA	NA	NA	NA
ENSG00000278725	NA	NA	NA	0	NA	NA	NA	NA	NA
ENSG00000278734	NA	NA	NA	0	NA	NA	NA	NA	NA
ENSG00000278736	NA	NA	NA	0	NA	NA	NA	NA	NA
ENSG00000278744	NA	NA	NA	0	NA	NA	NA	NA	NA
ENSG00000278745	NA	NA	NA	0	NA	NA	NA	NA	NA
ENSG00000278754	NA	NA	NA	0	NA	NA	NA	NA	NA
ENSG00000278763	NA	NA	NA	0,119673877	0,847101401	4,080456	0,207599697	0,835541541	NA
ENSG00000278790	DUX4L12	double homeobox 4 like 12 (pseudogene)	440014	0	NA	NA	NA	NA	NA
ENSG00000278797	NA	NA	NA	0	NA	NA	NA	NA	NA
ENSG00000278803	PWWP4	PWWP domain containing 4	728317	0	NA	NA	NA	NA	NA
ENSG00000278813	NA	NA	NA	0	NA	NA	NA	NA	NA
ENSG00000278817	DGCR6	DiGeorge syndrome critical region gene 6	8214	0,992377627	-1,206054019	3,424247	-0,352210005	0,724680774	NA
ENSG00000278819	NA	NA	NA	0	NA	NA	NA	NA	NA
ENSG00000278824	LOC100422013	catenin beta 1 pseudogene	100422013	0	NA	NA	NA	NA	NA
ENSG00000278828	H3C10	H3 clustered histone 10	8357	6,062700438	0,86547305	0,906339	0,954910534	0,339622942	NA
ENSG00000278830	NA	NA	NA	0	NA	NA	NA	NA	NA
ENSG00000278837	NA	NA	NA	0	NA	NA	NA	NA	NA
ENSG00000278842	NA	NA	NA	0	NA	NA	NA	NA	NA
ENSG00000278845	MRPL45	mitochondrial ribosomal protein L45	84311	3,354641768	0,928968146	1,589294	0,584516101	0,558873136	NA
ENSG00000278854	TRIM60P1Y	tripartite motif containing 60 pseudogene 1, Y-linked	100420362	0	NA	NA	NA	NA	NA
ENSG00000278857	IGKV1D-12	immunoglobulin kappa variable 1D-12	28903	0	NA	NA	NA	NA	NA
ENSG00000278870	OR51G1	olfactory receptor family 51 subfamily G member 1	79324	0	NA	NA	NA	NA	NA
ENSG00000278889	OR2S2	olfactory receptor family 2 subfamily S member 2	56656	0	NA	NA	NA	NA	NA
ENSG00000278931	NA	NA	NA	0,072165449	0,847101401	4,080456	0,207599697	0,835541541	NA
ENSG00000279012	OR51B2	olfactory receptor family 51 subfamily B member 2	79345	0	NA	NA	NA	NA	NA
ENSG00000279051	OR6Q1	olfactory receptor family 6 subfamily Q member 1	219952	0	NA	NA	NA	NA	NA
ENSG00000279058	NA	NA	NA	0	NA	NA	NA	NA	NA
ENSG00000279062	NA	NA	NA	0	NA	NA	NA	NA	NA
ENSG00000279073	NA	NA	NA	0,144330898	1,418786819	4,061262	0,349346267	0,726829368	NA
ENSG00000279081	EIF3FP2	eukaryotic translation initiation factor 3 subunit F pseudogene 2	83880	0	NA	NA	NA	NA	NA
ENSG00000279111	OR10X1	olfactory receptor family 10 subfamily X member 1	128367	0	NA	NA	NA	NA	NA
ENSG00000279116	OR8D2	olfactory receptor family 8 subfamily D member 2	283160	0	NA	NA	NA	NA	NA
ENSG00000279124	IGSF3P1	IGSF3 pseudogene 1	122965330	0	NA	NA	NA	NA	NA
ENSG00000279167	NA	NA	NA	0	NA	NA	NA	NA	NA
ENSG00000279170	TSTD3	thiosulfate sulfurtransferase like domain containing 3	100130890	10,33435195	0,759852244	0,62189	1,22184405	0,221766607	NA
ENSG00000279208	NA	NA	NA	0,209763681	-1,926644696	4,042043	-0,476651224	0,633610501	NA
ENSG00000279211	NA	NA	NA	0	NA	NA	NA	NA	NA
ENSG00000279213	NA	NA	NA	0	NA	NA	NA	NA	NA
ENSG00000279228	NA	NA	NA	0	NA	NA	NA	NA	NA
ENSG00000279231	NA	NA	NA	0	NA	NA	NA	NA	NA
ENSG00000279239	NA	NA	NA	0	NA	NA	NA	NA	NA

ENSG00000279263	OR2L8	olfactory receptor family 2 subfamily L member 8	391190	0,31672576	0	4,080456	0	1	NA
ENSG00000279270	OR52R1	olfactory receptor family 52 subfamily R member 1	119695	0	NA	NA	NA	NA	NA
ENSG00000279274	NA	NA	NA	0	NA	NA	NA	NA	NA
ENSG00000279301	OR2T11	olfactory receptor family 2 subfamily T member 11	127077	0	NA	NA	NA	NA	NA
ENSG00000279307	NDUFAF2P1	NDUFAF2 pseudogene 1	727919	0	NA	NA	NA	NA	NA
ENSG00000279381	NA	NA	NA	0	NA	NA	NA	NA	NA
ENSG00000279392	NA	NA	NA	0	NA	NA	NA	NA	NA
ENSG00000279395	OR5L1	olfactory receptor family 5 subfamily L member 1	219437	0	NA	NA	NA	NA	NA
ENSG00000279408	OR4N4C	olfactory receptor family 4 subfamily N member 4C	105369274	0	NA	NA	NA	NA	NA
ENSG00000279414	NA	NA	NA	0	NA	NA	NA	NA	NA
ENSG00000279445	OR4N3BP	olfactory receptor family 4 subfamily N member 3B pseudogene	118568823	0	NA	NA	NA	NA	NA
ENSG00000279461	NA	NA	NA	0	NA	NA	NA	NA	NA
ENSG00000279477	NA	NA	NA	0	NA	NA	NA	NA	NA
ENSG00000279486	OR2AG1	olfactory receptor family 2 subfamily AG member 1	144125	0,103998153	-1,076443944	4,080456	-0,263804824	0,791930317	NA
ENSG00000279514	OR4C16	olfactory receptor family 4 subfamily C member 16	219428	0	NA	NA	NA	NA	NA
ENSG00000279615	NA	NA	NA	0	NA	NA	NA	NA	NA
ENSG00000279626	DUX4L28	double homeobox 4 like 28 (pseudogene)	106481728	0	NA	NA	NA	NA	NA
ENSG00000279639	LOC107987217	olfactory receptor 11H12-like	107987217	0	NA	NA	NA	NA	NA
ENSG00000279647	NA	NA	NA	0	NA	NA	NA	NA	NA
ENSG00000279688	LOC112267859	putative aquaporin-7-like protein 3	112267859	0	NA	NA	NA	NA	NA
ENSG00000279709	NA	NA	NA	0	NA	NA	NA	NA	NA
ENSG00000279718	NA	NA	NA	0	NA	NA	NA	NA	NA
ENSG00000279720	NA	NA	NA	1,287959284	1,03616984	2,020522	0,512822898	0,608075211	NA
ENSG00000279728	NA	NA	NA	0	NA	NA	NA	NA	NA
ENSG00000279730	NA	NA	NA	0	NA	NA	NA	NA	NA
ENSG00000279761	OR5D13	olfactory receptor family 5 subfamily D member 13	390142	0	NA	NA	NA	NA	NA
ENSG00000279783	NA	NA	NA	0	NA	NA	NA	NA	NA
ENSG00000279788	NA	NA	NA	0	NA	NA	NA	NA	NA
ENSG00000279800	BCLAF1P2	BCL2 associated transcription factor 1 pseudogene 2	731605	0,310191842	1,671747863	3,839937	0,435358163	0,663302473	NA
ENSG00000279804	PRAMEF18	PRAME family member 18	391003	0	NA	NA	NA	NA	NA
ENSG00000279864	NA	NA	NA	0	NA	NA	NA	NA	NA
ENSG00000279895	NA	NA	NA	0	NA	NA	NA	NA	NA
ENSG00000279897	NA	NA	NA	0	NA	NA	NA	NA	NA
ENSG00000279924	NA	NA	NA	0	NA	NA	NA	NA	NA
ENSG00000279928	NA	NA	NA	0	NA	NA	NA	NA	NA
ENSG00000279956	NA	NA	NA	0	NA	NA	NA	NA	NA
ENSG00000279966	NA	NA	NA	0	NA	NA	NA	NA	NA
ENSG00000279990	NA	NA	NA	0	NA	NA	NA	NA	NA
ENSG00000280012	RPL23AP61	ribosomal protein L23a pseudogene 61	728484	0	NA	NA	NA	NA	NA
ENSG00000280019	NA	NA	NA	0	NA	NA	NA	NA	NA
ENSG00000280021	OR51F1	olfactory receptor family 51 subfamily F member 1	256892	0	NA	NA	NA	NA	NA
ENSG00000280081	LINC01667	long intergenic non-protein coding RNA 1667	441058	0	NA	NA	NA	NA	NA
ENSG00000280090	OR8B4	olfactory receptor family 8 subfamily B member 4	283162	0	NA	NA	NA	NA	NA
ENSG00000280094	OR1B1	olfactory receptor family 1 subfamily B member 1	347169	0,239347754	1,967469188	4,021404	0,489249276	0,624665227	NA
ENSG00000280114	NA	NA	NA	0	NA	NA	NA	NA	NA
ENSG00000280116	NA	NA	NA	0	NA	NA	NA	NA	NA
ENSG00000280148	NA	NA	NA	0	NA	NA	NA	NA	NA
ENSG00000280165	PCDH20	protocadherin 20	64881	0	NA	NA	NA	NA	NA
ENSG00000280171	NA	NA	NA	0	NA	NA	NA	NA	NA
ENSG00000280204	OR1S1	olfactory receptor family 1 subfamily S member 1	219959	0	NA	NA	NA	NA	NA
ENSG00000280208	NA	NA	NA	0	NA	NA	NA	NA	NA
ENSG00000280236	OR12D2	olfactory receptor family 12 subfamily D member 2	26529	0	NA	NA	NA	NA	NA
ENSG00000280243	NA	NA	NA	0	NA	NA	NA	NA	NA
ENSG00000280267	PRAMEF26	PRAME family member 26	645359	0	NA	NA	NA	NA	NA
ENSG00000280286	NA	NA	NA	0	NA	NA	NA	NA	NA
ENSG00000280330	NA	NA	NA	0	NA	NA	NA	NA	NA
ENSG00000280337	DUX4L25	double homeobox 4 like 25 (pseudogene)	102723423	0	NA	NA	NA	NA	NA

ENSG00000280411	IGHV1-69D	immunoglobulin heavy variable 1-69D	102723169	0	NA	NA	NA	NA	NA
ENSG00000280436	NA	NA	NA	0	NA	NA	NA	NA	NA
ENSG00000280537	NA	NA	NA	0	NA	NA	NA	NA	NA
ENSG00000280571	NA	NA	NA	0	NA	NA	NA	NA	NA
ENSG00000280646	RNA5SP196	RNA, 5S ribosomal pseudogene 196	100873458	0	NA	NA	NA	NA	NA
ENSG00000280655	NA	NA	NA	0,11808024	-1,076443944	4,080456	-0,263804824	0,791930317	NA
ENSG00000280670	CCDC163	CCDC163 homolog	126661	3,646210474	-0,216602839	1,366454	-0,158514543	0,874051362	NA
ENSG00000280709	LINC02203	long intergenic non-protein coding RNA 2203	727924	0	NA	NA	NA	NA	NA
ENSG00000280778	NA	NA	NA	0	NA	NA	NA	NA	NA
ENSG00000280852	LOC653653	adaptor related protein complex 1 subunit sigma 2 pseudogene	653653	0	NA	NA	NA	NA	NA
ENSG00000280893	NA	NA	NA	3,434744685	0,255790628	3,75968	0,068035213	0,945757604	NA
ENSG00000280913	CTSLP3	cathepsin L pseudogene 3	644021	0	NA	NA	NA	NA	NA
ENSG00000280969	RPS4Y2	ribosomal protein S4 Y-linked 2	140032	0	NA	NA	NA	NA	NA
ENSG00000280991	NA	NA	NA	0	NA	NA	NA	NA	NA
ENSG00000281039	NA	NA	NA	9,196160634	-0,665102764	0,741488	-0,896983565	0,369727687	NA
ENSG00000281058	DUX4L6	double homeobox 4 like 6 (pseudogene)	653544	0	NA	NA	NA	NA	NA
ENSG00000281087	LOC102724737	40S ribosomal protein S8-like	102724737	0,10543123	0	4,080456	0	1	NA
ENSG00000281106	TMEM272	transmembrane protein 272	283521	0	NA	NA	NA	NA	NA
ENSG00000281347	NA	NA	NA	0	NA	NA	NA	NA	NA
ENSG00000281348	NA	NA	NA	3,245799207	-0,446366541	1,305358	-0,34194962	0,732388807	NA
ENSG00000281379	NA	NA	NA	0,473635108	2,132277436	3,403815	0,626437585	0,531027964	NA
ENSG00000281406	BLACAT1	BLACAT1 overlapping LEMD1 locus	101669762	5,224895434	-0,071863472	0,772145	-0,093069906	0,925848025	NA
ENSG00000281490	NA	NA	NA	0,118286652	0	4,080456	0	1	NA
ENSG00000281550	NA	NA	NA	2,856750114	-1,194963583	2,152694	-0,555101366	0,578825329	NA
ENSG00000281593	NA	NA	NA	1,378915178	0,483420522	2,157541	0,224060865	0,822709943	NA
ENSG00000281613	NA	NA	NA	0	NA	NA	NA	NA	NA
ENSG00000281652	DUX4L7	double homeobox 4 like 7 (pseudogene)	653543	0	NA	NA	NA	NA	NA
ENSG00000281720	DUX4L8	double homeobox 4 like 8 (pseudogene)	26583	0	NA	NA	NA	NA	NA
ENSG00000281720	LOC124906452	double homeobox protein 4	124906452	0	NA	NA	NA	NA	NA
ENSG00000281720	LOC124906453	double homeobox protein 4	124906453	0	NA	NA	NA	NA	NA
ENSG00000281720	LOC124906454	double homeobox protein 4	124906454	0	NA	NA	NA	NA	NA
ENSG00000281720	LOC124906456	double homeobox protein 4	124906456	0	NA	NA	NA	NA	NA
ENSG00000281720	LOC124906457	double homeobox protein 4	124906457	0	NA	NA	NA	NA	NA
ENSG00000281720	LOC124906458	double homeobox protein 4	124906458	0	NA	NA	NA	NA	NA
ENSG00000281720	LOC124906459	double homeobox protein 4	124906459	0	NA	NA	NA	NA	NA
ENSG00000281720	LOC124906460	double homeobox protein 4	124906460	0	NA	NA	NA	NA	NA
ENSG00000281720	LOC124906461	double homeobox protein 4	124906461	0	NA	NA	NA	NA	NA
ENSG00000281720	LOC124906462	double homeobox protein 4	124906462	0	NA	NA	NA	NA	NA
ENSG00000281720	LOC124906463	double homeobox protein 4	124906463	0	NA	NA	NA	NA	NA
ENSG00000281720	LOC124906464	double homeobox protein 4	124906464	0	NA	NA	NA	NA	NA
ENSG00000281720	LOC124906465	double homeobox protein 4	124906465	0	NA	NA	NA	NA	NA
ENSG00000281720	LOC124906466	double homeobox protein 4	124906466	0	NA	NA	NA	NA	NA
ENSG00000281883	NA	NA	NA	1,160201026	0,37210129	2,179125	0,170757192	0,864414691	NA
ENSG00000281887	GIMAP1-GIMAP5	GIMAP1-GIMAP5 readthrough	100527949	6,655881791	-6,907393518	3,911111	-1,76609516	NA	NA
ENSG00000281896	NA	NA	NA	0	NA	NA	NA	NA	NA
ENSG00000281909	NA	NA	NA	0	NA	NA	NA	NA	NA
ENSG00000281938	NA	NA	NA	0	NA	NA	NA	NA	NA
ENSG00000281941	NA	NA	NA	0	NA	NA	NA	NA	NA
ENSG00000281955	NA	NA	NA	0	NA	NA	NA	NA	NA
ENSG00000281991	TMEM265	transmembrane protein 265	100862671	0	NA	NA	NA	NA	NA
ENSG00000282051	NA	NA	NA	0	NA	NA	NA	NA	NA
ENSG00000282059	NA	NA	NA	0	NA	NA	NA	NA	NA
ENSG00000282100	NA	NA	NA	4,674593531	0,578162324	0,850773	0,67957311	0,496774801	NA

ENSG00000282137	OR4G3P	olfactory receptor family 4 subfamily G member 3 pseudogene	26679	0	NA	NA	NA	NA	NA
ENSG00000282206	NA	NA	NA	0	NA	NA	NA	NA	NA
ENSG00000282218	NA	NA	NA	0	NA	NA	NA	NA	NA
ENSG00000282222	NA	NA	NA	0	NA	NA	NA	NA	NA
ENSG00000282277	CYP3A51P	cytochrome P450 family 3 subfamily A member 51, pseudogene	1578	0	NA	NA	NA	NA	NA
ENSG00000282278	NA	NA	NA	0	NA	NA	NA	NA	NA
ENSG00000282299	NA	NA	NA	0	NA	NA	NA	NA	NA
ENSG00000282300	NA	NA	NA	0	NA	NA	NA	NA	NA
ENSG00000282301	CYP3A7-CYP3A51P	CYP3A7-CYP3A51P readthrough	100861540	0	NA	NA	NA	NA	NA
ENSG00000282306	NA	NA	NA	0	NA	NA	NA	NA	NA
ENSG00000282308	DPRXP3	divergent-paired related homeobox pseudogene 3	503644	1,438390291	-0,727780522	1,874645	-0,38822321	0,697850858	NA
ENSG00000282321	MTND1P7	MT-ND1 pseudogene 7	100873172	0	NA	NA	NA	NA	NA
ENSG00000282327	NA	NA	NA	0	NA	NA	NA	NA	NA
ENSG00000282339	NA	NA	NA	0,321862036	0,962507344	4,025637	0,239094442	0,811032351	NA
ENSG00000282358	NA	NA	NA	0	NA	NA	NA	NA	NA
ENSG00000282413	NA	NA	NA	0,4048641	2,771090157	3,973045	0,697472658	0,485507045	NA
ENSG00000282416	NA	NA	NA	1,996128339	0,517124312	1,626312	0,317973656	0,75050492	NA
ENSG00000282419	TEX13D	TEX13 family member D	100132015	0	NA	NA	NA	NA	NA
ENSG00000282432	NA	NA	NA	0	NA	NA	NA	NA	NA
ENSG00000282458	WASH5P	WASP family homolog 5, pseudogene	375690	3,611704866	-0,044306938	1,037751	-0,042695147	0,965944548	NA
ENSG00000282499	TRBV25-1	T cell receptor beta variable 25-1	28562	0	NA	NA	NA	NA	NA
ENSG00000282501	NA	NA	NA	0	NA	NA	NA	NA	NA
ENSG00000282502	NA	NA	NA	0	NA	NA	NA	NA	NA
ENSG00000282511	CLUHP10	clustered mitochondria homolog pseudogene 10	107161230	0	NA	NA	NA	NA	NA
ENSG00000282520	NA	NA	NA	0	NA	NA	NA	NA	NA
ENSG00000282599	NA	NA	NA	0	NA	NA	NA	NA	NA
ENSG00000282600	IGHV3-69-1	immunoglobulin heavy variable 3-69-1 (pseudogene)	28402	0	NA	NA	NA	NA	NA
ENSG00000282608	ADORA3	adenosine A3 receptor	140	0	NA	NA	NA	NA	NA
ENSG00000282625	MRPL57P8	mitochondrial ribosomal protein L57 pseudogene 8	359732	0	NA	NA	NA	NA	NA
ENSG00000282639	IGHV3-64D	immunoglobulin heavy variable 3-64D	102723170	0	NA	NA	NA	NA	NA
ENSG00000282651	NA	NA	NA	0	NA	NA	NA	NA	NA
ENSG00000282668	NA	NA	NA	0	NA	NA	NA	NA	NA
ENSG00000282697	NA	NA	NA	0	NA	NA	NA	NA	NA
ENSG00000282732	NA	NA	NA	0	NA	NA	NA	NA	NA
ENSG00000282738	NA	NA	NA	0	NA	NA	NA	NA	NA
ENSG00000282757	DUXB	double homeobox B	100033411	0	NA	NA	NA	NA	NA
ENSG00000282780	TRBJ1-6	T cell receptor beta joining 1-6	28630	0	NA	NA	NA	NA	NA
ENSG00000282785	NA	NA	NA	0	NA	NA	NA	NA	NA
ENSG00000282804	NA	NA	NA	0,311570822	-2,496570387	3,998413	-0,624390293	0,532371299	NA
ENSG00000282815	TEX13C	TEX13 family member C	100129520	0	NA	NA	NA	NA	NA
ENSG00000282834	NA	NA	NA	0	NA	NA	NA	NA	NA
ENSG00000282842	NA	NA	NA	0	NA	NA	NA	NA	NA
ENSG00000282850	NA	NA	NA	0	NA	NA	NA	NA	NA
ENSG00000282852	NA	NA	NA	0	NA	NA	NA	NA	NA
ENSG00000282855	NA	NA	NA	0	NA	NA	NA	NA	NA
ENSG00000282870	NA	NA	NA	0,993276085	-0,23987479	1,746092	-0,137378101	0,89073194	NA
ENSG00000282872	C1orf232	chromosome 1 open reading frame 230	110806296	0	NA	NA	NA	NA	NA
ENSG00000282875	NA	NA	NA	0	NA	NA	NA	NA	NA
ENSG00000282879	NA	NA	NA	0	NA	NA	NA	NA	NA
ENSG00000282881	TMEM275	transmembrane protein 275	105378696	0,103998153	-1,076443944	4,080456	-0,263804824	0,791930317	NA
ENSG00000282886	NA	NA	NA	0	NA	NA	NA	NA	NA
ENSG00000282894	DUX4L33	double homeobox 4 like 33 (pseudogene)	107080551	0	NA	NA	NA	NA	NA
ENSG00000282909	NA	NA	NA	0	NA	NA	NA	NA	NA
ENSG00000282911	NA	NA	NA	0	NA	NA	NA	NA	NA
ENSG00000282912	NA	NA	NA	0	NA	NA	NA	NA	NA
ENSG00000282921	CFTRP2	CFTR pseudogene 2	107080633	0	NA	NA	NA	NA	NA
ENSG00000282924	NA	NA	NA	0	NA	NA	NA	NA	NA
ENSG00000282933	NA	NA	NA	0,421723323	1,427188195	3,595505	0,396936736	0,691414114	NA
ENSG00000282935	DUX4L34	double homeobox 4 like 34 (pseudogene)	105379479	0	NA	NA	NA	NA	NA
ENSG00000282939	TRBV7-2	T cell receptor beta variable 7-2	28596	0	NA	NA	NA	NA	NA
ENSG00000282946	NA	NA	NA	0	NA	NA	NA	NA	NA
ENSG00000282949	NA	NA	NA	0	NA	NA	NA	NA	NA
ENSG00000282968	NA	NA	NA	0	NA	NA	NA	NA	NA

ENSG00000282973	NA	NA	NA	0	NA	NA	NA	NA	NA	
ENSG00000282978	NA	NA	NA	0,526720021	0,819887845	3,432154	0,238884333	0,811195274	NA	
ENSG00000282980	NA	NA	NA	1,974037534	-0,097423746	1,255455	-0,077600325	0,938145983	NA	
ENSG00000282988	NA	NA	NA	3,446411474	0,626816654	1,500723	0,417676339	0,676183774	NA	
ENSG00000282989	NA	NA	NA	7,802268642	0,690560502	0,671164	1,028899705	0,303526807	NA	
ENSG00000282995	NA	NA	NA	5,015900553	-0,236821768	0,869772	-0,272280243	0,785406546	NA	
ENSG00000283005	NA	NA	NA	0	NA	NA	NA	NA	NA	
ENSG00000283016	NA	NA	NA	0	NA	NA	NA	NA	NA	
ENSG00000283020	DUX4L37	double homeobox 4 like 37 (pseudogene)	107080108	0,178083994	0,838066485	4,080139	0,20540147	0,837258472	NA	
ENSG00000283023	NA	NA	NA	6,733322804	-0,236415925	0,675314	-0,350082907	0,726276479	NA	
ENSG00000283039	KLF18	KLF transcription factor 18	105378952	0	NA	NA	NA	NA	NA	
ENSG00000283040	NA	NA	NA	0	NA	NA	NA	NA	NA	
ENSG00000283041	LOC729998	eukaryotic translation elongation factor 1 gamma pseudogene	729998	2,951739844	0,149990391	1,315792	0,113992495	0,909243742	NA	
ENSG00000283043	NA	NA	NA	0	NA	NA	NA	NA	NA	
ENSG00000283047	NA	NA	NA	0,326698585	-2,558324957	3,704267	-0,69064279	0,48979005	NA	
ENSG00000283050	NA	NA	NA	0	NA	NA	NA	NA	NA	
ENSG00000283057	NA	NA	NA	0,15836288	0	4,080456	0	1	NA	
ENSG00000283063	TRBV6-3	T cell receptor beta variable 6-3	28604	0	NA	NA	NA	NA	NA	
ENSG00000283065	NA	NA	NA	0	NA	NA	NA	NA	NA	
ENSG00000283069	DUX4L32	double homeobox 4 like 32 (pseudogene)	107075316	0	NA	NA	NA	NA	NA	
ENSG00000283071	LBHD2	LBH domain containing 2	107984640	0	NA	NA	NA	NA	NA	
ENSG00000283076	NA	NA	NA	0	NA	NA	NA	NA	NA	
ENSG00000283084	NA	NA	NA	0,449358356	1,902816143	4,02446	0,472812836	0,636346716	NA	
ENSG00000283093	CENPVL2	centromere protein V like 2	441495	0	NA	NA	NA	NA	NA	
ENSG00000283101	NA	NA	NA	0,072165449	0,847101401	4,080456	0,207599697	0,835541541	NA	
ENSG00000283110	NA	NA	NA	0	NA	NA	NA	NA	NA	
ENSG00000283123	NA	NA	NA	0	NA	NA	NA	NA	NA	
ENSG00000283128	NA	NA	NA	0	NA	NA	NA	NA	NA	
ENSG00000283130	NA	NA	NA	0	NA	NA	NA	NA	NA	
ENSG00000283131	MTND2P41	MT-ND2 pseudogene 41	109729121	0	NA	NA	NA	NA	NA	
ENSG00000283132	NA	NA	NA	0	NA	NA	NA	NA	NA	
ENSG00000283133	NA	NA	NA	0	NA	NA	NA	NA	NA	
ENSG00000283142	NA	NA	NA	0	NA	NA	NA	NA	NA	
ENSG00000283145	NA	NA	NA	0	NA	NA	NA	NA	NA	
ENSG00000283154	SCHIP1	schwannomin interacting protein 1	29970	0,103998153	-1,076443944	4,080456	-0,263804824	0,791930317	NA	
ENSG00000283154	IQCJ-SCHIP1	IQCJ-SCHIP1 readthrough	100505385	0,103998153	-1,076443944	4,080456	-0,263804824	0,791930317	NA	
ENSG00000283156	NA	NA	NA	0,117497108	0,847101401	4,080456	0,207599697	0,835541541	NA	
ENSG00000283162	NA	NA	NA	0	NA	NA	NA	NA	NA	
ENSG00000283166	MTND2P27	MT-ND2 pseudogene 27	100873232	0	NA	NA	NA	NA	NA	
ENSG00000283167	NA	NA	NA	0	NA	NA	NA	NA	NA	
ENSG00000283174	NA	NA	NA	0	NA	NA	NA	NA	NA	
ENSG00000283176	NA	NA	NA	0	NA	NA	NA	NA	NA	
ENSG00000283178	LOC100289206	chromosome X open reading frame 49 pseudogene	100289206	0	NA	NA	NA	NA	NA	
ENSG00000283189	NA	NA	NA	0	NA	NA	NA	NA	NA	
ENSG00000283195	IGHVII-43-1	immunoglobulin heavy variable (II)-43-1 (pseudogene)	28367	0	NA	NA	NA	NA	NA	
ENSG00000283196	NA	NA	NA	0,575481097	0,708383787	2,575428	0,275054773	0,783274158	NA	
ENSG00000283197	NA	NA	NA	0	NA	NA	NA	NA	NA	
ENSG00000283201	ZNF724	zinc finger protein 724	440519	7,394122872	0,906898213	0,896901	1,011146197	0,311946462	NA	
ENSG00000283202	NA	NA	NA	0	NA	NA	NA	NA	NA	
ENSG00000283205	LOC122394732	uncharacterized LOC122394732	122394732	0	NA	NA	NA	NA	NA	
ENSG00000283208	NA	NA	NA	0	NA	NA	NA	NA	NA	
ENSG00000283211	NA	NA	NA	0	NA	NA	NA	NA	NA	
ENSG00000283214	NA	NA	NA	0	NA	NA	NA	NA	NA	
ENSG00000283219	NA	NA	NA	0	NA	NA	NA	NA	NA	
ENSG00000283227	SPRR5	small proline rich protein 5	110806278	0	NA	NA	NA	NA	NA	
ENSG00000283228	NA	NA	NA	0	NA	NA	NA	NA	NA	
ENSG00000283230	NA	NA	NA	0	NA	NA	NA	NA	NA	
ENSG00000283232	CYP2C23P	cytochrome P450 family 2 subfamily C member 23, pseudogene	107436076	0	NA	NA	NA	NA	NA	
ENSG00000283234	NA	NA	NA	0	NA	NA	NA	NA	NA	
ENSG00000283236	NA	NA	NA	4,547132553	1,141312795	0,94789	1,204056639	0,228567688	NA	
ENSG00000283239	KBTBD11-OT1	KBTBD11 overlapping transcript 1	104266957	0,5904012	-3,405965431	3,955821	-0,861000872	0,389237563	NA	
ENSG00000283246	NA	NA	NA	0	NA	NA	NA	NA	NA	
ENSG00000283247	CCDC201	coiled-coil domain containing 201	114515518	0	NA	NA	NA	NA	NA	
ENSG00000283251	NA	NA	NA	0	NA	NA	NA	NA	NA	
ENSG00000283257	NA	NA	NA	0	NA	NA	NA	NA	NA	

ENSG00000283267	FAM237B	family with sequence similarity 237 member B	107986818	0	NA	NA	NA	NA	NA
ENSG00000283268	TEX54	testis expressed 54	111216277	0	NA	NA	NA	NA	NA
ENSG00000283269	NA	NA	NA	0	NA	NA	NA	NA	NA
ENSG00000283270	NA	NA	NA	0	NA	NA	NA	NA	NA
ENSG00000283273	NA	NA	NA	0	NA	NA	NA	NA	NA
ENSG00000283283	NA	NA	NA	0	NA	NA	NA	NA	NA
ENSG00000283285	NA	NA	NA	0	NA	NA	NA	NA	NA
ENSG00000283288	SMIM33	small integral membrane protein 33	111064649	0,117497108	0,847101401	4,080456	0,207599697	0,835541541	NA
ENSG00000283297	TEX52	testis expressed 52	101929469	0	NA	NA	NA	NA	NA
ENSG00000283307	OR5AK4P	olfactory receptor family 5 subfamily AK member 4 pseudogene	219525	0	NA	NA	NA	NA	NA
ENSG00000283311	NA	NA	NA	0	NA	NA	NA	NA	NA
ENSG00000283312	NA	NA	NA	0,832782519	0,760630654	2,232552	0,340700093	0,733329369	NA
ENSG00000283321	NA	NA	NA	0	NA	NA	NA	NA	NA
ENSG00000283324	CTXND2	cortexin domain containing 2	100996521	0	NA	NA	NA	NA	NA
ENSG00000283329	FAM240B	family with sequence similarity 240 member B	110806297	0	NA	NA	NA	NA	NA
ENSG00000283338	NA	NA	NA	0,10543123	0	4,080456	0	1	NA
ENSG00000283339	NA	NA	NA	0	NA	NA	NA	NA	NA
ENSG00000283342	NA	NA	NA	0	NA	NA	NA	NA	NA
ENSG00000283345	MPHOSPH10P3	MPHOSPH10 pseudogene 3	100652840	2,733837363	3,953708051	2,208913	1,789888679	0,073471809	NA
ENSG00000283349	PFN5P	profilin 5, pseudogene	112422909	0	NA	NA	NA	NA	NA
ENSG00000283356	NA	NA	NA	0	NA	NA	NA	NA	NA
ENSG00000283361	CFAP97D2	CFAP97 domain containing 2	101929355	0	NA	NA	NA	NA	NA
ENSG00000283365	NCOA5LP	nuclear receptor coactivator 5 like, pseudogene	118568822	0	NA	NA	NA	NA	NA
ENSG00000283366	NA	NA	NA	0	NA	NA	NA	NA	NA
ENSG00000283371	NA	NA	NA	0	NA	NA	NA	NA	NA
ENSG00000283376	NA	NA	NA	0	NA	NA	NA	NA	NA
ENSG00000283377	MTND1P25	MT-ND1 pseudogene 25	100288950	0	NA	NA	NA	NA	NA
ENSG00000283378	CNTNAP3	contactin associated protein family member 3	79937	0	NA	NA	NA	NA	NA
ENSG00000283378	CNTNAP3C	contactin associated protein family member 3C	100289279	0	NA	NA	NA	NA	NA
ENSG00000283381	NA	NA	NA	0	NA	NA	NA	NA	NA
ENSG00000283390	NA	NA	NA	0	NA	NA	NA	NA	NA
ENSG00000283393	NA	NA	NA	0,572054641	2,932620102	2,668507	1,098974114	0,271779357	NA
ENSG00000283399	LOC100887080	acyl-CoA synthetase medium chain family member 4 pseudogene	100887080	0	NA	NA	NA	NA	NA
ENSG00000283402	NA	NA	NA	0	NA	NA	NA	NA	NA
ENSG00000283421	NA	NA	NA	0	NA	NA	NA	NA	NA
ENSG00000283426	SMBD1P	somatomedin B domain containing 1, pseudogene	111674481	1,664313461	0,680312632	1,629047	0,417613947	0,676229398	NA
ENSG00000283427	NA	NA	NA	0	NA	NA	NA	NA	NA
ENSG00000283428	CCDC195	coiled-coil domain containing 195	110806281	0	NA	NA	NA	NA	NA
ENSG00000283431	NA	NA	NA	0	NA	NA	NA	NA	NA
ENSG00000283434	CSNKA2IP	casein kinase 2 subunit alpha' interacting protein	111064647	0	NA	NA	NA	NA	NA
ENSG00000283443	NA	NA	NA	0,10488184	-1,076443944	4,080456	-0,263804824	0,791930317	NA
ENSG00000283444	GPR141BP	G protein-coupled receptor 141B, pseudogene	100420413	0,488717015	0	3,373345	0	1	NA
ENSG00000283446	NA	NA	NA	0	NA	NA	NA	NA	NA
ENSG00000283451	NA	NA	NA	0	NA	NA	NA	NA	NA
ENSG00000283453	PRIM2BP	primase 2B, pseudogene	100996481	0,10488184	-1,076443944	4,080456	-0,263804824	0,791930317	NA
ENSG00000283456	MTND1P37	MT-ND1 pseudogene 37	109729170	0	NA	NA	NA	NA	NA
ENSG00000283463	HSFX4	heat shock transcription factor family, X-linked member 4	101927685	0,210313934	-1,075843829	4,080456	-0,263657754	0,792043652	NA
ENSG00000283464	NA	NA	NA	0	NA	NA	NA	NA	NA
ENSG00000283473	FAM240A	family with sequence similarity 240 member A	100132146	0	NA	NA	NA	NA	NA
ENSG00000283479	NA	NA	NA	0	NA	NA	NA	NA	NA
ENSG00000283481	MTND4LP32	MT-ND4L pseudogene 32	109729156	0	NA	NA	NA	NA	NA
ENSG00000283483	NA	NA	NA	0,328049653	-1,983707022	3,784357	-0,524185966	0,600149201	NA
ENSG00000283491	NA	NA	NA	0	NA	NA	NA	NA	NA
ENSG00000283496	ZNF511-PRAP1	ZNF511-PRAP1 readthrough	104326056	0	NA	NA	NA	NA	NA
ENSG00000283511	NA	NA	NA	0	NA	NA	NA	NA	NA
ENSG00000283516	LITAFD	LITAF domain containing	101929989	0	NA	NA	NA	NA	NA
ENSG00000283524	OR11J7P	olfactory receptor family 11 subfamily J member 7 pseudogene	100288112	0	NA	NA	NA	NA	NA
ENSG00000283525	NA	NA	NA	0	NA	NA	NA	NA	NA

ENSG00000283526	PRRT1B	proline rich transmembrane protein 1B	642515	0,118286652	0	4,080456	0	1	NA
ENSG00000283528	NA	NA	NA	0	NA	NA	NA	NA	NA
ENSG00000283529	NA	NA	NA	0	NA	NA	NA	NA	NA
ENSG00000283535	NA	NA	NA	0	NA	NA	NA	NA	NA
ENSG00000283536	LOC122455340	uncharacterized LOC122455340	122455340	0	NA	NA	NA	NA	NA
ENSG00000283537	NA	NA	NA	2,883241469	2,879583302	1,709479	1,68447991	0,092088959	NA
ENSG00000283541	LOC101930090	contactin associated protein family member 3B pseudogene	101930090	0	NA	NA	NA	NA	NA
ENSG00000283542	OASL2P	2'-5' oligoadenylate synthetase like 2, pseudogene	111216278	0	NA	NA	NA	NA	NA
ENSG00000283547	MTATP6P8	MT-ATP6 pseudogene 8	107131119	0	NA	NA	NA	NA	NA
ENSG00000283559	NA	NA	NA	0	NA	NA	NA	NA	NA
ENSG00000283562	NA	NA	NA	0	NA	NA	NA	NA	NA
ENSG00000283563	NA	NA	NA	0	NA	NA	NA	NA	NA
ENSG00000283566	LOC100996792	dual specificity mitogen-activated protein kinase kinase 3 pseudogene	100996792	0	NA	NA	NA	NA	NA
ENSG00000283567	C19orf85	chromosome 19 open reading frame 85	111064650	0	NA	NA	NA	NA	NA
ENSG00000283571	NA	NA	NA	0	NA	NA	NA	NA	NA
ENSG00000283576	NA	NA	NA	0	NA	NA	NA	NA	NA
ENSG00000283578	MTND5P42	MT-ND5 pseudogene 42	109729157	0	NA	NA	NA	NA	NA
ENSG00000283580	NA	NA	NA	0,917357722	2,77827968	3,531448	0,786725392	0,431442636	NA
ENSG00000283582	NA	NA	NA	0	NA	NA	NA	NA	NA
ENSG00000283583	NA	NA	NA	0	NA	NA	NA	NA	NA
ENSG00000283586	NA	NA	NA	0	NA	NA	NA	NA	NA
ENSG00000283587	NA	NA	NA	0	NA	NA	NA	NA	NA
ENSG00000283594	FAM236C	family with sequence similarity 236 member C	109729126	0	NA	NA	NA	NA	NA
ENSG00000283597	FAM169BP	protein FAM169B	283777	0	NA	NA	NA	NA	NA
ENSG00000283599	LOC101059915	chromosome X open reading frame 49-like	101059915	0,072165449	0,847101401	4,080456	0,207599697	0,835541541	NA
ENSG00000283601	MS4A19P	membrane spanning 4-domains A19, pseudogene	116435280	0	NA	NA	NA	NA	NA
ENSG00000283602	NA	NA	NA	0	NA	NA	NA	NA	NA
ENSG00000283607	IGHVII-30-21	immunoglobulin heavy variable (II)-30-21 (pseudogene)	107548099	0	NA	NA	NA	NA	NA
ENSG00000283611	TMEM274P	transmembrane protein 274, pseudogene	113391335	0	NA	NA	NA	NA	NA
ENSG00000283613	CUPIN1P	cupin superfamily member 1, pseudogene	116435282	0	NA	NA	NA	NA	NA
ENSG00000283625	NA	NA	NA	0	NA	NA	NA	NA	NA
ENSG00000283626	MTATP6P10	MT-ATP6 pseudogene 10	107403069	0	NA	NA	NA	NA	NA
ENSG00000283632	EXOC3L2	exocyst complex component 3 like 2	90332	0,117497108	0,847101401	4,080456	0,207599697	0,835541541	NA
ENSG00000283644	ETDC	embryonic testis differentiation homolog C	110806299	0	NA	NA	NA	NA	NA
ENSG00000283653	NA	NA	NA	0	NA	NA	NA	NA	NA
ENSG00000283654	CIROP	ciliated left-right organizer metallopeptidase	100128908	0	NA	NA	NA	NA	NA
ENSG00000283657	NA	NA	NA	0	NA	NA	NA	NA	NA
ENSG00000283659	NA	NA	NA	0	NA	NA	NA	NA	NA
ENSG00000283663	NA	NA	NA	0	NA	NA	NA	NA	NA
ENSG00000283669	NA	NA	NA	0	NA	NA	NA	NA	NA
ENSG00000283679	NA	NA	NA	0	NA	NA	NA	NA	NA
ENSG00000283680	OR11K1P	olfactory receptor family 11 subfamily K member 1 pseudogene	81109	0	NA	NA	NA	NA	NA
ENSG00000283683	MYOCOS	myocilin opposite strand	110806290	0,3360939	-0,120096611	3,793658	-0,031657202	0,974745425	NA
ENSG00000283686	NA	NA	NA	0	NA	NA	NA	NA	NA
ENSG00000283695	NA	NA	NA	0	NA	NA	NA	NA	NA
ENSG00000283697	HSFX3	heat shock transcription factor family, X-linked member 3	101928917	0	NA	NA	NA	NA	NA
ENSG00000283698	NA	NA	NA	0	NA	NA	NA	NA	NA
ENSG00000283703	VSIG10L2	V-set and immunoglobulin domain containing 10 like 2	338667	0	NA	NA	NA	NA	NA
ENSG00000283706	PRSS50	serine protease 50	29122	0	NA	NA	NA	NA	NA
ENSG00000283709	NA	NA	NA	0,10543123	0	4,080456	0	1	NA
ENSG00000283740	TAF11L11	TATA-box binding protein associated factor 11 like 11	112488746	0	NA	NA	NA	NA	NA
ENSG00000283752	NA	NA	NA	0	NA	NA	NA	NA	NA
ENSG00000283755	CPHXL	cytoplasmic polyadenylated homeobox like	105371346	0	NA	NA	NA	NA	NA

ENSG00000283758	PMIS2	PMIS2 transmembrane protein	111216276	0	NA	NA	NA	NA	NA
ENSG00000283761	NA	NA	NA	0,352491325	2,536807152	3,984503	0,636668335	0,524340903	NA
ENSG00000283765	NA	NA	NA	5,941040461	0,332413534	2,71298	0,122527108	0,902481578	NA
ENSG00000283776	TAF11L13	TATA-box binding protein associated factor 11 like 13	112488747	0	NA	NA	NA	NA	NA
ENSG00000283787	PRR33	proline rich 33	102724536	0,495522684	3,044227839	3,961617	0,768430627	0,44223139	NA
ENSG00000283809	NA	NA	NA	2,307892091	-2,362622041	1,538317	-1,535848568	0,124575525	NA
ENSG00000283865	MTND3P18	MT-ND3 pseudogene 18	107161164	0	NA	NA	NA	NA	NA
ENSG00000283873	SSU72L4	SSU72 like 4	441584	0	NA	NA	NA	NA	NA
ENSG00000283877	NA	NA	NA	0	NA	NA	NA	NA	NA
ENSG00000283879	MTND6P35	MT-ND6 pseudogene 35	112163686	0	NA	NA	NA	NA	NA
ENSG00000283900	TPTEP2-CSNK1E	TPTEP2-CSNK1E readthrough	102800317	5,26116924	0,121522853	1,116936	0,10880016	0,913360997	NA
ENSG00000283906	IGHV4-80	immunoglobulin heavy variable 4-80 (pseudogene)	28390	0	NA	NA	NA	NA	NA
ENSG00000283930	NA	NA	NA	8,247474343	-0,920209006	0,832756	-1,105015758	0,269152761	NA
ENSG00000283940	MTND3P15	MT-ND3 pseudogene 15	107161162	0	NA	NA	NA	NA	NA
ENSG00000283945	NA	NA	NA	0	NA	NA	NA	NA	NA
ENSG00000283948	IGHVIII-16-1	immunoglobulin heavy variable (III)-16-1 (pseudogene)	28350	0	NA	NA	NA	NA	NA
ENSG00000283952	NA	NA	NA	0	NA	NA	NA	NA	NA
ENSG00000283967	TAF11L8	TATA-box binding protein associated factor 11 like 8	112488737	0	NA	NA	NA	NA	NA
ENSG00000283967	LOC124906475	putative TAF11-like protein ENSP00000332601	124906475	0	NA	NA	NA	NA	NA
ENSG00000283967	LOC124906476	putative TAF11-like protein ENSP00000332601	124906476	0	NA	NA	NA	NA	NA
ENSG00000283967	LOC124906477	putative TAF11-like protein ENSP00000332601	124906477	0	NA	NA	NA	NA	NA
ENSG00000283967	LOC124906478	putative TAF11-like protein ENSP00000332601	124906478	0	NA	NA	NA	NA	NA
ENSG00000283967	LOC124906479	putative TAF11-like protein ENSP00000332601	124906479	0	NA	NA	NA	NA	NA
ENSG00000283967	LOC124906480	putative TAF11-like protein ENSP00000332601	124906480	0	NA	NA	NA	NA	NA
ENSG00000283967	LOC124906481	putative TAF11-like protein ENSP00000332601	124906481	0	NA	NA	NA	NA	NA
ENSG00000283967	LOC124906482	putative TAF11-like protein ENSP00000332601	124906482	0	NA	NA	NA	NA	NA
ENSG00000283967	LOC124906483	putative TAF11-like protein ENSP00000332601	124906483	0	NA	NA	NA	NA	NA
ENSG00000283967	LOC124906484	putative TAF11-like protein ENSP00000332601	124906484	0	NA	NA	NA	NA	NA
ENSG00000283967	LOC124906485	putative TAF11-like protein ENSP00000332601	124906485	0	NA	NA	NA	NA	NA
ENSG00000283967	LOC124906486	putative TAF11-like protein ENSP00000332601	124906486	0	NA	NA	NA	NA	NA
ENSG00000283967	LOC124906487	putative TAF11-like protein ENSP00000332601	124906487	0	NA	NA	NA	NA	NA
ENSG00000283967	LOC124906488	putative TAF11-like protein ENSP00000332601	124906488	0	NA	NA	NA	NA	NA
ENSG00000283967	LOC124906489	putative TAF11-like protein ENSP00000332601	124906489	0	NA	NA	NA	NA	NA
ENSG00000283967	LOC124906490	putative TAF11-like protein ENSP00000332601	124906490	0	NA	NA	NA	NA	NA
ENSG00000283967	LOC124906491	putative TAF11-like protein ENSP00000332601	124906491	0	NA	NA	NA	NA	NA
ENSG00000283972	NA	NA	NA	0	NA	NA	NA	NA	NA
ENSG00000283977	NA	NA	NA	0,473146607	0	4,080456	0	1	NA
ENSG00000283980	GNG14	G protein subunit gamma 14	105372280	0	NA	NA	NA	NA	NA
ENSG00000283988	TAF11L9	TATA-box binding protein associated factor 11 like 9	112488745	0	NA	NA	NA	NA	NA
ENSG00000283992	SLURP2	secreted LY6/PLAUR domain containing 2	432355	0	NA	NA	NA	NA	NA
ENSG00000284003	NA	NA	NA	0	NA	NA	NA	NA	NA
ENSG00000284018	SSU72L5	SSU72 like 5	196120	0	NA	NA	NA	NA	NA
ENSG00000284042	TAF11L6	TATA-box binding protein associated factor 11 like 6	391747	0	NA	NA	NA	NA	NA
ENSG00000284042	LOC124906475	putative TAF11-like protein ENSP00000332601	124906475	0	NA	NA	NA	NA	NA
ENSG00000284042	LOC124906476	putative TAF11-like protein ENSP00000332601	124906476	0	NA	NA	NA	NA	NA
ENSG00000284042	LOC124906477	putative TAF11-like protein ENSP00000332601	124906477	0	NA	NA	NA	NA	NA
ENSG00000284042	LOC124906478	putative TAF11-like protein ENSP00000332601	124906478	0	NA	NA	NA	NA	NA
ENSG00000284042	LOC124906479	putative TAF11-like protein ENSP00000332601	124906479	0	NA	NA	NA	NA	NA

ENSG00000284042	LOC124906480	putative TAF11-like protein ENSP00000332601	124906480	0	NA	NA	NA	NA	NA
ENSG00000284042	LOC124906481	putative TAF11-like protein ENSP00000332601	124906481	0	NA	NA	NA	NA	NA
ENSG00000284042	LOC124906482	putative TAF11-like protein ENSP00000332601	124906482	0	NA	NA	NA	NA	NA
ENSG00000284042	LOC124906483	putative TAF11-like protein ENSP00000332601	124906483	0	NA	NA	NA	NA	NA
ENSG00000284042	LOC124906484	putative TAF11-like protein ENSP00000332601	124906484	0	NA	NA	NA	NA	NA
ENSG00000284042	LOC124906485	putative TAF11-like protein ENSP00000332601	124906485	0	NA	NA	NA	NA	NA
ENSG00000284042	LOC124906486	putative TAF11-like protein ENSP00000332601	124906486	0	NA	NA	NA	NA	NA
ENSG00000284042	LOC124906487	putative TAF11-like protein ENSP00000332601	124906487	0	NA	NA	NA	NA	NA
ENSG00000284042	LOC124906488	putative TAF11-like protein ENSP00000332601	124906488	0	NA	NA	NA	NA	NA
ENSG00000284042	LOC124906489	putative TAF11-like protein ENSP00000332601	124906489	0	NA	NA	NA	NA	NA
ENSG00000284042	LOC124906490	putative TAF11-like protein ENSP00000332601	124906490	0	NA	NA	NA	NA	NA
ENSG00000284042	LOC124906491	putative TAF11-like protein ENSP00000332601	124906491	0	NA	NA	NA	NA	NA
ENSG00000284057	NA	NA	NA	4,391668402	4,449234153	3,927605	1,132811102	0,257293588	NA
ENSG00000284060	NA	NA	NA	0,533799114	-1,899522058	3,424423	-0,554698406	0,579100967	NA
ENSG00000284071	NA	NA	NA	0	NA	NA	NA	NA	NA
ENSG00000284098	NA	NA	NA	0	NA	NA	NA	NA	NA
ENSG00000284128	NA	NA	NA	0,327177357	-2,560241689	3,994461	-0,640947957	0,521556496	NA
ENSG00000284130	TUBA3FP	tubulin alpha 3f pseudogene	113691	0,933240469	2,664179186	2,449947	1,087443614	0,276840805	NA
ENSG00000284138	ATP6V0CP4	ATPase H+ transporting V0 subunit c pseudogene 4	112436690	1,753505023	-0,36105422	1,782937	-0,202505298	0,839521717	NA
ENSG00000284167	LOC124906080	ATP synthase subunit a-like	124906080	0	NA	NA	NA	NA	NA
ENSG00000284171	MTND4LP15	MT-ND4L pseudogene 15	107131123	0	NA	NA	NA	NA	NA
ENSG00000284188	C1orf202	chromosome 1 open reading frame 202	122455338	0	NA	NA	NA	NA	NA
ENSG00000284209	CCNYL1B	cyclin Y like 1B	102724485	0	NA	NA	NA	NA	NA
ENSG00000284221	MTND4LP4	MT-ND4L pseudogene 4	107131120	0	NA	NA	NA	NA	NA
ENSG00000284233	NA	NA	NA	0	NA	NA	NA	NA	NA
ENSG00000284234	TAF11L5	TATA-box binding protein associated factor 11 like 5	646066	0	NA	NA	NA	NA	NA
ENSG00000284234	LOC124906475	putative TAF11-like protein ENSP00000332601	124906475	0	NA	NA	NA	NA	NA
ENSG00000284234	LOC124906476	putative TAF11-like protein ENSP00000332601	124906476	0	NA	NA	NA	NA	NA
ENSG00000284234	LOC124906477	putative TAF11-like protein ENSP00000332601	124906477	0	NA	NA	NA	NA	NA
ENSG00000284234	LOC124906478	putative TAF11-like protein ENSP00000332601	124906478	0	NA	NA	NA	NA	NA
ENSG00000284234	LOC124906479	putative TAF11-like protein ENSP00000332601	124906479	0	NA	NA	NA	NA	NA
ENSG00000284234	LOC124906480	putative TAF11-like protein ENSP00000332601	124906480	0	NA	NA	NA	NA	NA
ENSG00000284234	LOC124906481	putative TAF11-like protein ENSP00000332601	124906481	0	NA	NA	NA	NA	NA
ENSG00000284234	LOC124906482	putative TAF11-like protein ENSP00000332601	124906482	0	NA	NA	NA	NA	NA
ENSG00000284234	LOC124906483	putative TAF11-like protein ENSP00000332601	124906483	0	NA	NA	NA	NA	NA
ENSG00000284234	LOC124906484	putative TAF11-like protein ENSP00000332601	124906484	0	NA	NA	NA	NA	NA
ENSG00000284234	LOC124906485	putative TAF11-like protein ENSP00000332601	124906485	0	NA	NA	NA	NA	NA
ENSG00000284234	LOC124906486	putative TAF11-like protein ENSP00000332601	124906486	0	NA	NA	NA	NA	NA
ENSG00000284234	LOC124906487	putative TAF11-like protein ENSP00000332601	124906487	0	NA	NA	NA	NA	NA
ENSG00000284234	LOC124906488	putative TAF11-like protein ENSP00000332601	124906488	0	NA	NA	NA	NA	NA
ENSG00000284234	LOC124906489	putative TAF11-like protein ENSP00000332601	124906489	0	NA	NA	NA	NA	NA
ENSG00000284234	LOC124906490	putative TAF11-like protein ENSP00000332601	124906490	0	NA	NA	NA	NA	NA
ENSG00000284234	LOC124906491	putative TAF11-like protein ENSP00000332601	124906491	0	NA	NA	NA	NA	NA
ENSG00000284242	NA	NA	NA	0	NA	NA	NA	NA	NA
ENSG00000284263	NA	NA	NA	0	NA	NA	NA	NA	NA
ENSG00000284283	TAF11L4	TATA-box binding protein associated factor 11 like 4	391746	0	NA	NA	NA	NA	NA

ENSG00000284291	MTCO2P10	MT-CO2 pseudogene 10	107161153	0	NA	NA	NA	NA	NA
ENSG00000284292	NA	NA	NA	1,480300249	3,47144754	2,718461	1,276989987	0,201605782	NA
ENSG00000284300	NA	NA	NA	0	NA	NA	NA	NA	NA
ENSG00000284306	SSU72L2	SSU72 like 2	390031	0	NA	NA	NA	NA	NA
ENSG00000284308	C2orf81	chromosome 2 open reading frame 81	388963	1,254191596	2,661163238	2,427509	1,09625286	0,272968133	NA
ENSG00000284327	NA	NA	NA	0	NA	NA	NA	NA	NA
ENSG00000284328	MTND4LP8	MT-ND4L pseudogene 8	107131124	0	NA	NA	NA	NA	NA
ENSG00000284337	LIMS3-LOC440895	LIMS3-LOC440895 readthrough	100271835	0,426503561	0,847970659	4,080456	0,207812726	0,835375195	NA
ENSG00000284341	NA	NA	NA	0	NA	NA	NA	NA	NA
ENSG00000284373	TAF11L2	TATA-box binding protein associated factor 11 like 2	391742	0	NA	NA	NA	NA	NA
ENSG00000284393	NA	NA	NA	0	NA	NA	NA	NA	NA
ENSG00000284395	PERCC1	proline and glutamate rich with coiled coil 1	105371045	0,93121738	-0,117526066	2,347108	-0,050072706	0,96006445	NA
ENSG00000284438	SSU72L1	SSU72 like 1	390033	0	NA	NA	NA	NA	NA
ENSG00000284439	TAF11L3	TATA-box binding protein associated factor 11 like 3	646103	0	NA	NA	NA	NA	NA
ENSG00000284461	NA	NA	NA	5,34236794	-1,042519196	1,364965	-0,763770164	0,44500422	NA
ENSG00000284465	TAF11L7	TATA-box binding protein associated factor 11 like 7	112488736	0	NA	NA	NA	NA	NA
ENSG00000284465	LOC124906475	putative TAF11-like protein ENSP00000332601	124906475	0	NA	NA	NA	NA	NA
ENSG00000284465	LOC124906476	putative TAF11-like protein ENSP00000332601	124906476	0	NA	NA	NA	NA	NA
ENSG00000284465	LOC124906477	putative TAF11-like protein ENSP00000332601	124906477	0	NA	NA	NA	NA	NA
ENSG00000284465	LOC124906478	putative TAF11-like protein ENSP00000332601	124906478	0	NA	NA	NA	NA	NA
ENSG00000284465	LOC124906479	putative TAF11-like protein ENSP00000332601	124906479	0	NA	NA	NA	NA	NA
ENSG00000284465	LOC124906480	putative TAF11-like protein ENSP00000332601	124906480	0	NA	NA	NA	NA	NA
ENSG00000284465	LOC124906481	putative TAF11-like protein ENSP00000332601	124906481	0	NA	NA	NA	NA	NA
ENSG00000284465	LOC124906482	putative TAF11-like protein ENSP00000332601	124906482	0	NA	NA	NA	NA	NA
ENSG00000284465	LOC124906483	putative TAF11-like protein ENSP00000332601	124906483	0	NA	NA	NA	NA	NA
ENSG00000284465	LOC124906484	putative TAF11-like protein ENSP00000332601	124906484	0	NA	NA	NA	NA	NA
ENSG00000284465	LOC124906485	putative TAF11-like protein ENSP00000332601	124906485	0	NA	NA	NA	NA	NA
ENSG00000284465	LOC124906486	putative TAF11-like protein ENSP00000332601	124906486	0	NA	NA	NA	NA	NA
ENSG00000284465	LOC124906487	putative TAF11-like protein ENSP00000332601	124906487	0	NA	NA	NA	NA	NA
ENSG00000284465	LOC124906488	putative TAF11-like protein ENSP00000332601	124906488	0	NA	NA	NA	NA	NA
ENSG00000284465	LOC124906489	putative TAF11-like protein ENSP00000332601	124906489	0	NA	NA	NA	NA	NA
ENSG00000284465	LOC124906490	putative TAF11-like protein ENSP00000332601	124906490	0	NA	NA	NA	NA	NA
ENSG00000284465	LOC124906491	putative TAF11-like protein ENSP00000332601	124906491	0	NA	NA	NA	NA	NA
ENSG00000284474	NA	NA	NA	0	NA	NA	NA	NA	NA
ENSG00000284479	SMIM39	small integral membrane protein 39	113523639	0	NA	NA	NA	NA	NA
ENSG00000284484	CPHXL2	cytoplasmic polyadenylated homeobox like 2	112268164	0	NA	NA	NA	NA	NA
ENSG00000284490	NA	NA	NA	0	NA	NA	NA	NA	NA
ENSG00000284491	THSD8	thrombospondin type 1 domain containing 8	111644133	0	NA	NA	NA	NA	NA
ENSG00000284505	LYNX1-SLURP2	LYNX1-SLURP2 readthrough	111188157	0	NA	NA	NA	NA	NA
ENSG00000284516	VAMP9P	vesicle associated membrane protein 9, pseudogene	105377307	0	NA	NA	NA	NA	NA
ENSG00000284522	NA	NA	NA	0	NA	NA	NA	NA	NA
ENSG00000284526	LOC122455342	testis-specific serine/proline-rich protein	122455342	0	NA	NA	NA	NA	NA
ENSG00000284546	SSU72L3	SSU72 like 3	143506	0	NA	NA	NA	NA	NA
ENSG00000284554	NA	NA	NA	0	NA	NA	NA	NA	NA
ENSG00000284558	NA	NA	NA	0	NA	NA	NA	NA	NA
ENSG00000284572	NA	NA	NA	0	NA	NA	NA	NA	NA
ENSG00000284591	NA	NA	NA	0	NA	NA	NA	NA	NA
ENSG00000284592	NA	NA	NA	0	NA	NA	NA	NA	NA
ENSG00000284603	NA	NA	NA	0	NA	NA	NA	NA	NA

ENSG00000284605	ENPP7P15	ectonucleotide pyrophosphatase/phosphodiesterase 7 pseudogene 15	121832813	0	NA	NA	NA	NA	NA
ENSG00000284609	OR8B3	olfactory receptor family 8 subfamily B member 3	390271	0	NA	NA	NA	NA	NA
ENSG00000284610	NA	NA	NA	0	NA	NA	NA	NA	NA
ENSG00000284611	NA	NA	NA	0	NA	NA	NA	NA	NA
ENSG00000284613	NA	NA	NA	0	NA	NA	NA	NA	NA
ENSG00000284614	NA	NA	NA	0	NA	NA	NA	NA	NA
ENSG00000284617	NA	NA	NA	0	NA	NA	NA	NA	NA
ENSG00000284620	NA	NA	NA	0	NA	NA	NA	NA	NA
ENSG00000284621	NA	NA	NA	0	NA	NA	NA	NA	NA
ENSG00000284622	SCYGR10	small cysteine and glycine repeat containing 10 (gene/pseudogene)	112441436	0	NA	NA	NA	NA	NA
ENSG00000284625	NA	NA	NA	0	NA	NA	NA	NA	NA
ENSG00000284626	NA	NA	NA	0,994116202	1,85034722	2,396404	0,772134798	0,440034594	NA
ENSG00000284627	NA	NA	NA	1,26540467	0,43061656	1,634989	0,263375767	0,792260966	NA
ENSG00000284629	SCYGR1	small cysteine and glycine repeat containing 1	112441428	0	NA	NA	NA	NA	NA
ENSG00000284630	NA	NA	NA	0,780448632	-1,588107037	2,163291	-0,734116293	0,462877864	NA
ENSG00000284631	SCYGR4	small cysteine and glycine repeat containing 4	112441430	0	NA	NA	NA	NA	NA
ENSG00000284635	SCYGR8	small cysteine and glycine repeat containing 8	112441432	0	NA	NA	NA	NA	NA
ENSG00000284636	NA	NA	NA	0	NA	NA	NA	NA	NA
ENSG00000284637	NA	NA	NA	0	NA	NA	NA	NA	NA
ENSG00000284638	SMIM44	small integral membrane protein 44	122405565	0,420014407	-2,924836807	3,975074	-0,735794247	0,461855923	NA
ENSG00000284639	NA	NA	NA	0	NA	NA	NA	NA	NA
ENSG00000284643	SCYGR2	small cysteine and glycine repeat containing 2	112441435	0	NA	NA	NA	NA	NA
ENSG00000284648	NA	NA	NA	0	NA	NA	NA	NA	NA
ENSG00000284649	NA	NA	NA	10,82720029	-0,151333041	0,586906	-0,257848958	0,796523479	NA
ENSG00000284651	NA	NA	NA	0	NA	NA	NA	NA	NA
ENSG00000284654	NA	NA	NA	0	NA	NA	NA	NA	NA
ENSG00000284655	NA	NA	NA	0	NA	NA	NA	NA	NA
ENSG00000284659	NA	NA	NA	0	NA	NA	NA	NA	NA
ENSG00000284660	NA	NA	NA	0	NA	NA	NA	NA	NA
ENSG00000284663	NA	NA	NA	0	NA	NA	NA	NA	NA
ENSG00000284665	NA	NA	NA	0	NA	NA	NA	NA	NA
ENSG00000284667	SCYGR5	small cysteine and glycine repeat containing 5	112441437	0	NA	NA	NA	NA	NA
ENSG00000284670	RPSAP56	ribosomal protein SA pseudogene 56	645656	0	NA	NA	NA	NA	NA
ENSG00000284673	NA	NA	NA	0	NA	NA	NA	NA	NA
ENSG00000284679	NA	NA	NA	0	NA	NA	NA	NA	NA
ENSG00000284680	OR8B2	olfactory receptor family 8 subfamily B member 2	26595	0	NA	NA	NA	NA	NA
ENSG00000284682	NA	NA	NA	0	NA	NA	NA	NA	NA
ENSG00000284684	LOC128125818	uncharacterized LOC128125818	128125818	0	NA	NA	NA	NA	NA
ENSG00000284686	NA	NA	NA	0	NA	NA	NA	NA	NA
ENSG00000284687	NA	NA	NA	0	NA	NA	NA	NA	NA
ENSG00000284689	NA	NA	NA	0	NA	NA	NA	NA	NA
ENSG00000284690	CD300H	CD300H molecule (gene/pseudogene)	100130520	0,118286652	0	4,080456	0	1	NA
ENSG00000284695	NA	NA	NA	0	NA	NA	NA	NA	NA
ENSG00000284698	NA	NA	NA	0	NA	NA	NA	NA	NA
ENSG00000284701	TMEM247	transmembrane protein 247	388946	0	NA	NA	NA	NA	NA
ENSG00000284704	SCYGR3	small cysteine and glycine repeat containing 3	112441429	0	NA	NA	NA	NA	NA
ENSG00000284706	LOC100420006	fatty acyl-CoA reductase 2 pseudogene	100420006	0	NA	NA	NA	NA	NA
ENSG00000284709	NA	NA	NA	0,072165449	0,847101401	4,080456	0,207599697	0,835541541	NA
ENSG00000284711	NA	NA	NA	0	NA	NA	NA	NA	NA
ENSG00000284713	SMIM38	small integral membrane protein 38	107984345	1,761067805	-2,815310852	2,037914	-1,38146727	0,167135335	NA
ENSG00000284715	NA	NA	NA	0	NA	NA	NA	NA	NA
ENSG00000284717	NA	NA	NA	0	NA	NA	NA	NA	NA
ENSG00000284718	SCYGR7	small cysteine and glycine repeat containing 7	112441438	0	NA	NA	NA	NA	NA
ENSG00000284723	OR8S1	olfactory receptor family 8 subfamily S member 1	341568	0	NA	NA	NA	NA	NA
ENSG00000284724	NA	NA	NA	0	NA	NA	NA	NA	NA
ENSG00000284725	SCYGR6	small cysteine and glycine repeat containing 6	112441431	0	NA	NA	NA	NA	NA
ENSG00000284727	NA	NA	NA	0	NA	NA	NA	NA	NA
ENSG00000284728	NA	NA	NA	0	NA	NA	NA	NA	NA

ENSG00000284730	TMDD1	transmembrane and death domain 1	112163659	0,209763681	-1,926644696	4,042043	-0,476651224	0,633610501	NA	
ENSG00000284731	LOC124906284	collagen alpha-1(III) chain-like	124906284	0	NA	NA	NA	NA	NA	
ENSG00000284733	OR4F29	olfactory receptor family 4 subfamily F member 29	729759	0,072165449	0,847101401	4,080456	0,207599697	0,835541541	NA	
ENSG00000284740	NA	NA	NA	0	NA	NA	NA	NA	NA	
ENSG00000284753	EEF1AKMT4	EEF1A lysine methyltransferase 4	110599564	7,603566132	-0,000572667	0,710779	-0,000805689	0,999357153	NA	
ENSG00000284762	PDE8B	phosphodiesterase 8B	8622	0	NA	NA	NA	NA	NA	
ENSG00000284763	NA	NA	NA	0	NA	NA	NA	NA	NA	
ENSG00000284772	NA	NA	NA	0	NA	NA	NA	NA	NA	
ENSG00000284776	NA	NA	NA	10,51665385	1,059054763	0,736046	1,438843312	0,150194922	NA	
ENSG00000284779	NA	NA	NA	0	NA	NA	NA	NA	NA	
ENSG00000284783	NA	NA	NA	0,11808024	-1,076443944	4,080456	-0,263804824	0,791930317	NA	
ENSG00000284788	OR11J1P	olfactory receptor family 11 subfamily J member 1 pseudogene	81111	0	NA	NA	NA	NA	NA	
ENSG00000284791	SMIM41	small integral membrane protein 41	113523638	0,119673877	0,847101401	4,080456	0,207599697	0,835541541	NA	
ENSG00000284797	CIST1	colon, intestine and stomach enriched 1	729966	0	NA	NA	NA	NA	NA	
ENSG00000284800	NA	NA	NA	0	NA	NA	NA	NA	NA	
ENSG00000284820	NA	NA	NA	7,995803036	0,571086554	1,18044	0,483791338	0,628533963	NA	
ENSG00000284834	NA	NA	NA	0	NA	NA	NA	NA	NA	
ENSG00000284840	NA	NA	NA	0	NA	NA	NA	NA	NA	
ENSG00000284844	TOMT	transmembrane O-methyltransferase	120356740	0	NA	NA	NA	NA	NA	
ENSG00000284847	NA	NA	NA	0	NA	NA	NA	NA	NA	
ENSG00000284858	LOC124901864	oocyte-secreted protein 4B-like	124901864	0	NA	NA	NA	NA	NA	
ENSG00000284862	CCDC39	coiled-coil domain containing 39	339829	0	NA	NA	NA	NA	NA	
ENSG00000284874	SEPT5-GP1BB	SEPT5-GP1BB readthrough	100526833	0	NA	NA	NA	NA	NA	
ENSG00000284883	NA	NA	NA	0	NA	NA	NA	NA	NA	
ENSG00000284892	NA	NA	NA	0	NA	NA	NA	NA	NA	
ENSG00000284895	LOC128125817	uncharacterized LOC128125817	128125817	0	NA	NA	NA	NA	NA	
ENSG00000284917	EEF1AKMT4-ECE2	EEF1AKMT4-ECE2 readthrough	110599583	0	NA	NA	NA	NA	NA	
ENSG00000284922	LRTOMT	leucine rich transmembrane and O-methyltransferase domain containing	220074	0	NA	NA	NA	NA	NA	
ENSG00000284931	NA	NA	NA	0	NA	NA	NA	NA	NA	
ENSG00000284932	NA	NA	NA	0	NA	NA	NA	NA	NA	
ENSG00000284934	LOC128125816	uncharacterized LOC128125816	128125816	0,729910405	1,159033047	2,699778	0,429306867	0,66769992	NA	
ENSG00000284951	NA	NA	NA	0	NA	NA	NA	NA	NA	
ENSG00000284952	NA	NA	NA	5,44888338	2,067597817	1,539888	1,342693311	0,179371302	NA	
ENSG00000284956	NA	NA	NA	0	NA	NA	NA	NA	NA	
ENSG00000284964	NA	NA	NA	0,833045481	1,114046971	3,056238	0,364515757	0,715472909	NA	
ENSG00000284969	NA	NA	NA	0	NA	NA	NA	NA	NA	
ENSG00000284976	LOC122513141	ring finger protein-like	122513141	0	NA	NA	NA	NA	NA	
ENSG00000284981	UPK3BL2	uroplakin 3B like 2	107983993	0,925191352	1,899128333	2,99721	0,633632051	0,526320979	NA	
ENSG00000284985	NA	NA	NA	0	NA	NA	NA	NA	NA	
ENSG00000284986	NA	NA	NA	0	NA	NA	NA	NA	NA	
ENSG00000284987	NA	NA	NA	0	NA	NA	NA	NA	NA	
ENSG00000284989	NA	NA	NA	4,995314421	-2,590436956	3,821316	-0,677891308	0,497840611	NA	
ENSG00000285000	NA	NA	NA	0	NA	NA	NA	NA	NA	
ENSG00000285010	OOSP4A	oocyte secreted protein family member 4A	112577462	0	NA	NA	NA	NA	NA	
ENSG00000285020	NA	NA	NA	0	NA	NA	NA	NA	NA	
ENSG00000285025	MIURF	mitochondrial elongation factor 1 upstream open reading frame	127898561	0	NA	NA	NA	NA	NA	
ENSG00000285030	NA	NA	NA	0,072165449	0,847101401	4,080456	0,207599697	0,835541541	NA	
ENSG00000285035	NA	NA	NA	0	NA	NA	NA	NA	NA	
ENSG00000285045	NA	NA	NA	0	NA	NA	NA	NA	NA	
ENSG00000285053	NA	NA	NA	5,98264449	-0,329153944	3,744876	-0,087894486	0,92996054	NA	
ENSG00000285057	NA	NA	NA	0	NA	NA	NA	NA	NA	
ENSG00000285062	NA	NA	NA	0,119673877	0,847101401	4,080456	0,207599697	0,835541541	NA	
ENSG00000285064	NA	NA	NA	0	NA	NA	NA	NA	NA	
ENSG00000285082	NA	NA	NA	0,222219638	-1,078728974	4,080456	-0,264364818	0,791498819	NA	
ENSG00000285085	NA	NA	NA	0	NA	NA	NA	NA	NA	
ENSG00000285102	NA	NA	NA	0	NA	NA	NA	NA	NA	
ENSG00000285115	NA	NA	NA	0	NA	NA	NA	NA	NA	

ENSG00000285116	OR4H6BP	olfactory receptor family 4 subfamily H member 6B pseudogene	105370722	0	NA	NA	NA	NA	NA
ENSG00000285117	NA	NA	NA	1,240704445	2,437631032	1,794164	1,35864453	0,174259257	NA
ENSG00000285130	NA	NA	NA	0,469988433	2,955860566	3,965096	0,745470098	0,455987581	NA
ENSG00000285133	LOC128125814	uncharacterized LOC128125814	128125814	0	NA	NA	NA	NA	NA
ENSG00000285135	OR4Q1P	olfactory receptor family 4 subfamily Q member 1 pseudogene	81104	0	NA	NA	NA	NA	NA
ENSG00000285142	NA	NA	NA	0	NA	NA	NA	NA	NA
ENSG00000285160	NA	NA	NA	1,869738625	2,142861442	1,63077	1,314018099	0,188840114	NA
ENSG00000285188	PDE4C	phosphodiesterase 4C	5143	0	NA	NA	NA	NA	NA
ENSG00000285215	NA	NA	NA	1,825259173	-0,097547086	1,852835	-0,052647463	0,958012799	NA
ENSG00000285218	NA	NA	NA	0	NA	NA	NA	NA	NA
ENSG00000285228	NA	NA	NA	0	NA	NA	NA	NA	NA
ENSG00000285231	OOSP3	oocyte secreted protein family member 3	112577461	0,11808024	-1,076443944	4,080456	-0,263804824	0,791930317	NA
ENSG00000285238	NA	NA	NA	1,174971082	4,285285414	3,930105	1,090374298	0,275548299	NA
ENSG00000285245	NA	NA	NA	1,286642909	3,974382601	2,655102	1,496885325	0,134423099	NA
ENSG00000285253	ZNF280D	zinc finger protein 280D	54816	9,076796319	-0,116776693	0,676994	-0,172493021	0,863049948	NA
ENSG00000285254	LOC124902392	uncharacterized LOC124902392	124902392	0	NA	NA	NA	NA	NA
ENSG00000285257	NA	NA	NA	0,605186905	2,443294605	3,31019	0,738113008	0,46044578	NA
ENSG00000285268	NA	NA	NA	0	NA	NA	NA	NA	NA
ENSG00000285269	SLC35D2	solute carrier family 35 member D2	11046	0	NA	NA	NA	NA	NA
ENSG00000285269	SLC35D2-HSD17B3	SLC35D2-HSD17B3 readthrough	127748585	0	NA	NA	NA	NA	NA
ENSG00000285283	NA	NA	NA	0	NA	NA	NA	NA	NA
ENSG00000285287	NA	NA	NA	0	NA	NA	NA	NA	NA
ENSG00000285303	NA	NA	NA	0	NA	NA	NA	NA	NA
ENSG00000285304	NA	NA	NA	9,975459854	-2,1133907	2,230837	-0,947353375	0,343458741	NA
ENSG00000285323	NA	NA	NA	0,754376908	-0,539172591	1,947211	-0,276894772	0,781860907	NA
ENSG00000285329	NA	NA	NA	0	NA	NA	NA	NA	NA
ENSG00000285330	NA	NA	NA	0,236573303	0	4,080456	0	1	NA
ENSG00000285382	NA	NA	NA	1,569413799	-2,983143513	2,181942	-1,367196746	0,171563645	NA
ENSG00000285388	NA	NA	NA	0	NA	NA	NA	NA	NA
ENSG00000285401	NA	NA	NA	0	NA	NA	NA	NA	NA
ENSG00000285404	NA	NA	NA	0	NA	NA	NA	NA	NA
ENSG00000285405	OR11J6P	olfactory receptor family 11 subfamily J member 6 pseudogene	100288148	0	NA	NA	NA	NA	NA
ENSG00000285416	NA	NA	NA	0	NA	NA	NA	NA	NA
ENSG00000285417	NA	NA	NA	0	NA	NA	NA	NA	NA
ENSG00000285420	PDGFRL2P	platelet derived growth factor receptor like 2, pseudogene	116435296	0,072165449	0,847101401	4,080456	0,207599697	0,835541541	NA
ENSG00000285444	NA	NA	NA	1,429095674	0,448110405	1,478256	0,30313451	0,761787356	NA
ENSG00000285445	TUSC2P2	TUSC2 pseudogene 2	112552163	0	NA	NA	NA	NA	NA
ENSG00000285446	NA	NA	NA	0,632587382	0	4,080456	0	1	NA
ENSG00000285458	C4orf36	chromosome 4 open reading frame 36	132989	3,175848436	0,47054501	1,0946	0,429878633	0,667283929	NA
ENSG00000285470	TUSC2P1	TUSC2 pseudogene 1	359794	0	NA	NA	NA	NA	NA
ENSG00000285471	NA	NA	NA	0	NA	NA	NA	NA	NA
ENSG00000285472	NA	NA	NA	0	NA	NA	NA	NA	NA
ENSG00000285480	H2BK1	H2B,K variant histone 1	114483833	0,144330898	1,418786819	4,061262	0,349346267	0,726829368	NA
ENSG00000285491	NA	NA	NA	0	NA	NA	NA	NA	NA
ENSG00000285505	NA	NA	NA	2,112976471	4,280101512	2,589149	1,653091916	0,098312158	NA
ENSG00000285509	NA	NA	NA	0,20943535	-1,074843093	4,080456	-0,263412503	0,792232655	NA
ENSG00000285522	NA	NA	NA	0	NA	NA	NA	NA	NA
ENSG00000285526	NA	NA	NA	0	NA	NA	NA	NA	NA
ENSG00000285528	NA	NA	NA	0	NA	NA	NA	NA	NA
ENSG00000285531	NA	NA	NA	2,78504958	0,903364157	1,102934	0,819055404	0,412754804	NA
ENSG00000285537	NA	NA	NA	0	NA	NA	NA	NA	NA
ENSG00000285542	NA	NA	NA	0,281949031	-0,125163167	3,990241	-0,031367322	0,974976601	NA
ENSG00000285547	NA	NA	NA	0,23616048	-2,075648354	4,028973	-0,515180498	0,606426895	NA
ENSG00000285558	NA	NA	NA	0	NA	NA	NA	NA	NA
ENSG00000285566	LOC647264	keratin-associated protein 21-1	647264	0	NA	NA	NA	NA	NA
ENSG00000285576	NA	NA	NA	0	NA	NA	NA	NA	NA
ENSG00000285585	NA	NA	NA	0	NA	NA	NA	NA	NA
ENSG00000285602	NA	NA	NA	0	NA	NA	NA	NA	NA
ENSG00000285607	FAM90A9	family with sequence similarity 90 member A9	441327	0	NA	NA	NA	NA	NA
ENSG00000285607	LOC124906754	putative protein FAM90A13P	124906754	0	NA	NA	NA	NA	NA
ENSG00000285607	LOC124906759	putative protein FAM90A13P	124906759	0	NA	NA	NA	NA	NA

ENSG00000285609	RNA5SP71	RNA, 5S ribosomal pseudogene 71	100873305	0	NA	NA	NA	NA	NA
ENSG00000285625	NA	NA	NA	0,144330898	1,418786819	4,061262	0,349346267	0,726829368	NA
ENSG00000285626	RNA5SP85	RNA, 5S ribosomal pseudogene 85	100873318	0	NA	NA	NA	NA	NA
ENSG00000285629	NA	NA	NA	0,11808024	-1,076443944	4,080456	-0,263804824	0,791930317	NA
ENSG00000285635	NA	NA	NA	0	NA	NA	NA	NA	NA
ENSG00000285641	NA	NA	NA	2,780343481	2,475668835	1,412596	1,75256677	0,079676399	NA
ENSG00000285645	NA	NA	NA	0	NA	NA	NA	NA	NA
ENSG00000285655	NA	NA	NA	0	NA	NA	NA	NA	NA
ENSG00000285665	NA	NA	NA	0,117497108	0,847101401	4,080456	0,207599697	0,835541541	NA
ENSG00000285674	RNA5SP463	RNA, 5S ribosomal pseudogene 463	100873707	0	NA	NA	NA	NA	NA
ENSG00000285687	FAM90A22	family with sequence similarity 90 member A22	645558	0	NA	NA	NA	NA	NA
ENSG00000285690	NA	NA	NA	0	NA	NA	NA	NA	NA
ENSG00000285697	NA	NA	NA	0	NA	NA	NA	NA	NA
ENSG00000285708	NA	NA	NA	0,736132874	-1,913340949	3,795611	-0,504093003	0,614196024	NA
ENSG00000285713	NA	NA	NA	2,407551351	2,700099974	1,700132	1,588170631	0,11224776	NA
ENSG00000285720	FAM90A17	family with sequence similarity 90 member A17	728746	0	NA	NA	NA	NA	NA
ENSG00000285733	NA	NA	NA	0	NA	NA	NA	NA	NA
ENSG00000285745	NA	NA	NA	0	NA	NA	NA	NA	NA
ENSG00000285746	LOC100129307	putative UPF0607 protein ENSP00000383144	100129307	0	NA	NA	NA	NA	NA
ENSG00000285749	NA	NA	NA	0	NA	NA	NA	NA	NA
ENSG00000285753	LCEP3	late cornified envelope pseudogene 3	450211	0	NA	NA	NA	NA	NA
ENSG00000285760	OR3A5P	olfactory receptor family 3 subfamily A member 5 pseudogene	100856809	0	NA	NA	NA	NA	NA
ENSG00000285765	FAM90A23	family with sequence similarity 90 member A23	645572	0	NA	NA	NA	NA	NA
ENSG00000285765	LOC124906757	putative protein FAM90A23P	124906757	0	NA	NA	NA	NA	NA
ENSG00000285772	NA	NA	NA	0,236573303	0	4,080456	0	1	NA
ENSG00000285776	RNA5SP506	RNA, 5S ribosomal pseudogene 506	100873557	0	NA	NA	NA	NA	NA
ENSG00000285777	NA	NA	NA	0	NA	NA	NA	NA	NA
ENSG00000285779	NA	NA	NA	0,598369386	3,305404915	3,952639	0,83625258	0,403012824	NA
ENSG00000285781	LOC100505502	POM121 transmembrane nucleoporin like, pseudogene	100505502	0	NA	NA	NA	NA	NA
ENSG00000285791	NA	NA	NA	3,298419126	-1,523494347	1,863903	-0,817367917	0,413718205	NA
ENSG00000285794	NA	NA	NA	3,523962473	0,832448702	1,091308	0,762799035	0,445583257	NA
ENSG00000285799	NA	NA	NA	0	NA	NA	NA	NA	NA
ENSG00000285814	FAM90A14	family with sequence similarity 90 member A14	645651	0	NA	NA	NA	NA	NA
ENSG00000285815	GET1-SH3BGR	GET1-SH3BGR readthrough	106865373	0,15836288	0	4,080456	0	1	NA
ENSG00000285816	NA	NA	NA	1,752777932	0,195445077	1,719975	0,113632527	0,909529102	NA
ENSG00000285818	LOC100131107	putative UPF0607 protein ENSP00000383783	100131107	0	NA	NA	NA	NA	NA
ENSG00000285827	NA	NA	NA	8,898457653	-1,08479431	2,194618	-0,494297455	0,621096122	NA
ENSG00000285837	NA	NA	NA	0	NA	NA	NA	NA	NA
ENSG00000285839	NA	NA	NA	0,736054145	-3,24837352	3,723081	-0,87249608	0,382937805	NA
ENSG00000285843	SCYGR9	small cysteine and glycine repeat containing 9 (gene/pseudogene)	117134595	0	NA	NA	NA	NA	NA
ENSG00000285854	NA	NA	NA	0,541958279	0,688370974	3,250819	0,211753113	0,832299649	NA
ENSG00000285862	NA	NA	NA	0	NA	NA	NA	NA	NA
ENSG00000285868	NA	NA	NA	0,576267415	-1,695570171	3,328566	-0,509399551	0,610472191	NA
ENSG00000285880	NA	NA	NA	0	NA	NA	NA	NA	NA
ENSG00000285891	MSANTD5	Myb/SANT DNA binding domain containing 5	102724657	0	NA	NA	NA	NA	NA
ENSG00000285897	NA	NA	NA	0,537690565	2,664422325	3,219961	0,82747044	0,40797047	NA
ENSG00000285901	NA	NA	NA	0	NA	NA	NA	NA	NA
ENSG00000285912	RNA5SP250	RNA, 5S ribosomal pseudogene 250	100873506	0	NA	NA	NA	NA	NA
ENSG00000285913	FAM90A18	family with sequence similarity 90 member A18	441326	0	NA	NA	NA	NA	NA
ENSG00000285920	NA	NA	NA	0	NA	NA	NA	NA	NA
ENSG00000285932	NA	NA	NA	0,548941018	-2,504346854	3,296656	-0,759662759	0,447456195	NA
ENSG00000285937	FAM90A8	family with sequence similarity 90 member A8	441324	0	NA	NA	NA	NA	NA
ENSG00000285942	NA	NA	NA	5,175445582	0,16846348	0,870118	0,193609835	0,846481397	NA
ENSG00000285944	H2AL1MP	H2A,L variant histone 1M, pseudogene	115482687	0	NA	NA	NA	NA	NA
ENSG00000285946	LCE7A	late cornified envelope 7A	122526777	0	NA	NA	NA	NA	NA
ENSG00000285947	NA	NA	NA	0	NA	NA	NA	NA	NA

ENSG00000285950	FAM90A10	family with sequence similarity 90 member A10	441328	0	NA	NA	NA	NA	NA
ENSG00000285950	LOC124906766	putative protein FAM90A10P	124906766	0	NA	NA	NA	NA	NA
ENSG00000285953	NA	NA	NA	0	NA	NA	NA	NA	NA
ENSG00000285975	FAM90A7	family with sequence similarity 90 member A7	441317	0	NA	NA	NA	NA	NA
ENSG00000285975	FAM90A9	family with sequence similarity 90 member A9	441327	0	NA	NA	NA	NA	NA
ENSG00000285978	NA	NA	NA	0,209763681	-1,926644696	4,042043	-0,476651224	0,633610501	NA
ENSG00000285982	NA	NA	NA	0	NA	NA	NA	NA	NA
ENSG00000285989	H2AQ1P	H2A,Q variant histone 1, pseudogene	115482715	0	NA	NA	NA	NA	NA
ENSG00000285990	NBEAP6	neurobeachin pseudogene 6	100418904	0	NA	NA	NA	NA	NA
ENSG00000285991	NA	NA	NA	1,096983979	3,375730454	2,848959	1,184899599	0,236057146	NA
ENSG00000286001	NA	NA	NA	4,765013546	3,402201356	1,544038	2,203443886	0,027563478	NA
ENSG00000286005	NA	NA	NA	0	NA	NA	NA	NA	NA
ENSG00000286014	SPDYE15	speedy/RINGO cell cycle regulator family member E15	105180391	0	NA	NA	NA	NA	NA
ENSG00000286015	NA	NA	NA	0	NA	NA	NA	NA	NA
ENSG00000286022	NA	NA	NA	0	NA	NA	NA	NA	NA
ENSG00000286025	FAM246C	family with sequence similarity 246 member C (gene/pseudogene)	117134596	0	NA	NA	NA	NA	NA
ENSG00000286030	NA	NA	NA	1,992754965	-5,168491681	3,396487	-1,521716727	0,128080075	NA
ENSG00000286038	SPDYE13	speedy/RINGO cell cycle regulator family member E13	105180390	0	NA	NA	NA	NA	NA
ENSG00000286053	ASDURF	ASNSD1 upstream open reading frame	110599588	9,078951238	-0,671311957	1,024883	-0,655013254	0,51245922	NA
ENSG00000286064	NA	NA	NA	0	NA	NA	NA	NA	NA
ENSG00000286070	NA	NA	NA	1,258569467	-3,825279298	2,736242	-1,398004883	0,162111601	NA
ENSG00000286073	NA	NA	NA	0	NA	NA	NA	NA	NA
ENSG00000286088	FAM24B-CUZD1	FAM24B-CUZD1 readthrough	100533195	0,632587382	0	4,080456	0	1	NA
ENSG00000286095	SPEGNB	SPEG neighbor	100996693	0	NA	NA	NA	NA	NA
ENSG00000286098	NA	NA	NA	3,263064847	2,548387348	1,22433	2,081454498	0,037392325	NA
ENSG00000286102	FAM246A	family with sequence similarity 246 member A	117134598	0	NA	NA	NA	NA	NA
ENSG00000286105	TMEM217B	transmembrane protein 217B	121725057	0,072165449	0,847101401	4,080456	0,207599697	0,835541541	NA
ENSG00000286112	KYAT1	kynurenine aminotransferase 1	883	2,958808432	-2,289123039	2,260906	-1,01248029	0,311308466	NA
ENSG00000286114	NA	NA	NA	0	NA	NA	NA	NA	NA
ENSG00000286120	NA	NA	NA	0	NA	NA	NA	NA	NA
ENSG00000286131	LOC128092250	uncharacterized LOC128092250	128092250	0	NA	NA	NA	NA	NA
ENSG00000286132	NA	NA	NA	0	NA	NA	NA	NA	NA
ENSG00000286135	SPADH	spermadhesin family member	112577516	0	NA	NA	NA	NA	NA
ENSG00000286143	NA	NA	NA	0	NA	NA	NA	NA	NA
ENSG00000286151	NA	NA	NA	0,11808024	-1,076443944	4,080456	-0,263804824	0,791930317	NA
ENSG00000286156	NA	NA	NA	0	NA	NA	NA	NA	NA
ENSG00000286165	NA	NA	NA	0,328236599	-0,991757253	3,782172	-0,262218966	0,793152632	NA
ENSG00000286175	FAM246B	family with sequence similarity 246 member B	117134597	0	NA	NA	NA	NA	NA
ENSG00000286192	NA	NA	NA	4,139456695	-0,678477676	3,404632	-0,199280769	0,842043122	NA
ENSG00000286203	NA	NA	NA	0	NA	NA	NA	NA	NA
ENSG00000286215	NA	NA	NA	0	NA	NA	NA	NA	NA
ENSG00000286220	NA	NA	NA	0	NA	NA	NA	NA	NA
ENSG00000286221	NA	NA	NA	0	NA	NA	NA	NA	NA
ENSG00000286224	LOC128092249	uncharacterized LOC128092249	128092249	0,118286652	0	4,080456	0	1	NA
ENSG00000286231	NA	NA	NA	0	NA	NA	NA	NA	NA
ENSG00000286235	NA	NA	NA	1,555760404	4,690183554	3,656278	1,282775519	0,199570733	NA
ENSG00000286237	ARMCX5-GPRASP2	ARMCX5-GPRASP2 readthrough	100528062	0,478695509	2,980701557	3,96411	0,751922061	0,45209793	NA
ENSG00000286239	NA	NA	NA	6,312981862	-0,401632621	0,838777	-0,478831319	0,632058634	NA
ENSG00000286243	NA	NA	NA	0	NA	NA	NA	NA	NA
ENSG00000286255	NA	NA	NA	0	NA	NA	NA	NA	NA
ENSG00000286268	NA	NA	NA	1,568528861	0,121880494	1,758099	0,069325165	0,944730795	NA
ENSG00000286396	NA	NA	NA	0	NA	NA	NA	NA	NA
ENSG00000286406	NA	NA	NA	0	NA	NA	NA	NA	NA
ENSG00000286522	H3C2	H3 clustered histone 2	8358	0,119673877	0,847101401	4,080456	0,207599697	0,835541541	NA
ENSG00000286614	NA	NA	NA	0,430737143	-2,958719508	3,376995	-0,87613978	0,380954049	NA
ENSG00000286651	PRSS43P	serine protease 43, pseudogene	100288960	0	NA	NA	NA	NA	NA
ENSG00000286676	NA	NA	NA	2,329137775	0,677166209	1,335254	0,507143983	0,612053796	NA
ENSG00000286704	NA	NA	NA	0	NA	NA	NA	NA	NA
ENSG00000286744	NA	NA	NA	0	NA	NA	NA	NA	NA
ENSG00000286793	NA	NA	NA	0	NA	NA	NA	NA	NA
ENSG00000286905	NA	NA	NA	1,840259294	2,511996654	3,167869	0,792961004	0,427800543	NA

ENSG00000286916	NA	NA	NA	0	NA	NA	NA	NA	NA
ENSG00000286920	SMIM40	small integral membrane protein 40	113523636	0	NA	NA	NA	NA	NA
ENSG00000286968	NA	NA	NA	0	NA	NA	NA	NA	NA
ENSG00000287002	NA	NA	NA	0	NA	NA	NA	NA	NA
ENSG00000287067	NA	NA	NA	0,679799856	-1,623552216	2,448893	-0,662973828	0,507347316	NA
ENSG00000287071	CDRT15P4	CDRT15 pseudogene 4	117314529	0	NA	NA	NA	NA	NA
ENSG00000287080	H3C3	H3 clustered histone 3	8352	0,117497108	0,847101401	4,080456	0,207599697	0,835541541	NA
ENSG00000287111	NA	NA	NA	0	NA	NA	NA	NA	NA
ENSG00000287194	NA	NA	NA	0	NA	NA	NA	NA	NA
ENSG00000287276	NA	NA	NA	0	NA	NA	NA	NA	NA
ENSG00000287328	NA	NA	NA	0	NA	NA	NA	NA	NA
ENSG00000287353	NA	NA	NA	0	NA	NA	NA	NA	NA
ENSG00000287363	CIMIP3	ciliary microtubule inner protein 3	114841037	0	NA	NA	NA	NA	NA
ENSG00000287450	NA	NA	NA	0	NA	NA	NA	NA	NA
ENSG00000287515	NA	NA	NA	0	NA	NA	NA	NA	NA
ENSG00000287541	NA	NA	NA	0	NA	NA	NA	NA	NA
ENSG00000287585	LOC122319696	uncharacterized LOC122319696	122319696	0	NA	NA	NA	NA	NA
ENSG00000287694	NA	NA	NA	0	NA	NA	NA	NA	NA
ENSG00000287725	NA	NA	NA	7,375677211	1,298901868	1,037743	1,251660738	0,210693512	NA
ENSG00000287751	NA	NA	NA	1,111311935	-1,004411257	2,212694	-0,453931379	0,649878217	NA
ENSG00000287770	NA	NA	NA	0	NA	NA	NA	NA	NA
ENSG00000287779	NA	NA	NA	0	NA	NA	NA	NA	NA
ENSG00000287794	ANCV1RP	ancient vomeronasal 1 receptor pseudogene	113939904	0	NA	NA	NA	NA	NA
ENSG00000287800	NA	NA	NA	0	NA	NA	NA	NA	NA
ENSG00000287810	NA	NA	NA	0	NA	NA	NA	NA	NA
ENSG00000287846	NA	NA	NA	0	NA	NA	NA	NA	NA
ENSG00000287856	NA	NA	NA	1,850846909	0,089089929	1,82371	0,048850943	0,961038084	NA
ENSG00000287908	NA	NA	NA	0	NA	NA	NA	NA	NA
ENSG00000287924	NA	NA	NA	0	NA	NA	NA	NA	NA
ENSG00000287977	NA	NA	NA	0	NA	NA	NA	NA	NA
ENSG00000288000	NA	NA	NA	0	NA	NA	NA	NA	NA
ENSG00000288014	NA	NA	NA	0	NA	NA	NA	NA	NA
ENSG00000288031	NA	NA	NA	0,381892022	0	3,632841	0	1	NA
ENSG00000288053	NA	NA	NA	0	NA	NA	NA	NA	NA
ENSG00000288139	NA	NA	NA	0	NA	NA	NA	NA	NA
ENSG00000288153	NA	NA	NA	0,117497108	0,847101401	4,080456	0,207599697	0,835541541	NA
ENSG00000288208	NA	NA	NA	0,505158144	3,09728805	3,959697	0,782203361	0,434095069	NA
ENSG00000288218	NA	NA	NA	0	NA	NA	NA	NA	NA
ENSG00000288225	NA	NA	NA	0	NA	NA	NA	NA	NA
ENSG00000288255	NA	NA	NA	0	NA	NA	NA	NA	NA
ENSG00000288258	NA	NA	NA	2,514121246	-4,224158585	2,566858	-1,645653556	0,099835106	NA
ENSG00000288262	ZNF73P	zinc finger protein 73, pseudogene	105379427	0	NA	NA	NA	NA	NA
ENSG00000288349	NA	NA	NA	0	NA	NA	NA	NA	NA
ENSG00000288393	NA	NA	NA	0	NA	NA	NA	NA	NA
ENSG00000288434	NA	NA	NA	0	NA	NA	NA	NA	NA
ENSG00000288437	LOC100419917	lysine-rich coiled-coil 1 pseudogene	100419917	0	NA	NA	NA	NA	NA
ENSG00000288460	SMIM42	small integral membrane protein 42	117981789	0	NA	NA	NA	NA	NA
ENSG00000288484	DYNAPP3	DYNAP pseuodgene 3	125078044	0	NA	NA	NA	NA	NA
ENSG00000288520	NA	NA	NA	0	NA	NA	NA	NA	NA
ENSG00000288525	NA	NA	NA	0	NA	NA	NA	NA	NA
ENSG00000288527	NA	NA	NA	0	NA	NA	NA	NA	NA
ENSG00000288529	LOC128092253	umcharacterized LOC128092253	128092253	0	NA	NA	NA	NA	NA
ENSG00000288534	TMX2-CTNND1	TMX2-CTNND1 readthrough (NMD candidate)	100528016	2,839199062	-4,924973761	2,966573	-1,660155653	0,096883143	NA
ENSG00000288550	NA	NA	NA	0	NA	NA	NA	NA	NA
ENSG00000288556	NA	NA	NA	0	NA	NA	NA	NA	NA
ENSG00000288561	LOC390614	relaxin family peptide receptor 3 pseudogene	390614	0	NA	NA	NA	NA	NA
ENSG00000288564	NA	NA	NA	0	NA	NA	NA	NA	NA
ENSG00000288567	NA	NA	NA	0	NA	NA	NA	NA	NA
ENSG00000288570	LOC128092248	umcharacterized LOC128092248	128092248	0	NA	NA	NA	NA	NA
ENSG00000288579	NA	NA	NA	0	NA	NA	NA	NA	NA
ENSG00000288591	NA	NA	NA	0	NA	NA	NA	NA	NA
ENSG00000288592	NA	NA	NA	0	NA	NA	NA	NA	NA
ENSG00000288593	NA	NA	NA	0,543772978	-0,126549654	3,427484	-0,036922027	0,970547177	NA
ENSG00000288601	RNA5SP424	RNA, 5S ribosomal pseudogene 424	100873675	0	NA	NA	NA	NA	NA
ENSG00000288602	C8orf44-SGK3	C8orf44-SGK3 readthrough	100533105	3,884033854	-3,91617773	3,941229	-0,993643829	0,320396298	NA
ENSG00000288604	NA	NA	NA	3,100147649	0,030300589	1,036943	0,029221073	0,976688274	NA

ENSG00000288607	ELOA3BP	elongin A3 family member B, pseudogene	728929	0	NA	NA	NA	NA	NA
ENSG00000288608	LOC128092247	uncharacterized LOC128092247	128092247	0	NA	NA	NA	NA	NA
ENSG00000288611	NPBWR1	neuropeptides B and W receptor 1	2831	0,518598333	-2,035041141	2,764189	-0,736216307	0,46159907	NA
ENSG00000288614	LOC128092246	uncharacterized LOC128092246	128092246	0	NA	NA	NA	NA	NA
ENSG00000288616	ELOA3DP	elongin A3 family member D, pseudogene	100506888	0	NA	NA	NA	NA	NA
ENSG00000288620	NA	NA	NA	10,29572894	-0,130745417	0,550398	-0,237547208	0,8122323	NA
ENSG00000288622	PDCD6-AHRR	PDCD6-AHRR readthrough (NMD candidate)	116412618	7,140770787	-5,50369447	3,004023	-1,832107712	0,066935375	NA
ENSG00000288623	LOC128071547	uncharacterized LOC128071547	128071547	0	NA	NA	NA	NA	NA
ENSG00000288625	LOC128071545	umcharacterized LOC128071545	128071545	0	NA	NA	NA	NA	NA
ENSG00000288626	LOC128071544	uncharacterized LOC128071544	128071544	0	NA	NA	NA	NA	NA
ENSG00000288627	NA	NA	NA	0	NA	NA	NA	NA	NA
ENSG00000288629	LOC128092251	uncharacterized LOC128092251	128092251	0	NA	NA	NA	NA	NA
ENSG00000288631	NA	NA	NA	0	NA	NA	NA	NA	NA
ENSG00000288633	NA	NA	NA	8,681406984	0,060086418	0,624634	0,096194558	0,923366052	NA
ENSG00000288634	LOC128031837	uncharacterized LOC128031837	128031837	0	NA	NA	NA	NA	NA
ENSG00000288635	NA	NA	NA	0,118286652	0	4,080456	0	1	NA
ENSG00000288636	NA	NA	NA	0,556962453	1,945774374	4,022415	0,483732834	0,628575488	NA
ENSG00000288637	NA	NA	NA	0	NA	NA	NA	NA	NA
ENSG00000288640	NA	NA	NA	3,630700173	-2,761010613	1,986638	-1,389790519	0,164592498	NA
ENSG00000288643	NA	NA	NA	0	NA	NA	NA	NA	NA
ENSG00000288644	LOC128031836	uncharacterized LOC128031836	128031836	0	NA	NA	NA	NA	NA
ENSG00000288645	LOC128092252	uncharacterized LOC128092252	128092252	0	NA	NA	NA	NA	NA
ENSG00000288646	LOC128031835	uncharacterized LOC128031835	128031835	0	NA	NA	NA	NA	NA
ENSG00000288649	ACTL10	actin like 10	170487	3,78694669	-1,342626244	1,056585	-1,27072295	0,203827228	NA
ENSG00000288654	LOC128031834	uncharacterized LOC128031834	128031834	0	NA	NA	NA	NA	NA
ENSG00000288658	LOC122319436	uncharacterized LOC122319436	122319436	0	NA	NA	NA	NA	NA
ENSG00000288661	LOC128031833	uncharacterized LOC128031833	128031833	0	NA	NA	NA	NA	NA
ENSG00000288669	NA	NA	NA	0	NA	NA	NA	NA	NA
ENSG00000288671	NA	NA	NA	0	NA	NA	NA	NA	NA
ENSG00000288675	PANO1	proapoptotic nucleolar protein 1	101927423	3,400222273	-0,923851965	0,950086	-0,972387967	0,330857576	NA
ENSG00000288677	HRURF	HR upstream open reading frame	120766137	0	NA	NA	NA	NA	NA
ENSG00000288678	LOC128031832	uncharacterized LOC128031832	128031832	0	NA	NA	NA	NA	NA
ENSG00000288680	NA	NA	NA	0,15836288	0	4,080456	0	1	NA
ENSG00000288681	NA	NA	NA	0	NA	NA	NA	NA	NA
ENSG00000288683	NA	NA	NA	7,963381126	-1,997383204	3,68534	-0,541980696	0,587831805	NA
ENSG00000288684	NA	NA	NA	1,509066912	4,663162909	2,831047	1,647151285	0,099526954	NA
ENSG00000288686	NA	NA	NA	0	NA	NA	NA	NA	NA
ENSG00000288690	NA	NA	NA	0,072165449	0,847101401	4,080456	0,207599697	0,835541541	NA
ENSG00000288694	NA	NA	NA	0	NA	NA	NA	NA	NA
ENSG00000288695	NA	NA	NA	0,732029312	1,957799181	2,670763	0,733048655	0,463528755	NA
ENSG00000288697	NA	NA	NA	0	NA	NA	NA	NA	NA
ENSG00000288698	NA	NA	NA	0	NA	NA	NA	NA	NA
ENSG00000288699	NA	NA	NA	0,144330898	1,418786819	4,061262	0,349346267	0,726829368	NA
ENSG00000288702	UGT1A3	UDP glucuronosyltransferase family 1 member A3	54659	0	NA	NA	NA	NA	NA
ENSG00000288705	UGT1A5	UDP glucuronosyltransferase family 1 member A5	54579	0	NA	NA	NA	NA	NA
ENSG00000288706	LOC127933115	uncharacterized LOC127933115	127933115	0	NA	NA	NA	NA	NA
ENSG00000288708	LOC127903862	uncharacterized LOC127903862	127903862	0	NA	NA	NA	NA	NA
ENSG00000288710	NA	NA	NA	1,1808024	-4,409019753	3,925764	-1,123098489	0,261395668	NA
ENSG00000288711	NA	NA	NA	0,415992612	-2,911962988	3,975679	-0,732444112	0,463897543	NA
ENSG00000288712	LOC127898563	uncharacterized LOC127898563	127898563	0	NA	NA	NA	NA	NA
ENSG00000288715	LOC128462377	uncharacterized LOC128462377	128462377	0	NA	NA	NA	NA	NA
ENSG00000288716	LOC127898562	uncharacterized LOC127898562	127898562	0	NA	NA	NA	NA	NA
ENSG00000288721	NA	NA	NA	4,820566671	0,492714648	1,653001	0,298072802	0,765647599	NA

ENSG00000288725	NA	NA	NA	1,177887507	0,002014661	3,340834	0,000603041	0,999518843	NA
ENSG00000288784	CTAGE4	CTAGE family member 4	100128553	0	NA	NA	NA	NA	NA
ENSG00000288796	NA	NA	NA	0	NA	NA	NA	NA	NA
ENSG00000288841	NA	NA	NA	4,253341776	1,441270231	3,760615	0,383253822	0,701531575	NA
ENSG00000288859	H2AC19	H2A clustered histone 19	723790	0	NA	NA	NA	NA	NA
ENSG00000288864	ARHGAP11A-SCG5	ARHGAP11A-SCG5 readthrough	114118903	2,682548577	1,505103883	1,233867	1,219826586	0,222530621	NA
ENSG00000288867	PPIAL4C	peptidylprolyl isomerase A like 4C	653598	5,800946316	-0,461030253	0,863203	-0,534092516	0,593277528	NA
ENSG00000288894	NA	NA	NA	0	NA	NA	NA	NA	NA
ENSG00000288914	NA	NA	NA	0	NA	NA	NA	NA	NA
ENSG00000289025	NA	NA	NA	0	NA	NA	NA	NA	NA
ENSG00000289027	NA	NA	NA	5,938752363	0,539113826	1,545643	0,34879584	0,727242587	NA
ENSG00000289051	NA	NA	NA	0	NA	NA	NA	NA	NA
ENSG00000289133	NA	NA	NA	0	NA	NA	NA	NA	NA
ENSG00000289258	NA	NA	NA	0	NA	NA	NA	NA	NA
ENSG00000289281	NA	NA	NA	0	NA	NA	NA	NA	NA
ENSG00000289282	NA	NA	NA	0	NA	NA	NA	NA	NA
ENSG00000289325	NA	NA	NA	0	NA	NA	NA	NA	NA
ENSG00000289360	NA	NA	NA	0	NA	NA	NA	NA	NA
ENSG00000289490	NA	NA	NA	0	NA	NA	NA	NA	NA
ENSG00000289491	NA	NA	NA	0	NA	NA	NA	NA	NA
ENSG00000289503	NA	NA	NA	0	NA	NA	NA	NA	NA
ENSG00000289517	NA	NA	NA	2,435289015	1,916344431	2,139157	0,895840932	0,370337725	NA
ENSG00000289549	PPIAL4D	peptidylprolyl isomerase A like 4D	645142	0	NA	NA	NA	NA	NA
ENSG00000289565	NA	NA	NA	0	NA	NA	NA	NA	NA
ENSG00000289568	NA	NA	NA	0	NA	NA	NA	NA	NA
ENSG00000289604	CTAGE8	CTAGE family member 8	100142659	0	NA	NA	NA	NA	NA
ENSG00000289653	NA	NA	NA	0	NA	NA	NA	NA	NA
ENSG00000289685	NA	NA	NA	0,35424072	-2,665130536	3,988406	-0,668219491	0,503993495	NA
ENSG00000289690	NA	NA	NA	0,525060894	-1,906914014	4,04357	-0,471591641	0,637218295	NA
ENSG00000289692	NA	NA	NA	0	NA	NA	NA	NA	NA
ENSG00000289695	NA	NA	NA	0	NA	NA	NA	NA	NA
ENSG00000289697	NA	NA	NA	0,234994216	1,944111989	4,02324	0,483220515	0,62893917	NA
ENSG00000289700	NA	NA	NA	1,430112836	0,054241085	3,164282	0,017141673	0,986323593	NA
ENSG00000289701	NA	NA	NA	4,19274975	-2,906808114	1,730173	-1,680068026	0,092944081	NA
ENSG00000289706	NA	NA	NA	0	NA	NA	NA	NA	NA
ENSG00000289708	NA	NA	NA	0	NA	NA	NA	NA	NA
ENSG00000289710	NA	NA	NA	0	NA	NA	NA	NA	NA
ENSG00000289711	NA	NA	NA	0	NA	NA	NA	NA	NA
ENSG00000289712	NA	NA	NA	0	NA	NA	NA	NA	NA
ENSG00000289713	NA	NA	NA	0	NA	NA	NA	NA	NA
ENSG00000289714	NA	NA	NA	0	NA	NA	NA	NA	NA
ENSG00000289715	NA	NA	NA	0	NA	NA	NA	NA	NA
ENSG00000289716	XNDC1N-ZNF705EP-ALG1L9P	XNDC1N-ZNF705EP-ALG1L9P readthrough	122319435	1,447125331	-1,562451901	2,155165	-0,724980057	0,468464265	NA
ENSG00000289718	XNDC1CP	XRCC1 N-terminal domain containing 1, C-terminal like pseudogene	115995503	0	NA	NA	NA	NA	NA
ENSG00000289720	ITCH	itchy E3 ubiquitin protein ligase	83737	0	NA	NA	NA	NA	NA
ENSG00000289721	PYDC5	pyrin domain containing 5	107181291	0,314645521	-2,509429468	3,997603	-0,627733573	0,530178488	NA
ENSG00000289723	NA	NA	NA	0	NA	NA	NA	NA	NA
ENSG00000289724	NA	NA	NA	0	NA	NA	NA	NA	NA
ENSG00000289729	NA	NA	NA	0	NA	NA	NA	NA	NA
ENSG00000289730	NA	NA	NA	0	NA	NA	NA	NA	NA
ENSG00000289731	NA	NA	NA	0	NA	NA	NA	NA	NA
ENSG00000289732	NA	NA	NA	0	NA	NA	NA	NA	NA
ENSG00000289742	CD200R1P1	CD200R1 pseudogene 1	127379738	0	NA	NA	NA	NA	NA
ENSG00000289747	NA	NA	NA	0	NA	NA	NA	NA	NA
ENSG00000289748	NA	NA	NA	0,330703262	-2,574456632	3,993581	-0,644648702	0,519154866	NA
ENSG00000289756	NA	NA	NA	0,590185399	-0,416170722	2,51155	-0,165702719	0,868390907	NA
ENSG00000289758	NA	NA	NA	0,612771516	3,348703192	3,734256	0,896752424	0,36985104	NA
ENSG00000289760	NA	NA	NA	0	NA	NA	NA	NA	NA
ENSG00000289761	NA	NA	NA	0	NA	NA	NA	NA	NA
ENSG00000289762	SMIM46	small integral membrane protein 46	127138866	0	NA	NA	NA	NA	NA
ENSG00000289763	NA	NA	NA	0	NA	NA	NA	NA	NA
ENSG00000289764	NA	NA	NA	0	NA	NA	NA	NA	NA
ENSG00000289766	NA	NA	NA	0	NA	NA	NA	NA	NA
ENSG00000289767	NA	NA	NA	0	NA	NA	NA	NA	NA
ENSG00000289768	NA	NA	NA	0	NA	NA	NA	NA	NA
ENSG00000289791	NA	NA	NA	0	NA	NA	NA	NA	NA
ENSG00000289809	NA	NA	NA	0	NA	NA	NA	NA	NA
ENSG00000290146	NA	NA	NA	9,322741356	-0,599996793	2,187555	-0,274277393	0,783871457	NA
ENSG00000290147	GUCA1ANB-GUCA1A	GUCA1ANB-GUCA1A readthrough	118142757	0	NA	NA	NA	NA	NA
ENSG00000290149	NA	NA	NA	0	NA	NA	NA	NA	NA

ENSG00000290162	NA	NA	NA	0	NA	NA	NA	NA	NA
ENSG00000290163	NA	NA	NA	0	NA	NA	NA	NA	NA
ENSG00000290164	NA	NA	NA	0	NA	NA	NA	NA	NA
ENSG00000290165	NA	NA	NA	0	NA	NA	NA	NA	NA
ENSG00000290180	NA	NA	NA	0	NA	NA	NA	NA	NA
ENSG00000290184	NA	NA	NA	0	NA	NA	NA	NA	NA
ENSG00000290203	NOX5	NADPH oxidase 5	79400	0	NA	NA	NA	NA	NA
ENSG00000290217	NA	NA	NA	0	NA	NA	NA	NA	NA
ENSG00000290230	NA	NA	NA	4,266578953	2,23650532	1,40814	1,588269005	0,112225523	NA
ENSG00000290263	NA	NA	NA	2,613742004	-0,350636213	2,64596	-0,132517582	0,894574917	NA
ENSG00000290315	NA	NA	NA	4,30825958	6,163297235	3,913117	1,575035312	0,115248294	NA
ENSG00000290316	NA	NA	NA	0,539181206	1,421606357	3,379607	0,420642547	0,67401612	NA
ENSG00000290317	NA	NA	NA	0	NA	NA	NA	NA	NA
ENSG00000290318	NA	NA	NA	7,955823146	1,88755381	1,541153	1,224767006	0,22066302	NA
ENSG00000290320	H2BN1	H2B,N variant histone 1	124188214	0,471022427	0	4,080456	0	1	NA
ENSG00000291239	NA	NA	NA	0	NA	NA	NA	NA	NA
ENSG00000291240	NA	NA	NA	0	NA	NA	NA	NA	NA
ENSG00000291292	NA	NA	NA	0	NA	NA	NA	NA	NA
ENSG00000291302	NA	NA	NA	0	NA	NA	NA	NA	NA
ENSG00000291303	NA	NA	NA	0	NA	NA	NA	NA	NA
ENSG00000291307	POLGARF	POLG alternative reading frame	125316803	0	NA	NA	NA	NA	NA
ENSG00000291308	NA	NA	NA	0	NA	NA	NA	NA	NA
ENSG00000291309	LY6S	lymphocyte antigen 6 family member S	105375795	0,263484924	-1,074315508	4,080456	-0,263283207	0,792332302	NA
ENSG00000291310	NA	NA	NA	0	NA	NA	NA	NA	NA
ENSG00000291312	NA	NA	NA	0	NA	NA	NA	NA	NA
ENSG00000291313	NA	NA	NA	3,134900221	0,611986412	3,816914	0,160335387	0,872616885	NA
ENSG00000291314	NA	NA	NA	0	NA	NA	NA	NA	NA
ENSG00000291315	NA	NA	NA	0	NA	NA	NA	NA	NA
ENSG00000291316	TMEM276-ZFTRAF1	TMEM276-ZFTRAF1 readthrough	157542	0	NA	NA	NA	NA	NA
ENSG00000292259	NA	NA	NA	0	NA	NA	NA	NA	NA
ENSG00000292271	TRBD2	T cell receptor beta diversity 2	28636	0	NA	NA	NA	NA	NA
ENSG00000292277	NA	NA	NA	5,885685173	-0,354886542	1,112823	-0,318906493	0,74979742	NA
ENSG00000292328	NA	NA	NA	0	NA	NA	NA	NA	NA
ENSG00000292329	NA	NA	NA	0,235301789	0,812226729	4,079033	0,199122372	0,842167021	NA
ENSG00000292332	IL3RA	interleukin 3 receptor subunit alpha	3563	0,488521686	-1,956555685	3,35895	-0,582490248	0,560236508	NA
ENSG00000292336	ASMT	acetylserotonin O-methyltransferase	438	2,006653173	0,088017226	1,366211	0,064424324	0,948632363	NA
ENSG00000292352	FABP5P13	fatty acid binding protein 5 pseudogene 13	106480712	0	NA	NA	NA	NA	NA
ENSG00000292353	KRT18P53	keratin 18 pseudogene 53	106478925	0	NA	NA	NA	NA	NA
ENSG00000292354	SHOX	SHOX homeobox	6473	0,10488184	-1,076443944	4,080456	-0,263804824	0,791930317	NA
ENSG00000292356	RNA5SP498	RNA, 5S ribosomal pseudogene 498	106480770	0	NA	NA	NA	NA	NA
ENSG00000292357	CSF2RA	colony stimulating factor 2 receptor subunit alpha	1438	0	NA	NA	NA	NA	NA
ENSG00000292361	NA	NA	NA	10,01533519	0,259247493	0,709313	0,365490963	0,714744951	NA
ENSG00000292363	CRLF2	cytokine receptor like factor 2	64109	0	NA	NA	NA	NA	NA
ENSG00000292364	NA	NA	NA	0	NA	NA	NA	NA	NA
ENSG00000292365	DPH3P2	diphthamide biosynthesis 3 pseudogene 2	106478926	0	NA	NA	NA	NA	NA
ENSG00000292367	ELOCP24	elongin C pseudogene 24	100287692	0	NA	NA	NA	NA	NA
ENSG00000292368	TRPC6P1	TRPC6 pseudogene 1	644218	0	NA	NA	NA	NA	NA
ENSG00000292371	NA	NA	NA	0	NA	NA	NA	NA	NA
ENSG00000293253	NA	NA	NA	0	NA	NA	NA	NA	NA
ENSG00000293542	NA	NA	NA	0	NA	NA	NA	NA	NA
ENSG00000293543	NA	NA	NA	0	NA	NA	NA	NA	NA
ENSG00000293553	NA	NA	NA	0,103998153	-1,076443944	4,080456	-0,263804824	0,791930317	NA
ENSG00000293554	NA	NA	NA	0	NA	NA	NA	NA	NA
ENSG00000293562	NA	NA	NA	0	NA	NA	NA	NA	NA
ENSG00000293563	NA	NA	NA	0	NA	NA	NA	NA	NA
ENSG00000293564	NA	NA	NA	0	NA	NA	NA	NA	NA
ENSG00000293565	NA	NA	NA	0	NA	NA	NA	NA	NA
ENSG00000293569	NA	NA	NA	0,072165449	0,847101401	4,080456	0,207599697	0,835541541	NA
ENSG00000293570	NA	NA	NA	0	NA	NA	NA	NA	NA
ENSG00000293575	NA	NA	NA	0	NA	NA	NA	NA	NA
ENSG00000293584	NA	NA	NA	0	NA	NA	NA	NA	NA
ENSG00000293594	NA	NA	NA	0	NA	NA	NA	NA	NA
